# Correction to: ASH2L drives proliferation and sensitivity to bleomycin and other genotoxins in Hodgkin’s lymphoma and testicular cancer cells

**DOI:** 10.1038/s41419-021-03386-4

**Published:** 2021-01-18

**Authors:** Daniel Constantin, Christian Widmann

**Affiliations:** Department of Biomedical Sciences, University of Lausanne, Bugnon 7, 1005 Lausanne, Switzerland

**Keywords:** Cancer, Cell biology

Correction to: *Cell Death & Disease*

10.1038/s41419-020-03231-0 published online 30 November 2020

The original version of this article unfortunately contained a mistake. The authors omitted to include the acknowledgement section. The authors apologize for the mistake. The original article has been corrected.

Acknowledgments.

We thank Mr. Gilles Dubuis for his expert technical assistance. We also thank, at the University of Lausanne, the Cellular Imaging Facility for its support in image acquisition, the Genomic Technologies Facility for its support in sequence generation and analyses, and the Biostatistics platform for its counseling and statistical method validation.

