# Supplementary material for: ASH2L drives proliferation and sensitivity to bleomycin and other genotoxins in Hodgkin’s lymphoma and testicular cancer cells
Source: Cell Death Dis. 2020 Nov 30;11(11):1019. doi: 10.1038/s41419-020-03231-0 (PMC7705021; doi:10.1038/s41419-020-03231-0)
Supplement: Supplementary file 1 — Table S1 [file 41419_2020_3231_MOESM1_ESM.pdf]

sgRNAs targetting these genes were detected as enriched in the 10-day bleomycin treated population, compared to the population left untreated for 10 days

id	num	pos|score	pos|p-value	pos|fdr	pos|rank	pos|goodsgrna	pos|lfc
ASH2L	6	1.99E-07	3.1455E-06	0.06436	1	6	1.4597
MYC	6	6.93E-06	0.000050086	0.24455	2	4	1.2104
LYRM1	6	7.42E-06	0.000052021	0.24455	3	5	1.3545
CNOT4	6	7.47E-06	0.000052505	0.24455	4	6	0.9488
HAUS3	6	8.72E-06	0.000059764	0.24455	5	6	0.8228
OR2T34	6	2.55E-05	0.00014058	0.36943	6	2	-0.04
AKAP9	6	2.6E-05	0.00014445	0.36943	7	5	0.6889
hsa-mir-708	3	2.66E-05	0.000084444	0.28795	8	3	1.7231
NUP107	6	2.92E-05	0.00016526	0.37569	9	6	0.7767
AHSA2	6	3.92E-05	0.00021897	0.44802	10	6	1.0896
CHD8	6	5.48E-05	0.00029882	0.4835	11	5	1.133
HIST1H2BL	6	5.81E-05	0.00031237	0.4835	12	6	1.3481
CEBPZ	6	5.98E-05	0.00032108	0.4835	13	5	0.9745
RPL18	6	6.39E-05	0.00033802	0.4835	14	6	0.9911
PARP8	6	7.45E-05	0.00038931	0.4835	15	3	0.1568
ACBD3	6	7.81E-05	0.00040528	0.4835	16	3	0.2312
UXT	6	7.93E-05	0.00041303	0.4835	17	4	1.3769
GATAD2B	6	8.29E-05	0.00043432	0.4835	18	5	1.3051
OGDH	6	8.56E-05	0.00045561	0.4835	19	4	0.5446
CCDC117	6	9.05E-05	0.00048513	0.4835	20	6	0.6383
DR1	6	9.19E-05	0.00049626	0.4835	21	6	0.5437
NDC80	6	0.000101	0.00054852	0.51013	22	4	1.2462
ADIG	6	0.000107	0.0005824	0.51808	23	5	1.174
GRAMD1A	6	0.00012	0.00065547	0.5429	24	4	0.8836
RPL28	6	0.000125	0.00067821	0.5429	25	6	0.7427
STARD9	6	0.00013	0.00070773	0.5429	26	2	-0.338
GFER	6	0.000132	0.00071644	0.5429	27	5	1.1809
CD59	6	0.000145	0.00079242	0.5775	28	6	0.9352
ASB7	6	0.000149	0.00081855	0.5775	29	4	0.8931
C18orf32	6	0.000175	0.0009584	0.58181	30	5	0.9053
LATS2	6	0.000182	0.0010131	0.58181	31	2	-0.338
AKAP2	6	0.000185	0.001031	0.58181	32	6	0.651
FAM84A	6	0.000188	0.0010426	0.58181	33	4	0.8029
KCNH3	6	0.00019	0.0010503	0.58181	34	4	0.9628
LAMC1	6	0.00019	0.0010518	0.58181	35	6	0.8102
GUSB	6	0.000191	0.0010533	0.58181	36	6	0.6287
GK	6	0.000202	0.0011041	0.58181	37	5	0.499
MIA	6	0.000205	0.001121	0.58181	38	5	0.9764
PGAP2	6	0.000208	0.0011375	0.58181	39	3	0.5482
C1orf162	6	0.000224	0.0012134	0.60362	40	4	0.7857
FOCAD	6	0.000234	0.0012662	0.60362	41	3	0.109
TLX2	6	0.000235	0.0012686	0.60362	42	5	0.7922
GPR158	6	0.000287	0.0015585	0.67843	43	3	0.2292
MKI67IP	6	0.000296	0.0016044	0.68038	44	4	1.3207
PLAC4	6	0.000302	0.0016306	0.68038	45	6	0.985
SLC52A3	6	0.000321	0.0017274	0.68038	46	6	0.7416
hsa-mir-3678	3	0.000331	0.0009734	0.58181	47	2	2.7563
PHYH	6	0.000332	0.001783	0.68038	48	6	0.578
FSHR	6	0.000335	0.0017995	0.68038	49	5	0.9386
C11orf87	6	0.000339	0.0018174	0.68038	50	2	-3E-04
BTD	6	0.000341	0.001829	0.68038	51	3	0.3418
NPM1	6	0.000356	0.0019049	0.68625	52	3	0.9293
PMF1	4	0.000358	0.0013562	0.63063	53	2	0.9195
GFM1	6	0.000358	0.0019185	0.68625	54	3	0.5215
NDUFB9	6	0.00037	0.00198	0.68625	55	5	0.9466
NDUFA13	6	0.000386	0.0020637	0.68625	56	5	0.6678
AFF1	6	0.000391	0.0020903	0.68625	57	3	0.2795
EIF1AX	6	0.000393	0.0021024	0.68625	58	4	1.1023
NDUFB7	4	0.000395	0.0014936	0.66434	59	4	0.9241
IQGAP1	6	0.000417	0.0022263	0.68625	60	5	0.567
SRFBP1	6	0.000426	0.0022674	0.68625	61	3	0.2911
CAPZB	6	0.000431	0.002294	0.68625	62	5	1.2598
ATP5C1	6	0.000435	0.0023143	0.68625	63	4	0.871
ATP12A	6	0.000443	0.0023531	0.68777	64	1	-0.585
hsa-mir-367	4	0.000444	0.001664	0.68038	65	3	1.1675
WFDC8	6	0.000475	0.0025098	0.71899	66	5	0.6002
DTNB	6	0.000479	0.0025302	0.71899	67	5	1.0125
CPA1	6	0.000495	0.0026173	0.73356	68	3	0.0914
PAK1IP1	6	0.000503	0.0026594	0.73528	69	6	0.7461
MAPKAP1	6	0.000526	0.0027673	0.73897	70	6	1.0268
MRPL39	6	0.000532	0.0027983	0.73897	71	5	0.6729
hsa-mir-4755	4	0.000538	0.0020056	0.68625	72	1	-0.192
UBL3	6	0.000547	0.0028786	0.73897	73	3	-0.158
CKAP5	6	0.000549	0.0028936	0.73897	74	6	0.4311
SLC7A9	6	0.000555	0.0029207	0.73897	75	3	-0.099
SS18L2	6	0.000588	0.003106	0.75595	76	4	0.6566
CHURC1	4	0.000596	0.0022176	0.68625	77	4	0.7649
RYK	6	0.000599	0.0031612	0.75595	78	2	-0.088
GMPR2	6	0.000608	0.0032052	0.75595	79	6	0.5387
PMAIP1	6	0.000609	0.003213	0.75595	80	5	0.6421

ID = gene name
num = number of sgRNAs targeting this gene (or microRNA) present in the library
pos|score = the RRA lo value of this gene
pos|p-value = the raw p-value (using permutation) of this gene
pos|fdr = the false discovery rate of this gene
pos|rank = rank of this gene in the list
pos|goodsgrna = number of sgRNAs targeting this gene found enriched in the selected population
pos|lfc = the log fold change of this gene

SNRPD3	6	0.000611	0.0032217	0.75595	81	3	0.2873
PHB	6	0.000613	0.0032343	0.75595	82	4	0.9205
FABP2	6	0.000629	0.0033112	0.75595	83	6	0.6292
AGRN	6	0.000631	0.0033267	0.75595	84	6	0.4168
CCDC83	6	0.000651	0.0034201	0.75595	85	5	0.5407
MRPS7	6	0.000665	0.0034893	0.75595	86	5	0.6785
TMC2	6	0.000676	0.0035522	0.75595	87	4	0.8273
MAD2L1	6	0.000678	0.0035667	0.75595	88	5	1.1351
SNX12	6	0.000685	0.0036006	0.75595	89	3	0.2501
GPC6	6	0.000699	0.0036688	0.75595	90	5	0.5991
GPHA2	6	0.000703	0.0036911	0.75595	91	3	0.0299
BRD4	6	0.000707	0.0037124	0.75595	92	6	0.5533
ZNF845	6	0.000708	0.0037165	0.75595	93	3	0.2895
XPO6	6	0.000717	0.0037661	0.75595	94	3	0.8403
HTN3	2	0.000721	0.0013993	0.63619	95	1	1.9553
MYBBP1A	6	0.000727	0.0038179	0.75595	96	6	0.5309
PKM	6	0.00073	0.0038348	0.75595	97	5	0.8388
PPIH	6	0.000731	0.0038397	0.75595	98	6	0.5492
VCP	6	0.000732	0.0038426	0.75595	99	4	0.7498
hsa-mir-454	4	0.000747	0.0027455	0.73897	100	2	0.5321
CTNNBIP1	6	0.000755	0.003965	0.7571	101	3	0.3256
hsa-mir-548ab	3	0.000768	0.0022011	0.68625	102	2	0.7783
RPLP0	6	0.000769	0.0040405	0.7571	103	6	0.7914
PAICS	6	0.00077	0.0040444	0.7571	104	6	0.7617
PITPNC1	6	0.000778	0.0040865	0.7571	105	4	0.387
TRIM41	6	0.000786	0.004129	0.7571	106	6	0.6787
hsa-mir-548m	4	0.000799	0.0029255	0.73897	107	1	-1.182
FBXO22	6	0.000814	0.0042699	0.7571	108	4	0.8531
LOC100653515	6	0.000825	0.004325	0.7571	109	3	0.3507
PIGU	6	0.000825	0.0043294	0.7571	110	3	0.4215
WDR33	6	0.000827	0.0043347	0.7571	111	6	0.3937
MGAT5	6	0.000832	0.0043657	0.7571	112	6	0.7634
ANO10	6	0.000839	0.004402	0.7571	113	5	0.7028
OR5L1	6	0.000859	0.0045142	0.7571	114	4	0.5883
37316	6	0.00088	0.0046091	0.7571	115	4	0.7177
KRT2	6	0.000896	0.0046962	0.7571	116	3	0.3724
SNIP1	6	0.000911	0.0047727	0.7571	117	4	0.4594
NPAS2	6	0.000912	0.0047741	0.7571	118	3	0.2681
HK2	6	0.000913	0.0047794	0.7571	119	5	0.6944
CCDC93	6	0.000916	0.0047954	0.7571	120	6	0.5136
SDHB	6	0.000931	0.0048602	0.7571	121	5	0.8758
DEFB118	6	0.000939	0.0049072	0.7571	122	4	0.8628
IL18	6	0.000948	0.0049522	0.7571	123	5	0.7093
TTC36	6	0.000963	0.0050219	0.7571	124	3	0.2694
ANP32E	6	0.000964	0.0050248	0.7571	125	5	1.0259
LCK	6	0.000993	0.0051554	0.7571	126	6	0.7525
CLDN19	6	0.000996	0.00517	0.7571	127	6	0.4785
RASGRF2	6	0.000997	0.0051762	0.7571	128	6	0.5859
OXNAD1	6	0.001004	0.0052053	0.7571	129	4	0.2906
RASSF1	4	0.001011	0.0036795	0.75595	130	4	0.7709
DDX4	6	0.001012	0.0052435	0.7571	131	6	0.6691
TRIM10	6	0.001016	0.0052609	0.7571	132	3	0.2097
HMGXB4	6	0.001023	0.0052929	0.7571	133	3	0.7041
ARL13B	6	0.001025	0.005305	0.7571	134	6	0.5815
RFK	6	0.001031	0.0053359	0.7571	135	4	1.1004
RBBP8	6	0.001066	0.0055019	0.7571	136	5	0.6796
CDAN1	6	0.001067	0.0055092	0.7571	137	4	0.6755
OXCT1	6	0.001068	0.0055131	0.7571	138	3	0.3562
KRT16	6	0.001093	0.0056505	0.7571	139	3	0.4131
PM20D1	6	0.001094	0.0056563	0.7571	140	3	0.484
CCDC110	6	0.001102	0.0056945	0.7571	141	6	0.4871
POLQ	6	0.001109	0.0057352	0.7571	142	4	0.8924
DARS	6	0.001109	0.0057381	0.7571	143	4	0.9459
OOEP	6	0.001117	0.0057753	0.7571	144	2	-0.075
MARCH9	6	0.001131	0.0058421	0.7571	145	5	0.7267
C2orf53	6	0.001132	0.0058523	0.7571	146	4	0.7866
RNF103	6	0.001139	0.0058818	0.7571	147	3	0.4568
MICU2	6	0.001157	0.005967	0.7571	148	4	0.7769
MILR1	6	0.001161	0.0059849	0.7571	149	3	0.4788
hsa-mir-4693	2	0.001182	0.0023051	0.68625	150	2	1.2475
TNFRSF10B	6	0.001198	0.0061533	0.7571	151	1	0.0888
FBXO7	6	0.001199	0.0061576	0.7571	152	4	0.7415
ADAM10	6	0.001211	0.0062162	0.7571	153	5	0.8154
AK9	6	0.001228	0.0062897	0.7571	154	5	0.5267
ORMDL1	6	0.00123	0.0063023	0.7571	155	5	0.6822
USP17L7	6	0.001234	0.006326	0.7571	156	4	0.9583
REC8	6	0.001241	0.0063633	0.7571	157	6	0.8341
LENG9	6	0.001245	0.0063836	0.7571	158	5	0.6349
LILRA3	5	0.001246	0.005589	0.7571	159	4	1.3231
OR6P1	6	0.001248	0.0064015	0.7571	160	5	0.8132
FDXR	6	0.00125	0.0064136	0.7571	161	5	0.7364
HEMGN	6	0.001269	0.0065046	0.7571	162	6	0.5614
APOBEC3B	6	0.001276	0.0065394	0.7571	163	3	-0.069
C4orf6	6	0.001278	0.0065491	0.7571	164	6	0.5045
SLC2A2	6	0.001283	0.0065825	0.7571	165	2	0.0212

NUDT5	6	0.001289	0.0066111	0.7571	166	6	0.4483
ELK3	6	0.001289	0.006614	0.7571	167	3	0.1319
RSL24D1	6	0.001294	0.0066343	0.7571	168	6	0.6282
PNLIPRP3	6	0.0013	0.0066607	0.7571	169	5	0.6955
STAC3	6	0.001316	0.0067359	0.76035	170	3	0.5098
CLDND1	6	0.001324	0.0067795	0.76035	171	6	0.8157
FAM96B	6	0.001328	0.0068008	0.76035	172	3	0.0762
ORMDL3	6	0.001351	0.0069111	0.76848	173	4	0.9355
hsa-mir-6500	4	0.001357	0.0049052	0.7571	174	2	1.0245
IL1R2	6	0.001367	0.0069784	0.76874	175	4	0.7351
TBPL2	6	0.001369	0.0069885	0.76874	176	6	0.6225
UBE2A	6	0.001388	0.0070766	0.77051	177	5	0.7456
FOXA2	6	0.001388	0.00708	0.77051	178	3	0.4556
CAMSAP2	6	0.001423	0.0072542	0.78067	179	3	0.5454
RFXAP	6	0.001427	0.0072735	0.78067	180	3	0.2573
RNASE12	6	0.001432	0.0072939	0.78067	181	1	-0.018
ARID3A	6	0.001441	0.0073398	0.78067	182	5	0.7318
FLOT1	6	0.001446	0.007364	0.78067	183	5	0.8539
RPL7	6	0.00146	0.0074197	0.78251	184	4	1.1933
MTL5	6	0.001479	0.0075136	0.78276	185	6	0.4465
ARPC5L	6	0.001484	0.0075368	0.78276	186	2	-0.208
TLL1	6	0.001513	0.0076747	0.78752	187	4	0.6484
KCNV1	6	0.001523	0.0077231	0.78752	188	3	0.23
PP2D1	6	0.001551	0.0078601	0.78752	189	6	0.3928
NOP16	6	0.001562	0.0079099	0.78752	190	5	1.0551
HSPA8	6	0.001566	0.0079249	0.78752	191	6	0.4657
RAB4B	6	0.001585	0.008028	0.78752	192	5	0.5244
SP9	6	0.001588	0.0080391	0.78752	193	3	-0.002
PRCD	6	0.001591	0.0080531	0.78752	194	5	0.905
F5	6	0.001594	0.0080652	0.78752	195	6	0.6522
hsa-mir-891b	4	0.001597	0.0057473	0.7571	196	1	0.0832
C1orf27	6	0.00161	0.0081335	0.78752	197	6	0.7175
BCCIP	6	0.001627	0.0082201	0.78752	198	4	0.6805
ATP6AP2	6	0.001629	0.0082293	0.78752	199	4	0.7306
YWHAB	6	0.00163	0.0082356	0.78752	200	5	0.5839
AMY2B	3	0.001637	0.004628	0.7571	201	3	1.1392
OR2V1	6	0.001639	0.008275	0.78752	202	5	0.8002
PPCS	6	0.00164	0.008282	0.78752	203	3	0.304
SRRD	6	0.001652	0.0083469	0.78752	204	6	0.3452
IDO1	6	0.00166	0.0083909	0.78752	205	6	0.44
hsa-mir-1277	4	0.00167	0.0059979	0.7571	206	3	1.3158
WDR12	6	0.001675	0.0084611	0.78752	207	3	0.6975
CDC42	6	0.001686	0.0085172	0.78752	208	4	0.3215
hsa-mir-4709	4	0.001713	0.0061489	0.7571	209	4	0.9561
hsa-mir-935	4	0.001715	0.0061533	0.7571	210	3	1.3645
RASL11B	6	0.001718	0.0086672	0.78752	211	3	0.1327
LOC100130480	6	0.001718	0.0086672	0.78752	212	4	0.5608
GRWD1	6	0.00172	0.0086779	0.78752	213	6	0.5999
hsa-mir-566	4	0.001735	0.0062263	0.7571	214	2	1.0684
PLEKHF1	6	0.001743	0.0087834	0.78752	215	5	0.6725
SHKBP1	6	0.001752	0.0088235	0.78752	216	5	0.6514
GANAB	6	0.00178	0.008946	0.78752	217	4	0.7119
CHIA	6	0.001787	0.008976	0.78752	218	5	0.7603
NAP1L1	6	0.001792	0.0090021	0.78752	219	5	0.5692
CSNK2A1	6	0.001796	0.0090239	0.78752	220	3	0.0256
AQP3	6	0.001803	0.0090534	0.78752	221	3	0.3165
CCL21	6	0.001806	0.0090694	0.78752	222	3	0.4707
hsa-mir-92b	4	0.001815	0.0064969	0.7571	223	4	1.9145
STXBP3	6	0.001821	0.0091473	0.78752	224	6	0.8603
NDUFS2	6	0.001848	0.0092775	0.78752	225	2	0.0903
CXCR4	6	0.001856	0.0093171	0.78752	226	4	0.6976
MOXD1	6	0.001886	0.0094701	0.78752	227	5	0.5506
ZNF267	6	0.0019	0.0095441	0.78752	228	3	0.3128
CPNE6	6	0.001911	0.009592	0.78752	229	5	0.6615
NPLOC4	6	0.001913	0.0096036	0.78752	230	4	0.7412
SPRED1	6	0.001916	0.0096186	0.78752	231	6	0.4128
RPL30	6	0.001932	0.0096873	0.78752	232	4	0.7725
hsa-mir-6088	3	0.001935	0.0054482	0.7571	233	3	1.3651
FAM221A	6	0.001945	0.0097532	0.78752	234	4	0.8765
EMG1	6	0.001952	0.009789	0.78752	235	2	0.0061
SRSF7	6	0.001962	0.0098432	0.78752	236	6	0.5559
NDUFB10	6	0.001987	0.009969	0.78752	237	5	0.6352
CCL22	6	0.001996	0.010003	0.78752	238	6	0.3075
WWC2	6	0.002004	0.010042	0.78752	239	2	0.0982
SLAMF6	6	0.002006	0.010055	0.78752	240	5	0.5707
OR2H2	6	0.002016	0.010105	0.78752	241	4	0.7859
FOPNL	6	0.002046	0.010221	0.78752	242	2	-0.22
ERP29	6	0.002049	0.010229	0.78752	243	5	0.4711
GTF2A2	6	0.002049	0.010232	0.78752	244	5	0.778
HIAT1	6	0.002056	0.010258	0.78752	245	2	-0.132
TRIM46	6	0.002058	0.010264	0.78752	246	5	0.8425
OR8D4	6	0.002058	0.010265	0.78752	247	5	0.5955
LSR	6	0.002059	0.010267	0.78752	248	5	0.7355
GGTLC1	6	0.002086	0.010361	0.78752	249	5	0.5506
JAG2	6	0.002108	0.010442	0.78752	250	2	-0.03

CYP4F22	6	0.002132	0.01054	0.78752	251	4	0.3104
ALDOA	6	0.002155	0.01063	0.78752	252	5	0.9857
PEX3	6	0.002184	0.010738	0.78752	253	5	0.7898
ECH1	6	0.002212	0.010844	0.78752	254	4	0.4456
POLRMT	6	0.00222	0.010875	0.78752	255	3	0.2552
PDGFRB	6	0.002236	0.010937	0.78752	256	2	-0.093
GPR156	6	0.002245	0.010971	0.78752	257	5	0.6428
ETV2	6	0.002248	0.010981	0.78752	258	5	0.5997
HFM1	6	0.002251	0.011	0.78752	259	4	0.7934
PLEKHH3	6	0.002264	0.01105	0.78752	260	2	0.2382
hsa-mir-1273f	4	0.002298	0.0082438	0.78752	261	2	0.3937
MRPL18	6	0.002301	0.011185	0.78752	262	5	0.6518
hsa-mir-548at	3	0.002304	0.0064412	0.7571	263	1	-0.325
GLIPR1L2	6	0.002316	0.011243	0.78752	264	3	0.4563
PHGDH	6	0.002322	0.011258	0.78752	265	3	0.2865
FANCM	6	0.002335	0.011301	0.78752	266	5	0.6213
TRIM65	6	0.002356	0.011392	0.78752	267	5	0.8087
DBF4B	6	0.00236	0.011405	0.78752	268	3	0.5075
TSTD2	6	0.002397	0.011536	0.78752	269	3	0.1547
OR2T1	6	0.002404	0.011563	0.78752	270	4	0.644
BTBD1	6	0.002408	0.011581	0.78752	271	4	0.663
LSMEM1	6	0.002449	0.011743	0.78752	272	5	0.6756
TATDN2	6	0.00245	0.011747	0.78752	273	4	0.6706
MPND	6	0.002459	0.011778	0.78752	274	3	0.3307
LYSMD4	6	0.002472	0.011833	0.78752	275	3	0.2101
TRRAP	4	0.002475	0.0088623	0.78752	276	2	0.6671
UTS2	6	0.002482	0.011879	0.78752	277	5	0.5743
hsa-mir-548s	4	0.002515	0.0089978	0.78752	278	3	1.7594
FGD5	6	0.002515	0.012016	0.78752	279	5	0.6472
KCNN2	6	0.002524	0.012059	0.78752	280	5	0.5146
LDLRAD2	6	0.002524	0.012059	0.78752	281	2	0.0737
MLLT10	6	0.002529	0.012074	0.78752	282	5	0.7544
DYRK1A	6	0.002543	0.012128	0.78752	283	4	0.6452
PARS2	6	0.002545	0.012133	0.78752	284	4	0.9509
CARS2	6	0.002549	0.012147	0.78752	285	3	0.5339
AMER3	6	0.002551	0.01215	0.78752	286	4	0.9057
SP1	6	0.002565	0.012209	0.78752	287	5	0.7181
FOLH1	6	0.002569	0.012229	0.78752	288	5	0.5038
TARBP2	6	0.002571	0.012236	0.78752	289	5	0.6422
VARS2	6	0.002573	0.012242	0.78752	290	3	0.6048
hsa-mir-520e	4	0.002585	0.0092262	0.78752	291	2	-0.502
YWHAH	6	0.002601	0.012348	0.78752	292	4	0.7506
S100A7A	6	0.002602	0.012351	0.78752	293	1	-0.419
TGOLN2	6	0.002609	0.012377	0.78752	294	5	0.711
SPINT2	6	0.002619	0.012415	0.78752	295	4	0.6208
FTSJD2	2	0.002646	0.0051835	0.7571	296	2	1.326
SRD5A2	6	0.002669	0.012608	0.78752	297	5	0.5954
IPP	6	0.00268	0.012649	0.78752	298	3	0.2035
PGP	6	0.00272	0.012798	0.78752	299	4	0.626
AAGAB	6	0.002732	0.012841	0.78752	300	3	0.0634
HELB	6	0.002733	0.012844	0.78752	301	3	0.5753
hsa-mir-4647	4	0.002737	0.009728	0.78752	302	3	1.1621
EMD	6	0.002743	0.012878	0.78752	303	4	0.7195
hsa-mir-4654	4	0.002749	0.0097735	0.78752	304	2	0.6649
B3GALTL	6	0.00275	0.012908	0.78752	305	5	0.4619
HDC	6	0.002767	0.012959	0.78752	306	2	-0.168
PRELID1	6	0.002772	0.012971	0.78752	307	3	0.4619
POGZ	6	0.00278	0.013005	0.78752	308	3	0.3657
MZF1	6	0.002783	0.013017	0.78752	309	4	0.6529
APCS	6	0.002784	0.01302	0.78752	310	2	0.1684
TMEM217	6	0.002785	0.013022	0.78752	311	5	0.9333
PRRC2C	6	0.002828	0.013178	0.78752	312	5	0.6098
FAM184A	6	0.002834	0.0132	0.78752	313	4	0.7046
TRIAP1	6	0.002836	0.013207	0.78752	314	5	0.6186
POLG	6	0.00284	0.013219	0.78752	315	5	0.4618
SWT1	5	0.00284	0.012275	0.78752	316	4	1.0134
MED30	6	0.002842	0.013229	0.78752	317	5	0.8694
IL34	6	0.002845	0.013241	0.78752	318	4	0.6348
hsa-mir-4463	4	0.002858	0.010143	0.78752	319	4	1.1727
SGOL1	6	0.002858	0.01328	0.78759	320	4	0.7734
TECTB	6	0.002888	0.013389	0.78944	321	2	0.129
hsa-mir-6087	4	0.002897	0.010273	0.78752	322	2	-0.179
CD84	6	0.002927	0.013534	0.79574	323	4	0.7242
hsa-mir-5586	4	0.002934	0.010398	0.78752	324	3	1.2931
ADCK3	6	0.002966	0.013685	0.79909	325	3	0.219
FOXF1	6	0.002966	0.013685	0.79909	326	3	0.0484
MFAP3L	6	0.002972	0.013709	0.79909	327	2	0.0022
SYT14	6	0.00299	0.013777	0.80079	328	4	1.0373
SYNC	6	0.003013	0.013862	0.80345	329	5	0.8817
RXRB	6	0.003089	0.014168	0.80991	330	3	0.4027
ARID5B	6	0.003096	0.014193	0.80991	331	2	0.1347
CHMP4C	6	0.003097	0.014196	0.80991	332	5	0.6767
CSN3	6	0.003131	0.014325	0.80991	333	5	0.4412
LELP1	6	0.003148	0.014379	0.80991	334	3	-0.241
MRPL13	6	0.003191	0.014533	0.80991	335	4	0.5595

POF1B	6	0.0032	0.014561	0.80991	336	3	0.0964
SLC6A20	4	0.0032	0.01133	0.78752	337	3	1.17
hsa-mir-92a-2	3	0.003205	0.0088811	0.78752	338	3	0.7577
hsa-mir-665	4	0.003209	0.011358	0.78752	339	2	0.1907
VWC2L	6	0.003226	0.014667	0.80991	340	4	0.9507
ZBTB26	4	0.003227	0.011421	0.78752	341	3	0.7274
PDZD9	6	0.003239	0.014711	0.80991	342	5	0.8594
NOC3L	6	0.003252	0.01476	0.80991	343	3	0.2165
DDX42	6	0.003261	0.014793	0.80991	344	4	0.5459
UPK1A	6	0.003274	0.014834	0.80991	345	4	0.4929
TM2D2	6	0.003283	0.014863	0.80991	346	4	0.7536
hsa-mir-649	4	0.003294	0.01165	0.78752	347	4	0.593
HIST1H3E	6	0.003304	0.014951	0.80991	348	2	-0.176
ZNF416	6	0.003304	0.014951	0.80991	349	4	0.8993
hsa-mir-4454	3	0.003304	0.0091555	0.78752	350	3	0.941
SERPINB5	6	0.00331	0.014967	0.80991	351	5	0.6013
SETD9	6	0.003312	0.014978	0.80991	352	3	0.1007
DNM2	6	0.003313	0.01498	0.80991	353	4	0.8878
ZNF721	5	0.00332	0.014189	0.80991	354	4	0.5769
ARR3	6	0.003333	0.015052	0.80991	355	5	0.6765
COMMD8	6	0.003346	0.015101	0.80991	356	2	-0.141
EPAS1	6	0.003356	0.015142	0.80991	357	2	0.0959
SUMO1	6	0.003356	0.015144	0.80991	358	5	0.7294
PET117	4	0.003362	0.011883	0.78752	359	3	0.7302
TOMM70A	4	0.003365	0.01189	0.78752	360	3	0.8401
IPO7	6	0.003368	0.015196	0.80991	361	3	0.4373
hsa-mir-660	4	0.003372	0.01191	0.78752	362	3	0.9483
AIM1	6	0.003382	0.015243	0.80991	363	4	0.5408
TYROBP	6	0.003398	0.01531	0.80991	364	3	0.1693
DHX58	6	0.003402	0.015325	0.80991	365	3	0.0731
ANTXR1	6	0.003404	0.015331	0.80991	366	5	0.5609
CDK6	6	0.003429	0.015436	0.80991	367	4	0.8669
SORBS2	6	0.00343	0.015438	0.80991	368	5	0.5933
KLK14	4	0.003447	0.012166	0.78752	369	3	0.7382
THBS3	6	0.00346	0.015551	0.81239	370	3	0.3467
EPCAM	6	0.003467	0.015581	0.81239	371	4	0.6066
ANK3	6	0.003484	0.015636	0.81239	372	4	0.488
FCGR2B	5	0.003484	0.014847	0.80991	373	5	0.3594
PRB3	5	0.00349	0.014867	0.80991	374	2	0.18
ITGA4	6	0.003501	0.015697	0.81239	375	3	0.5375
ZNF536	6	0.003511	0.015737	0.81239	376	2	-0.626
ZNF706	6	0.003516	0.015754	0.81239	377	3	0.1451
RPS27L	6	0.003524	0.015782	0.81239	378	4	0.6661
COPA	6	0.00353	0.015803	0.81239	379	4	1.0501
RAD51AP2	6	0.003563	0.015934	0.81708	380	3	0.167
MORN5	6	0.003615	0.016128	0.82291	381	3	0.0855
hsa-mir-548u	4	0.003625	0.012761	0.78752	382	2	-0.52
CALB1	6	0.003659	0.016273	0.82541	383	4	0.7758
MYO7B	6	0.003671	0.016319	0.82541	384	2	0.0384
TMEM202	6	0.003706	0.016452	0.82541	385	4	0.6552
CYP3A43	6	0.003719	0.016506	0.82541	386	3	0.2193
ABCC12	6	0.003725	0.016529	0.82541	387	3	0.443
DBR1	6	0.003726	0.016534	0.82541	388	5	0.5261
OR2AG1	6	0.003728	0.016542	0.82541	389	3	0.4656
PVRL2	6	0.003766	0.016671	0.82541	390	5	0.5492
RPL15	6	0.003767	0.016679	0.82541	391	4	0.9761
HIRIP3	6	0.003769	0.016685	0.82541	392	3	0.9193
TGIF2LX	6	0.003771	0.016702	0.82541	393	3	-0.002
hsa-mir-3658	4	0.003797	0.013345	0.78915	394	3	0.8296
ETV6	6	0.003823	0.016894	0.82736	395	2	-0.072
GRPEL1	6	0.003831	0.016921	0.82736	396	5	0.8452
PRR19	6	0.003838	0.01695	0.82736	397	5	0.6582
CCHCR1	6	0.003846	0.016984	0.82736	398	4	0.5298
hsa-mir-553	2	0.003858	0.0074947	0.78276	399	2	1.1324
SCGB1D1	5	0.003876	0.016386	0.82541	400	4	0.5488
RAB1A	6	0.003878	0.017095	0.82779	401	5	0.6205
FAM208B	6	0.003888	0.017134	0.82779	402	4	0.6668
MAT2A	6	0.003894	0.017156	0.82779	403	4	0.4157
ARNT2	6	0.003927	0.017283	0.82779	404	2	-0.323
SPANXN5	5	0.003945	0.016661	0.82541	405	3	0.2396
IL10RB	6	0.003945	0.01735	0.82779	406	5	0.3521
TIMM13	6	0.003979	0.017484	0.82779	407	3	-0.069
FGF1	6	0.003998	0.017546	0.82779	408	4	0.6789
PPP2R3A	6	0.004017	0.017624	0.82779	409	5	0.4909
FZD10	6	0.004031	0.017662	0.82779	410	3	0.1794
TADA3	6	0.004046	0.017723	0.82779	411	4	0.6451
ERGIC1	6	0.004048	0.017724	0.82779	412	5	0.4047
SF3A3	6	0.004071	0.017811	0.82779	413	3	0.3405
LCE3E	3	0.004071	0.01124	0.78752	414	2	1.4428
PQLC3	6	0.004077	0.017832	0.82779	415	5	0.415
PPP1R16A	6	0.004083	0.017853	0.82779	416	2	-0.368
GTF2F2	6	0.0041	0.017909	0.82779	417	3	0.2335
CRX	6	0.004135	0.018021	0.82779	418	3	0.2715
SRPX2	6	0.004147	0.018077	0.82779	419	4	0.6015
MIER1	6	0.004166	0.018144	0.82779	420	3	0.5977

hsa-mir-1286	4	0.004179	0.014611	0.80991	421	3	0.3393
INPP4A	6	0.004203	0.018276	0.82779	422	4	0.5633
PDCD2	6	0.004207	0.018294	0.82779	423	5	0.513
CCDC19	6	0.004218	0.018335	0.82779	424	4	0.7159
C1orf54	6	0.004238	0.018411	0.82779	425	3	-0.107
ATP5A1	6	0.004245	0.018436	0.82779	426	5	0.4217
EP400	6	0.004251	0.018466	0.82779	427	5	0.9376
hsa-mir-208b	4	0.004279	0.01496	0.80991	428	4	0.4962
FGB	6	0.004287	0.018613	0.82779	429	3	-0.141
CORO1A	6	0.00429	0.018623	0.82779	430	3	0.3206
INF2	6	0.004319	0.018726	0.82779	431	5	0.6631
DHX36	6	0.004346	0.018824	0.82779	432	4	0.6147
hsa-mir-4513	4	0.004355	0.015209	0.80991	433	4	0.5007
TRPC5OS	6	0.00436	0.018878	0.82779	434	3	0.2145
HNRNPUL1	6	0.004399	0.019018	0.82779	435	4	0.6542
ATP2A1	6	0.004409	0.019054	0.82779	436	3	0.0928
POU6F1	6	0.004431	0.019145	0.82779	437	5	0.524
COMMD4	6	0.004444	0.019187	0.82779	438	4	0.5918
LMTK2	6	0.004446	0.019199	0.82779	439	3	-0.032
ITFG3	6	0.004448	0.019204	0.82779	440	2	-0.106
FBXW9	6	0.004461	0.019257	0.82779	441	2	0.1189
DNAJC11	6	0.004465	0.019274	0.82779	442	4	0.4824
SLC25A46	6	0.00447	0.019294	0.82779	443	4	0.5879
ZNF660	6	0.004483	0.019343	0.82779	444	5	0.5819
LRRC42	6	0.004498	0.01939	0.82779	445	1	-0.309
KPRP	6	0.004508	0.019431	0.82779	446	5	0.608
DNAJC8	6	0.00455	0.019587	0.82779	447	2	0.0222
SBK2	6	0.004553	0.019598	0.82779	448	4	0.6148
HYDIN	6	0.004577	0.019677	0.82779	449	3	0.7308
GLRA4	5	0.004585	0.019206	0.82779	450	4	0.7528
SNX17	6	0.004602	0.019775	0.82779	451	5	0.6798
POLR3H	6	0.004602	0.019776	0.82779	452	4	0.7376
TDRKH	6	0.004612	0.019805	0.82779	453	5	0.4612
HTRA2	6	0.00462	0.019839	0.82779	454	3	0.1917
OR5P3	6	0.004626	0.019862	0.82779	455	4	0.6281
SLC29A4	6	0.004627	0.019866	0.82779	456	5	0.5918
ADSS	6	0.004654	0.019973	0.82779	457	5	0.4829
PGC	6	0.004655	0.019979	0.82779	458	3	0.2997
RPLP1	6	0.004668	0.020032	0.82779	459	4	0.7486
C6orf47	6	0.004686	0.020091	0.82779	460	5	0.5981
CAMLG	6	0.004713	0.020193	0.82779	461	5	0.5854
DYDC1	6	0.004713	0.020193	0.82779	462	5	0.6138
TCEAL2	6	0.004732	0.020266	0.82779	463	2	-0.022
FAM9B	6	0.004767	0.02039	0.82779	464	5	0.4534
MRPL33	6	0.004774	0.02041	0.82779	465	4	0.6539
HSD17B10	6	0.004774	0.02041	0.82779	466	4	0.6333
MBD4	6	0.004786	0.020455	0.82779	467	5	0.4081
BFSP2	6	0.004807	0.020539	0.82779	468	5	0.5008
TGDS	6	0.004809	0.020547	0.82779	469	4	0.4307
C16orf59	6	0.004817	0.020576	0.82779	470	5	0.6423
CCDC92	4	0.004836	0.016837	0.82736	471	2	0.4979
C8orf82	6	0.00484	0.020662	0.82779	472	4	0.5202
C3orf72	6	0.004849	0.020696	0.82779	473	5	0.5712
TRIM15	6	0.004861	0.02074	0.82779	474	4	0.3463
hsa-mir-610	4	0.004871	0.016961	0.82736	475	3	0.4564
DLG2	5	0.004887	0.020433	0.82779	476	3	0.2214
PIGC	6	0.004891	0.020849	0.82779	477	5	0.7356
MAX	6	0.00492	0.020944	0.82779	478	3	0.5549
hsa-mir-6880	4	0.00494	0.017178	0.82779	479	3	0.504
RCN3	5	0.004959	0.02073	0.82779	480	5	0.5608
RNPEP	6	0.004965	0.021108	0.82779	481	3	0.1476
GTPBP8	6	0.004972	0.021135	0.82779	482	5	0.4554
MCM10	6	0.004975	0.021147	0.82779	483	5	0.8443
DNM3	6	0.004982	0.021168	0.82779	484	3	0.447
ELAVL1	6	0.004985	0.021183	0.82779	485	5	0.64
hsa-mir-6892	4	0.00499	0.017334	0.82779	486	3	0.7413
PDE4A	6	0.004997	0.021226	0.82779	487	3	0.4966
CYP3A7-CYP3AP1	2	0.005004	0.0097004	0.78752	488	1	0.9385
ROMO1	6	0.005007	0.021271	0.82779	489	5	0.6704
FLOT2	6	0.005017	0.021309	0.82779	490	4	0.3576
STX18	6	0.00504	0.021389	0.82779	491	3	0.5948
CERCAM	6	0.005046	0.021409	0.82779	492	5	0.5664
hsa-mir-2110	4	0.0051	0.017702	0.82779	493	3	0.7807
MIR205HG	6	0.005102	0.021602	0.82779	494	3	0.4006
EWSR1	6	0.005114	0.021644	0.82779	495	5	0.4422
VOPP1	6	0.005116	0.021651	0.82779	496	2	-0.214
OR5M9	6	0.005119	0.02166	0.82779	497	5	0.7095
PHF3	6	0.005121	0.021667	0.82779	498	4	0.2963
PFN3	6	0.005135	0.021719	0.82779	499	5	0.6196
ATAD1	6	0.005136	0.021728	0.82779	500	3	0.1057
MANBA	6	0.00516	0.021814	0.82779	501	4	0.7484
ERMARD	6	0.005173	0.021855	0.82779	502	1	-0.244
SLC25A25	6	0.00518	0.021886	0.82779	503	3	0.4129
hsa-mir-3149	2	0.005181	0.010038	0.78752	504	2	2.0432
TMEM9	6	0.005182	0.021891	0.82779	505	4	0.7473

SCAF4	6	0.005195	0.02194	0.82779	506	5	0.4832
AAMP	6	0.005206	0.021979	0.82779	507	5	0.4423
hsa-mir-219a-1	4	0.005208	0.018063	0.82779	508	3	0.9259
ZNF408	6	0.005221	0.022031	0.82779	509	4	0.4268
hsa-mir-6820	4	0.005222	0.018108	0.82779	510	4	0.7351
SPINK14	6	0.005224	0.022042	0.82779	511	3	0.3574
DTWD1	6	0.00524	0.022089	0.82779	512	5	0.5325
PVRL3	6	0.005276	0.022211	0.82779	513	3	0.0544
DNAI1	6	0.005293	0.022266	0.82779	514	4	0.5481
PRR11	6	0.005302	0.022306	0.82779	515	5	0.5731
hsa-mir-3668	2	0.005304	0.010253	0.78752	516	2	0.7531
MRPS15	6	0.00531	0.022329	0.82779	517	5	0.4014
MMP3	5	0.00532	0.022141	0.82779	518	5	0.3373
C10orf82	6	0.005321	0.022368	0.82779	519	4	0.5528
PSMA7	6	0.005325	0.022384	0.82779	520	3	0.4177
STRC	6	0.005328	0.022395	0.82779	521	3	0.2016
NFX1	6	0.005328	0.022395	0.82779	522	2	-0.036
RPS10	2	0.005344	0.010331	0.78752	523	2	1.2607
DMRT3	6	0.00538	0.02259	0.82779	524	3	0.1255
GIMD1	6	0.005408	0.022696	0.82779	525	5	0.511
hsa-mir-6132	4	0.00543	0.018801	0.82779	526	4	0.8165
hsa-mir-4775	3	0.005457	0.015018	0.80991	527	3	1.0215
TBX3	6	0.005459	0.022887	0.82779	528	4	0.5065
MRPS24	5	0.005465	0.022706	0.82779	529	4	0.67
AREG	6	0.005473	0.02294	0.82779	530	2	-0.161
NUDCD2	6	0.005484	0.022974	0.82779	531	1	-0.039
LRRC61	6	0.005493	0.02301	0.82779	532	4	0.9687
CCL23	6	0.005497	0.023022	0.82779	533	2	-0.599
NUBPL	6	0.005516	0.023092	0.82779	534	3	0.3911
MCOLN2	6	0.00553	0.023138	0.82779	535	4	0.554
ANKRD17	6	0.005536	0.023157	0.82779	536	2	-0.216
RPS29	6	0.005537	0.023164	0.82779	537	3	0.666
hsa-mir-5787	4	0.00556	0.019223	0.82779	538	2	0.6367
OCIAD2	6	0.005562	0.023263	0.82779	539	4	0.6262
TMEM245	6	0.005585	0.023352	0.82779	540	4	0.6191
CCDC181	6	0.005599	0.023405	0.82779	541	4	0.5902
CSN1S1	6	0.005606	0.02343	0.82779	542	2	-0.264
COL6A2	6	0.005613	0.023455	0.82779	543	2	-0.123
C12orf29	6	0.005613	0.023455	0.82779	544	4	0.8444
HDHD3	6	0.005636	0.023537	0.82779	545	4	0.6697
DHRS7B	6	0.005659	0.023616	0.82779	546	4	0.5769
MND1	6	0.005666	0.023641	0.82779	547	3	0.5059
KIRREL	6	0.005669	0.023653	0.82779	548	4	0.5286
hsa-mir-3614	4	0.005674	0.019593	0.82779	549	4	0.7426
KAT2B	6	0.005675	0.023679	0.82779	550	4	0.5264
EGF	6	0.00568	0.023699	0.82779	551	4	0.6152
BIRC7	6	0.005685	0.023719	0.82779	552	3	0.1335
IL8	6	0.005691	0.023744	0.82779	553	2	-0.058
hsa-mir-4422	4	0.005701	0.019677	0.82779	554	2	0.5536
RALGDS	6	0.005726	0.023863	0.82779	555	3	0.6013
TESC	6	0.005741	0.023918	0.82779	556	3	0.4392
hsa-mir-651	4	0.005747	0.019836	0.82779	557	4	0.9039
GIMAP1	4	0.005754	0.019855	0.82779	558	4	0.531
SELV	6	0.005778	0.024052	0.82779	559	5	0.6065
KRTAP16-1	6	0.005795	0.024114	0.82779	560	2	0.1039
RAB40A	6	0.005796	0.02412	0.82779	561	3	0.2859
SKIV2L2	6	0.005803	0.024143	0.82779	562	4	0.6196
OR7D2	6	0.005828	0.02423	0.82779	563	2	-0.057
NR2E1	6	0.005847	0.024294	0.82779	564	3	0.1836
IVNS1ABP	6	0.005849	0.024298	0.82779	565	4	0.5735
ZP4	6	0.005862	0.024346	0.82779	566	3	0.1812
BUB3	6	0.005879	0.024416	0.82779	567	3	0.074
TKT	6	0.005886	0.024448	0.82779	568	4	0.3645
SLC13A4	6	0.005899	0.02449	0.82779	569	1	0.0353
HLA-DRB1	5	0.005918	0.0243	0.82779	570	4	0.6267
KCNH8	6	0.005928	0.024593	0.82779	571	5	0.4227
PRSS55	6	0.00595	0.024678	0.82779	572	4	0.1766
NOL4	6	0.005953	0.024687	0.82779	573	4	0.6881
LIAS	6	0.005955	0.024695	0.82779	574	4	0.5119
ATP6V1D	6	0.005965	0.024739	0.82779	575	4	0.6563
PHACTR2	6	0.005972	0.02476	0.82779	576	4	0.5925
PLA2G4D	6	0.006002	0.024872	0.82779	577	3	0.177
QSOX1	6	0.006004	0.024878	0.82779	578	3	0.2239
NAT8B	6	0.006014	0.024911	0.82779	579	5	0.3848
R3HCC1	6	0.006028	0.024971	0.82779	580	5	0.6498
EPT1	6	0.006054	0.02506	0.82779	581	3	0.149
CXCL10	6	0.006063	0.025087	0.82779	582	4	0.3864
PCDHA7	2	0.006078	0.011704	0.78752	583	2	0.8455
HOXC12	6	0.006079	0.025145	0.82779	584	3	0.4779
hsa-mir-1284	4	0.006082	0.020972	0.82779	585	3	0.9244
FAM60A	6	0.00609	0.025186	0.82779	586	4	0.6366
ELMO3	6	0.006104	0.025243	0.82779	587	4	0.5482
FAM83G	6	0.006106	0.025249	0.82779	588	3	0.3059
ACVRL1	6	0.006134	0.025347	0.82779	589	3	0.4228
SPDYE3	4	0.006151	0.021196	0.82779	590	3	0.5261

GPRIN2	6	0.006158	0.025434	0.82779	591	2	-0.113
TFPI	6	0.006188	0.025533	0.82779	592	2	-0.114
KRTAP3-3	6	0.00621	0.025611	0.82779	593	2	-0.12
KRBA2	6	0.00621	0.025614	0.82779	594	5	0.527
TBCB	6	0.006213	0.025628	0.82779	595	4	0.5523
PPA2	6	0.006214	0.025634	0.82779	596	5	0.6411
MLF1IP	6	0.006214	0.025634	0.82779	597	5	0.7101
CARTPT	6	0.00624	0.025734	0.82779	598	4	0.599
USP21	6	0.006245	0.02575	0.82779	599	4	0.7228
C9orf131	6	0.006278	0.025863	0.82779	600	3	0.4712
ORC6	6	0.00629	0.025907	0.82779	601	5	0.499
RANGRF	5	0.006291	0.025396	0.82779	602	3	0.3226
CNBD2	6	0.006307	0.025972	0.82779	603	4	0.7818
RPP40	6	0.006313	0.025994	0.82779	604	2	0.0209
DOCK3	6	0.00633	0.02606	0.82779	605	4	0.7493
DDX20	6	0.006351	0.026137	0.82779	606	5	0.7042
PRRT1	6	0.006365	0.026184	0.82779	607	2	0.0806
UQCR10	6	0.006368	0.026204	0.82779	608	5	0.7276
ZNF225	5	0.006372	0.025642	0.82779	609	4	0.9447
EARS2	6	0.006374	0.026224	0.82779	610	5	0.4001
NOL7	6	0.006382	0.026257	0.82779	611	3	0.1957
DNAJC19	6	0.006382	0.026259	0.82779	612	4	0.5755
ARMC3	6	0.006394	0.0263	0.82779	613	3	0.248
TMTC4	6	0.006412	0.026369	0.82779	614	5	0.5436
ASB11	6	0.006417	0.026388	0.82779	615	4	0.5172
ATF4	6	0.006417	0.02639	0.82779	616	4	0.5163
HPSE	6	0.006418	0.026391	0.82779	617	4	0.7536
MYSM1	6	0.00645	0.026508	0.82779	618	3	0.1221
OR2L3	6	0.006453	0.026517	0.82779	619	3	0.6079
LRRD1	6	0.006457	0.02653	0.82779	620	5	0.5062
MAPRE3	6	0.006463	0.026552	0.82779	621	4	0.6816
CD44	6	0.006469	0.026574	0.82779	622	2	0.0256
HUWE1	6	0.006469	0.026575	0.82779	623	4	0.7105
ARPC3	6	0.006479	0.026615	0.82779	624	4	0.6764
NCSTN	6	0.006479	0.026615	0.82779	625	4	0.5744
DOCK9	6	0.006514	0.026755	0.82779	626	4	0.5925
CRYGC	6	0.006514	0.026755	0.82779	627	3	0.3641
TUBA1A	5	0.006517	0.026065	0.82779	628	1	-0.133
DRG1	6	0.00652	0.026779	0.82779	629	3	-0.211
LYPD6	6	0.006545	0.026868	0.82779	630	3	0.2866
ARL2	6	0.006556	0.026907	0.82779	631	5	0.6031
GRIA1	6	0.006572	0.026971	0.82779	632	2	-0.003
FAM228A	6	0.006576	0.026984	0.82779	633	5	0.4459
MMS22L	6	0.006602	0.027073	0.82779	634	5	0.7028
RPS24	6	0.006624	0.027151	0.82779	635	2	-0.723
MTX3	6	0.006626	0.027155	0.82779	636	5	0.5484
MTA3	6	0.006634	0.027184	0.82779	637	4	0.7288
LAMA4	6	0.006634	0.027188	0.82779	638	5	0.3534
MRPS9	2	0.006666	0.012783	0.78752	639	1	0.1215
ZNF517	6	0.006695	0.027397	0.82881	640	4	0.6946
DHX29	6	0.006699	0.027409	0.82881	641	5	0.639
USP15	6	0.006707	0.027435	0.82881	642	4	0.5422
C12orf66	6	0.006708	0.027438	0.82881	643	5	0.3404
AKT3	6	0.006754	0.027611	0.82881	644	3	0.2772
SRSF10	6	0.006754	0.027611	0.82881	645	4	0.7866
PCYT2	6	0.006754	0.027611	0.82881	646	3	0.1684
RPL24	6	0.006768	0.02767	0.82881	647	4	0.6534
RPF1	6	0.006768	0.02767	0.82881	648	4	0.7236
ZNF208	3	0.006786	0.018513	0.82779	649	3	0.8142
DHDDS	6	0.006825	0.02787	0.83244	650	4	0.698
ETNK1	6	0.006841	0.027928	0.8328	651	4	0.393
GIGYF1	6	0.006871	0.028043	0.8328	652	5	0.7481
hsa-mir-6511a-4	2	0.006874	0.013172	0.78752	653	1	1.0817
LECT2	6	0.006883	0.028084	0.8328	654	3	0.2035
GMPR	6	0.006896	0.028131	0.8328	655	4	0.4827
PINX1	6	0.006899	0.028143	0.8328	656	4	0.5804
EHF	6	0.00693	0.028254	0.8328	657	4	0.526
OLFML1	6	0.006935	0.028273	0.8328	658	3	0.2031
hsa-mir-657	4	0.006938	0.023727	0.82779	659	3	0.8167
DPP7	6	0.006954	0.028335	0.8328	660	4	0.4729
SOGA3	6	0.006961	0.028363	0.8328	661	4	0.5998
SAE1	6	0.00697	0.028389	0.8328	662	5	0.6429
GHRH	6	0.006987	0.028446	0.8328	663	2	-0.564
SERTAD1	6	0.006988	0.028448	0.8328	664	5	0.522
SLC28A3	6	0.006996	0.028475	0.8328	665	2	-0.151
NDUFB8	6	0.007002	0.0285	0.8328	666	5	0.5584
THNSL2	6	0.007012	0.028541	0.8328	667	4	0.6087
SYNRG	4	0.007015	0.023982	0.82779	668	2	0.5601
hsa-mir-4268	4	0.007032	0.024034	0.82779	669	4	0.6052
CRNKL1	6	0.007034	0.028615	0.8328	670	5	0.6098
HOXC9	4	0.007035	0.024044	0.82779	671	4	0.5312
KRTAP1-3	5	0.007048	0.0276	0.82881	672	3	0.6541
GLRX5	6	0.007056	0.028705	0.83424	673	5	0.3951
C7orf49	6	0.007076	0.028777	0.83515	674	5	0.7857
OARD1	6	0.00709	0.028828	0.83517	675	2	0.0846

STT3A	6	0.007126	0.028951	0.83517	676	5	0.5281
UBASH3B	6	0.007136	0.028993	0.83517	677	3	0.2789
BECN1	6	0.007142	0.029009	0.83517	678	2	0.0494
FAM3C	3	0.007156	0.019488	0.82779	679	2	1.007
TNFRSF18	6	0.007162	0.029075	0.8355	680	4	0.6489
hsa-mir-4493	4	0.007169	0.024493	0.82779	681	4	0.9578
LUZP2	4	0.007187	0.024553	0.82779	682	1	-0.258
MOB3A	6	0.007194	0.029188	0.83613	683	2	0.0494
DDX39A	6	0.007204	0.029223	0.83613	684	3	0.1763
ART3	6	0.00722	0.029287	0.83613	685	3	0.2553
CCDC176	6	0.00727	0.029466	0.83613	686	4	0.6714
PEG10	6	0.007295	0.02956	0.83613	687	5	0.916
GLRX	6	0.007301	0.029579	0.83613	688	4	0.5028
RNF26	6	0.007306	0.0296	0.83613	689	5	0.4824
TIA1	6	0.00731	0.029621	0.83613	690	4	0.635
MSH3	6	0.007316	0.029642	0.83613	691	5	0.7745
CALU	6	0.007327	0.02968	0.83613	692	4	0.6005
ID2	6	0.007332	0.029694	0.83613	693	4	0.5728
HAUS8	6	0.007339	0.029724	0.83613	694	4	0.8415
AGBL2	6	0.007347	0.029746	0.83613	695	5	0.5626
LTB	6	0.007349	0.029755	0.83613	696	2	-0.181
TRIM43	4	0.00736	0.025079	0.82779	697	2	0.6771
RPP38	6	0.007374	0.029846	0.83613	698	4	0.6902
MCM7	6	0.007379	0.029864	0.83613	699	4	0.5283
AWAT2	6	0.007401	0.029946	0.83613	700	4	0.7311
KRTAP4-6	5	0.007402	0.028595	0.8328	701	1	0.0613
STK38	6	0.00741	0.029982	0.83613	702	4	0.6675
hsa-mir-3161	4	0.007429	0.025301	0.82779	703	1	0.108
KRTAP10-10	6	0.007453	0.03014	0.83613	704	2	-0.172
DHX9	6	0.007455	0.030146	0.83613	705	5	0.9782
OR4C16	6	0.007469	0.030192	0.83613	706	4	0.5539
ROCK2	6	0.007479	0.030228	0.83613	707	4	0.7338
hsa-mir-7154	4	0.007498	0.025532	0.82779	708	1	-0.223
INA	6	0.007505	0.030316	0.83613	709	3	0.1999
HOXD9	6	0.007511	0.030341	0.83613	710	5	0.492
EPC2	6	0.007519	0.030365	0.83613	711	2	-0.006
hsa-mir-568	4	0.007535	0.025642	0.82779	712	2	0.7046
WLS	6	0.007552	0.030492	0.83613	713	3	-0.045
BUB1B	6	0.007556	0.030507	0.83613	714	4	0.4785
ADD2	4	0.007558	0.025708	0.82779	715	3	0.5889
TRIM64B	3	0.007561	0.020525	0.82779	716	2	0.5164
MRPL14	6	0.007607	0.030684	0.83613	717	3	0.2133
PDZRN4	6	0.007608	0.030686	0.83613	718	3	0.3754
PPP1R15B	6	0.00761	0.030692	0.83613	719	5	0.4886
ZSWIM2	6	0.007616	0.030712	0.83613	720	3	0.4733
PDCD5	6	0.007631	0.03076	0.83613	721	5	0.4985
MTIF3	6	0.007639	0.030784	0.83613	722	4	0.569
LOC100130357	6	0.00766	0.030862	0.83613	723	2	-0.109
ABHD6	6	0.007687	0.030967	0.83613	724	4	0.668
NPHS1	6	0.007695	0.030994	0.83613	725	4	0.4373
CD19	6	0.007704	0.031028	0.83613	726	5	0.366
OSBPL10	6	0.007705	0.031032	0.83613	727	3	0.7271
TCF24	6	0.007712	0.031049	0.83613	728	2	-0.084
SERAC1	6	0.007712	0.031052	0.83613	729	5	0.5412
SNX2	6	0.007714	0.031059	0.83613	730	3	0.3176
WWC3	6	0.007741	0.031162	0.83723	731	5	0.4557
ECI1	6	0.007747	0.031181	0.83723	732	3	0.263
hsa-mir-6884	4	0.007751	0.026297	0.82779	733	3	0.8203
MRPS27	6	0.007776	0.031282	0.83884	734	5	0.4036
ATP5E	4	0.007809	0.026489	0.82779	735	2	0.2754
NAB2	6	0.007815	0.03142	0.84034	736	4	0.4472
ZNF544	6	0.007868	0.031601	0.84324	737	3	0.5825
SSX4B	3	0.007869	0.021332	0.82779	738	2	0.9726
hsa-mir-7843	4	0.007878	0.026705	0.82779	739	2	-0.749
CHST4	6	0.007919	0.03178	0.84664	740	3	0.1125
VEZT	6	0.007941	0.031864	0.84777	741	4	0.537
TSC22D2	6	0.00797	0.031967	0.84878	742	3	0.2591
hsa-mir-5685	4	0.007971	0.026997	0.82779	743	3	0.6442
ACOT4	6	0.007974	0.031985	0.84878	744	4	0.7146
KCMF1	6	0.007988	0.032027	0.8488	745	3	0.5157
C3orf55	6	0.008022	0.032155	0.85108	746	2	-0.364
TAS2R20	6	0.008057	0.032263	0.85226	747	5	0.3458
hsa-mir-3179-3	3	0.008062	0.021832	0.82779	748	3	0.5265
QRSL1	6	0.008074	0.032328	0.85226	749	4	0.5592
CRYGA	6	0.008087	0.032366	0.85226	750	5	0.5731
GABRP	6	0.008087	0.032366	0.85226	751	5	0.4508
SMN1	2	0.008096	0.015401	0.80991	752	2	1.328
GOLGA6A	6	0.0081	0.032412	0.85238	753	5	0.4915
GTSE1	4	0.008133	0.02752	0.82881	754	2	0.2998
SUSD3	6	0.008144	0.032574	0.854	755	4	0.5356
OGT	6	0.008174	0.032666	0.854	756	4	0.5993
WTH3DI	6	0.008177	0.032681	0.854	757	3	0.3004
EIF5AL1	6	0.00818	0.03269	0.854	758	3	0.5129
ANKRD10	6	0.008216	0.032815	0.854	759	5	0.4162
TSPYL5	6	0.008228	0.032862	0.854	760	5	0.3827

PIAS3	6	0.008229	0.032866	0.854	761	3	0.1788
C5	6	0.008237	0.032891	0.854	762	4	0.457
hsa-mir-4730	4	0.008278	0.027988	0.8328	763	4	0.4128
ODF1	6	0.0083	0.033122	0.85737	764	4	0.6638
ANKRD42	6	0.008303	0.033127	0.85737	765	5	0.525
CD74	6	0.008308	0.033147	0.85737	766	5	0.5097
FDFT1	6	0.008323	0.033198	0.85762	767	5	0.3864
FAM86C1	5	0.008329	0.031331	0.83904	768	3	0.764
NLE1	6	0.008384	0.033416	0.8577	769	5	0.45
ADAM30	6	0.008385	0.033417	0.8577	770	3	0.2439
ZNF792	6	0.008401	0.033476	0.8577	771	4	0.5495
C7orf29	3	0.008402	0.022689	0.82779	772	3	0.7356
MPHOSPH10	6	0.008404	0.033485	0.8577	773	4	0.5757
RBAK-RBAKDN	2	0.008419	0.01598	0.81736	774	2	1.9085
WNT7A	6	0.008436	0.033591	0.8577	775	4	0.5577
TMEM39B	6	0.00844	0.033608	0.8577	776	5	0.9302
DCAF5	6	0.008445	0.033629	0.8577	777	4	0.6087
SMG5	6	0.008454	0.033655	0.8577	778	2	-0.177
DNAJC24	6	0.008468	0.033704	0.8577	779	5	0.3822
UTP23	6	0.008475	0.033724	0.8577	780	3	0.4988
SIN3B	6	0.008488	0.033765	0.8577	781	4	0.4135
ELP3	6	0.008494	0.033788	0.8577	782	2	-0.168
RAB6C	6	0.008512	0.033849	0.85818	783	5	0.7798
GSDMA	6	0.008524	0.033891	0.85818	784	4	0.5108
hsa-mir-1205	4	0.008568	0.028907	0.83517	785	2	0.2497
HDAC7	6	0.008582	0.03409	0.86185	786	2	0.0431
CA13	6	0.008591	0.03412	0.86185	787	3	0.0864
hsa-mir-3606	4	0.008603	0.029023	0.83517	788	1	0.0148
DNAH8	6	0.008608	0.034184	0.8622	789	2	-0.153
SLC39A8	4	0.00863	0.029119	0.83558	790	3	0.7202
SEC16A	6	0.00864	0.034291	0.8622	791	4	0.3949
ATF7IP	6	0.008643	0.034302	0.8622	792	2	-0.068
RNF20	6	0.008674	0.034412	0.86257	793	4	0.5941
C5orf48	6	0.008678	0.034421	0.86257	794	5	0.6785
ZMYM3	6	0.008695	0.03448	0.86257	795	2	-0.425
CNGA1	6	0.008721	0.034572	0.86257	796	3	0.2705
POMGNT1	6	0.008726	0.034593	0.86257	797	5	0.478
MRPL23	6	0.008734	0.034619	0.86257	798	2	-0.15
ALYREF	6	0.008746	0.03466	0.86257	799	4	0.5098
CDH8	6	0.00877	0.034739	0.86257	800	5	0.5426
TFAP2A	6	0.008798	0.034839	0.86338	801	3	0.1778
ASB13	6	0.008804	0.034856	0.86338	802	4	0.5298
KRT8	6	0.008816	0.0349	0.86343	803	3	0.3751
PSMC6	6	0.008849	0.035	0.86381	804	3	0.2736
LCE3B	6	0.008876	0.035103	0.86532	805	4	0.3125
PPP2R1B	6	0.008942	0.035339	0.86798	806	3	0.4679
OR2F1	6	0.008948	0.035361	0.86798	807	4	0.6623
DLGAP4	6	0.008953	0.035381	0.86798	808	2	0.0392
SRP72	6	0.009002	0.035563	0.87141	809	5	0.4627
PKP2	6	0.009033	0.03567	0.87299	810	5	0.4461
SPESP1	5	0.009062	0.033428	0.8577	811	3	0.1785
GNRHR	6	0.009068	0.03579	0.87487	812	4	0.6693
KLHL42	6	0.009122	0.035983	0.87787	813	4	0.6432
SNX19	6	0.009128	0.035999	0.87787	814	2	-0.27
KCNK12	6	0.00916	0.036105	0.87943	815	3	-0.008
SLC30A4	6	0.009173	0.036153	0.87955	816	3	0.3632
hsa-mir-450b	4	0.009189	0.03091	0.83613	817	2	0.4006
hsa-mir-4524a	1	0.009191	0.0091512	0.78752	818	1	1.61
hsa-mir-92a-1	4	0.009207	0.030973	0.83613	819	4	0.458
BGN	6	0.009263	0.036468	0.8815	820	3	0.1792
KIAA1107	6	0.009279	0.036532	0.8815	821	5	0.5648
CLEC4M	6	0.009301	0.036608	0.8815	822	5	0.4623
PDCD1	6	0.009315	0.036655	0.8815	823	5	0.3059
MDM4	6	0.009346	0.03677	0.8815	824	4	0.5352
MRPS33	6	0.009346	0.03677	0.8815	825	4	0.6109
FXYD4	6	0.009364	0.036836	0.8815	826	4	0.5696
PRM1	6	0.009367	0.036843	0.8815	827	3	-0.017
EIF2B1	6	0.009384	0.036905	0.8815	828	4	0.686
TRPC1	6	0.009385	0.036909	0.8815	829	4	0.2964
UHRF1BP1	6	0.009388	0.036915	0.8815	830	4	0.6009
LENEP	6	0.009397	0.036946	0.8815	831	3	0.2331
TAF1A	6	0.009407	0.036982	0.8815	832	5	0.4845
FIGNL1	6	0.009408	0.036988	0.8815	833	4	0.5971
FLVCR2	6	0.009414	0.037007	0.8815	834	5	0.4403
GABRR1	6	0.009419	0.03702	0.8815	835	3	-0.095
AASDHPPT	6	0.009421	0.037027	0.8815	836	5	0.5556
EFHD1	6	0.009437	0.037074	0.8815	837	4	0.602
BTAF1	6	0.009444	0.037096	0.8815	838	5	0.6259
PARP3	6	0.00947	0.037191	0.88175	839	2	-0.046
C15orf26	6	0.00947	0.037192	0.88175	840	4	0.5982
COQ9	6	0.009522	0.037373	0.88286	841	1	-0.454
SAP130	6	0.009543	0.037452	0.88286	842	2	0.1924
HSPA6	6	0.009574	0.037552	0.88293	843	3	0.4015
OR6C74	6	0.009587	0.037605	0.88293	844	4	0.4709
RPAP2	6	0.009588	0.037609	0.88293	845	4	0.7093

PIGX	6	0.009595	0.03763	0.88293	846	4	0.5894
ZNF443	2	0.009658	0.018289	0.82779	847	1	1.3179
hsa-mir-759	4	0.009684	0.032514	0.85396	848	4	0.4574
BOD1	6	0.009701	0.037993	0.88735	849	4	0.6972
ZNF671	6	0.009716	0.038055	0.88735	850	3	0.5322
DAXX	6	0.009729	0.038105	0.88735	851	2	-0.009
SP3	6	0.009733	0.038125	0.88735	852	4	0.6474
KDM4E	6	0.009735	0.038132	0.88735	853	4	0.4881
OR4S1	6	0.009751	0.03819	0.88735	854	4	0.8584
hsa-mir-345	4	0.009776	0.032815	0.854	855	3	0.3484
LRTM2	6	0.009776	0.038279	0.88735	856	4	0.5958
AMOT	6	0.00978	0.038296	0.88735	857	2	-0.154
EIF3G	6	0.009832	0.038487	0.88894	858	2	-0.056
CYP2S1	6	0.009854	0.038574	0.88894	859	4	0.5859
SCN1B	6	0.009857	0.03858	0.88894	860	5	0.4042
GTPBP2	6	0.009877	0.038651	0.88894	861	3	0.1067
RAB3D	6	0.009898	0.038729	0.88894	862	5	0.3897
IMPDH2	6	0.009898	0.038729	0.88894	863	5	0.5474
P4HA1	6	0.009905	0.038749	0.88894	864	5	0.5024
LIFR	6	0.009923	0.0388	0.88894	865	5	0.4449
PYCARD	6	0.009935	0.038842	0.88894	866	5	0.3611
IFNA21	4	0.009948	0.033355	0.8577	867	1	-0.627
ZNF114	6	0.009999	0.039056	0.88959	868	5	0.5489
SMIM15	6	0.010017	0.039119	0.88959	869	5	0.4971
RAD51B	6	0.01002	0.039131	0.88959	870	2	0.0475
HOMER3	6	0.010039	0.039187	0.88959	871	2	-0.167
CENPA	6	0.010041	0.039191	0.88959	872	5	0.4467
ZCCHC6	6	0.010053	0.039231	0.88959	873	5	0.4551
NOP2	6	0.010058	0.039243	0.88959	874	4	0.449
CARD18	6	0.010059	0.039247	0.88959	875	4	0.5411
PLCL1	6	0.010063	0.039262	0.88959	876	5	0.625
SERTM1	6	0.01008	0.039322	0.88996	877	3	0.1187
S1PR3	6	0.01009	0.039368	0.89002	878	1	-0.161
MAN2B2	6	0.010118	0.03946	0.89009	879	4	0.704
hsa-mir-4435-2	3	0.010141	0.027166	0.82779	880	2	0.948
C2orf43	6	0.010142	0.039545	0.89009	881	2	-0.188
SIM2	6	0.010194	0.039714	0.89236	882	2	0.0831
NOP9	6	0.010199	0.039733	0.89236	883	4	0.6416
AAAS	6	0.010234	0.039847	0.89326	884	4	0.726
EDN3	6	0.010245	0.039886	0.89326	885	1	-0.415
CD48	6	0.010255	0.039909	0.89326	886	4	0.5942
PPP1R3C	6	0.010267	0.039948	0.89326	887	3	0.425
CHTF18	6	0.010297	0.040048	0.89373	888	3	0.1517
LHCGR	6	0.010317	0.040119	0.89373	889	4	0.5312
SSPN	6	0.010337	0.040193	0.89373	890	5	0.4313
RIN3	6	0.010349	0.040242	0.89373	891	3	0.3099
GDF3	6	0.01035	0.040246	0.89373	892	5	0.4344
GPATCH4	4	0.010362	0.034715	0.86257	893	2	-0.102
SCO1	6	0.010363	0.040301	0.89373	894	5	0.5186
MOB3C	6	0.010371	0.040324	0.89373	895	4	0.7158
CSK	6	0.010394	0.040403	0.89373	896	4	0.6011
DPH3	5	0.010398	0.037287	0.88271	897	2	0.0591
BEX4	6	0.0104	0.040419	0.89373	898	3	0.1422
HRSP12	6	0.01041	0.040449	0.89373	899	3	0.3983
hsa-mir-135b	4	0.010437	0.034966	0.86381	900	3	0.6898
USP33	6	0.010452	0.040595	0.89501	901	4	0.5675
hsa-mir-519a-1	2	0.010479	0.019789	0.82779	902	1	1.498
AGXT2L1	4	0.010535	0.035288	0.86798	903	4	0.6958
ZFR	6	0.010555	0.040941	0.90167	904	4	0.3099
COL19A1	6	0.010607	0.041112	0.9023	905	4	0.4689
GPR133	6	0.010608	0.041115	0.9023	906	4	0.4998
ART4	6	0.010618	0.041146	0.9023	907	5	0.4072
BACE2	6	0.010658	0.041285	0.90273	908	3	0.2157
ATP5B	6	0.010663	0.041298	0.90273	909	5	0.4324
FXYD3	6	0.01069	0.041388	0.90352	910	4	0.7389
RGS21	6	0.01071	0.041462	0.90352	911	3	0.2273
ARSB	6	0.010711	0.041466	0.90352	912	5	0.5397
AFM	6	0.010742	0.041566	0.90426	913	3	0.1868
C19orf44	6	0.010749	0.041589	0.90426	914	3	0.2992
HFE	6	0.010789	0.041712	0.9046	915	4	0.4754
KIF27	6	0.010801	0.041755	0.9046	916	4	0.5756
CTU2	6	0.010813	0.041801	0.9046	917	4	0.2728
WDR77	6	0.010815	0.041803	0.9046	918	4	0.4974
OIP5	6	0.010826	0.041848	0.9046	919	3	0.3078
MRPS28	6	0.010839	0.041883	0.9046	920	3	0.4366
WDR52	6	0.010851	0.041914	0.9046	921	4	0.3799
ARMCX5-GPRASP2	6	0.010917	0.042134	0.90663	922	3	0.1162
CEP68	6	0.010927	0.042166	0.90663	923	3	0.1448
hsa-mir-361	4	0.010961	0.036571	0.8815	924	3	0.7745
ARHGAP21	6	0.010968	0.042297	0.90712	925	2	0.0807
TET1	6	0.010986	0.042352	0.90735	926	2	0.1935
RPUSD2	6	0.01101	0.042435	0.90817	927	5	0.7078
PELI2	6	0.011071	0.042628	0.91047	928	1	-0.098
NUP62CL	6	0.011085	0.042684	0.91065	929	5	0.4887
VBP1	6	0.011099	0.042731	0.9107	930	5	0.5758

TMEM241	6	0.011141	0.042887	0.91118	931	5	0.3742
LARP1	6	0.011143	0.042894	0.91118	932	3	0.3573
SRY	6	0.011161	0.042957	0.91118	933	3	0.4846
CRP	6	0.011175	0.043002	0.91118	934	2	-0.05
KIF18A	6	0.011179	0.043015	0.91118	935	4	0.7024
CKS2	6	0.011183	0.043032	0.91118	936	4	0.4455
OR10V1	6	0.011192	0.043065	0.91118	937	4	0.6087
hsa-mir-3662	3	0.011276	0.03006	0.83613	938	2	0.4527
EAF2	6	0.011278	0.043365	0.91552	939	2	0.125
EFTUD2	6	0.011287	0.043392	0.91552	940	4	0.8675
OR5H14	6	0.011292	0.043404	0.91552	941	4	0.6307
TMEM208	6	0.011341	0.043578	0.91823	942	3	0.2756
hsa-mir-944	3	0.011353	0.030258	0.83613	943	1	0.1627
hsa-mir-126	4	0.011379	0.037885	0.88735	944	3	0.6818
CEP57L1	6	0.011381	0.04371	0.91826	945	3	0.3389
RIT2	4	0.011395	0.037929	0.88735	946	1	-0.22
RPL9	6	0.011419	0.043847	0.91826	947	4	0.4743
KIAA1045	6	0.011433	0.043886	0.91826	948	3	0.0947
NDUFS7	6	0.011439	0.043905	0.91826	949	4	0.6718
CAPN9	6	0.01147	0.044007	0.91826	950	5	0.4566
PRDM1	6	0.01148	0.044038	0.91826	951	3	0.3664
PLCXD3	6	0.011484	0.044057	0.91826	952	2	-0.347
CDK14	6	0.01149	0.044076	0.91826	953	4	0.4678
NACAD	6	0.011497	0.044094	0.91826	954	5	0.4255
SUN3	6	0.011497	0.044097	0.91826	955	3	-0.067
hsa-mir-576	4	0.011498	0.038236	0.88735	956	2	-0.235
LARP1B	6	0.011499	0.044101	0.91826	957	5	0.4275
MRPS30	6	0.011502	0.044118	0.91826	958	5	0.5593
ZSCAN21	6	0.011545	0.044251	0.91891	959	4	0.519
MCF2L2	6	0.011555	0.044292	0.91891	960	4	0.4563
PCDHB12	6	0.011568	0.044343	0.91891	961	4	0.3884
hsa-mir-190b	4	0.011583	0.038514	0.88894	962	2	0.6065
DOCK5	6	0.011585	0.04441	0.91891	963	3	0.2865
TMX3	6	0.011587	0.044418	0.91891	964	3	0.1909
ANP32A	6	0.011646	0.044616	0.92114	965	4	0.6942
hsa-mir-8084	4	0.011669	0.038773	0.88894	966	3	0.8092
SSB	6	0.011686	0.044748	0.9213	967	5	0.6416
PQLC2	6	0.011703	0.044802	0.9213	968	3	0.3145
NEDD1	6	0.011708	0.044819	0.9213	969	2	-0.085
IGFL1	6	0.011726	0.044877	0.9213	970	4	0.5759
PIK3C2B	6	0.011742	0.044935	0.9213	971	3	0.4644
C5orf47	6	0.01175	0.04497	0.9213	972	5	0.5939
KLRF2	6	0.011755	0.044984	0.9213	973	4	0.4695
CLINT1	6	0.011786	0.045082	0.92167	974	4	0.4993
NTRK2	6	0.011798	0.04512	0.92167	975	5	0.5173
KRTAP5-6	6	0.011803	0.045139	0.92167	976	3	-0.061
YWHAQ	6	0.011834	0.045246	0.92167	977	5	0.4574
AHR	6	0.011841	0.045262	0.92167	978	5	0.5567
FDX1L	6	0.011871	0.045366	0.92167	979	3	0.5062
PANK3	6	0.011871	0.045366	0.92167	980	3	0.0995
hsa-mir-7109	4	0.011887	0.039448	0.89009	981	3	0.8726
NUCKS1	6	0.011891	0.045431	0.92167	982	4	0.3809
LSM1	6	0.011895	0.045441	0.92167	983	5	0.4133
GCNT3	6	0.011899	0.045453	0.92167	984	5	0.3862
CENPH	3	0.011899	0.031611	0.84324	985	3	1.0619
hsa-mir-6878	4	0.011903	0.039507	0.89009	986	3	1.0295
DEFB104B	1	0.011943	0.011872	0.78752	987	1	1.3381
RBMX	6	0.011949	0.04561	0.92262	988	2	-0.01
RICTOR	6	0.011957	0.045638	0.92262	989	5	0.6204
ANAPC1	6	0.011961	0.045651	0.92262	990	5	0.443
STXBP6	6	0.012	0.045782	0.92287	991	3	0.179
FBXO5	6	0.012052	0.045956	0.92387	992	2	-0.146
RBX1	6	0.012054	0.045966	0.92387	993	3	0.0611
FARSA	6	0.012067	0.046013	0.92387	994	4	0.8191
RNF122	6	0.012067	0.046013	0.92387	995	4	0.7604
PDAP1	6	0.012093	0.046097	0.92392	996	4	0.6201
GON4L	6	0.012103	0.046132	0.92392	997	4	0.6068
CD5L	6	0.012118	0.046181	0.92392	998	4	0.4512
SLC39A5	6	0.012122	0.046196	0.92392	999	3	0.276
C17orf104	6	0.012181	0.046375	0.92659	1000	4	0.5986
C2orf16	6	0.012206	0.046461	0.92677	1001	1	-0.148
SPAG17	6	0.012235	0.046559	0.92677	1002	4	0.3214
hsa-mir-181c	4	0.012243	0.040557	0.89501	1003	3	1.1446
ATP5J2	5	0.012274	0.042631	0.91047	1004	4	0.5336
FAM211B	6	0.012293	0.046754	0.92913	1005	3	0.075
PYURF	6	0.01231	0.046809	0.92913	1006	3	0.1584
PIK3C2A	6	0.012323	0.04686	0.92913	1007	4	0.5561
APOO	6	0.012325	0.046865	0.92913	1008	5	0.4572
ANO9	6	0.012355	0.046954	0.92951	1009	3	0.0972
DAAM2	6	0.012361	0.046975	0.92951	1010	2	-0.296
EXOSC5	6	0.012381	0.047044	0.92973	1011	4	0.7248
LGI3	6	0.01239	0.047077	0.92973	1012	4	0.4595
ODF2	6	0.012413	0.047165	0.93056	1013	2	0.0656
hsa-mir-3618	4	0.012459	0.04123	0.90273	1014	3	0.6865
TMEM247	6	0.012466	0.047358	0.93143	1015	4	0.5329

MICU1	6	0.012466	0.047359	0.93143	1016	3	0.2349
SNTB1	6	0.012475	0.047391	0.93143	1017	5	0.4738
MFSD8	6	0.012493	0.047461	0.93143	1018	3	0.1672
CITED4	6	0.012498	0.047477	0.93143	1019	5	0.3849
RRP7A	6	0.012516	0.047542	0.93143	1020	4	0.6978
NOP10	6	0.012545	0.047642	0.93143	1021	4	0.5193
ZCWPW1	6	0.012562	0.0477	0.93143	1022	4	0.5268
POU2F1	6	0.012565	0.047712	0.93143	1023	4	0.7723
TBR1	6	0.012567	0.04772	0.93143	1024	2	-0.071
ASNSD1	6	0.01257	0.047728	0.93143	1025	4	0.3058
DIO1	6	0.012594	0.04781	0.93143	1026	2	-0.146
UNC50	6	0.012594	0.04781	0.93143	1027	4	0.4677
hsa-mir-329-2	1	0.012608	0.012526	0.78752	1028	1	2.7703
SH3BGRL	6	0.012619	0.047892	0.93143	1029	3	0.1046
DNM1L	6	0.012664	0.048051	0.93293	1030	4	0.5974
TNK2	6	0.012667	0.04806	0.93293	1031	5	0.4896
ANGPTL1	6	0.01271	0.04821	0.93496	1032	4	0.4441
CMTM2	6	0.012722	0.048256	0.93496	1033	4	0.2977
ZNF71	6	0.012763	0.048409	0.93703	1034	4	0.5079
hsa-mir-5186	4	0.012772	0.042185	0.90663	1035	2	-0.022
hsa-mir-4316	4	0.012802	0.042271	0.90712	1036	3	0.8498
SLAMF9	6	0.012814	0.048575	0.93764	1037	5	0.5907
CIC	6	0.012814	0.048575	0.93764	1038	3	0.2436
ZNHIT3	6	0.012828	0.048627	0.93764	1039	3	0.1523
VAPA	6	0.012829	0.048628	0.93764	1040	5	0.4097
KNOP1	6	0.012841	0.048674	0.93764	1041	4	0.5745
PIM1	6	0.012857	0.04872	0.93764	1042	4	0.6248
ATG4C	6	0.01293	0.048958	0.9396	1043	3	0.3346
MPI	6	0.012947	0.049019	0.9396	1044	3	0.1339
GPATCH1	6	0.012958	0.04906	0.9396	1045	4	0.5169
YY1	6	0.01298	0.049135	0.9396	1046	2	-0.356
UMPS	6	0.013004	0.049207	0.9396	1047	5	0.6291
ACTL9	6	0.013013	0.049237	0.9396	1048	4	0.4826
ZNF233	6	0.013032	0.049293	0.9396	1049	3	0.2353
TLL2	6	0.013051	0.049357	0.9396	1050	4	0.683
USP9Y	6	0.013072	0.049429	0.9396	1051	5	0.4281
PMP2	6	0.013074	0.049434	0.9396	1052	2	0.1083
PIH1D1	6	0.013076	0.049437	0.9396	1053	4	0.5771
OR1L4	6	0.013083	0.04946	0.9396	1054	3	0.188
P2RX6	6	0.013113	0.049564	0.94071	1055	4	0.561
TPR	6	0.013134	0.049638	0.94124	1056	2	-0.22
hsa-mir-4257	3	0.013141	0.034702	0.86257	1057	3	0.5133
SPHK1	6	0.013174	0.049765	0.94195	1058	3	0.0415
CYP51A1	6	0.013181	0.049783	0.94195	1059	5	0.5584
RASSF7	6	0.013189	0.049814	0.94195	1060	4	0.5533
RPL23A	6	0.013209	0.049881	0.94216	1061	3	0.5075
PIK3CB	6	0.01326	0.050045	0.94371	1062	5	0.4247
LACTB2	6	0.013289	0.050144	0.94417	1063	2	-0.545
ASCL4	6	0.013295	0.050163	0.94417	1064	5	0.5548
ICAM3	6	0.013328	0.050264	0.94417	1065	5	0.3742
MDGA1	6	0.013356	0.050366	0.94417	1066	5	0.4988
SRGAP1	6	0.013374	0.050422	0.94417	1067	4	0.5588
FER1L6	6	0.013392	0.050478	0.94417	1068	3	0.1687
AQP7	6	0.013416	0.050564	0.94417	1069	3	0.0564
NDUFA8	6	0.013431	0.050609	0.94417	1070	5	0.5191
LRP4	6	0.013443	0.050648	0.94417	1071	3	0.1371
hsa-mir-4782	4	0.013448	0.044202	0.91891	1072	3	0.6736
BCL3	6	0.013484	0.050789	0.94417	1073	5	0.4433
CENPL	6	0.013492	0.050815	0.94417	1074	5	0.4115
ZNF227	6	0.013514	0.050886	0.94417	1075	5	0.4836
KDM4A	6	0.013535	0.050952	0.94417	1076	4	0.6879
PDC	6	0.013546	0.050992	0.94417	1077	1	0.0627
ZNF565	6	0.013547	0.050994	0.94417	1078	5	0.608
SLC17A3	6	0.013557	0.051027	0.94417	1079	3	0.3399
UBAC1	6	0.013561	0.051042	0.94417	1080	5	0.4553
OCLM	4	0.013562	0.044561	0.92094	1081	4	0.3642
PAX3	6	0.013598	0.051169	0.94417	1082	1	0.0263
SAMHD1	6	0.013598	0.051171	0.94417	1083	4	0.5755
COX11	6	0.013629	0.05127	0.94417	1084	4	0.4419
OR2AG2	6	0.013649	0.051336	0.94417	1085	2	-0.484
ARID3C	6	0.013661	0.051373	0.94417	1086	4	0.5413
SNX32	6	0.013668	0.051397	0.94417	1087	5	0.4875
HTR1E	4	0.013684	0.044948	0.9213	1088	4	0.4646
SRSF5	6	0.013701	0.051503	0.94417	1089	2	-0.23
VN1R1	6	0.013708	0.051523	0.94417	1090	4	0.6733
TDRD12	6	0.013735	0.0516	0.94417	1091	5	0.3357
OSGIN2	6	0.013747	0.051648	0.94417	1092	5	0.4881
ATMIN	6	0.013747	0.051648	0.94417	1093	2	-0.142
KAT5	6	0.013774	0.051741	0.94417	1094	4	0.8045
FN3K	6	0.013804	0.051841	0.94417	1095	2	-0.066
TWISTNB	6	0.013827	0.051927	0.94417	1096	3	0.728
CLCN1	6	0.013834	0.051951	0.94417	1097	4	0.4971
SPATA31C1	6	0.013838	0.051961	0.94417	1098	3	0.2574
GBX2	6	0.013855	0.052013	0.94417	1099	4	0.531
AHCYL1	6	0.013883	0.052105	0.94417	1100	5	0.6481

DCN	6	0.013906	0.052197	0.94417	1101	4	0.3799
PTRH1	6	0.013907	0.052197	0.94417	1102	4	0.5121
hsa-mir-644a	4	0.013928	0.045706	0.92262	1103	3	0.641
hsa-mir-548j	4	0.013936	0.045725	0.92262	1104	3	0.683
AGK	6	0.013956	0.052365	0.94417	1105	4	0.3443
NAPSA	6	0.013958	0.052372	0.94417	1106	2	0.1746
EGFL7	6	0.013963	0.052392	0.94417	1107	4	0.5893
PRPF3	6	0.013973	0.052415	0.94417	1108	4	0.5124
PGD	6	0.013987	0.052465	0.94417	1109	3	0.0908
CPT1B	6	0.013993	0.052486	0.94417	1110	3	0.3177
FBXO28	6	0.013994	0.052488	0.94417	1111	4	0.5254
LOC100862671	6	0.01401	0.052539	0.94417	1112	3	0.1061
UBA52	6	0.014054	0.052679	0.94417	1113	5	0.4029
FLNC	6	0.014057	0.052685	0.94417	1114	4	0.6389
SARS2	6	0.01406	0.052696	0.94417	1115	3	0.5045
RECQL	6	0.01406	0.052697	0.94417	1116	3	0.2713
TMPRSS15	6	0.014061	0.0527	0.94417	1117	3	0.2799
MAP3K7CL	6	0.01412	0.052885	0.94583	1118	5	0.5944
DOCK4	5	0.014155	0.047849	0.93143	1119	4	0.4882
SKOR1	6	0.014164	0.053032	0.94673	1120	3	0.1217
NDUFA9	6	0.01417	0.053054	0.94673	1121	3	-0.029
UPK1B	6	0.014184	0.053107	0.94673	1122	3	0.1429
hsa-mir-142	4	0.014203	0.046547	0.92677	1123	3	0.8032
CCT6A	6	0.014203	0.05317	0.94673	1124	2	-0.009
hsa-mir-3609	4	0.014208	0.046565	0.92677	1125	3	0.4791
IL22	6	0.014216	0.053213	0.94673	1126	1	-0.146
PNPLA8	6	0.014268	0.053375	0.94734	1127	4	0.464
OR5AN1	6	0.014272	0.053396	0.94734	1128	2	0.0605
DLD	6	0.014293	0.053464	0.94734	1129	4	0.3744
TMEM177	6	0.014311	0.053528	0.94734	1130	5	0.3282
TMX4	6	0.014324	0.053572	0.94734	1131	2	0.2115
CALCRL	6	0.01437	0.053736	0.94743	1132	1	-0.43
XBP1	6	0.014398	0.053837	0.94743	1133	3	0.3537
RHOA	6	0.014398	0.053837	0.94743	1134	4	0.5227
LRRC41	6	0.014422	0.053914	0.94743	1135	2	-0.114
CRH	6	0.014445	0.053993	0.94743	1136	5	0.8412
AP3M2	6	0.014473	0.054075	0.94743	1137	2	-0.168
RASA2	6	0.014475	0.054083	0.94743	1138	5	0.4443
CC2D2A	6	0.014479	0.054097	0.94743	1139	3	0.1824
CDK11A	6	0.014522	0.054236	0.94743	1140	5	0.9155
GTF2H3	6	0.014531	0.054272	0.94743	1141	4	0.4563
RIMBP3	6	0.014539	0.054301	0.94743	1142	4	0.4471
CD8A	6	0.014551	0.054345	0.94743	1143	4	0.5645
ADAMTS13	6	0.014567	0.054394	0.94743	1144	5	0.3156
LRP8	6	0.014576	0.054424	0.94743	1145	1	-0.247
CCDC30	6	0.014587	0.054464	0.94743	1146	5	0.4814
ARL5C	6	0.014635	0.054629	0.94743	1147	3	0.509
ABHD16B	6	0.014679	0.054782	0.94743	1148	2	-0.218
EGFLAM	6	0.014691	0.054821	0.94743	1149	2	-0.215
FAM107B	6	0.014692	0.054822	0.94743	1150	5	0.4132
PDE10A	6	0.014701	0.054854	0.94743	1151	5	0.3356
SIPA1L3	6	0.014713	0.054896	0.94743	1152	3	0.2929
PNRC1	6	0.01473	0.054955	0.94743	1153	3	0.0669
IMP4	6	0.014732	0.054961	0.94743	1154	4	0.8444
RTCA	6	0.014754	0.055033	0.94743	1155	4	0.5315
LYPLA1	6	0.014763	0.055061	0.94743	1156	5	0.407
B3GNT7	6	0.014782	0.055124	0.94743	1157	3	0.2069
PMP22	6	0.014786	0.055143	0.94743	1158	4	0.697
YWHAG	6	0.014792	0.05516	0.94743	1159	3	0.0509
DCX	6	0.014797	0.055173	0.94743	1160	4	0.4751
GCSH	6	0.014826	0.055261	0.94743	1161	5	0.4956
ATP5J2-PTCD1	2	0.014829	0.027708	0.82881	1162	2	0.5516
BCAP31	6	0.014867	0.055399	0.94772	1163	4	0.7719
UGT2B7	5	0.014892	0.049917	0.94216	1164	4	0.5283
hsa-mir-8063	4	0.014939	0.048823	0.93795	1165	3	0.7488
CD70	6	0.014968	0.055733	0.94994	1166	4	0.461
EMX2	6	0.014979	0.055769	0.94994	1167	4	0.4213
MRPL4	6	0.01504	0.055993	0.94994	1168	4	0.4621
SLC39A14	6	0.015055	0.056041	0.94994	1169	4	0.6803
RSL1D1	6	0.015062	0.056066	0.94994	1170	4	0.5204
GOLGA8O	6	0.015065	0.056076	0.94994	1171	4	0.6102
RIOK1	6	0.015065	0.056076	0.94994	1172	2	0.1438
ANKRD61	6	0.015065	0.056076	0.94994	1173	3	0.32
AGFG2	6	0.015065	0.056077	0.94994	1174	5	0.5489
HAPLN1	6	0.015075	0.056104	0.94994	1175	5	0.5759
PARVB	6	0.015089	0.056152	0.94994	1176	5	0.348
SMG7	6	0.015153	0.056373	0.94994	1177	4	0.7881
ALAS1	6	0.015155	0.056379	0.94994	1178	5	0.4961
MCAM	6	0.015193	0.056503	0.94994	1179	3	-0.011
GGPS1	6	0.015195	0.056508	0.94994	1180	5	0.59
LRP3	6	0.015207	0.05655	0.94994	1181	4	0.4862
PNMA5	6	0.015231	0.056613	0.94994	1182	5	0.4176
KRCC1	6	0.015231	0.056613	0.94994	1183	5	0.3174
CHCHD1	6	0.015253	0.056682	0.94994	1184	5	0.4415
GGT5	6	0.015296	0.056822	0.94994	1185	2	-0.493

NUMB	6	0.015301	0.056838	0.94994	1186	4	0.5616
GNL3L	6	0.01536	0.057012	0.94994	1187	4	0.7099
hsa-mir-3942	3	0.015367	0.040265	0.89373	1188	2	0.8416
SPEM1	6	0.015396	0.057124	0.94994	1189	4	0.5017
SLC22A1	6	0.015419	0.057195	0.94994	1190	4	0.361
CBX3	6	0.015428	0.057228	0.94994	1191	4	0.4624
BCKDHA	6	0.015433	0.057244	0.94994	1192	4	0.4702
OTUD6A	6	0.015447	0.05729	0.94994	1193	5	0.5444
TBC1D15	6	0.015449	0.057296	0.94994	1194	2	-0.002
ASCC3	6	0.015451	0.057301	0.94994	1195	1	-0.143
MLYCD	6	0.015474	0.057377	0.94994	1196	4	0.6706
LEF1	6	0.015481	0.057402	0.94994	1197	3	0.1972
UBA1	6	0.015524	0.057562	0.94994	1198	3	0.1912
SCGB3A2	6	0.015531	0.05759	0.94994	1199	5	0.4086
ENAM	6	0.015553	0.057667	0.94994	1200	2	-0.033
APBA1	6	0.015613	0.05785	0.94994	1201	4	0.5401
hsa-mir-6128	4	0.015624	0.050904	0.94417	1202	4	0.5371
MMP24	6	0.015648	0.057952	0.94994	1203	3	-0.142
LZTR1	6	0.01565	0.057956	0.94994	1204	5	0.5197
ZCCHC5	6	0.015656	0.057976	0.94994	1205	2	-0.127
C18orf21	6	0.015657	0.057977	0.94994	1206	5	0.4918
JAG1	6	0.015668	0.058024	0.94994	1207	5	0.4687
CTSH	6	0.015699	0.058117	0.94994	1208	5	0.5291
FARS2	6	0.015701	0.058122	0.94994	1209	4	0.5647
UBE2I	6	0.015706	0.058138	0.94994	1210	3	0.3827
NEBL	6	0.015708	0.058144	0.94994	1211	4	0.3545
hsa-mir-124-2	3	0.01571	0.041115	0.9023	1212	2	0.8362
hsa-mir-6894	4	0.015727	0.051229	0.94417	1213	3	0.5105
NIPBL	6	0.015756	0.058298	0.94994	1214	3	0.4895
CARS	6	0.015757	0.058307	0.94994	1215	5	0.41
GUCA2B	6	0.015759	0.058314	0.94994	1216	1	-0.209
ATP8B4	6	0.015785	0.0584	0.94994	1217	4	0.6236
CEACAM20	6	0.015791	0.058416	0.94994	1218	5	0.3264
MASP1	6	0.015811	0.058474	0.94994	1219	1	0.0813
DBX2	6	0.015812	0.058475	0.94994	1220	5	0.5393
PRMT5	6	0.015819	0.058497	0.94994	1221	5	0.4703
PPIP5K2	6	0.015819	0.058497	0.94994	1222	5	0.661
TLR6	6	0.015828	0.05852	0.94994	1223	5	0.3274
HSPB11	6	0.015862	0.058633	0.94994	1224	2	-0.002
SEMA4C	6	0.015867	0.058648	0.94994	1225	4	0.4284
MOS	6	0.015871	0.058661	0.94994	1226	3	0.3901
NCKAP5	6	0.015913	0.05881	0.94994	1227	2	0.2291
DPY19L3	6	0.015918	0.058824	0.94994	1228	4	0.3515
RPL18A	6	0.015919	0.058833	0.94994	1229	5	0.6501
CCDC179	6	0.015925	0.058848	0.94994	1230	3	0.3505
hsa-mir-877	4	0.015933	0.05188	0.94417	1231	2	0.3137
OPALIN	6	0.015936	0.058883	0.94994	1232	5	0.3585
IL1RN	6	0.015984	0.059036	0.94994	1233	4	0.4346
SMG8	6	0.016016	0.05914	0.94994	1234	1	-0.545
hsa-mir-765	4	0.016053	0.052204	0.94417	1235	3	1.1171
LHX5	6	0.016068	0.0593	0.94994	1236	2	-0.101
BIRC6	6	0.016093	0.05937	0.94994	1237	4	0.5618
MBD3L3	2	0.0161	0.029971	0.83613	1238	2	0.8039
CELF4	6	0.016111	0.059423	0.94994	1239	5	0.3035
LDHD	6	0.016119	0.059455	0.94994	1240	1	-0.166
CHODL	6	0.016143	0.059535	0.94994	1241	3	-0.122
HOXC6	6	0.016147	0.059553	0.94994	1242	4	0.5504
PPIL4	6	0.01615	0.05956	0.94994	1243	5	0.3963
TMED9	6	0.016156	0.059573	0.94994	1244	3	0.3798
NUDT21	6	0.016162	0.059597	0.94994	1245	4	0.6426
ZFC3H1	6	0.016165	0.059608	0.94994	1246	5	0.5524
C9orf114	6	0.01617	0.059623	0.94994	1247	4	0.6065
NPAS3	6	0.016174	0.059636	0.94994	1248	5	0.2656
hsa-mir-3675	4	0.016193	0.052487	0.94417	1249	3	0.4556
CNTD1	6	0.016213	0.059754	0.94994	1250	4	0.6194
SASH1	6	0.016218	0.059771	0.94994	1251	5	0.4971
NR2E3	6	0.016222	0.059787	0.94994	1252	3	0.1907
ANKRD30A	6	0.016223	0.059792	0.94994	1253	4	0.455
MTOR	6	0.016238	0.059833	0.94994	1254	5	0.5423
hsa-mir-4258	4	0.016244	0.0526	0.94417	1255	2	0.5157
GNG4	6	0.016273	0.059953	0.94994	1256	1	-0.438
NOVA1	6	0.016304	0.060047	0.94994	1257	4	0.4216
TUBA1B	4	0.01631	0.052757	0.94437	1258	2	-0.171
ARHGAP26	6	0.016325	0.060121	0.94994	1259	4	0.5226
ATL2	6	0.01633	0.060136	0.94994	1260	2	-0.397
RPL5	6	0.016349	0.060201	0.94994	1261	5	0.7801
CHRNA1	6	0.016358	0.060237	0.94994	1262	5	0.367
RAB9A	6	0.016361	0.060248	0.94994	1263	4	0.4818
FIP1L1	6	0.016367	0.060268	0.94994	1264	5	0.527
SDHC	6	0.016369	0.060274	0.94994	1265	4	0.5927
PPP1R2	6	0.016382	0.060322	0.94994	1266	4	0.5228
QPCTL	6	0.016389	0.060346	0.94994	1267	4	0.4575
TXNRD3	6	0.016393	0.060356	0.94994	1268	5	0.4672
PLB1	6	0.016411	0.060416	0.94994	1269	4	0.4506
EBP	6	0.016427	0.060479	0.94994	1270	3	0.2377

NPM2	6	0.016463	0.060598	0.94994	1271	3	0.1795
GNB2L1	6	0.016479	0.060648	0.94994	1272	4	0.4804
hsa-mir-1267	4	0.016481	0.053129	0.94673	1273	1	-0.076
KIAA0922	6	0.016496	0.060708	0.94994	1274	4	0.432
THOC2	6	0.016526	0.060816	0.94994	1275	4	0.5293
APPBP2	6	0.016544	0.06087	0.94994	1276	5	0.4487
CHMP2A	6	0.016578	0.060961	0.94994	1277	4	0.5827
PAPSS1	6	0.016583	0.060978	0.94994	1278	5	0.4033
HYPK	6	0.016597	0.061024	0.94994	1279	4	0.4036
ACAN	6	0.016623	0.061105	0.94994	1280	3	0.4642
NDUFS8	6	0.016632	0.061135	0.94994	1281	4	0.6597
G6PC2	6	0.016633	0.061136	0.94994	1282	2	-0.061
hsa-mir-4670	4	0.016649	0.0535	0.94734	1283	3	0.6413
APLP1	6	0.016657	0.061223	0.94994	1284	4	0.4925
SEZ6L2	6	0.01667	0.061254	0.94994	1285	5	0.3809
hsa-mir-27b	4	0.01668	0.053558	0.94734	1286	3	0.7436
NIP7	6	0.016684	0.061301	0.94994	1287	4	0.3457
TTC21A	6	0.016736	0.061477	0.94994	1288	2	-0.031
HOXB4	6	0.016737	0.061483	0.94994	1289	4	0.7279
SPATA6L	6	0.016739	0.061493	0.94994	1290	4	0.3145
hsa-mir-15a	4	0.016739	0.053682	0.94743	1291	3	0.5973
CD55	6	0.016744	0.061518	0.94994	1292	3	0.3393
NEK1	5	0.016753	0.055203	0.94743	1293	3	0.3271
RAMP1	6	0.01677	0.0616	0.94994	1294	2	0.0754
CAPNS1	6	0.016771	0.0616	0.94994	1295	5	0.5724
SERP2	6	0.016772	0.061602	0.94994	1296	3	0.119
CSTF3	6	0.01678	0.061633	0.94994	1297	4	0.7952
RIN2	6	0.016787	0.061654	0.94994	1298	2	-0.138
RHEB	6	0.016792	0.061667	0.94994	1299	5	0.531
IFITM10	6	0.016806	0.061722	0.94994	1300	4	0.5422
NUP98	6	0.01681	0.061731	0.94994	1301	3	0.1129
MYO18B	6	0.016838	0.061818	0.95025	1302	1	-0.411
RPGR	6	0.016876	0.061942	0.95065	1303	5	0.515
MPP7	6	0.01689	0.061983	0.95065	1304	2	0.0921
TTC27	6	0.01692	0.062079	0.95092	1305	5	0.5032
SYMPK	6	0.016941	0.06215	0.95092	1306	2	0.0294
ANKRD13C	6	0.016957	0.062198	0.95092	1307	4	0.5869
SNRPD1	6	0.016991	0.062328	0.95092	1308	5	0.6108
ERLIN2	6	0.016992	0.062331	0.95092	1309	4	0.3615
hsa-mir-6069	4	0.01703	0.054326	0.94743	1310	2	-0.195
MED23	6	0.017061	0.062574	0.95241	1311	5	0.4397
PRRG2	6	0.01707	0.062604	0.95241	1312	4	0.5372
PIF1	6	0.017078	0.062634	0.95241	1313	5	0.7213
DCTPP1	6	0.017084	0.062656	0.95241	1314	5	0.2568
MN1	6	0.01712	0.062767	0.95332	1315	5	0.439
INSIG1	6	0.017134	0.062809	0.95332	1316	4	0.4041
HEYL	6	0.017171	0.062921	0.95387	1317	4	0.5053
USP43	6	0.017176	0.062938	0.95387	1318	3	0.419
hsa-mir-4711	4	0.017202	0.054701	0.94743	1319	2	0.1286
DENND4A	6	0.017209	0.063037	0.95465	1320	4	0.6227
EIF3K	4	0.017223	0.054735	0.94743	1321	2	0.1626
PRAMEF8	4	0.017228	0.054744	0.94743	1322	3	0.6941
SAR1B	6	0.017263	0.063205	0.95525	1323	5	0.5525
IMPDH1	6	0.017275	0.063238	0.95525	1324	3	0.2345
FGF7	6	0.017275	0.063238	0.95525	1325	2	-0.309
NOA1	6	0.017284	0.063263	0.95525	1326	5	0.3759
PION	1	0.017322	0.017298	0.82779	1327	1	1.3649
TFRC	6	0.017325	0.063398	0.95551	1328	3	0.1845
HGF	6	0.017337	0.063439	0.95551	1329	4	0.5666
KRT71	6	0.017352	0.063491	0.95551	1330	1	-0.498
FNTB	6	0.017371	0.06355	0.95551	1331	4	0.4118
C21orf62	6	0.017385	0.063594	0.95551	1332	4	0.4954
RPS16	6	0.017396	0.063628	0.95551	1333	4	0.7191
R3HDM1	6	0.017403	0.063654	0.95551	1334	5	0.3218
TNNT1	6	0.017403	0.063654	0.95551	1335	2	-0.495
C7orf43	6	0.017439	0.063769	0.95584	1336	4	0.5448
DDX49	6	0.017472	0.063891	0.95642	1337	4	0.7477
SIGLEC5	6	0.017475	0.063901	0.95642	1338	5	0.5629
SPSB4	4	0.01748	0.05529	0.94743	1339	2	0.4462
hsa-mir-3134	4	0.01751	0.055353	0.94772	1340	3	0.8736
SCGN	6	0.017512	0.064032	0.95667	1341	4	0.4714
TIMM22	6	0.017557	0.064172	0.95667	1342	4	0.5213
ANK2	6	0.01757	0.06422	0.95667	1343	3	-0.089
OR4A47	6	0.017597	0.064316	0.95667	1344	5	0.4273
TSACC	6	0.017609	0.064353	0.95667	1345	3	-0.186
NUP93	6	0.017647	0.064488	0.95667	1346	5	0.4797
MTO1	6	0.017651	0.064504	0.95667	1347	2	0.0177
MRPL45	6	0.01766	0.064524	0.95667	1348	4	0.53
ULBP1	6	0.01766	0.064524	0.95667	1349	2	-0.228
RCC2	6	0.017709	0.064693	0.95667	1350	5	0.4952
RNF222	6	0.017735	0.064767	0.95667	1351	5	0.5213
SLC22A17	6	0.017757	0.064836	0.95667	1352	5	0.613
PPM1A	6	0.017761	0.064859	0.95667	1353	4	0.4763
OS9	6	0.017763	0.064862	0.95667	1354	3	0.1437
PCYT1A	6	0.01777	0.064884	0.95667	1355	4	0.8003

SIGLEC8	6	0.017778	0.064909	0.95667	1356	5	0.3373
PPP1R12A	6	0.017794	0.064969	0.95667	1357	5	0.5002
LENG8	6	0.0178	0.064987	0.95667	1358	3	0.3094
SAA1	6	0.017814	0.065029	0.95667	1359	2	-0.287
DYNLL2	6	0.017858	0.065168	0.95667	1360	4	0.4241
ZBTB32	6	0.017865	0.065184	0.95667	1361	3	0.2987
hsa-mir-432	4	0.017887	0.056105	0.94994	1362	2	-0.2
PEBP1	6	0.017915	0.065353	0.95667	1363	4	0.4361
MYH13	6	0.017916	0.065358	0.95667	1364	2	-0.061
SPOCD1	6	0.017923	0.065376	0.95667	1365	3	0.4272
FUT2	6	0.017929	0.065396	0.95667	1366	5	0.414
OR4C6	6	0.017964	0.065509	0.95667	1367	4	0.5811
C12orf5	6	0.017993	0.06561	0.95667	1368	5	0.4316
COX7B	6	0.01801	0.065664	0.95667	1369	4	0.4586
ZNF600	6	0.018017	0.065682	0.95667	1370	3	0.1737
BCAS2	6	0.018019	0.065688	0.95667	1371	3	0.2443
PRSS23	6	0.018026	0.065707	0.95667	1372	3	0.2524
SPINK2	6	0.018049	0.065784	0.95667	1373	4	0.6456
PAXIP1	6	0.018076	0.065869	0.95667	1374	3	0.054
CASK	6	0.018099	0.065951	0.95667	1375	2	0.0354
PAPL	6	0.018107	0.065974	0.95667	1376	4	0.4628
EPG5	6	0.01814	0.066077	0.95667	1377	3	0.3555
CREBBP	6	0.018173	0.066183	0.95667	1378	4	0.6225
hsa-mir-1302-6	4	0.018218	0.056815	0.94994	1379	2	0.521
FAM198B	6	0.018222	0.066335	0.95667	1380	2	-0.11
IP6K2	6	0.018224	0.066346	0.95667	1381	1	-0.092
PIP5K1C	6	0.018258	0.066455	0.95667	1382	4	0.9164
MCTS1	6	0.018276	0.066506	0.95667	1383	3	0.2404
DEFB112	6	0.018284	0.066531	0.95667	1384	4	0.4944
CHRDL1	6	0.018288	0.066541	0.95667	1385	3	0.2853
CIB1	6	0.018296	0.066571	0.95667	1386	4	0.3923
ZNF559	6	0.018299	0.06658	0.95667	1387	5	0.5693
MUC7	6	0.018327	0.066685	0.95667	1388	1	-0.314
IRF2	6	0.018372	0.066826	0.95667	1389	5	0.4849
C2CD2	6	0.018378	0.066844	0.95667	1390	2	-0.232
C17orf62	6	0.018389	0.066871	0.95667	1391	4	0.4894
GFRA2	6	0.01843	0.067007	0.95667	1392	3	0.0405
CCL27	6	0.018437	0.067033	0.95667	1393	5	0.3021
EPB41L4B	6	0.018467	0.067138	0.95667	1394	4	0.4935
LHFPL2	6	0.018481	0.067187	0.95667	1395	3	0.4078
RPL35A	6	0.018511	0.067281	0.95667	1396	4	0.586
DENR	6	0.018515	0.067291	0.95667	1397	4	0.3021
LARP4B	6	0.018532	0.067341	0.95667	1398	2	0.1725
SH3BGRL2	6	0.01856	0.067436	0.95667	1399	4	0.455
GINS4	6	0.018568	0.06746	0.95667	1400	4	0.5855
ENDOU	6	0.018583	0.067507	0.95667	1401	2	-0.352
PMVK	6	0.018594	0.06754	0.95667	1402	4	0.3326
WDR31	6	0.018607	0.067588	0.95667	1403	4	0.4559
LSMD1	6	0.018613	0.067609	0.95667	1404	3	0.4577
MROH7	6	0.018614	0.067611	0.95667	1405	3	0.3493
FBXL20	6	0.018635	0.067678	0.95667	1406	1	-0.525
THBD	6	0.018686	0.067836	0.95667	1407	1	-0.381
NDRG1	6	0.018689	0.067842	0.95667	1408	4	0.5745
EP300	6	0.018692	0.067854	0.95667	1409	5	0.4222
SIPA1	6	0.018697	0.06787	0.95667	1410	3	0.0203
BRAP	6	0.018703	0.067889	0.95667	1411	3	0.5072
POLR3B	6	0.018716	0.067931	0.95667	1412	4	0.6238
RILP	6	0.018737	0.067998	0.95667	1413	3	0.3805
FAM53B	6	0.018737	0.067998	0.95667	1414	3	0.1925
ADTRP	6	0.018743	0.068019	0.95667	1415	3	-0.137
TMEM165	6	0.018745	0.068028	0.95667	1416	3	0.3476
NUPL2	6	0.018758	0.06807	0.95667	1417	3	0.1926
MCTP1	6	0.018789	0.06817	0.95667	1418	2	0.1518
KIAA0101	6	0.018796	0.068193	0.95667	1419	5	0.3924
TBC1D26	3	0.018818	0.048761	0.93764	1420	1	-0.419
HIPK3	6	0.01884	0.068335	0.95667	1421	2	-0.158
MPZL1	6	0.018843	0.068346	0.95667	1422	3	0.2896
NARS2	6	0.018849	0.068367	0.95667	1423	4	0.558
SUN5	6	0.018881	0.068461	0.95667	1424	4	0.5849
ZNF540	6	0.018891	0.068497	0.95667	1425	2	0.4249
TMEM126B	6	0.018914	0.068567	0.95667	1426	5	0.5175
C2orf69	6	0.018929	0.068621	0.95667	1427	5	0.3507
SFR1	6	0.018942	0.068669	0.95667	1428	2	0.0157
ZNF471	6	0.018963	0.068743	0.95667	1429	3	0.3388
HNF1B	4	0.018989	0.058441	0.94994	1430	3	0.5632
MRPL9	6	0.019	0.068858	0.95667	1431	4	0.5613
HDAC5	6	0.019	0.068858	0.95667	1432	4	0.4145
ZNF74	6	0.019002	0.068861	0.95667	1433	2	0.0043
C19orf43	6	0.019027	0.068942	0.95667	1434	5	0.4569
PET112	6	0.019045	0.069005	0.95667	1435	2	-0.212
HAUS4	6	0.01906	0.069051	0.95667	1436	4	0.6499
HMGA2	6	0.019065	0.069069	0.95667	1437	5	0.4105
SAV1	6	0.019077	0.069111	0.95667	1438	4	0.5987
TJAP1	6	0.019085	0.069136	0.95667	1439	4	0.5434
COPB2	6	0.019111	0.069224	0.95667	1440	3	0.3645

SENP6	6	0.019143	0.069311	0.95667	1441	5	0.7178
HERPUD2	6	0.019147	0.069325	0.95667	1442	4	0.4272
LYPD5	6	0.019175	0.069417	0.95667	1443	3	0.2793
CASP2	6	0.019199	0.069491	0.95667	1444	4	0.5173
AMMECR1L	6	0.019199	0.069491	0.95667	1445	2	-0.622
LONP1	6	0.019212	0.069541	0.95667	1446	3	0.2778
ACP2	6	0.019266	0.069728	0.95667	1447	4	0.4239
SOD1	6	0.019303	0.069839	0.95667	1448	3	0.535
hsa-mir-151b	4	0.019319	0.059186	0.94994	1449	3	0.7573
LAS1L	6	0.019321	0.069897	0.95667	1450	5	0.5672
TMEM67	6	0.019321	0.069897	0.95667	1451	5	0.3586
IL1R1	6	0.019327	0.069916	0.95667	1452	4	0.6612
SLC25A44	6	0.019335	0.069946	0.95667	1453	5	0.3516
PDILT	6	0.01935	0.070001	0.95667	1454	3	0.2905
NUDT16L1	6	0.019353	0.070016	0.95667	1455	3	0.1088
OGN	6	0.019353	0.070017	0.95667	1456	4	0.4416
CLEC7A	6	0.019368	0.070054	0.95667	1457	4	0.4464
BHLHE41	6	0.019371	0.070067	0.95667	1458	5	0.3898
CUL1	6	0.019404	0.070173	0.95667	1459	2	-0.283
CCDC59	6	0.019418	0.07022	0.95667	1460	5	0.6283
SYT3	6	0.019433	0.070275	0.95667	1461	4	0.5549
UBE2B	6	0.019455	0.070339	0.95667	1462	2	0.0069
NABP2	6	0.019458	0.070348	0.95667	1463	4	0.389
TCL1B	6	0.019499	0.070487	0.95667	1464	5	0.2937
BOC	6	0.019506	0.070509	0.95667	1465	2	-0.106
LRR1	6	0.019513	0.070523	0.95667	1466	5	0.444
ZNF440	6	0.019558	0.070667	0.95667	1467	2	-0.153
RBBP5	6	0.019575	0.070729	0.95667	1468	3	0.2258
FKBP11	6	0.019609	0.070833	0.95667	1469	2	-0.209
TAMM41	6	0.01966	0.071005	0.95667	1470	2	-0.633
TAAR6	6	0.019672	0.071047	0.95667	1471	2	0.0307
SLC5A1	6	0.01969	0.071106	0.95667	1472	3	0.3771
ZWILCH	6	0.019711	0.071181	0.95667	1473	3	0.0326
EXOC3L1	6	0.019755	0.071315	0.95667	1474	5	0.4145
UBN2	6	0.019763	0.071337	0.95667	1475	3	0.3009
PLEKHG2	6	0.019766	0.071345	0.95667	1476	3	0.3391
RAD21	6	0.019779	0.071388	0.95667	1477	4	0.6606
GABPB2	6	0.019787	0.07141	0.95667	1478	5	0.4416
NME3	6	0.019814	0.071495	0.95667	1479	3	0.2636
ZNF77	6	0.019835	0.071572	0.95667	1480	4	0.6328
KDM5A	6	0.019835	0.071572	0.95667	1481	3	0.2685
GLI4	6	0.019836	0.071576	0.95667	1482	4	0.4139
CLDN9	6	0.019881	0.07171	0.95667	1483	4	0.5765
POU3F4	6	0.019905	0.071784	0.95667	1484	5	0.4696
DUX2	6	0.019905	0.071784	0.95667	1485	5	0.4829
TGFBR3	6	0.019907	0.071789	0.95667	1486	4	0.3913
PPP1R3A	6	0.019914	0.071816	0.95667	1487	4	0.4744
HMGA1	6	0.019926	0.071862	0.95667	1488	5	0.3731
SPIRE2	6	0.019928	0.071869	0.95667	1489	3	0.2479
EPS8	6	0.019934	0.071885	0.95667	1490	5	0.3599
RXRG	6	0.019942	0.071914	0.95667	1491	3	0.3042
HARS	6	0.019968	0.072015	0.95733	1492	5	0.476
hsa-mir-3908	4	0.019988	0.060592	0.94994	1493	3	0.5637
hsa-mir-6516	4	0.019996	0.060603	0.94994	1494	2	0.5675
PCDHB14	6	0.02	0.072116	0.95733	1495	5	0.4568
TTC29	6	0.02	0.072116	0.95733	1496	5	0.393
SCN8A	6	0.020019	0.072184	0.95733	1497	3	0.3893
GRHL2	6	0.020022	0.072197	0.95733	1498	5	0.2831
C11orf70	6	0.020069	0.072341	0.95745	1499	3	0.1952
GPR97	6	0.020104	0.07245	0.95745	1500	4	0.4396
SSX2IP	6	0.020121	0.072504	0.95745	1501	4	0.3541
ZNF689	6	0.020138	0.07255	0.95745	1502	4	0.5549
METAP1D	6	0.020151	0.072587	0.95745	1503	5	0.5968
A2M	6	0.020155	0.0726	0.95745	1504	4	0.3727
SLC22A16	6	0.020156	0.072601	0.95745	1505	4	0.6431
PLD3	6	0.020171	0.072655	0.95745	1506	5	0.3096
TBC1D3F	2	0.020199	0.037319	0.88271	1507	2	1.0883
RIMKLB	6	0.020268	0.072937	0.95977	1508	5	0.3708
DDX58	6	0.020275	0.072958	0.95977	1509	5	0.5754
VPS26A	6	0.020323	0.073117	0.95977	1510	3	0.2322
HSPA9	6	0.020352	0.073223	0.95977	1511	2	-0.292
RPS27	6	0.020352	0.073223	0.95977	1512	3	0.2679
MRP63	6	0.020365	0.073258	0.95977	1513	4	0.6361
DPM1	6	0.020382	0.073315	0.95977	1514	5	0.3032
TRAF3IP1	6	0.020396	0.073363	0.95977	1515	3	0.3946
POP5	6	0.020409	0.073406	0.95977	1516	5	0.6133
PSMG2	6	0.020481	0.073634	0.95977	1517	5	0.41
NFKBIA	6	0.020527	0.073774	0.95977	1518	4	0.4376
SMC1A	6	0.020531	0.073783	0.95977	1519	2	0.0332
BTRC	6	0.02054	0.073818	0.95977	1520	5	0.2545
FOXI2	6	0.02054	0.073819	0.95977	1521	4	0.5541
hsa-mir-6887	4	0.020544	0.061751	0.94994	1522	2	0.2914
KIF5A	6	0.020552	0.07386	0.95977	1523	3	0.3797
CCND1	6	0.020553	0.073861	0.95977	1524	5	0.4169
ZNF185	6	0.020569	0.073916	0.95977	1525	5	0.5668

MITF	6	0.020582	0.073953	0.95977	1526	3	0.0407
TRIM62	4	0.020603	0.061878	0.95046	1527	2	0.2445
ZNF276	6	0.020613	0.074055	0.95977	1528	3	0.2086
SFXN5	6	0.020624	0.074087	0.95977	1529	4	0.585
RPL13A	6	0.020684	0.07428	0.95977	1530	2	0.0478
OSBPL5	6	0.020717	0.074387	0.95977	1531	2	-0.313
hsa-mir-181b-2	4	0.020727	0.062118	0.95092	1532	2	0.3386
FGF6	6	0.020745	0.07447	0.95977	1533	5	0.5818
IL17RC	6	0.020787	0.074585	0.95977	1534	3	0.2577
PDZD3	6	0.020804	0.074641	0.95977	1535	3	0.3143
BNIPL	6	0.020809	0.07465	0.95977	1536	4	0.4208
WNK1	6	0.020825	0.074701	0.95977	1537	4	0.5843
LRAT	6	0.020838	0.074731	0.95977	1538	2	-0.037
hsa-mir-198	4	0.020848	0.062372	0.95092	1539	2	0.0199
FRAT2	6	0.02085	0.074774	0.95977	1540	4	0.5995
GLI1	6	0.020874	0.074846	0.95977	1541	4	0.7666
SARS	6	0.020889	0.074893	0.95977	1542	2	0.1369
SUGP2	6	0.020901	0.074933	0.95977	1543	4	0.671
SCAMP3	6	0.020901	0.074933	0.95977	1544	3	0.4647
GTF3C3	6	0.020917	0.074985	0.95977	1545	5	0.3197
SDHA	6	0.020917	0.074985	0.95977	1546	5	0.3872
GJB2	6	0.020935	0.075049	0.95977	1547	5	0.6397
CALM3	6	0.020939	0.075062	0.95977	1548	2	0.194
MAGEE2	6	0.02094	0.075066	0.95977	1549	2	-0.069
TCEA2	6	0.020951	0.075097	0.95977	1550	5	0.4193
PLCL2	6	0.020992	0.075225	0.95977	1551	2	0.0952
TSPAN7	6	0.021043	0.0754	0.95977	1552	4	0.3486
ZNF639	6	0.021074	0.075503	0.95977	1553	3	0.1525
PNMA1	6	0.021088	0.075545	0.95977	1554	4	0.4806
PRCP	6	0.021094	0.075564	0.95977	1555	3	0.0898
BCL10	6	0.0211	0.075586	0.95977	1556	5	0.4373
ITSN2	6	0.02111	0.075619	0.95977	1557	3	0.4127
TRIO	6	0.021145	0.075736	0.95977	1558	4	0.3096
SLC25A45	6	0.021199	0.075897	0.95977	1559	5	0.2789
BIK	6	0.021215	0.075948	0.95977	1560	4	0.6117
LCMT2	6	0.021215	0.075948	0.95977	1561	3	0.4153
VCPIP1	6	0.021217	0.07595	0.95977	1562	4	0.5039
PPP1R15A	6	0.021222	0.075967	0.95977	1563	2	-0.019
SGCE	6	0.021227	0.075985	0.95977	1564	5	0.3573
TLE3	6	0.021233	0.076004	0.95977	1565	3	0.0778
THRAP3	6	0.021244	0.076039	0.95977	1566	4	0.6055
AGPAT1	6	0.021257	0.076078	0.95977	1567	4	0.5106
HIF3A	6	0.021268	0.076105	0.95977	1568	4	0.4543
GSKIP	6	0.021268	0.076106	0.95977	1569	2	-0.01
TNFSF13	6	0.021273	0.076123	0.95977	1570	5	0.2231
FGD4	6	0.021273	0.076123	0.95977	1571	5	0.2671
TAF2	6	0.021276	0.076131	0.95977	1572	4	0.5413
AGPAT2	6	0.02129	0.076183	0.95977	1573	4	0.458
SDF2L1	6	0.021298	0.07621	0.95977	1574	5	0.5316
KLRG1	6	0.021314	0.07626	0.95977	1575	5	0.3341
PABPC1	6	0.021325	0.076291	0.95977	1576	5	0.3379
TRMT1	6	0.021348	0.076366	0.95977	1577	5	0.3968
TBX5	6	0.021348	0.076366	0.95977	1578	5	0.3368
POU5F2	6	0.02135	0.076369	0.95977	1579	2	-0.276
RAP2C	6	0.021401	0.076532	0.96053	1580	2	-0.404
OR2T27	6	0.021452	0.076694	0.96053	1581	3	0.1726
PTPLB	6	0.021453	0.076699	0.96053	1582	4	0.5786
PLXNC1	6	0.021503	0.076852	0.96053	1583	2	-0.04
hsa-mir-3118-5	1	0.021521	0.021454	0.82779	1584	1	1.6109
IFNA2	6	0.021554	0.077003	0.96053	1585	3	0.1
HAT1	6	0.02159	0.077103	0.96053	1586	2	-0.065
C2orf49	6	0.021603	0.077154	0.96053	1587	3	0.1242
SLC17A5	6	0.021606	0.077166	0.96053	1588	2	-0.361
SEC31A	6	0.021633	0.077265	0.96053	1589	3	0.3613
P2RX5	6	0.021633	0.077265	0.96053	1590	4	0.5248
HIP1	6	0.021657	0.077345	0.96053	1591	2	-0.093
MLPH	6	0.021663	0.077364	0.96053	1592	4	0.4219
DDIT4	6	0.021665	0.077368	0.96053	1593	4	0.641
AGBL4	6	0.021665	0.077368	0.96053	1594	4	0.6093
SRSF8	6	0.021683	0.077428	0.96069	1595	3	0.3447
KLHL41	6	0.021754	0.077647	0.96114	1596	3	0.3813
CD151	6	0.021754	0.077647	0.96114	1597	3	0.2243
GLRA2	6	0.021759	0.077657	0.96114	1598	3	0.0982
SCN1A	6	0.02177	0.077699	0.96114	1599	3	0.0303
BSG	6	0.02181	0.077825	0.96154	1600	1	-0.05
TRMT2A	6	0.021831	0.077892	0.96179	1601	4	0.5077
LOC81691	6	0.021857	0.077967	0.96209	1602	3	0.3256
FHDC1	6	0.021882	0.078052	0.96209	1603	4	0.6624
TNNT2	6	0.021884	0.07806	0.96209	1604	5	0.5516
PIK3C3	6	0.021915	0.078148	0.96209	1605	2	-0.034
NDEL1	6	0.021937	0.078221	0.96209	1606	3	0.1592
EXOC4	6	0.02196	0.078301	0.96209	1607	5	0.4848
ALG6	6	0.021964	0.078312	0.96209	1608	3	0.1579
C22orf31	6	0.021973	0.07834	0.96209	1609	5	0.3822
PTCD1	3	0.021991	0.056498	0.94994	1610	2	0.3131

DDX21	6	0.022014	0.078474	0.96295	1611	4	0.5017
ZMYND15	6	0.022045	0.078566	0.96295	1612	3	0.4652
HERC6	6	0.022066	0.078629	0.96295	1613	2	0.2497
CSNK1A1	6	0.022067	0.078631	0.96295	1614	5	0.4651
PET100	6	0.022084	0.07868	0.96295	1615	4	0.6235
UTP6	6	0.022097	0.078729	0.96295	1616	5	0.4437
FAM206A	6	0.022114	0.078789	0.96295	1617	3	0.1699
SUV39H1	6	0.022138	0.078863	0.96295	1618	3	0.3254
GTF2E1	6	0.022168	0.078952	0.96295	1619	3	0.2024
CUL9	6	0.022196	0.079038	0.96295	1620	5	0.3113
AIFM1	6	0.022199	0.079046	0.96295	1621	4	0.453
SPP1	6	0.02223	0.079137	0.96295	1622	5	0.2259
UPK3BL	6	0.022251	0.079209	0.96295	1623	4	0.4633
CAMK2D	6	0.022257	0.079232	0.96295	1624	4	0.43
HYAL3	6	0.02227	0.079274	0.96295	1625	2	-0.073
KDELR1	6	0.022281	0.079311	0.96295	1626	3	0.0197
TNRC6A	5	0.022284	0.070292	0.95667	1627	4	0.4528
DPPA4	6	0.022304	0.079376	0.96295	1628	3	0.1983
TEK	6	0.022322	0.079439	0.96295	1629	1	-0.94
BBOX1	6	0.022323	0.079445	0.96295	1630	3	0.4027
TECTA	6	0.022364	0.079561	0.96321	1631	4	0.4871
GOLIM4	4	0.022365	0.06557	0.95667	1632	3	0.5256
CD1E	6	0.022386	0.079633	0.9635	1633	3	0.4348
SLC36A2	6	0.022439	0.079796	0.96402	1634	2	-0.072
ATP1A1	6	0.022444	0.079813	0.96402	1635	5	0.355
MNF1	4	0.022451	0.065737	0.95667	1636	3	0.5582
ZNF256	6	0.022465	0.079878	0.96402	1637	4	0.3834
TIMM44	6	0.022475	0.079906	0.96402	1638	2	0.1561
SKA1	6	0.022485	0.079944	0.96402	1639	3	0.36
HLA-E	6	0.02249	0.079958	0.96402	1640	5	0.5206
ARL3	6	0.022523	0.080071	0.96443	1641	5	0.3544
KDM3B	6	0.022526	0.080086	0.96443	1642	2	-0.472
SYN3	4	0.022539	0.065917	0.95667	1643	2	0.2457
GSDMD	6	0.022628	0.080399	0.96519	1644	2	-0.003
WASF1	6	0.022641	0.080429	0.96519	1645	5	0.2613
FLJ44313	6	0.022664	0.080503	0.96519	1646	5	0.3253
GJD2	6	0.022668	0.080514	0.96519	1647	4	0.4378
FCGBP	6	0.022679	0.080549	0.96519	1648	2	0.118
STON1	3	0.022683	0.058123	0.94994	1649	2	0.7984
EFCAB11	6	0.022693	0.080592	0.96519	1650	4	0.4846
NAA35	6	0.022729	0.080733	0.96519	1651	5	0.353
CBR4	6	0.022731	0.080734	0.96519	1652	1	0.0119
MT1X	4	0.022744	0.066346	0.95667	1653	1	-0.282
PROL1	6	0.022757	0.080827	0.96519	1654	5	0.408
SAMD13	6	0.022764	0.080854	0.96519	1655	3	0.1755
C17orf75	6	0.022782	0.080903	0.96519	1656	2	0.1132
LOC100505679	6	0.022785	0.080915	0.96519	1657	5	0.2577
SLC35E3	6	0.02279	0.08093	0.96519	1658	3	0.5482
hsa-mir-4721	4	0.022812	0.06649	0.95667	1659	1	-0.075
PXDNL	5	0.02282	0.07171	0.95667	1660	1	-0.195
WWC1	6	0.022859	0.08115	0.96519	1661	3	-0.057
LUZP6	5	0.022862	0.071837	0.95667	1662	4	0.542
SLC9A9	6	0.022877	0.081212	0.96519	1663	3	0.189
GPI	6	0.022897	0.081274	0.96519	1664	5	0.4746
ANKRD36	6	0.022935	0.081392	0.96519	1665	3	0.2998
GEN1	6	0.022935	0.081392	0.96519	1666	2	-0.466
PCBP2	6	0.022935	0.081392	0.96519	1667	2	-0.24
LDHAL6A	6	0.022959	0.081471	0.96519	1668	4	0.4931
OLIG3	6	0.022977	0.081528	0.96519	1669	3	0.4802
TECR	6	0.022977	0.081528	0.96519	1670	3	0.2655
GCGR	6	0.022995	0.08159	0.96519	1671	4	0.453
CRKL	6	0.02301	0.081639	0.96519	1672	4	0.3797
LOC730159	6	0.023017	0.08166	0.96519	1673	5	0.3523
SAMSN1	6	0.02302	0.08167	0.96519	1674	3	0.2857
NIPSNAP1	6	0.023037	0.08172	0.96519	1675	1	-0.091
UQCRFS1	6	0.023088	0.081874	0.96519	1676	2	0.2143
ADCY6	6	0.023096	0.081895	0.96519	1677	3	0.2513
FBXL14	6	0.0231	0.081913	0.96519	1678	3	0.4283
RPL34	6	0.023127	0.082	0.96519	1679	4	0.6913
SCN2A	6	0.023131	0.082015	0.96519	1680	5	0.3321
PAMR1	6	0.023139	0.082042	0.96519	1681	2	0.1255
SUDS3	6	0.023144	0.082063	0.96519	1682	5	0.4813
SPATA17	6	0.023167	0.082125	0.96519	1683	5	0.4527
FAM104A	6	0.023185	0.082182	0.96519	1684	4	0.6693
ANKS6	6	0.02319	0.082199	0.96519	1685	3	0.065
TBX22	6	0.023208	0.082253	0.96519	1686	4	0.477
DNLZ	6	0.023242	0.082358	0.96519	1687	3	0.0667
OSCAR	6	0.023246	0.08237	0.96519	1688	3	0.2582
RAD17	6	0.023258	0.082414	0.96519	1689	4	0.8978
hsa-mir-5008	4	0.023273	0.067424	0.95667	1690	2	0.4287
MIA2	6	0.023296	0.082523	0.96585	1691	5	0.4262
C1orf234	3	0.023325	0.059663	0.94994	1692	1	0.1181
TAF9B	6	0.023344	0.082683	0.96585	1693	3	0.2754
SGMS2	6	0.023351	0.082713	0.96585	1694	3	0.354
hsa-mir-3666	4	0.023358	0.067608	0.95667	1695	2	0.236

MRPL53	6	0.023375	0.082799	0.96585	1696	4	0.4094
FBXL6	4	0.02338	0.067654	0.95667	1697	3	0.6304
MTCH2	6	0.023392	0.082843	0.96585	1698	5	0.3337
SSBP2	6	0.023395	0.082848	0.96585	1699	1	-0.454
SRCAP	6	0.023446	0.083021	0.96721	1700	3	0.1792
COQ2	6	0.023459	0.083059	0.96721	1701	3	0.5598
SMC3	6	0.023536	0.083295	0.96722	1702	2	0.1624
FAM217A	6	0.023548	0.08333	0.96722	1703	2	0.0697
PSMD12	6	0.023586	0.083457	0.96722	1704	5	0.5162
TPBG	6	0.023594	0.083482	0.96722	1705	4	0.5958
DVL2	6	0.023599	0.083499	0.96722	1706	3	0.1886
KTI12	6	0.023613	0.083541	0.96722	1707	4	0.4846
BHMT2	6	0.02365	0.083651	0.96722	1708	4	0.4294
STARD7	6	0.023656	0.083667	0.96722	1709	5	0.5914
SLC1A5	6	0.023674	0.083718	0.96722	1710	5	0.5259
UBE4B	6	0.023674	0.083719	0.96722	1711	2	-0.145
UBE2NL	6	0.023691	0.083784	0.96722	1712	5	0.2648
NUFIP1	6	0.023728	0.08389	0.96722	1713	5	0.4239
RASGEF1C	6	0.02375	0.083959	0.96722	1714	4	0.4102
FAM228B	6	0.023752	0.083965	0.96722	1715	3	0.1298
RTN1	6	0.023766	0.084004	0.96722	1716	4	0.5232
CDCP1	6	0.023768	0.084008	0.96722	1717	5	0.3662
RAP1GAP	6	0.023806	0.084118	0.96722	1718	3	0.4589
RRP1	6	0.02383	0.0842	0.96722	1719	4	0.5486
TACO1	6	0.023831	0.084202	0.96722	1720	5	0.236
TMEM253	6	0.023839	0.084223	0.96722	1721	5	0.6666
GARS	6	0.02384	0.084229	0.96722	1722	4	0.5799
SLC45A1	6	0.023854	0.084277	0.96722	1723	3	0.221
PUS7	6	0.023891	0.084385	0.96722	1724	5	0.4801
GPR152	6	0.023957	0.084601	0.96722	1725	2	-0.278
EYA2	4	0.023963	0.068868	0.95667	1726	3	0.5618
CEBPG	6	0.023963	0.084622	0.96722	1727	5	0.4618
CDKN3	6	0.023982	0.08469	0.96722	1728	5	0.504
BPIFB2	6	0.024006	0.084762	0.96722	1729	4	0.4641
MTHFR	6	0.024008	0.084766	0.96722	1730	2	0.0021
ZNF345	6	0.02402	0.084806	0.96722	1731	4	0.4764
ZNF616	6	0.024059	0.084905	0.96722	1732	1	-0.535
OR4A16	6	0.024066	0.084926	0.96722	1733	3	0.201
ASB3	3	0.024069	0.061441	0.94994	1734	1	0.1145
PRMT6	6	0.02407	0.084937	0.96722	1735	4	0.4802
GTF2H1	6	0.024077	0.084949	0.96722	1736	4	0.3841
KIF24	6	0.024078	0.084951	0.96722	1737	4	0.4552
hsa-mir-484	4	0.024089	0.069129	0.95667	1738	3	0.548
RGP1	6	0.024111	0.085027	0.96722	1739	4	0.4148
LRRC19	6	0.024118	0.085044	0.96722	1740	3	0.3166
MBTD1	6	0.024135	0.085084	0.96722	1741	1	-0.172
PHC3	6	0.024135	0.085084	0.96722	1742	2	0.0641
TPX2	6	0.024151	0.085125	0.96722	1743	2	0.1949
HYAL2	6	0.024162	0.085153	0.96722	1744	4	0.5621
PTGR2	6	0.024179	0.085187	0.96722	1745	4	0.4239
OTUB2	6	0.024207	0.085248	0.96727	1746	2	-0.077
PSME1	6	0.024263	0.085364	0.96727	1747	3	0.2319
TMEM5	6	0.024288	0.085433	0.96727	1748	4	0.6044
LCE2D	5	0.02429	0.075611	0.95977	1749	1	0.1417
PYROXD1	6	0.024347	0.085562	0.96727	1750	4	0.4661
RAP1GDS1	6	0.024348	0.085563	0.96727	1751	4	0.5358
AP3D1	6	0.024352	0.085575	0.96727	1752	3	0.4245
ZBTB8OS	6	0.024357	0.085586	0.96727	1753	3	0.4878
MCM6	6	0.024416	0.08572	0.96727	1754	2	-0.562
hsa-mir-3936	4	0.024438	0.06984	0.95667	1755	3	0.5649
FTMT	6	0.024492	0.085901	0.96727	1756	4	0.526
TRPM8	6	0.024518	0.085974	0.96727	1757	4	0.2717
SAMD11	6	0.024529	0.085997	0.96727	1758	4	0.4604
CRIM1	6	0.024562	0.086082	0.96727	1759	4	0.5037
MAP3K10	6	0.024569	0.086099	0.96727	1760	2	-0.08
hsa-mir-4663	4	0.024586	0.070131	0.95667	1761	2	0.0737
ELP4	6	0.024605	0.086187	0.96727	1762	4	0.5781
CYHR1	6	0.024646	0.086278	0.96727	1763	3	0.1863
C6orf226	6	0.024655	0.086301	0.96727	1764	4	0.5309
SH3TC2	6	0.024675	0.08635	0.96727	1765	4	0.5299
RGL3	6	0.024683	0.08637	0.96727	1766	4	0.5398
REN	6	0.024688	0.086387	0.96727	1767	3	0.4603
PDLIM5	6	0.024697	0.086407	0.96727	1768	4	0.3862
CD164L2	6	0.024697	0.086407	0.96727	1769	3	0.3584
HMMR	6	0.024707	0.086433	0.96727	1770	3	0.1933
TRPM2	6	0.024727	0.086482	0.96727	1771	4	0.4229
SYT8	6	0.024752	0.086539	0.96727	1772	3	0.1158
ZYX	6	0.024773	0.086587	0.96727	1773	3	0.155
PRRX2	6	0.024797	0.086634	0.96727	1774	2	-0.775
MED15	6	0.024798	0.086636	0.96727	1775	4	0.5692
WDR93	6	0.024809	0.086654	0.96727	1776	4	0.5325
MTHFSD	6	0.024819	0.08668	0.96727	1777	4	0.3604
OR4C12	6	0.024847	0.08674	0.96727	1778	4	0.4604
DSE	6	0.024863	0.086775	0.96727	1779	3	0.2331
FAM210A	6	0.024871	0.086789	0.96727	1780	3	0.2099

CLEC11A	6	0.024875	0.086799	0.96727	1781	4	0.5225
FDX1	6	0.024901	0.086857	0.9674	1782	3	0.4902
KIAA1614	6	0.024933	0.086926	0.96763	1783	4	0.7363
RTP3	6	0.024977	0.087016	0.9678	1784	3	0.1601
ARHGEF6	6	0.025018	0.087106	0.9678	1785	2	0.0017
DPH1	6	0.025028	0.08713	0.9678	1786	4	0.5692
hsa-mir-199a-1	3	0.025042	0.06376	0.95584	1787	2	0.5309
OR4C11	6	0.025079	0.087254	0.9684	1788	3	0.3336
HUS1B	6	0.025141	0.087385	0.9684	1789	2	0.147
DEPDC5	6	0.025148	0.087403	0.9684	1790	4	0.5938
CKMT1B	4	0.025165	0.071324	0.95667	1791	3	0.3907
hsa-mir-6124	4	0.02518	0.071359	0.95667	1792	3	0.701
FAM155B	6	0.025181	0.087479	0.9684	1793	3	0.3462
hsa-mir-641	4	0.025191	0.071383	0.95667	1794	3	0.5743
FBXW7	6	0.025212	0.087558	0.9684	1795	3	0.1241
EPB41L3	6	0.025231	0.087599	0.9684	1796	2	-0.018
FAM169B	6	0.02526	0.087664	0.9684	1797	3	0.4033
MMRN1	6	0.025334	0.087846	0.9684	1798	3	0.2904
SH3GL3	6	0.025374	0.087938	0.9684	1799	4	0.5111
MRPS34	6	0.025385	0.087965	0.9684	1800	3	0.1016
C10orf35	6	0.025398	0.087991	0.9684	1801	4	0.4014
RASA3	6	0.025412	0.088026	0.9684	1802	3	0.1268
CDC42BPB	6	0.025412	0.088026	0.9684	1803	3	0.1433
MEF2BNB	6	0.025435	0.088085	0.9684	1804	4	0.3322
ECE2	6	0.025439	0.088094	0.9684	1805	3	0.4036
RTN2	6	0.025478	0.088181	0.9684	1806	2	0.0442
MAFA	6	0.025487	0.088198	0.9684	1807	2	-0.512
SEC24D	6	0.025504	0.088236	0.9684	1808	4	0.4789
NIPAL4	6	0.025524	0.08828	0.9684	1809	3	0.3724
INSC	6	0.025538	0.088314	0.9684	1810	3	0.2221
PRICKLE1	6	0.025548	0.088341	0.9684	1811	4	0.5589
C20orf195	6	0.025594	0.088445	0.9684	1812	4	0.4199
PLTP	6	0.025597	0.088451	0.9684	1813	2	0.0969
FIGN	6	0.025598	0.088453	0.9684	1814	3	0.474
TMCC2	6	0.025603	0.088462	0.9684	1815	4	0.4883
GNG11	5	0.02563	0.079163	0.96295	1816	2	0.1417
ASAH2B	6	0.025633	0.088538	0.96841	1817	3	0.0291
hsa-mir-3652	4	0.025654	0.072344	0.95745	1818	3	0.882
C19orf38	6	0.025702	0.088718	0.96841	1819	4	0.3535
ROS1	6	0.025742	0.0888	0.96841	1820	2	0.1303
C14orf28	6	0.025745	0.088802	0.96841	1821	4	0.5547
JPH4	6	0.025755	0.088824	0.96841	1822	4	0.5262
APOBEC3H	6	0.025793	0.088919	0.96841	1823	3	0.2208
SPPL3	4	0.025812	0.072674	0.95745	1824	1	-0.105
WDR37	6	0.025844	0.089039	0.96841	1825	2	0.0125
C8A	6	0.025871	0.089099	0.96841	1826	4	0.4364
GCDH	6	0.025871	0.0891	0.96841	1827	3	-0.172
CTCF	6	0.025895	0.089157	0.96841	1828	1	-0.011
KRTAP5-4	6	0.025999	0.089397	0.96841	1829	3	0.3116
SYNE2	6	0.026008	0.089416	0.96841	1830	4	0.5129
STAU1	6	0.026039	0.089482	0.96841	1831	4	0.3495
RBKS	6	0.026073	0.089561	0.96841	1832	2	0.177
DDX56	6	0.026129	0.089694	0.96841	1833	3	0.1753
LRRC8D	6	0.026201	0.089872	0.96841	1834	3	0.2897
DMKN	6	0.02623	0.089934	0.96841	1835	3	0.1184
ARHGEF3	6	0.02625	0.089985	0.96841	1836	2	0.1626
ANKRD34B	6	0.026283	0.090054	0.96841	1837	4	0.3388
OR8K5	6	0.026303	0.090107	0.96841	1838	2	-0.467
RNF111	6	0.026338	0.090176	0.96841	1839	4	0.4629
HOXD12	6	0.026354	0.090217	0.96841	1840	3	0.191
PROX1	6	0.02639	0.090302	0.96841	1841	2	-0.304
TMEM143	6	0.026419	0.090375	0.96841	1842	3	0.2272
OSER1	1	0.026448	0.026384	0.82779	1843	1	1.3456
GRIA2	6	0.026456	0.090447	0.96841	1844	2	0.1211
HENMT1	6	0.026462	0.09047	0.96841	1845	3	0.0907
DPM2	6	0.026533	0.090648	0.96841	1846	4	0.4773
SUPT16H	6	0.026545	0.090678	0.96841	1847	4	0.3675
PXK	6	0.026549	0.090689	0.96841	1848	4	0.3841
TBC1D19	6	0.026557	0.090707	0.96841	1849	2	-0.139
DENND2D	6	0.026608	0.09081	0.96841	1850	4	0.3248
SEMG1	5	0.026629	0.081791	0.96519	1851	2	0.2328
hsa-mir-4531	4	0.026629	0.074358	0.95977	1852	1	0.0543
POGLUT1	6	0.026632	0.090868	0.96841	1853	3	0.1848
CAPS2	6	0.026651	0.09092	0.96841	1854	2	0.1393
INHBA	6	0.026688	0.091011	0.96841	1855	4	0.4181
IL21	6	0.026693	0.091025	0.96841	1856	3	0.362
SLAIN1	6	0.026693	0.091025	0.96841	1857	3	0.1458
37865	3	0.026711	0.067693	0.95667	1858	3	1.1304
ZBTB40	6	0.02674	0.091131	0.96841	1859	4	0.4927
PPP3R1	6	0.02674	0.091131	0.96841	1860	3	0.0296
WDR70	6	0.026761	0.091185	0.96841	1861	3	0.0905
CNPPD1	6	0.026786	0.091247	0.96841	1862	3	0.1834
PFDN5	6	0.026812	0.091302	0.96841	1863	2	-0.069
NEFL	6	0.026863	0.091433	0.96841	1864	2	0.0525
VSIG10	6	0.026868	0.091445	0.96841	1865	4	0.4434

RPS3A	6	0.026875	0.091459	0.96841	1866	3	0.1955
PLA2G2F	6	0.026875	0.091459	0.96841	1867	3	0.2211
SLCO6A1	6	0.026885	0.091485	0.96841	1868	4	0.6385
CEACAM5	6	0.02691	0.09154	0.96841	1869	4	0.5667
C9orf89	6	0.026914	0.09155	0.96841	1870	2	-0.211
SF3B4	6	0.026919	0.091558	0.96841	1871	3	0.191
ATP13A1	6	0.026938	0.091604	0.96841	1872	2	-0.161
DDB1	6	0.026965	0.091663	0.96841	1873	4	0.4069
SNRPC	4	0.02697	0.075068	0.95977	1874	2	0.1873
ARMC5	6	0.026989	0.091721	0.96841	1875	4	0.4063
ERF	6	0.027067	0.091897	0.96841	1876	1	-0.142
APP	6	0.027118	0.092027	0.96841	1877	4	0.4413
ZNF551	6	0.027129	0.092056	0.96841	1878	4	0.4391
RNF121	6	0.027134	0.092065	0.96841	1879	3	0.5303
DNAJC9	6	0.027169	0.092145	0.96841	1880	2	-0.359
C22orf43	6	0.027193	0.092201	0.96841	1881	3	0.4628
ZNF90	4	0.027196	0.075521	0.95977	1882	3	0.5223
DLX3	6	0.027222	0.092266	0.96841	1883	4	0.4204
SNRNP27	6	0.027267	0.092367	0.96841	1884	4	0.6717
HES2	6	0.027274	0.092387	0.96841	1885	4	0.7581
ASB2	6	0.027372	0.092635	0.96841	1886	3	-0.42
EIF2B4	6	0.027389	0.092679	0.96841	1887	3	0.3293
HAS2	6	0.027485	0.092893	0.96841	1888	4	0.511
HS3ST3A1	4	0.027488	0.076116	0.95977	1889	2	0.238
MTR	6	0.027491	0.092912	0.96841	1890	4	0.3403
THYN1	6	0.027525	0.092989	0.96841	1891	3	0.1466
GCM2	6	0.027576	0.093109	0.96841	1892	4	0.486
BEAN1	6	0.02758	0.093118	0.96841	1893	4	0.4282
SMAD4	6	0.027595	0.093154	0.96841	1894	4	0.359
CAD	6	0.027606	0.093176	0.96841	1895	4	0.4475
FAM47C	6	0.027625	0.093222	0.96841	1896	3	0.5416
C9orf57	6	0.027659	0.093297	0.96841	1897	3	0.7445
ACE2	6	0.027683	0.093356	0.96841	1898	3	0.1234
ZSCAN18	6	0.027779	0.093578	0.96841	1899	4	0.4467
PNMAL1	6	0.027781	0.093581	0.96841	1900	3	0.5078
KIFC1	6	0.027799	0.093626	0.96841	1901	3	0.3908
OXSM	6	0.0278	0.093629	0.96841	1902	4	0.4078
RGS12	6	0.02783	0.093703	0.96841	1903	2	-4E-04
FOXI3	6	0.027877	0.093808	0.96841	1904	4	0.6534
MAP3K12	6	0.027881	0.093819	0.96841	1905	3	0.2258
GFPT2	6	0.027926	0.093929	0.96841	1906	2	-0.205
PHF19	6	0.02794	0.093958	0.96841	1907	4	0.5383
HILPDA	6	0.027983	0.094066	0.96841	1908	3	0.2046
hsa-mir-8054	4	0.027998	0.07718	0.96053	1909	3	0.8204
KCNJ15	6	0.028006	0.094114	0.96841	1910	3	0.2394
hsa-mir-4448	4	0.028027	0.077244	0.96053	1911	2	0.2433
CISD3	6	0.028034	0.094181	0.96841	1912	1	-0.542
LRRC6	6	0.02805	0.094224	0.96841	1913	2	-0.275
GPC2	6	0.02806	0.094255	0.96841	1914	4	0.5173
AP2A1	4	0.028071	0.077318	0.96053	1915	3	0.5217
hsa-mir-223	4	0.028075	0.077328	0.96053	1916	2	0.0522
TEX38	6	0.02809	0.094313	0.96841	1917	3	-0.188
MTRNR2L4	6	0.028136	0.094424	0.96841	1918	2	-0.442
PHRF1	6	0.028175	0.094515	0.96841	1919	2	-0.292
TOP1	6	0.028175	0.094515	0.96841	1920	3	0.0951
DQX1	6	0.028194	0.09457	0.96841	1921	4	0.513
SPN	6	0.02823	0.094656	0.96841	1922	4	0.605
PDXDC1	6	0.028287	0.09479	0.96841	1923	4	0.4656
MPHOSPH6	4	0.028304	0.077798	0.96154	1924	2	0.2801
RIMKLA	6	0.028306	0.094842	0.96841	1925	3	0.3326
PTCHD3	6	0.028339	0.094923	0.96841	1926	1	0.012
EVI5	6	0.02839	0.095045	0.96841	1927	1	-0.008
NETO1	6	0.028408	0.095089	0.96841	1928	4	0.4216
SNAI3	6	0.028463	0.095222	0.96841	1929	4	0.4178
SUGP1	6	0.028469	0.095236	0.96841	1930	4	0.4791
PABPC5	6	0.028482	0.095268	0.96841	1931	4	0.3827
MAML3	6	0.028492	0.095291	0.96841	1932	1	-0.1
MROH8	6	0.028502	0.095313	0.96841	1933	3	0.3977
TNFRSF17	6	0.028542	0.095399	0.96841	1934	1	-0.238
SIRT4	6	0.028559	0.095444	0.96841	1935	3	0.2234
OGFOD3	6	0.028574	0.095478	0.96841	1936	3	0.0483
NRG1	6	0.028593	0.09553	0.96841	1937	2	0.088
SSTR4	6	0.028619	0.095598	0.96841	1938	3	0.3024
FAM126B	6	0.028639	0.095635	0.96841	1939	4	0.5333
RACGAP1	6	0.028644	0.095646	0.96841	1940	1	-0.144
E2F1	6	0.028694	0.095771	0.96841	1941	3	0.349
AP3B2	6	0.028709	0.095807	0.96841	1942	4	0.4439
GSK3B	6	0.028712	0.095819	0.96841	1943	3	0.4109
LTB4R	6	0.028746	0.095892	0.96841	1944	2	-0.207
E4F1	6	0.02875	0.095904	0.96841	1945	3	0.4957
CDKL5	6	0.028774	0.095965	0.96841	1946	4	0.3715
RALYL	6	0.028788	0.095992	0.96841	1947	4	0.636
BIN1	6	0.028789	0.095994	0.96841	1948	4	0.5362
LLPH	6	0.028819	0.096065	0.96841	1949	2	-0.537
ELAVL2	6	0.028848	0.096134	0.96841	1950	4	0.2521

ANAPC16	6	0.028864	0.096169	0.96841	1951	3	0.3161
LRRC66	6	0.028898	0.09625	0.96841	1952	2	-0.264
ATF3	6	0.028949	0.096357	0.96841	1953	1	-0.224
PSMD13	6	0.02896	0.096383	0.96841	1954	2	-0.464
GP1BB	6	0.029	0.096469	0.96841	1955	4	0.4195
TAS2R30	6	0.029097	0.096694	0.96841	1956	4	0.451
SMIM21	4	0.029105	0.079477	0.96295	1957	2	0.0548
hsa-mir-6130	4	0.029112	0.079493	0.96295	1958	1	-0.309
SLC25A3	6	0.029122	0.096747	0.96841	1959	4	0.5523
RPP30	6	0.029153	0.096819	0.96841	1960	3	0.2916
LMX1A	6	0.029177	0.096876	0.96841	1961	4	0.5761
CELF1	6	0.029188	0.096902	0.96841	1962	4	0.5459
C20orf112	6	0.029192	0.096921	0.96841	1963	4	0.316
MRPL34	6	0.029193	0.096922	0.96841	1964	4	0.4902
PAX8	6	0.029222	0.096979	0.96841	1965	4	0.3531
PRKCA	6	0.029242	0.097015	0.96841	1966	4	0.6471
NOTCH3	6	0.029254	0.09705	0.96841	1967	4	0.44
JUNB	6	0.029305	0.097167	0.96841	1968	1	-0.394
RMND5B	6	0.029335	0.097245	0.96841	1969	3	0.1909
KCNIP4	6	0.029348	0.097269	0.96841	1970	4	0.4738
SPEN	6	0.029356	0.097287	0.96841	1971	2	-0.014
LRRC70	6	0.029364	0.097309	0.96841	1972	4	0.3629
UNC119B	6	0.029367	0.097317	0.96841	1973	4	0.5746
UGT2B10	3	0.029373	0.073907	0.95977	1974	2	0.8969
CAPN14	6	0.029407	0.097423	0.96841	1975	3	0.0571
FANCA	6	0.02945	0.097517	0.96841	1976	4	0.3127
PDP1	6	0.029458	0.097539	0.96841	1977	3	0.4347
hsa-mir-100	4	0.029467	0.080233	0.96519	1978	2	0.5965
CLN8	6	0.029475	0.09757	0.96841	1979	3	0.3331
JKAMP	6	0.029475	0.09757	0.96841	1980	4	0.5404
DTD2	6	0.029508	0.097645	0.96841	1981	4	0.3459
KRTAP8-1	6	0.029527	0.097697	0.96841	1982	4	0.4177
ARHGEF10	6	0.029533	0.097714	0.96841	1983	3	0.3994
HEPACAM2	6	0.029536	0.097719	0.96841	1984	2	-0.042
GALNT13	6	0.029547	0.097741	0.96841	1985	3	0.3215
PTDSS1	6	0.029564	0.09778	0.96841	1986	4	0.7408
C19orf60	6	0.02961	0.097881	0.96841	1987	2	-0.034
GTPBP4	6	0.029634	0.097932	0.96841	1988	4	0.5925
CACNB1	4	0.029655	0.080639	0.96519	1989	2	0.3933
ANKRD62	6	0.029661	0.098	0.96841	1990	2	0.19
FAM210B	6	0.029678	0.098046	0.96841	1991	4	0.4415
OR4B1	6	0.029709	0.09811	0.96841	1992	2	-0.189
CYTH1	6	0.029773	0.098262	0.96841	1993	4	0.7006
GBP1	6	0.02979	0.098304	0.96841	1994	3	0.3506
TP53INP2	6	0.029813	0.098353	0.96841	1995	2	0.0135
ARIH1	6	0.029831	0.098388	0.96841	1996	4	0.5785
ZAR1L	6	0.029841	0.09842	0.96841	1997	4	0.3219
hsa-mir-618	4	0.029843	0.081004	0.96519	1998	2	0.4759
DLG1	6	0.029864	0.098467	0.96841	1999	4	0.4684
HSD17B4	6	0.029872	0.098488	0.96841	2000	3	0.4066
PRRC2A	6	0.029915	0.098595	0.96841	2001	2	-0.083
hsa-mir-302e	4	0.029918	0.081163	0.96519	2002	3	0.7202
ZNF266	6	0.029943	0.098658	0.96841	2003	4	0.522
PCDHB3	6	0.029966	0.098705	0.96841	2004	4	0.3176
METTL21A	6	0.030015	0.098811	0.96841	2005	4	0.5982
UFSP1	6	0.030016	0.098815	0.96841	2006	1	-0.421
RIF1	6	0.030028	0.098845	0.96841	2007	4	0.4758
L3HYPDH	6	0.03004	0.098872	0.96841	2008	4	0.4201
hsa-mir-6743	4	0.030046	0.08144	0.96519	2009	1	-0.92
MYLK2	6	0.030067	0.098937	0.96841	2010	3	0.411
PHF1	6	0.030071	0.098943	0.96841	2011	2	-0.038
ASXL1	6	0.030096	0.099003	0.96841	2012	2	-0.829
MCM2	6	0.030127	0.099077	0.96841	2013	4	0.3089
ACTG1	6	0.030131	0.099084	0.96841	2014	4	0.5083
HS3ST5	6	0.030133	0.099088	0.96841	2015	3	0.4379
PLEKHD1	6	0.030156	0.099136	0.96841	2016	4	0.4678
TXNRD3NB	6	0.03022	0.099288	0.96841	2017	2	-0.09
PLG	6	0.03027	0.099406	0.96841	2018	2	-0.149
ARSK	6	0.030321	0.09953	0.96841	2019	2	-0.309
CIR1	6	0.030364	0.099629	0.96841	2020	3	0.393
CINP	6	0.030366	0.099633	0.96841	2021	4	0.4552
RASSF4	6	0.030372	0.099646	0.96841	2022	3	0.2383
LEPROT	6	0.030382	0.099674	0.96841	2023	4	0.3781
SLC16A7	6	0.030423	0.099763	0.96841	2024	1	-0.493
ENPEP	6	0.030423	0.099764	0.96841	2025	4	0.5422
hsa-mir-5706	4	0.03047	0.082311	0.96519	2026	1	-0.316
MAPK9	6	0.030476	0.09989	0.96841	2027	4	0.4245
CA2	6	0.030498	0.099939	0.96841	2028	4	0.4387
RUNDC3B	6	0.030517	0.099975	0.96841	2029	4	0.4581
PSMC3	6	0.030524	0.099992	0.96841	2030	2	0.1343
POLR2J2	1	0.030533	0.030417	0.83613	2031	1	1.2284
C17orf82	6	0.030566	0.1001	0.96841	2032	4	0.5795
ADAM9	6	0.030575	0.10012	0.96841	2033	2	-0.032
OR1D2	6	0.030583	0.10013	0.96841	2034	4	0.4407
MXRA7	6	0.030626	0.10024	0.96841	2035	4	0.3675

MRPS12	4	0.030673	0.082737	0.96585	2036	3	0.619
APOA1	6	0.030687	0.10039	0.96841	2037	4	0.3098
NOG	6	0.030727	0.10049	0.96841	2038	2	-0.058
CBL	6	0.030739	0.10052	0.96841	2039	4	0.3978
ATXN7L2	6	0.030741	0.10053	0.96841	2040	4	0.5363
RPS12	6	0.030748	0.10054	0.96841	2041	3	0.5742
TCP11L2	6	0.030771	0.10058	0.96841	2042	3	0.412
ICAM5	6	0.030778	0.1006	0.96841	2043	2	0.1524
WRAP53	6	0.030829	0.10071	0.96841	2044	2	-0.108
ATP8B2	6	0.030852	0.10077	0.96841	2045	3	0.4518
TLR4	6	0.030875	0.10084	0.96841	2046	4	0.3844
PLCB4	6	0.030878	0.10085	0.96841	2047	4	0.557
FZD7	6	0.030878	0.10085	0.96841	2048	4	0.472
STK39	6	0.03093	0.10096	0.96841	2049	3	0.0993
FAM207A	6	0.030971	0.10106	0.96841	2050	4	0.4479
ELMOD2	6	0.030981	0.10109	0.96841	2051	4	0.4465
hsa-mir-892b	4	0.030986	0.083405	0.96722	2052	2	0.6055
CBLB	6	0.031005	0.10115	0.96841	2053	3	0.0793
DOC2B	6	0.03103	0.1012	0.96841	2054	4	0.5122
PPAN-P2RY11	2	0.03104	0.056591	0.94994	2055	1	0.2136
LMNB1	6	0.031056	0.10125	0.96841	2056	4	0.4332
MYL6B	6	0.03107	0.10128	0.96841	2057	3	0.2353
LYAR	6	0.03109	0.10132	0.96841	2058	3	0.3867
SIGLEC14	6	0.031096	0.10133	0.96841	2059	2	-0.072
RNF182	6	0.031108	0.10135	0.96841	2060	2	-0.137
MKNK1	6	0.031127	0.1014	0.96841	2061	3	0.2349
RABEPK	6	0.031153	0.10146	0.96841	2062	4	0.3957
TUBGCP5	6	0.031203	0.10157	0.96841	2063	4	0.4145
PAK1	6	0.031235	0.10165	0.96841	2064	3	0.1838
PPP1R11	6	0.031236	0.10166	0.96841	2065	3	0.2583
RPL14	6	0.031266	0.10173	0.96841	2066	4	0.7665
ZNF793	6	0.031283	0.10177	0.96841	2067	4	0.6112
ENOPH1	6	0.031285	0.10177	0.96841	2068	3	0.4731
COG1	6	0.031285	0.10177	0.96841	2069	1	-0.448
CLTA	6	0.031303	0.1018	0.96841	2070	2	-0.178
RAB25	6	0.031314	0.10183	0.96841	2071	2	-0.197
DYNC2H1	6	0.031336	0.10188	0.96841	2072	1	-0.251
MAFF	6	0.031387	0.102	0.96841	2073	3	0.529
SF3B1	6	0.031387	0.102	0.96841	2074	3	0.2435
USP20	6	0.031404	0.10204	0.96841	2075	4	0.3634
PRAMEF2	6	0.031438	0.10212	0.96841	2076	2	0.0772
GSG2	6	0.031449	0.10214	0.96841	2077	2	0.0481
TSR1	6	0.03147	0.10219	0.96841	2078	4	0.7171
DDX39B	6	0.03147	0.10219	0.96841	2079	4	0.4689
C11orf65	6	0.031486	0.10224	0.96841	2080	2	0.0203
NUDT19	6	0.031488	0.10224	0.96841	2081	2	0.1232
KRTAP13-3	6	0.031501	0.10227	0.96841	2082	3	0.1777
RPL12	6	0.031512	0.1023	0.96841	2083	4	0.6664
ASF1A	6	0.031527	0.10234	0.96841	2084	3	0.4209
KIAA1598	6	0.03153	0.10235	0.96841	2085	4	0.5548
OR6C75	6	0.031539	0.10237	0.96841	2086	2	-0.026
ISM2	6	0.031621	0.10256	0.96841	2087	3	0.1891
SNUPN	6	0.031624	0.10256	0.96841	2088	4	0.4308
SPRN	6	0.03163	0.10258	0.96841	2089	3	0.2925
CCIN	6	0.031641	0.10261	0.96841	2090	2	-0.081
CNTD2	6	0.031679	0.10269	0.96841	2091	2	-0.103
EMILIN1	6	0.031691	0.10273	0.96841	2092	4	0.4077
POU4F1	6	0.031707	0.10277	0.96841	2093	4	0.5252
ZNF143	6	0.031722	0.1028	0.96841	2094	3	0.4146
AP2M1	6	0.031745	0.10287	0.96841	2095	4	0.5592
DCHS2	6	0.031752	0.10288	0.96841	2096	4	0.358
CYTIP	6	0.031767	0.10292	0.96841	2097	2	0.0223
CCNE2	6	0.031805	0.103	0.96841	2098	4	0.3679
MC2R	6	0.031869	0.10314	0.96841	2099	1	-0.267
LRRTM3	6	0.031891	0.10319	0.96841	2100	3	0.3022
USP40	6	0.031941	0.10329	0.96841	2101	3	0.2263
PTPLAD1	6	0.031945	0.1033	0.96841	2102	2	0.1546
TNFSF15	6	0.031951	0.10332	0.96841	2103	4	0.4824
RPS4X	6	0.031966	0.10335	0.96841	2104	3	0.292
ZNF470	6	0.031978	0.10338	0.96841	2105	3	0.3494
RASD2	6	0.032012	0.10345	0.96841	2106	4	0.3904
WNT3	6	0.032019	0.10346	0.96841	2107	4	0.3395
TNFSF9	6	0.032025	0.10347	0.96841	2108	4	0.5432
hsa-mir-5094	4	0.032031	0.085487	0.96727	2109	2	0.467
ISCA2	6	0.032049	0.10353	0.96841	2110	3	0.3716
GUCA2A	6	0.032054	0.10355	0.96841	2111	4	0.3877
PHF15	6	0.032077	0.10361	0.96841	2112	2	-0.237
ARMCX6	6	0.032111	0.10369	0.96841	2113	4	0.3794
ATAT1	6	0.032122	0.1037	0.96841	2114	1	0.0562
SYCE2	6	0.032157	0.10379	0.96841	2115	4	0.4843
CLPTM1	6	0.032162	0.1038	0.96841	2116	3	0.3013
ARHGEF26	6	0.032179	0.10384	0.96841	2117	4	0.4976
CHSY3	6	0.032198	0.10388	0.96841	2118	3	0.1017
ELANE	6	0.032198	0.10388	0.96841	2119	2	-0.083
BTBD16	6	0.032226	0.10396	0.96841	2120	3	0.4154

RARG	6	0.032249	0.10402	0.96841	2121	4	0.3729
ATP2B4	6	0.032281	0.10409	0.96841	2122	3	0.1431
TRMT5	6	0.032309	0.10415	0.96841	2123	3	0.44
FAN1	6	0.032326	0.10418	0.96841	2124	4	0.406
RBM26	6	0.03235	0.10424	0.96841	2125	2	-0.021
CREB3	6	0.032385	0.10431	0.96841	2126	4	0.4752
LEPREL2	6	0.032401	0.10435	0.96841	2127	2	-0.063
MGARP	6	0.032446	0.10445	0.96841	2128	3	0.0189
PREX1	6	0.032452	0.10447	0.96841	2129	4	0.346
EXOC3L4	6	0.032454	0.10448	0.96841	2130	3	0.4062
AP1S1	6	0.032478	0.10453	0.96841	2131	4	0.5893
RPS2	6	0.032495	0.10457	0.96841	2132	4	0.5284
CCNC	6	0.032507	0.10459	0.96841	2133	4	0.4588
KCP	6	0.03251	0.1046	0.96841	2134	3	0.1689
RDH5	6	0.03252	0.10462	0.96841	2135	3	0.2424
ZNF770	6	0.032573	0.10475	0.96841	2136	2	-0.334
IFIH1	6	0.032594	0.1048	0.96841	2137	3	0.4048
HMP19	6	0.032604	0.10483	0.96841	2138	3	0.2138
CDK1	6	0.032606	0.10483	0.96841	2139	3	0.2702
BROX	6	0.032644	0.10491	0.96841	2140	4	0.5337
TXNDC16	6	0.032644	0.10491	0.96841	2141	4	0.4914
CLDN11	6	0.03268	0.105	0.96841	2142	2	-0.033
LMAN1L	6	0.032713	0.10508	0.96841	2143	3	0.3749
CACTIN	6	0.032745	0.10515	0.96841	2144	3	0.2279
ZNF576	6	0.032756	0.10517	0.96841	2145	3	0.1128
TAOK3	6	0.032788	0.10525	0.96871	2146	4	0.3593
hsa-mir-1249	4	0.032809	0.087127	0.9678	2147	1	0.1925
SLC27A2	6	0.032908	0.10553	0.97003	2148	2	-0.114
APCDD1	6	0.032928	0.10558	0.97003	2149	4	0.3217
MED14	4	0.032973	0.087471	0.9684	2150	3	0.7412
NAPA	6	0.032987	0.10574	0.97015	2151	4	0.7347
UHRF1BP1L	6	0.032997	0.10576	0.97015	2152	3	0.2083
RPL32	6	0.033009	0.10579	0.97015	2153	1	-0.469
IQCB1	6	0.033035	0.10585	0.97015	2154	3	0.3406
STK17A	6	0.033061	0.10591	0.97015	2155	4	0.4896
NFKBIE	6	0.033135	0.10608	0.97015	2156	4	0.3913
ARMC12	6	0.033167	0.10615	0.97015	2157	3	0.364
PPP1R16B	6	0.033191	0.10621	0.97015	2158	4	0.3167
FKBP5	6	0.033212	0.10626	0.97015	2159	2	-0.187
ETNPPL	4	0.033215	0.087964	0.9684	2160	3	0.6775
CDC42BPA	6	0.033267	0.10638	0.97015	2161	4	0.443
41153	3	0.033268	0.08279	0.96585	2162	3	0.3441
DDX27	6	0.033276	0.1064	0.97015	2163	4	0.5161
DCAF13	6	0.033301	0.10646	0.97015	2164	3	0.4451
AGR2	6	0.033313	0.1065	0.97015	2165	4	0.4953
SETD4	6	0.033344	0.10657	0.97015	2166	3	0.4903
TEX37	6	0.033364	0.10661	0.97015	2167	4	0.3391
HIVEP1	6	0.033391	0.10667	0.97015	2168	4	0.5306
SHISA3	6	0.033414	0.10672	0.97015	2169	3	0.1464
TAF12	6	0.033421	0.10673	0.97015	2170	4	0.4195
PPP2R3C	6	0.033465	0.10683	0.97056	2171	4	0.241
AGPHD1	2	0.03347	0.060847	0.94994	2172	2	0.5886
FUT7	6	0.033516	0.10694	0.97095	2173	2	-0.691
CEP95	6	0.033525	0.10697	0.97095	2174	4	0.5476
CTAGE15	3	0.033546	0.083424	0.96722	2175	2	0.5602
MAST4	6	0.033604	0.10716	0.97102	2176	3	0.3973
CHP1	6	0.033648	0.10728	0.97102	2177	4	0.4684
COL6A3	6	0.033651	0.10729	0.97102	2178	3	0.3902
hsa-mir-1-2	3	0.033673	0.083712	0.96722	2179	1	0.0229
TMPRSS11E	6	0.033681	0.10736	0.97102	2180	2	-0.052
DNAJC27	6	0.033724	0.10745	0.97102	2181	4	0.3776
UBE2E2	6	0.033734	0.10748	0.97102	2182	3	0.3121
C10orf54	6	0.033741	0.10749	0.97102	2183	4	0.3782
CYP26C1	5	0.033767	0.10024	0.96841	2184	2	0.0144
FBXO4	6	0.033769	0.10755	0.97102	2185	3	0.168
MNS1	6	0.033773	0.10755	0.97102	2186	4	0.636
RAX2	6	0.033819	0.10768	0.97102	2187	2	-0.926
NUDT18	6	0.033835	0.10771	0.97102	2188	4	0.3613
SLURP1	6	0.033838	0.10772	0.97102	2189	4	0.6917
PGBD3	6	0.03387	0.10779	0.97102	2190	2	0.0145
IFI16	6	0.033871	0.10779	0.97102	2191	4	0.5433
hsa-mir-4466	4	0.033876	0.089303	0.96841	2192	1	-0.341
ADAMTSL3	4	0.033906	0.089373	0.96841	2193	2	0.2536
ETFB	6	0.033908	0.10788	0.97102	2194	4	0.8477
TGM5	6	0.033925	0.10792	0.97102	2195	3	0.1255
FBRSL1	6	0.033957	0.10798	0.97102	2196	4	0.6131
INSM1	6	0.033971	0.10802	0.97102	2197	4	0.4118
SESTD1	6	0.03402	0.10813	0.97102	2198	3	0.3281
CNTN1	6	0.034042	0.10819	0.97102	2199	2	0.0452
FBXO44	6	0.034062	0.10824	0.97102	2200	4	0.4476
LATS1	6	0.034123	0.10838	0.97102	2201	1	-0.028
ALDH1A1	6	0.034156	0.10845	0.97102	2202	4	0.3288
hsa-mir-4704	3	0.034165	0.084826	0.96722	2203	3	0.324
hsa-mir-9-1	4	0.034194	0.089987	0.96841	2204	2	0.4219
hsa-mir-664b	4	0.034197	0.089996	0.96841	2205	1	-0.197

CAPG	6	0.034198	0.10853	0.97102	2206	4	0.37
GALNT5	6	0.034224	0.10859	0.97102	2207	2	0.0448
TAF13	6	0.034275	0.10871	0.97102	2208	2	-0.083
SPAG7	6	0.034279	0.10872	0.97102	2209	4	0.4954
HS6ST3	6	0.034286	0.10874	0.97102	2210	3	0.2582
KPNA7	6	0.034307	0.10879	0.97102	2211	4	0.492
SPIN2A	1	0.03431	0.034224	0.8622	2212	1	2.4813
PCDHA10	2	0.034316	0.062289	0.95092	2213	2	0.4855
MIF	6	0.03432	0.10882	0.97102	2214	4	0.4296
ATP5J	6	0.034326	0.10883	0.97102	2215	4	0.3118
HOXC13	6	0.034376	0.10897	0.97102	2216	3	0.2154
PSMB6	6	0.034427	0.10907	0.97102	2217	1	-0.107
EHMT2	6	0.034427	0.10907	0.97102	2218	3	0.4965
CALCB	6	0.034477	0.10919	0.97102	2219	2	-0.406
MC4R	6	0.034528	0.10929	0.97102	2220	2	0.031
PRPSAP1	6	0.034557	0.10935	0.97102	2221	4	0.5166
CPT1C	6	0.034629	0.10952	0.97102	2222	1	-0.183
SLC25A37	6	0.034648	0.10957	0.97102	2223	4	0.392
ZDHHC13	6	0.034686	0.10965	0.97102	2224	3	0.2141
TBC1D16	6	0.034696	0.10967	0.97102	2225	4	0.6587
OR7A5	6	0.034696	0.10967	0.97102	2226	4	0.6201
ATP5O	6	0.034705	0.1097	0.97102	2227	3	0.2907
LPCAT3	6	0.03474	0.10978	0.97102	2228	3	0.4435
PHAX	6	0.034752	0.10981	0.97102	2229	3	0.3964
MAG	6	0.034781	0.10989	0.97102	2230	3	0.1952
BAX	6	0.034783	0.10989	0.97102	2231	4	0.4834
TC2N	6	0.034783	0.10989	0.97102	2232	3	0.2386
CCND2	6	0.034783	0.10989	0.97102	2233	3	0.3087
CCL8	6	0.034816	0.10996	0.97102	2234	4	0.6326
LAMB3	6	0.034839	0.11002	0.97102	2235	4	0.3581
IKZF2	6	0.034865	0.11008	0.97102	2236	4	0.3414
C19orf12	6	0.034881	0.11011	0.97102	2237	4	0.4851
NAA40	6	0.034882	0.11011	0.97102	2238	2	-0.068
BIN2	6	0.034903	0.11016	0.97102	2239	3	0.4696
CHD3	6	0.034925	0.1102	0.97102	2240	2	-0.376
EOGT	6	0.034933	0.11021	0.97102	2241	2	0.1059
SELP	4	0.034943	0.091495	0.96841	2242	2	0.0406
ADIPOR2	6	0.035034	0.11044	0.97102	2243	2	-0.137
RRH	6	0.03507	0.11053	0.97102	2244	4	0.6616
EXOC6B	6	0.035084	0.11056	0.97102	2245	3	0.2769
PPP1R10	6	0.035089	0.11057	0.97102	2246	2	-0.051
SNW1	6	0.035116	0.11063	0.97102	2247	4	0.6353
CTPS1	6	0.03516	0.11073	0.97102	2248	4	0.4079
FBXO43	6	0.035186	0.11079	0.97102	2249	2	-0.036
TNFAIP8	6	0.035189	0.11079	0.97102	2250	4	0.3521
WTIP	6	0.035217	0.11086	0.97102	2251	4	0.5745
SELO	6	0.035241	0.11092	0.97102	2252	4	0.5148
PLXND1	6	0.035247	0.11093	0.97102	2253	3	0.2908
ARHGEF40	6	0.03525	0.11094	0.97102	2254	4	0.4482
TTC9B	6	0.035272	0.11099	0.97102	2255	4	0.4327
RAD23A	6	0.035274	0.11099	0.97102	2256	3	-0.035
NADKD1	6	0.035287	0.11102	0.97102	2257	4	0.5188
C6orf57	6	0.035337	0.11114	0.97102	2258	2	-0.089
PHACTR3	6	0.035388	0.11124	0.97102	2259	2	-0.03
FZD5	6	0.035438	0.11134	0.97102	2260	2	-0.046
NLRP10	6	0.035454	0.11137	0.97102	2261	4	0.4478
ENPP5	6	0.035455	0.11137	0.97102	2262	2	-0.3
FAM195A	6	0.035472	0.11141	0.97102	2263	3	0.2739
IDH3A	6	0.035489	0.11145	0.97102	2264	4	0.4069
C10orf62	6	0.035518	0.11152	0.97102	2265	3	0.1969
MYH10	6	0.035546	0.11158	0.97102	2266	4	0.6334
MEGF8	6	0.035554	0.11161	0.97102	2267	3	-0.14
NUP205	6	0.035565	0.11164	0.97102	2268	3	0.1541
BRMS1	6	0.035565	0.11164	0.97102	2269	1	-0.069
KRTAP4-1	6	0.035567	0.11164	0.97102	2270	3	0.3393
CCDC42B	6	0.035616	0.11175	0.97102	2271	4	0.3265
CAND1	6	0.035636	0.11179	0.97102	2272	4	0.411
ZNF449	6	0.035669	0.11186	0.97102	2273	2	-0.322
PFKFB2	6	0.035702	0.11194	0.97102	2274	3	0.5121
WDTC1	6	0.03571	0.11196	0.97102	2275	4	0.3976
KRTAP12-1	6	0.035716	0.11198	0.97102	2276	3	0.1475
PPME1	6	0.035716	0.11198	0.97102	2277	2	0.0522
SSU72	6	0.03573	0.112	0.97102	2278	3	0.3915
FAP	4	0.035739	0.093115	0.96841	2279	2	0.4745
PDGFRL	6	0.035754	0.11206	0.97108	2280	4	0.4609
ATP5I	6	0.035792	0.11214	0.97114	2281	4	0.6599
RASIP1	6	0.035801	0.11216	0.97114	2282	4	0.4162
hsa-mir-4470	4	0.035814	0.093257	0.96841	2283	2	0.4405
RCOR3	6	0.035843	0.11227	0.97164	2284	4	0.3558
GOLGA6L4	3	0.035892	0.088752	0.96841	2285	3	0.8862
ZKSCAN3	6	0.035893	0.11239	0.97226	2286	2	-0.152
EPSTI1	6	0.035911	0.11243	0.97226	2287	3	0.4589
HDAC2	6	0.035985	0.11261	0.9726	2288	4	0.5297
SCAMP2	6	0.035994	0.11263	0.9726	2289	3	0.3511
ATIC	6	0.036013	0.11267	0.9726	2290	3	0.3343

SRI	6	0.036045	0.11275	0.9726	2291	1	-0.705
TRMT6	6	0.036055	0.11277	0.9726	2292	4	0.3166
UGGT2	6	0.036079	0.11282	0.9726	2293	4	0.3567
CBLL1	4	0.036091	0.093825	0.96841	2294	3	0.9891
PGBD4	6	0.036095	0.11287	0.9726	2295	2	-0.173
ACVR2B	6	0.036116	0.11292	0.9726	2296	3	0.0626
OR8G1	5	0.036159	0.10629	0.97015	2297	3	0.5131
ACVR1B	6	0.036196	0.11308	0.97292	2298	3	0.241
LY6G5C	6	0.036273	0.11325	0.97292	2299	4	0.3533
TTC32	6	0.036318	0.11337	0.97292	2300	3	0.1021
ENOSF1	6	0.036325	0.11338	0.97292	2301	4	0.5222
TCEB3CL	2	0.036332	0.065805	0.95667	2302	1	0.5069
ITGAL	6	0.036398	0.11354	0.97292	2303	3	0.0892
BRAT1	6	0.036405	0.11356	0.97292	2304	3	0.2885
hsa-mir-6868	4	0.036415	0.094469	0.96841	2305	3	0.4432
KNCN	6	0.03643	0.11361	0.97292	2306	4	0.32
TSEN34	6	0.036431	0.11362	0.97292	2307	3	0.1389
IFT52	6	0.036439	0.11364	0.97292	2308	4	0.3755
ARAP3	6	0.036499	0.11377	0.97292	2309	4	0.4543
PTPRM	6	0.036503	0.11378	0.97292	2310	4	0.3921
DBI	6	0.036526	0.11382	0.97292	2311	3	0.0997
MAD2L2	6	0.036526	0.11382	0.97292	2312	2	-0.203
PLXNB2	6	0.036553	0.11388	0.97292	2313	4	0.5483
SEBOX	6	0.036573	0.11392	0.97292	2314	3	0.4337
STOX2	6	0.036578	0.11393	0.97292	2315	4	0.5246
ELAVL4	6	0.036598	0.11397	0.97292	2316	2	-0.451
EXOSC6	6	0.0366	0.11398	0.97292	2317	3	0.1332
ZBTB22	6	0.036604	0.11398	0.97292	2318	3	0.4302
hsa-mir-4256	4	0.036615	0.094893	0.96841	2319	2	0.1725
ABCD2	6	0.03667	0.11413	0.97334	2320	4	0.4705
JTB	6	0.036691	0.11418	0.97334	2321	3	0.0404
FUNDC2	6	0.036719	0.11424	0.97348	2322	3	0.483
MTMR14	6	0.036752	0.11431	0.97371	2323	2	-0.422
TEX10	6	0.036802	0.11443	0.97434	2324	4	0.5724
CD177	6	0.036853	0.11455	0.97491	2325	3	0.3356
KIAA1407	6	0.03687	0.1146	0.97491	2326	3	0.0542
NOC4L	6	0.036916	0.1147	0.97534	2327	4	0.4994
hsa-mir-7159	4	0.036969	0.0956	0.96841	2328	2	0.1136
MCCC1	6	0.036972	0.11482	0.9754	2329	3	0.387
hsa-mir-2117	4	0.037036	0.095741	0.96841	2330	2	0.0683
FFAR1	6	0.037055	0.11503	0.9754	2331	2	0.1213
hsa-mir-4717	4	0.037096	0.095858	0.96841	2332	3	0.4436
FARSB	6	0.037105	0.11513	0.9754	2333	2	0.0889
MUCL1	6	0.037111	0.11514	0.9754	2334	4	0.4249
GXYLT2	6	0.037122	0.11517	0.9754	2335	2	-0.017
LGALS13	5	0.037124	0.10872	0.97102	2336	3	0.6557
VAMP2	6	0.037156	0.11525	0.9754	2337	2	-0.104
hsa-mir-1343	4	0.037171	0.096009	0.96841	2338	3	0.6124
LMTK3	4	0.037182	0.09603	0.96841	2339	2	0.3985
RDX	6	0.037188	0.11532	0.9754	2340	3	0.6325
RNASEL	6	0.037206	0.11536	0.9754	2341	2	-0.599
LYPLAL1	6	0.037209	0.11537	0.9754	2342	4	0.3423
TAF3	6	0.037352	0.11569	0.9754	2343	3	0.4125
DENND1B	6	0.037358	0.1157	0.9754	2344	4	0.4579
OXSR1	6	0.037379	0.11574	0.9754	2345	4	0.3125
NAGK	6	0.037384	0.11576	0.9754	2346	4	0.3559
hsa-mir-548ag-1	3	0.037385	0.092134	0.96841	2347	2	0.5752
RAD21L1	6	0.03739	0.11577	0.9754	2348	3	0.383
DGKE	6	0.03739	0.11577	0.9754	2349	3	0.1871
EBNA1BP2	6	0.037425	0.11584	0.9754	2350	4	0.4216
THRSP	6	0.037459	0.11592	0.9754	2351	3	0.1082
TEKT4	4	0.037476	0.09663	0.96841	2352	3	0.498
hsa-mir-6717	4	0.037486	0.096651	0.96841	2353	3	0.7221
hsa-mir-1185-2	1	0.037532	0.037455	0.88286	2354	1	1.186
MIPOL1	6	0.03756	0.11617	0.9754	2355	1	-0.06
COL7A1	6	0.037604	0.11626	0.9754	2356	3	0.0982
L1TD1	6	0.037661	0.11639	0.9754	2357	2	-0.009
OCM2	4	0.037661	0.096991	0.96841	2358	2	0.5915
SOCS3	6	0.03768	0.11644	0.9754	2359	4	0.4062
HIST1H4L	6	0.037753	0.11661	0.9754	2360	3	-0.164
KLHDC7A	6	0.037795	0.1167	0.9754	2361	3	0.2656
DACH2	6	0.037812	0.11673	0.9754	2362	3	0.3778
hsa-mir-548ag-2	3	0.037843	0.093167	0.96841	2363	1	-1.272
hsa-mir-7110	4	0.037868	0.097392	0.96841	2364	3	0.6503
ACLY	6	0.037913	0.11696	0.9754	2365	3	0.179
GATC	6	0.037941	0.11701	0.9754	2366	4	0.5523
IKBKAP	6	0.037952	0.11703	0.9754	2367	4	0.3826
GPR34	6	0.037952	0.11703	0.9754	2368	4	0.3229
PRAM1	6	0.037963	0.11706	0.9754	2369	3	0.032
EIF2B3	6	0.037972	0.11708	0.9754	2370	4	0.395
RAB12	6	0.03798	0.1171	0.9754	2371	4	0.4723
PRRT4	6	0.037998	0.11714	0.9754	2372	3	0.2497
MYH15	6	0.038065	0.1173	0.9754	2373	4	0.4155
KLF12	6	0.038115	0.11742	0.9754	2374	3	0.3811
NR2F2	6	0.038165	0.11754	0.9754	2375	3	0.2824

GK5	6	0.038185	0.11758	0.9754	2376	3	0.2303
ADAM7	4	0.038214	0.098077	0.96841	2377	3	0.7122
MTNR1A	6	0.038215	0.11765	0.9754	2378	3	0.2336
DPH6	2	0.038223	0.069095	0.95667	2379	2	0.9881
ABCF2	6	0.038258	0.11775	0.9754	2380	4	0.2726
hsa-mir-653	4	0.038265	0.098165	0.96841	2381	3	0.7978
ARF5	6	0.038266	0.11776	0.9754	2382	2	0.2615
B3GNT3	6	0.038274	0.11778	0.9754	2383	4	0.5393
PNOC	6	0.0383	0.11784	0.9754	2384	4	0.4427
SUOX	6	0.038316	0.11788	0.9754	2385	3	0.2284
TP73	6	0.03835	0.11796	0.9754	2386	4	0.4208
ERI1	6	0.038366	0.11799	0.9754	2387	4	0.3716
PRKD1	6	0.038367	0.11799	0.9754	2388	2	-0.048
SCP2	6	0.038392	0.11805	0.9754	2389	4	0.5442
SDHAF1	6	0.038412	0.11809	0.9754	2390	3	0.4145
DDX24	6	0.038417	0.1181	0.9754	2391	3	0.1268
KCNE1L	6	0.038492	0.11828	0.9754	2392	3	0.1403
HECW2	6	0.038518	0.11833	0.9754	2393	2	-0.064
KCTD15	6	0.038543	0.11838	0.9754	2394	4	0.332
ACER2	6	0.038559	0.11841	0.9754	2395	3	0.4175
SEH1L	6	0.038568	0.11843	0.9754	2396	3	0.285
hsa-mir-18b	4	0.038589	0.098827	0.96841	2397	1	-0.287
PPARGC1B	6	0.03859	0.11848	0.9754	2398	4	0.4418
USO1	6	0.038607	0.11852	0.9754	2399	3	0.0223
CDC37	6	0.038619	0.11855	0.9754	2400	2	-0.083
QRICH1	6	0.038632	0.11858	0.9754	2401	2	-0.102
CBLN1	6	0.038648	0.11862	0.9754	2402	4	0.3362
TAGLN	6	0.038655	0.11864	0.9754	2403	2	0.0439
OR6X1	6	0.038703	0.11874	0.9754	2404	3	0.4137
STRADA	6	0.038735	0.11881	0.9754	2405	3	0.2862
USF2	6	0.038745	0.11882	0.9754	2406	4	0.4625
ME3	6	0.038767	0.11887	0.9754	2407	4	0.3799
MDH2	6	0.038769	0.11887	0.9754	2408	2	-0.184
ARHGDIB	6	0.03882	0.11899	0.9754	2409	1	-0.521
CHIC1	6	0.038821	0.11899	0.9754	2410	4	0.4449
BRCA1	6	0.038832	0.11902	0.9754	2411	3	0.3808
MDM2	6	0.038871	0.11912	0.9754	2412	4	0.5905
DPH5	6	0.038896	0.11917	0.9754	2413	2	-0.144
STOM	6	0.038898	0.11918	0.9754	2414	4	0.4709
C4orf21	6	0.038909	0.11921	0.9754	2415	4	0.4546
H2AFV	6	0.03894	0.11927	0.9754	2416	2	0.1625
TIGD2	6	0.038956	0.11931	0.9754	2417	4	0.4382
CKLF	6	0.038962	0.11932	0.9754	2418	3	0.1872
SURF6	6	0.038962	0.11932	0.9754	2419	3	0.3114
SDC3	6	0.038962	0.11932	0.9754	2420	2	-0.054
MED22	6	0.038972	0.11934	0.9754	2421	3	0.1996
LOC100288814	6	0.038989	0.11938	0.9754	2422	4	0.4529
KIF9	6	0.03902	0.11944	0.9754	2423	4	0.3516
HESX1	6	0.03902	0.11944	0.9754	2424	4	0.3773
TRAF3IP3	6	0.039022	0.11945	0.9754	2425	2	0.1194
CNTRL	6	0.0391	0.11963	0.9754	2426	3	0.3718
LRIG2	6	0.039151	0.11973	0.9754	2427	3	-0.073
C10orf129	6	0.039173	0.11978	0.9754	2428	3	0.1433
USP10	6	0.039182	0.11981	0.9754	2429	4	0.5108
MEPCE	6	0.039217	0.11988	0.9754	2430	4	0.5187
FOXB1	6	0.039224	0.1199	0.9754	2431	1	-0.682
SPRED3	6	0.03925	0.11996	0.9754	2432	4	0.5503
HHLA3	6	0.039265	0.12	0.9754	2433	3	0.3826
C3orf14	6	0.039274	0.12002	0.9754	2434	2	0.0496
CYB5R1	6	0.039282	0.12003	0.9754	2435	4	0.2462
hsa-mir-4424	4	0.039296	0.10023	0.96841	2436	2	0.3876
USP11	6	0.039324	0.12012	0.9754	2437	2	-0.171
KANK2	6	0.039466	0.12044	0.9754	2438	3	0.6454
SLC38A2	6	0.039476	0.12047	0.9754	2439	2	0.0749
DOT1L	6	0.039517	0.12056	0.9754	2440	3	0.2981
DCXR	6	0.039526	0.12058	0.9754	2441	4	0.4597
NGF	6	0.039544	0.12062	0.9754	2442	4	0.3919
CLEC4E	6	0.039557	0.12065	0.9754	2443	3	0.1615
OSTM1	6	0.03956	0.12066	0.9754	2444	4	0.5872
RPL11	6	0.03956	0.12066	0.9754	2445	3	0.4536
SHF	6	0.039576	0.12069	0.9754	2446	2	-0.208
LYPD4	6	0.039638	0.12083	0.9754	2447	2	-0.181
TPO	6	0.039643	0.12084	0.9754	2448	4	0.3719
ATP6AP1	6	0.039657	0.12088	0.9754	2449	4	0.3689
INPP5A	6	0.039671	0.12091	0.9754	2450	3	0.3323
PTEN	6	0.039753	0.1211	0.9754	2451	4	0.4694
DDAH1	6	0.039778	0.12115	0.9754	2452	3	0.1245
hsa-mir-5591	2	0.039798	0.071776	0.95667	2453	1	-0.111
ZFYVE26	6	0.03981	0.12123	0.9754	2454	2	-0.168
GBGT1	6	0.039822	0.12126	0.9754	2455	4	0.3084
SPAG4	6	0.039828	0.12127	0.9754	2456	1	-0.127
LRRC37A2	6	0.039866	0.12136	0.9754	2457	4	0.4
ABHD17A	6	0.039876	0.12139	0.9754	2458	3	0.3136
PEX11G	6	0.039878	0.12139	0.9754	2459	4	0.3911
OR7A10	6	0.039948	0.12154	0.9754	2460	3	0.0497

ATP6V1G1	6	0.039979	0.12161	0.9754	2461	2	-0.697
ZNF782	6	0.039983	0.12161	0.9754	2462	4	0.3983
LZTS1	6	0.039987	0.12163	0.9754	2463	4	0.4086
SERTAD3	6	0.040035	0.12173	0.9754	2464	4	0.3343
CCDC13	6	0.040059	0.12179	0.9754	2465	4	0.3474
FCN2	6	0.04008	0.12184	0.9754	2466	1	-0.369
ZNF384	6	0.040082	0.12184	0.9754	2467	3	0.3851
HNRNPA3	5	0.040086	0.11613	0.9754	2468	3	0.1786
PRSS12	6	0.040128	0.12193	0.9754	2469	3	0.31
FBLN5	6	0.04013	0.12194	0.9754	2470	1	-1.372
ELL2	6	0.040133	0.12194	0.9754	2471	3	0.0304
CKAP2L	6	0.040177	0.12205	0.9754	2472	3	0.3121
ULK1	6	0.040214	0.12212	0.9754	2473	3	-0.039
SLC22A18AS	6	0.040217	0.12213	0.9754	2474	3	0.1405
ARHGEF33	6	0.040269	0.12225	0.9754	2475	4	0.4968
FANCL	6	0.040281	0.12229	0.9754	2476	3	0.2734
CMIP	4	0.040294	0.10224	0.96841	2477	2	0.4995
MTMR7	6	0.040306	0.12234	0.9754	2478	4	0.4311
hsa-mir-196a-1	4	0.040307	0.10227	0.96841	2479	3	0.4598
PAPOLG	6	0.040332	0.1224	0.9754	2480	1	-0.176
OSM	6	0.040335	0.1224	0.9754	2481	3	0.4603
ZNF774	6	0.040407	0.12257	0.9754	2482	3	0.2282
PODN	6	0.040407	0.12257	0.9754	2483	4	0.4509
IRF8	6	0.040432	0.12262	0.9754	2484	3	0.0846
PPP2R1A	6	0.040457	0.12267	0.9754	2485	3	0.3203
TAS2R60	6	0.040483	0.12273	0.9754	2486	2	-0.033
YIPF3	6	0.0405	0.12278	0.9754	2487	3	0.4473
SLC12A1	6	0.040533	0.12286	0.9754	2488	2	0.0476
VDAC2	5	0.040548	0.11728	0.9754	2489	2	0.135
HOXC8	6	0.040553	0.1229	0.9754	2490	4	0.5884
RENBP	6	0.040566	0.12293	0.9754	2491	4	0.4154
hsa-mir-6879	4	0.040577	0.10283	0.96841	2492	2	-0.073
ZNF729	6	0.040583	0.12296	0.9754	2493	1	-0.255
TRIM60	6	0.040601	0.123	0.9754	2494	4	0.4882
DRD2	6	0.040608	0.12302	0.9754	2495	2	-0.155
SLCO5A1	6	0.040671	0.12319	0.9754	2496	4	0.3882
ZNF84	6	0.040684	0.12321	0.9754	2497	3	0.1474
ANKFY1	6	0.040685	0.12322	0.9754	2498	4	0.3616
DCP1B	6	0.040712	0.12327	0.9754	2499	3	0.0564
GREB1L	6	0.040731	0.12332	0.9754	2500	4	0.3772
UBXN4	6	0.040785	0.12344	0.9754	2501	2	-0.327
hsa-mir-1234	4	0.040812	0.10329	0.96841	2502	2	0.1124
KCTD2	6	0.040835	0.12354	0.9754	2503	2	0.0807
COX6A2	6	0.04085	0.12358	0.9754	2504	3	0.2524
FAM229A	6	0.040869	0.12362	0.9754	2505	3	0.0452
ID1	6	0.040886	0.12365	0.9754	2506	2	-0.15
ZNF423	6	0.040923	0.12373	0.9754	2507	3	0.2142
GRSF1	6	0.040933	0.12375	0.9754	2508	4	0.4361
ZFP82	6	0.040978	0.12386	0.9754	2509	4	0.3571
MRPS18A	6	0.040986	0.12388	0.9754	2510	3	0.2133
LASP1	6	0.041003	0.12392	0.9754	2511	2	0.0047
hsa-mir-1237	4	0.041018	0.10373	0.96841	2512	3	0.5087
FBXL18	6	0.041019	0.12395	0.9754	2513	3	0.2334
ZNF32	6	0.041036	0.12399	0.9754	2514	3	0.2492
CHRNE	6	0.041044	0.12401	0.9754	2515	2	-0.609
CXorf48	6	0.041053	0.12403	0.9754	2516	4	0.5473
NDUFB2	6	0.041065	0.12405	0.9754	2517	4	0.4594
hsa-mir-4796	4	0.041182	0.10402	0.96841	2518	2	0.2525
CHIC2	6	0.041237	0.12445	0.9764	2519	4	0.3292
TMEM45A	6	0.041313	0.12462	0.9764	2520	4	0.4409
SPIN1	6	0.041338	0.12469	0.9764	2521	2	0.1044
GFRA1	6	0.041401	0.12483	0.9764	2522	4	0.3863
ARID3B	6	0.041423	0.12487	0.9764	2523	4	0.2561
GP2	6	0.041433	0.12489	0.9764	2524	4	0.6563
HPCAL1	6	0.041435	0.1249	0.9764	2525	4	0.4635
RNASET2	6	0.041439	0.12491	0.9764	2526	4	0.5259
SHC4	6	0.04147	0.12497	0.9764	2527	3	0.2207
CCDC140	6	0.041505	0.12506	0.9764	2528	3	0.2227
MBLAC1	6	0.041525	0.1251	0.9764	2529	4	0.5011
DDX52	6	0.041539	0.12513	0.9764	2530	3	0.3502
PIR	6	0.041552	0.12515	0.9764	2531	3	0.1371
SLC13A3	6	0.041564	0.12518	0.9764	2532	2	0.0072
RNF207	6	0.041657	0.12537	0.9764	2533	2	-0.193
SCARF1	6	0.04169	0.12544	0.9764	2534	4	0.2602
hsa-mir-150	4	0.041721	0.10511	0.96841	2535	2	0.1376
GID4	6	0.041722	0.12552	0.9764	2536	4	0.511
ZNF222	5	0.041723	0.12021	0.9754	2537	4	0.316
NEU4	6	0.04174	0.12556	0.9764	2538	2	-0.285
CCDC114	6	0.041749	0.12559	0.9764	2539	4	0.3309
NAT6	6	0.041763	0.12562	0.9764	2540	4	0.3917
CNN1	6	0.041773	0.12564	0.9764	2541	4	0.5908
PTPRB	6	0.04183	0.12576	0.9764	2542	4	0.4106
BPIFB6	6	0.041857	0.12582	0.9764	2543	4	0.3796
VHL	6	0.041943	0.12601	0.9764	2544	4	0.5339
hsa-mir-608	4	0.041945	0.10557	0.97003	2545	3	0.4212

STX4	6	0.041992	0.12612	0.9764	2546	1	-0.277
ZIM3	6	0.042004	0.12615	0.9764	2547	2	-0.034
ZEB2	6	0.042042	0.12623	0.9764	2548	2	-0.174
hsa-mir-1290	4	0.042078	0.10583	0.97015	2549	3	0.6735
AQP11	6	0.042092	0.12635	0.9764	2550	2	0.1651
HDHD2	6	0.042092	0.12635	0.9764	2551	1	-0.251
RBAK	2	0.042111	0.075736	0.95977	2552	1	0.3599
SKA3	6	0.042116	0.1264	0.9764	2553	4	0.4257
RASD1	6	0.042123	0.12641	0.9764	2554	3	0.0564
NME9	6	0.042139	0.12645	0.9764	2555	4	0.6544
NUP35	6	0.042175	0.12652	0.9764	2556	2	0.0276
RGL4	6	0.042193	0.12656	0.9764	2557	3	0.0029
PPP1R42	6	0.04221	0.1266	0.9764	2558	4	0.4067
hsa-mir-555	4	0.042213	0.10609	0.97015	2559	3	0.5293
hsa-mir-548ba	1	0.042229	0.042151	0.90663	2560	1	1.2259
SPATA5	4	0.042236	0.10613	0.97015	2561	2	0.0297
ERAP1	6	0.042237	0.12666	0.9764	2562	4	0.3022
CDK10	6	0.042243	0.12668	0.9764	2563	3	0.1717
EVC2	6	0.042256	0.1267	0.9764	2564	4	0.4391
OR6B3	6	0.042304	0.1268	0.9764	2565	4	0.4807
CMC4	6	0.042344	0.12688	0.9764	2566	1	-0.476
INO80B	6	0.042376	0.12695	0.9764	2567	3	0.2701
LIMA1	6	0.042376	0.12695	0.9764	2568	4	0.4984
TBC1D8B	4	0.042392	0.10644	0.97015	2569	3	0.3602
FBXO24	6	0.042425	0.12707	0.9764	2570	4	0.5156
CCKBR	6	0.04244	0.12711	0.9764	2571	3	0.127
CRYGS	6	0.042444	0.12712	0.9764	2572	4	0.2814
FKBPL	6	0.042487	0.12723	0.9764	2573	4	0.5203
COX10	6	0.042501	0.12726	0.9764	2574	4	0.3957
C19orf53	6	0.042501	0.12726	0.9764	2575	3	0.557
CD1C	6	0.042525	0.12732	0.9764	2576	4	0.3369
hsa-mir-7-3	4	0.042529	0.10671	0.97015	2577	3	0.597
RTN4IP1	6	0.042541	0.12736	0.9764	2578	3	0.3791
GTF3C4	6	0.042545	0.12737	0.9764	2579	1	-0.023
EMR1	6	0.042589	0.12746	0.9764	2580	4	0.3201
CCDC27	6	0.042595	0.12747	0.9764	2581	2	-0.282
PLCB3	6	0.042629	0.12753	0.9764	2582	3	0.173
C7orf72	6	0.042636	0.12754	0.9764	2583	4	0.4535
OR10J3	6	0.042695	0.12768	0.9764	2584	2	-0.085
CHMP7	6	0.042728	0.12776	0.9764	2585	4	0.5714
ABHD11	6	0.042732	0.12776	0.9764	2586	4	0.4228
RAN	6	0.042746	0.12779	0.9764	2587	3	-0.122
RP9	6	0.042766	0.12783	0.9764	2588	3	0.3009
USP32	6	0.042777	0.12785	0.9764	2589	2	-0.147
QSOX2	6	0.042783	0.12787	0.9764	2590	3	0.2371
PHYHIP	6	0.042796	0.1279	0.9764	2591	3	0.295
TMA16	6	0.042797	0.1279	0.9764	2592	3	0.604
VIP	6	0.042866	0.12805	0.9764	2593	3	0.2771
LAMTOR4	6	0.042896	0.12811	0.9764	2594	4	0.3026
TAS2R31	6	0.042896	0.12811	0.9764	2595	3	0.2789
CTSV	2	0.042943	0.077132	0.96053	2596	2	0.46
LMBR1	6	0.042946	0.12823	0.9764	2597	4	0.4867
TOR1AIP2	6	0.042977	0.1283	0.9764	2598	3	0.2785
HRASLS5	6	0.042977	0.1283	0.9764	2599	3	0.3096
BMPR1B	6	0.042983	0.12832	0.9764	2600	4	0.4065
PHTF1	6	0.043028	0.12843	0.9764	2601	4	0.3762
OR2A5	6	0.043028	0.12843	0.9764	2602	4	0.4328
GPRC5B	6	0.043032	0.12844	0.9764	2603	3	0.2988
KRTAP4-12	6	0.043047	0.12847	0.9764	2604	3	0.8583
hsa-mir-548ai	3	0.043099	0.10496	0.96841	2605	2	0.683
SLC18A1	6	0.043172	0.12874	0.9764	2606	4	0.4378
N4BP2L2	6	0.043198	0.1288	0.9764	2607	3	0.159
KCTD7	6	0.043235	0.12889	0.9764	2608	2	0.0555
hsa-mir-105-2	2	0.043241	0.077637	0.96114	2609	1	0.4771
hsa-mir-5192	4	0.043251	0.10817	0.97102	2610	2	0.21
WDR43	6	0.043295	0.12903	0.9764	2611	4	0.5019
hsa-mir-548o-2	3	0.043316	0.10544	0.97002	2612	1	0.1492
GCSAML	6	0.043332	0.12913	0.9764	2613	3	0.3833
CFL1	6	0.043339	0.12915	0.9764	2614	4	0.4983
TSSC1	6	0.04336	0.1292	0.9764	2615	2	0.0242
EMC9	4	0.043376	0.10841	0.97102	2616	2	-0.013
LGR5	6	0.043404	0.1293	0.9764	2617	4	0.315
LCNL1	6	0.043465	0.12943	0.9764	2618	4	0.27
BHMT	6	0.04348	0.12947	0.9764	2619	4	0.5893
MBIP	6	0.043485	0.12948	0.9764	2620	3	0.2181
ZNF557	6	0.043485	0.12948	0.9764	2621	2	-8E-04
ZNF157	6	0.043599	0.12974	0.9764	2622	2	-0.126
KLHL22	6	0.043614	0.12977	0.9764	2623	4	0.4438
RLTPR	6	0.043649	0.12985	0.9764	2624	3	0.2494
ILF2	5	0.043652	0.12494	0.9764	2625	2	0.0612
HCN1	6	0.043667	0.12989	0.9764	2626	4	0.4139
C7orf62	6	0.043676	0.12991	0.9764	2627	2	-0.283
APLN	6	0.0437	0.12997	0.9764	2628	2	-0.399
TRMT61B	6	0.043742	0.13006	0.9764	2629	2	0.1882
TRIM34	6	0.043768	0.13012	0.9764	2630	4	0.4134

PROKR1	6	0.043775	0.13013	0.9764	2631	2	-0.026
TOP3A	6	0.043775	0.13013	0.9764	2632	2	0.0593
DAW1	6	0.043793	0.13016	0.9764	2633	4	0.3467
PGAM1	6	0.043879	0.13035	0.9764	2634	4	0.6801
SLC7A7	6	0.043898	0.1304	0.9764	2635	3	0.0994
NEU1	6	0.043917	0.13044	0.9764	2636	3	0.3009
WDR4	6	0.043922	0.13045	0.9764	2637	4	0.5729
DTYMK	6	0.043951	0.1305	0.9764	2638	4	0.2838
BLOC1S4	4	0.043973	0.10964	0.97102	2639	2	0.185
PCDH11X	5	0.043987	0.12577	0.9764	2640	1	-0.56
RILPL2	6	0.044051	0.13074	0.9764	2641	1	-0.169
APBA3	6	0.044076	0.13079	0.9764	2642	3	0.3781
TMEM174	6	0.044086	0.13082	0.9764	2643	4	0.3289
PRR25	6	0.04409	0.13082	0.9764	2644	3	0.4515
KLK12	6	0.044138	0.13093	0.9764	2645	4	0.4561
hsa-mir-8074	2	0.044158	0.07923	0.96295	2646	2	0.8646
ELMOD3	6	0.044195	0.13105	0.9764	2647	4	0.363
PKHD1L1	6	0.044201	0.13107	0.9764	2648	1	0.0151
ZC3H8	6	0.044204	0.13107	0.9764	2649	4	0.5024
CNNM2	6	0.044218	0.13111	0.9764	2650	4	0.5257
PRDX1	6	0.04424	0.13116	0.9764	2651	2	0.1279
DENND5A	6	0.044252	0.13119	0.9764	2652	1	-0.009
TXNDC5	6	0.044269	0.13122	0.9764	2653	4	0.3435
LSG1	6	0.044282	0.13125	0.9764	2654	4	0.3703
MYH9	6	0.044327	0.13135	0.9764	2655	3	0.0448
EPS8L1	6	0.044327	0.13135	0.9764	2656	1	-0.544
MKKS	6	0.044353	0.13141	0.9764	2657	4	0.3238
D2HGDH	6	0.044356	0.13141	0.9764	2658	3	0.3837
CENPP	6	0.044414	0.13154	0.9764	2659	3	0.0135
GNA15	6	0.044432	0.13158	0.9764	2660	4	0.4029
GABBR1	6	0.04445	0.13162	0.9764	2661	4	0.4968
PGS1	6	0.044467	0.13165	0.9764	2662	4	0.3634
CCDC97	6	0.044499	0.13172	0.9764	2663	4	0.4627
CCNT1	6	0.044509	0.13175	0.9764	2664	4	0.4258
hsa-mir-6079	4	0.04451	0.11069	0.97102	2665	3	0.2988
NELFB	6	0.044516	0.13176	0.9764	2666	4	0.3422
ZNF599	6	0.044533	0.13179	0.9764	2667	3	0.2607
STK11IP	6	0.044596	0.13193	0.9764	2668	4	0.5626
NUSAP1	6	0.044603	0.13194	0.9764	2669	4	0.4571
C2orf80	6	0.044607	0.13195	0.9764	2670	4	0.4524
hsa-mir-4420	4	0.044623	0.11091	0.97102	2671	3	0.5194
SMIM18	6	0.044653	0.13205	0.9764	2672	3	-0.19
IFNG	6	0.044722	0.13219	0.9764	2673	4	0.4215
MAGEA3	6	0.044728	0.1322	0.9764	2674	1	-0.241
PRR5L	6	0.04474	0.13222	0.9764	2675	3	0.0875
CDH11	6	0.044752	0.13224	0.9764	2676	4	0.2702
hsa-mir-5739	4	0.044756	0.11117	0.97102	2677	3	0.3662
PIP4K2B	6	0.044758	0.13226	0.9764	2678	4	0.3226
TTBK2	6	0.044837	0.13243	0.9764	2679	4	0.5399
hsa-mir-30c-1	4	0.044838	0.11133	0.97102	2680	3	0.5198
MATN4	6	0.044873	0.13251	0.9764	2681	4	0.3696
TRAF2	6	0.044904	0.13258	0.9764	2682	3	0.1738
POLA1	6	0.044912	0.1326	0.9764	2683	4	0.665
SPIRE1	6	0.044933	0.13264	0.9764	2684	2	-0.242
GPD1	6	0.044958	0.1327	0.9764	2685	4	0.4697
GPX3	4	0.044983	0.11162	0.97102	2686	3	0.4477
MED9	6	0.044987	0.13276	0.9764	2687	4	0.5081
ITGA2B	6	0.045008	0.13281	0.9764	2688	4	0.4647
hsa-mir-1271	4	0.045013	0.11167	0.97102	2689	2	0.1338
YIPF7	6	0.045077	0.13296	0.9764	2690	4	0.3972
GPRASP1	6	0.045079	0.13296	0.9764	2691	1	0.1055
CDH10	6	0.045079	0.13296	0.9764	2692	2	-0.168
SCD	6	0.04509	0.13298	0.9764	2693	3	0.3372
NUDT13	6	0.045109	0.13303	0.9764	2694	3	0.4627
hsa-mir-199a-2	3	0.045114	0.10927	0.97102	2695	2	0.5538
hsa-mir-6810	4	0.045126	0.11189	0.97102	2696	3	0.6168
PTCD3	6	0.045162	0.13315	0.9764	2697	3	0.1933
C5orf64	6	0.045175	0.13318	0.9764	2698	3	0.0283
CNNM3	6	0.045193	0.13322	0.9764	2699	4	0.4782
UBL7	6	0.045199	0.13323	0.9764	2700	3	0.0785
REXO2	6	0.04525	0.13335	0.9764	2701	4	0.3213
KPNA3	6	0.045254	0.13336	0.9764	2702	2	-0.439
NEK7	6	0.045255	0.13336	0.9764	2703	2	-0.051
GHDC	6	0.045327	0.13352	0.9764	2704	3	0.163
CRTC3	6	0.045387	0.13364	0.9764	2705	4	0.5302
FAM167A	6	0.045405	0.13368	0.9764	2706	2	-0.082
DHX16	6	0.045406	0.13368	0.9764	2707	4	0.4764
HIPK1	6	0.045454	0.13381	0.9764	2708	3	0.2551
EGR2	5	0.045507	0.12955	0.9764	2709	4	0.3729
DNAJB1	6	0.04551	0.13393	0.9764	2710	4	0.4368
NEK5	6	0.04551	0.13393	0.9764	2711	4	0.6086
HOXD8	6	0.045538	0.13399	0.9764	2712	4	0.4162
GNPTG	6	0.045555	0.13403	0.9764	2713	3	0.0251
CD46	6	0.045567	0.13406	0.9764	2714	4	0.4305
OR2L8	4	0.045618	0.11287	0.9726	2715	3	0.4233

BTN1A1	6	0.045655	0.13426	0.9764	2716	2	0.0041
hsa-mir-3646	4	0.045658	0.11295	0.9726	2717	3	0.4081
GPR160	6	0.045694	0.13435	0.9764	2718	4	0.4037
AEBP2	6	0.04575	0.13446	0.9764	2719	3	0.4013
CCDC54	6	0.045756	0.13447	0.9764	2720	4	0.3188
GSK3A	6	0.045778	0.13451	0.9764	2721	3	0.2445
GPHN	6	0.045856	0.13468	0.9764	2722	4	0.2891
GRIA4	6	0.045887	0.13475	0.9764	2723	4	0.503
RETSAT	6	0.045906	0.13479	0.9764	2724	3	0.337
ANXA1	6	0.045917	0.13481	0.9764	2725	2	0.0384
PROCR	6	0.045932	0.13484	0.9764	2726	4	0.4346
UGT2B4	6	0.045972	0.13494	0.9764	2727	3	0.0097
SLC35G3	6	0.045972	0.13494	0.9764	2728	2	-0.03
OVCH2	6	0.04599	0.13497	0.9764	2729	2	0.1342
LHFPL1	6	0.046004	0.13501	0.9764	2730	3	0.2291
KCNAB3	6	0.046006	0.13501	0.9764	2731	2	-0.234
TMEM154	6	0.046056	0.13514	0.9764	2732	3	0.3583
TRMT61A	6	0.046076	0.13518	0.9764	2733	2	-0.15
TRMT11	6	0.046094	0.13521	0.9764	2734	4	0.4569
MBD5	6	0.046133	0.13529	0.9764	2735	4	0.2952
SOX21	6	0.046137	0.13531	0.9764	2736	3	0.2232
PSMB4	6	0.04618	0.13541	0.9764	2737	3	0.1164
RGS9	4	0.04622	0.11404	0.97302	2738	2	0.4131
TNFSF12	5	0.046222	0.13129	0.9764	2739	2	-0.642
ASZ1	6	0.046233	0.13551	0.9764	2740	4	0.4227
CRLS1	6	0.046233	0.13551	0.9764	2741	4	0.5127
PI4K2B	6	0.046238	0.13553	0.9764	2742	4	0.4035
LANCL3	6	0.046257	0.13557	0.9764	2743	1	-0.28
MRPS16	6	0.046296	0.13567	0.9764	2744	4	0.3202
TTC39B	6	0.046307	0.1357	0.9764	2745	3	0.2683
EIF3F	6	0.046327	0.13573	0.9764	2746	4	0.5401
RNMT	6	0.046375	0.13584	0.9764	2747	2	-0.12
IGFBPL1	6	0.046385	0.13586	0.9764	2748	4	0.4868
OR4E2	6	0.046416	0.13593	0.9764	2749	4	0.4116
TRAPPC4	6	0.046416	0.13593	0.9764	2750	4	0.6238
BCKDHB	6	0.046485	0.13607	0.9764	2751	4	0.381
F11	6	0.046491	0.13608	0.9764	2752	3	0.294
ADSSL1	6	0.046512	0.13612	0.9764	2753	4	0.3941
FMNL3	6	0.046523	0.13615	0.9764	2754	3	0.317
SETD3	6	0.046525	0.13616	0.9764	2755	4	0.3993
KIF23	6	0.046525	0.13616	0.9764	2756	4	0.424
OR1M1	6	0.046557	0.13623	0.9764	2757	4	0.2462
RRP8	6	0.046584	0.13629	0.9764	2758	2	0.1313
ZKSCAN5	6	0.046607	0.13634	0.9764	2759	4	0.4899
SMURF2	6	0.046612	0.13635	0.9764	2760	3	0.1786
ZNHIT6	6	0.046641	0.13641	0.9764	2761	3	0.3494
COL14A1	6	0.046641	0.13641	0.9764	2762	3	0.1426
ZCCHC18	6	0.046657	0.13644	0.9764	2763	2	0.0098
PREX2	6	0.046659	0.13645	0.9764	2764	3	0.2865
FAM180B	6	0.046676	0.13648	0.9764	2765	3	0.0751
KIAA1429	6	0.046704	0.13655	0.9764	2766	4	0.4394
ZNF485	6	0.046707	0.13656	0.9764	2767	3	0.3333
TMEM147	6	0.046757	0.13668	0.9764	2768	4	0.3121
hsa-mir-4735	4	0.04679	0.11516	0.9754	2769	2	0.2373
AKT2	6	0.046807	0.13678	0.97668	2770	3	0.1821
C17orf97	6	0.046857	0.1369	0.97668	2771	3	0.0453
BATF3	4	0.046879	0.11531	0.9754	2772	3	0.8137
RALGPS2	6	0.046907	0.13703	0.97668	2773	2	-0.03
LAMB1	6	0.04693	0.13707	0.97668	2774	3	0.3316
PAM16	6	0.046969	0.13715	0.97668	2775	4	0.3574
FECH	6	0.046995	0.13722	0.97668	2776	4	0.5006
GTF3C6	6	0.04703	0.1373	0.97668	2777	4	0.6173
KCND3	6	0.047133	0.13753	0.97668	2778	3	0.1048
ABCG1	6	0.047133	0.13753	0.97668	2779	3	0.1311
hsa-mir-378d-2	4	0.047159	0.11588	0.9754	2780	1	0.081
HORMAD2	6	0.047173	0.13761	0.97668	2781	4	0.4341
AMDHD1	6	0.047175	0.13761	0.97668	2782	2	-2E-05
SNTG1	6	0.047193	0.13765	0.97668	2783	2	0.0521
APOC2	6	0.047208	0.13768	0.97668	2784	4	0.2619
RGS4	6	0.04721	0.13768	0.97668	2785	4	0.3697
PWP1	6	0.047255	0.13778	0.97668	2786	2	-0.148
TTC38	6	0.047258	0.13778	0.97668	2787	4	0.274
PGK1	6	0.047261	0.13779	0.97668	2788	3	0.4766
ACTR3	6	0.047272	0.13781	0.97668	2789	4	0.4836
SEMA6C	6	0.047308	0.13787	0.97668	2790	4	0.361
ZNF573	5	0.047333	0.13395	0.9764	2791	2	-0.177
ADAMTS17	6	0.047354	0.13798	0.97668	2792	3	0.352
MTUS2	6	0.047354	0.13798	0.97668	2793	3	0.3271
SEMA3A	6	0.047365	0.138	0.97668	2794	4	0.4155
MRGPRX3	6	0.047408	0.13808	0.97668	2795	3	0.1863
BCO2	6	0.047428	0.13813	0.97668	2796	2	-0.036
CUEDC1	6	0.047437	0.13815	0.97668	2797	4	0.3628
MZB1	6	0.047458	0.1382	0.97668	2798	3	-0.022
TRUB2	6	0.047491	0.13827	0.97668	2799	4	0.5166
CDCA7	6	0.047505	0.13829	0.97668	2800	4	0.4727

LETM2	6	0.047581	0.13845	0.97713	2801	3	0.2805
EEF1B2	6	0.047608	0.1385	0.97713	2802	1	-0.179
RUNX2	6	0.047614	0.13851	0.97713	2803	4	0.3384
HSPA13	6	0.047658	0.1386	0.97713	2804	1	-0.645
BANP	6	0.047679	0.13864	0.97713	2805	3	0.3143
hsa-mir-1825	4	0.047695	0.11696	0.9754	2806	2	0.6838
ZNF585B	6	0.047708	0.13872	0.97731	2807	1	-0.411
SPACA7	6	0.047801	0.13893	0.97802	2808	4	0.443
ADAD2	6	0.047825	0.13899	0.97802	2809	4	0.4016
LAPTM4A	6	0.047844	0.13903	0.97802	2810	4	0.313
CHST10	6	0.047858	0.13907	0.97802	2811	1	-0.095
TRA2A	6	0.047861	0.13907	0.97802	2812	3	0.4556
hsa-mir-449c	4	0.047863	0.11726	0.9754	2813	3	0.5398
TJP3	6	0.047903	0.13915	0.97802	2814	3	0.146
CYTH3	6	0.047903	0.13915	0.97802	2815	4	0.3974
C1orf131	6	0.047927	0.13921	0.97811	2816	4	0.332
KCNN4	6	0.047968	0.13932	0.97828	2817	4	0.3921
HNRNPC	6	0.048008	0.1394	0.97828	2818	2	0.1462
DHH	6	0.048019	0.13943	0.97828	2819	4	0.572
GYPE	3	0.048026	0.1133	0.97292	2820	2	0.692
hsa-mir-4540	4	0.048051	0.11763	0.9754	2821	3	0.4256
RASAL2	6	0.048083	0.13957	0.97896	2822	1	-0.19
ATP11B	6	0.048127	0.13966	0.97926	2823	4	0.377
STXBP2	6	0.048208	0.13985	0.97935	2824	3	0.3178
TP53BP2	6	0.048228	0.1399	0.97935	2825	3	0.3176
MOB1A	6	0.048263	0.13997	0.97935	2826	4	0.41
CEPT1	6	0.048278	0.14	0.97935	2827	2	-0.073
SPC24	6	0.048284	0.14001	0.97935	2828	3	0.6503
SPRYD4	6	0.048342	0.14014	0.97935	2829	4	0.3661
EEF2K	6	0.048383	0.14023	0.97935	2830	4	0.3365
NSMF	6	0.048408	0.14029	0.97935	2831	2	0.0294
ALX4	5	0.048418	0.13663	0.9764	2832	3	0.4244
DNASE1L1	6	0.048471	0.14042	0.97935	2833	3	0.0934
CENPJ	6	0.048481	0.14044	0.97935	2834	4	0.407
ASB5	4	0.048482	0.11845	0.9754	2835	3	0.5204
ZNF22	6	0.04851	0.14051	0.97935	2836	3	0.2215
CNTNAP4	6	0.048545	0.14058	0.97935	2837	4	0.4199
C6orf222	6	0.048558	0.14061	0.97935	2838	2	0.136
ADRBK1	6	0.048565	0.14062	0.97935	2839	4	0.4408
IRG1	6	0.048613	0.14072	0.97935	2840	4	0.399
OR1D5	6	0.04862	0.14073	0.97935	2841	2	-0.336
DET1	4	0.048663	0.1188	0.9754	2842	2	0.3547
WTAP	5	0.048677	0.13723	0.97668	2843	4	0.3186
PARP10	6	0.048706	0.14093	0.97935	2844	4	0.4657
PCMT1	6	0.048708	0.14093	0.97935	2845	3	0.4116
GLUD2	6	0.048733	0.14098	0.97935	2846	2	-0.129
PPIE	6	0.048758	0.14104	0.97935	2847	1	-0.18
BUD31	6	0.048777	0.14108	0.97935	2848	3	0.2566
CYB5R2	6	0.048784	0.14109	0.97935	2849	4	0.4309
EVA1B	6	0.048833	0.14119	0.97935	2850	4	0.4131
NDOR1	6	0.048858	0.14125	0.97935	2851	2	0.1757
ISL2	6	0.048883	0.1413	0.97935	2852	3	0.3398
MUC17	6	0.048948	0.14145	0.97935	2853	4	0.3596
MCM8	6	0.048958	0.14147	0.97935	2854	2	0.0559
BCL2L12	6	0.048983	0.14153	0.97935	2855	3	0.2682
hsa-mir-5700	4	0.049011	0.11948	0.9754	2856	3	0.7394
NOD1	4	0.049033	0.11952	0.9754	2857	2	-0.236
CYP46A1	6	0.0491	0.14179	0.97935	2858	3	0.2928
CYP27C1	6	0.0491	0.14179	0.97935	2859	4	0.3901
TRMT2B	6	0.049121	0.14184	0.97935	2860	3	0.2054
SMEK1	6	0.049128	0.14185	0.97935	2861	4	0.3392
KRTAP20-3	6	0.049158	0.14192	0.97935	2862	2	0.0018
ISCA1	6	0.049195	0.14199	0.97935	2863	4	0.4446
hsa-mir-6786	4	0.049197	0.11986	0.9754	2864	3	0.5782
CLIP3	6	0.049207	0.14202	0.97935	2865	4	0.3952
C6orf52	6	0.049208	0.14202	0.97935	2866	2	-0.215
ESX1	6	0.049247	0.1421	0.97935	2867	4	0.336
PYCR1	6	0.049265	0.14214	0.97935	2868	4	0.3575
ATP6V0D1	6	0.049282	0.14218	0.97935	2869	3	0.1116
MURC	6	0.049295	0.14221	0.97935	2870	3	0.2853
GRK6	6	0.049295	0.14221	0.97935	2871	3	0.3521
SIK2	4	0.049301	0.12007	0.9754	2872	2	-0.034
PFN1	6	0.04932	0.14225	0.97935	2873	3	0.343
SLC16A11	6	0.049322	0.14226	0.97935	2874	4	0.3936
DCAF8	6	0.049347	0.14231	0.97935	2875	2	-0.164
ZNF526	6	0.049357	0.14233	0.97935	2876	2	-0.074
OR11H1	3	0.049358	0.11516	0.9754	2877	2	0.6041
ZNF558	6	0.049361	0.14234	0.97935	2878	4	0.3992
HNRNPA0	6	0.049387	0.1424	0.97935	2879	3	0.1607
TBX21	6	0.049404	0.14244	0.97935	2880	4	0.3604
CCDC137	6	0.049407	0.14244	0.97935	2881	2	-0.282
KRT84	6	0.049413	0.14245	0.97935	2882	4	0.3655
NANOS1	6	0.049413	0.14245	0.97935	2883	3	0.1644
CDS1	6	0.049454	0.14254	0.97935	2884	4	0.4172
FCRL4	6	0.049459	0.14255	0.97935	2885	4	0.3694

SLAMF1	6	0.049479	0.14259	0.97935	2886	3	-0.038
EEF2	6	0.049555	0.14276	0.97978	2887	4	0.4477
SNX22	6	0.049557	0.14277	0.97978	2888	1	-0.305
SPOPL	6	0.049573	0.1428	0.97978	2889	2	-0.068
DOPEY2	6	0.049643	0.14297	0.98059	2890	4	0.3791
RPS19BP1	6	0.049701	0.1431	0.98083	2891	3	0.3373
DENND1A	6	0.049741	0.1432	0.98083	2892	4	0.3708
hsa-mir-4483	4	0.049756	0.12098	0.9754	2893	3	0.7179
hsa-mir-216b	4	0.049766	0.12099	0.9754	2894	3	0.437
REV1	6	0.049775	0.14327	0.98083	2895	3	0.3599
COLEC12	6	0.049794	0.14331	0.98083	2896	4	0.3848
COL10A1	6	0.049808	0.14334	0.98083	2897	3	0.3187
VTN	6	0.049844	0.14343	0.98083	2898	4	0.4077
METTL10	6	0.04986	0.14346	0.98083	2899	3	0.4029
FRG1	6	0.049907	0.14355	0.98083	2900	4	0.3328
EDDM3A	6	0.049924	0.14359	0.98083	2901	4	0.5743
RPL8	6	0.049932	0.1436	0.98083	2902	2	-0.138
STAT4	6	0.049941	0.14362	0.98083	2903	4	0.3052
FAT4	6	0.049954	0.14365	0.98083	2904	4	0.3604
NDNF	6	0.049993	0.14373	0.98083	2905	4	0.5261
FAM76A	6	0.049999	0.14374	0.98083	2906	4	0.3643
VEGFC	6	0.050007	0.14377	0.98083	2907	3	0.189
TMEM205	6	0.050137	0.14405	0.98156	2908	4	0.6421
TMEM128	6	0.050149	0.14407	0.98156	2909	3	0.3069
EEF1A2	6	0.050156	0.14408	0.98156	2910	1	-0.343
hsa-mir-6895	4	0.050167	0.12174	0.9754	2911	3	0.5642
CHCHD4	6	0.050206	0.14421	0.98176	2912	2	-0.113
hsa-mir-1303	4	0.050261	0.12192	0.9754	2913	3	0.5221
EXOSC7	6	0.050299	0.14442	0.98266	2914	3	-0.158
hsa-let-7a-3	3	0.05031	0.11649	0.9754	2915	2	0.9205
ACYP1	5	0.050342	0.14121	0.97935	2916	3	0.5181
IPO4	6	0.050367	0.14458	0.98295	2917	4	0.4031
OR10A6	6	0.050381	0.14461	0.98295	2918	1	-0.072
OLFML3	6	0.050413	0.14468	0.98295	2919	3	0.23
C1orf111	6	0.050447	0.14475	0.98295	2920	3	0.1991
LTBP2	6	0.050451	0.14476	0.98295	2921	2	0.1361
ENG	6	0.050506	0.14489	0.98295	2922	4	0.2389
ANKRD23	6	0.050533	0.14495	0.98295	2923	4	0.3693
hsa-mir-1296	4	0.050537	0.12249	0.9754	2924	1	-0.058
GCHFR	6	0.050555	0.145	0.98295	2925	2	-0.452
FAM76B	6	0.050559	0.14501	0.98295	2926	4	0.3488
RPS13	6	0.050578	0.14507	0.98295	2927	3	0.2531
DOK6	6	0.050605	0.14514	0.98295	2928	3	0.2082
WASF2	6	0.050655	0.14524	0.98296	2929	2	0.0942
MRPS36	6	0.050704	0.14535	0.98296	2930	4	0.4603
FUCA2	6	0.050704	0.14535	0.98296	2931	4	0.4787
CDPF1	6	0.050755	0.14547	0.98296	2932	3	0.1221
hsa-mir-5682	4	0.05077	0.12297	0.9754	2933	2	0.4273
SAFB	6	0.050805	0.14558	0.98296	2934	2	-0.174
STAMBPL1	6	0.050849	0.14567	0.98296	2935	4	0.5486
NOM1	6	0.050881	0.14573	0.98296	2936	3	0.3662
TMEM41A	6	0.050905	0.14577	0.98296	2937	4	0.2945
SLC25A32	6	0.050926	0.14582	0.98296	2938	3	0.4094
C4orf29	6	0.050955	0.14589	0.98296	2939	3	0.1963
FXYD6-FXYD2	2	0.050977	0.090629	0.96841	2940	2	0.4648
DMXL1	6	0.050988	0.14596	0.98296	2941	4	0.4542
ING2	6	0.050997	0.14598	0.98296	2942	2	-0.03
hsa-mir-1307	4	0.05101	0.12344	0.9754	2943	3	0.5128
ANKRD32	6	0.051054	0.14613	0.98296	2944	3	-0.046
hsa-mir-6719	4	0.051091	0.12362	0.9754	2945	3	0.7686
CYP20A1	6	0.051098	0.14622	0.98296	2946	4	0.4255
PRUNE2	6	0.051104	0.14623	0.98296	2947	2	-0.075
FZD3	6	0.051126	0.14628	0.98296	2948	4	0.3011
HSPB8	6	0.051154	0.14635	0.98296	2949	2	-0.04
hsa-mir-6816	4	0.051172	0.12377	0.9754	2950	1	-0.222
FAHD2B	6	0.051188	0.14643	0.98296	2951	2	-0.131
ZNF683	6	0.051188	0.14643	0.98296	2952	3	0.1993
ACPT	6	0.05121	0.14647	0.98296	2953	4	0.4399
ICK	6	0.051211	0.14648	0.98296	2954	3	0.403
ISCU	6	0.051265	0.14659	0.98296	2955	3	0.7482
NIPAL3	6	0.051266	0.14659	0.98296	2956	4	0.4169
ARL13A	6	0.051286	0.14663	0.98296	2957	4	0.3027
ELP5	6	0.051304	0.14667	0.98296	2958	2	-0.092
hsa-mir-7855	4	0.051338	0.12409	0.9754	2959	2	0.4161
EXOC3	6	0.051405	0.14688	0.98311	2960	2	-0.163
AATK	6	0.051407	0.14689	0.98311	2961	4	0.3385
TCP10L2	5	0.051414	0.14384	0.98098	2962	2	-0.002
OR52D1	6	0.051449	0.14697	0.98311	2963	4	0.3702
GAPDH	6	0.051453	0.14698	0.98311	2964	3	0.3335
NAP1L4	6	0.051501	0.14709	0.98311	2965	3	0.1921
MUC6	6	0.051501	0.14709	0.98311	2966	3	0.4122
ZNF732	5	0.051536	0.14412	0.98156	2967	2	-0.066
C10orf32	6	0.051553	0.14721	0.98311	2968	2	-0.271
SPRR1B	5	0.051585	0.14424	0.98176	2969	1	-0.419
CCL24	6	0.051597	0.14729	0.98311	2970	3	0.1107

NAPEPLD	6	0.051629	0.14738	0.98311	2971	2	-0.263
LTC4S	6	0.051641	0.14741	0.98311	2972	3	0.2937
PPFIA1	6	0.051653	0.14744	0.98311	2973	2	-0.036
ZNF415	6	0.051654	0.14744	0.98311	2974	4	0.5303
EMC10	6	0.051667	0.14747	0.98311	2975	2	-0.38
CYB5D2	6	0.051702	0.14754	0.98311	2976	3	-0.275
FNIP2	6	0.051718	0.14757	0.98311	2977	4	0.3217
SCLT1	6	0.051769	0.14768	0.98311	2978	3	0.2782
OR2S2	6	0.05181	0.14777	0.98311	2979	4	0.2912
PPIAL4E	1	0.051811	0.051848	0.94417	2980	1	1.9787
FAM47B	6	0.051852	0.14788	0.98311	2981	2	-0.026
FFAR2	6	0.051856	0.14789	0.98311	2982	3	0.2507
ADAMTS20	6	0.051897	0.14799	0.98311	2983	3	0.2227
hsa-mir-554	4	0.05196	0.12531	0.9764	2984	3	0.5453
PIGS	6	0.051974	0.14817	0.98311	2985	4	0.3807
SLC6A11	6	0.051974	0.14817	0.98311	2986	3	0.135
CEP250	6	0.052001	0.14823	0.98311	2987	2	-0.061
SFTPC	4	0.052006	0.1254	0.9764	2988	3	0.3932
PTRH2	6	0.052013	0.14825	0.98311	2989	3	0.2096
LAMTOR2	6	0.052051	0.14833	0.98311	2990	1	0.0375
ZC3H7A	6	0.052103	0.14845	0.98311	2991	4	0.452
MLST8	6	0.052103	0.14845	0.98311	2992	4	0.5975
PSMC2	6	0.052107	0.14846	0.98311	2993	4	0.5487
CMTM4	6	0.052118	0.14848	0.98311	2994	3	0.1182
POP4	6	0.052201	0.14866	0.98311	2995	4	0.716
SLMO2	6	0.052221	0.1487	0.98311	2996	4	0.4578
PRR4	6	0.052283	0.14884	0.98311	2997	4	0.3778
PFDN2	6	0.052295	0.14887	0.98311	2998	4	0.5599
LRFN2	6	0.05232	0.14892	0.98311	2999	4	0.3765
NXPE2	6	0.052325	0.14892	0.98311	3000	3	0.2908
MAPK8IP1	6	0.052325	0.14892	0.98311	3001	1	-0.752
DFNB31	6	0.052338	0.14894	0.98311	3002	4	0.3
TOMM6	6	0.052339	0.14895	0.98311	3003	3	0.5014
SEC23B	6	0.052343	0.14896	0.98311	3004	4	0.4235
SPHK2	6	0.052372	0.14902	0.98311	3005	4	0.3513
IL11	6	0.052377	0.14903	0.98311	3006	4	0.2937
KIAA1244	6	0.052379	0.14903	0.98311	3007	3	0.2069
NLRP6	6	0.0524	0.14908	0.98311	3008	1	-0.285
hsa-mir-4745	4	0.052537	0.12645	0.9764	3009	3	0.7392
DNAJC14	6	0.052553	0.1494	0.98395	3010	2	-0.643
MBTPS2	6	0.052557	0.14941	0.98395	3011	4	0.436
COBLL1	6	0.052557	0.14941	0.98395	3012	4	0.4109
ELK4	6	0.052598	0.1495	0.98395	3013	3	0.3057
AFP	6	0.052617	0.14954	0.98395	3014	3	0.2263
OR6C65	6	0.052617	0.14954	0.98395	3015	3	0.2865
hsa-mir-4686	4	0.052635	0.12664	0.9764	3016	2	0.2971
HRK	6	0.052674	0.14966	0.98395	3017	4	0.3322
AGER	6	0.052677	0.14967	0.98395	3018	4	0.4332
TMSB10	6	0.052772	0.14987	0.98395	3019	3	0.2032
CD2	6	0.052798	0.14992	0.98395	3020	2	0.0228
PRLR	6	0.052814	0.14995	0.98395	3021	4	0.3965
ALPK2	6	0.052817	0.14995	0.98395	3022	3	0.1259
SPACA4	6	0.052829	0.14998	0.98395	3023	4	0.3171
KIF1B	6	0.052848	0.15002	0.98395	3024	3	0.2653
LIPJ	6	0.052861	0.15004	0.98395	3025	3	0.292
ALK	6	0.052861	0.15004	0.98395	3026	3	-0.033
RPL7L1	6	0.052862	0.15004	0.98395	3027	3	0.4576
SLC25A26	6	0.052932	0.1502	0.98459	3028	4	0.4414
ZNF24	6	0.052973	0.15029	0.98459	3029	2	0.137
CPXM2	6	0.053095	0.15055	0.98474	3030	2	0.0615
GOLGA5	6	0.053097	0.15055	0.98474	3031	3	0.1157
HLTF	6	0.053099	0.15056	0.98474	3032	3	0.299
PEX11A	6	0.053099	0.15056	0.98474	3033	3	0.2893
OR1S1	5	0.053103	0.14791	0.98311	3034	3	0.3314
KLHL33	6	0.053121	0.1506	0.98474	3035	3	0.0789
MAN2A1	6	0.053121	0.1506	0.98474	3036	3	0.1101
AQR	4	0.053143	0.12762	0.9764	3037	3	0.5569
SOX17	6	0.053222	0.15082	0.9851	3038	2	-0.133
BRWD1	6	0.053236	0.15085	0.9851	3039	4	0.427
PEX11B	6	0.053237	0.15085	0.9851	3040	3	0.3503
IFNL3	5	0.053248	0.14826	0.98311	3041	3	0.4879
DBT	6	0.053296	0.15098	0.98536	3042	4	0.1937
FAM20A	6	0.053334	0.15108	0.98536	3043	4	0.3488
RINL	6	0.053334	0.15108	0.98536	3044	2	-0.003
WFDC2	6	0.053334	0.15108	0.98536	3045	3	0.251
ARHGEF2	4	0.053339	0.12801	0.9764	3046	2	-0.058
INO80E	4	0.053391	0.12811	0.9764	3047	3	0.3601
MEFV	6	0.053444	0.15131	0.98538	3048	4	0.3801
ACSL1	6	0.053446	0.15132	0.98538	3049	1	-0.306
ADAL	6	0.053457	0.15134	0.98538	3050	3	0.2894
LAMB4	6	0.053495	0.15141	0.98538	3051	3	0.1617
SIGLEC10	5	0.053498	0.14887	0.98311	3052	1	-0.235
PRCC	6	0.053499	0.15143	0.98538	3053	3	0.364
C3orf17	6	0.053528	0.15149	0.98538	3054	4	0.3336
TMEM51	6	0.053545	0.15152	0.98538	3055	2	-0.674

DTX3	6	0.053574	0.15159	0.98538	3056	3	0.1929
RBPMS	6	0.053594	0.15162	0.98538	3057	2	-0.016
DUSP1	6	0.053595	0.15163	0.98538	3058	2	-0.085
PDZD11	6	0.053626	0.15169	0.98538	3059	2	-0.387
USP47	6	0.053655	0.15176	0.98538	3060	4	0.4795
TMEM194A	6	0.053655	0.15176	0.98538	3061	4	0.531
OR14A16	6	0.053695	0.15185	0.98568	3062	4	0.5764
MRTO4	6	0.053789	0.15204	0.98619	3063	2	-0.038
SPTAN1	6	0.053794	0.15205	0.98619	3064	2	-0.116
ARVCF	6	0.053805	0.15207	0.98619	3065	4	0.3671
ATPIF1	6	0.053873	0.15223	0.98619	3066	3	0.5094
CYP2F1	6	0.053873	0.15223	0.98619	3067	4	0.4786
ATP5F1	6	0.053901	0.1523	0.98619	3068	4	0.4161
hsa-mir-4252	4	0.053907	0.12913	0.9764	3069	1	0.1259
DNAI2	6	0.053943	0.15239	0.98619	3070	4	0.353
SLC22A31	6	0.053953	0.15241	0.98619	3071	4	0.4558
RPS6KB2	6	0.053955	0.15242	0.98619	3072	3	0.1386
UBOX5	6	0.053993	0.1525	0.98619	3073	2	0.2766
CENPV	6	0.053997	0.15251	0.98619	3074	3	0.222
ZNF559-ZNF177	2	0.054025	0.095739	0.96841	3075	2	0.3798
ARHGEF17	6	0.054029	0.15259	0.98639	3076	4	0.4185
FAM92A1	6	0.054055	0.15265	0.98648	3077	2	-0.242
GLTPD2	6	0.054142	0.15284	0.9871	3078	1	0.0352
NONO	6	0.054199	0.15295	0.98748	3079	3	0.305
FAM135A	6	0.054256	0.15308	0.98782	3080	4	0.3541
CAPRIN2	6	0.054401	0.15339	0.9892	3081	4	0.2515
PCBP1	6	0.054434	0.15345	0.9892	3082	2	0.1447
OR52K2	6	0.05444	0.15348	0.9892	3083	3	-0.059
WIPI1	6	0.054463	0.15353	0.9892	3084	4	0.3814
NR1H3	6	0.054499	0.15361	0.9892	3085	3	0.0595
ADSL	6	0.054506	0.15362	0.9892	3086	3	0.1168
RWDD1	6	0.05454	0.1537	0.9892	3087	4	0.4007
C6orf58	6	0.05459	0.15381	0.98963	3088	3	0.1153
SRR	6	0.054689	0.15402	0.99022	3089	2	-0.177
FOXD4L5	6	0.054702	0.15405	0.99022	3090	2	-0.332
SV2A	6	0.054775	0.15422	0.99035	3091	3	0.2141
hsa-mir-214	2	0.054784	0.097031	0.96841	3092	2	0.5788
CHEK1	6	0.054788	0.15425	0.99035	3093	2	-0.041
NDST2	6	0.054829	0.15435	0.99035	3094	4	0.3819
BAI2	6	0.054829	0.15435	0.99035	3095	4	0.4199
FBXO34	6	0.054829	0.15435	0.99035	3096	4	0.3602
SRL	6	0.054838	0.15436	0.99035	3097	2	-0.465
OR8J1	4	0.054884	0.13105	0.9764	3098	3	0.4795
hsa-mir-8068	4	0.054906	0.1311	0.9764	3099	2	0.1621
hsa-let-7e	4	0.054906	0.1311	0.9764	3100	1	-0.792
CKM	6	0.054918	0.15453	0.99045	3101	3	0.016
PPFIA4	6	0.054943	0.1546	0.99045	3102	4	0.4563
RHO	6	0.054957	0.15462	0.99045	3103	2	-0.078
SUCLG1	6	0.054983	0.15468	0.99045	3104	3	0.0899
CALML5	6	0.054987	0.15468	0.99045	3105	3	0.0875
C9orf24	6	0.055009	0.15474	0.99045	3106	3	0.3957
CCRL2	6	0.055023	0.15477	0.99045	3107	3	0.541
SSFA2	6	0.055048	0.15483	0.99045	3108	3	0.2925
BBS2	6	0.055062	0.15486	0.99045	3109	2	0.0671
MTX1	6	0.055091	0.15494	0.99045	3110	4	0.3898
SLC25A30	6	0.055103	0.15497	0.99045	3111	3	0.1759
GPR25	6	0.055134	0.15503	0.99045	3112	4	0.4762
PEX13	6	0.055146	0.15505	0.99045	3113	3	0.3438
WDR38	6	0.055246	0.1553	0.99127	3114	4	0.399
OR8G2	6	0.055285	0.15538	0.99127	3115	2	0.0019
CYP4X1	6	0.055285	0.15538	0.99127	3116	3	0.108
SLC38A9	6	0.055285	0.15538	0.99127	3117	3	-0.071
PDE12	6	0.055335	0.15549	0.9914	3118	4	0.396
POU3F1	6	0.055335	0.15549	0.9914	3119	2	-0.251
MTIF2	6	0.055376	0.15558	0.99141	3120	4	0.5887
FSIP1	6	0.055385	0.15559	0.99141	3121	1	-0.213
GALC	6	0.055409	0.15564	0.99144	3122	4	0.3417
CNIH3	6	0.055435	0.15571	0.99152	3123	2	0.0484
SPATA2L	4	0.055439	0.13212	0.9764	3124	2	0.1075
hsa-mir-339	4	0.055445	0.13213	0.9764	3125	3	0.425
BTK	6	0.055484	0.15582	0.99153	3126	1	-0.2
ZNF253	5	0.055485	0.1537	0.9892	3127	2	0.1461
AHSG	6	0.055496	0.15585	0.99153	3128	2	-0.463
hsa-mir-2054	3	0.055505	0.12365	0.9754	3129	2	1.1775
TMTC3	6	0.055534	0.15593	0.9916	3130	2	-0.231
hsa-mir-9-2	4	0.055605	0.13245	0.9764	3131	1	-0.099
CHRNA6	5	0.055607	0.15397	0.99022	3132	3	0.5262
PARP4	6	0.055627	0.15613	0.9916	3133	2	-0.024
BCL2L11	6	0.055633	0.15614	0.9916	3134	3	0.4011
TOMM34	6	0.055647	0.15617	0.9916	3135	3	0.2421
FNBP1L	6	0.055683	0.15625	0.9916	3136	2	-0.256
RAD18	6	0.0557	0.15628	0.9916	3137	4	0.4741
HHIP	6	0.055702	0.15629	0.9916	3138	4	0.3476
INS-IGF2	4	0.055705	0.13264	0.9764	3139	1	-0.227
ZNF224	6	0.055708	0.1563	0.9916	3140	3	0.3324

LOC149373	6	0.055732	0.15635	0.9916	3141	3	0.3201
BCDIN3D	6	0.055782	0.15646	0.99195	3142	2	-0.047
MAFB	6	0.055799	0.1565	0.99195	3143	3	-0.219
PFN4	6	0.055832	0.15657	0.99205	3144	3	0.0148
hsa-mir-4800	4	0.055839	0.1329	0.9764	3145	2	0.3383
VPS28	6	0.055882	0.15666	0.99228	3146	3	0.1451
TMEM190	6	0.055904	0.15671	0.99228	3147	3	0.1935
TSPAN11	6	0.055942	0.15679	0.99228	3148	4	0.6714
EML2	4	0.055959	0.13312	0.9764	3149	3	0.4335
MAGEA1	6	0.055968	0.15685	0.99228	3150	3	0.0473
SLC10A3	4	0.056005	0.13322	0.9764	3151	3	0.4263
CDKL4	6	0.05603	0.15698	0.99228	3152	2	0.0584
NOD2	6	0.0561	0.15712	0.99228	3153	4	0.3145
TMEM145	6	0.056135	0.1572	0.99228	3154	3	0.2085
hsa-mir-4270	2	0.056171	0.09936	0.96841	3155	2	0.4438
C5orf51	6	0.05618	0.1573	0.99228	3156	4	0.3436
KIAA0391	4	0.056191	0.13358	0.9764	3157	2	0.1156
CPSF2	4	0.056247	0.13369	0.9764	3158	2	0.436
hsa-mir-671	4	0.056262	0.13372	0.9764	3159	3	0.4626
DIO2	6	0.056281	0.15752	0.99228	3160	3	0.3506
KDELR3	6	0.056285	0.15752	0.99228	3161	4	0.3986
TRIML1	6	0.056314	0.15759	0.99228	3162	4	0.3375
ZNF175	6	0.056325	0.1576	0.99228	3163	3	0.3354
PRSS46	6	0.056332	0.15762	0.99228	3164	4	0.4259
PIK3CD	6	0.056367	0.15769	0.99228	3165	2	-0.002
LTV1	6	0.056377	0.15771	0.99228	3166	4	0.4173
TRIB2	6	0.056378	0.15771	0.99228	3167	2	-0.19
ALOX12	6	0.056388	0.15774	0.99228	3168	4	0.3633
TRPC6	6	0.056404	0.15778	0.99228	3169	4	0.3461
KRTAP10-4	6	0.056428	0.15783	0.99228	3170	2	0.2573
KCNN1	6	0.05646	0.15789	0.99228	3171	3	-0.009
CNTN6	6	0.056477	0.15793	0.99228	3172	2	0.1944
NKAP	6	0.056482	0.15794	0.99228	3173	4	0.4291
FBXO25	6	0.056482	0.15794	0.99228	3174	4	0.3995
ZNF622	6	0.056482	0.15794	0.99228	3175	4	0.4588
hsa-mir-7-2	2	0.056536	0.099966	0.96841	3176	2	0.765
NANP	6	0.056545	0.15809	0.99228	3177	4	0.3353
SACM1L	6	0.056545	0.15809	0.99228	3178	4	0.3384
SETD8	6	0.056552	0.1581	0.99228	3179	2	-0.168
C16orf11	6	0.056577	0.15816	0.99228	3180	2	0.1293
GIN1	6	0.056605	0.15821	0.99228	3181	3	-0.079
SCMH1	6	0.056616	0.15824	0.99228	3182	4	0.4751
ATP11A	6	0.056626	0.15826	0.99228	3183	4	0.4901
RELT	6	0.056664	0.15834	0.99228	3184	4	0.3494
NR1H2	6	0.056676	0.15836	0.99228	3185	2	0.1124
RET	6	0.056698	0.1584	0.99228	3186	3	0.2755
hsa-mir-663b	3	0.056765	0.12535	0.9764	3187	2	0.8722
C1orf35	6	0.056813	0.15865	0.99228	3188	3	0.4166
SMAD5	6	0.056818	0.15866	0.99228	3189	4	0.3719
SYCP2	6	0.056835	0.1587	0.99228	3190	4	0.4856
PRMT7	6	0.056865	0.15876	0.99228	3191	4	0.3841
DKC1	6	0.056865	0.15876	0.99228	3192	4	0.3776
TTC18	6	0.056895	0.15884	0.99228	3193	4	0.3246
LEMD2	6	0.056924	0.1589	0.99228	3194	4	0.2187
hsa-mir-6133	4	0.056963	0.13501	0.9764	3195	3	0.5627
NPS	6	0.056984	0.15904	0.99228	3196	4	0.4319
SPICE1	4	0.057039	0.13515	0.9764	3197	3	0.3773
ERP27	6	0.057064	0.15921	0.99228	3198	3	0.2314
RBP5	6	0.057073	0.15923	0.99228	3199	3	0.0255
CAB39	6	0.057077	0.15924	0.99228	3200	2	-1E-03
MAMDC2	6	0.05708	0.15925	0.99228	3201	3	0.2562
MCF2	6	0.057122	0.15934	0.99228	3202	2	-0.185
C3orf67	6	0.057136	0.15937	0.99228	3203	3	0.2608
SEMA6D	6	0.057144	0.15938	0.99228	3204	3	0.1247
hsa-mir-6739	4	0.057162	0.13536	0.9764	3205	3	0.5602
TMEM14E	6	0.05718	0.15946	0.99228	3206	4	0.4442
TMEM42	6	0.057218	0.15953	0.99228	3207	4	0.4218
C1orf185	6	0.057245	0.15959	0.99228	3208	4	0.4612
SPATA5L1	6	0.057246	0.15959	0.99228	3209	4	0.4218
ZBTB42	6	0.057246	0.15959	0.99228	3210	1	-0.721
hsa-mir-34c	2	0.057263	0.10119	0.96841	3211	2	0.3519
APBB1IP	6	0.057278	0.15965	0.99228	3212	4	0.5084
ARID4B	6	0.05728	0.15966	0.99228	3213	4	0.6091
C15orf56	6	0.05734	0.1598	0.9924	3214	3	0.2711
GPR116	6	0.057356	0.15984	0.9924	3215	4	0.5571
hsa-mir-26a-2	4	0.057367	0.13574	0.9764	3216	1	-0.214
SAMM50	6	0.057408	0.15994	0.9924	3217	4	0.4299
SNED1	6	0.057408	0.15994	0.9924	3218	4	0.5124
RALGPS1	6	0.05742	0.15997	0.9924	3219	3	0.472
ABCD3	6	0.057445	0.16002	0.9924	3220	2	-0.011
CAMK1	6	0.057492	0.16013	0.9925	3221	4	0.5146
FAS	6	0.057519	0.16018	0.9925	3222	2	-0.19
COPS7B	6	0.057569	0.16029	0.99276	3223	2	0.0962
hsa-mir-6745	4	0.057601	0.13618	0.9764	3224	3	0.5588
CACNG6	6	0.057668	0.1605	0.99276	3225	2	0.0459

TSG101	6	0.057725	0.16062	0.99276	3226	3	0.4619
MESDC2	6	0.057725	0.16062	0.99276	3227	3	0.3343
hsa-mir-6740	4	0.057799	0.13656	0.9764	3228	2	0.3137
hsa-mir-4328	4	0.057831	0.13662	0.9764	3229	2	0.2226
NRN1	6	0.057846	0.16088	0.99276	3230	4	0.3807
ACRBP	6	0.057859	0.1609	0.99276	3231	4	0.4259
MARCO	6	0.057859	0.1609	0.99276	3232	4	0.3325
KCTD5	6	0.057866	0.16091	0.99276	3233	1	0.1338
CGREF1	6	0.057877	0.16094	0.99276	3234	4	0.3362
OR10G2	6	0.05791	0.16102	0.99276	3235	3	0.2483
IL7	6	0.057916	0.16104	0.99276	3236	2	0.0938
CEBPA	5	0.057976	0.15899	0.99228	3237	2	-0.112
UFC1	6	0.058015	0.16125	0.99276	3238	4	0.322
FAM19A4	6	0.058032	0.16128	0.99276	3239	4	0.4097
hsa-mir-4696	4	0.058034	0.13701	0.97668	3240	3	0.4925
ZNF571	6	0.058058	0.16134	0.99276	3241	3	0.1223
RNF165	6	0.058066	0.16136	0.99276	3242	4	0.4012
KRT82	6	0.058089	0.1614	0.99276	3243	1	0.1696
NUBP1	6	0.058148	0.16152	0.99276	3244	4	0.2815
B3GNT1	6	0.058188	0.16159	0.99276	3245	3	0.2649
CFB	6	0.058204	0.16163	0.99276	3246	4	0.3944
METTL22	6	0.058219	0.16165	0.99276	3247	2	-0.296
ZNF134	6	0.058219	0.16165	0.99276	3248	2	-0.622
DNAJA2	6	0.058237	0.16169	0.99276	3249	3	0.3093
HIST1H3A	6	0.058263	0.16174	0.99276	3250	1	-0.767
CLTB	6	0.058273	0.16177	0.99276	3251	3	0.4374
PRAMEF16	4	0.058297	0.13751	0.97668	3252	2	0.1747
HMGN3	6	0.058312	0.16185	0.99276	3253	2	0.1449
CD28	6	0.05837	0.16197	0.99276	3254	3	0.29
ANGPTL6	6	0.058386	0.16201	0.99276	3255	4	0.3787
UBN1	6	0.058394	0.16202	0.99276	3256	2	0.2933
AP2S1	6	0.058411	0.16205	0.99276	3257	3	0.2729
LCE1D	6	0.058427	0.16208	0.99276	3258	3	0.3505
FCER1G	6	0.058524	0.16231	0.99276	3259	3	0.3969
OAS3	6	0.05856	0.16238	0.99276	3260	3	0.2382
ADAMTS4	6	0.05856	0.16238	0.99276	3261	3	0.3099
KLF9	6	0.058591	0.16245	0.99276	3262	4	0.4311
H1FNT	6	0.058595	0.16246	0.99276	3263	3	0.2086
GGA2	6	0.05861	0.16249	0.99276	3264	1	-0.191
TMEM204	6	0.058659	0.16259	0.99276	3265	3	0.1731
hsa-mir-323b	4	0.058703	0.13827	0.97668	3266	3	0.3941
ATP1A4	6	0.058723	0.16273	0.99276	3267	3	0.1739
APOM	6	0.058751	0.16279	0.99276	3268	3	0.2288
OR1E2	6	0.058844	0.16301	0.99276	3269	4	0.6532
BBS4	6	0.058857	0.16303	0.99276	3270	2	0.1117
C22orf28	4	0.058864	0.13857	0.97713	3271	2	0.4223
C21orf91	6	0.058901	0.16313	0.99276	3272	3	0.0738
DNAH3	6	0.058937	0.16319	0.99276	3273	3	0.2282
FBXO39	6	0.058956	0.16323	0.99276	3274	3	0.0762
SLC35B2	6	0.059027	0.16338	0.99276	3275	3	0.3804
GAB3	6	0.059053	0.16343	0.99276	3276	2	-0.314
RALB	6	0.059055	0.16343	0.99276	3277	2	0.1299
PADI3	6	0.059061	0.16344	0.99276	3278	4	0.3194
WNT2	6	0.059067	0.16345	0.99276	3279	3	0.1871
ABCA5	6	0.059079	0.16348	0.99276	3280	4	0.2818
NPIPL3	2	0.059091	0.10417	0.96841	3281	2	0.5792
SNX15	6	0.059113	0.16355	0.99276	3282	4	0.3002
CRYBB1	6	0.059114	0.16355	0.99276	3283	2	0.1966
CISD1	6	0.059138	0.1636	0.99276	3284	4	0.4582
MAP2K2	6	0.059193	0.1637	0.99276	3285	4	0.5189
RBBP4	6	0.059206	0.16373	0.99276	3286	4	0.4602
NCOA1	6	0.059246	0.16381	0.99276	3287	4	0.3966
EPHB3	6	0.059249	0.16382	0.99276	3288	2	-0.151
CDK5RAP2	6	0.059253	0.16383	0.99276	3289	1	-0.117
hsa-mir-3132	4	0.059256	0.13936	0.97828	3290	3	0.4377
OR8B4	6	0.059298	0.16393	0.99276	3291	4	0.4466
SLC17A2	6	0.059303	0.16394	0.99276	3292	2	0.1634
ARFRP1	6	0.059324	0.16399	0.99276	3293	3	0.1943
MARCH4	6	0.059352	0.16405	0.99276	3294	3	0.1353
DDX53	6	0.059381	0.16411	0.99276	3295	3	0.3471
CNST	6	0.059422	0.1642	0.99276	3296	4	0.4571
BCLAF1	6	0.059487	0.16434	0.99276	3297	4	0.4336
hsa-mir-7160	2	0.059495	0.1048	0.96841	3298	1	0.3775
ITSN1	6	0.059501	0.16437	0.99276	3299	3	0.2536
SETMAR	6	0.059524	0.16442	0.99276	3300	3	0.1947
RNF215	6	0.059527	0.16442	0.99276	3301	4	0.3614
hsa-mir-4327	4	0.05958	0.13997	0.97935	3302	2	0.3294
NCL	6	0.059589	0.16455	0.99276	3303	3	0.3238
GFM2	6	0.059593	0.16456	0.99276	3304	3	0.2063
PKMYT1	6	0.0596	0.16457	0.99276	3305	3	0.2561
EPRS	6	0.059626	0.16462	0.99276	3306	3	0.2684
CARF	6	0.059654	0.16468	0.99276	3307	4	0.2653
CYP4A11	6	0.059667	0.1647	0.99276	3308	4	0.8221
TIPARP	6	0.059699	0.16477	0.99276	3309	3	0.059
EPB49	6	0.059709	0.16479	0.99276	3310	3	0.1743

NBEA	6	0.059761	0.1649	0.99276	3311	4	0.4001
TMEM114	6	0.059798	0.16498	0.99276	3312	2	-0.195
BPIFA2	6	0.059823	0.16502	0.99276	3313	3	0.2492
TYW1B	6	0.059839	0.16506	0.99276	3314	4	0.5041
MAGEA10-MAGEA5	6	0.059897	0.16519	0.99276	3315	2	-0.088
SCG3	6	0.059993	0.16539	0.99276	3316	3	0.198
COX7C	6	0.059996	0.16539	0.99276	3317	3	0.21
LOC100287177	4	0.060036	0.14084	0.97935	3318	3	0.3816
NRXN3	6	0.060045	0.16551	0.99276	3319	4	0.3173
PAK6	6	0.060045	0.16551	0.99276	3320	4	0.3442
COPS7A	6	0.060045	0.16551	0.99276	3321	1	-0.191
DHCR7	6	0.060144	0.16571	0.99276	3322	2	0.0037
AGBL3	6	0.060149	0.16571	0.99276	3323	3	0.2375
PTPRK	6	0.060159	0.16574	0.99276	3324	3	0.3406
MYL9	6	0.060194	0.16582	0.99276	3325	2	0.0901
NANS	6	0.060238	0.1659	0.99276	3326	2	-0.11
hsa-mir-6068	4	0.060254	0.14127	0.97935	3327	1	-0.244
hsa-mir-4752	4	0.060258	0.14128	0.97935	3328	3	0.528
WBP2NL	6	0.060293	0.16603	0.99276	3329	2	0.1516
SNAI1	6	0.060316	0.16609	0.99276	3330	4	0.2999
IGLL5	6	0.060342	0.16615	0.99276	3331	4	0.4815
TTC22	6	0.060348	0.16617	0.99276	3332	4	0.4204
CTBP1	6	0.060382	0.16624	0.99276	3333	4	0.3099
FOXK1	6	0.060392	0.16626	0.99276	3334	2	-0.033
FNTA	6	0.060448	0.16638	0.99276	3335	3	0.1993
ZNF574	6	0.06046	0.1664	0.99276	3336	4	0.4163
KIF17	6	0.06046	0.1664	0.99276	3337	4	0.4236
hsa-mir-6833	4	0.060491	0.14174	0.97935	3338	3	0.6778
SCGB1D2	6	0.060507	0.16651	0.99276	3339	4	0.4549
AVPR1A	6	0.06054	0.16659	0.99276	3340	3	0.0858
GDF5	6	0.06056	0.16664	0.99276	3341	4	0.3964
IL33	6	0.060571	0.16665	0.99276	3342	3	0.2384
PIGY	6	0.060585	0.16668	0.99276	3343	4	0.3198
RPS23	6	0.060612	0.16674	0.99276	3344	4	0.7378
C16orf97	6	0.060612	0.16674	0.99276	3345	3	0.3818
WDR1	6	0.060625	0.16677	0.99276	3346	3	0.2174
NOX4	6	0.060639	0.1668	0.99276	3347	2	0.1559
hsa-mir-6875	4	0.060639	0.142	0.97935	3348	2	-0.583
COMMD9	6	0.060653	0.16683	0.99276	3349	3	0.1203
NELFCD	6	0.060666	0.16686	0.99276	3350	4	0.3995
KIAA0754	6	0.060674	0.16687	0.99276	3351	3	0.2099
SPRYD3	6	0.060688	0.1669	0.99276	3352	3	0.1762
NECAP2	6	0.060792	0.16712	0.99276	3353	4	0.294
IWS1	6	0.060803	0.16714	0.99276	3354	3	0.3031
DECR2	6	0.060831	0.1672	0.99276	3355	3	0.016
PARM1	6	0.060831	0.1672	0.99276	3356	3	0.3231
MTMR1	6	0.060837	0.16722	0.99276	3357	2	-0.152
DHTKD1	6	0.060843	0.16723	0.99276	3358	2	0.1853
C16orf58	6	0.060857	0.16727	0.99276	3359	3	0.3
GFRA3	6	0.060871	0.1673	0.99276	3360	3	0.0309
MEF2B	2	0.060875	0.10706	0.97102	3361	2	0.6901
DHRS7	6	0.060886	0.16734	0.99276	3362	3	0.3134
C9orf171	6	0.060958	0.16748	0.99276	3363	4	0.297
PGBD1	6	0.060966	0.16749	0.99276	3364	2	-0.119
CR1	6	0.060986	0.16754	0.99276	3365	4	0.3359
TCHH	6	0.061008	0.1676	0.99276	3366	4	0.3716
GATSL3	6	0.061034	0.16765	0.99283	3367	1	0.0745
LOC402160	6	0.061188	0.16796	0.99298	3368	4	0.4309
OTOF	6	0.061225	0.16804	0.99298	3369	4	0.3904
CACNA1G	6	0.061232	0.16806	0.99298	3370	3	0.1043
ANXA13	6	0.061282	0.16817	0.99298	3371	3	0.2676
RORC	5	0.061282	0.16444	0.99276	3372	1	-0.098
hsa-mir-144	4	0.0613	0.14324	0.98083	3373	3	0.7165
CLEC10A	6	0.061301	0.1682	0.99298	3374	4	0.5839
LRRC73	6	0.061301	0.1682	0.99298	3375	2	0.062
ZMYM1	6	0.061317	0.16824	0.99298	3376	4	0.3948
PRKAR1A	6	0.061331	0.16827	0.99298	3377	1	-0.585
NHSL1	6	0.061332	0.16827	0.99298	3378	4	0.4729
ZNF549	6	0.061339	0.16829	0.99298	3379	4	0.4145
C12orf79	5	0.06134	0.16453	0.99276	3380	2	-0.127
C6orf132	6	0.061405	0.16843	0.99298	3381	4	0.4468
GBP5	6	0.06143	0.16847	0.99298	3382	4	0.588
VPS33B	6	0.061431	0.16848	0.99298	3383	3	0.1913
C1orf186	6	0.061452	0.16852	0.99298	3384	4	0.3097
PHLDA2	6	0.061452	0.16852	0.99298	3385	4	0.4335
INTU	6	0.061452	0.16852	0.99298	3386	4	0.4049
NRDE2	6	0.061487	0.16859	0.99298	3387	4	0.4982
C5orf30	6	0.061511	0.16864	0.99298	3388	4	0.423
ARL6IP1	6	0.061535	0.16869	0.99298	3389	4	0.3085
RGS19	6	0.061568	0.16877	0.99298	3390	2	-0.744
RBM18	6	0.061578	0.16879	0.99298	3391	4	0.3721
ZNF165	6	0.061582	0.1688	0.99298	3392	4	0.4551
MYL10	6	0.06169	0.16903	0.99382	3393	4	0.3762
ZNF382	6	0.061744	0.16914	0.99382	3394	3	0.5608
UNC5C	6	0.061782	0.16922	0.99382	3395	3	0.4244

TNFSF13B	6	0.0618	0.16927	0.99382	3396	4	0.2947
PSME4	6	0.0618	0.16927	0.99382	3397	3	0.241
RPL19	6	0.061966	0.16963	0.99382	3398	4	0.3361
RTN4R	6	0.061994	0.16969	0.99382	3399	3	0.1316
MIS18BP1	6	0.062007	0.16972	0.99382	3400	2	0.1831
C12orf73	6	0.062023	0.16975	0.99382	3401	2	-0.495
LIMK2	6	0.062024	0.16975	0.99382	3402	3	0.2638
FAM110A	6	0.062072	0.16986	0.99382	3403	3	-0.019
CPLX3	6	0.062076	0.16987	0.99382	3404	2	-0.217
DSCR4	6	0.062076	0.16987	0.99382	3405	2	-0.051
PDLIM2	6	0.062121	0.16996	0.99382	3406	1	-0.061
C5orf22	6	0.062124	0.16997	0.99382	3407	3	0.2673
ZNF644	6	0.062186	0.1701	0.99382	3408	3	0.1251
LRRN2	6	0.0622	0.17013	0.99382	3409	3	0.1474
hsa-mir-3135b	4	0.062227	0.14507	0.98295	3410	2	0.2553
PSMG4	6	0.06227	0.17027	0.99382	3411	2	0.3841
TUBB2B	5	0.062273	0.16604	0.99276	3412	2	0.0798
HSPA1A	5	0.062273	0.16604	0.99276	3413	2	-0.419
HIST1H2BA	6	0.062279	0.17029	0.99382	3414	3	0.1745
ANKRD33B	6	0.062279	0.17029	0.99382	3415	4	0.5055
FRY	6	0.062289	0.17031	0.99382	3416	2	-0.454
hsa-mir-4718	4	0.06234	0.14529	0.98296	3417	2	0.4154
YBEY	6	0.062368	0.17048	0.99382	3418	3	-0.112
C1GALT1C1	6	0.062379	0.1705	0.99382	3419	3	0.1598
PLEKHA4	6	0.062379	0.1705	0.99382	3420	3	0.2232
CLDN20	6	0.062418	0.17059	0.99382	3421	3	-0.2
GPANK1	6	0.06243	0.17061	0.99382	3422	3	0.3045
ECSCR	6	0.062434	0.17062	0.99382	3423	3	0.4297
RGS6	6	0.062463	0.17067	0.99382	3424	3	0.2457
ITIH3	6	0.062463	0.17067	0.99382	3425	3	0.4386
CYLD	6	0.062516	0.17079	0.99382	3426	2	-0.35
MLANA	6	0.062529	0.17082	0.99382	3427	4	0.3517
BAGE	4	0.062533	0.14563	0.98296	3428	3	0.3263
GPR126	6	0.062544	0.17086	0.99382	3429	2	-0.084
EXOSC8	6	0.062566	0.1709	0.99382	3430	4	0.4689
SMO	6	0.062615	0.171	0.99382	3431	2	0.0776
KMT2A	4	0.062618	0.14579	0.98296	3432	2	0.4441
CHKA	6	0.06262	0.17101	0.99382	3433	2	-0.825
TMEM182	6	0.062628	0.17103	0.99382	3434	4	0.4172
ABCA4	6	0.062763	0.17131	0.99517	3435	3	0.2446
AREL1	4	0.062804	0.14613	0.98296	3436	2	0.3216
MRPS14	6	0.062826	0.17143	0.99543	3437	4	0.3239
CEACAM6	6	0.062837	0.17145	0.99543	3438	3	0.3356
BTG3	6	0.062878	0.17155	0.99568	3439	4	0.317
hsa-mir-6776	4	0.062887	0.14629	0.98296	3440	2	0.0997
GHSR	6	0.062896	0.17159	0.99568	3441	3	0.2066
CPA3	6	0.062924	0.17164	0.99571	3442	3	0.458
KRTAP4-9	4	0.063003	0.14652	0.98296	3443	1	-2.094
MVB12A	6	0.063007	0.17182	0.99589	3444	3	0.2056
MLLT3	6	0.06301	0.17182	0.99589	3445	3	0.282
PTGES	6	0.063039	0.17188	0.99596	3446	3	0.4071
hsa-mir-4508	4	0.063069	0.14665	0.98296	3447	2	0.7082
TIMM50	6	0.063109	0.17203	0.99654	3448	2	-0.025
ITGAX	6	0.063138	0.1721	0.99661	3449	3	0.2744
P2RX1	6	0.063158	0.17214	0.99661	3450	2	-0.151
ALG2	6	0.063187	0.1722	0.99661	3451	3	0.2316
ACTN4	6	0.063207	0.17224	0.99661	3452	3	0.2596
ELOF1	6	0.063247	0.17233	0.99671	3453	4	0.4297
PTPRF	6	0.063257	0.17235	0.99671	3454	2	-0.404
hsa-mir-4539	3	0.063257	0.13416	0.9764	3455	2	0.3284
TPI1	6	0.063279	0.1724	0.99671	3456	4	0.375
KIAA1468	6	0.063334	0.17251	0.99706	3457	4	0.4537
C20orf24	3	0.063352	0.13428	0.9764	3458	2	0.5718
FBXO33	6	0.063367	0.17258	0.99709	3459	3	0.1428
COQ4	6	0.063436	0.17273	0.99722	3460	3	0.1912
SPSB1	6	0.063454	0.17276	0.99722	3461	2	0.0251
RMND1	6	0.063482	0.17282	0.99722	3462	4	0.3455
NAT2	6	0.063503	0.17286	0.99722	3463	3	0.2251
PSEN2	6	0.063514	0.17288	0.99722	3464	4	0.4122
ST7L	6	0.063595	0.17304	0.9976	3465	2	-0.09
ZFP91	6	0.063627	0.17311	0.9976	3466	3	0.0357
PPP1R13L	6	0.063679	0.17322	0.9976	3467	2	-0.081
PUM1	6	0.063701	0.17327	0.9976	3468	4	0.4262
OR13D1	6	0.063748	0.17337	0.9976	3469	2	-0.237
THBS2	6	0.06375	0.17338	0.9976	3470	1	-0.153
CUBN	6	0.063762	0.1734	0.9976	3471	3	0.352
TLE6	6	0.063769	0.17341	0.9976	3472	4	0.2585
TMEM167B	6	0.063779	0.17343	0.9976	3473	4	0.2797
GALE	6	0.063799	0.17348	0.99761	3474	2	0.0981
KLRF1	6	0.063877	0.17367	0.99801	3475	3	0.4079
hsa-mir-212	4	0.063886	0.1482	0.98311	3476	3	0.5171
NUP133	6	0.063894	0.1737	0.99801	3477	2	0.0379
RORA	6	0.063922	0.17376	0.99805	3478	3	0.2099
C9orf78	6	0.063961	0.17384	0.99805	3479	4	0.3349
MAP2K5	6	0.063967	0.17385	0.99805	3480	4	0.2793

FAM65A	6	0.063997	0.1739	0.99806	3481	1	-0.051
SLC30A8	6	0.064051	0.17402	0.99842	3482	4	0.3539
ABHD15	6	0.064095	0.1741	0.99842	3483	1	0.0692
SLC46A1	6	0.064103	0.17411	0.99842	3484	4	0.3912
CST4	3	0.064203	0.1354	0.9764	3485	2	0.734
KCNJ14	6	0.064255	0.17443	0.99919	3486	3	0.417
LOC388849	6	0.064255	0.17443	0.99919	3487	4	0.403
TCP10L	6	0.064262	0.17445	0.99919	3488	4	0.2508
hsa-mir-3135a	4	0.064325	0.1491	0.98311	3489	2	0.2886
HNF4G	6	0.064332	0.1746	0.99919	3490	4	0.3685
HMGCL	6	0.064342	0.17461	0.99919	3491	2	-0.11
H2AFY2	6	0.064366	0.17466	0.99919	3492	4	0.4158
GGCX	6	0.064474	0.17488	0.99919	3493	4	0.2997
NPC1L1	6	0.064539	0.17502	0.99919	3494	4	0.3248
PRPF8	6	0.064561	0.17506	0.99919	3495	4	0.3592
MTFR2	6	0.064601	0.17515	0.99919	3496	4	0.4657
TEC	6	0.064627	0.17521	0.99919	3497	4	0.3692
YEATS2	6	0.064676	0.17532	0.99919	3498	4	0.3266
DPP10	6	0.064698	0.17536	0.99919	3499	4	0.418
EXD1	6	0.064723	0.17541	0.99919	3500	2	-0.38
ZNFX1	6	0.064738	0.17545	0.99919	3501	4	0.3798
PPP1R13B	6	0.064785	0.17554	0.99919	3502	3	0.1426
SLC44A1	6	0.064818	0.1756	0.99919	3503	3	0.2761
C1orf50	6	0.064884	0.17574	0.99919	3504	2	0.0013
MIS18A	6	0.064884	0.17574	0.99919	3505	3	0.2774
PRAMEF7	4	0.064925	0.15026	0.98459	3506	3	0.4437
CYB5R4	6	0.064933	0.17585	0.99919	3507	3	0.2007
TRAF3IP2	6	0.064934	0.17585	0.99919	3508	4	0.3706
VAT1	6	0.064975	0.17592	0.99919	3509	2	-0.324
OAF	6	0.064982	0.17593	0.99919	3510	3	0.0175
MBNL1	6	0.065031	0.17604	0.99919	3511	2	-0.132
GRB7	6	0.065081	0.17614	0.99919	3512	1	-0.178
OR2J2	6	0.065094	0.17616	0.99919	3513	3	0.1158
HLA-DRB5	6	0.065094	0.17616	0.99919	3514	4	0.409
HAPLN3	6	0.065129	0.17624	0.99919	3515	2	-0.098
CELF5	6	0.06513	0.17624	0.99919	3516	3	0.3161
hsa-mir-4480	4	0.065151	0.1507	0.98508	3517	1	-0.33
ATP5D	6	0.065171	0.17632	0.99919	3518	3	0.2323
CDC42EP4	6	0.065179	0.17634	0.99919	3519	3	0.2325
KLHDC4	6	0.065189	0.17636	0.99919	3520	4	0.3919
GSTO1	6	0.065221	0.17644	0.99919	3521	3	0.2768
PROP1	6	0.065222	0.17644	0.99919	3522	4	0.4386
ZNF491	6	0.065234	0.17646	0.99919	3523	2	-0.286
ERO1L	6	0.065278	0.17655	0.99919	3524	3	0.0021
hsa-mir-4481	4	0.065384	0.15116	0.98538	3525	3	0.4963
CFI	6	0.065475	0.17695	0.99919	3526	3	0.2652
COX8A	6	0.065483	0.17696	0.99919	3527	3	0.2209
EIF3H	6	0.065524	0.17706	0.99919	3528	3	0.2541
R3HDM4	6	0.065549	0.17712	0.99919	3529	4	0.4713
ATAD3A	6	0.065573	0.17717	0.99919	3530	2	-0.071
RPS3	6	0.065582	0.17719	0.99919	3531	4	0.4253
SLC43A3	6	0.065599	0.17723	0.99919	3532	2	-0.101
ECM1	6	0.065676	0.17739	0.99919	3533	3	0.0655
ESPL1	6	0.065694	0.17743	0.99919	3534	3	0.0894
BMP2	6	0.06577	0.17759	0.99919	3535	3	0.2026
CALB2	6	0.065811	0.17766	0.99919	3536	3	0.1835
SEC11C	6	0.065854	0.17776	0.99919	3537	4	0.5644
ACADSB	6	0.065868	0.17779	0.99919	3538	2	-0.057
PDE5A	6	0.06588	0.17781	0.99919	3539	4	0.3737
TLK1	6	0.065901	0.17785	0.99919	3540	3	0.1983
KIR2DL1	4	0.06591	0.15216	0.98619	3541	2	0.2602
TSPY3	1	0.065942	0.066001	0.95667	3542	1	0.795
EIF2S1	6	0.065978	0.17801	0.99919	3543	3	0.5265
OR1F1	6	0.066031	0.17813	0.99919	3544	3	0.3413
DESI1	6	0.066044	0.17816	0.99919	3545	3	0.2175
ABCB8	6	0.066061	0.1782	0.99919	3546	4	0.3588
OR2M2	6	0.06609	0.17826	0.99919	3547	1	-0.908
SC5D	6	0.06609	0.17826	0.99919	3548	1	-0.174
LCA5	6	0.066119	0.17831	0.99919	3549	3	0.2479
CCDC37	6	0.066182	0.17845	0.99919	3550	2	-0.395
hsa-mir-1302-7	4	0.06624	0.15275	0.98685	3551	2	0.4155
GPX7	6	0.066262	0.17862	0.99919	3552	2	0.0571
hsa-mir-1278	3	0.066271	0.13818	0.97668	3553	1	-0.101
C14orf79	6	0.066286	0.17867	0.99919	3554	3	0.2396
PPARD	6	0.066295	0.1787	0.99919	3555	3	0.0806
ATP5S	6	0.066311	0.17873	0.99919	3556	2	-0.436
HEXDC	6	0.066373	0.17886	0.99919	3557	2	-0.301
ERMAP	6	0.06641	0.17893	0.99919	3558	1	-0.138
TUFT1	6	0.066416	0.17893	0.99919	3559	4	0.3837
LTBP1	6	0.066426	0.17895	0.99919	3560	4	0.4591
hsa-let-7d	4	0.066428	0.1531	0.98782	3561	2	0.2335
FAM212A	6	0.066459	0.17903	0.99919	3562	2	-0.068
OR9A2	6	0.06649	0.17908	0.99919	3563	4	0.3489
TMEM216	6	0.066508	0.17911	0.99919	3564	3	0.0186
SLC30A1	6	0.066513	0.17913	0.99919	3565	4	0.3368

hsa-mir-3914-1	1	0.066533	0.066618	0.95667	3566	1	0.7911
DDX19A	6	0.066535	0.17917	0.99919	3567	2	-0.099
RHOG	6	0.066557	0.17922	0.99919	3568	3	0.1306
SPTB	6	0.066598	0.17931	0.99919	3569	2	0.0627
NTF4	6	0.066603	0.17932	0.99919	3570	4	0.4188
PRSS21	6	0.066603	0.17932	0.99919	3571	3	0.3616
SYNJ1	6	0.066607	0.17933	0.99919	3572	2	0.0539
RBMXL1	6	0.066616	0.17935	0.99919	3573	4	0.4435
MRGPRG	6	0.066625	0.17936	0.99919	3574	3	0.3267
HOXA3	6	0.066649	0.17941	0.99919	3575	4	0.4205
GSTCD	6	0.066656	0.17942	0.99919	3576	3	0.2033
TACC3	6	0.066689	0.17949	0.99919	3577	4	0.4016
C1orf101	6	0.066704	0.17951	0.99919	3578	2	0.1699
ELOVL3	6	0.066705	0.17951	0.99919	3579	3	0.3448
HAUS7	6	0.066711	0.17952	0.99919	3580	4	0.3923
THEM6	6	0.066747	0.1796	0.99919	3581	3	0.4236
PARP9	6	0.066762	0.17963	0.99919	3582	4	0.3539
HLA-A	6	0.066926	0.17998	0.99919	3583	2	-0.113
RPRD2	6	0.066933	0.17998	0.99919	3584	4	0.4577
EHD2	6	0.066972	0.18007	0.99919	3585	4	0.4497
ZNF746	6	0.066986	0.1801	0.99919	3586	4	0.337
STEAP1B	6	0.067	0.18014	0.99919	3587	2	-0.742
RBM15B	6	0.067009	0.18016	0.99919	3588	4	0.3229
SPATA20	6	0.067051	0.18023	0.99919	3589	3	0.1586
HIST1H4B	6	0.067115	0.18038	0.99919	3590	4	0.4574
ARHGEF4	6	0.067119	0.18039	0.99919	3591	4	0.2897
TSGA13	6	0.06719	0.18053	0.99919	3592	3	0.2874
NEXN	6	0.067197	0.18054	0.99919	3593	3	0.1586
LRFN4	6	0.067216	0.18059	0.99919	3594	4	0.2988
NR5A1	6	0.067235	0.18063	0.99919	3595	3	0.301
PLGLB1	1	0.067253	0.067326	0.95667	3596	1	1.202
CBLN3	6	0.067256	0.18068	0.99919	3597	3	0.2633
XPO4	6	0.067286	0.18074	0.99919	3598	4	0.487
CHEK2	6	0.067299	0.18077	0.99919	3599	2	-0.491
CD58	6	0.06732	0.18081	0.99919	3600	2	-0.097
FLCN	6	0.06732	0.18081	0.99919	3601	2	0.0527
NSUN2	6	0.067334	0.18085	0.99919	3602	2	-0.028
ZNF691	6	0.067343	0.18086	0.99919	3603	4	0.3313
TRPM7	6	0.067393	0.18097	0.99919	3604	1	-0.196
KBTBD4	6	0.067409	0.181	0.99919	3605	3	0.2589
STPG1	6	0.06741	0.181	0.99919	3606	4	0.3254
SPDL1	6	0.067442	0.18106	0.99919	3607	3	0.3995
NSUN6	6	0.067492	0.18117	0.99919	3608	1	-0.137
LARP4	6	0.067588	0.18137	0.99919	3609	3	0.0531
CYCS	6	0.067611	0.18142	0.99919	3610	4	0.4154
F2RL1	6	0.067662	0.18151	0.99919	3611	3	0.4019
KIRREL2	6	0.067689	0.18157	0.99919	3612	3	0.3389
ONECUT3	6	0.067713	0.18162	0.99919	3613	2	-0.201
SETD1A	6	0.067715	0.18162	0.99919	3614	3	0.4601
ARGFX	6	0.067794	0.18179	0.99919	3615	4	0.338
VPS35	6	0.067811	0.18183	0.99919	3616	3	0.4005
PLEKHM1	6	0.067826	0.18187	0.99919	3617	4	0.3255
HIGD1B	6	0.06786	0.18193	0.99919	3618	2	-0.273
FGF21	6	0.067881	0.18198	0.99919	3619	2	-0.068
hsa-mir-6504	4	0.067889	0.15585	0.99153	3620	2	0.1638
C11orf63	6	0.067895	0.182	0.99919	3621	3	0.0011
GPR107	6	0.067908	0.18204	0.99919	3622	3	0.0224
FAM192A	6	0.067934	0.18209	0.99919	3623	2	-0.095
CATSPERG	6	0.067952	0.18213	0.99919	3624	2	0.092
FN1	6	0.067968	0.18216	0.99919	3625	4	0.2778
KIAA0146	2	0.067973	0.11869	0.9754	3626	2	0.4347
LOC100506388	6	0.067983	0.18218	0.99919	3627	2	-0.261
CFLAR	6	0.068016	0.18225	0.99919	3628	3	0.2083
SMIM14	6	0.068032	0.18228	0.99919	3629	3	0.2106
TP53	6	0.068035	0.18228	0.99919	3630	4	0.3535
FGD6	6	0.068109	0.18244	0.99919	3631	4	0.2908
PHTF2	6	0.06813	0.18249	0.99919	3632	4	0.2516
PDE4C	6	0.068275	0.18278	0.99919	3633	4	0.3954
C11orf21	6	0.068277	0.18278	0.99919	3634	4	0.3553
ITGB1BP2	6	0.068376	0.18299	0.99919	3635	2	0.0275
TCF19	6	0.068378	0.18299	0.99919	3636	3	0.2972
CD180	6	0.06838	0.183	0.99919	3637	4	0.3646
SLC12A3	6	0.068425	0.18308	0.99919	3638	2	0.1914
BAHD1	6	0.068453	0.18314	0.99919	3639	3	0.3539
ZCCHC12	6	0.068453	0.18314	0.99919	3640	4	0.4324
PLCG2	6	0.068474	0.18318	0.99919	3641	2	0.1144
COX5A	6	0.068554	0.18336	0.99919	3642	3	0.1362
IGSF11	6	0.068572	0.18339	0.99919	3643	1	-0.644
ASRGL1	6	0.06861	0.18347	0.99919	3644	4	0.3201
COTL1	6	0.068622	0.1835	0.99919	3645	2	0.0905
SCN10A	6	0.068643	0.18354	0.99919	3646	3	0.2331
RPLP2	6	0.068644	0.18354	0.99919	3647	2	-0.125
SRD5A3	6	0.068644	0.18354	0.99919	3648	3	0.119
VIMP	6	0.068671	0.1836	0.99919	3649	1	-0.06
LTA4H	6	0.0687	0.18366	0.99919	3650	3	0.2836

PTMA	6	0.06872	0.1837	0.99919	3651	2	-0.408
HIBADH	6	0.068737	0.18373	0.99919	3652	3	0.168
FBXO15	6	0.068772	0.18381	0.99919	3653	4	0.2693
STMN2	6	0.068818	0.1839	0.99919	3654	3	0.3081
SLC26A10	6	0.068834	0.18393	0.99919	3655	4	0.324
LSM12	6	0.068867	0.184	0.99919	3656	4	0.4043
MROH9	6	0.068877	0.18403	0.99919	3657	3	0.2176
NAA15	6	0.068918	0.18411	0.99919	3658	4	0.4086
RNF219	6	0.068951	0.18418	0.99919	3659	2	0.083
SKIL	6	0.068951	0.18418	0.99919	3660	3	0.2002
FASTKD1	6	0.068951	0.18418	0.99919	3661	3	-0.095
CCDC24	6	0.068956	0.18419	0.99919	3662	4	0.3416
NME7	6	0.068956	0.18419	0.99919	3663	3	0.1747
MYRIP	6	0.069014	0.18432	0.99919	3664	4	0.338
STS	6	0.069015	0.18432	0.99919	3665	3	0.1345
ATE1	6	0.069036	0.18436	0.99919	3666	3	0.2381
INSR	6	0.069044	0.18437	0.99919	3667	4	0.3097
FMO3	6	0.069107	0.18449	0.99919	3668	4	0.3252
WASL	6	0.069133	0.18455	0.99919	3669	3	-0.119
MON1B	6	0.069151	0.18458	0.99919	3670	3	0.1595
GLRA3	6	0.069187	0.18467	0.99919	3671	3	0.1459
CCDC103	6	0.06926	0.18481	0.99919	3672	2	-0.153
PPP1R1A	6	0.069303	0.1849	0.99919	3673	4	0.3255
HDAC11	6	0.069382	0.18506	0.99919	3674	3	0.2403
ANKRD39	6	0.069395	0.18509	0.99919	3675	2	-0.447
KCNJ16	6	0.069456	0.18521	0.99919	3676	2	0.191
BBIP1	6	0.069484	0.18526	0.99919	3677	3	0.1058
CCDC170	6	0.069494	0.18529	0.99919	3678	4	0.3852
AGGF1	6	0.069505	0.18531	0.99919	3679	2	-0.025
IRX4	6	0.06951	0.18532	0.99919	3680	3	-0.03
RNF145	6	0.069533	0.18537	0.99919	3681	3	0.0671
FSCN2	6	0.069628	0.18555	0.99919	3682	2	0.0662
MTHFD2	6	0.069628	0.18555	0.99919	3683	2	0.1199
OR10S1	6	0.069646	0.18559	0.99919	3684	3	0.2545
MOV10L1	6	0.069662	0.18562	0.99919	3685	4	0.2665
FXYD6	4	0.069667	0.15924	0.99228	3686	2	-0.107
ARPC2	6	0.069668	0.18564	0.99919	3687	3	0.2656
SPPL2B	6	0.069672	0.18565	0.99919	3688	4	0.3029
TRIM13	6	0.069697	0.18569	0.99919	3689	3	0.1426
TSHB	6	0.069697	0.18569	0.99919	3690	4	0.3483
ZNF619	6	0.069725	0.18575	0.99919	3691	2	-0.121
NDUFA4L2	6	0.069747	0.1858	0.99919	3692	3	0.3768
ATP1A2	6	0.06975	0.1858	0.99919	3693	3	0.193
L2HGDH	6	0.069799	0.1859	0.99919	3694	3	0.2769
CACNA2D2	6	0.069833	0.18598	0.99919	3695	4	0.351
SFRP2	6	0.069893	0.18609	0.99919	3696	3	0.3448
HOXC5	6	0.069932	0.18618	0.99919	3697	4	0.4527
PLEKHS1	6	0.069995	0.18632	0.99919	3698	2	-0.097
FEZF2	6	0.070009	0.18634	0.99919	3699	4	0.3614
hsa-mir-3181	4	0.070009	0.15991	0.9924	3700	2	0.2658
OR4D10	6	0.070025	0.18639	0.99919	3701	4	0.4594
PTMS	6	0.070045	0.18643	0.99919	3702	2	0.0004
FAM129B	6	0.070073	0.18648	0.99919	3703	4	0.3388
MPPED1	6	0.070143	0.18664	0.99919	3704	3	-0.061
DAPK3	6	0.070169	0.18669	0.99919	3705	3	0.4288
SCNN1G	6	0.07018	0.18671	0.99919	3706	4	0.4575
ZNF827	6	0.070216	0.18678	0.99919	3707	3	0.2374
THY1	6	0.070216	0.18678	0.99919	3708	2	-0.284
FXYD5	6	0.070283	0.18692	0.99919	3709	4	0.2828
NPAT	6	0.070324	0.18701	0.99919	3710	4	0.324
PNLIPRP1	6	0.070339	0.18704	0.99919	3711	2	0.0226
hsa-mir-3682	4	0.070341	0.16053	0.99276	3712	2	0.3067
IARS	6	0.070388	0.18714	0.99919	3713	2	0.0065
ZNF697	6	0.070437	0.18726	0.99919	3714	1	-0.307
SP6	6	0.07051	0.18742	0.99919	3715	4	0.4286
MRPS22	6	0.07051	0.18742	0.99919	3716	4	0.462
RBM27	6	0.07051	0.18742	0.99919	3717	2	-0.238
GOLGA1	6	0.070532	0.18747	0.99919	3718	3	0.3102
RNASE6	6	0.070538	0.18748	0.99919	3719	4	0.3569
DDX60	6	0.070561	0.18752	0.99919	3720	2	0.1537
C4orf17	6	0.070567	0.18754	0.99919	3721	3	0.2615
C15orf32	6	0.070575	0.18755	0.99919	3722	2	0.0072
MDGA2	6	0.070584	0.18757	0.99919	3723	4	0.3753
MAGED2	6	0.070618	0.18765	0.99919	3724	3	-0.034
GPR89A	4	0.070643	0.16108	0.99276	3725	2	0.4002
ACOXL	6	0.070651	0.18771	0.99919	3726	4	0.3181
RGSL1	6	0.070724	0.18787	0.99919	3727	4	0.4715
RHOV	6	0.070731	0.18788	0.99919	3728	2	-0.195
CISD2	5	0.070737	0.17976	0.99919	3729	3	0.4574
JSRP1	6	0.070738	0.1879	0.99919	3730	3	0.2591
HCN3	6	0.070763	0.18794	0.99919	3731	2	-0.084
EIF4EBP1	6	0.07078	0.18798	0.99919	3732	2	-0.214
SUMF1	6	0.070824	0.18806	0.99919	3733	4	0.2754
DNAJB13	6	0.070829	0.18807	0.99919	3734	2	-0.196
RAB4A	6	0.070864	0.18814	0.99919	3735	3	0.0712

CD86	6	0.070873	0.18815	0.99919	3736	4	0.44
KIAA1324L	6	0.070878	0.18816	0.99919	3737	2	0.1618
PHGR1	6	0.070883	0.18818	0.99919	3738	4	0.3179
CDYL2	6	0.070917	0.18824	0.99919	3739	4	0.3286
CLCC1	6	0.070927	0.18826	0.99919	3740	2	-0.024
UQCRB	6	0.071001	0.18843	0.99919	3741	4	0.4038
WDR53	4	0.071048	0.16186	0.99276	3742	2	-0.023
hsa-mir-3183	4	0.071081	0.16192	0.99276	3743	2	-0.067
ROR1	6	0.071098	0.18864	0.99919	3744	2	-0.162
HSD17B14	6	0.071139	0.18873	0.99919	3745	2	0.1446
DTL	6	0.071153	0.18876	0.99919	3746	3	0.2718
ADAMTSL4	6	0.07117	0.1888	0.99919	3747	4	0.354
NBPF3	6	0.0712	0.18886	0.99919	3748	3	0.3052
NPR2	6	0.071219	0.1889	0.99919	3749	2	-0.289
SLC35B4	6	0.071221	0.1889	0.99919	3750	3	0.2084
MET	6	0.071241	0.18894	0.99919	3751	3	0.0064
ZNF43	6	0.071284	0.18903	0.99919	3752	3	0.2405
CST6	6	0.071294	0.18905	0.99919	3753	3	0.2225
CRIP2	6	0.071294	0.18905	0.99919	3754	3	0.2231
TAS2R7	6	0.071302	0.18907	0.99919	3755	4	0.3518
DBF4	5	0.071308	0.18067	0.99919	3756	3	0.4389
DNAJA4	6	0.071313	0.18909	0.99919	3757	2	-0.146
TRIM8	6	0.071344	0.18915	0.99919	3758	4	0.38
DDX31	6	0.071344	0.18915	0.99919	3759	4	0.5043
C7orf65	6	0.071349	0.18916	0.99919	3760	3	0.137
MAP9	6	0.071368	0.1892	0.99919	3761	2	0.0004
hsa-mir-129-1	4	0.071377	0.16248	0.99276	3762	3	0.2933
hsa-mir-548b	3	0.071388	0.14513	0.98295	3763	2	0.7686
DNAJC4	6	0.071414	0.18929	0.99919	3764	3	0.003
hsa-mir-605	4	0.071432	0.1626	0.99276	3765	3	0.5603
APOBEC3A	2	0.071439	0.12436	0.9764	3766	2	0.3952
PIAS1	6	0.071501	0.18946	0.99919	3767	3	0.3854
KIAA1432	6	0.071546	0.18954	0.99919	3768	4	0.5006
GTF2F1	4	0.071557	0.16281	0.99276	3769	2	0.1901
SSH1	6	0.071563	0.18958	0.99919	3770	4	0.4307
INSM2	6	0.071588	0.18963	0.99919	3771	2	-0.261
PANX2	6	0.071588	0.18963	0.99919	3772	1	-0.335
TXNL4B	6	0.071612	0.18967	0.99919	3773	4	0.3251
INTS12	6	0.071625	0.1897	0.99919	3774	3	-0.05
KANSL3	6	0.071678	0.18982	0.99919	3775	4	0.4941
LSM7	4	0.071701	0.1631	0.99276	3776	2	0.1424
GALNT1	6	0.071711	0.18989	0.99919	3777	3	-0.043
GEMIN5	6	0.07173	0.18992	0.99919	3778	4	0.421
SMARCD1	6	0.071748	0.18997	0.99919	3779	3	0.3784
MSL2	6	0.071787	0.19005	0.99919	3780	3	0.3011
COMMD2	6	0.071845	0.19018	0.99919	3781	4	0.3703
KPNA5	6	0.071845	0.19018	0.99919	3782	3	0.2268
TMEM47	6	0.07185	0.19018	0.99919	3783	2	-6E-04
MAFG	6	0.071877	0.19025	0.99919	3784	4	0.2357
hsa-mir-1976	4	0.071902	0.16348	0.99276	3785	3	0.6963
CCDC147	6	0.071906	0.19031	0.99919	3786	3	0.2798
TPT1	6	0.071918	0.19034	0.99919	3787	4	0.4878
HSPB6	6	0.071922	0.19035	0.99919	3788	2	-0.517
TRAPPC12	6	0.071936	0.19038	0.99919	3789	4	0.5399
PCDHGC3	2	0.071951	0.12518	0.9764	3790	2	0.3512
ZMAT2	6	0.071963	0.19044	0.99919	3791	3	0.2806
RTKN	6	0.071974	0.19046	0.99919	3792	4	0.3307
MGAT1	6	0.072002	0.19052	0.99919	3793	3	0.2104
DNHD1	6	0.072004	0.19052	0.99919	3794	2	0.0397
CWF19L2	6	0.07204	0.19059	0.99919	3795	3	-0.155
FRYL	4	0.072056	0.16379	0.99276	3796	3	0.5084
MPPED2	6	0.072217	0.19096	0.99919	3797	3	0.1605
CCDC71	6	0.072248	0.19103	0.99919	3798	3	0.2463
OTOG	6	0.072298	0.19113	0.99919	3799	3	0.2305
TRIM72	6	0.072301	0.19114	0.99919	3800	4	0.5028
H3F3B	6	0.072337	0.1912	0.99919	3801	3	0.1182
AGAP1	6	0.072352	0.19123	0.99919	3802	4	0.2444
KLRG2	4	0.072394	0.1644	0.99276	3803	2	0.1561
POTEC	4	0.072403	0.16442	0.99276	3804	2	-0.153
SMIM20	6	0.072407	0.19135	0.99919	3805	3	0.4474
TMEM70	6	0.072443	0.19141	0.99919	3806	4	0.3008
hsa-mir-4462	4	0.072461	0.16453	0.99276	3807	2	0.3052
OR52A5	6	0.07248	0.19149	0.99919	3808	3	0.0823
CACNB2	6	0.072521	0.19157	0.99919	3809	4	0.357
CHD2	6	0.072542	0.19161	0.99919	3810	4	0.3625
LTN1	6	0.072542	0.19161	0.99919	3811	4	0.3822
KLHL29	6	0.072543	0.19162	0.99919	3812	1	-0.091
GYS2	6	0.072543	0.19162	0.99919	3813	2	-0.151
hsa-mir-1470	4	0.072553	0.16471	0.99276	3814	2	0.5089
SEC61A2	6	0.072556	0.19164	0.99919	3815	3	0.2799
OR6C6	6	0.072578	0.19168	0.99919	3816	3	0.1629
hsa-mir-6075	4	0.072585	0.16478	0.99276	3817	3	0.4718
hsa-mir-1181	4	0.07262	0.16484	0.99276	3818	3	0.6388
CRYGN	6	0.072647	0.19183	0.99919	3819	4	0.3266
SCRN1	6	0.072672	0.19187	0.99919	3820	3	0.1341

ADRA1D	6	0.072694	0.19192	0.99919	3821	3	-0.035
ARHGAP12	6	0.072703	0.19194	0.99919	3822	4	0.2629
AGRP	6	0.07273	0.192	0.99919	3823	3	0.3304
RAB3GAP1	6	0.072738	0.19201	0.99919	3824	1	0.0329
PLCD3	6	0.072771	0.19208	0.99919	3825	4	0.4513
MB21D2	6	0.072787	0.1921	0.99919	3826	2	-0.061
DBH	6	0.072836	0.1922	0.99919	3827	3	0.0838
hsa-mir-4302	4	0.072842	0.16526	0.99276	3828	3	0.328
CLEC12A	6	0.072885	0.1923	0.99919	3829	3	0.0308
CCDC122	6	0.072934	0.19239	0.99919	3830	3	0.088
PCNX	6	0.072939	0.1924	0.99919	3831	4	0.269
GLUL	6	0.072994	0.19252	0.99919	3832	3	0.3894
TMEM161B	6	0.072995	0.19252	0.99919	3833	4	0.3931
CLCN6	6	0.072995	0.19252	0.99919	3834	4	0.2845
APOA2	6	0.073008	0.19255	0.99919	3835	3	-0.226
DPYSL4	6	0.073049	0.19264	0.99919	3836	3	0.2081
MAP3K13	6	0.073066	0.19267	0.99919	3837	4	0.3066
TAOK1	6	0.073097	0.19274	0.99919	3838	4	0.3859
MAPT	6	0.07314	0.19284	0.99919	3839	3	0.1982
FICD	6	0.073163	0.19288	0.99919	3840	3	0.3462
CCDC155	6	0.073227	0.19303	0.99919	3841	3	0.0975
PLEKHA6	6	0.073279	0.19312	0.99919	3842	3	0.0953
C6orf7	6	0.073336	0.19324	0.99919	3843	3	0.2859
FAM175B	6	0.073374	0.1933	0.99919	3844	1	-0.138
KIAA0319	6	0.073411	0.19338	0.99919	3845	2	-0.738
RNASE2	6	0.073419	0.19341	0.99919	3846	3	-0.024
SFXN2	6	0.073447	0.19346	0.99919	3847	3	0.1309
SLC26A6	6	0.07346	0.19349	0.99919	3848	3	0.1598
ERO1LB	6	0.073469	0.19351	0.99919	3849	2	0.064
OR8K3	6	0.073522	0.19362	0.99919	3850	4	0.4252
A1BG	6	0.073524	0.19362	0.99919	3851	3	0.2829
ZNF837	6	0.073583	0.19375	0.99919	3852	3	0.28
EEFSEC	4	0.073608	0.16666	0.99276	3853	1	-0.031
VAMP4	6	0.073619	0.19381	0.99919	3854	2	0.0142
RASGEF1B	6	0.073667	0.19393	0.99919	3855	2	-0.165
CLN6	6	0.073689	0.19397	0.99919	3856	3	0.1265
KRTAP5-7	5	0.073737	0.18464	0.99919	3857	2	-0.279
PKD2L2	6	0.073741	0.19408	0.99919	3858	2	0.1752
PIAS4	6	0.073748	0.19409	0.99919	3859	4	0.3375
RBBP8NL	6	0.073814	0.19422	0.99919	3860	3	0.2154
COL9A2	6	0.073843	0.19428	0.99919	3861	3	0.2044
PTPRE	6	0.073863	0.19432	0.99919	3862	3	0.2045
ADK	6	0.073865	0.19433	0.99919	3863	2	0.1059
KRT75	6	0.073908	0.19443	0.99919	3864	4	0.3967
INTS4	6	0.07399	0.19459	0.99919	3865	4	0.3826
GBP3	6	0.074065	0.19475	0.99919	3866	3	0.3975
ITGB3BP	5	0.074067	0.18513	0.99919	3867	3	0.4282
GPM6A	6	0.074068	0.19476	0.99919	3868	4	0.3894
OR56A1	6	0.074083	0.19479	0.99919	3869	3	0.28
EXOSC10	6	0.074086	0.1948	0.99919	3870	2	-0.44
hsa-mir-3659	4	0.074089	0.16755	0.99276	3871	3	0.638
hsa-mir-3648	4	0.074089	0.16755	0.99276	3872	3	0.8183
GALNT7	6	0.074157	0.19494	0.99919	3873	4	0.3553
YARS2	6	0.074217	0.19507	0.99919	3874	4	0.4818
DNAH14	6	0.074232	0.1951	0.99919	3875	4	0.2573
MYO1B	6	0.074254	0.19515	0.99919	3876	3	0.1366
hsa-mir-6759	4	0.074254	0.16783	0.99298	3877	2	0.2379
PIK3R6	6	0.074254	0.19515	0.99919	3878	2	-0.132
PNKP	6	0.074254	0.19515	0.99919	3879	2	0.0009
SIKE1	6	0.074387	0.19541	0.99919	3880	4	0.2371
BRF1	6	0.0744	0.19544	0.99919	3881	3	-0.018
PTPRQ	6	0.074421	0.19547	0.99919	3882	4	0.3167
S100A16	6	0.074439	0.19551	0.99919	3883	2	0.0021
SYVN1	6	0.074467	0.19557	0.99919	3884	4	0.28
VANGL2	6	0.074474	0.19558	0.99919	3885	1	-0.018
APOBEC2	6	0.074478	0.19559	0.99919	3886	4	0.3104
LGR4	6	0.074503	0.19565	0.99919	3887	4	0.3274
MOB1B	6	0.074511	0.19566	0.99919	3888	3	0.308
ZNF530	6	0.074542	0.19573	0.99919	3889	3	0.3248
GLTSCR1L	6	0.074544	0.19573	0.99919	3890	3	0.4061
GALNT11	6	0.074547	0.19574	0.99919	3891	1	-0.064
RIOK3	6	0.074571	0.19579	0.99919	3892	2	0.0507
SUV39H2	6	0.074595	0.19584	0.99919	3893	4	0.2833
CYB561	6	0.074596	0.19584	0.99919	3894	2	-0.194
SPPL2A	6	0.074638	0.19593	0.99919	3895	2	-0.198
MS4A8	6	0.074642	0.19593	0.99919	3896	4	0.3088
GALNT15	6	0.074645	0.19594	0.99919	3897	2	0.1
CNDP2	6	0.074667	0.19598	0.99919	3898	3	-0.262
PSMD14	6	0.074671	0.19599	0.99919	3899	4	0.4017
GPR123	6	0.074678	0.19601	0.99919	3900	3	0.2103
hsa-mir-1265	2	0.074708	0.12957	0.9764	3901	1	0.0648
FAM120B	6	0.074726	0.1961	0.99919	3902	3	0.1975
TCEANC2	6	0.074742	0.19614	0.99919	3903	4	0.6501
SLC4A1AP	6	0.074742	0.19614	0.99919	3904	4	0.3738
TAC1	6	0.074791	0.19623	0.99919	3905	3	-0.121

KCNAB1	6	0.074828	0.19631	0.99919	3906	4	0.3755
KRTAP9-1	6	0.074853	0.19637	0.99919	3907	4	0.4112
PPP1R21	6	0.074859	0.19639	0.99919	3908	2	0.0826
RHPN2	6	0.074889	0.19644	0.99919	3909	2	0.0734
PLA2G1B	6	0.074938	0.19655	0.99919	3910	3	0.0435
NUDT7	6	0.074964	0.19661	0.99919	3911	3	0.3279
C19orf81	6	0.074968	0.19661	0.99919	3912	4	0.3445
KMO	6	0.074986	0.19665	0.99919	3913	4	0.4561
BPGM	6	0.074986	0.19665	0.99919	3914	3	0.2174
SMARCC1	6	0.07501	0.19669	0.99919	3915	4	0.4176
PABPN1L	6	0.075021	0.1967	0.99919	3916	4	0.5132
HSPH1	6	0.075043	0.19675	0.99919	3917	2	-0.117
IPO11	6	0.075061	0.19679	0.99919	3918	4	0.3183
HIVEP3	6	0.075079	0.19683	0.99919	3919	4	0.3489
COL12A1	6	0.075133	0.19693	0.99919	3920	2	0.0338
ARF3	6	0.075136	0.19694	0.99919	3921	4	0.4028
PDCL3	6	0.075168	0.19701	0.99919	3922	4	0.3848
NARG2	6	0.075215	0.19711	0.99919	3923	4	0.3896
SLC24A1	6	0.075215	0.19711	0.99919	3924	4	0.4407
SLX4	6	0.075215	0.19711	0.99919	3925	4	0.3379
CASP10	6	0.075262	0.19722	0.99919	3926	4	0.3403
LOC729020	6	0.075265	0.19722	0.99919	3927	2	-0.265
IFI27L2	6	0.075274	0.19724	0.99919	3928	3	0.1299
TPM3	6	0.075279	0.19725	0.99919	3929	2	-0.314
SLC9C2	6	0.075291	0.19728	0.99919	3930	4	0.5145
MESDC1	6	0.075291	0.19728	0.99919	3931	4	0.3643
TIMM17A	6	0.075316	0.19733	0.99919	3932	4	0.3977
HTRA4	6	0.075398	0.1975	0.99919	3933	2	-0.123
LPAR5	6	0.075435	0.19758	0.99919	3934	3	0.2049
OSBPL6	6	0.075438	0.19759	0.99919	3935	4	0.4922
hsa-mir-593	4	0.075448	0.1701	0.99382	3936	3	0.4754
RPS6KA4	6	0.075463	0.19763	0.99919	3937	4	0.3402
GGA1	6	0.075469	0.19764	0.99919	3938	3	0.2593
IPCEF1	5	0.075476	0.18748	0.99919	3939	1	-0.112
hsa-mir-6769b	4	0.075476	0.17016	0.99382	3940	1	0.0875
MRPL54	6	0.075487	0.19768	0.99919	3941	4	0.3146
NSFL1C	6	0.075509	0.19773	0.99919	3942	2	-0.016
ZNF446	6	0.075523	0.19776	0.99919	3943	3	0.3615
IFFO1	6	0.075557	0.19783	0.99919	3944	4	0.563
USP1	6	0.075628	0.19799	0.99919	3945	3	0.292
SLC25A42	6	0.075701	0.19815	0.99919	3946	3	0.2755
ZNF91	5	0.075735	0.18789	0.99919	3947	3	0.3186
hsa-mir-1914	4	0.075735	0.17066	0.99382	3948	2	0.2938
ACAA2	6	0.075744	0.19824	0.99919	3949	4	0.3337
COQ3	6	0.075769	0.1983	0.99919	3950	4	0.3164
SPAG9	6	0.075795	0.19835	0.99919	3951	4	0.3227
ASIC5	6	0.075816	0.19839	0.99919	3952	2	0.1323
SLC5A3	6	0.07582	0.19839	0.99919	3953	4	0.2499
TMEM232	6	0.075835	0.19843	0.99919	3954	3	0.2218
CHID1	6	0.075865	0.19849	0.99919	3955	3	0.214
FBXO32	6	0.075902	0.19857	0.99919	3956	2	-0.178
IGSF23	6	0.075938	0.19862	0.99919	3957	2	0.046
C2orf73	6	0.075938	0.19862	0.99919	3958	3	-0.032
TMEM105	6	0.075939	0.19863	0.99919	3959	4	0.2929
PDE8A	6	0.075997	0.19874	0.99919	3960	4	0.2732
ZNF429	6	0.075998	0.19874	0.99919	3961	2	0.0271
UTP3	6	0.076011	0.19878	0.99919	3962	2	0.2289
BRDT	6	0.076015	0.19879	0.99919	3963	4	0.2781
APIP	6	0.076028	0.19882	0.99919	3964	2	-0.064
RPIA	6	0.076028	0.19882	0.99919	3965	4	0.374
CSF1	6	0.076055	0.19886	0.99919	3966	4	0.428
KIF4B	6	0.076065	0.19888	0.99919	3967	2	0.0432
INPP5K	6	0.076111	0.19898	0.99919	3968	3	0.2565
PAFAH2	6	0.076138	0.19903	0.99919	3969	4	0.2428
IKZF1	6	0.07628	0.19932	0.99919	3970	3	0.3415
PHOX2A	4	0.076319	0.17171	0.99578	3971	3	0.4779
GPRC6A	6	0.076328	0.19942	0.99919	3972	2	0.0818
C15orf43	6	0.07633	0.19942	0.99919	3973	3	-0.077
ACAP1	6	0.076339	0.19945	0.99919	3974	3	0.1275
MTERFD2	6	0.076366	0.19951	0.99919	3975	4	0.2778
RGS17	6	0.076423	0.19962	0.99919	3976	4	0.3038
CRB1	6	0.07645	0.19967	0.99919	3977	3	0.1048
TKTL1	6	0.076526	0.19981	0.99919	3978	4	0.4501
ELMO1	6	0.07655	0.19986	0.99919	3979	3	0.005
FBP2	6	0.076573	0.19991	0.99919	3980	3	0.3351
NPC2	6	0.076596	0.19996	0.99919	3981	2	0.0846
FRZB	6	0.076638	0.20004	0.99919	3982	4	0.3146
DPY19L2	6	0.076645	0.20006	0.99919	3983	2	-0.066
FLT1	6	0.076645	0.20006	0.99919	3984	4	0.227
COX16	6	0.076648	0.20006	0.99919	3985	3	0.1735
INTS9	6	0.076719	0.20022	0.99919	3986	2	0.3372
DEFB127	6	0.076743	0.20026	0.99919	3987	3	0.1584
TPSAB1	6	0.076764	0.2003	0.99919	3988	3	0.2621
CASP14	6	0.076784	0.20034	0.99919	3989	3	0.2405
LOC100507462	5	0.076821	0.18962	0.99919	3990	3	0.2956

CELSR3	6	0.076824	0.20044	0.99919	3991	4	0.431
HSF4	6	0.076831	0.20045	0.99919	3992	3	0.2808
SRRM1	6	0.076889	0.20055	0.99919	3993	3	0.1764
ABCC11	6	0.076913	0.20061	0.99919	3994	3	-0.12
CRISP2	6	0.076919	0.20062	0.99919	3995	3	-0.189
TLDC2	6	0.076937	0.20065	0.99919	3996	4	0.2803
OR8B12	6	0.076938	0.20066	0.99919	3997	3	0.2033
CASP12	6	0.076948	0.20068	0.99919	3998	4	0.3716
PTPRO	6	0.076965	0.20072	0.99919	3999	2	0.077
TCAIM	6	0.076965	0.20072	0.99919	4000	3	0.1483
KCNK6	6	0.077002	0.20078	0.99919	4001	3	0.2457
hsa-mir-7853	2	0.077033	0.1333	0.9764	4002	2	0.4586
GDE1	6	0.077084	0.20095	0.99919	4003	3	0.2004
ERLEC1	6	0.077084	0.20095	0.99919	4004	2	0.1476
CFHR3	6	0.077101	0.20099	0.99919	4005	4	0.3338
hsa-mir-548am	1	0.077134	0.077202	0.96053	4006	1	2.1024
GFRAL	6	0.077181	0.20115	0.99919	4007	3	0.1
FGF17	6	0.077189	0.20117	0.99919	4008	2	-0.29
hsa-mir-1272	4	0.077213	0.17337	0.9976	4009	2	0.2459
PGM2L1	6	0.077232	0.20125	0.99919	4010	4	0.3002
OR5AK2	6	0.077232	0.20125	0.99919	4011	4	0.5332
NRP1	6	0.077286	0.20135	0.99919	4012	2	-0.006
POLR2G	6	0.077327	0.20144	0.99919	4013	2	-0.51
NUAK2	6	0.077334	0.20146	0.99919	4014	4	0.4396
BZW2	4	0.077342	0.17363	0.99801	4015	3	0.5541
TMC3	6	0.077376	0.20156	0.99919	4016	1	-0.026
OR10A2	6	0.077398	0.2016	0.99919	4017	3	0.1633
TUBE1	6	0.077408	0.20162	0.99919	4018	4	0.3506
SYS1	6	0.077412	0.20163	0.99919	4019	3	0.3094
NCAPG	6	0.077425	0.20166	0.99919	4020	3	0.3408
NFATC3	6	0.077433	0.20167	0.99919	4021	4	0.2887
LRRCC1	6	0.077457	0.20172	0.99919	4022	2	0.13
ASAH2	6	0.077473	0.20175	0.99919	4023	1	-0.396
GNB1	6	0.077525	0.20185	0.99919	4024	4	0.2709
GPR65	6	0.077547	0.20189	0.99919	4025	3	0.1113
DNPEP	6	0.077547	0.20189	0.99919	4026	4	0.34
PRKAG2	6	0.077572	0.20194	0.99919	4027	4	0.25
LAMB2	6	0.077596	0.20199	0.99919	4028	3	0.1285
TMBIM1	6	0.077671	0.20212	0.99941	4029	4	0.4161
SLITRK6	6	0.077674	0.20213	0.99941	4030	3	0.2936
LIN52	6	0.077717	0.20223	0.9995	4031	2	0.1311
FTSJ2	6	0.077726	0.20224	0.9995	4032	2	0.1506
hsa-mir-1269b	2	0.077762	0.13447	0.9764	4033	1	0.3074
TMEM30A	6	0.077814	0.20242	0.9995	4034	3	0.0889
HCN2	6	0.077824	0.20244	0.9995	4035	2	0.104
TMEM14A	6	0.077824	0.20244	0.9995	4036	4	0.3933
CDKL2	6	0.077863	0.20253	0.9995	4037	2	-0.239
LOC643037	6	0.077869	0.20254	0.9995	4038	3	0.2082
CCDC14	6	0.077912	0.20263	0.9995	4039	2	-0.291
ATP6V0A2	6	0.077959	0.20273	0.9995	4040	3	0.1734
TATDN3	6	0.07796	0.20273	0.9995	4041	1	-0.135
FBL	6	0.078009	0.20283	0.9995	4042	3	0.461
FSD2	6	0.078056	0.20291	0.9995	4043	4	0.2805
hsa-mir-7978	1	0.078068	0.078156	0.96209	4044	1	0.8969
SLC30A5	4	0.078093	0.17506	0.99919	4045	2	0.2991
RHOXF1	6	0.078107	0.20301	0.9995	4046	2	-0.055
NRG2	6	0.078137	0.20308	0.9995	4047	4	0.3823
ARMCX4	6	0.078138	0.20308	0.9995	4048	2	0.0736
hsa-mir-6082	4	0.078142	0.17514	0.99919	4049	3	0.3355
ERMP1	6	0.078155	0.20312	0.9995	4050	2	0.0595
PSMC5	6	0.078163	0.20313	0.9995	4051	4	0.4224
ADAMTS7	6	0.078204	0.20323	0.9995	4052	3	0.3199
NFYC	6	0.078229	0.20328	0.9995	4053	4	0.4587
TRPT1	6	0.078253	0.20332	0.9995	4054	4	0.2405
hsa-mir-4786	4	0.078255	0.17535	0.99919	4055	3	0.295
BGLAP	5	0.078259	0.19194	0.99919	4056	3	0.5373
PRKAB2	6	0.078261	0.20333	0.9995	4057	3	0.3348
ZNF366	6	0.078301	0.20342	0.9995	4058	1	-0.475
CCDC33	6	0.078336	0.20349	0.9995	4059	4	0.2262
CLDN1	6	0.078388	0.20359	0.9995	4060	4	0.3223
PTPRN	6	0.078399	0.20361	0.9995	4061	3	0.2659
LINS	6	0.078446	0.20372	0.9995	4062	3	0.1562
NBN	6	0.078455	0.20374	0.9995	4063	3	0.0978
CALHM2	6	0.078468	0.20377	0.9995	4064	2	-0.058
SMIM7	6	0.078472	0.20378	0.9995	4065	2	-0.374
GBE1	6	0.078574	0.20396	0.9995	4066	3	0.1863
TSSK6	6	0.078576	0.20396	0.9995	4067	4	0.3353
B4GALT1	6	0.078576	0.20396	0.9995	4068	4	0.3167
GUCY2C	6	0.078616	0.20404	0.9995	4069	4	0.3682
CATSPERB	6	0.078616	0.20404	0.9995	4070	4	0.2743
CTSL	3	0.078634	0.1549	0.99045	4071	2	0.2494
STAR	6	0.078641	0.20409	0.9995	4072	4	0.2697
FAM53C	6	0.078655	0.20412	0.9995	4073	3	0.2847
PTAR1	6	0.078694	0.2042	0.9995	4074	4	0.5592
PDHA2	6	0.078715	0.20424	0.9995	4075	3	0.341

THNSL1	6	0.078746	0.2043	0.9995	4076	4	0.3733
COLEC10	6	0.07879	0.20439	0.9995	4077	4	0.2809
hsa-mir-1538	4	0.078815	0.17644	0.99919	4078	3	0.4706
SGOL2	6	0.078835	0.20447	0.9995	4079	4	0.3901
TCEB1	6	0.078835	0.20447	0.9995	4080	4	0.4065
RTFDC1	6	0.078853	0.20451	0.9995	4081	4	0.2584
TSC1	6	0.078873	0.20454	0.9995	4082	3	0.2511
ZNF432	6	0.078885	0.20456	0.9995	4083	2	-0.937
PPP1R14C	6	0.078886	0.20456	0.9995	4084	4	0.2637
URM1	6	0.078904	0.2046	0.9995	4085	3	0.1824
WDR88	6	0.078934	0.20466	0.9995	4086	2	0.1408
DCUN1D4	6	0.078954	0.2047	0.9995	4087	3	0.3586
MFSD2B	6	0.079047	0.2049	0.9995	4088	3	0.2273
PPIA	6	0.079058	0.20493	0.9995	4089	3	0.1867
GLIPR1	6	0.079092	0.20499	0.9995	4090	3	0.3322
C15orf53	6	0.0791	0.205	0.9995	4091	3	0.5111
EPO	6	0.07912	0.20504	0.9995	4092	4	0.3419
hsa-mir-630	4	0.079145	0.17707	0.99919	4093	3	0.2453
SMC2	6	0.079172	0.20515	0.9995	4094	3	0.3822
S100A6	6	0.079172	0.20515	0.9995	4095	4	0.3992
SIX1	6	0.079186	0.20518	0.9995	4096	4	0.4293
LRRC37B	5	0.079221	0.19346	0.99919	4097	1	-0.221
RBMXL3	6	0.079226	0.20527	0.9995	4098	1	-0.23
hsa-mir-6805	4	0.079228	0.17723	0.99919	4099	3	0.2433
ERI3	6	0.079274	0.20535	0.9995	4100	1	-0.066
TP53TG5	6	0.07928	0.20536	0.9995	4101	2	-0.139
TDO2	6	0.079282	0.20537	0.9995	4102	3	0.3174
ASTE1	6	0.07942	0.20563	0.9995	4103	2	-0.027
CWC15	6	0.079431	0.20565	0.9995	4104	2	-0.253
UFM1	6	0.079469	0.20572	0.9995	4105	2	-0.181
SERPINB8	6	0.079517	0.20583	0.9995	4106	4	0.2702
RRAGA	4	0.079591	0.17792	0.99919	4107	3	0.5348
GDF1	6	0.079597	0.20599	0.9995	4108	3	0.4947
MAGEH1	6	0.079597	0.20599	0.9995	4109	3	0.4902
SFTA3	6	0.079614	0.20603	0.9995	4110	4	0.3266
HSD3B2	6	0.079615	0.20603	0.9995	4111	4	0.2613
GSTM4	5	0.079637	0.19414	0.99919	4112	3	0.4315
CCSER1	6	0.079657	0.20611	0.9995	4113	2	0.0638
DCLK1	6	0.079733	0.20625	0.9995	4114	4	0.3011
CTSA	6	0.079749	0.20628	0.9995	4115	3	0.173
PIGV	6	0.079766	0.20631	0.9995	4116	4	0.3549
PPP1R3E	6	0.079861	0.20651	0.9995	4117	2	0.0055
SNRNP200	6	0.079863	0.20651	0.9995	4118	4	0.3121
DIAPH2	6	0.079906	0.20659	0.9995	4119	2	-0.036
42248	9	0.079959	0.23633	0.9995	4120	1	-0.183
ZMAT5	6	0.079983	0.20674	0.9995	4121	4	0.3208
OTOP1	6	0.080001	0.20678	0.9995	4122	4	0.3446
ABCG4	6	0.080012	0.2068	0.9995	4123	2	0.0276
GATS	6	0.080031	0.20685	0.9995	4124	3	0.1382
TBC1D10B	6	0.08005	0.20688	0.9995	4125	4	0.3606
ALDH6A1	6	0.080052	0.20688	0.9995	4126	3	-0.153
TSPAN33	4	0.080069	0.17876	0.99919	4127	3	0.3489
ZGLP1	6	0.080101	0.20698	0.9995	4128	2	-0.237
HJURP	6	0.080143	0.20706	0.9995	4129	4	0.2871
HDGF	6	0.080149	0.20707	0.9995	4130	2	-0.229
MUT	6	0.080151	0.20707	0.9995	4131	3	0.3214
LOC440563	5	0.080155	0.19496	0.99919	4132	2	-0.203
NFS1	6	0.080185	0.20715	0.9995	4133	3	0.3079
CEP19	6	0.080247	0.20728	0.9995	4134	3	0.2762
TIPRL	6	0.080278	0.20734	0.9995	4135	4	0.3069
GJD4	6	0.08029	0.20736	0.9995	4136	3	0.3009
CD69	6	0.0803	0.20738	0.9995	4137	2	-0.188
GADL1	6	0.080324	0.20743	0.9995	4138	3	0.2034
TBC1D23	6	0.080326	0.20744	0.9995	4139	4	0.3177
ZNF566	6	0.080326	0.20744	0.9995	4140	4	0.262
OR8D1	6	0.080344	0.20747	0.9995	4141	1	-0.364
ZNF431	6	0.080368	0.20752	0.9995	4142	2	-0.386
PKP1	6	0.080441	0.20766	0.9995	4143	2	-0.009
GJA9	6	0.08045	0.20767	0.9995	4144	4	0.2246
NOSTRIN	5	0.080455	0.19546	0.99919	4145	3	0.2491
DHX57	6	0.080482	0.20774	0.9995	4146	3	0.3121
RBBP9	6	0.080489	0.20776	0.9995	4147	1	-0.153
CTSF	6	0.080501	0.20779	0.9995	4148	3	0.3574
COMP	4	0.080509	0.17955	0.99919	4149	1	-0.215
OR6N1	6	0.080516	0.20782	0.9995	4150	3	0.1847
C1orf227	6	0.080535	0.20786	0.9995	4151	2	-0.161
CASQ2	6	0.080544	0.20788	0.9995	4152	4	0.4043
TYSND1	6	0.080544	0.20789	0.9995	4153	4	0.318
E2F4	6	0.080564	0.20793	0.9995	4154	3	0.0984
LRFN3	6	0.080587	0.20797	0.9995	4155	2	0.0611
TRMT10A	6	0.080656	0.20812	0.9995	4156	2	-0.024
hsa-mir-1264	4	0.080685	0.1799	0.99919	4157	3	0.3809
RECQL4	6	0.080693	0.20818	0.9995	4158	3	0.3218
IL13RA1	6	0.080701	0.2082	0.9995	4159	3	0.2611
PRPS2	6	0.080732	0.20826	0.9995	4160	3	0.2474

FCRLB	6	0.080809	0.2084	0.9995	4161	3	0.2381
PREPL	6	0.080829	0.20845	0.9995	4162	2	-0.148
DDX55	6	0.080884	0.20855	0.9995	4163	2	-0.047
ADAM23	6	0.080902	0.20859	0.9995	4164	4	0.3536
NME5	6	0.080949	0.20867	0.9995	4165	4	0.3365
RPRD1A	6	0.080975	0.20873	0.9995	4166	2	0.1605
CLDN5	6	0.081013	0.20882	0.9995	4167	2	0.1128
PIRT	6	0.081024	0.20884	0.9995	4168	2	0.024
PCDH19	6	0.081043	0.20888	0.9995	4169	4	0.2531
RBM4	3	0.081103	0.15825	0.99228	4170	2	0.5982
HNRNPH3	6	0.081115	0.20904	0.9995	4171	4	0.4458
hsa-mir-4999	4	0.081129	0.18075	0.99919	4172	2	0.2044
FAM187B	6	0.081145	0.20912	0.9995	4173	4	0.2819
CYR61	6	0.081209	0.20926	0.9995	4174	4	0.2871
USPL1	6	0.081266	0.20937	0.9995	4175	4	0.416
LILRA5	6	0.081315	0.20948	0.9995	4176	4	0.3228
CLPS	6	0.081332	0.20952	0.9995	4177	3	0.1319
ZBTB1	6	0.081341	0.20954	0.9995	4178	4	0.3343
SDF4	6	0.0814	0.20965	0.9995	4179	2	-0.24
CXorf66	6	0.081412	0.20967	0.9995	4180	3	0.2372
OR9G4	6	0.081431	0.20971	0.9995	4181	2	-0.099
RAB11FIP5	6	0.081455	0.20976	0.9995	4182	3	0.1311
38231	3	0.081467	0.15875	0.99228	4183	1	-0.183
MAU2	6	0.081485	0.20982	0.9995	4184	3	0.1432
USHBP1	6	0.081503	0.20985	0.9995	4185	4	0.2677
ZNF529	6	0.081537	0.20992	0.9995	4186	4	0.4982
TSC2	6	0.081552	0.20995	0.9995	4187	2	-0.05
ZNF607	6	0.081558	0.20996	0.9995	4188	1	-0.476
hsa-mir-1184-3	3	0.081615	0.15895	0.99228	4189	1	-0.8
GADD45A	6	0.081628	0.21011	0.9995	4190	3	0.1856
ARL8A	6	0.081667	0.21018	0.9995	4191	4	0.2512
ZBTB37	6	0.081735	0.21032	0.9995	4192	4	0.2882
DENND3	6	0.081752	0.21036	0.9995	4193	1	-0.176
DEFB4A	3	0.081787	0.15917	0.99228	4194	1	-0.262
LENG1	6	0.081788	0.21045	0.9995	4195	2	-0.106
FPR2	6	0.081813	0.2105	0.9995	4196	4	0.3071
CCDC111	6	0.081849	0.21057	0.9995	4197	1	0.0562
NIN	6	0.08188	0.21063	0.9995	4198	4	0.3585
PUS3	6	0.081926	0.21073	0.9995	4199	2	0.1291
IRF2BP2	6	0.081953	0.21079	0.9995	4200	4	0.4069
hsa-mir-617	4	0.081976	0.18236	0.99919	4201	3	0.3104
LRRC7	6	0.082132	0.21115	0.9995	4202	3	0.1137
NDUFA6	6	0.08214	0.21117	0.9995	4203	3	0.0611
OR6K2	6	0.082188	0.21127	0.9995	4204	2	-0.275
MRPL12	6	0.082193	0.21128	0.9995	4205	2	0.0729
CHRNB4	5	0.082209	0.19829	0.99919	4206	2	0.0326
DYNC1I1	6	0.082223	0.21134	0.9995	4207	4	0.4393
EPDR1	6	0.08223	0.21136	0.9995	4208	4	0.4059
SAMD4A	6	0.082312	0.21153	0.9995	4209	3	0.2258
CYLC2	6	0.082337	0.21158	0.9995	4210	4	0.365
HHATL	6	0.082338	0.21158	0.9995	4211	2	-0.016
PSMD1	6	0.08239	0.21169	0.9995	4212	3	0.2924
KDM2B	6	0.082409	0.21173	0.9995	4213	4	0.3146
RGS2	6	0.08242	0.21175	0.9995	4214	4	0.409
RAB11B	6	0.082431	0.21176	0.9995	4215	2	-0.208
PDE6A	6	0.082436	0.21177	0.9995	4216	4	0.2629
TERT	6	0.082436	0.21177	0.9995	4217	4	0.269
OPA1	6	0.082477	0.21186	0.9995	4218	4	0.3823
C6orf106	6	0.082504	0.21191	0.9995	4219	3	0.2309
MSC	6	0.082519	0.21194	0.9995	4220	4	0.229
hsa-mir-320c-1	3	0.082525	0.16015	0.9925	4221	2	0.2966
PPIB	6	0.082544	0.21198	0.9995	4222	2	0.0698
XPA	6	0.082576	0.21204	0.9995	4223	3	0.2247
FGF2	6	0.082599	0.21209	0.9995	4224	3	0.2808
VPS72	6	0.082618	0.21213	0.9995	4225	4	0.3159
GPR19	6	0.082625	0.21214	0.9995	4226	3	0.0913
WDR62	4	0.082625	0.18353	0.99919	4227	3	0.3932
SSX5	4	0.082627	0.18353	0.99919	4228	2	0.0454
RLBP1	6	0.082649	0.21218	0.9995	4229	4	0.3095
OR10H4	6	0.082698	0.21228	0.9995	4230	4	0.4402
DHRS9	6	0.082722	0.21233	0.9995	4231	3	0.1297
FKBP3	6	0.082763	0.21242	0.9995	4232	4	0.3056
PPT2	6	0.08277	0.21244	0.9995	4233	1	-0.001
A4GNT	6	0.082774	0.21244	0.9995	4234	2	-0.128
hsa-mir-7856	4	0.082805	0.18388	0.99919	4235	3	0.3736
COL4A3BP	6	0.082819	0.21253	0.9995	4236	3	0.3329
RASA1	6	0.082862	0.21262	0.9995	4237	4	0.3138
TBX20	6	0.082888	0.21267	0.9995	4238	3	0.1183
SLMAP	6	0.082912	0.21272	0.9995	4239	4	0.3686
PPM1M	6	0.082916	0.21272	0.9995	4240	1	-0.141
SDR39U1	6	0.08295	0.2128	0.9995	4241	4	0.2842
SLC8A2	6	0.082973	0.21285	0.9995	4242	2	-0.118
KATNAL2	6	0.082988	0.21289	0.9995	4243	3	-0.021
TCEB3	6	0.082988	0.21289	0.9995	4244	3	0.154
CSGALNACT1	6	0.083018	0.21293	0.9995	4245	3	0.1675

TRAF5	6	0.083122	0.21314	0.9995	4246	4	0.2988
SPIN4	6	0.083154	0.2132	0.9995	4247	3	0.331
DNAAF2	6	0.083248	0.2134	0.9995	4248	3	-0.095
PTBP1	6	0.083263	0.21342	0.9995	4249	4	0.2935
PIGK	6	0.08335	0.21358	0.9995	4250	3	0.3154
CIB2	6	0.083352	0.21359	0.9995	4251	3	0.2032
SLC11A2	6	0.0834	0.21368	0.9995	4252	3	0.1184
SOX13	6	0.083449	0.21377	0.9995	4253	4	0.3179
ITGAE	6	0.08347	0.21381	0.9995	4254	4	0.2911
ZNF507	6	0.083502	0.21387	0.9995	4255	3	0.0244
RBM17	6	0.083512	0.21389	0.9995	4256	4	0.4223
EMR2	6	0.08357	0.21401	0.9995	4257	4	0.2571
ABCC1	6	0.08357	0.21401	0.9995	4258	4	0.2825
COL4A4	6	0.08357	0.21401	0.9995	4259	4	0.3006
GRIN2D	6	0.083594	0.21406	0.9995	4260	2	-0.096
MEGF11	6	0.083601	0.21407	0.9995	4261	2	-0.439
SRSF4	6	0.083658	0.21418	0.9995	4262	4	0.3813
TCF7L1	6	0.083678	0.21422	0.9995	4263	3	0.1505
FLT4	6	0.083687	0.21424	0.9995	4264	3	0.0174
DDX51	6	0.083712	0.21429	0.9995	4265	4	0.4921
OR7E24	6	0.083715	0.21429	0.9995	4266	2	0.0833
WWP1	6	0.083763	0.2144	0.9995	4267	3	0.02
PGLYRP3	6	0.083766	0.21441	0.9995	4268	4	0.3199
PHF8	6	0.083789	0.21446	0.9995	4269	4	0.2216
AGAP3	6	0.08379	0.21446	0.9995	4270	3	0.1823
EYS	6	0.083797	0.21447	0.9995	4271	4	0.2905
AKR1E2	6	0.083816	0.21451	0.9995	4272	4	0.2661
B3GALT4	6	0.083836	0.21455	0.9995	4273	1	-0.473
DLL3	6	0.083878	0.21464	0.9995	4274	3	0.3381
TSPAN19	6	0.083885	0.21466	0.9995	4275	3	0.219
hsa-mir-3655	4	0.083908	0.18591	0.99919	4276	3	0.5399
SPRR3	6	0.083933	0.21476	0.9995	4277	3	0.1421
BTNL8	6	0.08394	0.21477	0.9995	4278	4	0.4262
TMEM248	6	0.083991	0.21488	0.9995	4279	3	-0.098
SLC2A6	6	0.083991	0.21488	0.9995	4280	3	0.4
LOC100144595	4	0.083994	0.18606	0.99919	4281	1	0.1498
hsa-mir-449a	4	0.084	0.18607	0.99919	4282	2	0.3464
HIST1H2BM	6	0.084017	0.21493	0.9995	4283	2	-0.037
CTNNAL1	6	0.08403	0.21496	0.9995	4284	1	-0.399
CES1	6	0.084039	0.21497	0.9995	4285	4	0.3295
MAP3K11	6	0.08407	0.21503	0.9995	4286	3	0.1424
OR56B4	6	0.08407	0.21503	0.9995	4287	2	0.023
GUCY1A3	6	0.084078	0.21505	0.9995	4288	2	-0.043
NEDD4L	6	0.084097	0.21509	0.9995	4289	4	0.2593
UNC5B	6	0.084109	0.21512	0.9995	4290	2	0.0713
LOC100130539	6	0.08414	0.21518	0.9995	4291	3	0.0676
CDC26	6	0.084149	0.21519	0.9995	4292	3	0.4027
DOM3Z	2	0.08415	0.14477	0.98295	4293	2	0.7429
RPL39L	5	0.084189	0.20138	0.99919	4294	3	0.3092
CBR3	6	0.084212	0.21533	0.9995	4295	4	0.4799
NWD1	6	0.084257	0.21543	0.9995	4296	3	0.2588
ASB6	6	0.084281	0.21547	0.9995	4297	3	0.2994
KRT24	6	0.084321	0.21556	0.9995	4298	2	-0.322
C2CD4C	6	0.08435	0.21563	0.9995	4299	3	-0.07
SLC22A20	6	0.084393	0.21573	0.9995	4300	4	0.3945
ZNF610	6	0.084404	0.21574	0.9995	4301	3	0.2614
MRPL15	6	0.084475	0.2159	0.9995	4302	4	0.2938
IFT122	6	0.084493	0.21594	0.9995	4303	3	0.2402
AGTRAP	6	0.084514	0.21598	0.9995	4304	2	0.021
PKP4	6	0.084556	0.21606	0.9995	4305	4	0.3846
CCL7	6	0.084563	0.21608	0.9995	4306	3	0.2104
hsa-mir-596	4	0.084611	0.18721	0.99919	4307	3	0.3185
CNBD1	6	0.08465	0.21625	0.9995	4308	3	0.3076
hsa-mir-4287	4	0.084758	0.18747	0.99919	4309	2	0.3947
AQP4	6	0.084803	0.21654	0.9995	4310	2	0.0126
PPP1R27	6	0.084853	0.21664	0.9995	4311	1	-0.074
PYGO2	6	0.084918	0.21678	0.9995	4312	2	-0.154
OSBPL11	6	0.084974	0.21689	0.9995	4313	4	0.3095
ZNF333	6	0.084991	0.21693	0.9995	4314	4	0.3974
CD79A	6	0.085001	0.21695	0.9995	4315	4	0.2414
LACC1	6	0.085046	0.21704	0.9995	4316	3	0.129
hsa-mir-4473	4	0.085164	0.18824	0.99919	4317	3	0.437
RUSC2	6	0.085191	0.21732	0.9995	4318	4	0.3801
EYA3	6	0.085191	0.21732	0.9995	4319	4	0.3858
TAS2R40	6	0.085228	0.21739	0.9995	4320	2	-0.459
ADCY4	6	0.08524	0.21742	0.9995	4321	2	-0.998
H1FOO	6	0.085274	0.21749	0.9995	4322	2	-1.173
KSR1	6	0.085274	0.21749	0.9995	4323	3	0.293
RPTOR	6	0.085288	0.21752	0.9995	4324	3	0.162
MAOB	6	0.085337	0.21762	0.9995	4325	3	0.0203
ITGB1BP1	6	0.085342	0.21763	0.9995	4326	3	0.2411
ADAMTS12	6	0.085346	0.21764	0.9995	4327	4	0.2586
MMADHC	6	0.085385	0.21772	0.9995	4328	4	0.3323
hsa-mir-3154	4	0.085391	0.18864	0.99919	4329	1	0.0187
ITLN2	4	0.085419	0.1887	0.99919	4330	3	0.2854

MTRNR2L3	4	0.085424	0.1887	0.99919	4331	1	-0.395
KLHL6	6	0.085431	0.21781	0.9995	4332	3	0.2177
ESCO2	6	0.085491	0.21793	0.9995	4333	2	0.1366
DTX3L	6	0.085498	0.21795	0.9995	4334	4	0.2581
ENTHD1	6	0.085533	0.21802	0.9995	4335	4	0.2986
UBE2U	6	0.085578	0.21811	0.9995	4336	2	0.0588
PLOD1	6	0.085607	0.21818	0.9995	4337	4	0.3464
SEZ6L	6	0.085607	0.21818	0.9995	4338	4	0.3267
SLC12A9	6	0.085607	0.21818	0.9995	4339	4	0.2994
IVD	6	0.085627	0.21822	0.9995	4340	3	0.1374
KRTAP4-3	6	0.085675	0.21831	0.9995	4341	2	0.0225
MOB3B	6	0.085748	0.21847	0.9995	4342	1	-0.16
LOC100129361	6	0.085753	0.21848	0.9995	4343	3	0.284
hsa-mir-4650-2	3	0.085817	0.16449	0.99276	4344	2	0.2309
BDH2	6	0.085824	0.21862	0.9995	4345	2	0.1168
SIRT2	6	0.085839	0.21865	0.9995	4346	2	0.0265
AGXT	6	0.085869	0.21872	0.9995	4347	3	-0.07
IRGQ	6	0.085877	0.21873	0.9995	4348	3	0.3195
COA5	6	0.085903	0.21878	0.9995	4349	4	0.4798
C12orf42	6	0.085932	0.21883	0.9995	4350	2	0.0176
FRMD7	6	0.085932	0.21883	0.9995	4351	2	-0.225
FTSJ3	6	0.085942	0.21885	0.9995	4352	3	0.4196
ASPH	6	0.085942	0.21885	0.9995	4353	4	0.3764
GNAZ	6	0.085963	0.21889	0.9995	4354	3	0.1956
TFB1M	6	0.085965	0.2189	0.9995	4355	3	0.0854
PDE4DIP	6	0.085996	0.21896	0.9995	4356	3	0.2518
FDPS	6	0.086014	0.219	0.9995	4357	3	0.2629
HN1	6	0.086016	0.219	0.9995	4358	4	0.3745
AMBN	6	0.086062	0.2191	0.9995	4359	4	0.3288
CWH43	6	0.086095	0.21917	0.9995	4360	3	0.1795
hsa-mir-218-1	2	0.086107	0.14787	0.98311	4361	1	-0.623
FAM118A	6	0.086111	0.2192	0.9995	4362	3	0.222
KRTAP21-2	6	0.08614	0.21926	0.9995	4363	3	0.0762
ST6GALNAC1	6	0.08615	0.21928	0.9995	4364	2	-0.111
WDR73	6	0.086173	0.21932	0.9995	4365	4	0.3226
CCDC180	6	0.086223	0.21943	0.9995	4366	4	0.2661
C2orf48	6	0.086239	0.21945	0.9995	4367	4	0.3763
DLAT	6	0.086263	0.2195	0.9995	4368	4	0.3637
CADPS2	6	0.086305	0.21959	0.9995	4369	3	0.3921
PPIAL4D	2	0.086306	0.14817	0.98311	4370	1	0.8338
hsa-mir-186	4	0.086322	0.19032	0.99919	4371	2	0.4389
IMPA2	6	0.086352	0.21968	0.9995	4372	2	0.0088
RCHY1	6	0.086369	0.21971	0.9995	4373	3	0.3278
KIAA0430	6	0.086372	0.21972	0.9995	4374	4	0.346
SLC25A18	6	0.0864	0.21977	0.9995	4375	4	0.2507
PEMT	6	0.0864	0.21977	0.9995	4376	3	0.1425
EFNA1	6	0.086448	0.21986	0.9995	4377	2	-0.171
IGFLR1	6	0.086497	0.21996	0.9995	4378	3	-0.122
DUSP22	6	0.086523	0.22	0.9995	4379	3	0.1071
SHCBP1L	6	0.086537	0.22003	0.9995	4380	4	0.3988
CCDC43	6	0.086545	0.22005	0.9995	4381	2	-0.008
CSGALNACT2	6	0.086574	0.2201	0.9995	4382	3	0.1927
OXLD1	6	0.086604	0.22015	0.9995	4383	3	0.3193
SLC22A25	6	0.086608	0.22016	0.9995	4384	3	-0.142
KRAS	6	0.086686	0.22032	0.9995	4385	4	0.3613
DDI2	6	0.086798	0.22054	0.9995	4386	3	0.3817
ESF1	6	0.086798	0.22054	0.9995	4387	4	0.3031
FATE1	6	0.086857	0.22066	0.9995	4388	2	-0.139
PVRIG	6	0.086883	0.22071	0.9995	4389	3	0.3392
BTC	6	0.086912	0.22076	0.9995	4390	3	0.22
PMS2	6	0.086958	0.22085	0.9995	4391	3	0.0949
ZPLD1	6	0.08698	0.22091	0.9995	4392	2	-0.244
IGFL2	6	0.087028	0.22101	0.9995	4393	3	0.1636
MRPL22	6	0.087052	0.22105	0.9995	4394	3	0.1798
C12orf74	6	0.087068	0.22108	0.9995	4395	3	0.2421
MTHFD1	6	0.087071	0.22109	0.9995	4396	4	0.3677
NUF2	6	0.087114	0.22117	0.9995	4397	3	0.2675
KIAA1467	6	0.087173	0.22128	0.9995	4398	2	-0.191
TMEM255B	6	0.087216	0.22137	0.9995	4399	2	0.0558
SKP2	6	0.087221	0.22139	0.9995	4400	2	0.0691
TCHP	6	0.087255	0.22146	0.9995	4401	3	0.3148
ABHD17C	6	0.08727	0.22148	0.9995	4402	2	0.1979
STRIP2	6	0.087286	0.22152	0.9995	4403	3	0.2359
hsa-mir-4479	4	0.087321	0.19216	0.99919	4404	2	0.5733
CXCR1	6	0.087347	0.22165	0.9995	4405	4	0.3883
SEL1L3	6	0.087366	0.22169	0.9995	4406	2	0.112
LAMTOR3	6	0.087383	0.22172	0.9995	4407	3	0.1346
HAO2	6	0.087395	0.22175	0.9995	4408	2	-0.269
HSCB	6	0.087463	0.22187	0.9995	4409	4	0.4178
hsa-mir-4490	4	0.087524	0.19255	0.99919	4410	2	0.2616
MTHFD1L	6	0.087559	0.22208	0.9995	4411	3	0.32
MUC1	6	0.087574	0.22211	0.9995	4412	3	0.2285
CELF6	6	0.087574	0.22211	0.9995	4413	3	0.0909
hsa-mir-4303	4	0.08763	0.19273	0.99919	4414	1	-0.115
DCLRE1A	6	0.087645	0.22224	0.9995	4415	2	0.0732

CCDC3	6	0.087659	0.22227	0.9995	4416	3	0.0961
PARP12	6	0.08766	0.22228	0.9995	4417	3	0.1618
NAT8L	4	0.087748	0.19294	0.99919	4418	3	0.7291
TMEM14B	3	0.087779	0.16708	0.99276	4419	1	-1.029
LRRC47	6	0.087794	0.22254	0.9995	4420	3	0.2455
FNDC7	6	0.08782	0.22259	0.9995	4421	3	0.3583
SEL1L	6	0.08784	0.22264	0.9995	4422	3	0.3048
CADM4	6	0.087848	0.22265	0.9995	4423	4	0.3226
CSN2	6	0.087849	0.22265	0.9995	4424	3	0.1647
MREG	6	0.087897	0.22275	0.9995	4425	2	-0.123
SLC37A1	6	0.087903	0.22277	0.9995	4426	2	-9E-04
FAM216B	6	0.087934	0.22284	0.9995	4427	2	-0.693
SCARB1	6	0.087994	0.22297	0.9995	4428	1	-0.435
TMEM144	6	0.088028	0.22304	0.9995	4429	2	0.0389
hsa-mir-4484	4	0.088036	0.19348	0.99919	4430	2	0.371
TCTA	6	0.088046	0.22308	0.9995	4431	3	0.19
TMEM25	6	0.088046	0.22308	0.9995	4432	4	0.2957
CDC42SE2	6	0.08809	0.22318	0.9995	4433	1	-0.283
UPF3A	6	0.08811	0.22323	0.9995	4434	4	0.4881
C6orf163	6	0.088146	0.22331	0.9995	4435	3	0.3486
KRTAP10-12	6	0.088235	0.22349	0.9995	4436	1	-0.356
LAMTOR1	6	0.088235	0.22349	0.9995	4437	4	0.3108
SST	6	0.088235	0.22349	0.9995	4438	1	-0.331
PC	6	0.08827	0.22357	0.9995	4439	3	0.0039
SMC6	6	0.088296	0.22362	0.9995	4440	4	0.3968
ATP1B2	6	0.088379	0.22378	0.9995	4441	3	0.1277
PARP6	5	0.08841	0.20813	0.9995	4442	3	0.4392
METTL14	6	0.088427	0.22386	0.9995	4443	2	0.009
OR52N5	6	0.088427	0.22386	0.9995	4444	2	0.0851
OR5P2	6	0.088452	0.22391	0.9995	4445	1	-0.341
PRR9	6	0.088463	0.22392	0.9995	4446	4	0.2897
OR10K2	6	0.088474	0.22394	0.9995	4447	2	-0.075
ZNF541	6	0.088507	0.22402	0.9995	4448	4	0.2877
SCAF8	6	0.088552	0.2241	0.9995	4449	2	0.0176
COG4	6	0.088596	0.22419	0.9995	4450	1	-0.833
ARID2	6	0.088599	0.2242	0.9995	4451	4	0.3736
ZNF506	6	0.08862	0.22424	0.9995	4452	3	0.5518
RP2	6	0.088639	0.22428	0.9995	4453	2	-0.173
SNRPB	6	0.088669	0.22433	0.9995	4454	3	-0.021
hsa-mir-3157	4	0.088679	0.19465	0.99919	4455	2	0.1662
AVPI1	6	0.088733	0.22447	0.9995	4456	3	0.2007
GEMIN2	6	0.088811	0.22463	0.9995	4457	2	0.1551
MDFIC	6	0.088811	0.22463	0.9995	4458	2	-0.133
KDR	6	0.088822	0.22465	0.9995	4459	4	0.4205
hsa-mir-4458	4	0.088825	0.19492	0.99919	4460	2	0.3436
hsa-mir-8081	4	0.088849	0.19496	0.99919	4461	3	0.4805
P4HA3	6	0.088862	0.22473	0.9995	4462	1	0.0049
PRKRIP1	6	0.088862	0.22473	0.9995	4463	2	-0.212
MTCP1	6	0.088862	0.22473	0.9995	4464	2	-0.123
CCT2	6	0.088872	0.22476	0.9995	4465	3	0.3738
TSPYL2	6	0.088909	0.22482	0.9995	4466	4	0.225
RNF113B	6	0.088929	0.22486	0.9995	4467	3	-0.005
NAALAD2	6	0.088933	0.22487	0.9995	4468	3	0.1762
PDK4	6	0.088941	0.22488	0.9995	4469	4	0.3285
SPRY1	6	0.088953	0.22491	0.9995	4470	3	0.1153
FIBP	6	0.088958	0.22491	0.9995	4471	1	-0.413
CSRP3	6	0.088992	0.22498	0.9995	4472	4	0.3613
ARPC1A	6	0.089015	0.22502	0.9995	4473	2	-0.04
CLVS2	6	0.089054	0.22511	0.9995	4474	2	0.009
OR5M11	6	0.089062	0.22513	0.9995	4475	2	0.0419
DENND2C	6	0.089062	0.22513	0.9995	4476	2	-0.186
RSPH1	6	0.089127	0.22524	0.9995	4477	1	-0.541
GUK1	6	0.08913	0.22525	0.9995	4478	4	0.4354
ADAT1	6	0.089152	0.2253	0.9995	4479	4	0.2991
UNC5A	6	0.089152	0.2253	0.9995	4480	4	0.2745
UQCRHL	6	0.089199	0.22541	0.9995	4481	2	0.0598
IFNAR2	6	0.08922	0.22545	0.9995	4482	3	0.1074
CHRNA10	6	0.089227	0.22546	0.9995	4483	3	0.1313
TRIP12	6	0.089247	0.2255	0.9995	4484	3	0.2004
hsa-mir-4763	4	0.089277	0.19576	0.99919	4485	3	0.4958
RPS4Y2	6	0.089295	0.22559	0.9995	4486	2	-0.055
OR8U1	3	0.089308	0.16909	0.99382	4487	2	0.4104
ITM2A	6	0.089358	0.22572	0.9995	4488	3	0.247
CPXCR1	6	0.08949	0.22599	0.9995	4489	3	0.0562
DNAJB9	6	0.089495	0.226	0.9995	4490	3	0.3346
PGLYRP4	3	0.089517	0.16937	0.99382	4491	2	0.3156
OASL	6	0.089536	0.22609	0.9995	4492	1	-0.165
MRPL46	6	0.089541	0.2261	0.9995	4493	3	0.3298
OR6N2	6	0.089574	0.22615	0.9995	4494	2	-0.352
SCLY	6	0.089581	0.22617	0.9995	4495	3	0.2172
CD164	6	0.08962	0.22624	0.9995	4496	4	0.2961
GJA1	6	0.089621	0.22624	0.9995	4497	2	0.0672
C16orf72	6	0.089633	0.22627	0.9995	4498	2	-0.129
OR4K5	6	0.089668	0.22635	0.9995	4499	4	0.2352
NEU3	6	0.089668	0.22635	0.9995	4500	3	0.0659

EIF2D	6	0.089681	0.22637	0.9995	4501	2	-0.526
CYP39A1	6	0.089729	0.22646	0.9995	4502	2	0.0695
ANO3	6	0.089774	0.22655	0.9995	4503	3	0.3742
ZNF300	6	0.089801	0.2266	0.9995	4504	2	0.0905
SYNJ2BP	1	0.089803	0.089808	0.96841	4505	1	0.7924
TARS2	6	0.08981	0.22662	0.9995	4506	2	-0.219
hsa-mir-8066	4	0.089919	0.19691	0.99919	4507	2	0.2324
NQO2	6	0.08992	0.22684	0.9995	4508	2	-0.222
FAM133B	5	0.089929	0.2106	0.9995	4509	3	0.5404
ITGA1	6	0.089944	0.22689	0.9995	4510	2	0.0343
MAPKAPK3	6	0.089949	0.2269	0.9995	4511	4	0.2669
UBB	6	0.08997	0.22695	0.9995	4512	1	-0.077
SOSTDC1	6	0.08997	0.22695	0.9995	4513	3	0.1654
FAM211A	6	0.08997	0.22695	0.9995	4514	3	0.4149
RAPH1	6	0.090081	0.22716	0.9995	4515	4	0.2551
RAB11FIP2	6	0.090114	0.22723	0.9995	4516	1	-0.284
POM121L12	6	0.090114	0.22723	0.9995	4517	2	-0.115
P2RY1	6	0.090125	0.22726	0.9995	4518	2	-0.272
NDST3	6	0.09013	0.22727	0.9995	4519	3	0.2838
DUSP8	6	0.090188	0.22737	0.9995	4520	2	0.1612
hsa-mir-4461	4	0.090205	0.19744	0.99919	4521	1	-0.633
WBP2	6	0.09022	0.22744	0.9995	4522	3	0.0506
LAMA2	6	0.090278	0.22756	0.9995	4523	3	0.2593
SNCA	6	0.090311	0.22763	0.9995	4524	4	0.3186
RDH16	6	0.090354	0.22771	0.9995	4525	3	0.2569
KIAA0895L	6	0.090386	0.22777	0.9995	4526	2	-0.113
CDCA7L	6	0.0905	0.22803	0.9995	4527	2	0.0631
ZNF569	6	0.090504	0.22804	0.9995	4528	4	0.3285
IL21R	6	0.090504	0.22804	0.9995	4529	4	0.4131
ZSCAN9	6	0.09054	0.22812	0.9995	4530	4	0.288
CXorf40A	3	0.090596	0.1708	0.99382	4531	2	0.3758
CCR8	6	0.090596	0.22823	0.9995	4532	1	0.0974
IL18BP	6	0.090644	0.22831	0.9995	4533	1	-0.117
SLC17A8	6	0.090673	0.22837	0.9995	4534	4	0.365
APOBEC4	6	0.090673	0.22837	0.9995	4535	4	0.2711
PTPMT1	6	0.090802	0.22866	0.9995	4536	4	0.4317
DHFR	6	0.090812	0.22867	0.9995	4537	3	0.1838
TFAP4	6	0.09088	0.2288	0.9995	4538	3	0.2729
HK3	6	0.090885	0.22881	0.9995	4539	2	-0.437
LOC389895	6	0.090907	0.22885	0.9995	4540	2	-0.146
ZNF580	6	0.09092	0.22888	0.9995	4541	4	0.3832
THOP1	6	0.090933	0.2289	0.9995	4542	1	-0.236
ZNF132	6	0.090946	0.22893	0.9995	4543	3	0.3676
NT5DC3	6	0.090982	0.22899	0.9995	4544	3	0.1354
SLC52A2	6	0.091044	0.22912	0.9995	4545	3	0.2747
PRKAR2A	6	0.091077	0.22918	0.9995	4546	3	0.2771
FABP5	6	0.091105	0.22923	0.9995	4547	3	0.4204
MAB21L1	6	0.091105	0.22923	0.9995	4548	3	0.4629
OR5AP2	6	0.091105	0.22923	0.9995	4549	3	0.1576
TAS2R10	6	0.091173	0.22937	0.9995	4550	2	-0.161
FAM135B	6	0.091224	0.22947	0.9995	4551	3	0.1229
hsa-mir-6866	4	0.091256	0.19941	0.99919	4552	1	-0.082
CTNNA2	6	0.091271	0.22957	0.9995	4553	2	0.1346
TPP1	6	0.091324	0.22967	0.9995	4554	4	0.4143
ANXA8L1	2	0.091361	0.15624	0.9916	4555	2	0.5383
CCDC136	6	0.091366	0.22977	0.9995	4556	2	-0.279
TOR1AIP1	6	0.091402	0.22985	0.9995	4557	4	0.438
SAMD9	6	0.091402	0.22985	0.9995	4558	3	0.2234
CPB2	6	0.091406	0.22985	0.9995	4559	3	0.2822
DAAM1	6	0.091426	0.22988	0.9995	4560	4	0.3914
MAN2C1	6	0.091438	0.2299	0.9995	4561	3	0.3106
PRDM7	4	0.091459	0.19975	0.99919	4562	3	0.241
CCDC172	6	0.091466	0.22995	0.9995	4563	4	0.3365
FAM20C	6	0.091515	0.23005	0.9995	4564	3	0.2521
RPE	6	0.091533	0.23008	0.9995	4565	3	0.206
MEF2C	6	0.091539	0.2301	0.9995	4566	4	0.4907
OR1J4	6	0.091558	0.23013	0.9995	4567	2	-0.046
hsa-mir-1204	4	0.09157	0.19995	0.99919	4568	3	0.5156
hsa-mir-634	4	0.091639	0.20007	0.99919	4569	3	0.3725
USP8	6	0.091707	0.23044	0.9995	4570	2	-0.192
TBX10	6	0.09175	0.23052	0.9995	4571	3	-0.077
ENC1	6	0.091799	0.23061	0.9995	4572	2	-0.188
BEST4	6	0.091864	0.23073	0.9995	4573	4	0.287
KLHL14	6	0.091871	0.23074	0.9995	4574	3	0.1787
TOMM22	6	0.091966	0.23092	0.9995	4575	4	0.3065
OPN1LW	6	0.092015	0.23102	0.9995	4576	4	0.2433
SCN9A	6	0.092096	0.23119	0.9995	4577	4	0.3161
KRTAP4-2	5	0.092113	0.21403	0.9995	4578	1	-0.617
AMHR2	6	0.09216	0.23133	0.9995	4579	3	-0.082
TMEM48	4	0.092197	0.20111	0.99919	4580	3	0.3908
GORASP1	6	0.092208	0.23143	0.9995	4581	3	0.1121
TRIP4	6	0.092215	0.23144	0.9995	4582	4	0.2161
FBXO18	6	0.092231	0.23147	0.9995	4583	2	0.1652
ADAD1	6	0.092264	0.23154	0.9995	4584	2	-0.327
TNFSF18	6	0.092271	0.23155	0.9995	4585	4	0.2775

MMGT1	6	0.092279	0.23158	0.9995	4586	2	-0.372
SPATA7	6	0.092345	0.2317	0.9995	4587	4	0.224
RRAGD	6	0.092345	0.2317	0.9995	4588	4	0.2114
RARRES1	6	0.092374	0.23176	0.9995	4589	4	0.3563
ACTR3C	5	0.092394	0.21447	0.9995	4590	3	0.3386
hsa-mir-328	4	0.092412	0.20149	0.99919	4591	3	0.404
MORN2	6	0.092414	0.23184	0.9995	4592	4	0.2379
EDA2R	6	0.092472	0.23194	0.9995	4593	4	0.3614
TRPM3	6	0.092472	0.23194	0.9995	4594	4	0.2436
hsa-mir-362	4	0.092475	0.20161	0.99919	4595	3	0.2538
hsa-mir-422a	4	0.092484	0.20162	0.99919	4596	3	0.4899
CALM2	6	0.092525	0.23204	0.9995	4597	4	0.273
GRIK5	6	0.092537	0.23207	0.9995	4598	4	0.316
NELFA	6	0.092568	0.23212	0.9995	4599	2	-0.043
CTAGE1	6	0.092623	0.23224	0.9995	4600	4	0.3602
MED12	6	0.092623	0.23224	0.9995	4601	4	0.3892
TAX1BP1	6	0.09263	0.23225	0.9995	4602	2	-0.395
FRMD6	6	0.092668	0.23233	0.9995	4603	4	0.3628
PAPPA	6	0.09276	0.23252	0.9995	4604	3	-0.11
TLR2	6	0.092803	0.23261	0.9995	4605	4	0.3238
DNER	6	0.092811	0.23263	0.9995	4606	4	0.2336
PEX26	6	0.092829	0.23267	0.9995	4607	2	-0.384
ZNF331	6	0.092831	0.23267	0.9995	4608	4	0.265
COL4A2	6	0.092843	0.2327	0.9995	4609	4	0.5699
ALS2CR8	6	0.092904	0.23282	0.9995	4610	1	-0.102
IGSF3	6	0.09295	0.23291	0.9995	4611	4	0.2769
POLR3K	6	0.092979	0.23296	0.9995	4612	4	0.3567
OGFRL1	6	0.092988	0.23298	0.9995	4613	3	0.0558
CA14	6	0.092995	0.23299	0.9995	4614	4	0.3446
SLBP	6	0.093048	0.23309	0.9995	4615	2	0.1453
POPDC3	6	0.093132	0.23325	0.9995	4616	3	-0.168
hsa-mir-4423	2	0.093157	0.15905	0.99228	4617	1	0.4414
TAPBP	6	0.093168	0.23331	0.9995	4618	2	0.0384
NDUFAF2	6	0.093168	0.23331	0.9995	4619	2	-0.189
DUSP15	6	0.09324	0.23345	0.9995	4620	3	0.0852
GPSM1	6	0.093242	0.23345	0.9995	4621	3	0.0648
CLLU1OS	6	0.093258	0.23349	0.9995	4622	4	0.2566
MAGEC3	6	0.093262	0.2335	0.9995	4623	3	0.1643
FAF1	6	0.093288	0.23355	0.9995	4624	3	0.2271
hsa-mir-548ac	1	0.09335	0.093342	0.96841	4625	1	0.8395
CIB3	6	0.093444	0.23387	0.9995	4626	4	0.4116
POLE2	6	0.09348	0.23394	0.9995	4627	1	0.092
SPINT1	6	0.09348	0.23394	0.9995	4628	2	-0.06
MS4A1	6	0.093539	0.23406	0.9995	4629	3	0.3024
CCDC94	6	0.093563	0.2341	0.9995	4630	2	-0.434
SIRT5	6	0.093563	0.2341	0.9995	4631	3	-0.041
PCDH20	6	0.0936	0.23418	0.9995	4632	3	0.3486
OR51B6	6	0.093607	0.23419	0.9995	4633	4	0.243
GLB1L	6	0.093636	0.23425	0.9995	4634	3	0.4092
PHB2	6	0.093667	0.23432	0.9995	4635	3	0.4197
RHOJ	6	0.093672	0.23432	0.9995	4636	2	-0.186
PDCD10	6	0.093681	0.23435	0.9995	4637	4	0.4122
STIL	6	0.093703	0.23439	0.9995	4638	3	0.3341
IL18R1	6	0.093764	0.23452	0.9995	4639	4	0.3551
MFAP5	6	0.093764	0.23452	0.9995	4640	4	0.2631
FAM133A	6	0.093805	0.23462	0.9995	4641	4	0.2632
FSD1	6	0.093816	0.23463	0.9995	4642	2	-0.302
TMOD4	6	0.093829	0.23465	0.9995	4643	4	0.3714
AKT1S1	6	0.093864	0.23472	0.9995	4644	4	0.3328
THUMPD2	6	0.093879	0.23476	0.9995	4645	4	0.3325
FRMD1	6	0.0939	0.2348	0.9995	4646	3	0.2632
SCML2	6	0.093916	0.23483	0.9995	4647	4	0.2987
LUM	6	0.093971	0.23494	0.9995	4648	3	0.0952
CENPF	6	0.093974	0.23495	0.9995	4649	4	0.52
RDH10	6	0.093978	0.23496	0.9995	4650	3	0.3131
MSRB3	6	0.093987	0.23497	0.9995	4651	2	-0.255
PRAMEF6	1	0.094005	0.094008	0.96841	4652	1	1.9898
SLC25A43	6	0.094019	0.23503	0.9995	4653	2	0.0475
HSF2	6	0.094035	0.23508	0.9995	4654	3	0.1965
RUFY1	6	0.094056	0.23511	0.9995	4655	2	0.082
CCDC88A	6	0.094056	0.23511	0.9995	4656	3	0.1333
DIRC1	6	0.094067	0.23513	0.9995	4657	3	0.2908
MSLN	6	0.094118	0.23524	0.9995	4658	4	0.2497
PON1	6	0.094118	0.23524	0.9995	4659	4	0.3803
ATP6V0A4	6	0.094147	0.2353	0.9995	4660	2	-0.484
hsa-mir-4662a	1	0.094214	0.09423	0.96841	4661	1	0.5938
CD200	6	0.09423	0.23546	0.9995	4662	4	0.2107
ERCC4	6	0.094248	0.23549	0.9995	4663	1	-0.026
DZIP1	6	0.094271	0.23554	0.9995	4664	4	0.3161
OR9Q1	6	0.094291	0.23558	0.9995	4665	2	-0.119
HAMP	6	0.094291	0.23558	0.9995	4666	3	0.0275
ATAD3C	6	0.094323	0.23564	0.9995	4667	4	0.2273
NRP2	6	0.094333	0.23566	0.9995	4668	4	0.3533
KIAA1841	6	0.094395	0.23579	0.9995	4669	4	0.335
GHRHR	6	0.094399	0.2358	0.9995	4670	4	0.2482

ANKRD13B	6	0.094436	0.23589	0.9995	4671	4	0.3606
VPS51	6	0.09444	0.23589	0.9995	4672	1	0.0214
PRKAA1	6	0.094468	0.23594	0.9995	4673	3	0.0271
GLYAT	6	0.094536	0.23607	0.9995	4674	3	0.1858
ZNF664	6	0.09454	0.23608	0.9995	4675	4	0.239
C1orf43	6	0.094608	0.23623	0.9995	4676	3	0.142
hsa-mir-4530	4	0.094618	0.20548	0.9995	4677	2	0.3358
SLC7A6OS	6	0.094636	0.23628	0.9995	4678	4	0.4227
NINJ1	6	0.094656	0.23633	0.9995	4679	4	0.2945
ADH7	6	0.094692	0.23641	0.9995	4680	3	0.4036
CCDC148	6	0.094708	0.23645	0.9995	4681	2	-0.111
GALNT3	6	0.094788	0.23662	0.9995	4682	4	0.2543
KCNU1	6	0.094833	0.23672	0.9995	4683	3	0.2059
hsa-mir-8085	4	0.094839	0.20587	0.9995	4684	3	0.3945
KIAA1147	6	0.09489	0.23684	0.9995	4685	3	0.1843
C19orf10	6	0.094949	0.23696	0.9995	4686	3	0.2115
GPR88	6	0.094991	0.23704	0.9995	4687	2	-0.171
KLHDC8B	6	0.094991	0.23704	0.9995	4688	2	-0.036
GPAA1	6	0.095021	0.2371	0.9995	4689	4	0.2788
ZBTB43	6	0.095037	0.23713	0.9995	4690	3	0.2717
SLC39A7	6	0.095079	0.23723	0.9995	4691	4	0.5334
PRKACA	6	0.095079	0.23723	0.9995	4692	4	0.3058
hsa-let-7g	4	0.095097	0.20631	0.9995	4693	1	-0.408
TMEM138	6	0.09511	0.23729	0.9995	4694	3	0.2536
IGFL3	6	0.095173	0.23741	0.9995	4695	3	0.0614
TRAPPC13	6	0.095187	0.23744	0.9995	4696	4	0.2284
GCK	6	0.095335	0.23773	0.9995	4697	2	0.1354
METTL15	6	0.095372	0.23781	0.9995	4698	3	0.3304
YLPM1	6	0.095383	0.23783	0.9995	4699	2	-0.299
hsa-mir-7977	4	0.095387	0.20682	0.9995	4700	2	0.4436
TTYH1	6	0.095399	0.23787	0.9995	4701	3	0.1736
EPB41L2	6	0.095445	0.23794	0.9995	4702	4	0.199
hsa-mir-6125	4	0.095485	0.20701	0.9995	4703	3	0.3917
LARGE	6	0.095494	0.23805	0.9995	4704	4	0.3012
MFSD10	6	0.095537	0.23811	0.9995	4705	4	0.3072
SCAPER	6	0.09555	0.23814	0.9995	4706	3	0.2721
BMP7	6	0.095603	0.23824	0.9995	4707	4	0.3036
FNBP4	6	0.095638	0.23831	0.9995	4708	2	-0.32
LCE2A	4	0.095645	0.2073	0.9995	4709	1	0.1197
NBEAL1	6	0.095662	0.23835	0.9995	4710	4	0.3386
UBD	6	0.095673	0.23837	0.9995	4711	2	-0.086
MMP27	6	0.095686	0.2384	0.9995	4712	2	-0.051
ADH4	6	0.095733	0.2385	0.9995	4713	4	0.3496
XPO1	6	0.095733	0.2385	0.9995	4714	4	0.4731
SNAPC5	6	0.095733	0.2385	0.9995	4715	4	0.3554
CASC4	6	0.095734	0.2385	0.9995	4716	2	0.0238
TYK2	6	0.095765	0.23856	0.9995	4717	3	0.2717
PPP4R1	6	0.095787	0.2386	0.9995	4718	4	0.3372
MICALL1	6	0.095797	0.23863	0.9995	4719	3	0.3271
NSD1	6	0.095962	0.23898	0.9995	4720	4	0.2855
MRPL47	6	0.096003	0.23905	0.9995	4721	2	0.1474
KLF1	6	0.096012	0.23907	0.9995	4722	4	0.3661
TNRC6C	6	0.096021	0.23908	0.9995	4723	3	0.1612
CTSD	6	0.096058	0.23917	0.9995	4724	4	0.236
C9orf153	6	0.09606	0.23917	0.9995	4725	2	-0.172
SLC23A1	6	0.096069	0.23919	0.9995	4726	1	-0.085
PPTC7	6	0.096088	0.23924	0.9995	4727	4	0.3331
NOSIP	6	0.096213	0.23948	0.9995	4728	2	-0.106
CHST15	6	0.096238	0.23953	0.9995	4729	3	0.2862
PPID	6	0.096238	0.23953	0.9995	4730	3	0.326
POLR1C	6	0.096255	0.23956	0.9995	4731	4	0.4757
GRIN3B	6	0.096285	0.23963	0.9995	4732	1	-0.48
PRSS8	6	0.096357	0.23976	0.9995	4733	2	-0.353
RCVRN	6	0.096404	0.23987	0.9995	4734	2	0.0073
PPP1R7	6	0.096431	0.23991	0.9995	4735	4	0.2984
CD53	6	0.096447	0.23994	0.9995	4736	2	-0.16
hsa-mir-2392	4	0.09645	0.20871	0.9995	4737	2	-0.253
TEX33	6	0.0965	0.24004	0.9995	4738	2	-0.317
ZNF213	6	0.096527	0.2401	0.9995	4739	4	0.4069
NBEAL2	4	0.09653	0.20886	0.9995	4740	1	-0.149
ODC1	6	0.096548	0.24014	0.9995	4741	2	0.1501
ELMOD1	6	0.096596	0.24024	0.9995	4742	2	0.0421
P2RY13	6	0.096609	0.24027	0.9995	4743	2	0.0388
C8B	4	0.096626	0.20904	0.9995	4744	3	0.2168
HS3ST6	6	0.096644	0.24033	0.9995	4745	1	-0.129
DHODH	6	0.096692	0.24042	0.9995	4746	3	0.3095
HSPE1	5	0.096703	0.22126	0.9995	4747	3	0.3332
RPS28	6	0.096707	0.24045	0.9995	4748	4	0.4436
ADAMTS18	6	0.096765	0.24057	0.9995	4749	3	0.2702
ATG4D	6	0.096787	0.24061	0.9995	4750	2	-0.03
KHK	6	0.096787	0.24061	0.9995	4751	2	0.1229
TMEM86B	4	0.096831	0.2094	0.9995	4752	3	0.3501
INCENP	6	0.096835	0.24072	0.9995	4753	2	0.0151
GPC3	6	0.09685	0.24075	0.9995	4754	3	0.0024
LIPF	6	0.096884	0.24082	0.9995	4755	4	0.2073

CNGA2	6	0.096902	0.24086	0.9995	4756	3	0.1073
H2AFB1	1	0.096931	0.096972	0.96841	4757	1	1.3362
DHDH	6	0.096943	0.24094	0.9995	4758	4	0.243
CRHR2	6	0.096979	0.24102	0.9995	4759	2	0.3114
RNF43	6	0.096997	0.24105	0.9995	4760	2	0.0097
MEDAG	6	0.097013	0.24109	0.9995	4761	2	0.0471
TFPT	6	0.097013	0.24109	0.9995	4762	4	0.3796
NDUFA1	6	0.097027	0.24112	0.9995	4763	4	0.291
RHBDF1	6	0.097051	0.24116	0.9995	4764	2	0.0066
XRCC6BP1	6	0.097051	0.24116	0.9995	4765	3	0.2424
KLF4	6	0.097077	0.24122	0.9995	4766	4	0.3245
KCNIP3	6	0.097077	0.24122	0.9995	4767	4	0.3304
COX6B1	6	0.097122	0.24131	0.9995	4768	3	0.303
FRMD5	6	0.09717	0.24139	0.9995	4769	2	-0.46
KRTDAP	5	0.097205	0.22208	0.9995	4770	3	0.1874
XKRX	6	0.097224	0.2415	0.9995	4771	3	0.2421
BICD2	6	0.097225	0.2415	0.9995	4772	4	0.3052
MUC15	6	0.097267	0.24157	0.9995	4773	4	0.4369
CCL4	5	0.097291	0.22223	0.9995	4774	3	0.4263
CDCA5	6	0.097346	0.24172	0.9995	4775	3	0.3658
ELAC1	6	0.097368	0.24175	0.9995	4776	4	0.3214
HMGCS2	6	0.097399	0.24181	0.9995	4777	3	0.2717
TTYH2	6	0.09744	0.24188	0.9995	4778	4	0.457
WNT7B	6	0.097499	0.24199	0.9995	4779	3	0.2448
AGAP2	6	0.09752	0.24202	0.9995	4780	4	0.3505
ZNF608	6	0.097653	0.2423	0.9995	4781	2	-0.098
NARS	6	0.097684	0.24238	0.9995	4782	4	0.3174
APH1B	6	0.097693	0.24239	0.9995	4783	4	0.2045
F12	6	0.097694	0.24239	0.9995	4784	2	-0.047
TMED4	6	0.097718	0.24243	0.9995	4785	4	0.2785
TAL2	6	0.097718	0.24243	0.9995	4786	4	0.303
NRGN	6	0.097742	0.24249	0.9995	4787	3	0.2566
NMUR1	6	0.097744	0.24249	0.9995	4788	1	-0.387
ADAMTS9	6	0.097749	0.2425	0.9995	4789	4	0.3491
hsa-mir-4308	4	0.097865	0.21121	0.9995	4790	1	-0.215
PLBD1	6	0.097882	0.24276	0.9995	4791	3	0.2798
DMC1	6	0.097883	0.24276	0.9995	4792	4	0.2056
BPIFB1	6	0.097888	0.24277	0.9995	4793	1	-0.253
ERAL1	6	0.097914	0.24282	0.9995	4794	3	0.2019
CMPK1	6	0.097935	0.24286	0.9995	4795	2	-0.004
ANO4	6	0.097961	0.24291	0.9995	4796	3	0.3289
hsa-mir-8078	4	0.098026	0.2115	0.9995	4797	2	0.3268
KDM3A	6	0.098034	0.24307	0.9995	4798	3	0.2481
TMEM117	6	0.098079	0.24315	0.9995	4799	2	0.0803
CDK18	6	0.098124	0.24324	0.9995	4800	2	-0.026
C12orf61	6	0.098175	0.24333	0.9995	4801	4	0.3319
YARS	6	0.098209	0.24339	0.9995	4802	4	0.3489
HTT	6	0.098264	0.2435	0.9995	4803	3	0.2266
UQCRQ	6	0.098298	0.24356	0.9995	4804	4	0.266
ZNF75A	6	0.098345	0.24367	0.9995	4805	4	0.3381
hsa-mir-612	4	0.098347	0.21206	0.9995	4806	1	-0.276
C4orf26	4	0.098353	0.21207	0.9995	4807	3	0.3312
LRGUK	6	0.098362	0.2437	0.9995	4808	4	0.2384
ZMYM4	6	0.098366	0.24371	0.9995	4809	2	0.1235
CDC123	6	0.098368	0.24371	0.9995	4810	2	0.1598
TMEM163	6	0.098381	0.24373	0.9995	4811	3	0.2652
C20orf144	6	0.098392	0.24375	0.9995	4812	2	0.1584
ATP4A	6	0.098466	0.24389	0.9995	4813	3	0.0845
SLC16A1	6	0.098466	0.24389	0.9995	4814	3	0.1674
TXNDC11	6	0.098493	0.24395	0.9995	4815	3	0.1414
TSPYL6	6	0.098533	0.24402	0.9995	4816	2	0.2881
ZFYVE20	6	0.098605	0.24416	0.9995	4817	2	-0.277
TTC7A	6	0.098605	0.24416	0.9995	4818	3	0.1943
SLC43A2	6	0.098652	0.24425	0.9995	4819	3	0.3848
ARFGEF1	6	0.098726	0.2444	0.9995	4820	2	0.0439
hsa-mir-4640	4	0.098735	0.21276	0.9995	4821	3	0.261
C1orf74	6	0.098786	0.24453	0.9995	4822	3	0.2709
C7orf66	6	0.098866	0.2447	0.9995	4823	4	0.4117
PARP2	6	0.098872	0.24471	0.9995	4824	3	0.1951
SLC22A9	6	0.098891	0.24475	0.9995	4825	2	-0.251
hsa-mir-496	4	0.098926	0.21311	0.9995	4826	1	-0.236
IL5RA	4	0.098927	0.21311	0.9995	4827	2	0.2276
ATP5H	6	0.098978	0.24494	0.9995	4828	2	-0.128
CDKL3	6	0.098987	0.24496	0.9995	4829	4	0.383
hsa-mir-924	4	0.09902	0.21327	0.9995	4830	3	0.4098
ZFYVE1	6	0.099034	0.24508	0.9995	4831	2	0.139
TM6SF1	6	0.09913	0.24527	0.9995	4832	1	-0.364
DCAF11	6	0.099144	0.2453	0.9995	4833	3	0.2294
KLF16	6	0.099176	0.24536	0.9995	4834	3	0.3547
FBXO38	6	0.099191	0.24539	0.9995	4835	4	0.2237
RGN	6	0.099202	0.24541	0.9995	4836	3	0.2771
NUDT15	6	0.099206	0.24542	0.9995	4837	3	0.1647
RBPJL	6	0.099226	0.24545	0.9995	4838	2	-0.157
G6PC3	6	0.099327	0.24565	0.9995	4839	4	0.289
PRR18	6	0.09934	0.24569	0.9995	4840	4	0.2033

BRK1	6	0.099361	0.24573	0.9995	4841	4	0.3192
CDKN2B	6	0.099391	0.24579	0.9995	4842	4	0.3269
RNF115	6	0.099434	0.24587	0.9995	4843	4	0.249
RABL5	6	0.099443	0.24588	0.9995	4844	3	0.0861
SLC3A2	6	0.099443	0.24588	0.9995	4845	3	0.3455
UCK2	6	0.099516	0.24603	0.9995	4846	3	0.232
KCNMB3	6	0.099516	0.24603	0.9995	4847	2	-0.068
SUN2	6	0.099528	0.24605	0.9995	4848	4	0.2918
SLC39A13	6	0.09955	0.24608	0.9995	4849	3	-0.104
RWDD4	6	0.099582	0.24614	0.9995	4850	3	0.4032
HOMER2	6	0.099607	0.24619	0.9995	4851	2	0.0185
SUSD4	6	0.099626	0.24623	0.9995	4852	4	0.3208
KRTAP4-5	6	0.099647	0.24628	0.9995	4853	2	-0.763
DDX5	6	0.099655	0.24629	0.9995	4854	2	-0.167
UCHL5	6	0.099682	0.24633	0.9995	4855	4	0.2788
LMLN	5	0.099696	0.22603	0.9995	4856	3	0.4418
ADRA1B	6	0.0997	0.24636	0.9995	4857	3	0.25
BOLL	6	0.099746	0.24645	0.9995	4858	4	0.2963
SECISBP2L	6	0.09978	0.2465	0.9995	4859	4	0.2554
AGPAT9	6	0.099786	0.24652	0.9995	4860	2	-0.358
IL2RB	6	0.099813	0.24657	0.9995	4861	3	0.2883
ARNTL	6	0.099827	0.2466	0.9995	4862	2	0.0552
AKAP10	6	0.099846	0.24664	0.9995	4863	2	0.0715
C11orf24	6	0.099859	0.24667	0.9995	4864	2	-0.021
INVS	6	0.099892	0.24672	0.9995	4865	3	0.2552
ZFAND5	6	0.099892	0.24672	0.9995	4866	4	0.2946
hsa-mir-4429	4	0.099912	0.21483	0.9995	4867	3	0.3382
FREM3	6	0.099914	0.24676	0.9995	4868	3	0.4247
MAK	6	0.099957	0.24684	0.9995	4869	3	0.2559
KCNIP2	6	0.099965	0.24686	0.9995	4870	2	-0.204
TMEM41B	6	0.10002	0.24696	0.9995	4871	3	0.2153
C8orf87	6	0.10004	0.247	0.9995	4872	3	0.0351
NAMPT	4	0.10008	0.21512	0.9995	4873	1	-0.561
DYNAP	6	0.10015	0.24722	0.9995	4874	4	0.3566
PPM1N	6	0.10016	0.24723	0.9995	4875	3	0.1921
TBL3	6	0.10017	0.24724	0.9995	4876	3	-0.072
LAIR1	4	0.10027	0.21542	0.9995	4877	2	0.0657
CTU1	6	0.10031	0.24753	0.9995	4878	3	0.1163
PLAC1	6	0.10032	0.24755	0.9995	4879	2	-0.142
NR1I3	4	0.10033	0.21553	0.9995	4880	3	0.2563
CDH12	6	0.10034	0.24759	0.9995	4881	4	0.2939
SPATA31E1	6	0.10039	0.24769	0.9995	4882	2	-0.188
hsa-mir-5096	4	0.1004	0.21566	0.9995	4883	1	-0.826
AP4S1	6	0.10046	0.2478	0.9995	4884	3	0.3011
IL27RA	6	0.10046	0.24783	0.9995	4885	2	-0.398
APLP2	6	0.10047	0.24783	0.9995	4886	2	-0.359
MLF1	6	0.10049	0.24788	0.9995	4887	4	0.2635
TCP11	6	0.10056	0.24801	0.9995	4888	1	-0.186
SUCO	6	0.10059	0.24807	0.9995	4889	3	0.2596
KRT20	6	0.10063	0.24816	0.9995	4890	2	-0.404
FBXO17	6	0.1007	0.24831	0.9995	4891	1	-0.151
hsa-mir-1246	4	0.10071	0.21623	0.9995	4892	3	0.3608
SOX11	6	0.10074	0.24837	0.9995	4893	2	-0.049
JAM2	6	0.10075	0.24841	0.9995	4894	3	0.1022
CLCF1	6	0.10077	0.24845	0.9995	4895	3	-0.145
CD200R1	6	0.10078	0.24846	0.9995	4896	2	-0.305
GPR4	6	0.1008	0.2485	0.9995	4897	4	0.2977
KLHL15	6	0.10087	0.24862	0.9995	4898	3	0.2304
LCN2	6	0.10093	0.24874	0.9995	4899	3	-0.011
CDK19	6	0.10096	0.24879	0.9995	4900	4	0.2288
ANKHD1	4	0.10096	0.21667	0.9995	4901	2	0.3137
CDH4	6	0.10101	0.24889	0.9995	4902	4	0.2878
SORBS1	6	0.10101	0.24889	0.9995	4903	4	0.2111
DCTN5	6	0.10104	0.24894	0.9995	4904	2	0.0527
CD163L1	6	0.10106	0.24899	0.9995	4905	3	0.342
CCDC67	6	0.10109	0.24905	0.9995	4906	2	0.0689
UBA7	6	0.10111	0.2491	0.9995	4907	4	0.2711
CUTC	6	0.10111	0.2491	0.9995	4908	4	0.3926
STX10	6	0.10111	0.2491	0.9995	4909	4	0.2519
CSNK1A1L	6	0.10113	0.24913	0.9995	4910	3	0.0781
SERPINA11	6	0.10115	0.24917	0.9995	4911	4	0.3018
S100A11	6	0.10116	0.24919	0.9995	4912	3	0.2919
OR1A1	6	0.1012	0.24927	0.9995	4913	3	0.1677
LCE3C	6	0.10122	0.2493	0.9995	4914	2	-0.384
CCDC149	6	0.10122	0.2493	0.9995	4915	4	0.4098
PGRMC2	6	0.10126	0.24937	0.9995	4916	4	0.3872
TTC24	6	0.10128	0.2494	0.9995	4917	2	0.0129
FOXP3	6	0.10131	0.24946	0.9995	4918	4	0.359
AQP2	6	0.10134	0.24952	0.9995	4919	3	0.1991
PRR22	6	0.10137	0.24956	0.9995	4920	2	-0.396
OR51G1	6	0.10147	0.24973	0.9995	4921	1	-0.376
RPS6KA5	6	0.1015	0.24979	0.9995	4922	3	0.3048
TMEM170A	6	0.10153	0.24985	0.9995	4923	3	0.189
SRGN	6	0.10154	0.24986	0.9995	4924	3	0.134
CDX2	6	0.10157	0.24993	0.9995	4925	3	0.2923

ZNF283	6	0.10158	0.24994	0.9995	4926	3	0.244
POLI	6	0.10168	0.25013	0.9995	4927	2	-0.331
hsa-mir-1248	4	0.10168	0.21796	0.9995	4928	3	0.309
L3MBTL3	6	0.10169	0.25015	0.9995	4929	4	0.2625
NME4	6	0.10169	0.25015	0.9995	4930	4	0.3232
FBXO27	6	0.10173	0.25024	0.9995	4931	4	0.2521
hsa-mir-4468	4	0.10175	0.21807	0.9995	4932	1	-0.295
AACS	6	0.10175	0.25028	0.9995	4933	2	0.0759
C3orf33	6	0.10176	0.2503	0.9995	4934	3	0.063
TMEM87B	6	0.1018	0.25038	0.9995	4935	1	0.0362
MEF2BNB-MEF2B	2	0.1018	0.17261	0.99709	4936	1	0.5325
KIAA1257	6	0.10186	0.25051	0.9995	4937	4	0.284
EIF3C	1	0.10188	0.10192	0.96841	4938	1	0.7119
LEKR1	6	0.10188	0.25053	0.9995	4939	4	0.3037
RAG2	6	0.10188	0.25053	0.9995	4940	3	0.2142
RDH11	6	0.10195	0.25069	0.9995	4941	3	0.234
AKAP8	6	0.10199	0.25079	0.9995	4942	4	0.3468
LOC284385	3	0.102	0.18598	0.99919	4943	2	0.5137
KIF20B	6	0.10202	0.25084	0.9995	4944	4	0.2466
CEACAM19	6	0.10203	0.25086	0.9995	4945	4	0.2632
IL6ST	6	0.10204	0.25088	0.9995	4946	3	0.3085
KCNK4	6	0.10206	0.25092	0.9995	4947	3	0.315
hsa-mir-29b-2	3	0.10209	0.18611	0.99919	4948	1	-0.291
PLA2G4A	6	0.1021	0.25101	0.9995	4949	4	0.2446
BLZF1	6	0.10216	0.2511	0.9995	4950	3	-0.069
MGST3	6	0.10216	0.25111	0.9995	4951	3	0.1833
NRBP1	6	0.10216	0.25111	0.9995	4952	3	0.397
APPL1	6	0.10216	0.25111	0.9995	4953	3	0.3679
ZC3HAV1	6	0.10217	0.25112	0.9995	4954	3	-0.119
hsa-mir-4703	1	0.10218	0.10219	0.96841	4955	1	0.6808
MYO1A	6	0.10219	0.25115	0.9995	4956	4	0.5109
UBE2L3	6	0.10223	0.25124	0.9995	4957	2	-0.123
GSDMC	6	0.10225	0.25128	0.9995	4958	4	0.321
NDRG2	6	0.10225	0.25128	0.9995	4959	4	0.2756
ACACB	6	0.10227	0.25132	0.9995	4960	4	0.2692
KRTAP3-2	4	0.1023	0.21904	0.9995	4961	3	0.495
SEMA4F	6	0.10232	0.25143	0.9995	4962	1	-0.063
BVES	6	0.10234	0.25146	0.9995	4963	3	-0.047
RSBN1	6	0.10237	0.25153	0.9995	4964	2	0.084
LUZP4	6	0.1024	0.25159	0.9995	4965	3	0.2781
MYO1H	6	0.10254	0.25186	0.9995	4966	3	0.078
C2orf42	6	0.10254	0.25187	0.9995	4967	4	0.3673
RAB14	6	0.10256	0.25191	0.9995	4968	2	0.1544
EIF2AK2	6	0.10258	0.25196	0.9995	4969	4	0.3356
EBAG9	6	0.10263	0.25204	0.9995	4970	4	0.3621
HSP90B1	6	0.10266	0.25211	0.9995	4971	3	0.1779
DNAJC10	6	0.10266	0.25211	0.9995	4972	3	0.0615
MAPK8IP3	6	0.10267	0.25212	0.9995	4973	2	0.0489
SH3D21	6	0.10267	0.25212	0.9995	4974	2	-0.147
ZNF396	6	0.10268	0.25214	0.9995	4975	4	0.4525
MIOS	6	0.10278	0.25234	0.9995	4976	4	0.2632
PIANP	6	0.10278	0.25234	0.9995	4977	3	0.2807
ZNF343	6	0.10279	0.25236	0.9995	4978	3	0.2579
PMCH	6	0.1028	0.25239	0.9995	4979	3	0.182
G6PD	6	0.10285	0.25246	0.9995	4980	3	0.1989
hsa-mir-629	4	0.10287	0.22005	0.9995	4981	3	0.3669
FRS2	6	0.1029	0.25256	0.9995	4982	3	0.041
MEIOB	6	0.1029	0.25257	0.9995	4983	2	0.0853
KRTAP5-3	6	0.10292	0.2526	0.9995	4984	2	0.0535
FAM168A	6	0.10292	0.2526	0.9995	4985	2	-0.026
DUSP11	6	0.10293	0.25262	0.9995	4986	4	0.4005
CEACAM4	6	0.10309	0.25293	0.9995	4987	2	0.0708
BBS7	6	0.10309	0.25294	0.9995	4988	4	0.3439
SYT17	6	0.10309	0.25294	0.9995	4989	4	0.2606
hsa-mir-6165	4	0.1031	0.22048	0.9995	4990	3	0.3843
TIAM2	6	0.10316	0.25308	0.9995	4991	4	0.2584
SYNE4	6	0.10318	0.25311	0.9995	4992	4	0.3167
VKORC1	6	0.1032	0.25314	0.9995	4993	4	0.3296
NDUFA11	6	0.1032	0.25315	0.9995	4994	3	-0.007
CD248	6	0.10325	0.25323	0.9995	4995	4	0.3234
RNF123	4	0.10327	0.22076	0.9995	4996	2	0.034
EPHA6	6	0.10328	0.25328	0.9995	4997	2	0.1645
BCL11A	6	0.10328	0.25328	0.9995	4998	4	0.2988
FBXL19	6	0.10335	0.25342	0.9995	4999	2	0.1344
hsa-mir-4419a	3	0.10337	0.18776	0.99919	5000	2	0.6006
ZIC4	6	0.10337	0.25346	0.9995	5001	4	0.3194
OR4K15	6	0.10339	0.25351	0.9995	5002	2	0.0839
C1QTNF9	2	0.1034	0.17509	0.99919	5003	2	0.8276
hsa-mir-6502	3	0.10341	0.18781	0.99919	5004	2	0.7233
hsa-mir-6823	4	0.10341	0.221	0.9995	5005	3	0.6003
TRAK2	6	0.10342	0.25357	0.9995	5006	3	0.0401
PDE6H	6	0.10345	0.25363	0.9995	5007	3	0.233
MMP26	6	0.10346	0.25365	0.9995	5008	3	0.4408
CARKD	6	0.10347	0.25367	0.9995	5009	3	0.1692
hsa-mir-3945	4	0.10348	0.22114	0.9995	5010	2	0.3668

ADD1	6	0.10349	0.2537	0.9995	5011	3	0.1951
DUSP14	6	0.10354	0.25381	0.9995	5012	4	0.2748
FPR1	6	0.10355	0.25382	0.9995	5013	3	0.3191
ITIH1	6	0.10359	0.2539	0.9995	5014	3	0.2191
SLC26A4	6	0.10359	0.2539	0.9995	5015	3	-0.192
ATP1B3	6	0.1036	0.25391	0.9995	5016	4	0.4157
C7orf73	5	0.10363	0.23211	0.9995	5017	1	-0.152
ELF2	6	0.10363	0.25398	0.9995	5018	3	0.4372
C10orf128	6	0.10363	0.25398	0.9995	5019	2	-0.127
TRAPPC3L	2	0.10365	0.17548	0.99919	5020	2	0.371
TCEAL8	6	0.10366	0.25404	0.9995	5021	4	0.3404
SMPD1	6	0.1037	0.25413	0.9995	5022	1	-0.187
hsa-mir-664a	4	0.10371	0.22156	0.9995	5023	3	0.2962
NUP155	6	0.10373	0.25418	0.9995	5024	3	-0.03
KIAA0226L	6	0.10373	0.25419	0.9995	5025	4	0.3104
SPTY2D1	6	0.10376	0.25424	0.9995	5026	3	0.0975
PCDHGA11	2	0.10379	0.1757	0.99919	5027	2	0.2583
KLHDC3	6	0.1038	0.25433	0.9995	5028	1	-0.304
KIAA0141	6	0.10381	0.25434	0.9995	5029	3	0.008
C16orf89	6	0.10382	0.25436	0.9995	5030	3	0.2274
NDUFC2-KCTD14	4	0.10383	0.22178	0.9995	5031	3	0.3683
CENPBD1	6	0.10384	0.25441	0.9995	5032	4	0.3254
C9orf135	6	0.10385	0.25441	0.9995	5033	3	0.2534
FAM189A1	6	0.10389	0.25449	0.9995	5034	4	0.3602
ZBTB38	6	0.1039	0.2545	0.9995	5035	2	0.0733
REEP5	6	0.10397	0.25466	0.9995	5036	1	-0.265
SCAP	6	0.104	0.25473	0.9995	5037	3	0.651
CCDC115	6	0.10401	0.25474	0.9995	5038	3	0.3143
SYNGR4	6	0.10402	0.25476	0.9995	5039	4	0.2298
EPN1	6	0.10404	0.2548	0.9995	5040	3	0.3877
LRP12	6	0.10405	0.25483	0.9995	5041	3	0.3088
ZBTB11	6	0.10408	0.25486	0.9995	5042	4	0.2805
HMGXB3	6	0.10409	0.25487	0.9995	5043	2	0.1433
COL6A6	6	0.10413	0.25496	0.9995	5044	3	0.0381
CASQ1	6	0.10413	0.25497	0.9995	5045	1	-0.006
WDFY1	6	0.10417	0.25506	0.9995	5046	2	-0.095
TSHZ1	6	0.10418	0.25508	0.9995	5047	2	-0.041
DARS2	6	0.10422	0.25516	0.9995	5048	4	0.2856
NLGN4X	6	0.10424	0.25519	0.9995	5049	3	-0.012
SLC26A5	6	0.10425	0.25521	0.9995	5050	4	0.2687
LIMS1	6	0.10427	0.25526	0.9995	5051	3	0.1048
SLC5A9	6	0.10428	0.25527	0.9995	5052	2	-0.254
ARHGEF9	6	0.10429	0.2553	0.9995	5053	4	0.2748
PACRGL	6	0.10432	0.25536	0.9995	5054	4	0.3231
UBXN7	6	0.10432	0.25536	0.9995	5055	1	-0.118
DNTT	6	0.10434	0.25538	0.9995	5056	3	0.192
LRMP	6	0.10436	0.25542	0.9995	5057	4	0.3687
SLC28A2	6	0.10439	0.25549	0.9995	5058	4	0.316
TMEM102	6	0.10439	0.25549	0.9995	5059	3	0.17
SH2D1A	6	0.10441	0.25553	0.9995	5060	3	-0.147
MAGEA4	6	0.10447	0.25562	0.9995	5061	1	-0.021
SGK2	6	0.10451	0.2557	0.9995	5062	4	0.2282
HIST1H2BB	6	0.10452	0.25574	0.9995	5063	4	0.4048
PRIM2	6	0.10454	0.25577	0.9995	5064	2	-0.053
CHTF8	6	0.10461	0.25592	0.9995	5065	3	0.1871
SLITRK5	6	0.10463	0.25598	0.9995	5066	4	0.2661
KBTBD2	6	0.10465	0.25601	0.9995	5067	4	0.2872
ZNF257	5	0.10467	0.23376	0.9995	5068	2	0.0962
hsa-mir-2277	4	0.10468	0.22327	0.9995	5069	3	0.223
WNT10A	6	0.1047	0.25611	0.9995	5070	4	0.3539
PRSS35	6	0.10473	0.25616	0.9995	5071	4	0.2342
RBM8A	6	0.10475	0.2562	0.9995	5072	3	-0.275
CCL2	6	0.10476	0.25621	0.9995	5073	4	0.3196
CFL2	6	0.10477	0.25624	0.9995	5074	2	-0.06
CNOT3	6	0.10477	0.25624	0.9995	5075	3	0.074
HINFP	4	0.10478	0.22345	0.9995	5076	3	0.4598
CXorf38	6	0.10484	0.25637	0.9995	5077	4	0.2966
SNRPG	6	0.10489	0.25646	0.9995	5078	3	0.1604
MUC16	6	0.1049	0.25647	0.9995	5079	4	0.193
SFRP1	6	0.10494	0.25656	0.9995	5080	3	0.4365
TBC1D14	6	0.105	0.25666	0.9995	5081	3	0.1712
WNT2B	6	0.10504	0.25673	0.9995	5082	2	-0.108
PALM2	1	0.10505	0.10499	0.96841	5083	1	1.3765
TRPS1	6	0.1051	0.25684	0.9995	5084	4	0.206
KRT81	4	0.10513	0.22405	0.9995	5085	3	0.3417
CYB561D2	6	0.10513	0.25691	0.9995	5086	1	-0.123
RPL17	1	0.10518	0.10513	0.96841	5087	1	1.2332
KPNA4	5	0.10522	0.23461	0.9995	5088	3	0.2259
HECA	6	0.10522	0.25706	0.9995	5089	4	0.4074
SEMA3G	6	0.10522	0.25706	0.9995	5090	4	0.3034
TTC19	4	0.10522	0.22424	0.9995	5091	3	0.322
WDR24	6	0.10526	0.25714	0.9995	5092	3	0.3384
TMEM132B	6	0.10526	0.25714	0.9995	5093	3	0.2442
COX7A2L	6	0.10527	0.25717	0.9995	5094	1	-0.432
MMP15	6	0.10532	0.25728	0.9995	5095	4	0.3235

LOXHD1	6	0.10532	0.25728	0.9995	5096	2	-0.099
EBF2	6	0.10535	0.25734	0.9995	5097	4	0.3196
TEDDM1	6	0.10537	0.25739	0.9995	5098	4	0.3742
LRWD1	6	0.10539	0.25742	0.9995	5099	2	-0.225
WDR65	6	0.10544	0.25752	0.9995	5100	3	0.3016
INPP5F	6	0.10551	0.25763	0.9995	5101	3	0.1538
GHR	6	0.10553	0.25767	0.9995	5102	4	0.2836
CSRNP1	6	0.10556	0.25772	0.9995	5103	2	-0.218
MMP21	6	0.10557	0.25774	0.9995	5104	4	0.3088
FABP7	6	0.1056	0.2578	0.9995	5105	2	-0.018
TNFRSF6B	6	0.10561	0.25781	0.9995	5106	3	0.1859
FTCD	6	0.10568	0.25794	0.9995	5107	4	0.2927
hsa-mir-4472-2	3	0.10569	0.19085	0.99919	5108	1	0.5444
CLCA1	6	0.1057	0.25797	0.9995	5109	2	-0.035
HEATR2	6	0.1057	0.25797	0.9995	5110	4	0.2086
FGF19	6	0.10574	0.25806	0.9995	5111	2	0.1183
LDLR	6	0.10575	0.25806	0.9995	5112	4	0.3566
GKN1	4	0.10578	0.22523	0.9995	5113	1	0.046
HIGD1C	6	0.1058	0.25816	0.9995	5114	3	0.3
SPINK1	6	0.1058	0.25816	0.9995	5115	4	0.2963
KLHDC10	6	0.10581	0.25817	0.9995	5116	2	-0.01
IAH1	6	0.10582	0.2582	0.9995	5117	3	-0.092
UQCC2	4	0.10586	0.22538	0.9995	5118	3	0.3754
MRPL24	6	0.10587	0.25829	0.9995	5119	1	-0.449
TMEM18	6	0.10587	0.25829	0.9995	5120	2	-0.084
IFRD1	6	0.10588	0.25832	0.9995	5121	2	-0.074
hsa-mir-933	4	0.10589	0.22542	0.9995	5122	3	0.3173
TRIM45	6	0.10594	0.25845	0.9995	5123	3	-0.056
TOX4	6	0.10599	0.25852	0.9995	5124	2	0.1283
NRBP2	6	0.10603	0.25861	0.9995	5125	1	-0.261
TM9SF3	6	0.10605	0.25866	0.9995	5126	4	0.2244
SMPDL3A	6	0.10608	0.2587	0.9995	5127	1	-0.023
RAET1G	6	0.10608	0.25871	0.9995	5128	2	0.0567
ZFAND3	6	0.10609	0.25872	0.9995	5129	3	0.3475
MYO9A	6	0.1061	0.25874	0.9995	5130	4	0.2877
RRN3	6	0.10613	0.25879	0.9995	5131	3	0.3268
GRM4	6	0.10615	0.25882	0.9995	5132	4	0.2854
SRP54	6	0.10615	0.25884	0.9995	5133	2	-0.025
APOBEC3C	6	0.10622	0.25897	0.9995	5134	2	0.0853
NTMT1	6	0.10623	0.25898	0.9995	5135	3	0.2521
PPP3R2	6	0.10627	0.25904	0.9995	5136	3	0.0784
PARN	6	0.10627	0.25904	0.9995	5137	3	0.2355
HNRNPA2B1	6	0.10627	0.25905	0.9995	5138	4	0.265
PHF6	6	0.10629	0.25908	0.9995	5139	2	-0.303
OR7D4	6	0.10629	0.25908	0.9995	5140	3	0.1559
ABCA6	6	0.10633	0.25917	0.9995	5141	3	0.1839
CTBP2	6	0.10633	0.25917	0.9995	5142	4	0.4375
HGSNAT	6	0.10634	0.25919	0.9995	5143	3	0.2578
PSG3	5	0.10637	0.23641	0.9995	5144	2	0.0093
ZBTB18	4	0.10639	0.22632	0.9995	5145	2	0.3378
ABHD1	6	0.10641	0.25933	0.9995	5146	3	-0.083
TMEM213	6	0.10643	0.25936	0.9995	5147	4	0.2714
MEP1B	6	0.10646	0.25941	0.9995	5148	3	0.2305
RAD54L2	4	0.10647	0.22646	0.9995	5149	2	0.2103
PDE4B	6	0.10648	0.25944	0.9995	5150	4	0.2625
RPUSD1	6	0.10649	0.25946	0.9995	5151	3	0.2407
PPARGC1A	6	0.10651	0.2595	0.9995	5152	3	0.3409
HCST	6	0.10652	0.25951	0.9995	5153	4	0.2633
GLRX3	6	0.10656	0.25958	0.9995	5154	4	0.2276
IFITM1	6	0.10659	0.25964	0.9995	5155	2	-0.045
VAPB	6	0.10665	0.25977	0.9995	5156	2	-0.398
NMBR	4	0.10669	0.22684	0.9995	5157	3	0.3354
METTL20	6	0.10672	0.2599	0.9995	5158	3	0.2942
ZUFSP	6	0.10672	0.2599	0.9995	5159	3	0.2483
RIMBP3B	2	0.10681	0.18028	0.99919	5160	1	0.2927
PRKD3	6	0.10684	0.26015	0.9995	5161	1	-0.299
HIST1H2AK	6	0.10689	0.26025	0.9995	5162	1	-0.374
ST6GALNAC3	6	0.10695	0.26035	0.9995	5163	3	0.2958
RGS16	6	0.10696	0.26037	0.9995	5164	4	0.4198
TPPP2	6	0.10697	0.2604	0.9995	5165	3	0.4371
CRYZL1	6	0.10698	0.26042	0.9995	5166	4	0.3168
PCDHB2	6	0.107	0.26045	0.9995	5167	3	-0.008
HAPLN2	6	0.107	0.26045	0.9995	5168	3	0.4203
VPS37C	6	0.10703	0.26051	0.9995	5169	2	-0.252
CADPS	6	0.10705	0.26056	0.9995	5170	2	0.0445
ABCC8	6	0.10709	0.26063	0.9995	5171	3	0.195
LOC100129216	3	0.10711	0.19273	0.99919	5172	1	-0.419
MLLT1	6	0.10712	0.26071	0.9995	5173	2	-0.067
hsa-mir-4276	4	0.10713	0.22763	0.9995	5174	3	0.2711
NCOA3	6	0.10714	0.26073	0.9995	5175	2	-0.154
ACACA	6	0.10717	0.26079	0.9995	5176	3	0.1242
SLC25A21	6	0.10722	0.2609	0.9995	5177	3	0.1318
DAG1	6	0.10731	0.26107	0.9995	5178	3	0.2137
PIGT	6	0.10731	0.26107	0.9995	5179	1	-0.294
PTH2R	6	0.10735	0.26113	0.9995	5180	4	0.277

PRSS56	6	0.10736	0.26115	0.9995	5181	3	0.0304
SLC24A3	6	0.1074	0.26122	0.9995	5182	3	-0.027
C1orf56	6	0.1074	0.26122	0.9995	5183	2	-0.754
PLOD2	6	0.10746	0.26134	0.9995	5184	3	0.2529
USP37	6	0.1075	0.26142	0.9995	5185	1	-0.28
SLC35D3	6	0.10755	0.26151	0.9995	5186	3	0.1234
CPSF3L	6	0.10756	0.26153	0.9995	5187	4	0.226
KLK6	4	0.10756	0.22837	0.9995	5188	3	0.1971
DDIT4L	6	0.10759	0.26159	0.9995	5189	4	0.3715
ZNF592	6	0.10759	0.26159	0.9995	5190	4	0.3215
TMC5	6	0.10759	0.26159	0.9995	5191	4	0.3036
CCDC91	6	0.10762	0.26165	0.9995	5192	2	-0.396
hsa-mir-563	4	0.10769	0.2286	0.9995	5193	3	0.3158
NDUFS1	6	0.1077	0.2618	0.9995	5194	4	0.2809
NMT1	6	0.10774	0.26187	0.9995	5195	2	0.0652
EVX1	6	0.10774	0.26187	0.9995	5196	1	-0.081
PLK4	6	0.10779	0.26198	0.9995	5197	3	0.2097
MORC3	6	0.10793	0.26225	0.9995	5198	3	-0.087
NOTCH2NL	6	0.10793	0.26225	0.9995	5199	3	0.2203
TIMP4	6	0.10796	0.2623	0.9995	5200	3	0.2874
ICAM4	6	0.10796	0.26232	0.9995	5201	4	0.2781
CCDC53	6	0.108	0.26239	0.9995	5202	3	0.4653
PAM	6	0.10803	0.26245	0.9995	5203	4	0.3475
RNASE7	6	0.10807	0.26252	0.9995	5204	3	-0.152
RBM38	6	0.10809	0.26256	0.9995	5205	4	0.3413
hsa-mir-1289-2	4	0.10814	0.2294	0.9995	5206	2	0.1712
WDR46	6	0.10814	0.26266	0.9995	5207	2	0.0447
MFSD2A	6	0.10818	0.26273	0.9995	5208	4	0.2501
NOL11	6	0.10819	0.26273	0.9995	5209	2	0.1259
SP2	6	0.10821	0.26279	0.9995	5210	4	0.3883
FAM229B	6	0.10821	0.26279	0.9995	5211	2	-0.059
GOT1L1	6	0.10825	0.26286	0.9995	5212	3	0.1057
CYP4A22	5	0.10827	0.23942	0.9995	5213	3	0.3827
hsa-mir-548h-2	2	0.10834	0.18264	0.99919	5214	2	0.3118
RRP36	6	0.10841	0.26316	0.9995	5215	4	0.2993
TRIM54	6	0.10845	0.26324	0.9995	5216	4	0.3987
XG	6	0.10845	0.26324	0.9995	5217	4	0.4003
NIF3L1	6	0.10845	0.26324	0.9995	5218	2	-0.107
AK6	6	0.10848	0.26331	0.9995	5219	4	0.5913
SLC25A19	6	0.1085	0.26334	0.9995	5220	3	0.2035
PCDHB4	6	0.10851	0.26336	0.9995	5221	3	0.1388
OR1I1	6	0.10852	0.26338	0.9995	5222	4	0.3086
C19orf80	6	0.10855	0.26344	0.9995	5223	4	0.2674
NCAPH	6	0.10855	0.26345	0.9995	5224	3	0.2462
hsa-mir-637	4	0.10856	0.23016	0.9995	5225	2	0.4259
FMR1	6	0.10857	0.26349	0.9995	5226	4	0.3231
TRMT112	6	0.10857	0.26349	0.9995	5227	4	0.4017
PTX3	6	0.10859	0.26352	0.9995	5228	2	-0.038
STMND1	6	0.10861	0.26356	0.9995	5229	4	0.286
ABR	6	0.10862	0.26357	0.9995	5230	2	-0.913
PTGIR	6	0.10869	0.26371	0.9995	5231	1	-0.263
C10orf68	6	0.10869	0.26371	0.9995	5232	4	0.3233
hsa-mir-5698	4	0.10872	0.23045	0.9995	5233	3	0.3231
NAPB	6	0.10874	0.26382	0.9995	5234	3	0.1653
DECR1	6	0.10874	0.26382	0.9995	5235	3	0.2789
TBX2	6	0.10876	0.26385	0.9995	5236	2	-0.051
SMAD6	6	0.10881	0.26396	0.9995	5237	3	0.35
hsa-mir-4459	3	0.10883	0.19502	0.99919	5238	1	-1.233
PPIF	6	0.10886	0.26403	0.9995	5239	3	0.2735
IGFN1	6	0.10887	0.26405	0.9995	5240	2	-0.189
C14orf183	6	0.1089	0.26411	0.9995	5241	3	0.1685
S100A9	6	0.10892	0.26416	0.9995	5242	4	0.3309
ARV1	6	0.10892	0.26417	0.9995	5243	3	0.023
SPTLC2	6	0.10892	0.26417	0.9995	5244	3	0.2034
PRAMEF13	5	0.10894	0.24046	0.9995	5245	2	-0.114
SETD6	6	0.10894	0.2642	0.9995	5246	4	0.2001
C20orf78	6	0.10897	0.26424	0.9995	5247	3	0.2113
C6orf15	6	0.10899	0.26428	0.9995	5248	4	0.2903
CPA5	6	0.10902	0.26433	0.9995	5249	2	-0.038
ZHX1	6	0.10903	0.26437	0.9995	5250	2	0.0138
CPLX1	6	0.10905	0.2644	0.9995	5251	4	0.2456
CFTR	6	0.10906	0.26443	0.9995	5252	3	0.2449
UFSP2	6	0.10907	0.26443	0.9995	5253	2	-0.377
C8orf44-SGK3	4	0.10908	0.23108	0.9995	5254	3	0.2042
ZNF425	6	0.1091	0.2645	0.9995	5255	4	0.2781
FYTTD1	6	0.10912	0.26454	0.9995	5256	3	0.1257
SLC41A3	6	0.10912	0.26454	0.9995	5257	3	0.033
DPPA3	6	0.10916	0.2646	0.9995	5258	3	0.1532
SPATA16	6	0.10916	0.2646	0.9995	5259	3	0.145
SNAP23	6	0.10917	0.26461	0.9995	5260	4	0.2482
MAPK12	5	0.10919	0.24085	0.9995	5261	3	0.3371
hsa-mir-4701	4	0.10919	0.23128	0.9995	5262	2	0.3225
GAREM	6	0.10921	0.2647	0.9995	5263	3	0.1635
MAP4K2	6	0.10921	0.26471	0.9995	5264	2	-0.17
TNFRSF19	6	0.10923	0.26474	0.9995	5265	3	0.0638

hsa-mir-4705	4	0.10924	0.23137	0.9995	5266	3	0.4616
PHLDA3	6	0.10925	0.2648	0.9995	5267	1	-0.021
WDR59	4	0.10926	0.23141	0.9995	5268	3	0.4772
TMCO5A	6	0.10934	0.26497	0.9995	5269	3	0.2794
GOT1	6	0.10935	0.26499	0.9995	5270	2	-0.155
SORCS3	6	0.10937	0.26503	0.9995	5271	2	-0.411
ADAM29	6	0.10939	0.26507	0.9995	5272	3	0.1845
RBFA	6	0.10944	0.26517	0.9995	5273	4	0.3249
TMEM150C	6	0.10946	0.26522	0.9995	5274	2	-0.604
ZNF350	6	0.10947	0.26523	0.9995	5275	3	0.2069
FAM177B	6	0.10949	0.26527	0.9995	5276	4	0.3181
ELK1	6	0.10955	0.26539	0.9995	5277	2	0.1741
PRAMEF21	2	0.10957	0.18457	0.99919	5278	1	-0.193
OAZ1	6	0.10962	0.26555	0.9995	5279	2	-0.345
ZBTB25	6	0.10964	0.26558	0.9995	5280	4	0.3931
PPP1R37	6	0.10965	0.26559	0.9995	5281	2	0.1062
CHRNA4	6	0.10969	0.26568	0.9995	5282	2	-0.024
ERC1	6	0.10972	0.26573	0.9995	5283	4	0.3909
FPR3	6	0.10973	0.26575	0.9995	5284	3	0.2816
SUMO2	6	0.10978	0.26584	0.9995	5285	3	-0.053
CCDC144A	5	0.1098	0.24178	0.9995	5286	2	0.1453
SHPRH	6	0.10982	0.26593	0.9995	5287	2	-0.097
TRIB1	6	0.10984	0.26598	0.9995	5288	2	-0.306
ADAMTS1	6	0.10989	0.26607	0.9995	5289	4	0.3001
CPN1	6	0.10991	0.26611	0.9995	5290	3	0.157
PHF20	6	0.10992	0.26612	0.9995	5291	3	0.1882
NCBP1	6	0.10998	0.26623	0.9995	5292	4	0.2172
PSG4	6	0.10999	0.26626	0.9995	5293	3	0.2796
LRRC16B	6	0.11006	0.2664	0.9995	5294	4	0.377
CXorf64	6	0.11006	0.2664	0.9995	5295	2	0.123
CLEC3A	6	0.11006	0.2664	0.9995	5296	2	-0.06
NPAP1	6	0.11006	0.2664	0.9995	5297	2	-0.166
CAP1	6	0.11007	0.26642	0.9995	5298	3	0.2767
FSD1L	6	0.11013	0.26653	0.9995	5299	3	0.2729
IDI1	6	0.11015	0.26656	0.9995	5300	4	0.3364
TAF8	6	0.11015	0.26656	0.9995	5301	2	-0.119
TROVE2	6	0.1102	0.26665	0.9995	5302	4	0.2966
SMUG1	6	0.11022	0.26668	0.9995	5303	3	0.1777
C19orf40	6	0.11029	0.26683	0.9995	5304	2	-0.13
ZNF891	6	0.1103	0.26684	0.9995	5305	2	0.0062
C2orf72	6	0.11036	0.26696	0.9995	5306	3	0.197
PI15	6	0.11041	0.26706	0.9995	5307	4	0.2778
DDN	6	0.11048	0.26721	0.9995	5308	2	-0.026
CHD1L	6	0.11051	0.26726	0.9995	5309	3	0.2674
MTPAP	6	0.11051	0.26726	0.9995	5310	4	0.4783
LOC730183	6	0.11053	0.2673	0.9995	5311	2	-0.204
C8orf86	6	0.1106	0.26744	0.9995	5312	4	0.3591
MBD2	6	0.11062	0.26748	0.9995	5313	4	0.2356
ZNF214	6	0.11072	0.26767	0.9995	5314	3	0.1428
B4GALT6	6	0.11072	0.26767	0.9995	5315	2	-0.19
FGD3	6	0.11076	0.26774	0.9995	5316	4	0.3753
B4GALT4	6	0.11077	0.26775	0.9995	5317	3	-0.006
OPRL1	6	0.11081	0.26785	0.9995	5318	3	0.2074
SGK196	4	0.11082	0.23424	0.9995	5319	3	0.3118
MS4A14	6	0.11091	0.26804	0.9995	5320	3	0.1759
PEX14	6	0.11092	0.26804	0.9995	5321	2	-0.117
FAM204A	6	0.11096	0.26812	0.9995	5322	2	0.2163
CLC	6	0.11101	0.26821	0.9995	5323	3	0.2334
SLC47A1	6	0.11104	0.26828	0.9995	5324	3	0.4588
CYP1A1	6	0.11105	0.26829	0.9995	5325	4	0.2417
ZNF229	6	0.11106	0.26832	0.9995	5326	3	0.3219
HS3ST4	6	0.11107	0.26834	0.9995	5327	4	0.3369
KDELR2	6	0.11108	0.26834	0.9995	5328	3	0.2138
OR5AU1	6	0.1111	0.26838	0.9995	5329	2	0.0384
DRAM2	6	0.1111	0.26838	0.9995	5330	4	0.3137
C20orf197	6	0.11121	0.2686	0.9995	5331	2	-0.158
C15orf41	6	0.11121	0.2686	0.9995	5332	4	0.246
RC3H2	6	0.11122	0.26861	0.9995	5333	3	0.3398
GJB7	6	0.11125	0.26867	0.9995	5334	3	0.3126
C16orf96	6	0.11129	0.26875	0.9995	5335	1	-0.315
CSPG4	6	0.11133	0.26882	0.9995	5336	3	0.377
CYS1	6	0.11133	0.26883	0.9995	5337	3	0.0819
EPHA5	6	0.11134	0.26883	0.9995	5338	4	0.2481
CEP57	6	0.11136	0.26889	0.9995	5339	4	0.2812
ING3	4	0.11138	0.23522	0.9995	5340	2	0.3486
FAR2	6	0.11138	0.26893	0.9995	5341	3	0.1988
KRTAP23-1	6	0.1114	0.26897	0.9995	5342	2	-0.114
hsa-mir-8056	4	0.11142	0.2353	0.9995	5343	1	0.0861
TLR10	6	0.11148	0.2691	0.9995	5344	2	-0.436
KCNT2	6	0.11149	0.26912	0.9995	5345	3	0.2081
AES	6	0.1115	0.26915	0.9995	5346	4	0.3208
CDKL1	6	0.11151	0.26919	0.9995	5347	3	0.2026
FAM110C	6	0.11155	0.26923	0.9995	5348	1	-0.235
WDR17	6	0.11155	0.26923	0.9995	5349	1	-0.036
KCNK16	6	0.11155	0.26924	0.9995	5350	2	-0.003

KRT3	6	0.11157	0.26928	0.9995	5351	3	0.1468
ANTXR2	6	0.1116	0.26933	0.9995	5352	4	0.2297
SLC16A14	6	0.1116	0.26934	0.9995	5353	3	0.3363
CCDC73	5	0.11161	0.24457	0.9995	5354	2	-0.333
PRPF38B	6	0.11164	0.26941	0.9995	5355	3	0.3459
TOP2B	6	0.11166	0.26946	0.9995	5356	4	0.2962
ZNF653	6	0.11167	0.26947	0.9995	5357	2	-0.291
ZNF804B	6	0.11167	0.26947	0.9995	5358	3	-0.078
ANO6	6	0.1117	0.26954	0.9995	5359	2	0.1412
NRBF2	6	0.11171	0.26956	0.9995	5360	2	0.1257
FGFR1OP	6	0.11175	0.26963	0.9995	5361	3	0.3383
DACT3	6	0.11176	0.26964	0.9995	5362	3	0.2188
SV2B	6	0.11176	0.26965	0.9995	5363	4	0.1986
hsa-mir-6084	4	0.1118	0.23598	0.9995	5364	1	-0.213
OLFM1	6	0.11181	0.26974	0.9995	5365	1	-0.146
ABHD10	6	0.11183	0.26978	0.9995	5366	3	0.4256
HBEGF	6	0.11185	0.26983	0.9995	5367	2	-0.183
NDUFC2	4	0.11186	0.2361	0.9995	5368	2	0.2182
ITPA	6	0.11194	0.26999	0.9995	5369	4	0.2453
PRSS54	6	0.11195	0.27001	0.9995	5370	2	-0.429
VTI1A	6	0.11195	0.27002	0.9995	5371	2	-0.089
AUP1	6	0.11198	0.27007	0.9995	5372	4	0.2809
DDX23	6	0.11199	0.27009	0.9995	5373	3	0.4107
CLMN	6	0.11204	0.27019	0.9995	5374	4	0.2644
ATRN	6	0.11204	0.27019	0.9995	5375	2	-0.252
CACNG2	6	0.11211	0.27033	0.9995	5376	4	0.353
HABP2	6	0.11212	0.27034	0.9995	5377	3	0.0923
PDGFA	6	0.11214	0.27038	0.9995	5378	3	-0.028
TBC1D8	6	0.11215	0.27039	0.9995	5379	2	0.1025
HOOK1	6	0.11215	0.2704	0.9995	5380	4	0.3757
VPS26B	6	0.11216	0.27043	0.9995	5381	3	0.2761
TUBB8	6	0.11223	0.27055	0.9995	5382	3	0.0267
MFN1	6	0.11226	0.27062	0.9995	5383	3	0.1532
LOC100130705	6	0.11226	0.27062	0.9995	5384	3	0.2826
hsa-mir-5095	4	0.11227	0.2368	0.9995	5385	2	0.2921
OTC	6	0.11228	0.27065	0.9995	5386	4	0.2673
HIST3H2BB	6	0.11231	0.27071	0.9995	5387	3	0.3716
IFIT1	6	0.11235	0.27079	0.9995	5388	3	0.2616
SIGLECL1	6	0.11235	0.27079	0.9995	5389	3	0.1252
hsa-mir-128-1	4	0.11238	0.237	0.9995	5390	2	0.2641
NALCN	6	0.11239	0.27087	0.9995	5391	3	0.2178
SLC9A2	6	0.11241	0.27091	0.9995	5392	4	0.3034
RSPO2	6	0.11242	0.27093	0.9995	5393	3	0.1724
C1orf229	6	0.11242	0.27093	0.9995	5394	2	-0.082
NID2	6	0.11247	0.27102	0.9995	5395	3	0.3748
UBQLNL	6	0.11251	0.2711	0.9995	5396	4	0.2703
FNDC1	6	0.11251	0.27111	0.9995	5397	2	-0.108
TSPAN4	6	0.11253	0.27115	0.9995	5398	3	0.1399
MTRF1	6	0.11256	0.2712	0.9995	5399	3	0.0877
NDUFC1	6	0.11264	0.27134	0.9995	5400	4	0.2274
C17orf78	6	0.11265	0.27137	0.9995	5401	3	0.124
PAAF1	6	0.11276	0.27161	0.9995	5402	4	0.2282
RBM19	6	0.11277	0.27162	0.9995	5403	4	0.3929
IFI30	6	0.11278	0.27163	0.9995	5404	3	0.287
ALS2CR11	6	0.1128	0.27166	0.9995	5405	3	0.0363
TMEM160	6	0.11284	0.27176	0.9995	5406	4	0.2294
SPAG11B	2	0.11286	0.18964	0.99919	5407	2	0.5429
ERMN	6	0.11288	0.27184	0.9995	5408	4	0.2021
EZH1	6	0.11291	0.27191	0.9995	5409	3	0.2385
PRTG	6	0.11292	0.27192	0.9995	5410	4	0.3552
RBM15	6	0.11296	0.27201	0.9995	5411	3	0.2317
FFAR3	6	0.11297	0.27202	0.9995	5412	4	0.3183
NRIP1	6	0.11308	0.27226	0.9995	5413	2	-0.021
TTC9	6	0.11312	0.27234	0.9995	5414	3	0.4058
ZDHHC6	6	0.1132	0.27248	0.9995	5415	3	0.2829
hsa-mir-6129	4	0.11322	0.23848	0.9995	5416	3	0.2905
RERG	6	0.11322	0.2725	0.9995	5417	4	0.1999
MAP3K8	6	0.11324	0.27254	0.9995	5418	4	0.3095
RPAIN	6	0.11324	0.27255	0.9995	5419	2	-0.07
FBXO46	6	0.11325	0.27256	0.9995	5420	2	-0.237
CBFB	6	0.11328	0.27261	0.9995	5421	4	0.2995
LGALS16	5	0.11331	0.24719	0.9995	5422	2	-0.185
C1orf21	6	0.11332	0.27269	0.9995	5423	2	-0.517
STPG2	6	0.11332	0.2727	0.9995	5424	4	0.3544
PCDHA11	2	0.11333	0.19035	0.99919	5425	1	0.7362
hsa-mir-299	4	0.11336	0.23874	0.9995	5426	1	-0.339
NSMAF	6	0.11338	0.27279	0.9995	5427	4	0.3423
COX7B2	6	0.11353	0.27308	0.9995	5428	4	0.2132
SLC43A1	6	0.11354	0.27311	0.9995	5429	4	0.1844
MYOZ2	6	0.11357	0.27315	0.9995	5430	1	-0.377
TAF6	6	0.11359	0.27318	0.9995	5431	4	0.2767
hsa-mir-3118-3	1	0.11366	0.11369	0.97292	5432	1	0.6693
CYP24A1	6	0.11369	0.27338	0.9995	5433	4	0.3742
FGF13	5	0.1138	0.24796	0.9995	5434	1	-0.146
OPCML	6	0.11383	0.27366	0.9995	5435	2	-0.013

FGF23	6	0.11387	0.27372	0.9995	5436	3	0.1055
TTC34	6	0.11394	0.27386	0.9995	5437	4	0.265
PLEKHA1	6	0.11398	0.27396	0.9995	5438	3	-0.012
SOD2	6	0.11402	0.27403	0.9995	5439	4	0.4125
FAM200B	6	0.11403	0.27404	0.9995	5440	3	0.31
IGDCC3	6	0.11407	0.27413	0.9995	5441	4	0.3208
SEC11A	6	0.1141	0.27419	0.9995	5442	4	0.2302
AOAH	6	0.11414	0.27426	0.9995	5443	1	-0.291
FAM163A	6	0.11419	0.27435	0.9995	5444	3	0.2959
FBN2	6	0.11426	0.27447	0.9995	5445	2	-0.444
MYH7B	6	0.1143	0.27455	0.9995	5446	3	0.2531
FUT4	6	0.1143	0.27456	0.9995	5447	1	-0.137
ADRB1	6	0.11431	0.27457	0.9995	5448	4	0.2783
NR2F1	6	0.11434	0.27462	0.9995	5449	3	0.2296
MFN2	6	0.11435	0.27464	0.9995	5450	3	0.436
DLL4	4	0.11437	0.2405	0.9995	5451	2	0.1865
NACC2	6	0.11443	0.27479	0.9995	5452	2	0.1341
HSPA5	6	0.11446	0.27483	0.9995	5453	4	0.3367
FGFBP2	6	0.11447	0.27485	0.9995	5454	2	-0.524
RDH12	6	0.1145	0.2749	0.9995	5455	4	0.3807
GCC1	6	0.11451	0.27492	0.9995	5456	2	0.0411
TRPC3	6	0.11456	0.27503	0.9995	5457	4	0.255
NUP85	4	0.11458	0.24085	0.9995	5458	3	0.327
RBL1	6	0.11459	0.27507	0.9995	5459	4	0.3512
TNNI3K	6	0.11463	0.27516	0.9995	5460	3	-0.021
AKAP7	6	0.11465	0.27519	0.9995	5461	2	-0.066
GGT7	6	0.11465	0.27519	0.9995	5462	3	0.0472
NTN3	6	0.11468	0.27524	0.9995	5463	4	0.3122
WDR63	6	0.1147	0.27528	0.9995	5464	3	0.2114
TMOD1	6	0.11478	0.27542	0.9995	5465	2	-0.114
GAS2L3	6	0.11481	0.27546	0.9995	5466	3	0.3833
AARS2	6	0.11483	0.27549	0.9995	5467	3	0.2121
ASAP1	6	0.11489	0.27563	0.9995	5468	3	0.083
HKR1	6	0.11492	0.27567	0.9995	5469	1	-0.46
MGRN1	6	0.11494	0.27572	0.9995	5470	3	0.2516
hsa-mir-6071	4	0.11494	0.24146	0.9995	5471	2	0.1901
LYST	6	0.115	0.27584	0.9995	5472	4	0.2689
PTPN3	6	0.11504	0.2759	0.9995	5473	4	0.2777
LMBR1L	6	0.11505	0.27593	0.9995	5474	3	-0.124
OAZ2	6	0.11505	0.27593	0.9995	5475	3	-0.052
PATE2	6	0.1151	0.27603	0.9995	5476	4	0.2816
VN1R4	6	0.11515	0.27613	0.9995	5477	2	-0.179
CCDC36	6	0.11522	0.27627	0.9995	5478	3	0.2655
FAM149B1	6	0.11525	0.27631	0.9995	5479	4	0.2035
SLC22A6	6	0.11527	0.27635	0.9995	5480	3	0.1395
MRPS11	6	0.11532	0.27645	0.9995	5481	4	0.2781
TTI1	6	0.11539	0.27658	0.9995	5482	3	0.1997
ALLC	6	0.1154	0.2766	0.9995	5483	4	0.2727
ANO8	6	0.11547	0.27674	0.9995	5484	4	0.2622
PDCD2L	6	0.11551	0.2768	0.9995	5485	4	0.346
MTMR12	6	0.11557	0.27692	0.9995	5486	4	0.2522
GPR157	6	0.11557	0.27692	0.9995	5487	4	0.3021
FYB	6	0.11557	0.27692	0.9995	5488	4	0.426
VPS41	6	0.11558	0.27693	0.9995	5489	2	-0.284
TUB	6	0.11559	0.27695	0.9995	5490	3	0.2399
RABGGTA	6	0.11563	0.27703	0.9995	5491	4	0.2786
MFSD7	6	0.11563	0.27704	0.9995	5492	3	0.2185
FANCD2	6	0.11566	0.27709	0.9995	5493	3	0.1687
CCT8	6	0.11567	0.27711	0.9995	5494	2	0.0772
ANAPC15	6	0.11571	0.27718	0.9995	5495	3	0.5059
CREG2	6	0.11572	0.2772	0.9995	5496	2	-0.775
USP34	6	0.11572	0.27721	0.9995	5497	3	0.2198
TCEB3B	6	0.11576	0.27731	0.9995	5498	3	0.3137
CPZ	6	0.11586	0.27748	0.9995	5499	2	-0.092
UBE2G1	6	0.11593	0.27763	0.9995	5500	3	0.2208
ZMYND19	6	0.11595	0.27766	0.9995	5501	1	-0.202
ENKD1	6	0.11601	0.27778	0.9995	5502	4	0.2518
PSENEN	6	0.11604	0.27784	0.9995	5503	4	0.256
DNAJC30	6	0.11607	0.27791	0.9995	5504	3	0.0711
ACBD4	6	0.11607	0.27791	0.9995	5505	3	0.2281
WIPF2	6	0.11619	0.27813	0.9995	5506	3	0.184
KCNMB4	6	0.11619	0.27813	0.9995	5507	3	0.3316
ACTL7B	6	0.11623	0.27821	0.9995	5508	1	-0.238
MTFMT	6	0.11625	0.27825	0.9995	5509	3	0.0544
TEX29	6	0.11628	0.27829	0.9995	5510	4	0.1726
CCDC58	6	0.11628	0.27829	0.9995	5511	3	0.0527
PITRM1	6	0.11633	0.27839	0.9995	5512	3	0.2386
HEATR3	6	0.11636	0.27846	0.9995	5513	3	0.183
TXNDC2	6	0.11639	0.27851	0.9995	5514	4	0.2323
DIXDC1	6	0.11644	0.2786	0.9995	5515	3	0.2674
EEF1A1	6	0.11649	0.27871	0.9995	5516	2	-0.224
LINGO4	6	0.1165	0.27872	0.9995	5517	4	0.21
DDRGK1	6	0.1165	0.27872	0.9995	5518	3	0.2143
SLC25A12	6	0.11652	0.27877	0.9995	5519	2	0.0855
C3AR1	6	0.11654	0.27881	0.9995	5520	3	0.1866

TPTE	6	0.11654	0.27881	0.9995	5521	3	0.2382
THAP11	6	0.11661	0.27894	0.9995	5522	3	0.3035
LILRB3	6	0.1167	0.27909	0.9995	5523	4	0.1955
DCTN2	6	0.11671	0.27912	0.9995	5524	2	0.0475
FANK1	6	0.11673	0.27915	0.9995	5525	2	-0.233
RDM1	6	0.11674	0.27917	0.9995	5526	3	0.2079
CCL15	6	0.11675	0.2792	0.9995	5527	4	0.2075
LRRC37A3	5	0.11676	0.2525	0.9995	5528	3	0.2618
GALNT2	6	0.1168	0.27928	0.9995	5529	3	0.2736
SIMC1	6	0.1168	0.27928	0.9995	5530	3	0.1363
STAT6	6	0.11682	0.27932	0.9995	5531	4	0.2828
HIST2H4A	1	0.11684	0.11682	0.9754	5532	1	0.5849
PHYHIPL	6	0.11688	0.27944	0.9995	5533	3	0.2862
EIF3D	6	0.11689	0.27945	0.9995	5534	4	0.3088
PLXNA3	6	0.11689	0.27945	0.9995	5535	4	0.2933
ZNF334	6	0.11699	0.27964	0.9995	5536	3	0.1701
RGPD4	5	0.11699	0.25284	0.9995	5537	2	0.1522
STARD3NL	6	0.11703	0.27972	0.9995	5538	2	-0.288
hsa-mir-4267	2	0.11707	0.19602	0.99919	5539	2	0.4377
FGA	6	0.11708	0.27979	0.9995	5540	3	0.0778
OR10T2	6	0.11708	0.2798	0.9995	5541	4	0.2733
CADM3	6	0.11708	0.2798	0.9995	5542	4	0.2975
hsa-mir-640	4	0.1171	0.24519	0.9995	5543	2	0.0565
CD7	6	0.11713	0.27989	0.9995	5544	2	0.2015
CRK	6	0.11713	0.2799	0.9995	5545	4	0.2183
hsa-mir-4442	4	0.11716	0.24529	0.9995	5546	2	0.2076
BMP10	6	0.11716	0.27995	0.9995	5547	3	0.2375
AKTIP	6	0.11716	0.27996	0.9995	5548	4	0.3113
ZNF26	6	0.11717	0.27998	0.9995	5549	4	0.2179
MMP28	4	0.11724	0.24542	0.9995	5550	2	0.3448
SYNDIG1	6	0.11725	0.28014	0.9995	5551	4	0.2451
hsa-mir-4777	4	0.11725	0.24546	0.9995	5552	2	-0.001
hsa-mir-4715	3	0.11726	0.20607	0.9995	5553	2	0.4989
VAC14	6	0.11727	0.28016	0.9995	5554	2	0.1545
CNTNAP1	6	0.11732	0.28026	0.9995	5555	3	0.226
GRAMD1C	6	0.11734	0.28031	0.9995	5556	4	0.2242
FRG2C	5	0.11734	0.25338	0.9995	5557	1	-0.177
FAM13C	6	0.11738	0.28038	0.9995	5558	4	0.2027
DNAJB8	6	0.11739	0.28039	0.9995	5559	3	0.416
FAM173A	6	0.1174	0.28041	0.9995	5560	4	0.3273
MYT1L	6	0.11743	0.28048	0.9995	5561	3	0.1808
B4GALNT3	6	0.11745	0.28052	0.9995	5562	3	0.3402
CLEC4C	6	0.11753	0.28065	0.9995	5563	2	-0.034
C19orf59	6	0.11755	0.28069	0.9995	5564	1	-0.362
PNO1	6	0.11755	0.28071	0.9995	5565	4	0.2375
AIFM2	6	0.11755	0.28071	0.9995	5566	4	0.2363
CFHR4	6	0.11756	0.28072	0.9995	5567	3	0.2708
ZNF160	6	0.1176	0.28078	0.9995	5568	1	-0.261
IBA57	6	0.1176	0.28079	0.9995	5569	2	-0.738
UBXN8	6	0.11763	0.28086	0.9995	5570	4	0.3092
C2orf83	6	0.11768	0.28095	0.9995	5571	4	0.2003
ZNF804A	6	0.11768	0.28096	0.9995	5572	3	0.0547
hsa-mir-3653	4	0.1177	0.24622	0.9995	5573	3	0.3676
hsa-mir-6733	4	0.1177	0.24623	0.9995	5574	2	-0.026
N6AMT1	6	0.11771	0.281	0.9995	5575	4	0.1879
PTPN6	6	0.11771	0.281	0.9995	5576	2	-0.004
ZP2	6	0.11774	0.28106	0.9995	5577	3	0.3093
PLXNA2	6	0.11776	0.28111	0.9995	5578	4	0.3545
HBB	6	0.11776	0.28111	0.9995	5579	4	0.2989
CRTAP	6	0.11776	0.28111	0.9995	5580	4	0.272
WFDC6	4	0.11778	0.24637	0.9995	5581	3	0.2917
GINM1	6	0.11779	0.28116	0.9995	5582	4	0.212
hsa-mir-548f-2	3	0.1178	0.20678	0.9995	5583	2	0.4061
ZNF322	6	0.11788	0.28131	0.9995	5584	4	0.3195
MUC5B	6	0.11788	0.28133	0.9995	5585	2	-0.039
NDUFB3	6	0.11789	0.28134	0.9995	5586	3	0.321
KMT2E	6	0.11795	0.28144	0.9995	5587	4	0.328
MICAL3	6	0.11795	0.28144	0.9995	5588	4	0.3598
GK2	6	0.11799	0.28153	0.9995	5589	3	0.1737
VIT	6	0.11802	0.28159	0.9995	5590	2	0.0971
HYAL4	6	0.11802	0.2816	0.9995	5591	3	0.3165
SGPP1	6	0.11802	0.2816	0.9995	5592	3	0.2862
C14orf159	6	0.11802	0.2816	0.9995	5593	4	0.2363
CCAR1	6	0.11806	0.28168	0.9995	5594	2	-0.101
RFWD2	6	0.11807	0.28169	0.9995	5595	4	0.2862
SMPD3	6	0.11811	0.28177	0.9995	5596	1	0.1211
NAV3	6	0.11813	0.2818	0.9995	5597	3	0.2228
BIRC2	6	0.11816	0.28185	0.9995	5598	2	-0.183
ATP9A	6	0.11821	0.28195	0.9995	5599	2	0.0457
SLA2	6	0.11824	0.28201	0.9995	5600	4	0.4426
RBMX2	6	0.11826	0.28206	0.9995	5601	2	0.1226
MTPN	6	0.11828	0.28209	0.9995	5602	4	0.208
CARD6	6	0.11831	0.28215	0.9995	5603	4	0.3648
ERCC6L	6	0.11831	0.28215	0.9995	5604	4	0.3563
GPX6	6	0.11833	0.28219	0.9995	5605	2	0.162

EVI2B	6	0.11835	0.28222	0.9995	5606	2	-0.036
NCOA6	6	0.11839	0.2823	0.9995	5607	3	0.2213
PCDHGA10	2	0.11846	0.19817	0.99919	5608	1	0.2779
FGL2	6	0.11849	0.28249	0.9995	5609	2	0.1107
FLJ27352	3	0.11851	0.2077	0.9995	5610	1	-0.253
TBC1D2B	4	0.11851	0.24765	0.9995	5611	3	0.2323
OSGIN1	6	0.11852	0.28254	0.9995	5612	3	0.3271
MLH1	6	0.11853	0.28257	0.9995	5613	3	0.1483
hsa-mir-492	4	0.11854	0.2477	0.9995	5614	2	0.2959
OR2A1	1	0.11854	0.11852	0.9754	5615	1	0.8987
P2RY12	6	0.11855	0.2826	0.9995	5616	4	0.2279
EDNRB	6	0.11855	0.28261	0.9995	5617	3	0.2614
ZNF829	6	0.11858	0.28267	0.9995	5618	3	0.1506
POFUT1	6	0.1186	0.2827	0.9995	5619	2	-0.278
METTL4	6	0.11862	0.28274	0.9995	5620	3	0.2255
GAST	6	0.11863	0.28275	0.9995	5621	2	-0.156
NDUFAF4	6	0.11867	0.28283	0.9995	5622	3	0.1107
ABRA	6	0.11871	0.2829	0.9995	5623	4	0.1778
SH3RF2	6	0.11872	0.28293	0.9995	5624	3	0.2539
ADCY1	6	0.11872	0.28294	0.9995	5625	4	0.2276
PRIM1	6	0.11875	0.28299	0.9995	5626	4	0.3628
DPP8	6	0.11881	0.2831	0.9995	5627	2	0.0727
hsa-mir-5585	4	0.11881	0.2482	0.9995	5628	3	0.401
MSANTD2	6	0.11882	0.28312	0.9995	5629	1	-0.257
FAM131A	6	0.11883	0.28314	0.9995	5630	4	0.2842
SLC11A1	6	0.11884	0.28317	0.9995	5631	3	0.0746
EPYC	6	0.11886	0.28321	0.9995	5632	2	-0.111
SRSF12	6	0.11889	0.28326	0.9995	5633	4	0.2535
STARD3	6	0.11891	0.2833	0.9995	5634	2	-0.055
CASS4	6	0.11901	0.2835	0.9995	5635	4	0.4699
RAC1	6	0.11907	0.28359	0.9995	5636	2	0.1018
MTMR10	6	0.1191	0.28364	0.9995	5637	4	0.2638
SLC5A6	6	0.11915	0.28376	0.9995	5638	2	-0.203
CCDC168	6	0.1192	0.28384	0.9995	5639	4	0.2937
TCF3	6	0.11921	0.28387	0.9995	5640	2	-0.098
ZNF285	6	0.11923	0.28391	0.9995	5641	3	0.2277
AK5	6	0.11925	0.28395	0.9995	5642	3	0.1861
GABRE	6	0.11928	0.284	0.9995	5643	3	0.0676
ARSD	6	0.11937	0.28417	0.9995	5644	3	0.1857
ATF7	6	0.11938	0.28419	0.9995	5645	3	0.1699
hsa-mir-4326	4	0.11942	0.24928	0.9995	5646	3	0.3589
ARL5A	6	0.11947	0.28437	0.9995	5647	3	0.1725
TMEM196	6	0.11949	0.2844	0.9995	5648	1	-0.456
LMBRD2	6	0.11952	0.28444	0.9995	5649	3	0.2946
NCMAP	6	0.11953	0.28447	0.9995	5650	3	0.0771
AGO4	6	0.11955	0.28451	0.9995	5651	4	0.5357
CTR9	6	0.11961	0.28463	0.9995	5652	3	0.2968
hsa-mir-3164	4	0.11963	0.24964	0.9995	5653	2	0.3426
NFU1	6	0.11969	0.28479	0.9995	5654	4	0.3212
DDX3Y	6	0.11971	0.28482	0.9995	5655	3	-0.003
C3orf37	4	0.11972	0.2498	0.9995	5656	1	-0.288
NLGN1	6	0.11976	0.28492	0.9995	5657	4	0.3163
ARG1	6	0.11976	0.28492	0.9995	5658	2	-0.131
SIRT1	6	0.11981	0.28502	0.9995	5659	4	0.215
LOC100129636	4	0.11982	0.24998	0.9995	5660	2	0.3567
RAB33B	6	0.11985	0.28508	0.9995	5661	3	0.1731
MORF4L2	6	0.11986	0.28511	0.9995	5662	4	0.2248
KCNA3	6	0.11989	0.28518	0.9995	5663	4	0.3832
SPRR2G	5	0.11993	0.25731	0.9995	5664	1	-0.106
PTTG1IP	6	0.11994	0.28527	0.9995	5665	4	0.2658
DCSTAMP	6	0.11994	0.28528	0.9995	5666	3	0.1952
AGPAT3	6	0.11995	0.28529	0.9995	5667	2	-0.059
THBS4	6	0.11996	0.28531	0.9995	5668	4	0.2287
CPA4	6	0.11999	0.28537	0.9995	5669	4	0.2817
CIRH1A	6	0.12	0.28538	0.9995	5670	4	0.2737
CYC1	6	0.12003	0.28543	0.9995	5671	2	-0.129
PPP1R32	6	0.12005	0.28548	0.9995	5672	4	0.2497
TRIOBP	6	0.12008	0.28553	0.9995	5673	1	-0.32
ZMPSTE24	6	0.12008	0.28554	0.9995	5674	3	0.1696
IL17C	6	0.1201	0.28556	0.9995	5675	4	0.2579
C1orf210	6	0.12013	0.28562	0.9995	5676	4	0.338
RGMB	6	0.12017	0.28568	0.9995	5677	3	0.2017
GPC4	6	0.12027	0.28588	0.9995	5678	1	-0.355
BNIP3	6	0.12033	0.28598	0.9995	5679	2	-0.226
TDRD7	6	0.12034	0.286	0.9995	5680	1	-0.082
TIFA	6	0.12034	0.286	0.9995	5681	2	-0.002
hsa-mir-6822	4	0.12036	0.2509	0.9995	5682	3	0.3145
HOMEZ	6	0.12036	0.28604	0.9995	5683	3	0.2463
DNAL1	3	0.12043	0.21023	0.9995	5684	1	-0.101
hsa-mir-3692	4	0.12046	0.25106	0.9995	5685	3	0.3425
SRMS	6	0.12046	0.28622	0.9995	5686	4	0.25
FARP1	6	0.12048	0.28626	0.9995	5687	2	-0.011
GNB2	6	0.1205	0.2863	0.9995	5688	2	-0.013
GYG2	6	0.1205	0.28631	0.9995	5689	4	0.2961
TRPV3	6	0.12052	0.28633	0.9995	5690	4	0.146

ZNF665	5	0.12056	0.25826	0.9995	5691	2	0.2991
NEFH	6	0.12058	0.28647	0.9995	5692	3	0.2819
CALHM3	6	0.1206	0.28649	0.9995	5693	2	-0.163
OR56A3	6	0.1206	0.2865	0.9995	5694	4	0.232
ABO	6	0.12061	0.28651	0.9995	5695	2	-0.324
ZIM2	1	0.12064	0.12057	0.9754	5696	1	1.0044
CCNB1	6	0.12068	0.28664	0.9995	5697	4	0.2962
C1orf189	6	0.12068	0.28664	0.9995	5698	4	0.3625
PAPOLA	6	0.12071	0.2867	0.9995	5699	4	0.2687
LAMTOR5	6	0.12071	0.28671	0.9995	5700	3	0.2634
NUPR1	6	0.12073	0.28674	0.9995	5701	4	0.3391
CTNNA3	6	0.1208	0.28686	0.9995	5702	3	0.2143
hsa-mir-4699	4	0.12082	0.25171	0.9995	5703	3	0.254
PAF1	6	0.12083	0.28692	0.9995	5704	3	0.1983
ZNF766	6	0.12087	0.28699	0.9995	5705	3	0.2476
NF1	6	0.12088	0.28701	0.9995	5706	3	0.0707
CYP4F8	6	0.12091	0.28706	0.9995	5707	4	0.3277
TMEM180	6	0.12094	0.28714	0.9995	5708	3	0.2282
RAB11FIP1	6	0.12097	0.28719	0.9995	5709	1	-0.272
DNAJC5G	6	0.12099	0.28722	0.9995	5710	4	0.3421
LRIT3	6	0.12104	0.28731	0.9995	5711	3	0.1906
DUSP27	6	0.12109	0.28741	0.9995	5712	4	0.2608
AADAT	6	0.12115	0.28751	0.9995	5713	3	0.2847
GJA5	6	0.12115	0.28751	0.9995	5714	3	0.2152
NUTF2	6	0.12119	0.28759	0.9995	5715	4	0.2636
RBBP7	6	0.1212	0.28761	0.9995	5716	2	0.1097
EIF2B5	6	0.12125	0.28769	0.9995	5717	4	0.2675
ITFG1	6	0.12125	0.28769	0.9995	5718	4	0.264
CBY3	6	0.12127	0.28773	0.9995	5719	3	0.3126
LPCAT4	6	0.12127	0.28773	0.9995	5720	2	-0.337
ACSBG1	6	0.1213	0.28778	0.9995	5721	2	-0.093
TST	6	0.12142	0.28801	0.9995	5722	4	0.2216
MRPL3	6	0.12144	0.28805	0.9995	5723	3	0.296
STX1B	6	0.12144	0.28805	0.9995	5724	3	0.069
OTOS	6	0.12146	0.28811	0.9995	5725	4	0.2646
DROSHA	6	0.1215	0.28818	0.9995	5726	3	0.0595
ZNF106	6	0.12152	0.2882	0.9995	5727	3	0.3294
COL5A3	6	0.12152	0.2882	0.9995	5728	3	0.2814
SMIM10	6	0.12152	0.28821	0.9995	5729	3	0.2718
H3F3A	6	0.12153	0.28823	0.9995	5730	2	-0.251
DIP2C	6	0.12155	0.28826	0.9995	5731	4	0.1607
PSKH2	6	0.12157	0.28831	0.9995	5732	4	0.2896
LOC200726	6	0.12165	0.28847	0.9995	5733	3	0.187
BEND4	6	0.12165	0.28847	0.9995	5734	1	-0.554
SELL	6	0.12171	0.28858	0.9995	5735	4	0.305
ZNF264	5	0.12172	0.25999	0.9995	5736	2	-0.066
ZNF587B	6	0.12176	0.28869	0.9995	5737	1	-0.162
SCAMP5	6	0.12177	0.2887	0.9995	5738	2	0.0026
hsa-mir-5683	2	0.12181	0.20317	0.9995	5739	2	0.3407
MKL2	6	0.12183	0.28882	0.9995	5740	4	0.2446
INTS8	6	0.1219	0.28895	0.9995	5741	4	0.3028
MAD1L1	6	0.12205	0.28923	0.9995	5742	3	0.2648
ALG11	6	0.12208	0.28929	0.9995	5743	4	0.408
PDE1B	6	0.12212	0.28936	0.9995	5744	3	0.3506
TNXB	6	0.12212	0.28936	0.9995	5745	3	0.0381
FETUB	6	0.12216	0.28946	0.9995	5746	4	0.2733
GJC3	6	0.12221	0.28954	0.9995	5747	4	0.2345
TMC1	6	0.12226	0.28964	0.9995	5748	4	0.452
ANKRD11	6	0.12226	0.28964	0.9995	5749	2	-0.233
KLHL1	6	0.12226	0.28964	0.9995	5750	3	0.0242
SLAMF7	6	0.12235	0.28982	0.9995	5751	3	0.1541
CPM	6	0.12235	0.28982	0.9995	5752	3	-0.052
SASH3	6	0.12236	0.28984	0.9995	5753	2	-0.101
hsa-mir-635	4	0.1224	0.25445	0.9995	5754	3	0.4991
ILKAP	6	0.12243	0.28996	0.9995	5755	2	-0.077
PSMA1	6	0.12246	0.29001	0.9995	5756	4	0.2673
hsa-mir-486	1	0.12249	0.12245	0.9754	5757	1	0.5816
OR2T10	6	0.12251	0.2901	0.9995	5758	3	0.2525
SULT1C2	6	0.12251	0.29011	0.9995	5759	2	-0.127
STMN3	6	0.12253	0.29014	0.9995	5760	4	0.254
TESK2	6	0.12261	0.29028	0.9995	5761	1	0.0581
ADPRHL2	6	0.12265	0.29036	0.9995	5762	2	0.1324
FAM73B	6	0.12265	0.29037	0.9995	5763	1	-0.144
PXT1	6	0.1227	0.29046	0.9995	5764	3	0.1259
ECD	6	0.12275	0.29055	0.9995	5765	3	-0.074
PSMD3	6	0.12279	0.29063	0.9995	5766	2	-0.486
AMPD2	6	0.12279	0.29064	0.9995	5767	4	0.2141
CAPZA2	6	0.12281	0.29068	0.9995	5768	3	0.0713
FAM19A2	6	0.12281	0.29068	0.9995	5769	4	0.1946
GRHL3	6	0.12289	0.29083	0.9995	5770	3	-0.012
SMC4	6	0.12291	0.29088	0.9995	5771	2	0.0749
PCDH8	6	0.12295	0.29095	0.9995	5772	3	0.2596
TMEM252	6	0.12295	0.29095	0.9995	5773	4	0.2046
hsa-mir-4802	4	0.12295	0.25538	0.9995	5774	3	0.258
NOLC1	6	0.12299	0.29102	0.9995	5775	3	0.27

ZNF324B	6	0.12302	0.29108	0.9995	5776	4	0.2707
TCP11L1	6	0.12304	0.29112	0.9995	5777	2	0.1096
AP1S2	4	0.12309	0.25563	0.9995	5778	1	0.16
RASGRP2	6	0.12311	0.29125	0.9995	5779	2	-0.237
OR5A2	6	0.12321	0.29142	0.9995	5780	1	-0.166
ZCCHC13	6	0.12324	0.29147	0.9995	5781	4	0.2707
SYAP1	6	0.12326	0.2915	0.9995	5782	3	-0.034
MYD88	6	0.1233	0.29158	0.9995	5783	2	-0.295
ACTR2	6	0.12331	0.2916	0.9995	5784	3	0.4388
MAZ	6	0.12333	0.29162	0.9995	5785	4	0.2863
TSEN2	6	0.12339	0.29176	0.9995	5786	2	0.0367
SMIM12	6	0.12343	0.29182	0.9995	5787	3	0.0733
hsa-mir-411	4	0.12344	0.25623	0.9995	5788	2	0.4085
UHRF2	6	0.12349	0.29195	0.9995	5789	1	-0.488
VMP1	4	0.1235	0.25633	0.9995	5790	3	0.2493
VASN	6	0.12351	0.29197	0.9995	5791	3	0.1502
HIST1H4G	6	0.12352	0.29199	0.9995	5792	4	0.2576
TP53I11	6	0.12356	0.29208	0.9995	5793	3	0.1826
FANCE	6	0.12356	0.29208	0.9995	5794	2	0.0296
CTNND1	6	0.12357	0.29209	0.9995	5795	2	-0.462
CCL26	6	0.12362	0.2922	0.9995	5796	2	-0.086
TNNT3	6	0.12363	0.29222	0.9995	5797	2	-0.091
TMED6	6	0.12364	0.29223	0.9995	5798	3	0.2146
SMTN	6	0.12367	0.29228	0.9995	5799	2	-0.129
SLC35G2	6	0.12368	0.29228	0.9995	5800	2	-0.246
NEUROG1	6	0.12373	0.29234	0.9995	5801	2	-0.227
OR5AC2	6	0.12373	0.29234	0.9995	5802	2	0.2913
UPP1	6	0.1238	0.29242	0.9995	5803	2	-0.01
SCML1	6	0.12389	0.29253	0.9995	5804	2	0.0023
TSPAN15	6	0.12393	0.29257	0.9995	5805	3	0.323
GSN	6	0.12394	0.29258	0.9995	5806	3	0.2655
KIF25	6	0.12396	0.29261	0.9995	5807	2	0.0398
SSR1	6	0.12401	0.29267	0.9995	5808	3	0.2463
TMEM240	6	0.12402	0.29268	0.9995	5809	2	-0.057
FCRL3	6	0.12405	0.29272	0.9995	5810	2	0.2282
COL5A2	6	0.12411	0.29278	0.9995	5811	2	0.0435
NELL2	6	0.12415	0.29284	0.9995	5812	2	0.0632
AMOTL2	6	0.12418	0.29289	0.9995	5813	2	0.0881
hsa-mir-4791	4	0.12419	0.25751	0.9995	5814	2	0.1697
hsa-mir-302d	4	0.12423	0.25757	0.9995	5815	2	0.3203
hsa-mir-4785	4	0.12427	0.25765	0.9995	5816	3	0.2558
OR7G1	6	0.12433	0.29307	0.9995	5817	2	-0.05
MRPL50	6	0.12445	0.29323	0.9995	5818	1	-0.124
ACTRT2	6	0.12445	0.29323	0.9995	5819	2	-0.205
ATP6AP1L	6	0.12445	0.29323	0.9995	5820	3	0.2322
ZNF711	6	0.12457	0.29337	0.9995	5821	2	-0.144
RNASE3	5	0.12457	0.26441	0.9995	5822	3	0.2009
BCAS3	6	0.12458	0.29339	0.9995	5823	2	-0.209
CSPP1	6	0.12465	0.29347	0.9995	5824	3	0.2586
MTMR8	6	0.12465	0.29347	0.9995	5825	3	0.2679
LY6D	6	0.12465	0.29347	0.9995	5826	3	0.3333
KAT6B	6	0.12466	0.29349	0.9995	5827	3	0.2104
KDM4B	6	0.1247	0.29355	0.9995	5828	3	0.2406
C11orf40	6	0.12471	0.29355	0.9995	5829	3	0.0875
hsa-mir-6825	4	0.12482	0.25862	0.9995	5830	2	0.0453
OR10A5	6	0.12491	0.2938	0.9995	5831	3	0.3033
DOLPP1	6	0.12492	0.29381	0.9995	5832	2	-0.098
INSL4	6	0.12499	0.29391	0.9995	5833	3	0.3129
DUSP21	6	0.12503	0.29396	0.9995	5834	1	-0.088
CASP6	6	0.12509	0.29403	0.9995	5835	2	-0.27
PSG6	6	0.12516	0.29414	0.9995	5836	2	0.0022
hsa-mir-658	4	0.1252	0.25925	0.9995	5837	1	-0.266
NFE2L3	6	0.12522	0.29422	0.9995	5838	2	0.026
GAL3ST4	6	0.12523	0.29423	0.9995	5839	2	-0.127
ANKRD35	6	0.12527	0.29429	0.9995	5840	3	0.0702
CCRN4L	6	0.12529	0.29432	0.9995	5841	3	0.1763
TREX1	6	0.12533	0.29436	0.9995	5842	3	-0.097
C17orf85	6	0.12543	0.29449	0.9995	5843	3	0.021
C5orf42	6	0.12545	0.29452	0.9995	5844	2	0.0207
CPT1A	4	0.12545	0.25969	0.9995	5845	3	0.2222
CD72	6	0.1255	0.29458	0.9995	5846	3	-0.078
CCL16	6	0.12552	0.2946	0.9995	5847	2	0.0356
TXN2	6	0.12555	0.29464	0.9995	5848	2	0.0063
IL22RA2	6	0.12559	0.29471	0.9995	5849	3	0.2506
GIPC3	6	0.1256	0.29472	0.9995	5850	3	0.0477
MRPL51	6	0.1256	0.29472	0.9995	5851	3	0.2197
OR51V1	6	0.12563	0.29476	0.9995	5852	3	0.2212
CD8B	6	0.12575	0.29492	0.9995	5853	2	0.0928
VGF	6	0.12575	0.29492	0.9995	5854	2	-0.02
SERPINB3	4	0.1258	0.2603	0.9995	5855	3	0.3079
SF3B14	6	0.12587	0.29507	0.9995	5856	3	0.1108
GDAP1L1	6	0.12588	0.29508	0.9995	5857	3	0.2637
TMEM74	6	0.12592	0.29514	0.9995	5858	3	-0.02
hsa-mir-23b	4	0.12595	0.26053	0.9995	5859	2	-0.014
hsa-mir-628	4	0.12598	0.26058	0.9995	5860	2	0.2946

FAM227B	6	0.12603	0.29527	0.9995	5861	3	-0.1
IGDCC4	6	0.12606	0.29531	0.9995	5862	3	0.2506
GSPT1	4	0.12612	0.26082	0.9995	5863	2	0.2329
NCKAP1L	6	0.12613	0.2954	0.9995	5864	2	-0.151
hsa-mir-151a	3	0.12618	0.21777	0.9995	5865	2	0.3451
CITED2	6	0.12622	0.29551	0.9995	5866	2	0.0649
DNAJC6	6	0.12632	0.29564	0.9995	5867	3	0.3373
CNOT8	6	0.12636	0.2957	0.9995	5868	2	0.0804
MAP3K19	6	0.12644	0.2958	0.9995	5869	2	0.1563
SULF1	6	0.12648	0.29584	0.9995	5870	1	0.0036
hsa-mir-6755	4	0.12653	0.26148	0.9995	5871	2	0.0901
RAX	6	0.12655	0.29593	0.9995	5872	3	0.303
PGAM5	6	0.12657	0.29596	0.9995	5873	3	0.15
PRMT1	6	0.12664	0.29605	0.9995	5874	3	0.3765
SRPK2	6	0.12664	0.29605	0.9995	5875	3	0.3195
GIMAP6	6	0.12667	0.2961	0.9995	5876	2	-0.549
ELMO2	6	0.12667	0.2961	0.9995	5877	2	0.1454
TMEM242	6	0.12671	0.29614	0.9995	5878	3	0.141
COL27A1	6	0.12673	0.29618	0.9995	5879	3	0.0055
PRPF40B	6	0.12677	0.29622	0.9995	5880	3	0.0818
HLA-DPA1	6	0.12682	0.29629	0.9995	5881	3	0.0672
hsa-mir-4310	4	0.12685	0.26202	0.9995	5882	2	0.2308
ATAD3B	6	0.12685	0.29632	0.9995	5883	3	-0.115
TFEC	6	0.12688	0.29636	0.9995	5884	3	0.1479
ESR2	6	0.12697	0.29646	0.9995	5885	3	0.2307
CSNK1G3	6	0.12699	0.29648	0.9995	5886	2	-0.009
GUCY2F	6	0.12703	0.29654	0.9995	5887	2	-0.087
DEPDC1	6	0.12708	0.29659	0.9995	5888	1	0.0519
SPA17	6	0.12713	0.29665	0.9995	5889	1	-0.314
MED12L	6	0.12716	0.29669	0.9995	5890	3	0.1373
RAP2B	6	0.12717	0.29672	0.9995	5891	3	0.1899
MAGEA8	4	0.12719	0.26259	0.9995	5892	3	0.4069
FCHO2	6	0.1272	0.29675	0.9995	5893	3	0.2799
SOX4	6	0.12722	0.29677	0.9995	5894	3	0.2845
SDE2	6	0.12726	0.29682	0.9995	5895	2	-0.093
ITFG2	6	0.12727	0.29683	0.9995	5896	2	-0.717
hsa-mir-487a	4	0.12729	0.26276	0.9995	5897	2	0.1932
CIB4	6	0.12731	0.29688	0.9995	5898	3	0.1225
NKX6-3	6	0.12734	0.29691	0.9995	5899	2	-0.889
TM4SF4	6	0.12739	0.29699	0.9995	5900	2	-0.111
FAM134B	6	0.12741	0.29701	0.9995	5901	1	-0.317
CRYZ	6	0.12745	0.29707	0.9995	5902	2	-0.192
PTGS2	6	0.12754	0.29717	0.9995	5903	2	-0.118
DOCK2	6	0.12755	0.29718	0.9995	5904	1	-0.487
KIAA1737	6	0.12759	0.29725	0.9995	5905	2	0.0117
EIF4G1	6	0.12764	0.29731	0.9995	5906	3	0.1361
NBAS	6	0.12764	0.29732	0.9995	5907	3	-0.036
hsa-mir-4525	4	0.12771	0.2635	0.9995	5908	2	0.1863
OR10A3	6	0.12773	0.29745	0.9995	5909	1	-0.283
hsa-mir-450a-1	2	0.12775	0.21103	0.9995	5910	1	0.3971
ZNF426	5	0.12782	0.26933	0.9995	5911	2	0.0832
MAPK6	6	0.1279	0.29766	0.9995	5912	2	0.0734
SLC19A3	6	0.1279	0.29766	0.9995	5913	2	-0.089
hsa-mir-2052	2	0.12791	0.21114	0.9995	5914	1	0.0998
UBAP2	6	0.12801	0.29781	0.9995	5915	3	0.1143
FAM46B	6	0.12803	0.29782	0.9995	5916	2	0.2086
KIF26B	6	0.12805	0.29787	0.9995	5917	3	0.0769
LGALS4	6	0.12806	0.29787	0.9995	5918	3	0.1493
RIPK3	6	0.12807	0.29788	0.9995	5919	3	0.3433
hsa-mir-937	3	0.12809	0.22024	0.9995	5920	1	-0.328
GUCY2D	6	0.12811	0.29793	0.9995	5921	2	0.0353
FAM20B	6	0.12816	0.298	0.9995	5922	3	0.2301
hsa-mir-1253	4	0.12827	0.26447	0.9995	5923	1	-0.06
hsa-mir-4643	3	0.12828	0.22048	0.9995	5924	2	0.4187
CHDC2	6	0.12832	0.29821	0.9995	5925	3	0.2717
ITGA9	6	0.12832	0.29821	0.9995	5926	2	0.1077
hsa-mir-3916	4	0.12835	0.26462	0.9995	5927	3	0.4986
hsa-mir-4660	4	0.12835	0.26462	0.9995	5928	3	0.5036
DEFB129	6	0.1284	0.29831	0.9995	5929	2	0.0558
TRABD	6	0.12843	0.29834	0.9995	5930	3	0.1129
MEF2D	6	0.12844	0.29834	0.9995	5931	3	0.3616
CHAMP1	6	0.12847	0.2984	0.9995	5932	3	0.1573
UCMA	6	0.12848	0.2984	0.9995	5933	2	-0.1
SLC31A2	6	0.12849	0.29842	0.9995	5934	2	-0.346
hsa-mir-4662b	1	0.12851	0.12844	0.9764	5935	1	1.0697
PPP2R2A	6	0.12851	0.29845	0.9995	5936	2	0.0135
SSX7	5	0.12854	0.27043	0.9995	5937	3	0.2961
TNFSF14	6	0.12857	0.29852	0.9995	5938	3	0.0455
KLHL21	6	0.12862	0.2986	0.9995	5939	3	0.2587
METAP2	6	0.12869	0.2987	0.9995	5940	3	0.0968
RHOD	6	0.12871	0.29872	0.9995	5941	1	-0.234
ACTL6B	6	0.12875	0.29879	0.9995	5942	3	0.1863
POLN	6	0.12876	0.2988	0.9995	5943	3	0.1251
ADARB2	6	0.12879	0.29883	0.9995	5944	2	-0.35
C11orf88	6	0.12882	0.29888	0.9995	5945	3	-0.022

GLT6D1	6	0.12884	0.2989	0.9995	5946	3	0.3588
KIN	6	0.12885	0.2989	0.9995	5947	2	-0.404
TMCO3	6	0.12887	0.29893	0.9995	5948	3	0.1627
hsa-mir-4317	4	0.12909	0.26587	0.9995	5949	3	0.3274
AP3S2	4	0.12912	0.26592	0.9995	5950	2	-0.04
KCNK5	6	0.12916	0.29932	0.9995	5951	3	0.3171
ARL9	6	0.12917	0.29933	0.9995	5952	2	-0.178
hsa-mir-6752	4	0.12919	0.26605	0.9995	5953	2	0.1347
SPC25	6	0.1292	0.29936	0.9995	5954	3	0.311
TYRP1	6	0.12927	0.29945	0.9995	5955	3	0.0836
IFT140	6	0.1293	0.29948	0.9995	5956	3	0.2458
JHDM1D	6	0.12933	0.29952	0.9995	5957	2	-0.178
S100A1	6	0.12942	0.29962	0.9995	5958	3	0.3382
FCHSD1	6	0.12953	0.29977	0.9995	5959	3	0.1455
RALA	6	0.12954	0.29979	0.9995	5960	2	-0.755
LCE1C	4	0.12956	0.26665	0.9995	5961	1	-0.625
PHOSPHO2	6	0.12957	0.29982	0.9995	5962	2	0.0071
C1orf109	6	0.12959	0.29985	0.9995	5963	1	-0.247
hsa-mir-6803	4	0.12965	0.26683	0.9995	5964	2	0.2759
GABPA	6	0.12965	0.29993	0.9995	5965	3	-0.129
EIF4H	6	0.12966	0.29993	0.9995	5966	3	0.1986
FEV	6	0.12969	0.29999	0.9995	5967	3	-0.008
ZWINT	6	0.12972	0.30003	0.9995	5968	3	0.3026
RAB9B	6	0.12973	0.30003	0.9995	5969	2	-0.134
RBM11	4	0.12976	0.26702	0.9995	5970	2	-0.142
TNFSF12-TNFSF13	5	0.12977	0.27228	0.9995	5971	1	-0.312
SUMO4	6	0.12978	0.30009	0.9995	5972	2	-0.01
ARC	6	0.12986	0.30021	0.9995	5973	2	0.0721
PDE3B	6	0.12988	0.30023	0.9995	5974	3	0.2766
BRIX1	6	0.12994	0.3003	0.9995	5975	2	-0.063
hsa-mir-30b	4	0.12998	0.2674	0.9995	5976	3	0.2735
RASSF2	6	0.12998	0.30036	0.9995	5977	2	-0.03
BCAP29	6	0.13005	0.30046	0.9995	5978	2	0.0199
GRXCR2	6	0.13006	0.30046	0.9995	5979	2	0.0628
HOXC4	6	0.13008	0.30048	0.9995	5980	3	0.3396
IFT74	6	0.1301	0.30051	0.9995	5981	2	0.1035
RARS2	6	0.13016	0.30057	0.9995	5982	2	-0.205
PRNP	6	0.13024	0.30068	0.9995	5983	3	0.1542
ANGPTL4	6	0.13026	0.30071	0.9995	5984	3	0.2496
CRCP	6	0.13026	0.30072	0.9995	5985	2	-0.547
NCF4	6	0.1303	0.30076	0.9995	5986	3	0.1314
BHLHA15	6	0.13034	0.3008	0.9995	5987	3	0.2701
hsa-mir-6762	4	0.13034	0.26801	0.9995	5988	1	0.0284
PCDHGB5	3	0.13038	0.22323	0.9995	5989	2	0.4211
FBXW4	6	0.13044	0.30094	0.9995	5990	3	0.294
GMDS	6	0.13049	0.30102	0.9995	5991	3	-0.002
FAM3B	6	0.13053	0.30105	0.9995	5992	3	0.2397
GYPA	5	0.13056	0.27354	0.9995	5993	3	0.3112
OR5H1	6	0.13059	0.30114	0.9995	5994	2	-0.198
DCPS	6	0.13059	0.30114	0.9995	5995	1	-0.347
C10orf111	6	0.13066	0.30122	0.9995	5996	2	-0.137
PPP1R8	6	0.13066	0.30122	0.9995	5997	3	0.0559
hsa-mir-7844	4	0.13071	0.26865	0.9995	5998	2	0.4535
VCAN	6	0.13075	0.30134	0.9995	5999	2	-0.114
CTAGE5	4	0.13075	0.26872	0.9995	6000	2	0.2296
FAM92B	6	0.13082	0.30143	0.9995	6001	3	0.2221
TTC7B	6	0.13083	0.30145	0.9995	6002	3	0.228
TULP4	6	0.13084	0.30146	0.9995	6003	3	0.3344
AMTN	6	0.13084	0.30146	0.9995	6004	2	-0.133
VAT1L	6	0.13089	0.30152	0.9995	6005	2	0.0969
PSMC4	6	0.13091	0.30155	0.9995	6006	3	0.1389
DRC1	6	0.13091	0.30155	0.9995	6007	3	-0.185
CDC7	6	0.13094	0.30159	0.9995	6008	3	0.0588
ITGAV	6	0.13107	0.30174	0.9995	6009	3	0.162
SLC41A2	6	0.13112	0.30181	0.9995	6010	2	-0.114
CDK15	6	0.13112	0.30181	0.9995	6011	3	0.3021
ARSH	6	0.13114	0.30184	0.9995	6012	3	0.2826
ADIPOQ	6	0.13119	0.30191	0.9995	6013	3	0.0862
HECTD3	6	0.13119	0.30191	0.9995	6014	2	-0.058
TMEM179	6	0.13123	0.30195	0.9995	6015	2	0.0725
PRH1	5	0.1313	0.27469	0.9995	6016	2	0.1326
RBM47	6	0.13133	0.30208	0.9995	6017	3	0.2582
ITPKA	6	0.13133	0.30208	0.9995	6018	2	-0.069
PPAT	6	0.13134	0.30209	0.9995	6019	3	0.2523
C9	6	0.13135	0.30212	0.9995	6020	1	-0.031
GLYATL1	6	0.13144	0.30223	0.9995	6021	2	-0.16
E2F8	6	0.13144	0.30223	0.9995	6022	2	-0.305
IL22RA1	6	0.13146	0.30225	0.9995	6023	3	0.2305
WDR5	6	0.13148	0.30228	0.9995	6024	3	0.1548
TMEM234	6	0.13158	0.30241	0.9995	6025	3	0.1301
GABRB3	6	0.13166	0.30251	0.9995	6026	3	0.2518
PDGFRA	6	0.13168	0.30253	0.9995	6027	1	-0.453
NOX1	6	0.1317	0.30257	0.9995	6028	2	-0.638
DEPTOR	6	0.13183	0.30276	0.9995	6029	3	0.044
LARS	6	0.13184	0.30276	0.9995	6030	2	-0.306

DRGX	4	0.13189	0.27066	0.9995	6031	2	0.2291
TEKT3	6	0.13191	0.30286	0.9995	6032	3	0.175
FAM181A	6	0.13193	0.30288	0.9995	6033	3	0.0719
CCBL1	6	0.13193	0.30289	0.9995	6034	3	-0.038
RWDD3	6	0.13193	0.30289	0.9995	6035	3	0.1131
PGGT1B	6	0.13205	0.30303	0.9995	6036	3	0.1437
OR10H1	5	0.13206	0.27583	0.9995	6037	1	-0.018
ACYP2	6	0.1321	0.3031	0.9995	6038	3	0.4612
CEP350	6	0.13212	0.30313	0.9995	6039	3	0.2579
HDAC1	6	0.13223	0.30327	0.9995	6040	3	0.058
PRR13	6	0.13223	0.30327	0.9995	6041	2	0.1197
SYBU	6	0.13239	0.30347	0.9995	6042	2	-0.075
NDUFB1	6	0.13244	0.30353	0.9995	6043	3	-0.136
MTMR4	6	0.13246	0.30357	0.9995	6044	2	0.0331
CYSTM1	6	0.13246	0.30357	0.9995	6045	3	0.2042
SLC39A3	6	0.13251	0.30363	0.9995	6046	3	0.1156
MEA1	6	0.13256	0.30369	0.9995	6047	1	0.0971
KRTAP5-8	5	0.13256	0.27658	0.9995	6048	1	-0.334
C3orf38	6	0.1326	0.30376	0.9995	6049	3	0.2898
POMP	6	0.13263	0.30378	0.9995	6050	1	-0.092
NGRN	6	0.13266	0.30383	0.9995	6051	2	-0.065
hsa-mir-6086	3	0.13273	0.22634	0.9995	6052	1	-0.797
hsa-mir-4321	4	0.13276	0.27157	0.9995	6053	2	0.3788
C7orf60	6	0.13286	0.30408	0.9995	6054	1	-0.173
ZNF420	6	0.13296	0.30422	0.9995	6055	3	0.2606
ATP13A2	6	0.13302	0.3043	0.9995	6056	3	0.1307
COL16A1	6	0.13306	0.30434	0.9995	6057	2	0.0605
SLC10A5	6	0.13307	0.30434	0.9995	6058	2	0.0875
ZNF675	4	0.13309	0.27191	0.9995	6059	2	0.3431
ICOS	6	0.1331	0.30438	0.9995	6060	2	-0.048
SNAPC3	6	0.13312	0.3044	0.9995	6061	3	0.1501
PON3	6	0.13312	0.30441	0.9995	6062	2	-0.156
WDSUB1	6	0.13323	0.30454	0.9995	6063	3	0.2449
RAB30	6	0.13334	0.30469	0.9995	6064	3	0.2072
SMARCA4	6	0.13334	0.30469	0.9995	6065	2	-0.1
TMEM59	6	0.13341	0.30478	0.9995	6066	2	-0.034
hsa-mir-183	4	0.13341	0.27225	0.9995	6067	2	-0.095
PLD1	4	0.13352	0.27237	0.9995	6068	2	0.3179
ANKS1A	6	0.13353	0.30494	0.9995	6069	2	-0.305
MACF1	6	0.13353	0.30494	0.9995	6070	3	0.1494
HRC	6	0.13354	0.30497	0.9995	6071	3	-0.02
CAB39L	6	0.13355	0.30497	0.9995	6072	3	0.1365
CCNH	6	0.13357	0.305	0.9995	6073	3	0.2629
MMACHC	6	0.13357	0.30501	0.9995	6074	2	-7E-04
POLR3F	6	0.13362	0.30506	0.9995	6075	3	0.0589
HIST1H2AM	6	0.13372	0.30521	0.9995	6076	3	-0.01
SDAD1	6	0.13379	0.30528	0.9995	6077	2	0.1291
SCN3A	6	0.13385	0.30537	0.9995	6078	2	0.0098
SMIM4	4	0.13387	0.27273	0.9995	6079	2	0.0418
GRHPR	6	0.13388	0.3054	0.9995	6080	3	0.3427
CNIH2	6	0.13395	0.30548	0.9995	6081	3	0.2976
OR4N2	6	0.13398	0.30552	0.9995	6082	2	-0.308
IL17RB	6	0.13401	0.30556	0.9995	6083	2	-0.134
LOC728819	6	0.13404	0.30559	0.9995	6084	3	0.1879
ZSWIM4	6	0.13417	0.30574	0.9995	6085	1	-0.586
DPH2	6	0.13422	0.30581	0.9995	6086	3	0.4042
CPAMD8	6	0.13422	0.30582	0.9995	6087	3	0.2157
PID1	6	0.13427	0.30587	0.9995	6088	2	-0.509
EMC3	6	0.13431	0.30593	0.9995	6089	2	0.0921
SUPT5H	6	0.13438	0.30602	0.9995	6090	3	0.3763
PTPRR	6	0.13438	0.30602	0.9995	6091	3	0.2351
SCGB2A1	6	0.13438	0.30602	0.9995	6092	2	-0.341
ACADL	6	0.13444	0.30611	0.9995	6093	2	-0.215
RAB3B	6	0.13445	0.30612	0.9995	6094	3	0.3209
RNF32	6	0.1345	0.30617	0.9995	6095	2	0.0728
FAM208A	6	0.13457	0.30627	0.9995	6096	1	-0.115
ERV3-1	6	0.13462	0.30635	0.9995	6097	3	0.2144
PABPC4	6	0.13464	0.30637	0.9995	6098	3	-0.016
FABP1	6	0.13466	0.3064	0.9995	6099	3	0.0964
BLOC1S3	6	0.13467	0.30641	0.9995	6100	2	-0.387
PWWP2B	6	0.13468	0.30643	0.9995	6101	3	-0.013
CRAMP1L	6	0.1347	0.30645	0.9995	6102	3	0.2867
SPP2	6	0.1347	0.30646	0.9995	6103	2	-0.023
IKBKE	6	0.13473	0.30649	0.9995	6104	1	-0.24
PLEKHJ1	6	0.13475	0.30653	0.9995	6105	3	0.2059
ZP3	6	0.1348	0.30659	0.9995	6106	3	0.1003
HIST2H2AC	6	0.13483	0.30663	0.9995	6107	3	0.5363
CCNF	6	0.13483	0.30663	0.9995	6108	3	0.3179
TMIE	6	0.13487	0.30667	0.9995	6109	2	0.0322
CHGA	6	0.13487	0.30668	0.9995	6110	2	-0.101
PI3	6	0.13496	0.30678	0.9995	6111	1	-0.467
RNF6	6	0.13497	0.3068	0.9995	6112	2	-0.178
USP27X	6	0.13497	0.3068	0.9995	6113	3	0.2545
hsa-mir-4774	4	0.13501	0.27395	0.9995	6114	1	-0.056
FAM110B	6	0.13504	0.30689	0.9995	6115	3	0.1583

SIRPA	6	0.13505	0.30691	0.9995	6116	1	-0.157
C2orf68	6	0.13512	0.307	0.9995	6117	2	0.0464
MCMBP	6	0.13514	0.30702	0.9995	6118	2	0.1854
CYSLTR2	6	0.13517	0.30705	0.9995	6119	1	0.0351
B3GALT1	6	0.13517	0.30705	0.9995	6120	2	0.0734
PSMD10	6	0.13533	0.30724	0.9995	6121	3	0.2821
SETD5	6	0.13536	0.30728	0.9995	6122	3	-0.135
ORC2	6	0.13538	0.3073	0.9995	6123	2	-0.175
FOXQ1	6	0.13542	0.30735	0.9995	6124	3	0.3016
HIST1H2BO	6	0.13547	0.30742	0.9995	6125	2	-0.652
IQCF1	6	0.13551	0.30747	0.9995	6126	3	0.1893
KDM6B	6	0.13551	0.30747	0.9995	6127	2	-0.265
hsa-mir-5003	4	0.1356	0.27455	0.9995	6128	2	0.2016
EIF4E1B	6	0.13565	0.30766	0.9995	6129	2	-0.067
CASP7	6	0.13565	0.30766	0.9995	6130	2	-0.212
IGFL4	6	0.13568	0.30769	0.9995	6131	3	0.2231
TBC1D9	6	0.13569	0.30772	0.9995	6132	3	0.2879
DEPDC1B	6	0.13573	0.30776	0.9995	6133	3	0.3629
EIF4EBP3	6	0.13579	0.30784	0.9995	6134	3	0.16
ZNF667	6	0.13584	0.30791	0.9995	6135	3	0.2122
NUGGC	6	0.13591	0.308	0.9995	6136	2	0.0562
RTF1	6	0.13595	0.30804	0.9995	6137	3	0.2844
TMEM88	6	0.13598	0.30808	0.9995	6138	3	0.2193
hsa-mir-302a	4	0.13602	0.27497	0.9995	6139	2	0.5074
BAI1	6	0.13602	0.30813	0.9995	6140	2	0.005
FAM188A	6	0.13616	0.30831	0.9995	6141	1	-0.601
DUSP28	6	0.13621	0.30838	0.9995	6142	1	-0.559
SGPP2	6	0.13623	0.30841	0.9995	6143	2	-0.061
HSPA2	6	0.13627	0.30844	0.9995	6144	3	0.3218
LRRC10	6	0.13634	0.30854	0.9995	6145	3	-0.13
USP39	6	0.13635	0.30855	0.9995	6146	3	0.2041
CTLA4	6	0.13643	0.30866	0.9995	6147	2	0.0331
CHST1	6	0.13644	0.30866	0.9995	6148	3	0.3376
hsa-mir-5687	4	0.13644	0.27541	0.9995	6149	2	-0.041
NR6A1	6	0.13648	0.30871	0.9995	6150	2	-0.17
TRPV4	6	0.13649	0.30872	0.9995	6151	2	-0.124
THSD1	6	0.13651	0.30874	0.9995	6152	2	0.0079
SMAP2	6	0.13658	0.30885	0.9995	6153	2	-0.32
ARFGAP3	6	0.13661	0.30889	0.9995	6154	3	0.2705
OR7C1	6	0.13666	0.30896	0.9995	6155	3	0.2245
PPP6R1	6	0.13666	0.30896	0.9995	6156	3	0.1494
PABPC1L	6	0.13666	0.30896	0.9995	6157	2	0.0665
CPNE5	6	0.1367	0.30901	0.9995	6158	2	-0.083
MPV17	6	0.13674	0.30906	0.9995	6159	3	-0.105
GIMAP7	6	0.13674	0.30907	0.9995	6160	3	0.2352
MAN1A1	6	0.13676	0.30909	0.9995	6161	2	-0.36
HRASLS	6	0.13683	0.30916	0.9995	6162	3	0.3236
CCDC169-SOHLH2	5	0.13685	0.28304	0.9995	6163	3	0.3361
SQLE	6	0.13685	0.30919	0.9995	6164	3	0.119
RALBP1	6	0.13688	0.30923	0.9995	6165	3	-0.116
LNP1	6	0.13692	0.30927	0.9995	6166	3	0.3517
PA2G4	6	0.13697	0.30933	0.9995	6167	1	-0.319
CLYBL	6	0.13697	0.30934	0.9995	6168	3	0.0926
ALOX15	6	0.13703	0.30941	0.9995	6169	3	0.2148
CD27	6	0.13708	0.30947	0.9995	6170	3	0.1375
ZNF548	6	0.13713	0.30953	0.9995	6171	2	-0.562
hsa-mir-4492	4	0.13722	0.27621	0.9995	6172	1	-0.705
hsa-mir-6807	4	0.13722	0.27621	0.9995	6173	1	-0.205
GDF11	6	0.13723	0.30965	0.9995	6174	3	0.0174
OR56A5	6	0.13727	0.30969	0.9995	6175	3	0.2491
RAD1	6	0.13731	0.30975	0.9995	6176	2	-0.002
BRPF3	6	0.13732	0.30976	0.9995	6177	3	0.2456
RFXANK	6	0.13732	0.30976	0.9995	6178	3	0.309
CCDC8	6	0.13734	0.30979	0.9995	6179	2	0.0898
UPP2	6	0.13745	0.30993	0.9995	6180	3	0.3113
RNF169	6	0.13752	0.31003	0.9995	6181	3	0.2686
ADIPOR1	6	0.13756	0.3101	0.9995	6182	1	-0.467
ZFP69	6	0.13766	0.31024	0.9995	6183	3	0.0969
hsa-mir-8073	4	0.13771	0.27674	0.9995	6184	2	0.0799
SORD	6	0.13773	0.31032	0.9995	6185	2	0.018
hsa-mir-4456	4	0.13774	0.27677	0.9995	6186	2	0.085
METTL7A	6	0.13774	0.31035	0.9995	6187	2	0.1868
OTUD1	6	0.13777	0.31039	0.9995	6188	3	0.0409
SYP	6	0.1378	0.31042	0.9995	6189	2	-0.167
IL2RG	6	0.13783	0.31045	0.9995	6190	3	0.1838
CEP78	6	0.13784	0.31047	0.9995	6191	2	-0.121
OSBPL8	6	0.13786	0.31049	0.9995	6192	2	0.2194
ATPAF2	6	0.13787	0.31051	0.9995	6193	3	0.0502
SWAP70	6	0.13796	0.31061	0.9995	6194	1	-0.329
CDK7	6	0.13808	0.31075	0.9995	6195	2	0.0327
LIPT2	6	0.13808	0.31075	0.9995	6196	2	0.0055
CSAD	6	0.13812	0.31081	0.9995	6197	2	-0.516
WBP1L	6	0.13819	0.31089	0.9995	6198	3	0.2551
GPS2	6	0.13829	0.31102	0.9995	6199	3	0.5269
DEF6	6	0.13831	0.31106	0.9995	6200	2	-0.642

ZBTB7C	6	0.13832	0.31107	0.9995	6201	3	0.1644
LZTS2	6	0.13837	0.31112	0.9995	6202	3	-0.016
NXNL2	5	0.13842	0.28547	0.9995	6203	3	0.367
SALL1	6	0.13846	0.31123	0.9995	6204	3	0.1366
CSNK1E	6	0.13857	0.31137	0.9995	6205	3	0.0906
OLAH	6	0.1386	0.31141	0.9995	6206	2	0.0251
RPA3	6	0.13861	0.31142	0.9995	6207	3	0.3567
SOX7	6	0.1387	0.31153	0.9995	6208	3	0.0003
LEAP2	6	0.13872	0.31155	0.9995	6209	3	-0.243
C11orf68	6	0.13875	0.3116	0.9995	6210	3	0.3301
CAV1	6	0.13876	0.31161	0.9995	6211	2	-0.218
FAM102A	6	0.13876	0.31161	0.9995	6212	3	0.1563
CCER1	6	0.13888	0.31176	0.9995	6213	1	-0.541
ASIC1	6	0.13891	0.31181	0.9995	6214	3	0.1349
TCF20	6	0.13896	0.31187	0.9995	6215	2	-0.019
HEXB	6	0.13897	0.31188	0.9995	6216	2	0.0416
SNAI2	6	0.13899	0.31191	0.9995	6217	2	-0.026
PRKCI	6	0.13906	0.31199	0.9995	6218	2	-0.055
IGFBP6	6	0.13923	0.31222	0.9995	6219	3	0.1027
FAM209A	6	0.13924	0.31224	0.9995	6220	3	0.1607
ZXDC	6	0.13937	0.31241	0.9995	6221	3	0.1291
hsa-mir-331	4	0.13937	0.27849	0.9995	6222	2	0.0089
GRIP1	6	0.13938	0.31243	0.9995	6223	2	-0.256
TROAP	6	0.13943	0.31249	0.9995	6224	2	-0.029
PTPN1	6	0.13946	0.31253	0.9995	6225	3	0.0861
ZMYND10	6	0.13946	0.31253	0.9995	6226	3	0.2
GPRC5D	6	0.13951	0.3126	0.9995	6227	3	0.0036
SFSWAP	6	0.13952	0.31261	0.9995	6228	2	-0.275
ZNF778	6	0.13975	0.3129	0.9995	6229	2	-0.043
GALR3	6	0.13984	0.31302	0.9995	6230	2	-0.415
HOXD4	6	0.13989	0.31306	0.9995	6231	3	-0.031
SCYL1	6	0.13989	0.31307	0.9995	6232	2	0.2122
NRL	6	0.1399	0.31308	0.9995	6233	3	-0.038
EFCAB6	6	0.13995	0.31315	0.9995	6234	3	0.2366
DGKI	4	0.13999	0.27913	0.9995	6235	2	0.1448
PCDH10	6	0.14	0.3132	0.9995	6236	3	0.2258
PPM1D	6	0.14005	0.31326	0.9995	6237	3	0.0885
hsa-mir-5680	4	0.14005	0.27921	0.9995	6238	2	0.2222
SPTLC3	6	0.14006	0.31327	0.9995	6239	3	0.1836
FHL2	6	0.14017	0.31341	0.9995	6240	2	-0.004
DRD4	6	0.14019	0.31344	0.9995	6241	3	0.1287
DEFB110	6	0.14021	0.31347	0.9995	6242	3	0.2696
CYFIP2	6	0.14026	0.31352	0.9995	6243	2	-0.196
SUSD2	6	0.14034	0.31363	0.9995	6244	2	-0.106
OR8K1	6	0.14035	0.31364	0.9995	6245	3	0.3247
C17orf51	6	0.14048	0.31382	0.9995	6246	3	0.0308
RAB32	6	0.14053	0.31388	0.9995	6247	3	0.1316
CCPG1	6	0.14053	0.31388	0.9995	6248	3	0.0629
SLC25A52	6	0.14058	0.31393	0.9995	6249	2	0.2117
RAB42	6	0.1406	0.31397	0.9995	6250	2	-0.22
STXBP5	6	0.14064	0.31401	0.9995	6251	3	0.1144
CAND2	5	0.14066	0.28887	0.9995	6252	3	0.2935
NBPF9	5	0.14066	0.28887	0.9995	6253	3	0.4402
TAX1BP3	6	0.14067	0.31405	0.9995	6254	2	0.1486
IGF1R	6	0.14068	0.31406	0.9995	6255	3	0.1807
hsa-mir-5692c-2	3	0.14068	0.23665	0.9995	6256	2	0.5773
LAMC3	6	0.14071	0.31411	0.9995	6257	3	0.1858
IFNB1	6	0.1409	0.31434	0.9995	6258	3	0.1288
C2CD4A	6	0.14091	0.31437	0.9995	6259	3	0.0208
PWP2	6	0.14094	0.31442	0.9995	6260	3	0.1896
EIF4E	6	0.14095	0.31443	0.9995	6261	3	0.4068
HSD17B13	6	0.14097	0.31444	0.9995	6262	3	0.2068
CGN	6	0.14099	0.31446	0.9995	6263	2	0.1779
NFE2L2	6	0.14101	0.3145	0.9995	6264	3	0.1149
SNCAIP	6	0.14106	0.31455	0.9995	6265	2	0.1639
BET1	6	0.14106	0.31455	0.9995	6266	2	-0.263
THAP10	6	0.1411	0.31459	0.9995	6267	3	0.1641
ZNF808	4	0.14111	0.28027	0.9995	6268	1	0.0382
CDS2	6	0.14115	0.31466	0.9995	6269	3	0.2182
FRK	6	0.14115	0.31466	0.9995	6270	3	0.0188
GSG1L	6	0.14122	0.31475	0.9995	6271	3	0.0794
MIB2	6	0.14126	0.3148	0.9995	6272	3	-0.002
NINJ2	6	0.14136	0.31493	0.9995	6273	1	-0.041
ZIC1	6	0.1414	0.31498	0.9995	6274	3	0.166
LPAR3	6	0.1415	0.3151	0.9995	6275	2	-0.013
CCDC174	6	0.1416	0.31523	0.9995	6276	3	0.1975
PDE6G	6	0.14162	0.31526	0.9995	6277	3	-0.036
PTGER2	6	0.14163	0.31527	0.9995	6278	1	-0.442
RXFP3	6	0.14172	0.31538	0.9995	6279	2	-0.289
hsa-mir-6514	4	0.14173	0.28091	0.9995	6280	1	-0.019
ATP13A3	6	0.14176	0.31543	0.9995	6281	3	0.0835
C6orf89	6	0.14182	0.31549	0.9995	6282	2	-0.341
ACO2	6	0.14186	0.31554	0.9995	6283	1	0.0892
PROK1	6	0.14188	0.31557	0.9995	6284	2	-0.128
KIF26A	6	0.14193	0.31563	0.9995	6285	3	0.226

MARCKS	6	0.14195	0.31566	0.9995	6286	1	-0.028
hsa-mir-3163	4	0.14202	0.28122	0.9995	6287	2	0.288
KCNK9	6	0.14204	0.31576	0.9995	6288	2	-0.227
LOC441155	6	0.14205	0.31577	0.9995	6289	3	0.0648
PBK	6	0.14206	0.31578	0.9995	6290	3	0.1859
PLD2	6	0.14206	0.31578	0.9995	6291	3	0.2554
HIBCH	6	0.14206	0.31578	0.9995	6292	3	0.1769
PPP2R2B	6	0.14209	0.31583	0.9995	6293	1	-0.353
ZNF563	5	0.14217	0.29119	0.9995	6294	3	0.3307
ATAD2B	6	0.14218	0.31595	0.9995	6295	2	-0.471
C1orf110	6	0.14218	0.31595	0.9995	6296	1	-0.428
OR10AG1	6	0.14223	0.31603	0.9995	6297	3	0.1676
RAI1	6	0.14228	0.31611	0.9995	6298	3	0.1886
ZNF239	6	0.14228	0.31611	0.9995	6299	2	-0.069
ZHX3	6	0.14232	0.31615	0.9995	6300	2	0.0097
FAM69C	6	0.14232	0.31616	0.9995	6301	2	-0.012
CLK2	6	0.14233	0.31616	0.9995	6302	3	0.3447
KIAA1967	4	0.14238	0.28157	0.9995	6303	1	-0.462
TUBGCP3	6	0.14252	0.31642	0.9995	6304	3	0.3635
PRR23B	6	0.14257	0.3165	0.9995	6305	2	0.0369
RFX7	6	0.14258	0.3165	0.9995	6306	3	0.1408
PKLR	6	0.14265	0.31659	0.9995	6307	3	0.2181
PLEKHA8	6	0.14269	0.31664	0.9995	6308	2	-0.063
LRRIQ1	6	0.14271	0.31668	0.9995	6309	2	0.0205
APH1A	6	0.14288	0.31687	0.9995	6310	3	0.1671
hsa-mir-1260a	4	0.14309	0.2823	0.9995	6311	2	-0.414
TDRD5	6	0.1431	0.31716	0.9995	6312	3	0.0839
NAA16	6	0.14314	0.31721	0.9995	6313	3	0.2862
UBQLN3	4	0.14318	0.28238	0.9995	6314	2	0.0722
VPS13A	6	0.14319	0.31728	0.9995	6315	2	0.0824
NAA25	6	0.14321	0.31731	0.9995	6316	3	0.2975
WIZ	6	0.14324	0.31735	0.9995	6317	1	-0.308
NUFIP2	6	0.14325	0.31736	0.9995	6318	3	0.1908
SLC39A6	6	0.14327	0.31738	0.9995	6319	2	0.0806
NSDHL	6	0.14338	0.31752	0.9995	6320	2	0.0639
PCSK5	6	0.14344	0.3176	0.9995	6321	1	-0.589
PTPRZ1	6	0.14348	0.31765	0.9995	6322	3	0.1532
TNS3	6	0.1435	0.31768	0.9995	6323	3	0.2322
S100A12	6	0.14355	0.31775	0.9995	6324	2	0.1079
CLCN7	6	0.14356	0.31776	0.9995	6325	1	-0.196
ATP6V1G3	6	0.14356	0.31777	0.9995	6326	3	-0.055
MAPK1	6	0.1436	0.31782	0.9995	6327	2	-0.151
METTL5	6	0.14365	0.31788	0.9995	6328	2	-0.18
LGI2	6	0.14372	0.31797	0.9995	6329	1	-0.382
RAB10	6	0.14372	0.31797	0.9995	6330	3	0.1128
THAP5	6	0.14382	0.3181	0.9995	6331	3	0.141
GBA2	6	0.14388	0.31819	0.9995	6332	3	0.1819
CRIPAK	6	0.1439	0.31821	0.9995	6333	3	-0.129
KALRN	6	0.14392	0.31824	0.9995	6334	3	0.2106
IGFBP5	6	0.14393	0.31825	0.9995	6335	3	0.319
PDE7A	6	0.14394	0.31826	0.9995	6336	3	0.2184
GART	6	0.14397	0.3183	0.9995	6337	3	0.2826
HECTD1	6	0.14398	0.31831	0.9995	6338	3	0.1929
SCGB1D4	6	0.14411	0.31847	0.9995	6339	3	0.1719
IL1F10	6	0.14413	0.31849	0.9995	6340	3	-0.058
SERPINC1	6	0.14415	0.31851	0.9995	6341	3	0.0113
38596	3	0.14418	0.24115	0.9995	6342	2	0.3885
C5orf45	6	0.14418	0.31855	0.9995	6343	3	0.3235
GPR124	4	0.1442	0.28344	0.9995	6344	2	0.0676
AURKC	6	0.14424	0.31862	0.9995	6345	3	0.2631
LYRM5	6	0.14425	0.31863	0.9995	6346	2	-0.024
ITIH4	6	0.14429	0.31868	0.9995	6347	3	0.1729
TEX19	6	0.14433	0.31873	0.9995	6348	2	-0.184
RAB44	6	0.14434	0.31874	0.9995	6349	1	-0.18
hsa-mir-6757	4	0.14434	0.28359	0.9995	6350	1	-0.069
ADH6	6	0.14438	0.31879	0.9995	6351	3	0.1266
ASIC2	6	0.14443	0.31885	0.9995	6352	2	-0.205
VSX2	6	0.14443	0.31885	0.9995	6353	1	-1.772
RAB3IL1	6	0.14447	0.3189	0.9995	6354	2	-0.154
SPAST	6	0.14452	0.31895	0.9995	6355	2	0.2896
CDK17	6	0.14453	0.31896	0.9995	6356	3	0.2265
NCALD	6	0.14456	0.319	0.9995	6357	2	-0.152
ID4	6	0.14461	0.31906	0.9995	6358	1	-0.171
MLL4	3	0.14474	0.24188	0.9995	6359	2	0.4506
CCR5	6	0.14476	0.31927	0.9995	6360	3	0.3077
AMICA1	6	0.14486	0.31939	0.9995	6361	3	-0.079
OXCT2	6	0.14487	0.3194	0.9995	6362	2	0.1876
TLR9	6	0.14494	0.31949	0.9995	6363	3	0.3928
VPS16	6	0.14498	0.31954	0.9995	6364	2	-0.606
ZNF48	6	0.14501	0.31958	0.9995	6365	2	0.1442
AIP	6	0.14514	0.31973	0.9995	6366	3	0.2085
NTN5	6	0.14515	0.31976	0.9995	6367	3	0.2331
hsa-mir-670	4	0.14516	0.28442	0.9995	6368	1	-0.052
XCL2	3	0.14521	0.24252	0.9995	6369	2	0.6334
SERHL2	6	0.14523	0.31986	0.9995	6370	3	0.2111

C10orf11	6	0.1453	0.31994	0.9995	6371	2	-0.145
COMMD7	6	0.14532	0.31997	0.9995	6372	3	0.0101
PPP6C	6	0.14536	0.32001	0.9995	6373	2	-0.08
LOC283403	6	0.14546	0.32014	0.9995	6374	2	-0.243
DCDC2	5	0.1455	0.29609	0.9995	6375	3	0.4726
ACR	6	0.1455	0.3202	0.9995	6376	2	-0.003
RMND5A	6	0.1455	0.3202	0.9995	6377	2	-0.073
DERL2	6	0.14554	0.32025	0.9995	6378	3	0.2796
HSD11B1	6	0.14567	0.32041	0.9995	6379	3	0.1072
hsa-mir-1291	4	0.14567	0.28492	0.9995	6380	2	-0.031
SLC26A8	6	0.14569	0.32044	0.9995	6381	3	0.1934
IL37	6	0.14579	0.32056	0.9995	6382	3	0.2457
PPP1CC	6	0.14594	0.32074	0.9995	6383	2	-0.22
KRBOX4	5	0.14596	0.29681	0.9995	6384	2	0.1069
SPRED2	6	0.14598	0.32081	0.9995	6385	2	-0.202
CGB1	3	0.146	0.24354	0.9995	6386	2	0.2975
TRIP11	6	0.14605	0.3209	0.9995	6387	3	0.0169
GABRR2	6	0.14612	0.32097	0.9995	6388	1	-0.174
VLDLR	6	0.14613	0.32099	0.9995	6389	3	0.025
SLFNL1	6	0.14613	0.32099	0.9995	6390	3	0.1967
SCNN1D	6	0.14617	0.32103	0.9995	6391	3	0.1517
AATF	6	0.14624	0.32112	0.9995	6392	3	0.0743
FBP1	6	0.14624	0.32112	0.9995	6393	3	0.2072
HSD17B7	6	0.14635	0.32125	0.9995	6394	3	0.2092
CHIT1	6	0.14639	0.32132	0.9995	6395	3	0.2631
RAVER2	6	0.14639	0.32132	0.9995	6396	2	0.1478
ZC3H14	6	0.14641	0.32134	0.9995	6397	3	0.2628
BTN3A2	6	0.14644	0.32137	0.9995	6398	1	-0.294
TWIST1	6	0.14648	0.32143	0.9995	6399	1	-0.573
IL6	4	0.14654	0.28579	0.9995	6400	1	-0.195
ZNF777	6	0.14654	0.3215	0.9995	6401	2	0.3467
PRR3	6	0.14655	0.32151	0.9995	6402	3	0.2727
CMTR2	4	0.14662	0.28588	0.9995	6403	2	-0.124
EGR4	6	0.14664	0.32163	0.9995	6404	2	0.0006
WAPAL	6	0.14668	0.32167	0.9995	6405	3	0.2539
EID3	6	0.14674	0.32177	0.9995	6406	2	-0.111
ARRB1	6	0.14674	0.32177	0.9995	6407	3	0.4599
NOMO3	2	0.14681	0.22433	0.9995	6408	1	0.5189
KLF3	6	0.14685	0.3219	0.9995	6409	3	0.2328
DDX1	6	0.14694	0.322	0.9995	6410	2	-0.002
HIST1H2AJ	3	0.14696	0.24476	0.9995	6411	2	0.3867
ROBO2	6	0.14699	0.32205	0.9995	6412	1	-0.14
UFL1	6	0.14702	0.32209	0.9995	6413	2	0.1927
hsa-mir-4708	4	0.14706	0.28633	0.9995	6414	2	-0.071
TMEM110-MUSTN1	4	0.14711	0.28638	0.9995	6415	1	-0.181
VPS13D	6	0.14717	0.3223	0.9995	6416	1	-0.129
POLR3GL	6	0.14719	0.32232	0.9995	6417	2	-0.078
OGDHL	6	0.14721	0.32234	0.9995	6418	3	0.0738
NLRP2	6	0.14722	0.32236	0.9995	6419	2	-0.049
XRN1	6	0.14727	0.32243	0.9995	6420	3	-0.088
SEC14L4	6	0.14728	0.32245	0.9995	6421	3	0.1712
FABP4	6	0.14731	0.32247	0.9995	6422	3	0.1182
ZFYVE21	6	0.14733	0.32251	0.9995	6423	3	0.1089
LRIT1	6	0.14734	0.32251	0.9995	6424	3	-0.099
SERPINE3	6	0.14735	0.32252	0.9995	6425	3	0.1862
TMEM246	6	0.14753	0.32276	0.9995	6426	2	0.0094
VSTM5	6	0.14757	0.32282	0.9995	6427	3	-0.01
NFKBIZ	6	0.14758	0.32284	0.9995	6428	3	0.1646
RC3H1	6	0.14765	0.32293	0.9995	6429	2	-0.178
RAPGEF6	6	0.14765	0.32293	0.9995	6430	3	0.2252
GPR101	6	0.14766	0.32294	0.9995	6431	3	0.3268
OR10H2	6	0.14771	0.32302	0.9995	6432	3	0.176
hsa-mir-4421	4	0.14775	0.28704	0.9995	6433	2	0.0664
NEURL	6	0.14776	0.32307	0.9995	6434	2	0.0768
REM1	6	0.14781	0.32314	0.9995	6435	3	0.2354
AAMDC	6	0.14786	0.32321	0.9995	6436	3	0.114
C14orf169	6	0.14792	0.32328	0.9995	6437	3	0.0456
XPC	6	0.14792	0.32328	0.9995	6438	1	-0.069
hsa-mir-5705	4	0.14802	0.28733	0.9995	6439	2	0.4633
SLFN12	6	0.14808	0.32347	0.9995	6440	2	0.1996
ENOX2	6	0.14812	0.32352	0.9995	6441	3	0.2665
KLHL25	6	0.14815	0.32357	0.9995	6442	3	0.2387
PLSCR3	6	0.14825	0.32369	0.9995	6443	2	-0.174
C2orf40	6	0.14835	0.3238	0.9995	6444	2	-0.08
ARPP21	6	0.14837	0.32382	0.9995	6445	3	0.3345
PIP4K2A	6	0.14839	0.32384	0.9995	6446	3	0.2703
CHFR	6	0.1484	0.32385	0.9995	6447	3	0.2007
DZIP3	6	0.14841	0.32386	0.9995	6448	3	0.2107
hsa-mir-200a	4	0.14841	0.28771	0.9995	6449	2	0.2557
GPN1	6	0.14849	0.32397	0.9995	6450	2	0.216
CEP89	6	0.14849	0.32397	0.9995	6451	3	0.2723
BSN	6	0.14852	0.324	0.9995	6452	2	0.0482
FAM65B	6	0.14852	0.32401	0.9995	6453	3	0.2129
ZRSR2	6	0.14861	0.32413	0.9995	6454	3	0.3527
TMX2	6	0.14865	0.32418	0.9995	6455	3	0.2331

ZNF596	6	0.14873	0.32428	0.9995	6456	2	-0.015
CLMP	6	0.14876	0.32433	0.9995	6457	2	-0.108
GPR182	6	0.14876	0.32433	0.9995	6458	3	0.1856
UBXN1	6	0.14876	0.32433	0.9995	6459	3	-0.74
BMP8B	3	0.14877	0.24708	0.9995	6460	1	-1.371
PPM1K	6	0.14881	0.3244	0.9995	6461	2	-0.168
PRSS53	6	0.14885	0.32445	0.9995	6462	1	-0.253
PRR15L	6	0.1489	0.32451	0.9995	6463	3	0.291
TRIM17	6	0.1489	0.32451	0.9995	6464	2	-0.204
GML	6	0.14891	0.32452	0.9995	6465	2	0.0189
CHAD	6	0.14893	0.32454	0.9995	6466	2	-0.202
TMEM255A	6	0.14894	0.32455	0.9995	6467	3	0.0789
CACFD1	6	0.14895	0.32456	0.9995	6468	1	-0.665
QARS	6	0.14897	0.3246	0.9995	6469	3	0.0733
hsa-mir-6729	4	0.14903	0.28833	0.9995	6470	1	-0.747
PIGW	6	0.14904	0.32467	0.9995	6471	1	-0.184
KIR3DL2	5	0.14906	0.30138	0.9995	6472	1	-0.333
PTAFR	6	0.14908	0.32473	0.9995	6473	2	-0.015
FAM78B	6	0.14913	0.32479	0.9995	6474	1	-0.264
PCNA	6	0.14917	0.32484	0.9995	6475	1	-0.425
NAA50	6	0.14918	0.32484	0.9995	6476	3	0.0859
GNMT	6	0.14922	0.3249	0.9995	6477	2	-0.39
ATG13	6	0.14922	0.32491	0.9995	6478	3	0.3497
TPD52L3	6	0.14926	0.32496	0.9995	6479	2	-0.039
RGS13	6	0.14927	0.32497	0.9995	6480	3	0.2393
RND1	6	0.14936	0.32508	0.9995	6481	3	0.2316
hsa-mir-130b	4	0.14937	0.28866	0.9995	6482	2	0.3388
UBQLN1	6	0.14938	0.32511	0.9995	6483	3	0.253
NOXO1	6	0.1494	0.32514	0.9995	6484	1	-0.188
LILRA6	4	0.14942	0.28872	0.9995	6485	2	0.1767
OR11L1	6	0.1495	0.32526	0.9995	6486	3	0.2208
CDKN1A	6	0.14952	0.32529	0.9995	6487	3	0.0986
hsa-mir-1183	4	0.14955	0.28885	0.9995	6488	2	0.2225
WDR5B	6	0.14959	0.32538	0.9995	6489	2	-0.261
MBD3	6	0.14966	0.32548	0.9995	6490	3	0.2115
SLC26A1	6	0.14966	0.32548	0.9995	6491	3	0.1909
hsa-mir-497	4	0.14969	0.28899	0.9995	6492	2	0.2821
ASIC4	6	0.14971	0.32554	0.9995	6493	3	0.1945
MYF6	6	0.14978	0.32563	0.9995	6494	3	0.2709
RIMS1	6	0.14981	0.32565	0.9995	6495	2	-0.159
TMEM175	6	0.14981	0.32565	0.9995	6496	3	0.0892
UBR4	6	0.14985	0.32571	0.9995	6497	3	0.1781
DDX19B	6	0.14991	0.32579	0.9995	6498	3	0.3455
hsa-mir-548al	2	0.14998	0.22649	0.9995	6499	1	0.6154
SCTR	6	0.14999	0.32588	0.9995	6500	2	0.1235
ZNF383	6	0.14999	0.32588	0.9995	6501	2	-0.657
RHBDF2	6	0.15002	0.32591	0.9995	6502	1	-0.025
MORC2	6	0.15002	0.32591	0.9995	6503	3	0.1892
AP1G1	6	0.15013	0.32606	0.9995	6504	2	0.0879
CTSL1	3	0.15015	0.24886	0.9995	6505	2	0.4236
CCNE1	6	0.15017	0.32612	0.9995	6506	3	-0.004
CLPSL2	6	0.1502	0.32615	0.9995	6507	2	0.1896
GPAM	6	0.15022	0.32618	0.9995	6508	2	0.0329
TNPO1	6	0.15027	0.32624	0.9995	6509	2	-0.133
KRTAP17-1	6	0.15031	0.32631	0.9995	6510	1	-0.159
SUB1	6	0.15033	0.32634	0.9995	6511	2	-0.238
FILIP1L	6	0.15034	0.32635	0.9995	6512	3	0.2554
TASP1	6	0.15036	0.32637	0.9995	6513	3	0.0105
CDC42EP2	6	0.1504	0.32643	0.9995	6514	1	-0.105
PRMT8	6	0.15056	0.32664	0.9995	6515	1	-0.095
MAP4K1	6	0.15057	0.32665	0.9995	6516	3	0.144
hsa-mir-5689	4	0.1506	0.28993	0.9995	6517	2	0.5033
KRTAP9-6	5	0.1507	0.30389	0.9995	6518	1	-1.074
NHP2L1	6	0.1507	0.32681	0.9995	6519	3	-0.59
POC5	6	0.15072	0.32685	0.9995	6520	3	-0.184
RAI14	6	0.15086	0.32703	0.9995	6521	2	-0.166
PDDC1	6	0.15088	0.32705	0.9995	6522	3	0.2084
MAP3K4	6	0.15096	0.32714	0.9995	6523	3	0.063
TEX12	6	0.15097	0.32715	0.9995	6524	2	0.0065
OR1K1	6	0.15097	0.32716	0.9995	6525	3	0.1514
hsa-mir-6131	4	0.151	0.29036	0.9995	6526	2	0.1062
PCDHGC4	2	0.15103	0.22721	0.9995	6527	1	-0.215
NEIL3	6	0.15105	0.32725	0.9995	6528	3	0.2534
NUDT10	3	0.1511	0.25008	0.9995	6529	1	-0.043
SLC1A6	6	0.15113	0.32736	0.9995	6530	2	-0.077
SFT2D2	6	0.15118	0.32742	0.9995	6531	2	-0.988
RBMS2	6	0.15122	0.32747	0.9995	6532	2	-0.248
MAGEB3	6	0.15127	0.32754	0.9995	6533	1	-0.207
RNASE13	6	0.15131	0.3276	0.9995	6534	2	-0.044
SEL1L2	4	0.15132	0.29069	0.9995	6535	2	0.3703
CSDC2	6	0.15136	0.32765	0.9995	6536	2	-0.09
ZNF625	5	0.15137	0.30494	0.9995	6537	2	-0.496
KCNT1	6	0.15138	0.32767	0.9995	6538	2	0.125
TLR8	6	0.15143	0.32775	0.9995	6539	3	0.1492
CNOT10	6	0.15147	0.3278	0.9995	6540	3	0.2362

LSM14A	6	0.15148	0.32781	0.9995	6541	3	0.2943
IFNA16	5	0.15151	0.30514	0.9995	6542	2	-0.076
IQCF2	6	0.15157	0.32792	0.9995	6543	3	0.1273
AURKB	6	0.15159	0.32795	0.9995	6544	2	0.1789
GDF15	6	0.15159	0.32795	0.9995	6545	2	-0.115
DDX28	6	0.15163	0.32801	0.9995	6546	2	0.1515
SLC25A47	6	0.15168	0.32806	0.9995	6547	1	-0.29
RIPK2	4	0.15175	0.29113	0.9995	6548	1	0.1017
LLGL2	6	0.15177	0.32817	0.9995	6549	3	0.1334
FAM166A	6	0.15179	0.3282	0.9995	6550	3	0.2527
ARHGAP23	6	0.15186	0.3283	0.9995	6551	1	-0.504
UQCRH	6	0.15188	0.32832	0.9995	6552	3	0.2305
CMTM8	6	0.15206	0.32857	0.9995	6553	2	0.1717
GRASP	6	0.15206	0.32857	0.9995	6554	3	0.2454
POU5F1B	6	0.15207	0.32858	0.9995	6555	2	-0.15
ADAM32	6	0.15217	0.32871	0.9995	6556	2	-0.08
TNFRSF10A	6	0.15219	0.32872	0.9995	6557	3	0.2308
BCAT2	6	0.15227	0.32883	0.9995	6558	1	-0.164
SLC7A2	6	0.15227	0.32884	0.9995	6559	3	0.0179
DUOXA2	6	0.1523	0.32887	0.9995	6560	3	0.2475
CHST13	6	0.15237	0.32896	0.9995	6561	2	-0.094
FAM157B	3	0.1524	0.25173	0.9995	6562	2	0.4699
PCDHB8	4	0.15243	0.29184	0.9995	6563	2	0.2248
WBSCR17	6	0.15248	0.3291	0.9995	6564	3	0.1674
MASTL	6	0.15249	0.32911	0.9995	6565	1	-0.28
HIST1H4I	6	0.15255	0.32917	0.9995	6566	3	-9E-04
ZBTB17	6	0.15266	0.32932	0.9995	6567	2	0.1221
FGF12	6	0.15267	0.32935	0.9995	6568	2	0.2134
C1orf53	6	0.15272	0.32941	0.9995	6569	3	0.4688
LINGO1	6	0.15281	0.32952	0.9995	6570	2	-0.083
C14orf166	6	0.15287	0.32961	0.9995	6571	3	-0.148
GRK7	6	0.15292	0.32967	0.9995	6572	3	-0.175
ALDH1A2	6	0.15295	0.32969	0.9995	6573	2	-0.296
hsa-mir-7641-1	2	0.15295	0.22858	0.9995	6574	1	0.8593
GALK2	6	0.15299	0.32974	0.9995	6575	2	0.1911
NDUFV1	6	0.15301	0.32978	0.9995	6576	3	0.0576
SIX5	6	0.15301	0.32978	0.9995	6577	2	-0.225
ADCY10	6	0.15308	0.32986	0.9995	6578	3	0.191
SENP8	6	0.15315	0.32995	0.9995	6579	1	-0.418
SUCLA2	6	0.15324	0.33007	0.9995	6580	2	-0.12
ANKRD28	6	0.15327	0.3301	0.9995	6581	3	0.2284
RBMS3	4	0.15332	0.29278	0.9995	6582	1	-0.113
NXN	6	0.15333	0.33019	0.9995	6583	2	-0.108
ARHGAP8	6	0.15337	0.33024	0.9995	6584	3	0.1603
FAM86A	5	0.15337	0.30791	0.9995	6585	3	0.268
GLMN	6	0.1534	0.33027	0.9995	6586	3	0.1529
hsa-mir-4426	3	0.15355	0.25322	0.9995	6587	2	0.3945
IGF2BP2	6	0.15357	0.33049	0.9995	6588	3	0.2701
TBC1D20	6	0.15358	0.3305	0.9995	6589	2	-0.229
GBAS	6	0.1536	0.33052	0.9995	6590	3	0.1329
TMEM116	4	0.15378	0.29324	0.9995	6591	2	0.1348
hsa-mir-9-3	4	0.15381	0.29328	0.9995	6592	2	0.1993
hsa-mir-4471	4	0.15388	0.29336	0.9995	6593	2	0.1537
IVL	6	0.15388	0.33087	0.9995	6594	3	0.2096
ERBB2IP	6	0.1539	0.33089	0.9995	6595	3	0.2729
EVI5L	6	0.15391	0.33091	0.9995	6596	2	-0.072
TMEM257	6	0.15394	0.33095	0.9995	6597	3	0.2351
KIAA0319L	6	0.15395	0.33095	0.9995	6598	3	0.2531
ZNF302	6	0.15402	0.33105	0.9995	6599	3	0.3137
hsa-mir-4523	4	0.15402	0.2935	0.9995	6600	1	-0.281
GATM	6	0.15409	0.33113	0.9995	6601	3	0.2332
NYAP2	6	0.15412	0.33117	0.9995	6602	3	0.0267
LIPH	6	0.15414	0.33118	0.9995	6603	3	0.1988
EBF3	6	0.15428	0.33139	0.9995	6604	3	0.1253
C20orf166	6	0.15429	0.3314	0.9995	6605	3	0.1302
SPG21	6	0.15438	0.33149	0.9995	6606	2	0.0771
ZNF484	6	0.1544	0.33152	0.9995	6607	3	0.2606
ZNF695	6	0.15446	0.33159	0.9995	6608	3	0.2601
FAM179A	6	0.15449	0.33163	0.9995	6609	2	-0.083
hsa-mir-6798	4	0.15455	0.29407	0.9995	6610	2	0.1142
ARHGAP39	6	0.15455	0.33171	0.9995	6611	2	-0.587
EFHC1	6	0.15457	0.33174	0.9995	6612	3	0.172
DMRTB1	6	0.15458	0.33175	0.9995	6613	1	-0.143
ATN1	6	0.1546	0.33178	0.9995	6614	3	0.2617
GATA6	6	0.15461	0.33179	0.9995	6615	3	0.0499
PEX1	6	0.15462	0.33181	0.9995	6616	3	-0.049
RAD51C	6	0.15473	0.33194	0.9995	6617	3	0.1959
OCEL1	6	0.15479	0.33201	0.9995	6618	3	0.2639
PDZK1IP1	6	0.15479	0.33201	0.9995	6619	3	0.2009
OR4S2	6	0.15481	0.33204	0.9995	6620	2	0.1061
KIF4A	6	0.1549	0.33216	0.9995	6621	2	0.1134
KRT27	6	0.15491	0.33217	0.9995	6622	2	0.0523
SHB	6	0.15491	0.33217	0.9995	6623	3	-0.028
LHX2	6	0.15501	0.33228	0.9995	6624	3	0.1682
LEO1	6	0.15508	0.33237	0.9995	6625	1	-0.699

CD160	6	0.15511	0.33241	0.9995	6626	2	-0.407
RAPGEF2	6	0.15526	0.3326	0.9995	6627	1	-0.193
NAA11	6	0.15526	0.3326	0.9995	6628	3	0.3912
TXNL1	6	0.15539	0.33276	0.9995	6629	3	0.115
hsa-mir-3922	4	0.15547	0.29504	0.9995	6630	2	-0.254
FAM171B	6	0.15551	0.3329	0.9995	6631	2	-0.245
hsa-mir-8058	4	0.15557	0.29516	0.9995	6632	1	0.083
BOP1	6	0.15561	0.33304	0.9995	6633	3	0.3894
hsa-mir-2116	4	0.15567	0.29527	0.9995	6634	1	-0.048
KRT14	5	0.15569	0.31131	0.9995	6635	3	0.3456
C9orf40	6	0.15571	0.33316	0.9995	6636	1	-0.664
NCAPD2	6	0.1558	0.33328	0.9995	6637	2	0.1578
PITX1	6	0.1558	0.33328	0.9995	6638	3	0.2751
SLC39A9	6	0.15589	0.33338	0.9995	6639	2	-0.3
GNPDA1	6	0.15594	0.33345	0.9995	6640	2	0.2666
GAGE10	4	0.15595	0.29557	0.9995	6641	1	-0.807
SLC7A1	6	0.15596	0.33347	0.9995	6642	1	-0.24
SOCS4	6	0.15598	0.33349	0.9995	6643	3	0.2472
EXOC3L2	6	0.15603	0.33356	0.9995	6644	2	0.0891
KRTAP7-1	6	0.1562	0.33378	0.9995	6645	3	0.1439
GPR84	6	0.15623	0.33382	0.9995	6646	3	0.131
EPS15L1	6	0.15631	0.33393	0.9995	6647	3	0.238
hsa-mir-4314	4	0.15644	0.29605	0.9995	6648	1	-0.535
ADAMTS14	6	0.1565	0.33417	0.9995	6649	2	0.0989
hsa-mir-604	4	0.15655	0.29616	0.9995	6650	2	0.4079
PHLDB2	6	0.15662	0.33432	0.9995	6651	3	0.0559
TAS2R14	6	0.15662	0.33432	0.9995	6652	2	0.0399
TAS1R3	6	0.15664	0.33435	0.9995	6653	1	-0.287
SAP30BP	6	0.15668	0.33441	0.9995	6654	3	0.315
MALL	6	0.15679	0.33455	0.9995	6655	3	0.1916
PMPCA	6	0.15686	0.33463	0.9995	6656	3	0.3107
KRTAP10-2	6	0.15689	0.33467	0.9995	6657	2	-0.092
FOXJ3	6	0.15693	0.33472	0.9995	6658	2	-0.187
ADAT2	6	0.15698	0.33477	0.9995	6659	2	-0.047
ALKBH8	6	0.15704	0.33486	0.9995	6660	3	0.2024
TMEM176B	6	0.1571	0.33493	0.9995	6661	3	0.2322
STH	6	0.15711	0.33494	0.9995	6662	1	-0.282
FSCN1	6	0.15716	0.33499	0.9995	6663	1	-0.306
HSPA1B	4	0.1572	0.29687	0.9995	6664	1	-0.197
SF3A1	6	0.15725	0.33512	0.9995	6665	2	0.0264
PHF7	6	0.15727	0.33514	0.9995	6666	3	0.3489
TAS2R19	6	0.15728	0.33515	0.9995	6667	2	-0.482
ZNF662	6	0.15738	0.33528	0.9995	6668	2	-0.155
COL3A1	6	0.15743	0.33535	0.9995	6669	3	0.2511
GOPC	6	0.15747	0.3354	0.9995	6670	3	0.1869
SLC9A1	6	0.15751	0.33544	0.9995	6671	2	-0.032
CD244	6	0.15752	0.33545	0.9995	6672	2	-0.136
VNN1	6	0.15754	0.33548	0.9995	6673	3	0.0606
ABCB5	6	0.15756	0.33552	0.9995	6674	3	0.1147
OR1S2	6	0.15759	0.33554	0.9995	6675	2	0.1057
KIF13A	6	0.15759	0.33554	0.9995	6676	2	-0.013
USP19	6	0.15761	0.33557	0.9995	6677	1	-0.219
C6orf223	6	0.15765	0.33563	0.9995	6678	2	0.0267
KCNH2	6	0.15768	0.33567	0.9995	6679	3	0.2325
AGR3	6	0.1578	0.33581	0.9995	6680	3	0.1358
SPECC1L	6	0.15781	0.33582	0.9995	6681	2	0.0113
ITGA11	6	0.15788	0.33591	0.9995	6682	1	-0.11
POLR1B	6	0.15791	0.33595	0.9995	6683	3	0.1254
LYG1	6	0.15797	0.33603	0.9995	6684	2	-0.149
SLC25A20	6	0.15801	0.33609	0.9995	6685	2	-0.453
hsa-mir-4650-1	3	0.15803	0.25905	0.9995	6686	1	-0.215
CACNA1D	6	0.15804	0.33611	0.9995	6687	3	0.0484
hsa-mir-8060	4	0.15806	0.29775	0.9995	6688	1	-0.103
PARVA	6	0.1582	0.33632	0.9995	6689	2	-0.357
RPP25	6	0.15821	0.33634	0.9995	6690	2	0.1401
ZW10	6	0.15826	0.33641	0.9995	6691	3	-0.018
TAGAP	6	0.15826	0.33641	0.9995	6692	3	0.3516
CUX1	6	0.1583	0.33645	0.9995	6693	3	0.2655
RNF220	6	0.15838	0.33656	0.9995	6694	3	0.2198
CCDC171	6	0.15838	0.33656	0.9995	6695	1	0.0778
NAT9	6	0.15846	0.33666	0.9995	6696	2	0.0526
NUDT3	6	0.15853	0.33674	0.9995	6697	2	-0.018
CENPT	6	0.15856	0.33677	0.9995	6698	1	-0.145
NPW	6	0.15862	0.33685	0.9995	6699	3	0.306
GTF3C5	6	0.15865	0.33688	0.9995	6700	1	-0.133
RAD52	6	0.15874	0.33701	0.9995	6701	2	-0.089
CD99L2	6	0.15876	0.33703	0.9995	6702	3	-0.08
LRPPRC	6	0.15883	0.33712	0.9995	6703	1	0.0901
RLN2	6	0.15885	0.33715	0.9995	6704	2	-0.294
REP15	6	0.15887	0.33717	0.9995	6705	2	-0.253
SVIP	6	0.15887	0.33718	0.9995	6706	3	0.1674
CLN3	6	0.1589	0.33721	0.9995	6707	3	0.2021
TMEM65	6	0.15892	0.33722	0.9995	6708	2	0.0455
C1orf51	6	0.15896	0.33728	0.9995	6709	2	-0.063
C1QB	6	0.15897	0.33729	0.9995	6710	2	-0.37

RAPGEF5	6	0.15904	0.33738	0.9995	6711	2	0.164
EDNRA	6	0.15904	0.33738	0.9995	6712	3	0.1643
CDC25A	6	0.1591	0.33744	0.9995	6713	1	0.0834
GLIS3	6	0.15921	0.33759	0.9995	6714	2	-0.166
SERPINF1	6	0.15923	0.33762	0.9995	6715	2	0.0309
ALG5	6	0.15923	0.33762	0.9995	6716	3	0.1878
FKBP9	6	0.15923	0.33762	0.9995	6717	3	0.1023
ALDH4A1	6	0.15935	0.33777	0.9995	6718	3	0.3052
SNCB	6	0.15937	0.33779	0.9995	6719	2	0.2563
PSORS1C2	6	0.15941	0.33784	0.9995	6720	1	-0.479
TMPRSS11D	6	0.15941	0.33784	0.9995	6721	3	0.198
WDR67	3	0.15943	0.26087	0.9995	6722	1	-0.147
hsa-mir-6806	4	0.15946	0.2992	0.9995	6723	1	-0.287
C1QTNF8	6	0.15966	0.33816	0.9995	6724	3	0.2768
hsa-mir-504	4	0.15968	0.29943	0.9995	6725	2	0.2373
GPSM2	6	0.15968	0.33819	0.9995	6726	3	0.1337
CPNE7	6	0.15971	0.33823	0.9995	6727	3	0.255
MRGPRX2	6	0.15973	0.33826	0.9995	6728	3	0.1831
ZNF768	6	0.15976	0.33829	0.9995	6729	2	-0.101
UGDH	6	0.15984	0.33839	0.9995	6730	2	-0.27
C1QL3	6	0.15984	0.33839	0.9995	6731	2	0.0627
ABHD13	6	0.15993	0.3385	0.9995	6732	2	0.0204
DFFA	6	0.15998	0.33855	0.9995	6733	3	0.1837
CIDEB	6	0.16	0.33857	0.9995	6734	3	0.2118
ALX1	6	0.16003	0.33861	0.9995	6735	3	0.1259
PSG1	5	0.16004	0.31768	0.9995	6736	3	0.4342
FAM129A	6	0.16007	0.33866	0.9995	6737	2	-0.218
SMDT1	6	0.16014	0.33875	0.9995	6738	2	0.0136
NDE1	6	0.16017	0.33879	0.9995	6739	3	0.2952
ACSF3	6	0.16018	0.3388	0.9995	6740	1	-0.705
LRRC34	6	0.16022	0.33885	0.9995	6741	2	0.045
PLIN5	6	0.16023	0.33886	0.9995	6742	3	0.339
NAT14	6	0.16025	0.33889	0.9995	6743	3	-0.209
HCLS1	6	0.16031	0.33896	0.9995	6744	2	-0.369
RTN4	6	0.16032	0.33897	0.9995	6745	2	-0.243
EFHC2	6	0.16034	0.33899	0.9995	6746	3	0.2658
PRSS36	6	0.16036	0.33902	0.9995	6747	2	-0.677
ARFGAP2	6	0.16038	0.33905	0.9995	6748	3	0.2728
EN2	6	0.1604	0.33907	0.9995	6749	2	-0.464
EME2	6	0.16043	0.33911	0.9995	6750	3	0.0084
ESRRG	6	0.16049	0.33919	0.9995	6751	2	-0.228
RAB19	6	0.16058	0.33931	0.9995	6752	2	-0.25
PIM2	6	0.16081	0.3396	0.9995	6753	2	0.1106
RPS6KB1	6	0.16082	0.33961	0.9995	6754	3	0.294
hsa-mir-548f-1	3	0.16086	0.26271	0.9995	6755	1	-0.235
OXGR1	6	0.16089	0.33971	0.9995	6756	3	0.1171
RING1	6	0.16098	0.3398	0.9995	6757	3	0.065
WFDC3	6	0.161	0.33983	0.9995	6758	3	0.0781
PTPRU	6	0.16103	0.33987	0.9995	6759	3	0.2201
TMEM243	6	0.16108	0.33992	0.9995	6760	2	-0.025
SUPV3L1	6	0.16108	0.33992	0.9995	6761	3	0.0524
TMEM201	6	0.16112	0.33998	0.9995	6762	1	0.0093
TMPRSS11A	6	0.16123	0.34011	0.9995	6763	2	-0.303
PARD6A	6	0.16123	0.34011	0.9995	6764	3	0.0598
hsa-mir-1252	3	0.16125	0.2632	0.9995	6765	1	-0.598
OPA3	6	0.16126	0.34014	0.9995	6766	2	0.0932
TPRKB	6	0.16131	0.34022	0.9995	6767	3	0.1776
CDH9	6	0.16135	0.34027	0.9995	6768	3	0.2973
VPS45	6	0.16138	0.34031	0.9995	6769	3	0.2094
RASGEF1A	6	0.16146	0.3404	0.9995	6770	3	0.0881
PHLDB3	6	0.16158	0.34055	0.9995	6771	3	0.1649
ZNF641	6	0.16162	0.34059	0.9995	6772	3	0.0951
KLK4	6	0.16162	0.3406	0.9995	6773	1	-0.363
CCDC60	6	0.16165	0.34064	0.9995	6774	3	0.2623
MYOD1	6	0.16165	0.34064	0.9995	6775	3	0.2233
GRAMD4	6	0.16165	0.34064	0.9995	6776	3	0.2161
MUS81	6	0.16173	0.34073	0.9995	6777	3	0.1542
RBM12	6	0.1618	0.34081	0.9995	6778	1	-0.234
hsa-mir-8079	4	0.16188	0.30167	0.9995	6779	2	0.3451
RAB26	6	0.16189	0.34093	0.9995	6780	2	0.1568
KDELC1	6	0.16189	0.34093	0.9995	6781	2	-0.032
IL31RA	6	0.16192	0.34097	0.9995	6782	3	0.2255
ATOH8	6	0.16195	0.34102	0.9995	6783	3	0.284
KLHL32	6	0.16198	0.34105	0.9995	6784	2	-0.171
CASP1	5	0.16199	0.32053	0.9995	6785	2	-0.296
FOXN2	6	0.162	0.34108	0.9995	6786	2	-0.107
FAM120AOS	6	0.162	0.34108	0.9995	6787	3	0.0316
ITPKC	6	0.16211	0.34122	0.9995	6788	2	0.1173
SVOPL	6	0.16211	0.34122	0.9995	6789	2	-0.006
MPEG1	6	0.16214	0.34127	0.9995	6790	3	0.3372
SULT1E1	6	0.16216	0.34129	0.9995	6791	3	0.087
HTR2A	6	0.16228	0.34144	0.9995	6792	3	0.2926
ORC4	6	0.16234	0.34152	0.9995	6793	3	0.297
UBE2T	6	0.16237	0.34156	0.9995	6794	3	0.2264
LEP	6	0.16248	0.34169	0.9995	6795	2	-0.154

ASCL1	6	0.16256	0.3418	0.9995	6796	2	-0.103
MRPL42	6	0.16262	0.34187	0.9995	6797	2	0.0976
IRF9	6	0.16264	0.34189	0.9995	6798	3	0.1866
TMPRSS3	6	0.16265	0.34191	0.9995	6799	1	-0.063
C11orf58	6	0.16269	0.34195	0.9995	6800	3	0.2774
OSTC	5	0.16274	0.32163	0.9995	6801	3	0.3185
ZNF14	6	0.16278	0.34207	0.9995	6802	3	0.3766
NAA20	6	0.16286	0.34217	0.9995	6803	1	-0.252
SART1	6	0.16304	0.3424	0.9995	6804	2	-0.439
EPHB1	6	0.16304	0.34241	0.9995	6805	3	0.3005
KCNK2	6	0.16311	0.3425	0.9995	6806	2	0.084
hsa-mir-3189	4	0.16311	0.30294	0.9995	6807	2	0.1848
WBSCR28	6	0.16319	0.3426	0.9995	6808	2	-0.091
TECRL	6	0.16324	0.34266	0.9995	6809	2	0.167
C11orf53	6	0.16325	0.34268	0.9995	6810	3	0.1801
ATP2B1	6	0.16325	0.34268	0.9995	6811	3	0.2675
FAM64A	6	0.16328	0.34272	0.9995	6812	3	-0.146
SLC25A35	5	0.16328	0.3224	0.9995	6813	3	0.2725
AMIGO3	6	0.16337	0.34284	0.9995	6814	3	0.2729
MTCH1	6	0.16342	0.34289	0.9995	6815	2	-0.368
PCDHGA12	2	0.16343	0.23595	0.9995	6816	1	0.2061
hsa-mir-548h-3	3	0.16344	0.26614	0.9995	6817	2	0.3712
OR5D13	6	0.16353	0.34303	0.9995	6818	2	0.1641
hsa-mir-548x-2	4	0.16354	0.30337	0.9995	6819	1	-0.189
CWC22	6	0.16357	0.34309	0.9995	6820	2	0.1468
NAP1L5	6	0.16362	0.34315	0.9995	6821	3	0.0735
TRIM14	6	0.16362	0.34315	0.9995	6822	3	0.3769
FAM109A	6	0.16365	0.34319	0.9995	6823	2	-0.293
GGH	6	0.16367	0.34322	0.9995	6824	3	0.1815
SEMA3C	6	0.16369	0.34324	0.9995	6825	3	0.2707
FUZ	6	0.16378	0.34335	0.9995	6826	2	-0.124
hsa-mir-934	4	0.1638	0.30366	0.9995	6827	2	0.2881
SYTL3	6	0.16382	0.34341	0.9995	6828	1	-0.292
YDJC	6	0.16384	0.34343	0.9995	6829	3	0.2463
hsa-mir-6747	4	0.16387	0.30373	0.9995	6830	2	-0.079
C17orf49	6	0.16391	0.34353	0.9995	6831	2	-0.105
CCDC158	6	0.16396	0.34358	0.9995	6832	2	0.1839
KLHL24	6	0.164	0.34364	0.9995	6833	2	0.1046
C1orf151-NBL1	3	0.164	0.26687	0.9995	6834	1	0.102
POLR2C	6	0.16402	0.34366	0.9995	6835	2	-0.395
SRSF2	6	0.1641	0.34376	0.9995	6836	3	0.408
CDH1	6	0.16414	0.3438	0.9995	6837	2	0.1229
FBXL17	6	0.16414	0.34381	0.9995	6838	3	0.2573
AGO3	6	0.16416	0.34383	0.9995	6839	2	-0.293
BAIAP2L2	6	0.16417	0.34384	0.9995	6840	3	0.1596
DIS3L	6	0.16422	0.34391	0.9995	6841	2	-0.07
IL1B	6	0.16431	0.34402	0.9995	6842	1	-0.288
hsa-mir-6797	4	0.16433	0.30421	0.9995	6843	2	0.3029
NNT	6	0.16445	0.34418	0.9995	6844	1	-0.676
ZBBX	6	0.16467	0.34447	0.9995	6845	2	0.2017
FSTL5	6	0.16469	0.34448	0.9995	6846	3	0.1052
IRF1	6	0.16469	0.34448	0.9995	6847	3	0.2261
CALN1	6	0.1647	0.3445	0.9995	6848	3	0.2291
GALNT18	6	0.16474	0.34455	0.9995	6849	1	-0.416
FOXN4	6	0.16476	0.34458	0.9995	6850	3	0.4103
SLC24A4	6	0.16481	0.34463	0.9995	6851	2	-0.046
TMEM178A	6	0.16481	0.34464	0.9995	6852	2	-0.036
ISG15	6	0.16498	0.34485	0.9995	6853	3	0.047
STK35	6	0.165	0.34486	0.9995	6854	3	-0.067
hsa-mir-106b	4	0.16507	0.30498	0.9995	6855	2	0.2465
FCGR2A	5	0.16516	0.3251	0.9995	6856	2	0.1536
CASD1	6	0.16525	0.34518	0.9995	6857	3	0.1755
BDKRB2	6	0.1653	0.34522	0.9995	6858	2	-0.207
PDSS1	6	0.16535	0.34528	0.9995	6859	2	-0.188
MX1	6	0.16539	0.34534	0.9995	6860	3	0.2419
SCN3B	6	0.16543	0.34539	0.9995	6861	3	0.0011
ARFGEF2	6	0.16544	0.34539	0.9995	6862	2	-0.257
OR2A2	6	0.16544	0.3454	0.9995	6863	3	0.0524
CATSPERD	6	0.1655	0.34546	0.9995	6864	2	-0.077
WISP1	6	0.16551	0.34547	0.9995	6865	3	0.2473
MYO5A	6	0.1656	0.34557	0.9995	6866	3	0.1555
DUSP5	6	0.16564	0.34563	0.9995	6867	3	-0.005
DUSP3	6	0.16565	0.34564	0.9995	6868	2	-0.432
RPS27A	6	0.16572	0.34575	0.9995	6869	3	0.1879
SPOCK1	6	0.16573	0.34577	0.9995	6870	3	0.2368
USP14	6	0.16576	0.34579	0.9995	6871	2	0.1803
MYO5B	6	0.16595	0.34604	0.9995	6872	3	0.2002
UBAP1L	6	0.16599	0.34608	0.9995	6873	2	-0.24
IGBP1	6	0.16602	0.34612	0.9995	6874	1	-0.064
ACSL3	6	0.16605	0.34615	0.9995	6875	3	0.2095
EIF3B	6	0.16607	0.34618	0.9995	6876	3	0.2244
GOSR2	6	0.16608	0.3462	0.9995	6877	1	-1.343
MAS1L	6	0.16608	0.3462	0.9995	6878	2	-0.222
THSD7B	6	0.16609	0.3462	0.9995	6879	3	0.1685
EFNB1	6	0.16615	0.34629	0.9995	6880	1	-0.447

MORC1	6	0.16617	0.3463	0.9995	6881	2	0.1265
MAPK8IP2	6	0.16617	0.34631	0.9995	6882	3	0.1288
PADI6	6	0.16621	0.34636	0.9995	6883	2	0.0398
SDHD	6	0.16622	0.34638	0.9995	6884	2	0.043
PAXBP1	6	0.16627	0.34644	0.9995	6885	3	-0.011
UGT3A2	6	0.16631	0.34647	0.9995	6886	2	0.0828
CACNA1S	6	0.16636	0.34655	0.9995	6887	3	0.4072
GCNT1	6	0.16642	0.34662	0.9995	6888	1	-0.671
NEIL2	6	0.16647	0.34668	0.9995	6889	3	0.3
PPAN	2	0.16654	0.23818	0.9995	6890	1	0.3069
SPDYE4	6	0.16654	0.34678	0.9995	6891	3	0.0965
CD207	6	0.16656	0.34679	0.9995	6892	1	-0.2
NPPC	6	0.1666	0.34684	0.9995	6893	3	0.275
ANKMY1	6	0.16664	0.34689	0.9995	6894	3	0.1537
ZNF251	6	0.16671	0.34699	0.9995	6895	3	0.2322
ETV1	6	0.16671	0.34699	0.9995	6896	3	0.4016
hsa-mir-4644	4	0.16674	0.30669	0.9995	6897	1	-0.37
PGR	6	0.16678	0.34708	0.9995	6898	1	-0.093
hsa-mir-6749	4	0.16685	0.30679	0.9995	6899	1	-0.096
PTPN11	6	0.16685	0.34716	0.9995	6900	2	0.14
OR51E1	6	0.16691	0.34724	0.9995	6901	1	-0.175
COL1A2	6	0.16692	0.34725	0.9995	6902	3	0.1925
ZNF839	6	0.16696	0.34729	0.9995	6903	2	0.0029
SMARCA5	6	0.16699	0.34734	0.9995	6904	3	0.2821
SLC27A5	6	0.16699	0.34734	0.9995	6905	3	0.2927
SUPT20HL1	6	0.167	0.34735	0.9995	6906	2	0.0088
MAPK11	6	0.16703	0.34738	0.9995	6907	3	0.1897
PTP4A1	6	0.16716	0.34754	0.9995	6908	3	-0.002
SYT15	6	0.16725	0.34765	0.9995	6909	2	-0.213
SPOCK2	6	0.16725	0.34766	0.9995	6910	3	0.2978
RUNDC1	6	0.16732	0.34775	0.9995	6911	1	-0.143
DNAH9	6	0.1674	0.34785	0.9995	6912	2	-0.201
CILP2	6	0.1674	0.34785	0.9995	6913	3	0.224
ASXL3	6	0.16745	0.34791	0.9995	6914	2	-0.574
NHLH1	6	0.16749	0.34796	0.9995	6915	2	0.2471
AKAP11	6	0.16749	0.34796	0.9995	6916	2	-0.506
hsa-mir-448	4	0.16751	0.30747	0.9995	6917	2	0.3063
LMO2	6	0.16754	0.34801	0.9995	6918	3	0.2097
BRINP3	3	0.16757	0.27145	0.9995	6919	2	0.398
ANKRD37	6	0.16758	0.34806	0.9995	6920	2	-0.256
C5orf20	6	0.16761	0.3481	0.9995	6921	2	-0.078
RAPGEF4	6	0.16765	0.34815	0.9995	6922	3	0.1169
SLC37A3	6	0.16767	0.34818	0.9995	6923	1	-0.267
BMP5	6	0.16774	0.34827	0.9995	6924	2	0.1592
SLC25A23	6	0.16781	0.34835	0.9995	6925	1	-0.319
SEPW1	6	0.16798	0.34855	0.9995	6926	3	0.2439
CAV2	6	0.16811	0.34871	0.9995	6927	3	0.0673
EIF5A	5	0.16812	0.32944	0.9995	6928	2	0.027
MTTP	6	0.16815	0.34875	0.9995	6929	3	0.2643
CCDC152	6	0.16819	0.3488	0.9995	6930	1	-0.107
SH2B1	6	0.16825	0.34888	0.9995	6931	2	-0.128
EGLN2	6	0.16825	0.34888	0.9995	6932	1	-0.005
hsa-mir-543	2	0.16828	0.23941	0.9995	6933	1	-0.142
RFX5	6	0.16841	0.34909	0.9995	6934	3	0.2024
HLA-DMA	6	0.16842	0.3491	0.9995	6935	3	0.2184
PCYOX1	6	0.16845	0.34913	0.9995	6936	3	0.0521
FAM83E	6	0.16846	0.34914	0.9995	6937	3	0.1734
SEC14L1	6	0.16847	0.34915	0.9995	6938	3	0.1906
SELK	6	0.16852	0.34922	0.9995	6939	1	0.0359
IGLL1	6	0.16857	0.34926	0.9995	6940	2	-0.385
C1orf105	4	0.16859	0.30859	0.9995	6941	2	0.3118
GPR39	6	0.16859	0.34929	0.9995	6942	3	0.2669
BARX1	6	0.16869	0.34941	0.9995	6943	3	0.0012
HES5	6	0.16869	0.34941	0.9995	6944	3	0.1697
STAB2	6	0.1687	0.34943	0.9995	6945	2	-0.288
SLC9A4	6	0.16875	0.34948	0.9995	6946	1	0.0269
hsa-mir-6072	4	0.16875	0.30877	0.9995	6947	1	-0.038
TTC30B	6	0.1688	0.34954	0.9995	6948	2	-0.078
DSG1	6	0.16892	0.3497	0.9995	6949	1	-0.293
NUCB2	6	0.16897	0.34975	0.9995	6950	2	0.1555
CD300C	6	0.169	0.34979	0.9995	6951	3	-0.041
HPN	6	0.16906	0.34987	0.9995	6952	3	0.143
SLC2A5	6	0.16906	0.34987	0.9995	6953	2	0.2313
C14orf37	6	0.16912	0.34996	0.9995	6954	2	0.1047
CDC42SE1	6	0.16917	0.35002	0.9995	6955	3	0.2272
hsa-mir-4761	4	0.16917	0.30921	0.9995	6956	2	0.1039
CUZD1	6	0.16926	0.35012	0.9995	6957	3	0.2692
hsa-mir-424	4	0.16932	0.30935	0.9995	6958	2	0.1826
SATL1	6	0.16939	0.35029	0.9995	6959	3	0.1422
ZNF586	6	0.16939	0.35029	0.9995	6960	3	0.1779
PAPSS2	6	0.16939	0.35029	0.9995	6961	3	0.2195
FKTN	6	0.16944	0.35036	0.9995	6962	3	0.2378
RAD51D	6	0.16946	0.35038	0.9995	6963	1	-0.191
TM6SF2	6	0.16951	0.35045	0.9995	6964	3	0.2553
hsa-mir-300	4	0.16955	0.30962	0.9995	6965	1	-0.135

GLYATL2	6	0.16957	0.35054	0.9995	6966	3	0.3616
TPGS2	6	0.16959	0.35056	0.9995	6967	3	0.1579
TMEM64	6	0.16966	0.35065	0.9995	6968	2	0.1911
AKIP1	6	0.16971	0.35071	0.9995	6969	2	-0.094
SELPLG	6	0.1698	0.35084	0.9995	6970	2	0.2269
IL3	6	0.1698	0.35084	0.9995	6971	2	0.0047
CHSY1	6	0.16986	0.35092	0.9995	6972	3	0.2674
SLC35A5	6	0.16991	0.35097	0.9995	6973	2	-0.435
VPS8	6	0.16991	0.35097	0.9995	6974	3	0.2859
ARID4A	6	0.16998	0.35106	0.9995	6975	3	0.1283
AQP6	6	0.17	0.35108	0.9995	6976	2	0.0601
NLRP3	6	0.17001	0.3511	0.9995	6977	3	0.2415
hsa-mir-539	4	0.17002	0.31011	0.9995	6978	2	0.3281
E2F6	6	0.17003	0.35112	0.9995	6979	2	-0.113
ANKRD20A1	1	0.17011	0.16992	0.99382	6980	1	0.7651
LOXL4	6	0.17015	0.35125	0.9995	6981	3	-0.274
C8orf74	6	0.17022	0.35135	0.9995	6982	3	0.1115
hsa-mir-3912	4	0.17023	0.31033	0.9995	6983	1	0.095
KIAA0020	6	0.17023	0.35137	0.9995	6984	3	0.0697
TAZ	6	0.17028	0.35143	0.9995	6985	2	-0.243
PDHA1	6	0.17038	0.35155	0.9995	6986	3	0.0588
POLE	6	0.1704	0.35157	0.9995	6987	1	-0.15
ALDH1B1	6	0.17047	0.35167	0.9995	6988	2	-0.012
NKAIN1	6	0.17055	0.35177	0.9995	6989	3	0.1479
HAVCR1	6	0.17055	0.35177	0.9995	6990	3	0.1124
IGFALS	6	0.17062	0.35185	0.9995	6991	2	-0.123
MOK	6	0.17063	0.35188	0.9995	6992	3	0.1906
RCAN1	6	0.17064	0.35188	0.9995	6993	3	0.1215
MYOCD	6	0.17065	0.35189	0.9995	6994	3	0.0973
MC3R	6	0.17073	0.352	0.9995	6995	3	0.2341
SNAPC2	6	0.17075	0.35202	0.9995	6996	3	0.0783
PUS7L	6	0.1708	0.35208	0.9995	6997	2	0.1295
HSPG2	6	0.17086	0.35217	0.9995	6998	1	0.068
OR10Z1	6	0.17088	0.35218	0.9995	6999	3	0.2964
ZNF583	6	0.17093	0.35225	0.9995	7000	3	0.1635
SCAND3	6	0.17106	0.3524	0.9995	7001	3	0.2611
NRAS	6	0.17114	0.35248	0.9995	7002	3	0.0621
MAGED4B	2	0.17117	0.24139	0.9995	7003	1	0.1613
FUCA1	6	0.17128	0.35265	0.9995	7004	2	-0.282
TRMT10C	6	0.17133	0.35271	0.9995	7005	2	0.0375
TKTL2	6	0.17134	0.35272	0.9995	7006	3	0.2419
TTC9C	6	0.17134	0.35272	0.9995	7007	3	0.2722
ETV5	6	0.17138	0.35277	0.9995	7008	3	0.0995
ARHGAP36	6	0.17139	0.35278	0.9995	7009	3	0.1998
DUSP12	6	0.17141	0.35279	0.9995	7010	3	0.0361
KTN1	6	0.17142	0.35281	0.9995	7011	2	0.0059
GABARAPL1	6	0.17147	0.35287	0.9995	7012	3	0.2532
hsa-mir-580	4	0.17147	0.31159	0.9995	7013	1	0.0009
hsa-mir-3660	3	0.17148	0.27638	0.9995	7014	1	-0.189
SNRPN	6	0.17151	0.35292	0.9995	7015	3	0.0063
WNT11	6	0.17156	0.35297	0.9995	7016	1	-0.577
MFSD6	6	0.17156	0.35297	0.9995	7017	2	0.0864
FHL5	6	0.17161	0.35304	0.9995	7018	3	0.2556
SEPSECS	6	0.17167	0.35311	0.9995	7019	2	0.1024
SPATA31D4	2	0.17168	0.24175	0.9995	7020	1	-0.019
GPR176	6	0.17184	0.35334	0.9995	7021	2	-0.074
hsa-mir-6734	4	0.17186	0.31201	0.9995	7022	2	0.3605
KCTD14	6	0.1719	0.35342	0.9995	7023	3	0.1558
ADHFE1	6	0.17191	0.35343	0.9995	7024	2	-0.242
ADI1	6	0.17198	0.35352	0.9995	7025	3	0.2347
TK2	6	0.17198	0.35353	0.9995	7026	3	0.3141
PCBD2	6	0.172	0.35354	0.9995	7027	2	-0.067
DDX26B	6	0.17213	0.3537	0.9995	7028	2	-0.249
ZNF488	6	0.17223	0.35382	0.9995	7029	2	-0.021
NMS	6	0.17226	0.35385	0.9995	7030	3	0.0922
HK1	6	0.17228	0.35388	0.9995	7031	3	-0.498
ANKZF1	6	0.17229	0.35389	0.9995	7032	3	0.1076
CASP8AP2	6	0.17229	0.35389	0.9995	7033	2	-0.533
CNTROB	6	0.17245	0.3541	0.9995	7034	3	-0.077
CLEC3B	6	0.17249	0.35415	0.9995	7035	2	0.0646
B9D1	6	0.17251	0.35416	0.9995	7036	3	0.1779
FMO1	6	0.17253	0.3542	0.9995	7037	1	-0.261
NRD1	6	0.17262	0.3543	0.9995	7038	2	0.196
EIF1AY	6	0.17265	0.35434	0.9995	7039	3	0.1852
CDCP2	6	0.17268	0.35438	0.9995	7040	2	-0.193
ZFP36	6	0.17268	0.35438	0.9995	7041	3	0.0622
CLP1	6	0.17276	0.35449	0.9995	7042	3	0.0329
ORAOV1	6	0.17291	0.35467	0.9995	7043	1	0.1869
CHAC2	6	0.17301	0.3548	0.9995	7044	3	0.228
HMCN1	6	0.17307	0.35488	0.9995	7045	2	0.0317
ENTPD4	6	0.17318	0.35503	0.9995	7046	3	-0.045
WFS1	6	0.1732	0.35505	0.9995	7047	2	0.0587
CCND3	6	0.17326	0.35512	0.9995	7048	3	0.1988
MYO9B	6	0.17327	0.35513	0.9995	7049	2	-0.481
hsa-mir-301a	3	0.17329	0.27875	0.9995	7050	2	0.351

TMEM87A	6	0.17331	0.35518	0.9995	7051	3	0.2481
hsa-mir-578	4	0.17331	0.31351	0.9995	7052	2	0.1426
RAB6B	6	0.17341	0.3553	0.9995	7053	3	0.1458
POP7	6	0.17342	0.35531	0.9995	7054	1	-0.492
hsa-mir-643	4	0.17349	0.31369	0.9995	7055	2	0.3596
PRRG4	4	0.17351	0.31372	0.9995	7056	2	-0.006
hsa-mir-4255	4	0.17352	0.31373	0.9995	7057	1	-0.273
RBM5	6	0.17358	0.35551	0.9995	7058	1	-0.334
ATP6V1B2	6	0.17363	0.35557	0.9995	7059	3	0.1871
NOL6	6	0.17367	0.35562	0.9995	7060	3	-0.033
SDC1	6	0.17367	0.35562	0.9995	7061	3	0.2362
EIF3J	6	0.17369	0.35565	0.9995	7062	1	-0.398
S1PR4	6	0.17376	0.35571	0.9995	7063	3	0.1644
hsa-mir-542	4	0.17378	0.31399	0.9995	7064	2	0.2785
PMFBP1	6	0.17379	0.35577	0.9995	7065	3	0.0112
ITGAD	6	0.17382	0.35582	0.9995	7066	1	-0.017
SLC2A3	5	0.17389	0.33786	0.9995	7067	3	0.2282
NKIRAS2	6	0.17391	0.35593	0.9995	7068	1	-0.038
AFF2	6	0.174	0.35604	0.9995	7069	3	0.0855
CNPY2	6	0.17409	0.35614	0.9995	7070	3	0.2308
C6	6	0.17414	0.3562	0.9995	7071	2	0.0916
ING4	6	0.17415	0.35621	0.9995	7072	3	0.2577
COL9A1	6	0.17415	0.35621	0.9995	7073	3	0.0612
DONSON	6	0.17416	0.35623	0.9995	7074	2	-0.159
HERC1	6	0.17422	0.35631	0.9995	7075	2	-0.236
SLC2A1	6	0.17433	0.35643	0.9995	7076	3	0.1549
JMY	6	0.17442	0.35654	0.9995	7077	3	-0.148
DEC1	6	0.17456	0.35672	0.9995	7078	3	0.1435
TINAG	6	0.17469	0.35687	0.9995	7079	2	0.1993
XKR9	6	0.17473	0.35691	0.9995	7080	3	0.115
EI24	6	0.17476	0.35695	0.9995	7081	2	-0.228
GSTT1	6	0.1748	0.35699	0.9995	7082	1	-0.337
MED11	6	0.17481	0.35701	0.9995	7083	3	0.4515
DNAH12	4	0.17484	0.31509	0.9995	7084	1	-0.1
hsa-mir-8080	4	0.17496	0.31521	0.9995	7085	2	0.3381
BANF1	6	0.17497	0.35722	0.9995	7086	2	-0.241
STK11	6	0.17498	0.35723	0.9995	7087	2	-0.335
ABCA12	6	0.175	0.35725	0.9995	7088	2	-0.162
STRA8	6	0.175	0.35726	0.9995	7089	3	0.2258
ATP6V0B	6	0.17507	0.35734	0.9995	7090	2	0.1462
C18orf25	6	0.17515	0.35744	0.9995	7091	3	0.2356
TNFSF10	6	0.17516	0.35745	0.9995	7092	2	-0.141
GCA	6	0.17516	0.35746	0.9995	7093	3	0.1663
MASP2	4	0.17517	0.31542	0.9995	7094	2	0.3368
SLITRK4	6	0.17522	0.35752	0.9995	7095	3	0.1231
NYX	6	0.17523	0.35754	0.9995	7096	2	-0.145
hsa-mir-573	2	0.17535	0.24436	0.9995	7097	1	0.0639
DCAF4	6	0.17542	0.35777	0.9995	7098	2	-0.519
C11orf84	6	0.17547	0.35782	0.9995	7099	3	0.1183
CALHM1	6	0.17551	0.35787	0.9995	7100	2	-0.063
NXPE3	6	0.17558	0.35797	0.9995	7101	3	0.3411
CCDC173	6	0.1756	0.358	0.9995	7102	3	0.0711
NLGN2	6	0.17563	0.35803	0.9995	7103	3	0.0389
PSPN	6	0.17576	0.3582	0.9995	7104	3	0.0681
C8orf58	6	0.17579	0.35824	0.9995	7105	2	-0.467
PDE6B	6	0.1758	0.35825	0.9995	7106	1	-0.107
PDIK1L	6	0.17583	0.35829	0.9995	7107	2	-0.202
KHDRBS2	6	0.17586	0.35832	0.9995	7108	2	-0.11
IFI35	6	0.17587	0.35833	0.9995	7109	2	-0.023
MRPS5	6	0.17592	0.35839	0.9995	7110	3	0.0895
SEMA3F	6	0.17602	0.35852	0.9995	7111	2	-0.186
PRRC2B	6	0.17606	0.35856	0.9995	7112	2	0.0258
NEDD4	6	0.17606	0.35856	0.9995	7113	3	-0.056
POTEG	3	0.17608	0.28237	0.9995	7114	2	0.2592
SLC2A11	6	0.17613	0.35866	0.9995	7115	2	-0.064
SEC14L3	6	0.17614	0.35868	0.9995	7116	2	-0.421
ZAR1	6	0.17621	0.35875	0.9995	7117	3	0.2314
RGS1	6	0.17624	0.35878	0.9995	7118	2	-0.087
CNEP1R1	6	0.17624	0.35878	0.9995	7119	1	-0.232
ZNF615	5	0.17629	0.34132	0.9995	7120	2	0.017
FAM111B	6	0.17631	0.35886	0.9995	7121	2	-0.184
DBNDD2	6	0.17634	0.3589	0.9995	7122	2	-0.074
MRPL28	6	0.17634	0.3589	0.9995	7123	3	0.2794
COL15A1	6	0.17634	0.3589	0.9995	7124	3	0.2968
HIST1H3H	5	0.17634	0.34141	0.9995	7125	2	0.1887
CXXC1	6	0.17635	0.35892	0.9995	7126	2	0.1306
S100PBP	6	0.17643	0.35901	0.9995	7127	3	0.2635
AOC3	6	0.17654	0.35914	0.9995	7128	2	-0.126
RPL41	4	0.17654	0.31679	0.9995	7129	2	0.5306
UBE2M	6	0.1766	0.35921	0.9995	7130	3	0.1677
hsa-mir-30d	4	0.17662	0.31686	0.9995	7131	2	0.2885
C3orf84	1	0.17663	0.17647	0.99919	7132	1	0.571
COPZ1	6	0.17676	0.35943	0.9995	7133	2	0.0814
GTF2H5	6	0.17681	0.35948	0.9995	7134	3	0.222
IKZF3	6	0.17682	0.35949	0.9995	7135	3	0.0819

ZKSCAN2	6	0.17683	0.3595	0.9995	7136	3	0.3135
ZRANB1	6	0.17686	0.35954	0.9995	7137	3	0.1102
OR4D5	6	0.17686	0.35954	0.9995	7138	1	-0.12
L3MBTL4	6	0.17686	0.35954	0.9995	7139	3	-0.055
SLC16A2	6	0.17693	0.35961	0.9995	7140	1	-0.39
CDKN2C	6	0.17702	0.35972	0.9995	7141	2	-0.063
C1orf64	6	0.17707	0.35978	0.9995	7142	2	-0.531
HSF1	6	0.17721	0.35996	0.9995	7143	3	0.0743
VSTM1	6	0.17731	0.36007	0.9995	7144	2	-0.507
SMARCA2	6	0.17736	0.36014	0.9995	7145	3	0.28
MIEN1	6	0.17736	0.36014	0.9995	7146	3	0.0048
ARL4A	6	0.17743	0.36023	0.9995	7147	3	0.2432
PLEKHH2	5	0.17744	0.343	0.9995	7148	2	0.0564
CNOT11	6	0.1775	0.36032	0.9995	7149	2	-0.189
GPR162	6	0.17751	0.36033	0.9995	7150	2	-0.102
CPED1	6	0.17751	0.36033	0.9995	7151	2	-0.02
TRMT1L	6	0.17753	0.36036	0.9995	7152	3	0.3082
BRCA2	6	0.17753	0.36036	0.9995	7153	3	0.2078
PCDH15	6	0.17757	0.3604	0.9995	7154	3	-0.164
FCRL2	6	0.1776	0.36044	0.9995	7155	3	0.1195
ARHGEF35	6	0.17762	0.36047	0.9995	7156	2	-0.756
KCNK1	6	0.17765	0.36051	0.9995	7157	3	0.0669
FITM1	6	0.17768	0.36055	0.9995	7158	2	-0.015
SLA	6	0.17768	0.36055	0.9995	7159	2	-0.04
PSMG1	6	0.17769	0.36057	0.9995	7160	3	0.3082
HNRNPUL2	6	0.17777	0.36066	0.9995	7161	1	-0.007
FEM1C	4	0.17782	0.31811	0.9995	7162	2	0.1455
ANKRD54	6	0.17783	0.36074	0.9995	7163	3	0.2445
CNBP	6	0.17786	0.36076	0.9995	7164	2	-0.547
ZNF2	6	0.17793	0.36085	0.9995	7165	2	-0.262
DZANK1	6	0.17802	0.36097	0.9995	7166	3	0.1098
JRK	6	0.17808	0.36103	0.9995	7167	3	0.2102
HIST1H1A	6	0.17808	0.36103	0.9995	7168	3	0.2178
PYCR2	6	0.17815	0.36111	0.9995	7169	3	-0.053
TSPAN31	6	0.17817	0.36113	0.9995	7170	3	0.179
KNSTRN	6	0.17819	0.36115	0.9995	7171	2	-0.352
PTP4A2	6	0.17821	0.36118	0.9995	7172	1	0.0411
SORBS3	6	0.17831	0.36132	0.9995	7173	3	0.1981
LTF	6	0.17839	0.36141	0.9995	7174	2	-0.247
BTBD7	6	0.17841	0.36144	0.9995	7175	3	0.0143
PSAPL1	6	0.17843	0.36147	0.9995	7176	3	0.1973
AKR7A2	6	0.17847	0.36151	0.9995	7177	3	0.1033
TM2D3	6	0.17851	0.36155	0.9995	7178	3	-0.106
KRTCAP2	6	0.17852	0.36157	0.9995	7179	2	0.0161
CERS4	6	0.17854	0.36158	0.9995	7180	3	0.1385
YBX1	5	0.17855	0.34458	0.9995	7181	3	0.372
hsa-mir-6505	4	0.17856	0.31888	0.9995	7182	2	0.3451
hsa-mir-1263	4	0.17859	0.31892	0.9995	7183	1	-0.642
LRRC4	6	0.17861	0.36168	0.9995	7184	2	-2E-04
PAIP1	6	0.17861	0.36168	0.9995	7185	3	0.2009
C12orf39	6	0.17868	0.36176	0.9995	7186	2	0.1001
RSF1	6	0.17874	0.36183	0.9995	7187	3	0.1813
TREH	6	0.17878	0.36189	0.9995	7188	2	-0.333
hsa-mir-802	4	0.17882	0.31914	0.9995	7189	1	-0.692
CBX6	6	0.17887	0.362	0.9995	7190	3	0.2849
hsa-mir-6858	4	0.17888	0.3192	0.9995	7191	2	0.1444
CPA2	6	0.17895	0.3621	0.9995	7192	2	0.064
ANGEL1	6	0.17896	0.36213	0.9995	7193	3	0.0322
HOGA1	6	0.17899	0.36217	0.9995	7194	2	-0.479
DCUN1D5	6	0.17901	0.36219	0.9995	7195	2	-0.068
SLC28A1	6	0.1791	0.36231	0.9995	7196	1	-0.201
hsa-mir-4518	4	0.17911	0.31944	0.9995	7197	2	0.346
UBALD1	6	0.17914	0.36236	0.9995	7198	2	-0.28
hsa-mir-1273a	3	0.17917	0.28635	0.9995	7199	1	0.3808
PROM2	6	0.17917	0.3624	0.9995	7200	3	0.1652
ZNF286A	6	0.17917	0.3624	0.9995	7201	3	0.2144
WNT16	6	0.17919	0.36242	0.9995	7202	2	0.016
CHGB	6	0.17923	0.36247	0.9995	7203	2	-0.425
POLR3C	4	0.17924	0.31956	0.9995	7204	1	-0.081
LCE5A	6	0.17944	0.36272	0.9995	7205	3	0.2272
EGFL6	6	0.17945	0.36273	0.9995	7206	2	-0.206
EPN3	6	0.17948	0.36277	0.9995	7207	3	-0.023
SFMBT1	6	0.1795	0.36279	0.9995	7208	2	0.099
ICT1	6	0.17952	0.36283	0.9995	7209	2	0.1704
SND1	6	0.17954	0.36285	0.9995	7210	3	-0.234
CPSF6	6	0.17954	0.36285	0.9995	7211	3	0.2548
hsa-mir-4671	3	0.17962	0.28698	0.9995	7212	2	0.2885
OR51E2	6	0.17963	0.36295	0.9995	7213	2	-0.024
LARP7	6	0.17966	0.36298	0.9995	7214	2	-0.237
C12orf50	6	0.17969	0.36304	0.9995	7215	2	0.0639
FEZ2	6	0.17971	0.36307	0.9995	7216	3	0.1849
WDR6	6	0.17975	0.36312	0.9995	7217	3	-0.061
DEF8	6	0.17984	0.36323	0.9995	7218	3	0.3239
RGAG1	6	0.17995	0.36336	0.9995	7219	3	0.2117
NUDCD1	4	0.17996	0.32029	0.9995	7220	2	0.3001

BCL2	6	0.17998	0.36339	0.9995	7221	2	0.0673
GOLT1B	6	0.18003	0.36346	0.9995	7222	2	0.0642
EPHX2	6	0.18009	0.36353	0.9995	7223	3	0.0357
CPA6	6	0.18009	0.36353	0.9995	7224	2	-0.117
ZNF705B	3	0.18013	0.28763	0.9995	7225	2	0.2704
SVIL	6	0.18019	0.36363	0.9995	7226	2	0.0676
PHACTR4	6	0.18042	0.36392	0.9995	7227	1	-0.199
HSF5	6	0.18053	0.36407	0.9995	7228	3	0.1592
OR11H12	3	0.18054	0.28817	0.9995	7229	2	0.3701
POU5F1	6	0.18056	0.3641	0.9995	7230	3	0.1238
SLC4A7	6	0.18078	0.36438	0.9995	7231	3	0.3848
PRKCQ	6	0.18083	0.36445	0.9995	7232	3	0.2562
RPN2	6	0.18097	0.36461	0.9995	7233	2	0.0323
TNFRSF4	6	0.18112	0.3648	0.9995	7234	3	0.3041
CRYBB2	6	0.18113	0.36481	0.9995	7235	1	-0.072
OR8H1	6	0.18117	0.36486	0.9995	7236	3	0.2168
FANCI	6	0.18119	0.36488	0.9995	7237	3	0.2552
M6PR	6	0.18126	0.36496	0.9995	7238	2	0.0745
ADA	6	0.18128	0.36498	0.9995	7239	3	0.1811
CAMK4	6	0.18131	0.36503	0.9995	7240	1	-0.339
FAM171A1	6	0.18135	0.36507	0.9995	7241	3	0.1629
EIF4A2	6	0.18143	0.36518	0.9995	7242	3	0.0059
PCDHB13	6	0.18153	0.36531	0.9995	7243	3	-0.133
CEP135	6	0.18162	0.36542	0.9995	7244	2	-0.158
CTRC	6	0.1817	0.36554	0.9995	7245	3	0.0339
RNF13	6	0.18174	0.36558	0.9995	7246	3	0.2927
C19orf57	6	0.18195	0.36583	0.9995	7247	3	0.0256
SETD7	6	0.18201	0.3659	0.9995	7248	2	0.0319
MRC2	6	0.1821	0.36601	0.9995	7249	3	0.1276
TSR3	6	0.18211	0.36602	0.9995	7250	3	0.3547
AMN	6	0.18211	0.36602	0.9995	7251	3	0.2541
TAB1	6	0.1822	0.36612	0.9995	7252	3	0.1682
YTHDF3	6	0.18223	0.36616	0.9995	7253	2	0.003
DUSP26	6	0.18223	0.36616	0.9995	7254	3	0.2174
hsa-mir-6865	4	0.18225	0.32268	0.9995	7255	2	-0.079
CHST7	6	0.18227	0.3662	0.9995	7256	3	-0.121
GMFB	5	0.18228	0.34997	0.9995	7257	3	0.2608
TRIM61	6	0.18232	0.36626	0.9995	7258	1	-0.142
MBL2	6	0.18241	0.36637	0.9995	7259	3	0.2678
SMYD4	6	0.18245	0.36643	0.9995	7260	2	-0.041
OR5T3	6	0.18251	0.3665	0.9995	7261	3	0.201
UBL4A	6	0.18256	0.36656	0.9995	7262	1	-0.262
SMAGP	5	0.18262	0.35046	0.9995	7263	1	-0.47
ERBB4	6	0.18263	0.36664	0.9995	7264	1	-0.482
HAS3	6	0.18267	0.3667	0.9995	7265	2	-0.115
SMIM17	6	0.18273	0.36676	0.9995	7266	3	0.2282
MALT1	6	0.18276	0.36679	0.9995	7267	2	-0.124
ANXA4	6	0.18277	0.36681	0.9995	7268	2	0.0961
RARRES3	6	0.1828	0.36685	0.9995	7269	2	-0.122
HSPB2	6	0.18284	0.3669	0.9995	7270	2	0.0966
ZNF439	4	0.18286	0.32328	0.9995	7271	2	-0.737
FAM196B	6	0.18294	0.367	0.9995	7272	3	0.0856
BEX2	6	0.18298	0.36706	0.9995	7273	1	-0.758
ERCC2	5	0.18307	0.35112	0.9995	7274	2	0.0753
39326	3	0.18308	0.2914	0.9995	7275	1	0.1838
RUNX3	6	0.18309	0.36719	0.9995	7276	2	-0.078
VPS18	6	0.18309	0.36719	0.9995	7277	2	-0.102
TTLL5	6	0.18312	0.36723	0.9995	7278	3	0.1923
BIRC3	6	0.18312	0.36723	0.9995	7279	3	0.2034
PITPNB	6	0.18315	0.36726	0.9995	7280	2	0.1559
UPF3B	6	0.1832	0.36733	0.9995	7281	2	0.1488
NEIL1	6	0.18322	0.36735	0.9995	7282	3	0.0381
PRDM14	6	0.18324	0.36737	0.9995	7283	3	0.0088
EIF2B2	6	0.18331	0.36746	0.9995	7284	3	0.2124
TACSTD2	6	0.18338	0.36754	0.9995	7285	1	-0.303
APOL1	6	0.18342	0.36761	0.9995	7286	1	-1.237
BBX	6	0.18343	0.36762	0.9995	7287	3	0.1961
NDUFAF7	6	0.18352	0.36774	0.9995	7288	3	0.2991
NFKBIB	6	0.18355	0.36777	0.9995	7289	2	-0.117
ZFP3	6	0.18357	0.36778	0.9995	7290	2	0.0522
E2F7	6	0.1836	0.36783	0.9995	7291	2	-0.475
NGDN	6	0.18363	0.36786	0.9995	7292	2	0.0806
ITPRIPL2	6	0.18366	0.36789	0.9995	7293	1	0.0272
PRND	6	0.18367	0.36791	0.9995	7294	3	0.1594
38231	3	0.18369	0.29217	0.9995	7295	1	-0.476
hsa-mir-3977	4	0.18383	0.32431	0.9995	7296	1	-0.326
CORO6	6	0.18384	0.36813	0.9995	7297	3	0.1675
BHLHB9	6	0.18392	0.36823	0.9995	7298	2	-0.042
POU4F2	6	0.18395	0.36826	0.9995	7299	2	-0.033
PKHD1	6	0.18399	0.36831	0.9995	7300	3	-0.056
CCDC90B	6	0.18409	0.36844	0.9995	7301	3	0.2046
PPP3CC	6	0.1841	0.36844	0.9995	7302	3	0.2096
MSR1	6	0.18413	0.36848	0.9995	7303	3	0.1199
HNRNPU	6	0.18415	0.36851	0.9995	7304	3	0.2703
MARCH6	6	0.18429	0.36868	0.9995	7305	3	0.2487

TBC1D10A	6	0.18437	0.36878	0.9995	7306	1	-0.154
MAP3K7	6	0.18437	0.36878	0.9995	7307	3	0.1016
MRGPRX4	6	0.18437	0.36878	0.9995	7308	3	0.1338
hsa-mir-6767	4	0.18437	0.32486	0.9995	7309	2	0.1845
TTPA	6	0.18438	0.3688	0.9995	7310	2	0.1444
FAM101B	6	0.18438	0.3688	0.9995	7311	2	0.0534
ZFAND1	6	0.1844	0.36882	0.9995	7312	3	0.276
DALRD3	6	0.18452	0.36898	0.9995	7313	1	-0.417
ACRC	5	0.18452	0.35321	0.9995	7314	3	0.2262
NXF3	6	0.18457	0.36904	0.9995	7315	3	0.2815
ACOT6	6	0.1846	0.36908	0.9995	7316	2	0.1776
GSTK1	6	0.18476	0.36928	0.9995	7317	3	0.1937
KCNA5	6	0.18477	0.36929	0.9995	7318	3	0.2755
EGLN3	6	0.18481	0.36933	0.9995	7319	3	0.0194
TRPV6	6	0.1849	0.36947	0.9995	7320	3	0.2164
NXNL1	6	0.1849	0.36947	0.9995	7321	3	-0.045
hsa-mir-376c	1	0.18491	0.18475	0.99919	7322	1	0.7159
TMEM133	6	0.18505	0.36964	0.9995	7323	3	0.1888
MANSC4	6	0.18508	0.36969	0.9995	7324	2	-0.315
FAM47A	6	0.18514	0.36975	0.9995	7325	2	0.0941
TOMM40	6	0.18519	0.36982	0.9995	7326	2	-0.365
SKAP1	6	0.1852	0.36983	0.9995	7327	1	-0.094
RNFT1	6	0.1852	0.36983	0.9995	7328	2	-0.12
SOX10	6	0.18524	0.36989	0.9995	7329	3	0.2219
NIPAL2	6	0.18524	0.36989	0.9995	7330	3	0.1299
RIC3	6	0.18527	0.36992	0.9995	7331	2	-0.302
hsa-let-7i	4	0.18532	0.32583	0.9995	7332	1	-0.462
KCNA1	6	0.18533	0.36998	0.9995	7333	2	-0.168
NAA10	6	0.18544	0.37013	0.9995	7334	2	-0.023
ANKRD44	6	0.18546	0.37015	0.9995	7335	3	0.2933
ERICH1	4	0.1855	0.32602	0.9995	7336	2	0.2788
hsa-mir-3975	4	0.18551	0.32603	0.9995	7337	1	0.1411
SPG7	6	0.18555	0.37026	0.9995	7338	2	-0.014
TRIM2	6	0.18559	0.37031	0.9995	7339	3	0.2691
UTP14C	6	0.18564	0.37039	0.9995	7340	3	-0.058
HPX	6	0.18566	0.37041	0.9995	7341	3	0.1723
PAX9	6	0.18568	0.37043	0.9995	7342	3	0.2416
MIDN	6	0.1857	0.37047	0.9995	7343	1	-0.187
TSPAN2	6	0.18577	0.37054	0.9995	7344	3	0.2556
RTCB	4	0.18578	0.32631	0.9995	7345	1	-0.224
ADAM22	6	0.18588	0.37068	0.9995	7346	3	0.1332
TNFRSF1B	6	0.18593	0.37074	0.9995	7347	2	-0.212
hsa-mir-6781	4	0.18603	0.32656	0.9995	7348	2	-0.003
SIX2	6	0.18603	0.37088	0.9995	7349	1	0.0646
TFAM	6	0.18614	0.37101	0.9995	7350	3	0.288
TBX19	6	0.18616	0.37102	0.9995	7351	3	0.2021
RGS3	6	0.1862	0.37107	0.9995	7352	3	0.1956
TPCN2	6	0.18621	0.37108	0.9995	7353	2	0.1104
OR51G2	6	0.1863	0.3712	0.9995	7354	3	-0.051
hsa-mir-1298	4	0.18634	0.32688	0.9995	7355	1	0.0069
CAPSL	6	0.1864	0.37133	0.9995	7356	2	-0.098
PUS10	6	0.18647	0.37143	0.9995	7357	3	-0.193
FCF1	6	0.18647	0.37143	0.9995	7358	2	0.052
HEG1	6	0.18656	0.37156	0.9995	7359	3	0.1168
DHCR24	6	0.18659	0.37158	0.9995	7360	3	0.3234
CALR3	6	0.18665	0.37166	0.9995	7361	2	-0.026
FBLN1	6	0.18668	0.37169	0.9995	7362	2	0.045
hsa-mir-128-2	4	0.1867	0.32724	0.9995	7363	2	0.1791
TLX3	6	0.18671	0.37172	0.9995	7364	3	0.215
TJP1	6	0.18671	0.37173	0.9995	7365	2	-0.041
ZNF581	6	0.18678	0.3718	0.9995	7366	2	-0.202
CNN3	6	0.18682	0.37184	0.9995	7367	3	0.0672
AKAP1	6	0.18683	0.37186	0.9995	7368	2	-0.093
AIPL1	6	0.18693	0.37198	0.9995	7369	1	0.0317
SPNS1	6	0.18694	0.37199	0.9995	7370	2	0.0813
DEFB124	6	0.18694	0.37199	0.9995	7371	3	0.1774
CUX2	6	0.18698	0.37203	0.9995	7372	2	0.1549
hsa-mir-6511b-1	1	0.18699	0.18688	0.99919	7373	1	0.4244
H2AFJ	4	0.18702	0.32758	0.9995	7374	2	0.2433
RPL17-C18orf32	1	0.18703	0.18691	0.99919	7375	1	0.829
NYAP1	6	0.18715	0.37225	0.9995	7376	2	-0.326
MMP16	6	0.18719	0.3723	0.9995	7377	3	0.0041
HCCS	6	0.1873	0.37242	0.9995	7378	2	0.0935
IGSF6	6	0.18742	0.37256	0.9995	7379	2	-0.069
CCDC146	4	0.18745	0.32801	0.9995	7380	2	0.2831
TMEM108	6	0.18746	0.37261	0.9995	7381	2	-0.137
CARD11	6	0.18753	0.3727	0.9995	7382	1	-0.159
C17orf64	6	0.18753	0.3727	0.9995	7383	2	-0.51
KIAA0556	6	0.18754	0.37271	0.9995	7384	2	-0.27
OR6C3	6	0.18763	0.37283	0.9995	7385	3	0.1626
TPK1	6	0.18763	0.37284	0.9995	7386	3	0.0268
PRPF4B	6	0.18767	0.37289	0.9995	7387	2	-0.163
FGF16	6	0.18769	0.37291	0.9995	7388	3	0.0952
HNRNPD	6	0.1877	0.37292	0.9995	7389	3	-0.176
hsa-mir-4284	4	0.18773	0.32831	0.9995	7390	1	0.1061

PRKD2	6	0.18774	0.37295	0.9995	7391	3	0.1996
MAP4	6	0.18777	0.37299	0.9995	7392	3	0.2262
WDR20	6	0.18779	0.37301	0.9995	7393	3	0.2061
C19orf47	6	0.18779	0.37301	0.9995	7394	2	-0.056
SLC25A11	6	0.1878	0.37303	0.9995	7395	2	-0.033
PRSS58	6	0.18785	0.37309	0.9995	7396	3	0.1308
SHROOM4	6	0.18788	0.37312	0.9995	7397	3	0.1686
OR1L1	6	0.18793	0.37318	0.9995	7398	3	0.2904
LHB	5	0.18804	0.35828	0.9995	7399	2	-0.036
CLIC2	6	0.18805	0.37334	0.9995	7400	3	0.1033
SAA4	3	0.18811	0.29783	0.9995	7401	2	0.438
DBP	6	0.18812	0.37342	0.9995	7402	1	-0.295
TINAGL1	6	0.18818	0.37349	0.9995	7403	3	0.05
R3HDM2	6	0.18831	0.37366	0.9995	7404	3	0.3759
RMDN2	6	0.18834	0.37369	0.9995	7405	2	-0.233
TES	6	0.18838	0.37374	0.9995	7406	2	-0.224
TPBGL	6	0.18839	0.37374	0.9995	7407	3	0.2242
ADPRHL1	6	0.18842	0.37379	0.9995	7408	1	-0.112
METTL25	6	0.18843	0.3738	0.9995	7409	3	0.0413
CALCA	6	0.18851	0.3739	0.9995	7410	1	-0.372
CCDC51	6	0.18855	0.37395	0.9995	7411	3	0.2273
REPS1	6	0.18858	0.37397	0.9995	7412	1	-0.398
MICB	6	0.18858	0.37397	0.9995	7413	1	-0.415
FAM57A	4	0.18864	0.32926	0.9995	7414	2	0.1765
FASTKD5	6	0.18864	0.37405	0.9995	7415	3	0.1603
BTBD6	6	0.18867	0.37407	0.9995	7416	3	0.1995
RNF212	6	0.18871	0.37412	0.9995	7417	2	0.0897
TIMM23	6	0.18873	0.37415	0.9995	7418	2	-0.09
CHRAC1	6	0.18874	0.37417	0.9995	7419	3	0.253
AR	6	0.18884	0.3743	0.9995	7420	2	-0.306
hsa-mir-4747	4	0.18897	0.32962	0.9995	7421	2	0.1786
TRIM50	6	0.18899	0.37448	0.9995	7422	2	-0.286
AVPR2	6	0.18904	0.37455	0.9995	7423	3	-0.092
PRAMEF18	4	0.18911	0.32975	0.9995	7424	2	0.1747
NLRP11	6	0.18915	0.37469	0.9995	7425	3	0.1896
AASS	6	0.18921	0.37475	0.9995	7426	3	0.2745
CORO7-PAM16	6	0.18921	0.37475	0.9995	7427	2	0.0457
RNF213	6	0.18934	0.3749	0.9995	7428	3	0.0784
FA2H	6	0.18938	0.37496	0.9995	7429	3	-6E-04
RSAD1	6	0.18939	0.37496	0.9995	7430	2	0.1978
RABIF	6	0.1894	0.37498	0.9995	7431	3	0.2561
FPGS	6	0.18943	0.37501	0.9995	7432	2	0.0111
hsa-mir-3713	4	0.18943	0.3301	0.9995	7433	2	0.1239
GPR149	6	0.18946	0.37504	0.9995	7434	3	0.2357
LRRC27	6	0.18947	0.37506	0.9995	7435	1	-0.104
AZGP1	6	0.18961	0.37524	0.9995	7436	3	0.1631
SPINK9	6	0.18966	0.3753	0.9995	7437	3	-0.159
RHOQ	6	0.18974	0.3754	0.9995	7438	1	-0.194
RARS	6	0.18974	0.3754	0.9995	7439	3	0.2782
MYH14	6	0.18974	0.3754	0.9995	7440	3	0.2297
CTTNBP2NL	6	0.18976	0.37543	0.9995	7441	3	0.1879
hsa-mir-320d-1	4	0.18983	0.33051	0.9995	7442	1	-0.31
VPS9D1	6	0.18989	0.37558	0.9995	7443	3	0.0897
IMPG1	6	0.18992	0.37563	0.9995	7444	3	0.1331
ZDHHC18	6	0.18998	0.3757	0.9995	7445	2	-0.14
CCDC121	6	0.19	0.37573	0.9995	7446	1	-0.363
IFNK	6	0.19004	0.37577	0.9995	7447	2	-0.07
GIPC1	6	0.19007	0.3758	0.9995	7448	3	0.1211
PLSCR4	6	0.19014	0.37589	0.9995	7449	2	-0.001
TBCA	6	0.19022	0.37601	0.9995	7450	3	-0.163
MYLK	6	0.19033	0.37613	0.9995	7451	3	0.1578
SH3TC1	6	0.19033	0.37613	0.9995	7452	2	-0.286
EFNA5	6	0.19034	0.37614	0.9995	7453	3	0.1689
DCAF6	6	0.1904	0.37622	0.9995	7454	2	-0.058
hsa-mir-7156	4	0.19041	0.33112	0.9995	7455	2	0.1995
PGBD2	6	0.19041	0.37624	0.9995	7456	2	0.0206
PDE11A	6	0.19047	0.3763	0.9995	7457	3	0.0122
MMAA	6	0.19048	0.37632	0.9995	7458	3	0.044
ZBTB4	6	0.19052	0.37638	0.9995	7459	1	-0.177
ZNF510	6	0.19055	0.3764	0.9995	7460	2	-0.14
GTDC1	6	0.19059	0.37645	0.9995	7461	3	0.3198
LRRC25	6	0.1907	0.37658	0.9995	7462	1	-0.255
VSTM2A	6	0.19083	0.37674	0.9995	7463	3	-0.042
40422	3	0.19085	0.30137	0.9995	7464	2	0.3596
BICC1	6	0.19088	0.37681	0.9995	7465	3	0.1764
SMARCB1	6	0.19089	0.37682	0.9995	7466	1	-0.816
PRDM10	6	0.19093	0.37687	0.9995	7467	3	0.0485
PRPF31	6	0.19098	0.37693	0.9995	7468	3	0.0542
HIC2	6	0.19104	0.37701	0.9995	7469	3	0.2173
PIK3R5	6	0.19116	0.37715	0.9995	7470	1	-0.117
FZR1	6	0.19122	0.37723	0.9995	7471	2	-0.489
NDNL2	6	0.19129	0.37731	0.9995	7472	2	-0.065
EIF1	6	0.19135	0.3774	0.9995	7473	2	0.0968
QKI	6	0.19137	0.37741	0.9995	7474	3	0.1638
FAM193A	6	0.19141	0.37745	0.9995	7475	3	-0.058

GP6	6	0.19146	0.37753	0.9995	7476	3	0.2029
WBSCR27	6	0.19153	0.37761	0.9995	7477	3	0.1539
EPM2A	6	0.19153	0.37761	0.9995	7478	2	-0.36
VEGFB	6	0.19157	0.37766	0.9995	7479	3	0.2926
RAB40C	6	0.1916	0.37769	0.9995	7480	2	-0.125
DACH1	6	0.19161	0.37771	0.9995	7481	1	0.0807
SLIT3	6	0.19165	0.37776	0.9995	7482	3	0.1105
ATP8B3	6	0.19168	0.3778	0.9995	7483	1	0.1232
FAM198A	6	0.19168	0.3778	0.9995	7484	3	0.2627
SLC2A13	6	0.19171	0.37783	0.9995	7485	3	0.2341
ACCSL	6	0.19171	0.37783	0.9995	7486	3	0.1236
RAB43	6	0.19174	0.37788	0.9995	7487	2	-0.153
PRRG3	6	0.19183	0.37798	0.9995	7488	3	0.0959
RSBN1L	6	0.19183	0.37799	0.9995	7489	2	0.0904
WEE1	6	0.19188	0.37804	0.9995	7490	2	-0.252
MIB1	6	0.19188	0.37805	0.9995	7491	3	-0.036
C8orf47	6	0.19192	0.3781	0.9995	7492	3	0.2647
SFXN3	6	0.19194	0.37812	0.9995	7493	2	-0.637
C7orf71	6	0.192	0.37821	0.9995	7494	3	0.1905
IFT80	6	0.19201	0.37821	0.9995	7495	3	0.2284
hsa-mir-5590	2	0.19203	0.25633	0.9995	7496	1	0.3178
ABL1	6	0.19207	0.37828	0.9995	7497	3	0.2073
TRIM22	6	0.19211	0.37834	0.9995	7498	3	0.1502
TARDBP	6	0.19211	0.37834	0.9995	7499	3	0.091
FOXR2	6	0.19222	0.37848	0.9995	7500	1	-0.094
hsa-mir-369	4	0.19226	0.33302	0.9995	7501	2	-0.077
ZMYM6NB	6	0.19227	0.37853	0.9995	7502	1	-0.251
RSPO3	6	0.19236	0.37864	0.9995	7503	3	0.2008
hsa-mir-6731	4	0.19236	0.33312	0.9995	7504	2	0.1734
hsa-mir-4641	4	0.19236	0.33312	0.9995	7505	2	0.197
YIPF6	6	0.19237	0.37867	0.9995	7506	3	0.2137
hsa-mir-6080	4	0.19241	0.33318	0.9995	7507	2	0.1885
BLM	6	0.19244	0.37875	0.9995	7508	1	-0.506
COL23A1	6	0.19249	0.37881	0.9995	7509	3	0.1657
hsa-mir-194-2	4	0.19251	0.33327	0.9995	7510	2	0.3442
PNMA3	6	0.19257	0.37891	0.9995	7511	1	-0.255
MAP7D2	6	0.1926	0.37895	0.9995	7512	2	-0.037
PTK2B	6	0.19266	0.37901	0.9995	7513	3	0.0179
RPH3A	6	0.19283	0.37922	0.9995	7514	2	0.2117
PROKR2	6	0.19292	0.37933	0.9995	7515	3	0.2346
C19orf77	6	0.19298	0.37938	0.9995	7516	2	-0.017
OR2L5	6	0.19306	0.37948	0.9995	7517	2	-0.088
ROGDI	6	0.1931	0.37954	0.9995	7518	3	0.2759
IFI44L	6	0.19313	0.37958	0.9995	7519	2	0.1076
SLC35C2	4	0.1932	0.33395	0.9995	7520	2	0.1561
MAF	6	0.19323	0.37969	0.9995	7521	2	-0.104
RAB11FIP3	6	0.19323	0.37969	0.9995	7522	3	0.1372
NR5A2	6	0.1933	0.37978	0.9995	7523	2	-0.093
CRYBA1	6	0.19331	0.37981	0.9995	7524	3	-0.041
HAO1	6	0.19344	0.37997	0.9995	7525	2	0.0042
HMGN1	6	0.19344	0.37997	0.9995	7526	2	0.1445
IFNA10	5	0.1935	0.36612	0.9995	7527	2	0.0671
TTC14	6	0.19353	0.38008	0.9995	7528	3	0.2028
CDON	6	0.19354	0.38009	0.9995	7529	2	0.0998
AUTS2	6	0.19358	0.38012	0.9995	7530	1	-0.099
ZNF554	6	0.19359	0.38015	0.9995	7531	3	0.1913
ANKRA2	6	0.19362	0.38017	0.9995	7532	2	0.2167
DPYS	6	0.19368	0.38025	0.9995	7533	2	0.1107
HIST1H1C	6	0.19368	0.38025	0.9995	7534	3	-0.052
SLC39A11	6	0.19374	0.38033	0.9995	7535	2	-0.444
LARS2	6	0.19379	0.38038	0.9995	7536	2	-0.78
SAMD4B	6	0.19381	0.3804	0.9995	7537	3	0.3388
hsa-mir-4634	4	0.19387	0.33464	0.9995	7538	1	-0.703
C2orf47	6	0.19396	0.38058	0.9995	7539	2	-0.25
CEBPD	6	0.19401	0.38065	0.9995	7540	2	-0.5
MRPS25	6	0.19405	0.3807	0.9995	7541	3	0.2611
DERL3	6	0.19409	0.38074	0.9995	7542	3	0.1609
C17orf105	6	0.19411	0.38077	0.9995	7543	2	-0.227
SPANXN1	5	0.19419	0.36712	0.9995	7544	3	0.221
WDR66	6	0.19427	0.38098	0.9995	7545	1	-0.035
RASSF9	6	0.1943	0.38101	0.9995	7546	3	0.1455
EIF5A2	6	0.19432	0.38103	0.9995	7547	1	-0.477
hsa-mir-3671	3	0.19432	0.30571	0.9995	7548	1	-0.174
PCNT	6	0.19433	0.38104	0.9995	7549	3	0.0748
hsa-mir-577	4	0.19433	0.3351	0.9995	7550	2	-0.017
INTS6	6	0.19436	0.38109	0.9995	7551	3	0.2286
WDR16	6	0.19436	0.38109	0.9995	7552	3	0.1469
RBM12B	6	0.1944	0.38113	0.9995	7553	3	0.2655
PTPRD	6	0.1944	0.38114	0.9995	7554	3	0.1031
NUMBL	6	0.19441	0.38114	0.9995	7555	3	0.0942
SART3	6	0.19451	0.38127	0.9995	7556	3	0.368
PNMAL2	6	0.19464	0.38143	0.9995	7557	3	-0.067
SLC4A9	6	0.19465	0.38144	0.9995	7558	3	0.2111
hsa-mir-4275	4	0.19467	0.33545	0.9995	7559	2	0.0511
UTP18	6	0.19468	0.38149	0.9995	7560	3	-0.016

CDHR1	6	0.19471	0.38152	0.9995	7561	2	-0.052
SLC13A5	6	0.19475	0.38157	0.9995	7562	2	-0.536
SP5	6	0.19478	0.38161	0.9995	7563	3	0.1921
SMAD9	6	0.19482	0.38165	0.9995	7564	2	-0.099
SPATA12	6	0.19484	0.38166	0.9995	7565	2	-0.286
KCTD19	6	0.1949	0.38175	0.9995	7566	3	-0.208
MSGN1	6	0.195	0.38187	0.9995	7567	2	0.1586
ZNF880	6	0.195	0.38187	0.9995	7568	3	0.1731
MAGEB2	6	0.19501	0.38188	0.9995	7569	2	-0.014
C20orf26	6	0.19506	0.38194	0.9995	7570	2	0.0607
PCGF5	6	0.19512	0.38201	0.9995	7571	2	-0.42
ABHD17B	6	0.19517	0.38207	0.9995	7572	2	0.097
hsa-mir-557	4	0.19522	0.33601	0.9995	7573	1	-0.441
CCL17	6	0.19523	0.38215	0.9995	7574	2	-0.263
MYPN	6	0.19526	0.38219	0.9995	7575	3	0.098
TTLL7	6	0.19526	0.38219	0.9995	7576	2	-0.068
ZNF703	6	0.19529	0.38223	0.9995	7577	2	-0.196
CCKAR	6	0.19534	0.38229	0.9995	7578	3	0.282
ARGLU1	6	0.19534	0.38229	0.9995	7579	2	-0.523
GLYCTK	4	0.1954	0.33619	0.9995	7580	1	-0.374
SLC24A6	2	0.19541	0.25878	0.9995	7581	1	-0.021
C12orf49	6	0.19545	0.38243	0.9995	7582	2	-0.102
ZNF780B	5	0.19547	0.36893	0.9995	7583	2	0.098
MORN3	6	0.19555	0.38256	0.9995	7584	3	0.1653
HOXA2	6	0.19565	0.38269	0.9995	7585	3	0.1465
KRT1	6	0.19566	0.3827	0.9995	7586	2	-0.005
FOXP4	6	0.19573	0.38279	0.9995	7587	2	0.0099
TEX28	6	0.19579	0.38285	0.9995	7588	2	0.1106
ZNF624	6	0.19584	0.38291	0.9995	7589	2	0.1441
DCAF8L2	6	0.19585	0.38292	0.9995	7590	2	0.0929
OR8S1	6	0.19588	0.38296	0.9995	7591	2	-0.328
BAK1	6	0.19594	0.38304	0.9995	7592	2	0.1053
ZNF394	6	0.19597	0.38307	0.9995	7593	1	0.1434
LOC554223	6	0.19599	0.3831	0.9995	7594	2	0.0222
LINGO2	6	0.19603	0.38315	0.9995	7595	3	0.1398
GCFC2	6	0.19607	0.38318	0.9995	7596	3	0.1874
hsa-mir-1282	4	0.19613	0.33694	0.9995	7597	1	-0.013
ALS2CL	6	0.19615	0.3833	0.9995	7598	2	-0.015
TARS	6	0.19618	0.38333	0.9995	7599	3	0.2409
THEM4	6	0.19618	0.38334	0.9995	7600	2	-0.279
UNG	6	0.19623	0.38341	0.9995	7601	3	0.2799
MOCS3	6	0.19625	0.38343	0.9995	7602	2	-0.209
MRM1	6	0.19631	0.38349	0.9995	7603	3	0.1486
TNP1	6	0.19631	0.38349	0.9995	7604	2	0.0655
IL9	6	0.19636	0.38355	0.9995	7605	3	0.1959
APOL2	5	0.19639	0.37024	0.9995	7606	2	-0.027
CNDP1	6	0.1964	0.38359	0.9995	7607	3	0.017
EFNA4	6	0.19646	0.38368	0.9995	7608	3	0.2072
hsa-mir-4279	4	0.19649	0.33731	0.9995	7609	2	-0.093
PTCHD4	6	0.19653	0.38376	0.9995	7610	2	-0.046
hsa-mir-4685	4	0.1966	0.33743	0.9995	7611	1	-0.453
SLC13A2	6	0.19661	0.38384	0.9995	7612	3	0.2408
GTF3A	6	0.19666	0.38391	0.9995	7613	3	0.413
ZDHHC11	6	0.19668	0.38393	0.9995	7614	2	-0.02
PARD6G	6	0.19675	0.38401	0.9995	7615	1	-0.34
SIGLEC1	6	0.19678	0.38405	0.9995	7616	2	-0.215
WDR34	6	0.19699	0.3843	0.9995	7617	2	-0.129
PRR5-ARHGAP8	4	0.19699	0.3378	0.9995	7618	1	-0.412
UTS2R	6	0.19703	0.38435	0.9995	7619	3	0.1468
XK	6	0.19704	0.38438	0.9995	7620	3	0.2319
MCOLN3	6	0.19707	0.38442	0.9995	7621	3	0.1754
ZNF234	6	0.19714	0.38451	0.9995	7622	1	-0.745
HSPA12A	6	0.19715	0.38453	0.9995	7623	2	-0.083
PRSS48	4	0.19717	0.33799	0.9995	7624	2	0.2171
TOPORS	6	0.19723	0.38461	0.9995	7625	1	-0.823
DEFB123	6	0.19731	0.38472	0.9995	7626	2	-0.407
PDP2	6	0.19732	0.38473	0.9995	7627	2	-0.019
OR52N1	6	0.19736	0.38478	0.9995	7628	3	0.1431
FAM83D	6	0.19736	0.38478	0.9995	7629	3	0.2278
MAP4K4	6	0.19751	0.38498	0.9995	7630	3	0.2723
hsa-mir-6844	1	0.19756	0.19733	0.99919	7631	1	0.4379
ATP6V1A	6	0.1977	0.38523	0.9995	7632	3	0.0874
CR2	6	0.19772	0.38525	0.9995	7633	3	0.2908
AP1G2	6	0.19773	0.38526	0.9995	7634	2	-0.066
CHN2	6	0.19777	0.3853	0.9995	7635	3	0.2175
MRPL27	6	0.19778	0.38532	0.9995	7636	2	-0.205
TNF	6	0.19786	0.38541	0.9995	7637	2	0.0314
SNX27	6	0.19788	0.38544	0.9995	7638	3	0.2027
SEZ6	6	0.19792	0.38549	0.9995	7639	2	0.0464
DCTN4	6	0.19797	0.38556	0.9995	7640	3	0.2612
OVCA2	6	0.19804	0.38563	0.9995	7641	3	0.3265
NOP56	6	0.19805	0.38565	0.9995	7642	2	-0.041
TM7SF3	6	0.19811	0.38572	0.9995	7643	3	0.201
MS4A10	6	0.19826	0.38591	0.9995	7644	3	0.1446
PITPNA	6	0.19828	0.38594	0.9995	7645	3	0.2748

LRBA	6	0.19829	0.38595	0.9995	7646	2	0.0213
hsa-mir-4742	4	0.1983	0.33916	0.9995	7647	2	0.0372
SLC26A7	4	0.19834	0.33921	0.9995	7648	2	0.2742
SLC8B1	2	0.19841	0.26091	0.9995	7649	1	0.2895
FOXH1	6	0.19846	0.38614	0.9995	7650	1	-0.017
hsa-mir-4799	4	0.19852	0.33941	0.9995	7651	2	0.0613
UPK2	6	0.19854	0.38624	0.9995	7652	3	0.1854
hsa-mir-6787	4	0.1986	0.3395	0.9995	7653	2	-0.181
NPAS4	6	0.19866	0.38638	0.9995	7654	3	0.065
IPO5	6	0.19868	0.3864	0.9995	7655	3	0.2578
hsa-mir-5093	4	0.19868	0.33958	0.9995	7656	2	-0.124
SLC6A16	6	0.1987	0.38643	0.9995	7657	2	-0.064
CAP2	6	0.19871	0.38646	0.9995	7658	3	0.1843
GDPD3	6	0.19884	0.38659	0.9995	7659	3	0.1945
RAB7L1	6	0.19884	0.38659	0.9995	7660	3	-0.095
FGF5	6	0.19888	0.38663	0.9995	7661	3	-0.197
PFDN6	6	0.19894	0.38672	0.9995	7662	2	0.0238
C7orf31	6	0.19902	0.38681	0.9995	7663	3	0.1839
NAGA	6	0.19909	0.38689	0.9995	7664	2	-0.655
C17orf53	6	0.19911	0.38691	0.9995	7665	3	0.262
DENND5B	6	0.19914	0.38694	0.9995	7666	3	0.1728
RNF114	6	0.19918	0.38698	0.9995	7667	3	0.3286
PLEKHA3	6	0.19918	0.387	0.9995	7668	2	-0.25
hsa-mir-376a-2	3	0.19922	0.31187	0.9995	7669	1	-0.183
KIF19	6	0.19926	0.38708	0.9995	7670	2	-0.52
UTP11L	6	0.19926	0.38709	0.9995	7671	3	0.1857
OR5B17	6	0.19933	0.38717	0.9995	7672	3	0.1683
PPP1R14A	6	0.19934	0.38718	0.9995	7673	3	0.174
KRT85	4	0.19935	0.34027	0.9995	7674	2	0.3723
ZNF98	5	0.19938	0.37453	0.9995	7675	3	0.6857
ANKRD6	6	0.19939	0.38724	0.9995	7676	1	-0.293
CDKN2AIP	6	0.19941	0.38727	0.9995	7677	2	-0.304
MGP	6	0.19944	0.38731	0.9995	7678	3	0.244
TTLL9	6	0.19946	0.38734	0.9995	7679	2	-0.595
TBC1D1	6	0.19955	0.38745	0.9995	7680	3	0.0831
SLC33A1	6	0.19961	0.38751	0.9995	7681	1	-0.317
METTL23	6	0.19963	0.38754	0.9995	7682	3	0.206
C10orf2	6	0.19963	0.38754	0.9995	7683	3	0.1144
ACSM5	6	0.19965	0.38757	0.9995	7684	2	-0.499
KRTAP21-1	6	0.19969	0.38761	0.9995	7685	2	-0.084
ARMCX3	6	0.19973	0.38768	0.9995	7686	3	0.237
C5AR1	6	0.19974	0.38768	0.9995	7687	2	0.006
G3BP1	6	0.19982	0.38776	0.9995	7688	3	0.1921
TFDP1	6	0.19984	0.38779	0.9995	7689	3	0.2049
GAP43	6	0.19984	0.38779	0.9995	7690	3	0.0679
RTDR1	6	0.19989	0.38785	0.9995	7691	3	0.1244
ZSCAN1	6	0.19993	0.3879	0.9995	7692	2	-0.036
DNAAF1	6	0.20013	0.38814	0.9995	7693	2	-0.37
CALM1	6	0.20015	0.38816	0.9995	7694	2	-0.116
FAM50A	6	0.20029	0.38836	0.9995	7695	3	0.2481
ENO4	6	0.20029	0.38836	0.9995	7696	3	-0.232
GTF2B	6	0.20034	0.38843	0.9995	7697	2	0.0862
TMEM132D	6	0.20039	0.38849	0.9995	7698	3	-0.082
CRIP1	6	0.20041	0.38851	0.9995	7699	3	0.1902
PLXNA4	6	0.20047	0.3886	0.9995	7700	2	-0.216
CLK3	6	0.20051	0.38864	0.9995	7701	3	0.3348
SPATS2	6	0.20058	0.38875	0.9995	7702	2	0.1121
SLC29A1	6	0.20076	0.38896	0.9995	7703	2	-0.024
PRKCD	6	0.20076	0.38896	0.9995	7704	3	0.1684
HEATR1	6	0.20082	0.38903	0.9995	7705	3	0.0756
MYO1D	6	0.20094	0.38918	0.9995	7706	2	-0.242
ZNF556	6	0.20098	0.38923	0.9995	7707	3	0.367
UTP14A	6	0.20101	0.38926	0.9995	7708	2	0.1355
SMCP	6	0.20112	0.3894	0.9995	7709	1	-0.376
NPFFR2	6	0.20117	0.38946	0.9995	7710	1	-0.341
MYLK4	6	0.2012	0.3895	0.9995	7711	3	0.0959
C14orf119	6	0.20125	0.38957	0.9995	7712	2	0.1188
GAPT	6	0.2013	0.38964	0.9995	7713	3	0.2491
IL1RL1	6	0.20149	0.38987	0.9995	7714	2	0.1854
PPP1R9B	6	0.20152	0.38992	0.9995	7715	3	0.281
SLC35D1	4	0.20155	0.34251	0.9995	7716	2	0.2505
ACTN1	6	0.20169	0.39012	0.9995	7717	3	0.2126
PTPRA	6	0.20171	0.39015	0.9995	7718	2	-0.053
KIAA1715	6	0.20171	0.39015	0.9995	7719	2	-0.034
ELOVL1	6	0.20181	0.39029	0.9995	7720	1	-0.183
CECR5	6	0.20181	0.39029	0.9995	7721	2	-0.201
FOXJ2	6	0.20189	0.39039	0.9995	7722	3	0.234
SEMA4B	6	0.20194	0.39045	0.9995	7723	2	-0.145
STARD10	6	0.20195	0.39046	0.9995	7724	3	-0.188
CARNS1	6	0.20203	0.39056	0.9995	7725	3	-0.025
HAND2	6	0.20214	0.39069	0.9995	7726	2	-0.441
HDAC10	6	0.20217	0.39071	0.9995	7727	2	-0.451
ACOX3	6	0.20218	0.39073	0.9995	7728	3	0.1191
SPATA6	6	0.20218	0.39073	0.9995	7729	2	0.0896
ST13	6	0.20218	0.39073	0.9995	7730	2	-0.013

B4GALNT2	6	0.2022	0.39075	0.9995	7731	2	-0.03
NHS	6	0.20227	0.39083	0.9995	7732	3	0.1841
SLC15A2	6	0.20227	0.39083	0.9995	7733	2	-0.081
IL5	6	0.20227	0.39083	0.9995	7734	3	0.2425
ZFP36L2	6	0.20231	0.39088	0.9995	7735	2	-0.172
EIF2S3	6	0.20241	0.39101	0.9995	7736	3	0.244
FAM169A	6	0.20256	0.39119	0.9995	7737	3	0.3854
USP35	6	0.20258	0.3912	0.9995	7738	2	0.0467
CAPN10	6	0.20277	0.39143	0.9995	7739	3	0.2236
OPRK1	6	0.20287	0.39156	0.9995	7740	3	-0.027
ZNF648	6	0.20287	0.39156	0.9995	7741	3	0.0844
COMMD1	6	0.20297	0.39168	0.9995	7742	2	-0.033
PCDHGB7	2	0.20298	0.26428	0.9995	7743	1	0.2985
OR52H1	6	0.203	0.39172	0.9995	7744	3	0.2279
ZC2HC1B	6	0.20306	0.3918	0.9995	7745	3	0.1482
C17orf47	6	0.20311	0.39185	0.9995	7746	2	-0.054
RPGRIP1L	6	0.20315	0.39191	0.9995	7747	3	-0.255
CHST3	6	0.20319	0.39195	0.9995	7748	3	0.2746
U2SURP	6	0.20328	0.39205	0.9995	7749	2	0.1133
PPOX	6	0.20332	0.39209	0.9995	7750	2	-0.223
POLH	6	0.20341	0.3922	0.9995	7751	3	0.2298
ARRDC5	6	0.20341	0.3922	0.9995	7752	1	-0.272
C3orf18	6	0.20344	0.39223	0.9995	7753	3	0.0926
NLGN3	6	0.20354	0.39235	0.9995	7754	1	-0.172
hsa-mir-190a	2	0.20357	0.26471	0.9995	7755	1	0.641
hsa-mir-4720	4	0.20357	0.34458	0.9995	7756	2	0.2508
LRP10	6	0.20358	0.39241	0.9995	7757	2	-0.193
ELSPBP1	6	0.20367	0.3925	0.9995	7758	3	0.2212
PKD2	6	0.20373	0.39256	0.9995	7759	3	0.1534
TBATA	6	0.20373	0.39256	0.9995	7760	2	0.1463
SNX24	6	0.20388	0.39274	0.9995	7761	2	0.0691
LAPTM4B	6	0.2041	0.393	0.9995	7762	2	0.0856
40057	3	0.20412	0.31807	0.9995	7763	1	-0.006
GABARAP	6	0.20413	0.39303	0.9995	7764	3	0.1565
ACSM1	4	0.20415	0.34519	0.9995	7765	1	-0.26
PLA2G4C	6	0.20417	0.39309	0.9995	7766	2	-0.01
NUP88	6	0.2042	0.39313	0.9995	7767	3	0.3742
HNRNPAB	6	0.20423	0.39315	0.9995	7768	3	-0.036
CASZ1	6	0.20436	0.39333	0.9995	7769	2	-0.234
OR52N4	6	0.20436	0.39333	0.9995	7770	2	-0.022
TMEM167A	6	0.20444	0.39343	0.9995	7771	1	-0.241
LMCD1	6	0.20449	0.39348	0.9995	7772	3	0.0818
TRPC4AP	6	0.20453	0.39353	0.9995	7773	3	0.2308
TRAM1	6	0.20453	0.39353	0.9995	7774	3	0.1834
ZCCHC9	6	0.20453	0.39353	0.9995	7775	3	0.2894
VPS37A	6	0.20462	0.39363	0.9995	7776	3	0.211
MTFR1	6	0.20466	0.39369	0.9995	7777	2	0.0436
COQ10B	6	0.2047	0.39375	0.9995	7778	3	-0.155
CYFIP1	6	0.20479	0.39385	0.9995	7779	2	-0.131
CLPSL1	6	0.20481	0.39389	0.9995	7780	2	0.0956
VEZF1	6	0.20486	0.39394	0.9995	7781	3	0.1752
hsa-mir-548w	1	0.20494	0.20462	0.9995	7782	1	1.1317
SUPT4H1	6	0.20494	0.39403	0.9995	7783	3	0.2626
FBXO8	6	0.20499	0.39409	0.9995	7784	2	0.1077
RAB24	6	0.20509	0.39421	0.9995	7785	2	0.1074
hsa-mir-4769	4	0.20521	0.34629	0.9995	7786	1	-0.63
RFX4	6	0.20528	0.39443	0.9995	7787	3	0.1013
ADCY5	6	0.20528	0.39443	0.9995	7788	3	0.2452
SIRT6	6	0.20537	0.39453	0.9995	7789	3	0.1972
MXRA5	6	0.20537	0.39453	0.9995	7790	1	-0.014
CSPG5	6	0.2054	0.39457	0.9995	7791	3	0.2107
CRYGB	6	0.20542	0.39459	0.9995	7792	3	0.1519
DRG2	6	0.20544	0.39462	0.9995	7793	2	-0.427
CHKB	6	0.20546	0.39464	0.9995	7794	3	0.1544
KLHL11	6	0.20549	0.39468	0.9995	7795	2	0.1849
STK25	6	0.20556	0.39476	0.9995	7796	3	0.1881
HMX2	6	0.20558	0.39479	0.9995	7797	1	-0.437
BRWD3	6	0.20562	0.39484	0.9995	7798	2	-0.17
HNRNPA1	6	0.2057	0.39493	0.9995	7799	3	0.2454
KRTAP11-1	6	0.20571	0.39495	0.9995	7800	3	0.1085
KRTAP6-3	6	0.20579	0.39504	0.9995	7801	3	0.1558
ZMYM2	6	0.20579	0.39505	0.9995	7802	2	0.0216
LRRC2	6	0.20579	0.39505	0.9995	7803	2	-0.105
FLNB	6	0.20586	0.39514	0.9995	7804	3	0.1321
LPCAT1	6	0.20592	0.39522	0.9995	7805	2	-0.12
hsa-mir-152	4	0.20596	0.34713	0.9995	7806	2	0.2396
SEC22A	6	0.206	0.39532	0.9995	7807	2	0.0032
CARD16	6	0.20606	0.3954	0.9995	7808	2	-0.349
HSD3B1	6	0.20607	0.3954	0.9995	7809	3	-0.011
CUL7	6	0.20612	0.39547	0.9995	7810	2	-0.077
TMC7	6	0.20612	0.39547	0.9995	7811	2	-0.028
CXCR3	6	0.20615	0.3955	0.9995	7812	3	0.2434
CCBE1	6	0.20615	0.3955	0.9995	7813	3	0.1554
FSTL1	6	0.20615	0.39551	0.9995	7814	2	0.1249
LIMD2	6	0.20616	0.39552	0.9995	7815	2	-0.342

TBL1Y	6	0.20617	0.39553	0.9995	7816	2	-0.056
MARC2	6	0.20619	0.39556	0.9995	7817	2	-0.075
DEFB125	6	0.20621	0.39557	0.9995	7818	3	0.0669
LOXL3	6	0.20624	0.39563	0.9995	7819	3	-0.199
NELFE	6	0.20631	0.39571	0.9995	7820	2	-0.069
OXER1	6	0.20631	0.39571	0.9995	7821	3	0.0777
LRRC4B	6	0.20633	0.39573	0.9995	7822	3	0.2175
SEMA4G	6	0.20639	0.39581	0.9995	7823	2	-0.15
PKN1	6	0.20647	0.39589	0.9995	7824	3	0.1034
GPR1	6	0.20653	0.39595	0.9995	7825	3	0.1204
C6orf62	6	0.20653	0.39596	0.9995	7826	3	0.2343
MPP2	6	0.20659	0.39602	0.9995	7827	3	0.1512
TMPRSS9	6	0.20659	0.39602	0.9995	7828	3	0.2441
MACC1	6	0.20666	0.39613	0.9995	7829	2	-0.128
GREM1	6	0.20666	0.39613	0.9995	7830	2	-0.151
NPTX2	4	0.20674	0.34793	0.9995	7831	2	0.2907
MANSC1	6	0.20676	0.39625	0.9995	7832	3	0.0874
CSRNP2	6	0.20678	0.39628	0.9995	7833	3	0.1211
SEC24B	6	0.20685	0.39635	0.9995	7834	3	0.2194
TSHR	6	0.20685	0.39635	0.9995	7835	3	0.2696
ZDHHC4	6	0.20694	0.39646	0.9995	7836	3	0.0676
SGTA	6	0.20698	0.39651	0.9995	7837	2	-0.134
OLFML2A	6	0.20698	0.39651	0.9995	7838	1	-0.093
SVOP	6	0.20698	0.39651	0.9995	7839	3	-0.083
FAM5C	3	0.20702	0.32171	0.9995	7840	2	0.2719
FOXD4	6	0.20706	0.39661	0.9995	7841	2	-0.406
UBQLN2	6	0.20715	0.39671	0.9995	7842	3	0.1243
EID2B	6	0.20715	0.39671	0.9995	7843	3	0.0796
APOF	6	0.2072	0.39676	0.9995	7844	3	0.1635
KLHL8	6	0.2074	0.397	0.9995	7845	3	-0.019
FAM180A	6	0.2074	0.397	0.9995	7846	3	0.2202
DCLK2	6	0.20754	0.39716	0.9995	7847	3	0.0291
CHN1	6	0.20755	0.39718	0.9995	7848	2	0.1316
ZDHHC16	4	0.20756	0.3488	0.9995	7849	2	0.2486
CACNA2D1	6	0.20763	0.39727	0.9995	7850	3	0.2526
SMLR1	6	0.20779	0.39747	0.9995	7851	2	-0.167
ITGA8	6	0.20781	0.39748	0.9995	7852	3	0.2696
PMPCB	6	0.20784	0.39753	0.9995	7853	2	-0.008
FAM86B2	3	0.20792	0.32281	0.9995	7854	1	-1.112
KRT26	6	0.20796	0.39767	0.9995	7855	2	-0.137
DNAJC5B	6	0.20805	0.39777	0.9995	7856	3	0.0113
hsa-mir-4469	4	0.20818	0.34943	0.9995	7857	1	-0.232
hsa-mir-591	4	0.20818	0.34943	0.9995	7858	1	-0.761
TSC22D3	6	0.20823	0.39798	0.9995	7859	2	0.0839
RCE1	6	0.20828	0.39805	0.9995	7860	2	-0.21
WIF1	6	0.20831	0.39809	0.9995	7861	3	0.1752
SHROOM1	6	0.20831	0.39809	0.9995	7862	2	-0.552
PELO	6	0.20839	0.39819	0.9995	7863	3	0.0981
RCC1	6	0.20839	0.39819	0.9995	7864	3	0.0119
TRIM6	4	0.2084	0.34966	0.9995	7865	1	-0.204
GJC1	6	0.20848	0.39831	0.9995	7866	3	0.3223
RAB18	6	0.20857	0.3984	0.9995	7867	2	0.1618
UBL4B	6	0.20874	0.3986	0.9995	7868	1	-0.328
TBC1D31	3	0.20876	0.32385	0.9995	7869	2	0.3164
ARPC1B	6	0.2088	0.39868	0.9995	7870	2	0.0842
HSBP1	6	0.2088	0.39868	0.9995	7871	2	0.1182
SLC5A4	6	0.20884	0.39872	0.9995	7872	3	0.2568
CA5B	6	0.20884	0.39872	0.9995	7873	3	-0.088
P2RY11	6	0.20891	0.39879	0.9995	7874	2	-0.188
YTHDC1	6	0.20895	0.39884	0.9995	7875	3	0.3019
C9orf43	6	0.20904	0.39894	0.9995	7876	2	-0.084
ASPHD2	6	0.20905	0.39895	0.9995	7877	2	-0.121
PAWR	6	0.20908	0.399	0.9995	7878	2	-0.062
ZMYM6	6	0.20911	0.39903	0.9995	7879	2	0.0428
LIN37	6	0.20921	0.39915	0.9995	7880	1	-0.476
FERMT2	6	0.20922	0.39917	0.9995	7881	2	-0.124
APOA4	6	0.20929	0.39925	0.9995	7882	3	0.2475
TEKT1	6	0.20933	0.39931	0.9995	7883	2	-0.032
MSH5	6	0.20934	0.39932	0.9995	7884	3	0.1915
C17orf74	6	0.20938	0.39937	0.9995	7885	2	-0.136
PARL	6	0.20938	0.39937	0.9995	7886	3	0.1848
ZNF816-ZNF321P	2	0.2094	0.26896	0.9995	7887	1	-0.15
PDCD11	6	0.2094	0.39939	0.9995	7888	3	0.1643
hsa-mir-6750	4	0.20952	0.35079	0.9995	7889	2	-0.015
CLTC	6	0.20952	0.39954	0.9995	7890	3	-0.156
hsa-mir-3621	4	0.20954	0.35082	0.9995	7891	2	0.3045
TMEM110	4	0.20968	0.35097	0.9995	7892	2	0.2797
KAT8	6	0.20968	0.39973	0.9995	7893	1	-0.437
CST11	6	0.20969	0.39974	0.9995	7894	3	0.2439
THSD7A	6	0.2097	0.39975	0.9995	7895	3	0.2721
FAM21A	6	0.20978	0.39985	0.9995	7896	3	0.1713
C8orf31	6	0.20984	0.39991	0.9995	7897	3	0.2259
GPR64	6	0.20987	0.39995	0.9995	7898	2	-0.015
MYOC	6	0.20993	0.40001	0.9995	7899	3	0.2084
OR5D14	6	0.21009	0.40021	0.9995	7900	2	0.079

IMP3	6	0.21009	0.40021	0.9995	7901	3	-0.018
ORMDL2	6	0.21011	0.40022	0.9995	7902	3	0.2868
PLEKHG3	6	0.2102	0.40034	0.9995	7903	2	-0.182
ZNF518B	6	0.21022	0.40037	0.9995	7904	2	-0.124
TPM1	6	0.21024	0.40039	0.9995	7905	2	-0.049
ACBD6	6	0.21026	0.40041	0.9995	7906	3	0.0795
MTERF	6	0.21028	0.40044	0.9995	7907	2	-0.142
NOB1	6	0.21036	0.40053	0.9995	7908	3	0.0663
CCZ1B	2	0.21039	0.26972	0.9995	7909	1	0.2126
AMPD1	6	0.2104	0.40059	0.9995	7910	3	0.0972
CCDC135	6	0.21042	0.40062	0.9995	7911	3	0.1984
ZBTB9	6	0.2105	0.40071	0.9995	7912	2	0.0583
TMEM98	6	0.21056	0.40079	0.9995	7913	1	-0.633
PPEF1	6	0.21062	0.40086	0.9995	7914	3	0.196
COQ7	6	0.21062	0.40086	0.9995	7915	3	-0.217
TPH2	6	0.21068	0.40093	0.9995	7916	3	0.0206
HNRNPH1	6	0.21071	0.40096	0.9995	7917	3	0.1877
ANO5	6	0.21071	0.40096	0.9995	7918	2	-0.021
SYNPO	6	0.21079	0.40107	0.9995	7919	2	-0.13
40422	3	0.21084	0.32642	0.9995	7920	1	-0.236
TCF23	4	0.21088	0.35216	0.9995	7921	2	-0.159
LEMD1	6	0.21088	0.40117	0.9995	7922	1	0.0878
HAP1	6	0.21089	0.40118	0.9995	7923	2	0.2646
DNAAF3	6	0.21095	0.40126	0.9995	7924	3	0.197
ERC2	6	0.21097	0.40127	0.9995	7925	2	-0.14
PCP4	6	0.21099	0.4013	0.9995	7926	3	0.2341
RRAS2	6	0.21101	0.40133	0.9995	7927	2	-0.082
MAD2L1BP	6	0.21104	0.40137	0.9995	7928	3	0.2234
IRF4	6	0.2111	0.40145	0.9995	7929	2	-0.081
DDI1	6	0.21114	0.40149	0.9995	7930	1	-0.414
EIF5	6	0.21116	0.40152	0.9995	7931	3	0.2188
ISG20L2	6	0.21116	0.40152	0.9995	7932	2	-0.214
SCN11A	6	0.21116	0.40152	0.9995	7933	2	0.0668
KANK1	6	0.21118	0.40153	0.9995	7934	2	-0.169
ANKRD34C	6	0.21134	0.40173	0.9995	7935	3	0.0782
CPNE1	6	0.21135	0.40175	0.9995	7936	1	-0.04
PVR	6	0.21137	0.40177	0.9995	7937	3	0.2273
OTUB1	6	0.21139	0.40178	0.9995	7938	3	0.025
DLX4	6	0.21139	0.40178	0.9995	7939	2	-0.527
MRPS10	6	0.21141	0.40181	0.9995	7940	3	-0.019
STAT2	6	0.21149	0.40191	0.9995	7941	3	-0.151
UGGT1	6	0.21151	0.40193	0.9995	7942	2	-0.356
TMEM179B	6	0.21154	0.40197	0.9995	7943	3	0.1471
IL18RAP	6	0.21154	0.40197	0.9995	7944	2	-0.197
ACSL5	6	0.21158	0.40201	0.9995	7945	3	0.1001
PAFAH1B3	6	0.21165	0.4021	0.9995	7946	3	0.2491
MFSD3	6	0.21167	0.40212	0.9995	7947	2	-0.126
TBCC	6	0.21167	0.40212	0.9995	7948	3	-0.029
SOCS2	6	0.21174	0.4022	0.9995	7949	2	-0.06
SLC35A1	6	0.21187	0.40236	0.9995	7950	2	0.0527
FURIN	6	0.21188	0.40237	0.9995	7951	3	0.1735
CSDE1	6	0.21191	0.4024	0.9995	7952	2	0.3236
LRP2	6	0.21201	0.40253	0.9995	7953	3	0.1778
ZNF391	6	0.21205	0.40259	0.9995	7954	3	0.0835
PMF1-BGLAP	3	0.21215	0.32807	0.9995	7955	2	0.2244
ABCG8	6	0.21215	0.4027	0.9995	7956	3	0.2362
EML4	6	0.21216	0.40272	0.9995	7957	2	0.2213
NCOA2	6	0.21221	0.40278	0.9995	7958	3	0.0653
hsa-mir-5000	4	0.21223	0.35352	0.9995	7959	2	0.2033
PEX19	6	0.21242	0.40302	0.9995	7960	2	0.0841
SLC12A6	6	0.21247	0.40309	0.9995	7961	3	-0.045
THAP1	6	0.2125	0.40312	0.9995	7962	3	0.207
GDPD4	4	0.21252	0.35383	0.9995	7963	2	0.1586
NAIP	6	0.21258	0.40321	0.9995	7964	3	0.1018
GRID2	6	0.21263	0.40327	0.9995	7965	2	-0.104
HTR3C	6	0.21265	0.40329	0.9995	7966	2	-0.044
CCK	6	0.21265	0.40329	0.9995	7967	2	0.11
TNFRSF25	6	0.21265	0.40329	0.9995	7968	3	0.1951
GRK4	6	0.2127	0.40334	0.9995	7969	3	0.3144
C16orf3	6	0.21271	0.40336	0.9995	7970	3	0.0046
ELN	6	0.21278	0.40345	0.9995	7971	3	0.0301
RAP1B	6	0.21285	0.40352	0.9995	7972	3	0.1645
C2orf61	6	0.21285	0.40352	0.9995	7973	2	0.0712
MBP	6	0.21286	0.40355	0.9995	7974	3	0.0473
CCDC132	6	0.2129	0.40359	0.9995	7975	3	0.2842
CDC45	6	0.21299	0.40372	0.9995	7976	1	-0.998
JMJD6	6	0.21299	0.40372	0.9995	7977	2	0.1301
MYL4	6	0.21301	0.40373	0.9995	7978	3	0.0851
ARHGDIA	6	0.21306	0.40378	0.9995	7979	3	0.1963
GDI1	6	0.2131	0.40384	0.9995	7980	2	-0.11
DGKG	6	0.21312	0.40386	0.9995	7981	2	-0.031
hsa-mir-562	4	0.21324	0.35456	0.9995	7982	1	-0.003
ABCA8	6	0.21328	0.40407	0.9995	7983	3	0.2008
REM2	6	0.21329	0.40408	0.9995	7984	3	-0.189
MYCBP	6	0.21331	0.4041	0.9995	7985	2	0.0403

OR12D2	6	0.21342	0.40423	0.9995	7986	2	-0.25
TSPAN3	6	0.21344	0.40426	0.9995	7987	2	-0.03
YTHDC2	6	0.21344	0.40426	0.9995	7988	3	0.1917
hsa-mir-627	4	0.21345	0.35478	0.9995	7989	2	0.0604
SDHAF2	6	0.2136	0.40446	0.9995	7990	3	0.2711
hsa-mir-6809	4	0.21364	0.355	0.9995	7991	2	0.1406
hsa-mir-4746	4	0.21364	0.355	0.9995	7992	2	0.2162
TMEM129	6	0.21366	0.40452	0.9995	7993	2	-0.162
RPAP1	6	0.21366	0.40452	0.9995	7994	2	-0.036
hsa-mir-3200	4	0.21373	0.35507	0.9995	7995	2	0.2967
PNPLA1	6	0.21374	0.40461	0.9995	7996	3	0.193
NCS1	6	0.21381	0.40471	0.9995	7997	3	0.1185
POU2F2	6	0.21385	0.40475	0.9995	7998	3	0.2192
hsa-mir-7705	2	0.21387	0.27222	0.9995	7999	1	-1.049
MTRF1L	6	0.214	0.40493	0.9995	8000	2	0.0902
ABI3BP	6	0.21402	0.40495	0.9995	8001	3	0.1624
NUMA1	6	0.21404	0.40498	0.9995	8002	2	-0.05
LRRIQ3	6	0.21408	0.40503	0.9995	8003	3	0.1513
C10orf131	5	0.21412	0.39526	0.9995	8004	3	0.3007
POLE4	6	0.21412	0.40507	0.9995	8005	2	0.057
PCMTD1	6	0.21418	0.40514	0.9995	8006	3	0.2669
CETN1	6	0.21421	0.40518	0.9995	8007	2	0.0281
DAO	6	0.21431	0.4053	0.9995	8008	3	0.2417
CEP41	6	0.21434	0.40533	0.9995	8009	2	-0.217
SLC1A2	6	0.21436	0.40535	0.9995	8010	2	-0.021
CD24	6	0.21436	0.40535	0.9995	8011	3	0.1012
TMEM71	6	0.21436	0.40535	0.9995	8012	3	0.3777
NIT2	6	0.21446	0.40548	0.9995	8013	3	0.2433
ULBP2	6	0.21458	0.40561	0.9995	8014	3	0.3211
GEMIN7	6	0.21466	0.40571	0.9995	8015	2	0.0296
TMPRSS2	6	0.2147	0.40577	0.9995	8016	3	0.2662
ICAM2	6	0.2147	0.40577	0.9995	8017	3	0.3013
TMEM91	6	0.21471	0.40578	0.9995	8018	3	0.2555
PSME3	6	0.21472	0.4058	0.9995	8019	3	0.1653
ATP4B	6	0.2148	0.40589	0.9995	8020	3	0.2122
KLHL12	6	0.21481	0.4059	0.9995	8021	1	-0.398
DYX1C1	6	0.21484	0.40594	0.9995	8022	3	-0.015
SNX21	6	0.21485	0.40595	0.9995	8023	1	0.146
CHERP	6	0.21497	0.40609	0.9995	8024	3	0.2146
BHLHE40	6	0.21506	0.40618	0.9995	8025	2	-0.066
hsa-mir-4778	4	0.21512	0.3565	0.9995	8026	2	0.4032
GREB1	6	0.21513	0.40627	0.9995	8027	3	0.0834
MADCAM1	6	0.21515	0.40629	0.9995	8028	2	-0.163
FAU	6	0.21531	0.40649	0.9995	8029	2	0.0561
TRAM1L1	6	0.21532	0.4065	0.9995	8030	1	-0.225
hsa-mir-4803	4	0.21537	0.35678	0.9995	8031	1	0.0913
OLIG1	6	0.21539	0.40658	0.9995	8032	3	0.3624
hsa-mir-6506	3	0.21541	0.33213	0.9995	8033	1	0.1956
LTA	6	0.21546	0.40666	0.9995	8034	2	-0.067
PIBF1	6	0.21551	0.40675	0.9995	8035	3	0.2459
TMEM173	6	0.21553	0.40677	0.9995	8036	2	0.0479
DUT	6	0.21557	0.40681	0.9995	8037	3	0.2108
hsa-mir-493	4	0.21559	0.35699	0.9995	8038	2	0.002
CIDEC	6	0.21564	0.40689	0.9995	8039	3	0.2702
SLN	5	0.21568	0.39734	0.9995	8040	2	-0.079
ESYT1	6	0.21578	0.40706	0.9995	8041	2	-0.051
SLCO2A1	6	0.21579	0.40706	0.9995	8042	2	0.1025
CCDC74A	5	0.21581	0.39753	0.9995	8043	3	0.2066
QRICH2	6	0.21584	0.40713	0.9995	8044	3	0.1242
LYPD2	6	0.21595	0.40725	0.9995	8045	3	-0.152
TCTEX1D1	6	0.21601	0.40733	0.9995	8046	3	0.1588
HLA-DQA2	6	0.21605	0.40736	0.9995	8047	2	0.299
VWC2	4	0.21607	0.35749	0.9995	8048	2	0.1883
CEL	6	0.21611	0.40742	0.9995	8049	3	0.1698
KRTAP19-2	6	0.21615	0.40746	0.9995	8050	2	-0.158
KPNB1	6	0.21615	0.40746	0.9995	8051	3	0.3057
CXCL13	6	0.21622	0.40756	0.9995	8052	3	0.1202
STXBP5L	6	0.21622	0.40756	0.9995	8053	3	0.0946
FRRS1L	6	0.21622	0.40756	0.9995	8054	3	0.1834
GPR63	6	0.21627	0.40762	0.9995	8055	2	0.0102
PIWIL2	6	0.21633	0.40768	0.9995	8056	2	0.0782
NDST4	6	0.21642	0.40779	0.9995	8057	3	0.1008
NEURL4	6	0.21655	0.40796	0.9995	8058	1	-0.212
MRE11A	6	0.21658	0.40798	0.9995	8059	3	0.2353
CLEC4D	6	0.21659	0.408	0.9995	8060	3	0.1381
PTP4A3	6	0.21659	0.408	0.9995	8061	3	0.145
RAD50	6	0.21662	0.40803	0.9995	8062	3	0.0996
USP53	6	0.21664	0.40806	0.9995	8063	1	-0.284
RTN4RL2	6	0.21664	0.40807	0.9995	8064	3	0.0664
RASL12	6	0.21665	0.40807	0.9995	8065	2	0.1037
MGAT2	6	0.21667	0.4081	0.9995	8066	3	0.0468
PCYT1B	6	0.2167	0.40813	0.9995	8067	3	0.216
ZC3H7B	6	0.2168	0.40824	0.9995	8068	3	0.0444
PHF23	6	0.21685	0.40831	0.9995	8069	3	0.2329
OR12D3	6	0.21693	0.40842	0.9995	8070	2	0.1728

RGS8	6	0.21693	0.40842	0.9995	8071	2	0.1829
ZNF136	6	0.21704	0.40854	0.9995	8072	3	0.1818
KNG1	6	0.21706	0.40857	0.9995	8073	2	0.0055
OR5L2	6	0.2171	0.40862	0.9995	8074	2	0.0973
OR2T11	6	0.21721	0.40875	0.9995	8075	2	-0.132
TMEM219	6	0.21723	0.40878	0.9995	8076	1	-0.279
SSBP3	6	0.2173	0.40886	0.9995	8077	3	0.1224
TXNDC12	6	0.21734	0.4089	0.9995	8078	2	-0.174
FUT9	6	0.21737	0.40894	0.9995	8079	2	-0.009
ZNF620	6	0.21745	0.40904	0.9995	8080	2	0.0545
UNC5D	6	0.21748	0.40907	0.9995	8081	3	-0.051
hsa-mir-4293	4	0.2175	0.35896	0.9995	8082	2	0.197
SLC25A40	6	0.21753	0.40913	0.9995	8083	3	0.2091
hsa-mir-6742	4	0.21759	0.35906	0.9995	8084	1	-0.565
JOSD1	6	0.21765	0.40925	0.9995	8085	3	0.1826
ITGB7	6	0.21765	0.40925	0.9995	8086	3	0.2379
hsa-mir-326	4	0.21768	0.35915	0.9995	8087	2	0.3982
SGCA	6	0.21769	0.40931	0.9995	8088	3	0.084
VPRBP	6	0.21774	0.40938	0.9995	8089	3	0.2509
SNTA1	6	0.21778	0.40943	0.9995	8090	1	-0.459
FSIP2	6	0.21791	0.40959	0.9995	8091	2	-0.296
GPR31	6	0.21791	0.40959	0.9995	8092	3	-0.133
SLC8A1	6	0.21795	0.40964	0.9995	8093	3	-0.221
ABCC10	4	0.21796	0.35944	0.9995	8094	1	0.1631
HPS1	6	0.218	0.40969	0.9995	8095	2	-0.038
PAPD5	6	0.21804	0.40976	0.9995	8096	3	0.2296
ESYT3	6	0.21804	0.40976	0.9995	8097	2	-0.145
SPIC	6	0.21821	0.40994	0.9995	8098	2	0.026
C16orf54	6	0.21825	0.40999	0.9995	8099	3	0.1612
INPP5B	6	0.21831	0.41006	0.9995	8100	3	0.2273
C1orf168	6	0.21833	0.41008	0.9995	8101	2	-0.056
KLHL26	6	0.21842	0.4102	0.9995	8102	2	-0.048
TTC37	6	0.21843	0.41021	0.9995	8103	2	0.0766
AKT1	6	0.21846	0.41025	0.9995	8104	2	-0.253
TBRG4	6	0.21854	0.41034	0.9995	8105	3	0.1768
CLDN22	6	0.21855	0.41035	0.9995	8106	2	0.0532
CLK1	6	0.21855	0.41035	0.9995	8107	3	0.111
HMHB1	5	0.21855	0.40137	0.9995	8108	2	0.106
FKBP15	6	0.21857	0.41037	0.9995	8109	3	0.2055
ZNF692	6	0.21863	0.41044	0.9995	8110	3	0.1988
PRKAG1	6	0.21866	0.41048	0.9995	8111	3	0.2246
OR10A4	6	0.21872	0.41053	0.9995	8112	3	0.3833
COMT	6	0.21876	0.41058	0.9995	8113	2	0.1843
CNTN2	6	0.21886	0.41071	0.9995	8114	2	-0.01
NXF1	6	0.21886	0.41071	0.9995	8115	2	0.0898
ZNF100	6	0.21889	0.41074	0.9995	8116	2	0.2206
RFTN1	6	0.21889	0.41075	0.9995	8117	3	0.254
hsa-mir-548ae-2	1	0.21892	0.21857	0.9995	8118	1	0.4919
HMGN2	6	0.21903	0.41089	0.9995	8119	3	0.2128
FAM154B	6	0.21903	0.41089	0.9995	8120	2	0.0884
TMEM17	6	0.21911	0.41098	0.9995	8121	3	0.3387
IBTK	6	0.21914	0.41101	0.9995	8122	3	0.3513
INSIG2	6	0.21914	0.41102	0.9995	8123	2	-0.07
SMG6	6	0.21918	0.41106	0.9995	8124	2	0.1311
MKS1	6	0.21927	0.41116	0.9995	8125	3	-0.175
ANKRD13A	6	0.21931	0.41121	0.9995	8126	2	0.1094
SIDT2	6	0.21933	0.41125	0.9995	8127	2	0.0365
THEMIS2	6	0.21933	0.41125	0.9995	8128	2	0.0479
ZNF714	5	0.21935	0.40249	0.9995	8129	3	0.2444
PRG4	6	0.21938	0.41131	0.9995	8130	2	-0.015
DHX40	6	0.21939	0.41132	0.9995	8131	3	0.2283
SLC22A8	6	0.21946	0.41141	0.9995	8132	3	0.224
TMEM169	6	0.21948	0.41143	0.9995	8133	2	-0.082
ETV4	6	0.21971	0.4117	0.9995	8134	2	0.0546
AHRR	6	0.21971	0.4117	0.9995	8135	2	-0.21
ADAMTSL2	6	0.21971	0.4117	0.9995	8136	3	0.0501
WDHD1	6	0.21973	0.41174	0.9995	8137	2	-0.042
DEK	6	0.21978	0.4118	0.9995	8138	2	-0.584
OR5D16	6	0.2199	0.41193	0.9995	8139	3	0.106
OR1Q1	6	0.2199	0.41193	0.9995	8140	2	-0.276
S1PR1	6	0.21995	0.41198	0.9995	8141	3	-0.051
RPL10	6	0.21995	0.41199	0.9995	8142	3	0.1497
SCRG1	6	0.21999	0.41204	0.9995	8143	2	0.1016
hsa-mir-4443	4	0.22004	0.36156	0.9995	8144	1	-0.153
ACTR10	6	0.22007	0.41213	0.9995	8145	2	0.0188
ALDH1L2	6	0.22012	0.41218	0.9995	8146	1	-0.374
ATXN7	6	0.22016	0.41222	0.9995	8147	2	-0.176
FAM160A1	6	0.22018	0.41225	0.9995	8148	2	-0.059
ZFAND4	4	0.22031	0.36183	0.9995	8149	2	-0.024
ATG16L1	6	0.22035	0.41245	0.9995	8150	2	0.1402
ARHGEF12	6	0.22035	0.41245	0.9995	8151	2	0.0544
hsa-mir-1910	4	0.22045	0.36197	0.9995	8152	2	0.3547
C2orf66	6	0.22046	0.41259	0.9995	8153	2	0.0798
hsa-mir-548o	3	0.2205	0.33843	0.9995	8154	2	0.3356
SPCS2	6	0.22052	0.41265	0.9995	8155	3	-0.143

hsa-mir-6513	4	0.22058	0.36209	0.9995	8156	2	0.282
TAS2R42	6	0.22058	0.41275	0.9995	8157	2	-0.108
SMIM13	6	0.22075	0.41295	0.9995	8158	3	-0.189
SLC47A2	6	0.22081	0.41301	0.9995	8159	3	0.1368
TRHDE	6	0.22082	0.41303	0.9995	8160	3	-0.01
TIGD1	6	0.22088	0.41311	0.9995	8161	1	0.0195
ZNF786	6	0.22089	0.41313	0.9995	8162	3	0.1472
ZAP70	6	0.22091	0.41314	0.9995	8163	3	0.267
ARL10	6	0.22097	0.41322	0.9995	8164	3	0.1683
hsa-mir-597	4	0.22098	0.36252	0.9995	8165	2	0.2277
ACVR1C	6	0.22098	0.41323	0.9995	8166	3	0.0716
EBF1	6	0.22111	0.41338	0.9995	8167	3	-0.262
CLDN25	6	0.22117	0.41346	0.9995	8168	3	0.1465
CCNY	6	0.22122	0.4135	0.9995	8169	1	-0.161
NDUFS5	6	0.22123	0.41352	0.9995	8170	3	0.2551
NLRP4	6	0.22125	0.41355	0.9995	8171	3	0.2564
PRR12	6	0.22126	0.41356	0.9995	8172	2	-0.109
OR6F1	6	0.22126	0.41356	0.9995	8173	1	0.0727
CNTLN	6	0.22136	0.41367	0.9995	8174	2	-0.192
DMRTC2	6	0.22138	0.4137	0.9995	8175	2	0.1384
RPL26	5	0.2214	0.40532	0.9995	8176	2	0.0912
URGCP-MRPS24	1	0.2215	0.22115	0.9995	8177	1	0.8073
hsa-mir-378c	4	0.22158	0.36314	0.9995	8178	2	0.0343
REST	6	0.22158	0.41392	0.9995	8179	2	-0.264
KCNJ9	6	0.22164	0.41399	0.9995	8180	1	-0.999
IFT57	6	0.22164	0.41399	0.9995	8181	3	0.1272
CXCL14	6	0.22172	0.41409	0.9995	8182	3	0.1931
PNRC2	6	0.22179	0.41418	0.9995	8183	2	-0.014
ZBTB45	6	0.22182	0.41422	0.9995	8184	3	0.2638
GAK	6	0.22186	0.41427	0.9995	8185	2	0.1016
DLST	6	0.22189	0.41432	0.9995	8186	3	0.1614
HEXIM1	6	0.22192	0.41435	0.9995	8187	2	-0.214
PVRL4	6	0.22197	0.41442	0.9995	8188	2	0.0071
OR51B5	6	0.22202	0.41447	0.9995	8189	1	-0.734
MAP3K5	6	0.2221	0.41458	0.9995	8190	1	-0.432
PPP1R12B	6	0.22214	0.41462	0.9995	8191	3	0.2284
MANEAL	6	0.22219	0.41469	0.9995	8192	1	-0.423
TM4SF18	6	0.22228	0.41479	0.9995	8193	3	0.1693
COX8C	6	0.22231	0.41484	0.9995	8194	3	0.2069
FAM122A	6	0.22231	0.41484	0.9995	8195	1	-0.614
DHPS	6	0.22242	0.41497	0.9995	8196	3	0.2346
TEP1	6	0.22242	0.41498	0.9995	8197	2	-0.113
ZCCHC2	6	0.22248	0.41504	0.9995	8198	2	-0.511
TRAK1	6	0.22253	0.41508	0.9995	8199	2	-0.167
KPTN	6	0.22257	0.41516	0.9995	8200	3	0.0677
CRTAM	6	0.22259	0.41518	0.9995	8201	2	0.1974
ELP2	6	0.22261	0.41521	0.9995	8202	3	0.2414
GLG1	6	0.22263	0.41523	0.9995	8203	3	0.2474
CRLF1	6	0.22265	0.41525	0.9995	8204	1	-0.357
HS6ST2	6	0.22269	0.41529	0.9995	8205	3	0.2199
GCKR	6	0.22278	0.4154	0.9995	8206	2	-0.471
GLT8D2	6	0.22284	0.41548	0.9995	8207	2	-0.081
ATP6V1E2	6	0.22284	0.41548	0.9995	8208	1	-0.303
CACNA2D4	6	0.22285	0.41549	0.9995	8209	2	-0.04
hsa-mir-4264	4	0.22299	0.36461	0.9995	8210	1	-0.127
OTUD7B	6	0.223	0.41568	0.9995	8211	2	-0.14
TMEM236	6	0.223	0.41568	0.9995	8212	2	-0.087
hsa-mir-217	4	0.223	0.36462	0.9995	8213	2	0.0022
ARL14EPL	6	0.2231	0.4158	0.9995	8214	3	0.0153
BEND5	6	0.22312	0.41583	0.9995	8215	2	0.2065
UBE2S	6	0.22316	0.41587	0.9995	8216	2	-0.364
EIF3A	6	0.22333	0.41607	0.9995	8217	3	0.0946
WIBG	6	0.22337	0.41612	0.9995	8218	2	-0.134
SLC27A1	6	0.2235	0.41628	0.9995	8219	3	0.1867
hsa-mir-4499	4	0.22359	0.36522	0.9995	8220	1	-0.095
IL20RB	6	0.22362	0.41643	0.9995	8221	2	-0.065
PSG9	6	0.22368	0.4165	0.9995	8222	3	0.2146
USP24	4	0.22371	0.36534	0.9995	8223	2	0.2838
WNT9A	6	0.22375	0.41657	0.9995	8224	3	0.1252
PTS	6	0.22377	0.4166	0.9995	8225	1	-0.144
SLC27A6	6	0.22383	0.41667	0.9995	8226	3	0.0012
P2RY14	6	0.22383	0.41667	0.9995	8227	3	0.1016
PSMB3	6	0.22383	0.41667	0.9995	8228	2	0.082
CHMP3	3	0.2239	0.34263	0.9995	8229	2	0.402
TDRD3	6	0.22392	0.41677	0.9995	8230	1	-0.056
GAR1	6	0.22402	0.4169	0.9995	8231	3	0.3223
ORC1	6	0.22411	0.417	0.9995	8232	2	-0.496
hsa-mir-5690	4	0.22413	0.36575	0.9995	8233	2	0.3745
GIMAP8	6	0.22413	0.41703	0.9995	8234	3	0.0861
AHCTF1	6	0.22415	0.41705	0.9995	8235	3	0.1392
hsa-mir-503	4	0.22415	0.36578	0.9995	8236	1	0.1053
NDUFB5	6	0.22428	0.41722	0.9995	8237	3	0.1853
LIPC	6	0.2243	0.41724	0.9995	8238	2	-0.175
ACOX2	6	0.22438	0.41734	0.9995	8239	3	0.0613
FRMPD2	6	0.22447	0.41744	0.9995	8240	2	-0.142

ABCC5	6	0.22448	0.41745	0.9995	8241	2	0.1367
C3orf27	6	0.22448	0.41745	0.9995	8242	3	0.246
hsa-mir-598	4	0.2245	0.36612	0.9995	8243	2	0.2646
VPS53	6	0.2245	0.41748	0.9995	8244	3	0.3066
CSTB	6	0.22457	0.41756	0.9995	8245	3	0.2144
BTNL9	6	0.22459	0.41759	0.9995	8246	2	0.0287
CSH1	3	0.22464	0.34353	0.9995	8247	1	0.0589
SEC24A	6	0.22466	0.41767	0.9995	8248	3	-0.069
C4orf46	6	0.22473	0.41777	0.9995	8249	3	0.052
EPHX4	6	0.22487	0.41793	0.9995	8250	2	-0.051
C1QTNF1	6	0.22489	0.41797	0.9995	8251	2	-0.11
ROBO3	6	0.22495	0.41804	0.9995	8252	2	0.1532
PEX2	6	0.22495	0.41804	0.9995	8253	1	-0.219
PRLHR	6	0.225	0.4181	0.9995	8254	3	-0.037
HSPB1	6	0.22501	0.41813	0.9995	8255	3	0.3195
TCEA1	6	0.2251	0.41822	0.9995	8256	2	-0.494
C16orf52	6	0.22514	0.41826	0.9995	8257	2	-0.141
CPB1	6	0.22518	0.41831	0.9995	8258	3	0.24
CCDC177	6	0.22518	0.41832	0.9995	8259	3	0.232
PATL2	6	0.22522	0.41837	0.9995	8260	2	-0.29
ST8SIA3	6	0.22527	0.41843	0.9995	8261	3	0.161
POLR2L	6	0.22531	0.41848	0.9995	8262	2	-0.739
hsa-mir-873	4	0.22534	0.36698	0.9995	8263	2	0.2097
LIN28A	6	0.22544	0.41863	0.9995	8264	3	0.0102
MDM1	6	0.22545	0.41864	0.9995	8265	3	0.1025
LGMN	6	0.22546	0.41864	0.9995	8266	1	-0.332
TMPO	6	0.22552	0.41872	0.9995	8267	3	0.2703
HSD11B1L	6	0.22554	0.41874	0.9995	8268	2	-0.468
CREM	4	0.22555	0.36721	0.9995	8269	2	0.3927
ANXA2	6	0.22555	0.41875	0.9995	8270	3	-0.069
OR5C1	6	0.22557	0.41878	0.9995	8271	2	-0.296
hsa-mir-1587	4	0.22562	0.36727	0.9995	8272	2	-0.321
HDDC2	6	0.22563	0.41885	0.9995	8273	3	0.2629
GMFG	6	0.22565	0.41886	0.9995	8274	3	0.0325
BCL6	6	0.22569	0.41892	0.9995	8275	3	0.086
ZC3HC1	6	0.22579	0.41905	0.9995	8276	1	-0.055
KLC2	6	0.22586	0.41914	0.9995	8277	3	0.1778
UBE3B	6	0.22588	0.41916	0.9995	8278	2	0.0289
TAB3	6	0.22597	0.41926	0.9995	8279	2	-0.489
hsa-mir-4678	4	0.22598	0.36763	0.9995	8280	2	0.2658
hsa-mir-4323	4	0.22599	0.36763	0.9995	8281	2	0.0394
RRS1	6	0.22601	0.41931	0.9995	8282	3	0.2299
CARD8	6	0.22604	0.41936	0.9995	8283	3	0.0905
LST1	6	0.22606	0.41938	0.9995	8284	3	0.2401
hsa-mir-1293	4	0.22606	0.3677	0.9995	8285	2	0.3745
DPYSL3	6	0.22609	0.41941	0.9995	8286	2	-0.077
HP1BP3	6	0.22611	0.41943	0.9995	8287	2	-0.387
GPR27	6	0.22623	0.41959	0.9995	8288	2	-0.283
FAM150B	6	0.22625	0.41961	0.9995	8289	2	-0.102
PRAMEF1	6	0.22632	0.4197	0.9995	8290	2	-0.49
RNF185	6	0.22634	0.41972	0.9995	8291	3	0.2105
HTR2B	6	0.22636	0.41974	0.9995	8292	2	-0.086
KDM5B	6	0.2264	0.41979	0.9995	8293	2	-0.061
RBM41	6	0.22645	0.41984	0.9995	8294	1	-0.246
ASTN1	6	0.22657	0.42	0.9995	8295	3	0.217
DIS3L2	6	0.22658	0.42001	0.9995	8296	3	0.0796
ZNF304	6	0.2267	0.42017	0.9995	8297	1	-0.454
DCP2	6	0.22678	0.42027	0.9995	8298	3	0.1882
hsa-mir-6852	4	0.22682	0.36847	0.9995	8299	1	-0.086
STRADB	6	0.22687	0.42038	0.9995	8300	2	-0.116
TMEM150B	6	0.22691	0.42044	0.9995	8301	3	-0.146
KDM5D	6	0.22693	0.42047	0.9995	8302	3	0.1568
MTBP	6	0.22695	0.42048	0.9995	8303	1	-0.095
SEMA7A	6	0.22697	0.4205	0.9995	8304	3	0.15
SHROOM3	6	0.22697	0.4205	0.9995	8305	3	0.1336
GALNS	6	0.22705	0.42061	0.9995	8306	2	0.3124
LSP1	6	0.2271	0.42066	0.9995	8307	3	-0.721
PDCL2	6	0.22715	0.42073	0.9995	8308	3	0.2518
OR52R1	6	0.2272	0.42078	0.9995	8309	3	0.0225
MAF1	6	0.2272	0.42079	0.9995	8310	1	-0.291
CAPN8	5	0.22728	0.41361	0.9995	8311	2	-0.423
COL4A6	6	0.22728	0.42089	0.9995	8312	3	0.0298
RNF224	6	0.2273	0.42092	0.9995	8313	2	-0.109
C8orf4	6	0.22737	0.42099	0.9995	8314	2	-0.241
FTSJD1	4	0.22744	0.36912	0.9995	8315	2	0.0741
LCE3A	6	0.22746	0.42109	0.9995	8316	2	-0.302
EXOC2	6	0.22746	0.42109	0.9995	8317	2	-0.394
GMPS	6	0.22754	0.42119	0.9995	8318	3	0.3304
FLJ25363	6	0.22754	0.42119	0.9995	8319	3	0.3178
LGALS2	6	0.2276	0.42127	0.9995	8320	2	-0.075
TTYH3	6	0.22764	0.42132	0.9995	8321	3	0.2696
hsa-mir-143	4	0.22767	0.36935	0.9995	8322	1	-0.11
UGT2B17	4	0.22767	0.36935	0.9995	8323	1	-0.58
LRRC45	6	0.22768	0.42137	0.9995	8324	3	-0.048
NR2C2AP	6	0.22771	0.4214	0.9995	8325	2	-0.066

IPO9	6	0.22774	0.42144	0.9995	8326	3	0.1906
CDC27	6	0.22775	0.42146	0.9995	8327	3	-0.133
MSANTD4	6	0.22779	0.4215	0.9995	8328	2	-0.015
C22orf15	6	0.22789	0.42163	0.9995	8329	3	0.1841
COL4A1	6	0.2279	0.42164	0.9995	8330	2	-0.043
hsa-mir-1909	4	0.2279	0.36957	0.9995	8331	2	0.3343
hsa-mir-5197	4	0.22795	0.36962	0.9995	8332	1	-0.391
SLC26A11	6	0.22796	0.42172	0.9995	8333	1	-0.187
MACROD2	6	0.22796	0.42172	0.9995	8334	3	0.0024
SLC30A3	6	0.22796	0.42172	0.9995	8335	3	0.2645
CACNB4	6	0.22796	0.42172	0.9995	8336	3	0.1683
COX20	6	0.22818	0.42199	0.9995	8337	3	0.2395
JAKMIP2	6	0.22821	0.42203	0.9995	8338	3	-0.149
RPS8	6	0.2283	0.42213	0.9995	8339	2	-0.661
CCDC86	6	0.2284	0.42226	0.9995	8340	1	-0.806
TFDP2	6	0.22857	0.42243	0.9995	8341	2	-0.242
RGS10	6	0.2286	0.42248	0.9995	8342	3	0.0939
COX15	6	0.22871	0.42261	0.9995	8343	2	-0.088
ENDOG	6	0.22876	0.42267	0.9995	8344	3	0.1791
C16orf86	6	0.22889	0.42281	0.9995	8345	2	0.1589
IL15RA	6	0.2289	0.42281	0.9995	8346	3	0.1718
GPLD1	6	0.22899	0.42292	0.9995	8347	1	-0.292
DMBT1	6	0.22903	0.42297	0.9995	8348	3	0.2154
ARID5A	6	0.22903	0.42297	0.9995	8349	3	0.0244
CLEC17A	6	0.22924	0.42321	0.9995	8350	2	-0.069
UBXN6	6	0.22926	0.42323	0.9995	8351	3	0.2132
SNURF	6	0.22926	0.42323	0.9995	8352	3	0.1986
CDC42EP1	6	0.22932	0.4233	0.9995	8353	3	0.2188
ATP6V1C2	6	0.22932	0.4233	0.9995	8354	1	0.0358
GPR37	6	0.22936	0.42333	0.9995	8355	3	0.2746
DCAF7	6	0.22941	0.42339	0.9995	8356	2	0.0781
C5orf38	6	0.22942	0.42341	0.9995	8357	3	0.2054
CCDC157	6	0.22945	0.42344	0.9995	8358	2	-0.27
SRSF1	6	0.22945	0.42344	0.9995	8359	2	0.1889
KIF7	6	0.22946	0.42345	0.9995	8360	2	-0.084
NCOR1	6	0.22953	0.42354	0.9995	8361	2	-0.125
FAM78A	6	0.22965	0.42368	0.9995	8362	2	-0.091
KCNV2	6	0.22971	0.42376	0.9995	8363	2	-0.059
GPC5	6	0.22985	0.42393	0.9995	8364	1	-0.327
OTX2	6	0.2299	0.424	0.9995	8365	3	0.0268
PLCH1	6	0.22993	0.42404	0.9995	8366	2	-0.302
GPR153	6	0.23004	0.42417	0.9995	8367	1	-0.621
LOC152586	6	0.23004	0.42417	0.9995	8368	2	-0.065
LIG4	6	0.2301	0.42425	0.9995	8369	2	0.0016
FER	6	0.23016	0.42433	0.9995	8370	3	0.2945
KAT7	6	0.23017	0.42434	0.9995	8371	3	0.284
LGALS3	6	0.23017	0.42434	0.9995	8372	3	0.162
NPBWR2	6	0.23027	0.42445	0.9995	8373	1	-0.35
C2CD4B	5	0.23027	0.41775	0.9995	8374	2	0.2201
EPS8L2	6	0.23034	0.42455	0.9995	8375	3	0.1799
RPRML	6	0.23039	0.42459	0.9995	8376	1	-0.504
STOML2	6	0.23039	0.42459	0.9995	8377	1	-0.448
hsa-mir-3654	4	0.23041	0.37212	0.9995	8378	2	-0.55
ILDR1	6	0.23043	0.42463	0.9995	8379	2	-0.46
JAKMIP3	6	0.23051	0.42472	0.9995	8380	3	0.007
USP31	6	0.23054	0.42476	0.9995	8381	1	-0.003
KRTAP24-1	6	0.23054	0.42476	0.9995	8382	2	0.036
CACNG8	6	0.23062	0.42484	0.9995	8383	3	0.1741
ZNF594	6	0.23062	0.42484	0.9995	8384	2	-0.082
ARMC10	6	0.23068	0.42492	0.9995	8385	2	0.0483
ACAD11	6	0.23073	0.42497	0.9995	8386	3	0.1178
TAF9	6	0.23077	0.42502	0.9995	8387	3	0.141
AKR1B15	5	0.23088	0.41861	0.9995	8388	2	-0.306
GADD45GIP1	6	0.23094	0.42523	0.9995	8389	3	0.1612
SOHLH1	6	0.23098	0.42527	0.9995	8390	3	0.1006
MRPS2	6	0.23123	0.42557	0.9995	8391	3	0.0313
FST	6	0.23127	0.42562	0.9995	8392	2	-0.137
KRTAP13-1	6	0.23129	0.42565	0.9995	8393	2	-0.181
ZNF727	5	0.2313	0.41918	0.9995	8394	2	0.024
NT5C1B	3	0.23133	0.35188	0.9995	8395	2	0.243
LRRFIP1	6	0.2314	0.42576	0.9995	8396	2	-0.278
RNF150	6	0.23144	0.42582	0.9995	8397	3	0.0652
TP63	6	0.23144	0.42582	0.9995	8398	3	0.2074
DCLRE1B	6	0.23153	0.42593	0.9995	8399	2	-0.017
GNPAT	6	0.23153	0.42593	0.9995	8400	2	-0.107
GINS1	6	0.23161	0.42601	0.9995	8401	2	0.2162
hsa-mir-4739	4	0.23166	0.37341	0.9995	8402	1	-0.216
SCAI	6	0.23166	0.42609	0.9995	8403	3	0.0768
CCM2	6	0.2317	0.42614	0.9995	8404	3	0.2239
SEMG2	5	0.23172	0.41979	0.9995	8405	2	0.1964
HOXA11	6	0.23177	0.42623	0.9995	8406	2	-0.363
ST3GAL6	6	0.23194	0.42642	0.9995	8407	3	-0.434
MFSD9	6	0.23194	0.42643	0.9995	8408	1	0.0209
CTNND2	6	0.23195	0.42644	0.9995	8409	3	0.2606
RASA4	2	0.23199	0.2855	0.9995	8410	1	-0.167

DERA	6	0.232	0.42649	0.9995	8411	2	-0.086
ZDHHC7	6	0.23204	0.42654	0.9995	8412	2	-0.278
YPEL4	6	0.23207	0.42656	0.9995	8413	2	-0.214
MT1G	3	0.23217	0.35294	0.9995	8414	1	-0.419
TBL1X	6	0.23219	0.42671	0.9995	8415	3	0.0681
FAR1	6	0.23226	0.42679	0.9995	8416	3	0.1976
C14orf182	6	0.23227	0.42681	0.9995	8417	2	-0.11
ANAPC10	6	0.2323	0.42684	0.9995	8418	2	-0.188
MTFR1L	6	0.23232	0.42686	0.9995	8419	2	-0.367
FANCF	6	0.23242	0.42698	0.9995	8420	1	-0.438
FAM196A	6	0.23251	0.42708	0.9995	8421	3	0.2418
CACNG3	6	0.23256	0.42714	0.9995	8422	3	-0.01
WHSC1	6	0.23258	0.42717	0.9995	8423	3	0.1786
UBA5	6	0.23258	0.42717	0.9995	8424	3	0.153
C8orf22	6	0.23258	0.42717	0.9995	8425	3	-0.023
CAPN13	6	0.23265	0.42727	0.9995	8426	3	0.1073
LYG2	6	0.23265	0.42727	0.9995	8427	2	0.0591
hsa-mir-423	1	0.23277	0.23243	0.9995	8428	1	0.5304
hsa-mir-8053	2	0.23278	0.28611	0.9995	8429	1	0.0989
ACTRT3	4	0.23279	0.37453	0.9995	8430	2	0.2014
USP50	6	0.23281	0.42747	0.9995	8431	3	0.0795
TBC1D21	6	0.23286	0.42752	0.9995	8432	1	-0.585
ITGA7	6	0.23289	0.42757	0.9995	8433	3	0.1243
FAM222B	6	0.23292	0.4276	0.9995	8434	2	-0.006
hsa-mir-4417	4	0.23293	0.37468	0.9995	8435	2	0.383
PRODH	6	0.23295	0.42764	0.9995	8436	3	0.2827
SET	6	0.23295	0.42764	0.9995	8437	3	0.2052
NCOR2	6	0.23298	0.42767	0.9995	8438	3	0.1404
MT3	6	0.23312	0.42784	0.9995	8439	3	0.2578
MAT1A	6	0.23322	0.42795	0.9995	8440	3	0.1013
ADAMTS5	6	0.23322	0.42795	0.9995	8441	3	0.0118
SMS	6	0.23326	0.42797	0.9995	8442	3	0.1789
PTCHD1	6	0.23332	0.42805	0.9995	8443	3	0.2746
VIM	6	0.23344	0.42819	0.9995	8444	3	0.1474
MAGT1	6	0.23349	0.42822	0.9995	8445	2	-0.142
MAP1A	6	0.23359	0.42834	0.9995	8446	3	0.1554
CYP4F11	6	0.23359	0.42835	0.9995	8447	3	0.28
TMEM229B	6	0.23363	0.42839	0.9995	8448	2	-0.133
PAH	6	0.23369	0.42846	0.9995	8449	3	0.3515
CYB561D1	6	0.23374	0.42852	0.9995	8450	1	-0.179
C1orf95	6	0.23374	0.42852	0.9995	8451	3	0.1609
IL20	6	0.23383	0.42863	0.9995	8452	3	0.267
SPINK13	6	0.23394	0.42877	0.9995	8453	2	-0.225
MAP2K6	6	0.23399	0.42881	0.9995	8454	3	0.0477
LRRC30	6	0.23405	0.42889	0.9995	8455	1	-0.25
BTBD11	6	0.23413	0.42898	0.9995	8456	2	0.1345
ERVFRD-1	6	0.23413	0.42898	0.9995	8457	3	0.0765
FAM46A	6	0.23421	0.42908	0.9995	8458	3	0.0563
FAM46D	6	0.23421	0.42908	0.9995	8459	3	0.142
CETN2	6	0.23432	0.42921	0.9995	8460	2	0.0756
MVB12B	6	0.23432	0.42921	0.9995	8461	3	0.195
COX14	6	0.23434	0.42923	0.9995	8462	3	0.1224
CUTA	6	0.23442	0.42933	0.9995	8463	3	-0.104
WDR44	6	0.23442	0.42933	0.9995	8464	3	0.0403
CXorf36	6	0.23446	0.42937	0.9995	8465	3	0.0637
CUL2	6	0.23447	0.42938	0.9995	8466	2	0.0024
PTGDS	6	0.23453	0.42946	0.9995	8467	1	-0.003
IREB2	6	0.23459	0.42953	0.9995	8468	2	-0.258
FNDC4	6	0.23471	0.42968	0.9995	8469	3	0.0222
EDC4	6	0.23476	0.42973	0.9995	8470	2	0.079
ST6GAL1	6	0.23482	0.42981	0.9995	8471	1	-0.194
RPF2	5	0.23492	0.42423	0.9995	8472	2	-0.507
PPP1R17	6	0.23496	0.42998	0.9995	8473	3	0.1836
TDRD10	6	0.235	0.43003	0.9995	8474	3	0.0584
FIGF	6	0.23515	0.43022	0.9995	8475	2	-0.055
CDK5RAP3	4	0.23518	0.377	0.9995	8476	2	0.2062
TYW5	6	0.23526	0.43034	0.9995	8477	3	0.121
MED21	6	0.2353	0.4304	0.9995	8478	3	0.0594
CPPED1	6	0.23534	0.43044	0.9995	8479	3	-0.086
R3HDML	6	0.23534	0.43044	0.9995	8480	2	-0.37
IRS1	6	0.2354	0.43053	0.9995	8481	2	0.0311
ALKBH3	6	0.23542	0.43056	0.9995	8482	3	0.0045
SLC2A14	3	0.23542	0.35701	0.9995	8483	1	-0.083
B2M	6	0.23555	0.43069	0.9995	8484	3	0.1177
WDR47	6	0.23557	0.43072	0.9995	8485	3	0.2539
SLC6A17	6	0.23561	0.43076	0.9995	8486	2	-0.268
DEFB115	6	0.23571	0.43089	0.9995	8487	3	0.088
ZNF814	5	0.23571	0.42534	0.9995	8488	2	-0.719
CFD	6	0.23574	0.43091	0.9995	8489	2	-0.099
GATAD1	6	0.23577	0.43096	0.9995	8490	3	-0.037
HCN4	6	0.23577	0.43096	0.9995	8491	3	-0.285
ACSL4	6	0.2358	0.43099	0.9995	8492	3	0.1227
PRICKLE3	6	0.23583	0.43102	0.9995	8493	3	0.1358
TGM1	6	0.23583	0.43102	0.9995	8494	3	-0.041
NFYB	6	0.23589	0.4311	0.9995	8495	3	0.0334

FAM32A	6	0.236	0.43124	0.9995	8496	3	-0.142
DNAJB2	6	0.23608	0.43134	0.9995	8497	2	0.0685
SLC18A3	6	0.23609	0.43135	0.9995	8498	3	0.1751
C9orf142	6	0.23631	0.43164	0.9995	8499	3	0.2111
LAMC2	6	0.23638	0.43172	0.9995	8500	3	0.0878
FAM209B	6	0.23646	0.4318	0.9995	8501	3	0.2062
H2BFM	6	0.23647	0.4318	0.9995	8502	3	0.1595
NLRP5	4	0.2365	0.37832	0.9995	8503	1	0.0089
SLFN11	6	0.23665	0.43202	0.9995	8504	2	-0.057
OR52M1	6	0.23668	0.43206	0.9995	8505	3	0.0413
TBK1	6	0.23678	0.43219	0.9995	8506	2	-0.331
TMED7	4	0.23681	0.37863	0.9995	8507	2	0.2982
CD247	6	0.23683	0.43222	0.9995	8508	2	-0.24
SPINK7	6	0.23686	0.43226	0.9995	8509	2	0.1423
XRCC5	6	0.23691	0.43232	0.9995	8510	3	0.0513
ALDH9A1	6	0.23693	0.43234	0.9995	8511	2	0.1442
TMEM184C	6	0.237	0.43244	0.9995	8512	2	-0.031
LAP3	6	0.23703	0.43248	0.9995	8513	2	-0.14
TMEM225	6	0.23713	0.43259	0.9995	8514	3	0.092
NARFL	6	0.23715	0.43262	0.9995	8515	2	-0.918
MSH6	6	0.23718	0.43265	0.9995	8516	3	0.2067
CUL4A	6	0.23724	0.43272	0.9995	8517	3	0.1715
SPZ1	6	0.23736	0.43287	0.9995	8518	3	-0.055
KRTAP25-1	6	0.2374	0.43291	0.9995	8519	3	0.1305
SRRT	6	0.23757	0.43312	0.9995	8520	2	0.1958
ACAD10	6	0.23763	0.43318	0.9995	8521	2	-0.144
VSTM2B	6	0.23776	0.43335	0.9995	8522	2	-0.008
IL1A	6	0.23776	0.43335	0.9995	8523	3	0.0487
TRAPPC9	6	0.23782	0.43341	0.9995	8524	1	-0.035
NEU2	6	0.23783	0.43342	0.9995	8525	3	0.0633
TNS1	6	0.23786	0.43345	0.9995	8526	1	-0.797
OR8U8	3	0.23793	0.36012	0.9995	8527	2	0.3928
TCEA3	6	0.23794	0.43354	0.9995	8528	2	0.022
USP17L5	1	0.23795	0.23767	0.9995	8529	1	0.7846
LNX1	6	0.23795	0.43355	0.9995	8530	3	0.284
NCDN	6	0.23798	0.43359	0.9995	8531	2	-0.428
LOC100505478	6	0.23805	0.43365	0.9995	8532	3	0.16
YME1L1	6	0.23815	0.43377	0.9995	8533	1	0.0276
CACHD1	6	0.23819	0.43383	0.9995	8534	2	0.1799
METTL9	6	0.2382	0.43384	0.9995	8535	3	-0.053
HARBI1	6	0.23822	0.43386	0.9995	8536	3	0.0804
TSNAX	6	0.23822	0.43386	0.9995	8537	3	0.0865
hsa-mir-7107	4	0.2383	0.38015	0.9995	8538	2	-0.09
EDARADD	6	0.23833	0.43398	0.9995	8539	3	0.1101
MRGPRF	6	0.23836	0.43402	0.9995	8540	2	-0.41
TRIL	6	0.23851	0.43419	0.9995	8541	2	-0.148
MAP2K1	6	0.23852	0.4342	0.9995	8542	2	-0.388
hsa-mir-500b	3	0.23861	0.36098	0.9995	8543	2	0.31
ASB10	6	0.23863	0.43433	0.9995	8544	2	0.0414
ZXDA	6	0.23867	0.43438	0.9995	8545	3	0.2068
CHMP1A	6	0.23867	0.43438	0.9995	8546	3	0.1947
KCNQ1	5	0.23868	0.42942	0.9995	8547	3	0.3186
TIMM21	6	0.23871	0.43443	0.9995	8548	2	-0.395
SLC15A4	6	0.23881	0.43457	0.9995	8549	2	-0.31
GOLGB1	6	0.23883	0.43458	0.9995	8550	3	0.1529
CD109	4	0.23892	0.38079	0.9995	8551	2	0.2628
CLCN5	6	0.23894	0.43472	0.9995	8552	3	0.302
ZNF677	6	0.23897	0.43476	0.9995	8553	2	-0.002
UVRAG	6	0.23906	0.43488	0.9995	8554	3	0.0855
NCOA4	6	0.23917	0.435	0.9995	8555	1	-0.125
PHOSPHO1	6	0.23926	0.43513	0.9995	8556	3	0.0791
MESP2	6	0.23937	0.43526	0.9995	8557	2	-0.188
SIRPD	6	0.2394	0.43529	0.9995	8558	3	0.2441
PPIL1	6	0.23945	0.43534	0.9995	8559	2	-0.024
ARIH2	6	0.23947	0.43536	0.9995	8560	2	0.1119
SNX14	6	0.23963	0.43556	0.9995	8561	3	0.2575
KLHL10	6	0.23968	0.43565	0.9995	8562	2	-0.148
GUF1	6	0.23979	0.43577	0.9995	8563	2	-0.031
CYB5R3	6	0.23991	0.43592	0.9995	8564	2	0.0442
SENP5	6	0.23991	0.43592	0.9995	8565	2	-0.276
GLTSCR2	6	0.24	0.43602	0.9995	8566	1	0.0092
ZNF81	6	0.24	0.43603	0.9995	8567	3	0.3265
C4orf32	6	0.24003	0.43607	0.9995	8568	2	0.0041
hsa-mir-3118-2	1	0.24006	0.2398	0.9995	8569	1	1.2128
TIRAP	6	0.24008	0.43612	0.9995	8570	2	-0.336
BTNL3	6	0.24008	0.43612	0.9995	8571	2	0.0056
TSPAN32	6	0.24018	0.43625	0.9995	8572	2	-0.096
USP17L4	3	0.24021	0.36294	0.9995	8573	1	-1.233
MVP	6	0.24041	0.43652	0.9995	8574	3	0.2404
MBNL2	6	0.24041	0.43652	0.9995	8575	3	-0.098
GRXCR1	6	0.24051	0.43664	0.9995	8576	2	0.1259
SAR1A	6	0.2406	0.43675	0.9995	8577	3	0.2112
CD33	6	0.24061	0.43676	0.9995	8578	3	0.2581
SLC10A7	6	0.24061	0.43676	0.9995	8579	3	0.1328
MYEF2	6	0.24064	0.43679	0.9995	8580	2	-0.236

ZNF395	4	0.24064	0.3825	0.9995	8581	2	-0.005
SCN5A	6	0.24069	0.43686	0.9995	8582	3	0.0895
SNCG	6	0.24078	0.43698	0.9995	8583	2	0.066
PDXK	6	0.24089	0.43712	0.9995	8584	3	0.1122
CDIPT	6	0.24092	0.43717	0.9995	8585	2	-0.036
SLC35A4	6	0.24095	0.4372	0.9995	8586	2	0.0038
LUC7L3	6	0.24098	0.43723	0.9995	8587	2	-0.013
CD1B	6	0.24101	0.43726	0.9995	8588	2	-0.575
NR3C2	6	0.24103	0.43729	0.9995	8589	3	0.2804
LCMT1	6	0.24106	0.43733	0.9995	8590	3	0.1558
hsa-mir-548p	2	0.24109	0.29227	0.9995	8591	1	-0.051
ARMCX5	6	0.24109	0.43736	0.9995	8592	2	-0.226
PIK3R3	6	0.2411	0.43737	0.9995	8593	3	0.1092
hsa-mir-101-2	3	0.24114	0.36406	0.9995	8594	2	0.2012
FRRS1	6	0.24118	0.43747	0.9995	8595	2	0.0748
DUSP2	6	0.24122	0.43752	0.9995	8596	2	-0.033
ADAM2	6	0.24126	0.43757	0.9995	8597	3	0.2316
C17orf59	6	0.2413	0.4376	0.9995	8598	2	-0.026
CST3	6	0.24138	0.4377	0.9995	8599	1	-0.16
GLTPD1	6	0.24144	0.43778	0.9995	8600	3	-0.071
NCKIPSD	6	0.24144	0.43778	0.9995	8601	3	-0.042
LOC728392	6	0.24144	0.43778	0.9995	8602	3	0.0067
DEFB114	6	0.24154	0.43789	0.9995	8603	3	0.0781
UBXN10	6	0.24156	0.43792	0.9995	8604	3	0.1529
EXO1	6	0.24157	0.43793	0.9995	8605	1	-0.489
ZBTB12	6	0.24165	0.43803	0.9995	8606	3	-0.025
SLC14A1	6	0.24166	0.43803	0.9995	8607	2	-0.031
ANXA8L2	2	0.24166	0.29268	0.9995	8608	1	0.3548
KLHL38	6	0.24181	0.43822	0.9995	8609	2	-0.28
EIF6	6	0.24185	0.43827	0.9995	8610	3	0.0975
OR7G2	6	0.24186	0.43827	0.9995	8611	3	0.0256
hsa-mir-6073	4	0.24186	0.38375	0.9995	8612	2	-0.142
MYADML2	6	0.24192	0.43835	0.9995	8613	1	-0.89
RSPH3	6	0.24193	0.43836	0.9995	8614	3	0.1383
GPRASP2	6	0.24198	0.43842	0.9995	8615	1	-0.293
TAF7	6	0.2421	0.43856	0.9995	8616	3	0.0783
TGFBR2	6	0.24217	0.43865	0.9995	8617	2	0.1639
hsa-mir-4726	4	0.24217	0.38406	0.9995	8618	2	0.2217
PSMA5	6	0.24219	0.43867	0.9995	8619	2	-0.46
MRPL32	6	0.24241	0.43895	0.9995	8620	2	0.0813
SYNPR	6	0.24246	0.43901	0.9995	8621	3	0.2554
MED16	6	0.24248	0.43902	0.9995	8622	2	0.0404
MAP1B	6	0.24251	0.43907	0.9995	8623	2	-0.133
FLYWCH1	6	0.24262	0.43921	0.9995	8624	3	0.2073
IZUMO1	6	0.24268	0.43927	0.9995	8625	3	0.0171
ZNF365	6	0.24271	0.43932	0.9995	8626	2	-0.531
UHMK1	6	0.2428	0.43943	0.9995	8627	2	-0.208
ARL14	6	0.24281	0.43944	0.9995	8628	3	0.2257
HORMAD1	4	0.2429	0.38482	0.9995	8629	2	0.2975
OLFM3	6	0.24291	0.43958	0.9995	8630	1	-0.242
ZBP1	6	0.24294	0.43961	0.9995	8631	3	0.1612
CHTOP	6	0.24304	0.43972	0.9995	8632	2	-0.202
SH3PXD2B	6	0.24305	0.43972	0.9995	8633	3	0.1115
ENTPD8	6	0.24308	0.43975	0.9995	8634	3	0.0617
CACUL1	6	0.24312	0.43981	0.9995	8635	2	0.0924
FAM96A	6	0.24325	0.43996	0.9995	8636	3	0.1813
POM121C	4	0.24327	0.3852	0.9995	8637	2	0.3728
hsa-mir-3133	3	0.24349	0.36702	0.9995	8638	2	0.2104
AKR7A3	6	0.24349	0.44024	0.9995	8639	3	0.0294
KRTAP10-7	6	0.24353	0.4403	0.9995	8640	3	0.2131
ATP10A	6	0.24353	0.4403	0.9995	8641	3	0.2907
ANKRD46	6	0.24353	0.44031	0.9995	8642	2	-0.002
SCGB2A2	5	0.24364	0.43612	0.9995	8643	2	0.0144
GZMK	6	0.24367	0.44048	0.9995	8644	2	0.0056
SAMD1	6	0.24368	0.4405	0.9995	8645	3	-0.007
PPAPDC3	6	0.24368	0.4405	0.9995	8646	3	0.2632
SWSAP1	6	0.2437	0.44052	0.9995	8647	2	-0.172
ZKSCAN8	6	0.24371	0.44054	0.9995	8648	2	0.1158
CLEC12B	6	0.24374	0.44056	0.9995	8649	3	0.2051
RRM2B	6	0.24378	0.4406	0.9995	8650	1	0.1204
DPT	6	0.24386	0.4407	0.9995	8651	2	-0.031
PPP5D1	6	0.24386	0.4407	0.9995	8652	3	-0.014
MAPK13	6	0.24401	0.44087	0.9995	8653	2	-0.117
C11orf16	6	0.24406	0.44092	0.9995	8654	2	-0.14
SEC62	6	0.24407	0.44094	0.9995	8655	2	0.1922
SCCPDH	6	0.2442	0.44109	0.9995	8656	3	0.1913
SLC25A39	6	0.24421	0.44111	0.9995	8657	2	-0.578
hsa-mir-3180-1	1	0.24424	0.24402	0.9995	8658	1	0.5307
hsa-mir-516b-1	2	0.24426	0.29462	0.9995	8659	1	-0.213
HSD17B12	6	0.2443	0.4412	0.9995	8660	3	0.2248
ICA1L	6	0.2444	0.44133	0.9995	8661	2	0.0966
ICMT	6	0.24445	0.44138	0.9995	8662	3	0.0809
STAG1	6	0.24448	0.44142	0.9995	8663	3	0.1971
TRPM4	6	0.24453	0.44148	0.9995	8664	3	-0.02
TAF11	6	0.24462	0.44158	0.9995	8665	3	0.1782

S1PR2	6	0.24462	0.44158	0.9995	8666	2	0.0323
INHBC	6	0.24462	0.44159	0.9995	8667	3	0.1548
EMID1	6	0.24465	0.44161	0.9995	8668	2	-0.289
PACS2	6	0.24469	0.44166	0.9995	8669	3	0.1
PRELP	6	0.24481	0.44182	0.9995	8670	2	-0.137
CDC20	6	0.24481	0.44182	0.9995	8671	3	0.1137
ECI2	6	0.24481	0.44182	0.9995	8672	3	0.2652
SERP1	6	0.24485	0.44186	0.9995	8673	2	0.0594
DFNA5	6	0.24492	0.44195	0.9995	8674	3	0.3762
INHA	6	0.24493	0.44196	0.9995	8675	1	-0.45
RPS21	6	0.24493	0.44196	0.9995	8676	1	-0.363
BAG4	6	0.24495	0.44197	0.9995	8677	3	0.3375
hsa-mir-585	4	0.24495	0.38692	0.9995	8678	2	-0.886
SNAPIN	6	0.24496	0.44199	0.9995	8679	3	0.1805
HIPK4	6	0.24506	0.44211	0.9995	8680	3	0.2115
ALS2CR12	6	0.24516	0.44222	0.9995	8681	3	0.2545
ZNRD1	6	0.24516	0.44222	0.9995	8682	3	0.2821
PIAS2	6	0.24516	0.44222	0.9995	8683	3	0.2408
FAM161B	6	0.24526	0.44233	0.9995	8684	3	0.1361
hsa-mir-3064	4	0.24537	0.38734	0.9995	8685	2	0.1834
HRNR	6	0.2454	0.44251	0.9995	8686	2	-0.36
DPY19L4	6	0.24547	0.44259	0.9995	8687	1	-0.047
LANCL1	6	0.24549	0.4426	0.9995	8688	3	0.2089
KIDINS220	6	0.24554	0.44266	0.9995	8689	2	0.0753
IKBIP	6	0.24554	0.44266	0.9995	8690	3	-0.046
NR4A3	6	0.24561	0.44274	0.9995	8691	3	0.1179
AKIRIN2	6	0.24566	0.44281	0.9995	8692	2	0.0013
IP6K3	6	0.24588	0.44307	0.9995	8693	1	-0.161
GALNT9	6	0.24589	0.44308	0.9995	8694	2	0.0982
ATG14	6	0.24594	0.44314	0.9995	8695	2	-0.095
NBPF8	4	0.24601	0.38803	0.9995	8696	1	-0.244
C9orf37	6	0.24606	0.44327	0.9995	8697	2	-0.08
hsa-mir-3173	4	0.24609	0.38812	0.9995	8698	2	0.3891
TAAR2	6	0.2461	0.44332	0.9995	8699	3	0.2557
NBL1	3	0.24615	0.3703	0.9995	8700	2	0.2824
TBKBP1	6	0.24625	0.44349	0.9995	8701	1	-0.14
WBSCR16	6	0.24636	0.44363	0.9995	8702	2	0.0959
MORN4	6	0.24642	0.44369	0.9995	8703	2	0.1608
TLX1	6	0.24647	0.44375	0.9995	8704	2	-0.018
TRIM27	6	0.24658	0.44388	0.9995	8705	2	-0.24
SCUBE2	6	0.24658	0.44388	0.9995	8706	3	0.1986
ZSWIM3	6	0.24663	0.44393	0.9995	8707	3	0.0536
CREB3L4	6	0.24669	0.444	0.9995	8708	3	0.0109
IFNGR1	6	0.24677	0.4441	0.9995	8709	3	0.1407
HCFC2	6	0.24685	0.4442	0.9995	8710	2	0.0003
RPL27A	6	0.24685	0.4442	0.9995	8711	2	-0.473
IFIT5	6	0.24686	0.44421	0.9995	8712	3	0.152
COMMD10	6	0.24695	0.44433	0.9995	8713	2	-0.165
CCP110	6	0.24703	0.44442	0.9995	8714	2	0.0401
PSPC1	6	0.24706	0.44445	0.9995	8715	3	0.2844
HERC5	6	0.24708	0.44447	0.9995	8716	2	-0.197
hsa-mir-1251	4	0.24714	0.38921	0.9995	8717	2	0.0415
KIFAP3	6	0.24714	0.44454	0.9995	8718	2	-0.046
TXNRD2	6	0.24714	0.44454	0.9995	8719	2	-0.49
hsa-mir-4724	4	0.24715	0.38922	0.9995	8720	2	0.2291
GAN	6	0.24719	0.4446	0.9995	8721	3	0.2141
FCGR1B	4	0.2472	0.38928	0.9995	8722	2	0.0068
ZNF461	6	0.24729	0.44472	0.9995	8723	3	0.1752
GPR128	6	0.24729	0.44472	0.9995	8724	2	0.1136
hsa-mir-3127	4	0.24733	0.3894	0.9995	8725	2	0.1412
CTSO	6	0.24747	0.44493	0.9995	8726	1	-0.582
TCF21	6	0.24747	0.44493	0.9995	8727	1	-0.093
MUL1	6	0.24747	0.44494	0.9995	8728	3	0.2232
USP18	6	0.24749	0.44495	0.9995	8729	2	0.1765
TGFBR1	6	0.24757	0.44505	0.9995	8730	3	0.1548
FAM63A	6	0.2476	0.44508	0.9995	8731	2	-0.042
hsa-mir-135a-1	4	0.24761	0.38972	0.9995	8732	1	0.0667
SLC35A3	6	0.24764	0.44514	0.9995	8733	2	-0.071
PRDX5	6	0.24773	0.44524	0.9995	8734	2	-0.51
SULT2B1	6	0.24776	0.44527	0.9995	8735	3	-0.037
CEACAM1	6	0.24779	0.44529	0.9995	8736	2	-0.414
hsa-mir-4748	4	0.24788	0.38997	0.9995	8737	2	0.2903
PRKCH	6	0.24789	0.4454	0.9995	8738	3	0.0702
STK24	6	0.2479	0.44542	0.9995	8739	2	-0.181
C6orf118	6	0.24791	0.44543	0.9995	8740	3	0.2142
GAPDHS	6	0.24798	0.4455	0.9995	8741	3	0.1781
hsa-mir-1972-2	1	0.24812	0.2479	0.9995	8742	1	0.4193
SULT4A1	6	0.24819	0.44577	0.9995	8743	1	0.0048
FAM81B	6	0.24832	0.44597	0.9995	8744	3	0.014
ZFYVE27	6	0.24843	0.44609	0.9995	8745	3	-0.036
LIN7A	6	0.24844	0.44611	0.9995	8746	3	0.2618
ALDH1A3	6	0.24849	0.44616	0.9995	8747	2	-0.032
ST3GAL5	6	0.24851	0.44619	0.9995	8748	1	-0.246
KIF3C	6	0.24859	0.44628	0.9995	8749	3	-0.157
CPEB2	6	0.24865	0.44637	0.9995	8750	2	-0.253

FAM104B	6	0.24868	0.4464	0.9995	8751	3	0.1814
ASB17	6	0.24868	0.4464	0.9995	8752	3	0.2323
KCNG2	6	0.2487	0.44643	0.9995	8753	3	0.3706
FANCC	4	0.24872	0.39085	0.9995	8754	2	0.1195
POU3F3	6	0.24873	0.44646	0.9995	8755	2	0.0625
RCN1	6	0.24876	0.44649	0.9995	8756	1	-0.297
RNASE4	6	0.2488	0.44654	0.9995	8757	3	0.1276
CAMK2G	6	0.24882	0.44656	0.9995	8758	3	-0.167
KCNJ2	6	0.24882	0.44656	0.9995	8759	3	0.1678
SELENBP1	6	0.24892	0.44668	0.9995	8760	2	-0.058
TMEM52B	6	0.24897	0.44672	0.9995	8761	2	-0.077
LMOD3	6	0.24901	0.44679	0.9995	8762	2	-0.097
RAB17	6	0.24903	0.4468	0.9995	8763	2	-0.065
UTF1	6	0.24913	0.44693	0.9995	8764	2	-0.281
YY2	6	0.24914	0.44694	0.9995	8765	3	0.0903
FAM63B	6	0.24914	0.44694	0.9995	8766	2	-0.052
TUBD1	6	0.24921	0.44702	0.9995	8767	2	-0.108
USP6NL	6	0.24934	0.44718	0.9995	8768	2	-0.069
NUP62	6	0.24942	0.44728	0.9995	8769	3	0.1571
LHX6	6	0.24947	0.44734	0.9995	8770	3	0.3939
PCED1B	6	0.24947	0.44734	0.9995	8771	3	-0.026
RRM1	6	0.24947	0.44734	0.9995	8772	3	0.1665
TAF1C	6	0.24947	0.44734	0.9995	8773	3	0.1305
DAZL	6	0.24947	0.44734	0.9995	8774	3	0.0785
LOR	6	0.24948	0.44735	0.9995	8775	3	0.1256
CRYAB	6	0.24954	0.44741	0.9995	8776	2	-0.099
CKLF-CMTM1	4	0.24966	0.39178	0.9995	8777	2	0.0406
EDC3	6	0.24966	0.44755	0.9995	8778	2	0.073
CLDN18	6	0.24967	0.44757	0.9995	8779	2	-0.3
OR5H6	6	0.2497	0.44762	0.9995	8780	1	0.0278
UBTD2	6	0.24975	0.44766	0.9995	8781	2	-0.171
RNF10	6	0.24978	0.4477	0.9995	8782	3	0.1782
CKAP4	6	0.24982	0.44776	0.9995	8783	2	0.066
FAM83A	4	0.24985	0.39196	0.9995	8784	2	0.2328
FZD2	6	0.24987	0.44781	0.9995	8785	2	0.0817
C13orf35	6	0.24993	0.44787	0.9995	8786	2	0.0222
XKR8	6	0.24995	0.44789	0.9995	8787	3	0.1624
BYSL	6	0.24998	0.44795	0.9995	8788	3	0.2145
ACTL8	6	0.24999	0.44796	0.9995	8789	1	-0.09
PODXL	6	0.25	0.44797	0.9995	8790	3	-0.062
CTPS2	6	0.25005	0.44803	0.9995	8791	2	-0.409
hsa-mir-3934	4	0.25006	0.39218	0.9995	8792	2	0.2092
LOC729059	3	0.25012	0.37524	0.9995	8793	1	0.0376
CCDC78	6	0.25024	0.44825	0.9995	8794	2	0.0843
TRIM29	6	0.2503	0.44833	0.9995	8795	2	0.1307
HSP90AA1	6	0.2503	0.44833	0.9995	8796	3	-0.064
hsa-mir-3139	4	0.25039	0.39251	0.9995	8797	1	-0.03
SLC12A5	6	0.25042	0.44848	0.9995	8798	2	-0.025
HOXC11	6	0.25045	0.44851	0.9995	8799	3	0.1994
hsa-mir-762	4	0.25046	0.39258	0.9995	8800	1	-0.489
P4HA2	6	0.25052	0.44862	0.9995	8801	3	-0.126
HTR7	6	0.25055	0.44865	0.9995	8802	3	0.0803
SPTBN5	6	0.25059	0.4487	0.9995	8803	3	0.1508
CCDC96	6	0.25065	0.44878	0.9995	8804	3	0.0431
LHPP	6	0.25069	0.44882	0.9995	8805	1	-0.261
GNA14	6	0.25071	0.44884	0.9995	8806	2	-0.117
PIDD	6	0.25072	0.44886	0.9995	8807	3	0.0283
BCORL1	6	0.25074	0.44888	0.9995	8808	3	0.2462
IGIP	6	0.25079	0.44895	0.9995	8809	1	-0.171
LZTFL1	6	0.25088	0.44907	0.9995	8810	2	-0.494
SAP18	6	0.25089	0.44907	0.9995	8811	3	-0.058
GLOD5	6	0.2509	0.44909	0.9995	8812	2	-0.013
MYOM2	6	0.25093	0.44912	0.9995	8813	1	-0.256
CENPO	6	0.25094	0.44913	0.9995	8814	3	0.1289
DNM1	6	0.25097	0.44916	0.9995	8815	2	-0.082
ZBTB39	6	0.25101	0.44921	0.9995	8816	2	-0.44
ZNF860	6	0.25104	0.44925	0.9995	8817	3	-0.071
ITGB5	6	0.25104	0.44925	0.9995	8818	3	0.1701
PTPN2	6	0.25116	0.44939	0.9995	8819	3	0.0393
DIS3	6	0.25116	0.44939	0.9995	8820	3	0.3504
OR51A2	6	0.2512	0.44944	0.9995	8821	2	-0.832
KRTAP5-2	6	0.2512	0.44944	0.9995	8822	1	-0.13
LPGAT1	6	0.25124	0.44948	0.9995	8823	3	0.2401
RASEF	6	0.25124	0.44948	0.9995	8824	2	-0.415
DES	6	0.2513	0.44957	0.9995	8825	3	0.0889
ACY1	6	0.25132	0.44959	0.9995	8826	2	-0.334
RABGGTB	6	0.25142	0.44971	0.9995	8827	3	0.2589
PLIN2	6	0.25145	0.44974	0.9995	8828	2	-0.177
SLC17A7	4	0.25151	0.39362	0.9995	8829	1	-0.236
GPR3	6	0.25152	0.44982	0.9995	8830	3	0.1393
GLDN	6	0.25155	0.44985	0.9995	8831	1	-0.282
POLL	6	0.2516	0.44992	0.9995	8832	3	-0.336
C9orf163	6	0.25165	0.45	0.9995	8833	1	0.0487
MMP19	6	0.25171	0.45007	0.9995	8834	2	-0.082
ZNF354B	5	0.25173	0.44285	0.9995	8835	2	-0.132

POSTN	6	0.25173	0.45009	0.9995	8836	3	0.2254
ZFPL1	6	0.25176	0.45014	0.9995	8837	3	-0.044
TBCK	6	0.25179	0.45016	0.9995	8838	3	0.0504
MAGEB6	6	0.25183	0.45022	0.9995	8839	3	0.1915
HSPA14	6	0.2519	0.45028	0.9995	8840	2	-0.021
OR4L1	6	0.25192	0.45033	0.9995	8841	3	0.1417
C16orf95	6	0.25198	0.45041	0.9995	8842	3	0.0968
CABLES2	4	0.25199	0.39412	0.9995	8843	2	0.0135
TCP11X2	6	0.252	0.45043	0.9995	8844	2	-0.133
PLBD2	6	0.25208	0.45054	0.9995	8845	2	0.1091
OR5B2	6	0.25211	0.45057	0.9995	8846	3	0.0153
hsa-mir-7706	4	0.25222	0.39437	0.9995	8847	2	0.2704
RNF139	6	0.25224	0.45073	0.9995	8848	2	-0.049
ESRRA	6	0.25225	0.45075	0.9995	8849	3	0.0323
LYPD6B	6	0.25228	0.45078	0.9995	8850	3	0.1931
FLVCR1	6	0.25234	0.45086	0.9995	8851	2	0.0671
FXN	6	0.25244	0.45099	0.9995	8852	3	0.3702
CYP2A13	5	0.25249	0.44345	0.9995	8853	2	-0.475
FHL1	6	0.25249	0.45103	0.9995	8854	2	0.1081
COL6A5	6	0.25253	0.45109	0.9995	8855	3	0.12
DNALI1	6	0.25254	0.45111	0.9995	8856	3	0.0428
OR4P4	6	0.25263	0.45123	0.9995	8857	2	-0.013
FRMPD4	6	0.25263	0.45123	0.9995	8858	2	0.025
PGPEP1L	6	0.25267	0.45127	0.9995	8859	3	0.2087
CNOT6L	6	0.25271	0.45132	0.9995	8860	3	0.3013
CABIN1	6	0.25275	0.45136	0.9995	8861	2	-0.303
RD3L	6	0.25277	0.45138	0.9995	8862	3	0.1609
DLX6	6	0.25277	0.45139	0.9995	8863	3	0.1877
GOLT1A	6	0.25285	0.4515	0.9995	8864	3	0.0615
HIATL1	6	0.25288	0.45152	0.9995	8865	2	0.1151
PCDH18	6	0.25296	0.45163	0.9995	8866	3	0.14
C9orf152	6	0.253	0.45168	0.9995	8867	2	-0.103
PHF13	6	0.25309	0.45177	0.9995	8868	3	0.2317
NDUFB11	6	0.2532	0.45191	0.9995	8869	3	0.1498
FAM13A	6	0.2532	0.45191	0.9995	8870	3	0.1283
OR8H3	6	0.25322	0.45194	0.9995	8871	1	-0.113
HOPX	6	0.25335	0.45209	0.9995	8872	2	-0.181
KIF2B	6	0.25349	0.45228	0.9995	8873	3	0.0837
OCA2	6	0.25353	0.45233	0.9995	8874	3	0.1783
HDLBP	6	0.25355	0.45234	0.9995	8875	1	-0.229
GJB5	6	0.25357	0.45236	0.9995	8876	2	-0.37
CACNB3	6	0.25359	0.45239	0.9995	8877	1	-0.117
LOC100288255	1	0.25361	0.25342	0.9995	8878	1	0.4996
ZNF263	6	0.25368	0.45249	0.9995	8879	3	0.2708
CYP2U1	6	0.25368	0.45249	0.9995	8880	3	0.1859
CPS1	6	0.25399	0.45288	0.9995	8881	3	0.2121
PREB	6	0.25406	0.45296	0.9995	8882	3	0.128
AAK1	6	0.25408	0.45299	0.9995	8883	2	-0.587
COPRS	6	0.25409	0.453	0.9995	8884	3	0.1293
IGFBP1	6	0.2542	0.45316	0.9995	8885	2	0.0653
SALL4	6	0.25426	0.45321	0.9995	8886	3	-0.006
IL36A	6	0.25427	0.45323	0.9995	8887	2	0.0647
USP29	6	0.25427	0.45323	0.9995	8888	2	-0.155
hsa-mir-1302-5	3	0.25431	0.38052	0.9995	8889	1	0.0272
SPATA18	6	0.25433	0.4533	0.9995	8890	1	-0.116
CXCR6	6	0.25433	0.45331	0.9995	8891	3	0.2375
hsa-mir-181a-2	4	0.25434	0.39656	0.9995	8892	2	0.1244
EMC1	6	0.25434	0.45332	0.9995	8893	3	0.1469
ZDHHC23	6	0.25434	0.45332	0.9995	8894	2	0.0192
CEP290	6	0.25443	0.45344	0.9995	8895	3	0.2352
CHST8	6	0.25445	0.45345	0.9995	8896	1	-0.13
SLC6A6	6	0.2545	0.45351	0.9995	8897	3	0.2163
LRRC8B	6	0.25451	0.45352	0.9995	8898	2	-0.173
RGS22	6	0.25453	0.45354	0.9995	8899	2	0.08
CST9L	6	0.25457	0.45359	0.9995	8900	2	-0.165
NANOS3	6	0.25465	0.45368	0.9995	8901	3	0.1071
ZCCHC7	6	0.25465	0.45368	0.9995	8902	3	0.2656
ARMS2	6	0.25471	0.45376	0.9995	8903	2	-0.568
ZNF805	6	0.25471	0.45376	0.9995	8904	2	-0.48
hsa-mir-181b-1	4	0.25475	0.39695	0.9995	8905	2	-0.148
ENPP4	6	0.25475	0.4538	0.9995	8906	2	-0.062
VPS25	6	0.25482	0.4539	0.9995	8907	2	-0.36
ANGPT1	6	0.25485	0.45394	0.9995	8908	3	0.0175
SLC9A8	4	0.25486	0.39708	0.9995	8909	2	0.2428
NUP43	6	0.25494	0.45404	0.9995	8910	1	-0.19
ODAM	6	0.25496	0.45406	0.9995	8911	3	0.1553
GOLPH3	6	0.25498	0.45409	0.9995	8912	1	-0.238
hsa-mir-4274	4	0.25501	0.39723	0.9995	8913	1	0.1509
PRR15	6	0.25501	0.45413	0.9995	8914	3	0.0186
MXD3	6	0.25502	0.45414	0.9995	8915	1	-0.555
AQP9	6	0.25505	0.45418	0.9995	8916	3	0.1362
PGM5	4	0.25509	0.39731	0.9995	8917	2	0.2059
LCN8	6	0.2551	0.45424	0.9995	8918	3	0.0536
DTX1	6	0.2551	0.45424	0.9995	8919	3	0.0136
PLCG1	6	0.25511	0.45424	0.9995	8920	3	0.2011

SNTG2	6	0.25522	0.45436	0.9995	8921	1	-0.168
CAMSAP3	6	0.25524	0.45437	0.9995	8922	2	-0.195
ECHDC3	6	0.2553	0.45445	0.9995	8923	3	0.2172
hsa-mir-4320	4	0.25548	0.39772	0.9995	8924	2	0.1622
MEOX1	6	0.25553	0.45472	0.9995	8925	3	0.1423
ARHGEF37	6	0.25555	0.45475	0.9995	8926	2	0.0689
LOC391322	5	0.25559	0.44591	0.9995	8927	2	-0.173
ZNF428	6	0.25561	0.45482	0.9995	8928	3	0.1036
MFGE8	6	0.25563	0.45485	0.9995	8929	2	-0.172
SEPP1	6	0.25565	0.45488	0.9995	8930	3	0.0951
CST1	4	0.25566	0.3979	0.9995	8931	1	-0.367
ZNF133	6	0.25567	0.4549	0.9995	8932	3	0.138
LONRF2	6	0.25581	0.45508	0.9995	8933	2	0.0681
CCDC150	6	0.25588	0.45517	0.9995	8934	3	0.1951
B4GALNT4	6	0.25592	0.45521	0.9995	8935	1	-0.031
HEPACAM	6	0.25592	0.45523	0.9995	8936	3	0.1729
STT3B	6	0.25599	0.45529	0.9995	8937	3	0.2309
TANK	6	0.2561	0.45542	0.9995	8938	2	-0.058
hsa-mir-5010	4	0.25612	0.39837	0.9995	8939	2	0.1319
LOC100130301	6	0.25625	0.45561	0.9995	8940	3	0.2237
SLC6A1	6	0.25628	0.45565	0.9995	8941	2	-0.126
CCT4	6	0.25632	0.4557	0.9995	8942	2	0.0971
NTN4	6	0.25636	0.45574	0.9995	8943	2	-0.174
DSC3	6	0.2564	0.45578	0.9995	8944	3	-0.017
VWA8	6	0.2564	0.45578	0.9995	8945	3	0.188
TTC4	6	0.25652	0.45593	0.9995	8946	3	0.2497
PAFAH1B1	4	0.25661	0.3989	0.9995	8947	1	-0.019
RBPJ	6	0.25665	0.45608	0.9995	8948	3	0.1873
FADS2	6	0.25667	0.4561	0.9995	8949	3	0.0827
NEB	6	0.25669	0.45612	0.9995	8950	2	-0.169
EXOC7	6	0.25674	0.45619	0.9995	8951	3	0.1667
NOL12	6	0.25674	0.45619	0.9995	8952	3	0.1199
PECAM1	6	0.25674	0.45619	0.9995	8953	3	0.2382
IFIT1B	6	0.2568	0.45626	0.9995	8954	3	0.0508
CHPT1	6	0.25683	0.4563	0.9995	8955	2	0.0227
MRPL49	6	0.25685	0.45632	0.9995	8956	2	0.0573
FTSJ1	6	0.25697	0.45645	0.9995	8957	3	0.0706
hsa-mir-601	4	0.25704	0.39934	0.9995	8958	1	0.1636
CIAO1	6	0.25711	0.45663	0.9995	8959	3	-0.107
ASCL3	6	0.25722	0.45675	0.9995	8960	3	-0.121
PBX1	6	0.25728	0.45682	0.9995	8961	3	0.2285
MYBPC1	6	0.25732	0.45688	0.9995	8962	3	0.1588
TM4SF1	6	0.25738	0.45695	0.9995	8963	2	0.1061
SEMA6B	6	0.25743	0.457	0.9995	8964	2	-0.278
KLK5	6	0.25745	0.45701	0.9995	8965	3	0.0159
VCX3B	2	0.25747	0.30454	0.9995	8966	1	0.2605
TMPPE	6	0.25754	0.45713	0.9995	8967	2	0.055
RRAS	6	0.25757	0.45717	0.9995	8968	3	0.1443
C1orf141	6	0.25762	0.45723	0.9995	8969	2	0.0116
TAF1	6	0.25763	0.45723	0.9995	8970	3	0.2737
hsa-mir-4779	4	0.25768	0.40001	0.9995	8971	1	-1.09
GSC2	6	0.25771	0.45734	0.9995	8972	1	-0.484
DACT1	6	0.25776	0.4574	0.9995	8973	2	0.1363
GJA4	6	0.25778	0.45742	0.9995	8974	3	-7E-04
MSH2	6	0.25782	0.45746	0.9995	8975	3	0.0893
EAPP	6	0.25785	0.4575	0.9995	8976	3	0.2116
TIMELESS	6	0.25787	0.45752	0.9995	8977	3	-0.029
MBTPS1	6	0.25798	0.45765	0.9995	8978	2	0.0597
SDCCAG3	6	0.25801	0.45769	0.9995	8979	2	-0.436
RPP21	6	0.25801	0.45769	0.9995	8980	1	0.0965
ECE1	6	0.25803	0.45771	0.9995	8981	3	0.2534
ANKRD7	6	0.25811	0.4578	0.9995	8982	2	-0.174
SIL1	6	0.25811	0.4578	0.9995	8983	3	0.1767
HIST1H2BN	6	0.25811	0.4578	0.9995	8984	2	0.0174
DPPA5	6	0.25815	0.45785	0.9995	8985	3	-0.199
MTMR2	6	0.25818	0.45789	0.9995	8986	2	0.0973
PIGF	6	0.25824	0.45796	0.9995	8987	3	0.0256
CAPNS2	6	0.25824	0.45796	0.9995	8988	2	-0.528
RTKN2	6	0.25834	0.45809	0.9995	8989	3	-0.023
KLK10	6	0.25835	0.45809	0.9995	8990	2	-0.09
CASP8	6	0.2584	0.45816	0.9995	8991	3	0.0705
SAYSD1	6	0.25852	0.4583	0.9995	8992	1	-0.28
INS	4	0.25868	0.40104	0.9995	8993	1	0.1535
BTLA	6	0.25868	0.45851	0.9995	8994	1	-0.197
KIF15	6	0.25879	0.45864	0.9995	8995	2	-0.269
RNH1	6	0.25881	0.45866	0.9995	8996	3	-0.182
IRF2BPL	6	0.25884	0.45869	0.9995	8997	2	-0.196
GPER	4	0.25894	0.40131	0.9995	8998	1	-0.593
JRKL	6	0.25907	0.45896	0.9995	8999	1	-0.041
hsa-mir-5087	3	0.25924	0.38661	0.9995	9000	1	-0.44
MYO19	6	0.25925	0.45919	0.9995	9001	2	0.0401
RBM44	6	0.25926	0.45919	0.9995	9002	3	0.2043
DOHH	6	0.25935	0.45929	0.9995	9003	3	0.0811
MSTO1	6	0.25935	0.45929	0.9995	9004	3	0.3158
SIGLEC9	6	0.25935	0.4593	0.9995	9005	2	-0.263

HPSE2	6	0.25942	0.45938	0.9995	9006	3	0.2205
PLS3	6	0.25945	0.45942	0.9995	9007	2	-0.248
GLE1	6	0.25948	0.45946	0.9995	9008	2	-0.006
hsa-mir-1227	4	0.25951	0.40193	0.9995	9009	2	0.1851
NKD1	6	0.25953	0.45952	0.9995	9010	2	-0.09
SLC4A2	6	0.25954	0.45953	0.9995	9011	3	0.1802
HDX	6	0.25965	0.45967	0.9995	9012	2	-0.078
hsa-mir-340	4	0.2597	0.40213	0.9995	9013	1	-0.423
SBNO2	6	0.25974	0.45977	0.9995	9014	2	-0.246
TOMM20L	6	0.25979	0.45983	0.9995	9015	3	0.1338
FBXO42	6	0.25996	0.46003	0.9995	9016	2	-0.162
UROC1	6	0.25998	0.46006	0.9995	9017	3	0.0367
ATP11C	6	0.26003	0.46014	0.9995	9018	2	0.1732
USP16	6	0.26006	0.46016	0.9995	9019	3	0.2135
APOA1BP	6	0.26008	0.46019	0.9995	9020	3	0.1977
BLID	6	0.26008	0.46019	0.9995	9021	3	0.1923
CDK5RAP1	6	0.26012	0.46024	0.9995	9022	2	0.1129
CLASP1	6	0.26019	0.46031	0.9995	9023	3	0.2066
SNX25	6	0.26023	0.46037	0.9995	9024	2	-0.004
PTGFR	6	0.26033	0.46049	0.9995	9025	2	0.0685
ELFN1	6	0.26041	0.4606	0.9995	9026	3	0.0724
CEMP1	6	0.26042	0.46062	0.9995	9027	1	-0.315
RNF183	6	0.26046	0.46065	0.9995	9028	2	-0.027
hsa-mir-4687	4	0.26052	0.40294	0.9995	9029	2	0.1921
CXXC4	6	0.26055	0.46075	0.9995	9030	1	-0.029
URB1	6	0.26056	0.46077	0.9995	9031	3	0.0867
CD3G	6	0.2607	0.46095	0.9995	9032	3	0.067
MAS1	6	0.26071	0.46096	0.9995	9033	2	-0.084
FREM1	6	0.26077	0.46103	0.9995	9034	3	0.1869
TFAP2E	6	0.26077	0.46103	0.9995	9035	3	0.2104
hsa-mir-3674	4	0.26085	0.40329	0.9995	9036	2	0.2413
PI16	6	0.26091	0.46121	0.9995	9037	3	0.2242
hsa-mir-4269	4	0.26092	0.40336	0.9995	9038	2	0.2306
hsa-mir-5694	4	0.26094	0.40338	0.9995	9039	1	-0.096
PPARG	6	0.26094	0.46124	0.9995	9040	2	0.0936
CEP170B	6	0.261	0.46132	0.9995	9041	3	0.067
HUNK	6	0.26103	0.46135	0.9995	9042	3	0.0902
DERL1	6	0.26103	0.46135	0.9995	9043	3	0.2679
TSEN54	4	0.26107	0.40352	0.9995	9044	1	-0.431
DUS3L	6	0.26108	0.46141	0.9995	9045	2	-0.086
DPPA2	6	0.26112	0.46145	0.9995	9046	3	0.0624
MFSD11	6	0.26119	0.46156	0.9995	9047	3	0.1492
POC1B-GALNT4	1	0.26122	0.26112	0.9995	9048	1	0.5331
CD320	6	0.26128	0.46165	0.9995	9049	2	-0.104
GNS	6	0.26128	0.46165	0.9995	9050	2	0.1094
PLIN3	6	0.26134	0.46171	0.9995	9051	3	-0.094
KIF2C	6	0.26144	0.46182	0.9995	9052	2	0.2149
DEFB121	6	0.26148	0.46189	0.9995	9053	3	0.2058
PANK4	6	0.2615	0.4619	0.9995	9054	1	-0.185
WDR45B	6	0.26158	0.462	0.9995	9055	2	-0.109
XRCC6	6	0.26158	0.462	0.9995	9056	2	-0.026
HDAC9	6	0.26168	0.46212	0.9995	9057	1	-0.411
CASC5	6	0.26176	0.46221	0.9995	9058	2	-0.004
FBXO40	6	0.2618	0.46225	0.9995	9059	2	-0.211
RNASEK	6	0.26182	0.46227	0.9995	9060	2	0.0915
TTF2	6	0.26186	0.46232	0.9995	9061	3	-0.14
hsa-mir-498	4	0.26188	0.40433	0.9995	9062	1	-0.17
KRT33A	6	0.26192	0.46238	0.9995	9063	2	-0.153
hsa-mir-3174	4	0.26194	0.40439	0.9995	9064	2	0.2291
SLC5A10	6	0.26194	0.4624	0.9995	9065	3	0.3279
hsa-mir-4427	4	0.26197	0.40443	0.9995	9066	2	0.3297
COPS3	6	0.262	0.46246	0.9995	9067	3	-0.122
ATXN7L3B	6	0.26202	0.46249	0.9995	9068	2	-0.161
CD3E	6	0.26207	0.46255	0.9995	9069	2	-0.256
NKG7	6	0.26208	0.46257	0.9995	9070	2	-0.244
ECT2	6	0.26218	0.46268	0.9995	9071	2	-0.136
OSR2	6	0.26218	0.46268	0.9995	9072	2	-0.55
EML5	6	0.2622	0.4627	0.9995	9073	3	0.0509
IFNA7	6	0.26225	0.46278	0.9995	9074	2	0.2936
PHF20L1	6	0.26225	0.46278	0.9995	9075	2	-0.218
USP45	6	0.26229	0.46281	0.9995	9076	1	-0.316
LHFPL4	6	0.26232	0.46285	0.9995	9077	3	0.1336
NUTM1	6	0.26232	0.46285	0.9995	9078	3	0.0405
CCDC25	6	0.26233	0.46286	0.9995	9079	2	-0.081
GPSM3	6	0.26242	0.46298	0.9995	9080	3	0.1243
RPL21	6	0.26251	0.46308	0.9995	9081	1	-0.352
POLDIP2	6	0.26254	0.46312	0.9995	9082	3	0.1226
hsa-mir-6886	2	0.26258	0.30833	0.9995	9083	1	-0.305
CERK	6	0.26261	0.4632	0.9995	9084	1	-0.345
hsa-mir-29c	4	0.26269	0.40517	0.9995	9085	1	-0.003
SDR42E1	6	0.26277	0.46338	0.9995	9086	2	-0.259
ETV3L	6	0.26279	0.46342	0.9995	9087	1	0.0288
B3GNT6	6	0.26282	0.46346	0.9995	9088	2	-0.316
SMC5	6	0.26284	0.46348	0.9995	9089	3	0.2382
ZYG11A	6	0.26293	0.46359	0.9995	9090	1	-0.126

FAT2	6	0.26297	0.46363	0.9995	9091	3	0.1177
ANKS1B	6	0.26299	0.46365	0.9995	9092	2	0.0701
hsa-mir-4657	4	0.26314	0.40565	0.9995	9093	2	0.292
TMEM107	6	0.26315	0.46384	0.9995	9094	2	-0.137
SYNPO2L	6	0.26322	0.46393	0.9995	9095	2	0.0186
TSSK1B	6	0.2633	0.46401	0.9995	9096	3	0.2043
JAK1	6	0.26331	0.46403	0.9995	9097	3	0.0584
ACBD5	6	0.26332	0.46404	0.9995	9098	2	-0.091
LEPREL4	6	0.26338	0.4641	0.9995	9099	1	-0.305
C12orf52	6	0.26338	0.4641	0.9995	9100	3	0.2145
MYO6	6	0.26344	0.46418	0.9995	9101	3	0.156
ZNF630	6	0.26354	0.46429	0.9995	9102	2	-0.301
CER1	6	0.26363	0.46439	0.9995	9103	3	0.0353
SHPK	6	0.26375	0.46455	0.9995	9104	3	0.1061
METRN	6	0.26386	0.46469	0.9995	9105	1	-0.303
ZNF30	6	0.26388	0.46471	0.9995	9106	2	0.1162
PDE1A	6	0.26388	0.46472	0.9995	9107	3	0.0463
BRE	6	0.26399	0.46486	0.9995	9108	3	0.1311
LYSMD3	6	0.26401	0.46487	0.9995	9109	2	-0.108
NCK1	6	0.26408	0.46496	0.9995	9110	2	-0.071
MYH3	6	0.26408	0.46496	0.9995	9111	3	0.2376
ATCAY	6	0.26408	0.46496	0.9995	9112	3	0.3185
SIPA1L1	6	0.26412	0.46501	0.9995	9113	2	-0.151
KIAA0930	6	0.26426	0.46519	0.9995	9114	2	0.0595
RIMS2	6	0.26442	0.46537	0.9995	9115	3	0.2062
ALG14	6	0.26446	0.46543	0.9995	9116	2	-0.1
PAQR9	6	0.26455	0.46552	0.9995	9117	1	-0.322
TLR3	6	0.26455	0.46552	0.9995	9118	2	-0.502
CLIC4	6	0.26457	0.46555	0.9995	9119	3	0.1696
GPR174	6	0.2646	0.46557	0.9995	9120	2	0.16
KCTD20	6	0.26461	0.46558	0.9995	9121	2	-0.051
ABCC3	6	0.2647	0.46569	0.9995	9122	2	-0.293
SH3GLB2	6	0.26471	0.4657	0.9995	9123	2	-0.19
RRAGC	6	0.26477	0.46577	0.9995	9124	1	0.0547
RFPL3	6	0.26483	0.46585	0.9995	9125	2	-0.584
GCAT	6	0.26487	0.46589	0.9995	9126	3	0.1485
MEPE	6	0.26488	0.46591	0.9995	9127	2	-0.042
C3orf20	6	0.26493	0.46597	0.9995	9128	2	-0.209
BAZ2B	6	0.26494	0.46598	0.9995	9129	2	-0.296
hsa-mir-642a	1	0.26498	0.26485	0.9995	9130	1	0.2657
AP5Z1	6	0.26511	0.46617	0.9995	9131	2	0.0504
LRCH2	6	0.26514	0.4662	0.9995	9132	3	0.1155
CUL4B	6	0.26515	0.46621	0.9995	9133	2	-0.103
CCL13	6	0.26525	0.46633	0.9995	9134	3	-0.066
NUS1	6	0.26529	0.46638	0.9995	9135	3	0.3466
RGPD1	4	0.26532	0.40786	0.9995	9136	2	0.4266
DSTN	6	0.26535	0.46644	0.9995	9137	2	-0.102
SLITRK2	6	0.26541	0.46652	0.9995	9138	3	-0.063
ZNF292	6	0.26541	0.46652	0.9995	9139	2	0.1486
hsa-mir-4727	4	0.26543	0.40796	0.9995	9140	2	0.0507
hsa-mir-429	4	0.26543	0.40796	0.9995	9141	2	0.1029
SPSB3	6	0.26543	0.46654	0.9995	9142	3	0.0396
DCUN1D3	6	0.26549	0.46662	0.9995	9143	2	0.094
TRIM32	6	0.2656	0.46674	0.9995	9144	1	-0.54
HIST1H2AC	6	0.2656	0.46674	0.9995	9145	2	-0.335
GAB1	6	0.26567	0.46684	0.9995	9146	2	0.0748
ENGASE	6	0.26572	0.46689	0.9995	9147	2	0.0426
PNISR	6	0.26573	0.4669	0.9995	9148	3	0.3083
GEM	6	0.26579	0.46697	0.9995	9149	3	0.2652
TMEM106B	6	0.26583	0.46703	0.9995	9150	3	-0.046
CTDSP1	6	0.26598	0.46721	0.9995	9151	2	-0.043
hsa-mir-3907	4	0.26607	0.40858	0.9995	9152	2	0.1574
WDR11	6	0.26612	0.46737	0.9995	9153	2	-0.063
AARS	6	0.2663	0.46756	0.9995	9154	3	0.1326
FAM83F	6	0.26632	0.46758	0.9995	9155	2	-0.029
MROH1	6	0.26635	0.46761	0.9995	9156	3	-0.093
ZAK	6	0.26638	0.46765	0.9995	9157	3	0.2263
LGALS14	6	0.2664	0.46768	0.9995	9158	3	0.1816
ADAMTS16	6	0.26646	0.46774	0.9995	9159	2	-4E-04
BTF3	6	0.2665	0.46779	0.9995	9160	2	0.0342
LY86	6	0.2665	0.4678	0.9995	9161	3	0.1367
NFKBID	6	0.26656	0.46786	0.9995	9162	3	0.1852
UQCRC1	6	0.26656	0.46786	0.9995	9163	2	-0.033
CX3CR1	6	0.26661	0.46791	0.9995	9164	3	0.068
FSCB	6	0.26676	0.46809	0.9995	9165	2	-0.079
RRM2	6	0.26678	0.46811	0.9995	9166	3	0.3634
ZC3H12B	6	0.2669	0.46825	0.9995	9167	3	0.1302
GLCCI1	6	0.2669	0.46825	0.9995	9168	3	0.0573
SPDYE6	1	0.26695	0.26687	0.9995	9169	1	0.4048
C22orf29	6	0.26695	0.46831	0.9995	9170	2	0.1073
ERRFI1	6	0.26695	0.46831	0.9995	9171	3	-0.134
ADAM17	6	0.26699	0.46836	0.9995	9172	2	-0.205
HLA-DQB2	6	0.267	0.46838	0.9995	9173	2	0.2231
HYOU1	6	0.26718	0.4686	0.9995	9174	2	-0.107
SLC6A15	6	0.26719	0.4686	0.9995	9175	3	0.2111

hsa-mir-378h	4	0.2672	0.40971	0.9995	9176	1	-0.117
TXLNG	6	0.26725	0.46868	0.9995	9177	2	-0.1
SALL2	6	0.26729	0.46873	0.9995	9178	1	-0.518
ECM2	6	0.26738	0.46884	0.9995	9179	3	0.2891
PRAC	4	0.26743	0.40993	0.9995	9180	2	-0.209
DPCD	6	0.26745	0.46893	0.9995	9181	1	-0.139
OR11H6	6	0.26747	0.46895	0.9995	9182	2	0.1026
TJP2	6	0.26747	0.46895	0.9995	9183	2	-0.028
UNC79	6	0.26755	0.46905	0.9995	9184	2	-0.099
YPEL1	6	0.26757	0.46907	0.9995	9185	1	-0.301
CTNNB1	6	0.26761	0.46912	0.9995	9186	1	-0.127
hsa-mir-3687	4	0.26769	0.41019	0.9995	9187	2	0.448
RPL36AL	6	0.26769	0.46921	0.9995	9188	2	-0.192
OSBP	6	0.26773	0.46927	0.9995	9189	2	-0.153
KL	6	0.26781	0.46937	0.9995	9190	3	0.2612
C10orf55	3	0.2679	0.39713	0.9995	9191	1	-0.205
hsa-mir-5572	4	0.26791	0.4104	0.9995	9192	2	0.2411
COX7A2	6	0.26792	0.46951	0.9995	9193	3	0.111
TSTD3	6	0.26792	0.46951	0.9995	9194	3	0.2145
MRPL16	6	0.26793	0.46952	0.9995	9195	1	0.1532
ZNF329	6	0.26806	0.46968	0.9995	9196	2	0.0551
OR6A2	6	0.26809	0.46972	0.9995	9197	3	0.2221
STARD8	6	0.26811	0.46974	0.9995	9198	3	0.1939
C3orf36	6	0.26817	0.46981	0.9995	9199	3	-0.036
IDH1	6	0.26818	0.46982	0.9995	9200	3	0.1099
FAM150A	6	0.26831	0.46998	0.9995	9201	2	-0.384
TNPO3	6	0.26842	0.47011	0.9995	9202	3	0.1658
TCTEX1D2	6	0.26851	0.47024	0.9995	9203	3	0.2182
PMEL	6	0.26855	0.47028	0.9995	9204	2	0.062
UBE2L6	6	0.26862	0.47037	0.9995	9205	3	0.2144
DHRS11	6	0.26866	0.47041	0.9995	9206	2	-0.101
MYT1	6	0.26866	0.47041	0.9995	9207	2	0.0305
RNASE1	6	0.26869	0.47045	0.9995	9208	3	0.1682
IDE	6	0.26877	0.47055	0.9995	9209	2	-0.186
NMRK1	6	0.26883	0.47061	0.9995	9210	3	0.1692
POLR2K	6	0.26893	0.47071	0.9995	9211	3	0.2576
IQCF3	6	0.26893	0.47071	0.9995	9212	3	0.3374
L3MBTL2	6	0.26919	0.47102	0.9995	9213	2	-0.034
KLK15	6	0.26925	0.47109	0.9995	9214	1	-0.54
FAM5B	4	0.26925	0.41177	0.9995	9215	2	0.1843
MATN2	6	0.26926	0.4711	0.9995	9216	3	0.0367
ADAM11	6	0.26926	0.4711	0.9995	9217	3	0.194
PTPN4	6	0.26931	0.47116	0.9995	9218	2	-0.196
ARL6	6	0.26932	0.47117	0.9995	9219	2	-0.129
MCM9	6	0.26935	0.47121	0.9995	9220	2	-0.01
FMO5	6	0.26935	0.47121	0.9995	9221	2	0.1407
ZNF254	4	0.2694	0.41192	0.9995	9222	1	-0.211
ZBED4	6	0.26962	0.47153	0.9995	9223	2	0.0977
MCUR1	6	0.26965	0.47156	0.9995	9224	3	0.2734
SLC22A18	6	0.26973	0.47167	0.9995	9225	2	-0.201
AIF1	6	0.26977	0.47171	0.9995	9226	3	-0.245
CCSAP	6	0.26979	0.47173	0.9995	9227	2	0.1545
SLCO1C1	6	0.26981	0.47176	0.9995	9228	2	0.0561
hsa-mir-4751	4	0.26986	0.4124	0.9995	9229	2	0.0033
CLDN6	6	0.26992	0.47189	0.9995	9230	3	0.0406
NAT1	6	0.26993	0.47191	0.9995	9231	2	-0.054
OR10G3	6	0.26996	0.47193	0.9995	9232	3	0.1518
ADAM8	6	0.26999	0.47197	0.9995	9233	3	0.1283
hsa-mir-5191	4	0.27012	0.41268	0.9995	9234	2	-0.211
PRAMEF17	2	0.27012	0.31392	0.9995	9235	1	-0.299
RBP4	6	0.27016	0.47215	0.9995	9236	1	-0.269
GEMIN6	6	0.27026	0.47225	0.9995	9237	2	0.0826
GTF2E2	6	0.27027	0.47228	0.9995	9238	2	-0.789
FIGLA	6	0.27027	0.47228	0.9995	9239	2	-0.239
DCST1	6	0.27042	0.47244	0.9995	9240	1	-0.244
HERC4	6	0.27044	0.47247	0.9995	9241	3	0.2212
hsa-mir-5187	4	0.27054	0.4131	0.9995	9242	2	0.3323
GFOD2	6	0.27056	0.47259	0.9995	9243	2	-0.099
LUC7L2	3	0.27059	0.40051	0.9995	9244	2	0.2637
DHX8	6	0.27059	0.47263	0.9995	9245	3	0.1798
RAB33A	6	0.27067	0.47274	0.9995	9246	2	0.0739
E2F5	6	0.27072	0.47279	0.9995	9247	3	0.0006
PHKG1	6	0.27073	0.4728	0.9995	9248	2	-0.037
GSTA3	6	0.27078	0.47286	0.9995	9249	1	0.0846
ICAM1	6	0.27078	0.47286	0.9995	9250	2	-0.074
NOTCH2	6	0.27078	0.47286	0.9995	9251	1	-0.251
OR4D11	6	0.27079	0.47287	0.9995	9252	2	-0.059
ACAT2	6	0.27079	0.47287	0.9995	9253	3	0.2055
HDGFRP3	6	0.27085	0.47294	0.9995	9254	3	-0.081
GLCE	6	0.27089	0.47301	0.9995	9255	2	-0.203
hsa-mir-4419b	4	0.27097	0.41355	0.9995	9256	2	0.4147
hsa-mir-622	4	0.27104	0.41361	0.9995	9257	2	0.1007
LRRN3	6	0.27105	0.47319	0.9995	9258	3	0.1662
RAB31	6	0.27111	0.47326	0.9995	9259	3	-0.016
TMEM151B	6	0.27114	0.47329	0.9995	9260	1	-0.08

SALL3	6	0.27116	0.47332	0.9995	9261	3	0.0729
FAM218A	6	0.27122	0.47338	0.9995	9262	2	0.1234
PLEKHM2	6	0.27124	0.47341	0.9995	9263	3	0.1697
FAM151A	6	0.2713	0.47347	0.9995	9264	2	-0.096
DESI2	6	0.27134	0.47351	0.9995	9265	2	0.0528
KAAG1	5	0.27138	0.45865	0.9995	9266	2	0.0379
WDR36	6	0.2714	0.47358	0.9995	9267	2	-0.241
SRP14	6	0.27157	0.47378	0.9995	9268	3	0.2208
VN1R2	6	0.27159	0.47379	0.9995	9269	3	0.056
SPRYD7	6	0.27162	0.47382	0.9995	9270	1	-0.228
MKRN2	6	0.27168	0.47389	0.9995	9271	2	0.0017
MAP7D1	6	0.27168	0.47389	0.9995	9272	3	0.1152
YWHAE	6	0.27174	0.47397	0.9995	9273	3	0.1867
GRM3	6	0.27174	0.47397	0.9995	9274	3	0.005
MAN1C1	6	0.27178	0.47401	0.9995	9275	2	0.0949
POLD1	6	0.27179	0.47402	0.9995	9276	2	0.0178
MAGI1	6	0.27197	0.47423	0.9995	9277	3	0.1382
CTXN1	6	0.27204	0.47429	0.9995	9278	1	-0.283
ZZZ3	6	0.27204	0.47429	0.9995	9279	2	-0.096
SHH	6	0.27209	0.47436	0.9995	9280	3	0.1354
ACSF2	6	0.2721	0.47436	0.9995	9281	1	-1.048
ZFHX4	6	0.27214	0.47443	0.9995	9282	3	0.1977
HMSD	6	0.2723	0.4746	0.9995	9283	2	-0.228
ZNF700	5	0.27239	0.45944	0.9995	9284	2	0.1121
VPS54	6	0.27241	0.47474	0.9995	9285	3	0.1442
GTSF1L	6	0.27246	0.4748	0.9995	9286	3	0.1462
GPR85	6	0.2726	0.47496	0.9995	9287	2	0.049
JPH3	6	0.27262	0.47499	0.9995	9288	3	0.1152
GBP7	6	0.27267	0.47505	0.9995	9289	3	-0.154
TAS2R5	6	0.27269	0.47507	0.9995	9290	3	0.1068
PSEN1	6	0.27269	0.47508	0.9995	9291	2	0.02
NMNAT1	6	0.27272	0.47511	0.9995	9292	1	-0.292
ADRA1A	6	0.27275	0.47514	0.9995	9293	2	-0.045
C10orf76	6	0.2728	0.47521	0.9995	9294	3	0.1829
OR1A2	6	0.27281	0.47521	0.9995	9295	2	0.2332
FUNDC1	6	0.27288	0.4753	0.9995	9296	3	0.117
TTC25	6	0.273	0.47543	0.9995	9297	2	-0.377
UEVLD	6	0.27302	0.47545	0.9995	9298	1	-0.209
NR1D2	6	0.27305	0.47549	0.9995	9299	3	0.1764
hsa-mir-4535	4	0.27306	0.4157	0.9995	9300	2	0.2338
TAF5L	6	0.27306	0.4755	0.9995	9301	1	-0.304
PEA15	6	0.2731	0.47554	0.9995	9302	3	0.2076
HSPB7	6	0.27313	0.47559	0.9995	9303	2	0.1828
P2RY6	6	0.27314	0.47559	0.9995	9304	3	0.3182
FBXO30	6	0.27314	0.47559	0.9995	9305	3	-0.002
KRTAP5-1	6	0.27319	0.47566	0.9995	9306	3	0.3205
SPEF1	6	0.27324	0.47571	0.9995	9307	2	-0.172
YIPF4	6	0.27328	0.47576	0.9995	9308	2	0.0592
DPYSL5	6	0.27329	0.47578	0.9995	9309	2	-0.069
UBE2D3	6	0.27331	0.4758	0.9995	9310	3	0.3914
DAP3	6	0.27333	0.47582	0.9995	9311	3	0.1383
SHANK1	6	0.27348	0.47599	0.9995	9312	3	-0.09
MDH1B	6	0.27357	0.4761	0.9995	9313	3	0.1106
NODAL	6	0.27358	0.47611	0.9995	9314	2	-0.119
NSMCE4A	6	0.27358	0.47611	0.9995	9315	2	-0.168
ALDOB	6	0.27363	0.47617	0.9995	9316	3	0.1969
SON	6	0.27365	0.47619	0.9995	9317	2	-0.074
AMT	6	0.27365	0.47619	0.9995	9318	1	0.0266
KRT39	6	0.2737	0.47625	0.9995	9319	3	-0.048
USP28	6	0.27374	0.4763	0.9995	9320	2	-0.213
ZSCAN4	6	0.2738	0.47635	0.9995	9321	3	0.0055
GOLGA7B	6	0.27381	0.47639	0.9995	9322	3	0.0807
ZDHHC20	6	0.27381	0.47639	0.9995	9323	1	0.0622
SPEF2	6	0.27381	0.47639	0.9995	9324	2	0.1122
NES	6	0.27388	0.47646	0.9995	9325	2	-0.043
STIM1	6	0.27389	0.47647	0.9995	9326	3	0.0921
VAV2	6	0.27397	0.47656	0.9995	9327	1	-0.149
ITGB4	6	0.27398	0.47656	0.9995	9328	3	0.1545
ICA1	6	0.27398	0.47656	0.9995	9329	3	0.1097
C9orf41	6	0.27398	0.47656	0.9995	9330	3	0.2232
OR5M8	6	0.27398	0.47656	0.9995	9331	3	0.0931
EIF4G3	6	0.27399	0.47658	0.9995	9332	2	0.07
FHOD1	6	0.2741	0.47673	0.9995	9333	3	0.1459
PTCD2	6	0.27411	0.47674	0.9995	9334	2	-0.018
hsa-mir-375	4	0.27416	0.41682	0.9995	9335	1	-0.273
hsa-mir-4691	4	0.27416	0.41682	0.9995	9336	1	-0.166
GLUD1	6	0.27416	0.4768	0.9995	9337	3	0.1852
PDCD7	6	0.27424	0.4769	0.9995	9338	2	-0.135
SNX6	6	0.27425	0.47691	0.9995	9339	3	-0.088
ARHGAP17	6	0.27425	0.47691	0.9995	9340	3	0.1637
hsa-mir-1911	4	0.27427	0.41693	0.9995	9341	1	-0.632
C5orf49	6	0.27429	0.47696	0.9995	9342	3	0.1764
COL20A1	6	0.27432	0.47699	0.9995	9343	3	0.1284
RCBTB2	6	0.27435	0.47702	0.9995	9344	3	0.1186
hsa-mir-5193	4	0.27439	0.41706	0.9995	9345	2	-0.029

hsa-mir-6774	4	0.2745	0.41715	0.9995	9346	2	-0.03
KCNRG	6	0.27453	0.47724	0.9995	9347	2	-0.493
FEM1B	6	0.27454	0.47725	0.9995	9348	3	0.2672
PYGO1	6	0.27454	0.47725	0.9995	9349	3	0.1285
BARHL2	6	0.27456	0.47728	0.9995	9350	2	-0.195
VPS29	6	0.27459	0.47731	0.9995	9351	3	0.1599
SHC3	6	0.27462	0.47734	0.9995	9352	3	0.2176
CRYBB3	6	0.27466	0.47738	0.9995	9353	3	0.1557
CDHR3	6	0.27467	0.47741	0.9995	9354	3	0.1832
RCOR2	6	0.27469	0.47743	0.9995	9355	2	0.1202
hsa-mir-20b	4	0.27479	0.41746	0.9995	9356	2	0.1713
FAM102B	6	0.27481	0.47756	0.9995	9357	3	0.191
WDR87	6	0.27487	0.47763	0.9995	9358	2	0.1565
SEPN1	6	0.27496	0.47773	0.9995	9359	2	0.0011
UBALD2	6	0.27504	0.47783	0.9995	9360	3	0.1445
WDYHV1	6	0.27508	0.47788	0.9995	9361	2	-0.12
FKBP14	6	0.2751	0.4779	0.9995	9362	2	-0.03
ATP6V0E1	6	0.27511	0.47791	0.9995	9363	2	-0.05
PLEKHA5	6	0.27516	0.47798	0.9995	9364	2	0.052
PIP	6	0.27523	0.47805	0.9995	9365	3	0.0577
hsa-mir-4537	4	0.27523	0.41791	0.9995	9366	1	-0.195
PLSCR5	6	0.27531	0.47814	0.9995	9367	3	0.1806
hsa-mir-6773	4	0.27538	0.41804	0.9995	9368	2	0.0194
UFD1L	6	0.27538	0.47823	0.9995	9369	2	-0.192
OR13H1	6	0.27545	0.47831	0.9995	9370	3	-0.037
LOC100506422	6	0.27545	0.47831	0.9995	9371	1	-0.562
hsa-mir-7641-2	1	0.27549	0.27544	0.9995	9372	1	0.2842
SLC6A13	6	0.27557	0.47845	0.9995	9373	1	0.0535
KATNBL1	6	0.2756	0.47848	0.9995	9374	3	0.2805
BID	6	0.2756	0.47848	0.9995	9375	3	0.1813
KCNA7	6	0.2756	0.47848	0.9995	9376	3	-0.025
C10orf118	6	0.27561	0.4785	0.9995	9377	2	-0.16
RXFP1	6	0.27571	0.47862	0.9995	9378	3	-0.083
PTN	6	0.27581	0.47874	0.9995	9379	3	0.1153
AGTPBP1	6	0.27587	0.47881	0.9995	9380	3	0.1993
CC2D2B	6	0.27597	0.47892	0.9995	9381	3	0.0514
TRIP6	6	0.27601	0.47896	0.9995	9382	2	-0.066
SMARCAD1	6	0.27609	0.47905	0.9995	9383	2	0.2584
TAC3	6	0.27628	0.47928	0.9995	9384	2	-0.24
MYL6	6	0.2763	0.47931	0.9995	9385	3	-0.163
XKR7	6	0.27633	0.47934	0.9995	9386	3	0.1115
hsa-mir-4305	4	0.27642	0.41917	0.9995	9387	2	0.3148
SMEK2	6	0.27644	0.47946	0.9995	9388	2	-0.119
hsa-mir-4732	4	0.27648	0.41923	0.9995	9389	2	0.1455
CCNL2	6	0.27648	0.47951	0.9995	9390	2	-0.161
FAM179B	6	0.27656	0.4796	0.9995	9391	2	0.0468
KLRC1	6	0.27658	0.47963	0.9995	9392	3	-0.038
RUNX1	6	0.2766	0.47965	0.9995	9393	2	-0.042
GIP	6	0.27665	0.47971	0.9995	9394	3	0.2427
PLEKHG1	5	0.27668	0.46293	0.9995	9395	2	0.0832
LOC100130370	6	0.2767	0.47977	0.9995	9396	3	0.0095
PNPLA4	6	0.27676	0.47984	0.9995	9397	1	-0.497
hsa-mir-1537	3	0.27687	0.40825	0.9995	9398	1	-0.607
ATP1B4	6	0.27691	0.48001	0.9995	9399	3	0.1651
CLEC6A	6	0.27696	0.48007	0.9995	9400	2	0.0917
AASDH	6	0.27696	0.48008	0.9995	9401	2	0.2134
PRSS3	6	0.27696	0.48008	0.9995	9402	2	-0.11
OR52E2	6	0.27702	0.48014	0.9995	9403	3	0.1556
ACBD7	6	0.27704	0.48016	0.9995	9404	2	0.1311
NECAB1	6	0.27704	0.48016	0.9995	9405	2	-0.527
HSP90AB1	6	0.27705	0.48018	0.9995	9406	3	0.3555
CCNDBP1	6	0.27705	0.48018	0.9995	9407	2	-0.307
RPL10L	6	0.2771	0.48023	0.9995	9408	3	0.134
hsa-mir-4478	4	0.27711	0.41988	0.9995	9409	2	0.1542
ST18	6	0.27712	0.48025	0.9995	9410	3	0.1275
CCDC69	6	0.27724	0.4804	0.9995	9411	3	0.0351
RYR3	6	0.27728	0.48044	0.9995	9412	2	-0.138
ATF6	6	0.27729	0.48045	0.9995	9413	3	0.0957
hsa-mir-28	4	0.27738	0.42017	0.9995	9414	2	0.0037
EDA	6	0.27743	0.48063	0.9995	9415	3	-0.006
SLC31A1	6	0.27762	0.48085	0.9995	9416	2	-0.187
PIK3R1	6	0.27766	0.4809	0.9995	9417	3	0.1779
C19orf82	3	0.27768	0.40922	0.9995	9418	1	-0.24
ARHGAP35	6	0.27776	0.48102	0.9995	9419	3	0.1626
SH2B3	6	0.2778	0.48105	0.9995	9420	2	-0.066
CLDN3	6	0.2778	0.48106	0.9995	9421	2	-0.313
AGPS	6	0.27785	0.48111	0.9995	9422	3	0.2557
hsa-mir-6788	4	0.27802	0.42086	0.9995	9423	1	-0.612
ROBO4	6	0.27803	0.48134	0.9995	9424	2	-0.191
C19orf66	6	0.27807	0.48138	0.9995	9425	1	-0.128
NOVA2	6	0.27815	0.48148	0.9995	9426	3	0.1065
RAMP3	6	0.27816	0.48149	0.9995	9427	3	0.2002
TNFRSF10C	6	0.2782	0.48153	0.9995	9428	2	-0.261
FLJ22184	6	0.27823	0.48157	0.9995	9429	3	0.2129
C1orf233	6	0.27831	0.48166	0.9995	9430	1	-0.675

NT5M	6	0.27843	0.4818	0.9995	9431	2	-0.249
FBXO36	6	0.27844	0.48181	0.9995	9432	2	0.0232
LCN6	6	0.27844	0.48181	0.9995	9433	2	-0.044
SPIB	6	0.27851	0.48191	0.9995	9434	2	-0.014
GPR32	6	0.27854	0.48194	0.9995	9435	3	-0.004
S100A4	6	0.2786	0.482	0.9995	9436	3	0.249
CENPC	2	0.27861	0.32037	0.9995	9437	1	-0.117
DDX10	6	0.27863	0.48203	0.9995	9438	2	0.0858
SPATC1L	6	0.27866	0.48206	0.9995	9439	2	-0.038
AGAP9	2	0.27879	0.32051	0.9995	9440	1	-0.572
ZBTB10	6	0.27879	0.48222	0.9995	9441	2	0.0394
TCTEX1D4	6	0.27885	0.48229	0.9995	9442	1	-0.198
HTR3A	6	0.27886	0.48231	0.9995	9443	3	0.1542
STK32C	6	0.27891	0.48235	0.9995	9444	1	-0.583
PRR23C	6	0.27895	0.48241	0.9995	9445	3	0.0089
MAPK7	6	0.27905	0.48254	0.9995	9446	3	0.2581
GTF2H2	2	0.27912	0.32075	0.9995	9447	1	0.2915
ARNTL2	6	0.27912	0.48261	0.9995	9448	3	0.0884
GGTLC2	6	0.27914	0.48264	0.9995	9449	1	0.112
ACTR5	6	0.27922	0.48273	0.9995	9450	2	-0.032
IL1RL2	6	0.27922	0.48273	0.9995	9451	2	0.0554
GPR108	6	0.27927	0.48278	0.9995	9452	3	0.2382
hsa-mir-6720	4	0.27935	0.42225	0.9995	9453	1	-0.62
ITPR3	6	0.27944	0.48298	0.9995	9454	2	0.0973
BNIP3L	6	0.27946	0.483	0.9995	9455	1	-0.002
IL6R	6	0.27946	0.483	0.9995	9456	1	-0.45
ADAM28	6	0.27969	0.48327	0.9995	9457	2	-0.448
JAK3	6	0.27977	0.48337	0.9995	9458	2	0.0048
MTERFD1	6	0.27992	0.48355	0.9995	9459	3	-0.05
EIF2AK3	6	0.27996	0.48359	0.9995	9460	1	-0.117
GAS7	6	0.28004	0.48368	0.9995	9461	3	0.2365
NAV1	6	0.28004	0.48368	0.9995	9462	3	0.2862
PDLIM1	6	0.28008	0.48373	0.9995	9463	1	-0.15
DEFB132	6	0.28009	0.48374	0.9995	9464	3	-0.091
hsa-mir-4772	3	0.2801	0.4122	0.9995	9465	2	0.3719
SH3YL1	6	0.28014	0.48378	0.9995	9466	2	0.1956
SAPCD1	6	0.28014	0.48379	0.9995	9467	2	-0.255
RTP1	6	0.28022	0.48388	0.9995	9468	2	0.1326
hsa-mir-5100	4	0.28022	0.42312	0.9995	9469	2	0.0476
KAT2A	6	0.28022	0.48388	0.9995	9470	3	-0.049
SETD2	6	0.28022	0.48388	0.9995	9471	2	-0.058
37500	3	0.28026	0.41238	0.9995	9472	1	-1.102
TEAD4	6	0.28028	0.48397	0.9995	9473	2	-0.195
NKX2-1	6	0.28029	0.48398	0.9995	9474	2	-0.21
TXNDC15	6	0.28033	0.48402	0.9995	9475	2	-0.137
KIAA1210	6	0.28041	0.48412	0.9995	9476	3	0.2201
GPR33	6	0.28041	0.48412	0.9995	9477	1	-0.497
PYHIN1	6	0.28044	0.48415	0.9995	9478	3	0.054
CD1A	6	0.28045	0.48417	0.9995	9479	2	-0.305
TXK	6	0.28045	0.48417	0.9995	9480	2	-0.381
PEPD	6	0.28051	0.48423	0.9995	9481	2	0.029
POLR2M	6	0.28051	0.48423	0.9995	9482	2	-0.283
hsa-mir-6789	4	0.28055	0.42347	0.9995	9483	2	0.3672
GNAI2	6	0.28057	0.4843	0.9995	9484	3	-0.25
hsa-mir-4646	4	0.28059	0.42352	0.9995	9485	1	-0.581
TEX11	6	0.28065	0.48439	0.9995	9486	3	0.0088
hsa-mir-3182	2	0.28077	0.32201	0.9995	9487	1	0.3127
CSF2RB	6	0.28077	0.48454	0.9995	9488	2	0.0847
PWWP2A	6	0.2808	0.48457	0.9995	9489	2	-0.544
CCDC130	6	0.28089	0.48466	0.9995	9490	1	-0.209
PREP	6	0.28092	0.48469	0.9995	9491	3	0.1433
SHCBP1	6	0.28093	0.48471	0.9995	9492	3	0.1743
TMEM214	6	0.28098	0.48477	0.9995	9493	2	-0.492
SELM	6	0.28101	0.48481	0.9995	9494	3	-0.009
RYR1	6	0.28109	0.48489	0.9995	9495	1	-0.11
hsa-mir-4432	4	0.2811	0.42407	0.9995	9496	2	0.0132
LGI1	6	0.28111	0.48492	0.9995	9497	3	0.089
LDHA	6	0.28116	0.48497	0.9995	9498	3	0.0146
BCAR3	6	0.28117	0.48498	0.9995	9499	1	-0.068
SLC35B3	6	0.28121	0.48503	0.9995	9500	3	0.0437
VGLL1	6	0.28121	0.48503	0.9995	9501	3	-0.125
KPNA6	6	0.28122	0.48505	0.9995	9502	2	-0.097
LZIC	6	0.28125	0.48508	0.9995	9503	3	0.288
GNLY	6	0.28129	0.48511	0.9995	9504	2	0.0263
PRR14	6	0.28134	0.48518	0.9995	9505	3	0.1647
CCDC85C	6	0.28136	0.4852	0.9995	9506	1	-0.368
PLA2G16	6	0.28136	0.4852	0.9995	9507	1	-0.193
DLGAP3	6	0.28145	0.4853	0.9995	9508	3	0.1598
PSMF1	6	0.28151	0.48538	0.9995	9509	3	0.1964
CETP	6	0.28152	0.48539	0.9995	9510	1	-0.359
KRTAP12-2	6	0.28167	0.48556	0.9995	9511	2	-0.262
CTDNEP1	6	0.28176	0.48566	0.9995	9512	2	0.1351
COL1A1	6	0.28182	0.48574	0.9995	9513	3	0.1773
ZNF707	6	0.28182	0.48574	0.9995	9514	3	-0.038
TMEM207	6	0.2819	0.48583	0.9995	9515	2	-0.067

hsa-mir-6800	4	0.28194	0.42491	0.9995	9516	1	-0.093
KAL1	4	0.28198	0.42494	0.9995	9517	1	-0.334
FOSB	6	0.28204	0.48598	0.9995	9518	3	0.2477
CCT5	6	0.28213	0.4861	0.9995	9519	2	-0.14
OTUD7A	4	0.28221	0.42517	0.9995	9520	1	-0.52
PRC1	6	0.28225	0.48624	0.9995	9521	3	0.0604
ANKRD22	6	0.28225	0.48625	0.9995	9522	2	-0.101
CHCHD3	6	0.28225	0.48625	0.9995	9523	2	-0.108
hsa-mir-4277	4	0.28228	0.42524	0.9995	9524	1	-0.652
FHL3	6	0.28232	0.48634	0.9995	9525	3	0.1335
CDC40	6	0.28235	0.48637	0.9995	9526	1	0.0282
CCM2L	6	0.28236	0.48637	0.9995	9527	3	0.2397
PLEKHN1	6	0.28246	0.48649	0.9995	9528	2	-0.082
C19orf73	6	0.28246	0.48649	0.9995	9529	3	0.0434
SOWAHD	6	0.28253	0.48657	0.9995	9530	3	0.1057
CEACAM8	6	0.28253	0.48658	0.9995	9531	2	0.1033
hsa-mir-6754	4	0.28261	0.42558	0.9995	9532	1	0.0622
MPDU1	6	0.28262	0.48667	0.9995	9533	3	0.1843
DCUN1D2	6	0.28271	0.48678	0.9995	9534	3	0.0621
KRTAP20-1	6	0.28273	0.48681	0.9995	9535	3	0.2648
NCR2	6	0.28276	0.48683	0.9995	9536	3	-0.032
SSMEM1	6	0.28276	0.48683	0.9995	9537	3	0.2419
hsa-mir-889	4	0.28281	0.42579	0.9995	9538	2	0.3222
ZNF614	6	0.28282	0.48689	0.9995	9539	3	0.0613
MORC4	6	0.28284	0.48692	0.9995	9540	3	-6E-04
ZC3H3	6	0.28285	0.48693	0.9995	9541	2	0.0056
hsa-mir-8052	4	0.28293	0.42592	0.9995	9542	2	-0.072
TDRD1	6	0.28298	0.48708	0.9995	9543	3	0.0282
FOXO4	6	0.28308	0.48719	0.9995	9544	3	0.0109
C20orf27	6	0.28308	0.48719	0.9995	9545	2	-0.057
PPP1CB	6	0.28308	0.48719	0.9995	9546	2	-0.494
RIN1	6	0.28313	0.48726	0.9995	9547	2	-0.22
TOMM5	6	0.28317	0.4873	0.9995	9548	2	-0.346
hsa-mir-3195	4	0.28321	0.42621	0.9995	9549	1	-0.647
PSMB11	6	0.28324	0.48739	0.9995	9550	3	-0.006
OCIAD1	6	0.28324	0.48739	0.9995	9551	3	0.1413
hsa-mir-6848	4	0.28326	0.42626	0.9995	9552	2	0.1163
ARHGAP1	6	0.28329	0.48744	0.9995	9553	3	0.2251
ATP10B	4	0.2833	0.42629	0.9995	9554	2	0.1694
GOLGA6B	2	0.28334	0.32398	0.9995	9555	1	0.4228
KDM1B	6	0.28338	0.48755	0.9995	9556	3	0.1386
UNCX	6	0.28349	0.48768	0.9995	9557	3	0.1818
TTLL6	6	0.2835	0.48769	0.9995	9558	1	-0.404
SDCCAG8	6	0.2836	0.48781	0.9995	9559	3	0.2675
FAM109B	6	0.28361	0.48782	0.9995	9560	2	0.0111
HPGDS	6	0.28367	0.48788	0.9995	9561	2	-0.141
ADAM20	6	0.28373	0.48796	0.9995	9562	2	-0.091
ACO1	6	0.28381	0.48804	0.9995	9563	2	-0.018
ASB12	6	0.28383	0.48807	0.9995	9564	2	0.0809
VSTM2L	6	0.28385	0.48809	0.9995	9565	2	-0.05
YRDC	6	0.28385	0.48809	0.9995	9566	2	-0.152
LBX1	6	0.28394	0.4882	0.9995	9567	3	0.1469
BIRC5	6	0.28397	0.48824	0.9995	9568	2	-0.705
EXD3	6	0.28402	0.48828	0.9995	9569	3	0.1367
CA3	6	0.28403	0.48831	0.9995	9570	2	-0.246
PEX6	6	0.28409	0.48838	0.9995	9571	3	0.3508
ARPC5	6	0.28426	0.48858	0.9995	9572	3	0.1755
ZNF835	6	0.28426	0.48859	0.9995	9573	2	-0.001
LOC100507003	6	0.28429	0.48861	0.9995	9574	2	-0.077
KRT13	6	0.28436	0.48871	0.9995	9575	3	0.0489
ZDHHC21	6	0.2844	0.48875	0.9995	9576	3	0.0274
hsa-mir-548h-5	3	0.28441	0.41744	0.9995	9577	2	0.4813
LRRC3B	6	0.28442	0.48876	0.9995	9578	3	0.0718
POLR3A	6	0.28442	0.48876	0.9995	9579	3	0.1396
TMEFF2	6	0.28445	0.48881	0.9995	9580	3	0.1669
CA5A	6	0.28447	0.48883	0.9995	9581	3	0.2931
LRRC29	6	0.28448	0.48884	0.9995	9582	3	0.1224
FAM105B	6	0.28452	0.48889	0.9995	9583	3	0.1367
UBXN11	6	0.28452	0.48889	0.9995	9584	3	0.124
PIPOX	6	0.28464	0.48903	0.9995	9585	1	-0.181
TRAPPC6B	6	0.28468	0.48906	0.9995	9586	1	-0.251
HSPA4L	6	0.28472	0.48911	0.9995	9587	2	-0.114
GNL3	6	0.28474	0.48913	0.9995	9588	3	0.1236
SPINT4	6	0.28474	0.48913	0.9995	9589	3	0.1376
STAP2	6	0.28481	0.4892	0.9995	9590	2	-0.28
LIPE	6	0.28485	0.48924	0.9995	9591	3	0.2083
hsa-mir-581	2	0.28485	0.32517	0.9995	9592	1	0.5405
SIGIRR	6	0.28491	0.48932	0.9995	9593	2	-0.473
MPP6	6	0.28492	0.48933	0.9995	9594	1	-0.106
RPL29	6	0.28506	0.4895	0.9995	9595	3	0.2118
hsa-mir-2114	4	0.28506	0.42807	0.9995	9596	2	-0.059
ACOT8	6	0.28518	0.48966	0.9995	9597	3	0.1367
hsa-mir-6876	4	0.28521	0.42823	0.9995	9598	2	0.1616
EIF3CL	1	0.28522	0.28503	0.9995	9599	1	0.3078
PLD5	6	0.28523	0.48971	0.9995	9600	1	-0.169

GATA1	6	0.2853	0.4898	0.9995	9601	3	0.0916
hsa-mir-1908	3	0.28542	0.41862	0.9995	9602	1	0.6306
NAA38	6	0.28551	0.49002	0.9995	9603	3	0.0462
C12orf40	6	0.28556	0.49011	0.9995	9604	2	0.1455
ANPEP	6	0.28557	0.49012	0.9995	9605	2	-0.303
VAX1	6	0.28557	0.49012	0.9995	9606	3	0.1984
TSFM	6	0.28562	0.49018	0.9995	9607	3	-0.025
TARM1	6	0.2857	0.49027	0.9995	9608	2	-0.118
CDH7	6	0.28571	0.49029	0.9995	9609	2	-0.166
SLC38A3	6	0.28573	0.49031	0.9995	9610	3	0.0366
LIG3	6	0.2858	0.49039	0.9995	9611	2	-0.221
ALG1L	6	0.28581	0.4904	0.9995	9612	3	0.199
PDS5B	6	0.28581	0.4904	0.9995	9613	3	-0.177
ZNF513	6	0.28581	0.4904	0.9995	9614	3	0.2359
RAB3C	6	0.28581	0.4904	0.9995	9615	3	0.1143
ADM5	6	0.28583	0.49042	0.9995	9616	3	0.4314
hsa-mir-4506	4	0.28588	0.4289	0.9995	9617	1	0.1391
OR5M10	4	0.28597	0.42899	0.9995	9618	2	0.0353
CCDC154	6	0.28598	0.49059	0.9995	9619	1	-0.048
hsa-mir-4283-1	4	0.2861	0.42912	0.9995	9620	2	0.246
BRD7	6	0.28615	0.49081	0.9995	9621	3	0.1304
hsa-mir-3186	4	0.28623	0.42925	0.9995	9622	2	-0.174
RASL11A	6	0.28625	0.49094	0.9995	9623	3	-0.178
BPIFA1	6	0.28625	0.49094	0.9995	9624	3	0.1743
LHX9	6	0.28633	0.49103	0.9995	9625	2	-0.005
OCLN	6	0.28635	0.49105	0.9995	9626	3	0.1304
CYP2D6	6	0.28641	0.49113	0.9995	9627	1	-0.427
SH3GL2	6	0.28645	0.49116	0.9995	9628	3	0.1749
NREP	6	0.28651	0.49125	0.9995	9629	1	-0.287
CPO	6	0.28651	0.49125	0.9995	9630	1	-0.055
TMX1	6	0.28651	0.49125	0.9995	9631	2	-0.081
HMGB3	6	0.28651	0.49125	0.9995	9632	2	-0.112
C4orf27	6	0.28655	0.49128	0.9995	9633	2	-7E-04
ZDHHC15	6	0.2866	0.49133	0.9995	9634	3	0.1818
NT5C3A	6	0.28664	0.49138	0.9995	9635	3	0.0875
GPR20	6	0.28665	0.49139	0.9995	9636	2	0.1212
hsa-mir-342	4	0.28666	0.42969	0.9995	9637	1	-0.247
hsa-mir-6779	4	0.28666	0.42969	0.9995	9638	1	0.0254
ANGPT4	6	0.2867	0.49147	0.9995	9639	3	0.1944
TRIM31	6	0.2867	0.49147	0.9995	9640	2	-0.133
TMCO6	6	0.28673	0.49149	0.9995	9641	2	-0.313
CUEDC2	6	0.28682	0.4916	0.9995	9642	2	-0.711
ANGPTL2	6	0.28682	0.4916	0.9995	9643	2	0.1739
KCNS1	6	0.28689	0.49168	0.9995	9644	2	0.0066
C11orf30	6	0.28692	0.4917	0.9995	9645	3	0.1767
FRMD4A	6	0.28695	0.49173	0.9995	9646	2	-0.047
CTDSPL2	6	0.28696	0.49175	0.9995	9647	3	0.1377
SH3GL1	6	0.28698	0.49177	0.9995	9648	3	0.0637
DGCR2	6	0.28702	0.49181	0.9995	9649	2	-0.273
P2RX3	6	0.28705	0.49183	0.9995	9650	3	0.2515
MAPKBP1	6	0.28706	0.49185	0.9995	9651	3	0.1189
BCL11B	6	0.28706	0.49185	0.9995	9652	2	0.1338
TMED8	6	0.28712	0.49192	0.9995	9653	2	-0.213
PRG2	5	0.28713	0.47136	0.9995	9654	2	-0.126
NCCRP1	6	0.28715	0.49196	0.9995	9655	2	-0.014
LYN	6	0.28716	0.49197	0.9995	9656	3	-0.04
SNAP91	5	0.28722	0.47142	0.9995	9657	1	0.0127
OR13C5	6	0.28726	0.4921	0.9995	9658	2	-0.167
RANBP1	6	0.28734	0.49219	0.9995	9659	2	0.095
C15orf59	4	0.28737	0.43041	0.9995	9660	2	0.1809
MRPS18C	6	0.28737	0.49222	0.9995	9661	3	0.1245
PCSK2	6	0.28744	0.49232	0.9995	9662	3	0.2755
SENP3	6	0.28747	0.49235	0.9995	9663	2	-2E-05
TRMT10B	6	0.28753	0.49242	0.9995	9664	2	-0.172
PRM3	6	0.28762	0.49253	0.9995	9665	3	0.1115
OR2G3	6	0.2877	0.4926	0.9995	9666	3	0.0931
PPT1	6	0.28771	0.49262	0.9995	9667	2	-0.261
MAST3	6	0.28779	0.49271	0.9995	9668	3	0.1496
TSSK2	6	0.28787	0.4928	0.9995	9669	1	0.0837
APOPT1	6	0.28791	0.49286	0.9995	9670	2	0.1106
POMT1	6	0.28797	0.49293	0.9995	9671	2	-0.138
hsa-mir-548av	4	0.28799	0.43107	0.9995	9672	2	0.1025
SEMA6A	6	0.28803	0.49298	0.9995	9673	3	0.2049
C6orf99	6	0.28803	0.49298	0.9995	9674	3	0.2971
hsa-mir-887	4	0.28804	0.43112	0.9995	9675	2	0.0223
CCDC112	6	0.28814	0.49311	0.9995	9676	3	-0.043
MAP2	6	0.28816	0.49314	0.9995	9677	1	-0.107
ATP13A4	6	0.28816	0.49314	0.9995	9678	2	-0.031
TINF2	6	0.28817	0.49315	0.9995	9679	3	-0.233
MAGEB1	6	0.28826	0.49325	0.9995	9680	3	0.04
hsa-mir-524	3	0.28828	0.42208	0.9995	9681	2	0.2424
SERPINA1	6	0.28836	0.49337	0.9995	9682	3	0.2306
CLEC9A	6	0.28836	0.49337	0.9995	9683	3	0.1795
CNOT2	6	0.28836	0.49337	0.9995	9684	3	0.1159
PYDC1	6	0.28842	0.49342	0.9995	9685	1	0.1402

MTA2	6	0.28848	0.49352	0.9995	9686	2	0.0686
DCTN3	6	0.28849	0.49353	0.9995	9687	1	-0.724
HACL1	6	0.28849	0.49353	0.9995	9688	2	-0.121
PARP11	6	0.28853	0.49357	0.9995	9689	2	0.0769
CNTNAP5	6	0.28853	0.49357	0.9995	9690	2	0.093
LBP	6	0.28855	0.49358	0.9995	9691	3	0.1686
TADA1	6	0.28865	0.49371	0.9995	9692	2	-0.159
ECT2L	6	0.28868	0.49375	0.9995	9693	3	0.2246
RNF34	6	0.2887	0.49377	0.9995	9694	3	0.2313
FAH	6	0.28874	0.49383	0.9995	9695	2	-0.276
KRT6B	4	0.28887	0.43193	0.9995	9696	2	0.0058
HIVEP2	6	0.28888	0.49399	0.9995	9697	2	-0.171
FOXL1	6	0.28889	0.494	0.9995	9698	3	0.2632
PYY	6	0.28892	0.49405	0.9995	9699	2	-0.113
EMILIN2	6	0.289	0.49415	0.9995	9700	1	-0.065
PDK1	6	0.28908	0.49424	0.9995	9701	2	0.1728
SGIP1	6	0.28922	0.49441	0.9995	9702	3	0.3441
UBTF	6	0.28926	0.49445	0.9995	9703	3	0.187
DYNLRB2	6	0.28933	0.49454	0.9995	9704	2	0.0951
TBC1D22B	6	0.28935	0.49456	0.9995	9705	3	0.0759
hsa-mir-517c	1	0.2894	0.28925	0.9995	9706	1	0.2915
TBX15	6	0.28952	0.49478	0.9995	9707	2	-0.3
ESAM	6	0.28969	0.49497	0.9995	9708	2	-0.237
PAQR6	6	0.28972	0.495	0.9995	9709	3	0.033
CHI3L1	6	0.2899	0.49521	0.9995	9710	2	-0.005
TAF4B	6	0.28997	0.4953	0.9995	9711	3	0.1986
ENTPD2	6	0.29004	0.49537	0.9995	9712	3	0.2369
C2orf82	6	0.29004	0.49537	0.9995	9713	3	-0.066
SMCO2	6	0.29017	0.49552	0.9995	9714	3	0.1935
hsa-mir-199b	4	0.29028	0.43338	0.9995	9715	2	0.3958
KPNA2	6	0.29034	0.49572	0.9995	9716	3	0.2808
OR4K13	6	0.29034	0.49572	0.9995	9717	3	0.2065
ASXL2	6	0.29035	0.49573	0.9995	9718	2	-0.013
CLCN2	6	0.29042	0.4958	0.9995	9719	3	0.0905
PPP2R5B	6	0.29046	0.49585	0.9995	9720	2	-0.391
PQLC1	6	0.29047	0.49587	0.9995	9721	3	0.0872
CELA1	6	0.29047	0.49587	0.9995	9722	3	-0.111
RNF146	6	0.29047	0.49587	0.9995	9723	2	-0.057
SIGLEC7	6	0.29052	0.49594	0.9995	9724	2	-0.141
INSL6	6	0.29055	0.49596	0.9995	9725	3	0.2507
HIF1A	6	0.29057	0.49598	0.9995	9726	2	0.1629
C6orf195	6	0.29063	0.49606	0.9995	9727	3	0.2165
CDKN2D	6	0.29074	0.49619	0.9995	9728	3	0.187
ATP6V1F	6	0.2908	0.49627	0.9995	9729	1	-0.264
SERTAD2	6	0.29081	0.49627	0.9995	9730	3	0.2628
TMEM11	6	0.29084	0.4963	0.9995	9731	2	0.1173
FAM151B	6	0.29084	0.49631	0.9995	9732	1	-0.06
DEFB103B	1	0.29086	0.29071	0.9995	9733	1	0.4214
TAPBPL	6	0.2909	0.49638	0.9995	9734	2	-0.017
hsa-mir-196b	4	0.29093	0.43404	0.9995	9735	2	0.2471
DNASE1L2	6	0.29096	0.49643	0.9995	9736	2	0.1201
UGT1A1	2	0.29105	0.3299	0.9995	9737	1	0.1955
MED26	6	0.29108	0.49657	0.9995	9738	2	0.029
LSM4	6	0.29109	0.49659	0.9995	9739	3	0.1693
AK2	6	0.29115	0.49665	0.9995	9740	2	-0.077
NCAN	6	0.29118	0.49669	0.9995	9741	3	0.1536
MCM5	6	0.29126	0.4968	0.9995	9742	3	-0.047
GCLM	6	0.2913	0.49684	0.9995	9743	3	-0.016
JAKMIP1	6	0.2913	0.49684	0.9995	9744	3	0.1247
OR5K4	6	0.29143	0.49698	0.9995	9745	2	-0.261
APPL2	6	0.29152	0.49708	0.9995	9746	2	-0.077
TNFAIP3	6	0.29159	0.49716	0.9995	9747	1	-0.117
PURA	6	0.29163	0.49721	0.9995	9748	1	-0.124
SFMBT2	6	0.29167	0.49726	0.9995	9749	2	-0.161
ARL16	6	0.29169	0.49729	0.9995	9750	3	0.2412
LEMD3	4	0.29169	0.43478	0.9995	9751	2	-0.022
PDGFC	6	0.29171	0.49731	0.9995	9752	3	-0.136
PANX3	4	0.29171	0.4348	0.9995	9753	1	-0.151
DLEU7	6	0.29173	0.49734	0.9995	9754	3	0.0845
RBP3	6	0.29176	0.49738	0.9995	9755	1	-0.143
PLEKHG6	6	0.29177	0.49738	0.9995	9756	3	0.0966
SLC10A6	6	0.29182	0.49745	0.9995	9757	2	-0.224
APOC3	6	0.29184	0.49747	0.9995	9758	3	0.1587
ENO1	6	0.29196	0.49762	0.9995	9759	3	0.0941
NANOGNB	6	0.29196	0.49762	0.9995	9760	3	0.203
PLA2G4E	6	0.29198	0.49764	0.9995	9761	1	-0.355
AXIN1	6	0.29198	0.49764	0.9995	9762	1	0.1214
hsa-mir-544a	4	0.29198	0.43507	0.9995	9763	2	0.0539
hsa-mir-611	4	0.29209	0.43516	0.9995	9764	1	-0.61
PNKD	6	0.29216	0.49786	0.9995	9765	3	0.0672
MYH6	6	0.29217	0.49787	0.9995	9766	1	0.1547
MEIG1	6	0.29217	0.49787	0.9995	9767	2	0.005
TMPRSS11B	6	0.29223	0.49795	0.9995	9768	2	-0.021
PTPRN2	6	0.29234	0.49808	0.9995	9769	3	0.1635
PAQR8	6	0.29236	0.4981	0.9995	9770	2	-0.55

TTC31	6	0.29236	0.4981	0.9995	9771	2	-0.052
FGGY	6	0.29244	0.49819	0.9995	9772	3	0.1917
COG3	6	0.29252	0.49828	0.9995	9773	2	-0.099
RIMBP2	6	0.29256	0.49834	0.9995	9774	1	-0.064
OR4C45	6	0.29268	0.49846	0.9995	9775	2	-0.064
MS4A15	6	0.29272	0.4985	0.9995	9776	2	-0.211
FAM131C	6	0.29276	0.49855	0.9995	9777	2	0.0968
KRTAP10-9	6	0.2928	0.49859	0.9995	9778	3	-0.025
hsa-mir-6070	4	0.29282	0.43595	0.9995	9779	1	-0.244
FOXJ1	6	0.29283	0.49862	0.9995	9780	3	0.1527
hsa-mir-412	4	0.29287	0.43599	0.9995	9781	2	-0.081
HOXB3	6	0.2929	0.4987	0.9995	9782	3	0.0406
OR2C3	6	0.29293	0.49874	0.9995	9783	2	0.0284
MAML2	6	0.29296	0.49877	0.9995	9784	2	0.0844
PLK3	6	0.29299	0.4988	0.9995	9785	3	0.2369
EMP2	6	0.29303	0.49884	0.9995	9786	1	-0.35
hsa-mir-548ap	3	0.29309	0.42542	0.9995	9787	1	0.0283
OR4F15	6	0.29309	0.4989	0.9995	9788	3	0.0512
FAM120A	6	0.29322	0.49906	0.9995	9789	3	0.1309
ZDHHC9	6	0.29323	0.49906	0.9995	9790	2	-0.043
PRSS2	6	0.29325	0.49908	0.9995	9791	3	0.1412
PTGIS	6	0.29331	0.49915	0.9995	9792	2	0.1268
DDX59	6	0.29333	0.49918	0.9995	9793	2	0.1178
PTCHD2	6	0.29333	0.49918	0.9995	9794	2	-0.137
TOX	6	0.29337	0.49922	0.9995	9795	3	0.1349
hsa-mir-378a	4	0.29346	0.43658	0.9995	9796	2	0.305
MYH8	6	0.29353	0.49942	0.9995	9797	3	-0.213
CCNT2	6	0.29354	0.49943	0.9995	9798	2	0.1624
TMCO4	6	0.29357	0.49946	0.9995	9799	3	0.1861
PXDC1	6	0.2936	0.4995	0.9995	9800	2	-0.015
ZNF337	6	0.29364	0.49954	0.9995	9801	3	0.159
KISS1R	6	0.29365	0.49954	0.9995	9802	2	-0.272
NKTR	6	0.29369	0.4996	0.9995	9803	3	0.2556
RCBTB1	4	0.29369	0.43681	0.9995	9804	1	-0.223
SGK223	6	0.29372	0.49963	0.9995	9805	3	0.1961
TMEM37	6	0.29375	0.49967	0.9995	9806	2	0.0067
WAC	6	0.29375	0.49967	0.9995	9807	1	-0.224
ERN2	6	0.29387	0.49983	0.9995	9808	3	0.1305
ACTRT1	6	0.29397	0.49995	0.9995	9809	2	-0.048
FIGNL2	6	0.29404	0.50003	0.9995	9810	3	0.2148
HDAC3	6	0.29405	0.50003	0.9995	9811	1	-0.159
GCC2	6	0.29405	0.50003	0.9995	9812	3	-0.12
C15orf38	2	0.29422	0.33238	0.9995	9813	1	0.2579
hsa-mir-124-1	4	0.29427	0.4374	0.9995	9814	2	0.1654
NTAN1	6	0.29428	0.50031	0.9995	9815	2	0.0202
ANGPTL5	6	0.29431	0.50034	0.9995	9816	3	0.1494
ADRA2A	6	0.29431	0.50034	0.9995	9817	2	-0.232
CANX	6	0.29436	0.50039	0.9995	9818	2	-0.139
RBM6	6	0.29442	0.50046	0.9995	9819	3	0.0025
ZNF169	6	0.29444	0.50048	0.9995	9820	2	0.041
DUSP4	6	0.29448	0.50053	0.9995	9821	2	0.0396
TTC40	6	0.29451	0.50056	0.9995	9822	2	0.1013
ETHE1	6	0.29452	0.50058	0.9995	9823	3	0.2425
ARF4	6	0.29467	0.50076	0.9995	9824	2	-0.167
MRPL17	6	0.29467	0.50076	0.9995	9825	1	-0.036
KIAA1586	5	0.29475	0.47753	0.9995	9826	2	0.4222
MAN1B1	6	0.29477	0.50088	0.9995	9827	2	-0.235
TOM1	6	0.29479	0.5009	0.9995	9828	1	-0.043
OMD	6	0.29483	0.50094	0.9995	9829	3	-0.247
SMARCAL1	6	0.29488	0.501	0.9995	9830	3	0.1423
PAK7	6	0.29492	0.50105	0.9995	9831	3	0.0558
CXADR	6	0.29492	0.50106	0.9995	9832	2	-0.125
BSDC1	6	0.29492	0.50106	0.9995	9833	1	-0.21
MYL2	6	0.29498	0.50112	0.9995	9834	1	0.1314
SERPINA9	6	0.295	0.50115	0.9995	9835	3	0.2719
GPR110	6	0.29506	0.50121	0.9995	9836	2	-0.174
hsa-mir-3188	4	0.29509	0.43821	0.9995	9837	2	0.296
ZBTB20	6	0.29513	0.50129	0.9995	9838	3	0.096
MSX2	6	0.29516	0.50133	0.9995	9839	3	0.1266
HMBS	6	0.29517	0.50134	0.9995	9840	3	0.2034
MBD6	6	0.29526	0.50142	0.9995	9841	3	0.2343
LMAN2L	6	0.29531	0.50148	0.9995	9842	3	0.1521
CCDC15	6	0.29534	0.50151	0.9995	9843	3	0.1884
LTBP3	6	0.29542	0.5016	0.9995	9844	2	-0.04
WDR72	6	0.29547	0.50167	0.9995	9845	2	-0.222
hsa-mir-8077	4	0.29547	0.43859	0.9995	9846	1	-0.307
AIF1L	6	0.29551	0.5017	0.9995	9847	3	0.1183
RASSF6	6	0.29551	0.5017	0.9995	9848	1	-0.157
MST1	6	0.29552	0.50172	0.9995	9849	2	-0.288
PLA2G2E	6	0.29556	0.50178	0.9995	9850	2	0.0316
CARHSP1	6	0.29557	0.50178	0.9995	9851	3	0.0277
DUS1L	6	0.2957	0.50194	0.9995	9852	2	0.0608
TRPM6	6	0.2958	0.50206	0.9995	9853	2	0.1289
PPP1R1B	6	0.29582	0.5021	0.9995	9854	3	0.1422
ZNF326	6	0.29583	0.50211	0.9995	9855	2	-0.021

SPDEF	6	0.29595	0.50223	0.9995	9856	1	-0.574
BMPER	6	0.29604	0.50234	0.9995	9857	3	0.1508
SLC7A10	6	0.29615	0.50246	0.9995	9858	2	0.1088
C11orf80	6	0.29619	0.50251	0.9995	9859	1	-0.258
DYNLT3	6	0.29627	0.50261	0.9995	9860	3	0.0979
NUDT17	6	0.2964	0.50275	0.9995	9861	3	0.1671
MADD	6	0.2964	0.50275	0.9995	9862	3	0.2744
IZUMO3	6	0.2964	0.50275	0.9995	9863	3	0.1048
CLEC2A	4	0.29646	0.43963	0.9995	9864	2	0.2179
ACOT9	6	0.29648	0.50283	0.9995	9865	2	-0.398
MX2	6	0.2965	0.50285	0.9995	9866	2	-0.299
ALDH8A1	6	0.29652	0.50287	0.9995	9867	2	-0.102
hsa-mir-6780a	4	0.29658	0.43976	0.9995	9868	1	-0.13
TAS2R3	6	0.2966	0.50297	0.9995	9869	1	0.1026
CENPN	6	0.29662	0.503	0.9995	9870	3	0.118
TIAF1	6	0.29662	0.503	0.9995	9871	3	-0.015
PCDP1	6	0.29662	0.503	0.9995	9872	3	0.2452
HOXB9	4	0.29663	0.43981	0.9995	9873	2	0.0718
HOXD10	6	0.29665	0.50303	0.9995	9874	2	0.0123
ISLR2	6	0.29669	0.50307	0.9995	9875	1	-0.231
SRSF6	6	0.29675	0.50314	0.9995	9876	3	0.2898
OR52E8	6	0.29675	0.50314	0.9995	9877	2	0.0518
SH3RF3	6	0.29678	0.50318	0.9995	9878	3	0.2105
SUV420H1	6	0.29685	0.50325	0.9995	9879	3	0.2447
APLF	6	0.29691	0.50332	0.9995	9880	3	0.1369
CTC1	6	0.29697	0.50339	0.9995	9881	3	0.1568
EGLN1	6	0.297	0.50344	0.9995	9882	2	0.1165
CNP	6	0.29701	0.50344	0.9995	9883	3	0.0683
MSL1	6	0.29708	0.50352	0.9995	9884	3	0.0168
CENPM	6	0.29714	0.5036	0.9995	9885	3	0.2284
PDE2A	6	0.29714	0.5036	0.9995	9886	3	0.1462
PTPLA	6	0.29714	0.5036	0.9995	9887	2	-0.166
STON2	6	0.29715	0.50362	0.9995	9888	2	-0.093
C9orf66	6	0.29729	0.50377	0.9995	9889	3	-0.005
GPM6B	6	0.29731	0.5038	0.9995	9890	2	-0.273
C6orf1	6	0.29731	0.5038	0.9995	9891	1	-0.01
TOX2	6	0.29737	0.50387	0.9995	9892	2	-0.388
TRAPPC11	4	0.29742	0.4406	0.9995	9893	2	0.1025
PIK3C2G	6	0.29742	0.50393	0.9995	9894	3	0.1114
CNR1	6	0.29747	0.50398	0.9995	9895	2	0.0147
MGEA5	6	0.29748	0.50399	0.9995	9896	2	0.1311
MAGEL2	6	0.29753	0.50404	0.9995	9897	2	-0.266
CYP2J2	6	0.29759	0.50412	0.9995	9898	2	-0.06
ZNF226	6	0.2976	0.50413	0.9995	9899	3	0.1982
hsa-mir-137	4	0.29763	0.44083	0.9995	9900	2	-0.357
OR2C1	6	0.2977	0.50426	0.9995	9901	2	-0.121
ZNF280D	6	0.29781	0.50438	0.9995	9902	3	0.1715
NRK	6	0.29793	0.50453	0.9995	9903	3	0.1358
ETAA1	6	0.29793	0.50453	0.9995	9904	2	-0.082
hsa-mir-548ae-1	2	0.29809	0.33536	0.9995	9905	1	0.3317
ZNF680	6	0.29809	0.50473	0.9995	9906	3	0.0207
NECAB3	6	0.2981	0.50473	0.9995	9907	3	-0.012
LIPN	6	0.29811	0.50475	0.9995	9908	2	-0.035
CTSC	6	0.29817	0.50481	0.9995	9909	2	0.1129
KATNB1	6	0.29826	0.50492	0.9995	9910	2	-0.102
EPB42	6	0.29832	0.50499	0.9995	9911	3	0.3277
ECHDC2	6	0.29832	0.50499	0.9995	9912	3	-0.034
RFC5	6	0.29832	0.50499	0.9995	9913	3	-0.013
GPRIN3	6	0.29838	0.50505	0.9995	9914	2	-0.329
CCDC62	6	0.29852	0.5052	0.9995	9915	1	-0.334
ATXN3L	6	0.29858	0.50527	0.9995	9916	2	-0.052
DSC2	6	0.29863	0.50535	0.9995	9917	1	-0.235
C2CD5	6	0.29865	0.50537	0.9995	9918	3	0.1426
ITPRIPL1	6	0.29865	0.50537	0.9995	9919	3	0.0859
hsa-mir-613	4	0.29873	0.44194	0.9995	9920	2	0.3204
C15orf40	6	0.29886	0.50562	0.9995	9921	3	0.2074
OCRL	6	0.29896	0.50575	0.9995	9922	3	0.1343
GLP2R	6	0.29904	0.50583	0.9995	9923	2	-0.172
SENP1	6	0.29909	0.5059	0.9995	9924	3	-0.121
IRX3	6	0.2991	0.50591	0.9995	9925	1	-0.614
TMEM40	6	0.29914	0.50597	0.9995	9926	3	0.137
MLN	6	0.29916	0.50599	0.9995	9927	2	0.076
MRPL41	6	0.29922	0.50605	0.9995	9928	3	-0.124
CILP	4	0.29931	0.44251	0.9995	9929	2	0.1524
PHKB	6	0.29933	0.50618	0.9995	9930	3	0.0319
MEX3C	6	0.29933	0.50618	0.9995	9931	2	0.0378
DNAJB5	6	0.29936	0.50623	0.9995	9932	3	0.0943
C10orf10	6	0.29937	0.50623	0.9995	9933	1	-0.177
OR6C1	6	0.2995	0.50638	0.9995	9934	3	0.1736
SARNP	6	0.29952	0.50639	0.9995	9935	3	-0.062
CRISPLD1	6	0.29955	0.50643	0.9995	9936	3	0.1883
PSCA	6	0.29958	0.50647	0.9995	9937	2	-0.532
hsa-mir-875	4	0.29964	0.44287	0.9995	9938	2	-0.071
TLR1	6	0.29964	0.50655	0.9995	9939	3	0.1432
PLP1	6	0.29964	0.50655	0.9995	9940	3	0.1732

TBCCD1	6	0.2997	0.50662	0.9995	9941	2	0.0139
MST4	6	0.2997	0.50662	0.9995	9942	2	-0.175
STRA6	6	0.29987	0.50682	0.9995	9943	2	-0.388
FAM49A	6	0.29991	0.50687	0.9995	9944	3	0.1119
ZC3H6	6	0.29999	0.50696	0.9995	9945	3	0.1676
RHBDD1	6	0.3	0.50697	0.9995	9946	2	0.1207
AFAP1	6	0.30001	0.50699	0.9995	9947	3	0.2084
ZNF570	6	0.30007	0.50706	0.9995	9948	3	-0.346
RPS6KC1	6	0.30007	0.50706	0.9995	9949	2	0.1069
BEND3	6	0.30009	0.50709	0.9995	9950	2	0.1072
PLGRKT	6	0.3001	0.50711	0.9995	9951	2	-0.196
TMEM61	6	0.30015	0.50715	0.9995	9952	3	-0.182
NMUR2	6	0.30019	0.5072	0.9995	9953	3	0.0401
PPM1L	6	0.30022	0.50723	0.9995	9954	1	-0.041
UACA	6	0.30034	0.50735	0.9995	9955	2	-0.021
TAGLN2	6	0.30034	0.50735	0.9995	9956	2	0.0361
WT1	6	0.30034	0.50735	0.9995	9957	2	0.1697
EFNA3	6	0.30034	0.50735	0.9995	9958	3	0.0884
KLC4	6	0.30036	0.50738	0.9995	9959	2	-0.177
NEDD9	6	0.30037	0.5074	0.9995	9960	3	0.1939
MAK16	6	0.30042	0.50746	0.9995	9961	3	0.2825
ASB8	6	0.30049	0.50754	0.9995	9962	2	-0.189
AHCYL2	6	0.30052	0.50757	0.9995	9963	2	0.1104
PACSIN2	6	0.30055	0.50761	0.9995	9964	2	0.0996
F3	6	0.30061	0.50768	0.9995	9965	2	-0.054
C11orf82	6	0.30065	0.50773	0.9995	9966	2	-0.061
FTO	6	0.30066	0.50775	0.9995	9967	3	0.1188
HIST1H2AA	6	0.30072	0.50781	0.9995	9968	3	0.1103
TANC1	6	0.30072	0.50781	0.9995	9969	3	0.0149
ASAP2	6	0.30078	0.50787	0.9995	9970	3	0.2743
BSCL2	6	0.30078	0.50787	0.9995	9971	3	0.1395
DNAJC15	6	0.3008	0.50789	0.9995	9972	1	-0.46
SLC34A2	6	0.30085	0.50796	0.9995	9973	3	0.1488
hsa-mir-650	4	0.30086	0.44409	0.9995	9974	2	0.2585
APOA5	6	0.30092	0.50803	0.9995	9975	1	-0.357
MNAT1	6	0.30099	0.50813	0.9995	9976	2	-0.106
HDGFRP2	6	0.30104	0.50818	0.9995	9977	3	0.2597
OR5M3	6	0.30107	0.50821	0.9995	9978	1	-0.168
hsa-mir-4491	4	0.30108	0.4443	0.9995	9979	1	-0.125
ADCK4	6	0.30112	0.50828	0.9995	9980	3	0.1732
ACCS	6	0.30118	0.50834	0.9995	9981	3	-0.119
LYSMD1	6	0.3012	0.50835	0.9995	9982	2	-0.017
EML6	6	0.30121	0.50837	0.9995	9983	3	0.173
CTBS	6	0.30123	0.50839	0.9995	9984	3	0.1618
MUSTN1	6	0.3013	0.50849	0.9995	9985	3	0.2032
KLF15	6	0.3013	0.50849	0.9995	9986	3	0.0735
ZRANB2	6	0.30132	0.50851	0.9995	9987	2	-0.021
GAL	6	0.30137	0.50858	0.9995	9988	3	0.056
HLA-DMB	6	0.30153	0.50877	0.9995	9989	2	-0.247
hsa-mir-1281	4	0.30161	0.44484	0.9995	9990	2	0.0934
FLYWCH2	6	0.30165	0.50891	0.9995	9991	3	-0.048
SPAG16	6	0.30171	0.50897	0.9995	9992	2	-0.008
hsa-mir-532	4	0.30171	0.44496	0.9995	9993	1	0.0997
ZNF674	5	0.30178	0.4832	0.9995	9994	1	-0.648
C1QTNF3	6	0.30182	0.5091	0.9995	9995	3	0.102
GSC	6	0.30187	0.50916	0.9995	9996	2	0.0404
PCDHA8	2	0.30196	0.33833	0.9995	9997	1	-0.303
C7orf55-LUC7L2	2	0.30202	0.33838	0.9995	9998	1	0.3406
TAF10	6	0.30213	0.50946	0.9995	9999	3	0.1168
MRAP2	6	0.30213	0.50946	0.9995	10000	3	0.176
DYTN	6	0.30218	0.50952	0.9995	10001	3	0.2658
C8orf37	6	0.30218	0.50952	0.9995	10002	3	0.1712
AGAP11	6	0.30222	0.50956	0.9995	10003	3	-0.038
hsa-mir-19b-2	3	0.30224	0.43029	0.9995	10004	1	0.0703
COL4A5	4	0.30226	0.44547	0.9995	10005	1	-0.396
TMEM194B	6	0.30228	0.50962	0.9995	10006	3	0.1414
LFNG	6	0.30231	0.50966	0.9995	10007	1	-0.297
C17orf98	6	0.30234	0.5097	0.9995	10008	3	0.2571
ZNF418	6	0.30234	0.5097	0.9995	10009	3	0.0853
CH25H	6	0.30234	0.5097	0.9995	10010	3	0.2328
B3GALNT2	6	0.30246	0.50984	0.9995	10011	2	-0.169
TMC4	6	0.30246	0.50984	0.9995	10012	2	-0.17
ERH	6	0.30252	0.50991	0.9995	10013	3	0.1461
LMO1	6	0.30271	0.51014	0.9995	10014	2	0.0992
SLPI	6	0.3028	0.51026	0.9995	10015	3	-0.125
PIGG	6	0.30282	0.51027	0.9995	10016	3	-0.144
ANXA3	6	0.30296	0.51043	0.9995	10017	3	0.1349
SLC2A9	6	0.30303	0.51051	0.9995	10018	3	0.1785
USH2A	6	0.30306	0.51055	0.9995	10019	3	0.1903
hsa-mir-4780	4	0.30319	0.44648	0.9995	10020	2	-0.029
LITAF	6	0.30319	0.5107	0.9995	10021	2	0.075
RNF38	6	0.30333	0.51085	0.9995	10022	2	-0.268
HMGB2	6	0.30338	0.5109	0.9995	10023	2	0.0428
DCST2	6	0.30339	0.51091	0.9995	10024	3	0.1799
LOC100131094	6	0.30359	0.51116	0.9995	10025	3	0.0603

CD80	6	0.30362	0.51118	0.9995	10026	3	-0.053
TPPP3	6	0.30373	0.51131	0.9995	10027	2	0.044
JOSD2	6	0.30375	0.51132	0.9995	10028	3	-0.02
A1CF	6	0.30385	0.51147	0.9995	10029	2	0.0716
PSMA8	6	0.30387	0.51149	0.9995	10030	2	-0.263
AADACL3	6	0.3039	0.51152	0.9995	10031	2	0.0349
DIRAS1	6	0.30406	0.51171	0.9995	10032	2	0.1156
CACNG5	6	0.30408	0.51173	0.9995	10033	1	-0.054
UTP20	6	0.30412	0.51177	0.9995	10034	1	-0.044
TSHZ3	6	0.30416	0.51182	0.9995	10035	3	0.2498
CD6	4	0.30418	0.4475	0.9995	10036	2	0.2089
ZNF567	6	0.3042	0.51186	0.9995	10037	1	-0.404
VGLL2	6	0.30425	0.51194	0.9995	10038	3	0.1894
THOC7	6	0.30431	0.51201	0.9995	10039	3	0.1218
DCK	6	0.30431	0.51201	0.9995	10040	2	-0.014
RBMS1	6	0.30439	0.51209	0.9995	10041	3	0.1624
OC90	6	0.30447	0.51218	0.9995	10042	3	0.1664
C12orf65	6	0.30466	0.5124	0.9995	10043	3	0.2351
NCK2	6	0.3047	0.51245	0.9995	10044	1	0.0545
hsa-mir-6760	4	0.30471	0.44802	0.9995	10045	2	0.3328
TTC8	6	0.30471	0.51246	0.9995	10046	3	0.0713
PRAMEF12	6	0.30484	0.51259	0.9995	10047	2	-0.153
MAST1	6	0.30489	0.51267	0.9995	10048	1	-0.449
ROM1	6	0.30499	0.51279	0.9995	10049	1	-0.081
TMEM206	6	0.30505	0.51286	0.9995	10050	2	0.0857
SOWAHA	6	0.30506	0.51287	0.9995	10051	2	-0.465
DOK4	6	0.30513	0.51295	0.9995	10052	3	0.245
MAP4K5	6	0.30516	0.51299	0.9995	10053	2	-0.137
SCNN1A	6	0.30522	0.51305	0.9995	10054	2	0.1222
GCH1	6	0.30524	0.51308	0.9995	10055	3	0.1859
FAM134C	6	0.3053	0.51314	0.9995	10056	3	0.1864
TUBB4B	6	0.3053	0.51314	0.9995	10057	3	-0.002
ACRV1	6	0.30538	0.51323	0.9995	10058	3	-0.14
RAB27B	6	0.30539	0.51324	0.9995	10059	3	0.0581
C7orf63	6	0.30539	0.51324	0.9995	10060	3	0.1882
DBNL	6	0.3054	0.51326	0.9995	10061	2	-0.369
SPTBN4	6	0.30542	0.51329	0.9995	10062	2	0.0265
RLIM	6	0.3055	0.51339	0.9995	10063	1	-0.322
SFT2D3	6	0.30555	0.51344	0.9995	10064	3	0.3058
TBXAS1	6	0.30555	0.51344	0.9995	10065	3	0.3161
PML	6	0.30557	0.51346	0.9995	10066	3	0.2157
hsa-mir-135a-2	4	0.30558	0.44891	0.9995	10067	2	0.0812
GLYR1	6	0.30561	0.51352	0.9995	10068	2	0.0677
USH1G	6	0.30561	0.51352	0.9995	10069	2	0.092
TRIM43B	5	0.30568	0.48634	0.9995	10070	2	-0.103
PDZD2	6	0.3057	0.51362	0.9995	10071	2	-0.013
FAM159A	6	0.30588	0.51384	0.9995	10072	2	-0.1
PER2	6	0.30592	0.51389	0.9995	10073	3	0.079
RIC8B	6	0.30595	0.51393	0.9995	10074	2	-0.271
PRPF4	6	0.30597	0.51394	0.9995	10075	2	-0.062
HM13	6	0.30602	0.51399	0.9995	10076	3	-0.144
FBXO47	6	0.30612	0.51412	0.9995	10077	3	0.0019
SIRPG	6	0.30618	0.51418	0.9995	10078	2	0.0539
ACTBL2	6	0.30618	0.51418	0.9995	10079	3	-0.055
DFFB	6	0.30618	0.51418	0.9995	10080	3	0.1784
hsa-mir-6871	4	0.30619	0.44956	0.9995	10081	1	-0.091
C9orf156	6	0.3063	0.51432	0.9995	10082	3	0.1966
CMA1	6	0.30631	0.51434	0.9995	10083	2	-0.121
ATP2A2	6	0.30631	0.51434	0.9995	10084	2	-0.079
ATF7IP2	6	0.3064	0.51444	0.9995	10085	3	0.1374
RFX1	6	0.30647	0.51451	0.9995	10086	3	-0.011
WFDC11	6	0.30651	0.51456	0.9995	10087	3	0.1886
ODF4	6	0.30657	0.51464	0.9995	10088	2	-0.038
OMG	6	0.30664	0.51473	0.9995	10089	2	-0.092
hsa-mir-6832	4	0.30672	0.45011	0.9995	10090	1	-0.365
DGKB	6	0.30673	0.51485	0.9995	10091	1	-0.039
ARHGAP6	6	0.30673	0.51485	0.9995	10092	1	-0.734
APOBEC3D	6	0.30691	0.51505	0.9995	10093	2	-0.007
C11orf1	6	0.30692	0.51506	0.9995	10094	3	0.1766
CLN5	6	0.30692	0.51506	0.9995	10095	3	0.0053
HCRTR2	6	0.30696	0.51511	0.9995	10096	2	-0.004
FAM43B	6	0.30699	0.51515	0.9995	10097	3	0.2506
ERVMER34-1	6	0.30704	0.5152	0.9995	10098	3	0.2456
RPL31	6	0.30708	0.51524	0.9995	10099	2	0.1304
DGKH	6	0.30717	0.51534	0.9995	10100	3	0.1764
C3orf79	6	0.30721	0.51539	0.9995	10101	2	0.0684
RPS14	6	0.30727	0.51545	0.9995	10102	1	-0.161
hsa-mir-4436a	3	0.30729	0.43302	0.9995	10103	1	-1.233
CHST5	6	0.30731	0.51549	0.9995	10104	1	-0.275
RPGRIP1	6	0.30732	0.5155	0.9995	10105	2	-0.092
SLC25A29	6	0.30733	0.51552	0.9995	10106	3	0.1331
PCGF2	6	0.30742	0.51561	0.9995	10107	1	-0.293
TAB2	6	0.30746	0.51566	0.9995	10108	3	0.0909
PRKG2	6	0.3075	0.51569	0.9995	10109	2	0.1245
ARHGDIG	6	0.30756	0.51576	0.9995	10110	3	0.1543

SNX9	6	0.30767	0.51589	0.9995	10111	2	0.0704
TMEM45B	6	0.30767	0.51589	0.9995	10112	2	0.1162
MAPKAPK5	6	0.30782	0.51606	0.9995	10113	3	0.1592
MAGI2	6	0.30783	0.51607	0.9995	10114	2	-0.041
MYCN	6	0.30806	0.51638	0.9995	10115	3	0.2218
IFNL4	6	0.30808	0.5164	0.9995	10116	2	-0.036
TEX264	6	0.30822	0.51655	0.9995	10117	3	0.0724
C11orf35	6	0.30824	0.51658	0.9995	10118	2	0.0872
TMF1	6	0.30836	0.51673	0.9995	10119	2	-0.049
TRIM77	6	0.30839	0.51675	0.9995	10120	3	0.2293
hsa-mir-329-1	1	0.30839	0.30839	0.9995	10121	1	0.9323
C1R	6	0.30846	0.51684	0.9995	10122	3	-0.11
CLEC4F	6	0.30846	0.51684	0.9995	10123	3	0.2186
NSRP1	6	0.30848	0.51686	0.9995	10124	1	-0.172
GSTA4	6	0.30848	0.51686	0.9995	10125	2	-0.153
MPPE1	6	0.30851	0.5169	0.9995	10126	3	0.0282
hsa-mir-582	4	0.30852	0.45195	0.9995	10127	1	-0.053
PQBP1	6	0.30854	0.51693	0.9995	10128	2	0.0846
hsa-mir-433	4	0.30856	0.45198	0.9995	10129	2	0.0343
REEP1	6	0.30858	0.51697	0.9995	10130	3	0.1985
GNAT1	6	0.30866	0.51706	0.9995	10131	3	-0.094
hsa-mir-34b	4	0.30872	0.45215	0.9995	10132	2	-0.294
ZNF497	6	0.30873	0.51714	0.9995	10133	2	-0.149
SLC6A18	6	0.30873	0.51714	0.9995	10134	3	0.0243
SUPT20H	4	0.30881	0.45224	0.9995	10135	2	0.1584
CD5	6	0.30884	0.51726	0.9995	10136	2	-0.017
MBOAT7	6	0.30889	0.51732	0.9995	10137	3	-0.052
HIST1H4C	6	0.30893	0.51737	0.9995	10138	3	0.2426
ZNF281	6	0.30896	0.5174	0.9995	10139	2	-0.102
P2RY4	6	0.30906	0.5175	0.9995	10140	3	0.2009
hsa-mir-3941	4	0.30911	0.45256	0.9995	10141	2	0.1303
ORC3	6	0.30911	0.51758	0.9995	10142	1	-0.044
UBA2	6	0.30915	0.51764	0.9995	10143	2	0.2031
LYPD8	6	0.30915	0.51764	0.9995	10144	2	-0.051
DNAJB14	6	0.30925	0.51775	0.9995	10145	2	-0.652
hsa-mir-141	4	0.30928	0.45275	0.9995	10146	2	0.1916
TMEM261	4	0.30931	0.45277	0.9995	10147	2	0.0091
SLC38A7	6	0.30942	0.51795	0.9995	10148	2	0.1058
OR5B12	6	0.30956	0.51811	0.9995	10149	3	0.0624
BTN2A1	6	0.30957	0.51811	0.9995	10150	2	0.1329
YWHAZ	6	0.30971	0.51827	0.9995	10151	3	0.2568
ETV3	6	0.30976	0.51833	0.9995	10152	3	0.0983
PRPH2	6	0.30982	0.5184	0.9995	10153	3	0.1108
MRPL19	6	0.30986	0.51844	0.9995	10154	2	-0.004
CCDC88C	6	0.3099	0.51849	0.9995	10155	3	0.2695
hsa-mir-4652	4	0.3099	0.4534	0.9995	10156	1	0.0636
CCL25	6	0.30993	0.51853	0.9995	10157	3	0.092
KCNA2	6	0.30995	0.51855	0.9995	10158	2	-0.166
LAIR2	5	0.30998	0.48981	0.9995	10159	1	-0.318
COL2A1	6	0.30999	0.51859	0.9995	10160	3	-0.146
SAC3D1	6	0.31003	0.51864	0.9995	10161	3	0.1972
ZNF407	6	0.31006	0.51867	0.9995	10162	3	0.1078
CNR2	6	0.3101	0.51872	0.9995	10163	2	0.1373
RHBDD3	6	0.31014	0.51876	0.9995	10164	3	0.239
HLCS	6	0.3102	0.51884	0.9995	10165	2	0.0867
LGALSL	6	0.31022	0.51885	0.9995	10166	3	0.1395
CDKAL1	6	0.31026	0.51891	0.9995	10167	2	-0.214
TTC3	6	0.31027	0.51891	0.9995	10168	3	0.0728
C8orf34	4	0.31031	0.45382	0.9995	10169	2	-0.03
hsa-mir-3926-1	1	0.31033	0.31042	0.9995	10170	1	0.4079
CMTM1	4	0.31036	0.45387	0.9995	10171	1	-0.689
TRDN	6	0.31039	0.51905	0.9995	10172	3	0.243
FKBP1B	6	0.31045	0.51912	0.9995	10173	3	0.1212
EFCAB9	6	0.3106	0.51931	0.9995	10174	1	-0.11
hsa-mir-6855	4	0.31072	0.45423	0.9995	10175	2	0.1624
ISLR	6	0.31079	0.51953	0.9995	10176	3	-0.078
CLASP2	6	0.31079	0.51953	0.9995	10177	1	-0.055
MRPS17	6	0.31083	0.51957	0.9995	10178	3	-0.124
PPP1R14D	6	0.31092	0.51969	0.9995	10179	3	0.2089
CCR2	6	0.31096	0.51974	0.9995	10180	2	-0.053
PDE6C	6	0.31102	0.51981	0.9995	10181	1	-0.303
SH3PXD2A	6	0.31103	0.51981	0.9995	10182	2	-0.187
ALDH2	6	0.31107	0.51986	0.9995	10183	3	0.0355
CDH5	6	0.31119	0.52	0.9995	10184	3	0.0028
CCNO	6	0.31131	0.52014	0.9995	10185	2	-0.383
MRPL37	6	0.3114	0.52024	0.9995	10186	2	-0.284
TSPAN18	6	0.31144	0.52028	0.9995	10187	3	0.2366
FLRT3	6	0.31144	0.52028	0.9995	10188	1	-0.236
OPN5	6	0.31151	0.52035	0.9995	10189	3	0.0332
CABP4	6	0.31151	0.52035	0.9995	10190	3	0.0923
STX12	6	0.31154	0.52038	0.9995	10191	2	0.1047
NFE2	6	0.3116	0.52045	0.9995	10192	2	0.0447
FLT3LG	6	0.31171	0.52059	0.9995	10193	2	0.0094
UGCG	6	0.31171	0.52059	0.9995	10194	3	0.2162
CLCNKB	6	0.31183	0.52074	0.9995	10195	2	-0.194

GPAT2	6	0.31183	0.52074	0.9995	10196	2	0.0397
UNC5CL	6	0.31184	0.52074	0.9995	10197	2	-0.193
TEX15	6	0.31194	0.52085	0.9995	10198	1	0.0616
NOTUM	6	0.31202	0.52094	0.9995	10199	1	-0.14
RFC4	6	0.31203	0.52095	0.9995	10200	2	-0.179
GRAMD3	6	0.31203	0.52095	0.9995	10201	3	0.1079
THUMPD1	6	0.31203	0.52096	0.9995	10202	3	0.0326
AFG3L2	6	0.31206	0.52099	0.9995	10203	3	-0.057
HTR3B	6	0.31206	0.52099	0.9995	10204	3	0.19
UBASH3A	6	0.31209	0.52102	0.9995	10205	3	0.0365
TRIM25	6	0.31211	0.52104	0.9995	10206	3	0.1141
hsa-mir-548ao	4	0.31215	0.45568	0.9995	10207	2	0.4134
FBLN7	6	0.31219	0.52115	0.9995	10208	3	0.1904
ZFP62	6	0.31224	0.5212	0.9995	10209	1	-0.27
SLC9A3R1	6	0.31226	0.52122	0.9995	10210	2	0.012
RBM7	6	0.31226	0.52122	0.9995	10211	2	-0.365
EFNB2	6	0.31228	0.52124	0.9995	10212	3	0.1802
IFIT2	6	0.31231	0.52126	0.9995	10213	2	-0.137
FAM222A	6	0.31234	0.52129	0.9995	10214	3	0.2165
CLGN	6	0.31237	0.52133	0.9995	10215	2	-0.044
SAGE1	6	0.31242	0.52138	0.9995	10216	1	-0.079
TFCP2	6	0.31242	0.52139	0.9995	10217	3	0.1812
hsa-mir-744	4	0.3125	0.45605	0.9995	10218	2	0.339
ZNF362	6	0.31251	0.52149	0.9995	10219	3	0.0456
CAPN2	6	0.3126	0.52161	0.9995	10220	3	0.247
RFFL	6	0.31268	0.52169	0.9995	10221	2	0.0241
TOP3B	6	0.31268	0.5217	0.9995	10222	3	0.1357
ELOVL6	6	0.31268	0.5217	0.9995	10223	2	-0.584
ZNF627	3	0.31275	0.43596	0.9995	10224	1	0.3719
CRCT1	6	0.31276	0.52178	0.9995	10225	2	-0.02
hsa-mir-6127	3	0.31288	0.43604	0.9995	10226	1	-0.077
EIF1B	6	0.31301	0.52206	0.9995	10227	3	0.1871
ECHS1	6	0.31308	0.52212	0.9995	10228	3	-0.183
DUXA	6	0.31318	0.52223	0.9995	10229	3	0.06
TMEM134	6	0.31322	0.52228	0.9995	10230	2	-0.008
hsa-mir-3663	4	0.31328	0.45686	0.9995	10231	1	-0.111
C1orf146	6	0.31329	0.52237	0.9995	10232	1	-0.292
TLN2	6	0.31335	0.52244	0.9995	10233	2	-0.278
BCS1L	6	0.31341	0.5225	0.9995	10234	2	0.144
ZBTB21	6	0.31352	0.52263	0.9995	10235	2	-0.154
CREBZF	6	0.31356	0.52268	0.9995	10236	3	0.2336
PI4KB	6	0.31361	0.52272	0.9995	10237	3	-0.337
CECR2	6	0.31363	0.52276	0.9995	10238	3	0.1683
KLHL7	6	0.31368	0.52281	0.9995	10239	3	0.2019
OR2T6	6	0.31369	0.52283	0.9995	10240	2	-0.308
CHD5	6	0.31373	0.52288	0.9995	10241	2	-0.076
TP53RK	6	0.31378	0.52294	0.9995	10242	2	0.1494
GSPT2	6	0.31381	0.52296	0.9995	10243	2	-0.153
ZNF141	4	0.31381	0.45741	0.9995	10244	2	0.1177
SURF2	6	0.31385	0.523	0.9995	10245	2	0.0717
hsa-mir-552	4	0.31388	0.45748	0.9995	10246	2	-0.042
PHF14	6	0.3139	0.52305	0.9995	10247	2	0.057
PHYHD1	6	0.31393	0.52309	0.9995	10248	3	0.0639
hsa-mir-548f-4	1	0.31396	0.31408	0.9995	10249	1	0.6544
CEACAM16	6	0.31413	0.52333	0.9995	10250	3	0.1545
SLIRP	6	0.31417	0.52339	0.9995	10251	3	0.0693
SNX5	6	0.31423	0.52346	0.9995	10252	2	-0.108
C4orf19	6	0.31432	0.52358	0.9995	10253	3	0.0098
KIF3A	6	0.31436	0.52363	0.9995	10254	2	-0.108
TAF5	6	0.31448	0.52376	0.9995	10255	3	0.0166
PDE9A	6	0.31455	0.52383	0.9995	10256	3	0.1107
TRIM38	4	0.31459	0.45822	0.9995	10257	1	-0.406
PLAC8L1	6	0.31461	0.5239	0.9995	10258	2	-0.31
NGB	6	0.31461	0.52391	0.9995	10259	2	-0.102
MSMP	6	0.31463	0.52394	0.9995	10260	3	0.1006
KCNJ13	6	0.31466	0.52397	0.9995	10261	3	0.147
hsa-mir-922	4	0.31466	0.45828	0.9995	10262	2	0.1018
CTXN2	6	0.31472	0.52403	0.9995	10263	2	0.1177
KIAA1671	6	0.3149	0.52423	0.9995	10264	3	0.1016
VILL	6	0.31499	0.52434	0.9995	10265	1	-0.292
TNNI2	6	0.31501	0.52437	0.9995	10266	3	0.061
OR2L2	6	0.31505	0.52441	0.9995	10267	3	0.0138
hsa-mir-572	4	0.3151	0.45873	0.9995	10268	2	0.1375
FBLN2	6	0.31518	0.52456	0.9995	10269	2	-0.023
LOC100130348	6	0.31523	0.52463	0.9995	10270	2	-0.192
FAM193B	4	0.31533	0.45897	0.9995	10271	1	-1.085
SPEG	6	0.31537	0.52479	0.9995	10272	3	0.0978
NUP188	6	0.31543	0.52485	0.9995	10273	2	-0.306
NOTCH1	6	0.31543	0.52486	0.9995	10274	3	-0.083
KRTAP13-4	6	0.31544	0.52486	0.9995	10275	1	0.0617
ALDH3B1	5	0.31545	0.49432	0.9995	10276	2	0.1355
APOE	6	0.31559	0.52505	0.9995	10277	1	-0.266
TP53INP1	6	0.31561	0.52506	0.9995	10278	3	0.3232
AXL	6	0.31566	0.52512	0.9995	10279	3	0.1084
DMD	6	0.31574	0.52523	0.9995	10280	3	0.0683

SLC9A3	6	0.31577	0.52525	0.9995	10281	2	0.0961
WHAMM	6	0.31581	0.5253	0.9995	10282	3	0.1765
TMEM260	6	0.31585	0.52535	0.9995	10283	3	0.2686
COPS8	6	0.31585	0.52535	0.9995	10284	3	0.1956
SLAIN2	6	0.31585	0.52535	0.9995	10285	3	0.0447
VAV3	6	0.3159	0.5254	0.9995	10286	3	0.0968
GPR55	6	0.31598	0.5255	0.9995	10287	3	0.1648
ZSWIM5	6	0.31609	0.52563	0.9995	10288	3	0.1221
ZNF274	6	0.31615	0.52568	0.9995	10289	2	-0.857
TRIM74	2	0.31618	0.34931	0.9995	10290	1	0.0145
ZKSCAN7	6	0.31621	0.52575	0.9995	10291	3	0.0591
TERF2IP	6	0.31621	0.52575	0.9995	10292	3	0.1282
ZC3H11A	6	0.31626	0.5258	0.9995	10293	1	-0.073
BRMS1L	6	0.31628	0.52583	0.9995	10294	3	0.1567
CENPI	6	0.31631	0.52587	0.9995	10295	3	-0.374
DTWD2	5	0.31639	0.4951	0.9995	10296	2	-0.481
METTL2A	5	0.31647	0.49516	0.9995	10297	2	0.0846
KIAA2022	6	0.31651	0.52608	0.9995	10298	3	-0.048
MSRB1	6	0.31658	0.52616	0.9995	10299	2	-0.132
PATE1	6	0.31662	0.5262	0.9995	10300	3	0.0477
hsa-mir-4667	4	0.31666	0.46033	0.9995	10301	2	0.2034
FGFR4	6	0.31669	0.52629	0.9995	10302	1	-0.243
DYSF	6	0.3167	0.52629	0.9995	10303	2	0.1583
RNF138	6	0.31673	0.52634	0.9995	10304	1	-0.068
EME1	6	0.31674	0.52635	0.9995	10305	3	0.2062
RTL1	6	0.31674	0.52635	0.9995	10306	3	-0.16
GOLGA8A	5	0.31678	0.49543	0.9995	10307	1	-0.156
VPS37D	6	0.31684	0.52645	0.9995	10308	3	0.0531
ATP7B	6	0.31685	0.52646	0.9995	10309	3	0.1851
KIAA1524	6	0.31686	0.52648	0.9995	10310	1	-0.046
FAM157A	3	0.31687	0.43814	0.9995	10311	1	-0.979
TNIP2	6	0.31696	0.52659	0.9995	10312	2	-0.131
KIFC2	6	0.31696	0.52659	0.9995	10313	2	0.2243
DYNC1I2	6	0.31713	0.52678	0.9995	10314	3	0.1033
SLC29A3	6	0.31717	0.52683	0.9995	10315	3	0.0488
COL22A1	6	0.31724	0.5269	0.9995	10316	1	-0.217
hsa-mir-4694	4	0.31725	0.46092	0.9995	10317	2	0.1914
MAPK8	6	0.31733	0.527	0.9995	10318	3	0.1754
EFCAB12	6	0.31747	0.52717	0.9995	10319	1	-0.268
NETO2	6	0.31755	0.52726	0.9995	10320	2	-0.551
SCIN	4	0.31755	0.46125	0.9995	10321	1	-0.306
GJC2	6	0.31763	0.52735	0.9995	10322	3	0.187
KIAA1456	6	0.31763	0.52735	0.9995	10323	2	-0.096
NXT2	6	0.31763	0.52735	0.9995	10324	2	-0.245
ZNF57	6	0.31766	0.52739	0.9995	10325	1	-0.323
DOLK	6	0.31772	0.52746	0.9995	10326	3	0.1343
PCDHGC5	2	0.31773	0.35049	0.9995	10327	1	-0.327
IFRD2	6	0.31779	0.52755	0.9995	10328	2	-0.106
PCSK1N	6	0.31786	0.52763	0.9995	10329	2	-0.194
TAS2R50	6	0.31791	0.5277	0.9995	10330	3	0.0309
POR	6	0.31793	0.52772	0.9995	10331	3	0.0769
KHNYN	6	0.31798	0.52778	0.9995	10332	2	0.1122
METTL16	6	0.31805	0.52786	0.9995	10333	2	-0.138
OR10J1	6	0.31806	0.52787	0.9995	10334	2	-0.014
KCNA10	6	0.31817	0.528	0.9995	10335	2	0.1272
FASLG	6	0.31821	0.52804	0.9995	10336	3	-0.054
CCDC11	6	0.31821	0.52804	0.9995	10337	3	-0.085
ZC3H15	6	0.31821	0.52804	0.9995	10338	2	0.0298
OR2K2	6	0.31822	0.52805	0.9995	10339	2	-0.128
GPD2	6	0.31829	0.52812	0.9995	10340	2	-0.122
TMEM8A	6	0.31839	0.52823	0.9995	10341	2	0.0304
LNPEP	6	0.31844	0.52829	0.9995	10342	2	0.0519
PRPF40A	6	0.31848	0.52833	0.9995	10343	3	0.0996
PCDHB11	6	0.31854	0.5284	0.9995	10344	3	0.1899
YIF1A	6	0.31857	0.52843	0.9995	10345	3	0.0974
SERGEF	6	0.31859	0.52846	0.9995	10346	3	0.1209
BAALC	6	0.31866	0.52854	0.9995	10347	2	0.0541
YAF2	6	0.3187	0.52859	0.9995	10348	3	0.1574
DEPDC4	6	0.31882	0.52873	0.9995	10349	1	-0.106
LPPR1	6	0.31883	0.52874	0.9995	10350	2	-0.082
ISX	6	0.31888	0.5288	0.9995	10351	3	-0.035
TRIM56	6	0.31894	0.52888	0.9995	10352	3	0.098
ARRB2	6	0.319	0.52896	0.9995	10353	3	0.1759
TRIM16L	5	0.31904	0.49722	0.9995	10354	2	0.1013
MTSS1	6	0.31905	0.52901	0.9995	10355	2	-0.635
TCEB2	6	0.31908	0.52905	0.9995	10356	1	-0.299
UBE3D	6	0.31919	0.52916	0.9995	10357	2	0.1222
THRA	6	0.31923	0.52921	0.9995	10358	2	-0.08
TSLP	6	0.31925	0.52924	0.9995	10359	2	-0.309
DEFA6	6	0.31927	0.52926	0.9995	10360	1	-0.137
TBC1D29	6	0.31934	0.52934	0.9995	10361	3	0.1137
CYP4F12	5	0.31941	0.49752	0.9995	10362	2	-0.221
IQCH	6	0.3195	0.52953	0.9995	10363	3	0.1271
EFCAB2	6	0.31953	0.52957	0.9995	10364	1	-0.212
C2orf78	6	0.3196	0.52964	0.9995	10365	3	0.2153

GTF2A1	6	0.3196	0.52964	0.9995	10366	3	0.1765
MBLAC2	6	0.31963	0.52969	0.9995	10367	2	-0.115
WNT5A	6	0.31969	0.52976	0.9995	10368	3	0.1157
CHDH	6	0.31978	0.52986	0.9995	10369	3	0.0347
TMEM126A	6	0.31978	0.52987	0.9995	10370	1	-0.115
ZSWIM1	6	0.31984	0.52993	0.9995	10371	2	-0.255
hsa-mir-7703	4	0.31985	0.46357	0.9995	10372	2	0.1758
UBP1	6	0.31996	0.53008	0.9995	10373	3	0.0144
PDCD6IP	6	0.31999	0.53011	0.9995	10374	2	-0.23
MCM4	6	0.32005	0.53018	0.9995	10375	3	0.1626
AGXT2	6	0.32006	0.5302	0.9995	10376	1	0.069
MAML1	6	0.32006	0.5302	0.9995	10377	2	0.0448
RRP9	6	0.32017	0.53031	0.9995	10378	3	0.2028
ZNF850	6	0.32018	0.53032	0.9995	10379	2	-0.104
SLC4A1	6	0.32021	0.53036	0.9995	10380	3	0.1408
SHMT2	6	0.32027	0.53042	0.9995	10381	2	0.0591
PRPF38A	6	0.32027	0.53042	0.9995	10382	2	-0.5
hsa-mir-302c	4	0.32028	0.46398	0.9995	10383	2	0.1677
FUS	6	0.32039	0.53055	0.9995	10384	3	0.1423
FBXO21	6	0.3204	0.53057	0.9995	10385	2	-0.161
HSPA1L	6	0.3205	0.53067	0.9995	10386	3	0.2227
ZFAT	6	0.3205	0.53067	0.9995	10387	3	0.0622
OR7A17	6	0.3205	0.53068	0.9995	10388	1	-0.165
PXN	6	0.32071	0.53092	0.9995	10389	3	-0.045
KDSR	6	0.32078	0.531	0.9995	10390	2	0.1153
MED28	6	0.3208	0.53103	0.9995	10391	1	-0.176
HINT2	6	0.3208	0.53103	0.9995	10392	2	-0.18
PLA2G2A	6	0.32094	0.53118	0.9995	10393	2	-0.339
RLN3	4	0.32097	0.46466	0.9995	10394	2	0.1578
MUC20	4	0.32099	0.46468	0.9995	10395	2	0.1366
HERC2	6	0.32103	0.53127	0.9995	10396	1	-0.051
GJB3	6	0.32104	0.53129	0.9995	10397	3	0.2136
OR5T2	6	0.32117	0.53143	0.9995	10398	2	-0.375
CHRM5	6	0.32126	0.53152	0.9995	10399	2	-0.122
RUVBL1	6	0.32126	0.53152	0.9995	10400	2	-0.205
TAS1R2	6	0.32131	0.53157	0.9995	10401	3	0.1786
FAM21C	4	0.32135	0.46503	0.9995	10402	1	-0.077
STAT1	6	0.3214	0.53167	0.9995	10403	1	-0.645
C1orf216	6	0.32143	0.5317	0.9995	10404	3	0.0941
OR5H2	6	0.32149	0.53177	0.9995	10405	2	-0.267
BCAT1	6	0.32156	0.53185	0.9995	10406	3	0.1039
TTC5	6	0.32156	0.53185	0.9995	10407	3	0.0403
HIC1	6	0.32157	0.53187	0.9995	10408	2	0.1532
GZMH	6	0.32164	0.53195	0.9995	10409	2	0.087
HAPLN4	6	0.32176	0.53209	0.9995	10410	2	-0.395
RNASE11	6	0.32195	0.53232	0.9995	10411	2	0.0304
GGT1	6	0.32197	0.53234	0.9995	10412	1	-0.187
SEC31B	6	0.32197	0.53234	0.9995	10413	1	-0.218
BAIAP3	6	0.32202	0.5324	0.9995	10414	3	0.1134
NLRC3	6	0.32202	0.5324	0.9995	10415	3	-0.128
TMEM100	6	0.32214	0.53255	0.9995	10416	2	0.0924
FYN	6	0.32221	0.53262	0.9995	10417	2	-0.112
ABHD2	6	0.32227	0.5327	0.9995	10418	2	-0.443
CCNI	6	0.32239	0.53283	0.9995	10419	2	-0.422
CASP9	6	0.32242	0.53287	0.9995	10420	2	-0.231
METTL21B	4	0.32249	0.46617	0.9995	10421	1	0.0775
STX7	6	0.3225	0.53295	0.9995	10422	3	0.0398
PLAC9	6	0.32251	0.53297	0.9995	10423	2	0.0712
hsa-mir-7155	4	0.32254	0.46621	0.9995	10424	1	-0.084
BBS5	6	0.32255	0.53302	0.9995	10425	3	0.1241
CYP2E1	6	0.32258	0.53305	0.9995	10426	2	0.0918
INMT	6	0.32262	0.5331	0.9995	10427	2	0.0377
SLC35F5	6	0.32262	0.5331	0.9995	10428	2	-0.121
MSX1	6	0.32263	0.5331	0.9995	10429	2	-0.314
ZIC3	6	0.32264	0.53311	0.9995	10430	3	0.1978
TSN	6	0.32277	0.53327	0.9995	10431	3	0.1271
hsa-mir-626	4	0.32278	0.46646	0.9995	10432	2	0.127
HOXB5	6	0.32278	0.53328	0.9995	10433	2	-0.235
HECW1	6	0.32283	0.53333	0.9995	10434	3	0.0107
hsa-mir-6746	4	0.32293	0.46661	0.9995	10435	2	0.0971
KIAA0317	4	0.32293	0.46661	0.9995	10436	2	0.1068
EGR1	6	0.32294	0.53344	0.9995	10437	3	0.1469
ADAMTS15	6	0.32297	0.53348	0.9995	10438	1	-0.615
ZNF296	6	0.323	0.53352	0.9995	10439	2	-0.546
DMP1	6	0.32306	0.53359	0.9995	10440	1	-0.128
DEFB119	6	0.32313	0.53367	0.9995	10441	2	0.0859
COLEC11	6	0.32314	0.53367	0.9995	10442	1	-0.283
RPS25	6	0.32324	0.53379	0.9995	10443	3	0.2501
FAXDC2	6	0.32327	0.53382	0.9995	10444	3	0.0783
FOXA3	6	0.32327	0.53382	0.9995	10445	2	-0.047
PNMA2	6	0.32334	0.53391	0.9995	10446	2	-0.055
SERINC4	6	0.32342	0.53399	0.9995	10447	3	0.0273
GDPD1	6	0.32353	0.53412	0.9995	10448	3	0.0963
HNRNPM	6	0.32362	0.53422	0.9995	10449	3	0.2966
AQP8	6	0.32368	0.5343	0.9995	10450	2	-0.133

C17orf77	6	0.32371	0.53432	0.9995	10451	2	-0.404
C15orf65	3	0.32374	0.44188	0.9995	10452	1	0.351
CDK2AP1	6	0.32375	0.53438	0.9995	10453	3	0.1692
RPS9	6	0.32377	0.5344	0.9995	10454	3	-0.078
ARHGAP15	6	0.32377	0.5344	0.9995	10455	2	0.1005
FAM69B	6	0.32384	0.53449	0.9995	10456	3	-0.032
ATP9B	6	0.32398	0.53465	0.9995	10457	3	0.2275
TAS2R38	6	0.32409	0.53478	0.9995	10458	3	0.1761
ZNF23	6	0.32409	0.53478	0.9995	10459	2	-0.153
N4BP1	6	0.3241	0.53478	0.9995	10460	2	-0.11
DNAJC7	6	0.32421	0.53493	0.9995	10461	2	-0.213
COX18	6	0.32422	0.53494	0.9995	10462	2	0.0737
ACE	6	0.3244	0.53514	0.9995	10463	1	-0.291
CABP1	6	0.32443	0.53517	0.9995	10464	2	-0.299
JAK2	6	0.32448	0.53522	0.9995	10465	2	-0.372
RELL1	6	0.32448	0.53523	0.9995	10466	3	-0.04
MIA3	6	0.32448	0.53523	0.9995	10467	3	0.0101
SBDS	6	0.32451	0.53525	0.9995	10468	1	-0.368
FNDC3A	6	0.32457	0.53533	0.9995	10469	2	-0.237
C20orf194	6	0.32464	0.5354	0.9995	10470	2	-0.267
ITGB1	6	0.32467	0.53543	0.9995	10471	3	0.0872
SRRM2	6	0.32467	0.53543	0.9995	10472	3	0.2416
PRPS1L1	6	0.3247	0.53546	0.9995	10473	1	-0.162
COL17A1	6	0.32473	0.5355	0.9995	10474	1	-0.321
SCUBE3	6	0.32485	0.53563	0.9995	10475	1	-0.032
SLC1A4	6	0.32487	0.53566	0.9995	10476	3	0.1737
PTGDR	6	0.32496	0.53576	0.9995	10477	1	-0.521
PDZD7	6	0.32499	0.53579	0.9995	10478	2	-0.395
FAM72D	1	0.32502	0.32522	0.9995	10479	1	0.2008
TACR3	6	0.32503	0.53584	0.9995	10480	2	0.1128
RPUSD3	6	0.32505	0.53586	0.9995	10481	2	0.0814
PIWIL3	6	0.32509	0.53591	0.9995	10482	3	-0.099
TIMD4	6	0.32511	0.53593	0.9995	10483	2	0.0468
TMEM218	6	0.32516	0.53599	0.9995	10484	3	0.1705
DPY30	6	0.32516	0.53599	0.9995	10485	3	0.1573
GPR50	6	0.32518	0.53601	0.9995	10486	2	-0.354
TSGA10	6	0.32525	0.53608	0.9995	10487	2	0.0447
FAIM2	6	0.32526	0.53609	0.9995	10488	3	0.1259
BRPF1	6	0.32532	0.53615	0.9995	10489	2	0.2547
IQCA1	6	0.32532	0.53615	0.9995	10490	2	0.0282
TMEM106C	6	0.32537	0.53621	0.9995	10491	1	-0.563
CRYM	6	0.32539	0.53622	0.9995	10492	2	0.1483
HSD17B8	6	0.32547	0.53631	0.9995	10493	2	-0.093
RHBDD2	6	0.32563	0.53649	0.9995	10494	3	0.121
SH2D3A	6	0.32574	0.53662	0.9995	10495	3	0.2753
MTMR9	6	0.32575	0.53663	0.9995	10496	1	-0.255
STX6	6	0.32585	0.53674	0.9995	10497	2	-0.22
TP53I3	6	0.32585	0.53674	0.9995	10498	2	0.0051
hsa-mir-3128	4	0.32585	0.46965	0.9995	10499	2	0.0806
PRR24	6	0.32588	0.53678	0.9995	10500	3	0.07
HOXD11	6	0.32603	0.53697	0.9995	10501	3	0.021
RLN1	5	0.32607	0.50291	0.9995	10502	1	-0.357
C1orf63	6	0.3261	0.53705	0.9995	10503	2	0.2457
CTAGE4	4	0.32611	0.46994	0.9995	10504	2	-0.035
DGUOK	6	0.32616	0.53713	0.9995	10505	3	0.054
COG6	6	0.32616	0.53713	0.9995	10506	3	-0.057
SFT2D1	6	0.32622	0.5372	0.9995	10507	2	-0.121
MAP1LC3B2	6	0.32623	0.53721	0.9995	10508	2	0.0578
WDR82	6	0.32633	0.53732	0.9995	10509	2	-0.119
NUDT6	6	0.32646	0.53747	0.9995	10510	2	-0.155
PLEK	6	0.32648	0.53749	0.9995	10511	3	0.1673
hsa-mir-22	4	0.32651	0.47034	0.9995	10512	2	0.1803
AWAT1	6	0.32652	0.53753	0.9995	10513	1	-0.216
NANOS2	6	0.32654	0.53756	0.9995	10514	2	0.0575
KCTD3	4	0.32655	0.47038	0.9995	10515	1	0.0965
hsa-mir-431	4	0.32664	0.47046	0.9995	10516	2	0.0953
SPATA4	6	0.32668	0.53773	0.9995	10517	2	0.0132
PKN3	6	0.32674	0.5378	0.9995	10518	2	0.0992
SBSPON	6	0.32674	0.5378	0.9995	10519	1	-0.276
NDUFA10	6	0.32675	0.53781	0.9995	10520	3	0.1456
GSG1	6	0.32691	0.53799	0.9995	10521	2	0.0841
hsa-mir-147b	3	0.32691	0.4436	0.9995	10522	1	-0.141
UGT2A2	1	0.32708	0.32727	0.9995	10523	1	0.2652
hsa-mir-508	4	0.3271	0.47093	0.9995	10524	1	-0.038
AKAP6	6	0.32713	0.53824	0.9995	10525	2	-0.017
NAA30	6	0.32713	0.53825	0.9995	10526	2	-0.047
KLF2	6	0.32717	0.5383	0.9995	10527	1	-0.362
GMPPB	6	0.32719	0.53831	0.9995	10528	3	0.2912
GPER1	4	0.32721	0.47105	0.9995	10529	1	-0.203
AGMAT	6	0.32723	0.53835	0.9995	10530	2	-0.329
XYLT1	6	0.32731	0.53844	0.9995	10531	3	0.2038
hsa-mir-1287	4	0.32733	0.47118	0.9995	10532	2	0.1169
CTNNA1	6	0.32743	0.53859	0.9995	10533	3	0.1876
CCDC64B	6	0.32747	0.53863	0.9995	10534	3	0.018
SLAMF8	6	0.32755	0.53871	0.9995	10535	3	0.2096

FAM71C	6	0.32762	0.53881	0.9995	10536	2	-0.275
FAM162B	6	0.3277	0.53889	0.9995	10537	2	-0.07
hsa-mir-4435-1	3	0.32774	0.44405	0.9995	10538	1	-0.199
GAREML	6	0.32777	0.53898	0.9995	10539	3	0.0783
GNL2	6	0.32784	0.53906	0.9995	10540	3	0.1547
EEF1E1	6	0.32785	0.53908	0.9995	10541	2	-0.055
PLEKHF2	6	0.3279	0.53913	0.9995	10542	1	-0.727
SOWAHC	6	0.32791	0.53913	0.9995	10543	3	0.2209
KCNK3	6	0.32806	0.53931	0.9995	10544	2	-0.165
CAMTA1	6	0.32807	0.53932	0.9995	10545	2	-0.185
HOXA9	6	0.32807	0.53932	0.9995	10546	1	-0.166
HSD17B11	6	0.32833	0.53963	0.9995	10547	2	-0.59
CNGA3	6	0.32837	0.53967	0.9995	10548	2	0.1264
PLCH2	6	0.32838	0.53968	0.9995	10549	2	-0.003
TCTN2	6	0.32843	0.53974	0.9995	10550	3	0.1745
C1QC	6	0.32855	0.53988	0.9995	10551	3	0.201
PIGR	6	0.32856	0.53988	0.9995	10552	1	-0.025
YES1	6	0.32863	0.53996	0.9995	10553	3	0.1398
CCDC151	6	0.32865	0.53998	0.9995	10554	2	-0.124
ACTR1B	6	0.32877	0.54012	0.9995	10555	2	0.2978
TRAPPC8	6	0.32889	0.54025	0.9995	10556	2	-0.132
POLM	6	0.32889	0.54025	0.9995	10557	2	-0.101
CD97	6	0.32891	0.54027	0.9995	10558	2	0.1563
EPS15	6	0.32906	0.54044	0.9995	10559	3	0.156
ADAMTS6	6	0.32917	0.54057	0.9995	10560	2	0.1431
NUDT4	6	0.3292	0.5406	0.9995	10561	3	0.0086
ASB15	6	0.32926	0.54066	0.9995	10562	3	0.133
CRY2	6	0.32926	0.54066	0.9995	10563	3	0.0357
hsa-mir-8069	4	0.3293	0.47323	0.9995	10564	1	-0.21
HEATR4	6	0.3293	0.54072	0.9995	10565	2	0.0713
WDR27	6	0.32937	0.5408	0.9995	10566	3	-0.2
KLHL9	6	0.32937	0.5408	0.9995	10567	3	-0.121
POLR2A	6	0.32949	0.54093	0.9995	10568	1	-0.047
CYBA	6	0.32952	0.54096	0.9995	10569	2	0.1478
TNFAIP8L2	6	0.32955	0.54099	0.9995	10570	3	0.116
RSAD2	6	0.32962	0.54108	0.9995	10571	3	0.1964
PASK	6	0.32966	0.54112	0.9995	10572	3	0.2335
hsa-mir-3944	4	0.32968	0.47364	0.9995	10573	2	0.064
MS4A13	6	0.32974	0.54122	0.9995	10574	2	-0.162
UCP2	6	0.32977	0.54124	0.9995	10575	3	0.0286
DDX43	6	0.32985	0.54133	0.9995	10576	2	0.0662
OR1J1	6	0.32986	0.54134	0.9995	10577	3	0.2076
FXYD7	6	0.32988	0.54137	0.9995	10578	2	0.036
ZMAT1	6	0.33001	0.54153	0.9995	10579	2	-0.176
C18orf8	6	0.33005	0.54155	0.9995	10580	3	0.1675
KIAA0226	6	0.33014	0.54167	0.9995	10581	3	0.1158
TIMMDC1	6	0.33016	0.5417	0.9995	10582	2	0.1111
MAP10	6	0.33021	0.54176	0.9995	10583	2	-0.321
INO80	6	0.33027	0.54183	0.9995	10584	2	-0.065
CCDC40	6	0.33042	0.542	0.9995	10585	1	-0.357
FBXW11	6	0.33044	0.54203	0.9995	10586	3	0.0909
TNMD	6	0.33045	0.54203	0.9995	10587	2	-0.016
SFTA2	6	0.33045	0.54203	0.9995	10588	3	0.1238
TAPT1	6	0.33046	0.54205	0.9995	10589	1	0.1424
RSU1	6	0.33052	0.54211	0.9995	10590	2	0.1292
F2RL2	6	0.33055	0.54215	0.9995	10591	3	0.0301
MGAM	6	0.33057	0.54218	0.9995	10592	2	0.0861
SPG11	6	0.33057	0.54218	0.9995	10593	3	0.1741
ANKRD24	6	0.33072	0.54234	0.9995	10594	1	-0.455
hsa-mir-1266	4	0.33086	0.47485	0.9995	10595	2	0.4337
YBX3	6	0.33091	0.54255	0.9995	10596	1	-0.276
SLITRK1	6	0.331	0.54266	0.9995	10597	3	0.089
C9orf69	6	0.33103	0.54269	0.9995	10598	3	0.0724
RABAC1	6	0.33109	0.54278	0.9995	10599	3	0.1533
BOK	6	0.33109	0.54278	0.9995	10600	3	-0.005
ZNF701	5	0.33109	0.50709	0.9995	10601	2	0.3269
EID1	6	0.33114	0.54284	0.9995	10602	3	0.1473
hsa-mir-548au	1	0.33115	0.33133	0.9995	10603	1	0.3029
C14orf39	6	0.33117	0.54287	0.9995	10604	2	-0.183
NLRP8	6	0.33119	0.54289	0.9995	10605	1	-0.4
JAGN1	6	0.33126	0.54297	0.9995	10606	2	0.185
ODF2L	5	0.33127	0.50723	0.9995	10607	1	0.2292
hsa-mir-4260	4	0.33135	0.47536	0.9995	10608	2	-0.061
CSNK2B	6	0.33142	0.54315	0.9995	10609	3	0.2308
RAB39B	6	0.33147	0.5432	0.9995	10610	1	-0.74
NCR1	6	0.33152	0.54327	0.9995	10611	3	0.135
CDRT15	6	0.33159	0.54335	0.9995	10612	2	-0.233
SFXN4	4	0.33161	0.47563	0.9995	10613	1	-0.034
ZNF568	6	0.33173	0.54351	0.9995	10614	2	0.0615
hsa-mir-3610	4	0.33173	0.47575	0.9995	10615	2	0.4063
MECR	4	0.33184	0.47587	0.9995	10616	2	-0.348
MRPL38	6	0.33189	0.5437	0.9995	10617	2	-0.075
CYP7B1	6	0.33191	0.5437	0.9995	10618	3	0.1925
RANGAP1	6	0.33195	0.54376	0.9995	10619	2	-0.029
GALM	6	0.33197	0.54378	0.9995	10620	1	-0.173

hsa-mir-208a	4	0.332	0.47604	0.9995	10621	2	0.1124
ARHGAP44	6	0.33217	0.544	0.9995	10622	2	0.0813
SESN1	6	0.33219	0.54402	0.9995	10623	3	0.0176
hsa-mir-6877	4	0.33221	0.47625	0.9995	10624	2	0.2724
KCTD10	6	0.33223	0.54406	0.9995	10625	2	0.2354
IDH3G	6	0.33227	0.54411	0.9995	10626	2	-0.196
PTBP3	6	0.33229	0.54412	0.9995	10627	1	-0.252
HOXB6	6	0.33235	0.54419	0.9995	10628	3	0.0793
hsa-mir-3917	4	0.33244	0.47647	0.9995	10629	2	-0.09
BCOR	6	0.33245	0.54431	0.9995	10630	2	-0.363
FOLR1	6	0.33251	0.54437	0.9995	10631	2	-0.412
ALOX5AP	6	0.33251	0.54437	0.9995	10632	2	0.1006
SMAP1	6	0.33253	0.5444	0.9995	10633	3	0.1023
PER1	6	0.33256	0.54443	0.9995	10634	2	-0.365
SLC35G6	6	0.33262	0.5445	0.9995	10635	3	0.1735
PGM3	6	0.33287	0.5448	0.9995	10636	3	0.133
C14orf2	6	0.33293	0.54486	0.9995	10637	3	0.0423
GOLM1	6	0.33298	0.54491	0.9995	10638	3	0.174
ZNF879	6	0.33305	0.54499	0.9995	10639	3	-0.023
hsa-mir-4524b	1	0.3331	0.33324	0.9995	10640	1	0.1904
COLCA2	6	0.3331	0.54506	0.9995	10641	3	0.1845
PCDHB9	6	0.33313	0.54509	0.9995	10642	3	0.2063
PSKH1	6	0.33314	0.54511	0.9995	10643	2	-0.521
DOK2	6	0.33318	0.54514	0.9995	10644	3	0.086
TCIRG1	6	0.33331	0.54528	0.9995	10645	3	0.1945
EFHB	6	0.33331	0.54528	0.9995	10646	3	0.2569
MED17	6	0.33335	0.54532	0.9995	10647	2	-0.061
BPIFC	6	0.33341	0.5454	0.9995	10648	2	0.1412
MEMO1	6	0.33342	0.54541	0.9995	10649	3	0.258
PLEKHO2	6	0.33347	0.54547	0.9995	10650	2	-0.098
GNAL	6	0.33369	0.54571	0.9995	10651	3	0.1786
THEGL	6	0.33369	0.54571	0.9995	10652	3	0.2279
KIAA0586	4	0.3337	0.47773	0.9995	10653	2	0.1254
ZNF681	6	0.33379	0.54581	0.9995	10654	1	-0.28
CTDP1	6	0.33379	0.54581	0.9995	10655	2	-0.271
FXYD1	6	0.33383	0.54585	0.9995	10656	2	0.0758
SETX	6	0.33396	0.546	0.9995	10657	1	-0.609
MAN1A2	6	0.33409	0.54614	0.9995	10658	3	0.239
C16orf87	6	0.33411	0.54617	0.9995	10659	1	-0.51
UST	6	0.33422	0.54629	0.9995	10660	3	0.0903
POFUT2	6	0.33422	0.54629	0.9995	10661	3	0.1103
ATG4A	6	0.33424	0.54631	0.9995	10662	2	-0.065
C1orf122	6	0.33424	0.54631	0.9995	10663	2	0.108
S100Z	6	0.33429	0.54637	0.9995	10664	2	-0.288
RGPD3	6	0.33433	0.54642	0.9995	10665	1	-0.072
EDEM2	6	0.33438	0.54647	0.9995	10666	3	0.2227
VASP	6	0.33443	0.54653	0.9995	10667	2	-0.048
PYROXD2	6	0.33443	0.54653	0.9995	10668	2	-0.183
SLC44A5	6	0.33449	0.54658	0.9995	10669	3	-0.008
DTNA	6	0.33451	0.54661	0.9995	10670	2	-0.24
GAS8	6	0.33457	0.54668	0.9995	10671	2	-0.015
FIBCD1	6	0.33463	0.54675	0.9995	10672	2	0.0699
BSPH1	5	0.33464	0.50996	0.9995	10673	2	0.1508
TTC39C	6	0.33464	0.54677	0.9995	10674	1	0.0139
VSTM4	6	0.33477	0.54693	0.9995	10675	1	-0.183
RAB28	6	0.3348	0.54696	0.9995	10676	3	0.0019
TANGO6	6	0.33489	0.54706	0.9995	10677	2	0.0499
SERPINI2	6	0.33491	0.54709	0.9995	10678	3	0.1777
NMD3	6	0.335	0.54719	0.9995	10679	3	0.3222
NIM1	6	0.33505	0.54725	0.9995	10680	1	-0.42
OTOA	6	0.33505	0.54725	0.9995	10681	2	0.2047
SLC5A7	6	0.33513	0.54733	0.9995	10682	3	0.0641
PLEKHO1	6	0.33517	0.54739	0.9995	10683	2	0.0927
CBR1	6	0.33521	0.54744	0.9995	10684	3	0.1559
PZP	6	0.33521	0.54744	0.9995	10685	3	0.1991
C4orf40	6	0.33527	0.54751	0.9995	10686	3	0.1574
KCND1	6	0.33535	0.54759	0.9995	10687	2	-0.115
MIEF2	1	0.33536	0.33551	0.9995	10688	1	0.365
STMN4	6	0.33536	0.5476	0.9995	10689	3	0.1629
ZNRF4	6	0.33539	0.54764	0.9995	10690	2	-0.252
TOPAZ1	4	0.33544	0.47957	0.9995	10691	1	-0.304
IRF6	6	0.33547	0.54772	0.9995	10692	3	0.1559
AZIN1	6	0.33552	0.54777	0.9995	10693	1	-0.01
SSRP1	6	0.33563	0.54789	0.9995	10694	1	-0.041
DEDD	6	0.33564	0.5479	0.9995	10695	2	0.0547
EDIL3	6	0.33571	0.54799	0.9995	10696	3	-0.018
PRDM8	6	0.33578	0.54807	0.9995	10697	2	-0.049
ACOT12	6	0.33579	0.54809	0.9995	10698	2	-0.37
LYRM2	6	0.33582	0.54813	0.9995	10699	3	0.0384
MARK3	6	0.33585	0.54816	0.9995	10700	2	-0.166
KDM4C	6	0.33593	0.54826	0.9995	10701	3	0.2829
DDOST	6	0.33598	0.5483	0.9995	10702	2	-0.116
CALML3	6	0.33611	0.54847	0.9995	10703	2	-0.226
hsa-mir-3976	4	0.33613	0.48029	0.9995	10704	2	0.1156
FAM91A1	6	0.33618	0.54854	0.9995	10705	3	0.2321

RFX8	6	0.33625	0.54862	0.9995	10706	3	0.0845
GPR180	6	0.33629	0.54868	0.9995	10707	1	-0.923
BET1L	6	0.3364	0.54881	0.9995	10708	2	0.0607
GNA12	6	0.33648	0.5489	0.9995	10709	1	-0.183
MPC2	6	0.33648	0.5489	0.9995	10710	2	0.1193
UBAC2	6	0.33652	0.54893	0.9995	10711	3	0.0711
LGALS9C	2	0.33654	0.36514	0.9995	10712	1	0.5072
PDE8B	6	0.33659	0.54902	0.9995	10713	1	0.0388
MMP13	6	0.33663	0.54906	0.9995	10714	2	-0.049
LPPR3	6	0.33672	0.54918	0.9995	10715	3	-0.016
HPCAL4	6	0.33678	0.54924	0.9995	10716	3	0.0866
CD274	6	0.33678	0.54924	0.9995	10717	3	0.0964
ZMAT3	6	0.33684	0.54931	0.9995	10718	3	0.1697
FBXO31	6	0.33685	0.54933	0.9995	10719	2	0.1184
BMP15	6	0.33689	0.54937	0.9995	10720	3	0.0634
GADD45G	6	0.33695	0.54945	0.9995	10721	2	-0.087
CLDN16	6	0.33707	0.54958	0.9995	10722	1	-0.245
ARNT	6	0.33711	0.54963	0.9995	10723	1	-0.51
CORIN	6	0.33727	0.54981	0.9995	10724	3	-0.018
ANKRD33	6	0.33731	0.54986	0.9995	10725	3	0.1972
PLA2G2D	6	0.33737	0.54993	0.9995	10726	2	-0.095
NXPH1	6	0.33737	0.54994	0.9995	10727	3	0.0463
TTC26	6	0.33749	0.55005	0.9995	10728	2	-0.36
OR6Y1	6	0.33751	0.55009	0.9995	10729	3	0.2119
PARD3B	6	0.33751	0.55009	0.9995	10730	3	0.2009
SYT13	6	0.33751	0.55009	0.9995	10731	3	0.088
RGS18	6	0.33751	0.55009	0.9995	10732	1	-0.141
OR2G2	6	0.33755	0.55014	0.9995	10733	2	-0.456
CCNI2	6	0.33761	0.55019	0.9995	10734	1	-0.904
CMTM6	6	0.33766	0.55025	0.9995	10735	1	-0.15
SLC23A3	6	0.3377	0.5503	0.9995	10736	2	-0.115
C15orf48	6	0.3377	0.5503	0.9995	10737	2	0.1386
STRIP1	6	0.3377	0.5503	0.9995	10738	2	-0.026
DNAH5	6	0.33774	0.55034	0.9995	10739	1	-0.05
hsa-mir-3937	4	0.33777	0.48198	0.9995	10740	2	0.4226
OR14J1	6	0.33781	0.55042	0.9995	10741	3	-0.176
UBL5	6	0.33784	0.55045	0.9995	10742	3	0.2439
ZBTB7A	6	0.33796	0.55059	0.9995	10743	2	-0.073
PNMT	6	0.33798	0.55062	0.9995	10744	3	0.1319
TEAD2	6	0.33801	0.55065	0.9995	10745	2	-0.217
PIWIL1	4	0.33802	0.48222	0.9995	10746	1	0.0857
TMEM150A	6	0.33812	0.55076	0.9995	10747	3	0.0918
ART1	6	0.33812	0.55076	0.9995	10748	3	0.0861
PLAGL2	6	0.33819	0.55085	0.9995	10749	3	0.0379
ETF1	6	0.33825	0.55093	0.9995	10750	2	0.0363
MDK	6	0.33829	0.55096	0.9995	10751	3	0.2112
hsa-mir-6799	4	0.33831	0.48252	0.9995	10752	2	0.151
SYCP3	6	0.33833	0.55101	0.9995	10753	2	0.1434
ABCA2	6	0.33837	0.55105	0.9995	10754	3	-0.169
DNMBP	6	0.33842	0.55112	0.9995	10755	2	0.1657
ATRAID	6	0.33844	0.55113	0.9995	10756	3	0.1934
SLC26A3	6	0.33851	0.55123	0.9995	10757	3	0.0741
SLC30A2	6	0.33876	0.5515	0.9995	10758	3	0.1877
FAAH2	6	0.33877	0.55152	0.9995	10759	1	0.0122
BTF3L4	6	0.3388	0.55155	0.9995	10760	2	0.1979
NSUN4	6	0.33885	0.55161	0.9995	10761	2	0.0608
OSMR	6	0.33899	0.55178	0.9995	10762	1	0.0176
CCR4	6	0.33901	0.5518	0.9995	10763	2	-1.151
CD302	6	0.33904	0.55184	0.9995	10764	2	0.0129
ELF4	6	0.33909	0.55188	0.9995	10765	3	0.2083
MYCL	2	0.33917	0.36725	0.9995	10766	1	0.2807
REPS2	6	0.33919	0.55199	0.9995	10767	2	0.0153
DSTYK	6	0.33927	0.55207	0.9995	10768	3	-0.035
ZNF598	6	0.33929	0.55209	0.9995	10769	1	-1.573
EIF2AK4	6	0.33932	0.55212	0.9995	10770	3	0.3382
KARS	6	0.33938	0.55219	0.9995	10771	3	-0.011
hsa-mir-3657	4	0.3394	0.48366	0.9995	10772	2	0.1881
FAM172A	6	0.33943	0.55224	0.9995	10773	2	-0.173
PARP15	6	0.33944	0.55226	0.9995	10774	3	0.156
INO80D	6	0.33949	0.55233	0.9995	10775	2	-0.107
SBF1	6	0.33956	0.55239	0.9995	10776	2	-0.033
RNF125	6	0.33958	0.55241	0.9995	10777	2	-0.037
TRIM69	6	0.33962	0.55246	0.9995	10778	2	0.1612
PSD4	6	0.33964	0.55249	0.9995	10779	3	0.1626
ZC3H10	6	0.33966	0.55251	0.9995	10780	2	0.0548
hsa-mir-4296	4	0.33967	0.48393	0.9995	10781	2	0.2442
SOCS1	6	0.33979	0.55265	0.9995	10782	2	-0.296
MS4A12	6	0.33981	0.55266	0.9995	10783	3	0.1617
AIG1	6	0.33982	0.55268	0.9995	10784	2	-0.28
SMOX	6	0.33991	0.55278	0.9995	10785	3	0.0781
POU2F3	6	0.33994	0.55282	0.9995	10786	3	0.1788
C10orf137	6	0.34013	0.55304	0.9995	10787	2	-0.339
LARP6	6	0.34015	0.55306	0.9995	10788	3	0.1266
VRK3	6	0.34027	0.5532	0.9995	10789	2	-0.026
SERPINB13	6	0.34039	0.55333	0.9995	10790	3	0.1011

THOC5	6	0.34043	0.55337	0.9995	10791	2	-0.16
ABHD5	6	0.34047	0.55342	0.9995	10792	3	-0.076
COPG2	6	0.34054	0.5535	0.9995	10793	3	0.1389
SSBP4	6	0.3406	0.55355	0.9995	10794	3	0.1831
TNFAIP8L2-SCNM1	1	0.34077	0.34105	0.9995	10795	1	0.3685
HSPB9	6	0.34077	0.55376	0.9995	10796	3	0.1358
hsa-mir-489	4	0.3408	0.48509	0.9995	10797	2	0.0123
KCNE1	6	0.34085	0.55384	0.9995	10798	3	0.1719
TNFRSF9	6	0.34089	0.55389	0.9995	10799	2	0.0443
CIITA	6	0.341	0.554	0.9995	10800	1	-0.457
hsa-mir-27a	4	0.34109	0.48538	0.9995	10801	2	0.0372
AKAP14	6	0.34109	0.5541	0.9995	10802	2	-0.42
C6orf203	6	0.34115	0.55417	0.9995	10803	3	0.2338
SHFM1	6	0.34115	0.55417	0.9995	10804	3	-0.176
CA7	6	0.34127	0.5543	0.9995	10805	2	-0.094
MPST	6	0.3413	0.55433	0.9995	10806	3	0.0441
EHMT1	6	0.34137	0.5544	0.9995	10807	2	-0.019
RPS7	6	0.34138	0.55441	0.9995	10808	2	0.0847
CD300LF	6	0.34147	0.55452	0.9995	10809	3	0.136
TXNDC8	6	0.34147	0.55452	0.9995	10810	3	-0.039
MYL7	6	0.34156	0.55462	0.9995	10811	3	0.0687
GNAO1	6	0.34156	0.55462	0.9995	10812	2	-0.267
HNRPLL	4	0.34167	0.486	0.9995	10813	1	-0.271
GALNTL5	6	0.34175	0.55484	0.9995	10814	2	-0.493
hsa-mir-615	4	0.34182	0.48616	0.9995	10815	1	-0.712
CNPY1	6	0.34185	0.55494	0.9995	10816	3	0.1078
EPN2	6	0.3419	0.55499	0.9995	10817	3	0.1705
OPN4	6	0.3419	0.555	0.9995	10818	2	-0.043
NFIB	6	0.34199	0.5551	0.9995	10819	2	2E-05
hsa-mir-6830	4	0.342	0.48634	0.9995	10820	1	0.1024
AMN1	6	0.34203	0.55515	0.9995	10821	3	0.1806
CXCL12	6	0.34209	0.55522	0.9995	10822	3	0.1468
GSX2	6	0.34209	0.55522	0.9995	10823	3	-0.242
hsa-mir-5092	4	0.34211	0.48645	0.9995	10824	2	-0.123
CEACAM21	6	0.34216	0.55529	0.9995	10825	3	0.1253
SPTLC1	6	0.34222	0.55536	0.9995	10826	3	-0.096
IL23A	6	0.34225	0.55539	0.9995	10827	1	-0.16
HHAT	6	0.34225	0.5554	0.9995	10828	3	-0.11
PHEX	6	0.34231	0.55546	0.9995	10829	3	0.018
KRTAP4-7	4	0.34236	0.48672	0.9995	10830	2	0.0577
hsa-mir-6882	4	0.34236	0.48672	0.9995	10831	2	0.4533
GIMAP1-GIMAP5	3	0.34238	0.45201	0.9995	10832	1	0.0783
MBD3L4	1	0.34244	0.34276	0.9995	10833	1	0.273
COL25A1	6	0.34245	0.55565	0.9995	10834	2	0.0084
HIGD1A	6	0.34253	0.55574	0.9995	10835	1	-0.553
CYB5RL	6	0.34254	0.55575	0.9995	10836	2	-0.125
C9orf84	6	0.34257	0.55578	0.9995	10837	3	0.205
ADRA2C	6	0.34267	0.5559	0.9995	10838	1	-0.87
GABRQ	6	0.34271	0.55594	0.9995	10839	1	-0.058
IFNA14	5	0.34277	0.51659	0.9995	10840	2	-0.968
C1QTNF7	6	0.3428	0.55605	0.9995	10841	2	-0.164
ZNF572	6	0.34285	0.5561	0.9995	10842	3	0.1923
TMEM159	6	0.34285	0.5561	0.9995	10843	3	0.1595
FLJ45513	6	0.34287	0.55612	0.9995	10844	1	-0.164
hsa-mir-365a	4	0.34291	0.48727	0.9995	10845	2	0.3595
TBC1D22A	6	0.34301	0.55629	0.9995	10846	3	0.1759
A2ML1	6	0.34304	0.55632	0.9995	10847	2	-0.075
TULP3	6	0.34308	0.55637	0.9995	10848	3	0.0403
SAMD12	6	0.3431	0.55639	0.9995	10849	3	0.143
hsa-mir-652	4	0.34311	0.48747	0.9995	10850	2	0.2552
LOC646862	6	0.34317	0.55647	0.9995	10851	3	-0.059
SULT1B1	6	0.34319	0.55649	0.9995	10852	2	0.0465
OR3A2	6	0.34326	0.55657	0.9995	10853	2	0.127
KDM6A	6	0.34332	0.55663	0.9995	10854	1	-0.241
DUSP18	6	0.34332	0.55663	0.9995	10855	3	0.1514
DMBX1	6	0.34337	0.55668	0.9995	10856	3	0.0558
CSNK2A2	6	0.34337	0.55668	0.9995	10857	3	0.098
PEX7	6	0.34354	0.55689	0.9995	10858	2	-0.22
C11orf42	6	0.34357	0.55692	0.9995	10859	2	0.0443
COPS2	6	0.34359	0.55694	0.9995	10860	3	0.2499
COPS5	6	0.34359	0.55694	0.9995	10861	3	0.4036
SPATA3	6	0.34368	0.55704	0.9995	10862	2	-0.456
SMR3A	4	0.34371	0.48807	0.9995	10863	1	-0.119
USP12	6	0.34374	0.5571	0.9995	10864	2	0.0107
IMMP1L	6	0.34377	0.55714	0.9995	10865	3	-0.245
LAX1	6	0.34377	0.55714	0.9995	10866	3	-0.05
SNRNP40	6	0.34377	0.55714	0.9995	10867	2	-0.216
OPRM1	6	0.34381	0.55719	0.9995	10868	1	-0.431
CDH16	6	0.34386	0.55725	0.9995	10869	3	0.1733
RHEBL1	6	0.34397	0.55738	0.9995	10870	3	0.3923
GMCL1	6	0.34407	0.55748	0.9995	10871	3	0.1338
BMPR1A	6	0.34409	0.5575	0.9995	10872	2	-0.033
PCYOX1L	6	0.34414	0.55757	0.9995	10873	1	-0.704
hsa-mir-3973	4	0.34426	0.48863	0.9995	10874	1	-0.227
MZT1	6	0.34432	0.55777	0.9995	10875	1	-0.168

hsa-mir-384	4	0.34437	0.48874	0.9995	10876	2	0.1912
TMSB4X	4	0.34442	0.48879	0.9995	10877	2	0.2749
PALLD	4	0.34447	0.48884	0.9995	10878	1	-0.277
PRSS45	6	0.34451	0.55799	0.9995	10879	2	-0.153
CBWD2	2	0.34452	0.37158	0.9995	10880	1	0.0616
OR4N5	6	0.34456	0.55805	0.9995	10881	3	0.0426
SMARCC2	6	0.34461	0.55811	0.9995	10882	2	-0.365
AMBRA1	6	0.34464	0.55814	0.9995	10883	2	-0.042
GUCY1B3	6	0.34467	0.55817	0.9995	10884	3	0.1561
KATNAL1	6	0.3447	0.55821	0.9995	10885	3	0.2629
PTGR1	6	0.34476	0.55828	0.9995	10886	3	-0.008
RGMA	6	0.34476	0.55828	0.9995	10887	3	0.1159
hsa-mir-4325	4	0.34479	0.48916	0.9995	10888	2	0.1258
ZFP64	6	0.3448	0.55833	0.9995	10889	2	0.0342
SHISA4	6	0.3448	0.55833	0.9995	10890	2	-0.507
HCAR3	6	0.34483	0.55836	0.9995	10891	3	0.1918
MT1E	4	0.34483	0.48919	0.9995	10892	2	0.4943
NUDCD3	6	0.34493	0.55849	0.9995	10893	2	-0.061
C20orf173	6	0.34495	0.5585	0.9995	10894	3	0.1166
SEMA4A	6	0.34495	0.5585	0.9995	10895	3	0.2131
DTD1	6	0.345	0.55857	0.9995	10896	3	-0.135
CSNK2A3	5	0.34502	0.51846	0.9995	10897	2	-0.123
CMYA5	6	0.34503	0.5586	0.9995	10898	3	0.2186
ZBTB14	6	0.34508	0.55865	0.9995	10899	2	-0.302
NOL9	6	0.3451	0.55867	0.9995	10900	3	0.0757
hsa-mir-7845	4	0.34519	0.48956	0.9995	10901	1	-0.077
ZNF735	5	0.34523	0.51862	0.9995	10902	1	-0.65
TEAD3	6	0.34523	0.55882	0.9995	10903	2	0.1648
EID2	6	0.34523	0.55882	0.9995	10904	3	0.3117
CDHR5	6	0.34528	0.55889	0.9995	10905	2	-0.597
hsa-mir-4712	4	0.34536	0.48974	0.9995	10906	2	0.2504
HMX3	6	0.34537	0.55899	0.9995	10907	2	-0.044
SCO2	6	0.34537	0.55899	0.9995	10908	1	0.076
RANBP3	6	0.34538	0.55901	0.9995	10909	2	-0.16
SLC39A10	6	0.34544	0.55907	0.9995	10910	1	-0.163
OR5F1	6	0.34544	0.55907	0.9995	10911	2	-0.237
OR52L1	6	0.34548	0.5591	0.9995	10912	3	0.0366
NUDT16	6	0.34548	0.5591	0.9995	10913	3	0.1199
ZFP30	6	0.34553	0.55916	0.9995	10914	2	-0.115
RBM33	6	0.34557	0.55919	0.9995	10915	3	0.176
NCAM1	6	0.34568	0.55931	0.9995	10916	2	0.1009
SGK1	6	0.34573	0.55938	0.9995	10917	2	0.1615
C15orf27	6	0.34574	0.55939	0.9995	10918	3	0.1076
ABCC2	6	0.34574	0.55939	0.9995	10919	3	0.1735
DLK1	6	0.34584	0.5595	0.9995	10920	2	0.2138
KRTAP29-1	6	0.34584	0.5595	0.9995	10921	2	-0.095
MESP1	6	0.34593	0.55962	0.9995	10922	3	0.2637
SEMA4D	6	0.34593	0.55962	0.9995	10923	1	-0.175
C19orf18	6	0.34594	0.55962	0.9995	10924	2	-0.463
hsa-mir-4282	4	0.34605	0.49044	0.9995	10925	2	0.1921
C11orf93	6	0.34617	0.55986	0.9995	10926	2	-0.198
SAP25	6	0.34622	0.55991	0.9995	10927	3	0.2002
CHM	6	0.34624	0.55993	0.9995	10928	3	-0.067
MDN1	6	0.34624	0.55993	0.9995	10929	2	0.0896
ZNF3	6	0.34626	0.55995	0.9995	10930	2	-0.148
CALR	6	0.34627	0.55996	0.9995	10931	3	0.1603
ZNF862	6	0.34638	0.56007	0.9995	10932	3	-0.089
FILIP1	6	0.34642	0.56012	0.9995	10933	2	-0.694
PMEPA1	6	0.34642	0.56012	0.9995	10934	2	-0.37
OAZ3	6	0.34647	0.56019	0.9995	10935	3	0.0263
NPSR1	6	0.34659	0.56034	0.9995	10936	2	-0.187
GBF1	6	0.34662	0.56036	0.9995	10937	2	-0.281
ERCC1	6	0.34662	0.56036	0.9995	10938	2	0.0135
ZNF174	6	0.3467	0.56046	0.9995	10939	3	0.0538
TMEM230	6	0.3467	0.56046	0.9995	10940	2	0.0006
KRTAP5-5	6	0.34681	0.56059	0.9995	10941	3	0.4306
RGCC	6	0.34694	0.56074	0.9995	10942	1	-0.451
FAM114A2	6	0.34695	0.56074	0.9995	10943	3	0.0506
HUS1	6	0.34703	0.56084	0.9995	10944	3	0.0899
FIG4	6	0.34704	0.56085	0.9995	10945	2	-0.741
SSTR1	6	0.34707	0.56087	0.9995	10946	2	-0.207
PHPT1	6	0.3471	0.56092	0.9995	10947	3	-0.01
GPR139	6	0.34724	0.5611	0.9995	10948	3	0.0955
FOXP1	6	0.34735	0.56122	0.9995	10949	2	0.0474
OR13A1	6	0.34737	0.56125	0.9995	10950	3	-0.042
USP22	6	0.34737	0.56125	0.9995	10951	3	-0.052
TTC21B	6	0.34742	0.5613	0.9995	10952	2	-0.497
RNF168	6	0.34749	0.56139	0.9995	10953	2	-0.864
SLC48A1	6	0.34754	0.56145	0.9995	10954	1	-0.369
TGFBRAP1	6	0.34757	0.56147	0.9995	10955	3	0.048
MAP3K2	6	0.34765	0.56157	0.9995	10956	3	0.0561
SLC35E1	6	0.3477	0.56163	0.9995	10957	3	-0.208
GNPDA2	6	0.3477	0.56163	0.9995	10958	3	-0.042
SRGAP2	4	0.34773	0.49218	0.9995	10959	2	0.2578
LUC7L	6	0.34779	0.56174	0.9995	10960	3	0.2875

MTUS1	6	0.3478	0.56175	0.9995	10961	2	0.0584
hsa-mir-4697	4	0.34787	0.49232	0.9995	10962	1	-0.108
ABAT	6	0.34788	0.56183	0.9995	10963	2	-0.025
HBM	6	0.34794	0.5619	0.9995	10964	2	-0.461
NPBWR1	6	0.34799	0.56196	0.9995	10965	3	-0.017
DHRS3	6	0.34805	0.56203	0.9995	10966	1	0.0809
SMARCA1	6	0.34809	0.56207	0.9995	10967	3	0.2076
hsa-mir-3148	4	0.34826	0.49274	0.9995	10968	2	0.2158
PRR7	6	0.34827	0.56226	0.9995	10969	2	-0.213
MARS	6	0.34831	0.56231	0.9995	10970	3	-0.029
ZNF710	6	0.34846	0.56248	0.9995	10971	3	0.1055
GPR148	6	0.34857	0.5626	0.9995	10972	3	0.0059
hsa-mir-3938	4	0.34857	0.49306	0.9995	10973	2	0.1688
FBXL3	6	0.34868	0.56273	0.9995	10974	3	0.0446
OR10AD1	6	0.34879	0.56286	0.9995	10975	2	0.1806
TIGD4	6	0.34882	0.56289	0.9995	10976	3	0.0267
ERCC8	6	0.34882	0.56289	0.9995	10977	3	0.2394
ZBED2	6	0.34885	0.56294	0.9995	10978	3	0.0442
VPS36	4	0.34892	0.49342	0.9995	10979	2	-0.045
DENND4C	6	0.34899	0.56309	0.9995	10980	3	0.0035
hsa-mir-518e	2	0.34902	0.37518	0.9995	10981	1	-0.018
CHRM3	6	0.34907	0.56318	0.9995	10982	3	0.0948
TUBGCP6	6	0.34909	0.56321	0.9995	10983	2	-0.305
EFCAB1	4	0.34912	0.49361	0.9995	10984	2	0.0154
SLC10A1	6	0.34918	0.56331	0.9995	10985	1	-0.412
PHF2	4	0.34922	0.49372	0.9995	10986	1	-0.823
ST20	4	0.34922	0.49372	0.9995	10987	1	-0.079
IFNA17	5	0.34923	0.52182	0.9995	10988	2	-0.21
TMEM63A	6	0.34923	0.56336	0.9995	10989	3	0.0345
ARHGAP4	6	0.34925	0.56339	0.9995	10990	2	-0.238
PPP4R4	6	0.34929	0.56345	0.9995	10991	2	-0.176
UTRN	6	0.34935	0.56352	0.9995	10992	2	-0.275
PEX16	6	0.34935	0.56352	0.9995	10993	3	-0.124
SSR2	6	0.34958	0.56377	0.9995	10994	1	0.2027
hsa-mir-4251	4	0.34967	0.49417	0.9995	10995	2	0.3695
TMEM89	6	0.3497	0.5639	0.9995	10996	3	0.1268
GCG	6	0.3497	0.56391	0.9995	10997	2	0.045
PPP1R35	6	0.3497	0.56391	0.9995	10998	2	-0.254
C4orf3	6	0.34978	0.564	0.9995	10999	2	0.0376
JUP	6	0.34984	0.56405	0.9995	11000	2	-0.237
NLRC5	6	0.34984	0.56406	0.9995	11001	2	-0.317
OR4F5	2	0.34986	0.37587	0.9995	11002	1	0.6171
RP1L1	6	0.34993	0.56415	0.9995	11003	1	0.036
SMCO3	6	0.34994	0.56417	0.9995	11004	3	0.1576
ZNF763	4	0.34995	0.49446	0.9995	11005	1	0.1266
EREG	6	0.34997	0.56421	0.9995	11006	2	0.1531
TRPC4	6	0.35002	0.56425	0.9995	11007	2	-0.003
IQCC	6	0.35003	0.56427	0.9995	11008	3	0.0409
IFITM3	6	0.35004	0.56427	0.9995	11009	1	-0.593
WIPI2	6	0.35015	0.5644	0.9995	11010	3	0.1484
LY75	4	0.35018	0.49471	0.9995	11011	1	-0.258
LLGL1	6	0.35021	0.56447	0.9995	11012	3	0.0313
EDEM3	6	0.35022	0.56449	0.9995	11013	2	0.0275
CELF2	6	0.35031	0.56459	0.9995	11014	2	0.176
TPRG1L	6	0.35031	0.56459	0.9995	11015	3	0.0659
HPGD	6	0.35034	0.56462	0.9995	11016	3	0.1453
MED13	6	0.35037	0.56465	0.9995	11017	3	0.042
TYMP	6	0.35041	0.56469	0.9995	11018	3	0.083
INPP4B	6	0.35048	0.56477	0.9995	11019	2	-0.488
C1QTNF4	6	0.35049	0.56479	0.9995	11020	1	-0.486
CNKSR3	6	0.35054	0.56485	0.9995	11021	3	0.0307
TMEM63B	6	0.35055	0.56486	0.9995	11022	2	-0.05
hsa-mir-675	4	0.3506	0.49515	0.9995	11023	2	0.1409
C16orf74	6	0.35063	0.56494	0.9995	11024	3	0.219
SDCBP2	6	0.35064	0.56497	0.9995	11025	2	-0.058
EIF4A1	6	0.35068	0.56502	0.9995	11026	2	-0.024
LDLRAP1	6	0.35079	0.56514	0.9995	11027	2	0.091
C3orf35	6	0.3508	0.56516	0.9995	11028	3	0.1393
ENHO	6	0.3508	0.56516	0.9995	11029	2	0.153
IDH3B	6	0.35087	0.56523	0.9995	11030	3	0.1464
MSANTD3	6	0.35096	0.56533	0.9995	11031	3	0.1942
NKX6-2	6	0.35098	0.56536	0.9995	11032	2	-0.132
SLC6A14	6	0.35098	0.56536	0.9995	11033	2	0.0229
CAST	6	0.35101	0.56541	0.9995	11034	2	-0.13
TAF1B	6	0.35107	0.56546	0.9995	11035	2	-0.05
NOS3	6	0.35118	0.5656	0.9995	11036	2	0.06
IQSEC2	6	0.35127	0.56568	0.9995	11037	3	0.0633
HTR3E	6	0.35127	0.56568	0.9995	11038	3	0.1968
CLEC16A	6	0.35133	0.56575	0.9995	11039	3	-0.198
DCAF12	6	0.35137	0.56579	0.9995	11040	2	0.0243
LOC100132146	6	0.35142	0.56585	0.9995	11041	3	0.0933
TSGA10IP	6	0.35144	0.56587	0.9995	11042	2	-0.316
ASTN2	6	0.35146	0.56589	0.9995	11043	2	0.0296
GPR35	6	0.35147	0.56589	0.9995	11044	3	0.1991
TRAF7	6	0.3515	0.56593	0.9995	11045	2	0.0135

SLC26A2	6	0.35152	0.56595	0.9995	11046	3	0.0956
ABCA1	6	0.35155	0.56599	0.9995	11047	2	-0.039
LRRK2	6	0.35156	0.566	0.9995	11048	3	0.154
KCNG3	6	0.35162	0.56606	0.9995	11049	2	0.1351
GPR68	6	0.35162	0.56607	0.9995	11050	2	-0.224
TRIM28	6	0.35166	0.5661	0.9995	11051	1	-0.152
SCAND1	6	0.35175	0.56621	0.9995	11052	2	-0.27
GLDC	6	0.35176	0.56622	0.9995	11053	3	0.1234
CLUAP1	6	0.35184	0.5663	0.9995	11054	3	0.1881
GPR115	6	0.35186	0.56632	0.9995	11055	2	-0.26
TMEM43	6	0.35192	0.56639	0.9995	11056	3	0.2416
VCX3A	1	0.35195	0.35217	0.9995	11057	1	0.7783
ELOVL2	6	0.35196	0.56642	0.9995	11058	2	0.0055
ATP6V1E1	6	0.35198	0.56644	0.9995	11059	2	0.2844
DMTF1	6	0.35209	0.56658	0.9995	11060	2	-0.258
FAM111A	6	0.35211	0.5666	0.9995	11061	3	0.1362
hsa-mir-4455	4	0.35222	0.49682	0.9995	11062	2	0.2441
EPS8L3	6	0.35223	0.56674	0.9995	11063	3	0.1108
SLC3A1	6	0.35227	0.56679	0.9995	11064	1	-0.005
LHFPL5	6	0.35228	0.5668	0.9995	11065	3	0.0708
CCNB3	6	0.35229	0.56682	0.9995	11066	2	-0.194
ARL4C	6	0.35231	0.56684	0.9995	11067	2	-0.102
ARHGAP18	6	0.35233	0.56686	0.9995	11068	3	0.1006
COQ10A	6	0.35258	0.56716	0.9995	11069	2	0.1836
NUP160	6	0.35259	0.56716	0.9995	11070	2	0.2371
GATA2	6	0.3526	0.56716	0.9995	11071	3	0.1118
ATP6V1G2	6	0.3526	0.56716	0.9995	11072	3	0.1731
CHST6	6	0.3526	0.56716	0.9995	11073	3	0.102
KCNJ10	6	0.35267	0.56726	0.9995	11074	1	-0.138
AS3MT	6	0.35271	0.56729	0.9995	11075	3	0.1603
TGFB3	6	0.35273	0.56732	0.9995	11076	2	0.0544
OR2M7	6	0.3528	0.56739	0.9995	11077	2	-0.396
RSC1A1	6	0.35282	0.56742	0.9995	11078	2	-0.27
PURG	6	0.35287	0.56748	0.9995	11079	2	-0.119
ARHGAP29	6	0.35287	0.56748	0.9995	11080	1	-0.162
FOXRED1	6	0.35289	0.5675	0.9995	11081	3	0.06
TSEN15	6	0.35293	0.56755	0.9995	11082	3	0.1154
C1D	6	0.35296	0.56758	0.9995	11083	2	-0.327
SNX30	6	0.35301	0.56764	0.9995	11084	2	0.1436
OR3A3	6	0.35302	0.56765	0.9995	11085	1	-0.046
hsa-mir-6812	4	0.35306	0.49769	0.9995	11086	2	0.2294
ASIP	6	0.35308	0.56773	0.9995	11087	2	-0.343
SOX14	6	0.35323	0.56789	0.9995	11088	2	-0.038
UBXN2A	6	0.35323	0.56789	0.9995	11089	1	-0.298
ZSCAN30	5	0.35328	0.52517	0.9995	11090	2	0.0859
KCNJ12	4	0.35336	0.498	0.9995	11091	1	-0.442
MGAT4C	6	0.35339	0.56805	0.9995	11092	3	0.212
hsa-mir-4633	4	0.35345	0.49809	0.9995	11093	2	-0.219
DPEP3	6	0.35346	0.56812	0.9995	11094	2	-0.406
CHCHD6	6	0.35346	0.56813	0.9995	11095	3	0.0184
C15orf54	6	0.35354	0.56822	0.9995	11096	1	-0.144
TTC13	6	0.35354	0.56822	0.9995	11097	2	-0.197
LRG1	6	0.35365	0.56833	0.9995	11098	1	-0.105
CYP21A2	6	0.35367	0.56835	0.9995	11099	3	0.1143
SCGB1C1	3	0.35374	0.45832	0.9995	11100	1	0.0223
EYA1	6	0.35375	0.56844	0.9995	11101	3	0.2015
KRT10	6	0.35387	0.56856	0.9995	11102	2	0.0374
TMEM82	6	0.35387	0.56856	0.9995	11103	2	0.1442
PSMB7	6	0.35387	0.56856	0.9995	11104	2	0.0693
SLCO1A2	6	0.3539	0.56859	0.9995	11105	1	-0.346
LGR6	6	0.35394	0.56863	0.9995	11106	2	-0.187
NAALADL2	6	0.35399	0.56868	0.9995	11107	3	0.0727
RELL2	6	0.35401	0.56871	0.9995	11108	2	-0.507
PAK4	6	0.35401	0.56871	0.9995	11109	2	0.0704
HRG	6	0.35403	0.56873	0.9995	11110	2	-0.071
SLC1A1	6	0.35407	0.56877	0.9995	11111	3	0.0159
STIP1	6	0.35416	0.56886	0.9995	11112	2	0.0145
DBNDD1	6	0.3543	0.56903	0.9995	11113	3	-0.187
hsa-mir-607	4	0.35439	0.49907	0.9995	11114	2	0.1463
OR2D2	6	0.35445	0.56921	0.9995	11115	2	0.1265
ABLIM2	6	0.35445	0.56921	0.9995	11116	1	-0.402
STX1A	6	0.35445	0.56921	0.9995	11117	1	-0.224
ZNF623	6	0.35447	0.56924	0.9995	11118	2	-0.045
CLEC4G	6	0.35448	0.56925	0.9995	11119	3	0.0814
IQCD	6	0.35455	0.56933	0.9995	11120	1	-0.415
TSPAN13	6	0.35463	0.56943	0.9995	11121	3	-0.076
hsa-mir-219a-2	4	0.35466	0.49933	0.9995	11122	2	0.0299
PDZRN3	6	0.35469	0.56949	0.9995	11123	3	0.17
hsa-mir-378d-1	4	0.35475	0.49942	0.9995	11124	1	-1.323
FOXD4L3	1	0.35477	0.35498	0.9995	11125	1	0.5589
ZC3H13	6	0.3548	0.56961	0.9995	11126	3	-0.225
C16orf46	6	0.3548	0.56961	0.9995	11127	3	0.2215
HS6ST1	6	0.3548	0.56961	0.9995	11128	3	-0.065
BTN3A1	5	0.35486	0.52645	0.9995	11129	1	-0.174
HCFC1R1	6	0.35487	0.56971	0.9995	11130	3	0.0462

CELA3B	5	0.35493	0.52651	0.9995	11131	1	-1.134
FXR2	6	0.35493	0.56979	0.9995	11132	1	0.0998
SMAD2	6	0.355	0.56987	0.9995	11133	3	0.0992
TTLL4	6	0.3551	0.56997	0.9995	11134	3	0.2115
FLG	6	0.35511	0.56997	0.9995	11135	3	0.2152
hsa-mir-8088	4	0.35521	0.49987	0.9995	11136	1	-0.534
NUPR1L	6	0.35522	0.5701	0.9995	11137	1	-0.077
VWA1	6	0.35522	0.5701	0.9995	11138	2	-0.169
ZFHX2	6	0.35539	0.57029	0.9995	11139	2	0.0801
UBIAD1	6	0.35539	0.57029	0.9995	11140	2	-0.08
UIMC1	6	0.35548	0.5704	0.9995	11141	1	-0.265
hsa-mir-711	4	0.35548	0.50015	0.9995	11142	2	-0.063
PRPSAP2	6	0.35552	0.57044	0.9995	11143	3	0.096
RFC1	6	0.35552	0.57044	0.9995	11144	3	0.3173
KCNC4	6	0.35553	0.57045	0.9995	11145	2	0.1196
hsa-mir-520c	2	0.35554	0.38047	0.9995	11146	1	0.7411
PSME2	6	0.35556	0.57048	0.9995	11147	2	-0.011
IL2	6	0.35557	0.57049	0.9995	11148	2	0.0579
THADA	6	0.35559	0.57051	0.9995	11149	3	0.1991
hsa-mir-632	4	0.35566	0.50033	0.9995	11150	2	-0.276
ZDHHC22	6	0.35566	0.57059	0.9995	11151	3	0.0356
OR51S1	6	0.35582	0.57077	0.9995	11152	3	0.2115
hsa-mir-6791	4	0.35591	0.50058	0.9995	11153	1	-0.243
GLT8D1	6	0.35591	0.57085	0.9995	11154	2	-0.167
MTAP	6	0.35595	0.57089	0.9995	11155	3	0.2704
VMO1	6	0.35595	0.57089	0.9995	11156	3	0.1735
hsa-mir-1468	4	0.35598	0.50065	0.9995	11157	1	-0.411
INCA1	6	0.356	0.57094	0.9995	11158	2	0.1204
LURAP1	6	0.35602	0.57096	0.9995	11159	2	-0.272
UBE2Z	6	0.35602	0.57096	0.9995	11160	2	-0.002
hsa-mir-8064	4	0.35603	0.5007	0.9995	11161	1	-0.297
ING1	6	0.35615	0.57111	0.9995	11162	3	0.1223
IFLTD1	6	0.3562	0.57117	0.9995	11163	2	-0.054
hsa-mir-125a	4	0.35622	0.5009	0.9995	11164	2	0.1164
GPR26	6	0.35629	0.57126	0.9995	11165	3	0.1616
PRKCSH	6	0.35632	0.5713	0.9995	11166	3	0.201
RNLS	6	0.35633	0.5713	0.9995	11167	2	-0.291
B3GNT8	6	0.35636	0.57134	0.9995	11168	3	0.2155
SNX31	6	0.35639	0.57138	0.9995	11169	3	0.0465
HLA-DRA	6	0.3564	0.57138	0.9995	11170	2	-0.21
ADAM21	6	0.35643	0.57142	0.9995	11171	2	-0.417
IRF7	6	0.35645	0.57144	0.9995	11172	3	-0.011
KRTAP10-5	6	0.35652	0.57152	0.9995	11173	2	-0.213
ABHD3	6	0.35653	0.57152	0.9995	11174	3	0.1744
CEP164	6	0.35658	0.57158	0.9995	11175	1	-0.361
PDLIM7	6	0.35669	0.57171	0.9995	11176	1	-0.239
TBC1D5	6	0.35676	0.57178	0.9995	11177	2	0.1644
S100A13	6	0.35677	0.5718	0.9995	11178	2	-0.251
GRIK1	6	0.35679	0.57184	0.9995	11179	2	-0.537
KCTD16	6	0.35683	0.57187	0.9995	11180	1	-0.938
ZNF669	6	0.35685	0.57189	0.9995	11181	3	0.2055
MYLPF	6	0.35685	0.57189	0.9995	11182	3	0.2854
NAAA	6	0.35685	0.57189	0.9995	11183	3	0.0354
ZNF585A	6	0.35685	0.57189	0.9995	11184	3	0.0218
IGF1	6	0.35685	0.57189	0.9995	11185	3	0.2815
RHD	6	0.35694	0.57199	0.9995	11186	1	-0.259
IK	6	0.35696	0.57201	0.9995	11187	2	0.0215
hsa-mir-4642	4	0.35698	0.50169	0.9995	11188	1	0.0491
DNAJC18	6	0.357	0.57206	0.9995	11189	2	-0.406
FTHL17	6	0.35705	0.57212	0.9995	11190	2	-0.026
USP36	6	0.35708	0.57216	0.9995	11191	2	0.0797
WNT6	6	0.35712	0.5722	0.9995	11192	2	-0.184
VWA3B	6	0.35719	0.57227	0.9995	11193	2	0.0245
ELMSAN1	6	0.35723	0.57232	0.9995	11194	3	-0.023
hsa-mir-1292	4	0.35726	0.50197	0.9995	11195	2	0.142
C1GALT1	6	0.35731	0.5724	0.9995	11196	2	-0.205
GSS	6	0.35733	0.57242	0.9995	11197	3	0.1264
N4BP3	6	0.35741	0.57252	0.9995	11198	3	0.041
PCP4L1	6	0.35743	0.57253	0.9995	11199	2	0.1678
OBSCN	6	0.35747	0.57259	0.9995	11200	3	-0.063
TCEAL7	6	0.35747	0.57259	0.9995	11201	3	0.1911
hsa-mir-4771-2	1	0.3576	0.35782	0.9995	11202	0	0.2172
GLP1R	6	0.35766	0.57278	0.9995	11203	3	-0.005
STATH	6	0.3577	0.57282	0.9995	11204	2	0.035
SCFD1	6	0.35781	0.57295	0.9995	11205	3	0.1191
TCEAL1	6	0.35781	0.57295	0.9995	11206	3	0.1117
HIST1H2BJ	6	0.35784	0.57298	0.9995	11207	2	-0.129
SERPINA4	6	0.35791	0.57307	0.9995	11208	2	-0.166
SPATS2L	6	0.35802	0.57318	0.9995	11209	3	0.0322
VIPR2	6	0.35805	0.57321	0.9995	11210	3	0.082
NDUFS6	6	0.35806	0.57323	0.9995	11211	3	0.1954
TOP1MT	6	0.35809	0.57325	0.9995	11212	2	0.1317
STRBP	6	0.35809	0.57326	0.9995	11213	2	-0.403
STK40	6	0.3581	0.57327	0.9995	11214	3	0.2183
VIL1	6	0.35814	0.57332	0.9995	11215	2	-0.102

RAF1	6	0.35818	0.57337	0.9995	11216	2	0.1277
C12orf10	6	0.35822	0.57341	0.9995	11217	1	-0.185
AGMO	6	0.35825	0.57344	0.9995	11218	2	-0.626
SERTAD4	6	0.35831	0.57352	0.9995	11219	3	-8E-04
ASCL5	6	0.35831	0.57352	0.9995	11220	3	0.052
C1QL2	6	0.35847	0.5737	0.9995	11221	2	-0.281
FNIP1	6	0.35861	0.57387	0.9995	11222	2	-0.08
IL1RAP	6	0.35867	0.57393	0.9995	11223	2	-0.256
PSMG3	6	0.35867	0.57394	0.9995	11224	2	-0.05
hsa-mir-3138	4	0.35874	0.50352	0.9995	11225	1	-0.076
CD3EAP	6	0.35885	0.57416	0.9995	11226	2	0.1018
FCN3	6	0.35885	0.57416	0.9995	11227	2	0.1648
STAM2	6	0.35888	0.5742	0.9995	11228	2	0.1722
CLRN3	6	0.35897	0.57429	0.9995	11229	3	0.0924
CLU	6	0.35913	0.57447	0.9995	11230	3	0.11
VANGL1	6	0.35929	0.57463	0.9995	11231	3	0.2051
C2CD4D	6	0.35933	0.57468	0.9995	11232	1	-0.122
PTF1A	6	0.35933	0.57468	0.9995	11233	1	-0.25
CCNB1IP1	6	0.3594	0.57476	0.9995	11234	3	0.1259
PEAR1	6	0.35949	0.57486	0.9995	11235	3	0.0925
RFX6	6	0.35951	0.57489	0.9995	11236	2	-0.031
hsa-mir-4638	4	0.35952	0.50433	0.9995	11237	2	-0.045
YY1AP1	6	0.35972	0.57512	0.9995	11238	2	0.0358
ENO2	6	0.35972	0.57512	0.9995	11239	2	0.0421
VSIG1	6	0.35977	0.57517	0.9995	11240	3	0.0207
UBE2Q1	6	0.35981	0.57522	0.9995	11241	3	0.0359
ALDH5A1	6	0.35987	0.57528	0.9995	11242	3	0.1071
SCRN3	6	0.35987	0.57528	0.9995	11243	2	0.0068
ECEL1	6	0.36005	0.57549	0.9995	11244	3	0.0924
NSMCE1	6	0.36018	0.57562	0.9995	11245	3	0.0878
RIPPLY1	6	0.36025	0.57571	0.9995	11246	2	-0.008
DEFB135	6	0.36033	0.57579	0.9995	11247	2	-0.121
hsa-mir-23a	4	0.36035	0.50519	0.9995	11248	2	-0.334
ATG9A	6	0.36043	0.5759	0.9995	11249	3	0.2726
ZNF501	6	0.36081	0.57633	0.9995	11250	3	0.0201
CEACAM3	6	0.36081	0.57633	0.9995	11251	3	-0.049
RABGAP1L	6	0.36081	0.57633	0.9995	11252	3	0.1919
C17orf58	6	0.36081	0.57633	0.9995	11253	3	0.1189
ADAT3	6	0.36082	0.57634	0.9995	11254	2	-0.198
MBOAT4	6	0.3609	0.57643	0.9995	11255	3	0.1933
SRP19	6	0.36093	0.57646	0.9995	11256	1	-0.211
SDPR	6	0.36093	0.57646	0.9995	11257	2	0.003
SRSF11	6	0.36103	0.57657	0.9995	11258	3	0.1794
SMYD2	6	0.36116	0.57673	0.9995	11259	3	0.1532
SPECC1	6	0.36116	0.57673	0.9995	11260	3	0.0851
RRP12	6	0.36122	0.5768	0.9995	11261	2	-0.164
hsa-mir-939	4	0.36126	0.50612	0.9995	11262	1	-0.224
KCNC2	6	0.36127	0.57686	0.9995	11263	3	0.0605
IL24	6	0.36127	0.57686	0.9995	11264	3	-0.053
HLA-DPB1	6	0.36129	0.57687	0.9995	11265	2	-0.002
ATP13A5	6	0.36138	0.57697	0.9995	11266	3	0.0646
ATOH7	6	0.36143	0.57704	0.9995	11267	2	-0.137
ASPHD1	6	0.36148	0.57708	0.9995	11268	3	-0.094
POTEI	1	0.36152	0.36177	0.9995	11269	0	0.5826
HPS6	4	0.36157	0.50644	0.9995	11270	1	0.0298
FAM189A2	6	0.36163	0.57726	0.9995	11271	3	-0.306
LILRB5	6	0.36168	0.57733	0.9995	11272	2	-0.222
OSBP2	6	0.36172	0.57738	0.9995	11273	2	-0.026
SHC1	6	0.36172	0.57738	0.9995	11274	3	-0.099
CSE1L	6	0.36179	0.57747	0.9995	11275	2	-0.274
PRDX4	6	0.36179	0.57747	0.9995	11276	1	-0.193
PSTPIP2	6	0.3618	0.57748	0.9995	11277	3	-0.027
TOR4A	6	0.36183	0.57752	0.9995	11278	2	-0.178
DEFB108B	6	0.36194	0.57766	0.9995	11279	3	0.1482
SLC12A7	6	0.36197	0.57769	0.9995	11280	1	-0.721
MED29	6	0.36202	0.57774	0.9995	11281	2	0.1379
WHSC1L1	6	0.36206	0.57779	0.9995	11282	1	-0.032
LCE2B	6	0.36206	0.57779	0.9995	11283	2	0.0661
BLVRB	6	0.36218	0.57792	0.9995	11284	2	-0.349
KLHDC8A	6	0.36235	0.57811	0.9995	11285	2	0.0584
GPR137	6	0.36242	0.57819	0.9995	11286	2	0.1002
CNN2	6	0.36244	0.57822	0.9995	11287	2	-0.309
ANO7	6	0.36261	0.5784	0.9995	11288	2	-0.06
LDHAL6B	6	0.36261	0.5784	0.9995	11289	2	0.1698
BCL2L2	6	0.36277	0.57857	0.9995	11290	2	0.0826
MAGEB5	6	0.36284	0.57865	0.9995	11291	2	-0.076
SLC2A4	6	0.36288	0.57869	0.9995	11292	3	-0.108
POU2AF1	6	0.36288	0.5787	0.9995	11293	2	-0.141
GOT2	6	0.36303	0.57887	0.9995	11294	3	0.3325
EFR3B	6	0.36312	0.57896	0.9995	11295	1	-0.545
RHOBTB1	6	0.36316	0.57901	0.9995	11296	2	-0.339
HR	6	0.36325	0.57911	0.9995	11297	3	0.1486
MFRP	6	0.36332	0.5792	0.9995	11298	3	0.0557
AMDHD2	6	0.36333	0.5792	0.9995	11299	2	-0.07
TRIM52	6	0.36335	0.57923	0.9995	11300	2	-0.179

UQCRC2	6	0.3634	0.57928	0.9995	11301	1	-0.132
ZXDB	6	0.36343	0.57931	0.9995	11302	3	0.035
IL4I1	6	0.36346	0.57933	0.9995	11303	3	0.0877
IL20RA	6	0.36347	0.57935	0.9995	11304	1	-0.34
OR4D9	6	0.36353	0.57942	0.9995	11305	2	-0.049
PCCA	5	0.36355	0.53362	0.9995	11306	2	-0.128
TMEM178B	6	0.36358	0.57948	0.9995	11307	2	-0.32
THAP7	6	0.36358	0.57948	0.9995	11308	2	-0.241
LRIG3	6	0.36362	0.57952	0.9995	11309	2	0.1118
NRXN2	6	0.36367	0.57959	0.9995	11310	2	0.0275
PRSS42	6	0.36373	0.57966	0.9995	11311	2	0.1325
TM4SF20	6	0.36378	0.57972	0.9995	11312	2	-0.32
KIAA1211	6	0.36378	0.57972	0.9995	11313	2	-0.095
MSH4	6	0.36383	0.57978	0.9995	11314	1	-0.047
LYZL4	6	0.36385	0.5798	0.9995	11315	3	0.1608
C6orf120	6	0.36389	0.57984	0.9995	11316	2	-0.015
CDK13	6	0.36389	0.57984	0.9995	11317	2	-0.092
hsa-mir-449b	4	0.36389	0.50884	0.9995	11318	2	0.1289
PPM1E	6	0.36389	0.57984	0.9995	11319	3	-0.013
KCNE3	6	0.36389	0.57984	0.9995	11320	3	0.1789
TDG	6	0.36394	0.57989	0.9995	11321	2	0.1049
hsa-mir-548az	3	0.36396	0.46406	0.9995	11322	1	-0.541
SNRNP48	6	0.36397	0.57993	0.9995	11323	3	0.2747
REEP4	6	0.36397	0.57993	0.9995	11324	3	0.0984
AHI1	6	0.36413	0.58012	0.9995	11325	2	0.1132
SLC25A27	6	0.36415	0.58014	0.9995	11326	3	0.1866
NFAT5	6	0.36422	0.58022	0.9995	11327	2	-0.193
TFF3	6	0.36429	0.58029	0.9995	11328	2	0.0273
PTPN18	6	0.3643	0.5803	0.9995	11329	1	-0.134
SH3D19	6	0.36442	0.58039	0.9995	11330	2	0.0016
SOX12	6	0.36449	0.58043	0.9995	11331	1	-0.494
KRTAP5-11	4	0.36473	0.50971	0.9995	11332	2	0.1754
LUZP1	6	0.36476	0.58061	0.9995	11333	1	-0.217
SULF2	6	0.36481	0.58063	0.9995	11334	2	0.0568
TMEM119	6	0.36481	0.58063	0.9995	11335	2	0.1436
MUM1L1	6	0.3649	0.58068	0.9995	11336	1	-0.084
PIWIL4	6	0.36505	0.58076	0.9995	11337	2	0.1275
OR11A1	6	0.36513	0.58082	0.9995	11338	1	-0.27
ITGB2	6	0.36529	0.58092	0.9995	11339	2	-0.328
ZNF799	2	0.3653	0.38834	0.9995	11340	1	-0.021
SYF2	6	0.36537	0.58096	0.9995	11341	2	0.148
CTSL2	2	0.36551	0.38852	0.9995	11342	1	-0.25
IL17REL	6	0.36552	0.58106	0.9995	11343	2	-0.158
SLC19A2	6	0.36552	0.58106	0.9995	11344	2	-0.129
TSTA3	6	0.36566	0.58113	0.9995	11345	2	0.0265
MED7	6	0.36586	0.58127	0.9995	11346	2	0.0254
TDGF1	6	0.36594	0.58131	0.9995	11347	2	-0.546
hsa-mir-6509	4	0.36602	0.51102	0.9995	11348	1	-0.247
GJA3	6	0.36607	0.5814	0.9995	11349	2	-0.211
ZFP42	6	0.36626	0.58152	0.9995	11350	2	0.0655
CDH3	6	0.36644	0.58164	0.9995	11351	1	-0.245
OPTC	6	0.36651	0.58168	0.9995	11352	2	-0.124
SYN1	6	0.36654	0.58171	0.9995	11353	2	-0.224
AMELX	5	0.36655	0.53608	0.9995	11354	2	-0.181
DDHD2	4	0.36656	0.51155	0.9995	11355	2	0.2307
hsa-mir-8057	4	0.36658	0.51158	0.9995	11356	1	-0.147
ZNF511	6	0.36658	0.58173	0.9995	11357	2	-0.165
AKR1C1	5	0.3666	0.53612	0.9995	11358	2	0.1531
EIF4A3	6	0.36661	0.58175	0.9995	11359	2	-0.661
LYZL6	6	0.36667	0.58178	0.9995	11360	2	-0.074
CLCNKA	6	0.36673	0.58182	0.9995	11361	2	0.0749
hsa-mir-4521	4	0.36676	0.51178	0.9995	11362	2	0.0481
FKBP2	6	0.3669	0.58193	0.9995	11363	2	-0.174
GATA3	6	0.3669	0.58193	0.9995	11364	2	-0.395
N4BP2L1	6	0.3669	0.58193	0.9995	11365	2	-0.572
MAVS	6	0.36712	0.58206	0.9995	11366	2	-0.361
PRKRA	4	0.36713	0.51214	0.9995	11367	2	0.2303
NCF1	6	0.36738	0.58222	0.9995	11368	1	-0.289
hsa-mir-5196	4	0.36741	0.51243	0.9995	11369	1	0.0287
KLK7	6	0.3675	0.5823	0.9995	11370	1	0.1466
IGSF9	6	0.36751	0.5823	0.9995	11371	2	-0.009
ZNF385A	6	0.36765	0.58238	0.9995	11372	1	-0.904
MRGPRD	6	0.36768	0.5824	0.9995	11373	2	-0.156
MGST2	6	0.36773	0.58244	0.9995	11374	2	-0.151
FBXO45	4	0.36779	0.51282	0.9995	11375	2	0.0245
TMEM233	6	0.36779	0.58248	0.9995	11376	1	-0.268
BMP3	6	0.36801	0.58262	0.9995	11377	2	-0.075
ZMYND12	6	0.36811	0.58268	0.9995	11378	2	-0.097
ZSCAN5B	6	0.36837	0.58285	0.9995	11379	2	-0.113
WSCD2	6	0.36864	0.58302	0.9995	11380	2	-0.076
OPHN1	6	0.36868	0.58305	0.9995	11381	2	0.1767
IFNE	6	0.36871	0.58307	0.9995	11382	1	0.0379
TRIB3	6	0.36876	0.5831	0.9995	11383	1	-0.304
WDR18	6	0.36882	0.58313	0.9995	11384	1	-0.208
HIST2H4B	1	0.36885	0.36903	0.9995	11385	0	0.528

KLRC2	5	0.36889	0.53802	0.9995	11386	2	-0.209
LOC283710	6	0.36898	0.58323	0.9995	11387	2	-0.253
CRMP1	6	0.36908	0.5833	0.9995	11388	1	0.0402
CHD6	6	0.3692	0.58337	0.9995	11389	2	-0.087
AMY2A	2	0.36922	0.39156	0.9995	11390	1	0.0295
hsa-mir-1306	4	0.36936	0.51447	0.9995	11391	2	-0.006
hsa-mir-6718	4	0.36949	0.51459	0.9995	11392	2	-0.163
hsa-mir-4514	4	0.36962	0.51471	0.9995	11393	2	0.0711
PACSIN3	6	0.36965	0.58366	0.9995	11394	1	-0.679
PABPC3	6	0.36974	0.58371	0.9995	11395	2	-0.281
hsa-mir-1302-1	4	0.36974	0.51485	0.9995	11396	1	-0.73
NGFRAP1	6	0.36993	0.58383	0.9995	11397	2	-0.342
hsa-mir-4798	4	0.37009	0.51519	0.9995	11398	2	0.1586
MLF2	6	0.37011	0.58395	0.9995	11399	2	-0.088
MAPK15	6	0.37017	0.58398	0.9995	11400	2	-0.061
SLC44A3	6	0.37018	0.58399	0.9995	11401	2	-0.138
SLC25A22	6	0.37038	0.58411	0.9995	11402	1	0.0812
GXYLT1	6	0.37042	0.58413	0.9995	11403	2	-0.012
WFDC10B	5	0.37042	0.53933	0.9995	11404	1	-1.537
OR9K2	6	0.37061	0.58425	0.9995	11405	2	0.1474
KIAA2018	6	0.37063	0.58426	0.9995	11406	2	0.0356
TMEM99	6	0.37075	0.58433	0.9995	11407	2	0.0869
P4HTM	6	0.37075	0.58433	0.9995	11408	2	-0.079
ABCC4	6	0.37083	0.58438	0.9995	11409	2	-0.119
HGD	6	0.37087	0.58441	0.9995	11410	1	-0.164
DTNBP1	6	0.37087	0.58441	0.9995	11411	1	-0.015
HES7	6	0.37089	0.58442	0.9995	11412	2	-0.119
AHCY	6	0.37093	0.58444	0.9995	11413	2	-0.267
FADS6	6	0.37095	0.58446	0.9995	11414	2	-0.243
KATNA1	6	0.3711	0.58456	0.9995	11415	2	-0.067
SLC7A6	6	0.37113	0.58458	0.9995	11416	2	-0.068
BARX2	6	0.37127	0.58466	0.9995	11417	2	0.0946
ADRBK2	6	0.37133	0.5847	0.9995	11418	1	-0.347
ZNF181	5	0.37139	0.54015	0.9995	11419	2	0.2096
ZEB1	4	0.3718	0.51696	0.9995	11420	2	0.1054
SOAT1	6	0.37181	0.58499	0.9995	11421	2	0.0714
CCDC38	6	0.37187	0.58503	0.9995	11422	2	-0.013
FBXL4	6	0.37189	0.58505	0.9995	11423	2	-0.228
MAP2K4	6	0.37189	0.58505	0.9995	11424	2	0.1347
OST4	5	0.37207	0.54073	0.9995	11425	2	-0.347
LBX2	6	0.37208	0.58515	0.9995	11426	2	-0.092
HLA-DOB	6	0.37208	0.58515	0.9995	11427	2	0.0886
WDR49	6	0.37208	0.58515	0.9995	11428	2	0.0829
C6orf136	6	0.37218	0.58522	0.9995	11429	2	-0.043
hsa-mir-3974	4	0.37244	0.5176	0.9995	11430	2	-0.077
HTR1D	6	0.37246	0.58539	0.9995	11431	2	-0.118
GTDC2	4	0.37249	0.51766	0.9995	11432	2	0.0275
TBX4	6	0.37255	0.58545	0.9995	11433	2	-0.087
CDC34	6	0.37259	0.58548	0.9995	11434	2	-0.06
OR2A14	6	0.37264	0.58552	0.9995	11435	2	-0.053
UNC13A	6	0.37265	0.58553	0.9995	11436	2	-0.299
TSPY8	1	0.37274	0.37289	0.9995	11437	0	0.1497
ZNF367	6	0.37282	0.58564	0.9995	11438	1	-0.155
KRTAP4-8	4	0.37289	0.51808	0.9995	11439	1	-0.419
SNRPB2	6	0.37296	0.58572	0.9995	11440	2	-0.298
ZBTB33	6	0.3731	0.5858	0.9995	11441	2	0.1678
KIAA1239	6	0.37313	0.58583	0.9995	11442	2	-0.279
hsa-mir-541	4	0.37324	0.51843	0.9995	11443	2	-0.134
NCKAP5L	6	0.37329	0.58592	0.9995	11444	2	0.088
hsa-mir-1288	4	0.37336	0.51855	0.9995	11445	2	0.2519
TMEM220	6	0.37337	0.58597	0.9995	11446	1	-0.187
hsa-mir-3928	4	0.37358	0.51878	0.9995	11447	1	-1.033
TCN1	6	0.37374	0.58621	0.9995	11448	2	-0.094
ODF3B	6	0.3739	0.58633	0.9995	11449	1	0.0577
CDRT1	6	0.3739	0.58633	0.9995	11450	2	-0.379
PLCXD2	6	0.37397	0.58638	0.9995	11451	2	-0.216
hsa-mir-559	4	0.37401	0.51925	0.9995	11452	2	0.1424
ARHGAP11A	3	0.37403	0.46981	0.9995	11453	1	0.1446
C6orf201	6	0.37415	0.58649	0.9995	11454	2	-0.416
SORCS2	6	0.37424	0.58654	0.9995	11455	2	0.082
DEXI	6	0.37425	0.58655	0.9995	11456	1	-0.294
RGS7BP	4	0.37426	0.51951	0.9995	11457	1	-0.165
ABHD12B	6	0.3743	0.58658	0.9995	11458	2	0.0114
hsa-mir-378j	4	0.3744	0.51966	0.9995	11459	1	-0.498
KIAA0895	4	0.37442	0.51968	0.9995	11460	2	-0.067
ING5	6	0.37443	0.58665	0.9995	11461	2	-0.094
GFPT1	6	0.37448	0.58669	0.9995	11462	2	-0.032
POLR2J	2	0.37453	0.39586	0.9995	11463	1	0.1082
EIF3I	6	0.37462	0.58678	0.9995	11464	2	0.1052
CCL3	6	0.37472	0.58684	0.9995	11465	2	-0.494
TGM3	6	0.37472	0.58685	0.9995	11466	1	0.1308
KLF10	6	0.37476	0.58688	0.9995	11467	2	-0.118
RABEP2	6	0.37483	0.58692	0.9995	11468	1	-0.016
ZNF704	4	0.37484	0.52008	0.9995	11469	2	0.2873
PTPN5	6	0.3749	0.58697	0.9995	11470	1	-0.048

KRTAP21-3	4	0.37491	0.52016	0.9995	11471	1	-0.421
NOXA1	6	0.37497	0.587	0.9995	11472	1	-0.363
DEFB136	6	0.37502	0.58704	0.9995	11473	2	-0.015
SLC9B2	6	0.37502	0.58704	0.9995	11474	2	0.0538
SPI1	6	0.37506	0.58707	0.9995	11475	2	-0.273
HOXD1	6	0.37506	0.58707	0.9995	11476	2	-0.078
hsa-mir-3151	4	0.37509	0.52035	0.9995	11477	1	-0.468
KRT79	4	0.37513	0.52038	0.9995	11478	2	-0.026
hsa-mir-2909	4	0.37517	0.52043	0.9995	11479	1	-0.123
RTN3	6	0.37519	0.58714	0.9995	11480	2	-0.265
PRAME	6	0.37529	0.5872	0.9995	11481	2	0.1596
C1orf52	6	0.3754	0.58729	0.9995	11482	2	-0.287
COA3	6	0.37549	0.58734	0.9995	11483	2	-0.435
ZNF773	5	0.37563	0.54367	0.9995	11484	2	-0.384
hsa-mir-1262	4	0.37568	0.52095	0.9995	11485	2	0.133
PLEKHG5	6	0.37581	0.58755	0.9995	11486	2	-0.005
CCDC12	6	0.37611	0.58775	0.9995	11487	1	-0.233
FKBP6	6	0.37617	0.58779	0.9995	11488	2	-0.095
COX19	6	0.37617	0.58779	0.9995	11489	2	0.1756
FOXL2	6	0.37624	0.58783	0.9995	11490	2	-0.023
NVL	6	0.37624	0.58783	0.9995	11491	2	0.0194
DLX5	6	0.37627	0.58785	0.9995	11492	2	-0.265
hsa-mir-770	4	0.37641	0.52167	0.9995	11493	1	0.0651
HYKK	2	0.37648	0.39744	0.9995	11494	1	-0.166
DXO	2	0.3765	0.39745	0.9995	11495	1	0.0746
hsa-mir-4707	4	0.37657	0.52183	0.9995	11496	2	-0.374
PTH	6	0.37658	0.58804	0.9995	11497	2	-0.008
SLC6A3	6	0.37659	0.58804	0.9995	11498	1	-0.185
AMD1	6	0.37666	0.58809	0.9995	11499	2	0.157
hsa-mir-127	4	0.37666	0.52193	0.9995	11500	2	0.0246
CCDC84	6	0.37667	0.58809	0.9995	11501	2	-0.167
ATRIP	6	0.37686	0.58821	0.9995	11502	2	0.0779
MAGEA6	5	0.37687	0.54471	0.9995	11503	2	-0.419
KIAA1731	6	0.37692	0.58826	0.9995	11504	1	-0.556
TBL2	6	0.37718	0.58842	0.9995	11505	2	-0.205
SIGLEC11	6	0.37722	0.58844	0.9995	11506	2	0.0943
TMEM35	6	0.37733	0.58852	0.9995	11507	1	-0.022
hsa-mir-4515	4	0.37739	0.52269	0.9995	11508	2	0.2313
C17orf107	6	0.37741	0.58857	0.9995	11509	1	-0.195
hsa-mir-7852	2	0.37746	0.39824	0.9995	11510	1	0.1623
RTN4RL1	6	0.37748	0.58862	0.9995	11511	2	-0.065
HOXB2	4	0.37768	0.52297	0.9995	11512	2	0.1589
PLIN4	6	0.37771	0.58876	0.9995	11513	1	-0.267
hsa-mir-3978	4	0.37775	0.52304	0.9995	11514	1	0.072
hsa-mir-3607	1	0.3778	0.37795	0.9995	11515	0	0.2248
ATOH1	6	0.37784	0.58885	0.9995	11516	2	-0.047
hsa-mir-6083	4	0.37792	0.5232	0.9995	11517	2	0.2486
PKNOX1	6	0.37792	0.58891	0.9995	11518	2	-0.111
C19orf35	6	0.3781	0.58902	0.9995	11519	2	-0.605
ZDHHC14	6	0.37817	0.58906	0.9995	11520	2	0.1466
KIAA1804	6	0.37821	0.58909	0.9995	11521	2	-0.199
MUM1	6	0.37826	0.58913	0.9995	11522	2	0.0061
OLIG2	6	0.37834	0.58917	0.9995	11523	2	-0.906
MAPRE2	6	0.37839	0.5892	0.9995	11524	2	0.1213
C3orf58	6	0.3784	0.5892	0.9995	11525	1	-1.219
hsa-mir-1276	4	0.37852	0.52381	0.9995	11526	1	-0.116
COX6A1	6	0.37854	0.58929	0.9995	11527	2	0.0775
ITGBL1	6	0.37862	0.58935	0.9995	11528	1	-0.173
ST7	6	0.37865	0.58936	0.9995	11529	2	-0.136
AP1M2	6	0.37877	0.58945	0.9995	11530	2	-0.539
CLIP4	6	0.37882	0.58947	0.9995	11531	2	-0.083
SPANXN2	6	0.37894	0.58957	0.9995	11532	2	-0.462
SYNGR3	6	0.37908	0.58965	0.9995	11533	1	-0.343
LOC154872	6	0.37918	0.58973	0.9995	11534	2	-0.157
RNF25	6	0.37925	0.58978	0.9995	11535	2	0.0896
PTGES3L	2	0.37934	0.39977	0.9995	11536	1	-0.234
SH2D5	6	0.37937	0.58985	0.9995	11537	2	0.2047
NARF	6	0.37946	0.5899	0.9995	11538	2	-0.015
CASP3	6	0.37946	0.5899	0.9995	11539	2	0.0016
LDHB	6	0.37955	0.58996	0.9995	11540	2	-0.007
GARNL3	6	0.37965	0.59003	0.9995	11541	2	-0.07
C1orf87	6	0.37966	0.59004	0.9995	11542	2	-0.037
RNPS1	6	0.3797	0.59006	0.9995	11543	2	0.2674
IL15	6	0.37987	0.59017	0.9995	11544	2	-0.108
WDFY3	6	0.37993	0.59021	0.9995	11545	2	0.2164
CCNB2	6	0.37997	0.59023	0.9995	11546	2	0.2182
TRIM21	6	0.38013	0.59032	0.9995	11547	2	-0.175
OR4C15	6	0.38016	0.59035	0.9995	11548	1	-0.318
hsa-mir-4673	4	0.38017	0.5255	0.9995	11549	1	-0.495
BMP2K	6	0.38018	0.59036	0.9995	11550	2	0.0827
NPTN	6	0.38025	0.5904	0.9995	11551	2	0.0266
TAS2R16	6	0.38033	0.59046	0.9995	11552	2	-0.122
S100B	6	0.3805	0.59056	0.9995	11553	2	0.0161
PPIAL4G	1	0.3806	0.38073	0.9995	11554	0	0.1721
GPR137B	6	0.38062	0.59065	0.9995	11555	2	-0.056

PPP1R3B	6	0.38072	0.5907	0.9995	11556	1	-0.164
GPHB5	6	0.38072	0.5907	0.9995	11557	1	-0.24
DLX1	6	0.38079	0.59075	0.9995	11558	2	-0.068
LMF2	6	0.3809	0.59082	0.9995	11559	2	-0.014
CD4	6	0.38093	0.59084	0.9995	11560	2	0.035
KHDRBS1	6	0.38099	0.59087	0.9995	11561	1	-0.198
hsa-mir-197	4	0.38108	0.52642	0.9995	11562	2	0.204
CDK8	6	0.38109	0.59095	0.9995	11563	1	-0.072
OTOL1	6	0.38109	0.59095	0.9995	11564	1	-0.2
TUBG2	4	0.38111	0.52646	0.9995	11565	1	-0.107
TMEM200B	6	0.3812	0.591	0.9995	11566	2	0.0639
KRTAP15-1	6	0.38125	0.59103	0.9995	11567	2	-0.031
GTF2A1L	6	0.38132	0.59107	0.9995	11568	2	0.1272
ANG	6	0.38133	0.59108	0.9995	11569	2	0.042
PCDHGA1	2	0.38135	0.40137	0.9995	11570	1	-0.276
IFNL2	5	0.38142	0.54845	0.9995	11571	1	-0.224
EPC1	6	0.38144	0.59115	0.9995	11572	2	-0.185
OR5B3	6	0.38144	0.59115	0.9995	11573	1	-0.135
RPS18	6	0.3815	0.59119	0.9995	11574	2	-1.129
RALY	6	0.38161	0.59127	0.9995	11575	1	-0.23
hsa-mir-335	4	0.38169	0.52705	0.9995	11576	1	0.033
HGC6.3	4	0.38177	0.52712	0.9995	11577	1	0.0806
AXIN2	6	0.38181	0.59139	0.9995	11578	1	0.0334
PANK1	6	0.38184	0.59141	0.9995	11579	1	0.1058
hsa-mir-6846	2	0.38187	0.4018	0.9995	11580	1	0.3592
KIAA1024L	6	0.38188	0.59142	0.9995	11581	1	-0.133
OGG1	6	0.3822	0.59165	0.9995	11582	2	0.0693
ACTC1	6	0.38222	0.59166	0.9995	11583	1	-0.292
CDADC1	6	0.38229	0.59171	0.9995	11584	2	0.2055
hsa-mir-6863	4	0.38231	0.52766	0.9995	11585	2	0.2379
DSN1	4	0.38243	0.5278	0.9995	11586	1	-0.096
hsa-mir-3125	4	0.38253	0.5279	0.9995	11587	1	-0.243
GFOD1	6	0.38268	0.59197	0.9995	11588	2	-0.321
hsa-mir-4280	4	0.38269	0.52805	0.9995	11589	2	0.1826
SF3B3	6	0.38291	0.59213	0.9995	11590	2	-0.071
C14orf132	6	0.38291	0.59213	0.9995	11591	2	-0.054
CD200R1L	5	0.38297	0.54975	0.9995	11592	2	0.1875
CDC14A	4	0.383	0.52834	0.9995	11593	1	-0.255
ZFPM2	6	0.3831	0.59225	0.9995	11594	2	-0.036
ADM2	6	0.38315	0.59228	0.9995	11595	2	-0.141
HPS3	6	0.38318	0.5923	0.9995	11596	2	-0.224
hsa-mir-6885	4	0.38343	0.52879	0.9995	11597	2	-0.125
TOE1	6	0.38346	0.59248	0.9995	11598	2	0.2006
SEMA3E	6	0.38353	0.59253	0.9995	11599	1	-0.591
COPS6	6	0.38353	0.59253	0.9995	11600	1	-0.094
PBRM1	6	0.38366	0.5926	0.9995	11601	2	-0.144
UCN3	6	0.38369	0.59263	0.9995	11602	2	-0.072
AZU1	4	0.38371	0.5291	0.9995	11603	1	-0.048
NR4A1	6	0.38377	0.59269	0.9995	11604	1	-0.301
ANXA10	6	0.38381	0.5927	0.9995	11605	2	0.0606
SORT1	6	0.38386	0.59274	0.9995	11606	2	-0.006
hsa-mir-1469	4	0.38393	0.52932	0.9995	11607	2	0.0664
SERPINA12	6	0.384	0.59284	0.9995	11608	1	-0.525
RNF11	6	0.38404	0.59286	0.9995	11609	1	-0.244
hsa-mir-4503	2	0.38411	0.4037	0.9995	11610	1	0.2649
IRGC	6	0.38412	0.59291	0.9995	11611	2	-0.219
DPCR1	6	0.38428	0.59302	0.9995	11612	2	-0.021
XAF1	6	0.38431	0.59304	0.9995	11613	2	0.154
EFNB3	6	0.38445	0.59313	0.9995	11614	1	-0.067
URB2	6	0.38459	0.59321	0.9995	11615	2	0.134
hsa-mir-3165	4	0.38468	0.53008	0.9995	11616	2	0.1582
CAPRIN1	6	0.38482	0.59336	0.9995	11617	2	-0.18
SCEL	6	0.38484	0.59336	0.9995	11618	2	0.1724
CDCA3	6	0.38486	0.59338	0.9995	11619	2	-0.352
ZNF184	6	0.38487	0.59338	0.9995	11620	1	-0.058
GRIK4	6	0.38502	0.5935	0.9995	11621	2	0.0155
NRARP	6	0.38508	0.59353	0.9995	11622	2	0.0317
hsa-mir-513a-1	1	0.38516	0.38539	0.9995	11623	0	0.1821
CXXC5	6	0.38521	0.5936	0.9995	11624	2	-0.281
ARHGEF1	6	0.38537	0.59371	0.9995	11625	2	0.008
hsa-mir-3617	4	0.38544	0.53084	0.9995	11626	2	0.2384
MPC1	6	0.38564	0.5939	0.9995	11627	2	0.0256
RPS15A	6	0.38564	0.5939	0.9995	11628	2	-0.135
CERS5	6	0.38564	0.5939	0.9995	11629	2	-0.302
XKR3	6	0.38573	0.59395	0.9995	11630	2	0.1474
UNC119	6	0.38583	0.59402	0.9995	11631	2	0.0073
RPS6KA3	6	0.38596	0.5941	0.9995	11632	2	0.1377
DIABLO	6	0.38609	0.59419	0.9995	11633	1	-0.061
RAG1	6	0.38609	0.5942	0.9995	11634	2	-0.571
hsa-mir-3651	4	0.38614	0.53155	0.9995	11635	2	-0.263
SERPINI1	6	0.38618	0.59426	0.9995	11636	2	-0.307
RBM10	6	0.38619	0.59426	0.9995	11637	1	-0.316
ZNF764	5	0.38625	0.55252	0.9995	11638	2	-0.314
KRT83	4	0.3863	0.5317	0.9995	11639	1	-0.592
DCAF8L1	6	0.38631	0.59434	0.9995	11640	2	-0.054

PPEF2	6	0.38631	0.59434	0.9995	11641	2	0.0382
FAM26E	6	0.38634	0.59437	0.9995	11642	2	-0.192
AP4B1	6	0.38634	0.59437	0.9995	11643	2	0.0822
TSPAN16	6	0.3864	0.59441	0.9995	11644	2	0.0277
ALKBH5	6	0.38643	0.59443	0.9995	11645	1	-0.341
hsa-mir-325	4	0.38646	0.53188	0.9995	11646	1	0.078
INTS7	6	0.38664	0.59456	0.9995	11647	1	0.0782
KIAA1024	6	0.38666	0.59458	0.9995	11648	2	0.0489
TIMM10	6	0.38666	0.59458	0.9995	11649	2	-0.213
RPL39	4	0.38667	0.53211	0.9995	11650	2	-0.074
LRRC40	6	0.38668	0.59459	0.9995	11651	2	-0.236
DLGAP2	6	0.38671	0.59461	0.9995	11652	2	0.18
TRIM55	6	0.38676	0.59464	0.9995	11653	2	0.0451
PAGE5	4	0.38676	0.5322	0.9995	11654	1	-0.537
GIPR	6	0.38684	0.5947	0.9995	11655	2	-0.302
GABRA4	6	0.38701	0.5948	0.9995	11656	2	-0.032
hsa-mir-619	4	0.38708	0.53253	0.9995	11657	1	-0.856
RASA4B	1	0.38713	0.38732	0.9995	11658	0	0.1354
ZNF200	6	0.38718	0.5949	0.9995	11659	1	-0.208
TLE4	6	0.38744	0.59506	0.9995	11660	1	-0.384
SLC38A5	6	0.38749	0.59509	0.9995	11661	1	-0.018
UTY	5	0.38752	0.55357	0.9995	11662	2	0.0744
TACR2	6	0.38761	0.59517	0.9995	11663	2	0.1363
SH3BGRL3	6	0.38764	0.59519	0.9995	11664	2	0.0769
TBPL1	6	0.38765	0.59519	0.9995	11665	2	-0.046
BRI3	6	0.38793	0.59536	0.9995	11666	2	-0.699
MUC2	6	0.38794	0.59537	0.9995	11667	1	-0.418
ZBTB2	6	0.38802	0.59543	0.9995	11668	2	-0.175
TNK1	6	0.3881	0.59547	0.9995	11669	2	0.0624
INTS3	6	0.38815	0.59551	0.9995	11670	2	0.261
APOL5	6	0.38816	0.59552	0.9995	11671	2	-0.547
ZNF318	6	0.38822	0.59556	0.9995	11672	2	-0.338
ZNF479	4	0.38832	0.53383	0.9995	11673	2	0.0547
CHML	6	0.38838	0.59567	0.9995	11674	2	-0.31
ZNF287	6	0.38841	0.59568	0.9995	11675	2	-0.202
AZI1	6	0.38847	0.59573	0.9995	11676	2	-0.4
TMEM203	6	0.38854	0.59578	0.9995	11677	1	-0.286
GPRC5A	6	0.38863	0.59583	0.9995	11678	1	-0.165
ANGPTL3	6	0.38871	0.5959	0.9995	11679	2	-0.275
PRSS33	6	0.38871	0.5959	0.9995	11680	2	0.1111
hsa-mir-606	4	0.38877	0.53431	0.9995	11681	1	-0.401
AMMECR1	6	0.38882	0.59598	0.9995	11682	2	-0.14
CBX5	6	0.38897	0.59607	0.9995	11683	2	-0.141
TFCP2L1	6	0.38923	0.59625	0.9995	11684	2	-0.111
CELSR2	6	0.38936	0.59633	0.9995	11685	1	-0.244
OR10H3	6	0.38937	0.59634	0.9995	11686	2	-0.241
ZNRF3	6	0.38941	0.59636	0.9995	11687	1	0.0878
KRTAP9-2	5	0.38958	0.55531	0.9995	11688	1	-1.233
STEAP2	6	0.38963	0.5965	0.9995	11689	2	-0.588
NAPRT1	6	0.38981	0.59661	0.9995	11690	1	-0.222
hsa-mir-6085	4	0.38992	0.53551	0.9995	11691	1	-0.245
CLUL1	6	0.38993	0.59669	0.9995	11692	2	-0.337
POTED	2	0.39	0.40847	0.9995	11693	1	-1.098
RNF141	6	0.3901	0.59678	0.9995	11694	2	-0.461
PAX5	6	0.3901	0.59678	0.9995	11695	2	-0.067
OR11H2	6	0.39036	0.59695	0.9995	11696	2	-0.374
hsa-mir-4500	4	0.39051	0.53613	0.9995	11697	2	0.0443
KIF5B	6	0.39055	0.59708	0.9995	11698	2	-0.523
BEX1	6	0.39059	0.59711	0.9995	11699	2	-0.215
PHYKPL	1	0.39062	0.39081	0.9995	11700	0	0.131
hsa-mir-6814	4	0.39063	0.53625	0.9995	11701	1	-0.684
CRADD	6	0.39075	0.59721	0.9995	11702	2	-0.056
RAB15	6	0.39084	0.59726	0.9995	11703	2	0.1047
hsa-mir-31	4	0.39087	0.5365	0.9995	11704	2	0.0555
BMPR2	6	0.39093	0.59732	0.9995	11705	2	0.0971
hsa-mir-4425	4	0.39096	0.5366	0.9995	11706	2	0.2008
CYP2R1	6	0.39103	0.59739	0.9995	11707	2	0.106
SLC25A53	6	0.3911	0.59743	0.9995	11708	2	0.209
HSDL1	6	0.3911	0.59743	0.9995	11709	2	-0.136
CLVS1	6	0.3911	0.59743	0.9995	11710	2	0.0501
LGALS1	6	0.3912	0.59749	0.9995	11711	1	-0.502
ITGB3	6	0.39124	0.59751	0.9995	11712	1	0.0466
HIST1H2BH	6	0.39132	0.59757	0.9995	11713	2	-0.9
GALNT14	4	0.39137	0.537	0.9995	11714	2	0.2712
IL7R	6	0.39141	0.59763	0.9995	11715	2	-0.128
TNFSF4	6	0.39148	0.59768	0.9995	11716	2	0.0892
GMIP	6	0.39148	0.59769	0.9995	11717	2	-0.042
OR3A1	6	0.39153	0.59772	0.9995	11718	2	-0.005
RBM4B	6	0.39168	0.59781	0.9995	11719	2	-0.23
S100A10	6	0.39182	0.59789	0.9995	11720	2	0.1144
MYH1	5	0.39193	0.55728	0.9995	11721	2	-0.017
PPIL6	6	0.39208	0.59807	0.9995	11722	1	-0.197
KIAA0195	6	0.39214	0.5981	0.9995	11723	2	0.0936
TTPAL	6	0.39219	0.59814	0.9995	11724	2	-0.195
ZMIZ2	6	0.39225	0.59818	0.9995	11725	1	-0.129

SOWAHB	6	0.3923	0.59821	0.9995	11726	2	-0.353
YEATS4	6	0.39232	0.59823	0.9995	11727	2	-0.063
RCSD1	6	0.39241	0.59828	0.9995	11728	2	-0.054
RPL36	6	0.39241	0.59828	0.9995	11729	2	0.0337
SLC26A9	6	0.39248	0.59833	0.9995	11730	2	-0.14
ADCK5	6	0.39251	0.59835	0.9995	11731	2	0.1868
NUCB1	6	0.39259	0.59841	0.9995	11732	2	0.0515
NR1H4	6	0.3926	0.59842	0.9995	11733	2	-0.18
BAIAP2	6	0.39265	0.59844	0.9995	11734	1	-0.65
KIF11	6	0.39272	0.59848	0.9995	11735	1	-0.215
hsa-mir-3921	4	0.39272	0.5384	0.9995	11736	1	-0.358
CENPQ	6	0.39283	0.59856	0.9995	11737	2	-0.207
hsa-mir-4765	4	0.39283	0.53852	0.9995	11738	1	-0.443
BHLHA9	6	0.39285	0.59858	0.9995	11739	2	0.1057
SYTL4	6	0.39294	0.59863	0.9995	11740	2	-0.459
LRTOMT	6	0.39308	0.59871	0.9995	11741	2	0.0401
RBBP6	4	0.39308	0.53878	0.9995	11742	2	0.1489
SPHAR	1	0.39316	0.39338	0.9995	11743	0	0.2665
hsa-mir-3685	4	0.39346	0.53916	0.9995	11744	1	0.2592
ARL8B	6	0.3937	0.59908	0.9995	11745	2	-0.834
OR51Q1	6	0.39374	0.59911	0.9995	11746	2	0.0901
RNF126	6	0.39375	0.59911	0.9995	11747	1	-0.367
hsa-mir-5089	4	0.39375	0.53946	0.9995	11748	1	-0.008
hsa-mir-187	4	0.39382	0.53953	0.9995	11749	2	-0.022
NMNAT2	6	0.39385	0.59918	0.9995	11750	2	-0.946
LOC653486	3	0.39399	0.4812	0.9995	11751	1	0.1066
hsa-mir-4322	4	0.39402	0.53973	0.9995	11752	2	0.2506
C16orf45	6	0.39403	0.59931	0.9995	11753	2	-0.064
TMEM211	6	0.39409	0.59935	0.9995	11754	2	-0.209
EVL	6	0.39433	0.5995	0.9995	11755	2	-0.031
AP2A2	6	0.39433	0.5995	0.9995	11756	2	0.0283
OR51B4	6	0.39433	0.5995	0.9995	11757	2	0.0442
TIGD6	4	0.39435	0.54008	0.9995	11758	1	0.163
RPAP3	6	0.39445	0.59956	0.9995	11759	2	-0.205
CRYBA4	6	0.39466	0.59969	0.9995	11760	1	-0.443
KLF6	6	0.39474	0.59975	0.9995	11761	2	-0.35
hsa-mir-548ax	1	0.39478	0.39501	0.9995	11762	0	0.1745
NDUFAF6	6	0.39494	0.59987	0.9995	11763	2	0.0264
C20orf96	6	0.39503	0.59993	0.9995	11764	2	-0.033
PLRG1	6	0.39504	0.59993	0.9995	11765	2	0.0201
ZNF579	6	0.3951	0.59997	0.9995	11766	1	0.1214
PDIA5	6	0.39529	0.60011	0.9995	11767	1	-0.09
ANLN	6	0.39549	0.60024	0.9995	11768	2	-0.192
KCNE2	6	0.39555	0.60028	0.9995	11769	2	-0.311
PRDM5	6	0.39558	0.60029	0.9995	11770	2	-0.026
NKAIN2	6	0.39568	0.60035	0.9995	11771	2	0.1873
LRRC17	6	0.39574	0.60039	0.9995	11772	2	-0.159
RAB7A	6	0.39582	0.60044	0.9995	11773	1	-0.344
LCE6A	6	0.39591	0.6005	0.9995	11774	2	-0.034
BAG1	6	0.39601	0.60056	0.9995	11775	2	0.0612
RBP2	6	0.39604	0.60059	0.9995	11776	2	-0.084
MOGAT1	6	0.3961	0.60062	0.9995	11777	2	-0.193
ANO1	6	0.3962	0.60068	0.9995	11778	1	-0.417
GPR171	4	0.39644	0.54225	0.9995	11779	2	0.124
RANBP2	3	0.39644	0.48263	0.9995	11780	1	0.0865
SSX2B	2	0.39652	0.41387	0.9995	11781	1	-0.692
SHISA8	6	0.39665	0.60094	0.9995	11782	2	-0.288
FAM205A	6	0.39665	0.60094	0.9995	11783	2	-0.022
VRK2	6	0.39666	0.60095	0.9995	11784	2	-0.009
SLCO4C1	6	0.39684	0.60107	0.9995	11785	2	-0.024
hsa-mir-4453	4	0.39692	0.54274	0.9995	11786	1	-0.198
FOS	6	0.397	0.60117	0.9995	11787	1	-0.414
EBLN2	6	0.39711	0.60124	0.9995	11788	2	-0.252
HOXD3	6	0.39719	0.6013	0.9995	11789	1	0.0057
LAMA5	6	0.39731	0.60137	0.9995	11790	1	0.011
MINOS1	6	0.39736	0.60141	0.9995	11791	2	0.0499
VASH1	6	0.39741	0.60144	0.9995	11792	2	-0.25
SPTA1	6	0.39745	0.60147	0.9995	11793	2	-0.04
CASKIN1	6	0.39751	0.60151	0.9995	11794	2	-0.196
hsa-mir-365b	4	0.39771	0.54355	0.9995	11795	2	0.1721
RBM34	6	0.39782	0.60172	0.9995	11796	1	-0.098
RTP2	6	0.39799	0.60183	0.9995	11797	2	-0.017
hsa-mir-4737	4	0.39804	0.54386	0.9995	11798	2	0.162
ATP6V0A1	6	0.3982	0.60195	0.9995	11799	1	-0.104
ZNF790	6	0.39828	0.602	0.9995	11800	2	0.1006
DRD1	6	0.3983	0.60201	0.9995	11801	2	0.0388
MIP	6	0.39837	0.60206	0.9995	11802	2	-0.04
SLC12A4	6	0.3985	0.60215	0.9995	11803	2	-0.116
TARBP1	6	0.39853	0.60217	0.9995	11804	2	-0.094
MRPL2	6	0.39867	0.60225	0.9995	11805	2	-0.25
MIS12	6	0.39869	0.60226	0.9995	11806	2	0.0321
IL17RE	6	0.39895	0.60241	0.9995	11807	1	-0.259
41883	3	0.39895	0.48408	0.9995	11808	1	0.1996
CHRM1	4	0.39908	0.54493	0.9995	11809	2	-0.039
TAF4	6	0.39913	0.60252	0.9995	11810	2	0.1028

TSTD1	6	0.39917	0.60254	0.9995	11811	2	-0.028
TNPO2	6	0.39922	0.60258	0.9995	11812	1	-0.434
EHHADH	6	0.39929	0.60262	0.9995	11813	2	-0.242
CCDC108	6	0.39936	0.60266	0.9995	11814	2	-0.202
EMB	6	0.39936	0.60267	0.9995	11815	2	-0.135
PYCRL	6	0.39946	0.60273	0.9995	11816	2	-0.172
DEAF1	6	0.39947	0.60273	0.9995	11817	2	-0.073
hsa-mir-378e	4	0.39956	0.54541	0.9995	11818	2	0.2223
GDNF	6	0.39961	0.60282	0.9995	11819	1	0.0593
RPL3	6	0.39971	0.60287	0.9995	11820	2	-0.234
INTS1	6	0.3998	0.60293	0.9995	11821	2	-0.074
HIST1H2BG	6	0.39985	0.60296	0.9995	11822	2	-0.021
SLC2A10	6	0.39985	0.60296	0.9995	11823	1	-0.172
HTR1F	6	0.40004	0.60308	0.9995	11824	2	-0.101
STAT3	6	0.40005	0.60309	0.9995	11825	1	-0.262
C15orf39	6	0.40011	0.60313	0.9995	11826	2	-0.008
LINGO3	6	0.40012	0.60314	0.9995	11827	2	0.0089
hsa-mir-505	4	0.40018	0.54605	0.9995	11828	1	-0.071
TREML4	6	0.40021	0.60319	0.9995	11829	2	0.1906
NMU	6	0.40023	0.6032	0.9995	11830	2	-0.407
hsa-mir-4460	4	0.40025	0.54614	0.9995	11831	2	0.019
SLC35G5	6	0.40038	0.6033	0.9995	11832	1	-0.279
DNAJC12	6	0.40044	0.60335	0.9995	11833	2	0.0235
PPP4C	6	0.40058	0.60344	0.9995	11834	2	0.0228
VPS4B	6	0.40066	0.60349	0.9995	11835	2	-0.02
ISYNA1	6	0.40072	0.60353	0.9995	11836	2	0.055
GM2A	6	0.40096	0.60368	0.9995	11837	1	-0.065
THEMIS	6	0.40101	0.60371	0.9995	11838	2	-0.252
CST7	6	0.40103	0.60372	0.9995	11839	2	-0.074
ELOVL7	6	0.40108	0.60375	0.9995	11840	2	-0.068
TNNC2	6	0.40122	0.60385	0.9995	11841	2	-0.342
C1orf85	6	0.40143	0.60398	0.9995	11842	1	-0.663
EVC	6	0.40151	0.60403	0.9995	11843	2	-0.116
hsa-mir-3620	4	0.40163	0.54759	0.9995	11844	1	-0.335
GLIS1	6	0.40167	0.60412	0.9995	11845	2	-0.081
ZBTB47	6	0.40175	0.60419	0.9995	11846	1	-0.422
RFC2	6	0.40176	0.60419	0.9995	11847	2	-0.059
LOC100652824	6	0.40184	0.60424	0.9995	11848	2	0.1107
CTAG2	5	0.40201	0.56571	0.9995	11849	1	-0.297
CSTA	6	0.4022	0.60448	0.9995	11850	2	-0.172
AMPD3	6	0.40237	0.60459	0.9995	11851	2	-0.403
HSPB3	6	0.40238	0.6046	0.9995	11852	2	0.0417
PSG11	6	0.40238	0.6046	0.9995	11853	1	0.0566
IMMP2L	6	0.40246	0.60465	0.9995	11854	2	-0.23
PHLDB1	6	0.40246	0.60465	0.9995	11855	2	-0.224
TUSC3	6	0.4025	0.60467	0.9995	11856	2	-0.082
SLC10A2	6	0.40259	0.60473	0.9995	11857	2	-0.096
hsa-mir-26b	4	0.40266	0.54861	0.9995	11858	2	0.0361
OR6K3	6	0.40272	0.60482	0.9995	11859	1	-0.097
PSMA3	6	0.40289	0.60493	0.9995	11860	2	-0.184
SPATA32	6	0.40294	0.60497	0.9995	11861	2	-0.407
WDR61	6	0.40309	0.60507	0.9995	11862	2	0.2511
MLC1	6	0.40324	0.60517	0.9995	11863	2	-0.267
OR5R1	6	0.4034	0.60527	0.9995	11864	1	-0.329
KBTBD7	5	0.40345	0.56696	0.9995	11865	1	-0.246
hsa-mir-4430	4	0.40347	0.54942	0.9995	11866	2	0.2475
hsa-mir-7704	4	0.40353	0.54948	0.9995	11867	2	0.2726
TNFRSF12A	6	0.40359	0.6054	0.9995	11868	2	-0.335
C1QBP	6	0.4036	0.60541	0.9995	11869	1	-0.222
MALSU1	6	0.40369	0.60546	0.9995	11870	2	0.0243
hsa-mir-125b-1	4	0.40376	0.54971	0.9995	11871	1	-0.06
TMBIM4	6	0.40378	0.60552	0.9995	11872	2	0.0607
TMEM161A	6	0.40382	0.60555	0.9995	11873	1	-0.359
PCDHB7	6	0.40386	0.60556	0.9995	11874	2	0.1422
hsa-mir-583	4	0.40395	0.54991	0.9995	11875	1	0.2758
EMC4	6	0.40401	0.60566	0.9995	11876	2	-0.62
PLVAP	6	0.40411	0.60573	0.9995	11877	2	0.0204
hsa-mir-6804	4	0.40418	0.55013	0.9995	11878	1	0.2402
VWA2	6	0.40421	0.60579	0.9995	11879	2	-0.231
MYO7A	6	0.40442	0.60592	0.9995	11880	2	-0.078
KDM2A	6	0.40442	0.60592	0.9995	11881	2	-0.525
hsa-mir-1268b	4	0.40447	0.55042	0.9995	11882	2	0.2038
ME2	6	0.40452	0.60598	0.9995	11883	2	0.0028
hsa-mir-4653	4	0.40453	0.55048	0.9995	11884	2	-0.141
DKK4	6	0.4046	0.60603	0.9995	11885	2	-0.101
THEG	6	0.40468	0.60608	0.9995	11886	2	0.0171
hsa-mir-551b	4	0.40475	0.5507	0.9995	11887	1	-0.147
MPV17L2	6	0.40479	0.60615	0.9995	11888	2	-0.137
SPATA31A2	2	0.40487	0.42081	0.9995	11889	1	-0.345
CXCL1	6	0.40496	0.60625	0.9995	11890	2	-0.268
TMEM130	6	0.40497	0.60626	0.9995	11891	2	0.2142
hsa-mir-30a	4	0.40499	0.55094	0.9995	11892	1	-0.108
hsa-mir-6512	4	0.40504	0.55098	0.9995	11893	2	0.2387
PTOV1	6	0.40514	0.60636	0.9995	11894	2	0.0148
ST3GAL4	6	0.40541	0.60654	0.9995	11895	1	-0.094

COX4I1	6	0.4055	0.6066	0.9995	11896	2	-0.227
MAP1LC3B	5	0.40554	0.56871	0.9995	11897	2	-0.325
TUBGCP2	6	0.40555	0.60663	0.9995	11898	2	-0.02
HOXB1	6	0.40562	0.60668	0.9995	11899	2	0.0571
TOMM7	6	0.40568	0.60672	0.9995	11900	2	-0.013
SLC37A4	6	0.40585	0.60682	0.9995	11901	2	-0.183
FASTKD3	6	0.4059	0.60685	0.9995	11902	2	-0.19
FAM122C	6	0.40592	0.60687	0.9995	11903	2	-0.095
SLK	4	0.40605	0.55201	0.9995	11904	1	-0.286
ACTA1	6	0.4061	0.60698	0.9995	11905	2	0.016
ZMYM5	6	0.40615	0.60702	0.9995	11906	1	0.0754
MON2	6	0.40623	0.60706	0.9995	11907	2	0.0632
hsa-mir-4736	4	0.40624	0.55222	0.9995	11908	1	-0.448
FAM127B	6	0.40626	0.60708	0.9995	11909	1	-0.174
MOGAT3	6	0.40637	0.60715	0.9995	11910	2	-0.116
STAC	6	0.40654	0.60727	0.9995	11911	2	0.1108
LAMP3	6	0.40667	0.60734	0.9995	11912	2	0.0092
LY6K	6	0.40681	0.60744	0.9995	11913	2	-0.163
ZNF207	6	0.40683	0.60745	0.9995	11914	2	-0.308
LRRC24	4	0.40686	0.55285	0.9995	11915	2	0.2752
FAHD1	6	0.40691	0.6075	0.9995	11916	1	-0.077
hsa-mir-129-2	4	0.40693	0.55291	0.9995	11917	1	-0.247
KCNK7	6	0.40733	0.60777	0.9995	11918	2	-0.198
KIF16B	6	0.4074	0.60781	0.9995	11919	2	0.1015
LOC388813	5	0.40744	0.57031	0.9995	11920	2	0.2768
GYPB	2	0.40744	0.42291	0.9995	11921	1	0.3421
CHMP6	6	0.40752	0.60789	0.9995	11922	2	-0.02
ZNF260	6	0.40752	0.60789	0.9995	11923	1	-0.01
COX5B	6	0.40767	0.60799	0.9995	11924	2	-0.28
FBXL12	6	0.40772	0.60803	0.9995	11925	2	-0.07
ENTPD3	6	0.40791	0.60815	0.9995	11926	2	-0.275
OR8J3	5	0.40796	0.57076	0.9995	11927	2	0.0731
SETDB2	6	0.40802	0.60824	0.9995	11928	2	-0.045
hsa-mir-4639	4	0.40805	0.55404	0.9995	11929	2	0.0985
ACOX1	6	0.40806	0.60825	0.9995	11930	2	0.0932
MTMR6	6	0.40824	0.60837	0.9995	11931	1	-0.49
SULT1C3	6	0.40824	0.60837	0.9995	11932	1	-0.453
OTOR	6	0.40827	0.60839	0.9995	11933	2	0.1174
C18orf42	6	0.40836	0.60844	0.9995	11934	2	0.2074
DCC	6	0.40839	0.60846	0.9995	11935	2	0.0546
GNG10	6	0.40839	0.60846	0.9995	11936	2	-0.297
ZPBP2	6	0.40855	0.60855	0.9995	11937	2	0.0293
GEMIN8	6	0.40865	0.60862	0.9995	11938	2	0.0139
FAM149A	6	0.40865	0.60862	0.9995	11939	2	-0.052
SGTB	6	0.40875	0.60868	0.9995	11940	2	-0.311
hsa-mir-570	4	0.40876	0.55478	0.9995	11941	1	-0.024
NPM3	6	0.40896	0.60883	0.9995	11942	2	-0.4
TYMS	6	0.40896	0.60883	0.9995	11943	2	-0.124
CSF3	4	0.40897	0.55498	0.9995	11944	1	-0.498
CIDEA	6	0.40909	0.60891	0.9995	11945	2	-0.114
ESRP2	6	0.40913	0.60894	0.9995	11946	2	-0.018
ERG	6	0.4092	0.60897	0.9995	11947	1	-0.261
UCK1	6	0.40924	0.609	0.9995	11948	2	0.1811
UCHL1	6	0.4093	0.60904	0.9995	11949	2	0.0024
TMOD2	6	0.40938	0.60908	0.9995	11950	2	-0.213
VSIG2	6	0.40938	0.60908	0.9995	11951	2	0.1084
hsa-mir-297	1	0.40945	0.40967	0.9995	11952	0	0.1305
XYLT2	6	0.40971	0.60929	0.9995	11953	1	-0.498
NSUN3	6	0.4098	0.60935	0.9995	11954	2	0.1207
SLC9A5	6	0.41006	0.60953	0.9995	11955	2	0.2429
HNMT	6	0.41014	0.60957	0.9995	11956	2	-0.573
C19orf71	6	0.41019	0.60961	0.9995	11957	2	0.0128
MITD1	6	0.41028	0.60967	0.9995	11958	2	0.1567
ATP1A3	6	0.41029	0.60967	0.9995	11959	2	-0.424
EPHA1	6	0.41044	0.60977	0.9995	11960	1	-0.644
DEGS2	6	0.41044	0.60977	0.9995	11961	1	-0.137
EXTL3	6	0.41053	0.60982	0.9995	11962	2	-0.117
hsa-mir-561	4	0.41053	0.55661	0.9995	11963	1	-0.058
DNASE2	6	0.41058	0.60986	0.9995	11964	1	0.0398
EFEMP2	6	0.4107	0.60993	0.9995	11965	1	-0.4
hsa-mir-3918	4	0.41082	0.55692	0.9995	11966	2	-0.046
DNTTIP1	6	0.41085	0.61003	0.9995	11967	1	-0.255
hsa-mir-6768	4	0.41088	0.55699	0.9995	11968	1	-0.16
MLKL	6	0.41088	0.61006	0.9995	11969	1	-0.608
IMPAD1	6	0.41091	0.61007	0.9995	11970	2	0.0372
FITM2	6	0.41091	0.61007	0.9995	11971	2	-0.099
FGF18	6	0.41095	0.61009	0.9995	11972	1	-1.072
TMEM95	6	0.41098	0.61012	0.9995	11973	2	0.0894
SP140L	6	0.41118	0.61025	0.9995	11974	2	0.0026
TPD52	6	0.41132	0.61033	0.9995	11975	2	-0.151
hsa-mir-3940	4	0.41138	0.55749	0.9995	11976	2	0.2323
C7orf10	6	0.41152	0.61045	0.9995	11977	2	0.083
OR2B3	6	0.41162	0.61052	0.9995	11978	1	-0.2
TTC17	6	0.41168	0.61057	0.9995	11979	1	-1.125
CCR7	6	0.41185	0.61069	0.9995	11980	2	-0.673

ARSA	6	0.41192	0.61074	0.9995	11981	2	0.209
OR5J2	6	0.41193	0.61075	0.9995	11982	1	-0.22
C1orf112	6	0.41195	0.61076	0.9995	11983	2	-0.3
OR52N2	6	0.41198	0.61079	0.9995	11984	1	-0.219
hsa-mir-6872	4	0.41213	0.55825	0.9995	11985	2	-0.049
OR13C8	6	0.41222	0.61092	0.9995	11986	2	0.0019
hsa-mir-324	4	0.41224	0.55836	0.9995	11987	2	-0.171
GPT2	6	0.41245	0.61109	0.9995	11988	2	-0.183
hsa-mir-760	4	0.41248	0.55859	0.9995	11989	1	-0.024
KIAA1704	3	0.41249	0.49204	0.9995	11990	1	-0.951
DEFB104A	1	0.41267	0.41295	0.9995	11991	0	0.1123
GPR89B	4	0.41276	0.55886	0.9995	11992	1	-0.069
SLC32A1	6	0.4128	0.61132	0.9995	11993	2	-0.206
TP53TG3D	4	0.41283	0.55894	0.9995	11994	1	-0.676
B4GALT7	6	0.41299	0.61144	0.9995	11995	2	-0.009
PCDH7	6	0.41305	0.61147	0.9995	11996	2	0.0976
BMF	6	0.41312	0.61152	0.9995	11997	2	0.0398
FLI1	6	0.4132	0.61155	0.9995	11998	2	0.0425
CPEB4	6	0.41327	0.6116	0.9995	11999	2	-0.484
HPD	6	0.41336	0.61166	0.9995	12000	2	-0.212
TBC1D12	6	0.41341	0.61169	0.9995	12001	2	-0.098
ARHGAP10	4	0.41348	0.55959	0.9995	12002	1	-0.044
HIST3H3	6	0.41351	0.61175	0.9995	12003	2	0.1513
hsa-mir-5006	4	0.41362	0.55973	0.9995	12004	1	0.2225
F9	6	0.41369	0.61187	0.9995	12005	2	-0.203
NHLRC3	6	0.41389	0.61199	0.9995	12006	2	0.0968
CGB7	1	0.41389	0.41415	0.9995	12007	0	0.4779
LMO7	6	0.41392	0.61201	0.9995	12008	2	-0.001
TMCC3	6	0.41412	0.61213	0.9995	12009	2	0.1102
CDKN1B	6	0.41417	0.61216	0.9995	12010	2	-0.184
hsa-mir-4313	4	0.4142	0.56036	0.9995	12011	2	0.2194
BCKDK	6	0.41426	0.61221	0.9995	12012	1	-0.502
CBWD5	2	0.41429	0.42867	0.9995	12013	1	0.5668
TGFA	6	0.41434	0.61226	0.9995	12014	1	-0.076
RPS6KA2	6	0.41442	0.61231	0.9995	12015	2	0.1693
MLXIP	6	0.41444	0.61232	0.9995	12016	1	-0.25
BIVM	2	0.4145	0.42885	0.9995	12017	1	0.2202
CASC3	6	0.41452	0.61237	0.9995	12018	1	-0.047
ARSI	6	0.41459	0.61242	0.9995	12019	2	2E-05
SLC25A36	6	0.41459	0.61243	0.9995	12020	1	-0.08
KIRREL3	6	0.41467	0.61247	0.9995	12021	2	0.0071
TNKS2	5	0.41471	0.57629	0.9995	12022	2	0.1244
METTL21D	6	0.41475	0.61253	0.9995	12023	2	0.1959
CATSPER3	4	0.41512	0.56128	0.9995	12024	2	-0.098
AK8	6	0.41518	0.6128	0.9995	12025	2	-0.045
C16orf13	6	0.41526	0.61286	0.9995	12026	2	-0.054
KCNG4	6	0.41527	0.61287	0.9995	12027	2	-0.123
MRPS6	6	0.41527	0.61287	0.9995	12028	2	0.0081
hsa-mir-548aj-2	4	0.41532	0.56147	0.9995	12029	2	0.2097
ZNF83	6	0.41554	0.61304	0.9995	12030	2	-0.403
COL4A3	6	0.41559	0.61307	0.9995	12031	1	-0.479
OR1B1	6	0.41564	0.6131	0.9995	12032	2	0.0666
FCGRT	6	0.41566	0.61312	0.9995	12033	2	0.0305
hsa-mir-4635	4	0.4157	0.56187	0.9995	12034	2	0.2985
H1FX	6	0.41576	0.61317	0.9995	12035	2	-0.26
BATF2	6	0.41584	0.61324	0.9995	12036	2	-0.417
RPA2	6	0.41591	0.61328	0.9995	12037	1	-0.347
hsa-mir-499a	4	0.41592	0.56209	0.9995	12038	1	-0.529
PFKFB3	6	0.41596	0.6133	0.9995	12039	2	0.0434
hsa-mir-7111	4	0.41606	0.56223	0.9995	12040	2	0.1318
CP	6	0.41609	0.61339	0.9995	12041	1	-0.091
SLC22A2	6	0.41612	0.6134	0.9995	12042	2	0.0229
PCIF1	6	0.41616	0.61344	0.9995	12043	2	-0.14
C1orf198	6	0.41616	0.61344	0.9995	12044	2	-0.063
ACVR2A	6	0.41619	0.61345	0.9995	12045	1	-0.311
KLRK1	6	0.41629	0.61351	0.9995	12046	2	-0.012
SLC39A1	6	0.4164	0.61358	0.9995	12047	2	-0.083
SNX29	6	0.41643	0.61359	0.9995	12048	2	0.066
ZCCHC3	6	0.41652	0.61365	0.9995	12049	1	-0.173
LMBRD1	6	0.41653	0.61365	0.9995	12050	2	-0.04
UBE2V1	6	0.41667	0.61376	0.9995	12051	2	0.0889
SYNJ2BP-COX16	1	0.41671	0.41693	0.9995	12052	0	0.1311
PRPF18	6	0.41689	0.6139	0.9995	12053	2	-0.243
TMEM53	6	0.41697	0.61395	0.9995	12054	1	-0.516
FEZ1	6	0.41705	0.614	0.9995	12055	1	-0.433
ZNF480	6	0.4171	0.61403	0.9995	12056	2	-0.446
AP1M1	6	0.4171	0.61404	0.9995	12057	1	-0.19
CATSPER2	4	0.41718	0.56338	0.9995	12058	2	0.0014
PIGH	6	0.41721	0.61411	0.9995	12059	2	-0.118
MKL1	6	0.41737	0.61422	0.9995	12060	1	-0.464
LILRB1	3	0.41739	0.49499	0.9995	12061	1	-0.754
PRMT10	6	0.41745	0.61427	0.9995	12062	2	-0.066
LSAMP	6	0.41745	0.61427	0.9995	12063	2	-0.175
DBN1	6	0.41754	0.61432	0.9995	12064	2	-0.014
PTPDC1	6	0.41758	0.61436	0.9995	12065	2	0.1085

SERINC1	6	0.41789	0.61457	0.9995	12066	2	0.1277
ADAP1	6	0.41793	0.6146	0.9995	12067	1	-0.018
TCP1	6	0.41798	0.61463	0.9995	12068	2	0.2663
SCRN2	6	0.41805	0.61467	0.9995	12069	1	-0.204
LDLRAD3	6	0.41807	0.61469	0.9995	12070	2	-0.16
TRIM59	6	0.41808	0.6147	0.9995	12071	1	-0.196
OR6C68	6	0.41827	0.61481	0.9995	12072	2	-0.027
RREB1	6	0.41834	0.61485	0.9995	12073	2	-0.042
RASL10A	6	0.41834	0.61485	0.9995	12074	2	0.1458
BORA	6	0.41838	0.61489	0.9995	12075	1	-0.101
ZSCAN23	6	0.41841	0.61491	0.9995	12076	1	-0.456
C5orf28	6	0.41845	0.61493	0.9995	12077	1	-0.264
NKAIN4	6	0.41864	0.61507	0.9995	12078	2	-0.269
DISP2	6	0.41866	0.61509	0.9995	12079	2	-0.024
TTC30A	5	0.41881	0.57967	0.9995	12080	1	-0.444
LCE1B	6	0.41909	0.61537	0.9995	12081	1	-0.037
PFAS	4	0.41922	0.56543	0.9995	12082	2	0.1216
ZSCAN10	6	0.41933	0.61552	0.9995	12083	2	-0.02
SPINK5	6	0.41951	0.61564	0.9995	12084	2	-0.286
C12orf4	6	0.41951	0.61564	0.9995	12085	1	-0.208
KCNK17	6	0.41967	0.61575	0.9995	12086	1	-0.449
ZNF124	6	0.41974	0.61579	0.9995	12087	2	-0.019
hsa-mir-3129	3	0.41977	0.49643	0.9995	12088	1	0.2743
PKD1	6	0.41989	0.61589	0.9995	12089	2	-0.399
hsa-mir-136	4	0.41991	0.56614	0.9995	12090	2	-0.298
STK17B	6	0.41997	0.61595	0.9995	12091	2	0.1828
OR4D2	6	0.42009	0.61602	0.9995	12092	1	-0.239
CHORDC1	6	0.42019	0.61609	0.9995	12093	1	0.2276
PDE4D	6	0.42022	0.61611	0.9995	12094	2	0.1834
BTBD2	6	0.42029	0.61614	0.9995	12095	1	-0.159
KRT78	6	0.42044	0.61624	0.9995	12096	2	0.1887
PROB1	6	0.42047	0.61625	0.9995	12097	1	-0.178
HTR3D	6	0.42049	0.61627	0.9995	12098	2	-0.067
PRKCE	6	0.42083	0.6165	0.9995	12099	2	-0.069
OR10W1	6	0.42085	0.61651	0.9995	12100	2	-0.005
AP4E1	6	0.42086	0.61651	0.9995	12101	1	-0.503
hsa-mir-6828	4	0.42089	0.56717	0.9995	12102	2	0.1523
IMMT	6	0.42098	0.6166	0.9995	12103	2	-0.54
ACTL10	6	0.42118	0.61673	0.9995	12104	2	0.1472
CHD9	6	0.42129	0.6168	0.9995	12105	2	0.0575
KIF1A	6	0.42137	0.61686	0.9995	12106	2	-0.24
VWA7	6	0.42159	0.61701	0.9995	12107	2	-0.099
hsa-mir-4445	2	0.42159	0.43487	0.9995	12108	1	-0.129
UBE4A	6	0.42172	0.61709	0.9995	12109	2	-0.488
ZFP69B	6	0.42195	0.61724	0.9995	12110	2	-0.087
GNE	6	0.42197	0.61726	0.9995	12111	2	0.1433
ZNF404	6	0.42199	0.61727	0.9995	12112	2	0.1661
GBP2	6	0.42205	0.61732	0.9995	12113	2	-0.137
UTS2B	6	0.42207	0.61733	0.9995	12114	2	-0.28
PAIP2B	6	0.42209	0.61735	0.9995	12115	2	-0.026
PRPF19	6	0.42219	0.61742	0.9995	12116	2	-0.671
COX7A1	6	0.42242	0.61757	0.9995	12117	2	-0.456
PFDN1	6	0.42247	0.61761	0.9995	12118	2	-0.08
HMHA1	6	0.42247	0.61761	0.9995	12119	2	-0.484
CASKIN2	6	0.42263	0.61771	0.9995	12120	2	-0.281
IFNA6	6	0.42263	0.61771	0.9995	12121	2	-0.041
UBE2D2	6	0.42275	0.61779	0.9995	12122	2	-0.392
PLA2G5	6	0.42275	0.61779	0.9995	12123	2	0.0763
DENND6B	6	0.42288	0.61788	0.9995	12124	1	-0.296
ZNF516	6	0.42288	0.61788	0.9995	12125	2	-0.111
DST	6	0.42294	0.61792	0.9995	12126	2	-0.074
TUFM	6	0.423	0.61795	0.9995	12127	2	0.0082
ZNF503	6	0.42308	0.61801	0.9995	12128	2	-0.043
IFI27L1	6	0.42321	0.61809	0.9995	12129	1	-0.037
PEX5	6	0.42326	0.61813	0.9995	12130	2	-0.409
SULT2A1	6	0.42334	0.61819	0.9995	12131	2	-0.389
KRTAP2-2	1	0.42345	0.4237	0.9995	12132	0	0.1874
SATB1	6	0.42356	0.61832	0.9995	12133	2	-0.262
TOX3	6	0.42366	0.61838	0.9995	12134	2	0.1015
ST3GAL1	6	0.42375	0.61844	0.9995	12135	2	-0.179
NUP210	6	0.42418	0.61872	0.9995	12136	1	0.1431
C8orf42	3	0.42428	0.49909	0.9995	12137	1	-0.5
CHP2	6	0.42429	0.61879	0.9995	12138	2	0.0094
SPATA13	6	0.42429	0.61879	0.9995	12139	2	-0.105
MYO1F	6	0.42433	0.61882	0.9995	12140	2	-0.144
ABTB2	6	0.42433	0.61882	0.9995	12141	2	0.1984
OMP	6	0.42436	0.61884	0.9995	12142	2	0.0064
hsa-mir-1224	4	0.42467	0.5711	0.9995	12143	1	-0.376
CXorf61	6	0.42475	0.6191	0.9995	12144	2	-0.488
LIN9	6	0.42486	0.61918	0.9995	12145	2	-0.096
hsa-mir-188	4	0.42491	0.57135	0.9995	12146	2	0.2747
LRRC18	6	0.42525	0.61941	0.9995	12147	2	-0.14
EPHA2	6	0.4253	0.61944	0.9995	12148	2	0.1402
FAM47E	2	0.42532	0.43803	0.9995	12149	1	0.1748
ZNF737	5	0.42545	0.58519	0.9995	12150	2	-0.419

SDR16C5	6	0.42548	0.61955	0.9995	12151	2	0.1648
OR9A4	6	0.42551	0.61957	0.9995	12152	1	-0.322
BOLA3	6	0.42559	0.61961	0.9995	12153	2	0.0927
TPH1	6	0.42562	0.61963	0.9995	12154	2	0.1063
HBZ	4	0.42562	0.5721	0.9995	12155	2	-0.183
DTHD1	6	0.42571	0.6197	0.9995	12156	2	-0.063
HBG2	2	0.42572	0.43839	0.9995	12157	1	-0.328
XRCC1	6	0.4258	0.61975	0.9995	12158	1	-0.28
RFESD	6	0.42584	0.61977	0.9995	12159	2	0.1412
hsa-mir-1913	4	0.42589	0.57238	0.9995	12160	2	0.0869
HSD17B2	6	0.42602	0.61988	0.9995	12161	2	-0.243
DHRS7C	6	0.42626	0.62003	0.9995	12162	2	-0.002
KIAA1377	6	0.4263	0.62005	0.9995	12163	2	-0.04
C1orf204	6	0.42633	0.62008	0.9995	12164	1	-0.057
FABP12	6	0.42658	0.62024	0.9995	12165	2	-0.053
TARSL2	6	0.42659	0.62024	0.9995	12166	1	-0.281
ZNF19	6	0.42666	0.62028	0.9995	12167	2	0.2905
hsa-mir-602	4	0.42666	0.57316	0.9995	12168	1	-0.273
DOK5	6	0.42669	0.6203	0.9995	12169	2	-0.033
PNP	6	0.42676	0.62035	0.9995	12170	2	-0.011
DMRTC1B	2	0.42679	0.43934	0.9995	12171	1	0.2906
MECOM	6	0.42689	0.62043	0.9995	12172	2	-0.184
IL31	6	0.42689	0.62043	0.9995	12173	1	-0.486
hsa-mir-6780b	4	0.42694	0.57347	0.9995	12174	1	-0.674
GIGYF2	6	0.42713	0.6206	0.9995	12175	2	-0.194
hsa-mir-4788	4	0.42719	0.57374	0.9995	12176	1	-0.055
SIM1	6	0.42723	0.62065	0.9995	12177	1	-0.375
MID1IP1	6	0.4273	0.6207	0.9995	12178	1	-0.434
SPCS3	6	0.4273	0.6207	0.9995	12179	2	0.0422
C12orf77	6	0.4273	0.6207	0.9995	12180	2	0.1866
SPATA31C2	5	0.42738	0.58681	0.9995	12181	2	-0.495
SRGAP3	6	0.42739	0.62077	0.9995	12182	1	-0.2
RYR2	6	0.42746	0.62081	0.9995	12183	2	-0.23
BZW1	6	0.42747	0.62083	0.9995	12184	2	0.0234
SAMD5	6	0.42752	0.62086	0.9995	12185	2	-0.095
CHRDL2	6	0.42754	0.62087	0.9995	12186	2	-0.047
IGFBP7	6	0.42764	0.62093	0.9995	12187	2	-0.094
KRT34	6	0.4277	0.62097	0.9995	12188	2	0.0741
ABCA7	6	0.4277	0.62097	0.9995	12189	2	-0.229
C20orf202	6	0.4277	0.62097	0.9995	12190	2	-0.072
DPH7	3	0.42781	0.50122	0.9995	12191	1	0.1018
FAM19A1	6	0.42782	0.62106	0.9995	12192	2	-0.283
C16orf55	2	0.42798	0.44035	0.9995	12193	1	0.1547
hsa-mir-3156-3	3	0.42798	0.50132	0.9995	12194	1	0.0798
RNF44	6	0.42805	0.62122	0.9995	12195	2	-0.304
KLHL20	6	0.42808	0.62124	0.9995	12196	2	-0.172
C20orf196	6	0.42815	0.62127	0.9995	12197	2	0.1106
ESRP1	6	0.4282	0.62131	0.9995	12198	1	-0.026
CSRP2	6	0.4284	0.62143	0.9995	12199	2	-0.014
hsa-mir-4299	4	0.42842	0.57503	0.9995	12200	2	0.1721
THAP4	6	0.42846	0.62148	0.9995	12201	2	-0.302
FES	6	0.42867	0.62162	0.9995	12202	2	-0.151
TDP1	6	0.42875	0.62167	0.9995	12203	1	-0.587
MOBP	6	0.42879	0.62168	0.9995	12204	2	-0.099
ITCH	6	0.42889	0.62175	0.9995	12205	2	-0.078
CLEC18B	4	0.42899	0.5756	0.9995	12206	2	0.0165
PSPH	6	0.42901	0.62182	0.9995	12207	2	-0.185
C10orf113	6	0.42907	0.62187	0.9995	12208	2	-0.012
RNF128	6	0.42913	0.62191	0.9995	12209	1	-0.034
ZNF441	6	0.42915	0.62193	0.9995	12210	2	-0.169
ATP8A2	6	0.42931	0.62203	0.9995	12211	2	-0.203
NDUFA3	6	0.42935	0.62206	0.9995	12212	1	-0.381
hsa-mir-3136	4	0.42937	0.576	0.9995	12213	2	0.2091
hsa-mir-5581	4	0.42957	0.5762	0.9995	12214	2	0.2523
GTSF1	6	0.4297	0.6223	0.9995	12215	2	0.105
KCNJ11	4	0.42971	0.57636	0.9995	12216	2	-0.167
SLC25A15	6	0.42982	0.62237	0.9995	12217	2	-0.034
C12orf60	6	0.4299	0.62243	0.9995	12218	2	-0.265
HYI	6	0.4299	0.62243	0.9995	12219	2	0.0492
METTL11B	6	0.43007	0.62255	0.9995	12220	2	0.1727
SYNM	6	0.43012	0.62258	0.9995	12221	2	-0.497
CAPZA3	6	0.43025	0.62267	0.9995	12222	2	-0.197
GLT1D1	6	0.43046	0.62281	0.9995	12223	2	-0.164
EHBP1	6	0.43054	0.62286	0.9995	12224	2	0.0287
OIT3	6	0.43054	0.62286	0.9995	12225	2	0.0853
DNASE1L3	6	0.43058	0.62288	0.9995	12226	2	-0.189
CERS6	6	0.43066	0.62293	0.9995	12227	2	-0.069
GABRG1	6	0.43068	0.62294	0.9995	12228	1	-0.644
PLCE1	6	0.43074	0.62298	0.9995	12229	2	-0.126
TRIQK	6	0.4309	0.62308	0.9995	12230	2	-0.308
SLC2A8	6	0.43092	0.62309	0.9995	12231	2	0.1723
MINPP1	6	0.43098	0.62313	0.9995	12232	2	0.0219
FOSL2	6	0.43103	0.62316	0.9995	12233	2	-0.074
hsa-mir-7850	4	0.43113	0.57773	0.9995	12234	1	0.0108
FAM98C	6	0.43114	0.62324	0.9995	12235	2	-0.059

SYPL1	6	0.43114	0.62324	0.9995	12236	2	-0.206
ELL	6	0.43131	0.62335	0.9995	12237	1	-0.26
SPANXC	1	0.43135	0.43157	0.9995	12238	0	0.0707
RBFOX1	6	0.43136	0.62338	0.9995	12239	2	0.0794
CCDC125	6	0.43175	0.62363	0.9995	12240	2	0.1016
hsa-mir-373	4	0.43176	0.57837	0.9995	12241	2	0.2179
hsa-mir-3661	3	0.43188	0.50365	0.9995	12242	1	-0.012
EEF1G	6	0.43191	0.62373	0.9995	12243	1	-0.152
PES1	6	0.43206	0.62383	0.9995	12244	2	-0.002
N6AMT2	6	0.43212	0.62388	0.9995	12245	2	-0.112
TMEM50A	6	0.43212	0.62388	0.9995	12246	2	-0.21
JMJD8	6	0.4323	0.62399	0.9995	12247	2	-0.121
TRDMT1	6	0.43234	0.62402	0.9995	12248	2	-0.17
TRIM47	6	0.43235	0.62403	0.9995	12249	2	0.0527
CSF2	6	0.43244	0.62408	0.9995	12250	2	-0.066
hsa-mir-4457	4	0.43245	0.57908	0.9995	12251	2	-0.053
TRIM33	6	0.43258	0.62417	0.9995	12252	2	-0.105
CALD1	6	0.43266	0.62422	0.9995	12253	1	-0.682
FUBP3	6	0.43271	0.62426	0.9995	12254	2	0.1446
hsa-mir-8086	2	0.43272	0.44441	0.9995	12255	1	0.311
LGALS7B	2	0.43272	0.44441	0.9995	12256	1	-0.72
GRM5	6	0.43287	0.62437	0.9995	12257	2	-0.284
OR5K3	6	0.43294	0.62441	0.9995	12258	2	-0.216
FAM160A2	6	0.43297	0.62443	0.9995	12259	2	-0.283
C11orf52	6	0.43308	0.6245	0.9995	12260	1	-0.083
hsa-mir-155	4	0.43309	0.57973	0.9995	12261	2	0.1865
UBE2D1	6	0.43329	0.62464	0.9995	12262	2	-0.162
GTF3C2	6	0.43353	0.62479	0.9995	12263	2	0.0793
REG4	6	0.43357	0.62482	0.9995	12264	1	-0.063
KIAA0355	6	0.43377	0.62496	0.9995	12265	2	-0.415
ANAPC5	6	0.43401	0.62513	0.9995	12266	2	-0.358
hsa-mir-764	4	0.43414	0.58079	0.9995	12267	1	0.0567
ARHGEF38	6	0.4342	0.62526	0.9995	12268	1	-0.124
FCER1A	4	0.43421	0.58086	0.9995	12269	1	-0.391
LRRC8A	6	0.43425	0.62529	0.9995	12270	1	0.1473
HADHB	6	0.4344	0.62539	0.9995	12271	1	-0.295
FAM183A	6	0.43454	0.62548	0.9995	12272	2	-0.18
LRCH1	6	0.43466	0.62556	0.9995	12273	2	-0.026
LOC643669	6	0.43467	0.62556	0.9995	12274	1	0.0264
EZR	6	0.43474	0.6256	0.9995	12275	2	-0.103
KRTAP10-6	6	0.4348	0.62564	0.9995	12276	2	-0.159
NFASC	6	0.43487	0.62569	0.9995	12277	2	-0.143
CFH	6	0.43488	0.6257	0.9995	12278	1	-0.597
NMRK2	6	0.43489	0.62571	0.9995	12279	2	-0.151
AICDA	6	0.43496	0.62576	0.9995	12280	2	-0.223
ZDHHC8	6	0.43496	0.62576	0.9995	12281	2	-0.001
TGM2	6	0.43498	0.62576	0.9995	12282	2	0.0396
LGALS9	4	0.43501	0.58168	0.9995	12283	2	0.0461
ZNF776	5	0.43502	0.59323	0.9995	12284	2	-0.173
SYNJ2	6	0.43504	0.62581	0.9995	12285	2	0.0199
HAUS6	6	0.43508	0.62583	0.9995	12286	2	0.1194
FAM105A	6	0.43519	0.62589	0.9995	12287	2	-0.309
FUT3	6	0.43525	0.62593	0.9995	12288	2	-0.342
PCDH1	6	0.43527	0.62594	0.9995	12289	1	0.178
HNRNPH2	4	0.4353	0.58197	0.9995	12290	1	-0.017
PNPLA2	6	0.43531	0.62597	0.9995	12291	2	-0.052
NMNAT3	6	0.4355	0.62609	0.9995	12292	2	-0.1
LILRB4	6	0.4355	0.6261	0.9995	12293	2	-0.768
OSTN	6	0.4356	0.62616	0.9995	12294	2	0.043
ZNF740	6	0.43561	0.62617	0.9995	12295	1	-0.178
ACHE	6	0.43574	0.62625	0.9995	12296	2	-0.239
hsa-mir-3117	4	0.43588	0.58256	0.9995	12297	2	0.1392
FUT10	6	0.43589	0.62634	0.9995	12298	2	0.0787
DNMT3L	6	0.43606	0.62646	0.9995	12299	1	0.0148
TAS2R43	6	0.43609	0.62648	0.9995	12300	1	-0.023
TENM3	6	0.43614	0.62652	0.9995	12301	1	0.0041
GAGE12J	1	0.43621	0.43642	0.9995	12302	0	0.0675
QRFPR	6	0.43626	0.62659	0.9995	12303	2	-0.077
CCDC126	6	0.43629	0.62661	0.9995	12304	2	-0.311
AGTR2	6	0.43635	0.62666	0.9995	12305	2	-0.297
ZFP57	6	0.4365	0.62675	0.9995	12306	2	-0.024
UBAP1	6	0.43651	0.62676	0.9995	12307	2	-0.372
OR2M5	6	0.4366	0.62682	0.9995	12308	2	-0.148
hsa-mir-4665	4	0.43663	0.5833	0.9995	12309	1	-1E-04
MCCD1	6	0.43667	0.62685	0.9995	12310	2	0.026
C1QTNF9B-AS1	6	0.43671	0.62688	0.9995	12311	1	-0.524
IFITM2	6	0.43674	0.6269	0.9995	12312	2	-0.133
LY75-CD302	4	0.43676	0.58344	0.9995	12313	1	-0.026
OR10R2	6	0.43683	0.62695	0.9995	12314	2	-0.145
TUSC1	6	0.43683	0.62695	0.9995	12315	2	-0.122
hsa-mir-381	4	0.43689	0.58358	0.9995	12316	2	0.0216
RAP1GAP2	6	0.43689	0.62699	0.9995	12317	1	-0.038
ZNF16	6	0.437	0.62706	0.9995	12318	2	-0.126
KIAA1549	6	0.43725	0.62722	0.9995	12319	2	0.0734
STAG2	6	0.43745	0.62735	0.9995	12320	1	-0.413

OR6V1	6	0.43751	0.62739	0.9995	12321	2	-0.082
hsa-mir-7161	4	0.43753	0.58425	0.9995	12322	1	-1.084
SLC5A5	6	0.43776	0.62755	0.9995	12323	2	-0.193
AFTPH	6	0.43779	0.62758	0.9995	12324	2	0.1033
MFSD12	6	0.43783	0.62761	0.9995	12325	2	0.0348
hsa-mir-2681	4	0.43783	0.58457	0.9995	12326	1	-0.525
NPC1	6	0.43792	0.62766	0.9995	12327	2	-0.572
METTL12	6	0.43798	0.6277	0.9995	12328	1	-0.528
SH3RF1	6	0.43798	0.6277	0.9995	12329	2	0.103
CAPN11	6	0.43805	0.62775	0.9995	12330	2	-0.151
FAIM3	6	0.43808	0.62776	0.9995	12331	1	0.0098
SIN3A	6	0.43808	0.62776	0.9995	12332	2	-0.087
H2AFY	5	0.4381	0.59578	0.9995	12333	1	-0.5
CRHBP	6	0.43835	0.62796	0.9995	12334	2	0.0006
FN3KRP	6	0.43835	0.62796	0.9995	12335	1	-0.365
OR2AP1	6	0.43861	0.62814	0.9995	12336	2	0.0333
OR6K6	6	0.43865	0.62816	0.9995	12337	2	0.07
PIM3	6	0.43865	0.62816	0.9995	12338	2	-0.005
COLGALT1	6	0.43883	0.62829	0.9995	12339	2	0.1053
ANKDD1B	6	0.43885	0.6283	0.9995	12340	1	0.0159
ANKRD1	6	0.43903	0.62843	0.9995	12341	2	0.1286
GPX8	6	0.43908	0.62846	0.9995	12342	1	-0.102
hsa-mir-517b	2	0.4391	0.44995	0.9995	12343	1	0.0488
DAD1	6	0.43912	0.62849	0.9995	12344	2	-0.185
XPOT	4	0.43912	0.58591	0.9995	12345	2	0.0847
hsa-mir-3180-5	2	0.4392	0.45004	0.9995	12346	1	0.3587
PKIB	6	0.43927	0.62859	0.9995	12347	2	-0.178
GPR155	4	0.43937	0.58618	0.9995	12348	2	0.1607
CABP5	6	0.43938	0.62866	0.9995	12349	1	-0.593
STK16	6	0.43946	0.62871	0.9995	12350	2	-0.187
NHLH2	6	0.43951	0.62875	0.9995	12351	1	-0.716
RNF144B	6	0.43956	0.62879	0.9995	12352	2	-0.557
hsa-mir-4757	4	0.43957	0.5864	0.9995	12353	2	-0.067
SSH2	6	0.43969	0.62887	0.9995	12354	1	0.0326
ST5	6	0.43974	0.62891	0.9995	12355	2	-0.371
ATG3	6	0.43974	0.62891	0.9995	12356	2	-0.063
NPL	6	0.43977	0.62892	0.9995	12357	2	-0.354
CKB	6	0.43983	0.62897	0.9995	12358	1	-0.163
METTL18	6	0.43984	0.62897	0.9995	12359	2	-0.026
WFIKKN2	6	0.43992	0.62902	0.9995	12360	2	-0.426
PRSS22	6	0.43996	0.62905	0.9995	12361	1	-0.358
STARD13	6	0.43999	0.62907	0.9995	12362	2	0.1021
CD81	6	0.44006	0.62911	0.9995	12363	1	0.0328
SGSH	6	0.44025	0.62925	0.9995	12364	1	-0.193
PCM1	6	0.44038	0.62934	0.9995	12365	2	-0.255
LPIN1	6	0.44064	0.62951	0.9995	12366	2	-0.069
ALPK1	6	0.44068	0.62954	0.9995	12367	1	-0.015
C11orf31	6	0.44081	0.62963	0.9995	12368	2	-0.697
FOXR1	6	0.44107	0.62981	0.9995	12369	2	-0.108
IZUMO4	6	0.4411	0.62984	0.9995	12370	2	-0.277
UGT2A3	6	0.44115	0.62987	0.9995	12371	1	-0.399
HES1	6	0.44121	0.62991	0.9995	12372	1	-0.385
RAB2B	6	0.44139	0.63002	0.9995	12373	2	-0.042
NFATC1	4	0.4414	0.58829	0.9995	12374	1	-0.099
hsa-mir-3194	4	0.4414	0.58829	0.9995	12375	1	-0.01
KMT2C	4	0.44141	0.5883	0.9995	12376	2	0.1191
hsa-mir-4298	4	0.44148	0.58837	0.9995	12377	1	-0.41
SUGT1	4	0.44148	0.58837	0.9995	12378	1	-0.59
IPPK	6	0.44156	0.63013	0.9995	12379	2	0.037
SEMA3B	6	0.44165	0.63019	0.9995	12380	2	-0.106
TSKS	6	0.44182	0.6303	0.9995	12381	2	-0.123
SKI	6	0.44182	0.6303	0.9995	12382	2	-0.092
MIOX	6	0.44186	0.63032	0.9995	12383	1	-0.074
OR51M1	6	0.44189	0.63034	0.9995	12384	2	0.1449
KRTCAP3	6	0.44191	0.63035	0.9995	12385	2	0.0984
HSDL2	6	0.44194	0.63038	0.9995	12386	2	-0.068
SLC7A4	6	0.44211	0.63049	0.9995	12387	1	-0.161
TNFRSF11A	6	0.44213	0.6305	0.9995	12388	2	-0.112
ENPP1	6	0.4423	0.63062	0.9995	12389	2	-0.139
KRTAP5-9	6	0.44232	0.63063	0.9995	12390	2	-0.146
OR52E4	6	0.44254	0.63077	0.9995	12391	1	-0.601
IMPG2	6	0.44257	0.63079	0.9995	12392	2	-0.02
TLX1NB	6	0.44267	0.63087	0.9995	12393	2	0.0904
MRPL55	6	0.44276	0.63093	0.9995	12394	2	-0.046
hsa-mir-2115	4	0.44278	0.58969	0.9995	12395	2	0.1745
XPR1	6	0.44284	0.63098	0.9995	12396	2	-0.026
UPF2	6	0.44288	0.63101	0.9995	12397	2	0.0446
hsa-mir-148a	3	0.44295	0.51031	0.9995	12398	1	0.1874
SFRP4	6	0.44298	0.63108	0.9995	12399	2	-0.205
MED8	6	0.44306	0.63113	0.9995	12400	1	-0.188
hsa-mir-4666b	4	0.44332	0.59021	0.9995	12401	2	-0.169
LPP	6	0.44333	0.63132	0.9995	12402	2	-0.113
TUBA8	6	0.44334	0.63132	0.9995	12403	1	-0.108
AQP1	6	0.44339	0.63136	0.9995	12404	2	-0.067
TNP2	6	0.44342	0.63138	0.9995	12405	2	-0.569

hsa-mir-3613	4	0.44344	0.59033	0.9995	12406	1	-0.751
SYT1	6	0.44347	0.63141	0.9995	12407	2	0.0404
OR4D1	6	0.44347	0.63141	0.9995	12408	2	0.1163
INSL3	6	0.44357	0.63147	0.9995	12409	1	-0.763
TRIM6-TRIM34	4	0.44371	0.59061	0.9995	12410	2	0.1822
C14orf177	6	0.44377	0.6316	0.9995	12411	2	0.0651
DCBLD2	6	0.44378	0.63161	0.9995	12412	2	0.1564
CDC42BPG	4	0.44385	0.59075	0.9995	12413	1	-0.109
SYNGAP1	6	0.44393	0.63171	0.9995	12414	2	-0.606
PIK3R4	6	0.44393	0.63171	0.9995	12415	2	-0.082
MBD3L1	6	0.44403	0.63177	0.9995	12416	1	-0.163
ANKDD1A	6	0.44425	0.6319	0.9995	12417	1	-0.061
MRI1	6	0.44456	0.6321	0.9995	12418	2	-0.453
SAMD8	6	0.44461	0.63214	0.9995	12419	2	-0.14
ADAM33	6	0.44461	0.63214	0.9995	12420	2	-0.13
SETBP1	6	0.44464	0.63216	0.9995	12421	1	-0.281
SLC25A4	6	0.44464	0.63216	0.9995	12422	2	0.1144
THUMPD3	6	0.44481	0.63227	0.9995	12423	1	-0.227
DYNLL1	6	0.44483	0.63228	0.9995	12424	2	-0.073
OR5K1	3	0.4449	0.51153	0.9995	12425	1	-0.315
MAST2	6	0.44491	0.63233	0.9995	12426	2	0.0604
SLC39A2	6	0.44491	0.63233	0.9995	12427	2	-0.012
FAM184B	6	0.44498	0.63237	0.9995	12428	2	-0.199
GLOD4	6	0.44505	0.63242	0.9995	12429	1	-0.049
hsa-mir-6793	4	0.44511	0.59209	0.9995	12430	1	-0.189
CHMP2B	6	0.44515	0.63249	0.9995	12431	2	-0.003
GALR1	6	0.44521	0.63254	0.9995	12432	2	-0.091
BAGE2	2	0.44523	0.45526	0.9995	12433	1	0.0332
JUN	6	0.44528	0.63258	0.9995	12434	1	0.0377
LRIT2	6	0.44534	0.63261	0.9995	12435	1	-0.23
LETMD1	6	0.44544	0.63268	0.9995	12436	1	-0.006
TEFM	6	0.44549	0.6327	0.9995	12437	2	0.0938
RGS11	6	0.44549	0.6327	0.9995	12438	2	0.0014
MDH1	6	0.4455	0.63271	0.9995	12439	2	-0.114
LAT	4	0.44556	0.59253	0.9995	12440	1	-0.27
OR13G1	6	0.4456	0.63278	0.9995	12441	2	0.0251
GNGT1	4	0.44562	0.5926	0.9995	12442	2	-0.08
SLMO1	6	0.44569	0.63284	0.9995	12443	2	-0.368
RAD23B	6	0.44574	0.63286	0.9995	12444	2	0.2376
MARCH8	6	0.44582	0.63291	0.9995	12445	1	-0.388
CD163	6	0.44585	0.63294	0.9995	12446	2	-0.017
PHC1	6	0.44586	0.63294	0.9995	12447	2	-0.869
FCHSD2	6	0.44586	0.63294	0.9995	12448	2	-0.075
KLHL3	6	0.44593	0.63299	0.9995	12449	1	-0.011
BLNK	6	0.44598	0.63302	0.9995	12450	1	-0.525
SLFN14	6	0.44609	0.63308	0.9995	12451	2	-0.585
APC	6	0.44618	0.63315	0.9995	12452	2	-0.357
TCEAL4	6	0.44625	0.63319	0.9995	12453	2	0.1521
SYCP1	5	0.44629	0.60264	0.9995	12454	2	0.1368
hsa-mir-3679	4	0.44638	0.59336	0.9995	12455	1	-0.482
SETDB1	6	0.44642	0.6333	0.9995	12456	1	-0.054
ATP7A	6	0.44651	0.63337	0.9995	12457	2	-0.021
GNG8	6	0.44655	0.63339	0.9995	12458	2	0.0022
WSB2	6	0.44668	0.63348	0.9995	12459	2	0.0034
CR1L	5	0.44671	0.60302	0.9995	12460	2	0.1284
SRGAP2D	5	0.44675	0.60305	0.9995	12461	2	0.0613
CYBRD1	4	0.44699	0.59399	0.9995	12462	2	0.1147
GOLGA4	6	0.44725	0.63387	0.9995	12463	1	-0.18
ZNF44	6	0.44728	0.63389	0.9995	12464	1	-0.113
TBX18	6	0.44732	0.6339	0.9995	12465	2	-0.356
NRCAM	6	0.44734	0.63392	0.9995	12466	1	-0.245
CEBPE	6	0.44754	0.63404	0.9995	12467	1	-0.13
SLCO2B1	6	0.44754	0.63404	0.9995	12468	2	0.1131
NEO1	6	0.44774	0.63419	0.9995	12469	2	-0.035
AP2B1	6	0.44779	0.63421	0.9995	12470	2	0.1716
CLTCL1	6	0.44782	0.63424	0.9995	12471	2	0.1465
THAP8	6	0.44785	0.63426	0.9995	12472	2	-0.036
SMOC2	6	0.44792	0.6343	0.9995	12473	2	-0.138
MICALL2	6	0.44801	0.63437	0.9995	12474	1	-0.386
HPS5	6	0.44815	0.63445	0.9995	12475	2	-0.103
TNFAIP2	6	0.44822	0.63449	0.9995	12476	2	-0.12
CADM2	6	0.44839	0.63461	0.9995	12477	1	0.117
ANKRD49	6	0.44842	0.63463	0.9995	12478	2	0.0512
SLC25A41	6	0.44854	0.63471	0.9995	12479	2	-0.275
FAM159B	6	0.44863	0.63478	0.9995	12480	2	0.0904
CEP72	6	0.44874	0.63486	0.9995	12481	1	-0.23
PCOLCE2	6	0.44874	0.63486	0.9995	12482	2	-0.208
RFX3	6	0.44885	0.63494	0.9995	12483	1	-0.249
ABCE1	6	0.44889	0.63497	0.9995	12484	2	-0.105
RPS15	6	0.44906	0.6351	0.9995	12485	2	-0.573
KSR2	6	0.44906	0.6351	0.9995	12486	2	0.0861
WDR55	6	0.44934	0.63528	0.9995	12487	1	-0.105
DCTN1	6	0.44968	0.63549	0.9995	12488	2	0.1491
RHAG	6	0.44979	0.63556	0.9995	12489	2	-0.189
hsa-mir-495	4	0.44982	0.59518	0.9995	12490	1	-0.198

RAB40B	6	0.44987	0.63562	0.9995	12491	2	-0.146
PRSS50	6	0.44988	0.63563	0.9995	12492	2	-0.307
KBTBD11	6	0.44989	0.63563	0.9995	12493	2	0.1872
MED31	6	0.45009	0.63576	0.9995	12494	2	0.128
RUFY2	6	0.45019	0.63582	0.9995	12495	2	-0.118
EZH2	6	0.45035	0.63593	0.9995	12496	2	-0.053
TUBA3E	3	0.45047	0.51493	0.9995	12497	1	-0.549
RDH13	6	0.4505	0.63602	0.9995	12498	1	-0.525
ITM2C	6	0.45054	0.63605	0.9995	12499	2	-0.07
MAL	6	0.45056	0.63606	0.9995	12500	2	-0.128
FGF14	6	0.45056	0.63606	0.9995	12501	1	-0.304
CLIP1	6	0.45061	0.6361	0.9995	12502	1	-0.201
SH3KBP1	6	0.45068	0.63614	0.9995	12503	2	-0.082
hsa-mir-6888	4	0.45073	0.59553	0.9995	12504	1	0.0707
NXPH2	6	0.45083	0.63625	0.9995	12505	2	0.1229
GRIN2C	6	0.45093	0.63632	0.9995	12506	1	-0.076
DNAJC28	6	0.45093	0.63632	0.9995	12507	2	-0.273
hsa-mir-29b-1	1	0.45096	0.45111	0.9995	12508	0	0.0503
MNX1	6	0.45107	0.63641	0.9995	12509	1	-0.504
BTBD8	6	0.45121	0.63651	0.9995	12510	2	0.1546
SNRPA1	6	0.45129	0.63657	0.9995	12511	2	-0.345
TACC1	6	0.45129	0.63657	0.9995	12512	2	-0.319
ARHGAP5	6	0.45138	0.63662	0.9995	12513	1	-0.011
LHX4	6	0.4514	0.63663	0.9995	12514	2	0.1219
PNPLA6	6	0.45144	0.63666	0.9995	12515	2	0.0229
WRB	6	0.45151	0.63671	0.9995	12516	1	-0.097
NPHP3	6	0.45159	0.63676	0.9995	12517	2	0.1853
CHRNB3	6	0.45159	0.63676	0.9995	12518	1	-0.2
COL9A3	6	0.45162	0.63677	0.9995	12519	2	-0.099
ANKRD18B	5	0.45166	0.60716	0.9995	12520	2	0.2121
ENSA	6	0.45172	0.63683	0.9995	12521	2	0.1376
ALKBH7	6	0.45184	0.63691	0.9995	12522	2	-0.135
NEK11	6	0.45191	0.63696	0.9995	12523	2	-0.164
WBP5	6	0.45191	0.63696	0.9995	12524	1	-0.079
CNOT6	6	0.45191	0.63696	0.9995	12525	1	-0.935
RECQL5	6	0.45194	0.63698	0.9995	12526	2	-0.025
hsa-mir-296	4	0.45209	0.59614	0.9995	12527	1	-0.319
TMEM164	6	0.45214	0.63711	0.9995	12528	2	-0.045
SGSM1	6	0.45234	0.63724	0.9995	12529	2	-0.158
GSTA1	4	0.45235	0.59625	0.9995	12530	1	-0.394
KIAA1984	6	0.45235	0.63724	0.9995	12531	2	-0.003
CMBL	6	0.45249	0.63734	0.9995	12532	2	-0.216
RNASE10	6	0.45249	0.63734	0.9995	12533	2	-0.024
PSD3	6	0.45262	0.63742	0.9995	12534	2	0.1884
BMS1	6	0.45266	0.63746	0.9995	12535	2	0.1992
AGBL5	6	0.45269	0.63749	0.9995	12536	1	-0.146
SUMO3	6	0.45273	0.63751	0.9995	12537	1	-0.527
AMZ2	6	0.45279	0.63755	0.9995	12538	2	0.0527
POGK	6	0.45284	0.63757	0.9995	12539	1	-0.116
TBX6	6	0.45295	0.63766	0.9995	12540	2	0.0637
TMEM68	6	0.45296	0.63766	0.9995	12541	1	-0.407
OR2A25	6	0.45304	0.63771	0.9995	12542	2	-0.178
XAGE2	6	0.45307	0.63774	0.9995	12543	2	-0.795
ZNF99	5	0.45309	0.60833	0.9995	12544	1	-0.498
DAB2	6	0.45323	0.63784	0.9995	12545	1	-0.234
VDR	6	0.45341	0.63794	0.9995	12546	2	-0.342
KRTAP19-5	6	0.45343	0.63796	0.9995	12547	1	-0.37
GLTSCR1	6	0.45364	0.6381	0.9995	12548	2	-0.197
MYOF	6	0.45391	0.63828	0.9995	12549	2	0.0659
PTRF	6	0.45411	0.63841	0.9995	12550	2	0.1189
C9orf9	6	0.45413	0.63842	0.9995	12551	2	0.1323
CD40LG	6	0.45419	0.63845	0.9995	12552	2	-0.033
PPP2R5E	6	0.4543	0.63852	0.9995	12553	2	0.0976
ZNF682	6	0.4543	0.63852	0.9995	12554	1	-0.619
PRSS37	6	0.45446	0.63863	0.9995	12555	2	0.0217
PYGB	6	0.45449	0.63864	0.9995	12556	2	-0.131
MT4	6	0.45449	0.63864	0.9995	12557	2	0.0992
SPON1	6	0.45455	0.63868	0.9995	12558	2	0.0259
TCHHL1	6	0.45455	0.63868	0.9995	12559	2	-0.427
TMEM132E	6	0.45475	0.63882	0.9995	12560	2	-0.005
TAS2R39	6	0.45482	0.63886	0.9995	12561	2	-0.221
ZNF117	5	0.45484	0.60981	0.9995	12562	1	-0.406
MED19	6	0.4549	0.63891	0.9995	12563	2	-0.041
DEFB116	6	0.45499	0.63897	0.9995	12564	2	-0.181
MID1	6	0.45501	0.63898	0.9995	12565	2	-0.14
CDH26	6	0.45509	0.63903	0.9995	12566	2	-0.286
ZMAT4	6	0.4552	0.6391	0.9995	12567	2	-0.345
FAM127A	6	0.45543	0.63926	0.9995	12568	2	-0.007
ANP32D	6	0.45543	0.63926	0.9995	12569	2	-0.161
KIF3B	6	0.45553	0.63932	0.9995	12570	1	-0.443
TVP23A	6	0.45557	0.63937	0.9995	12571	1	0.0795
SPATA31A4	5	0.45561	0.61047	0.9995	12572	2	0.1752
DUPD1	6	0.45565	0.63941	0.9995	12573	1	-0.028
hsa-mir-4434	4	0.4557	0.59769	0.9995	12574	1	-0.269
LRRC36	6	0.45576	0.6395	0.9995	12575	1	-0.196

OSR1	6	0.45586	0.63957	0.9995	12576	2	-0.004
hsa-mir-648	4	0.45588	0.59777	0.9995	12577	1	0.006
SDK1	6	0.45595	0.63962	0.9995	12578	1	0.0471
EXOG	6	0.45595	0.63962	0.9995	12579	1	-0.268
FGD2	6	0.45601	0.63966	0.9995	12580	2	-0.166
CPEB1	6	0.45603	0.63967	0.9995	12581	2	0.2212
DIMT1	6	0.45614	0.63974	0.9995	12582	2	-0.466
hsa-mir-487b	4	0.45616	0.59788	0.9995	12583	1	-0.215
RNF4	6	0.4562	0.63978	0.9995	12584	1	-0.468
HAUS5	6	0.45637	0.6399	0.9995	12585	2	0.0832
HTR5A	6	0.4564	0.63992	0.9995	12586	1	-0.666
hsa-mir-452	2	0.45647	0.4651	0.9995	12587	1	0.2744
FSHB	6	0.45655	0.64002	0.9995	12588	2	-0.405
hsa-mir-7515	4	0.45662	0.59807	0.9995	12589	1	0.0058
C2orf54	6	0.45663	0.64007	0.9995	12590	2	-0.421
SCGB1A1	6	0.45669	0.64011	0.9995	12591	1	-0.017
ENKUR	6	0.45675	0.64014	0.9995	12592	2	-0.408
SLC15A3	6	0.45675	0.64014	0.9995	12593	2	-0.058
TCEB3C	3	0.4568	0.51888	0.9995	12594	1	-0.493
OR13J1	6	0.45686	0.64021	0.9995	12595	2	0.2875
GNB1L	6	0.457	0.6403	0.9995	12596	1	-0.533
MECP2	6	0.457	0.6403	0.9995	12597	2	0.0667
MYOM1	6	0.45705	0.64034	0.9995	12598	2	-0.271
FRMD4B	6	0.45719	0.64041	0.9995	12599	1	0.074
ANKRD40	6	0.45731	0.6405	0.9995	12600	1	0.141
ABCF1	6	0.45736	0.64053	0.9995	12601	2	0.0396
REXO1L1	6	0.45742	0.64057	0.9995	12602	2	-0.376
CWF19L1	6	0.45752	0.64063	0.9995	12603	1	0.0997
PNPLA5	6	0.45759	0.64069	0.9995	12604	2	-0.167
DPP4	6	0.45759	0.64069	0.9995	12605	1	-0.081
C2orf50	6	0.45771	0.64076	0.9995	12606	2	-0.443
FAM220A	6	0.45779	0.64082	0.9995	12607	2	-0.438
DEFB128	6	0.45794	0.64093	0.9995	12608	2	-0.231
ARHGAP22	6	0.45799	0.64095	0.9995	12609	1	-0.356
CSNK1G2	6	0.458	0.64096	0.9995	12610	2	-0.435
MCM3	6	0.45805	0.641	0.9995	12611	1	-0.069
SKIDA1	6	0.4581	0.64103	0.9995	12612	2	0.0819
VAX2	6	0.45811	0.64104	0.9995	12613	1	-0.639
GZMB	6	0.4585	0.64128	0.9995	12614	2	-0.07
C4orf36	6	0.4585	0.64128	0.9995	12615	2	-0.512
CAMK1G	6	0.4585	0.64128	0.9995	12616	2	-0.037
HIST2H2BF	6	0.4585	0.64128	0.9995	12617	2	-0.087
ZER1	6	0.4585	0.64128	0.9995	12618	2	-0.163
hsa-mir-5707	2	0.45867	0.46706	0.9995	12619	1	0.3716
hsa-mir-941-1	2	0.45867	0.46706	0.9995	12620	1	-0.178
FAM185A	6	0.45872	0.64142	0.9995	12621	2	-0.568
SOX30	6	0.45875	0.64144	0.9995	12622	1	-0.116
COL11A2	6	0.45888	0.64153	0.9995	12623	2	-0.007
PITX2	6	0.45891	0.64155	0.9995	12624	2	-0.129
hsa-mir-3673	1	0.45896	0.45918	0.9995	12625	0	0.1531
WFDC5	6	0.45897	0.64158	0.9995	12626	2	0.0987
ETNK2	6	0.459	0.6416	0.9995	12627	2	0.0836
HIST2H2BE	6	0.459	0.6416	0.9995	12628	2	-0.539
CXCL2	3	0.45921	0.52037	0.9995	12629	1	-1.233
CNIH4	6	0.45923	0.64177	0.9995	12630	2	0.1345
TMEM209	6	0.45933	0.64183	0.9995	12631	1	-0.343
PDS5A	6	0.45938	0.64188	0.9995	12632	2	-0.177
TXNDC17	6	0.45945	0.64192	0.9995	12633	2	-0.145
ARHGAP25	6	0.45948	0.64194	0.9995	12634	1	-0.087
FAM189B	6	0.45956	0.642	0.9995	12635	2	0.0408
EVA1C	6	0.45963	0.64205	0.9995	12636	1	-0.358
GZMA	6	0.45976	0.64214	0.9995	12637	2	0.0725
TAOK2	4	0.45983	0.59946	0.9995	12638	1	-0.349
PPBP	6	0.45984	0.64218	0.9995	12639	2	0.0351
GAB4	6	0.45984	0.64218	0.9995	12640	2	-0.059
WRNIP1	6	0.4601	0.64236	0.9995	12641	2	0.0495
XRN2	6	0.46014	0.64239	0.9995	12642	2	-0.199
SYDE2	6	0.46019	0.64242	0.9995	12643	2	-0.254
YJEFN3	6	0.46022	0.64245	0.9995	12644	2	-0.246
SDC4	6	0.46026	0.64247	0.9995	12645	2	-0.399
NEURL1B	6	0.46028	0.64249	0.9995	12646	2	-0.128
LPAR6	6	0.46039	0.64255	0.9995	12647	2	-0.044
DHFRL1	6	0.46042	0.64257	0.9995	12648	2	0.1295
HS3ST2	6	0.4605	0.64262	0.9995	12649	2	-0.165
BFAR	6	0.46053	0.64263	0.9995	12650	2	-0.121
CST5	6	0.46056	0.64266	0.9995	12651	1	-0.284
PFKM	6	0.46058	0.64267	0.9995	12652	2	0.0004
C7	6	0.46065	0.64273	0.9995	12653	2	-0.194
AIMP2	6	0.46072	0.64276	0.9995	12654	2	-0.323
PFN2	6	0.46085	0.64286	0.9995	12655	2	0.2001
LYZ	6	0.46092	0.6429	0.9995	12656	1	-0.462
hsa-mir-1185-1	1	0.46093	0.46117	0.9995	12657	0	0.1514
OVOL2	6	0.46106	0.64299	0.9995	12658	2	-0.816
hsa-mir-569	4	0.46116	0.60002	0.9995	12659	1	0.1309
PFKL	6	0.46121	0.64308	0.9995	12660	2	0.0004

CTH	6	0.46123	0.64309	0.9995	12661	2	-0.021
STC1	6	0.46137	0.64318	0.9995	12662	1	-0.19
NOP14	6	0.46143	0.64322	0.9995	12663	2	-0.319
CCBL2	6	0.46145	0.64324	0.9995	12664	2	-0.036
LOC650293	6	0.4615	0.64327	0.9995	12665	2	0.0512
FAM117A	6	0.46153	0.64329	0.9995	12666	2	-0.325
KIAA0947	6	0.46159	0.64333	0.9995	12667	2	-0.019
OR4A5	6	0.4616	0.64334	0.9995	12668	2	0.1134
USP17L13	1	0.46167	0.46189	0.9995	12669	0	0.0452
MRPL35	6	0.46168	0.6434	0.9995	12670	2	-0.093
hsa-mir-659	4	0.46203	0.60039	0.9995	12671	1	-0.686
ZNF527	5	0.46211	0.61591	0.9995	12672	2	0.1166
RFNG	6	0.4622	0.64373	0.9995	12673	2	-0.292
ZNF354C	6	0.46232	0.6438	0.9995	12674	2	0.1368
PACSIN1	6	0.46241	0.64387	0.9995	12675	1	-0.176
IQCG	6	0.4625	0.64393	0.9995	12676	2	0.0812
CCDC89	6	0.46261	0.644	0.9995	12677	1	-0.058
GDF7	6	0.46261	0.644	0.9995	12678	1	-0.194
ZNF442	6	0.46263	0.64401	0.9995	12679	2	-0.027
KRT31	6	0.46266	0.64404	0.9995	12680	2	-0.637
ABL2	6	0.46266	0.64404	0.9995	12681	2	0.0594
hsa-mir-6775	4	0.46279	0.60069	0.9995	12682	1	-0.146
GLB1	6	0.46283	0.64416	0.9995	12683	2	0.0897
ZNF555	6	0.46286	0.64418	0.9995	12684	2	-0.019
RSPH4A	6	0.4629	0.64421	0.9995	12685	2	0.1085
CDK2AP2	6	0.46303	0.6443	0.9995	12686	2	0.129
OLR1	6	0.46314	0.64437	0.9995	12687	2	-0.177
NUDC	6	0.46314	0.64437	0.9995	12688	2	-0.172
NTRK3	6	0.4632	0.64441	0.9995	12689	2	-0.014
EXTL2	6	0.46333	0.64449	0.9995	12690	2	0.0931
KRTAP19-7	5	0.4634	0.61701	0.9995	12691	2	-0.09
ZBED3	6	0.46342	0.64456	0.9995	12692	2	-0.264
ZNF668	6	0.46347	0.64459	0.9995	12693	2	0.0596
BLOC1S5	6	0.46353	0.64463	0.9995	12694	2	-0.012
GRM6	6	0.46374	0.64478	0.9995	12695	2	-0.227
hsa-mir-4532	4	0.46395	0.60116	0.9995	12696	1	0.195
FZD6	6	0.46402	0.64496	0.9995	12697	2	-0.203
IFI44	6	0.46402	0.64497	0.9995	12698	2	-0.255
ALB	6	0.46417	0.64507	0.9995	12699	2	-0.58
GDF9	6	0.46418	0.64508	0.9995	12700	1	0.0558
ZNF189	6	0.46423	0.64511	0.9995	12701	2	-0.174
ZNF177	2	0.46445	0.47219	0.9995	12702	1	-0.069
PTK6	6	0.4645	0.64529	0.9995	12703	1	-0.557
SMPD4	6	0.4646	0.64536	0.9995	12704	1	-0.38
SREK1IP1	6	0.46462	0.64538	0.9995	12705	2	0.182
OMA1	6	0.46466	0.64541	0.9995	12706	1	-0.463
TUBG1	5	0.46483	0.61822	0.9995	12707	2	-0.084
CDYL	6	0.46508	0.64569	0.9995	12708	1	-0.087
KLRD1	6	0.46526	0.64581	0.9995	12709	2	0.0289
hsa-mir-3611	1	0.46533	0.46554	0.9995	12710	0	0.0471
FADS3	6	0.46545	0.64594	0.9995	12711	1	-0.121
ZNF486	5	0.46548	0.61875	0.9995	12712	2	0.098
RNF166	6	0.46554	0.64601	0.9995	12713	2	0.0788
SCAMP4	6	0.46555	0.64601	0.9995	12714	2	0.0056
FAM213A	6	0.46572	0.64611	0.9995	12715	2	-0.278
CCDC64	6	0.46583	0.64619	0.9995	12716	2	-0.035
BRD8	6	0.46598	0.64628	0.9995	12717	2	-0.09
KRTAP19-6	6	0.46603	0.64632	0.9995	12718	2	-0.085
SNAPC4	6	0.46603	0.64632	0.9995	12719	1	-0.112
FANCG	6	0.46628	0.64648	0.9995	12720	2	-0.511
SHC2	6	0.46638	0.64655	0.9995	12721	2	-0.161
hsa-mir-4475	4	0.46638	0.60217	0.9995	12722	1	-0.065
GABBR2	6	0.46641	0.64657	0.9995	12723	2	0.0066
NPPB	6	0.46641	0.64657	0.9995	12724	2	-0.106
RAB5B	6	0.46648	0.64663	0.9995	12725	2	-0.011
UBE2J1	6	0.46655	0.64667	0.9995	12726	2	0.2266
NFIA	4	0.46657	0.60225	0.9995	12727	1	-0.296
PIFO	6	0.46667	0.64674	0.9995	12728	1	-0.153
F2R	6	0.46673	0.64677	0.9995	12729	2	0.1653
hsa-mir-631	4	0.46675	0.60232	0.9995	12730	1	-0.187
WDR19	6	0.46679	0.64681	0.9995	12731	2	-0.245
RELN	6	0.46707	0.64698	0.9995	12732	2	-0.221
DNAJC17	6	0.4671	0.64699	0.9995	12733	2	-0.243
BPIFB3	6	0.46719	0.64704	0.9995	12734	2	-0.268
CCNG1	6	0.46748	0.64723	0.9995	12735	2	-0.129
RELA	6	0.46766	0.64735	0.9995	12736	2	0.0593
TRIM16	4	0.46767	0.60271	0.9995	12737	1	-1.121
PIK3CA	6	0.4678	0.64745	0.9995	12738	2	-0.435
FASTK	6	0.46784	0.64748	0.9995	12739	2	-0.037
ASL	6	0.46791	0.64753	0.9995	12740	2	0.0307
DUSP19	6	0.46816	0.64769	0.9995	12741	2	-0.285
MCPH1	6	0.46825	0.64776	0.9995	12742	2	-0.387
HMG20B	6	0.46833	0.64782	0.9995	12743	1	-0.392
CDC5L	6	0.46836	0.64782	0.9995	12744	2	0.0238
DYRK1B	6	0.46878	0.64812	0.9995	12745	2	-0.176

STON1-GTF2A1L	3	0.4688	0.52639	0.9995	12746	1	-0.489
HLA-F	6	0.46882	0.64816	0.9995	12747	2	-0.285
C14orf142	6	0.46889	0.6482	0.9995	12748	1	-0.071
XKR4	6	0.46891	0.64823	0.9995	12749	2	-0.019
TUSC2	6	0.46895	0.64824	0.9995	12750	2	-0.332
HDDC3	6	0.46898	0.64827	0.9995	12751	2	0.1164
hsa-mir-146a	4	0.469	0.60331	0.9995	12752	1	-0.021
MARCH10	6	0.46905	0.64831	0.9995	12753	2	-0.455
C4B_2	6	0.46908	0.64833	0.9995	12754	2	-0.075
PASD1	6	0.4691	0.64834	0.9995	12755	2	0.0806
SH2D4A	6	0.46925	0.64845	0.9995	12756	2	-0.145
FABP3	6	0.46925	0.64845	0.9995	12757	1	-0.394
RAB11A	6	0.46944	0.64858	0.9995	12758	2	0.0015
CD22	6	0.4695	0.64862	0.9995	12759	2	0.2127
GCM1	6	0.46958	0.64867	0.9995	12760	1	-0.253
PRH2	5	0.46964	0.62229	0.9995	12761	1	-0.19
RNF113A	6	0.46984	0.64883	0.9995	12762	2	-0.258
ZNF534	6	0.46996	0.64891	0.9995	12763	2	-0.227
C15orf62	6	0.46998	0.64893	0.9995	12764	2	-0.551
GLO1	6	0.47002	0.64895	0.9995	12765	2	-0.463
CDK5R2	6	0.47013	0.64903	0.9995	12766	1	-0.04
OR51T1	6	0.47021	0.64908	0.9995	12767	2	-0.073
POLD2	6	0.47024	0.6491	0.9995	12768	2	-0.009
MARCKSL1	6	0.47031	0.64915	0.9995	12769	2	-0.37
SELE	6	0.47038	0.64919	0.9995	12770	2	-0.089
KRTAP3-1	6	0.47038	0.64919	0.9995	12771	2	-0.247
HIST1H2BD	6	0.47051	0.64927	0.9995	12772	2	-0.037
TMEM27	6	0.47051	0.64927	0.9995	12773	1	-0.178
VPREB3	6	0.47055	0.64929	0.9995	12774	2	-0.054
C2orf76	6	0.47067	0.64938	0.9995	12775	1	-0.023
C12orf54	4	0.47073	0.60406	0.9995	12776	1	-0.093
ARID1B	6	0.47091	0.64954	0.9995	12777	1	-0.205
ALPI	6	0.47096	0.64956	0.9995	12778	2	0.1699
GAL3ST1	6	0.47112	0.64967	0.9995	12779	2	-0.339
SPRTN	6	0.47114	0.64969	0.9995	12780	2	0.1092
SPDYE1	2	0.47115	0.47816	0.9995	12781	1	-0.061
ESRRB	6	0.47131	0.64979	0.9995	12782	2	0.0682
INHBE	6	0.47136	0.64983	0.9995	12783	1	-0.786
TWIST2	6	0.4714	0.64985	0.9995	12784	2	0.1291
TSPO	6	0.47165	0.65	0.9995	12785	2	0.0821
ABCB7	6	0.47165	0.65	0.9995	12786	2	0.156
hsa-mir-567	4	0.47168	0.60446	0.9995	12787	1	0.0095
OR2L13	6	0.47177	0.65009	0.9995	12788	1	-0.307
QDPR	6	0.47184	0.65013	0.9995	12789	2	-0.084
TF	6	0.47224	0.65038	0.9995	12790	1	-0.042
CHST2	6	0.47228	0.65042	0.9995	12791	2	-0.047
CYP17A1	6	0.47242	0.65052	0.9995	12792	2	-0.67
TH	6	0.47246	0.65053	0.9995	12793	2	0.0872
hsa-mir-139	4	0.47249	0.60483	0.9995	12794	1	-0.084
GRIK2	6	0.47252	0.65058	0.9995	12795	2	0.0797
CACNA1A	6	0.4726	0.65063	0.9995	12796	2	0.0163
TMEM249	6	0.47263	0.65064	0.9995	12797	2	0.1507
TMEM140	6	0.47269	0.65069	0.9995	12798	1	-0.105
VKORC1L1	6	0.4727	0.6507	0.9995	12799	2	0.0986
PTH1R	6	0.4727	0.6507	0.9995	12800	2	-0.052
TIMM10B	6	0.47275	0.65073	0.9995	12801	1	-0.377
MTRNR2L5	4	0.47276	0.60494	0.9995	12802	1	0.23
WBSCR22	6	0.47292	0.65083	0.9995	12803	2	-0.003
STAB1	6	0.47303	0.6509	0.9995	12804	2	-0.433
UBE2E1	6	0.47308	0.65094	0.9995	12805	1	-0.367
WFDC13	5	0.47317	0.62524	0.9995	12806	2	-0.105
SLC34A1	6	0.47321	0.65102	0.9995	12807	1	-0.032
ZNF142	6	0.47337	0.65113	0.9995	12808	1	-0.136
TBXA2R	6	0.47356	0.65124	0.9995	12809	1	-0.133
GRK1	6	0.47358	0.65127	0.9995	12810	2	0.0818
AJUBA	6	0.47379	0.65141	0.9995	12811	2	0.146
LYRM4	6	0.47388	0.65148	0.9995	12812	2	0.0788
C8orf33	6	0.47394	0.65152	0.9995	12813	2	-0.49
PKN2	6	0.47406	0.65159	0.9995	12814	2	-0.115
hsa-mir-370	4	0.47408	0.60553	0.9995	12815	1	-0.161
TRNT1	6	0.47417	0.65167	0.9995	12816	2	-0.168
ZIC5	6	0.47424	0.65172	0.9995	12817	2	-0.021
ST8SIA6	6	0.47442	0.65186	0.9995	12818	2	0.0461
MSANTD1	6	0.47444	0.65187	0.9995	12819	1	-0.161
KCTD13	6	0.47451	0.65192	0.9995	12820	2	-0.294
hsa-mir-378i	3	0.47457	0.5301	0.9995	12821	1	-0.123
C5AR2	6	0.47458	0.65197	0.9995	12822	1	-0.354
CYP4V2	6	0.4747	0.65205	0.9995	12823	2	-0.363
SYCE3	6	0.47477	0.65211	0.9995	12824	2	0.1501
CEP152	6	0.47478	0.65211	0.9995	12825	1	-0.154
MACROD1	6	0.4751	0.65233	0.9995	12826	1	-0.213
OR8D2	6	0.47511	0.65235	0.9995	12827	2	-0.138
EXOSC9	6	0.47513	0.65236	0.9995	12828	2	0.0648
DYRK4	6	0.47517	0.65239	0.9995	12829	1	0.004
EFCAB3	6	0.47519	0.65241	0.9995	12830	2	0.1315

ANKRD26	6	0.47523	0.65243	0.9995	12831	2	-0.151
ITGB8	6	0.47528	0.65245	0.9995	12832	2	-0.438
DNAJC16	6	0.4753	0.65246	0.9995	12833	2	-0.236
BDKRB1	6	0.47531	0.65247	0.9995	12834	1	-0.125
RPS6KL1	6	0.47534	0.6525	0.9995	12835	2	0.0198
ZNF750	6	0.47535	0.6525	0.9995	12836	1	-0.573
TFEB	6	0.47542	0.65254	0.9995	12837	2	0.0829
ZCCHC10	6	0.47553	0.65262	0.9995	12838	2	0.0649
NDUFB6	6	0.4756	0.65268	0.9995	12839	2	-0.256
CLNK	5	0.47573	0.62738	0.9995	12840	2	-0.065
BRSK2	6	0.47574	0.65277	0.9995	12841	1	-0.192
PPP3CA	6	0.47578	0.6528	0.9995	12842	2	-0.004
MRPL48	6	0.47595	0.6529	0.9995	12843	2	0.1658
EXOC8	6	0.4761	0.65299	0.9995	12844	1	-0.21
FAM72B	2	0.47619	0.48256	0.9995	12845	1	0.1741
DCAF10	6	0.47634	0.65316	0.9995	12846	1	-0.142
PPP6R3	6	0.47643	0.65323	0.9995	12847	2	-0.172
BAMBI	6	0.47654	0.65329	0.9995	12848	2	-0.561
FGFR3	6	0.47659	0.65333	0.9995	12849	2	-0.052
GPX1	6	0.47663	0.65335	0.9995	12850	2	-0.749
MARCH3	6	0.47668	0.65338	0.9995	12851	2	-0.069
KRT80	6	0.47678	0.65345	0.9995	12852	2	0.117
CD300LD	6	0.47687	0.65351	0.9995	12853	2	0.185
RHOU	6	0.47692	0.65355	0.9995	12854	1	-0.138
IFNA8	6	0.47693	0.65355	0.9995	12855	2	-0.05
THRB	6	0.47704	0.65363	0.9995	12856	2	-0.107
hsa-mir-5697	4	0.47707	0.60684	0.9995	12857	1	-0.107
GPALPP1	3	0.4771	0.53173	0.9995	12858	1	-0.842
SAT1	6	0.47711	0.65367	0.9995	12859	2	-0.161
C9orf129	6	0.47716	0.65371	0.9995	12860	2	-0.105
CDR1	6	0.47718	0.65372	0.9995	12861	2	-0.092
MGME1	6	0.4773	0.65381	0.9995	12862	2	-0.054
PUS1	6	0.4774	0.65387	0.9995	12863	2	0.1261
ABHD14B	6	0.47741	0.65387	0.9995	12864	2	-0.229
SFN	4	0.47791	0.60721	0.9995	12865	1	0.2373
CCDC169	5	0.47791	0.62926	0.9995	12866	1	0.0178
SYNE1	6	0.478	0.65427	0.9995	12867	2	-0.566
SPACA1	6	0.478	0.65427	0.9995	12868	2	-0.051
ZNF502	6	0.47806	0.65431	0.9995	12869	2	-0.098
TCERG1L	6	0.47808	0.65433	0.9995	12870	2	-0.176
PPM1F	6	0.47821	0.6544	0.9995	12871	2	0.1393
MFAP4	6	0.47845	0.65458	0.9995	12872	1	-0.086
hsa-mir-206	4	0.47853	0.60749	0.9995	12873	1	0.112
OLA1	6	0.4786	0.65467	0.9995	12874	2	0.1556
UPK3B	6	0.47881	0.6548	0.9995	12875	2	-0.169
ARMC7	6	0.47895	0.65488	0.9995	12876	1	-0.043
KIAA1328	6	0.47898	0.65489	0.9995	12877	2	-0.348
COL28A1	6	0.47904	0.65493	0.9995	12878	2	-0.784
PNLIPRP2	6	0.47922	0.65505	0.9995	12879	2	0.0384
SPATA9	6	0.47924	0.65507	0.9995	12880	2	-0.191
SLCO1B1	6	0.4794	0.65519	0.9995	12881	2	-0.063
hsa-mir-4452	4	0.47954	0.60795	0.9995	12882	1	-0.229
SH3BP5L	6	0.4796	0.65532	0.9995	12883	1	-0.101
CSTL1	6	0.47963	0.65533	0.9995	12884	2	-0.369
LIN28B	6	0.47963	0.65533	0.9995	12885	2	-0.372
PTPN9	6	0.47965	0.65535	0.9995	12886	1	-0.325
C10orf91	6	0.47974	0.65542	0.9995	12887	2	-0.034
GPKOW	6	0.47978	0.65545	0.9995	12888	1	0.0774
VSIG4	6	0.47983	0.65549	0.9995	12889	1	-0.199
SLC16A4	6	0.47989	0.65552	0.9995	12890	2	-0.146
NDUFS4	4	0.48	0.60814	0.9995	12891	1	0.0803
TMED1	6	0.4801	0.65566	0.9995	12892	1	-0.476
HAAO	6	0.48018	0.65572	0.9995	12893	1	-0.076
TAGLN3	6	0.48018	0.65572	0.9995	12894	2	-0.241
AGBL1	6	0.48028	0.65578	0.9995	12895	1	-0.149
ZNF417	2	0.48033	0.48623	0.9995	12896	1	-0.424
FAM212B	6	0.4805	0.65593	0.9995	12897	2	-0.11
GPC1	6	0.48058	0.65597	0.9995	12898	2	0.1307
ARHGAP31	6	0.48078	0.65611	0.9995	12899	2	-0.261
FAM186A	6	0.48078	0.65611	0.9995	12900	1	-0.128
GCSAM	6	0.48078	0.65611	0.9995	12901	2	-0.401
ULBP3	6	0.48088	0.65618	0.9995	12902	2	0.1641
PATE4	6	0.48102	0.65627	0.9995	12903	2	-0.013
TELO2	6	0.48102	0.65627	0.9995	12904	2	-0.27
NTF3	6	0.48102	0.65627	0.9995	12905	2	-0.062
ARMC2	6	0.4811	0.65632	0.9995	12906	1	-0.465
SEMA5A	6	0.4811	0.65632	0.9995	12907	2	-0.14
SPANXF1	5	0.48119	0.63206	0.9995	12908	2	0.0607
UBR1	6	0.48135	0.6565	0.9995	12909	1	-0.307
LGALS8	6	0.48156	0.65663	0.9995	12910	2	-0.003
TNIP3	6	0.48158	0.65664	0.9995	12911	2	-0.388
PPWD1	6	0.48158	0.65664	0.9995	12912	2	0.0701
LGALS7	1	0.48164	0.48172	0.9995	12913	0	0.098
C6orf48	6	0.48168	0.6567	0.9995	12914	2	0.0843
FYCO1	6	0.48168	0.6567	0.9995	12915	2	-0.222

PTPN22	6	0.48176	0.65675	0.9995	12916	1	-0.247
LOC147646	6	0.48203	0.65692	0.9995	12917	2	0.096
NFAM1	6	0.48211	0.65699	0.9995	12918	2	-0.103
DYNC1H1	6	0.48211	0.65699	0.9995	12919	2	0.0853
ZSCAN5A	6	0.48224	0.65708	0.9995	12920	1	-0.591
IL19	6	0.48227	0.6571	0.9995	12921	2	-0.141
TFB2M	6	0.48257	0.65729	0.9995	12922	2	-0.551
PPP2R5C	6	0.48269	0.65737	0.9995	12923	2	0.0544
SEPHS1	6	0.48269	0.65737	0.9995	12924	2	0.0437
SPATC1	6	0.48269	0.65737	0.9995	12925	2	-0.005
hsa-mir-4649	4	0.48271	0.60933	0.9995	12926	1	-0.045
EHD1	6	0.48284	0.65747	0.9995	12927	2	0.1641
IER5L	6	0.48284	0.65747	0.9995	12928	2	0.134
ST6GALNAC6	6	0.48287	0.65749	0.9995	12929	2	-0.026
POTEF	2	0.4829	0.48857	0.9995	12930	1	0.4361
KCNA6	6	0.48302	0.65759	0.9995	12931	2	-0.849
ZBED6	6	0.48308	0.65763	0.9995	12932	2	0.0384
KRT38	6	0.48314	0.65768	0.9995	12933	1	-0.522
CHCHD5	6	0.48323	0.65774	0.9995	12934	1	-0.041
LDOC1	6	0.48345	0.6579	0.9995	12935	2	0.0537
MYO10	6	0.48375	0.65811	0.9995	12936	2	-0.161
PTTG2	6	0.48376	0.65812	0.9995	12937	1	-0.059
SMIM1	6	0.48389	0.65821	0.9995	12938	2	-0.388
OR2A12	6	0.48408	0.65834	0.9995	12939	2	-0.346
PRKCZ	6	0.48408	0.65834	0.9995	12940	2	-0.268
IGHMBP2	6	0.48415	0.65838	0.9995	12941	2	0.0125
NRF1	6	0.48415	0.65838	0.9995	12942	2	0.0932
ATOX1	6	0.48428	0.65847	0.9995	12943	2	-0.06
PPAP2A	6	0.48469	0.65873	0.9995	12944	2	-0.234
IL12B	6	0.48476	0.65879	0.9995	12945	2	-0.124
PRRT2	6	0.48485	0.65885	0.9995	12946	1	-0.309
TRIM68	6	0.48491	0.6589	0.9995	12947	2	-0.086
SPATA21	6	0.48501	0.65895	0.9995	12948	2	0.1151
CDY2B	1	0.48506	0.48514	0.9995	12949	0	0.0195
HRAS	6	0.48519	0.65908	0.9995	12950	2	-0.014
ARIH2OS	6	0.48528	0.65914	0.9995	12951	2	-0.14
SI	6	0.48532	0.65916	0.9995	12952	2	0.2122
OR2A42	2	0.48536	0.49077	0.9995	12953	1	-0.921
CCDC28B	6	0.48544	0.65924	0.9995	12954	2	-0.009
ADAMTS10	6	0.48544	0.65924	0.9995	12955	1	-0.235
SLC22A4	6	0.48548	0.65927	0.9995	12956	2	-0.303
TRAPPC2	6	0.48557	0.65933	0.9995	12957	1	-0.015
CCNL1	6	0.48558	0.65934	0.9995	12958	2	-0.25
ZNF146	6	0.48567	0.65941	0.9995	12959	2	-0.482
TUBB6	6	0.48572	0.65944	0.9995	12960	1	-0.349
GDAP2	6	0.48582	0.65951	0.9995	12961	2	0.025
hsa-mir-4285	4	0.48598	0.61076	0.9995	12962	1	-0.058
CYP2A6	5	0.48613	0.6363	0.9995	12963	1	-0.069
AMPH	6	0.48614	0.65973	0.9995	12964	2	-0.312
hsa-mir-551a	4	0.48617	0.61085	0.9995	12965	1	-0.369
ARMC6	6	0.48618	0.65975	0.9995	12966	2	0.0547
KRTAP10-1	6	0.48619	0.65976	0.9995	12967	1	-0.557
TPRN	6	0.48619	0.65976	0.9995	12968	2	-0.022
BLK	6	0.48619	0.65976	0.9995	12969	1	-0.112
TMEM104	6	0.48622	0.65978	0.9995	12970	2	-0.268
APOL6	6	0.48624	0.65979	0.9995	12971	2	0.1337
PROSC	6	0.48629	0.65983	0.9995	12972	2	-0.075
CYP2W1	6	0.48635	0.65986	0.9995	12973	2	-0.171
SLC35E2	2	0.48657	0.49185	0.9995	12974	1	-0.045
PDIA4	6	0.48658	0.66002	0.9995	12975	2	-0.175
TRMU	6	0.4868	0.66017	0.9995	12976	2	0.0459
TRIM24	6	0.48693	0.66026	0.9995	12977	2	0.0127
GPX2	6	0.48708	0.66037	0.9995	12978	2	-0.61
MYO3A	6	0.48709	0.66037	0.9995	12979	2	-0.016
hsa-mir-4520a	1	0.4871	0.48724	0.9995	12980	0	0.0218
ATP2C1	6	0.48717	0.66042	0.9995	12981	1	-0.142
LOC100996485	5	0.48718	0.63716	0.9995	12982	1	-0.35
hsa-mir-1256	4	0.48723	0.61131	0.9995	12983	1	-0.163
TRAT1	5	0.48729	0.63727	0.9995	12984	2	0.1499
MMP17	6	0.48738	0.66056	0.9995	12985	2	-0.056
LOC649330	1	0.48747	0.48763	0.9995	12986	0	0.0132
PNPLA7	6	0.48747	0.66062	0.9995	12987	1	-0.116
TMEM44	6	0.48749	0.66063	0.9995	12988	2	0.1876
WWOX	6	0.48754	0.66067	0.9995	12989	2	0.0857
ZNF696	6	0.48757	0.66069	0.9995	12990	2	-0.012
USP25	6	0.48759	0.66071	0.9995	12991	1	-1E-04
KIF12	6	0.48766	0.66076	0.9995	12992	2	0.0101
MYBPC3	6	0.48766	0.66076	0.9995	12993	2	-0.172
PRDM11	6	0.4879	0.66093	0.9995	12994	1	0.0363
SRBD1	6	0.48792	0.66095	0.9995	12995	2	-0.167
EPHA3	6	0.48796	0.66097	0.9995	12996	2	-0.205
TFE3	6	0.48803	0.66102	0.9995	12997	2	-0.183
DNPH1	6	0.48808	0.66105	0.9995	12998	1	-0.167
PGF	6	0.48817	0.6611	0.9995	12999	1	-0.417
H1F0	6	0.48833	0.66121	0.9995	13000	2	-0.041

C9orf62	6	0.48839	0.66126	0.9995	13001	2	-0.019
EPGN	6	0.4884	0.66127	0.9995	13002	1	-0.33
RB1	6	0.48862	0.66141	0.9995	13003	1	-0.038
SLC35E4	6	0.48869	0.66146	0.9995	13004	2	0.1079
TOB1	6	0.48869	0.66146	0.9995	13005	2	-0.116
SLC51B	6	0.48878	0.66153	0.9995	13006	1	-0.207
CLSPN	6	0.48879	0.66153	0.9995	13007	2	0.0193
FERD3L	6	0.48892	0.66162	0.9995	13008	1	-0.234
UBE2V2	6	0.48923	0.66183	0.9995	13009	1	-0.259
38961	3	0.48936	0.53959	0.9995	13010	1	0.0453
EXTL1	6	0.48938	0.66192	0.9995	13011	2	-0.394
BTBD17	6	0.48957	0.66204	0.9995	13012	1	-0.074
NUPL1	6	0.48958	0.66205	0.9995	13013	2	0.1445
OR6S1	6	0.48958	0.66205	0.9995	13014	2	0.0845
AP1B1	6	0.48958	0.66205	0.9995	13015	2	0.0561
PPIC	6	0.4896	0.66206	0.9995	13016	2	0.0205
TMEM185A	6	0.48966	0.66209	0.9995	13017	2	-0.378
WDR83OS	6	0.48966	0.66209	0.9995	13018	2	-0.294
FAM21B	4	0.48984	0.61241	0.9995	13019	1	0.0903
SERPINB9	6	0.48988	0.66224	0.9995	13020	2	-0.144
C3	6	0.49005	0.66235	0.9995	13021	1	-0.217
TBC1D9B	6	0.49005	0.66235	0.9995	13022	2	-0.067
LSM11	6	0.49021	0.66246	0.9995	13023	2	-0.349
NME1	3	0.49023	0.54017	0.9995	13024	1	-0.498
RAB8B	6	0.49032	0.66254	0.9995	13025	2	-0.228
C12orf45	6	0.49058	0.66272	0.9995	13026	1	-0.11
MAL2	6	0.49063	0.66274	0.9995	13027	2	-0.071
PRDM16	6	0.49075	0.66283	0.9995	13028	2	-0.199
PSMD8	6	0.49075	0.66283	0.9995	13029	1	0.1146
RASSF3	6	0.49075	0.66283	0.9995	13030	2	0.0997
LGALS9B	6	0.49087	0.66291	0.9995	13031	2	0.1589
AJAP1	6	0.49087	0.66291	0.9995	13032	2	-0.048
USP44	6	0.4909	0.66294	0.9995	13033	2	0.0926
GBX1	6	0.49096	0.66298	0.9995	13034	2	-0.289
NMT2	6	0.49097	0.66299	0.9995	13035	2	-0.356
TENM4	6	0.49099	0.663	0.9995	13036	2	-0.17
TOMM20	6	0.49108	0.66306	0.9995	13037	2	-0.137
C1orf172	6	0.49115	0.66311	0.9995	13038	2	-0.043
GINS2	6	0.49121	0.66314	0.9995	13039	1	0.0422
OLFM2	6	0.49124	0.66317	0.9995	13040	2	-0.414
CYP26A1	6	0.49125	0.66318	0.9995	13041	2	0.1322
RNASE9	6	0.49144	0.6633	0.9995	13042	2	-0.017
VRK1	6	0.49149	0.66332	0.9995	13043	2	-0.753
STAMBP	6	0.49155	0.66337	0.9995	13044	1	-0.145
FAM160B1	6	0.49161	0.66341	0.9995	13045	2	-0.181
SIRT3	6	0.49175	0.66349	0.9995	13046	2	-0.225
MKX	6	0.49177	0.6635	0.9995	13047	2	-0.021
STRA13	6	0.49183	0.66354	0.9995	13048	2	-0.197
KLHDC1	6	0.49195	0.66361	0.9995	13049	2	-0.241
FBXO48	6	0.49199	0.66364	0.9995	13050	2	0.1577
SLC22A11	6	0.49205	0.66368	0.9995	13051	1	-0.713
hsa-mir-761	3	0.4926	0.54173	0.9995	13052	1	0.0778
OR4X2	6	0.49262	0.66408	0.9995	13053	2	-0.281
ERBB3	6	0.49291	0.66429	0.9995	13054	2	0.0433
TAC4	6	0.49315	0.66445	0.9995	13055	2	-0.225
C1orf106	6	0.49315	0.66445	0.9995	13056	1	-0.458
KEL	6	0.49329	0.66456	0.9995	13057	1	-0.081
DDX54	6	0.4933	0.66457	0.9995	13058	2	0.0876
SPDYA	6	0.49338	0.66462	0.9995	13059	1	-0.115
BTG4	6	0.49338	0.66462	0.9995	13060	1	-0.404
ADCK1	6	0.49343	0.66465	0.9995	13061	2	-0.279
NUDT12	6	0.49349	0.66468	0.9995	13062	1	-0.892
ACADS	6	0.49355	0.66473	0.9995	13063	2	-0.216
GABRD	6	0.4936	0.66476	0.9995	13064	2	-0.264
RABL3	6	0.49386	0.66493	0.9995	13065	1	-0.487
BRAF	6	0.4939	0.66496	0.9995	13066	2	-0.047
CDH18	6	0.4939	0.66496	0.9995	13067	2	-0.045
HTRA3	6	0.49403	0.66504	0.9995	13068	2	-0.099
DCAF17	6	0.49408	0.66507	0.9995	13069	1	-0.158
PLEKHA7	6	0.49408	0.66507	0.9995	13070	1	-0.094
ABCG5	6	0.49408	0.66507	0.9995	13071	2	-0.097
hsa-mir-3972	4	0.49409	0.61436	0.9995	13072	1	-0.024
TLK2	6	0.49412	0.6651	0.9995	13073	2	0.1498
MYEOV2	6	0.49417	0.66514	0.9995	13074	1	-0.287
FEM1A	6	0.49419	0.66516	0.9995	13075	2	-0.278
SHBG	6	0.49434	0.66526	0.9995	13076	1	-0.28
MTF1	6	0.49436	0.66527	0.9995	13077	2	0.084
ASB16	6	0.49449	0.66536	0.9995	13078	1	-0.139
INTS10	6	0.49451	0.66537	0.9995	13079	2	0.0497
SMG9	6	0.49465	0.66546	0.9995	13080	2	0.0486
CBY1	6	0.49474	0.66552	0.9995	13081	2	-0.214
LRRC8C	6	0.49478	0.66556	0.9995	13082	2	-0.076
TMEM151A	6	0.49483	0.66559	0.9995	13083	2	-0.807
HLA-G	6	0.49483	0.66559	0.9995	13084	2	-0.163
ABCA10	6	0.49487	0.66562	0.9995	13085	2	0.0031

AHNAK2	6	0.49487	0.66562	0.9995	13086	1	-0.754
SOX2	6	0.49492	0.66565	0.9995	13087	2	-0.385
RPSA	6	0.49504	0.66574	0.9995	13088	2	-0.601
STIM2	6	0.49509	0.66578	0.9995	13089	2	0.045
C9orf170	6	0.49512	0.6658	0.9995	13090	1	-0.309
LRFN1	6	0.49518	0.66584	0.9995	13091	2	-0.171
PROC	6	0.49521	0.66586	0.9995	13092	1	-0.323
TMPRSS13	6	0.49535	0.66595	0.9995	13093	2	-0.028
hsa-mir-134	4	0.49536	0.61492	0.9995	13094	1	-0.333
CALML4	6	0.49537	0.66596	0.9995	13095	1	-0.464
GRAMD2	6	0.4956	0.66611	0.9995	13096	2	-0.08
MANBAL	6	0.49566	0.66615	0.9995	13097	2	0.0529
SSX1	6	0.49571	0.66619	0.9995	13098	2	0.2892
PRB4	6	0.4958	0.66625	0.9995	13099	1	-0.564
DIP2B	6	0.49585	0.66629	0.9995	13100	2	-0.219
TACC2	6	0.49596	0.66637	0.9995	13101	2	-0.285
hsa-mir-4431	4	0.49608	0.61526	0.9995	13102	1	-0.309
ZNF101	6	0.4961	0.66646	0.9995	13103	2	0.0962
CYP2C19	3	0.49611	0.54403	0.9995	13104	1	-0.218
DHX35	6	0.49611	0.66647	0.9995	13105	2	0.3085
METTL1	6	0.49622	0.66653	0.9995	13106	2	-0.276
RGPD6	2	0.49628	0.50066	0.9995	13107	1	-0.103
NFIC	6	0.4963	0.66659	0.9995	13108	1	0.0741
SPTSSB	6	0.4963	0.66659	0.9995	13109	1	-0.483
ENPP6	6	0.49646	0.6667	0.9995	13110	1	-0.231
MRPL21	6	0.49657	0.66677	0.9995	13111	2	-0.044
CCS	6	0.49658	0.66677	0.9995	13112	1	-0.252
NAA60	6	0.49659	0.66678	0.9995	13113	2	-0.175
MORF4L1	6	0.49671	0.66686	0.9995	13114	2	0.0756
SLC2A4RG	6	0.49679	0.66691	0.9995	13115	2	-0.15
COASY	6	0.49693	0.66702	0.9995	13116	1	-0.356
KAT6A	6	0.49695	0.66704	0.9995	13117	2	-0.294
CCNA2	6	0.49706	0.66711	0.9995	13118	2	-0.33
hsa-mir-4283-2	4	0.49712	0.61571	0.9995	13119	1	0.048
TPD52L2	6	0.49717	0.6672	0.9995	13120	2	-0.097
SAMD14	6	0.49727	0.66726	0.9995	13121	2	-0.006
NRN1L	6	0.49737	0.66734	0.9995	13122	1	0.0657
ZNF816	2	0.49755	0.50185	0.9995	13123	1	0.0768
LRSAM1	6	0.49761	0.6675	0.9995	13124	2	-0.128
TMEM244	6	0.49761	0.6675	0.9995	13125	2	-0.092
HOXB8	6	0.49781	0.66764	0.9995	13126	2	0.1227
SIRT7	6	0.49793	0.66772	0.9995	13127	1	-0.285
hsa-mir-6716	4	0.49797	0.61611	0.9995	13128	1	-0.265
ZCCHC11	6	0.498	0.66776	0.9995	13129	2	0.0644
KLC1	6	0.49809	0.66782	0.9995	13130	2	0.045
ITM2B	6	0.49812	0.66784	0.9995	13131	1	-0.234
PFKFB1	6	0.49821	0.66791	0.9995	13132	2	0.0989
SLC5A2	6	0.49821	0.66792	0.9995	13133	1	-0.315
SNX3	6	0.49821	0.66792	0.9995	13134	2	-0.096
hsa-mir-4301	4	0.49824	0.61623	0.9995	13135	1	0.0995
NUDT14	6	0.49829	0.66797	0.9995	13136	2	0.052
KCNQ4	6	0.4984	0.66805	0.9995	13137	2	0.0561
DNAJC25	6	0.49853	0.66813	0.9995	13138	2	-0.464
RBPMS2	6	0.49858	0.66817	0.9995	13139	1	-0.152
MAEL	6	0.49859	0.66818	0.9995	13140	2	0.0211
CRYGD	6	0.49881	0.66832	0.9995	13141	2	0.111
USP38	6	0.4989	0.66838	0.9995	13142	2	-0.2
MYO1C	6	0.49892	0.66839	0.9995	13143	2	0.1475
PMM1	6	0.49928	0.66863	0.9995	13144	2	0.0548
KLHL2	6	0.49931	0.66866	0.9995	13145	2	0.0502
EQTN	6	0.49953	0.6688	0.9995	13146	2	-0.168
SAT2	6	0.49953	0.6688	0.9995	13147	2	-0.33
RFPL1	5	0.49959	0.64784	0.9995	13148	1	0.0975
RIPK1	6	0.4996	0.66885	0.9995	13149	1	-0.393
CAV3	6	0.49965	0.66889	0.9995	13150	1	-0.336
RPS20	6	0.49987	0.66904	0.9995	13151	2	-0.085
OR2T12	6	0.49988	0.66905	0.9995	13152	1	-0.447
TRAPPC5	6	0.49988	0.66905	0.9995	13153	2	0.125
ELAC2	6	0.49994	0.66909	0.9995	13154	2	0.0383
FHIT	6	0.50011	0.66922	0.9995	13155	2	0.2434
CCR3	6	0.50022	0.66929	0.9995	13156	2	0.0237
GNAI3	5	0.5003	0.64841	0.9995	13157	2	0.158
FAM47E-STBD1	2	0.50032	0.50435	0.9995	13158	1	0.1056
LACE1	6	0.50036	0.66939	0.9995	13159	2	-0.05
HTATIP2	6	0.50046	0.66946	0.9995	13160	2	-0.158
KRT7	6	0.50049	0.66946	0.9995	13161	2	0.0484
ABCB11	6	0.50054	0.6695	0.9995	13162	2	-0.204
CMTM3	6	0.50079	0.66968	0.9995	13163	1	0.0222
ATP6V0D2	6	0.50093	0.66978	0.9995	13164	1	-0.04
ZNF821	6	0.50093	0.66978	0.9995	13165	1	-0.076
AKR1C4	6	0.50096	0.66979	0.9995	13166	2	-0.211
hsa-mir-548v	4	0.50108	0.61749	0.9995	13167	1	-0.311
hsa-mir-5708	3	0.50113	0.54734	0.9995	13168	1	-0.031
RABGAP1	6	0.50124	0.66998	0.9995	13169	2	-0.343
RHOXF2B	3	0.50128	0.54745	0.9995	13170	1	-1.233

SPAM1	6	0.50129	0.67002	0.9995	13171	2	0.1352
DFNB59	6	0.50136	0.67006	0.9995	13172	2	-0.192
CCL1	6	0.5014	0.67009	0.9995	13173	1	-0.094
TIAM1	6	0.50145	0.67012	0.9995	13174	2	-0.072
ZFR2	6	0.50168	0.67027	0.9995	13175	2	-0.29
MYCL1	2	0.5018	0.50573	0.9995	13176	1	0.007
SRRM4	6	0.50207	0.67054	0.9995	13177	2	-0.106
CCDC7	6	0.5021	0.67056	0.9995	13178	2	-0.06
hsa-mir-1182	4	0.50216	0.61795	0.9995	13179	1	0.0692
TSSK3	6	0.50217	0.67061	0.9995	13180	2	-0.101
TEPP	6	0.5022	0.67062	0.9995	13181	2	-0.023
EVI2A	6	0.5022	0.67062	0.9995	13182	2	-0.413
WDR13	6	0.50242	0.67076	0.9995	13183	2	-0.142
TIMM8B	6	0.50249	0.67082	0.9995	13184	1	0.0355
TMEM185B	6	0.50254	0.67085	0.9995	13185	2	0.0029
CRTAC1	6	0.50259	0.67089	0.9995	13186	2	0.0944
SCN7A	6	0.5027	0.67095	0.9995	13187	1	0.0641
CMSS1	6	0.50274	0.67099	0.9995	13188	1	-0.745
EXOSC1	6	0.50301	0.67117	0.9995	13189	2	-0.09
CGGBP1	6	0.50301	0.67117	0.9995	13190	2	-0.244
DSEL	6	0.50309	0.67122	0.9995	13191	1	-0.292
ADAMTS8	6	0.50312	0.67125	0.9995	13192	2	-0.001
LRRC43	6	0.50312	0.67125	0.9995	13193	2	0.1005
HES4	6	0.50312	0.67125	0.9995	13194	2	0.1867
PPP2R5A	6	0.50313	0.67126	0.9995	13195	2	-0.143
C17orf96	6	0.50318	0.67129	0.9995	13196	2	0.088
ATL3	6	0.50339	0.67143	0.9995	13197	2	0.1015
FAM114A1	6	0.50344	0.67147	0.9995	13198	2	0.0868
hsa-mir-548an	3	0.50354	0.54895	0.9995	13199	1	-0.419
MEP1A	6	0.50362	0.67158	0.9995	13200	2	-0.19
CA1	6	0.50367	0.67162	0.9995	13201	2	0.0022
POMK	4	0.5038	0.6187	0.9995	13202	1	0.0549
HSFX2	1	0.50392	0.50394	0.9995	13203	0	-0.015
BCL7B	6	0.50396	0.67181	0.9995	13204	2	-0.271
OR13C9	5	0.504	0.65168	0.9995	13205	2	-0.007
NEUROD1	6	0.50421	0.672	0.9995	13206	2	-0.231
GNGT2	6	0.50421	0.672	0.9995	13207	2	-0.732
PRKG1	6	0.50421	0.672	0.9995	13208	1	-0.18
hsa-mir-3120	2	0.50434	0.50802	0.9995	13209	1	0.1895
ZNF582	6	0.50435	0.6721	0.9995	13210	2	-0.264
RAB8A	6	0.50438	0.67212	0.9995	13211	2	0.1795
GRAPL	2	0.50439	0.50806	0.9995	13212	1	0.18
DEFB126	6	0.50441	0.67214	0.9995	13213	2	-0.364
hsa-mir-6795	2	0.50448	0.50815	0.9995	13214	1	-0.011
ZBTB6	6	0.5045	0.6722	0.9995	13215	2	-0.365
PRKRIR	6	0.50456	0.67223	0.9995	13216	2	-0.01
hsa-mir-4291	4	0.50462	0.61905	0.9995	13217	1	-0.914
TFAP2B	6	0.5047	0.67234	0.9995	13218	1	-0.187
MOGS	6	0.5047	0.67234	0.9995	13219	2	0.1168
FUOM	6	0.5048	0.6724	0.9995	13220	2	-0.402
VMAC	6	0.50492	0.67248	0.9995	13221	1	-0.242
CEBPB	3	0.50511	0.54995	0.9995	13222	1	0.0805
OR5B21	6	0.50511	0.67261	0.9995	13223	2	-3E-04
ZNF284	5	0.50514	0.6527	0.9995	13224	2	0.1731
BANK1	6	0.50518	0.67266	0.9995	13225	1	-0.186
GPR82	6	0.50542	0.67284	0.9995	13226	2	-0.119
CPSF4L	6	0.50557	0.67293	0.9995	13227	2	-0.193
LAD1	6	0.50568	0.673	0.9995	13228	2	-0.212
PDK3	6	0.50568	0.673	0.9995	13229	2	-0.296
AMOTL1	6	0.50572	0.67303	0.9995	13230	1	0.0197
TGS1	6	0.50572	0.67303	0.9995	13231	2	0.022
UCKL1	6	0.50574	0.67304	0.9995	13232	2	-0.489
GPR113	6	0.50579	0.67308	0.9995	13233	2	-0.029
ZNHIT1	6	0.50579	0.67308	0.9995	13234	1	-0.175
MBOAT1	6	0.50585	0.67312	0.9995	13235	2	0.127
WFDC9	6	0.50596	0.6732	0.9995	13236	2	-6E-04
GPR114	6	0.50601	0.67323	0.9995	13237	1	-0.484
PI4K2A	6	0.50604	0.67326	0.9995	13238	2	0.1163
TCN2	6	0.50638	0.67347	0.9995	13239	2	-0.126
ZDHHC19	6	0.50641	0.6735	0.9995	13240	1	-0.325
PDHX	6	0.50649	0.67355	0.9995	13241	2	-0.032
LOC440243	2	0.5067	0.51015	0.9995	13242	1	-0.455
ZBTB3	6	0.50671	0.67372	0.9995	13243	1	-0.481
ZNF212	6	0.50673	0.67373	0.9995	13244	2	0.1431
OR2T4	6	0.50676	0.67376	0.9995	13245	2	-0.122
CXCL17	6	0.50709	0.67399	0.9995	13246	2	0.0099
PAPD4	6	0.50728	0.67412	0.9995	13247	2	-0.278
BTG2	6	0.50731	0.67414	0.9995	13248	2	-0.178
C7orf57	6	0.50736	0.67418	0.9995	13249	2	-0.262
CASP4	6	0.50742	0.67421	0.9995	13250	2	0.0698
EHBP1L1	6	0.5077	0.6744	0.9995	13251	1	0.0531
ALKBH4	6	0.50776	0.67444	0.9995	13252	2	0.0079
C5orf46	6	0.50786	0.67452	0.9995	13253	2	-0.657
CHADL	6	0.50792	0.67456	0.9995	13254	2	-0.221
TNC	6	0.50807	0.67466	0.9995	13255	2	-0.46

DCTN6	6	0.50819	0.67474	0.9995	13256	2	0.1448
LRRIQ4	6	0.50827	0.6748	0.9995	13257	1	-0.076
SLC27A3	6	0.50832	0.67482	0.9995	13258	2	-0.288
CCDC42	6	0.50842	0.6749	0.9995	13259	2	-0.113
CA10	6	0.50862	0.67503	0.9995	13260	1	-0.426
CDO1	6	0.50865	0.67505	0.9995	13261	2	-0.222
NAPG	4	0.50868	0.62089	0.9995	13262	1	0.1583
TBC1D10C	6	0.50873	0.6751	0.9995	13263	2	-0.097
SMPX	6	0.50876	0.67513	0.9995	13264	2	0.0471
TMIGD2	6	0.50892	0.67522	0.9995	13265	2	0.171
WNT8A	6	0.50895	0.67525	0.9995	13266	1	-0.057
FAM117B	6	0.50908	0.67534	0.9995	13267	1	-0.053
TRPC5	6	0.50911	0.67536	0.9995	13268	2	0.2514
FAM57B	6	0.50922	0.67543	0.9995	13269	2	0.1155
FAM203A	6	0.5093	0.67549	0.9995	13270	1	0.0782
FLJ44635	6	0.50934	0.67552	0.9995	13271	1	-0.101
BRINP2	4	0.5094	0.62121	0.9995	13272	1	0.0482
PIH1D3	6	0.50944	0.67559	0.9995	13273	2	0.0401
MRPS21	6	0.5096	0.67569	0.9995	13274	2	0.1983
PSIP1	6	0.5096	0.67569	0.9995	13275	2	0.0216
FERMT3	6	0.5096	0.67569	0.9995	13276	2	-0.302
ITGAM	6	0.50961	0.67569	0.9995	13277	2	-0.362
TBCD	6	0.50976	0.67581	0.9995	13278	2	0.0065
TRAF3	6	0.50976	0.67581	0.9995	13279	2	-0.326
ASS1	6	0.50995	0.67594	0.9995	13280	2	-0.2
OR6C76	6	0.50995	0.67594	0.9995	13281	2	-0.08
TMEM238	6	0.51012	0.67607	0.9995	13282	2	-0.542
AURKAIP1	6	0.51027	0.67619	0.9995	13283	1	0.0171
MZT2B	3	0.51028	0.55332	0.9995	13284	1	-1.074
CDK16	6	0.51033	0.67623	0.9995	13285	1	0.1009
NOS1AP	6	0.51045	0.67628	0.9995	13286	2	0.1164
DNAJB7	6	0.51052	0.67634	0.9995	13287	2	-0.147
UTP15	6	0.5106	0.67639	0.9995	13288	2	-0.167
SENP7	6	0.51072	0.67646	0.9995	13289	2	0.097
BLOC1S1	6	0.51077	0.6765	0.9995	13290	2	-0.15
C6orf10	6	0.51085	0.67655	0.9995	13291	2	-0.027
TCTN1	6	0.51092	0.67661	0.9995	13292	2	-0.076
PCGF6	6	0.51092	0.67661	0.9995	13293	2	0.138
RNF40	6	0.51105	0.67669	0.9995	13294	2	0.0861
ITK	6	0.51135	0.67688	0.9995	13295	2	-0.128
hsa-mir-655	4	0.51136	0.62211	0.9995	13296	1	-0.101
ACTN2	6	0.51141	0.67692	0.9995	13297	2	-0.009
SATB2	4	0.51164	0.62224	0.9995	13298	1	-0.144
TRPM1	6	0.51166	0.67709	0.9995	13299	2	0.083
ZNF546	6	0.51173	0.67713	0.9995	13300	2	-0.279
SKA2	6	0.51178	0.67716	0.9995	13301	2	-0.209
hsa-mir-1321	4	0.5118	0.62232	0.9995	13302	1	-0.606
XPNPEP3	6	0.51199	0.67731	0.9995	13303	2	-0.049
FAM84B	6	0.51215	0.67742	0.9995	13304	2	-0.018
OR6Q1	4	0.51221	0.6225	0.9995	13305	1	-0.849
ATP1B1	6	0.51235	0.67756	0.9995	13306	2	-0.304
GRP	6	0.51257	0.67772	0.9995	13307	2	-0.164
HLA-DQB1	6	0.51267	0.6778	0.9995	13308	2	0.1383
SAP30	6	0.51274	0.67786	0.9995	13309	2	-0.241
CREBL2	6	0.5128	0.6779	0.9995	13310	2	-0.025
IQCK	6	0.51299	0.67803	0.9995	13311	2	-0.046
STRN3	6	0.51328	0.67822	0.9995	13312	2	-0.066
CYSLTR1	6	0.51334	0.67826	0.9995	13313	1	-0.062
DEDD2	6	0.51344	0.67834	0.9995	13314	1	-0.096
SH2D6	6	0.51353	0.67841	0.9995	13315	2	-0.015
IRAK1BP1	6	0.51375	0.67857	0.9995	13316	1	-0.322
AVEN	6	0.51379	0.67859	0.9995	13317	2	-0.196
IL13RA2	6	0.51386	0.67864	0.9995	13318	2	-0.075
SLC9C1	6	0.51402	0.67874	0.9995	13319	2	-0.263
ZFP2	6	0.51404	0.67875	0.9995	13320	2	0.1224
CRBN	6	0.51407	0.67877	0.9995	13321	1	-0.145
SH2D1B	6	0.51411	0.67881	0.9995	13322	2	0.0461
AEBP1	6	0.51417	0.67885	0.9995	13323	1	-0.063
NYNRIN	6	0.51418	0.67886	0.9995	13324	2	-0.102
PCDHGB1	2	0.51421	0.51705	0.9995	13325	1	0.1884
SIGLEC6	6	0.51428	0.67893	0.9995	13326	1	-0.284
EIF3M	6	0.51445	0.67903	0.9995	13327	2	-0.104
AK4	6	0.51452	0.67908	0.9995	13328	2	-0.122
KLHL5	6	0.51455	0.67911	0.9995	13329	1	-0.228
FAM53A	6	0.51458	0.67913	0.9995	13330	2	0.1266
SEC61G	6	0.51463	0.67916	0.9995	13331	1	-0.186
KRTAP19-8	6	0.51465	0.67918	0.9995	13332	2	-0.505
PSMB1	6	0.51466	0.67918	0.9995	13333	2	-0.503
MLXIPL	6	0.51476	0.67925	0.9995	13334	2	-0.072
NRIP2	6	0.51478	0.67926	0.9995	13335	2	-0.387
MCL1	6	0.51485	0.67932	0.9995	13336	2	-0.159
CDKN1C	6	0.51488	0.67934	0.9995	13337	1	0.0364
hsa-mir-5691	4	0.51493	0.62378	0.9995	13338	1	-0.027
hsa-mir-371a	1	0.51504	0.515	0.9995	13339	0	-0.023
JAM3	6	0.51504	0.67946	0.9995	13340	2	0.1118

CPLX4	6	0.51504	0.67946	0.9995	13341	2	0.0125
TRMT13	3	0.51513	0.55662	0.9995	13342	1	-0.368
C1orf123	6	0.51516	0.67954	0.9995	13343	2	0.0468
OR6C2	6	0.51516	0.67954	0.9995	13344	2	0.0457
C7orf61	6	0.51528	0.67961	0.9995	13345	2	0.0919
CAPN3	6	0.51535	0.67967	0.9995	13346	2	-0.231
ELOVL5	6	0.5154	0.67971	0.9995	13347	2	0.1481
DPM3	6	0.51542	0.67972	0.9995	13348	1	-0.374
CXorf58	6	0.51562	0.67985	0.9995	13349	1	-0.114
MAPKAPK2	6	0.51569	0.6799	0.9995	13350	2	-0.304
NAB1	6	0.51578	0.67996	0.9995	13351	2	-0.287
TRHR	6	0.51578	0.67996	0.9995	13352	1	-0.002
GPR142	6	0.51599	0.68012	0.9995	13353	1	-0.394
IGSF5	6	0.51612	0.68022	0.9995	13354	2	0.0692
hsa-mir-4278	4	0.51612	0.62431	0.9995	13355	1	0.0362
MRPS18B	6	0.51618	0.68027	0.9995	13356	2	-0.076
C2orf27B	3	0.51623	0.55738	0.9995	13357	1	0.1613
RBM14-RBM4	1	0.51624	0.5162	0.9995	13358	0	-0.046
HIST1H1T	6	0.51635	0.68038	0.9995	13359	2	-0.19
ZNF454	6	0.51652	0.68049	0.9995	13360	2	0.0285
FRMPD1	6	0.51652	0.6805	0.9995	13361	1	-0.389
MPZ	6	0.51656	0.68052	0.9995	13362	2	0.0851
TNFSF11	6	0.51656	0.68052	0.9995	13363	2	0.1004
ZC3H12C	6	0.51661	0.68056	0.9995	13364	2	-0.22
ILDR2	6	0.51665	0.68058	0.9995	13365	2	0.0003
PLEKHM3	6	0.51665	0.68058	0.9995	13366	2	0.0573
TET2	6	0.5169	0.68075	0.9995	13367	1	-0.526
ZNF550	6	0.51693	0.68078	0.9995	13368	2	0.0655
PLK2	6	0.51696	0.6808	0.9995	13369	2	-0.155
NEDD8	5	0.51698	0.66289	0.9995	13370	2	-0.017
SFTPB	6	0.51707	0.68088	0.9995	13371	2	0.036
hsa-mir-4312	4	0.51709	0.62476	0.9995	13372	1	-0.097
SH3BP2	6	0.51724	0.68099	0.9995	13373	1	0.0023
TRA2B	6	0.51727	0.681	0.9995	13374	2	-0.215
CCDC159	6	0.51735	0.68106	0.9995	13375	2	-0.057
ADCYAP1	6	0.51758	0.68122	0.9995	13376	1	-0.013
LOC401052	6	0.51758	0.68122	0.9995	13377	2	-0.106
hsa-mir-4781	4	0.51761	0.625	0.9995	13378	1	0.1209
CCL19	6	0.51772	0.68132	0.9995	13379	2	0.1628
MRFAP1L1	6	0.51773	0.68133	0.9995	13380	2	0.1395
PDXP	6	0.51775	0.68135	0.9995	13381	1	-0.348
SLFN12L	6	0.51776	0.68135	0.9995	13382	2	-0.222
NXPH4	6	0.51807	0.68155	0.9995	13383	1	-0.196
SNRNP70	6	0.51812	0.68158	0.9995	13384	2	-0.033
DENND2A	6	0.51818	0.68162	0.9995	13385	2	0.0779
CDH17	6	0.51831	0.68172	0.9995	13386	2	0.0974
ELL3	6	0.51832	0.68173	0.9995	13387	1	0.1244
GOLGA2	6	0.51843	0.6818	0.9995	13388	2	-0.434
hsa-mir-30e	4	0.51846	0.6254	0.9995	13389	1	-0.282
DNTTIP2	6	0.51848	0.68184	0.9995	13390	1	-0.568
ADCY9	6	0.51849	0.68184	0.9995	13391	2	0.0891
DISC1	4	0.51852	0.62542	0.9995	13392	1	0.0166
EVPLL	6	0.51872	0.68201	0.9995	13393	2	-0.147
TFG	6	0.51882	0.68207	0.9995	13394	1	0.0215
CLEC18C	2	0.51885	0.52138	0.9995	13395	1	-0.148
METTL6	6	0.51886	0.6821	0.9995	13396	2	0.0667
COPS4	6	0.51894	0.68215	0.9995	13397	2	0.1364
ATG10	6	0.51894	0.68215	0.9995	13398	2	-0.326
GIT2	6	0.51911	0.68226	0.9995	13399	2	-0.015
IRX2	6	0.51936	0.68242	0.9995	13400	1	-0.25
ARHGAP11B	2	0.51937	0.52187	0.9995	13401	1	-0.338
RAB1B	6	0.51938	0.68244	0.9995	13402	2	0.0951
TSPAN8	6	0.5196	0.68258	0.9995	13403	2	0.1076
BCL2A1	6	0.51968	0.68264	0.9995	13404	2	0.0856
OAT	6	0.51978	0.6827	0.9995	13405	1	-0.494
RASAL1	6	0.51983	0.68273	0.9995	13406	2	0.0848
OR2M4	6	0.51983	0.68273	0.9995	13407	2	-0.118
KIAA1143	6	0.51999	0.68286	0.9995	13408	2	0.0965
PDLIM4	6	0.52005	0.68289	0.9995	13409	2	-0.017
CYP11A1	6	0.52015	0.68296	0.9995	13410	2	-0.083
C15orf61	6	0.52054	0.68325	0.9995	13411	2	0.0526
MGST1	6	0.52058	0.68328	0.9995	13412	1	-0.597
PPHLN1	6	0.52058	0.68328	0.9995	13413	1	0.0385
DMPK	6	0.52073	0.68337	0.9995	13414	1	-0.412
SCML4	6	0.52081	0.68343	0.9995	13415	2	0.2304
VTA1	6	0.52091	0.6835	0.9995	13416	2	-0.598
RPL37	6	0.52106	0.68361	0.9995	13417	1	-0.366
CORO1B	6	0.52112	0.68365	0.9995	13418	2	0.1399
WNT10B	6	0.52115	0.68367	0.9995	13419	1	-0.119
AIDA	6	0.52125	0.68373	0.9995	13420	2	0.1263
CD79B	6	0.52125	0.68374	0.9995	13421	1	-0.615
KIF2A	6	0.52129	0.68376	0.9995	13422	2	0.1869
hsa-mir-603	4	0.52131	0.62676	0.9995	13423	1	-0.181
SLC37A2	6	0.52133	0.68378	0.9995	13424	2	-0.221
MAGEB16	6	0.52139	0.68382	0.9995	13425	2	0.0706

DTX2	6	0.52147	0.68389	0.9995	13426	2	0.0367
hsa-mir-1193	4	0.5216	0.62689	0.9995	13427	1	-0.23
CSAG1	6	0.52168	0.68404	0.9995	13428	1	-0.085
USMG5	6	0.52169	0.68405	0.9995	13429	2	0.1173
ALS2	6	0.52188	0.68417	0.9995	13430	1	-0.06
SH2D3C	6	0.52191	0.68419	0.9995	13431	2	0.0254
ANKS3	6	0.52191	0.68419	0.9995	13432	2	0.2017
ADAM15	6	0.5223	0.68445	0.9995	13433	2	0.0176
METTL24	4	0.52235	0.62724	0.9995	13434	1	-0.364
ZNF385D	6	0.5224	0.68453	0.9995	13435	2	-0.209
FLT3	6	0.52243	0.68455	0.9995	13436	1	-0.035
CCDC17	6	0.52247	0.68458	0.9995	13437	1	-0.285
PCCB	6	0.52254	0.68462	0.9995	13438	2	-0.07
CERKL	6	0.52259	0.68466	0.9995	13439	2	-0.134
TMOD3	6	0.52275	0.68478	0.9995	13440	2	-0.132
TMEM191C	5	0.5229	0.66805	0.9995	13441	2	-0.133
NUDT1	6	0.52291	0.68487	0.9995	13442	2	0.0753
hsa-mir-4527	4	0.52293	0.62752	0.9995	13443	1	0.0994
FAM72A	1	0.52302	0.52294	0.9995	13444	0	-0.036
SPAG1	6	0.52303	0.68496	0.9995	13445	2	0.0909
PHF21A	6	0.52306	0.68499	0.9995	13446	2	0.0321
PTPLAD2	6	0.52311	0.68502	0.9995	13447	2	-0.006
TADA2A	6	0.52317	0.68506	0.9995	13448	2	0.0902
ARHGEF25	6	0.52327	0.68513	0.9995	13449	2	-0.07
EXOSC3	6	0.5234	0.68522	0.9995	13450	1	-0.771
B4GALT2	6	0.52344	0.68525	0.9995	13451	1	-0.251
TXLNB	6	0.52347	0.68527	0.9995	13452	2	-0.053
PAN3	4	0.5235	0.62779	0.9995	13453	1	-0.459
RTEL1	6	0.5236	0.68536	0.9995	13454	2	-0.214
TWSG1	6	0.52386	0.68554	0.9995	13455	1	-0.321
FCHO1	6	0.5244	0.6859	0.9995	13456	2	-0.117
RMDN3	6	0.52454	0.68601	0.9995	13457	1	-0.372
ADCYAP1R1	6	0.52459	0.68605	0.9995	13458	2	0.0963
ADAMTSL5	6	0.52464	0.68608	0.9995	13459	1	0.1357
NDN	6	0.52475	0.68616	0.9995	13460	2	0.0509
PAOX	6	0.52481	0.68621	0.9995	13461	2	-0.413
ARFIP1	6	0.52501	0.68635	0.9995	13462	2	0.0541
TM9SF2	6	0.52506	0.68638	0.9995	13463	2	0.0465
RHOXF2	2	0.52533	0.52745	0.9995	13464	1	-0.473
CCL14	6	0.52536	0.68661	0.9995	13465	2	-0.117
hsa-mir-548h-4	2	0.52539	0.52751	0.9995	13466	1	0.0418
LRRC14	6	0.52539	0.68663	0.9995	13467	1	-0.559
B9D2	6	0.52541	0.68664	0.9995	13468	2	-0.058
SNRNP25	4	0.52544	0.6287	0.9995	13469	1	-0.332
NME2	3	0.52548	0.56367	0.9995	13470	1	-0.3
SH2D4B	6	0.52552	0.68672	0.9995	13471	2	-0.137
TRANK1	6	0.52555	0.68673	0.9995	13472	1	-0.238
PLA2G4F	6	0.52555	0.68673	0.9995	13473	2	-0.25
ZCCHC24	6	0.52555	0.68674	0.9995	13474	2	-0.52
SNX8	6	0.52571	0.68685	0.9995	13475	2	0.1074
ACY3	6	0.52573	0.68686	0.9995	13476	2	-0.095
C7orf50	6	0.5259	0.68698	0.9995	13477	1	-0.124
NPHS2	6	0.52596	0.68702	0.9995	13478	2	0.0888
VIPAS39	6	0.52616	0.68716	0.9995	13479	2	-0.547
PDHB	6	0.52624	0.68721	0.9995	13480	2	-0.32
ZFY	6	0.52624	0.68721	0.9995	13481	2	-0.306
SLU7	6	0.52632	0.68727	0.9995	13482	1	-0.891
TSPAN6	6	0.52638	0.68731	0.9995	13483	2	-0.023
ZNF500	6	0.52639	0.68731	0.9995	13484	1	-0.373
TAF1L	6	0.52652	0.68741	0.9995	13485	2	-0.365
UBE2F	6	0.52666	0.68752	0.9995	13486	2	-0.3
SLC25A31	6	0.5267	0.68755	0.9995	13487	2	0.112
IER2	6	0.52671	0.68755	0.9995	13488	2	0.2545
TMEM109	6	0.52673	0.68757	0.9995	13489	1	0.108
CAPN5	6	0.52675	0.68759	0.9995	13490	2	-0.032
GAS6	6	0.52679	0.68762	0.9995	13491	2	0.0213
TBC1D17	6	0.5269	0.6877	0.9995	13492	2	-0.204
KRT72	6	0.52701	0.68777	0.9995	13493	2	-0.764
RPL23	6	0.52712	0.68785	0.9995	13494	2	-0.363
PALD1	6	0.52721	0.68791	0.9995	13495	2	-0.14
SP100	6	0.52721	0.68791	0.9995	13496	2	0.058
PRX	6	0.52742	0.68805	0.9995	13497	1	-0.647
LOC100505841	4	0.52751	0.62963	0.9995	13498	1	-0.049
SMC1B	6	0.52755	0.68815	0.9995	13499	2	-0.176
TOMM40L	6	0.5276	0.68819	0.9995	13500	2	-0.107
MSMO1	6	0.52762	0.68821	0.9995	13501	2	0.206
CEP55	6	0.52772	0.68827	0.9995	13502	2	-0.075
TRAM2	6	0.52786	0.68837	0.9995	13503	1	-0.198
FAM186B	6	0.52786	0.68837	0.9995	13504	2	-0.1
CTSK	6	0.52793	0.68842	0.9995	13505	2	-0.112
FAM45A	6	0.52803	0.68849	0.9995	13506	2	-0.106
SPANXE	2	0.52806	0.53	0.9995	13507	1	0.2621
SGPL1	6	0.5281	0.68854	0.9995	13508	1	-0.202
PPP2R5D	6	0.5281	0.68854	0.9995	13509	2	-0.186
EVX2	6	0.52823	0.68863	0.9995	13510	2	0.0446

PLCD1	6	0.52827	0.68865	0.9995	13511	2	-0.194
ANKS4B	6	0.52828	0.68866	0.9995	13512	2	0.1834
ZNF131	6	0.52837	0.68874	0.9995	13513	1	-0.313
BCL2L14	6	0.52845	0.6888	0.9995	13514	1	-0.356
DIP2A	6	0.52856	0.68889	0.9995	13515	2	-0.113
EBF4	6	0.52859	0.68891	0.9995	13516	1	-0.285
NAALADL1	6	0.5288	0.68907	0.9995	13517	1	-0.005
TRIM7	6	0.52885	0.6891	0.9995	13518	2	-0.041
CNPY4	6	0.52895	0.68918	0.9995	13519	2	0.027
FAM178A	6	0.52895	0.68918	0.9995	13520	1	-0.429
TM9SF4	6	0.52899	0.6892	0.9995	13521	2	0.0239
SGCG	6	0.52906	0.68925	0.9995	13522	2	0.0612
RAD9A	6	0.52914	0.68931	0.9995	13523	2	-0.142
C1orf94	6	0.52914	0.68931	0.9995	13524	2	-0.062
RIOK2	6	0.5292	0.68936	0.9995	13525	2	0.0397
SAMD3	6	0.52934	0.68945	0.9995	13526	2	-0.377
MIF4GD	6	0.52935	0.68946	0.9995	13527	2	-0.035
NDRG3	6	0.5294	0.68948	0.9995	13528	1	-0.048
GORAB	4	0.52951	0.63054	0.9995	13529	1	-0.15
KIFC3	6	0.52955	0.6896	0.9995	13530	2	0.065
CTNNBL1	6	0.52965	0.68967	0.9995	13531	2	0.038
hsa-mir-661	4	0.52973	0.63063	0.9995	13532	1	0.1826
ZNF324	6	0.52981	0.68979	0.9995	13533	2	-0.122
GOLPH3L	6	0.52984	0.6898	0.9995	13534	2	0.1773
GPR112	6	0.52991	0.68986	0.9995	13535	1	-0.257
PIN4	6	0.52994	0.68988	0.9995	13536	2	0.1325
PM20D2	6	0.53002	0.68994	0.9995	13537	2	0.069
UCN2	6	0.53003	0.68995	0.9995	13538	1	-0.219
OR52E6	6	0.53003	0.68995	0.9995	13539	1	-0.081
KYNU	6	0.5301	0.68999	0.9995	13540	1	-0.109
ZSCAN22	6	0.53016	0.69003	0.9995	13541	1	-0.027
PTPN21	6	0.53023	0.69008	0.9995	13542	1	-0.057
HNRNPL	6	0.53023	0.69008	0.9995	13543	2	-0.081
hsa-mir-4428	4	0.5303	0.63089	0.9995	13544	1	0.0041
ACSS2	4	0.53046	0.63098	0.9995	13545	1	-0.095
TRAP1	6	0.53053	0.69029	0.9995	13546	2	-0.072
FRA10AC1	3	0.53053	0.56715	0.9995	13547	1	0.4271
AGPAT4	6	0.53062	0.69035	0.9995	13548	2	0.0881
HERPUD1	6	0.53074	0.69044	0.9995	13549	2	-0.143
MPLKIP	6	0.53081	0.69049	0.9995	13550	2	-0.245
GPR61	6	0.53083	0.6905	0.9995	13551	1	-0.237
SMTNL2	6	0.53085	0.69052	0.9995	13552	2	-0.016
NRROS	3	0.53088	0.5674	0.9995	13553	1	0.0003
FAM131B	6	0.53088	0.69054	0.9995	13554	2	0.0419
RFC3	6	0.53091	0.69056	0.9995	13555	1	0.1466
APMAP	6	0.53101	0.69063	0.9995	13556	2	-0.119
HEMK1	6	0.53107	0.69066	0.9995	13557	2	-0.261
B4GALT5	6	0.53118	0.69075	0.9995	13558	2	0.1822
NUB1	5	0.53125	0.67542	0.9995	13559	1	0.2667
INPP5E	6	0.53127	0.6908	0.9995	13560	1	-0.367
TEKT2	6	0.53127	0.6908	0.9995	13561	2	0.0385
TNFRSF10D	6	0.53131	0.69083	0.9995	13562	2	-0.417
POP1	6	0.53135	0.69086	0.9995	13563	1	-0.169
FBXL16	6	0.53141	0.69091	0.9995	13564	1	-0.082
CLSTN2	6	0.53148	0.69097	0.9995	13565	2	0.0951
OR4A15	6	0.53152	0.69099	0.9995	13566	2	-0.218
RAB5A	6	0.53156	0.69102	0.9995	13567	2	-0.078
KRTAP10-3	6	0.53165	0.69108	0.9995	13568	2	-0.68
POTEH	2	0.53168	0.53338	0.9995	13569	1	0.1615
KRTAP2-3	2	0.53174	0.53343	0.9995	13570	1	-0.042
SPDYE5	2	0.53185	0.53354	0.9995	13571	1	0.0731
STXBP1	6	0.5319	0.69125	0.9995	13572	2	0.0902
HOXA13	6	0.53191	0.69125	0.9995	13573	2	-0.061
hsa-mir-7846	4	0.53201	0.63168	0.9995	13574	1	-0.144
SLC6A9	6	0.53203	0.69134	0.9995	13575	2	-0.06
ZNF512	6	0.53203	0.69134	0.9995	13576	2	-0.15
GTPBP10	6	0.53207	0.69138	0.9995	13577	2	-0.016
hsa-mir-4510	3	0.53207	0.56821	0.9995	13578	1	0.1954
PROSER2	6	0.5321	0.69139	0.9995	13579	1	-0.463
AKR1D1	6	0.5321	0.69139	0.9995	13580	2	-0.233
ARG2	6	0.53217	0.69144	0.9995	13581	2	0.0167
NPTXR	6	0.53227	0.69152	0.9995	13582	2	0.0071
IMPACT	6	0.53232	0.69155	0.9995	13583	2	-0.238
KLK11	6	0.53243	0.69163	0.9995	13584	2	-0.229
PHF16	6	0.53254	0.6917	0.9995	13585	2	0.0867
ZNF595	6	0.53257	0.69172	0.9995	13586	2	0.0658
PRSS57	6	0.53281	0.69189	0.9995	13587	2	-0.034
INADL	6	0.53286	0.69193	0.9995	13588	2	-0.079
hsa-mir-7975	4	0.53295	0.63211	0.9995	13589	1	-0.247
hsa-mir-4713	4	0.53311	0.63218	0.9995	13590	1	-0.27
USF1	6	0.53312	0.6921	0.9995	13591	2	-0.005
DHRS2	6	0.53312	0.6921	0.9995	13592	2	-0.319
WFDC12	6	0.53323	0.69217	0.9995	13593	1	-0.338
UMODL1	6	0.53356	0.69241	0.9995	13594	2	-0.364
MS4A4A	6	0.53362	0.69245	0.9995	13595	2	0.0487

SLC25A34	6	0.53362	0.69245	0.9995	13596	2	0.0521
MTERFD3	6	0.53376	0.69254	0.9995	13597	2	0.0333
hsa-mir-4706	4	0.5338	0.6325	0.9995	13598	1	0.1754
ZNF430	4	0.53394	0.63256	0.9995	13599	1	0.2418
NCEH1	6	0.53404	0.69272	0.9995	13600	2	-0.005
ZNF645	6	0.53418	0.69282	0.9995	13601	1	-0.511
HCAR2	6	0.53422	0.69283	0.9995	13602	2	0.1028
ROR2	6	0.53422	0.69283	0.9995	13603	2	-0.096
hsa-mir-4795	2	0.53425	0.53582	0.9995	13604	1	0.2771
TMEM155	6	0.53425	0.69286	0.9995	13605	1	-0.73
PAQR3	6	0.53434	0.69291	0.9995	13606	2	-0.145
TNFAIP8L1	6	0.53439	0.69294	0.9995	13607	2	-0.11
MAP3K3	6	0.53444	0.69298	0.9995	13608	2	-0.408
C19orf33	6	0.53444	0.69298	0.9995	13609	2	-0.32
ZNF514	6	0.53446	0.693	0.9995	13610	1	-0.412
NME8	6	0.5345	0.69304	0.9995	13611	2	0.218
CTGF	5	0.53454	0.67828	0.9995	13612	1	-0.203
TRIM26	6	0.53465	0.69314	0.9995	13613	1	-0.749
MMD	6	0.53465	0.69314	0.9995	13614	1	-0.07
RAB39A	6	0.53485	0.69326	0.9995	13615	2	0.0058
hsa-mir-614	4	0.53486	0.633	0.9995	13616	1	0.0875
OR5AS1	6	0.53491	0.6933	0.9995	13617	2	-0.221
PTHLH	6	0.53511	0.69344	0.9995	13618	2	-0.173
LTK	6	0.53515	0.69348	0.9995	13619	1	-0.366
JARID2	6	0.53531	0.69359	0.9995	13620	1	-0.279
GSTA5	5	0.53532	0.67895	0.9995	13621	2	0.1234
MYBPH	6	0.53535	0.69362	0.9995	13622	1	-0.39
SNX1	6	0.53547	0.69369	0.9995	13623	2	0.0603
hsa-mir-3935	4	0.5355	0.6333	0.9995	13624	1	0.0986
MEAF6	6	0.53554	0.69373	0.9995	13625	2	0.0382
hsa-mir-1244-1	1	0.53561	0.53548	0.9995	13626	0	-0.079
ADCY8	6	0.53565	0.69381	0.9995	13627	2	-0.457
MFSD4	6	0.53575	0.69388	0.9995	13628	2	0.0637
OXA1L	6	0.53595	0.69401	0.9995	13629	2	-0.076
HLA-DQA1	6	0.536	0.69405	0.9995	13630	1	-0.346
PPAP2B	6	0.53602	0.69406	0.9995	13631	2	-0.129
UNC13C	6	0.53629	0.69426	0.9995	13632	1	-0.026
RHCG	6	0.53631	0.69428	0.9995	13633	2	-0.183
CSRP2BP	6	0.53655	0.69445	0.9995	13634	1	-0.205
PRKCG	6	0.53674	0.69459	0.9995	13635	2	-0.282
TAL1	6	0.53682	0.69463	0.9995	13636	2	0.1871
CYP3A5	4	0.5369	0.634	0.9995	13637	1	-0.942
HPCA	6	0.53693	0.69473	0.9995	13638	1	-0.218
HMGCS1	6	0.53697	0.69474	0.9995	13639	2	0.1186
LOC100289187	4	0.53697	0.63403	0.9995	13640	1	-0.257
NCAPD3	6	0.53699	0.69477	0.9995	13641	1	0.0644
PLEC	6	0.53704	0.69481	0.9995	13642	2	-0.156
TNFRSF13C	6	0.53704	0.69481	0.9995	13643	2	-0.039
OR6M1	6	0.53709	0.69483	0.9995	13644	2	0.09
KCND2	6	0.53712	0.69486	0.9995	13645	2	-0.026
CHCHD10	6	0.53714	0.69487	0.9995	13646	1	-0.124
CDK4	6	0.53743	0.69508	0.9995	13647	2	0.0213
PINLYP	6	0.53744	0.6951	0.9995	13648	2	0.0344
RABL2B	4	0.53746	0.63425	0.9995	13649	1	-0.446
TCTE1	6	0.53755	0.69517	0.9995	13650	1	-0.234
ADH5	6	0.53761	0.6952	0.9995	13651	1	-0.254
RASGRP3	6	0.53773	0.6953	0.9995	13652	2	0.0365
MZT2A	2	0.53779	0.53911	0.9995	13653	1	0.2231
EPHX1	6	0.53789	0.69541	0.9995	13654	2	0.1154
NINL	6	0.53817	0.69561	0.9995	13655	2	-0.43
MPP4	6	0.53841	0.69578	0.9995	13656	2	-0.393
hsa-mir-4522	4	0.53841	0.63471	0.9995	13657	1	-0.27
MMAB	6	0.53845	0.69581	0.9995	13658	2	-0.453
hsa-mir-409	4	0.53852	0.63477	0.9995	13659	1	-0.132
CDCA2	6	0.53858	0.6959	0.9995	13660	2	-0.093
HRH1	6	0.53862	0.69593	0.9995	13661	2	-0.212
ETV7	6	0.53884	0.69609	0.9995	13662	2	-0.266
ACAT1	6	0.53885	0.69609	0.9995	13663	2	0.17
CCDC153	6	0.53889	0.69613	0.9995	13664	2	-0.594
TMEM198	6	0.53889	0.69613	0.9995	13665	2	-0.003
PIEZO1	6	0.53893	0.69615	0.9995	13666	2	-0.6
KCNQ5	6	0.53896	0.69618	0.9995	13667	2	0.0871
FLAD1	6	0.53907	0.69625	0.9995	13668	2	-0.34
CABP2	6	0.53912	0.69628	0.9995	13669	2	-0.536
FBXO11	6	0.53917	0.69632	0.9995	13670	2	-0.219
ARHGEF15	6	0.53923	0.69636	0.9995	13671	2	-0.114
DNAJB11	6	0.5393	0.69641	0.9995	13672	2	-0.226
S100G	4	0.53938	0.63519	0.9995	13673	1	-0.091
OR5A1	6	0.5394	0.69649	0.9995	13674	2	0.1068
AHNAK	6	0.53944	0.69651	0.9995	13675	1	-0.035
FAM227A	6	0.53952	0.69658	0.9995	13676	1	-0.055
ISY1-RAB43	1	0.53954	0.53941	0.9995	13677	0	-0.139
ARHGEF7	6	0.53962	0.69664	0.9995	13678	2	0.0562
hsa-mir-6134	4	0.53964	0.63531	0.9995	13679	1	-0.547
SPINK6	6	0.53965	0.69666	0.9995	13680	2	0.0052

PRADC1	6	0.53974	0.69672	0.9995	13681	2	-0.043
IL17F	6	0.5398	0.69676	0.9995	13682	2	0.0029
EIF3E	6	0.53986	0.6968	0.9995	13683	2	-0.084
hsa-mir-609	3	0.54006	0.57376	0.9995	13684	1	-0.353
TMEM256	6	0.54011	0.69698	0.9995	13685	2	0.1665
LRRN4	6	0.54016	0.69701	0.9995	13686	1	-0.31
ABRACL	6	0.54036	0.69716	0.9995	13687	2	0.0205
SOHLH2	6	0.54042	0.69721	0.9995	13688	1	-0.321
ZNF445	6	0.54044	0.69723	0.9995	13689	2	0.0169
C4orf22	6	0.54054	0.6973	0.9995	13690	2	-0.069
KLHDC9	6	0.5406	0.69735	0.9995	13691	2	0.1405
TTLL10	6	0.54077	0.69747	0.9995	13692	2	-0.193
DACT2	6	0.5409	0.69756	0.9995	13693	1	-0.05
NCAPH2	6	0.54095	0.69759	0.9995	13694	2	-0.248
ABCB4	6	0.54096	0.6976	0.9995	13695	2	0.0712
ZNF878	6	0.541	0.69763	0.9995	13696	2	0.1316
SLC1A7	6	0.54121	0.69779	0.9995	13697	1	-0.081
PPP1R14B	6	0.54136	0.69789	0.9995	13698	1	-0.28
CAMK2N1	6	0.54136	0.69789	0.9995	13699	1	0.0547
C20orf141	6	0.54149	0.698	0.9995	13700	2	-0.247
TRIM42	6	0.54151	0.69801	0.9995	13701	2	-0.188
ZNF223	6	0.54158	0.69806	0.9995	13702	2	-0.442
CD37	6	0.54158	0.69806	0.9995	13703	2	0.0464
ALDH18A1	6	0.54181	0.69821	0.9995	13704	2	-0.016
WDR7	4	0.54182	0.63635	0.9995	13705	1	-0.133
CD2BP2	6	0.54182	0.69822	0.9995	13706	1	0.0339
KIT	6	0.5419	0.69827	0.9995	13707	2	-0.109
RSPO4	6	0.5419	0.69827	0.9995	13708	2	-0.564
OR4K2	6	0.54199	0.69833	0.9995	13709	2	-0.084
PLD4	6	0.54213	0.69844	0.9995	13710	2	-0.144
ITLN1	6	0.54215	0.69846	0.9995	13711	2	-0.021
HIST1H2AB	6	0.54217	0.69846	0.9995	13712	2	-0.891
CXCR2	6	0.54226	0.69853	0.9995	13713	1	-0.473
TWF2	6	0.54237	0.6986	0.9995	13714	1	-0.258
DHRS12	6	0.54258	0.69876	0.9995	13715	2	-0.085
BCAS1	6	0.5426	0.69877	0.9995	13716	1	-0.309
ZNF830	6	0.54267	0.69882	0.9995	13717	2	-0.197
MTG1	6	0.54267	0.69882	0.9995	13718	2	-0.127
CCDC77	6	0.54276	0.69888	0.9995	13719	2	-0.092
SERPINB1	6	0.543	0.69906	0.9995	13720	2	0.0538
GPCPD1	6	0.54304	0.69909	0.9995	13721	2	-0.052
OR2T2	6	0.5431	0.69913	0.9995	13722	1	-1.514
BARHL1	6	0.5431	0.69913	0.9995	13723	1	-0.115
P2RY2	6	0.5431	0.69913	0.9995	13724	2	-0.007
FAM90A1	6	0.54318	0.69919	0.9995	13725	2	-0.033
ZNF688	6	0.54325	0.69923	0.9995	13726	2	-0.276
ADC	6	0.54327	0.69925	0.9995	13727	2	-0.098
RFTN2	6	0.54341	0.69933	0.9995	13728	2	-0.078
CRYAA	6	0.54341	0.69933	0.9995	13729	1	-0.079
SIGLEC15	6	0.54348	0.69939	0.9995	13730	2	0.1368
SRP68	6	0.54348	0.69939	0.9995	13731	1	-0.427
TMEM254	6	0.54362	0.69949	0.9995	13732	2	-0.03
COL6A1	6	0.54365	0.69951	0.9995	13733	2	-0.473
BST2	6	0.54369	0.69954	0.9995	13734	2	-0.067
CYP2C8	6	0.54371	0.69956	0.9995	13735	2	0.208
NDST1	6	0.54373	0.69957	0.9995	13736	2	-0.111
TMEM259	6	0.5438	0.69963	0.9995	13737	1	-0.375
KNTC1	6	0.54388	0.69969	0.9995	13738	2	-0.116
TSR2	6	0.54394	0.69974	0.9995	13739	2	0.1501
GRPR	6	0.54401	0.69977	0.9995	13740	1	-0.009
SLC40A1	6	0.54405	0.6998	0.9995	13741	2	-0.209
GATA4	6	0.54413	0.69986	0.9995	13742	1	0.0426
DAND5	6	0.54413	0.69986	0.9995	13743	1	-0.361
FOXM1	6	0.54416	0.69988	0.9995	13744	2	-0.206
KCNK13	6	0.54437	0.70002	0.9995	13745	2	-0.037
TCOF1	6	0.54437	0.70002	0.9995	13746	2	0.1234
RARB	6	0.54443	0.70006	0.9995	13747	2	0.0962
ACAD9	6	0.5445	0.7001	0.9995	13748	2	-0.004
HCK	6	0.5446	0.70019	0.9995	13749	1	-0.214
hsa-mir-3147	4	0.54462	0.63766	0.9995	13750	1	0.0217
GOLGA6L10	2	0.54462	0.54563	0.9995	13751	1	0.2113
WWP2	6	0.54462	0.70019	0.9995	13752	2	0.1547
TTBK1	6	0.54465	0.70022	0.9995	13753	2	-0.297
TVP23B	4	0.54468	0.63769	0.9995	13754	1	-0.236
CTIF	6	0.5447	0.70025	0.9995	13755	2	-0.076
KMT2B	3	0.54489	0.57713	0.9995	13756	1	-0.192
MOV10	6	0.54493	0.70041	0.9995	13757	2	-0.129
CDRT15L2	6	0.54501	0.70046	0.9995	13758	1	-0.205
SCG5	6	0.54502	0.70047	0.9995	13759	2	0.0259
C16orf70	6	0.54509	0.70052	0.9995	13760	2	-0.681
GPD1L	6	0.54522	0.70063	0.9995	13761	2	-0.119
OVOL3	6	0.54524	0.70064	0.9995	13762	2	0.1431
HABP4	6	0.54524	0.70064	0.9995	13763	2	-0.265
IGSF22	6	0.5453	0.70069	0.9995	13764	2	-0.165
hsa-mir-4645	4	0.54542	0.63805	0.9995	13765	1	-1.595

LIX1L	6	0.54543	0.70077	0.9995	13766	2	0.0995
IL36RN	6	0.54543	0.70077	0.9995	13767	2	-0.217
NAP1L2	6	0.54547	0.7008	0.9995	13768	1	-0.071
ARSJ	6	0.54553	0.70085	0.9995	13769	1	-0.197
RBM46	6	0.54557	0.70088	0.9995	13770	2	0.0466
KRT74	6	0.54559	0.70089	0.9995	13771	2	-0.334
ATXN2	6	0.5457	0.70096	0.9995	13772	1	-0.454
BTBD18	6	0.5457	0.70096	0.9995	13773	2	-0.192
UBA3	6	0.54576	0.70102	0.9995	13774	1	-0.481
hsa-mir-181a-1	4	0.54583	0.63826	0.9995	13775	1	-0.031
BIN3	6	0.54606	0.7012	0.9995	13776	2	-0.212
C17orf89	6	0.54615	0.70127	0.9995	13777	1	0.0731
GSAP	1	0.54623	0.54619	0.9995	13778	0	-0.059
C17orf100	6	0.54626	0.70135	0.9995	13779	2	-0.288
BEND6	6	0.54634	0.70141	0.9995	13780	1	-0.406
SLC17A1	6	0.54643	0.70146	0.9995	13781	2	-0.13
USP13	6	0.54645	0.70148	0.9995	13782	1	-0.089
SLC4A5	6	0.54671	0.70167	0.9995	13783	1	0.1928
hsa-mir-1197	4	0.54672	0.63869	0.9995	13784	1	-0.004
EEPD1	6	0.54685	0.70176	0.9995	13785	2	-0.101
DMRT1	6	0.54685	0.70176	0.9995	13786	2	0.0554
TUBGCP4	6	0.54685	0.70176	0.9995	13787	2	-0.013
CDX4	6	0.5469	0.7018	0.9995	13788	1	-0.268
ZNF791	6	0.54695	0.70184	0.9995	13789	2	-0.157
SERINC3	6	0.54699	0.70186	0.9995	13790	1	-0.254
BCL2L13	6	0.54716	0.70197	0.9995	13791	1	0.0534
GALNT6	6	0.54723	0.70202	0.9995	13792	1	-0.231
SNRPE	6	0.54727	0.70205	0.9995	13793	2	-0.023
SDCBP	6	0.54729	0.70206	0.9995	13794	2	0.1796
GDF6	6	0.54734	0.7021	0.9995	13795	2	0.1044
GJA8	6	0.54738	0.70214	0.9995	13796	1	-0.222
ZNF451	6	0.54739	0.70214	0.9995	13797	2	-0.257
UBE2O	6	0.54739	0.70214	0.9995	13798	2	-0.424
ANKRD30B	6	0.54742	0.70217	0.9995	13799	1	-0.274
MGAT5B	6	0.54747	0.7022	0.9995	13800	1	-0.137
CA9	6	0.5475	0.70222	0.9995	13801	2	-0.018
PPFIBP2	6	0.5475	0.70222	0.9995	13802	2	-0.044
SHQ1	6	0.54753	0.70224	0.9995	13803	1	-0.111
USH1C	6	0.5476	0.70228	0.9995	13804	1	-0.227
GSDMB	6	0.5476	0.70229	0.9995	13805	2	0.1307
SLC25A51	6	0.54768	0.70235	0.9995	13806	2	-0.379
CLRN1	6	0.54771	0.70236	0.9995	13807	2	-0.026
OR2B11	6	0.54773	0.70237	0.9995	13808	2	-0.026
COMMD3	2	0.54774	0.54859	0.9995	13809	1	-0.144
PKD1L1	6	0.54784	0.70245	0.9995	13810	2	-0.282
KCNIP1	6	0.54794	0.70252	0.9995	13811	1	0.0805
CD96	6	0.54797	0.70253	0.9995	13812	2	-0.182
NFYA	6	0.54807	0.7026	0.9995	13813	2	0.0695
CPSF4	6	0.5481	0.70261	0.9995	13814	2	-0.248
TM7SF2	6	0.54821	0.70269	0.9995	13815	2	-0.141
TIGD5	6	0.54825	0.70273	0.9995	13816	2	-0.262
XRCC4	6	0.54825	0.70273	0.9995	13817	2	0.0488
TIMP2	6	0.54825	0.70273	0.9995	13818	1	-0.133
C3orf43	6	0.54825	0.70273	0.9995	13819	1	-0.107
PCDHB10	6	0.54831	0.70277	0.9995	13820	2	-0.599
MRO	6	0.54836	0.70281	0.9995	13821	1	-0.249
TTL	6	0.54837	0.70281	0.9995	13822	2	0.1606
ZBTB24	6	0.54837	0.70281	0.9995	13823	2	-0.017
NKX2-8	6	0.54854	0.70293	0.9995	13824	2	0.1157
RPH3AL	6	0.54858	0.70295	0.9995	13825	1	-0.354
C1orf194	6	0.5486	0.70297	0.9995	13826	2	0.0445
SLC41A1	6	0.54866	0.70302	0.9995	13827	2	0.0669
PTGES3L-AARSD1	2	0.54866	0.54948	0.9995	13828	1	-0.377
GPR56	6	0.54867	0.70302	0.9995	13829	1	-0.094
AP3S1	6	0.54871	0.70306	0.9995	13830	1	-0.576
C16orf80	6	0.54896	0.70325	0.9995	13831	2	-0.548
RFPL2	6	0.54897	0.70326	0.9995	13832	2	-0.217
ZNF521	6	0.54902	0.7033	0.9995	13833	2	-0.485
C9orf116	6	0.54903	0.7033	0.9995	13834	2	-0.068
THEM5	6	0.54903	0.7033	0.9995	13835	2	-0.037
MAPK1IP1L	6	0.54916	0.7034	0.9995	13836	2	0.1076
DENND4B	6	0.54925	0.70345	0.9995	13837	2	0.054
MFHAS1	6	0.54929	0.70349	0.9995	13838	2	-0.067
TMEM120A	6	0.54945	0.70359	0.9995	13839	2	-0.114
NFRKB	6	0.54958	0.7037	0.9995	13840	1	-0.003
RPN1	6	0.54961	0.70372	0.9995	13841	2	0.0486
ZNF528	6	0.54961	0.70372	0.9995	13842	2	-0.388
P2RY10	6	0.54964	0.70373	0.9995	13843	1	-0.174
HMOX1	6	0.54972	0.70379	0.9995	13844	1	-0.011
NDUFA7	6	0.54977	0.70383	0.9995	13845	1	-0.384
CBX2	6	0.5498	0.70386	0.9995	13846	2	-0.283
POLA2	6	0.54986	0.7039	0.9995	13847	2	-0.035
DNAH11	6	0.55004	0.70402	0.9995	13848	2	0.1328
CYP3A7	2	0.5501	0.55087	0.9995	13849	1	-0.089
T	6	0.55014	0.70409	0.9995	13850	2	-0.264

AGTR1	6	0.55014	0.70409	0.9995	13851	2	-0.003
NTSR1	6	0.55025	0.70418	0.9995	13852	1	-0.131
TDRD6	6	0.55035	0.70425	0.9995	13853	2	-0.025
ZNF319	6	0.55035	0.70425	0.9995	13854	2	0.0271
RBM22	6	0.55035	0.70425	0.9995	13855	2	0.2161
C12orf76	6	0.55036	0.70425	0.9995	13856	1	-0.209
hsa-mir-1206	1	0.55045	0.55038	0.9995	13857	0	-0.14
LRP1B	6	0.55047	0.70433	0.9995	13858	2	-0.728
ABLIM3	6	0.55057	0.70441	0.9995	13859	1	-0.324
FAM69A	6	0.55061	0.70444	0.9995	13860	2	-0.114
SSH3	6	0.55064	0.70446	0.9995	13861	2	-0.517
KIF18B	6	0.55069	0.70449	0.9995	13862	2	0.029
EPB41L4A	6	0.55087	0.7046	0.9995	13863	2	-0.607
ANKMY2	6	0.55101	0.70472	0.9995	13864	2	-0.314
FMO4	6	0.55102	0.70472	0.9995	13865	2	-0.309
S100A14	6	0.55111	0.70478	0.9995	13866	2	-0.097
ZNF575	6	0.55123	0.70485	0.9995	13867	2	-0.434
F7	6	0.55124	0.70486	0.9995	13868	1	-0.195
TGFBI	6	0.55133	0.70493	0.9995	13869	2	-0.34
ANKFN1	4	0.55135	0.64093	0.9995	13870	1	-0.182
DGCR6L	5	0.5514	0.69316	0.9995	13871	1	-0.034
MLLT4	6	0.55144	0.705	0.9995	13872	2	0.0306
hsa-mir-718	4	0.55147	0.64098	0.9995	13873	1	-0.203
OR6B1	6	0.55148	0.70504	0.9995	13874	2	-0.194
SRPK3	6	0.55155	0.70509	0.9995	13875	2	-0.217
ARRDC4	6	0.55155	0.70509	0.9995	13876	2	0.0486
OR11G2	6	0.5516	0.70513	0.9995	13877	1	0.0593
LRRC46	6	0.55179	0.70526	0.9995	13878	2	0.0395
GNAQ	6	0.55186	0.70531	0.9995	13879	1	-0.048
QTRT1	6	0.55194	0.70536	0.9995	13880	2	0.1255
hsa-mir-647	4	0.55201	0.64125	0.9995	13881	1	-0.128
HIST1H4D	6	0.5521	0.70548	0.9995	13882	1	-0.034
XDH	6	0.55226	0.70557	0.9995	13883	1	-0.126
hsa-mir-6862-2	2	0.55232	0.55296	0.9995	13884	1	0.1618
SHOX2	6	0.55252	0.70575	0.9995	13885	1	-0.331
SSBP1	6	0.55257	0.70579	0.9995	13886	2	-0.119
C2	6	0.55283	0.70596	0.9995	13887	2	-0.164
TNFRSF8	6	0.55283	0.70596	0.9995	13888	2	-0.394
WNK3	6	0.55304	0.70612	0.9995	13889	2	-0.138
MIIP	6	0.55328	0.70629	0.9995	13890	2	0.005
TSPO2	6	0.55337	0.70636	0.9995	13891	2	0.1325
OGFOD2	4	0.55344	0.64198	0.9995	13892	1	0.0073
SLC8A3	6	0.55345	0.70641	0.9995	13893	1	0.0444
HELZ2	6	0.55358	0.7065	0.9995	13894	2	-0.03
hsa-mir-548aq	1	0.55363	0.55357	0.9995	13895	0	-0.419
OR2A7	1	0.55363	0.55357	0.9995	13896	0	-0.419
hsa-mir-548aj-1	1	0.55363	0.55357	0.9995	13897	0	-0.419
TRIM49D1	1	0.55363	0.55357	0.9995	13898	0	-0.419
hsa-mir-3198-1	1	0.55363	0.55357	0.9995	13899	0	-0.419
hsa-mir-3669	1	0.55363	0.55357	0.9995	13900	0	-0.419
MAGEF1	6	0.5537	0.7066	0.9995	13901	2	-0.131
MOB4	2	0.55397	0.55457	0.9995	13902	1	0.1753
TMEM237	6	0.55399	0.70679	0.9995	13903	1	0.0698
ZNF248	6	0.55404	0.70682	0.9995	13904	1	-0.52
ZC2HC1C	6	0.55408	0.70685	0.9995	13905	2	-0.053
ZNF713	6	0.55414	0.70689	0.9995	13906	2	-0.086
hsa-mir-6784	4	0.55414	0.6423	0.9995	13907	1	-0.17
LY6G6F	6	0.55424	0.70697	0.9995	13908	2	0.0372
GLRA1	6	0.55437	0.70706	0.9995	13909	1	-0.074
TUBB4A	6	0.55437	0.70706	0.9995	13910	1	-0.123
BRF2	6	0.55457	0.7072	0.9995	13911	2	0.089
MKRN3	6	0.55457	0.7072	0.9995	13912	1	-0.56
hsa-mir-4716	4	0.55465	0.64256	0.9995	13913	1	-0.691
RSPH10B2	6	0.55472	0.7073	0.9995	13914	1	-0.656
RADIL	6	0.55478	0.70734	0.9995	13915	2	0.019
SLIT1	6	0.55488	0.7074	0.9995	13916	2	0.1117
HSH2D	6	0.55494	0.70745	0.9995	13917	2	-0.174
KLRC4-KLRK1	2	0.55507	0.55564	0.9995	13918	1	0.2097
RDH14	6	0.55508	0.70755	0.9995	13919	2	0.2005
RAB37	6	0.55508	0.70755	0.9995	13920	2	-0.143
NDUFS3	6	0.55521	0.70764	0.9995	13921	2	-0.134
UVSSA	6	0.55533	0.70774	0.9995	13922	2	0.0586
PODNL1	6	0.55544	0.70781	0.9995	13923	2	0.0013
ANAPC4	6	0.55561	0.70794	0.9995	13924	1	0.0732
CCDC178	6	0.55632	0.70844	0.9995	13925	2	0.1747
DNAJC13	6	0.55634	0.70845	0.9995	13926	2	0.0423
SMR3B	4	0.55638	0.6434	0.9995	13927	1	-0.256
LPPR2	6	0.55643	0.70851	0.9995	13928	1	-0.209
PRSS1	6	0.55644	0.70852	0.9995	13929	2	-0.213
hsa-mir-5684	4	0.55645	0.64344	0.9995	13930	1	-0.668
GSTP1	6	0.55649	0.70855	0.9995	13931	2	-0.008
IKZF4	6	0.55659	0.70862	0.9995	13932	1	-0.11
SLC16A9	6	0.55659	0.70863	0.9995	13933	2	-0.016
RXFP4	6	0.55664	0.70866	0.9995	13934	1	-0.198
hsa-mir-26a-1	4	0.55671	0.64355	0.9995	13935	1	-0.019

NEUROD6	6	0.55672	0.70873	0.9995	13936	2	-0.193
SLC16A5	6	0.55682	0.70879	0.9995	13937	2	-0.295
C7orf69	6	0.55692	0.70887	0.9995	13938	1	0.1443
CLEC1A	6	0.55718	0.70906	0.9995	13939	2	-0.11
GNPTAB	6	0.5573	0.70914	0.9995	13940	2	0.0795
CTSW	6	0.55731	0.70915	0.9995	13941	2	0.2032
COG8	6	0.55731	0.70915	0.9995	13942	1	-0.115
PICK1	6	0.55733	0.70916	0.9995	13943	2	-0.213
MST1L	6	0.55741	0.70922	0.9995	13944	2	-0.288
RND3	6	0.55745	0.70924	0.9995	13945	2	-0.042
hsa-mir-4661	3	0.55751	0.586	0.9995	13946	1	-0.41
SBSN	6	0.55764	0.70938	0.9995	13947	1	-0.045
C4BPB	6	0.55775	0.70946	0.9995	13948	1	-0.303
DGCR14	4	0.5578	0.64412	0.9995	13949	1	-0.208
hsa-mir-4467	4	0.5578	0.64412	0.9995	13950	1	-0.357
EFNA2	6	0.55782	0.70951	0.9995	13951	2	-0.332
TTLL11	6	0.55796	0.7096	0.9995	13952	2	-0.04
INTS5	6	0.55799	0.70963	0.9995	13953	2	0.1105
FARP2	6	0.558	0.70963	0.9995	13954	2	0.0312
XAGE3	6	0.55808	0.70968	0.9995	13955	2	-0.108
CES3	6	0.55808	0.70968	0.9995	13956	2	-0.012
CATSPER4	6	0.55814	0.70973	0.9995	13957	2	-0.411
NPTX1	6	0.55816	0.70974	0.9995	13958	2	-0.482
MYCT1	6	0.55828	0.70982	0.9995	13959	2	0.04
CYGB	6	0.55834	0.70986	0.9995	13960	2	0.0603
CDHR2	6	0.55842	0.70992	0.9995	13961	2	0.1243
EPHA10	6	0.55854	0.71	0.9995	13962	1	-0.071
SOAT2	6	0.55855	0.71001	0.9995	13963	2	0.1799
NLRX1	6	0.55861	0.71005	0.9995	13964	1	-0.514
AKAP13	6	0.55861	0.71005	0.9995	13965	1	-0.395
TICAM2	4	0.55861	0.64452	0.9995	13966	1	-0.61
PITPNM2	6	0.55876	0.71015	0.9995	13967	2	-0.079
TEX22	6	0.55885	0.7102	0.9995	13968	2	-0.093
FAM168B	6	0.55885	0.7102	0.9995	13969	2	0.1242
ARHGEF18	6	0.55886	0.71021	0.9995	13970	1	-0.697
PIP5KL1	6	0.55891	0.71024	0.9995	13971	2	-0.452
MOSPD2	6	0.55897	0.71029	0.9995	13972	2	-0.375
HIST1H1D	6	0.55926	0.71049	0.9995	13973	2	-0.281
TMEM101	6	0.55926	0.71049	0.9995	13974	1	-0.158
MAPK14	6	0.55934	0.71055	0.9995	13975	2	0.2252
NEK3	6	0.55934	0.71055	0.9995	13976	2	-0.059
TAAR9	6	0.55939	0.71059	0.9995	13977	2	-0.013
hsa-mir-450a-2	2	0.55941	0.55987	0.9995	13978	1	-0.191
ATXN2L	6	0.55945	0.71063	0.9995	13979	2	0.1987
RNF208	6	0.55963	0.71076	0.9995	13980	1	-0.632
GATSL1	2	0.55967	0.56014	0.9995	13981	1	0.1741
ERGIC2	6	0.55969	0.7108	0.9995	13982	2	0.0016
BMP6	6	0.55971	0.71081	0.9995	13983	1	-0.319
JMJD4	6	0.55983	0.71089	0.9995	13984	2	-0.419
MRPL30	6	0.55985	0.71091	0.9995	13985	2	-0.153
ZNF34	6	0.55985	0.71091	0.9995	13986	2	0.1581
LRRC16A	6	0.55996	0.71098	0.9995	13987	2	-0.669
TEX261	6	0.56005	0.71106	0.9995	13988	2	-0.135
PPCDC	6	0.56005	0.71106	0.9995	13989	2	-0.371
CAGE1	6	0.56005	0.71106	0.9995	13990	2	-0.035
TAF1D	6	0.56014	0.71112	0.9995	13991	2	0.0184
C1orf116	6	0.56031	0.71123	0.9995	13992	2	-0.024
STRN	6	0.56051	0.71137	0.9995	13993	2	-0.342
NOL8	6	0.56061	0.71145	0.9995	13994	2	0.2069
AHSP	6	0.56061	0.71145	0.9995	13995	2	-0.058
TOB2	6	0.56069	0.71149	0.9995	13996	2	-0.087
DAGLB	6	0.56081	0.71159	0.9995	13997	2	-0.714
RAPSN	6	0.56088	0.71165	0.9995	13998	1	-0.273
TPCN1	6	0.56093	0.71168	0.9995	13999	2	0.0802
DEFB1	6	0.56095	0.71171	0.9995	14000	2	-0.015
LOC100289561	6	0.56098	0.71173	0.9995	14001	2	-0.173
DHRS4L1	6	0.561	0.71174	0.9995	14002	2	-0.014
FAT1	6	0.56106	0.71179	0.9995	14003	2	-0.459
SUPT7L	6	0.56109	0.71181	0.9995	14004	2	0.0754
EML1	6	0.56116	0.71185	0.9995	14005	2	-0.057
hsa-mir-23c	4	0.56125	0.64583	0.9995	14006	1	-0.367
KRT17	6	0.56142	0.71204	0.9995	14007	2	-0.016
PCBD1	6	0.56149	0.71209	0.9995	14008	2	-0.389
hsa-mir-6081	4	0.56163	0.64602	0.9995	14009	1	-0.127
PPA1	6	0.56173	0.71226	0.9995	14010	2	0.1694
DNAH7	6	0.56173	0.71226	0.9995	14011	2	0.0001
CAPN7	6	0.56184	0.71234	0.9995	14012	1	-0.488
GPN3	6	0.5619	0.71239	0.9995	14013	2	0.1252
HHLA2	6	0.56203	0.7125	0.9995	14014	1	-0.647
UNKL	6	0.56204	0.71251	0.9995	14015	2	-0.115
EMR3	6	0.5621	0.71254	0.9995	14016	1	-0.351
BTG1	6	0.56211	0.71255	0.9995	14017	2	0.1319
UBE2K	6	0.56215	0.71258	0.9995	14018	2	-0.266
ZNF398	6	0.56222	0.71263	0.9995	14019	2	-0.236
hsa-mir-33a	4	0.56223	0.64632	0.9995	14020	1	-0.251

RNGTT	6	0.56236	0.71275	0.9995	14021	2	-0.263
CYLC1	5	0.56243	0.70294	0.9995	14022	2	-0.094
MRPL10	6	0.56245	0.71281	0.9995	14023	2	-0.117
hsa-mir-4651	4	0.56246	0.64642	0.9995	14024	1	-0.458
L3MBTL1	6	0.56257	0.71289	0.9995	14025	2	-0.166
PBX4	6	0.5627	0.71298	0.9995	14026	2	-0.081
CYP8B1	4	0.56272	0.64653	0.9995	14027	1	-0.565
SLC38A1	6	0.56275	0.71303	0.9995	14028	2	0.0359
ULK4	6	0.56275	0.71303	0.9995	14029	2	-0.2
hsa-mir-514b	4	0.56288	0.64662	0.9995	14030	1	-0.643
IL36B	6	0.56299	0.7132	0.9995	14031	2	-0.129
IQCF6	6	0.56303	0.71323	0.9995	14032	1	-0.256
TNFAIP6	6	0.56314	0.71332	0.9995	14033	2	0.0114
DKKL1	6	0.56314	0.71332	0.9995	14034	2	0.1951
BUD13	6	0.56318	0.71335	0.9995	14035	1	-0.214
PALM2-AKAP2	1	0.5632	0.56314	0.9995	14036	0	-0.235
ATF5	6	0.56333	0.71345	0.9995	14037	2	-0.002
MKLN1	6	0.56342	0.71352	0.9995	14038	1	-0.282
HSD11B2	6	0.56348	0.71356	0.9995	14039	2	-0.026
KIAA0368	6	0.56352	0.71359	0.9995	14040	2	-0.229
KCNK10	6	0.56352	0.71359	0.9995	14041	1	-0.065
CABLES1	4	0.56361	0.64701	0.9995	14042	1	0.0088
SLC46A2	6	0.56377	0.71377	0.9995	14043	2	-0.167
CELSR1	6	0.56384	0.71382	0.9995	14044	1	-0.092
SERPINA10	6	0.56388	0.71385	0.9995	14045	2	-0.036
RNF17	6	0.56388	0.71385	0.9995	14046	2	0.0642
PELI3	6	0.56403	0.71396	0.9995	14047	1	-0.032
UBE2C	6	0.56404	0.71396	0.9995	14048	2	-0.002
TRIM67	6	0.56412	0.71401	0.9995	14049	2	-0.278
GPS1	6	0.56412	0.71401	0.9995	14050	2	-0.221
OR4X1	6	0.56412	0.71402	0.9995	14051	2	0.0269
RMDN1	6	0.56419	0.71406	0.9995	14052	1	0.0251
HSPBP1	6	0.56423	0.71409	0.9995	14053	2	0.1542
ATP5G1	6	0.56425	0.7141	0.9995	14054	2	0.1513
OR11H4	6	0.56446	0.71425	0.9995	14055	2	-0.152
IQUB	6	0.56446	0.71425	0.9995	14056	2	-0.194
B3GNT9	6	0.56453	0.71429	0.9995	14057	1	-0.775
ZNF823	6	0.56457	0.71432	0.9995	14058	2	-0.079
CADM1	6	0.56461	0.71435	0.9995	14059	2	0.0884
MTRR	6	0.56462	0.71435	0.9995	14060	2	-0.031
BSND	6	0.5647	0.71442	0.9995	14061	2	-0.427
ANP32B	6	0.5647	0.71442	0.9995	14062	2	-0.04
TRIM11	6	0.56487	0.71455	0.9995	14063	2	-0.024
MXRA8	6	0.56494	0.71459	0.9995	14064	1	-0.237
UNC93B1	6	0.56501	0.71464	0.9995	14065	2	-0.368
ZNF438	6	0.56501	0.71464	0.9995	14066	2	0.0714
NUP214	6	0.56507	0.71468	0.9995	14067	2	-0.132
OBFC1	6	0.56507	0.71468	0.9995	14068	2	0.0854
ACTR6	6	0.56511	0.71471	0.9995	14069	1	-0.274
CLHC1	6	0.56525	0.71482	0.9995	14070	2	0.1119
GALNT16	6	0.5653	0.71485	0.9995	14071	2	-0.07
ZNF180	6	0.56536	0.71489	0.9995	14072	2	-0.012
CTTN	6	0.5654	0.71493	0.9995	14073	1	-0.216
hsa-mir-7114	4	0.56544	0.6479	0.9995	14074	1	-0.623
AGFG1	6	0.56559	0.71507	0.9995	14075	2	0.1299
TMEM239	6	0.56559	0.71507	0.9995	14076	2	0.0376
MAGEE1	6	0.5657	0.71514	0.9995	14077	1	-0.03
FUT5	6	0.56579	0.71521	0.9995	14078	1	-0.187
ELAVL3	6	0.56581	0.71523	0.9995	14079	2	-0.324
M1AP	6	0.56582	0.71523	0.9995	14080	1	-0.151
SDK2	6	0.56608	0.71541	0.9995	14081	2	-0.239
APOBEC3G	6	0.56614	0.71547	0.9995	14082	2	0.0552
HSBP1L1	6	0.56622	0.71553	0.9995	14083	2	-0.073
RSPH6A	6	0.56626	0.71556	0.9995	14084	1	-0.23
SNRPF	6	0.56628	0.71558	0.9995	14085	2	-0.085
CDC23	6	0.56628	0.71558	0.9995	14086	1	-0.096
CSNK1G1	6	0.56632	0.7156	0.9995	14087	1	-0.516
VPS13B	6	0.56641	0.71567	0.9995	14088	2	0.1452
IFT172	6	0.56653	0.71575	0.9995	14089	2	-0.225
IGSF21	6	0.56673	0.71591	0.9995	14090	1	-0.184
DIO3	6	0.56678	0.71595	0.9995	14091	1	-0.129
EOMES	6	0.5668	0.71597	0.9995	14092	2	0.154
ZNF433	6	0.5668	0.71597	0.9995	14093	2	-0.163
EFHD2	6	0.5668	0.71597	0.9995	14094	2	-0.416
CDSN	6	0.56684	0.716	0.9995	14095	1	0.1025
BTBD10	6	0.56686	0.71602	0.9995	14096	2	0.1466
GPR135	6	0.56702	0.71612	0.9995	14097	2	0.099
FAM160B2	6	0.56715	0.71623	0.9995	14098	1	-0.41
CNTN4	6	0.56722	0.71627	0.9995	14099	1	-0.297
GC	6	0.56724	0.71628	0.9995	14100	1	-0.208
CD93	6	0.56736	0.71637	0.9995	14101	2	-0.073
SYT12	6	0.56737	0.71637	0.9995	14102	2	-0.447
KDM5C	6	0.56737	0.71637	0.9995	14103	2	-0.354
OR5T1	6	0.56745	0.71643	0.9995	14104	1	-0.381
ASCC1	6	0.56754	0.7165	0.9995	14105	2	-0.03

hsa-mir-4754	4	0.56761	0.64897	0.9995	14106	1	-1.743
DUSP9	6	0.56763	0.71656	0.9995	14107	1	-0.31
TRIM71	6	0.56769	0.7166	0.9995	14108	2	-0.133
SCYL3	6	0.56769	0.7166	0.9995	14109	2	0.0633
RAB6A	6	0.56769	0.7166	0.9995	14110	2	-0.183
hsa-mir-5695	4	0.5678	0.64906	0.9995	14111	1	-0.989
RHOBTB2	6	0.56783	0.71671	0.9995	14112	2	-0.065
PCBP3	6	0.56812	0.71694	0.9995	14113	2	-0.258
RGS14	6	0.56829	0.71706	0.9995	14114	1	-0.047
SP110	6	0.56833	0.71709	0.9995	14115	2	-0.508
EMP1	6	0.56833	0.71709	0.9995	14116	2	-0.079
SPATA31A5	1	0.56839	0.56828	0.9995	14117	0	-0.051
CEP170	6	0.56846	0.71719	0.9995	14118	1	-0.622
RXFP2	6	0.56846	0.71719	0.9995	14119	2	-0.483
UBTD1	6	0.56846	0.71719	0.9995	14120	2	-0.063
ZNF79	6	0.56856	0.71726	0.9995	14121	2	-0.014
ZFP41	6	0.56864	0.71731	0.9995	14122	2	0.0576
TMEM66	6	0.56864	0.71731	0.9995	14123	2	-0.03
SHMT1	6	0.56872	0.71737	0.9995	14124	1	-0.147
ANXA2R	6	0.56872	0.71737	0.9995	14125	2	-0.037
hsa-mir-1279	2	0.56876	0.56902	0.9995	14126	1	-0.463
KIAA1279	6	0.56879	0.71742	0.9995	14127	2	-0.298
FOXF2	6	0.56886	0.71748	0.9995	14128	2	-0.404
CLNS1A	6	0.56892	0.71752	0.9995	14129	2	-0.007
FAM26F	6	0.56899	0.71758	0.9995	14130	2	-0.259
LOC643355	6	0.56904	0.71761	0.9995	14131	2	-0.16
hsa-mir-758	4	0.56914	0.64974	0.9995	14132	1	-0.326
NAT10	6	0.56924	0.71775	0.9995	14133	2	0.0826
SH2D7	6	0.56934	0.71783	0.9995	14134	2	-0.204
RPA4	6	0.56935	0.71784	0.9995	14135	1	-0.28
hsa-mir-1207	4	0.56938	0.64984	0.9995	14136	1	-0.425
NAIF1	6	0.56943	0.71789	0.9995	14137	1	-0.042
SERPINA5	6	0.56944	0.71791	0.9995	14138	2	-0.046
BDH1	6	0.56954	0.71798	0.9995	14139	2	-0.428
FAM155A	6	0.56958	0.71801	0.9995	14140	1	-0.448
SPIN3	6	0.56964	0.71805	0.9995	14141	2	-0.436
PHOX2B	6	0.56964	0.71805	0.9995	14142	2	0.2225
PTPRT	6	0.5698	0.71818	0.9995	14143	2	-0.06
NR1I2	6	0.56988	0.71823	0.9995	14144	1	-0.418
TEX36	6	0.56988	0.71823	0.9995	14145	1	-0.221
hsa-mir-8087	4	0.56996	0.65014	0.9995	14146	1	0.2335
C18orf63	6	0.57001	0.71831	0.9995	14147	1	-0.209
APOC1	6	0.57001	0.71831	0.9995	14148	2	-0.052
LOC100506688	6	0.57009	0.71836	0.9995	14149	2	0.0029
SVEP1	6	0.57015	0.71841	0.9995	14150	1	-0.173
SIX4	6	0.57028	0.7185	0.9995	14151	2	-0.153
C14orf105	6	0.57048	0.71865	0.9995	14152	2	-0.217
LTB4R2	6	0.57065	0.71877	0.9995	14153	1	-0.123
NCOA7	6	0.57076	0.71884	0.9995	14154	2	0.1861
hsa-mir-499b	3	0.57089	0.59567	0.9995	14155	1	0.0546
TEN1	6	0.5709	0.71895	0.9995	14156	2	-0.079
C1orf100	6	0.57094	0.71897	0.9995	14157	1	-0.427
GPR179	6	0.57097	0.71899	0.9995	14158	2	0.096
TPRA1	6	0.57101	0.71902	0.9995	14159	1	-0.224
GALNT8	6	0.57129	0.71923	0.9995	14160	2	-0.095
ZNF221	4	0.57132	0.65083	0.9995	14161	1	0.0335
METRNL	6	0.57142	0.71931	0.9995	14162	2	-0.221
C14orf80	6	0.57158	0.71942	0.9995	14163	2	0.0544
hsa-mir-302b	4	0.57159	0.65095	0.9995	14164	1	-0.474
C10orf114	3	0.57159	0.59618	0.9995	14165	1	0.1925
ZNF605	6	0.57174	0.71953	0.9995	14166	2	-0.276
ZNF76	6	0.5719	0.71967	0.9995	14167	2	0.0442
TMEM229A	6	0.57194	0.7197	0.9995	14168	2	-0.133
AKR1A1	6	0.57208	0.7198	0.9995	14169	2	-0.418
OR51A4	5	0.57208	0.71156	0.9995	14170	2	0.0254
WDR60	6	0.57212	0.71982	0.9995	14171	1	-0.16
ASB14	6	0.57242	0.72004	0.9995	14172	1	-0.031
ZNF330	6	0.57245	0.72007	0.9995	14173	1	-0.503
ZNF354A	6	0.57251	0.72011	0.9995	14174	2	0.0071
OR13C4	6	0.57251	0.72011	0.9995	14175	2	0.0755
TMEM212	6	0.57251	0.72011	0.9995	14176	2	-0.241
HRH3	6	0.57251	0.72011	0.9995	14177	2	-0.234
ELF3	6	0.57258	0.72016	0.9995	14178	1	-0.169
ADD3	6	0.57261	0.72018	0.9995	14179	1	-0.211
KLHL18	6	0.57272	0.72025	0.9995	14180	1	-0.062
hsa-mir-4436b-1	3	0.57274	0.59704	0.9995	14181	1	0.098
ORM2	3	0.57274	0.59704	0.9995	14182	1	0.148
hsa-mir-3929	3	0.57274	0.59704	0.9995	14183	1	-1.233
hsa-mir-526b	3	0.57274	0.59704	0.9995	14184	1	-0.177
GNG7	6	0.57282	0.72033	0.9995	14185	2	0.3248
XIRP2	6	0.57282	0.72033	0.9995	14186	2	-0.08
STX2	6	0.57287	0.72036	0.9995	14187	2	-0.663
PKD2L1	6	0.57303	0.72048	0.9995	14188	2	-0.164
hsa-mir-4294	4	0.57307	0.65172	0.9995	14189	1	-0.047
PERP	6	0.57308	0.72052	0.9995	14190	1	-0.449

FNBP1	4	0.57317	0.65177	0.9995	14191	1	-0.279
SURF4	6	0.57325	0.72065	0.9995	14192	2	-0.07
GPR87	6	0.57342	0.72076	0.9995	14193	1	-0.411
BSX	6	0.57344	0.72078	0.9995	14194	2	0.0508
hsa-mir-5195	4	0.57358	0.65195	0.9995	14195	1	-0.33
CACNA1E	6	0.57367	0.72094	0.9995	14196	2	0.0249
LRRC1	6	0.57371	0.72097	0.9995	14197	2	-0.041
ASPRV1	6	0.57371	0.72097	0.9995	14198	1	-0.285
ZHX2	6	0.57377	0.72102	0.9995	14199	1	-0.145
TMEM136	6	0.57391	0.72112	0.9995	14200	2	-0.269
GCLC	6	0.57413	0.72127	0.9995	14201	1	-0.273
POPDC2	6	0.57413	0.72127	0.9995	14202	2	-0.395
AK1	6	0.57414	0.72127	0.9995	14203	2	-0.103
KNDC1	6	0.57414	0.72127	0.9995	14204	2	-0.092
hsa-mir-3201	2	0.57419	0.57426	0.9995	14205	1	-0.057
SF3A2	6	0.57428	0.72138	0.9995	14206	2	-0.078
VCX	2	0.57429	0.57435	0.9995	14207	1	0.241
OR56A4	6	0.57434	0.72141	0.9995	14208	1	-0.17
MIXL1	6	0.57437	0.72144	0.9995	14209	2	-0.439
CRYBA2	6	0.57441	0.72147	0.9995	14210	1	-0.167
SLC22A10	6	0.57444	0.72148	0.9995	14211	2	-0.242
hsa-mir-3192	4	0.57447	0.65241	0.9995	14212	1	-0.619
ARTN	6	0.57451	0.72154	0.9995	14213	2	-0.15
NLRP1	6	0.57458	0.72159	0.9995	14214	1	-0.466
MFSD5	6	0.5746	0.7216	0.9995	14215	1	-0.362
TMED7-TICAM2	2	0.57476	0.57484	0.9995	14216	1	0.1751
AK7	6	0.57487	0.72179	0.9995	14217	1	-0.159
TMUB1	6	0.57493	0.72183	0.9995	14218	2	-0.081
B3GNT5	6	0.57493	0.72183	0.9995	14219	2	0.1765
LEPREL1	6	0.57495	0.72185	0.9995	14220	1	0.0297
CBLN4	6	0.57495	0.72185	0.9995	14221	1	-0.367
OSGEPL1	6	0.57508	0.72195	0.9995	14222	1	-0.609
PRAMEF10	4	0.57522	0.65281	0.9995	14223	1	-0.295
DSG2	6	0.57545	0.7222	0.9995	14224	1	0.0527
NGEF	6	0.57547	0.72221	0.9995	14225	1	-0.154
FAM173B	6	0.57574	0.72241	0.9995	14226	2	-0.382
ITGA3	6	0.57588	0.72251	0.9995	14227	1	-0.248
SLC16A12	6	0.576	0.7226	0.9995	14228	1	-0.036
SREBF2	6	0.57602	0.72262	0.9995	14229	2	0.1447
CXCL3	4	0.57608	0.65328	0.9995	14230	1	-1.962
EFCAB4A	6	0.57619	0.72274	0.9995	14231	1	-0.577
ACAD8	6	0.57622	0.72277	0.9995	14232	2	-0.068
CT45A5	3	0.57629	0.59966	0.9995	14233	1	-0.436
TPST2	6	0.57638	0.72287	0.9995	14234	1	-0.652
hsa-mir-219b	4	0.57657	0.65353	0.9995	14235	1	-0.146
RBM43	6	0.57657	0.72301	0.9995	14236	2	-0.123
BEST1	6	0.57661	0.72304	0.9995	14237	1	-0.069
HBS1L	6	0.57663	0.72305	0.9995	14238	2	-0.245
SLC7A8	6	0.57669	0.7231	0.9995	14239	2	-0.248
PLXNA1	6	0.57672	0.72313	0.9995	14240	1	-0.359
PSMD6	6	0.57678	0.72317	0.9995	14241	2	-0.029
ABHD12	6	0.57695	0.72328	0.9995	14242	1	-0.105
GPR125	6	0.57703	0.72334	0.9995	14243	1	-0.135
MEX3D	6	0.57714	0.72342	0.9995	14244	1	-0.51
LEFTY2	6	0.57715	0.72343	0.9995	14245	2	0.1164
PDCD6	6	0.57715	0.72343	0.9995	14246	2	-0.198
GRB10	6	0.5772	0.72346	0.9995	14247	2	-0.002
GLIS2	6	0.5772	0.72347	0.9995	14248	1	0.0309
UNC93A	6	0.57724	0.72349	0.9995	14249	2	-0.248
CERS3	6	0.57726	0.7235	0.9995	14250	2	-0.256
BCAS4	6	0.57726	0.7235	0.9995	14251	2	-0.069
PLA2G15	6	0.57738	0.72359	0.9995	14252	2	-0.1
MEST	6	0.57738	0.72359	0.9995	14253	2	-0.338
FOXD1	6	0.57742	0.72361	0.9995	14254	2	-0.318
DAPK2	6	0.57746	0.72364	0.9995	14255	1	-0.259
VSIG10L	6	0.57747	0.72365	0.9995	14256	2	-0.106
TREM2	6	0.57755	0.7237	0.9995	14257	2	0.1053
RESP18	6	0.57766	0.72378	0.9995	14258	2	-0.025
NXPE1	6	0.57768	0.72379	0.9995	14259	1	-0.06
SSUH2	6	0.57803	0.72403	0.9995	14260	1	-0.425
SLC6A4	6	0.57803	0.72403	0.9995	14261	1	-0.875
ZNF785	6	0.57823	0.72418	0.9995	14262	2	-0.198
TRIM9	6	0.57831	0.72423	0.9995	14263	1	-0.562
SRSF3	6	0.57836	0.72428	0.9995	14264	2	0.2398
HAVCR2	6	0.57847	0.72436	0.9995	14265	2	-0.241
CKMT1A	4	0.57869	0.65462	0.9995	14266	1	0.0642
GPR52	6	0.57885	0.72465	0.9995	14267	2	-0.185
CXCR7	3	0.57888	0.60154	0.9995	14268	1	-0.402
KLF8	6	0.57891	0.72469	0.9995	14269	2	-0.294
PHKA2	6	0.57899	0.72476	0.9995	14270	1	-0.342
PIGQ	6	0.57908	0.72484	0.9995	14271	2	-0.361
ARPC4-TTLL3	1	0.57913	0.57895	0.9995	14272	0	-0.142
GABARAPL2	6	0.57921	0.72494	0.9995	14273	2	-0.165
PAPPA2	6	0.57924	0.72496	0.9995	14274	2	-0.166
ALAD	6	0.57932	0.72503	0.9995	14275	1	-0.351

ASH1L	6	0.57946	0.72513	0.9995	14276	2	0.1286
ODF3L1	6	0.57956	0.7252	0.9995	14277	1	-0.158
SPG20	6	0.57961	0.72523	0.9995	14278	2	-0.058
MUC22	6	0.57966	0.72527	0.9995	14279	2	-0.471
UBE2W	6	0.57969	0.7253	0.9995	14280	1	-0.286
LYPD3	6	0.57978	0.72537	0.9995	14281	2	0.1185
PILRA	6	0.57988	0.72543	0.9995	14282	2	-0.027
LBH	6	0.57993	0.72548	0.9995	14283	1	0.1126
GNA11	6	0.57993	0.72548	0.9995	14284	2	0.2403
RPL36A	2	0.58003	0.58007	0.9995	14285	1	0.4893
hsa-mir-1283-2	2	0.58003	0.58007	0.9995	14286	1	-0.227
CACNA1H	6	0.58017	0.72566	0.9995	14287	1	-0.328
AMER1	6	0.58023	0.72571	0.9995	14288	1	-0.417
DHX34	6	0.58032	0.72577	0.9995	14289	2	-0.499
MDC1	6	0.58035	0.72579	0.9995	14290	1	-0.203
C11orf49	6	0.58042	0.72584	0.9995	14291	2	-0.215
GPR151	6	0.58048	0.72588	0.9995	14292	2	0.1516
ZNF789	6	0.58052	0.72591	0.9995	14293	2	-0.028
UGT3A1	6	0.58068	0.72601	0.9995	14294	1	-0.248
ATG7	6	0.58079	0.7261	0.9995	14295	2	-0.396
NLN	6	0.58098	0.72623	0.9995	14296	2	-0.017
FOXD2	6	0.58098	0.72623	0.9995	14297	2	-0.073
ZNF230	6	0.58105	0.72628	0.9995	14298	2	0.097
PRKCB	6	0.58115	0.72635	0.9995	14299	2	-0.089
LXN	6	0.58116	0.72635	0.9995	14300	1	-0.483
IL10RA	6	0.58122	0.72639	0.9995	14301	2	-0.439
DBC1	2	0.5813	0.58137	0.9995	14302	1	-0.27
ALPK3	6	0.5814	0.72652	0.9995	14303	2	0.2357
GLS	6	0.58148	0.72659	0.9995	14304	2	-0.029
ROBO1	6	0.58151	0.72661	0.9995	14305	1	-0.228
CCDC120	6	0.58162	0.72669	0.9995	14306	1	-0.314
GRIA3	6	0.58166	0.72672	0.9995	14307	2	0.1009
ZBTB46	6	0.58167	0.72673	0.9995	14308	1	-0.151
C2orf27A	3	0.58172	0.60362	0.9995	14309	1	-0.126
SLC25A38	6	0.58172	0.72677	0.9995	14310	2	0.054
OSTF1	6	0.58187	0.72687	0.9995	14311	2	0.0936
NISCH	6	0.58187	0.72687	0.9995	14312	2	0.1109
COQ6	6	0.58199	0.72694	0.9995	14313	2	0.0367
ZFX	6	0.58208	0.72701	0.9995	14314	2	-0.14
DCLK3	6	0.58211	0.72704	0.9995	14315	1	-0.342
IQSEC3	6	0.58239	0.72722	0.9995	14316	1	-0.115
DUOXA1	6	0.58255	0.72733	0.9995	14317	2	-0.132
VSIG8	6	0.58255	0.72733	0.9995	14318	2	0.0123
PAQR5	6	0.58255	0.72733	0.9995	14319	2	0.0171
TRIM63	6	0.58264	0.72741	0.9995	14320	1	-0.109
C17orf102	6	0.58266	0.72741	0.9995	14321	2	0.0754
MAMLD1	6	0.58269	0.72744	0.9995	14322	2	-0.162
hsa-mir-4744	4	0.58272	0.65675	0.9995	14323	1	0.2075
ISL1	6	0.58276	0.72749	0.9995	14324	2	-0.126
GAD1	6	0.58286	0.72755	0.9995	14325	2	0.106
CCT8L2	6	0.58286	0.72755	0.9995	14326	2	-0.055
IRF3	6	0.58299	0.72765	0.9995	14327	1	-0.333
KIAA0825	6	0.58309	0.72772	0.9995	14328	2	0.1713
ZNF195	6	0.58309	0.72772	0.9995	14329	2	-0.085
CASR	6	0.58309	0.72772	0.9995	14330	2	0.0885
hsa-mir-147a	3	0.58316	0.60468	0.9995	14331	1	0.0407
CNNM4	6	0.58323	0.72783	0.9995	14332	2	0.1578
hsa-mir-633	4	0.58329	0.65704	0.9995	14333	1	-0.21
IL26	6	0.58341	0.72796	0.9995	14334	2	0.0611
PPP2R2C	6	0.58341	0.72796	0.9995	14335	2	-0.098
SUMF2	6	0.58349	0.72802	0.9995	14336	2	-0.148
C22orf23	6	0.58355	0.72806	0.9995	14337	1	-0.403
ITIH6	6	0.58372	0.72819	0.9995	14338	1	-0.532
ZSCAN29	6	0.58373	0.7282	0.9995	14339	2	-0.259
MBOAT2	6	0.58373	0.7282	0.9995	14340	2	-0.018
CHRNB1	6	0.58386	0.7283	0.9995	14341	1	-0.321
hsa-mir-5194	4	0.58389	0.65736	0.9995	14342	1	-0.295
NR0B2	6	0.5839	0.72833	0.9995	14343	2	0.0288
RAB5C	6	0.58397	0.72838	0.9995	14344	2	0.0481
ZNF202	6	0.58397	0.72838	0.9995	14345	2	-0.817
RARA	6	0.58399	0.72839	0.9995	14346	1	0.0143
TIPIN	6	0.58407	0.72846	0.9995	14347	2	-0.274
CBX7	6	0.5842	0.72856	0.9995	14348	2	-0.224
GLYATL3	6	0.58423	0.72858	0.9995	14349	2	0.0354
hsa-mir-4750	4	0.58428	0.65757	0.9995	14350	1	0.2383
HS3ST1	6	0.58441	0.72871	0.9995	14351	2	-0.003
RIMBP3C	2	0.58447	0.58452	0.9995	14352	1	0.0065
hsa-mir-4512	3	0.58472	0.60587	0.9995	14353	1	0.0782
ASPDH	6	0.58479	0.72899	0.9995	14354	1	-0.101
COG2	6	0.5848	0.729	0.9995	14355	2	-0.282
RBMY1J	2	0.58493	0.58498	0.9995	14356	0	-0.616
PDE3A	6	0.58497	0.72911	0.9995	14357	2	-0.022
GREM2	6	0.58498	0.72913	0.9995	14358	2	-0.029
ZNF25	6	0.58517	0.72927	0.9995	14359	2	-0.194
FJX1	6	0.58525	0.72931	0.9995	14360	2	-0.076

NIPA1	6	0.58525	0.72931	0.9995	14361	2	0.0871
PAPLN	6	0.58553	0.72953	0.9995	14362	1	-0.139
DEFB134	5	0.58553	0.72173	0.9995	14363	1	-0.018
PLAG1	6	0.58558	0.72957	0.9995	14364	2	-0.6
ANKH	6	0.58561	0.7296	0.9995	14365	1	-0.59
CANT1	6	0.58564	0.72961	0.9995	14366	2	-0.059
IGLON5	6	0.58565	0.72962	0.9995	14367	1	0.0411
CHST11	6	0.58568	0.72964	0.9995	14368	1	-0.455
SCN2B	6	0.58581	0.72974	0.9995	14369	2	0.1013
RPL22L1	6	0.58585	0.72978	0.9995	14370	1	-0.725
USP26	6	0.5859	0.72981	0.9995	14371	2	-0.047
HTATSF1	6	0.58599	0.72989	0.9995	14372	2	0.1155
MED27	6	0.58605	0.72994	0.9995	14373	1	-0.88
ZNF17	6	0.58614	0.73	0.9995	14374	1	0.1898
MMS19	6	0.58617	0.73003	0.9995	14375	2	-0.365
CBFA2T2	6	0.58619	0.73004	0.9995	14376	1	0.0206
SMKR1	6	0.58624	0.73008	0.9995	14377	2	-0.376
ALG13	6	0.58636	0.73015	0.9995	14378	2	-0.011
DAB2IP	6	0.58638	0.73017	0.9995	14379	1	-0.9
RBM48	6	0.58643	0.73019	0.9995	14380	1	-0.217
NDUFAF5	6	0.58645	0.73022	0.9995	14381	2	-0.355
LMAN2	6	0.58658	0.73031	0.9995	14382	1	-0.151
C1orf174	6	0.58669	0.73038	0.9995	14383	2	-0.412
GCN1L1	6	0.58672	0.7304	0.9995	14384	2	-0.996
PIK3AP1	6	0.58697	0.7306	0.9995	14385	1	-0.074
FAM162A	6	0.58706	0.73067	0.9995	14386	2	-0.068
CUL3	6	0.58718	0.73075	0.9995	14387	2	-0.137
TMEM97	6	0.58725	0.73079	0.9995	14388	2	-0.016
hsa-mir-518d	1	0.58744	0.58721	0.9995	14389	0	-0.342
RHOBTB3	6	0.58749	0.73097	0.9995	14390	2	-0.25
ATRX	6	0.58749	0.73097	0.9995	14391	2	-0.046
DNAJA1	6	0.58758	0.73103	0.9995	14392	2	-0.122
CHRFAM7A	4	0.58765	0.65932	0.9995	14393	1	0.0601
FAM214A	6	0.58768	0.7311	0.9995	14394	2	0.065
UBLCP1	6	0.58768	0.73111	0.9995	14395	2	-0.016
SLC14A2	6	0.58768	0.73111	0.9995	14396	2	-0.019
CALML6	6	0.58771	0.73113	0.9995	14397	2	-0.248
OR13F1	6	0.58775	0.73116	0.9995	14398	2	-0.281
MYEOV	6	0.58777	0.73117	0.9995	14399	1	-0.199
GTF2IRD1	6	0.58787	0.73124	0.9995	14400	2	-0.328
SUSD1	6	0.58792	0.73128	0.9995	14401	2	-0.545
EDDM3B	6	0.58798	0.73132	0.9995	14402	2	-0.119
UGP2	6	0.58802	0.73135	0.9995	14403	2	-0.377
ZBTB5	6	0.58804	0.73136	0.9995	14404	2	-0.203
LIPT1	6	0.58814	0.73144	0.9995	14405	2	0.1101
RAP2A	6	0.58817	0.73146	0.9995	14406	2	-0.148
CEP112	6	0.58822	0.73149	0.9995	14407	2	-0.07
WNT4	6	0.58824	0.73151	0.9995	14408	2	0.0561
SPINK8	6	0.5883	0.73156	0.9995	14409	2	-0.038
CYP27B1	6	0.58836	0.7316	0.9995	14410	2	0.2125
SECISBP2	6	0.58839	0.73162	0.9995	14411	2	-0.294
IFI6	6	0.58842	0.73165	0.9995	14412	2	-0.072
GPR173	6	0.58852	0.73171	0.9995	14413	2	-0.077
C2orf44	6	0.58854	0.73173	0.9995	14414	2	-0.211
LEPR	6	0.58856	0.73174	0.9995	14415	2	-0.226
PHF21B	6	0.58863	0.7318	0.9995	14416	2	-0.078
USP17L10	5	0.58866	0.72286	0.9995	14417	1	-0.408
GNAI1	6	0.5887	0.73184	0.9995	14418	2	0.1491
BAP1	6	0.58878	0.7319	0.9995	14419	1	-0.106
ZNF646	6	0.58878	0.7319	0.9995	14420	2	-0.432
CENPK	6	0.589	0.73205	0.9995	14421	2	0.1508
hsa-mir-7848	4	0.58907	0.66005	0.9995	14422	1	-0.294
YIF1B	6	0.58914	0.73216	0.9995	14423	2	-0.044
FGFRL1	6	0.58931	0.7323	0.9995	14424	1	-0.278
PPM1J	6	0.58935	0.73232	0.9995	14425	2	0.0678
MS4A5	6	0.58939	0.73235	0.9995	14426	1	-0.22
TMEM2	6	0.58947	0.73242	0.9995	14427	2	-0.088
COQ5	6	0.58947	0.73243	0.9995	14428	2	-0.057
QTRTD1	6	0.58967	0.73259	0.9995	14429	2	-0.279
ESCO1	6	0.58967	0.73259	0.9995	14430	2	0.0891
CCL5	6	0.58987	0.73272	0.9995	14431	2	-0.1
NPRL2	6	0.59001	0.73283	0.9995	14432	2	-0.19
MFAP3	6	0.59001	0.73283	0.9995	14433	2	-0.182
ILK	6	0.59001	0.73283	0.9995	14434	2	-0.11
OVOL1	6	0.59001	0.73283	0.9995	14435	2	-0.234
NSUN5	6	0.59019	0.73296	0.9995	14436	1	-0.274
EPHB4	6	0.59021	0.73298	0.9995	14437	2	-0.009
PHOSPHO2-KLHL23	2	0.59029	0.59034	0.9995	14438	0	0.0459
PTER	6	0.59033	0.73307	0.9995	14439	2	0.0066
CNTN3	6	0.59038	0.73311	0.9995	14440	2	0.0791
FAM199X	6	0.59045	0.73316	0.9995	14441	1	-0.069
hsa-mir-4656	4	0.59047	0.6608	0.9995	14442	1	-0.389
DOCK8	6	0.59055	0.73325	0.9995	14443	2	-0.788
SCIMP	6	0.59071	0.73337	0.9995	14444	2	-0.02
SAMD9L	6	0.59071	0.73337	0.9995	14445	1	-0.714

NR2F6	6	0.59077	0.7334	0.9995	14446	2	-0.291
MAP2K3	6	0.59082	0.73345	0.9995	14447	2	-0.233
KLHDC2	6	0.59083	0.73346	0.9995	14448	2	-0.227
FAM25C	1	0.5909	0.59068	0.9995	14449	0	-0.626
SLC35F2	6	0.59098	0.73358	0.9995	14450	2	-0.032
HOXA1	6	0.59105	0.73362	0.9995	14451	2	0.131
CDC73	6	0.59111	0.73367	0.9995	14452	1	-0.211
LCN10	6	0.59121	0.73375	0.9995	14453	1	-0.209
hsa-mir-3124	4	0.59124	0.66122	0.9995	14454	1	-0.764
PTPN13	6	0.59127	0.73379	0.9995	14455	2	0.0056
hsa-mir-3691	4	0.5913	0.66125	0.9995	14456	1	-0.844
GRB14	6	0.59173	0.73413	0.9995	14457	2	0.0905
PAK2	6	0.5918	0.73418	0.9995	14458	2	-0.144
ADH1B	6	0.59184	0.7342	0.9995	14459	1	-0.291
LYL1	6	0.59189	0.73424	0.9995	14460	1	-0.517
CNTFR	6	0.59194	0.73428	0.9995	14461	1	-0.087
C11orf48	6	0.59202	0.73435	0.9995	14462	1	-0.849
IL13	6	0.59218	0.73447	0.9995	14463	2	0.0081
LCN1	6	0.59221	0.73449	0.9995	14464	1	-0.065
SH3BGR	6	0.59227	0.73453	0.9995	14465	2	-0.15
RASGRP4	6	0.59229	0.73454	0.9995	14466	2	0.121
IGF2BP1	6	0.59239	0.73461	0.9995	14467	2	0.1012
ERCC5	6	0.59251	0.73472	0.9995	14468	2	-0.298
ARL6IP6	6	0.59251	0.73472	0.9995	14469	2	-0.355
STX8	6	0.59253	0.73473	0.9995	14470	1	-0.251
KLHL35	6	0.59266	0.73483	0.9995	14471	1	-0.112
GPR132	6	0.59275	0.73489	0.9995	14472	1	-0.05
GABRA3	6	0.59279	0.73492	0.9995	14473	1	0.0682
FADS1	6	0.59279	0.73492	0.9995	14474	2	0.1471
ALG8	6	0.59292	0.735	0.9995	14475	2	-0.092
PDIA3	6	0.59301	0.73506	0.9995	14476	2	0.0356
BAG5	6	0.59309	0.73512	0.9995	14477	2	-0.199
ANK1	6	0.59309	0.73512	0.9995	14478	2	-0.39
hsa-mir-885	4	0.59314	0.66223	0.9995	14479	1	-0.579
MSI1	6	0.59318	0.73519	0.9995	14480	2	0.0776
ADPRH	6	0.59318	0.73519	0.9995	14481	2	-0.06
EBPL	6	0.59319	0.7352	0.9995	14482	1	-0.708
SULT1A4	4	0.59321	0.66226	0.9995	14483	1	-1.418
RBM25	6	0.59327	0.73525	0.9995	14484	2	-0.176
HVCN1	6	0.59332	0.73529	0.9995	14485	2	-0.108
CYP2C9	4	0.59337	0.66234	0.9995	14486	1	-0.595
FGL1	6	0.59337	0.73532	0.9995	14487	1	-0.234
ACOT11	6	0.59352	0.73543	0.9995	14488	2	-0.006
TGIF1	6	0.59355	0.73545	0.9995	14489	1	-0.037
TCF7	6	0.59369	0.73555	0.9995	14490	1	-0.791
CCDC107	6	0.59372	0.73558	0.9995	14491	2	-0.1
MPO	6	0.59376	0.7356	0.9995	14492	1	-0.502
RNF7	6	0.59391	0.73571	0.9995	14493	1	-0.351
ZNF613	6	0.59409	0.73586	0.9995	14494	1	-0.673
LIMS2	6	0.59409	0.73586	0.9995	14495	1	-0.062
hsa-mir-3529	2	0.59418	0.59418	0.9995	14496	0	0.0723
PEX12	6	0.59421	0.73594	0.9995	14497	1	-0.288
TTR	6	0.59431	0.73602	0.9995	14498	1	-0.541
SMCR8	6	0.59452	0.73618	0.9995	14499	1	-0.396
TLN1	6	0.59454	0.73619	0.9995	14500	2	-0.288
C1QTNF6	6	0.59467	0.73628	0.9995	14501	2	-0.309
JDP2	6	0.59467	0.73628	0.9995	14502	2	-0.479
ZNF33B	6	0.59472	0.73632	0.9995	14503	1	-0.4
ZNF490	6	0.59472	0.73632	0.9995	14504	1	-0.733
HIST1H4H	6	0.59472	0.73632	0.9995	14505	1	-0.285
hsa-mir-4319	4	0.59478	0.66309	0.9995	14506	1	-0.184
APITD1-CORT	2	0.59482	0.59483	0.9995	14507	0	-0.004
MGMT	6	0.5949	0.73644	0.9995	14508	2	-0.32
OR10G7	6	0.59496	0.73649	0.9995	14509	1	-0.292
SIAH1	6	0.59501	0.73653	0.9995	14510	2	-0.295
ZNRF1	6	0.59516	0.73663	0.9995	14511	2	-0.186
UBR7	6	0.59521	0.73667	0.9995	14512	1	-0.264
KCNJ6	6	0.59524	0.73669	0.9995	14513	2	-0.268
hsa-mir-483	4	0.59525	0.66335	0.9995	14514	1	-0.084
OR4F6	6	0.59528	0.73672	0.9995	14515	2	-0.036
FOXB2	6	0.59533	0.73677	0.9995	14516	2	-0.178
TXNL4A	6	0.59536	0.73678	0.9995	14517	1	-0.605
hsa-mir-4760	2	0.59536	0.59538	0.9995	14518	0	-0.109
LOH12CR1	6	0.59542	0.73683	0.9995	14519	2	-0.126
CYP2C18	6	0.59547	0.73687	0.9995	14520	2	-0.238
TTN	6	0.59557	0.73694	0.9995	14521	2	-0.086
C5orf55	6	0.59563	0.73699	0.9995	14522	2	-0.347
SP8	6	0.59574	0.73707	0.9995	14523	2	0.061
FZD4	6	0.59574	0.73707	0.9995	14524	2	-0.313
HIST1H2AD	6	0.59582	0.73713	0.9995	14525	2	-0.035
ZNF655	6	0.59582	0.73713	0.9995	14526	2	-0.067
TRIML2	6	0.59591	0.7372	0.9995	14527	2	0.0195
FGFBP3	6	0.59593	0.73721	0.9995	14528	1	-0.116
IFNGR2	6	0.59602	0.73728	0.9995	14529	2	-0.077
NLRP7	6	0.59603	0.73728	0.9995	14530	1	-0.389

STOML3	6	0.5961	0.73732	0.9995	14531	1	-0.005
hsa-mir-4495	2	0.59613	0.59615	0.9995	14532	0	-0.264
TBRG1	6	0.59619	0.73739	0.9995	14533	2	-0.258
C11orf86	6	0.59619	0.73739	0.9995	14534	1	-0.172
SCOC	6	0.59621	0.7374	0.9995	14535	2	-0.067
FHOD3	6	0.59639	0.73753	0.9995	14536	2	0.0187
CNOT7	6	0.59648	0.73759	0.9995	14537	2	-0.506
NTN1	6	0.59655	0.73765	0.9995	14538	1	-0.236
SRRM5	6	0.59662	0.73769	0.9995	14539	1	-0.01
GPN2	6	0.5967	0.73775	0.9995	14540	2	0.1939
TRIM44	6	0.59671	0.73776	0.9995	14541	2	0.1971
RASAL3	6	0.59675	0.73779	0.9995	14542	2	-0.145
ROPN1	5	0.59676	0.72578	0.9995	14543	1	-1.475
THAP6	6	0.59681	0.73783	0.9995	14544	2	0.0699
METTL2B	6	0.59688	0.73789	0.9995	14545	2	-0.251
SLC45A4	6	0.5969	0.73789	0.9995	14546	2	-0.138
MAP3K14	6	0.59698	0.73795	0.9995	14547	1	0.0461
MATK	6	0.59707	0.73801	0.9995	14548	1	-0.078
GOSR1	6	0.5971	0.73803	0.9995	14549	1	-0.07
AADACL2	6	0.59723	0.73812	0.9995	14550	2	-0.341
SFRP5	6	0.59762	0.73841	0.9995	14551	2	-0.056
KDM4D	6	0.59767	0.73845	0.9995	14552	1	-0.311
PTCRA	6	0.59778	0.73853	0.9995	14553	2	-0.212
ZNF358	6	0.59787	0.7386	0.9995	14554	1	-0.322
FCRL1	6	0.59794	0.73864	0.9995	14555	2	0.0055
MARK4	6	0.59804	0.73872	0.9995	14556	1	-0.892
API5	6	0.5981	0.73877	0.9995	14557	2	0.0635
RPUSD4	6	0.59818	0.73882	0.9995	14558	2	-0.02
RSRC2	6	0.59831	0.73891	0.9995	14559	2	0.0273
hsa-let-7a-1	4	0.59833	0.665	0.9995	14560	1	-0.469
DAOA	6	0.59843	0.73899	0.9995	14561	2	0.0103
PHF17	6	0.5986	0.73911	0.9995	14562	1	-0.027
PTPRG	6	0.59871	0.73919	0.9995	14563	2	-0.296
DPF3	6	0.59873	0.7392	0.9995	14564	1	-0.094
AKAP4	6	0.59873	0.7392	0.9995	14565	1	-0.301
FOXD3	6	0.59873	0.7392	0.9995	14566	1	-0.757
hsa-mir-3180-4	2	0.59877	0.59877	0.9995	14567	0	-0.359
hsa-mir-4329	2	0.59877	0.59877	0.9995	14568	0	0.1142
SP7	6	0.59885	0.73929	0.9995	14569	2	-0.192
BAZ1B	6	0.59887	0.73931	0.9995	14570	2	-0.237
C21orf2	6	0.59892	0.73935	0.9995	14571	2	0.1431
OR13C3	6	0.59907	0.73945	0.9995	14572	1	-0.738
REG1A	6	0.5991	0.73948	0.9995	14573	2	0.047
ATG16L2	6	0.5991	0.73948	0.9995	14574	2	0.117
LAMP2	6	0.59913	0.7395	0.9995	14575	1	-0.321
DDX17	6	0.59913	0.7395	0.9995	14576	1	-0.215
KLLN	6	0.59933	0.73966	0.9995	14577	1	0.0158
NUDT8	6	0.59936	0.73967	0.9995	14578	2	-0.153
LSS	6	0.59955	0.73981	0.9995	14579	1	-0.05
IFI27	6	0.59965	0.73989	0.9995	14580	2	0.0739
ZBTB49	6	0.59972	0.73995	0.9995	14581	1	-0.658
OR1N1	6	0.59978	0.73999	0.9995	14582	1	-0.446
C17orf66	6	0.59984	0.74004	0.9995	14583	1	-0.178
U2AF2	6	0.59989	0.74008	0.9995	14584	2	0.0721
PIGP	6	0.59993	0.74011	0.9995	14585	1	0.0735
NEUROG3	6	0.60001	0.74018	0.9995	14586	1	-0.361
ENTPD7	6	0.60006	0.74021	0.9995	14587	2	-0.05
CNIH	2	0.60008	0.60005	0.9995	14588	0	0.1409
SLC17A6	6	0.60009	0.74023	0.9995	14589	1	-0.12
FAM26D	6	0.60012	0.74026	0.9995	14590	2	0.1096
AGAP6	2	0.60013	0.60009	0.9995	14591	0	-0.478
NAT16	6	0.60016	0.74028	0.9995	14592	2	-0.02
hsa-mir-6777	4	0.60019	0.666	0.9995	14593	1	-0.086
KRT15	6	0.60019	0.74031	0.9995	14594	2	-0.226
CMKLR1	6	0.60022	0.74033	0.9995	14595	1	-0.36
RARRES2	6	0.60024	0.74034	0.9995	14596	2	-0.027
ATG12	6	0.60031	0.7404	0.9995	14597	2	-0.053
MR1	6	0.60033	0.74042	0.9995	14598	2	-0.067
CELF3	6	0.60046	0.7405	0.9995	14599	1	-0.313
CREB5	6	0.60051	0.74054	0.9995	14600	1	-0.155
MED25	6	0.60051	0.74054	0.9995	14601	2	-0.322
RQCD1	6	0.60067	0.74065	0.9995	14602	2	-0.046
PATZ1	6	0.60076	0.74071	0.9995	14603	2	-0.358
hsa-mir-4281	4	0.60095	0.66641	0.9995	14604	1	-0.144
NDUFAF3	6	0.60096	0.74085	0.9995	14605	1	0.0389
SH3GLB1	6	0.60109	0.74094	0.9995	14606	1	-0.407
RAPGEF1	6	0.60109	0.74094	0.9995	14607	1	-0.195
PHF11	6	0.60117	0.74101	0.9995	14608	2	0.0774
IYD	6	0.60122	0.74104	0.9995	14609	2	-0.027
hsa-mir-502	4	0.60129	0.6666	0.9995	14610	1	-0.576
hsa-mir-599	4	0.60129	0.6666	0.9995	14611	1	-0.033
SLC34A3	6	0.6013	0.7411	0.9995	14612	2	-0.176
C9orf72	6	0.60138	0.74116	0.9995	14613	2	-0.453
hsa-mir-371b	1	0.60141	0.60116	0.9995	14614	0	-0.153
HDHD1	6	0.60142	0.74118	0.9995	14615	1	-0.522

OR52W1	6	0.60152	0.74126	0.9995	14616	2	-0.127
FAM86B1	3	0.60157	0.61877	0.9995	14617	1	-0.089
SEC13	6	0.60165	0.74137	0.9995	14618	1	-0.323
DNMT3B	6	0.60186	0.74152	0.9995	14619	2	-0.451
UROS	6	0.60186	0.74152	0.9995	14620	2	0.045
KRT35	6	0.602	0.74162	0.9995	14621	2	0.1146
PRB1	3	0.60217	0.61922	0.9995	14622	1	0.2441
PSMB5	6	0.60223	0.74179	0.9995	14623	2	-0.084
TCF25	6	0.60239	0.74191	0.9995	14624	2	0.0658
ZP1	6	0.60239	0.74191	0.9995	14625	2	-0.486
ATP8A1	6	0.6024	0.74192	0.9995	14626	1	-0.476
IFT43	6	0.60247	0.74197	0.9995	14627	1	-0.04
HAUS2	6	0.60265	0.74208	0.9995	14628	1	-0.145
OR10G4	6	0.60271	0.74212	0.9995	14629	1	-0.954
hsa-mir-3168	4	0.60282	0.66743	0.9995	14630	1	0.1119
C16orf78	6	0.60292	0.74227	0.9995	14631	1	0.1063
DLX2	6	0.60303	0.74235	0.9995	14632	2	0.0964
DNAH1	6	0.60316	0.74246	0.9995	14633	2	-0.29
LMOD1	6	0.60317	0.74246	0.9995	14634	2	0.1096
FOXC1	6	0.60317	0.74246	0.9995	14635	2	0.1597
CORO2A	6	0.60335	0.74259	0.9995	14636	2	0.0477
REEP6	6	0.6034	0.74263	0.9995	14637	1	-0.082
KCNB1	6	0.60351	0.74271	0.9995	14638	2	0.0856
KRT40	6	0.60352	0.74271	0.9995	14639	2	-0.042
ALKBH6	6	0.60352	0.74271	0.9995	14640	2	-0.124
HMGCR	6	0.60357	0.74276	0.9995	14641	1	-0.025
GATA5	6	0.60357	0.74276	0.9995	14642	1	-0.24
KY	6	0.60362	0.74279	0.9995	14643	1	-0.707
CCDC34	6	0.60364	0.74281	0.9995	14644	2	0.2305
METTL3	6	0.60369	0.74285	0.9995	14645	2	0.0448
ISG20	6	0.60369	0.74286	0.9995	14646	1	0.0389
CAMKK2	6	0.60374	0.74289	0.9995	14647	2	-0.141
IQGAP2	6	0.60381	0.74294	0.9995	14648	2	-0.071
hsa-mir-1973	3	0.60385	0.6205	0.9995	14649	1	-0.814
TERF1	6	0.60393	0.74302	0.9995	14650	2	0.0425
ANKLE2	6	0.60405	0.74313	0.9995	14651	1	-0.255
STMN1	6	0.60426	0.74328	0.9995	14652	2	-0.142
STARD4	6	0.60434	0.74335	0.9995	14653	2	0.092
CDK12	6	0.60444	0.74342	0.9995	14654	1	-0.444
HIST1H2BI	5	0.60447	0.72858	0.9995	14655	1	-0.058
IHH	6	0.60458	0.74352	0.9995	14656	1	-0.204
TECPR2	6	0.60464	0.74356	0.9995	14657	2	-0.062
NDFIP2	6	0.60467	0.74358	0.9995	14658	1	-0.246
WEE2	6	0.6048	0.74368	0.9995	14659	1	-0.137
NRSN2	6	0.60491	0.74375	0.9995	14660	2	0.0376
CPNE8	3	0.60492	0.62131	0.9995	14661	1	0.2915
ITPKB	6	0.60496	0.74379	0.9995	14662	2	-0.213
hsa-mir-584	4	0.60497	0.66859	0.9995	14663	1	-0.244
CD63	6	0.605	0.74381	0.9995	14664	2	-0.026
RCL1	6	0.60504	0.74385	0.9995	14665	2	0.0643
SLC5A11	6	0.60508	0.74389	0.9995	14666	2	-0.192
GSTM5	5	0.60511	0.72882	0.9995	14667	1	-0.667
KIAA1324	6	0.60527	0.74401	0.9995	14668	1	0.0506
FSCN3	6	0.6053	0.74403	0.9995	14669	1	-0.109
DKK2	6	0.60544	0.74413	0.9995	14670	2	-0.185
ID3	6	0.60552	0.74419	0.9995	14671	2	0.0291
SUPT3H	6	0.60552	0.74419	0.9995	14672	2	0.0491
EFEMP1	6	0.60558	0.74423	0.9995	14673	2	-0.013
AUNIP	6	0.60558	0.74423	0.9995	14674	2	-0.036
CXorf23	6	0.6059	0.74448	0.9995	14675	1	-0.027
MEGF10	6	0.60599	0.74455	0.9995	14676	2	0.0371
HRH4	6	0.60605	0.74459	0.9995	14677	1	-0.127
RPS10-NUDT3	2	0.60619	0.60611	0.9995	14678	0	0.0035
ITGB6	6	0.60622	0.74471	0.9995	14679	2	-0.267
C6orf25	6	0.60629	0.74476	0.9995	14680	2	-0.029
NTNG2	6	0.60639	0.74484	0.9995	14681	2	0.0079
MMP8	6	0.60644	0.74488	0.9995	14682	2	0.125
SH3BP1	6	0.60648	0.74492	0.9995	14683	2	-0.594
HAUS1	6	0.60656	0.74496	0.9995	14684	1	0.0333
STARD6	6	0.60656	0.74496	0.9995	14685	2	-0.195
GLRX2	6	0.60656	0.74496	0.9995	14686	2	-0.226
PPP3CB	6	0.60665	0.74502	0.9995	14687	2	0.0083
RAB20	6	0.60667	0.74503	0.9995	14688	2	-0.064
TMEM52	6	0.60669	0.74505	0.9995	14689	2	0.0777
MRPL52	6	0.60674	0.74509	0.9995	14690	2	0.0734
URGCP	1	0.60695	0.60664	0.9995	14691	0	-0.188
CLDN17	6	0.60703	0.7453	0.9995	14692	2	-0.127
SRCIN1	6	0.60705	0.74531	0.9995	14693	1	-0.065
GRK5	6	0.6072	0.74543	0.9995	14694	2	-0.179
SLC7A5	6	0.60723	0.74545	0.9995	14695	2	0.0333
ANXA6	6	0.60733	0.74552	0.9995	14696	1	-0.341
C4orf51	6	0.60737	0.74556	0.9995	14697	1	-0.189
CLEC2B	6	0.60747	0.74564	0.9995	14698	2	0.0901
KCNH1	6	0.60752	0.74568	0.9995	14699	2	-0.116
hsa-mir-549a	4	0.60753	0.66996	0.9995	14700	1	-0.281

CEP44	6	0.60782	0.74592	0.9995	14701	2	-0.06
C19orf25	6	0.60784	0.74594	0.9995	14702	1	-0.346
SLC20A1	6	0.60789	0.74598	0.9995	14703	1	-0.378
MRPS23	6	0.60796	0.74603	0.9995	14704	2	-0.071
NADK	6	0.60803	0.74608	0.9995	14705	1	-0.225
RNF14	6	0.60815	0.74617	0.9995	14706	2	0.186
C14orf93	6	0.60818	0.74619	0.9995	14707	2	-0.041
MISP	1	0.6084	0.60808	0.9995	14708	0	-0.175
MYO15A	6	0.60851	0.74643	0.9995	14709	2	-0.222
SCARB2	6	0.60856	0.74646	0.9995	14710	2	0.0524
OR10C1	6	0.60866	0.74653	0.9995	14711	2	0.0697
ADORA2A	6	0.60877	0.74661	0.9995	14712	1	0.0442
hsa-mir-106a	2	0.60887	0.60884	0.9995	14713	0	0.1887
C7orf33	6	0.60906	0.74681	0.9995	14714	1	-0.147
MS4A2	6	0.60908	0.74683	0.9995	14715	1	-0.531
NDUFA5	6	0.60924	0.74695	0.9995	14716	2	0.0701
MMP12	6	0.60932	0.74701	0.9995	14717	2	0.1616
CRB2	6	0.60932	0.74701	0.9995	14718	2	-0.546
NECAP1	6	0.6094	0.74706	0.9995	14719	2	0.0275
ACTR3B	5	0.60946	0.73037	0.9995	14720	1	-0.158
UBE2D4	6	0.60957	0.74719	0.9995	14721	2	-0.207
TAF15	6	0.60957	0.74719	0.9995	14722	2	-0.179
VWA3A	6	0.6096	0.74722	0.9995	14723	1	-0.249
TOR3A	6	0.60964	0.74724	0.9995	14724	2	-0.246
C11orf74	6	0.6097	0.74729	0.9995	14725	1	-0.316
RNF170	6	0.60977	0.74734	0.9995	14726	2	0.0391
MVD	6	0.6099	0.74745	0.9995	14727	1	-0.138
APOH	6	0.60991	0.74746	0.9995	14728	2	-0.323
TLR7	6	0.61003	0.74756	0.9995	14729	1	-0.013
CCDC71L	6	0.61003	0.74756	0.9995	14730	1	-0.264
ANKRD34A	6	0.61003	0.74756	0.9995	14731	1	-0.063
GNA13	6	0.61008	0.74759	0.9995	14732	2	-0.052
HGS	6	0.61017	0.74767	0.9995	14733	2	0.1214
hsa-mir-3622b	1	0.61028	0.60996	0.9995	14734	0	-0.179
WDR81	6	0.61042	0.74785	0.9995	14735	2	-0.45
B3GALT5	6	0.61047	0.74789	0.9995	14736	1	-0.193
GABRG2	6	0.61048	0.74789	0.9995	14737	2	0.1297
hsa-mir-8070	2	0.6105	0.61042	0.9995	14738	0	-0.362
TMEM8B	6	0.61053	0.74793	0.9995	14739	2	-0.439
UBC	6	0.61053	0.74793	0.9995	14740	2	-0.105
BAG3	6	0.61059	0.74797	0.9995	14741	2	-0.19
HBD	6	0.6107	0.74807	0.9995	14742	2	-0.198
OR1L3	6	0.61073	0.7481	0.9995	14743	1	-0.231
SLC30A7	6	0.61076	0.74812	0.9995	14744	2	-0.087
PSD	6	0.61098	0.74827	0.9995	14745	2	0.0109
SLC25A13	6	0.61101	0.7483	0.9995	14746	1	-0.365
TIE1	6	0.61107	0.74835	0.9995	14747	2	-0.122
STEAP1	6	0.61112	0.74838	0.9995	14748	2	-0.058
GTF2H2D	3	0.61116	0.62605	0.9995	14749	1	-0.147
hsa-mir-4418	4	0.61117	0.67197	0.9995	14750	1	0.1323
CERS1	6	0.61122	0.74846	0.9995	14751	2	0.0617
ATR	6	0.61127	0.7485	0.9995	14752	2	-0.057
SPOP	6	0.61128	0.7485	0.9995	14753	1	-0.542
FBXL7	6	0.61128	0.7485	0.9995	14754	2	-0.01
CPOX	6	0.61128	0.7485	0.9995	14755	1	-0.052
MYO18A	4	0.61136	0.67208	0.9995	14756	1	-0.229
NDUFB4	6	0.61139	0.74858	0.9995	14757	2	0.0618
ABCA3	6	0.61141	0.7486	0.9995	14758	1	0.0622
IFFO2	6	0.61143	0.74862	0.9995	14759	2	0.005
HKDC1	6	0.61156	0.74872	0.9995	14760	2	-0.226
hsa-mir-767	4	0.61159	0.6722	0.9995	14761	1	0.023
GIF	6	0.61172	0.74884	0.9995	14762	2	-0.293
LILRA4	6	0.61176	0.74888	0.9995	14763	2	-0.118
FAM219B	6	0.61176	0.74888	0.9995	14764	2	-0.08
POLR1A	6	0.61181	0.74891	0.9995	14765	2	-0.07
PLEKHG4B	6	0.61188	0.74896	0.9995	14766	2	0.1445
ACTB	6	0.61198	0.74904	0.9995	14767	2	0.0595
BLOC1S2	6	0.61215	0.74917	0.9995	14768	2	-0.121
DDA1	4	0.61219	0.67252	0.9995	14769	1	-0.118
hsa-mir-6507	2	0.61226	0.61219	0.9995	14770	0	0.0904
ZNF277	6	0.61236	0.74932	0.9995	14771	2	0.1379
PALM3	6	0.61244	0.74938	0.9995	14772	2	-0.017
TAP2	6	0.61251	0.74942	0.9995	14773	2	-0.253
LRRC3C	6	0.61262	0.7495	0.9995	14774	2	-0.099
CTXN3	6	0.61264	0.74952	0.9995	14775	1	-0.202
ETS2	6	0.61272	0.74958	0.9995	14776	1	-0.022
APRT	6	0.61278	0.74963	0.9995	14777	1	-0.256
QRFP	6	0.6128	0.74964	0.9995	14778	2	0.1322
C1orf228	6	0.6128	0.74964	0.9995	14779	2	-0.007
POLD3	6	0.6128	0.74964	0.9995	14780	2	-0.053
ARRDC3	6	0.6128	0.74964	0.9995	14781	2	-0.1
hsa-mir-6831	4	0.61284	0.67288	0.9995	14782	1	-0.558
hsa-mir-558	4	0.61287	0.6729	0.9995	14783	1	-0.288
S100P	6	0.61288	0.74969	0.9995	14784	1	-0.343
CSTF2	6	0.61289	0.7497	0.9995	14785	2	-0.812

RILPL1	6	0.61292	0.74973	0.9995	14786	2	-0.083
CARD9	6	0.61296	0.74975	0.9995	14787	1	-0.282
DOCK6	6	0.61311	0.74987	0.9995	14788	2	0.0035
STK36	6	0.61312	0.74988	0.9995	14789	2	-0.434
SECTM1	6	0.61316	0.74991	0.9995	14790	2	0.0899
TAS2R1	6	0.6134	0.75009	0.9995	14791	2	-0.287
HDAC4	6	0.61342	0.75011	0.9995	14792	1	-0.296
TCF7L2	6	0.61354	0.7502	0.9995	14793	1	-0.5
WDR35	6	0.61354	0.75021	0.9995	14794	2	-0.026
PADI2	6	0.61367	0.75031	0.9995	14795	2	-0.582
CCDC74B	5	0.61368	0.73193	0.9995	14796	1	-0.36
B3GAT2	4	0.61369	0.67334	0.9995	14797	1	0.0335
LHX8	6	0.61372	0.75035	0.9995	14798	1	-0.213
PPFIA3	6	0.61381	0.7504	0.9995	14799	2	0.1132
TBL1XR1	6	0.61387	0.75044	0.9995	14800	2	0.0475
FDXACB1	6	0.61391	0.75047	0.9995	14801	1	-0.044
MAP3K6	6	0.61397	0.75051	0.9995	14802	2	0.0768
MAP2K7	6	0.61397	0.75051	0.9995	14803	1	-0.206
IARS2	6	0.6141	0.7506	0.9995	14804	2	0.0072
MPP5	6	0.6141	0.7506	0.9995	14805	1	-0.01
WASF3	6	0.6145	0.7509	0.9995	14806	2	-0.009
hsa-mir-3622a	1	0.6145	0.61422	0.9995	14807	0	-0.213
FCAMR	6	0.61456	0.75095	0.9995	14808	2	-0.506
IPO13	6	0.61461	0.75098	0.9995	14809	1	-0.381
MRPL44	6	0.61463	0.751	0.9995	14810	2	-0.54
ZNF45	6	0.61463	0.751	0.9995	14811	2	-0.047
SCAMP1	6	0.61468	0.75104	0.9995	14812	1	-0.062
RHPN1	6	0.61468	0.75104	0.9995	14813	2	-0.162
BCR	6	0.61477	0.75111	0.9995	14814	2	0.1339
hsa-mir-6862-1	2	0.61483	0.61465	0.9995	14815	0	0.1888
hsa-mir-491	4	0.61495	0.67403	0.9995	14816	1	-0.306
ZNF709	6	0.61502	0.7513	0.9995	14817	1	-0.463
HADHA	6	0.61514	0.75138	0.9995	14818	1	-0.409
KIAA0408	6	0.61517	0.7514	0.9995	14819	2	-0.189
TEX26	6	0.61523	0.75144	0.9995	14820	1	-0.113
PAN2	6	0.61524	0.75146	0.9995	14821	2	0.048
BATF	6	0.61524	0.75146	0.9995	14822	2	-0.106
ZNF474	6	0.61529	0.75149	0.9995	14823	1	0.1401
IER5	6	0.61529	0.75149	0.9995	14824	2	-0.068
SLC25A24	6	0.61535	0.75154	0.9995	14825	2	-0.249
SLC7A14	6	0.61542	0.75159	0.9995	14826	2	0.0251
DSC1	6	0.61559	0.75172	0.9995	14827	2	-0.566
IFT27	6	0.6156	0.75173	0.9995	14828	1	-0.02
COLQ	6	0.61567	0.75179	0.9995	14829	2	-0.124
GMPPA	6	0.61571	0.75183	0.9995	14830	2	-0.034
JUND	6	0.6158	0.7519	0.9995	14831	2	0.1438
GGA3	6	0.61587	0.75196	0.9995	14832	2	-0.155
STAM	6	0.61587	0.75196	0.9995	14833	2	-0.051
STK10	6	0.6159	0.75198	0.9995	14834	1	-0.082
MSTN	6	0.61595	0.75201	0.9995	14835	2	-0.418
PNCK	6	0.61605	0.75211	0.9995	14836	2	-0.213
SLC4A8	6	0.61606	0.75212	0.9995	14837	2	-0.191
MAGI3	6	0.61606	0.75212	0.9995	14838	2	-0.81
SMCO4	6	0.61611	0.75215	0.9995	14839	1	0.0078
U2AF1L4	4	0.61634	0.67482	0.9995	14840	1	-0.343
TEF	6	0.61641	0.75237	0.9995	14841	1	-0.143
FAM89B	6	0.61646	0.75241	0.9995	14842	2	-0.293
OR5D18	6	0.61653	0.75247	0.9995	14843	2	-0.109
C1RL	6	0.61653	0.75247	0.9995	14844	2	0.0122
hsa-mir-4729	4	0.61655	0.67495	0.9995	14845	1	-0.713
CNRIP1	6	0.61666	0.75257	0.9995	14846	2	-0.158
PAX4	6	0.61685	0.75271	0.9995	14847	1	-0.345
ANKHD1-EIF4EBP3	4	0.61687	0.67512	0.9995	14848	1	0.0908
CIAPIN1	6	0.61692	0.75276	0.9995	14849	1	-0.2
FAM3D	6	0.61697	0.75279	0.9995	14850	1	-0.298
GLB1L3	5	0.61704	0.73321	0.9995	14851	1	-0.209
NABP1	6	0.61708	0.75287	0.9995	14852	2	0.144
TAAR8	6	0.61723	0.75297	0.9995	14853	2	0.0362
UPF1	6	0.61731	0.75303	0.9995	14854	2	0.032
DDX3X	6	0.61733	0.75305	0.9995	14855	1	-0.432
UBFD1	6	0.61733	0.75305	0.9995	14856	1	-0.555
C12orf71	6	0.61739	0.75309	0.9995	14857	1	-0.232
PYGM	6	0.61745	0.75314	0.9995	14858	2	-0.126
TTC39A	6	0.61753	0.75321	0.9995	14859	2	-0.13
OR51I2	6	0.61757	0.75324	0.9995	14860	2	-0.302
hsa-mir-6764	4	0.61757	0.67552	0.9995	14861	1	-0.134
PTPN14	6	0.61761	0.75327	0.9995	14862	1	-0.118
ISY1	1	0.61794	0.6177	0.9995	14863	0	-0.245
CD82	6	0.61798	0.75354	0.9995	14864	2	-0.702
CIZ1	6	0.61798	0.75354	0.9995	14865	2	-0.308
LPA	6	0.61816	0.75368	0.9995	14866	1	-0.152
PCDHAC2	2	0.61818	0.61794	0.9995	14867	0	0.0433
ZC3H4	6	0.61822	0.75373	0.9995	14868	2	-0.295
SIGMAR1	6	0.61822	0.75373	0.9995	14869	2	0.1091
WDR86	6	0.6183	0.75379	0.9995	14870	2	0.0996

RIPPLY2	6	0.61832	0.75383	0.9995	14871	1	-0.337
SORL1	6	0.61836	0.75385	0.9995	14872	2	0.0701
ABCC6	6	0.61855	0.754	0.9995	14873	2	-0.278
ATRNL1	6	0.61855	0.754	0.9995	14874	2	-0.344
hsa-mir-181d	4	0.61876	0.67618	0.9995	14875	1	-0.113
TSHZ2	6	0.61877	0.75417	0.9995	14876	2	-0.166
ATXN3	6	0.6188	0.75419	0.9995	14877	1	0.0316
FCN1	6	0.61891	0.75426	0.9995	14878	1	-0.278
SLC16A13	6	0.61898	0.75431	0.9995	14879	2	0.012
ZNF775	6	0.619	0.75433	0.9995	14880	2	-0.021
PPP2R4	6	0.61905	0.75437	0.9995	14881	1	-0.249
DEFA5	6	0.61909	0.75441	0.9995	14882	1	-0.059
HES6	6	0.61913	0.75443	0.9995	14883	1	-0.288
LNX2	6	0.61925	0.75452	0.9995	14884	2	-0.135
hsa-mir-6802	4	0.61944	0.67656	0.9995	14885	1	-0.343
CDH6	6	0.61949	0.7547	0.9995	14886	2	-0.295
RIMS3	6	0.61953	0.75473	0.9995	14887	2	0.0452
ZDHHC3	6	0.61957	0.75476	0.9995	14888	2	-0.416
BPIFB4	6	0.61966	0.75482	0.9995	14889	2	-0.346
hsa-mir-6873	4	0.6197	0.67671	0.9995	14890	1	-0.258
OTUD5	6	0.61971	0.75486	0.9995	14891	1	-0.244
CCT3	4	0.61974	0.67673	0.9995	14892	1	-0.179
MAPK10	6	0.61974	0.75489	0.9995	14893	2	0.2051
hsa-mir-5579	4	0.61979	0.67675	0.9995	14894	1	0.0015
FAM9C	6	0.61979	0.75492	0.9995	14895	1	-0.434
OR8H2	6	0.61993	0.75502	0.9995	14896	2	-0.436
PIN1	6	0.61993	0.75502	0.9995	14897	2	0.0551
CACNA1I	6	0.61993	0.75502	0.9995	14898	2	-0.087
GAL3ST2	6	0.61997	0.75505	0.9995	14899	1	-0.249
C17orf112	6	0.62011	0.75516	0.9995	14900	1	-0.599
ACIN1	6	0.62015	0.75519	0.9995	14901	2	-0.159
SBF2	6	0.6202	0.75523	0.9995	14902	2	-0.029
ZNF182	6	0.62023	0.75525	0.9995	14903	1	-0.418
HERC3	6	0.62027	0.75528	0.9995	14904	2	-0.284
CDK9	6	0.62027	0.75528	0.9995	14905	2	0.1484
ASTL	6	0.62065	0.75556	0.9995	14906	2	-0.145
C3orf52	6	0.62072	0.75561	0.9995	14907	1	-0.206
FH	6	0.62073	0.75561	0.9995	14908	2	-0.156
PPAPDC1A	6	0.62073	0.75561	0.9995	14909	2	-0.009
WBP4	6	0.62079	0.75566	0.9995	14910	2	0.0804
hsa-mir-4286	4	0.62088	0.67736	0.9995	14911	1	-0.279
ATXN1L	6	0.6209	0.75575	0.9995	14912	2	-0.155
DDX50	6	0.62097	0.75581	0.9995	14913	1	-0.261
TET3	6	0.62107	0.75587	0.9995	14914	2	0.0747
TMEM30B	6	0.62112	0.75591	0.9995	14915	2	-0.564
CCL20	6	0.62117	0.75595	0.9995	14916	2	-0.478
PDCD4	6	0.62119	0.75596	0.9995	14917	1	-0.268
USP42	6	0.62121	0.75598	0.9995	14918	1	0.0866
C10orf67	6	0.62122	0.75598	0.9995	14919	2	0.1564
STAP1	6	0.62132	0.75604	0.9995	14920	2	-0.031
TRAF1	6	0.62132	0.75604	0.9995	14921	2	-0.007
ARAP2	6	0.62137	0.75608	0.9995	14922	2	0.0708
CNTN5	6	0.62141	0.75611	0.9995	14923	2	-0.051
ARFGAP1	6	0.6216	0.75627	0.9995	14924	1	-0.234
SMYD1	6	0.62165	0.7563	0.9995	14925	2	-0.044
OLFM4	6	0.62165	0.7563	0.9995	14926	1	-0.008
RRP1B	6	0.62186	0.75646	0.9995	14927	2	0.2206
UCHL3	6	0.62191	0.75649	0.9995	14928	2	-0.1
RIIAD1	6	0.62199	0.75655	0.9995	14929	2	-0.15
FMNL2	6	0.62204	0.75659	0.9995	14930	2	0.1617
LRP5L	6	0.62207	0.75661	0.9995	14931	1	-0.515
FAM126A	6	0.62218	0.7567	0.9995	14932	1	-0.368
GIT1	6	0.62219	0.7567	0.9995	14933	2	-0.031
PAQR4	6	0.62237	0.75684	0.9995	14934	2	-0.27
LCN9	6	0.62257	0.75697	0.9995	14935	2	-0.137
hsa-mir-513c	2	0.62294	0.62274	0.9995	14936	0	0.1963
CRIPT	6	0.62297	0.75727	0.9995	14937	1	-0.212
KRTAP1-4	6	0.62322	0.75745	0.9995	14938	1	-0.003
DOK7	6	0.62328	0.75749	0.9995	14939	1	-0.435
SLC30A10	6	0.62336	0.75756	0.9995	14940	2	0.0409
MLH3	6	0.62344	0.75761	0.9995	14941	2	0.1264
C9orf117	6	0.62351	0.75766	0.9995	14942	2	-0.335
SP4	6	0.62357	0.75771	0.9995	14943	2	0.0817
SMARCE1	6	0.62363	0.75775	0.9995	14944	1	-1.01
PLA2G6	6	0.62371	0.75782	0.9995	14945	2	-0.553
GRPEL2	6	0.62378	0.75788	0.9995	14946	2	0.1165
RBM28	6	0.62381	0.7579	0.9995	14947	2	-0.306
CNGA4	6	0.62381	0.7579	0.9995	14948	1	-0.159
TBC1D13	6	0.62386	0.75793	0.9995	14949	2	-0.347
ECSIT	6	0.62394	0.75799	0.9995	14950	1	-0.007
NMB	6	0.62404	0.75807	0.9995	14951	2	0.0132
hsa-mir-4801	3	0.62404	0.63625	0.9995	14952	1	0.1419
C9orf172	6	0.62414	0.75814	0.9995	14953	1	-0.079
CDKN2A	6	0.62423	0.75821	0.9995	14954	2	-0.004
BAHCC1	6	0.62426	0.75823	0.9995	14955	1	0.0081

DSP	6	0.62429	0.75826	0.9995	14956	2	-0.263
MPDZ	6	0.62429	0.75826	0.9995	14957	2	0.0526
ELP6	6	0.62438	0.75833	0.9995	14958	2	-0.515
DPYD	6	0.62447	0.75839	0.9995	14959	1	-0.339
AKAP8L	6	0.62456	0.75845	0.9995	14960	2	-0.126
PIK3R2	6	0.62456	0.75845	0.9995	14961	2	-0.059
hsa-mir-4476	4	0.62482	0.67958	0.9995	14962	1	-0.44
ZNF589	6	0.62486	0.75868	0.9995	14963	2	-0.392
NT5E	6	0.62486	0.75868	0.9995	14964	1	-0.092
FAM24A	6	0.62486	0.75868	0.9995	14965	2	-0.128
PPIG	6	0.6249	0.75872	0.9995	14966	2	-0.185
TCL1A	6	0.62494	0.75875	0.9995	14967	2	-0.192
C11orf73	6	0.62502	0.75881	0.9995	14968	1	0.0019
hsa-mir-4509-2	2	0.62511	0.62492	0.9995	14969	0	-0.111
SIDT1	6	0.62522	0.75895	0.9995	14970	2	-0.192
SYT2	6	0.62529	0.75901	0.9995	14971	2	-0.044
BUB1	6	0.62534	0.75904	0.9995	14972	2	0.0382
CRTC1	6	0.62538	0.75906	0.9995	14973	1	-0.024
WDFY2	6	0.62546	0.75912	0.9995	14974	2	0.023
HARS2	6	0.62551	0.75915	0.9995	14975	2	0.0324
COL8A2	6	0.62552	0.75916	0.9995	14976	1	-0.094
KIF21A	6	0.62556	0.75919	0.9995	14977	1	0.0271
KCNH6	6	0.62559	0.75921	0.9995	14978	2	0.0035
hsa-mir-4668	4	0.62563	0.68003	0.9995	14979	1	-0.556
NXT1	5	0.62569	0.73642	0.9995	14980	1	-0.058
KCNH4	6	0.62576	0.75933	0.9995	14981	2	0.2094
NUDT2	6	0.62588	0.75942	0.9995	14982	2	-0.339
HMGCLL1	6	0.62592	0.75946	0.9995	14983	2	0.1284
CASC10	3	0.62594	0.63775	0.9995	14984	1	-0.439
MYO1E	6	0.62598	0.7595	0.9995	14985	2	-0.042
FTH1	6	0.62598	0.7595	0.9995	14986	2	0.0621
RB1CC1	6	0.62598	0.7595	0.9995	14987	2	0.0021
FCRL6	6	0.62606	0.75957	0.9995	14988	2	-0.245
CD3D	6	0.62608	0.75958	0.9995	14989	1	-0.361
KRBOX1	6	0.62615	0.75963	0.9995	14990	2	-0.094
PAGE2B	1	0.62619	0.62608	0.9995	14991	0	-1.074
KRTAP2-4	1	0.62619	0.62608	0.9995	14992	0	-1.074
LCOR	6	0.62625	0.75968	0.9995	14993	2	-0.176
RSPRY1	6	0.62625	0.75968	0.9995	14994	2	-0.366
OSBPL2	6	0.62626	0.75969	0.9995	14995	2	-0.113
MARK2	6	0.62631	0.75973	0.9995	14996	1	0.0265
TMEM8C	6	0.62634	0.75975	0.9995	14997	2	-0.098
ZNF771	6	0.62634	0.75975	0.9995	14998	2	-0.033
C1orf158	6	0.62634	0.75975	0.9995	14999	2	0.0941
MAP1S	6	0.62644	0.75983	0.9995	15000	2	0.177
HAL	6	0.62645	0.75983	0.9995	15001	2	-0.01
ZNF679	6	0.62647	0.75985	0.9995	15002	2	-0.276
BEST2	6	0.62652	0.75988	0.9995	15003	2	0.045
APOD	6	0.62681	0.7601	0.9995	15004	1	-0.45
LRP11	6	0.62701	0.76024	0.9995	15005	2	-0.022
DIAPH3	6	0.62703	0.76026	0.9995	15006	1	-0.013
CAPN6	6	0.6271	0.76031	0.9995	15007	2	0.1101
IQCF5	6	0.62727	0.76044	0.9995	15008	1	-0.126
OR52K1	6	0.62736	0.76051	0.9995	15009	2	-0.226
hsa-mir-1203	4	0.62736	0.68096	0.9995	15010	1	-0.765
APBA2	6	0.62741	0.76054	0.9995	15011	1	-0.276
DEFB113	6	0.62742	0.76055	0.9995	15012	2	-0.115
MAOA	6	0.62752	0.76063	0.9995	15013	2	-0.163
NTM	6	0.62763	0.76071	0.9995	15014	2	0.1193
HIST1H4A	6	0.62775	0.7608	0.9995	15015	2	-0.454
SPOCK3	6	0.62782	0.76085	0.9995	15016	2	0.0607
KRTAP12-4	6	0.62782	0.76085	0.9995	15017	1	-0.204
APOL4	6	0.62795	0.76095	0.9995	15018	2	0.0331
CREB1	6	0.62795	0.76095	0.9995	15019	2	-0.066
SPATA33	2	0.6281	0.62791	0.9995	15020	0	-0.756
LEPRE1	6	0.62818	0.76112	0.9995	15021	2	-0.013
RUNX1T1	6	0.62821	0.76114	0.9995	15022	2	0.0088
USP30	6	0.62834	0.76124	0.9995	15023	1	-0.4
C3orf30	6	0.62839	0.76127	0.9995	15024	2	-0.178
LOC643802	6	0.62839	0.76127	0.9995	15025	2	0.0328
PLXNB1	6	0.62841	0.76129	0.9995	15026	1	-0.097
RIT1	6	0.62849	0.76135	0.9995	15027	2	-0.041
NELL1	6	0.62854	0.76139	0.9995	15028	1	0.1155
PRKAR2B	6	0.62854	0.76139	0.9995	15029	1	-0.087
MYOM3	6	0.62857	0.7614	0.9995	15030	2	-0.102
CC2D1B	6	0.6286	0.76142	0.9995	15031	1	-0.487
ASB1	6	0.62867	0.76149	0.9995	15032	2	-0.524
LRRC26	6	0.62882	0.7616	0.9995	15033	1	-0.52
POLE3	6	0.62884	0.76161	0.9995	15034	2	-0.155
DENND6A	6	0.62898	0.76171	0.9995	15035	2	-0.145
GKAP1	6	0.62922	0.76189	0.9995	15036	1	-0.167
OR2W1	4	0.62923	0.68204	0.9995	15037	1	-0.315
MINK1	6	0.62933	0.76197	0.9995	15038	2	-0.017
C3orf62	6	0.62934	0.76197	0.9995	15039	2	-0.144
C2orf57	6	0.62952	0.7621	0.9995	15040	2	-0.11

CKMT2	6	0.62958	0.76214	0.9995	15041	2	-0.042
TRIM23	6	0.62958	0.76214	0.9995	15042	2	0.0643
DCAF4L1	6	0.62964	0.7622	0.9995	15043	2	-0.45
COBL	6	0.62967	0.76221	0.9995	15044	1	-0.283
PRDX2	6	0.62971	0.76225	0.9995	15045	2	-0.258
PPP1R3D	6	0.62976	0.76228	0.9995	15046	2	-0.033
hsa-mir-4324	4	0.62997	0.68245	0.9995	15047	1	-0.913
ANKRD66	6	0.63026	0.76266	0.9995	15048	2	0.0195
LRRC28	4	0.63035	0.68265	0.9995	15049	1	-0.217
C20orf111	2	0.63038	0.6302	0.9995	15050	0	-0.055
LRP2BP	6	0.63046	0.76281	0.9995	15051	2	-0.027
ERAP2	6	0.6306	0.7629	0.9995	15052	2	-0.046
FNDC9	6	0.63077	0.76304	0.9995	15053	2	-0.005
XCR1	6	0.63077	0.76304	0.9995	15054	2	-0.628
IRF5	6	0.63093	0.76316	0.9995	15055	2	0.0486
ZNF747	6	0.63105	0.76326	0.9995	15056	1	-0.434
NKX1-2	6	0.63105	0.76326	0.9995	15057	1	-0.124
TAAR1	6	0.63112	0.76331	0.9995	15058	2	0.0415
ZNF436	6	0.63117	0.76335	0.9995	15059	1	-0.282
TOR1B	6	0.63125	0.76341	0.9995	15060	1	-0.132
KRTAP20-2	6	0.63129	0.76344	0.9995	15061	2	-0.208
MFF	6	0.6314	0.76353	0.9995	15062	2	-0.19
TRIP13	6	0.63177	0.76381	0.9995	15063	2	-0.022
ARHGAP28	6	0.63181	0.76383	0.9995	15064	2	-0.166
TBC1D3H	1	0.63187	0.63181	0.9995	15065	0	-1.233
hsa-mir-3156-1	1	0.63187	0.63181	0.9995	15066	0	-1.233
hsa-mir-527	1	0.63187	0.63181	0.9995	15067	0	-1.233
AGAP5	1	0.63187	0.63181	0.9995	15068	0	-1.233
hsa-mir-513a-2	1	0.63187	0.63181	0.9995	15069	0	-1.233
RBMY1F	1	0.63187	0.63181	0.9995	15070	0	-1.233
C4B	1	0.63187	0.63181	0.9995	15071	0	-1.233
SPRR2D	1	0.63187	0.63181	0.9995	15072	0	-1.233
hsa-mir-6511b-2	1	0.63187	0.63181	0.9995	15073	0	-1.233
hsa-mir-518f	1	0.63187	0.63181	0.9995	15074	0	-1.233
POTEJ	1	0.63187	0.63181	0.9995	15075	0	-1.233
hsa-mir-4441	1	0.63187	0.63181	0.9995	15076	0	-1.233
CCL4L2	1	0.63187	0.63181	0.9995	15077	0	-1.233
SPRR2F	1	0.63187	0.63181	0.9995	15078	0	-1.233
hsa-mir-516a-1	1	0.63187	0.63181	0.9995	15079	0	-1.233
GOLGA6L6	1	0.63187	0.63181	0.9995	15080	0	-1.233
hsa-mir-516a-2	1	0.63187	0.63181	0.9995	15081	0	-1.233
SPRR2A	1	0.63187	0.63181	0.9995	15082	0	-1.233
hsa-mir-103b-1	1	0.63187	0.63181	0.9995	15083	0	-1.233
SPRR2E	1	0.63187	0.63181	0.9995	15084	0	-1.233
FAM25G	1	0.63187	0.63181	0.9995	15085	0	-1.233
SLFN13	6	0.6319	0.76389	0.9995	15086	2	0.1291
GLI3	6	0.63198	0.76397	0.9995	15087	2	0.1736
BTBD3	6	0.63202	0.76399	0.9995	15088	1	-0.31
MATR3	6	0.63202	0.764	0.9995	15089	2	-0.256
NTRK1	6	0.63202	0.764	0.9995	15090	2	0.0946
hsa-mir-3145	3	0.63206	0.6427	0.9995	15091	1	-0.137
BBS12	6	0.63214	0.76409	0.9995	15092	2	0.0318
CATSPER1	6	0.63233	0.76424	0.9995	15093	2	-0.153
C1orf65	6	0.63241	0.76431	0.9995	15094	1	-0.356
TXNDC9	6	0.63247	0.76435	0.9995	15095	2	0.2449
CCR1	6	0.63247	0.76435	0.9995	15096	2	-0.146
FAM163B	6	0.6325	0.76438	0.9995	15097	1	-0.517
LEFTY1	6	0.63255	0.7644	0.9995	15098	1	-0.193
SPAG11A	2	0.63255	0.63231	0.9995	15099	0	-0.01
ZNF10	6	0.63259	0.76444	0.9995	15100	2	0.1463
TULP2	6	0.63271	0.76453	0.9995	15101	1	-0.333
PCDHGB3	2	0.63276	0.63253	0.9995	15102	0	0.0707
RGAG4	6	0.63294	0.76472	0.9995	15103	2	-0.022
KCNMB2	6	0.63331	0.76498	0.9995	15104	1	-0.019
SLC38A6	6	0.63331	0.76498	0.9995	15105	1	-0.077
BTBD9	6	0.63334	0.76502	0.9995	15106	1	-0.479
SNTB2	6	0.63343	0.76508	0.9995	15107	2	-0.168
MKNK2	6	0.6335	0.76513	0.9995	15108	2	0.1714
PARPBP	6	0.6335	0.76513	0.9995	15109	2	-0.39
TAS2R9	6	0.63351	0.76514	0.9995	15110	1	-0.167
SNX10	6	0.63351	0.76514	0.9995	15111	1	-0.123
PTX4	6	0.63361	0.76522	0.9995	15112	1	-0.81
GPBP1L1	6	0.63363	0.76522	0.9995	15113	2	0.1224
CDH22	6	0.63368	0.76526	0.9995	15114	1	-0.511
BNC2	6	0.63379	0.76535	0.9995	15115	2	-0.177
VTI1B	6	0.63379	0.76535	0.9995	15116	2	-0.056
MCOLN1	6	0.63388	0.76543	0.9995	15117	1	-0.692
APEX2	6	0.63397	0.76549	0.9995	15118	2	-0.282
MIPEP	6	0.63403	0.76553	0.9995	15119	2	-0.073
OTP	6	0.63412	0.76559	0.9995	15120	2	-0.577
hsa-mir-6811	4	0.6342	0.6848	0.9995	15121	1	0.1039
SGCD	6	0.63421	0.76566	0.9995	15122	2	-0.48
MAP7D3	6	0.63424	0.76569	0.9995	15123	1	-0.449
ABCG2	6	0.63437	0.76579	0.9995	15124	2	-0.05
PDGFB	6	0.63439	0.7658	0.9995	15125	1	-0.32

OAS1	6	0.63439	0.7658	0.9995	15126	2	-0.469
C19orf67	6	0.63445	0.76585	0.9995	15127	2	0.221
CDC42EP5	6	0.63449	0.76587	0.9995	15128	1	-0.713
hsa-mir-548ah	1	0.63487	0.6348	0.9995	15129	0	-0.485
TFDP3	6	0.63488	0.76616	0.9995	15130	2	-0.091
TICAM1	6	0.63497	0.76622	0.9995	15131	2	0.0515
NUP37	6	0.63502	0.76624	0.9995	15132	2	-0.095
RAB11FIP4	6	0.63504	0.76627	0.9995	15133	1	-0.064
PAG1	6	0.63519	0.76637	0.9995	15134	2	-0.297
LIPM	6	0.63519	0.76637	0.9995	15135	2	-0.24
BAI3	6	0.63519	0.76637	0.9995	15136	2	-0.086
LMNB2	6	0.63519	0.76637	0.9995	15137	1	-0.608
ESR1	6	0.63541	0.76655	0.9995	15138	2	0.1061
CEP85L	6	0.63553	0.76664	0.9995	15139	2	0.1319
hsa-mir-6126	4	0.63561	0.68556	0.9995	15140	1	-0.71
SCARA3	6	0.63572	0.76677	0.9995	15141	1	-0.136
BACE1	6	0.63577	0.76681	0.9995	15142	1	-0.295
hsa-mir-574	4	0.63582	0.68567	0.9995	15143	1	-0.419
ZNF552	6	0.63592	0.76693	0.9995	15144	1	-0.041
OR10J5	6	0.63595	0.76695	0.9995	15145	2	-0.288
PIGA	6	0.63603	0.76701	0.9995	15146	2	-0.168
C5orf60	6	0.63603	0.76701	0.9995	15147	2	-0.288
hsa-mir-8062	4	0.63614	0.68586	0.9995	15148	1	-0.099
AP5M1	6	0.63622	0.76716	0.9995	15149	1	0.0586
TEX101	6	0.63624	0.76717	0.9995	15150	2	-0.052
TPSD1	6	0.63625	0.76718	0.9995	15151	2	-0.11
NOX3	6	0.63625	0.76718	0.9995	15152	1	-0.388
hsa-mir-5088	4	0.63631	0.68596	0.9995	15153	1	0.0679
SOX15	6	0.63657	0.76742	0.9995	15154	1	-0.041
PPDPF	6	0.63667	0.7675	0.9995	15155	1	-0.308
TBC1D4	6	0.63669	0.76751	0.9995	15156	2	-0.143
KIAA1551	6	0.63678	0.76759	0.9995	15157	2	-0.03
MRPS31	6	0.63691	0.76768	0.9995	15158	2	-0.049
ENO3	6	0.63696	0.76773	0.9995	15159	2	-0.31
GTF2IRD2	2	0.63697	0.63684	0.9995	15160	0	-0.247
SNRNP35	6	0.63705	0.76779	0.9995	15161	2	-0.116
FXYD2	4	0.63705	0.68641	0.9995	15162	1	-0.097
UCN	6	0.63711	0.76783	0.9995	15163	1	-0.139
SCARA5	6	0.63711	0.76783	0.9995	15164	2	0.0914
CDX1	6	0.63713	0.76785	0.9995	15165	2	-0.161
DYNLT1	6	0.63713	0.76785	0.9995	15166	2	-0.073
hsa-mir-4664	4	0.6372	0.68651	0.9995	15167	1	-0.143
A3GALT2	6	0.63727	0.76794	0.9995	15168	1	0.041
MPRIP	6	0.63727	0.76794	0.9995	15169	1	0.0908
SNAP25	6	0.63728	0.76795	0.9995	15170	2	-0.066
SOX9	6	0.63746	0.76808	0.9995	15171	2	-0.011
IER3	6	0.63748	0.76809	0.9995	15172	1	-0.097
CHRM2	6	0.6375	0.76812	0.9995	15173	2	0.0162
hsa-mir-1972-1	1	0.63752	0.6375	0.9995	15174	0	-0.568
LY6G6C	6	0.63752	0.76813	0.9995	15175	1	0.0528
CCNG2	6	0.63762	0.76821	0.9995	15176	2	-0.033
NDP	6	0.6377	0.76826	0.9995	15177	1	-0.625
TLCD1	6	0.63774	0.7683	0.9995	15178	2	-0.131
NKX2-5	6	0.63774	0.7683	0.9995	15179	1	-0.418
FAM65C	6	0.63794	0.76846	0.9995	15180	1	-0.1
HEXIM2	6	0.63797	0.76848	0.9995	15181	2	0.0043
BBS10	6	0.63809	0.76857	0.9995	15182	2	-0.033
TSC22D4	6	0.63819	0.76865	0.9995	15183	1	-0.777
CDT1	6	0.63819	0.76865	0.9995	15184	2	-0.028
PLAT	6	0.6382	0.76866	0.9995	15185	2	0.095
FAM98B	6	0.63843	0.76885	0.9995	15186	2	0.0658
TMEM171	6	0.63844	0.76885	0.9995	15187	2	-0.011
HRH2	6	0.63863	0.76899	0.9995	15188	1	-0.122
DIRAS2	6	0.6387	0.76905	0.9995	15189	2	-0.105
SMAD1	6	0.63874	0.76907	0.9995	15190	2	-0.113
MPG	6	0.63877	0.76908	0.9995	15191	2	-0.492
LRP6	6	0.63883	0.76914	0.9995	15192	2	0.1314
AKR1B1	6	0.63883	0.76914	0.9995	15193	1	-0.002
PATE3	6	0.63886	0.76917	0.9995	15194	2	-0.029
SPNS3	6	0.63889	0.76919	0.9995	15195	2	-0.128
FXR1	6	0.63893	0.76921	0.9995	15196	2	0.0028
RABL2A	4	0.63893	0.68753	0.9995	15197	1	-0.118
ATP6V1C1	6	0.63908	0.76932	0.9995	15198	2	0.1247
hsa-mir-6076	4	0.63911	0.68763	0.9995	15199	1	-1.124
ARCN1	6	0.63926	0.76947	0.9995	15200	2	0.017
USP17L15	6	0.63943	0.76961	0.9995	15201	2	0.1432
CPSF1	6	0.63975	0.76983	0.9995	15202	2	-0.303
LMNA	6	0.6398	0.76987	0.9995	15203	1	-0.57
hsa-mir-6843	4	0.64004	0.68817	0.9995	15204	1	-0.112
CLPP	6	0.64031	0.77025	0.9995	15205	1	-0.382
WBP1	6	0.64043	0.77034	0.9995	15206	2	-0.102
ROCK1	6	0.64048	0.77039	0.9995	15207	1	-0.236
PDE7B	6	0.64054	0.77043	0.9995	15208	2	-0.226
TYRO3	6	0.64061	0.77047	0.9995	15209	1	-0.395
FTH1P18	6	0.64061	0.77047	0.9995	15210	1	-0.254

STUB1	6	0.64061	0.77047	0.9995	15211	1	-0.245
NDC1	4	0.64066	0.68856	0.9995	15212	1	-0.266
SRC	6	0.64071	0.77055	0.9995	15213	1	0.064
CORT	6	0.64077	0.7706	0.9995	15214	2	0.074
MED10	6	0.6408	0.77062	0.9995	15215	2	-0.204
AMELY	5	0.64093	0.74223	0.9995	15216	1	-0.629
NUBP2	6	0.64093	0.77072	0.9995	15217	2	-0.032
LONRF1	6	0.64097	0.77076	0.9995	15218	2	-0.064
LRRC52	6	0.64104	0.77081	0.9995	15219	2	0.0791
AKAP3	6	0.64104	0.77081	0.9995	15220	2	-0.037
UGT2B15	4	0.64106	0.68879	0.9995	15221	1	0.0485
SYT10	6	0.64111	0.77085	0.9995	15222	1	-0.536
USP2	6	0.64111	0.77086	0.9995	15223	2	-0.131
ZNF618	6	0.64115	0.77088	0.9995	15224	2	-0.704
ST6GAL2	6	0.64117	0.7709	0.9995	15225	1	-0.686
BARD1	6	0.64127	0.77099	0.9995	15226	1	-0.327
CPSF3	6	0.64132	0.77102	0.9995	15227	1	-0.04
ZC3H12A	6	0.64149	0.77116	0.9995	15228	2	-0.271
DLK2	6	0.64167	0.77129	0.9995	15229	2	0.1086
ABI3	6	0.64167	0.77129	0.9995	15230	2	-0.13
C1orf127	6	0.64176	0.77135	0.9995	15231	2	-0.205
C2orf70	6	0.64176	0.77135	0.9995	15232	2	-0.372
FCAR	6	0.64183	0.77141	0.9995	15233	2	-0.245
KIAA1211L	6	0.64186	0.77142	0.9995	15234	2	-0.126
ZNF80	6	0.64187	0.77143	0.9995	15235	2	-0.761
LRRK1	6	0.64197	0.77151	0.9995	15236	1	-0.228
CST8	6	0.64209	0.7716	0.9995	15237	2	0.1131
GABRB1	6	0.64212	0.77161	0.9995	15238	2	-0.05
ERGIC3	6	0.64221	0.77169	0.9995	15239	1	-0.25
FBXO2	6	0.64228	0.77175	0.9995	15240	2	-0.129
LDLRAD4	6	0.64237	0.77181	0.9995	15241	1	-0.29
RDH8	6	0.64252	0.77193	0.9995	15242	2	-0.043
HIST1H1B	6	0.64252	0.77193	0.9995	15243	2	0.0439
GLB1L2	4	0.64256	0.68967	0.9995	15244	1	-0.26
DNASE1	6	0.64259	0.77199	0.9995	15245	2	-0.074
GPRC5C	6	0.64263	0.77202	0.9995	15246	1	0.037
PRKX	6	0.64274	0.77209	0.9995	15247	1	-0.126
hsa-mir-6808	4	0.6429	0.68987	0.9995	15248	1	-0.279
CCDC85B	6	0.64306	0.77235	0.9995	15249	1	0.1384
CABYR	6	0.64313	0.7724	0.9995	15250	1	-0.13
C8orf48	6	0.64316	0.77242	0.9995	15251	2	-0.475
TYW3	6	0.64322	0.77247	0.9995	15252	2	-0.075
TNFRSF11B	6	0.64329	0.77251	0.9995	15253	2	-0.018
NOXRED1	6	0.64334	0.77256	0.9995	15254	2	-0.108
IGSF8	6	0.64338	0.7726	0.9995	15255	1	-0.219
LRRC49	6	0.64341	0.77262	0.9995	15256	2	-0.022
MMP14	6	0.64346	0.77266	0.9995	15257	2	0.1269
AVIL	6	0.64349	0.77268	0.9995	15258	2	-0.124
SNAPC1	6	0.64381	0.77295	0.9995	15259	2	-0.227
TBCEL	6	0.64383	0.77296	0.9995	15260	1	-0.077
C21orf59	6	0.64386	0.77299	0.9995	15261	2	0.1352
WISP3	6	0.64394	0.77305	0.9995	15262	1	-0.422
SREBF1	6	0.644	0.7731	0.9995	15263	1	-0.413
CMPK2	6	0.644	0.7731	0.9995	15264	2	-0.215
TMPRSS11F	6	0.64405	0.77313	0.9995	15265	2	-0.037
CAMKMT	6	0.64409	0.77317	0.9995	15266	2	0.1391
IRAK3	6	0.64429	0.77333	0.9995	15267	1	-0.044
TEX13A	6	0.64457	0.77354	0.9995	15268	2	-0.198
TTF1	6	0.64457	0.77354	0.9995	15269	2	-0.143
PSMB10	6	0.64463	0.77359	0.9995	15270	1	-0.334
ARHGEF11	6	0.64476	0.77369	0.9995	15271	1	-0.465
GFI1B	6	0.64482	0.77373	0.9995	15272	1	-0.26
ZFAND2B	6	0.64494	0.77382	0.9995	15273	2	0.0787
ZC3H18	6	0.64494	0.77382	0.9995	15274	2	0.0264
IMPA1	6	0.64512	0.77396	0.9995	15275	2	0.1137
FAM216A	6	0.64515	0.77397	0.9995	15276	1	-0.27
NFATC4	6	0.64515	0.77397	0.9995	15277	1	-0.142
NR1D1	6	0.64524	0.77405	0.9995	15278	1	-0.499
TSPAN5	6	0.64544	0.77419	0.9995	15279	2	0.0624
CD47	6	0.64556	0.77428	0.9995	15280	2	0.0799
NHLRC1	6	0.6456	0.7743	0.9995	15281	2	0.1213
hsa-mir-4655	4	0.64562	0.69145	0.9995	15282	1	-0.364
LY9	6	0.64562	0.77433	0.9995	15283	2	-0.147
LRRC31	6	0.64566	0.77436	0.9995	15284	1	-0.207
BEX5	6	0.64572	0.77441	0.9995	15285	2	0.0595
ATP5G3	6	0.64578	0.77445	0.9995	15286	2	-0.049
FAM175A	6	0.64588	0.77453	0.9995	15287	2	0.0696
SLC10A4	6	0.64595	0.77459	0.9995	15288	2	-0.117
VCAM1	6	0.64605	0.77466	0.9995	15289	2	0.0476
TMPRSS12	6	0.64605	0.77466	0.9995	15290	2	-0.042
DCAF12L1	6	0.64608	0.7747	0.9995	15291	1	-0.208
CCDC23	4	0.6461	0.69173	0.9995	15292	1	-0.162
SLC25A2	6	0.64612	0.77473	0.9995	15293	2	-0.022
AHDC1	6	0.64618	0.77477	0.9995	15294	1	-0.09
HNF1A	6	0.64623	0.77481	0.9995	15295	2	-0.35

CPNE9	6	0.64634	0.77489	0.9995	15296	2	-0.897
RNF130	6	0.64634	0.77489	0.9995	15297	2	-0.02
PSORS1C1	6	0.64634	0.77489	0.9995	15298	2	-0.462
MMP9	6	0.64634	0.77489	0.9995	15299	1	-0.184
SNX18	6	0.64638	0.77492	0.9995	15300	2	-0.536
SPAG6	6	0.64639	0.77492	0.9995	15301	1	-0.251
VCL	6	0.64648	0.775	0.9995	15302	2	-0.485
LIN54	6	0.64652	0.77503	0.9995	15303	1	-0.234
MSMB	6	0.6466	0.7751	0.9995	15304	2	0.0813
SRA1	6	0.6466	0.7751	0.9995	15305	2	-0.581
CAMK1D	6	0.64666	0.77513	0.9995	15306	2	0.1954
FGG	6	0.64666	0.77513	0.9995	15307	2	0.0062
MLLT11	6	0.64666	0.77513	0.9995	15308	2	0.0588
BCAN	6	0.64671	0.77518	0.9995	15309	2	-0.302
SAFB2	6	0.64676	0.77522	0.9995	15310	2	-0.094
BPHL	6	0.64701	0.7754	0.9995	15311	1	-0.143
MEIS1	6	0.64701	0.7754	0.9995	15312	2	0.1226
C5orf34	6	0.64706	0.77545	0.9995	15313	2	-0.133
PTK2	6	0.6471	0.77547	0.9995	15314	2	-0.088
DSCR6	2	0.64712	0.64688	0.9995	15315	0	-0.031
BAZ1A	6	0.64715	0.77551	0.9995	15316	2	-0.262
ZNF853	6	0.64715	0.77551	0.9995	15317	2	-0.031
ZKSCAN1	6	0.64727	0.7756	0.9995	15318	1	-0.473
VWCE	6	0.64728	0.7756	0.9995	15319	2	-0.04
hsa-mir-548aw	3	0.64745	0.65528	0.9995	15320	1	0.1427
ESM1	6	0.64747	0.77576	0.9995	15321	2	-0.338
hsa-mir-616	4	0.64752	0.69259	0.9995	15322	1	0.0335
TRIT1	6	0.64765	0.77591	0.9995	15323	2	0.0597
DUS2	2	0.64765	0.64739	0.9995	15324	0	0.0189
ST14	6	0.64792	0.77611	0.9995	15325	1	-0.104
SLFN5	6	0.64797	0.77615	0.9995	15326	1	-0.48
LOXL1	6	0.64797	0.77615	0.9995	15327	2	0.023
ZMYND8	6	0.64808	0.77624	0.9995	15328	1	-0.146
ATAD2	6	0.64823	0.77634	0.9995	15329	2	0.1094
ALOX15B	6	0.64844	0.77651	0.9995	15330	2	-0.274
TENM2	6	0.64849	0.77655	0.9995	15331	2	-0.042
DHRS13	6	0.64852	0.77657	0.9995	15332	2	0.0556
NFATC2	6	0.64858	0.77662	0.9995	15333	2	-0.432
RANBP17	6	0.64865	0.77667	0.9995	15334	2	-0.167
GLI2	6	0.64867	0.77668	0.9995	15335	1	-0.069
ZFPM1	6	0.64872	0.77673	0.9995	15336	2	0.1008
HIST1H2AG	6	0.64876	0.77675	0.9995	15337	1	-0.195
KLK2	6	0.64883	0.77681	0.9995	15338	1	-0.709
PROS1	6	0.64896	0.7769	0.9995	15339	2	-0.139
FAM174B	4	0.64902	0.69344	0.9995	15340	1	0.0506
BICD1	6	0.64904	0.77696	0.9995	15341	1	-0.275
ASF1B	6	0.64907	0.77698	0.9995	15342	2	-0.676
DPF1	6	0.64926	0.77713	0.9995	15343	1	-0.358
FGD1	6	0.64928	0.77715	0.9995	15344	1	-0.151
LGI4	6	0.64939	0.77723	0.9995	15345	2	0.0119
G3BP2	6	0.64942	0.77726	0.9995	15346	1	0.0952
MXI1	6	0.64951	0.77733	0.9995	15347	2	0.0852
ASGR1	6	0.64951	0.77733	0.9995	15348	2	0.0597
ENPP3	6	0.64965	0.77744	0.9995	15349	2	-0.125
ZNF69	5	0.64966	0.74567	0.9995	15350	1	-0.244
BBC3	6	0.64967	0.77746	0.9995	15351	2	-0.335
hsa-mir-3126	4	0.64975	0.69389	0.9995	15352	1	-0.027
DNAJB12	4	0.64975	0.69389	0.9995	15353	1	-1.044
EFS	6	0.64976	0.77753	0.9995	15354	2	-0.327
MYL12B	4	0.64981	0.69392	0.9995	15355	1	-0.933
hsa-mir-4794	3	0.64986	0.65726	0.9995	15356	1	0.0801
KCNN3	6	0.6499	0.77763	0.9995	15357	2	-0.083
GJA10	6	0.6499	0.77763	0.9995	15358	2	0.1623
GANC	6	0.64997	0.77769	0.9995	15359	2	0.1606
SNRPD2	6	0.64997	0.77769	0.9995	15360	1	-0.284
CPE	6	0.64997	0.77769	0.9995	15361	1	-0.025
hsa-mir-625	4	0.64999	0.69403	0.9995	15362	1	-0.276
ARF6	4	0.65005	0.69406	0.9995	15363	1	-0.314
HNRNPLL	4	0.65005	0.69406	0.9995	15364	1	-0.108
TMEM50B	6	0.65006	0.77776	0.9995	15365	2	-0.062
hsa-mir-6857	2	0.65014	0.64987	0.9995	15366	0	0.1384
CSH2	3	0.65018	0.65752	0.9995	15367	1	-0.81
NFXL1	6	0.65021	0.77788	0.9995	15368	2	-0.021
TEAD1	6	0.65021	0.77788	0.9995	15369	2	-0.053
SYNGR1	6	0.65024	0.7779	0.9995	15370	2	-0.016
ESYT2	6	0.65029	0.77794	0.9995	15371	1	-0.103
STOX1	6	0.65047	0.77806	0.9995	15372	2	-0.076
ENAH	6	0.65056	0.77814	0.9995	15373	2	-0.289
FOXN3	6	0.65058	0.77816	0.9995	15374	2	-0.112
MAN2B1	6	0.65061	0.77819	0.9995	15375	1	-0.218
ADAMDEC1	6	0.65065	0.77822	0.9995	15376	2	-0.204
RPL4	6	0.65066	0.77822	0.9995	15377	1	-0.098
NAGS	6	0.65068	0.77824	0.9995	15378	2	-0.255
SMIM6	6	0.65077	0.77832	0.9995	15379	2	-0.29
LALBA	6	0.65079	0.77833	0.9995	15380	1	-0.264

PCDHA2	2	0.65083	0.65056	0.9995	15381	0	-0.035
OSGEP	6	0.65094	0.77844	0.9995	15382	2	0.0338
USB1	6	0.65095	0.77844	0.9995	15383	1	0.0083
hsa-mir-6847	4	0.65097	0.69462	0.9995	15384	1	0.1255
ADCY7	6	0.651	0.77849	0.9995	15385	1	-0.803
PCID2	6	0.65107	0.77854	0.9995	15386	2	-0.35
RNASEH2A	6	0.65121	0.77864	0.9995	15387	1	0.0231
GBA	6	0.65126	0.77868	0.9995	15388	1	-0.403
SCNN1B	6	0.65128	0.77869	0.9995	15389	2	-0.098
DOK3	6	0.6513	0.77871	0.9995	15390	2	0.0708
C16orf90	6	0.65136	0.77875	0.9995	15391	2	0.0956
ZNF670	6	0.65143	0.77879	0.9995	15392	1	-0.18
PPP6R2	6	0.65145	0.77882	0.9995	15393	2	0.0788
BLVRA	6	0.65147	0.77884	0.9995	15394	1	-0.153
ITPR2	6	0.65149	0.77885	0.9995	15395	1	-0.245
CRTC2	6	0.65156	0.7789	0.9995	15396	2	0.2181
FKBP7	6	0.65158	0.77892	0.9995	15397	1	-0.034
G2E3	6	0.65171	0.779	0.9995	15398	2	0.033
FAM71A	6	0.65183	0.77909	0.9995	15399	2	-0.306
RRP15	6	0.65183	0.77909	0.9995	15400	2	-0.202
KRT76	6	0.65183	0.77909	0.9995	15401	1	-0.046
PRICKLE4	6	0.65198	0.77919	0.9995	15402	1	-0.336
LHX3	6	0.6521	0.77929	0.9995	15403	1	-0.411
STX19	6	0.65217	0.77934	0.9995	15404	2	-0.13
BACH1	6	0.65226	0.77941	0.9995	15405	2	-0.188
SESN2	6	0.65235	0.77949	0.9995	15406	2	-0.15
MAGIX	6	0.65244	0.77955	0.9995	15407	2	-0.208
DOC2A	6	0.65244	0.77955	0.9995	15408	2	0.0195
APOLD1	6	0.65248	0.77957	0.9995	15409	1	-0.603
CDR2	6	0.65248	0.77957	0.9995	15410	1	-0.505
hsa-mir-1229	4	0.65257	0.69553	0.9995	15411	1	0.1784
TNN	6	0.65282	0.77984	0.9995	15412	2	-0.176
BRICD5	6	0.65283	0.77984	0.9995	15413	1	-0.127
TK1	6	0.6529	0.7799	0.9995	15414	2	-0.038
APEX1	6	0.65298	0.77996	0.9995	15415	1	-0.665
AHSA1	6	0.65314	0.78007	0.9995	15416	2	-0.057
OGFOD1	6	0.65317	0.7801	0.9995	15417	2	-0.27
XCL1	3	0.65329	0.66014	0.9995	15418	1	-1.811
hsa-mir-513b	4	0.6533	0.69594	0.9995	15419	1	-0.964
TMEM31	5	0.65332	0.74711	0.9995	15420	1	0.2056
NACA2	6	0.65334	0.78023	0.9995	15421	2	0.1965
VPS39	6	0.65342	0.78027	0.9995	15422	2	0.153
ENPP7	6	0.65347	0.78031	0.9995	15423	2	0.0273
LIG1	6	0.65347	0.78031	0.9995	15424	2	0.0461
LOC100129924	6	0.65356	0.78038	0.9995	15425	2	0.0348
INSRR	6	0.65368	0.78047	0.9995	15426	2	0.1287
IL16	6	0.65385	0.7806	0.9995	15427	2	-0.113
KCNH7	6	0.65406	0.78076	0.9995	15428	2	-0.003
hsa-mir-1231	4	0.65406	0.6964	0.9995	15429	1	-0.335
G6PC	6	0.65412	0.7808	0.9995	15430	1	-0.309
H2AFB3	1	0.65413	0.65423	0.9995	15431	0	-0.242
hsa-mir-4265	4	0.65415	0.69644	0.9995	15432	1	0.0475
HNRNPA1L2	6	0.65418	0.78084	0.9995	15433	2	0.0339
KRTAP6-1	6	0.65423	0.78088	0.9995	15434	2	-0.065
BMX	6	0.65427	0.78091	0.9995	15435	2	-0.193
CGRRF1	6	0.65435	0.78097	0.9995	15436	2	0.012
WARS	6	0.65435	0.78097	0.9995	15437	2	0.1028
KHDRBS3	6	0.65438	0.78099	0.9995	15438	2	-0.092
UGT2B28	3	0.65441	0.66107	0.9995	15439	1	0.1461
TRERF1	6	0.65444	0.78104	0.9995	15440	2	-0.325
UBR3	6	0.65446	0.78105	0.9995	15441	1	-0.272
RSRC1	6	0.6545	0.78108	0.9995	15442	2	0.1057
GP5	6	0.65486	0.78134	0.9995	15443	2	-0.008
RXRA	6	0.65488	0.78135	0.9995	15444	1	-0.181
MCAT	6	0.65498	0.78143	0.9995	15445	2	-0.179
ZNF638	6	0.65502	0.78146	0.9995	15446	1	-0.244
FEZF1	6	0.65507	0.7815	0.9995	15447	2	0.0083
AMY1C	2	0.65509	0.65488	0.9995	15448	0	0.0433
ADRB3	6	0.65511	0.78153	0.9995	15449	2	0.0721
USP48	6	0.65521	0.7816	0.9995	15450	2	-0.118
40787	3	0.65521	0.66172	0.9995	15451	1	0.1336
GPR18	6	0.65524	0.78163	0.9995	15452	1	-0.015
ACSM4	6	0.65526	0.78164	0.9995	15453	2	0.031
ADAR	6	0.65526	0.78164	0.9995	15454	2	-0.378
SLC6A5	6	0.65528	0.78166	0.9995	15455	2	0.1229
LOC100130880	6	0.65533	0.78169	0.9995	15456	1	-0.226
TIMM9	6	0.65536	0.78172	0.9995	15457	1	-0.186
GNAS	6	0.65542	0.78177	0.9995	15458	1	-0.223
HAGH	6	0.65544	0.78178	0.9995	15459	2	0.0953
MAGED1	6	0.65554	0.78187	0.9995	15460	1	-0.015
CBWD3	2	0.65558	0.65536	0.9995	15461	0	-0.048
FAM134A	6	0.65562	0.78193	0.9995	15462	1	-0.018
GOLGA8B	4	0.6557	0.6974	0.9995	15463	1	-0.17
DEPDC7	6	0.65573	0.78203	0.9995	15464	1	0.0245
DPH3P1	6	0.65577	0.78204	0.9995	15465	2	0.1272

SLC30A6	6	0.65577	0.78204	0.9995	15466	2	0.051
HSD17B1	6	0.65582	0.78209	0.9995	15467	2	-0.009
IGJ	6	0.65583	0.7821	0.9995	15468	1	-0.009
TMEM106A	6	0.65594	0.78218	0.9995	15469	1	-0.09
XIAP	6	0.65605	0.78226	0.9995	15470	2	-0.042
hsa-mir-7849	2	0.65612	0.65589	0.9995	15471	0	-0.162
SOX18	6	0.65622	0.78239	0.9995	15472	2	-0.163
C17orf72	6	0.65634	0.78248	0.9995	15473	2	-0.146
hsa-mir-548f-5	4	0.65645	0.69784	0.9995	15474	1	0.2397
SASS6	6	0.65646	0.78257	0.9995	15475	2	-0.003
CFC1	2	0.65648	0.65625	0.9995	15476	0	-0.237
KLK8	6	0.6565	0.7826	0.9995	15477	2	-0.051
C4orf33	6	0.65651	0.78261	0.9995	15478	1	-0.422
hsa-mir-7108	4	0.65656	0.69791	0.9995	15479	1	-0.217
LAMP5	6	0.65657	0.78265	0.9995	15480	1	-0.315
TPRG1	6	0.65666	0.78273	0.9995	15481	1	-0.062
OR14C36	6	0.65668	0.78274	0.9995	15482	2	0.0692
RNF216	6	0.65681	0.78285	0.9995	15483	2	-0.157
PRR14L	6	0.65686	0.78289	0.9995	15484	2	0.1562
CHL1	6	0.657	0.78299	0.9995	15485	1	-0.104
NPDC1	6	0.65705	0.78303	0.9995	15486	2	-0.398
MARCH11	6	0.6571	0.78306	0.9995	15487	1	-0.38
MEGF6	6	0.65722	0.78316	0.9995	15488	2	-0.108
FAM49B	6	0.65722	0.78316	0.9995	15489	1	-0.385
B3GAT3	6	0.65727	0.7832	0.9995	15490	2	-0.164
hsa-mir-4516	4	0.65739	0.69841	0.9995	15491	1	-0.06
GGNBP2	4	0.65743	0.69843	0.9995	15492	1	0.1003
HADH	6	0.65756	0.78342	0.9995	15493	2	0.1174
ERAS	6	0.65765	0.78349	0.9995	15494	2	-0.056
PTGS1	6	0.65772	0.78355	0.9995	15495	2	-0.222
SHOC2	6	0.65777	0.78359	0.9995	15496	2	0.0908
KLF13	6	0.65783	0.78363	0.9995	15497	1	-0.377
NFKB2	6	0.65788	0.78368	0.9995	15498	2	0.1747
TSPYL1	6	0.6579	0.78369	0.9995	15499	1	-0.182
KCNS2	6	0.65809	0.78384	0.9995	15500	2	0.1061
hsa-mir-510	4	0.65811	0.69882	0.9995	15501	1	0.1001
DLGAP1	6	0.65816	0.7839	0.9995	15502	1	-0.033
C22orf39	6	0.65832	0.78401	0.9995	15503	2	-0.108
PSG5	6	0.65832	0.78401	0.9995	15504	1	-0.079
ACSS1	6	0.65837	0.78406	0.9995	15505	2	0.1916
ZNF419	6	0.65842	0.7841	0.9995	15506	1	-0.132
DMRT2	6	0.65848	0.78414	0.9995	15507	2	-0.206
CYTL1	6	0.65848	0.78414	0.9995	15508	2	-0.052
ERVV-1	6	0.65857	0.78421	0.9995	15509	2	0.1062
SNX13	6	0.65868	0.7843	0.9995	15510	2	0.147
ZNF205	6	0.65871	0.78433	0.9995	15511	2	0.1203
SLC16A10	6	0.65882	0.78441	0.9995	15512	2	-0.377
KRT25	6	0.65887	0.78444	0.9995	15513	2	-0.415
SLC25A5	6	0.65896	0.78451	0.9995	15514	2	0.0288
ACTL7A	6	0.65901	0.78454	0.9995	15515	1	0.0244
GIMAP5	5	0.65908	0.74943	0.9995	15516	1	0.1091
PIP5K1A	6	0.65918	0.78467	0.9995	15517	2	-0.46
AFMID	6	0.65929	0.78477	0.9995	15518	2	-0.142
BPI	6	0.65929	0.78477	0.9995	15519	2	-0.465
KANSL1L	6	0.65933	0.7848	0.9995	15520	2	0.0614
ARHGEF19	6	0.65934	0.78481	0.9995	15521	1	0.1325
IRX5	6	0.65939	0.78485	0.9995	15522	2	0.1004
CCNA1	6	0.65941	0.78487	0.9995	15523	2	-0.14
DAP	6	0.65944	0.78489	0.9995	15524	2	-0.335
ELFN2	6	0.65945	0.7849	0.9995	15525	2	-0.072
ACSS3	6	0.65949	0.78492	0.9995	15526	1	-0.356
SS18L1	6	0.6595	0.78493	0.9995	15527	2	-0.328
CCL18	6	0.65956	0.78498	0.9995	15528	2	-0.289
LY6G6D	6	0.65961	0.78503	0.9995	15529	2	-0.241
USP49	6	0.65969	0.78509	0.9995	15530	2	-0.226
BOD1L2	6	0.65972	0.78511	0.9995	15531	2	-0.103
CENPE	4	0.65974	0.6998	0.9995	15532	1	-0.087
DCUN1D1	6	0.65977	0.78515	0.9995	15533	1	-0.219
ALOXE3	6	0.65978	0.78515	0.9995	15534	2	0.0399
SERBP1	6	0.65982	0.78518	0.9995	15535	2	-0.182
CPQ	6	0.65992	0.78525	0.9995	15536	2	-0.025
ADAM12	6	0.65999	0.7853	0.9995	15537	2	-0.51
hsa-mir-1255b-1	1	0.66001	0.66008	0.9995	15538	0	-0.332
hsa-mir-2276	4	0.66002	0.69995	0.9995	15539	1	0.0213
U2AF1	6	0.66003	0.78533	0.9995	15540	2	0.1445
GAL3ST3	6	0.66003	0.78533	0.9995	15541	2	-0.269
HSD17B6	6	0.6601	0.78538	0.9995	15542	2	-0.043
SAMD10	6	0.66019	0.78545	0.9995	15543	1	-0.989
RAPGEF3	6	0.66028	0.78553	0.9995	15544	2	-0.236
MED20	6	0.66028	0.78553	0.9995	15545	2	0.0392
TSPAN12	6	0.66038	0.78561	0.9995	15546	2	0.1357
ARHGAP19	6	0.66041	0.78564	0.9995	15547	1	-0.159
WNT1	6	0.66046	0.78568	0.9995	15548	1	0.1788
CEP85	6	0.66055	0.78574	0.9995	15549	1	-0.321
PVRL1	6	0.66058	0.78576	0.9995	15550	2	-0.116

hsa-mir-587	2	0.66059	0.66033	0.9995	15551	0	-0.613
PCDH12	6	0.66061	0.78579	0.9995	15552	2	-0.13
hsa-mir-3159	4	0.66062	0.70031	0.9995	15553	1	-0.347
MMP25	6	0.66065	0.78582	0.9995	15554	2	0.2037
NLRP13	6	0.6607	0.78586	0.9995	15555	2	-0.221
hsa-mir-4262	4	0.6608	0.70043	0.9995	15556	1	0.0637
TMEM57	6	0.66088	0.78601	0.9995	15557	2	-0.263
LACRT	6	0.66088	0.78601	0.9995	15558	2	-0.072
C1orf177	6	0.661	0.7861	0.9995	15559	2	-0.271
MUC12	6	0.66107	0.78616	0.9995	15560	2	-0.14
CACNG7	6	0.66113	0.78621	0.9995	15561	2	-0.127
HIST1H2AL	5	0.6613	0.75032	0.9995	15562	1	-0.279
ZNF705A	2	0.66131	0.66105	0.9995	15563	0	-0.236
RNF175	6	0.6615	0.78649	0.9995	15564	2	0.1242
hsa-mir-1305	4	0.66152	0.70084	0.9995	15565	1	0.2021
TMEM63C	6	0.66152	0.78651	0.9995	15566	1	0.1117
ADO	6	0.66162	0.78659	0.9995	15567	2	-0.126
FAM195B	6	0.66173	0.78668	0.9995	15568	1	-0.128
ZNF259	6	0.66176	0.7867	0.9995	15569	2	-0.324
GABRA2	6	0.6619	0.78681	0.9995	15570	2	0.1226
FBXW8	6	0.66194	0.78683	0.9995	15571	1	0.0543
FAM177A1	6	0.66195	0.78683	0.9995	15572	2	0.1218
RNF187	6	0.66206	0.78693	0.9995	15573	2	-0.04
FAM98A	6	0.66209	0.78695	0.9995	15574	1	-0.659
ABHD4	6	0.66216	0.787	0.9995	15575	1	0.0057
FAM171A2	6	0.66219	0.78702	0.9995	15576	2	-0.128
EXT2	6	0.66232	0.78713	0.9995	15577	2	-0.16
PLSCR1	6	0.66243	0.78722	0.9995	15578	2	-0.324
MRPL43	6	0.66248	0.78726	0.9995	15579	2	-0.3
PPP1R3F	6	0.66258	0.78733	0.9995	15580	2	0.0941
GPR98	6	0.66258	0.78733	0.9995	15581	2	-0.325
NLRC4	6	0.66258	0.78733	0.9995	15582	2	0.1473
TRIM5	6	0.66258	0.78733	0.9995	15583	2	-0.184
XIRP1	6	0.66264	0.78739	0.9995	15584	1	-0.197
DDHD1	6	0.66269	0.78742	0.9995	15585	2	-0.213
FOXE1	6	0.66275	0.78747	0.9995	15586	1	-0.236
hsa-mir-1225	4	0.66276	0.70156	0.9995	15587	1	-1.32
HOXC10	6	0.66276	0.78747	0.9995	15588	2	-0.098
OR4K17	6	0.66281	0.78751	0.9995	15589	1	-0.077
APBB3	6	0.66289	0.78757	0.9995	15590	1	0.118
FAM214B	6	0.66297	0.78763	0.9995	15591	2	-0.179
hsa-mir-6839	4	0.66309	0.70175	0.9995	15592	1	-0.213
hsa-mir-193a	4	0.66326	0.70185	0.9995	15593	1	-0.042
ZNF7	6	0.66346	0.78801	0.9995	15594	2	0.0847
hsa-mir-6758	4	0.66346	0.70198	0.9995	15595	1	0.0406
ACP5	6	0.6636	0.78812	0.9995	15596	2	-0.258
LRRC71	6	0.66364	0.78815	0.9995	15597	1	-0.377
ACSM2A	5	0.66368	0.7513	0.9995	15598	1	-0.689
TUBB1	6	0.66368	0.7882	0.9995	15599	2	-0.1
WDR74	6	0.66374	0.78824	0.9995	15600	1	-0.225
ZACN	6	0.66378	0.78827	0.9995	15601	2	-0.165
FBXO41	6	0.66383	0.78831	0.9995	15602	2	0.0987
hsa-mir-564	4	0.66385	0.70221	0.9995	15603	1	-0.341
EBI3	6	0.66387	0.78834	0.9995	15604	2	0.1331
CCDC82	6	0.6639	0.78836	0.9995	15605	1	-0.02
SLC1A3	6	0.66395	0.7884	0.9995	15606	2	-0.123
RGL2	6	0.66395	0.7884	0.9995	15607	2	0.011
SLC5A12	6	0.66402	0.78846	0.9995	15608	2	-0.188
hsa-mir-3689d-1	3	0.66406	0.6691	0.9995	15609	1	0.1582
TOM1L1	6	0.66408	0.7885	0.9995	15610	2	-0.309
PCDHGA6	2	0.66417	0.66391	0.9995	15611	0	-0.349
LIMD1	6	0.6643	0.78867	0.9995	15612	2	0.1189
DOCK11	6	0.66435	0.78871	0.9995	15613	1	-0.383
KRR1	6	0.66441	0.78875	0.9995	15614	2	-0.312
SPIDR	2	0.6645	0.66424	0.9995	15615	0	-0.029
hsa-mir-10b	4	0.66453	0.70263	0.9995	15616	1	-0.189
hsa-mir-6735	4	0.66453	0.70263	0.9995	15617	1	-0.128
MUSK	6	0.66454	0.78887	0.9995	15618	2	-0.133
hsa-mir-6515	4	0.66477	0.70277	0.9995	15619	1	-0.346
YTHDF1	6	0.6648	0.78907	0.9995	15620	1	0.03
MYBL1	6	0.66491	0.78916	0.9995	15621	1	-0.392
CYP2B6	6	0.66509	0.78929	0.9995	15622	2	-0.092
GTF2I	6	0.66515	0.78934	0.9995	15623	1	-0.093
GRM1	6	0.66516	0.78934	0.9995	15624	2	-0.128
FBXW10	4	0.66516	0.70303	0.9995	15625	1	-0.263
CRISP1	6	0.6653	0.78947	0.9995	15626	1	-0.253
TTC1	6	0.66546	0.7896	0.9995	15627	2	-0.044
CLCN3	6	0.66549	0.78962	0.9995	15628	1	0.1546
PRODH2	6	0.6656	0.7897	0.9995	15629	1	-0.012
CHAT	6	0.66564	0.78973	0.9995	15630	1	-0.076
hsa-mir-6763	4	0.66565	0.70333	0.9995	15631	1	-0.004
hsa-mir-6751	4	0.66585	0.70345	0.9995	15632	1	-0.005
UNC13B	6	0.66612	0.79012	0.9995	15633	2	-0.25
EIF2S2	6	0.66612	0.79012	0.9995	15634	2	-0.114
hsa-mir-3684	3	0.66613	0.67088	0.9995	15635	1	0.108

ZNRF2	6	0.6662	0.79018	0.9995	15636	1	-0.06
HNRNPR	6	0.66622	0.7902	0.9995	15637	2	-0.053
DHX38	6	0.66629	0.79026	0.9995	15638	2	-0.193
PCDH17	6	0.66648	0.79041	0.9995	15639	2	-0.254
MBD1	6	0.66648	0.79041	0.9995	15640	2	-0.249
TOP2A	6	0.66657	0.79047	0.9995	15641	2	-0.02
COMMD6	6	0.66657	0.79047	0.9995	15642	2	-0.019
NCAM2	6	0.66658	0.79048	0.9995	15643	1	-0.321
EYA4	6	0.66665	0.79055	0.9995	15644	1	-0.221
DNASE2B	6	0.66668	0.79058	0.9995	15645	2	-0.217
RPS6	6	0.66688	0.79072	0.9995	15646	2	-0.083
KIAA1109	6	0.66697	0.79081	0.9995	15647	1	0.0216
KCNAB2	6	0.66705	0.79086	0.9995	15648	1	0.0155
CTHRC1	6	0.66719	0.79097	0.9995	15649	1	-0.102
JPH2	6	0.66726	0.79102	0.9995	15650	2	-0.189
hsa-mir-6859-2	2	0.66727	0.66707	0.9995	15651	0	-0.414
SMYD5	6	0.66728	0.79105	0.9995	15652	2	0.124
ARL5B	6	0.66733	0.79109	0.9995	15653	2	-0.059
ADNP2	6	0.66734	0.7911	0.9995	15654	1	-0.117
SBK1	6	0.66739	0.79114	0.9995	15655	2	-0.118
CA4	6	0.66744	0.79117	0.9995	15656	2	-0.436
PCSK9	6	0.66746	0.79119	0.9995	15657	1	-0.097
HIGD2A	6	0.66746	0.79119	0.9995	15658	1	-0.258
RORB	6	0.66747	0.7912	0.9995	15659	2	-0.156
FAM132A	6	0.66751	0.79123	0.9995	15660	2	0.0203
PDLIM3	6	0.66754	0.79125	0.9995	15661	2	0.0601
CD1D	6	0.66759	0.79129	0.9995	15662	1	-0.142
FCGR1A	3	0.66776	0.67226	0.9995	15663	1	0.1419
WIPF1	6	0.66777	0.79144	0.9995	15664	2	-0.26
DAZAP1	6	0.66782	0.79148	0.9995	15665	2	-0.103
OR2B6	6	0.66785	0.7915	0.9995	15666	1	-0.117
SH2D2A	6	0.66788	0.79153	0.9995	15667	2	-0.052
ALPPL2	5	0.66797	0.75308	0.9995	15668	1	-0.245
GNB3	6	0.668	0.79161	0.9995	15669	2	0.2088
CALY	6	0.66816	0.79176	0.9995	15670	1	-0.452
ALG9	6	0.66821	0.79179	0.9995	15671	2	-0.271
RALGAPA2	6	0.6683	0.79186	0.9995	15672	2	-0.467
ANKRD53	6	0.66833	0.7919	0.9995	15673	2	-0.153
hsa-mir-590	3	0.66858	0.67294	0.9995	15674	1	0.0713
EMCN	6	0.66864	0.79214	0.9995	15675	1	0.0601
FAAH	6	0.66873	0.79222	0.9995	15676	1	-0.257
ALDOC	6	0.66896	0.7924	0.9995	15677	2	0.1221
PLXDC1	6	0.66896	0.7924	0.9995	15678	2	-0.106
BRIP1	4	0.66897	0.70537	0.9995	15679	1	-0.126
FOLR2	6	0.66905	0.79248	0.9995	15680	1	-0.234
GGT6	6	0.66911	0.79252	0.9995	15681	2	0.009
NUAK1	6	0.66915	0.79254	0.9995	15682	1	-0.404
hsa-mir-218-2	3	0.66919	0.67347	0.9995	15683	1	-0.067
CCDC9	6	0.6692	0.79258	0.9995	15684	2	-0.035
C9orf50	6	0.6692	0.79258	0.9995	15685	2	-0.519
ZNF250	6	0.66932	0.79268	0.9995	15686	2	0.0651
CREB3L3	6	0.66937	0.79271	0.9995	15687	2	0.0618
ST6GALNAC4	6	0.66937	0.79271	0.9995	15688	2	-0.222
RBMXL2	6	0.6694	0.79274	0.9995	15689	1	-0.167
FAM166B	6	0.66949	0.79281	0.9995	15690	1	-0.201
CCDC66	6	0.66949	0.79282	0.9995	15691	2	-0.154
PAGE4	6	0.66957	0.79288	0.9995	15692	2	0.0848
ACTR1A	6	0.66964	0.79293	0.9995	15693	2	0.0922
ARSG	6	0.66964	0.79293	0.9995	15694	2	-0.171
C1orf115	6	0.6697	0.79297	0.9995	15695	1	0.0374
SNAP29	6	0.66972	0.79298	0.9995	15696	1	-0.195
MPP3	6	0.66973	0.79299	0.9995	15697	2	0.0976
SPTBN2	6	0.66983	0.79307	0.9995	15698	1	-0.355
TRIM39	2	0.67	0.66981	0.9995	15699	0	-0.637
SLC12A8	6	0.67007	0.79326	0.9995	15700	2	0.1161
IGFBP3	6	0.67009	0.79327	0.9995	15701	2	0.0618
SMIM19	6	0.67012	0.79329	0.9995	15702	2	0.0729
LDOC1L	6	0.67013	0.7933	0.9995	15703	1	-0.122
IL1RAPL2	6	0.67024	0.79339	0.9995	15704	2	-0.142
DGAT1	6	0.67027	0.79341	0.9995	15705	2	-0.133
EXD2	6	0.67033	0.79346	0.9995	15706	2	-0.002
POLR1E	6	0.67045	0.79355	0.9995	15707	2	0.0977
IL23R	6	0.67045	0.79355	0.9995	15708	2	-0.039
AGL	6	0.67045	0.79355	0.9995	15709	2	-0.067
ELF5	6	0.67055	0.79364	0.9995	15710	2	0.0302
SOX3	6	0.67073	0.79378	0.9995	15711	1	-0.37
KBTBD3	6	0.67081	0.79384	0.9995	15712	2	0.052
HIST4H4	6	0.67082	0.79385	0.9995	15713	1	-0.524
TLCD2	6	0.67087	0.79388	0.9995	15714	2	-0.152
FAM124A	6	0.67107	0.79405	0.9995	15715	2	0.1397
IFNA5	6	0.67108	0.79407	0.9995	15716	1	-0.132
MEF2A	6	0.67108	0.79407	0.9995	15717	1	-0.217
ZNF524	6	0.67114	0.79412	0.9995	15718	2	0.0321
SOST	6	0.67132	0.79426	0.9995	15719	2	-0.611
CIT	6	0.67144	0.79434	0.9995	15720	1	-0.469

SMAD3	6	0.67144	0.79434	0.9995	15721	1	-0.144
RD3	6	0.67152	0.7944	0.9995	15722	2	0.0885
hsa-mir-5002	4	0.67154	0.70697	0.9995	15723	1	0.1288
CD2AP	6	0.67156	0.79443	0.9995	15724	1	-0.94
SYPL2	6	0.67161	0.79447	0.9995	15725	1	-0.238
CHAC1	6	0.67162	0.79448	0.9995	15726	2	0.0502
hsa-mir-522	2	0.67168	0.67153	0.9995	15727	0	-0.128
RNF5	6	0.67177	0.7946	0.9995	15728	2	0.1812
DDC	6	0.67186	0.79466	0.9995	15729	2	-0.264
FMNL1	6	0.67187	0.79468	0.9995	15730	1	-0.163
PFDN4	6	0.67191	0.79471	0.9995	15731	2	-0.157
C10orf105	6	0.67201	0.79479	0.9995	15732	2	-0.146
IGFBP2	6	0.67205	0.79481	0.9995	15733	2	0.1702
ANAPC11	6	0.67208	0.79484	0.9995	15734	1	-0.372
KRTAP19-1	6	0.67208	0.79484	0.9995	15735	1	-0.69
LSM6	6	0.67217	0.79491	0.9995	15736	2	-0.499
SFTPD	6	0.6723	0.79502	0.9995	15737	2	-0.138
LAG3	5	0.67239	0.75495	0.9995	15738	1	0.1577
SNX7	6	0.67243	0.79511	0.9995	15739	2	-0.094
hsa-mir-4496	2	0.67243	0.67227	0.9995	15740	0	-0.027
PROZ	6	0.6725	0.79517	0.9995	15741	2	0.1343
C6orf165	6	0.67266	0.7953	0.9995	15742	2	0.1963
KIAA1958	6	0.67275	0.79536	0.9995	15743	1	-0.316
MPHOSPH8	6	0.67279	0.79539	0.9995	15744	2	-0.101
F13A1	6	0.67279	0.79539	0.9995	15745	2	0.1537
HEATR6	6	0.673	0.79556	0.9995	15746	2	0.1203
hsa-mir-548ad	3	0.67303	0.67679	0.9995	15747	1	-0.076
PRRG1	6	0.67309	0.79563	0.9995	15748	1	-0.541
CARD14	6	0.67309	0.79563	0.9995	15749	1	0.0163
LYNX1	6	0.67315	0.79568	0.9995	15750	1	-0.026
ANGPT2	6	0.67315	0.79569	0.9995	15751	2	-0.009
FBXL22	6	0.67318	0.7957	0.9995	15752	2	0.0215
COIL	6	0.67338	0.79586	0.9995	15753	2	-0.168
hsa-mir-4464	4	0.6734	0.70811	0.9995	15754	1	-0.198
LIM2	6	0.67341	0.79588	0.9995	15755	1	-0.213
PITX3	6	0.67346	0.79591	0.9995	15756	2	0.0105
ZADH2	6	0.6736	0.79602	0.9995	15757	1	-0.454
ZFP112	6	0.6736	0.79602	0.9995	15758	1	-0.85
GPR75-ASB3	3	0.67375	0.67744	0.9995	15759	1	-0.168
CC2D1A	6	0.67383	0.7962	0.9995	15760	2	0.2098
LRRC3	6	0.67393	0.79627	0.9995	15761	1	-0.017
KRTAP9-9	4	0.67395	0.70846	0.9995	15762	1	-1.56
PIGM	6	0.67396	0.79629	0.9995	15763	2	-0.129
ARSE	6	0.67404	0.79635	0.9995	15764	2	-0.044
FANCB	6	0.67412	0.79642	0.9995	15765	2	-0.158
CSMD2	6	0.67439	0.79662	0.9995	15766	2	-0.114
MAFK	6	0.67449	0.7967	0.9995	15767	2	-0.261
ST8SIA2	6	0.67462	0.7968	0.9995	15768	2	-0.409
CA11	6	0.67463	0.79681	0.9995	15769	1	-0.135
CENPW	6	0.67463	0.79681	0.9995	15770	1	-0.388
TGFB1	6	0.67473	0.79689	0.9995	15771	1	-0.164
CXCL11	6	0.67479	0.79694	0.9995	15772	1	-0.382
XPO7	6	0.67484	0.79698	0.9995	15773	2	0.03
MDFI	6	0.67489	0.79702	0.9995	15774	2	0.078
NKX6-1	6	0.67489	0.79702	0.9995	15775	2	0.1401
C1orf86	6	0.67494	0.79706	0.9995	15776	2	-0.022
RND2	6	0.675	0.79711	0.9995	15777	2	-0.031
VAMP8	6	0.67507	0.79717	0.9995	15778	2	0.0112
PRIMA1	6	0.67522	0.7973	0.9995	15779	2	0.0351
OR52A1	6	0.67522	0.7973	0.9995	15780	2	-0.002
SNX16	6	0.6753	0.79736	0.9995	15781	1	-0.094
CSTF2T	6	0.67545	0.79746	0.9995	15782	2	0.0762
RPL13	6	0.67546	0.79747	0.9995	15783	1	-0.126
OR1J2	6	0.67546	0.79748	0.9995	15784	2	0.0894
NAP1L3	6	0.67552	0.79751	0.9995	15785	2	0.0614
DUOX1	6	0.67569	0.79766	0.9995	15786	2	-0.177
ADARB1	6	0.67569	0.79766	0.9995	15787	2	-0.101
SARDH	6	0.67573	0.79769	0.9995	15788	1	-0.113
hsa-mir-3185	4	0.67579	0.7096	0.9995	15789	1	-1.229
ENTPD6	6	0.67581	0.79775	0.9995	15790	2	-0.215
KLB	6	0.6759	0.79783	0.9995	15791	1	-0.103
MCCC2	6	0.67617	0.79804	0.9995	15792	2	-0.039
FBXO9	6	0.67623	0.79808	0.9995	15793	2	-0.031
TRIM48	4	0.6763	0.70992	0.9995	15794	1	-0.826
IZUMO2	6	0.67633	0.79816	0.9995	15795	2	-0.274
PIGL	4	0.67638	0.70997	0.9995	15796	1	-0.062
ITPK1	6	0.67638	0.79819	0.9995	15797	2	-0.171
KHDC1	6	0.67645	0.79824	0.9995	15798	2	0.0121
B3GALT2	6	0.67647	0.79826	0.9995	15799	1	0.0498
LHFP	6	0.67662	0.79839	0.9995	15800	2	-0.237
NOC2L	6	0.67673	0.79848	0.9995	15801	1	-1.015
ARL17B	1	0.67674	0.67678	0.9995	15802	0	-0.341
LSM14B	6	0.67682	0.79855	0.9995	15803	2	0.0651
XXYLT1	6	0.67683	0.79856	0.9995	15804	1	-0.183
40787	3	0.67684	0.68017	0.9995	15805	1	-0.026

IGFBP4	6	0.67697	0.79866	0.9995	15806	2	-0.238
CLK4	6	0.67697	0.79866	0.9995	15807	2	0.097
CMTM5	6	0.67697	0.79866	0.9995	15808	2	-0.06
RAET1E	6	0.67706	0.79874	0.9995	15809	2	0.0048
PRPF6	6	0.6774	0.79899	0.9995	15810	2	-0.031
CXorf21	6	0.67742	0.79901	0.9995	15811	1	-0.432
EMC7	6	0.67753	0.7991	0.9995	15812	2	-0.171
RPL37A	6	0.67759	0.79914	0.9995	15813	2	0.174
CAMKV	6	0.67759	0.79914	0.9995	15814	2	0.1529
ACMSD	6	0.67759	0.79914	0.9995	15815	2	0.0982
DUS2L	2	0.67765	0.67754	0.9995	15816	0	-0.102
UNK	6	0.67767	0.7992	0.9995	15817	1	-0.177
ZBTB41	6	0.6778	0.79932	0.9995	15818	2	-0.31
NDFIP1	6	0.67784	0.79934	0.9995	15819	2	0.0255
ARL2BP	6	0.67786	0.79936	0.9995	15820	1	-0.216
SNX11	6	0.67792	0.79941	0.9995	15821	2	0.0749
YBX2	6	0.67795	0.79943	0.9995	15822	1	-0.428
USP9X	6	0.67795	0.79943	0.9995	15823	1	-0.444
NIPSNAP3A	6	0.67799	0.79946	0.9995	15824	1	-0.111
LOC646498	1	0.678	0.67805	0.9995	15825	0	-0.357
MLNR	6	0.67801	0.79947	0.9995	15826	2	0.0341
CCDC127	6	0.67803	0.79949	0.9995	15827	1	-0.163
ACAA1	6	0.67809	0.79954	0.9995	15828	2	0.1141
ZMIZ1	6	0.67821	0.79965	0.9995	15829	1	-0.061
OR51D1	6	0.67828	0.7997	0.9995	15830	2	0.1467
GSTM2	5	0.67829	0.75745	0.9995	15831	1	-0.197
ATP6V0C	6	0.67837	0.79977	0.9995	15832	1	-1.053
EMC8	6	0.67843	0.7998	0.9995	15833	2	0.1496
PAIP2	6	0.67847	0.79983	0.9995	15834	2	0.1524
CAPN1	6	0.67853	0.79988	0.9995	15835	1	-0.185
hsa-mir-621	4	0.67855	0.71134	0.9995	15836	1	-1.081
OTUD6B	6	0.67855	0.7999	0.9995	15837	2	0.0163
SLC35F6	6	0.67863	0.79995	0.9995	15838	2	0.07
NOS2	6	0.67865	0.79997	0.9995	15839	1	-0.418
NDRG4	6	0.67871	0.80002	0.9995	15840	1	-0.685
RIPPLY3	2	0.67875	0.67866	0.9995	15841	0	-0.055
PCSK7	6	0.67898	0.80023	0.9995	15842	1	0.0332
TREX2	6	0.67902	0.80025	0.9995	15843	2	-0.244
NCR3LG1	6	0.67903	0.80026	0.9995	15844	1	-0.576
SLC22A3	6	0.67913	0.80034	0.9995	15845	2	-0.566
FMN1	6	0.67913	0.80034	0.9995	15846	2	0.0107
C12orf68	6	0.67926	0.80045	0.9995	15847	1	0.1019
ZNF496	6	0.67928	0.80047	0.9995	15848	1	-0.152
MTSS1L	6	0.67939	0.80054	0.9995	15849	2	-0.424
RER1	6	0.67954	0.80065	0.9995	15850	1	-0.131
NPNT	6	0.67961	0.80071	0.9995	15851	2	0.0575
SPTSSA	6	0.67964	0.80073	0.9995	15852	1	-0.074
PNLIP	6	0.67966	0.80075	0.9995	15853	2	0.0052
AKR1C2	5	0.67975	0.75808	0.9995	15854	1	-0.16
CRB3	6	0.67985	0.80091	0.9995	15855	1	0.0368
ATPBD4	2	0.67994	0.67988	0.9995	15856	0	0.0237
DEFA4	6	0.68	0.80103	0.9995	15857	2	-0.061
WDR91	6	0.68003	0.80105	0.9995	15858	1	-0.404
LIPA	6	0.68011	0.80111	0.9995	15859	2	-0.306
DICER1	6	0.68012	0.80112	0.9995	15860	2	-0.093
GRINA	6	0.68017	0.80115	0.9995	15861	2	-0.059
NSMCE2	6	0.68021	0.80118	0.9995	15862	2	-0.149
RAB3A	6	0.68045	0.80138	0.9995	15863	2	0.0639
EIF1AD	6	0.68046	0.80138	0.9995	15864	1	0.1837
POU4F3	6	0.6805	0.80142	0.9995	15865	2	-0.431
IQCE	6	0.68052	0.80143	0.9995	15866	2	-0.578
SOCS5	6	0.68062	0.80151	0.9995	15867	2	-0.472
FAM213B	6	0.68062	0.80151	0.9995	15868	2	-0.211
SOS2	6	0.68078	0.80163	0.9995	15869	2	-0.031
KRT18	6	0.68082	0.80166	0.9995	15870	1	-0.371
S100A5	6	0.68086	0.8017	0.9995	15871	2	0.0394
NDUFA4	6	0.68105	0.80184	0.9995	15872	2	0.0829
CLDN24	6	0.68109	0.80187	0.9995	15873	2	-0.07
TLR5	6	0.6811	0.80188	0.9995	15874	1	-0.261
LDB2	6	0.6811	0.80188	0.9995	15875	1	-0.054
FBXW5	6	0.68115	0.80192	0.9995	15876	2	-0.073
PLN	1	0.68117	0.68118	0.9995	15877	0	-1.888
SPRR2B	1	0.68117	0.68118	0.9995	15878	0	-1.888
hsa-mir-518a-1	1	0.68117	0.68118	0.9995	15879	0	-1.888
C11orf57	6	0.6812	0.80196	0.9995	15880	2	0.0467
MCM3AP	6	0.68128	0.80203	0.9995	15881	2	-0.091
KIR2DL3	5	0.68132	0.75874	0.9995	15882	1	0.0727
DCD	6	0.68136	0.80208	0.9995	15883	2	0.0154
ARSF	6	0.68153	0.80222	0.9995	15884	1	-0.162
hsa-mir-5047	4	0.68165	0.7133	0.9995	15885	1	-0.179
ATL1	6	0.6817	0.80237	0.9995	15886	2	-0.238
UBQLN4	6	0.6817	0.80237	0.9995	15887	2	0.0852
CLDN12	6	0.68177	0.80243	0.9995	15888	2	0.0568
PLA2G4B	6	0.6818	0.80245	0.9995	15889	1	-0.382
FAM194B	6	0.6819	0.80253	0.9995	15890	1	-0.432

PPP1R26	6	0.6819	0.80253	0.9995	15891	1	-0.278
CSTF1	6	0.68192	0.80254	0.9995	15892	2	-0.144
LOC100130451	6	0.68201	0.80262	0.9995	15893	1	0.0352
C9orf3	6	0.68214	0.80271	0.9995	15894	2	0.0758
RBM24	6	0.68214	0.80271	0.9995	15895	2	-0.034
THOC6	4	0.68218	0.71363	0.9995	15896	1	-0.235
ADM	6	0.6822	0.80277	0.9995	15897	2	-0.126
KLF17	6	0.68224	0.8028	0.9995	15898	2	0.061
RPL3L	6	0.68226	0.80281	0.9995	15899	2	-0.317
LRRN1	6	0.6824	0.80293	0.9995	15900	1	0.0368
ZNF784	6	0.68245	0.80296	0.9995	15901	2	0.1293
FAM153B	2	0.68253	0.68245	0.9995	15902	0	-0.348
FUT11	6	0.68257	0.80306	0.9995	15903	2	-0.066
PEF1	6	0.68262	0.8031	0.9995	15904	1	-0.066
TSPYL4	6	0.68269	0.80316	0.9995	15905	1	-0.228
CNFN	6	0.68273	0.80318	0.9995	15906	2	-0.023
VWA5B2	6	0.68282	0.80326	0.9995	15907	1	-0.3
PFKFB4	6	0.68285	0.80327	0.9995	15908	2	-0.192
SHARPIN	6	0.68285	0.80327	0.9995	15909	2	-0.522
FBXO6	6	0.6829	0.80331	0.9995	15910	1	-0.603
PCNXL3	6	0.68303	0.80341	0.9995	15911	2	-0.21
TMEM184A	6	0.68303	0.80341	0.9995	15912	2	-0.366
POLR1D	6	0.68305	0.80343	0.9995	15913	1	-0.299
S100A7	5	0.68318	0.75955	0.9995	15914	1	-0.451
ADCY3	6	0.6832	0.80355	0.9995	15915	2	0.0868
DDTL	2	0.6833	0.68323	0.9995	15916	0	-0.343
COL11A1	6	0.68342	0.8037	0.9995	15917	1	-0.741
LRRC58	6	0.68353	0.80379	0.9995	15918	2	-0.191
LDLRAD1	6	0.68353	0.80379	0.9995	15919	2	-0.161
SEC14L5	6	0.68361	0.80386	0.9995	15920	2	0.0325
MIEF1	2	0.68362	0.68352	0.9995	15921	0	-0.324
IRAK4	6	0.68379	0.80401	0.9995	15922	2	0.0206
C7orf25	6	0.68379	0.80401	0.9995	15923	2	-0.045
RS1	6	0.68386	0.80406	0.9995	15924	2	-0.349
LPIN2	6	0.68391	0.80411	0.9995	15925	2	-0.097
SUV420H2	6	0.68398	0.80415	0.9995	15926	1	-0.152
hsa-mir-8059	4	0.6841	0.71489	0.9995	15927	1	-0.028
MYL1	6	0.68411	0.80427	0.9995	15928	2	-0.088
VTCN1	6	0.68419	0.80433	0.9995	15929	2	-0.195
PRR23A	6	0.68456	0.80461	0.9995	15930	2	-0.106
KRT5	6	0.68456	0.80461	0.9995	15931	2	-0.139
BOLA1	6	0.68456	0.80461	0.9995	15932	2	-0.26
RGS5	6	0.68459	0.80464	0.9995	15933	1	-0.103
OR51A7	6	0.68464	0.80467	0.9995	15934	1	-0.219
SCYL2	6	0.68483	0.80482	0.9995	15935	1	0.0136
ACTG2	5	0.685	0.76034	0.9995	15936	1	-0.133
FRS3	6	0.68503	0.80497	0.9995	15937	2	0.0183
TNKS1BP1	6	0.68504	0.80499	0.9995	15938	2	-0.164
SPATA31D3	2	0.6851	0.68499	0.9995	15939	0	-0.258
SLC20A2	6	0.6852	0.80511	0.9995	15940	1	-0.59
hsa-mir-382	3	0.68534	0.68781	0.9995	15941	1	-0.252
BBS1	6	0.68535	0.80521	0.9995	15942	1	-0.064
GP9	6	0.68542	0.80527	0.9995	15943	1	-0.544
STK4	6	0.68545	0.8053	0.9995	15944	1	-0.337
SF3B2	6	0.68554	0.80537	0.9995	15945	2	-0.214
hsa-mir-421	4	0.6857	0.71587	0.9995	15946	1	-0.197
GTPBP1	6	0.6858	0.80559	0.9995	15947	2	-0.119
SELT	6	0.6858	0.80559	0.9995	15948	2	-0.215
TMEM19	6	0.68585	0.80563	0.9995	15949	1	-0.111
CST9	6	0.68587	0.80564	0.9995	15950	2	-0.023
RAB22A	6	0.6859	0.80566	0.9995	15951	2	-0.239
KLF14	6	0.68593	0.80568	0.9995	15952	2	-0.106
NT5C3B	6	0.68598	0.80572	0.9995	15953	2	-0.009
LRPAP1	6	0.68603	0.80576	0.9995	15954	2	-0.088
PSMA6	6	0.68609	0.80581	0.9995	15955	2	-0.671
MAB21L3	6	0.68609	0.80581	0.9995	15956	2	-0.336
F2RL3	6	0.68617	0.80587	0.9995	15957	2	-0.027
GLS2	6	0.68623	0.80592	0.9995	15958	2	-0.029
KHDC3L	6	0.68629	0.80597	0.9995	15959	2	0.0492
CCDC175	6	0.68646	0.8061	0.9995	15960	1	0.0707
PTPRC	6	0.68653	0.80615	0.9995	15961	1	-0.185
SPTBN1	6	0.6866	0.8062	0.9995	15962	2	0.1467
EIF5B	6	0.68671	0.80628	0.9995	15963	2	-0.327
C12orf44	6	0.68671	0.80628	0.9995	15964	2	0.1013
KLHL4	6	0.68686	0.8064	0.9995	15965	1	-0.272
SPINT3	6	0.6869	0.80643	0.9995	15966	2	-0.205
PPP2CB	6	0.6869	0.80643	0.9995	15967	2	-0.177
MOB2	6	0.68697	0.80648	0.9995	15968	1	-0.971
CGA	6	0.68698	0.80649	0.9995	15969	2	0.1038
ZNF547	6	0.68706	0.80655	0.9995	15970	2	-0.091
DNAJC22	6	0.68716	0.80663	0.9995	15971	2	-0.096
DOCK7	6	0.68721	0.80668	0.9995	15972	2	-0.191
hsa-mir-6841	2	0.68733	0.68719	0.9995	15973	0	-0.265
COL13A1	6	0.68756	0.80697	0.9995	15974	1	-0.706
NDUFA2	6	0.68778	0.80715	0.9995	15975	2	-0.079

TAS2R4	6	0.68783	0.80719	0.9995	15976	1	-0.261
CAAP1	6	0.68784	0.8072	0.9995	15977	2	-0.055
SERPING1	6	0.68789	0.80724	0.9995	15978	2	0.1251
hsa-mir-6881	4	0.68792	0.71722	0.9995	15979	1	-0.079
SERPINB2	6	0.68804	0.80736	0.9995	15980	2	0.1896
ARL14EP	6	0.6881	0.8074	0.9995	15981	2	-0.391
KRTAP19-4	4	0.68812	0.71737	0.9995	15982	1	-1.785
TOR1A	6	0.68819	0.80748	0.9995	15983	1	-0.544
CCDC47	6	0.68827	0.80756	0.9995	15984	2	-0.502
SYCE1	6	0.68827	0.80756	0.9995	15985	2	-0.136
hsa-mir-192	4	0.6883	0.71748	0.9995	15986	1	0.0467
PCED1A	6	0.68841	0.80766	0.9995	15987	1	-0.495
RPS6KA1	6	0.68844	0.80768	0.9995	15988	1	-0.466
DNAJC2	6	0.68851	0.80775	0.9995	15989	2	0.0976
FAF2	6	0.68853	0.80777	0.9995	15990	1	0.0956
MOCS1	6	0.68868	0.80788	0.9995	15991	2	0.1327
CLCA4	6	0.68873	0.80792	0.9995	15992	2	0.0732
GALNT12	6	0.68886	0.80804	0.9995	15993	2	-0.1
NCF2	6	0.68888	0.80805	0.9995	15994	1	-0.703
CS	6	0.68888	0.80805	0.9995	15995	1	-0.076
LOC158434	6	0.68903	0.80819	0.9995	15996	1	-0.539
MAEA	6	0.68907	0.80821	0.9995	15997	1	-0.318
LRRC38	6	0.68925	0.80836	0.9995	15998	1	-0.476
ZNF462	6	0.68925	0.80836	0.9995	15999	1	-0.379
NOTO	6	0.68928	0.80838	0.9995	16000	2	-0.618
SERPINH1	6	0.68943	0.80851	0.9995	16001	2	0.0957
KIAA1033	6	0.68947	0.80854	0.9995	16002	2	-0.036
hsa-mir-548as	4	0.6895	0.71827	0.9995	16003	1	-0.806
KRT33B	6	0.68955	0.80861	0.9995	16004	2	-0.47
KLKB1	6	0.68955	0.80861	0.9995	16005	2	-0.078
KLHL17	6	0.68969	0.80873	0.9995	16006	2	-0.233
B3GALT6	6	0.68975	0.80877	0.9995	16007	1	-0.104
MYO3B	6	0.68982	0.80882	0.9995	16008	2	0.0032
PEX10	6	0.68984	0.80883	0.9995	16009	2	-0.023
MTM1	6	0.68986	0.80885	0.9995	16010	2	-0.036
hsa-mir-372	4	0.68995	0.71854	0.9995	16011	1	-0.844
hsa-mir-1297	3	0.68997	0.69194	0.9995	16012	1	-0.341
DPP3	6	0.69003	0.80898	0.9995	16013	2	0.0854
SLC35F1	6	0.69011	0.80905	0.9995	16014	2	0.0223
CA6	6	0.69018	0.80911	0.9995	16015	2	-0.196
C10orf53	6	0.69018	0.80911	0.9995	16016	1	-0.199
CRIP3	6	0.69021	0.80914	0.9995	16017	2	0.0882
PRDX3	6	0.69023	0.80916	0.9995	16018	1	0.004
CTTNBP2	6	0.69025	0.80916	0.9995	16019	2	-0.127
SCN4B	6	0.69036	0.80925	0.9995	16020	2	-0.307
MAN2A2	6	0.6904	0.80928	0.9995	16021	2	0.0406
EBLN1	6	0.69057	0.8094	0.9995	16022	1	-0.029
ACER1	6	0.69061	0.80943	0.9995	16023	1	-0.161
C22orf24	6	0.69066	0.80949	0.9995	16024	1	-0.134
HAGHL	6	0.69072	0.80953	0.9995	16025	2	-0.051
KPNA1	6	0.69072	0.80953	0.9995	16026	2	0.006
OR5W2	6	0.69078	0.80957	0.9995	16027	2	-0.023
KRT32	6	0.69081	0.8096	0.9995	16028	2	0.0253
NEDD8-MDP1	3	0.69089	0.6928	0.9995	16029	1	-0.028
HOOK3	6	0.69091	0.80967	0.9995	16030	2	0.099
IGF2BP3	6	0.69093	0.80969	0.9995	16031	1	-0.335
RNF2	6	0.69102	0.80975	0.9995	16032	1	-0.196
OLFML2B	6	0.6911	0.80983	0.9995	16033	1	-0.368
CABS1	6	0.69113	0.80986	0.9995	16034	1	-0.018
DARC	6	0.69123	0.80994	0.9995	16035	2	-0.019
SCAF11	6	0.69136	0.81003	0.9995	16036	2	-0.011
AOC2	6	0.69147	0.81006	0.9995	16037	1	0.0786
OR8B3	6	0.69187	0.81017	0.9995	16038	1	-0.419
OVGP1	6	0.69195	0.81019	0.9995	16039	1	-0.245
KRT222	6	0.69202	0.81022	0.9995	16040	1	-0.259
hsa-mir-1304	4	0.69218	0.72	0.9995	16041	1	0.0776
OR10G9	5	0.69224	0.76357	0.9995	16042	1	-0.222
UXS1	6	0.69236	0.81032	0.9995	16043	1	-0.371
GEMIN4	6	0.69257	0.81039	0.9995	16044	1	-0.251
THG1L	6	0.69267	0.81042	0.9995	16045	1	-0.029
TPP2	6	0.69292	0.8105	0.9995	16046	1	-0.332
RNF181	6	0.69297	0.81051	0.9995	16047	1	0.0591
hsa-mir-1-1	4	0.69316	0.72066	0.9995	16048	1	-0.946
CDY2A	1	0.69327	0.69333	0.9995	16049	0	-0.234
HOXA5	6	0.69333	0.81063	0.9995	16050	1	-0.244
DAB1	6	0.69333	0.81063	0.9995	16051	1	-0.23
GAMT	6	0.69351	0.81068	0.9995	16052	1	-0.07
hsa-mir-6845	4	0.69355	0.72092	0.9995	16053	1	0.1782
SEC24C	6	0.69365	0.81072	0.9995	16054	1	-0.377
ALKBH1	6	0.69369	0.81074	0.9995	16055	1	-0.984
OR4D6	6	0.69373	0.81074	0.9995	16056	1	-0.158
KIF5C	6	0.69373	0.81074	0.9995	16057	1	-0.088
MOGAT2	6	0.69379	0.81076	0.9995	16058	1	-0.206
PADI1	6	0.69384	0.81077	0.9995	16059	1	-0.237
PILRB	6	0.69392	0.8108	0.9995	16060	1	-0.077

MED1	6	0.69404	0.81082	0.9995	16061	1	0.116
CUL5	6	0.69421	0.81087	0.9995	16062	1	-0.495
MGLL	6	0.69451	0.81096	0.9995	16063	1	-0.415
ASCL2	6	0.69451	0.81096	0.9995	16064	1	-0.131
WBP11	6	0.69462	0.811	0.9995	16065	1	-0.057
MYL5	6	0.69509	0.81113	0.9995	16066	1	-0.441
hsa-mir-1539	4	0.69516	0.72192	0.9995	16067	1	-0.163
KCNMA1	6	0.69538	0.81122	0.9995	16068	1	-0.213
EHD3	4	0.69554	0.72216	0.9995	16069	1	-0.381
CCDC28A	4	0.69561	0.7222	0.9995	16070	1	-0.301
MBD3L2	4	0.69569	0.72224	0.9995	16071	1	-1.382
ZCWPW2	6	0.6958	0.81135	0.9995	16072	1	-0.408
ZNF843	6	0.69628	0.8115	0.9995	16073	1	0.0424
CRY1	6	0.69636	0.81152	0.9995	16074	1	-0.816
hsa-mir-6860	4	0.69637	0.72267	0.9995	16075	1	-0.302
PGK2	6	0.6964	0.81154	0.9995	16076	1	-0.627
hsa-mir-4733	4	0.69662	0.72283	0.9995	16077	1	0.0516
PTGER4	6	0.69705	0.81173	0.9995	16078	1	0.0032
ZNF687	6	0.69781	0.812	0.9995	16079	1	-0.025
ATG4B	6	0.69794	0.81204	0.9995	16080	1	-0.158
CXCL6	6	0.69802	0.81207	0.9995	16081	1	-0.815
GDAP1	6	0.69802	0.81207	0.9995	16082	1	0.0542
SUPT6H	6	0.69828	0.81215	0.9995	16083	1	-0.414
MGAT4B	6	0.69838	0.81219	0.9995	16084	1	-0.228
STARD5	6	0.69845	0.81221	0.9995	16085	1	-0.295
hsa-mir-124-3	3	0.69849	0.69986	0.9995	16086	1	0.0688
MARCH7	6	0.69886	0.81233	0.9995	16087	1	-0.208
PIP5K1B	6	0.69886	0.81233	0.9995	16088	1	-0.288
WDR96	6	0.69936	0.81248	0.9995	16089	1	-0.451
KEAP1	6	0.69941	0.81249	0.9995	16090	1	-0.237
NIPA2	6	0.69945	0.81251	0.9995	16091	1	-0.107
SLC13A1	6	0.69948	0.81252	0.9995	16092	1	-0.126
SEMA5B	6	0.69961	0.81255	0.9995	16093	1	0.0513
OTOP2	6	0.69969	0.81258	0.9995	16094	1	-0.191
ZNF460	6	0.69987	0.81262	0.9995	16095	1	-0.004
GPATCH8	6	0.69995	0.81265	0.9995	16096	1	-0.378
XAB2	6	0.70002	0.81267	0.9995	16097	1	-0.206
MAGEA11	6	0.70015	0.81271	0.9995	16098	1	-0.161
LMF1	6	0.7002	0.81273	0.9995	16099	1	-0.227
TUBB2A	5	0.70021	0.76713	0.9995	16100	1	0.1183
PSD2	6	0.70029	0.81275	0.9995	16101	1	-0.036
LSM10	6	0.70039	0.81278	0.9995	16102	1	-0.407
ATXN10	6	0.70076	0.8129	0.9995	16103	1	0.1023
CFC1B	2	0.70142	0.70115	0.9995	16104	0	-1.719
C1QA	6	0.70148	0.81311	0.9995	16105	1	0.0131
DMRTC1	2	0.70155	0.70127	0.9995	16106	0	-0.43
TREML1	6	0.70184	0.81323	0.9995	16107	1	-0.276
PGAP1	6	0.70203	0.81328	0.9995	16108	1	-0.063
hsa-mir-4451	4	0.70216	0.72648	0.9995	16109	1	0.1533
IRS2	6	0.70235	0.81338	0.9995	16110	1	-0.125
LMX1B	6	0.70264	0.81346	0.9995	16111	1	-0.153
OR13C2	5	0.70277	0.7683	0.9995	16112	1	-0.071
CHD4	6	0.70294	0.81356	0.9995	16113	1	-0.181
FASN	6	0.70298	0.81358	0.9995	16114	1	-0.045
DCT	6	0.70309	0.81361	0.9995	16115	1	-0.31
ZNF597	6	0.70317	0.81364	0.9995	16116	1	-0.191
MERTK	6	0.70319	0.81364	0.9995	16117	1	-0.209
KCTD8	6	0.70322	0.81365	0.9995	16118	1	-0.428
LYSMD2	6	0.70327	0.81367	0.9995	16119	1	-0.458
PDK2	6	0.70337	0.8137	0.9995	16120	1	-0.057
NRTN	6	0.70373	0.81381	0.9995	16121	1	-0.158
TRPA1	6	0.70379	0.81382	0.9995	16122	1	-0.161
MROH2B	4	0.70408	0.72772	0.9995	16123	1	-0.291
GPR119	6	0.70414	0.81392	0.9995	16124	1	-0.522
SPR	6	0.7042	0.81394	0.9995	16125	1	-0.296
hsa-mir-5090	4	0.70421	0.7278	0.9995	16126	1	0.2398
MLL2	2	0.70431	0.70408	0.9995	16127	0	-0.055
PARP1	6	0.70435	0.81398	0.9995	16128	1	-0.587
SLC7A13	6	0.70439	0.814	0.9995	16129	1	-0.312
OPN3	6	0.70439	0.814	0.9995	16130	1	-0.236
CCR9	6	0.70442	0.81401	0.9995	16131	1	-0.528
MMP10	5	0.70448	0.7691	0.9995	16132	1	-0.023
hsa-mir-6864	4	0.7045	0.72801	0.9995	16133	1	-0.434
LRRC57	6	0.70456	0.81405	0.9995	16134	1	-0.261
NOL10	6	0.70472	0.8141	0.9995	16135	1	-0.198
PPAP2C	4	0.70564	0.72877	0.9995	16136	1	-0.422
FOXRED2	6	0.70568	0.81438	0.9995	16137	1	-0.164
RLF	6	0.706	0.81448	0.9995	16138	1	-0.427
KBTBD6	6	0.70608	0.8145	0.9995	16139	1	-0.37
MEX3A	6	0.70613	0.81451	0.9995	16140	1	-0.492
hsa-mir-4511	4	0.70618	0.72914	0.9995	16141	1	-0.279
hsa-mir-6796	4	0.70631	0.72923	0.9995	16142	1	-0.127
GDPD5	6	0.70634	0.81458	0.9995	16143	1	-0.021
EPM2AIP1	6	0.70645	0.81461	0.9995	16144	1	-0.407
ANKRD13D	6	0.70676	0.8147	0.9995	16145	1	-0.282

hsa-mir-4669	4	0.70751	0.73004	0.9995	16146	1	-0.233
hsa-mir-3123	4	0.70757	0.73008	0.9995	16147	1	-0.196
GJB1	6	0.70763	0.81497	0.9995	16148	1	-0.175
EXOSC4	6	0.70768	0.81498	0.9995	16149	1	0.0164
hsa-mir-6756	4	0.70794	0.73033	0.9995	16150	1	-0.332
DCAKD	6	0.70803	0.81508	0.9995	16151	1	-0.265
NT5C1B-RDH14	3	0.70823	0.70894	0.9995	16152	1	0.083
EPHA4	6	0.70854	0.81524	0.9995	16153	1	-0.403
LCE1F	5	0.70867	0.77101	0.9995	16154	1	-1.168
GJD3	6	0.70878	0.8153	0.9995	16155	1	-0.133
HSPE1-MOB4	1	0.70879	0.70894	0.9995	16156	0	-0.268
CLEC2L	6	0.70886	0.81532	0.9995	16157	1	-0.275
PCLO	6	0.70886	0.81532	0.9995	16158	1	-0.35
TMEM139	6	0.70891	0.81533	0.9995	16159	1	-0.138
ZNF865	6	0.70909	0.81539	0.9995	16160	1	-0.465
KLHL28	6	0.70923	0.81544	0.9995	16161	1	-0.344
hsa-mir-10a	4	0.70934	0.73126	0.9995	16162	1	-0.65
SLC9B1	5	0.70981	0.77155	0.9995	16163	1	-0.161
ATP5L	4	0.70991	0.73161	0.9995	16164	1	0.2783
ST6GALNAC5	6	0.70998	0.81567	0.9995	16165	1	-0.167
RINT1	6	0.71012	0.81571	0.9995	16166	1	-0.377
ZFAND2A	6	0.71014	0.81572	0.9995	16167	1	-0.275
ZNF467	6	0.71016	0.81573	0.9995	16168	1	-0.166
hsa-mir-200c	4	0.71031	0.73187	0.9995	16169	1	-0.668
C18orf54	6	0.7104	0.81579	0.9995	16170	1	-0.217
hsa-mir-32	2	0.71049	0.7103	0.9995	16171	0	-0.04
PCMTD2	6	0.71053	0.81583	0.9995	16172	1	0.1665
hsa-mir-195	4	0.71081	0.73221	0.9995	16173	1	-0.122
hsa-mir-2861	4	0.71096	0.7323	0.9995	16174	1	-0.355
CD14	6	0.71096	0.81595	0.9995	16175	1	0.1705
PARK7	6	0.711	0.81597	0.9995	16176	1	-0.33
PTH2	6	0.71105	0.81598	0.9995	16177	1	-0.454
TIFAB	6	0.71126	0.81605	0.9995	16178	1	-0.345
XKR6	6	0.71138	0.81609	0.9995	16179	1	-0.321
38961	3	0.71147	0.712	0.9995	16180	1	-0.481
ARHGEF5	6	0.71167	0.81618	0.9995	16181	1	-0.212
VAMP1	6	0.71172	0.8162	0.9995	16182	1	-0.895
CPN2	6	0.71178	0.81621	0.9995	16183	1	-0.336
MTG2	2	0.71185	0.71167	0.9995	16184	0	-0.259
LPL	6	0.71186	0.81624	0.9995	16185	1	0.0012
TMEM56	1	0.71186	0.71205	0.9995	16186	0	-0.296
PRL	6	0.71205	0.8163	0.9995	16187	1	-0.326
KIF22	6	0.7121	0.81631	0.9995	16188	1	-0.411
CPXM1	6	0.7121	0.81631	0.9995	16189	1	-0.379
CACNA1F	6	0.71219	0.81635	0.9995	16190	1	-0.132
LDHC	6	0.71242	0.81642	0.9995	16191	1	-0.508
DDX18	6	0.71278	0.81652	0.9995	16192	1	0.1893
RAE1	6	0.71278	0.81652	0.9995	16193	1	-0.35
hsa-mir-523	2	0.71298	0.71277	0.9995	16194	0	-0.57
MICALCL	6	0.71336	0.81669	0.9995	16195	1	-0.126
NPAS1	6	0.71347	0.81673	0.9995	16196	1	0.0296
hsa-mir-3667	4	0.71347	0.73401	0.9995	16197	1	-0.894
KCTD9	6	0.71371	0.81681	0.9995	16198	1	-0.306
UAP1	6	0.71375	0.81682	0.9995	16199	1	-0.209
SOCS7	6	0.71381	0.81683	0.9995	16200	1	0.0075
hsa-mir-514a-1	1	0.71391	0.71408	0.9995	16201	0	-0.456
SRD5A1	6	0.71401	0.81689	0.9995	16202	1	-0.646
RNF217	6	0.71407	0.81691	0.9995	16203	1	-0.497
SDR9C7	6	0.7143	0.81697	0.9995	16204	1	0.0785
GRAMD1B	6	0.71442	0.81701	0.9995	16205	1	-0.336
GPATCH2L	6	0.71457	0.81705	0.9995	16206	1	-0.652
hsa-mir-215	4	0.71491	0.735	0.9995	16207	1	-0.042
RTP4	6	0.71516	0.81726	0.9995	16208	1	-0.267
PRMT2	6	0.71526	0.81729	0.9995	16209	1	-0.074
CD36	4	0.71535	0.73532	0.9995	16210	1	-0.195
CYP4Z1	6	0.71542	0.81735	0.9995	16211	1	-0.636
ANGPTL7	6	0.71542	0.81735	0.9995	16212	1	-0.124
VGLL3	6	0.71547	0.81736	0.9995	16213	1	-0.455
CRLF3	6	0.71574	0.81746	0.9995	16214	1	-0.263
BRD9	6	0.71577	0.81747	0.9995	16215	1	-0.052
hsa-mir-4804	4	0.71606	0.73581	0.9995	16216	1	-0.389
LRRC15	4	0.71609	0.73584	0.9995	16217	1	-0.898
RBM14	4	0.71627	0.73596	0.9995	16218	1	-0.583
hsa-mir-7106	4	0.71643	0.73608	0.9995	16219	1	-0.027
BEST3	6	0.7166	0.81774	0.9995	16220	1	-0.262
hsa-mir-8055	4	0.71662	0.73621	0.9995	16221	1	0.0111
hsa-mir-7974	4	0.71712	0.73656	0.9995	16222	1	-0.444
hsa-let-7f-1	3	0.71714	0.71743	0.9995	16223	1	-0.347
PPP1R12C	6	0.7173	0.81796	0.9995	16224	1	-0.29
hsa-mir-4793	4	0.71784	0.73707	0.9995	16225	1	-0.467
FAM122B	6	0.71788	0.81815	0.9995	16226	1	-0.01
AIFM3	6	0.71807	0.81822	0.9995	16227	1	-0.302
MAP6	6	0.71824	0.81828	0.9995	16228	1	0.1197
hsa-mir-6837	4	0.71867	0.73767	0.9995	16229	1	-0.103
KLRC3	5	0.71876	0.7758	0.9995	16230	1	-0.72

BTN3A3	2	0.71881	0.71861	0.9995	16231	0	0.004
TMEM55B	6	0.71885	0.81849	0.9995	16232	1	-0.017
PRSS38	6	0.71909	0.81855	0.9995	16233	1	-0.538
RASGRP1	6	0.71933	0.8186	0.9995	16234	1	-0.189
CDC37L1	6	0.71942	0.81863	0.9995	16235	1	-0.042
DLG3	6	0.71959	0.81868	0.9995	16236	1	-0.523
DGKD	6	0.71998	0.81882	0.9995	16237	1	-0.184
hsa-mir-3184	1	0.72009	0.72029	0.9995	16238	0	-0.409
PCDHB6	6	0.72009	0.81885	0.9995	16239	1	-0.541
PTRHD1	6	0.72028	0.8189	0.9995	16240	1	-0.465
CCDC167	6	0.72041	0.81895	0.9995	16241	1	-0.129
TANC2	6	0.72053	0.81898	0.9995	16242	1	-0.153
LILRA2	6	0.72053	0.81898	0.9995	16243	1	-0.77
POC1B	4	0.72056	0.73898	0.9995	16244	1	-0.009
PLEKHA2	6	0.72115	0.81918	0.9995	16245	1	0.0734
SPATA31D1	6	0.72119	0.8192	0.9995	16246	1	-0.837
BIVM-ERCC5	2	0.7212	0.721	0.9995	16247	0	-0.018
EPPIN	2	0.72127	0.72107	0.9995	16248	0	-0.032
hsa-mir-8072	4	0.72132	0.7395	0.9995	16249	1	-0.22
hsa-mir-600	4	0.7216	0.73969	0.9995	16250	1	-0.445
ENPP2	6	0.72168	0.81936	0.9995	16251	1	-0.066
TUBA1C	4	0.72184	0.73987	0.9995	16252	1	-0.722
FAIM	6	0.72188	0.81942	0.9995	16253	1	-0.216
hsa-mir-1294	4	0.72195	0.73995	0.9995	16254	1	-0.158
NEUROG2	6	0.72228	0.81955	0.9995	16255	1	-0.38
CLDN7	6	0.72228	0.81955	0.9995	16256	1	0.0483
PJA2	6	0.72266	0.81967	0.9995	16257	1	0.003
HAS1	6	0.72305	0.81979	0.9995	16258	1	-0.342
CDC25C	6	0.72332	0.81986	0.9995	16259	1	0.0932
hsa-mir-4753	4	0.72335	0.74093	0.9995	16260	1	-0.487
CLIP2	6	0.72335	0.81988	0.9995	16261	1	-0.042
HIST1H4K	1	0.72351	0.72378	0.9995	16262	0	-0.363
OR10H5	4	0.72371	0.74119	0.9995	16263	1	-0.431
FAM124B	6	0.72388	0.82003	0.9995	16264	1	-0.599
ZNF268	6	0.72389	0.82004	0.9995	16265	1	-0.103
ACPP	6	0.72396	0.82005	0.9995	16266	1	0.1601
PAGR1	6	0.72405	0.82008	0.9995	16267	1	-0.02
FAM71F2	6	0.72405	0.82008	0.9995	16268	1	-0.6
SERPINA3	6	0.72405	0.82008	0.9995	16269	1	0.058
hsa-mir-93	4	0.72429	0.7416	0.9995	16270	1	-0.624
REV3L	6	0.7243	0.82016	0.9995	16271	1	-0.516
PARP14	6	0.72441	0.8202	0.9995	16272	1	0.0305
PTPRCAP	6	0.72451	0.82023	0.9995	16273	1	-0.218
UBE2E3	6	0.72504	0.8204	0.9995	16274	1	-0.245
PLEKHG4	6	0.72507	0.82041	0.9995	16275	1	-0.204
hsa-mir-153-1	3	0.72518	0.72531	0.9995	16276	1	0.0673
CLDN23	6	0.72527	0.82047	0.9995	16277	1	-0.055
NEK6	6	0.72533	0.82049	0.9995	16278	1	-0.51
JMJD1C	6	0.72541	0.82051	0.9995	16279	1	-0.017
S100A8	6	0.72549	0.82053	0.9995	16280	1	-0.611
MAPK4	6	0.72555	0.82055	0.9995	16281	1	-0.035
NDUFAB1	6	0.72561	0.82058	0.9995	16282	1	-0.096
P2RX7	4	0.72562	0.74256	0.9995	16283	1	-0.203
LRRC33	3	0.72562	0.72575	0.9995	16284	1	0.1757
ERVW-1	6	0.72576	0.82063	0.9995	16285	1	-0.162
MGA	6	0.72586	0.82066	0.9995	16286	1	-0.36
PKDCC	6	0.72586	0.82066	0.9995	16287	1	0.0175
CMC1	6	0.72613	0.82075	0.9995	16288	1	-0.028
TMEM59L	6	0.7262	0.82077	0.9995	16289	1	-0.068
PEBP4	6	0.72629	0.8208	0.9995	16290	1	-0.127
PXDN	6	0.72634	0.82082	0.9995	16291	1	-0.104
H2AFB2	1	0.72642	0.7267	0.9995	16292	0	-0.508
FIZ1	6	0.72647	0.82087	0.9995	16293	1	-0.157
ALOX5	6	0.72666	0.82093	0.9995	16294	1	-0.475
C2orf88	6	0.72666	0.82093	0.9995	16295	1	0.043
CTSS	6	0.72666	0.82093	0.9995	16296	1	-0.086
MGAT3	6	0.72666	0.82093	0.9995	16297	1	-0.375
MST1R	6	0.7268	0.82097	0.9995	16298	1	-0.303
CEP63	6	0.72685	0.82098	0.9995	16299	1	-0.02
CES5A	6	0.72685	0.82098	0.9995	16300	1	-0.087
HOMER1	6	0.72696	0.82102	0.9995	16301	1	-0.202
MAGED4	2	0.72699	0.72673	0.9995	16302	0	-0.34
MICA	6	0.72702	0.82103	0.9995	16303	1	-0.781
hsa-mir-6874	4	0.72715	0.74363	0.9995	16304	1	-0.261
MCEE	6	0.72724	0.82111	0.9995	16305	1	-0.061
PPP5C	6	0.72735	0.82115	0.9995	16306	1	-0.128
LCE2C	3	0.72736	0.72745	0.9995	16307	1	0.0682
CAMK2N2	6	0.7274	0.82116	0.9995	16308	1	-0.15
INTS2	6	0.7274	0.82116	0.9995	16309	1	-0.009
hsa-mir-4289	4	0.72741	0.7438	0.9995	16310	1	-0.31
C1QL1	6	0.72754	0.82121	0.9995	16311	1	-0.488
hsa-mir-942	4	0.72756	0.7439	0.9995	16312	1	0.1294
UNC13D	6	0.72789	0.8213	0.9995	16313	1	-0.079
hsa-mir-1285-1	3	0.72813	0.72822	0.9995	16314	1	-0.19
hsa-mir-6074	4	0.72815	0.74431	0.9995	16315	1	-0.348

ADORA3	6	0.72825	0.82142	0.9995	16316	1	-0.486
OR52B2	6	0.72833	0.82144	0.9995	16317	1	-0.409
STYX	6	0.7286	0.82152	0.9995	16318	1	-0.069
AKAP5	6	0.72864	0.82154	0.9995	16319	1	-0.584
IPO8	6	0.72879	0.8216	0.9995	16320	1	-0.409
OTOP3	6	0.72895	0.82165	0.9995	16321	1	-0.442
MRPL36	6	0.72898	0.82165	0.9995	16322	1	0.0888
CHD7	6	0.72912	0.8217	0.9995	16323	1	-0.294
ODF3L2	6	0.72932	0.82176	0.9995	16324	1	-0.146
DNAH10	6	0.72946	0.82181	0.9995	16325	1	-0.155
RAB36	6	0.72974	0.82191	0.9995	16326	1	-0.169
MYL12A	4	0.72978	0.74549	0.9995	16327	1	-0.611
RAPGEFL1	6	0.72985	0.82195	0.9995	16328	1	-0.158
C1QL4	6	0.72997	0.82199	0.9995	16329	1	-0.388
C9orf91	6	0.73011	0.82203	0.9995	16330	1	0.0132
ALOX12B	6	0.73032	0.8221	0.9995	16331	1	0.0895
GAS2L2	6	0.73045	0.82214	0.9995	16332	1	-0.082
PSMB8	6	0.73045	0.82214	0.9995	16333	1	-0.302
C5orf58	6	0.73083	0.82227	0.9995	16334	1	-0.343
RPRD1B	6	0.73083	0.82227	0.9995	16335	1	-0.318
ABT1	6	0.73092	0.8223	0.9995	16336	1	-0.066
LOX	6	0.7311	0.82237	0.9995	16337	1	-0.042
hsa-mir-378g	4	0.73116	0.74647	0.9995	16338	1	-0.058
CELA2B	6	0.7313	0.82243	0.9995	16339	1	-0.212
hsa-mir-520d	2	0.7313	0.73113	0.9995	16340	0	-1.203
hsa-mir-519b	2	0.7313	0.73113	0.9995	16341	0	-0.079
hsa-mir-320d-2	2	0.7313	0.73113	0.9995	16342	0	-0.839
DUSP10	6	0.73138	0.82246	0.9995	16343	1	-0.197
CDH13	6	0.73148	0.82249	0.9995	16344	1	-0.134
GRM8	6	0.73167	0.82256	0.9995	16345	1	-0.229
ASB18	6	0.73171	0.82257	0.9995	16346	1	-0.736
MNDA	6	0.73174	0.82258	0.9995	16347	1	0.0722
CTSE	6	0.73181	0.82261	0.9995	16348	1	-0.11
SAPCD2	6	0.73202	0.82269	0.9995	16349	1	-0.203
APITD1	2	0.73222	0.73206	0.9995	16350	0	-0.034
FAM71F1	6	0.73234	0.82279	0.9995	16351	1	-0.191
hsa-mir-575	4	0.73238	0.74735	0.9995	16352	1	-0.24
PCDHGB4	2	0.7324	0.73223	0.9995	16353	0	-0.208
PRAC1	4	0.73243	0.74738	0.9995	16354	1	0.1215
LIX1	6	0.73284	0.82296	0.9995	16355	1	-0.382
WFDC1	6	0.73303	0.82302	0.9995	16356	1	-0.195
RAB34	3	0.73323	0.73324	0.9995	16357	0	0.1369
OTUD4	6	0.73324	0.82309	0.9995	16358	1	0.0597
TBC1D30	6	0.73329	0.82311	0.9995	16359	1	-0.201
NXPH3	6	0.73332	0.82312	0.9995	16360	1	-0.444
TP53AIP1	6	0.73339	0.82314	0.9995	16361	1	-0.038
OR2J3	6	0.73342	0.82315	0.9995	16362	1	0.048
DPY19L1	5	0.73357	0.78287	0.9995	16363	1	-0.066
ZFYVE16	6	0.7338	0.82327	0.9995	16364	1	-0.137
hsa-mir-1254-2	4	0.7343	0.74867	0.9995	16365	1	-0.791
PCDHA5	2	0.73489	0.7348	0.9995	16366	0	-0.558
DYNLRB1	6	0.73494	0.82365	0.9995	16367	1	0.0636
IFNLR1	6	0.73499	0.82366	0.9995	16368	1	-0.458
hsa-mir-6783	4	0.735	0.74915	0.9995	16369	1	0.1549
PEG3	1	0.73515	0.73537	0.9995	16370	0	-0.361
PIK3CG	6	0.73519	0.82373	0.9995	16371	1	-0.129
LETM1	6	0.73538	0.82379	0.9995	16372	1	-0.128
PCTP	6	0.7355	0.82384	0.9995	16373	1	-0.226
GAD2	6	0.73561	0.82387	0.9995	16374	1	-0.497
hsa-mir-548q	3	0.73595	0.73601	0.9995	16375	0	-0.419
hsa-mir-148b	4	0.73602	0.74989	0.9995	16376	1	0.1443
ZGPAT	6	0.73604	0.82403	0.9995	16377	1	-0.503
LIME1	6	0.73613	0.82406	0.9995	16378	1	-0.623
CHRNA2	6	0.73625	0.8241	0.9995	16379	1	-0.744
FGFR1	6	0.7363	0.82412	0.9995	16380	1	-0.466
RAD54B	6	0.73638	0.82416	0.9995	16381	1	-0.474
ANKEF1	6	0.73649	0.82419	0.9995	16382	1	0.0121
hsa-mir-4675	4	0.73653	0.75026	0.9995	16383	1	-0.41
C14orf166B	6	0.73653	0.8242	0.9995	16384	1	-0.082
C7orf41	6	0.73678	0.82428	0.9995	16385	1	-0.552
hsa-mir-6721	4	0.73697	0.75059	0.9995	16386	1	0.1533
TMEM187	6	0.73706	0.82438	0.9995	16387	1	0.1342
NPIPA5	2	0.7371	0.737	0.9995	16388	0	-0.75
hsa-mir-6842	4	0.73715	0.75073	0.9995	16389	1	-0.528
CKS1B	6	0.73728	0.82445	0.9995	16390	1	-0.017
LYVE1	6	0.73736	0.82447	0.9995	16391	1	-0.457
SRPK1	6	0.73744	0.82449	0.9995	16392	1	-0.328
DGKZ	6	0.73766	0.82457	0.9995	16393	1	-0.409
KIF14	6	0.73766	0.82457	0.9995	16394	1	-0.114
MTRNR2L10	3	0.7377	0.73779	0.9995	16395	0	-0.419
TNS4	6	0.7379	0.82464	0.9995	16396	1	-0.718
TCEAL3	5	0.73802	0.78513	0.9995	16397	1	-1.233
CCDC102A	6	0.73804	0.82468	0.9995	16398	1	-0.056
TMEM72	6	0.73807	0.82469	0.9995	16399	1	-1.036
hsa-mir-320b-1	4	0.73813	0.75146	0.9995	16400	1	-0.794

OXR1	6	0.7384	0.8248	0.9995	16401	1	-0.229
AFAP1L2	6	0.73842	0.82482	0.9995	16402	1	-0.161
hsa-mir-518a-2	1	0.73848	0.73876	0.9995	16403	0	-2.141
GPR141	6	0.73857	0.82487	0.9995	16404	1	-0.124
PSMD7	6	0.73866	0.82491	0.9995	16405	1	-0.252
TEKT5	6	0.73872	0.82493	0.9995	16406	1	-0.326
SHD	6	0.73875	0.82494	0.9995	16407	1	-0.204
ZNF728	4	0.73875	0.75189	0.9995	16408	1	-0.451
NT5C1A	6	0.73877	0.82494	0.9995	16409	1	-0.009
FAM9A	5	0.73921	0.78571	0.9995	16410	1	0.0127
PTDSS2	6	0.73923	0.82509	0.9995	16411	1	-0.367
HACE1	6	0.73932	0.82511	0.9995	16412	1	-0.15
hsa-mir-7851	3	0.73938	0.73944	0.9995	16413	0	-0.22
LECT1	6	0.73966	0.82525	0.9995	16414	1	-0.213
hsa-mir-6728	4	0.73971	0.75258	0.9995	16415	1	0.155
hsa-mir-526a-2	3	0.73972	0.73978	0.9995	16416	0	-1.074
RNF133	6	0.73997	0.82535	0.9995	16417	1	-0.357
hsa-mir-5704	4	0.74	0.75281	0.9995	16418	1	-0.319
SPATA24	6	0.74021	0.82544	0.9995	16419	1	-0.075
SPCS1	6	0.74028	0.82546	0.9995	16420	1	-0.477
YAP1	6	0.74104	0.82571	0.9995	16421	1	-0.378
GPR62	6	0.74104	0.82571	0.9995	16422	1	0.0004
KRT6A	5	0.74106	0.78665	0.9995	16423	1	-0.948
HLX	6	0.74116	0.82575	0.9995	16424	1	-0.522
hsa-mir-3196	4	0.74143	0.7539	0.9995	16425	1	-0.27
KMT2D	2	0.74143	0.74131	0.9995	16426	0	-0.041
hsa-mir-548h-1	3	0.74157	0.74161	0.9995	16427	0	-0.162
hsa-mir-1289-1	4	0.7416	0.75404	0.9995	16428	1	0.0026
TBC1D25	6	0.74171	0.82595	0.9995	16429	1	-0.38
hsa-mir-4487	2	0.74173	0.7416	0.9995	16430	0	-0.294
GPATCH2	6	0.74178	0.82598	0.9995	16431	1	-0.581
ORM1	2	0.74213	0.74199	0.9995	16432	0	-0.203
COX4I2	6	0.74233	0.82615	0.9995	16433	1	-0.343
HPRT1	6	0.74238	0.82617	0.9995	16434	1	0.0642
hsa-mir-3923	4	0.74255	0.75473	0.9995	16435	1	-0.054
hsa-mir-1258	4	0.74273	0.75486	0.9995	16436	1	-0.578
ENDOV	6	0.7428	0.82631	0.9995	16437	1	-0.423
KRTAP5-10	4	0.7428	0.75491	0.9995	16438	1	-0.337
CLEC2D	6	0.74296	0.82636	0.9995	16439	1	-0.357
hsa-mir-6813	4	0.74299	0.75505	0.9995	16440	1	-0.399
KRT19	6	0.74318	0.82644	0.9995	16441	1	-0.299
OR1C1	6	0.74325	0.82646	0.9995	16442	1	0.008
ZNF883	6	0.74333	0.82648	0.9995	16443	1	0.0566
DSCC1	6	0.74389	0.82667	0.9995	16444	1	-0.778
IGF2R	6	0.74408	0.82674	0.9995	16445	1	0.1281
ADORA1	6	0.74426	0.82681	0.9995	16446	1	-0.172
ANXA11	6	0.74442	0.82687	0.9995	16447	1	-0.189
UBR5	6	0.74467	0.82696	0.9995	16448	1	-0.231
hsa-mir-1200	2	0.74477	0.7447	0.9995	16449	0	-0.297
AKNA	6	0.74483	0.82702	0.9995	16450	1	-0.07
TMTC1	6	0.74492	0.82705	0.9995	16451	1	-0.042
VPS13C	6	0.74495	0.82706	0.9995	16452	1	-0.215
C1S	6	0.74508	0.82711	0.9995	16453	1	-0.003
GPR143	6	0.74521	0.82716	0.9995	16454	1	-0.335
CLSTN1	6	0.74524	0.82717	0.9995	16455	1	-0.465
LPPR5	6	0.74534	0.8272	0.9995	16456	1	0.0293
C11orf54	6	0.74541	0.82723	0.9995	16457	1	-0.024
hsa-mir-200b	4	0.74541	0.75685	0.9995	16458	1	-0.242
hsa-mir-133a-1	2	0.74564	0.74556	0.9995	16459	0	-0.215
SEC16B	6	0.74585	0.82739	0.9995	16460	1	-0.707
PPFIBP1	6	0.74592	0.82741	0.9995	16461	1	-0.711
TESPA1	6	0.74592	0.82741	0.9995	16462	1	0.0434
hsa-mir-3121	4	0.74608	0.75735	0.9995	16463	1	0.1352
SPATA31A3	2	0.74643	0.74632	0.9995	16464	0	-0.181
hsa-mir-4436b-2	3	0.74655	0.74666	0.9995	16465	0	-0.31
LPHN3	6	0.74674	0.8277	0.9995	16466	1	-0.316
COG7	6	0.74698	0.82778	0.9995	16467	1	-0.247
TMEM125	6	0.74698	0.82778	0.9995	16468	1	-0.165
SYNE3	6	0.74716	0.82785	0.9995	16469	1	0.0399
PRKACB	6	0.74731	0.8279	0.9995	16470	1	0.0764
LYPD1	6	0.74731	0.8279	0.9995	16471	1	-0.038
S1PR5	6	0.74734	0.82791	0.9995	16472	1	-1.148
hsa-mir-363	4	0.74767	0.75857	0.9995	16473	1	-0.122
NEUROD2	6	0.74778	0.82806	0.9995	16474	1	-0.445
KLK9	4	0.74779	0.75866	0.9995	16475	1	-0.174
GH2	6	0.74824	0.82822	0.9995	16476	1	-0.348
OR1E1	6	0.74824	0.82822	0.9995	16477	1	-0.024
USP17L3	3	0.74838	0.74852	0.9995	16478	0	-0.419
IL17RA	6	0.74843	0.82829	0.9995	16479	1	-0.231
IFT46	6	0.74843	0.82829	0.9995	16480	1	-0.12
RALGAPA1	6	0.74861	0.82834	0.9995	16481	1	-0.309
DHX37	6	0.74867	0.82836	0.9995	16482	1	-0.184
hsa-mir-6817	4	0.74883	0.7594	0.9995	16483	1	0.0196
hsa-mir-6782	4	0.74898	0.75951	0.9995	16484	1	0.0442
RANBP3L	4	0.74919	0.75969	0.9995	16485	1	-0.095

PNPLA3	6	0.7492	0.82854	0.9995	16486	1	0.0806
ACOT2	5	0.74926	0.79091	0.9995	16487	1	0.053
hsa-mir-6794	4	0.74941	0.75984	0.9995	16488	1	0.0979
CKAP2	6	0.74948	0.82864	0.9995	16489	1	-0.406
MED4	6	0.7496	0.82868	0.9995	16490	1	-0.363
MATN3	6	0.74963	0.82869	0.9995	16491	1	-0.265
MCC	6	0.74977	0.82874	0.9995	16492	1	-0.222
DSCAM	6	0.75008	0.82885	0.9995	16493	1	-0.027
AP4M1	6	0.75008	0.82885	0.9995	16494	1	-0.149
ZNF621	6	0.75026	0.82892	0.9995	16495	1	-0.405
AK3	6	0.75033	0.82895	0.9995	16496	1	-0.446
SMIM9	6	0.75048	0.82899	0.9995	16497	1	-0.115
hsa-mir-520b	2	0.75056	0.7504	0.9995	16498	0	-1.385
TRIM36	6	0.75063	0.82905	0.9995	16499	1	-0.512
UPRT	6	0.75069	0.82907	0.9995	16500	1	-0.344
PAX1	4	0.75073	0.76086	0.9995	16501	1	-0.22
FFAR4	6	0.7508	0.82911	0.9995	16502	1	-0.287
OR2F2	6	0.75096	0.82917	0.9995	16503	1	-0.26
YIPF1	6	0.75101	0.82919	0.9995	16504	1	-0.104
IQSEC1	6	0.75101	0.82919	0.9995	16505	1	-0.282
CASC1	6	0.75133	0.82931	0.9995	16506	1	0.0882
CCR6	6	0.75139	0.82934	0.9995	16507	1	-0.082
CCDC138	6	0.75143	0.82935	0.9995	16508	1	-0.395
AVL9	6	0.75146	0.82936	0.9995	16509	1	-0.281
hsa-mir-509-3	2	0.75154	0.75137	0.9995	16510	0	-0.381
TUBA4A	6	0.75196	0.82954	0.9995	16511	1	-0.131
hsa-mir-4330	4	0.75215	0.76197	0.9995	16512	1	-0.171
ISM1	6	0.75242	0.8297	0.9995	16513	1	-0.678
FNDC3B	6	0.75265	0.82979	0.9995	16514	1	-0.276
BCL7C	6	0.75271	0.82981	0.9995	16515	1	-0.275
ATP5G2	6	0.75306	0.82994	0.9995	16516	1	-0.17
NKX3-2	6	0.75323	0.83	0.9995	16517	1	-0.128
ABCB1	6	0.75353	0.83011	0.9995	16518	1	-0.463
hsa-mir-216a	4	0.75358	0.76305	0.9995	16519	1	-0.104
TMEM26	6	0.75362	0.83014	0.9995	16520	1	0.1265
ASNA1	6	0.75394	0.83026	0.9995	16521	1	-0.679
NXPE4	6	0.75394	0.83026	0.9995	16522	1	-0.258
hsa-mir-1302-8	4	0.75401	0.76338	0.9995	16523	1	-0.033
hsa-mir-4482	4	0.75421	0.76356	0.9995	16524	1	-0.607
NDUFAF1	6	0.75431	0.83038	0.9995	16525	1	0.0992
C4BPA	6	0.75434	0.83039	0.9995	16526	1	-0.056
TSPAN14	6	0.75442	0.83042	0.9995	16527	1	-0.49
EPB41L5	6	0.75451	0.83045	0.9995	16528	1	-0.219
hsa-mir-4534	4	0.75464	0.76387	0.9995	16529	1	-0.019
DEFB103A	1	0.75473	0.75506	0.9995	16530	0	-0.354
PPL	6	0.75474	0.83053	0.9995	16531	1	-0.424
AIM2	6	0.75489	0.83057	0.9995	16532	1	-0.716
G0S2	6	0.75493	0.83059	0.9995	16533	1	-0.028
TCEB3CL2	2	0.75494	0.75472	0.9995	16534	0	-0.254
MS4A7	6	0.75499	0.83062	0.9995	16535	1	-0.673
hsa-mir-4449	4	0.75499	0.76413	0.9995	16536	1	-0.708
KIAA0753	6	0.75505	0.83064	0.9995	16537	1	-0.069
DCAF4L2	6	0.75512	0.83067	0.9995	16538	1	-0.461
EFCAB14	6	0.75522	0.83071	0.9995	16539	1	-0.094
ZNF587	4	0.75524	0.76431	0.9995	16540	1	0.0616
GDPGP1	6	0.75538	0.83076	0.9995	16541	1	-0.221
ADAMTS3	6	0.75555	0.83082	0.9995	16542	1	-0.87
hsa-mir-506	4	0.7558	0.76474	0.9995	16543	1	-0.368
hsa-mir-6769a	4	0.75589	0.76481	0.9995	16544	1	-0.382
SYT16	6	0.75597	0.83098	0.9995	16545	1	-0.085
CEACAM7	6	0.75619	0.83107	0.9995	16546	1	-0.631
SSSCA1	6	0.75671	0.83126	0.9995	16547	1	-0.248
CCDC61	6	0.75696	0.83136	0.9995	16548	1	0.1541
SEC61A1	6	0.75708	0.8314	0.9995	16549	1	-0.4
SLC22A5	6	0.75708	0.8314	0.9995	16550	1	-0.327
NASP	6	0.75716	0.83143	0.9995	16551	1	-0.615
38596	3	0.75726	0.7574	0.9995	16552	0	-0.826
hsa-mir-5692c-1	2	0.7575	0.75723	0.9995	16553	0	-0.551
PCDH11Y	5	0.75762	0.79528	0.9995	16554	1	0.0893
BPNT1	6	0.7578	0.83164	0.9995	16555	1	-0.063
ZNF765	4	0.75796	0.76637	0.9995	16556	1	-0.619
TMPRSS5	6	0.75805	0.83173	0.9995	16557	1	-0.993
MFNG	6	0.75814	0.83177	0.9995	16558	1	-0.474
PLD6	6	0.75824	0.83181	0.9995	16559	1	-0.13
GAS1	6	0.75827	0.83182	0.9995	16560	1	-0.288
ILF3	6	0.7583	0.83183	0.9995	16561	1	-0.016
hsa-mir-548g	2	0.75834	0.75804	0.9995	16562	0	-0.804
hsa-mir-6738	2	0.75844	0.75814	0.9995	16563	0	-0.282
OR2AK2	6	0.75846	0.83188	0.9995	16564	1	-0.493
hsa-mir-4446	4	0.7586	0.76682	0.9995	16565	1	-0.663
SLC45A3	6	0.7588	0.83201	0.9995	16566	1	-0.22
PCK1	6	0.75889	0.83205	0.9995	16567	1	-0.185
EFCAB13	6	0.75897	0.83208	0.9995	16568	1	-0.302
DNAH6	6	0.75904	0.8321	0.9995	16569	1	-0.236
KRT73	6	0.7592	0.83215	0.9995	16570	1	-0.373

hsa-mir-1322	1	0.75943	0.7598	0.9995	16571	0	-0.485
MAB21L2	6	0.75945	0.83226	0.9995	16572	1	-0.304
ACTA2	6	0.75955	0.83229	0.9995	16573	1	-0.364
WNT9B	6	0.75955	0.83229	0.9995	16574	1	-0.01
hsa-mir-4488	4	0.75961	0.76763	0.9995	16575	1	0.1053
APLNR	6	0.75963	0.83232	0.9995	16576	1	-0.351
SCRIB	6	0.75977	0.83237	0.9995	16577	1	-0.34
TCTN3	6	0.75986	0.83241	0.9995	16578	1	-0.629
LTBR	6	0.76009	0.8325	0.9995	16579	1	-0.725
SACS	6	0.76025	0.83256	0.9995	16580	1	-0.149
RNASEH2C	6	0.7603	0.83258	0.9995	16581	1	-0.063
hsa-mir-21	4	0.76043	0.76827	0.9995	16582	1	-0.018
HMGN5	6	0.7605	0.83266	0.9995	16583	1	-0.21
DPYSL2	6	0.76061	0.8327	0.9995	16584	1	-0.102
CAMK2B	6	0.76072	0.83274	0.9995	16585	1	-0.435
GRID1	6	0.76072	0.83274	0.9995	16586	1	-0.182
KXD1	6	0.76072	0.83274	0.9995	16587	1	-0.212
hsa-mir-205	4	0.76074	0.76852	0.9995	16588	1	-0.084
ALKBH2	6	0.76076	0.83276	0.9995	16589	1	-0.357
39692	3	0.76095	0.76113	0.9995	16590	0	0.0658
AARD	6	0.7611	0.83287	0.9995	16591	1	-0.342
CSB-PGBD3	2	0.76118	0.76083	0.9995	16592	0	-0.388
KCTD21	6	0.76126	0.83292	0.9995	16593	1	0.0037
PCF11	6	0.76133	0.83294	0.9995	16594	1	-0.094
DRAP1	6	0.76133	0.83294	0.9995	16595	1	-0.006
C5orf52	6	0.76142	0.83297	0.9995	16596	1	-0.19
CNTF	6	0.76142	0.83297	0.9995	16597	1	-0.314
OPTN	6	0.76142	0.83297	0.9995	16598	1	-0.076
SGSM3	4	0.76147	0.7691	0.9995	16599	1	-0.093
SEC63	6	0.76178	0.8331	0.9995	16600	1	-0.515
SLC45A2	6	0.76186	0.83312	0.9995	16601	1	0.1256
PRDM15	4	0.7619	0.76945	0.9995	16602	1	-0.068
NACA	6	0.76214	0.83323	0.9995	16603	1	-0.215
CPVL	6	0.76219	0.83324	0.9995	16604	1	-0.169
VWF	6	0.76223	0.83326	0.9995	16605	1	-0.296
CLOCK	4	0.76225	0.76974	0.9995	16606	1	-0.528
RHBDL2	6	0.76227	0.83327	0.9995	16607	1	-0.426
NFATC2IP	6	0.7623	0.83328	0.9995	16608	1	-0.908
GSTT2B	1	0.76232	0.76272	0.9995	16609	0	-2.372
CBX8	6	0.76248	0.83334	0.9995	16610	1	-0.057
ZNF676	2	0.7626	0.76222	0.9995	16611	0	-0.245
GDA	6	0.76268	0.8334	0.9995	16612	1	-0.675
C2orf91	6	0.76277	0.83343	0.9995	16613	1	-0.336
CHAF1B	6	0.76285	0.83347	0.9995	16614	1	0.1374
KCNH5	6	0.76297	0.83352	0.9995	16615	1	-0.15
CTCFL	6	0.76311	0.83358	0.9995	16616	1	0.0343
TOR2A	6	0.76311	0.83358	0.9995	16617	1	0.1556
FKBP4	6	0.7633	0.83364	0.9995	16618	1	-0.152
MLIP	6	0.76346	0.8337	0.9995	16619	1	-0.192
PPM1B	6	0.76346	0.8337	0.9995	16620	1	-0.18
GALNT4	3	0.76365	0.76384	0.9995	16621	0	-0.042
ADRB2	6	0.76375	0.83381	0.9995	16622	1	-0.057
DLG4	6	0.76382	0.83383	0.9995	16623	1	-0.27
GPBAR1	6	0.76408	0.83393	0.9995	16624	1	-0.035
CTSG	6	0.76413	0.83395	0.9995	16625	1	-0.408
CPNE2	6	0.76416	0.83396	0.9995	16626	1	0.0702
THBS1	6	0.76416	0.83396	0.9995	16627	1	-0.172
PAQR7	6	0.76423	0.83399	0.9995	16628	1	-0.111
OR8B2	5	0.76438	0.79898	0.9995	16629	1	-1.233
C11orf91	6	0.7644	0.83405	0.9995	16630	1	0.0005
LOC100129083	5	0.76475	0.79917	0.9995	16631	1	-0.346
BLCAP	6	0.76496	0.83425	0.9995	16632	1	-0.159
hsa-mir-16-1	4	0.76499	0.77188	0.9995	16633	1	-0.051
hsa-mir-5693	3	0.76513	0.7653	0.9995	16634	0	-0.285
IFNAR1	6	0.76547	0.83447	0.9995	16635	1	-0.218
CAPN12	6	0.76593	0.83464	0.9995	16636	1	-0.29
hsa-mir-1255b-2	2	0.76602	0.7656	0.9995	16637	0	-0.301
TBC1D24	6	0.7662	0.83474	0.9995	16638	1	-0.419
hsa-mir-4505	4	0.76629	0.77291	0.9995	16639	1	-0.744
hsa-mir-525	3	0.76639	0.76656	0.9995	16640	0	-0.419
WNK4	6	0.76641	0.83481	0.9995	16641	1	-0.194
RHNO1	6	0.76641	0.83481	0.9995	16642	1	-0.265
EXOSC2	6	0.76647	0.83484	0.9995	16643	1	-0.171
PRDM13	6	0.76657	0.83487	0.9995	16644	1	-0.008
hsa-mir-4789	4	0.76667	0.77325	0.9995	16645	1	0.0453
CRISPLD2	6	0.76669	0.83492	0.9995	16646	1	-0.303
ZSCAN20	6	0.76681	0.83496	0.9995	16647	1	-0.256
POT1	6	0.7669	0.83501	0.9995	16648	1	-0.344
B3GALNT1	6	0.76727	0.83515	0.9995	16649	1	0.0226
ERBB2	6	0.76736	0.83519	0.9995	16650	1	-0.12
APBB2	6	0.76746	0.83522	0.9995	16651	1	-0.258
CFHR2	6	0.76748	0.83523	0.9995	16652	1	-0.015
NSUN7	6	0.76771	0.83532	0.9995	16653	1	-0.354
NF2	6	0.76807	0.83546	0.9995	16654	1	-0.106
OR2D3	6	0.76823	0.83551	0.9995	16655	1	-0.057

GPR22	6	0.76854	0.83564	0.9995	16656	1	0.0545
TMEM131	6	0.76864	0.83567	0.9995	16657	1	-0.599
KLK13	6	0.76898	0.83579	0.9995	16658	1	-0.52
LRRC4C	6	0.76898	0.83579	0.9995	16659	1	-0.345
hsa-mir-6790	4	0.769	0.77511	0.9995	16660	1	-0.019
DKK1	6	0.7691	0.83584	0.9995	16661	1	0.0871
FAM24B	6	0.76915	0.83586	0.9995	16662	1	-0.592
NACC1	6	0.76923	0.83589	0.9995	16663	1	-0.248
ZNF320	4	0.76934	0.77538	0.9995	16664	1	-0.811
WRAP73	6	0.76961	0.83605	0.9995	16665	1	-0.098
VPREB1	6	0.76963	0.83606	0.9995	16666	1	0.0783
PCDHA1	2	0.76972	0.76939	0.9995	16667	0	-0.328
NMI	6	0.76987	0.83615	0.9995	16668	1	-0.271
SAAL1	6	0.76998	0.83619	0.9995	16669	1	-0.067
TMEM88B	6	0.77013	0.83625	0.9995	16670	1	-0.124
DHX15	6	0.77013	0.83625	0.9995	16671	1	0.1404
hsa-mir-620	2	0.77035	0.77003	0.9995	16672	0	-0.636
IL4R	6	0.77038	0.83635	0.9995	16673	1	0.0907
MSS51	6	0.77043	0.83638	0.9995	16674	1	-0.071
DUS4L	6	0.77051	0.83641	0.9995	16675	1	0.0913
ACP6	6	0.77055	0.83643	0.9995	16676	1	-0.007
hsa-mir-548a-3	4	0.77065	0.77642	0.9995	16677	1	-0.01
HIST1H3B	6	0.77078	0.83652	0.9995	16678	1	-0.215
HMX1	6	0.77096	0.83657	0.9995	16679	1	-0.425
ARFIP2	6	0.77101	0.8366	0.9995	16680	1	0.1049
RPL35	6	0.77101	0.8366	0.9995	16681	1	-0.088
ZNF335	6	0.77115	0.83665	0.9995	16682	1	-0.074
TMEM33	6	0.77135	0.83673	0.9995	16683	1	-0.22
SPAG8	6	0.77137	0.83674	0.9995	16684	1	-0.009
LAT2	6	0.77141	0.83676	0.9995	16685	1	-0.01
GNL1	6	0.77148	0.83678	0.9995	16686	1	0.0064
USP17L8	6	0.77162	0.83683	0.9995	16687	1	-0.377
EFR3A	6	0.77165	0.83684	0.9995	16688	1	-0.331
MEIS2	6	0.77172	0.83687	0.9995	16689	1	-0.286
C9orf85	6	0.77179	0.83689	0.9995	16690	1	-0.386
TPRX1	6	0.77199	0.83697	0.9995	16691	1	-0.147
hsa-mir-656	4	0.77204	0.77753	0.9995	16692	1	0.0086
STX3	6	0.77207	0.837	0.9995	16693	1	-0.225
hsa-mir-105-1	2	0.77228	0.77197	0.9995	16694	0	-0.662
NCR3	6	0.77235	0.83711	0.9995	16695	1	-0.13
TACR1	6	0.77287	0.8373	0.9995	16696	1	-0.083
hsa-mir-501	4	0.77289	0.77822	0.9995	16697	1	-0.394
TXNIP	6	0.77302	0.83735	0.9995	16698	1	-0.209
HIST1H3C	6	0.77302	0.83735	0.9995	16699	1	-0.235
NOP58	6	0.77308	0.83738	0.9995	16700	1	-0.244
HELQ	6	0.77313	0.8374	0.9995	16701	1	0.0338
CHD1	6	0.77313	0.8374	0.9995	16702	1	0.0529
SP140	6	0.77332	0.83748	0.9995	16703	1	-0.122
NICN1	6	0.77332	0.83748	0.9995	16704	1	-0.215
MMP7	6	0.77343	0.83751	0.9995	16705	1	-0.24
C3orf22	6	0.7737	0.83763	0.9995	16706	1	0.0369
hsa-mir-7157	4	0.77376	0.77892	0.9995	16707	1	0.1062
TLDC1	6	0.77376	0.83764	0.9995	16708	1	-0.077
GSE1	6	0.77398	0.83772	0.9995	16709	1	-0.191
hsa-mir-6801	4	0.77399	0.7791	0.9995	16710	1	0.0434
hsa-mir-224	4	0.7743	0.77936	0.9995	16711	1	-0.552
CLIC1	6	0.77432	0.83784	0.9995	16712	1	-0.256
hsa-mir-194-1	4	0.77449	0.77951	0.9995	16713	1	0.1493
LGALS3BP	6	0.77458	0.83795	0.9995	16714	1	-0.038
NGLY1	6	0.77462	0.83796	0.9995	16715	1	-0.132
LANCL2	6	0.77478	0.83802	0.9995	16716	1	-0.06
AP5B1	6	0.77484	0.83804	0.9995	16717	1	-0.361
UCP3	6	0.77499	0.83811	0.9995	16718	1	-0.243
SCN4A	6	0.77499	0.83811	0.9995	16719	1	0.0324
NPR3	6	0.77508	0.83814	0.9995	16720	1	-0.241
PPIL3	6	0.77514	0.83816	0.9995	16721	1	0.0651
ZNF564	6	0.77526	0.83821	0.9995	16722	1	-0.257
ADRA2B	6	0.77532	0.83824	0.9995	16723	1	-0.122
HDGFL1	6	0.7755	0.83831	0.9995	16724	1	0.01
NIPSNAP3B	6	0.7758	0.83841	0.9995	16725	1	-0.484
hsa-mir-8067	4	0.77582	0.78062	0.9995	16726	1	-0.357
SF1	6	0.77601	0.83849	0.9995	16727	1	-0.095
TCP10	4	0.77603	0.78077	0.9995	16728	1	-0.066
BASP1	6	0.77621	0.83856	0.9995	16729	1	-0.01
DDX60L	6	0.77635	0.8386	0.9995	16730	1	-0.427
TRAPPC1	6	0.77639	0.83862	0.9995	16731	1	0.065
UAP1L1	6	0.7766	0.83871	0.9995	16732	1	-0.637
TTC33	6	0.77666	0.83874	0.9995	16733	1	-0.154
RUVBL2	6	0.77666	0.83874	0.9995	16734	1	-0.189
ZNF577	6	0.77674	0.83877	0.9995	16735	1	-0.235
BPIFA3	6	0.77681	0.83879	0.9995	16736	1	-0.255
TAF7L	4	0.77697	0.78151	0.9995	16737	1	-0.417
PLCZ1	6	0.77698	0.83886	0.9995	16738	1	-0.223
hsa-mir-1250	4	0.77701	0.78154	0.9995	16739	1	0.0299
LYRM9	6	0.77709	0.8389	0.9995	16740	1	-0.226

SCNM1	1	0.77722	0.77763	0.9995	16741	0	-0.322
hsa-mir-490	4	0.77738	0.78186	0.9995	16742	1	-0.185
hsa-mir-182	4	0.7774	0.78188	0.9995	16743	1	-0.409
HLA-C	6	0.7775	0.83907	0.9995	16744	1	-0.032
LCP2	6	0.77761	0.83911	0.9995	16745	1	-0.005
PCDHA9	2	0.77762	0.77724	0.9995	16746	0	-0.074
CSF3R	5	0.77772	0.80652	0.9995	16747	1	-0.988
COA1	6	0.77779	0.83918	0.9995	16748	1	-0.323
TMEM156	6	0.77816	0.83934	0.9995	16749	1	-0.118
AAED1	6	0.77816	0.83934	0.9995	16750	1	-0.359
hsa-mir-4263	4	0.77826	0.78257	0.9995	16751	1	-0.074
hsa-mir-638	4	0.77828	0.78258	0.9995	16752	1	-0.897
hsa-mir-3178	4	0.77848	0.78276	0.9995	16753	1	-0.638
AMACR	6	0.77855	0.8395	0.9995	16754	1	-0.043
RAC2	6	0.77889	0.83963	0.9995	16755	1	-0.247
TMEM141	6	0.77899	0.83967	0.9995	16756	1	-0.187
hsa-mir-645	4	0.77906	0.78323	0.9995	16757	1	-0.07
PRR21	6	0.7791	0.83973	0.9995	16758	1	-0.078
SIAE	6	0.77918	0.83976	0.9995	16759	1	0.1179
METTL17	4	0.77919	0.78334	0.9995	16760	1	0.1743
hsa-mir-7-1	4	0.77936	0.78347	0.9995	16761	1	-0.004
ENOX1	6	0.77937	0.83984	0.9995	16762	1	-0.768
USP51	6	0.77945	0.83986	0.9995	16763	1	0.0547
PLK1S1	6	0.77956	0.83991	0.9995	16764	1	0.0008
SZT2	6	0.77967	0.83995	0.9995	16765	1	-0.379
HMCES	4	0.7797	0.78376	0.9995	16766	1	-0.067
SNX4	6	0.77978	0.83999	0.9995	16767	1	0.0244
UBE2H	6	0.78007	0.84011	0.9995	16768	1	-0.278
LIPK	6	0.78018	0.84015	0.9995	16769	1	-0.064
ASAP3	6	0.78047	0.84026	0.9995	16770	1	-0.363
PLXNB3	6	0.78059	0.84031	0.9995	16771	1	-0.128
GYLTL1B	6	0.7807	0.84035	0.9995	16772	1	-0.07
DLEC1	6	0.78072	0.84035	0.9995	16773	1	-0.491
PLCD4	6	0.78084	0.8404	0.9995	16774	1	-0.369
SLC22A15	6	0.78084	0.8404	0.9995	16775	1	-0.129
AXDND1	6	0.78104	0.84048	0.9995	16776	1	-0.358
GCNT4	6	0.78142	0.84062	0.9995	16777	1	-0.823
GSTA2	2	0.78152	0.78116	0.9995	16778	0	-0.113
VPS11	6	0.78153	0.84066	0.9995	16779	1	-0.353
TULP1	6	0.78156	0.84067	0.9995	16780	1	-0.448
EIF4ENIF1	6	0.78169	0.84072	0.9995	16781	1	-0.249
hsa-mir-518c	4	0.78172	0.78551	0.9995	16782	1	-0.419
ITGA10	6	0.78178	0.84076	0.9995	16783	1	-0.025
NNMT	6	0.78183	0.84078	0.9995	16784	1	-0.597
GNRH2	6	0.78201	0.84085	0.9995	16785	1	-0.264
GNG12	6	0.78201	0.84085	0.9995	16786	1	-0.502
FAM194A	6	0.78221	0.84092	0.9995	16787	1	-0.156
TAS1R1	6	0.78223	0.84093	0.9995	16788	1	-0.206
PLIN1	6	0.78243	0.84101	0.9995	16789	1	-0.266
MTRNR2L2	6	0.78243	0.84101	0.9995	16790	1	-0.268
ANAPC2	6	0.78254	0.84105	0.9995	16791	1	-0.107
ARHGAP33	6	0.7826	0.84108	0.9995	16792	1	0.1383
CHMP4A	6	0.7826	0.84108	0.9995	16793	1	-0.031
ZNF672	6	0.78272	0.84113	0.9995	16794	1	-0.245
SV2C	6	0.78308	0.84127	0.9995	16795	1	-0.11
TMEM135	6	0.78314	0.84129	0.9995	16796	1	-0.27
hsa-mir-17	3	0.78335	0.78339	0.9995	16797	0	-0.194
TMCC1	6	0.78342	0.84141	0.9995	16798	1	-0.371
LGSN	6	0.78342	0.84141	0.9995	16799	1	-0.122
hsa-mir-320a	3	0.78342	0.78347	0.9995	16800	0	-0.423
hsa-mir-196a-2	4	0.78363	0.78713	0.9995	16801	1	0.0847
hsa-mir-1269a	4	0.78377	0.78724	0.9995	16802	1	-0.355
BCAM	6	0.78393	0.84163	0.9995	16803	1	-0.23
hsa-mir-337	4	0.78397	0.78741	0.9995	16804	1	-1.364
IP6K1	6	0.78417	0.84172	0.9995	16805	1	-0.222
POLR2B	6	0.78428	0.84176	0.9995	16806	1	-0.203
MARVELD2	6	0.78428	0.84176	0.9995	16807	1	-0.005
BCMO1	6	0.78442	0.84182	0.9995	16808	1	-0.177
LGALS12	6	0.78478	0.84196	0.9995	16809	1	-0.271
GRTP1	6	0.78481	0.84197	0.9995	16810	1	-0.325
GNAT3	6	0.7849	0.842	0.9995	16811	1	-0.467
NT5C2	6	0.78491	0.84201	0.9995	16812	1	-0.48
hsa-mir-920	4	0.78514	0.78843	0.9995	16813	1	0.1448
VMA21	6	0.78515	0.8421	0.9995	16814	1	-0.624
COPZ2	6	0.78524	0.84213	0.9995	16815	1	-0.191
BMP8A	3	0.78525	0.78528	0.9995	16816	0	-0.19
PRPH	6	0.78532	0.84216	0.9995	16817	1	-0.051
DAPK1	6	0.78537	0.84219	0.9995	16818	1	-0.322
MOG	6	0.78548	0.84222	0.9995	16819	1	-0.167
ZNF155	6	0.78557	0.84226	0.9995	16820	1	-0.506
CCDC129	4	0.7856	0.78883	0.9995	16821	1	-0.251
hsa-mir-6778	4	0.7856	0.78883	0.9995	16822	1	-0.223
SPATA2	6	0.78579	0.84235	0.9995	16823	1	-0.353
TBP	6	0.78585	0.84238	0.9995	16824	1	-0.588
IL11RA	6	0.78595	0.84242	0.9995	16825	1	-0.222

KAZALD1	6	0.78595	0.84242	0.9995	16826	1	-0.187
CHRNA9	6	0.78595	0.84242	0.9995	16827	1	-0.001
FUT6	6	0.786	0.84244	0.9995	16828	1	0.0761
hsa-mir-519a-2	2	0.78613	0.78577	0.9995	16829	0	-0.576
MORN1	6	0.78618	0.84251	0.9995	16830	1	-0.348
FTL	6	0.78624	0.84253	0.9995	16831	1	-0.262
SLC52A1	6	0.78627	0.84254	0.9995	16832	1	-0.264
PAPD7	6	0.78631	0.84256	0.9995	16833	1	-0.394
PTBP2	6	0.78665	0.8427	0.9995	16834	1	0.0313
NLRP12	6	0.78676	0.84274	0.9995	16835	1	-0.241
PRKDC	6	0.78685	0.84278	0.9995	16836	1	-0.01
HAND1	6	0.78728	0.84295	0.9995	16837	1	-0.415
hsa-mir-3689b	1	0.7873	0.7877	0.9995	16838	0	-3.186
BCL6B	6	0.78733	0.84297	0.9995	16839	1	-0.298
COL8A1	6	0.78736	0.84298	0.9995	16840	1	-0.118
ZNF121	6	0.78751	0.84305	0.9995	16841	1	-0.279
PLAGL1	6	0.7879	0.84322	0.9995	16842	1	-0.013
ZNF140	6	0.78795	0.84323	0.9995	16843	1	-0.373
FAM107A	6	0.78811	0.84331	0.9995	16844	1	-0.543
PHF10	6	0.78826	0.84337	0.9995	16845	1	0.0001
LRCOL1	6	0.78829	0.84337	0.9995	16846	1	-0.064
C8orf46	6	0.78834	0.8434	0.9995	16847	1	0.0424
BRSK1	6	0.78853	0.84348	0.9995	16848	1	-0.795
CENPB	6	0.78855	0.84349	0.9995	16849	1	0.0643
HECTD2	6	0.78867	0.84353	0.9995	16850	1	-0.755
C22orf26	6	0.78867	0.84353	0.9995	16851	1	-0.588
ZFYVE19	6	0.78884	0.8436	0.9995	16852	1	-0.372
CHRNG	6	0.78884	0.8436	0.9995	16853	1	-0.153
ZNF584	6	0.78892	0.84364	0.9995	16854	1	-0.116
KCTD12	6	0.78894	0.84365	0.9995	16855	1	-0.252
hsa-mir-6078	4	0.78895	0.79166	0.9995	16856	1	-0.322
hsa-mir-874	4	0.78912	0.79181	0.9995	16857	1	-1.298
CHAF1A	6	0.78918	0.84374	0.9995	16858	1	-0.22
YTHDF2	6	0.78918	0.84374	0.9995	16859	1	-0.11
ZNF720	6	0.78939	0.84383	0.9995	16860	1	-0.024
ERN1	6	0.78943	0.84384	0.9995	16861	1	-0.07
CCDC109B	6	0.78952	0.84388	0.9995	16862	1	-0.359
hsa-mir-378b	4	0.78979	0.79238	0.9995	16863	1	0.1065
IRX6	6	0.78985	0.84401	0.9995	16864	1	-0.123
OR51F2	6	0.7899	0.84403	0.9995	16865	1	-0.458
ERCC6	2	0.79008	0.78973	0.9995	16866	0	-0.246
TRPM5	6	0.7901	0.8441	0.9995	16867	1	-0.243
RRNAD1	6	0.79014	0.84413	0.9995	16868	1	-0.11
SLC39A12	4	0.79017	0.79267	0.9995	16869	1	0.0057
ACAP2	6	0.79019	0.84415	0.9995	16870	1	-0.489
ZNF347	6	0.79019	0.84415	0.9995	16871	1	-0.158
PIH1D2	6	0.79024	0.84417	0.9995	16872	1	-0.07
ZNF841	6	0.79026	0.84418	0.9995	16873	1	-0.637
LILRA1	5	0.79039	0.81391	0.9995	16874	1	-0.419
LCN15	6	0.79043	0.84425	0.9995	16875	1	-0.168
OR5K2	5	0.79046	0.81395	0.9995	16876	1	-0.685
LCT	6	0.79048	0.84427	0.9995	16877	1	-0.445
IFT20	6	0.79052	0.84429	0.9995	16878	1	0.0449
PF4V1	5	0.79054	0.814	0.9995	16879	1	-0.419
ZBED6CL	3	0.79058	0.79068	0.9995	16880	0	0.0616
GPRIN1	6	0.79066	0.84435	0.9995	16881	1	-0.753
BRINP1	2	0.7907	0.79038	0.9995	16882	0	-0.15
GUCD1	6	0.79082	0.84441	0.9995	16883	1	0.1093
BPTF	6	0.79113	0.84453	0.9995	16884	1	-0.128
CDH23	6	0.79127	0.84459	0.9995	16885	1	0.0332
GALT	6	0.7914	0.84465	0.9995	16886	1	-0.588
CLIC5	6	0.79159	0.84472	0.9995	16887	1	-0.057
SLC27A4	6	0.79166	0.84475	0.9995	16888	1	-0.268
OTX1	6	0.79169	0.84476	0.9995	16889	1	-0.228
hsa-mir-19a	3	0.79189	0.79197	0.9995	16890	0	-0.102
ALG1	6	0.79191	0.84485	0.9995	16891	1	-0.355
hsa-mir-221	4	0.79194	0.79422	0.9995	16892	1	-0.144
HBQ1	4	0.79194	0.79422	0.9995	16893	1	0.1191
hsa-mir-320c-2	3	0.79203	0.7921	0.9995	16894	0	-0.37
hsa-mir-16-2	2	0.79204	0.79173	0.9995	16895	0	-0.214
hsa-mir-1471	4	0.79205	0.79433	0.9995	16896	1	0.1692
PSMA4	6	0.79219	0.84496	0.9995	16897	1	-0.013
MFSD1	6	0.79227	0.845	0.9995	16898	1	-0.032
FANCD2OS	6	0.79231	0.84501	0.9995	16899	1	-0.465
PRKAA2	4	0.79245	0.79468	0.9995	16900	1	-0.15
FAM178B	6	0.79253	0.84509	0.9995	16901	1	-0.113
NPRL3	6	0.79266	0.84514	0.9995	16902	1	-0.369
C19orf24	6	0.79271	0.84516	0.9995	16903	1	-0.19
PCDHGA2	2	0.79284	0.79252	0.9995	16904	0	-0.737
PITPNM1	6	0.79285	0.84522	0.9995	16905	1	-0.045
ZNF8	6	0.79289	0.84524	0.9995	16906	1	-0.274
LCP1	6	0.79303	0.84529	0.9995	16907	1	-0.076
hsa-mir-6770-3	2	0.7931	0.79278	0.9995	16908	0	-0.626
TMUB2	6	0.79312	0.84532	0.9995	16909	1	-0.247
HNRNPK	6	0.79315	0.84534	0.9995	16910	1	-0.403

hsa-mir-4438	4	0.79319	0.79531	0.9995	16911	1	-0.112
PKP3	6	0.79324	0.84538	0.9995	16912	1	0.0776
RCAN2	6	0.7933	0.8454	0.9995	16913	1	-4E-04
SIRPB1	6	0.79338	0.84543	0.9995	16914	1	-0.447
NIPAL1	6	0.79338	0.84543	0.9995	16915	1	-0.145
RIPK4	6	0.79341	0.84545	0.9995	16916	1	-0.431
ZNF219	6	0.79357	0.84551	0.9995	16917	1	-0.128
ZIK1	6	0.79374	0.84558	0.9995	16918	1	-0.592
THTPA	6	0.79403	0.8457	0.9995	16919	1	-0.023
GNAT2	5	0.79424	0.81627	0.9995	16920	1	-0.054
VDAC1	6	0.79435	0.84583	0.9995	16921	1	-0.251
TRPV2	6	0.79435	0.84583	0.9995	16922	1	-0.373
ERP44	6	0.79446	0.84587	0.9995	16923	1	-0.08
ZG16	6	0.79459	0.84592	0.9995	16924	1	-0.09
hsa-mir-4684	4	0.79467	0.79662	0.9995	16925	1	0.059
TGFB2	6	0.79468	0.84596	0.9995	16926	1	-0.262
hsa-mir-3115	4	0.79486	0.79678	0.9995	16927	1	-0.286
hsa-mir-4307	4	0.79489	0.79682	0.9995	16928	1	0.0024
LAMP1	6	0.79493	0.84607	0.9995	16929	1	-0.136
BNIP1	6	0.79493	0.84607	0.9995	16930	1	-0.004
SLC18B1	6	0.79509	0.84612	0.9995	16931	1	-0.248
TTK	6	0.7952	0.84616	0.9995	16932	1	-0.254
ETS1	6	0.79525	0.84618	0.9995	16933	1	-0.041
MTDH	6	0.79536	0.84623	0.9995	16934	1	0.0561
HSD17B3	6	0.79541	0.84625	0.9995	16935	1	-0.219
ERVV-2	6	0.7955	0.84629	0.9995	16936	1	0.0055
hsa-mir-2113	4	0.79556	0.7974	0.9995	16937	1	0.0982
RCN2	6	0.7957	0.84637	0.9995	16938	1	-0.341
OR5AR1	6	0.79603	0.84652	0.9995	16939	1	-0.484
COL24A1	6	0.7962	0.84659	0.9995	16940	1	-0.029
hsa-mir-5011	4	0.79631	0.79804	0.9995	16941	1	-0.034
GPR150	4	0.79634	0.79807	0.9995	16942	1	-0.036
DPRX	6	0.79636	0.84666	0.9995	16943	1	-0.272
CLPB	6	0.79636	0.84666	0.9995	16944	1	-0.203
EIF4E3	6	0.79646	0.8467	0.9995	16945	1	-0.312
hsa-mir-3677	4	0.79655	0.79825	0.9995	16946	1	-0.142
UBAP2L	6	0.7966	0.84676	0.9995	16947	1	-0.276
FOXO3	6	0.79667	0.84679	0.9995	16948	1	0.0732
ZCCHC4	6	0.79678	0.84684	0.9995	16949	1	-0.177
RAB40AL	5	0.79718	0.81808	0.9995	16950	1	-0.19
hsa-mir-185	4	0.79722	0.79886	0.9995	16951	1	-0.752
GKN2	6	0.79741	0.8471	0.9995	16952	1	-0.269
hsa-mir-4734	4	0.7975	0.79911	0.9995	16953	1	-0.221
C19orf70	6	0.79759	0.84718	0.9995	16954	1	-0.278
GABRG3	6	0.79764	0.8472	0.9995	16955	1	-0.539
ZNF518A	6	0.79764	0.8472	0.9995	16956	1	-0.246
PCDHGB2	2	0.7977	0.79728	0.9995	16957	0	-0.462
hsa-mir-5188	4	0.79782	0.79938	0.9995	16958	1	-0.476
hsa-mir-8061	2	0.79784	0.79741	0.9995	16959	0	-0.198
EIF3L	6	0.79809	0.84738	0.9995	16960	1	-0.392
CX3CL1	6	0.79813	0.8474	0.9995	16961	1	-0.556
AP3M1	6	0.79814	0.84741	0.9995	16962	1	-0.154
C19orf54	6	0.7982	0.84743	0.9995	16963	1	-0.504
APOBEC3A_B	2	0.79826	0.79785	0.9995	16964	0	-0.388
KIAA1009	6	0.79838	0.8475	0.9995	16965	1	0.0192
KCNC3	6	0.79852	0.84757	0.9995	16966	1	-0.31
RHCE	5	0.79857	0.81895	0.9995	16967	1	-0.01
TAS2R13	6	0.79859	0.84758	0.9995	16968	1	-0.168
PRAMEF22	3	0.79866	0.79866	0.9995	16969	0	0.1874
hsa-mir-3686	3	0.79866	0.79866	0.9995	16970	0	-0.265
TRPV5	6	0.79868	0.84762	0.9995	16971	1	0.0776
MYNN	6	0.79875	0.84765	0.9995	16972	1	-0.457
ZDBF2	6	0.79895	0.84773	0.9995	16973	1	-0.063
BAG2	6	0.79936	0.8479	0.9995	16974	1	-0.043
PPP1R18	6	0.79946	0.84794	0.9995	16975	1	0.1511
TMEM170B	6	0.79964	0.84801	0.9995	16976	1	0.1099
hsa-mir-3683	4	0.79971	0.80107	0.9995	16977	1	-0.307
PAFAH1B2	6	0.79978	0.84807	0.9995	16978	1	-0.008
hsa-mir-4311	4	0.79979	0.80114	0.9995	16979	1	-0.646
FAM136A	6	0.79987	0.84812	0.9995	16980	1	-0.243
PTPRJ	6	0.79991	0.84813	0.9995	16981	1	-0.209
SIX3	6	0.80018	0.84825	0.9995	16982	1	-0.167
KHDC1L	6	0.80022	0.84826	0.9995	16983	1	-0.586
DIDO1	6	0.80032	0.8483	0.9995	16984	1	-0.39
SPINK4	6	0.80045	0.84835	0.9995	16985	1	-0.193
PSMA2	6	0.80045	0.84835	0.9995	16986	1	-0.241
POLDIP3	6	0.8005	0.84837	0.9995	16987	1	-0.196
UNC45A	6	0.80064	0.84843	0.9995	16988	1	-0.055
RGS20	6	0.80068	0.84845	0.9995	16989	1	-0.159
TMSB15A	2	0.80075	0.80039	0.9995	16990	0	-0.826
RGPD2	2	0.80075	0.80039	0.9995	16991	0	-1.99
OR2A4	2	0.80075	0.80039	0.9995	16992	0	-0.826
HBG1	2	0.80075	0.80039	0.9995	16993	0	-1.073
SSX2	2	0.80075	0.80039	0.9995	16994	0	-0.419
hsa-mir-520f	2	0.80075	0.80039	0.9995	16995	0	-0.826

TUT1	6	0.80084	0.84851	0.9995	16996	1	-0.521
PHLPP1	6	0.80095	0.84856	0.9995	16997	1	-0.402
SIAH2	6	0.80124	0.84868	0.9995	16998	1	-0.391
hsa-mir-138-2	4	0.80127	0.80248	0.9995	16999	1	-0.181
hsa-mir-5587	4	0.80127	0.80248	0.9995	17000	1	0.0245
hsa-mir-668	4	0.80133	0.80252	0.9995	17001	1	-0.25
RPP14	6	0.80138	0.84874	0.9995	17002	1	-0.429
HIST1H3D	6	0.80147	0.84878	0.9995	17003	1	-0.37
DNAJA3	4	0.80153	0.8027	0.9995	17004	1	-0.114
CDR2L	6	0.80192	0.84896	0.9995	17005	1	-0.21
FBXL5	6	0.80202	0.849	0.9995	17006	1	0.0967
COMMD5	6	0.80217	0.84907	0.9995	17007	1	-0.693
hsa-mir-6819	4	0.8024	0.80347	0.9995	17008	1	0.1789
KCNQ3	6	0.80245	0.8492	0.9995	17009	1	-0.118
PIGN	6	0.80254	0.84924	0.9995	17010	1	-0.953
WDR54	6	0.80259	0.84927	0.9995	17011	1	-0.227
RUNDC3A	6	0.8029	0.8494	0.9995	17012	1	-0.317
OCM	4	0.80293	0.80395	0.9995	17013	1	-0.826
hsa-mir-769	4	0.80307	0.80408	0.9995	17014	1	-0.409
EDN1	6	0.80311	0.8495	0.9995	17015	1	-0.032
LMO3	6	0.80314	0.84951	0.9995	17016	1	-0.105
HIPK2	6	0.80319	0.84954	0.9995	17017	1	0.0208
DRAM1	6	0.80328	0.84958	0.9995	17018	1	-0.218
NAE1	6	0.80346	0.84967	0.9995	17019	1	-0.416
RPL27	6	0.80346	0.84967	0.9995	17020	1	-0.026
PTGES3	6	0.80346	0.84967	0.9995	17021	1	-0.507
TTC12	6	0.80351	0.84968	0.9995	17022	1	-0.491
CCAR2	6	0.80358	0.84971	0.9995	17023	1	-0.38
hsa-mir-4507	4	0.80381	0.80476	0.9995	17024	1	-0.297
hsa-mir-6824	4	0.80398	0.8049	0.9995	17025	1	-0.256
FKRP	6	0.80403	0.84991	0.9995	17026	1	-0.006
ZNF215	6	0.80403	0.84991	0.9995	17027	1	-0.047
C9orf139	6	0.80406	0.84992	0.9995	17028	1	-0.177
CD300LG	6	0.80409	0.84993	0.9995	17029	1	-0.083
MYLIP	6	0.80411	0.84994	0.9995	17030	1	-0.054
CDA	6	0.80415	0.84996	0.9995	17031	1	0.0948
CCDC113	6	0.80434	0.85004	0.9995	17032	1	0.1259
IQCJ	2	0.80443	0.80406	0.9995	17033	0	-0.186
hsa-mir-1827	4	0.80452	0.80539	0.9995	17034	1	-0.14
PRR5	4	0.8048	0.80565	0.9995	17035	1	-0.082
KLRB1	6	0.80485	0.85027	0.9995	17036	1	-0.328
SGCZ	6	0.80485	0.85027	0.9995	17037	1	-0.136
DZIP1L	6	0.8053	0.85046	0.9995	17038	1	-0.014
C17orf70	6	0.8053	0.85046	0.9995	17039	1	-0.119
hsa-mir-4689	4	0.80537	0.80616	0.9995	17040	1	0.0628
MAP1LC3C	6	0.80541	0.85051	0.9995	17041	1	-0.127
HTR1A	6	0.80544	0.85052	0.9995	17042	1	-0.252
SLC17A4	6	0.8055	0.85054	0.9995	17043	1	-0.202
hsa-mir-202	4	0.8056	0.80637	0.9995	17044	1	-0.043
POMC	6	0.80562	0.85059	0.9995	17045	1	-0.354
hsa-mir-203a	1	0.80576	0.80598	0.9995	17046	0	-3.386
hsa-mir-6503	4	0.80577	0.80653	0.9995	17047	1	-0.016
SERPINE1	4	0.80591	0.80667	0.9995	17048	1	-0.744
hsa-mir-3144	4	0.80596	0.80672	0.9995	17049	1	-0.124
hsa-mir-6827	4	0.80602	0.80678	0.9995	17050	1	0.0345
CCDC102B	6	0.80606	0.8508	0.9995	17051	1	-0.176
KIR3DL3	6	0.8061	0.85081	0.9995	17052	1	-0.19
FGR	6	0.80619	0.85085	0.9995	17053	1	-0.129
hsa-mir-379	4	0.80624	0.80697	0.9995	17054	1	-0.878
TSPAN1	6	0.80628	0.85089	0.9995	17055	1	-0.257
hsa-mir-7158	4	0.80639	0.80711	0.9995	17056	1	-0.551
hsa-mir-595	4	0.80649	0.8072	0.9995	17057	1	0.0051
SNRPA	6	0.80686	0.85115	0.9995	17058	1	-0.257
PLEK2	6	0.8069	0.85116	0.9995	17059	1	-0.098
DNMT3A	6	0.80702	0.85122	0.9995	17060	1	-0.155
ZIC2	6	0.80709	0.85124	0.9995	17061	1	0.0612
SPATA31A1	3	0.80747	0.80738	0.9995	17062	0	-0.016
HLA-DOA	6	0.80749	0.85141	0.9995	17063	1	0.1644
hsa-mir-6792	4	0.80761	0.80822	0.9995	17064	1	-0.026
hsa-mir-642b	1	0.80763	0.80785	0.9995	17065	0	-0.494
hsa-mir-3171	2	0.80776	0.80742	0.9995	17066	0	-0.255
PCDHGA5	2	0.80791	0.80758	0.9995	17067	0	-0.531
CDNF	6	0.80793	0.85159	0.9995	17068	1	0.1495
TMED3	4	0.80794	0.80851	0.9995	17069	1	-0.292
SURF1	6	0.80833	0.85176	0.9995	17070	1	-0.1
DUSP16	6	0.80837	0.85177	0.9995	17071	1	-0.151
UBE2G2	6	0.80855	0.85185	0.9995	17072	1	-0.353
EXPH5	6	0.80867	0.85189	0.9995	17073	1	-0.311
ZNF286B	6	0.80887	0.85198	0.9995	17074	1	-0.033
ZFHX3	6	0.80897	0.85203	0.9995	17075	1	-0.456
SMCR7L	2	0.80914	0.80881	0.9995	17076	0	-0.825
CAPN15	5	0.80924	0.82573	0.9995	17077	1	0.1265
PRSS16	6	0.80931	0.85218	0.9995	17078	1	-0.006
CALCR	6	0.8095	0.85227	0.9995	17079	1	-0.1
ALG3	6	0.80976	0.85239	0.9995	17080	1	-0.171

STEAP4	6	0.80986	0.85243	0.9995	17081	1	0.0905
FBXL13	6	0.8099	0.85244	0.9995	17082	1	-0.011
C13orf45	6	0.81008	0.85252	0.9995	17083	1	-0.265
TONSL	6	0.81015	0.85256	0.9995	17084	1	-0.322
RAB2A	6	0.81021	0.85258	0.9995	17085	1	0.0021
SPHKAP	6	0.81021	0.85258	0.9995	17086	1	-0.136
CYTH4	4	0.81027	0.8107	0.9995	17087	1	-0.175
hsa-mir-639	4	0.81033	0.81075	0.9995	17088	1	-0.565
MYH4	6	0.81034	0.85263	0.9995	17089	1	-0.396
MS4A3	6	0.81048	0.8527	0.9995	17090	1	-0.192
CCDC41	6	0.81048	0.8527	0.9995	17091	1	-0.324
LRRC59	6	0.81053	0.85273	0.9995	17092	1	-0.55
PLAC8	6	0.8106	0.85277	0.9995	17093	1	-0.261
PIK3IP1	4	0.81094	0.81132	0.9995	17094	1	-0.354
EXT1	6	0.81096	0.85293	0.9995	17095	1	-0.288
ZRANB3	6	0.81096	0.85293	0.9995	17096	1	-0.219
CLEC19A	6	0.81105	0.85297	0.9995	17097	1	-0.331
USP17L1P	3	0.81108	0.81093	0.9995	17098	0	-0.504
CAMSAP1	6	0.8111	0.85299	0.9995	17099	1	-0.873
APOC4	6	0.81116	0.85302	0.9995	17100	1	-0.079
DCAF15	6	0.81135	0.85309	0.9995	17101	1	-0.2
TICRR	6	0.81135	0.85309	0.9995	17102	1	-0.417
KIAA1522	6	0.81146	0.85314	0.9995	17103	1	-0.108
ATP10D	6	0.81154	0.85317	0.9995	17104	1	0.0274
SLC35E2B	2	0.81176	0.81145	0.9995	17105	0	-0.673
IRS4	6	0.81182	0.8533	0.9995	17106	1	-0.175
ATM	6	0.81188	0.85332	0.9995	17107	1	-0.113
hsa-mir-4676	4	0.81198	0.81229	0.9995	17108	1	-0.426
RFX2	6	0.81217	0.85346	0.9995	17109	1	-0.694
CCDC142	4	0.81229	0.81258	0.9995	17110	1	0.1714
POLD4	6	0.81239	0.85357	0.9995	17111	1	-0.04
CITED1	6	0.81276	0.85374	0.9995	17112	1	-0.447
CMTR1	2	0.81313	0.81282	0.9995	17113	0	-0.138
ZSCAN16	6	0.81331	0.854	0.9995	17114	1	-0.063
hsa-mir-4766	4	0.81331	0.81356	0.9995	17115	1	-0.077
EPHA8	6	0.81348	0.85408	0.9995	17116	1	-0.636
hsa-mir-4437	4	0.81349	0.81372	0.9995	17117	1	-0.45
RPL7A	6	0.81353	0.85411	0.9995	17118	1	-0.062
WDR45	6	0.8137	0.85418	0.9995	17119	1	-0.272
hsa-mir-103a-1	1	0.8138	0.8141	0.9995	17120	0	-0.51
MSRB2	6	0.81382	0.85424	0.9995	17121	1	-0.132
ZNF232	6	0.81382	0.85424	0.9995	17122	1	-0.627
REG3G	6	0.81395	0.8543	0.9995	17123	1	-0.454
C21orf58	6	0.81403	0.85434	0.9995	17124	1	-0.085
hsa-mir-3943	4	0.81413	0.81431	0.9995	17125	1	0.044
RNF167	6	0.81417	0.85439	0.9995	17126	1	-0.4
PNPT1	6	0.81419	0.85441	0.9995	17127	1	-0.547
AKR1C3	6	0.81425	0.85443	0.9995	17128	1	0.0264
hsa-mir-623	4	0.81459	0.81473	0.9995	17129	1	-0.335
PRDM12	6	0.81471	0.85466	0.9995	17130	1	-0.565
hsa-mir-425	4	0.81477	0.8149	0.9995	17131	1	-0.364
MAMDC4	6	0.81492	0.85474	0.9995	17132	1	-0.072
PUSL1	6	0.81492	0.85474	0.9995	17133	1	-0.016
GTF3C1	6	0.81492	0.85474	0.9995	17134	1	-0.015
C5orf24	6	0.81509	0.85482	0.9995	17135	1	0.0446
SLC4A3	6	0.81519	0.85486	0.9995	17136	1	-0.284
F2	6	0.81519	0.85486	0.9995	17137	1	-0.318
FAM101A	4	0.81563	0.81575	0.9995	17138	1	-0.061
hsa-mir-6867	4	0.8157	0.81582	0.9995	17139	1	-0.446
RNF214	6	0.81576	0.85514	0.9995	17140	1	0.0681
ATP2B2	6	0.81614	0.85532	0.9995	17141	1	-0.08
FOXK2	6	0.81614	0.85532	0.9995	17142	1	-0.864
RNMTL1	6	0.8162	0.85535	0.9995	17143	1	-0.004
MDP1	4	0.81623	0.81631	0.9995	17144	1	-1.06
hsa-mir-4261	2	0.81626	0.81594	0.9995	17145	0	-0.127
TDP2	6	0.81654	0.85551	0.9995	17146	1	-0.235
VSNL1	6	0.81696	0.85569	0.9995	17147	1	-0.39
MYADM	6	0.81707	0.85574	0.9995	17148	1	-0.635
STAG3	5	0.8172	0.83103	0.9995	17149	1	-0.497
ZNF844	6	0.81745	0.85592	0.9995	17150	1	-0.374
A4GALT	4	0.8176	0.81763	0.9995	17151	1	-0.052
LY6G5B	6	0.81765	0.85601	0.9995	17152	1	-0.282
APAF1	6	0.81775	0.85606	0.9995	17153	1	-0.238
CARM1	6	0.81775	0.85606	0.9995	17154	1	-0.275
CA8	6	0.81775	0.85606	0.9995	17155	1	-0.106
C1QTNF9B	2	0.81779	0.81747	0.9995	17156	0	-0.75
PCNP	6	0.81782	0.8561	0.9995	17157	1	-0.061
NPFFR1	6	0.81786	0.85611	0.9995	17158	1	-0.127
SMCHD1	6	0.8179	0.85613	0.9995	17159	1	-0.165
PLA2G12A	6	0.81794	0.85615	0.9995	17160	1	0.0843
GSTT2	1	0.81794	0.81823	0.9995	17161	0	-2.904
hsa-mir-526a-1	1	0.81794	0.81823	0.9995	17162	0	-2.904
NBPF24	1	0.81794	0.81823	0.9995	17163	0	-2.904
RNF152	6	0.81803	0.85618	0.9995	17164	1	-0.734
TMSB4Y	4	0.81805	0.81804	0.9995	17165	1	-0.187

LIMCH1	6	0.81807	0.8562	0.9995	17166	1	-0.213
ADRM1	6	0.81808	0.8562	0.9995	17167	1	-0.243
ICOSLG	6	0.81814	0.85623	0.9995	17168	1	-0.339
MMEL1	6	0.81814	0.85623	0.9995	17169	1	-0.537
METAP1	4	0.81832	0.81832	0.9995	17170	1	-0.014
ZNF341	6	0.81837	0.85634	0.9995	17171	1	-0.071
GPR12	6	0.81862	0.85645	0.9995	17172	1	-0.704
YPEL2	6	0.81902	0.85664	0.9995	17173	1	-0.393
PARG	6	0.81906	0.85666	0.9995	17174	1	-0.252
FAM19A5	6	0.81909	0.85667	0.9995	17175	1	-0.476
GBP6	6	0.81922	0.85673	0.9995	17176	1	-0.082
TSKU	6	0.81927	0.85675	0.9995	17177	1	-0.294
TRMT44	6	0.81939	0.85681	0.9995	17178	1	-0.328
LOC100505549	6	0.81944	0.85683	0.9995	17179	1	-0.119
PCDHB16	6	0.81955	0.85687	0.9995	17180	1	-0.184
TCEANC	5	0.8196	0.83261	0.9995	17181	1	0.0062
SZRD1	6	0.81969	0.85695	0.9995	17182	1	-0.039
C14orf178	6	0.81969	0.85695	0.9995	17183	1	-0.057
HSPD1	6	0.81991	0.85706	0.9995	17184	1	-0.185
hsa-mir-3197	4	0.81998	0.81993	0.9995	17185	1	-0.492
hsa-mir-6850	4	0.81998	0.81993	0.9995	17186	1	-0.552
hsa-mir-7976	4	0.82001	0.81996	0.9995	17187	1	0.0677
TMEM56-RWDD3	1	0.82008	0.82034	0.9995	17188	0	-0.365
hsa-mir-5582	2	0.82009	0.8198	0.9995	17189	0	-0.895
SCARF2	6	0.82012	0.85717	0.9995	17190	1	-0.026
TPTE2	6	0.82016	0.85719	0.9995	17191	1	-0.201
PRRX1	6	0.82024	0.85723	0.9995	17192	1	-0.47
RECK	6	0.82027	0.85725	0.9995	17193	1	-0.258
VGLL4	6	0.82034	0.85728	0.9995	17194	1	-0.167
hsa-mir-145	4	0.82046	0.8204	0.9995	17195	1	-0.453
RAET1L	6	0.82062	0.8574	0.9995	17196	1	-0.025
RRBP1	6	0.82077	0.85747	0.9995	17197	1	-0.359
PCDHA13	2	0.82085	0.82056	0.9995	17198	0	-0.334
ZCCHC8	6	0.82105	0.85759	0.9995	17199	1	0.0115
ACADVL	6	0.82105	0.85759	0.9995	17200	1	-0.107
OXT	6	0.8211	0.85761	0.9995	17201	1	-0.324
WDR89	6	0.82112	0.85761	0.9995	17202	1	-0.047
hsa-mir-3919	2	0.82114	0.82086	0.9995	17203	0	-0.799
ZNF652	6	0.8212	0.85766	0.9995	17204	1	-0.273
hsa-mir-548t	3	0.82135	0.82131	0.9995	17205	0	-0.056
IQGAP3	6	0.82139	0.85775	0.9995	17206	1	-0.221
ZNF519	6	0.8215	0.8578	0.9995	17207	1	-0.766
UBE2QL1	6	0.82154	0.85782	0.9995	17208	1	-0.087
ASUN	6	0.82163	0.85786	0.9995	17209	1	0.0116
hsa-mir-579	4	0.82171	0.8216	0.9995	17210	1	-0.287
CDH2	6	0.82179	0.85794	0.9995	17211	1	-0.141
hsa-mir-7152	4	0.8218	0.82169	0.9995	17212	1	-0.769
PNPO	6	0.82185	0.85796	0.9995	17213	1	-0.196
FBXO3	6	0.82193	0.85799	0.9995	17214	1	0.0957
KRTAP6-2	6	0.82197	0.85801	0.9995	17215	1	-0.098
LIPG	6	0.82214	0.8581	0.9995	17216	1	-0.61
hsa-mir-4266	4	0.82218	0.82208	0.9995	17217	1	0.0651
FCER2	6	0.82221	0.85814	0.9995	17218	1	0.0652
TMEM181	6	0.82221	0.85814	0.9995	17219	1	-0.295
hsa-mir-624	4	0.82224	0.82213	0.9995	17220	1	0.0116
hsa-mir-3180-3	4	0.82231	0.8222	0.9995	17221	1	0.1944
DNAJB6	6	0.82231	0.85819	0.9995	17222	1	-0.718
hsa-mir-3672	1	0.82253	0.8228	0.9995	17223	0	-0.668
hsa-mir-5009	4	0.82255	0.82244	0.9995	17224	1	-0.063
NSG1	6	0.8226	0.85831	0.9995	17225	1	-0.531
MYL3	6	0.8228	0.85841	0.9995	17226	1	-0.585
GULP1	6	0.82292	0.85846	0.9995	17227	1	-0.469
SSX4	1	0.82298	0.82324	0.9995	17228	0	-0.545
ASPM	6	0.823	0.8585	0.9995	17229	1	0.0566
TMEM121	5	0.8231	0.83491	0.9995	17230	1	-0.229
ZNF92	6	0.82315	0.85856	0.9995	17231	1	-0.115
SOBP	6	0.82336	0.85866	0.9995	17232	1	-0.071
hsa-mir-204	4	0.82343	0.82332	0.9995	17233	1	0.0862
hsa-mir-548ay	4	0.82362	0.8235	0.9995	17234	1	-0.335
KRTAP9-8	4	0.82362	0.8235	0.9995	17235	1	-0.482
hsa-mir-3689f	4	0.82362	0.8235	0.9995	17236	1	-0.443
hsa-mir-380	4	0.82362	0.8235	0.9995	17237	1	-0.5
TRAPPC10	6	0.82363	0.85879	0.9995	17238	1	-0.02
NSF	6	0.82363	0.85879	0.9995	17239	1	0.0409
MTMR3	6	0.82372	0.85884	0.9995	17240	1	-0.063
hsa-mir-4632	4	0.82383	0.8237	0.9995	17241	1	-1.65
TMEM54	6	0.82388	0.85892	0.9995	17242	1	-0.438
NUDT22	6	0.82392	0.85894	0.9995	17243	1	-0.106
SLC6A8	6	0.82396	0.85896	0.9995	17244	1	-0.429
BDNF	6	0.82399	0.85897	0.9995	17245	1	-0.307
MRC1	6	0.82401	0.85898	0.9995	17246	1	0.0956
HNRNPF	6	0.82416	0.85905	0.9995	17247	1	-0.253
ANKRD27	6	0.82432	0.85913	0.9995	17248	1	-0.747
SLIT2	4	0.82466	0.82452	0.9995	17249	1	-0.67
KIAA0513	6	0.8248	0.85935	0.9995	17250	1	-0.09

MFI2	6	0.82484	0.85938	0.9995	17251	1	-0.393
ACSBG2	6	0.82504	0.85946	0.9995	17252	1	-0.217
TRUB1	6	0.82509	0.85949	0.9995	17253	1	0.112
PLA2G3	6	0.82512	0.8595	0.9995	17254	1	0.0045
OR2Z1	6	0.8252	0.85954	0.9995	17255	1	-0.324
hsa-mir-1257	4	0.82541	0.82523	0.9995	17256	1	-0.079
TMEM132A	6	0.82546	0.85965	0.9995	17257	1	-0.441
SLCO4A1	6	0.82556	0.8597	0.9995	17258	1	-0.117
NRAP	6	0.82559	0.85971	0.9995	17259	1	-0.261
RGR	6	0.82566	0.85974	0.9995	17260	1	-0.078
RPP25L	6	0.82566	0.85974	0.9995	17261	1	-0.782
MANF	6	0.82566	0.85974	0.9995	17262	1	-0.059
GABRB2	6	0.82573	0.85977	0.9995	17263	1	-0.121
RAB41	6	0.82594	0.85988	0.9995	17264	1	-0.375
MMRN2	6	0.82602	0.85993	0.9995	17265	1	-0.135
PJA1	6	0.82602	0.85993	0.9995	17266	1	-0.331
CD276	6	0.8261	0.85996	0.9995	17267	1	-0.009
CCR10	4	0.82618	0.826	0.9995	17268	1	-0.173
SNTN	6	0.82629	0.86006	0.9995	17269	1	0.0608
hsa-mir-377	4	0.82634	0.82616	0.9995	17270	1	-0.219
GPIHBP1	6	0.82646	0.86014	0.9995	17271	1	-0.238
SETD1B	6	0.82646	0.86014	0.9995	17272	1	-0.249
hsa-mir-6737	4	0.82647	0.82629	0.9995	17273	1	-1.18
PTCH1	6	0.8266	0.8602	0.9995	17274	1	0.0133
PDPK1	6	0.82677	0.86028	0.9995	17275	1	0.0348
SH2B2	6	0.82683	0.8603	0.9995	17276	1	-0.152
MROH6	6	0.82688	0.86032	0.9995	17277	1	-0.299
PRR16	6	0.82704	0.86039	0.9995	17278	1	-0.472
TPGS1	4	0.82707	0.8269	0.9995	17279	1	-0.562
TTC23	6	0.82712	0.86043	0.9995	17280	1	-0.373
ZFYVE28	6	0.8272	0.86046	0.9995	17281	1	-0.49
hsa-mir-4784	4	0.82722	0.82704	0.9995	17282	1	-0.036
GIPC2	6	0.82726	0.8605	0.9995	17283	1	-0.376
FBXW2	6	0.82734	0.86053	0.9995	17284	1	-0.185
SLC4A11	6	0.82747	0.8606	0.9995	17285	1	-0.332
LBR	6	0.82752	0.86062	0.9995	17286	1	-0.352
ZNF28	5	0.82784	0.8382	0.9995	17287	1	-0.419
OR5H15	5	0.82784	0.8382	0.9995	17288	1	-0.419
GLRB	6	0.82803	0.86089	0.9995	17289	1	-0.327
TMIGD1	6	0.82818	0.86096	0.9995	17290	1	-0.173
LRCH4	6	0.82818	0.86096	0.9995	17291	1	-0.388
MED13L	6	0.82832	0.86103	0.9995	17292	1	-0.106
TADA2B	6	0.82838	0.86105	0.9995	17293	1	0.0529
FUBP1	5	0.82845	0.83863	0.9995	17294	1	-0.29
B4GALT3	6	0.82859	0.86116	0.9995	17295	1	-0.509
CCNYL1	6	0.82864	0.86119	0.9995	17296	1	-0.45
KRBA1	6	0.82864	0.86119	0.9995	17297	1	-0.118
hsa-mir-3915	4	0.82864	0.82848	0.9995	17298	0	-0.007
WRN	6	0.82868	0.86121	0.9995	17299	1	-1.023
BCHE	6	0.82876	0.86124	0.9995	17300	1	-0.252
NRSN1	6	0.82881	0.86126	0.9995	17301	1	-0.009
BAZ2A	6	0.82884	0.86128	0.9995	17302	1	-0.724
TTC23L	6	0.82886	0.86128	0.9995	17303	1	-0.112
TAF6L	6	0.8289	0.8613	0.9995	17304	1	-0.262
HES3	6	0.8289	0.8613	0.9995	17305	1	0.0645
TMEFF1	3	0.82899	0.82891	0.9995	17306	0	-0.209
FLNA	6	0.829	0.86135	0.9995	17307	1	-0.698
CLECL1	6	0.82907	0.86138	0.9995	17308	1	-0.328
CCNK	6	0.82931	0.86149	0.9995	17309	1	-0.198
FGF22	6	0.82936	0.86151	0.9995	17310	1	-0.149
RBM45	6	0.82954	0.86159	0.9995	17311	1	-0.311
CLRN2	6	0.82958	0.86161	0.9995	17312	1	-0.064
TM2D1	6	0.82966	0.86164	0.9995	17313	1	0.1678
SLC38A4	6	0.8298	0.86171	0.9995	17314	1	-0.237
POM121	4	0.82992	0.82978	0.9995	17315	0	-0.487
KIF21B	6	0.83004	0.86185	0.9995	17316	1	-0.649
SOD3	6	0.8301	0.86188	0.9995	17317	1	-0.339
SIPA1L2	6	0.83033	0.86199	0.9995	17318	1	-0.283
SLC17A9	6	0.83039	0.86201	0.9995	17319	1	-0.506
UGT2B11	3	0.83051	0.83048	0.9995	17320	0	0.003
RIBC1	6	0.83067	0.86214	0.9995	17321	1	-0.469
MTRNR2L1	1	0.83072	0.83105	0.9995	17322	0	-2.656
DCDC1	6	0.83112	0.86235	0.9995	17323	1	-0.007
hsa-mir-33b	2	0.83116	0.83092	0.9995	17324	0	-0.226
MMP2	6	0.83117	0.86238	0.9995	17325	1	-0.285
CNIH1	2	0.83147	0.83123	0.9995	17326	0	-0.461
hsa-mir-5190	4	0.83152	0.8314	0.9995	17327	0	-0.742
SPAG5	6	0.83169	0.86265	0.9995	17328	1	-0.107
GPR17	6	0.83176	0.86267	0.9995	17329	1	0.0731
hsa-mir-6723	4	0.83199	0.83184	0.9995	17330	0	-0.031
NEMF	6	0.83204	0.8628	0.9995	17331	1	-0.188
GPR75	6	0.83213	0.86284	0.9995	17332	1	-0.074
MYOG	6	0.83213	0.86284	0.9995	17333	1	-0.297
OVCH1	6	0.83213	0.86284	0.9995	17334	1	-0.084
PON2	6	0.83231	0.86293	0.9995	17335	1	0.0344

SLC25A33	6	0.83245	0.863	0.9995	17336	1	-0.277
ST3GAL2	6	0.83252	0.86304	0.9995	17337	1	0.0561
FLRT2	4	0.83291	0.83275	0.9995	17338	0	-0.382
MAP1LC3A	4	0.83302	0.83286	0.9995	17339	0	-0.26
hsa-mir-101-1	3	0.83316	0.83314	0.9995	17340	0	-0.02
ZNF783	6	0.83317	0.86338	0.9995	17341	1	-0.064
CHMP5	6	0.83321	0.86341	0.9995	17342	1	-0.33
hsa-mir-3920	4	0.83322	0.83306	0.9995	17343	0	-0.278
UQCR11	6	0.83335	0.86348	0.9995	17344	1	-0.356
ZBTB44	6	0.83348	0.86354	0.9995	17345	1	-0.177
hsa-mir-3187	4	0.8335	0.83333	0.9995	17346	0	-0.237
CETN3	6	0.83361	0.86361	0.9995	17347	1	-0.228
TPD52L1	6	0.83366	0.86362	0.9995	17348	1	0.0392
CLPTM1L	6	0.83368	0.86364	0.9995	17349	1	-0.007
PAGE1	6	0.83384	0.86372	0.9995	17350	1	-0.707
ME1	6	0.83389	0.86375	0.9995	17351	1	-0.688
LRRFIP2	6	0.83392	0.86376	0.9995	17352	1	-0.122
hsa-mir-1226	4	0.8341	0.83395	0.9995	17353	0	-0.005
PSMD5	6	0.83415	0.86388	0.9995	17354	1	-0.376
TAT	6	0.8343	0.86395	0.9995	17355	1	-0.308
PPP1R36	6	0.83457	0.8641	0.9995	17356	1	-0.019
CPLX2	6	0.83476	0.8642	0.9995	17357	1	-0.672
CYP1A2	6	0.8348	0.86421	0.9995	17358	1	-0.122
NTS	4	0.83488	0.83475	0.9995	17359	0	0.1876
SLC22A23	6	0.83492	0.86428	0.9995	17360	1	-0.274
GDF2	6	0.83498	0.86431	0.9995	17361	1	-0.028
SMYD3	6	0.83526	0.86444	0.9995	17362	1	-0.229
DAPL1	6	0.83531	0.86447	0.9995	17363	1	-0.322
MRRF	6	0.83534	0.86448	0.9995	17364	1	-0.148
HCFC1	6	0.83541	0.86451	0.9995	17365	1	-0.433
WDR3	6	0.83544	0.86452	0.9995	17366	1	0.0862
SLC51A	6	0.8355	0.86455	0.9995	17367	1	-0.12
CELA3A	5	0.83555	0.8437	0.9995	17368	1	-0.026
HOXA10	6	0.83558	0.86459	0.9995	17369	1	-0.281
ALG10	6	0.83565	0.86463	0.9995	17370	1	0.0146
HTR2C	6	0.83565	0.86463	0.9995	17371	1	-0.096
DEFB131	4	0.83573	0.83557	0.9995	17372	0	-0.951
DAPP1	6	0.83584	0.86473	0.9995	17373	1	-0.37
NUP54	6	0.83592	0.86477	0.9995	17374	1	-0.025
TSPAN9	6	0.83592	0.86477	0.9995	17375	1	-0.16
hsa-mir-6836	4	0.83593	0.83576	0.9995	17376	0	-0.064
PTTG1	6	0.836	0.86482	0.9995	17377	1	-0.544
hsa-mir-6826	4	0.83602	0.83585	0.9995	17378	0	-0.182
TMC6	6	0.83607	0.86484	0.9995	17379	1	-0.067
MYO16	6	0.8362	0.86491	0.9995	17380	1	-0.092
TGM4	6	0.83622	0.86492	0.9995	17381	1	-0.292
TAS2R46	6	0.83623	0.86492	0.9995	17382	1	-0.719
DND1	6	0.83647	0.86504	0.9995	17383	1	-0.35
CREB3L2	4	0.83653	0.83639	0.9995	17384	0	-0.089
SEC22C	6	0.83675	0.86519	0.9995	17385	1	-0.177
RABL6	6	0.83681	0.86522	0.9995	17386	1	-0.226
ANKRD12	6	0.83685	0.86523	0.9995	17387	1	0.0368
NPHP4	6	0.83692	0.86527	0.9995	17388	1	-0.16
MAGEB17	6	0.83701	0.86531	0.9995	17389	1	-0.344
TXN	6	0.83704	0.86533	0.9995	17390	1	-0.945
LCAT	6	0.83714	0.86538	0.9995	17391	1	-0.417
YIPF2	6	0.83718	0.86541	0.9995	17392	1	-0.323
GUCA1B	6	0.83723	0.86543	0.9995	17393	1	-0.153
KRT36	6	0.8373	0.86546	0.9995	17394	1	-0.199
ACER3	6	0.83738	0.86549	0.9995	17395	1	-0.158
GPR137C	6	0.83747	0.86554	0.9995	17396	1	-0.365
hsa-mir-24-2	4	0.83754	0.83736	0.9995	17397	0	-0.775
hsa-mir-6508	4	0.83754	0.83736	0.9995	17398	0	0.0033
DEGS1	6	0.83765	0.86563	0.9995	17399	1	-0.491
MTRNR2L8	2	0.83767	0.83759	0.9995	17400	0	-1.026
MKRN1	6	0.83772	0.86567	0.9995	17401	1	-0.313
hsa-mir-4749	4	0.83785	0.83766	0.9995	17402	0	-0.631
CLEC18A	2	0.83795	0.83789	0.9995	17403	0	-0.718
LYPLA2	6	0.83797	0.8658	0.9995	17404	1	-0.277
hsa-mir-4728	4	0.83801	0.83781	0.9995	17405	0	-0.276
UGT8	6	0.83816	0.86591	0.9995	17406	1	-0.39
SMCR7	1	0.83824	0.8386	0.9995	17407	0	-0.451
ALMS1	6	0.83829	0.86597	0.9995	17408	1	-0.178
NUP210L	6	0.83837	0.86601	0.9995	17409	1	-0.438
FAM19A3	6	0.83844	0.86605	0.9995	17410	1	-0.082
NCOA5	6	0.83853	0.86608	0.9995	17411	1	-0.461
GPR83	6	0.83877	0.86621	0.9995	17412	1	-0.095
TMPRSS7	6	0.83878	0.86621	0.9995	17413	1	-0.077
ATF1	6	0.83889	0.86626	0.9995	17414	1	-0.461
DRD5	6	0.83899	0.86632	0.9995	17415	1	-0.481
TGIF2	4	0.83901	0.83879	0.9995	17416	0	-0.208
KIAA2013	6	0.83913	0.86639	0.9995	17417	1	-0.217
SLC24A2	6	0.83913	0.86639	0.9995	17418	1	-0.419
CXCL9	6	0.83919	0.86642	0.9995	17419	1	-0.075
SLC22A12	6	0.83923	0.86644	0.9995	17420	1	-0.006

NPVF	6	0.83942	0.86653	0.9995	17421	1	-0.132
PARVG	6	0.83948	0.86657	0.9995	17422	1	-0.228
hsa-mir-3616	4	0.8395	0.83931	0.9995	17423	0	0.1421
INPP5D	6	0.83952	0.86658	0.9995	17424	1	0.0024
LOXL2	6	0.83976	0.86671	0.9995	17425	1	-0.246
ONECUT1	6	0.83983	0.86674	0.9995	17426	1	-0.422
LIN7C	6	0.83983	0.86674	0.9995	17427	1	-0.22
PLGLB2	1	0.83984	0.84019	0.9995	17428	0	-0.799
hsa-mir-6829	4	0.84002	0.8398	0.9995	17429	0	0.1512
LY6H	6	0.84003	0.86684	0.9995	17430	1	0.0586
SULT1C4	6	0.84003	0.86684	0.9995	17431	1	0.0821
NLK	6	0.84006	0.86686	0.9995	17432	1	-0.283
DNAJB4	6	0.8401	0.86687	0.9995	17433	1	-0.353
CDK11B	6	0.84015	0.8669	0.9995	17434	1	0.1009
CSNK1D	4	0.84018	0.83994	0.9995	17435	0	0.043
NSA2	6	0.8403	0.86697	0.9995	17436	1	-0.267
KLHDC7B	6	0.8403	0.86697	0.9995	17437	1	-0.111
ADAP2	6	0.84036	0.867	0.9995	17438	1	-0.207
DMRTA1	6	0.84036	0.867	0.9995	17439	1	-0.38
KCNJ5	6	0.84036	0.867	0.9995	17440	1	-0.165
TMEM258	6	0.8406	0.86711	0.9995	17441	1	0.0953
OFD1	6	0.84074	0.86718	0.9995	17442	1	-0.168
hsa-mir-4740	4	0.84077	0.8405	0.9995	17443	0	-0.069
NKX2-4	6	0.84079	0.86721	0.9995	17444	1	-0.291
hsa-mir-4666a	4	0.84088	0.84063	0.9995	17445	0	0.1183
hsa-mir-4764	4	0.84101	0.84076	0.9995	17446	0	-0.751
C19orf26	6	0.84103	0.86734	0.9995	17447	1	0.1063
EFCAB4B	6	0.84108	0.86737	0.9995	17448	1	-0.102
ZFP90	6	0.84113	0.8674	0.9995	17449	1	-0.153
DIEXF	6	0.84113	0.8674	0.9995	17450	1	-0.111
THOC1	5	0.84123	0.84787	0.9995	17451	1	-0.684
FGF11	6	0.8413	0.86749	0.9995	17452	1	-0.159
hsa-mir-4273	4	0.84143	0.84118	0.9995	17453	0	-0.146
hsa-mir-548i-2	1	0.84145	0.84179	0.9995	17454	0	-1.314
ADNP	6	0.84146	0.86756	0.9995	17455	1	0.0161
hsa-mir-589	4	0.84155	0.84132	0.9995	17456	0	-0.65
CTRL	6	0.84162	0.86764	0.9995	17457	1	-0.16
COL18A1	6	0.84162	0.86764	0.9995	17458	1	-0.503
GRAP	2	0.84169	0.84161	0.9995	17459	0	-0.329
TRIM3	6	0.84169	0.86768	0.9995	17460	1	0.0407
DGKK	6	0.84193	0.8678	0.9995	17461	1	-0.303
BBS9	6	0.84204	0.86786	0.9995	17462	1	-0.049
PHKA1	6	0.8421	0.8679	0.9995	17463	1	-0.387
SCD5	6	0.8421	0.8679	0.9995	17464	1	-0.015
hsa-mir-4309	4	0.84214	0.84189	0.9995	17465	0	-0.195
hsa-mir-3939	4	0.84221	0.84195	0.9995	17466	0	-0.195
CHCHD2	6	0.84221	0.86796	0.9995	17467	1	-0.297
AQP10	6	0.84222	0.86797	0.9995	17468	1	0.0262
POM121L2	6	0.84224	0.86798	0.9995	17469	1	-0.55
DLL1	6	0.84227	0.868	0.9995	17470	1	-0.246
C1QTNF5	6	0.84235	0.86804	0.9995	17471	1	-0.383
ERI2	6	0.84239	0.86807	0.9995	17472	1	-0.119
THPO	6	0.84244	0.86809	0.9995	17473	1	-0.091
RMI1	6	0.84252	0.86814	0.9995	17474	1	-0.048
CD68	6	0.84258	0.86816	0.9995	17475	1	0.0849
hsa-mir-6753	4	0.84259	0.84233	0.9995	17476	0	-0.457
SERINC2	6	0.84259	0.86817	0.9995	17477	1	-0.149
TMEM168	6	0.84263	0.86819	0.9995	17478	1	-0.215
PSMB9	6	0.84268	0.86821	0.9995	17479	1	-0.163
RAP1A	6	0.84289	0.86833	0.9995	17480	1	-0.341
ADPRM	6	0.84322	0.8685	0.9995	17481	1	-0.438
RAB38	6	0.84324	0.86852	0.9995	17482	1	0.133
CDH24	6	0.84347	0.86864	0.9995	17483	1	-0.101
SLC5A8	6	0.84347	0.86864	0.9995	17484	1	-0.16
NNAT	6	0.84361	0.8687	0.9995	17485	1	-0.204
IPMK	6	0.84368	0.86874	0.9995	17486	1	-0.372
RFT1	6	0.84382	0.86881	0.9995	17487	1	-0.129
SSNA1	6	0.84382	0.86881	0.9995	17488	1	-0.169
KIAA1919	6	0.84382	0.86881	0.9995	17489	1	-0.335
TOPBP1	6	0.84391	0.86886	0.9995	17490	1	-0.316
MCMDC2	6	0.844	0.8689	0.9995	17491	1	-0.288
hsa-mir-4254	2	0.84408	0.84403	0.9995	17492	0	-0.2
MME	6	0.8441	0.86896	0.9995	17493	1	-0.293
ALDH1L1	6	0.84412	0.86897	0.9995	17494	1	-0.06
SRCRB4D	6	0.84431	0.86907	0.9995	17495	1	-0.247
hsa-mir-5588	2	0.84467	0.84463	0.9995	17496	0	-0.408
KLRC4	2	0.845	0.84495	0.9995	17497	0	-0.259
LACTB	6	0.84508	0.86945	0.9995	17498	1	-0.63
PAX6	6	0.84511	0.86946	0.9995	17499	1	-0.091
hsa-mir-4306	4	0.84524	0.84497	0.9995	17500	0	-0.151
hsa-mir-636	4	0.84524	0.84497	0.9995	17501	0	-0.733
RAC3	6	0.84525	0.86955	0.9995	17502	1	-0.151
CTDSP2	6	0.84552	0.86969	0.9995	17503	1	0.0892
DGKQ	6	0.84566	0.86976	0.9995	17504	1	-0.355
RCAN3	6	0.84571	0.86978	0.9995	17505	1	-0.425

TNNI1	6	0.84582	0.86983	0.9995	17506	1	-0.132
ZFP1	6	0.84593	0.8699	0.9995	17507	1	-0.329
SRF	6	0.84606	0.86996	0.9995	17508	1	-0.348
EFTUD1	6	0.84618	0.87003	0.9995	17509	1	-0.136
hsa-mir-7847	4	0.84621	0.84597	0.9995	17510	0	-0.253
MTRNR2L6	6	0.84625	0.87007	0.9995	17511	1	-0.705
CLEC5A	6	0.84632	0.8701	0.9995	17512	1	-0.413
SSR3	6	0.84659	0.87024	0.9995	17513	1	-0.167
HOOK2	6	0.8466	0.87024	0.9995	17514	1	-0.191
ELOVL4	6	0.8467	0.87029	0.9995	17515	1	-0.203
DHRS1	6	0.84678	0.87033	0.9995	17516	1	-0.127
hsa-mir-4722	4	0.84683	0.84657	0.9995	17517	0	0.0734
ADH1A	6	0.847	0.87043	0.9995	17518	1	-0.469
TCERG1	6	0.847	0.87043	0.9995	17519	1	-0.002
PRKACG	6	0.84713	0.87051	0.9995	17520	1	-0.257
MB21D1	6	0.84725	0.87057	0.9995	17521	1	-0.59
MINA	6	0.84727	0.87058	0.9995	17522	1	-0.138
NDUFA12	6	0.84729	0.8706	0.9995	17523	1	-0.142
SIAH3	6	0.84734	0.87062	0.9995	17524	1	-0.816
hsa-mir-4797	2	0.84739	0.84732	0.9995	17525	0	-0.432
SPATA31A7	2	0.84739	0.84732	0.9995	17526	0	-0.595
CDV3	6	0.84741	0.87065	0.9995	17527	1	-0.193
HOXA7	6	0.84774	0.87084	0.9995	17528	1	-0.313
PTPRS	6	0.84793	0.87095	0.9995	17529	1	0.16
CCDC106	6	0.84793	0.87095	0.9995	17530	1	0.0261
hsa-mir-15b	4	0.84793	0.84768	0.9995	17531	0	0.0769
LRRC37A	6	0.84821	0.8711	0.9995	17532	1	-0.562
LPAR4	6	0.84829	0.87114	0.9995	17533	1	-0.296
ZYG11B	6	0.84832	0.87117	0.9995	17534	1	-0.203
SYNCRIP	6	0.84837	0.87119	0.9995	17535	1	-0.188
FIBIN	6	0.84837	0.87119	0.9995	17536	1	-0.173
ATXN7L1	6	0.84845	0.87124	0.9995	17537	1	-0.25
RPS6KA6	6	0.84848	0.87125	0.9995	17538	1	-0.388
TMEM231	6	0.8485	0.87127	0.9995	17539	1	-0.237
CYB561A3	6	0.8485	0.87127	0.9995	17540	1	-0.192
AGA	6	0.84867	0.87136	0.9995	17541	1	-0.057
GATSL2	2	0.8487	0.8486	0.9995	17542	0	-0.235
DGAT2L6	6	0.8488	0.87144	0.9995	17543	1	-0.509
PRAMEF20	1	0.84881	0.84915	0.9995	17544	0	-3.858
SKP1	6	0.84886	0.87147	0.9995	17545	1	-0.05
HEY1	6	0.84894	0.87151	0.9995	17546	1	-0.235
NRG4	6	0.849	0.87153	0.9995	17547	1	-0.099
hsa-mir-3909	4	0.84901	0.84875	0.9995	17548	0	-0.073
MLL	4	0.84912	0.84886	0.9995	17549	0	-0.405
KRT23	4	0.84914	0.84888	0.9995	17550	0	-0.049
ABCA13	6	0.8495	0.8718	0.9995	17551	1	-0.355
FUT8	6	0.84953	0.87181	0.9995	17552	1	-0.061
hsa-mir-99a	4	0.84953	0.84927	0.9995	17553	0	-0.46
SIRPB2	6	0.84966	0.87189	0.9995	17554	1	-0.435
COMMD3-BMI1	2	0.8497	0.84957	0.9995	17555	0	-0.209
GAA	6	0.84973	0.87192	0.9995	17556	1	-0.044
BNC1	6	0.84976	0.87194	0.9995	17557	1	-0.016
FAM43A	6	0.84983	0.87197	0.9995	17558	1	-0.363
IL17A	6	0.84986	0.87199	0.9995	17559	1	-0.267
SLCO1B7	6	0.85008	0.87211	0.9995	17560	1	-0.137
KIAA1683	6	0.85018	0.87216	0.9995	17561	1	-0.612
ACSM2B	5	0.85034	0.85483	0.9995	17562	1	0.0362
ZNF280C	6	0.85035	0.87225	0.9995	17563	1	-0.386
MTFP1	4	0.85057	0.85032	0.9995	17564	0	0.0482
LPO	6	0.85059	0.87238	0.9995	17565	1	-0.307
FGFR1OP2	6	0.85059	0.87238	0.9995	17566	1	-0.232
HIST1H2BK	5	0.85066	0.8551	0.9995	17567	1	-0.434
NEK8	6	0.85078	0.87248	0.9995	17568	1	-0.177
DCAF12L2	6	0.85083	0.87251	0.9995	17569	1	-0.402
CCSER2	6	0.8509	0.87255	0.9995	17570	1	0.0297
CREG1	6	0.85094	0.87257	0.9995	17571	1	-0.37
hsa-mir-455	4	0.85096	0.85069	0.9995	17572	0	-0.077
BNIP2	6	0.85098	0.87259	0.9995	17573	1	-0.406
CTDSPL	6	0.851	0.87261	0.9995	17574	1	-0.047
LIN7B	6	0.85107	0.87264	0.9995	17575	1	-0.01
GJB4	6	0.85117	0.87269	0.9995	17576	1	-0.235
ELTD1	6	0.85117	0.87269	0.9995	17577	1	-0.785
hsa-mir-146b	4	0.85124	0.85095	0.9995	17578	0	0.0454
NENF	6	0.85127	0.87275	0.9995	17579	1	-0.122
PSG2	6	0.8514	0.87281	0.9995	17580	1	-0.261
SLC7A3	6	0.85156	0.87289	0.9995	17581	1	-0.474
ILVBL	6	0.85158	0.8729	0.9995	17582	1	0.0733
ZDHHC12	6	0.8516	0.87291	0.9995	17583	1	-0.478
hsa-mir-4472-1	4	0.85164	0.85134	0.9995	17584	0	-0.51
SLC19A1	6	0.85167	0.87297	0.9995	17585	1	-0.28
CD34	6	0.852	0.87316	0.9995	17586	1	-0.622
KANK4	6	0.85212	0.87321	0.9995	17587	1	-0.603
CAMP	6	0.85221	0.87326	0.9995	17588	1	-0.192
WASH1	6	0.85226	0.87329	0.9995	17589	1	-0.491
DPEP1	6	0.85247	0.8734	0.9995	17590	1	-0.241

ARID1A	6	0.85263	0.87349	0.9995	17591	1	-0.525
MYB	6	0.85294	0.87367	0.9995	17592	1	-0.405
TMA7	6	0.85304	0.87372	0.9995	17593	1	-0.03
BTN2A2	6	0.85304	0.87372	0.9995	17594	1	-0.369
TCAP	6	0.85304	0.87372	0.9995	17595	1	0.0631
NR2C1	6	0.85321	0.8738	0.9995	17596	1	-0.356
PTPN23	6	0.8533	0.87385	0.9995	17597	1	-0.067
hsa-mir-4692	4	0.85333	0.85309	0.9995	17598	0	-0.068
CEP76	6	0.85341	0.87392	0.9995	17599	1	-0.074
WSB1	6	0.85364	0.87404	0.9995	17600	1	-0.452
BLOC1S6	4	0.85365	0.8534	0.9995	17601	0	-0.026
SDC2	6	0.85368	0.87406	0.9995	17602	1	-0.061
CMTM7	6	0.85368	0.87406	0.9995	17603	1	-0.18
HTN1	2	0.85368	0.8536	0.9995	17604	0	-0.584
hsa-mir-3143	4	0.8537	0.85345	0.9995	17605	0	-0.432
PPIP5K1	6	0.85375	0.87411	0.9995	17606	1	-0.287
ZCCHC16	6	0.85375	0.87411	0.9995	17607	1	-0.182
PDZD8	6	0.85395	0.87422	0.9995	17608	1	-0.379
ABI1	6	0.85398	0.87424	0.9995	17609	1	-0.121
NTPCR	6	0.85419	0.87436	0.9995	17610	1	-0.549
EPHB2	6	0.8543	0.87441	0.9995	17611	1	-0.606
hsa-mir-301b	4	0.85438	0.85411	0.9995	17612	0	-0.509
ERLIN1	6	0.85442	0.87448	0.9995	17613	1	-0.794
EED	6	0.85453	0.87454	0.9995	17614	1	-0.008
hsa-mir-6748	4	0.85461	0.85437	0.9995	17615	0	-0.749
STBD1	6	0.8547	0.87462	0.9995	17616	1	-0.282
CLDN15	6	0.8549	0.87474	0.9995	17617	1	-0.008
TMED5	6	0.85506	0.87483	0.9995	17618	1	-0.061
KBTBD13	6	0.85506	0.87483	0.9995	17619	1	-0.138
C16orf92	6	0.85509	0.87484	0.9995	17620	1	-0.144
hsa-mir-943	4	0.85509	0.85483	0.9995	17621	0	0.1804
RPA1	6	0.85513	0.87486	0.9995	17622	1	-0.5
ARRDC1	6	0.85539	0.87502	0.9995	17623	1	-0.103
TENM1	6	0.85542	0.87504	0.9995	17624	1	-0.024
PELI1	6	0.85545	0.87505	0.9995	17625	1	-0.069
RNF31	6	0.85574	0.87524	0.9995	17626	1	-0.578
ABCB10	6	0.85574	0.87524	0.9995	17627	1	-0.42
WIPF3	6	0.85577	0.87525	0.9995	17628	1	-0.106
TECPR1	6	0.85586	0.87531	0.9995	17629	1	0.0064
TBX1	6	0.85596	0.87536	0.9995	17630	1	-0.488
PRM2	6	0.856	0.87539	0.9995	17631	1	-0.233
CCZ1	2	0.85608	0.85601	0.9995	17632	0	-0.837
MFAP1	6	0.85628	0.87554	0.9995	17633	1	-0.006
HBP1	6	0.85639	0.87561	0.9995	17634	1	-0.178
MAGEB10	6	0.85642	0.87563	0.9995	17635	1	-0.043
AANAT	6	0.85645	0.87564	0.9995	17636	1	-0.047
ANKRD9	6	0.85647	0.87565	0.9995	17637	1	-0.171
MYRF	6	0.8565	0.87566	0.9995	17638	1	-0.297
GLTP	6	0.8565	0.87566	0.9995	17639	1	-0.234
ST6GALNAC2	4	0.85652	0.85629	0.9995	17640	0	-0.058
AUH	6	0.85659	0.87572	0.9995	17641	1	-0.054
TBC1D2	6	0.85676	0.8758	0.9995	17642	1	-0.025
hsa-mir-1180	4	0.85692	0.85668	0.9995	17643	0	0.0283
GALNT10	6	0.85726	0.87609	0.9995	17644	1	-0.56
hsa-mir-3649	4	0.85739	0.85714	0.9995	17645	0	-0.112
USP7	6	0.85745	0.87621	0.9995	17646	1	0.0248
VPS37B	6	0.8575	0.87624	0.9995	17647	1	-0.361
TMTC2	6	0.8575	0.87624	0.9995	17648	1	-0.564
MAGEC2	6	0.8575	0.87624	0.9995	17649	1	0.0023
HTR1B	6	0.85754	0.87626	0.9995	17650	1	-0.12
hsa-mir-4683	4	0.85768	0.85742	0.9995	17651	0	-0.071
LRRC39	6	0.85774	0.87637	0.9995	17652	1	-0.177
CT47B1	4	0.85783	0.85756	0.9995	17653	0	-0.105
LCE3D	4	0.85783	0.85756	0.9995	17654	0	0.0294
OR2W3	6	0.8579	0.87647	0.9995	17655	1	-0.598
RRAD	6	0.85805	0.87656	0.9995	17656	1	-0.436
SLC15A1	6	0.8581	0.87658	0.9995	17657	1	-0.213
C9orf173	6	0.85813	0.8766	0.9995	17658	1	-0.785
MEX3B	6	0.85813	0.8766	0.9995	17659	1	-0.271
PDIA2	4	0.85829	0.85799	0.9995	17660	0	-0.478
SUPT20HL2	6	0.85833	0.8767	0.9995	17661	1	-0.17
ZNF626	4	0.85842	0.85812	0.9995	17662	0	-0.419
AVPR1B	6	0.85848	0.87679	0.9995	17663	1	-0.285
SQSTM1	6	0.85849	0.8768	0.9995	17664	1	-0.195
FAM161A	6	0.85852	0.87681	0.9995	17665	1	-0.088
RASGRF1	6	0.85857	0.87684	0.9995	17666	1	0.0764
NDUFV3	6	0.8586	0.87687	0.9995	17667	1	-0.095
SHISA6	6	0.85862	0.87687	0.9995	17668	1	0.0389
CARD10	6	0.85893	0.87705	0.9995	17669	1	-0.573
TFPI2	6	0.859	0.87709	0.9995	17670	1	0.0663
ZNF317	6	0.85901	0.87709	0.9995	17671	1	-0.169
DSG3	6	0.85904	0.87712	0.9995	17672	1	-0.114
ARHGAP32	6	0.85904	0.87712	0.9995	17673	1	-0.148
hsa-mir-4688	4	0.85905	0.85872	0.9995	17674	0	-0.267
SLC6A2	6	0.8591	0.87715	0.9995	17675	1	-0.267

RAMP2	6	0.8591	0.87715	0.9995	17676	1	-0.154
hsa-mir-4771-1	1	0.85922	0.85956	0.9995	17677	0	-0.62
CNGB1	6	0.85923	0.87722	0.9995	17678	1	-0.808
EPHA7	6	0.85932	0.87727	0.9995	17679	1	-0.382
GSTZ1	6	0.85954	0.87739	0.9995	17680	1	-0.456
NFIL3	6	0.85955	0.87739	0.9995	17681	1	-0.288
R3HCC1L	6	0.8596	0.87741	0.9995	17682	1	-0.003
hsa-mir-4767	4	0.85961	0.85927	0.9995	17683	0	-0.188
ALCAM	6	0.85963	0.87743	0.9995	17684	1	-0.467
CEP70	6	0.85973	0.87749	0.9995	17685	1	-0.057
NBPF1	6	0.85976	0.8775	0.9995	17686	1	-0.6
TNFRSF1A	6	0.85976	0.8775	0.9995	17687	1	-0.018
NPEPL1	6	0.85987	0.87756	0.9995	17688	1	-0.024
CXorf56	6	0.85992	0.87758	0.9995	17689	1	-0.128
HIST1H2BE	5	0.85996	0.86258	0.9995	17690	1	-1.038
POC1A	6	0.85997	0.87762	0.9995	17691	1	-0.366
hsa-mir-1273d	3	0.86006	0.86011	0.9995	17692	0	-1.233
SMG1	6	0.86011	0.8777	0.9995	17693	1	-0.143
C1orf192	6	0.8602	0.87776	0.9995	17694	1	-0.168
F10	6	0.86023	0.87777	0.9995	17695	1	-0.826
hsa-mir-548ar	2	0.86027	0.86025	0.9995	17696	0	-1.911
DHRS4	2	0.86027	0.86025	0.9995	17697	0	-1.153
LRRTM4	6	0.86046	0.87789	0.9995	17698	1	-0.122
PIKFYVE	6	0.86048	0.87791	0.9995	17699	1	-0.297
hsa-mir-6785	4	0.86051	0.86019	0.9995	17700	0	0.031
ADCY2	6	0.86053	0.87793	0.9995	17701	1	-0.161
TARP	4	0.86068	0.86035	0.9995	17702	0	-0.224
CTAGE9	3	0.86071	0.86075	0.9995	17703	0	-0.733
hsa-mir-4474	3	0.86071	0.86075	0.9995	17704	0	-0.203
GNG3	6	0.86075	0.87806	0.9995	17705	1	-0.146
TTI2	6	0.86079	0.87807	0.9995	17706	1	-0.482
SYT9	6	0.86079	0.87807	0.9995	17707	1	-0.347
DPP6	6	0.86079	0.87807	0.9995	17708	1	-0.12
HSD3B7	6	0.86087	0.87812	0.9995	17709	1	-0.616
PLCB1	6	0.86093	0.87815	0.9995	17710	1	-0.294
ANKRD29	6	0.86102	0.87819	0.9995	17711	1	-0.562
LOC100127983	6	0.86115	0.87828	0.9995	17712	1	-0.127
TBCE	6	0.86121	0.87832	0.9995	17713	1	0.1311
hsa-mir-193b	4	0.86129	0.86096	0.9995	17714	0	-0.026
hsa-mir-138-1	4	0.8614	0.86107	0.9995	17715	0	-0.201
C1orf173	6	0.86142	0.87843	0.9995	17716	1	-0.573
OR52I2	4	0.8615	0.86118	0.9995	17717	0	-0.362
CREBRF	6	0.86151	0.87849	0.9995	17718	1	-0.153
TAP1	6	0.86163	0.87857	0.9995	17719	1	-0.028
LY6E	6	0.8617	0.87861	0.9995	17720	1	-0.303
AVP	6	0.8619	0.87872	0.9995	17721	1	-0.567
hsa-mir-3167	4	0.86193	0.86164	0.9995	17722	0	-0.087
FUT1	6	0.86198	0.87877	0.9995	17723	1	-0.647
PPM1H	6	0.86204	0.87879	0.9995	17724	1	-0.27
DAK	6	0.86209	0.87882	0.9995	17725	1	-0.737
ZNF397	5	0.86214	0.86444	0.9995	17726	1	-0.38
PSMD4	6	0.86223	0.87891	0.9995	17727	1	-0.228
LRIF1	6	0.86229	0.87894	0.9995	17728	1	-0.208
ZNF85	4	0.86238	0.86212	0.9995	17729	0	0.1754
ZNF93	4	0.8627	0.86245	0.9995	17730	0	-0.786
LRRN4CL	6	0.86274	0.87919	0.9995	17731	1	-0.047
CHPF	6	0.86286	0.87926	0.9995	17732	1	-0.367
APOB	6	0.86289	0.87929	0.9995	17733	1	-0.044
hsa-mir-592	3	0.86294	0.86295	0.9995	17734	0	-0.204
SLC25A10	6	0.86304	0.87937	0.9995	17735	1	-0.163
IRAK1	6	0.86313	0.87942	0.9995	17736	1	-0.282
KCNQ2	6	0.86315	0.87944	0.9995	17737	1	0.0164
MED18	6	0.8633	0.87953	0.9995	17738	1	-0.133
PRPF39	6	0.8633	0.87953	0.9995	17739	1	-0.307
ZNF543	6	0.86336	0.87957	0.9995	17740	1	0.0634
OR10P1	4	0.86338	0.86314	0.9995	17741	0	-0.759
hsa-mir-376b	3	0.8634	0.86338	0.9995	17742	0	-0.12
hsa-mir-4648	4	0.86349	0.86325	0.9995	17743	0	-0.68
NCBP2	6	0.86352	0.87966	0.9995	17744	1	-0.463
SAP30L	6	0.86352	0.87966	0.9995	17745	1	-0.202
FGF4	6	0.86352	0.87966	0.9995	17746	1	-0.232
LRP1	6	0.86363	0.87972	0.9995	17747	1	-0.477
OR6T1	6	0.86373	0.87977	0.9995	17748	1	-0.23
HMG20A	6	0.86381	0.87983	0.9995	17749	1	-0.042
CDC6	6	0.86395	0.87991	0.9995	17750	1	0.1142
DVL3	6	0.86397	0.87991	0.9995	17751	1	-0.227
CD38	6	0.86399	0.87992	0.9995	17752	1	-0.188
VPS4A	4	0.86399	0.86374	0.9995	17753	0	-0.163
RBFOX2	6	0.86407	0.87997	0.9995	17754	1	0.0629
SLC36A1	6	0.86415	0.88002	0.9995	17755	1	-0.329
KRT6C	4	0.86415	0.86391	0.9995	17756	0	-0.192
ZNF726	5	0.86423	0.86622	0.9995	17757	1	0.0045
CASP5	6	0.86428	0.8801	0.9995	17758	1	-0.175
HTR6	6	0.86439	0.88016	0.9995	17759	1	-0.19
HIST1H2AI	2	0.86448	0.86449	0.9995	17760	0	-1.233

hsa-mir-521-1	2	0.86448	0.86449	0.9995	17761	0	-1.56
hsa-mir-3689e	2	0.86448	0.86449	0.9995	17762	0	-1.233
TRIM64	2	0.86448	0.86449	0.9995	17763	0	-2.397
FRG2B	2	0.86448	0.86449	0.9995	17764	0	-0.742
PPIAL4F	2	0.86448	0.86449	0.9995	17765	0	-1.902
LYZL1	2	0.86448	0.86449	0.9995	17766	0	-1.058
GAGE12H	2	0.86448	0.86449	0.9995	17767	0	-1.2
GAGE2A	2	0.86448	0.86449	0.9995	17768	0	-1.56
DHRS4L2	2	0.86448	0.86449	0.9995	17769	0	-1.56
hsa-mir-3689a	2	0.86448	0.86449	0.9995	17770	0	-1.103
TMSB15B	2	0.86448	0.86449	0.9995	17771	0	-2.309
PAGE2	2	0.86448	0.86449	0.9995	17772	0	-0.992
hsa-mir-500a	2	0.86448	0.86449	0.9995	17773	0	-2.61
ELF1	6	0.86454	0.88025	0.9995	17774	1	-0.141
TGIF2-C20orf24	1	0.86459	0.86492	0.9995	17775	0	-0.823
ANHX	6	0.86469	0.88034	0.9995	17776	1	-0.175
OR14I1	6	0.86493	0.88047	0.9995	17777	1	-0.198
SRPR	6	0.86498	0.88051	0.9995	17778	1	-0.166
hsa-mir-30c-2	4	0.86498	0.86475	0.9995	17779	0	-0.071
PARD6B	6	0.86519	0.88064	0.9995	17780	1	-0.064
OR5I1	6	0.86524	0.88067	0.9995	17781	1	-0.088
ZKSCAN4	6	0.86531	0.88071	0.9995	17782	1	0.0462
RPS4Y1	6	0.86534	0.88072	0.9995	17783	1	-0.446
SHISA7	6	0.86535	0.88073	0.9995	17784	1	0.0397
CHST12	6	0.86539	0.88075	0.9995	17785	1	-0.176
hsa-mir-4538	4	0.86583	0.86562	0.9995	17786	0	-0.351
HIST1H3F	6	0.86583	0.88101	0.9995	17787	1	-0.4
KRTAP9-3	5	0.86593	0.86773	0.9995	17788	1	-1.233
TRNAU1AP	6	0.86594	0.88108	0.9995	17789	1	0.07
IDH2	6	0.86599	0.88112	0.9995	17790	1	-0.102
hsa-mir-4695	4	0.86604	0.86582	0.9995	17791	0	-0.12
ATF6B	6	0.86604	0.88114	0.9995	17792	1	-0.085
hsa-mir-4465	4	0.86605	0.86583	0.9995	17793	0	0.0624
DPAGT1	6	0.8661	0.88117	0.9995	17794	1	-0.092
METTL7B	6	0.86615	0.8812	0.9995	17795	1	-0.567
ISOC2	6	0.86618	0.88122	0.9995	17796	1	-0.205
XRCC2	6	0.86624	0.88126	0.9995	17797	1	-0.34
PIP4K2C	6	0.8663	0.88129	0.9995	17798	1	-0.44
RANBP10	6	0.86639	0.88134	0.9995	17799	1	-0.421
SPDYE2L	2	0.86649	0.86649	0.9995	17800	0	-0.644
hsa-mir-5584	4	0.86662	0.86636	0.9995	17801	0	-0.416
NR0B1	6	0.86677	0.88158	0.9995	17802	1	-0.423
KCTD6	6	0.86681	0.8816	0.9995	17803	1	-0.285
hsa-mir-1208	4	0.86693	0.86666	0.9995	17804	0	0.0351
SEMA3D	6	0.86705	0.88175	0.9995	17805	1	-0.518
REEP3	6	0.86715	0.88181	0.9995	17806	1	0.0444
CTSZ	6	0.86715	0.88181	0.9995	17807	1	-0.242
PGRMC1	6	0.8672	0.88183	0.9995	17808	1	-0.319
ZNF311	4	0.86746	0.8672	0.9995	17809	0	-0.336
MT2A	3	0.86746	0.8675	0.9995	17810	0	-1.006
hsa-mir-921	4	0.8676	0.86734	0.9995	17811	0	-0.143
ABI2	6	0.86761	0.88207	0.9995	17812	1	-0.082
ANKRD50	6	0.86763	0.88208	0.9995	17813	1	-0.229
HS1BP3	6	0.86777	0.88218	0.9995	17814	1	-0.236
PDF	6	0.86783	0.88221	0.9995	17815	1	-0.655
UPK3A	6	0.86808	0.88236	0.9995	17816	1	-0.052
ATG2A	6	0.86814	0.88241	0.9995	17817	1	-0.642
COG5	6	0.8682	0.88243	0.9995	17818	1	-0.034
EN1	6	0.86823	0.88246	0.9995	17819	1	-0.229
TRMT12	6	0.86825	0.88248	0.9995	17820	1	-0.453
SHE	6	0.86852	0.88263	0.9995	17821	1	-0.142
hsa-mir-5688	4	0.86857	0.86832	0.9995	17822	0	-0.94
MPZL3	6	0.86858	0.88267	0.9995	17823	1	-0.293
KLC3	6	0.86865	0.88271	0.9995	17824	1	-0.397
ARPC4	3	0.86868	0.86877	0.9995	17825	0	-0.335
COPG1	4	0.86883	0.86859	0.9995	17826	0	-0.168
HOXB7	6	0.86889	0.88284	0.9995	17827	1	-0.072
MGAT4A	6	0.86904	0.88294	0.9995	17828	1	-0.328
TM9SF1	6	0.86914	0.88299	0.9995	17829	1	0.1422
HID1	6	0.86929	0.88307	0.9995	17830	1	-0.406
MAGEB18	6	0.86937	0.88312	0.9995	17831	1	0.0359
FZD9	6	0.86937	0.88312	0.9995	17832	1	-0.175
BCAR1	6	0.86937	0.88312	0.9995	17833	1	-0.191
RPL22	6	0.86947	0.88318	0.9995	17834	1	-0.539
CDH20	6	0.86961	0.88329	0.9995	17835	1	-0.241
PDZK1	6	0.86977	0.88338	0.9995	17836	1	-0.79
LRRC55	6	0.8698	0.88341	0.9995	17837	1	-0.339
MT1A	6	0.86993	0.88348	0.9995	17838	1	-0.492
RFPL4B	6	0.87003	0.88355	0.9995	17839	1	-0.027
STRAP	6	0.87007	0.88358	0.9995	17840	1	-0.29
hsa-mir-6861	4	0.87032	0.87009	0.9995	17841	0	0.0028
HPDL	6	0.87045	0.88379	0.9995	17842	1	-0.538
hsa-mir-676	4	0.87061	0.87038	0.9995	17843	0	0.08
NT5DC2	6	0.87066	0.88394	0.9995	17844	1	-0.464
FASTKD2	6	0.87069	0.88396	0.9995	17845	1	-0.073

hsa-mir-4501	2	0.8707	0.87072	0.9995	17846	0	-0.438
NTNG1	6	0.87071	0.88397	0.9995	17847	1	-0.268
GRAP2	6	0.87073	0.884	0.9995	17848	1	-0.465
RBFOX3	4	0.87074	0.8705	0.9995	17849	0	-0.26
DMAP1	6	0.87076	0.88401	0.9995	17850	1	-0.15
CXCR5	6	0.87096	0.88414	0.9995	17851	1	-0.241
FAM46C	6	0.87109	0.8842	0.9995	17852	1	-0.821
JAZF1	6	0.87113	0.88422	0.9995	17853	1	-0.398
KIF13B	6	0.8712	0.88426	0.9995	17854	1	-0.065
ZSWIM6	6	0.87123	0.88428	0.9995	17855	1	-0.541
INPP5J	6	0.87129	0.88432	0.9995	17856	1	-0.127
MELK	6	0.87137	0.88436	0.9995	17857	1	-0.049
SREK1	6	0.87138	0.88436	0.9995	17858	1	0.0451
ABHD14A	6	0.87145	0.8844	0.9995	17859	1	-0.255
KRTAP1-5	6	0.87148	0.88442	0.9995	17860	1	-0.113
LYRM7	6	0.87154	0.88445	0.9995	17861	1	-0.413
MEI1	6	0.87165	0.88453	0.9995	17862	1	-0.2
ARL17A	1	0.87167	0.87195	0.9995	17863	0	-1.409
C6orf141	6	0.87169	0.88456	0.9995	17864	1	-0.179
CRNN	6	0.87172	0.88457	0.9995	17865	1	-0.366
hsa-mir-938	4	0.87176	0.87154	0.9995	17866	0	-0.891
HRASLS2	6	0.87181	0.88463	0.9995	17867	1	0.1256
ZNF749	6	0.87184	0.88465	0.9995	17868	1	-0.3
LURAP1L	6	0.87187	0.88467	0.9995	17869	1	-0.207
OR4C46	6	0.87205	0.88478	0.9995	17870	1	-0.203
DDX41	6	0.8723	0.88493	0.9995	17871	1	-0.503
GYG1	6	0.87235	0.88496	0.9995	17872	1	-0.405
DGCR6	6	0.87253	0.88507	0.9995	17873	1	-0.884
PDSS2	6	0.87253	0.88507	0.9995	17874	1	-0.244
ZNF780A	6	0.87263	0.88514	0.9995	17875	1	-0.114
LOC256021	5	0.87279	0.87385	0.9995	17876	1	-0.171
DSCR3	6	0.87299	0.88535	0.9995	17877	1	0.1144
OR8I2	6	0.87305	0.88539	0.9995	17878	1	-0.487
hsa-mir-1299	4	0.87308	0.87287	0.9995	17879	0	-1.487
SFI1	6	0.87309	0.88542	0.9995	17880	1	-0.328
hsa-mir-548n	2	0.87312	0.87321	0.9995	17881	0	-0.338
SPSB2	6	0.87315	0.88545	0.9995	17882	1	-0.025
DYRK3	6	0.87328	0.88554	0.9995	17883	1	-0.402
hsa-mir-383	4	0.87342	0.87321	0.9995	17884	0	-0.126
FOXE3	6	0.87345	0.88565	0.9995	17885	1	-1.098
DNAJC21	6	0.87347	0.88566	0.9995	17886	1	-0.1
hsa-mir-520h	1	0.87355	0.87386	0.9995	17887	0	-0.64
PLEKHB2	6	0.87357	0.88572	0.9995	17888	1	-0.364
hsa-mir-7151	4	0.87364	0.87342	0.9995	17889	0	-1.026
FRMD3	6	0.87364	0.88577	0.9995	17890	1	-0.088
TRAF6	6	0.87366	0.88577	0.9995	17891	1	-0.335
GID8	6	0.87377	0.88584	0.9995	17892	1	-0.207
ACOT7	6	0.87383	0.88589	0.9995	17893	1	-0.518
ZBTB16	6	0.87397	0.88597	0.9995	17894	1	0.0681
UPB1	6	0.87403	0.88601	0.9995	17895	1	0.025
hsa-mir-4636	4	0.87412	0.87392	0.9995	17896	0	-0.079
BRD2	6	0.87414	0.88608	0.9995	17897	1	0.0373
hsa-mir-1261	4	0.87431	0.87413	0.9995	17898	0	-0.275
hsa-mir-4741	4	0.87458	0.87438	0.9995	17899	0	-0.29
RNFT2	6	0.87468	0.88642	0.9995	17900	1	-0.114
IL17RD	6	0.8747	0.88643	0.9995	17901	1	-0.066
hsa-mir-488	4	0.87484	0.87465	0.9995	17902	0	0.0482
SLCO1B3	5	0.87492	0.8758	0.9995	17903	1	-0.068
LOC440335	5	0.87499	0.87585	0.9995	17904	1	-0.282
EPHX3	6	0.87513	0.88669	0.9995	17905	1	-0.336
PTGFRN	6	0.87515	0.88671	0.9995	17906	1	-0.18
LMO4	6	0.87521	0.88673	0.9995	17907	1	0.1149
hsa-mir-6890	4	0.87537	0.87521	0.9995	17908	0	-0.378
CCL11	6	0.87544	0.88689	0.9995	17909	1	-0.342
TLE2	6	0.87557	0.88696	0.9995	17910	1	-0.513
KIAA1199	6	0.87564	0.887	0.9995	17911	1	-0.613
DYNC2LI1	6	0.87566	0.88701	0.9995	17912	1	0.1105
KLF5	6	0.87566	0.88701	0.9995	17913	1	-0.335
hsa-mir-4439	4	0.87572	0.87555	0.9995	17914	0	-0.084
WFIKKN1	6	0.87579	0.8871	0.9995	17915	1	-0.138
IFNL1	6	0.87582	0.88712	0.9995	17916	1	-0.478
ENTPD1	6	0.87582	0.88712	0.9995	17917	1	-0.291
hsa-mir-302f	3	0.87583	0.87599	0.9995	17918	0	-0.217
FOXI1	6	0.87587	0.88714	0.9995	17919	1	-0.011
hsa-let-7a-2	4	0.87591	0.87574	0.9995	17920	0	-0.337
ERCC3	6	0.87595	0.8872	0.9995	17921	1	-0.382
PALB2	6	0.87596	0.88721	0.9995	17922	1	-0.284
STK31	6	0.87609	0.8873	0.9995	17923	1	0.0527
hsa-mir-7854	4	0.87626	0.8761	0.9995	17924	0	-0.206
hsa-mir-6854	2	0.87632	0.87638	0.9995	17925	0	-0.259
SMARCD3	6	0.87635	0.88745	0.9995	17926	1	-0.583
FAM89A	6	0.87646	0.88753	0.9995	17927	1	-0.161
PRDM9	5	0.87653	0.87723	0.9995	17928	1	0.2938
hsa-mir-2355	4	0.87673	0.87659	0.9995	17929	0	-0.099
CDC42EP3	4	0.87688	0.87673	0.9995	17930	0	-0.104

FLG2	6	0.87689	0.88781	0.9995	17931	1	-0.272
HINT1	6	0.87689	0.88781	0.9995	17932	1	-0.406
SYT6	6	0.87703	0.88789	0.9995	17933	1	-0.338
OBP2A	2	0.87715	0.8772	0.9995	17934	0	-1.445
OR2B2	6	0.87724	0.88802	0.9995	17935	1	-0.358
STX17	6	0.87728	0.88803	0.9995	17936	1	-0.253
ZNF410	6	0.8773	0.88804	0.9995	17937	1	-0.219
TRPC7	6	0.87735	0.88807	0.9995	17938	1	0.0603
DNAJC3	6	0.87745	0.88814	0.9995	17939	1	-0.427
hsa-mir-6869	4	0.87749	0.87731	0.9995	17940	0	-0.085
E2F2	6	0.87751	0.88818	0.9995	17941	1	0.06
NAF1	6	0.87755	0.8882	0.9995	17942	1	0.034
KCNK18	6	0.87758	0.88822	0.9995	17943	1	-0.427
NFKBIL1	6	0.87758	0.88822	0.9995	17944	1	-0.106
MAMSTR	6	0.87762	0.88825	0.9995	17945	1	-0.247
hsa-mir-5189	4	0.87764	0.87747	0.9995	17946	0	-0.236
hsa-mir-298	4	0.87771	0.87755	0.9995	17947	0	-0.294
C21orf33	6	0.87772	0.88831	0.9995	17948	1	-0.076
NRIP3	6	0.87783	0.88839	0.9995	17949	1	-0.049
DSG4	6	0.87789	0.88843	0.9995	17950	1	-0.409
HOXA4	6	0.87789	0.88843	0.9995	17951	1	-0.083
ABHD8	6	0.87789	0.88843	0.9995	17952	1	0.0353
SEC61B	6	0.87796	0.88847	0.9995	17953	1	-0.098
TAAR5	6	0.87799	0.8885	0.9995	17954	1	-0.504
SCFD2	6	0.87811	0.88857	0.9995	17955	1	0.0238
SOS1	6	0.8783	0.88868	0.9995	17956	1	-0.171
FEN1	6	0.87837	0.88873	0.9995	17957	1	0.123
GSTM3	6	0.87841	0.88876	0.9995	17958	1	-0.152
hsa-mir-548i-3	2	0.87845	0.87849	0.9995	17959	0	-1.327
USP3	6	0.87854	0.88885	0.9995	17960	1	-0.199
RCCD1	6	0.87858	0.88888	0.9995	17961	1	-0.421
ATP6V1B1	6	0.87862	0.8889	0.9995	17962	1	0.1059
OR2AT4	6	0.87866	0.88893	0.9995	17963	1	0.0983
WWTR1	6	0.87873	0.88897	0.9995	17964	1	-0.605
SCPEP1	6	0.87874	0.88898	0.9995	17965	1	-0.16
OR7C2	4	0.8788	0.87867	0.9995	17966	0	-0.143
SGMS1	6	0.87891	0.88908	0.9995	17967	1	-0.142
SPATA31A6	6	0.87891	0.88908	0.9995	17968	1	-0.419
NECAB2	6	0.87891	0.88908	0.9995	17969	1	-0.41
DNAH2	6	0.87916	0.88924	0.9995	17970	1	0.0309
VNN2	6	0.87927	0.88932	0.9995	17971	1	-0.266
MXD4	6	0.87933	0.88935	0.9995	17972	1	-0.389
NFKB1	6	0.87951	0.88946	0.9995	17973	1	-0.03
FBF1	6	0.87955	0.88948	0.9995	17974	1	0.1077
PCDHGA7	2	0.8796	0.87965	0.9995	17975	0	-0.283
METTL21C	6	0.87964	0.88955	0.9995	17976	1	-0.627
MOCS2	6	0.87968	0.88958	0.9995	17977	1	-0.183
WNT8B	6	0.8797	0.88959	0.9995	17978	1	-0.354
MPL	6	0.87972	0.8896	0.9995	17979	1	0.0275
RNF144A	6	0.87979	0.88965	0.9995	17980	1	-0.008
NAV2	6	0.88	0.88978	0.9995	17981	1	-0.33
SQRDL	6	0.88005	0.88981	0.9995	17982	1	-0.126
STAU2	6	0.88005	0.88981	0.9995	17983	1	-0.133
GPR78	6	0.88019	0.88991	0.9995	17984	1	-0.097
EPOR	6	0.88021	0.88993	0.9995	17985	1	-0.132
CSF1R	6	0.88035	0.89002	0.9995	17986	1	-0.15
TMEM158	6	0.88049	0.89011	0.9995	17987	1	-0.183
AFF3	6	0.88057	0.89016	0.9995	17988	1	-0.079
LMOD2	6	0.88057	0.89016	0.9995	17989	1	-0.053
hsa-mir-3619	4	0.88062	0.88051	0.9995	17990	0	-0.106
CDK2	6	0.8808	0.89033	0.9995	17991	1	-0.224
C11orf96	6	0.8808	0.89033	0.9995	17992	1	-0.055
RALGAPB	6	0.88088	0.89038	0.9995	17993	1	-0.057
hsa-mir-3176	4	0.88094	0.88081	0.9995	17994	0	0.0418
IL17B	6	0.88107	0.89049	0.9995	17995	1	-0.328
CBFA2T3	6	0.88118	0.89057	0.9995	17996	1	-0.407
OR2M3	5	0.88124	0.88171	0.9995	17997	1	-0.765
KCNJ18	4	0.88125	0.88111	0.9995	17998	0	-0.245
DRD3	6	0.88129	0.89065	0.9995	17999	1	-0.223
CABP7	6	0.88139	0.8907	0.9995	18000	1	-0.169
LAMA3	6	0.88166	0.89088	0.9995	18001	1	-0.253
DEFB133	3	0.88169	0.88179	0.9995	18002	0	-0.089
hsa-mir-4698	4	0.88171	0.88159	0.9995	18003	0	-0.051
ZSCAN2	6	0.88171	0.89092	0.9995	18004	1	-0.217
CSHL1	6	0.88184	0.89101	0.9995	18005	1	-0.018
RYBP	6	0.88189	0.89104	0.9995	18006	1	-0.082
HPS4	6	0.88195	0.89108	0.9995	18007	1	-0.42
EIF4E2	6	0.88195	0.89108	0.9995	18008	1	-0.105
SPRR1A	4	0.88199	0.88188	0.9995	18009	0	-1.065
AKIRIN1	6	0.88203	0.89113	0.9995	18010	1	0.0667
TMED10	6	0.8821	0.89118	0.9995	18011	1	-0.279
QSER1	6	0.88221	0.89124	0.9995	18012	1	-0.054
CROT	6	0.88221	0.89124	0.9995	18013	1	-0.22
ARL1	6	0.88225	0.89126	0.9995	18014	1	-0.455
RPL6	6	0.88227	0.89128	0.9995	18015	1	-0.152

IKBKG	6	0.88229	0.8913	0.9995	18016	1	-0.18
PTGDR2	6	0.88231	0.8913	0.9995	18017	1	-0.338
SMPDL3B	6	0.88246	0.89141	0.9995	18018	1	-0.029
DDX46	6	0.88254	0.89146	0.9995	18019	1	-0.397
ZSCAN32	6	0.8826	0.8915	0.9995	18020	1	-0.272
FAM174A	6	0.88266	0.89155	0.9995	18021	1	-0.108
hsa-mir-550a-3	3	0.88275	0.88286	0.9995	18022	0	-0.26
hsa-mir-4790	3	0.88275	0.88286	0.9995	18023	0	-0.419
HIST1H4J	1	0.88275	0.88312	0.9995	18024	0	-0.769
PTPRH	6	0.88292	0.89172	0.9995	18025	1	-0.391
TRIP10	6	0.88292	0.89172	0.9995	18026	1	-0.879
hsa-mir-4526	4	0.88299	0.8829	0.9995	18027	0	-0.491
RUFY3	6	0.883	0.89177	0.9995	18028	1	-0.156
MTF2	5	0.88317	0.88357	0.9995	18029	1	-0.101
NPEPPS	6	0.88327	0.89196	0.9995	18030	1	0.0399
PLA2G10	6	0.88334	0.892	0.9995	18031	1	-0.512
RNF135	6	0.88345	0.89208	0.9995	18032	1	-0.427
NUP50	6	0.88345	0.89208	0.9995	18033	1	-0.683
hsa-mir-1273g	4	0.8836	0.8835	0.9995	18034	0	-0.583
CYP1B1	6	0.88362	0.89219	0.9995	18035	1	-0.174
VARS	6	0.88369	0.89224	0.9995	18036	1	-0.048
CEP104	6	0.88374	0.89227	0.9995	18037	1	-0.724
NKRF	6	0.88374	0.89227	0.9995	18038	1	-0.258
ETFA	6	0.88376	0.89229	0.9995	18039	1	-0.57
TANGO2	6	0.88396	0.89244	0.9995	18040	1	-0.123
RNF186	6	0.88408	0.8925	0.9995	18041	1	-0.249
COX6B2	6	0.8841	0.89251	0.9995	18042	1	-0.082
STOML1	6	0.88417	0.89257	0.9995	18043	1	-0.198
C11orf83	6	0.88421	0.8926	0.9995	18044	1	-0.077
DDX47	6	0.88436	0.8927	0.9995	18045	1	-0.056
BHLHE22	6	0.88446	0.89275	0.9995	18046	1	-0.163
SLC38A8	6	0.8845	0.89278	0.9995	18047	1	-0.375
CCDC22	6	0.8845	0.89278	0.9995	18048	1	-0.241
POLR2E	6	0.88458	0.89283	0.9995	18049	1	-0.18
ABTB1	6	0.88466	0.89287	0.9995	18050	1	0.0032
GJB6	6	0.88475	0.89294	0.9995	18051	1	-0.051
ACOT13	6	0.8848	0.89297	0.9995	18052	1	0.0786
GRM2	6	0.88499	0.8931	0.9995	18053	1	-0.041
UGT2A1	1	0.88521	0.88559	0.9995	18054	0	-1.009
SPARC	6	0.88524	0.89327	0.9995	18055	1	-0.241
hsa-mir-410	4	0.88526	0.88516	0.9995	18056	0	-0.555
MAP6D1	6	0.88533	0.89332	0.9995	18057	1	-0.219
OSCP1	4	0.88538	0.88528	0.9995	18058	0	-0.335
KRTAP27-1	6	0.88545	0.8934	0.9995	18059	1	-0.079
HEPH	6	0.88545	0.8934	0.9995	18060	1	-0.114
DCTD	6	0.88547	0.89341	0.9995	18061	1	-0.175
ARMC8	6	0.88553	0.89346	0.9995	18062	1	0.0203
SEPHS2	6	0.88555	0.89348	0.9995	18063	1	-0.315
TRAPPC6A	6	0.88556	0.89349	0.9995	18064	1	0.013
SLCO3A1	6	0.88561	0.89353	0.9995	18065	1	-0.037
PLEKHG7	6	0.88566	0.89356	0.9995	18066	1	0.0805
ZNF211	6	0.8857	0.89358	0.9995	18067	1	-0.161
CDC14B	6	0.88574	0.8936	0.9995	18068	1	-0.232
PROCA1	6	0.88577	0.89362	0.9995	18069	1	-0.215
hsa-mir-132	4	0.88581	0.88571	0.9995	18070	0	-0.037
TRIM35	6	0.88581	0.89365	0.9995	18071	1	-1.073
IL36G	6	0.88584	0.89366	0.9995	18072	1	-0.115
PDX1	6	0.88586	0.89367	0.9995	18073	1	-0.464
BCL9	6	0.88597	0.89375	0.9995	18074	1	-0.143
LPAR2	6	0.88609	0.89384	0.9995	18075	1	-0.167
TRAPPC2L	6	0.88612	0.89386	0.9995	18076	1	-0.257
TNRC18	6	0.88619	0.89392	0.9995	18077	1	-0.148
FAM181B	6	0.88621	0.89392	0.9995	18078	1	-0.085
ADAMTS19	6	0.88629	0.89398	0.9995	18079	1	-0.017
BAIAP2L1	6	0.8863	0.89399	0.9995	18080	1	-0.625
SLC22A14	6	0.88632	0.89401	0.9995	18081	1	-0.069
STX5	6	0.88635	0.89402	0.9995	18082	1	-0.281
PSMB2	6	0.88642	0.89408	0.9995	18083	1	-0.189
LAMA1	6	0.88667	0.89424	0.9995	18084	1	-0.151
CDC16	6	0.88668	0.89425	0.9995	18085	1	-0.199
PCDHB1	6	0.88672	0.89427	0.9995	18086	1	-0.586
CFHR5	6	0.88686	0.89436	0.9995	18087	1	-0.595
KLF11	6	0.88686	0.89436	0.9995	18088	1	-0.287
PCDHGB6	2	0.88691	0.88689	0.9995	18089	0	-0.615
SIK1	6	0.88692	0.89441	0.9995	18090	1	-0.134
PLA2G12B	6	0.88694	0.89441	0.9995	18091	1	-0.228
CDC20B	6	0.88708	0.89451	0.9995	18092	1	-0.197
ZMYND11	6	0.88709	0.89452	0.9995	18093	1	-0.628
PGAM4	6	0.88714	0.89456	0.9995	18094	1	-0.235
hsa-mir-3665	4	0.88722	0.88714	0.9995	18095	0	-0.31
hsa-mir-6883	4	0.88733	0.88726	0.9995	18096	0	0.0308
VAMP3	6	0.88734	0.8947	0.9995	18097	1	-0.211
PFKP	6	0.88734	0.8947	0.9995	18098	1	-0.29
PHF12	6	0.88738	0.89473	0.9995	18099	1	-0.167
CMAS	6	0.88745	0.89478	0.9995	18100	1	-0.155

PRMT3	6	0.88749	0.89482	0.9995	18101	1	-0.058
REXO4	6	0.88758	0.89487	0.9995	18102	1	-0.461
SMIM11	6	0.88758	0.89487	0.9995	18103	1	-0.171
PXMP2	6	0.88765	0.89492	0.9995	18104	1	-0.264
ST8SIA1	6	0.88771	0.89496	0.9995	18105	1	-0.38
hsa-mir-3166	4	0.88772	0.88765	0.9995	18106	0	-0.353
SMU1	6	0.88773	0.89498	0.9995	18107	1	-0.253
PSRC1	6	0.88773	0.89498	0.9995	18108	1	-0.003
SERPINF2	6	0.88786	0.89506	0.9995	18109	1	-0.753
ANGEL2	6	0.88794	0.89512	0.9995	18110	1	-0.375
CCDC104	6	0.88797	0.89514	0.9995	18111	1	-0.16
KRT9	6	0.88813	0.89524	0.9995	18112	1	-0.034
hsa-mir-662	4	0.88819	0.88812	0.9995	18113	0	-0.283
hsa-mir-6838	4	0.8883	0.88824	0.9995	18114	0	-0.29
WDR25	6	0.88832	0.89537	0.9995	18115	1	-0.569
SYN2	6	0.88848	0.89549	0.9995	18116	1	-0.047
PHF5A	6	0.88853	0.89552	0.9995	18117	1	-0.126
LAPTM5	6	0.88861	0.89558	0.9995	18118	1	-0.194
PDYN	6	0.88861	0.89558	0.9995	18119	1	-0.266
TP53I13	6	0.88868	0.89563	0.9995	18120	1	-0.561
FLRT1	6	0.88868	0.89563	0.9995	18121	1	-0.168
ZDHHC1	6	0.88878	0.89568	0.9995	18122	1	-0.116
KLK1	6	0.88884	0.89574	0.9995	18123	1	0.007
OR4Q3	6	0.88884	0.89574	0.9995	18124	1	-0.518
MCFD2	6	0.88886	0.89575	0.9995	18125	1	-0.253
IRGM	6	0.88889	0.89577	0.9995	18126	1	-0.281
RNF103-CHMP3	1	0.88898	0.88937	0.9995	18127	0	-1.952
TMEM222	6	0.88901	0.89586	0.9995	18128	1	0.0399
TMEM123	6	0.88906	0.89589	0.9995	18129	1	-0.163
hsa-mir-3141	4	0.88907	0.88898	0.9995	18130	0	-0.614
CCDC160	6	0.88911	0.89592	0.9995	18131	1	0.0766
C9orf96	6	0.88914	0.89595	0.9995	18132	1	-0.193
CLDN2	6	0.8892	0.89599	0.9995	18133	1	-0.114
GDI2	6	0.88922	0.896	0.9995	18134	1	-0.142
OR2T29	3	0.88924	0.88932	0.9995	18135	0	-0.419
GCNT2	6	0.88925	0.89602	0.9995	18136	1	-0.051
CLCA2	6	0.88932	0.89607	0.9995	18137	1	-0.039
HDAC6	6	0.88932	0.89607	0.9995	18138	1	-0.157
DSPP	6	0.88932	0.89607	0.9995	18139	1	-0.328
hsa-mir-3612	4	0.88934	0.88925	0.9995	18140	0	-0.258
MPC1L	6	0.88939	0.89612	0.9995	18141	1	-0.234
RPS11	6	0.88939	0.89612	0.9995	18142	1	0.0538
KRTAP10-8	6	0.88942	0.89615	0.9995	18143	1	-0.197
hsa-mir-99b	4	0.88943	0.88933	0.9995	18144	0	-0.067
CYP11B1	5	0.88954	0.88985	0.9995	18145	0	0.1853
HINT3	6	0.88954	0.89623	0.9995	18146	1	-0.172
NUP153	6	0.88962	0.89628	0.9995	18147	1	-0.41
CACYBP	6	0.88969	0.89633	0.9995	18148	1	-0.082
ANAPC7	6	0.88973	0.89637	0.9995	18149	1	-0.365
ZC2HC1A	6	0.88978	0.8964	0.9995	18150	1	-0.131
INO80C	6	0.88987	0.89648	0.9995	18151	1	-0.111
PBDC1	6	0.88989	0.8965	0.9995	18152	1	-0.1
ORAI1	6	0.88989	0.8965	0.9995	18153	1	-0.268
LRCH3	6	0.88992	0.89652	0.9995	18154	1	-0.1
hsa-mir-1285-2	4	0.88993	0.88984	0.9995	18155	0	-0.454
KLF7	6	0.89003	0.8966	0.9995	18156	1	-0.39
FAXC	6	0.89005	0.89662	0.9995	18157	1	0.0548
CHUK	6	0.89005	0.89662	0.9995	18158	1	-0.632
ALX3	6	0.89027	0.89677	0.9995	18159	1	-0.151
MOSPD3	6	0.89034	0.89683	0.9995	18160	1	-0.191
FCGR3A	6	0.89036	0.89684	0.9995	18161	1	-0.775
NPY1R	6	0.89038	0.89686	0.9995	18162	1	-0.091
CPEB3	6	0.8904	0.89687	0.9995	18163	1	-0.1
HSFX1	1	0.89041	0.89078	0.9995	18164	0	-0.6
FAHD2A	6	0.89041	0.89688	0.9995	18165	1	-0.434
POLR3G	6	0.89045	0.89691	0.9995	18166	1	-0.637
SSPO	6	0.89049	0.89693	0.9995	18167	1	-0.459
ONECUT2	6	0.89049	0.89693	0.9995	18168	1	-0.439
BCL2L1	6	0.89049	0.89693	0.9995	18169	1	-0.916
USP6	6	0.89057	0.89698	0.9995	18170	1	-0.251
ODF3	6	0.89057	0.89698	0.9995	18171	1	0.0565
SORCS1	6	0.89057	0.89698	0.9995	18172	1	-0.126
ZNF280A	6	0.89057	0.89698	0.9995	18173	1	-0.512
HEBP1	6	0.89068	0.89706	0.9995	18174	1	-0.422
TXNRD1	6	0.89071	0.89708	0.9995	18175	1	-0.082
PSTK	6	0.89076	0.89711	0.9995	18176	1	-0.194
hsa-mir-4504	4	0.89078	0.89068	0.9995	18177	0	-0.773
LPCAT2	6	0.89081	0.89714	0.9995	18178	1	-0.267
FGF9	6	0.89099	0.89728	0.9995	18179	1	-0.648
PITHD1	6	0.89104	0.89731	0.9995	18180	1	-0.208
PRAMEF4	6	0.89111	0.89736	0.9995	18181	1	-0.103
TEX2	6	0.8912	0.89744	0.9995	18182	1	-0.319
GAS2	6	0.89122	0.89746	0.9995	18183	1	-0.185
EDEM1	6	0.89127	0.89749	0.9995	18184	1	-0.154
hsa-mir-7150	4	0.89138	0.89128	0.9995	18185	0	-0.724

MRAS	6	0.89154	0.89769	0.9995	18186	1	-0.179
hsa-mir-5692b	3	0.89157	0.89166	0.9995	18187	0	-0.717
SEC14L6	6	0.89161	0.89775	0.9995	18188	1	-0.301
SULT1A3	4	0.89204	0.89194	0.9995	18189	0	-0.08
AMIGO1	6	0.89214	0.89815	0.9995	18190	1	-0.148
KBTBD8	6	0.89214	0.89815	0.9995	18191	1	-0.062
hsa-mir-6766	4	0.89223	0.89213	0.9995	18192	0	-0.047
PTPN12	5	0.89224	0.89254	0.9995	18193	0	0.0223
hsa-mir-3664	4	0.89226	0.89218	0.9995	18194	0	-0.274
EAF1	6	0.89232	0.89828	0.9995	18195	1	-0.005
GPR161	6	0.89239	0.89833	0.9995	18196	1	-0.159
TOLLIP	6	0.89239	0.89833	0.9995	18197	1	-0.042
TSNAXIP1	6	0.89239	0.89833	0.9995	18198	1	-0.091
FAM129C	6	0.89248	0.89839	0.9995	18199	1	-0.388
hsa-mir-556	3	0.89249	0.89259	0.9995	18200	0	-0.075
LOC100129520	6	0.89255	0.89845	0.9995	18201	1	-0.161
PDZD4	6	0.89262	0.8985	0.9995	18202	1	-0.056
SPRY4	6	0.89262	0.8985	0.9995	18203	1	-0.403
hsa-mir-3175	4	0.89278	0.89266	0.9995	18204	0	-0.361
ANXA9	6	0.89279	0.89862	0.9995	18205	1	-0.36
OSBPL9	4	0.89297	0.89285	0.9995	18206	0	-0.33
PALMD	6	0.89305	0.89882	0.9995	18207	1	-0.803
GNPNAT1	6	0.89305	0.89882	0.9995	18208	1	-0.188
hsa-mir-3650	4	0.89312	0.893	0.9995	18209	0	-0.246
TNFAIP8L3	6	0.89324	0.89896	0.9995	18210	1	-0.254
WDR76	6	0.89324	0.89896	0.9995	18211	1	-0.481
PARK2	6	0.89329	0.89899	0.9995	18212	1	0.0161
C12orf43	6	0.89331	0.89901	0.9995	18213	1	-0.268
LOC339862	6	0.89336	0.89905	0.9995	18214	1	-0.749
NAGPA	6	0.89343	0.8991	0.9995	18215	1	-0.467
C11orf45	4	0.89354	0.89343	0.9995	18216	0	-0.213
HCRT	6	0.89365	0.89925	0.9995	18217	1	-0.526
NKX2-3	6	0.89365	0.89925	0.9995	18218	1	-0.656
PEAK1	6	0.89369	0.89928	0.9995	18219	1	-0.217
LSM5	6	0.89376	0.89933	0.9995	18220	1	-0.164
MAGEA10	6	0.89379	0.89935	0.9995	18221	1	-0.138
MC5R	6	0.8939	0.89943	0.9995	18222	1	-0.469
ZNF813	3	0.894	0.8941	0.9995	18223	0	-0.119
THAP2	6	0.89401	0.89951	0.9995	18224	1	0.0949
STYXL1	6	0.89406	0.89954	0.9995	18225	1	-1.061
SMOC1	6	0.89409	0.89956	0.9995	18226	1	-0.476
FBXL15	6	0.89422	0.89964	0.9995	18227	1	-0.025
KISS1	6	0.89423	0.89965	0.9995	18228	1	-0.476
hsa-mir-516b-2	2	0.89429	0.89419	0.9995	18229	0	-1.227
HN1L	6	0.89435	0.89973	0.9995	18230	1	-0.071
TIMM17B	6	0.89435	0.89973	0.9995	18231	1	-0.243
ACKR4	5	0.89435	0.89467	0.9995	18232	0	-0.36
ARL6IP5	6	0.89449	0.89983	0.9995	18233	1	-0.133
hsa-mir-1912	2	0.89459	0.89453	0.9995	18234	0	-0.384
PPARA	6	0.8946	0.89991	0.9995	18235	1	-0.439
DENND1C	6	0.89463	0.89993	0.9995	18236	1	-0.444
C17orf99	6	0.89465	0.89993	0.9995	18237	1	0.0798
FNDC8	6	0.89467	0.89995	0.9995	18238	1	-0.037
SLITRK3	6	0.89473	0.89999	0.9995	18239	1	0.0153
PROK2	6	0.89479	0.90003	0.9995	18240	1	0.0114
GNG2	6	0.89479	0.90003	0.9995	18241	1	-0.037
ABCC9	6	0.89482	0.90005	0.9995	18242	1	-0.589
CAT	6	0.89487	0.90009	0.9995	18243	1	-0.152
FAM167B	6	0.89491	0.90012	0.9995	18244	1	0.0925
RAI2	6	0.89494	0.90013	0.9995	18245	1	-0.37
TNRC6B	6	0.89502	0.90019	0.9995	18246	1	-0.187
TENC1	6	0.89508	0.90024	0.9995	18247	1	-0.161
GHITM	6	0.89523	0.90035	0.9995	18248	1	-0.063
SLC25A17	6	0.89525	0.90036	0.9995	18249	1	-0.03
TPPP	6	0.89529	0.9004	0.9995	18250	1	-0.014
MARK1	6	0.89541	0.90048	0.9995	18251	1	-0.402
PDCD1LG2	6	0.8955	0.90056	0.9995	18252	1	-0.489
OR10A7	6	0.89553	0.90059	0.9995	18253	1	-0.044
SLC16A6	6	0.89562	0.90066	0.9995	18254	1	-0.372
COL5A1	6	0.89578	0.90079	0.9995	18255	1	-0.29
DDR2	6	0.89591	0.90087	0.9995	18256	1	-0.258
FGFR2	6	0.89602	0.90096	0.9995	18257	1	-0.023
POLB	6	0.89605	0.90098	0.9995	18258	1	-0.347
CHRD	6	0.89622	0.90109	0.9995	18259	1	-0.756
PROSER1	6	0.89625	0.90112	0.9995	18260	1	-0.265
SOX6	6	0.89642	0.90123	0.9995	18261	1	-0.08
PRDM4	6	0.89655	0.90133	0.9995	18262	1	-0.293
SRPRB	6	0.89655	0.90133	0.9995	18263	1	-0.086
hsa-mir-936	4	0.89668	0.89656	0.9995	18264	0	-0.281
hsa-mir-4271	4	0.89677	0.89664	0.9995	18265	0	-0.41
RAD54L	6	0.89684	0.90154	0.9995	18266	1	-0.15
CYB5B	6	0.89688	0.90158	0.9995	18267	1	-0.627
PBLD	6	0.89688	0.90158	0.9995	18268	1	-0.358
SCGB3A1	6	0.89694	0.90161	0.9995	18269	1	-0.334
CXorf27	6	0.89696	0.90163	0.9995	18270	1	0.0282

CFHR1	6	0.897	0.90166	0.9995	18271	1	-0.512
MRGPRX1	6	0.897	0.90166	0.9995	18272	1	0.0433
ZNF781	6	0.89702	0.90167	0.9995	18273	1	-0.109
SLC2A12	6	0.89716	0.90177	0.9995	18274	1	-0.091
HIST1H2AE	6	0.89728	0.90186	0.9995	18275	1	-0.209
hsa-mir-571	4	0.89729	0.89717	0.9995	18276	0	-0.945
CCDC134	6	0.89733	0.90189	0.9995	18277	1	-0.388
GRIPAP1	6	0.89736	0.90192	0.9995	18278	1	-0.34
CISH	6	0.8974	0.90195	0.9995	18279	1	-0.365
LRRC14B	6	0.89745	0.90199	0.9995	18280	1	-0.039
NT5DC1	6	0.89762	0.90213	0.9995	18281	1	-0.411
C17orf50	6	0.89767	0.90216	0.9995	18282	1	-0.028
TRH	6	0.89779	0.90226	0.9995	18283	1	-0.253
KANK3	6	0.89782	0.90228	0.9995	18284	1	-0.521
PTGER1	6	0.89782	0.90228	0.9995	18285	1	-0.104
hsa-mir-6726	4	0.89786	0.89775	0.9995	18286	0	0.0308
ASB9	6	0.8979	0.90233	0.9995	18287	1	-0.191
DGAT2	6	0.89802	0.90242	0.9995	18288	1	-0.127
hsa-mir-8089	4	0.89803	0.89792	0.9995	18289	0	-0.729
GYS1	6	0.89809	0.90248	0.9995	18290	1	-0.065
hsa-mir-4529	3	0.8982	0.89835	0.9995	18291	0	-0.991
hsa-mir-519c	2	0.89835	0.8983	0.9995	18292	0	-1.72
FOLR3	6	0.89835	0.90267	0.9995	18293	1	-0.329
KCNMB1	6	0.89849	0.90276	0.9995	18294	1	-0.1
hsa-mir-4533	4	0.89852	0.8984	0.9995	18295	0	-0.495
MIER2	6	0.89852	0.90279	0.9995	18296	1	-0.013
OR8G5	5	0.89855	0.89885	0.9995	18297	0	0.0306
ITPRIP	6	0.89868	0.90291	0.9995	18298	1	-0.106
CNPY3	6	0.89869	0.90293	0.9995	18299	1	-0.37
DKK3	6	0.8988	0.90301	0.9995	18300	1	-0.16
hsa-mir-4486	4	0.89887	0.89877	0.9995	18301	0	-0.503
MAPRE1	6	0.89891	0.9031	0.9995	18302	1	-0.127
GOLGA7	6	0.89893	0.90311	0.9995	18303	1	-0.152
H2AFZ	6	0.89897	0.90313	0.9995	18304	1	-1.045
PRTFDC1	6	0.89897	0.90313	0.9995	18305	1	-0.051
GPATCH3	6	0.89904	0.90318	0.9995	18306	1	-0.659
ADAMTSL1	6	0.89913	0.90324	0.9995	18307	1	-0.107
SERPINB6	6	0.8993	0.90336	0.9995	18308	1	-0.051
KCTD4	6	0.89938	0.90344	0.9995	18309	1	-0.387
RFWD3	6	0.89943	0.90348	0.9995	18310	1	-0.419
TCEAL6	6	0.89947	0.90351	0.9995	18311	1	-0.576
hsa-mir-323a	4	0.89949	0.89941	0.9995	18312	0	-0.314
hsa-mir-4731	4	0.89962	0.89953	0.9995	18313	0	-0.144
GRB2	6	0.89962	0.90362	0.9995	18314	1	0.0374
C5orf63	6	0.89964	0.90364	0.9995	18315	1	-0.13
NUDT11	6	0.89969	0.90367	0.9995	18316	1	-0.826
CAPS	4	0.8997	0.89961	0.9995	18317	0	-0.201
OR51F1	6	0.89971	0.90369	0.9995	18318	1	-0.069
ROPN1L	6	0.89974	0.90371	0.9995	18319	1	-0.122
hsa-mir-19b-1	4	0.89975	0.89966	0.9995	18320	0	-0.484
RTTN	6	0.89984	0.90379	0.9995	18321	1	-0.071
TRPV1	6	0.89988	0.90383	0.9995	18322	1	-0.492
hsa-mir-519e	2	0.89989	0.89983	0.9995	18323	0	-1.27
ZNF705D	1	0.89992	0.90037	0.9995	18324	0	-0.654
CCDC63	6	0.89994	0.90387	0.9995	18325	1	0.0136
TMEM60	6	0.90005	0.90396	0.9995	18326	1	-0.438
UBE2Q2	6	0.90005	0.90396	0.9995	18327	1	-0.301
IGF2	6	0.90023	0.90408	0.9995	18328	1	-0.044
ACSL6	6	0.9003	0.90414	0.9995	18329	1	-0.553
GP1BA	6	0.90046	0.90425	0.9995	18330	1	0.0513
PCDHB15	6	0.90058	0.90434	0.9995	18331	1	-0.09
C2CD3	6	0.90073	0.90445	0.9995	18332	1	-0.415
ZFP36L1	6	0.90073	0.90445	0.9995	18333	1	-0.11
hsa-mir-3153	4	0.9008	0.90071	0.9995	18334	0	-0.229
PISD	6	0.9009	0.90458	0.9995	18335	1	-0.59
hsa-mir-4723	4	0.90091	0.90081	0.9995	18336	0	-0.312
NLRP14	6	0.90092	0.90459	0.9995	18337	1	-0.313
ZNF664-FAM101A	4	0.90098	0.90089	0.9995	18338	0	-0.565
POU6F2	6	0.90099	0.90464	0.9995	18339	1	-0.274
RGS9BP	6	0.90103	0.90467	0.9995	18340	1	-0.705
MTMR11	6	0.90106	0.90469	0.9995	18341	1	-0.132
KLHL31	6	0.90108	0.90471	0.9995	18342	1	-0.337
KIAA0196	6	0.90108	0.90471	0.9995	18343	1	-0.345
hsa-mir-29a	4	0.9011	0.90102	0.9995	18344	0	-0.178
RAD51	6	0.90122	0.90482	0.9995	18345	1	-0.27
TFAP2D	6	0.90125	0.90483	0.9995	18346	1	-0.193
MFAP2	6	0.90131	0.90488	0.9995	18347	1	-0.398
CDK3	6	0.90131	0.90488	0.9995	18348	1	-0.058
MKI67	6	0.90133	0.9049	0.9995	18349	1	0.0777
hsa-mir-34a	2	0.90134	0.90131	0.9995	18350	0	-0.301
hsa-mir-1179	4	0.90138	0.90132	0.9995	18351	0	-0.047
NEK2	6	0.90144	0.90498	0.9995	18352	1	-0.249
DLGAP5	6	0.90147	0.90501	0.9995	18353	1	-0.097
LDB3	6	0.90147	0.90501	0.9995	18354	1	-0.335
GFI1	6	0.90151	0.90503	0.9995	18355	1	-0.216

KRT28	6	0.90159	0.90509	0.9995	18356	1	-0.335
GALNTL6	6	0.90166	0.90515	0.9995	18357	1	-0.076
SLC25A14	6	0.90168	0.90516	0.9995	18358	1	0.046
hsa-mir-3656	4	0.90168	0.90162	0.9995	18359	0	-0.337
SYCP2L	4	0.90168	0.90162	0.9995	18360	0	-0.123
BSPRY	6	0.90169	0.90517	0.9995	18361	1	-0.161
UBA6	6	0.90172	0.90518	0.9995	18362	1	-0.227
SOX5	6	0.90176	0.90522	0.9995	18363	1	-0.056
hsa-mir-3146	4	0.90177	0.9017	0.9995	18364	0	-1.059
CNTNAP2	6	0.90178	0.90524	0.9995	18365	1	-0.499
AQPEP	6	0.90181	0.90526	0.9995	18366	1	-0.072
CCNJL	6	0.90185	0.9053	0.9995	18367	1	-0.507
MLL3	4	0.90196	0.9019	0.9995	18368	0	-0.744
ANKRD2	6	0.90198	0.9054	0.9995	18369	1	-0.25
CTF1	6	0.90207	0.90547	0.9995	18370	1	-0.117
hsa-mir-1273c	4	0.90212	0.90207	0.9995	18371	0	-0.276
CLEC4A	6	0.90215	0.90554	0.9995	18372	1	0.0948
SERPINB4	4	0.90216	0.9021	0.9995	18373	0	-0.169
KRTAP13-2	6	0.90219	0.90557	0.9995	18374	1	-0.241
LPXN	6	0.90221	0.9056	0.9995	18375	1	-0.236
ERCC6L2	6	0.90229	0.90567	0.9995	18376	1	0.0141
BMI1	6	0.90232	0.90568	0.9995	18377	1	-0.067
MPP1	6	0.90232	0.90568	0.9995	18378	1	-0.176
ARHGEF10L	6	0.90238	0.90573	0.9995	18379	1	-0.084
SMCR9	6	0.90242	0.90577	0.9995	18380	1	-0.075
TRO	6	0.90243	0.90578	0.9995	18381	1	-0.658
LRRC23	6	0.90246	0.90579	0.9995	18382	1	0.0128
hsa-mir-6732	4	0.90259	0.90254	0.9995	18383	0	0.0322
SULT1A2	5	0.9026	0.90286	0.9995	18384	0	0.0282
SULT1A1	5	0.90263	0.9029	0.9995	18385	0	0.1786
CST2	5	0.90263	0.9029	0.9995	18386	0	-0.056
GMEB2	6	0.90266	0.90594	0.9995	18387	1	-0.034
SSX3	3	0.90269	0.90278	0.9995	18388	0	-1.233
MRPL11	6	0.90276	0.90602	0.9995	18389	1	-0.441
PTGES2	6	0.90278	0.90604	0.9995	18390	1	-0.167
SLC25A16	6	0.90286	0.9061	0.9995	18391	1	-0.111
CD101	6	0.90291	0.90614	0.9995	18392	1	-0.44
ZCRB1	6	0.90294	0.90616	0.9995	18393	1	-0.217
MRPL1	6	0.90294	0.90616	0.9995	18394	1	-0.242
GMNN	6	0.90312	0.90631	0.9995	18395	1	-0.128
TPMT	6	0.90316	0.90634	0.9995	18396	1	-0.066
GNG13	6	0.90319	0.90636	0.9995	18397	1	-0.388
hsa-mir-588	4	0.90319	0.90312	0.9995	18398	0	-0.013
GZF1	6	0.90322	0.90639	0.9995	18399	1	-0.1
SPANXN4	6	0.90333	0.90648	0.9995	18400	1	-0.228
SERPINE2	6	0.90348	0.90658	0.9995	18401	1	-0.012
SLC36A3	6	0.90352	0.90662	0.9995	18402	1	-0.546
NADSYN1	6	0.90357	0.90666	0.9995	18403	1	0.0299
SLC9A7	6	0.90362	0.90671	0.9995	18404	1	-0.139
hsa-mir-4743	4	0.90364	0.90357	0.9995	18405	0	-1.206
PACS1	6	0.90365	0.90673	0.9995	18406	1	-0.027
ZNF148	6	0.90374	0.90679	0.9995	18407	1	-0.06
APOOL	6	0.90374	0.90679	0.9995	18408	1	-0.144
hsa-mir-3180-2	1	0.90376	0.90418	0.9995	18409	0	-0.768
TCTE3	6	0.90391	0.90693	0.9995	18410	1	-0.199
SFTPA1	5	0.90397	0.90424	0.9995	18411	0	-0.637
OR10G8	5	0.90399	0.90426	0.9995	18412	0	0.0857
SDSL	6	0.90418	0.90715	0.9995	18413	1	-0.061
OR10K1	6	0.90423	0.90719	0.9995	18414	1	-0.306
KRTAP22-2	6	0.90423	0.90719	0.9995	18415	1	0.0414
ABCA9	6	0.90423	0.90719	0.9995	18416	1	-0.379
PRAMEF11	5	0.90426	0.90453	0.9995	18417	0	-0.513
ASCC2	6	0.90429	0.90724	0.9995	18418	1	-0.269
TMEM221	6	0.90434	0.90727	0.9995	18419	1	-0.347
SPRR4	6	0.90444	0.90735	0.9995	18420	1	-0.225
ABCF3	6	0.9045	0.9074	0.9995	18421	1	-0.299
RBP7	6	0.90454	0.90743	0.9995	18422	1	-0.309
LOC100287036	6	0.90467	0.90754	0.9995	18423	1	-0.235
hsa-mir-4700	4	0.90471	0.90459	0.9995	18424	0	-0.099
CD226	6	0.90478	0.90763	0.9995	18425	1	-0.32
hsa-mir-210	4	0.9048	0.90469	0.9995	18426	0	-0.451
PSAT1	6	0.90483	0.90767	0.9995	18427	1	-0.085
PDRG1	6	0.90483	0.90767	0.9995	18428	1	-0.137
ZNF33A	6	0.90488	0.9077	0.9995	18429	1	-0.333
POLG2	6	0.9049	0.90772	0.9995	18430	1	-0.066
hsa-mir-544b	4	0.90497	0.90486	0.9995	18431	0	-0.256
AP5S1	6	0.90513	0.9079	0.9995	18432	1	-0.216
CCDC39	6	0.90517	0.90795	0.9995	18433	1	-0.182
GINS3	6	0.90517	0.90795	0.9995	18434	1	-0.304
PECR	6	0.9052	0.90797	0.9995	18435	1	-0.079
ZSWIM8	4	0.90524	0.90515	0.9995	18436	0	-0.053
C10orf107	6	0.90531	0.90805	0.9995	18437	1	-0.456
SOGA2	6	0.90535	0.90808	0.9995	18438	1	-0.225
UBXN2B	6	0.9054	0.90812	0.9995	18439	1	0.0386
C19orf48	6	0.90544	0.90815	0.9995	18440	1	-0.822

APOBR	6	0.90558	0.90825	0.9995	18441	1	0.0409
SMIM5	6	0.90567	0.90832	0.9995	18442	1	-0.41
BCL2L15	6	0.90571	0.90836	0.9995	18443	1	-0.42
EPB41L1	6	0.90576	0.9084	0.9995	18444	1	0.1931
RANBP6	6	0.90577	0.90841	0.9995	18445	1	-0.236
RHBDL3	6	0.90582	0.90844	0.9995	18446	1	-0.423
PIGB	6	0.90582	0.90844	0.9995	18447	1	-0.048
ACN9	6	0.90587	0.90848	0.9995	18448	1	0.0491
FBN3	6	0.90591	0.90851	0.9995	18449	1	-0.23
PKDREJ	6	0.90591	0.90851	0.9995	18450	1	-0.141
XRCC3	6	0.90598	0.90857	0.9995	18451	1	-0.006
PBX3	6	0.906	0.90859	0.9995	18452	1	-0.359
NPR1	6	0.90613	0.9087	0.9995	18453	1	-0.254
PCGF3	6	0.90623	0.90878	0.9995	18454	1	0.0629
PPAPDC2	4	0.90631	0.90623	0.9995	18455	0	-0.118
KDELC2	6	0.90633	0.90887	0.9995	18456	1	-0.271
ZNF578	3	0.90633	0.90643	0.9995	18457	0	-1.233
NDUFV2	6	0.9064	0.90892	0.9995	18458	1	-0.124
LRRTM2	6	0.90643	0.90894	0.9995	18459	1	0.0742
OCSTAMP	6	0.90645	0.90897	0.9995	18460	1	-0.274
GYPC	6	0.90647	0.90898	0.9995	18461	1	0.0455
PALM	6	0.90649	0.909	0.9995	18462	1	-0.458
AGT	6	0.90649	0.909	0.9995	18463	1	-0.309
ASPG	6	0.90656	0.90904	0.9995	18464	1	0.0262
CBS	4	0.90666	0.90656	0.9995	18465	0	-0.161
SLC35F3	6	0.90671	0.90918	0.9995	18466	1	-0.198
PCDHGA8	2	0.90672	0.90668	0.9995	18467	0	-1.137
RFPL4A	4	0.90673	0.90663	0.9995	18468	0	-0.826
hsa-mir-4783	4	0.90683	0.90673	0.9995	18469	0	-0.411
QPCT	6	0.90685	0.90929	0.9995	18470	1	-0.17
ASAH1	6	0.9069	0.90933	0.9995	18471	1	-0.972
BRD1	6	0.9069	0.90933	0.9995	18472	1	-0.304
KRTAP9-7	5	0.90699	0.9072	0.9995	18473	0	-0.325
PAX7	6	0.90712	0.9095	0.9995	18474	1	-0.156
SYT4	6	0.9072	0.90957	0.9995	18475	1	-0.015
USP46	6	0.90732	0.90966	0.9995	18476	1	-0.007
KRTAP26-1	6	0.90732	0.90966	0.9995	18477	1	-0.381
TMEM69	6	0.90735	0.90968	0.9995	18478	1	-0.087
HIST3H2A	6	0.90746	0.90978	0.9995	18479	1	-0.486
SOX1	6	0.90746	0.90978	0.9995	18480	1	-0.342
hsa-mir-6727	4	0.9075	0.9074	0.9995	18481	0	-0.085
TESK1	6	0.90753	0.90983	0.9995	18482	1	-0.357
SNX33	6	0.90768	0.90994	0.9995	18483	1	-0.133
WDR78	6	0.90774	0.90999	0.9995	18484	1	-0.094
AGPAT6	6	0.90781	0.91004	0.9995	18485	1	-0.168
hsa-mir-4674	4	0.90784	0.90775	0.9995	18486	0	-0.238
PRF1	6	0.90786	0.91007	0.9995	18487	1	-0.033
UHRF1	6	0.90792	0.91012	0.9995	18488	1	-0.066
ZNF138	6	0.90798	0.91017	0.9995	18489	1	-0.826
TNFRSF14	6	0.90805	0.91022	0.9995	18490	1	-0.974
MAT2B	6	0.90805	0.91022	0.9995	18491	1	-0.024
SHISA5	6	0.90818	0.91033	0.9995	18492	1	-0.444
GPR6	6	0.90835	0.91046	0.9995	18493	1	-0.344
HIST1H2BC	6	0.90835	0.91046	0.9995	18494	1	-0.957
GLIPR2	6	0.90841	0.91051	0.9995	18495	1	-0.217
LMAN1	6	0.90848	0.91057	0.9995	18496	1	-0.243
RNASEH2B	6	0.90851	0.91059	0.9995	18497	1	-0.015
hsa-mir-548i-4	3	0.90856	0.90862	0.9995	18498	0	-1.074
EFCC1	6	0.90872	0.91077	0.9995	18499	1	0.1091
hsa-mir-3162	4	0.90879	0.90868	0.9995	18500	0	-0.276
OBP2B	3	0.9088	0.90885	0.9995	18501	0	-1.233
RAB23	6	0.90881	0.91084	0.9995	18502	1	-0.631
FCRL5	6	0.90893	0.91093	0.9995	18503	1	-0.025
TWF1	6	0.90902	0.911	0.9995	18504	1	-0.131
PENK	6	0.90906	0.91104	0.9995	18505	1	-0.23
CSMD3	6	0.90917	0.91113	0.9995	18506	1	-0.293
NKX3-1	6	0.90925	0.91119	0.9995	18507	1	-0.043
ALDH3A1	6	0.90927	0.9112	0.9995	18508	1	-0.099
VEPH1	6	0.90932	0.91124	0.9995	18509	1	-0.03
MFSD6L	6	0.90936	0.91127	0.9995	18510	1	-0.186
ABCD1	6	0.90936	0.91127	0.9995	18511	1	-0.412
hsa-mir-1915	4	0.90942	0.9093	0.9995	18512	0	-0.348
hsa-mir-8082	4	0.90943	0.90932	0.9995	18513	0	-0.518
KCNJ3	6	0.90945	0.91135	0.9995	18514	1	-0.37
CYB5D1	6	0.90953	0.91141	0.9995	18515	1	-0.239
MANEA	6	0.90956	0.91144	0.9995	18516	1	-0.279
NEK10	6	0.90962	0.91149	0.9995	18517	1	-0.148
CLDN14	6	0.90969	0.91155	0.9995	18518	1	-0.369
LRRC10B	6	0.90973	0.91156	0.9995	18519	1	-0.048
SLC36A4	6	0.90979	0.91162	0.9995	18520	1	-0.086
NKAIN3	6	0.90983	0.91165	0.9995	18521	1	-0.328
ATP2A3	6	0.90987	0.91168	0.9995	18522	1	-0.059
GRM7	6	0.9099	0.91171	0.9995	18523	1	0.0716
ZNF468	5	0.90993	0.91014	0.9995	18524	0	-0.535
NEK9	6	0.90994	0.91174	0.9995	18525	1	-0.231

DHX33	6	0.91002	0.91181	0.9995	18526	1	-0.411
PLAU	3	0.91002	0.91011	0.9995	18527	0	-0.562
ATHL1	6	0.91024	0.91199	0.9995	18528	1	-0.198
POLR3E	6	0.91032	0.91206	0.9995	18529	1	-0.485
RMI2	4	0.91036	0.91022	0.9995	18530	0	-0.07
ZNF20	5	0.91046	0.91067	0.9995	18531	0	-1.233
CCDC70	6	0.91052	0.91224	0.9995	18532	1	-0.135
PRTN3	6	0.91056	0.91226	0.9995	18533	1	-0.17
IRX1	6	0.91059	0.91229	0.9995	18534	1	-0.375
hsa-mir-663a	4	0.91062	0.91048	0.9995	18535	0	-0.767
hsa-mir-654	4	0.91063	0.91049	0.9995	18536	0	-0.521
MRPS35	6	0.91065	0.91234	0.9995	18537	1	-0.333
FZD1	6	0.91065	0.91234	0.9995	18538	1	-0.387
PSMC3IP	6	0.91073	0.91241	0.9995	18539	1	-0.197
hsa-mir-4288	4	0.91075	0.91062	0.9995	18540	0	-0.221
TMCO1	6	0.91076	0.91243	0.9995	18541	1	-0.237
BEND2	6	0.91076	0.91243	0.9995	18542	1	0.0377
hsa-mir-4719	1	0.9108	0.91116	0.9995	18543	0	-1.458
hsa-mir-4292	4	0.9108	0.91066	0.9995	18544	0	-0.567
MUTYH	6	0.91092	0.91256	0.9995	18545	1	-0.004
OR51L1	6	0.91098	0.91263	0.9995	18546	1	-0.595
FAM115A	6	0.91102	0.91267	0.9995	18547	1	-0.04
KRTAP4-11	3	0.91106	0.91113	0.9995	18548	0	-1.233
hsa-mir-4759	3	0.91106	0.91113	0.9995	18549	0	-0.419
hsa-mir-520a	3	0.91106	0.91113	0.9995	18550	0	-0.715
CXorf40B	3	0.91106	0.91113	0.9995	18551	0	-0.584
hsa-mir-320e	3	0.91106	0.91113	0.9995	18552	0	-1.233
RFPL4AL1	3	0.91106	0.91113	0.9995	18553	0	-0.419
hsa-mir-548y	3	0.91106	0.91113	0.9995	18554	0	-0.799
hsa-mir-521-2	3	0.91106	0.91113	0.9995	18555	0	-1.233
CNOT1	6	0.91109	0.91273	0.9995	18556	1	0.0854
GRHL1	6	0.91111	0.91275	0.9995	18557	1	-0.372
NSL1	6	0.91112	0.91276	0.9995	18558	1	-0.563
CEP192	6	0.91114	0.91277	0.9995	18559	1	0.1256
APOBEC1	6	0.91118	0.91282	0.9995	18560	1	0.0196
SFXN1	6	0.91125	0.91287	0.9995	18561	1	-0.245
BRI3BP	6	0.91126	0.91288	0.9995	18562	1	-0.193
BRCC3	6	0.91129	0.9129	0.9995	18563	1	-0.307
hsa-let-7f-2	4	0.9113	0.91115	0.9995	18564	0	-0.277
EIF4G2	6	0.91134	0.91295	0.9995	18565	1	0.0163
KRT12	6	0.91145	0.91305	0.9995	18566	1	-0.352
CBX1	6	0.91148	0.91306	0.9995	18567	1	0.088
RBM42	6	0.91156	0.91312	0.9995	18568	1	-0.485
GDPD2	6	0.91162	0.91317	0.9995	18569	1	-0.3
hsa-mir-1273h	4	0.91165	0.91151	0.9995	18570	0	-0.919
CDIP1	6	0.91168	0.91323	0.9995	18571	1	0.0733
VIPR1	6	0.9117	0.91323	0.9995	18572	1	-0.209
C14orf180	6	0.91171	0.91325	0.9995	18573	1	-0.133
INHBB	6	0.91175	0.91328	0.9995	18574	1	-0.116
hsa-mir-191	4	0.91175	0.91162	0.9995	18575	0	-0.169
RNF180	6	0.91182	0.91335	0.9995	18576	1	-0.045
OR1N2	6	0.91184	0.91336	0.9995	18577	1	-0.558
WSCD1	6	0.91199	0.9135	0.9995	18578	1	-0.605
MAPK3	6	0.91222	0.91371	0.9995	18579	1	-0.63
CACNG1	6	0.91224	0.91372	0.9995	18580	1	-0.273
KIAA1549L	6	0.91226	0.91374	0.9995	18581	1	-0.074
hsa-mir-374a	4	0.9123	0.91218	0.9995	18582	0	-0.726
CNKSR1	6	0.91232	0.9138	0.9995	18583	1	0.0251
TRNP1	6	0.91236	0.91383	0.9995	18584	1	-0.426
AIMP1	6	0.9124	0.91386	0.9995	18585	1	-0.294
ECHDC1	6	0.9124	0.91386	0.9995	18586	1	-0.098
MSL3	6	0.91241	0.91388	0.9995	18587	1	-0.059
RBM23	6	0.91244	0.9139	0.9995	18588	1	-0.237
PCSK4	6	0.91256	0.914	0.9995	18589	1	0.0476
TNFRSF21	6	0.9127	0.91412	0.9995	18590	1	0.0973
MON1A	6	0.91275	0.91415	0.9995	18591	1	0.0566
RNF39	6	0.91282	0.91421	0.9995	18592	1	-0.207
PI4KA	6	0.91284	0.91423	0.9995	18593	1	-9E-04
ZCCHC14	6	0.91286	0.91424	0.9995	18594	1	0.0925
F13B	6	0.91288	0.91427	0.9995	18595	1	-0.679
hsa-mir-330	4	0.913	0.91287	0.9995	18596	0	-0.223
ORAI3	6	0.91303	0.91437	0.9995	18597	1	-0.227
TMEM199	6	0.91305	0.91439	0.9995	18598	1	-0.48
SERPINB10	6	0.91305	0.91439	0.9995	18599	1	-0.103
CAMKK1	6	0.91305	0.91439	0.9995	18600	1	0.0133
APTX	6	0.9131	0.91444	0.9995	18601	1	-0.429
PRICKLE2	6	0.9133	0.9146	0.9995	18602	1	-0.111
KCNA4	6	0.91342	0.91471	0.9995	18603	1	-0.11
MYCBPAP	6	0.91346	0.91475	0.9995	18604	1	-0.249
NPY4R	6	0.91348	0.91477	0.9995	18605	1	-0.179
SKAP2	6	0.91351	0.9148	0.9995	18606	1	-0.287
RHOT1	5	0.91361	0.91387	0.9995	18607	0	-0.418
hsa-mir-3177	4	0.91361	0.91349	0.9995	18608	0	-0.087
OR4K1	6	0.91368	0.91493	0.9995	18609	1	-0.29
L1CAM	6	0.91368	0.91493	0.9995	18610	1	-0.313

CRISP3	6	0.91376	0.915	0.9995	18611	1	-0.276
SLC24A5	6	0.914	0.91521	0.9995	18612	1	-0.438
PUF60	6	0.91404	0.91524	0.9995	18613	1	-0.244
DDAH2	6	0.91408	0.91527	0.9995	18614	1	-0.227
TAS2R8	6	0.91408	0.91527	0.9995	18615	1	-0.512
C2CD2L	6	0.91413	0.91532	0.9995	18616	1	0.0892
TSPAN17	6	0.91413	0.91532	0.9995	18617	1	-0.1
hsa-mir-133a-2	4	0.91427	0.91417	0.9995	18618	0	-0.199
hsa-mir-7162	4	0.91434	0.91424	0.9995	18619	0	-0.398
TP53BP1	6	0.91462	0.91574	0.9995	18620	1	-0.129
TFR2	6	0.91465	0.91576	0.9995	18621	1	-0.301
ALG10B	5	0.91471	0.91497	0.9995	18622	0	-0.549
ADAM19	6	0.91471	0.91581	0.9995	18623	1	0.0653
CCDC116	6	0.91479	0.91587	0.9995	18624	1	-0.426
SRP9	6	0.91487	0.91594	0.9995	18625	1	-0.91
INIP	6	0.91491	0.91597	0.9995	18626	1	-0.21
hsa-mir-3131	4	0.91492	0.91483	0.9995	18627	0	-0.397
PDCL	6	0.91502	0.91607	0.9995	18628	1	-0.165
IKZF5	6	0.91507	0.91611	0.9995	18629	1	-0.193
KHSRP	6	0.91522	0.91625	0.9995	18630	1	-0.641
PSAP	6	0.91533	0.91634	0.9995	18631	1	-0.375
TNKS	6	0.91537	0.91637	0.9995	18632	1	-0.204
SIVA1	6	0.91539	0.91639	0.9995	18633	1	-0.142
RSG1	6	0.91551	0.91651	0.9995	18634	1	-0.095
CRHR1	4	0.91562	0.91556	0.9995	18635	0	-0.518
P2RX4	6	0.91567	0.91665	0.9995	18636	1	-0.765
KIAA0232	6	0.91573	0.9167	0.9995	18637	1	-0.402
VSX1	6	0.91573	0.9167	0.9995	18638	1	-0.168
ZSCAN25	6	0.91583	0.91679	0.9995	18639	1	-0.156
GAB2	6	0.9159	0.91683	0.9995	18640	1	0.0283
C2orf71	6	0.91592	0.91686	0.9995	18641	1	-0.735
DMXL2	6	0.91595	0.91688	0.9995	18642	1	-0.269
DUSP13	6	0.91608	0.91698	0.9995	18643	1	0.052
UNC45B	6	0.91612	0.91701	0.9995	18644	1	0.009
TEX35	6	0.91621	0.91709	0.9995	18645	1	-0.118
RPTN	6	0.9163	0.91718	0.9995	18646	1	-0.46
CMC2	5	0.9163	0.91656	0.9995	18647	0	0.0631
RELB	6	0.91632	0.9172	0.9995	18648	1	-0.207
SLC35G1	6	0.91637	0.91725	0.9995	18649	1	-0.127
PTCH2	6	0.91638	0.91726	0.9995	18650	1	-0.26
GBP4	6	0.91646	0.91733	0.9995	18651	1	-0.068
ANKRD65	6	0.91651	0.91737	0.9995	18652	1	-0.56
OR2AE1	6	0.91657	0.91743	0.9995	18653	1	-0.01
hsa-mir-8083	4	0.91662	0.91659	0.9995	18654	0	-1.36
TRIM40	6	0.91679	0.91761	0.9995	18655	1	-0.12
VWA9	6	0.91692	0.91773	0.9995	18656	1	-0.109
F11R	6	0.91696	0.91776	0.9995	18657	1	-0.465
hsa-mir-4637	3	0.91698	0.91704	0.9995	18658	0	-0.361
CACNA2D3	6	0.91699	0.91778	0.9995	18659	1	-0.073
UMOD	6	0.91704	0.91783	0.9995	18660	1	-0.663
hsa-mir-1247	4	0.91706	0.91703	0.9995	18661	0	-0.371
CXorf57	6	0.91707	0.91785	0.9995	18662	1	-0.028
C4orf48	6	0.9171	0.91788	0.9995	18663	1	0.0694
SNPH	6	0.9171	0.91788	0.9995	18664	1	0.0426
GMEB1	6	0.9171	0.91788	0.9995	18665	1	-0.36
ZNF469	6	0.91714	0.91793	0.9995	18666	1	-0.842
LSM3	6	0.91714	0.91793	0.9995	18667	1	-0.797
DIRAS3	6	0.91726	0.91802	0.9995	18668	1	-0.089
CPNE4	6	0.91729	0.91805	0.9995	18669	1	-0.249
HOXD13	6	0.91747	0.91821	0.9995	18670	1	-0.427
ARHGEF16	6	0.91752	0.91825	0.9995	18671	1	-0.196
MOCOS	6	0.91752	0.91825	0.9995	18672	1	-0.29
CCDC87	6	0.91758	0.9183	0.9995	18673	1	0.0416
AAR2	6	0.91758	0.9183	0.9995	18674	1	-0.008
FAM127C	5	0.91761	0.91785	0.9995	18675	0	-0.419
KCNJ4	6	0.91765	0.91835	0.9995	18676	1	-0.6
PDE6D	6	0.91766	0.91837	0.9995	18677	1	-0.403
hsa-mir-4681	4	0.91769	0.91765	0.9995	18678	0	-0.09
ASGR2	6	0.91771	0.91841	0.9995	18679	1	-0.448
ACTL6A	6	0.91771	0.91841	0.9995	18680	1	-0.23
MCRS1	6	0.91773	0.91843	0.9995	18681	1	0.0206
RTBDN	6	0.91775	0.91845	0.9995	18682	1	-0.357
GNB5	6	0.91779	0.91849	0.9995	18683	1	-0.479
RERE	6	0.91779	0.91849	0.9995	18684	1	-0.177
POMZP3	5	0.91788	0.91813	0.9995	18685	0	-0.595
FSTL4	6	0.91796	0.91864	0.9995	18686	1	-0.228
DUOX2	6	0.91806	0.91872	0.9995	18687	1	-0.158
LAGE3	6	0.9181	0.91876	0.9995	18688	1	-0.194
FCGR3B	6	0.91812	0.91877	0.9995	18689	1	-0.339
KLK3	6	0.91815	0.9188	0.9995	18690	1	-0.274
KRTAP12-3	6	0.91822	0.91887	0.9995	18691	1	-0.307
hsa-mir-4447	4	0.91823	0.91818	0.9995	18692	0	-0.375
TPSG1	6	0.91831	0.91894	0.9995	18693	1	-0.019
ZNF493	5	0.91834	0.91859	0.9995	18694	0	-1.185
GORASP2	6	0.91838	0.919	0.9995	18695	1	-0.465

hsa-mir-3925	3	0.91839	0.91847	0.9995	18696	0	-1.233
C6orf70	6	0.9185	0.91912	0.9995	18697	1	-0.003
C16orf71	6	0.91853	0.91914	0.9995	18698	1	-0.363
MC1R	6	0.91855	0.91916	0.9995	18699	1	0.0192
KITLG	5	0.91858	0.91883	0.9995	18700	0	-0.469
DDX25	6	0.91867	0.91928	0.9995	18701	1	0.0269
LRIG1	6	0.91867	0.91928	0.9995	18702	1	-0.314
CDH19	6	0.91889	0.9195	0.9995	18703	1	-0.051
LIPI	6	0.91889	0.9195	0.9995	18704	1	-0.169
HIST1H3I	5	0.91889	0.91917	0.9995	18705	0	-0.552
PKNOX2	6	0.91893	0.91954	0.9995	18706	1	-0.393
TNNC1	6	0.91897	0.91957	0.9995	18707	1	-0.227
TMEM38A	6	0.91901	0.91961	0.9995	18708	1	-1.022
ESPN	6	0.91911	0.91969	0.9995	18709	1	-0.123
hsa-mir-4725	4	0.9192	0.91916	0.9995	18710	0	-0.268
CAMTA2	6	0.9192	0.91977	0.9995	18711	1	-0.683
CEP120	6	0.91928	0.91983	0.9995	18712	1	-0.093
PORCN	6	0.91931	0.91986	0.9995	18713	1	-0.016
hsa-mir-140	4	0.91937	0.91933	0.9995	18714	0	-0.644
CALCOCO1	6	0.91948	0.92	0.9995	18715	1	0.0182
ITGA2	6	0.91948	0.92	0.9995	18716	1	-0.186
TXLNA	6	0.91955	0.92006	0.9995	18717	1	-0.508
PHC2	6	0.91956	0.92008	0.9995	18718	1	-0.457
TMEM14C	5	0.91956	0.91987	0.9995	18719	0	-0.001
DCLRE1C	6	0.91957	0.92009	0.9995	18720	1	0.0341
ARHGAP20	6	0.91964	0.92014	0.9995	18721	1	-0.125
CREB3L1	4	0.91967	0.91967	0.9995	18722	0	-0.144
FAM8A1	6	0.91968	0.92018	0.9995	18723	1	-0.043
CTAGE6	4	0.91981	0.91982	0.9995	18724	0	-0.324
CCDC68	6	0.91985	0.92034	0.9995	18725	1	-0.226
GTPBP3	6	0.91987	0.92035	0.9995	18726	1	-0.069
ARAF	6	0.9199	0.92039	0.9995	18727	1	-0.413
E2F3	6	0.91993	0.92042	0.9995	18728	1	-0.11
FAM71E1	6	0.91998	0.92046	0.9995	18729	1	-0.25
CLEC14A	6	0.92009	0.92057	0.9995	18730	1	-0.123
NEGR1	6	0.9201	0.92058	0.9995	18731	1	0.0719
FAM110D	6	0.9201	0.92058	0.9995	18732	1	-0.372
hsa-mir-4295	4	0.9202	0.92021	0.9995	18733	0	-0.512
RNF157	6	0.92021	0.92068	0.9995	18734	1	-0.401
FBXO10	6	0.92023	0.92071	0.9995	18735	1	-0.164
PAX2	6	0.92037	0.92084	0.9995	18736	1	-0.002
TIGIT	6	0.92037	0.92084	0.9995	18737	1	-0.299
OSBPL7	6	0.92039	0.92087	0.9995	18738	1	-0.155
FUK	6	0.92051	0.92097	0.9995	18739	1	-0.548
C3orf80	6	0.92051	0.92097	0.9995	18740	1	-0.374
ZNF562	5	0.92073	0.92106	0.9995	18741	0	-0.014
INPPL1	6	0.92085	0.92127	0.9995	18742	1	-0.031
TMEM200A	6	0.92088	0.9213	0.9995	18743	1	-0.196
BIRC8	6	0.92088	0.9213	0.9995	18744	1	-0.235
VASH2	6	0.92091	0.92133	0.9995	18745	1	-0.044
ZSWIM7	6	0.92106	0.92147	0.9995	18746	1	-0.526
CD9	6	0.92109	0.92149	0.9995	18747	1	-0.132
DYM	6	0.92118	0.92157	0.9995	18748	1	-0.274
MICU3	6	0.92124	0.92163	0.9995	18749	1	0.0026
hsa-mir-3170	4	0.92125	0.92127	0.9995	18750	0	-0.141
TNIK	6	0.92127	0.92166	0.9995	18751	1	-0.341
NLGN4Y	6	0.9213	0.92169	0.9995	18752	1	-0.466
ULK2	6	0.92135	0.92173	0.9995	18753	1	-0.18
AQP5	6	0.92138	0.92176	0.9995	18754	1	0.047
OR9Q2	6	0.92142	0.9218	0.9995	18755	1	-0.15
SERINC5	6	0.92142	0.9218	0.9995	18756	1	-0.251
TREM1	6	0.92149	0.92186	0.9995	18757	1	-0.41
OR2T35	5	0.92161	0.92193	0.9995	18758	0	-1.233
PLK5	6	0.92161	0.92197	0.9995	18759	1	0.0631
HSF2BP	6	0.92165	0.922	0.9995	18760	1	-0.151
CCDC65	6	0.9217	0.92204	0.9995	18761	1	0.0386
GABRA1	6	0.92172	0.92205	0.9995	18762	1	-0.2
RASSF8	6	0.92174	0.92207	0.9995	18763	1	0.0239
BACH2	6	0.92186	0.92217	0.9995	18764	1	0.1095
TGFB1I1	6	0.92189	0.9222	0.9995	18765	1	-0.451
EMILIN3	6	0.92192	0.92223	0.9995	18766	1	-0.073
TTC28	4	0.92196	0.92203	0.9995	18767	0	-0.288
THSD4	6	0.92197	0.92228	0.9995	18768	1	-0.355
NKIRAS1	6	0.92209	0.92238	0.9995	18769	1	0.0541
TNNI3	6	0.92214	0.92242	0.9995	18770	1	-0.126
MRPL20	6	0.92214	0.92242	0.9995	18771	1	-0.51
HMGN4	6	0.92214	0.92242	0.9995	18772	1	-0.253
OSBPL1A	6	0.92219	0.92248	0.9995	18773	1	-0.304
ARMCX2	6	0.92226	0.92253	0.9995	18774	1	-0.138
EXOC6	6	0.92227	0.92255	0.9995	18775	1	-0.101
MYOZ3	6	0.92231	0.92259	0.9995	18776	1	-0.516
ADORA2B	6	0.92239	0.92266	0.9995	18777	1	0.1396
PROM1	6	0.92248	0.92275	0.9995	18778	1	-0.338
SLC29A2	6	0.9225	0.92277	0.9995	18779	1	-0.296
SGSM2	6	0.92253	0.92281	0.9995	18780	1	-0.401

hsa-mir-1324	4	0.92257	0.92262	0.9995	18781	0	-1
hsa-mir-1243	4	0.92266	0.92271	0.9995	18782	0	-0.15
SLC12A2	6	0.92271	0.92299	0.9995	18783	1	-0.613
SYTL1	6	0.92275	0.92302	0.9995	18784	1	-0.13
hsa-mir-6849	4	0.92279	0.92284	0.9995	18785	0	-0.499
MYH2	6	0.92279	0.92306	0.9995	18786	1	-0.188
hsa-mir-6835	2	0.92281	0.92273	0.9995	18787	0	-0.599
YKT6	6	0.92281	0.92307	0.9995	18788	1	-0.71
C10orf95	6	0.92283	0.92309	0.9995	18789	1	-0.238
ARHGAP27	6	0.92292	0.92318	0.9995	18790	1	-0.249
IL2RA	6	0.92292	0.92318	0.9995	18791	1	-0.921
NXF5	6	0.92298	0.92323	0.9995	18792	1	-0.142
OR4K14	6	0.92298	0.92323	0.9995	18793	1	-0.484
KIR2DL4	2	0.92306	0.923	0.9995	18794	0	-0.365
NEK4	6	0.92307	0.92332	0.9995	18795	1	-0.021
CORO1C	6	0.92314	0.92339	0.9995	18796	1	-0.113
CCT6B	6	0.92314	0.92339	0.9995	18797	1	-0.331
HS3ST3B1	4	0.92321	0.92331	0.9995	18798	0	-0.403
SLC23A2	6	0.92322	0.92346	0.9995	18799	1	-0.358
PLSCR2	6	0.92324	0.92348	0.9995	18800	1	-0.066
TMEM86A	6	0.92327	0.9235	0.9995	18801	1	-0.502
URI1	6	0.92327	0.9235	0.9995	18802	1	0.0724
ZNF70	6	0.92336	0.92359	0.9995	18803	1	-0.153
NEUROD4	6	0.92344	0.92368	0.9995	18804	1	0.069
OR4N4	6	0.92353	0.92376	0.9995	18805	1	-0.6
ETFDH	6	0.92358	0.92381	0.9995	18806	1	-0.119
KIAA1161	6	0.92367	0.9239	0.9995	18807	1	-0.114
MEOX2	6	0.92374	0.92397	0.9995	18808	1	-0.098
CLDN4	6	0.92379	0.92401	0.9995	18809	1	0.0723
FAM221B	6	0.92383	0.92404	0.9995	18810	1	-0.449
HIF1AN	6	0.92383	0.92404	0.9995	18811	1	-0.035
HRCT1	4	0.92384	0.92392	0.9995	18812	0	-0.484
PRUNE	6	0.92385	0.92407	0.9995	18813	1	-0.059
XAGE5	6	0.92387	0.92408	0.9995	18814	1	-0.515
GSR	6	0.92389	0.9241	0.9995	18815	1	-0.137
CNTNAP3B	4	0.92393	0.92402	0.9995	18816	0	-1.233
MCTP2	6	0.92398	0.9242	0.9995	18817	1	-0.103
PLAA	6	0.92401	0.92422	0.9995	18818	1	-0.109
HELZ	6	0.92401	0.92422	0.9995	18819	1	0.1708
C15orf57	6	0.92404	0.92426	0.9995	18820	1	-0.256
ASIC3	6	0.92415	0.92436	0.9995	18821	1	-0.069
hsa-mir-891a	4	0.9242	0.92428	0.9995	18822	0	-1.178
HSPBAP1	6	0.92426	0.92447	0.9995	18823	1	-0.339
KCNB2	6	0.92428	0.9245	0.9995	18824	1	-0.319
PRB2	3	0.92433	0.92442	0.9995	18825	0	-0.341
RASL10B	6	0.92434	0.92455	0.9995	18826	1	-0.31
NMRAL1	6	0.92447	0.92469	0.9995	18827	1	-0.106
TBC1D7	6	0.92461	0.92483	0.9995	18828	1	-0.352
CLPX	6	0.92463	0.92484	0.9995	18829	1	-0.369
hsa-mir-2682	4	0.92465	0.92472	0.9995	18830	0	-0.225
FGF8	6	0.92466	0.92488	0.9995	18831	1	-0.307
PYGL	6	0.92474	0.92495	0.9995	18832	1	-0.222
LRRC8E	6	0.92492	0.92512	0.9995	18833	1	-0.005
FSBP	6	0.92492	0.92512	0.9995	18834	1	-0.411
RP1	6	0.92494	0.92515	0.9995	18835	1	-0.29
IRAK2	6	0.925	0.92521	0.9995	18836	1	-0.433
GTF2H4	6	0.92516	0.92537	0.9995	18837	1	-0.027
hsa-mir-4758	4	0.92517	0.92525	0.9995	18838	0	-0.398
SLC6A12	6	0.92525	0.92544	0.9995	18839	1	-0.462
RERGL	6	0.9253	0.92548	0.9995	18840	1	-0.353
SLC22A13	6	0.9253	0.92548	0.9995	18841	1	0.0547
TPM4	6	0.92536	0.92555	0.9995	18842	1	0.0554
TMCO2	6	0.92536	0.92555	0.9995	18843	1	-0.479
ATP5SL	6	0.92536	0.92555	0.9995	18844	1	-0.106
PRAP1	6	0.92544	0.92563	0.9995	18845	1	-0.311
hsa-mir-8076	4	0.92545	0.92552	0.9995	18846	0	-0.465
VN1R5	6	0.9255	0.92568	0.9995	18847	1	-0.044
TYW1	6	0.92552	0.9257	0.9995	18848	1	0.1406
PLCB2	6	0.92553	0.92571	0.9995	18849	1	-0.142
CENPC1	4	0.92565	0.92573	0.9995	18850	0	-0.794
SYT5	6	0.92567	0.92585	0.9995	18851	1	-0.072
LOC100287534	2	0.9257	0.9256	0.9995	18852	0	-0.354
hsa-mir-1260b	4	0.92571	0.92579	0.9995	18853	0	-0.45
TTLL12	6	0.92574	0.92593	0.9995	18854	1	-0.051
hsa-mir-3714	4	0.92579	0.92587	0.9995	18855	0	-0.376
hsa-mir-5696	3	0.92592	0.92598	0.9995	18856	0	-0.748
PSG8	6	0.92593	0.9261	0.9995	18857	1	-0.708
TCFL5	6	0.92593	0.9261	0.9995	18858	1	-0.2
MUC21	6	0.92593	0.9261	0.9995	18859	1	0.1123
IL12RB2	6	0.92606	0.92622	0.9995	18860	1	-0.193
BABAM1	6	0.9262	0.92636	0.9995	18861	1	-0.779
hsa-mir-5004	4	0.9263	0.92637	0.9995	18862	0	-0.76
CELA2A	6	0.92631	0.92645	0.9995	18863	1	-0.133
hsa-mir-7702	3	0.92635	0.92643	0.9995	18864	0	-0.74
SENP2	6	0.92636	0.9265	0.9995	18865	1	-0.195

hsa-mir-548k	4	0.92639	0.92647	0.9995	18866	0	-0.204
ZNF836	6	0.9264	0.92655	0.9995	18867	1	-0.948
KCNK15	6	0.92644	0.92659	0.9995	18868	1	-0.294
ANKRD45	6	0.92658	0.92674	0.9995	18869	1	0.0913
CSRNP3	6	0.9266	0.92676	0.9995	18870	1	-0.251
PSMD9	6	0.92668	0.92683	0.9995	18871	1	-0.186
PDPN	6	0.92668	0.92683	0.9995	18872	1	-0.285
DDX6	6	0.92672	0.92686	0.9995	18873	1	-0.165
ARHGEF39	6	0.92672	0.92686	0.9995	18874	1	-0.161
HAX1	6	0.92672	0.92686	0.9995	18875	1	0.0412
MYBL2	6	0.92675	0.9269	0.9995	18876	1	-0.128
ASPA	6	0.92677	0.92691	0.9995	18877	1	-0.058
GPR21	6	0.92688	0.92703	0.9995	18878	1	-0.466
B3GNTL1	6	0.92692	0.92706	0.9995	18879	1	-0.398
ATP2B3	6	0.92694	0.92708	0.9995	18880	1	-0.213
hsa-mir-1268a	4	0.92697	0.92707	0.9995	18881	0	-0.257
ATF2	6	0.92697	0.92712	0.9995	18882	1	-0.193
hsa-mir-4517	4	0.92704	0.92714	0.9995	18883	0	-0.53
SPATA8	6	0.92705	0.92718	0.9995	18884	1	-0.564
C10orf12	6	0.92705	0.92718	0.9995	18885	1	0.0788
XYLB	6	0.92719	0.92732	0.9995	18886	1	-0.22
FMOD	6	0.92722	0.92736	0.9995	18887	1	-0.124
C10orf90	6	0.92737	0.92749	0.9995	18888	1	-0.19
CNNM1	6	0.9274	0.92751	0.9995	18889	1	-0.183
PKIA	6	0.92743	0.92754	0.9995	18890	1	-0.384
TPM2	6	0.92747	0.92758	0.9995	18891	1	-0.1
OSBPL3	6	0.92753	0.92765	0.9995	18892	1	-0.211
P4HB	4	0.92754	0.92763	0.9995	18893	0	-0.493
RNF41	6	0.92756	0.92768	0.9995	18894	1	-0.229
IGSF10	6	0.92761	0.92772	0.9995	18895	1	-0.042
COLGALT2	6	0.92761	0.92772	0.9995	18896	1	-0.14
DOCK1	6	0.92767	0.92779	0.9995	18897	1	-0.16
FAM81A	6	0.92767	0.92779	0.9995	18898	1	-0.284
PRAF2	6	0.92769	0.9278	0.9995	18899	1	-0.285
MYF5	6	0.92775	0.92787	0.9995	18900	1	-0.44
CCL28	6	0.92777	0.92789	0.9995	18901	1	0.1097
PUM2	6	0.92779	0.92791	0.9995	18902	1	-0.183
hsa-mir-548a-1	4	0.9278	0.92791	0.9995	18903	0	-0.374
LCE1E	4	0.9278	0.92791	0.9995	18904	0	-0.419
hsa-mir-3681	4	0.9278	0.92791	0.9995	18905	0	-1.638
ZDHHC2	6	0.92782	0.92793	0.9995	18906	1	-0.219
TTC16	6	0.92783	0.92794	0.9995	18907	1	-0.307
IER3IP1	6	0.92784	0.92795	0.9995	18908	1	-0.257
APEH	6	0.92787	0.92798	0.9995	18909	1	-0.473
STX11	6	0.92796	0.92809	0.9995	18910	1	-0.378
UBE2J2	6	0.92798	0.9281	0.9995	18911	1	-0.502
GADD45B	4	0.92809	0.9282	0.9995	18912	0	-0.436
HS2ST1	6	0.92811	0.92821	0.9995	18913	1	-0.568
EMC2	6	0.92818	0.92828	0.9995	18914	1	-0.495
hsa-mir-4259	4	0.92828	0.92838	0.9995	18915	0	-0.404
APOL3	6	0.92841	0.92853	0.9995	18916	1	-0.075
hsa-mir-507	4	0.92849	0.92858	0.9995	18917	0	-0.042
THAP3	6	0.92851	0.92862	0.9995	18918	0	-0.51
KCTD18	6	0.92851	0.92862	0.9995	18919	0	-0.157
CDCA4	6	0.92853	0.92864	0.9995	18920	0	-0.724
FREM2	6	0.92856	0.92868	0.9995	18921	0	0.0543
ZNF483	6	0.92858	0.92869	0.9995	18922	0	-0.212
hsa-mir-1236	4	0.92859	0.92867	0.9995	18923	0	-0.599
SHISA9	6	0.92862	0.92874	0.9995	18924	0	-0.065
COA6	6	0.92862	0.92874	0.9995	18925	0	-0.26
GOLGA3	6	0.92871	0.92883	0.9995	18926	0	-0.162
hsa-mir-6834	4	0.92878	0.92885	0.9995	18927	0	-0.474
STAT5A	6	0.92881	0.92893	0.9995	18928	0	-0.414
SRXN1	6	0.92892	0.92904	0.9995	18929	0	-0.027
COPB1	6	0.92895	0.92907	0.9995	18930	0	-0.362
KLHL34	6	0.92901	0.92913	0.9995	18931	0	-0.253
hsa-mir-4450	4	0.92903	0.92909	0.9995	18932	0	-0.044
GUCA1C	6	0.92908	0.92921	0.9995	18933	0	-0.62
OR4M2	5	0.92909	0.92943	0.9995	18934	0	-0.127
hsa-mir-1275	4	0.92916	0.92922	0.9995	18935	0	-0.503
ZNF736	6	0.9292	0.92934	0.9995	18936	0	-0.419
C1orf61	6	0.92927	0.9294	0.9995	18937	0	-0.07
ATG5	6	0.92933	0.92947	0.9995	18938	0	-0.144
OR5V1	6	0.92937	0.92951	0.9995	18939	0	-0.087
SYTL5	6	0.92948	0.92961	0.9995	18940	0	-0.029
FOXG1	4	0.92949	0.92953	0.9995	18941	0	-0.342
ZFP92	6	0.92955	0.92967	0.9995	18942	0	-0.131
PSMC1	6	0.9296	0.92973	0.9995	18943	0	-0.139
SYNPO2	6	0.9296	0.92973	0.9995	18944	0	-0.08
TMEM235	6	0.92973	0.92985	0.9995	18945	0	-0.272
C10orf120	6	0.92977	0.92989	0.9995	18946	0	-0.099
TEX13B	6	0.92977	0.92989	0.9995	18947	0	-0.247
DMWD	6	0.92977	0.92989	0.9995	18948	0	0.0384
CNKSR2	4	0.92981	0.92984	0.9995	18949	0	-0.172
SPNS2	6	0.92991	0.93003	0.9995	18950	0	-0.213

NPFF	6	0.93002	0.93013	0.9995	18951	0	-0.326
ANKIB1	6	0.93003	0.93014	0.9995	18952	0	-0.192
MRVI1	6	0.9301	0.93021	0.9995	18953	0	-0.45
ANKRD18A	6	0.93014	0.93025	0.9995	18954	0	-0.584
EMC6	6	0.93014	0.93025	0.9995	18955	0	-0.784
PARP16	6	0.93019	0.93029	0.9995	18956	0	-0.131
MRPS26	6	0.93027	0.93037	0.9995	18957	0	-0.348
IST1	6	0.93029	0.93041	0.9995	18958	0	-0.735
ZNF654	6	0.93034	0.93045	0.9995	18959	0	-0.691
ZFP14	6	0.93036	0.93047	0.9995	18960	0	0.0935
KIAA1191	6	0.93038	0.93049	0.9995	18961	0	-0.37
C17orf80	6	0.93043	0.93054	0.9995	18962	0	0.0271
FBXL2	6	0.9305	0.9306	0.9995	18963	0	-0.059
NOX5	5	0.9306	0.9309	0.9995	18964	0	-0.424
GABPB1	6	0.9306	0.9307	0.9995	18965	0	-0.243
PTPN7	6	0.9306	0.93071	0.9995	18966	0	-0.256
ORC5	6	0.93061	0.93072	0.9995	18967	0	-0.181
CCT7	6	0.93062	0.93073	0.9995	18968	0	-0.087
HIRA	6	0.93064	0.93074	0.9995	18969	0	-0.263
MCIDAS	6	0.93066	0.93077	0.9995	18970	0	-0.404
ST8SIA4	6	0.9308	0.93091	0.9995	18971	0	-0.265
CHRNB2	5	0.93083	0.93114	0.9995	18972	0	-0.111
C20orf201	6	0.93087	0.93098	0.9995	18973	0	-0.467
SLC25A48	6	0.93088	0.93099	0.9995	18974	0	-0.102
hsa-mir-3911	4	0.93088	0.93092	0.9995	18975	0	-0.344
MYH11	6	0.93091	0.93104	0.9995	18976	0	-0.505
TTLL3	4	0.93094	0.93097	0.9995	18977	0	-0.538
KRT86	4	0.93103	0.93106	0.9995	18978	0	-0.301
FOXN1	6	0.93111	0.93123	0.9995	18979	0	-0.365
ZFP106	6	0.93117	0.93129	0.9995	18980	0	-0.155
HFE2	6	0.93128	0.9314	0.9995	18981	0	-0.178
C1QTNF2	6	0.93138	0.93149	0.9995	18982	0	0.0478
DLG5	6	0.93139	0.93151	0.9995	18983	0	0.0528
SRM	6	0.93142	0.93153	0.9995	18984	0	-0.344
LCLAT1	6	0.93146	0.93157	0.9995	18985	0	-0.278
ZC3HAV1L	6	0.93146	0.93157	0.9995	18986	0	-0.019
C5orf15	6	0.93146	0.93157	0.9995	18987	0	-0.061
RAD9B	6	0.93146	0.93157	0.9995	18988	0	0.0446
hsa-mir-7153	4	0.93151	0.93153	0.9995	18989	0	-0.468
C10orf25	6	0.93161	0.93173	0.9995	18990	0	-0.401
CXXC11	6	0.93161	0.93173	0.9995	18991	0	-0.208
TSPEAR	6	0.93162	0.93174	0.9995	18992	0	-0.425
MED24	6	0.93164	0.93176	0.9995	18993	0	-0.182
IL12RB1	4	0.93167	0.93168	0.9995	18994	0	-0.277
CBLC	6	0.9317	0.93182	0.9995	18995	0	0.0728
DGCR8	6	0.93184	0.93198	0.9995	18996	0	-0.039
CYP19A1	6	0.93187	0.93201	0.9995	18997	0	-0.064
LYZL2	1	0.9319	0.93211	0.9995	18998	0	-0.861
EPX	6	0.93193	0.93206	0.9995	18999	0	-0.711
BMP1	6	0.932	0.93212	0.9995	19000	0	-0.214
NOV	6	0.932	0.93212	0.9995	19001	0	-0.145
hsa-mir-4297	4	0.93202	0.93201	0.9995	19002	0	-0.559
MB	6	0.93202	0.93214	0.9995	19003	0	-0.284
YAE1D1	6	0.93202	0.93214	0.9995	19004	0	-0.065
GABRA6	6	0.93219	0.93231	0.9995	19005	0	-0.036
DAZAP2	5	0.93219	0.93247	0.9995	19006	0	-0.292
SOGA1	6	0.93221	0.93233	0.9995	19007	0	-0.379
ZC4H2	6	0.93225	0.93238	0.9995	19008	0	-0.223
CDHR4	6	0.93225	0.93238	0.9995	19009	0	-0.426
COMTD1	6	0.93225	0.93238	0.9995	19010	0	-0.424
hsa-mir-6889	4	0.93228	0.93227	0.9995	19011	0	-0.105
37500	3	0.93233	0.93242	0.9995	19012	0	-0.482
FAM153A	2	0.93242	0.93235	0.9995	19013	0	-0.394
DCDC2B	6	0.93246	0.93257	0.9995	19014	0	-0.006
FGF10	6	0.93254	0.93264	0.9995	19015	0	-0.028
ZFYVE9	6	0.93256	0.93267	0.9995	19016	0	-0.323
hsa-mir-4489	4	0.93257	0.93254	0.9995	19017	0	-1.933
TMBIM6	6	0.93259	0.93269	0.9995	19018	0	-0.136
NFIX	6	0.9326	0.93271	0.9995	19019	0	-0.378
C4orf47	6	0.93262	0.93274	0.9995	19020	0	-0.659
CCDC88B	6	0.93264	0.93277	0.9995	19021	0	-0.212
KDM8	6	0.93264	0.93277	0.9995	19022	0	-0.124
FAM103A1	6	0.93266	0.93278	0.9995	19023	0	-0.454
ZNF35	6	0.93272	0.93283	0.9995	19024	0	-0.212
hsa-mir-940	4	0.93275	0.93272	0.9995	19025	0	-0.434
MAP3K1	6	0.93278	0.93289	0.9995	19026	0	-0.022
ARMCX1	6	0.93287	0.93299	0.9995	19027	0	-0.054
RABEP1	6	0.93294	0.93305	0.9995	19028	0	0.0112
DMGDH	6	0.93295	0.93306	0.9995	19029	0	-0.342
CLDN8	6	0.93305	0.93315	0.9995	19030	0	-0.335
EEF1D	6	0.93334	0.93344	0.9995	19031	0	-0.218
AEN	6	0.93351	0.93361	0.9995	19032	0	-0.19
hsa-mir-133b	4	0.93361	0.9336	0.9995	19033	0	-0.211
TGFBR3L	6	0.93365	0.93375	0.9995	19034	0	-0.11
NEFM	6	0.93372	0.93383	0.9995	19035	0	-0.194

SLC35A2	6	0.93386	0.93398	0.9995	19036	0	-0.329
C8G	6	0.93386	0.93398	0.9995	19037	0	-0.482
PAK3	6	0.93391	0.93403	0.9995	19038	0	-0.255
VEGFA	6	0.93391	0.93403	0.9995	19039	0	-0.129
NCAPG2	6	0.93394	0.93406	0.9995	19040	0	-0.238
RBM3	6	0.93395	0.93407	0.9995	19041	0	-0.497
PLEKHB1	6	0.934	0.93411	0.9995	19042	0	-0.905
FDCSP	6	0.93408	0.93419	0.9995	19043	0	-0.189
CCBP2	4	0.93411	0.93408	0.9995	19044	0	-0.368
KCNC1	6	0.93419	0.9343	0.9995	19045	0	-0.179
MTX2	6	0.93423	0.93433	0.9995	19046	0	-0.281
UROD	6	0.93425	0.93435	0.9995	19047	0	-0.4
TMEM74B	6	0.93427	0.93438	0.9995	19048	0	-0.159
LCE4A	6	0.9343	0.93441	0.9995	19049	0	-0.171
ARL15	6	0.93435	0.93445	0.9995	19050	0	-0.009
SPERT	6	0.93442	0.93453	0.9995	19051	0	-0.315
SYK	6	0.93448	0.93458	0.9995	19052	0	-0.555
CHRNA5	6	0.93452	0.93462	0.9995	19053	0	-0.305
FABP6	6	0.93452	0.93462	0.9995	19054	0	-0.22
IL4	6	0.93461	0.93471	0.9995	19055	0	-0.352
KAZN	6	0.9347	0.9348	0.9995	19056	0	-0.328
IFIT3	6	0.93472	0.93483	0.9995	19057	0	-0.064
C12orf23	6	0.93475	0.93485	0.9995	19058	0	-0.07
MCHR2	6	0.93479	0.93489	0.9995	19059	0	-0.062
MYO5C	6	0.93484	0.93494	0.9995	19060	0	-0.246
CTNS	6	0.93492	0.93502	0.9995	19061	0	-0.056
SUZ12	6	0.93494	0.93504	0.9995	19062	0	-0.871
OR51I1	6	0.93494	0.93504	0.9995	19063	0	-0.321
AP3B1	6	0.93496	0.93505	0.9995	19064	0	-0.297
CWC25	6	0.935	0.93509	0.9995	19065	0	-0.263
ARHGAP24	6	0.93505	0.93513	0.9995	19066	0	-0.119
NPY5R	6	0.93505	0.93513	0.9995	19067	0	-0.065
GHRL	6	0.9352	0.93527	0.9995	19068	0	-0.325
VWDE	6	0.93527	0.93535	0.9995	19069	0	0.0169
KLHL13	6	0.93527	0.93535	0.9995	19070	0	-0.217
PRDM6	6	0.93535	0.93543	0.9995	19071	0	-0.571
WISP2	6	0.93537	0.93544	0.9995	19072	0	-0.149
hsa-mir-184	4	0.93539	0.93533	0.9995	19073	0	-0.691
PCBP4	6	0.93548	0.93556	0.9995	19074	0	-0.419
MMP20	6	0.93559	0.93566	0.9995	19075	0	-0.491
CRAT	6	0.9357	0.93577	0.9995	19076	0	-0.235
ABHD16A	6	0.93573	0.9358	0.9995	19077	0	-0.194
hsa-mir-876	2	0.93573	0.93571	0.9995	19078	0	-0.461
VRTN	6	0.9358	0.93587	0.9995	19079	0	-0.224
HMBOX1	6	0.9358	0.93587	0.9995	19080	0	-0.48
TSC22D1	6	0.93584	0.9359	0.9995	19081	0	-0.115
EPPK1	6	0.93585	0.93592	0.9995	19082	0	-0.267
PDPR	6	0.93586	0.93592	0.9995	19083	0	-0.181
hsa-mir-4787	4	0.93588	0.93582	0.9995	19084	0	-0.281
GPX5	6	0.93591	0.93598	0.9995	19085	0	-0.07
SLC35D2	6	0.93595	0.93602	0.9995	19086	0	-0.07
POLR2F	6	0.93597	0.93604	0.9995	19087	0	-0.385
EGFR	6	0.93599	0.93606	0.9995	19088	0	-0.14
ZBTB48	6	0.93613	0.9362	0.9995	19089	0	-0.018
C11orf71	6	0.93615	0.93622	0.9995	19090	0	-0.244
ARMC9	6	0.9362	0.93627	0.9995	19091	0	-0.197
UBR2	6	0.93624	0.93632	0.9995	19092	0	-0.038
ABCB9	6	0.93625	0.93633	0.9995	19093	0	-0.176
hsa-mir-6730	4	0.93625	0.93617	0.9995	19094	0	-0.79
GFAP	6	0.93631	0.93637	0.9995	19095	0	-0.325
CSMD1	6	0.93632	0.93639	0.9995	19096	0	-0.65
TIGD7	6	0.93646	0.93653	0.9995	19097	0	-0.467
hsa-mir-3605	4	0.93647	0.93639	0.9995	19098	0	-0.972
MIER3	6	0.93649	0.93656	0.9995	19099	0	-0.13
ZNF561	5	0.9365	0.93675	0.9995	19100	0	-0.055
CHST14	6	0.93653	0.93661	0.9995	19101	0	-0.272
CDCA8	6	0.93656	0.93664	0.9995	19102	0	-0.12
CYP26B1	6	0.93672	0.9368	0.9995	19103	0	-0.536
OTUD3	6	0.93672	0.9368	0.9995	19104	0	-0.015
EFCAB7	6	0.93675	0.93682	0.9995	19105	0	-0.323
MS4A6A	6	0.93679	0.93687	0.9995	19106	0	-0.055
CCDC144NL	5	0.93684	0.93709	0.9995	19107	0	-0.406
UBE3A	6	0.93687	0.93696	0.9995	19108	0	-0.295
CHPF2	6	0.93688	0.93696	0.9995	19109	0	-0.099
PLS1	6	0.93688	0.93696	0.9995	19110	0	-0.358
ZNF532	6	0.9369	0.93697	0.9995	19111	0	-0.061
KLHL23	2	0.9369	0.93692	0.9995	19112	0	-0.792
AP1AR	4	0.93693	0.93687	0.9995	19113	0	-0.348
hsa-mir-6891	4	0.93693	0.93687	0.9995	19114	0	-1.672
PODXL2	6	0.93702	0.9371	0.9995	19115	0	0.0036
OR9I1	6	0.93704	0.93712	0.9995	19116	0	-0.249
TMEM120B	6	0.93707	0.93715	0.9995	19117	0	-0.574
CPSF7	6	0.93712	0.9372	0.9995	19118	0	0.0901
PAPOLB	6	0.93712	0.9372	0.9995	19119	0	0.0421
TRABD2A	6	0.93721	0.9373	0.9995	19120	0	-0.488

RHOC	6	0.9373	0.93739	0.9995	19121	0	-0.239
RIMS4	6	0.93732	0.93741	0.9995	19122	0	-0.682
OAS2	6	0.93732	0.93741	0.9995	19123	0	-0.532
GIMAP4	6	0.93737	0.93746	0.9995	19124	0	-0.375
PPAPDC1B	6	0.93743	0.93753	0.9995	19125	0	0.0129
TMEM62	6	0.93746	0.93757	0.9995	19126	0	-0.25
FERMT1	6	0.93748	0.93757	0.9995	19127	0	-0.039
HMGB1	6	0.93751	0.93761	0.9995	19128	0	-0.305
ZNF75D	6	0.93755	0.93765	0.9995	19129	0	-0.167
SPATA22	6	0.93761	0.93771	0.9995	19130	0	-0.108
FAM120C	6	0.93768	0.93778	0.9995	19131	0	-0.199
GNRH1	6	0.93771	0.93782	0.9995	19132	0	-0.127
ANKRD16	6	0.93774	0.93785	0.9995	19133	0	0.0179
CFP	6	0.93774	0.93785	0.9995	19134	0	-0.23
hsa-mir-888	4	0.93778	0.93771	0.9995	19135	0	-0.119
CACNA1B	6	0.93778	0.93789	0.9995	19136	0	-0.602
FAM73A	6	0.93783	0.93794	0.9995	19137	0	-0.073
TRIM37	6	0.93796	0.93807	0.9995	19138	0	-0.281
WDPCP	6	0.93796	0.93807	0.9995	19139	0	-0.448
MYO1G	6	0.93808	0.93819	0.9995	19140	0	-0.346
MYLK3	4	0.93808	0.938	0.9995	19141	0	-1.119
ADPGK	6	0.93809	0.9382	0.9995	19142	0	-0.329
PPP4R2	6	0.9381	0.93821	0.9995	19143	0	-0.36
PCDH9	6	0.93812	0.93823	0.9995	19144	0	-0.16
HIST2H2AB	6	0.93815	0.93826	0.9995	19145	0	-0.113
FRAS1	6	0.93815	0.93826	0.9995	19146	0	-0.029
C16orf62	6	0.93822	0.93832	0.9995	19147	0	-0.102
hsa-mir-1301	4	0.93829	0.9382	0.9995	19148	0	-0.178
hsa-mir-1254-1	3	0.9383	0.93851	0.9995	19149	0	-0.229
MRGBP	6	0.93832	0.93843	0.9995	19150	0	-0.632
TOM1L2	6	0.93834	0.93844	0.9995	19151	0	-0.283
NME6	6	0.93841	0.9385	0.9995	19152	0	-0.684
hsa-mir-548a-2	2	0.93863	0.93865	0.9995	19153	0	-0.441
ALDH3A2	6	0.93864	0.93874	0.9995	19154	0	0.0303
CES2	6	0.9387	0.9388	0.9995	19155	0	-0.337
PXMP4	6	0.93885	0.93894	0.9995	19156	0	-0.191
AADAC	6	0.93885	0.93894	0.9995	19157	0	0.0415
MSANTD3-TMEFF1	3	0.93889	0.93909	0.9995	19158	0	-0.293
DDR1	6	0.93891	0.939	0.9995	19159	0	-0.472
DUSP7	6	0.93895	0.93905	0.9995	19160	0	-0.228
SUN1	6	0.93899	0.93909	0.9995	19161	0	-0.117
CYP27A1	6	0.93901	0.93911	0.9995	19162	0	-0.497
HIP1R	6	0.93904	0.93913	0.9995	19163	0	-0.615
DNAJB3	6	0.93906	0.93915	0.9995	19164	0	-0.23
EDN2	6	0.93906	0.93915	0.9995	19165	0	-0.13
ATP8B1	4	0.93909	0.93903	0.9995	19166	0	-0.726
hsa-mir-3169	4	0.93909	0.93903	0.9995	19167	0	-0.315
EVPL	5	0.93919	0.9394	0.9995	19168	0	-0.419
SLC25A1	6	0.9392	0.93927	0.9995	19169	0	-0.522
PGAM2	6	0.9392	0.93927	0.9995	19170	0	-0.245
PRPS1	6	0.93925	0.93931	0.9995	19171	0	-0.118
TMPRSS4	6	0.93934	0.93941	0.9995	19172	0	-0.139
hsa-mir-1199	4	0.93935	0.93931	0.9995	19173	0	-0.644
CYP7A1	6	0.93943	0.93949	0.9995	19174	0	-0.569
PRKCDBP	6	0.93944	0.9395	0.9995	19175	0	-1.046
OPLAH	6	0.93946	0.93951	0.9995	19176	0	-0.009
CD52	6	0.9395	0.93956	0.9995	19177	0	-0.737
CORO2B	6	0.93951	0.93956	0.9995	19178	0	-0.728
HIST1H2AH	6	0.93954	0.93959	0.9995	19179	0	-0.255
ZNF787	6	0.93954	0.93959	0.9995	19180	0	-0.369
ARHGAP30	6	0.93964	0.93968	0.9995	19181	0	-0.054
TRIM58	6	0.93966	0.93971	0.9995	19182	0	0.0591
EIF2A	6	0.93969	0.93974	0.9995	19183	0	-0.464
LHX1	6	0.93969	0.93974	0.9995	19184	0	-0.487
PARD3	6	0.93972	0.93978	0.9995	19185	0	-0.121
OR1L6	6	0.93976	0.93982	0.9995	19186	0	-0.091
TRAPPC3	6	0.93985	0.93991	0.9995	19187	0	-0.112
UBE3C	6	0.93991	0.93998	0.9995	19188	0	-0.071
GUCY1A2	6	0.94002	0.94009	0.9995	19189	0	-0.28
hsa-mir-5007	4	0.9401	0.94005	0.9995	19190	0	-0.134
TRAFD1	6	0.94018	0.94023	0.9995	19191	0	-0.011
ZNF512B	6	0.9402	0.94025	0.9995	19192	0	-0.118
CLEC1B	6	0.9402	0.94025	0.9995	19193	0	-0.13
ATP2C2	6	0.9402	0.94025	0.9995	19194	0	-0.301
ZNF708	6	0.94025	0.9403	0.9995	19195	0	-1.287
TRIM64C	5	0.94028	0.94048	0.9995	19196	0	-0.17
ATP6V1H	6	0.9403	0.94035	0.9995	19197	0	-0.601
KIAA1755	6	0.94033	0.94038	0.9995	19198	0	-0.43
CRABP1	6	0.94039	0.94044	0.9995	19199	0	-0.341
hsa-mir-153-2	3	0.94041	0.94058	0.9995	19200	0	-0.254
PICALM	6	0.94042	0.94047	0.9995	19201	0	-0.325
hsa-mir-6870	4	0.94043	0.94038	0.9995	19202	0	-0.642
MUC4	6	0.94049	0.94054	0.9995	19203	0	-0.209
SLX4IP	6	0.94051	0.94055	0.9995	19204	0	-0.472
MAGOH	6	0.94052	0.94057	0.9995	19205	0	-0.116

DRAXIN	6	0.94055	0.94059	0.9995	19206	0	-0.617
HTRA1	6	0.94056	0.94061	0.9995	19207	0	-0.518
40057	3	0.94058	0.94073	0.9995	19208	0	-0.418
FBXL8	6	0.94058	0.94062	0.9995	19209	0	-0.097
SCAF1	6	0.94061	0.94066	0.9995	19210	0	-0.03
PDGFD	6	0.94083	0.94087	0.9995	19211	0	-0.435
C12orf57	6	0.94085	0.9409	0.9995	19212	0	-0.315
RAD51AP1	6	0.94089	0.94093	0.9995	19213	0	-0.393
STAC2	6	0.94101	0.94105	0.9995	19214	0	-0.248
PCP2	6	0.94105	0.9411	0.9995	19215	0	0.008
BCL2L10	6	0.94116	0.94123	0.9995	19216	0	-0.617
RNF112	6	0.94119	0.94126	0.9995	19217	0	-0.122
SMAD7	6	0.94138	0.94146	0.9995	19218	0	-0.245
EVA1A	6	0.94167	0.94175	0.9995	19219	0	-0.407
TMEM9B	6	0.94173	0.94181	0.9995	19220	0	-0.311
ACKR2	4	0.94177	0.94173	0.9995	19221	0	-0.126
EIF4B	6	0.9418	0.94187	0.9995	19222	0	-0.332
OR2Y1	6	0.94183	0.9419	0.9995	19223	0	-0.479
TSSK4	6	0.94187	0.94193	0.9995	19224	0	0.0167
LAYN	6	0.94195	0.94201	0.9995	19225	0	-0.336
GDF10	6	0.94198	0.94204	0.9995	19226	0	-0.384
CLASRP	6	0.9421	0.94216	0.9995	19227	0	0.0236
STK32B	6	0.94224	0.94229	0.9995	19228	0	-0.024
F8	6	0.94229	0.94234	0.9995	19229	0	-0.475
hsa-mir-96	4	0.94229	0.94224	0.9995	19230	0	-0.167
DNAJC5	6	0.94231	0.94236	0.9995	19231	0	-0.142
POLK	6	0.94241	0.94248	0.9995	19232	0	-0.47
PATL1	6	0.94244	0.9425	0.9995	19233	0	-0.381
MT1H	4	0.94249	0.94245	0.9995	19234	0	-0.567
ZNF812	5	0.9425	0.94268	0.9995	19235	0	-0.345
GPA33	6	0.94253	0.9426	0.9995	19236	0	-0.368
RAB35	6	0.94253	0.9426	0.9995	19237	0	0.0103
COCH	6	0.94255	0.94261	0.9995	19238	0	-0.069
HEXA	6	0.94259	0.94265	0.9995	19239	0	-0.123
ZNF649	6	0.94275	0.9428	0.9995	19240	0	-0.253
RPL10A	6	0.94282	0.94287	0.9995	19241	0	0.0512
AQP12B	6	0.94286	0.94291	0.9995	19242	0	-0.285
BCL7A	6	0.94288	0.94292	0.9995	19243	0	-0.493
TGM7	6	0.94289	0.94294	0.9995	19244	0	-0.395
VPS52	6	0.943	0.94304	0.9995	19245	0	-0.321
TYR	6	0.94303	0.94307	0.9995	19246	0	-0.265
MID2	6	0.94315	0.9432	0.9995	19247	0	-0.005
C15orf52	6	0.94328	0.94332	0.9995	19248	0	-0.658
DDO	6	0.94342	0.94345	0.9995	19249	0	-0.484
RHOF	6	0.94352	0.94355	0.9995	19250	0	-0.064
KANSL2	4	0.94369	0.94366	0.9995	19251	0	-0.468
NHLRC4	6	0.94371	0.94373	0.9995	19252	0	0.0201
POU1F1	6	0.94378	0.94381	0.9995	19253	0	-0.153
C10orf88	6	0.94378	0.94381	0.9995	19254	0	-0.606
PLP2	6	0.9438	0.94383	0.9995	19255	0	-0.323
SCP2D1	6	0.94384	0.94388	0.9995	19256	0	-0.175
TUBB	6	0.94389	0.94392	0.9995	19257	0	-0.44
hsa-mir-8065	4	0.94397	0.94394	0.9995	19258	0	-0.263
MS4A6E	6	0.94406	0.94408	0.9995	19259	0	-0.031
KCTD1	6	0.94429	0.94431	0.9995	19260	0	-0.277
FIS1	6	0.94429	0.94431	0.9995	19261	0	-1.029
ANXA7	6	0.94443	0.94444	0.9995	19262	0	-0.089
H3F3C	6	0.94451	0.94452	0.9995	19263	0	-0.335
DDX11	6	0.94461	0.94464	0.9995	19264	0	0.0774
OR52B4	6	0.94467	0.94469	0.9995	19265	0	-0.32
TMEM251	6	0.94469	0.94471	0.9995	19266	0	-0.251
PTGER3	6	0.94474	0.94477	0.9995	19267	0	-0.013
ACP1	6	0.94476	0.94479	0.9995	19268	0	-0.194
MXD1	6	0.94486	0.94488	0.9995	19269	0	-0.008
hsa-mir-545	4	0.94493	0.94484	0.9995	19270	0	-0.296
GET4	6	0.94494	0.94496	0.9995	19271	0	0.0509
SH3BP4	6	0.94494	0.94496	0.9995	19272	0	0.117
AMZ1	4	0.945	0.94491	0.9995	19273	0	-0.203
hsa-mir-18a	4	0.94502	0.94493	0.9995	19274	0	-0.228
HEATR5B	6	0.94506	0.94508	0.9995	19275	0	-0.222
IL17D	6	0.94512	0.94513	0.9995	19276	0	-0.305
TMPRSS6	6	0.94532	0.94532	0.9995	19277	0	0.0135
TRIM4	6	0.9454	0.9454	0.9995	19278	0	-0.762
ZDHHC24	6	0.94541	0.94541	0.9995	19279	0	-0.228
KIAA1462	6	0.94543	0.94543	0.9995	19280	0	-0.224
TEX30	6	0.94543	0.94543	0.9995	19281	0	-0.216
ALDH7A1	6	0.94549	0.94549	0.9995	19282	0	-0.249
HYAL1	6	0.94551	0.94551	0.9995	19283	0	-0.264
hsa-mir-6741	4	0.94551	0.9454	0.9995	19284	0	-0.22
IBSP	6	0.94553	0.94553	0.9995	19285	0	-0.519
ARRDC2	6	0.94557	0.94557	0.9995	19286	0	-0.34
SYT7	6	0.94571	0.9457	0.9995	19287	0	-0.07
ACTR8	6	0.94574	0.94573	0.9995	19288	0	-0.905
ATXN1	6	0.94577	0.94576	0.9995	19289	0	-0.679
hsa-mir-4792	4	0.94582	0.94571	0.9995	19290	0	-0.945

SDF2	6	0.94582	0.94581	0.9995	19291	0	-0.435
XPNPEP2	6	0.94583	0.94583	0.9995	19292	0	-0.032
NAGLU	6	0.94589	0.94587	0.9995	19293	0	-0.29
hsa-mir-320b-2	3	0.94592	0.94612	0.9995	19294	0	-0.207
TFF1	6	0.94593	0.94591	0.9995	19295	0	-0.526
LONP2	6	0.94602	0.94601	0.9995	19296	0	-0.127
C9orf123	4	0.94611	0.94601	0.9995	19297	0	-0.335
C19orf21	1	0.94614	0.94638	0.9995	19298	0	-0.721
PCDHGA3	2	0.94615	0.9462	0.9995	19299	0	-0.418
NOL3	6	0.94617	0.94616	0.9995	19300	0	-0.101
GGACT	6	0.94622	0.9462	0.9995	19301	0	-0.517
FAM83C	6	0.94637	0.94635	0.9995	19302	0	-0.363
hsa-mir-1238	4	0.94647	0.94637	0.9995	19303	0	-0.376
MAATS1	6	0.94657	0.94657	0.9995	19304	0	-0.238
SFTPA2	5	0.94659	0.94679	0.9995	19305	0	-0.304
RNF148	6	0.94671	0.9467	0.9995	19306	0	-0.206
OR6C4	6	0.94676	0.94674	0.9995	19307	0	-0.206
ADCK2	6	0.9468	0.94679	0.9995	19308	0	-0.046
INSL5	6	0.9468	0.94679	0.9995	19309	0	-0.441
GFRA4	6	0.94682	0.94681	0.9995	19310	0	-0.326
hsa-mir-6840	4	0.94689	0.94679	0.9995	19311	0	-0.104
SPRY2	6	0.94689	0.94687	0.9995	19312	0	-0.214
ZNF154	6	0.9469	0.94688	0.9995	19313	0	-0.237
IDS	6	0.94693	0.9469	0.9995	19314	0	-0.187
DLC1	6	0.94694	0.94692	0.9995	19315	0	-0.189
CHRNA3	5	0.94695	0.94716	0.9995	19316	0	-0.015
37865	3	0.94696	0.94716	0.9995	19317	0	-0.715
PVALB	6	0.94697	0.94696	0.9995	19318	0	-0.16
C11orf85	6	0.94713	0.94711	0.9995	19319	0	-0.295
HMGB4	6	0.94715	0.94714	0.9995	19320	0	-0.398
RBP1	6	0.94718	0.94717	0.9995	19321	0	-0.162
MTA1	6	0.94722	0.94721	0.9995	19322	0	-0.205
OPN1SW	6	0.94727	0.94726	0.9995	19323	0	-0.12
SELRC1	6	0.94731	0.9473	0.9995	19324	0	-0.195
hsa-let-7c	4	0.94739	0.94733	0.9995	19325	0	-0.309
APCDD1L	6	0.9475	0.9475	0.9995	19326	0	-0.046
EIF4EBP2	6	0.94754	0.94755	0.9995	19327	0	-0.497
HELLS	6	0.94754	0.94755	0.9995	19328	0	-0.33
STK33	6	0.94759	0.94759	0.9995	19329	0	-0.158
CCDC50	6	0.94762	0.94762	0.9995	19330	0	-0.048
CLSTN3	6	0.94769	0.94769	0.9995	19331	0	-0.187
CAMK2A	6	0.94775	0.94777	0.9995	19332	0	-0.172
ZNF385B	6	0.94779	0.9478	0.9995	19333	0	-0.222
LOC286238	6	0.94781	0.94782	0.9995	19334	0	-0.075
RETN	6	0.94789	0.9479	0.9995	19335	0	-0.06
SLC50A1	6	0.94793	0.94794	0.9995	19336	0	-0.97
PPP1R9A	6	0.94793	0.94795	0.9995	19337	0	-0.158
CAPZA1	5	0.94796	0.94816	0.9995	19338	0	-0.012
RNF151	6	0.94797	0.94798	0.9995	19339	0	-0.678
ZDHHC17	6	0.94803	0.94805	0.9995	19340	0	-0.427
FAM154A	6	0.94803	0.94805	0.9995	19341	0	-0.186
SMURF1	6	0.94806	0.94808	0.9995	19342	0	-0.03
RRAGB	6	0.94809	0.9481	0.9995	19343	0	-0.224
NR2C2	6	0.94815	0.94816	0.9995	19344	0	-0.709
CTSB	6	0.9482	0.94821	0.9995	19345	0	-0.171
hsa-mir-4440	3	0.94823	0.94843	0.9995	19346	0	-0.359
TMEM215	6	0.94826	0.94827	0.9995	19347	0	-0.021
SERPINA7	6	0.94827	0.94828	0.9995	19348	0	-0.544
hsa-mir-1302-4	4	0.94829	0.94821	0.9995	19349	0	-0.774
SERF2	6	0.94834	0.94836	0.9995	19350	0	-0.046
CXCL16	6	0.94835	0.94837	0.9995	19351	0	-0.295
hsa-mir-5589	4	0.94841	0.94833	0.9995	19352	0	-0.186
RBCK1	6	0.94846	0.94847	0.9995	19353	0	-0.1
TSPY2	2	0.94847	0.9486	0.9995	19354	0	-0.661
HCAR1	6	0.94848	0.9485	0.9995	19355	0	-0.048
EXOC5	4	0.94853	0.94845	0.9995	19356	0	-0.39
OXTR	6	0.94859	0.9486	0.9995	19357	0	-0.297
PRKAG3	6	0.94872	0.94874	0.9995	19358	0	-0.452
PKIG	6	0.94874	0.94875	0.9995	19359	0	0.0233
C12orf75	6	0.94874	0.94875	0.9995	19360	0	-0.07
C10orf99	6	0.94897	0.94897	0.9995	19361	0	-0.563
SCUBE1	6	0.94902	0.94902	0.9995	19362	0	-0.03
LCTL	6	0.94902	0.94902	0.9995	19363	0	-0.231
SUCNR1	6	0.94913	0.94913	0.9995	19364	0	-0.132
MSN	6	0.94916	0.94917	0.9995	19365	0	-0.227
THOC3	6	0.94922	0.94922	0.9995	19366	0	-0.397
RSPO1	6	0.94925	0.94925	0.9995	19367	0	-0.465
hsa-mir-211	4	0.94927	0.9492	0.9995	19368	0	-0.656
CDH15	6	0.94939	0.94939	0.9995	19369	0	-0.424
FMR1NB	6	0.9495	0.9495	0.9995	19370	0	0.0062
ARL11	6	0.94953	0.94952	0.9995	19371	0	-0.198
PPFIA2	6	0.94956	0.94954	0.9995	19372	0	-0.369
ASNS	6	0.94958	0.94956	0.9995	19373	0	-0.239
CLUH	6	0.94961	0.9496	0.9995	19374	0	-0.024
ZZEF1	6	0.94971	0.9497	0.9995	19375	0	-0.343

INPP1	6	0.94974	0.94973	0.9995	19376	0	-0.194
DDT	2	0.94975	0.9499	0.9995	19377	0	-0.5
SSTR3	6	0.94976	0.94975	0.9995	19378	0	-0.402
REEP2	6	0.94976	0.94975	0.9995	19379	0	-0.033
RBM20	6	0.94991	0.94991	0.9995	19380	0	-0.086
PNN	6	0.94995	0.94995	0.9995	19381	0	-0.211
RNPC3	6	0.94997	0.94996	0.9995	19382	0	0.0311
WDR92	6	0.95001	0.95001	0.9995	19383	0	-0.951
KERA	6	0.95002	0.95002	0.9995	19384	0	-0.019
hsa-mir-3152	4	0.95004	0.94996	0.9995	19385	0	-0.534
NGFR	6	0.95006	0.95006	0.9995	19386	0	-0.113
ANKRD52	6	0.9501	0.9501	0.9995	19387	0	-0.203
hsa-mir-3927	3	0.95011	0.95034	0.9995	19388	0	-1.233
OR2T5	3	0.95011	0.95034	0.9995	19389	0	-1.888
OR2T3	3	0.95011	0.95034	0.9995	19390	0	-1.233
hsa-mir-376a-1	3	0.95011	0.95034	0.9995	19391	0	-1.861
GGN	6	0.95023	0.95024	0.9995	19392	0	-0.058
ARX	6	0.95028	0.95031	0.9995	19393	0	-0.071
POLR3D	6	0.95033	0.95035	0.9995	19394	0	-0.54
SHROOM2	6	0.95034	0.95037	0.9995	19395	0	-0.074
CLDND2	6	0.95048	0.9505	0.9995	19396	0	0.0273
TTLL2	6	0.95055	0.95056	0.9995	19397	0	-0.151
TGIF2LY	6	0.95055	0.95056	0.9995	19398	0	-0.544
POU3F2	6	0.95061	0.95062	0.9995	19399	0	-0.015
AARSD1	6	0.95061	0.95062	0.9995	19400	0	-0.209
BAD	6	0.95061	0.95062	0.9995	19401	0	-0.388
PLOD3	6	0.95063	0.95065	0.9995	19402	0	-0.172
RAVER1	6	0.95064	0.95066	0.9995	19403	0	-0.035
VDAC3	6	0.95064	0.95066	0.9995	19404	0	-0.675
hsa-mir-6499	4	0.95068	0.95059	0.9995	19405	0	-0.744
H6PD	6	0.95076	0.95078	0.9995	19406	0	-0.228
TIAL1	6	0.95086	0.95086	0.9995	19407	0	-0.134
CXCL5	6	0.95097	0.95098	0.9995	19408	0	-0.747
VWA5A	6	0.95098	0.95099	0.9995	19409	0	-0.029
hsa-mir-6821	4	0.951	0.95091	0.9995	19410	0	-0.22
KCTD11	6	0.95103	0.95105	0.9995	19411	0	-0.192
hsa-mir-6856	4	0.95105	0.95097	0.9995	19412	0	-1.023
B4GALNT1	6	0.95106	0.95108	0.9995	19413	0	-0.442
POMGNT2	4	0.95107	0.95098	0.9995	19414	0	-0.475
SNRK	6	0.95116	0.95119	0.9995	19415	0	-0.774
MSRA	6	0.95116	0.95119	0.9995	19416	0	-0.396
NPPA	6	0.95119	0.95123	0.9995	19417	0	-0.226
ENTHD2	6	0.9512	0.95124	0.9995	19418	0	-0.021
FAM118B	4	0.95125	0.95117	0.9995	19419	0	-0.217
PGLYRP2	6	0.95133	0.95138	0.9995	19420	0	-0.504
hsa-mir-6736	4	0.95141	0.95133	0.9995	19421	0	-0.768
CHRNA7	4	0.95149	0.95142	0.9995	19422	0	-0.436
CCDC81	6	0.95155	0.95158	0.9995	19423	0	0.0978
B3GNT2	6	0.95161	0.95165	0.9995	19424	0	-0.269
C19orf45	6	0.95165	0.95168	0.9995	19425	0	-0.656
SLC35C1	6	0.95165	0.95168	0.9995	19426	0	-0.272
TMEM192	6	0.95171	0.95173	0.9995	19427	0	-0.199
MPV17L	6	0.95173	0.95176	0.9995	19428	0	-0.316
SLC4A4	6	0.95181	0.95184	0.9995	19429	0	-0.272
ST3GAL3	6	0.95191	0.95194	0.9995	19430	0	-0.321
ZBTB8A	6	0.95192	0.95195	0.9995	19431	0	-0.174
TNIP1	6	0.95194	0.95197	0.9995	19432	0	-0.016
SPARCL1	6	0.95196	0.95199	0.9995	19433	0	-0.574
WNT5B	6	0.95198	0.95201	0.9995	19434	0	-0.153
BFSP1	6	0.95199	0.95203	0.9995	19435	0	-0.275
SIGLEC12	6	0.952	0.95204	0.9995	19436	0	-0.626
VCX2	4	0.95205	0.95199	0.9995	19437	0	-0.417
CYBB	6	0.95206	0.95209	0.9995	19438	0	-0.521
CCNJ	6	0.95208	0.95211	0.9995	19439	0	-0.81
H2AFX	6	0.95209	0.95213	0.9995	19440	0	-0.471
MLLT6	6	0.95212	0.95215	0.9995	19441	0	-0.175
ZNF846	6	0.95213	0.95216	0.9995	19442	0	-0.237
OGFR	6	0.95223	0.95226	0.9995	19443	0	-0.201
DPP9	6	0.9523	0.95232	0.9995	19444	0	-0.255
BRD3	5	0.95241	0.95258	0.9995	19445	0	-0.198
hsa-mir-4672	4	0.95242	0.95235	0.9995	19446	0	-0.817
MTHFD2L	6	0.95243	0.95246	0.9995	19447	0	-0.371
ZNF346	6	0.95245	0.95249	0.9995	19448	0	-0.154
hsa-mir-4528	2	0.95245	0.95258	0.9995	19449	0	-0.966
BOD1L1	6	0.95248	0.95252	0.9995	19450	0	-0.306
KCNJ8	6	0.95259	0.95262	0.9995	19451	0	-0.211
PGAP3	6	0.95262	0.95265	0.9995	19452	0	-0.64
YPEL3	6	0.95262	0.95265	0.9995	19453	0	-0.662
MCU	6	0.95263	0.95267	0.9995	19454	0	-0.023
C22orf46	6	0.95264	0.95268	0.9995	19455	0	-0.432
BAAT	6	0.95271	0.95275	0.9995	19456	0	-0.005
HNF4A	6	0.95285	0.95288	0.9995	19457	0	-0.186
CCDC6	6	0.95285	0.95288	0.9995	19458	0	-0.106
MRAP	6	0.95287	0.9529	0.9995	19459	0	-0.277
ZNF658	6	0.95289	0.95292	0.9995	19460	0	-0.324

PMS1	6	0.95302	0.95305	0.9995	19461	0	-0.227
TUBA3C	6	0.95309	0.95312	0.9995	19462	0	-0.347
FOXC2	6	0.95313	0.95316	0.9995	19463	0	-0.628
NCLN	6	0.95314	0.95316	0.9995	19464	0	-0.666
hsa-mir-203b	1	0.95321	0.95341	0.9995	19465	0	-0.798
ZNF12	6	0.95329	0.95332	0.9995	19466	0	-0.422
TGM6	6	0.9533	0.95333	0.9995	19467	0	-0.513
BZRAP1	6	0.95345	0.95349	0.9995	19468	0	-0.169
hsa-mir-6853	4	0.95347	0.95337	0.9995	19469	0	-0.446
SFPQ	6	0.95363	0.95365	0.9995	19470	0	-0.315
GATAD2A	6	0.95366	0.95368	0.9995	19471	0	-0.124
VAV1	6	0.95376	0.95379	0.9995	19472	0	-0.431
FKBP8	6	0.95383	0.95385	0.9995	19473	0	-0.812
SPO11	6	0.95393	0.95395	0.9995	19474	0	-0.37
FRAT1	6	0.95393	0.95395	0.9995	19475	0	-0.235
DUSP23	6	0.95396	0.95397	0.9995	19476	0	0.0187
CLDN10	6	0.95396	0.95397	0.9995	19477	0	-0.225
PURB	6	0.95396	0.95397	0.9995	19478	0	-0.302
S100A2	6	0.95408	0.9541	0.9995	19479	0	0.0271
MICAL2	6	0.95408	0.9541	0.9995	19480	0	-0.041
FAM115C	6	0.95426	0.95429	0.9995	19481	0	-0.474
SS18	6	0.9543	0.95433	0.9995	19482	0	-0.368
SNX20	6	0.95442	0.95443	0.9995	19483	0	-0.086
MAP4K3	6	0.95443	0.95445	0.9995	19484	0	-0.2
STRN4	6	0.95445	0.95447	0.9995	19485	0	-0.338
HHIPL1	6	0.95449	0.95451	0.9995	19486	0	-0.156
TCF4	6	0.95453	0.95455	0.9995	19487	0	-0.17
hsa-mir-4690	4	0.95463	0.95451	0.9995	19488	0	-0.15
hsa-mir-4682	4	0.95466	0.95454	0.9995	19489	0	-0.172
TTLL1	6	0.95468	0.9547	0.9995	19490	0	-0.292
FAM170A	6	0.95473	0.95474	0.9995	19491	0	-0.374
NUTM2A	5	0.95474	0.95493	0.9995	19492	0	-0.62
CNTNAP3	4	0.95475	0.95463	0.9995	19493	0	-0.332
NCKAP1	6	0.95493	0.95493	0.9995	19494	0	0.0387
GRIN2A	6	0.95498	0.95499	0.9995	19495	0	-0.093
SPATA25	6	0.955	0.95501	0.9995	19496	0	-0.053
hsa-mir-3122	4	0.95505	0.95492	0.9995	19497	0	-0.404
POLR2I	6	0.95508	0.9551	0.9995	19498	0	-0.155
hsa-mir-6818	4	0.95509	0.95496	0.9995	19499	0	-0.736
GABRA5	6	0.9551	0.95512	0.9995	19500	0	-0.41
DEFB4B	3	0.95511	0.95537	0.9995	19501	0	-0.537
LPAR1	6	0.95527	0.95528	0.9995	19502	0	-0.294
ALPP	5	0.95538	0.95554	0.9995	19503	0	-0.316
WDFY4	6	0.95539	0.95538	0.9995	19504	0	-3E-04
RCOR1	6	0.95547	0.95546	0.9995	19505	0	-0.021
CDK20	6	0.95547	0.95546	0.9995	19506	0	-0.244
MT1B	4	0.95551	0.9554	0.9995	19507	0	-1.52
C12orf56	6	0.95552	0.95551	0.9995	19508	0	-0.169
MICAL1	6	0.95552	0.95551	0.9995	19509	0	-0.302
SLC38A11	6	0.95558	0.95556	0.9995	19510	0	-0.213
GZMM	6	0.95562	0.95561	0.9995	19511	0	-0.14
BLMH	6	0.95564	0.95563	0.9995	19512	0	-0.274
ZNF236	6	0.95568	0.95567	0.9995	19513	0	-0.215
CDC25B	6	0.95574	0.95572	0.9995	19514	0	-0.256
YPEL5	6	0.95578	0.95577	0.9995	19515	0	-0.072
ZSCAN31	6	0.95581	0.95579	0.9995	19516	0	-0.448
EEA1	6	0.9559	0.95588	0.9995	19517	0	-0.143
MAGEA12	6	0.95596	0.95593	0.9995	19518	0	-0.38
FAM71D	6	0.956	0.95598	0.9995	19519	0	-0.545
KANSL1	6	0.95602	0.956	0.9995	19520	0	-0.068
ABCB6	6	0.95604	0.95602	0.9995	19521	0	-0.138
C22orf42	6	0.95609	0.95606	0.9995	19522	0	-0.157
CRELD1	6	0.95613	0.9561	0.9995	19523	0	-0.246
FGF20	6	0.95619	0.95616	0.9995	19524	0	-0.014
GPR37L1	6	0.95623	0.95621	0.9995	19525	0	-0.102
hsa-mir-766	4	0.95625	0.95616	0.9995	19526	0	-0.818
OR8A1	6	0.95627	0.95624	0.9995	19527	0	-0.177
RSPH10B	6	0.95635	0.95632	0.9995	19528	0	-0.229
hsa-mir-125b-2	4	0.95642	0.95632	0.9995	19529	0	-0.414
CORO7	4	0.95644	0.95635	0.9995	19530	0	-0.361
GMNC	6	0.95646	0.95644	0.9995	19531	0	-0.211
GIMAP2	6	0.95656	0.95654	0.9995	19532	0	-0.569
ACAP3	6	0.95658	0.95656	0.9995	19533	0	-0.714
OR52I1	6	0.9566	0.95658	0.9995	19534	0	-0.338
PPRC1	6	0.95669	0.95666	0.9995	19535	0	-0.216
KIF6	6	0.95675	0.95673	0.9995	19536	0	-0.159
AKNAD1	6	0.95677	0.95675	0.9995	19537	0	-0.169
SNF8	6	0.95678	0.95676	0.9995	19538	0	-0.204
MSI2	6	0.95683	0.9568	0.9995	19539	0	-0.543
SLC4A10	6	0.95691	0.95688	0.9995	19540	0	-0.267
ZCCHC17	6	0.95694	0.95691	0.9995	19541	0	-0.155
AIRE	6	0.95706	0.95701	0.9995	19542	0	-0.774
CD83	6	0.95706	0.95701	0.9995	19543	0	-0.239
GSX1	6	0.95708	0.95702	0.9995	19544	0	-0.292
ANXA5	6	0.95711	0.95706	0.9995	19545	0	-0.709

DCBLD1	6	0.95712	0.95708	0.9995	19546	0	-0.252
TMEM79	6	0.95715	0.9571	0.9995	19547	0	-0.324
PCSK1	6	0.95719	0.95714	0.9995	19548	0	-0.351
MAGOHB	6	0.95723	0.95719	0.9995	19549	0	-0.288
LRRTM1	6	0.95728	0.95723	0.9995	19550	0	-0.611
hsa-mir-1178	4	0.9573	0.9572	0.9995	19551	0	-0.429
ZPBP	6	0.9573	0.95725	0.9995	19552	0	0.02
GRIN3A	6	0.95735	0.95729	0.9995	19553	0	-0.123
KIAA0247	6	0.95737	0.95731	0.9995	19554	0	-0.262
SERPINA6	6	0.95737	0.95731	0.9995	19555	0	-0.405
MAP7	6	0.9574	0.95735	0.9995	19556	0	-0.397
GUCA1A	6	0.95748	0.95744	0.9995	19557	0	-0.164
MOSPD1	6	0.95755	0.9575	0.9995	19558	0	-0.107
ALDH3B2	6	0.95756	0.95751	0.9995	19559	0	-0.512
TMEM200C	6	0.95762	0.95758	0.9995	19560	0	-0.276
ACKR3	3	0.9578	0.95801	0.9995	19561	0	-0.43
LTBP4	6	0.95785	0.9578	0.9995	19562	0	-0.274
LRRC72	6	0.9579	0.95785	0.9995	19563	0	-0.249
ZFAND6	6	0.95794	0.95789	0.9995	19564	0	-0.125
TUBAL3	6	0.95797	0.95792	0.9995	19565	0	-0.602
COA4	4	0.95798	0.95793	0.9995	19566	0	-0.164
IDUA	6	0.95803	0.95798	0.9995	19567	0	-0.128
TDRD9	6	0.95811	0.95805	0.9995	19568	0	-0.038
ZNF611	4	0.95811	0.95805	0.9995	19569	0	-0.396
FBLIM1	6	0.95814	0.95808	0.9995	19570	0	-0.258
ALG12	6	0.95816	0.9581	0.9995	19571	0	-0.176
CFDP1	6	0.95822	0.95816	0.9995	19572	0	-0.229
KIAA1430	6	0.95825	0.95819	0.9995	19573	0	-0.064
REL	6	0.95826	0.9582	0.9995	19574	0	-0.207
PF4	4	0.95833	0.95827	0.9995	19575	0	-0.284
AMIGO2	6	0.95835	0.9583	0.9995	19576	0	-0.476
GALP	6	0.95844	0.95839	0.9995	19577	0	-0.126
TATDN1	4	0.95845	0.95839	0.9995	19578	0	-0.29
HPR	6	0.95846	0.95841	0.9995	19579	0	-0.254
AFF4	6	0.95852	0.95846	0.9995	19580	0	-0.161
RAB3IP	6	0.95854	0.95849	0.9995	19581	0	-0.548
hsa-mir-7113	4	0.95856	0.95849	0.9995	19582	0	-1.538
39326	3	0.95859	0.95881	0.9995	19583	0	-0.589
hsa-mir-378f	4	0.9586	0.95852	0.9995	19584	0	-0.204
DCHS1	6	0.95868	0.95864	0.9995	19585	0	-0.041
HOXA6	6	0.95872	0.95869	0.9995	19586	0	-0.079
hsa-mir-4768	4	0.95875	0.95868	0.9995	19587	0	-0.207
PER3	6	0.95879	0.95875	0.9995	19588	0	-0.186
FAM188B	6	0.95881	0.95878	0.9995	19589	0	-0.09
USE1	6	0.95885	0.95882	0.9995	19590	0	-0.326
WFDC10A	5	0.95888	0.95902	0.9995	19591	0	-0.148
LRRC20	6	0.95896	0.95891	0.9995	19592	0	0.024
ARHGEF28	6	0.95898	0.95893	0.9995	19593	0	-0.127
C14orf1	6	0.959	0.95895	0.9995	19594	0	-0.46
PINK1	6	0.95902	0.95896	0.9995	19595	0	-0.155
KREMEN1	6	0.95905	0.959	0.9995	19596	0	-0.388
TAS2R41	6	0.95909	0.95903	0.9995	19597	0	-0.281
hsa-mir-346	4	0.95914	0.95907	0.9995	19598	0	-0.295
ABCD4	6	0.9592	0.95915	0.9995	19599	0	-0.58
IL32	6	0.95922	0.95917	0.9995	19600	0	-0.038
STYK1	6	0.95923	0.95918	0.9995	19601	0	-0.044
HMOX2	6	0.95933	0.95928	0.9995	19602	0	-0.201
CYP11B2	6	0.95946	0.9594	0.9995	19603	0	0.098
ZNF41	6	0.95946	0.9594	0.9995	19604	0	-0.154
SMNDC1	6	0.95946	0.9594	0.9995	19605	0	-0.062
HIST1H4E	6	0.95946	0.9594	0.9995	19606	0	-0.472
TNFSF8	6	0.95962	0.95955	0.9995	19607	0	-0.458
AGO1	6	0.95969	0.95963	0.9995	19608	0	-0.242
ALAS2	6	0.95982	0.95975	0.9995	19609	0	-0.18
LRRC48	6	0.96014	0.9601	0.9995	19610	0	-0.553
FAM25A	5	0.9602	0.9603	0.9995	19611	0	-0.22
ACOT1	6	0.96022	0.96019	0.9995	19612	0	-0.32
JPH1	6	0.96024	0.9602	0.9995	19613	0	-0.305
MT1F	4	0.9603	0.96021	0.9995	19614	0	-1.14
hsa-mir-1283-1	4	0.9603	0.96021	0.9995	19615	0	-1.618
TUBA3D	4	0.9603	0.96021	0.9995	19616	0	-1.227
MTRNR2L7	4	0.9603	0.96021	0.9995	19617	0	-0.826
GSTM1	4	0.9603	0.96021	0.9995	19618	0	-1.062
ROPN1B	4	0.9603	0.96021	0.9995	19619	0	-0.826
TLE1	4	0.9603	0.96021	0.9995	19620	0	-0.561
hsa-mir-3689d-2	4	0.9603	0.96021	0.9995	19621	0	-1.153
BET3L	2	0.96057	0.96071	0.9995	19622	0	-1.247
LSM2	6	0.96058	0.96055	0.9995	19623	0	-0.085
AADACL4	6	0.96062	0.96059	0.9995	19624	0	-0.12
PCDHA6	2	0.96076	0.96091	0.9995	19625	0	-0.379
MMD2	6	0.96083	0.96081	0.9995	19626	0	-0.071
PHIP	6	0.96087	0.96084	0.9995	19627	0	-0.727
DYRK2	6	0.96102	0.961	0.9995	19628	0	-0.089
ITPR1	6	0.96103	0.96102	0.9995	19629	0	-0.345
RNASEH1	6	0.9612	0.96118	0.9995	19630	0	-0.514

CHST9	6	0.9612	0.96118	0.9995	19631	0	-0.404
PLAC1L	6	0.96125	0.96124	0.9995	19632	0	-0.167
PCDHA4	2	0.96127	0.96142	0.9995	19633	0	-1.053
RAB21	6	0.96141	0.96141	0.9995	19634	0	-0.193
IRF2BP1	6	0.96144	0.96143	0.9995	19635	0	-0.18
CLCN4	6	0.96151	0.9615	0.9995	19636	0	-0.123
SLC6A7	6	0.96155	0.96154	0.9995	19637	0	-0.916
C2orf62	6	0.96163	0.96163	0.9995	19638	0	-1.099
NHLRC2	6	0.96175	0.96175	0.9995	19639	0	-0.746
RGL1	6	0.96183	0.96184	0.9995	19640	0	-0.006
SYTL2	6	0.96185	0.96186	0.9995	19641	0	-0.068
SYCN	6	0.96185	0.96186	0.9995	19642	0	-0.219
SSTR5	6	0.96191	0.96192	0.9995	19643	0	-0.109
hsa-mir-154	4	0.96195	0.96188	0.9995	19644	0	-0.532
LILRB2	6	0.96198	0.96199	0.9995	19645	0	-0.373
GGCT	6	0.96201	0.96202	0.9995	19646	0	-0.296
SGCB	6	0.96203	0.96204	0.9995	19647	0	-0.102
FAM217B	6	0.96205	0.96206	0.9995	19648	0	-0.123
HIST1H2BF	5	0.96207	0.9622	0.9995	19649	0	-1.094
hsa-mir-3137	4	0.96212	0.96205	0.9995	19650	0	-0.368
SEC14L2	6	0.96218	0.96218	0.9995	19651	0	-0.386
CECR1	6	0.96225	0.96225	0.9995	19652	0	-0.721
SF3B5	4	0.96227	0.96221	0.9995	19653	0	-0.38
hsa-mir-4253	4	0.96233	0.96227	0.9995	19654	0	-0.643
ZNF609	6	0.96237	0.96238	0.9995	19655	0	0.0525
ZNF18	6	0.96243	0.96243	0.9995	19656	0	-0.22
BDP1	6	0.96245	0.96245	0.9995	19657	0	-0.341
PAEP	6	0.9625	0.96249	0.9995	19658	0	-0.43
DNAJC1	6	0.96257	0.96256	0.9995	19659	0	-0.114
CYP4F2	5	0.96257	0.96271	0.9995	19660	0	-0.316
IFNA4	5	0.96257	0.96271	0.9995	19661	0	-1.233
STXBP4	6	0.96259	0.96259	0.9995	19662	0	-0.163
CD40	6	0.96268	0.96268	0.9995	19663	0	-0.162
LPHN1	6	0.96273	0.96272	0.9995	19664	0	-0.179
IL1RAPL1	6	0.96275	0.96274	0.9995	19665	0	-0.185
WDR75	6	0.96281	0.96279	0.9995	19666	0	-0.249
FOXP2	6	0.96283	0.96282	0.9995	19667	0	-0.204
PPP1CA	6	0.96294	0.96293	0.9995	19668	0	-0.181
hsa-mir-98	4	0.96295	0.9629	0.9995	19669	0	-0.373
RPRM	6	0.96296	0.96295	0.9995	19670	0	-0.022
hsa-mir-2467	4	0.96312	0.96307	0.9995	19671	0	-0.996
HNRNPCL1	1	0.96318	0.96338	0.9995	19672	0	-0.955
USP17L2	6	0.96319	0.96319	0.9995	19673	0	-0.218
PANK2	6	0.96336	0.96337	0.9995	19674	0	-0.221
hsa-mir-5703	4	0.96338	0.96333	0.9995	19675	0	-0.42
CLIC3	6	0.96339	0.9634	0.9995	19676	0	-0.546
BEGAIN	6	0.96344	0.96344	0.9995	19677	0	-0.152
DGKA	4	0.96344	0.96338	0.9995	19678	0	-0.529
hsa-mir-6851	4	0.96346	0.96339	0.9995	19679	0	-0.672
PROX2	6	0.96356	0.96356	0.9995	19680	0	-0.18
AGPAT5	6	0.96359	0.96359	0.9995	19681	0	-0.159
LRTM1	6	0.96361	0.96361	0.9995	19682	0	-0.391
MPZL2	6	0.96366	0.96368	0.9995	19683	0	-0.118
EHD4	6	0.96371	0.96373	0.9995	19684	0	-0.358
THAP9	6	0.96373	0.96375	0.9995	19685	0	-0.15
hsa-mir-4497	4	0.96375	0.96371	0.9995	19686	0	-0.588
UNC80	6	0.96392	0.96394	0.9995	19687	0	-0.322
EGR3	6	0.96394	0.96395	0.9995	19688	0	0.0594
ARMC4	6	0.96405	0.96405	0.9995	19689	0	-0.229
ACSM3	6	0.96416	0.96417	0.9995	19690	0	-0.377
AMER2	6	0.9642	0.96421	0.9995	19691	0	-0.645
NRG3	6	0.96421	0.96422	0.9995	19692	0	-0.372
hsa-mir-6772	4	0.96436	0.96433	0.9995	19693	0	-0.134
ALDH16A1	6	0.96437	0.96438	0.9995	19694	0	-1.027
REG1B	6	0.9644	0.9644	0.9995	19695	0	-0.002
OR51B2	6	0.96445	0.96446	0.9995	19696	0	-0.026
HEBP2	6	0.96451	0.96452	0.9995	19697	0	-0.391
SEC23A	6	0.9646	0.96462	0.9995	19698	0	-0.213
RNF223	4	0.96463	0.96459	0.9995	19699	0	-0.598
CHI3L2	3	0.96465	0.96475	0.9995	19700	0	-0.801
TMC8	6	0.96465	0.96466	0.9995	19701	0	-0.464
ZNF606	6	0.96467	0.96468	0.9995	19702	0	-0.57
CA12	6	0.96468	0.96469	0.9995	19703	0	-0.44
SNN	6	0.96471	0.96473	0.9995	19704	0	-0.695
IL27	6	0.96483	0.96483	0.9995	19705	0	-0.259
IDNK	6	0.96492	0.96493	0.9995	19706	0	-0.299
REG3A	6	0.96501	0.96502	0.9995	19707	0	-0.747
DDB2	6	0.96507	0.96508	0.9995	19708	0	-0.308
ZNF831	6	0.96514	0.96515	0.9995	19709	0	-0.391
hsa-mir-3615	4	0.96521	0.96517	0.9995	19710	0	-0.547
YIPF5	6	0.96529	0.96528	0.9995	19711	0	-0.134
WARS2	6	0.96531	0.96531	0.9995	19712	0	-0.403
SRPX	6	0.96549	0.96549	0.9995	19713	0	-0.369
TFF2	6	0.96559	0.96559	0.9995	19714	0	-0.168
RPS5	6	0.96568	0.96569	0.9995	19715	0	-0.414

MCHR1	6	0.96571	0.96572	0.9995	19716	0	-0.325
TIMM8A	6	0.96571	0.96572	0.9995	19717	0	-0.361
C9orf169	6	0.96573	0.96574	0.9995	19718	0	-0.453
PRG3	6	0.96575	0.96575	0.9995	19719	0	-0.285
HCRTR1	6	0.96576	0.96577	0.9995	19720	0	-0.226
CDKN2AIPNL	6	0.96578	0.96578	0.9995	19721	0	-0.281
SLC22A7	6	0.96578	0.96579	0.9995	19722	0	-0.601
PLXDC2	6	0.9658	0.9658	0.9995	19723	0	-0.169
DNAL4	6	0.96581	0.96581	0.9995	19724	0	-0.172
SPDYC	6	0.96586	0.96586	0.9995	19725	0	-0.286
TBC1D28	3	0.96592	0.96602	0.9995	19726	0	-1.348
RETNLB	6	0.96597	0.96598	0.9995	19727	0	-0.071
CES4A	6	0.96608	0.96611	0.9995	19728	0	-0.462
PSMD11	6	0.96609	0.96612	0.9995	19729	0	-0.158
KBTBD12	6	0.96616	0.96619	0.9995	19730	0	-0.328
SERPINB12	6	0.96619	0.96622	0.9995	19731	0	-0.591
IFITM5	6	0.96622	0.96625	0.9995	19732	0	-0.258
KLHL36	6	0.96623	0.96626	0.9995	19733	0	-0.293
GRIN1	6	0.96626	0.96628	0.9995	19734	0	-0.476
PEX5L	6	0.96629	0.96632	0.9995	19735	0	-0.314
FBXO16	6	0.96629	0.96632	0.9995	19736	0	-0.598
STAT5B	6	0.96653	0.96656	0.9995	19737	0	-0.458
SYCE1L	6	0.96654	0.96656	0.9995	19738	0	-0.014
CERS2	6	0.96664	0.96666	0.9995	19739	0	-0.164
STK19	6	0.96672	0.96675	0.9995	19740	0	-0.127
XRRA1	6	0.96675	0.96678	0.9995	19741	0	-0.236
NPHP1	6	0.96675	0.96678	0.9995	19742	0	-0.154
CHRND	6	0.96676	0.96679	0.9995	19743	0	-0.516
CEP128	6	0.96678	0.9668	0.9995	19744	0	-0.106
NLRP9	6	0.96684	0.96687	0.9995	19745	0	-0.521
BMP4	6	0.96688	0.9669	0.9995	19746	0	-0.391
CHMP1B	6	0.96696	0.96697	0.9995	19747	0	-0.438
KRT77	6	0.96715	0.96718	0.9995	19748	0	-0.347
STK3	6	0.96715	0.96718	0.9995	19749	0	0.0092
ISOC1	6	0.96723	0.96726	0.9995	19750	0	-0.459
ZNF699	6	0.96725	0.96728	0.9995	19751	0	-0.207
SLC2A7	6	0.9674	0.96745	0.9995	19752	0	-0.181
B3GAT1	6	0.96742	0.96746	0.9995	19753	0	-0.429
hsa-mir-4770	4	0.96745	0.96738	0.9995	19754	0	-0.254
CRYL1	6	0.9675	0.96753	0.9995	19755	0	-0.144
SAA2	3	0.96759	0.96768	0.9995	19756	0	-1.175
SCG2	6	0.96763	0.96766	0.9995	19757	0	-0.074
hsa-mir-892c	4	0.96763	0.96758	0.9995	19758	0	-0.866
hsa-mir-4502	4	0.96768	0.96763	0.9995	19759	0	-0.384
FMN2	6	0.96774	0.96778	0.9995	19760	0	-0.138
ZNF730	6	0.96782	0.96785	0.9995	19761	0	-0.169
ATP6V0E2	6	0.9679	0.96794	0.9995	19762	0	-0.397
STX16	6	0.96793	0.96797	0.9995	19763	0	-0.608
GRIK3	6	0.96795	0.96799	0.9995	19764	0	-0.459
FAM13B	6	0.96802	0.96805	0.9995	19765	0	-0.13
SMN2	2	0.96808	0.96825	0.9995	19766	0	-2.142
TMEM184B	6	0.96813	0.96816	0.9995	19767	0	-0.193
ZBTB8B	6	0.96814	0.96817	0.9995	19768	0	-0.124
WDR90	6	0.96818	0.96821	0.9995	19769	0	-1.027
CIRBP	6	0.96829	0.96832	0.9995	19770	0	-0.378
PACRG	6	0.96854	0.96857	0.9995	19771	0	-0.164
WDR85	3	0.9686	0.96871	0.9995	19772	0	-0.484
NPY	6	0.96866	0.96869	0.9995	19773	0	-0.494
SEC23IP	6	0.96869	0.96872	0.9995	19774	0	-0.869
CRABP2	6	0.96871	0.96875	0.9995	19775	0	-0.146
TMEM223	6	0.96887	0.96889	0.9995	19776	0	-0.084
HIST1H1E	6	0.96892	0.96895	0.9995	19777	0	-0.071
hsa-mir-4756	4	0.96894	0.96888	0.9995	19778	0	-0.572
HLF	6	0.969	0.96903	0.9995	19779	0	-0.228
FCRLA	6	0.96909	0.96913	0.9995	19780	0	-0.231
hsa-mir-2053	4	0.96911	0.96905	0.9995	19781	0	-0.575
PGPEP1	6	0.96916	0.9692	0.9995	19782	0	-0.032
COX17	6	0.96929	0.96933	0.9995	19783	0	-0.425
ZNF800	6	0.96929	0.96933	0.9995	19784	0	-0.258
KRT4	6	0.9693	0.96935	0.9995	19785	0	-0.53
RAB27A	6	0.96938	0.96942	0.9995	19786	0	-0.217
LCA5L	6	0.96939	0.96944	0.9995	19787	0	-0.02
SHISA2	6	0.96942	0.96947	0.9995	19788	0	-0.1
HSPA12B	6	0.96949	0.96955	0.9995	19789	0	-0.403
EMP3	6	0.96954	0.9696	0.9995	19790	0	-0.532
TRIM39-RPP21	2	0.96966	0.96982	0.9995	19791	0	-0.648
LONRF3	6	0.96967	0.96973	0.9995	19792	0	-0.384
COL21A1	6	0.96969	0.96974	0.9995	19793	0	0.0126
TMEM186	6	0.96974	0.96979	0.9995	19794	0	-0.251
hsa-mir-5702	4	0.96984	0.96978	0.9995	19795	0	-0.367
FBN1	6	0.96984	0.96989	0.9995	19796	0	-0.59
FADD	6	0.96986	0.96991	0.9995	19797	0	-0.309
DHX32	6	0.96987	0.96991	0.9995	19798	0	-0.072
OR8B8	6	0.96993	0.96997	0.9995	19799	0	-0.503
VAMP5	6	0.96995	0.96998	0.9995	19800	0	-0.39

SCHIP1	4	0.96995	0.96989	0.9995	19801	0	-0.369
RWDD2A	6	0.97	0.97003	0.9995	19802	0	-0.631
OR52J3	6	0.97007	0.97011	0.9995	19803	0	-0.159
ZFP28	6	0.97007	0.97011	0.9995	19804	0	-0.242
NIT1	6	0.97009	0.97012	0.9995	19805	0	-0.296
C7orf34	6	0.97016	0.97019	0.9995	19806	0	-0.194
SLC16A8	6	0.9703	0.97033	0.9995	19807	0	-0.302
ACPL2	6	0.97037	0.9704	0.9995	19808	0	-0.105
GPBP1	6	0.9704	0.97043	0.9995	19809	0	-0.029
PLA2R1	6	0.97042	0.97045	0.9995	19810	0	-0.115
FZD8	6	0.97044	0.97048	0.9995	19811	0	-0.411
YOD1	6	0.97044	0.97048	0.9995	19812	0	-0.155
ZNF593	6	0.97047	0.9705	0.9995	19813	0	-0.262
NR4A2	6	0.97048	0.97052	0.9995	19814	0	-0.311
hsa-mir-2278	4	0.9706	0.97055	0.9995	19815	0	-0.375
PPP1R1C	6	0.9706	0.97066	0.9995	19816	0	-0.195
SGK110	6	0.97067	0.97072	0.9995	19817	0	-0.049
CBX4	6	0.97071	0.97076	0.9995	19818	0	-0.444
PGLS	6	0.97079	0.97085	0.9995	19819	0	-0.816
RABGEF1	6	0.97081	0.97086	0.9995	19820	0	-0.368
RASSF5	6	0.97082	0.97087	0.9995	19821	0	-0.156
hsa-mir-6893	4	0.97087	0.97083	0.9995	19822	0	-0.467
PCNXL2	6	0.97089	0.97095	0.9995	19823	0	-0.285
PLA1A	6	0.9709	0.97096	0.9995	19824	0	-0.13
C11orf94	6	0.97095	0.97101	0.9995	19825	0	-0.223
APBB1	6	0.97101	0.97108	0.9995	19826	0	-0.193
DPEP2	6	0.97103	0.97109	0.9995	19827	0	-0.468
GCNT7	6	0.97103	0.97109	0.9995	19828	0	-0.223
RPL26L1	6	0.97107	0.97114	0.9995	19829	0	-0.481
DYNC1LI1	6	0.97116	0.97123	0.9995	19830	0	-0.395
WAS	6	0.97133	0.97141	0.9995	19831	0	-0.436
ATAD5	6	0.97142	0.9715	0.9995	19832	0	-0.178
hsa-mir-20a	1	0.97146	0.97152	0.9995	19833	0	-1.857
SPPL2C	6	0.97147	0.97156	0.9995	19834	0	-0.336
41153	3	0.97156	0.97163	0.9995	19835	0	-0.425
ARPP19	6	0.97159	0.97168	0.9995	19836	0	-0.11
TMEM55A	6	0.97161	0.9717	0.9995	19837	0	-0.121
DOPEY1	6	0.97162	0.97171	0.9995	19838	0	-0.241
DOCK10	6	0.97163	0.97172	0.9995	19839	0	-0.229
KRIT1	6	0.97168	0.97177	0.9995	19840	0	-0.312
HSPA4	6	0.97179	0.97189	0.9995	19841	0	-0.153
TMEM176A	6	0.97184	0.97192	0.9995	19842	0	-0.236
AURKA	6	0.97189	0.97197	0.9995	19843	0	-0.589
VPS33A	6	0.97202	0.9721	0.9995	19844	0	-0.421
VENTX	6	0.97203	0.97211	0.9995	19845	0	-0.233
OPRD1	6	0.97206	0.97214	0.9995	19846	0	-0.482
SLC25A28	6	0.97208	0.97215	0.9995	19847	0	-0.686
BCL9L	6	0.97208	0.97216	0.9995	19848	0	-0.607
hsa-mir-4290	4	0.97209	0.9721	0.9995	19849	0	-0.441
SPANXN3	6	0.97214	0.97221	0.9995	19850	0	-0.61
ENTPD5	6	0.9722	0.97226	0.9995	19851	0	-0.268
MMP1	6	0.97222	0.97228	0.9995	19852	0	-0.095
FKBP1A	6	0.97223	0.97229	0.9995	19853	0	-0.094
CYP4B1	6	0.97224	0.9723	0.9995	19854	0	-0.421
NRM	6	0.97236	0.97242	0.9995	19855	0	-0.272
UCP1	6	0.97242	0.97247	0.9995	19856	0	-0.288
GALK1	6	0.97242	0.97247	0.9995	19857	0	-0.32
TSNARE1	6	0.97252	0.97258	0.9995	19858	0	-0.325
ART5	6	0.97252	0.97258	0.9995	19859	0	-0.93
AMH	6	0.9726	0.97267	0.9995	19860	0	-0.057
SUCLG2	6	0.97266	0.97272	0.9995	19861	0	-0.435
hsa-mir-6722	4	0.9728	0.97283	0.9995	19862	0	-0.613
PIGO	6	0.97284	0.97289	0.9995	19863	0	0.0336
RAB3GAP2	6	0.97293	0.973	0.9995	19864	0	-0.562
MMP11	6	0.97296	0.97302	0.9995	19865	0	-0.418
ARHGAP9	6	0.97299	0.97307	0.9995	19866	0	-0.355
PRRT3	6	0.97304	0.97311	0.9995	19867	0	-0.52
hsa-mir-548e	4	0.97308	0.9731	0.9995	19868	0	-0.527
PLAUR	6	0.97314	0.97321	0.9995	19869	0	-0.443
PSMD2	6	0.97319	0.97326	0.9995	19870	0	-0.149
KCTD17	6	0.97322	0.97329	0.9995	19871	0	-0.552
TMLHE	4	0.97323	0.97326	0.9995	19872	0	-0.192
KIF1C	6	0.97323	0.9733	0.9995	19873	0	-0.374
NUDT9	6	0.97324	0.97331	0.9995	19874	0	-0.134
SLC9A6	6	0.97325	0.97332	0.9995	19875	0	-0.13
COX6C	6	0.97326	0.97333	0.9995	19876	0	-0.157
MRFAP1	6	0.97331	0.97338	0.9995	19877	0	-0.607
CGNL1	6	0.97338	0.97346	0.9995	19878	0	-0.357
ZNF280B	6	0.97338	0.97346	0.9995	19879	0	-0.011
LPPR4	6	0.97343	0.9735	0.9995	19880	0	-0.99
KREMEN2	6	0.97347	0.97355	0.9995	19881	0	-0.2
CYP3A4	5	0.97348	0.97351	0.9995	19882	0	-0.325
CEND1	6	0.97353	0.97362	0.9995	19883	0	-0.51
FGF3	6	0.97356	0.97365	0.9995	19884	0	-0.179
IL10	6	0.97358	0.97368	0.9995	19885	0	-0.041

NET1	6	0.97366	0.97375	0.9995	19886	0	-0.501
H2BFWT	6	0.97367	0.97376	0.9995	19887	0	-0.129
FOXO1	6	0.97375	0.97384	0.9995	19888	0	-0.195
FOXD4L1	5	0.97387	0.97389	0.9995	19889	0	-0.619
UBE2R2	6	0.97393	0.97403	0.9995	19890	0	-0.064
MEGF9	6	0.97405	0.97414	0.9995	19891	0	-0.073
CPNE3	6	0.97409	0.97418	0.9995	19892	0	-0.499
CPD	6	0.9741	0.97419	0.9995	19893	0	-0.133
SLC44A4	6	0.9741	0.97419	0.9995	19894	0	-0.258
MYH7	6	0.97418	0.97427	0.9995	19895	0	-0.369
C8orf59	6	0.97423	0.97432	0.9995	19896	0	-0.608
PDE1C	6	0.97426	0.97436	0.9995	19897	0	-0.24
OR5M1	4	0.97429	0.97433	0.9995	19898	0	-0.873
AP1S3	6	0.97438	0.97449	0.9995	19899	0	-0.401
hsa-mir-4485	3	0.97448	0.97457	0.9995	19900	0	-0.81
CECR6	6	0.97455	0.97465	0.9995	19901	0	-0.22
C7orf55	5	0.97455	0.9746	0.9995	19902	0	-0.383
PHACTR1	6	0.97459	0.97469	0.9995	19903	0	-0.452
ZBTB7B	6	0.9748	0.97492	0.9995	19904	0	-0.349
CHMP4B	6	0.97481	0.97493	0.9995	19905	0	-0.522
C15orf60	6	0.9749	0.97502	0.9995	19906	0	-0.116
NR3C1	6	0.97494	0.97507	0.9995	19907	0	-0.125
HP	6	0.97496	0.97509	0.9995	19908	0	-0.216
MATN1	6	0.97496	0.97509	0.9995	19909	0	-0.594
C3orf70	6	0.97498	0.97511	0.9995	19910	0	-0.036
EML3	6	0.97498	0.97511	0.9995	19911	0	-0.704
IFNW1	6	0.97502	0.97515	0.9995	19912	0	-0.182
POMT2	6	0.97512	0.97525	0.9995	19913	0	-0.388
FLII	6	0.97514	0.97526	0.9995	19914	0	-0.471
SMIM22	5	0.97515	0.97521	0.9995	19915	0	-0.415
C2orf15	6	0.97524	0.97536	0.9995	19916	0	-0.616
MLEC	6	0.97525	0.97537	0.9995	19917	0	-0.319
QPRT	6	0.97528	0.9754	0.9995	19918	0	-0.253
ARAP1	6	0.97532	0.97544	0.9995	19919	0	-0.213
hsa-mir-95	4	0.97536	0.97544	0.9995	19920	0	-0.795
MEN1	6	0.9754	0.97552	0.9995	19921	0	-0.266
HTR4	6	0.9754	0.97552	0.9995	19922	0	-0.207
TMEM191B	6	0.9754	0.97552	0.9995	19923	0	-0.289
OR4C3	6	0.9755	0.97563	0.9995	19924	0	-0.481
ZNF716	5	0.97552	0.97558	0.9995	19925	0	-0.72
FAM50B	6	0.97557	0.9757	0.9995	19926	0	-0.461
C8orf44	5	0.97561	0.97567	0.9995	19927	0	-0.472
PRRC1	6	0.97563	0.97577	0.9995	19928	0	-0.097
CCDC85A	6	0.97573	0.97587	0.9995	19929	0	-0.837
NKX2-2	6	0.97584	0.97598	0.9995	19930	0	-0.449
hsa-mir-1228	4	0.97589	0.97598	0.9995	19931	0	-0.503
PDIA6	6	0.97594	0.97608	0.9995	19932	0	-0.255
ACVR1	6	0.97603	0.97616	0.9995	19933	0	-0.183
NPY2R	6	0.97608	0.97622	0.9995	19934	0	-0.138
DISP1	6	0.97608	0.97622	0.9995	19935	0	-0.473
EXOC1	6	0.97613	0.97626	0.9995	19936	0	-0.591
PKD1L3	6	0.97614	0.97627	0.9995	19937	0	-0.141
SULT6B1	6	0.97615	0.97628	0.9995	19938	0	-0.328
CNGB3	6	0.97616	0.97629	0.9995	19939	0	-0.333
HHEX	6	0.9762	0.97633	0.9995	19940	0	-0.225
ZNF473	6	0.9763	0.97643	0.9995	19941	0	-0.37
HOXB13	6	0.97638	0.97651	0.9995	19942	0	-0.392
EMX1	6	0.9764	0.97652	0.9995	19943	0	-0.233
LPIN3	6	0.97642	0.97654	0.9995	19944	0	-0.52
NRXN1	6	0.97644	0.97656	0.9995	19945	0	-0.504
GPNMB	6	0.97651	0.97664	0.9995	19946	0	-0.907
TUSC5	6	0.97653	0.97665	0.9995	19947	0	-0.361
RPS19	6	0.97658	0.97671	0.9995	19948	0	-0.359
PADI4	6	0.97661	0.97674	0.9995	19949	0	-0.298
RNF19B	6	0.97665	0.97678	0.9995	19950	0	-0.229
PCDHAC1	2	0.97665	0.97681	0.9995	19951	0	-0.844
MYPOP	6	0.97666	0.97679	0.9995	19952	0	-0.19
KIF20A	6	0.97672	0.97686	0.9995	19953	0	-0.193
MRPL40	6	0.97678	0.97692	0.9995	19954	0	-0.51
HIST1H3J	5	0.97683	0.97688	0.9995	19955	0	-0.581
PCK2	6	0.97684	0.97699	0.9995	19956	0	-0.312
SERPINB7	6	0.97684	0.97699	0.9995	19957	0	-0.606
DSCAML1	6	0.97687	0.97702	0.9995	19958	0	-0.427
AZI2	6	0.97689	0.97704	0.9995	19959	0	-0.225
OR1L8	6	0.97694	0.97708	0.9995	19960	0	-0.395
AGO2	6	0.977	0.97712	0.9995	19961	0	-0.405
ARMC1	6	0.97701	0.97714	0.9995	19962	0	-0.372
GPT	6	0.97702	0.97715	0.9995	19963	0	-0.456
PLA2G7	6	0.97705	0.97718	0.9995	19964	0	-0.419
NQO1	6	0.97709	0.97721	0.9995	19965	0	-0.267
BAG6	6	0.97714	0.97726	0.9995	19966	0	-0.619
S100A3	6	0.97715	0.97727	0.9995	19967	0	-0.192
FOXA1	6	0.97724	0.97737	0.9995	19968	0	-0.365
hsa-mir-107	4	0.97726	0.97738	0.9995	19969	0	-0.82
ATG2B	6	0.97738	0.97751	0.9995	19970	0	-0.141

PMM2	6	0.97738	0.97751	0.9995	19971	0	-0.111
IGSF1	6	0.97742	0.97756	0.9995	19972	0	-0.196
ZFP37	6	0.97752	0.97767	0.9995	19973	0	-0.383
IFT81	6	0.97755	0.97769	0.9995	19974	0	-0.989
MCF2L	6	0.97757	0.97771	0.9995	19975	0	-0.553
ZNF37A	6	0.9776	0.97775	0.9995	19976	0	-0.635
KDM1A	6	0.97761	0.97776	0.9995	19977	0	-0.402
CALCOCO2	6	0.97763	0.97777	0.9995	19978	0	-0.053
NKAPL	6	0.97772	0.97786	0.9995	19979	0	-0.581
hsa-mir-4304	4	0.97776	0.97786	0.9995	19980	0	-0.64
B3GNT4	6	0.97776	0.97789	0.9995	19981	0	-0.401
CD300A	6	0.97779	0.97792	0.9995	19982	0	-0.267
AKAP12	6	0.97791	0.97804	0.9995	19983	0	-0.594
hsa-mir-6090	4	0.97811	0.97819	0.9995	19984	0	-1.135
hsa-mir-130a	4	0.97813	0.9782	0.9995	19985	0	-0.893
ZNF107	5	0.97822	0.97829	0.9995	19986	0	-0.111
SNAP47	6	0.97825	0.97839	0.9995	19987	0	-0.596
DNMT1	6	0.97835	0.97848	0.9995	19988	0	-0.309
FOXS1	6	0.97835	0.97849	0.9995	19989	0	-0.309
OR4M1	6	0.97838	0.97852	0.9995	19990	0	-0.64
WDR48	6	0.9784	0.97854	0.9995	19991	0	-0.425
KCNJ1	6	0.97841	0.97854	0.9995	19992	0	-0.442
MBNL3	6	0.97843	0.97857	0.9995	19993	0	-0.235
PBXIP1	6	0.97854	0.97868	0.9995	19994	0	-0.674
NKD2	6	0.97859	0.97872	0.9995	19995	0	-0.199
TCEAL5	5	0.97859	0.97865	0.9995	19996	0	-0.123
RWDD2B	6	0.97862	0.97877	0.9995	19997	0	-0.252
SSTR2	6	0.97867	0.97882	0.9995	19998	0	-0.263
DYDC2	6	0.97869	0.97883	0.9995	19999	0	-0.295
DMTN	6	0.97869	0.97883	0.9995	20000	0	-0.194
CRELD2	6	0.9787	0.97885	0.9995	20001	0	-0.162
ZNF772	6	0.97872	0.97886	0.9995	20002	0	-0.287
CYTH2	6	0.97874	0.97888	0.9995	20003	0	-0.303
SPATA19	6	0.97876	0.9789	0.9995	20004	0	-0.868
TM4SF5	6	0.97883	0.97896	0.9995	20005	0	-0.448
KLHL30	6	0.97895	0.97909	0.9995	20006	0	-0.366
CCRL1	5	0.97895	0.97902	0.9995	20007	0	-0.375
LIMK1	6	0.97897	0.97911	0.9995	20008	0	-0.493
HEY2	6	0.97907	0.97921	0.9995	20009	0	-0.613
TMEM115	6	0.97907	0.97921	0.9995	20010	0	-0.373
USP5	6	0.97909	0.97923	0.9995	20011	0	-0.194
SAMD15	6	0.97911	0.97925	0.9995	20012	0	-0.567
TIGD3	6	0.97914	0.97928	0.9995	20013	0	-1.445
MYBPC2	6	0.97914	0.97928	0.9995	20014	0	-0.346
TEX14	6	0.97915	0.97929	0.9995	20015	0	-0.474
PHLPP2	6	0.97916	0.9793	0.9995	20016	0	-0.126
BRS3	6	0.97919	0.97933	0.9995	20017	0	-0.235
CYP4F3	6	0.97932	0.97946	0.9995	20018	0	-0.605
TMEM38B	6	0.97934	0.97948	0.9995	20019	0	-0.407
EDAR	6	0.97948	0.97962	0.9995	20020	0	-0.039
KRTAP1-1	6	0.97955	0.9797	0.9995	20021	0	-0.37
CT62	6	0.97972	0.97988	0.9995	20022	0	-0.206
PLLP	6	0.97989	0.98005	0.9995	20023	0	-0.203
WDR83	6	0.97995	0.98011	0.9995	20024	0	-0.518
PSTPIP1	6	0.97995	0.98011	0.9995	20025	0	-0.135
CDK5	6	0.97996	0.98011	0.9995	20026	0	-0.443
PCDHA3	2	0.98001	0.98012	0.9995	20027	0	-0.675
OR10X1	6	0.98011	0.98026	0.9995	20028	0	-0.15
PPP1R3G	6	0.98015	0.9803	0.9995	20029	0	-0.331
SYT11	6	0.98015	0.9803	0.9995	20030	0	-0.254
PCDHA12	2	0.98018	0.98031	0.9995	20031	0	-0.719
PHKG2	6	0.98021	0.98037	0.9995	20032	0	-0.189
C5orf54	6	0.98024	0.9804	0.9995	20033	0	-0.344
hsa-mir-6744	4	0.98029	0.9803	0.9995	20034	0	-0.279
hsa-mir-3960	4	0.98029	0.9803	0.9995	20035	0	-0.895
WNT3A	6	0.98033	0.98048	0.9995	20036	0	-0.364
SPATS1	6	0.98036	0.98051	0.9995	20037	0	-0.312
UBE2N	6	0.98039	0.98055	0.9995	20038	0	-0.389
PPY	6	0.98043	0.98059	0.9995	20039	0	-0.258
TREML2	6	0.9806	0.98077	0.9995	20040	0	-0.113
DIRC2	6	0.98072	0.98089	0.9995	20041	0	-0.26
NANOG	6	0.98072	0.98089	0.9995	20042	0	-0.625
ENY2	6	0.98077	0.98094	0.9995	20043	0	-0.19
RPE65	6	0.98081	0.98098	0.9995	20044	0	-0.152
DCAF16	6	0.98083	0.98099	0.9995	20045	0	-0.205
PANX1	6	0.98085	0.98102	0.9995	20046	0	-0.193
TNR	6	0.98089	0.98105	0.9995	20047	0	-0.719
METTL13	6	0.98093	0.98109	0.9995	20048	0	-0.236
SPACA3	6	0.98094	0.9811	0.9995	20049	0	-0.13
WDR41	6	0.98097	0.98113	0.9995	20050	0	-0.161
C9orf47	6	0.98098	0.98114	0.9995	20051	0	-0.497
FKBP10	6	0.981	0.98117	0.9995	20052	0	-0.237
GPR15	6	0.98101	0.98118	0.9995	20053	0	-0.387
FAM83H	6	0.98109	0.98126	0.9995	20054	0	-0.31
OR2T8	6	0.98117	0.98134	0.9995	20055	0	-0.285

MED6	6	0.98119	0.98136	0.9995	20056	0	-0.705
STEAP3	6	0.98122	0.98139	0.9995	20057	0	-0.188
ACD	6	0.98136	0.98153	0.9995	20058	0	-0.305
SH3BP5	6	0.98136	0.98154	0.9995	20059	0	-0.508
HHIPL2	6	0.98137	0.98155	0.9995	20060	0	-0.228
ESD	6	0.98141	0.98159	0.9995	20061	0	-0.511
HGFAC	6	0.98145	0.98162	0.9995	20062	0	-0.283
GTF2IRD2B	2	0.98151	0.98163	0.9995	20063	0	-1.047
CCDC105	6	0.98151	0.98168	0.9995	20064	0	-0.29
hsa-mir-5001	4	0.98163	0.98158	0.9995	20065	0	-0.892
SYNGR2	4	0.98163	0.98158	0.9995	20066	0	-0.953
hsa-mir-4658	4	0.98163	0.98158	0.9995	20067	0	-0.796
hsa-let-7b	4	0.98163	0.98158	0.9995	20068	0	-0.924
hsa-mir-519d	4	0.98163	0.98158	0.9995	20069	0	-2.713
hsa-mir-494	4	0.98163	0.98158	0.9995	20070	0	-1.203
LCE1A	4	0.98163	0.98158	0.9995	20071	0	-1.233
hsa-mir-548l	4	0.98163	0.98158	0.9995	20072	0	-0.423
AGXT2L2	1	0.98168	0.98173	0.9995	20073	0	-1.044
39692	3	0.98174	0.98186	0.9995	20074	0	-0.655
SLC18A2	6	0.98185	0.98203	0.9995	20075	0	-0.412
CACNG4	6	0.98196	0.98214	0.9995	20076	0	-0.756
IL12A	6	0.98197	0.98215	0.9995	20077	0	-0.307
SYDE1	6	0.9821	0.98228	0.9995	20078	0	-0.399
CDK5R1	6	0.98211	0.98229	0.9995	20079	0	-0.325
TRABD2B	6	0.9822	0.98236	0.9995	20080	0	-0.998
SCRT1	6	0.98221	0.98237	0.9995	20081	0	-0.202
MAP3K9	6	0.98222	0.98238	0.9995	20082	0	-0.325
KRT37	5	0.98228	0.98235	0.9995	20083	0	-1.233
ZNF492	5	0.98228	0.98235	0.9995	20084	0	-0.419
PCGF1	6	0.9823	0.98247	0.9995	20085	0	-0.548
CHRM4	6	0.98231	0.98248	0.9995	20086	0	-0.085
hsa-mir-222	4	0.98234	0.98229	0.9995	20087	0	-0.745
hsa-mir-5091	4	0.98242	0.98238	0.9995	20088	0	-0.372
HYLS1	6	0.98242	0.98258	0.9995	20089	0	-0.232
ANKK1	6	0.98246	0.98262	0.9995	20090	0	-0.286
XPO5	6	0.98249	0.98264	0.9995	20091	0	-0.106
SOLH	5	0.98261	0.98265	0.9995	20092	0	-0.246
OR6C70	6	0.98275	0.9829	0.9995	20093	0	-0.163
CEP97	6	0.98285	0.983	0.9995	20094	0	-0.216
ZNF684	6	0.98288	0.98303	0.9995	20095	0	-0.279
HEPN1	6	0.98289	0.98304	0.9995	20096	0	-0.298
SOX8	6	0.98297	0.98312	0.9995	20097	0	-0.376
POLR2D	6	0.98297	0.98312	0.9995	20098	0	-0.44
NAT8	6	0.98305	0.9832	0.9995	20099	0	-0.532
PRELID2	6	0.98306	0.98322	0.9995	20100	0	-0.456
ITGA6	6	0.98308	0.98323	0.9995	20101	0	-0.334
PBX2	6	0.98316	0.98332	0.9995	20102	0	-0.734
NEURL2	6	0.98319	0.98334	0.9995	20103	0	-0.456
TCF12	6	0.98324	0.9834	0.9995	20104	0	-0.173
SMARCD2	6	0.98324	0.9834	0.9995	20105	0	-0.471
FRMD8	6	0.98327	0.98343	0.9995	20106	0	-0.212
XPNPEP1	6	0.98329	0.98345	0.9995	20107	0	-0.529
ANKAR	6	0.98344	0.9836	0.9995	20108	0	-0.267
TUBB3	6	0.98345	0.98361	0.9995	20109	0	-0.358
OR4C13	6	0.98351	0.98366	0.9995	20110	0	-0.288
CCDC101	6	0.98354	0.98369	0.9995	20111	0	-0.528
EXO5	6	0.98354	0.98369	0.9995	20112	0	-0.26
hsa-mir-646	4	0.98357	0.98351	0.9995	20113	0	-0.775
PCDHGA9	2	0.98357	0.98367	0.9995	20114	0	-0.896
TMEM39A	6	0.98364	0.98378	0.9995	20115	0	-0.617
hsa-mir-1255a	4	0.98365	0.9836	0.9995	20116	0	-0.89
GPR146	6	0.98365	0.98378	0.9995	20117	0	-0.367
C4orf45	6	0.98366	0.98379	0.9995	20118	0	-0.113
C19orf52	6	0.98366	0.9838	0.9995	20119	0	-0.707
TRAIP	6	0.98368	0.98381	0.9995	20120	0	-0.658
APC2	6	0.98368	0.98381	0.9995	20121	0	-0.673
hsa-mir-4762	2	0.98378	0.98388	0.9995	20122	0	-1.365
ZNF560	6	0.98383	0.98395	0.9995	20123	0	-0.679
C9orf92	6	0.98386	0.98399	0.9995	20124	0	-0.578
TPST1	6	0.98391	0.98403	0.9995	20125	0	-0.256
PRKAB1	6	0.98396	0.98408	0.9995	20126	0	-0.501
ADAMTS2	6	0.98398	0.98411	0.9995	20127	0	-0.242
SLC16A3	6	0.98405	0.98418	0.9995	20128	0	-0.223
OR56B1	6	0.98414	0.98427	0.9995	20129	0	-0.693
MAGEC1	6	0.98416	0.98429	0.9995	20130	0	-0.444
TEX9	6	0.9842	0.98434	0.9995	20131	0	-0.401
GRID2IP	6	0.98421	0.98435	0.9995	20132	0	-0.262
hsa-mir-4677	4	0.98425	0.9842	0.9995	20133	0	-0.435
ZNF629	6	0.9843	0.98443	0.9995	20134	0	-0.206
MTNR1B	6	0.98438	0.98451	0.9995	20135	0	-0.432
DMRTA2	6	0.9844	0.98452	0.9995	20136	0	-0.878
DVL1	6	0.98453	0.98466	0.9995	20137	0	-0.346
hsa-mir-4714	4	0.98455	0.9845	0.9995	20138	0	-0.569
RSPH9	6	0.98458	0.98471	0.9995	20139	0	-0.282
SLC30A9	6	0.9846	0.98473	0.9995	20140	0	-0.258

ZDHHC5	6	0.98469	0.98482	0.9995	20141	0	-0.558
OR10Q1	6	0.98471	0.98484	0.9995	20142	0	-0.1
ADAM18	6	0.98482	0.98495	0.9995	20143	0	-0.579
ABLIM1	6	0.98482	0.98496	0.9995	20144	0	-0.417
TIMP3	6	0.98492	0.98506	0.9995	20145	0	-0.147
PBOV1	6	0.98494	0.98509	0.9995	20146	0	-0.59
SSR4	6	0.98503	0.98517	0.9995	20147	0	-0.083
HELT	6	0.98506	0.9852	0.9995	20148	0	-0.237
CYYR1	6	0.98514	0.98526	0.9995	20149	0	-0.438
LRFN5	6	0.98515	0.98527	0.9995	20150	0	-0.215
DIAPH1	6	0.98515	0.98527	0.9995	20151	0	-0.115
hsa-mir-4318	4	0.98521	0.98518	0.9995	20152	0	-0.388
MMP23B	6	0.98526	0.98539	0.9995	20153	0	-0.592
GPR45	6	0.98534	0.98547	0.9995	20154	0	-0.25
KCNS3	6	0.98535	0.98548	0.9995	20155	0	-0.588
hsa-mir-485	4	0.98538	0.98534	0.9995	20156	0	-0.519
ATXN7L3	6	0.98538	0.98551	0.9995	20157	0	-0.401
DRP2	6	0.98538	0.98551	0.9995	20158	0	-0.456
C16orf93	6	0.9854	0.98552	0.9995	20159	0	-0.317
AFAP1L1	6	0.9854	0.98552	0.9995	20160	0	-0.401
GSTO2	6	0.9854	0.98552	0.9995	20161	0	-0.22
MVK	6	0.98547	0.9856	0.9995	20162	0	-0.129
PLEKHH1	6	0.98553	0.98566	0.9995	20163	0	-0.251
TSSC4	6	0.98556	0.98567	0.9995	20164	0	-0.438
KCNG1	6	0.9856	0.98572	0.9995	20165	0	-0.625
SMIM8	6	0.98562	0.98574	0.9995	20166	0	-0.412
EGFL8	6	0.98574	0.98586	0.9995	20167	0	-0.543
TFIP11	6	0.98575	0.98587	0.9995	20168	0	-0.538
TRAF4	6	0.98582	0.98593	0.9995	20169	0	-0.395
IFT88	6	0.98583	0.98595	0.9995	20170	0	-0.373
RBL2	6	0.98596	0.98608	0.9995	20171	0	-0.344
CCDC124	6	0.98596	0.98608	0.9995	20172	0	-0.66
ZNF217	6	0.98597	0.98609	0.9995	20173	0	-0.624
MLX	6	0.98603	0.98614	0.9995	20174	0	-0.177
OR2T33	6	0.98607	0.98617	0.9995	20175	0	-0.759
SMPD2	6	0.98611	0.98622	0.9995	20176	0	-0.605
DOK1	6	0.98614	0.98624	0.9995	20177	0	-0.43
NTHL1	6	0.98616	0.98627	0.9995	20178	0	-0.28
LIF	6	0.98622	0.98632	0.9995	20179	0	-0.199
C7orf26	6	0.98624	0.98634	0.9995	20180	0	-0.167
SMIM3	6	0.98628	0.98637	0.9995	20181	0	-0.343
GLIPR1L1	6	0.98629	0.98638	0.9995	20182	0	-0.245
LOC100288524	6	0.98635	0.98644	0.9995	20183	0	-0.395
TM4SF19	6	0.98637	0.98645	0.9995	20184	0	-0.266
IL25	6	0.98639	0.98647	0.9995	20185	0	-0.146
RIC8A	6	0.98646	0.98653	0.9995	20186	0	-0.52
LDB1	6	0.98647	0.98654	0.9995	20187	0	-0.357
hsa-mir-6771	4	0.98648	0.98642	0.9995	20188	0	-0.44
EPB41	6	0.98651	0.98658	0.9995	20189	0	-0.215
RHOH	6	0.98657	0.98663	0.9995	20190	0	-0.517
CYB5A	6	0.98665	0.98671	0.9995	20191	0	-0.35
PGM1	6	0.98671	0.98677	0.9995	20192	0	-0.649
NT5C	6	0.98672	0.98677	0.9995	20193	0	-0.619
ZNF135	6	0.98674	0.9868	0.9995	20194	0	-0.434
SCGB2B2	6	0.98685	0.98691	0.9995	20195	0	-0.579
KIAA1217	6	0.98693	0.98699	0.9995	20196	0	-0.626
hsa-mir-25	4	0.98705	0.987	0.9995	20197	0	-0.572
hsa-mir-4300	2	0.98707	0.98714	0.9995	20198	0	-1.258
ARF1	6	0.98707	0.98713	0.9995	20199	0	-0.7
SGK3	5	0.98708	0.98707	0.9995	20200	0	-0.339
KIAA0907	6	0.98717	0.98723	0.9995	20201	0	-0.438
TMEM81	6	0.98718	0.98724	0.9995	20202	0	-0.554
KRTAP10-11	6	0.98721	0.98727	0.9995	20203	0	-0.613
RGS7	6	0.98737	0.98743	0.9995	20204	0	-0.451
MEIS3	6	0.98741	0.98747	0.9995	20205	0	-0.523
FGFBP1	6	0.98742	0.98749	0.9995	20206	0	-0.556
C6orf211	6	0.9875	0.98757	0.9995	20207	0	-0.222
DPF2	6	0.98754	0.98762	0.9995	20208	0	-0.592
PCOLCE	6	0.98762	0.98769	0.9995	20209	0	-0.445
ZNHIT2	6	0.98763	0.9877	0.9995	20210	0	-0.85
NARR	3	0.98764	0.98779	0.9995	20211	0	-0.521
RNF24	6	0.98765	0.98772	0.9995	20212	0	-0.288
FMO2	6	0.98767	0.98774	0.9995	20213	0	-0.368
SLC9A3R2	6	0.98769	0.98775	0.9995	20214	0	-0.344
KIAA1644	6	0.9877	0.98776	0.9995	20215	0	-0.36
ASPSCR1	6	0.98775	0.98781	0.9995	20216	0	-0.31
MARVELD3	6	0.98775	0.98781	0.9995	20217	0	-0.4
hsa-mir-6501	4	0.98778	0.98777	0.9995	20218	0	-0.884
hsa-mir-892a	4	0.98779	0.98779	0.9995	20219	0	-1.153
ORAI2	6	0.9878	0.98787	0.9995	20220	0	-0.335
DHX30	6	0.98782	0.98789	0.9995	20221	0	-0.417
RUSC1	6	0.98794	0.98801	0.9995	20222	0	-0.398
TDRP	3	0.98794	0.98811	0.9995	20223	0	-0.573
C1orf159	6	0.98795	0.98802	0.9995	20224	0	-0.216
CARD17	6	0.98803	0.98811	0.9995	20225	0	-0.3

RNASE8	6	0.98803	0.98811	0.9995	20226	0	-0.261
SCRT2	6	0.98804	0.98812	0.9995	20227	0	-0.654
PRDX6	6	0.98821	0.98829	0.99961	20228	0	-0.196
ENDOD1	6	0.98824	0.98832	0.99961	20229	0	-0.765
GPR183	6	0.98844	0.98852	0.99965	20230	0	-0.793
CPT2	6	0.98853	0.98861	0.99965	20231	0	-0.465
N4BP2	6	0.98858	0.98866	0.99965	20232	0	-0.337
MTHFS	2	0.98861	0.98862	0.99965	20233	0	-2.093
PGLYRP1	6	0.98864	0.98872	0.99965	20234	0	-0.327
GALR2	6	0.98866	0.98874	0.99965	20235	0	-0.934
CXorf30	6	0.98868	0.98876	0.99965	20236	0	-0.564
CACNA1C	6	0.9887	0.98878	0.99965	20237	0	-0.6
RNF19A	6	0.98878	0.98887	0.99965	20238	0	-0.751
EIF2AK1	6	0.98883	0.98891	0.99965	20239	0	-0.788
ESPNL	6	0.98887	0.98895	0.99965	20240	0	-0.302
hsa-mir-518b	4	0.98895	0.98894	0.99965	20241	0	-1.908
HLA-B	6	0.98899	0.98907	0.99972	20242	0	-0.503
DUSP6	6	0.98909	0.98915	0.99972	20243	0	-0.62
RPS26	6	0.98912	0.98918	0.99972	20244	0	-0.701
ITIH2	6	0.98916	0.98922	0.99972	20245	0	-0.177
TMED2	6	0.98929	0.98934	0.99973	20246	0	-0.831
TNFRSF13B	6	0.98936	0.98943	0.99973	20247	0	-0.24
HEPHL1	6	0.98947	0.98956	0.99973	20248	0	-0.147
TALDO1	6	0.98948	0.98957	0.99973	20249	0	-0.472
ARHGAP40	6	0.9895	0.98959	0.99973	20250	0	-0.554
hsa-mir-3140	4	0.98955	0.98953	0.99973	20251	0	-0.577
ACADM	6	0.98959	0.98968	0.99973	20252	0	-0.593
DAGLA	6	0.9896	0.98969	0.99973	20253	0	-0.658
PRDM2	6	0.98962	0.98971	0.99973	20254	0	-0.281
PTK7	6	0.9897	0.98979	0.99973	20255	0	-0.292
ATPAF1	6	0.98977	0.98985	0.99973	20256	0	-0.446
SLC38A10	6	0.98991	0.98998	0.99973	20257	0	-0.352
NOS1	6	0.98993	0.98999	0.99973	20258	0	-0.534
TMEM92	6	0.99	0.99006	0.99973	20259	0	-0.188
TG	6	0.99009	0.99016	0.99973	20260	0	-0.656
OR2H1	6	0.99017	0.99023	0.99973	20261	0	-0.347
hsa-mir-6765	4	0.99019	0.99016	0.99973	20262	0	-0.695
SUFU	6	0.99021	0.99027	0.99973	20263	0	-0.403
LSMEM2	6	0.99036	0.99042	0.99973	20264	0	-0.189
hsa-mir-4498	4	0.99037	0.99036	0.99973	20265	0	-0.477
PPM1G	6	0.9904	0.99046	0.99973	20266	0	-0.463
C1orf68	6	0.9905	0.99055	0.99973	20267	0	-0.488
STK38L	6	0.99069	0.99073	0.99973	20268	0	-0.702
ZBTB34	6	0.99076	0.99079	0.99973	20269	0	-0.593
TMEM127	6	0.99079	0.99083	0.99973	20270	0	-0.653
hsa-mir-4738	4	0.99081	0.99079	0.99973	20271	0	-0.452
hsa-mir-548f-3	3	0.99083	0.99093	0.99973	20272	0	-0.735
COPE	6	0.99083	0.99086	0.99973	20273	0	-0.64
SPON2	6	0.99085	0.99088	0.99973	20274	0	-0.413
CXorf65	6	0.99087	0.9909	0.99973	20275	0	-0.336
OR6B2	6	0.99093	0.99097	0.99973	20276	0	-0.328
FOSL1	6	0.99146	0.99149	0.99973	20277	0	-0.585
FAM3A	6	0.99147	0.9915	0.99973	20278	0	-0.43
hsa-mir-6510	4	0.99147	0.99149	0.99973	20279	0	-1.248
LRP5	6	0.9915	0.99154	0.99973	20280	0	-0.223
FSTL3	6	0.99157	0.99162	0.99973	20281	0	-0.556
RAB13	6	0.9916	0.99165	0.99973	20282	0	-0.836
C20orf85	6	0.99168	0.99172	0.99973	20283	0	-0.23
OR7G3	6	0.99169	0.99173	0.99973	20284	0	-0.462
SCT	6	0.99191	0.99196	0.99973	20285	0	-0.172
hsa-mir-4710	4	0.99199	0.99199	0.99973	20286	0	-1.305
hsa-mir-5571	4	0.99206	0.99205	0.99973	20287	0	-0.784
AKR1B10	6	0.99209	0.99215	0.99973	20288	0	-0.275
KRTAP9-4	6	0.99209	0.99215	0.99973	20289	0	-0.826
ZNF273	6	0.99209	0.99215	0.99973	20290	0	-0.488
ZNF235	6	0.99209	0.99215	0.99973	20291	0	-0.543
HIST2H3D	6	0.99209	0.99215	0.99973	20292	0	-1.359
GH1	6	0.99209	0.99215	0.99973	20293	0	-0.794
NOTCH4	6	0.99225	0.99231	0.99973	20294	0	-0.556
SLC46A3	6	0.9923	0.99236	0.99973	20295	0	-0.417
P2RX2	6	0.99233	0.9924	0.99973	20296	0	-0.23
MOAP1	4	0.99236	0.99237	0.99973	20297	0	-0.766
NPB	6	0.99237	0.99243	0.99973	20298	0	-0.841
CHCHD7	6	0.99239	0.99244	0.99973	20299	0	-0.546
RHOT2	6	0.99244	0.99249	0.99973	20300	0	-0.47
RPL38	6	0.99252	0.99258	0.99973	20301	0	-0.83
BANF2	6	0.99253	0.99259	0.99973	20302	0	-0.902
OR1G1	6	0.99257	0.99263	0.99973	20303	0	-0.875
CXorf22	6	0.99264	0.9927	0.99973	20304	0	-0.653
ATP5L2	5	0.99265	0.99263	0.99973	20305	0	-0.804
SLC22A24	4	0.99272	0.99272	0.99973	20306	0	-0.8
MRS2	6	0.99274	0.9928	0.99973	20307	0	-0.757
USP54	6	0.99276	0.99282	0.99973	20308	0	-0.284
PGM2	6	0.9928	0.99286	0.99973	20309	0	-0.737
ALPL	6	0.99283	0.9929	0.99973	20310	0	-0.499

ANAPC13	6	0.99287	0.99294	0.99973	20311	0	-0.605
PRLH	6	0.99289	0.99296	0.99973	20312	0	-0.757
FAM83B	6	0.99291	0.99298	0.99973	20313	0	-0.398
NHEJ1	6	0.99295	0.99302	0.99973	20314	0	-0.485
ZNF282	6	0.99299	0.99306	0.99973	20315	0	-0.62
HDAC8	6	0.99302	0.9931	0.99973	20316	0	-0.638
ZNF444	6	0.99314	0.99321	0.99973	20317	0	-0.368
MT1M	5	0.99324	0.9932	0.99973	20318	0	-1.233
CYP2A7	5	0.99324	0.9932	0.99973	20319	0	-1.233
FAM170B	6	0.99336	0.99343	0.99973	20320	0	-0.455
SAMD7	6	0.99343	0.99349	0.99973	20321	0	-0.604
hsa-mir-149	4	0.99346	0.99348	0.99973	20322	0	-0.963
IAPP	6	0.9935	0.99355	0.99973	20323	0	-0.853
AOX1	6	0.9935	0.99356	0.99973	20324	0	-0.721
GRN	6	0.99351	0.99357	0.99973	20325	0	-0.684
ARL4D	6	0.99353	0.99359	0.99973	20326	0	-0.498
SIX6	6	0.99354	0.99359	0.99973	20327	0	-0.258
RBAK-LOC389458	2	0.99356	0.99357	0.99973	20328	0	-1.094
RNF8	6	0.99358	0.99364	0.99973	20329	0	-0.215
CROCC	6	0.99358	0.99365	0.99973	20330	0	-0.438
STK32A	6	0.99362	0.99368	0.99973	20331	0	-0.556
MNT	6	0.99362	0.99368	0.99973	20332	0	-0.603
hsa-mir-890	4	0.99371	0.99374	0.99973	20333	0	-0.891
ITGA5	6	0.99372	0.99377	0.99973	20334	0	-0.673
TNFAIP1	6	0.99393	0.99398	0.99973	20335	0	-0.421
LHFPL3	6	0.99394	0.99398	0.99973	20336	0	-0.682
DTX4	6	0.99395	0.994	0.99973	20337	0	-0.33
ZG16B	6	0.99408	0.99413	0.99973	20338	0	-0.506
APOBEC3F	6	0.99412	0.99415	0.99973	20339	0	-0.287
SBNO1	6	0.99414	0.99418	0.99973	20340	0	-0.395
PLK1	6	0.99422	0.99425	0.99973	20341	0	-0.69
CCDC80	6	0.99431	0.99434	0.99973	20342	0	-0.589
EDF1	6	0.99439	0.99442	0.99973	20343	0	-0.291
NID1	6	0.9944	0.99444	0.99973	20344	0	-0.253
RBM39	6	0.99442	0.99445	0.99973	20345	0	-1.225
PITPNM3	6	0.99444	0.99447	0.99973	20346	0	-0.42
hsa-mir-1202	4	0.99447	0.99448	0.99973	20347	0	-0.751
LCN12	6	0.9945	0.99453	0.99973	20348	0	-0.45
FAM200A	6	0.99453	0.99456	0.99973	20349	0	-0.334
C10orf71	6	0.9946	0.99463	0.99973	20350	0	-0.463
OR2V2	6	0.99467	0.99471	0.99973	20351	0	-0.321
PHLDA1	6	0.99472	0.99475	0.99973	20352	0	-0.362
TRIM73	2	0.99478	0.99476	0.99973	20353	0	-1.013
PCSK6	6	0.99489	0.99493	0.99973	20354	0	-0.403
SOCS6	6	0.99493	0.99496	0.99973	20355	0	-0.336
OBSL1	4	0.99497	0.99497	0.99973	20356	0	-0.403
TFAP2C	6	0.99499	0.99502	0.99973	20357	0	-0.229
RANBP9	6	0.99507	0.99511	0.99973	20358	0	-0.282
NADK2	6	0.9952	0.99525	0.99973	20359	0	-0.726
NBR1	6	0.9952	0.99525	0.99973	20360	0	-0.632
HIST1H4F	6	0.99535	0.9954	0.99973	20361	0	-0.455
C9orf64	6	0.99538	0.99544	0.99973	20362	0	-0.447
GNG5	6	0.99546	0.99552	0.99973	20363	0	-0.418
OR2G6	6	0.99547	0.99554	0.99973	20364	0	-0.447
MYBPHL	6	0.99548	0.99555	0.99973	20365	0	-0.333
SAG	6	0.99566	0.99572	0.99973	20366	0	-0.86
LPHN2	6	0.9957	0.99576	0.99973	20367	0	-1.096
C9orf16	6	0.9957	0.99576	0.99973	20368	0	-0.269
SLC44A2	6	0.99578	0.99583	0.99973	20369	0	-0.292
TIMP1	6	0.99578	0.99583	0.99973	20370	0	-0.42
LRRC56	6	0.99579	0.99585	0.99973	20371	0	-0.699
BST1	6	0.99587	0.99592	0.99973	20372	0	-0.29
AMBP	6	0.99588	0.99593	0.99973	20373	0	-0.589
C1orf226	6	0.9959	0.99595	0.99973	20374	0	-1.033
LRRC32	6	0.99596	0.99601	0.99973	20375	0	-0.187
USP4	6	0.99596	0.99601	0.99973	20376	0	-0.428
FAM219A	6	0.99607	0.99611	0.99973	20377	0	-0.367
SLC7A11	6	0.99608	0.99613	0.99973	20378	0	-0.353
WDR26	6	0.99609	0.99614	0.99973	20379	0	-0.706
KRTAP22-1	6	0.9961	0.99614	0.99973	20380	0	-0.503
SYNDIG1L	6	0.99614	0.99619	0.99973	20381	0	-0.346
SERPIND1	6	0.99622	0.99626	0.99973	20382	0	-0.41
ZNF414	6	0.99633	0.99638	0.99973	20383	0	-0.538
KIR3DL1	6	0.99637	0.99642	0.99973	20384	0	-0.655
CLIC6	6	0.9964	0.99645	0.99973	20385	0	-0.515
SUSD5	6	0.9964	0.99645	0.99973	20386	0	-0.292
POLR2H	6	0.99641	0.99645	0.99973	20387	0	-0.456
ZNF197	6	0.99644	0.99648	0.99973	20388	0	-0.275
IKBKB	6	0.99648	0.99652	0.99973	20389	0	-0.423
ASB4	6	0.99651	0.99656	0.99973	20390	0	-0.37
SKIV2L	6	0.99655	0.99659	0.99973	20391	0	-0.622
SSC5D	6	0.99667	0.99672	0.99973	20392	0	-0.504
LEPROTL1	6	0.99673	0.99678	0.99973	20393	0	-0.437
MYOZ1	6	0.99674	0.99679	0.99973	20394	0	-0.466
PPP2CA	5	0.99676	0.99673	0.99973	20395	0	-0.54

SIT1	6	0.99679	0.99684	0.99973	20396	0	-0.573
PCDHB5	6	0.99691	0.99696	0.99973	20397	0	-0.468
ANKRD55	6	0.99691	0.99697	0.99973	20398	0	-0.448
hsa-mir-6761	4	0.99693	0.99694	0.99973	20399	0	-0.892
AMFR	6	0.99694	0.99699	0.99973	20400	0	-0.732
SESN3	6	0.99701	0.99707	0.99973	20401	0	-0.243
NFE2L1	6	0.99704	0.99709	0.99973	20402	0	-0.369
hsa-mir-4519	4	0.99706	0.99707	0.99973	20403	0	-0.589
MLL5	6	0.99711	0.99716	0.99973	20404	0	-0.36
SLC6A19	6	0.99727	0.9973	0.99973	20405	0	-0.658
HBE1	6	0.99727	0.9973	0.99973	20406	0	-1.035
KCNF1	6	0.99729	0.99731	0.99973	20407	0	-0.47
KRTAP4-4	6	0.99751	0.99753	0.99973	20408	0	-1.436
KRTAP19-3	6	0.99751	0.99753	0.99973	20409	0	-1.233
HIST1H3G	6	0.99761	0.99766	0.99973	20410	0	-0.523
STC2	6	0.9977	0.99774	0.99973	20411	0	-0.372
CD209	5	0.99771	0.99768	0.99973	20412	0	-0.483
CBLN2	6	0.99771	0.99774	0.99973	20413	0	-0.753
BEND7	6	0.99776	0.99779	0.99973	20414	0	-0.32
PRKAR1B	6	0.9978	0.99783	0.99973	20415	0	-0.396
GNB4	6	0.99782	0.99785	0.99973	20416	0	-0.367
CWC27	6	0.99789	0.99791	0.99973	20417	0	-0.541
GLA	6	0.99796	0.99798	0.99973	20418	0	-0.527
hsa-mir-4494	4	0.99799	0.99799	0.99973	20419	0	-0.713
RHOB	6	0.99802	0.99803	0.99973	20420	0	-0.579
GRIN2B	6	0.99803	0.99804	0.99973	20421	0	-0.419
OR52B6	6	0.99811	0.99811	0.99973	20422	0	-0.471
DDIT3	6	0.99814	0.99814	0.99973	20423	0	-0.432
PPIL2	6	0.99815	0.99815	0.99973	20424	0	-1.151
GPX4	6	0.99822	0.99823	0.99973	20425	0	-0.887
IDI2	6	0.99831	0.99831	0.99973	20426	0	-0.487
FBXW12	6	0.99832	0.99833	0.99973	20427	0	-0.558
DYNC1LI2	6	0.99838	0.99838	0.99973	20428	0	-0.693
LCORL	6	0.9984	0.99839	0.99973	20429	0	-0.45
CCDC166	6	0.99846	0.99846	0.99973	20430	0	-0.62
hsa-mir-6815	4	0.99849	0.99846	0.99973	20431	0	-0.59
TCF15	6	0.9985	0.99849	0.99973	20432	0	-0.662
hsa-mir-5699	4	0.99851	0.99848	0.99973	20433	0	-1.063
ADIRF	6	0.99855	0.99853	0.99973	20434	0	-0.804
FAM71B	6	0.99868	0.99867	0.99973	20435	0	-0.788
CSRP1	6	0.99868	0.99867	0.99973	20436	0	-0.663
PRSS27	6	0.99872	0.99871	0.99973	20437	0	-0.452
SLTM	3	0.99874	0.99877	0.99973	20438	0	-1.607
NTSR2	6	0.99883	0.99882	0.99973	20439	0	-0.381
LY96	6	0.99885	0.99883	0.99973	20440	0	-0.45
hsa-mir-1323	4	0.99886	0.99885	0.99973	20441	0	-1.045
PIGZ	6	0.99893	0.99892	0.99973	20442	0	-0.518
RNF149	6	0.99893	0.99892	0.99973	20443	0	-0.476
REPIN1	6	0.99898	0.99897	0.99973	20444	0	-0.477
REXO1	6	0.99903	0.99901	0.99973	20445	0	-0.468
KLHL40	6	0.99908	0.99908	0.99973	20446	0	-0.539
SLC39A4	6	0.9991	0.9991	0.99973	20447	0	-0.77
hsa-mir-3193	4	0.99917	0.99917	0.99974	20448	0	-1.724
EFCAB5	4	0.9992	0.99921	0.99974	20449	0	-1.044
ZNF628	6	0.99932	0.99933	0.99976	20450	0	-0.742
SRSF9	6	0.99945	0.99944	0.99976	20451	0	-0.809
KIAA0100	6	0.99946	0.99945	0.99976	20452	0	-0.601
FAM35A	6	0.99948	0.99948	0.99976	20453	0	-1.276
MYOT	6	0.99955	0.99954	0.99976	20454	0	-0.571
MARCH5	6	0.99959	0.99959	0.99976	20455	0	-0.838
MAGEB4	6	0.9996	0.99959	0.99976	20456	0	-0.83
NHP2	6	0.99962	0.99961	0.99976	20457	0	-0.553
ST8SIA5	6	0.99971	0.99972	0.99981	20458	0	-0.721
SDS	6	0.99981	0.9998	0.99985	20459	0	-0.668
TRADD	6	0.99999	0.99999	0.99999	20460	0	-1.07
