# Supplementary material for: ASH2L drives proliferation and sensitivity to bleomycin and other genotoxins in Hodgkin’s lymphoma and testicular cancer cells
Source: Cell Death Dis. 2020 Nov 30;11(11):1019. doi: 10.1038/s41419-020-03231-0 (PMC7705021; doi:10.1038/s41419-020-03231-0)
Supplement: Supplementary file 2 — Table S2 [file 41419_2020_3231_MOESM2_ESM.pdf]

sgRNAs targetting these genes were detected as depleted in the 10-day bleomycin treated population, compared to the population left untreated for 10 days

id	num	neg|score	neg|p-value	neg|fdr	neg|rank	neg|lfc
PFN1	6	6.079E-11	2.4196E-07	0.00495	1	-1.172
PPP4C	6	1.334E-07	2.1776E-06	0.01114	2	-1.2844
ASH2L	6	1.596E-07	2.1776E-06	0.01114	3	-1.1476
DUT	6	1.607E-07	2.1776E-06	0.01114	4	-1.2822
RPLP0	6	2.056E-07	3.1455E-06	0.01287	5	-0.7017
CPSF4	6	9.68E-07	9.4364E-06	0.02475	6	-0.8633
POLE4	6	1.027E-06	9.9203E-06	0.02475	7	-0.589
FAM50A	6	1.202E-06	9.9203E-06	0.02475	8	-1.1328
CCNA2	6	1.512E-06	0.000010888	0.02475	9	-1.0708
ARIH1	6	1.705E-06	0.000012824	0.02624	10	-1.2357
RFC5	6	2.039E-06	0.000014276	0.02655	11	-0.6747
WDR82	6	2.844E-06	0.00002105	0.03313	12	-0.8233
CSTF3	6	2.844E-06	0.00002105	0.03313	13	-0.9253
LSM11	6	3.763E-06	0.000027825	0.04067	14	-0.641
SART3	6	4.828E-06	0.000035084	0.04786	15	-1.0513
UBE2I	6	5.585E-06	0.000040891	0.05154	16	-0.4575
RPL23A	6	5.849E-06	0.000042827	0.05154	17	-0.9397
NCBP1	6	6.559E-06	0.000048634	0.05528	18	-0.7557
LONP1	6	7.65E-06	0.000053957	0.0581	19	-0.9558
UTP18	6	8.899E-06	0.000060248	0.06163	20	-0.5826
UFD1L	6	1.007E-05	0.000069442	0.06766	21	-0.5863
WDR4	6	1.53E-05	0.00009509	0.08359	22	-0.4852
CDC20	6	1.675E-05	0.00010138	0.08359	23	-1.2536
GAPDH	6	1.687E-05	0.00010187	0.08359	24	-1.0913
PPIE	6	1.758E-05	0.00010525	0.08359	25	-1.0067
TMEM199	6	1.803E-05	0.00010622	0.08359	26	-0.7896
POLR3K	6	2.102E-05	0.00012025	0.0907	27	-1.1208
GFER	6	2.179E-05	0.00012413	0.0907	28	-0.8562
SDHD	6	2.286E-05	0.00013235	0.09236	29	-0.912
NOP16	6	2.399E-05	0.00013719	0.09236	30	-1.0188
TRIAP1	6	2.555E-05	0.00014058	0.09236	31	-0.6764
C16orf95	6	2.605E-05	0.00014445	0.09236	32	-0.1152
GMPPB	6	2.69E-05	0.00015268	0.09466	33	-0.8767
ANAPC5	6	3.033E-05	0.000173	0.09666	34	-0.7787
POLR3B	6	3.2E-05	0.00018316	0.09666	35	-0.7531
KIF23	6	3.317E-05	0.00018897	0.09666	36	-0.3916
CMPK1	6	3.352E-05	0.00019091	0.09666	37	-0.6083
SS18L2	6	3.473E-05	0.00019913	0.09666	38	-0.7214
YARS	6	3.629E-05	0.00020591	0.09666	39	-0.6165
NELFB	6	3.743E-05	0.00021317	0.09666	40	-0.803
PTAR1	6	3.792E-05	0.0002151	0.09666	41	-0.2628
TRMT112	6	3.899E-05	0.00021849	0.09666	42	-0.6231
EIF3D	6	3.929E-05	0.00021897	0.09666	43	-0.8373
SLC7A6OS	6	4.012E-05	0.00022333	0.09666	44	-0.8617
DCAF13	6	4.11E-05	0.00022962	0.09666	45	-0.5865
PKMYT1	6	4.21E-05	0.00023494	0.09666	46	-0.7791
RNMT	6	4.244E-05	0.00023688	0.09666	47	-0.8137
SLC30A1	6	4.312E-05	0.00024123	0.09666	48	-0.868
MED9	6	4.59E-05	0.00025527	0.09666	49	-0.5979
AAMP	6	4.61E-05	0.00025624	0.09666	50	-0.3107
SYMPK	6	4.662E-05	0.0002589	0.09666	51	-0.803
BIRC5	6	4.87E-05	0.0002664	0.09666	52	-0.7446
POP5	6	4.883E-05	0.0002664	0.09666	53	-0.9861
FLCN	6	4.956E-05	0.00027075	0.09666	54	-0.6707
NDOR1	6	5.016E-05	0.00027559	0.09666	55	-0.7362
PIP5K1C	6	5.091E-05	0.0002785	0.09666	56	-0.7497
hsa-mir-622	4	5.193E-05	0.00019962	0.09666	57	-1.1214
UXT	6	5.222E-05	0.00028479	0.09666	58	-0.8361
ATP5I	6	5.222E-05	0.00028479	0.09666	59	-1.1024
C9orf114	6	5.274E-05	0.00028817	0.09666	60	-0.4066
HSPE1	5	5.314E-05	0.00024656	0.09666	61	-0.7809
TADA1	6	5.446E-05	0.00029834	0.0972	62	-0.5881
CAPZB	6	5.49E-05	0.0002993	0.0972	63	-1.1223
FDX1L	6	5.721E-05	0.00030995	0.09863	64	-0.586
EMC3	6	5.835E-05	0.00031334	0.09863	65	-0.7726
MDN1	6	5.962E-05	0.0003206	0.09882	66	-0.3862
HAUS3	6	6.307E-05	0.0003356	0.09882	67	-0.8204
MCM2	6	6.344E-05	0.00033657	0.09882	68	-0.4493
EMC6	6	6.451E-05	0.00034286	0.09882	69	-0.7219
DARS	6	6.497E-05	0.00034576	0.09882	70	-0.6324
PHB2	6	6.678E-05	0.00035447	0.09882	71	-0.8413
SCAP	6	6.692E-05	0.00035495	0.09882	72	-1.0452
ALG2	6	6.899E-05	0.00036608	0.09882	73	-0.9239
SYVN1	6	6.934E-05	0.00036899	0.09882	74	-0.7224
GINS2	6	6.941E-05	0.00036899	0.09882	75	-0.5897
ALDOA	6	6.956E-05	0.00036996	0.09882	76	-0.6825
DHX36	6	7.006E-05	0.00037189	0.09882	77	-0.8558
MAP3K7CL	6	7.134E-05	0.00037818	0.09907	78	-0.7295

ID = gene name
num = number of sgRNAs targeting this gene (or microRNA) present in the library
neg|score = the RRA lo value of this gene
neg|p-value = the raw p-value (using permutation) of this gene
neg|fdr = the false discovery rate of this gene
neg|rank = rank of this gene in the list
neg|lfc = the log fold change of this gene

SOD2	6	7.34E-05	0.00038254	0.09907	79	-0.5957
EOMES	6	7.508E-05	0.00039173	0.10019	80	-0.5178
MMP27	6	7.814E-05	0.00040528	0.10229	81	-0.2194
USP14	6	7.94E-05	0.00041351	0.10229	82	-0.6947
SPC24	6	7.973E-05	0.00041496	0.10229	83	-1.2672
CIRH1A	6	8.162E-05	0.00042754	0.10291	84	-0.7271
WDR77	6	8.162E-05	0.00042754	0.10291	85	-0.4778
NUBP1	6	8.84E-05	0.00047013	0.11159	86	-0.1614
CHUK	6	8.891E-05	0.00047448	0.11159	87	-0.7056
ARL2	6	9.286E-05	0.0005011	0.1156	88	-0.5935
MBTPS2	6	9.372E-05	0.00050449	0.1156	89	-0.8189
CCDC115	6	9.512E-05	0.00051174	0.1156	90	-0.5803
GPN2	6	9.537E-05	0.00051416	0.1156	91	-0.6982
MCM4	6	9.71E-05	0.00052239	0.11618	92	-0.0841
ATP5A1	6	9.94E-05	0.00053449	0.11759	93	-0.3595
REXO1L1	6	0.0001093	0.00059691	0.12695	94	-0.8333
PSMD12	6	0.0001103	0.00060321	0.12695	95	-0.5046
PSMB7	6	0.0001111	0.00060804	0.12695	96	-0.3821
RPL15	6	0.0001165	0.00063853	0.13001	97	-0.785
CENPM	6	0.0001185	0.00065015	0.13001	98	-0.6376
CHORDC1	6	0.0001198	0.0006545	0.13001	99	-0.698
C21orf59	6	0.0001264	0.00068402	0.13352	100	-0.6651
NAA20	6	0.0001272	0.00069079	0.13352	101	-0.4959
DIAPH1	6	0.0001276	0.00069176	0.13352	102	-0.3958
HIST1H2BM	6	0.0001304	0.00070822	0.13542	103	-0.3591
BGLAP	5	0.0001417	0.00064918	0.13001	104	-0.5044
hsa-mir-4419l	4	0.0001421	0.00056788	0.12272	105	-1.0362
hsa-mir-449c	4	0.0001428	0.00056982	0.12272	106	-0.5682
SFSWAP	6	0.0001475	0.00081032	0.15351	107	-0.6901
MLF1IP	6	0.0001514	0.00083307	0.15391	108	-0.7974
NCAPG	6	0.0001514	0.00083355	0.15391	109	-0.163
HMGXB4	6	0.0001518	0.000835	0.15391	110	-0.4352
SUDS3	6	0.0001548	0.00084855	0.15501	111	-0.7727
ACTR3	6	0.0001571	0.00086549	0.15671	112	-0.443
hsa-mir-142	4	0.0001585	0.00062692	0.12956	113	-0.6266
RPS27	6	0.0001613	0.00088678	0.1571	114	-0.9664
EIF5AL1	6	0.0001641	0.0008984	0.1571	115	-1.2418
TEN1	6	0.0001641	0.0008984	0.1571	116	-0.567
DIMT1	6	0.0001677	0.00091824	0.15854	117	-0.771
RPL19	6	0.0001686	0.00092356	0.15854	118	-0.8247
MAD2L1	6	0.0001707	0.00093517	0.15854	119	-0.6596
NUPL1	6	0.0001712	0.00093759	0.15854	120	-0.567
PRPF19	6	0.0001725	0.00094582	0.15862	121	-0.4606
COPS2	6	0.000176	0.00096905	0.16119	122	-0.6341
CHD8	6	0.0001807	0.00099857	0.16408	123	-0.4876
MTO1	6	0.0001813	0.0010024	0.16408	124	-0.3713
RPN1	6	0.0001823	0.0010131	0.1643	125	-0.2196
MAD2L2	6	0.0001833	0.0010199	0.1643	126	-0.628
RPS28	6	0.000188	0.0010426	0.16665	127	-0.5165
CTR9	6	0.0001925	0.0010629	0.16821	128	-0.5181
MST1L	6	0.0001943	0.0010707	0.16821	129	-0.4844
BRIX1	6	0.0001961	0.0010828	0.16821	130	-0.5982
C2orf16	6	0.0001985	0.0010934	0.16821	131	-0.6811
CHMP7	6	0.0001985	0.0010934	0.16821	132	-0.6066
POLR3H	6	0.0002044	0.0011162	0.16916	133	-1.0749
RPL11	6	0.0002044	0.0011162	0.16916	134	-0.5784
COMMD2	6	0.0002082	0.0011399	0.17148	135	-0.5271
PRPF3	6	0.0002101	0.001151	0.17189	136	-0.6771
UBE2M	6	0.000214	0.0011704	0.17352	137	-0.5295
ACVR2B	6	0.0002171	0.0011849	0.17441	138	-0.4259
ARMC7	6	0.0002235	0.0012134	0.17733	139	-0.5661
MCM6	6	0.0002277	0.0012318	0.17832	140	-0.6603
C22orf28	4	0.0002286	0.00088581	0.1571	141	-0.7027
RPP21	6	0.0002332	0.0012575	0.17832	142	-0.7629
RPS19	6	0.0002333	0.0012579	0.17832	143	-0.3918
GPN3	6	0.0002341	0.0012638	0.17832	144	-0.648
RPA3	6	0.0002347	0.0012667	0.17832	145	-0.5716
HMGA1	6	0.0002424	0.0013063	0.18051	146	-0.2521
POLG	6	0.0002439	0.0013146	0.18051	147	-0.3329
SF3A1	6	0.0002641	0.0014297	0.19502	148	-0.345
ESPL1	6	0.0002683	0.001451	0.19661	149	-0.4877
SRSF10	6	0.0002776	0.0015086	0.20242	150	-0.6744
HMGB1	6	0.00028	0.0015231	0.20242	151	-0.7412
NPLOC4	6	0.0002802	0.0015236	0.20242	152	-0.7844
TRAPPC2	6	0.0002865	0.0015585	0.20567	153	-0.1499
CCT4	6	0.0002912	0.0015822	0.20567	154	-0.4315
TECR	6	0.0002913	0.0015827	0.20567	155	-0.4518
TOMM22	6	0.0002963	0.0016054	0.20567	156	-0.527
TSR2	6	0.0003008	0.0016267	0.20567	157	-0.6175
PDE4DIP	6	0.0003009	0.0016267	0.20567	158	-0.3571
G6PD	6	0.000302	0.0016325	0.20567	159	-0.3359

ZNHIT6	6	0.000305	0.0016485	0.20567	160	-0.1804
SUPT16H	6	0.0003057	0.0016557	0.20567	161	-0.5247
IARS	6	0.0003057	0.0016557	0.20567	162	-0.3834
HSPA5	6	0.0003063	0.0016586	0.20567	163	-0.3968
CDK12	6	0.0003085	0.0016693	0.20574	164	-0.4943
KIAA1432	6	0.0003251	0.0017511	0.21453	165	-0.5246
LSM7	4	0.0003363	0.0012725	0.17832	166	-0.5418
BUB1B	6	0.0003386	0.0018174	0.21835	167	-0.653
UQCR10	6	0.00034	0.0018251	0.21835	168	-0.5381
GTF3C6	6	0.0003421	0.0018333	0.21835	169	-0.7024
ROMO1	6	0.0003434	0.0018387	0.21835	170	-0.376
SRP54	6	0.0003445	0.0018435	0.21835	171	-0.2262
hsa-mir-6165	4	0.0003446	0.0013039	0.18051	172	-1.136
CCDC168	6	0.0003457	0.0018517	0.21835	173	0.0541
MYC	6	0.0003495	0.0018691	0.21835	174	-0.6949
SKP1	6	0.0003507	0.0018793	0.21835	175	-0.3945
RAN	6	0.0003539	0.0018962	0.21835	176	-0.3945
TRAPPC8	6	0.0003545	0.0018982	0.21835	177	-0.6649
RPL5	6	0.0003555	0.0019011	0.21835	178	-0.5164
NOP2	6	0.0003607	0.0019282	0.21835	179	-0.4428
EIF3F	6	0.0003645	0.0019432	0.21835	180	-0.5661
DCTN5	6	0.0003658	0.001947	0.21835	181	-0.1948
SNRPF	6	0.0003667	0.0019538	0.21835	182	-1.3462
CPSF3	6	0.0003696	0.0019737	0.21835	183	-0.2064
SARS2	6	0.0003715	0.001985	0.21835	184	-0.2766
SMC6	6	0.0003812	0.002038	0.22298	185	-0.2097
GTF2A2	6	0.000393	0.0021014	0.22806	186	-0.5975
CDS2	6	0.0003943	0.0021067	0.22806	187	-0.4507
THOC2	6	0.0004041	0.0021551	0.23207	188	-0.1663
NUP93	6	0.0004142	0.0022118	0.23692	189	-0.5679
POLR2I	6	0.0004203	0.0022442	0.23779	190	-0.7872
CRKL	6	0.0004233	0.0022587	0.23779	191	-0.5902
SDHB	6	0.0004253	0.0022645	0.23779	192	-0.681
R3HDM2	6	0.0004269	0.0022732	0.23779	193	-0.4949
NUMA1	6	0.000432	0.0022969	0.23779	194	-0.078
ZNRD1	6	0.0004333	0.0023066	0.23779	195	-0.7689
GRINA	6	0.0004358	0.0023177	0.23779	196	-0.3327
IMP4	6	0.0004381	0.0023284	0.23779	197	-0.7801
TBL1XR1	6	0.0004428	0.0023531	0.23779	198	0.193
POLR1C	6	0.0004474	0.0023763	0.23779	199	-0.6754
SRSF1	6	0.0004474	0.0023763	0.23779	200	-0.7463
GPKOW	6	0.0004486	0.0023816	0.23779	201	-0.4905
DNM2	6	0.0004493	0.002386	0.23779	202	-0.9678
GINS1	6	0.0004508	0.0023932	0.23779	203	-0.6906
CARS	6	0.0004509	0.0023942	0.23779	204	-0.4189
CDC23	6	0.0004527	0.0024077	0.23798	205	-0.7968
TMTC3	6	0.0004661	0.0024736	0.24331	206	-0.4247
NAA50	6	0.0004707	0.0024915	0.24349	207	-0.4294
ISCA2	6	0.0004725	0.0024992	0.24349	208	-0.4649
DPM3	6	0.0004757	0.0025157	0.24388	209	-0.3814
ARHGEF25	6	0.0004786	0.0025297	0.24388	210	-0.6225
PRIM1	6	0.0004807	0.0025389	0.24388	211	-0.7882
EWSR1	6	0.0005144	0.002717	0.25881	212	-0.3264
AHCY	6	0.0005147	0.0027196	0.25881	213	-0.493
SF3B5	4	0.0005191	0.0019383	0.21835	214	-0.5574
PSMG4	6	0.0005215	0.0027504	0.25891	215	-1.0608
PRR23C	6	0.0005269	0.0027712	0.25891	216	-0.3741
C19orf43	6	0.0005294	0.0027862	0.25891	217	-0.3947
MRPS21	6	0.0005359	0.0028147	0.25891	218	-0.5642
MED31	6	0.0005369	0.0028181	0.25891	219	-0.7046
ZDHHC9	6	0.0005376	0.002822	0.25891	220	-0.3251
RPL18	6	0.0005417	0.0028486	0.26019	221	-0.5881
TINF2	6	0.0005469	0.0028786	0.26087	222	-0.1922
SRP14	6	0.0005475	0.0028815	0.26087	223	-0.4705
COA5	6	0.0005534	0.002911	0.26225	224	-0.6351
FAM210A	6	0.0005555	0.0029231	0.26225	225	-0.5885
CEBPZ	6	0.000558	0.0029352	0.26225	226	-0.4531
MKI67IP	6	0.0005804	0.0030605	0.26995	227	-0.8049
PFDN2	6	0.0005804	0.003061	0.26995	228	-0.6273
PHB	6	0.0005885	0.0031114	0.27161	229	-0.6569
RANBP1	6	0.0005901	0.0031191	0.27161	230	-0.2012
ATP6V1B2	6	0.0005966	0.0031506	0.27161	231	-0.4075
VPS51	6	0.0005981	0.0031588	0.27161	232	-0.5107
RPL13A	6	0.0005986	0.0031602	0.27161	233	-0.4295
DAD1	6	0.000599	0.0031612	0.27161	234	-0.3556
ECD	6	0.0006008	0.0031728	0.27161	235	-0.5013
NLE1	6	0.0006126	0.0032319	0.27552	236	-0.5229
HSD17B7	6	0.0006194	0.0032696	0.27676	237	-0.3164
HYPK	6	0.000621	0.0032735	0.27676	238	-0.3009
PAK1IP1	6	0.0006329	0.0033354	0.27841	239	-0.5204
GEMIN5	6	0.0006354	0.0033441	0.27841	240	-0.6941

SCYL1	6	0.0006374	0.0033538	0.27841	241	-0.8513
NOP14	6	0.0006409	0.0033712	0.27841	242	-0.705
SCUBE3	6	0.000641	0.0033717	0.27841	243	-0.1971
PRPF38B	6	0.0006417	0.0033746	0.27841	244	-0.5622
PPIL4	6	0.000646	0.003392	0.27872	245	-0.4796
RUVBL1	6	0.0006494	0.0034099	0.27879	246	-0.3856
LAMTOR2	6	0.000651	0.0034201	0.27879	247	-0.0308
DCK	6	0.0006574	0.0034511	0.28019	248	-0.3593
HSP90AB1	6	0.000664	0.0034869	0.28198	249	-0.5985
EMC4	6	0.000668	0.0035101	0.28272	250	-0.0698
STRAP	6	0.0006706	0.0035237	0.28272	251	-0.365
TAF1	6	0.0006842	0.0035929	0.28552	252	-0.5037
GLI1	6	0.0006842	0.0035929	0.28552	253	-0.8472
FXN	6	0.0006889	0.003618	0.28552	254	-0.5412
OR10K2	6	0.0006917	0.0036296	0.28552	255	-0.0834
TACC3	6	0.0006941	0.0036422	0.28552	256	-0.615
ORC6	6	0.0007026	0.0036882	0.28715	257	-0.7205
CCDC71L	6	0.0007031	0.0036911	0.28715	258	0.1348
SYT14	6	0.0007118	0.0037385	0.28974	259	-0.6442
NOM1	6	0.0007166	0.0037666	0.29077	260	-0.3131
PSMG1	6	0.000721	0.0037884	0.29077	261	-0.3702
TPI1	6	0.0007261	0.0038155	0.29077	262	-0.2982
DTYMK	6	0.0007308	0.0038382	0.29077	263	-0.4752
NLGN2	6	0.0007363	0.0038697	0.29077	264	-0.4459
PTDSS1	6	0.0007444	0.0039147	0.29077	265	-0.4099
PRPF8	6	0.0007471	0.0039263	0.29077	266	-0.4094
KANK2	6	0.0007471	0.0039263	0.29077	267	-1.0298
NCOR1	6	0.0007483	0.0039326	0.29077	268	-0.4702
NUTF2	6	0.0007493	0.0039386	0.29077	269	-0.8359
UNC50	6	0.0007497	0.0039408	0.29077	270	-0.8286
PARS2	6	0.0007523	0.0039548	0.29077	271	-0.5897
hsa-mir-3188	4	0.0007531	0.0027716	0.25891	272	-0.5243
RER1	6	0.0007551	0.003965	0.29077	273	-0.5173
GALNT1	6	0.0007552	0.003965	0.29077	274	-0.2686
NAMPT	4	0.0007592	0.002792	0.25891	275	-0.7428
ZFC3H1	6	0.000778	0.0040865	0.29826	276	-0.1472
PSMB8	6	0.0007808	0.0041005	0.29826	277	-0.4574
NRF1	6	0.0007865	0.004129	0.29826	278	-0.6765
SNRPD1	6	0.0007909	0.0041494	0.29826	279	-1.016
KIAA1257	6	0.0007941	0.0041673	0.29826	280	-0.4455
ATP6V0C	6	0.0007943	0.0041678	0.29826	281	-0.8604
NOP56	6	0.0007946	0.0041692	0.29826	282	-0.3621
FAM96B	6	0.0008058	0.0042188	0.30076	283	-0.6332
MYBBP1A	6	0.0008208	0.0043028	0.30568	284	-0.4037
PPP4R2	6	0.0008275	0.004341	0.30647	285	-0.5084
SUGP1	6	0.0008284	0.0043439	0.30647	286	-0.4339
LETM1	6	0.0008354	0.0043763	0.3077	287	-0.6932
hsa-mir-4709	4	0.0008366	0.003061	0.26995	288	-0.7003
PAICS	6	0.0008479	0.0044431	0.31132	289	-0.4484
PRKD2	6	0.0008524	0.0044726	0.31232	290	-0.2857
CDK9	6	0.0008593	0.0045142	0.31415	291	0.0386
RRP36	6	0.0008648	0.0045404	0.31422	292	-0.2638
URB2	6	0.0008725	0.0045709	0.31422	293	-0.2495
NAA30	6	0.0008725	0.0045723	0.31422	294	-0.7322
RIMKLB	6	0.0008732	0.0045767	0.31422	295	-0.2878
RSL1D1	6	0.0008813	0.0046149	0.3157	296	-0.6105
CAD	6	0.0008887	0.004658	0.3157	297	-0.5633
ASUN	6	0.0008888	0.0046584	0.3157	298	-0.3972
C9orf142	6	0.0008891	0.0046599	0.3157	299	-0.3615
ADAMTS2	6	0.0009114	0.0047727	0.32227	300	-0.3019
WDR38	6	0.0009206	0.0048138	0.32378	301	-0.5232
EIF1AX	6	0.0009279	0.0048457	0.32378	302	-0.5137
SSU72	6	0.0009289	0.0048506	0.32378	303	-0.4566
COQ2	6	0.0009305	0.0048583	0.32378	304	-0.7113
KRT8	6	0.0009409	0.0049149	0.32576	305	-0.3427
IKZF1	6	0.0009423	0.0049198	0.32576	306	-0.5406
KIF18A	6	0.0009474	0.004942	0.32617	307	-0.475
C19orf53	6	0.0009542	0.0049677	0.32681	308	-0.3774
CCT8	6	0.0009598	0.0049865	0.32691	309	-0.5254
MMGT1	6	0.0009634	0.0050011	0.32691	310	-0.0608
PGS1	6	0.0009731	0.0050364	0.32817	311	-0.3981
MED11	6	0.000986	0.0050819	0.33008	312	-0.7606
hsa-mir-9-2	4	0.0009882	0.003604	0.28552	313	-0.5884
hsa-mir-6859-	2	0.0010196	0.0019756	0.21835	314	-1.1671
PGBD2	6	0.0010257	0.0052406	0.33527	315	-0.4111
GFM1	6	0.0010266	0.0052454	0.33527	316	-0.4817
TAF2	6	0.0010283	0.0052508	0.33527	317	-0.4946
RBM19	6	0.0010283	0.0052513	0.33527	318	-0.6333
EBNA1BP2	6	0.0010328	0.0052682	0.33527	319	-0.4485
POLR2C	6	0.001034	0.0052764	0.33527	320	-0.6599
EIF6	6	0.0010408	0.0053035	0.33594	321	-0.6049

hsa-mir-765	4	0.0010569	0.0038522	0.29077	322	-0.618
CDCA8	6	0.0010585	0.0053674	0.33894	323	0.2804
TELO2	6	0.0010675	0.005408	0.34046	324	-0.1663
MRPL43	6	0.0010907	0.0055043	0.34495	325	-0.4376
TMEM106C	6	0.0010925	0.0055131	0.34495	326	-0.5504
NELFCD	6	0.0010965	0.0055305	0.34498	327	-0.4238
MTOR	6	0.0011094	0.0055852	0.34643	328	-0.6303
UTP23	6	0.0011105	0.0055895	0.34643	329	-0.6563
WDR74	6	0.001117	0.0056137	0.34643	330	-0.895
ERCC8	6	0.0011412	0.0057071	0.35065	331	-0.4227
PRR19	6	0.001155	0.0057613	0.35292	332	-0.5983
RFK	6	0.0011797	0.0058595	0.35787	333	-0.8951
RPP38	6	0.0011885	0.005891	0.35872	334	-0.544
SUPT4H1	6	0.0012237	0.0060226	0.36456	335	-0.3625
RPL36	6	0.0012312	0.0060541	0.36539	336	-0.476
ABCD2	6	0.0012428	0.0061025	0.36723	337	-0.4256
SF3B1	6	0.0012472	0.0061247	0.36748	338	-0.5149
COPB2	6	0.0012509	0.0061446	0.3676	339	-0.4546
RPTOR	6	0.00129	0.0063052	0.37261	340	-0.3993
MRPL13	6	0.0012923	0.0063125	0.37261	341	-0.1815
CHMP4C	6	0.0012944	0.0063193	0.37261	342	-0.7723
SNRPA1	6	0.001297	0.0063299	0.37261	343	-0.5408
CTDP1	6	0.0012988	0.0063376	0.37261	344	-0.6613
GINS4	6	0.0013111	0.0063798	0.37396	345	-0.4438
MRPL3	6	0.0013159	0.0063972	0.37396	346	-0.3548
PPP1R37	6	0.0013199	0.0064185	0.37406	347	-0.3877
MAS1	6	0.0013277	0.0064509	0.37406	348	0.033
PES1	6	0.0013291	0.0064538	0.37406	349	-0.3027
BIN1	6	0.0013419	0.006507	0.37608	350	-0.3684
CENPW	6	0.0013572	0.0065661	0.37843	351	-0.5225
RSL24D1	6	0.0013834	0.0066599	0.3821	352	-0.0479
TTC25	6	0.0013854	0.0066672	0.3821	353	-0.3308
POLD3	6	0.0013981	0.0067156	0.38334	354	-0.6844
INTS9	6	0.0014008	0.0067262	0.38334	355	-0.4432
SLC4A1AP	6	0.001413	0.0067824	0.3847	356	-0.3595
ACTL6A	6	0.0014144	0.0067877	0.3847	357	-0.3484
SPATA5	4	0.0014406	0.005199	0.33527	358	-0.3483
RPS29	6	0.0014562	0.0069358	0.39093	359	-0.5827
DDX42	6	0.00147	0.0069929	0.39106	360	-0.3588
37316	6	0.001471	0.0069982	0.39106	361	-0.6727
PSMC2	6	0.0014801	0.0070282	0.39106	362	-0.6306
DDX20	6	0.0014839	0.0070461	0.39106	363	-0.2579
RBMX	6	0.0014855	0.0070529	0.39106	364	-0.6574
EXOC3	6	0.0014977	0.0070979	0.39249	365	-0.2219
OAZ1	6	0.0015067	0.0071313	0.39324	366	-0.647
WARS	6	0.0015193	0.0071821	0.39324	367	-0.2744
EXOSC5	6	0.0015194	0.0071821	0.39324	368	-0.4528
DDX10	6	0.0015232	0.0071971	0.39324	369	-0.645
PCF11	6	0.0015334	0.0072348	0.39324	370	-0.1945
POLA2	6	0.0015359	0.007246	0.39324	371	-0.2282
BIRC6	6	0.0015488	0.0072973	0.39456	372	-0.3622
TP53RK	6	0.0015511	0.0073089	0.39456	373	-0.5658
hsa-mir-6873	4	0.0015615	0.0056214	0.34643	374	-0.6256
NAA38	6	0.0015631	0.00735	0.39463	375	-0.6076
GTF3C1	6	0.0015653	0.0073587	0.39463	376	-0.4896
PITPNB	6	0.0015756	0.007409	0.39579	377	-0.4203
C12orf79	5	0.0015828	0.0070108	0.39106	378	-0.6303
VPS11	6	0.0015879	0.007457	0.39598	379	-0.1956
RPL22	6	0.001593	0.0074749	0.39598	380	-0.4124
FBL	6	0.0015942	0.0074787	0.39598	381	-0.1651
FZD7	6	0.0015967	0.0074899	0.39598	382	-0.4728
LARS	6	0.0016399	0.0076626	0.40303	383	-0.5614
RPRD2	6	0.0016488	0.0077091	0.40373	384	-0.4132
AATF	6	0.0016508	0.0077154	0.40373	385	-0.1292
hsa-mir-5096	4	0.0016515	0.0059389	0.36056	386	-1.5966
YBX1	5	0.001665	0.0073679	0.39463	387	-0.3453
RASGRP3	6	0.0017015	0.007914	0.41306	388	-0.4301
PSMA6	6	0.0017208	0.0079873	0.41433	389	-0.6972
CENPN	6	0.0017252	0.0079989	0.41433	390	-0.2698
PTPMT1	6	0.0017473	0.0080836	0.41693	391	-0.6373
WDR12	6	0.0017485	0.0080899	0.41693	392	-0.4986
KRTAP3-2	4	0.0017528	0.0062893	0.37261	393	-0.3369
ATP6V1G1	6	0.0017862	0.0082249	0.42125	394	-0.4552
NUDT4	6	0.0017881	0.0082322	0.42125	395	-0.4499
MCM5	6	0.0017918	0.0082525	0.42125	396	-0.2783
UPF1	6	0.001796	0.0082714	0.42125	397	-0.0006
C12orf52	6	0.0017973	0.0082767	0.42125	398	-0.4053
RPAIN	6	0.001821	0.0083759	0.42495	399	-0.5961
BEND5	6	0.0018245	0.0083909	0.42495	400	-0.3167
HIST1H2BL	6	0.001832	0.0084195	0.42529	401	-0.7997
CDON	6	0.0018419	0.0084504	0.42529	402	-0.2475

DHRS7	6	0.001846	0.0084698	0.42529	403	-0.2862
OR4K17	6	0.0018716	0.0085763	0.42798	404	-0.4535
EXOSC4	6	0.0018716	0.0085763	0.42798	405	-0.4284
RPS25	6	0.0018798	0.0086126	0.42813	406	-0.4038
FARSB	6	0.0018876	0.0086421	0.42813	407	-0.6286
SMC4	6	0.0018876	0.0086421	0.42813	408	-0.6254
JUNB	6	0.0018999	0.0086977	0.42984	409	-0.3989
WDR59	4	0.0019092	0.0068482	0.38706	410	-0.6179
PNO1	6	0.0019241	0.0087935	0.43351	411	-0.5358
CHAF1B	6	0.0019292	0.0088143	0.43351	412	-0.5284
TUBGCP3	6	0.0019401	0.0088569	0.43456	413	-0.4857
MRPL33	6	0.0019521	0.0089063	0.43575	414	-0.1783
AIP	6	0.001957	0.0089266	0.43575	415	-0.4278
PTPN23	6	0.0019612	0.008945	0.43575	416	-0.4337
LIPT2	6	0.0019678	0.0089794	0.43624	417	-0.3464
WASH1	6	0.0019839	0.0090495	0.43772	418	-0.3185
TNPO3	6	0.0020041	0.0091197	0.43795	419	-0.252
MED22	6	0.0020083	0.0091395	0.43795	420	-0.407
MED17	6	0.0020087	0.00914	0.43795	421	-0.4902
ZNF837	6	0.0020184	0.0091783	0.43852	422	-0.2848
hsa-mir-4469	4	0.0020187	0.0072426	0.39324	423	-0.452
FARSA	6	0.0020225	0.0091947	0.43852	424	-0.4668
ARFGEF1	6	0.0020409	0.0092654	0.44011	425	-0.4423
CPA3	6	0.0020448	0.0092779	0.44011	426	-0.5327
TRMT5	6	0.0020561	0.009321	0.44011	427	-0.0604
LOC10050567	6	0.002085	0.0094212	0.44011	428	-0.2596
TTC27	6	0.0020851	0.0094217	0.44011	429	-0.5036
INTS10	6	0.0020902	0.0094444	0.44011	430	-0.301
TOR1AIP2	6	0.0020996	0.0094783	0.44011	431	-0.3041
HAUS1	6	0.0021017	0.0094836	0.44011	432	-0.3818
NOP9	6	0.0021022	0.0094851	0.44011	433	-0.3082
SQLE	6	0.0021025	0.009486	0.44011	434	-0.491
DENND1B	6	0.002105	0.0094943	0.44011	435	-0.456
TSG101	6	0.0021081	0.0095078	0.44011	436	-0.4661
hsa-mir-4428	4	0.0021315	0.007652	0.40303	437	-0.4967
MED30	6	0.0021363	0.0096293	0.4428	438	-0.7188
ALG11	6	0.0021366	0.0096307	0.4428	439	-0.1694
RPAP2	6	0.0021455	0.0096665	0.44345	440	-0.3931
RAB25	6	0.002172	0.0097662	0.44503	441	-0.6039
SYT1	6	0.0021917	0.0098453	0.44757	442	-0.571
CHEK1	6	0.0022049	0.0098945	0.44757	443	-0.5276
C7orf26	6	0.0022121	0.0099182	0.44757	444	-0.3811
PICK1	6	0.0022176	0.0099399	0.44757	445	-0.2331
SAE1	6	0.0022238	0.0099675	0.44757	446	-0.4343
URB1	6	0.002227	0.0099782	0.44757	447	-0.4645
HDAC3	6	0.002231	0.009997	0.44757	448	-0.293
ABCA4	6	0.0022494	0.010068	0.44791	449	0.0493
DONSON	6	0.0022571	0.010096	0.44791	450	-0.29
XRCC5	6	0.0022641	0.010126	0.44791	451	-0.865
NUP43	6	0.0022664	0.010136	0.44791	452	-0.2192
APBB3	6	0.0022757	0.010169	0.44839	453	-0.2715
TSFM	6	0.0022813	0.010191	0.44839	454	-0.4158
MGAT2	6	0.0022943	0.010239	0.44953	455	-0.3165
WTAP	5	0.0023095	0.010126	0.44791	456	-0.6571
TRAPPC1	6	0.0023161	0.010324	0.4523	457	-0.522
NCAPH	6	0.0023392	0.010399	0.45461	458	-0.2839
ADAMTSL4	6	0.0023489	0.010438	0.45537	459	-0.3941
RPL30	6	0.0023589	0.010486	0.45598	460	-0.7393
ATP6AP1	6	0.002363	0.010501	0.45598	461	-0.5548
hsa-mir-3159	4	0.002366	0.0084809	0.42529	462	-0.5998
WDR61	6	0.0023681	0.010519	0.45598	463	-0.4893
SBNO1	6	0.0023798	0.010561	0.4566	464	-0.4874
RNF222	6	0.0023915	0.010601	0.4566	465	-0.4531
LAMTOR5	6	0.0023918	0.010601	0.4566	466	-0.4522
USH2A	6	0.0024042	0.010651	0.4566	467	-0.3386
SNRNP27	6	0.0024061	0.010662	0.4566	468	-0.5848
SRP9	6	0.0024079	0.010667	0.4566	469	-0.5289
LRP11	6	0.0024157	0.010701	0.45707	470	-0.236
SEMA6A	6	0.0024312	0.010767	0.4588	471	-0.2882
PISD	6	0.0024421	0.010801	0.4588	472	-0.3598
NELFA	6	0.002448	0.010828	0.4588	473	-0.5205
RPUSD4	6	0.0024485	0.010831	0.4588	474	-0.2478
DHDDS	6	0.0024721	0.010921	0.46093	475	-0.282
PRMT1	6	0.0024735	0.010926	0.46093	476	-0.2076
THUMPD2	6	0.0024879	0.010992	0.46275	477	-0.4582
CRIPAK	6	0.0025079	0.011079	0.4636	478	-0.352
SHFM1	6	0.0025088	0.01108	0.4636	479	-0.5933
hsa-mir-6776	4	0.002515	0.0089978	0.43624	480	-0.6921
PSMD3	6	0.00255	0.011243	0.46722	481	-0.1528
RBBP4	6	0.0025537	0.011258	0.46722	482	-0.3498
HSPA8	6	0.0025579	0.011278	0.46722	483	-0.3832

LSM4	6	0.0025648	0.011301	0.46722	484	-0.3319
BRF1	6	0.0025652	0.011304	0.46722	485	-0.4763
RPS21	6	0.0025723	0.011335	0.46725	486	-0.4786
DEPDC1	6	0.0025765	0.01135	0.46725	487	-0.3336
EIF5A	5	0.0025805	0.011219	0.46722	488	0.108
SNX32	6	0.0025972	0.011431	0.46895	489	-0.4084
hsa-mir-4448	4	0.0026049	0.0093031	0.44011	490	-0.7957
BTAF1	6	0.002635	0.011575	0.46895	491	-0.3998
THOC3	6	0.0026392	0.011592	0.46895	492	-0.2657
DNPEP	6	0.0026397	0.011594	0.46895	493	-0.4684
ITGA7	6	0.0026436	0.011608	0.46895	494	-0.4115
KAT8	6	0.0026442	0.01161	0.46895	495	-0.4466
DHX16	6	0.0026497	0.011632	0.46895	496	-0.5391
SPTSSA	6	0.002651	0.011638	0.46895	497	-0.4977
PSMB1	6	0.0026569	0.011661	0.46895	498	-0.6766
RBM8A	6	0.0026573	0.011663	0.46895	499	-0.6787
MAX	6	0.0026592	0.01167	0.46895	500	-0.4854
BCAS2	6	0.0026699	0.011711	0.46895	501	-0.4785
SOD1	6	0.002676	0.011735	0.46895	502	-0.4554
CDC7	6	0.0026761	0.011735	0.46895	503	-0.5629
DDOST	6	0.0026761	0.011735	0.46895	504	-0.4066
RRM2	6	0.0026943	0.011812	0.47109	505	-0.7061
hsa-mir-3937	4	0.0027306	0.0097019	0.44407	506	-0.6211
hsa-mir-135b	4	0.0027396	0.0097348	0.44458	507	-0.4019
AP2M1	6	0.0027639	0.012053	0.47978	508	-0.5013
CDC27	6	0.0027744	0.012089	0.48026	509	-0.3929
PSMD7	6	0.0027924	0.012156	0.48201	510	-0.7944
CYCS	6	0.0028015	0.012193	0.48252	511	-0.657
PELO	6	0.0028202	0.012257	0.48283	512	-0.4216
PGBD4	6	0.0028221	0.012264	0.48283	513	0.2559
WDR43	6	0.0028324	0.012307	0.48283	514	-0.4949
IP6K2	6	0.0028359	0.012318	0.48283	515	-0.0683
GART	6	0.0028444	0.012351	0.48319	516	-0.5138
FSHR	6	0.0028606	0.012402	0.48367	517	-0.2696
hsa-mir-4435-	3	0.0028636	0.0079651	0.41433	518	-0.8724
CAB39	6	0.002865	0.012424	0.48367	519	-0.6286
TRIM28	6	0.0028749	0.01245	0.48367	520	-0.3497
NARFL	6	0.0028774	0.012458	0.48367	521	0.1953
RCE1	6	0.0028921	0.012518	0.48507	522	-0.5279
ADSL	6	0.0029166	0.012609	0.48507	523	-0.2405
NUS1	6	0.0029166	0.01261	0.48507	524	-0.6624
ACOT13	6	0.0029169	0.01261	0.48507	525	-0.3736
RRP9	6	0.0029179	0.012613	0.48507	526	-0.3621
CLNS1A	6	0.0029399	0.012705	0.48686	527	0.1075
KPNB1	6	0.0029471	0.012731	0.48686	528	-0.3559
SYS1	6	0.0029543	0.012758	0.48699	529	-0.3192
PPP1R10	6	0.0029719	0.012827	0.48848	530	-0.3544
ACTR6	6	0.002979	0.012857	0.48848	531	-0.4224
AURKB	6	0.0029819	0.012869	0.48848	532	-0.4941
NDUFB2	6	0.0029959	0.012917	0.48856	533	-0.3761
ARL2BP	6	0.0029966	0.012918	0.48856	534	-0.4732
FAM204A	6	0.0030049	0.01295	0.48885	535	-0.4095
SNAPC5	6	0.0030297	0.013053	0.49003	536	-0.6041
SNAPC4	6	0.0030299	0.013053	0.49003	537	-0.3334
KIF26A	6	0.0030482	0.013127	0.49116	538	-0.0866
SELO	6	0.0030538	0.01315	0.49116	539	-0.2928
PRMT5	6	0.0030554	0.013155	0.49116	540	-0.5213
MRPL39	6	0.0030643	0.013193	0.49167	541	-0.3097
WDR65	6	0.0030779	0.013247	0.49188	542	-0.3405
PCDHB14	6	0.0030965	0.013321	0.49287	543	-0.4777
RGS16	6	0.0030966	0.013322	0.49287	544	-0.1647
hsa-mir-8078	4	0.0031329	0.011078	0.4636	545	-0.8754
STAC3	6	0.0031726	0.013608	0.50075	546	-0.3371
EIF2B4	6	0.0032246	0.013797	0.50569	547	-0.4435
PVRL4	6	0.0032256	0.013801	0.50569	548	-0.0617
UBXN7	6	0.0032317	0.013822	0.50569	549	-0.4212
NDUFAF3	6	0.0032532	0.013901	0.50569	550	-0.4899
INTS2	6	0.0032584	0.013922	0.50569	551	-0.4201
TWISTNB	6	0.003272	0.013965	0.50569	552	-0.6005
GTF3A	6	0.003272	0.013965	0.50569	553	-0.6342
RBKS	6	0.003272	0.013965	0.50569	554	-0.3873
BRF2	6	0.0032762	0.013976	0.50569	555	-0.4735
UBA2	6	0.003282	0.013998	0.50569	556	-0.6272
HCFC1	6	0.0032913	0.014037	0.50569	557	-0.3871
CHMP6	6	0.003292	0.014039	0.50569	558	-0.3942
RPS24	6	0.0033422	0.014226	0.51031	559	-0.3151
SNX30	6	0.0033488	0.014254	0.51031	560	-0.3095
CAMLG	6	0.0033508	0.014262	0.51031	561	-0.4875
CNOT11	6	0.0033525	0.014267	0.51031	562	-0.5882
CDC45	6	0.0033602	0.014304	0.51072	563	-0.5837
ERH	6	0.0033662	0.014328	0.51072	564	-0.8677

COPS4	6	0.0033908	0.014418	0.51304	565	-0.1949
SLC3A2	6	0.0033984	0.014452	0.51333	566	-0.2758
PCBP1	6	0.0034078	0.01449	0.51381	567	-0.5487
HTRA2	6	0.0034202	0.014546	0.51489	568	-0.6731
UGT2B10	3	0.0034474	0.0095393	0.44057	569	-0.7729
PGC	6	0.0034595	0.014694	0.51875	570	0.1851
XPO6	6	0.0034626	0.014706	0.51875	571	-0.3922
RPL24	6	0.0034823	0.014775	0.5189	572	-0.7142
BET1	6	0.0034851	0.014784	0.5189	573	-0.4269
COQ6	6	0.0034854	0.014786	0.5189	574	-0.1873
CDC42EP1	6	0.0035121	0.014887	0.52018	575	-0.0075
RPL14	6	0.0035414	0.015002	0.52197	576	-0.4891
IPO13	6	0.0035486	0.015026	0.52197	577	-0.4282
PSMD13	6	0.0035562	0.015056	0.52213	578	-0.2024
PPME1	6	0.0035634	0.015086	0.52227	579	-0.0306
ARFRP1	6	0.0035716	0.015114	0.52237	580	-0.6159
CSTF1	6	0.003589	0.015178	0.52368	581	-0.2196
TBCB	6	0.0035998	0.015217	0.52375	582	-0.3651
NUP153	6	0.003604	0.015231	0.52375	583	-0.3305
AP2S1	6	0.0036153	0.015282	0.52387	584	-0.1272
CDC16	6	0.0036296	0.01533	0.52449	585	-0.2983
DCUN1D4	6	0.0036545	0.015413	0.52471	586	-0.6626
hsa-mir-5692c	3	0.0036554	0.010126	0.44791	587	-0.3074
LAIR2	5	0.0036631	0.014961	0.52147	588	-0.1161
AARS	6	0.0036672	0.015465	0.5256	589	-0.4337
HUS1	6	0.0036895	0.015553	0.52714	590	-0.3574
GUK1	6	0.0036913	0.015562	0.52714	591	-0.4986
hsa-mir-450b	4	0.0036917	0.012996	0.48968	592	-0.7647
GTF2E2	6	0.0037106	0.015632	0.52863	593	-0.3194
HNRNPL	6	0.0037192	0.015667	0.52863	594	-0.1582
ITIH1	6	0.0037225	0.015683	0.52863	595	-0.345
SLC11A2	6	0.0037371	0.015734	0.52894	596	-0.1576
MAPKAP1	6	0.0037395	0.015744	0.52894	597	-0.4569
NDUFA13	6	0.0037487	0.015776	0.52916	598	-0.4219
KANSL3	6	0.0037711	0.015867	0.53132	599	-0.2426
hsa-mir-3135b	4	0.0038093	0.013384	0.49427	600	-2.7768
GNL2	6	0.0038123	0.01603	0.53396	601	0.0037
WDR1	6	0.0038192	0.016049	0.53396	602	-0.5184
TAF10	6	0.0038307	0.01609	0.53396	603	-0.2764
FAM163A	6	0.0038356	0.016115	0.53396	604	-0.5805
hsa-mir-3691	4	0.003843	0.013503	0.49779	605	-0.5442
RPL35A	6	0.0038538	0.016182	0.53396	606	-0.8032
FAM120AOS	6	0.003854	0.016183	0.53396	607	-0.3515
DBX2	6	0.0038719	0.016248	0.53396	608	-0.2951
RPL31	6	0.0038741	0.016254	0.53396	609	-0.0638
TPR	6	0.0038861	0.016301	0.53396	610	-0.116
ZC2HC1C	6	0.0038865	0.016301	0.53396	611	-0.384
CDT1	6	0.0038892	0.016311	0.53396	612	-0.6419
MCM10	6	0.0039172	0.016422	0.53564	613	-0.4591
C10orf76	6	0.0039223	0.016441	0.53564	614	-0.1533
ABT1	6	0.0039529	0.016561	0.53808	615	-0.0164
SRXN1	6	0.0039612	0.016595	0.53808	616	-0.3776
KLHDC8A	6	0.0039809	0.016672	0.53839	617	-0.3196
HARS	6	0.0039825	0.016676	0.53839	618	-0.5333
CPT1C	6	0.0039967	0.016721	0.53839	619	-0.5277
TSGA10IP	6	0.0039991	0.016733	0.53839	620	-0.1577
ANAPC4	6	0.0040072	0.016761	0.53839	621	-0.7292
NAA25	6	0.0040073	0.016762	0.53839	622	-0.4257
STT3B	6	0.0040198	0.016811	0.53882	623	-0.4595
RPL3	6	0.0040312	0.016846	0.53882	624	-0.4606
AIPL1	6	0.004034	0.016855	0.53882	625	-0.2326
RPS16	6	0.0040465	0.016907	0.5395	626	-0.6105
RXFP4	6	0.0040585	0.016953	0.5395	627	-0.345
CARS2	6	0.0040596	0.016955	0.5395	628	-0.4407
KRTAP5-7	5	0.0040959	0.016305	0.53396	629	-0.3034
DAP3	6	0.0041125	0.017141	0.54357	630	-0.3431
AQP7	6	0.0041159	0.017149	0.54357	631	-0.2102
MED20	6	0.0041242	0.017176	0.54357	632	-0.3015
HOXC13	6	0.0041432	0.017247	0.54357	633	-0.2
PHF5A	6	0.004149	0.01727	0.54357	634	-0.2377
DENR	6	0.0041531	0.017283	0.54357	635	-0.0436
PSORS1C2	6	0.0041696	0.01735	0.54357	636	-0.033
TFDP1	6	0.0041754	0.017373	0.54357	637	-0.3327
CINP	6	0.0041865	0.017415	0.54357	638	0.076
DNM1L	6	0.0041947	0.017441	0.54357	639	-0.3775
LRR1	6	0.0041988	0.017455	0.54357	640	-0.2562
HAUS7	6	0.0042055	0.017478	0.54357	641	-0.3389
TAF12	6	0.0042181	0.017531	0.54357	642	-0.2749
MRPL23	6	0.0042196	0.017535	0.54357	643	-0.1206
CHKB	6	0.0042384	0.017606	0.54495	644	0.0746
hsa-mir-483	4	0.0042607	0.014899	0.52018	645	-0.9508

DEXI	6	0.0042872	0.017813	0.54867	646	-0.3494
TMEM108	6	0.0042987	0.01786	0.54867	647	-0.397
NFKB2	6	0.0042987	0.01786	0.54867	648	-0.3761
PTPLB	6	0.0043266	0.017956	0.55032	649	-0.2386
CSN3	6	0.0043315	0.017973	0.55032	650	-0.2545
C3orf38	6	0.0043422	0.018016	0.55032	651	-0.4021
NOL11	6	0.0043498	0.018045	0.55032	652	-0.3065
THEM6	6	0.0043507	0.018048	0.55032	653	-0.2839
NUDT21	6	0.0043747	0.018137	0.55221	654	-0.5104
hsa-mir-650	4	0.0043812	0.015286	0.52387	655	-0.6728
QRSL1	6	0.0043977	0.01822	0.5539	656	-0.3771
TUBGCP4	6	0.0044135	0.018277	0.55483	657	-0.2937
WDR7	4	0.0044209	0.015403	0.52471	658	-0.1575
KIAA0586	4	0.0044224	0.015407	0.52471	659	-0.3143
TXN	6	0.004434	0.018365	0.55573	660	-0.4474
RTFDC1	6	0.0044349	0.018367	0.55573	661	-0.3051
PSMA3	6	0.0044461	0.01841	0.55573	662	-0.4568
ELP3	6	0.0044539	0.018441	0.55573	663	-0.4129
ADAM10	6	0.0044612	0.018471	0.55573	664	-0.3841
ABCB7	6	0.0044678	0.018497	0.55573	665	-0.3376
MFSD4	6	0.0045166	0.01868	0.55723	666	-0.2671
TIMM22	6	0.0045288	0.018728	0.55723	667	-0.4086
SCFD1	6	0.0045288	0.018728	0.55723	668	-0.5141
HSD17B10	6	0.004542	0.018773	0.55723	669	-0.3723
DHH	6	0.0045521	0.018811	0.55723	670	-0.2469
KCTD5	6	0.0045534	0.018819	0.55723	671	-0.2761
OR6C65	6	0.0045534	0.018819	0.55723	672	-0.4458
RAB5C	6	0.0045754	0.018889	0.55792	673	-0.4547
SLURP1	6	0.004577	0.018897	0.55792	674	-0.3322
MAP4K4	6	0.0045879	0.018947	0.55857	675	-0.7832
PARG	6	0.0046018	0.018998	0.55899	676	-0.3246
LMAN2L	6	0.0046073	0.019015	0.55899	677	-0.245
VPS4A	4	0.0046124	0.016056	0.53396	678	-0.4388
SRSF3	6	0.0046491	0.019176	0.56151	679	-0.4626
ATP5B	6	0.0046537	0.019198	0.56151	680	0.074
HEPACAM2	6	0.0046542	0.0192	0.56151	681	-0.3768
hsa-mir-3183	4	0.0046632	0.016223	0.53396	682	0.0512
DUX2	6	0.0046776	0.01929	0.56151	683	-0.5287
MAK16	6	0.0046792	0.019293	0.56151	684	-0.2752
SKIDA1	6	0.0046792	0.019293	0.56151	685	-0.4066
TSR1	6	0.0046903	0.019335	0.56151	686	-0.5651
SMN1	2	0.0046919	0.0091003	0.43795	687	-1.1676
EPM2AIP1	6	0.0046941	0.019348	0.56151	688	-0.2627
DDX27	6	0.0047195	0.019448	0.56254	689	-0.343
CYP11A1	6	0.0047241	0.019466	0.56254	690	-0.4891
SCD	6	0.0047276	0.019487	0.56254	691	-0.4808
SLC9A3R2	6	0.0047315	0.019499	0.56254	692	0.4016
EXOSC9	6	0.0047474	0.019549	0.56254	693	-0.4559
LDLR	6	0.0047474	0.019549	0.56254	694	-0.1605
C11orf45	4	0.0047625	0.016588	0.53808	695	-0.4059
ZRSR2	6	0.0047654	0.019622	0.56318	696	-0.324
TONSL	6	0.0047673	0.019626	0.56318	697	-0.399
ATP1B3	6	0.0047899	0.019703	0.56459	698	-0.2601
VPRBP	6	0.004805	0.019766	0.56536	699	-0.2541
OSBP	6	0.0048093	0.019785	0.56536	700	-0.3201
AGRN	6	0.0048338	0.019878	0.56617	701	-0.1021
PMS2	6	0.004853	0.019948	0.56617	702	-0.4511
ABCE1	6	0.004854	0.019953	0.56617	703	-0.3197
GPANK1	6	0.0048545	0.019954	0.56617	704	-0.1819
SLC23A1	6	0.0048561	0.019963	0.56617	705	-0.2129
EIF4G1	6	0.0048612	0.019979	0.56617	706	-0.1927
HCLS1	6	0.004874	0.020026	0.56672	707	0.1518
TMEM123	6	0.004888	0.020081	0.56748	708	-0.3544
LSM2	6	0.0049098	0.020155	0.56877	709	-0.2341
ZNF227	6	0.0049336	0.020243	0.56923	710	-0.2776
RFWD2	6	0.0049344	0.020246	0.56923	711	0.0101
C2orf68	6	0.0049366	0.020254	0.56923	712	-0.4117
MSMO1	6	0.004965	0.020353	0.56992	713	-0.4451
hsa-mir-3138	4	0.004965	0.017251	0.54357	714	-0.6534
OS9	6	0.0049659	0.020355	0.56992	715	-0.0572
SMDT1	6	0.0050016	0.020501	0.57224	716	-0.3666
SMG5	6	0.0050178	0.020565	0.57325	717	-0.3263
hsa-mir-6891	4	0.0050182	0.01742	0.54357	718	-1.0892
RRAGA	4	0.0050358	0.017487	0.54357	719	-0.4394
TTI2	6	0.0050542	0.020689	0.57514	720	-0.3054
HLA-A	6	0.0050688	0.020743	0.57585	721	0.1278
hsa-mir-1273h	4	0.0051148	0.017754	0.54867	722	-1.0838
TUBD1	6	0.0051206	0.020924	0.5801	723	-0.0787
H3F3B	6	0.0051347	0.020975	0.58048	724	-0.5561
hsa-mir-128-2	4	0.0051378	0.017816	0.54867	725	-0.4738
TMEM9	6	0.0051418	0.021003	0.58048	726	-0.3528

TBC1D3F	2	0.0051423	0.0099666	0.44757	727	-1.3391
DGCR8	6	0.0051478	0.021023	0.58048	728	-0.5282
MRPL18	6	0.0052137	0.021267	0.58464	729	-0.3917
MPP2	6	0.0052137	0.021267	0.58464	730	-0.2878
CNIH4	6	0.0052214	0.021295	0.58464	731	-0.2588
PRPF4	6	0.0052244	0.021306	0.58464	732	-0.2104
CHD4	6	0.005228	0.021317	0.58464	733	-0.3285
AQP1	6	0.0052762	0.021478	0.58754	734	0.2206
PSMD14	6	0.0052771	0.02148	0.58754	735	-0.3765
STARD7	6	0.0053007	0.021569	0.58918	736	-0.4687
SARS	6	0.0053281	0.021663	0.59098	737	-0.2503
FBLN2	6	0.00537	0.021819	0.59444	738	-0.4321
HJURP	6	0.005412	0.021986	0.59684	739	-0.4231
RPL28	6	0.0054122	0.021986	0.59684	740	-0.3318
hsa-mir-151b	4	0.0054126	0.018733	0.55723	741	-0.8644
TPT1	6	0.0054201	0.022012	0.59684	742	-0.3634
DPH3	5	0.0054206	0.02047	0.57214	743	-0.0581
ARPC3	6	0.005431	0.02205	0.59684	744	-0.4902
TEX11	6	0.0054318	0.022053	0.59684	745	0.0324
UTP11L	6	0.0054478	0.022114	0.59691	746	-0.3047
RPS11	6	0.0054594	0.022159	0.59734	747	-0.6586
LOR	6	0.005469	0.022197	0.59756	748	-0.3854
YY1	6	0.0054837	0.022248	0.59815	749	-0.3217
DDX6	6	0.0055002	0.022309	0.59875	750	-0.4148
SUPT6H	6	0.0055065	0.022329	0.59875	751	-0.2207
PFKFB1	6	0.0055277	0.022409	0.60012	752	-0.3689
LITAF	6	0.0055524	0.02251	0.60152	753	-0.4328
BARD1	6	0.0055563	0.022523	0.60152	754	-0.2838
VPS29	6	0.0055801	0.022613	0.60152	755	-0.3063
hsa-mir-1181	4	0.0055845	0.019297	0.56151	756	-0.6535
PLA2G2A	6	0.0055874	0.022639	0.60152	757	0.3567
VPS26A	6	0.0055967	0.022673	0.60152	758	-0.367
QTRT1	6	0.0056002	0.02269	0.60152	759	-0.4141
UPP2	6	0.0056021	0.022697	0.60152	760	-0.4209
RAB27B	6	0.0056532	0.022884	0.60482	761	-0.2839
GDF11	6	0.0056633	0.022917	0.60482	762	-0.2218
NSRP1	6	0.0056709	0.022949	0.60482	763	-0.4447
XIAP	6	0.005679	0.022986	0.60482	764	-0.387
FSD2	6	0.0056845	0.023005	0.60482	765	-0.2412
PRPF40B	6	0.0056899	0.023028	0.60482	766	-0.3794
SUMO1	6	0.0056997	0.023064	0.60492	767	-0.1257
AKAP9	6	0.0057078	0.023091	0.60492	768	-0.4528
KIF17	6	0.0057482	0.023237	0.60719	769	-0.2526
RBP2	6	0.0057931	0.023408	0.6096	770	-0.3358
RTF1	6	0.0057984	0.023427	0.6096	771	-0.3564
SLC20A2	6	0.0058052	0.023449	0.6096	772	-0.48
SUPV3L1	6	0.0058138	0.023484	0.60976	773	-0.2613
TSTA3	6	0.0058279	0.023529	0.61014	774	-0.4826
VIT	6	0.005848	0.0236	0.61043	775	-0.3373
BMP7	6	0.0058625	0.023652	0.61095	776	-0.2511
LGALS9B	6	0.005869	0.023679	0.61095	777	-0.1707
RBX1	6	0.0058985	0.023797	0.61244	778	-0.3757
hsa-mir-8066	4	0.0059046	0.020362	0.56992	779	-0.4051
PPWD1	6	0.0059059	0.023827	0.61244	780	-0.1998
AKAP7	6	0.0059059	0.023827	0.61244	781	0.0714
MIA	6	0.0059366	0.023939	0.61272	782	-0.358
SMG7	6	0.0059497	0.023984	0.61272	783	-0.5912
SPACA1	6	0.00595	0.023986	0.61272	784	-0.0486
RPL10	6	0.0059503	0.023988	0.61272	785	0.0313
NDUFS8	6	0.0059789	0.024104	0.61418	786	-0.055
KRT2	6	0.0059819	0.024115	0.61418	787	0.0407
GYPE	3	0.0059858	0.016417	0.53564	788	-0.6105
hsa-mir-566	4	0.0059859	0.020648	0.57476	789	-2.1093
AGO4	6	0.005987	0.024135	0.61418	790	-0.388
TBCC	6	0.0060069	0.024198	0.61501	791	-0.4934
ELMOD2	6	0.006028	0.024284	0.61509	792	-0.1912
SLMAP	6	0.0060299	0.024291	0.61509	793	-0.3766
RPSA	6	0.0060755	0.024447	0.61827	794	-1.1242
FAM47C	6	0.00609	0.024503	0.61892	795	-0.3834
CDC123	6	0.0061058	0.024567	0.61978	796	-0.1674
C1orf189	6	0.0061244	0.02463	0.62005	797	-0.2678
RPL12	6	0.0061289	0.024651	0.62005	798	-0.7336
FAM180B	6	0.0061348	0.024669	0.62005	799	-0.3051
NRD1	6	0.0061753	0.024822	0.62239	800	-0.0589
TXNL4A	6	0.0061917	0.024873	0.62288	801	-0.6492
CKS2	6	0.0062058	0.024921	0.62333	802	-0.3549
RNF41	6	0.0062128	0.024954	0.62338	803	-0.0179
COPS8	6	0.0062304	0.025021	0.6242	804	-0.429
EXOSC6	6	0.0062386	0.025057	0.6242	805	-0.4215
NBPF1	6	0.0062479	0.025084	0.6242	806	-0.4145
DDX21	6	0.0062558	0.025108	0.6242	807	-0.4817

RASL12	6	0.006298	0.025268	0.62529	808	-0.2567
DBR1	6	0.0063074	0.025304	0.62529	809	-0.3535
GNB2L1	6	0.0063173	0.025341	0.62529	810	-0.3517
PKM	6	0.0063256	0.025378	0.62529	811	-0.398
CELA1	6	0.0063257	0.025378	0.62529	812	-0.3594
GABPA	6	0.0063273	0.025384	0.62529	813	-0.4837
MRPS30	6	0.0063273	0.025384	0.62529	814	-0.5011
TIMELESS	6	0.0063361	0.025414	0.62529	815	-0.3041
GDF6	6	0.0063391	0.025427	0.62529	816	0.2214
SETD1A	6	0.0063792	0.025584	0.62738	817	-0.3552
SPIRE2	6	0.0063933	0.025634	0.62738	818	-0.2424
MIS18BP1	6	0.0063991	0.025655	0.62738	819	-0.3243
MBD5	6	0.0064123	0.025709	0.62738	820	-0.3265
ZNF565	6	0.006416	0.025723	0.62738	821	-0.4701
GPR64	6	0.0064168	0.025727	0.62738	822	0.0203
CPSF2	4	0.0064298	0.022094	0.59691	823	-0.5085
JAK1	6	0.0064403	0.025815	0.62835	824	-0.409
SNRPE	6	0.0064692	0.025918	0.62835	825	-0.3864
ANKRD24	6	0.0064728	0.025934	0.62835	826	-0.4481
EXOC3L4	6	0.0064836	0.025978	0.62835	827	-0.2651
HNRNPK	6	0.00649	0.026007	0.62835	828	-0.3905
RAB36	6	0.006503	0.026054	0.62835	829	-0.2483
XRN2	6	0.0065065	0.026065	0.62835	830	-0.3285
NUDC	6	0.0065117	0.026087	0.62835	831	0.0405
TUT1	6	0.0065204	0.026124	0.62835	832	0.0975
ATP5J	6	0.0065254	0.026141	0.62835	833	-0.5149
MTPAP	6	0.0065254	0.026141	0.62835	834	-0.375
VDAC2	5	0.0065373	0.023869	0.61272	835	-1.1932
POLR3A	6	0.0065437	0.026211	0.62835	836	-0.4317
ATAD3A	6	0.0065722	0.026319	0.62835	837	-0.5112
KDM3B	6	0.006581	0.026347	0.62835	838	-0.119
DHX33	6	0.0065821	0.02635	0.62835	839	-0.3708
ORAOV1	6	0.0065958	0.026402	0.62886	840	-0.2282
THAP11	6	0.00663	0.026519	0.6309	841	-0.2264
OLA1	6	0.0066926	0.026744	0.63552	842	-0.5345
hsa-mir-6513	4	0.0066944	0.022962	0.60482	843	-0.1249
TIMM13	6	0.0067094	0.026799	0.63609	844	-0.2601
GOLGA6A	6	0.0067509	0.026956	0.63727	845	-0.3526
XPO5	6	0.0067605	0.027	0.63727	846	-0.3412
hsa-mir-4419a	3	0.006763	0.018444	0.55573	847	-0.8254
NOC3L	6	0.0067795	0.027069	0.63727	848	-0.4587
RPS18	6	0.006793	0.027117	0.63727	849	-0.4382
HOXB6	6	0.006793	0.027117	0.63727	850	-0.3855
NDUFB9	6	0.0067963	0.027129	0.63727	851	-0.2093
hsa-mir-4716	4	0.0068056	0.023338	0.60904	852	-0.7183
WDR76	6	0.0068146	0.027197	0.63814	853	-0.3461
RPA1	6	0.0068329	0.02726	0.63888	854	-0.3579
OR4D10	6	0.0068606	0.027367	0.63959	855	-0.3388
SLTM	3	0.0068787	0.018772	0.55723	856	-0.5413
CDC26	6	0.0068831	0.027451	0.64041	857	-0.2534
NANOGNB	6	0.0068946	0.027493	0.64067	858	-0.2035
SPDYE5	2	0.0069083	0.013234	0.49188	859	0.0089
REV3L	6	0.0069486	0.027686	0.64411	860	-0.3827
PRPF6	6	0.0069609	0.027735	0.64411	861	-0.356
TAGAP	6	0.0069609	0.027735	0.64411	862	-0.249
SRSF7	6	0.0069909	0.02783	0.64514	863	-0.389
OSBPL10	6	0.0069948	0.027843	0.64514	864	-0.3811
POGK	6	0.0070142	0.027925	0.64601	865	-0.4076
SRFBP1	6	0.0070253	0.027964	0.64601	866	-0.5615
ELF2	6	0.0070333	0.027989	0.64601	867	-0.3944
POLR2L	6	0.0070385	0.028007	0.64601	868	-0.1688
CRCP	6	0.0070903	0.028205	0.64809	869	-0.4826
ZMIZ1	6	0.0070918	0.02821	0.64809	870	0.2571
UQCRQ	6	0.0070943	0.028223	0.64809	871	-0.4357
hsa-mir-6856	4	0.0071027	0.024272	0.61509	872	-0.4903
SHMT1	6	0.007142	0.028389	0.65018	873	-0.1443
TMEM182	6	0.0071452	0.0284	0.65018	874	-0.1553
RASGEF1C	6	0.0071481	0.02841	0.65018	875	-0.269
TAF11	6	0.0071774	0.028506	0.65118	876	-0.0657
THOC5	6	0.0071807	0.028517	0.65118	877	-0.1851
NDE1	6	0.0072076	0.028618	0.65203	878	-0.3023
ABHD17C	6	0.0072076	0.028618	0.65203	879	-0.3736
TAF5	6	0.0072406	0.02875	0.65431	880	-0.4406
NADK	6	0.007293	0.028943	0.65797	881	0.0784
SAP18	6	0.007309	0.029005	0.65865	882	-0.4574
NCOA1	6	0.0073336	0.029092	0.65989	883	-0.3336
THOC1	5	0.0073779	0.026332	0.62835	884	-0.0354
SFPQ	6	0.0073824	0.029273	0.66014	885	-0.3987
KRR1	6	0.007386	0.029288	0.66014	886	-0.3281
LSMD1	6	0.0073953	0.029324	0.66014	887	-0.4846
ATP8B4	6	0.0074009	0.029341	0.66014	888	0.0699

PPP2R1A	6	0.0074096	0.029376	0.66014	889	-0.6222
SAYSD1	6	0.0074239	0.02943	0.66014	890	-0.1502
C18orf21	6	0.0074276	0.02945	0.66014	891	-0.2772
LARP4	6	0.0074314	0.029465	0.66014	892	-0.4215
WAS	6	0.0074335	0.02947	0.66014	893	-0.3761
PIP4K2A	6	0.0074406	0.02949	0.66014	894	-0.1367
GRXCR2	6	0.0074645	0.029574	0.66071	895	-0.4762
AKT1S1	6	0.0074717	0.0296	0.66071	896	-0.5218
CAND1	6	0.0074799	0.029627	0.66071	897	-0.2413
ACE	6	0.007486	0.029645	0.66071	898	-0.1953
FLOT1	6	0.0075028	0.02971	0.66083	899	-0.336
TMEM230	6	0.0075045	0.029715	0.66083	900	-0.231
hsa-mir-1304	4	0.0075089	0.025567	0.62738	901	-0.5101
RPL7	6	0.0075197	0.029767	0.66127	902	-0.3721
FAM83G	6	0.0075669	0.029946	0.66355	903	-0.3923
RPL35	6	0.0075702	0.029958	0.66355	904	-0.3152
CRIM1	6	0.0075992	0.030058	0.66486	905	-0.2764
KIR3DL2	5	0.0076179	0.027059	0.63727	906	-0.3286
SORD	6	0.0076301	0.030166	0.66561	907	-0.4399
PHF12	6	0.0076598	0.030271	0.66592	908	-0.0187
UQCRC2	6	0.0076648	0.030292	0.66592	909	-0.2445
hsa-mir-1299	4	0.0076758	0.026068	0.62835	910	-0.8292
PFDN6	6	0.0076812	0.030359	0.66592	911	-0.2166
KDM4A	6	0.0076842	0.030367	0.66592	912	-0.2534
ICT1	6	0.0077111	0.030461	0.66726	913	-0.1914
MRPL34	6	0.0077283	0.030529	0.66805	914	-0.2477
hsa-mir-181b-	4	0.0077348	0.026244	0.62835	915	-1.5757
ASB6	6	0.0077398	0.030573	0.66829	916	-0.3519
DOCK6	6	0.0077916	0.030755	0.66951	917	-0.4358
UBOX5	6	0.0077923	0.03076	0.66951	918	-0.0935
DNAJC8	6	0.0078648	0.031017	0.67352	919	-0.5088
INTS8	6	0.0078816	0.031071	0.67352	920	-0.5479
VHL	6	0.0078904	0.031105	0.67352	921	-0.4237
ID2	6	0.0078919	0.031109	0.67352	922	-0.397
ZNF692	6	0.0079186	0.031208	0.67496	923	0.0605
NDUFV3	6	0.0079395	0.031289	0.676	924	-0.3404
hsa-mir-625	4	0.0079409	0.026899	0.63727	925	-0.6792
GNL3L	6	0.0079652	0.031379	0.67723	926	-0.4355
hsa-mir-146a	4	0.0079788	0.027023	0.63727	927	-0.3083
FAM131A	6	0.0080314	0.031619	0.67991	928	-0.2652
ASNA1	6	0.0080317	0.03162	0.67991	929	-0.3146
GNB1L	6	0.0080455	0.031666	0.67991	930	-0.3435
C1orf109	6	0.0080545	0.031696	0.67991	931	-0.373
POLE	6	0.008059	0.031709	0.67991	932	-0.2262
CRTC3	6	0.0080738	0.031769	0.67991	933	-0.263
MRTO4	6	0.0080738	0.031769	0.67991	934	0.1247
hsa-mir-4735	4	0.0080783	0.027349	0.63959	935	-0.3624
DDX41	6	0.0081256	0.031954	0.68237	936	-0.3162
NRG2	6	0.0081338	0.031984	0.68237	937	-0.3302
FZD3	6	0.008177	0.032127	0.68384	938	-0.3275
KIAA1429	6	0.0081786	0.032132	0.68384	939	-0.2974
EIF2B5	6	0.008185	0.032153	0.68384	940	-0.3757
SDAD1	6	0.0081953	0.032188	0.68387	941	-0.479
NIPBL	6	0.0082291	0.032317	0.68394	942	-0.1427
PPP2R4	6	0.008241	0.032362	0.68394	943	-0.3626
SUPT7L	6	0.0082753	0.032484	0.68394	944	-0.4362
PGD	6	0.0082808	0.032504	0.68394	945	-0.0973
PCNA	6	0.0082836	0.032514	0.68394	946	-0.8973
C1orf95	6	0.008306	0.032593	0.68394	947	-0.33
NSA2	6	0.008306	0.032593	0.68394	948	-0.201
PSMG3	6	0.0083097	0.032607	0.68394	949	-0.0347
PARN	6	0.0083155	0.032626	0.68394	950	-0.2662
VMP1	4	0.0083306	0.028158	0.64809	951	-0.5328
GCKR	6	0.0083598	0.03278	0.68507	952	-0.3097
HYOU1	6	0.0083598	0.03278	0.68507	953	-0.3605
C2orf27B	3	0.0083613	0.022592	0.60152	954	-0.4153
SRP72	6	0.0083791	0.032849	0.68562	955	-0.3958
NDUFAB1	6	0.0083848	0.032878	0.68562	956	-0.4179
MAP4K2	6	0.0083928	0.032907	0.68562	957	-0.2275
YEATS4	6	0.0084191	0.032998	0.68658	958	-0.1318
MAD1L1	6	0.0084486	0.033104	0.68658	959	-0.3357
KIAA0947	6	0.0084611	0.033147	0.68658	960	-0.3579
EBP	6	0.0084626	0.033152	0.68658	961	-0.5464
RPL6	6	0.0084626	0.033152	0.68658	962	-0.4416
ICK	6	0.0084738	0.033188	0.68658	963	-0.2189
MAP3K5	6	0.0084938	0.033251	0.68719	964	-0.3641
GRPEL1	6	0.008516	0.03333	0.68813	965	-0.3621
PMPCA	6	0.0085305	0.033379	0.68826	966	-0.3297
ZNF512	6	0.0085395	0.03341	0.68826	967	0.0698
SCAMP4	6	0.0085459	0.033438	0.68826	968	-0.1897
C16orf62	6	0.0085714	0.033526	0.68939	969	-0.3212

OR2T27	6	0.0085912	0.033591	0.68965	970	0.1044
RINT1	6	0.0085984	0.033618	0.68965	971	-0.283
PAIP2B	6	0.0086049	0.03364	0.68965	972	-0.3222
VPS8	6	0.0086218	0.0337	0.6902	973	-0.3766
RPL32	6	0.0086312	0.033742	0.69035	974	-0.4287
RPL26	5	0.0086312	0.030128	0.66561	975	-0.2605
LCE4A	6	0.0086429	0.033775	0.69035	976	-0.0324
RIOK1	6	0.0086648	0.033855	0.69046	977	-0.5525
TMEM258	6	0.0086946	0.033955	0.69127	978	0.208
hsa-mir-1306	4	0.0087188	0.029421	0.66014	979	-0.5016
OR2C3	6	0.0087316	0.034091	0.69265	980	-0.4418
EME1	6	0.0087723	0.034226	0.69386	981	-0.2858
UVRAG	6	0.0087836	0.034269	0.69386	982	-0.4714
SLC25A3	6	0.0087901	0.034292	0.69386	983	-0.659
DOT1L	6	0.0087905	0.034293	0.69386	984	-0.3771
MIS12	6	0.0087981	0.03432	0.69386	985	-0.1155
FANCM	6	0.0088191	0.03439	0.6946	986	-0.3886
CCDC144A	5	0.0088565	0.030754	0.66951	987	-0.4614
CCDC53	6	0.0088831	0.034612	0.69839	988	-0.4609
hsa-mir-6749	4	0.0088964	0.029967	0.66355	989	-0.4055
NARS2	6	0.0089015	0.034689	0.69852	990	-0.1994
CSPG4	6	0.0089202	0.034753	0.69852	991	-0.4
PET100	6	0.0089211	0.034755	0.69852	992	-0.5976
ZNF491	6	0.0089382	0.034817	0.69908	993	-0.2572
ADTRP	6	0.0089539	0.034877	0.69949	994	-0.2087
USP21	6	0.0089606	0.034906	0.69949	995	-0.4046
hsa-mir-361	4	0.0089652	0.03019	0.66561	996	-0.426
hsa-mir-4738	4	0.0090169	0.030353	0.66592	997	0.0745
NSF	6	0.0090258	0.035141	0.70306	998	-0.4564
DOC2A	6	0.0090709	0.035304	0.70401	999	-0.126
NEDD8	5	0.0091054	0.031465	0.67836	1000	-0.271
HEATR3	6	0.0091083	0.035441	0.70497	1001	-0.0128
UQCRFS1	6	0.0091101	0.035445	0.70497	1002	-0.3018
NPHS1	6	0.0091296	0.035509	0.70497	1003	-0.295
MAT2A	6	0.0091345	0.035525	0.70497	1004	-0.375
SAFB2	6	0.009142	0.035556	0.70497	1005	-0.4853
SLC52A3	6	0.0091437	0.035559	0.70497	1006	-0.3524
hsa-mir-1184-	3	0.00919	0.024713	0.62039	1007	-0.6921
CARM1	6	0.0091986	0.035756	0.70797	1008	-0.2772
CYP21A2	6	0.0092117	0.035801	0.70797	1009	-0.22
hsa-mir-943	4	0.0092238	0.031027	0.67352	1010	-0.5352
NCL	6	0.0092316	0.035872	0.70797	1011	-0.3679
TMEM248	6	0.0092368	0.035894	0.70797	1012	-0.3441
GPR107	6	0.0092457	0.035924	0.70797	1013	-0.2412
FLNC	6	0.0092615	0.035978	0.70797	1014	-0.7577
TFAM	6	0.0092634	0.035983	0.70797	1015	-0.2499
COL4A2	6	0.0092789	0.036046	0.70797	1016	-0.2863
COQ4	6	0.0092816	0.036056	0.70797	1017	-0.4547
AP2B1	6	0.0093228	0.036197	0.71007	1018	-0.2976
NXPH4	6	0.0093627	0.036347	0.71164	1019	-0.3773
CCRL2	6	0.0093968	0.036464	0.71325	1020	-0.4565
RNF40	6	0.0094212	0.036547	0.7141	1021	-0.2688
UHRF1BP1	6	0.0094303	0.036578	0.7141	1022	-0.2988
SSX1	6	0.0094508	0.036639	0.71448	1023	-0.396
TMEM255A	6	0.0094819	0.03675	0.71448	1024	-0.3925
hsa-mir-2276	4	0.0094851	0.031864	0.68124	1025	-0.394
SCRN3	6	0.0094945	0.036807	0.71448	1026	-0.221
XAGE5	6	0.0095051	0.036842	0.71448	1027	-0.2796
SMNDC1	6	0.0095051	0.036842	0.71448	1028	-0.4485
SSRP1	6	0.0095373	0.036959	0.7154	1029	0.027
VPS33A	6	0.0095373	0.036959	0.7154	1030	0.2099
FGFR1OP	6	0.0095735	0.037079	0.71656	1031	-0.034
RRP1B	6	0.0095765	0.037089	0.71656	1032	-0.4303
YME1L1	6	0.0095986	0.037176	0.71725	1033	-0.2112
ATP6V1E1	6	0.0096044	0.037195	0.71725	1034	-0.2932
PDCD11	6	0.0096252	0.037268	0.71799	1035	-0.2733
MMS22L	6	0.0096354	0.037304	0.718	1036	-0.0819
SNRNP25	4	0.0096377	0.032341	0.68394	1037	-0.3982
MED8	6	0.0096503	0.037361	0.71813	1038	-0.3945
RPL27A	6	0.0096556	0.037381	0.71813	1039	-0.4212
RWDD4	6	0.0096727	0.037432	0.71844	1040	-0.5278
DLST	6	0.0096816	0.037469	0.71848	1041	-0.009
GRB7	6	0.009705	0.037544	0.71924	1042	-0.2691
HOXD12	6	0.0097286	0.037639	0.7204	1043	-0.0678
GTF2F1	4	0.0097756	0.032676	0.6843	1044	-0.2723
RPL37A	6	0.0097787	0.037827	0.72161	1045	-0.4475
CBX5	6	0.0097803	0.037834	0.72161	1046	0.0433
MVB12A	6	0.0097842	0.037844	0.72161	1047	-0.1966
hsa-mir-548ap	3	0.0098012	0.026299	0.62835	1048	-0.1425
LHFPL1	6	0.0098207	0.037981	0.72308	1049	-0.0485
ISCU	6	0.0098306	0.038021	0.72308	1050	-0.6731

TCEB2	6	0.0098319	0.038027	0.72308	1051	-0.0629
RNF14	6	0.009846	0.038085	0.7235	1052	-0.422
CBWD2	2	0.0098481	0.018645	0.55723	1053	-0.9105
TMCC2	6	0.0098641	0.03815	0.72407	1054	-0.092
SPCS3	6	0.0098836	0.038225	0.72481	1055	-0.0976
RP2	6	0.0099134	0.038319	0.72541	1056	-0.4448
NPM1	6	0.0099383	0.038396	0.72541	1057	-0.486
NR5A2	6	0.0099386	0.038398	0.72541	1058	-0.5184
TRIM47	6	0.0099611	0.03847	0.72544	1059	-0.1138
SNW1	6	0.0099967	0.038598	0.72545	1060	-0.3681
LGALS3BP	6	0.0099989	0.038608	0.72545	1061	-0.2359
OR2A5	6	0.010014	0.038663	0.72545	1062	-0.2041
RPL10A	6	0.01002	0.038682	0.72545	1063	-0.0915
TOX	6	0.010021	0.038683	0.72545	1064	-0.2361
TMED2	6	0.010039	0.038738	0.72581	1065	-0.3575
BANF1	6	0.010061	0.038809	0.72646	1066	-0.2656
MRC2	6	0.01008	0.038876	0.72659	1067	-0.3585
CNOT2	6	0.010089	0.038915	0.72659	1068	-0.37
CORO1A	6	0.01009	0.038922	0.72659	1069	-0.3422
FAHD2B	6	0.010136	0.039074	0.72669	1070	-0.3128
FAM101B	6	0.010136	0.039075	0.72669	1071	-0.1722
FIP1L1	6	0.010142	0.0391	0.72669	1072	-0.0402
PLA2G12A	6	0.010155	0.039141	0.72669	1073	-0.0568
RPLP1	6	0.010155	0.039141	0.72669	1074	-0.2183
RBBP5	6	0.010183	0.039235	0.72718	1075	-0.3032
DDX39B	6	0.010194	0.039273	0.72718	1076	-0.1653
LMBR1L	6	0.010216	0.039348	0.72779	1077	-0.3568
C3orf27	6	0.010226	0.039378	0.72779	1078	-0.2917
GOLGA6L4	3	0.010226	0.027384	0.63959	1079	-1.021
ATP5E	4	0.010279	0.033856	0.69046	1080	-0.3742
CTAGE5	4	0.010291	0.033882	0.69046	1081	-0.4292
MAP3K11	6	0.010307	0.039657	0.73229	1082	-0.3485
hsa-mir-219a-	4	0.010346	0.034021	0.69192	1083	-0.6046
NDUFS5	6	0.010351	0.039821	0.73453	1084	-0.1821
LOC149373	6	0.010361	0.039859	0.73453	1085	-0.3998
EIF3H	6	0.010373	0.039907	0.73453	1086	-0.0928
GHRHR	6	0.010379	0.039922	0.73453	1087	-0.2523
CTC1	6	0.010437	0.040118	0.73616	1088	-0.1245
FABP5	6	0.010452	0.040172	0.73648	1089	-0.3002
KCNH6	6	0.010488	0.040297	0.73746	1090	0.0602
ALG1	6	0.010503	0.040353	0.73783	1091	0.1777
GTF2IRD1	6	0.010531	0.04045	0.73828	1092	-0.2622
SUSD3	6	0.010591	0.040654	0.74068	1093	-0.3154
ATP5D	6	0.010613	0.040715	0.74113	1094	-0.1802
TRAPPC12	6	0.010645	0.040835	0.74134	1095	-0.3534
TMEM48	4	0.010647	0.034735	0.69852	1096	-0.4511
CHKA	6	0.010658	0.040876	0.74134	1097	-0.4417
NUDCD2	6	0.010662	0.040885	0.74134	1098	-0.2979
HSCB	6	0.010669	0.040908	0.74134	1099	-0.4497
ANP32E	6	0.010729	0.04112	0.7433	1100	-0.2681
CACTIN	6	0.010737	0.041144	0.7433	1101	-0.513
KNSTRN	6	0.010742	0.041161	0.7433	1102	-0.4642
EXOSC8	6	0.010764	0.041233	0.74393	1103	-0.4133
WASF2	6	0.010792	0.041327	0.74475	1104	-0.6312
HMGN3	6	0.010801	0.041351	0.74475	1105	-0.2693
NDUFS1	6	0.010813	0.041399	0.7448	1106	-0.0685
TRAPPC4	6	0.010823	0.041436	0.7448	1107	-0.6741
PFAS	4	0.010844	0.035153	0.70306	1108	-0.2475
SKIV2L2	6	0.010852	0.041516	0.7448	1109	-0.2491
HIST1H4H	6	0.01086	0.041542	0.7448	1110	-0.528
RNF113A	6	0.010862	0.041554	0.7448	1111	-0.1187
TXNDC16	6	0.010868	0.041572	0.7448	1112	-0.2715
hsa-mir-583	4	0.010869	0.035212	0.70356	1113	-0.4148
HNRNPM	6	0.010896	0.041665	0.74569	1114	-0.3658
hsa-mir-2117	4	0.01091	0.035299	0.70401	1115	-0.2815
PLK5	6	0.010925	0.041763	0.74619	1116	-0.2523
CNOT4	6	0.010982	0.041945	0.74822	1117	-0.264
YPEL5	6	0.011001	0.042011	0.74874	1118	-0.2494
UBTF	6	0.011024	0.042089	0.74946	1119	-0.2971
C9orf78	6	0.011057	0.042189	0.75059	1120	-0.1315
EPRS	6	0.011071	0.042243	0.75088	1121	-0.112
OSGEP	6	0.011081	0.042278	0.75088	1122	-0.7001
RTN4IP1	6	0.011118	0.042416	0.75239	1123	-0.2241
POLA1	6	0.011123	0.042437	0.75239	1124	-0.4876
NDUFS3	6	0.01117	0.042608	0.75442	1125	-0.2627
MARCKSL1	6	0.011178	0.042635	0.75442	1126	-0.2871
ELAVL1	6	0.011186	0.042662	0.75442	1127	-0.3268
PMF1	4	0.011223	0.03602	0.70797	1128	-0.7504
ABHD6	6	0.01123	0.042822	0.7566	1129	-0.4058
C2CD3	6	0.011272	0.042964	0.7578	1130	-0.2848
PSMB2	6	0.011286	0.043014	0.75802	1131	-0.3249

C3orf17	6	0.011315	0.043107	0.75804	1132	0.0711
EIF1	6	0.01132	0.043126	0.75804	1133	-0.3413
UCK2	6	0.01132	0.043126	0.75804	1134	-0.3307
hsa-mir-555	4	0.011326	0.036242	0.71026	1135	-0.1156
DDX59	6	0.011358	0.043262	0.75977	1136	-0.3488
PPP1R11	6	0.011395	0.043391	0.76023	1137	-0.3856
FBXO27	6	0.011395	0.043391	0.76023	1138	-0.2935
MTSS1	6	0.011407	0.043434	0.76023	1139	-0.1062
AK6	6	0.011407	0.043436	0.76023	1140	-0.6104
BOP1	6	0.011444	0.043553	0.76162	1141	-0.4091
RDH16	6	0.01148	0.043672	0.76207	1142	-0.2363
ALDH3A2	6	0.011484	0.04369	0.76207	1143	-0.1949
NAA10	6	0.011497	0.04373	0.76212	1144	-0.3996
KRT7	6	0.011522	0.043813	0.76291	1145	-0.2896
RFC4	6	0.011542	0.043879	0.7634	1146	-0.3058
hsa-mir-7111	4	0.011549	0.036748	0.71448	1147	-0.5784
hsa-mir-635	4	0.011549	0.036748	0.71448	1148	-0.5677
FABP1	6	0.011553	0.043919	0.76344	1149	-0.1895
EIF2B2	6	0.011564	0.043968	0.76344	1150	-0.2073
TOP2A	6	0.011587	0.044056	0.76344	1151	-0.3496
ENOSF1	6	0.011588	0.044058	0.76344	1152	-0.3123
DAND5	6	0.011591	0.044067	0.76344	1153	-0.0895
KRT75	6	0.011613	0.04414	0.76402	1154	-0.352
ARHGEF4	6	0.011623	0.044176	0.76402	1155	-0.4003
TMPRSS11D	6	0.01165	0.044266	0.76429	1156	-0.2813
SLC33A1	6	0.01165	0.044266	0.76429	1157	-0.2929
CUL3	6	0.011729	0.044531	0.76691	1158	-0.3381
DCLK1	6	0.011729	0.044531	0.76691	1159	-0.3392
SLC17A2	6	0.011747	0.044603	0.76751	1160	-0.3069
FAM86C1	5	0.011755	0.03927	0.72718	1161	-0.4918
NMT1	6	0.011782	0.044715	0.76862	1162	-0.1338
PEX10	6	0.0118	0.044781	0.76862	1163	-0.2929
NFS1	6	0.011812	0.044817	0.76862	1164	-0.2648
POLR2D	6	0.011828	0.044875	0.76896	1165	-0.3993
P2RX6	6	0.011856	0.044972	0.76979	1166	-0.4123
BRWD1	6	0.011898	0.045108	0.76979	1167	-0.299
MPLKIP	6	0.011901	0.045118	0.76979	1168	-0.409
PUF60	6	0.011913	0.045154	0.76979	1169	-0.3432
SIGLEC5	6	0.01195	0.045273	0.76979	1170	-0.3781
FSD1	6	0.01195	0.045273	0.76979	1171	-0.36
TGFBI	6	0.011961	0.045311	0.76979	1172	-0.379
FAM192A	6	0.011994	0.045425	0.76979	1173	-0.2767
DPPA3	6	0.012	0.045444	0.76979	1174	-0.0474
NXT1	5	0.012007	0.039991	0.73514	1175	0.2009
BCAP31	6	0.012015	0.045495	0.76979	1176	-0.2601
RPS23	6	0.012015	0.045496	0.76979	1177	-0.4904
KAT7	6	0.012024	0.045525	0.76979	1178	-0.346
EXOSC7	6	0.012103	0.0458	0.77334	1179	0.0029
IARS2	6	0.012116	0.045847	0.77334	1180	-0.5246
PABPC1	6	0.012122	0.045865	0.77334	1181	-0.2928
DNLZ	6	0.012129	0.04589	0.77334	1182	-0.2211
hsa-mir-3929	3	0.012145	0.032223	0.6839	1183	-2.44
PPT2	6	0.012161	0.045985	0.77334	1184	-0.3535
IWS1	6	0.012165	0.04599	0.77334	1185	-0.2627
TBCD	6	0.012177	0.046034	0.77334	1186	-0.2745
SMYD2	6	0.012189	0.046075	0.77334	1187	-0.3617
LSM14A	6	0.012201	0.046122	0.77348	1188	-0.2292
hsa-mir-6127	3	0.012207	0.03238	0.68394	1189	-0.5702
LAS1L	6	0.012242	0.046261	0.77477	1190	-0.054
SIN3A	6	0.012247	0.046274	0.77477	1191	-0.1392
ATP2A2	6	0.012274	0.046366	0.77568	1192	-0.4434
SPDYE3	4	0.01229	0.038439	0.72544	1193	-0.1051
NOTCH3	6	0.0123	0.046461	0.77663	1194	-0.4253
TICRR	6	0.012322	0.04654	0.77731	1195	-0.5183
PGK1	6	0.012361	0.046658	0.77802	1196	0.1964
MBD3L4	1	0.012369	0.012296	0.48283	1197	-0.8449
TGFBR2	6	0.012379	0.046719	0.77829	1198	-0.2245
EXOSC1	6	0.012392	0.046767	0.77829	1199	-0.1945
VPS25	6	0.012397	0.046788	0.77829	1200	-0.1853
PRC1	6	0.012444	0.046971	0.78018	1201	-0.2793
TTF1	6	0.012464	0.04704	0.78018	1202	-0.1694
VEZT	6	0.012466	0.047047	0.78018	1203	-0.3057
SORT1	6	0.012474	0.047075	0.78018	1204	-0.1891
ASB13	6	0.012477	0.047093	0.78018	1205	-0.2574
FNDC3B	6	0.012516	0.047234	0.78188	1206	0.0282
AGAP6	2	0.012542	0.02359	0.61043	1207	-1.0551
C2orf42	6	0.012567	0.047414	0.78422	1208	0.0339
METTL21A	6	0.012611	0.047569	0.78555	1209	-0.2123
PIK3C2A	6	0.012613	0.04757	0.78555	1210	-0.4253
CENPI	6	0.012627	0.047618	0.7857	1211	-0.3372
PSMC6	6	0.012647	0.047685	0.78602	1212	-0.0973

OR6C3	6	0.012675	0.047781	0.78602	1213	-0.2583
SGOL2	6	0.01269	0.047835	0.78602	1214	-0.3095
BROX	6	0.012696	0.047854	0.78602	1215	-0.3379
DTNB	6	0.012702	0.047878	0.78602	1216	-0.3383
FBXL16	6	0.012706	0.047895	0.78602	1217	-0.3257
GMPR2	6	0.012727	0.047977	0.78655	1218	-0.2647
NFYA	6	0.01274	0.048027	0.78674	1219	-0.3044
DAGLB	6	0.012759	0.048093	0.78707	1220	-0.4204
CNOT3	6	0.01281	0.048265	0.78874	1221	-0.4299
RNF103-CHMI	1	0.012812	0.01273	0.48686	1222	-1.3132
PLIN5	6	0.012825	0.048321	0.78903	1223	-0.326
TRMT61A	6	0.012839	0.04837	0.7892	1224	-0.0972
ZEB2	6	0.012877	0.048487	0.79005	1225	-0.3481
IL1RL2	6	0.012885	0.048518	0.79005	1226	-0.3653
IFT46	6	0.012895	0.048545	0.79005	1227	-0.2088
CAP1	6	0.012904	0.048577	0.79005	1228	-0.2287
THOC7	6	0.012921	0.048638	0.79014	1229	-0.2557
GTF3C5	6	0.012928	0.048662	0.79014	1230	-0.0373
NUPL2	6	0.012938	0.048698	0.79014	1231	-0.2014
CYP2F1	6	0.012969	0.048813	0.79137	1232	-0.1667
CDK11B	6	0.013005	0.048925	0.79166	1233	-0.2133
SEC31B	6	0.013016	0.048956	0.79166	1234	-0.2405
C5orf38	6	0.013029	0.048999	0.79166	1235	-0.2848
RPS15	6	0.013031	0.049005	0.79166	1236	-0.0055
DDX24	6	0.013046	0.049058	0.79166	1237	-0.3839
SACM1L	6	0.013083	0.049179	0.79245	1238	-0.1083
hsa-mir-935	4	0.013098	0.040223	0.73676	1239	-0.6471
SCCPDH	6	0.0131	0.049241	0.79245	1240	-0.379
BLOC1S2	6	0.013134	0.049362	0.79245	1241	0.1559
SLA	6	0.013142	0.049385	0.79245	1242	-0.1783
ATL2	6	0.013161	0.04945	0.79245	1243	-0.5453
GTF2B	6	0.013161	0.04945	0.79245	1244	-0.3925
C19orf12	6	0.013161	0.04945	0.79245	1245	-0.2897
SLFN12	6	0.013169	0.049473	0.79245	1246	-0.3482
DMAP1	6	0.01317	0.04948	0.79245	1247	-0.2948
COPB1	6	0.013191	0.049554	0.79245	1248	-0.2753
C16orf59	6	0.013191	0.049554	0.79245	1249	-0.2402
SPATA2L	4	0.013197	0.040437	0.73828	1250	-0.435
OR2H2	6	0.013199	0.049577	0.79245	1251	-0.3977
C5orf30	6	0.013243	0.04972	0.79297	1252	-0.2616
DNAH14	6	0.013255	0.049762	0.79297	1253	-0.0433
STIP1	6	0.013264	0.049793	0.79297	1254	-0.3395
PGLYRP3	6	0.013272	0.049821	0.79297	1255	-0.4386
EIF3G	6	0.013295	0.049899	0.79297	1256	-0.2492
KLHL2	6	0.013307	0.049942	0.79297	1257	-0.2716
MRPL42	6	0.013341	0.050049	0.79297	1258	-0.1154
OTUD6B	6	0.013359	0.050112	0.79297	1259	-0.1642
SCAF4	6	0.01336	0.050113	0.79297	1260	-0.3576
BIK	6	0.01336	0.050113	0.79297	1261	-0.7514
TIMM23	6	0.013443	0.050325	0.7951	1262	-0.0807
hsa-mir-4430	4	0.013494	0.041105	0.7433	1263	-0.4107
SF3B3	6	0.013522	0.050535	0.79707	1264	-0.3386
WNK1	6	0.013535	0.050567	0.79707	1265	-0.3705
MAP1LC3C	6	0.013609	0.050762	0.79892	1266	-0.3556
MRPL52	6	0.013639	0.050832	0.7994	1267	-0.1868
CYTH3	6	0.013682	0.050947	0.80009	1268	-0.3646
SHKBP1	6	0.013693	0.050974	0.80009	1269	-0.1817
FTSJ3	6	0.013701	0.050993	0.80009	1270	-0.2536
FBXL6	4	0.013741	0.041695	0.74569	1271	-0.4527
THAP1	6	0.013754	0.051119	0.80145	1272	-0.2175
EIF3A	6	0.013782	0.051188	0.80192	1273	-0.3534
hsa-mir-323a	4	0.013788	0.041795	0.74619	1274	-0.2974
WBP4	6	0.013804	0.051249	0.80226	1275	0.0315
UQCRB	6	0.013855	0.051384	0.80341	1276	-0.3384
BOLA3	6	0.013862	0.051401	0.80341	1277	-0.2814
NDC80	6	0.013921	0.051571	0.80538	1278	-0.4894
SLC5A10	6	0.013936	0.051605	0.80538	1279	-0.3794
TUFM	6	0.014111	0.052043	0.81008	1280	0.0218
ATP6V0D2	6	0.01417	0.052197	0.81008	1281	-0.2721
LAMTOR4	6	0.01417	0.052197	0.81008	1282	-0.3651
SHROOM1	6	0.014177	0.052218	0.81008	1283	-0.3105
RBM18	6	0.014177	0.052218	0.81008	1284	-0.6662
MLST8	6	0.014179	0.052223	0.81008	1285	-0.3882
PAQR5	6	0.014214	0.052319	0.81094	1286	-0.2849
ECSCR	6	0.014263	0.05244	0.81127	1287	-0.3174
UBAP1L	6	0.014267	0.052449	0.81127	1288	0.1073
SUGT1	4	0.014287	0.042934	0.7578	1289	-0.3706
WDR24	6	0.014293	0.052519	0.81127	1290	-0.2694
NAT9	6	0.014316	0.052589	0.81127	1291	-0.2428
NAP1L3	6	0.014317	0.05259	0.81127	1292	-0.4324
hsa-mir-708	3	0.014338	0.03773	0.72145	1293	-0.4267

ARHGAP22	6	0.01436	0.052714	0.81197	1294	-0.2581
RPP30	6	0.01437	0.052743	0.81197	1295	-0.1728
TRIM4	6	0.014394	0.052806	0.81235	1296	-0.3687
NUDT15	6	0.014432	0.052908	0.81285	1297	-0.1759
SEC61A1	6	0.014452	0.052958	0.81285	1298	-0.408
EMG1	6	0.014452	0.052958	0.81285	1299	-0.415
SNUPN	6	0.014487	0.053043	0.81354	1300	-0.2877
ECH1	6	0.014513	0.053103	0.81385	1301	-0.3521
RELB	6	0.014538	0.053177	0.81409	1302	-0.4153
C9orf50	6	0.014568	0.053255	0.81409	1303	-0.167
POLR3E	6	0.014579	0.053288	0.81409	1304	-0.6831
TFEB	6	0.014582	0.053291	0.81409	1305	-0.2119
RPL7L1	6	0.014594	0.053318	0.81409	1306	-0.2539
SRP19	6	0.014679	0.053558	0.81679	1307	-0.256
EPHA3	6	0.014699	0.053614	0.81679	1308	-0.2982
SETD4	6	0.014699	0.053614	0.81679	1309	-0.263
CENPF	6	0.014737	0.053709	0.81763	1310	-0.4541
MFN2	6	0.014754	0.053755	0.81772	1311	-0.6394
POU5F1	6	0.014801	0.053877	0.8181	1312	-0.3721
RAB6A	6	0.014833	0.05395	0.8181	1313	0.2921
PLA2G4A	6	0.014848	0.053983	0.8181	1314	-0.3196
DHX32	6	0.014857	0.054007	0.8181	1315	-0.2545
ARHGEF33	6	0.014874	0.05406	0.8181	1316	-0.3096
TFR2	6	0.014874	0.05406	0.8181	1317	-0.3888
PIP4K2C	6	0.014874	0.05406	0.8181	1318	-0.2357
FAM20C	6	0.01493	0.054203	0.81847	1319	-0.3813
ELP5	6	0.014936	0.05422	0.81847	1320	-0.1462
EIF3C	1	0.014939	0.014853	0.52018	1321	-0.6636
C8orf33	6	0.014948	0.05425	0.81847	1322	-0.2635
SUPT3H	6	0.014953	0.054263	0.81847	1323	-0.3693
TBP	6	0.014962	0.054284	0.81847	1324	-0.3816
SF3A3	6	0.015006	0.054415	0.8197	1325	-0.0311
METTL16	6	0.015047	0.054523	0.8197	1326	-0.004
CHMP1A	6	0.015062	0.054556	0.8197	1327	-0.1867
EPAS1	6	0.015069	0.054572	0.8197	1328	-0.1569
DDI2	6	0.015141	0.054754	0.8197	1329	-0.1799
TOE1	6	0.015168	0.054826	0.8197	1330	-0.2497
DACT1	6	0.015168	0.054826	0.8197	1331	-0.0412
PRIM2	6	0.015181	0.054857	0.8197	1332	-0.1216
GDAP1	6	0.015189	0.054878	0.8197	1333	-0.2303
RABGGTA	6	0.015226	0.054965	0.8197	1334	-0.2814
PGM2L1	6	0.015232	0.054979	0.8197	1335	0.0589
RRAS2	6	0.015245	0.055011	0.8197	1336	-0.156
ZUFSP	6	0.015257	0.055047	0.8197	1337	-0.3081
hsa-mir-4506	4	0.015262	0.045147	0.76979	1338	-0.3883
CLDND2	6	0.015273	0.055088	0.81971	1339	-0.2733
SPG11	6	0.015294	0.05514	0.81989	1340	-0.3206
METTL3	6	0.015354	0.055272	0.82064	1341	-0.1639
RANGRF	5	0.015378	0.049625	0.79261	1342	-0.248
LAPTM5	6	0.015384	0.055357	0.82064	1343	-0.3642
hsa-mir-449a	4	0.015418	0.045483	0.76979	1344	-0.2323
C4orf6	6	0.015449	0.055514	0.82064	1345	-0.4015
PSMF1	6	0.015451	0.055518	0.82064	1346	-0.0109
OR9K2	6	0.015463	0.055551	0.82064	1347	-0.2443
MARCH9	6	0.015488	0.055616	0.82064	1348	-0.3581
SDHA	6	0.015491	0.055625	0.82064	1349	-0.1849
RRP1	6	0.015502	0.05566	0.82064	1350	-0.2515
DHX15	6	0.015504	0.055667	0.82064	1351	-0.3993
APBB1IP	6	0.01553	0.055742	0.82064	1352	-0.5609
TSC22D2	6	0.015553	0.055806	0.82064	1353	-0.1119
USP10	6	0.015567	0.055832	0.82064	1354	-0.2512
CUL1	6	0.015567	0.055832	0.82064	1355	-0.2301
PPP1R18	6	0.015568	0.055832	0.82064	1356	-0.1465
C15orf39	6	0.015626	0.05599	0.8222	1357	-0.3243
PARD6B	6	0.015644	0.056032	0.8222	1358	-0.1756
TRAP1	6	0.015656	0.056059	0.8222	1359	0.0246
hsa-mir-6717	4	0.015658	0.046038	0.77334	1360	-0.1353
POLE3	6	0.015817	0.056474	0.82747	1361	-0.3234
RAB5A	6	0.015837	0.056522	0.82747	1362	-0.0919
EIF2S3	6	0.015844	0.05654	0.82747	1363	-0.2036
MINOS1	6	0.015879	0.05663	0.82792	1364	-0.1679
AHSA2	6	0.015886	0.056651	0.82792	1365	-0.5356
IPMK	6	0.015913	0.056732	0.82851	1366	0.0151
TPK1	6	0.015965	0.056857	0.82974	1367	-0.1483
INTS3	6	0.016012	0.056976	0.83088	1368	-0.3312
ALG12	6	0.016068	0.057109	0.83222	1369	-0.2619
FCHSD2	6	0.016108	0.0572	0.83251	1370	-0.1791
NRDE2	6	0.016131	0.057262	0.83251	1371	-0.403
OR4S1	6	0.01616	0.057345	0.83251	1372	-0.426
ANO6	6	0.01617	0.057368	0.83251	1373	0.1989
FAM3D	6	0.016175	0.05738	0.83251	1374	-0.2775

LAMB2	6	0.0162	0.057441	0.83251	1375	-0.2589
CNTLN	6	0.016206	0.057454	0.83251	1376	-0.1283
GOLT1A	6	0.016206	0.057454	0.83251	1377	-0.2984
AMBRA1	6	0.016244	0.057546	0.8326	1378	-0.2518
HYLS1	6	0.016258	0.057582	0.8326	1379	-0.194
AURKA	6	0.016273	0.057626	0.83265	1380	0.0005
RNGTT	6	0.01632	0.05774	0.8337	1381	-0.3411
HEATR6	6	0.016347	0.057809	0.83412	1382	-0.5009
PRR13	6	0.016361	0.057853	0.83416	1383	-0.2439
NEK1	5	0.016411	0.052617	0.81127	1384	-0.4861
TXNDC11	6	0.016455	0.058113	0.83614	1385	-0.2221
hsa-mir-543	2	0.016473	0.03062	0.66861	1386	-1.1756
hsa-mir-4784	4	0.016476	0.047906	0.78602	1387	-0.5647
SMC5	6	0.016522	0.058287	0.83672	1388	-0.2593
NOP58	6	0.01653	0.058311	0.83672	1389	-0.4063
LEMD1	6	0.016535	0.058318	0.83672	1390	-0.3309
EEF1B2	6	0.016561	0.058369	0.83672	1391	-0.2702
PPP1R27	6	0.016623	0.058519	0.83672	1392	-0.2993
USO1	6	0.016623	0.058519	0.83672	1393	-0.2987
ATP6V0D1	6	0.016633	0.058544	0.83672	1394	-0.0315
MRPS7	6	0.016672	0.058637	0.83672	1395	-0.1522
MRPL24	6	0.016684	0.058668	0.83672	1396	-0.0614
C1orf87	6	0.016687	0.058681	0.83672	1397	-0.1047
EED	6	0.016687	0.058681	0.83672	1398	-0.2836
TAF1B	6	0.016701	0.058715	0.83672	1399	-0.3318
ZNF609	6	0.016711	0.058739	0.83672	1400	-0.3239
KCTD21	6	0.016736	0.058804	0.83672	1401	0.23
MAPKBP1	6	0.016745	0.05884	0.83672	1402	-0.2918
GALNT9	6	0.016751	0.058849	0.83672	1403	-0.3269
SGPP1	6	0.016838	0.059074	0.83779	1404	-0.1445
SLU7	6	0.016842	0.059083	0.83779	1405	-0.4327
SRPR	6	0.01689	0.059211	0.83869	1406	-0.3078
CYB561	6	0.016923	0.05929	0.83869	1407	-0.2998
NFIL3	6	0.016923	0.05929	0.83869	1408	-0.4219
EHF	6	0.016933	0.059315	0.83869	1409	-0.1957
NARG2	6	0.016987	0.059466	0.83953	1410	-0.2946
DHODH	6	0.016987	0.059466	0.83953	1411	-0.2614
RGS7BP	4	0.016996	0.049063	0.79166	1412	-0.3088
NOP10	6	0.016997	0.059497	0.83953	1413	-0.4164
CHIT1	6	0.017043	0.059626	0.84024	1414	-0.3224
PPP2R1B	6	0.017078	0.05972	0.84024	1415	-0.2937
SCAMP2	6	0.017078	0.05972	0.84024	1416	-0.3299
SPR	6	0.017125	0.059838	0.84024	1417	-0.3754
AHNAK	6	0.017146	0.059894	0.84024	1418	0.1595
LMNA	6	0.01716	0.059928	0.84024	1419	-0.2384
C8orf87	6	0.017172	0.059952	0.84024	1420	0.0907
SH2D3A	6	0.017174	0.059956	0.84024	1421	-0.2594
MED26	6	0.017198	0.060012	0.84024	1422	-0.5019
GOLGA2	6	0.01722	0.060068	0.84024	1423	0.0378
SMAD5	6	0.017248	0.060144	0.84024	1424	-0.1487
RPUSD2	6	0.017249	0.060145	0.84024	1425	-0.2449
AK4	6	0.017249	0.060146	0.84024	1426	0.1822
MSANTD4	6	0.017258	0.060166	0.84024	1427	-0.2993
BCL2L1	6	0.017301	0.060261	0.84045	1428	-0.3179
POTEG	3	0.017302	0.045031	0.76979	1429	-0.3223
FDPS	6	0.017349	0.060394	0.84069	1430	-0.3729
ENPP5	6	0.017352	0.060402	0.84069	1431	0.1856
CEBPB	3	0.017377	0.045222	0.76979	1432	-0.0378
RAD17	6	0.017405	0.060536	0.84069	1433	-0.135
TMCO6	6	0.01741	0.060548	0.84069	1434	-0.3118
TPD52L2	6	0.017417	0.060566	0.84069	1435	-0.3139
hsa-mir-6763	4	0.017423	0.050026	0.79297	1436	-0.0412
ZMAT5	6	0.017455	0.06067	0.84156	1437	-0.124
CTSL2	2	0.017509	0.032459	0.68394	1438	-0.5574
CMIP	4	0.017512	0.050209	0.79387	1439	-0.4557
ARID5A	6	0.017518	0.060849	0.84291	1440	-0.4718
HIST1H3D	6	0.017518	0.060849	0.84291	1441	-0.4291
SCAMP3	6	0.017539	0.060899	0.84303	1442	-0.3249
ZNF580	6	0.017582	0.061024	0.84419	1443	-0.1901
THEM4	6	0.017609	0.061091	0.8444	1444	-0.0709
HERC2	6	0.017646	0.061193	0.8444	1445	-0.4139
NOTCH2NL	6	0.017646	0.061193	0.8444	1446	-0.8264
RIPPLY2	6	0.01766	0.061224	0.8444	1447	-0.1323
RIF1	6	0.017667	0.061246	0.8444	1448	-0.0895
SMC1A	6	0.017692	0.061313	0.84463	1449	-0.2583
INO80E	4	0.017707	0.050606	0.79707	1450	-0.1526
VPS72	6	0.017736	0.061416	0.84463	1451	-0.1694
CAPS2	6	0.017743	0.061428	0.84463	1452	-0.332
LRRN4CL	6	0.017777	0.061519	0.84532	1453	-0.2834
PTPRQ	6	0.017808	0.061607	0.84596	1454	-0.3188
BTD	6	0.017865	0.061744	0.84728	1455	0.2112

hsa-mir-7641-	2	0.017902	0.033178	0.68658	1456	-1.7845
RELL2	6	0.017914	0.061876	0.84844	1457	-0.1624
MEI1	6	0.017951	0.061965	0.84844	1458	-0.1857
ZNF671	6	0.017962	0.061995	0.84844	1459	-0.2789
C14orf37	6	0.018	0.062099	0.84917	1460	-0.2524
ACTR1A	6	0.018013	0.062132	0.84917	1461	-0.0281
AUP1	6	0.018044	0.062209	0.84924	1462	-0.3232
CCDC94	6	0.01807	0.062277	0.84924	1463	-0.4064
COA6	6	0.01808	0.062302	0.84924	1464	-0.3406
OR10J1	6	0.018109	0.062381	0.84939	1465	-0.1716
LARS2	6	0.018116	0.062396	0.84939	1466	-0.4301
SMC2	6	0.01816	0.062497	0.84958	1467	-0.3707
RNF20	6	0.018177	0.062535	0.84958	1468	-0.1492
EIF3K	4	0.0182	0.051676	0.80586	1469	-0.3618
GJA5	6	0.0182	0.062595	0.84983	1470	-0.281
APOBEC3H	6	0.018264	0.062755	0.85065	1471	-0.0924
NCAN	6	0.018267	0.062763	0.85065	1472	0.0009
RPS4X	6	0.018276	0.06278	0.85065	1473	-0.0319
GLRX5	6	0.018304	0.062856	0.85112	1474	-0.3166
ANKS6	6	0.018325	0.062909	0.85127	1475	-0.3147
hsa-mir-3135a	4	0.018367	0.052039	0.81008	1476	-0.298
CENPL	6	0.018386	0.063066	0.85283	1477	-0.2766
ZBTB49	6	0.01843	0.063185	0.85331	1478	-0.2403
HPSE2	6	0.018486	0.063337	0.85424	1479	-0.2182
AGAP2	6	0.018536	0.063452	0.85472	1480	-0.1915
IL8	6	0.018537	0.063457	0.85472	1481	-0.2888
DHX35	6	0.018597	0.063604	0.85615	1482	-0.3035
BTN2A1	6	0.01863	0.063692	0.85635	1483	-0.2858
COPE	6	0.018737	0.063967	0.85879	1484	-0.2464
MUC17	6	0.018781	0.06408	0.85879	1485	-0.2584
OXSM	6	0.018781	0.06408	0.85879	1486	-0.2244
ADCY1	6	0.018786	0.064094	0.85879	1487	-0.3867
TSSK6	6	0.018831	0.064208	0.85945	1488	-0.357
ZBTB34	6	0.01884	0.064227	0.85945	1489	-0.1766
MSTO1	6	0.018885	0.064331	0.85971	1490	-0.3474
PRKRIR	6	0.018885	0.064331	0.85971	1491	-0.2091
CCND2	6	0.018904	0.064379	0.85979	1492	-0.324
ANAPC15	6	0.018931	0.064451	0.86007	1493	-0.3047
METTL14	6	0.018942	0.064484	0.86007	1494	-0.0682
BTBD8	6	0.018982	0.064587	0.86088	1495	-0.2061
ARHGEF10	6	0.019035	0.064731	0.86224	1496	-0.087
WRAP53	6	0.019096	0.0649	0.86392	1497	-0.0133
SPESP1	5	0.019099	0.060205	0.84024	1498	-0.2357
MBD6	6	0.019117	0.064945	0.86396	1499	-0.2916
GTF2H3	6	0.019144	0.065004	0.86418	1500	-0.2654
OR11H6	6	0.019239	0.065258	0.86643	1501	-0.1418
VIPR1	6	0.019258	0.065306	0.86651	1502	-0.4337
PAF1	6	0.019309	0.065444	0.86655	1503	-0.364
NCOA6	6	0.019336	0.06551	0.86655	1504	-0.3333
EIF2B1	6	0.019353	0.065563	0.86655	1505	-0.1799
IFIH1	6	0.019354	0.065566	0.86655	1506	-0.1399
COG3	6	0.01938	0.065626	0.86655	1507	-0.1579
IDI1	6	0.019393	0.065655	0.86655	1508	-0.4896
PARVG	6	0.019427	0.065756	0.86655	1509	-0.2019
BRPF3	6	0.019455	0.065825	0.86655	1510	-0.1005
MLH3	6	0.019465	0.065848	0.86655	1511	-0.1992
HSPD1	6	0.019481	0.0659	0.86655	1512	-0.3092
DOLPP1	6	0.01952	0.065996	0.86655	1513	-0.1997
MTIF3	6	0.019583	0.066163	0.86655	1514	-0.1548
CLCN5	6	0.019596	0.066198	0.86655	1515	-0.1744
GPRC5B	6	0.019598	0.066204	0.86655	1516	-0.2267
IMPDH2	6	0.01963	0.066287	0.86655	1517	-0.2282
HIC2	6	0.019642	0.06632	0.86655	1518	-0.3382
TAF5L	6	0.019642	0.06632	0.86655	1519	-0.3375
ZNF274	6	0.019647	0.066331	0.86655	1520	-0.3432
STX18	6	0.019647	0.066331	0.86655	1521	-0.417
CCDC78	6	0.019647	0.066331	0.86655	1522	-0.2578
hsa-mir-508	4	0.019651	0.054859	0.8197	1523	-0.4561
TACR3	6	0.01966	0.066363	0.86655	1524	0.0621
ACLY	6	0.01966	0.066365	0.86655	1525	-0.0745
MYH9	6	0.019763	0.066622	0.86826	1526	-0.2581
ETV5	6	0.019814	0.066753	0.86826	1527	-0.0467
hsa-mir-5095	4	0.019836	0.055277	0.82064	1528	-0.5348
LRRC8D	6	0.019838	0.066826	0.86826	1529	0.0277
SLC39A14	6	0.01985	0.066858	0.86826	1530	-0.4286
ONECUT1	6	0.019858	0.06688	0.86826	1531	-0.3729
RPS5	6	0.019908	0.066993	0.86917	1532	-0.3122
CCDC19	6	0.019928	0.067052	0.86938	1533	-0.0359
NEK3	6	0.019958	0.067138	0.86964	1534	-0.058
ATAD3B	6	0.01997	0.067176	0.86964	1535	-0.3415
FPGS	6	0.019988	0.067217	0.86964	1536	0.0365

MRPS14	6	0.02004	0.067361	0.86964	1537	-0.221
EIF2AK1	6	0.020055	0.067397	0.86964	1538	-0.3193
MED23	6	0.020061	0.06741	0.86964	1539	-0.3832
SLC9C2	6	0.02007	0.067425	0.86964	1540	0.0225
FAM84A	6	0.020079	0.067454	0.86964	1541	-0.3559
TOR1AIP1	6	0.020121	0.067544	0.86971	1542	-0.2195
SNX17	6	0.020121	0.067545	0.86971	1543	-0.168
MCM3AP	6	0.020151	0.067613	0.86992	1544	-0.2796
ATP6V0B	6	0.020191	0.067713	0.86992	1545	-0.2292
CCNL1	6	0.020224	0.067782	0.86992	1546	0.0324
XDH	6	0.020225	0.067788	0.86992	1547	-0.2866
CCDC59	6	0.020261	0.067873	0.86992	1548	-0.5298
NAE1	6	0.020272	0.067897	0.86992	1549	-0.2452
LTA	6	0.020275	0.067907	0.86992	1550	0.0544
HSD3B2	6	0.020289	0.067944	0.86992	1551	-0.0723
PRKACA	6	0.020326	0.068042	0.87002	1552	-0.3086
NANOS2	6	0.020352	0.068114	0.87002	1553	-0.2743
WDR70	6	0.02037	0.068162	0.87002	1554	-0.0533
PSENEN	6	0.020374	0.06817	0.87002	1555	-0.175
CPSF3L	6	0.020377	0.06818	0.87002	1556	-0.2441
MFSD2A	6	0.020388	0.068207	0.87002	1557	-0.2936
EFTUD1	6	0.020428	0.068315	0.87005	1558	-0.0426
GSDMD	6	0.020474	0.068431	0.87005	1559	-0.2617
KLHDC10	6	0.02048	0.068444	0.87005	1560	0.0114
DLGAP3	6	0.020483	0.068452	0.87005	1561	-0.2773
CD2BP2	6	0.020489	0.068464	0.87005	1562	-0.3156
STIL	6	0.020531	0.068569	0.87017	1563	-0.2694
TWF2	6	0.020542	0.068603	0.87017	1564	-0.2956
DUSP11	6	0.020542	0.068603	0.87017	1565	-0.3171
TSPAN2	6	0.020596	0.068745	0.87017	1566	-0.4793
WDR16	6	0.020596	0.068745	0.87017	1567	-0.295
MAGEA12	6	0.020639	0.068852	0.87017	1568	-0.0501
LENG9	6	0.02064	0.068859	0.87017	1569	-0.3415
C10orf105	6	0.02064	0.068859	0.87017	1570	-0.2812
PHIP	6	0.020656	0.068899	0.87017	1571	-0.2052
PRDX1	6	0.02072	0.069052	0.87103	1572	-0.3362
RPS10	2	0.020765	0.03837	0.72541	1573	-0.7979
SIX1	6	0.020774	0.069179	0.87122	1574	-0.2285
FADS1	6	0.020821	0.069291	0.87122	1575	-0.4103
INA	6	0.020838	0.069325	0.87122	1576	-0.2059
MPI	6	0.020848	0.069355	0.87122	1577	-0.1673
REC8	6	0.020849	0.069356	0.87122	1578	-0.3072
hsa-mir-5591	2	0.020858	0.03853	0.72545	1579	-0.7224
CCT6A	6	0.020889	0.069453	0.87122	1580	-0.2421
MRPL54	6	0.020891	0.069459	0.87122	1581	-0.0675
hsa-mir-664b	4	0.020904	0.057576	0.8326	1582	-0.2582
DDX49	6	0.020918	0.069531	0.87122	1583	-0.3735
PAPL	6	0.020937	0.069582	0.87122	1584	-0.1324
RAB33B	6	0.02094	0.06959	0.87122	1585	-0.1363
PRMT2	6	0.020955	0.069621	0.87122	1586	-0.3413
MRPS18C	6	0.020955	0.069621	0.87122	1587	-0.3054
ORAI2	6	0.020955	0.069621	0.87122	1588	-0.3616
hsa-mir-1302-	4	0.021077	0.057956	0.83506	1589	-0.0557
hsa-mir-562	4	0.021111	0.058029	0.83552	1590	-0.2921
RPF1	6	0.021158	0.070153	0.87496	1591	-0.335
CCL19	6	0.021194	0.070235	0.87496	1592	-0.2092
ARL5C	6	0.021224	0.070316	0.87496	1593	-0.2136
C1orf35	6	0.021246	0.070366	0.87496	1594	-0.3176
TMEM69	6	0.021247	0.070369	0.87496	1595	0.0001
IL4R	6	0.021251	0.070378	0.87496	1596	-0.4304
MDK	6	0.021268	0.070416	0.87496	1597	-0.0946
FASLG	6	0.021275	0.070433	0.87496	1598	-0.2808
ECE1	6	0.021299	0.0705	0.87525	1599	0.0858
OR4K2	6	0.021316	0.070542	0.87525	1600	-0.1638
hsa-mir-378d-	4	0.021343	0.058525	0.83672	1601	0.5203
NAF1	6	0.021411	0.070775	0.87723	1602	-0.232
RPL21	6	0.021451	0.070874	0.87728	1603	-0.83
EIF4G2	6	0.021452	0.070877	0.87728	1604	-0.101
CYP26C1	5	0.021472	0.066788	0.86826	1605	-0.6519
UBE2E2	6	0.021478	0.070944	0.87756	1606	-0.2803
HMGCS1	6	0.021506	0.071013	0.87756	1607	-0.0862
hsa-mir-7976	4	0.021521	0.058916	0.8371	1608	-0.3289
OLFM1	6	0.021577	0.071184	0.87895	1609	-0.0704
SLC17A7	4	0.0216	0.059087	0.83779	1610	-0.0164
HSDL1	6	0.0216	0.071241	0.87913	1611	-0.2281
NAT10	6	0.021648	0.071382	0.88034	1612	-0.2997
CHMP1B	6	0.021688	0.071486	0.8808	1613	0.2604
TRIM15	6	0.021697	0.071506	0.8808	1614	-0.4221
GTF2H2	2	0.021718	0.040086	0.73616	1615	-0.2565
DNAJC22	6	0.021828	0.071828	0.88371	1616	-0.4083
RHOD	6	0.021861	0.071914	0.88375	1617	0.2705

NUFIP2	6	0.021899	0.072011	0.88375	1618	-0.2849
RGS22	6	0.021901	0.072016	0.88375	1619	-0.274
MYO5A	6	0.02191	0.072038	0.88375	1620	0.0105
AGXT	6	0.021923	0.07207	0.88375	1621	-0.3868
RGPD6	2	0.021963	0.040539	0.73923	1622	-0.5695
CCDC130	6	0.021964	0.072171	0.88375	1623	-0.27
COX7B	6	0.021964	0.072171	0.88375	1624	-0.3176
DCLRE1A	6	0.021966	0.072177	0.88375	1625	-0.0049
TREML4	6	0.022017	0.072314	0.88424	1626	-0.1168
AKIP1	6	0.022077	0.072442	0.88424	1627	-0.2911
XPO1	6	0.022132	0.072586	0.88424	1628	-0.3178
KIAA1147	6	0.022168	0.072678	0.88424	1629	-0.1365
HNRNPA0	6	0.022177	0.072698	0.88424	1630	-0.2158
TMX2	6	0.022196	0.072754	0.88424	1631	-0.3498
GEMIN2	6	0.022205	0.072773	0.88424	1632	-0.4285
KIF5A	6	0.022217	0.072806	0.88424	1633	-0.2843
ASS1	6	0.022219	0.072811	0.88424	1634	-0.0022
VPS41	6	0.022244	0.072877	0.88424	1635	-0.282
IER5L	6	0.02225	0.072893	0.88424	1636	0.202
RELA	6	0.02225	0.072893	0.88424	1637	-0.2644
PTTG1	6	0.02227	0.072942	0.88424	1638	-0.1377
hsa-mir-641	4	0.02228	0.060566	0.84069	1639	-0.4243
KAT5	6	0.022299	0.073003	0.88424	1640	-0.399
GTF3C4	6	0.022351	0.073142	0.88424	1641	-0.3413
ACTL6B	6	0.02236	0.073162	0.88424	1642	-0.2411
BUB3	6	0.02238	0.073209	0.88424	1643	-0.0399
CHST13	6	0.0224	0.073259	0.88424	1644	-0.2055
ZFYVE20	6	0.022424	0.07331	0.88424	1645	-0.0803
ABHD1	6	0.022472	0.073432	0.88424	1646	-0.48
ZC3H18	6	0.022501	0.073508	0.88424	1647	0.1563
RPE	6	0.022501	0.073508	0.88424	1648	-0.0368
TIMM17A	6	0.022509	0.073526	0.88424	1649	-0.3581
ZNF236	6	0.022545	0.073618	0.88424	1650	-0.2567
KCNA10	6	0.022552	0.073635	0.88424	1651	-0.2111
TRIM40	6	0.022562	0.07366	0.88424	1652	-0.103
ENKD1	6	0.022565	0.073665	0.88424	1653	-0.3939
NUP107	6	0.022592	0.073745	0.88424	1654	-0.3717
TESC	6	0.022603	0.073773	0.88424	1655	0.1635
ATPAF2	6	0.022603	0.073773	0.88424	1656	0.0974
hsa-mir-199b	4	0.022647	0.061349	0.84463	1657	-0.3675
TPPP2	6	0.022656	0.073902	0.88483	1658	0.0196
EFNB3	6	0.022679	0.073956	0.88483	1659	0.1522
FBN3	6	0.022691	0.073985	0.88483	1660	-0.0929
ORC3	6	0.022723	0.074085	0.8851	1661	-0.2265
PSMB4	6	0.022731	0.074104	0.8851	1662	0.2463
NELL1	6	0.022782	0.074238	0.88618	1663	0.1142
PPP5C	6	0.022828	0.074345	0.88652	1664	-0.2307
TAF1C	6	0.022846	0.074397	0.88652	1665	-0.2503
PCYT1A	6	0.022935	0.074627	0.88669	1666	-0.4608
POP4	6	0.022937	0.074631	0.88669	1667	-0.4164
KRTAP22-1	6	0.022937	0.074631	0.88669	1668	-0.1428
GTSE1	4	0.022948	0.061963	0.84844	1669	-0.2501
TSHR	6	0.022951	0.07467	0.88669	1670	-0.3393
LAMTOR3	6	0.022951	0.07467	0.88669	1671	-0.2615
ATF6B	6	0.022989	0.074763	0.88701	1672	-0.316
SRRM2	6	0.022996	0.074785	0.88701	1673	-0.0234
ERCC3	6	0.023037	0.074883	0.88767	1674	-0.1729
VPS28	6	0.023088	0.075012	0.88807	1675	-0.116
LMOD3	6	0.023137	0.075137	0.88807	1676	0.0859
POLE2	6	0.023139	0.075142	0.88807	1677	-0.5061
UFM1	6	0.023155	0.075177	0.88807	1678	-0.2803
CMTR2	4	0.023171	0.062459	0.84958	1679	-0.3775
RPL17-C18orf	1	0.023209	0.023156	0.60585	1680	-1.211
NDUFB10	6	0.023253	0.075422	0.88992	1681	-0.4923
GABPB1	6	0.023253	0.075422	0.88992	1682	-0.2035
ZNF20	5	0.02327	0.071711	0.88279	1683	-0.9223
VIM	6	0.023284	0.07549	0.88992	1684	-0.2648
LNX1	6	0.023311	0.075552	0.88992	1685	-0.2695
INHBE	6	0.023311	0.075552	0.88992	1686	-0.2169
FMO1	6	0.023349	0.075664	0.89073	1687	-0.3072
FBXW2	6	0.023385	0.075754	0.89127	1688	-0.2138
DDX55	6	0.023471	0.075978	0.89275	1689	0.0345
RPL34	6	0.023471	0.075978	0.89275	1690	-0.2556
hsa-mir-877	4	0.023483	0.06313	0.85313	1691	-0.9334
ZBTB42	6	0.023502	0.076052	0.89275	1692	-0.3054
CRH	6	0.023504	0.076054	0.89275	1693	-0.4047
SLBP	6	0.023531	0.076114	0.89283	1694	-0.0504
hsa-mir-6878	4	0.023536	0.063241	0.8535	1695	-0.6122
VAMP3	6	0.023547	0.076148	0.89283	1696	-0.2593
ATAD1	6	0.023625	0.076348	0.89443	1697	-0.1507
NCOA3	6	0.023661	0.076435	0.89443	1698	-0.267

CSNK1A1	6	0.023701	0.076549	0.89447	1699	-0.3325
GOLGA6L10	2	0.023707	0.04367	0.76207	1700	-1.6333
hsa-mir-6847	4	0.02375	0.063703	0.85635	1701	-0.3524
OR5D13	6	0.023765	0.0767	0.8957	1702	-0.235
GRIP1	6	0.023845	0.076896	0.89697	1703	0.1478
DEGS2	6	0.023877	0.076978	0.89742	1704	-0.1814
CXXC1	6	0.023914	0.077068	0.89786	1705	-0.1195
EMC9	4	0.023922	0.064071	0.85879	1706	-0.354
OGT	6	0.023931	0.077104	0.89786	1707	-0.0334
SNRPB	6	0.02397	0.077214	0.89824	1708	-0.2597
PTGES3	6	0.023974	0.077224	0.89824	1709	-0.3835
TUFT1	6	0.024059	0.077456	0.89854	1710	-0.1562
ATG3	6	0.02406	0.077459	0.89854	1711	-0.4678
ZDHHC12	6	0.024081	0.077506	0.89854	1712	-0.0985
SPATA31D4	2	0.024081	0.044327	0.76469	1713	-0.4939
IPO7	6	0.0241	0.077557	0.89854	1714	-0.2311
PRRG1	6	0.02411	0.077578	0.89854	1715	-0.1064
SP2	6	0.02412	0.077604	0.89854	1716	-0.2765
CSK	6	0.024137	0.077645	0.89854	1717	-0.1215
RRP15	6	0.024161	0.077712	0.89881	1718	0.0135
SGCA	6	0.024269	0.077954	0.9011	1719	-0.1703
CDKN2AIP	6	0.024307	0.078054	0.90138	1720	-0.2421
SPOP	6	0.024314	0.07807	0.90138	1721	-0.2786
RABGAP1L	6	0.024361	0.07819	0.90138	1722	-0.2991
C16orf72	6	0.024365	0.078196	0.90138	1723	0.1632
AQP11	6	0.024408	0.078295	0.90138	1724	-0.3772
NARS	6	0.024423	0.078337	0.90138	1725	-0.484
KARS	6	0.024441	0.078375	0.90138	1726	-0.2542
CYBRD1	4	0.024445	0.065196	0.86617	1727	-0.3034
SETMAR	6	0.024545	0.078656	0.90208	1728	-0.2916
LOC728819	6	0.024545	0.078656	0.90208	1729	-0.2875
POLDIP2	6	0.024545	0.078656	0.90208	1730	-0.2816
HAPLN2	6	0.024545	0.078656	0.90208	1731	-0.3948
GALNT15	6	0.024545	0.078656	0.90208	1732	-0.4674
TRIP12	6	0.024569	0.078714	0.90218	1733	-0.0514
ADH1B	6	0.024627	0.07886	0.90218	1734	-0.8177
CCDC28B	6	0.024633	0.078876	0.90218	1735	-0.1026
CES2	6	0.024633	0.078876	0.90218	1736	-0.1861
RAC3	6	0.024671	0.078968	0.90218	1737	-0.1128
INCENP	6	0.024684	0.079002	0.90218	1738	-0.1564
GAS2L3	6	0.024695	0.079033	0.90218	1739	-0.2853
ANTXR2	6	0.024714	0.079084	0.90218	1740	-0.3278
SLC27A5	6	0.024714	0.079084	0.90218	1741	-0.3532
RAD9A	6	0.024722	0.079106	0.90218	1742	-0.2446
FANCE	6	0.024741	0.079156	0.90225	1743	-0.3673
HOXC9	4	0.024793	0.065921	0.86655	1744	-0.3222
IRF4	6	0.024799	0.079282	0.90261	1745	-0.1205
CCDC84	6	0.024826	0.07934	0.90261	1746	-0.3858
C17orf85	6	0.024875	0.079454	0.90261	1747	-0.1128
APOM	6	0.024888	0.079489	0.90261	1748	-0.2357
MBNL1	6	0.024904	0.079524	0.90261	1749	-0.2815
TCF7L1	6	0.024909	0.079537	0.90261	1750	-0.2994
SLC4A8	6	0.024936	0.079588	0.90265	1751	-0.3566
ANKLE2	6	0.024966	0.079664	0.903	1752	-0.1789
hsa-mir-5689	4	0.024998	0.066367	0.86655	1753	-0.4114
TRIM43	4	0.025015	0.066411	0.86656	1754	-0.5618
ZMYM5	6	0.025028	0.079801	0.90364	1755	-0.116
DPY30	6	0.025034	0.079815	0.90364	1756	-0.2822
UPF3B	6	0.025079	0.079931	0.90364	1757	-0.0508
GEMIN8	6	0.02513	0.080047	0.90389	1758	-0.2251
ADCK2	6	0.025132	0.080052	0.90389	1759	0.0242
hsa-mir-6758	4	0.025163	0.066702	0.86826	1760	-0.4129
PCMTD1	6	0.025179	0.080165	0.90423	1761	-0.2751
SLC7A5	6	0.025181	0.080171	0.90423	1762	-0.119
RIMKLA	6	0.025212	0.080253	0.90423	1763	-0.2058
hsa-mir-6866	4	0.025218	0.066829	0.86826	1764	-0.7062
OR11H4	6	0.025221	0.080272	0.90423	1765	-0.2598
MAU2	6	0.025232	0.080302	0.90423	1766	-0.3567
ZFR2	6	0.025296	0.080454	0.90478	1767	-0.3203
SPINK8	6	0.025302	0.080468	0.90478	1768	-0.1628
BRK1	6	0.025309	0.080484	0.90478	1769	-0.0241
NIP7	6	0.025385	0.080682	0.90511	1770	-0.1019
hsa-mir-7852	2	0.025408	0.046653	0.77802	1771	-0.1528
TTC9C	6	0.025414	0.080754	0.90511	1772	-0.2768
TAF1D	6	0.025436	0.080809	0.90511	1773	-0.0928
ACAT1	6	0.02545	0.080842	0.90511	1774	-0.1643
M6PR	6	0.025451	0.080846	0.90511	1775	-0.2522
TAPBP	6	0.025538	0.081048	0.90511	1776	-0.2336
BRD2	6	0.025554	0.08109	0.90511	1777	-0.2648
ETV1	6	0.025555	0.081091	0.90511	1778	-0.4349
PLK4	6	0.025582	0.081159	0.90511	1779	-0.2841

UBXN4	6	0.025589	0.081172	0.90511	1780	0.0615
HIST3H2BB	6	0.02564	0.081301	0.90511	1781	-0.3784
PDE2A	6	0.02564	0.081302	0.90511	1782	-0.1173
PCDH8	6	0.025663	0.081367	0.90511	1783	-0.2088
hsa-mir-3682	4	0.025668	0.06779	0.86992	1784	-0.4614
TYK2	6	0.025683	0.08142	0.90511	1785	-0.2383
TSNAX	6	0.025691	0.081443	0.90511	1786	0.1912
PEX11B	6	0.025706	0.081472	0.90511	1787	-0.3723
CDC42BPA	6	0.025736	0.081538	0.90511	1788	-0.2313
PDCD6IP	6	0.025742	0.081555	0.90511	1789	-0.0441
CRLS1	6	0.025759	0.081586	0.90511	1790	-0.2456
PFDN1	6	0.025775	0.081623	0.90511	1791	-0.265
DYNC1I1	6	0.025807	0.081716	0.90511	1792	-0.3803
ARSI	6	0.02581	0.081726	0.90511	1793	-0.2673
PVR	6	0.025877	0.081881	0.90511	1794	-0.2703
OR5K4	6	0.025889	0.081914	0.90511	1795	-0.4491
GPR89B	4	0.025915	0.068299	0.87005	1796	0.2326
C6orf57	6	0.025919	0.082	0.90511	1797	-0.3009
ZNF503	6	0.025919	0.082	0.90511	1798	-0.3521
DLGAP2	6	0.02594	0.082051	0.90511	1799	-0.1522
RAB6C	6	0.025946	0.082065	0.90511	1800	-0.3797
AFF3	6	0.025947	0.082067	0.90511	1801	-0.2433
TERT	6	0.025948	0.082067	0.90511	1802	-0.1534
SETD5	6	0.025963	0.082109	0.90511	1803	-0.2857
FCF1	6	0.025997	0.082183	0.90511	1804	-0.1518
ZMYND10	6	0.026003	0.082194	0.90511	1805	-0.3906
TRIB2	6	0.026031	0.082266	0.90523	1806	-0.2823
HIST2H2AB	6	0.026058	0.082328	0.90523	1807	-0.2032
SULT1C2	6	0.026062	0.082338	0.90523	1808	-0.2532
PIBF1	6	0.026109	0.082446	0.90547	1809	-0.2708
RNF169	6	0.026182	0.082632	0.9069	1810	0.2006
CD200R1L	5	0.026183	0.079541	0.90261	1811	-0.2507
hsa-mir-4778	4	0.026187	0.068884	0.87017	1812	-0.5167
FANCA	6	0.026201	0.082682	0.9069	1813	-0.1188
ASIC3	6	0.026279	0.082862	0.9069	1814	-0.2928
TMEM43	6	0.026338	0.083	0.9069	1815	-0.3372
GSDMB	6	0.026354	0.083043	0.9069	1816	-0.0613
WFDC9	6	0.026356	0.08305	0.9069	1817	-0.0143
ALLC	6	0.026411	0.083191	0.9069	1818	-0.1678
LAPTM4A	6	0.026449	0.08327	0.9069	1819	-0.3334
DUS3L	6	0.026449	0.08327	0.9069	1820	-0.2896
IQGAP3	6	0.026504	0.083429	0.9069	1821	-0.3193
EVL	6	0.026506	0.083436	0.9069	1822	-0.2308
SLC35C1	6	0.026535	0.0835	0.9069	1823	-0.0936
C2orf49	6	0.026557	0.083557	0.9069	1824	-0.2761
hsa-mir-4260	4	0.02656	0.069678	0.87141	1825	-0.2952
USE1	6	0.026582	0.083611	0.9069	1826	-0.2403
HLA-DMB	6	0.026588	0.083625	0.9069	1827	-0.1206
USP39	6	0.026589	0.083629	0.9069	1828	-0.0526
CACNB3	6	0.026626	0.083717	0.9069	1829	-0.3484
KCNJ16	6	0.026648	0.083771	0.9069	1830	-0.2486
ELF4	6	0.026659	0.083806	0.9069	1831	-0.298
GPR124	4	0.026667	0.069921	0.8739	1832	-0.2513
CFL1	6	0.02669	0.083885	0.9069	1833	-0.4574
TMEM205	6	0.026701	0.083914	0.9069	1834	-0.2857
AHCYL1	6	0.026714	0.083943	0.9069	1835	-0.3044
CHD1	6	0.026761	0.084062	0.9069	1836	-0.2517
RPS3A	6	0.026782	0.084122	0.9069	1837	-0.3386
PSME1	6	0.026795	0.084149	0.9069	1838	-0.3043
MAPK8	6	0.026799	0.084155	0.9069	1839	-0.3624
PPARD	6	0.026812	0.084185	0.9069	1840	-0.1566
hsa-mir-576	4	0.026844	0.07029	0.87496	1841	-0.374
WDR18	6	0.026846	0.084275	0.9069	1842	-0.3371
GLUD1	6	0.026863	0.084321	0.9069	1843	0.0099
GNRHR	6	0.02688	0.084362	0.9069	1844	-0.1642
SGOL1	6	0.026924	0.084463	0.9069	1845	-0.3977
RRP7A	6	0.026929	0.084478	0.9069	1846	-0.2456
MTHFD1	6	0.026952	0.084535	0.9069	1847	-0.3068
TMCO5A	6	0.026952	0.084535	0.9069	1848	-0.2958
PPP2R2A	6	0.026958	0.08455	0.9069	1849	0.0112
TJP2	6	0.02696	0.084555	0.9069	1850	-0.1201
SERPINE3	6	0.026968	0.084573	0.9069	1851	-0.1902
DHX38	6	0.026987	0.084617	0.9069	1852	-0.2153
LAMTOR1	6	0.027007	0.084672	0.90701	1853	-0.2954
HNRPLL	4	0.027084	0.070787	0.87723	1854	-0.2985
LTBP3	6	0.027086	0.084857	0.90778	1855	-0.2853
TNK2	6	0.027118	0.084943	0.90778	1856	-0.3678
NDUFV1	6	0.027167	0.085064	0.90778	1857	-0.1176
NUBP2	6	0.027169	0.085066	0.90778	1858	-0.1755
KRTAP11-1	6	0.027191	0.085123	0.90778	1859	-0.2377
hsa-mir-1343	4	0.027191	0.071028	0.87756	1860	-0.3706

CD9	6	0.027197	0.085134	0.90778	1861	0.0228
FAM114A2	6	0.027215	0.085172	0.90778	1862	-0.4063
SPATA5L1	6	0.027215	0.085172	0.90778	1863	-0.2456
AIFM1	6	0.027217	0.085182	0.90778	1864	-0.1752
GLTPD1	6	0.02722	0.085187	0.90778	1865	-0.0678
LRRC33	3	0.027256	0.068962	0.87043	1866	-0.2194
INSM1	6	0.027261	0.085284	0.908	1867	0.0091
DHRS2	6	0.027266	0.085297	0.908	1868	-0.2255
CNNM3	6	0.027314	0.085423	0.90887	1869	-0.3326
CCDC90B	6	0.027349	0.085514	0.90889	1870	-0.197
NUMB	6	0.027405	0.085662	0.90961	1871	-0.282
DPH1	6	0.027408	0.08567	0.90961	1872	-0.1801
SDR9C7	6	0.027482	0.085842	0.91003	1873	-0.2171
KRTAP24-1	6	0.027531	0.085965	0.91003	1874	-0.3328
NAPSA	6	0.027548	0.086007	0.91003	1875	-0.3645
FOXA2	6	0.027548	0.086007	0.91003	1876	-0.3887
ENDOV	6	0.027554	0.086022	0.91003	1877	-0.2549
FTSJD2	2	0.027558	0.050474	0.79683	1878	-0.582
MPHOSPH8	6	0.027584	0.086091	0.9103	1879	-0.3665
ABHD11	6	0.027608	0.086149	0.91043	1880	-0.2457
PRRT1	6	0.027645	0.086241	0.91048	1881	-0.256
ASGR2	6	0.027645	0.086241	0.91048	1882	-0.2546
CDK13	6	0.027701	0.086378	0.9111	1883	-0.2972
RPRD1B	6	0.027716	0.086413	0.9111	1884	-0.3276
ACBD6	6	0.027729	0.086434	0.9111	1885	-0.0353
CDC5L	6	0.027759	0.086521	0.91154	1886	-0.2288
MAPK8IP1	6	0.027803	0.086627	0.91219	1887	0.2843
PROK1	6	0.027826	0.086677	0.91225	1888	-0.1947
NDUFS7	6	0.027927	0.086936	0.91251	1889	-0.2301
F11	6	0.02794	0.086967	0.91251	1890	-0.091
SLC35C2	4	0.027944	0.072624	0.88424	1891	-0.5898
KRTAP19-2	6	0.02795	0.086989	0.91251	1892	-0.4621
TMEM180	6	0.027971	0.08705	0.91251	1893	-0.3005
OR52W1	6	0.027983	0.08708	0.91251	1894	-0.2365
SHB	6	0.028001	0.087121	0.91251	1895	0.0531
XRCC6BP1	6	0.028001	0.087121	0.91251	1896	-0.0759
SH3TC1	6	0.028004	0.087125	0.91251	1897	-0.2736
IL34	6	0.028034	0.087196	0.91251	1898	-0.0701
SYNGR2	4	0.028054	0.07286	0.88424	1899	-0.4928
TECTB	6	0.02808	0.087316	0.91251	1900	0.2497
HLA-DQB2	6	0.02808	0.087316	0.91251	1901	-0.0786
PPEF2	6	0.028119	0.08741	0.91251	1902	-0.3529
DUSP4	6	0.028119	0.08741	0.91251	1903	-0.2308
CDK5RAP1	6	0.028136	0.087452	0.91251	1904	0.0667
SLC9A8	4	0.028136	0.073026	0.88424	1905	-0.0935
PLCH2	6	0.02814	0.08746	0.91251	1906	0.175
hsa-mir-5001	4	0.028185	0.073132	0.88424	1907	-0.4503
C1QC	6	0.028262	0.087774	0.91499	1908	-0.257
hsa-mir-6070	4	0.028265	0.073297	0.88424	1909	-0.2295
GPAT2	6	0.028279	0.08782	0.91499	1910	-0.2563
EIF4EBP3	6	0.028288	0.087843	0.91499	1911	-0.0148
CAPN9	6	0.028339	0.087977	0.91508	1912	0.0049
PDSS2	6	0.028399	0.088124	0.91508	1913	-0.339
LRRC37A3	5	0.028405	0.085496	0.90889	1914	-0.2843
USPL1	6	0.028413	0.088166	0.91508	1915	-0.3792
HGS	6	0.028413	0.088166	0.91508	1916	-0.323
MYO1H	6	0.028419	0.088182	0.91508	1917	-0.1376
TMEM169	6	0.028449	0.088253	0.91508	1918	-0.0143
ONECUT3	6	0.028461	0.088287	0.91508	1919	-0.289
TMEM102	6	0.028519	0.088425	0.91557	1920	-0.1323
KIAA1522	6	0.028577	0.088571	0.91662	1921	-0.1866
SRGAP2D	5	0.02859	0.085977	0.91003	1922	-0.355
hsa-mir-30c-2	4	0.028596	0.073995	0.88483	1923	-0.3698
ARHGAP31	6	0.028623	0.088694	0.91743	1924	-0.2647
DBT	6	0.028659	0.088775	0.91781	1925	-0.1905
hsa-mir-6846	2	0.028699	0.052491	0.81127	1926	-0.6772
ARSD	6	0.02872	0.088936	0.91841	1927	0.1584
KRT24	6	0.02875	0.089005	0.91841	1928	-0.0758
RNF157	6	0.028787	0.089101	0.91886	1929	-0.3225
hsa-mir-4769	4	0.028813	0.074477	0.88669	1930	-0.5464
TXNL4B	6	0.028898	0.089371	0.91944	1931	-0.029
NEK10	6	0.02892	0.089414	0.91944	1932	0.098
FAF2	6	0.028978	0.089547	0.91944	1933	-0.2438
ZNF516	6	0.029019	0.089641	0.91944	1934	-0.2761
RAB41	6	0.029026	0.089658	0.91944	1935	0.0015
POLR1E	6	0.029026	0.089658	0.91944	1936	-0.2077
GSPT1	4	0.029033	0.074945	0.88788	1937	-0.3855
VWC2L	6	0.029103	0.089846	0.91944	1938	-0.2613
NUP50	6	0.029116	0.089876	0.91944	1939	0.3895
TEKT2	6	0.029116	0.089876	0.91944	1940	0.0138
KRT16	6	0.029124	0.089896	0.91944	1941	-0.2548

MYL7	6	0.029127	0.089906	0.91944	1942	-0.0006
RBM17	6	0.029139	0.089933	0.91944	1943	-0.311
LST1	6	0.029139	0.089933	0.91944	1944	-0.36
KIFC1	6	0.029191	0.090069	0.92002	1945	-0.462
hsa-mir-1972-	1	0.029196	0.029128	0.65997	1946	-1.5489
OR6P1	6	0.029262	0.090228	0.9212	1947	-0.1575
SLC17A4	6	0.029305	0.090336	0.92183	1948	-0.4127
C12orf40	6	0.029356	0.09046	0.92203	1949	-0.1462
CNTNAP5	6	0.029364	0.090484	0.92203	1950	-0.4149
MYOC	6	0.029398	0.090579	0.92203	1951	-0.2241
HIST1H2BH	6	0.029407	0.090602	0.92203	1952	-0.1067
PSMA7	6	0.029423	0.090636	0.92203	1953	-0.2848
NKX6-3	6	0.029453	0.090711	0.92203	1954	-0.4729
C9orf131	6	0.029492	0.090794	0.92203	1955	-0.1666
ILF2	5	0.029499	0.088348	0.91525	1956	-0.2602
CERS2	6	0.029525	0.090882	0.92203	1957	-0.1903
SCARB1	6	0.029529	0.090893	0.92203	1958	-0.2797
PITRM1	6	0.029556	0.090955	0.92203	1959	-0.1443
COX7C	6	0.029559	0.090961	0.92203	1960	-0.0657
MYH10	6	0.029569	0.090986	0.92203	1961	-0.3036
PSPC1	6	0.02963	0.09113	0.92303	1962	-0.3169
CNST	6	0.029712	0.091328	0.92339	1963	-0.2808
hsa-mir-4649	4	0.029733	0.076459	0.89443	1964	-0.46
MED14	4	0.029733	0.076459	0.89443	1965	-0.5486
VGLL3	6	0.029749	0.091416	0.92339	1966	-0.2897
ACO2	6	0.029762	0.091451	0.92339	1967	-0.0539
hsa-mir-6868	4	0.029771	0.07655	0.89447	1968	-0.4422
TBC1D20	6	0.02979	0.091522	0.92339	1969	-0.2524
DNAJC12	6	0.029813	0.091574	0.92339	1970	-0.176
SEC31A	6	0.029831	0.09161	0.92339	1971	0.0901
FECH	6	0.029839	0.091638	0.92339	1972	-0.2323
LPPR3	6	0.029855	0.09167	0.92339	1973	-0.2586
KCMF1	6	0.029857	0.091675	0.92339	1974	-0.3116
PCID2	6	0.029897	0.09178	0.92339	1975	-0.1413
hsa-mir-6514	4	0.029913	0.07684	0.89683	1976	-0.1116
DENND3	6	0.029915	0.091827	0.92339	1977	0.0703
ZNF132	6	0.029918	0.091833	0.92339	1978	-0.3222
GRAPL	2	0.029928	0.054629	0.8197	1979	-0.3088
IL19	6	0.029966	0.091942	0.92394	1980	-0.1649
ISG20L2	6	0.030016	0.092057	0.92463	1981	-0.2891
PLK1	6	0.03008	0.09222	0.92487	1982	-0.2907
ARHGAP11B	2	0.030099	0.054939	0.8197	1983	-0.9506
SEC61A2	6	0.030133	0.092343	0.92487	1984	-0.3954
SCML2	6	0.030137	0.092351	0.92487	1985	-0.2365
TBL3	6	0.03016	0.092403	0.92494	1986	-0.3294
ZIM3	6	0.030202	0.092517	0.92553	1987	-0.3235
FCGR3B	6	0.03022	0.092553	0.92553	1988	-0.0482
hsa-mir-4763	4	0.030241	0.077535	0.89854	1989	-0.3203
HIST1H3F	6	0.030332	0.092824	0.92643	1990	-0.672
PARP14	6	0.030332	0.092824	0.92643	1991	-0.1857
MMACHC	6	0.030388	0.092971	0.92654	1992	-0.3984
SGMS2	6	0.030388	0.092971	0.92654	1993	-0.4034
TAF3	6	0.030388	0.092971	0.92654	1994	-0.3984
SEBOX	6	0.030496	0.093227	0.92819	1995	-0.2263
WDR88	6	0.030496	0.093227	0.92819	1996	-0.3082
DYNLL1	6	0.03055	0.093365	0.92854	1997	-0.2673
hsa-mir-1267	4	0.030572	0.078237	0.90138	1998	0.1185
CHIC1	6	0.030575	0.093417	0.92854	1999	-0.2975
OR2AG1	6	0.030575	0.093417	0.92854	2000	-0.4113
SDF2L1	6	0.030605	0.093495	0.92854	2001	-0.0739
METTL17	4	0.030606	0.078317	0.90138	2002	-0.5127
AK9	6	0.030639	0.09358	0.92854	2003	-0.1986
EHMT2	6	0.030676	0.093679	0.92854	2004	0.0641
GNGT2	6	0.030689	0.093706	0.92854	2005	-0.3763
COX14	6	0.030692	0.093713	0.92854	2006	-0.0093
C10orf82	6	0.030816	0.094005	0.93037	2007	-0.2468
MKL1	6	0.030816	0.094006	0.93037	2008	0.1725
NOC4L	6	0.030829	0.094037	0.93037	2009	-0.1854
CYP1A2	6	0.030851	0.094093	0.93047	2010	-0.2792
SGCE	6	0.030875	0.094172	0.9308	2011	-0.1967
NAPA	6	0.03093	0.0943	0.93146	2012	-0.1811
RAC1	6	0.030945	0.094335	0.93146	2013	-0.135
UBE2G2	6	0.03096	0.094375	0.93146	2014	-0.1483
CXADR	6	0.031008	0.094501	0.93226	2015	-0.3693
CD3D	6	0.031054	0.094594	0.93244	2016	-0.2406
C1QL3	6	0.031082	0.094658	0.93244	2017	-0.2665
hsa-mir-129-2	4	0.031097	0.079388	0.90261	2018	-0.3637
IFT172	6	0.031125	0.09475	0.93244	2019	-0.2429
FOSL2	6	0.031125	0.09475	0.93244	2020	-0.3297
HIST1H3H	5	0.031142	0.092676	0.92631	2021	-0.2249
BMS1	6	0.031174	0.094862	0.93244	2022	-0.3578

ZNF16	6	0.031175	0.094864	0.93244	2023	-0.2716
DEFB118	6	0.031175	0.094864	0.93244	2024	-0.2659
GTPBP4	6	0.031194	0.094911	0.93244	2025	-0.3383
ZC3H4	6	0.031235	0.095019	0.93244	2026	-0.2288
HLA-DRA	6	0.031284	0.09514	0.93244	2027	-0.3991
PLEKHA1	6	0.031317	0.095207	0.93244	2028	-0.3099
KNTC1	6	0.031325	0.095232	0.93244	2029	-0.2356
FLT3LG	6	0.03134	0.095269	0.93244	2030	-0.1739
FRYL	4	0.031369	0.07994	0.90364	2031	-0.1799
ZC3H12B	6	0.031373	0.095351	0.93244	2032	-0.2898
ANAPC10	6	0.031373	0.095351	0.93244	2033	-0.2831
RBP5	6	0.031373	0.095351	0.93244	2034	-0.3184
DUSP23	6	0.031388	0.095386	0.93244	2035	-0.281
TMEM165	6	0.031539	0.09576	0.93298	2036	-0.2203
TFRC	6	0.03159	0.09588	0.93298	2037	0.0889
MILR1	6	0.031593	0.095884	0.93298	2038	-0.4852
HCAR3	6	0.031593	0.095884	0.93298	2039	-0.5412
SMPDL3A	6	0.031593	0.095884	0.93298	2040	-0.3302
RAP2B	6	0.031599	0.095898	0.93298	2041	-0.2141
HAO1	6	0.031641	0.096013	0.93366	2042	0.2054
ASIC1	6	0.031689	0.09613	0.93374	2043	-0.2305
PSMD1	6	0.031693	0.096141	0.93374	2044	-0.2876
SLC16A13	6	0.031742	0.096275	0.93438	2045	0.1132
JMJD6	6	0.031757	0.096315	0.93438	2046	-0.1728
ALG1L	6	0.031878	0.096595	0.93496	2047	0.1451
HINFP	4	0.031887	0.080996	0.90511	2048	-0.4712
ACSL4	6	0.031922	0.09669	0.93496	2049	-0.3382
SMARCAL1	6	0.031925	0.096696	0.93496	2050	-0.0396
SCN8A	6	0.031949	0.09675	0.93496	2051	-0.2219
ZNF471	6	0.031974	0.096807	0.93496	2052	-0.2934
hsa-mir-3649	4	0.031996	0.081208	0.90511	2053	-0.0082
NTMT1	6	0.032024	0.096917	0.93496	2054	-0.3621
RPL39	4	0.03203	0.081285	0.90511	2055	-0.1205
SNRPC	4	0.032034	0.081296	0.90511	2056	-0.3301
SYF2	6	0.032058	0.097007	0.93496	2057	-0.4163
GUF1	6	0.032058	0.097007	0.93496	2058	-0.3193
ATP6V1A	6	0.032075	0.097052	0.93496	2059	-0.5613
ITGB7	6	0.032097	0.097106	0.93496	2060	-0.2661
GTF2A1	6	0.032109	0.097135	0.93496	2061	-0.2189
IFNK	6	0.032124	0.097167	0.93496	2062	-0.0841
MAP3K19	6	0.032224	0.097418	0.9362	2063	0.018
FAM195A	6	0.03226	0.097507	0.93661	2064	-0.3025
LOC10013048	6	0.03232	0.097646	0.93752	2065	-0.2562
DSTYK	6	0.03239	0.097811	0.93789	2066	-0.3846
CCDC132	6	0.032408	0.097857	0.93789	2067	-0.1953
hsa-mir-135a-	4	0.032409	0.082118	0.90511	2068	-0.2966
NXN	6	0.032424	0.097897	0.93789	2069	-0.2343
IBTK	6	0.032425	0.0979	0.93789	2070	0.0074
TADA2B	6	0.032452	0.097967	0.93789	2071	-0.0826
ATF4	6	0.032478	0.098031	0.93789	2072	-0.4444
TOMM20	6	0.032478	0.098031	0.93789	2073	-0.532
PDZK1IP1	6	0.032553	0.098215	0.93807	2074	0.0419
hsa-mir-5094	4	0.032562	0.082448	0.90547	2075	-0.282
C12orf74	6	0.032594	0.098321	0.93807	2076	-0.3205
MXD1	6	0.032603	0.098346	0.93807	2077	-0.3311
OARD1	6	0.032642	0.098432	0.93807	2078	-0.6913
HNRNPH1	6	0.032642	0.098432	0.93807	2079	-0.474
KIAA0319	6	0.032658	0.098465	0.93807	2080	0.2038
COPS6	6	0.03268	0.098531	0.93807	2081	0.0212
POU2F1	6	0.032699	0.098575	0.93807	2082	-0.4146
FSTL1	6	0.032699	0.098575	0.93807	2083	-0.2729
TUBA1B	4	0.032809	0.082979	0.9069	2084	-0.2764
PRKAR1A	6	0.032845	0.098924	0.93959	2085	-0.0439
ZZEF1	6	0.032857	0.098957	0.93959	2086	0.0601
LPXN	6	0.032874	0.098991	0.93959	2087	-0.1736
SEH1L	6	0.032908	0.099079	0.93959	2088	-0.227
HS1BP3	6	0.032909	0.099082	0.93959	2089	-0.2438
hsa-mir-6802	4	0.032945	0.083274	0.9069	2090	-0.1182
GSK3B	6	0.032959	0.099214	0.93959	2091	-0.2822
MGLL	6	0.032974	0.099258	0.93959	2092	-0.2735
GDAP2	6	0.033029	0.0994	0.93959	2093	-0.1849
hsa-mir-449b	4	0.033035	0.083459	0.9069	2094	-0.4143
MED10	6	0.03304	0.099421	0.93959	2095	-0.0429
TMEM260	6	0.033041	0.099423	0.93959	2096	-0.1327
LRG1	6	0.033041	0.099424	0.93959	2097	-0.2392
PEX13	6	0.03306	0.099466	0.93959	2098	-0.2246
KIAA1524	6	0.033061	0.09947	0.93959	2099	-0.1225
hsa-mir-339	4	0.033087	0.083564	0.9069	2100	-0.3073
POLD4	6	0.033126	0.099622	0.94043	2101	-0.5384
ZNF782	6	0.033136	0.099651	0.94043	2102	-0.0192
CACNB1	4	0.033199	0.083806	0.9069	2103	0.2017

ZNF573	5	0.033206	0.098164	0.93807	2104	-0.0747
CHRNA4	6	0.033212	0.099829	0.94045	2105	0.0156
FAM179B	6	0.033224	0.099852	0.94045	2106	-0.2436
POR	6	0.033224	0.099852	0.94045	2107	-0.2292
CS	6	0.033232	0.099872	0.94045	2108	-0.3079
hsa-mir-4531	4	0.033236	0.083878	0.9069	2109	-0.536
CCZ1B	2	0.033237	0.060434	0.84069	2110	-0.4773
NUP85	4	0.03324	0.083885	0.9069	2111	-0.355
UPK3BL	6	0.033264	0.099947	0.94045	2112	-0.3215
NUP98	6	0.033285	0.1	0.94045	2113	-0.1189
NPFF	6	0.033296	0.10003	0.94045	2114	-0.2452
UBA52	6	0.033297	0.10003	0.94045	2115	-0.2789
CDIPT	6	0.033313	0.10008	0.94045	2116	-0.1352
PRSS46	6	0.033326	0.10011	0.94045	2117	-0.1358
UTS2	6	0.033429	0.10035	0.94149	2118	-0.2295
GPR123	6	0.033435	0.10036	0.94149	2119	-0.2515
PRKAG2	6	0.033459	0.10041	0.94156	2120	-0.2128
COPS5	6	0.03349	0.10048	0.94162	2121	-0.1217
PRPF31	6	0.033504	0.10051	0.94162	2122	0.0202
CWF19L1	6	0.033539	0.1006	0.9419	2123	-0.287
UTP14A	6	0.033585	0.10072	0.94191	2124	-0.3268
ESRRG	6	0.033606	0.10078	0.94191	2125	0.1486
PLA2G2D	6	0.033617	0.10081	0.94191	2126	0.0518
ELP6	6	0.033652	0.1009	0.94191	2127	-0.2863
C12orf49	6	0.033693	0.101	0.94191	2128	-0.0482
CTPS1	6	0.033693	0.101	0.94191	2129	-0.1234
SH2D2A	6	0.033716	0.10105	0.94191	2130	-0.2791
EIF3I	6	0.03375	0.10113	0.94191	2131	-0.4011
RNASE7	6	0.03375	0.10113	0.94191	2132	-0.2841
YPEL3	6	0.033769	0.10117	0.94191	2133	0.4315
GIMD1	6	0.033776	0.10119	0.94191	2134	-0.3973
SCGB3A2	6	0.033795	0.10124	0.94191	2135	-0.2078
ALYREF	6	0.033819	0.10131	0.94191	2136	-0.1819
LIMA1	6	0.033829	0.10133	0.94191	2137	-0.3043
C14orf80	6	0.033829	0.10133	0.94191	2138	-0.3075
FAM89B	6	0.03387	0.10142	0.94239	2139	-0.1311
OR51M1	6	0.033936	0.10159	0.94259	2140	-0.3186
GCSH	6	0.033936	0.10159	0.94259	2141	-0.3175
TUBB	6	0.033949	0.10161	0.94259	2142	-0.207
LYRM4	6	0.034022	0.10179	0.94259	2143	0.1466
ZNF185	6	0.034025	0.1018	0.94259	2144	0.0056
PTPRN	6	0.034025	0.1018	0.94259	2145	-0.128
PLCG1	6	0.034041	0.10184	0.94259	2146	-0.1624
SCN1A	6	0.034085	0.10196	0.94259	2147	-0.2475
KRTAP5-1	6	0.034085	0.10196	0.94259	2148	-0.4459
MEF2BNB	6	0.034085	0.10196	0.94259	2149	-0.2954
DGAT2L6	6	0.034123	0.10204	0.94259	2150	-0.2308
TMEM71	6	0.034123	0.10204	0.94259	2151	-0.3967
hsa-mir-3167	4	0.034163	0.085856	0.91003	2152	0.5525
TNIP1	6	0.034235	0.1023	0.94349	2153	-0.2474
DEPDC5	6	0.034247	0.10233	0.94349	2154	-0.2711
PDE6C	6	0.034275	0.10239	0.94349	2155	0.0464
CIAO1	6	0.034326	0.10252	0.94397	2156	-0.1736
INO80B	6	0.034363	0.10262	0.94442	2157	-0.2649
MYO1C	6	0.034376	0.10266	0.94442	2158	0.0978
SPC25	6	0.034441	0.1028	0.9447	2159	0.1437
TRA2B	6	0.034453	0.10283	0.9447	2160	-0.2
URI1	6	0.034479	0.10289	0.94478	2161	-0.3325
NONO	6	0.034562	0.10308	0.94478	2162	-0.2209
INHBC	6	0.034599	0.10316	0.94478	2163	-0.1206
CXorf64	6	0.034599	0.10316	0.94478	2164	-0.5486
BSN	6	0.034604	0.10317	0.94478	2165	-0.1079
EP400	6	0.034604	0.10317	0.94478	2166	-0.0971
TMEM144	6	0.034616	0.10321	0.94478	2167	-0.2337
GPR182	6	0.034616	0.10321	0.94478	2168	-0.2955
RPF2	5	0.034713	0.10203	0.94259	2169	-0.5345
hsa-mir-3943	4	0.034722	0.086996	0.91251	2170	-0.1477
STARD4	6	0.034725	0.10348	0.94533	2171	-0.2507
ALG13	6	0.03473	0.10349	0.94533	2172	-0.1629
LSS	6	0.03473	0.10349	0.94533	2173	0.0483
CNGA1	6	0.034759	0.10357	0.94533	2174	-0.3387
FBRSL1	6	0.034824	0.10372	0.94533	2175	-0.238
TOB1	6	0.034832	0.10374	0.94533	2176	-0.1627
RAB40A	6	0.034843	0.10377	0.94533	2177	-0.1888
COX15	6	0.034864	0.10382	0.94533	2178	-0.3754
ERP44	6	0.03487	0.10383	0.94533	2179	-0.3629
REM2	6	0.03487	0.10383	0.94533	2180	-0.3001
PAX5	6	0.034936	0.10398	0.94533	2181	-0.3905
PPP1R9B	6	0.034948	0.10401	0.94533	2182	-0.3601
ENO3	6	0.034974	0.10407	0.94533	2183	0.0989
FAM78B	6	0.034974	0.10407	0.94533	2184	-0.3212

YIPF3	6	0.03504	0.10422	0.94533	2185	-0.3103
ABCF1	6	0.035059	0.10428	0.94533	2186	0.1627
SF3B14	6	0.035059	0.10428	0.94533	2187	-0.2532
PSMD2	6	0.035061	0.10428	0.94533	2188	-0.0811
MRP63	6	0.035061	0.10428	0.94533	2189	-0.3116
GOT1L1	6	0.035101	0.10437	0.94533	2190	-0.2596
FBXO7	6	0.035101	0.10437	0.94533	2191	-0.1945
hsa-mir-432	4	0.035132	0.087877	0.91499	2192	-0.197
TSC2	6	0.035136	0.10446	0.94567	2193	-0.2847
BRD4	6	0.035167	0.10452	0.94583	2194	-0.2522
ZNF572	6	0.035224	0.10466	0.94588	2195	-0.3002
FBXW11	6	0.035254	0.10474	0.94588	2196	-0.3224
STRC	6	0.035261	0.10476	0.94588	2197	-0.0847
USP24	4	0.035281	0.088203	0.91508	2198	-0.302
PRKCH	6	0.035316	0.10488	0.94588	2199	-0.23
IGFBPL1	6	0.035337	0.10493	0.94588	2200	0.1277
NUP35	6	0.035388	0.10504	0.94588	2201	-0.3478
LRP4	6	0.035395	0.10505	0.94588	2202	-0.1549
GLIPR2	6	0.035399	0.10505	0.94588	2203	-0.1474
IL23R	6	0.035413	0.10508	0.94588	2204	-0.164
NAA15	6	0.035437	0.10513	0.94593	2205	-0.4085
RNF183	6	0.035515	0.10533	0.94649	2206	-0.0733
JPH4	6	0.035536	0.10537	0.94649	2207	-0.3078
ZFP91	6	0.035537	0.10537	0.94649	2208	-0.0646
JKAMP	6	0.035539	0.10538	0.94649	2209	-0.0991
GATA4	6	0.035587	0.10551	0.94654	2210	0.1415
TAAR9	6	0.035589	0.10552	0.94654	2211	-0.2434
RBM25	6	0.035601	0.10554	0.94654	2212	-0.3459
hsa-mir-6783	4	0.035618	0.088874	0.91836	2213	-0.2795
CRBN	6	0.035651	0.10565	0.94654	2214	-0.3073
NMD3	6	0.035656	0.10566	0.94654	2215	-0.4206
VARS2	6	0.035656	0.10566	0.94654	2216	-0.581
hsa-mir-6789	4	0.035685	0.089013	0.91841	2217	-0.3917
WDR5B	6	0.035716	0.10582	0.94682	2218	-0.2095
ISLR	6	0.035724	0.10583	0.94682	2219	0.0217
CORO2A	6	0.035748	0.10589	0.94686	2220	-0.2353
ZC3H14	6	0.035782	0.10596	0.9471	2221	-0.1088
MTRNR2L3	4	0.03582	0.089284	0.91944	2222	-0.9923
hsa-mir-5698	4	0.035859	0.08936	0.91944	2223	-0.2914
ARHGDIA	6	0.035868	0.10619	0.94841	2224	-0.0692
ZSCAN21	6	0.035982	0.10647	0.94863	2225	-0.1204
GFRAL	6	0.03601	0.10653	0.94863	2226	-0.2535
ZNF79	6	0.03601	0.10653	0.94863	2227	-0.2779
TADA3	6	0.036016	0.10655	0.94863	2228	-0.1288
STXBP2	6	0.036045	0.10662	0.94871	2229	0.1164
FGD5	6	0.036059	0.10665	0.94871	2230	-0.1814
ZNF226	6	0.036095	0.10675	0.94916	2231	-0.1365
hsa-mir-4692	4	0.036097	0.089867	0.91944	2232	-0.2824
ZBTB22	6	0.036127	0.10682	0.94932	2233	-0.2999
NDUFA9	6	0.036146	0.10686	0.94932	2234	-0.0493
hsa-mir-6726	4	0.036151	0.089967	0.91944	2235	-0.3693
EME2	6	0.036168	0.10691	0.94939	2236	-0.2469
TCEB3CL	2	0.036169	0.065512	0.86655	2237	-0.4782
SDF4	6	0.036188	0.10696	0.94942	2238	-0.1184
SEPSECS	6	0.036294	0.10721	0.94993	2239	-0.3357
IGF2BP2	6	0.036298	0.10722	0.94993	2240	-0.1581
INPP5A	6	0.036348	0.10735	0.94993	2241	-0.1071
C1orf94	6	0.036354	0.10737	0.94993	2242	-0.3491
GCM1	6	0.036362	0.10738	0.94993	2243	-0.3638
P2RY2	6	0.036415	0.1075	0.94993	2244	-0.2813
ELOVL3	6	0.036449	0.10758	0.94993	2245	0.1846
SELP	4	0.036488	0.090686	0.92203	2246	-0.4
MRPL10	6	0.036492	0.10768	0.94993	2247	-0.2874
RABGGTB	6	0.036492	0.10768	0.94993	2248	-0.4671
CHMP2A	6	0.036508	0.10771	0.94993	2249	-0.3491
RASGRP4	6	0.036533	0.10777	0.94993	2250	-0.2499
CSRP2	6	0.03655	0.10781	0.94993	2251	-0.1055
KISS1R	6	0.036551	0.10781	0.94993	2252	-0.2583
hsa-mir-6820	4	0.036597	0.090919	0.92203	2253	-0.4004
DYNC1H1	6	0.0366	0.10792	0.95011	2254	-0.1903
LCK	6	0.036668	0.10807	0.95013	2255	-0.3554
SNRNP40	6	0.036676	0.10809	0.95013	2256	-0.168
PIP4K2B	6	0.036701	0.10815	0.95013	2257	-0.2244
KLHL42	6	0.036744	0.10825	0.95013	2258	-0.3157
ARHGEF26	6	0.036778	0.10834	0.95013	2259	-0.3517
RPS19BP1	6	0.036778	0.10834	0.95013	2260	-0.2449
MRPL14	6	0.036778	0.10834	0.95013	2261	-0.2401
C12orf45	6	0.036785	0.10836	0.95013	2262	-0.0905
NOS1	6	0.036828	0.10847	0.95013	2263	-0.2145
PTPRB	6	0.036847	0.1085	0.95013	2264	-0.2501
FDX1	6	0.036848	0.10851	0.95013	2265	-0.2213

TTC1	6	0.036853	0.10852	0.95013	2266	0.013
HAUS4	6	0.036893	0.10862	0.95013	2267	-0.1688
FGR	6	0.036903	0.10864	0.95013	2268	0.0907
PAXBP1	6	0.036912	0.10866	0.95013	2269	-0.3311
MMP13	6	0.036979	0.10881	0.95056	2270	-0.0058
SMG6	6	0.036979	0.10881	0.95056	2271	-0.145
NMI	6	0.036994	0.10885	0.95056	2272	-0.2406
hsa-mir-3973	4	0.036999	0.09175	0.92339	2273	-0.2223
hsa-mir-4781	4	0.037003	0.091757	0.92339	2274	0.0111
hsa-mir-6836	4	0.037043	0.091843	0.92339	2275	-0.5287
MRPL53	6	0.037047	0.109	0.95138	2276	-0.3658
RNF34	6	0.037079	0.10906	0.95157	2277	-0.0799
KIF2A	6	0.037141	0.10922	0.95231	2278	-0.2922
OPA1	6	0.037154	0.10924	0.95231	2279	-0.2689
GTF2H4	6	0.037206	0.10937	0.95297	2280	0.171
hsa-mir-4462	4	0.037215	0.092198	0.92487	2281	-0.2584
RPS6KA4	6	0.037222	0.10941	0.95297	2282	-0.2586
hsa-mir-6771	4	0.037249	0.092278	0.92487	2283	-0.3234
PSMD8	6	0.037267	0.10951	0.95341	2284	-0.2791
TTC30B	6	0.037293	0.10957	0.95341	2285	-0.2786
GPSM3	6	0.03732	0.10964	0.95341	2286	-0.2525
IL13RA1	6	0.037408	0.10984	0.95341	2287	-0.2334
ASRGL1	6	0.037409	0.10984	0.95341	2288	-0.2981
AACS	6	0.037441	0.10992	0.95341	2289	-0.0942
SLC6A16	6	0.037502	0.11007	0.95341	2290	-0.0197
PAQR8	6	0.037502	0.11007	0.95341	2291	-0.2666
OR3A3	6	0.037531	0.11014	0.95341	2292	-0.1603
TMEM126B	6	0.037553	0.1102	0.95341	2293	-0.2551
CA1	6	0.037554	0.11021	0.95341	2294	0.0191
SLC25A53	6	0.037658	0.11044	0.95506	2295	-0.3607
HAP1	6	0.037734	0.11063	0.95608	2296	-0.2958
ZNF496	6	0.037761	0.1107	0.95608	2297	0.0573
C17orf49	6	0.037778	0.11073	0.95608	2298	-0.1238
KRT76	6	0.03781	0.1108	0.95608	2299	-0.2797
KTI12	6	0.037812	0.1108	0.95608	2300	-0.1352
TRNT1	6	0.037888	0.11099	0.95608	2301	0.2143
MRPL45	6	0.037888	0.11099	0.95608	2302	-0.3559
GPD1L	6	0.037896	0.11101	0.95608	2303	-0.3404
hsa-mir-6888	4	0.037897	0.093583	0.92854	2304	-0.3174
CTDSPL2	6	0.037941	0.1111	0.95608	2305	-0.1245
HTR1E	4	0.037967	0.093716	0.92854	2306	-0.1807
GPD1	6	0.037975	0.11118	0.95617	2307	-0.0588
CCL27	6	0.038051	0.11136	0.95693	2308	-0.1917
SVEP1	6	0.038056	0.11137	0.95693	2309	-0.2256
API5	6	0.038115	0.11153	0.95694	2310	-0.0104
CHRNA1	6	0.038215	0.11177	0.95694	2311	-0.1162
FAR1	6	0.038268	0.1119	0.95694	2312	-0.2221
PXK	6	0.038335	0.11206	0.95694	2313	-0.2673
DIDO1	6	0.038371	0.11214	0.95694	2314	-0.1398
GEMIN4	6	0.038371	0.11214	0.95694	2315	-0.0701
VEPH1	6	0.038399	0.11221	0.95694	2316	-0.3062
UBALD2	6	0.038399	0.11221	0.95694	2317	-0.2203
DDX23	6	0.038403	0.11222	0.95694	2318	-0.4244
CCDC105	6	0.038433	0.11228	0.95694	2319	0.0388
ZNF2	6	0.038447	0.11231	0.95694	2320	-0.035
ADRB2	6	0.038468	0.11236	0.95694	2321	0.1116
ARCN1	6	0.038469	0.11236	0.95694	2322	-0.2259
C5orf51	6	0.038499	0.11244	0.95694	2323	-0.2134
GPR87	6	0.038518	0.11249	0.95694	2324	-0.0109
TIMMDC1	6	0.03853	0.11251	0.95694	2325	-0.2349
HECW2	6	0.03853	0.11251	0.95694	2326	-0.4208
EXTL2	6	0.038531	0.11251	0.95694	2327	-0.1786
C17orf51	6	0.038597	0.11266	0.95694	2328	-0.2627
EIF1AD	6	0.038601	0.11267	0.95694	2329	-0.3609
ACSL3	6	0.038601	0.11267	0.95694	2330	-0.2475
IER3IP1	6	0.038601	0.11267	0.95694	2331	-0.2491
hsa-mir-6880	4	0.038618	0.095064	0.93244	2332	-0.7879
SLC45A1	6	0.038669	0.11284	0.95738	2333	-0.2768
PARD6G	6	0.038701	0.11292	0.95738	2334	-0.2967
GRSF1	6	0.038707	0.11293	0.95738	2335	-0.2065
ARL8A	6	0.03872	0.11296	0.95738	2336	-0.158
PCDHGC5	2	0.038743	0.069982	0.87413	2337	-0.5347
hsa-mir-4699	4	0.038755	0.095342	0.93244	2338	-0.2453
RPS8	6	0.038793	0.11311	0.95824	2339	-0.3769
PTH1R	6	0.038845	0.11325	0.95904	2340	-0.2203
hsa-mir-34b	4	0.038875	0.095581	0.93298	2341	-0.467
hsa-mir-4451	4	0.038877	0.095586	0.93298	2342	-0.2906
hsa-mir-1231	4	0.038926	0.095689	0.93298	2343	-0.3209
ETF1	6	0.038972	0.11355	0.96039	2344	-0.09
PIK3IP1	4	0.038993	0.095817	0.93298	2345	-0.1201
KCNG4	6	0.039048	0.11372	0.96096	2346	-0.2827

SCFD2	6	0.039096	0.11384	0.96096	2347	-0.1977
FICD	6	0.039123	0.11389	0.96096	2348	-0.0085
SLC16A1	6	0.039128	0.1139	0.96096	2349	-0.2542
HSFX1	1	0.039129	0.039047	0.72669	2350	-0.4358
hsa-mir-671	4	0.039164	0.096157	0.93374	2351	-0.1804
LRRC1	6	0.039173	0.11401	0.96096	2352	0.0377
SLC13A2	6	0.039208	0.1141	0.96096	2353	0.0172
SSH1	6	0.03922	0.11413	0.96096	2354	-0.4035
S100A12	6	0.039224	0.11414	0.96096	2355	-0.2327
OR10H2	6	0.039262	0.11423	0.96096	2356	-0.2737
OR2J3	6	0.039274	0.11425	0.96096	2357	-0.1178
SRSF2	6	0.039352	0.11442	0.96096	2358	-0.465
TMEM242	6	0.039375	0.11448	0.96096	2359	0.0381
hsa-mir-6822	4	0.039421	0.096711	0.93496	2360	-0.0365
KLF11	6	0.039425	0.11461	0.96096	2361	0.0365
DDX26B	6	0.039446	0.11466	0.96096	2362	-0.2164
SYCE2	6	0.039446	0.11466	0.96096	2363	-0.1975
DYSF	6	0.039454	0.11467	0.96096	2364	-0.1214
FANCC	4	0.039459	0.096786	0.93496	2365	-0.3004
PDE12	6	0.039495	0.11476	0.96096	2366	-0.2348
PDILT	6	0.039506	0.11478	0.96096	2367	0.1415
RAG1	6	0.039526	0.11483	0.96096	2368	-0.0644
CSF1	6	0.039545	0.11488	0.96096	2369	-0.2703
JPH1	6	0.039545	0.11488	0.96096	2370	-0.2908
C20orf144	6	0.039547	0.11489	0.96096	2371	-0.0757
hsa-mir-183	4	0.039556	0.096988	0.93496	2372	-0.3724
ELOF1	6	0.039576	0.11496	0.96096	2373	-0.1622
EIF4E3	6	0.039583	0.11498	0.96096	2374	-0.1817
hsa-mir-601	4	0.039605	0.097097	0.93496	2375	-0.4585
FAM26E	6	0.039607	0.11503	0.96097	2376	-0.2419
COMMD4	6	0.039627	0.11507	0.96098	2377	-0.0836
SDHC	6	0.039648	0.11512	0.96101	2378	-0.2564
hsa-mir-646	4	0.039755	0.097415	0.9362	2379	-0.4469
NUBPL	6	0.039755	0.11538	0.96196	2380	-0.4463
NUP88	6	0.039783	0.11544	0.9621	2381	-0.3621
CLK3	6	0.039828	0.11556	0.96268	2382	-0.0595
HELZ	6	0.039868	0.11565	0.96294	2383	-0.2658
DNAJB13	6	0.039879	0.11569	0.96294	2384	-0.0695
PRR3	6	0.039935	0.11582	0.96301	2385	-0.1549
KCNMB3	6	0.039943	0.11583	0.96301	2386	-0.036
HSPB7	6	0.039943	0.11583	0.96301	2387	-0.1896
FAM32A	6	0.039979	0.11591	0.96329	2388	0.1743
CDC42SE1	6	0.040116	0.11623	0.96504	2389	-0.3389
VASN	6	0.04013	0.11626	0.96504	2390	0.1666
MACC1	6	0.040133	0.11627	0.96504	2391	-0.2349
EFHD2	6	0.040151	0.11631	0.96504	2392	-0.22
hsa-mir-3945	4	0.040189	0.098295	0.93807	2393	0.1343
PROCA1	6	0.04021	0.11645	0.96525	2394	-0.1713
AIM2	6	0.040231	0.11649	0.96525	2395	-0.2261
UBP1	6	0.040299	0.11667	0.96525	2396	-0.1716
UCHL5	6	0.040332	0.11674	0.96525	2397	-0.1344
HTRA3	6	0.040338	0.11675	0.96525	2398	-0.2138
NDUFB8	6	0.040381	0.11685	0.96525	2399	-0.3253
hsa-mir-7162	4	0.040392	0.098711	0.93893	2400	-0.2077
ECT2	6	0.040407	0.11692	0.96525	2401	-0.019
ZDHHC8	6	0.040407	0.11692	0.96525	2402	0.0253
LRRD1	6	0.040424	0.11695	0.96525	2403	-0.2902
hsa-mir-631	4	0.040433	0.098783	0.93918	2404	-0.33
HENMT1	6	0.040533	0.11722	0.96588	2405	-0.1607
TMEM167B	6	0.040536	0.11723	0.96588	2406	-0.2933
NOL8	6	0.040536	0.11723	0.96588	2407	-0.3266
SPRR1B	5	0.040578	0.11366	0.96095	2408	-0.9148
TSHZ2	6	0.040596	0.11736	0.96588	2409	-0.0347
CLDN1	6	0.040609	0.1174	0.96588	2410	-0.0989
RBM15B	6	0.040609	0.1174	0.96588	2411	0.0005
RARG	6	0.040613	0.11741	0.96588	2412	-0.2539
TBPL2	6	0.040655	0.11752	0.9664	2413	-0.3137
hsa-mir-6721	4	0.040659	0.09927	0.93959	2414	-0.2473
CCDC27	6	0.040689	0.1176	0.96663	2415	0.082
C6orf106	6	0.040705	0.11764	0.96663	2416	-0.2726
GFRA3	6	0.040746	0.11775	0.96688	2417	-0.2265
PPIH	6	0.04076	0.11778	0.96688	2418	-0.2166
PPP1R13L	6	0.0408	0.11786	0.96688	2419	-0.1649
OR4L1	6	0.0408	0.11786	0.96688	2420	-0.1156
SLC30A4	6	0.040822	0.11791	0.96688	2421	-0.0144
hsa-mir-4662b	1	0.040905	0.040785	0.74134	2422	-1.1224
RNF31	6	0.040919	0.11813	0.96735	2423	-0.193
HBB	6	0.040921	0.11813	0.96735	2424	-0.0479
PSMC3	6	0.040961	0.11823	0.96735	2425	-0.1007
IL1RAP	6	0.040969	0.11825	0.96735	2426	-0.4649
PNPLA6	6	0.040988	0.11829	0.96735	2427	-0.2414

HOGA1	6	0.041029	0.11839	0.96735	2428	-0.1433
PIM2	6	0.041087	0.11853	0.96775	2429	-0.0527
HOXD8	6	0.041178	0.11874	0.96775	2430	-0.278
KIAA1009	6	0.041178	0.11874	0.96775	2431	-0.3416
RNLS	6	0.041178	0.11874	0.96775	2432	-0.169
MAGEA1	6	0.041213	0.11882	0.96775	2433	0.0055
FAM114A1	6	0.041236	0.11887	0.96775	2434	-0.0875
KPNA6	6	0.041238	0.11888	0.96775	2435	-0.3052
SPERT	6	0.041248	0.11891	0.96775	2436	-0.2836
PTBP1	6	0.041284	0.119	0.96775	2437	-0.265
KCNT2	6	0.041288	0.11901	0.96775	2438	0.0607
COX17	6	0.041288	0.11901	0.96775	2439	-0.164
ZNF787	6	0.041288	0.11901	0.96775	2440	0.2029
BRINP1	2	0.041308	0.074368	0.88652	2441	-0.3487
WBSCR27	6	0.041318	0.11907	0.96791	2442	-0.1695
hsa-mir-6894	4	0.04133	0.10064	0.9419	2443	-0.3162
H3F3A	6	0.041372	0.11922	0.96868	2444	-0.3266
C6orf58	6	0.041429	0.11934	0.96915	2445	-0.0307
TNFSF14	6	0.041452	0.11939	0.96915	2446	-0.2739
NMNAT3	6	0.041464	0.11942	0.96915	2447	-0.2329
BCL2L12	6	0.041499	0.11951	0.96915	2448	-0.1701
PTCD1	3	0.041549	0.09274	0.92643	2449	-0.3305
STK38	6	0.041552	0.11962	0.96915	2450	-0.2176
NACC2	6	0.04159	0.1197	0.96915	2451	-0.1196
IL18BP	6	0.041609	0.11975	0.96915	2452	-0.041
HIST3H3	6	0.041611	0.11975	0.96915	2453	-0.2845
ADAM22	6	0.041639	0.11981	0.96915	2454	-0.2929
LTV1	6	0.041693	0.11993	0.9695	2455	-0.1603
MYO1A	6	0.041737	0.12003	0.96962	2456	-0.3265
NLRP13	6	0.04174	0.12004	0.96962	2457	0.0279
ATP5J2-PTCD1	2	0.041797	0.075178	0.88807	2458	-0.2853
LIMK2	6	0.041817	0.12022	0.96994	2459	-0.0505
HIST1H2AD	6	0.04188	0.12037	0.96994	2460	0.1074
POLR2E	6	0.04188	0.12037	0.96994	2461	-0.301
C14orf132	6	0.041906	0.12042	0.96994	2462	-0.286
RBBP7	6	0.041928	0.12048	0.96994	2463	-0.1795
CDC37L1	6	0.041941	0.12051	0.96994	2464	-0.2179
AGFG2	6	0.042051	0.12077	0.97154	2465	-0.1671
OR2J2	6	0.042067	0.1208	0.97154	2466	-0.1672
PCDP1	6	0.042113	0.12091	0.972	2467	-0.2205
HOXA11	6	0.042139	0.12097	0.97213	2468	-0.3116
ARL14EP	6	0.042163	0.12102	0.97215	2469	-0.1783
hsa-mir-3687	4	0.042167	0.10237	0.94349	2470	-0.761
hsa-mir-191	4	0.042167	0.10237	0.94349	2471	-0.5277
PLD1	4	0.042192	0.10242	0.94349	2472	-0.1517
ADIPOR1	6	0.042204	0.12112	0.97251	2473	-0.3752
OR5AC2	6	0.042268	0.12125	0.97251	2474	-0.3072
FAM20A	6	0.042269	0.12125	0.97251	2475	-0.2612
TMEM219	6	0.042293	0.12131	0.97251	2476	-0.131
TIMM10	6	0.042344	0.12142	0.97251	2477	-0.0317
PDCD2L	6	0.042356	0.12145	0.97251	2478	-0.3075
MBOAT1	6	0.04237	0.12148	0.97251	2479	-0.2292
LEFTY1	6	0.042411	0.12158	0.97251	2480	0.0486
KIF19	6	0.042423	0.12162	0.97251	2481	-0.3483
NRIP3	6	0.042428	0.12163	0.97251	2482	-0.2043
IKBKE	6	0.042428	0.12163	0.97251	2483	0.1897
PALMD	6	0.042444	0.12167	0.97251	2484	0.0503
C17orf105	6	0.042476	0.12176	0.97251	2485	-0.1631
HSPA9	6	0.042494	0.1218	0.97251	2486	-0.1204
MYH7B	6	0.042504	0.12182	0.97251	2487	-0.213
C19orf81	6	0.042505	0.12183	0.97251	2488	0.0064
BTNL9	6	0.042535	0.1219	0.97258	2489	-0.1274
GSG2	6	0.042645	0.12213	0.97273	2490	-0.0099
WNT1	6	0.042654	0.12215	0.97273	2491	-0.2713
KCNA3	6	0.042654	0.12215	0.97273	2492	-0.1401
KRCC1	6	0.042666	0.12219	0.97273	2493	-0.252
TXNDC17	6	0.042695	0.12225	0.97286	2494	-0.0361
RGL1	6	0.04273	0.12233	0.97315	2495	-0.1583
PRAMEF7	4	0.042781	0.10363	0.94533	2496	-0.5541
ASCL1	6	0.042795	0.12248	0.97355	2497	-0.3126
BORA	6	0.042868	0.12264	0.97358	2498	-0.2278
USP43	6	0.042871	0.12265	0.97358	2499	-0.1391
ZFP36L2	6	0.042902	0.12272	0.97358	2500	-0.0965
MED27	6	0.042908	0.12273	0.97358	2501	-0.2205
CSNK1D	4	0.042937	0.10396	0.94533	2502	-0.5427
TAF13	6	0.042979	0.12291	0.97358	2503	-0.1068
EIF4ENIF1	6	0.042997	0.12295	0.97358	2504	0.0386
PLXNC1	6	0.043028	0.12303	0.97358	2505	-0.0772
CMTM3	6	0.043028	0.12303	0.97358	2506	-0.2982
TRIM65	6	0.04304	0.12305	0.97358	2507	-0.263
MDC1	6	0.04304	0.12305	0.97358	2508	-0.2498

SGCG	6	0.043097	0.12318	0.97379	2509	-0.1652
SPEF1	6	0.043134	0.12326	0.97395	2510	0.1217
NMNAT1	6	0.043147	0.12329	0.97395	2511	-0.2564
PCDHA11	2	0.043153	0.077495	0.89854	2512	-0.4864
TPM1	6	0.043187	0.12338	0.97432	2513	0.019
OR1D2	6	0.043241	0.12352	0.97477	2514	-0.0171
FUCA2	6	0.043266	0.12358	0.97477	2515	-0.2373
CDAN1	6	0.043266	0.12358	0.97477	2516	-0.2343
GPER	4	0.04329	0.10468	0.94588	2517	-0.3366
hsa-mir-4706	4	0.043352	0.10481	0.94588	2518	-0.8298
KIAA1841	6	0.043362	0.12383	0.97492	2519	-0.2488
hsa-mir-137	4	0.043402	0.10491	0.94588	2520	-0.2222
ELANE	6	0.043423	0.12398	0.97492	2521	0.1205
CCNE1	6	0.043431	0.124	0.97492	2522	-0.1827
ZIC5	6	0.043431	0.124	0.97492	2523	0.0471
CCT2	6	0.043452	0.12405	0.97492	2524	-0.1955
KCNH3	6	0.04347	0.12409	0.97492	2525	-0.3269
ETFDH	6	0.043479	0.12411	0.97492	2526	-0.0404
ATG12	6	0.043487	0.12413	0.97492	2527	-0.1423
TIPARP	6	0.043524	0.12422	0.97492	2528	-0.0199
ZNF484	6	0.043538	0.12426	0.97492	2529	-0.2568
EGR1	6	0.043546	0.12427	0.97492	2530	-0.2511
POLR1B	6	0.04369	0.12461	0.97643	2531	-0.2041
FOXR1	6	0.0437	0.12464	0.97643	2532	0.0765
FAM90A1	6	0.043748	0.12475	0.97643	2533	-0.1818
NCAPD3	6	0.043758	0.12477	0.97643	2534	-0.2047
DCTN3	6	0.043775	0.12481	0.97643	2535	0.0008
CC2D2B	6	0.043806	0.12487	0.97643	2536	-0.1447
DNAJC18	6	0.043814	0.12489	0.97643	2537	-0.0378
BLOC1S4	4	0.043825	0.10583	0.94682	2538	-0.3197
SLC5A9	6	0.043889	0.12507	0.97697	2539	-0.1683
FBXL12	6	0.0439	0.1251	0.97697	2540	-0.2507
L2HGDH	6	0.043948	0.12519	0.97697	2541	-0.287
AQP5	6	0.043951	0.1252	0.97697	2542	-0.111
VPS13D	6	0.043951	0.1252	0.97697	2543	-0.2164
NKAP	6	0.043994	0.12531	0.97742	2544	-0.3274
hsa-mir-7515	4	0.044048	0.10628	0.94863	2545	-0.2001
RAB1A	6	0.044051	0.12544	0.97811	2546	-0.2131
hsa-mir-6741	4	0.044084	0.10637	0.94863	2547	-0.098
OR10A6	6	0.044101	0.12557	0.97826	2548	-0.02
YKT6	6	0.04412	0.12561	0.97826	2549	-0.0971
DR1	6	0.044151	0.12567	0.97842	2550	-0.0823
hsa-mir-4257	3	0.044192	0.096594	0.93496	2551	-0.5953
AMMECR1L	6	0.044226	0.12585	0.9787	2552	0.1647
CHERP	6	0.044226	0.12585	0.9787	2553	-0.0458
APRT	6	0.044336	0.1261	0.9788	2554	-0.1839
RRAGC	6	0.044371	0.12619	0.9788	2555	-0.2187
GAL3ST2	6	0.044371	0.12619	0.9788	2556	-0.3663
SFT2D3	6	0.044372	0.12619	0.9788	2557	-0.2871
PFDN5	6	0.044412	0.12629	0.9788	2558	-0.2979
ACPT	6	0.044427	0.12632	0.9788	2559	-0.1531
WASF3	6	0.044458	0.12638	0.9788	2560	-0.2103
hsa-mir-2110	4	0.044461	0.10712	0.94993	2561	-0.3321
CLK2	6	0.044488	0.12645	0.9788	2562	-0.1571
C6orf222	6	0.044488	0.12645	0.9788	2563	-0.257
C4orf17	6	0.044502	0.12649	0.9788	2564	-0.3135
TM9SF1	6	0.044522	0.12653	0.9788	2565	-0.1622
NPIPA5	2	0.044548	0.079891	0.90364	2566	0.1201
FAS	6	0.044553	0.1266	0.9788	2567	0.0705
ANXA2	6	0.044622	0.12676	0.9788	2568	-0.1772
SRMS	6	0.044622	0.12676	0.9788	2569	-0.2456
hsa-mir-3621	4	0.044627	0.10745	0.94993	2570	-0.1734
LACC1	6	0.044669	0.12686	0.9788	2571	-0.0795
HFM1	6	0.044694	0.12691	0.9788	2572	-0.3096
CYC1	6	0.044694	0.12691	0.9788	2573	-0.1291
ARPP21	6	0.044706	0.12694	0.9788	2574	-0.2269
TRPM5	6	0.044753	0.12704	0.9788	2575	-0.2925
GPBP1L1	6	0.044755	0.12704	0.9788	2576	-0.2581
hsa-mir-3178	4	0.044761	0.10772	0.94993	2577	0.4597
PALM2	1	0.044765	0.044742	0.76862	2578	-1.4438
ODF3B	6	0.044788	0.12711	0.9788	2579	-0.0838
SIDT2	6	0.044788	0.12711	0.9788	2580	-0.1788
SLC6A20	4	0.044828	0.10785	0.94993	2581	-0.6387
PSMC5	6	0.044842	0.12724	0.97942	2582	-0.3068
NUP188	6	0.044904	0.12738	0.97982	2583	-0.1345
CEP152	6	0.044945	0.12748	0.97982	2584	-0.1663
PSTK	6	0.044946	0.12748	0.97982	2585	-0.3261
AHCYL2	6	0.044946	0.12748	0.97982	2586	-0.2902
PTGIR	6	0.044979	0.12756	0.98003	2587	-0.0979
HELLS	6	0.045039	0.12769	0.98025	2588	-0.2373
SNAPC3	6	0.045104	0.12784	0.98073	2589	-0.1228

ZNF701	5	0.045128	0.12193	0.97258	2590	-0.2762
NOL4	6	0.045134	0.1279	0.98086	2591	-0.2595
DENND6A	6	0.04525	0.12819	0.98168	2592	-0.2198
PHGDH	6	0.045255	0.1282	0.98168	2593	-0.109
LCMT1	6	0.045355	0.12843	0.98168	2594	0.0899
LRRC31	6	0.045405	0.12854	0.98168	2595	-0.1708
NRIP2	6	0.045406	0.12854	0.98168	2596	-0.1226
UQCRH	6	0.045436	0.12862	0.98168	2597	-0.7642
SCUBE1	6	0.045436	0.12862	0.98168	2598	-0.3881
STMN3	6	0.045439	0.12863	0.98168	2599	-0.2376
SRCIN1	6	0.045454	0.12867	0.98168	2600	-0.0067
NYAP1	6	0.045455	0.12867	0.98168	2601	-0.1439
SMAD2	6	0.045496	0.12876	0.98168	2602	-0.138
SEC24D	6	0.045524	0.12882	0.98168	2603	-0.221
STRIP2	6	0.045584	0.12896	0.98235	2604	-0.212
CNOT1	6	0.045605	0.12902	0.98241	2605	-0.0348
ERI2	6	0.04563	0.12909	0.98255	2606	-0.2956
PNPLA1	6	0.045655	0.12914	0.98261	2607	-0.2638
MED7	6	0.04571	0.12926	0.98294	2608	-0.1988
RAE1	6	0.045756	0.12936	0.98294	2609	-0.006
SLC1A4	6	0.045771	0.12939	0.98294	2610	-0.2271
ARID4A	6	0.045771	0.12939	0.98294	2611	-0.2762
CCR10	4	0.045788	0.10978	0.95341	2612	-0.3186
ZNF639	6	0.045789	0.12942	0.98294	2613	-0.2335
hsa-mir-7109	4	0.045852	0.10991	0.95341	2614	0.2003
GMPS	6	0.045859	0.12958	0.98378	2615	-0.3615
MED12	6	0.045894	0.12967	0.98389	2616	-0.2576
IKBIP	6	0.045906	0.12969	0.98389	2617	0.0279
hsa-mir-4691	4	0.045921	0.11005	0.95341	2618	-0.1719
CHODL	6	0.045956	0.12982	0.98418	2619	-0.0413
GGCT	6	0.045963	0.12983	0.98418	2620	-0.2856
ZBTB8OS	6	0.046031	0.13	0.98475	2621	-0.2707
GRAMD1A	6	0.046088	0.13012	0.98475	2622	-0.3223
ZHX2	6	0.046096	0.13013	0.98475	2623	-0.3414
H2AFZ	6	0.046118	0.13018	0.98475	2624	-0.4957
RGN	6	0.046149	0.13026	0.98487	2625	-0.0884
DACT3	6	0.046283	0.13058	0.98692	2626	-0.3263
USP8	6	0.046357	0.13073	0.98738	2627	-0.0523
hsa-mir-6500	4	0.046399	0.11103	0.95608	2628	-0.5147
TRIM43B	5	0.046405	0.1242	0.97492	2629	0.0387
ATF7	6	0.046422	0.13089	0.98798	2630	-0.1577
hsa-mir-188	4	0.046447	0.11112	0.95608	2631	-0.4925
NAA16	6	0.046456	0.13096	0.98798	2632	-0.2628
YIPF5	6	0.046457	0.13096	0.98798	2633	0.1362
GNAO1	6	0.046557	0.13119	0.9894	2634	-0.0452
hsa-mir-4467	4	0.046589	0.11141	0.95693	2635	-0.7208
GNAI2	6	0.046601	0.13129	0.98951	2636	-0.3827
TBC1D9	6	0.046773	0.1317	0.98951	2637	-0.2846
CCND3	6	0.046776	0.13171	0.98951	2638	-0.2444
GON4L	6	0.046776	0.13171	0.98951	2639	-0.2551
FUNDC2	6	0.046776	0.13171	0.98951	2640	-0.3294
BATF3	4	0.046779	0.11179	0.95694	2641	-0.3296
EEF2	6	0.04678	0.13171	0.98951	2642	-0.1711
CNTD2	6	0.04678	0.13171	0.98951	2643	-0.0417
MRPL38	6	0.046807	0.13178	0.98951	2644	-0.1844
TECTA	6	0.046811	0.13178	0.98951	2645	-0.0325
hsa-mir-4653	4	0.046837	0.11189	0.95694	2646	-0.5706
C17orf97	6	0.046857	0.1319	0.98951	2647	0.0811
FAM89A	6	0.046873	0.13193	0.98951	2648	0.0519
TCF23	4	0.046902	0.11202	0.95694	2649	-0.3483
TNF	6	0.046907	0.13203	0.98956	2650	-0.0496
PLA2G4F	6	0.046916	0.13204	0.98956	2651	-0.1932
DDX56	6	0.046945	0.13211	0.98971	2652	-0.1441
TFB1M	6	0.047008	0.13226	0.98972	2653	0.0988
PRELID1	6	0.047011	0.13227	0.98972	2654	-0.2639
C19orf57	6	0.047054	0.13236	0.98972	2655	-0.2397
OLFML2A	6	0.047057	0.13237	0.98972	2656	-0.04
C9orf16	6	0.047147	0.13258	0.98972	2657	-0.2086
DTX2	6	0.04715	0.13259	0.98972	2658	-0.0187
PUM1	6	0.047156	0.13259	0.98972	2659	-0.0837
PHC1	6	0.047156	0.13259	0.98972	2660	0.0495
PSMA4	6	0.047192	0.13267	0.98972	2661	-0.1983
IFI30	6	0.047192	0.13267	0.98972	2662	-0.3567
OR5A1	6	0.047208	0.13271	0.98972	2663	-0.1223
HOXB8	6	0.047258	0.13282	0.98972	2664	-0.1268
MSL1	6	0.047264	0.13283	0.98972	2665	-0.2054
PUS10	6	0.047308	0.13291	0.98972	2666	-0.0599
ICAM3	6	0.047339	0.13299	0.98972	2667	-0.2199
DNAJC30	6	0.047358	0.13303	0.98972	2668	-0.2712
IFT80	6	0.047358	0.13303	0.98972	2669	-0.2709
MTX3	6	0.047405	0.13313	0.99012	2670	-0.3595

STAM	6	0.047437	0.1332	0.99022	2671	-0.2031
VN1R2	6	0.047458	0.13325	0.99022	2672	0.0897
CNPPD1	6	0.047491	0.13332	0.99022	2673	-0.3288
ZNF438	6	0.047558	0.13347	0.99022	2674	-0.1154
AWAT2	6	0.047563	0.13348	0.99022	2675	-0.233
DNAJB8	6	0.047574	0.1335	0.99022	2676	-0.4006
COX4I1	6	0.047603	0.13356	0.99022	2677	-0.1964
PLEKHG3	6	0.047617	0.13359	0.99022	2678	-0.3204
CLEC18B	4	0.047623	0.11351	0.96039	2679	-0.2468
ATP5F1	6	0.047667	0.13369	0.99034	2680	-0.2461
CLHC1	6	0.047686	0.13374	0.99034	2681	-0.295
DMRTA1	6	0.04774	0.13387	0.99061	2682	-0.3362
ZNF691	6	0.04774	0.13387	0.99061	2683	-0.2461
NEDD1	6	0.047805	0.13403	0.9912	2684	-0.2906
HMGCR	6	0.047833	0.1341	0.9912	2685	0.1274
CD3EAP	6	0.047833	0.1341	0.9912	2686	-0.0195
COX8C	6	0.04789	0.13422	0.99172	2687	-0.3636
JSRP1	6	0.047911	0.13426	0.99172	2688	-0.0976
ENHO	6	0.047928	0.13432	0.99174	2689	0.0309
hsa-mir-4508	4	0.047936	0.11411	0.96096	2690	-0.4899
PSG9	6	0.047958	0.1344	0.992	2691	0.1892
SLC1A6	6	0.047979	0.13445	0.992	2692	-0.2169
ALMS1	6	0.048026	0.13455	0.99209	2693	-0.2858
ARL14	6	0.048066	0.13465	0.99209	2694	-0.2509
CSRP3	6	0.04807	0.13465	0.99209	2695	-0.4404
COPG1	4	0.048071	0.11441	0.96096	2696	-0.2416
POC1B-GALNT	1	0.048108	0.048125	0.78707	2697	-0.8345
DGCR2	6	0.048215	0.13499	0.99312	2698	0.1249
LRRC58	6	0.04823	0.13503	0.99312	2699	-0.065
OR14A16	6	0.04823	0.13503	0.99312	2700	-0.4878
SPAG7	6	0.048234	0.13504	0.99312	2701	-0.2538
LGSN	6	0.048234	0.13504	0.99312	2702	-0.3102
TARS2	6	0.048258	0.13509	0.99316	2703	0.0438
SIPA1L2	6	0.04835	0.13529	0.99397	2704	-0.0613
CD84	6	0.048353	0.1353	0.99397	2705	-0.2558
CADM3	6	0.048408	0.13543	0.99397	2706	-0.1458
SMARCB1	6	0.048408	0.13543	0.99397	2707	-0.1893
PDCD2	6	0.048452	0.13553	0.99397	2708	-0.2748
TJAP1	6	0.048508	0.13565	0.99397	2709	-0.121
hsa-mir-124-2	3	0.048509	0.10282	0.9447	2710	-0.3587
FBXW7	6	0.04854	0.13573	0.99397	2711	-0.257
TBCA	6	0.04854	0.13573	0.99397	2712	-0.2971
BATF	6	0.04854	0.13573	0.99397	2713	-0.2501
IMP3	6	0.048633	0.13593	0.99397	2714	-0.2075
U2AF2	6	0.04867	0.13602	0.99397	2715	-0.0287
SNX9	6	0.048708	0.13611	0.99397	2716	0.1516
HAUS5	6	0.04872	0.13613	0.99397	2717	-0.0916
STK17A	6	0.048749	0.1362	0.99397	2718	-0.1762
FSIP2	6	0.048788	0.13629	0.99397	2719	-0.3556
WASL	6	0.048789	0.13629	0.99397	2720	-0.0075
COQ3	6	0.048795	0.13631	0.99397	2721	-0.225
GAB3	6	0.048843	0.13641	0.99397	2722	-0.1918
B4GALT1	6	0.048858	0.13644	0.99397	2723	-0.0869
PKN3	6	0.048908	0.13656	0.99397	2724	-0.2495
SAR1B	6	0.048918	0.13658	0.99397	2725	-0.2144
TCP10L2	5	0.048939	0.12871	0.98168	2726	-0.0586
GRIN3B	6	0.048958	0.13667	0.99397	2727	0.3657
BCKDHA	6	0.048971	0.13671	0.99397	2728	-0.1979
OAS2	6	0.04898	0.13672	0.99397	2729	-0.2297
TMEM247	6	0.04898	0.13672	0.99397	2730	-0.242
ITGB2	6	0.048983	0.13673	0.99397	2731	0.3794
SLC41A2	6	0.049008	0.1368	0.99397	2732	-0.1729
ITFG3	6	0.049045	0.13689	0.99397	2733	-0.1021
PTGR2	6	0.04907	0.13694	0.99397	2734	-0.2177
KDELR2	6	0.049081	0.13697	0.99397	2735	-0.3424
ZNF154	6	0.049083	0.13697	0.99397	2736	-0.2497
ATP6V0A4	6	0.049083	0.13697	0.99397	2737	-0.1053
PPP4R4	6	0.049114	0.13704	0.99397	2738	-0.1879
C5orf45	6	0.049177	0.13719	0.99397	2739	-0.154
APOF	6	0.049208	0.13725	0.99397	2740	-0.1822
hsa-mir-3134	4	0.04923	0.11673	0.96525	2741	-0.6076
hsa-mir-4298	4	0.049277	0.11682	0.96525	2742	-0.2401
MYO5B	6	0.049282	0.13742	0.99397	2743	0.2529
CYP4F8	6	0.049282	0.13742	0.99397	2744	-0.0109
hsa-mir-5579	4	0.049317	0.11691	0.96525	2745	0.1107
CXCL17	6	0.049382	0.13764	0.99397	2746	-0.237
AMER3	6	0.049445	0.13777	0.99397	2747	-0.2844
METAP1D	6	0.049445	0.13777	0.99397	2748	-0.2826
SLCO1B7	6	0.049446	0.13777	0.99397	2749	-0.2587
TRNAU1AP	6	0.049457	0.1378	0.99397	2750	-0.1515
KCNJ15	6	0.049489	0.13788	0.99397	2751	-0.2438

HOXB4	6	0.04949	0.13788	0.99397	2752	-0.3809
ROM1	6	0.04949	0.13788	0.99397	2753	-0.1745
PPM1K	6	0.04952	0.13795	0.99397	2754	-0.2403
SSX2B	2	0.049524	0.088179	0.91508	2755	-0.2407
OR4P4	6	0.049525	0.13796	0.99397	2756	-0.189
hsa-mir-760	4	0.049535	0.11735	0.96588	2757	0.2778
SMIM13	6	0.049598	0.13813	0.99397	2758	-0.2848
KLHL11	6	0.049607	0.13815	0.99397	2759	-0.2559
SF3B4	6	0.049629	0.13821	0.99397	2760	-0.4372
SNCAIP	6	0.049657	0.13827	0.99397	2761	0.1896
HIST2H2BE	6	0.049709	0.1384	0.99397	2762	-0.5221
MIS18A	6	0.049731	0.13846	0.99397	2763	-0.2828
MXRA7	6	0.049783	0.13858	0.99402	2764	-0.4232
GET4	6	0.049813	0.13863	0.99402	2765	0.1167
MUCL1	6	0.049816	0.13864	0.99402	2766	-0.2187
TNFAIP8L2-SC	1	0.049905	0.049982	0.79297	2767	-0.8834
KRTAP5-11	4	0.049905	0.11809	0.96735	2768	-0.7887
GLE1	6	0.049938	0.13892	0.99402	2769	-0.2331
hsa-mir-181a-	4	0.049944	0.11817	0.96735	2770	-0.3472
ESF1	6	0.049977	0.139	0.99402	2771	0.0399
CH25H	6	0.049982	0.13902	0.99402	2772	-0.1181
CRYGS	6	0.049995	0.13904	0.99402	2773	-0.2094
ZNF22	6	0.050012	0.13909	0.99402	2774	0.0001
RGS9	4	0.050047	0.11836	0.96735	2775	-0.6334
ZNF664	6	0.050104	0.13929	0.99402	2776	-0.282
TCERG1	6	0.050106	0.1393	0.99402	2777	-0.1756
NTRK1	6	0.050106	0.1393	0.99402	2778	-0.1225
RIMBP3C	2	0.050114	0.089199	0.9194	2779	-0.2238
NCKAP5	6	0.050141	0.13937	0.99402	2780	-0.0515
RAC2	6	0.050145	0.13938	0.99402	2781	-0.143
PIN1	6	0.05016	0.13942	0.99402	2782	-0.1656
CSNK2B	6	0.050191	0.1395	0.99402	2783	-0.0452
UQCRC1	6	0.050206	0.13954	0.99402	2784	-0.1334
IL2	6	0.050229	0.1396	0.99402	2785	-0.24
DIEXF	6	0.05025	0.13965	0.99402	2786	-0.2999
BCL2A1	6	0.050256	0.13966	0.99402	2787	-0.0328
GLS2	6	0.050284	0.13973	0.99402	2788	-0.2233
DEFB112	6	0.050284	0.13973	0.99402	2789	-0.3109
RIOK2	6	0.050305	0.13978	0.99402	2790	-0.3021
WNT4	6	0.050305	0.13978	0.99402	2791	-0.2115
GABPB2	6	0.050356	0.1399	0.99409	2792	-0.2169
KEAP1	6	0.050362	0.13992	0.99409	2793	-0.021
UBE2E1	6	0.050406	0.14002	0.99409	2794	-0.1019
PEX1	6	0.050434	0.14008	0.99409	2795	-0.2235
ALOX15B	6	0.050434	0.14008	0.99409	2796	-0.2193
ITGA10	6	0.050434	0.14008	0.99409	2797	-0.2699
AXIN1	6	0.05055	0.14037	0.99494	2798	-0.532
IPO11	6	0.050614	0.14052	0.99494	2799	-0.2054
hsa-mir-92b	4	0.050646	0.1196	0.96915	2800	-1.2259
TARS	6	0.05066	0.14063	0.99494	2801	-0.261
CDC73	6	0.050705	0.14074	0.99494	2802	-0.3695
TRMT10A	6	0.050717	0.14077	0.99494	2803	-0.3966
VCX2	4	0.050758	0.11984	0.96915	2804	-0.3211
PAXIP1	6	0.05081	0.14098	0.99494	2805	-0.1362
ICOS	6	0.050844	0.14106	0.99494	2806	-0.0405
UBE2Z	6	0.050854	0.14108	0.99494	2807	-0.2055
FAM86B2	3	0.050858	0.1062	0.94841	2808	-0.326
DIS3	6	0.050885	0.14114	0.99494	2809	-0.223
CYP2S1	6	0.050905	0.14118	0.99494	2810	0.1235
RNF144A	6	0.050907	0.14119	0.99494	2811	-0.208
FGD1	6	0.050927	0.14123	0.99494	2812	-0.1533
ANXA4	6	0.050969	0.14132	0.99494	2813	-0.2631
HSD17B12	6	0.050969	0.14132	0.99494	2814	-0.2462
CRHR1	4	0.050974	0.12027	0.96994	2815	-0.3493
WDR55	6	0.050989	0.14137	0.99494	2816	-0.0603
TMEM45A	6	0.050989	0.14137	0.99494	2817	-0.1989
BIN2	6	0.051004	0.14141	0.99494	2818	0.036
CSRNP2	6	0.051004	0.14141	0.99494	2819	-0.298
hsa-mir-3942	3	0.051022	0.10644	0.94863	2820	-0.0232
MBTPS1	6	0.051068	0.14158	0.99526	2821	-0.2697
EIF2AK4	6	0.051081	0.1416	0.99526	2822	-0.2238
ZFAND2B	6	0.051081	0.1416	0.99526	2823	-0.2579
NEU3	6	0.051104	0.14165	0.99526	2824	-0.091
ACTR8	6	0.051154	0.14178	0.99579	2825	-0.0283
SPEF2	6	0.051214	0.14191	0.99631	2826	-0.0077
RGPD3	6	0.051229	0.14195	0.99631	2827	-0.0314
ANXA13	6	0.051304	0.14212	0.99646	2828	-0.101
BLZF1	6	0.051313	0.14214	0.99646	2829	-0.2763
ZMYM6NB	6	0.051365	0.14226	0.99646	2830	-0.0752
FBXW12	6	0.051415	0.14236	0.99646	2831	-0.203
COX7A2	6	0.051468	0.14247	0.99646	2832	-0.2516

ARHGEF11	6	0.051501	0.14256	0.99646	2833	0.0096
ERI3	6	0.051553	0.14268	0.99646	2834	-0.0139
SMAD6	6	0.051565	0.14271	0.99646	2835	0.0073
MED1	6	0.051587	0.14275	0.99646	2836	-0.289
HTR3B	6	0.051588	0.14275	0.99646	2837	-0.2357
POMGNT1	6	0.051588	0.14275	0.99646	2838	-0.1187
IGSF3	6	0.051588	0.14275	0.99646	2839	-0.2108
C2CD4D	6	0.051628	0.14285	0.99674	2840	0.0138
hsa-mir-761	3	0.051679	0.10737	0.94993	2841	-0.2689
ACSF2	6	0.051702	0.14302	0.99705	2842	0.2067
TMEM143	6	0.051728	0.14308	0.99705	2843	-0.2507
SLC43A1	6	0.051833	0.14332	0.99773	2844	-0.0899
LIN52	6	0.051834	0.14332	0.99773	2845	-0.1079
PNMA1	6	0.051883	0.14346	0.99792	2846	-0.2462
WDR44	6	0.051883	0.14346	0.99792	2847	-0.2188
RPL36A	2	0.051893	0.092155	0.92487	2848	-1.7231
EXOSC10	6	0.051902	0.14351	0.99792	2849	-0.1907
IL1R1	6	0.051913	0.14354	0.99792	2850	-0.2987
SLC45A2	6	0.051989	0.14371	0.99815	2851	-0.259
ABAT	6	0.052001	0.14374	0.99815	2852	-0.3486
hsa-mir-6753	4	0.052023	0.12238	0.97315	2853	-0.3554
BTBD17	6	0.052076	0.1439	0.9983	2854	-0.1761
MYBPC3	6	0.052096	0.14395	0.9983	2855	-0.2303
WDR73	6	0.052109	0.14398	0.9983	2856	-0.084
CHAC2	6	0.052116	0.14399	0.9983	2857	-0.2912
OGFOD2	4	0.052123	0.12258	0.97358	2858	0.0187
FA2H	6	0.052164	0.1441	0.9983	2859	-0.1911
COL14A1	6	0.052201	0.14419	0.9983	2860	-0.0163
SPTB	6	0.052241	0.14428	0.9983	2861	-0.1584
UBE2V1	6	0.052244	0.14429	0.9983	2862	0.1387
WWC2	6	0.052244	0.14429	0.9983	2863	-0.2505
MYL5	6	0.052258	0.14433	0.9983	2864	-0.1966
BUD31	6	0.052276	0.14437	0.9983	2865	-0.2075
TREM1	6	0.052411	0.14465	0.99851	2866	0.1731
NFKB1	6	0.05245	0.14474	0.99851	2867	0.0003
MTHFD2	6	0.052466	0.14477	0.99851	2868	-0.3018
CLTA	6	0.052514	0.14488	0.99851	2869	0.1493
ZNF259	6	0.052517	0.14488	0.99851	2870	-0.2333
TUBA3E	3	0.052589	0.10867	0.95013	2871	-0.3882
NLRP3	6	0.052617	0.14511	0.99851	2872	0.0338
FZR1	6	0.052624	0.14513	0.99851	2873	-0.147
CCNB2	6	0.052678	0.14525	0.99851	2874	-0.2445
ATP5C1	6	0.052682	0.14526	0.99851	2875	-0.1074
MMP28	4	0.052701	0.12375	0.97492	2876	-0.2814
FAM188A	6	0.05272	0.14534	0.99851	2877	-0.0389
NPPA	6	0.052798	0.14551	0.99851	2878	-0.2779
KLHL22	6	0.052798	0.14551	0.99851	2879	-0.3612
UBE2D3	6	0.052798	0.14551	0.99851	2880	-0.4381
RPL23	6	0.052798	0.14551	0.99851	2881	-0.206
ZNF600	6	0.052798	0.14552	0.99851	2882	-0.1629
SSBP3	6	0.052816	0.14554	0.99851	2883	-0.2649
GNAT2	5	0.052876	0.13572	0.99397	2884	-0.2758
hsa-mir-518c	4	0.052879	0.12411	0.97492	2885	-0.8376
NPR3	6	0.052888	0.14569	0.99851	2886	-0.1274
MFAP1	6	0.052898	0.14571	0.99851	2887	-0.1322
ZNF276	6	0.052915	0.14576	0.99851	2888	-0.177
EIF4E	6	0.05293	0.1458	0.99851	2889	-0.1273
ANKRD27	6	0.05293	0.1458	0.99851	2890	-0.2179
GPR84	6	0.052955	0.14585	0.99851	2891	-0.2352
TRAK1	6	0.053075	0.14611	0.99851	2892	-0.2799
DDN	6	0.053075	0.14611	0.99851	2893	-0.2474
hsa-mir-27b	4	0.053088	0.12453	0.97643	2894	-0.4282
ZNF529	6	0.053147	0.14628	0.99851	2895	-0.2654
ALDH1L2	6	0.053173	0.14635	0.99851	2896	-0.2195
ZNF770	6	0.053173	0.14635	0.99851	2897	-0.1755
HS6ST2	6	0.053176	0.14635	0.99851	2898	-0.2636
GDF3	6	0.053237	0.14648	0.99851	2899	-0.1371
SUMO2	6	0.053248	0.14651	0.99851	2900	-0.2043
SMEK1	6	0.053248	0.14651	0.99851	2901	-0.1827
hsa-mir-3169	4	0.053257	0.12487	0.97643	2902	0.378
ITGAX	6	0.053302	0.14664	0.99851	2903	-0.0714
GLRA4	5	0.053321	0.13654	0.99397	2904	-0.3442
TNFAIP8L3	6	0.05334	0.14673	0.99851	2905	-0.2609
MADD	6	0.05334	0.14673	0.99851	2906	-0.2793
CCDC160	6	0.05334	0.14673	0.99851	2907	-0.2458
EPS8	6	0.053356	0.14676	0.99851	2908	-0.1365
NR5A1	6	0.053373	0.14679	0.99851	2909	-0.0899
RFC3	6	0.053403	0.14686	0.99851	2910	-0.2513
CCDC117	6	0.053487	0.14706	0.99851	2911	-0.2076
ARMC10	6	0.053487	0.14706	0.99851	2912	-0.222
CHMP3	3	0.053497	0.11002	0.95341	2913	-0.1749

COG6	6	0.053507	0.14711	0.99851	2914	-0.1901
NDNL2	6	0.053507	0.14711	0.99851	2915	-0.2632
HAX1	6	0.053528	0.14715	0.99851	2916	-0.1628
TMPRSS11B	6	0.053539	0.14718	0.99851	2917	-0.1606
PAPSS1	6	0.053568	0.14724	0.99851	2918	-0.1649
VGLL1	6	0.053595	0.1473	0.99851	2919	0.0151
POM121C	4	0.053607	0.12557	0.97826	2920	-0.3701
UBE2A	6	0.053642	0.1474	0.99851	2921	-0.25
NUB1	5	0.053644	0.13714	0.99397	2922	-0.2048
TACR2	6	0.053655	0.14743	0.99851	2923	-0.2724
C14orf166	6	0.053694	0.14753	0.99851	2924	-0.0154
hsa-mir-663a	4	0.053707	0.12578	0.9787	2925	-0.3805
PRG2	5	0.053724	0.13728	0.99397	2926	-0.148
KRT77	6	0.053733	0.14761	0.99851	2927	-0.2907
SMIM17	6	0.053773	0.1477	0.99851	2928	-0.2679
LRRC52	6	0.053823	0.14781	0.99851	2929	-0.2606
ICAM1	6	0.053823	0.14781	0.99851	2930	-0.2303
PRUNE2	6	0.05386	0.14789	0.99851	2931	-0.1549
MED21	6	0.053869	0.14791	0.99851	2932	-0.2002
TMEM191C	5	0.053871	0.13755	0.99397	2933	-0.0305
REG1B	6	0.053913	0.14803	0.99851	2934	-0.2382
C19orf67	6	0.053925	0.14806	0.99851	2935	-0.1977
RNF168	6	0.053925	0.14806	0.99851	2936	-0.1113
FAM136A	6	0.053943	0.1481	0.99851	2937	0.0437
PRSS55	6	0.053977	0.14818	0.99851	2938	-0.1844
ANO3	6	0.053986	0.1482	0.99851	2939	-0.2818
GGPS1	6	0.053993	0.14821	0.99851	2940	-0.0567
ADI1	6	0.054036	0.14832	0.99851	2941	-0.0909
TRHR	6	0.054067	0.1484	0.99851	2942	-0.1555
LPCAT3	6	0.054068	0.1484	0.99851	2943	-0.1505
ZNF614	6	0.054069	0.1484	0.99851	2944	-0.2225
OFD1	6	0.054069	0.1484	0.99851	2945	-0.2431
AGBL2	6	0.054092	0.14846	0.99851	2946	0.2199
C6orf62	6	0.054114	0.14851	0.99851	2947	-0.088
TBATA	6	0.054142	0.14857	0.99851	2948	0.1031
TMEM158	6	0.054173	0.14864	0.99851	2949	-0.0964
EID3	6	0.054217	0.14873	0.99851	2950	0.2125
FAM21C	4	0.054268	0.12691	0.9788	2951	-0.3044
ANKHD1	4	0.054309	0.12699	0.9788	2952	-0.2385
C10orf2	6	0.054341	0.149	0.99851	2953	-0.0654
hsa-mir-6813	4	0.054357	0.12709	0.9788	2954	-0.4617
SNCG	6	0.054381	0.1491	0.99851	2955	-0.1785
CEP89	6	0.054414	0.14918	0.99851	2956	-0.0545
ABCG8	6	0.05444	0.14924	0.99851	2957	-0.1185
HEXIM2	6	0.05446	0.14929	0.99851	2958	-0.36
MYD88	6	0.05446	0.14929	0.99851	2959	-0.4831
HIST1H4I	6	0.05446	0.14929	0.99851	2960	-0.3844
SNRNP35	6	0.05446	0.14929	0.99851	2961	-0.287
EIF4A3	6	0.05446	0.14929	0.99851	2962	-0.2766
STEAP1B	6	0.05446	0.14929	0.99851	2963	-0.8386
H1F0	6	0.054475	0.14933	0.99851	2964	-0.1476
ZNF839	6	0.05449	0.14936	0.99851	2965	-0.042
ATP2A3	6	0.054525	0.14944	0.99851	2966	-0.1644
EML4	6	0.054545	0.14948	0.99851	2967	-0.2531
STAG3	5	0.054548	0.13879	0.99402	2968	-0.2049
MSR1	6	0.054639	0.14971	0.99968	2969	0.1567
hsa-mir-328	4	0.05464	0.12765	0.98025	2970	-0.3406
OR2L8	4	0.054678	0.12773	0.98025	2971	-0.2357
SLMO1	6	0.054689	0.14981	0.99969	2972	0.0329
PHACTR3	6	0.054689	0.14981	0.99969	2973	0.0594
ACBD4	6	0.054788	0.15005	0.99998	2974	-0.1273
TIMM44	6	0.054872	0.15024	0.99998	2975	-0.0388
OR8B4	6	0.054872	0.15024	0.99998	2976	-0.0415
HIST1H4J	1	0.054919	0.054875	0.8197	2977	-0.5074
PIGL	4	0.054923	0.12822	0.98168	2978	0.1555
ACP2	6	0.054954	0.15042	0.99998	2979	-0.2318
WNT10B	6	0.055013	0.15056	0.99998	2980	-0.1506
SET	6	0.055026	0.1506	0.99998	2981	-0.2207
PIGS	6	0.055026	0.1506	0.99998	2982	-0.249
PCBD2	6	0.055037	0.15063	0.99998	2983	-0.1376
hsa-mir-1973	3	0.055104	0.11231	0.95694	2984	-0.4632
MICALCL	6	0.055108	0.1508	0.99998	2985	-0.1787
BTBD1	6	0.05512	0.15083	0.99998	2986	-0.2499
hsa-mir-616	4	0.055172	0.12872	0.98168	2987	-0.0732
CLEC16A	6	0.055178	0.15095	0.99998	2988	-0.118
ROR1	6	0.055178	0.15095	0.99998	2989	-0.1867
hsa-mir-6775	4	0.055229	0.12883	0.98168	2990	-0.2993
RPRM	6	0.055232	0.15109	0.99998	2991	-0.2364
KIAA0195	6	0.055232	0.15109	0.99998	2992	-0.3374
UPF2	6	0.055307	0.15127	0.99998	2993	-0.1464
TMEM208	6	0.055325	0.15132	0.99998	2994	-0.0106

HELQ	6	0.055338	0.15135	0.99998	2995	-0.2487
EEF1G	6	0.05536	0.1514	0.99998	2996	0.1278
BHLHE40	6	0.05536	0.1514	0.99998	2997	-0.038
ADAM11	6	0.055376	0.15142	0.99998	2998	-0.2023
C1QTNF9B	2	0.055387	0.098052	0.93789	2999	-1.4809
OTUD5	6	0.055393	0.15146	0.99998	3000	-0.2792
SLC2A7	6	0.055435	0.15156	0.99998	3001	-0.1007
CCDC179	6	0.055456	0.15161	0.99998	3002	-0.1615
CKAP5	6	0.055484	0.15168	0.99998	3003	-0.195
C19orf38	6	0.055497	0.15171	0.99998	3004	-0.2395
BHMT2	6	0.055548	0.15183	0.99998	3005	-0.0364
RNF130	6	0.055575	0.15189	0.99998	3006	0.0318
ECHDC3	6	0.055584	0.15191	0.99998	3007	-0.3327
RPL17	1	0.055584	0.055538	0.82064	3008	-1.1328
VPS54	6	0.055595	0.15194	0.99998	3009	-0.3985
MCL1	6	0.055614	0.15197	0.99998	3010	-0.102
ANGPTL6	6	0.055614	0.15197	0.99998	3011	-0.3382
LGALS2	6	0.055648	0.15205	0.99998	3012	-0.1837
GIPC1	6	0.055648	0.15205	0.99998	3013	-0.2551
C2orf54	6	0.055661	0.15207	0.99998	3014	-0.395
AKIRIN2	6	0.055696	0.15216	0.99998	3015	-0.2018
TP53INP2	6	0.055696	0.15216	0.99998	3016	-0.2391
AGR2	6	0.055716	0.1522	0.99998	3017	-0.2709
hsa-mir-1302-	4	0.055822	0.13002	0.98475	3018	-0.4531
H2AFX	6	0.055865	0.15253	0.99998	3019	-0.197
hsa-mir-6788	4	0.055915	0.13019	0.98475	3020	-0.2994
POLQ	6	0.055927	0.15267	0.99998	3021	-0.2386
TUBG1	5	0.055946	0.14128	0.99494	3022	-0.0151
OR13C4	6	0.055981	0.15279	0.99998	3023	-0.0842
KLHL26	6	0.055983	0.1528	0.99998	3024	0.0207
PNPLA3	6	0.05603	0.1529	0.99998	3025	-0.1922
MYH4	6	0.056031	0.15291	0.99998	3026	-0.378
FGF19	6	0.056055	0.15296	0.99998	3027	-0.1259
TAF7	6	0.056055	0.15296	0.99998	3028	0.0593
STAU1	6	0.056079	0.153	0.99998	3029	-0.2271
PTGES	6	0.05613	0.15313	0.99998	3030	-0.0298
POP7	6	0.056138	0.15314	0.99998	3031	-0.2519
BCLAF1	6	0.056138	0.15314	0.99998	3032	-0.294
hsa-mir-5090	4	0.056169	0.1307	0.98738	3033	-0.3713
LDB2	6	0.056228	0.15334	0.99998	3034	0.0785
LIN9	6	0.056229	0.15335	0.99998	3035	-0.0626
DENND4B	6	0.056273	0.15345	0.99998	3036	-0.2737
GPR52	6	0.056301	0.15351	0.99998	3037	-0.3965
CMTM2	6	0.056301	0.15351	0.99998	3038	-0.144
OR8K1	6	0.056359	0.15363	0.99998	3039	-0.2711
TTC39B	6	0.056364	0.15364	0.99998	3040	-0.1839
GOSR1	6	0.056364	0.15364	0.99998	3041	-0.3093
MX1	6	0.056419	0.15377	0.99998	3042	-0.2196
SMYD5	6	0.056436	0.1538	0.99998	3043	-0.2541
SFMBT1	6	0.056449	0.15384	0.99998	3044	-0.2267
ZMYND12	6	0.056477	0.1539	0.99998	3045	0.4176
hsa-mir-1262	4	0.056503	0.13136	0.98951	3046	-0.1137
C2orf69	6	0.056518	0.154	0.99998	3047	-0.2021
TGM5	6	0.056601	0.15419	0.99998	3048	0.2437
DYRK1A	6	0.056616	0.15422	0.99998	3049	-0.2724
USP5	6	0.056722	0.15445	0.99998	3050	-0.4859
PKP1	6	0.056722	0.15445	0.99998	3051	-0.2716
hsa-mir-8077	4	0.056746	0.13184	0.98951	3052	-0.4401
NT5C1B	3	0.056868	0.11482	0.96096	3053	-0.2783
ZNF517	6	0.056899	0.15486	0.99998	3054	-0.1825
PIK3R6	6	0.056907	0.15488	0.99998	3055	-0.2396
ANKRD33	6	0.056969	0.15503	0.99998	3056	-0.1821
DICER1	6	0.056974	0.15504	0.99998	3057	-0.1561
TAMM41	6	0.056974	0.15504	0.99998	3058	-0.2159
TLR5	6	0.056974	0.15504	0.99998	3059	-0.2991
OPCML	6	0.057034	0.15517	0.99998	3060	-0.1651
TLR9	6	0.05707	0.15526	0.99998	3061	-0.1859
PDK1	6	0.057088	0.1553	0.99998	3062	-0.2259
hsa-mir-5682	4	0.057101	0.13248	0.98972	3063	-0.102
ADAMTS1	6	0.057118	0.15537	0.99998	3064	-0.1721
YTHDC1	6	0.057124	0.15538	0.99998	3065	-0.1482
THUMPD3	6	0.057147	0.15543	0.99998	3066	-0.189
ARPC4	3	0.057184	0.11526	0.9614	3067	-0.3016
PDZRN4	6	0.057277	0.1557	0.99998	3068	-0.103
SIKE1	6	0.057308	0.15578	0.99998	3069	-0.0414
LRFN1	6	0.057326	0.15582	0.99998	3070	-0.2261
MAST4	6	0.057326	0.15582	0.99998	3071	-0.1918
SSX7	5	0.057339	0.14373	0.99815	3072	-0.1976
SRCAP	6	0.057393	0.15598	0.99998	3073	-0.2522
ARHGEF17	6	0.057393	0.15598	0.99998	3074	-0.2574
GUCY2C	6	0.057495	0.15621	0.99998	3075	-0.2469

SMIM20	6	0.057495	0.15621	0.99998	3076	-0.2258
RTL1	6	0.057538	0.1563	0.99998	3077	-0.1856
CNTROB	6	0.057569	0.15637	0.99998	3078	0.0426
hsa-mir-499a	4	0.057614	0.13345	0.99022	3079	-0.2099
C19orf54	6	0.057621	0.15648	0.99998	3080	-0.1699
UPB1	6	0.057657	0.15656	0.99998	3081	-0.11
hsa-mir-3648	4	0.057704	0.13363	0.99022	3082	-0.4409
GMFB	5	0.057728	0.14438	0.9983	3083	-0.1188
DDX18	6	0.057732	0.15673	0.99998	3084	-0.3323
NOTUM	6	0.057777	0.15683	0.99998	3085	-0.2444
GLYATL2	6	0.057777	0.15683	0.99998	3086	-0.0739
DNAJC24	6	0.057859	0.15701	0.99998	3087	-0.2322
ZBTB20	6	0.057866	0.15703	0.99998	3088	0.1624
SORCS1	6	0.057884	0.15707	0.99998	3089	-0.0941
SERPINA5	6	0.057923	0.15718	0.99998	3090	-0.2776
CASP14	6	0.057945	0.15722	0.99998	3091	-0.1865
XYLT2	6	0.058038	0.15743	0.99998	3092	-0.0296
ADAMTS19	6	0.058066	0.15749	0.99998	3093	-0.2002
WDR36	6	0.058066	0.15749	0.99998	3094	-0.2601
TRIP4	6	0.058079	0.15751	0.99998	3095	0.0443
CYP2C19	3	0.058139	0.11665	0.96525	3096	-0.401
SENP8	6	0.058152	0.15766	0.99998	3097	0.2265
SLC25A41	6	0.058164	0.15768	0.99998	3098	0.0003
DHX37	6	0.058164	0.15768	0.99998	3099	-0.2934
MLPH	6	0.058213	0.15779	0.99998	3100	-0.1527
ZFP57	6	0.058267	0.1579	0.99998	3101	-0.1841
HBS1L	6	0.058273	0.15792	0.99998	3102	0.0071
FBXO33	6	0.058312	0.15801	0.99998	3103	-0.0617
EXOSC2	6	0.058312	0.15801	0.99998	3104	-0.1716
UPK3B	6	0.058316	0.15802	0.99998	3105	-0.1867
CCDC65	6	0.058316	0.15802	0.99998	3106	-0.2068
CLCN6	6	0.058382	0.15816	0.99998	3107	-0.3006
BRWD3	6	0.058382	0.15816	0.99998	3108	-0.3023
SOX14	6	0.058383	0.15816	0.99998	3109	-0.2951
ISL2	6	0.058484	0.15838	0.99998	3110	-0.2463
MIB1	6	0.05851	0.15845	0.99998	3111	0.1095
PSCA	6	0.058602	0.15865	0.99998	3112	-0.1834
SRPX2	6	0.058634	0.15872	0.99998	3113	0.0895
MEF2B	2	0.058695	0.10351	0.94533	3114	-0.153
CASP8	6	0.058702	0.15888	0.99998	3115	-0.1752
RPL22L1	6	0.058702	0.15888	0.99998	3116	-0.3843
ABR	6	0.05875	0.15899	0.99998	3117	-0.0193
PFN4	6	0.058758	0.15901	0.99998	3118	-0.094
PCIF1	6	0.058766	0.15902	0.99998	3119	-0.1926
C1orf27	6	0.058783	0.15907	0.99998	3120	-0.1894
MRPL21	6	0.058851	0.15922	0.99998	3121	-0.2387
NETO2	6	0.058852	0.15922	0.99998	3122	-0.2401
RFXANK	6	0.058918	0.15937	0.99998	3123	-0.1167
PRPH2	6	0.058919	0.15937	0.99998	3124	-0.2437
MCAM	6	0.058956	0.15945	0.99998	3125	-0.0828
ECI1	6	0.058962	0.15946	0.99998	3126	-0.1925
ZDHHC18	6	0.059002	0.15955	0.99998	3127	-0.1762
EZR	6	0.059039	0.15963	0.99998	3128	-0.1741
DDX54	6	0.059055	0.15966	0.99998	3129	0.0932
AMH	6	0.059128	0.15981	0.99998	3130	-0.193
EMC10	6	0.059154	0.15986	0.99998	3131	0.063
LIPN	6	0.059316	0.16022	0.99998	3132	-0.1794
ACTL10	6	0.059352	0.16031	0.99998	3133	-0.162
CHTF18	6	0.059413	0.16044	0.99998	3134	-0.2575
hsa-mir-4285	4	0.05942	0.13702	0.99397	3135	-0.2265
BBS9	6	0.059427	0.16047	0.99998	3136	-0.1991
SRD5A2	6	0.059487	0.16061	0.99998	3137	-0.2176
POU4F3	6	0.059501	0.16065	0.99998	3138	0.0043
ABCB8	6	0.059512	0.16067	0.99998	3139	0.1034
BNIP2	6	0.059516	0.16068	0.99998	3140	-0.107
AGBL3	6	0.05953	0.1607	0.99998	3141	-0.1702
NDUFC2-KCTD	4	0.059542	0.13725	0.99397	3142	0.0024
RCOR2	6	0.059583	0.16082	0.99998	3143	-0.2429
LATS2	6	0.0596	0.16085	0.99998	3144	-0.051
LIN7C	6	0.05961	0.16088	0.99998	3145	-0.2434
ZNF28	5	0.059626	0.14769	0.99851	3146	-0.6257
RPP14	6	0.059654	0.16097	0.99998	3147	-0.2911
MMP1	6	0.059708	0.16109	0.99998	3148	-0.1995
MRPS25	6	0.059745	0.16117	0.99998	3149	-0.1168
NUDT12	6	0.059745	0.16117	0.99998	3150	-0.1599
NT5M	6	0.059745	0.16117	0.99998	3151	-0.1493
CACNA1I	6	0.059765	0.16121	0.99998	3152	-0.202
PRKAB1	6	0.05979	0.16127	0.99998	3153	-0.0324
TOP1MT	6	0.059806	0.1613	0.99998	3154	-0.1865
SIX2	6	0.059843	0.16138	0.99998	3155	-0.3136
NRBP1	6	0.059897	0.16151	0.99998	3156	-0.1177

OSER1	1	0.059903	0.059915	0.84024	3157	-0.6757
KRTAP1-4	6	0.059921	0.16156	0.99998	3158	-0.2102
hsa-mir-219a-	4	0.059923	0.138	0.99397	3159	0.1443
hsa-mir-7106	4	0.059974	0.1381	0.99397	3160	-0.2704
C10orf113	6	0.059995	0.16172	0.99998	3161	-0.3361
RHPN1	6	0.059995	0.16172	0.99998	3162	-0.2019
MALT1	6	0.06004	0.16183	0.99998	3163	-0.3343
FBXW9	6	0.060061	0.16187	0.99998	3164	-0.0737
hsa-mir-617	4	0.06009	0.13834	0.99397	3165	-0.2763
MAPK4	6	0.060095	0.16193	0.99998	3166	0.0128
hsa-mir-143	4	0.060138	0.13845	0.99397	3167	0.0423
TOMM70A	4	0.060141	0.13845	0.99397	3168	-0.2838
PPP4R1	6	0.060145	0.16205	0.99998	3169	-0.2257
TRAPPC5	6	0.060182	0.16214	0.99998	3170	-0.2886
GAK	6	0.060182	0.16214	0.99998	3171	-0.2124
MLLT10	6	0.060183	0.16214	0.99998	3172	-0.1414
ZNF117	5	0.060224	0.14878	0.99851	3173	-0.4153
RXRA	6	0.060243	0.16226	0.99998	3174	-0.03
hsa-mir-4722	4	0.060255	0.13867	0.99402	3175	-0.353
COL15A1	6	0.060257	0.16229	0.99998	3176	-0.2899
FZD1	6	0.060295	0.1624	0.99998	3177	-0.161
NXPE4	6	0.060466	0.16279	0.99998	3178	-0.0145
TAS2R7	6	0.060475	0.16281	0.99998	3179	-0.206
hsa-mir-8069	4	0.060509	0.13919	0.99402	3180	-0.2602
PYROXD2	6	0.060544	0.16298	0.99998	3181	-0.2314
MIER3	6	0.060565	0.16303	0.99998	3182	-0.1015
BSG	6	0.060578	0.16305	0.99998	3183	-0.0041
SH3TC2	6	0.0606	0.1631	0.99998	3184	-0.1931
TMEM59	6	0.060622	0.16315	0.99998	3185	-0.1776
GALNT7	6	0.060622	0.16315	0.99998	3186	-0.2318
SCO2	6	0.060682	0.16329	0.99998	3187	-0.3011
CRYBA1	6	0.060682	0.16329	0.99998	3188	-0.3089
SMIM6	6	0.060688	0.1633	0.99998	3189	0.3156
C2orf27A	3	0.060773	0.12039	0.96994	3190	-0.1489
SPHK2	6	0.060782	0.16351	0.99998	3191	-0.1073
UBA5	6	0.060785	0.16351	0.99998	3192	-0.3479
DESI1	6	0.060785	0.16351	0.99998	3193	-0.2689
E2F1	6	0.060787	0.16352	0.99998	3194	-0.2197
RGL3	6	0.060794	0.16353	0.99998	3195	-0.363
KIF14	6	0.060837	0.16363	0.99998	3196	-0.1958
NENF	6	0.060864	0.1637	0.99998	3197	-0.1499
DCLRE1B	6	0.060864	0.1637	0.99998	3198	-0.1458
RPS6KA2	6	0.06087	0.16371	0.99998	3199	-0.2238
HIST1H2BG	6	0.060958	0.1639	0.99998	3200	-0.4194
AEBP1	6	0.060958	0.1639	0.99998	3201	-0.3509
BTBD18	6	0.061035	0.16408	0.99998	3202	-0.0926
ARAP3	6	0.061083	0.16418	0.99998	3203	-0.0726
KIAA0141	6	0.061089	0.16419	0.99998	3204	-0.1313
SLC35F6	6	0.061089	0.16419	0.99998	3205	-0.1138
SF3B2	6	0.061109	0.16423	0.99998	3206	0.0118
CDA	6	0.061134	0.16429	0.99998	3207	-0.2703
C19orf40	6	0.061134	0.16429	0.99998	3208	-0.2118
hsa-mir-4646	4	0.061149	0.14042	0.99494	3209	-0.403
PTCHD1	6	0.061171	0.16436	0.99998	3210	-0.1584
NAP1L1	6	0.061216	0.16447	0.99998	3211	-0.2158
CACNA1S	6	0.061253	0.16455	0.99998	3212	-0.3642
IRF2BPL	6	0.061304	0.16466	0.99998	3213	-0.17
SPTLC3	6	0.061316	0.16469	0.99998	3214	-0.1006
H2AFJ	4	0.061351	0.14082	0.99494	3215	-0.3627
SASS6	6	0.061357	0.16479	0.99998	3216	-0.1175
NFYC	6	0.06138	0.16484	0.99998	3217	-0.253
RBFA	6	0.061386	0.16485	0.99998	3218	0.0173
IQCA1	6	0.061411	0.1649	0.99998	3219	-0.0072
hsa-mir-202	4	0.061422	0.14097	0.99494	3220	-0.2035
SDE2	6	0.06143	0.16494	0.99998	3221	-0.0096
SIGLEC1	6	0.061443	0.16496	0.99998	3222	-0.1633
SLC25A11	6	0.061478	0.16504	0.99998	3223	-0.2965
hsa-mir-8067	4	0.061497	0.14113	0.99494	3224	0.2484
IGFL1	6	0.061529	0.16515	0.99998	3225	0.0653
FIGNL1	6	0.061552	0.1652	0.99998	3226	-0.2127
PCGF6	6	0.061552	0.1652	0.99998	3227	-0.2041
NBPF3	6	0.061627	0.16538	0.99998	3228	0.0899
CYFIP1	6	0.061657	0.16544	0.99998	3229	-0.2562
PDE1B	6	0.061677	0.16549	0.99998	3230	-0.0751
POLR3GL	6	0.061677	0.16549	0.99998	3231	-0.2179
PPM1D	6	0.061702	0.16554	0.99998	3232	-0.2056
MTX1	6	0.061719	0.16558	0.99998	3233	-0.1389
C19orf10	6	0.061746	0.16565	0.99998	3234	0.0427
SLAMF8	6	0.061762	0.16568	0.99998	3235	-0.2062
C4orf32	6	0.0618	0.16577	0.99998	3236	0.0607
RCC2	6	0.0618	0.16577	0.99998	3237	-0.0854

EIF3J	6	0.061807	0.16579	0.99998	3238	-0.3357
ELMO2	6	0.061816	0.1658	0.99998	3239	-0.2277
RPS12	6	0.061822	0.16582	0.99998	3240	-0.0462
MROH8	6	0.061932	0.16607	0.99998	3241	-0.1606
37865	3	0.06196	0.12205	0.97273	3242	0.089
MMP26	6	0.061978	0.16617	0.99998	3243	-0.3529
MESP1	6	0.061987	0.16619	0.99998	3244	-0.0714
IL15RA	6	0.062047	0.16633	0.99998	3245	-0.2134
GDPD4	4	0.062081	0.14231	0.99646	3246	-0.2122
GLUL	6	0.062083	0.16641	0.99998	3247	-0.3274
CCT3	4	0.062236	0.1426	0.99646	3248	-0.1702
SPDYE6	1	0.062265	0.062269	0.84924	3249	-0.5608
SCML4	6	0.062269	0.16681	0.99998	3250	-0.0658
RMI1	6	0.062269	0.16681	0.99998	3251	0.1473
OR51E1	6	0.06227	0.16681	0.99998	3252	-0.0794
SYT17	6	0.06227	0.16681	0.99998	3253	-0.0711
HSPB1	6	0.06228	0.16683	0.99998	3254	-0.219
KIF4A	6	0.062283	0.16684	0.99998	3255	-0.2507
hsa-mir-6787	4	0.062293	0.1427	0.99646	3256	-0.2758
TPP1	6	0.062379	0.16705	0.99998	3257	-0.1597
hsa-mir-6800	4	0.062381	0.14289	0.99674	3258	-0.3446
GRB2	6	0.062434	0.16718	0.99998	3259	-0.1533
SLC34A3	6	0.062434	0.16718	0.99998	3260	-0.0789
ZNF624	6	0.062475	0.16726	0.99998	3261	-0.1281
PVRL2	6	0.062476	0.16726	0.99998	3262	-0.1792
PGLYRP4	3	0.062484	0.12278	0.97358	3263	-0.0197
MAML1	6	0.062509	0.16734	0.99998	3264	-0.1767
FRMD6	6	0.06251	0.16734	0.99998	3265	-0.2043
PRPH	6	0.06251	0.16734	0.99998	3266	-0.1831
PLEKHJ1	6	0.062544	0.16742	0.99998	3267	-0.1522
RAP1GAP	6	0.06257	0.16748	0.99998	3268	-0.2744
EHBP1	6	0.062585	0.1675	0.99998	3269	-0.2092
SLIT2	4	0.062614	0.14332	0.99773	3270	-0.2508
EFNA1	6	0.062654	0.16766	0.99998	3271	-0.1636
TMEM217	6	0.062689	0.16774	0.99998	3272	-0.335
PTGS2	6	0.062689	0.16774	0.99998	3273	0.2667
RARRES1	6	0.062703	0.16777	0.99998	3274	-0.2354
EIF2S2	6	0.062728	0.16781	0.99998	3275	-0.1752
RXRB	6	0.062728	0.16781	0.99998	3276	-0.2765
CEACAM1	6	0.062737	0.16784	0.99998	3277	-0.1399
OR11H12	3	0.062758	0.12317	0.97379	3278	-0.0476
C19orf66	6	0.062763	0.1679	0.99998	3279	-0.0434
HESX1	6	0.062766	0.1679	0.99998	3280	-0.2053
GGT7	6	0.062766	0.1679	0.99998	3281	-0.1671
MTRNR2L4	6	0.062801	0.16797	0.99998	3282	-0.2018
KPNA4	5	0.062818	0.15328	0.99998	3283	-0.2114
HSF1	6	0.06285	0.16808	0.99998	3284	-0.1923
RGS10	6	0.06285	0.16808	0.99998	3285	-0.2351
hsa-mir-6810	4	0.062854	0.14377	0.99815	3286	-0.1397
MPP1	6	0.062862	0.16811	0.99998	3287	0.0098
METTL7A	6	0.062889	0.16818	0.99998	3288	-0.298
WSB2	6	0.062889	0.16818	0.99998	3289	0.1169
C17orf58	6	0.062946	0.16829	0.99998	3290	-0.2363
GNG7	6	0.062951	0.1683	0.99998	3291	-0.0716
UBA7	6	0.063035	0.16848	0.99998	3292	0.2329
ZHX3	6	0.063048	0.16851	0.99998	3293	-0.226
MDM4	6	0.063154	0.16876	0.99998	3294	-0.1838
PRSS23	6	0.063207	0.16886	0.99998	3295	0.2072
SHBG	6	0.063235	0.16893	0.99998	3296	0.062
FBXL19	6	0.063235	0.16893	0.99998	3297	-0.2156
HLA-DMA	6	0.063247	0.16896	0.99998	3298	-0.2229
AAMDC	6	0.063257	0.16898	0.99998	3299	0.1431
ST6GALNAC1	6	0.06328	0.16903	0.99998	3300	-0.0827
UTP6	6	0.063302	0.16908	0.99998	3301	-0.288
MRPS9	2	0.063305	0.1111	0.95608	3302	-0.2341
ZBTB39	6	0.063306	0.16909	0.99998	3303	-0.1424
MRFAP1L1	6	0.063311	0.1691	0.99998	3304	-0.1487
LTB4R	6	0.06334	0.16916	0.99998	3305	-0.3604
hsa-mir-3180-	4	0.063352	0.14476	0.99851	3306	-0.4665
RCC1	6	0.063374	0.16923	0.99998	3307	-0.0959
COL8A1	6	0.063443	0.16939	0.99998	3308	-0.0988
SOAT2	6	0.063446	0.16939	0.99998	3309	-0.2193
CKAP2L	6	0.063454	0.16941	0.99998	3310	-0.0961
CMTM8	6	0.063466	0.16943	0.99998	3311	-0.2421
TRIM67	6	0.063471	0.16945	0.99998	3312	-0.182
MED19	6	0.063495	0.16949	0.99998	3313	-0.2537
PUS7L	6	0.063495	0.16949	0.99998	3314	-0.2079
KRT81	4	0.063499	0.14504	0.99851	3315	-0.1198
RRN3	6	0.063511	0.16953	0.99998	3316	-0.2446
ZBTB17	6	0.063511	0.16953	0.99998	3317	-0.1722
hsa-mir-3922	4	0.063556	0.14516	0.99851	3318	-0.2473

hsa-mir-7158	4	0.063565	0.14517	0.99851	3319	-0.1723
F10	6	0.063577	0.16966	0.99998	3320	0.2193
ZNF574	6	0.063651	0.16982	0.99998	3321	0.0134
hsa-mir-589	4	0.063665	0.14537	0.99851	3322	-0.3094
hsa-mir-302a	4	0.063665	0.14537	0.99851	3323	-0.2587
ABTB1	6	0.06369	0.16991	0.99998	3324	-0.2523
IP6K3	6	0.063718	0.16998	0.99998	3325	-0.1965
LRTM1	6	0.063824	0.17023	0.99998	3326	-0.0573
HTR3C	6	0.063859	0.17032	0.99998	3327	0.0705
FIGN	6	0.063859	0.17032	0.99998	3328	-0.0556
CALCB	6	0.063898	0.17039	0.99998	3329	0.1382
hsa-mir-7705	2	0.063927	0.11209	0.95694	3330	-0.305
MCEE	6	0.063928	0.17045	0.99998	3331	-0.2754
EMILIN1	6	0.063928	0.17045	0.99998	3332	-0.41
KIF21A	6	0.06395	0.17051	0.99998	3333	0.0406
RALB	6	0.064046	0.17071	0.99998	3334	0.1408
C7orf49	6	0.064059	0.17074	0.99998	3335	-0.158
CCDC37	6	0.06414	0.1709	0.99998	3336	-0.2528
LSM6	6	0.06414	0.1709	0.99998	3337	-0.1132
NKTR	6	0.064144	0.17091	0.99998	3338	0.1694
DLX4	6	0.064178	0.17099	0.99998	3339	-0.2475
TMEM52B	6	0.064178	0.17099	0.99998	3340	-0.2191
SRRM1	6	0.064194	0.17102	0.99998	3341	0.2554
C16orf3	6	0.064225	0.17108	0.99998	3342	-0.1049
TSTD2	6	0.064243	0.17112	0.99998	3343	-0.0869
NIT1	6	0.064306	0.17127	0.99998	3344	-0.3227
TGFBR3L	6	0.064337	0.17133	0.99998	3345	-0.3039
RHBDD3	6	0.064337	0.17133	0.99998	3346	-0.2538
RPL7A	6	0.064342	0.17134	0.99998	3347	0.0753
ZNF816	2	0.064355	0.1128	0.95738	3348	-0.2302
HS3ST4	6	0.064451	0.17157	0.99998	3349	-0.238
HELB	6	0.064468	0.17161	0.99998	3350	-0.0965
SMG9	6	0.064489	0.17166	0.99998	3351	-0.1311
HSH2D	6	0.064545	0.17178	0.99998	3352	-0.1778
FOXR2	6	0.064563	0.17182	0.99998	3353	0.1716
CACNG8	6	0.064604	0.17191	0.99998	3354	-0.1495
OLIG3	6	0.064627	0.17197	0.99998	3355	-0.2692
PIN4	6	0.064687	0.1721	0.99998	3356	0.0463
PPAN	2	0.064708	0.11336	0.95963	3357	-0.1253
hsa-mir-6858	4	0.064738	0.14757	0.99851	3358	-0.3834
IL3	6	0.064786	0.17232	0.99998	3359	-0.1831
GPS1	6	0.064793	0.17233	0.99998	3360	-0.0912
PIK3CB	6	0.064796	0.17234	0.99998	3361	-0.1783
hsa-mir-892b	4	0.064824	0.14773	0.99851	3362	-0.3255
COL6A6	6	0.064826	0.17239	0.99998	3363	-0.1948
SCN4B	6	0.064834	0.17241	0.99998	3364	0.0288
C11orf80	6	0.06484	0.17242	0.99998	3365	-0.1778
SAMD13	6	0.064872	0.17249	0.99998	3366	-0.2001
DERL3	6	0.064877	0.1725	0.99998	3367	0.0137
AASDHPPT	6	0.064884	0.17252	0.99998	3368	-0.0426
LMBR1	6	0.06501	0.17278	0.99998	3369	-0.1066
hsa-mir-1234	4	0.065019	0.1481	0.99851	3370	0.1173
PPP1R35	6	0.065028	0.17282	0.99998	3371	0.143
PRSS35	6	0.065081	0.17293	0.99998	3372	-0.064
MARS	6	0.065101	0.17297	0.99998	3373	0.0802
TMCC3	6	0.065101	0.17297	0.99998	3374	-0.1309
HIGD1C	6	0.065127	0.17304	0.99998	3375	-0.1898
HNRNPUL1	6	0.06513	0.17304	0.99998	3376	-0.1448
DQX1	6	0.065163	0.17311	0.99998	3377	-0.2067
SYDE2	6	0.065196	0.17318	0.99998	3378	-0.2483
DHX29	6	0.065204	0.1732	0.99998	3379	0.0798
MPEG1	6	0.065235	0.17327	0.99998	3380	-0.2881
TRRAP	4	0.065247	0.14857	0.99851	3381	-0.3343
RMND5B	6	0.065251	0.17331	0.99998	3382	-0.156
hsa-mir-3181	4	0.065258	0.14859	0.99851	3383	-0.4882
hsa-mir-4470	4	0.065258	0.14859	0.99851	3384	-0.3176
SESTD1	6	0.065302	0.17341	0.99998	3385	0.1494
FAM3C	3	0.06533	0.12678	0.9788	3386	-0.6494
OIP5	6	0.065389	0.1736	0.99998	3387	-0.1956
LTB	6	0.065399	0.17361	0.99998	3388	-0.298
UHMK1	6	0.06545	0.17372	0.99998	3389	0.1091
UROD	6	0.065535	0.17393	0.99998	3390	-0.1641
LOC10028956	6	0.065535	0.17393	0.99998	3391	-0.2366
hsa-mir-3653	4	0.065546	0.14917	0.99851	3392	-0.2633
ANKRD34B	6	0.065564	0.17398	0.99998	3393	-0.1802
ETV3	6	0.065593	0.17405	0.99998	3394	-0.2204
TCF25	6	0.065598	0.17407	0.99998	3395	0.0943
SPIN1	6	0.065598	0.17407	0.99998	3396	-0.1651
CNOT7	6	0.065636	0.17415	0.99998	3397	-0.1745
hsa-mir-6742	4	0.065677	0.14942	0.99851	3398	-0.2895
ATG4D	6	0.065679	0.17425	0.99998	3399	-0.1767

NPAT	6	0.065732	0.17437	0.99998	3400	-0.2763
CNOT8	6	0.065752	0.17441	0.99998	3401	-0.2161
QARS	6	0.065789	0.17448	0.99998	3402	-0.1046
OR10R2	6	0.065801	0.1745	0.99998	3403	-0.1611
LRRC30	6	0.065809	0.17452	0.99998	3404	0.0177
TCP1	6	0.065819	0.17455	0.99998	3405	-0.351
ERMP1	6	0.065824	0.17456	0.99998	3406	-0.0572
PIGU	6	0.065841	0.1746	0.99998	3407	-0.2117
TSPY2	2	0.065841	0.1152	0.96123	3408	-0.0453
BBS2	6	0.065897	0.17472	0.99998	3409	-0.1617
VAMP4	6	0.065908	0.17474	0.99998	3410	0.0877
ABHD15	6	0.065908	0.17474	0.99998	3411	-0.1169
DDX4	6	0.065908	0.17474	0.99998	3412	-0.2272
CFDP1	6	0.065979	0.17489	0.99998	3413	-0.0399
RBM22	6	0.065992	0.17492	0.99998	3414	-0.263
OR51I2	6	0.06607	0.1751	0.99998	3415	-0.1704
TYROBP	6	0.066113	0.17519	0.99998	3416	-0.1871
BAK1	6	0.066113	0.17519	0.99998	3417	-0.069
GTF2F2	6	0.066115	0.17519	0.99998	3418	-0.2482
EDC3	6	0.066251	0.1755	0.99998	3419	-0.236
SNED1	6	0.066251	0.1755	0.99998	3420	-0.2533
UBA1	6	0.066316	0.17565	0.99998	3421	-0.0881
hsa-mir-767	4	0.066342	0.15068	0.99998	3422	-0.2526
hsa-mir-8082	4	0.066342	0.15068	0.99998	3423	-0.2708
RBM24	6	0.066394	0.17581	0.99998	3424	0.0802
XRN1	6	0.066476	0.17598	0.99998	3425	-0.2929
OR3A2	6	0.066476	0.17598	0.99998	3426	-0.238
PIH1D3	6	0.066486	0.176	0.99998	3427	-0.2849
PPIB	6	0.066508	0.17604	0.99998	3428	0.0282
PPP1R1B	6	0.066528	0.17609	0.99998	3429	-0.1412
DZIP1L	6	0.066546	0.17613	0.99998	3430	-0.1702
CALML3	6	0.066579	0.1762	0.99998	3431	-0.2217
TMTC1	6	0.066632	0.17632	0.99998	3432	-0.2558
RND2	6	0.066656	0.17636	0.99998	3433	-0.0782
IL1RL1	6	0.066751	0.17655	0.99998	3434	-0.2273
ROR2	6	0.066751	0.17655	0.99998	3435	-0.046
HIST1H1D	6	0.066789	0.17665	0.99998	3436	-0.3933
WDR33	6	0.066803	0.17668	0.99998	3437	-0.2859
TTC18	6	0.066828	0.17673	0.99998	3438	0.172
SP9	6	0.066828	0.17673	0.99998	3439	0.0117
KRTAP4-3	6	0.066851	0.17678	0.99998	3440	-0.1617
SHROOM4	6	0.066859	0.1768	0.99998	3441	-0.0084
KCNA2	6	0.066879	0.17684	0.99998	3442	-0.1998
ZNF57	6	0.066879	0.17684	0.99998	3443	-0.5421
CLCA4	6	0.066888	0.17686	0.99998	3444	-0.1633
ACOT7	6	0.066902	0.17689	0.99998	3445	-0.0328
GIGYF2	6	0.066902	0.17689	0.99998	3446	0.0876
UBQLN1	6	0.066925	0.17694	0.99998	3447	-0.1649
JTB	6	0.066926	0.17694	0.99998	3448	-0.2385
CAMKK1	6	0.066926	0.17694	0.99998	3449	-0.2332
SNX21	6	0.066938	0.17696	0.99998	3450	-0.2042
LRRC72	6	0.066951	0.17699	0.99998	3451	0.0543
GNG13	6	0.067	0.17711	0.99998	3452	-0.0433
AMHR2	6	0.067049	0.17721	0.99998	3453	-0.149
PPP2CB	6	0.067055	0.17722	0.99998	3454	-0.1144
OR56B4	6	0.067112	0.17736	0.99998	3455	-0.0683
SH2B1	6	0.067123	0.17739	0.99998	3456	-0.2885
HK3	6	0.067126	0.17739	0.99998	3457	-0.1847
BTF3L4	6	0.067136	0.17742	0.99998	3458	-0.0384
ETV2	6	0.067183	0.17751	0.99998	3459	-0.2248
TOLLIP	6	0.067197	0.17754	0.99998	3460	-0.2901
C16orf93	6	0.06723	0.17762	0.99998	3461	-0.1667
hsa-mir-431	4	0.067233	0.15243	0.99998	3462	-0.191
RECQL	6	0.067246	0.17766	0.99998	3463	0.0261
KRTAP2-2	1	0.067262	0.067338	0.86964	3464	-0.8547
THOP1	6	0.067295	0.17777	0.99998	3465	0.2483
FAM57B	6	0.06733	0.17784	0.99998	3466	-0.1825
APOC1	6	0.067369	0.17793	0.99998	3467	0.2326
AFAP1	6	0.067369	0.17794	0.99998	3468	-0.2041
ASF1A	6	0.067391	0.17798	0.99998	3469	-0.2342
MRPL12	6	0.067393	0.17799	0.99998	3470	-0.3167
OVCA2	6	0.067487	0.17819	0.99998	3471	-0.2902
ZZZ3	6	0.06749	0.17819	0.99998	3472	-0.225
ATAT1	6	0.067514	0.17824	0.99998	3473	-0.1895
PWP2	6	0.067541	0.17829	0.99998	3474	-0.2488
MUC5B	6	0.067541	0.17829	0.99998	3475	-0.1555
XIRP2	6	0.067541	0.17829	0.99998	3476	0.1067
NOS2	6	0.067566	0.17835	0.99998	3477	-0.2166
EMID1	6	0.067585	0.1784	0.99998	3478	-0.2274
ZBTB12	6	0.067639	0.1785	0.99998	3479	-0.0965
BCKDHB	6	0.067654	0.17853	0.99998	3480	-0.2495

MRPL47	6	0.067662	0.17855	0.99998	3481	-0.1955
TSR3	6	0.067702	0.17864	0.99998	3482	-0.2447
hsa-mir-3125	4	0.067734	0.1534	0.99998	3483	-0.2382
ASB17	6	0.067746	0.17873	0.99998	3484	-0.2837
HAT1	6	0.067746	0.17873	0.99998	3485	-0.2778
SSB	6	0.067773	0.1788	0.99998	3486	-0.1896
APPBP2	6	0.067773	0.1788	0.99998	3487	-0.2295
DLX6	6	0.067782	0.17882	0.99998	3488	-0.1825
LEO1	6	0.067782	0.17882	0.99998	3489	-0.0131
SH3BP2	6	0.067836	0.17895	0.99998	3490	-0.0815
EMC7	6	0.067836	0.17895	0.99998	3491	0.0565
PSPN	6	0.067836	0.17895	0.99998	3492	0.3928
HMG20A	6	0.067925	0.17913	0.99998	3493	-0.0594
CBR3	6	0.067996	0.17927	0.99998	3494	-0.2071
KIAA0101	6	0.068023	0.17933	0.99998	3495	-0.2081
TMEM114	6	0.068032	0.17935	0.99998	3496	0.1313
REEP5	6	0.068106	0.1795	0.99998	3497	-0.1927
B4GALT3	6	0.068116	0.17953	0.99998	3498	0.0261
hsa-mir-4747	4	0.068126	0.15416	0.99998	3499	-0.3565
SOWAHC	6	0.06818	0.17968	0.99998	3500	-0.104
SKA1	6	0.06818	0.17968	0.99998	3501	-0.19
CCDC12	6	0.068278	0.17988	0.99998	3502	-0.2265
SERPINA11	6	0.068363	0.18007	0.99998	3503	-0.1773
TMEM184C	6	0.068376	0.18009	0.99998	3504	-0.2685
RGL2	6	0.0684	0.18014	0.99998	3505	-0.2241
CACNA2D4	6	0.068415	0.18017	0.99998	3506	-0.2378
RPL37	6	0.06844	0.18023	0.99998	3507	-0.3142
KIAA1107	6	0.068444	0.18023	0.99998	3508	-0.1582
hsa-mir-496	4	0.068449	0.15479	0.99998	3509	0.2042
HIST1H2BN	6	0.068474	0.18029	0.99998	3510	0.0431
LETM2	6	0.068491	0.18033	0.99998	3511	-0.1818
GUSB	6	0.068508	0.18037	0.99998	3512	-0.1832
HFE	6	0.068522	0.1804	0.99998	3513	-0.0184
SPECC1	6	0.068537	0.18043	0.99998	3514	-0.0631
hsa-mir-4268	4	0.068555	0.15499	0.99998	3515	-0.163
SERPINB5	6	0.068597	0.18057	0.99998	3516	-0.2038
hsa-mir-5683	2	0.068609	0.11972	0.96915	3517	-0.3856
LSMEM2	6	0.0687	0.18079	0.99998	3518	-0.1427
DSP	6	0.068722	0.18084	0.99998	3519	-0.0934
FANCL	6	0.068744	0.18088	0.99998	3520	0.0405
ADIPOR2	6	0.068751	0.1809	0.99998	3521	-0.0825
LSR	6	0.06883	0.18106	0.99998	3522	-0.1939
RBM11	4	0.068835	0.15556	0.99998	3523	-0.2421
SFTPC	4	0.068848	0.15558	0.99998	3524	-0.2815
MAPK14	6	0.068898	0.18122	0.99998	3525	-0.2025
CPZ	6	0.068915	0.18125	0.99998	3526	0.1029
MYO7B	6	0.068916	0.18125	0.99998	3527	0.0048
NDST2	6	0.068944	0.18131	0.99998	3528	0.0245
CDC6	6	0.068965	0.18136	0.99998	3529	-0.014
ZNF816-ZNF3;	2	0.068969	0.12032	0.96994	3530	-0.2012
SLC7A1	6	0.068969	0.18137	0.99998	3531	-0.3002
PRKRIP1	6	0.068969	0.18137	0.99998	3532	-0.3596
hsa-mir-4674	4	0.069013	0.15589	0.99998	3533	-0.0659
CPNE9	6	0.069051	0.18155	0.99998	3534	-0.1675
HES2	6	0.06908	0.18161	0.99998	3535	0.0005
C2orf61	6	0.06908	0.18161	0.99998	3536	-0.1743
SRI	6	0.069102	0.18165	0.99998	3537	-0.2148
EFTUD2	6	0.069112	0.18168	0.99998	3538	-0.2754
CCDC62	6	0.06913	0.18172	0.99998	3539	-0.2689
RPS15A	6	0.069195	0.18185	0.99998	3540	-0.147
ZSCAN23	6	0.069225	0.18192	0.99998	3541	-0.2496
SLC1A5	6	0.069225	0.18192	0.99998	3542	-0.269
EYA4	6	0.069231	0.18193	0.99998	3543	-0.1862
TMEM259	6	0.069284	0.18204	0.99998	3544	-0.0378
DCTN2	6	0.069284	0.18204	0.99998	3545	-0.0018
PPP1CB	6	0.069309	0.1821	0.99998	3546	-0.0545
GP1BB	6	0.069364	0.18222	0.99998	3547	-0.3215
TPX2	6	0.069381	0.18225	0.99998	3548	0.0041
CNNM2	6	0.069457	0.18241	0.99998	3549	-0.1815
CCDC40	6	0.06947	0.18244	0.99998	3550	-0.0927
MEF2C	6	0.069504	0.18251	0.99998	3551	-0.4051
DHX8	6	0.069504	0.18251	0.99998	3552	-0.4184
TK1	6	0.06951	0.18253	0.99998	3553	-0.0016
KLHL7	6	0.069603	0.18272	0.99998	3554	0.0585
POU4F1	6	0.069604	0.18272	0.99998	3555	-0.1709
PSORS1C1	6	0.06961	0.18273	0.99998	3556	-0.3207
TSPAN11	6	0.06961	0.18273	0.99998	3557	-0.383
OR2D3	6	0.06961	0.18273	0.99998	3558	-0.255
LOC401052	6	0.069661	0.18284	0.99998	3559	-0.2263
C4orf29	6	0.069665	0.18285	0.99998	3560	-0.1531
ISCA1	6	0.069677	0.18287	0.99998	3561	0.0086

HIST1H2AJ	3	0.069738	0.13298	0.98972	3562	-0.2077
PTGDR2	6	0.06975	0.18302	0.99998	3563	0.1207
POMC	6	0.069764	0.18306	0.99998	3564	-0.1939
ACR	6	0.069764	0.18306	0.99998	3565	-0.2482
hsa-mir-4744	4	0.069832	0.15748	0.99998	3566	-0.236
KLHL41	6	0.069848	0.18324	0.99998	3567	0.1234
MRPS33	6	0.069876	0.18329	0.99998	3568	0.0185
hsa-mir-4803	4	0.069885	0.15759	0.99998	3569	-0.1993
SRD5A3	6	0.069888	0.18332	0.99998	3570	-0.2302
BRAT1	6	0.069922	0.18339	0.99998	3571	-0.1663
LRP5	6	0.069942	0.18344	0.99998	3572	-0.1696
LCOR	6	0.069943	0.18344	0.99998	3573	-0.2191
MYEOV2	6	0.069943	0.18344	0.99998	3574	-0.2498
LEF1	6	0.069943	0.18344	0.99998	3575	-0.2137
RIT1	6	0.069963	0.18348	0.99998	3576	-0.1168
hsa-mir-4688	4	0.069979	0.15779	0.99998	3577	0.0256
DTX3	6	0.069984	0.18354	0.99998	3578	-0.0533
SLC25A31	6	0.069995	0.18356	0.99998	3579	-0.1339
hsa-mir-4475	4	0.070098	0.15802	0.99998	3580	-0.2087
TPM3	6	0.070099	0.18379	0.99998	3581	-0.1715
G6PC	6	0.070118	0.18383	0.99998	3582	-0.0711
PTH	6	0.070125	0.18385	0.99998	3583	-0.1322
TTYH1	6	0.070183	0.18397	0.99998	3584	-0.1512
PTCH1	6	0.070193	0.184	0.99998	3585	-0.0524
TDO2	6	0.070232	0.18408	0.99998	3586	-0.2105
NR1H2	6	0.070255	0.18413	0.99998	3587	-0.1888
VMA21	6	0.070265	0.18415	0.99998	3588	0.0475
DLD	6	0.07031	0.18426	0.99998	3589	-0.1822
ALG14	6	0.070323	0.18428	0.99998	3590	-0.2021
PCSK5	6	0.070323	0.18428	0.99998	3591	0.125
PAK4	6	0.070363	0.18437	0.99998	3592	0.0123
FARS2	6	0.070375	0.18439	0.99998	3593	-0.3073
CISD3	6	0.070417	0.18449	0.99998	3594	-0.2223
KCNC3	6	0.070476	0.18462	0.99998	3595	-0.1612
SPATA17	6	0.070476	0.18462	0.99998	3596	-0.1535
HEATR1	6	0.070486	0.18465	0.99998	3597	-0.2412
NAIP	6	0.07051	0.1847	0.99998	3598	-0.0381
GBAS	6	0.07051	0.1847	0.99998	3599	-0.0527
STK4	6	0.070531	0.18475	0.99998	3600	-0.1731
COPA	6	0.070532	0.18475	0.99998	3601	-0.4915
GJD4	6	0.070582	0.18486	0.99998	3602	-0.2438
C17orf70	6	0.070597	0.18489	0.99998	3603	-0.1621
CSDE1	6	0.070608	0.18492	0.99998	3604	-0.1429
ZNF446	6	0.070691	0.1851	0.99998	3605	-0.3
MCF2L2	6	0.070715	0.18515	0.99998	3606	-0.1326
GCK	6	0.07078	0.18529	0.99998	3607	-0.0188
FAT4	6	0.070785	0.1853	0.99998	3608	0.2679
GPBP1	6	0.070824	0.18537	0.99998	3609	-0.1831
LAP3	6	0.070836	0.18539	0.99998	3610	-0.2904
CCHCR1	6	0.070853	0.18543	0.99998	3611	-0.1541
CLPP	6	0.070886	0.1855	0.99998	3612	-0.2654
TTN	6	0.070886	0.1855	0.99998	3613	-0.2865
hsa-mir-663b	3	0.070917	0.13465	0.99209	3614	-0.704
CCL14	6	0.07098	0.18571	0.99998	3615	-0.1057
CRY2	6	0.071049	0.18586	0.99998	3616	-0.0528
CD58	6	0.071049	0.18586	0.99998	3617	-0.0197
WBSCR22	6	0.071059	0.18588	0.99998	3618	-0.1825
PHKB	6	0.071123	0.18603	0.99998	3619	0.0095
HRCT1	4	0.071196	0.16013	0.99998	3620	0.1105
ASXL1	6	0.071245	0.1863	0.99998	3621	0.0178
ZNF585B	6	0.071245	0.1863	0.99998	3622	0.5272
ZNF608	6	0.071257	0.18632	0.99998	3623	-0.3324
TFF1	6	0.071308	0.18643	0.99998	3624	-0.1807
RABL2B	4	0.071341	0.16043	0.99998	3625	-0.2061
LUZP1	6	0.071342	0.1865	0.99998	3626	-0.1601
G3BP1	6	0.071342	0.1865	0.99998	3627	0.0705
MRPS2	6	0.071343	0.1865	0.99998	3628	-0.0194
hsa-mir-6778	4	0.071365	0.16047	0.99998	3629	-0.2264
PEAK1	6	0.071417	0.18666	0.99998	3630	0.3174
TLL2	6	0.071452	0.18672	0.99998	3631	-0.1935
SPATS1	6	0.071452	0.18672	0.99998	3632	-0.1941
CDK1	6	0.071472	0.18677	0.99998	3633	-0.2255
TEX33	6	0.071504	0.18683	0.99998	3634	-0.1918
ARRB2	6	0.071507	0.18683	0.99998	3635	-0.1781
hsa-mir-4458	4	0.071623	0.16097	0.99998	3636	-0.3388
CRNKL1	6	0.071662	0.18717	0.99998	3637	-0.0616
SUCLG1	6	0.071702	0.18726	0.99998	3638	-0.2362
C12orf44	6	0.071737	0.18733	0.99998	3639	-0.2049
NPRL3	6	0.07176	0.18738	0.99998	3640	-0.0283
C9orf41	6	0.071762	0.18739	0.99998	3641	-0.2277
EXOC3L1	6	0.071792	0.18745	0.99998	3642	0.0039

GFI1	6	0.071793	0.18745	0.99998	3643	-0.1834
HIST1H2BJ	6	0.071859	0.1876	0.99998	3644	-0.5209
ESR2	6	0.071882	0.18765	0.99998	3645	-0.002
RPL13	6	0.071939	0.18779	0.99998	3646	-0.3126
HYAL3	6	0.071986	0.18789	0.99998	3647	-0.121
CHCHD6	6	0.071995	0.18791	0.99998	3648	0.0969
BTBD11	6	0.072029	0.18798	0.99998	3649	-0.0742
SLC35E1	6	0.072034	0.18799	0.99998	3650	-0.2084
LCNL1	6	0.072094	0.18813	0.99998	3651	-0.2008
AGRP	6	0.0721	0.18814	0.99998	3652	-0.2122
SAP30BP	6	0.072102	0.18814	0.99998	3653	-0.1781
C10orf128	6	0.072104	0.18815	0.99998	3654	-0.0088
PRDM14	6	0.072206	0.18836	0.99998	3655	-0.0988
IL13RA2	6	0.07224	0.18844	0.99998	3656	-0.2406
RHEB	6	0.072275	0.18851	0.99998	3657	-0.2013
ESPN	6	0.072298	0.18857	0.99998	3658	-0.1093
OR4B1	6	0.072321	0.18861	0.99998	3659	-0.2968
HAVCR1	6	0.072363	0.1887	0.99998	3660	-0.1604
SPDL1	6	0.072366	0.1887	0.99998	3661	-0.2547
ZNF335	6	0.07242	0.18881	0.99998	3662	-0.0329
CALM2	6	0.072462	0.1889	0.99998	3663	-0.3647
PRLHR	6	0.072494	0.18897	0.99998	3664	-0.1914
FKBPL	6	0.072521	0.18903	0.99998	3665	-0.1147
CHTOP	6	0.072592	0.18917	0.99998	3666	-0.1341
hsa-mir-6744	4	0.072652	0.16298	0.99998	3667	-0.2082
GLI2	6	0.072703	0.18941	0.99998	3668	-0.1778
RDH8	6	0.072738	0.18948	0.99998	3669	-0.1935
MAGI2	6	0.072738	0.18948	0.99998	3670	-0.1852
RBM34	6	0.072738	0.18948	0.99998	3671	-0.2119
hsa-mir-1302-	3	0.072751	0.13724	0.99397	3672	-0.4026
FDXR	6	0.072753	0.18952	0.99998	3673	-0.1553
hsa-mir-6131	4	0.072764	0.16319	0.99998	3674	-0.2131
PRELP	6	0.072787	0.18958	0.99998	3675	0.0655
LOC10013035	6	0.072797	0.1896	0.99998	3676	0.0108
ZC3H13	6	0.072821	0.18965	0.99998	3677	-0.2394
OR51Q1	6	0.072879	0.18977	0.99998	3678	-0.2837
PDRG1	6	0.072879	0.18977	0.99998	3679	-0.2722
CTRL	6	0.072885	0.18979	0.99998	3680	-0.1299
GAS6	6	0.072912	0.18983	0.99998	3681	-0.1328
PCNXL3	6	0.072932	0.18988	0.99998	3682	-0.2215
RBM14-RBM4	1	0.072958	0.073005	0.88424	3683	-0.7081
hsa-mir-3163	4	0.07296	0.16357	0.99998	3684	-0.239
PTPRT	6	0.072979	0.18999	0.99998	3685	-0.0527
PLEK	6	0.072979	0.18999	0.99998	3686	-0.167
RNF215	6	0.072999	0.19003	0.99998	3687	-0.0635
ZNF205	6	0.072999	0.19003	0.99998	3688	-0.1801
AP1M1	6	0.073052	0.19015	0.99998	3689	0.2524
SYNGAP1	6	0.073062	0.19017	0.99998	3690	-0.1846
FOLR1	6	0.073103	0.19027	0.99998	3691	-0.0812
IGSF21	6	0.073103	0.19027	0.99998	3692	0.0525
CISD1	6	0.073179	0.19044	0.99998	3693	-0.1685
CEP95	6	0.073235	0.19057	0.99998	3694	-0.2018
ZNF155	6	0.073276	0.19064	0.99998	3695	0.1483
SF3A2	6	0.073277	0.19064	0.99998	3696	-0.1075
ST3GAL3	6	0.073325	0.19075	0.99998	3697	0.1482
MRPS24	5	0.073412	0.17164	0.99998	3698	-0.3475
IKZF4	6	0.073422	0.19095	0.99998	3699	-0.2078
EXT1	6	0.073423	0.19095	0.99998	3700	-0.1624
USP17L15	6	0.073521	0.19117	0.99998	3701	0.16
LYPD6	6	0.073521	0.19117	0.99998	3702	-0.1975
ZXDC	6	0.073524	0.19117	0.99998	3703	-0.1095
ACVRL1	6	0.073543	0.19122	0.99998	3704	-0.2069
RSAD1	6	0.07357	0.19128	0.99998	3705	-0.008
CCDC73	5	0.073579	0.17194	0.99998	3706	-0.2249
OR10Z1	6	0.073594	0.19132	0.99998	3707	0.0571
DLG1	6	0.073653	0.19145	0.99998	3708	-0.2631
MLC1	6	0.073667	0.19149	0.99998	3709	-0.1099
hsa-mir-1254-	4	0.073693	0.16496	0.99998	3710	-0.5096
DBI	6	0.073703	0.19156	0.99998	3711	-0.3384
IGFL2	6	0.073711	0.19158	0.99998	3712	0.1376
hsa-mir-4523	4	0.073714	0.16499	0.99998	3713	-0.3963
BVES	6	0.073771	0.19171	0.99998	3714	-0.2775
CPLX1	6	0.073773	0.19171	0.99998	3715	-0.2058
NAGK	6	0.07379	0.19175	0.99998	3716	0.1022
GSK3A	6	0.073799	0.19177	0.99998	3717	0.035
MRPL19	6	0.073799	0.19177	0.99998	3718	0.0288
MMP14	6	0.073883	0.19195	0.99998	3719	-0.1752
MAMDC2	6	0.073883	0.19195	0.99998	3720	-0.1923
CTNND1	6	0.073912	0.19201	0.99998	3721	-0.1446
LNP1	6	0.073912	0.19201	0.99998	3722	-0.0424
AKIRIN1	6	0.073951	0.19209	0.99998	3723	-0.2872

TUBE1	6	0.073954	0.1921	0.99998	3724	-0.045
CYB5D2	6	0.074	0.19221	0.99998	3725	-0.2269
hsa-mir-5692c	2	0.074093	0.12859	0.98168	3726	-0.6291
C15orf32	6	0.074093	0.19241	0.99998	3727	-0.1886
PITPNA	6	0.074093	0.19241	0.99998	3728	-0.244
DHFR	6	0.07413	0.1925	0.99998	3729	-0.2872
DNASE1L1	6	0.074148	0.19253	0.99998	3730	-0.2113
DHX9	6	0.074171	0.19258	0.99998	3731	-0.2075
SLC35A2	6	0.07418	0.1926	0.99998	3732	-0.2209
PSAP	6	0.074181	0.1926	0.99998	3733	0.0733
SDCCAG8	6	0.074225	0.1927	0.99998	3734	-0.2373
MRPS22	6	0.074225	0.1927	0.99998	3735	-0.1288
NINL	6	0.074225	0.1927	0.99998	3736	0.0958
NANOS1	6	0.074226	0.1927	0.99998	3737	-0.1597
LRIT2	6	0.074309	0.19287	0.99998	3738	-0.2457
PRSS12	6	0.074446	0.19316	0.99998	3739	-0.1097
ARG2	6	0.074446	0.19316	0.99998	3740	-0.0908
FBXO4	6	0.074456	0.19318	0.99998	3741	-0.2004
RERG	6	0.074495	0.19327	0.99998	3742	-0.2028
TRIM31	6	0.074502	0.19328	0.99998	3743	-0.1885
CCDC102A	6	0.074513	0.19331	0.99998	3744	-0.1952
CD8A	6	0.074539	0.19337	0.99998	3745	-0.1075
PPM1L	6	0.074571	0.19344	0.99998	3746	-0.0332
NFAM1	6	0.074571	0.19344	0.99998	3747	-0.1634
LRTM2	6	0.074594	0.19349	0.99998	3748	-0.2414
IFT52	6	0.074594	0.19349	0.99998	3749	-0.1711
ELOVL2	6	0.074594	0.19349	0.99998	3750	-0.2031
TEC	6	0.074601	0.1935	0.99998	3751	-0.2522
HIST1H4E	6	0.074671	0.19365	0.99998	3752	-0.507
PHAX	6	0.074697	0.1937	0.99998	3753	-0.0696
CERCAM	6	0.074735	0.19379	0.99998	3754	-0.2877
MIOS	6	0.07483	0.19399	0.99998	3755	-0.2017
OPALIN	6	0.074832	0.194	0.99998	3756	-0.1748
SAPCD2	6	0.074837	0.19401	0.99998	3757	-0.1708
COX6A2	6	0.07484	0.19401	0.99998	3758	-0.0149
SRSF4	6	0.074913	0.19417	0.99998	3759	0.2874
TUBB3	6	0.074932	0.19422	0.99998	3760	-0.4617
hsa-mir-5002	4	0.074954	0.16734	0.99998	3761	-0.3029
SNIP1	6	0.074968	0.1943	0.99998	3762	-0.1745
RFC2	6	0.075011	0.19438	0.99998	3763	-0.0007
ITPRIPL1	6	0.075029	0.19441	0.99998	3764	-0.2321
DPYS	6	0.075107	0.19459	0.99998	3765	-0.1929
FOLR2	6	0.075117	0.1946	0.99998	3766	-0.1449
RPS6	6	0.075133	0.19463	0.99998	3767	0.0176
CACNA2D2	6	0.07519	0.19477	0.99998	3768	-0.2065
SNAPC2	6	0.07519	0.19477	0.99998	3769	-0.1934
SLC7A9	6	0.07519	0.19477	0.99998	3770	-0.4569
INTS4	6	0.07519	0.19477	0.99998	3771	-0.2149
PAM	6	0.075273	0.19495	0.99998	3772	-0.3073
TMEM232	6	0.075304	0.19502	0.99998	3773	0.0784
GHRL	6	0.075304	0.19502	0.99998	3774	0.0264
hsa-mir-6777	4	0.075362	0.16814	0.99998	3775	-0.0605
FES	6	0.075371	0.19517	0.99998	3776	-0.3059
ST20	4	0.075411	0.16825	0.99998	3777	-0.3395
GATAD2B	6	0.075438	0.19532	0.99998	3778	-0.3512
IRGQ	6	0.075438	0.19532	0.99998	3779	-0.2279
hsa-mir-7150	4	0.075441	0.16831	0.99998	3780	-0.0688
PAH	6	0.075441	0.19532	0.99998	3781	-0.1618
IL20RB	6	0.075497	0.19544	0.99998	3782	-0.2166
C16orf86	6	0.075528	0.19551	0.99998	3783	-0.2056
POLR2F	6	0.075608	0.19568	0.99998	3784	-0.0865
KRT10	6	0.075611	0.19569	0.99998	3785	-0.2409
NHSL1	6	0.075611	0.19569	0.99998	3786	-0.2606
PNN	6	0.075621	0.19572	0.99998	3787	0.2528
DTNA	6	0.07567	0.19583	0.99998	3788	0.2223
INSC	6	0.075676	0.19584	0.99998	3789	-0.1744
KLRC1	6	0.075676	0.19584	0.99998	3790	-0.1789
hsa-mir-1827	4	0.075698	0.16883	0.99998	3791	-0.3268
U2SURP	6	0.075718	0.19594	0.99998	3792	-0.101
C1orf52	6	0.075739	0.19599	0.99998	3793	0.0074
SEPHS1	6	0.075816	0.19615	0.99998	3794	0.0228
hsa-mir-5006	4	0.07582	0.16907	0.99998	3795	-0.1703
ECI2	6	0.075889	0.1963	0.99998	3796	0.1668
PLEKHM1	6	0.075892	0.19631	0.99998	3797	-0.1606
CXCL6	6	0.07592	0.19637	0.99998	3798	-0.2486
HIST1H3A	6	0.075957	0.19643	0.99998	3799	-0.2116
MYO1F	6	0.075976	0.19647	0.99998	3800	-0.1467
hsa-mir-6839	4	0.07601	0.16941	0.99998	3801	-0.0065
DDX47	6	0.076011	0.19656	0.99998	3802	-0.1164
RNASEK	6	0.076011	0.19656	0.99998	3803	-0.1627
MS4A3	6	0.076011	0.19656	0.99998	3804	-0.0697

PPP1R2	6	0.076041	0.19662	0.99998	3805	-0.214
CLEC18C	2	0.076084	0.13183	0.98951	3806	-0.2513
OR5V1	6	0.076095	0.19674	0.99998	3807	0.0294
CYS1	6	0.076109	0.19676	0.99998	3808	-0.0765
ZFHX3	6	0.076139	0.19683	0.99998	3809	-0.0814
MTHFS	2	0.076144	0.13192	0.98951	3810	0.2629
LNPEP	6	0.076185	0.19692	0.99998	3811	-0.2422
FABP6	6	0.076206	0.19697	0.99998	3812	0.2003
TTC31	6	0.076241	0.19703	0.99998	3813	-0.1561
EPHA2	6	0.076391	0.19737	0.99998	3814	-0.2666
PHKG1	6	0.076391	0.19737	0.99998	3815	-0.2623
PIGM	6	0.07645	0.1975	0.99998	3816	-0.1144
DMP1	6	0.076471	0.19754	0.99998	3817	-0.2346
SMIM15	6	0.076499	0.19759	0.99998	3818	-0.0872
MAP3K12	6	0.076508	0.1976	0.99998	3819	-0.1665
IGSF5	6	0.076529	0.19765	0.99998	3820	-0.1508
ARID5B	6	0.076548	0.19769	0.99998	3821	-0.0623
TMEM30A	6	0.076548	0.19769	0.99998	3822	0.1548
TMEM177	6	0.076562	0.19772	0.99998	3823	-0.1703
hsa-mir-205	4	0.076589	0.17051	0.99998	3824	-0.0451
MGAT1	6	0.076613	0.19783	0.99998	3825	-0.2164
MGEA5	6	0.076652	0.19791	0.99998	3826	0.0889
hsa-mir-3975	4	0.076668	0.17065	0.99998	3827	-0.1883
XPO4	6	0.076694	0.19801	0.99998	3828	0.2699
hsa-mir-6728	4	0.076758	0.17083	0.99998	3829	-0.2788
ARHGAP28	6	0.076771	0.19816	0.99998	3830	0.3748
FBP1	6	0.076846	0.19833	0.99998	3831	-0.2296
RRP8	6	0.076868	0.19837	0.99998	3832	-0.2042
CCNT2	6	0.076868	0.19837	0.99998	3833	-0.2308
GALR1	6	0.076868	0.19837	0.99998	3834	-0.1912
ANKRD26	6	0.076868	0.19837	0.99998	3835	-0.2439
SEMA3B	6	0.076889	0.19841	0.99998	3836	-0.2137
hsa-mir-3127	4	0.076941	0.1712	0.99998	3837	-0.1655
RCN1	6	0.076985	0.19862	0.99998	3838	-0.0521
OR52E4	6	0.077017	0.19869	0.99998	3839	0.3507
EXOC7	6	0.077035	0.19872	0.99998	3840	0.0943
hsa-mir-378b	4	0.077047	0.17141	0.99998	3841	0.0752
LCAT	6	0.077121	0.1989	0.99998	3842	-0.0969
OTOA	6	0.07713	0.19891	0.99998	3843	-0.1278
M1AP	6	0.077206	0.19908	0.99998	3844	-0.1342
hsa-mir-4698	4	0.077265	0.17182	0.99998	3845	-0.3233
FN3K	6	0.077285	0.19924	0.99998	3846	-0.1732
RBM27	6	0.077285	0.19924	0.99998	3847	-0.2881
ADIPOQ	6	0.077371	0.19944	0.99998	3848	-0.1815
hsa-mir-6889	4	0.077407	0.1721	0.99998	3849	-0.1081
hsa-mir-3908	4	0.077418	0.17212	0.99998	3850	-0.4658
hsa-mir-1277	4	0.077418	0.17212	0.99998	3851	-0.4933
TRPV4	6	0.077498	0.1997	0.99998	3852	-0.1624
MUC1	6	0.077565	0.19983	0.99998	3853	-0.2997
SLC6A6	6	0.077565	0.19983	0.99998	3854	-0.2197
UNC80	6	0.077571	0.19984	0.99998	3855	0.145
MRPS6	6	0.077601	0.1999	0.99998	3856	-0.1772
GGTLC2	6	0.07762	0.19994	0.99998	3857	-0.3319
POU3F2	6	0.077622	0.19994	0.99998	3858	-0.347
RAB11FIP1	6	0.077622	0.19994	0.99998	3859	0.0289
hsa-mir-4768	4	0.077799	0.17284	0.99998	3860	0.2177
PM20D1	6	0.077863	0.20046	0.99998	3861	0.0571
SEMA6C	6	0.077869	0.20047	0.99998	3862	-0.098
UBXN6	6	0.077869	0.20047	0.99998	3863	-0.2345
NCBP2	6	0.07791	0.20056	0.99998	3864	-0.3436
hsa-mir-4442	4	0.077924	0.17309	0.99998	3865	-0.2932
VEGFC	6	0.07796	0.20067	0.99998	3866	0.2029
PLAC8L1	6	0.078001	0.20076	0.99998	3867	-0.0755
PER1	6	0.078033	0.20082	0.99998	3868	-0.0016
HSDL2	6	0.078033	0.20082	0.99998	3869	-0.0642
RTN2	6	0.078078	0.20091	0.99998	3870	-0.1831
APP	6	0.07809	0.20093	0.99998	3871	-0.2446
UNC93A	6	0.07809	0.20093	0.99998	3872	-0.1805
KRT86	4	0.078094	0.17343	0.99998	3873	-0.2994
SPRN	6	0.078096	0.20094	0.99998	3874	-0.0863
FBXO43	6	0.078096	0.20094	0.99998	3875	-0.1794
LANCL3	6	0.078131	0.20103	0.99998	3876	0.0543
ADRA1A	6	0.078131	0.20103	0.99998	3877	0.2369
CNTNAP4	6	0.078161	0.20109	0.99998	3878	-0.1389
ABCC5	6	0.078161	0.20109	0.99998	3879	-0.0665
GCLC	6	0.078189	0.20116	0.99998	3880	-0.2141
TM9SF3	6	0.078228	0.20125	0.99998	3881	0.0601
CYP2J2	6	0.078253	0.20129	0.99998	3882	-0.2492
POLRMT	6	0.078273	0.20133	0.99998	3883	0.0397
CREB5	6	0.078301	0.2014	0.99998	3884	0.2106
ENDOG	6	0.078318	0.20142	0.99998	3885	-0.1745

TMEM161A	6	0.078369	0.20154	0.99998	3886	-0.2427
MTERF	6	0.078399	0.2016	0.99998	3887	-0.2003
PHF23	6	0.078403	0.20161	0.99998	3888	-0.1186
RTP4	6	0.078446	0.20171	0.99998	3889	0.0242
KIF22	6	0.078447	0.20171	0.99998	3890	-0.135
CPO	6	0.078496	0.20182	0.99998	3891	0.1553
AAED1	6	0.078573	0.20196	0.99998	3892	0.0516
BEX1	6	0.078602	0.20202	0.99998	3893	-0.1461
USP18	6	0.078602	0.20202	0.99998	3894	-0.2534
ALKBH7	6	0.078602	0.20202	0.99998	3895	-0.1876
SPRED3	6	0.078633	0.20209	0.99998	3896	0.1269
DKKL1	6	0.078642	0.2021	0.99998	3897	-1E-05
SNRPD3	6	0.078679	0.20219	0.99998	3898	-0.5221
C11orf65	6	0.078701	0.20223	0.99998	3899	0.1479
FAM222A	6	0.07873	0.20229	0.99998	3900	-0.2873
hsa-mir-6782	4	0.078771	0.17476	0.99998	3901	-0.2492
MUS81	6	0.078772	0.20238	0.99998	3902	-0.2405
FBXO40	6	0.078776	0.20238	0.99998	3903	-0.0411
WDR31	6	0.078812	0.20246	0.99998	3904	-0.0439
CA2	6	0.078874	0.20258	0.99998	3905	-0.1466
KLK3	6	0.078909	0.20264	0.99998	3906	-0.287
C12orf56	6	0.079001	0.20284	0.99998	3907	-0.2192
PPA2	6	0.079007	0.20285	0.99998	3908	0.0733
NGRN	6	0.079007	0.20285	0.99998	3909	-0.0007
H2AFV	6	0.079009	0.20286	0.99998	3910	0.0622
ACSM2A	5	0.079061	0.18129	0.99998	3911	-0.2272
RASSF1	4	0.079069	0.17534	0.99998	3912	-0.1874
RANGAP1	6	0.079107	0.20307	0.99998	3913	-0.1199
PCSK9	6	0.07912	0.20309	0.99998	3914	-0.1957
SP3	6	0.079157	0.20318	0.99998	3915	-0.3263
PTGER3	6	0.079177	0.20323	0.99998	3916	-0.1337
CMTM4	6	0.079291	0.20345	0.99998	3917	-0.2092
MCM9	6	0.079301	0.20347	0.99998	3918	-0.2068
VDAC3	6	0.079301	0.20347	0.99998	3919	-0.2041
UBE2L6	6	0.079323	0.20352	0.99998	3920	0.1729
C1D	6	0.079323	0.20352	0.99998	3921	0.2788
PPIL1	6	0.079325	0.20352	0.99998	3922	-0.2791
NTM	6	0.079476	0.20383	0.99998	3923	0.0119
PARP9	6	0.079498	0.20388	0.99998	3924	-0.2115
SERPINA10	6	0.079507	0.2039	0.99998	3925	0.147
KRT33B	6	0.079606	0.20411	0.99998	3926	-0.1742
hsa-mir-1224	4	0.079661	0.1765	0.99998	3927	-0.2266
ZWINT	6	0.079663	0.20423	0.99998	3928	-0.1083
GJA10	6	0.079677	0.20426	0.99998	3929	-0.215
HES5	6	0.079687	0.20428	0.99998	3930	-0.1515
PPM1M	6	0.079725	0.20435	0.99998	3931	0.0004
THRAP3	6	0.079729	0.20436	0.99998	3932	-0.3255
OR2A25	6	0.079729	0.20436	0.99998	3933	-0.2286
SPATC1L	6	0.079774	0.20444	0.99998	3934	-0.2132
TAOK3	6	0.079774	0.20444	0.99998	3935	-0.2012
TSC22D3	6	0.07985	0.20461	0.99998	3936	-0.1187
UBR2	6	0.079917	0.20474	0.99998	3937	-0.2789
KRTAP22-2	6	0.079968	0.20485	0.99998	3938	-0.3202
hsa-mir-141	4	0.079971	0.17705	0.99998	3939	-0.1731
EDN2	6	0.079979	0.20488	0.99998	3940	-0.2542
LOC650293	6	0.080005	0.20493	0.99998	3941	-0.2394
PPP1R16B	6	0.080111	0.20515	0.99998	3942	0.0603
ATP1A4	6	0.080111	0.20515	0.99998	3943	-0.2549
BRI3BP	6	0.080118	0.20516	0.99998	3944	-0.1559
CHN1	6	0.080118	0.20516	0.99998	3945	-0.1186
CD59	6	0.080161	0.20524	0.99998	3946	-0.1122
SOX11	6	0.080213	0.20537	0.99998	3947	-0.1705
OR6N2	6	0.080217	0.20537	0.99998	3948	-0.0017
PXT1	6	0.080222	0.20538	0.99998	3949	0.0129
CPPED1	6	0.080225	0.20539	0.99998	3950	-0.1426
RNF115	6	0.080225	0.20539	0.99998	3951	-0.2162
EIF3E	6	0.080225	0.20539	0.99998	3952	-0.2048
SPRED1	6	0.080267	0.20548	0.99998	3953	-0.1977
KLHDC7A	6	0.080285	0.20551	0.99998	3954	-0.0719
ERVMER34-1	6	0.080285	0.20551	0.99998	3955	-0.2004
ERO1LB	6	0.080334	0.20562	0.99998	3956	-0.1756
ATAD2	6	0.080372	0.20569	0.99998	3957	-0.3408
COX5A	6	0.080406	0.20577	0.99998	3958	0.1438
FRAT2	6	0.080417	0.20579	0.99998	3959	0.1188
RICTOR	6	0.080417	0.20579	0.99998	3960	-0.203
ZNF98	5	0.080419	0.18364	0.99998	3961	-1.1932
CILP2	6	0.080496	0.20595	0.99998	3962	-0.2751
LRRC70	6	0.080538	0.20605	0.99998	3963	-0.0789
ZIC2	6	0.080573	0.20612	0.99998	3964	-0.1224
GAMT	6	0.080592	0.20617	0.99998	3965	-0.1908
PCNP	6	0.080631	0.20625	0.99998	3966	-0.1602

ITPK1	6	0.080645	0.20628	0.99998	3967	-0.1806
KIR2DL1	4	0.080669	0.17838	0.99998	3968	-0.6832
ADCY8	6	0.080679	0.20636	0.99998	3969	0.0017
AFTPH	6	0.080679	0.20636	0.99998	3970	-0.0939
MAL	6	0.08069	0.20638	0.99998	3971	-0.2169
OTUB1	6	0.08069	0.20638	0.99998	3972	-0.2063
GSN	6	0.080732	0.20646	0.99998	3973	0.0357
hsa-mir-6882	4	0.080759	0.17856	0.99998	3974	-0.3591
TMEM40	6	0.080781	0.20657	0.99998	3975	-0.0394
BEST1	6	0.080815	0.20663	0.99998	3976	-0.0922
POTED	2	0.080829	0.13945	0.99402	3977	-0.4586
PEX6	6	0.080871	0.20675	0.99998	3978	-0.3651
SF1	6	0.080878	0.20676	0.99998	3979	-0.3192
GATA2	6	0.080919	0.20685	0.99998	3980	-0.181
CDH24	6	0.080919	0.20685	0.99998	3981	-0.2029
PTGER1	6	0.080959	0.20693	0.99998	3982	0.043
hsa-mir-1268a	4	0.080996	0.17902	0.99998	3983	-0.3241
NHLRC2	6	0.081073	0.20719	0.99998	3984	-0.1282
KLHL36	6	0.081126	0.20731	0.99998	3985	-0.2124
LIAS	6	0.081126	0.20731	0.99998	3986	-0.2591
GPHN	6	0.081131	0.20732	0.99998	3987	-0.1078
SLC12A9	6	0.081131	0.20732	0.99998	3988	-0.1003
BIRC7	6	0.081201	0.20748	0.99998	3989	-0.1569
IKBKG	6	0.08124	0.20757	0.99998	3990	-0.0049
UBE2C	6	0.08124	0.20757	0.99998	3991	-0.1456
KRTAP16-1	6	0.081242	0.20757	0.99998	3992	-0.0011
SLC25A19	6	0.081299	0.2077	0.99998	3993	-0.2166
ELSPBP1	6	0.081344	0.20781	0.99998	3994	-0.1348
U2AF1	6	0.081385	0.20789	0.99998	3995	0.1229
SLC26A6	6	0.081388	0.20789	0.99998	3996	-0.1368
MAP2K4	6	0.081431	0.20797	0.99998	3997	-0.1717
FAM71B	6	0.081461	0.20802	0.99998	3998	0.2822
hsa-mir-5787	4	0.081487	0.18001	0.99998	3999	-0.2195
TC2N	6	0.081498	0.20807	0.99998	4000	-0.0954
CLN3	6	0.081509	0.20808	0.99998	4001	0.0952
ABCG5	6	0.081537	0.20812	0.99998	4002	-0.1794
SPATA4	6	0.08159	0.20821	0.99998	4003	-0.0822
hsa-mir-497	4	0.081613	0.18024	0.99998	4004	-0.3692
TFPT	6	0.081624	0.20825	0.99998	4005	-0.1991
PDPR	6	0.081636	0.20827	0.99998	4006	-0.0265
TRIM55	6	0.081677	0.20833	0.99998	4007	-0.1184
MASP1	6	0.081677	0.20833	0.99998	4008	-0.1321
CD96	6	0.081712	0.20839	0.99998	4009	0.0278
CR2	6	0.081712	0.20839	0.99998	4010	-0.1679
hsa-mir-1273f	4	0.081743	0.18048	0.99998	4011	-0.2088
ZBTB26	4	0.081766	0.18052	0.99998	4012	-0.2154
hsa-mir-3182	2	0.081777	0.14098	0.99494	4013	-0.4282
TBX3	6	0.0818	0.20852	0.99998	4014	0.0518
AGMAT	6	0.081804	0.20852	0.99998	4015	-0.0789
hsa-mir-3179-	3	0.081815	0.15	0.99998	4016	-0.2764
DNMT3A	6	0.081873	0.20863	0.99998	4017	-0.2787
GPR68	6	0.081873	0.20863	0.99998	4018	0.4844
NDUFS6	6	0.082006	0.20883	0.99998	4019	-0.2156
PEX3	6	0.082091	0.20896	0.99998	4020	0.0025
FOXC1	6	0.082113	0.209	0.99998	4021	-0.0302
OR8H1	6	0.082116	0.209	0.99998	4022	-0.1305
LOC643669	6	0.082166	0.20909	0.99998	4023	-0.0837
H2BFM	6	0.082261	0.20922	0.99998	4024	-0.0895
BMP5	6	0.082261	0.20922	0.99998	4025	-0.0836
ZBTB21	6	0.082345	0.20935	0.99998	4026	-0.0041
TIRAP	6	0.08238	0.2094	0.99998	4027	-0.1339
CHSY3	6	0.08238	0.2094	0.99998	4028	-0.136
ORMDL2	6	0.08238	0.2094	0.99998	4029	-0.2322
THBS1	6	0.082407	0.20944	0.99998	4030	-0.0699
ITGA1	6	0.08246	0.20951	0.99998	4031	-0.0196
LSAMP	6	0.082528	0.20961	0.99998	4032	0.1019
SYNC	6	0.08257	0.20966	0.99998	4033	-0.2274
FAU	6	0.08257	0.20966	0.99998	4034	-0.2483
REEP6	6	0.082625	0.20973	0.99998	4035	-0.0933
TMEM44	6	0.082659	0.20978	0.99998	4036	-0.1556
TMEM179	6	0.082722	0.20988	0.99998	4037	-0.1061
SPRYD3	6	0.082769	0.20995	0.99998	4038	-0.2132
C3orf36	6	0.08277	0.20995	0.99998	4039	-0.0348
ANGEL1	6	0.082867	0.21009	0.99998	4040	-0.1854
MTERFD2	6	0.082871	0.2101	0.99998	4041	-0.1735
SNRPA	6	0.082873	0.2101	0.99998	4042	-0.102
DUSP15	6	0.083019	0.21031	0.99998	4043	0.0986
HIST1H4B	6	0.083019	0.21031	0.99998	4044	-0.1617
MOB4	2	0.083053	0.14305	0.99705	4045	-0.4394
hsa-mir-4771-	1	0.083087	0.083103	0.9069	4046	-0.4518
XRCC4	6	0.083187	0.21054	0.99998	4047	-0.0437

hsa-mir-6512	4	0.083197	0.18323	0.99998	4048	-0.2495
hsa-mir-924	4	0.083197	0.18323	0.99998	4049	-0.2424
OR5R1	6	0.083207	0.21057	0.99998	4050	0.1499
KCNN2	6	0.083218	0.21059	0.99998	4051	-0.0909
LHCGR	6	0.083267	0.21065	0.99998	4052	-0.2168
FBXO28	6	0.083279	0.21067	0.99998	4053	-0.0906
OR5P3	6	0.083316	0.21072	0.99998	4054	-0.163
KATNB1	6	0.083325	0.21074	0.99998	4055	-0.1736
NLK	6	0.083376	0.2108	0.99998	4056	0.1428
DIO2	6	0.083376	0.2108	0.99998	4057	-0.1062
hsa-mir-4539	3	0.08346	0.15229	0.99998	4058	0.1932
BCAS4	6	0.083471	0.21093	0.99998	4059	0.1937
SRSF11	6	0.083472	0.21093	0.99998	4060	-0.1163
HOXC8	6	0.083473	0.21093	0.99998	4061	-0.1549
HOXC5	6	0.083526	0.21101	0.99998	4062	-0.1837
DHCR24	6	0.08358	0.21108	0.99998	4063	-0.1739
PCYT2	6	0.083601	0.21111	0.99998	4064	0.0555
SIGLEC8	6	0.083618	0.21113	0.99998	4065	-0.0871
EPHX1	6	0.083683	0.21123	0.99998	4066	-0.1965
KIAA1324L	6	0.083766	0.21137	0.99998	4067	-0.1378
HYAL4	6	0.083766	0.21137	0.99998	4068	-0.186
RC3H2	6	0.083766	0.21137	0.99998	4069	-0.1374
hsa-mir-298	4	0.083771	0.18431	0.99998	4070	-0.1217
RMND5A	6	0.08384	0.21148	0.99998	4071	-0.2517
CCNO	6	0.083861	0.21151	0.99998	4072	-0.1014
DNAJC19	6	0.083861	0.21151	0.99998	4073	-0.1707
CNFN	6	0.083898	0.21157	0.99998	4074	-0.2017
COL4A3BP	6	0.083898	0.21157	0.99998	4075	-0.2606
TCEAL1	6	0.083917	0.2116	0.99998	4076	-0.0207
TNFRSF11B	6	0.083917	0.2116	0.99998	4077	0.1632
CNOT6	6	0.083917	0.2116	0.99998	4078	0.1168
REXO2	6	0.083917	0.2116	0.99998	4079	-0.0831
ZNF513	6	0.083982	0.2117	0.99998	4080	-0.148
KCNK5	6	0.08403	0.21178	0.99998	4081	0.0158
ARHGAP4	6	0.08404	0.21178	0.99998	4082	0.1739
DCPS	6	0.08404	0.21178	0.99998	4083	-0.0713
CSNK1G3	6	0.084041	0.21179	0.99998	4084	-0.2084
C5orf47	6	0.084041	0.21179	0.99998	4085	-0.3186
hsa-mir-627	4	0.084096	0.18492	0.99998	4086	-0.3535
CEACAM7	6	0.084132	0.21192	0.99998	4087	-0.0532
hsa-let-7c	4	0.084156	0.18504	0.99998	4088	-0.564
SLC10A7	6	0.084168	0.21197	0.99998	4089	-0.1429
FEN1	6	0.084199	0.21201	0.99998	4090	-0.0323
AGPAT3	6	0.084286	0.21215	0.99998	4091	-0.2417
DDX46	6	0.084309	0.21219	0.99998	4092	-0.2415
SPTBN5	6	0.084402	0.21233	0.99998	4093	0.0269
MBTD1	6	0.084402	0.21233	0.99998	4094	-0.1925
FNTB	6	0.084417	0.21236	0.99998	4095	-0.1273
hsa-mir-25	4	0.08443	0.18555	0.99998	4096	-0.2587
TM6SF2	6	0.084453	0.21241	0.99998	4097	-0.0351
THAP6	6	0.084502	0.21249	0.99998	4098	0.0973
SPHK1	6	0.084514	0.21251	0.99998	4099	-0.2589
RFX5	6	0.084587	0.21261	0.99998	4100	-0.04
MYL9	6	0.084587	0.21261	0.99998	4101	-0.1125
WFDC13	5	0.084651	0.19079	0.99998	4102	-0.1973
TAF8	6	0.084659	0.21272	0.99998	4103	-0.1214
TUBA1C	4	0.084676	0.18605	0.99998	4104	-0.4445
GRIA4	6	0.084708	0.2128	0.99998	4105	0.0474
ATP6V1E2	6	0.084741	0.21283	0.99998	4106	-0.093
hsa-mir-7156	4	0.084806	0.18628	0.99998	4107	-0.1092
OSGIN1	6	0.084826	0.21296	0.99998	4108	-0.2038
ZNF3	6	0.084843	0.21298	0.99998	4109	-0.0594
CCDC147	6	0.084853	0.21299	0.99998	4110	0.2083
ABHD14B	6	0.084927	0.21311	0.99998	4111	-0.1588
CLVS1	6	0.08495	0.21315	0.99998	4112	0.0789
RHOXF2	2	0.085229	0.14649	0.99851	4113	-0.8253
ASCC3	6	0.085288	0.21363	0.99998	4114	0.0329
AMN	6	0.08532	0.21368	0.99998	4115	-0.0668
VN1R4	6	0.085385	0.21377	0.99998	4116	0.2088
SERBP1	6	0.085385	0.21377	0.99998	4117	-0.0044
ZG16	6	0.085441	0.21384	0.99998	4118	-0.1868
PLEKHN1	6	0.085467	0.21389	0.99998	4119	0.0304
TULP1	6	0.085482	0.21391	0.99998	4120	-0.2919
FIGLA	6	0.08553	0.21399	0.99998	4121	-0.1595
SMARCC2	6	0.085565	0.21402	0.99998	4122	-0.2321
NANP	6	0.085651	0.21416	0.99998	4123	-0.1346
PCDHAC2	2	0.085709	0.14725	0.99851	4124	-0.2318
ACTR3C	5	0.085711	0.19261	0.99998	4125	-0.221
PCOLCE2	6	0.085724	0.21427	0.99998	4126	0.0467
hsa-mir-1470	4	0.085725	0.188	0.99998	4127	-0.2207
CRYZ	6	0.085731	0.21428	0.99998	4128	0.1517

KDM6B	6	0.08582	0.21441	0.99998	4129	-0.1526
hsa-mir-3658	4	0.085911	0.18837	0.99998	4130	-0.0631
TPRKB	6	0.085917	0.21454	0.99998	4131	0.0913
XRCC6	6	0.085957	0.21461	0.99998	4132	-0.4319
GPR114	6	0.086014	0.21468	0.99998	4133	-0.0503
IGHMBP2	6	0.086062	0.21476	0.99998	4134	0.0025
PLXNB1	6	0.086072	0.21477	0.99998	4135	0.0406
NLRC4	6	0.086072	0.21477	0.99998	4136	-0.0421
IFI16	6	0.086086	0.21479	0.99998	4137	-0.162
PET117	4	0.086106	0.18872	0.99998	4138	-0.0047
hsa-mir-1913	4	0.086199	0.18889	0.99998	4139	-0.1044
PDGFRA	6	0.08622	0.21499	0.99998	4140	0.0316
ZKSCAN4	6	0.08624	0.21502	0.99998	4141	-0.1277
CWC25	6	0.086304	0.21511	0.99998	4142	-0.1823
MSH4	6	0.086305	0.21511	0.99998	4143	0.202
SCGB1D2	6	0.086305	0.21511	0.99998	4144	-0.3499
TSEN34	6	0.086305	0.21511	0.99998	4145	-0.1912
HOXC4	6	0.086473	0.21533	0.99998	4146	-0.2585
CEP19	6	0.086494	0.21536	0.99998	4147	-0.1997
hsa-mir-130b	4	0.086502	0.18946	0.99998	4148	-0.3009
CDH13	6	0.086569	0.21547	0.99998	4149	0.1257
TRPM1	6	0.086584	0.21548	0.99998	4150	-0.1749
TNFRSF18	6	0.086631	0.21555	0.99998	4151	0.3662
PSD3	6	0.086631	0.21555	0.99998	4152	-0.194
hsa-mir-4323	4	0.086663	0.18975	0.99998	4153	-0.1765
CENPP	6	0.086666	0.21561	0.99998	4154	0.258
hsa-mir-4705	4	0.08672	0.18986	0.99998	4155	-0.2547
hsa-mir-4326	4	0.08686	0.19013	0.99998	4156	-0.2905
CCNL2	6	0.086873	0.2159	0.99998	4157	-0.1038
NANOS3	6	0.086883	0.21591	0.99998	4158	-0.0791
CD40	6	0.086943	0.21599	0.99998	4159	0.1564
ATP1B4	6	0.08698	0.21605	0.99998	4160	0.0085
USP31	6	0.086998	0.21607	0.99998	4161	-0.2541
hsa-mir-638	4	0.087001	0.19039	0.99998	4162	-0.4162
hsa-mir-422a	4	0.087047	0.19048	0.99998	4163	-0.2147
hsa-mir-3137	4	0.087047	0.19048	0.99998	4164	0.2592
DCAF11	6	0.087076	0.21618	0.99998	4165	-0.2545
MED28	6	0.087192	0.21635	0.99998	4166	-0.0973
hsa-mir-3180-	1	0.087198	0.087202	0.91251	4167	-0.6219
OR2T35	5	0.0872	0.19517	0.99998	4168	-0.9148
PPP1R14C	6	0.087221	0.2164	0.99998	4169	0.0425
hsa-mir-6739	4	0.087233	0.19084	0.99998	4170	-0.0656
SMARCD2	6	0.087247	0.21644	0.99998	4171	-0.1002
CAPN2	6	0.08727	0.21647	0.99998	4172	-0.1279
41883	3	0.087313	0.15757	0.99998	4173	-0.1725
ADCY4	6	0.087333	0.21657	0.99998	4174	0.0697
C8orf34	4	0.087403	0.19117	0.99998	4175	0.0283
SIPA1L3	6	0.087511	0.21684	0.99998	4176	-0.0258
C11orf86	6	0.087535	0.21687	0.99998	4177	-0.1647
SLC25A26	6	0.08768	0.21708	0.99998	4178	-0.1386
CYTIP	6	0.087774	0.21721	0.99998	4179	-0.2215
ZCCHC2	6	0.087776	0.21722	0.99998	4180	0.0927
OR2L2	6	0.087885	0.21738	0.99998	4181	-0.4145
INVS	6	0.087885	0.21738	0.99998	4182	-0.1056
DNAL4	6	0.087903	0.21741	0.99998	4183	0.0227
SYNRG	4	0.087917	0.19213	0.99998	4184	-0.2328
RPL18A	6	0.087969	0.21752	0.99998	4185	-0.2807
hsa-mir-5091	4	0.088025	0.19234	0.99998	4186	-0.2897
CXCR3	6	0.088096	0.21772	0.99998	4187	-0.2288
UBE2Q1	6	0.088169	0.21784	0.99998	4188	-0.1043
TTBK1	6	0.088187	0.21786	0.99998	4189	-0.2114
hsa-mir-4522	4	0.088248	0.19273	0.99998	4190	-0.1841
KDM2A	6	0.088267	0.21798	0.99998	4191	-0.1766
NTRK3	6	0.088307	0.21804	0.99998	4192	0.0536
EFCAB6	6	0.088307	0.21804	0.99998	4193	0.2325
CPSF6	6	0.088379	0.21813	0.99998	4194	-0.1237
VPS37C	6	0.088428	0.2182	0.99998	4195	0.0561
AFAP1L1	6	0.088479	0.21826	0.99998	4196	-0.0436
NOB1	6	0.088599	0.21843	0.99998	4197	0.1114
hsa-mir-3909	4	0.088658	0.19353	0.99998	4198	-0.1701
ZNF625	5	0.088667	0.19764	0.99998	4199	-0.248
IFNL3	5	0.088667	0.19764	0.99998	4200	-0.33
AP3B1	6	0.088827	0.21876	0.99998	4201	-0.0996
DNAJA4	6	0.088827	0.21876	0.99998	4202	-0.022
C16orf80	6	0.088827	0.21876	0.99998	4203	0.1397
MMP10	5	0.088946	0.19813	0.99998	4204	-0.3851
SMIM11	6	0.088958	0.21894	0.99998	4205	-0.1298
hsa-mir-4311	4	0.088991	0.19416	0.99998	4206	-0.23
PDGFD	6	0.089006	0.219	0.99998	4207	0.358
PDHA1	6	0.089102	0.21915	0.99998	4208	-0.0062
DMRT3	6	0.089102	0.21915	0.99998	4209	-0.2286

SWT1	5	0.089135	0.19846	0.99998	4210	-0.3534
ZNF302	6	0.089151	0.21921	0.99998	4211	-0.3772
TMEM255B	6	0.089271	0.21939	0.99998	4212	-0.0048
POMP	6	0.089368	0.21954	0.99998	4213	0.0816
FMO3	6	0.0894	0.21959	0.99998	4214	-0.1022
hsa-mir-6757	4	0.089461	0.19506	0.99998	4215	-0.1952
SFXN4	4	0.089479	0.19509	0.99998	4216	-0.1612
STH	6	0.089536	0.21979	0.99998	4217	0.0067
ADC	6	0.089585	0.21985	0.99998	4218	0.1738
MSH2	6	0.089668	0.21997	0.99998	4219	-0.1223
POLR1D	6	0.089705	0.22002	0.99998	4220	-0.1957
SPOCD1	6	0.089774	0.22013	0.99998	4221	-0.2788
SENP6	6	0.089774	0.22013	0.99998	4222	-0.3039
hsa-mir-4712	4	0.089878	0.19579	0.99998	4223	-0.2706
OR6C2	6	0.089944	0.22037	0.99998	4224	-0.102
RAPGEF2	6	0.089944	0.22037	0.99998	4225	-0.3098
PYCR1	6	0.089944	0.22037	0.99998	4226	-0.0474
FAM186A	6	0.089946	0.22038	0.99998	4227	0.1114
RPL41	4	0.089951	0.19593	0.99998	4228	-0.6942
ZBTB8A	6	0.089983	0.22043	0.99998	4229	-0.2043
IKZF5	6	0.089983	0.22043	0.99998	4230	-0.1799
NIPAL3	6	0.090059	0.22053	0.99998	4231	-0.2099
CNKSR3	6	0.090109	0.22061	0.99998	4232	0.1275
SPANXN1	5	0.090129	0.20016	0.99998	4233	-0.3591
hsa-mir-4299	4	0.090129	0.19627	0.99998	4234	-0.1709
TMOD1	6	0.090163	0.22069	0.99998	4235	0.0067
C17orf89	6	0.09018	0.22071	0.99998	4236	-0.0477
NUP160	6	0.090307	0.22091	0.99998	4237	-0.223
ATXN7L3B	6	0.090386	0.22102	0.99998	4238	-0.0773
TUBGCP5	6	0.090403	0.22106	0.99998	4239	-0.1286
PTPRO	6	0.090451	0.22114	0.99998	4240	-0.0504
FAM101A	4	0.090513	0.197	0.99998	4241	-0.2137
RFX2	6	0.090524	0.22126	0.99998	4242	0.0515
PPIP5K1	6	0.090596	0.22137	0.99998	4243	0.2624
BARX1	6	0.090599	0.22137	0.99998	4244	0.3467
PTPRD	6	0.090644	0.22142	0.99998	4245	0.1031
CNTNAP3B	4	0.090708	0.19737	0.99998	4246	-0.2515
CALCOCO1	6	0.090778	0.22162	0.99998	4247	-0.1643
SERP2	6	0.090812	0.22167	0.99998	4248	0.1386
MYH6	6	0.090844	0.22172	0.99998	4249	-0.1765
NFX1	6	0.090947	0.22187	0.99998	4250	0.1396
FAM160A1	6	0.091028	0.22199	0.99998	4251	-0.026
hsa-mir-4739	4	0.09103	0.19798	0.99998	4252	-0.0488
CPVL	6	0.091125	0.22212	0.99998	4253	0.1299
CHMP4B	6	0.091145	0.22215	0.99998	4254	0.1044
KANK4	6	0.091208	0.22224	0.99998	4255	-0.0872
OR2T4	6	0.091208	0.22224	0.99998	4256	0.0237
SLC17A8	6	0.091294	0.22237	0.99998	4257	0.0245
SEC13	6	0.091359	0.22246	0.99998	4258	-0.1206
GABBR1	6	0.09139	0.22251	0.99998	4259	-0.1112
PTEN	6	0.091486	0.22265	0.99998	4260	0.0763
FLVCR1	6	0.091557	0.22275	0.99998	4261	-0.1471
NR1I2	6	0.091702	0.22297	0.99998	4262	-0.1959
CLTCL1	6	0.091763	0.22304	0.99998	4263	-0.1265
PARP2	6	0.091763	0.22304	0.99998	4264	-0.013
HNRNPU	6	0.091838	0.22315	0.99998	4265	-0.3222
OR5P2	6	0.091838	0.22315	0.99998	4266	-0.2798
OR2G6	6	0.091906	0.22324	0.99998	4267	-0.0167
PLSCR5	6	0.09191	0.22324	0.99998	4268	-0.1117
RPS20	6	0.091919	0.22325	0.99998	4269	-0.1838
OAZ3	6	0.091991	0.22336	0.99998	4270	0.0859
hsa-mir-4427	4	0.092027	0.19986	0.99998	4271	-0.264
TPD52	6	0.092039	0.22344	0.99998	4272	0.1113
RIMBP3	6	0.092065	0.22348	0.99998	4273	0.0586
ZNF613	6	0.092065	0.22348	0.99998	4274	-0.4658
CGRRF1	6	0.092111	0.22354	0.99998	4275	0.1067
ABCG4	6	0.092231	0.22372	0.99998	4276	-0.1155
MKL2	6	0.092248	0.22374	0.99998	4277	-0.2025
hsa-mir-6505	4	0.09228	0.20033	0.99998	4278	-0.2507
TRIP10	6	0.092317	0.22385	0.99998	4279	-0.2475
MRPS31	6	0.092379	0.22393	0.99998	4280	-0.2119
ADO	6	0.092431	0.22401	0.99998	4281	0.0254
IFT57	6	0.092568	0.22419	0.99998	4282	-0.2249
YIPF7	6	0.092568	0.22419	0.99998	4283	-0.0336
SSBP4	6	0.092662	0.22434	0.99998	4284	-0.1645
hsa-mir-6756	4	0.092673	0.20106	0.99998	4285	-0.3564
ZNF133	6	0.092704	0.22439	0.99998	4286	-0.018
LSMEM1	6	0.092712	0.2244	0.99998	4287	-0.2156
SCAND3	6	0.092725	0.22443	0.99998	4288	-0.1762
NPC1L1	6	0.092755	0.22448	0.99998	4289	-0.104
SETD9	6	0.092776	0.22451	0.99998	4290	-0.0821

LUZP2	4	0.092791	0.20129	0.99998	4291	0.2025
EFCAB12	6	0.09288	0.22466	0.99998	4292	0.2086
FUT1	6	0.092967	0.22479	0.99998	4293	-0.0758
TMEM229B	6	0.092967	0.22479	0.99998	4294	-0.1535
UNC45B	6	0.092967	0.22479	0.99998	4295	-0.1441
39326	3	0.093012	0.16551	0.99998	4296	-0.1921
MRPL51	6	0.093045	0.2249	0.99998	4297	-0.2425
CHST7	6	0.093048	0.2249	0.99998	4298	0.3772
hsa-mir-1205	4	0.093114	0.20189	0.99998	4299	-0.1511
USF2	6	0.093143	0.22503	0.99998	4300	-0.2121
ARMCX1	6	0.093143	0.22503	0.99998	4301	-0.141
IGJ	6	0.093143	0.22503	0.99998	4302	-0.158
NFYB	6	0.093143	0.22503	0.99998	4303	-0.252
MTRF1	6	0.093216	0.22514	0.99998	4304	-0.2649
MPV17	6	0.093216	0.22514	0.99998	4305	0.0939
hsa-mir-155	4	0.093364	0.20235	0.99998	4306	-0.2985
ASPRV1	6	0.093449	0.22548	0.99998	4307	-0.1836
CDC42BPB	6	0.093659	0.22579	0.99998	4308	-0.192
ERBB3	6	0.093734	0.22589	0.99998	4309	-0.267
NBAS	6	0.093734	0.22589	0.99998	4310	-0.1145
ITSN1	6	0.093803	0.22601	0.99998	4311	-0.1258
KIAA0754	6	0.093899	0.22615	0.99998	4312	-0.1062
TFDP3	6	0.093912	0.22616	0.99998	4313	0.0839
hsa-mir-5700	4	0.093924	0.20337	0.99998	4314	-0.5179
CCT7	6	0.09396	0.22624	0.99998	4315	-0.0564
LBP	6	0.094008	0.2263	0.99998	4316	-0.0981
hsa-mir-1273a	3	0.094012	0.16691	0.99998	4317	0.1932
CORO7-PAM1	6	0.094015	0.22631	0.99998	4318	-0.2457
hsa-mir-5688	4	0.094065	0.20358	0.99998	4319	0.5155
hsa-mir-606	4	0.094121	0.20364	0.99998	4320	-0.1493
DDX52	6	0.094129	0.22649	0.99998	4321	-0.1944
PPP2R5D	6	0.094176	0.22655	0.99998	4322	0.1346
MTFP1	4	0.094319	0.20387	0.99998	4323	-0.2411
MMP17	6	0.094344	0.22679	0.99998	4324	0.0771
S100A11	6	0.094387	0.22686	0.99998	4325	0.0422
NKX2-3	6	0.09439	0.22686	0.99998	4326	-0.1569
STEAP4	6	0.094403	0.22688	0.99998	4327	0.1256
PDXP	6	0.09444	0.22694	0.99998	4328	0.0481
OPLAH	6	0.094488	0.22701	0.99998	4329	-0.087
AP2A1	4	0.094533	0.20411	0.99998	4330	-0.1804
ADAMTS7	6	0.094536	0.22707	0.99998	4331	-0.312
MAP2K6	6	0.094536	0.22707	0.99998	4332	-0.1625
CPEB1	6	0.094536	0.22707	0.99998	4333	-0.3594
OR5H2	6	0.094536	0.22707	0.99998	4334	0.0835
FAH	6	0.094608	0.22719	0.99998	4335	-0.1302
PLTP	6	0.09462	0.2272	0.99998	4336	-0.0764
ADD3	6	0.09468	0.2273	0.99998	4337	0.2855
SPATA31A2	2	0.094685	0.16144	0.99998	4338	-0.1651
ITGA8	6	0.094708	0.22735	0.99998	4339	-0.0232
CEP112	6	0.094718	0.22737	0.99998	4340	-0.2003
BTG3	6	0.094718	0.22737	0.99998	4341	-0.209
FSIP1	6	0.094864	0.22759	0.99998	4342	-0.2149
C14orf119	6	0.094864	0.22759	0.99998	4343	-0.1931
MOCS3	6	0.094871	0.22761	0.99998	4344	0.2586
F5	6	0.094877	0.22762	0.99998	4345	-0.2485
CENPJ	6	0.094965	0.22774	0.99998	4346	-0.1243
ZNF500	6	0.094967	0.22775	0.99998	4347	0.0765
NYX	6	0.095026	0.22784	0.99998	4348	-0.202
ADAMDEC1	6	0.095111	0.22797	0.99998	4349	0.0311
LRP2	6	0.095206	0.2281	0.99998	4350	-0.0742
hsa-mir-6829	4	0.095242	0.20491	0.99998	4351	-0.1659
C8orf59	6	0.095263	0.2282	0.99998	4352	0.0193
ZSCAN1	6	0.095263	0.2282	0.99998	4353	0.127
OR4E2	6	0.095267	0.22821	0.99998	4354	-0.2075
ASPHD1	6	0.095279	0.22823	0.99998	4355	0.0083
UMPS	6	0.095279	0.22823	0.99998	4356	-0.145
PPP1CA	6	0.095335	0.2283	0.99998	4357	-0.1294
IRGM	6	0.095393	0.2284	0.99998	4358	-0.1621
FRA10AC1	3	0.095414	0.16884	0.99998	4359	0.2221
IFIT1	6	0.095508	0.22857	0.99998	4360	-0.1777
SMC3	6	0.095508	0.22857	0.99998	4361	-0.0786
HOXD10	6	0.095601	0.22868	0.99998	4362	-0.1682
MRPL11	6	0.09571	0.22884	0.99998	4363	0.1471
SHC4	6	0.09583	0.22902	0.99998	4364	0.0292
FRY	6	0.09606	0.22937	0.99998	4365	-0.1457
METTL1	6	0.096069	0.22939	0.99998	4366	-0.1493
ZDHHC15	6	0.096117	0.22945	0.99998	4367	-0.0604
NPB	6	0.096138	0.22948	0.99998	4368	-0.1928
BRMS1	6	0.096189	0.22956	0.99998	4369	0.1619
CYP4F2	5	0.096209	0.21033	0.99998	4370	-0.4267
IQCK	6	0.096262	0.22965	0.99998	4371	-0.0156

FAM124B	6	0.096301	0.22972	0.99998	4372	-0.1269
EXTL3	6	0.096302	0.22973	0.99998	4373	-0.0385
TYW1B	6	0.096309	0.22973	0.99998	4374	-0.0439
SEC23A	6	0.096357	0.22979	0.99998	4375	0.2862
TRIM37	6	0.096415	0.22989	0.99998	4376	-0.0866
DNAH8	6	0.096455	0.22994	0.99998	4377	0.0043
FXYD3	6	0.096476	0.22997	0.99998	4378	-0.2042
ZC3H11A	6	0.096476	0.22997	0.99998	4379	-0.099
BIRC3	6	0.096522	0.23003	0.99998	4380	-0.1181
TTI1	6	0.096644	0.23021	0.99998	4381	-0.1926
RPS13	6	0.096673	0.23026	0.99998	4382	-0.1468
ITGB1BP1	6	0.096673	0.23026	0.99998	4383	-0.0631
S100A9	6	0.096692	0.23028	0.99998	4384	0.0178
HLX	6	0.096755	0.23037	0.99998	4385	0.0339
HCAR2	6	0.096787	0.23042	0.99998	4386	-0.0368
hsa-mir-4510	3	0.096817	0.17077	0.99998	4387	-0.3705
POLR2H	6	0.096828	0.23048	0.99998	4388	-0.1118
MCCC1	6	0.096931	0.23063	0.99998	4389	0.099
TMED6	6	0.096997	0.23074	0.99998	4390	-0.1136
XCR1	6	0.097003	0.23075	0.99998	4391	-0.0616
GABRB2	6	0.097027	0.23078	0.99998	4392	0.1977
DENND4A	6	0.097061	0.23083	0.99998	4393	-0.1136
RAD51AP2	6	0.097075	0.23085	0.99998	4394	0.2534
ANKRD34A	6	0.097078	0.23086	0.99998	4395	-0.1168
LOC10028825	1	0.097162	0.097197	0.93496	4396	-0.5776
SH2D5	6	0.097172	0.23098	0.99998	4397	-0.249
LRTOMT	6	0.097218	0.23105	0.99998	4398	-0.0708
GRM2	6	0.097224	0.23106	0.99998	4399	0.1398
CELF1	6	0.097224	0.23106	0.99998	4400	-0.1903
TSPAN5	6	0.097266	0.23111	0.99998	4401	-0.316
LRIT3	6	0.097314	0.23118	0.99998	4402	-0.1427
FCGR2A	5	0.097371	0.21234	0.99998	4403	-0.2116
KIAA0226L	6	0.097394	0.23128	0.99998	4404	0.3012
PRPF40A	6	0.097452	0.23136	0.99998	4405	-0.1695
DMBX1	6	0.097481	0.23139	0.99998	4406	0.1775
USP51	6	0.097481	0.23139	0.99998	4407	0.3178
LOC152586	6	0.097511	0.23143	0.99998	4408	-0.0922
C9orf129	6	0.097511	0.23143	0.99998	4409	-0.0505
TECPR2	6	0.097553	0.23149	0.99998	4410	-0.211
hsa-mir-411	4	0.097576	0.20749	0.99998	4411	0.0765
hsa-mir-541	4	0.097744	0.20767	0.99998	4412	-0.2321
FAM13B	6	0.097775	0.23182	0.99998	4413	-0.1045
HOXB5	6	0.097876	0.23197	0.99998	4414	0.119
CLEC2D	6	0.097924	0.23203	0.99998	4415	-0.1865
FAM109B	6	0.098007	0.23215	0.99998	4416	0.204
FAM159B	6	0.098007	0.23215	0.99998	4417	-0.0504
TLX2	6	0.098094	0.23228	0.99998	4418	-0.2758
AKR7A2	6	0.098127	0.23232	0.99998	4419	0.0282
USP12	6	0.098174	0.23239	0.99998	4420	-0.0382
ATP5G1	6	0.098211	0.23244	0.99998	4421	-0.2262
FAM171A2	6	0.098222	0.23246	0.99998	4422	-0.0292
SLC17A9	6	0.09827	0.23253	0.99998	4423	0.4043
LY6G6F	6	0.098294	0.23256	0.99998	4424	0.1904
EPO	6	0.098317	0.23259	0.99998	4425	-0.2378
TMEM147	6	0.098317	0.23259	0.99998	4426	0.0226
NOL12	6	0.098351	0.23265	0.99998	4427	-0.1368
FOXE1	6	0.098351	0.23265	0.99998	4428	-0.0391
hsa-mir-548u	4	0.098406	0.2084	0.99998	4429	-0.3931
AVPR1B	6	0.098437	0.23276	0.99998	4430	0.1966
OVGP1	6	0.098441	0.23276	0.99998	4431	-0.1508
SNX15	6	0.098441	0.23276	0.99998	4432	0.1569
WNT5B	6	0.098509	0.23286	0.99998	4433	0.0285
EEF1A1	6	0.098557	0.23293	0.99998	4434	0.0691
hsa-mir-3674	4	0.098604	0.20862	0.99998	4435	0.0794
RFX1	6	0.098626	0.23303	0.99998	4436	-0.1249
CARHSP1	6	0.098626	0.23303	0.99998	4437	-0.2591
TMEM245	6	0.098701	0.23315	0.99998	4438	0.0746
RBM28	6	0.098791	0.23329	0.99998	4439	-0.0683
TCEB3C	3	0.098796	0.17348	0.99998	4440	0.06
TRIM11	6	0.09882	0.23334	0.99998	4441	0.0067
GBP5	6	0.099003	0.23365	0.99998	4442	-0.2401
DNAJB12	4	0.099025	0.20911	0.99998	4443	-0.0142
ZCCHC6	6	0.099058	0.23374	0.99998	4444	-0.1669
DCN	6	0.099058	0.23374	0.99998	4445	0.0698
METTL2A	5	0.099059	0.21519	0.99998	4446	-0.4599
LOC10050642	6	0.099117	0.23382	0.99998	4447	0.115
MATN2	6	0.099178	0.2339	0.99998	4448	-0.0393
BTRC	6	0.099213	0.23395	0.99998	4449	-0.0842
TM2D2	6	0.099213	0.23395	0.99998	4450	-0.1124
RAB4A	6	0.099272	0.23403	0.99998	4451	-0.1539
C13orf35	6	0.099272	0.23403	0.99998	4452	-0.0841

TMEM252	6	0.099297	0.23407	0.99998	4453	-0.1983
GORASP1	6	0.099345	0.23415	0.99998	4454	-0.1201
LAT2	6	0.099352	0.23416	0.99998	4455	-0.156
SEPHS2	6	0.099369	0.23419	0.99998	4456	-0.0066
hsa-mir-4295	4	0.099439	0.20957	0.99998	4457	0.2683
TREX1	6	0.099451	0.2343	0.99998	4458	-0.0744
SPINK7	6	0.099539	0.23443	0.99998	4459	-0.1273
KBTBD4	6	0.099565	0.23446	0.99998	4460	0.0273
OR5M9	6	0.099565	0.23446	0.99998	4461	-0.2631
GPR116	6	0.099663	0.2346	0.99998	4462	-0.1917
QPRT	6	0.099663	0.2346	0.99998	4463	-0.1932
SMAD4	6	0.099663	0.2346	0.99998	4464	-0.214
ETNK1	6	0.099751	0.23472	0.99998	4465	-0.0907
CUZD1	6	0.099829	0.23484	0.99998	4466	-0.1068
HLA-E	6	0.09987	0.2349	0.99998	4467	-0.0399
SNRPD2	6	0.099877	0.23491	0.99998	4468	-0.0301
hsa-mir-4258	4	0.099889	0.21007	0.99998	4469	-0.1602
C1orf110	6	0.099942	0.235	0.99998	4470	0.0022
TBX21	6	0.10004	0.23514	0.99998	4471	-0.0212
ABHD16B	6	0.10004	0.23514	0.99998	4472	-0.1817
GUCA1B	6	0.10008	0.23521	0.99998	4473	-0.0239
KLK8	6	0.10008	0.23521	0.99998	4474	-0.0063
DPCR1	6	0.10013	0.23527	0.99998	4475	-0.2209
CD180	6	0.10013	0.23527	0.99998	4476	-0.2395
ANKRD20A1	1	0.10015	0.10021	0.94093	4477	-0.6415
hsa-mir-4519	4	0.10019	0.21039	0.99998	4478	-0.0906
WDR3	6	0.10024	0.23542	0.99998	4479	-0.2316
REEP4	6	0.10024	0.23542	0.99998	4480	-0.2608
MAPK9	6	0.10028	0.23548	0.99998	4481	0.1511
C3orf55	6	0.10037	0.23561	0.99998	4482	-0.036
KRTAP8-1	6	0.10037	0.23562	0.99998	4483	0.032
C13orf45	6	0.10042	0.2357	0.99998	4484	0.2544
EXOC2	6	0.10043	0.23571	0.99998	4485	-0.2028
PPP1R15B	6	0.10046	0.23574	0.99998	4486	-0.0789
CEBPG	6	0.10055	0.23588	0.99998	4487	-0.1219
CCDC88C	6	0.10055	0.23588	0.99998	4488	-0.0215
IL10RB	6	0.10057	0.2359	0.99998	4489	-0.1777
CHD7	6	0.10061	0.23597	0.99998	4490	0.224
DENND1A	6	0.10069	0.23607	0.99998	4491	-0.0626
TLR6	6	0.1007	0.23611	0.99998	4492	0.0296
ZMYND15	6	0.10078	0.23622	0.99998	4493	0.004
FANCD2OS	6	0.1008	0.23625	0.99998	4494	0.098
UCHL3	6	0.10094	0.23644	0.99998	4495	0.1739
MMP24	6	0.10106	0.23661	0.99998	4496	0.0632
STK39	6	0.10124	0.23687	0.99998	4497	0.0336
DMRTC1	2	0.10128	0.16906	0.99998	4498	-0.4116
DHPS	6	0.10131	0.23696	0.99998	4499	-0.1847
KHSRP	6	0.10137	0.23705	0.99998	4500	-0.057
hsa-mir-1256	4	0.10138	0.21176	0.99998	4501	0.287
FAM170A	6	0.10161	0.23736	0.99998	4502	-0.1986
SLC29A2	6	0.10161	0.23736	0.99998	4503	-0.201
hsa-mir-147b	3	0.10165	0.17742	0.99998	4504	-0.2913
HDDC3	6	0.10166	0.23744	0.99998	4505	-0.0465
hsa-mir-140	4	0.10168	0.21208	0.99998	4506	-0.0678
ABCC8	6	0.10187	0.23777	0.99998	4507	0.0196
OR14I1	6	0.10187	0.23777	0.99998	4508	0.1178
ZNF100	6	0.10191	0.23784	0.99998	4509	-0.7454
ATP6AP2	6	0.10191	0.23784	0.99998	4510	-0.1232
ZNF45	6	0.10191	0.23784	0.99998	4511	-0.1851
C1orf172	6	0.10202	0.23801	0.99998	4512	-0.14
hsa-mir-367	4	0.10205	0.2125	0.99998	4513	-0.2889
PPP2R5A	6	0.10207	0.23807	0.99998	4514	-0.1148
ATRN	6	0.10221	0.23826	0.99998	4515	-0.1389
hsa-mir-30c-1	4	0.10221	0.21269	0.99998	4516	-0.0644
INADL	6	0.10226	0.23833	0.99998	4517	0.0061
LDLRAD3	6	0.10226	0.23833	0.99998	4518	-0.0784
LMOD1	6	0.10232	0.23843	0.99998	4519	-0.0769
ITCH	6	0.10233	0.23843	0.99998	4520	-0.2241
PCCB	6	0.10233	0.23843	0.99998	4521	-0.0182
hsa-mir-4767	4	0.10235	0.21285	0.99998	4522	-0.3038
ARGLU1	6	0.10244	0.23861	0.99998	4523	-0.1405
HAGH	6	0.10245	0.23862	0.99998	4524	-0.1398
SIPA1	6	0.10252	0.2387	0.99998	4525	0.0062
RABAC1	6	0.10256	0.23879	0.99998	4526	-0.0757
CCBL2	6	0.10258	0.23881	0.99998	4527	-0.225
BCAS3	6	0.10263	0.23888	0.99998	4528	0.017
MCTP2	6	0.10267	0.23893	0.99998	4529	-0.1195
WNT3	6	0.10267	0.23894	0.99998	4530	-0.1903
LTF	6	0.10275	0.23905	0.99998	4531	0.047
CYFIP2	6	0.1028	0.23913	0.99998	4532	0.3077
ZNF850	6	0.10287	0.23922	0.99998	4533	-0.0353

MAPKAPK5	6	0.103	0.2394	0.99998	4534	-0.0602
VASP	6	0.10312	0.23957	0.99998	4535	-0.1202
IL36G	6	0.10314	0.23961	0.99998	4536	-0.1405
OR13H1	6	0.10323	0.23973	0.99998	4537	-0.0152
OCIAD2	6	0.10325	0.23976	0.99998	4538	-0.0124
OR13C9	5	0.10326	0.22211	0.99998	4539	-0.3035
NRG4	6	0.10333	0.23986	0.99998	4540	-0.2158
C11orf73	6	0.10337	0.23993	0.99998	4541	-0.0562
G0S2	6	0.10347	0.24007	0.99998	4542	-6E-05
TXN2	6	0.10353	0.24015	0.99998	4543	-0.1333
PRSS2	6	0.10353	0.24015	0.99998	4544	-0.1875
PON1	6	0.10358	0.24022	0.99998	4545	-0.1769
LCE3C	6	0.10359	0.24023	0.99998	4546	0.0432
PRRC2A	6	0.10359	0.24023	0.99998	4547	-0.1647
PNKP	6	0.10362	0.24029	0.99998	4548	-0.1911
LRCH1	6	0.10362	0.24029	0.99998	4549	-0.2444
hsa-mir-4320	4	0.10369	0.21439	0.99998	4550	-0.0063
MAS1L	6	0.10372	0.24044	0.99998	4551	0.0911
ARHGEF37	6	0.1038	0.24056	0.99998	4552	0.0213
NOTCH1	6	0.1039	0.24068	0.99998	4553	-0.1005
LHPP	6	0.10401	0.24087	0.99998	4554	0.2025
CREB3L4	6	0.10401	0.24087	0.99998	4555	0.2384
hsa-mir-4450	4	0.10404	0.21479	0.99998	4556	-0.1633
JDP2	6	0.10411	0.241	0.99998	4557	-0.0363
SDC2	6	0.10411	0.241	0.99998	4558	-0.0352
ZKSCAN7	6	0.10418	0.24112	0.99998	4559	-0.1018
RILP	6	0.10422	0.24117	0.99998	4560	-0.1726
SLC22A18AS	6	0.10423	0.24118	0.99998	4561	0.1472
hsa-mir-6719	4	0.10425	0.215	0.99998	4562	0.139
DCP2	6	0.1043	0.2413	0.99998	4563	0.175
KDELC2	6	0.10437	0.24139	0.99998	4564	0.1119
HERC3	6	0.10437	0.24139	0.99998	4565	-0.0598
GNL3	6	0.10438	0.2414	0.99998	4566	-0.2184
MNDA	6	0.10445	0.24151	0.99998	4567	0.0361
TSPO2	6	0.10449	0.24156	0.99998	4568	-0.138
USP7	6	0.10451	0.24159	0.99998	4569	0.0063
FRG1	6	0.10463	0.24177	0.99998	4570	0.0974
OR10C1	6	0.10467	0.24183	0.99998	4571	-0.0656
CTSG	6	0.10473	0.24191	0.99998	4572	0.0658
PLOD3	6	0.1048	0.24201	0.99998	4573	0.3242
PFKM	6	0.1048	0.24202	0.99998	4574	-0.1395
hsa-mir-649	4	0.10485	0.21569	0.99998	4575	-0.28
TTC40	6	0.10487	0.2421	0.99998	4576	0.2867
SLMO2	6	0.10491	0.24214	0.99998	4577	-0.3418
LDLRAD1	6	0.10499	0.24226	0.99998	4578	-0.1622
FAM102A	6	0.10502	0.2423	0.99998	4579	-0.0904
TNP1	6	0.10508	0.24239	0.99998	4580	0.036
CXorf56	6	0.10513	0.24246	0.99998	4581	0.1228
CLPX	6	0.10516	0.2425	0.99998	4582	-0.0581
PTCD3	6	0.1053	0.2427	0.99998	4583	-0.1999
MFI2	6	0.10536	0.2428	0.99998	4584	-0.1746
DRAP1	6	0.10536	0.2428	0.99998	4585	0.0193
PPAT	6	0.10544	0.24291	0.99998	4586	-0.1847
NOL9	6	0.10545	0.24292	0.99998	4587	-0.1671
TNMD	6	0.10553	0.24302	0.99998	4588	-0.0514
hsa-mir-146b	4	0.10557	0.2165	0.99998	4589	-0.1056
C6orf165	6	0.10562	0.24314	0.99998	4590	-0.1562
ZNF737	5	0.10573	0.22623	0.99998	4591	0.1932
GCM2	6	0.10585	0.24346	0.99998	4592	-0.1764
SPRTN	6	0.10587	0.24349	0.99998	4593	0.0363
RTKN	6	0.106	0.24367	0.99998	4594	-0.135
ST14	6	0.10604	0.24373	0.99998	4595	0.0304
NPRL2	6	0.10608	0.24379	0.99998	4596	-0.2042
KCNJ13	6	0.10608	0.24379	0.99998	4597	-0.1512
ELAC1	6	0.10612	0.24383	0.99998	4598	-0.1546
FAM126B	6	0.10617	0.24392	0.99998	4599	0.0954
CHAD	6	0.10623	0.24399	0.99998	4600	-0.0826
GBGT1	6	0.10634	0.24415	0.99998	4601	-0.115
AMACR	6	0.10636	0.24419	0.99998	4602	-0.0662
SMURF1	6	0.10641	0.24427	0.99998	4603	-0.1008
CCDC167	6	0.10644	0.24431	0.99998	4604	-0.1172
SUV420H1	6	0.10644	0.24432	0.99998	4605	-0.1531
ADSS	6	0.10644	0.24432	0.99998	4606	0.0155
PTPN1	6	0.10649	0.24437	0.99998	4607	-0.1534
AP3B2	6	0.10656	0.24447	0.99998	4608	-0.159
PDE8B	6	0.10658	0.24449	0.99998	4609	0.1182
CTU2	6	0.10658	0.24449	0.99998	4610	-0.1016
MRI1	6	0.10659	0.24452	0.99998	4611	-0.2112
DRD3	6	0.10664	0.2446	0.99998	4612	-0.115
DDX51	6	0.10665	0.24461	0.99998	4613	-0.3333
GSE1	6	0.10665	0.24461	0.99998	4614	0.0207

LAMC1	6	0.10672	0.24469	0.99998	4615	-0.2035
TNFAIP8	6	0.10678	0.2448	0.99998	4616	-0.1719
TMEM17	6	0.10678	0.2448	0.99998	4617	-0.2401
ZPLD1	6	0.10684	0.24488	0.99998	4618	0.044
TMEM211	6	0.10692	0.24498	0.99998	4619	-0.2446
SYNDIG1L	6	0.10694	0.24501	0.99998	4620	0.0382
PHLPP2	6	0.10696	0.24504	0.99998	4621	-0.0136
PRCD	6	0.10705	0.24516	0.99998	4622	-0.1316
ORMDL3	6	0.10708	0.24521	0.99998	4623	-0.1532
PARP6	5	0.10711	0.22849	0.99998	4624	-0.2277
ZNF721	5	0.10711	0.22849	0.99998	4625	-0.2728
ATP6V1C1	6	0.10717	0.24533	0.99998	4626	-0.0511
C9orf43	6	0.10717	0.24533	0.99998	4627	0.0945
PARP3	6	0.10717	0.24534	0.99998	4628	-0.1146
OR51A7	6	0.10717	0.24534	0.99998	4629	-0.2336
B4GALT4	6	0.10722	0.24541	0.99998	4630	-0.0575
hsa-mir-1287	4	0.10724	0.21839	0.99998	4631	-0.2121
ZNF229	6	0.10727	0.24547	0.99998	4632	0.2351
APOBEC3B	6	0.1073	0.24553	0.99998	4633	-0.017
CDK2	6	0.10734	0.24557	0.99998	4634	-0.2177
FAM13C	6	0.10734	0.24558	0.99998	4635	0.1727
hsa-mir-4425	4	0.10735	0.21853	0.99998	4636	0.0386
SLC35F3	6	0.1075	0.24581	0.99998	4637	0.2427
TMEM194A	6	0.10754	0.24586	0.99998	4638	-0.2972
ARF1	6	0.1076	0.24594	0.99998	4639	0.383
C6orf25	6	0.10766	0.24603	0.99998	4640	-0.0683
RNF145	6	0.10767	0.24605	0.99998	4641	-0.2002
MAOA	6	0.10769	0.24609	0.99998	4642	0.0764
BEST2	6	0.10775	0.24615	0.99998	4643	-0.1118
DAK	6	0.10779	0.24623	0.99998	4644	-0.0133
GPR65	6	0.10784	0.24629	0.99998	4645	-0.1516
OMP	6	0.10791	0.2464	0.99998	4646	0.1862
TMEM74B	6	0.10793	0.24643	0.99998	4647	0.0588
ARL9	6	0.10793	0.24643	0.99998	4648	-0.0927
FAM20B	6	0.10797	0.24649	0.99998	4649	-0.1922
ABCC11	6	0.10797	0.24649	0.99998	4650	-0.226
MED18	6	0.10802	0.24657	0.99998	4651	0.0416
IFI27L1	6	0.10803	0.24658	0.99998	4652	-0.0893
EHD2	6	0.10805	0.24659	0.99998	4653	-0.1876
DHRS13	6	0.10816	0.24677	0.99998	4654	-0.0512
E4F1	6	0.10819	0.2468	0.99998	4655	-0.1904
SULT1E1	6	0.10819	0.2468	0.99998	4656	0.0706
KDM8	6	0.10835	0.24703	0.99998	4657	-0.1151
FADS3	6	0.1084	0.24709	0.99998	4658	0.0668
C12orf10	6	0.1085	0.24724	0.99998	4659	-0.1488
LYPD8	6	0.1085	0.24724	0.99998	4660	-0.108
C15orf65	3	0.1086	0.18698	0.99998	4661	-0.2653
WBP2NL	6	0.10864	0.24744	0.99998	4662	-0.0382
SKP2	6	0.10871	0.24755	0.99998	4663	-0.222
OR4K15	6	0.10872	0.24757	0.99998	4664	0.154
hsa-mir-6853	4	0.10873	0.22011	0.99998	4665	0.0324
CRCT1	6	0.10873	0.24759	0.99998	4666	-0.1939
PRODH	6	0.10873	0.24759	0.99998	4667	0.0101
DUSP1	6	0.10878	0.24765	0.99998	4668	-0.0356
FAR2	6	0.10878	0.24765	0.99998	4669	-0.1564
GZMH	6	0.10883	0.24771	0.99998	4670	-0.1473
CDC42EP4	6	0.1089	0.24781	0.99998	4671	-0.1233
DNAJB5	6	0.10891	0.24782	0.99998	4672	-0.1387
GRP	6	0.10891	0.24782	0.99998	4673	-0.0828
RACGAP1	6	0.10897	0.24791	0.99998	4674	0.0634
hsa-mir-3197	4	0.10902	0.22044	0.99998	4675	0.0787
C1orf226	6	0.10908	0.24807	0.99998	4676	-0.329
SLC50A1	6	0.10908	0.24807	0.99998	4677	-0.0761
FGF1	6	0.10914	0.24816	0.99998	4678	-0.0795
WAC	6	0.10914	0.24816	0.99998	4679	-0.1362
hsa-mir-6516	4	0.10922	0.22068	0.99998	4680	0.2393
AOC3	6	0.1093	0.2484	0.99998	4681	-0.2118
ZBTB38	6	0.1093	0.2484	0.99998	4682	-0.0408
DNAJC27	6	0.10934	0.24845	0.99998	4683	-0.1231
MTA1	6	0.10935	0.24848	0.99998	4684	-0.1943
OR1J2	6	0.10939	0.24853	0.99998	4685	-0.0567
OBP2A	2	0.1094	0.17493	0.99998	4686	-0.8286
HIST1H1B	6	0.10948	0.24867	0.99998	4687	0.0946
DIRC1	6	0.10954	0.24874	0.99998	4688	0.0967
LCE5A	6	0.10954	0.24875	0.99998	4689	-0.0945
ULK1	6	0.10961	0.24886	0.99998	4690	0.0606
USP45	6	0.10968	0.24895	0.99998	4691	0.1011
NAT8B	6	0.10973	0.24903	0.99998	4692	-0.1451
NCR1	6	0.10977	0.24907	0.99998	4693	-0.103
SIGLEC11	6	0.10977	0.24909	0.99998	4694	-0.1126
hsa-mir-3622a	1	0.1098	0.1098	0.95341	4695	-0.4612

ZBTB33	6	0.10982	0.24916	0.99998	4696	0.0041
NUFIP1	6	0.10989	0.24927	0.99998	4697	-0.1673
ITM2C	6	0.10994	0.24932	0.99998	4698	0.1322
hsa-mir-3136	4	0.11004	0.2216	0.99998	4699	-0.9026
PTP4A3	6	0.11006	0.24951	0.99998	4700	-0.0184
NAT6	6	0.11018	0.24967	0.99998	4701	0.1035
CIR1	6	0.11022	0.24973	0.99998	4702	0.0116
ARHGAP12	6	0.11027	0.24981	0.99998	4703	0.2259
NHP2L1	6	0.11027	0.24981	0.99998	4704	0.3649
POU2F2	6	0.1104	0.25	0.99998	4705	-0.177
MTX2	6	0.11041	0.25002	0.99998	4706	-0.1183
PIGX	6	0.11045	0.25009	0.99998	4707	-0.2389
SNX12	6	0.11045	0.25009	0.99998	4708	-0.1369
TUSC2	6	0.11052	0.25018	0.99998	4709	0.0586
UBN1	6	0.11055	0.25022	0.99998	4710	-0.164
ADCY5	6	0.11063	0.25034	0.99998	4711	0.0467
TRIM9	6	0.11071	0.25047	0.99998	4712	0.2091
MAGIX	6	0.11072	0.25048	0.99998	4713	0.0437
MTDH	6	0.11072	0.25048	0.99998	4714	0.1454
BRSK1	6	0.11073	0.2505	0.99998	4715	-0.036
SLC25A21	6	0.11073	0.2505	0.99998	4716	-0.0883
PDHB	6	0.11073	0.2505	0.99998	4717	-0.0619
C12orf39	6	0.11077	0.25055	0.99998	4718	-0.0227
STON2	6	0.11077	0.25055	0.99998	4719	-0.0476
NUP62	6	0.11083	0.25064	0.99998	4720	-0.2135
SMARCD1	6	0.11083	0.25064	0.99998	4721	-0.1004
PTMA	6	0.11083	0.25064	0.99998	4722	-0.1051
BAG4	6	0.11089	0.25071	0.99998	4723	0.085
MAGEE1	6	0.11096	0.25082	0.99998	4724	-0.1128
AAR2	6	0.11102	0.25091	0.99998	4725	-0.0922
CADPS	6	0.1111	0.25103	0.99998	4726	-0.1388
RPS14	6	0.11117	0.25113	0.99998	4727	-0.1773
N4BP3	6	0.11118	0.25115	0.99998	4728	-0.0078
hsa-mir-6743	4	0.1112	0.223	0.99998	4729	-0.2303
CABP1	6	0.11131	0.25133	0.99998	4730	0.0616
OR5C1	6	0.11131	0.25133	0.99998	4731	0.0391
SERPINE2	6	0.11132	0.25135	0.99998	4732	-0.1802
CD247	6	0.11132	0.25135	0.99998	4733	-0.0781
S100A13	6	0.11143	0.2515	0.99998	4734	-0.2346
BSDC1	6	0.11155	0.25166	0.99998	4735	0.2127
PGM3	6	0.11158	0.25171	0.99998	4736	0.1412
GCA	6	0.11158	0.25171	0.99998	4737	-0.0429
C4orf36	6	0.11164	0.2518	0.99998	4738	-0.1599
HTR3E	6	0.11167	0.25185	0.99998	4739	-0.1706
PIGT	6	0.11176	0.25198	0.99998	4740	-0.1468
SRPK1	6	0.11176	0.25198	0.99998	4741	-0.112
DDHD1	6	0.11181	0.25204	0.99998	4742	0.1291
NUDT22	6	0.11181	0.25204	0.99998	4743	0.1756
hsa-mir-4262	4	0.11185	0.22374	0.99998	4744	0.1215
ZC3H15	6	0.11187	0.25214	0.99998	4745	-0.1861
ISYNA1	6	0.11195	0.25225	0.99998	4746	-0.0915
UBE2U	6	0.11195	0.25225	0.99998	4747	0.1213
ABI1	6	0.11206	0.25241	0.99998	4748	-0.1862
CEP170	6	0.11209	0.25246	0.99998	4749	0.2159
TRIM48	4	0.1121	0.22403	0.99998	4750	-0.4871
TUBGCP6	6	0.11225	0.25269	0.99998	4751	-0.1276
SPTBN1	6	0.11225	0.25269	0.99998	4752	-0.1738
BCL3	6	0.11226	0.25271	0.99998	4753	-0.0963
hsa-mir-433	4	0.11233	0.22428	0.99998	4754	-0.24
FCGR2B	5	0.11242	0.23731	0.99998	4755	-0.2436
hsa-mir-6833	4	0.11245	0.2244	0.99998	4756	-0.2716
OR6B3	6	0.11251	0.25308	0.99998	4757	-0.1031
HNRNPH2	4	0.11255	0.22452	0.99998	4758	-0.1159
LRRC8A	6	0.11266	0.25327	0.99998	4759	0.0554
IL1B	6	0.11266	0.25328	0.99998	4760	-0.0291
hsa-mir-548o	3	0.11271	0.19265	0.99998	4761	-0.3027
RPP25L	6	0.11275	0.25342	0.99998	4762	-0.2623
SPATA20	6	0.11275	0.25342	0.99998	4763	-0.1031
GOPC	6	0.11276	0.25344	0.99998	4764	0.0002
OSBPL7	6	0.1128	0.25348	0.99998	4765	0.0123
CCBP2	4	0.1128	0.22482	0.99998	4766	-0.0315
ARHGAP35	6	0.1128	0.2535	0.99998	4767	-0.1577
UNC13A	6	0.11287	0.25361	0.99998	4768	-0.0986
ZW10	6	0.11292	0.25368	0.99998	4769	-0.2396
ANAPC2	6	0.11293	0.25369	0.99998	4770	-0.2159
ZNF593	6	0.11293	0.25369	0.99998	4771	-0.2415
C22orf43	6	0.11294	0.25371	0.99998	4772	-0.1137
SERAC1	6	0.11303	0.25385	0.99998	4773	0.0558
TTC30A	5	0.11308	0.23838	0.99998	4774	-0.3084
CPNE8	3	0.1131	0.19316	0.99998	4775	-0.5313
KLF5	6	0.11311	0.25396	0.99998	4776	0.1832

hsa-mir-4654	4	0.11317	0.22523	0.99998	4777	0.0593
OR8D2	6	0.11319	0.25407	0.99998	4778	-0.2037
HIST1H2AE	6	0.11322	0.25411	0.99998	4779	-0.305
S100B	6	0.11333	0.25426	0.99998	4780	-0.2247
BTNL8	6	0.11333	0.25426	0.99998	4781	-0.1019
GLCE	6	0.11334	0.25426	0.99998	4782	-0.1001
LY6K	6	0.11337	0.2543	0.99998	4783	-0.0826
hsa-mir-215	4	0.11337	0.22545	0.99998	4784	-0.2198
NKAIN2	6	0.11339	0.25434	0.99998	4785	-0.1908
PPHLN1	6	0.11341	0.25437	0.99998	4786	0.0488
ATP2C2	6	0.11341	0.25437	0.99998	4787	-0.0396
OSMR	6	0.11345	0.25443	0.99998	4788	-0.1528
SLC25A46	6	0.11348	0.25447	0.99998	4789	0.0115
ETS1	6	0.11348	0.25447	0.99998	4790	-0.1797
hsa-mir-6087	4	0.11355	0.22567	0.99998	4791	-0.1455
PAN2	6	0.11359	0.25462	0.99998	4792	0.0028
ZNF324	6	0.1136	0.25463	0.99998	4793	0.1737
LGALS9	4	0.11363	0.22576	0.99998	4794	0.1412
PICALM	6	0.11367	0.25473	0.99998	4795	0.4109
MOCS1	6	0.11373	0.25482	0.99998	4796	-0.077
ACOX2	6	0.11374	0.25482	0.99998	4797	-0.1024
LBH	6	0.11383	0.25496	0.99998	4798	-0.1823
CACNA1D	6	0.11383	0.25496	0.99998	4799	-0.0746
ELL2	6	0.11397	0.25517	0.99998	4800	0.0381
ZNF263	6	0.11397	0.25517	0.99998	4801	-0.0913
IGLL5	6	0.11399	0.25519	0.99998	4802	-0.1991
GOLGA8O	6	0.11399	0.25519	0.99998	4803	-0.3454
hsa-mir-4743	4	0.11404	0.2262	0.99998	4804	-0.2195
ZBTB48	6	0.11407	0.25532	0.99998	4805	-0.0972
hsa-mir-4662a	1	0.11411	0.11412	0.96096	4806	-0.2751
RAX2	6	0.11411	0.25538	0.99998	4807	0.1751
IBA57	6	0.11419	0.25548	0.99998	4808	0.0737
MARCH10	6	0.11419	0.25548	0.99998	4809	0.068
KLHL29	6	0.11427	0.25558	0.99998	4810	-0.2196
PTGER4	6	0.11435	0.2557	0.99998	4811	-0.0464
FBXL3	6	0.11438	0.25574	0.99998	4812	-0.0875
TOR2A	6	0.11439	0.25576	0.99998	4813	-0.0615
POT1	6	0.11445	0.25583	0.99998	4814	0.0773
TRIM38	4	0.11447	0.22667	0.99998	4815	-0.2505
EYA3	6	0.11453	0.25594	0.99998	4816	-0.0553
TKT	6	0.11454	0.25595	0.99998	4817	0.057
ABL2	6	0.11456	0.25598	0.99998	4818	-0.065
MAZ	6	0.1146	0.25602	0.99998	4819	-0.2073
RAB22A	6	0.11472	0.25618	0.99998	4820	-0.0375
OR6C6	6	0.11478	0.25627	0.99998	4821	-0.0657
ZNF75A	6	0.11478	0.25627	0.99998	4822	-0.1747
PLXDC2	6	0.11478	0.25627	0.99998	4823	-0.2202
EVC2	6	0.11487	0.25638	0.99998	4824	-0.1965
CIB3	6	0.11487	0.25639	0.99998	4825	-0.199
PLCB2	6	0.11491	0.25644	0.99998	4826	-0.0881
TMEM125	6	0.11494	0.25649	0.99998	4827	0.3167
hsa-mir-326	4	0.11497	0.2272	0.99998	4828	-0.2118
PSD2	6	0.11499	0.25657	0.99998	4829	-0.0991
TRIM29	6	0.11508	0.2567	0.99998	4830	-0.0399
XKR7	6	0.11515	0.2568	0.99998	4831	-0.0248
P2RY4	6	0.11516	0.2568	0.99998	4832	-0.1668
NAT8L	4	0.11518	0.22744	0.99998	4833	-0.2594
FAM174A	6	0.11529	0.25699	0.99998	4834	0.1117
TUBB8	6	0.11529	0.25699	0.99998	4835	-0.0021
CLEC3B	6	0.11532	0.25703	0.99998	4836	0.0386
LRRC42	6	0.11532	0.25703	0.99998	4837	0.3147
TMEM159	6	0.11538	0.25712	0.99998	4838	-0.1501
PLEKHG4	6	0.11544	0.2572	0.99998	4839	0.1148
ZNF644	6	0.11546	0.25723	0.99998	4840	-0.0978
hsa-mir-4732	4	0.11547	0.22776	0.99998	4841	-0.0632
CCDC176	6	0.11557	0.25738	0.99998	4842	-0.1551
SLC2A4	6	0.11559	0.25741	0.99998	4843	-0.2103
GDPGP1	6	0.11562	0.25745	0.99998	4844	0.0912
SH3BGRL	6	0.11565	0.2575	0.99998	4845	-0.061
TASP1	6	0.11567	0.25752	0.99998	4846	0.0013
ZNF727	5	0.11569	0.24266	0.99998	4847	-0.3183
UPK1A	6	0.11572	0.2576	0.99998	4848	-0.116
PMM2	6	0.11579	0.25771	0.99998	4849	0.0827
DAG1	6	0.11584	0.25777	0.99998	4850	-0.131
MAN1A1	6	0.1159	0.25787	0.99998	4851	-0.1285
HIRA	6	0.1159	0.25787	0.99998	4852	0.0223
KIAA1737	6	0.116	0.25801	0.99998	4853	-0.0958
AJUBA	6	0.11601	0.25802	0.99998	4854	-0.2006
SNX11	6	0.11601	0.25802	0.99998	4855	0.045
YWHAG	6	0.11605	0.25808	0.99998	4856	-0.2153
RHOU	6	0.11609	0.25815	0.99998	4857	0.0031

ZNF556	6	0.1161	0.25816	0.99998	4858	-0.4171
TEX28	6	0.1161	0.25816	0.99998	4859	-0.2948
PPTC7	6	0.11618	0.25828	0.99998	4860	-0.0464
TMEM106B	6	0.11619	0.25829	0.99998	4861	-0.0564
BTG2	6	0.11625	0.25837	0.99998	4862	-0.1129
CHDC2	6	0.11631	0.25845	0.99998	4863	-0.2303
ZNF292	6	0.11634	0.25851	0.99998	4864	-0.1114
hsa-mir-1255b	2	0.11637	0.18003	0.99998	4865	-1.1706
LOC10050668	6	0.11637	0.25855	0.99998	4866	-0.0343
ADPGK	6	0.11642	0.25862	0.99998	4867	-0.1563
SLC45A3	6	0.11649	0.25872	0.99998	4868	-0.0344
RETSAT	6	0.11649	0.25872	0.99998	4869	-0.0749
ZNF799	2	0.11654	0.18015	0.99998	4870	-0.057
PLAGL2	6	0.11656	0.25883	0.99998	4871	-0.0455
SEMA6B	6	0.11659	0.25885	0.99998	4872	-0.4571
SLIT3	6	0.11659	0.25885	0.99998	4873	-0.0331
SLC5A7	6	0.1167	0.259	0.99998	4874	-0.2762
PCSK7	6	0.11673	0.25904	0.99998	4875	0.0732
OR52E2	6	0.11685	0.25921	0.99998	4876	-0.1005
hsa-mir-3661	3	0.11685	0.19826	0.99998	4877	-0.211
OR4A5	6	0.11694	0.25932	0.99998	4878	-0.2522
IL23A	6	0.11697	0.25937	0.99998	4879	0.0144
ZNF257	5	0.11703	0.2448	0.99998	4880	0.1932
CFB	6	0.1171	0.25955	0.99998	4881	0.1178
DUXA	6	0.1171	0.25955	0.99998	4882	0.0215
TMEM150B	6	0.11713	0.2596	0.99998	4883	-0.0198
SLC51A	6	0.11724	0.25978	0.99998	4884	-0.1463
WRB	6	0.11732	0.25989	0.99998	4885	-0.0924
KRTAP4-5	6	0.11736	0.25995	0.99998	4886	-0.0656
SEC62	6	0.11744	0.26006	0.99998	4887	-0.0652
FAM169A	6	0.11744	0.26006	0.99998	4888	-0.1504
hsa-mir-1251	4	0.11746	0.23	0.99998	4889	-0.112
ADCY2	6	0.11752	0.26018	0.99998	4890	-0.1457
PRL	6	0.11753	0.26018	0.99998	4891	0.0813
TMEM238	6	0.11753	0.26018	0.99998	4892	-0.1258
KCNH1	6	0.11753	0.26018	0.99998	4893	-0.1886
ARPC2	6	0.11765	0.26036	0.99998	4894	-0.0036
COL5A3	6	0.11765	0.26036	0.99998	4895	0.0011
TTC23	6	0.11767	0.26039	0.99998	4896	0.0634
GOLPH3L	6	0.11774	0.26048	0.99998	4897	0.0481
FLYWCH2	6	0.11775	0.2605	0.99998	4898	-0.0436
LY86	6	0.11775	0.2605	0.99998	4899	-0.1411
SLC37A1	6	0.11778	0.26056	0.99998	4900	0.1318
CCDC34	6	0.11783	0.26062	0.99998	4901	-0.2401
ITIH3	6	0.11783	0.26062	0.99998	4902	-0.2564
SPATA31C1	6	0.11785	0.26065	0.99998	4903	0.5652
DNAL1	3	0.11792	0.19972	0.99998	4904	0.3613
XPNPEP3	6	0.11792	0.26075	0.99998	4905	0.0796
NDRG1	6	0.11797	0.26081	0.99998	4906	0.0285
TMEM175	6	0.11799	0.26084	0.99998	4907	-0.1979
GEMIN7	6	0.11804	0.26092	0.99998	4908	0.1083
SAMD9	6	0.11804	0.26092	0.99998	4909	-0.1487
PHTF2	6	0.11806	0.26094	0.99998	4910	0.0175
SPATA24	6	0.11806	0.26094	0.99998	4911	-0.0045
ZNF225	5	0.11823	0.24677	0.99998	4912	-0.4514
RAB3C	6	0.11825	0.26122	0.99998	4913	-0.5215
ZNF560	6	0.11825	0.26122	0.99998	4914	-0.2692
ACO1	6	0.11828	0.26125	0.99998	4915	-0.0142
ANK1	6	0.11842	0.26146	0.99998	4916	0.1846
C1orf198	6	0.11845	0.26151	0.99998	4917	-0.0112
BLOC1S1	6	0.11849	0.26156	0.99998	4918	-0.0547
AKT3	6	0.11855	0.26163	0.99998	4919	-0.2713
IRF6	6	0.11855	0.26163	0.99998	4920	0.1036
SCARF1	6	0.11856	0.26165	0.99998	4921	-0.014
NAALADL2	6	0.1186	0.26171	0.99998	4922	-0.2931
TUBGCP2	6	0.1187	0.26184	0.99998	4923	-0.1395
AVIL	6	0.11872	0.26187	0.99998	4924	-0.1787
HHIP	6	0.11873	0.26188	0.99998	4925	0.0344
OLFML2B	6	0.11881	0.262	0.99998	4926	-0.1889
LPP	6	0.11881	0.262	0.99998	4927	-0.1723
AMIGO3	6	0.11881	0.262	0.99998	4928	-0.173
PIGV	6	0.11884	0.26204	0.99998	4929	-0.1323
IL33	6	0.11884	0.26204	0.99998	4930	-0.1609
LIX1L	6	0.11884	0.26204	0.99998	4931	-0.1526
hsa-mir-17	3	0.11887	0.20101	0.99998	4932	-0.5798
FAM86B1	3	0.11887	0.20101	0.99998	4933	-0.7324
hsa-mir-3175	4	0.1189	0.23166	0.99998	4934	0.2139
ZSWIM4	6	0.11891	0.26215	0.99998	4935	0.1121
TPH1	6	0.119	0.26228	0.99998	4936	-0.0108
ZFR	6	0.11901	0.26228	0.99998	4937	-0.1975
HTR4	6	0.11905	0.26234	0.99998	4938	-0.0709

ZNF415	6	0.11908	0.26238	0.99998	4939	-0.4548
PDCD5	6	0.1191	0.2624	0.99998	4940	-0.1465
KLF2	6	0.11914	0.26247	0.99998	4941	0.1124
CD86	6	0.11915	0.26248	0.99998	4942	-0.1188
TOMM40	6	0.11931	0.26269	0.99998	4943	-0.1821
STARD10	6	0.11935	0.26275	0.99998	4944	-0.0151
CXXC4	6	0.11953	0.26301	0.99998	4945	-0.117
CNGB3	6	0.11959	0.26311	0.99998	4946	0.126
PMFBP1	6	0.11968	0.26325	0.99998	4947	-0.036
FAM26D	6	0.11968	0.26325	0.99998	4948	-0.0953
MEIS1	6	0.1197	0.26329	0.99998	4949	-0.1467
YDJC	6	0.1197	0.26329	0.99998	4950	-0.1304
CHRNB1	6	0.11973	0.26331	0.99998	4951	-0.0349
ALOX5	6	0.11982	0.26346	0.99998	4952	-0.288
RNF38	6	0.11983	0.26347	0.99998	4953	-0.2345
STT3A	6	0.1199	0.26357	0.99998	4954	0.1205
CD1B	6	0.11994	0.26363	0.99998	4955	-0.0951
hsa-mir-3925	3	0.12006	0.20264	0.99998	4956	-0.9148
hsa-mir-4482	4	0.12011	0.23301	0.99998	4957	-0.1929
VWC2	4	0.12011	0.23301	0.99998	4958	-0.1737
EMILIN2	6	0.12022	0.26402	0.99998	4959	0.1088
KCTD16	6	0.12027	0.26409	0.99998	4960	-0.1328
BANP	6	0.12032	0.26415	0.99998	4961	-0.1268
C9orf96	6	0.12032	0.26416	0.99998	4962	-0.0797
NFASC	6	0.12043	0.26431	0.99998	4963	0.0119
LRRC26	6	0.1205	0.26441	0.99998	4964	0.2442
CNGA3	6	0.12051	0.26442	0.99998	4965	-0.0305
HTRA4	6	0.12053	0.26445	0.99998	4966	-0.16
PIF1	6	0.12065	0.26462	0.99998	4967	-0.0475
TMC1	6	0.12074	0.26474	0.99998	4968	-0.2705
CCNJ	6	0.12081	0.26484	0.99998	4969	-0.0985
SPINT3	6	0.12094	0.26503	0.99998	4970	-0.3961
LOC643355	6	0.12099	0.2651	0.99998	4971	0.0122
NCK1	6	0.12099	0.2651	0.99998	4972	0.1159
ZNF146	6	0.12105	0.26517	0.99998	4973	0.1667
HHATL	6	0.12125	0.26544	0.99998	4974	-0.1389
MAGEB6	6	0.12132	0.26553	0.99998	4975	-0.1598
TAPBPL	6	0.12133	0.26554	0.99998	4976	-0.1115
ANKFY1	6	0.12137	0.26561	0.99998	4977	0.0368
FLOT2	6	0.12137	0.26561	0.99998	4978	0.1551
RNF121	6	0.12137	0.26561	0.99998	4979	-0.1116
NSMCE1	6	0.12151	0.26581	0.99998	4980	-0.0458
ACTN2	6	0.12151	0.26581	0.99998	4981	-0.0821
SLC39A5	6	0.12151	0.26581	0.99998	4982	0.3447
SLC2A3	5	0.12152	0.25203	0.99998	4983	-0.7555
NICN1	6	0.12156	0.26589	0.99998	4984	-0.0308
SCP2	6	0.12162	0.26598	0.99998	4985	0.0438
CRLF1	6	0.1218	0.26625	0.99998	4986	-0.1635
CARTPT	6	0.1218	0.26625	0.99998	4987	-0.0234
KMT2E	6	0.12187	0.26634	0.99998	4988	-0.056
ITLN2	4	0.12194	0.23508	0.99998	4989	0.1273
ALDH16A1	6	0.12198	0.2665	0.99998	4990	-0.1593
C1orf186	6	0.12217	0.26678	0.99998	4991	-0.1816
SPINT1	6	0.12217	0.26678	0.99998	4992	-0.1612
C2orf50	6	0.12217	0.26678	0.99998	4993	-0.1526
HNRNPA1L2	6	0.12219	0.26682	0.99998	4994	-0.1943
ATP1A3	6	0.12219	0.26682	0.99998	4995	-0.0571
IKBKB	6	0.12226	0.26692	0.99998	4996	-0.1851
DPPA4	6	0.12229	0.26698	0.99998	4997	-0.0603
DAZAP2	5	0.12234	0.25334	0.99998	4998	-0.2202
MAPK3	6	0.12237	0.26708	0.99998	4999	-0.1014
RPL8	6	0.12237	0.26708	0.99998	5000	-0.0618
LOC200726	6	0.12237	0.26708	0.99998	5001	-0.0739
C9orf57	6	0.1224	0.26711	0.99998	5002	-0.1638
PAGR1	6	0.1224	0.26711	0.99998	5003	-0.0536
SLC2A6	6	0.12246	0.2672	0.99998	5004	-0.2245
MORF4L1	6	0.12246	0.26721	0.99998	5005	-0.2049
WDR25	6	0.12251	0.26728	0.99998	5006	-0.0297
PTGES3L-AAR!	2	0.12255	0.1845	0.99998	5007	-0.541
OR2T29	3	0.12258	0.20608	0.99998	5008	-0.9148
S100A16	6	0.12258	0.26737	0.99998	5009	0.1454
CD47	6	0.12259	0.26738	0.99998	5010	0.0711
PEX11A	6	0.12259	0.26738	0.99998	5011	0.0116
hsa-mir-181c	4	0.12264	0.23584	0.99998	5012	-0.3955
NDST3	6	0.12265	0.26747	0.99998	5013	-0.0395
TCEB1	6	0.12266	0.26748	0.99998	5014	-0.2213
PRSS38	6	0.12273	0.26758	0.99998	5015	-0.038
GRWD1	6	0.12287	0.26779	0.99998	5016	-0.1013
ZNF224	6	0.12287	0.26779	0.99998	5017	-0.2305
OCLM	4	0.12287	0.2361	0.99998	5018	-0.0762
TSC1	6	0.12293	0.26789	0.99998	5019	-0.1911

PKIB	6	0.12298	0.26795	0.99998	5020	0.1295
ZNF784	6	0.12307	0.26808	0.99998	5021	-0.0356
FAM134C	6	0.12307	0.26808	0.99998	5022	-0.2031
NAA11	6	0.12307	0.26808	0.99998	5023	0.3347
KBTBD2	6	0.12318	0.26823	0.99998	5024	-0.1884
OR2H1	6	0.12321	0.26827	0.99998	5025	0.27
HHIPL1	6	0.12322	0.26828	0.99998	5026	-0.1001
MCMBP	6	0.12328	0.26837	0.99998	5027	-8E-05
DAPK3	6	0.12335	0.26846	0.99998	5028	-0.2236
ZNF582	6	0.12335	0.26846	0.99998	5029	0.0802
KRT78	6	0.12335	0.26846	0.99998	5030	0.0432
OR10J5	6	0.12337	0.26848	0.99998	5031	-0.1828
FAM134A	6	0.12337	0.26848	0.99998	5032	-0.1939
NEDD4	6	0.12354	0.26873	0.99998	5033	-0.2031
LY6G6D	6	0.12355	0.26875	0.99998	5034	-0.3968
HPD	6	0.12358	0.26879	0.99998	5035	-0.0453
PC	6	0.12358	0.26879	0.99998	5036	0.0538
HMGXB3	6	0.1237	0.26897	0.99998	5037	-0.2536
SYCN	6	0.1237	0.26897	0.99998	5038	-0.0774
KIAA1551	6	0.1237	0.26898	0.99998	5039	0.1097
ENPP4	6	0.12374	0.26902	0.99998	5040	0.026
MED13	6	0.12377	0.26907	0.99998	5041	-0.0386
TPBGL	6	0.12377	0.26907	0.99998	5042	-0.0271
TDRD10	6	0.12384	0.26917	0.99998	5043	0.2308
EIF4B	6	0.12384	0.26917	0.99998	5044	0.0452
SOCS5	6	0.12384	0.26917	0.99998	5045	-0.0115
E2F8	6	0.12384	0.26917	0.99998	5046	0.1389
SLC35D3	6	0.12387	0.26919	0.99998	5047	0.0204
IFFO2	6	0.12387	0.26919	0.99998	5048	-0.1045
LRRFIP1	6	0.12387	0.26919	0.99998	5049	-0.1099
CD5L	6	0.12396	0.26931	0.99998	5050	-0.0264
ING3	4	0.12397	0.23733	0.99998	5051	0.0005
PEX7	6	0.124	0.26936	0.99998	5052	-0.185
HSD17B1	6	0.12403	0.2694	0.99998	5053	-0.004
ATR	6	0.12403	0.26941	0.99998	5054	-0.1411
RAB6B	6	0.1241	0.2695	0.99998	5055	-0.2137
HRG	6	0.1241	0.2695	0.99998	5056	-0.148
KLHL1	6	0.12412	0.26952	0.99998	5057	-0.0891
MMP21	6	0.12423	0.26969	0.99998	5058	-0.1293
PTGR1	6	0.12424	0.26969	0.99998	5059	-0.0945
YPEL1	6	0.12425	0.26971	0.99998	5060	0.1018
FOXD4L1	5	0.12436	0.25667	0.99998	5061	0.0215
DBN1	6	0.12437	0.26988	0.99998	5062	-0.1021
CECR2	6	0.12438	0.2699	0.99998	5063	-0.3773
SGK196	4	0.12441	0.23784	0.99998	5064	-0.08
OR5T3	6	0.12447	0.27002	0.99998	5065	-0.0006
CALHM3	6	0.12447	0.27002	0.99998	5066	-0.1763
HSD11B1	6	0.12447	0.27002	0.99998	5067	-0.0952
PCSK2	6	0.12448	0.27005	0.99998	5068	-0.0004
ZNF174	6	0.12461	0.27023	0.99998	5069	-0.1272
LRP8	6	0.12467	0.2703	0.99998	5070	0.0246
PHYHD1	6	0.12467	0.2703	0.99998	5071	-0.2039
RGPD1	4	0.12468	0.23815	0.99998	5072	-0.0257
MUTYH	6	0.12473	0.27039	0.99998	5073	-0.0111
TIPIN	6	0.12473	0.27039	0.99998	5074	-0.1275
PKD2L2	6	0.12473	0.27039	0.99998	5075	0.103
APOA1	6	0.12485	0.27055	0.99998	5076	-0.1825
ZNF559-ZNF1	2	0.12487	0.18618	0.99998	5077	-0.1812
NUGGC	6	0.12491	0.27064	0.99998	5078	-0.0193
ZNF322	6	0.12491	0.27064	0.99998	5079	-0.2197
TMEM151A	6	0.125	0.27077	0.99998	5080	-0.2405
TNNI3	6	0.12502	0.27079	0.99998	5081	-0.2306
TMA16	6	0.12503	0.27081	0.99998	5082	-0.1727
NCAPD2	6	0.12514	0.27098	0.99998	5083	-0.1385
STMND1	6	0.12517	0.27103	0.99998	5084	0.0499
hsa-mir-4463	4	0.12519	0.23869	0.99998	5085	-0.256
MTA3	6	0.1253	0.27122	0.99998	5086	0.1364
RPS3	6	0.12531	0.27123	0.99998	5087	-0.2628
SRRD	6	0.12534	0.27127	0.99998	5088	-0.0195
TMEM231	6	0.12539	0.27134	0.99998	5089	0.0525
ATP13A2	6	0.12545	0.27142	0.99998	5090	-0.0819
HIST1H1C	6	0.12545	0.27142	0.99998	5091	0.071
TRPC5OS	6	0.1255	0.2715	0.99998	5092	-0.0957
IL21	6	0.12559	0.27164	0.99998	5093	-0.0334
LGALS4	6	0.12561	0.27167	0.99998	5094	0.0677
hsa-mir-7975	4	0.12562	0.23917	0.99998	5095	-0.522
FAM171A1	6	0.12565	0.27172	0.99998	5096	-0.2411
DOK5	6	0.12568	0.27177	0.99998	5097	-0.0427
ADAMTS6	6	0.12573	0.27184	0.99998	5098	-0.0993
RND1	6	0.12576	0.27187	0.99998	5099	-0.099
TBX6	6	0.12582	0.27197	0.99998	5100	-0.0849

LENEP	6	0.12586	0.27202	0.99998	5101	-0.2198
INTS6	6	0.12587	0.27204	0.99998	5102	-0.0969
RLF	6	0.12594	0.27213	0.99998	5103	-0.1338
GDF7	6	0.12596	0.27216	0.99998	5104	0.0207
DCBLD1	6	0.12596	0.27216	0.99998	5105	-0.0864
SLC48A1	6	0.12596	0.27216	0.99998	5106	0.0465
HECA	6	0.12605	0.27229	0.99998	5107	-0.0752
ARMCX2	6	0.12615	0.27242	0.99998	5108	-0.0513
SETD2	6	0.12624	0.27255	0.99998	5109	0.0943
CPA4	6	0.12626	0.27257	0.99998	5110	-0.1157
hsa-mir-5690	4	0.12627	0.23987	0.99998	5111	-0.0694
ZNF675	4	0.12634	0.23995	0.99998	5112	-0.3943
MS4A14	6	0.12636	0.27272	0.99998	5113	-0.1172
hsa-mir-6132	4	0.12639	0.24	0.99998	5114	-0.1282
XAF1	6	0.12641	0.27278	0.99998	5115	0.0563
CPA6	6	0.12643	0.2728	0.99998	5116	-0.201
DNAJB14	6	0.12643	0.2728	0.99998	5117	0.1236
NCOR2	6	0.12649	0.27289	0.99998	5118	-0.1373
SLC37A4	6	0.12658	0.27302	0.99998	5119	-0.1247
COL4A6	6	0.12674	0.27325	0.99998	5120	-0.2584
PLEKHO1	6	0.1268	0.27333	0.99998	5121	0.0872
RGS7	6	0.12695	0.27354	0.99998	5122	-0.0755
hsa-mir-4711	4	0.12699	0.24066	0.99998	5123	-0.0992
PDCD6	6	0.12699	0.27358	0.99998	5124	-0.0242
hsa-mir-7155	4	0.12704	0.24072	0.99998	5125	-0.1712
DEFB108B	6	0.12705	0.27366	0.99998	5126	-0.0064
DKC1	6	0.12708	0.2737	0.99998	5127	-0.1273
FAM13A	6	0.1271	0.27372	0.99998	5128	-0.0884
MYB	6	0.12713	0.27378	0.99998	5129	-0.1439
ZPBP2	6	0.12723	0.27391	0.99998	5130	-0.2395
USP19	6	0.12723	0.27391	0.99998	5131	-0.1741
ITGB4	6	0.12724	0.27393	0.99998	5132	0.0757
MTR	6	0.12726	0.27395	0.99998	5133	0.0194
UNC119B	6	0.12726	0.27395	0.99998	5134	0.0364
UGGT1	6	0.12736	0.27411	0.99998	5135	0.1536
NAB2	6	0.12738	0.27414	0.99998	5136	-0.1429
SLC4A1	6	0.12739	0.27415	0.99998	5137	-0.0989
PCDHA8	2	0.12739	0.18802	0.99998	5138	0.0098
KLRC4-KLRK1	2	0.12743	0.18805	0.99998	5139	-0.1963
CTSH	6	0.1275	0.2743	0.99998	5140	-0.0296
ING2	6	0.1275	0.2743	0.99998	5141	0.0223
DCTN4	6	0.12752	0.27432	0.99998	5142	-0.4714
PKHD1L1	6	0.12755	0.27437	0.99998	5143	0.0574
SYTL3	6	0.12763	0.27449	0.99998	5144	-0.0249
ZCCHC18	6	0.12772	0.27463	0.99998	5145	0.1361
SSBP2	6	0.12772	0.27463	0.99998	5146	0.1426
HNRNPD	6	0.12772	0.27463	0.99998	5147	-0.2971
RFPL1	5	0.12773	0.26214	0.99998	5148	-0.1883
RRM1	6	0.12782	0.27478	0.99998	5149	-0.1546
SLC20A1	6	0.12784	0.27482	0.99998	5150	0.0874
RSPRY1	6	0.12787	0.27486	0.99998	5151	-0.0276
SDK1	6	0.12787	0.27486	0.99998	5152	-0.1291
FAM78A	6	0.12792	0.27492	0.99998	5153	-0.1605
TAS2R20	6	0.12799	0.27502	0.99998	5154	0.0967
hsa-mir-5011	4	0.12802	0.24184	0.99998	5155	-0.1142
C6orf132	6	0.12808	0.27515	0.99998	5156	-0.1754
SBDS	6	0.12808	0.27516	0.99998	5157	-0.1089
PIAS1	6	0.12818	0.2753	0.99998	5158	-0.1321
MYH15	6	0.12818	0.2753	0.99998	5159	-0.2007
hsa-mir-198	4	0.12824	0.24209	0.99998	5160	0.1056
KLF15	6	0.12825	0.27542	0.99998	5161	-0.0254
ZNF281	6	0.12828	0.27546	0.99998	5162	-0.0621
ORC1	6	0.12828	0.27546	0.99998	5163	0.0598
PPP1R16A	6	0.12834	0.27552	0.99998	5164	0.1572
PPP1R15A	6	0.12834	0.27552	0.99998	5165	-0.0092
MECP2	6	0.12834	0.27552	0.99998	5166	0.1809
NFRKB	6	0.12834	0.27552	0.99998	5167	0.0825
hsa-mir-6886	2	0.12836	0.18871	0.99998	5168	-0.0216
hsa-mir-340	4	0.12838	0.24225	0.99998	5169	-0.1529
LYSMD4	6	0.12838	0.27559	0.99998	5170	-0.0456
RIMBP2	6	0.12841	0.27561	0.99998	5171	0.0559
CTSV	2	0.12851	0.18882	0.99998	5172	-0.1512
hsa-mir-484	4	0.12852	0.24241	0.99998	5173	-0.4104
CPLX2	6	0.12857	0.27583	0.99998	5174	0.0215
GPSM2	6	0.12862	0.27592	0.99998	5175	0.1594
STOML3	6	0.12866	0.27599	0.99998	5176	0.1723
C16orf46	6	0.1287	0.27604	0.99998	5177	-0.2847
ATP5J2	5	0.1287	0.26369	0.99998	5178	-0.1741
TSPAN33	4	0.12871	0.24263	0.99998	5179	0.0007
CSE1L	6	0.12882	0.27623	0.99998	5180	0.0716
FAM161B	6	0.12882	0.27623	0.99998	5181	0.0361

PROL1	6	0.12887	0.27628	0.99998	5182	-0.1849
AMER1	6	0.12887	0.27628	0.99998	5183	-0.0562
LPHN1	6	0.12894	0.2764	0.99998	5184	-0.1167
FOXO1	6	0.12899	0.27646	0.99998	5185	-0.0939
OR51G1	6	0.12899	0.27646	0.99998	5186	-0.1827
SLC7A7	6	0.12899	0.27646	0.99998	5187	-0.2721
CSNK2A1	6	0.12903	0.27653	0.99998	5188	-0.409
LCTL	6	0.12905	0.27656	0.99998	5189	-0.21
YAE1D1	6	0.12913	0.27666	0.99998	5190	-0.1922
RGS2	6	0.12913	0.27666	0.99998	5191	-0.0117
hsa-mir-655	4	0.12915	0.24313	0.99998	5192	-0.3516
CTAG2	5	0.12922	0.26453	0.99998	5193	-0.1578
PCDHA4	2	0.12924	0.18935	0.99998	5194	0.1552
ELL	6	0.12927	0.27686	0.99998	5195	-0.0192
OR10AG1	6	0.12927	0.27687	0.99998	5196	-0.0073
UNG	6	0.12934	0.27695	0.99998	5197	-0.0401
ISM2	6	0.12934	0.27695	0.99998	5198	-0.1238
OR4X2	6	0.12939	0.27704	0.99998	5199	0.1812
MMD2	6	0.1294	0.27705	0.99998	5200	0.0583
RBP7	6	0.12945	0.27712	0.99998	5201	0.1152
CCP110	6	0.12949	0.27718	0.99998	5202	-0.0573
ABCA1	6	0.12954	0.27725	0.99998	5203	0.0124
BNIPL	6	0.12959	0.27731	0.99998	5204	-0.0197
SMOX	6	0.12963	0.27737	0.99998	5205	-0.0831
MFSD11	6	0.12971	0.27748	0.99998	5206	0.0008
SRRT	6	0.12978	0.27758	0.99998	5207	-0.0467
MYCBP	6	0.12983	0.27767	0.99998	5208	-0.0049
TRMT6	6	0.12985	0.2777	0.99998	5209	-0.2523
LIMS1	6	0.12985	0.2777	0.99998	5210	-0.2071
DUSP18	6	0.12985	0.2777	0.99998	5211	-0.2038
RAB18	6	0.12987	0.27773	0.99998	5212	-0.0261
OR52E8	6	0.12994	0.27782	0.99998	5213	-0.0236
PHLDA3	6	0.13007	0.27799	0.99998	5214	-0.0717
LRFN5	6	0.13015	0.27811	0.99998	5215	-0.1018
hsa-mir-4695	4	0.13021	0.24433	0.99998	5216	-0.2979
JRKL	6	0.13022	0.27821	0.99998	5217	0.2426
CRMP1	6	0.13022	0.27821	0.99998	5218	-0.2625
ACER1	6	0.13026	0.27827	0.99998	5219	-0.1706
CCDC135	6	0.13026	0.27827	0.99998	5220	-0.161
LPCAT2	6	0.13033	0.27837	0.99998	5221	0.0672
CFD	6	0.13036	0.27841	0.99998	5222	0.0112
VAX1	6	0.13047	0.27858	0.99998	5223	-0.0625
RAB3IP	6	0.13047	0.27858	0.99998	5224	-0.0996
PIANP	6	0.13059	0.27875	0.99998	5225	-0.063
OGDHL	6	0.13059	0.27875	0.99998	5226	0.5092
IVL	6	0.13065	0.27882	0.99998	5227	-0.0576
TMSB4X	4	0.13065	0.24486	0.99998	5228	-0.1435
RERE	6	0.13066	0.27884	0.99998	5229	-0.2698
RFX3	6	0.13066	0.27884	0.99998	5230	0.0909
UBE2B	6	0.13072	0.27894	0.99998	5231	-0.1375
RPAP1	6	0.13073	0.27894	0.99998	5232	0.0206
MEF2D	6	0.13084	0.27911	0.99998	5233	-0.24
STXBP3	6	0.13087	0.27915	0.99998	5234	-0.2049
FBLN7	6	0.13091	0.2792	0.99998	5235	-0.1353
EID1	6	0.13098	0.2793	0.99998	5236	-0.0115
CDH19	6	0.13101	0.27934	0.99998	5237	0.0628
GDF9	6	0.13101	0.27934	0.99998	5238	-0.2048
TRIM22	6	0.13107	0.27942	0.99998	5239	-0.0664
SMIM22	5	0.13113	0.26763	0.99998	5240	-0.1619
KLF6	6	0.13114	0.27954	0.99998	5241	0.0927
ZNF474	6	0.13114	0.27954	0.99998	5242	-0.0261
KCNJ14	6	0.13118	0.27959	0.99998	5243	-0.1405
PRAC1	4	0.13143	0.24573	0.99998	5244	-0.0176
SLC22A31	6	0.13153	0.28009	0.99998	5245	-0.1422
RAB13	6	0.13155	0.2801	0.99998	5246	-0.0189
PLXNB3	6	0.13163	0.28022	0.99998	5247	0.2924
PFN3	6	0.13167	0.28028	0.99998	5248	-0.1612
hsa-mir-19b-1	4	0.13169	0.24603	0.99998	5249	-0.2503
SMCR8	6	0.13173	0.28036	0.99998	5250	0.0189
FAM19A1	6	0.13175	0.2804	0.99998	5251	-0.1176
GLT8D2	6	0.13175	0.2804	0.99998	5252	-0.168
SEC23B	6	0.13188	0.2806	0.99998	5253	0.2388
LRPPRC	6	0.13205	0.28085	0.99998	5254	-0.0141
SIN3B	6	0.13206	0.28085	0.99998	5255	-0.134
TBC1D10B	6	0.13216	0.28102	0.99998	5256	-0.0985
KIRREL3	6	0.13216	0.28102	0.99998	5257	-0.1798
ADCY10	6	0.13228	0.28118	0.99998	5258	-0.0554
hsa-mir-548l	4	0.13231	0.24669	0.99998	5259	-0.2189
FBXL5	6	0.13239	0.28133	0.99998	5260	0.0048
GDF1	6	0.13242	0.28138	0.99998	5261	-0.0135
GNPAT	6	0.13242	0.28138	0.99998	5262	0.1629

NHS	6	0.13251	0.2815	0.99998	5263	-0.1355
PRDM8	6	0.13251	0.28151	0.99998	5264	-0.1412
SDCBP	6	0.13252	0.28152	0.99998	5265	-0.033
DAO	6	0.13252	0.28152	0.99998	5266	-0.1701
KMT2B	3	0.13259	0.21978	0.99998	5267	-0.3918
IMMT	6	0.1326	0.28165	0.99998	5268	-0.2127
LIPA	6	0.13265	0.28171	0.99998	5269	-0.0691
C21orf2	6	0.13267	0.28174	0.99998	5270	-0.1585
BICD1	6	0.13267	0.28174	0.99998	5271	-0.2082
FBXO3	6	0.13276	0.28187	0.99998	5272	0.1708
RPS26	6	0.1328	0.28192	0.99998	5273	-0.1304
HOOK3	6	0.13282	0.28195	0.99998	5274	-0.0609
CCDC170	6	0.13283	0.28197	0.99998	5275	0.0026
hsa-mir-4755	4	0.13287	0.24734	0.99998	5276	0.1985
GPR151	6	0.13288	0.28205	0.99998	5277	0.1987
STEAP2	6	0.13289	0.28207	0.99998	5278	-0.1202
hsa-mir-6805	4	0.13289	0.24737	0.99998	5279	-0.1757
RGS12	6	0.13293	0.28212	0.99998	5280	-0.1241
SP6	6	0.13295	0.28214	0.99998	5281	-0.2989
RIN2	6	0.13296	0.28216	0.99998	5282	-0.2155
FGF23	6	0.13304	0.28227	0.99998	5283	-0.0715
CCDC60	6	0.13309	0.28233	0.99998	5284	-0.1335
C6orf1	6	0.13311	0.28235	0.99998	5285	-0.297
CCBE1	6	0.13314	0.2824	0.99998	5286	-0.1144
DOPEY1	6	0.13317	0.28244	0.99998	5287	-0.1282
CDKL5	6	0.1332	0.28249	0.99998	5288	0.1997
RIMBP3B	2	0.13321	0.19235	0.99998	5289	-0.2899
TMEM198	6	0.13325	0.28255	0.99998	5290	0.0648
C6orf203	6	0.13333	0.28266	0.99998	5291	-0.0967
OR8U1	3	0.1334	0.22089	0.99998	5292	-0.2149
ACYP2	6	0.13345	0.28284	0.99998	5293	-0.1672
RAD54L2	4	0.13346	0.248	0.99998	5294	-0.1449
RPS4Y1	6	0.13348	0.28289	0.99998	5295	-0.0719
ZNF853	6	0.13348	0.28289	0.99998	5296	-0.0473
F13B	6	0.1335	0.28292	0.99998	5297	-0.2093
OR1A2	6	0.1335	0.28292	0.99998	5298	0.1751
AGER	6	0.13357	0.28302	0.99998	5299	-0.1175
KIAA1244	6	0.13362	0.28308	0.99998	5300	-0.0502
PRAMEF11	5	0.13367	0.27174	0.99998	5301	-0.1125
PSMD9	6	0.13368	0.28318	0.99998	5302	-0.05
TET2	6	0.13368	0.28318	0.99998	5303	-0.0774
HLA-F	6	0.13369	0.2832	0.99998	5304	-0.2273
MYEF2	6	0.13369	0.2832	0.99998	5305	-0.1992
GTF2I	6	0.13381	0.28337	0.99998	5306	0.0596
CAPN6	6	0.13383	0.28338	0.99998	5307	0.0347
HMSD	6	0.13383	0.28338	0.99998	5308	0.1186
TNFRSF1A	6	0.13391	0.2835	0.99998	5309	-0.1638
ATP11B	6	0.13395	0.28355	0.99998	5310	-0.0914
BMPR1B	6	0.13406	0.28368	0.99998	5311	-0.1695
ORC5	6	0.13406	0.28368	0.99998	5312	0.0444
hsa-mir-4532	4	0.13409	0.24874	0.99998	5313	-0.0164
38961	3	0.13415	0.22194	0.99998	5314	-0.1937
LRRC7	6	0.13417	0.28383	0.99998	5315	-0.0372
ABCB1	6	0.13417	0.28383	0.99998	5316	-0.2261
HRASLS	6	0.13422	0.28391	0.99998	5317	-0.023
hsa-mir-4280	4	0.13425	0.24892	0.99998	5318	-0.0035
KMT2A	4	0.13434	0.24902	0.99998	5319	0.0481
RPS6KC1	6	0.13434	0.28407	0.99998	5320	0.0181
MRM1	6	0.13445	0.28424	0.99998	5321	0.0502
EBI3	6	0.13445	0.28424	0.99998	5322	-0.0302
CDKN2C	6	0.1345	0.28431	0.99998	5323	-0.027
SCAF1	6	0.1345	0.28431	0.99998	5324	-0.1124
OR2B3	6	0.13458	0.28444	0.99998	5325	-0.1233
WNK4	6	0.13458	0.28444	0.99998	5326	-0.2144
DDX3X	6	0.1346	0.28447	0.99998	5327	0.0886
APBA1	6	0.13468	0.28459	0.99998	5328	-0.1823
SEMA4A	6	0.13468	0.28459	0.99998	5329	-0.2056
LIFR	6	0.13468	0.2846	0.99998	5330	0.0746
AMICA1	6	0.13482	0.28479	0.99998	5331	-0.1498
SLC35B2	6	0.13482	0.28479	0.99998	5332	0.2353
CMSS1	6	0.13484	0.28481	0.99998	5333	0.1541
ZNF157	6	0.13484	0.28481	0.99998	5334	0.1681
ABTB2	6	0.13503	0.28508	0.99998	5335	-0.0591
RAB3GAP1	6	0.13503	0.28508	0.99998	5336	-0.0382
LIN28B	6	0.1351	0.28518	0.99998	5337	-0.0654
EIF4H	6	0.13512	0.28521	0.99998	5338	-0.1279
CDNF	6	0.13515	0.28525	0.99998	5339	-0.0552
TBC1D31	3	0.13516	0.22325	0.99998	5340	-0.1521
ZFP30	6	0.13538	0.28554	0.99998	5341	0.1019
ZNF233	6	0.13539	0.28556	0.99998	5342	-0.0983
GTF2E1	6	0.13539	0.28556	0.99998	5343	-0.147

RPL39L	5	0.1354	0.27449	0.99998	5344	-0.5413
ZNF562	5	0.1354	0.27449	0.99998	5345	-0.2802
RENBP	6	0.13542	0.2856	0.99998	5346	-0.1544
GTSF1	6	0.13551	0.28573	0.99998	5347	-0.0917
MAP3K14	6	0.13551	0.28573	0.99998	5348	0.0067
TMEM212	6	0.13555	0.2858	0.99998	5349	-0.2431
NR0B1	6	0.13555	0.2858	0.99998	5350	-0.0593
VBP1	6	0.13565	0.28594	0.99998	5351	-0.0618
USP17L2	6	0.1357	0.28601	0.99998	5352	0.0283
ZNF267	6	0.13574	0.28606	0.99998	5353	-0.4392
ADHFE1	6	0.13574	0.28606	0.99998	5354	-0.3579
UBXN2A	6	0.13574	0.28606	0.99998	5355	-0.189
SELRC1	6	0.13581	0.28618	0.99998	5356	-0.1526
OR5A2	6	0.13593	0.28634	0.99998	5357	-0.091
ABHD16A	6	0.13593	0.28634	0.99998	5358	-0.1584
GPR153	6	0.13595	0.28636	0.99998	5359	0.1306
P2RX3	6	0.13602	0.28647	0.99998	5360	-0.1242
PPIL2	6	0.13611	0.28661	0.99998	5361	-0.0657
ZCCHC11	6	0.13613	0.28663	0.99998	5362	-0.1322
PASK	6	0.13619	0.28671	0.99998	5363	-0.1603
ZNF672	6	0.13621	0.28674	0.99998	5364	0.1572
LCN1	6	0.13623	0.28677	0.99998	5365	0.0996
PMAIP1	6	0.13627	0.28681	0.99998	5366	-0.14
hsa-mir-302e	4	0.13628	0.25122	0.99998	5367	-0.2477
hsa-mir-4700	4	0.13628	0.25122	0.99998	5368	0.1063
IL15	6	0.13631	0.28687	0.99998	5369	0.0756
CITED2	6	0.13639	0.28699	0.99998	5370	-0.1712
SNRNP70	6	0.13642	0.28704	0.99998	5371	-0.0702
BTN3A2	6	0.13642	0.28704	0.99998	5372	-0.2531
ADAMTS9	6	0.13643	0.28705	0.99998	5373	-0.0796
ALKBH3	6	0.13643	0.28705	0.99998	5374	-0.1503
YAP1	6	0.13652	0.28717	0.99998	5375	-0.1939
hsa-mir-4432	4	0.13655	0.25153	0.99998	5376	-0.0553
SPAG5	6	0.13669	0.28743	0.99998	5377	0.0273
ILF3	6	0.13672	0.28747	0.99998	5378	-0.037
C6orf226	6	0.13678	0.28754	0.99998	5379	-0.0464
C11orf53	6	0.13678	0.28754	0.99998	5380	0.0143
ANKRD40	6	0.13681	0.28758	0.99998	5381	-0.1787
TCEB3B	6	0.13681	0.28758	0.99998	5382	-0.1704
CD68	6	0.13687	0.28767	0.99998	5383	0.0204
CASP7	6	0.13687	0.28767	0.99998	5384	0.0242
TMEM129	6	0.13688	0.2877	0.99998	5385	-0.1082
NEBL	6	0.13699	0.28783	0.99998	5386	-0.0613
OGFRL1	6	0.137	0.28785	0.99998	5387	-0.0768
CATSPERG	6	0.13707	0.28794	0.99998	5388	-0.3652
OSM	6	0.13708	0.28795	0.99998	5389	0.208
POU2F3	6	0.13709	0.28797	0.99998	5390	-0.0308
hsa-mir-4421	4	0.13712	0.25217	0.99998	5391	-0.4208
C1orf64	6	0.13717	0.28808	0.99998	5392	-0.0503
AP4E1	6	0.13718	0.28809	0.99998	5393	-0.1515
hsa-mir-1909	4	0.1372	0.25225	0.99998	5394	-0.0893
OR7A17	6	0.13722	0.28815	0.99998	5395	0.0206
HIF3A	6	0.13733	0.28829	0.99998	5396	-0.0997
TMEM52	6	0.13742	0.28843	0.99998	5397	0.0196
GMNN	6	0.13745	0.28848	0.99998	5398	-0.2502
SLITRK3	6	0.13745	0.28848	0.99998	5399	-0.2353
FLJ25363	6	0.13745	0.28848	0.99998	5400	-0.188
ASB10	6	0.13759	0.28868	0.99998	5401	-0.1337
CBWD5	2	0.1376	0.19563	0.99998	5402	-0.9455
AATK	6	0.13766	0.28879	0.99998	5403	-0.0402
GLYAT	6	0.13767	0.28881	0.99998	5404	-0.1925
RABEPK	6	0.13777	0.28897	0.99998	5405	0.0197
ICAM4	6	0.13788	0.28911	0.99998	5406	-0.2257
ZBTB24	6	0.13788	0.28911	0.99998	5407	-0.2065
EGR2	5	0.13791	0.27853	0.99998	5408	0.1277
OR6V1	6	0.13793	0.28917	0.99998	5409	-3E-05
SAMM50	6	0.13798	0.28924	0.99998	5410	-0.0585
AP3D1	6	0.13798	0.28924	0.99998	5411	0.1726
hsa-mir-6081	4	0.13821	0.25343	0.99998	5412	-0.0399
KRTAP9-7	5	0.13822	0.27905	0.99998	5413	-0.7622
SGSM1	6	0.13822	0.28958	0.99998	5414	0.0267
MGST1	6	0.13823	0.28959	0.99998	5415	-0.1549
MAP2K7	6	0.13838	0.28979	0.99998	5416	-0.1166
MRE11A	6	0.13839	0.28982	0.99998	5417	0.0774
BCL9L	6	0.13844	0.28987	0.99998	5418	0.0133
CCNB1IP1	6	0.13849	0.28994	0.99998	5419	0.0765
RBBP6	4	0.13849	0.25375	0.99998	5420	-0.007
C3orf84	1	0.13849	0.13836	0.99397	5421	-0.3799
TIMM17B	6	0.13851	0.28997	0.99998	5422	-0.0218
FOXI2	6	0.1386	0.29009	0.99998	5423	0.0282
NADKD1	6	0.13869	0.29022	0.99998	5424	0.0083

STIM1	6	0.13872	0.29026	0.99998	5425	0.1883
C20orf111	2	0.13872	0.19647	0.99998	5426	-0.1589
TAP1	6	0.13878	0.29035	0.99998	5427	-0.1391
BAHD1	6	0.13881	0.29039	0.99998	5428	-0.0264
hsa-mir-6893	4	0.13887	0.2542	0.99998	5429	0.1358
VSNL1	6	0.13888	0.29049	0.99998	5430	0.0573
HDHD2	6	0.13897	0.29062	0.99998	5431	0.1042
NPPC	6	0.13897	0.29062	0.99998	5432	0.0913
BECN1	6	0.139	0.29067	0.99998	5433	-0.1019
ABCD3	6	0.13904	0.29072	0.99998	5434	-0.1307
CIDEB	6	0.13906	0.29074	0.99998	5435	-0.1147
ANKHD1-EIF4I	4	0.13916	0.25451	0.99998	5436	-0.0394
hsa-mir-7977	4	0.13918	0.25453	0.99998	5437	-0.0869
PDCL3	6	0.1392	0.29095	0.99998	5438	-0.0013
TTC14	6	0.13936	0.29119	0.99998	5439	-0.0763
NEMF	6	0.13941	0.29126	0.99998	5440	0.0194
hsa-mir-3920	4	0.13964	0.25505	0.99998	5441	-0.1163
GLRA2	6	0.13966	0.2916	0.99998	5442	-0.1534
ADM2	6	0.13977	0.29177	0.99998	5443	0.0625
hsa-mir-6737	4	0.1398	0.25523	0.99998	5444	-0.0835
CLEC11A	6	0.13981	0.29183	0.99998	5445	-0.1835
LSM1	6	0.13985	0.29188	0.99998	5446	-0.1076
ZSCAN25	6	0.13987	0.2919	0.99998	5447	0.0593
OLFM3	6	0.13988	0.29192	0.99998	5448	-0.1066
CLTB	6	0.13995	0.29201	0.99998	5449	-0.1387
LOX	6	0.13995	0.29202	0.99998	5450	0.0057
CENPC1	4	0.13996	0.25541	0.99998	5451	-0.1836
FCRLB	6	0.14003	0.29211	0.99998	5452	0.0201
hsa-mir-544a	4	0.14004	0.25551	0.99998	5453	-0.1521
hsa-mir-199a-	3	0.14009	0.22993	0.99998	5454	-0.5938
ATP5G2	6	0.14013	0.29225	0.99998	5455	0.0496
ALDH3B2	6	0.14016	0.2923	0.99998	5456	0.0184
hsa-mir-939	4	0.14026	0.25575	0.99998	5457	-0.0408
WDR20	6	0.14037	0.2926	0.99998	5458	-0.0657
MICALL2	6	0.14037	0.2926	0.99998	5459	0.1365
PTRH1	6	0.1404	0.29265	0.99998	5460	-0.0898
SLC35A4	6	0.14053	0.29283	0.99998	5461	-0.1257
DNTTIP1	6	0.14054	0.29284	0.99998	5462	0.381
PLOD1	6	0.14054	0.29285	0.99998	5463	-0.0874
SYNJ2BP	1	0.14062	0.14047	0.99494	5464	-0.3591
SCARB2	6	0.14067	0.29301	0.99998	5465	-0.0791
PTER	6	0.14069	0.29305	0.99998	5466	-0.0575
C6orf15	6	0.1407	0.29305	0.99998	5467	-0.0244
TMEM41A	6	0.14076	0.29315	0.99998	5468	-0.0351
hsa-mir-4310	4	0.14083	0.25637	0.99998	5469	-0.1848
HTR5A	6	0.14085	0.29329	0.99998	5470	-0.1273
MRGPRG	6	0.14085	0.29329	0.99998	5471	-0.0122
RPS27A	6	0.14085	0.29329	0.99998	5472	0.0042
ZNF287	6	0.14091	0.29336	0.99998	5473	-0.1536
RP9	6	0.14094	0.29341	0.99998	5474	0.2373
DNAAF3	6	0.14094	0.29341	0.99998	5475	0.014
LOC81691	6	0.14104	0.29354	0.99998	5476	-0.1121
ZNF616	6	0.14117	0.29372	0.99998	5477	-0.0737
GNA11	6	0.14117	0.29372	0.99998	5478	-0.2912
MOB2	6	0.14117	0.29372	0.99998	5479	0.0515
CCDC30	6	0.14123	0.2938	0.99998	5480	-0.1731
EBF4	6	0.14127	0.29386	0.99998	5481	-0.0575
CENPQ	6	0.14128	0.29387	0.99998	5482	-0.1556
PTPLA	6	0.14135	0.29397	0.99998	5483	0.094
ZNFX1	6	0.14146	0.29413	0.99998	5484	-0.1342
ALDOB	6	0.14146	0.29413	0.99998	5485	-0.121
UXS1	6	0.14152	0.29421	0.99998	5486	-0.1481
NXF1	6	0.14152	0.29421	0.99998	5487	-0.1543
MNF1	4	0.14153	0.25716	0.99998	5488	-0.098
NPC2	6	0.14154	0.29423	0.99998	5489	0.1958
NCAM2	6	0.14154	0.29424	0.99998	5490	-0.0856
CELSR2	6	0.14159	0.2943	0.99998	5491	-0.0392
FAM198A	6	0.14163	0.29437	0.99998	5492	-0.1364
HNRNPA1	6	0.14169	0.29445	0.99998	5493	-0.4801
SLC12A2	6	0.14172	0.29449	0.99998	5494	0.0249
MRGPRX3	6	0.14172	0.29449	0.99998	5495	-0.0929
ATP1A1	6	0.1418	0.29459	0.99998	5496	-0.1548
C14orf169	6	0.14184	0.29465	0.99998	5497	0.0346
HSD17B3	6	0.1419	0.29473	0.99998	5498	-0.13
FCGR1A	3	0.14194	0.23243	0.99998	5499	-0.2656
IQCJ	2	0.14197	0.19885	0.99998	5500	-0.1096
ZFPL1	6	0.142	0.29487	0.99998	5501	0.1674
IGFBP7	6	0.142	0.29487	0.99998	5502	-0.0763
GLT1D1	6	0.142	0.29487	0.99998	5503	-0.1394
SH3BGRL3	6	0.14214	0.29507	0.99998	5504	0.0831
ISG15	6	0.14216	0.2951	0.99998	5505	0.1153

MZF1	6	0.14218	0.29513	0.99998	5506	-0.1957
C4orf46	6	0.14223	0.29522	0.99998	5507	-0.0319
LCE1D	6	0.14234	0.29538	0.99998	5508	0.0979
MDGA2	6	0.14238	0.29544	0.99998	5509	-0.034
TMEM253	6	0.14242	0.2955	0.99998	5510	-0.1695
HIST1H2AH	6	0.14242	0.2955	0.99998	5511	-0.2029
OR2A14	6	0.14246	0.29556	0.99998	5512	0.0781
hsa-mir-4760	2	0.14253	0.19927	0.99998	5513	-0.1259
TMED7	4	0.14253	0.25828	0.99998	5514	-0.1555
MCRS1	6	0.14255	0.29567	0.99998	5515	-0.0881
GPR176	6	0.14255	0.29567	0.99998	5516	-0.166
FNTA	6	0.14263	0.29581	0.99998	5517	0.0851
ARL17B	1	0.14269	0.14256	0.99646	5518	-0.4154
DEF8	6	0.1427	0.29592	0.99998	5519	-0.1484
QDPR	6	0.14273	0.29596	0.99998	5520	-0.3995
TTC9	6	0.14281	0.29606	0.99998	5521	-0.0703
ANKRD36	6	0.14282	0.29608	0.99998	5522	-0.4182
TBCE	6	0.14287	0.29613	0.99998	5523	-0.0844
ABCC12	6	0.1429	0.29617	0.99998	5524	-0.1801
ST3GAL6	6	0.1429	0.29617	0.99998	5525	-0.1998
GPATCH4	4	0.14291	0.2587	0.99998	5526	-0.1142
SOS2	6	0.14298	0.2963	0.99998	5527	-0.0729
MAGEB1	6	0.14298	0.2963	0.99998	5528	0.2119
DPM1	6	0.14305	0.29639	0.99998	5529	-0.1121
MAN2A2	6	0.14305	0.29639	0.99998	5530	-0.0266
ZNF524	6	0.14309	0.29644	0.99998	5531	0.108
HIST1H4F	6	0.14309	0.29645	0.99998	5532	-0.094
SLC14A2	6	0.14318	0.29658	0.99998	5533	-0.0826
GLI4	6	0.14319	0.2966	0.99998	5534	-0.091
ZSWIM3	6	0.14322	0.29664	0.99998	5535	-0.1378
FUT8	6	0.14322	0.29664	0.99998	5536	-0.1457
C12orf29	6	0.14322	0.29664	0.99998	5537	-0.3567
PSME2	6	0.14322	0.29664	0.99998	5538	-0.0438
WDR37	6	0.14322	0.29664	0.99998	5539	-0.2864
CEP135	6	0.14332	0.29677	0.99998	5540	0.0296
IL1R2	6	0.14347	0.29698	0.99998	5541	-0.0609
GPRIN2	6	0.14347	0.29698	0.99998	5542	0.2319
CHGA	6	0.14348	0.297	0.99998	5543	0.241
CLN8	6	0.14348	0.297	0.99998	5544	0.0092
KCNJ10	6	0.14348	0.297	0.99998	5545	-0.0884
BMP15	6	0.14351	0.29704	0.99998	5546	-0.1429
RAB5B	6	0.14356	0.29712	0.99998	5547	0.07
SRD5A1	6	0.1436	0.29717	0.99998	5548	-0.0225
ASB7	6	0.1436	0.29717	0.99998	5549	-0.1973
OCSTAMP	6	0.1436	0.29717	0.99998	5550	-0.2182
GAGE2A	2	0.14361	0.20006	0.99998	5551	-1.042
MYBL2	6	0.14367	0.29728	0.99998	5552	-0.0681
AQP3	6	0.1437	0.29732	0.99998	5553	0.159
LRRC34	6	0.14379	0.29745	0.99998	5554	-0.0392
PHTF1	6	0.14381	0.29746	0.99998	5555	0.1752
CBR1	6	0.14384	0.29751	0.99998	5556	-0.1322
DNMT3B	6	0.14388	0.29757	0.99998	5557	0.0651
VGLL2	6	0.1439	0.29761	0.99998	5558	-0.0967
LEFTY2	6	0.14399	0.29772	0.99998	5559	-0.2164
MORC2	6	0.14411	0.29788	0.99998	5560	0.0381
KCNN3	6	0.14424	0.29805	0.99998	5561	-0.1741
ERVV-1	6	0.14428	0.29811	0.99998	5562	-0.1712
CADM4	6	0.14433	0.29818	0.99998	5563	-0.0498
CIRBP	6	0.14437	0.29822	0.99998	5564	-0.1393
NCALD	6	0.14438	0.29824	0.99998	5565	0.0987
SPRED2	6	0.14442	0.29829	0.99998	5566	0.0263
NPSR1	6	0.14442	0.29829	0.99998	5567	0.0136
ATG14	6	0.14445	0.29834	0.99998	5568	-0.0505
RBBP8	6	0.14447	0.29837	0.99998	5569	-0.1681
PRKACG	6	0.14452	0.29843	0.99998	5570	-0.0718
UPK1B	6	0.14458	0.2985	0.99998	5571	-0.0585
hsa-mir-651	4	0.14466	0.26066	0.99998	5572	-0.1931
C3orf52	6	0.14469	0.29867	0.99998	5573	0.0256
hsa-mir-567	4	0.1447	0.26072	0.99998	5574	-0.0009
LOC10065351	6	0.1447	0.29868	0.99998	5575	-0.0292
ZIC4	6	0.14475	0.29875	0.99998	5576	0.0864
ST3GAL1	6	0.14485	0.2989	0.99998	5577	-0.1285
C9orf84	6	0.14486	0.29891	0.99998	5578	-0.1385
ZNHIT2	6	0.14486	0.29891	0.99998	5579	-0.2339
MZB1	6	0.14498	0.29907	0.99998	5580	0.4443
CHCHD5	6	0.14503	0.29914	0.99998	5581	-0.0481
CP	6	0.14511	0.29925	0.99998	5582	-0.1645
C5orf24	6	0.14518	0.29935	0.99998	5583	-0.0098
hsa-mir-518e	2	0.1452	0.20127	0.99998	5584	-0.1522
ID1	6	0.14522	0.29941	0.99998	5585	-0.0759
HOXB3	6	0.14522	0.29941	0.99998	5586	-0.0587

PCGF3	6	0.14525	0.29945	0.99998	5587	0.006
KL	6	0.14525	0.29945	0.99998	5588	0.3823
KDSR	6	0.14534	0.29956	0.99998	5589	-0.049
CCDC149	6	0.14541	0.29968	0.99998	5590	-0.2646
ACAA1	6	0.14541	0.29968	0.99998	5591	-0.1774
hsa-mir-8060	4	0.14542	0.26151	0.99998	5592	-0.1143
PIGC	6	0.14549	0.29978	0.99998	5593	-0.1207
LOC440243	2	0.14558	0.20154	0.99998	5594	-0.243
C1orf21	6	0.14558	0.29991	0.99998	5595	-0.2803
UNC5D	6	0.14566	0.30003	0.99998	5596	-0.2602
GADD45GIP1	6	0.14566	0.30003	0.99998	5597	0.0524
IGSF6	6	0.14576	0.30017	0.99998	5598	-0.0719
RAB15	6	0.14576	0.30017	0.99998	5599	-0.1483
PF4V1	5	0.14582	0.29117	0.99998	5600	-0.3121
RGPD4	5	0.14588	0.29128	0.99998	5601	-0.0953
ROS1	6	0.14592	0.30038	0.99998	5602	-0.0776
MIEN1	6	0.14592	0.30038	0.99998	5603	0.1577
FLII	6	0.14592	0.30038	0.99998	5604	-0.1059
RALYL	6	0.14592	0.3004	0.99998	5605	-0.2051
OR6C76	6	0.14606	0.3006	0.99998	5606	-0.3237
MGAT5B	6	0.14607	0.30061	0.99998	5607	0.0799
PSMG2	6	0.14612	0.30067	0.99998	5608	-0.0575
hsa-mir-1207	4	0.14612	0.26227	0.99998	5609	-0.3498
GARS	6	0.14616	0.30073	0.99998	5610	0.1334
GPR75-ASB3	3	0.14617	0.2381	0.99998	5611	-0.1381
CBL	6	0.14618	0.30077	0.99998	5612	-0.1178
PDE6B	6	0.14621	0.3008	0.99998	5613	-0.005
PVRL1	6	0.14632	0.30095	0.99998	5614	0.1769
APOBEC2	6	0.14635	0.30098	0.99998	5615	0.008
RCHY1	6	0.14635	0.30098	0.99998	5616	-0.105
MFSD8	6	0.14639	0.30104	0.99998	5617	0.0436
MLXIP	6	0.14646	0.30114	0.99998	5618	0.3181
MID1IP1	6	0.14647	0.30114	0.99998	5619	-0.0646
SYCP1	5	0.14648	0.29223	0.99998	5620	0.1834
HTR6	6	0.14648	0.30117	0.99998	5621	-0.2042
NPS	6	0.14655	0.30126	0.99998	5622	0.0217
HMGB3	6	0.14655	0.30126	0.99998	5623	0.2728
CEACAM5	6	0.1466	0.30133	0.99998	5624	0.0053
LAMC3	6	0.14678	0.30159	0.99998	5625	-0.0562
JMJD8	6	0.14689	0.30173	0.99998	5626	-0.0859
TMEM196	6	0.14692	0.30177	0.99998	5627	-0.2109
PLEKHG2	6	0.14695	0.30181	0.99998	5628	-0.173
RASL10A	6	0.14695	0.30181	0.99998	5629	-0.3022
FOLH1	6	0.14698	0.30185	0.99998	5630	0.0101
CCR3	6	0.14699	0.30186	0.99998	5631	0.0369
TMEM35	6	0.14703	0.30192	0.99998	5632	-0.0706
hsa-mir-4798	4	0.14712	0.26341	0.99998	5633	-0.6385
DIRAS2	6	0.14717	0.30213	0.99998	5634	-0.2227
ULBP1	6	0.14726	0.30226	0.99998	5635	-0.1716
hsa-mir-1260a	4	0.14734	0.26366	0.99998	5636	-0.1693
RNF146	6	0.14734	0.30238	0.99998	5637	0.0002
MRPS28	6	0.14737	0.30242	0.99998	5638	-0.077
ZDHHC19	6	0.14749	0.30259	0.99998	5639	0.1958
RPL9	6	0.14755	0.30268	0.99998	5640	-0.2088
hsa-mir-7703	4	0.14757	0.26392	0.99998	5641	-0.1389
hsa-mir-4656	4	0.14764	0.264	0.99998	5642	-0.0696
SAA4	3	0.14775	0.24021	0.99998	5643	-0.2407
SIRPA	6	0.14788	0.30315	0.99998	5644	-0.1026
KIAA0100	6	0.14788	0.30315	0.99998	5645	0.1633
TUB	6	0.14788	0.30315	0.99998	5646	-0.1317
IFNA16	5	0.14797	0.29457	0.99998	5647	0.1932
LOC728392	6	0.14799	0.30331	0.99998	5648	-0.1713
ZMYM4	6	0.14803	0.30337	0.99998	5649	-0.0528
EBF1	6	0.14806	0.30341	0.99998	5650	-0.1545
ERAP1	6	0.14808	0.30343	0.99998	5651	-0.1466
CDKN1C	6	0.14808	0.30343	0.99998	5652	-0.0934
DMRTC2	6	0.14808	0.30343	0.99998	5653	-0.1807
TBC1D5	6	0.14815	0.30353	0.99998	5654	0.0294
ARID4B	6	0.14815	0.30353	0.99998	5655	-0.0703
ATG5	6	0.14819	0.30357	0.99998	5656	-0.1635
ARMC3	6	0.14819	0.30357	0.99998	5657	0.0261
PRR24	6	0.14819	0.30357	0.99998	5658	-0.1666
ATM	6	0.14827	0.30369	0.99998	5659	-0.2058
LRWD1	6	0.14831	0.30373	0.99998	5660	0.2375
CIT	6	0.14832	0.30375	0.99998	5661	-0.2205
AGGF1	6	0.14842	0.30389	0.99998	5662	-0.1094
SYTL4	6	0.14842	0.30389	0.99998	5663	0.2317
hsa-mir-6801	4	0.14848	0.26493	0.99998	5664	-0.0835
AGAP3	6	0.14849	0.30398	0.99998	5665	0.3565
RALGPS1	6	0.14856	0.30408	0.99998	5666	-0.1311
BID	6	0.14856	0.30408	0.99998	5667	-0.1594

PTPRS	6	0.14856	0.30408	0.99998	5668	-0.0544
PCNX	6	0.14867	0.30423	0.99998	5669	-0.0057
ALKBH8	6	0.14867	0.30425	0.99998	5670	-0.0826
BTN2A2	6	0.14871	0.3043	0.99998	5671	-0.1351
TNFRSF13C	6	0.14871	0.3043	0.99998	5672	-0.2522
PMF1-BGLAP	3	0.14872	0.2415	0.99998	5673	-0.388
TMEM170B	6	0.14876	0.30437	0.99998	5674	-0.0362
CNKSR2	4	0.14878	0.26525	0.99998	5675	-0.0576
ATF7IP	6	0.14879	0.30441	0.99998	5676	0.0111
TMEM173	6	0.14882	0.30446	0.99998	5677	-0.0298
HLA-B	6	0.14885	0.30451	0.99998	5678	0.3009
RNF207	6	0.14895	0.30463	0.99998	5679	0.0769
SLC22A13	6	0.14904	0.30475	0.99998	5680	0.0128
hsa-mir-3148	4	0.14904	0.26554	0.99998	5681	-0.0339
LDLRAD2	6	0.14915	0.30491	0.99998	5682	-0.0422
PTPRU	6	0.14918	0.30494	0.99998	5683	-0.1536
SLC47A1	6	0.14918	0.30494	0.99998	5684	-0.2072
GBP3	6	0.14923	0.30502	0.99998	5685	-0.0173
CTBP2	6	0.14923	0.30502	0.99998	5686	-0.099
HP	6	0.14926	0.30507	0.99998	5687	-0.3952
hsa-mir-4693	2	0.14932	0.20428	0.99998	5688	-0.208
TAS2R39	6	0.14933	0.30516	0.99998	5689	-0.1767
MCM7	6	0.14933	0.30517	0.99998	5690	-0.045
DZIP1	6	0.14938	0.30523	0.99998	5691	-0.162
ZFP3	6	0.14954	0.30547	0.99998	5692	0.0172
ROBO3	6	0.14967	0.30566	0.99998	5693	-0.1642
MGRN1	6	0.14967	0.30566	0.99998	5694	-0.1439
MDH2	6	0.14967	0.30566	0.99998	5695	0.0403
OSBPL6	6	0.14967	0.30568	0.99998	5696	-0.1505
HMGCL	6	0.14969	0.3057	0.99998	5697	-0.1983
ABCC2	6	0.14969	0.3057	0.99998	5698	-0.2703
ENTPD2	6	0.14972	0.30572	0.99998	5699	0.0549
NOMO3	2	0.14974	0.20457	0.99998	5700	-0.0075
VAC14	6	0.14974	0.30576	0.99998	5701	-0.0868
HUWE1	6	0.14982	0.30586	0.99998	5702	-0.0146
NPY1R	6	0.14989	0.30595	0.99998	5703	0.1246
CDK6	6	0.14995	0.30602	0.99998	5704	-0.2225
OTUD4	6	0.15004	0.30615	0.99998	5705	-0.1256
ACBD5	6	0.15007	0.3062	0.99998	5706	-0.0259
BARHL1	6	0.15013	0.30628	0.99998	5707	-0.2003
KCNA5	6	0.1502	0.3064	0.99998	5708	0.1368
AGFG1	6	0.15025	0.30646	0.99998	5709	-0.0971
C22orf39	6	0.15027	0.30648	0.99998	5710	0.0843
NT5DC1	6	0.15029	0.30652	0.99998	5711	-0.096
FGF11	6	0.15036	0.30662	0.99998	5712	0.2331
AZGP1	6	0.15049	0.30681	0.99998	5713	-0.0805
TUBA3C	6	0.15054	0.30689	0.99998	5714	0.1245
TAS2R3	6	0.15057	0.30692	0.99998	5715	-0.0954
hsa-mir-1307	4	0.15057	0.26723	0.99998	5716	-0.0547
TDP2	6	0.15058	0.30694	0.99998	5717	-0.0148
MEIOB	6	0.1506	0.30697	0.99998	5718	-0.0515
ITGAD	6	0.15068	0.30707	0.99998	5719	-0.1276
WISP1	6	0.1507	0.30711	0.99998	5720	-0.0111
RAVER2	6	0.15072	0.30713	0.99998	5721	0.197
CCDC171	6	0.15073	0.30715	0.99998	5722	0.0076
LGALS13	5	0.15074	0.299	0.99998	5723	0.1759
EXOC3L2	6	0.15074	0.30717	0.99998	5724	0.0721
GAPT	6	0.15077	0.30721	0.99998	5725	0.041
PLCD3	6	0.15085	0.30731	0.99998	5726	0.0697
PROP1	6	0.15092	0.30741	0.99998	5727	-0.0815
PIGN	6	0.15102	0.30755	0.99998	5728	0.2912
SMOC2	6	0.15103	0.30757	0.99998	5729	-0.1702
C17orf62	6	0.15103	0.30757	0.99998	5730	0.0997
COMMD1	6	0.15113	0.30771	0.99998	5731	-0.0718
HRK	6	0.15115	0.30774	0.99998	5732	-0.1445
ZSCAN5B	6	0.15125	0.30787	0.99998	5733	-0.1539
ELOVL4	6	0.15125	0.30787	0.99998	5734	-0.259
CHAF1A	6	0.15134	0.308	0.99998	5735	-0.0346
MAPK10	6	0.15138	0.30806	0.99998	5736	-0.0107
CELF6	6	0.15138	0.30806	0.99998	5737	-0.2258
hsa-mir-570	4	0.15141	0.2682	0.99998	5738	0.0114
CDSN	6	0.15143	0.30813	0.99998	5739	0.0487
SLC2A5	6	0.15148	0.3082	0.99998	5740	-0.0982
C12orf68	6	0.15163	0.30842	0.99998	5741	-0.0562
PHYHIP	6	0.15166	0.30845	0.99998	5742	-0.151
HDAC4	6	0.1517	0.30851	0.99998	5743	-0.0795
IL2RB	6	0.1517	0.30851	0.99998	5744	0.0212
VWA7	6	0.15177	0.30859	0.99998	5745	-0.155
CDC42EP2	6	0.15178	0.30862	0.99998	5746	-0.009
RASGRF1	6	0.15186	0.30874	0.99998	5747	0.0077
CCL23	6	0.15186	0.30874	0.99998	5748	-0.2988

LRFN4	6	0.15189	0.30877	0.99998	5749	-0.1161
ADNP	6	0.15192	0.30882	0.99998	5750	0.0335
TRIM8	6	0.15192	0.30882	0.99998	5751	0.1152
TNS1	6	0.15192	0.30882	0.99998	5752	0.0919
DEFB110	6	0.15192	0.30882	0.99998	5753	-0.0423
SUZ12	6	0.15192	0.30882	0.99998	5754	0.0845
ACCS	6	0.15195	0.30886	0.99998	5755	-0.1354
MICAL3	6	0.152	0.30893	0.99998	5756	-0.1738
C2CD2L	6	0.152	0.30893	0.99998	5757	-0.2074
ALPP	5	0.15202	0.30103	0.99998	5758	-0.5127
SDC4	6	0.15204	0.30898	0.99998	5759	-0.0495
CYB5R3	6	0.15204	0.30898	0.99998	5760	-0.1521
hsa-mir-1293	4	0.15207	0.26896	0.99998	5761	-0.2669
CGNL1	6	0.15214	0.30913	0.99998	5762	-0.1559
hsa-mir-4327	4	0.1522	0.26911	0.99998	5763	-0.1853
DSCC1	6	0.15229	0.30935	0.99998	5764	0.1394
EFCC1	6	0.1523	0.30935	0.99998	5765	0.0117
MGARP	6	0.15234	0.30942	0.99998	5766	-0.0365
ENDOU	6	0.1524	0.30949	0.99998	5767	-0.0842
FUBP3	6	0.15247	0.30959	0.99998	5768	-0.1763
MSTN	6	0.15247	0.30959	0.99998	5769	-0.1492
hsa-mir-6780a	4	0.1525	0.26944	0.99998	5770	0.092
ZIK1	6	0.15251	0.30965	0.99998	5771	-0.0681
GATAD2A	6	0.15253	0.30967	0.99998	5772	-0.1464
SMARCA1	6	0.15263	0.3098	0.99998	5773	-0.2296
CD160	6	0.15263	0.3098	0.99998	5774	-0.1853
GABRR2	6	0.15272	0.30995	0.99998	5775	-0.1207
NOD2	6	0.15272	0.30995	0.99998	5776	-0.1112
hsa-mir-30b	4	0.15278	0.26978	0.99998	5777	0.0293
TBRG1	6	0.15279	0.31005	0.99998	5778	-0.1418
CDC42BPG	4	0.15282	0.26982	0.99998	5779	-0.1059
MTFR1	6	0.15286	0.31014	0.99998	5780	-0.0184
hsa-mir-212	4	0.15295	0.26997	0.99998	5781	-0.3171
ASCL2	6	0.15296	0.31028	0.99998	5782	-0.1653
PALB2	6	0.15301	0.31036	0.99998	5783	-0.0692
PRLH	6	0.15307	0.31043	0.99998	5784	-0.2287
CUL9	6	0.15309	0.31045	0.99998	5785	0.013
ZNF554	6	0.15315	0.31055	0.99998	5786	-0.1998
GPR139	6	0.15315	0.31055	0.99998	5787	-0.1469
CHD5	6	0.15326	0.31071	0.99998	5788	-0.1479
YWHAZ	6	0.15326	0.31071	0.99998	5789	-0.1771
G6PC3	6	0.15326	0.31071	0.99998	5790	0.0402
TCEAL3	5	0.15333	0.30311	0.99998	5791	-0.9148
hsa-mir-766	4	0.15344	0.27052	0.99998	5792	0.3692
MSH6	6	0.15348	0.31101	0.99998	5793	-0.0617
DDX5	6	0.15351	0.31105	0.99998	5794	-0.1686
SIGMAR1	6	0.15351	0.31105	0.99998	5795	0.1146
CHRNB2	5	0.15351	0.3034	0.99998	5796	0.1781
PECAM1	6	0.15351	0.31105	0.99998	5797	-0.0732
IL22	6	0.15351	0.31105	0.99998	5798	-0.1111
SEMG1	5	0.15356	0.30348	0.99998	5799	-0.2117
DNAJB7	6	0.15357	0.31114	0.99998	5800	-0.0477
SULT1A1	5	0.15364	0.30359	0.99998	5801	0.1932
RNF216	6	0.15365	0.31125	0.99998	5802	0.0076
MSX1	6	0.15372	0.31134	0.99998	5803	-0.0292
ZNF14	6	0.15372	0.31134	0.99998	5804	-0.2384
CD46	6	0.15372	0.31134	0.99998	5805	-0.0165
SLC13A1	6	0.15383	0.31148	0.99998	5806	-0.0027
CSNK1G1	6	0.15383	0.31148	0.99998	5807	-0.2182
KRT6C	4	0.15385	0.27098	0.99998	5808	-0.3494
C22orf29	6	0.15394	0.31164	0.99998	5809	-0.0482
LOC554223	6	0.15394	0.31164	0.99998	5810	0.1649
CPT1A	4	0.154	0.27114	0.99998	5811	-0.119
SERPINC1	6	0.15402	0.31175	0.99998	5812	-0.1458
PGRMC2	6	0.15414	0.3119	0.99998	5813	-0.0746
CCL24	6	0.15417	0.31195	0.99998	5814	0.1748
HIST1H4G	6	0.15417	0.31195	0.99998	5815	-0.1991
hsa-mir-6867	4	0.15422	0.27137	0.99998	5816	0.1423
LEPRE1	6	0.15424	0.31205	0.99998	5817	0.0031
LYPD2	6	0.1543	0.31214	0.99998	5818	-0.1514
GLP2R	6	0.1543	0.31214	0.99998	5819	-0.1592
hsa-mir-150	4	0.15431	0.27148	0.99998	5820	0.0286
CRIP1	6	0.15437	0.31223	0.99998	5821	0.0797
SLC25A35	5	0.15442	0.30482	0.99998	5822	-0.0755
FCHSD1	6	0.15445	0.31235	0.99998	5823	0.0549
NEK4	6	0.15446	0.31235	0.99998	5824	-0.2688
KLRG1	6	0.15449	0.31239	0.99998	5825	0.0006
CST4	3	0.15449	0.24922	0.99998	5826	-0.3031
hsa-mir-2054	3	0.15449	0.24922	0.99998	5827	-0.5849
HMOX2	6	0.15458	0.31253	0.99998	5828	0.1537
TP53BP1	6	0.15458	0.31253	0.99998	5829	-0.0917

CHIC2	6	0.15458	0.31253	0.99998	5830	0.189
MATN4	6	0.15473	0.31274	0.99998	5831	-0.1269
EHHADH	6	0.15473	0.31274	0.99998	5832	-0.2072
40422	3	0.15475	0.24956	0.99998	5833	0.2072
TPPP3	6	0.15481	0.31284	0.99998	5834	-0.1203
GNG4	6	0.15483	0.31287	0.99998	5835	0.1137
POLR3F	6	0.15483	0.31287	0.99998	5836	-0.1278
MTUS1	6	0.1549	0.31297	0.99998	5837	0.0459
C6orf120	6	0.15505	0.31317	0.99998	5838	-0.1198
TMEM201	6	0.15505	0.31317	0.99998	5839	-0.1619
AGO1	6	0.15508	0.3132	0.99998	5840	0.0548
C17orf47	6	0.15515	0.31331	0.99998	5841	-0.1542
OBSCN	6	0.1552	0.31339	0.99998	5842	0.0791
ZBTB32	6	0.1552	0.31339	0.99998	5843	0.0966
TBX5	6	0.15521	0.3134	0.99998	5844	-0.0013
C1orf216	6	0.15522	0.31341	0.99998	5845	-0.1344
OR2F1	6	0.15526	0.31346	0.99998	5846	0.0261
CNTNAP2	6	0.15535	0.31357	0.99998	5847	0.0644
NSMF	6	0.15536	0.3136	0.99998	5848	0.025
RAB2B	6	0.15539	0.31363	0.99998	5849	-0.0677
STRADA	6	0.15551	0.3138	0.99998	5850	-0.142
LRRN4	6	0.15551	0.3138	0.99998	5851	-0.1225
NAT16	6	0.15552	0.31382	0.99998	5852	-0.0626
OXT	6	0.15552	0.31382	0.99998	5853	-0.1742
MZT2B	3	0.1556	0.25073	0.99998	5854	-0.898
KDELR3	6	0.15562	0.31396	0.99998	5855	0.266
FAM63A	6	0.15568	0.31404	0.99998	5856	-0.1199
hsa-mir-378a	4	0.15572	0.27313	0.99998	5857	0.0556
KPNA5	6	0.15578	0.31417	0.99998	5858	0.0336
ZNF581	6	0.15578	0.31417	0.99998	5859	-0.0655
COL1A2	6	0.1558	0.31421	0.99998	5860	0.0279
CCDC24	6	0.15585	0.31427	0.99998	5861	-0.0053
PGR	6	0.15591	0.31436	0.99998	5862	0.1467
RFESD	6	0.15591	0.31436	0.99998	5863	-0.0608
SLC25A23	6	0.15594	0.3144	0.99998	5864	-0.1414
PCDHA2	2	0.15603	0.2093	0.99998	5865	-0.2889
EIF2S1	6	0.15607	0.31458	0.99998	5866	0.1578
UGT3A2	6	0.15607	0.31458	0.99998	5867	0.0531
IPO4	6	0.15613	0.31467	0.99998	5868	-0.2815
CBFB	6	0.15613	0.31467	0.99998	5869	-0.2704
GSS	6	0.15622	0.3148	0.99998	5870	-0.1072
KLK4	6	0.15625	0.31485	0.99998	5871	0.0175
NBPF24	1	0.15634	0.15611	0.99998	5872	-0.9647
TNRC6A	5	0.15637	0.30782	0.99998	5873	-0.296
ZNF558	6	0.15643	0.31509	0.99998	5874	-0.044
VAPA	6	0.1565	0.31519	0.99998	5875	0.0593
ATRAID	6	0.15657	0.31528	0.99998	5876	-0.0254
ZNF417	2	0.1566	0.2097	0.99998	5877	-0.1621
hsa-mir-4468	4	0.15664	0.27417	0.99998	5878	0.0438
INHBA	6	0.15665	0.3154	0.99998	5879	-0.1803
SPATS2	6	0.15673	0.31551	0.99998	5880	0.1098
NUDT5	6	0.15674	0.31554	0.99998	5881	-0.1477
HIATL1	6	0.1568	0.31561	0.99998	5882	0.0164
ALKBH5	6	0.15686	0.31569	0.99998	5883	0.1062
REPS1	6	0.15686	0.31569	0.99998	5884	-0.1574
PNCK	6	0.15694	0.31581	0.99998	5885	-0.2346
ACER3	6	0.15695	0.31583	0.99998	5886	0.2322
CHEK2	6	0.15695	0.31583	0.99998	5887	0.1996
IL1RAPL2	6	0.15697	0.31585	0.99998	5888	0.1156
EHD3	4	0.157	0.27459	0.99998	5889	0.0517
ASB2	6	0.15704	0.31595	0.99998	5890	0.2152
ROGDI	6	0.15704	0.31595	0.99998	5891	0.182
CPNE1	6	0.15706	0.31597	0.99998	5892	-0.0559
BCL7C	6	0.15712	0.31605	0.99998	5893	-0.1498
EVA1B	6	0.15718	0.31613	0.99998	5894	-0.1175
FBXO46	6	0.15718	0.31613	0.99998	5895	0.1666
hsa-mir-378e	4	0.15719	0.27481	0.99998	5896	-0.0301
GRID1	6	0.15726	0.31625	0.99998	5897	-0.0532
FOSB	6	0.15731	0.31633	0.99998	5898	-0.1285
GTDC1	6	0.15731	0.31633	0.99998	5899	0.0253
PRLR	6	0.15731	0.31633	0.99998	5900	-0.1061
HLA-DRB1	5	0.15734	0.30935	0.99998	5901	-0.2249
BBOX1	6	0.15735	0.31638	0.99998	5902	0.0238
RAB42	6	0.15738	0.31641	0.99998	5903	-0.0815
ZNF384	6	0.15742	0.31647	0.99998	5904	-0.1274
TRPM2	6	0.15742	0.31647	0.99998	5905	0.0107
CPA5	6	0.15748	0.31655	0.99998	5906	0.0577
SLC19A1	6	0.1575	0.31658	0.99998	5907	-0.1019
DUSP19	6	0.15756	0.31668	0.99998	5908	0.0012
RET	6	0.1576	0.31674	0.99998	5909	0.2045
PNPLA4	6	0.15761	0.31674	0.99998	5910	0.1158

MYH13	6	0.15776	0.31696	0.99998	5911	-0.0706
PAOX	6	0.15779	0.31698	0.99998	5912	0.1107
HES4	6	0.15786	0.31707	0.99998	5913	-0.1327
STMN4	6	0.15786	0.31707	0.99998	5914	0.0368
LILRA1	5	0.15788	0.31017	0.99998	5915	-0.3079
MFSD3	6	0.15791	0.31715	0.99998	5916	-0.0384
LRRTM2	6	0.15795	0.31721	0.99998	5917	0.012
NUDT17	6	0.15795	0.31721	0.99998	5918	-0.0504
PI16	6	0.15807	0.31738	0.99998	5919	-0.1568
R3HCC1L	6	0.15807	0.31738	0.99998	5920	-0.2353
SPEN	6	0.15808	0.31739	0.99998	5921	-0.0461
PHRF1	6	0.15808	0.3174	0.99998	5922	-0.1809
AK2	6	0.15811	0.31744	0.99998	5923	-0.0558
hsa-mir-6124	4	0.15814	0.27589	0.99998	5924	-0.2195
PSMD5	6	0.1582	0.31755	0.99998	5925	0.2962
MUT	6	0.1582	0.31755	0.99998	5926	-0.1584
PCLO	6	0.1582	0.31755	0.99998	5927	-0.176
DNAJC11	6	0.15824	0.31761	0.99998	5928	-0.1405
NUP205	6	0.15826	0.31765	0.99998	5929	0.1426
hsa-mir-1910	4	0.15829	0.27605	0.99998	5930	-0.2155
RSU1	6	0.1583	0.31771	0.99998	5931	0.0894
COX8A	6	0.15832	0.31774	0.99998	5932	-0.16
TEX35	6	0.15842	0.31789	0.99998	5933	-0.2636
CCDC61	6	0.15842	0.31789	0.99998	5934	-0.1187
FFAR1	6	0.15842	0.31789	0.99998	5935	-0.1859
ABCD4	6	0.15842	0.31789	0.99998	5936	-0.1118
TMEM18	6	0.15844	0.31791	0.99998	5937	-0.1217
PANX3	4	0.15861	0.2764	0.99998	5938	0.0236
EPSTI1	6	0.15865	0.31818	0.99998	5939	-0.3274
ZNF800	6	0.15865	0.31818	0.99998	5940	-0.1399
LUC7L3	6	0.1587	0.31826	0.99998	5941	-0.1498
ZNF662	6	0.15871	0.31828	0.99998	5942	0.1446
hsa-mir-1179	4	0.15876	0.27656	0.99998	5943	-0.2369
IFT20	6	0.15878	0.31836	0.99998	5944	-0.1068
APC2	6	0.1588	0.3184	0.99998	5945	-0.0708
CTCF	6	0.15884	0.31845	0.99998	5946	0.0632
CPSF7	6	0.15884	0.31845	0.99998	5947	0.0569
SYN3	4	0.15888	0.27669	0.99998	5948	-0.2617
SOLH	5	0.15889	0.31171	0.99998	5949	-0.0304
CCDC80	6	0.1589	0.31853	0.99998	5950	-0.0302
SLC37A2	6	0.15898	0.31864	0.99998	5951	0.0324
LRRC29	6	0.15905	0.31874	0.99998	5952	0.0081
PCTP	6	0.15906	0.31875	0.99998	5953	-0.1313
SIRPB2	6	0.1591	0.3188	0.99998	5954	0.2302
ARHGEF19	6	0.15917	0.3189	0.99998	5955	-0.0827
ARPC5L	6	0.15917	0.3189	0.99998	5956	-0.1013
hsa-mir-670	4	0.15933	0.27721	0.99998	5957	-0.1393
APITD1	2	0.15934	0.21176	0.99998	5958	-0.2182
RBM4B	6	0.15937	0.31919	0.99998	5959	-0.2879
FAM92B	6	0.15937	0.31919	0.99998	5960	-0.0827
TMEM167A	6	0.15938	0.31919	0.99998	5961	-0.1023
ABCF3	6	0.15938	0.31919	0.99998	5962	0.0024
SLC22A18	6	0.15938	0.31919	0.99998	5963	0.1329
PHF13	6	0.15939	0.31921	0.99998	5964	-0.012
CST7	6	0.15949	0.31936	0.99998	5965	0.0485
MPHOSPH6	4	0.15953	0.27744	0.99998	5966	-0.1482
STARD5	6	0.15956	0.31947	0.99998	5967	-0.1631
hsa-mir-98	4	0.15966	0.27757	0.99998	5968	0.1372
ZBTB6	6	0.15968	0.31963	0.99998	5969	-0.0747
PQBP1	6	0.15972	0.31967	0.99998	5970	0.0989
MESDC2	6	0.15972	0.31967	0.99998	5971	0.1291
SYCP3	6	0.15972	0.31967	0.99998	5972	0.0319
CYP19A1	6	0.15995	0.31999	0.99998	5973	0.0654
SYNPO	6	0.16002	0.32008	0.99998	5974	-0.0526
MAP4K1	6	0.16002	0.32008	0.99998	5975	-0.1982
IDH3A	6	0.16004	0.32012	0.99998	5976	0.1168
PDE4A	6	0.16018	0.3203	0.99998	5977	-0.0543
CPNE7	6	0.16018	0.3203	0.99998	5978	-0.1163
GATS	6	0.16023	0.32036	0.99998	5979	-0.0531
CHM	6	0.16027	0.32042	0.99998	5980	-0.0356
RAB3GAP2	6	0.16035	0.32053	0.99998	5981	-0.1252
RHOH	6	0.16035	0.32053	0.99998	5982	-0.1692
FGA	6	0.16035	0.32053	0.99998	5983	-0.0665
ERN2	6	0.1604	0.32059	0.99998	5984	0.2324
40057	3	0.16042	0.25722	0.99998	5985	-0.1086
hsa-mir-5087	3	0.16042	0.25722	0.99998	5986	-0.1065
SVOP	6	0.16049	0.32072	0.99998	5987	-0.0063
UBIAD1	6	0.16056	0.32082	0.99998	5988	-0.121
ZNF598	6	0.16057	0.32084	0.99998	5989	-0.0013
hsa-mir-4748	4	0.16068	0.27872	0.99998	5990	-0.1708
ZKSCAN8	6	0.16069	0.32102	0.99998	5991	0.0168

RDH5	6	0.16069	0.32102	0.99998	5992	-0.187
KRT31	6	0.1607	0.32103	0.99998	5993	-0.05
LOC10050638	6	0.1607	0.32103	0.99998	5994	-0.0209
hsa-mir-4731	4	0.16083	0.27888	0.99998	5995	0.0165
GIMAP1	4	0.16083	0.27888	0.99998	5996	0.0628
IL17B	6	0.1609	0.32131	0.99998	5997	0.0063
TPGS2	6	0.16091	0.32132	0.99998	5998	-0.1323
THADA	6	0.16099	0.32143	0.99998	5999	-0.1764
PRRX2	6	0.16103	0.32148	0.99998	6000	0.1121
HBG2	2	0.16108	0.21307	0.99998	6001	-0.806
RBM6	6	0.16118	0.32168	0.99998	6002	0.1201
GRM6	6	0.16118	0.32168	0.99998	6003	-0.0963
PEX16	6	0.16127	0.3218	0.99998	6004	-0.1591
CDH1	6	0.16127	0.3218	0.99998	6005	-0.2246
MAEA	6	0.16133	0.3219	0.99998	6006	0.0784
CAMKK2	6	0.16136	0.32194	0.99998	6007	-0.019
NSDHL	6	0.16136	0.32194	0.99998	6008	-0.0432
C21orf62	6	0.16138	0.32196	0.99998	6009	-0.015
RNF103	6	0.16149	0.32211	0.99998	6010	0.1239
CTXN3	6	0.16155	0.3222	0.99998	6011	-0.209
GRASP	6	0.16155	0.3222	0.99998	6012	-0.2254
LRRC61	6	0.16155	0.3222	0.99998	6013	0.2701
RPP25	6	0.16168	0.32237	0.99998	6014	-0.2386
SLC22A20	6	0.16168	0.32237	0.99998	6015	-0.1553
FBN2	6	0.16171	0.3224	0.99998	6016	0.0121
PPP1R3A	6	0.16171	0.3224	0.99998	6017	-0.0544
hsa-mir-1292	4	0.16175	0.2799	0.99998	6018	0.1545
PPCDC	6	0.16182	0.32256	0.99998	6019	-0.1493
SYTL1	6	0.16185	0.32259	0.99998	6020	-0.0315
SLC10A1	6	0.16185	0.32259	0.99998	6021	-0.1345
ANKRD13B	6	0.16185	0.32259	0.99998	6022	-0.1105
PLCXD3	6	0.16189	0.32266	0.99998	6023	0.2227
ERCC6L	6	0.16191	0.32268	0.99998	6024	-0.0456
UBASH3B	6	0.16191	0.32268	0.99998	6025	0.1743
SH2B2	6	0.16193	0.32272	0.99998	6026	-0.09
LIPK	6	0.16198	0.32279	0.99998	6027	-0.0695
DUSP22	6	0.16198	0.32279	0.99998	6028	-0.0113
SELPLG	6	0.16206	0.32291	0.99998	6029	-0.0702
RCVRN	6	0.16214	0.32302	0.99998	6030	-0.1331
USF1	6	0.16222	0.32314	0.99998	6031	-0.0908
PSRC1	6	0.16225	0.32317	0.99998	6032	0.1386
LELP1	6	0.16229	0.32324	0.99998	6033	0.0566
GGT1	6	0.1624	0.32338	0.99998	6034	0.0418
SEL1L	6	0.16245	0.32345	0.99998	6035	-0.0693
AEBP2	6	0.16251	0.32353	0.99998	6036	-0.1529
EPGN	6	0.16251	0.32353	0.99998	6037	-0.1076
HRNR	6	0.1626	0.32365	0.99998	6038	0.0446
MAP3K7	6	0.1626	0.32365	0.99998	6039	-0.1549
ATP6V1G3	6	0.16273	0.32384	0.99998	6040	-0.175
PITPNM2	6	0.16274	0.32385	0.99998	6041	-0.0734
CDC42SE2	6	0.16274	0.32385	0.99998	6042	-0.0094
CAMKV	6	0.16277	0.3239	0.99998	6043	-0.1115
CLPB	6	0.16293	0.32414	0.99998	6044	-0.0967
ZFYVE21	6	0.16293	0.32414	0.99998	6045	-0.037
PLXNA4	6	0.16297	0.32418	0.99998	6046	-0.1721
HHLA3	6	0.1631	0.32437	0.99998	6047	-0.0569
hsa-mir-3122	4	0.16313	0.28146	0.99998	6048	-0.1889
KCNK12	6	0.16318	0.32448	0.99998	6049	-0.0248
IRS4	6	0.16318	0.32448	0.99998	6050	-0.1802
hsa-mir-6881	4	0.16326	0.2816	0.99998	6051	-0.3377
ZBP1	6	0.16328	0.32464	0.99998	6052	0.0426
hsa-mir-425	4	0.16331	0.28166	0.99998	6053	-0.2717
BEX5	6	0.16334	0.32473	0.99998	6054	-0.1831
RPGRIP1	6	0.16334	0.32473	0.99998	6055	-0.1313
MIR205HG	6	0.16337	0.32476	0.99998	6056	0.157
KRTCAP3	6	0.16343	0.32484	0.99998	6057	-0.102
HLA-DOA	6	0.16346	0.32487	0.99998	6058	-0.1618
SUV39H1	6	0.16348	0.32493	0.99998	6059	0.043
ART3	6	0.16356	0.32502	0.99998	6060	-0.0318
PLXNA2	6	0.16359	0.32509	0.99998	6061	-0.1176
ATP6V1H	6	0.16362	0.32511	0.99998	6062	-0.0531
CDCA5	6	0.16362	0.32511	0.99998	6063	0.0203
RPL27	6	0.1637	0.32523	0.99998	6064	-0.1082
GPR179	6	0.16373	0.32526	0.99998	6065	-0.1589
RAD21	6	0.16387	0.32546	0.99998	6066	-0.2253
EVI5	6	0.16397	0.3256	0.99998	6067	-0.0649
PLCG2	6	0.16398	0.32561	0.99998	6068	-0.2427
LCT	6	0.164	0.32564	0.99998	6069	0.1572
ABCB6	6	0.164	0.32564	0.99998	6070	-0.1297
COLEC10	6	0.16405	0.3257	0.99998	6071	0.0179
SEC22A	6	0.16406	0.32572	0.99998	6072	-0.1791

hsa-mir-6817	4	0.16407	0.28254	0.99998	6073	0.0706
FBXO22	6	0.16414	0.32583	0.99998	6074	-0.0763
SHCBP1	6	0.16418	0.32588	0.99998	6075	-0.0787
MRPL2	6	0.16418	0.32588	0.99998	6076	0.2566
LOC10028881	6	0.16422	0.32594	0.99998	6077	0.1969
BPHL	6	0.16427	0.326	0.99998	6078	-0.1156
MYO18B	6	0.16431	0.32606	0.99998	6079	0.1265
ARL15	6	0.16431	0.32606	0.99998	6080	0.0871
ZNF429	6	0.16431	0.32606	0.99998	6081	0.0999
NUDT16	6	0.16443	0.32621	0.99998	6082	-0.0375
ANLN	6	0.16452	0.32633	0.99998	6083	-0.0361
hsa-mir-921	4	0.16453	0.28302	0.99998	6084	-0.1747
RPH3A	6	0.16453	0.32634	0.99998	6085	-0.1746
RAB3A	6	0.16463	0.32649	0.99998	6086	-0.0685
USP1	6	0.16463	0.32649	0.99998	6087	-0.0743
CACFD1	6	0.16463	0.32649	0.99998	6088	-0.1688
CLEC10A	6	0.16463	0.32649	0.99998	6089	-0.1185
CCR1	6	0.16465	0.32652	0.99998	6090	-0.0589
USP16	6	0.16479	0.3267	0.99998	6091	0.1407
ERLIN2	6	0.16484	0.32678	0.99998	6092	-0.1279
IK	6	0.16484	0.32678	0.99998	6093	-0.1822
MED29	6	0.16502	0.32702	0.99998	6094	-0.0617
METTL11B	6	0.16503	0.32703	0.99998	6095	-0.0112
LRRC36	6	0.16507	0.32709	0.99998	6096	-0.2084
SGMS1	6	0.16521	0.32729	0.99998	6097	-0.0412
FN1	6	0.16524	0.32732	0.99998	6098	-0.1252
EIF4E1B	6	0.16524	0.32732	0.99998	6099	-0.0701
DDIT4	6	0.16526	0.32736	0.99998	6100	-0.1416
MTRNR2L5	4	0.16538	0.284	0.99998	6101	-0.4046
IMPA2	6	0.16539	0.32752	0.99998	6102	-0.1123
ARPP19	6	0.1654	0.32753	0.99998	6103	-0.1713
OR52B2	6	0.1654	0.32753	0.99998	6104	-0.125
SLC39A13	6	0.16548	0.32764	0.99998	6105	-0.1663
TLDC2	6	0.1655	0.32766	0.99998	6106	0.0073
MAPK15	6	0.1655	0.32766	0.99998	6107	0.0095
ANAPC11	6	0.16556	0.32774	0.99998	6108	-0.1913
CLUL1	6	0.16558	0.32775	0.99998	6109	0.3704
FABP2	6	0.16573	0.32799	0.99998	6110	-0.1023
hsa-mir-4696	4	0.16574	0.28441	0.99998	6111	-0.1491
TRPC4AP	6	0.16579	0.32808	0.99998	6112	-0.0268
IFNGR2	6	0.16581	0.32811	0.99998	6113	-0.0826
SAMD11	6	0.16584	0.32816	0.99998	6114	-0.1567
TRPV2	6	0.16587	0.3282	0.99998	6115	-0.153
CTAGE1	6	0.16587	0.3282	0.99998	6116	-0.1741
ARL1	6	0.16592	0.32826	0.99998	6117	-0.1338
RASL10B	6	0.16595	0.3283	0.99998	6118	0.1463
DUOX2	6	0.16601	0.32838	0.99998	6119	-0.1342
ACP1	6	0.16615	0.32857	0.99998	6120	-0.1762
JAM2	6	0.16628	0.32875	0.99998	6121	-0.1904
HRH2	6	0.16628	0.32875	0.99998	6122	-0.0086
CBX2	6	0.16635	0.32885	0.99998	6123	-0.1195
PALD1	6	0.16635	0.32885	0.99998	6124	-0.1353
TGS1	6	0.16642	0.32894	0.99998	6125	0.0864
POP1	6	0.16647	0.329	0.99998	6126	-0.096
NDUFA2	6	0.16647	0.32901	0.99998	6127	-0.1812
CACNG7	6	0.16653	0.3291	0.99998	6128	-0.0628
ECE2	6	0.16653	0.3291	0.99998	6129	0.0284
ARL17A	1	0.16655	0.1664	0.99998	6130	-0.5173
C9orf156	6	0.16662	0.3292	0.99998	6131	0.0448
SDR16C5	6	0.16662	0.32921	0.99998	6132	0.1892
MKNK1	6	0.16662	0.32921	0.99998	6133	-0.0544
CST6	6	0.16663	0.32922	0.99998	6134	-0.1662
ADAM32	6	0.1668	0.32946	0.99998	6135	0.127
TLE1	4	0.16681	0.28558	0.99998	6136	-0.09
FAM104A	6	0.16687	0.32954	0.99998	6137	-0.1678
hsa-mir-1273g	4	0.16689	0.28568	0.99998	6138	-0.0725
SSH2	6	0.16689	0.32958	0.99998	6139	-0.192
ASB15	6	0.16693	0.32963	0.99998	6140	-0.0402
POU5F1B	6	0.16694	0.32964	0.99998	6141	0.1284
C15orf41	6	0.16696	0.32967	0.99998	6142	0.0003
HIST2H2BF	6	0.16697	0.32968	0.99998	6143	-0.4759
CCL15	6	0.16697	0.32968	0.99998	6144	-0.263
SPEG	6	0.16697	0.32968	0.99998	6145	-0.1653
PRB1	3	0.16718	0.26639	0.99998	6146	-0.102
KCTD12	6	0.16729	0.33014	0.99998	6147	-0.0169
HBZ	4	0.16731	0.28612	0.99998	6148	-0.1802
NSL1	6	0.16731	0.33016	0.99998	6149	-0.0618
SLC6A14	6	0.16737	0.33024	0.99998	6150	-0.1629
COX19	6	0.16744	0.33034	0.99998	6151	-0.1687
TICAM2	4	0.16759	0.28644	0.99998	6152	0.1037
TATDN3	6	0.16763	0.33059	0.99998	6153	0.1177

GOLGA8A	5	0.16766	0.32519	0.99998	6154	-0.2636
SELL	6	0.16767	0.33065	0.99998	6155	0.2586
MCFD2	6	0.16774	0.33074	0.99998	6156	0.1794
PARM1	6	0.16779	0.3308	0.99998	6157	-0.1148
TMUB2	6	0.1678	0.33082	0.99998	6158	-0.0268
PAPOLB	6	0.16788	0.33092	0.99998	6159	0.2258
BAI3	6	0.16788	0.33092	0.99998	6160	-0.0668
CLCN2	6	0.16792	0.33098	0.99998	6161	-0.06
OAS1	6	0.16803	0.33112	0.99998	6162	-0.0761
KIFAP3	6	0.16803	0.33112	0.99998	6163	-0.0768
PPFIA1	6	0.16803	0.33112	0.99998	6164	0.0132
C19orf80	6	0.16803	0.33112	0.99998	6165	-0.106
USP20	6	0.16805	0.33115	0.99998	6166	-0.0912
ZNF502	6	0.1681	0.33121	0.99998	6167	0.0608
ADIG	6	0.1681	0.33121	0.99998	6168	-0.1468
BCL7A	6	0.16815	0.33128	0.99998	6169	-0.0195
FAM214A	6	0.16822	0.33138	0.99998	6170	0.0659
DCAF6	6	0.16822	0.33138	0.99998	6171	-0.0365
LGR4	6	0.16823	0.33139	0.99998	6172	0.1495
TMC6	6	0.16827	0.33145	0.99998	6173	-0.1432
AQPEP	6	0.1683	0.33149	0.99998	6174	-0.0871
UBXN1	6	0.16832	0.33151	0.99998	6175	-0.185
DMTF1	6	0.16835	0.33155	0.99998	6176	-0.1559
FAXDC2	6	0.16835	0.33155	0.99998	6177	-0.1718
P4HTM	6	0.16835	0.33155	0.99998	6178	-0.0114
EMD	6	0.16837	0.33158	0.99998	6179	-0.0993
COL3A1	6	0.16837	0.33158	0.99998	6180	0.2242
KRTCAP2	6	0.16852	0.33179	0.99998	6181	-0.2287
AQP12B	6	0.16852	0.33179	0.99998	6182	-0.1174
MT1A	6	0.16852	0.33179	0.99998	6183	-0.4467
GEMIN6	6	0.16853	0.3318	0.99998	6184	0.0684
LRRC2	6	0.16853	0.3318	0.99998	6185	0.2937
FAM129A	6	0.16857	0.33185	0.99998	6186	0.1522
CHIA	6	0.16857	0.33185	0.99998	6187	-0.1519
hsa-mir-4473	4	0.16859	0.28759	0.99998	6188	-0.1573
SRSF12	6	0.16866	0.33196	0.99998	6189	-0.074
TEP1	6	0.16869	0.33201	0.99998	6190	-0.1369
TNFRSF21	6	0.16871	0.33204	0.99998	6191	-0.0356
FAM221A	6	0.16872	0.33206	0.99998	6192	0.11
INTS1	6	0.16882	0.33219	0.99998	6193	-0.111
IGSF11	6	0.16884	0.33221	0.99998	6194	-0.1563
BCAR1	6	0.16887	0.33224	0.99998	6195	-0.0801
LOC10013214	6	0.16887	0.33224	0.99998	6196	-0.1777
NXNL1	6	0.16892	0.33233	0.99998	6197	-0.1876
FOXM1	6	0.16892	0.33233	0.99998	6198	-0.0873
PI4KA	6	0.16903	0.33247	0.99998	6199	0.0176
ELK1	6	0.1691	0.33259	0.99998	6200	0.11
COX10	6	0.1691	0.33259	0.99998	6201	0.1362
TFF2	6	0.16915	0.33265	0.99998	6202	-0.1581
hsa-mir-4685	4	0.1693	0.28838	0.99998	6203	-0.1083
EGR4	6	0.16943	0.33303	0.99998	6204	-0.1341
NUTM1	6	0.16944	0.33306	0.99998	6205	-0.2097
RAB39B	6	0.16948	0.33311	0.99998	6206	-0.0424
ENTPD3	6	0.1695	0.33313	0.99998	6207	-0.1039
C3orf72	6	0.16962	0.33331	0.99998	6208	-0.1591
ELK4	6	0.16962	0.33331	0.99998	6209	-0.1315
hsa-mir-3944	4	0.1697	0.28887	0.99998	6210	-0.1612
CERKL	6	0.16971	0.33344	0.99998	6211	-0.1077
VCPIP1	6	0.16971	0.33344	0.99998	6212	0.051
DSCAM	6	0.16971	0.33344	0.99998	6213	-0.1205
NLRP9	6	0.16974	0.33351	0.99998	6214	0.1219
ACOXL	6	0.16979	0.33357	0.99998	6215	-0.0386
RPS9	6	0.16979	0.33357	0.99998	6216	0.0078
PTCRA	6	0.16979	0.33358	0.99998	6217	-0.0305
HTR1D	6	0.1698	0.33358	0.99998	6218	0.24
CLPS	6	0.1698	0.33358	0.99998	6219	0.0126
SP100	6	0.16986	0.33366	0.99998	6220	-0.3583
hsa-mir-8052	4	0.16987	0.28907	0.99998	6221	-0.0822
RPRD1A	6	0.16995	0.33378	0.99998	6222	-0.0699
hsa-mir-3613	4	0.16998	0.28918	0.99998	6223	-0.056
BRD9	6	0.17002	0.33387	0.99998	6224	-0.1327
TMEM261	4	0.17005	0.28927	0.99998	6225	-0.3258
ANKZF1	6	0.17022	0.33414	0.99998	6226	0.0059
WDR53	4	0.17029	0.28954	0.99998	6227	0.0497
TAF4	6	0.1703	0.33427	0.99998	6228	-0.0815
OAZ2	6	0.17043	0.33444	0.99998	6229	-0.1974
MYH14	6	0.17045	0.33446	0.99998	6230	-0.0445
hsa-mir-656	4	0.17046	0.28973	0.99998	6231	-0.1035
ETNPPL	4	0.1705	0.28979	0.99998	6232	-0.1383
PIP	6	0.17054	0.3346	0.99998	6233	-0.0251
PABPC4	6	0.17061	0.33469	0.99998	6234	-0.0766

CRTC2	6	0.17061	0.33469	0.99998	6235	-0.0039
BLM	6	0.17061	0.33469	0.99998	6236	-0.0267
OR10W1	6	0.17062	0.3347	0.99998	6237	-0.0124
HDGF	6	0.1707	0.33481	0.99998	6238	-0.1549
ADARB1	6	0.17073	0.33486	0.99998	6239	-0.0545
PHF20L1	6	0.17081	0.33497	0.99998	6240	-0.1806
CCAR1	6	0.17088	0.33507	0.99998	6241	-0.1514
REN	6	0.17094	0.33515	0.99998	6242	-0.2272
AGTR1	6	0.17098	0.33519	0.99998	6243	0.2629
GLOD5	6	0.17103	0.33526	0.99998	6244	-0.1166
OSGEPL1	6	0.17108	0.33532	0.99998	6245	-0.1174
TTC28	4	0.17115	0.2905	0.99998	6246	0.1075
KPNA7	6	0.17116	0.33542	0.99998	6247	-0.0448
TRAF3IP2	6	0.17119	0.33546	0.99998	6248	0.0615
ATG13	6	0.17119	0.33546	0.99998	6249	-0.1805
TADA2A	6	0.1712	0.33547	0.99998	6250	-0.0501
SCNN1D	6	0.1712	0.33547	0.99998	6251	0.0262
ETFA	6	0.1712	0.33547	0.99998	6252	0.0678
KDM4C	6	0.1712	0.33547	0.99998	6253	-0.0599
ADAP2	6	0.17129	0.3356	0.99998	6254	-0.0084
C7orf60	6	0.17149	0.33588	0.99998	6255	0.2134
SIAH1	6	0.17156	0.33596	0.99998	6256	-0.0909
IQCC	6	0.17156	0.33596	0.99998	6257	0.1539
PA2G4	6	0.1717	0.33615	0.99998	6258	-0.1603
FBXO9	6	0.1717	0.33615	0.99998	6259	-0.0805
hsa-mir-6849	4	0.17171	0.29113	0.99998	6260	-0.0737
ADAM17	6	0.17174	0.33621	0.99998	6261	-0.0858
PTGER2	6	0.17176	0.33623	0.99998	6262	0.0166
HERC6	6	0.17184	0.33635	0.99998	6263	-0.1715
C7orf66	6	0.17184	0.33636	0.99998	6264	-0.0993
TRAPPC10	6	0.17187	0.3364	0.99998	6265	-0.1543
THSD1	6	0.17198	0.33655	0.99998	6266	0.0683
NTRK2	6	0.17198	0.33655	0.99998	6267	-0.0588
EGLN1	6	0.17198	0.33656	0.99998	6268	-0.1625
hsa-mir-516b-	2	0.17201	0.22124	0.99998	6269	-0.7473
PSMB5	6	0.17203	0.33662	0.99998	6270	-0.0879
PI4K2B	6	0.17203	0.33662	0.99998	6271	-0.1207
RBM4	3	0.17205	0.27281	0.99998	6272	0.0017
UBQLN2	6	0.17209	0.3367	0.99998	6273	0.0595
OR10H1	5	0.17215	0.33216	0.99998	6274	-0.372
CSPP1	6	0.17218	0.33682	0.99998	6275	0.1608
SLC38A7	6	0.17219	0.33682	0.99998	6276	-0.066
APOBEC3A	2	0.17221	0.22139	0.99998	6277	-0.3157
LPA	6	0.17233	0.33701	0.99998	6278	-0.1643
CITED4	6	0.17243	0.33716	0.99998	6279	-0.1972
KIAA0391	4	0.17251	0.29204	0.99998	6280	0.0708
COPS7B	6	0.17251	0.33727	0.99998	6281	0.0199
HECTD3	6	0.17251	0.33727	0.99998	6282	-0.1541
DMRTC1B	2	0.17256	0.22165	0.99998	6283	-0.2204
SLN	5	0.17258	0.33284	0.99998	6284	-0.1159
RBMX2	6	0.1726	0.33738	0.99998	6285	-0.0101
RPA2	6	0.1726	0.33739	0.99998	6286	0.0263
SYNDIG1	6	0.1726	0.33739	0.99998	6287	-0.0722
HSD17B14	6	0.1726	0.33739	0.99998	6288	0.036
TCP11L1	6	0.1728	0.33766	0.99998	6289	-0.1335
INSR	6	0.17285	0.33773	0.99998	6290	-0.1198
TRIM58	6	0.17294	0.33786	0.99998	6291	0.0064
UTP3	6	0.17296	0.3379	0.99998	6292	-0.1869
INMT	6	0.17298	0.33793	0.99998	6293	-0.1691
HIBADH	6	0.17308	0.33805	0.99998	6294	-0.2075
TNFSF10	6	0.17309	0.33807	0.99998	6295	-0.0994
TBX10	6	0.17309	0.33807	0.99998	6296	0.0392
VPS35	6	0.17321	0.33823	0.99998	6297	0.1672
ZDHHC13	6	0.17322	0.33825	0.99998	6298	0.0447
TMEM50A	6	0.17326	0.3383	0.99998	6299	-0.1175
APOBEC3D	6	0.17331	0.33837	0.99998	6300	0.203
RSAD2	6	0.17336	0.33844	0.99998	6301	-0.0163
EVPL	5	0.17343	0.33417	0.99998	6302	-0.1815
N6AMT2	6	0.17347	0.33857	0.99998	6303	-0.0895
METTL25	6	0.17349	0.33862	0.99998	6304	0.1523
RWDD1	6	0.1735	0.33862	0.99998	6305	-0.1597
WDHD1	6	0.17357	0.33872	0.99998	6306	-0.1112
MAFK	6	0.1736	0.33876	0.99998	6307	0.1656
CA5A	6	0.17367	0.33885	0.99998	6308	0.0796
hsa-mir-214	2	0.17371	0.22253	0.99998	6309	-0.3429
P2RY10	6	0.17382	0.33907	0.99998	6310	-0.0995
GLTSCR1L	6	0.17382	0.33907	0.99998	6311	-0.0128
MS4A6E	6	0.17385	0.3391	0.99998	6312	0.0706
TMEM187	6	0.17388	0.33914	0.99998	6313	-0.1147
LY6E	6	0.17389	0.33916	0.99998	6314	-0.0937
ATP2B4	6	0.17395	0.33924	0.99998	6315	-0.0577

NOL7	6	0.17395	0.33924	0.99998	6316	0.066
WIBG	6	0.17396	0.33925	0.99998	6317	0.106
EXOC4	6	0.17403	0.33935	0.99998	6318	-0.0931
ALDH18A1	6	0.17403	0.33935	0.99998	6319	-0.1183
PAK2	6	0.17411	0.33946	0.99998	6320	0.1173
SLC25A5	6	0.17418	0.33955	0.99998	6321	0.1035
PLA2G12B	6	0.17418	0.33955	0.99998	6322	-0.1007
SYK	6	0.17426	0.33966	0.99998	6323	-0.0886
MAP3K13	6	0.17431	0.33973	0.99998	6324	0.0857
GJD3	6	0.17439	0.33982	0.99998	6325	-0.0601
TAF9B	6	0.17439	0.33982	0.99998	6326	-0.219
C1GALT1C1	6	0.17439	0.33982	0.99998	6327	-0.1215
SELE	6	0.17443	0.33988	0.99998	6328	-0.0483
CLOCK	4	0.17445	0.29422	0.99998	6329	-0.0232
ST13	6	0.17449	0.33997	0.99998	6330	-0.0214
CCDC177	6	0.17451	0.34	0.99998	6331	-0.0665
hsa-mir-4689	4	0.17451	0.29429	0.99998	6332	0.3181
ZNF697	6	0.17452	0.34001	0.99998	6333	0.0899
GIPC3	6	0.17455	0.34007	0.99998	6334	-0.1691
MORN1	6	0.17457	0.34008	0.99998	6335	0.0043
EPN2	6	0.17465	0.34018	0.99998	6336	0.0872
TRAF3IP3	6	0.17465	0.34018	0.99998	6337	-0.0851
C11orf48	6	0.17465	0.34018	0.99998	6338	-0.0813
RGP1	6	0.17467	0.34021	0.99998	6339	-0.0195
CCDC54	6	0.17482	0.3404	0.99998	6340	-0.0849
DLL3	6	0.17494	0.34058	0.99998	6341	0.0843
BAG1	6	0.17496	0.34059	0.99998	6342	-0.1142
C10orf129	6	0.17496	0.34061	0.99998	6343	-0.1301
RADIL	6	0.17503	0.3407	0.99998	6344	-0.0257
PLA2G16	6	0.17509	0.34079	0.99998	6345	0.0022
CHRAC1	6	0.17509	0.34079	0.99998	6346	-0.2121
HMBS	6	0.17509	0.34079	0.99998	6347	0.029
LOC10012908	5	0.1751	0.33673	0.99998	6348	0.1527
BZRAP1	6	0.17512	0.34081	0.99998	6349	-0.0914
SLC4A2	6	0.17514	0.34084	0.99998	6350	0.0144
KATNAL1	6	0.17519	0.34091	0.99998	6351	-0.0958
UCN	6	0.17522	0.34095	0.99998	6352	-0.0855
MCM3	6	0.17529	0.34105	0.99998	6353	-0.0775
hsa-mir-6760	4	0.17533	0.29518	0.99998	6354	-0.0807
DLK2	6	0.17536	0.34114	0.99998	6355	0.5125
FLJ44313	6	0.1754	0.3412	0.99998	6356	-0.1214
DLGAP1	6	0.1754	0.3412	0.99998	6357	-0.1383
SERTAD1	6	0.17547	0.34128	0.99998	6358	0.0091
IFITM1	6	0.17551	0.34133	0.99998	6359	0.2615
PLGLB2	1	0.17561	0.17542	0.99998	6360	-0.3704
VAV2	6	0.17578	0.34173	0.99998	6361	-0.0184
INTS7	6	0.17582	0.34179	0.99998	6362	-0.0801
SELV	6	0.17583	0.34179	0.99998	6363	-0.1784
LRRIQ1	6	0.17588	0.34187	0.99998	6364	-0.1611
UTP20	6	0.17588	0.34187	0.99998	6365	-0.2497
HPGD	6	0.17595	0.34196	0.99998	6366	0.0464
DPYSL2	6	0.17599	0.34201	0.99998	6367	0.008
ZNF444	6	0.176	0.34202	0.99998	6368	0.1563
MLH1	6	0.17609	0.34214	0.99998	6369	0.1258
CCK	6	0.17609	0.34215	0.99998	6370	-0.046
SLC16A2	6	0.17609	0.34215	0.99998	6371	-0.1555
hsa-mir-548ar	1	0.17621	0.17603	0.99998	6372	-2.44
ARHGEF1	6	0.17623	0.34233	0.99998	6373	0.0717
TAS2R13	6	0.1764	0.34255	0.99998	6374	-0.1173
DBF4	5	0.17642	0.33869	0.99998	6375	-0.2541
KRTAP6-1	6	0.17642	0.34257	0.99998	6376	-0.04
ADCK4	6	0.17642	0.34258	0.99998	6377	0.0355
NT5DC2	6	0.17646	0.34264	0.99998	6378	-0.0728
KRTAP10-1	6	0.17653	0.34273	0.99998	6379	-0.5072
RNF10	6	0.17659	0.34281	0.99998	6380	-0.1448
GSDMC	6	0.17666	0.34292	0.99998	6381	0.0769
PDLIM5	6	0.17672	0.34301	0.99998	6382	-0.0809
ISOC1	6	0.1768	0.34311	0.99998	6383	0.1122
AMTN	6	0.17681	0.34313	0.99998	6384	-0.0776
FRMPD2	6	0.17681	0.34313	0.99998	6385	-0.1797
CLYBL	6	0.17681	0.34313	0.99998	6386	-0.0933
FAP	4	0.17685	0.29685	0.99998	6387	-0.2352
KLK9	4	0.17686	0.29685	0.99998	6388	0.0309
RTN4	6	0.17687	0.3432	0.99998	6389	0.0779
RAB3D	6	0.17694	0.3433	0.99998	6390	0.0142
PRR25	6	0.17702	0.34338	0.99998	6391	-0.0693
CRISP2	6	0.17704	0.34341	0.99998	6392	-0.0946
SURF2	6	0.17704	0.34341	0.99998	6393	-0.051
SLC25A48	6	0.17713	0.34353	0.99998	6394	-0.0927
AAAS	6	0.17716	0.34358	0.99998	6395	-0.2238
C1orf210	6	0.17716	0.34358	0.99998	6396	-0.149

ZCCHC9	6	0.17716	0.34358	0.99998	6397	-0.1077
ATP8B2	6	0.17722	0.34367	0.99998	6398	-0.0905
ANKRD34C	6	0.17722	0.34367	0.99998	6399	-0.0614
NT5C3B	6	0.17722	0.34367	0.99998	6400	-0.0275
SLC12A8	6	0.1773	0.34377	0.99998	6401	-0.1489
DNAI2	6	0.1773	0.34377	0.99998	6402	-0.1073
hsa-mir-302d	4	0.17735	0.29741	0.99998	6403	-0.3093
MRPL37	6	0.17737	0.34387	0.99998	6404	0.0132
OR10A2	6	0.17742	0.34394	0.99998	6405	-0.1899
TRIM60	6	0.17745	0.34397	0.99998	6406	-0.2235
RFX7	6	0.17746	0.34399	0.99998	6407	-0.1078
OR2B2	6	0.17751	0.34406	0.99998	6408	-0.1481
AHR	6	0.17754	0.3441	0.99998	6409	-0.1661
SMAGP	5	0.17754	0.34043	0.99998	6410	-0.2147
TMEM215	6	0.17757	0.34414	0.99998	6411	-0.0587
FAM81A	6	0.17758	0.34416	0.99998	6412	-0.044
hsa-mir-4726	4	0.17764	0.29772	0.99998	6413	0.0524
hsa-mir-5008	4	0.17764	0.29772	0.99998	6414	-0.0707
DEFB136	6	0.17767	0.34427	0.99998	6415	-0.1265
RALGDS	6	0.17773	0.34436	0.99998	6416	-0.2077
PDIA6	6	0.17776	0.34441	0.99998	6417	0.0217
SIDT1	6	0.17783	0.3445	0.99998	6418	-0.1664
GAP43	6	0.17783	0.3445	0.99998	6419	-0.0172
DPH2	6	0.1779	0.34459	0.99998	6420	-0.2683
GPR128	6	0.1779	0.3446	0.99998	6421	0.0118
hsa-mir-19b-2	3	0.17796	0.28077	0.99998	6422	-0.1949
ANAPC16	6	0.17803	0.34477	0.99998	6423	-0.2304
TBC1D15	6	0.17808	0.34484	0.99998	6424	0.0452
HSPA12A	6	0.17815	0.34492	0.99998	6425	-0.0052
hsa-mir-7110	4	0.17823	0.29842	0.99998	6426	-0.1186
PRAMEF13	5	0.17824	0.34146	0.99998	6427	-0.2285
GPR63	6	0.17825	0.34506	0.99998	6428	-0.0264
RPLP2	6	0.17832	0.34516	0.99998	6429	-0.1765
ZXDA	6	0.17833	0.34518	0.99998	6430	-0.2175
TM2D1	6	0.17841	0.34528	0.99998	6431	-0.0191
TBC1D7	6	0.17861	0.34554	0.99998	6432	0.1672
MFAP4	6	0.17861	0.34554	0.99998	6433	-0.0014
ZNF619	6	0.17872	0.34569	0.99998	6434	-0.0471
DDB2	6	0.17873	0.3457	0.99998	6435	-0.113
RTBDN	6	0.17873	0.3457	0.99998	6436	-0.0267
INS-IGF2	4	0.17876	0.29901	0.99998	6437	0.329
STAM2	6	0.17888	0.34592	0.99998	6438	-0.0397
GTPBP8	6	0.17888	0.34592	0.99998	6439	-0.0775
OGDH	6	0.17888	0.34592	0.99998	6440	-0.2273
TPD52L1	6	0.17895	0.34602	0.99998	6441	-0.1395
ATXN1L	6	0.17895	0.34602	0.99998	6442	-0.1525
PFKL	6	0.17897	0.34606	0.99998	6443	-0.1467
TMC7	6	0.17906	0.34619	0.99998	6444	-0.152
DLK1	6	0.17906	0.34619	0.99998	6445	-0.0438
PVALB	6	0.17906	0.34619	0.99998	6446	-0.1109
LOC283403	6	0.17908	0.34621	0.99998	6447	-0.2015
GAD1	6	0.17917	0.34634	0.99998	6448	-0.0529
NOD1	4	0.17922	0.29952	0.99998	6449	0.0474
ARHGAP21	6	0.17927	0.34648	0.99998	6450	-0.2321
CCDC104	6	0.17927	0.34648	0.99998	6451	-0.1026
MAB21L1	6	0.17927	0.34648	0.99998	6452	-0.1167
NR1D2	6	0.17927	0.34648	0.99998	6453	0.0439
INSL4	6	0.17929	0.34651	0.99998	6454	-0.0328
NDUFB7	4	0.1793	0.29961	0.99998	6455	-0.1146
URGCP-MRPS:	1	0.1793	0.17911	0.99998	6456	-0.6712
LIMK1	6	0.17938	0.34662	0.99998	6457	-0.0412
NCSTN	6	0.17941	0.34666	0.99998	6458	-0.0876
TPRN	6	0.17944	0.3467	0.99998	6459	-0.0903
OPA3	6	0.17954	0.34684	0.99998	6460	-0.1269
ATP1A2	6	0.17963	0.34695	0.99998	6461	-0.1473
C3orf22	6	0.17963	0.34696	0.99998	6462	-0.0091
PSG8	6	0.17963	0.34696	0.99998	6463	-0.0343
hsa-mir-26b	4	0.17982	0.30018	0.99998	6464	-0.291
SPOCK2	6	0.17998	0.34743	0.99998	6465	-0.0788
ZNF331	6	0.17998	0.34743	0.99998	6466	-0.142
SCARA5	6	0.17998	0.34743	0.99998	6467	-0.1353
ZCCHC12	6	0.17999	0.34745	0.99998	6468	-0.0251
FBXO15	6	0.18	0.34747	0.99998	6469	0.1846
HMGN1	6	0.18	0.34747	0.99998	6470	0.0437
DPH6	2	0.18009	0.22736	0.99998	6471	-0.3676
TMEM200A	6	0.18011	0.34761	0.99998	6472	-0.2318
LMNB1	6	0.18019	0.34769	0.99998	6473	-0.2347
C2orf48	6	0.18019	0.34769	0.99998	6474	-0.178
FAM168B	6	0.1802	0.34771	0.99998	6475	-0.026
MLLT3	6	0.1802	0.34771	0.99998	6476	-0.09
PVRIG	6	0.18032	0.34787	0.99998	6477	-0.1117

hsa-mir-29b-2	3	0.18038	0.28407	0.99998	6478	-0.276
SLC38A9	6	0.18038	0.34797	0.99998	6479	-0.1931
ZNF213	6	0.18047	0.34808	0.99998	6480	0.1604
FIS1	6	0.18047	0.34808	0.99998	6481	0.0292
hsa-mir-9-1	4	0.18048	0.30091	0.99998	6482	0.104
C7orf72	6	0.18055	0.3482	0.99998	6483	-0.1631
FAM207A	6	0.18055	0.3482	0.99998	6484	-0.1942
LIPJ	6	0.18059	0.34826	0.99998	6485	0.0116
ZNF286A	6	0.18065	0.34834	0.99998	6486	-0.1062
KIF2C	6	0.18067	0.34836	0.99998	6487	-0.0755
hsa-mir-7159	4	0.18071	0.30119	0.99998	6488	-0.2492
TCTEX1D1	6	0.18073	0.34845	0.99998	6489	-0.101
TXNRD1	6	0.18073	0.34845	0.99998	6490	0.2608
hsa-mir-6075	4	0.18088	0.30139	0.99998	6491	-0.233
WNT7B	6	0.1809	0.34869	0.99998	6492	-0.0881
ZMYM3	6	0.1809	0.34869	0.99998	6493	-0.1256
TEX10	6	0.1809	0.34869	0.99998	6494	-0.0273
NBR1	6	0.18108	0.34894	0.99998	6495	-0.0902
GPHB5	6	0.18108	0.34894	0.99998	6496	0.2002
C14orf159	6	0.18108	0.34894	0.99998	6497	0.0948
ELAVL2	6	0.18108	0.34894	0.99998	6498	0.0442
SLC3A1	6	0.18113	0.34899	0.99998	6499	0.004
EPX	6	0.18113	0.34899	0.99998	6500	-0.1135
MATR3	6	0.18133	0.34927	0.99998	6501	-0.0242
GPR98	6	0.18142	0.34938	0.99998	6502	-0.0231
SNX27	6	0.18144	0.34941	0.99998	6503	-0.068
TNFRSF25	6	0.1816	0.34964	0.99998	6504	-0.1281
TSGA13	6	0.1816	0.34964	0.99998	6505	-0.1789
RAB24	6	0.18164	0.34969	0.99998	6506	0.3789
CTSL1	3	0.18175	0.2859	0.99998	6507	-0.2235
SLC45A4	6	0.18195	0.35012	0.99998	6508	-0.1854
TAX1BP1	6	0.18197	0.35014	0.99998	6509	-0.0585
IZUMO2	6	0.18197	0.35014	0.99998	6510	0.0801
CDKN1A	6	0.18197	0.35014	0.99998	6511	-0.1314
APOPT1	6	0.18197	0.35014	0.99998	6512	0.2166
hsa-mir-185	4	0.18201	0.30267	0.99998	6513	0.0501
MAGEB5	6	0.18203	0.35024	0.99998	6514	-0.1667
EYS	6	0.18209	0.3503	0.99998	6515	-0.2237
CAPNS1	6	0.1821	0.35032	0.99998	6516	-0.0975
hsa-mir-3186	4	0.18214	0.30283	0.99998	6517	-0.178
RAB27A	6	0.18215	0.35039	0.99998	6518	-0.0226
ZBTB16	6	0.18215	0.35039	0.99998	6519	-0.1253
GSTM4	5	0.18216	0.34668	0.99998	6520	-0.028
LIG4	6	0.18219	0.35043	0.99998	6521	-0.1322
HBP1	6	0.18225	0.35051	0.99998	6522	0.018
ALDH6A1	6	0.18225	0.35051	0.99998	6523	0.1804
HBQ1	4	0.18228	0.30298	0.99998	6524	-0.1774
SLC44A4	6	0.18231	0.35059	0.99998	6525	-0.1211
FAM218A	6	0.18231	0.35059	0.99998	6526	-0.138
ACTR2	6	0.18231	0.35059	0.99998	6527	-0.2127
ILDR1	6	0.18242	0.35075	0.99998	6528	-0.0327
PTCHD4	6	0.18242	0.35075	0.99998	6529	0.1277
CDO1	6	0.18245	0.35078	0.99998	6530	0.0791
SUV39H2	6	0.18252	0.35087	0.99998	6531	0.0337
CSRNP3	6	0.18253	0.35088	0.99998	6532	-0.1211
GADD45B	4	0.18262	0.30334	0.99998	6533	0.1281
MTTP	6	0.18262	0.351	0.99998	6534	-0.1067
HLA-DOB	6	0.18263	0.35101	0.99998	6535	-0.0026
NHLRC3	6	0.18264	0.35104	0.99998	6536	-0.1124
CMAS	6	0.18269	0.3511	0.99998	6537	-0.0686
BCKDK	6	0.18271	0.35112	0.99998	6538	-0.0776
PLA2G4C	6	0.18271	0.35112	0.99998	6539	-0.1538
WDR83OS	6	0.18271	0.35112	0.99998	6540	-0.1704
ZNF746	6	0.18274	0.35115	0.99998	6541	0.1278
RHOQ	6	0.18276	0.35118	0.99998	6542	-0.0531
GTF3C3	6	0.18281	0.35125	0.99998	6543	-0.179
LRRC71	6	0.18285	0.3513	0.99998	6544	0.2595
PCDHGA8	2	0.18287	0.2295	0.99998	6545	-0.0304
HAO2	6	0.18294	0.35141	0.99998	6546	-0.0288
CDCA2	6	0.18295	0.35142	0.99998	6547	-0.1435
CYP4F11	6	0.18295	0.35142	0.99998	6548	-0.1605
hsa-mir-4746	4	0.18296	0.30373	0.99998	6549	0.0686
ADAMTS18	6	0.18301	0.35152	0.99998	6550	-0.0684
UCN3	6	0.18313	0.35167	0.99998	6551	0.0445
GNS	6	0.18313	0.35167	0.99998	6552	-0.1487
SHC1	6	0.18323	0.3518	0.99998	6553	-0.0953
RNASEL	6	0.18324	0.35182	0.99998	6554	-0.0542
IGFBP5	6	0.18327	0.35186	0.99998	6555	-0.0902
hsa-mir-4266	4	0.18341	0.30424	0.99998	6556	-0.08
SUCLA2	6	0.18343	0.35208	0.99998	6557	-0.1173
CD27	6	0.18343	0.35208	0.99998	6558	0.0415

TOPBP1	6	0.18353	0.35223	0.99998	6559	-0.1742
TRAPPC3	6	0.18354	0.35224	0.99998	6560	-0.0339
SLC12A6	6	0.18355	0.35225	0.99998	6561	0.0053
hsa-mir-6748	4	0.18356	0.30441	0.99998	6562	-0.3571
SLC6A3	6	0.1836	0.35232	0.99998	6563	0.209
MRPS11	6	0.1836	0.35232	0.99998	6564	-0.081
PDCD7	6	0.1836	0.35233	0.99998	6565	-0.1914
TP53BP2	6	0.1837	0.35245	0.99998	6566	-0.1113
PCDH20	6	0.1837	0.35245	0.99998	6567	-0.2624
NUDT13	6	0.18375	0.35252	0.99998	6568	-0.1072
RNASE4	6	0.18375	0.35252	0.99998	6569	-0.0026
EMR1	6	0.18386	0.35267	0.99998	6570	-0.034
ST18	6	0.1839	0.35274	0.99998	6571	-0.0851
HIST1H2AC	6	0.18395	0.3528	0.99998	6572	0.1456
IGLL1	6	0.18395	0.3528	0.99998	6573	-0.0715
NUP155	6	0.18395	0.3528	0.99998	6574	-0.1103
ZNF880	6	0.18396	0.35282	0.99998	6575	-0.19
C9orf66	6	0.18401	0.35289	0.99998	6576	-0.0499
HIRIP3	6	0.18403	0.35291	0.99998	6577	-0.1608
CCDC15	6	0.18403	0.35291	0.99998	6578	-0.0811
MRPL4	6	0.18403	0.35291	0.99998	6579	-0.1359
GALNTL5	6	0.18411	0.35302	0.99998	6580	-0.0536
SAA1	6	0.18411	0.35302	0.99998	6581	-0.5122
ACTR10	6	0.18411	0.35302	0.99998	6582	-0.2495
hsa-mir-502	4	0.18419	0.30512	0.99998	6583	-0.6845
KRTAP4-7	4	0.18419	0.30512	0.99998	6584	-1.5489
KRT6B	4	0.1842	0.30513	0.99998	6585	0.075
C16orf97	6	0.1843	0.35327	0.99998	6586	-0.0821
AGO2	6	0.1843	0.35327	0.99998	6587	-0.0336
POF1B	6	0.18439	0.35341	0.99998	6588	-0.0413
CRLF3	6	0.18441	0.35343	0.99998	6589	-0.06
GRIK5	6	0.18441	0.35343	0.99998	6590	-0.1437
TIMM50	6	0.18443	0.35346	0.99998	6591	-0.0812
DUPD1	6	0.18445	0.3535	0.99998	6592	-0.0951
RAMP1	6	0.18445	0.3535	0.99998	6593	-0.1144
MPPED2	6	0.1845	0.35356	0.99998	6594	0.0385
WDR67	3	0.18464	0.28972	0.99998	6595	-0.2041
HOXC6	6	0.18465	0.35376	0.99998	6596	-0.2015
hsa-mir-4665	4	0.18466	0.30566	0.99998	6597	-0.0858
ZNF165	6	0.18467	0.3538	0.99998	6598	-0.1302
DNAJB1	6	0.18467	0.3538	0.99998	6599	-0.0065
APOA4	6	0.18485	0.35404	0.99998	6600	-0.061
E2F3	6	0.18485	0.35404	0.99998	6601	-0.1327
SCO1	6	0.18488	0.35409	0.99998	6602	-0.1574
TREH	6	0.18488	0.35409	0.99998	6603	0.1308
MFSD6L	6	0.18499	0.35424	0.99998	6604	0.0024
SIL1	6	0.18499	0.35424	0.99998	6605	-0.1682
CACHD1	6	0.185	0.35426	0.99998	6606	0.1864
AARS2	6	0.18502	0.35428	0.99998	6607	-0.2904
ERBB2	6	0.18512	0.35442	0.99998	6608	-0.1191
OTUD7B	6	0.18513	0.35443	0.99998	6609	-0.0867
GOLGA7B	6	0.18521	0.35454	0.99998	6610	0.0474
IRX5	6	0.18522	0.35455	0.99998	6611	-0.121
LGI3	6	0.18522	0.35456	0.99998	6612	-0.0448
RQCD1	6	0.18522	0.35456	0.99998	6613	0.2121
CRYBB3	6	0.18538	0.35477	0.99998	6614	-0.1228
CALU	6	0.18562	0.3551	0.99998	6615	-0.0677
RD3L	6	0.18565	0.35516	0.99998	6616	-0.2362
hsa-mir-3614	4	0.18567	0.30678	0.99998	6617	-0.2272
KBTBD6	6	0.18571	0.35523	0.99998	6618	-0.2481
PHLDA2	6	0.18571	0.35523	0.99998	6619	-0.1195
OASL	6	0.18579	0.35535	0.99998	6620	0.184
SVIL	6	0.1858	0.35536	0.99998	6621	-0.1127
hsa-mir-4454	3	0.18585	0.29136	0.99998	6622	-0.2551
CFHR3	6	0.18595	0.35555	0.99998	6623	-0.0507
EQTN	6	0.18598	0.3556	0.99998	6624	0.0394
38231	3	0.18607	0.29167	0.99998	6625	0.1695
KCNJ18	4	0.18612	0.30728	0.99998	6626	0.0836
C16orf89	6	0.18614	0.35581	0.99998	6627	-0.1429
SOSTDC1	6	0.18614	0.35582	0.99998	6628	-0.0949
BSND	6	0.18614	0.35582	0.99998	6629	0.076
ZSCAN10	6	0.18625	0.35597	0.99998	6630	0.0654
STK10	6	0.18632	0.35606	0.99998	6631	-0.2442
MMP19	6	0.18632	0.35606	0.99998	6632	0.0998
UGT2B7	5	0.18635	0.35051	0.99998	6633	-0.2891
CLDN19	6	0.18637	0.35614	0.99998	6634	-0.0569
A4GNT	6	0.18641	0.35619	0.99998	6635	-0.0256
FADD	6	0.18641	0.35619	0.99998	6636	0.2652
FAM199X	6	0.18641	0.35619	0.99998	6637	0.1378
TTF2	6	0.18644	0.35624	0.99998	6638	-0.0293
HIPK1	6	0.18654	0.35638	0.99998	6639	0.0152

FAM206A	6	0.18656	0.35641	0.99998	6640	-0.2302
TCP10L	6	0.18656	0.35641	0.99998	6641	-0.0533
RPUSD3	6	0.18658	0.35644	0.99998	6642	-0.0797
FOXB2	6	0.18665	0.35653	0.99998	6643	-0.1511
MIEF1	2	0.18665	0.23237	0.99998	6644	-0.2286
DNAJA2	6	0.18668	0.35656	0.99998	6645	-0.1759
KLHL20	6	0.18668	0.35656	0.99998	6646	-0.1734
PRCC	6	0.18677	0.35669	0.99998	6647	-0.221
ZBED2	6	0.18684	0.35677	0.99998	6648	-0.209
MTCP1	6	0.18684	0.35677	0.99998	6649	0.0631
EPHA7	6	0.18684	0.35677	0.99998	6650	-0.2339
FNDC8	6	0.18685	0.35679	0.99998	6651	-0.0267
RBMXL1	6	0.18685	0.35679	0.99998	6652	-0.0415
FRMPD1	6	0.18698	0.35695	0.99998	6653	-0.0272
DOCK4	5	0.18702	0.35109	0.99998	6654	-0.037
SLX4	6	0.18706	0.35707	0.99998	6655	-0.0936
PHLPP1	6	0.18707	0.35708	0.99998	6656	0.0517
hsa-mir-4302	4	0.1871	0.30837	0.99998	6657	-0.1675
TSSC1	6	0.18711	0.35713	0.99998	6658	-0.1323
IGIP	6	0.18718	0.35723	0.99998	6659	-0.0797
OTOP1	6	0.18718	0.35723	0.99998	6660	-0.219
CD300LD	6	0.18724	0.35731	0.99998	6661	0.0526
ADCYAP1	6	0.18728	0.35737	0.99998	6662	0.159
TBC1D3H	1	0.1873	0.1872	0.99998	6663	-1.7394
INHA	6	0.18736	0.35747	0.99998	6664	-0.0222
SRC	6	0.18737	0.35749	0.99998	6665	-0.1054
PCDHGA12	2	0.1874	0.23293	0.99998	6666	-0.1499
LRP5L	6	0.18753	0.35769	0.99998	6667	0.0201
DDX1	6	0.18753	0.35769	0.99998	6668	0.0758
CDPF1	6	0.18764	0.35785	0.99998	6669	-0.1196
CEP57	6	0.18768	0.3579	0.99998	6670	0.0713
TBCK	6	0.1877	0.35793	0.99998	6671	-0.1811
DFFA	6	0.1877	0.35793	0.99998	6672	-0.1247
PARP15	6	0.1877	0.35793	0.99998	6673	-0.1452
FOXRED1	6	0.1877	0.35793	0.99998	6674	-0.1581
hsa-mir-4486	4	0.18782	0.30917	0.99998	6675	-0.1732
CCR7	6	0.18786	0.35813	0.99998	6676	0.1633
RGS17	6	0.18789	0.35818	0.99998	6677	-0.1033
GPATCH2L	6	0.1879	0.35818	0.99998	6678	0.311
SLC39A11	6	0.188	0.35833	0.99998	6679	-0.0455
PEX5L	6	0.18801	0.35835	0.99998	6680	-0.0057
ZNF382	6	0.18801	0.35835	0.99998	6681	0.2113
ACP5	6	0.1881	0.35847	0.99998	6682	-0.3209
C1orf162	6	0.1881	0.35847	0.99998	6683	-0.1914
VDR	6	0.1881	0.35847	0.99998	6684	-0.1111
PSMB9	6	0.18818	0.35857	0.99998	6685	0.0562
ACOX1	6	0.18825	0.35867	0.99998	6686	0.2065
LANCL1	6	0.18828	0.35871	0.99998	6687	-0.0743
MAGEA10-MA	6	0.18834	0.35879	0.99998	6688	0.037
STK32B	6	0.18835	0.35882	0.99998	6689	0.089
DAXX	6	0.18847	0.35896	0.99998	6690	0.0331
GPR97	6	0.18854	0.35906	0.99998	6691	-0.0814
CDKN3	6	0.18854	0.35906	0.99998	6692	-0.0165
NKX2-1	6	0.18862	0.35915	0.99998	6693	0.0416
GCNT7	6	0.18862	0.35915	0.99998	6694	0.0247
FGF4	6	0.18862	0.35915	0.99998	6695	0.0058
DCTPP1	6	0.18876	0.35935	0.99998	6696	0.1166
CYB5R4	6	0.18891	0.35956	0.99998	6697	-0.0765
hsa-mir-7154	4	0.18893	0.31045	0.99998	6698	0.2708
RHEBL1	6	0.18894	0.35961	0.99998	6699	-0.2911
MYCL1	2	0.18899	0.23417	0.99998	6700	-0.1746
GPS2	6	0.1891	0.35983	0.99998	6701	-0.2034
CXorf58	6	0.1891	0.35983	0.99998	6702	-0.1856
ALS2CL	6	0.18915	0.35989	0.99998	6703	0.0917
ATP10D	6	0.18924	0.36	0.99998	6704	0.0182
KANK1	6	0.18924	0.36	0.99998	6705	-0.1788
MSGN1	6	0.18924	0.36	0.99998	6706	-0.2069
hsa-mir-8074	2	0.18928	0.23441	0.99998	6707	-0.3788
CCL28	6	0.18934	0.36014	0.99998	6708	0.1242
EVI2B	6	0.18935	0.36015	0.99998	6709	-0.08
SIX6	6	0.18935	0.36015	0.99998	6710	-0.2118
hsa-mir-4307	4	0.1894	0.31098	0.99998	6711	0.1447
hsa-mir-7850	4	0.1894	0.31098	0.99998	6712	-0.086
NEUROD1	6	0.18948	0.36031	0.99998	6713	-0.0566
PIEZO1	6	0.18948	0.36031	0.99998	6714	0.3267
CTLA4	6	0.18956	0.36044	0.99998	6715	0.0697
SLC25A29	6	0.1896	0.36049	0.99998	6716	-0.1251
PGGT1B	6	0.1896	0.36049	0.99998	6717	-0.0987
TRIP13	6	0.1896	0.36049	0.99998	6718	-0.0626
KRTAP10-12	6	0.18964	0.36054	0.99998	6719	-0.2176
CD320	6	0.18964	0.36054	0.99998	6720	-0.1012

hsa-mir-542	4	0.18967	0.31128	0.99998	6721	-0.161
PCDHGA5	2	0.18968	0.23472	0.99998	6722	-0.1341
LILRA6	4	0.18975	0.31136	0.99998	6723	-0.0637
UGCG	6	0.18978	0.36074	0.99998	6724	0.3482
PEX2	6	0.18978	0.36074	0.99998	6725	-0.0267
ANXA10	6	0.18981	0.36077	0.99998	6726	-0.0948
C1QA	6	0.18991	0.36091	0.99998	6727	-0.0954
CYP7B1	6	0.18991	0.36091	0.99998	6728	-0.0872
APCS	6	0.19	0.36104	0.99998	6729	-0.0904
C1QBP	6	0.19007	0.36112	0.99998	6730	-0.0816
TLCD2	6	0.19007	0.36112	0.99998	6731	-0.0064
hsa-mir-6872	4	0.19012	0.31179	0.99998	6732	0.1054
FIG4	6	0.19018	0.36128	0.99998	6733	-0.152
OR11A1	6	0.19024	0.36136	0.99998	6734	0.1692
ST6GALNAC3	6	0.19025	0.36138	0.99998	6735	0.187
ZNF777	6	0.19025	0.36138	0.99998	6736	0.002
TEF	6	0.19025	0.36138	0.99998	6737	0.1464
SCN2A	6	0.1903	0.36145	0.99998	6738	0.2728
AVPI1	6	0.19034	0.36148	0.99998	6739	-0.137
STOM	6	0.19039	0.36156	0.99998	6740	0.3125
MARVELD3	6	0.19049	0.3617	0.99998	6741	-0.1422
CCM2	6	0.19052	0.36175	0.99998	6742	0.0544
XPA	6	0.19052	0.36175	0.99998	6743	0.1406
CHST4	6	0.19061	0.36186	0.99998	6744	0.1577
ATP9B	6	0.19072	0.36201	0.99998	6745	0.0589
TAF1A	6	0.19074	0.36204	0.99998	6746	0.0019
TBC1D16	6	0.19074	0.36204	0.99998	6747	0.0633
KRTAP26-1	6	0.19074	0.36204	0.99998	6748	-0.1415
LAMP1	6	0.19076	0.36206	0.99998	6749	0.2465
C1QTNF4	6	0.19076	0.36206	0.99998	6750	0.0344
TMEM86A	6	0.19089	0.36224	0.99998	6751	-0.0652
PCDHB8	4	0.19093	0.31269	0.99998	6752	-0.0543
NOA1	6	0.19099	0.36238	0.99998	6753	-0.1839
PANK2	6	0.19108	0.36249	0.99998	6754	0.1198
hsa-mir-3671	3	0.19109	0.29835	0.99998	6755	-0.2217
ZNF653	6	0.19111	0.36253	0.99998	6756	0.1336
hsa-mir-1244-	1	0.19111	0.19096	0.99998	6757	-0.3791
BFSP2	6	0.19115	0.36259	0.99998	6758	0.1382
KRTAP1-5	6	0.19117	0.36262	0.99998	6759	-0.2894
SGTA	6	0.19117	0.36262	0.99998	6760	-0.0196
DPP4	6	0.19141	0.36293	0.99998	6761	-0.0982
MYBPH	6	0.19144	0.36298	0.99998	6762	0.0484
GSX2	6	0.19146	0.36301	0.99998	6763	-0.1212
ATP5S	6	0.19148	0.36304	0.99998	6764	-0.1407
hsa-mir-329-1	1	0.19151	0.19137	0.99998	6765	-1.4375
IL17REL	6	0.19155	0.36313	0.99998	6766	0.2539
C5orf52	6	0.19157	0.36316	0.99998	6767	-0.1056
ERGIC1	6	0.19157	0.36316	0.99998	6768	-0.0619
hsa-mir-4750	4	0.19164	0.31351	0.99998	6769	-0.2264
hsa-mir-6732	4	0.19164	0.31351	0.99998	6770	-0.201
KCNS2	6	0.19174	0.3634	0.99998	6771	0.1188
COG7	6	0.19182	0.3635	0.99998	6772	-0.0693
PURA	6	0.19182	0.3635	0.99998	6773	-0.0995
DALRD3	6	0.19182	0.3635	0.99998	6774	-0.1309
CYP1B1	6	0.1919	0.36361	0.99998	6775	-0.0857
C6orf201	6	0.1919	0.36361	0.99998	6776	-0.0671
PLP1	6	0.19199	0.36374	0.99998	6777	-0.1561
RASSF4	6	0.19215	0.36395	0.99998	6778	0.0114
PSEN2	6	0.19215	0.36395	0.99998	6779	-0.1017
DFNA5	6	0.19224	0.36407	0.99998	6780	-0.0857
NGEF	6	0.19236	0.36424	0.99998	6781	-0.0182
GAL3ST1	6	0.19236	0.36424	0.99998	6782	0.1865
HMGN2	6	0.1925	0.36444	0.99998	6783	-0.0489
MAMDC4	6	0.19261	0.36458	0.99998	6784	0.0113
PLAA	6	0.19263	0.3646	0.99998	6785	-0.0823
hsa-mir-1538	4	0.19264	0.31462	0.99998	6786	-0.1031
INSIG1	6	0.19288	0.36493	0.99998	6787	0.0375
hsa-mir-29c	4	0.19293	0.31493	0.99998	6788	-0.293
PRKAG1	6	0.19297	0.36504	0.99998	6789	-0.0426
LRCH2	6	0.19297	0.36504	0.99998	6790	0.1379
OR2AG2	6	0.19297	0.36504	0.99998	6791	0.0358
SH3RF2	6	0.19303	0.36514	0.99998	6792	-0.1585
AK7	6	0.19303	0.36514	0.99998	6793	-0.2005
CTSF	6	0.19316	0.36532	0.99998	6794	-0.0896
RTKN2	6	0.1932	0.36537	0.99998	6795	-0.1117
PEX5	6	0.1932	0.36537	0.99998	6796	-0.1856
FKBP11	6	0.19325	0.36543	0.99998	6797	0.1694
IL5	6	0.19337	0.3656	0.99998	6798	-0.1149
KCNK16	6	0.19337	0.3656	0.99998	6799	-0.156
CD1E	6	0.19337	0.3656	0.99998	6800	-0.0736
hsa-mir-345	4	0.1934	0.31545	0.99998	6801	0.1674

PATE3	6	0.19344	0.36571	0.99998	6802	0.0815
MCHR2	6	0.19346	0.36573	0.99998	6803	0.0449
SMLR1	6	0.19351	0.36579	0.99998	6804	0.0143
SPANXN2	6	0.19355	0.36586	0.99998	6805	-0.3143
DPH5	6	0.19355	0.36586	0.99998	6806	-0.2035
MOB3C	6	0.19355	0.36586	0.99998	6807	-0.183
UBN2	6	0.19364	0.36597	0.99998	6808	0.0768
TAOK1	6	0.19364	0.36597	0.99998	6809	-0.0419
PAQR6	6	0.19366	0.366	0.99998	6810	-0.1207
PINX1	6	0.19373	0.36608	0.99998	6811	-0.2118
CFLAR	6	0.19373	0.36608	0.99998	6812	-0.2103
PRSS21	6	0.19373	0.36608	0.99998	6813	-0.1961
FAM111B	6	0.19379	0.36616	0.99998	6814	0.2458
APOBR	6	0.19382	0.36619	0.99998	6815	-0.1148
MCF2L	6	0.1939	0.3663	0.99998	6816	-0.0849
ARMCX6	6	0.19393	0.36634	0.99998	6817	-0.177
SWSAP1	6	0.19393	0.36634	0.99998	6818	-0.1827
MBD4	6	0.19395	0.36637	0.99998	6819	0.0195
GIT1	6	0.194	0.36644	0.99998	6820	0.0919
FGFR4	6	0.19403	0.36648	0.99998	6821	-0.0166
NDP	6	0.19405	0.36651	0.99998	6822	0.1411
TLR1	6	0.19408	0.36655	0.99998	6823	-0.1398
PSKH1	6	0.19408	0.36655	0.99998	6824	-0.0757
CST8	6	0.1941	0.36657	0.99998	6825	-0.2022
hsa-mir-296	4	0.19412	0.31624	0.99998	6826	-0.1595
SCG3	6	0.19415	0.36666	0.99998	6827	-0.2672
NDUFA3	6	0.19419	0.36671	0.99998	6828	0.0788
FFAR4	6	0.19419	0.36671	0.99998	6829	-0.1557
SHISA8	6	0.19432	0.36688	0.99998	6830	0.0101
FAM213B	6	0.19444	0.36705	0.99998	6831	-0.1345
DNAJC3	6	0.19448	0.36712	0.99998	6832	-0.0491
SASH1	6	0.19453	0.36718	0.99998	6833	-0.0047
hsa-mir-629	4	0.19453	0.31671	0.99998	6834	-0.1087
METTL21D	6	0.1946	0.36727	0.99998	6835	-0.0572
MFN1	6	0.19463	0.36731	0.99998	6836	-0.1967
HNRNPAB	6	0.19463	0.36731	0.99998	6837	0.0021
PHC3	6	0.19463	0.36731	0.99998	6838	-0.1431
CYP2U1	6	0.19463	0.36731	0.99998	6839	0.0016
ANKRD16	6	0.19463	0.36731	0.99998	6840	-0.1506
MFGE8	6	0.19474	0.36746	0.99998	6841	0.0754
ELFN2	6	0.19475	0.36747	0.99998	6842	-0.0902
RAB4B	6	0.19485	0.3676	0.99998	6843	-0.1458
REV1	6	0.1949	0.36768	0.99998	6844	0.0173
ZNF345	6	0.19494	0.36773	0.99998	6845	-0.134
TRPC5	6	0.19494	0.36773	0.99998	6846	0.0356
PRR22	6	0.19494	0.36773	0.99998	6847	-0.047
GPR61	6	0.195	0.36781	0.99998	6848	-0.1053
ANKRD13D	6	0.19505	0.36787	0.99998	6849	-0.031
COTL1	6	0.19506	0.36788	0.99998	6850	0.0116
PADI6	6	0.19506	0.36788	0.99998	6851	-0.0828
FOXJ2	6	0.19512	0.36796	0.99998	6852	-0.0851
hsa-mir-8076	4	0.19513	0.31736	0.99998	6853	-0.0085
GHDC	6	0.19518	0.36804	0.99998	6854	0.1138
APIP	6	0.1952	0.36807	0.99998	6855	-0.1225
TRAF3	6	0.1952	0.36807	0.99998	6856	-0.1885
DIO3	6	0.19523	0.3681	0.99998	6857	-0.0871
hsa-mir-8056	4	0.19528	0.31752	0.99998	6858	0.0854
KPTN	6	0.19532	0.36824	0.99998	6859	-0.089
DNM1	6	0.19532	0.36824	0.99998	6860	-0.1833
RNASE13	6	0.19532	0.36824	0.99998	6861	-0.229
TMEM233	6	0.19543	0.36839	0.99998	6862	0.0724
IL36A	6	0.1955	0.36849	0.99998	6863	-0.2291
C8orf82	6	0.19551	0.3685	0.99998	6864	0.0513
HEG1	6	0.19566	0.36869	0.99998	6865	-0.1233
WT1	6	0.19566	0.36869	0.99998	6866	-0.1017
RUNX3	6	0.19567	0.36871	0.99998	6867	0.0159
PPARG	6	0.19567	0.36871	0.99998	6868	-0.0504
hsa-mir-4527	4	0.19571	0.31799	0.99998	6869	-0.1034
VN1R1	6	0.19571	0.36875	0.99998	6870	0.0522
RSPH1	6	0.1958	0.36887	0.99998	6871	0.0362
R3HDML	6	0.19593	0.36905	0.99998	6872	-0.0998
KRT3	6	0.19595	0.36908	0.99998	6873	0.0154
KIAA0930	6	0.19595	0.36908	0.99998	6874	-0.1557
NSFL1C	6	0.19595	0.36908	0.99998	6875	0.2478
ST6GALNAC4	6	0.19596	0.3691	0.99998	6876	-0.1421
ZNRF1	6	0.19605	0.36922	0.99998	6877	-0.1518
SLC22A17	6	0.1961	0.36929	0.99998	6878	0.0264
SRGN	6	0.19614	0.36935	0.99998	6879	-0.1095
TMPRSS11E	6	0.19616	0.36937	0.99998	6880	-0.0895
DDR1	6	0.19616	0.36937	0.99998	6881	0.192
USP17L8	6	0.19621	0.36943	0.99998	6882	0.1919

FGGY	6	0.19625	0.36949	0.99998	6883	-0.1687
NEURL4	6	0.19631	0.36957	0.99998	6884	0.3163
ALOX5AP	6	0.19636	0.36962	0.99998	6885	-0.0711
ADH1A	6	0.19638	0.36964	0.99998	6886	-0.3952
GAL	6	0.19638	0.36964	0.99998	6887	0.2368
SMR3A	4	0.19642	0.3188	0.99998	6888	-0.0371
hsa-mir-577	4	0.1965	0.31888	0.99998	6889	0.1604
CACNA1A	6	0.19657	0.36991	0.99998	6890	-0.0485
SEZ6L2	6	0.19657	0.36991	0.99998	6891	0.012
IGFBP6	6	0.19657	0.36991	0.99998	6892	-0.1286
P4HA3	6	0.19661	0.36995	0.99998	6893	0.0866
PACS1	6	0.19662	0.36997	0.99998	6894	0.2711
NMB	6	0.19665	0.37001	0.99998	6895	-0.1494
hsa-mir-7856	4	0.19674	0.31914	0.99998	6896	0.0997
FGF2	6	0.19677	0.37018	0.99998	6897	0.0915
ARFGAP2	6	0.19677	0.37018	0.99998	6898	0.0562
SNX33	6	0.19677	0.37018	0.99998	6899	0.0519
ZNF831	6	0.19677	0.37018	0.99998	6900	-0.0233
GALNT4	3	0.19683	0.30576	0.99998	6901	-0.1305
ATP9A	6	0.19689	0.37034	0.99998	6902	-0.0085
C5orf22	6	0.19691	0.37036	0.99998	6903	0.1177
PDE6A	6	0.19702	0.37051	0.99998	6904	-0.1754
hsa-mir-204	4	0.19708	0.3195	0.99998	6905	-0.0929
R3HCC1	6	0.19712	0.37065	0.99998	6906	-0.078
COL1A1	6	0.19726	0.37084	0.99998	6907	-0.1579
TMPRSS7	6	0.1973	0.37091	0.99998	6908	0.2225
WHSC1L1	6	0.1973	0.37091	0.99998	6909	0.1248
RNF25	6	0.19742	0.37107	0.99998	6910	-0.142
ALDH1A1	6	0.19742	0.37107	0.99998	6911	-0.1546
NAA35	6	0.19742	0.37108	0.99998	6912	0.1799
SOX13	6	0.19742	0.37108	0.99998	6913	0.0985
TP73	6	0.19749	0.37118	0.99998	6914	-0.0604
TOMM20L	6	0.19753	0.37124	0.99998	6915	-0.2717
CCNT1	6	0.19753	0.37124	0.99998	6916	-0.1039
AIMP2	6	0.19753	0.37124	0.99998	6917	-0.1364
GPR89A	4	0.19754	0.32003	0.99998	6918	-0.2263
FLAD1	6	0.19757	0.3713	0.99998	6919	0.2072
OBFC1	6	0.19757	0.3713	0.99998	6920	0.2145
RAB1B	6	0.19763	0.37137	0.99998	6921	-0.2817
ABCA12	6	0.19763	0.37137	0.99998	6922	-0.0867
TMEM179B	6	0.19775	0.37153	0.99998	6923	-0.0359
TSPAN31	6	0.19777	0.37156	0.99998	6924	-0.1824
C10orf88	6	0.19783	0.37163	0.99998	6925	0.1564
PKD1	6	0.19797	0.37183	0.99998	6926	-0.054
hsa-mir-3147	4	0.19802	0.32057	0.99998	6927	-0.0884
KCNQ3	6	0.19809	0.37199	0.99998	6928	-0.0507
CREB3L2	4	0.19815	0.32071	0.99998	6929	-0.0196
CNTF	6	0.1982	0.37214	0.99998	6930	0.2395
POU3F1	6	0.1982	0.37214	0.99998	6931	0.1308
ERICH1	4	0.19821	0.32077	0.99998	6932	-0.2045
HIST1H3B	6	0.19824	0.37219	0.99998	6933	0.0318
APBA2	6	0.19824	0.37219	0.99998	6934	0.0081
ADAMTSL5	6	0.19835	0.37235	0.99998	6935	0.0906
DSN1	4	0.19839	0.321	0.99998	6936	0.0801
CETN3	6	0.1984	0.3724	0.99998	6937	-0.124
SLC22A6	6	0.19851	0.37256	0.99998	6938	0.0909
EDARADD	6	0.19851	0.37256	0.99998	6939	-0.1121
CLDN24	6	0.19857	0.37263	0.99998	6940	0.0561
FGL1	6	0.19859	0.37265	0.99998	6941	0.0531
ZBTB18	4	0.19863	0.32129	0.99998	6942	-0.1046
JRK	6	0.19865	0.37273	0.99998	6943	-0.1354
NXPH3	6	0.19866	0.37274	0.99998	6944	0.1301
hsa-mir-492	4	0.19869	0.32135	0.99998	6945	0.0186
KLF3	6	0.19874	0.37285	0.99998	6946	-0.1007
RPL36AL	6	0.19875	0.37287	0.99998	6947	-0.2185
ASPM	6	0.19875	0.37287	0.99998	6948	-0.0437
SFTPD	6	0.19879	0.37291	0.99998	6949	0.174
CCIN	6	0.19889	0.37305	0.99998	6950	0.1003
PDE3B	6	0.19889	0.37305	0.99998	6951	-0.059
LCMT2	6	0.199	0.37318	0.99998	6952	-0.2521
UBTD1	6	0.19902	0.37321	0.99998	6953	-0.0215
MINK1	6	0.19902	0.37321	0.99998	6954	-0.1183
OMD	6	0.1991	0.3733	0.99998	6955	-0.0966
NCR2	6	0.19915	0.37337	0.99998	6956	0.1242
KRTAP5-4	6	0.19917	0.3734	0.99998	6957	0.0164
PYDC1	6	0.1992	0.37344	0.99998	6958	-0.2616
MMP11	6	0.19929	0.37355	0.99998	6959	-0.01
TTC22	6	0.19933	0.3736	0.99998	6960	0.004
BTF3	6	0.19935	0.37363	0.99998	6961	-0.0836
EIF5	6	0.19935	0.37363	0.99998	6962	-0.2212
OR4C15	6	0.19935	0.37363	0.99998	6963	-0.0648

FAM211B	6	0.19936	0.37365	0.99998	6964	-0.0083
COL7A1	6	0.19941	0.37372	0.99998	6965	-0.3132
CCDC174	6	0.19951	0.37385	0.99998	6966	-0.1055
NDUFAF1	6	0.19951	0.37385	0.99998	6967	-0.1504
ZRANB1	6	0.19959	0.37395	0.99998	6968	-0.0543
MRPL46	6	0.19959	0.37395	0.99998	6969	0.1387
HES3	6	0.19959	0.37395	0.99998	6970	-0.2608
RXFP3	6	0.1997	0.37409	0.99998	6971	-0.006
SLC16A10	6	0.19973	0.37415	0.99998	6972	0.0272
KIAA1324	6	0.19985	0.37429	0.99998	6973	-0.0288
NTN4	6	0.19985	0.37429	0.99998	6974	0.2212
TSSK2	6	0.19995	0.37442	0.99998	6975	-0.0166
LCLAT1	6	0.19996	0.37443	0.99998	6976	-0.0169
C1QTNF9	2	0.19997	0.24264	0.99998	6977	-0.0692
CNDP2	6	0.20006	0.37457	0.99998	6978	0.0589
TMOD3	6	0.20006	0.37457	0.99998	6979	0.1232
ZNF646	6	0.20012	0.37465	0.99998	6980	-0.2376
ZNF620	6	0.20012	0.37465	0.99998	6981	-0.1314
SMC1B	6	0.20012	0.37465	0.99998	6982	-0.1202
IQUB	6	0.20012	0.37465	0.99998	6983	-0.1709
FAM216B	6	0.20012	0.37465	0.99998	6984	0.0068
MLF1	6	0.20012	0.37465	0.99998	6985	-0.1378
MT1X	4	0.20016	0.32303	0.99998	6986	-0.0531
ZFAND4	4	0.20022	0.32309	0.99998	6987	0.0544
RPS7	6	0.20026	0.37486	0.99998	6988	-0.0976
KIAA1143	6	0.20032	0.37492	0.99998	6989	-0.0794
LCE3E	3	0.20039	0.31045	0.99998	6990	-0.1537
SNX7	6	0.2004	0.37505	0.99998	6991	0.2266
PLEKHA8	6	0.2004	0.37506	0.99998	6992	-0.0143
FKRP	6	0.20045	0.37512	0.99998	6993	0.2246
MBLAC1	6	0.20047	0.37515	0.99998	6994	-0.0792
HBE1	6	0.20052	0.37521	0.99998	6995	0.0091
SRR	6	0.2006	0.37535	0.99998	6996	-0.0484
C12orf5	6	0.2006	0.37535	0.99998	6997	0.0043
MITF	6	0.20063	0.37539	0.99998	6998	-0.1753
CDKL3	6	0.20069	0.37548	0.99998	6999	-0.1342
ZCCHC7	6	0.20069	0.37548	0.99998	7000	-0.0572
PSMD10	6	0.20076	0.37555	0.99998	7001	0.092
PRR11	6	0.20082	0.37563	0.99998	7002	-0.1182
MYOG	6	0.20082	0.37563	0.99998	7003	0.0265
HERPUD1	6	0.20087	0.3757	0.99998	7004	0.3567
PSAT1	6	0.20087	0.3757	0.99998	7005	0.0119
hsa-mir-6797	4	0.20092	0.32385	0.99998	7006	-0.0942
CEP290	6	0.20097	0.37584	0.99998	7007	-0.1462
C2orf71	6	0.20097	0.37584	0.99998	7008	-0.1909
ADAM2	6	0.20097	0.37584	0.99998	7009	-0.2407
ZNF648	6	0.20103	0.37591	0.99998	7010	0.0211
BCL6	6	0.20112	0.37603	0.99998	7011	-0.1126
HAUS8	6	0.20112	0.37603	0.99998	7012	-0.2144
OR5B17	6	0.2012	0.37614	0.99998	7013	0.0266
EFNA4	6	0.2013	0.37629	0.99998	7014	0.1588
B3GAT1	6	0.20131	0.37631	0.99998	7015	0.0174
ZBTB3	6	0.20135	0.37636	0.99998	7016	0.0057
hsa-mir-4788	4	0.20137	0.32434	0.99998	7017	0.4949
FPR2	6	0.20142	0.37646	0.99998	7018	-0.0857
CYP26B1	6	0.20152	0.37659	0.99998	7019	-0.2398
FKBP6	6	0.20153	0.37661	0.99998	7020	0.0302
DROSHA	6	0.20155	0.37662	0.99998	7021	-0.1128
MYO6	6	0.2016	0.37671	0.99998	7022	-0.0334
MEGF11	6	0.20164	0.37675	0.99998	7023	-0.0666
hsa-mir-8054	4	0.20175	0.32476	0.99998	7024	-0.2104
OR2L5	6	0.2018	0.37697	0.99998	7025	0.1323
TMA7	6	0.20191	0.37712	0.99998	7026	-0.2647
KALRN	6	0.20191	0.37712	0.99998	7027	-0.1177
hsa-mir-4789	4	0.20196	0.325	0.99998	7028	0.3576
MCTP1	6	0.20196	0.37719	0.99998	7029	0.1278
C8orf37	6	0.20202	0.37727	0.99998	7030	-0.1292
hsa-mir-1286	4	0.20209	0.32516	0.99998	7031	-0.1168
ACAD9	6	0.2021	0.37737	0.99998	7032	-0.0481
CEP68	6	0.2021	0.37737	0.99998	7033	-0.1016
PCDH10	6	0.20212	0.37739	0.99998	7034	0.0936
PTPRM	6	0.20219	0.37748	0.99998	7035	-0.096
CMTM6	6	0.20219	0.37748	0.99998	7036	-0.1337
CPNE6	6	0.2022	0.3775	0.99998	7037	0.2793
VCX3A	1	0.2022	0.20191	0.99998	7038	-2.1831
CGB7	1	0.2022	0.20191	0.99998	7039	-2.1831
GALT	6	0.20227	0.37757	0.99998	7040	0.1202
THBS3	6	0.20235	0.37769	0.99998	7041	0.0314
SUFU	6	0.20241	0.37777	0.99998	7042	-0.0385
TMEM168	6	0.20241	0.37777	0.99998	7043	0.0468
WNT16	6	0.20244	0.37781	0.99998	7044	-0.1871

MS4A8	6	0.20252	0.3779	0.99998	7045	0.1095
OR7A10	6	0.20253	0.37792	0.99998	7046	0.0046
NBL1	3	0.20253	0.31325	0.99998	7047	-0.158
PIPOX	6	0.20257	0.37797	0.99998	7048	-0.0981
TFCP2L1	6	0.20258	0.37798	0.99998	7049	-0.0794
hsa-mir-4533	4	0.20259	0.32571	0.99998	7050	-0.0964
REXO4	6	0.20263	0.37806	0.99998	7051	0.2427
OR1I1	6	0.20269	0.37813	0.99998	7052	-0.0771
TMEM61	6	0.20281	0.37829	0.99998	7053	-0.1064
PEMT	6	0.20283	0.37832	0.99998	7054	-0.1472
HMX2	6	0.20283	0.37833	0.99998	7055	-0.1424
CALM1	6	0.20294	0.37846	0.99998	7056	0.0029
PARL	6	0.20298	0.37852	0.99998	7057	0.0565
SLC35F1	6	0.20306	0.37863	0.99998	7058	0.0336
ADAMTSL2	6	0.20306	0.37863	0.99998	7059	0.0368
TNPO2	6	0.20311	0.37869	0.99998	7060	0.0417
FOXK1	6	0.20315	0.37875	0.99998	7061	-0.126
LDHD	6	0.20322	0.37882	0.99998	7062	0.0132
NFATC1	4	0.20332	0.3265	0.99998	7063	-0.1293
PRKCZ	6	0.20332	0.37895	0.99998	7064	-0.0712
SCARF2	6	0.20345	0.37914	0.99998	7065	0.098
LHX5	6	0.20347	0.37917	0.99998	7066	0.0951
AHSP	6	0.20358	0.3793	0.99998	7067	-0.0297
PDE5A	6	0.20358	0.3793	0.99998	7068	-0.0947
RFX6	6	0.20365	0.37938	0.99998	7069	0.0752
EID2	6	0.20365	0.37938	0.99998	7070	-0.1412
LOC649330	1	0.20365	0.20333	0.99998	7071	-0.2397
HIST1H2AK	6	0.20379	0.37955	0.99998	7072	-0.2088
NKX2-5	6	0.20383	0.37959	0.99998	7073	-0.0791
UQCRHL	6	0.20404	0.37987	0.99998	7074	-0.1342
ERF	6	0.20415	0.38002	0.99998	7075	0.0618
WDR26	6	0.20416	0.38005	0.99998	7076	-0.0985
MTG1	6	0.20423	0.38013	0.99998	7077	-0.1444
GRHPR	6	0.20425	0.38018	0.99998	7078	-0.0552
RARS2	6	0.20429	0.38024	0.99998	7079	-0.1011
CRX	6	0.20438	0.38035	0.99998	7080	-0.0541
ITGA9	6	0.20438	0.38035	0.99998	7081	0.0002
MROH6	6	0.20438	0.38035	0.99998	7082	0.0576
hsa-mir-4671	3	0.2044	0.31573	0.99998	7083	-0.1936
FAM149B1	6	0.20441	0.38039	0.99998	7084	-0.0446
NUDT3	6	0.20442	0.38041	0.99998	7085	0.1097
PPP2CA	5	0.20444	0.36699	0.99998	7086	0.0114
IL12RB2	6	0.20449	0.38049	0.99998	7087	-0.1272
PPIP5K2	6	0.20449	0.38049	0.99998	7088	-0.0381
FGF17	6	0.20459	0.38064	0.99998	7089	-0.0504
HRASLS2	6	0.20462	0.38067	0.99998	7090	-0.1255
SPATA6	6	0.20462	0.38067	0.99998	7091	-0.0401
hsa-mir-3689f	4	0.20464	0.32802	0.99998	7092	-0.6647
HS6ST3	6	0.20465	0.3807	0.99998	7093	-0.2
hsa-mir-581	2	0.20467	0.24631	0.99998	7094	-0.249
DBNDD2	6	0.20477	0.38087	0.99998	7095	0.0609
C11orf87	6	0.20477	0.38087	0.99998	7096	0.2608
SUPT5H	6	0.20489	0.38103	0.99998	7097	-0.0589
RFC1	6	0.20489	0.38103	0.99998	7098	-0.1871
CUL4B	6	0.20498	0.38115	0.99998	7099	0.0286
SLC35D2	6	0.20498	0.38115	0.99998	7100	0.1673
hsa-mir-3618	4	0.20502	0.32845	0.99998	7101	-0.2245
hsa-mir-4264	4	0.20502	0.32845	0.99998	7102	-0.2277
SOCS6	6	0.20502	0.3812	0.99998	7103	-0.1799
SH3BGRL2	6	0.20507	0.38126	0.99998	7104	-0.0206
BSCL2	6	0.20513	0.38134	0.99998	7105	-0.1171
TAS1R2	6	0.20526	0.3815	0.99998	7106	-0.0603
MROH9	6	0.20526	0.3815	0.99998	7107	-0.1186
SYBU	6	0.2053	0.38157	0.99998	7108	-0.0245
NAAA	6	0.2053	0.38157	0.99998	7109	-0.0087
ZNF428	6	0.20534	0.3816	0.99998	7110	-0.1548
CT45A5	3	0.20538	0.31702	0.99998	7111	-0.4495
SH2D1B	6	0.20552	0.38184	0.99998	7112	-0.0891
GSTO1	6	0.20552	0.38184	0.99998	7113	0.1541
FUCA1	6	0.20558	0.38191	0.99998	7114	0.2738
SRSF6	6	0.2057	0.38208	0.99998	7115	-0.0048
ZNF830	6	0.20573	0.38212	0.99998	7116	0.0255
MRPS10	6	0.20574	0.38213	0.99998	7117	-0.0638
ADAMTS3	6	0.20586	0.3823	0.99998	7118	-0.2549
CTSE	6	0.20586	0.3823	0.99998	7119	0.125
GOSR2	6	0.20605	0.38258	0.99998	7120	-0.1976
TMEM145	6	0.20605	0.38258	0.99998	7121	-0.0402
SGSM3	4	0.20607	0.32967	0.99998	7122	-0.0577
DCAF12	6	0.20616	0.38272	0.99998	7123	0.1212
hsa-mir-4283-	4	0.20623	0.32983	0.99998	7124	-0.1537
MAPK13	6	0.20623	0.38282	0.99998	7125	-0.1695

IHH	6	0.20631	0.38293	0.99998	7126	0.2154
VSIG1	6	0.20638	0.38302	0.99998	7127	-0.0235
ARMC9	6	0.20641	0.38307	0.99998	7128	0.0401
RIMS2	6	0.2065	0.38317	0.99998	7129	-0.0561
SLFNL1	6	0.2065	0.38317	0.99998	7130	-0.101
MED15	6	0.20651	0.38318	0.99998	7131	0.0137
MCCD1	6	0.20651	0.38318	0.99998	7132	0.0324
LEPREL1	6	0.20659	0.38328	0.99998	7133	-0.0652
PLD3	6	0.20661	0.3833	0.99998	7134	-0.1989
TTC5	6	0.20666	0.38339	0.99998	7135	-0.1712
COL4A3	6	0.20671	0.38345	0.99998	7136	0.0821
RPL4	6	0.20671	0.38345	0.99998	7137	-0.1293
ZNF607	6	0.20679	0.38357	0.99998	7138	-0.0383
SV2C	6	0.20681	0.3836	0.99998	7139	0.1615
TMEM64	6	0.20682	0.38361	0.99998	7140	-0.0362
DDHD2	4	0.20683	0.33052	0.99998	7141	0.1899
hsa-mir-4707	4	0.20685	0.33055	0.99998	7142	-0.1471
CCDC110	6	0.20689	0.3837	0.99998	7143	-0.0257
LMF1	6	0.20694	0.38377	0.99998	7144	-0.1711
PLCB4	6	0.20694	0.38377	0.99998	7145	-0.1343
PRR7	6	0.20699	0.38382	0.99998	7146	0.0088
hsa-mir-4790	3	0.20707	0.31922	0.99998	7147	-0.2554
CASP8AP2	6	0.20717	0.38407	0.99998	7148	-0.2294
H1FNT	6	0.20722	0.38412	0.99998	7149	0.0019
IKBKAP	6	0.20722	0.38412	0.99998	7150	-0.1479
NGDN	6	0.20728	0.38421	0.99998	7151	-0.1321
NDST1	6	0.2073	0.38423	0.99998	7152	0.0354
SLCO5A1	6	0.20731	0.38425	0.99998	7153	-0.1199
PNMAL1	6	0.20737	0.38432	0.99998	7154	-0.0743
RAF1	6	0.20747	0.38445	0.99998	7155	-0.0591
TMPRSS13	6	0.20747	0.38445	0.99998	7156	-0.0508
C10orf10	6	0.20757	0.38459	0.99998	7157	0.0461
RIMS4	6	0.20764	0.38467	0.99998	7158	-0.0017
GBP4	6	0.20764	0.38467	0.99998	7159	-0.1749
ANKRD13C	6	0.20766	0.38471	0.99998	7160	-0.0097
GDPD5	6	0.20769	0.38475	0.99998	7161	-0.0324
MRAP	6	0.20776	0.38484	0.99998	7162	-0.0147
ATP13A3	6	0.20785	0.38496	0.99998	7163	0.1131
hsa-mir-598	4	0.20789	0.33172	0.99998	7164	-0.0494
hsa-mir-1468	4	0.20789	0.33172	0.99998	7165	-0.2228
GABARAP	6	0.20794	0.38507	0.99998	7166	-0.1824
TCEAL2	6	0.20794	0.38507	0.99998	7167	-0.0026
GLO1	6	0.20794	0.38507	0.99998	7168	-0.0783
PDP2	6	0.208	0.38515	0.99998	7169	-0.117
ASAP3	6	0.208	0.38515	0.99998	7170	-0.0271
CCDC140	6	0.208	0.38515	0.99998	7171	-0.0562
NUCKS1	6	0.208	0.38515	0.99998	7172	0.0268
RHOF	6	0.20818	0.38537	0.99998	7173	-0.2097
TAAR2	6	0.20823	0.38543	0.99998	7174	-0.1184
PRKG2	6	0.20837	0.38563	0.99998	7175	-0.1366
EIF5A2	6	0.20844	0.38573	0.99998	7176	0.1877
APOL4	6	0.20844	0.38573	0.99998	7177	0.1171
KRTAP13-4	6	0.20844	0.38573	0.99998	7178	0.2356
RLIM	6	0.20844	0.38573	0.99998	7179	0.4566
ZNF652	6	0.2085	0.38581	0.99998	7180	-0.1168
TNNC1	6	0.20857	0.3859	0.99998	7181	0.0991
ALOX12	6	0.20859	0.38592	0.99998	7182	-0.0494
AFAP1L2	6	0.20859	0.38592	0.99998	7183	0.107
ARHGAP29	6	0.20863	0.38597	0.99998	7184	0.1866
KLHDC9	6	0.20865	0.38599	0.99998	7185	-0.0992
CA11	6	0.20867	0.38602	0.99998	7186	-0.0064
TREML2	6	0.20879	0.38618	0.99998	7187	-0.0565
BPIFC	6	0.20879	0.38618	0.99998	7188	-0.1047
TJP1	6	0.20883	0.38623	0.99998	7189	-0.0827
INTS5	6	0.20883	0.38623	0.99998	7190	-0.0713
JAKMIP1	6	0.20889	0.3863	0.99998	7191	-0.1101
FHIT	6	0.209	0.38644	0.99998	7192	0.1125
CES1	6	0.20903	0.38648	0.99998	7193	-0.0874
OR1J4	6	0.20905	0.3865	0.99998	7194	0.2053
AKAP6	6	0.20917	0.38666	0.99998	7195	-0.0094
CLDN4	6	0.20918	0.38668	0.99998	7196	-0.1527
hsa-mir-548f-4	1	0.20923	0.20889	0.99998	7197	-0.7813
hsa-mir-7844	4	0.20923	0.33322	0.99998	7198	-0.0876
CC2D1A	6	0.20925	0.38677	0.99998	7199	-0.0444
hsa-mir-3617	4	0.20932	0.33332	0.99998	7200	-0.0721
hsa-mir-4301	4	0.20932	0.33332	0.99998	7201	0.0398
CKMT1A	4	0.20945	0.33347	0.99998	7202	-0.1675
LDHB	6	0.20961	0.38726	0.99998	7203	-0.1126
TRAM1	6	0.20972	0.38741	0.99998	7204	-0.0051
C12orf66	6	0.20974	0.38742	0.99998	7205	-0.1898
UGT2B11	3	0.20974	0.32263	0.99998	7206	-0.2049

GPX7	6	0.2098	0.38749	0.99998	7207	-0.1456
SLC22A4	6	0.20982	0.38753	0.99998	7208	-0.0371
GPR162	6	0.20987	0.38759	0.99998	7209	0.1861
ZNF76	6	0.20993	0.38767	0.99998	7210	-0.0322
NLGN3	6	0.20994	0.38768	0.99998	7211	-0.0392
AURKC	6	0.20994	0.38768	0.99998	7212	-0.0803
ZFYVE28	6	0.20996	0.3877	0.99998	7213	-0.0206
HOXD13	6	0.21011	0.3879	0.99998	7214	0.0583
PPM1G	6	0.21011	0.3879	0.99998	7215	0.0589
YWHAE	6	0.21015	0.38796	0.99998	7216	0.1287
IGF1R	6	0.21028	0.38814	0.99998	7217	-0.0572
ZCCHC5	6	0.21029	0.38814	0.99998	7218	-0.1428
OR4C13	6	0.2103	0.38816	0.99998	7219	0.1253
EVI2A	6	0.21039	0.38828	0.99998	7220	0.0859
MRPL36	6	0.21041	0.38831	0.99998	7221	-0.1683
CALB2	6	0.21049	0.38842	0.99998	7222	0.0162
PIGK	6	0.21054	0.38849	0.99998	7223	0.0125
MAST1	6	0.21059	0.38854	0.99998	7224	-0.0381
PDZD2	6	0.21077	0.38878	0.99998	7225	-0.0648
C11orf88	6	0.21079	0.38883	0.99998	7226	0.2077
UBE4B	6	0.21089	0.38894	0.99998	7227	0.0537
OPN4	6	0.21089	0.38894	0.99998	7228	-0.1027
MLXIPL	6	0.21089	0.38894	0.99998	7229	-0.0057
ARID3B	6	0.21093	0.389	0.99998	7230	-0.0776
FAM110A	6	0.21094	0.38902	0.99998	7231	0.1317
TRIM45	6	0.21094	0.38902	0.99998	7232	-0.0047
PELI3	6	0.21096	0.38904	0.99998	7233	-0.1249
ENOX2	6	0.21101	0.3891	0.99998	7234	-0.1949
PHACTR2	6	0.21104	0.38916	0.99998	7235	-0.1131
SLC5A1	6	0.21113	0.38928	0.99998	7236	-0.125
MYO15A	6	0.21113	0.38928	0.99998	7237	-0.1535
FAM107A	6	0.21113	0.38928	0.99998	7238	-0.1039
IGSF23	6	0.21114	0.38929	0.99998	7239	0.1939
HAPLN3	6	0.2112	0.38936	0.99998	7240	-0.1338
LIMCH1	6	0.21127	0.38946	0.99998	7241	-0.2115
CXCL5	6	0.21127	0.38946	0.99998	7242	-0.2006
C6orf52	6	0.21136	0.38958	0.99998	7243	-0.0329
RRAGD	6	0.21136	0.38959	0.99998	7244	-0.0198
SLC25A52	6	0.21147	0.38973	0.99998	7245	-0.0807
CTSC	6	0.21148	0.38974	0.99998	7246	-0.0717
DNASE1	6	0.21158	0.38986	0.99998	7247	-0.0211
CD48	6	0.21165	0.38995	0.99998	7248	-0.0627
PTAFR	6	0.21169	0.39001	0.99998	7249	-0.0856
SKA3	6	0.21169	0.39001	0.99998	7250	-0.1879
WIPF3	6	0.21169	0.39001	0.99998	7251	-0.1142
GJA9	6	0.21169	0.39001	0.99998	7252	-0.1755
CAPN3	6	0.21169	0.39001	0.99998	7253	-0.2094
L3MBTL3	6	0.21184	0.39021	0.99998	7254	-0.0944
BTBD6	6	0.21192	0.39031	0.99998	7255	-0.1685
CSMD3	6	0.21192	0.39031	0.99998	7256	-0.1647
GJB3	6	0.21192	0.39031	0.99998	7257	-0.0627
MEX3A	6	0.21192	0.39031	0.99998	7258	-0.0808
CLDN9	6	0.21192	0.39031	0.99998	7259	-0.1307
C3orf67	6	0.21196	0.39037	0.99998	7260	-0.07
MBLAC2	6	0.2121	0.39055	0.99998	7261	-0.1998
CAV1	6	0.2121	0.39055	0.99998	7262	-0.0342
CCL16	6	0.21211	0.39057	0.99998	7263	0.1134
CRISPLD1	6	0.21211	0.39057	0.99998	7264	0.0317
TNFSF4	6	0.21211	0.39057	0.99998	7265	0.0319
ZNF829	6	0.21218	0.39067	0.99998	7266	0.0418
DKK1	6	0.21219	0.39067	0.99998	7267	-0.2014
NTN1	6	0.21219	0.39067	0.99998	7268	-0.2399
TCEANC2	6	0.21219	0.39067	0.99998	7269	-0.1839
SSX4B	3	0.21225	0.32588	0.99998	7270	-0.188
AKNA	6	0.21232	0.39086	0.99998	7271	-0.0326
MC3R	6	0.21235	0.3909	0.99998	7272	0.0757
FAM26F	6	0.21242	0.39097	0.99998	7273	-0.1442
FCER2	6	0.21242	0.39097	0.99998	7274	-0.1704
NLRC5	6	0.21266	0.39128	0.99998	7275	0.0874
SPAST	6	0.21274	0.39137	0.99998	7276	-0.1935
CEP55	6	0.21276	0.3914	0.99998	7277	-0.1091
FAM179A	6	0.21286	0.39154	0.99998	7278	-0.0061
AGTR2	6	0.21289	0.39159	0.99998	7279	-0.1873
hsa-mir-4666a	4	0.21289	0.33727	0.99998	7280	-0.2011
SPA17	6	0.21293	0.39164	0.99998	7281	-0.0508
TSHZ3	6	0.21293	0.39164	0.99998	7282	-0.0333
KIAA0232	6	0.21301	0.39175	0.99998	7283	0.0556
MEPCE	6	0.21301	0.39175	0.99998	7284	-0.0316
LHFPL3	6	0.21306	0.39181	0.99998	7285	0.0069
PROSER2	6	0.21306	0.39181	0.99998	7286	0.0421
TAB2	6	0.21312	0.39189	0.99998	7287	0.0396

GALNT14	4	0.21319	0.33758	0.99998	7288	0.1561
TSN	6	0.21319	0.39199	0.99998	7289	-0.145
hsa-mir-7853	2	0.21321	0.25295	0.99998	7290	-0.2011
USP38	6	0.21323	0.39204	0.99998	7291	0.1164
IRF5	6	0.21323	0.39204	0.99998	7292	0.1472
NUDT18	6	0.21323	0.39204	0.99998	7293	0.0625
PLXNA3	6	0.21326	0.39209	0.99998	7294	-0.0055
CCDC28A	4	0.21329	0.33771	0.99998	7295	-0.1916
MBOAT2	6	0.21332	0.39216	0.99998	7296	-0.1351
S100A1	6	0.21332	0.39216	0.99998	7297	-0.1967
ZBTB43	6	0.21335	0.39222	0.99998	7298	-0.102
SLC10A6	6	0.21342	0.39231	0.99998	7299	-0.0095
GHR	6	0.21343	0.39232	0.99998	7300	-0.1153
RNF185	6	0.21343	0.39232	0.99998	7301	-0.1363
LARP4B	6	0.21346	0.39236	0.99998	7302	-0.1195
hsa-mir-4516	4	0.21346	0.33791	0.99998	7303	-0.2981
C1orf50	6	0.21351	0.39243	0.99998	7304	-0.1061
hsa-mir-133a-	4	0.21355	0.33801	0.99998	7305	0.0382
FAM189A1	6	0.21358	0.39253	0.99998	7306	0.1095
LMTK2	6	0.21359	0.39255	0.99998	7307	0.1453
SH3YL1	6	0.21359	0.39255	0.99998	7308	-0.0466
DDRGK1	6	0.2137	0.39269	0.99998	7309	-0.0112
RCSD1	6	0.21378	0.3928	0.99998	7310	-0.0463
hsa-mir-6074	4	0.2139	0.33841	0.99998	7311	-0.1639
hsa-mir-6885	4	0.2139	0.33841	0.99998	7312	-0.2192
PCP4	6	0.21392	0.39298	0.99998	7313	-0.045
SFRP2	6	0.21403	0.39311	0.99998	7314	-0.1435
IL11	6	0.21408	0.39319	0.99998	7315	0.1453
YJEFN3	6	0.21412	0.39323	0.99998	7316	-0.064
GALNT11	6	0.21423	0.39337	0.99998	7317	0.1271
SPTLC1	6	0.21423	0.39337	0.99998	7318	-0.0917
VRK3	6	0.21423	0.39337	0.99998	7319	0.1589
BACE1	6	0.21434	0.39352	0.99998	7320	0.1597
YARS2	6	0.21446	0.39368	0.99998	7321	-0.0879
MON2	6	0.21446	0.39368	0.99998	7322	-0.0225
TRPV6	6	0.21447	0.39369	0.99998	7323	0.0881
KRTDAP	5	0.21455	0.37614	0.99998	7324	-0.0461
C10orf12	6	0.21456	0.3938	0.99998	7325	-0.2377
CCDC88A	6	0.21457	0.39382	0.99998	7326	0.0844
SIMC1	6	0.21457	0.39382	0.99998	7327	0.0625
hsa-mir-27a	4	0.21464	0.33923	0.99998	7328	-0.4648
HID1	6	0.21468	0.39397	0.99998	7329	0.0174
DGKZ	6	0.21468	0.39397	0.99998	7330	-0.0804
SMIM1	6	0.21468	0.39397	0.99998	7331	0.0933
HEBP1	6	0.21468	0.39397	0.99998	7332	-0.0273
KIAA0247	6	0.21469	0.39398	0.99998	7333	-0.1608
KCTD2	6	0.21469	0.39398	0.99998	7334	-0.2255
GPR45	6	0.21469	0.39398	0.99998	7335	-0.1544
LDLRAP1	6	0.21477	0.39409	0.99998	7336	-0.2261
CHCHD4	6	0.21483	0.39416	0.99998	7337	-0.1037
MLX	6	0.21483	0.39416	0.99998	7338	-0.1657
ZRANB3	6	0.21483	0.39416	0.99998	7339	0.1413
LRRC37A2	6	0.21487	0.39422	0.99998	7340	-0.0573
CASD1	6	0.21489	0.39424	0.99998	7341	0.0484
RPS6KB1	6	0.21492	0.39428	0.99998	7342	-0.2221
MEA1	6	0.21492	0.39428	0.99998	7343	-0.1539
MBP	6	0.21493	0.39429	0.99998	7344	-0.0199
ATP8B1	4	0.21494	0.33958	0.99998	7345	0.332
hsa-mir-3131	4	0.21499	0.33964	0.99998	7346	-0.0789
ABCC1	6	0.21501	0.3944	0.99998	7347	0.0829
TTLL4	6	0.2151	0.39451	0.99998	7348	-0.0438
PPAPDC1A	6	0.21511	0.39451	0.99998	7349	0.1831
HADH	6	0.21513	0.39454	0.99998	7350	-0.2792
KIAA2022	6	0.21513	0.39454	0.99998	7351	-0.1097
HLA-DPB1	6	0.21519	0.39462	0.99998	7352	0.0038
SLC25A40	6	0.21521	0.39465	0.99998	7353	0.0645
PLA2G4D	6	0.21521	0.39465	0.99998	7354	-0.0719
NQO2	6	0.21534	0.39482	0.99998	7355	0.0417
MRPS16	6	0.21534	0.39482	0.99998	7356	-0.0392
FAM131C	6	0.21561	0.39519	0.99998	7357	-0.0742
ASPHD2	6	0.21561	0.39519	0.99998	7358	-0.0643
TBX22	6	0.21561	0.39519	0.99998	7359	-0.0692
CASC5	6	0.21561	0.39519	0.99998	7360	-0.0731
hsa-mir-133b	4	0.21562	0.34035	0.99998	7361	-0.2204
KIAA1731	6	0.21566	0.39524	0.99998	7362	0.1111
hsa-mir-4704	3	0.21571	0.33039	0.99998	7363	0.0855
ANKH	6	0.21587	0.39553	0.99998	7364	0.1397
RNASE6	6	0.21606	0.39575	0.99998	7365	0.0176
TRIM72	6	0.21609	0.39579	0.99998	7366	0.1002
HIST1H2BB	6	0.21609	0.39579	0.99998	7367	-0.1032
CD200R1	6	0.21635	0.39612	0.99998	7368	0.0029

GNAT1	6	0.21635	0.39612	0.99998	7369	-0.1679
OR2L3	6	0.21635	0.39612	0.99998	7370	-0.3941
HLA-G	6	0.21635	0.39612	0.99998	7371	-0.2552
ZNF92	6	0.21635	0.39612	0.99998	7372	-0.2271
HIST1H2BD	6	0.21635	0.39612	0.99998	7373	-0.4751
KRT17	6	0.21635	0.39612	0.99998	7374	-0.3078
BEND2	6	0.21635	0.39612	0.99998	7375	-0.1403
KCNRG	6	0.21635	0.39612	0.99998	7376	-0.1189
ZNF736	6	0.21635	0.39612	0.99998	7377	-1.1925
CDKL4	6	0.21636	0.39613	0.99998	7378	0.0204
SNRNP200	6	0.21637	0.39614	0.99998	7379	-0.0411
NNMT	6	0.21637	0.39614	0.99998	7380	0.2896
NLRP6	6	0.21637	0.39614	0.99998	7381	-0.2353
TMEM19	6	0.21642	0.39622	0.99998	7382	0.0524
LRP6	6	0.21647	0.39627	0.99998	7383	0.0172
hsa-mir-4251	4	0.21658	0.34143	0.99998	7384	0.1271
NELL2	6	0.21662	0.39647	0.99998	7385	-0.0989
SCN5A	6	0.21662	0.39647	0.99998	7386	0.0401
SLC25A30	6	0.21671	0.3966	0.99998	7387	-0.0031
CHSY1	6	0.21672	0.39662	0.99998	7388	-0.0289
PRKX	6	0.2168	0.39671	0.99998	7389	0.0965
GAB1	6	0.21693	0.39691	0.99998	7390	-0.1669
NANS	6	0.21712	0.39716	0.99998	7391	-0.0381
hsa-mir-4330	4	0.2172	0.3421	0.99998	7392	0.0859
KCNB1	6	0.2172	0.39726	0.99998	7393	-0.0936
SOAT1	6	0.21721	0.39727	0.99998	7394	-0.0501
DNASE2B	6	0.21721	0.39727	0.99998	7395	-0.1743
DNAJC15	6	0.21727	0.39735	0.99998	7396	0.083
BIN3	6	0.21731	0.3974	0.99998	7397	-0.1612
SVIP	6	0.21743	0.39756	0.99998	7398	-0.0062
KLF1	6	0.21743	0.39756	0.99998	7399	-0.2029
MYO1D	6	0.21744	0.39758	0.99998	7400	0.0261
hsa-mir-3689c	3	0.21746	0.33261	0.99998	7401	-0.9148
SPINK2	6	0.21747	0.39761	0.99998	7402	-0.1503
ZNF143	6	0.21749	0.39762	0.99998	7403	-0.1323
PRTFDC1	6	0.21759	0.39775	0.99998	7404	-0.0973
OR2M5	6	0.2176	0.39776	0.99998	7405	0.1355
EHBP1L1	6	0.21762	0.39779	0.99998	7406	-0.1534
KCNU1	6	0.21764	0.39782	0.99998	7407	-0.0619
ALDOC	6	0.2177	0.3979	0.99998	7408	-0.0949
CYP27B1	6	0.21776	0.39798	0.99998	7409	0.0301
C11orf68	6	0.21776	0.39798	0.99998	7410	0.0528
AKAP13	6	0.21778	0.39801	0.99998	7411	0.11
IFIT5	6	0.21781	0.39806	0.99998	7412	0.0005
FKBP15	6	0.21781	0.39806	0.99998	7413	-0.119
SYT10	6	0.21781	0.39806	0.99998	7414	-0.1076
FAM47A	6	0.21789	0.39817	0.99998	7415	0.0247
CEP72	6	0.21789	0.39817	0.99998	7416	0.0379
FKBP5	6	0.21789	0.39817	0.99998	7417	-0.0479
KRT79	4	0.21791	0.3429	0.99998	7418	-0.1538
hsa-mir-329-2	1	0.21796	0.21759	0.99998	7419	-1.2876
ATAD5	6	0.21804	0.39837	0.99998	7420	-0.0249
HEY2	6	0.21808	0.39842	0.99998	7421	0.3381
ARHGAP19	6	0.21808	0.39842	0.99998	7422	-0.0899
SLITRK4	6	0.21808	0.39842	0.99998	7423	0.1072
CPA1	6	0.21819	0.39855	0.99998	7424	0.4296
KLF7	6	0.21819	0.39855	0.99998	7425	-0.154
GTF2H2D	3	0.21819	0.33351	0.99998	7426	-0.1615
AMD1	6	0.21833	0.39874	0.99998	7427	0.183
ROPN1L	6	0.21833	0.39874	0.99998	7428	0.0356
HCFC1R1	6	0.21834	0.39876	0.99998	7429	-0.161
GPSM1	6	0.21834	0.39876	0.99998	7430	-0.1587
APOL1	6	0.2184	0.39883	0.99998	7431	0.176
PCYOX1	6	0.2184	0.39883	0.99998	7432	-0.0856
CNNM1	6	0.2185	0.39897	0.99998	7433	0.037
CALD1	6	0.21853	0.399	0.99998	7434	0.0306
DEFB104B	1	0.21854	0.21821	0.99998	7435	-0.2655
SERPINF2	6	0.21855	0.39903	0.99998	7436	-0.006
PROCR	6	0.21858	0.39907	0.99998	7437	-0.1544
SIT1	6	0.21863	0.39913	0.99998	7438	0.1179
C15orf38	2	0.21863	0.25715	0.99998	7439	-0.11
PRAM1	6	0.21872	0.39923	0.99998	7440	-0.0917
hsa-mir-6088	3	0.21902	0.33458	0.99998	7441	-0.3315
RAB14	6	0.21903	0.39962	0.99998	7442	-0.1611
ZPBP	6	0.21903	0.39962	0.99998	7443	-0.0269
LRRCC1	6	0.21904	0.39964	0.99998	7444	-0.0675
UBXN8	6	0.2192	0.39984	0.99998	7445	-0.0479
NDUFA4L2	6	0.2192	0.39984	0.99998	7446	-0.276
MSRB2	6	0.2192	0.39984	0.99998	7447	0.0501
TMEM178A	6	0.21928	0.39994	0.99998	7448	-0.3191
TTC23L	6	0.21946	0.40018	0.99998	7449	0.1899

KIAA0895L	6	0.21951	0.40024	0.99998	7450	-0.1425
CD5	6	0.21956	0.40031	0.99998	7451	0.2616
DCBLD2	6	0.21963	0.4004	0.99998	7452	-0.0609
NOLC1	6	0.21965	0.40043	0.99998	7453	0.0288
RABIF	6	0.21974	0.40055	0.99998	7454	-0.1214
IL36RN	6	0.21974	0.40056	0.99998	7455	-0.0398
TBCEL	6	0.21974	0.40056	0.99998	7456	0.0639
TSHB	6	0.2198	0.40064	0.99998	7457	-0.0041
TCP11L2	6	0.2198	0.40064	0.99998	7458	-0.0712
TRDN	6	0.21988	0.40074	0.99998	7459	-0.1683
CSGALNACT2	6	0.22003	0.40092	0.99998	7460	-0.0645
FRMD4A	6	0.22007	0.40098	0.99998	7461	0.0173
hsa-mir-7-2	2	0.22011	0.25829	0.99998	7462	-0.3488
METTL23	6	0.22012	0.40103	0.99998	7463	-0.0134
ADCK3	6	0.22014	0.40105	0.99998	7464	-0.1549
AGT	6	0.22014	0.40105	0.99998	7465	-0.1889
H2AFY	5	0.22021	0.38124	0.99998	7466	-0.0447
HIST1H2BI	5	0.22021	0.38124	0.99998	7467	0.0057
YWHAH	6	0.22031	0.40127	0.99998	7468	-0.0297
CAPN11	6	0.22031	0.40127	0.99998	7469	0.0956
VCP	6	0.22037	0.40137	0.99998	7470	-0.171
SAP25	6	0.22037	0.40137	0.99998	7471	-0.0509
KCNE3	6	0.22037	0.40137	0.99998	7472	0.0618
ZNF451	6	0.2204	0.4014	0.99998	7473	-0.0657
HOXA4	6	0.2204	0.4014	0.99998	7474	-0.1773
C12orf54	4	0.22054	0.34579	0.99998	7475	-0.0456
LY9	6	0.22056	0.40161	0.99998	7476	0.0354
BRI3	6	0.22056	0.40161	0.99998	7477	-0.24
DND1	6	0.22059	0.40167	0.99998	7478	-0.1168
RAPGEFL1	6	0.22067	0.40177	0.99998	7479	-0.0131
TUBB4A	6	0.22072	0.40183	0.99998	7480	-0.056
MAFF	6	0.22072	0.40183	0.99998	7481	-0.1852
HAAO	6	0.22078	0.40192	0.99998	7482	0.0248
PON2	6	0.22078	0.40192	0.99998	7483	-0.1206
hsa-mir-553	2	0.22089	0.25889	0.99998	7484	-0.1426
BST2	6	0.22102	0.40224	0.99998	7485	-0.0674
MTCH2	6	0.22111	0.40235	0.99998	7486	-0.0923
SETX	6	0.22124	0.40253	0.99998	7487	-0.0398
PUS3	6	0.22124	0.40253	0.99998	7488	-0.0535
GATSL2	2	0.22128	0.25918	0.99998	7489	-0.1492
ZNF891	6	0.22134	0.40265	0.99998	7490	-0.0268
DOM3Z	2	0.22145	0.25932	0.99998	7491	-0.5088
TGM2	6	0.22147	0.40281	0.99998	7492	0.1789
CUTC	6	0.22147	0.40281	0.99998	7493	-0.0738
OR8D4	6	0.22153	0.40288	0.99998	7494	-0.0787
POLR2K	6	0.22155	0.40291	0.99998	7495	-0.3704
hsa-mir-4308	4	0.22171	0.34713	0.99998	7496	-0.0443
HIST1H3I	5	0.22172	0.38262	0.99998	7497	0.1892
KDM5D	6	0.22179	0.40323	0.99998	7498	-0.0938
MFSD12	6	0.2219	0.4034	0.99998	7499	-0.0292
TP53TG5	6	0.2219	0.4034	0.99998	7500	-0.1091
FEM1C	4	0.22201	0.34745	0.99998	7501	0.0141
MMD	6	0.22204	0.40357	0.99998	7502	-0.0127
ZNF706	6	0.22208	0.40363	0.99998	7503	-0.1459
SLCO4A1	6	0.22208	0.40363	0.99998	7504	-0.2142
UBL5	6	0.22217	0.40376	0.99998	7505	-0.0969
GAR1	6	0.22221	0.40382	0.99998	7506	-0.1554
LILRB5	6	0.22221	0.40382	0.99998	7507	-0.189
CXorf38	6	0.22223	0.40384	0.99998	7508	-0.1482
PTGFR	6	0.22223	0.40384	0.99998	7509	-0.1134
ANP32A	6	0.22231	0.40395	0.99998	7510	-0.1207
SH2D6	6	0.22231	0.40395	0.99998	7511	-0.1175
TGM6	6	0.22236	0.40401	0.99998	7512	-0.0704
DEFB124	6	0.22244	0.40412	0.99998	7513	-0.1785
DIAPH2	6	0.22244	0.40412	0.99998	7514	-0.0377
hsa-mir-3936	4	0.22247	0.34798	0.99998	7515	-0.0875
OR10G8	5	0.22255	0.38337	0.99998	7516	0.0309
MAP1B	6	0.22256	0.40428	0.99998	7517	-0.2002
TNRC18	6	0.22261	0.40435	0.99998	7518	0.0618
PRSS45	6	0.22264	0.40439	0.99998	7519	0.0201
FIGNL2	6	0.22278	0.40457	0.99998	7520	0.1032
RHAG	6	0.22278	0.40457	0.99998	7521	-0.0592
FLJ22184	6	0.22278	0.40457	0.99998	7522	-0.0068
GRB14	6	0.22295	0.40481	0.99998	7523	-0.0391
CEBPE	6	0.223	0.40488	0.99998	7524	-0.1021
MPHOSPH10	6	0.22303	0.4049	0.99998	7525	-0.0741
hsa-mir-7848	4	0.22308	0.34866	0.99998	7526	0.1145
CLU	6	0.22316	0.40508	0.99998	7527	-0.0194
LACRT	6	0.22316	0.40508	0.99998	7528	-0.1013
LCORL	6	0.2232	0.40514	0.99998	7529	0.0884
ADAD1	6	0.22336	0.40534	0.99998	7530	-0.178

CYBB	6	0.22336	0.40534	0.99998	7531	-0.1786
SAMD5	6	0.22336	0.40534	0.99998	7532	-0.1036
KHDRBS1	6	0.22348	0.4055	0.99998	7533	0.1555
P4HA1	6	0.22348	0.4055	0.99998	7534	-0.1009
MYL6B	6	0.22348	0.4055	0.99998	7535	0.0935
ZNF396	6	0.22357	0.40561	0.99998	7536	-0.0869
GADL1	6	0.22357	0.40561	0.99998	7537	0.0329
OR5AU1	6	0.22366	0.40573	0.99998	7538	-0.144
SPATA18	6	0.22367	0.40575	0.99998	7539	0.0793
ELP4	6	0.22377	0.40589	0.99998	7540	0.0632
PRDM12	6	0.22378	0.40589	0.99998	7541	-0.1352
SPACA7	6	0.22382	0.40594	0.99998	7542	-0.13
RAI1	6	0.22388	0.40603	0.99998	7543	-0.1487
hsa-mir-125b-	4	0.2239	0.34959	0.99998	7544	-0.1395
SPATA25	6	0.22393	0.40609	0.99998	7545	0.0737
EPS8L1	6	0.22394	0.4061	0.99998	7546	0.0152
TBCCD1	6	0.22395	0.40611	0.99998	7547	0.1237
ARMCX5-GPR	6	0.22404	0.40623	0.99998	7548	-0.1818
ACTL7A	6	0.22419	0.40644	0.99998	7549	-0.1084
NR2E1	6	0.22424	0.40651	0.99998	7550	0.0062
PON3	6	0.22424	0.40651	0.99998	7551	-0.0684
CACNG4	6	0.2243	0.40661	0.99998	7552	-0.1928
SLC10A2	6	0.2243	0.40661	0.99998	7553	0.0125
MIPOL1	6	0.2243	0.40661	0.99998	7554	-0.0984
hsa-mir-6877	4	0.22432	0.35001	0.99998	7555	-0.1325
SLC25A24	6	0.22437	0.40669	0.99998	7556	-0.2013
EFCAB2	6	0.22437	0.40669	0.99998	7557	-0.1734
NFKBIL1	6	0.22437	0.40669	0.99998	7558	-0.1419
DEFB113	6	0.22438	0.40671	0.99998	7559	0.3093
PHF1	6	0.2245	0.40686	0.99998	7560	-0.0991
CWF19L2	6	0.2245	0.40686	0.99998	7561	-0.0795
CCDC6	6	0.22451	0.40688	0.99998	7562	0.1345
CAPN14	6	0.22452	0.40689	0.99998	7563	-0.1342
ATF3	6	0.22452	0.40689	0.99998	7564	-0.2303
C12orf65	6	0.22465	0.40706	0.99998	7565	-0.1697
KCTD15	6	0.2247	0.40714	0.99998	7566	0.0736
STMN1	6	0.2247	0.40714	0.99998	7567	-0.08
MOGS	6	0.2247	0.40714	0.99998	7568	-0.0478
NR2C1	6	0.22474	0.40719	0.99998	7569	-0.0964
CATSPERD	6	0.22485	0.40734	0.99998	7570	-0.1718
CCDC42	6	0.22491	0.40742	0.99998	7571	-2E-05
MED24	6	0.22491	0.40743	0.99998	7572	-0.1017
PANK1	6	0.22491	0.40743	0.99998	7573	0.0492
C1orf63	6	0.22491	0.40743	0.99998	7574	0.1345
GK5	6	0.22491	0.40743	0.99998	7575	-0.0258
KRT71	6	0.22506	0.40762	0.99998	7576	0.0673
ID3	6	0.22512	0.4077	0.99998	7577	-0.0606
TPCN1	6	0.22513	0.40771	0.99998	7578	-0.0864
RC3H1	6	0.22522	0.40784	0.99998	7579	-0.0745
SDR39U1	6	0.22522	0.40784	0.99998	7580	-0.0927
SOWAHD	6	0.22522	0.40784	0.99998	7581	0.011
OR2A2	6	0.22523	0.40784	0.99998	7582	0.0556
GAS1	6	0.22525	0.40787	0.99998	7583	0.1397
OXA1L	6	0.2254	0.40807	0.99998	7584	-0.128
PCYOX1L	6	0.2255	0.4082	0.99998	7585	-0.0318
CALR	6	0.22558	0.4083	0.99998	7586	-0.1748
KIF13A	6	0.22558	0.4083	0.99998	7587	-0.203
hsa-mir-1266	4	0.22561	0.35146	0.99998	7588	-0.2384
LRCH4	6	0.22567	0.40842	0.99998	7589	-0.053
MMP15	6	0.22567	0.40842	0.99998	7590	0.0328
GJB7	6	0.22567	0.40842	0.99998	7591	0.0127
RAB23	6	0.22581	0.40863	0.99998	7592	0.1793
PLA2G1B	6	0.22581	0.40863	0.99998	7593	-0.0241
RBM39	6	0.22581	0.40863	0.99998	7594	-0.065
NFKBID	6	0.22589	0.40873	0.99998	7595	-0.1544
STARD3	6	0.226	0.40887	0.99998	7596	0.0242
COX20	6	0.22603	0.40891	0.99998	7597	-0.0511
NUDT14	6	0.22609	0.40899	0.99998	7598	-0.0567
INPP5B	6	0.22609	0.40899	0.99998	7599	0.1361
SCD5	6	0.22609	0.40899	0.99998	7600	-0.1075
FAM21A	6	0.22609	0.40899	0.99998	7601	-0.1678
SLC17A3	6	0.22609	0.40899	0.99998	7602	-0.1421
FRMD1	6	0.22609	0.40899	0.99998	7603	-0.0946
FAM155A	6	0.22615	0.40907	0.99998	7604	0.1879
POM121L12	6	0.22622	0.40916	0.99998	7605	-0.1502
LOC10028852	6	0.22624	0.40919	0.99998	7606	-0.0539
UBE2F	6	0.22626	0.40921	0.99998	7607	0.0223
ENO1	6	0.22626	0.40921	0.99998	7608	-0.0044
C2orf44	6	0.22637	0.40936	0.99998	7609	-0.0326
ENG	6	0.22638	0.40938	0.99998	7610	0.0613
PPARGC1A	6	0.22639	0.40938	0.99998	7611	-0.2534

BNIP1	6	0.22645	0.40946	0.99998	7612	-0.0894
CORO1B	6	0.22661	0.40969	0.99998	7613	-0.0802
HS3ST1	6	0.22664	0.40973	0.99998	7614	-0.1783
NLGN1	6	0.22664	0.40973	0.99998	7615	-0.0252
RBM33	6	0.22664	0.40973	0.99998	7616	-0.1428
WDR62	4	0.22668	0.35261	0.99998	7617	-0.0243
IYD	6	0.22674	0.40988	0.99998	7618	0.1198
IFT122	6	0.22695	0.41016	0.99998	7619	0.035
SLC2A4RG	6	0.22696	0.41018	0.99998	7620	-0.1441
TYR	6	0.22697	0.41018	0.99998	7621	0.0678
TCEAL7	6	0.22697	0.41018	0.99998	7622	-0.3603
CCDC106	6	0.22701	0.41023	0.99998	7623	-0.0293
CTRC	6	0.2271	0.41035	0.99998	7624	-0.1854
ALDH1L1	6	0.22719	0.41047	0.99998	7625	-0.1311
ZFP106	6	0.22721	0.4105	0.99998	7626	0.1003
TPTE	6	0.22727	0.41058	0.99998	7627	0.2133
GMDS	6	0.22727	0.41058	0.99998	7628	-0.1153
ICA1L	6	0.22727	0.41058	0.99998	7629	-0.0886
OTUD6A	6	0.22738	0.41073	0.99998	7630	-0.0337
ERI1	6	0.2274	0.41075	0.99998	7631	-0.0858
RCAN1	6	0.2274	0.41075	0.99998	7632	-0.0873
ANGPT4	6	0.22759	0.41101	0.99998	7633	-0.0187
RHBDD1	6	0.22767	0.41111	0.99998	7634	0.0328
MYL2	6	0.22774	0.41121	0.99998	7635	-0.1213
KNOP1	6	0.22774	0.41121	0.99998	7636	-0.1745
NXNL2	5	0.22774	0.38812	0.99998	7637	0.166
STRA13	6	0.22775	0.41122	0.99998	7638	-0.1708
FAM5B	4	0.22782	0.35387	0.99998	7639	-0.1706
PRICKLE4	6	0.22783	0.41135	0.99998	7640	-0.0995
KRTAP6-3	6	0.22788	0.41141	0.99998	7641	-0.1421
RCOR3	6	0.22789	0.41142	0.99998	7642	-0.0457
DNMBP	6	0.22804	0.41162	0.99998	7643	-0.1501
PRTG	6	0.22804	0.41163	0.99998	7644	-0.0948
CYP3A5	4	0.22807	0.35414	0.99998	7645	0.0285
KRTAP5-5	6	0.22808	0.41167	0.99998	7646	0.2562
ATXN10	6	0.22813	0.41175	0.99998	7647	0.0721
hsa-mir-4499	4	0.22823	0.35432	0.99998	7648	-0.162
SESN2	6	0.22825	0.41191	0.99998	7649	0.047
LEP	6	0.22832	0.41201	0.99998	7650	-0.1329
PGAM1	6	0.22836	0.41205	0.99998	7651	-0.2736
KAT6A	6	0.22836	0.41205	0.99998	7652	-0.0406
PCBP2	6	0.22838	0.41209	0.99998	7653	-0.0753
THSD7A	6	0.22845	0.41217	0.99998	7654	-0.1333
SLC38A5	6	0.22846	0.41218	0.99998	7655	-0.0191
DGKQ	6	0.22853	0.41226	0.99998	7656	-0.0136
hsa-mir-3201	2	0.22853	0.26491	0.99998	7657	0.1418
CNOT6L	6	0.22869	0.41247	0.99998	7658	0.0315
C3orf14	6	0.22882	0.41263	0.99998	7659	0.1273
FAF1	6	0.22883	0.41264	0.99998	7660	-0.0946
ATG16L2	6	0.22898	0.41282	0.99998	7661	-0.1472
MFSD9	6	0.22898	0.41282	0.99998	7662	0.0758
hsa-mir-920	4	0.22899	0.35516	0.99998	7663	-0.0457
OR14C36	6	0.22916	0.41305	0.99998	7664	-0.061
FTH1	6	0.22916	0.41305	0.99998	7665	-0.1356
FAM45A	6	0.22916	0.41305	0.99998	7666	-0.1438
MAPK1	6	0.22916	0.41305	0.99998	7667	0.0545
NARF	6	0.22922	0.41313	0.99998	7668	0.003
hsa-mir-4459	3	0.22925	0.3478	0.99998	7669	-0.181
MSX2	6	0.22927	0.41318	0.99998	7670	-0.1401
ETHE1	6	0.2294	0.41335	0.99998	7671	-0.1253
hsa-mir-1271	4	0.22941	0.35562	0.99998	7672	-0.245
ODF1	6	0.22943	0.41338	0.99998	7673	-0.1615
BGN	6	0.22955	0.41354	0.99998	7674	0.2437
DYNLT1	6	0.22955	0.41354	0.99998	7675	0.1293
RNF39	6	0.22958	0.41358	0.99998	7676	-0.135
SPDYE2L	2	0.22961	0.26577	0.99998	7677	-0.2858
MAGEB4	6	0.22973	0.41379	0.99998	7678	-0.0947
ZNF365	6	0.22973	0.41379	0.99998	7679	-0.0247
C12orf61	6	0.22973	0.41379	0.99998	7680	-0.2148
LMAN1L	6	0.22979	0.41386	0.99998	7681	-0.0419
OR1C1	6	0.22986	0.41395	0.99998	7682	-0.0945
hsa-mir-5187	4	0.22993	0.35618	0.99998	7683	-0.0899
SOX9	6	0.22996	0.41409	0.99998	7684	-0.1487
hsa-mir-4455	4	0.23008	0.35633	0.99998	7685	-0.0188
SNRPB2	6	0.2301	0.41429	0.99998	7686	0.014
CTNS	6	0.23014	0.41435	0.99998	7687	0.0405
TMEM256	6	0.23018	0.4144	0.99998	7688	-0.0791
OLAH	6	0.23018	0.4144	0.99998	7689	-0.0002
KPNA3	6	0.23018	0.4144	0.99998	7690	0.0444
GKAP1	6	0.23028	0.41452	0.99998	7691	-0.0928
CCL21	6	0.23055	0.41487	0.99998	7692	-0.0699

DTX1	6	0.23068	0.41503	0.99998	7693	0.0644
SLC18A3	6	0.23069	0.41504	0.99998	7694	-0.1931
SMPD3	6	0.23077	0.41514	0.99998	7695	0.0654
KCTD4	6	0.23077	0.41514	0.99998	7696	-0.0859
ANKRD30A	6	0.23077	0.41514	0.99998	7697	-0.0665
IRS1	6	0.23089	0.41531	0.99998	7698	0.1256
RNASE1	6	0.2309	0.41531	0.99998	7699	-0.2531
KLHL25	6	0.23091	0.41533	0.99998	7700	-0.0705
hsa-mir-5585	4	0.23103	0.3574	0.99998	7701	-0.1044
TIFAB	6	0.23108	0.41555	0.99998	7702	-0.1222
BCAT2	6	0.23108	0.41555	0.99998	7703	0.032
CPA2	6	0.23141	0.41597	0.99998	7704	-0.0976
NFIA	4	0.23141	0.35783	0.99998	7705	0.169
WDR86	6	0.23145	0.41602	0.99998	7706	-0.1356
ITFG1	6	0.23148	0.41606	0.99998	7707	0.2335
PHGR1	6	0.23148	0.41606	0.99998	7708	0.0171
BMF	6	0.23152	0.41612	0.99998	7709	-0.0563
ZCWPW1	6	0.23155	0.41616	0.99998	7710	-0.0759
SECISBP2	6	0.23163	0.41626	0.99998	7711	-0.0492
C18orf25	6	0.23167	0.41632	0.99998	7712	-0.0443
GIMAP8	6	0.23171	0.41638	0.99998	7713	-0.1048
GNG10	6	0.23171	0.41638	0.99998	7714	-0.0309
FAM19A2	6	0.2318	0.41651	0.99998	7715	-0.1899
DBH	6	0.23181	0.41652	0.99998	7716	0.0862
OR52K1	6	0.23181	0.41652	0.99998	7717	0.0745
KIR2DL4	2	0.23183	0.26749	0.99998	7718	-0.0724
CACNA1F	6	0.23191	0.41664	0.99998	7719	-0.0872
hsa-mir-323b	4	0.23193	0.3584	0.99998	7720	-0.0357
PPP1R21	6	0.23194	0.41669	0.99998	7721	0.1134
AURKAIP1	6	0.23194	0.41669	0.99998	7722	-0.123
ANKRD17	6	0.23194	0.41669	0.99998	7723	0.233
PUS7	6	0.23199	0.41675	0.99998	7724	-0.0959
DNAH2	6	0.23199	0.41675	0.99998	7725	-0.1248
WBP2	6	0.23213	0.41692	0.99998	7726	-0.2161
DOCK2	6	0.23219	0.41699	0.99998	7727	0.0025
DNAJC2	6	0.23224	0.41707	0.99998	7728	-0.0861
EPHA6	6	0.23227	0.4171	0.99998	7729	0.0197
GRIN2A	6	0.23227	0.4171	0.99998	7730	-0.1696
CASR	6	0.23234	0.4172	0.99998	7731	0.0974
ARHGAP25	6	0.2324	0.41727	0.99998	7732	0.003
TRPV3	6	0.23269	0.41767	0.99998	7733	0.3584
HN1L	6	0.23269	0.41767	0.99998	7734	0.144
JOSD1	6	0.23269	0.41767	0.99998	7735	0.0914
LGALS14	6	0.23282	0.41785	0.99998	7736	-0.2428
DUSP3	6	0.23282	0.41785	0.99998	7737	-0.0167
PRMT6	6	0.23288	0.41791	0.99998	7738	0.1492
OR1E1	6	0.23288	0.41791	0.99998	7739	-0.3185
ZNF358	6	0.23288	0.41791	0.99998	7740	-0.1062
CDH17	6	0.23288	0.41792	0.99998	7741	-0.052
OR11G2	6	0.23288	0.41792	0.99998	7742	-0.0117
B3GNT3	6	0.23296	0.41803	0.99998	7743	0.0208
OR7E24	6	0.23303	0.41812	0.99998	7744	-0.1283
SCN2B	6	0.23304	0.41814	0.99998	7745	-0.0839
CAPN1	6	0.23313	0.41825	0.99998	7746	0.1209
SNX8	6	0.23326	0.41839	0.99998	7747	0.0174
HM13	6	0.23326	0.41839	0.99998	7748	0.1102
TYW5	6	0.23326	0.41839	0.99998	7749	0.0159
SLC4A5	6	0.23326	0.41839	0.99998	7750	-0.2158
DSTN	6	0.23326	0.41839	0.99998	7751	-0.415
EEF1A2	6	0.23326	0.41839	0.99998	7752	-0.3695
SH3D21	6	0.23332	0.41847	0.99998	7753	-0.1214
C1orf61	6	0.23346	0.41864	0.99998	7754	-0.0511
SERP1	6	0.23352	0.41871	0.99998	7755	-0.123
IGFALS	6	0.23353	0.41872	0.99998	7756	-0.0062
C1orf158	6	0.23369	0.41893	0.99998	7757	-0.0418
RNASE3	5	0.23384	0.39376	0.99998	7758	-0.0904
DUSP12	6	0.2339	0.41921	0.99998	7759	0.0038
ARHGAP39	6	0.23412	0.41948	0.99998	7760	-0.0894
POGZ	6	0.23412	0.41948	0.99998	7761	0.2418
CDR2L	6	0.23413	0.4195	0.99998	7762	-0.3015
CER1	6	0.23413	0.4195	0.99998	7763	-0.3058
PRR21	6	0.23413	0.4195	0.99998	7764	-0.2208
DTL	6	0.23417	0.41955	0.99998	7765	-0.0728
SNX10	6	0.23417	0.41955	0.99998	7766	0.2877
PLD4	6	0.23424	0.41964	0.99998	7767	0.1549
LOXL3	6	0.23426	0.41967	0.99998	7768	-0.1528
ADH7	6	0.23426	0.41967	0.99998	7769	-0.1034
STK35	6	0.2343	0.41973	0.99998	7770	-0.0204
PAM16	6	0.2343	0.41973	0.99998	7771	-0.101
THAP7	6	0.23437	0.4198	0.99998	7772	-0.1876
TRIM32	6	0.23437	0.4198	0.99998	7773	-0.1355

THG1L	6	0.23437	0.4198	0.99998	7774	-0.1214
FUNDC1	6	0.23442	0.41988	0.99998	7775	0.0811
EPCAM	6	0.23448	0.41995	0.99998	7776	-0.1964
ALKBH4	6	0.23453	0.42002	0.99998	7777	0.1529
SAMHD1	6	0.23457	0.42007	0.99998	7778	0.1563
MAATS1	6	0.23457	0.42007	0.99998	7779	0.1641
FAM196B	6	0.23467	0.42021	0.99998	7780	0.0408
ZC3HAV1	6	0.23467	0.42021	0.99998	7781	0.0254
CDS1	6	0.23473	0.42029	0.99998	7782	-0.1408
hsa-mir-29a	4	0.23473	0.36153	0.99998	7783	-0.2273
PER2	6	0.23488	0.42048	0.99998	7784	0.025
CCSER1	6	0.23488	0.42048	0.99998	7785	0.0686
PI4K2A	6	0.23488	0.42048	0.99998	7786	0.015
ADAMTS12	6	0.23499	0.42063	0.99998	7787	-0.1001
OR7D4	6	0.23509	0.42075	0.99998	7788	0.058
VPS26B	6	0.23509	0.42075	0.99998	7789	-0.0518
SAV1	6	0.23509	0.42076	0.99998	7790	-0.1562
AGK	6	0.23512	0.42081	0.99998	7791	-0.0316
EDEM1	6	0.23512	0.42081	0.99998	7792	-0.1521
DIAPH3	6	0.23512	0.42081	0.99998	7793	-0.2275
ZAR1L	6	0.23526	0.42099	0.99998	7794	-0.0004
ETV6	6	0.23526	0.42099	0.99998	7795	0.333
C2orf78	6	0.23532	0.42108	0.99998	7796	-0.0535
FAN1	6	0.23532	0.42108	0.99998	7797	0.0749
PRPF18	6	0.23534	0.4211	0.99998	7798	-0.2801
CACNA1C	6	0.2354	0.42119	0.99998	7799	-0.0206
ANKRD37	6	0.23548	0.42128	0.99998	7800	-0.0401
SPN	6	0.23555	0.42137	0.99998	7801	-0.0913
ZNF763	4	0.23556	0.36244	0.99998	7802	0.0153
FAT1	6	0.23559	0.42142	0.99998	7803	0.099
LHX9	6	0.2356	0.42144	0.99998	7804	-0.2217
DAB2IP	6	0.23565	0.4215	0.99998	7805	-0.0073
DNAJC4	6	0.23569	0.42156	0.99998	7806	0.0749
BDP1	6	0.23578	0.42168	0.99998	7807	-0.1674
WNT2B	6	0.23578	0.42168	0.99998	7808	-0.02
FRK	6	0.2359	0.42183	0.99998	7809	-0.1226
OR1B1	6	0.23595	0.42189	0.99998	7810	-0.1631
FAM53C	6	0.23599	0.42194	0.99998	7811	0.0417
SNX13	6	0.23599	0.42195	0.99998	7812	0.0903
hsa-mir-5693	3	0.23601	0.35428	0.99998	7813	0.0616
PDE8A	6	0.23609	0.42208	0.99998	7814	-0.1381
NOL6	6	0.23613	0.42214	0.99998	7815	-0.0715
POLR2G	6	0.23613	0.42214	0.99998	7816	0.053
CHAT	6	0.23617	0.4222	0.99998	7817	-0.1856
LOXHD1	6	0.23619	0.42224	0.99998	7818	0.1626
KCNIP1	6	0.23621	0.42227	0.99998	7819	0.0686
SMIM3	6	0.23624	0.4223	0.99998	7820	0.1666
MYT1	6	0.23624	0.42231	0.99998	7821	-0.0037
ZFAND6	6	0.23633	0.42243	0.99998	7822	-0.0166
CKLF-CMTM1	4	0.23638	0.36332	0.99998	7823	-0.12
CNIH2	6	0.2364	0.4225	0.99998	7824	-0.1376
ASB11	6	0.23641	0.42252	0.99998	7825	0.1023
CCM2L	6	0.23654	0.42269	0.99998	7826	-0.1325
MSL3	6	0.23655	0.42269	0.99998	7827	-0.0765
VN1R5	6	0.23664	0.42281	0.99998	7828	0.1318
TYMP	6	0.23665	0.42282	0.99998	7829	-0.1557
ITPR3	6	0.23674	0.42294	0.99998	7830	-0.1059
CHTF8	6	0.23684	0.42306	0.99998	7831	-0.1339
ERV3-1	6	0.23692	0.42316	0.99998	7832	-0.1333
ANKRD52	6	0.23694	0.42318	0.99998	7833	-0.1415
ANAPC13	6	0.23696	0.42321	0.99998	7834	-0.0349
TIPRL	6	0.23698	0.42324	0.99998	7835	-0.1476
ACVR1	6	0.23699	0.42326	0.99998	7836	-0.0423
B3GNT8	6	0.23709	0.4234	0.99998	7837	0.1571
EPHA1	6	0.23709	0.4234	0.99998	7838	-0.0523
ITPKA	6	0.23709	0.4234	0.99998	7839	-0.0423
LMX1A	6	0.23711	0.42343	0.99998	7840	-0.2145
MOGAT1	6	0.23711	0.42343	0.99998	7841	0.0357
MUC22	6	0.23719	0.42351	0.99998	7842	0.0833
hsa-mir-5695	4	0.23725	0.36429	0.99998	7843	-0.5968
KLHL13	6	0.23728	0.42363	0.99998	7844	-0.0769
TLX1	6	0.23728	0.42363	0.99998	7845	0.0801
OXNAD1	6	0.23728	0.42363	0.99998	7846	0.0322
hsa-mir-8086	2	0.23731	0.27189	0.99998	7847	-0.9911
MARCH5	6	0.23736	0.42374	0.99998	7848	-0.0837
ARPC1B	6	0.2374	0.42379	0.99998	7849	-0.1171
DEFB134	5	0.23741	0.39709	0.99998	7850	-0.0755
ECEL1	6	0.23743	0.42382	0.99998	7851	-0.0167
STX17	6	0.23747	0.42387	0.99998	7852	-0.1661
TTC9B	6	0.23754	0.42397	0.99998	7853	-0.1391
ANKRD45	6	0.23761	0.42407	0.99998	7854	-0.0982

LSP1	6	0.23776	0.42426	0.99998	7855	0.1081
OR2T8	6	0.23776	0.42426	0.99998	7856	-0.1772
PRPF39	6	0.23776	0.42426	0.99998	7857	-0.2284
CTNNBL1	6	0.23776	0.42426	0.99998	7858	-0.0993
UMODL1	6	0.23776	0.42426	0.99998	7859	-0.0919
hsa-mir-1199	4	0.23792	0.36506	0.99998	7860	-0.2029
LPO	6	0.23802	0.42458	0.99998	7861	0.0171
TTYH3	6	0.23802	0.42458	0.99998	7862	-0.158
ADAMTS8	6	0.23804	0.4246	0.99998	7863	-0.0331
OTUB2	6	0.23805	0.42461	0.99998	7864	-0.0702
hsa-mir-6784	4	0.23813	0.36529	0.99998	7865	-0.165
IGF1	6	0.23823	0.42484	0.99998	7866	-0.0174
FAM83E	6	0.23828	0.4249	0.99998	7867	-0.0735
SREBF2	6	0.23828	0.4249	0.99998	7868	0.0581
SPCS1	6	0.23833	0.42496	0.99998	7869	-0.1072
COL18A1	6	0.23833	0.42496	0.99998	7870	-0.2076
OR56A1	6	0.23845	0.42512	0.99998	7871	-0.0804
ARRB1	6	0.23851	0.42519	0.99998	7872	-0.1022
ACADS	6	0.23861	0.42533	0.99998	7873	0.1335
IFI27L2	6	0.23861	0.42533	0.99998	7874	-0.0228
hsa-mir-4322	4	0.23862	0.36583	0.99998	7875	-0.1619
PITX3	6	0.23873	0.42549	0.99998	7876	4E-06
TOM1L2	6	0.23875	0.42554	0.99998	7877	-0.0496
THY1	6	0.23877	0.42557	0.99998	7878	-0.0095
UTP14C	6	0.23877	0.42557	0.99998	7879	0.0216
OR2Z1	6	0.23877	0.42557	0.99998	7880	-0.1088
UBE2N	6	0.23883	0.42563	0.99998	7881	0.1964
BTBD10	6	0.23885	0.42566	0.99998	7882	-0.1377
AQP2	6	0.23892	0.42575	0.99998	7883	-0.0208
TARBP1	6	0.23896	0.42581	0.99998	7884	0.155
hsa-mir-6862-	2	0.23897	0.27319	0.99998	7885	-0.1888
hsa-mir-3171	2	0.23913	0.2733	0.99998	7886	-0.2265
SIM1	6	0.23915	0.42607	0.99998	7887	0.3103
LENG1	6	0.23923	0.42619	0.99998	7888	-0.0971
MRPL30	6	0.23927	0.42625	0.99998	7889	0.1877
WDR96	6	0.23933	0.42632	0.99998	7890	-0.1554
C9orf152	6	0.23933	0.42633	0.99998	7891	0.0358
CABIN1	6	0.23935	0.42636	0.99998	7892	-0.0835
MC4R	6	0.23948	0.42652	0.99998	7893	-0.1832
GDAP1L1	6	0.23948	0.42652	0.99998	7894	-0.0919
LRRC10	6	0.23958	0.42666	0.99998	7895	0.0584
SLC39A7	6	0.23962	0.42671	0.99998	7896	-0.1113
GALP	6	0.23965	0.42676	0.99998	7897	0.0392
DRP2	6	0.23973	0.42686	0.99998	7898	-0.1899
COL5A2	6	0.23979	0.42695	0.99998	7899	-0.0229
ANKRD39	6	0.23979	0.42695	0.99998	7900	-0.0312
ZP2	6	0.24002	0.42724	0.99998	7901	0.1409
SLC24A2	6	0.24002	0.42724	0.99998	7902	0.2099
hsa-mir-4457	4	0.24007	0.3674	0.99998	7903	-0.1329
CSAG1	6	0.24011	0.42736	0.99998	7904	-0.1876
MYLPF	6	0.24011	0.42736	0.99998	7905	-0.2052
FSCN2	6	0.24011	0.42736	0.99998	7906	-0.1755
CAMK1G	6	0.24011	0.42736	0.99998	7907	-0.0533
SLC25A18	6	0.24023	0.42752	0.99998	7908	0.1501
FASN	6	0.24026	0.42756	0.99998	7909	0.1511
FOCAD	6	0.24037	0.4277	0.99998	7910	0.2555
GNPTAB	6	0.24047	0.42784	0.99998	7911	-0.0197
AQP6	6	0.24047	0.42784	0.99998	7912	-0.0443
PMP22	6	0.24064	0.42806	0.99998	7913	0.1455
B4GALNT1	6	0.24072	0.42819	0.99998	7914	-0.1283
DBP	6	0.24072	0.42819	0.99998	7915	-0.1126
KRTAP10-9	6	0.24087	0.42841	0.99998	7916	0.0996
AADACL3	6	0.24087	0.42841	0.99998	7917	0.0668
DYNLRB1	6	0.24087	0.42841	0.99998	7918	0.1108
MARC2	6	0.24087	0.42841	0.99998	7919	0.0035
SMCR9	6	0.24099	0.42855	0.99998	7920	0.037
HSF5	6	0.24105	0.42864	0.99998	7921	0.182
FAM149A	6	0.24111	0.42871	0.99998	7922	-0.1778
FAM181A	6	0.24111	0.42871	0.99998	7923	-0.1548
LIPC	6	0.24111	0.42871	0.99998	7924	-0.1961
IL6R	6	0.24111	0.42871	0.99998	7925	-0.1609
NBEA	6	0.24111	0.42871	0.99998	7926	-0.0364
GKN1	4	0.24119	0.36863	0.99998	7927	-0.0211
CCDC103	6	0.24126	0.42891	0.99998	7928	-0.0674
hsa-mir-6830	4	0.24128	0.36873	0.99998	7929	0.0379
CEACAM21	6	0.24132	0.42897	0.99998	7930	-0.0606
PPP1R3B	6	0.24136	0.42904	0.99998	7931	-0.1408
EDEM2	6	0.24138	0.42906	0.99998	7932	-0.1588
FAM46B	6	0.24147	0.42918	0.99998	7933	0.1737
SORBS2	6	0.24147	0.42918	0.99998	7934	-0.0318
LAMC2	6	0.24149	0.42921	0.99998	7935	0.1656

hsa-mir-181b-	4	0.24155	0.36904	0.99998	7936	-0.1417
SMIM18	6	0.24161	0.42937	0.99998	7937	0.1482
MEIS3	6	0.24163	0.42939	0.99998	7938	0.2191
FLRT2	4	0.24167	0.36916	0.99998	7939	-0.0434
XYLT1	6	0.24171	0.42949	0.99998	7940	-0.1704
PLEKHF1	6	0.24179	0.4296	0.99998	7941	0.0333
KLK7	6	0.24179	0.4296	0.99998	7942	0.1251
RHOT1	5	0.2418	0.40102	0.99998	7943	0.3993
CALN1	6	0.24181	0.42962	0.99998	7944	-0.1289
TPSD1	6	0.24189	0.42973	0.99998	7945	0.0237
hsa-mir-7704	4	0.24193	0.36946	0.99998	7946	0.0078
C1orf127	6	0.24197	0.42983	0.99998	7947	-0.1505
C1orf100	6	0.24201	0.42988	0.99998	7948	0.0445
FOXQ1	6	0.24201	0.42988	0.99998	7949	-0.0146
ZNF785	6	0.24212	0.43003	0.99998	7950	-0.0586
MLLT6	6	0.24212	0.43003	0.99998	7951	-0.137
CENPA	6	0.24215	0.43006	0.99998	7952	-0.1232
ACSM5	6	0.24219	0.43012	0.99998	7953	0.1887
GREM1	6	0.24222	0.43016	0.99998	7954	-0.1161
TMEM121	5	0.24226	0.40144	0.99998	7955	0.0237
STAC	6	0.24229	0.43026	0.99998	7956	0.0424
C14orf178	6	0.2424	0.43039	0.99998	7957	0.0051
AUH	6	0.2424	0.43039	0.99998	7958	0.122
hsa-mir-4417	4	0.24255	0.37014	0.99998	7959	0.1222
TCOF1	6	0.24259	0.43065	0.99998	7960	-0.0788
LZTS1	6	0.24263	0.43071	0.99998	7961	0.0163
SLC8A1	6	0.24263	0.43071	0.99998	7962	-0.1026
ZNF443	2	0.24273	0.27613	0.99998	7963	-0.581
MTNR1B	6	0.24277	0.43089	0.99998	7964	0.1895
PLK2	6	0.24277	0.43089	0.99998	7965	0.1182
PLEKHA4	6	0.2428	0.43093	0.99998	7966	-0.0965
MKRN2	6	0.2428	0.43093	0.99998	7967	-0.0423
OR13F1	6	0.2428	0.43093	0.99998	7968	0.0569
NME8	6	0.24287	0.43103	0.99998	7969	0.2395
TNFSF18	6	0.24287	0.43103	0.99998	7970	0.1022
SPHAR	1	0.24294	0.24268	0.99998	7971	-0.4267
HCRTR2	6	0.24299	0.43119	0.99998	7972	-0.177
POU3F4	6	0.24309	0.43132	0.99998	7973	-0.1288
NEUROG1	6	0.2431	0.43133	0.99998	7974	-0.0922
HSPA4	6	0.2431	0.43133	0.99998	7975	-0.0197
LAIR1	4	0.24311	0.37077	0.99998	7976	0.0219
SCIN	4	0.24318	0.37086	0.99998	7977	0.0871
RBM47	6	0.24328	0.43155	0.99998	7978	-0.0588
RAB3IL1	6	0.24328	0.43155	0.99998	7979	0.0319
DGKK	6	0.24339	0.43171	0.99998	7980	-0.1013
ZSCAN2	6	0.24349	0.43183	0.99998	7981	-0.0696
NME5	6	0.24349	0.43183	0.99998	7982	-0.0538
C16orf45	6	0.24354	0.43191	0.99998	7983	0.1442
OR7G2	6	0.24366	0.43206	0.99998	7984	0.0526
TSSK3	6	0.24378	0.43223	0.99998	7985	-0.0508
SIGLEC7	6	0.24378	0.43223	0.99998	7986	0.0343
ZNF645	6	0.24378	0.43223	0.99998	7987	-0.2772
hsa-mir-8085	4	0.24381	0.37157	0.99998	7988	-0.0715
APMAP	6	0.24389	0.43238	0.99998	7989	-0.0461
TAGLN	6	0.2439	0.4324	0.99998	7990	0.1239
NOVA2	6	0.24397	0.43247	0.99998	7991	-0.0468
C15orf43	6	0.24405	0.43257	0.99998	7992	-0.1092
DSC2	6	0.24409	0.43263	0.99998	7993	0.2377
OR5B21	6	0.24411	0.43266	0.99998	7994	0.0038
hsa-mir-4284	4	0.24414	0.37194	0.99998	7995	-0.0973
TCP11	6	0.24416	0.43273	0.99998	7996	-0.1469
ARRDC4	6	0.24422	0.43279	0.99998	7997	-0.0502
CBY1	6	0.24422	0.43279	0.99998	7998	-0.1095
PREPL	6	0.24423	0.43281	0.99998	7999	0.0213
C16orf87	6	0.24423	0.43281	0.99998	8000	-0.1747
ATP8B3	6	0.24424	0.43282	0.99998	8001	0.0076
KIF1B	6	0.24424	0.43282	0.99998	8002	-0.0549
CCDC136	6	0.24424	0.43282	0.99998	8003	-0.1158
CYB5R2	6	0.24436	0.43296	0.99998	8004	0.2678
CDH12	6	0.2444	0.43302	0.99998	8005	0.0734
hsa-mir-3914-	1	0.24441	0.24421	0.99998	8006	-0.1995
hsa-mir-675	4	0.24446	0.37228	0.99998	8007	-0.126
USP17L5	1	0.24448	0.24428	0.99998	8008	-0.5411
PCBP4	6	0.24452	0.43319	0.99998	8009	-0.1615
APAF1	6	0.24452	0.43319	0.99998	8010	-0.1226
RFFL	6	0.24452	0.43319	0.99998	8011	0.3153
hsa-mir-597	4	0.24458	0.37243	0.99998	8012	-0.0313
ALOX12B	6	0.24473	0.43345	0.99998	8013	0.0979
IGF2BP1	6	0.24473	0.43345	0.99998	8014	0.2063
LRRC24	4	0.24479	0.37266	0.99998	8015	-0.2918
AASS	6	0.24489	0.43366	0.99998	8016	-0.2213

UBR4	6	0.24496	0.43374	0.99998	8017	-0.0019
PMCH	6	0.245	0.43381	0.99998	8018	-0.1319
SPINK9	6	0.24502	0.43383	0.99998	8019	0.0016
CERS6	6	0.24504	0.43385	0.99998	8020	0.0806
NPW	6	0.24504	0.43385	0.99998	8021	-0.1588
PMS1	6	0.24514	0.43398	0.99998	8022	-0.061
DPEP3	6	0.24514	0.43398	0.99998	8023	-0.018
CTSW	6	0.24522	0.43408	0.99998	8024	-0.2148
EPB41L2	6	0.24522	0.43409	0.99998	8025	0.0078
ACTR5	6	0.24524	0.4341	0.99998	8026	0.1973
OR2B6	6	0.24547	0.43442	0.99998	8027	-0.087
NPPB	6	0.24547	0.43442	0.99998	8028	-0.0599
TRIM69	6	0.24553	0.43448	0.99998	8029	-0.1016
RSPO1	6	0.24553	0.43448	0.99998	8030	-0.0657
LOC10013030	6	0.24553	0.43448	0.99998	8031	0.1919
CCDC51	6	0.24553	0.43448	0.99998	8032	-0.0967
RGS3	6	0.24555	0.43452	0.99998	8033	0.0039
ABCA9	6	0.24558	0.43455	0.99998	8034	-0.1408
CASC1	6	0.24568	0.43468	0.99998	8035	0.0211
TMEM97	6	0.24569	0.4347	0.99998	8036	0.0456
NFKBIE	6	0.24572	0.43473	0.99998	8037	0.019
DNAJB9	6	0.24578	0.43482	0.99998	8038	-0.0267
LTC4S	6	0.24578	0.43482	0.99998	8039	0.0715
DRAM1	6	0.24587	0.43493	0.99998	8040	0.0568
ZNF398	6	0.24587	0.43493	0.99998	8041	-0.0143
PDE6G	6	0.24588	0.43495	0.99998	8042	0.0141
SHOX2	6	0.246	0.43511	0.99998	8043	0.1013
IL17A	6	0.246	0.43511	0.99998	8044	-0.0811
JAK2	6	0.24603	0.43514	0.99998	8045	-0.1256
UBL7	6	0.24607	0.43518	0.99998	8046	0.1426
NAPG	4	0.24618	0.37422	0.99998	8047	-0.2767
FMO5	6	0.24623	0.43538	0.99998	8048	0.0153
GPR142	6	0.24626	0.43542	0.99998	8049	-0.0708
ZBTB5	6	0.24629	0.43548	0.99998	8050	0.0634
hsa-mir-4521	4	0.24637	0.37445	0.99998	8051	-0.2168
OR52D1	6	0.24642	0.43562	0.99998	8052	-0.0176
C1orf227	6	0.24642	0.43563	0.99998	8053	-0.1133
CPXCR1	6	0.24642	0.43563	0.99998	8054	-0.0455
TMIE	6	0.24654	0.43578	0.99998	8055	0.0028
C5orf48	6	0.24654	0.43578	0.99998	8056	0.1486
ATP5H	6	0.24655	0.4358	0.99998	8057	-0.1757
OR10A7	6	0.24655	0.4358	0.99998	8058	0.0834
AVP	6	0.24662	0.43588	0.99998	8059	0.2614
E2F4	6	0.24663	0.43589	0.99998	8060	-0.0328
AP4S1	6	0.24663	0.43589	0.99998	8061	-0.09
AGPAT2	6	0.24669	0.43597	0.99998	8062	0.1333
C20orf27	6	0.24674	0.43605	0.99998	8063	-0.0046
OR2Y1	6	0.24674	0.43605	0.99998	8064	-0.1568
BCL11A	6	0.24679	0.4361	0.99998	8065	-0.0288
KCTD11	6	0.24679	0.4361	0.99998	8066	-0.2063
RPL29	6	0.24681	0.43613	0.99998	8067	0.1182
SERHL2	6	0.24681	0.43613	0.99998	8068	-0.0768
SOX21	6	0.24695	0.43632	0.99998	8069	0.1594
RAB11FIP3	6	0.247	0.43638	0.99998	8070	-0.1343
FAM180A	6	0.247	0.43638	0.99998	8071	-0.0057
ARIH2OS	6	0.247	0.43638	0.99998	8072	-0.1464
CRELD1	6	0.24702	0.4364	0.99998	8073	0.086
APOC4	6	0.24705	0.43645	0.99998	8074	-0.0774
CD1D	6	0.24708	0.43647	0.99998	8075	-0.0776
ACTRT1	6	0.2472	0.43663	0.99998	8076	0.0213
SLIRP	6	0.2472	0.43663	0.99998	8077	-0.148
CXXC5	6	0.24722	0.43666	0.99998	8078	-0.1185
LILRB2	6	0.24726	0.43671	0.99998	8079	0.0017
CYB561D1	6	0.24726	0.43671	0.99998	8080	0.3129
PLRG1	6	0.24726	0.43671	0.99998	8081	-0.1041
MAP3K6	6	0.24728	0.43674	0.99998	8082	0.0375
TOP3B	6	0.24728	0.43674	0.99998	8083	0.0895
SNRPN	6	0.2474	0.43689	0.99998	8084	-0.0348
RASGRP1	6	0.24749	0.437	0.99998	8085	-0.1609
CLDND1	6	0.24749	0.437	0.99998	8086	-0.1029
UBD	6	0.24749	0.43701	0.99998	8087	0.0479
SALL2	6	0.24749	0.43701	0.99998	8088	-0.0704
RNPS1	6	0.2475	0.43703	0.99998	8089	-0.2179
EFNB1	6	0.2475	0.43703	0.99998	8090	0.0505
hsa-mir-139	4	0.24767	0.37592	0.99998	8091	0.0862
NDUFA10	6	0.24772	0.43729	0.99998	8092	0.0191
HEXDC	6	0.24772	0.43729	0.99998	8093	0.0685
CCR8	6	0.24781	0.43739	0.99998	8094	-0.1442
ZNF346	6	0.24781	0.43739	0.99998	8095	-0.0281
NPHS2	6	0.24781	0.43739	0.99998	8096	-0.0166
BNIP3L	6	0.24782	0.4374	0.99998	8097	0.2254

TP63	6	0.24782	0.4374	0.99998	8098	0.1333
ZDHHC16	4	0.24783	0.37608	0.99998	8099	-0.0055
CGN	6	0.24791	0.43753	0.99998	8100	0.0849
SENP1	6	0.24798	0.43761	0.99998	8101	-0.0648
STMN2	6	0.24804	0.43769	0.99998	8102	-0.1346
TNFRSF19	6	0.24804	0.43769	0.99998	8103	-0.1628
RAPH1	6	0.24804	0.43769	0.99998	8104	-0.0851
VSIG8	6	0.2481	0.43778	0.99998	8105	0.0093
CKMT1B	4	0.24814	0.37643	0.99998	8106	-0.1249
hsa-mir-4675	4	0.24814	0.37643	0.99998	8107	-0.0171
CRIPT	6	0.24816	0.43786	0.99998	8108	-0.2178
CMA1	6	0.24816	0.43786	0.99998	8109	-0.2331
GNG3	6	0.24817	0.43788	0.99998	8110	-0.213
NDUFA6	6	0.24829	0.43805	0.99998	8111	-0.0327
KIAA0317	4	0.24832	0.37663	0.99998	8112	-0.1654
PRPSAP1	6	0.24836	0.43815	0.99998	8113	-0.049
SFTA2	6	0.24839	0.43818	0.99998	8114	0.0109
LY6G6C	6	0.24839	0.43818	0.99998	8115	-0.1253
PRRT3	6	0.24839	0.43818	0.99998	8116	-0.0285
SPRR2D	1	0.24843	0.24823	0.99998	8117	-1.332
AGAP5	1	0.24843	0.24823	0.99998	8118	-1.332
CPM	6	0.24849	0.43832	0.99998	8119	-0.0807
CXCL9	6	0.24852	0.43835	0.99998	8120	-0.113
hsa-mir-7641-	1	0.24862	0.24842	0.99998	8121	-0.1741
ATP6V1G2	6	0.2487	0.4386	0.99998	8122	-0.0524
MAN1A2	6	0.2487	0.4386	0.99998	8123	-0.0346
MS4A4A	6	0.24878	0.4387	0.99998	8124	-0.0056
PLB1	6	0.24878	0.4387	0.99998	8125	0.0898
SLC36A3	6	0.2489	0.43886	0.99998	8126	-0.0633
DNAAF2	6	0.2489	0.43886	0.99998	8127	0.0131
CLMN	6	0.24899	0.43896	0.99998	8128	0.2974
hsa-mir-6884	4	0.249	0.3774	0.99998	8129	0.1535
NUDT1	6	0.24908	0.43908	0.99998	8130	-0.1983
ABHD17B	6	0.24908	0.43908	0.99998	8131	-0.1696
TMEM192	6	0.24909	0.4391	0.99998	8132	-0.0573
hsa-mir-148a	3	0.24913	0.36175	0.99998	8133	0.1737
OR12D2	6	0.24919	0.43923	0.99998	8134	0.0071
ZNF285	6	0.24919	0.43923	0.99998	8135	-0.1608
HEXIM1	6	0.24925	0.43932	0.99998	8136	0.05
HIST1H3E	6	0.24925	0.43932	0.99998	8137	-0.0819
TTYH2	6	0.24934	0.43945	0.99998	8138	-0.1325
CD40LG	6	0.24938	0.43949	0.99998	8139	-0.0424
PBX3	6	0.24938	0.43949	0.99998	8140	0.0247
GPR78	6	0.24938	0.43949	0.99998	8141	0.1011
PHKA2	6	0.24941	0.43953	0.99998	8142	0.0861
FAM195B	6	0.24953	0.43968	0.99998	8143	0.0019
AP1S1	6	0.24953	0.43968	0.99998	8144	-0.0532
TMCC1	6	0.24954	0.43969	0.99998	8145	-0.1147
hsa-mir-4269	4	0.24965	0.37808	0.99998	8146	0.0064
MLL3	4	0.24966	0.37809	0.99998	8147	-0.121
GCC1	6	0.24966	0.43984	0.99998	8148	0.0714
PDK2	6	0.24966	0.43985	0.99998	8149	-0.1427
hsa-mir-4329	2	0.2497	0.28163	0.99998	8150	-0.5754
OSR1	6	0.24987	0.44012	0.99998	8151	-0.1148
C9orf153	6	0.24992	0.44018	0.99998	8152	0.048
GAA	6	0.24999	0.44029	0.99998	8153	-0.061
SLC26A2	6	0.25001	0.44031	0.99998	8154	0.0054
SPATA31D3	2	0.25002	0.28188	0.99998	8155	-0.0546
ITIH4	6	0.25009	0.44041	0.99998	8156	-0.1177
TLN2	6	0.25013	0.44047	0.99998	8157	0.0975
DRG1	6	0.25021	0.44057	0.99998	8158	0.0394
SRCRB4D	6	0.25021	0.44058	0.99998	8159	-0.0126
PAAF1	6	0.25021	0.44058	0.99998	8160	-0.0962
KDM5A	6	0.25029	0.44068	0.99998	8161	-0.0193
DNAJC16	6	0.25032	0.44071	0.99998	8162	0.1869
PLGLB1	1	0.25033	0.25009	0.99998	8163	-0.3702
CACNA1G	6	0.25037	0.4408	0.99998	8164	0.1309
PARP12	6	0.25037	0.4408	0.99998	8165	0.0296
SAMD4A	6	0.25041	0.44084	0.99998	8166	-0.1271
ZNF599	6	0.25054	0.44103	0.99998	8167	-0.043
HBM	6	0.25056	0.44105	0.99998	8168	-0.103
SNTG2	6	0.25056	0.44105	0.99998	8169	0.0094
DPP6	6	0.25056	0.44105	0.99998	8170	-0.1237
MORC4	6	0.25071	0.44125	0.99998	8171	0.179
PGM5	4	0.25075	0.3793	0.99998	8172	-0.1516
NDFIP1	6	0.25079	0.44137	0.99998	8173	-0.053
SUMF1	6	0.25082	0.44141	0.99998	8174	-0.0762
RAD54L	6	0.25083	0.44142	0.99998	8175	-0.128
KRTAP12-2	6	0.25087	0.44148	0.99998	8176	0.2664
ANKEF1	6	0.25093	0.44156	0.99998	8177	-0.0868
HORMAD2	6	0.25093	0.44156	0.99998	8178	-0.1763

SSTR2	6	0.25096	0.44159	0.99998	8179	-0.1003
MUC21	6	0.25108	0.44174	0.99998	8180	-0.1143
NIT2	6	0.25111	0.44177	0.99998	8181	-0.0956
CCT5	6	0.2512	0.4419	0.99998	8182	-0.0017
BAD	6	0.25124	0.44196	0.99998	8183	-0.0219
TTLL11	6	0.2513	0.44204	0.99998	8184	0.0774
LOC10012992	6	0.2513	0.44204	0.99998	8185	0.0076
RAD21L1	6	0.25138	0.44214	0.99998	8186	-0.0371
IFT43	6	0.25138	0.44214	0.99998	8187	-0.1122
FAM173B	6	0.25138	0.44214	0.99998	8188	-0.107
HIST1H3G	6	0.25138	0.44214	0.99998	8189	0.0642
FTMT	6	0.25138	0.44215	0.99998	8190	-0.0838
NPTX2	4	0.25145	0.38004	0.99998	8191	0.0885
hsa-mir-887	4	0.25146	0.38005	0.99998	8192	-0.1121
C16orf52	6	0.25157	0.44239	0.99998	8193	0.0088
ARHGAP30	6	0.2516	0.44242	0.99998	8194	-0.1921
PTK6	6	0.2516	0.44242	0.99998	8195	-0.0962
hsa-mir-5587	4	0.25166	0.38026	0.99998	8196	-0.0756
NXPE2	6	0.25166	0.44252	0.99998	8197	0.0074
HOXD3	6	0.25166	0.44252	0.99998	8198	-0.012
TIGD7	6	0.25176	0.44265	0.99998	8199	-0.1525
hsa-mir-1253	4	0.25182	0.38047	0.99998	8200	-0.2793
CEP41	6	0.25184	0.44275	0.99998	8201	-0.0813
PSG6	6	0.25185	0.44277	0.99998	8202	-0.0147
CASP6	6	0.25185	0.44277	0.99998	8203	0.1382
LOC646862	6	0.25185	0.44277	0.99998	8204	0.5153
ATF6	6	0.25191	0.44285	0.99998	8205	-0.1695
RPN2	6	0.25191	0.44285	0.99998	8206	-0.0907
TIA1	6	0.25191	0.44285	0.99998	8207	-0.2106
C8A	6	0.252	0.44299	0.99998	8208	-0.1097
IL17RC	6	0.25206	0.44307	0.99998	8209	0.0065
hsa-mir-548au	1	0.25207	0.2519	0.99998	8210	-0.2707
GBP2	6	0.25208	0.44309	0.99998	8211	0.0828
ZNF696	6	0.25217	0.44321	0.99998	8212	-0.2287
GJB2	6	0.25217	0.44321	0.99998	8213	-0.1609
HEATR4	6	0.25217	0.44321	0.99998	8214	-0.3149
TCF20	6	0.25218	0.44322	0.99998	8215	-0.2638
PLEKHG1	5	0.25228	0.41071	0.99998	8216	0.0297
CASQ1	6	0.25232	0.44341	0.99998	8217	-0.1474
ZNF280B	6	0.25232	0.44341	0.99998	8218	0.0513
HTN1	2	0.25237	0.28381	0.99998	8219	-0.0534
PCDHGB4	2	0.25237	0.28381	0.99998	8220	-0.0494
ENTPD6	6	0.25246	0.4436	0.99998	8221	-0.0489
GRAMD4	6	0.25257	0.44375	0.99998	8222	0.0004
TRIM25	6	0.25257	0.44375	0.99998	8223	0.0645
ZNF695	6	0.25258	0.44376	0.99998	8224	-0.1206
CLPSL2	6	0.25265	0.44387	0.99998	8225	-0.0949
MAN2C1	6	0.25275	0.444	0.99998	8226	-0.1204
CEACAM3	6	0.25275	0.444	0.99998	8227	-0.1339
ANKRD44	6	0.25277	0.44402	0.99998	8228	-0.0411
WDR34	6	0.25294	0.44426	0.99998	8229	-0.0444
LECT1	6	0.25298	0.4443	0.99998	8230	-0.1144
RAB39A	6	0.25298	0.4443	0.99998	8231	-0.2224
CDK16	6	0.253	0.44433	0.99998	8232	0.1333
hsa-mir-564	4	0.25306	0.38186	0.99998	8233	-0.3411
SDPR	6	0.2531	0.44445	0.99998	8234	-0.033
USP44	6	0.25314	0.4445	0.99998	8235	0.1968
PPP1CC	6	0.25316	0.44453	0.99998	8236	0.116
RYR2	6	0.25316	0.44453	0.99998	8237	0.1022
KLRF1	6	0.25317	0.44455	0.99998	8238	-0.0599
NLRP8	6	0.25317	0.44455	0.99998	8239	-0.2429
STK11	6	0.25353	0.44503	0.99998	8240	-0.1254
FDXACB1	6	0.25353	0.44503	0.99998	8241	0.1962
CBX6	6	0.25354	0.44503	0.99998	8242	0.0147
GPR158	6	0.25354	0.44504	0.99998	8243	-0.0242
VCL	6	0.25366	0.44519	0.99998	8244	0.0587
STRN4	6	0.25366	0.44519	0.99998	8245	0.1528
DOCK7	6	0.25368	0.44521	0.99998	8246	-0.1228
CASKIN2	6	0.25368	0.44521	0.99998	8247	-0.0137
SON	6	0.25368	0.44521	0.99998	8248	-0.2085
SLCO2B1	6	0.25369	0.44524	0.99998	8249	0.0611
TPPP	6	0.25369	0.44524	0.99998	8250	0.1865
H2AFB2	1	0.25384	0.25365	0.99998	8251	-0.2929
NMS	6	0.25386	0.44546	0.99998	8252	-0.027
SENP2	6	0.25386	0.44546	0.99998	8253	0.048
PCDHGA10	2	0.25386	0.28501	0.99998	8254	-0.0615
C1QTNF6	6	0.25389	0.44552	0.99998	8255	-0.0619
STIM2	6	0.25404	0.44571	0.99998	8256	-0.0558
RBFOX1	6	0.25404	0.44571	0.99998	8257	-0.1029
PRDM15	4	0.25412	0.38306	0.99998	8258	0.1604
NCKAP1L	6	0.25422	0.44596	0.99998	8259	-0.1493

SMG1	6	0.25424	0.44599	0.99998	8260	-0.0006
KHK	6	0.25424	0.44599	0.99998	8261	-0.0708
TBC1D8	6	0.25427	0.44602	0.99998	8262	0.0049
hsa-mir-92a-1	4	0.25429	0.38324	0.99998	8263	-0.1325
ALCAM	6	0.25444	0.44627	0.99998	8264	0.1274
FAM71E1	6	0.25446	0.44628	0.99998	8265	-0.0373
ARL16	6	0.25446	0.44628	0.99998	8266	-0.0739
COL11A2	6	0.25453	0.44637	0.99998	8267	0.0418
CLEC2B	6	0.25459	0.44646	0.99998	8268	-0.1142
RTN4RL2	6	0.25467	0.44657	0.99998	8269	-0.0366
CPSF4L	6	0.25467	0.44657	0.99998	8270	-0.1737
hsa-mir-8083	4	0.25469	0.38366	0.99998	8271	-0.1903
C6orf163	6	0.25475	0.44667	0.99998	8272	-0.0161
ADRBK1	6	0.25478	0.44671	0.99998	8273	-0.0862
INO80C	6	0.25487	0.44684	0.99998	8274	-0.0814
NEIL3	6	0.25487	0.44684	0.99998	8275	-0.2293
LYPD4	6	0.25487	0.44684	0.99998	8276	-0.0737
hsa-mir-1257	4	0.2549	0.3839	0.99998	8277	0.038
D2HGDH	6	0.25491	0.44689	0.99998	8278	-0.0095
AIDA	6	0.25491	0.44689	0.99998	8279	-0.0811
C6orf136	6	0.25519	0.44725	0.99998	8280	-0.1124
CORIN	6	0.25519	0.44725	0.99998	8281	-0.1106
MECR	4	0.25519	0.38423	0.99998	8282	-0.0779
CCND1	6	0.25524	0.4473	0.99998	8283	0.0426
LDOC1L	6	0.25524	0.44731	0.99998	8284	-0.0425
LSM10	6	0.2554	0.44751	0.99998	8285	0.001
hsa-mir-132	4	0.25545	0.38452	0.99998	8286	0.149
SH2D4A	6	0.25557	0.44774	0.99998	8287	-0.0756
MCUR1	6	0.25559	0.44776	0.99998	8288	-0.0088
SPSB2	6	0.25559	0.44776	0.99998	8289	-0.0563
WSCD2	6	0.2557	0.44792	0.99998	8290	-0.0967
FAM115C	6	0.2557	0.44792	0.99998	8291	-0.1548
MGP	6	0.25577	0.44802	0.99998	8292	-0.0034
CD74	6	0.25577	0.44802	0.99998	8293	0.0219
HSPG2	6	0.25577	0.44802	0.99998	8294	0.2216
ITGA2B	6	0.25578	0.44803	0.99998	8295	-0.0275
TNXB	6	0.25579	0.44804	0.99998	8296	-0.1088
DSG2	6	0.25581	0.44806	0.99998	8297	0.0035
RCBTB1	4	0.25584	0.38494	0.99998	8298	-0.0639
S100A2	6	0.25592	0.44821	0.99998	8299	0.1512
FGL2	6	0.25595	0.44826	0.99998	8300	-0.1385
NCS1	6	0.25595	0.44826	0.99998	8301	-0.2299
APOL5	6	0.25595	0.44826	0.99998	8302	-0.1919
CCT8L2	6	0.256	0.44831	0.99998	8303	0.3996
RAET1G	6	0.25608	0.44842	0.99998	8304	0.0307
ZNF507	6	0.25608	0.44842	0.99998	8305	-0.104
C17orf59	6	0.25608	0.44842	0.99998	8306	0.0652
hsa-mir-6793	4	0.25627	0.38544	0.99998	8307	0.135
ZNF567	6	0.25632	0.44874	0.99998	8308	0.5182
SAMD1	6	0.25632	0.44874	0.99998	8309	0.1877
NDUFAF7	6	0.25634	0.44876	0.99998	8310	-0.0934
GBP6	6	0.25634	0.44877	0.99998	8311	0.0399
CCNC	6	0.2564	0.44883	0.99998	8312	-0.0955
ACTR1B	6	0.2564	0.44883	0.99998	8313	-0.0862
KIF11	6	0.25646	0.44891	0.99998	8314	-0.1366
PRICKLE3	6	0.25647	0.44892	0.99998	8315	0.1108
OR6X1	6	0.25652	0.449	0.99998	8316	-0.1051
hsa-mir-718	4	0.25658	0.38579	0.99998	8317	-0.1481
LEKR1	6	0.25666	0.44918	0.99998	8318	-0.1763
ACBD3	6	0.25666	0.44918	0.99998	8319	-0.0391
EXOC6B	6	0.25666	0.44918	0.99998	8320	-0.0421
COMMD10	6	0.25673	0.44927	0.99998	8321	-0.0199
CALHM1	6	0.25673	0.44927	0.99998	8322	-0.0845
PELI2	6	0.25681	0.44938	0.99998	8323	0.0402
hsa-mir-875	4	0.25684	0.38609	0.99998	8324	-0.1805
MRGBP	6	0.25685	0.44942	0.99998	8325	-0.01
LRIG2	6	0.25692	0.44951	0.99998	8326	-0.1643
PARVB	6	0.25694	0.44955	0.99998	8327	-0.101
DIRAS3	6	0.25694	0.44955	0.99998	8328	-0.0347
CCR5	6	0.25699	0.44962	0.99998	8329	0.0416
CCL17	6	0.25701	0.44963	0.99998	8330	0.0964
SERF2	6	0.25701	0.44963	0.99998	8331	-0.2178
EPHB1	6	0.25701	0.44963	0.99998	8332	-0.0349
RNF150	6	0.25706	0.44969	0.99998	8333	-0.1082
TMEM251	6	0.25715	0.44981	0.99998	8334	-0.0771
TOR3A	6	0.25716	0.44982	0.99998	8335	0.1372
hsa-mir-4314	4	0.25719	0.3865	0.99998	8336	0.3631
COMMD7	6	0.2572	0.44988	0.99998	8337	-0.1079
SMPD4	6	0.25723	0.44991	0.99998	8338	-0.06
SLC5A11	6	0.25726	0.44994	0.99998	8339	0.0621
SLC38A2	6	0.25729	0.45	0.99998	8340	0.0508

INPP4B	6	0.25729	0.45	0.99998	8341	-0.1493
OR5F1	6	0.25734	0.45005	0.99998	8342	0.1848
WBSCR16	6	0.25738	0.4501	0.99998	8343	-0.0852
PHF8	6	0.2574	0.45013	0.99998	8344	-0.0807
NTF3	6	0.25742	0.45016	0.99998	8345	0.0368
hsa-mir-96	4	0.25748	0.38682	0.99998	8346	0.0734
DRG2	6	0.25752	0.45029	0.99998	8347	-0.0592
CUEDC2	6	0.25752	0.45029	0.99998	8348	-0.2421
SRSF8	6	0.25752	0.45029	0.99998	8349	-0.1591
EEPD1	6	0.25752	0.45029	0.99998	8350	-0.0526
CDH4	6	0.25754	0.45031	0.99998	8351	-0.0401
UHRF1	6	0.25764	0.45044	0.99998	8352	0.2873
TGIF2LX	6	0.25765	0.45045	0.99998	8353	0.0361
ZKSCAN5	6	0.25765	0.45045	0.99998	8354	0.0179
LTBP4	6	0.25765	0.45045	0.99998	8355	-0.2095
CCDC86	6	0.25775	0.45059	0.99998	8356	0.0816
hsa-mir-3692	4	0.25776	0.38713	0.99998	8357	-0.0573
COL22A1	6	0.25776	0.45061	0.99998	8358	-0.0328
MEGF9	6	0.25776	0.45061	0.99998	8359	0.0833
APOO	6	0.25784	0.45071	0.99998	8360	-0.1329
SURF4	6	0.25791	0.4508	0.99998	8361	0.0512
NR4A3	6	0.25797	0.45088	0.99998	8362	-0.132
RNF4	6	0.25797	0.45088	0.99998	8363	-0.2023
LOC10012936	6	0.25809	0.45104	0.99998	8364	0.183
HADHA	6	0.25809	0.45104	0.99998	8365	-0.0562
C1orf146	6	0.25809	0.45104	0.99998	8366	0.0008
SLC24A4	6	0.25821	0.45119	0.99998	8367	0.0286
MUC7	6	0.25831	0.45133	0.99998	8368	0.097
THNSL2	6	0.25832	0.45134	0.99998	8369	-0.0897
DIS3L2	6	0.25832	0.45134	0.99998	8370	-0.1581
ERCC4	6	0.25832	0.45134	0.99998	8371	-0.1255
GPX4	6	0.25832	0.45134	0.99998	8372	-0.0309
SPATA22	6	0.25832	0.45134	0.99998	8373	-0.0434
APOH	6	0.25834	0.45136	0.99998	8374	0.0173
RETNLB	6	0.25842	0.45146	0.99998	8375	-0.0342
WHAMM	6	0.25844	0.4515	0.99998	8376	-0.0958
ANKRD49	6	0.25846	0.45152	0.99998	8377	-0.1347
LRP1	6	0.25846	0.45152	0.99998	8378	-0.054
hsa-mir-940	4	0.25855	0.38801	0.99998	8379	0.0841
ZNF592	6	0.25855	0.45165	0.99998	8380	-0.0647
KCNA7	6	0.25855	0.45165	0.99998	8381	-0.1094
RHOXF1	6	0.25855	0.45165	0.99998	8382	-0.1076
ATG9A	6	0.25864	0.45176	0.99998	8383	-0.0787
CHPF	6	0.25866	0.45179	0.99998	8384	0.0976
IFNL1	6	0.25882	0.452	0.99998	8385	0.0344
IL6	4	0.25882	0.38831	0.99998	8386	-0.0731
hsa-mir-892c	4	0.25882	0.38831	0.99998	8387	-0.1969
DGKE	6	0.25892	0.45214	0.99998	8388	0.0347
CABP4	6	0.25905	0.45229	0.99998	8389	-0.0963
GRIN2D	6	0.25905	0.45229	0.99998	8390	0.0355
RUFY2	6	0.2591	0.45237	0.99998	8391	-0.1115
PSMA1	6	0.2591	0.45237	0.99998	8392	-0.1529
USP28	6	0.2591	0.45237	0.99998	8393	-0.1569
FCRL2	6	0.2591	0.45237	0.99998	8394	-0.1178
HECTD1	6	0.2591	0.45237	0.99998	8395	-0.2063
TAS2R50	6	0.2591	0.45237	0.99998	8396	-0.0228
CHADL	6	0.25919	0.45247	0.99998	8397	0.0783
TM2D3	6	0.25919	0.45247	0.99998	8398	0.0136
LRCH3	6	0.25919	0.45247	0.99998	8399	0.2149
SHISA6	6	0.25932	0.45264	0.99998	8400	0.0104
KCNQ5	6	0.25932	0.45264	0.99998	8401	-0.0491
hsa-mir-412	4	0.25943	0.38903	0.99998	8402	0.3357
COL9A3	6	0.25947	0.45285	0.99998	8403	-0.0258
CCNYL1	6	0.25947	0.45285	0.99998	8404	0.3253
FAM162B	6	0.25948	0.45286	0.99998	8405	-0.0328
ACTB	6	0.25955	0.45297	0.99998	8406	0.1315
SNAP29	6	0.25955	0.45297	0.99998	8407	-0.0233
IPO9	6	0.25966	0.45311	0.99998	8408	-0.1376
ZYX	6	0.25978	0.45325	0.99998	8409	-0.112
DEFB119	6	0.25979	0.45327	0.99998	8410	0.1739
UBXN11	6	0.25988	0.45339	0.99998	8411	-0.0639
TXNRD3	6	0.25988	0.45339	0.99998	8412	-0.1388
ZNF514	6	0.2599	0.45342	0.99998	8413	0.1557
ZNF461	6	0.25995	0.45348	0.99998	8414	-0.1412
CYP1A1	6	0.26002	0.45359	0.99998	8415	-0.1074
N6AMT1	6	0.26012	0.45372	0.99998	8416	-0.0669
TIMM9	6	0.26018	0.45378	0.99998	8417	-0.1739
SMAP2	6	0.2602	0.45382	0.99998	8418	-0.0205
RIPK3	6	0.26024	0.45386	0.99998	8419	-0.1329
LOC10050547	6	0.26033	0.45399	0.99998	8420	-0.0887
PRPF4B	6	0.2604	0.45409	0.99998	8421	0.1768

EPS8L3	6	0.2604	0.45409	0.99998	8422	0.2651
CRB3	6	0.2606	0.45435	0.99998	8423	0.0217
PFN2	6	0.26065	0.45441	0.99998	8424	0.1199
OR4N2	6	0.26065	0.45441	0.99998	8425	-0.1242
YIF1A	6	0.26073	0.45452	0.99998	8426	0.0105
SMU1	6	0.26079	0.45461	0.99998	8427	-0.2316
MOG	6	0.26079	0.45461	0.99998	8428	-0.1399
EFHC1	6	0.26079	0.45461	0.99998	8429	-0.0319
MOB1A	6	0.26083	0.45466	0.99998	8430	0.4476
RWDD2B	6	0.26095	0.45482	0.99998	8431	-0.092
TANGO6	6	0.26095	0.45482	0.99998	8432	-0.0856
hsa-mir-1202	4	0.26095	0.39072	0.99998	8433	0.4433
PCMT1	6	0.26101	0.45491	0.99998	8434	-0.0058
hsa-mir-548i-4	3	0.26105	0.36853	0.99998	8435	0.257
PTGIS	6	0.26109	0.45502	0.99998	8436	-0.0826
hsa-mir-26a-2	4	0.26111	0.39091	0.99998	8437	-0.296
KCNJ12	4	0.26112	0.39091	0.99998	8438	0.2595
SPAG8	6	0.26115	0.4551	0.99998	8439	0.2428
POLM	6	0.26117	0.45513	0.99998	8440	-0.073
ABCA7	6	0.26127	0.45525	0.99998	8441	-0.0665
LRRC8E	6	0.26129	0.45527	0.99998	8442	-0.0852
PRX	6	0.26129	0.45527	0.99998	8443	-0.0629
ACSM1	4	0.26131	0.39113	0.99998	8444	-0.0286
hsa-mir-5188	4	0.26131	0.39113	0.99998	8445	-0.2696
ERC2	6	0.26139	0.45539	0.99998	8446	-0.0171
EEFSEC	4	0.26142	0.39124	0.99998	8447	0.0817
TRIP11	6	0.26148	0.45551	0.99998	8448	-0.0876
MUSTN1	6	0.26154	0.45559	0.99998	8449	0.1003
HN1	6	0.26162	0.45568	0.99998	8450	-0.1248
FAM212B	6	0.26166	0.45574	0.99998	8451	0.0979
TBC1D1	6	0.26166	0.45574	0.99998	8452	-0.0158
ARMC12	6	0.26168	0.45577	0.99998	8453	-0.0946
B3GALT5	6	0.26179	0.4559	0.99998	8454	0.0119
SEC14L5	6	0.26179	0.4559	0.99998	8455	-0.0827
DDX25	6	0.26182	0.45593	0.99998	8456	-0.0294
ZSCAN16	6	0.26182	0.45593	0.99998	8457	0.0335
hsa-mir-1976	4	0.26191	0.39178	0.99998	8458	-0.286
RAB3B	6	0.26192	0.45605	0.99998	8459	0.0654
LYRM5	6	0.26202	0.45618	0.99998	8460	0.035
POTEI	1	0.26203	0.26192	0.99998	8461	-0.7783
PCDH7	6	0.26208	0.45626	0.99998	8462	0.023
FAM171B	6	0.26224	0.45646	0.99998	8463	-0.0367
FOXP4	6	0.26227	0.4565	0.99998	8464	0.0866
ASF1B	6	0.26227	0.4565	0.99998	8465	0.0595
SLC7A8	6	0.26227	0.4565	0.99998	8466	-0.1438
CD207	6	0.26232	0.45657	0.99998	8467	-0.0761
GRIK2	6	0.26232	0.45657	0.99998	8468	-0.0879
HTR3D	6	0.26233	0.45658	0.99998	8469	0.0594
DCP1B	6	0.26245	0.45675	0.99998	8470	0.026
FTSJ2	6	0.26247	0.45678	0.99998	8471	0.0038
MACROD2	6	0.26257	0.4569	0.99998	8472	0.066
GBE1	6	0.26258	0.45691	0.99998	8473	-0.1328
CALM3	6	0.26258	0.45691	0.99998	8474	-0.0866
hsa-mir-3652	4	0.26265	0.3926	0.99998	8475	-0.2637
BPIFB3	6	0.26268	0.45704	0.99998	8476	-0.0188
MNAT1	6	0.26268	0.45704	0.99998	8477	-0.0785
CYP2B6	6	0.26268	0.45704	0.99998	8478	-0.113
ADAP1	6	0.26269	0.45705	0.99998	8479	-0.0038
hsa-mir-4466	4	0.26273	0.3927	0.99998	8480	-0.0852
TTC7A	6	0.26279	0.45718	0.99998	8481	-0.0225
ZP3	6	0.26279	0.45719	0.99998	8482	-0.1105
C5AR2	6	0.26283	0.45725	0.99998	8483	0.1805
ZNHIT3	6	0.26283	0.45725	0.99998	8484	0.0425
SUCO	6	0.26295	0.45738	0.99998	8485	-0.1458
hsa-mir-4422	4	0.26297	0.39298	0.99998	8486	-0.2022
STRADB	6	0.26299	0.45745	0.99998	8487	-0.0091
ATP6V1D	6	0.26305	0.45751	0.99998	8488	-0.053
hsa-mir-4294	4	0.26307	0.3931	0.99998	8489	-0.1903
KIF7	6	0.26322	0.45774	0.99998	8490	-0.0533
PSMB10	6	0.26326	0.45778	0.99998	8491	0.0389
GFRA1	6	0.2633	0.45783	0.99998	8492	-0.055
ZC3H3	6	0.26332	0.45787	0.99998	8493	-0.0483
ZNF200	6	0.26334	0.45788	0.99998	8494	-0.109
POLR3C	4	0.26337	0.39342	0.99998	8495	-0.1009
WIPI2	6	0.26349	0.45808	0.99998	8496	0.1922
OR2M2	6	0.2635	0.45809	0.99998	8497	0.2266
HDAC9	6	0.2635	0.4581	0.99998	8498	-0.1456
CLIP2	6	0.2635	0.4581	0.99998	8499	0.0022
RFPL2	6	0.26357	0.45818	0.99998	8500	-0.1898
DNAJA3	4	0.26358	0.39365	0.99998	8501	-0.2036
hsa-mir-1237	4	0.26366	0.39374	0.99998	8502	-0.1503

ZMYM6	6	0.26378	0.45847	0.99998	8503	-0.0708
FKBP1B	6	0.26378	0.45848	0.99998	8504	-0.0528
OR6C74	6	0.26384	0.45855	0.99998	8505	-0.157
hsa-mir-6134	4	0.26391	0.39402	0.99998	8506	-0.1464
IGFBP4	6	0.26397	0.45873	0.99998	8507	0.3613
KIAA1211L	6	0.26397	0.45873	0.99998	8508	-0.0697
LOC10099648	5	0.26402	0.42152	0.99998	8509	0.0415
OLIG1	6	0.26405	0.45884	0.99998	8510	0.0636
TMEM53	6	0.26408	0.45888	0.99998	8511	0.0769
CEP104	6	0.26408	0.45888	0.99998	8512	0.065
DPT	6	0.26408	0.45888	0.99998	8513	0.0254
hsa-mir-4745	4	0.26414	0.39427	0.99998	8514	-0.1662
hsa-mir-4259	4	0.2642	0.39433	0.99998	8515	-0.0549
CETP	6	0.26422	0.45906	0.99998	8516	-0.0937
SLC43A2	6	0.26423	0.45907	0.99998	8517	-0.0434
PALM3	6	0.26433	0.4592	0.99998	8518	-0.0718
PDK4	6	0.26445	0.45937	0.99998	8519	0.0173
LEAP2	6	0.26447	0.45939	0.99998	8520	-0.0238
CCR6	6	0.26448	0.4594	0.99998	8521	0.0288
SLC35D1	4	0.26455	0.39472	0.99998	8522	0.0051
BRMS1L	6	0.2646	0.45954	0.99998	8523	-0.1367
MROH1	6	0.2646	0.45954	0.99998	8524	-0.058
TRIB1	6	0.26471	0.45968	0.99998	8525	-0.1841
OR2A12	6	0.26471	0.45968	0.99998	8526	-0.0289
BRD7	6	0.26482	0.45982	0.99998	8527	-0.094
FAM49A	6	0.26487	0.45989	0.99998	8528	0.0536
VPS33B	6	0.26487	0.45989	0.99998	8529	-0.0138
BOLL	6	0.26487	0.45989	0.99998	8530	-0.0307
SCGB1A1	6	0.26487	0.4599	0.99998	8531	-0.0287
NDRG3	6	0.26487	0.4599	0.99998	8532	0.0244
CAPRIN1	6	0.26489	0.45992	0.99998	8533	-0.0042
RASA4B	1	0.2652	0.26508	0.99998	8534	-0.162
XPC	6	0.26523	0.46035	0.99998	8535	0.2587
TNFSF13	6	0.26524	0.46036	0.99998	8536	0.0252
RIBC1	6	0.26527	0.4604	0.99998	8537	0.0233
EGFL6	6	0.26531	0.46045	0.99998	8538	-0.0612
TAS2R4	6	0.26531	0.46045	0.99998	8539	-0.0532
UNK	6	0.26537	0.46052	0.99998	8540	-0.1244
EAF2	6	0.26541	0.46059	0.99998	8541	0.0014
MRPL20	6	0.26541	0.46059	0.99998	8542	0.1018
TRPC1	6	0.26552	0.46073	0.99998	8543	-0.1233
KCNN4	6	0.26552	0.46073	0.99998	8544	-0.2005
FOXN4	6	0.26557	0.46079	0.99998	8545	0.1094
TMEM110-MU	4	0.26571	0.39598	0.99998	8546	0.0362
LTBP2	6	0.26574	0.46101	0.99998	8547	-0.0498
HPCAL1	6	0.26584	0.46115	0.99998	8548	-0.1111
OR2A42	2	0.26594	0.2946	0.99998	8549	0.0102
MADCAM1	6	0.26594	0.46128	0.99998	8550	-0.1361
hsa-mir-423	1	0.26598	0.2659	0.99998	8551	-0.2761
OPTC	6	0.266	0.46137	0.99998	8552	0.1613
RGSL1	6	0.266	0.46137	0.99998	8553	-0.1822
hsa-mir-5010	4	0.26604	0.39631	0.99998	8554	-0.2236
FOXD2	6	0.26606	0.46145	0.99998	8555	-0.0596
CCDC116	6	0.26607	0.46145	0.99998	8556	-0.2122
PPAPDC3	6	0.26612	0.46152	0.99998	8557	0.0651
ARID1B	6	0.26612	0.46152	0.99998	8558	-0.0208
TTC26	6	0.2662	0.46161	0.99998	8559	-0.0336
PLEK2	6	0.26622	0.46162	0.99998	8560	0.0884
MSLN	6	0.26623	0.46164	0.99998	8561	0.1243
TRPV1	6	0.26636	0.4618	0.99998	8562	-0.0765
EOGT	6	0.26644	0.4619	0.99998	8563	0.216
FAM211A	6	0.26644	0.4619	0.99998	8564	-0.1271
COL6A2	6	0.26644	0.4619	0.99998	8565	-0.0893
hsa-mir-5680	4	0.26646	0.39676	0.99998	8566	0.1668
FAM200A	6	0.26655	0.46204	0.99998	8567	-0.1207
OR12D3	6	0.26659	0.46209	0.99998	8568	0.078
hsa-mir-6823	4	0.2666	0.39691	0.99998	8569	-0.1917
MSANTD1	6	0.26663	0.46214	0.99998	8570	0.0981
RAB7A	6	0.26674	0.46227	0.99998	8571	-0.1116
OR2G2	6	0.26674	0.46227	0.99998	8572	-0.0028
LMBRD2	6	0.26674	0.46227	0.99998	8573	-0.1104
MAGI1	6	0.26684	0.4624	0.99998	8574	0.0845
SOCS3	6	0.26684	0.4624	0.99998	8575	0.0908
GTF2H1	6	0.26685	0.46241	0.99998	8576	-0.0669
ANKRD32	6	0.26692	0.46251	0.99998	8577	-0.1179
MAGEC3	6	0.26692	0.46251	0.99998	8578	-0.0277
GGT6	6	0.26692	0.46251	0.99998	8579	-0.181
EPS15L1	6	0.26695	0.46254	0.99998	8580	-0.0379
CDHR2	6	0.26695	0.46254	0.99998	8581	-0.1346
NLN	6	0.26702	0.46264	0.99998	8582	0.0947
CYP3A43	6	0.26702	0.46264	0.99998	8583	0.207

KRT80	6	0.26702	0.46264	0.99998	8584	-0.0112
PDE9A	6	0.26704	0.46268	0.99998	8585	-0.0046
hsa-mir-373	4	0.26707	0.39741	0.99998	8586	-0.0935
PPRC1	6	0.26711	0.46278	0.99998	8587	-0.104
INSL5	6	0.26713	0.46279	0.99998	8588	0.059
SLC26A10	6	0.26724	0.46294	0.99998	8589	0.1004
KCTD13	6	0.26732	0.46304	0.99998	8590	-0.0827
RNF123	4	0.26736	0.39773	0.99998	8591	0.1465
ZFP64	6	0.26737	0.46311	0.99998	8592	0.0293
SMTNL2	6	0.26738	0.46312	0.99998	8593	0.0197
STXBP6	6	0.26751	0.46329	0.99998	8594	-0.1799
CADM1	6	0.26757	0.46338	0.99998	8595	0.2003
NLRP11	6	0.26767	0.46351	0.99998	8596	0.0533
MARCH3	6	0.26779	0.46368	0.99998	8597	0.0632
ANKRD7	6	0.26783	0.46374	0.99998	8598	0.0684
PTPN18	6	0.26783	0.46374	0.99998	8599	-0.1285
BCAP29	6	0.26783	0.46374	0.99998	8600	-0.0949
SLC6A9	6	0.26783	0.46374	0.99998	8601	-0.1223
CWH43	6	0.26791	0.46386	0.99998	8602	-0.0756
RCN3	5	0.26792	0.42511	0.99998	8603	-0.1444
hsa-mir-5586	4	0.26805	0.39847	0.99998	8604	-0.1563
TAT	6	0.26809	0.46408	0.99998	8605	0.0172
NME9	6	0.26814	0.46415	0.99998	8606	-0.1634
LY75-CD302	4	0.26822	0.39866	0.99998	8607	-0.0815
MAP3K8	6	0.26824	0.46428	0.99998	8608	0.1353
FAM103A1	6	0.26835	0.46441	0.99998	8609	-0.0542
SDHAF2	6	0.26835	0.46441	0.99998	8610	-0.1449
HOXD9	6	0.26837	0.46444	0.99998	8611	-0.0536
PEG10	6	0.26837	0.46444	0.99998	8612	-0.0845
hsa-mir-6128	4	0.26838	0.39885	0.99998	8613	-0.2263
CYP26A1	6	0.26845	0.46455	0.99998	8614	-0.0991
NPR1	6	0.26857	0.46472	0.99998	8615	-0.0396
KIF3A	6	0.26859	0.46475	0.99998	8616	-0.0302
hsa-mir-1229	4	0.2686	0.39908	0.99998	8617	-0.1183
MME	6	0.26865	0.46482	0.99998	8618	-0.015
COASY	6	0.26865	0.46482	0.99998	8619	-0.1546
TIMP4	6	0.26867	0.46486	0.99998	8620	0.0321
SULT1A4	4	0.26875	0.39924	0.99998	8621	0.0643
LYL1	6	0.2688	0.46503	0.99998	8622	-0.0131
IMMP1L	6	0.2688	0.46503	0.99998	8623	-0.2343
MICA	6	0.26891	0.46514	0.99998	8624	0.1346
MUC16	6	0.26899	0.46525	0.99998	8625	0.0967
MFSD1	6	0.26901	0.46527	0.99998	8626	-0.1009
FIBIN	6	0.26901	0.46527	0.99998	8627	-0.1285
SP1	6	0.26903	0.46529	0.99998	8628	-0.1272
hsa-mir-3677	4	0.26904	0.39958	0.99998	8629	-0.0165
CDK19	6	0.26911	0.46539	0.99998	8630	0.0014
CCDC181	6	0.26917	0.46548	0.99998	8631	-0.037
SSFA2	6	0.26925	0.46557	0.99998	8632	-0.0893
PCDHB5	6	0.26925	0.46557	0.99998	8633	-0.0589
TDP1	6	0.26931	0.46566	0.99998	8634	0.0829
DDX17	6	0.26931	0.46566	0.99998	8635	0.2555
C17orf72	6	0.26931	0.46566	0.99998	8636	0.0088
DARS2	6	0.26937	0.46573	0.99998	8637	-0.0727
SCTR	6	0.26949	0.46589	0.99998	8638	-0.0755
RAB33A	6	0.26953	0.46594	0.99998	8639	-0.2082
NMRAL1	6	0.26958	0.466	0.99998	8640	0.0182
EXOSC3	6	0.2696	0.46604	0.99998	8641	0.1979
SLC22A16	6	0.26969	0.46614	0.99998	8642	-0.0382
TMEM200B	6	0.26971	0.46618	0.99998	8643	0.0539
ZNF667	6	0.26977	0.46625	0.99998	8644	0.0292
TCN1	6	0.26981	0.46632	0.99998	8645	-0.0111
TMEM141	6	0.26984	0.46636	0.99998	8646	-0.0272
GCSAM	6	0.26986	0.46637	0.99998	8647	0.0777
GATA6	6	0.2699	0.46642	0.99998	8648	-0.1086
hsa-mir-320b-	3	0.27003	0.3737	0.99998	8649	0.2028
MIA3	6	0.2701	0.46668	0.99998	8650	-0.1732
PKIG	6	0.2701	0.46668	0.99998	8651	-0.1695
CENPT	6	0.27013	0.46672	0.99998	8652	0.2851
TMEM25	6	0.27013	0.46672	0.99998	8653	-0.0905
NT5DC3	6	0.27026	0.46687	0.99998	8654	0.0744
RAB37	6	0.27026	0.46687	0.99998	8655	-0.0052
ZNF462	6	0.27036	0.46699	0.99998	8656	0.0552
CCDC113	6	0.27036	0.46699	0.99998	8657	-0.1387
TMBIM4	6	0.27042	0.46707	0.99998	8658	-0.0955
GOLGA7	6	0.27054	0.4672	0.99998	8659	0.1295
TSPAN1	6	0.27056	0.46722	0.99998	8660	-0.0717
RREB1	6	0.27056	0.46722	0.99998	8661	0.1834
hsa-mir-6735	4	0.27076	0.40151	0.99998	8662	-0.1271
VENTX	6	0.27082	0.46756	0.99998	8663	-0.1722
GPX1	6	0.27082	0.46756	0.99998	8664	-0.1355

SNAPIN	6	0.27097	0.46778	0.99998	8665	-0.1487
hsa-mir-4426	3	0.27098	0.37425	0.99998	8666	-0.1262
OC90	6	0.27113	0.46797	0.99998	8667	-0.321
PIK3C2B	6	0.27113	0.46797	0.99998	8668	0.0471
AANAT	6	0.27116	0.46801	0.99998	8669	0.019
HAUS6	6	0.27129	0.46817	0.99998	8670	0.32
CGGBP1	6	0.27131	0.46819	0.99998	8671	0.0215
FAM200B	6	0.27144	0.46836	0.99998	8672	-0.0358
FAM50B	6	0.27144	0.46836	0.99998	8673	-0.103
hsa-mir-6733	4	0.27156	0.40241	0.99998	8674	-0.1558
RAPGEF3	6	0.27158	0.46852	0.99998	8675	-0.1214
TGOLN2	6	0.2716	0.46854	0.99998	8676	-0.0645
CBX1	6	0.2716	0.46854	0.99998	8677	-0.0251
FCGBP	6	0.2716	0.46854	0.99998	8678	-0.1358
PCDHA1	2	0.27163	0.29912	0.99998	8679	-0.0152
PROC	6	0.27177	0.46876	0.99998	8680	-0.1308
TEX264	6	0.27182	0.46883	0.99998	8681	-0.0035
GCDH	6	0.27191	0.46893	0.99998	8682	-0.1034
MS4A1	6	0.27191	0.46893	0.99998	8683	-0.1267
EDDM3A	6	0.27191	0.46893	0.99998	8684	-0.1145
BAZ2B	6	0.27191	0.46893	0.99998	8685	-0.1833
NAT2	6	0.27196	0.469	0.99998	8686	-0.1407
TTLL7	6	0.272	0.46904	0.99998	8687	-0.0099
C11orf58	6	0.2721	0.46916	0.99998	8688	0.0096
CDK18	6	0.27212	0.4692	0.99998	8689	-0.1772
CAMP	6	0.27212	0.4692	0.99998	8690	-0.0816
OSBPL2	6	0.27213	0.46922	0.99998	8691	-0.0845
KLHDC8B	6	0.27218	0.46927	0.99998	8692	-0.1655
TRPV5	6	0.27228	0.4694	0.99998	8693	-0.1432
PRAMEF8	4	0.2723	0.40324	0.99998	8694	-0.2158
CHPT1	6	0.27233	0.46947	0.99998	8695	-0.0902
JAKMIP2	6	0.27233	0.46947	0.99998	8696	0.3981
hsa-mir-4799	4	0.27241	0.40336	0.99998	8697	-0.1303
DEPTOR	6	0.27252	0.46972	0.99998	8698	0.1238
SLC52A2	6	0.27264	0.46986	0.99998	8699	-0.0927
RALGPS2	6	0.27268	0.46991	0.99998	8700	-0.1857
WDR6	6	0.27268	0.46991	0.99998	8701	-0.26
TRIM39	2	0.27271	0.30001	0.99998	8702	-0.0286
RPS2	6	0.27274	0.46998	0.99998	8703	-0.1019
NUTM2A	5	0.27274	0.42949	0.99998	8704	-0.1631
NEK5	6	0.27283	0.4701	0.99998	8705	0.2509
YRDC	6	0.27284	0.47011	0.99998	8706	0.0859
PIM1	6	0.27298	0.47029	0.99998	8707	-0.0574
DSCR3	6	0.27298	0.47029	0.99998	8708	-0.0034
CCDC82	6	0.27308	0.47043	0.99998	8709	-0.0946
hsa-mir-4690	4	0.27309	0.40412	0.99998	8710	-0.3532
ADCY6	6	0.27311	0.47046	0.99998	8711	0.2479
PGK2	6	0.27311	0.47046	0.99998	8712	0.3388
DPPA5	6	0.27326	0.47065	0.99998	8713	-0.0754
NSUN3	6	0.2733	0.4707	0.99998	8714	-0.0621
KIF5C	6	0.2733	0.47071	0.99998	8715	-0.1262
TMEM237	6	0.2733	0.47071	0.99998	8716	-0.075
FGD6	6	0.27346	0.47091	0.99998	8717	-0.0061
FABP12	6	0.27351	0.47097	0.99998	8718	-0.1132
AADACL2	6	0.27359	0.47108	0.99998	8719	0.2165
SLC38A1	6	0.27359	0.47108	0.99998	8720	0.0273
C4orf21	6	0.27364	0.47114	0.99998	8721	-0.1308
ORAI1	6	0.27364	0.47114	0.99998	8722	-0.0673
GALK1	6	0.27382	0.47136	0.99998	8723	0.0284
ATP6AP1L	6	0.27382	0.47136	0.99998	8724	-0.0903
C6orf7	6	0.27382	0.47136	0.99998	8725	-0.0669
IRF2BP2	6	0.27384	0.47138	0.99998	8726	-0.178
LGALS1	6	0.27384	0.47138	0.99998	8727	-0.029
SMS	6	0.27384	0.47138	0.99998	8728	-0.1592
TMEM87A	6	0.27387	0.47143	0.99998	8729	0.1228
POMK	4	0.27402	0.40515	0.99998	8730	-0.1055
LILRA4	6	0.27404	0.47166	0.99998	8731	0.1259
NT5C3A	6	0.27404	0.47166	0.99998	8732	0.035
ZNF511	6	0.27405	0.47167	0.99998	8733	-0.1395
ARL5A	6	0.27405	0.47167	0.99998	8734	-0.0102
HAS3	6	0.27428	0.47198	0.99998	8735	-0.1788
HLF	6	0.27439	0.47212	0.99998	8736	-0.0924
ZCCHC13	6	0.27439	0.47212	0.99998	8737	-0.1317
ZFP2	6	0.27441	0.47214	0.99998	8738	0.0386
TCF24	6	0.27451	0.47228	0.99998	8739	-0.116
FITM2	6	0.2746	0.4724	0.99998	8740	-0.1635
PNPO	6	0.2746	0.4724	0.99998	8741	-0.1398
MYOZ2	6	0.27463	0.47243	0.99998	8742	-0.0275
DCT	6	0.27465	0.47246	0.99998	8743	-0.0645
EYA2	4	0.27474	0.40593	0.99998	8744	0.1393
NAGPA	6	0.27483	0.47269	0.99998	8745	0.0743

CACNG3	6	0.27483	0.47269	0.99998	8746	-0.0251
RANBP10	6	0.2749	0.47279	0.99998	8747	-0.2224
KIF3C	6	0.2749	0.47279	0.99998	8748	-0.1121
LLPH	6	0.2749	0.47279	0.99998	8749	-0.1676
NMNAT2	6	0.2749	0.47279	0.99998	8750	-0.0783
ZBTB1	6	0.2749	0.47279	0.99998	8751	-0.226
AGPAT4	6	0.2749	0.47279	0.99998	8752	-0.1482
hsa-mir-4701	4	0.27493	0.40616	0.99998	8753	0.1091
LRRC14	6	0.27494	0.47283	0.99998	8754	-0.0557
TRABD	6	0.27495	0.47284	0.99998	8755	0.0104
SMARCD3	6	0.27509	0.47303	0.99998	8756	0.2752
OAS3	6	0.27522	0.47319	0.99998	8757	-0.0956
DSE	6	0.27522	0.47319	0.99998	8758	-0.1923
SSPN	6	0.27522	0.47319	0.99998	8759	-0.156
ZFX	6	0.27535	0.47336	0.99998	8760	-0.0079
ZNF605	6	0.27536	0.47338	0.99998	8761	0.1255
SFR1	6	0.27536	0.47338	0.99998	8762	-0.0033
NEU4	6	0.27546	0.4735	0.99998	8763	0.0059
PLA2G2F	6	0.27546	0.4735	0.99998	8764	-0.076
FOXK2	6	0.27546	0.4735	0.99998	8765	-0.0099
TLR7	6	0.27546	0.4735	0.99998	8766	-0.0688
B2M	6	0.27549	0.47352	0.99998	8767	0.0183
hsa-mir-557	4	0.27554	0.40682	0.99998	8768	-0.0095
EBAG9	6	0.27555	0.47361	0.99998	8769	-0.0466
TMEM134	6	0.2756	0.47368	0.99998	8770	0.0141
CYP4A11	6	0.2756	0.47368	0.99998	8771	-0.2431
KCNG1	6	0.27566	0.47376	0.99998	8772	-0.0823
FKBP9	6	0.2757	0.47381	0.99998	8773	-0.1027
RNASEH2B	6	0.27594	0.47413	0.99998	8774	-0.0071
ATP5L	4	0.27598	0.40733	0.99998	8775	-0.2105
FCER1G	6	0.27598	0.47417	0.99998	8776	0.0945
JAG1	6	0.27604	0.47423	0.99998	8777	-0.0979
ATPBD4	2	0.27608	0.30278	0.99998	8778	-0.4307
hsa-mir-4672	4	0.27609	0.40746	0.99998	8779	0.3507
RB1CC1	6	0.27617	0.47439	0.99998	8780	0.0952
LRRIQ4	6	0.27617	0.47439	0.99998	8781	0.1188
PTCHD2	6	0.27621	0.47445	0.99998	8782	-0.0742
C15orf48	6	0.27621	0.47445	0.99998	8783	-0.1543
C9orf47	6	0.27622	0.47446	0.99998	8784	-0.0818
SORL1	6	0.27632	0.4746	0.99998	8785	-0.045
LRP2BP	6	0.27638	0.47466	0.99998	8786	-0.2043
AKNAD1	6	0.2764	0.47469	0.99998	8787	-0.0423
PAGE1	6	0.27656	0.4749	0.99998	8788	0.3847
CDKAL1	6	0.27656	0.4749	0.99998	8789	-0.0526
GMPR	6	0.27672	0.47511	0.99998	8790	-0.0505
KIN	6	0.27672	0.47511	0.99998	8791	-1E-05
MVB12B	6	0.2768	0.47521	0.99998	8792	0.1971
OR5D18	6	0.27684	0.47525	0.99998	8793	-0.0611
ADM	6	0.2769	0.47534	0.99998	8794	0.0507
MOV10	6	0.27691	0.47535	0.99998	8795	0.1109
PRSS36	6	0.27691	0.47535	0.99998	8796	-0.0124
hsa-mir-618	4	0.27697	0.40846	0.99998	8797	0.0231
TOX2	6	0.27698	0.47544	0.99998	8798	0.1737
TCL1B	6	0.27699	0.47545	0.99998	8799	-0.0144
CHRND	6	0.27701	0.47548	0.99998	8800	0.099
ZNF184	6	0.27701	0.47548	0.99998	8801	-0.0234
WDR5	6	0.27706	0.47555	0.99998	8802	-0.1589
GABARAPL1	6	0.27708	0.47557	0.99998	8803	0.0283
hsa-mir-7847	4	0.2771	0.40862	0.99998	8804	-0.1723
HCST	6	0.27715	0.47565	0.99998	8805	-0.1151
MAP2K3	6	0.27715	0.47565	0.99998	8806	0.035
UPF3A	6	0.27715	0.47565	0.99998	8807	-0.0526
hsa-mir-551b	4	0.27722	0.40876	0.99998	8808	0.1063
OSBPL5	6	0.27722	0.47574	0.99998	8809	-0.0571
KRT6A	5	0.27723	0.43366	0.99998	8810	-0.0571
GOLM1	6	0.27725	0.47579	0.99998	8811	0.0072
C17orf99	6	0.2773	0.47584	0.99998	8812	-0.0222
TIMP2	6	0.2773	0.47584	0.99998	8813	0.2271
METAP2	6	0.27733	0.4759	0.99998	8814	-0.1441
CAMKMT	6	0.27733	0.4759	0.99998	8815	-0.1713
ANP32D	6	0.27741	0.47599	0.99998	8816	-0.1079
PREX1	6	0.27741	0.47599	0.99998	8817	0.0875
ITM2A	6	0.27741	0.476	0.99998	8818	-0.0656
CCDC108	6	0.27741	0.476	0.99998	8819	-0.0422
SS18	6	0.27749	0.47609	0.99998	8820	-0.146
NDUFAF2	6	0.27761	0.47625	0.99998	8821	-0.2856
KLC3	6	0.27761	0.47625	0.99998	8822	-0.0934
ACACB	6	0.27761	0.47625	0.99998	8823	-0.0066
CCDC85B	6	0.27765	0.47631	0.99998	8824	0.1651
SNX20	6	0.27765	0.47631	0.99998	8825	0.0369
ANO5	6	0.27765	0.47631	0.99998	8826	-0.02

PPP2R2B	6	0.2777	0.47637	0.99998	8827	-0.0829
KLK14	4	0.27775	0.40938	0.99998	8828	-0.127
PRDM2	6	0.27789	0.47661	0.99998	8829	0.0189
CDC20B	6	0.27798	0.47673	0.99998	8830	-0.0498
LAMA5	6	0.27809	0.47688	0.99998	8831	-0.1391
FBXL22	6	0.27814	0.47695	0.99998	8832	-0.1568
SPIN2A	1	0.27815	0.27802	0.99998	8833	-1.4702
hsa-mir-516a-	1	0.27815	0.27802	0.99998	8834	-1.4702
GOLGA6L6	1	0.27815	0.27802	0.99998	8835	-1.4702
SPRR2E	1	0.27815	0.27802	0.99998	8836	-1.4702
hsa-mir-1303	4	0.27815	0.40984	0.99998	8837	0.1211
ZNF768	6	0.27823	0.47706	0.99998	8838	0.1528
DGKB	6	0.27823	0.47706	0.99998	8839	-0.0598
IL37	6	0.27827	0.47711	0.99998	8840	-0.0291
MLLT11	6	0.27837	0.47723	0.99998	8841	-0.0769
AMOT	6	0.27837	0.47723	0.99998	8842	-0.0593
FOXB1	6	0.27839	0.47726	0.99998	8843	-0.0253
CABP7	6	0.27847	0.47737	0.99998	8844	-0.0423
hsa-mir-5703	4	0.27849	0.41023	0.99998	8845	0.0101
hsa-mir-623	4	0.27849	0.41023	0.99998	8846	-0.0599
ZNF821	6	0.27853	0.47745	0.99998	8847	-0.0082
CCL13	6	0.27853	0.47745	0.99998	8848	-0.1671
CLEC12B	6	0.27863	0.47756	0.99998	8849	-0.175
FAM98A	6	0.27863	0.47756	0.99998	8850	-0.0256
TNFRSF12A	6	0.27869	0.47763	0.99998	8851	-0.0083
XKR8	6	0.27875	0.4777	0.99998	8852	-0.0951
SPIN4	6	0.27875	0.47771	0.99998	8853	0.1153
SPATA31A4	5	0.2788	0.4351	0.99998	8854	0.0823
PPP3CA	6	0.27887	0.47787	0.99998	8855	0.0237
MAPK8IP2	6	0.27889	0.4779	0.99998	8856	-0.0669
MEX3D	6	0.27889	0.4779	0.99998	8857	-0.0524
B4GALNT4	6	0.27895	0.47798	0.99998	8858	0.2456
KLF9	6	0.27896	0.47801	0.99998	8859	-0.155
NTN3	6	0.27896	0.47801	0.99998	8860	-0.1537
LGMN	6	0.27905	0.47812	0.99998	8861	-0.1183
SNX5	6	0.27912	0.47821	0.99998	8862	-0.0971
PEX14	6	0.27914	0.47823	0.99998	8863	0.0279
CENPK	6	0.27914	0.47823	0.99998	8864	-0.0852
CD34	6	0.27914	0.47823	0.99998	8865	-0.1171
ZNF423	6	0.27916	0.47826	0.99998	8866	0.0602
COL21A1	6	0.27925	0.47837	0.99998	8867	0.2424
ASB1	6	0.27926	0.47838	0.99998	8868	-0.0771
hsa-mir-6887	4	0.27932	0.41115	0.99998	8869	-0.0469
PDGFRL	6	0.27935	0.47848	0.99998	8870	-0.0509
LRRC14B	6	0.27936	0.4785	0.99998	8871	0.0374
CCL20	6	0.27939	0.47852	0.99998	8872	0.0019
APOE	6	0.27948	0.47864	0.99998	8873	0.0262
PNP	6	0.2795	0.47866	0.99998	8874	0.0392
PYCR2	6	0.2795	0.47866	0.99998	8875	-0.0575
MTA2	6	0.27956	0.47874	0.99998	8876	0.1589
SMARCA2	6	0.27966	0.47888	0.99998	8877	0.1944
PILRB	6	0.2797	0.47893	0.99998	8878	-0.0168
PPP1R7	6	0.2798	0.47907	0.99998	8879	-0.1593
COX6B1	6	0.2798	0.47907	0.99998	8880	-0.2202
USP17L10	5	0.27982	0.43604	0.99998	8881	-0.0642
SNX31	6	0.27982	0.4791	0.99998	8882	0.0775
EIF4EBP2	6	0.27988	0.47916	0.99998	8883	0.0415
KIFC3	6	0.27999	0.4793	0.99998	8884	-0.1446
SCN3A	6	0.28003	0.47935	0.99998	8885	-0.0372
hsa-mir-4312	4	0.28003	0.41194	0.99998	8886	0.0973
TTC39C	6	0.28004	0.47936	0.99998	8887	0.0622
CYP27C1	6	0.28004	0.47936	0.99998	8888	0.0161
ISM1	6	0.28005	0.47938	0.99998	8889	-0.0197
THEM5	6	0.28016	0.4795	0.99998	8890	-0.1177
SGSM2	6	0.28019	0.47954	0.99998	8891	-0.1758
ZMAT3	6	0.28026	0.47965	0.99998	8892	-0.032
RGS4	6	0.28027	0.47966	0.99998	8893	0.1266
MAP3K3	6	0.28027	0.47966	0.99998	8894	-0.0738
CRISP1	6	0.28039	0.47982	0.99998	8895	0.0879
FMNL3	6	0.28039	0.47982	0.99998	8896	-0.1366
FGFBP2	6	0.28053	0.47994	0.99998	8897	0.3895
KCTD3	4	0.28058	0.41258	0.99998	8898	-0.106
CTF1	6	0.28069	0.48006	0.99998	8899	0.0484
FBXL14	6	0.28075	0.48011	0.99998	8900	-0.1334
GTDC2	4	0.28079	0.41283	0.99998	8901	-0.0775
NDUFS2	6	0.28081	0.48015	0.99998	8902	-0.0369
CRB1	6	0.28082	0.48016	0.99998	8903	-0.0228
DUOXA1	6	0.28091	0.48021	0.99998	8904	-0.0702
OR4S2	6	0.28103	0.4803	0.99998	8905	-0.0807
EFHD1	6	0.28104	0.48031	0.99998	8906	-0.1102
VASH2	6	0.28113	0.48037	0.99998	8907	-0.0383

MS4A13	6	0.28113	0.48037	0.99998	8908	0.1941
hsa-mir-548p	2	0.28115	0.30687	0.99998	8909	-0.1282
DLL4	4	0.28123	0.41331	0.99998	8910	-0.1241
ZNF273	6	0.28126	0.48045	0.99998	8911	0.3439
RASL11B	6	0.28126	0.48045	0.99998	8912	0.0338
MMP12	6	0.28129	0.48047	0.99998	8913	-0.0815
FAM65B	6	0.28129	0.48047	0.99998	8914	0.1843
FLJ44635	6	0.28136	0.48052	0.99998	8915	0.0153
VWA3B	6	0.28148	0.4806	0.99998	8916	-0.1704
DEFA4	6	0.2815	0.48063	0.99998	8917	-0.048
MRPL48	6	0.28152	0.48064	0.99998	8918	0.3072
hsa-mir-1322	1	0.28163	0.28146	0.99998	8919	-0.2104
CASP12	6	0.28165	0.48072	0.99998	8920	0.2468
ANTXR1	6	0.28165	0.48072	0.99998	8921	-0.112
SCUBE2	6	0.28172	0.48079	0.99998	8922	0.09
RABL6	6	0.2818	0.48084	0.99998	8923	0.1286
GALNT3	6	0.28185	0.48087	0.99998	8924	0.0953
NR1H3	6	0.28198	0.48097	0.99998	8925	-0.0158
FOXO4	6	0.28198	0.48097	0.99998	8926	-0.0434
ZNF622	6	0.28198	0.48097	0.99998	8927	-0.0761
hsa-mir-575	4	0.28201	0.41416	0.99998	8928	-0.1009
POU1F1	6	0.28237	0.48128	0.99998	8929	-0.03
SEL1L3	6	0.28253	0.48139	0.99998	8930	-0.042
AGPAT9	6	0.28257	0.48142	0.99998	8931	0.0723
OR1E2	6	0.28271	0.48152	0.99998	8932	-0.0602
SEMA4B	6	0.2829	0.48166	0.99998	8933	-0.0138
BLOC1S6	4	0.28294	0.41521	0.99998	8934	-0.1419
DVL1	6	0.28324	0.48191	0.99998	8935	0.0983
TICAM1	6	0.28324	0.48191	0.99998	8936	0.3068
REEP2	6	0.28324	0.48191	0.99998	8937	0.0219
ASTN2	6	0.28324	0.48191	0.99998	8938	0.0021
KRTAP10-4	6	0.28367	0.48221	0.99998	8939	0.2974
P2RY14	6	0.28396	0.48243	0.99998	8940	-0.0259
hsa-mir-7851	3	0.28398	0.38182	0.99998	8941	-0.1224
USP15	6	0.28411	0.48253	0.99998	8942	-0.0237
RMDN3	6	0.28411	0.48253	0.99998	8943	0.0808
SDK2	6	0.28419	0.48259	0.99998	8944	-0.1285
VSIG10	6	0.28421	0.48261	0.99998	8945	-0.0683
HIST1H2AI	2	0.28423	0.30939	0.99998	8946	-1.5101
PLXDC1	6	0.2843	0.48267	0.99998	8947	0.1802
FLI1	6	0.28431	0.48269	0.99998	8948	-0.1796
CCL25	6	0.28447	0.4828	0.99998	8949	-0.0655
CYLD	6	0.28447	0.4828	0.99998	8950	0.0695
MASTL	6	0.28454	0.48286	0.99998	8951	0.0497
FOXD3	6	0.28468	0.48295	0.99998	8952	0.0339
AES	6	0.2847	0.48297	0.99998	8953	-0.1643
ART4	6	0.2847	0.48297	0.99998	8954	-0.0316
hsa-mir-642a	1	0.28472	0.28454	0.99998	8955	-0.1274
PTGS1	6	0.2848	0.48303	0.99998	8956	0.2161
hsa-mir-23a	4	0.28481	0.41727	0.99998	8957	0.042
OR4K1	6	0.28492	0.48312	0.99998	8958	0.1226
TIGD5	6	0.28505	0.48323	0.99998	8959	0.1235
CCDC151	6	0.28507	0.48324	0.99998	8960	-0.1378
SPOPL	6	0.28523	0.48337	0.99998	8961	-0.0541
RPS10-NUDT3	2	0.28533	0.31031	0.99998	8962	-0.0954
ATG2B	6	0.28545	0.48352	0.99998	8963	0.0872
SLAMF1	6	0.28545	0.48352	0.99998	8964	0.1102
TPRX1	6	0.28553	0.48359	0.99998	8965	0.1045
KLHL31	6	0.28564	0.48368	0.99998	8966	0.1003
hsa-mir-187	4	0.28571	0.41826	0.99998	8967	-0.1084
ACAD11	6	0.28584	0.48382	0.99998	8968	0.0314
THBS4	6	0.28592	0.48388	0.99998	8969	0.0207
VPS45	6	0.28592	0.48388	0.99998	8970	-0.068
ZNF700	5	0.28601	0.44175	0.99998	8971	0.1799
PSMD11	6	0.28604	0.48397	0.99998	8972	-0.0185
ALAS2	6	0.28607	0.48399	0.99998	8973	-0.0068
hsa-mir-124-3	3	0.28612	0.38304	0.99998	8974	0.084
CDH10	6	0.28623	0.48412	0.99998	8975	0.0892
ROBO2	6	0.28635	0.4842	0.99998	8976	0.3398
RAD9B	6	0.28635	0.4842	0.99998	8977	-0.1204
TAS2R30	6	0.28636	0.48421	0.99998	8978	-0.1488
ADRM1	6	0.28643	0.48426	0.99998	8979	0.1265
LRRN2	6	0.28659	0.48438	0.99998	8980	0.0721
CD28	6	0.28665	0.48442	0.99998	8981	0.053
MAGED4B	2	0.28668	0.3114	0.99998	8982	-0.1147
C15orf60	6	0.2868	0.48453	0.99998	8983	0.1906
RECQL5	6	0.28691	0.4846	0.99998	8984	0.1796
GBP1	6	0.28691	0.4846	0.99998	8985	-0.1784
STAB2	6	0.28698	0.48466	0.99998	8986	0.0406
TTPA	6	0.28704	0.4847	0.99998	8987	-0.136
OR5L1	6	0.28724	0.48485	0.99998	8988	-0.073

hsa-mir-342	4	0.28734	0.42003	0.99998	8989	-0.179
ZNF549	6	0.28745	0.48503	0.99998	8990	0.1587
hsa-mir-2277	4	0.28749	0.42021	0.99998	8991	0.1087
CHST5	6	0.28761	0.48515	0.99998	8992	0.0361
ITPR2	6	0.28773	0.48523	0.99998	8993	0.0386
DYNC1LI1	6	0.28785	0.48532	0.99998	8994	0.1596
TSEN54	4	0.28787	0.42063	0.99998	8995	-0.2279
MN1	6	0.2879	0.48537	0.99998	8996	-0.1699
HTT	6	0.2879	0.48537	0.99998	8997	0.0045
DNAJA1	6	0.2879	0.48537	0.99998	8998	-0.0072
C10orf114	3	0.28795	0.38411	0.99998	8999	-0.1198
RGS18	6	0.28796	0.48542	0.99998	9000	0.0511
NGFR	6	0.28802	0.48546	0.99998	9001	-0.1326
VTN	6	0.28812	0.48552	0.99998	9002	-0.0747
DPY19L4	6	0.28828	0.48564	0.99998	9003	-0.0885
MRPL41	6	0.28832	0.48567	0.99998	9004	-0.0717
hsa-mir-6759	4	0.28836	0.42117	0.99998	9005	0.0927
PPIA	6	0.28847	0.48579	0.99998	9006	0.1448
TNNI2	6	0.2886	0.48587	0.99998	9007	0.2977
PAPD7	6	0.2886	0.48587	0.99998	9008	0.3068
MPDZ	6	0.28865	0.48591	0.99998	9009	-0.0785
ESRP1	6	0.28869	0.48594	0.99998	9010	-0.1404
DMXL1	6	0.28876	0.48599	0.99998	9011	-0.0412
MAP7D3	6	0.28881	0.48602	0.99998	9012	0.1335
hsa-mir-3132	4	0.28896	0.4218	0.99998	9013	-0.1754
ZNF75D	6	0.28896	0.48615	0.99998	9014	0.1938
HARS2	6	0.28896	0.48615	0.99998	9015	-0.0349
RBBP8NL	6	0.28896	0.48615	0.99998	9016	0.0594
FBXO30	6	0.28916	0.48631	0.99998	9017	0.2194
SRPK2	6	0.28916	0.48631	0.99998	9018	-0.125
SPATA7	6	0.28916	0.48631	0.99998	9019	-0.0419
YIPF4	6	0.2892	0.48634	0.99998	9020	-0.0063
PAX9	6	0.28924	0.48637	0.99998	9021	0.0435
PRAME	6	0.2893	0.48642	0.99998	9022	-0.0132
COL2A1	6	0.28952	0.48659	0.99998	9023	0.1845
BMP4	6	0.28971	0.48672	0.99998	9024	0.0867
MRPS26	6	0.28971	0.48672	0.99998	9025	-0.0403
ZFP36L1	6	0.28971	0.48672	0.99998	9026	0.0221
hsa-mir-4443	4	0.28972	0.42264	0.99998	9027	-0.0596
FAM228B	6	0.28978	0.48678	0.99998	9028	-0.1823
SLC39A9	6	0.28999	0.48693	0.99998	9029	-0.1535
YBX3	6	0.2902	0.48707	0.99998	9030	-0.0713
ELMO3	6	0.2902	0.48707	0.99998	9031	0.0861
hsa-mir-3174	4	0.29028	0.42328	0.99998	9032	-0.1305
OR8G1	5	0.29032	0.44573	0.99998	9033	-0.1295
ADAMTS13	6	0.29041	0.48722	0.99998	9034	-0.0238
TBC1D23	6	0.29044	0.48725	0.99998	9035	-0.0803
PDCL2	6	0.29064	0.4874	0.99998	9036	-0.0851
SCN1B	6	0.29077	0.48749	0.99998	9037	0.4346
GCAT	6	0.2908	0.48751	0.99998	9038	-0.0505
PBRM1	6	0.2908	0.48752	0.99998	9039	0.0925
ACOT6	6	0.29092	0.48761	0.99998	9040	0.0808
CEP350	6	0.29113	0.48775	0.99998	9041	-0.1505
ZNF354A	6	0.29113	0.48775	0.99998	9042	-0.0862
C20orf78	6	0.29113	0.48775	0.99998	9043	-0.0635
NRBF2	6	0.29122	0.48782	0.99998	9044	0.2231
GPR161	6	0.29122	0.48782	0.99998	9045	-0.0053
C7orf61	6	0.29137	0.48792	0.99998	9046	0.076
RUNDC3B	6	0.29144	0.48797	0.99998	9047	-0.058
RDH13	6	0.29149	0.488	0.99998	9048	0.0472
LEPREL2	6	0.29155	0.48805	0.99998	9049	-0.2225
39692	3	0.29166	0.38631	0.99998	9050	-0.052
SERPINI1	6	0.29167	0.48814	0.99998	9051	-0.1106
KRTAP5-2	6	0.29167	0.48814	0.99998	9052	0.3155
LSM3	6	0.29167	0.48814	0.99998	9053	0.1475
hsa-mir-210	4	0.29167	0.4248	0.99998	9054	-0.0827
BRD3	5	0.29179	0.44712	0.99998	9055	-0.059
UNC5CL	6	0.29182	0.48826	0.99998	9056	0.3056
GPR3	6	0.29185	0.48828	0.99998	9057	-0.0757
SLC4A7	6	0.2919	0.48833	0.99998	9058	0.1925
ZNF90	4	0.29193	0.42507	0.99998	9059	-0.1042
LMX1B	6	0.29204	0.48843	0.99998	9060	0.3405
CAPG	6	0.29204	0.48843	0.99998	9061	0.0911
MVD	6	0.29219	0.48855	0.99998	9062	-0.0955
THBD	6	0.29235	0.48865	0.99998	9063	0.0656
TRMT2A	6	0.29235	0.48865	0.99998	9064	-0.0707
TTR	6	0.29238	0.48867	0.99998	9065	-0.0631
ADAMTS5	6	0.29245	0.48872	0.99998	9066	-0.0318
hsa-mir-7114	4	0.29248	0.42568	0.99998	9067	0.2078
PRKAR2A	6	0.29251	0.48876	0.99998	9068	0.0339
LCN8	6	0.29268	0.48889	0.99998	9069	0.0067

CIDEC	6	0.29268	0.48889	0.99998	9070	0.0331
DNAJC6	6	0.29296	0.48908	0.99998	9071	-0.0701
SDCCAG3	6	0.29297	0.48909	0.99998	9072	-0.0731
hsa-mir-124-1	4	0.29305	0.42633	0.99998	9073	-0.1125
MFSD5	6	0.29315	0.4892	0.99998	9074	-0.1723
MAP7D2	6	0.29315	0.4892	0.99998	9075	-0.1544
NPIPL3	2	0.29321	0.31676	0.99998	9076	-0.0477
PET112	6	0.29334	0.48935	0.99998	9077	0.2118
hsa-mir-6874	4	0.29335	0.42666	0.99998	9078	-0.2042
LOC10050584	4	0.29337	0.42669	0.99998	9079	0.0592
HTR3A	6	0.29352	0.48949	0.99998	9080	0.045
WDR75	6	0.2936	0.48953	0.99998	9081	-0.1309
MTFR2	6	0.29369	0.4896	0.99998	9082	-0.0087
RAI14	6	0.29369	0.4896	0.99998	9083	0.1829
ZNF780B	5	0.29383	0.44897	0.99998	9084	-0.5261
NFATC2IP	6	0.29389	0.48977	0.99998	9085	0.2993
OR5T2	6	0.29404	0.48987	0.99998	9086	-0.0882
hsa-mir-217	4	0.29406	0.42743	0.99998	9087	0.0996
PLA2G5	6	0.29422	0.49	0.99998	9088	0.0693
C20orf195	6	0.29424	0.49003	0.99998	9089	-0.0416
WNT8A	6	0.29432	0.49009	0.99998	9090	-0.1603
IP6K1	6	0.29444	0.49017	0.99998	9091	0.0754
TTC32	6	0.29444	0.49017	0.99998	9092	0.1341
hsa-mir-6126	4	0.29447	0.42788	0.99998	9093	0.0521
RNF182	6	0.29447	0.49019	0.99998	9094	0.0742
NCAPH2	6	0.29463	0.49032	0.99998	9095	0.3477
FABP7	6	0.29463	0.49032	0.99998	9096	-0.0864
CPN1	6	0.29469	0.49037	0.99998	9097	-0.0797
OR4C12	6	0.29479	0.49044	0.99998	9098	0.1118
PDIA2	4	0.2949	0.42835	0.99998	9099	-0.1296
OR10V1	6	0.29493	0.49054	0.99998	9100	0.0829
GJB1	6	0.2951	0.49067	0.99998	9101	0.1708
LPCAT1	6	0.29511	0.49068	0.99998	9102	0.0214
hsa-mir-3126	4	0.29518	0.42866	0.99998	9103	-0.1286
SPIRE1	6	0.29529	0.4908	0.99998	9104	-0.0133
ABCB5	6	0.29539	0.49086	0.99998	9105	0.0018
SEZ6	6	0.29539	0.49086	0.99998	9106	0.029
NEO1	6	0.29539	0.49086	0.99998	9107	0.2422
JAK3	6	0.29572	0.49111	0.99998	9108	-0.0568
VOPP1	6	0.29572	0.49111	0.99998	9109	-0.0797
PSMD4	6	0.29572	0.49111	0.99998	9110	-0.0494
hsa-mir-4802	4	0.29574	0.42928	0.99998	9111	-0.1051
TRAPPC2L	6	0.29576	0.49115	0.99998	9112	0.0876
FOXN2	6	0.29586	0.49122	0.99998	9113	-0.0773
RAB34	3	0.29596	0.38888	0.99998	9114	-0.0056
LAMA3	6	0.296	0.49132	0.99998	9115	0.2046
HILPDA	6	0.296	0.49132	0.99998	9116	-0.1843
KDR	6	0.29603	0.49135	0.99998	9117	0.3007
TUSC1	6	0.29603	0.49135	0.99998	9118	0.136
POMZP3	5	0.29609	0.45111	0.99998	9119	-0.2164
CRYL1	6	0.29623	0.49149	0.99998	9120	0.1051
TMBIM6	6	0.29634	0.49156	0.99998	9121	0.1806
LRRC19	6	0.2964	0.49161	0.99998	9122	-0.0398
STXBP1	6	0.2965	0.49168	0.99998	9123	-0.0241
RBM46	6	0.29654	0.49171	0.99998	9124	-0.1103
ADH4	6	0.2966	0.49177	0.99998	9125	-0.0748
hsa-mir-193a	4	0.29663	0.43031	0.99998	9126	-0.1749
C19orf26	6	0.29665	0.4918	0.99998	9127	0.0142
ARHGAP36	6	0.29665	0.4918	0.99998	9128	0.1722
OR10H5	4	0.29668	0.43035	0.99998	9129	-0.2661
KCNK4	6	0.29679	0.49191	0.99998	9130	0.0578
KIAA0226	6	0.29679	0.49191	0.99998	9131	0.1127
CAMK2B	6	0.29679	0.49191	0.99998	9132	-0.0298
WRNIP1	6	0.297	0.49206	0.99998	9133	0.0009
FAM73B	6	0.29706	0.4921	0.99998	9134	0.0453
NMBR	4	0.29707	0.43078	0.99998	9135	-0.1564
EBF3	6	0.29721	0.49222	0.99998	9136	0.2361
C5orf34	6	0.29732	0.49232	0.99998	9137	0.0146
hsa-mir-4652	4	0.2974	0.43114	0.99998	9138	-0.5251
MAGEA8	4	0.2974	0.43114	0.99998	9139	-0.1705
TRMT44	6	0.29741	0.49238	0.99998	9140	0.2197
hsa-mir-7854	4	0.29742	0.43116	0.99998	9141	-0.007
MAP4	6	0.29743	0.49239	0.99998	9142	0.0717
FPR3	6	0.29743	0.49239	0.99998	9143	-0.1353
GLTP	6	0.29755	0.49247	0.99998	9144	-0.0701
PRKDC	6	0.29763	0.49254	0.99998	9145	-0.0769
C8orf44	5	0.29767	0.45255	0.99998	9146	-0.231
CHRNA6	5	0.29767	0.45255	0.99998	9147	0.102
DOCK3	6	0.29768	0.49258	0.99998	9148	-0.0228
OPHN1	6	0.29768	0.49258	0.99998	9149	0.0081
hsa-mir-3921	4	0.29777	0.43156	0.99998	9150	-0.1868

SRP68	6	0.29786	0.4927	0.99998	9151	0.0743
ASXL2	6	0.29786	0.4927	0.99998	9152	0.1519
BRINP2	4	0.29799	0.43181	0.99998	9153	0.0253
PRRG2	6	0.29805	0.49285	0.99998	9154	0.0489
SMCO3	6	0.29805	0.49285	0.99998	9155	-0.0579
ACCSL	6	0.29825	0.49299	0.99998	9156	0.1646
FBXO24	6	0.29825	0.49299	0.99998	9157	0.0209
ZNF492	5	0.29832	0.45315	0.99998	9158	-0.5071
LCE3B	6	0.29832	0.49304	0.99998	9159	0.1327
CIZ1	6	0.29847	0.49314	0.99998	9160	-0.0528
PHF2	4	0.2985	0.43236	0.99998	9161	0.0636
PPP2R3C	6	0.29857	0.49321	0.99998	9162	0.1083
ARSG	6	0.29857	0.49321	0.99998	9163	0.1038
PRPS2	6	0.29873	0.49333	0.99998	9164	0.0365
NME4	6	0.299	0.49355	0.99998	9165	0.1241
PCDHGA6	2	0.29901	0.32156	0.99998	9166	-0.0622
B4GALNT3	6	0.29904	0.49358	0.99998	9167	-0.2425
MRPL16	6	0.29904	0.49358	0.99998	9168	-0.1127
TUBA4A	6	0.29904	0.49358	0.99998	9169	-0.0121
LY6D	6	0.29904	0.49358	0.99998	9170	-0.0943
BLID	6	0.29904	0.49358	0.99998	9171	0.0026
RHNO1	6	0.29908	0.49361	0.99998	9172	-0.0085
CPNE5	6	0.29918	0.49369	0.99998	9173	-0.0622
PION	1	0.29921	0.29913	0.99998	9174	-0.2126
FXYD6	4	0.29926	0.43317	0.99998	9175	0.5128
CPLX3	6	0.29931	0.49379	0.99998	9176	0.044
NAIF1	6	0.29931	0.49379	0.99998	9177	-0.1393
SULT1C3	6	0.29935	0.49382	0.99998	9178	0.0193
DEC1	6	0.29948	0.49391	0.99998	9179	0.042
STK11IP	6	0.29949	0.49391	0.99998	9180	-0.0288
NDUFB3	6	0.29949	0.49391	0.99998	9181	0.3385
hsa-mir-934	4	0.29967	0.43366	0.99998	9182	0.0097
hsa-mir-6845	4	0.29968	0.43368	0.99998	9183	-0.3195
MLANA	6	0.29972	0.4941	0.99998	9184	0.3484
KIAA0513	6	0.29979	0.49416	0.99998	9185	-0.1105
DIS3L	6	0.30003	0.49433	0.99998	9186	0.1724
ASCL4	6	0.30014	0.49441	0.99998	9187	-0.2156
PLAUR	6	0.30034	0.49455	0.99998	9188	-0.127
TMEM132E	6	0.30038	0.49459	0.99998	9189	0.0593
PTS	6	0.30064	0.49479	0.99998	9190	0.0521
PGAP1	6	0.30064	0.49479	0.99998	9191	-0.0265
CNTFR	6	0.30065	0.49479	0.99998	9192	0.0193
hsa-mir-1208	4	0.30065	0.43471	0.99998	9193	0.06
ALDH5A1	6	0.30092	0.49499	0.99998	9194	0.2141
SUMF2	6	0.30092	0.49499	0.99998	9195	-0.0857
WFDC11	6	0.30099	0.49506	0.99998	9196	-0.0219
WEE1	6	0.30099	0.49506	0.99998	9197	0.0586
hsa-mir-6808	4	0.30111	0.4352	0.99998	9198	-0.1307
CAGE1	6	0.30123	0.49522	0.99998	9199	0.1225
DEDD2	6	0.30123	0.49522	0.99998	9200	-0.0549
PXDN	6	0.30124	0.49523	0.99998	9201	-0.0701
SSX5	4	0.30145	0.43557	0.99998	9202	-0.2991
NPM2	6	0.30157	0.4955	0.99998	9203	0.0282
KIR3DL3	6	0.30167	0.49556	0.99998	9204	0.0805
RUSC2	6	0.30167	0.49556	0.99998	9205	-0.0568
NASP	6	0.30167	0.49556	0.99998	9206	0.1614
ORM1	2	0.30169	0.32374	0.99998	9207	-0.7891
SLC35F2	6	0.30178	0.49564	0.99998	9208	0.2026
hsa-mir-4265	4	0.30186	0.43602	0.99998	9209	-0.023
CYBA	6	0.30187	0.49571	0.99998	9210	-0.0207
TPH2	6	0.30196	0.49577	0.99998	9211	0.1677
RNH1	6	0.30196	0.49577	0.99998	9212	-0.131
KIAA1377	6	0.302	0.4958	0.99998	9213	-0.048
ALAS1	6	0.30207	0.49585	0.99998	9214	-0.0443
PNPLA2	6	0.30219	0.49594	0.99998	9215	-0.0217
PRRG3	6	0.30227	0.49599	0.99998	9216	0.2143
CREG2	6	0.30235	0.49605	0.99998	9217	0.1925
FBLN5	6	0.30235	0.49605	0.99998	9218	0.5527
CD44	6	0.30235	0.49605	0.99998	9219	0.0717
ARHGEF9	6	0.30242	0.49611	0.99998	9220	0.0422
FLNA	6	0.30242	0.49611	0.99998	9221	-0.0955
hsa-mir-1185-	1	0.30256	0.30253	0.99998	9222	-0.5595
hsa-mir-4749	4	0.30264	0.43687	0.99998	9223	-0.0201
hsa-mir-1825	4	0.30266	0.4369	0.99998	9224	0.2442
TOP3A	6	0.30267	0.4963	0.99998	9225	-0.1381
C2CD4B	5	0.30292	0.45739	0.99998	9226	0.0696
GABRB1	6	0.30294	0.49651	0.99998	9227	0.1492
FAM217A	6	0.30294	0.49651	0.99998	9228	-0.0425
SPINK4	6	0.30308	0.4966	0.99998	9229	0.1553
TEAD1	6	0.30308	0.4966	0.99998	9230	0.1077
OR8K3	6	0.3032	0.49669	0.99998	9231	-0.0762

ALDH8A1	6	0.3032	0.49669	0.99998	9232	0.2577
ADAMTSL3	4	0.30327	0.43762	0.99998	9233	0.0137
hsa-mir-6825	4	0.30338	0.43774	0.99998	9234	-0.0164
hsa-mir-4697	4	0.3036	0.43798	0.99998	9235	-0.1636
BET3L	2	0.30361	0.32532	0.99998	9236	-0.053
LXN	6	0.30365	0.49703	0.99998	9237	-0.1059
SLITRK1	6	0.30366	0.49703	0.99998	9238	0.2162
SFI1	6	0.30372	0.49707	0.99998	9239	0.1333
ZBTB7B	6	0.30375	0.49711	0.99998	9240	0.3089
SERPINB2	6	0.30375	0.49711	0.99998	9241	-0.1502
THAP10	6	0.30385	0.49719	0.99998	9242	0.2284
CSNK1E	6	0.30389	0.49722	0.99998	9243	-0.0224
hsa-mir-614	4	0.30394	0.43839	0.99998	9244	-0.0472
KLK6	4	0.30417	0.43864	0.99998	9245	-0.0623
MTL5	6	0.3042	0.49746	0.99998	9246	0.1056
VPS18	6	0.3042	0.49747	0.99998	9247	-0.1539
UTY	5	0.30441	0.45875	0.99998	9248	0.2076
TMEM236	6	0.30446	0.49766	0.99998	9249	0.1029
SV2A	6	0.30462	0.49779	0.99998	9250	0.2233
ERCC1	6	0.30471	0.49786	0.99998	9251	-0.0644
ACADSB	6	0.30481	0.49792	0.99998	9252	0.1857
SBSPON	6	0.30482	0.49792	0.99998	9253	0.0291
GCGR	6	0.30489	0.49799	0.99998	9254	0.2052
PLD5	6	0.30499	0.49806	0.99998	9255	-0.0565
SLC30A8	6	0.30504	0.4981	0.99998	9256	0.1891
TNFAIP8L2	6	0.30505	0.49811	0.99998	9257	0.1661
ELOVL1	6	0.3051	0.49814	0.99998	9258	0.1119
CCDC69	6	0.30516	0.49819	0.99998	9259	0.0338
PRRX1	6	0.30527	0.49828	0.99998	9260	0.265
RANBP3L	4	0.3053	0.43988	0.99998	9261	0.2369
DYNLT3	6	0.30531	0.4983	0.99998	9262	-0.0011
TSPYL2	6	0.30531	0.4983	0.99998	9263	0.1061
OR6N1	6	0.30537	0.49835	0.99998	9264	0.1378
hsa-mir-548a:	3	0.3054	0.39458	0.99998	9265	0.2138
OR52A1	6	0.3055	0.49846	0.99998	9266	-0.0641
SEC14L6	6	0.30572	0.49862	0.99998	9267	0.0692
hsa-mir-626	4	0.30575	0.44038	0.99998	9268	-0.1688
PPM1H	6	0.30577	0.49866	0.99998	9269	-0.0652
KIAA1683	6	0.306	0.49883	0.99998	9270	0.3108
TMED8	6	0.30602	0.49884	0.99998	9271	-0.0148
hsa-mir-4296	4	0.30608	0.44077	0.99998	9272	-0.0437
RAPSN	6	0.30612	0.49891	0.99998	9273	-0.1409
GPRASP1	6	0.30622	0.49899	0.99998	9274	0.1739
PCGF2	6	0.30629	0.49905	0.99998	9275	0.0332
NDUFAF4	6	0.30629	0.49905	0.99998	9276	0.1364
HIST1H2AA	6	0.30629	0.49905	0.99998	9277	-0.088
NXPH2	6	0.30635	0.4991	0.99998	9278	0.0561
SNAP25	6	0.30657	0.49925	0.99998	9279	-0.0815
RHOJ	6	0.30657	0.49925	0.99998	9280	-0.1052
AMIGO1	6	0.30657	0.49925	0.99998	9281	-0.0045
FAM53A	6	0.30671	0.49938	0.99998	9282	-0.1748
AHDC1	6	0.30683	0.49945	0.99998	9283	0.1977
RANBP6	6	0.30701	0.49959	0.99998	9284	-0.0981
RASD2	6	0.30707	0.49963	0.99998	9285	-0.1528
FAM213A	6	0.3071	0.49966	0.99998	9286	-0.0672
hsa-mir-7843	4	0.30715	0.44197	0.99998	9287	-0.097
TPM4	6	0.3074	0.49986	0.99998	9288	-0.0887
DMGDH	6	0.30741	0.49986	0.99998	9289	0.1362
SH3BGR	6	0.30741	0.49986	0.99998	9290	0.1022
hsa-mir-4305	4	0.30756	0.44241	0.99998	9291	0.0285
RECK	6	0.3076	0.5	0.99998	9292	0.0461
KIF24	6	0.30771	0.50008	0.99998	9293	0.0118
POTEH	2	0.30776	0.32872	0.99998	9294	-0.3836
CLDN16	6	0.30777	0.50012	0.99998	9295	0.159
SYNE3	6	0.30777	0.50012	0.99998	9296	-0.0059
PLAC8	6	0.30777	0.50012	0.99998	9297	0.3024
NBPF9	5	0.30787	0.46193	0.99998	9298	-0.5787
SLC25A22	6	0.30787	0.5002	0.99998	9299	-0.0755
GFPT1	6	0.30787	0.5002	0.99998	9300	-0.0241
PIH1D1	6	0.30794	0.50025	0.99998	9301	-0.083
HIST1H2BK	5	0.30797	0.46201	0.99998	9302	0.0829
LCN9	6	0.30802	0.50032	0.99998	9303	0.2136
PSME3	6	0.30802	0.50032	0.99998	9304	0.0902
FAM47E	2	0.30806	0.32895	0.99998	9305	0.0543
OR2AP1	6	0.30806	0.50035	0.99998	9306	-0.073
KIAA1586	5	0.30823	0.46226	0.99998	9307	-0.0611
VPS52	6	0.30838	0.50059	0.99998	9308	-0.0635
PCMTD2	6	0.30838	0.50059	0.99998	9309	0.0816
GNG2	6	0.30842	0.50062	0.99998	9310	-0.0107
CD177	6	0.30848	0.50066	0.99998	9311	-0.0412
DSC3	6	0.3085	0.50068	0.99998	9312	0.1071

SEMA3G	6	0.30874	0.50087	0.99998	9313	-0.1353
TMEM42	6	0.30898	0.50103	0.99998	9314	0.0745
hsa-mir-2115	4	0.309	0.44401	0.99998	9315	-0.1112
SMARCC1	6	0.30909	0.50112	0.99998	9316	0.0495
SRSF5	6	0.30912	0.50114	0.99998	9317	-0.1127
BLOC1S3	6	0.30926	0.50126	0.99998	9318	-0.022
OVCH2	6	0.3093	0.50129	0.99998	9319	0.1399
CEP57L1	6	0.30932	0.5013	0.99998	9320	-0.1316
MPPED1	6	0.30932	0.5013	0.99998	9321	0.1036
MATN1	6	0.30942	0.50138	0.99998	9322	0.0155
PWWP2A	6	0.30946	0.50141	0.99998	9323	-0.0068
C14orf79	6	0.30949	0.50143	0.99998	9324	-0.078
FAM219B	6	0.30953	0.50146	0.99998	9325	0.1177
ABCD1	6	0.30968	0.50156	0.99998	9326	-0.0084
SLAMF7	6	0.30968	0.50156	0.99998	9327	-0.105
ARHGEF7	6	0.3097	0.50157	0.99998	9328	0.0064
TIAM1	6	0.3097	0.50157	0.99998	9329	0.0225
BPIFB2	6	0.3097	0.50157	0.99998	9330	-0.001
JUND	6	0.30989	0.50173	0.99998	9331	-0.0799
TUBA8	6	0.30989	0.50173	0.99998	9332	-0.0351
DDB1	6	0.30989	0.50173	0.99998	9333	0.0861
OGFOD3	6	0.30989	0.50173	0.99998	9334	-0.0507
APOBEC1	6	0.30993	0.50176	0.99998	9335	-0.0725
LCE1A	4	0.31001	0.44518	0.99998	9336	-1.1925
hsa-mir-107	4	0.31001	0.44518	0.99998	9337	-0.3142
hsa-mir-3911	4	0.31001	0.44518	0.99998	9338	-0.2861
LCE1E	4	0.31001	0.44518	0.99998	9339	-1.1925
LOC10028703	6	0.31014	0.5019	0.99998	9340	0.0006
EIF4A2	6	0.31014	0.5019	0.99998	9341	-0.1962
FAM96A	6	0.31028	0.50201	0.99998	9342	-0.1199
KIAA1199	6	0.31028	0.50201	0.99998	9343	0.0685
TCEA2	6	0.31056	0.50223	0.99998	9344	-0.0974
KRT74	6	0.31056	0.50223	0.99998	9345	0.0739
hsa-mir-4736	4	0.31061	0.44584	0.99998	9346	-0.1529
KPRP	6	0.31079	0.5024	0.99998	9347	0.0196
hsa-mir-4782	4	0.31088	0.44613	0.99998	9348	-0.0946
hsa-mir-6769a	4	0.31088	0.44613	0.99998	9349	0.1405
LDHA	6	0.31091	0.50248	0.99998	9350	-0.0673
ENY2	6	0.31091	0.50248	0.99998	9351	0.0149
LRRC8C	6	0.31112	0.50265	0.99998	9352	0.0433
PLAC4	6	0.31122	0.50272	0.99998	9353	0.2523
PCYT1B	6	0.31122	0.50272	0.99998	9354	0.0244
TSGA10	6	0.31137	0.50284	0.99998	9355	-0.1078
STOML2	6	0.31139	0.50286	0.99998	9356	0.0105
ZNF569	6	0.31156	0.50298	0.99998	9357	-0.0929
F13A1	6	0.31157	0.50298	0.99998	9358	0.0489
HDAC5	6	0.31157	0.50298	0.99998	9359	-0.0678
SFXN3	6	0.31165	0.50305	0.99998	9360	-0.0885
S1PR4	6	0.31165	0.50305	0.99998	9361	-0.059
PPA1	6	0.31174	0.50312	0.99998	9362	-0.0782
RAD52	6	0.31175	0.50313	0.99998	9363	-0.0199
hsa-mir-610	4	0.31181	0.44716	0.99998	9364	-0.1375
PXMP2	6	0.31192	0.50326	0.99998	9365	-0.0734
ULBP2	6	0.31198	0.50329	0.99998	9366	-0.0947
MOSPD2	6	0.31198	0.50329	0.99998	9367	-0.0909
hsa-mir-1289-	4	0.31204	0.44743	0.99998	9368	-0.0396
BTN1A1	6	0.31215	0.50341	0.99998	9369	0.1707
TAS2R16	6	0.31228	0.50351	0.99998	9370	0.1006
GRK6	6	0.31229	0.50351	0.99998	9371	0.0704
STARD3NL	6	0.31242	0.5036	0.99998	9372	0.0601
GLI3	6	0.31242	0.5036	0.99998	9373	0.1255
ZNF470	6	0.31243	0.50361	0.99998	9374	0.1034
HIST1H3J	5	0.31249	0.46627	0.99998	9375	-0.0194
OR52L1	6	0.31256	0.50372	0.99998	9376	0.0874
TRIM61	6	0.31259	0.50374	0.99998	9377	0.0858
INTS12	6	0.31263	0.50377	0.99998	9378	-0.047
hsa-mir-6716	4	0.31267	0.44813	0.99998	9379	-0.1729
USP36	6	0.31276	0.50386	0.99998	9380	0.3016
ZNF532	6	0.31276	0.50386	0.99998	9381	0.0055
CXCL13	6	0.3128	0.5039	0.99998	9382	0.148
OR52B4	6	0.31291	0.50398	0.99998	9383	-0.0353
TEKT3	6	0.31293	0.504	0.99998	9384	0.0075
COL8A2	6	0.31297	0.50401	0.99998	9385	0.0008
SCOC	6	0.31298	0.50403	0.99998	9386	0.02
TNN	6	0.31322	0.50419	0.99998	9387	-0.1417
AKR1B10	6	0.31345	0.50437	0.99998	9388	0.061
CCL7	6	0.31345	0.50437	0.99998	9389	-0.2019
PSMC1	6	0.31352	0.50442	0.99998	9390	-0.063
ZNF354B	5	0.31359	0.46728	0.99998	9391	-0.1324
SLC16A4	6	0.31365	0.50452	0.99998	9392	0.1452
FAM194A	6	0.31365	0.50452	0.99998	9393	0.1115

hsa-mir-605	4	0.31377	0.44937	0.99998	9394	-0.0784
SELENBP1	6	0.31389	0.50469	0.99998	9395	-0.0845
SEC14L3	6	0.31394	0.50473	0.99998	9396	0.0917
GNGT1	4	0.31394	0.44957	0.99998	9397	0.1391
hsa-mir-3681	4	0.31404	0.44967	0.99998	9398	-0.5952
hsa-mir-4507	4	0.31404	0.44967	0.99998	9399	-0.7842
CKMT2	6	0.31404	0.5048	0.99998	9400	-0.0124
PRNP	6	0.31404	0.5048	0.99998	9401	-0.0199
SEC16B	6	0.31404	0.5048	0.99998	9402	-0.0231
LIMD1	6	0.31404	0.5048	0.99998	9403	0.0259
GTF2H5	6	0.31404	0.5048	0.99998	9404	0.0035
ZFYVE16	6	0.31417	0.50491	0.99998	9405	0.2444
RTDR1	6	0.31417	0.50491	0.99998	9406	-0.0788
NCF1	6	0.31417	0.50491	0.99998	9407	0.0464
SATB1	6	0.31429	0.50502	0.99998	9408	0.1054
VPS39	6	0.31429	0.50502	0.99998	9409	0.0794
ALPK3	6	0.3144	0.5051	0.99998	9410	0.071
UROC1	6	0.31449	0.50516	0.99998	9411	0.0403
SPNS2	6	0.3145	0.50516	0.99998	9412	0.1546
BICD2	6	0.31461	0.50524	0.99998	9413	-0.0855
DCAF4L1	6	0.31461	0.50525	0.99998	9414	-0.0256
CARD11	6	0.31468	0.5053	0.99998	9415	0.1466
LNX2	6	0.31477	0.50536	0.99998	9416	0.1018
TMC8	6	0.31478	0.50537	0.99998	9417	-0.0269
MPC1L	6	0.31478	0.50537	0.99998	9418	0.1822
hsa-mir-6071	4	0.31487	0.45059	0.99998	9419	-0.0468
EN2	6	0.31487	0.50544	0.99998	9420	0.2989
F12	6	0.31492	0.50549	0.99998	9421	0.1452
FZD2	6	0.31497	0.50553	0.99998	9422	-0.0893
PACRG	6	0.31497	0.50553	0.99998	9423	0.3861
LOC10065282	6	0.31504	0.50557	0.99998	9424	0.0646
hsa-mir-3927	3	0.31516	0.4005	0.99998	9425	0.1158
FARP1	6	0.31519	0.5057	0.99998	9426	-0.0507
hsa-mir-3655	4	0.31527	0.45103	0.99998	9427	-0.0487
CCKAR	6	0.31542	0.50587	0.99998	9428	0.1106
SPATA32	6	0.3155	0.50592	0.99998	9429	-0.0006
ATOH8	6	0.3155	0.50592	0.99998	9430	-0.0669
CCDC127	6	0.3155	0.50593	0.99998	9431	-0.1227
CCDC146	4	0.31555	0.45136	0.99998	9432	0.0535
KRT18	6	0.31565	0.50603	0.99998	9433	-0.1358
CA3	6	0.31574	0.5061	0.99998	9434	-0.0219
PTPDC1	6	0.31595	0.50626	0.99998	9435	-0.0357
PKN1	6	0.31595	0.50626	0.99998	9436	0.2219
IGFL4	6	0.31612	0.50639	0.99998	9437	-0.0736
MMP25	6	0.31628	0.50651	0.99998	9438	-0.1308
hsa-mir-8089	4	0.31636	0.45226	0.99998	9439	0.2622
TMEM133	6	0.31637	0.50657	0.99998	9440	0.0405
EPB42	6	0.31658	0.50672	0.99998	9441	0.0127
PAMR1	6	0.31658	0.50672	0.99998	9442	-0.0611
ARPC5	6	0.31663	0.50676	0.99998	9443	0.004
hsa-mir-3657	4	0.31671	0.45262	0.99998	9444	-0.1671
hsa-mir-6890	4	0.31671	0.45262	0.99998	9445	0.0835
RIN1	6	0.31672	0.50683	0.99998	9446	-0.0098
C15orf27	6	0.31694	0.50698	0.99998	9447	0.1011
OIT3	6	0.31709	0.50711	0.99998	9448	-0.1155
hsa-mir-4514	4	0.31713	0.4531	0.99998	9449	0.2532
ZNF48	6	0.31727	0.50724	0.99998	9450	-0.102
ANG	6	0.31739	0.50731	0.99998	9451	0.1068
USH1G	6	0.31745	0.50737	0.99998	9452	-0.0246
hsa-mir-4328	4	0.31763	0.45367	0.99998	9453	0.0144
NIPSNAP1	6	0.31766	0.50752	0.99998	9454	0.1618
TSPAN6	6	0.31767	0.50753	0.99998	9455	0.3689
RESP18	6	0.31779	0.50763	0.99998	9456	0.0956
PRSS57	6	0.31779	0.50763	0.99998	9457	-0.069
ZNF253	5	0.31787	0.47125	0.99998	9458	0.5776
ZSCAN30	5	0.31811	0.47145	0.99998	9459	0.0674
KLLN	6	0.31817	0.50792	0.99998	9460	-0.1084
HPCA	6	0.31829	0.50801	0.99998	9461	0.2819
SOX17	6	0.31829	0.50801	0.99998	9462	-0.0571
FBXO31	6	0.31835	0.50805	0.99998	9463	-0.0114
C3orf79	6	0.31847	0.50812	0.99998	9464	-0.0146
ECHDC2	6	0.3186	0.50822	0.99998	9465	-0.2224
PCDHA13	2	0.31861	0.33764	0.99998	9466	-0.0499
C4orf3	6	0.31874	0.50832	0.99998	9467	0.0476
RBM26	6	0.31879	0.50836	0.99998	9468	0.052
CFHR2	6	0.31879	0.50836	0.99998	9469	-0.0439
hsa-mir-4642	4	0.31888	0.45501	0.99998	9470	-0.1123
VTI1B	6	0.31891	0.50846	0.99998	9471	0.069
LRRC16B	6	0.31895	0.5085	0.99998	9472	0.1844
C3orf37	4	0.31916	0.45531	0.99998	9473	-0.053
PITX1	6	0.31923	0.50871	0.99998	9474	-0.0292

PPP1R36	6	0.31923	0.50871	0.99998	9475	0.036
EPB41L4B	6	0.31923	0.50871	0.99998	9476	-0.1375
hsa-mir-7107	4	0.31934	0.45552	0.99998	9477	-0.0916
hsa-mir-223	4	0.31934	0.45552	0.99998	9478	-0.025
SYT8	6	0.3194	0.50883	0.99998	9479	0.075
TMEM65	6	0.31961	0.50899	0.99998	9480	0.0069
ANKRD61	6	0.31961	0.50899	0.99998	9481	0.0778
GALNT8	6	0.31963	0.50902	0.99998	9482	0.088
hsa-mir-604	4	0.31966	0.45587	0.99998	9483	0.0198
FAM186B	6	0.3197	0.50907	0.99998	9484	0.0027
VASH1	6	0.31974	0.50909	0.99998	9485	0.1171
TRPC6	6	0.31981	0.50914	0.99998	9486	0.0245
TMEM243	6	0.31982	0.50915	0.99998	9487	0.2461
C2orf43	6	0.31994	0.50924	0.99998	9488	-0.0678
PUM2	6	0.31994	0.50924	0.99998	9489	-0.127
MANBA	6	0.31994	0.50924	0.99998	9490	-0.0262
hsa-mir-548f-!	4	0.31994	0.45617	0.99998	9491	-0.1452
KCNMB1	6	0.32012	0.50937	0.99998	9492	-0.1615
CPAMD8	6	0.3202	0.50943	0.99998	9493	0.1173
MGST3	6	0.32025	0.50947	0.99998	9494	0.1629
TSPAN8	6	0.32052	0.50968	0.99998	9495	0.2186
PPP3R1	6	0.32052	0.50968	0.99998	9496	-0.1348
hsa-mir-106a	2	0.32063	0.33928	0.99998	9497	-0.0863
CNIH	2	0.32063	0.33928	0.99998	9498	0.0369
TLR4	6	0.32074	0.50984	0.99998	9499	-0.1479
C15orf40	6	0.32089	0.50995	0.99998	9500	0.0347
BLVRA	6	0.3211	0.5101	0.99998	9501	0.1093
NKAIN1	6	0.32114	0.51013	0.99998	9502	0.0469
DEFB135	6	0.32123	0.5102	0.99998	9503	0.2824
TMPRSS9	6	0.32123	0.5102	0.99998	9504	0.1428
CKS1B	6	0.32135	0.51028	0.99998	9505	0.04
hsa-mir-522	2	0.32137	0.33989	0.99998	9506	-1.3114
hsa-mir-1272	4	0.3214	0.45775	0.99998	9507	-0.0703
TDG	6	0.32142	0.51033	0.99998	9508	0.202
MRPL55	6	0.32146	0.51035	0.99998	9509	0.0704
SPOCK3	6	0.3215	0.51038	0.99998	9510	-0.0146
EPB41L4A	6	0.32161	0.51048	0.99998	9511	0.1981
GPX6	6	0.32161	0.51048	0.99998	9512	-0.06
TSTD1	6	0.32177	0.51061	0.99998	9513	0.1434
TSKS	6	0.32177	0.51061	0.99998	9514	0.0445
ACAP3	6	0.32203	0.5108	0.99998	9515	-0.064
TMEM136	6	0.32203	0.5108	0.99998	9516	0.005
hsa-mir-3121	4	0.32219	0.4586	0.99998	9517	-0.2308
TYRO3	6	0.32225	0.51097	0.99998	9518	-0.1515
ACHE	6	0.32231	0.51101	0.99998	9519	-0.023
DEFA5	6	0.32231	0.51101	0.99998	9520	0.1067
GPATCH8	6	0.32242	0.5111	0.99998	9521	0.0586
OCEL1	6	0.32267	0.51128	0.99998	9522	0.2666
hsa-mir-331	4	0.32269	0.45914	0.99998	9523	-0.0542
DHRS1	6	0.32281	0.51138	0.99998	9524	-0.0624
LCN2	6	0.323	0.51151	0.99998	9525	0.0716
TRIM34	6	0.323	0.51151	0.99998	9526	-0.0294
ZBED3	6	0.32306	0.51156	0.99998	9527	0.0423
XCL2	3	0.32308	0.40536	0.99998	9528	-0.4558
CD97	6	0.32315	0.51162	0.99998	9529	0.0591
NOL10	6	0.32315	0.51162	0.99998	9530	-0.1003
hsa-mir-33b	2	0.32319	0.3414	0.99998	9531	-0.1893
EML2	4	0.32323	0.45973	0.99998	9532	-0.1596
USB1	6	0.32327	0.51171	0.99998	9533	-0.0661
hsa-let-7i	4	0.32328	0.45978	0.99998	9534	0.0532
ZNF584	6	0.32336	0.51179	0.99998	9535	-0.0441
RBM41	6	0.32346	0.51187	0.99998	9536	0.0735
NDUFV2	6	0.32354	0.51192	0.99998	9537	-0.2014
ANKRD66	6	0.32364	0.512	0.99998	9538	0.2829
SLAMF6	6	0.32377	0.5121	0.99998	9539	-0.0893
PPP2R5E	6	0.32377	0.5121	0.99998	9540	0.2196
CBX4	6	0.32378	0.5121	0.99998	9541	-0.1487
GPA33	6	0.32383	0.51215	0.99998	9542	0.0431
DDX60L	6	0.32396	0.51225	0.99998	9543	-0.1595
PAK6	6	0.32396	0.51225	0.99998	9544	-0.0761
DNAJB6	6	0.32404	0.51231	0.99998	9545	-0.0988
LYZL4	6	0.32404	0.51231	0.99998	9546	0.149
TEKT1	6	0.32423	0.51245	0.99998	9547	-0.1185
KCNJ9	6	0.32423	0.51245	0.99998	9548	0.2166
C14orf183	6	0.32428	0.51249	0.99998	9549	-0.0507
FPR1	6	0.32458	0.51269	0.99998	9550	0.0917
hsa-mir-3917	4	0.3246	0.46128	0.99998	9551	0.1807
C2orf53	6	0.32462	0.51272	0.99998	9552	-0.0729
CMTR1	2	0.32472	0.34267	0.99998	9553	-0.0088
RBBP9	6	0.32481	0.51285	0.99998	9554	-0.0405
IMPDH1	6	0.32488	0.51291	0.99998	9555	-0.0217

KCNV2	6	0.32488	0.51291	0.99998	9556	-0.0964
PRDM16	6	0.32509	0.51307	0.99998	9557	0.0015
hsa-mir-5572	4	0.32517	0.46191	0.99998	9558	0.0448
IER5	6	0.3252	0.51315	0.99998	9559	0.1375
KRTAP4-6	5	0.32534	0.47811	0.99998	9560	-0.024
FHL3	6	0.32535	0.51325	0.99998	9561	-0.0412
CCDC42B	6	0.32535	0.51325	0.99998	9562	0.1449
TMEM150C	6	0.32535	0.51325	0.99998	9563	-0.2073
FUT7	6	0.32537	0.51327	0.99998	9564	0.1527
ZNF23	6	0.32545	0.51332	0.99998	9565	-0.0228
CD24	6	0.32556	0.5134	0.99998	9566	0.014
TMEM206	6	0.32571	0.51351	0.99998	9567	0.0255
OR8H3	6	0.32577	0.51355	0.99998	9568	-0.0741
AXL	6	0.3258	0.51358	0.99998	9569	-0.0518
ARRDC2	6	0.3258	0.51358	0.99998	9570	0.0352
hsa-mir-8068	4	0.32582	0.46266	0.99998	9571	0.2468
KERA	6	0.32593	0.51368	0.99998	9572	0.0374
GUCY1A3	6	0.32599	0.51373	0.99998	9573	0.0778
GAL3ST4	6	0.32599	0.51373	0.99998	9574	-0.0735
BTK	6	0.32605	0.51377	0.99998	9575	0.0306
EFNB2	6	0.32605	0.51377	0.99998	9576	0.142
CD6	4	0.32611	0.46299	0.99998	9577	0.3182
CLTC	6	0.32618	0.51387	0.99998	9578	-0.0221
PLSCR1	6	0.32624	0.51391	0.99998	9579	0.0221
SDF2	6	0.32624	0.51391	0.99998	9580	0.3037
VKORC1	6	0.32624	0.51391	0.99998	9581	0.0676
YBEY	6	0.32624	0.51391	0.99998	9582	0.0854
RHBDL3	6	0.32653	0.51414	0.99998	9583	-0.0828
TMEM5	6	0.32659	0.51418	0.99998	9584	0.0234
hsa-mir-1197	4	0.32667	0.46358	0.99998	9585	-0.0796
CDK11A	6	0.32674	0.51429	0.99998	9586	0.0668
OR2M3	5	0.32683	0.47952	0.99998	9587	0.5478
hsa-mir-6511a	2	0.32685	0.34442	0.99998	9588	-0.8618
POLR1A	6	0.32685	0.51438	0.99998	9589	0.043
FJX1	6	0.32697	0.51447	0.99998	9590	0.0388
MFSD2B	6	0.32725	0.51467	0.99998	9591	-0.0713
CNTN1	6	0.32729	0.5147	0.99998	9592	0.008
SCN11A	6	0.32729	0.5147	0.99998	9593	-0.0058
ZFP90	6	0.32729	0.5147	0.99998	9594	0.0265
STAT6	6	0.3273	0.51471	0.99998	9595	-0.1306
DEFB116	6	0.32743	0.51482	0.99998	9596	-0.0271
TRAF5	6	0.32749	0.51486	0.99998	9597	-0.0056
LYPD5	6	0.32762	0.51496	0.99998	9598	0.0672
CNRIP1	6	0.3278	0.5151	0.99998	9599	0.067
LRRC4	6	0.32781	0.5151	0.99998	9600	0.0342
KLHL38	6	0.32814	0.51537	0.99998	9601	0.2265
TMEM241	6	0.32814	0.51537	0.99998	9602	-0.0168
EVX2	6	0.32822	0.51542	0.99998	9603	-0.0793
IFNA10	5	0.32826	0.48087	0.99998	9604	-0.3622
OR4D11	6	0.32831	0.51549	0.99998	9605	-0.1033
OR1M1	6	0.32837	0.51554	0.99998	9606	0.1294
SLC22A7	6	0.32839	0.51555	0.99998	9607	-0.0719
RHO	6	0.32841	0.51557	0.99998	9608	-0.0624
KIF9	6	0.32845	0.5156	0.99998	9609	-0.0428
C11orf30	6	0.32846	0.51561	0.99998	9610	0.0103
CAMK1D	6	0.32846	0.51561	0.99998	9611	0.111
MGAT5	6	0.32861	0.51573	0.99998	9612	-0.0631
NR2E3	6	0.32865	0.51576	0.99998	9613	0.1049
ZNF280C	6	0.3288	0.51586	0.99998	9614	0.2132
LRPAP1	6	0.32889	0.51594	0.99998	9615	0.0777
NHEJ1	6	0.32889	0.51594	0.99998	9616	-0.0056
GNAI1	6	0.32895	0.51599	0.99998	9617	0.2325
ADK	6	0.32919	0.51616	0.99998	9618	0.0071
HS6ST1	6	0.3292	0.51616	0.99998	9619	-0.021
GPR135	6	0.3292	0.51616	0.99998	9620	-0.0499
NEK2	6	0.32928	0.51623	0.99998	9621	-0.0236
CDK17	6	0.32934	0.51627	0.99998	9622	0.3033
RINL	6	0.32938	0.5163	0.99998	9623	-0.019
PPAPDC1B	6	0.32942	0.51633	0.99998	9624	0.0691
TMEM216	6	0.32942	0.51633	0.99998	9625	0.0452
PYGO2	6	0.32955	0.51643	0.99998	9626	-0.0824
TULP4	6	0.32958	0.51645	0.99998	9627	0.0124
IKZF3	6	0.32958	0.51645	0.99998	9628	0.0998
KLHL24	6	0.32977	0.5166	0.99998	9629	0.2639
ZNF526	6	0.32977	0.5166	0.99998	9630	0.0478
ETV4	6	0.32987	0.51667	0.99998	9631	0.0355
GPC5	6	0.32997	0.51675	0.99998	9632	-0.0577
OXTR	6	0.33	0.51677	0.99998	9633	0.009
BAGE2	2	0.33009	0.34713	0.99998	9634	-0.1912
B3GALTL	6	0.33012	0.51687	0.99998	9635	-0.0736
GRIK1	6	0.33016	0.5169	0.99998	9636	-0.0283

FGF18	6	0.3303	0.517	0.99998	9637	0.078
MKKS	6	0.33046	0.51713	0.99998	9638	0.0051
ZNF414	6	0.33046	0.51713	0.99998	9639	-0.0058
MRPS12	4	0.3306	0.46802	0.99998	9640	-0.1397
EFHC2	6	0.33066	0.51727	0.99998	9641	0.0844
IDO1	6	0.33066	0.51727	0.99998	9642	0.0365
LTBP1	6	0.33088	0.51743	0.99998	9643	0.0331
BCR	6	0.33088	0.51743	0.99998	9644	0.0037
TMEM89	6	0.33088	0.51743	0.99998	9645	-0.0947
SUSD4	6	0.33088	0.51743	0.99998	9646	0.1614
HIST1H2AG	6	0.33088	0.51743	0.99998	9647	-0.0136
CCDC87	6	0.33117	0.51767	0.99998	9648	-0.0428
FKTN	6	0.33132	0.51778	0.99998	9649	-0.0281
RBMS2	6	0.33132	0.51778	0.99998	9650	0.0343
PRADC1	6	0.3315	0.51792	0.99998	9651	-0.0396
C2orf80	6	0.33153	0.51795	0.99998	9652	0.1177
RSRC1	6	0.33163	0.51803	0.99998	9653	0.0064
PLEKHG6	6	0.33182	0.51817	0.99998	9654	0.0879
PAFAH1B3	6	0.33182	0.51817	0.99998	9655	0.3013
MRPL17	6	0.33186	0.5182	0.99998	9656	-0.0867
ASAH2	6	0.33186	0.5182	0.99998	9657	0.0922
SUSD2	6	0.33186	0.5182	0.99998	9658	0.2024
hsa-mir-888	4	0.33186	0.46943	0.99998	9659	-0.0924
UBL4A	6	0.33195	0.51827	0.99998	9660	-0.0192
KRTAP10-11	6	0.33195	0.51827	0.99998	9661	-0.0624
TENM2	6	0.33198	0.51829	0.99998	9662	0.0632
DCUN1D2	6	0.33208	0.51835	0.99998	9663	0.0105
GORASP2	6	0.33209	0.51836	0.99998	9664	-0.0518
hsa-mir-363	4	0.33212	0.46972	0.99998	9665	-0.1736
TESK2	6	0.3323	0.51852	0.99998	9666	0.1507
OAT	6	0.33245	0.51863	0.99998	9667	0.1636
PTBP3	6	0.33245	0.51863	0.99998	9668	0.1274
SLC9A5	6	0.33246	0.51863	0.99998	9669	-0.2207
SLC9A2	6	0.33271	0.51882	0.99998	9670	0.1507
hsa-mir-3157	4	0.33273	0.47036	0.99998	9671	0.0467
hsa-mir-3153	4	0.33273	0.47037	0.99998	9672	-0.0716
hsa-mir-4710	4	0.33273	0.47037	0.99998	9673	-0.2945
NEUROG2	6	0.33297	0.51901	0.99998	9674	0.2527
IL6ST	6	0.33318	0.51917	0.99998	9675	0.0054
CCDC97	6	0.33321	0.51919	0.99998	9676	0.1237
GLYATL1	6	0.33321	0.51919	0.99998	9677	0.1294
ZSWIM5	6	0.33321	0.51919	0.99998	9678	0.0948
hsa-mir-548ac	4	0.33334	0.47103	0.99998	9679	-0.5745
hsa-mir-3185	4	0.33334	0.47103	0.99998	9680	-0.2502
GSTM1	4	0.33334	0.47103	0.99998	9681	-0.5349
MTRNR2L8	2	0.3336	0.35007	0.99998	9682	0.1138
hsa-mir-4460	4	0.33373	0.47146	0.99998	9683	-0.0061
RNF220	6	0.33374	0.51957	0.99998	9684	-0.1294
DNAJC21	6	0.33385	0.51965	0.99998	9685	0.1131
WFDC3	6	0.33398	0.51974	0.99998	9686	-0.0732
PLSCR4	6	0.33401	0.51977	0.99998	9687	0.1588
ZDHHC14	6	0.33418	0.51989	0.99998	9688	0.2555
FZD9	6	0.33418	0.51989	0.99998	9689	0.0047
AMPD1	6	0.33418	0.51989	0.99998	9690	0.058
UBE2K	6	0.33435	0.52001	0.99998	9691	0.2143
PTPN9	6	0.33457	0.52017	0.99998	9692	0.171
ZNF208	3	0.3346	0.41237	0.99998	9693	-0.0587
P2RY11	6	0.33491	0.52045	0.99998	9694	-0.0607
PLEKHG4B	6	0.33502	0.52052	0.99998	9695	0.0506
MISP	1	0.33506	0.33521	0.99998	9696	-0.1439
BYSL	6	0.3351	0.52059	0.99998	9697	0.0307
PPP2R5C	6	0.33519	0.52065	0.99998	9698	-0.097
NUP62CL	6	0.33519	0.52065	0.99998	9699	0.0895
TARDBP	6	0.3352	0.52066	0.99998	9700	0.0341
SLC16A11	6	0.33535	0.52078	0.99998	9701	-0.1143
NEIL2	6	0.33542	0.52083	0.99998	9702	0.2978
SLC25A14	6	0.33542	0.52083	0.99998	9703	0.2084
HMMR	6	0.33555	0.52091	0.99998	9704	0.0692
PIP5KL1	6	0.33555	0.52091	0.99998	9705	-0.0851
RNPC3	6	0.33555	0.52091	0.99998	9706	0.2016
KRT5	6	0.33605	0.52129	0.99998	9707	0.0601
CCDC96	6	0.33612	0.52135	0.99998	9708	0.0592
USP48	6	0.33613	0.52135	0.99998	9709	0.0511
hsa-mir-6750	4	0.33615	0.47421	0.99998	9710	0.129
TUBA1A	5	0.3363	0.48839	0.99998	9711	-0.1067
HIVEP2	6	0.33631	0.52149	0.99998	9712	0.3088
ZNF391	6	0.33637	0.52153	0.99998	9713	0.1423
EMILIN3	6	0.33637	0.52153	0.99998	9714	-0.0421
CLDN18	6	0.33648	0.52162	0.99998	9715	-0.0469
FAM178A	6	0.33648	0.52162	0.99998	9716	0.0506
C1orf56	6	0.33653	0.52164	0.99998	9717	-0.0726

RAD54B	6	0.33653	0.52164	0.99998	9718	0.0767
VGF	6	0.33661	0.52171	0.99998	9719	0.1418
ETAA1	6	0.33666	0.52175	0.99998	9720	-0.1207
CABLES2	4	0.33667	0.47481	0.99998	9721	0.0247
CMC2	5	0.33669	0.48874	0.99998	9722	-0.0207
ASTL	6	0.3367	0.52178	0.99998	9723	0.1233
TNFRSF1B	6	0.33672	0.5218	0.99998	9724	-0.0282
LRRC37A	6	0.33686	0.52191	0.99998	9725	0.139
NKX1-2	6	0.33686	0.52191	0.99998	9726	0.2776
DNAH9	6	0.3369	0.52195	0.99998	9727	0.0668
ASAH2B	6	0.33705	0.52206	0.99998	9728	0.1077
ZNF32	6	0.33705	0.52206	0.99998	9729	0.0049
STRN	6	0.3371	0.5221	0.99998	9730	-0.0239
CDH5	6	0.3371	0.5221	0.99998	9731	0.0247
FCN3	6	0.3371	0.5221	0.99998	9732	-0.0325
FATE1	6	0.33716	0.52214	0.99998	9733	-0.0236
DCUN1D5	6	0.33725	0.5222	0.99998	9734	-0.0241
C2orf91	6	0.33725	0.5222	0.99998	9735	-0.1661
ANXA6	6	0.33735	0.52228	0.99998	9736	0.0104
ZNF835	6	0.33739	0.52231	0.99998	9737	-0.0479
SLC5A2	6	0.33739	0.52231	0.99998	9738	0.0727
ATXN7L2	6	0.33749	0.52238	0.99998	9739	0.0746
S100A4	6	0.33749	0.52238	0.99998	9740	-0.0607
CXorf40B	3	0.33776	0.41437	0.99998	9741	-0.3622
hsa-mir-362	4	0.33781	0.47608	0.99998	9742	-0.1243
VAMP8	6	0.33784	0.52265	0.99998	9743	-0.1593
FAM49B	6	0.33784	0.52265	0.99998	9744	-0.1363
PSMA8	6	0.33799	0.52276	0.99998	9745	0.0837
CHP2	6	0.33808	0.52282	0.99998	9746	0.006
LGALS12	6	0.33823	0.52295	0.99998	9747	0.2559
hsa-mir-4497	4	0.33827	0.47658	0.99998	9748	-0.2157
HOXB1	6	0.33831	0.523	0.99998	9749	-0.025
MTFMT	6	0.33831	0.523	0.99998	9750	-0.0132
NSUN4	6	0.33831	0.523	0.99998	9751	-0.0043
OR5AK2	6	0.33855	0.52318	0.99998	9752	0.0528
HS3ST5	6	0.33855	0.52318	0.99998	9753	0.3092
TBX2	6	0.33855	0.52318	0.99998	9754	0.1475
IL13	6	0.33858	0.52322	0.99998	9755	-0.104
hsa-mir-7845	4	0.33862	0.477	0.99998	9756	-0.0504
APOC2	6	0.33873	0.52332	0.99998	9757	-0.077
PPP5D1	6	0.33881	0.52338	0.99998	9758	0.0611
C20orf201	6	0.33887	0.52343	0.99998	9759	0.0137
C11orf52	6	0.33887	0.52343	0.99998	9760	-0.1304
CYP3A7	2	0.33906	0.3547	0.99998	9761	-0.5904
LIPH	6	0.33912	0.52361	0.99998	9762	0.0555
OR9A4	6	0.33912	0.52361	0.99998	9763	0.1891
hsa-mir-6769b	4	0.33916	0.47759	0.99998	9764	0.0134
STAT1	6	0.33929	0.52373	0.99998	9765	0.091
GATA3	6	0.33942	0.52382	0.99998	9766	-0.0311
PKIA	6	0.33945	0.52384	0.99998	9767	0.0789
FAM161A	6	0.33945	0.52384	0.99998	9768	-0.0911
FAM107B	6	0.33947	0.52386	0.99998	9769	0.0987
FASTKD5	6	0.33958	0.52395	0.99998	9770	0.1068
MED16	6	0.33958	0.52395	0.99998	9771	0.1516
PKD1L3	6	0.33959	0.52395	0.99998	9772	0.0963
FAM169B	6	0.33969	0.52403	0.99998	9773	0.0801
RNF114	6	0.33969	0.52403	0.99998	9774	0.011
SPRR2A	1	0.33971	0.33996	0.99998	9775	-0.9148
RBMY1F	1	0.33971	0.33996	0.99998	9776	-0.9148
CCL4L2	1	0.33971	0.33996	0.99998	9777	-0.9148
TRIM49D1	1	0.33971	0.33996	0.99998	9778	-0.9148
hsa-mir-548aj	1	0.33971	0.33996	0.99998	9779	-0.9148
hsa-mir-3156-	1	0.33971	0.33996	0.99998	9780	-0.9148
PRAMEF6	1	0.33971	0.33996	0.99998	9781	-0.9148
POTEJ	1	0.33971	0.33996	0.99998	9782	-0.9148
FAM25G	1	0.33971	0.33996	0.99998	9783	-0.9148
LGALS7	1	0.33971	0.33996	0.99998	9784	-0.9148
hsa-mir-6511b	1	0.33971	0.33996	0.99998	9785	-0.9148
hsa-mir-4441	1	0.33971	0.33996	0.99998	9786	-0.9148
C4B	1	0.33971	0.33996	0.99998	9787	-0.9148
hsa-mir-548ac	1	0.33971	0.33996	0.99998	9788	-0.9148
hsa-mir-527	1	0.33971	0.33996	0.99998	9789	-0.9148
CD3G	6	0.3399	0.52418	0.99998	9790	-0.0168
KCNK1	6	0.33997	0.52424	0.99998	9791	0.1059
hsa-mir-8057	4	0.33999	0.47853	0.99998	9792	-0.2155
hsa-mir-585	4	0.33999	0.47853	0.99998	9793	-0.301
hsa-mir-4650-	3	0.34004	0.41581	0.99998	9794	-0.2158
SENP5	6	0.34026	0.52447	0.99998	9795	0.0486
DGUOK	6	0.34026	0.52447	0.99998	9796	0.1598
FAM189B	6	0.34028	0.52449	0.99998	9797	0.1975
hsa-mir-4252	4	0.34044	0.47902	0.99998	9798	-0.0639

GPAA1	6	0.34052	0.52466	0.99998	9799	-0.0108
STAT2	6	0.34069	0.52479	0.99998	9800	-0.0757
hsa-mir-4728	4	0.34076	0.47937	0.99998	9801	-0.0016
C14orf2	6	0.34078	0.52487	0.99998	9802	0.0438
ARHGEF38	6	0.34087	0.52494	0.99998	9803	0.2188
hsa-mir-6773	4	0.34089	0.4795	0.99998	9804	-0.0606
hsa-mir-4493	4	0.34098	0.4796	0.99998	9805	-0.1306
FAM122A	6	0.34098	0.52502	0.99998	9806	-0.0336
P2RY12	6	0.34113	0.52513	0.99998	9807	0.1693
C2orf70	6	0.34129	0.52525	0.99998	9808	0.0028
SBNO2	6	0.34129	0.52525	0.99998	9809	-0.012
GP1BA	6	0.34129	0.52525	0.99998	9810	0.0568
ACAT2	6	0.34132	0.52527	0.99998	9811	0.0802
TMEM62	6	0.34137	0.5253	0.99998	9812	0.013
hsa-mir-4754	4	0.34137	0.48006	0.99998	9813	-0.2605
TMEM151B	6	0.3415	0.52541	0.99998	9814	-0.0248
OR5L2	6	0.34154	0.52544	0.99998	9815	0.0361
RBFOX2	6	0.3416	0.52549	0.99998	9816	0.1618
DNALI1	6	0.34168	0.52554	0.99998	9817	-0.0096
C16orf96	6	0.34171	0.52556	0.99998	9818	0.3233
RASSF5	6	0.34177	0.52561	0.99998	9819	-0.0549
hsa-mir-644a	4	0.34181	0.48054	0.99998	9820	-0.1148
EEF2K	6	0.34183	0.52565	0.99998	9821	0.1356
TACSTD2	6	0.34189	0.52569	0.99998	9822	-0.0529
ZNHIT1	6	0.3419	0.5257	0.99998	9823	0.0362
hsa-mir-6819	4	0.34198	0.48074	0.99998	9824	-0.0185
HOXA1	6	0.34203	0.52581	0.99998	9825	-0.0737
PPP1R26	6	0.34212	0.52588	0.99998	9826	-0.0324
TLE2	6	0.34221	0.52595	0.99998	9827	0.0487
GPR115	6	0.34221	0.52595	0.99998	9828	0.0775
ATP1B2	6	0.34223	0.52596	0.99998	9829	-0.0544
SUN2	6	0.34235	0.52606	0.99998	9830	0.1387
CCR4	6	0.34256	0.52622	0.99998	9831	0.1041
DAAM2	6	0.3428	0.5264	0.99998	9832	-0.0613
MRPL40	6	0.34286	0.52643	0.99998	9833	0.1617
PTCHD3	6	0.34292	0.52648	0.99998	9834	-0.0197
FOXF2	6	0.34317	0.52666	0.99998	9835	0.2561
PPAP2C	4	0.3434	0.48227	0.99998	9836	-0.3255
LOC286238	6	0.34341	0.52685	0.99998	9837	-0.0052
BPI	6	0.34343	0.52686	0.99998	9838	0.2643
KLRK1	6	0.34351	0.52694	0.99998	9839	0.049
RAB8B	6	0.34363	0.52702	0.99998	9840	0.335
C2orf47	6	0.34368	0.52706	0.99998	9841	-0.0128
MOB3A	6	0.34373	0.52709	0.99998	9842	0.006
PTPRN2	6	0.34373	0.52709	0.99998	9843	0.0994
COL5A1	6	0.34373	0.52709	0.99998	9844	-0.2156
DPM2	6	0.34383	0.52718	0.99998	9845	0.1372
PYGB	6	0.34383	0.52718	0.99998	9846	0.0228
CCDC112	6	0.34383	0.52718	0.99998	9847	-0.0535
DNAH10	6	0.34405	0.52736	0.99998	9848	0.0878
TRIM46	6	0.34405	0.52736	0.99998	9849	-0.0349
EFCAB4A	6	0.34405	0.52736	0.99998	9850	0.0824
TEAD2	6	0.34413	0.52743	0.99998	9851	-0.0658
ALOXE3	6	0.3442	0.52747	0.99998	9852	-0.0371
ARMC4	6	0.34431	0.52755	0.99998	9853	0.0316
hsa-mir-5706	4	0.34439	0.48338	0.99998	9854	-0.1012
hsa-mir-4650-	3	0.3444	0.41855	0.99998	9855	0.1726
GFPT2	6	0.34452	0.52772	0.99998	9856	0.1532
NSUN7	6	0.34454	0.52774	0.99998	9857	-0.0611
APOC3	6	0.34477	0.52791	0.99998	9858	0.1703
HYAL1	6	0.34477	0.52791	0.99998	9859	-0.0856
ZBED6	6	0.3448	0.52793	0.99998	9860	-0.0489
RSRC2	6	0.3448	0.52793	0.99998	9861	0.0208
CCDC121	6	0.3448	0.52793	0.99998	9862	0.0464
SAPCD1	6	0.3448	0.52793	0.99998	9863	-0.0319
LZTFL1	6	0.34502	0.5281	0.99998	9864	0.0087
SPANXE	2	0.34526	0.36006	0.99998	9865	-0.1567
PLCB3	6	0.34531	0.52832	0.99998	9866	-0.064
NKX6-2	6	0.34551	0.52847	0.99998	9867	0.2136
hsa-mir-1268b	4	0.3459	0.48504	0.99998	9868	0.1193
KLB	6	0.34595	0.52881	0.99998	9869	0.101
FRS2	6	0.34596	0.52881	0.99998	9870	0.1783
SUN3	6	0.34604	0.52887	0.99998	9871	0.1188
hsa-mir-448	4	0.34636	0.48554	0.99998	9872	0.3947
ZNF91	5	0.34643	0.4978	0.99998	9873	0.073
SCGB2A2	5	0.34674	0.4981	0.99998	9874	0.1201
PDAP1	6	0.34679	0.52941	0.99998	9875	0.0251
RHOBTB3	6	0.34687	0.52948	0.99998	9876	0.2053
NPEPL1	6	0.34687	0.52948	0.99998	9877	-0.0716
EIF3L	6	0.34688	0.52949	0.99998	9878	-0.0925
UHRF1BP1L	6	0.34701	0.5296	0.99998	9879	-0.0111

hsa-mir-4635	4	0.34704	0.48629	0.99998	9880	-0.2082
VNN1	6	0.34705	0.52962	0.99998	9881	-0.0688
GPR149	6	0.34716	0.52972	0.99998	9882	-0.031
SLC8B1	2	0.34718	0.3617	0.99998	9883	-0.1072
hsa-mir-4418	4	0.34733	0.48663	0.99998	9884	-0.151
SYNPO2L	6	0.34735	0.52987	0.99998	9885	-0.1518
hsa-mir-1228	4	0.34752	0.48684	0.99998	9886	-0.204
MFAP5	6	0.34759	0.53005	0.99998	9887	0.0147
TCEAL8	6	0.34759	0.53005	0.99998	9888	-0.0466
hsa-mir-513a-	1	0.34759	0.34787	0.99998	9889	-0.7622
CHRNE	6	0.34773	0.53016	0.99998	9890	-0.078
INPP4A	6	0.34787	0.53026	0.99998	9891	0.1466
HIST1H2BF	5	0.3479	0.49915	0.99998	9892	1.1736
TIMM8B	6	0.34802	0.53037	0.99998	9893	0.0504
PHYHIPL	6	0.34816	0.53048	0.99998	9894	-0.108
ZDHHC6	6	0.34824	0.53053	0.99998	9895	-0.1661
PNMA2	6	0.34828	0.53056	0.99998	9896	0.1254
hsa-mir-6791	4	0.34845	0.48787	0.99998	9897	0.177
ELOVL6	6	0.3486	0.53081	0.99998	9898	0.0131
HIVEP1	6	0.3486	0.53081	0.99998	9899	-0.0759
hsa-mir-3672	1	0.34869	0.34897	0.99998	9900	-0.149
ESRP2	6	0.34876	0.53093	0.99998	9901	0.137
N4BP2L1	6	0.34876	0.53093	0.99998	9902	0.1182
PRSS50	6	0.34881	0.53095	0.99998	9903	-0.008
DCUN1D1	6	0.34881	0.53095	0.99998	9904	0.1122
MAGOH	6	0.34896	0.53108	0.99998	9905	0.0306
DGKI	4	0.34896	0.48845	0.99998	9906	0.1757
KCTD7	6	0.34905	0.53114	0.99998	9907	-0.1052
ETNK2	6	0.34918	0.53125	0.99998	9908	0.0138
MFRP	6	0.34918	0.53125	0.99998	9909	0.1337
THBS2	6	0.34936	0.53139	0.99998	9910	-0.0364
RBPMS2	6	0.34936	0.53139	0.99998	9911	0.1283
hsa-mir-4682	4	0.3494	0.48894	0.99998	9912	-0.1535
hsa-mir-3196	4	0.34947	0.48901	0.99998	9913	-0.1623
RSBN1	6	0.34951	0.53149	0.99998	9914	0.0121
LCE6A	6	0.3496	0.53156	0.99998	9915	-0.0826
DDX39A	6	0.35005	0.53189	0.99998	9916	-0.0531
IFNA7	6	0.35005	0.53189	0.99998	9917	-0.1482
RORB	6	0.35005	0.53189	0.99998	9918	-0.0199
ZNF234	6	0.35005	0.53189	0.99998	9919	0.0062
ZNF404	6	0.35005	0.53189	0.99998	9920	0.1003
BOLA1	6	0.35005	0.53189	0.99998	9921	-0.1304
37500	3	0.35009	0.42208	0.99998	9922	0.0824
DPP7	6	0.35013	0.53195	0.99998	9923	0.0537
RUNDC1	6	0.35013	0.53195	0.99998	9924	-0.1076
CD81	6	0.35013	0.53195	0.99998	9925	0.1005
TXK	6	0.35033	0.5321	0.99998	9926	0.0155
SBSN	6	0.35038	0.53214	0.99998	9927	0.1651
IQCD	6	0.35049	0.53223	0.99998	9928	0.1015
SLC36A1	6	0.35066	0.53237	0.99998	9929	0.0513
SARNP	6	0.35071	0.53241	0.99998	9930	0.3309
MRPL49	6	0.35088	0.53253	0.99998	9931	0.0315
SEC14L1	6	0.35098	0.53261	0.99998	9932	0.0469
MYPOP	6	0.35098	0.53261	0.99998	9933	0.1545
THAP2	6	0.35098	0.53261	0.99998	9934	0.0337
hsa-mir-6838	4	0.35102	0.49075	0.99998	9935	-0.1643
ZSWIM1	6	0.35107	0.53267	0.99998	9936	-0.0918
ZNF347	6	0.35112	0.53272	0.99998	9937	0.044
TEKT5	6	0.35146	0.53297	0.99998	9938	0.1582
GABRG1	6	0.35147	0.53298	0.99998	9939	0.0364
HOXA9	6	0.3516	0.53308	0.99998	9940	0.0262
MS4A7	6	0.35163	0.5331	0.99998	9941	0.1673
RAD51	6	0.35163	0.5331	0.99998	9942	-0.1063
HSPB2	6	0.35163	0.5331	0.99998	9943	-0.0198
RHOBTB2	6	0.35169	0.53315	0.99998	9944	-0.0366
MAPRE1	6	0.35172	0.53316	0.99998	9945	-0.0308
TMPRSS11A	6	0.35175	0.53318	0.99998	9946	0.3499
UNC5B	6	0.35182	0.53322	0.99998	9947	0.0893
ARL6IP5	6	0.35182	0.53322	0.99998	9948	0.094
SPATA6L	6	0.35193	0.5333	0.99998	9949	0.0662
COL23A1	6	0.35193	0.5333	0.99998	9950	0.0527
GNAZ	6	0.35195	0.53332	0.99998	9951	-0.1294
FNDC9	6	0.35195	0.53332	0.99998	9952	0.0008
hsa-mir-510	4	0.35207	0.49194	0.99998	9953	-0.0676
SIGLEC6	6	0.35209	0.53343	0.99998	9954	0.0879
C4orf26	4	0.35214	0.49202	0.99998	9955	0.2741
SMARCAD1	6	0.35216	0.53347	0.99998	9956	-0.0487
ATP2B1	6	0.35235	0.53361	0.99998	9957	0.0659
LYG1	6	0.35235	0.53361	0.99998	9958	-0.0013
TRPC4	6	0.35236	0.53363	0.99998	9959	-0.0609
C22orf42	6	0.35245	0.53369	0.99998	9960	0.0463

B3GALT2	6	0.35247	0.53371	0.99998	9961	-0.0047
SMAD1	6	0.35247	0.53371	0.99998	9962	0.0813
CRY1	6	0.35254	0.53375	0.99998	9963	-0.0158
PYGM	6	0.35254	0.53375	0.99998	9964	0.0112
SELM	6	0.35261	0.53381	0.99998	9965	0.1338
PLEKHM2	6	0.3528	0.53395	0.99998	9966	0.0712
CCDC175	6	0.3528	0.53395	0.99998	9967	0.2153
hsa-mir-1302-	4	0.35281	0.49275	0.99998	9968	-0.4609
OR52J3	6	0.35287	0.53401	0.99998	9969	0.1981
KPNA2	6	0.35287	0.53401	0.99998	9970	-0.1116
GALM	6	0.35287	0.53401	0.99998	9971	0.1402
hsa-mir-2681	4	0.35299	0.49295	0.99998	9972	-0.0125
TRIM68	6	0.35312	0.5342	0.99998	9973	0.1174
C8orf48	6	0.35312	0.5342	0.99998	9974	0.2896
hsa-mir-514b	4	0.35314	0.49312	0.99998	9975	-0.0394
CEP70	6	0.35325	0.5343	0.99998	9976	-0.0068
hsa-mir-370	4	0.35329	0.49329	0.99998	9977	-0.0978
PDGFC	6	0.35341	0.53441	0.99998	9978	0.0335
PMPCB	6	0.35344	0.53443	0.99998	9979	0.0966
COG5	6	0.35347	0.53445	0.99998	9980	-0.0447
OR4C3	6	0.35351	0.53448	0.99998	9981	0.121
SCGB1D1	5	0.35369	0.50457	0.99998	9982	-0.1591
UBL4B	6	0.35372	0.53463	0.99998	9983	-0.0425
CELF3	6	0.35383	0.5347	0.99998	9984	-0.0965
hsa-mir-4718	4	0.35385	0.49392	0.99998	9985	0.603
ARHGAP11A	3	0.35386	0.42449	0.99998	9986	0.1742
MBD3L3	2	0.35394	0.36744	0.99998	9987	-0.0475
RCOR1	6	0.35394	0.53479	0.99998	9988	0.1527
GPM6A	6	0.35394	0.53479	0.99998	9989	0.0791
PACSIN1	6	0.35394	0.53479	0.99998	9990	0.1101
HPSE	6	0.35416	0.53495	0.99998	9991	-0.04
TARBP2	6	0.35416	0.53495	0.99998	9992	-0.1652
PCGF1	6	0.35432	0.53507	0.99998	9993	0.0749
GZMK	6	0.35439	0.53513	0.99998	9994	0.2623
MIB2	6	0.35445	0.53517	0.99998	9995	-0.025
TNR	6	0.35452	0.53523	0.99998	9996	0.1114
USP2	6	0.35472	0.53541	0.99998	9997	-0.0473
CCDC36	6	0.35481	0.53548	0.99998	9998	0.074
IL17RD	6	0.35493	0.53558	0.99998	9999	0.0486
SLC40A1	6	0.35495	0.5356	0.99998	10000	0.128
RBM12	6	0.35495	0.5356	0.99998	10001	-0.0469
hsa-mir-548ac	1	0.35497	0.35519	0.99998	10002	-0.1462
FAM57A	4	0.35498	0.49514	0.99998	10003	0.2638
hsa-mir-6745	4	0.35504	0.49521	0.99998	10004	-0.1006
TNNT3	6	0.3551	0.53571	0.99998	10005	0.0078
ARHGEF3	6	0.3551	0.53571	0.99998	10006	-0.0916
USP30	6	0.35515	0.53574	0.99998	10007	0.0796
KCNQ4	6	0.35531	0.53587	0.99998	10008	0.0731
FAM83F	6	0.35531	0.53587	0.99998	10009	-0.0148
C14orf180	6	0.35543	0.53597	0.99998	10010	0.2433
CLDN2	6	0.35548	0.536	0.99998	10011	0.0408
GSTM2	5	0.35554	0.50625	0.99998	10012	0.0147
hsa-mir-4291	4	0.35554	0.49576	0.99998	10013	0.0571
TBC1D12	6	0.35566	0.53614	0.99998	10014	0.1451
GKN2	6	0.35566	0.53614	0.99998	10015	0.0746
hsa-mir-7-3	4	0.3557	0.49593	0.99998	10016	-0.0437
NTPCR	6	0.3558	0.53624	0.99998	10017	-0.0979
C10orf131	5	0.35586	0.50657	0.99998	10018	0.0849
OR1L3	6	0.35595	0.53635	0.99998	10019	0.02
SERINC4	6	0.35597	0.53636	0.99998	10020	-0.0379
CHML	6	0.35597	0.53636	0.99998	10021	0.1018
EXPH5	6	0.35604	0.53641	0.99998	10022	-0.0677
CBY3	6	0.3563	0.53658	0.99998	10023	0.2052
TMEM27	6	0.3563	0.53658	0.99998	10024	0.0289
RBM43	6	0.3563	0.53658	0.99998	10025	0.0213
PLEKHA6	6	0.35631	0.53658	0.99998	10026	0.1985
GNLY	6	0.35631	0.53658	0.99998	10027	-0.0431
CWC15	6	0.35631	0.53658	0.99998	10028	-0.1667
ABCF2	6	0.35667	0.53685	0.99998	10029	0.1227
MFAP2	6	0.35667	0.53685	0.99998	10030	0.3618
ZNF77	6	0.35668	0.53686	0.99998	10031	-0.0815
hsa-mir-6879	4	0.3567	0.49705	0.99998	10032	0.0769
FOXRED2	6	0.35681	0.53696	0.99998	10033	0.0954
ZMYND8	6	0.35681	0.53696	0.99998	10034	0.0638
C2orf82	6	0.35683	0.53697	0.99998	10035	-0.0843
CD164	6	0.35687	0.53701	0.99998	10036	-0.0048
LMO3	6	0.35694	0.53706	0.99998	10037	0.1347
TMEM81	6	0.35696	0.53707	0.99998	10038	0.061
CLN5	6	0.35715	0.53723	0.99998	10039	0.0127
hsa-mir-5191	4	0.35721	0.49762	0.99998	10040	-0.1343
FBXO34	6	0.35723	0.53727	0.99998	10041	-0.002

hsa-mir-3170	4	0.35728	0.49769	0.99998	10042	0.0143
DTWD1	6	0.35732	0.53733	0.99998	10043	0.1203
FGF7	6	0.35737	0.53737	0.99998	10044	0.1579
C3AR1	6	0.35744	0.53742	0.99998	10045	-0.0511
hsa-mir-3622b	1	0.35763	0.35784	0.99998	10046	-0.1277
IAH1	6	0.35771	0.53762	0.99998	10047	0.1403
SMYD1	6	0.35789	0.53776	0.99998	10048	-0.1573
PAPOLA	6	0.35791	0.53778	0.99998	10049	0.0739
SLC24A1	6	0.35797	0.53782	0.99998	10050	0.0617
LRRC28	4	0.35801	0.49849	0.99998	10051	-0.0504
ISY1	1	0.3581	0.3583	0.99998	10052	-0.1636
SAMSN1	6	0.35816	0.53796	0.99998	10053	-0.0041
DIP2B	6	0.35816	0.53796	0.99998	10054	-0.0821
LSM5	6	0.35822	0.53801	0.99998	10055	0.0885
HIPK4	6	0.35822	0.53801	0.99998	10056	0.0107
MAPT	6	0.35833	0.5381	0.99998	10057	0.2134
CCZ1	2	0.35833	0.37128	0.99998	10058	-0.2159
PRRC1	6	0.35837	0.53814	0.99998	10059	0.0131
hsa-mir-1178	4	0.35841	0.49895	0.99998	10060	-0.1328
LRRC16A	6	0.35849	0.53823	0.99998	10061	0.1072
ELF5	6	0.35874	0.53841	0.99998	10062	0.3977
AGXT2L1	4	0.35876	0.49937	0.99998	10063	0.0238
JMJD1C	6	0.35887	0.53852	0.99998	10064	-0.0206
PDZD3	6	0.35887	0.53852	0.99998	10065	0.0009
ANKRD46	6	0.35907	0.53867	0.99998	10066	0.0017
hsa-mir-320b-	4	0.35924	0.49988	0.99998	10067	-0.6464
TSPAN18	6	0.35928	0.53883	0.99998	10068	-0.1374
ACN9	6	0.35928	0.53883	0.99998	10069	-0.0689
hsa-mir-95	4	0.35931	0.49997	0.99998	10070	-0.0675
hsa-mir-4765	4	0.35944	0.50012	0.99998	10071	0.2289
EFCAB14	6	0.35947	0.53898	0.99998	10072	-0.0197
CBLL1	4	0.35949	0.50018	0.99998	10073	0.0135
ZCCHC8	6	0.35962	0.53908	0.99998	10074	-0.034
ZNF283	6	0.35966	0.53911	0.99998	10075	0.0173
ANXA1	6	0.35971	0.53915	0.99998	10076	0.0627
AEN	6	0.35977	0.53919	0.99998	10077	0.3005
CARKD	6	0.3598	0.53922	0.99998	10078	0.0362
hsa-mir-548s	4	0.36009	0.50084	0.99998	10079	-0.7042
PNRC2	6	0.36013	0.53947	0.99998	10080	0.193
CRP	6	0.36013	0.53947	0.99998	10081	0.0018
GSTA4	6	0.36018	0.5395	0.99998	10082	0.1217
AKAP1	6	0.36018	0.5395	0.99998	10083	0.0319
BZW2	4	0.36029	0.50108	0.99998	10084	0.0288
ARPC4-TTLL3	1	0.36047	0.36071	0.99998	10085	-0.1403
CLDN25	6	0.36054	0.53978	0.99998	10086	0.1
hsa-mir-6754	4	0.36071	0.50157	0.99998	10087	0.0438
OR2T12	6	0.36072	0.53993	0.99998	10088	0.3986
HNRNPA2B1	6	0.36075	0.53995	0.99998	10089	0.1085
hsa-mir-6730	4	0.36079	0.50165	0.99998	10090	0.3479
VEGFB	6	0.36089	0.54006	0.99998	10091	0.0703
DYDC1	6	0.36089	0.54006	0.99998	10092	-0.0559
GNPTG	6	0.36089	0.54006	0.99998	10093	0.0028
GGNBP2	4	0.361	0.50187	0.99998	10094	0.0153
OR4C16	6	0.3611	0.54023	0.99998	10095	0.038
MIEF2	1	0.36113	0.36138	0.99998	10096	-0.1963
PRSS8	6	0.3612	0.5403	0.99998	10097	0.1225
TRUB1	6	0.36125	0.54034	0.99998	10098	0.2155
hsa-mir-8088	4	0.36131	0.50222	0.99998	10099	-0.121
ASTN1	6	0.36133	0.5404	0.99998	10100	-0.046
HPRT1	6	0.36134	0.54041	0.99998	10101	0.2785
PNLIPRP2	6	0.36158	0.54058	0.99998	10102	-0.0855
TMEM41B	6	0.36158	0.54058	0.99998	10103	-0.0457
GRAP	2	0.36163	0.3741	0.99998	10104	-0.1785
ATG4C	6	0.36168	0.54068	0.99998	10105	0.0653
ZBTB9	6	0.36168	0.54068	0.99998	10106	0.0818
TFF3	6	0.36172	0.54071	0.99998	10107	0.0411
UGT2A2	1	0.36179	0.36202	0.99998	10108	-0.1233
hsa-mir-552	4	0.36179	0.50276	0.99998	10109	0.0937
GPC3	6	0.3619	0.54085	0.99998	10110	0.0172
ZNF566	6	0.3619	0.54085	0.99998	10111	0.3389
ZNF689	6	0.3619	0.54085	0.99998	10112	-0.0208
TP53AIP1	6	0.36213	0.54102	0.99998	10113	-0.02
KIF12	6	0.36213	0.54102	0.99998	10114	0.0871
RAP1GDS1	6	0.36213	0.54102	0.99998	10115	0.0142
CARD10	6	0.36213	0.54102	0.99998	10116	0.0838
PGM2	6	0.36213	0.54102	0.99998	10117	0.2082
SPRY1	6	0.36216	0.54104	0.99998	10118	0.1054
KLK12	6	0.36216	0.54104	0.99998	10119	0.1341
hsa-mir-548n	2	0.36219	0.3746	0.99998	10120	-0.002
FRG2C	5	0.36224	0.51258	0.99998	10121	-0.2897
hsa-mir-619	4	0.36225	0.50327	0.99998	10122	-0.1079

HSPA4L	6	0.36235	0.54119	0.99998	10123	0.188
SLC9B1	5	0.36242	0.51276	0.99998	10124	0.183
hsa-mir-580	4	0.36244	0.50347	0.99998	10125	-0.0226
SAGE1	6	0.36251	0.54131	0.99998	10126	0.0954
KRT19	6	0.36256	0.54136	0.99998	10127	0.0748
hsa-mir-378d-	4	0.36274	0.50382	0.99998	10128	0.0447
SCNN1A	6	0.36279	0.54152	0.99998	10129	0.1728
PPP2R3A	6	0.36288	0.54159	0.99998	10130	-0.0623
OPN1SW	6	0.36297	0.54166	0.99998	10131	0.1406
GATAD1	6	0.36297	0.54166	0.99998	10132	-0.0166
hsa-mir-4666l	4	0.36306	0.50417	0.99998	10133	-0.1544
IGLON5	6	0.36315	0.54179	0.99998	10134	0.1543
SMCO2	6	0.36326	0.54186	0.99998	10135	0.084
HORMAD1	4	0.36333	0.50447	0.99998	10136	-0.1676
CFHR4	6	0.36335	0.54192	0.99998	10137	0.0031
SLC25A13	6	0.36335	0.54192	0.99998	10138	-0.0275
IL4I1	6	0.36335	0.54192	0.99998	10139	-0.0043
ARL5B	6	0.36346	0.542	0.99998	10140	0.2291
hsa-mir-520f	2	0.36352	0.37576	0.99998	10141	-1.5489
POTEF	2	0.36352	0.37576	0.99998	10142	-1.1461
BAZ1A	6	0.36358	0.5421	0.99998	10143	0.1176
ERMARD	6	0.36358	0.5421	0.99998	10144	0.024
PLCB1	6	0.36364	0.54214	0.99998	10145	-0.0677
CCL3	6	0.36364	0.54214	0.99998	10146	-0.3312
CCDC38	6	0.36371	0.5422	0.99998	10147	-0.0523
FAM84B	6	0.3638	0.54227	0.99998	10148	0.0155
MOS	6	0.3638	0.54227	0.99998	10149	-0.0033
NDUFAF6	6	0.36382	0.54229	0.99998	10150	-0.0849
DLG2	5	0.36383	0.51407	0.99998	10151	0.0771
IFI44	6	0.36396	0.54239	0.99998	10152	-0.019
VMAC	6	0.36396	0.54239	0.99998	10153	-0.0041
SLC7A11	6	0.36397	0.5424	0.99998	10154	0.2808
SELT	6	0.36405	0.54246	0.99998	10155	0.1254
CRHBP	6	0.36415	0.54255	0.99998	10156	0.0279
WNT8B	6	0.36421	0.54259	0.99998	10157	0.0519
hsa-mir-4645	4	0.36422	0.50547	0.99998	10158	0.5201
KNG1	6	0.36424	0.54262	0.99998	10159	0.0978
POU3F3	6	0.36424	0.54262	0.99998	10160	0.0034
CDK8	6	0.3644	0.54274	0.99998	10161	-0.0059
hsa-mir-6804	4	0.36444	0.50572	0.99998	10162	0.0727
TPRG1L	6	0.36451	0.54282	0.99998	10163	0.0239
GLIPR1	6	0.36451	0.54282	0.99998	10164	0.1015
hsa-mir-5088	4	0.3646	0.50591	0.99998	10165	-0.1262
INTU	6	0.36466	0.54294	0.99998	10166	0.0829
TMEM127	6	0.36472	0.54299	0.99998	10167	0.083
LOXL1	6	0.36474	0.543	0.99998	10168	-0.0244
WWC1	6	0.36479	0.54303	0.99998	10169	0.203
hsa-mir-5190	4	0.3648	0.50611	0.99998	10170	0.0092
NEFL	6	0.36487	0.54309	0.99998	10171	0.0939
PPP1R13B	6	0.36487	0.54309	0.99998	10172	-0.0607
SLC9A3	6	0.36496	0.54315	0.99998	10173	-0.072
hsa-mir-4517	4	0.36504	0.50637	0.99998	10174	-0.0594
MRGPRX1	6	0.36505	0.54321	0.99998	10175	-0.059
C1QB	6	0.36512	0.54326	0.99998	10176	-0.0449
S100P	6	0.36526	0.54337	0.99998	10177	0.1514
MAGI3	6	0.36526	0.54337	0.99998	10178	0.1202
SNAP47	6	0.36529	0.54339	0.99998	10179	-0.1155
C4orf47	6	0.36529	0.54339	0.99998	10180	0.0236
FMO2	6	0.36542	0.54348	0.99998	10181	0.1552
hsa-mir-4279	4	0.36542	0.50679	0.99998	10182	-0.0609
hsa-mir-873	4	0.36543	0.50679	0.99998	10183	-0.0584
LHFPL5	6	0.36549	0.54354	0.99998	10184	-0.0031
PSD	6	0.36553	0.54357	0.99998	10185	0.1223
LACTB2	6	0.36553	0.54357	0.99998	10186	0.1
MC5R	6	0.36572	0.5437	0.99998	10187	0.1625
YWHAB	6	0.36587	0.54382	0.99998	10188	0.2111
hsa-mir-6090	4	0.36595	0.50739	0.99998	10189	-0.1639
TSPAN3	6	0.36601	0.54392	0.99998	10190	-0.0244
VPS9D1	6	0.3662	0.54408	0.99998	10191	-0.072
C6orf99	6	0.3662	0.54408	0.99998	10192	-0.1279
UBE2V2	6	0.3662	0.54408	0.99998	10193	-0.0322
hsa-mir-4488	4	0.3663	0.50778	0.99998	10194	-0.1629
ZC3HC1	6	0.36642	0.54424	0.99998	10195	0.0584
CCDC148	6	0.36654	0.54434	0.99998	10196	0.086
STPG1	6	0.36654	0.54434	0.99998	10197	0.2994
ACYP1	5	0.36656	0.51662	0.99998	10198	-0.0702
RLBP1	6	0.36671	0.54446	0.99998	10199	-0.0694
PPP6C	6	0.36683	0.54455	0.99998	10200	0.1927
SSX4	1	0.36689	0.36708	0.99998	10201	-0.1093
FAM124A	6	0.36695	0.54463	0.99998	10202	0.0573
SDS	6	0.36695	0.54463	0.99998	10203	0.284

ELP2	6	0.36695	0.54463	0.99998	10204	-0.1023
BRINP3	3	0.36699	0.43285	0.99998	10205	-0.1282
NUDT10	3	0.36699	0.43285	0.99998	10206	-0.373
hsa-mir-4478	4	0.36708	0.50864	0.99998	10207	-0.0509
OR4N5	6	0.36717	0.54479	0.99998	10208	0.0727
TRIL	6	0.36732	0.54491	0.99998	10209	0.0676
hsa-mir-5004	4	0.36734	0.50894	0.99998	10210	0.1245
OR8J3	5	0.36742	0.51742	0.99998	10211	0.0064
STEAP3	6	0.36752	0.54507	0.99998	10212	0.1497
ACADVL	6	0.36753	0.54507	0.99998	10213	-0.1271
hsa-mir-4261	2	0.36771	0.37938	0.99998	10214	-0.0651
CNGA4	6	0.36777	0.54526	0.99998	10215	0.0222
CHCHD2	6	0.36777	0.54526	0.99998	10216	0.0619
RASGRF2	6	0.36777	0.54526	0.99998	10217	0.0101
hsa-mir-197	4	0.3678	0.50943	0.99998	10218	-0.2481
TSPAN7	6	0.36788	0.54535	0.99998	10219	-0.0923
LOC10028753	2	0.3679	0.37954	0.99998	10220	-0.1833
C7orf55	5	0.36797	0.51793	0.99998	10221	-0.1848
TMEM130	6	0.36807	0.5455	0.99998	10222	-0.1221
TAPT1	6	0.36814	0.54555	0.99998	10223	0.2601
NR2C2AP	6	0.36814	0.54555	0.99998	10224	-0.1207
FBXO5	6	0.36836	0.54571	0.99998	10225	-0.0482
COX7B2	6	0.36836	0.54571	0.99998	10226	-0.0542
ZBTB2	6	0.36839	0.54575	0.99998	10227	0.1473
hsa-mir-4286	4	0.36843	0.51016	0.99998	10228	0.0519
MYH3	6	0.36849	0.54582	0.99998	10229	0.1142
DYM	6	0.36862	0.54591	0.99998	10230	0.0507
L3MBTL4	6	0.36878	0.54604	0.99998	10231	0.043
LRRTM3	6	0.36884	0.54609	0.99998	10232	0.1719
ZNF426	5	0.36886	0.51878	0.99998	10233	-0.0373
SYNJ2BP-COX	1	0.36891	0.36909	0.99998	10234	-0.124
ZNF687	6	0.36894	0.54616	0.99998	10235	0.0644
GEM	6	0.36901	0.54623	0.99998	10236	-0.0454
COCH	6	0.36905	0.54625	0.99998	10237	-0.0685
FEV	6	0.36905	0.54625	0.99998	10238	0.0277
NBPF8	4	0.3691	0.51092	0.99998	10239	-0.2729
RNF165	6	0.36912	0.5463	0.99998	10240	0.2196
CYGB	6	0.36918	0.54635	0.99998	10241	-0.0917
ATP6V0A2	6	0.36918	0.54635	0.99998	10242	-0.0521
TMEM30B	6	0.3693	0.54642	0.99998	10243	0.048
PDGFRB	6	0.3693	0.54642	0.99998	10244	0.0262
TBL2	6	0.3693	0.54642	0.99998	10245	-0.0001
PTPN4	6	0.36932	0.54645	0.99998	10246	0.0211
hsa-mir-6762	4	0.36945	0.51129	0.99998	10247	-0.0818
KRT85	4	0.36945	0.51129	0.99998	10248	0.1267
ZCCHC14	6	0.36951	0.5466	0.99998	10249	-0.1911
DLEC1	6	0.36955	0.54661	0.99998	10250	-0.1038
SGTB	6	0.36955	0.54661	0.99998	10251	0.0817
EPHA8	6	0.36955	0.54661	0.99998	10252	0.0699
SOCS2	6	0.36955	0.54661	0.99998	10253	-0.0368
hsa-mir-500b	3	0.36957	0.43453	0.99998	10254	-0.3622
ASB9	6	0.36961	0.54667	0.99998	10255	-0.0065
ZNF750	6	0.36969	0.54674	0.99998	10256	0.0247
SFRP5	6	0.36978	0.54681	0.99998	10257	0.0476
EPPK1	6	0.36978	0.54681	0.99998	10258	0.0517
TRIM41	6	0.36985	0.54686	0.99998	10259	0.0387
ZNF776	5	0.36992	0.51981	0.99998	10260	0.0263
OR2M4	6	0.36994	0.54692	0.99998	10261	0.0561
CLDN5	6	0.37008	0.54703	0.99998	10262	-0.0994
PTOV1	6	0.37028	0.54718	0.99998	10263	0.2033
IFNE	6	0.37034	0.54723	0.99998	10264	0.1782
PRKACB	6	0.37048	0.54734	0.99998	10265	-0.0182
NSUN5	6	0.37048	0.54734	0.99998	10266	0.093
PURG	6	0.37051	0.54736	0.99998	10267	-0.0857
HSPA2	6	0.37051	0.54736	0.99998	10268	-0.0124
BIVM-ERCC5	2	0.37054	0.38179	0.99998	10269	-0.2023
FGFBP3	6	0.37064	0.54745	0.99998	10270	-0.0176
TMEM86B	4	0.37071	0.51267	0.99998	10271	-0.0734
RNF223	4	0.37071	0.51267	0.99998	10272	0.0944
DOCK5	6	0.37083	0.54759	0.99998	10273	-0.0791
COLEC11	6	0.37083	0.54759	0.99998	10274	0.0117
NIF3L1	6	0.37103	0.54775	0.99998	10275	0.0943
LOC158434	6	0.37103	0.54775	0.99998	10276	-0.0353
TXNL1	6	0.37106	0.54777	0.99998	10277	-0.02
hsa-mir-6083	4	0.37109	0.51309	0.99998	10278	-0.0698
MOV10L1	6	0.37121	0.54788	0.99998	10279	0.1725
CIB1	6	0.37123	0.5479	0.99998	10280	0.0547
hsa-mir-6795	2	0.37125	0.38241	0.99998	10281	-0.0061
DEDD	6	0.37129	0.54795	0.99998	10282	-0.0187
IGDCC4	6	0.3714	0.54802	0.99998	10283	0.2761
HHLA2	6	0.3714	0.54802	0.99998	10284	0.1859

AMZ1	4	0.37154	0.51363	0.99998	10285	-0.0966
PARPBP	6	0.37156	0.54815	0.99998	10286	0.1226
IFI35	6	0.37156	0.54815	0.99998	10287	-0.0211
CPT1B	6	0.37156	0.54815	0.99998	10288	0.0228
ZNF440	6	0.37174	0.54827	0.99998	10289	0.4564
CRYGC	6	0.37179	0.54831	0.99998	10290	0.1079
ZNF583	6	0.37179	0.54831	0.99998	10291	-0.0534
APOL3	6	0.37204	0.54849	0.99998	10292	0.2268
DUSP8	6	0.3721	0.54853	0.99998	10293	0.0498
TRMT10B	6	0.37215	0.54857	0.99998	10294	0.2157
PKP2	6	0.37222	0.54863	0.99998	10295	-0.0858
ZNF266	6	0.37239	0.54875	0.99998	10296	-0.0193
SPACA3	6	0.37239	0.54875	0.99998	10297	0.0415
FAM118A	6	0.37239	0.54875	0.99998	10298	-0.0725
SLC5A3	6	0.37248	0.54882	0.99998	10299	0.0111
DBNL	6	0.37254	0.54886	0.99998	10300	-0.0124
TFEC	6	0.37265	0.54896	0.99998	10301	0.1337
hsa-mir-4321	4	0.37276	0.51419	0.99998	10302	-0.1605
PRSS58	6	0.37276	0.54905	0.99998	10303	-0.0394
VEGFA	6	0.37285	0.54912	0.99998	10304	0.1465
ACTL9	6	0.37285	0.54912	0.99998	10305	-0.2092
PLCL2	6	0.37291	0.54916	0.99998	10306	0.177
C7orf71	6	0.37291	0.54916	0.99998	10307	0.2108
DEFA6	6	0.37291	0.54916	0.99998	10308	0.0972
PTPN21	6	0.37291	0.54916	0.99998	10309	0.0185
SLCO6A1	6	0.37291	0.54916	0.99998	10310	-0.1616
KIAA0895	4	0.37292	0.51427	0.99998	10311	0.1821
CACNB4	6	0.3731	0.5493	0.99998	10312	0.0997
PRSS27	6	0.37316	0.54934	0.99998	10313	-0.0604
OPRL1	6	0.37316	0.54934	0.99998	10314	-0.0521
DERL2	6	0.37324	0.54941	0.99998	10315	-0.0386
GOLGA8B	4	0.37325	0.51442	0.99998	10316	0.0496
POLR2J2	1	0.37336	0.3735	0.99998	10317	-0.1376
CHL1	6	0.37338	0.54952	0.99998	10318	0.1367
SPINT2	6	0.3734	0.54954	0.99998	10319	-0.0524
FAM129B	6	0.37351	0.54963	0.99998	10320	0.1443
PCP4L1	6	0.37351	0.54963	0.99998	10321	-0.1248
APOLD1	6	0.37351	0.54963	0.99998	10322	-0.1
ECSIT	6	0.37351	0.54963	0.99998	10323	0.1888
CXCR6	6	0.37351	0.54963	0.99998	10324	0.0128
MTERFD1	6	0.37351	0.54963	0.99998	10325	-0.0495
C10orf99	6	0.37355	0.54967	0.99998	10326	0.0528
hsa-mir-4793	4	0.37357	0.51457	0.99998	10327	-0.0581
ANKMY1	6	0.37372	0.54979	0.99998	10328	0.0724
CHP1	6	0.37376	0.54982	0.99998	10329	0.052
MKNK2	6	0.37379	0.54984	0.99998	10330	0.0682
C6	6	0.37382	0.54987	0.99998	10331	0.0154
TAS2R38	6	0.37388	0.54992	0.99998	10332	0.0682
hsa-mir-548ax	1	0.37391	0.37406	0.99998	10333	-0.127
IL12B	6	0.37395	0.54997	0.99998	10334	0.0248
NPAP1	6	0.37403	0.55004	0.99998	10335	0.1348
YAF2	6	0.37403	0.55004	0.99998	10336	0.0107
ENPP3	6	0.37449	0.55039	0.99998	10337	0.1058
OR4D6	6	0.37449	0.55039	0.99998	10338	0.2422
WDR66	6	0.3745	0.55039	0.99998	10339	0.0896
ZC3H12A	6	0.37457	0.55045	0.99998	10340	-0.0613
MDGA1	6	0.37469	0.55055	0.99998	10341	0.0539
GALNT10	6	0.37477	0.55061	0.99998	10342	0.1236
NME6	6	0.37477	0.55061	0.99998	10343	0.1113
CXorf40A	3	0.37482	0.43794	0.99998	10344	-0.2259
PROZ	6	0.37486	0.55068	0.99998	10345	0.2215
GNA14	6	0.3749	0.55071	0.99998	10346	-0.1169
PDE7B	6	0.3749	0.55071	0.99998	10347	0.0005
B3GALT1	6	0.3749	0.55071	0.99998	10348	-0.0867
PODXL	6	0.37499	0.55078	0.99998	10349	0.2491
TMEM178B	6	0.37504	0.55081	0.99998	10350	-0.132
SYNE2	6	0.37504	0.55081	0.99998	10351	0.0016
AARD	6	0.37508	0.55084	0.99998	10352	-0.0937
LIN28A	6	0.37515	0.55089	0.99998	10353	0.0272
hsa-mir-1537	3	0.37524	0.43821	0.99998	10354	0.3655
C17orf104	6	0.37524	0.55096	0.99998	10355	-0.0786
ARR3	6	0.37538	0.55107	0.99998	10356	0.0595
RNF126	6	0.37554	0.55119	0.99998	10357	0.0761
MAGEH1	6	0.37562	0.55125	0.99998	10358	0.1879
CORT	6	0.37562	0.55125	0.99998	10359	0.1301
ZNF138	6	0.37562	0.55125	0.99998	10360	0.2272
MUM1L1	6	0.37568	0.55129	0.99998	10361	0.0945
CCDC124	6	0.37574	0.55135	0.99998	10362	0.0269
HSD17B11	6	0.37582	0.55141	0.99998	10363	-0.0121
hsa-mir-3156-	3	0.37585	0.43861	0.99998	10364	0.1374
TNFAIP6	6	0.37585	0.55144	0.99998	10365	0.1303

POLH	6	0.37593	0.5515	0.99998	10366	-0.0633
HVCN1	6	0.37593	0.5515	0.99998	10367	-0.0118
APOD	6	0.37601	0.55157	0.99998	10368	0.0383
FOXD4L3	1	0.3761	0.37625	0.99998	10369	-0.355
LONRF1	6	0.37616	0.55169	0.99998	10370	-0.0587
C11orf16	6	0.37616	0.55169	0.99998	10371	0.0307
HIGD2A	6	0.37632	0.5518	0.99998	10372	-0.0733
PTHLH	6	0.37652	0.55195	0.99998	10373	0.1626
GAREM	6	0.37673	0.55211	0.99998	10374	0.0749
NF2	6	0.37674	0.55212	0.99998	10375	-0.0094
SLC5A6	6	0.37674	0.55212	0.99998	10376	0.0207
EIF3M	6	0.37674	0.55212	0.99998	10377	-0.0298
hsa-mir-4777	4	0.3768	0.51605	0.99998	10378	0.2838
HDHD3	6	0.37685	0.5522	0.99998	10379	-0.0226
RHBDF1	6	0.37685	0.5522	0.99998	10380	0.0011
MRPL1	6	0.37693	0.55227	0.99998	10381	0.0353
ALS2CR12	6	0.37701	0.55233	0.99998	10382	-0.0469
F8	6	0.37701	0.55233	0.99998	10383	0.2523
KNCN	6	0.37706	0.55236	0.99998	10384	-0.0719
CITED1	6	0.37711	0.5524	0.99998	10385	-0.1358
ALOX15	6	0.37717	0.55244	0.99998	10386	-0.0373
CLDN22	6	0.37747	0.55268	0.99998	10387	0.2574
PSMC4	6	0.37762	0.5528	0.99998	10388	-0.077
GLRB	6	0.37762	0.5528	0.99998	10389	0.2379
UBLCP1	6	0.37783	0.55296	0.99998	10390	0.0587
C9orf69	6	0.37784	0.55297	0.99998	10391	0.0085
GALNS	6	0.37796	0.55306	0.99998	10392	0.0217
USP37	6	0.37806	0.55314	0.99998	10393	0.0604
SLC2A11	6	0.37806	0.55314	0.99998	10394	0.0312
CHST10	6	0.37806	0.55314	0.99998	10395	0.1283
MKRN3	6	0.37806	0.55314	0.99998	10396	0.0005
DNAH6	6	0.37813	0.55319	0.99998	10397	0.0139
KDM6A	6	0.37813	0.55319	0.99998	10398	-0.1585
RHBDD2	6	0.37813	0.55319	0.99998	10399	0.0247
COPS3	6	0.37813	0.55319	0.99998	10400	-0.169
TMPO	6	0.37839	0.55339	0.99998	10401	-0.0075
EIF4A1	6	0.37839	0.55339	0.99998	10402	-0.1533
SCGN	6	0.37839	0.55339	0.99998	10403	-0.0313
RAB12	6	0.37839	0.55339	0.99998	10404	0.0748
OR3A1	6	0.37857	0.55353	0.99998	10405	-0.0893
TRPM3	6	0.37869	0.55363	0.99998	10406	0.0328
HIBCH	6	0.37873	0.55365	0.99998	10407	-0.0668
MANEA	6	0.37873	0.55365	0.99998	10408	0.3901
MTMR9	6	0.37879	0.5537	0.99998	10409	0.0403
CREB3	6	0.37879	0.5537	0.99998	10410	-0.044
CEP250	6	0.37887	0.55377	0.99998	10411	-0.0271
NPTX1	6	0.37887	0.55377	0.99998	10412	0.0655
hsa-mir-548ae	2	0.37893	0.38909	0.99998	10413	-0.0866
ARHGAP9	6	0.379	0.55387	0.99998	10414	-0.1759
SNX25	6	0.37907	0.55393	0.99998	10415	-0.0605
SLC26A5	6	0.37907	0.55393	0.99998	10416	0.1779
APTX	6	0.37918	0.55403	0.99998	10417	0.1466
MICU1	6	0.37922	0.55405	0.99998	10418	0.0745
SPATA16	6	0.37929	0.55411	0.99998	10419	-0.0285
GPR32	6	0.37929	0.55411	0.99998	10420	0.0361
SERINC5	6	0.37941	0.55421	0.99998	10421	-0.0414
hsa-mir-3145	3	0.37942	0.44094	0.99998	10422	-0.1911
BHLHB9	6	0.37964	0.55437	0.99998	10423	0.1743
TDRD1	6	0.37972	0.55443	0.99998	10424	0.0031
PFKFB2	6	0.37972	0.55443	0.99998	10425	-0.0148
MMP16	6	0.37985	0.55453	0.99998	10426	0.1231
BCDIN3D	6	0.37986	0.55454	0.99998	10427	0.0201
AK5	6	0.37988	0.55456	0.99998	10428	-0.0283
FUK	6	0.37992	0.55459	0.99998	10429	0.2859
FBXO18	6	0.3802	0.5548	0.99998	10430	0.2794
SLC44A3	6	0.3802	0.5548	0.99998	10431	0.2056
WWP1	6	0.3802	0.5548	0.99998	10432	-0.0166
hsa-mir-18a	4	0.3802	0.5176	0.99998	10433	0.2726
CAPZA2	6	0.38034	0.55491	0.99998	10434	0.0451
GPR141	6	0.38034	0.55491	0.99998	10435	-0.0341
GIP	6	0.38046	0.555	0.99998	10436	-0.0902
OR5M8	6	0.3805	0.55503	0.99998	10437	0.1967
CCDC129	4	0.38054	0.51775	0.99998	10438	-0.0161
SLC38A8	6	0.38057	0.55509	0.99998	10439	0.0952
RNF133	6	0.38059	0.55511	0.99998	10440	-0.2669
ASAP2	6	0.38083	0.5553	0.99998	10441	-0.108
GLT8D1	6	0.38086	0.55532	0.99998	10442	0.0672
CLECL1	6	0.38111	0.5555	0.99998	10443	-0.046
STRA6	6	0.38111	0.5555	0.99998	10444	0.1298
COA3	6	0.38114	0.55552	0.99998	10445	-0.0436
PTRHD1	6	0.38121	0.55557	0.99998	10446	-0.0236

DHRS7B	6	0.38128	0.55563	0.99998	10447	-0.0046
MGAT4B	6	0.38147	0.55577	0.99998	10448	0.1649
SLC25A17	6	0.38147	0.55577	0.99998	10449	-0.0893
FGF5	6	0.38147	0.55577	0.99998	10450	0.2809
BATF2	6	0.38163	0.55589	0.99998	10451	-0.0231
GIPR	6	0.38163	0.55589	0.99998	10452	-0.0027
NPAS2	6	0.38163	0.55589	0.99998	10453	-0.1726
GEN1	6	0.38163	0.55589	0.99998	10454	-0.1724
GSTA3	6	0.38163	0.55589	0.99998	10455	-0.2963
FOLR3	6	0.38172	0.55596	0.99998	10456	-0.031
TMEM119	6	0.38186	0.55607	0.99998	10457	-0.1082
TAC4	6	0.38198	0.55615	0.99998	10458	0.0147
CYP2A7	5	0.38238	0.5316	0.99998	10459	0.0843
hsa-mir-10a	4	0.38245	0.51864	0.99998	10460	0.0165
hsa-mir-647	4	0.38245	0.51864	0.99998	10461	-0.0088
EPB41L3	6	0.38248	0.55656	0.99998	10462	0.0202
TXNRD2	6	0.38257	0.55663	0.99998	10463	0.0372
RNF113B	6	0.38265	0.55669	0.99998	10464	-0.0872
SERPINA1	6	0.38283	0.55685	0.99998	10465	0.1283
KCNIP4	6	0.38283	0.55685	0.99998	10466	0.1174
CBX3	6	0.383	0.55698	0.99998	10467	0.0744
REEP3	6	0.38303	0.55699	0.99998	10468	-0.059
GINS3	6	0.38303	0.55699	0.99998	10469	-0.0503
IL7R	6	0.38313	0.55707	0.99998	10470	0.0485
ATP6V0E2	6	0.3832	0.55713	0.99998	10471	-0.002
OR5H1	6	0.38332	0.55722	0.99998	10472	0.1374
SALL3	6	0.38332	0.55722	0.99998	10473	0.0881
KRT83	4	0.38338	0.51907	0.99998	10474	0.0356
TWIST1	6	0.38357	0.55741	0.99998	10475	0.1001
SLC25A27	6	0.38367	0.55749	0.99998	10476	-0.0526
CCER1	6	0.38369	0.55751	0.99998	10477	0.328
HOXC12	6	0.38391	0.55768	0.99998	10478	-0.0819
CADPS2	6	0.38395	0.55771	0.99998	10479	-0.0516
FOXL2	6	0.38407	0.55781	0.99998	10480	0.0749
VRK2	6	0.38426	0.55796	0.99998	10481	0.1033
PABPC1L	6	0.38426	0.55796	0.99998	10482	-0.0889
STX12	6	0.3844	0.55807	0.99998	10483	-0.0555
LMLN	5	0.38464	0.53377	0.99998	10484	-0.0624
hsa-mir-6799	4	0.38464	0.51967	0.99998	10485	0.0669
PEAR1	6	0.38474	0.55831	0.99998	10486	0.0847
PTTG2	6	0.38474	0.55831	0.99998	10487	-0.0842
FGF3	6	0.38474	0.55831	0.99998	10488	-0.0259
hsa-mir-6814	4	0.38485	0.51975	0.99998	10489	0.1245
C11orf71	6	0.38489	0.55844	0.99998	10490	0.098
GPR25	6	0.38514	0.55863	0.99998	10491	-0.0251
ARFIP2	6	0.38515	0.55864	0.99998	10492	0.0733
DLX1	6	0.3853	0.55876	0.99998	10493	-0.0648
SLC39A12	4	0.38533	0.51999	0.99998	10494	0.2364
IRX2	6	0.38545	0.55887	0.99998	10495	0.1045
NIPAL2	6	0.38551	0.55893	0.99998	10496	0.0561
MZT1	6	0.38551	0.55893	0.99998	10497	-0.0734
SIRPG	6	0.3856	0.559	0.99998	10498	0.1105
CCDC43	6	0.3856	0.559	0.99998	10499	-0.1353
COX6C	6	0.38572	0.55909	0.99998	10500	0.0598
KLHDC2	6	0.38572	0.55909	0.99998	10501	0.0765
FBXO25	6	0.38576	0.55913	0.99998	10502	-0.1144
RNASEH1	6	0.38576	0.55913	0.99998	10503	-0.0681
WNT2	6	0.38588	0.55922	0.99998	10504	0.022
KRTAP19-5	6	0.38609	0.5594	0.99998	10505	-0.281
RABEP2	6	0.38609	0.5594	0.99998	10506	-0.1199
PLXND1	6	0.38613	0.55943	0.99998	10507	-0.1008
OCLN	6	0.38613	0.55943	0.99998	10508	-0.0351
C9orf64	6	0.38613	0.55943	0.99998	10509	0.186
CHMP4A	6	0.38632	0.55958	0.99998	10510	-0.0874
hsa-mir-5702	4	0.38658	0.52055	0.99998	10511	0.1303
OR5B3	6	0.3868	0.55996	0.99998	10512	0.0001
ESM1	6	0.3869	0.56003	0.99998	10513	-0.1166
CSH1	3	0.38697	0.44592	0.99998	10514	-0.4149
KIAA0430	6	0.38706	0.56015	0.99998	10515	-0.0971
SLC13A4	6	0.38714	0.56022	0.99998	10516	0.0108
ZNF121	6	0.38726	0.5603	0.99998	10517	0.1787
INPP5D	6	0.3873	0.56033	0.99998	10518	0.114
PHF15	6	0.38735	0.56036	0.99998	10519	-0.0719
C10orf120	6	0.3874	0.56041	0.99998	10520	0.24
CCDC8	6	0.38762	0.56057	0.99998	10521	-0.0027
VSTM2B	6	0.38763	0.56058	0.99998	10522	-0.0201
TSPAN32	6	0.38763	0.56058	0.99998	10523	-0.0519
DXO	2	0.38772	0.39675	0.99998	10524	-0.0738
RCAN3	6	0.38776	0.56067	0.99998	10525	0.0374
CLPTM1	6	0.38776	0.56067	0.99998	10526	-0.0019
SULF1	6	0.38786	0.56075	0.99998	10527	0.0008

EDA2R	6	0.38789	0.56078	0.99998	10528	-0.1967
TMEM164	6	0.38805	0.5609	0.99998	10529	-0.1262
HERC5	6	0.38805	0.5609	0.99998	10530	0.1283
ZNF44	6	0.38805	0.5609	0.99998	10531	0.1951
DHTKD1	6	0.38805	0.5609	0.99998	10532	0.1681
KRTAP1-3	5	0.38823	0.53718	0.99998	10533	-0.2353
FOXP1	6	0.38833	0.56113	0.99998	10534	0.1174
TAC1	6	0.38839	0.56117	0.99998	10535	0.3519
PODNL1	6	0.38839	0.56117	0.99998	10536	0.0431
HIST1H2AM	6	0.38846	0.56123	0.99998	10537	-0.0954
PIWIL4	6	0.38846	0.56123	0.99998	10538	0.1236
KRTAP20-2	6	0.38851	0.56127	0.99998	10539	-0.18
FGFR1	6	0.3886	0.56133	0.99998	10540	0.0989
SEC24C	6	0.3887	0.56142	0.99998	10541	-0.0107
MYH7	6	0.3887	0.56142	0.99998	10542	0.0612
NUAK2	6	0.3887	0.56142	0.99998	10543	0.1142
TBXA2R	6	0.3887	0.56142	0.99998	10544	0.1326
USP6	6	0.3887	0.56142	0.99998	10545	-0.2754
FGFR1OP2	6	0.38882	0.56152	0.99998	10546	0.3429
CCL1	6	0.38892	0.5616	0.99998	10547	0.0034
hsa-mir-4306	4	0.38896	0.52169	0.99998	10548	0.0843
ZNF589	6	0.38897	0.56163	0.99998	10549	0.2205
hsa-mir-3926-	1	0.38903	0.38923	0.99998	10550	-0.1488
MINA	6	0.38906	0.56171	0.99998	10551	-0.0273
ZNF80	6	0.38906	0.56171	0.99998	10552	-0.0464
SERPINA9	6	0.3891	0.56174	0.99998	10553	0.036
DCAF10	6	0.38923	0.56185	0.99998	10554	-0.0125
YTHDF1	6	0.38924	0.56185	0.99998	10555	0.0517
HPS6	4	0.38931	0.52185	0.99998	10556	0.1596
GRIN2B	6	0.38936	0.56194	0.99998	10557	0.11
PDE1A	6	0.38936	0.56194	0.99998	10558	0.1603
YY1AP1	6	0.38943	0.562	0.99998	10559	-0.0723
C17orf77	6	0.38948	0.56203	0.99998	10560	0.0113
OMG	6	0.38958	0.5621	0.99998	10561	0.0439
TMEM244	6	0.38971	0.5622	0.99998	10562	0.0093
OSTC	5	0.38981	0.53868	0.99998	10563	0.0856
FAM220A	6	0.38983	0.56227	0.99998	10564	0.039
LIPM	6	0.38983	0.56227	0.99998	10565	-0.0598
COLGALT2	6	0.38984	0.56228	0.99998	10566	0.0532
ZNF681	6	0.38984	0.56228	0.99998	10567	0.0863
FRMD7	6	0.38998	0.56239	0.99998	10568	-0.0781
SLC34A1	6	0.39006	0.56245	0.99998	10569	0.3166
C15orf62	6	0.39006	0.56245	0.99998	10570	-0.0393
NUP133	6	0.39006	0.56245	0.99998	10571	0.1436
ACPP	6	0.39014	0.56251	0.99998	10572	-0.0393
ZFHX2	6	0.39032	0.56264	0.99998	10573	-0.0235
LRIG1	6	0.39048	0.56278	0.99998	10574	0.0872
TAGLN2	6	0.39048	0.56278	0.99998	10575	-0.0699
TPCN2	6	0.39054	0.56282	0.99998	10576	-0.165
ARGFX	6	0.39065	0.56291	0.99998	10577	0.0605
hsa-mir-4535	4	0.39079	0.52258	0.99998	10578	-0.0505
ATP5SL	6	0.39086	0.56307	0.99998	10579	-0.0546
HUS1B	6	0.39086	0.56307	0.99998	10580	0.055
AP5Z1	6	0.39086	0.56307	0.99998	10581	-0.0567
FAM71F2	6	0.39089	0.56309	0.99998	10582	0.2487
PXMP4	6	0.39089	0.56309	0.99998	10583	0.1175
H3F3C	6	0.39089	0.56309	0.99998	10584	0.0553
HRAS	6	0.39089	0.56309	0.99998	10585	0.1304
VCAM1	6	0.39112	0.56326	0.99998	10586	-0.0467
MIPEP	6	0.39122	0.56333	0.99998	10587	-0.0639
hsa-mir-1-1	4	0.39141	0.52285	0.99998	10588	0.2714
F2RL1	6	0.39162	0.56366	0.99998	10589	0.1017
hsa-mir-203b	1	0.39162	0.39183	0.99998	10590	-0.0713
CRADD	6	0.39162	0.56366	0.99998	10591	0.4009
NUDT19	6	0.39165	0.56368	0.99998	10592	-0.0598
THRSP	6	0.39184	0.56383	0.99998	10593	0.0523
MRPL35	6	0.39194	0.56391	0.99998	10594	0.2252
FAM127A	6	0.39216	0.56409	0.99998	10595	-0.054
CYP7A1	6	0.39216	0.56409	0.99998	10596	0.0988
GMEB2	6	0.39216	0.56409	0.99998	10597	0.2125
DEFB103A	1	0.39224	0.39243	0.99998	10598	-0.0783
SPPL2B	6	0.39233	0.56423	0.99998	10599	-0.0912
CD226	6	0.39236	0.56425	0.99998	10600	0.1922
LOC10086267	6	0.39236	0.56425	0.99998	10601	-0.1282
EGFL7	6	0.3924	0.56427	0.99998	10602	-0.1413
SPRR2B	1	0.3924	0.39259	0.99998	10603	-0.4558
hsa-mir-4498	4	0.39248	0.52336	0.99998	10604	-0.2074
ZNF594	6	0.3925	0.56436	0.99998	10605	0.0017
TEDDM1	6	0.3925	0.56436	0.99998	10606	0.0921
ZAR1	6	0.39256	0.5644	0.99998	10607	0.1354
THYN1	6	0.39265	0.56447	0.99998	10608	0.0707

ENAM	6	0.3928	0.56458	0.99998	10609	0.226
DEAF1	6	0.39292	0.56467	0.99998	10610	-0.0302
TAF1L	6	0.39313	0.56481	0.99998	10611	0.1302
MRPS35	6	0.39317	0.56485	0.99998	10612	-0.0976
NDUFC1	6	0.39317	0.56485	0.99998	10613	0.1332
CIB2	6	0.39317	0.56485	0.99998	10614	0.0117
hsa-mir-4276	4	0.39323	0.52371	0.99998	10615	0.008
SETD7	6	0.39327	0.56491	0.99998	10616	0.1746
CKAP4	6	0.39342	0.56503	0.99998	10617	0.0575
NEK9	6	0.39342	0.56503	0.99998	10618	0.1903
HEPN1	6	0.39342	0.56503	0.99998	10619	0.1311
KLHL12	6	0.39358	0.56515	0.99998	10620	0.0339
EMP2	6	0.39358	0.56515	0.99998	10621	0.1169
TNFRSF9	6	0.39358	0.56515	0.99998	10622	0.0363
CFHR1	6	0.39359	0.56516	0.99998	10623	0.1829
LAPTM4B	6	0.39359	0.56516	0.99998	10624	0.0101
ADAM20	6	0.39378	0.56531	0.99998	10625	-0.099
OR10H4	6	0.39385	0.56537	0.99998	10626	-0.0327
hsa-mir-7706	4	0.39386	0.52402	0.99998	10627	-0.0436
RBMS1	6	0.39388	0.5654	0.99998	10628	0.063
ZP4	6	0.39388	0.5654	0.99998	10629	0.0613
REM1	6	0.39388	0.5654	0.99998	10630	0.1402
CSTB	6	0.3939	0.56541	0.99998	10631	0.0815
CEP76	6	0.39411	0.56558	0.99998	10632	-0.0918
C1R	6	0.39411	0.56558	0.99998	10633	-0.0716
CALR3	6	0.39411	0.56558	0.99998	10634	0.1719
GALNT6	6	0.39411	0.56558	0.99998	10635	0.1658
TSPY3	1	0.3942	0.39444	0.99998	10636	-0.0817
hsa-mir-3189	4	0.39428	0.52421	0.99998	10637	0.0313
SPP1	6	0.3943	0.56572	0.99998	10638	0.0618
PCED1B	6	0.3943	0.56573	0.99998	10639	-0.0787
RHOXF2B	3	0.39444	0.45073	0.99998	10640	-0.0668
SSX3	3	0.39444	0.45073	0.99998	10641	-0.9148
MIER2	6	0.39444	0.56582	0.99998	10642	-0.1121
ZMAT2	6	0.39468	0.566	0.99998	10643	0.0118
TAS2R42	6	0.39468	0.566	0.99998	10644	0.1479
COMT	6	0.39468	0.566	0.99998	10645	-0.0583
TINAGL1	6	0.39471	0.56603	0.99998	10646	0.1399
MYLK2	6	0.39491	0.56618	0.99998	10647	0.0599
TIAL1	6	0.39491	0.56618	0.99998	10648	0.038
VPS16	6	0.39511	0.56634	0.99998	10649	-0.1094
NFKBIA	6	0.39527	0.56647	0.99998	10650	-0.0781
C11orf63	6	0.39528	0.56648	0.99998	10651	0.0048
PLCE1	6	0.39528	0.56648	0.99998	10652	0.0072
ADORA2A	6	0.39546	0.56662	0.99998	10653	0.0433
PPP1R3E	6	0.39546	0.56662	0.99998	10654	0.2687
PATL2	6	0.39555	0.56669	0.99998	10655	-0.0666
ZNF677	6	0.39555	0.56669	0.99998	10656	0.0327
EIF5B	6	0.39555	0.56669	0.99998	10657	0.1987
DIO1	6	0.39591	0.56695	0.99998	10658	-0.0092
hsa-mir-4277	4	0.39591	0.52501	0.99998	10659	0.0636
UNKL	6	0.39606	0.56707	0.99998	10660	0.1268
FLT1	6	0.39606	0.56707	0.99998	10661	0.0236
GJB6	6	0.39615	0.56713	0.99998	10662	0.0878
CCDC58	6	0.39621	0.56718	0.99998	10663	-0.1459
CLIC1	6	0.39635	0.56728	0.99998	10664	0.0883
KCND3	6	0.39638	0.5673	0.99998	10665	-0.0354
HIST2H4B	1	0.3964	0.39665	0.99998	10666	-0.3127
KIAA0020	6	0.39644	0.56735	0.99998	10667	0.3415
hsa-mir-3146	4	0.39654	0.52531	0.99998	10668	0.2761
LPAR2	6	0.39655	0.56742	0.99998	10669	0.0003
SEC11A	6	0.39659	0.56746	0.99998	10670	0.0661
ZFYVE27	6	0.39659	0.56746	0.99998	10671	0.0537
EPG5	6	0.39669	0.56753	0.99998	10672	-0.0495
YIPF1	6	0.39673	0.56756	0.99998	10673	0.0112
GUCD1	6	0.39673	0.56756	0.99998	10674	0.0748
MYCL	2	0.39678	0.40472	0.99998	10675	-0.1805
RASA2	6	0.39684	0.56764	0.99998	10676	-0.0723
SLC12A4	6	0.39684	0.56764	0.99998	10677	-0.1731
FAM193B	4	0.39686	0.52547	0.99998	10678	0.0821
MYSM1	6	0.39688	0.56767	0.99998	10679	0.0784
TMPRSS2	6	0.39688	0.56767	0.99998	10680	0.0094
DPP8	6	0.39698	0.56774	0.99998	10681	-0.0996
NMT2	6	0.39705	0.5678	0.99998	10682	0.1915
GLTPD2	6	0.39705	0.5678	0.99998	10683	0.0348
SLC29A3	6	0.39706	0.56781	0.99998	10684	-0.1137
KRTAP12-3	6	0.39717	0.5679	0.99998	10685	0.3115
C3orf58	6	0.3972	0.56792	0.99998	10686	0.2404
hsa-mir-424	4	0.3973	0.52567	0.99998	10687	-0.0578
NDC1	4	0.39742	0.52573	0.99998	10688	0.2239
KLF8	6	0.39742	0.5681	0.99998	10689	-0.0741

SCAMP5	6	0.39743	0.5681	0.99998	10690	0.2689
AMPD3	6	0.39743	0.5681	0.99998	10691	0.1448
GLB1L2	4	0.39744	0.52574	0.99998	10692	-0.1062
NUP210	6	0.39751	0.56817	0.99998	10693	0.3611
ACOT11	6	0.39758	0.56822	0.99998	10694	-0.0962
TMEM131	6	0.39769	0.5683	0.99998	10695	-0.0567
RASA4	2	0.39769	0.4055	0.99998	10696	0.2633
hsa-mir-4644	4	0.39779	0.5259	0.99998	10697	-0.0524
YEATS2	6	0.39797	0.56852	0.99998	10698	0.0369
PLEKHA7	6	0.39797	0.56852	0.99998	10699	-0.029
SLITRK5	6	0.39797	0.56852	0.99998	10700	0.2043
C1orf112	6	0.39813	0.56863	0.99998	10701	-0.0422
SLC26A11	6	0.39813	0.56863	0.99998	10702	-0.0092
GARNL3	6	0.3983	0.56876	0.99998	10703	0.2094
hsa-mir-196a-	4	0.39841	0.52621	0.99998	10704	-0.0075
hsa-mir-3665	4	0.39841	0.52621	0.99998	10705	0.0448
MLN	6	0.39847	0.56891	0.99998	10706	0.2664
DIRAS1	6	0.3985	0.56893	0.99998	10707	-0.0209
CA8	6	0.3985	0.56893	0.99998	10708	-0.0823
CXorf61	6	0.39864	0.56904	0.99998	10709	0.1288
PACS2	6	0.39874	0.56911	0.99998	10710	-0.072
GPX5	6	0.39879	0.56915	0.99998	10711	-0.1191
CXorf66	6	0.39879	0.56915	0.99998	10712	0.0108
APC	6	0.39889	0.56922	0.99998	10713	-0.038
C1orf229	6	0.39889	0.56922	0.99998	10714	-0.0144
VPS37A	6	0.39902	0.5693	0.99998	10715	0.0167
FHL1	6	0.39902	0.56931	0.99998	10716	-0.0639
USP6NL	6	0.39918	0.56941	0.99998	10717	-0.027
LILRA2	6	0.39918	0.56941	0.99998	10718	-0.0474
EIF2AK2	6	0.39918	0.56941	0.99998	10719	0.0458
SPG21	6	0.3992	0.56943	0.99998	10720	0.1215
hsa-mir-6780b	4	0.39929	0.52664	0.99998	10721	0.0111
hsa-mir-3200	4	0.39929	0.52664	0.99998	10722	0.0703
ARID1A	6	0.39937	0.56957	0.99998	10723	-0.1037
FAM160B1	6	0.39946	0.56963	0.99998	10724	0.0225
FOXP3	6	0.39954	0.56968	0.99998	10725	0.0648
CKB	6	0.3997	0.5698	0.99998	10726	0.1481
ADH6	6	0.3997	0.5698	0.99998	10727	-0.0142
FAIM3	6	0.3997	0.5698	0.99998	10728	0.1506
MAP4K5	6	0.3999	0.56995	0.99998	10729	0.4301
PRMT7	6	0.3999	0.56995	0.99998	10730	-0.1085
hsa-mir-590	3	0.39993	0.45434	0.99998	10731	0.0563
CUBN	6	0.4	0.57002	0.99998	10732	-0.0038
RFWD3	6	0.4	0.57002	0.99998	10733	-0.0746
DOC2B	6	0.4	0.57002	0.99998	10734	-0.0228
OR2T1	6	0.40007	0.57007	0.99998	10735	0.102
ZNF540	6	0.40012	0.57012	0.99998	10736	-0.0429
FAM126A	6	0.40026	0.57022	0.99998	10737	0.0657
FREM2	6	0.40031	0.57026	0.99998	10738	-0.0168
SERPINB9	6	0.40031	0.57026	0.99998	10739	-0.0149
PCDH9	6	0.40056	0.57046	0.99998	10740	0.0469
FGF21	6	0.40073	0.5706	0.99998	10741	0.1368
POM121	4	0.40075	0.52736	0.99998	10742	0.052
TMEM171	6	0.40087	0.57069	0.99998	10743	0.0268
CLRN2	6	0.40087	0.57069	0.99998	10744	-0.0291
TMEM11	6	0.40087	0.57069	0.99998	10745	-0.0456
TSEN2	6	0.40087	0.57069	0.99998	10746	-0.043
FAM5C	3	0.40089	0.45497	0.99998	10747	-0.052
ARL4D	6	0.4009	0.57072	0.99998	10748	0.1896
DNAH7	6	0.4009	0.57072	0.99998	10749	0.0648
ACRC	5	0.40106	0.54935	0.99998	10750	0.0328
DEFB126	6	0.40111	0.57088	0.99998	10751	-0.0076
FAM151A	6	0.40111	0.57088	0.99998	10752	-0.0888
FAM46A	6	0.40135	0.57106	0.99998	10753	0.0487
HCRT	6	0.40142	0.57112	0.99998	10754	0.0629
TGIF2LY	6	0.40142	0.57112	0.99998	10755	0.1141
IL11RA	6	0.4015	0.57118	0.99998	10756	-0.0046
SYNE1	6	0.40155	0.57122	0.99998	10757	0.2856
ERVV-2	6	0.40155	0.57122	0.99998	10758	0.0692
OR4N4	6	0.40156	0.57123	0.99998	10759	0.1159
SMAP1	6	0.40184	0.57144	0.99998	10760	0.2509
AZI2	6	0.40202	0.57158	0.99998	10761	-0.0058
RNF112	6	0.40209	0.57165	0.99998	10762	0.0484
IL36B	6	0.40227	0.57177	0.99998	10763	-0.0378
KLHL30	6	0.40251	0.57197	0.99998	10764	-0.0291
MARCO	6	0.40251	0.57197	0.99998	10765	0.1235
ETV3L	6	0.4026	0.57205	0.99998	10766	0.2089
TBC1D22A	6	0.40278	0.57219	0.99998	10767	-0.0109
SLC1A7	6	0.40278	0.57219	0.99998	10768	-0.0566
NKAIN4	6	0.40278	0.57219	0.99998	10769	0.0132
hsa-mir-6508	4	0.40279	0.52833	0.99998	10770	-0.2569

ERP29	6	0.40282	0.57222	0.99998	10771	-0.0001
FAM166A	6	0.40286	0.57225	0.99998	10772	-0.0296
ZC3H8	6	0.40292	0.5723	0.99998	10773	-0.102
UBXN2B	6	0.40292	0.5723	0.99998	10774	-0.0976
H1FOO	6	0.40312	0.57246	0.99998	10775	-0.1807
ZNF781	6	0.4032	0.57251	0.99998	10776	0.0993
NHLRC1	6	0.40346	0.57272	0.99998	10777	-0.1651
GPCPD1	6	0.40357	0.57281	0.99998	10778	0.0042
LOC10012798	6	0.40357	0.57281	0.99998	10779	-0.056
TRPM4	6	0.40357	0.57281	0.99998	10780	0.4138
TRPC7	6	0.40357	0.57281	0.99998	10781	-0.0547
PMEPA1	6	0.4036	0.57284	0.99998	10782	0.0066
ZNF530	6	0.40377	0.57298	0.99998	10783	-0.0351
GRTP1	6	0.40386	0.57303	0.99998	10784	0.2496
BTG1	6	0.40391	0.57307	0.99998	10785	-0.0784
C1QL1	6	0.40394	0.5731	0.99998	10786	0.1183
PIDD	6	0.40411	0.57323	0.99998	10787	0.0409
UBE2G1	6	0.40418	0.57328	0.99998	10788	0.0915
SPP2	6	0.40419	0.57329	0.99998	10789	0.1208
CDX2	6	0.40438	0.57344	0.99998	10790	0.1618
C20orf166	6	0.40438	0.57344	0.99998	10791	0.0709
RASA3	6	0.40438	0.57344	0.99998	10792	-0.0627
FAM65C	6	0.40443	0.57348	0.99998	10793	0.0623
COMTD1	6	0.40448	0.57351	0.99998	10794	0.0442
HUNK	6	0.40459	0.57359	0.99998	10795	-0.0015
KLC4	6	0.40459	0.57359	0.99998	10796	-0.0206
MCOLN2	6	0.40459	0.57359	0.99998	10797	0.0478
PBX1	6	0.40459	0.57359	0.99998	10798	-0.1314
PKD1L1	6	0.40465	0.57364	0.99998	10799	0.1065
ARVCF	6	0.40465	0.57364	0.99998	10800	-0.0043
MUC15	6	0.40473	0.57369	0.99998	10801	0.0231
hsa-mir-5192	4	0.40483	0.52926	0.99998	10802	-0.0838
RASAL1	6	0.40487	0.57379	0.99998	10803	0.0796
TPP2	6	0.40488	0.5738	0.99998	10804	-0.1591
ADAT3	6	0.40488	0.5738	0.99998	10805	0.0328
SLC25A36	6	0.40488	0.5738	0.99998	10806	0.0709
APCDD1	6	0.40488	0.5738	0.99998	10807	-0.0668
CYB5A	6	0.40504	0.57392	0.99998	10808	-0.1314
hsa-mir-8070	2	0.40515	0.41214	0.99998	10809	-0.0333
ZNF860	6	0.40531	0.57411	0.99998	10810	-0.0712
CETN2	6	0.40531	0.57411	0.99998	10811	0.1972
hsa-mir-1288	4	0.40543	0.52952	0.99998	10812	-0.0234
TSACC	6	0.4055	0.57427	0.99998	10813	0.178
SOX15	6	0.40555	0.57431	0.99998	10814	-0.0479
NRK	6	0.40555	0.57431	0.99998	10815	0.0125
FRAS1	6	0.40575	0.57447	0.99998	10816	0.0671
COG2	6	0.40575	0.57447	0.99998	10817	0.0085
TBR1	6	0.40581	0.57451	0.99998	10818	0.0198
ERN1	6	0.406	0.57467	0.99998	10819	0.15
PCDHGA7	2	0.40605	0.41291	0.99998	10820	0.0256
TP53I3	6	0.40616	0.57479	0.99998	10821	0.0595
GSTK1	6	0.40622	0.57483	0.99998	10822	0.007
TENC1	6	0.40636	0.57494	0.99998	10823	-0.0383
CACYBP	6	0.40649	0.57504	0.99998	10824	0.0049
GZF1	6	0.40649	0.57504	0.99998	10825	0.0481
GAS2L2	6	0.40654	0.57508	0.99998	10826	0.0128
POC1A	6	0.40666	0.57517	0.99998	10827	0.0909
PTMS	6	0.40666	0.57517	0.99998	10828	0.0367
CD4	6	0.40676	0.57524	0.99998	10829	0.0459
KLHDC3	6	0.40676	0.57524	0.99998	10830	0.2002
MAST3	6	0.40676	0.57524	0.99998	10831	0.1415
SEMA4D	6	0.40685	0.57532	0.99998	10832	0.2243
RSPH6A	6	0.40698	0.57541	0.99998	10833	0.0296
SUGP2	6	0.40702	0.57543	0.99998	10834	-0.0911
GLG1	6	0.40702	0.57543	0.99998	10835	-0.1167
RYR3	6	0.40702	0.57543	0.99998	10836	-0.115
hsa-mir-3907	4	0.4071	0.53031	0.99998	10837	0.0302
HES7	6	0.40722	0.5756	0.99998	10838	0.124
ZNF418	6	0.40724	0.57562	0.99998	10839	-0.1821
GPR143	6	0.40739	0.57574	0.99998	10840	-0.0377
MPC2	6	0.40752	0.57584	0.99998	10841	0.2369
RRS1	6	0.40765	0.57595	0.99998	10842	-0.1063
GNG5	6	0.40774	0.57602	0.99998	10843	-0.1062
CAMK4	6	0.40774	0.57602	0.99998	10844	0.0748
TNFSF8	6	0.40782	0.57609	0.99998	10845	0.0741
SNAI3	6	0.40792	0.57617	0.99998	10846	0.1557
EGFL8	6	0.40802	0.57626	0.99998	10847	0.1511
hsa-mir-5189	4	0.40813	0.53079	0.99998	10848	0.0544
RASSF8	6	0.40821	0.57639	0.99998	10849	0.2286
ADIRF	6	0.40821	0.57639	0.99998	10850	0.1109
SLC32A1	6	0.40827	0.57644	0.99998	10851	0.0267

SLC25A45	6	0.40834	0.57649	0.99998	10852	0.0565
SLC22A14	6	0.40847	0.57658	0.99998	10853	0.204
FGFR2	6	0.40847	0.57658	0.99998	10854	0.2089
RGL4	6	0.40851	0.57661	0.99998	10855	0.2285
GPIHBP1	6	0.40851	0.57661	0.99998	10856	0.1528
DTHD1	6	0.40864	0.57671	0.99998	10857	-0.0498
MET	6	0.40869	0.57675	0.99998	10858	-0.0289
DNAAF1	6	0.40869	0.57675	0.99998	10859	0.2329
TNFRSF10A	6	0.40875	0.57679	0.99998	10860	-0.0379
C5orf55	6	0.40878	0.57682	0.99998	10861	0.1666
DPH3P1	6	0.40878	0.57682	0.99998	10862	-0.0879
BPIFB6	6	0.40889	0.57691	0.99998	10863	0.0066
GGCX	6	0.40898	0.57699	0.99998	10864	0.094
RAP1B	6	0.40898	0.57699	0.99998	10865	-0.182
TNFRSF11A	6	0.40898	0.57699	0.99998	10866	0.048
OR5H15	5	0.40906	0.55699	0.99998	10867	-0.1821
TMEM45B	6	0.40912	0.57709	0.99998	10868	-0.0257
hsa-mir-548h-	2	0.40913	0.4156	0.99998	10869	-0.0418
EIF2B3	6	0.40918	0.57712	0.99998	10870	0.0361
EFR3B	6	0.40924	0.57717	0.99998	10871	0.1304
CD93	6	0.40925	0.57717	0.99998	10872	0.0219
hsa-mir-7157	4	0.40931	0.53134	0.99998	10873	0.2273
STAT5A	6	0.40933	0.57724	0.99998	10874	-0.0859
CCDC107	6	0.40957	0.57741	0.99998	10875	0.0628
FSCN1	6	0.40958	0.57741	0.99998	10876	0.1311
KSR2	6	0.40971	0.57752	0.99998	10877	-0.0331
CYP51A1	6	0.40971	0.57752	0.99998	10878	-0.1317
SH3GLB2	6	0.40984	0.57761	0.99998	10879	0.0936
COQ9	6	0.40989	0.57766	0.99998	10880	0.0985
hsa-mir-630	4	0.40993	0.53164	0.99998	10881	0.0265
MYOF	6	0.40997	0.57772	0.99998	10882	0.0779
CCDC144NL	5	0.40998	0.55788	0.99998	10883	0.0067
hsa-mir-4318	4	0.40999	0.53167	0.99998	10884	0.0481
IRS2	6	0.41	0.57775	0.99998	10885	0.1339
DDX19B	6	0.41	0.57775	0.99998	10886	-0.0696
MICB	6	0.41011	0.57784	0.99998	10887	0.124
DCTN6	6	0.41015	0.57787	0.99998	10888	0.0471
SLC28A3	6	0.41027	0.57797	0.99998	10889	-0.0103
SEC16A	6	0.41031	0.57799	0.99998	10890	0.0533
TTPAL	6	0.41031	0.57799	0.99998	10891	0.4648
TIGD3	6	0.41031	0.57799	0.99998	10892	0.1734
C20orf85	6	0.41041	0.57807	0.99998	10893	-0.0366
KAT6B	6	0.41058	0.5782	0.99998	10894	-0.0358
POLD2	6	0.41058	0.5782	0.99998	10895	-0.2253
ZAK	6	0.41066	0.57826	0.99998	10896	0.12
CD7	6	0.41066	0.57826	0.99998	10897	0.1541
hsa-mir-4727	4	0.4107	0.53203	0.99998	10898	0.1497
PSMD6	6	0.41082	0.57838	0.99998	10899	-0.2074
ST7	6	0.41082	0.57838	0.99998	10900	0.1063
GPR75	6	0.41082	0.57838	0.99998	10901	-0.0798
CACNA1B	6	0.41088	0.57843	0.99998	10902	0.0906
hsa-mir-4472-	3	0.41102	0.4618	0.99998	10903	-0.4501
PIGQ	6	0.4111	0.57859	0.99998	10904	0.1298
NAPEPLD	6	0.41113	0.57862	0.99998	10905	0.0251
GSTM5	5	0.41117	0.55898	0.99998	10906	-0.7622
NBEAL2	4	0.41121	0.53227	0.99998	10907	0.0944
TAAR8	6	0.4113	0.57874	0.99998	10908	-0.0158
TBC1D29	6	0.4113	0.57874	0.99998	10909	0.0212
LACE1	6	0.4113	0.57874	0.99998	10910	0.1358
BAGE	4	0.41131	0.53232	0.99998	10911	0.109
FOXJ3	6	0.41138	0.5788	0.99998	10912	0.1667
PTDSS2	6	0.41142	0.57882	0.99998	10913	0.0435
LDLRAD4	6	0.41147	0.57886	0.99998	10914	0.1161
GSR	6	0.41147	0.57886	0.99998	10915	0.0859
SYNCRIP	6	0.41155	0.57893	0.99998	10916	0.0187
SH2D3C	6	0.41157	0.57894	0.99998	10917	0.1461
NDUFA11	6	0.41163	0.57901	0.99998	10918	-0.0118
CALHM2	6	0.41173	0.57909	0.99998	10919	-0.0141
TTC29	6	0.41173	0.57909	0.99998	10920	-0.1306
POLR2B	6	0.41189	0.57923	0.99998	10921	-0.0576
MRPS36	6	0.41202	0.57934	0.99998	10922	-0.0837
CST11	6	0.41202	0.57934	0.99998	10923	0.0744
PNPT1	6	0.41202	0.57934	0.99998	10924	-0.1105
CCR2	6	0.41202	0.57934	0.99998	10925	0.0357
ADM5	6	0.41216	0.57943	0.99998	10926	-0.1657
C20orf194	6	0.41222	0.57948	0.99998	10927	0.196
EPHA5	6	0.41222	0.57948	0.99998	10928	0.0266
hsa-mir-6798	4	0.41228	0.53278	0.99998	10929	0.0411
C8orf22	6	0.41237	0.57961	0.99998	10930	0.1067
FBXO17	6	0.41237	0.57961	0.99998	10931	0.0606
BCCIP	6	0.41245	0.57968	0.99998	10932	0.1639

TCTE1	6	0.41245	0.57968	0.99998	10933	-0.1088
HAPLN1	6	0.41245	0.57968	0.99998	10934	-0.1624
ADAM18	6	0.41245	0.57968	0.99998	10935	0.0044
LRFN3	6	0.4127	0.57987	0.99998	10936	0.0174
TEX9	6	0.4127	0.57987	0.99998	10937	0.0683
RNF19A	6	0.4127	0.57987	0.99998	10938	-0.1911
MYO3B	6	0.4127	0.57987	0.99998	10939	-0.0105
TP53INP1	6	0.4127	0.57987	0.99998	10940	-0.0234
CWC22	6	0.4127	0.57987	0.99998	10941	-0.0429
N4BP2L2	6	0.41279	0.57994	0.99998	10942	0.1606
MAGEB17	6	0.41279	0.57994	0.99998	10943	0.0583
TM4SF1	6	0.41279	0.57994	0.99998	10944	0.311
ALKBH6	6	0.41299	0.58009	0.99998	10945	0.0214
C19orf60	6	0.41313	0.58019	0.99998	10946	0.0221
TREX2	6	0.41319	0.58023	0.99998	10947	0.1769
PYHIN1	6	0.41326	0.58028	0.99998	10948	-0.0433
TRIM23	6	0.41327	0.58029	0.99998	10949	0.0908
CEACAM6	6	0.41331	0.58033	0.99998	10950	0.2198
ATXN2L	6	0.41341	0.58039	0.99998	10951	-0.0762
PAIP2	6	0.41351	0.58047	0.99998	10952	0.1244
CYP27A1	6	0.41354	0.5805	0.99998	10953	-0.0019
ARNT	6	0.41354	0.5805	0.99998	10954	0.0353
ITGB1	6	0.41369	0.5806	0.99998	10955	0.1702
ZSWIM2	6	0.4137	0.58061	0.99998	10956	-0.0927
OR11L1	6	0.4137	0.58061	0.99998	10957	-0.0676
RAB40B	6	0.4137	0.58061	0.99998	10958	-0.13
RTP1	6	0.41419	0.58097	0.99998	10959	-0.102
MMP3	5	0.41438	0.56193	0.99998	10960	-0.1224
AP4B1	6	0.41439	0.58112	0.99998	10961	0.0504
FAM166B	6	0.41446	0.58116	0.99998	10962	0.1034
EI24	6	0.41446	0.58116	0.99998	10963	0.0171
LINGO4	6	0.41446	0.58116	0.99998	10964	-0.0314
SETDB1	6	0.41446	0.58116	0.99998	10965	-0.029
IREB2	6	0.41446	0.58116	0.99998	10966	9E-05
FUT9	6	0.41461	0.58128	0.99998	10967	0.0779
TTC37	6	0.41481	0.58144	0.99998	10968	0.1132
BABAM1	6	0.41481	0.58144	0.99998	10969	0.0055
CGA	6	0.41481	0.58144	0.99998	10970	-0.0118
GSTCD	6	0.41491	0.58152	0.99998	10971	0.043
ELK3	6	0.41498	0.58158	0.99998	10972	0.0412
PSEN1	6	0.41498	0.58158	0.99998	10973	0.0859
SPAG6	6	0.41499	0.58159	0.99998	10974	0.0016
SCNN1G	6	0.41514	0.58169	0.99998	10975	-0.0031
ARHGEF16	6	0.41529	0.58181	0.99998	10976	-0.0788
CYP24A1	6	0.41529	0.58181	0.99998	10977	-0.0799
DHRS4	2	0.4153	0.42117	0.99998	10978	-0.8066
GAGE12H	2	0.4153	0.42117	0.99998	10979	-0.8441
VCX3B	2	0.4153	0.42117	0.99998	10980	-0.8139
LOC391322	5	0.41554	0.56302	0.99998	10981	0.0697
CBLB	6	0.41559	0.58205	0.99998	10982	-0.0475
RASSF7	6	0.41564	0.58208	0.99998	10983	0.1699
hsa-mir-4774	4	0.41565	0.53441	0.99998	10984	0.0691
H6PD	6	0.41584	0.58225	0.99998	10985	0.0458
OR1K1	6	0.41589	0.58228	0.99998	10986	0.0532
PRSS16	6	0.41589	0.58228	0.99998	10987	-0.0994
ZNF195	6	0.41589	0.58228	0.99998	10988	0.2513
MS4A5	6	0.41589	0.58228	0.99998	10989	0.0422
CD300A	6	0.41607	0.58241	0.99998	10990	0.1938
hsa-mir-1185-	1	0.41622	0.41649	0.99998	10991	-0.0931
MAGEB18	6	0.41626	0.58256	0.99998	10992	0.1948
HMHA1	6	0.41632	0.5826	0.99998	10993	0.0898
C14orf28	6	0.41639	0.58265	0.99998	10994	-0.05
FAM135A	6	0.41639	0.58265	0.99998	10995	0.0157
RHOBTB1	6	0.41639	0.58265	0.99998	10996	-0.0677
SLCO2A1	6	0.41639	0.58265	0.99998	10997	-0.0804
ADARB2	6	0.41649	0.58272	0.99998	10998	0.0076
DUSP28	6	0.41659	0.58281	0.99998	10999	-0.0237
PRUNE	6	0.41671	0.58291	0.99998	11000	-0.1521
CAPZA3	6	0.41685	0.58302	0.99998	11001	-0.0309
MMRN1	6	0.41685	0.58302	0.99998	11002	0.0428
NSUN2	6	0.41685	0.58302	0.99998	11003	-0.0261
C1orf106	6	0.41685	0.58302	0.99998	11004	0.2111
hsa-mir-3659	4	0.41704	0.53508	0.99998	11005	0.0099
IDUA	6	0.4171	0.5832	0.99998	11006	0.0455
TMEM55B	6	0.4171	0.5832	0.99998	11007	0.0134
PTK2	6	0.4171	0.58321	0.99998	11008	-0.0938
GALE	6	0.4171	0.58321	0.99998	11009	-0.0539
TSNAXIP1	6	0.41726	0.58334	0.99998	11010	0.0714
AGPHD1	2	0.41733	0.42304	0.99998	11011	-0.0518
RAD51D	6	0.41739	0.58344	0.99998	11012	0.0571
EPOR	6	0.41739	0.58344	0.99998	11013	0.166

PTGES3L	2	0.41739	0.42309	0.99998	11014	0.0613
FHDC1	6	0.41739	0.58344	0.99998	11015	-0.2092
BAG5	6	0.41751	0.58353	0.99998	11016	-0.1065
HBG1	2	0.41759	0.42328	0.99998	11017	-0.7773
GAREML	6	0.41771	0.58369	0.99998	11018	-0.1059
UGDH	6	0.41771	0.58369	0.99998	11019	-0.0062
OR52N2	6	0.41771	0.58369	0.99998	11020	0.0481
WDSUB1	6	0.41782	0.58378	0.99998	11021	0.288
UBE2J1	6	0.41788	0.58383	0.99998	11022	-0.1545
ST8SIA6	6	0.41797	0.58389	0.99998	11023	-0.069
KANSL1L	6	0.41797	0.58389	0.99998	11024	-0.0299
hsa-mir-6855	4	0.41802	0.53555	0.99998	11025	0.4005
WNT3A	6	0.4181	0.58399	0.99998	11026	-0.0403
EPHB4	6	0.4181	0.58399	0.99998	11027	-0.1609
KRT25	6	0.41824	0.5841	0.99998	11028	-0.1925
CCDC93	6	0.41833	0.58416	0.99998	11029	-0.0084
hsa-mir-6502	3	0.41846	0.46687	0.99998	11030	0.0183
OTP	6	0.41858	0.58438	0.99998	11031	0.0163
CLLU1OS	6	0.41858	0.58438	0.99998	11032	0.0306
ANXA8L2	2	0.41868	0.42426	0.99998	11033	-0.0068
ZDHHC1	6	0.41883	0.58459	0.99998	11034	0.0305
GHSR	6	0.41883	0.58459	0.99998	11035	0.0508
MAMLD1	6	0.41897	0.58469	0.99998	11036	0.0495
HIST1H2BA	6	0.41906	0.58476	0.99998	11037	-0.0892
NT5C1A	6	0.41914	0.58482	0.99998	11038	-0.0563
LRRC3C	6	0.41914	0.58482	0.99998	11039	0.1938
ATXN2	6	0.41928	0.58493	0.99998	11040	0.1317
SH3PXD2A	6	0.4193	0.58494	0.99998	11041	-0.0514
PIR	6	0.4193	0.58494	0.99998	11042	-0.1064
SHOC2	6	0.41935	0.58498	0.99998	11043	0.1122
MX2	6	0.41946	0.58507	0.99998	11044	0.1049
RUNDC3A	6	0.41957	0.58517	0.99998	11045	-0.0922
hsa-mir-1290	4	0.41959	0.53627	0.99998	11046	0.0409
hsa-mir-6861	4	0.41959	0.53627	0.99998	11047	-0.1886
PLBD2	6	0.41965	0.58523	0.99998	11048	0.1947
ZNF445	6	0.41984	0.58537	0.99998	11049	0.1392
TNS3	6	0.41991	0.58543	0.99998	11050	-0.0181
ZNF430	4	0.42003	0.53649	0.99998	11051	0.1559
SAP130	6	0.42021	0.58568	0.99998	11052	-0.1224
STX5	6	0.42021	0.58568	0.99998	11053	-0.1597
NT5E	6	0.42027	0.58571	0.99998	11054	0.0483
SLC38A4	6	0.42036	0.58579	0.99998	11055	0.1552
MACROD1	6	0.42039	0.58581	0.99998	11056	0.0369
MON1B	6	0.4204	0.58582	0.99998	11057	0.0813
XPR1	6	0.42044	0.58585	0.99998	11058	0.1217
PPIAL4E	1	0.42045	0.42069	0.99998	11059	-0.2541
KREMEN2	6	0.42048	0.58588	0.99998	11060	0.1397
PDHA2	6	0.4206	0.58598	0.99998	11061	0.1956
DNTTIP2	6	0.4206	0.58598	0.99998	11062	0.0306
STK38L	6	0.4206	0.58598	0.99998	11063	-0.0038
CTDSP2	6	0.42077	0.5861	0.99998	11064	-0.0987
GRIK3	6	0.42085	0.58616	0.99998	11065	0.1873
POU6F1	6	0.42096	0.58625	0.99998	11066	-0.0599
LPPR1	6	0.42098	0.58627	0.99998	11067	0.1975
YLPM1	6	0.42098	0.58627	0.99998	11068	0.061
SLC22A1	6	0.42098	0.58627	0.99998	11069	-0.0166
REG1A	6	0.42105	0.58632	0.99998	11070	-0.1672
TBL1Y	6	0.42109	0.58635	0.99998	11071	0.0952
hsa-mir-4491	4	0.42123	0.53711	0.99998	11072	0.2685
RNASET2	6	0.42124	0.58648	0.99998	11073	-0.0295
SLFN5	6	0.42124	0.58648	0.99998	11074	0.1569
TRIB3	6	0.42145	0.58664	0.99998	11075	0.2249
SNRPG	6	0.42145	0.58664	0.99998	11076	-0.0602
RUNX2	6	0.42161	0.58677	0.99998	11077	0.1729
FNBP1	4	0.42162	0.53731	0.99998	11078	0.0818
C16orf74	6	0.42166	0.58681	0.99998	11079	0.0014
UBE2O	6	0.42167	0.58682	0.99998	11080	0.2659
CBLN3	6	0.42167	0.58682	0.99998	11081	0.0259
hsa-mir-7846	4	0.42173	0.53736	0.99998	11082	-0.1034
ASB14	6	0.42184	0.58695	0.99998	11083	-0.059
NOX3	6	0.42202	0.5871	0.99998	11084	0.1797
OR8S1	6	0.42207	0.58714	0.99998	11085	0.209
NDUFA12	6	0.42207	0.58714	0.99998	11086	0.1539
DCAF17	6	0.42207	0.58714	0.99998	11087	0.2079
MAP1A	6	0.42212	0.58718	0.99998	11088	-0.0021
CCDC157	6	0.42212	0.58718	0.99998	11089	0.1021
SMEK2	6	0.4222	0.58725	0.99998	11090	-0.0928
ACADM	6	0.42222	0.58726	0.99998	11091	-0.1143
S100PBP	6	0.42236	0.58737	0.99998	11092	-0.1296
DEFB103B	1	0.42253	0.42279	0.99998	11093	-0.0952
TREM2	6	0.42253	0.58752	0.99998	11094	0.0943

ORC4	6	0.42253	0.58752	0.99998	11095	0.0809
NCR3	6	0.42271	0.58765	0.99998	11096	-0.0895
KCTD10	6	0.42271	0.58765	0.99998	11097	-0.023
TECRL	6	0.42287	0.58779	0.99998	11098	0.0906
hsa-mir-1278	3	0.42288	0.46985	0.99998	11099	0.4616
ENTPD7	6	0.42293	0.58783	0.99998	11100	-0.0317
FAM69B	6	0.42302	0.5879	0.99998	11101	0.0789
hsa-mir-516a-	1	0.42307	0.42333	0.99998	11102	-0.3622
hsa-mir-3669	1	0.42307	0.42333	0.99998	11103	-0.3622
hsa-mir-518f	1	0.42307	0.42333	0.99998	11104	-0.3622
FUT6	6	0.42311	0.58798	0.99998	11105	-0.1339
AVL9	6	0.42317	0.58802	0.99998	11106	-0.0625
TSLP	6	0.42329	0.58812	0.99998	11107	0.0978
OPRM1	6	0.4235	0.58828	0.99998	11108	0.178
C11orf74	6	0.4235	0.58828	0.99998	11109	0.1512
LOC730183	6	0.42357	0.58833	0.99998	11110	0.4371
HCCS	6	0.42363	0.58838	0.99998	11111	-0.0346
ARHGEF10L	6	0.42363	0.58838	0.99998	11112	0.0733
COL13A1	6	0.4237	0.58843	0.99998	11113	0.3909
DCST1	6	0.42377	0.58847	0.99998	11114	0.0079
A3GALT2	6	0.42388	0.58856	0.99998	11115	0.0835
GP6	6	0.424	0.58865	0.99998	11116	-0.1471
CDK20	6	0.42403	0.58868	0.99998	11117	0.1721
GSTT1	6	0.42403	0.58868	0.99998	11118	0.2054
NACAD	6	0.4241	0.58873	0.99998	11119	0.0195
GABRA3	6	0.4242	0.58882	0.99998	11120	-0.1481
RIOK3	6	0.42434	0.58893	0.99998	11121	-0.1529
ADAMTS17	6	0.42436	0.58894	0.99998	11122	0.0534
MRPL44	6	0.42436	0.58894	0.99998	11123	-0.0809
CDHR4	6	0.42436	0.58894	0.99998	11124	0.1043
BAIAP2L2	6	0.42456	0.5891	0.99998	11125	0.322
DAW1	6	0.42462	0.58916	0.99998	11126	-0.0018
SPPL2A	6	0.42464	0.58918	0.99998	11127	-0.0806
TRAPPC13	6	0.42467	0.5892	0.99998	11128	0.1278
AREL1	4	0.42469	0.53882	0.99998	11129	-0.0368
hsa-mir-136	4	0.42484	0.53889	0.99998	11130	-0.1586
TLE6	6	0.42488	0.58937	0.99998	11131	0.0129
IL24	6	0.42495	0.58942	0.99998	11132	-0.0084
ZNF10	6	0.42495	0.58942	0.99998	11133	0.0265
OR2L13	6	0.42519	0.58958	0.99998	11134	0.0466
ZKSCAN3	6	0.42519	0.58958	0.99998	11135	0.0752
ING5	6	0.4252	0.58959	0.99998	11136	0.0452
hsa-mir-378i	3	0.42526	0.47145	0.99998	11137	0.0304
ACTA1	6	0.42527	0.58964	0.99998	11138	0.3545
hsa-mir-5694	4	0.42528	0.53909	0.99998	11139	0.1481
SMIM19	6	0.42535	0.5897	0.99998	11140	0.045
TMCO4	6	0.4257	0.58997	0.99998	11141	0.1522
C2CD2	6	0.4257	0.58997	0.99998	11142	0.335
LURAP1	6	0.42582	0.59006	0.99998	11143	0.0429
FAM120A	6	0.42584	0.59007	0.99998	11144	-0.0135
TMEM239	6	0.42598	0.59017	0.99998	11145	-0.0919
ENO4	6	0.426	0.59019	0.99998	11146	-0.0303
EBF2	6	0.426	0.59019	0.99998	11147	0.2543
TCERG1L	6	0.42605	0.59022	0.99998	11148	0.1073
hsa-mir-5186	4	0.42623	0.53956	0.99998	11149	-0.064
AKAP8L	6	0.42624	0.59036	0.99998	11150	0.1553
CEP128	6	0.42624	0.59036	0.99998	11151	0.1492
PPP6R1	6	0.42624	0.59036	0.99998	11152	-0.0567
KBTBD7	5	0.4263	0.57317	0.99998	11153	0.0809
AIM1	6	0.42639	0.59049	0.99998	11154	0.001
ACTR3B	5	0.42641	0.57328	0.99998	11155	-0.0616
ATP5L2	5	0.42641	0.57328	0.99998	11156	-0.1902
GSTA5	5	0.42641	0.57328	0.99998	11157	-0.2409
ZNF99	5	0.42641	0.57328	0.99998	11158	-0.136
ROPN1	5	0.42641	0.57328	0.99998	11159	-0.143
PRDM9	5	0.42641	0.57328	0.99998	11160	-0.0581
LYZL2	1	0.42642	0.42668	0.99998	11161	-0.0583
KBTBD12	6	0.42643	0.59051	0.99998	11162	2E-05
SKAP2	6	0.42643	0.59051	0.99998	11163	0.1095
KCNAB1	6	0.42666	0.59069	0.99998	11164	0.1095
METAP1	4	0.4267	0.53978	0.99998	11165	0.1138
ARHGDIG	6	0.42677	0.59078	0.99998	11166	-0.0757
MLLT1	6	0.42692	0.5909	0.99998	11167	0.0908
NLRP7	6	0.42694	0.59092	0.99998	11168	-0.0569
COX7A2L	6	0.42694	0.59092	0.99998	11169	0.0971
KIAA1024	6	0.42694	0.59092	0.99998	11170	0.1673
UPRT	6	0.42694	0.59092	0.99998	11171	0.095
RPS6KL1	6	0.42705	0.59102	0.99998	11172	0.1686
A2ML1	6	0.42718	0.59111	0.99998	11173	0.1422
HEPH	6	0.4273	0.5912	0.99998	11174	0.2375
PLLP	6	0.42746	0.59133	0.99998	11175	-0.0257

FLT4	6	0.42774	0.59155	0.99998	11176	-0.0269
ACTG1	6	0.42774	0.59155	0.99998	11177	-0.0161
TAX1BP3	6	0.42775	0.59156	0.99998	11178	0.0021
FAM81B	6	0.4278	0.59162	0.99998	11179	0.0768
ARHGAP24	6	0.42783	0.59164	0.99998	11180	0.0708
CCDC153	6	0.42789	0.5917	0.99998	11181	0.0072
GYS2	6	0.42793	0.59172	0.99998	11182	0.0034
AFP	6	0.42798	0.59177	0.99998	11183	-0.1136
SLCO1A2	6	0.42798	0.59177	0.99998	11184	-0.0046
hsa-mir-1281	4	0.42799	0.54043	0.99998	11185	0.264
TRDMT1	6	0.42815	0.59189	0.99998	11186	0.0714
UNC13C	6	0.42815	0.59189	0.99998	11187	0.012
C7orf31	6	0.42815	0.59189	0.99998	11188	-0.0195
SCG5	6	0.42815	0.59189	0.99998	11189	0.049
ITGA3	6	0.42827	0.59199	0.99998	11190	-0.0674
TMEM39B	6	0.42829	0.59201	0.99998	11191	-0.0251
C1orf111	6	0.42829	0.59201	0.99998	11192	0.0735
CASC10	3	0.42835	0.47357	0.99998	11193	-0.108
CARD14	6	0.42848	0.59216	0.99998	11194	-0.1202
RASA1	6	0.42848	0.59216	0.99998	11195	-0.0088
GPR101	6	0.4286	0.59225	0.99998	11196	0.0658
SPDYC	6	0.42862	0.59226	0.99998	11197	-0.0757
TRMT10C	6	0.42867	0.59231	0.99998	11198	-0.0731
SLC25A42	6	0.42867	0.59231	0.99998	11199	-0.0155
MTSS1L	6	0.42874	0.59236	0.99998	11200	0.3139
OR10S1	6	0.42874	0.59236	0.99998	11201	-0.062
FYCO1	6	0.4288	0.59239	0.99998	11202	0.1607
LGALS16	5	0.4288	0.57553	0.99998	11203	-0.0002
BHLHE41	6	0.42883	0.59242	0.99998	11204	-0.0275
MEAF6	6	0.42883	0.59242	0.99998	11205	-0.0512
TIMM8A	6	0.42883	0.59242	0.99998	11206	0.1274
RAB28	6	0.42883	0.59242	0.99998	11207	0.1685
DPYSL3	6	0.42892	0.59249	0.99998	11208	-0.1385
C8orf47	6	0.42905	0.59259	0.99998	11209	0.0179
KIF16B	6	0.42905	0.59259	0.99998	11210	-0.0709
IL10RA	6	0.42909	0.59262	0.99998	11211	0.0531
SPAG11A	2	0.42918	0.43387	0.99998	11212	-0.2198
PAPOLG	6	0.42931	0.59279	0.99998	11213	0.1225
OVOL3	6	0.42931	0.59279	0.99998	11214	0.0833
NHP2	6	0.42931	0.59279	0.99998	11215	0.2578
GGA3	6	0.42954	0.59298	0.99998	11216	-0.0765
TMEM163	6	0.42954	0.59298	0.99998	11217	0.047
KY	6	0.42958	0.59301	0.99998	11218	0.0811
ARFIP1	6	0.42976	0.59316	0.99998	11219	0.0509
FDFT1	6	0.42976	0.59316	0.99998	11220	0.0646
ZNF169	6	0.42977	0.59316	0.99998	11221	-0.1341
NUP54	6	0.42984	0.59323	0.99998	11222	0.101
CRK	6	0.4299	0.59327	0.99998	11223	0.1405
KRT82	6	0.42991	0.59328	0.99998	11224	-0.1853
KRTAP4-2	5	0.42991	0.57657	0.99998	11225	0.1809
MYCT1	6	0.42993	0.5933	0.99998	11226	0.1564
FAM83A	4	0.42999	0.54142	0.99998	11227	-0.043
CALML6	6	0.43	0.59336	0.99998	11228	0.2127
GPR33	6	0.43	0.59336	0.99998	11229	0.1063
TSTD3	6	0.43012	0.59345	0.99998	11230	0.0807
UBR5	6	0.43012	0.59345	0.99998	11231	0.0663
ZNF660	6	0.43022	0.59353	0.99998	11232	-0.0132
MRPL22	6	0.43022	0.59353	0.99998	11233	-0.0031
RBM20	6	0.43022	0.59353	0.99998	11234	-0.0501
TMEM120A	6	0.43028	0.59358	0.99998	11235	-0.0622
TFPI	6	0.43036	0.59363	0.99998	11236	-0.115
FAM73A	6	0.43037	0.59364	0.99998	11237	0.0887
MUC12	6	0.43041	0.59367	0.99998	11238	0.1972
hsa-mir-4651	4	0.43047	0.54166	0.99998	11239	0.1035
CT47B1	4	0.43047	0.54166	0.99998	11240	0.1975
ZNF764	5	0.43049	0.57713	0.99998	11241	0.1607
PCDHGC3	2	0.43058	0.43516	0.99998	11242	-0.1155
TNFSF12	5	0.43065	0.57729	0.99998	11243	-0.0798
MS4A2	6	0.43068	0.59388	0.99998	11244	0.1324
PRSS37	6	0.43087	0.59402	0.99998	11245	-0.1118
DDX19A	6	0.43098	0.5941	0.99998	11246	-0.1578
TCFL5	6	0.43098	0.5941	0.99998	11247	-0.2443
ZNF844	6	0.43098	0.5941	0.99998	11248	0.261
METTL2B	6	0.43098	0.5941	0.99998	11249	-0.3407
ABCA8	6	0.43098	0.5941	0.99998	11250	-0.0905
ZNF33B	6	0.43098	0.5941	0.99998	11251	-0.3605
S100A7A	6	0.43098	0.5941	0.99998	11252	-0.6385
FCGR3A	6	0.43098	0.5941	0.99998	11253	0.0759
CARF	6	0.43116	0.59424	0.99998	11254	0.1216
ZBTB7A	6	0.43157	0.59456	0.99998	11255	0.0527
MVK	6	0.43157	0.59456	0.99998	11256	-0.0115

HSPH1	6	0.43157	0.59456	0.99998	11257	-0.0824
SDCBP2	6	0.43157	0.59456	0.99998	11258	-0.0857
TNFSF13B	6	0.43167	0.59463	0.99998	11259	-0.1296
TRAF2	6	0.43175	0.59469	0.99998	11260	0.1163
PDX1	6	0.43175	0.59469	0.99998	11261	0.0415
ZNF134	6	0.43185	0.59478	0.99998	11262	0.1537
OTOS	6	0.43195	0.59485	0.99998	11263	-0.047
NUF2	6	0.43196	0.59485	0.99998	11264	-0.103
TBC1D14	6	0.43196	0.59485	0.99998	11265	0.2008
OR8A1	6	0.43196	0.59485	0.99998	11266	-0.0968
DUS1L	6	0.43196	0.59485	0.99998	11267	-0.004
LACTB	6	0.43196	0.59485	0.99998	11268	-0.0643
ATAD3C	6	0.43223	0.59507	0.99998	11269	-0.2264
IQCF3	6	0.43234	0.59516	0.99998	11270	-0.0241
CATSPER1	6	0.43256	0.59534	0.99998	11271	0.291
CD3E	6	0.43256	0.59534	0.99998	11272	-0.042
hsa-mir-573	2	0.43271	0.43706	0.99998	11273	-0.0143
hsa-mir-1289-	4	0.43274	0.54275	0.99998	11274	-0.0395
ABHD4	6	0.43281	0.59553	0.99998	11275	0.1595
WDR35	6	0.43281	0.59553	0.99998	11276	0.0268
DFFB	6	0.43281	0.59553	0.99998	11277	-0.033
SEMA5B	6	0.43299	0.59567	0.99998	11278	-0.0228
TRABD2A	6	0.43303	0.59571	0.99998	11279	0.2291
RHOT2	6	0.43303	0.59571	0.99998	11280	0.2707
ADAMTS14	6	0.43321	0.59585	0.99998	11281	-0.0011
ELAC2	6	0.43331	0.59593	0.99998	11282	0.0285
hsa-mir-192	4	0.43355	0.54313	0.99998	11283	0.099
SFTA3	6	0.43358	0.59615	0.99998	11284	-0.2094
SPI1	6	0.43375	0.59628	0.99998	11285	0.1039
RGS21	6	0.43375	0.59628	0.99998	11286	-0.1095
ARHGAP26	6	0.43396	0.59647	0.99998	11287	0.0894
SOHLH1	6	0.43396	0.59647	0.99998	11288	0.1463
ZNF843	6	0.43409	0.59656	0.99998	11289	0.1288
SREK1IP1	6	0.43409	0.59656	0.99998	11290	-0.042
GRIN3A	6	0.43419	0.59665	0.99998	11291	0.0879
ACRV1	6	0.43419	0.59665	0.99998	11292	0.1076
INSIG2	6	0.43419	0.59665	0.99998	11293	0.0467
AKT2	6	0.43426	0.59671	0.99998	11294	-0.0534
PPP6R2	6	0.43434	0.59678	0.99998	11295	0.1294
ARFGEF2	6	0.43436	0.59679	0.99998	11296	0.1202
RBP3	6	0.43444	0.59686	0.99998	11297	0.0496
KRT84	6	0.43444	0.59686	0.99998	11298	-0.0048
CTXN2	6	0.43453	0.59691	0.99998	11299	0.0059
SPIN3	6	0.43457	0.59695	0.99998	11300	0.0731
LYSMD2	6	0.43459	0.59697	0.99998	11301	-0.1432
ZIC3	6	0.43459	0.59697	0.99998	11302	-0.0794
OR7D2	6	0.43459	0.59697	0.99998	11303	-0.0433
SLC22A2	6	0.43459	0.59697	0.99998	11304	0.2607
ACOX3	6	0.43459	0.59697	0.99998	11305	0.0718
BLOC1S5	6	0.43461	0.59698	0.99998	11306	-0.0688
KLHL32	6	0.43464	0.59701	0.99998	11307	-0.0009
ADORA1	6	0.43475	0.59709	0.99998	11308	0.1124
KLK1	6	0.43483	0.59714	0.99998	11309	-0.035
DDAH2	6	0.43485	0.59716	0.99998	11310	-0.0403
DPP3	6	0.43498	0.59727	0.99998	11311	0.1458
PIGG	6	0.43498	0.59727	0.99998	11312	0.1128
LYZL1	2	0.43515	0.43932	0.99998	11313	-0.6011
HGD	6	0.43519	0.59742	0.99998	11314	0.1081
GABRP	6	0.43528	0.59749	0.99998	11315	-0.1286
DDIT3	6	0.43542	0.5976	0.99998	11316	-0.1219
EPB41L1	6	0.43542	0.5976	0.99998	11317	-0.1321
TMEM154	6	0.43542	0.5976	0.99998	11318	-0.2024
OTC	6	0.43543	0.59762	0.99998	11319	0.1752
EPT1	6	0.43554	0.5977	0.99998	11320	0.0718
NLRP4	6	0.43554	0.5977	0.99998	11321	-0.0495
PCK2	6	0.43577	0.59788	0.99998	11322	0.1481
B9D2	6	0.43592	0.59798	0.99998	11323	0.1763
ZGLP1	6	0.436	0.59805	0.99998	11324	0.1651
C12orf23	6	0.436	0.59805	0.99998	11325	-0.0072
C19orf35	6	0.43612	0.59816	0.99998	11326	0.0673
DLG4	6	0.43612	0.59816	0.99998	11327	-0.1319
WNT9B	6	0.43612	0.59816	0.99998	11328	-0.0343
FGD2	6	0.43612	0.59816	0.99998	11329	-0.0406
OSBPL11	6	0.43629	0.5983	0.99998	11330	0.0078
RTCA	6	0.43631	0.59832	0.99998	11331	0.0212
INO80D	6	0.4364	0.59838	0.99998	11332	0.0093
SPNS1	6	0.43645	0.59842	0.99998	11333	-0.1266
MORC3	6	0.43661	0.59855	0.99998	11334	0.1238
NUDT16L1	6	0.43663	0.59856	0.99998	11335	0.1525
TCEB3CL2	2	0.43683	0.44085	0.99998	11336	-0.1838
FCRL5	6	0.43695	0.5988	0.99998	11337	0.1355

TAS2R14	6	0.43695	0.5988	0.99998	11338	0.3336
CCNI	6	0.43695	0.5988	0.99998	11339	-0.0575
PHF7	6	0.43695	0.5988	0.99998	11340	0.0725
CHST15	6	0.43713	0.59895	0.99998	11341	0.0453
NKAIN3	6	0.43719	0.59899	0.99998	11342	0.149
PINLYP	6	0.43725	0.59903	0.99998	11343	-0.0519
OPTN	6	0.43729	0.59907	0.99998	11344	-0.1181
MAGEC2	6	0.43736	0.59913	0.99998	11345	0.034
PLAG1	6	0.43745	0.5992	0.99998	11346	0.2273
CDR2	6	0.4375	0.59923	0.99998	11347	0.15
ATP2A1	6	0.4375	0.59923	0.99998	11348	-0.0901
SKIL	6	0.4375	0.59923	0.99998	11349	-0.0206
SLC9A4	6	0.43753	0.59926	0.99998	11350	0.0178
NPM3	6	0.43763	0.59934	0.99998	11351	-0.017
PRR4	6	0.43773	0.59942	0.99998	11352	0.0704
NNT	6	0.43774	0.59943	0.99998	11353	-0.0183
hsa-mir-8073	4	0.43784	0.54523	0.99998	11354	0.0845
CSPG5	6	0.43806	0.59968	0.99998	11355	-0.0222
ATOX1	6	0.43812	0.59974	0.99998	11356	-0.0265
KCNK9	6	0.43818	0.59977	0.99998	11357	-0.0434
hsa-mir-23c	4	0.43821	0.54543	0.99998	11358	0.2476
KHDC3L	6	0.43826	0.59985	0.99998	11359	0.2559
RAD18	6	0.43831	0.59989	0.99998	11360	-0.1912
SPAG4	6	0.43831	0.59989	0.99998	11361	0.1372
LYG2	6	0.43838	0.59995	0.99998	11362	0.0762
KIAA1715	6	0.43842	0.59998	0.99998	11363	-0.0992
CD33	6	0.43856	0.6001	0.99998	11364	0.0372
OR8J1	4	0.43863	0.54564	0.99998	11365	-0.0437
JUP	6	0.43871	0.60021	0.99998	11366	0.0626
EFR3A	6	0.43874	0.60024	0.99998	11367	-0.0921
SLC38A11	6	0.43874	0.60024	0.99998	11368	0.0484
KCNJ11	4	0.43884	0.54574	0.99998	11369	0.0294
SALL4	6	0.43893	0.60039	0.99998	11370	-0.0265
DYRK1B	6	0.43893	0.60039	0.99998	11371	0.3497
TRIM5	6	0.43903	0.60048	0.99998	11372	-0.0206
SLC16A14	6	0.4391	0.60053	0.99998	11373	-0.0501
ANGPTL2	6	0.43913	0.60055	0.99998	11374	0.3243
TFAP4	6	0.43926	0.60065	0.99998	11375	-0.0045
FNDC1	6	0.4394	0.60076	0.99998	11376	0.0707
PNISR	6	0.4394	0.60076	0.99998	11377	0.1307
VWA2	6	0.4394	0.60076	0.99998	11378	0.1179
ATOH1	6	0.4394	0.60076	0.99998	11379	0.0404
TF	6	0.43946	0.6008	0.99998	11380	-0.0494
GGA1	6	0.43958	0.60092	0.99998	11381	-0.0324
FAM43B	6	0.43962	0.60094	0.99998	11382	-0.074
RGR	6	0.43982	0.6011	0.99998	11383	0.0551
CCDC81	6	0.43982	0.6011	0.99998	11384	-0.0533
hsa-mir-3173	4	0.43995	0.5463	0.99998	11385	0.519
OR1G1	6	0.43995	0.6012	0.99998	11386	-0.0562
SP4	6	0.43995	0.6012	0.99998	11387	-0.1355
SEZ6L	6	0.44004	0.60127	0.99998	11388	0.0566
MARCH7	6	0.44011	0.60132	0.99998	11389	0.2066
LOC643037	6	0.44011	0.60132	0.99998	11390	0.2119
OR4D1	6	0.44016	0.60136	0.99998	11391	-0.0545
CRYAB	6	0.44035	0.60152	0.99998	11392	0.2069
ADSSL1	6	0.44035	0.60152	0.99998	11393	0.0972
CECR1	6	0.44039	0.60156	0.99998	11394	0.0002
MAN2A1	6	0.44039	0.60156	0.99998	11395	0.0055
C3orf43	6	0.44039	0.60156	0.99998	11396	0.1357
ITGAE	6	0.44049	0.60164	0.99998	11397	0.2841
CYSTM1	6	0.44064	0.60175	0.99998	11398	-0.0204
hsa-mir-4785	4	0.44067	0.54667	0.99998	11399	-0.1206
MAGEA11	6	0.4407	0.6018	0.99998	11400	0.0667
RRAS	6	0.44079	0.60188	0.99998	11401	0.0422
TRH	6	0.44094	0.602	0.99998	11402	0.0522
IQGAP1	6	0.44094	0.602	0.99998	11403	-0.0568
FBXO36	6	0.44111	0.60214	0.99998	11404	-0.0903
CHRNA10	6	0.44112	0.60215	0.99998	11405	0.1268
NINJ1	6	0.4412	0.60222	0.99998	11406	-0.1068
OR5B2	6	0.4412	0.60222	0.99998	11407	0.1322
hsa-mir-3193	4	0.44125	0.54696	0.99998	11408	0.3314
OSBPL8	6	0.4413	0.60229	0.99998	11409	0.0817
UBQLN4	6	0.44146	0.60241	0.99998	11410	-0.1214
VANGL1	6	0.44146	0.60241	0.99998	11411	-0.072
RAB11B	6	0.44146	0.60241	0.99998	11412	0.0815
USP9X	6	0.44161	0.60252	0.99998	11413	-0.0288
CXXC11	6	0.44173	0.60263	0.99998	11414	-0.0636
CMTM1	4	0.44175	0.54719	0.99998	11415	0.0429
ENPP7	6	0.44194	0.60278	0.99998	11416	0.1576
MEPE	6	0.44194	0.60278	0.99998	11417	0.1366
PRAMEF4	6	0.44214	0.60293	0.99998	11418	0.1558

TRIM26	6	0.44214	0.60293	0.99998	11419	-0.0705
FAM168A	6	0.44227	0.60305	0.99998	11420	0.0008
hsa-mir-4456	4	0.44232	0.5475	0.99998	11421	0.0293
GIGYF1	6	0.44232	0.60309	0.99998	11422	-0.0426
BCL9	6	0.44232	0.60309	0.99998	11423	-0.0637
TRUB2	6	0.44232	0.60309	0.99998	11424	-0.0656
TCTN1	6	0.44237	0.60312	0.99998	11425	-0.0232
PPBP	6	0.44237	0.60312	0.99998	11426	0.2448
JAM3	6	0.4425	0.60323	0.99998	11427	0.1918
MUSK	6	0.44277	0.60345	0.99998	11428	-0.0548
AJAP1	6	0.44286	0.60352	0.99998	11429	0.0506
MRAS	6	0.44286	0.60352	0.99998	11430	0.031
hsa-mir-582	4	0.44288	0.54775	0.99998	11431	-0.0248
OR51B5	6	0.44301	0.60364	0.99998	11432	-0.0518
DNMT1	6	0.44301	0.60364	0.99998	11433	-0.0263
CTSO	6	0.4431	0.60373	0.99998	11434	0.14
SH2D4B	6	0.44319	0.60379	0.99998	11435	-0.0882
FRZB	6	0.44329	0.60387	0.99998	11436	0.0762
SARDH	6	0.44331	0.60389	0.99998	11437	0.023
MOAP1	4	0.44338	0.548	0.99998	11438	-0.0714
C3orf18	6	0.44344	0.60399	0.99998	11439	-0.1284
RUFY1	6	0.44344	0.60399	0.99998	11440	0.1018
GADD45G	6	0.44354	0.60407	0.99998	11441	0.2232
OVOL1	6	0.44357	0.6041	0.99998	11442	0.0051
hsa-mir-4487	2	0.44358	0.44711	0.99998	11443	-0.0784
CAMK2G	6	0.44364	0.60415	0.99998	11444	0.377
TSC22D4	6	0.44365	0.60415	0.99998	11445	0.167
NEUROG3	6	0.44365	0.60415	0.99998	11446	0.1048
LOC10012963	4	0.44372	0.54818	0.99998	11447	-0.0136
KRTAP19-8	6	0.44386	0.60431	0.99998	11448	-0.1502
ERRFI1	6	0.44386	0.60431	0.99998	11449	0.077
ZNF408	6	0.44386	0.60431	0.99998	11450	0.0556
CCL8	6	0.44394	0.60437	0.99998	11451	-0.0045
ZNF214	6	0.44405	0.60445	0.99998	11452	0.0957
MYO9B	6	0.44405	0.60445	0.99998	11453	0.02
AKAP3	6	0.44405	0.60445	0.99998	11454	0.3162
RBM3	6	0.44414	0.60452	0.99998	11455	-0.0969
TAS2R19	6	0.44422	0.60458	0.99998	11456	0.0967
TMEM132A	6	0.44436	0.60469	0.99998	11457	-0.0154
hsa-mir-3714	4	0.44442	0.54854	0.99998	11458	-0.0939
MLIP	6	0.44453	0.60482	0.99998	11459	0.0854
ELOVL7	6	0.44465	0.60492	0.99998	11460	0.0091
NOTCH4	6	0.44468	0.60494	0.99998	11461	-0.1934
MPC1	6	0.44472	0.60497	0.99998	11462	0.1705
BTNL3	6	0.44478	0.60501	0.99998	11463	0.1689
TMEM74	6	0.44482	0.60504	0.99998	11464	-0.0056
hsa-mir-4734	4	0.44482	0.54876	0.99998	11465	0.016
POC1B	4	0.44482	0.54876	0.99998	11466	0.0112
FMR1NB	6	0.44486	0.60507	0.99998	11467	0.1721
B3GAT3	6	0.44497	0.60514	0.99998	11468	-0.02
FOXO3	6	0.44517	0.60531	0.99998	11469	-0.0395
TFAP2E	6	0.44517	0.60531	0.99998	11470	-0.0831
RNF166	6	0.44518	0.60532	0.99998	11471	-0.0229
GCFC2	6	0.44531	0.60542	0.99998	11472	-0.1046
PRH1	5	0.44541	0.59129	0.99998	11473	0.1701
PRAP1	6	0.4455	0.60555	0.99998	11474	-0.0425
PSIP1	6	0.4455	0.60555	0.99998	11475	0.0164
MLF2	6	0.4455	0.60555	0.99998	11476	0.0592
SIRT5	6	0.4455	0.60555	0.99998	11477	-0.0422
BRPF1	6	0.44582	0.60579	0.99998	11478	0.1321
ZNF493	5	0.44583	0.59169	0.99998	11479	0.094
ARID2	6	0.44588	0.60584	0.99998	11480	0.0623
HIPK2	6	0.44588	0.60584	0.99998	11481	-0.1133
CASP3	6	0.4461	0.60601	0.99998	11482	0.0319
CHST3	6	0.4461	0.60601	0.99998	11483	0.0516
hsa-mir-933	4	0.44615	0.54942	0.99998	11484	0.0346
ZNF432	6	0.44631	0.60619	0.99998	11485	0.1082
GNAI3	5	0.44634	0.59217	0.99998	11486	0.0463
C6orf10	6	0.44644	0.60629	0.99998	11487	0.1344
MGA	6	0.44644	0.60629	0.99998	11488	0.0021
PXDC1	6	0.44657	0.60639	0.99998	11489	0.011
ZFP28	6	0.44657	0.60639	0.99998	11490	-0.053
OR10A4	6	0.4468	0.60657	0.99998	11491	0.1568
TNPO1	6	0.4468	0.60657	0.99998	11492	-0.0965
hsa-mir-4684	4	0.44682	0.54976	0.99998	11493	0.0328
EDF1	6	0.44687	0.60663	0.99998	11494	0.1485
CPT2	6	0.44703	0.60675	0.99998	11495	0.0752
TAL2	6	0.44722	0.60691	0.99998	11496	0.2243
HSFX2	1	0.44722	0.44737	0.99998	11497	-0.0623
BCAR3	6	0.44741	0.60705	0.99998	11498	0.0543
C11orf35	6	0.44742	0.60706	0.99998	11499	-0.019

C10orf35	6	0.44742	0.60706	0.99998	11500	0.0728
DAPL1	6	0.44742	0.60706	0.99998	11501	0.0132
ATPIF1	6	0.44742	0.60706	0.99998	11502	-0.0889
NKIRAS1	6	0.44749	0.60711	0.99998	11503	0.0923
IQCF1	6	0.4476	0.6072	0.99998	11504	-0.0498
hsa-mir-3607	1	0.44761	0.44775	0.99998	11505	-0.0607
CDK2AP1	6	0.44771	0.60729	0.99998	11506	0.0389
NUDT9	6	0.44776	0.60733	0.99998	11507	-0.0298
SACS	6	0.44804	0.60757	0.99998	11508	-0.0121
LYPD1	6	0.44804	0.60757	0.99998	11509	-0.0317
ZNF19	6	0.44804	0.60758	0.99998	11510	0.1329
SPRR2G	5	0.44813	0.5939	0.99998	11511	-0.1202
FBXO39	6	0.44817	0.60766	0.99998	11512	0.0623
MGST2	6	0.44817	0.60766	0.99998	11513	0.028
MDH1B	6	0.44825	0.60773	0.99998	11514	-0.1513
PTRH2	6	0.4483	0.60776	0.99998	11515	0.0712
CDH6	6	0.44838	0.60783	0.99998	11516	-0.1225
VARS	6	0.44838	0.60783	0.99998	11517	-0.0465
HIST1H4C	6	0.44838	0.60783	0.99998	11518	0.2169
MID1	6	0.44843	0.60788	0.99998	11519	0.0765
C9orf40	6	0.44843	0.60788	0.99998	11520	0.2652
TBX1	6	0.4486	0.60802	0.99998	11521	-0.0013
HK1	6	0.44868	0.60809	0.99998	11522	-0.0534
hsa-mir-4484	4	0.44879	0.55074	0.99998	11523	0.4624
ANGPTL1	6	0.44882	0.6082	0.99998	11524	0.1176
EXOC1	6	0.44882	0.6082	0.99998	11525	0.1425
hsa-mir-548a-	4	0.44891	0.55081	0.99998	11526	-0.1279
SETBP1	6	0.44894	0.60832	0.99998	11527	-0.0127
F2	6	0.44894	0.60832	0.99998	11528	-0.0608
SH3GL2	6	0.44898	0.60835	0.99998	11529	0.1559
RBFOX3	4	0.44915	0.55093	0.99998	11530	0.0586
CD70	6	0.44915	0.60849	0.99998	11531	-0.132
SOX4	6	0.44928	0.60858	0.99998	11532	0.0082
ZNF467	6	0.44938	0.60867	0.99998	11533	0.1883
SERTAD4	6	0.44967	0.6089	0.99998	11534	-0.0398
PLA1A	6	0.44967	0.6089	0.99998	11535	-0.0967
hsa-mir-1324	4	0.44982	0.55129	0.99998	11536	0.206
SCYL3	6	0.44995	0.60912	0.99998	11537	-0.0638
TDGF1	6	0.44997	0.60914	0.99998	11538	0.2024
FIBP	6	0.45022	0.60934	0.99998	11539	0.1012
FBXO21	6	0.45022	0.60934	0.99998	11540	0.071
PPCS	6	0.45023	0.60935	0.99998	11541	0.0356
ZFAND1	6	0.45023	0.60935	0.99998	11542	-0.079
AP3M2	6	0.45039	0.60947	0.99998	11543	-0.0689
CTU1	6	0.45051	0.60955	0.99998	11544	-0.077
JAKMIP3	6	0.45055	0.60958	0.99998	11545	0.1553
LOC643802	6	0.45071	0.60972	0.99998	11546	0.146
hsa-mir-5590	2	0.45081	0.45386	0.99998	11547	-0.0667
FUT5	6	0.45094	0.60992	0.99998	11548	0.2917
CHRNG	6	0.45101	0.60997	0.99998	11549	0.1891
THRB	6	0.45126	0.61018	0.99998	11550	0.2376
KRAS	6	0.45126	0.61018	0.99998	11551	-0.1306
CA14	6	0.45133	0.61022	0.99998	11552	-0.0757
KIAA1328	6	0.45144	0.61031	0.99998	11553	0.0867
GRAMD2	6	0.45144	0.61031	0.99998	11554	0.0515
C16orf92	6	0.45144	0.61031	0.99998	11555	0.0022
EGFLAM	6	0.45152	0.61038	0.99998	11556	-0.1212
C4orf33	6	0.45171	0.61052	0.99998	11557	0.0517
ALG6	6	0.45171	0.61052	0.99998	11558	0.0842
IL1F10	6	0.45171	0.61052	0.99998	11559	0.0234
CLEC3A	6	0.45173	0.61053	0.99998	11560	0.0253
HELT	6	0.45173	0.61053	0.99998	11561	0.1833
PLEKHG7	6	0.45173	0.61053	0.99998	11562	0.0524
ATP7B	6	0.45188	0.61065	0.99998	11563	-0.1515
IER3	6	0.45188	0.61065	0.99998	11564	0.0258
OR2T11	6	0.45188	0.61065	0.99998	11565	0.0993
NDUFA5	6	0.45194	0.61069	0.99998	11566	0.2514
hsa-mir-1273c	4	0.45207	0.55244	0.99998	11567	-0.2881
VPS53	6	0.45222	0.61092	0.99998	11568	-0.0724
ZFYVE26	6	0.45232	0.61098	0.99998	11569	0.1827
FOXD4	6	0.45235	0.61101	0.99998	11570	0.1167
UCN2	6	0.45243	0.61107	0.99998	11571	0.0451
EPYC	6	0.45251	0.61113	0.99998	11572	0.075
ACSL6	6	0.45263	0.61123	0.99998	11573	0.1349
GIT2	6	0.45268	0.61127	0.99998	11574	0.0201
C21orf33	6	0.45276	0.61134	0.99998	11575	-0.0736
C10orf67	6	0.45282	0.61139	0.99998	11576	-0.0422
OR4F15	6	0.45291	0.61145	0.99998	11577	-0.0482
STAP2	6	0.45291	0.61145	0.99998	11578	0.0361
OVCH1	6	0.45291	0.61145	0.99998	11579	-0.1605
APLP2	6	0.45295	0.61149	0.99998	11580	0.1215

hsa-mir-4647	4	0.45297	0.55289	0.99998	11581	-0.0699
LRRC59	6	0.45321	0.61169	0.99998	11582	0.2117
UFSP2	6	0.45344	0.61186	0.99998	11583	0.0966
SLFN12L	6	0.45351	0.61192	0.99998	11584	-0.0285
ARHGAP17	6	0.45364	0.61202	0.99998	11585	0.0293
SSTR1	6	0.45364	0.61202	0.99998	11586	0.0257
APBA3	6	0.4537	0.61207	0.99998	11587	0.1224
SUN1	6	0.4537	0.61207	0.99998	11588	0.0715
VAMP2	6	0.4537	0.61207	0.99998	11589	0.0712
NRTN	6	0.45405	0.61235	0.99998	11590	-0.0276
VANGL2	6	0.45408	0.61237	0.99998	11591	-0.048
LPAR6	6	0.45408	0.61237	0.99998	11592	-0.008
RASL11A	6	0.45408	0.61237	0.99998	11593	0.0594
ADCY9	6	0.45408	0.61237	0.99998	11594	0.0894
FAM86A	5	0.45422	0.59968	0.99998	11595	0.153
hsa-let-7a-1	4	0.45425	0.55355	0.99998	11596	0.046
ZNF141	4	0.45425	0.55355	0.99998	11597	0.2711
LAMP3	6	0.45427	0.61251	0.99998	11598	0.1217
NEUROD6	6	0.45441	0.61262	0.99998	11599	0.2517
PASD1	6	0.45454	0.61272	0.99998	11600	0.1544
DKK3	6	0.45458	0.61276	0.99998	11601	0.1168
CD79A	6	0.45458	0.61276	0.99998	11602	0.2411
CLDN23	6	0.45468	0.61283	0.99998	11603	0.089
FAM163B	6	0.45468	0.61283	0.99998	11604	-0.028
PCDHB11	6	0.4547	0.61285	0.99998	11605	-0.0423
GPN1	6	0.45476	0.6129	0.99998	11606	0.0586
MCC	6	0.45478	0.61292	0.99998	11607	-0.0019
LHX6	6	0.45483	0.61295	0.99998	11608	-0.0318
AKR1D1	6	0.45509	0.61315	0.99998	11609	0.3865
ACKR2	4	0.45511	0.554	0.99998	11610	-0.0023
hsa-mir-762	4	0.45511	0.554	0.99998	11611	0.0603
OTOF	6	0.45512	0.61317	0.99998	11612	-0.0119
CLDN14	6	0.45512	0.61317	0.99998	11613	0.0385
UCK1	6	0.4552	0.61323	0.99998	11614	-0.2238
hsa-mir-6781	4	0.45523	0.55406	0.99998	11615	0.0663
CCDC3	6	0.45529	0.6133	0.99998	11616	0.3198
hsa-mir-8063	4	0.45535	0.55413	0.99998	11617	-0.1872
hsa-mir-922	4	0.45535	0.55413	0.99998	11618	0.2164
ZNF793	6	0.45539	0.61338	0.99998	11619	-0.0474
SNRNP48	6	0.45553	0.61349	0.99998	11620	-0.1368
FUT4	6	0.45555	0.61352	0.99998	11621	-0.079
TCN2	6	0.45555	0.61352	0.99998	11622	-0.0875
ASB16	6	0.45555	0.61352	0.99998	11623	0.0406
TRAPPC6A	6	0.45568	0.61363	0.99998	11624	0.0025
ATP2C1	6	0.45568	0.61363	0.99998	11625	0.1521
KRT39	6	0.45578	0.61371	0.99998	11626	0.1385
GPR173	6	0.45578	0.61371	0.99998	11627	0.0719
ZNF383	6	0.45589	0.61379	0.99998	11628	-0.1228
KLHL15	6	0.45595	0.61384	0.99998	11629	0.2567
CCDC64B	6	0.456	0.61388	0.99998	11630	0.2665
AMBN	6	0.45615	0.614	0.99998	11631	0.0775
FAM155B	6	0.45626	0.61409	0.99998	11632	-0.1329
KRTAP7-1	6	0.45626	0.61409	0.99998	11633	0.0116
SMUG1	6	0.45626	0.61409	0.99998	11634	0.0167
MAPK6	6	0.45633	0.61414	0.99998	11635	0.0832
RBM7	6	0.45637	0.61418	0.99998	11636	0.0849
LMO2	6	0.45637	0.61418	0.99998	11637	0.0571
GYLTL1B	6	0.45637	0.61418	0.99998	11638	0.126
HOXC10	6	0.45644	0.61423	0.99998	11639	-0.0531
RABL3	6	0.45653	0.61431	0.99998	11640	0.0692
RIN3	6	0.45659	0.61435	0.99998	11641	0.043
hsa-mir-182	4	0.45681	0.55486	0.99998	11642	0.1164
PIGF	6	0.45681	0.61452	0.99998	11643	0.1123
PREP	6	0.45684	0.61454	0.99998	11644	-0.0493
MMAB	6	0.45695	0.61463	0.99998	11645	0.0986
SLC25A44	6	0.45698	0.61465	0.99998	11646	-0.0477
CCNB1	6	0.457	0.61467	0.99998	11647	0.0661
MARCKS	6	0.45705	0.61471	0.99998	11648	-0.1346
SMAD3	6	0.45711	0.61474	0.99998	11649	-0.1353
EHD4	6	0.45712	0.61476	0.99998	11650	0.0002
MPST	6	0.45712	0.61476	0.99998	11651	0.0711
SATL1	6	0.45712	0.61476	0.99998	11652	-0.021
ZNF107	5	0.45718	0.60254	0.99998	11653	0.0132
MYO5C	6	0.45738	0.61495	0.99998	11654	0.0787
FSCB	6	0.45738	0.61495	0.99998	11655	0.2166
CYP4V2	6	0.45738	0.61495	0.99998	11656	0.091
HNF1A	6	0.45738	0.61495	0.99998	11657	0.0906
ZYG11A	6	0.45764	0.61517	0.99998	11658	0.183
hsa-mir-337	4	0.45771	0.55531	0.99998	11659	0.4504
LZTR1	6	0.45772	0.61523	0.99998	11660	-0.0692
OLFM2	6	0.45776	0.61526	0.99998	11661	0.0473

LY75	4	0.45777	0.55534	0.99998	11662	0.0494
ARF4	6	0.45778	0.61528	0.99998	11663	0.0615
CD151	6	0.45778	0.61528	0.99998	11664	-0.0089
STK36	6	0.45784	0.61533	0.99998	11665	-0.0174
hsa-mir-645	4	0.45791	0.55541	0.99998	11666	0.1422
ICA1	6	0.45792	0.61539	0.99998	11667	0.1699
KIAA1024L	6	0.45792	0.61539	0.99998	11668	0.1177
38596	3	0.45795	0.49408	0.99998	11669	-0.1031
C2CD5	6	0.45805	0.61549	0.99998	11670	0.0683
PPM1E	6	0.45805	0.61549	0.99998	11671	0.1051
NUDT8	6	0.45808	0.61551	0.99998	11672	-0.0546
RSF1	6	0.45816	0.61557	0.99998	11673	0.0696
C6orf47	6	0.4582	0.6156	0.99998	11674	-0.0227
GOLPH3	6	0.45826	0.61565	0.99998	11675	0.1137
GIMAP4	6	0.45826	0.61565	0.99998	11676	0.1537
PPM1J	6	0.45826	0.61565	0.99998	11677	-0.0448
hsa-mir-3620	4	0.45827	0.55561	0.99998	11678	-0.2282
SHF	6	0.45842	0.61577	0.99998	11679	-0.0046
AGPS	6	0.45842	0.61577	0.99998	11680	-0.0368
SNX18	6	0.45842	0.61577	0.99998	11681	0.2277
ZHX1	6	0.45849	0.61583	0.99998	11682	0.0749
SLC25A20	6	0.45849	0.61583	0.99998	11683	0.0194
YOD1	6	0.45849	0.61583	0.99998	11684	0.1082
IPPK	6	0.45849	0.61583	0.99998	11685	-0.1004
CXorf22	6	0.45863	0.61593	0.99998	11686	0.1479
TMEM202	6	0.45865	0.61594	0.99998	11687	-0.1224
HSPA1L	6	0.45875	0.61602	0.99998	11688	0.0047
MC1R	6	0.45875	0.61602	0.99998	11689	0.1295
FBXL13	6	0.4588	0.61606	0.99998	11690	0.1175
GJB5	6	0.45895	0.61618	0.99998	11691	0.0008
RMDN2	6	0.45895	0.61618	0.99998	11692	0.0022
hsa-mir-100	4	0.45896	0.55599	0.99998	11693	0.0665
PCDHGB5	3	0.45897	0.49478	0.99998	11694	-0.1415
hsa-mir-5696	3	0.45897	0.49478	0.99998	11695	-0.0531
CEP170B	6	0.45898	0.6162	0.99998	11696	0.0349
CRTAP	6	0.45912	0.61632	0.99998	11697	-0.0368
AIRE	6	0.45923	0.61642	0.99998	11698	0.3491
LONRF3	6	0.45923	0.61642	0.99998	11699	-0.0495
VRTN	6	0.45932	0.61649	0.99998	11700	0.0368
GTF2IRD2	2	0.45934	0.46185	0.99998	11701	-0.1943
WISP3	6	0.45954	0.61667	0.99998	11702	0.2174
LMO7	6	0.45954	0.61667	0.99998	11703	0.1628
TMF1	6	0.45972	0.61683	0.99998	11704	-0.0562
C1orf151-NBL	3	0.4598	0.49536	0.99998	11705	0.0271
PITPNC1	6	0.46001	0.61706	0.99998	11706	0.0088
C22orf26	6	0.46001	0.61706	0.99998	11707	0.317
TSNARE1	6	0.46001	0.61706	0.99998	11708	0.1854
FBXW8	6	0.46005	0.6171	0.99998	11709	-0.0989
RASSF6	6	0.46005	0.6171	0.99998	11710	-0.1
HCN4	6	0.46005	0.6171	0.99998	11711	0.2281
DDAH1	6	0.46022	0.61722	0.99998	11712	0.1478
ZNF35	6	0.46022	0.61722	0.99998	11713	0.2189
PPP1R8	6	0.46022	0.61722	0.99998	11714	0.0002
SEMA3C	6	0.46036	0.61733	0.99998	11715	-0.0342
FURIN	6	0.46036	0.61733	0.99998	11716	-0.1202
GNAL	6	0.46048	0.61742	0.99998	11717	0.0937
KCNA1	6	0.46067	0.61757	0.99998	11718	0.2402
MINPP1	6	0.46067	0.61757	0.99998	11719	0.0451
WDR45B	6	0.46089	0.61774	0.99998	11720	0.1601
SCN10A	6	0.46089	0.61774	0.99998	11721	-0.0273
PSMC3IP	6	0.4609	0.61775	0.99998	11722	-0.028
JMJD4	6	0.4609	0.61775	0.99998	11723	0.1266
SPATA31A7	2	0.46106	0.46344	0.99998	11724	-0.3064
WTH3DI	6	0.46111	0.61792	0.99998	11725	0.2743
DDX28	6	0.46142	0.61815	0.99998	11726	0.1942
ATP7A	6	0.46145	0.61818	0.99998	11727	-0.0387
C9orf89	6	0.46145	0.61818	0.99998	11728	0.0313
HADHB	6	0.46145	0.61818	0.99998	11729	0.0193
hsa-mir-6767	4	0.46147	0.55728	0.99998	11730	0.0589
hsa-mir-16-2	2	0.46147	0.46382	0.99998	11731	-0.1502
LOC729059	3	0.46155	0.49661	0.99998	11732	-0.0539
EPS8L2	6	0.46156	0.61826	0.99998	11733	-0.0588
KRBOX1	6	0.46159	0.61829	0.99998	11734	0.0118
DNAJC14	6	0.46174	0.61841	0.99998	11735	0.093
EIF1AY	6	0.46174	0.61841	0.99998	11736	0.2356
CTDNEP1	6	0.46174	0.61841	0.99998	11737	-0.0503
ERCC2	5	0.46183	0.60692	0.99998	11738	-0.1409
C15orf57	6	0.46184	0.61849	0.99998	11739	-0.1753
ARID3A	6	0.46184	0.61849	0.99998	11740	0.1505
FBXW4	6	0.46184	0.61849	0.99998	11741	0.0706
VAV3	6	0.46202	0.61862	0.99998	11742	0.0722

MT1M	5	0.46205	0.60713	0.99998	11743	0.3037
hsa-mir-1587	4	0.46232	0.55772	0.99998	11744	0.1171
CACNG2	6	0.46232	0.61885	0.99998	11745	-0.0328
SLITRK2	6	0.46232	0.61885	0.99998	11746	-0.0272
ACTC1	6	0.46235	0.61887	0.99998	11747	0.1222
FAM47E-STBD	2	0.46244	0.46475	0.99998	11748	-0.1401
C9orf171	6	0.46249	0.61898	0.99998	11749	-0.1253
TNFAIP1	6	0.46255	0.61903	0.99998	11750	0.0388
TMPRSS15	6	0.46261	0.61908	0.99998	11751	-0.074
NETO1	6	0.46286	0.6193	0.99998	11752	0.0639
BOC	6	0.46286	0.6193	0.99998	11753	-0.088
C2orf40	6	0.46296	0.61937	0.99998	11754	-0.0644
BAZ1B	6	0.46311	0.61949	0.99998	11755	-0.0012
NEK8	6	0.46324	0.61961	0.99998	11756	-0.0068
MLKL	6	0.46324	0.61961	0.99998	11757	0.1747
PPP1R14A	6	0.46331	0.61966	0.99998	11758	-0.034
PHYH	6	0.46331	0.61966	0.99998	11759	-0.1326
PEPD	6	0.46342	0.61975	0.99998	11760	0.0094
GRIK4	6	0.46342	0.61975	0.99998	11761	-0.0077
FERMT3	6	0.46342	0.61975	0.99998	11762	0.3298
BBS4	6	0.46342	0.61975	0.99998	11763	0.009
hsa-mir-6803	4	0.46356	0.55834	0.99998	11764	0.0061
HIPK3	6	0.46358	0.61988	0.99998	11765	-0.1324
NUDT6	6	0.46371	0.61997	0.99998	11766	0.2006
RIMS1	6	0.46371	0.61997	0.99998	11767	0.0637
hsa-mir-516b-	2	0.46378	0.46601	0.99998	11768	-0.221
WFDC12	6	0.46382	0.62006	0.99998	11769	0.0112
OR1Q1	6	0.46389	0.62012	0.99998	11770	0.0365
AKAP2	6	0.46389	0.62012	0.99998	11771	-0.1456
PTPN13	6	0.46389	0.62012	0.99998	11772	0.0836
METTL9	6	0.46389	0.62012	0.99998	11773	0.0747
PEBP1	6	0.46399	0.6202	0.99998	11774	0.2407
ZNF317	6	0.46418	0.62036	0.99998	11775	-0.0097
SPG20	6	0.46426	0.62041	0.99998	11776	-0.0986
NACA	6	0.46435	0.6205	0.99998	11777	-0.1154
PDZD9	6	0.46439	0.62052	0.99998	11778	0.174
C12orf76	6	0.46457	0.62067	0.99998	11779	0.1131
TIGD2	6	0.46469	0.62077	0.99998	11780	-0.051
PDS5A	6	0.46469	0.62077	0.99998	11781	0.0748
FAM217B	6	0.46491	0.62094	0.99998	11782	-0.0871
MMP7	6	0.46492	0.62096	0.99998	11783	0.2198
LOC388849	6	0.46504	0.62105	0.99998	11784	0.1687
ST7L	6	0.46509	0.6211	0.99998	11785	-0.0207
PLAC9	6	0.46509	0.6211	0.99998	11786	0.0951
C1orf86	6	0.46521	0.62119	0.99998	11787	0.0486
TACC1	6	0.46521	0.62119	0.99998	11788	0.0082
SPECC1L	6	0.46528	0.62124	0.99998	11789	0.1445
MEX3C	6	0.46528	0.62124	0.99998	11790	0.1046
KRT72	6	0.46549	0.62141	0.99998	11791	-0.0008
hsa-mir-6815	4	0.46549	0.55928	0.99998	11792	0.1931
HMGCS2	6	0.46575	0.62161	0.99998	11793	0.0128
DYRK2	6	0.46576	0.62161	0.99998	11794	0.0925
LOC10028717	4	0.4658	0.55944	0.99998	11795	-0.0533
SMIM12	6	0.46584	0.62167	0.99998	11796	0.0985
HSP90AA1	6	0.46597	0.62177	0.99998	11797	0.1525
BAG2	6	0.46597	0.62177	0.99998	11798	0.0673
ABCB9	6	0.46613	0.62191	0.99998	11799	-0.078
EPC2	6	0.46634	0.62207	0.99998	11800	-0.0408
PRMT10	6	0.46634	0.62207	0.99998	11801	0.3527
EPN3	6	0.46634	0.62207	0.99998	11802	-0.0226
HCAR1	6	0.46647	0.62218	0.99998	11803	0.1387
PHF14	6	0.46659	0.62227	0.99998	11804	-0.096
RNF170	6	0.46659	0.62227	0.99998	11805	0.0963
IRF3	6	0.46677	0.6224	0.99998	11806	-0.0012
OR4D9	6	0.46688	0.62247	0.99998	11807	0.2032
DHFRL1	6	0.46688	0.62247	0.99998	11808	0.0757
XG	6	0.46708	0.62261	0.99998	11809	-0.072
CDKL1	6	0.46708	0.62261	0.99998	11810	-0.0323
ELAVL3	6	0.46708	0.62261	0.99998	11811	0.2214
EAPP	6	0.46715	0.62265	0.99998	11812	0.0303
hsa-mir-3662	3	0.46717	0.50061	0.99998	11813	-0.143
FOSL1	6	0.46719	0.62268	0.99998	11814	-0.0975
OR4K5	6	0.46735	0.62282	0.99998	11815	-0.0134
TMEM223	6	0.46737	0.62283	0.99998	11816	-0.125
BCS1L	6	0.46737	0.62283	0.99998	11817	0.0457
LRAT	6	0.46737	0.62283	0.99998	11818	0.1568
DTWD2	5	0.46739	0.61222	0.99998	11819	0.0578
RTN4RL1	6	0.46755	0.62296	0.99998	11820	-0.0434
SYT16	6	0.46755	0.62296	0.99998	11821	0.1074
TMEM139	6	0.46755	0.62296	0.99998	11822	-0.0368
VCX	2	0.46778	0.46983	0.99998	11823	-0.155

MAP2K2	6	0.46779	0.62317	0.99998	11824	0.0835
GRHL3	6	0.46796	0.6233	0.99998	11825	0.0396
ZNF354C	6	0.46812	0.62342	0.99998	11826	-0.0552
UNC5C	6	0.46812	0.62342	0.99998	11827	-0.1697
LZTS2	6	0.46829	0.62355	0.99998	11828	-0.0231
CHD1L	6	0.46832	0.62358	0.99998	11829	0.1929
RARB	6	0.46852	0.62375	0.99998	11830	-0.0723
SLC27A4	6	0.46853	0.62376	0.99998	11831	0.2063
SLC7A4	6	0.46879	0.62397	0.99998	11832	-0.0127
TMEM63C	6	0.46879	0.62397	0.99998	11833	-0.1294
TRIM17	6	0.46879	0.62397	0.99998	11834	0.1041
KPNA1	6	0.46879	0.62397	0.99998	11835	0.4519
WBSCR28	6	0.46879	0.62397	0.99998	11836	0.0738
C4orf22	6	0.46883	0.62401	0.99998	11837	0.0275
C9orf135	6	0.46895	0.6241	0.99998	11838	-0.0616
METRNL	6	0.46895	0.6241	0.99998	11839	-0.0331
USP9Y	6	0.46914	0.62425	0.99998	11840	0.0517
ZDHHC24	6	0.46914	0.62425	0.99998	11841	-0.0041
FNBP4	6	0.46914	0.62425	0.99998	11842	0.1522
FUT10	6	0.46928	0.62438	0.99998	11843	0.0621
LAMP2	6	0.46928	0.62438	0.99998	11844	-0.0966
AIMP1	6	0.46946	0.62451	0.99998	11845	0.3435
FASTKD1	6	0.46946	0.62451	0.99998	11846	0.0902
GLIPR1L2	6	0.46946	0.62451	0.99998	11847	0.0035
ADRA2B	6	0.46948	0.62453	0.99998	11848	0.0506
AVPR1A	6	0.46948	0.62453	0.99998	11849	0.0893
DEFB133	3	0.46966	0.50242	0.99998	11850	0.2478
SPACA4	6	0.46966	0.62468	0.99998	11851	0.0068
IL17RB	6	0.46966	0.62468	0.99998	11852	0.0292
CHRM5	6	0.46966	0.62468	0.99998	11853	-0.0619
B4GALT7	6	0.46971	0.62472	0.99998	11854	0.4787
ZAP70	6	0.46987	0.62483	0.99998	11855	-0.1314
TTLL2	6	0.46987	0.62483	0.99998	11856	0.1855
PRKAG3	6	0.46987	0.62483	0.99998	11857	0.111
KIAA1191	6	0.46987	0.62483	0.99998	11858	0.0911
AKAP11	6	0.46987	0.62483	0.99998	11859	0.092
OR6B1	6	0.46987	0.62483	0.99998	11860	0.0119
ESAM	6	0.47003	0.62496	0.99998	11861	0.006
MMP9	6	0.47011	0.62503	0.99998	11862	0.3158
PTPN12	5	0.47015	0.61486	0.99998	11863	-0.1572
OR5M10	4	0.47016	0.56171	0.99998	11864	0.3423
TIFA	6	0.47023	0.62512	0.99998	11865	0.195
COMMD6	6	0.47023	0.62512	0.99998	11866	-0.0568
LPIN1	6	0.47025	0.62514	0.99998	11867	0.3191
SYT2	6	0.47025	0.62514	0.99998	11868	-0.0662
LRRC25	6	0.47025	0.62514	0.99998	11869	0.1052
GPR148	6	0.47033	0.6252	0.99998	11870	0.0437
BRE	6	0.47038	0.62524	0.99998	11871	-0.0265
TEKT4	4	0.47046	0.56186	0.99998	11872	0.0409
SLC38A3	6	0.47054	0.62536	0.99998	11873	-0.0376
YTHDF2	6	0.47056	0.62538	0.99998	11874	-0.1792
CUX2	6	0.47066	0.62546	0.99998	11875	0.0292
CRABP2	6	0.47067	0.62547	0.99998	11876	-0.0422
ZMYM1	6	0.47076	0.62554	0.99998	11877	0.0553
SERGEF	6	0.47083	0.6256	0.99998	11878	0.0156
EIF4EBP1	6	0.47098	0.62571	0.99998	11879	-0.1733
PSAPL1	6	0.47098	0.62571	0.99998	11880	0.0651
OR13G1	6	0.47098	0.62571	0.99998	11881	-0.0732
hsa-mir-6727	4	0.47105	0.56219	0.99998	11882	0.0771
FAM91A1	6	0.47109	0.6258	0.99998	11883	0.0379
RBCK1	6	0.47123	0.62591	0.99998	11884	0.0521
RNF152	6	0.47123	0.62591	0.99998	11885	0.1122
NXF5	6	0.47126	0.62594	0.99998	11886	0.136
NTF4	6	0.47126	0.62594	0.99998	11887	0.3496
PIGB	6	0.47126	0.62594	0.99998	11888	-0.0086
ZNF638	6	0.47142	0.62605	0.99998	11889	-0.0006
CD163L1	6	0.47152	0.62613	0.99998	11890	0.0318
DENND4C	6	0.47152	0.62613	0.99998	11891	0.0723
PMP2	6	0.47168	0.62625	0.99998	11892	0.1697
TM9SF4	6	0.47177	0.62632	0.99998	11893	-0.1697
COX16	6	0.47177	0.62632	0.99998	11894	0.083
MSL2	6	0.47183	0.62637	0.99998	11895	0.213
NDN	6	0.47187	0.62639	0.99998	11896	0.0236
ATG4A	6	0.47192	0.62644	0.99998	11897	-0.1213
CCDC50	6	0.47201	0.6265	0.99998	11898	0.1675
KDELR1	6	0.47211	0.62658	0.99998	11899	0.1031
LARP1	6	0.47212	0.62659	0.99998	11900	0.0557
CBX8	6	0.47212	0.62659	0.99998	11901	0.0742
S100A5	6	0.47212	0.62659	0.99998	11902	0.047
RCN2	6	0.47221	0.62666	0.99998	11903	0.2235
IQSEC1	6	0.47221	0.62666	0.99998	11904	0.0987

SPANXF1	5	0.47227	0.61689	0.99998	11905	0.1797
FAM19A4	6	0.4723	0.62673	0.99998	11906	-0.0919
NRL	6	0.47241	0.62683	0.99998	11907	-0.0247
PTCD2	6	0.47253	0.62692	0.99998	11908	0.1014
DENND6B	6	0.47253	0.62692	0.99998	11909	-0.0814
IDH3B	6	0.47254	0.62692	0.99998	11910	0.1499
SCMH1	6	0.47272	0.62707	0.99998	11911	0.0517
CLK1	6	0.47291	0.62721	0.99998	11912	-0.048
CRYGB	6	0.47291	0.62721	0.99998	11913	0.0916
RNASE11	6	0.47296	0.62724	0.99998	11914	0.1502
MEIS2	6	0.47296	0.62724	0.99998	11915	0.0055
hsa-mir-4655	4	0.473	0.56322	0.99998	11916	-0.0589
hsa-mir-518a-	1	0.47306	0.47315	0.99998	11917	-0.0867
AGPAT1	6	0.47311	0.62736	0.99998	11918	-0.0553
RBL2	6	0.47311	0.62736	0.99998	11919	-0.0709
PARP1	6	0.47318	0.62741	0.99998	11920	0.0421
ATF1	6	0.47339	0.62757	0.99998	11921	0.1406
DIABLO	6	0.47343	0.62761	0.99998	11922	0.146
XBP1	6	0.47343	0.62761	0.99998	11923	-0.0275
COLCA2	6	0.47351	0.62767	0.99998	11924	0.0045
CDH3	6	0.47356	0.62771	0.99998	11925	-0.0438
RGS14	6	0.47362	0.62776	0.99998	11926	0.0548
DGKA	4	0.47372	0.56359	0.99998	11927	0.0841
STK32C	6	0.47382	0.62793	0.99998	11928	-0.0755
PTPRK	6	0.47382	0.62794	0.99998	11929	0.0977
PBX4	6	0.47389	0.628	0.99998	11930	0.2638
PARD3B	6	0.47406	0.62812	0.99998	11931	-0.0323
MCAT	6	0.47406	0.62812	0.99998	11932	0.2913
GPR157	6	0.47406	0.62812	0.99998	11933	-0.0624
MAPK7	6	0.47408	0.62814	0.99998	11934	-0.0053
ADAM8	6	0.47408	0.62814	0.99998	11935	0.0435
EPHX3	6	0.47421	0.62824	0.99998	11936	0.0088
CTTNBP2	6	0.47421	0.62824	0.99998	11937	0.2258
TMEM254	6	0.47429	0.62832	0.99998	11938	-0.0833
WTIP	6	0.47438	0.6284	0.99998	11939	-0.0661
TMC2	6	0.47438	0.62841	0.99998	11940	0.1539
NCOA7	6	0.47451	0.6285	0.99998	11941	0.145
KCTD1	6	0.47456	0.62855	0.99998	11942	0.0142
TSPYL6	6	0.47466	0.62864	0.99998	11943	-0.1538
C1QTNF1	6	0.47466	0.62864	0.99998	11944	-0.0327
TRIM56	6	0.47466	0.62864	0.99998	11945	0.0474
SCHIP1	4	0.47488	0.56422	0.99998	11946	0.2739
OTX2	6	0.4749	0.62882	0.99998	11947	0.1781
TM6SF1	6	0.4749	0.62882	0.99998	11948	0.0201
VILL	6	0.4749	0.62882	0.99998	11949	0.1894
OGG1	6	0.47493	0.62885	0.99998	11950	0.0086
hsa-mir-4270	2	0.47506	0.47666	0.99998	11951	-0.0564
SRA1	6	0.47507	0.62897	0.99998	11952	0.3709
BACH1	6	0.47515	0.62904	0.99998	11953	-0.0423
VWA8	6	0.47515	0.62904	0.99998	11954	0.1018
MARCH6	6	0.47531	0.62916	0.99998	11955	-0.0891
TMEM87B	6	0.47531	0.62916	0.99998	11956	0.0888
VPS13A	6	0.47531	0.62916	0.99998	11957	0.2394
PDSS1	6	0.47539	0.62922	0.99998	11958	-0.0347
ALX4	5	0.47539	0.61982	0.99998	11959	0.1779
ANKS1B	6	0.47555	0.62935	0.99998	11960	0.0283
COL10A1	6	0.4757	0.62948	0.99998	11961	0.3416
UNC13D	6	0.4758	0.62955	0.99998	11962	0.0267
PLEKHB2	6	0.47594	0.62966	0.99998	11963	0.0058
FAM3B	6	0.47601	0.62971	0.99998	11964	-0.0096
GPR18	6	0.47609	0.62976	0.99998	11965	0.1938
SLC6A15	6	0.4761	0.62977	0.99998	11966	0.0576
KRTAP13-2	6	0.47618	0.62984	0.99998	11967	-0.0459
SLC4A9	6	0.47621	0.62987	0.99998	11968	-0.0517
ADD1	6	0.47621	0.62987	0.99998	11969	-0.0158
OR4A47	6	0.4763	0.62993	0.99998	11970	-0.0412
DEFB125	6	0.47633	0.62996	0.99998	11971	0.0186
ROCK2	6	0.4765	0.6301	0.99998	11972	0.0791
PPM1A	6	0.47659	0.63018	0.99998	11973	-0.1338
HK2	6	0.47659	0.63018	0.99998	11974	0.1134
RBM5	6	0.47669	0.63026	0.99998	11975	-0.0984
ZNF555	6	0.47672	0.63029	0.99998	11976	-0.034
HSD17B4	6	0.47682	0.63036	0.99998	11977	-0.0155
OR5D14	6	0.47691	0.63043	0.99998	11978	0.1124
WDR60	6	0.47691	0.63043	0.99998	11979	0.1503
SIGLEC10	5	0.47696	0.62132	0.99998	11980	-0.1878
DUSP14	6	0.477	0.63052	0.99998	11981	-0.0479
N4BP1	6	0.477	0.63052	0.99998	11982	-0.1249
ZNF284	5	0.47703	0.6214	0.99998	11983	-0.3079
NTN5	6	0.47727	0.63073	0.99998	11984	0.0814
LGI1	6	0.47732	0.63078	0.99998	11985	-0.0316

PSG1	5	0.47742	0.62178	0.99998	11986	-0.3042
hsa-mir-3960	4	0.47745	0.56557	0.99998	11987	0.0839
CDKN2D	6	0.47751	0.63093	0.99998	11988	-0.1257
GPR183	6	0.47751	0.63093	0.99998	11989	0.063
CEP192	6	0.47751	0.63093	0.99998	11990	-0.0641
OSR2	6	0.47751	0.63093	0.99998	11991	0.038
PIGR	6	0.47763	0.63102	0.99998	11992	-0.0451
MED4	6	0.47769	0.63106	0.99998	11993	0.0507
IL1A	6	0.47771	0.63108	0.99998	11994	-0.0654
GPR17	6	0.47771	0.63108	0.99998	11995	-0.0055
CST2	5	0.47775	0.62213	0.99998	11996	0.0037
RAB19	6	0.47777	0.63112	0.99998	11997	0.0055
HDAC2	6	0.478	0.63131	0.99998	11998	-0.0934
OR2T6	6	0.478	0.63131	0.99998	11999	0.1138
ALDH1A2	6	0.47812	0.63141	0.99998	12000	-0.1364
UBL3	6	0.47812	0.63141	0.99998	12001	0.042
NCF4	6	0.47812	0.63141	0.99998	12002	0.1155
ARL10	6	0.47812	0.63141	0.99998	12003	-0.0332
MS4A15	6	0.47816	0.63144	0.99998	12004	-0.0359
CAPN5	6	0.47827	0.63151	0.99998	12005	0.0137
FBXL18	6	0.47831	0.63155	0.99998	12006	0.115
LAG3	5	0.4784	0.62277	0.99998	12007	-0.0938
H1FX	6	0.47841	0.63164	0.99998	12008	-0.003
SNX2	6	0.47848	0.63169	0.99998	12009	0.0526
MDP1	4	0.47851	0.56615	0.99998	12010	0.0686
ZC3H7A	6	0.47854	0.63175	0.99998	12011	0.1306
TDRD9	6	0.47862	0.6318	0.99998	12012	0.2425
WDR87	6	0.47865	0.63183	0.99998	12013	-0.1061
ASIP	6	0.47871	0.63187	0.99998	12014	0.0888
IDI2	6	0.47871	0.63187	0.99998	12015	-0.0809
CDHR1	6	0.47871	0.63187	0.99998	12016	0.1491
CLIC5	6	0.47878	0.63192	0.99998	12017	-0.0025
STX4	6	0.47878	0.63192	0.99998	12018	-0.0048
GATSL3	6	0.47878	0.63192	0.99998	12019	0.312
hsa-mir-519a-	2	0.47894	0.48032	0.99998	12020	-0.5802
AMY2A	2	0.47894	0.48032	0.99998	12021	-0.113
hsa-mir-4509-	2	0.47894	0.48032	0.99998	12022	-0.5993
CFC1	2	0.47894	0.48032	0.99998	12023	-0.545
EPB41L5	6	0.47894	0.63203	0.99998	12024	-0.0418
SIRT7	6	0.47894	0.63203	0.99998	12025	-0.01
ZNF518A	6	0.47898	0.63206	0.99998	12026	0.2018
hsa-mir-615	4	0.47902	0.56643	0.99998	12027	-0.1567
NYNRIN	6	0.47904	0.63211	0.99998	12028	0.1448
OR51V1	6	0.47904	0.63211	0.99998	12029	0.1472
C11orf82	6	0.47915	0.6322	0.99998	12030	0.0893
DDA1	4	0.47923	0.56655	0.99998	12031	0.0686
UBE3C	6	0.47928	0.63231	0.99998	12032	0.0911
SLCO3A1	6	0.47931	0.63233	0.99998	12033	0.0003
ALDH7A1	6	0.47944	0.63244	0.99998	12034	0.1419
RS1	6	0.4795	0.63248	0.99998	12035	-0.0047
CDH16	6	0.47963	0.63259	0.99998	12036	0.0632
HAL	6	0.47963	0.63259	0.99998	12037	0.0475
ASH1L	6	0.47963	0.63259	0.99998	12038	-0.0662
ESX1	6	0.4798	0.63275	0.99998	12039	-0.0102
CEP44	6	0.47983	0.63278	0.99998	12040	-0.0415
ST6GALNAC5	6	0.47983	0.63278	0.99998	12041	0.042
CATSPER3	4	0.47985	0.56686	0.99998	12042	0.0457
RIC8B	6	0.47995	0.63287	0.99998	12043	-0.009
ZFP69	6	0.48015	0.63302	0.99998	12044	0.0593
LAMB4	6	0.48027	0.63312	0.99998	12045	0.015
COL19A1	6	0.4803	0.63314	0.99998	12046	0.0199
MKS1	6	0.4803	0.63314	0.99998	12047	0.1229
MYT1L	6	0.48037	0.63321	0.99998	12048	-0.1225
OR51A4	5	0.48055	0.62484	0.99998	12049	-0.0818
AOC2	6	0.48059	0.63337	0.99998	12050	-0.0117
SLC35B3	6	0.4806	0.63339	0.99998	12051	0.1432
HOXA3	6	0.48072	0.63348	0.99998	12052	-0.0192
AGXT2	6	0.48072	0.63348	0.99998	12053	0.0129
PLIN4	6	0.48072	0.63348	0.99998	12054	0.1632
hsa-mir-642b	1	0.48076	0.48082	0.99998	12055	-0.0158
ZCCHC4	6	0.48082	0.63356	0.99998	12056	0.0891
SPG7	6	0.48085	0.63359	0.99998	12057	0.0524
MED6	6	0.4812	0.63387	0.99998	12058	0.0285
KCNH4	6	0.48134	0.634	0.99998	12059	-0.0454
KIAA1549	6	0.48141	0.63405	0.99998	12060	-0.0131
FAM63B	6	0.48146	0.63408	0.99998	12061	-0.0277
GNB4	6	0.48149	0.6341	0.99998	12062	-0.0475
ZNF222	5	0.48152	0.62578	0.99998	12063	0.0711
GRAMD1C	6	0.48161	0.63419	0.99998	12064	0.117
NODAL	6	0.48161	0.63419	0.99998	12065	0.0347
MARK4	6	0.48168	0.63424	0.99998	12066	0.1823

PSTPIP2	6	0.48168	0.63424	0.99998	12067	0.0683
hsa-mir-548ba	1	0.4817	0.48178	0.99998	12068	-0.023
hsa-mir-3978	4	0.48175	0.56786	0.99998	12069	0.0712
PRR9	6	0.48185	0.63436	0.99998	12070	-0.0256
TPSAB1	6	0.48188	0.63438	0.99998	12071	0.078
C1orf53	6	0.48191	0.63442	0.99998	12072	-0.0156
FFAR3	6	0.48191	0.63442	0.99998	12073	-0.091
TBC1D10C	6	0.48206	0.63455	0.99998	12074	0.0035
RAB11A	6	0.48207	0.63455	0.99998	12075	0.0384
KDM3A	6	0.48215	0.63461	0.99998	12076	0.1356
PRDM4	6	0.48219	0.63464	0.99998	12077	0.0437
hsa-mir-376a-	3	0.48221	0.51151	0.99998	12078	-0.0116
TMSB4Y	4	0.48224	0.56812	0.99998	12079	0.2548
LGALS9C	2	0.48227	0.4835	0.99998	12080	-0.3804
THRA	6	0.4823	0.63473	0.99998	12081	0.1319
STPG2	6	0.48233	0.63476	0.99998	12082	-0.1158
DARC	6	0.48243	0.63484	0.99998	12083	0.0236
OR6C4	6	0.48251	0.6349	0.99998	12084	0.0671
RSPO3	6	0.48259	0.63496	0.99998	12085	-0.0283
MC2R	6	0.48259	0.63496	0.99998	12086	0.1457
GABRA2	6	0.48268	0.63504	0.99998	12087	-0.1136
hsa-mir-1915	4	0.48287	0.56847	0.99998	12088	-0.0611
DNAH3	6	0.48293	0.63525	0.99998	12089	-0.1642
ANP32B	6	0.48294	0.63526	0.99998	12090	0.188
BAI2	6	0.48294	0.63526	0.99998	12091	0.0055
CRYGD	6	0.48294	0.63526	0.99998	12092	0.0975
TEX13A	6	0.48313	0.63541	0.99998	12093	-0.0161
ACTL7B	6	0.48317	0.63544	0.99998	12094	0.2243
SP140	6	0.4832	0.63547	0.99998	12095	0.3242
MYO1E	6	0.4832	0.63547	0.99998	12096	0.1214
hsa-mir-4742	4	0.48326	0.56866	0.99998	12097	0.0842
LTN1	6	0.48329	0.63555	0.99998	12098	0.1275
RIC3	6	0.48338	0.63563	0.99998	12099	-0.0132
SLC35F5	6	0.48356	0.63578	0.99998	12100	-0.0294
CYTH2	6	0.48356	0.63578	0.99998	12101	-0.0641
SPNS3	6	0.48368	0.63589	0.99998	12102	-0.065
CCDC67	6	0.48368	0.63589	0.99998	12103	0.1047
SPAG17	6	0.48368	0.63589	0.99998	12104	0.1881
FGF14	6	0.48368	0.63589	0.99998	12105	-0.1129
PRDM11	6	0.48368	0.63589	0.99998	12106	0.0207
RNF139	6	0.48372	0.63592	0.99998	12107	0.0402
IFFO1	6	0.48372	0.63592	0.99998	12108	0.7651
hsa-mir-3612	4	0.48383	0.56896	0.99998	12109	0.0537
hsa-mir-6752	4	0.48383	0.56896	0.99998	12110	0.0935
hsa-mir-6844	1	0.4839	0.48397	0.99998	12111	-0.0125
AMPD2	6	0.48395	0.63611	0.99998	12112	0.07
REG4	6	0.48403	0.63618	0.99998	12113	0.1892
SLAIN1	6	0.48403	0.63618	0.99998	12114	0.3383
E2F6	6	0.48403	0.63618	0.99998	12115	0.0002
HGC6.3	4	0.48404	0.56908	0.99998	12116	0.0185
CSNK1A1L	6	0.48412	0.63626	0.99998	12117	-0.0796
KRTAP17-1	6	0.48426	0.63637	0.99998	12118	0.1192
CTSK	6	0.48426	0.63637	0.99998	12119	-0.015
CLVS2	6	0.48428	0.63639	0.99998	12120	0.1194
HDAC1	6	0.48445	0.63652	0.99998	12121	0.0261
ASIC5	6	0.48445	0.63652	0.99998	12122	-0.0152
NGF	6	0.48466	0.63667	0.99998	12123	-0.0501
PLA2G10	6	0.48466	0.63667	0.99998	12124	0.1547
ZNF280D	6	0.48473	0.63674	0.99998	12125	0.2166
PDIA5	6	0.48481	0.63681	0.99998	12126	-0.0248
CLEC1B	6	0.48485	0.63684	0.99998	12127	0.0151
C20orf202	6	0.48497	0.63695	0.99998	12128	-0.0594
KCNJ4	6	0.48497	0.63695	0.99998	12129	0.1026
ZSCAN5A	6	0.48497	0.63695	0.99998	12130	0.1704
GPR132	6	0.485	0.63696	0.99998	12131	-0.142
CAP2	6	0.485	0.63696	0.99998	12132	-0.033
GLTSCR2	6	0.48509	0.63703	0.99998	12133	-0.0321
HNRNPR	6	0.48517	0.6371	0.99998	12134	0.1603
NUSAP1	6	0.48529	0.6372	0.99998	12135	0.0355
METTL21C	6	0.48529	0.6372	0.99998	12136	0.1628
C14orf166B	6	0.48529	0.6372	0.99998	12137	-0.0085
FASTKD3	6	0.48535	0.63724	0.99998	12138	0.0389
LPL	6	0.48535	0.63724	0.99998	12139	0.1264
ZNF814	5	0.48546	0.62962	0.99998	12140	-0.0808
BMP10	6	0.48554	0.63739	0.99998	12141	0.155
KIT	6	0.48568	0.63752	0.99998	12142	0.1398
KLK10	6	0.48568	0.63752	0.99998	12143	0.1507
SYT13	6	0.48589	0.6377	0.99998	12144	-0.1587
C1orf173	6	0.48589	0.6377	0.99998	12145	-0.0205
PUSL1	6	0.48589	0.6377	0.99998	12146	-0.0365
CHST12	6	0.48625	0.63798	0.99998	12147	0.2398

PLEKHH3	6	0.48625	0.63798	0.99998	12148	0.1101
SLC6A11	6	0.4863	0.63802	0.99998	12149	-0.0459
LIG1	6	0.48644	0.63814	0.99998	12150	0.0911
DMXL2	6	0.48644	0.63814	0.99998	12151	0.2211
ZBTB45	6	0.48645	0.63814	0.99998	12152	0.1043
TRIM52	6	0.48645	0.63814	0.99998	12153	-0.1832
hsa-mir-4524a	1	0.48654	0.48666	0.99998	12154	-0.0169
KHNYN	6	0.4866	0.63827	0.99998	12155	-0.1232
ALKBH1	6	0.4866	0.63827	0.99998	12156	0.1221
COL6A5	6	0.48664	0.6383	0.99998	12157	0.027
B3GNT7	6	0.48664	0.6383	0.99998	12158	-0.01
GUCA1A	6	0.48672	0.63836	0.99998	12159	0.3398
BTN3A3	2	0.48673	0.48779	0.99998	12160	0.1092
SFT2D1	6	0.48686	0.63847	0.99998	12161	0.393
ZNF8	6	0.48697	0.63855	0.99998	12162	0.016
HMGCLL1	6	0.48708	0.63866	0.99998	12163	0.1367
BMX	6	0.48725	0.63879	0.99998	12164	-0.0254
ODF3L1	6	0.48725	0.63879	0.99998	12165	0.1679
NAP1L5	6	0.48725	0.63879	0.99998	12166	0.0428
CCSAP	6	0.48743	0.63894	0.99998	12167	-0.0167
ZNF778	6	0.48756	0.63905	0.99998	12168	0.1036
SLC35B4	6	0.48762	0.6391	0.99998	12169	0.0739
MAGEL2	6	0.48767	0.63913	0.99998	12170	0.0438
CALML4	6	0.48767	0.63913	0.99998	12171	0.0587
hsa-mir-1294	4	0.48768	0.571	0.99998	12172	-0.0268
hsa-mir-3916	4	0.48768	0.571	0.99998	12173	0.1642
hsa-mir-130a	4	0.48768	0.571	0.99998	12174	0.1568
MTMR14	6	0.48774	0.6392	0.99998	12175	-0.0743
NHLH1	6	0.48783	0.63927	0.99998	12176	-0.094
SAAL1	6	0.48789	0.63932	0.99998	12177	0.2626
OXCT1	6	0.4879	0.63933	0.99998	12178	-0.0279
CATSPER4	6	0.48804	0.63944	0.99998	12179	-0.0437
LHX4	6	0.48804	0.63944	0.99998	12180	0.295
EIF4G3	6	0.48808	0.63947	0.99998	12181	0.2157
CYB561A3	6	0.48832	0.63966	0.99998	12182	-0.0878
RNF8	6	0.48832	0.63966	0.99998	12183	0.0261
TET1	6	0.48832	0.63966	0.99998	12184	-0.0821
TANC2	6	0.4885	0.63982	0.99998	12185	-0.074
SHPK	6	0.4886	0.63989	0.99998	12186	-0.1346
PWWP2B	6	0.4886	0.63989	0.99998	12187	0.0016
ZNF805	6	0.48872	0.63998	0.99998	12188	0.0556
UGT1A1	2	0.48874	0.48973	0.99998	12189	-0.0189
hsa-mir-6870	4	0.48895	0.57166	0.99998	12190	0.1358
TOPAZ1	4	0.48895	0.57166	0.99998	12191	0.1848
TTC34	6	0.489	0.64022	0.99998	12192	0.1206
RWDD3	6	0.48913	0.64032	0.99998	12193	0.0415
SRSF9	6	0.48918	0.64036	0.99998	12194	0.0133
PCED1A	6	0.48918	0.64036	0.99998	12195	0.0568
ATRX	6	0.4893	0.64045	0.99998	12196	0.144
TRAM2	6	0.48935	0.64049	0.99998	12197	-0.063
MTRR	6	0.48935	0.64049	0.99998	12198	0.2039
hsa-mir-6796	4	0.48941	0.5719	0.99998	12199	0.1262
IGFBP2	6	0.48953	0.64062	0.99998	12200	-0.0084
RAVER1	6	0.48953	0.64062	0.99998	12201	0.0106
AMDHD2	6	0.48953	0.64062	0.99998	12202	-0.0813
NDRG4	6	0.48954	0.64064	0.99998	12203	0.0601
hsa-mir-208a	4	0.48956	0.57197	0.99998	12204	0.0007
AFG3L2	6	0.48968	0.64073	0.99998	12205	-0.063
MTMR7	6	0.48974	0.64078	0.99998	12206	0.0889
SPRY2	6	0.48983	0.64086	0.99998	12207	0.1633
KXD1	6	0.48984	0.64086	0.99998	12208	0.0763
CPXM1	6	0.48984	0.64086	0.99998	12209	-0.0528
FAM183A	6	0.48997	0.64097	0.99998	12210	0.0479
LRRC43	6	0.49	0.64099	0.99998	12211	0.032
TMEM229A	6	0.49	0.64099	0.99998	12212	0.0181
RSC1A1	6	0.49015	0.64111	0.99998	12213	0.1343
GSTA1	4	0.49017	0.57231	0.99998	12214	-0.3065
CREBRF	6	0.49029	0.64121	0.99998	12215	0.2924
NT5C1B-RDH1	3	0.4903	0.51748	0.99998	12216	0.0372
HS3ST3A1	4	0.49031	0.57239	0.99998	12217	0.219
C19orf77	6	0.49054	0.64142	0.99998	12218	0.13
FBXL20	6	0.49054	0.64142	0.99998	12219	0.1166
MPRIP	6	0.4906	0.64148	0.99998	12220	-0.0721
COPG2	6	0.4906	0.64148	0.99998	12221	-0.0916
C4BPB	6	0.4907	0.64155	0.99998	12222	0.1043
OR1S1	5	0.49074	0.63464	0.99998	12223	-0.015
OLFML1	6	0.49088	0.6417	0.99998	12224	-0.0528
hsa-mir-4429	4	0.49093	0.57274	0.99998	12225	0.051
UBAP1	6	0.49104	0.64184	0.99998	12226	-0.0223
MYL10	6	0.49113	0.64191	0.99998	12227	0.0687
CCL22	6	0.49113	0.64191	0.99998	12228	0.0326

FYB	6	0.49113	0.64191	0.99998	12229	0.0794
OR2AE1	6	0.49144	0.64217	0.99998	12230	-0.0532
PCDHAC1	2	0.49147	0.49234	0.99998	12231	-0.0522
NUDCD1	4	0.49151	0.57306	0.99998	12232	0.0967
WBP1L	6	0.49153	0.64224	0.99998	12233	0.1021
SLC7A13	6	0.49168	0.64235	0.99998	12234	0.1367
C16orf78	6	0.49168	0.64235	0.99998	12235	0.0962
CCNE2	6	0.49175	0.6424	0.99998	12236	-0.1051
PDYN	6	0.49175	0.6424	0.99998	12237	0.0566
hsa-mir-548w	1	0.49178	0.49186	0.99998	12238	-0.027
GANAB	6	0.4919	0.64252	0.99998	12239	0.0798
ABHD10	6	0.4919	0.64252	0.99998	12240	-0.067
LOC440563	5	0.49199	0.63592	0.99998	12241	0.0601
TXNDC5	6	0.49201	0.64259	0.99998	12242	-0.001
hsa-mir-4464	4	0.49213	0.5734	0.99998	12243	-0.1109
EEA1	6	0.4922	0.64275	0.99998	12244	0.1253
PLCXD2	6	0.4922	0.64275	0.99998	12245	-0.061
hsa-mir-320e	3	0.49222	0.51894	0.99998	12246	0.1932
hsa-mir-548ar	2	0.49229	0.49313	0.99998	12247	-0.2108
GOLGB1	6	0.49233	0.64285	0.99998	12248	0.1853
THEG	6	0.49235	0.64287	0.99998	12249	-0.0017
IST1	6	0.49235	0.64287	0.99998	12250	0.3097
STRBP	6	0.49245	0.64297	0.99998	12251	0.0159
ALG9	6	0.49245	0.64297	0.99998	12252	-0.1058
KRT14	5	0.4925	0.63641	0.99998	12253	0.166
SOST	6	0.4926	0.64308	0.99998	12254	-0.0079
C1QTNF7	6	0.4926	0.64308	0.99998	12255	-0.0486
TRMT61B	6	0.4926	0.64308	0.99998	12256	0.017
DNAJC5B	6	0.49266	0.64313	0.99998	12257	0.2269
PCDHB3	6	0.49281	0.64326	0.99998	12258	-0.0185
hsa-mir-4686	4	0.49283	0.57378	0.99998	12259	0.1284
KIAA1109	6	0.49285	0.6433	0.99998	12260	0.001
TBC1D19	6	0.49285	0.6433	0.99998	12261	0.0553
RHOG	6	0.49293	0.64335	0.99998	12262	0.0791
FTSJD1	4	0.493	0.57388	0.99998	12263	0.1104
EFCAB11	6	0.49302	0.64343	0.99998	12264	0.0916
KLHL10	6	0.49306	0.64346	0.99998	12265	0.2826
hsa-mir-579	4	0.49318	0.57397	0.99998	12266	-0.4631
ZFP69B	6	0.49343	0.64377	0.99998	12267	-0.1091
RAX	6	0.49346	0.64379	0.99998	12268	0.2225
CD55	6	0.49347	0.6438	0.99998	12269	-0.0383
SOX12	6	0.49366	0.64396	0.99998	12270	-0.077
SEMA5A	6	0.49375	0.64403	0.99998	12271	0.0247
SCLY	6	0.49375	0.64403	0.99998	12272	0.2329
CEP78	6	0.49375	0.64403	0.99998	12273	0.1288
FHL2	6	0.49395	0.64419	0.99998	12274	-0.0227
MIF4GD	6	0.49395	0.64419	0.99998	12275	0.0291
PTX3	6	0.49403	0.64425	0.99998	12276	0.0376
OTOL1	6	0.49436	0.64452	0.99998	12277	0.0522
GPR56	6	0.49436	0.64452	0.99998	12278	0.0425
ARHGAP15	6	0.49439	0.64454	0.99998	12279	-0.0364
C7orf73	5	0.49449	0.63833	0.99998	12280	0.3711
NFKBIB	6	0.49453	0.64466	0.99998	12281	-0.0112
USP33	6	0.4947	0.64479	0.99998	12282	0.2275
TMSB10	6	0.4947	0.64479	0.99998	12283	0.0457
PRDX6	6	0.49483	0.6449	0.99998	12284	-0.0741
hsa-mir-5739	4	0.49483	0.57485	0.99998	12285	0.0415
GBX2	6	0.49496	0.64502	0.99998	12286	0.1968
TMEM184B	6	0.49509	0.64512	0.99998	12287	0.0523
PFKFB3	6	0.49514	0.64517	0.99998	12288	-0.0165
APEX1	6	0.49514	0.64517	0.99998	12289	0.0035
BCL2L10	6	0.49514	0.64517	0.99998	12290	0.1338
UBA3	6	0.49514	0.64517	0.99998	12291	-0.0341
PKNOX2	6	0.49514	0.64517	0.99998	12292	0.0034
LYRM7	6	0.4953	0.64528	0.99998	12293	-0.0322
SURF6	6	0.4954	0.64536	0.99998	12294	-0.1209
OGFR	6	0.4954	0.64536	0.99998	12295	0.0699
ALG3	6	0.49542	0.64537	0.99998	12296	0.1363
SAT2	6	0.49561	0.64553	0.99998	12297	-0.0002
PDZK1	6	0.49573	0.64563	0.99998	12298	0.091
ELN	6	0.49577	0.64566	0.99998	12299	0.0476
FAM98B	6	0.49584	0.64572	0.99998	12300	0.1599
DHRS12	6	0.4959	0.64578	0.99998	12301	0.2312
NIPSNAP3A	6	0.4959	0.64578	0.99998	12302	0.0565
NAP1L4	6	0.49593	0.6458	0.99998	12303	0.0108
FMNL1	6	0.49605	0.6459	0.99998	12304	-0.0544
AHSA1	6	0.49605	0.6459	0.99998	12305	0.0208
HERC1	6	0.49627	0.64606	0.99998	12306	0.0422
TOP1	6	0.49627	0.64606	0.99998	12307	0.1967
SCEL	6	0.49635	0.64612	0.99998	12308	0.048
CA13	6	0.49648	0.64623	0.99998	12309	-0.0443

GXYLT1	6	0.49648	0.64623	0.99998	12310	0.1589
PDZD8	6	0.49658	0.64631	0.99998	12311	-0.1004
TMEM213	6	0.49658	0.64631	0.99998	12312	-0.0207
VRK1	6	0.49658	0.64631	0.99998	12313	0.0344
EIF3B	6	0.49658	0.64631	0.99998	12314	-0.0805
SGCB	6	0.49667	0.64638	0.99998	12315	0.1982
TIMM10B	6	0.49679	0.64648	0.99998	12316	0.2576
LYRM2	6	0.4969	0.64658	0.99998	12317	-0.1205
SERPINA3	6	0.49692	0.6466	0.99998	12318	0.1927
STAT4	6	0.49709	0.64675	0.99998	12319	-0.0363
AIFM3	6	0.49718	0.64683	0.99998	12320	0.0511
C18orf8	6	0.49729	0.64691	0.99998	12321	0.0157
SYAP1	6	0.49729	0.64691	0.99998	12322	-0.0869
GPR152	6	0.49729	0.64691	0.99998	12323	0.0198
PRKG1	6	0.49729	0.64691	0.99998	12324	0.0693
WIPF1	6	0.49737	0.64698	0.99998	12325	0.2246
HOXB7	6	0.49754	0.64712	0.99998	12326	0.0914
MTMR1	6	0.49765	0.64722	0.99998	12327	0.1714
TPTE2	6	0.49772	0.64727	0.99998	12328	-0.1879
NUDCD3	6	0.49772	0.64727	0.99998	12329	0.0516
CDH20	6	0.49776	0.6473	0.99998	12330	0.017
KIRREL2	6	0.49787	0.6474	0.99998	12331	0.024
GSTM3	6	0.49787	0.6474	0.99998	12332	-0.0103
NME7	6	0.49795	0.64745	0.99998	12333	-0.0527
DHRS4L2	2	0.49803	0.49868	0.99998	12334	-0.1313
HTN3	2	0.49803	0.49868	0.99998	12335	-0.5492
hsa-mir-523	2	0.49803	0.49868	0.99998	12336	-0.4458
C17orf74	6	0.49809	0.64756	0.99998	12337	0.1855
hsa-mir-4504	4	0.49821	0.57668	0.99998	12338	-0.0114
GID4	6	0.49823	0.6477	0.99998	12339	0.1397
FAM65A	6	0.49826	0.64772	0.99998	12340	-0.0426
TRAM1L1	6	0.49835	0.6478	0.99998	12341	0.1014
CTNND2	6	0.49835	0.6478	0.99998	12342	0.1125
MTRNR2L2	6	0.49842	0.64786	0.99998	12343	0.0547
ZNF595	6	0.49855	0.64797	0.99998	12344	0.245
TNIP2	6	0.49855	0.64797	0.99998	12345	-0.0754
HAUS2	6	0.49855	0.64797	0.99998	12346	0.0789
HTR2C	6	0.49857	0.64799	0.99998	12347	-0.1667
KCNH2	6	0.49869	0.64809	0.99998	12348	0.2048
ETV7	6	0.49883	0.6482	0.99998	12349	-0.1231
SNX24	6	0.49883	0.6482	0.99998	12350	0.015
VPS37B	6	0.49883	0.6482	0.99998	12351	-0.0373
KATNAL2	6	0.49883	0.6482	0.99998	12352	-0.0228
PAPPA2	6	0.49889	0.64824	0.99998	12353	0.0506
RAP2A	6	0.49889	0.64824	0.99998	12354	-0.0901
DCSTAMP	6	0.49903	0.64834	0.99998	12355	0.0815
GAB4	6	0.49909	0.64839	0.99998	12356	-0.1036
ADAMTS15	6	0.49917	0.64846	0.99998	12357	0.1428
EIF2D	6	0.49924	0.64851	0.99998	12358	0.0302
hsa-mir-607	4	0.49929	0.57726	0.99998	12359	0.0798
ZSWIM7	6	0.49934	0.6486	0.99998	12360	-0.0871
OST4	5	0.49936	0.64268	0.99998	12361	0.0782
AKT1	6	0.49954	0.64876	0.99998	12362	-0.1211
UBXN10	6	0.49957	0.64877	0.99998	12363	0.0205
ITGA5	6	0.49957	0.64877	0.99998	12364	-0.078
KIAA1468	6	0.49957	0.64877	0.99998	12365	-0.0203
CDC40	6	0.49969	0.64888	0.99998	12366	0.0225
SH3BP5L	6	0.49988	0.64903	0.99998	12367	0.1826
FAM178B	6	0.49988	0.64903	0.99998	12368	-0.066
HIST1H1T	6	0.4999	0.64904	0.99998	12369	0.168
hsa-mir-518d	1	0.4999	0.49998	0.99998	12370	-0.0026
hsa-mir-1193	4	0.50007	0.57768	0.99998	12371	-0.0288
IFT81	6	0.50009	0.64921	0.99998	12372	0.2324
LYVE1	6	0.50013	0.64925	0.99998	12373	-0.0203
SPTAN1	6	0.50013	0.64925	0.99998	12374	0.0659
CBLN1	6	0.50042	0.64948	0.99998	12375	-0.1095
CLCN1	6	0.50047	0.64952	0.99998	12376	0.233
SLC1A2	6	0.50047	0.64952	0.99998	12377	-0.0099
EPPIN	2	0.50049	0.50109	0.99998	12378	-0.0724
FAM212A	6	0.50058	0.6496	0.99998	12379	0.0098
ABCG2	6	0.50072	0.64972	0.99998	12380	-0.0831
BBS7	6	0.50072	0.64972	0.99998	12381	-0.0323
PHYKPL	1	0.50074	0.50081	0.99998	12382	-0.0003
SMURF2	6	0.50086	0.64984	0.99998	12383	-0.1244
SIGLEC15	6	0.50086	0.64984	0.99998	12384	-0.0448
OR51S1	6	0.50086	0.64984	0.99998	12385	-0.1163
SYNJ1	6	0.50097	0.64993	0.99998	12386	-0.0597
SIM2	6	0.50098	0.64994	0.99998	12387	0.06
ROCK1	6	0.50099	0.64995	0.99998	12388	-0.0588
PIK3CA	6	0.50108	0.65003	0.99998	12389	0.2256
KDM4B	6	0.5011	0.65004	0.99998	12390	-0.0759

FAM170B	6	0.50121	0.65013	0.99998	12391	0.0147
OR6Y1	6	0.50137	0.65026	0.99998	12392	-0.1463
MPP5	6	0.50137	0.65026	0.99998	12393	0.117
EVX1	6	0.50137	0.65026	0.99998	12394	-0.0259
RD3	6	0.50141	0.65029	0.99998	12395	0.1242
hsa-mir-4278	4	0.50171	0.57855	0.99998	12396	0.1202
MDM1	6	0.50173	0.65055	0.99998	12397	0.0883
KIF27	6	0.50192	0.65069	0.99998	12398	0.0749
HRASLS5	6	0.50209	0.65084	0.99998	12399	0.0407
MRPL50	6	0.50209	0.65084	0.99998	12400	-0.0538
hsa-mir-6503	4	0.50211	0.57876	0.99998	12401	-0.0716
SOX2	6	0.50211	0.65086	0.99998	12402	0.0852
NR3C1	6	0.50222	0.65093	0.99998	12403	-0.0696
SLC15A4	6	0.50233	0.65102	0.99998	12404	-0.0244
FAM104B	6	0.50233	0.65102	0.99998	12405	0.179
HIST1H4K	1	0.50244	0.5025	0.99998	12406	0.0014
ZNF71	6	0.50258	0.65123	0.99998	12407	-0.0009
RYK	6	0.50268	0.65131	0.99998	12408	0.2195
C19orf52	6	0.50268	0.65131	0.99998	12409	0.1465
ASIC2	6	0.50268	0.65131	0.99998	12410	0.0733
NFATC4	6	0.50272	0.65134	0.99998	12411	-0.0691
KRTAP4-1	6	0.50281	0.65142	0.99998	12412	-0.0817
hsa-mir-4683	4	0.50282	0.57915	0.99998	12413	-0.1054
PSMA2	6	0.50294	0.65153	0.99998	12414	-0.0381
OR4C6	6	0.50299	0.65156	0.99998	12415	0.1258
hsa-mir-4420	4	0.50307	0.5793	0.99998	12416	-0.0457
NSMAF	6	0.50313	0.65169	0.99998	12417	-0.1134
EFCAB9	6	0.5033	0.65182	0.99998	12418	0.0768
hsa-mir-5708	3	0.50339	0.52727	0.99998	12419	0.104
LRP3	6	0.50345	0.65195	0.99998	12420	0.01
RNFT1	6	0.50356	0.65202	0.99998	12421	0.1947
SLC9C1	6	0.50356	0.65202	0.99998	12422	0.0721
EPHB2	6	0.50361	0.65206	0.99998	12423	0.0309
APCDD1L	6	0.50396	0.65234	0.99998	12424	-0.0393
RARRES2	6	0.50396	0.65234	0.99998	12425	0.1496
KAAG1	5	0.50396	0.64453	0.99998	12426	-0.098
BNIP3	6	0.50399	0.65237	0.99998	12427	-0.0135
ZNF705A	2	0.5041	0.50459	0.99998	12428	0.0378
hsa-mir-301b	4	0.50414	0.57985	0.99998	12429	-0.1161
CHDH	6	0.50449	0.65279	0.99998	12430	0.038
CHST14	6	0.50458	0.65284	0.99998	12431	0.1011
CSTF2T	6	0.50486	0.65309	0.99998	12432	0.0053
PSMA5	6	0.50511	0.65328	0.99998	12433	-0.0886
ATE1	6	0.50513	0.6533	0.99998	12434	-0.0873
TAS2R40	6	0.50524	0.6534	0.99998	12435	-0.0849
hsa-mir-4643	3	0.5053	0.52867	0.99998	12436	0.0238
FOXJ1	6	0.50531	0.65346	0.99998	12437	-0.0128
NABP2	6	0.50537	0.65351	0.99998	12438	0.0167
PRSS54	6	0.50537	0.65351	0.99998	12439	-0.0131
CCL18	6	0.50537	0.65351	0.99998	12440	0.0297
NDRG2	6	0.5054	0.65353	0.99998	12441	0.0062
MYL1	6	0.5054	0.65353	0.99998	12442	0.1903
ANGPTL4	6	0.50565	0.65372	0.99998	12443	0.1592
MYO10	6	0.50569	0.65375	0.99998	12444	0.0036
C1QTNF5	6	0.50569	0.65375	0.99998	12445	0.0609
HCN1	6	0.50574	0.65379	0.99998	12446	-0.1645
C11orf93	6	0.50582	0.65385	0.99998	12447	-0.0745
CPEB2	6	0.50586	0.65389	0.99998	12448	-9E-05
MAG	6	0.50586	0.65389	0.99998	12449	0.2178
C8orf74	6	0.50597	0.65398	0.99998	12450	0.0027
ANKRD2	6	0.50597	0.65398	0.99998	12451	-0.1431
RBM15	6	0.50597	0.65398	0.99998	12452	0.0257
MANEAL	6	0.50598	0.65399	0.99998	12453	-0.0302
PLA2G2E	6	0.50598	0.65399	0.99998	12454	-0.1308
RMI2	4	0.50606	0.58088	0.99998	12455	-0.0617
PRPSAP2	6	0.5061	0.65409	0.99998	12456	-0.0155
FSD1L	6	0.5061	0.65409	0.99998	12457	0.3531
YIPF6	6	0.50616	0.65414	0.99998	12458	0.0199
DUSP27	6	0.50616	0.65414	0.99998	12459	0.1215
AHCTF1	6	0.50619	0.65416	0.99998	12460	0.0027
IFNAR1	6	0.50635	0.65429	0.99998	12461	-0.0635
PNPLA5	6	0.50635	0.65429	0.99998	12462	0.0728
MXRA5	6	0.50635	0.65429	0.99998	12463	0.029
C8orf86	6	0.50635	0.65429	0.99998	12464	-0.1161
ARFGAP3	6	0.50644	0.65436	0.99998	12465	-0.1246
SYCP2	6	0.50648	0.65439	0.99998	12466	0.0255
GRID2	6	0.50652	0.65442	0.99998	12467	0.0799
DISP1	6	0.50667	0.65455	0.99998	12468	-0.0009
C12orf77	6	0.50667	0.65455	0.99998	12469	-0.0674
STX3	6	0.5069	0.65476	0.99998	12470	0.1045
AGAP1	6	0.50717	0.65498	0.99998	12471	0.0712

ACOT1	6	0.50723	0.65503	0.99998	12472	0.1932
hsa-mir-550a-	3	0.50727	0.53013	0.99998	12473	-0.2571
hsa-mir-4687	4	0.5073	0.58158	0.99998	12474	-0.0623
ATRIP	6	0.50733	0.65511	0.99998	12475	-0.0844
VPS13C	6	0.50733	0.65511	0.99998	12476	-0.1545
KRBA2	6	0.50742	0.65518	0.99998	12477	0.1181
SCN4A	6	0.50742	0.65518	0.99998	12478	-0.0237
WNT5A	6	0.5076	0.65532	0.99998	12479	-0.2
GP2	6	0.50761	0.65533	0.99998	12480	-0.0516
GLDN	6	0.50775	0.65544	0.99998	12481	0.1758
ACTBL2	6	0.50785	0.65553	0.99998	12482	0.0587
PRDM6	6	0.50788	0.65555	0.99998	12483	-0.0177
GTPBP1	6	0.50788	0.65555	0.99998	12484	0.0528
hsa-mir-203a	1	0.50794	0.50792	0.99998	12485	0.0193
TMED10	6	0.50803	0.65566	0.99998	12486	-0.0506
KLK5	6	0.50803	0.65566	0.99998	12487	0.3755
RASGRP2	6	0.50803	0.65566	0.99998	12488	0.009
PLXNB2	6	0.50816	0.65577	0.99998	12489	-0.0205
OXLD1	6	0.50816	0.65577	0.99998	12490	-0.0562
CARNS1	6	0.50816	0.65577	0.99998	12491	0.0353
DPYSL5	6	0.50816	0.65577	0.99998	12492	-0.0749
RNF217	6	0.50816	0.65577	0.99998	12493	0.1749
NLGN4Y	6	0.50816	0.65577	0.99998	12494	0.0521
FBXO32	6	0.50829	0.65588	0.99998	12495	0.0773
ALPK1	6	0.50829	0.65588	0.99998	12496	0.1347
ZNF101	6	0.50837	0.65595	0.99998	12497	-0.1357
GNB3	6	0.50837	0.65595	0.99998	12498	-0.0172
EXD2	6	0.50858	0.65611	0.99998	12499	0.1746
ZNF528	6	0.50862	0.65615	0.99998	12500	0.2116
ZDBF2	6	0.50862	0.65615	0.99998	12501	0.2372
GMIP	6	0.50871	0.65622	0.99998	12502	-0.0177
ARSF	6	0.50883	0.65632	0.99998	12503	0.1672
ID4	6	0.50885	0.65633	0.99998	12504	0.0066
SCRG1	6	0.50891	0.65639	0.99998	12505	0.1019
ST3GAL2	6	0.50891	0.65639	0.99998	12506	-0.0394
hsa-mir-4534	4	0.50898	0.58247	0.99998	12507	0.0561
SH2B3	6	0.50904	0.65649	0.99998	12508	0.2962
CAAP1	6	0.50904	0.65649	0.99998	12509	0.1542
TTC7B	6	0.50925	0.65667	0.99998	12510	0.1555
SUMO4	6	0.50933	0.65674	0.99998	12511	0.0055
DENND2D	6	0.50935	0.65676	0.99998	12512	-0.0007
hsa-mir-6511l	1	0.50938	0.50935	0.99998	12513	0.0057
CD200	6	0.50943	0.65681	0.99998	12514	0.1029
TST	6	0.50945	0.65683	0.99998	12515	0.1001
USP42	6	0.50947	0.65685	0.99998	12516	-0.0022
TMOD2	6	0.50953	0.65689	0.99998	12517	0.0886
RASGEF1A	6	0.50955	0.65691	0.99998	12518	0.2816
BRCA1	6	0.50965	0.65699	0.99998	12519	-0.0051
FAM154B	6	0.50965	0.65699	0.99998	12520	-0.0178
TCAP	6	0.50975	0.65707	0.99998	12521	-0.0085
LOC10013045	6	0.50983	0.65714	0.99998	12522	-0.0815
DECR2	6	0.50983	0.65714	0.99998	12523	0.0227
TKTL1	6	0.50983	0.65714	0.99998	12524	-0.0989
JARID2	6	0.50983	0.65714	0.99998	12525	-0.0496
ASAP1	6	0.50989	0.65718	0.99998	12526	0.12
MOGAT3	6	0.50989	0.65718	0.99998	12527	-0.057
CNP	6	0.50989	0.65718	0.99998	12528	0.1484
PILRA	6	0.51009	0.65736	0.99998	12529	0.1415
TCTA	6	0.51009	0.65736	0.99998	12530	0.1797
PCSK6	6	0.51009	0.65736	0.99998	12531	0.1167
hsa-mir-4300	2	0.5102	0.51048	0.99998	12532	-0.0606
PROX1	6	0.51027	0.65753	0.99998	12533	0.2596
SERPINF1	6	0.51035	0.65759	0.99998	12534	-0.0586
H2AFB1	1	0.51038	0.51034	0.99998	12535	0.0204
FSHB	6	0.5104	0.65762	0.99998	12536	-0.1081
CACNA1E	6	0.51045	0.65765	0.99998	12537	0.0159
MCPH1	6	0.5105	0.6577	0.99998	12538	0.3357
INSRR	6	0.5105	0.6577	0.99998	12539	0.1553
RIC8A	6	0.51059	0.65777	0.99998	12540	0.025
NPNT	6	0.5107	0.65785	0.99998	12541	0.2411
NFATC3	6	0.5107	0.65785	0.99998	12542	0.0341
C1GALT1	6	0.51088	0.65799	0.99998	12543	0.0047
IFITM3	6	0.51088	0.65799	0.99998	12544	0.2675
RNASEH2C	6	0.51088	0.65799	0.99998	12545	0.0275
IFIT2	6	0.51098	0.65807	0.99998	12546	-0.1281
KRTAP19-4	4	0.51098	0.58358	0.99998	12547	-0.2013
HNRNPC	6	0.51104	0.65812	0.99998	12548	-0.075
TCL1A	6	0.51109	0.65816	0.99998	12549	0.0245
CRHR2	6	0.51109	0.65816	0.99998	12550	0.1637
SULF2	6	0.51124	0.65826	0.99998	12551	0.0543
LDHAL6B	6	0.51124	0.65826	0.99998	12552	0.019

BLK	6	0.51124	0.65826	0.99998	12553	0.2273
SLC16A5	6	0.51128	0.6583	0.99998	12554	0.0357
PREB	6	0.51131	0.65833	0.99998	12555	-0.0055
COG8	6	0.51152	0.6585	0.99998	12556	-0.1392
AMBP	6	0.51152	0.6585	0.99998	12557	0.1406
MRPL28	6	0.51152	0.6585	0.99998	12558	-0.1322
C9orf85	6	0.51155	0.65853	0.99998	12559	0.2473
ZNF688	6	0.51167	0.65862	0.99998	12560	0.145
PCDHB10	6	0.51177	0.65869	0.99998	12561	0.2342
GLRX	6	0.5119	0.65879	0.99998	12562	-0.0933
TVP23B	4	0.51199	0.58413	0.99998	12563	-0.5318
UFC1	6	0.51205	0.65893	0.99998	12564	0.0762
HCN3	6	0.5122	0.65905	0.99998	12565	0.018
AHSG	6	0.5122	0.65905	0.99998	12566	0.0286
F7	6	0.5122	0.65905	0.99998	12567	-0.0345
OR56A3	6	0.5122	0.65905	0.99998	12568	0.344
ZNF34	6	0.51222	0.65907	0.99998	12569	0.2649
hsa-mir-4677	4	0.51225	0.58429	0.99998	12570	0.2957
SLC17A1	6	0.51239	0.6592	0.99998	12571	-0.0308
C3	6	0.51245	0.65926	0.99998	12572	-0.2013
NFIX	6	0.51245	0.65926	0.99998	12573	-0.0243
hsa-mir-4648	4	0.51255	0.58446	0.99998	12574	0.0863
hsa-mir-3144	4	0.51263	0.5845	0.99998	12575	0.1656
RBL1	6	0.51269	0.65947	0.99998	12576	-0.0962
TTC21B	6	0.51278	0.65955	0.99998	12577	0.0244
OR7G1	6	0.51279	0.65956	0.99998	12578	0.1188
TDRD7	6	0.51279	0.65956	0.99998	12579	-0.0518
UGT3A1	6	0.51296	0.65969	0.99998	12580	-0.0895
B4GALT6	6	0.51296	0.65969	0.99998	12581	-0.1417
GLP1R	6	0.51321	0.6599	0.99998	12582	0.1856
AGTRAP	6	0.51324	0.65991	0.99998	12583	0.056
ODF2	6	0.51324	0.65991	0.99998	12584	0.1496
hsa-mir-4529	3	0.51325	0.53468	0.99998	12585	-0.0302
PCDHB16	6	0.51336	0.66001	0.99998	12586	-0.0331
WDYHV1	6	0.51336	0.66001	0.99998	12587	0.2099
RAB10	6	0.51336	0.66001	0.99998	12588	0.0588
ACER2	6	0.51336	0.66001	0.99998	12589	0.1823
PLA2G3	6	0.5134	0.66004	0.99998	12590	0.1379
RRNAD1	6	0.5134	0.66004	0.99998	12591	0.1274
hsa-mir-3149	2	0.51357	0.51381	0.99998	12592	-0.3381
hsa-mir-5707	2	0.51357	0.51381	0.99998	12593	-0.1385
CLCNKA	6	0.51358	0.66021	0.99998	12594	0.0382
hsa-mir-3124	4	0.51369	0.58508	0.99998	12595	-0.1896
hsa-mir-532	4	0.51381	0.58514	0.99998	12596	0.0089
ADRA1D	6	0.51387	0.66045	0.99998	12597	-0.1287
OR7G3	6	0.51389	0.66047	0.99998	12598	-0.0143
DCXR	6	0.51389	0.66047	0.99998	12599	0.0033
CYP4F3	6	0.51389	0.66047	0.99998	12600	0.0388
C6orf89	6	0.51405	0.66059	0.99998	12601	0.1022
UBE2J2	6	0.51418	0.66071	0.99998	12602	-0.0135
PPDPF	6	0.51444	0.6609	0.99998	12603	-0.1033
HMX3	6	0.51444	0.6609	0.99998	12604	0.0902
IL27	6	0.51444	0.6609	0.99998	12605	0.1872
EVC	6	0.51456	0.66101	0.99998	12606	-0.0585
SSMEM1	6	0.5146	0.66103	0.99998	12607	0.2213
hsa-mir-4479	4	0.5146	0.5856	0.99998	12608	0.0507
BTBD2	6	0.5147	0.66111	0.99998	12609	0.058
OR52I1	6	0.51483	0.66122	0.99998	12610	0.2065
SLC25A15	6	0.51483	0.66122	0.99998	12611	0.1632
IL32	6	0.51483	0.66122	0.99998	12612	0.2293
hsa-mir-3177	4	0.51484	0.58574	0.99998	12613	0.036
ATP10B	4	0.51484	0.58574	0.99998	12614	0.0522
SOGA2	6	0.51485	0.66124	0.99998	12615	0.0864
DOCK9	6	0.51485	0.66124	0.99998	12616	-0.0801
LANCL2	6	0.51492	0.66132	0.99998	12617	0.0416
TMC4	6	0.51501	0.66139	0.99998	12618	0.0607
SYP	6	0.51514	0.66149	0.99998	12619	0.1053
KIAA2018	6	0.51514	0.66149	0.99998	12620	0.1516
RASD1	6	0.51514	0.66149	0.99998	12621	0.0965
SCPEP1	6	0.51524	0.66156	0.99998	12622	0.3818
WASF1	6	0.51528	0.6616	0.99998	12623	0.0579
EARS2	6	0.51534	0.66166	0.99998	12624	-0.0058
ZNF775	6	0.51534	0.66166	0.99998	12625	0.1048
OTX1	6	0.51534	0.66166	0.99998	12626	0.0137
CYP2R1	6	0.51542	0.66172	0.99998	12627	-0.0431
LOC402160	6	0.51552	0.6618	0.99998	12628	-0.0727
TMEM95	6	0.51558	0.66185	0.99998	12629	-0.0399
RALGAPA1	6	0.51596	0.66217	0.99998	12630	0.1457
SEPN1	6	0.51597	0.66217	0.99998	12631	-0.008
WDTC1	6	0.51597	0.66217	0.99998	12632	-0.014
DET1	4	0.51598	0.58637	0.99998	12633	0.0956

hsa-mir-4465	4	0.5162	0.5865	0.99998	12634	-0.1037
PPIF	6	0.51628	0.66246	0.99998	12635	-0.0918
JMY	6	0.51633	0.6625	0.99998	12636	0.228
ISY1-RAB43	1	0.51637	0.51633	0.99998	12637	0.0292
TBC1D26	3	0.51639	0.53711	0.99998	12638	0.0952
DUSP16	6	0.51643	0.66258	0.99998	12639	0.0229
NXF3	6	0.51653	0.66267	0.99998	12640	0.0562
ST5	6	0.51653	0.66267	0.99998	12641	0.0407
hsa-mir-640	4	0.51659	0.58672	0.99998	12642	-0.057
SIAH2	6	0.51671	0.66281	0.99998	12643	0.0056
DOCK11	6	0.51671	0.66281	0.99998	12644	-0.0368
CNGB1	6	0.51671	0.66281	0.99998	12645	0.0775
hsa-mir-32	2	0.51684	0.51704	0.99998	12646	0.0405
PTGDR	6	0.51703	0.66308	0.99998	12647	0.1511
MICU2	6	0.51711	0.66314	0.99998	12648	-0.0756
ABRA	6	0.51726	0.66326	0.99998	12649	0.0678
TBC1D2B	4	0.51733	0.58713	0.99998	12650	0.0565
SLITRK6	6	0.51746	0.66341	0.99998	12651	-0.1036
TSPYL1	6	0.51746	0.66341	0.99998	12652	0.0862
LRRC73	6	0.51753	0.66347	0.99998	12653	0.2498
hsa-mir-153-1	3	0.51761	0.53808	0.99998	12654	-0.0926
GPATCH1	6	0.51771	0.66364	0.99998	12655	0.0335
LTB4R2	6	0.51788	0.66377	0.99998	12656	-0.0233
MTHFD1L	6	0.51788	0.66377	0.99998	12657	0.1727
SBK2	6	0.51788	0.66377	0.99998	12658	-0.0415
TMEM117	6	0.51788	0.66377	0.99998	12659	-0.0419
HPGDS	6	0.51811	0.66396	0.99998	12660	-0.107
CRYAA	6	0.5182	0.66402	0.99998	12661	0.0789
GC	6	0.51821	0.66403	0.99998	12662	-0.0479
hsa-mir-548ar	3	0.51823	0.53855	0.99998	12663	-0.9148
SLC27A1	6	0.51833	0.66413	0.99998	12664	0.0046
SC5D	6	0.51841	0.66419	0.99998	12665	0.269
C21orf58	6	0.51849	0.66426	0.99998	12666	-0.0576
HIST1H1A	6	0.51852	0.66429	0.99998	12667	0.1433
hsa-mir-889	4	0.51863	0.58786	0.99998	12668	0.168
STOX2	6	0.51865	0.6644	0.99998	12669	-0.1472
ASCL3	6	0.51882	0.66453	0.99998	12670	-0.0145
PHF21A	6	0.51886	0.66457	0.99998	12671	-0.0158
FAM193A	6	0.51899	0.66466	0.99998	12672	-0.0694
GRIN1	6	0.51899	0.66466	0.99998	12673	0.116
CALY	6	0.51907	0.66472	0.99998	12674	0.1468
PRKCDBP	6	0.51907	0.66472	0.99998	12675	-0.0217
R3HDM1	6	0.51907	0.66472	0.99998	12676	-0.0499
TLR3	6	0.51907	0.66472	0.99998	12677	0.062
COPZ1	6	0.51928	0.66489	0.99998	12678	0.1931
NRARP	6	0.51934	0.66494	0.99998	12679	-0.0809
RBM42	6	0.51937	0.66497	0.99998	12680	0.0995
INCA1	6	0.51937	0.66497	0.99998	12681	0.1887
PANX2	6	0.51956	0.66511	0.99998	12682	0.0779
WNT10A	6	0.51957	0.66512	0.99998	12683	0.0855
CTNNBIP1	6	0.51977	0.66528	0.99998	12684	0.0336
hsa-mir-4787	4	0.52	0.58865	0.99998	12685	0.0636
MORN4	6	0.52009	0.66556	0.99998	12686	-0.0085
SGK110	6	0.52013	0.66559	0.99998	12687	0.183
FOXI3	6	0.52013	0.66559	0.99998	12688	-0.0663
TMTC2	6	0.52037	0.66582	0.99998	12689	0.0575
HTR1A	6	0.52037	0.66582	0.99998	12690	0.0287
RAET1L	6	0.5204	0.66585	0.99998	12691	0.0371
PHF10	6	0.52071	0.66609	0.99998	12692	0.1409
CACNA2D1	6	0.5208	0.66616	0.99998	12693	0.0167
GALC	6	0.52099	0.66633	0.99998	12694	-0.1109
MAGOHB	6	0.52104	0.66637	0.99998	12695	0.0687
HHAT	6	0.52104	0.66637	0.99998	12696	0.0184
PCDHGA1	2	0.52108	0.52124	0.99998	12697	0.3095
ACY1	6	0.52114	0.66645	0.99998	12698	-0.1555
XPNPEP2	6	0.52114	0.66645	0.99998	12699	-0.0312
C1RL	6	0.52114	0.66645	0.99998	12700	-0.0244
GOLGA3	6	0.52114	0.66645	0.99998	12701	-0.0791
C14orf177	6	0.52114	0.66645	0.99998	12702	0.0092
SIGLECL1	6	0.52126	0.66655	0.99998	12703	0.0508
KIAA1279	6	0.52126	0.66655	0.99998	12704	-0.0821
GCG	6	0.52134	0.6666	0.99998	12705	0.0098
CHMP5	6	0.52134	0.6666	0.99998	12706	-0.0531
hsa-mir-668	4	0.52148	0.58952	0.99998	12707	0.1923
GFOD1	6	0.52153	0.66675	0.99998	12708	0.1749
PRG4	6	0.52153	0.66675	0.99998	12709	0.1537
PTPN22	6	0.52155	0.66678	0.99998	12710	-0.1163
ISOC2	6	0.52155	0.66678	0.99998	12711	-0.0239
TMED1	6	0.52172	0.66692	0.99998	12712	-0.0858
DDX58	6	0.52176	0.66695	0.99998	12713	0.1453
BMP1	6	0.52176	0.66695	0.99998	12714	0.0809

CNN3	6	0.5219	0.66706	0.99998	12715	0.0256
PNPLA8	6	0.52205	0.66718	0.99998	12716	0.1543
EDN3	6	0.52205	0.66718	0.99998	12717	0.1901
RAB9A	6	0.52205	0.66718	0.99998	12718	0.0215
UBR3	6	0.52208	0.66721	0.99998	12719	-0.036
KIAA0196	6	0.52208	0.66721	0.99998	12720	0.1683
KRTAP4-8	4	0.52215	0.58989	0.99998	12721	-0.3608
RBM12B	6	0.52219	0.66729	0.99998	12722	-0.1428
FXYD6-FXYD2	2	0.52231	0.52247	0.99998	12723	-0.0362
ZNF416	6	0.52236	0.66744	0.99998	12724	-0.1027
ZNF823	6	0.52238	0.66746	0.99998	12725	0.1243
MYO1B	6	0.52238	0.66746	0.99998	12726	0.0975
hsa-mir-5697	4	0.52239	0.59002	0.99998	12727	0.0812
hsa-mir-3923	4	0.52254	0.59011	0.99998	12728	0.3107
hsa-mir-7113	4	0.52254	0.59011	0.99998	12729	0.0686
POSTN	6	0.52257	0.66762	0.99998	12730	-0.0784
GLUD2	6	0.52259	0.66764	0.99998	12731	0.1085
GPT2	6	0.52277	0.66779	0.99998	12732	0.4878
LRRN3	6	0.52278	0.6678	0.99998	12733	-0.0117
SSNA1	6	0.52286	0.66787	0.99998	12734	0.1896
hsa-mir-548g	2	0.52292	0.52306	0.99998	12735	-0.0238
hsa-mir-874	4	0.52325	0.59051	0.99998	12736	0.1554
TNFRSF13B	6	0.52327	0.6682	0.99998	12737	-0.0652
FAM24A	6	0.52327	0.6682	0.99998	12738	-0.1323
ACD	6	0.52341	0.66832	0.99998	12739	0.1504
CTSD	6	0.52344	0.66835	0.99998	12740	0.3028
C14orf142	6	0.52344	0.66835	0.99998	12741	0.0996
hsa-mir-196a-	4	0.52349	0.59065	0.99998	12742	0.0413
XKR4	6	0.52378	0.66863	0.99998	12743	-0.0502
SLC17A6	6	0.52378	0.66863	0.99998	12744	-0.0358
GALNTL6	6	0.5239	0.66873	0.99998	12745	0.0318
EVA1A	6	0.52397	0.66878	0.99998	12746	0.0949
SLC13A5	6	0.524	0.6688	0.99998	12747	0.0379
hsa-mir-3651	4	0.52406	0.59099	0.99998	12748	0.0534
ARAP1	6	0.52414	0.66891	0.99998	12749	-0.0327
SLC6A4	6	0.52414	0.66891	0.99998	12750	0.1991
MAP6	6	0.52417	0.66893	0.99998	12751	0.2624
KRT27	6	0.52417	0.66893	0.99998	12752	0.0557
CTAGE6	4	0.52427	0.59111	0.99998	12753	-0.1236
SNF8	6	0.52431	0.66904	0.99998	12754	0.0819
LRRC3B	6	0.52446	0.66918	0.99998	12755	0.0038
SPON1	6	0.52451	0.66922	0.99998	12756	0.0892
METTL10	6	0.52462	0.66932	0.99998	12757	0.216
CELSR3	6	0.52462	0.66932	0.99998	12758	-0.0163
hsa-mir-105-1	2	0.52465	0.52479	0.99998	12759	-0.0983
SLC5A12	6	0.52472	0.6694	0.99998	12760	-0.0863
EDC4	6	0.52473	0.6694	0.99998	12761	0.0075
PRRC2C	6	0.5248	0.66947	0.99998	12762	-0.0614
SLCO4C1	6	0.52496	0.66961	0.99998	12763	0.1425
GAST	6	0.52496	0.66961	0.99998	12764	-0.0719
BEST4	6	0.52507	0.66969	0.99998	12765	-0.1025
CDX4	6	0.52507	0.66969	0.99998	12766	0.1505
C10orf137	6	0.52508	0.66971	0.99998	12767	0.1126
FAM98C	6	0.52522	0.66983	0.99998	12768	0.0987
SLC30A3	6	0.52522	0.66983	0.99998	12769	0.1879
hsa-mir-639	4	0.52525	0.59168	0.99998	12770	0.0722
NR6A1	6	0.52536	0.66994	0.99998	12771	-0.0289
TRPT1	6	0.52536	0.66994	0.99998	12772	-0.0232
FBXL17	6	0.52541	0.66998	0.99998	12773	0.1175
ZNF260	6	0.52564	0.67017	0.99998	12774	-0.0071
MFF	6	0.52566	0.67018	0.99998	12775	0.0944
CCNF	6	0.52566	0.67018	0.99998	12776	-0.1114
FAM153A	2	0.52571	0.52587	0.99998	12777	0.0081
SZT2	6	0.52577	0.67028	0.99998	12778	0.2071
PAFAH1B2	6	0.52587	0.67036	0.99998	12779	-0.0834
MAPKAPK2	6	0.52587	0.67036	0.99998	12780	0.1209
NAPB	6	0.52587	0.67036	0.99998	12781	0.1602
MBD3	6	0.52588	0.67037	0.99998	12782	0.1545
DGAT1	6	0.52601	0.67047	0.99998	12783	0.1683
MYLK	6	0.52601	0.67047	0.99998	12784	-0.0326
ZNF682	6	0.52608	0.67053	0.99998	12785	0.1398
LUC7L	6	0.52612	0.67056	0.99998	12786	-0.0677
LEMD2	6	0.52618	0.6706	0.99998	12787	0.0332
STX2	6	0.52618	0.6706	0.99998	12788	0.1135
EEF1D	6	0.52618	0.6706	0.99998	12789	0.0642
PRAMEF17	2	0.52621	0.52638	0.99998	12790	-0.5434
hsa-mir-6826	4	0.52623	0.59222	0.99998	12791	-0.0759
SGPL1	6	0.52631	0.67071	0.99998	12792	-0.1338
TMEM184A	6	0.52631	0.67071	0.99998	12793	0.1609
ESYT2	6	0.52636	0.67075	0.99998	12794	0.0047
CPQ	6	0.52636	0.67075	0.99998	12795	0.062

IQCE	6	0.52636	0.67075	0.99998	12796	0.0893
RPAP3	6	0.52655	0.67092	0.99998	12797	0.1804
ASCC1	6	0.52673	0.67107	0.99998	12798	-0.0472
C15orf53	6	0.52673	0.67107	0.99998	12799	0.1741
SRRM4	6	0.52673	0.67108	0.99998	12800	0.1777
BOD1L1	6	0.52687	0.6712	0.99998	12801	0.2084
PDCD4	6	0.52697	0.67128	0.99998	12802	0.1494
GLB1L	6	0.52707	0.67135	0.99998	12803	-0.077
C20orf197	6	0.52707	0.67135	0.99998	12804	-0.0765
C4orf51	6	0.52707	0.67136	0.99998	12805	-0.0613
AR	6	0.52707	0.67136	0.99998	12806	-0.0298
C5orf42	6	0.52715	0.67143	0.99998	12807	0.3157
EMP3	6	0.52715	0.67143	0.99998	12808	0.0664
PIGH	6	0.52732	0.67156	0.99998	12809	0.2784
KDM4E	6	0.52735	0.67159	0.99998	12810	0.0749
APOA1BP	6	0.52735	0.67159	0.99998	12811	-0.052
GP5	6	0.52735	0.67159	0.99998	12812	-0.0116
ZDHHC7	6	0.52763	0.67184	0.99998	12813	0.1
TCTEX1D2	6	0.52764	0.67185	0.99998	12814	-0.1111
C9orf163	6	0.52764	0.67185	0.99998	12815	0.0107
SPSB1	6	0.52778	0.67196	0.99998	12816	0.066
NFE2L1	6	0.52779	0.67196	0.99998	12817	0.095
C17orf66	6	0.52779	0.67196	0.99998	12818	0.2252
CETN1	6	0.52779	0.67196	0.99998	12819	0.1117
AS3MT	6	0.52791	0.67206	0.99998	12820	0.3267
OR6T1	6	0.52791	0.67206	0.99998	12821	-0.0635
hsa-mir-22	4	0.52802	0.59321	0.99998	12822	0.2407
hsa-mir-1238	4	0.52802	0.59321	0.99998	12823	0.1542
GGN	6	0.52809	0.67221	0.99998	12824	0.1296
GPR156	6	0.52809	0.67221	0.99998	12825	0.1032
CSN2	6	0.52809	0.67221	0.99998	12826	-0.0864
CABLES1	4	0.52809	0.59325	0.99998	12827	0.1618
RNF122	6	0.52814	0.67226	0.99998	12828	0.0602
C21orf91	6	0.52814	0.67226	0.99998	12829	-0.0744
CLEC4E	6	0.52828	0.67237	0.99998	12830	0.0179
ADRB3	6	0.52831	0.67239	0.99998	12831	0.1329
TGFBR3	6	0.52832	0.6724	0.99998	12832	0.086
PLAC1	6	0.52839	0.67247	0.99998	12833	0.0805
FAM229A	6	0.52851	0.67259	0.99998	12834	0.0887
TFG	6	0.52871	0.67278	0.99998	12835	0.1992
SERTAD3	6	0.52878	0.67284	0.99998	12836	0.0425
GOLT1B	6	0.5291	0.6731	0.99998	12837	0.1272
C9orf3	6	0.5291	0.6731	0.99998	12838	0.0551
DNASE1L2	6	0.52912	0.67311	0.99998	12839	0.0298
EPHX4	6	0.52924	0.67322	0.99998	12840	0.0905
SFXN2	6	0.52928	0.67326	0.99998	12841	0.0293
TTC36	6	0.52945	0.67338	0.99998	12842	0.1476
C9orf92	6	0.52946	0.67339	0.99998	12843	0.4083
UQCR11	6	0.52959	0.67351	0.99998	12844	0.071
SYTL5	6	0.52959	0.67351	0.99998	12845	0.1579
APOA2	6	0.52967	0.67358	0.99998	12846	0.1462
PRKCSH	6	0.52967	0.67358	0.99998	12847	-0.0394
RTN4R	6	0.52967	0.67358	0.99998	12848	0.0973
HTR7	6	0.52971	0.67361	0.99998	12849	-0.0642
CNPY2	6	0.52986	0.67374	0.99998	12850	-0.0475
hsa-mir-378h	4	0.53002	0.59432	0.99998	12851	-0.1663
HPS1	6	0.5301	0.67395	0.99998	12852	0.1718
VIPAS39	6	0.53012	0.67395	0.99998	12853	0.0667
NPHP1	6	0.53012	0.67395	0.99998	12854	0.0076
DGKH	6	0.53012	0.67395	0.99998	12855	-0.0366
ZNF26	6	0.53019	0.67402	0.99998	12856	0.1611
HINT3	6	0.53019	0.67402	0.99998	12857	0.0698
hsa-mir-4472-	4	0.53022	0.59443	0.99998	12858	0.139
hsa-mir-548al	1	0.53039	0.53031	0.99998	12859	0.059
hsa-mir-454	4	0.53043	0.59456	0.99998	12860	0.1987
C14orf1	6	0.53048	0.67426	0.99998	12861	0.0783
SLC30A9	6	0.53048	0.67426	0.99998	12862	0.0681
LRP12	6	0.53048	0.67426	0.99998	12863	-0.0705
PRB2	3	0.53066	0.54817	0.99998	12864	0.0589
CXCR2	6	0.53099	0.67469	0.99998	12865	0.0624
CTSB	6	0.53121	0.67487	0.99998	12866	0.1706
C11orf31	6	0.53121	0.67487	0.99998	12867	0.0718
NTSR2	6	0.53131	0.67494	0.99998	12868	0.2109
HOMEZ	6	0.53141	0.67503	0.99998	12869	-0.0051
GYPC	6	0.53144	0.67506	0.99998	12870	0.0368
SPATA31A6	6	0.53144	0.67506	0.99998	12871	-0.9148
KIAA1614	6	0.53144	0.67506	0.99998	12872	-0.2408
CXCL16	6	0.53144	0.67506	0.99998	12873	-0.1126
HMGN5	6	0.53144	0.67506	0.99998	12874	0.031
KRT38	6	0.53144	0.67506	0.99998	12875	-0.1075
ZNF189	6	0.53144	0.67506	0.99998	12876	-0.0044

ITGAL	6	0.53144	0.67506	0.99998	12877	0.044
BCL2L2	6	0.53144	0.67506	0.99998	12878	-0.1022
TMX4	6	0.53144	0.67506	0.99998	12879	0.0318
IFNA5	6	0.53144	0.67506	0.99998	12880	-0.1611
RNPEP	6	0.53159	0.67519	0.99998	12881	0.1006
LILRA3	5	0.53164	0.6555	0.99998	12882	-0.4663
FKBP3	6	0.53182	0.6754	0.99998	12883	0.0982
TAAR5	6	0.53182	0.6754	0.99998	12884	0.0103
IGBP1	6	0.53182	0.6754	0.99998	12885	-0.028
COX18	6	0.53182	0.6754	0.99998	12886	-0.0104
AP1S3	6	0.53182	0.6754	0.99998	12887	-0.0881
STK19	6	0.5321	0.67563	0.99998	12888	0.2073
ANKRD6	6	0.5321	0.67563	0.99998	12889	0.0809
SREK1	6	0.53229	0.67579	0.99998	12890	-0.0309
ZFP92	6	0.53235	0.67584	0.99998	12891	-0.0491
C11orf54	6	0.53276	0.67618	0.99998	12892	-0.1336
GK2	6	0.5328	0.67621	0.99998	12893	0.2173
STK17B	6	0.5329	0.67628	0.99998	12894	0.2063
PCDHB13	6	0.53294	0.67632	0.99998	12895	0.0122
SLC4A3	6	0.53305	0.6764	0.99998	12896	0.0653
ARHGDIB	6	0.53308	0.67642	0.99998	12897	0.1379
AGL	6	0.53318	0.67649	0.99998	12898	0.0303
METTL6	6	0.5332	0.67652	0.99998	12899	0.1052
FAM110C	6	0.53326	0.67657	0.99998	12900	0.0694
SH2D1A	6	0.53334	0.67663	0.99998	12901	-0.0237
BEAN1	6	0.53334	0.67663	0.99998	12902	0.0317
CCDC138	6	0.5334	0.67668	0.99998	12903	0.0188
TARM1	6	0.53365	0.6769	0.99998	12904	-0.067
ANAPC7	6	0.53375	0.67698	0.99998	12905	0.0338
TRIM63	6	0.53375	0.67698	0.99998	12906	-0.013
LINS	6	0.53384	0.67705	0.99998	12907	0.1545
MYOCD	6	0.53384	0.67705	0.99998	12908	0.134
RNF175	6	0.53384	0.67705	0.99998	12909	-0.0363
NDUFA8	6	0.53387	0.67706	0.99998	12910	0.0166
COG4	6	0.53395	0.67713	0.99998	12911	0.0838
CEACAM4	6	0.53402	0.67719	0.99998	12912	-0.0839
PAX1	4	0.53403	0.59656	0.99998	12913	0.0143
XYLB	6	0.53406	0.67722	0.99998	12914	0.086
HSP90B1	6	0.53415	0.67729	0.99998	12915	0.2437
OR2G3	6	0.53419	0.67732	0.99998	12916	0.0449
STX16	6	0.53421	0.67733	0.99998	12917	0.1095
SERTAD2	6	0.53421	0.67733	0.99998	12918	0.0956
HMGA2	6	0.53425	0.67736	0.99998	12919	-0.0627
ATP2B3	6	0.53433	0.67743	0.99998	12920	0.0234
DCTD	6	0.53442	0.67749	0.99998	12921	0.069
LRRC4C	6	0.53451	0.67758	0.99998	12922	-0.1881
TDRD12	6	0.53451	0.67758	0.99998	12923	-0.0232
CCDC92	4	0.53456	0.59685	0.99998	12924	0.3647
hsa-mir-599	4	0.53456	0.59685	0.99998	12925	-0.2321
ZMYND11	6	0.53461	0.67766	0.99998	12926	0.1938
CDCP2	6	0.53468	0.67772	0.99998	12927	0.0659
BTBD7	6	0.53468	0.67772	0.99998	12928	0.0112
CLEC4D	6	0.53468	0.67772	0.99998	12929	-0.0577
ZFP41	6	0.53472	0.67775	0.99998	12930	0.0292
C17orf64	6	0.53472	0.67775	0.99998	12931	0.05
IRAK3	6	0.53491	0.67789	0.99998	12932	-0.02
ZNF114	6	0.53491	0.67789	0.99998	12933	-0.0169
hsa-mir-6863	4	0.535	0.5971	0.99998	12934	-0.0418
QSOX1	6	0.53507	0.67803	0.99998	12935	-0.0606
ZNF547	6	0.53508	0.67804	0.99998	12936	0.2096
RALGAPB	6	0.53508	0.67804	0.99998	12937	0.0659
FUT3	6	0.5352	0.67815	0.99998	12938	-0.0067
GPRC5C	6	0.5354	0.6783	0.99998	12939	0.0815
UTF1	6	0.5354	0.6783	0.99998	12940	-0.057
PIAS3	6	0.5354	0.6783	0.99998	12941	0.1288
ELL3	6	0.53555	0.67842	0.99998	12942	0.0547
ITGB1BP2	6	0.53556	0.67844	0.99998	12943	-0.0185
ZWILCH	6	0.53556	0.67844	0.99998	12944	-0.0547
FRRS1	6	0.53557	0.67845	0.99998	12945	0.0741
ADORA2B	6	0.53572	0.67857	0.99998	12946	0.0672
FTHL17	6	0.53582	0.67865	0.99998	12947	-0.0898
KLRF2	6	0.53585	0.67867	0.99998	12948	0.0998
hsa-mir-6860	4	0.53588	0.59762	0.99998	12949	0.1126
OR10H3	6	0.53596	0.67876	0.99998	12950	0.2798
DOCK10	6	0.53601	0.6788	0.99998	12951	0.0165
FAM115A	6	0.53613	0.67891	0.99998	12952	0.1306
TBRG4	6	0.53613	0.67891	0.99998	12953	0.0005
GRM3	6	0.53615	0.67892	0.99998	12954	-0.0321
SAC3D1	6	0.53633	0.67908	0.99998	12955	-0.154
hsa-mir-3180-	2	0.5364	0.53651	0.99998	12956	-0.1401
C5orf64	6	0.53647	0.67919	0.99998	12957	-0.0053

TUSC5	6	0.53648	0.6792	0.99998	12958	0.001
ANKMY2	6	0.53667	0.67937	0.99998	12959	-0.1084
SPRY4	6	0.53667	0.67937	0.99998	12960	-0.089
RAB17	6	0.53674	0.67943	0.99998	12961	0.1474
TMUB1	6	0.53674	0.67943	0.99998	12962	0.0564
KCNAB2	6	0.53674	0.67943	0.99998	12963	0.0114
VWDE	6	0.53674	0.67943	0.99998	12964	-0.0569
RASSF9	6	0.53686	0.67952	0.99998	12965	0.0825
TMEM14E	6	0.53691	0.67956	0.99998	12966	0.1203
hsa-mir-569	4	0.53699	0.59828	0.99998	12967	-0.0424
SLC5A5	6	0.53699	0.67965	0.99998	12968	-0.0791
GPR39	6	0.53706	0.67971	0.99998	12969	0.0192
ADPRH	6	0.53716	0.6798	0.99998	12970	-0.0658
hsa-mir-26a-1	4	0.53726	0.59842	0.99998	12971	0.2225
ANGPTL3	6	0.53729	0.67991	0.99998	12972	-0.0361
NRM	6	0.53729	0.67991	0.99998	12973	0.0034
STRN3	6	0.53734	0.67995	0.99998	12974	-0.1387
SSBP1	6	0.53746	0.68006	0.99998	12975	0.2321
FAM172A	6	0.53746	0.68006	0.99998	12976	0.0017
NKIRAS2	6	0.53746	0.68006	0.99998	12977	-0.0365
PLEKHH1	6	0.53746	0.68006	0.99998	12978	-0.1013
HPDL	6	0.53756	0.68014	0.99998	12979	0.0028
PTPRE	6	0.53756	0.68014	0.99998	12980	0.1094
DHX30	6	0.53756	0.68014	0.99998	12981	0.1689
ATXN3L	6	0.53756	0.68014	0.99998	12982	-0.0634
RAD23A	6	0.53771	0.68028	0.99998	12983	-0.1174
OR52I2	4	0.53773	0.5987	0.99998	12984	0.0068
SALL1	6	0.53775	0.6803	0.99998	12985	-0.1298
NCF2	6	0.53775	0.6803	0.99998	12986	0.0913
ABCB11	6	0.53775	0.6803	0.99998	12987	0.1276
DACH2	6	0.53775	0.6803	0.99998	12988	0.0384
FRMPD4	6	0.53778	0.68034	0.99998	12989	-0.0696
SPAG9	6	0.53793	0.68046	0.99998	12990	0.0407
GNAS	6	0.53793	0.68046	0.99998	12991	-0.0627
C10orf11	6	0.53793	0.68046	0.99998	12992	0.0646
hsa-mir-2113	4	0.53794	0.59882	0.99998	12993	0.1573
RNF167	6	0.53804	0.68055	0.99998	12994	-0.1668
RALA	6	0.53827	0.68074	0.99998	12995	-0.0096
MST1	6	0.53837	0.68083	0.99998	12996	-0.0495
UFL1	6	0.53837	0.68083	0.99998	12997	-0.068
SLC9A1	6	0.53848	0.68091	0.99998	12998	0.1667
HCK	6	0.53848	0.68091	0.99998	12999	0.2012
FMR1	6	0.53848	0.68091	0.99998	13000	-0.1034
LARP7	6	0.53859	0.68101	0.99998	13001	-0.0213
FAM167B	6	0.53859	0.68101	0.99998	13002	0.0629
KITLG	5	0.53866	0.6584	0.99998	13003	0.1142
CFL2	6	0.53871	0.68112	0.99998	13004	0.0088
NRN1	6	0.53871	0.68112	0.99998	13005	0.1116
PPAP2A	6	0.53882	0.68121	0.99998	13006	0.0665
COX6A1	6	0.53889	0.68127	0.99998	13007	-0.0045
IMPACT	6	0.53889	0.68127	0.99998	13008	0.1146
ARAP2	6	0.53889	0.68127	0.99998	13009	-0.0511
OR10X1	6	0.53898	0.68134	0.99998	13010	0.0465
MITD1	6	0.53906	0.68141	0.99998	13011	0.1686
MXI1	6	0.53911	0.68145	0.99998	13012	-0.0554
C1orf101	6	0.53914	0.68148	0.99998	13013	0.2112
PARVA	6	0.53918	0.68151	0.99998	13014	0.1042
OTOP3	6	0.53928	0.68159	0.99998	13015	-0.0633
ADRA2C	6	0.53928	0.68159	0.99998	13016	-0.017
ECHDC1	6	0.53928	0.68159	0.99998	13017	-0.0589
hsa-mir-520a	3	0.5393	0.55499	0.99998	13018	-0.8481
OAF	6	0.53934	0.68164	0.99998	13019	0.1018
hsa-mir-4660	4	0.53946	0.59972	0.99998	13020	0.0494
TMEM185A	6	0.53954	0.68182	0.99998	13021	0.158
FAM177B	6	0.53954	0.68182	0.99998	13022	-0.0889
MCOLN1	6	0.53954	0.68182	0.99998	13023	0.0889
SLC25A12	6	0.53957	0.68184	0.99998	13024	0.0807
CRYBA4	6	0.53972	0.68196	0.99998	13025	0.1137
C5orf28	6	0.53972	0.68196	0.99998	13026	0.0777
TMEM79	6	0.53972	0.68196	0.99998	13027	-0.0645
PDCD10	6	0.53975	0.68199	0.99998	13028	0.0823
RNF2	6	0.53988	0.6821	0.99998	13029	-0.0503
CAPN13	6	0.53988	0.6821	0.99998	13030	-0.1587
PDIA4	6	0.53988	0.6821	0.99998	13031	0.0551
CUL7	6	0.53995	0.68214	0.99998	13032	0.0709
hsa-mir-4492	4	0.53997	0.60002	0.99998	13033	0.2575
KSR1	6	0.54	0.68218	0.99998	13034	0.0755
AZI1	6	0.54	0.68218	0.99998	13035	0.1265
SLC12A1	6	0.54	0.68218	0.99998	13036	0.0139
C9orf117	6	0.54	0.68218	0.99998	13037	0.1027
MRPS34	6	0.54012	0.68229	0.99998	13038	-0.1762

NKG7	6	0.54012	0.68229	0.99998	13039	0.1686
MND1	6	0.54032	0.68246	0.99998	13040	0.122
ZSCAN31	6	0.54034	0.68247	0.99998	13041	0.0782
CREBBP	6	0.54043	0.68257	0.99998	13042	-0.0373
COQ5	6	0.54044	0.68258	0.99998	13043	0.1578
CAT	6	0.54044	0.68258	0.99998	13044	0.1321
SLC7A2	6	0.54054	0.68266	0.99998	13045	-0.0765
COL11A1	6	0.54067	0.68277	0.99998	13046	-0.1044
TMEM138	6	0.54067	0.68277	0.99998	13047	0.0709
APEH	6	0.54067	0.68277	0.99998	13048	0.1228
OR5J2	6	0.54081	0.68289	0.99998	13049	0.0379
MYO9A	6	0.54089	0.68297	0.99998	13050	-0.0516
BARX2	6	0.54089	0.68297	0.99998	13051	0.0108
TSPAN17	6	0.54111	0.68315	0.99998	13052	-0.115
hsa-mir-4501	2	0.54115	0.54127	0.99998	13053	-0.2553
FASTKD2	6	0.5412	0.68323	0.99998	13054	0.148
PCDH15	6	0.54131	0.68333	0.99998	13055	0.0547
TMEM140	6	0.54144	0.68344	0.99998	13056	-0.0358
VWA3A	6	0.54144	0.68344	0.99998	13057	-0.0067
NAA60	6	0.54151	0.68351	0.99998	13058	-0.076
CTSZ	6	0.54154	0.68353	0.99998	13059	0.088
DLX5	6	0.54154	0.68353	0.99998	13060	0.1897
SOX5	6	0.54154	0.68353	0.99998	13061	0.0898
PDIA3	6	0.54158	0.68357	0.99998	13062	0.0163
GPR174	6	0.54173	0.68369	0.99998	13063	0.0865
FCER1A	4	0.54186	0.6011	0.99998	13064	0.0679
FERD3L	6	0.54189	0.68382	0.99998	13065	0.112
CRYBB2	6	0.54192	0.68384	0.99998	13066	-0.1116
ERGIC2	6	0.54198	0.68389	0.99998	13067	-0.0353
HSPB9	6	0.54202	0.68392	0.99998	13068	-0.0023
NAT1	6	0.54211	0.68399	0.99998	13069	-0.0487
SLC28A2	6	0.54219	0.68407	0.99998	13070	-0.058
CPN2	6	0.54219	0.68407	0.99998	13071	0.0828
DOK1	6	0.5422	0.68408	0.99998	13072	0.0287
hsa-mir-6751	4	0.54226	0.60132	0.99998	13073	0.1104
C17orf96	6	0.54234	0.68419	0.99998	13074	0.2349
MED12L	6	0.54234	0.68419	0.99998	13075	-0.041
GAS7	6	0.54234	0.68419	0.99998	13076	-0.0446
CA6	6	0.54235	0.6842	0.99998	13077	0.0283
GTPBP3	6	0.54235	0.6842	0.99998	13078	0.0411
SRBD1	6	0.54235	0.6842	0.99998	13079	-0.0025
MALSU1	6	0.54252	0.68435	0.99998	13080	-0.0951
GALNT12	6	0.54252	0.68435	0.99998	13081	0.0442
hsa-mir-8059	4	0.5426	0.60151	0.99998	13082	-0.2958
SENP7	6	0.54261	0.68442	0.99998	13083	-0.0115
FIGF	6	0.54261	0.68442	0.99998	13084	0.1248
FAM133A	6	0.54277	0.68455	0.99998	13085	0.0297
FAM132A	6	0.54281	0.68458	0.99998	13086	0.0985
C11orf91	6	0.54281	0.68458	0.99998	13087	0.0341
AQP4	6	0.54304	0.68478	0.99998	13088	-0.0999
hsa-mir-4255	4	0.54305	0.60177	0.99998	13089	0.0842
IRAK2	6	0.54323	0.68495	0.99998	13090	0.1594
MNS1	6	0.54323	0.68495	0.99998	13091	0.2019
DNER	6	0.54327	0.68497	0.99998	13092	0.149
DCUN1D3	6	0.54327	0.68497	0.99998	13093	0.0528
RIPPLY3	2	0.54331	0.54339	0.99998	13094	0.0084
BEX2	6	0.54346	0.68513	0.99998	13095	0.2375
PAX8	6	0.54346	0.68513	0.99998	13096	-0.0127
OCIAD1	6	0.54346	0.68513	0.99998	13097	0.0669
LIN7A	6	0.5435	0.68517	0.99998	13098	-0.1444
RBM45	6	0.54361	0.68525	0.99998	13099	-0.0211
RING1	6	0.54376	0.68539	0.99998	13100	-0.0016
ANO9	6	0.54376	0.6854	0.99998	13101	0.2801
PGPEP1	6	0.54376	0.6854	0.99998	13102	0.1037
FNDC4	6	0.5439	0.68552	0.99998	13103	0.2563
RMND1	6	0.5439	0.68552	0.99998	13104	-0.054
HIAT1	6	0.54407	0.68565	0.99998	13105	-0.0007
KAZN	6	0.54409	0.68567	0.99998	13106	0.07
CRTAM	6	0.54409	0.68567	0.99998	13107	-0.0045
ZDHHC20	6	0.54428	0.68583	0.99998	13108	0.1027
FCRL3	6	0.54428	0.68583	0.99998	13109	-0.2067
ADAM19	6	0.54445	0.68597	0.99998	13110	0.0662
TTBK2	6	0.54445	0.68597	0.99998	13111	0.0259
REXO1	6	0.54447	0.68598	0.99998	13112	0.1551
S100A6	6	0.5446	0.68611	0.99998	13113	-0.0956
CNOT10	6	0.54466	0.68615	0.99998	13114	0.0043
MYBL1	6	0.54466	0.68615	0.99998	13115	0.1427
GATC	6	0.54466	0.68615	0.99998	13116	0.0498
CELSR1	6	0.54466	0.68615	0.99998	13117	0.1832
PLS3	6	0.54501	0.68644	0.99998	13118	0.1568
DYTN	6	0.54501	0.68644	0.99998	13119	-0.1251

KRT26	6	0.54509	0.68651	0.99998	13120	0.1869
MAML3	6	0.54529	0.6867	0.99998	13121	0.0339
SMYD3	6	0.5454	0.68678	0.99998	13122	0.009
MBNL2	6	0.54546	0.68683	0.99998	13123	-0.1354
LRRC18	6	0.54549	0.68686	0.99998	13124	0.1907
SNCB	6	0.54556	0.68693	0.99998	13125	0.2514
GNPDA1	6	0.54556	0.68693	0.99998	13126	-0.0424
CCNG2	6	0.54571	0.68705	0.99998	13127	0.0375
PRDM10	6	0.54571	0.68705	0.99998	13128	-0.1041
OR5H14	6	0.54571	0.68705	0.99998	13129	0.1959
hsa-mir-4638	4	0.54574	0.60333	0.99998	13130	0.2184
BBS12	6	0.54579	0.68712	0.99998	13131	0.0614
PGAM5	6	0.54598	0.68727	0.99998	13132	-0.1077
RUFY3	6	0.54598	0.68727	0.99998	13133	0.0212
BRSK2	6	0.54602	0.68729	0.99998	13134	0.1603
TVP23A	6	0.54617	0.68741	0.99998	13135	0.0583
MYADML2	6	0.54622	0.68746	0.99998	13136	0.2137
CLCN7	6	0.5463	0.68752	0.99998	13137	0.0946
IDNK	6	0.5463	0.68752	0.99998	13138	0.0757
TRHDE	6	0.54641	0.68761	0.99998	13139	0.0829
ASB12	6	0.54646	0.68766	0.99998	13140	0.0975
PALM2-AKAP2	1	0.54654	0.54649	0.99998	13141	0.0978
SERPINB8	6	0.54656	0.68774	0.99998	13142	0.0758
ALB	6	0.5466	0.68779	0.99998	13143	-0.1319
FAM203A	6	0.54664	0.68782	0.99998	13144	-0.1348
CEACAM20	6	0.54676	0.68791	0.99998	13145	-0.0786
PHF17	6	0.54703	0.68812	0.99998	13146	0.1786
UTP15	6	0.54703	0.68812	0.99998	13147	-0.1253
SDHAF1	6	0.54716	0.68822	0.99998	13148	0.0199
HMP19	6	0.54716	0.68822	0.99998	13149	-0.1334
SLC2A12	6	0.54716	0.68822	0.99998	13150	0.1048
hsa-mir-6736	4	0.54718	0.60416	0.99998	13151	0.0158
TCEAL6	6	0.54723	0.68828	0.99998	13152	0.1932
PHOX2A	4	0.54735	0.60428	0.99998	13153	-0.0143
hsa-mir-652	4	0.54735	0.60428	0.99998	13154	0.1023
FANCI	6	0.54739	0.68842	0.99998	13155	0.0757
TRIM3	6	0.54754	0.68854	0.99998	13156	-0.0211
GCSAML	6	0.54754	0.68854	0.99998	13157	0.1765
ARHGEF5	6	0.54754	0.68854	0.99998	13158	-0.0349
ZBBX	6	0.54754	0.68854	0.99998	13159	0.2988
SMG8	6	0.54754	0.68854	0.99998	13160	0.0045
hsa-mir-381	4	0.5476	0.60443	0.99998	13161	0.1865
RGAG1	6	0.54777	0.68873	0.99998	13162	0.042
REL	6	0.54777	0.68873	0.99998	13163	-0.0467
NADSYN1	6	0.54807	0.68898	0.99998	13164	-0.0255
ARHGEF39	6	0.54807	0.68898	0.99998	13165	0.0509
MMS19	6	0.54809	0.68899	0.99998	13166	-0.0096
HOXB13	6	0.5482	0.68908	0.99998	13167	0.1363
KIF5B	6	0.5482	0.68908	0.99998	13168	0.0237
TTLL10	6	0.54829	0.68916	0.99998	13169	0.1184
SEC14L4	6	0.54829	0.68916	0.99998	13170	0.0167
CDKN1B	6	0.54832	0.68919	0.99998	13171	0.0687
C5orf63	6	0.54839	0.68925	0.99998	13172	0.2197
CA7	6	0.54849	0.68933	0.99998	13173	-0.0921
hsa-mir-3689b	1	0.54852	0.54846	0.99998	13174	0.1404
ZNF483	6	0.54864	0.68946	0.99998	13175	0.1461
GFAP	6	0.54864	0.68946	0.99998	13176	-0.1664
HFE2	6	0.54876	0.68957	0.99998	13177	0.1812
H2AFB3	1	0.54879	0.54875	0.99998	13178	0.0421
hsa-mir-1911	4	0.54896	0.60521	0.99998	13179	0.004
HIC1	6	0.54897	0.68976	0.99998	13180	0.0972
EXTL1	6	0.54916	0.68993	0.99998	13181	-0.0328
hsa-mir-548f-3	3	0.5497	0.5633	0.99998	13182	0.2244
RELT	6	0.54982	0.69049	0.99998	13183	0.0687
GNG11	5	0.54985	0.66306	0.99998	13184	-0.0696
KRTAP25-1	6	0.54994	0.69059	0.99998	13185	-0.0374
ZNF394	6	0.55	0.69064	0.99998	13186	0.4231
BLMH	6	0.55	0.69064	0.99998	13187	0.1262
OR2A7	1	0.55013	0.55007	0.99998	13188	0.1932
hsa-mir-103b-	1	0.55013	0.55007	0.99998	13189	0.1932
hsa-mir-3198-	1	0.55013	0.55007	0.99998	13190	0.1932
SPRR2F	1	0.55013	0.55007	0.99998	13191	0.1932
SHISA5	6	0.55017	0.69079	0.99998	13192	0.1314
DYDC2	6	0.55017	0.69079	0.99998	13193	0.1003
LPHN2	6	0.55017	0.69079	0.99998	13194	-0.021
COIL	6	0.55021	0.69082	0.99998	13195	0.1144
TAL1	6	0.55039	0.69098	0.99998	13196	0.0603
C1orf131	6	0.55043	0.69102	0.99998	13197	-0.0251
CREB1	6	0.55043	0.69102	0.99998	13198	-0.0948
NR0B2	6	0.55047	0.69105	0.99998	13199	0.0193
KRTAP29-1	6	0.55052	0.6911	0.99998	13200	0.0996

CEBPD	6	0.55055	0.69112	0.99998	13201	0.0631
TRMT2B	6	0.55062	0.69118	0.99998	13202	0.2221
LRRN1	6	0.55063	0.69118	0.99998	13203	-0.0119
SUOX	6	0.55071	0.69125	0.99998	13204	0.1683
DNAJC25	6	0.55075	0.69127	0.99998	13205	0.1198
LILRA5	6	0.55075	0.69127	0.99998	13206	0.0937
C19orf71	6	0.55087	0.69137	0.99998	13207	0.0289
TMEM60	6	0.55087	0.69137	0.99998	13208	0.1457
LGALSL	6	0.55106	0.69153	0.99998	13209	-0.1302
MAB21L2	6	0.55119	0.69163	0.99998	13210	-0.0245
S100Z	6	0.55121	0.69165	0.99998	13211	0.0802
SEMA3E	6	0.5513	0.69172	0.99998	13212	0.0068
ZNF341	6	0.5513	0.69172	0.99998	13213	0.11
hsa-mir-4717	4	0.55137	0.60664	0.99998	13214	-0.0524
LBX1	6	0.55147	0.69187	0.99998	13215	0.2235
KNDC1	6	0.55148	0.69188	0.99998	13216	0.1332
CHRNA2	6	0.55148	0.69188	0.99998	13217	0.1956
ZFHX4	6	0.55148	0.69188	0.99998	13218	-0.0161
CSF2RB	6	0.55158	0.69197	0.99998	13219	0.0749
TMEM126A	6	0.55172	0.69208	0.99998	13220	0.2957
hsa-let-7f-2	4	0.55177	0.60688	0.99998	13221	0.0131
NFE2L2	6	0.5518	0.69214	0.99998	13222	0.0598
FBN1	6	0.55182	0.69216	0.99998	13223	0.1716
hsa-mir-133a-	2	0.55193	0.55197	0.99998	13224	-0.1177
SSTR4	6	0.55196	0.69227	0.99998	13225	0.0199
DDX3Y	6	0.55196	0.69228	0.99998	13226	0.1377
CYB5B	6	0.55196	0.69228	0.99998	13227	0.0769
C1orf234	3	0.55203	0.56513	0.99998	13228	-0.0931
IGFBP1	6	0.55206	0.69236	0.99998	13229	0.0578
NOV	6	0.55216	0.69245	0.99998	13230	0.1901
39326	3	0.5522	0.56526	0.99998	13231	-0.1491
TMEM116	4	0.55242	0.60726	0.99998	13232	0.207
NMUR2	6	0.55248	0.69271	0.99998	13233	0.2165
NKX3-2	6	0.55248	0.69271	0.99998	13234	0.1502
SLC24A6	2	0.55256	0.5526	0.99998	13235	0.4934
hsa-mir-3154	4	0.55273	0.60746	0.99998	13236	-0.0471
CYTH4	4	0.55273	0.60746	0.99998	13237	-0.0627
OPN3	6	0.55276	0.69294	0.99998	13238	0.1589
SDC1	6	0.55276	0.69294	0.99998	13239	0.1167
MYCN	6	0.55276	0.69294	0.99998	13240	-0.0464
KIDINS220	6	0.55276	0.69294	0.99998	13241	0.0698
LPAR1	6	0.55287	0.69304	0.99998	13242	0.1063
hsa-mir-7702	3	0.55291	0.56583	0.99998	13243	-0.0798
hsa-mir-548ag	3	0.55291	0.56583	0.99998	13244	-0.5237
hsa-mir-4512	3	0.55291	0.56583	0.99998	13245	0.0267
hsa-mir-521-2	3	0.55291	0.56583	0.99998	13246	-0.9148
MAP4K3	6	0.55294	0.6931	0.99998	13247	0.0382
APOBEC3F	6	0.5531	0.69324	0.99998	13248	0.0639
SNX4	6	0.55319	0.69332	0.99998	13249	0.1377
EFS	6	0.55319	0.69332	0.99998	13250	0.1731
TTC38	6	0.55327	0.69339	0.99998	13251	0.0529
HAS1	6	0.55327	0.69339	0.99998	13252	0.2187
IAPP	6	0.55331	0.69342	0.99998	13253	-0.1155
RDX	6	0.55331	0.69342	0.99998	13254	-0.061
KRIT1	6	0.55339	0.6935	0.99998	13255	0.3323
PYURF	6	0.55339	0.6935	0.99998	13256	-0.078
SETD1B	6	0.55339	0.6935	0.99998	13257	0.0504
TAB1	6	0.55339	0.6935	0.99998	13258	-0.1136
FLRT3	6	0.55349	0.69358	0.99998	13259	-0.1076
OR8U8	3	0.55362	0.56642	0.99998	13260	-0.0477
SUSD5	6	0.55366	0.69372	0.99998	13261	-0.0947
hsa-mir-661	4	0.55369	0.60805	0.99998	13262	0.1499
EXOC8	6	0.55374	0.69378	0.99998	13263	-0.0674
CHI3L1	6	0.55382	0.69385	0.99998	13264	0.1279
DEFB104A	1	0.55384	0.55377	0.99998	13265	0.0391
SLC29A4	6	0.55395	0.69395	0.99998	13266	0.0043
GFM2	6	0.55395	0.69395	0.99998	13267	-0.1064
DBNDD1	6	0.554	0.69399	0.99998	13268	0.1121
FBLN1	6	0.554	0.69399	0.99998	13269	0.0057
PSTPIP1	6	0.554	0.69399	0.99998	13270	0.2957
CERS4	6	0.55411	0.69408	0.99998	13271	-0.2029
CEP97	6	0.55411	0.69408	0.99998	13272	-0.0029
hsa-mir-7-1	4	0.55423	0.60836	0.99998	13273	0.01
DAAM1	6	0.55425	0.6942	0.99998	13274	0.0276
AGBL5	6	0.55439	0.69431	0.99998	13275	0.1492
FRMD4B	6	0.55448	0.69438	0.99998	13276	0.3706
PSME4	6	0.55449	0.6944	0.99998	13277	-0.0369
IQCB1	6	0.55462	0.6945	0.99998	13278	-0.1058
OR5AR1	6	0.55462	0.6945	0.99998	13279	0.0644
ZNF239	6	0.55469	0.69457	0.99998	13280	0.0612
GHITM	6	0.55479	0.69464	0.99998	13281	0.1741

CYP46A1	6	0.5549	0.69474	0.99998	13282	0.1686
DGAT2	6	0.55495	0.69479	0.99998	13283	0.1792
GIMAP7	6	0.55504	0.69486	0.99998	13284	0.1029
KRTAP10-2	6	0.55513	0.69494	0.99998	13285	-0.2102
EMC2	6	0.55517	0.69497	0.99998	13286	0.1838
PPP1R12A	6	0.55517	0.69497	0.99998	13287	-0.0247
KCNK17	6	0.55517	0.69497	0.99998	13288	-0.1739
hsa-mir-4657	4	0.55521	0.60894	0.99998	13289	0.1552
BET1L	6	0.55542	0.69519	0.99998	13290	0.2096
PABPN1L	6	0.55542	0.69519	0.99998	13291	0.0542
RAB7L1	6	0.55545	0.69522	0.99998	13292	0.0101
KIAA1217	6	0.55545	0.69522	0.99998	13293	0.073
FMNL2	6	0.55545	0.69522	0.99998	13294	-0.0901
CCDC91	6	0.55545	0.69522	0.99998	13295	0.0798
FZD5	6	0.55545	0.69522	0.99998	13296	0.3253
SLC30A6	6	0.55545	0.69522	0.99998	13297	0.0287
IL5RA	4	0.5556	0.60918	0.99998	13298	0.1475
COX7A1	6	0.55561	0.69537	0.99998	13299	0.2006
PDZD11	6	0.55563	0.69539	0.99998	13300	-0.0671
CLPTM1L	6	0.55576	0.6955	0.99998	13301	-0.0492
TIMP1	6	0.55576	0.6955	0.99998	13302	0.0441
CBFA2T2	6	0.55576	0.6955	0.99998	13303	0.0464
MOXD1	6	0.55576	0.6955	0.99998	13304	-0.0239
ADAMTS10	6	0.55576	0.6955	0.99998	13305	0.0034
KCNE2	6	0.55576	0.6955	0.99998	13306	-0.0255
TCEAL4	6	0.55576	0.6955	0.99998	13307	-0.1188
PI3	6	0.55598	0.69568	0.99998	13308	0.0288
TXNIP	6	0.55598	0.69568	0.99998	13309	0.0614
RAB31	6	0.55622	0.69587	0.99998	13310	0.12
KRT13	6	0.55624	0.69588	0.99998	13311	-0.0244
hsa-mir-1258	4	0.55631	0.60959	0.99998	13312	0.1727
MAN1C1	6	0.55635	0.69597	0.99998	13313	0.1572
SPINK14	6	0.55635	0.69597	0.99998	13314	-0.0099
MTAP	6	0.55635	0.69597	0.99998	13315	0.2645
SH3D19	6	0.55635	0.69597	0.99998	13316	-0.0631
PCSK1N	6	0.55636	0.69598	0.99998	13317	0.0452
TUBG2	4	0.55645	0.60967	0.99998	13318	0.0963
CLASP1	6	0.55655	0.69614	0.99998	13319	-0.1087
ZNF223	6	0.55665	0.69623	0.99998	13320	0.1192
S100A8	6	0.55674	0.69631	0.99998	13321	-0.1401
ZNF136	6	0.5568	0.69636	0.99998	13322	-0.0869
PIGZ	6	0.55684	0.6964	0.99998	13323	0.1036
SLC12A3	6	0.55688	0.69643	0.99998	13324	0.2219
hsa-mir-6510	4	0.55695	0.60997	0.99998	13325	0.0099
SH2D7	6	0.55705	0.69657	0.99998	13326	0.0387
FHOD3	6	0.55705	0.69657	0.99998	13327	0.0087
PTPRJ	6	0.55705	0.69657	0.99998	13328	-0.0174
OR1F1	6	0.55711	0.69662	0.99998	13329	0.0596
RRAD	6	0.55723	0.69673	0.99998	13330	0.0986
PIK3C3	6	0.55723	0.69673	0.99998	13331	-0.1547
DPP10	6	0.55725	0.69674	0.99998	13332	0.0979
FBP2	6	0.55725	0.69674	0.99998	13333	-0.0261
SNAP91	5	0.55738	0.66627	0.99998	13334	0.2484
TNFSF9	6	0.55745	0.69691	0.99998	13335	-0.1822
MSI2	6	0.55752	0.69696	0.99998	13336	-0.0942
CD300C	6	0.55752	0.69696	0.99998	13337	0.2001
SLC25A47	6	0.55759	0.69702	0.99998	13338	0.0282
FMN2	6	0.55759	0.69702	0.99998	13339	0.252
MARK3	6	0.55759	0.69702	0.99998	13340	-0.2243
PATE2	6	0.55763	0.69705	0.99998	13341	0.0847
hsa-mir-3683	4	0.5577	0.61045	0.99998	13342	0.1889
PPM1N	6	0.55781	0.69721	0.99998	13343	0.0308
TMEM194B	6	0.55782	0.69722	0.99998	13344	-0.1491
C5AR1	6	0.55797	0.69734	0.99998	13345	-0.1176
SNPH	6	0.55804	0.6974	0.99998	13346	0.1791
MUC4	6	0.55818	0.69751	0.99998	13347	0.2176
NELFE	6	0.55818	0.69751	0.99998	13348	0.074
NCCRP1	6	0.55841	0.69769	0.99998	13349	0.0944
BPIFA2	6	0.55841	0.69769	0.99998	13350	0.1793
GPATCH2	6	0.55841	0.69769	0.99998	13351	0.0689
ZXDB	6	0.55841	0.69769	0.99998	13352	0.141
EPC1	6	0.55841	0.69769	0.99998	13353	-0.1567
hsa-let-7d	4	0.55843	0.61088	0.99998	13354	0.1784
hsa-mir-770	4	0.55843	0.61088	0.99998	13355	-0.0901
YPEL4	6	0.55843	0.69771	0.99998	13356	0.183
ITGB3	6	0.55843	0.69771	0.99998	13357	0.0501
SLC35G2	6	0.55861	0.69787	0.99998	13358	0.0689
TUSC3	6	0.55861	0.69787	0.99998	13359	0.0957
LAD1	6	0.55876	0.69798	0.99998	13360	-0.0738
TMEM181	6	0.55876	0.69798	0.99998	13361	-0.0656
CMC4	6	0.5589	0.69808	0.99998	13362	0.0655

SMO	6	0.55891	0.69808	0.99998	13363	-0.1319
TAF7L	4	0.55894	0.61117	0.99998	13364	0.4246
SHH	6	0.55898	0.69815	0.99998	13365	-0.0631
AMY2B	3	0.55901	0.57077	0.99998	13366	-0.0671
ACRBP	6	0.55911	0.69825	0.99998	13367	-0.0662
YIPF2	6	0.55911	0.69825	0.99998	13368	0.1581
hsa-mir-1200	2	0.55914	0.55924	0.99998	13369	-0.0057
GSG1L	6	0.55926	0.69838	0.99998	13370	0.037
CPEB3	6	0.55926	0.69838	0.99998	13371	0.3208
DEFB123	6	0.55938	0.69849	0.99998	13372	0.0056
ERBB2IP	6	0.55942	0.69852	0.99998	13373	0.0008
PEX26	6	0.55942	0.69852	0.99998	13374	-0.1039
SBK1	6	0.55968	0.69874	0.99998	13375	0.0032
C17orf100	6	0.55968	0.69874	0.99998	13376	-0.0394
CCDC85C	6	0.55974	0.69879	0.99998	13377	-0.0388
CSN1S1	6	0.55974	0.69879	0.99998	13378	0.0299
PEA15	6	0.55991	0.69892	0.99998	13379	-0.0428
DNAJC13	6	0.55991	0.69892	0.99998	13380	0.1114
CDH15	6	0.55991	0.69892	0.99998	13381	-0.0036
MTF1	6	0.55991	0.69892	0.99998	13382	-0.0659
KAZALD1	6	0.55991	0.69892	0.99998	13383	0.0394
OR10G3	6	0.55991	0.69892	0.99998	13384	-0.0348
CXCR5	6	0.55991	0.69892	0.99998	13385	-0.1468
ELMSAN1	6	0.56004	0.69904	0.99998	13386	-0.0716
MANF	6	0.56004	0.69904	0.99998	13387	0.0466
MS4A6A	6	0.56004	0.69904	0.99998	13388	-0.0852
ZNF282	6	0.56013	0.69912	0.99998	13389	0.1278
hsa-mir-6837	4	0.56013	0.61188	0.99998	13390	0.0978
CORO1C	6	0.56025	0.6992	0.99998	13391	-0.1035
FBXL8	6	0.56026	0.69921	0.99998	13392	-0.0245
ITIH2	6	0.56032	0.69926	0.99998	13393	0.2301
CCDC83	6	0.56032	0.69926	0.99998	13394	0.1001
CLUH	6	0.56051	0.69943	0.99998	13395	0.072
ZNF232	6	0.56051	0.69943	0.99998	13396	0.2535
hsa-mir-378j	4	0.56063	0.61219	0.99998	13397	0.2852
DCC	6	0.56064	0.69954	0.99998	13398	0.0422
RUNX1	6	0.56064	0.69954	0.99998	13399	0.0862
PXN	6	0.56064	0.69954	0.99998	13400	0.0069
DHRS7C	6	0.56079	0.69966	0.99998	13401	-0.0529
MMEL1	6	0.56081	0.69968	0.99998	13402	-0.0531
ZP1	6	0.56088	0.69976	0.99998	13403	0.3898
LRCOL1	6	0.56105	0.69991	0.99998	13404	0.1869
ADH5	6	0.56105	0.69991	0.99998	13405	-0.0177
SCRT2	6	0.56118	0.70003	0.99998	13406	-0.015
NUP37	6	0.56118	0.70003	0.99998	13407	0.0883
TRPA1	6	0.56128	0.70011	0.99998	13408	-0.1313
OR2T34	6	0.56128	0.70011	0.99998	13409	-0.9148
MEGF10	6	0.56128	0.70011	0.99998	13410	-0.0868
SUPT20HL2	6	0.56128	0.70011	0.99998	13411	-0.014
USP17L7	6	0.56128	0.70011	0.99998	13412	0.0184
ZNF729	6	0.56128	0.70011	0.99998	13413	-0.1071
KRTAP19-6	6	0.56128	0.70011	0.99998	13414	0.2183
HNRNPH3	6	0.56131	0.70014	0.99998	13415	0.0813
NR4A1	6	0.56131	0.70014	0.99998	13416	0.0511
hsa-mir-548h-	3	0.56162	0.57289	0.99998	13417	-0.3622
FAM153B	2	0.56169	0.56177	0.99998	13418	-0.0549
HIF1A	6	0.56176	0.7005	0.99998	13419	0.1472
CKM	6	0.56176	0.7005	0.99998	13420	-0.0091
LILRB3	6	0.56181	0.70055	0.99998	13421	0.065
IFI44L	6	0.56181	0.70055	0.99998	13422	0.0221
UBAP2	6	0.56188	0.70061	0.99998	13423	0.2499
LDHC	6	0.56202	0.70074	0.99998	13424	0.0407
DTD2	6	0.56217	0.70087	0.99998	13425	-0.1175
LPAR4	6	0.56222	0.70091	0.99998	13426	0.0225
STX8	6	0.56235	0.70103	0.99998	13427	0.0279
UBE2L3	6	0.56243	0.70109	0.99998	13428	-0.0445
ZBTB25	6	0.56248	0.70114	0.99998	13429	0.0748
RAPGEF1	6	0.56248	0.70114	0.99998	13430	0.0694
IL9	6	0.56248	0.70114	0.99998	13431	-0.135
PLEKHF2	6	0.56267	0.7013	0.99998	13432	0.1137
TXNDC15	6	0.56282	0.70144	0.99998	13433	-0.0977
COLEC12	6	0.56282	0.70144	0.99998	13434	-0.222
ZDHHC17	6	0.56282	0.70144	0.99998	13435	0.0799
hsa-mir-3663	4	0.56291	0.61357	0.99998	13436	0.2255
SULT1C4	6	0.56296	0.70156	0.99998	13437	-0.0528
UTRN	6	0.56329	0.70184	0.99998	13438	0.1813
IL18	6	0.56333	0.70187	0.99998	13439	-0.0373
FAM157A	3	0.56352	0.57445	0.99998	13440	0.0902
KRT40	6	0.56353	0.70205	0.99998	13441	0.153
SP110	6	0.56353	0.70205	0.99998	13442	0.0684
STK3	6	0.56353	0.70205	0.99998	13443	-0.215

BUB1	6	0.56371	0.7022	0.99998	13444	0.0895
SFN	4	0.56376	0.61411	0.99998	13445	-0.0617
DCAKD	6	0.56376	0.70225	0.99998	13446	-0.0545
CRIP3	6	0.56383	0.70231	0.99998	13447	-0.1045
hsa-mir-5582	2	0.56401	0.5641	0.99998	13448	-0.3289
hsa-mir-3689a	2	0.56401	0.5641	0.99998	13449	-0.3916
KRTAP2-3	2	0.56401	0.5641	0.99998	13450	-0.5374
RGPD2	2	0.56401	0.5641	0.99998	13451	-0.1081
RBAK-RBAKDN	2	0.56401	0.5641	0.99998	13452	-0.4266
hsa-mir-105-2	2	0.56401	0.5641	0.99998	13453	-0.4293
hsa-mir-520d	2	0.56401	0.5641	0.99998	13454	0.0362
SSX2	2	0.56401	0.5641	0.99998	13455	-0.6385
POLR2J	2	0.56401	0.5641	0.99998	13456	-0.5559
OR2A4	2	0.56401	0.5641	0.99998	13457	-0.3608
hsa-mir-1283-	2	0.56401	0.5641	0.99998	13458	-0.3608
FRG2B	2	0.56401	0.5641	0.99998	13459	-0.4855
TMSB15A	2	0.56401	0.5641	0.99998	13460	-0.9148
SLC35E2	2	0.56401	0.5641	0.99998	13461	-0.4588
MSH5	6	0.56406	0.70249	0.99998	13462	0.0967
CCDC150	6	0.56421	0.70262	0.99998	13463	-0.049
hsa-mir-6734	4	0.56427	0.61441	0.99998	13464	-0.1241
TEAD3	6	0.56428	0.70267	0.99998	13465	0.037
NEDD4L	6	0.56453	0.70287	0.99998	13466	0.0498
ACE2	6	0.56464	0.70297	0.99998	13467	0.1704
PRPF38A	6	0.56469	0.70302	0.99998	13468	0.0718
FIBCD1	6	0.56469	0.70302	0.99998	13469	-0.1123
C3orf80	6	0.56469	0.70302	0.99998	13470	-0.0119
ZNF30	6	0.56479	0.70311	0.99998	13471	-0.0916
SYN1	6	0.5649	0.7032	0.99998	13472	-0.0675
ABCC6	6	0.5649	0.7032	0.99998	13473	-0.0805
PNOC	6	0.56511	0.70337	0.99998	13474	-0.0891
HMOX1	6	0.56511	0.70337	0.99998	13475	0.0893
ROBO1	6	0.56511	0.70337	0.99998	13476	0.0077
PAIP1	6	0.56511	0.70337	0.99998	13477	-0.0592
CTBP1	6	0.56519	0.70345	0.99998	13478	0.0473
NCKAP1	6	0.56537	0.70361	0.99998	13479	0.055
THAP3	6	0.56539	0.70362	0.99998	13480	0.1242
LPAR3	6	0.56552	0.70374	0.99998	13481	-0.04
WDFY4	6	0.56572	0.70391	0.99998	13482	0.0891
MEN1	6	0.56572	0.70391	0.99998	13483	-0.0806
PLCD1	6	0.56574	0.70393	0.99998	13484	-0.0514
ZBTB11	6	0.56585	0.70402	0.99998	13485	0.2536
hsa-mir-4741	4	0.56595	0.61539	0.99998	13486	0.0144
ZNF710	6	0.56605	0.70418	0.99998	13487	0.1556
LMF2	6	0.56605	0.70418	0.99998	13488	0.1978
APH1A	6	0.56605	0.70418	0.99998	13489	0.0262
VSIG10L	6	0.56611	0.70423	0.99998	13490	0.0383
C2orf76	6	0.56635	0.70444	0.99998	13491	0.0092
CELF5	6	0.56643	0.70451	0.99998	13492	0.0117
PRODH2	6	0.56648	0.70456	0.99998	13493	0.0574
ZNF735	5	0.56656	0.67023	0.99998	13494	-0.0951
GNA13	6	0.5666	0.70468	0.99998	13495	0.1413
TOMM6	6	0.5666	0.70468	0.99998	13496	-0.0505
RNF208	6	0.56666	0.70472	0.99998	13497	0.2417
EIF3CL	1	0.5667	0.56665	0.99998	13498	0.0523
WNK3	6	0.56676	0.70482	0.99998	13499	-0.0391
SBF2	6	0.56686	0.70492	0.99998	13500	0.0141
CHCHD1	6	0.56686	0.70492	0.99998	13501	0.0944
LRRC41	6	0.56686	0.70492	0.99998	13502	0.2163
ZNF148	6	0.56686	0.70492	0.99998	13503	-0.0674
MTRF1L	6	0.56701	0.70504	0.99998	13504	0.0473
TNNC2	6	0.56701	0.70504	0.99998	13505	0.2555
H2BFWT	6	0.56701	0.70504	0.99998	13506	0.1116
LOC10013088	6	0.56705	0.70507	0.99998	13507	0.0303
ABHD12B	6	0.56705	0.70507	0.99998	13508	0.1788
ATL1	6	0.56705	0.70507	0.99998	13509	0.2367
C3orf70	6	0.56705	0.70507	0.99998	13510	0.0639
RHCG	6	0.56718	0.70519	0.99998	13511	-0.0828
SMCP	6	0.56718	0.70519	0.99998	13512	-0.0052
PLA2G4B	6	0.56739	0.70535	0.99998	13513	0.1488
VWF	6	0.56739	0.70535	0.99998	13514	-0.0255
KDM5C	6	0.56744	0.7054	0.99998	13515	-0.0729
RASAL3	6	0.56744	0.7054	0.99998	13516	-0.0801
CDK10	6	0.56758	0.70552	0.99998	13517	0.2748
CABP5	6	0.56758	0.70552	0.99998	13518	0.1165
ESCO2	6	0.56781	0.70572	0.99998	13519	0.0676
NCAPG2	6	0.56781	0.70572	0.99998	13520	0.17
LCE2A	4	0.56788	0.61654	0.99998	13521	0.1646
PGAP2	6	0.56788	0.70577	0.99998	13522	0.1221
SULT4A1	6	0.56788	0.70577	0.99998	13523	-0.0706
EML5	6	0.56788	0.70577	0.99998	13524	-0.0213

KRTAP9-8	4	0.56803	0.61663	0.99998	13525	-0.367
PLA2G4E	6	0.56804	0.70593	0.99998	13526	0.0883
KCNMA1	6	0.56816	0.70605	0.99998	13527	-0.0163
GPRIN1	6	0.56817	0.70606	0.99998	13528	-0.1344
ESYT1	6	0.56817	0.70606	0.99998	13529	0.2638
hsa-mir-6895	4	0.56826	0.61676	0.99998	13530	-0.0248
RHOA	6	0.56833	0.70618	0.99998	13531	0.0967
hsa-mir-7855	4	0.56841	0.61686	0.99998	13532	-0.0171
ITGB5	6	0.56842	0.70626	0.99998	13533	-0.032
MICAL2	6	0.56842	0.70626	0.99998	13534	-0.0802
MAGEB2	6	0.56842	0.70626	0.99998	13535	-0.0117
PLD6	6	0.56842	0.70626	0.99998	13536	0.1318
MGAT4C	6	0.56846	0.7063	0.99998	13537	0.0576
ART1	6	0.56846	0.7063	0.99998	13538	0.0597
ANKRD18B	5	0.56855	0.67112	0.99998	13539	-0.0499
OR13C8	6	0.56856	0.70639	0.99998	13540	0.1312
TOMM7	6	0.56862	0.70643	0.99998	13541	0.0459
MCU	6	0.56867	0.70646	0.99998	13542	0.1929
HLCS	6	0.56868	0.70648	0.99998	13543	0.0045
TLK1	6	0.56868	0.70648	0.99998	13544	-0.0125
KCNA4	6	0.56868	0.70648	0.99998	13545	0.0643
NOXO1	6	0.56875	0.70654	0.99998	13546	-0.0229
IZUMO3	6	0.56875	0.70654	0.99998	13547	0.0831
ATP6V1B1	6	0.56886	0.70664	0.99998	13548	0.0123
FAM117B	6	0.56888	0.70666	0.99998	13549	0.0797
BEND6	6	0.56895	0.70673	0.99998	13550	0.1169
FITM1	6	0.56895	0.70673	0.99998	13551	0.2026
KRTAP2-4	1	0.56902	0.5689	0.99998	13552	0.257
MLL2	2	0.56903	0.56912	0.99998	13553	-0.1376
CHRNA9	6	0.56913	0.70687	0.99998	13554	-0.1346
hsa-mir-490	4	0.56924	0.61735	0.99998	13555	0.243
SLC2A13	6	0.56926	0.70699	0.99998	13556	-0.1354
F9	6	0.56933	0.70705	0.99998	13557	0.1804
hsa-mir-8081	4	0.56941	0.61745	0.99998	13558	0.3694
KRTAP19-3	6	0.56958	0.70726	0.99998	13559	-0.2259
ZNF570	6	0.56958	0.70726	0.99998	13560	-0.0201
GFOD2	6	0.56958	0.70726	0.99998	13561	0.0121
FKBP7	6	0.56958	0.70726	0.99998	13562	-0.0316
CD79B	6	0.56958	0.70726	0.99998	13563	0.0936
C9orf139	6	0.56958	0.70726	0.99998	13564	0.0311
MYH8	6	0.56958	0.70726	0.99998	13565	-0.0453
TBKBP1	6	0.56965	0.70732	0.99998	13566	0.0129
hsa-mir-568	4	0.56992	0.61777	0.99998	13567	0.0713
hsa-mir-4446	4	0.56992	0.61777	0.99998	13568	0.4333
ZNF7	6	0.56997	0.70759	0.99998	13569	-0.0347
PDE3A	6	0.56997	0.70759	0.99998	13570	-0.0014
OR5B12	6	0.56998	0.70761	0.99998	13571	0.2575
TRIM27	6	0.5701	0.7077	0.99998	13572	-0.0198
hsa-mir-7152	4	0.57017	0.61793	0.99998	13573	0.0292
BEND4	6	0.57019	0.70779	0.99998	13574	0.0885
DUS2	2	0.57043	0.57052	0.99998	13575	0.0992
RDM1	6	0.57053	0.70808	0.99998	13576	-0.108
HTR1B	6	0.57058	0.70811	0.99998	13577	-0.1032
UGT2A3	6	0.57064	0.70816	0.99998	13578	-0.0061
ABHD3	6	0.57064	0.70816	0.99998	13579	0.0206
AFMID	6	0.57069	0.70822	0.99998	13580	-0.0492
SAG	6	0.57074	0.70826	0.99998	13581	0.0081
TOMM5	6	0.57074	0.70826	0.99998	13582	0.0884
FAM60A	6	0.57081	0.70831	0.99998	13583	-0.1444
LRRC4B	6	0.57081	0.70831	0.99998	13584	0.0155
BDH1	6	0.57081	0.70831	0.99998	13585	0.0948
TMEM72	6	0.57086	0.70835	0.99998	13586	0.1229
hsa-mir-320a	3	0.57087	0.58048	0.99998	13587	0.4168
SPDYE4	6	0.57095	0.70842	0.99998	13588	-0.0083
RXFP2	6	0.57105	0.70851	0.99998	13589	0.0011
TAS2R10	6	0.57105	0.70851	0.99998	13590	0.0376
TCHP	6	0.57105	0.70851	0.99998	13591	0.1017
DMBT1	6	0.57126	0.70869	0.99998	13592	0.002
SERPINA4	6	0.57129	0.70872	0.99998	13593	-0.0127
TM7SF2	6	0.57139	0.7088	0.99998	13594	0.114
GABRD	6	0.5714	0.7088	0.99998	13595	0.026
DMTN	6	0.5715	0.70888	0.99998	13596	0.3238
MAPK1IP1L	6	0.57163	0.70899	0.99998	13597	0.0213
TXNDC8	6	0.57163	0.70899	0.99998	13598	0.1923
IVNS1ABP	6	0.57164	0.70901	0.99998	13599	-0.0485
TFB2M	6	0.57164	0.70901	0.99998	13600	0.2976
CCDC169-SOH	5	0.57182	0.67254	0.99998	13601	0.3715
TBC1D25	6	0.57183	0.70917	0.99998	13602	0.2154
TLE4	6	0.57183	0.70917	0.99998	13603	-0.0714
hsa-mir-3619	4	0.5719	0.61899	0.99998	13604	0.181
PHLDB2	6	0.57204	0.70935	0.99998	13605	0.024

APLN	6	0.57204	0.70935	0.99998	13606	0.1181
MID2	6	0.57205	0.70936	0.99998	13607	0.128
ERCC5	6	0.57205	0.70936	0.99998	13608	0.0422
hsa-mir-2861	4	0.57211	0.61911	0.99998	13609	0.0429
C19orf24	6	0.57225	0.70953	0.99998	13610	0.1611
MAK	6	0.57225	0.70953	0.99998	13611	0.1957
GNB5	6	0.57236	0.70962	0.99998	13612	-0.0201
SAMD12	6	0.57236	0.70962	0.99998	13613	0.0097
HSD17B13	6	0.57236	0.70962	0.99998	13614	-0.0134
STON1	3	0.57257	0.58189	0.99998	13615	0.1491
hsa-mir-548av	4	0.57272	0.6195	0.99998	13616	-0.111
ANKRD35	6	0.57274	0.70994	0.99998	13617	0.0952
ACVR1B	6	0.57274	0.70994	0.99998	13618	0.102
TMEM174	6	0.57276	0.70996	0.99998	13619	0.1545
RNF13	6	0.57284	0.71003	0.99998	13620	-0.0169
TNFRSF8	6	0.57284	0.71003	0.99998	13621	-0.0587
SLC6A12	6	0.57298	0.71015	0.99998	13622	-0.1314
CCDC173	6	0.57298	0.71015	0.99998	13623	-0.0366
FAIM	6	0.57298	0.71015	0.99998	13624	-0.1639
DMD	6	0.57298	0.71015	0.99998	13625	0.0952
GGACT	6	0.57311	0.71025	0.99998	13626	0.0682
UBE2H	6	0.57327	0.7104	0.99998	13627	0.0619
KIAA1210	6	0.57327	0.7104	0.99998	13628	0.0815
TRANK1	6	0.57334	0.71046	0.99998	13629	-0.1577
TRIM16L	5	0.57345	0.67322	0.99998	13630	0.257
hsa-mir-6892	4	0.5735	0.61998	0.99998	13631	0.03
PSKH2	6	0.5735	0.71059	0.99998	13632	-0.0264
SLC39A1	6	0.5735	0.71059	0.99998	13633	-0.0796
MAGT1	6	0.5735	0.71059	0.99998	13634	-0.0699
AFM	6	0.5735	0.71059	0.99998	13635	0.0181
CORO6	6	0.57367	0.71074	0.99998	13636	0.1704
MAP3K1	6	0.5738	0.71084	0.99998	13637	0.0447
SOX10	6	0.5738	0.71084	0.99998	13638	-0.0119
PREX2	6	0.5738	0.71084	0.99998	13639	0.0343
RUSC1	6	0.5738	0.71084	0.99998	13640	0.2302
SMIM5	6	0.57384	0.71087	0.99998	13641	0.1687
SNX3	6	0.57397	0.71098	0.99998	13642	0.0478
C5orf15	6	0.57401	0.71102	0.99998	13643	0.3589
GFRA2	6	0.57401	0.71102	0.99998	13644	-0.079
DHRS11	6	0.57408	0.71107	0.99998	13645	0.1162
TET3	6	0.57408	0.71107	0.99998	13646	0.0877
PAX2	6	0.57412	0.7111	0.99998	13647	0.0686
HAVCR2	6	0.57422	0.7112	0.99998	13648	0.27
NCKIPSD	6	0.57424	0.71122	0.99998	13649	-0.1249
TMEM132D	6	0.57424	0.71122	0.99998	13650	0.0553
MT1B	4	0.57426	0.62044	0.99998	13651	-0.0703
EFNA2	6	0.57435	0.7113	0.99998	13652	0.2615
hsa-mir-521-1	2	0.57437	0.57437	0.99998	13653	0.0251
hsa-mir-3689e	2	0.57437	0.57437	0.99998	13654	-0.2845
hsa-mir-620	2	0.57437	0.57437	0.99998	13655	-0.4458
FOXN3	6	0.57437	0.71132	0.99998	13656	0.0265
HBEGF	6	0.57445	0.71138	0.99998	13657	0.081
ADA	6	0.57445	0.71138	0.99998	13658	-0.1148
NDUFAF5	6	0.57453	0.71146	0.99998	13659	-0.1167
RAB32	6	0.57476	0.71164	0.99998	13660	0.111
TXNDC9	6	0.57476	0.71164	0.99998	13661	0.0079
SYNM	6	0.57476	0.71164	0.99998	13662	0.0951
MB21D1	6	0.57477	0.71165	0.99998	13663	-0.1845
C8orf42	3	0.57494	0.5839	0.99998	13664	-0.0934
TNFRSF10B	6	0.57498	0.71182	0.99998	13665	-0.147
AKR1B1	6	0.57521	0.71204	0.99998	13666	0.0675
FNDC7	6	0.57523	0.71205	0.99998	13667	-0.1584
DOHH	6	0.57523	0.71205	0.99998	13668	-0.0855
OR51B6	6	0.57523	0.71205	0.99998	13669	0.0873
CYB5D1	6	0.57523	0.71205	0.99998	13670	0.1563
MAGEB16	6	0.57531	0.71211	0.99998	13671	-0.0444
RPGR	6	0.57533	0.71213	0.99998	13672	0.072
DUSP5	6	0.57547	0.71224	0.99998	13673	0.0366
hsa-mir-548q	3	0.57548	0.58434	0.99998	13674	-0.9148
RNF148	6	0.5756	0.71235	0.99998	13675	0.1268
GREB1L	6	0.57565	0.71239	0.99998	13676	0.1754
CLUAP1	6	0.5757	0.71243	0.99998	13677	0.0302
TRA2A	6	0.5757	0.71243	0.99998	13678	0.2042
B3GNT9	6	0.5757	0.71243	0.99998	13679	0.1671
HDC	6	0.5757	0.71243	0.99998	13680	0.1443
IFNW1	6	0.5757	0.71243	0.99998	13681	0.1274
BCL7B	6	0.57574	0.71247	0.99998	13682	-0.0961
TBC1D17	6	0.57588	0.71259	0.99998	13683	0.0869
GRK5	6	0.57589	0.7126	0.99998	13684	0.04
MUL1	6	0.576	0.71271	0.99998	13685	-0.0416
GPRASP2	6	0.57607	0.71276	0.99998	13686	-0.0753

POLR2M	6	0.57611	0.7128	0.99998	13687	-0.0959
PRND	6	0.57618	0.71287	0.99998	13688	-0.0692
NPBWR2	6	0.57618	0.71287	0.99998	13689	0.0412
FOXL1	6	0.57626	0.71293	0.99998	13690	0.1195
POU5F2	6	0.5764	0.71305	0.99998	13691	0.2474
PPP1R12B	6	0.5764	0.71305	0.99998	13692	0.0925
CDHR5	6	0.5764	0.71305	0.99998	13693	0.0856
C7orf33	6	0.57641	0.71306	0.99998	13694	0.0302
SLC7A3	6	0.57641	0.71306	0.99998	13695	0.0334
KIAA0922	6	0.57658	0.7132	0.99998	13696	0.1234
ADRA1B	6	0.57665	0.71326	0.99998	13697	0.0527
SOCS4	6	0.57665	0.71326	0.99998	13698	0.0669
RNF24	6	0.5767	0.7133	0.99998	13699	0.1412
MEIG1	6	0.57674	0.71334	0.99998	13700	-0.1091
E2F5	6	0.57677	0.71337	0.99998	13701	0.1498
HNRNPUL2	6	0.57681	0.7134	0.99998	13702	0.1064
TMEM100	6	0.57688	0.71345	0.99998	13703	0.0714
CLEC4M	6	0.57688	0.71345	0.99998	13704	-0.1789
TESK1	6	0.57688	0.71345	0.99998	13705	-0.0627
RPIA	6	0.57688	0.71345	0.99998	13706	-0.0971
LRRC55	6	0.57689	0.71346	0.99998	13707	0.028
TPD52L3	6	0.57695	0.7135	0.99998	13708	0.0887
PRRT4	6	0.57701	0.71356	0.99998	13709	0.1994
ZNF420	6	0.57705	0.71359	0.99998	13710	0.2675
GNE	6	0.57705	0.71359	0.99998	13711	-0.0926
OR6S1	6	0.57705	0.71359	0.99998	13712	0.0094
PDPK1	6	0.57718	0.7137	0.99998	13713	-0.0859
FMN1	6	0.57736	0.71384	0.99998	13714	0.0782
CC2D2A	6	0.57736	0.71384	0.99998	13715	-0.051
B3GAT2	4	0.57738	0.6224	0.99998	13716	0.2484
TGFBR1	6	0.57747	0.71394	0.99998	13717	-0.0276
SETD8	6	0.57747	0.71394	0.99998	13718	0.2194
HLA-DQA2	6	0.57747	0.71394	0.99998	13719	-0.1332
TMC5	6	0.57747	0.71394	0.99998	13720	-0.0675
KRT34	6	0.57747	0.71394	0.99998	13721	-0.0734
TSC22D1	6	0.57747	0.71394	0.99998	13722	0.1321
OR2T2	6	0.57761	0.71406	0.99998	13723	0.4239
OR2V1	6	0.57774	0.71416	0.99998	13724	0.1232
ZNF783	6	0.57774	0.71416	0.99998	13725	-0.0631
hsa-mir-4730	4	0.5778	0.62266	0.99998	13726	0.0287
CROT	6	0.5779	0.71428	0.99998	13727	-0.0078
TRIM24	6	0.5779	0.71429	0.99998	13728	-0.0148
CISD2	5	0.57794	0.67518	0.99998	13729	0.3626
ABCA5	6	0.57797	0.71435	0.99998	13730	0.0782
COQ10B	6	0.57803	0.7144	0.99998	13731	0.1075
P2RY13	6	0.57811	0.71447	0.99998	13732	-0.1151
KIF4B	6	0.57825	0.71458	0.99998	13733	0.0473
MFSD10	6	0.57838	0.71471	0.99998	13734	0.0199
GCNT2	6	0.57851	0.71483	0.99998	13735	0.0599
DLG5	6	0.57851	0.71483	0.99998	13736	-0.0985
hsa-mir-3118-	1	0.57863	0.57843	0.99998	13737	0.0644
SSUH2	6	0.57864	0.71494	0.99998	13738	0.0631
hsa-mir-4520a	1	0.57874	0.57855	0.99998	13739	0.1019
KBTBD11	6	0.57876	0.71505	0.99998	13740	0.0432
CNTN2	6	0.57876	0.71505	0.99998	13741	-0.1134
PROKR2	6	0.57879	0.71507	0.99998	13742	0.0656
ALAD	6	0.57879	0.71507	0.99998	13743	0.0686
GPRC5A	6	0.57892	0.71519	0.99998	13744	0.2141
TNRC6C	6	0.57897	0.71523	0.99998	13745	0.1043
G3BP2	6	0.57897	0.71523	0.99998	13746	0.1894
STX1A	6	0.57906	0.71531	0.99998	13747	0.1401
CHST6	6	0.57908	0.71534	0.99998	13748	0.0487
HSD11B2	6	0.57908	0.71534	0.99998	13749	0.0347
XKRX	6	0.57908	0.71534	0.99998	13750	0.0576
PRKCQ	6	0.57924	0.71549	0.99998	13751	-0.1391
NR4A2	6	0.57926	0.71551	0.99998	13752	0.1502
RFT1	6	0.57939	0.71562	0.99998	13753	0.0823
NDUFA1	6	0.57952	0.71574	0.99998	13754	0.2124
MRPL27	6	0.57952	0.71574	0.99998	13755	-0.0315
SGK1	6	0.57968	0.71588	0.99998	13756	0.1791
TCHH	6	0.57983	0.71601	0.99998	13757	0.0099
GLB1	6	0.57983	0.71601	0.99998	13758	-0.0031
COG1	6	0.57984	0.71603	0.99998	13759	-0.0102
ZER1	6	0.57996	0.71612	0.99998	13760	-0.0241
hsa-mir-5100	4	0.57996	0.62404	0.99998	13761	0.139
hsa-mir-99b	4	0.58015	0.62416	0.99998	13762	0.3292
hsa-mir-658	4	0.58044	0.62432	0.99998	13763	-0.1322
PAPSS2	6	0.58046	0.71656	0.99998	13764	-0.1229
ZSCAN9	6	0.58046	0.71656	0.99998	13765	0.0912
OXSR1	6	0.58053	0.71662	0.99998	13766	-0.0436
SHC3	6	0.58058	0.71665	0.99998	13767	0.1465

ZFP36	6	0.58058	0.71665	0.99998	13768	-0.1375
G2E3	6	0.58058	0.71665	0.99998	13769	-0.0843
KCNJ5	6	0.58066	0.71673	0.99998	13770	-0.066
hsa-mir-603	4	0.58082	0.62458	0.99998	13771	-0.0021
NME2	3	0.58083	0.5888	0.99998	13772	-0.0558
GHRH	6	0.58088	0.71692	0.99998	13773	-0.092
PSMB3	6	0.58096	0.71699	0.99998	13774	-0.0989
HRC	6	0.58096	0.71699	0.99998	13775	0.3734
WDR47	6	0.58096	0.71699	0.99998	13776	-0.0508
PIK3R4	6	0.58117	0.71716	0.99998	13777	0.0957
TRADD	6	0.58128	0.71725	0.99998	13778	0.0958
TWF1	6	0.58151	0.71744	0.99998	13779	0.0733
ACOT12	6	0.58156	0.71749	0.99998	13780	0.0908
NUPR1	6	0.58169	0.7176	0.99998	13781	0.1622
IFNLR1	6	0.58169	0.7176	0.99998	13782	-0.1054
TAF4B	6	0.58169	0.7176	0.99998	13783	0.1898
CLDN8	6	0.58175	0.71766	0.99998	13784	0.1255
L3MBTL1	6	0.58177	0.71767	0.99998	13785	0.1999
TEX15	6	0.58196	0.71782	0.99998	13786	0.0104
INO80	6	0.58214	0.71797	0.99998	13787	-0.1672
ZNF543	6	0.5822	0.71802	0.99998	13788	0.0248
GNA12	6	0.58227	0.71807	0.99998	13789	-0.0234
FABP3	6	0.58234	0.71814	0.99998	13790	0.0374
CX3CR1	6	0.58234	0.71814	0.99998	13791	0.0847
GPR37L1	6	0.58248	0.71825	0.99998	13792	-0.1824
PCBP3	6	0.5825	0.71827	0.99998	13793	0.0894
MTF2	5	0.58251	0.6772	0.99998	13794	0.1409
SLC39A4	6	0.58262	0.71839	0.99998	13795	-0.061
WFDC8	6	0.58262	0.71839	0.99998	13796	0.2053
ABHD17A	6	0.58264	0.7184	0.99998	13797	-0.0608
ASPDH	6	0.58264	0.7184	0.99998	13798	0.056
hsa-mir-6768	4	0.58278	0.62581	0.99998	13799	0.0055
hsa-mir-34a	2	0.58282	0.58284	0.99998	13800	0.0584
TBC1D13	6	0.58288	0.7186	0.99998	13801	-0.0837
DUOXA2	6	0.58289	0.7186	0.99998	13802	0.0792
GPR137C	6	0.58289	0.7186	0.99998	13803	0.3592
UPP1	6	0.58301	0.7187	0.99998	13804	0.0476
PRAC	4	0.58303	0.62598	0.99998	13805	-0.0437
MTBP	6	0.58307	0.71876	0.99998	13806	0.1546
NDUFB5	6	0.58307	0.71876	0.99998	13807	-0.1514
FZD6	6	0.58319	0.71887	0.99998	13808	-0.0122
FAM92A1	6	0.58329	0.71895	0.99998	13809	-0.026
KIAA1804	6	0.58335	0.71901	0.99998	13810	0.0885
PPOX	6	0.58335	0.71901	0.99998	13811	0.1821
SLC25A43	6	0.5834	0.71905	0.99998	13812	-0.1473
SKI	6	0.58353	0.71917	0.99998	13813	0.2673
SPHKAP	6	0.58357	0.7192	0.99998	13814	0.1056
AZIN1	6	0.58387	0.71948	0.99998	13815	0.2218
ANKRD11	6	0.58387	0.71948	0.99998	13816	0.1363
TRAPPC3L	2	0.58394	0.58397	0.99998	13817	0.1773
FBXO41	6	0.58404	0.71961	0.99998	13818	0.1618
IL7	6	0.58404	0.71961	0.99998	13819	0.0187
ACAN	6	0.58404	0.71962	0.99998	13820	-0.1337
THEGL	6	0.58404	0.71962	0.99998	13821	-0.0869
SLAIN2	6	0.58404	0.71962	0.99998	13822	0.1374
C6orf211	6	0.58416	0.71973	0.99998	13823	0.2698
CYB561D2	6	0.58422	0.71978	0.99998	13824	-0.1297
LIMD2	6	0.58422	0.71978	0.99998	13825	0.1348
C1QL4	6	0.58432	0.71988	0.99998	13826	-0.1111
MAB21L3	6	0.5844	0.71994	0.99998	13827	0.1326
ZFPM1	6	0.5844	0.71994	0.99998	13828	-0.041
CCNK	6	0.5844	0.71994	0.99998	13829	0.109
NRCAM	6	0.5844	0.71994	0.99998	13830	-0.0035
C9orf170	6	0.5844	0.71994	0.99998	13831	0.0494
ENO2	6	0.58448	0.72001	0.99998	13832	-0.0003
ZNF623	6	0.58453	0.72004	0.99998	13833	0.1612
hsa-mir-4772	3	0.58457	0.59198	0.99998	13834	-0.1232
LMAN1	6	0.58469	0.72018	0.99998	13835	-0.1049
SESN1	6	0.58469	0.72018	0.99998	13836	-0.0133
P2RX1	6	0.58481	0.72029	0.99998	13837	-0.0776
PLSCR2	6	0.58489	0.72036	0.99998	13838	0.073
ZNF596	6	0.58489	0.72036	0.99998	13839	0.2114
CCRL1	5	0.58489	0.67829	0.99998	13840	-0.072
hsa-mir-539	4	0.5851	0.62731	0.99998	13841	-0.0306
C11orf84	6	0.58519	0.72062	0.99998	13842	0.1929
CNPY4	6	0.58519	0.72062	0.99998	13843	-0.029
CDHR3	6	0.58538	0.72079	0.99998	13844	0.0582
MLL	4	0.58546	0.62757	0.99998	13845	-0.1402
CHST1	6	0.58555	0.72094	0.99998	13846	0.0019
RANBP9	6	0.58555	0.72094	0.99998	13847	-0.1018
PSD4	6	0.58555	0.72094	0.99998	13848	-0.1013

TES	6	0.58555	0.72094	0.99998	13849	-0.043
ATN1	6	0.58555	0.72094	0.99998	13850	0.0483
CTNNA1	6	0.58556	0.72095	0.99998	13851	0.1126
SHISA4	6	0.58556	0.72095	0.99998	13852	0.0672
SREBF1	6	0.58556	0.72095	0.99998	13853	-0.0915
ANKRD18A	6	0.58556	0.72095	0.99998	13854	0.3361
hsa-mir-6506	3	0.58559	0.59287	0.99998	13855	0.4556
SOCS1	6	0.58573	0.72109	0.99998	13856	0.2432
CSRNP1	6	0.58608	0.72142	0.99998	13857	-0.0224
FAM76A	6	0.58608	0.72142	0.99998	13858	0.0425
CLEC7A	6	0.5861	0.72143	0.99998	13859	0.0747
WWTR1	6	0.58623	0.72155	0.99998	13860	0.1482
FAM71D	6	0.58643	0.7217	0.99998	13861	0.177
RSPH9	6	0.58643	0.7217	0.99998	13862	-0.0144
SPATA19	6	0.58648	0.72175	0.99998	13863	0.1114
TERF2IP	6	0.58649	0.72176	0.99998	13864	0.1787
ADAM23	6	0.58663	0.72187	0.99998	13865	0.221
DNAJB11	6	0.58663	0.72187	0.99998	13866	0.0015
ZNF182	6	0.58663	0.72187	0.99998	13867	-0.0108
HIST1H4D	6	0.58672	0.72194	0.99998	13868	-0.1023
CTNNB1	6	0.58676	0.72199	0.99998	13869	-0.1133
RGS1	6	0.58676	0.72199	0.99998	13870	0.1859
AADAT	6	0.58676	0.72199	0.99998	13871	0.0172
GSC	6	0.58689	0.7221	0.99998	13872	0.0618
KCNH8	6	0.58689	0.7221	0.99998	13873	-0.0113
MYO1G	6	0.58696	0.72216	0.99998	13874	0.019
PFKFB4	6	0.58696	0.72216	0.99998	13875	-0.1342
FAM3A	6	0.58705	0.72224	0.99998	13876	0.1208
TNRC6B	6	0.58709	0.72227	0.99998	13877	0.1244
SLC22A11	6	0.58719	0.72235	0.99998	13878	0.1019
SMARCE1	6	0.58719	0.72235	0.99998	13879	0.1224
BBC3	6	0.58739	0.72253	0.99998	13880	0.2473
PDE6H	6	0.5874	0.72254	0.99998	13881	-0.0152
FOS	6	0.5874	0.72254	0.99998	13882	0.066
LRRC66	6	0.58751	0.72263	0.99998	13883	0.1431
NOSIP	6	0.58751	0.72263	0.99998	13884	0.0339
GOLGA1	6	0.58756	0.72266	0.99998	13885	0.3523
AKTIP	6	0.58763	0.72272	0.99998	13886	0.1518
ATP6V0A1	6	0.58763	0.72273	0.99998	13887	-0.1353
GAS8	6	0.5878	0.72286	0.99998	13888	0.0944
ITPKB	6	0.58782	0.72288	0.99998	13889	0.4751
hsa-mir-200b	4	0.58791	0.62912	0.99998	13890	-0.0565
MSANTD3	6	0.58791	0.72296	0.99998	13891	0.0797
ZNF385A	6	0.58792	0.72297	0.99998	13892	0.1947
ZNF804A	6	0.58797	0.72301	0.99998	13893	0.4316
FRAT1	6	0.58804	0.72307	0.99998	13894	-0.0592
CERS3	6	0.58833	0.72331	0.99998	13895	0.2411
PAX6	6	0.58847	0.72344	0.99998	13896	-0.0289
TACO1	6	0.58857	0.72352	0.99998	13897	-0.0624
NME3	6	0.58857	0.72352	0.99998	13898	0.0336
KEL	6	0.58864	0.72359	0.99998	13899	-0.0118
SI	6	0.58864	0.72359	0.99998	13900	0.0826
ABHD2	6	0.58864	0.72359	0.99998	13901	0.3242
PHACTR4	6	0.58911	0.72399	0.99998	13902	-0.0137
OGN	6	0.58911	0.72399	0.99998	13903	0.2026
PRDM1	6	0.58928	0.72413	0.99998	13904	0.1499
CYTH1	6	0.58928	0.72413	0.99998	13905	-0.076
GCNT4	6	0.58928	0.72413	0.99998	13906	0.1863
PNRC1	6	0.58928	0.72413	0.99998	13907	-0.0312
ZNF774	6	0.5893	0.72416	0.99998	13908	0.0348
GOLGA6B	2	0.58935	0.58941	0.99998	13909	-0.0633
SLC25A10	6	0.58935	0.7242	0.99998	13910	-0.0565
ZNF135	6	0.58935	0.7242	0.99998	13911	0.0407
SHARPIN	6	0.58948	0.72432	0.99998	13912	-0.1825
PLIN1	6	0.58948	0.72432	0.99998	13913	0.1306
SNURF	6	0.58948	0.72432	0.99998	13914	0.0721
IL2RG	6	0.58949	0.72434	0.99998	13915	-0.0419
CKLF	6	0.58955	0.7244	0.99998	13916	-0.0961
PRR15L	6	0.58968	0.72451	0.99998	13917	0.171
GRHL1	6	0.58973	0.72455	0.99998	13918	0.0464
IGDCC3	6	0.58982	0.72462	0.99998	13919	-0.1047
ZSCAN4	6	0.58997	0.72475	0.99998	13920	-0.0908
LECT2	6	0.59006	0.72483	0.99998	13921	0.1375
FSCN3	6	0.59006	0.72483	0.99998	13922	0.0458
hsa-mir-944	3	0.59007	0.59671	0.99998	13923	-0.03
CHFR	6	0.5901	0.72486	0.99998	13924	-0.0937
hsa-mir-6076	4	0.59015	0.63051	0.99998	13925	-0.0632
METTL12	6	0.59023	0.72498	0.99998	13926	-0.1374
CNGA2	6	0.59023	0.72498	0.99998	13927	0.0901
GDPD3	6	0.59027	0.72503	0.99998	13928	-0.0595
SP8	6	0.59035	0.7251	0.99998	13929	-0.0561

SULT2B1	6	0.59035	0.7251	0.99998	13930	0.0714
PNLIPRP1	6	0.59035	0.7251	0.99998	13931	0.1527
NOXRED1	6	0.5904	0.72515	0.99998	13932	0.0212
IRF9	6	0.5904	0.72515	0.99998	13933	0.0402
AGTPBP1	6	0.59043	0.72517	0.99998	13934	-0.0808
ZNF519	6	0.59047	0.72521	0.99998	13935	-0.0674
hsa-mir-4800	4	0.59051	0.63074	0.99998	13936	-0.0504
TSPAN16	6	0.59051	0.72526	0.99998	13937	-0.1371
OR10A3	6	0.5906	0.72534	0.99998	13938	0.0875
PNLIP	6	0.59067	0.72539	0.99998	13939	-0.0362
CHRM1	4	0.59071	0.63089	0.99998	13940	-0.1429
GNMT	6	0.59076	0.72547	0.99998	13941	-0.0155
hsa-mir-24-2	4	0.59078	0.63093	0.99998	13942	0.148
PIGA	6	0.5908	0.72552	0.99998	13943	-0.1294
RIPK2	4	0.59085	0.63097	0.99998	13944	-0.1005
DMRTA2	6	0.5909	0.7256	0.99998	13945	0.1398
RORC	5	0.59091	0.68101	0.99998	13946	0.1495
MSC	6	0.59105	0.72574	0.99998	13947	-0.0835
CLEC19A	6	0.59115	0.72583	0.99998	13948	0.0189
PRMT8	6	0.59115	0.72583	0.99998	13949	-0.1072
VIP	6	0.59115	0.72583	0.99998	13950	-0.0649
CDH7	6	0.59115	0.72583	0.99998	13951	-0.0557
HTR2A	6	0.59127	0.72593	0.99998	13952	0.126
KCNQ1	5	0.59131	0.6812	0.99998	13953	0.2649
SLC10A3	4	0.59132	0.63127	0.99998	13954	0.1426
CCNB3	6	0.5914	0.72604	0.99998	13955	-0.147
UBE2D2	6	0.5914	0.72604	0.99998	13956	-0.1154
ZC3H10	6	0.5914	0.72604	0.99998	13957	-0.0318
ATXN7	6	0.5914	0.72604	0.99998	13958	-0.0835
RNF125	6	0.59141	0.72605	0.99998	13959	-0.0874
CCNI2	6	0.59141	0.72605	0.99998	13960	0.2205
hsa-let-7f-1	3	0.59172	0.59811	0.99998	13961	-0.0878
CLEC1A	6	0.59176	0.72637	0.99998	13962	0.2289
TRERF1	6	0.59182	0.72642	0.99998	13963	-0.0502
RYBP	6	0.59182	0.72642	0.99998	13964	0.1524
MOCOS	6	0.59193	0.72651	0.99998	13965	-0.1108
SRGAP1	6	0.59193	0.72651	0.99998	13966	-0.1199
DNAJC5	6	0.59202	0.7266	0.99998	13967	0.298
PPP1R3D	6	0.59211	0.72667	0.99998	13968	-0.092
hsa-let-7a-3	3	0.59215	0.59849	0.99998	13969	0.1008
ZNF668	6	0.59217	0.72673	0.99998	13970	0.0506
ATP8A1	6	0.59232	0.72685	0.99998	13971	-0.0596
CLEC4A	6	0.59232	0.72685	0.99998	13972	-0.0326
KLF17	6	0.59232	0.72685	0.99998	13973	-0.004
ACKR3	3	0.59233	0.59866	0.99998	13974	0.1372
C17orf82	6	0.59242	0.72693	0.99998	13975	0.0606
PQLC2	6	0.59242	0.72693	0.99998	13976	-0.0611
PLD2	6	0.59254	0.72706	0.99998	13977	0.0692
CENPH	3	0.59254	0.59885	0.99998	13978	-0.0775
TEFM	6	0.59259	0.7271	0.99998	13979	-0.0946
hsa-mir-4453	4	0.59266	0.63212	0.99998	13980	-0.0116
HTR2B	6	0.59274	0.72722	0.99998	13981	0.1759
ARMCX4	6	0.59274	0.72723	0.99998	13982	-0.1135
PCDHGA3	2	0.5928	0.59285	0.99998	13983	-0.0563
NRAS	6	0.59296	0.7274	0.99998	13984	0.014
C12orf50	6	0.59296	0.7274	0.99998	13985	0.0552
DDX60	6	0.59296	0.7274	0.99998	13986	-0.1205
UGT2B4	6	0.59319	0.72759	0.99998	13987	-0.0853
UTS2R	6	0.59319	0.72759	0.99998	13988	0.1616
hsa-mir-758	4	0.5932	0.63246	0.99998	13989	0.1696
FGF6	6	0.5934	0.72778	0.99998	13990	-0.0355
ZNF575	6	0.5934	0.72778	0.99998	13991	-0.1243
PNMA5	6	0.5934	0.72778	0.99998	13992	-0.1022
TMEM8B	6	0.59343	0.7278	0.99998	13993	0.2509
LRP10	6	0.59357	0.72792	0.99998	13994	0.0355
GJB4	6	0.59357	0.72792	0.99998	13995	-0.0472
MREG	6	0.59368	0.72802	0.99998	13996	0.0512
PJA2	6	0.5937	0.72803	0.99998	13997	0.0285
AICDA	6	0.59377	0.7281	0.99998	13998	0.1261
MAVS	6	0.59389	0.7282	0.99998	13999	0.0564
INPP1	6	0.59393	0.72824	0.99998	14000	-0.0785
BDKRB2	6	0.59412	0.72841	0.99998	14001	0.0503
KRTAP20-1	6	0.59412	0.72841	0.99998	14002	-0.0255
CREBL2	6	0.59412	0.72841	0.99998	14003	0.0319
OR1L4	6	0.59412	0.72841	0.99998	14004	0.0556
CNN2	6	0.59419	0.72847	0.99998	14005	0.0201
CASP4	6	0.59419	0.72847	0.99998	14006	0.0233
AMPH	6	0.59426	0.72854	0.99998	14007	0.2223
OR7C2	4	0.59432	0.63317	0.99998	14008	0.1637
hsa-mir-3915	4	0.59432	0.63317	0.99998	14009	0.0514
C1orf122	6	0.59442	0.72867	0.99998	14010	0.0009

FKBP2	6	0.59454	0.72878	0.99998	14011	-0.0017
OSBP2	6	0.59454	0.72878	0.99998	14012	0.2863
LIPT1	6	0.59454	0.72878	0.99998	14013	0.0883
HIST1H4A	6	0.59454	0.72878	0.99998	14014	0.214
ABCA2	6	0.59454	0.72878	0.99998	14015	0.0276
OR7A5	6	0.59459	0.72883	0.99998	14016	0.2777
IPP	6	0.59483	0.72902	0.99998	14017	-0.0161
KIAA0355	6	0.59487	0.72905	0.99998	14018	0.0136
PAGE5	4	0.59489	0.63353	0.99998	14019	-0.4727
SDC3	6	0.59491	0.72909	0.99998	14020	-0.1413
KDM1A	6	0.59502	0.7292	0.99998	14021	0.0551
AP3M1	6	0.59504	0.72921	0.99998	14022	-0.0485
COLGALT1	6	0.5951	0.72926	0.99998	14023	-0.1076
CATSPERB	6	0.5951	0.72926	0.99998	14024	-0.0573
XKR6	6	0.59515	0.7293	0.99998	14025	0.1523
MAGEA10	6	0.59516	0.72931	0.99998	14026	-0.0139
AGO3	6	0.59526	0.72941	0.99998	14027	0.0008
GPR160	6	0.59526	0.72941	0.99998	14028	-0.0636
KDM5B	6	0.59529	0.72944	0.99998	14029	0.1461
CHST2	6	0.59542	0.72954	0.99998	14030	0.0075
TMEM161B	6	0.5956	0.72971	0.99998	14031	0.0107
PDGFB	6	0.59567	0.72977	0.99998	14032	0.3165
C7orf65	6	0.59567	0.72977	0.99998	14033	0.1112
IPO5	6	0.59583	0.72991	0.99998	14034	-0.001
SNX14	6	0.59594	0.73001	0.99998	14035	0.0317
TUBB2B	5	0.59595	0.68336	0.99998	14036	-0.0779
IL2RA	6	0.59596	0.73003	0.99998	14037	0.0533
CEP164	6	0.59596	0.73003	0.99998	14038	-0.1298
EIF1B	6	0.59605	0.7301	0.99998	14039	-0.0706
GTF2A1L	6	0.59611	0.73014	0.99998	14040	-0.0798
TSPAN13	6	0.59626	0.73028	0.99998	14041	0.0028
hsa-mir-4771-	1	0.59628	0.59604	0.99998	14042	0.076
KRT28	6	0.59633	0.73033	0.99998	14043	0.2359
MARCH11	6	0.59657	0.73053	0.99998	14044	0.1064
CCL26	6	0.5967	0.73065	0.99998	14045	0.0405
SCN9A	6	0.5967	0.73066	0.99998	14046	0.2432
KIAA1462	6	0.5967	0.73066	0.99998	14047	0.2116
WIF1	6	0.5967	0.73066	0.99998	14048	-0.1109
HR	6	0.59682	0.73077	0.99998	14049	0.1653
THUMPD1	6	0.59682	0.73077	0.99998	14050	0.1316
DTNBP1	6	0.59699	0.73091	0.99998	14051	0.0387
NBEAL1	6	0.59699	0.73091	0.99998	14052	0.11
SFXN5	6	0.59714	0.73103	0.99998	14053	-0.1486
IRAK4	6	0.59714	0.73103	0.99998	14054	0.1621
MARK2	6	0.59714	0.73103	0.99998	14055	-0.1079
ACMSD	6	0.59714	0.73103	0.99998	14056	0.0202
BIVM	2	0.5973	0.59731	0.99998	14057	-0.0259
TTC13	6	0.5973	0.73116	0.99998	14058	0.199
ZFAT	6	0.59731	0.73117	0.99998	14059	0.175
NLRP10	6	0.59731	0.73117	0.99998	14060	0.0357
TTK	6	0.59737	0.73121	0.99998	14061	0.0218
HMCES	4	0.59743	0.63518	0.99998	14062	0.0492
MEF2A	6	0.59749	0.73132	0.99998	14063	-0.0112
TCTE3	6	0.59769	0.73151	0.99998	14064	0.075
LRRIQ3	6	0.59769	0.73151	0.99998	14065	-0.1362
KCNJ6	6	0.59787	0.73166	0.99998	14066	-0.0785
SLC51B	6	0.59804	0.73181	0.99998	14067	0.057
NEK7	6	0.59804	0.73181	0.99998	14068	-0.0071
SLC28A1	6	0.59829	0.73202	0.99998	14069	0.0343
PRSS56	6	0.59836	0.73208	0.99998	14070	-0.0396
QPCTL	6	0.59836	0.73208	0.99998	14071	-0.0549
C8orf31	6	0.59842	0.73213	0.99998	14072	0.2114
C2orf66	6	0.59842	0.73213	0.99998	14073	0.1425
TLR8	6	0.59859	0.73228	0.99998	14074	-0.0035
BOD1L2	6	0.59859	0.73228	0.99998	14075	0.1767
SSR4	6	0.5986	0.73228	0.99998	14076	-0.0537
KIF3B	6	0.5986	0.73228	0.99998	14077	-0.0206
hsa-mir-4779	4	0.59879	0.63605	0.99998	14078	0.0712
ASNSD1	6	0.5989	0.73254	0.99998	14079	-0.0775
AVEN	6	0.5989	0.73254	0.99998	14080	-0.1039
SAMD14	6	0.5989	0.73254	0.99998	14081	0.0907
LOC653486	3	0.59893	0.60444	0.99998	14082	-0.0997
SELK	6	0.59913	0.73274	0.99998	14083	-0.0222
PCDHB7	6	0.59916	0.73277	0.99998	14084	0.1082
COL6A1	6	0.59922	0.73282	0.99998	14085	0.1498
BZW1	6	0.59931	0.7329	0.99998	14086	0.2669
LMCD1	6	0.5994	0.73298	0.99998	14087	0.0838
OR6A2	6	0.5994	0.73298	0.99998	14088	-0.0843
TCTN2	6	0.5994	0.73298	0.99998	14089	-0.0673
GPR85	6	0.5994	0.73298	0.99998	14090	-0.1029
hsa-mir-1265	2	0.59948	0.59948	0.99998	14091	0.0883

DHX57	6	0.59953	0.73309	0.99998	14092	0.0019
AK1	6	0.59966	0.73322	0.99998	14093	0.1907
NR2F2	6	0.59967	0.73322	0.99998	14094	0.0444
IFT140	6	0.59975	0.7333	0.99998	14095	0.0904
hsa-mir-609	3	0.59988	0.60526	0.99998	14096	0.1932
NOS1AP	6	0.59991	0.73345	0.99998	14097	0.1178
OTOG	6	0.59992	0.73346	0.99998	14098	-0.1138
NLGN4X	6	0.59992	0.73346	0.99998	14099	-0.0913
TNFSF15	6	0.59992	0.73346	0.99998	14100	-0.1245
ENSA	6	0.60016	0.73368	0.99998	14101	0.0433
ENTHD1	6	0.6002	0.73371	0.99998	14102	-0.0797
TEX13B	6	0.60029	0.7338	0.99998	14103	0.2289
ACSL1	6	0.60029	0.7338	0.99998	14104	-0.0022
SERINC3	6	0.60029	0.7338	0.99998	14105	0.0066
GAD2	6	0.60044	0.73392	0.99998	14106	0.0593
ATP8A2	6	0.60044	0.73392	0.99998	14107	0.2738
GLIS2	6	0.60051	0.73398	0.99998	14108	0.1407
hsa-mir-369	4	0.60074	0.63733	0.99998	14109	-0.2868
TCF7L2	6	0.60075	0.73417	0.99998	14110	0.0509
CDCA3	6	0.60101	0.7344	0.99998	14111	0.0294
ESD	6	0.60106	0.73443	0.99998	14112	-0.0315
KLHDC1	6	0.60106	0.73443	0.99998	14113	0.1725
GPR126	6	0.60114	0.7345	0.99998	14114	-0.0457
TAZ	6	0.60114	0.7345	0.99998	14115	-0.0549
hsa-mir-612	4	0.60115	0.6376	0.99998	14116	-0.0877
C10orf118	6	0.60127	0.73461	0.99998	14117	0.0526
ZNF670	6	0.60127	0.73461	0.99998	14118	0.0971
DENND2C	6	0.60137	0.7347	0.99998	14119	0.0163
hsa-mir-6720	4	0.60147	0.63781	0.99998	14120	0.0268
ZBED4	6	0.60151	0.73482	0.99998	14121	0.0232
FAM69C	6	0.60151	0.73482	0.99998	14122	0.0382
KLK13	6	0.60151	0.73482	0.99998	14123	0.1675
CXCL10	6	0.6017	0.73501	0.99998	14124	-0.0971
IRF8	6	0.6017	0.73501	0.99998	14125	0.1958
TMEM150A	6	0.6017	0.73501	0.99998	14126	0.0599
SLC22A9	6	0.6017	0.73501	0.99998	14127	0.3259
ZNF564	6	0.6017	0.73501	0.99998	14128	0.1585
SMIM9	6	0.60182	0.73512	0.99998	14129	0.2716
NFE2	6	0.60192	0.73521	0.99998	14130	-0.1009
hsa-mir-4525	4	0.60202	0.63816	0.99998	14131	0.1223
FSTL3	6	0.60205	0.73531	0.99998	14132	0.0822
PAPLN	6	0.60205	0.73531	0.99998	14133	-0.0297
TNFAIP2	6	0.60205	0.73531	0.99998	14134	0.0155
C11orf42	6	0.60216	0.73541	0.99998	14135	0.2732
SPRR4	6	0.60216	0.73541	0.99998	14136	0.0756
KLHL34	6	0.60218	0.73543	0.99998	14137	-0.0981
C10orf68	6	0.60232	0.73554	0.99998	14138	0.1047
KRT35	6	0.60232	0.73555	0.99998	14139	0.0119
MPP7	6	0.60232	0.73555	0.99998	14140	0.1416
TRMT1L	6	0.60242	0.73564	0.99998	14141	0.1167
KREMEN1	6	0.60242	0.73564	0.99998	14142	0.1315
MIF	6	0.60248	0.73569	0.99998	14143	0.2571
LTA4H	6	0.60267	0.73584	0.99998	14144	0.0423
FCRL4	6	0.60267	0.73584	0.99998	14145	-0.0873
FKBP4	6	0.60268	0.73585	0.99998	14146	0.1181
ZNF732	5	0.6027	0.68656	0.99998	14147	0.0336
hsa-mir-517b	2	0.6028	0.60277	0.99998	14148	0.3727
MLYCD	6	0.60282	0.73597	0.99998	14149	-0.1266
BOD1	6	0.60296	0.73608	0.99998	14150	0.078
METTL7B	6	0.60297	0.73609	0.99998	14151	0.0968
THTPA	6	0.60297	0.73609	0.99998	14152	-0.1065
PRDX2	6	0.60297	0.73609	0.99998	14153	0.0704
ZNF497	6	0.60316	0.73626	0.99998	14154	0.2483
ZNF84	6	0.60316	0.73626	0.99998	14155	0.0949
ING1	6	0.60324	0.73634	0.99998	14156	0.1337
GUCY1A2	6	0.60324	0.73634	0.99998	14157	0.3066
LYST	6	0.60334	0.73642	0.99998	14158	-0.0074
hsa-mir-30d	4	0.60338	0.63904	0.99998	14159	-0.0214
DRD1	6	0.60349	0.73654	0.99998	14160	0.0849
ABHD13	6	0.60349	0.73654	0.99998	14161	-0.1246
NFIB	6	0.60349	0.73654	0.99998	14162	0.0464
DDC	6	0.60358	0.73663	0.99998	14163	0.2368
ITGA4	6	0.6036	0.73664	0.99998	14164	-0.0714
RTEL1	6	0.60375	0.73678	0.99998	14165	-0.0016
S1PR1	6	0.60375	0.73678	0.99998	14166	0.0398
UVSSA	6	0.60394	0.73694	0.99998	14167	0.0005
UHRF2	6	0.60394	0.73694	0.99998	14168	0.2131
BTC	6	0.60396	0.73695	0.99998	14169	0.0224
hsa-mir-30a	4	0.60411	0.63951	0.99998	14170	-0.0545
TAF6	6	0.60412	0.73711	0.99998	14171	-0.0542
OR2V2	6	0.60412	0.73711	0.99998	14172	0.1012

TRAPPC9	6	0.60419	0.73717	0.99998	14173	-0.0794
DHRS3	6	0.60419	0.73717	0.99998	14174	-0.1544
HAND2	6	0.60427	0.73724	0.99998	14175	0.0916
KMT2C	4	0.60434	0.63967	0.99998	14176	0.0704
UNC5A	6	0.60436	0.73733	0.99998	14177	0.1214
ZNF367	6	0.60441	0.73737	0.99998	14178	-0.0061
TAS1R1	6	0.60452	0.73746	0.99998	14179	0.1366
HIST4H4	6	0.60452	0.73747	0.99998	14180	0.4165
SLC26A8	6	0.60453	0.73748	0.99998	14181	-0.0502
DNAH12	4	0.60455	0.6398	0.99998	14182	-0.0317
ZSCAN32	6	0.60457	0.73751	0.99998	14183	0.0296
PPAN-P2RY11	2	0.60462	0.60457	0.99998	14184	0.2169
C8orf46	6	0.6048	0.7377	0.99998	14185	0.1487
RSPO4	6	0.6048	0.7377	0.99998	14186	0.1451
42248	9	0.60481	0.78504	0.99998	14187	0.1441
METRN	6	0.60494	0.73782	0.99998	14188	0.1487
USP53	6	0.60504	0.7379	0.99998	14189	0.1592
CTXN1	6	0.6052	0.73803	0.99998	14190	-0.0963
SLC9B2	6	0.6052	0.73803	0.99998	14191	-0.1326
HEXA	6	0.6052	0.73803	0.99998	14192	-0.0672
GPR21	6	0.60554	0.73832	0.99998	14193	-0.0488
CACUL1	6	0.60554	0.73832	0.99998	14194	-0.0084
QTRTD1	6	0.60554	0.73832	0.99998	14195	0.1385
PHF19	6	0.60554	0.73832	0.99998	14196	0.0016
TOMM40L	6	0.60554	0.73832	0.99998	14197	-0.0728
KLHL18	6	0.60554	0.73832	0.99998	14198	0.0489
PDE11A	6	0.60559	0.73836	0.99998	14199	0.2481
hsa-mir-4481	4	0.6057	0.64056	0.99998	14200	-0.1345
SIRT1	6	0.60573	0.7385	0.99998	14201	0.0672
SENP3	6	0.60583	0.73858	0.99998	14202	0.151
OR5H6	6	0.60583	0.73858	0.99998	14203	-0.1339
MURC	6	0.60585	0.73859	0.99998	14204	0.2412
PCSK4	6	0.60606	0.73879	0.99998	14205	0.0345
C5orf60	6	0.60606	0.73879	0.99998	14206	0.0017
CEND1	6	0.60619	0.73889	0.99998	14207	0.1079
SYNPO2	6	0.60629	0.73896	0.99998	14208	0.0083
ANXA8L1	2	0.60632	0.60625	0.99998	14209	0.1222
DHX40	6	0.60639	0.73906	0.99998	14210	-0.0317
CLDN12	6	0.60643	0.7391	0.99998	14211	0.0419
hsa-mir-4316	4	0.60647	0.64103	0.99998	14212	0.3122
B3GALT4	6	0.60647	0.73915	0.99998	14213	-0.0908
HMGB2	6	0.6065	0.73917	0.99998	14214	0.2606
MXD4	6	0.6065	0.73917	0.99998	14215	0.1051
SLC35G6	6	0.6065	0.73917	0.99998	14216	0.1368
ENOX1	6	0.60685	0.73945	0.99998	14217	0.3204
CAMK2D	6	0.60688	0.73948	0.99998	14218	0.0878
RAB26	6	0.60697	0.73955	0.99998	14219	-0.1376
TMEM82	6	0.60697	0.73955	0.99998	14220	-0.0982
SLPI	6	0.6071	0.73967	0.99998	14221	0.1427
KATNA1	6	0.60712	0.73969	0.99998	14222	-0.049
RLN2	6	0.60712	0.73969	0.99998	14223	0.0023
CLEC17A	6	0.60712	0.73969	0.99998	14224	-0.0143
IQCF2	6	0.60712	0.73969	0.99998	14225	-0.1135
TRAFD1	6	0.6073	0.73985	0.99998	14226	0.048
ITGA2	6	0.6073	0.73985	0.99998	14227	-0.0438
HGFAC	6	0.60741	0.73995	0.99998	14228	-0.0863
TMEM56-RWI	1	0.60748	0.60718	0.99998	14229	0.0609
MAFB	6	0.60753	0.74006	0.99998	14230	0.3615
RABL5	6	0.60753	0.74006	0.99998	14231	0.1909
FAM21B	4	0.60754	0.64173	0.99998	14232	0.0093
PHLDB1	6	0.60754	0.74007	0.99998	14233	0.0182
OR10G2	6	0.60754	0.74007	0.99998	14234	0.011
SIRPD	6	0.60768	0.74021	0.99998	14235	-0.0221
hsa-mir-218-1	2	0.6077	0.60765	0.99998	14236	0.1449
KIAA1644	6	0.60776	0.74027	0.99998	14237	0.2084
ATP13A1	6	0.60776	0.74027	0.99998	14238	0.0708
PDE4B	6	0.60776	0.74028	0.99998	14239	0.0076
CCDC63	6	0.60798	0.74038	0.99998	14240	0.2779
ACOT8	6	0.60798	0.74038	0.99998	14241	0.0507
CEP85	6	0.60809	0.74042	0.99998	14242	0.2393
BHLHE22	6	0.60823	0.74046	0.99998	14243	0.1766
PLAT	6	0.60851	0.74055	0.99998	14244	0.154
FAM209B	6	0.60856	0.74056	0.99998	14245	0.0459
MAPKAPK3	6	0.60856	0.74056	0.99998	14246	0.1989
ZNF683	6	0.60866	0.74059	0.99998	14247	0.0073
CNIH3	6	0.60878	0.74063	0.99998	14248	-0.0045
hsa-mir-330	4	0.60898	0.6427	0.99998	14249	0.2261
ZBTB37	6	0.60935	0.74081	0.99998	14250	0.0474
TGFBRAP1	6	0.60968	0.74091	0.99998	14251	0.0495
hsa-mir-19a	3	0.60974	0.61381	0.99998	14252	-0.167
OMA1	6	0.60982	0.74096	0.99998	14253	0.2448

PANX1	6	0.60982	0.74096	0.99998	14254	0.2345
PPIAL4D	2	0.61028	0.61021	0.99998	14255	-0.0909
ARL13A	6	0.61029	0.74112	0.99998	14256	0.2159
OR2T33	6	0.61044	0.74117	0.99998	14257	0.2987
SEC24A	6	0.61044	0.74117	0.99998	14258	-0.0528
RNF111	6	0.61086	0.74131	0.99998	14259	0.0174
SLC9A7	6	0.61086	0.74131	0.99998	14260	0.2101
TROAP	6	0.61098	0.74135	0.99998	14261	0.4236
LIN37	6	0.61121	0.74142	0.99998	14262	0.0969
CHAMP1	6	0.6114	0.74149	0.99998	14263	0.0655
TMEM106A	6	0.6114	0.74149	0.99998	14264	0.0167
hsa-mir-324	4	0.61155	0.64438	0.99998	14265	0.2045
MATK	6	0.6116	0.74156	0.99998	14266	0.1128
FAM227A	6	0.6116	0.74156	0.99998	14267	-0.0515
PIK3R2	6	0.6118	0.74163	0.99998	14268	0.1337
MAP1S	6	0.61204	0.74171	0.99998	14269	0.0465
CMTM7	6	0.61204	0.74171	0.99998	14270	0.0467
IRF2	6	0.61204	0.74171	0.99998	14271	0.1515
WAPAL	6	0.61204	0.74171	0.99998	14272	0.1194
hsa-mir-1285-	3	0.61219	0.61598	0.99998	14273	0.1232
RHCE	5	0.61229	0.69105	0.99998	14274	0.0085
USP17L4	3	0.61239	0.61615	0.99998	14275	-0.8095
SLC52A1	6	0.61245	0.74185	0.99998	14276	0.0727
KIAA0368	6	0.61245	0.74185	0.99998	14277	-0.115
ESRRA	6	0.61269	0.74192	0.99998	14278	0.1933
hsa-mir-3143	4	0.61282	0.64523	0.99998	14279	0.4189
FAM210B	6	0.61291	0.742	0.99998	14280	0.0536
LFNG	6	0.61308	0.74206	0.99998	14281	0.3847
INSL3	6	0.61318	0.74209	0.99998	14282	0.1821
OR1L1	6	0.61318	0.74209	0.99998	14283	0.3806
HDGFL1	6	0.61318	0.74209	0.99998	14284	0.0443
CDYL	6	0.6133	0.74213	0.99998	14285	-0.0834
SLK	4	0.61342	0.64562	0.99998	14286	0.1173
IQCH	6	0.61344	0.74218	0.99998	14287	0.0834
WFIKKN2	6	0.61358	0.74222	0.99998	14288	-0.1196
NKX2-4	6	0.61446	0.74249	0.99998	14289	-0.004
CATSPER2	4	0.61457	0.64638	0.99998	14290	0.0472
LRRC57	6	0.61466	0.74255	0.99998	14291	0.1034
GPX8	6	0.61466	0.74255	0.99998	14292	-0.1018
OR51G2	6	0.61488	0.74263	0.99998	14293	0.2174
CC2D1B	6	0.61488	0.74263	0.99998	14294	0.1755
RPL3L	6	0.61488	0.74263	0.99998	14295	0.0761
LAYN	6	0.6151	0.74271	0.99998	14296	0.148
IFT88	6	0.61543	0.74283	0.99998	14297	0.0627
LOC10014459	4	0.61549	0.64698	0.99998	14298	-0.1066
hsa-mir-1908	3	0.61552	0.61895	0.99998	14299	-0.2638
WDR83	6	0.61562	0.7429	0.99998	14300	0.0122
NAB1	6	0.61588	0.74301	0.99998	14301	0.0752
AP1M2	6	0.61608	0.74308	0.99998	14302	0.2044
C7	6	0.61608	0.74308	0.99998	14303	0.1566
TMEM249	6	0.61626	0.74314	0.99998	14304	0.068
hsa-mir-584	4	0.61627	0.64753	0.99998	14305	0.042
hsa-mir-4658	4	0.61627	0.64753	0.99998	14306	0.0746
EREG	6	0.61642	0.74319	0.99998	14307	0.0685
SLC19A3	6	0.61663	0.74325	0.99998	14308	0.2755
MAD2L1BP	6	0.61683	0.74332	0.99998	14309	0.0517
GPRC5D	6	0.61683	0.74332	0.99998	14310	0.0601
VSTM2A	6	0.61683	0.74332	0.99998	14311	0.0932
hsa-mir-3918	4	0.61688	0.64795	0.99998	14312	0.1477
CCKBR	6	0.61708	0.74341	0.99998	14313	0.0627
LBX2	6	0.61734	0.74349	0.99998	14314	0.067
SIK2	4	0.61739	0.64829	0.99998	14315	-0.0687
LY6H	6	0.6179	0.74367	0.99998	14316	0.0439
HARBI1	6	0.61821	0.74378	0.99998	14317	0.0302
hsa-mir-548ac	3	0.6185	0.62161	0.99998	14318	-0.0958
OR6F1	6	0.61855	0.74392	0.99998	14319	0.0049
MBD1	6	0.61855	0.74392	0.99998	14320	0.0455
ZNF527	5	0.61866	0.69414	0.99998	14321	-0.0883
MAN2B2	6	0.61873	0.74399	0.99998	14322	0.1245
PKP3	6	0.61873	0.74399	0.99998	14323	0.1537
HRH4	6	0.61888	0.74404	0.99998	14324	0.2864
SNX6	6	0.61888	0.74404	0.99998	14325	-0.0807
SOGA3	6	0.61904	0.74408	0.99998	14326	-0.0331
PLA2G15	6	0.61904	0.74408	0.99998	14327	0.1593
HEYL	6	0.61927	0.74415	0.99998	14328	0.2769
ADAMTS4	6	0.61927	0.74415	0.99998	14329	0.102
NIPAL1	6	0.61947	0.74421	0.99998	14330	0.3237
KIAA1033	6	0.61947	0.74421	0.99998	14331	0.0926
TCEB3	6	0.61966	0.74429	0.99998	14332	0.0145
hsa-mir-662	4	0.62001	0.65004	0.99998	14333	0.1638
hsa-mir-6809	4	0.62001	0.65004	0.99998	14334	-0.073

SLC26A3	6	0.62003	0.74441	0.99998	14335	0.1513
SOD3	6	0.62005	0.74442	0.99998	14336	0.1271
PNMT	6	0.62016	0.74447	0.99998	14337	-0.069
ERLIN1	6	0.62016	0.74447	0.99998	14338	0.2477
GIMAP1-GIMA	3	0.62033	0.62325	0.99998	14339	0.0161
TM4SF19	6	0.62048	0.74457	0.99998	14340	0.3087
hsa-mir-301a	3	0.62062	0.62349	0.99998	14341	0.0661
KLF4	6	0.62062	0.74461	0.99998	14342	0.0791
CDCA7	6	0.62062	0.74461	0.99998	14343	0.2483
LCE1B	6	0.62083	0.7447	0.99998	14344	-0.0904
SYT12	6	0.62083	0.7447	0.99998	14345	0.0109
DOCK1	6	0.62083	0.7447	0.99998	14346	0.2024
hsa-mir-4489	4	0.62089	0.65064	0.99998	14347	-0.0481
CR1	6	0.62104	0.74478	0.99998	14348	-0.1171
hsa-mir-1284	4	0.62107	0.65076	0.99998	14349	-0.2119
RNMTL1	6	0.62123	0.74484	0.99998	14350	-0.0037
PCBD1	6	0.6214	0.74488	0.99998	14351	0.2777
DTD1	6	0.6214	0.74488	0.99998	14352	0.0892
LY6G5C	6	0.62165	0.74498	0.99998	14353	0.1204
MARVELD2	6	0.62177	0.74501	0.99998	14354	0.2595
PRDX4	6	0.62194	0.74507	0.99998	14355	-0.024
C2CD4C	6	0.62194	0.74507	0.99998	14356	0.0801
RXRG	6	0.62213	0.74513	0.99998	14357	-0.0969
hsa-mir-4518	4	0.62214	0.65145	0.99998	14358	0.0978
hsa-mir-587	2	0.62224	0.62203	0.99998	14359	-0.1117
UBE3A	6	0.62254	0.74528	0.99998	14360	0.2691
EID2B	6	0.62254	0.74528	0.99998	14361	0.0789
ARL4A	6	0.6227	0.74534	0.99998	14362	0.0605
OR5M11	6	0.62291	0.7454	0.99998	14363	-0.0082
LIN7B	6	0.62291	0.7454	0.99998	14364	0.0773
MTMR3	6	0.62291	0.7454	0.99998	14365	0.1487
MYBPC1	6	0.62317	0.74548	0.99998	14366	0.1428
SNTN	6	0.62341	0.74556	0.99998	14367	0.1403
MUM1	6	0.62374	0.74567	0.99998	14368	0.1912
hsa-mir-4436a	3	0.62387	0.62648	0.99998	14369	0.1932
OR2T3	3	0.62387	0.62648	0.99998	14370	-0.9148
UGT2B28	3	0.62387	0.62648	0.99998	14371	0.2103
KRTAP4-11	3	0.62387	0.62648	0.99998	14372	-0.9148
hsa-mir-1273c	3	0.62387	0.62648	0.99998	14373	-0.3622
hsa-mir-548y	3	0.62387	0.62648	0.99998	14374	0.024
hsa-mir-194-2	4	0.62394	0.6527	0.99998	14375	-0.0689
NACA2	6	0.62419	0.74582	0.99998	14376	-0.1148
HEATR5B	6	0.62442	0.7459	0.99998	14377	0.1627
ZNF544	6	0.62442	0.7459	0.99998	14378	0.0345
PRB4	6	0.62442	0.7459	0.99998	14379	0.3283
ODF4	6	0.62442	0.7459	0.99998	14380	-0.0314
CFC1B	2	0.62449	0.62432	0.99998	14381	0.5801
PIGW	6	0.6246	0.74596	0.99998	14382	-0.0131
FAM227B	6	0.6246	0.74596	0.99998	14383	0.1739
C1QTNF3	6	0.62473	0.74601	0.99998	14384	0.081
C20orf196	6	0.62481	0.74603	0.99998	14385	-0.0224
ZBTB47	6	0.62504	0.74611	0.99998	14386	0.2902
SPDYE1	2	0.62529	0.62509	0.99998	14387	-0.0612
OR11H2	6	0.62529	0.7462	0.99998	14388	-0.0171
C6orf70	6	0.62529	0.7462	0.99998	14389	0.3714
CNTN6	6	0.62529	0.7462	0.99998	14390	0.1624
hsa-mir-556	3	0.62565	0.62808	0.99998	14391	-0.0056
BARHL2	6	0.62569	0.74632	0.99998	14392	0.3303
hsa-mir-4723	4	0.62618	0.65423	0.99998	14393	-0.0334
PIK3R5	6	0.62626	0.74652	0.99998	14394	0.0065
POMT2	6	0.62626	0.74652	0.99998	14395	-0.0028
KIAA0146	2	0.62628	0.6261	0.99998	14396	0.0634
MCMDC2	6	0.62645	0.74657	0.99998	14397	0.1979
FAM109A	6	0.62645	0.74657	0.99998	14398	0.1749
L1TD1	6	0.62661	0.74662	0.99998	14399	0.2047
RARRES3	6	0.62661	0.74662	0.99998	14400	0.1642
CBX7	6	0.62661	0.74662	0.99998	14401	0.3578
GPR137	6	0.62661	0.74662	0.99998	14402	0.0727
GLT6D1	6	0.62674	0.74667	0.99998	14403	0.0013
CSTA	6	0.62674	0.74667	0.99998	14404	0.251
CYP2W1	6	0.62685	0.7467	0.99998	14405	-0.1134
hsa-mir-1248	4	0.62695	0.65472	0.99998	14406	-0.1401
SLC46A1	6	0.62701	0.74676	0.99998	14407	0.032
hsa-mir-4502	4	0.62718	0.65487	0.99998	14408	0.204
ELMOD3	6	0.62744	0.74691	0.99998	14409	0.2987
SYT15	6	0.62744	0.74691	0.99998	14410	-0.0308
RNF128	6	0.62744	0.74691	0.99998	14411	-0.005
FBXO6	6	0.62761	0.74697	0.99998	14412	-0.0151
ZNF268	6	0.62777	0.74703	0.99998	14413	0.1303
KRTAP10-8	6	0.62777	0.74703	0.99998	14414	0.0822
CTSA	6	0.62798	0.74711	0.99998	14415	0.0907

DIXDC1	6	0.62824	0.74719	0.99998	14416	-0.0554
ADCY7	6	0.62832	0.74722	0.99998	14417	0.154
RNF32	6	0.62844	0.74726	0.99998	14418	0.0759
hsa-mir-6854	2	0.62848	0.62829	0.99998	14419	0.1838
TIGD1	6	0.62898	0.74745	0.99998	14420	-0.0672
GGT5	6	0.62898	0.74745	0.99998	14421	0.1134
EMC1	6	0.62898	0.74745	0.99998	14422	0.0076
TCP10	4	0.62905	0.65615	0.99998	14423	-0.0249
SCGB1C1	3	0.62907	0.63119	0.99998	14424	0.0339
CLNK	5	0.62938	0.69924	0.99998	14425	0.0069
SP7	6	0.62949	0.74763	0.99998	14426	0.1998
hsa-mir-6779	4	0.62962	0.65657	0.99998	14427	0.0722
PPP3R2	6	0.62988	0.74775	0.99998	14428	0.2091
CTNNAL1	6	0.62988	0.74775	0.99998	14429	0.1429
FAM219A	6	0.62988	0.74775	0.99998	14430	-0.0065
DCAF8L2	6	0.63032	0.74791	0.99998	14431	0.0208
LHFPL2	6	0.63032	0.74791	0.99998	14432	-0.0928
GDE1	6	0.63032	0.74791	0.99998	14433	0.1476
hsa-mir-3673	1	0.6304	0.63031	0.99998	14434	0.3419
PAPD4	6	0.63062	0.74801	0.99998	14435	0.2021
TMEM221	6	0.63062	0.74801	0.99998	14436	0.1017
LINGO3	6	0.63062	0.74801	0.99998	14437	-0.0223
SRM	6	0.63097	0.74813	0.99998	14438	0.1591
SPCS2	6	0.63097	0.74813	0.99998	14439	-0.1064
SLC1A1	6	0.63097	0.74813	0.99998	14440	0.1747
RDH11	6	0.63113	0.74819	0.99998	14441	0.2053
NALCN	6	0.63128	0.74825	0.99998	14442	-0.0242
MELK	6	0.63143	0.74829	0.99998	14443	0.0101
AGBL4	6	0.63143	0.74829	0.99998	14444	-0.0513
SHISA2	6	0.63143	0.74829	0.99998	14445	0.0813
MTMR10	6	0.63143	0.74829	0.99998	14446	0.1598
RETN	6	0.63168	0.74838	0.99998	14447	0.0826
MAGED4	2	0.63187	0.63167	0.99998	14448	0.1269
FBXO8	6	0.63195	0.74847	0.99998	14449	0.1593
AADACL4	6	0.63195	0.74847	0.99998	14450	0.0251
ZNF264	5	0.63224	0.70065	0.99998	14451	-0.0004
hsa-mir-1243	4	0.63229	0.65836	0.99998	14452	0.283
KCNC4	6	0.63229	0.7486	0.99998	14453	0.035
CYHR1	6	0.63244	0.74865	0.99998	14454	0.0155
STOML1	6	0.63263	0.74872	0.99998	14455	0.2378
DNAH11	6	0.63278	0.74877	0.99998	14456	0.1037
LOC646498	1	0.63287	0.63281	0.99998	14457	0.1465
hsa-mir-1276	4	0.63313	0.65893	0.99998	14458	0.1162
LCE3A	6	0.6333	0.74896	0.99998	14459	0.2571
CD209	5	0.63344	0.70127	0.99998	14460	0.1986
hsa-mir-505	4	0.63357	0.65922	0.99998	14461	0.1607
MFAP3L	6	0.63394	0.7492	0.99998	14462	-0.0299
GREB1	6	0.63419	0.74929	0.99998	14463	0.0062
ERMN	6	0.63419	0.74929	0.99998	14464	0.2028
hsa-mir-126	4	0.63424	0.65966	0.99998	14465	-0.2138
SLC25A34	6	0.63436	0.74936	0.99998	14466	0.0018
ZC2HC1B	6	0.63442	0.74938	0.99998	14467	0.1231
WIPI1	6	0.63442	0.74938	0.99998	14468	0.04
DYNC2LI1	6	0.63486	0.74953	0.99998	14469	0.0528
LDHAL6A	6	0.63499	0.74956	0.99998	14470	0.0142
ST3GAL4	6	0.63499	0.74956	0.99998	14471	-0.0442
AP1G1	6	0.63499	0.74956	0.99998	14472	0.1949
EGF	6	0.63508	0.74959	0.99998	14473	0.0086
RASEF	6	0.63531	0.74968	0.99998	14474	-0.0477
CD2	6	0.63531	0.74968	0.99998	14475	0.1632
ANKFN1	4	0.6354	0.66044	0.99998	14476	0.0628
TAB3	6	0.63549	0.74975	0.99998	14477	0.0377
hsa-mir-3660	3	0.6356	0.63721	0.99998	14478	0.1459
CLSTN3	6	0.63572	0.74983	0.99998	14479	0.2307
BPGM	6	0.63597	0.74992	0.99998	14480	0.3094
TNFRSF4	6	0.63612	0.74997	0.99998	14481	0.0512
TMEM207	6	0.63636	0.75005	0.99998	14482	0.0555
KCNT1	6	0.6365	0.7501	0.99998	14483	0.2674
RILPL1	6	0.6366	0.75014	0.99998	14484	-0.0642
hsa-mir-520b	2	0.63688	0.63675	0.99998	14485	-0.0822
hsa-mir-450a-	2	0.63688	0.63675	0.99998	14486	-0.0838
USP49	6	0.63694	0.75026	0.99998	14487	0.1525
CPNE4	6	0.63731	0.7504	0.99998	14488	0.0144
hsa-mir-3675	4	0.63763	0.662	0.99998	14489	-0.0263
HRH1	6	0.63768	0.75053	0.99998	14490	0.0148
ARHGAP32	6	0.63768	0.75053	0.99998	14491	0.0703
SAMD15	6	0.63768	0.75053	0.99998	14492	-0.047
hsa-mir-4720	4	0.63776	0.66208	0.99998	14493	-0.0698
hsa-mir-6862-	2	0.6379	0.63775	0.99998	14494	-0.0626
KIAA1045	6	0.63792	0.75062	0.99998	14495	-0.0038
RECQL4	6	0.63807	0.75066	0.99998	14496	0.1032

DEFB4A	3	0.63823	0.63968	0.99998	14497	0.1377
hsa-mir-376a-	3	0.63823	0.63968	0.99998	14498	0.1276
FAM216A	6	0.63827	0.75074	0.99998	14499	0.0961
PTN	6	0.63827	0.75074	0.99998	14500	0.2236
GLYCTK	4	0.63836	0.66252	0.99998	14501	0.0562
SRPK3	6	0.63958	0.7512	0.99998	14502	0.0325
B3GALT6	6	0.6398	0.75128	0.99998	14503	0.2527
C10orf55	3	0.6398	0.64117	0.99998	14504	0.166
MAGEA6	5	0.63987	0.70445	0.99998	14505	-0.0624
PLP2	6	0.63992	0.75133	0.99998	14506	0.1865
NEURL	6	0.63992	0.75133	0.99998	14507	0.1986
LETMD1	6	0.63992	0.75133	0.99998	14508	0.0278
ZNF676	2	0.63992	0.63974	0.99998	14509	0.017
CLIC4	6	0.64012	0.75141	0.99998	14510	0.1373
SFXN1	6	0.64012	0.75141	0.99998	14511	0.0387
CCDC154	6	0.64012	0.75141	0.99998	14512	0.0775
CPNE2	6	0.64034	0.75148	0.99998	14513	0.0467
KIAA1919	6	0.64044	0.75152	0.99998	14514	0.1061
PMEL	6	0.64051	0.75154	0.99998	14515	0.0164
GPR119	6	0.64063	0.75158	0.99998	14516	0.255
PECR	6	0.64063	0.75158	0.99998	14517	-0.0096
CCAR2	6	0.64095	0.7517	0.99998	14518	0.3038
CECR6	6	0.64095	0.7517	0.99998	14519	0.0167
OR9A2	6	0.64095	0.7517	0.99998	14520	0.2033
DAPK1	6	0.64106	0.75174	0.99998	14521	0.1911
PLEKHM3	6	0.64115	0.75177	0.99998	14522	0.1821
CD82	6	0.64115	0.75177	0.99998	14523	0.154
WDR46	6	0.64128	0.75182	0.99998	14524	-0.0006
WLS	6	0.64158	0.75192	0.99998	14525	0.0027
HIST3H2A	6	0.64158	0.75192	0.99998	14526	0.1281
SIPA1L1	6	0.64158	0.75192	0.99998	14527	0.0249
GDA	6	0.64168	0.75196	0.99998	14528	0.0192
hsa-mir-429	4	0.64188	0.66505	0.99998	14529	0.1654
SERPINB13	6	0.64197	0.75205	0.99998	14530	-0.0438
ARRDC5	6	0.64197	0.75205	0.99998	14531	0.0429
ABCC3	6	0.64197	0.75205	0.99998	14532	-0.0994
CLSPN	6	0.64197	0.75205	0.99998	14533	0.0759
NCOA2	6	0.64227	0.75216	0.99998	14534	0.2925
POFUT1	6	0.64227	0.75216	0.99998	14535	0.1238
DHX58	6	0.64227	0.75216	0.99998	14536	-0.0322
CMBL	6	0.64227	0.75216	0.99998	14537	0.1871
hsa-mir-2116	4	0.64238	0.6654	0.99998	14538	0.1084
PCM1	6	0.64245	0.75222	0.99998	14539	0.2329
SGIP1	6	0.6425	0.75224	0.99998	14540	-0.1002
PCDHGC4	2	0.64258	0.64237	0.99998	14541	0.0101
FHOD1	6	0.64284	0.75236	0.99998	14542	0.2163
TRMT13	3	0.64287	0.64396	0.99998	14543	-0.0739
hsa-mir-6766	4	0.64301	0.66584	0.99998	14544	-0.1436
CHURC1	4	0.64335	0.66607	0.99998	14545	0.0054
TMEM31	5	0.64348	0.70628	0.99998	14546	-0.0273
PODXL2	6	0.64348	0.75259	0.99998	14547	0.0219
OR2C1	6	0.64348	0.75259	0.99998	14548	0.0802
RAB43	6	0.64367	0.75266	0.99998	14549	0.1453
hsa-mir-372	4	0.6437	0.66632	0.99998	14550	0.2655
AKR1A1	6	0.64392	0.75276	0.99998	14551	0.1376
SLC7A6	6	0.644	0.75279	0.99998	14552	0.0075
ADD2	4	0.644	0.66651	0.99998	14553	0.1737
SHD	6	0.64412	0.75284	0.99998	14554	0.3248
A1BG	6	0.64423	0.75288	0.99998	14555	0.1304
BAZ2A	6	0.64434	0.75293	0.99998	14556	0.0597
HIP1R	6	0.64434	0.75293	0.99998	14557	0.11
hsa-mir-611	4	0.64443	0.66682	0.99998	14558	0.2134
hsa-mir-3615	4	0.64443	0.66682	0.99998	14559	-0.0744
ADAM7	4	0.64443	0.66682	0.99998	14560	-0.047
TMCO1	6	0.64454	0.753	0.99998	14561	0.2386
ANKRD13A	6	0.64454	0.753	0.99998	14562	0.2197
PTPLAD1	6	0.64482	0.75309	0.99998	14563	0.1988
ORMDL1	6	0.64482	0.75309	0.99998	14564	0.1253
SNAPC1	6	0.64482	0.75309	0.99998	14565	0.2226
hsa-mir-4267	2	0.64499	0.64477	0.99998	14566	0.22
COMMD8	6	0.6451	0.75318	0.99998	14567	0.1981
NMUR1	6	0.64529	0.75325	0.99998	14568	0.0098
QRFPR	6	0.6454	0.75329	0.99998	14569	0.1048
SNX1	6	0.6454	0.75329	0.99998	14570	0.4459
KRTAP9-2	5	0.64547	0.70728	0.99998	14571	-0.9148
ZFYVE1	6	0.64569	0.75341	0.99998	14572	-0.0872
BCL2L11	6	0.64569	0.75341	0.99998	14573	0.1488
LIPI	6	0.64569	0.75341	0.99998	14574	-0.0437
GLCCI1	6	0.64586	0.75347	0.99998	14575	-0.0299
LGALS3	6	0.64602	0.75354	0.99998	14576	0.1822
ATP4A	6	0.64602	0.75354	0.99998	14577	0.2887

LHX2	6	0.64602	0.75354	0.99998	14578	0.1106
hsa-mir-548h-	3	0.6461	0.64702	0.99998	14579	0.23
EP300	6	0.64622	0.75361	0.99998	14580	0.0937
ZNF142	6	0.64652	0.75371	0.99998	14581	0.2495
hsa-mir-3685	4	0.64653	0.66833	0.99998	14582	0.1386
TRPM8	6	0.64668	0.75378	0.99998	14583	0.2803
CDC14B	6	0.64668	0.75378	0.99998	14584	0.0965
TAGLN3	6	0.64674	0.7538	0.99998	14585	-0.0622
FRMD5	6	0.64692	0.75386	0.99998	14586	0.1807
ZNF559	6	0.64692	0.75386	0.99998	14587	0.0969
LONP2	6	0.6471	0.75392	0.99998	14588	0.0864
KLF12	6	0.64717	0.75395	0.99998	14589	0.0164
BCL2	6	0.64728	0.75399	0.99998	14590	0.065
hsa-mir-6133	4	0.64737	0.66894	0.99998	14591	-0.126
DNMT3L	6	0.64742	0.75404	0.99998	14592	-0.0047
IDE	6	0.64758	0.75411	0.99998	14593	-0.0122
PSG2	6	0.64766	0.75414	0.99998	14594	0.1374
RPGRIP1L	6	0.64782	0.7542	0.99998	14595	0.3733
PPP1R1A	6	0.64782	0.7542	0.99998	14596	-0.0128
hsa-mir-632	4	0.64788	0.6693	0.99998	14597	-0.1157
PHLDA1	6	0.648	0.75428	0.99998	14598	-0.0521
CDCA7L	6	0.64825	0.75438	0.99998	14599	-0.0604
NIPA1	6	0.64841	0.75443	0.99998	14600	0.1625
CELA3B	5	0.64851	0.70885	0.99998	14601	0.2371
TTLL1	6	0.64853	0.75447	0.99998	14602	0.1602
SERPINB12	6	0.64898	0.75464	0.99998	14603	-0.002
CLEC4C	6	0.64898	0.75464	0.99998	14604	-0.0176
WFDC6	4	0.64907	0.67011	0.99998	14605	-0.0829
CEP120	6	0.64922	0.75471	0.99998	14606	0.0449
CLCC1	6	0.64943	0.7548	0.99998	14607	0.0845
TEX2	6	0.6496	0.75486	0.99998	14608	0.2433
TAC3	6	0.6496	0.75486	0.99998	14609	-0.0484
KANSL1	6	0.64993	0.75498	0.99998	14610	0.0935
BBS5	6	0.64993	0.75498	0.99998	14611	0.1426
THPO	6	0.6501	0.75504	0.99998	14612	0.0893
C17orf80	6	0.65026	0.7551	0.99998	14613	0.1164
ST6GALNAC2	4	0.65036	0.67105	0.99998	14614	0.102
NPTN	6	0.65038	0.75513	0.99998	14615	0.2389
hsa-mir-4673	4	0.65047	0.67112	0.99998	14616	0.187
SFT2D2	6	0.65067	0.75524	0.99998	14617	0.3629
ZNF280A	6	0.65083	0.7553	0.99998	14618	0.131
CNN1	6	0.65083	0.7553	0.99998	14619	0.291
CRYBB1	6	0.65083	0.7553	0.99998	14620	0.2594
UBE2Q2	6	0.65112	0.75541	0.99998	14621	-0.0116
ABCG1	6	0.65112	0.75541	0.99998	14622	0.1449
ANXA5	6	0.65125	0.75545	0.99998	14623	0.2985
SPATC1	6	0.65152	0.75554	0.99998	14624	0.0359
FAM157B	3	0.65156	0.65216	0.99998	14625	0.072
hsa-mir-4636	4	0.65171	0.67199	0.99998	14626	0.1507
ZNF304	6	0.65183	0.75565	0.99998	14627	0.1779
hsa-mir-8062	4	0.65184	0.67209	0.99998	14628	-0.1101
IFNG	6	0.65191	0.75567	0.99998	14629	0.327
BAIAP2L1	6	0.652	0.75571	0.99998	14630	0.1439
EXOG	6	0.6526	0.75592	0.99998	14631	0.0674
SLC35E4	6	0.6526	0.75592	0.99998	14632	0.1195
CENPE	4	0.65262	0.67265	0.99998	14633	0.2025
ARL3	6	0.65273	0.75597	0.99998	14634	0.1559
IPCEF1	5	0.6528	0.71105	0.99998	14635	-0.0093
ACTA2	6	0.65285	0.75602	0.99998	14636	0.0286
PPP3CC	6	0.65285	0.75602	0.99998	14637	0.1077
C1orf116	6	0.65285	0.75602	0.99998	14638	0.0734
hsa-mir-1183	4	0.65309	0.67296	0.99998	14639	0.0789
C9orf9	6	0.6531	0.75611	0.99998	14640	0.052
TSPO	6	0.6531	0.75611	0.99998	14641	0.1301
hsa-mir-3928	4	0.6533	0.67312	0.99998	14642	0.1018
NPR2	6	0.65334	0.7562	0.99998	14643	-0.0588
hsa-mir-181a-	4	0.6536	0.67335	0.99998	14644	-0.1692
hsa-mir-561	4	0.65382	0.6735	0.99998	14645	0.0131
hsa-mir-4766	4	0.65395	0.67359	0.99998	14646	-0.0171
NUPR1L	6	0.65416	0.7565	0.99998	14647	0.1256
RNF212	6	0.65416	0.7565	0.99998	14648	0.0279
IL18RAP	6	0.65416	0.7565	0.99998	14649	0.1214
POU4F2	6	0.65436	0.75657	0.99998	14650	0.0945
GPD2	6	0.65466	0.75668	0.99998	14651	-0.0266
hsa-mir-375	4	0.65472	0.67414	0.99998	14652	0.0082
RNF224	6	0.65489	0.75676	0.99998	14653	0.0994
hsa-mir-4505	4	0.65496	0.6743	0.99998	14654	0.098
SNAI1	6	0.65505	0.75681	0.99998	14655	0.2437
TM4SF4	6	0.65505	0.75681	0.99998	14656	-0.062
DMC1	6	0.65518	0.75686	0.99998	14657	0.1203
UBFD1	6	0.65554	0.75701	0.99998	14658	0.0637

UNC13B	6	0.65554	0.75701	0.99998	14659	0.2562
SYTL2	6	0.65554	0.75701	0.99998	14660	0.0028
DNAI1	6	0.65554	0.75701	0.99998	14661	0.1742
hsa-mir-637	4	0.65561	0.67479	0.99998	14662	-0.0227
VPREB1	6	0.6557	0.75708	0.99998	14663	0.0354
OSTM1	6	0.65578	0.7571	0.99998	14664	0.1845
GPR55	6	0.65583	0.75712	0.99998	14665	0.077
FAM133B	5	0.65592	0.71267	0.99998	14666	-0.0895
ATHL1	6	0.65611	0.75723	0.99998	14667	0.2311
RAD23B	6	0.65611	0.75723	0.99998	14668	-0.0677
GAGE10	4	0.65628	0.6753	0.99998	14669	-0.1066
RBM44	6	0.65629	0.75729	0.99998	14670	-0.0285
WDR27	6	0.65629	0.75729	0.99998	14671	0.1663
TMEM170A	6	0.65629	0.75729	0.99998	14672	0.0616
hsa-mir-486	1	0.65634	0.65642	0.99998	14673	0.1158
ATP6V1F	6	0.65644	0.75733	0.99998	14674	0.098
DCAF15	6	0.65702	0.75755	0.99998	14675	0.1677
TSHZ1	6	0.65702	0.75755	0.99998	14676	0.127
SRF	6	0.65702	0.75755	0.99998	14677	-0.0279
VMO1	6	0.65702	0.75755	0.99998	14678	0.1535
BNC1	6	0.65729	0.75766	0.99998	14679	-0.0802
SLC46A2	6	0.65737	0.75769	0.99998	14680	0.1615
ZNF703	6	0.65749	0.75773	0.99998	14681	-0.0597
ZNF615	5	0.6576	0.71351	0.99998	14682	0.0021
SLC22A15	6	0.65772	0.75782	0.99998	14683	0.2088
38231	3	0.65799	0.65826	0.99998	14684	0.0445
hsa-mir-548ae	1	0.65803	0.6581	0.99998	14685	0.16
HSPA1B	4	0.65813	0.67659	0.99998	14686	0.407
SQRDL	6	0.65815	0.75798	0.99998	14687	0.1805
OR51F2	6	0.65828	0.75803	0.99998	14688	-0.0028
ZCCHC3	6	0.65845	0.7581	0.99998	14689	0.0778
CAV2	6	0.65845	0.7581	0.99998	14690	0.0869
hsa-mir-7160	2	0.65851	0.65827	0.99998	14691	0.006
PTP4A2	6	0.65908	0.75833	0.99998	14692	0.0092
XAB2	6	0.65908	0.75833	0.99998	14693	0.0528
UGGT2	6	0.65908	0.75833	0.99998	14694	0.0924
LOC256021	5	0.65925	0.71437	0.99998	14695	0.0016
ZNF131	6	0.65931	0.75842	0.99998	14696	-0.1511
CCDC22	6	0.65959	0.75853	0.99998	14697	0.0505
KMO	6	0.65959	0.75853	0.99998	14698	-0.0861
FLG2	6	0.65987	0.75863	0.99998	14699	0.1483
CSTL1	6	0.65995	0.75866	0.99998	14700	0.1717
XK	6	0.66005	0.75869	0.99998	14701	0.1495
KRTAP21-2	6	0.66005	0.75869	0.99998	14702	-0.1945
SLC39A10	6	0.6603	0.75879	0.99998	14703	0.1014
AGA	6	0.6603	0.75879	0.99998	14704	0.1785
TMED7-TICAM	2	0.66038	0.66012	0.99998	14705	-0.0076
LMBRD1	6	0.66046	0.75885	0.99998	14706	-0.0075
NLRP5	4	0.66056	0.67835	0.99998	14707	-0.0614
WNT6	6	0.66066	0.75893	0.99998	14708	0.2567
HMGB4	6	0.66066	0.75893	0.99998	14709	0.2512
JHDM1D	6	0.66066	0.75893	0.99998	14710	0.2125
ALX1	6	0.66066	0.75893	0.99998	14711	0.1018
hsa-mir-376b	3	0.66074	0.66085	0.99998	14712	-0.1189
C7orf55-LUC7	2	0.6608	0.66052	0.99998	14713	-0.0427
KIAA0907	6	0.66086	0.75901	0.99998	14714	-0.0876
ITIH6	6	0.66086	0.75901	0.99998	14715	0.1393
TCIRG1	6	0.66103	0.75907	0.99998	14716	0.0684
FAM127B	6	0.6611	0.75909	0.99998	14717	0.305
SLC4A4	6	0.66115	0.75911	0.99998	14718	0.0448
hsa-mir-6740	4	0.66117	0.67878	0.99998	14719	0.5632
ARHGAP10	4	0.66117	0.67878	0.99998	14720	0.0766
TMEM222	6	0.66121	0.75913	0.99998	14721	0.1718
DGKD	6	0.66121	0.75913	0.99998	14722	0.0324
TRIM21	6	0.66133	0.75918	0.99998	14723	0.0403
CRYGA	6	0.66133	0.75918	0.99998	14724	-0.1144
AKR1C1	5	0.66136	0.71549	0.99998	14725	-0.3622
CLCA1	6	0.66165	0.7593	0.99998	14726	0.1905
hsa-mir-6722	4	0.66193	0.67932	0.99998	14727	0.2434
REG3G	6	0.66207	0.75945	0.99998	14728	0.0123
SEPW1	6	0.66228	0.75954	0.99998	14729	-0.0939
AHNAK2	6	0.66228	0.75954	0.99998	14730	0.2733
ZC3H7B	6	0.66247	0.75961	0.99998	14731	0.2412
TTLL6	6	0.66266	0.75969	0.99998	14732	0.0257
C17orf107	6	0.66284	0.75975	0.99998	14733	0.0555
THAP5	6	0.66284	0.75975	0.99998	14734	0.086
hsa-mir-6831	4	0.66303	0.68009	0.99998	14735	0.0532
COX11	6	0.66326	0.75989	0.99998	14736	-0.0583
hsa-mir-578	4	0.6634	0.68037	0.99998	14737	-0.1524
EEF1E1	6	0.66342	0.75995	0.99998	14738	0.0594
SUPT20H	4	0.66365	0.68055	0.99998	14739	-0.0974

CCDC120	6	0.66369	0.76007	0.99998	14740	0.2417
DLG3	6	0.66374	0.76008	0.99998	14741	0.07
SLC6A17	6	0.6639	0.76014	0.99998	14742	0.273
ARL14EPL	6	0.6639	0.76014	0.99998	14743	0.039
SMCO4	6	0.6639	0.76014	0.99998	14744	-0.0933
URGCP	1	0.6643	0.66437	0.99998	14745	0.1651
ARF6	4	0.6645	0.68118	0.99998	14746	-0.0496
WSB1	6	0.66452	0.76037	0.99998	14747	0.2942
hsa-mir-5588	2	0.66452	0.66427	0.99998	14748	0.1364
MTFR1L	6	0.66467	0.76043	0.99998	14749	-0.0021
KCNJ1	6	0.66467	0.76043	0.99998	14750	0.1131
SLC35A5	6	0.66467	0.76043	0.99998	14751	-0.0834
CHRNA3	5	0.66468	0.71721	0.99998	14752	0.0292
PERP	6	0.66482	0.76049	0.99998	14753	0.1122
GALNT5	6	0.66493	0.76053	0.99998	14754	0.0531
C4BPA	6	0.6651	0.76059	0.99998	14755	0.0643
PDXK	6	0.6653	0.76066	0.99998	14756	-0.0577
F2R	6	0.66547	0.76072	0.99998	14757	0.0414
FOPNL	6	0.66547	0.76072	0.99998	14758	0.0541
TRIM6-TRIM3	4	0.66559	0.68201	0.99998	14759	0.2023
TRIP6	6	0.66566	0.76079	0.99998	14760	0.0089
GPR15	6	0.66574	0.76083	0.99998	14761	0.0311
WFDC10B	5	0.66578	0.71781	0.99998	14762	0.4854
TGIF2-C20orf2	1	0.66582	0.6659	0.99998	14763	0.1707
SERPINA7	6	0.66585	0.76086	0.99998	14764	0.0357
DMRTB1	6	0.66585	0.76086	0.99998	14765	0.0547
RPRML	6	0.66603	0.76094	0.99998	14766	0.0141
PAX4	6	0.6662	0.761	0.99998	14767	0.1139
FAM72D	1	0.6663	0.66637	0.99998	14768	0.1078
OR5K1	3	0.66671	0.66664	0.99998	14769	-0.0981
MKI67	6	0.66675	0.76121	0.99998	14770	0.0646
PCNT	6	0.66675	0.76121	0.99998	14771	0.1498
DRGX	4	0.6668	0.6829	0.99998	14772	0.1331
hsa-mir-634	4	0.66698	0.68303	0.99998	14773	0.1836
SPPL3	4	0.66708	0.68311	0.99998	14774	0.2589
SLC35E2B	2	0.66715	0.66693	0.99998	14775	-0.2068
hsa-mir-941-1	2	0.66715	0.66693	0.99998	14776	0.4758
OR4F5	2	0.66715	0.66693	0.99998	14777	0.1909
PARD6A	6	0.6672	0.7614	0.99998	14778	-0.1294
FAM83D	6	0.6672	0.7614	0.99998	14779	-0.0692
TAS2R31	6	0.6672	0.7614	0.99998	14780	-0.0583
GPER1	4	0.66744	0.68336	0.99998	14781	0.0379
SEC22C	6	0.66744	0.7615	0.99998	14782	0.057
ANXA2R	6	0.66757	0.76156	0.99998	14783	0.2125
hsa-mir-190a	2	0.66764	0.66747	0.99998	14784	0.1172
CLIP1	6	0.6677	0.76161	0.99998	14785	0.4956
hsa-mir-942	4	0.66775	0.68359	0.99998	14786	0.1014
hsa-mir-4513	4	0.66775	0.68359	0.99998	14787	0.0349
C19orf73	6	0.6678	0.76165	0.99998	14788	0.1383
USHBP1	6	0.66793	0.7617	0.99998	14789	0.0916
SYCE1L	6	0.66793	0.7617	0.99998	14790	0.1377
hsa-mir-1291	4	0.66797	0.68375	0.99998	14791	0.0915
PRSS3	6	0.6681	0.76177	0.99998	14792	0.1381
TAS2R43	6	0.66829	0.76184	0.99998	14793	-0.0508
hsa-mir-6871	4	0.66841	0.68409	0.99998	14794	0.1788
ASB3	3	0.66846	0.66831	0.99998	14795	-0.1951
hsa-mir-4663	4	0.66869	0.68429	0.99998	14796	0.0956
GPR4	6	0.66871	0.76202	0.99998	14797	0.0232
IFRD1	6	0.66871	0.76202	0.99998	14798	0.184
PINK1	6	0.6688	0.76206	0.99998	14799	0.0154
TCEANC	5	0.66887	0.71944	0.99998	14800	0.2649
PHOSPHO2	6	0.66898	0.76213	0.99998	14801	0.1499
hsa-mir-676	4	0.66914	0.68464	0.99998	14802	-0.0838
ADAM9	6	0.66918	0.76219	0.99998	14803	0.0166
C7orf25	6	0.66923	0.76221	0.99998	14804	0.0535
SRGAP2	4	0.66923	0.6847	0.99998	14805	-0.0131
CD109	4	0.66923	0.6847	0.99998	14806	-0.052
PAQR9	6	0.66948	0.7623	0.99998	14807	0.0673
ZNF878	6	0.66967	0.76237	0.99998	14808	0.1503
hsa-mir-4756	4	0.66972	0.68506	0.99998	14809	-0.177
QSOX2	6	0.67029	0.76261	0.99998	14810	0.1837
GPC6	6	0.67029	0.76261	0.99998	14811	0.1109
C19orf45	6	0.67041	0.76266	0.99998	14812	-0.0003
SLC2A2	6	0.67047	0.76269	0.99998	14813	-0.0248
SCLT1	6	0.67047	0.76269	0.99998	14814	0.0848
CDK4	6	0.67054	0.76272	0.99998	14815	-0.0678
ZNRF3	6	0.67054	0.76272	0.99998	14816	0.1456
FAM177A1	6	0.67059	0.76274	0.99998	14817	0.2083
CCDC47	6	0.67067	0.76277	0.99998	14818	0.0201
VTCN1	6	0.67067	0.76277	0.99998	14819	0.1082
NPBWR1	6	0.67082	0.76283	0.99998	14820	-0.0005

SMIM4	4	0.67093	0.68596	0.99998	14821	0.1929
hsa-mir-4435-	3	0.67121	0.67102	0.99998	14822	0.0448
DNAJB2	6	0.67126	0.76299	0.99998	14823	0.0831
hsa-mir-6084	4	0.6713	0.68625	0.99998	14824	0.1815
PHACTR1	6	0.67133	0.76303	0.99998	14825	0.1497
KLK15	6	0.67166	0.76315	0.99998	14826	0.0378
UBE2R2	6	0.67166	0.76315	0.99998	14827	0.0056
LURAP1L	6	0.67166	0.76315	0.99998	14828	0.2716
ZFAND3	6	0.67166	0.76315	0.99998	14829	0.0715
LRRC17	6	0.67166	0.76315	0.99998	14830	-0.0222
TFCP2	6	0.67182	0.76321	0.99998	14831	0.0141
PPAP2B	6	0.67193	0.76325	0.99998	14832	0.1539
SMPDL3B	6	0.67207	0.76329	0.99998	14833	0.004
GLOD4	6	0.67207	0.76329	0.99998	14834	0.0271
R3HDM4	6	0.67222	0.76335	0.99998	14835	-0.0482
KCNS3	6	0.67222	0.76335	0.99998	14836	-0.0788
PORCN	6	0.67239	0.76341	0.99998	14837	-0.0306
TRIM77	6	0.67251	0.76345	0.99998	14838	-0.0057
KRT73	6	0.67251	0.76345	0.99998	14839	-0.1493
FBXL4	6	0.67269	0.76352	0.99998	14840	0.1174
MAP7D1	6	0.67269	0.76352	0.99998	14841	0.062
hsa-mir-653	4	0.67279	0.68739	0.99998	14842	0.3329
OR52K2	6	0.67289	0.7636	0.99998	14843	0.1235
IL31	6	0.67289	0.7636	0.99998	14844	0.0501
ACKR4	5	0.67297	0.72168	0.99998	14845	0.1785
ZNF85	4	0.67331	0.68776	0.99998	14846	0.0374
hsa-mir-129-1	4	0.67358	0.68796	0.99998	14847	0.1522
C16orf54	6	0.67364	0.76389	0.99998	14848	0.0977
hsa-mir-211	4	0.67367	0.68803	0.99998	14849	0.1969
ZIM2	1	0.67374	0.67381	0.99998	14850	0.2788
TOX4	6	0.67377	0.76393	0.99998	14851	-0.0531
ERGIC3	6	0.67377	0.76393	0.99998	14852	0.1671
KIAA1671	6	0.6739	0.76398	0.99998	14853	0.1123
KLHL40	6	0.6739	0.76398	0.99998	14854	0.1372
SRY	6	0.67409	0.76405	0.99998	14855	0.0785
COMP	4	0.67439	0.68856	0.99998	14856	-0.0392
RNF219	6	0.67457	0.76423	0.99998	14857	0.0147
DDTL	2	0.67458	0.6744	0.99998	14858	0.2287
PCDHA7	2	0.67473	0.67455	0.99998	14859	0.2474
CASC4	6	0.67508	0.76443	0.99998	14860	-0.0252
MAPK8IP3	6	0.6751	0.76444	0.99998	14861	0.0061
hsa-mir-5003	4	0.67516	0.68915	0.99998	14862	0.124
NID1	6	0.67516	0.76447	0.99998	14863	0.0944
OR9I1	6	0.67516	0.76447	0.99998	14864	0.2123
MPZL1	6	0.67537	0.76455	0.99998	14865	0.1876
CEP85L	6	0.67553	0.76461	0.99998	14866	0.0148
STX19	6	0.67553	0.76461	0.99998	14867	0.1224
TRIML1	6	0.67553	0.76461	0.99998	14868	-0.006
TSPAN15	6	0.67553	0.76461	0.99998	14869	0.2078
SEL1L2	4	0.67586	0.68967	0.99998	14870	-0.0274
CD244	6	0.67587	0.76475	0.99998	14871	0.1042
ZNF665	5	0.67623	0.72348	0.99998	14872	-0.0027
PROK2	6	0.67625	0.76489	0.99998	14873	-0.0369
FARP2	6	0.67643	0.76495	0.99998	14874	0.195
RNF213	6	0.67662	0.76503	0.99998	14875	0.0672
LOC10050746	5	0.67681	0.7238	0.99998	14876	-0.0891
CMKLR1	6	0.67703	0.7652	0.99998	14877	0.0644
GSDMA	6	0.67703	0.7652	0.99998	14878	0.1061
ARHGEF6	6	0.67703	0.7652	0.99998	14879	0.2878
ZNF160	6	0.67735	0.76532	0.99998	14880	0.2169
hsa-mir-624	4	0.67739	0.69082	0.99998	14881	0.0658
hsa-mir-135a-	4	0.67739	0.69082	0.99998	14882	0.1257
hsa-mir-8079	4	0.67758	0.69097	0.99998	14883	0.3992
hsa-mir-548ag	3	0.67772	0.67753	0.99998	14884	-0.1246
RIPK4	6	0.67781	0.76549	0.99998	14885	0.4527
TMEFF1	3	0.67794	0.67774	0.99998	14886	0.0254
BLCAP	6	0.67801	0.76557	0.99998	14887	0.0326
FAM185A	6	0.67812	0.76561	0.99998	14888	0.0887
PHF3	6	0.67838	0.76571	0.99998	14889	0.2483
SHISA9	6	0.67838	0.76571	0.99998	14890	0.1083
ALS2CR8	6	0.67838	0.76571	0.99998	14891	0.2343
FAM83C	6	0.67838	0.76571	0.99998	14892	0.4453
LIPE	6	0.67838	0.76571	0.99998	14893	-0.0076
CYB5RL	6	0.67852	0.76576	0.99998	14894	-0.0781
hsa-mir-4445	2	0.67865	0.67856	0.99998	14895	0.1236
ARHGAP44	6	0.67874	0.76585	0.99998	14896	0.0846
ATP10A	6	0.67899	0.76594	0.99998	14897	0.0595
CCDC71	6	0.67899	0.76594	0.99998	14898	-0.013
CXCL12	6	0.67922	0.76604	0.99998	14899	0.0157
PKD2	6	0.67922	0.76604	0.99998	14900	0.0593
BACE2	6	0.67922	0.76604	0.99998	14901	0.0399

TNNT1	6	0.67922	0.76604	0.99998	14902	-0.0284
CREB3L1	4	0.6793	0.6923	0.99998	14903	0.286
FAM25A	5	0.67933	0.72519	0.99998	14904	-0.0387
KIAA1984	6	0.67946	0.76612	0.99998	14905	-0.0353
CTAGE4	4	0.6795	0.69244	0.99998	14906	-0.0144
HNRNPLL	4	0.67961	0.69251	0.99998	14907	0.1906
ERCC6L2	6	0.6797	0.76622	0.99998	14908	0.0886
MZT2A	2	0.67973	0.67967	0.99998	14909	0.1265
IRF7	6	0.67982	0.76626	0.99998	14910	0.0848
C19orf48	6	0.67982	0.76626	0.99998	14911	0.0521
RASAL2	6	0.67982	0.76626	0.99998	14912	0.0757
PGF	6	0.67995	0.76632	0.99998	14913	0.0824
TCF7	6	0.68007	0.76637	0.99998	14914	-0.0429
PCDH12	6	0.68027	0.76645	0.99998	14915	0.0965
PUS1	6	0.68041	0.7665	0.99998	14916	0.3426
GUCY2D	6	0.68041	0.7665	0.99998	14917	-0.0485
FXR2	6	0.68041	0.7665	0.99998	14918	-0.0944
TMEM63B	6	0.68041	0.7665	0.99998	14919	0.1653
NECAB3	6	0.68041	0.7665	0.99998	14920	0.3203
ZNF254	4	0.68058	0.69327	0.99998	14921	0.1041
40057	3	0.68075	0.68061	0.99998	14922	-0.1572
C16orf90	6	0.68076	0.76664	0.99998	14923	-0.0365
NSMCE4A	6	0.68076	0.76664	0.99998	14924	0.0214
hsa-mir-513b	4	0.68094	0.69355	0.99998	14925	-0.6356
hsa-mir-18b	4	0.68094	0.69355	0.99998	14926	-0.046
FASTK	6	0.68099	0.76673	0.99998	14927	0.0636
AGAP11	6	0.68106	0.76675	0.99998	14928	0.1628
C19orf18	6	0.68112	0.76677	0.99998	14929	0.2675
TRAT1	5	0.68121	0.72622	0.99998	14930	0.2327
ULK4	6	0.68146	0.76691	0.99998	14931	0.1364
GOT2	6	0.68162	0.76698	0.99998	14932	0.1734
CACNG6	6	0.68172	0.76702	0.99998	14933	0.0621
ECM1	6	0.68192	0.7671	0.99998	14934	-0.0355
CDH26	6	0.68212	0.76717	0.99998	14935	-0.0088
LIM2	6	0.68218	0.76719	0.99998	14936	0.1646
KIAA1467	6	0.68246	0.7673	0.99998	14937	-0.0128
ZNF865	6	0.68259	0.76735	0.99998	14938	0.089
TMEM9B	6	0.68259	0.76735	0.99998	14939	-0.0181
TUBA3D	4	0.68261	0.69482	0.99998	14940	0.1932
PRAMEF18	4	0.68261	0.69482	0.99998	14941	-0.4061
MT1F	4	0.68261	0.69482	0.99998	14942	-0.9148
hsa-mir-371b	1	0.68277	0.68284	0.99998	14943	0.1587
FAM64A	6	0.6828	0.76743	0.99998	14944	0.1535
PENK	6	0.68287	0.76746	0.99998	14945	0.004
LRRK1	6	0.68296	0.76749	0.99998	14946	0.2557
hsa-mir-7974	4	0.68299	0.69511	0.99998	14947	0.0739
ANXA9	6	0.68322	0.76759	0.99998	14948	0.0642
hsa-mir-6072	4	0.68329	0.69534	0.99998	14949	0.2727
hsa-mir-4721	4	0.68337	0.69539	0.99998	14950	0.0404
ENPEP	6	0.68351	0.76771	0.99998	14951	0.1056
FADS6	6	0.68363	0.76777	0.99998	14952	0.0529
LRRC27	6	0.68363	0.76777	0.99998	14953	0.0952
ATG7	6	0.68363	0.76777	0.99998	14954	0.0029
MCM8	6	0.68378	0.76782	0.99998	14955	-0.0135
KANSL2	4	0.68397	0.69586	0.99998	14956	0.1279
NPAS4	6	0.68399	0.7679	0.99998	14957	0.0022
MESP2	6	0.68437	0.76805	0.99998	14958	-0.0377
GSTT2B	1	0.6844	0.68448	0.99998	14959	0.5478
SPAG11B	2	0.68442	0.68431	0.99998	14960	0.0924
USP54	6	0.68454	0.76811	0.99998	14961	0.0676
LGI4	6	0.68454	0.76811	0.99998	14962	0.2826
SMKR1	6	0.68471	0.76818	0.99998	14963	0.2439
TENM4	6	0.68495	0.76827	0.99998	14964	0.0036
B3GNT5	6	0.68495	0.76827	0.99998	14965	-0.0922
PDPN	6	0.68495	0.76827	0.99998	14966	0.0799
CSB-PGBD3	2	0.68501	0.6849	0.99998	14967	-0.0244
hsa-mir-6774	4	0.68505	0.69667	0.99998	14968	0.0282
WDR93	6	0.68522	0.76838	0.99998	14969	-0.0145
GATSL1	2	0.68531	0.68521	0.99998	14970	-0.01
hsa-mir-936	4	0.68556	0.69706	0.99998	14971	0.2071
TMEM176B	6	0.6857	0.76857	0.99998	14972	0.2139
GUCA2B	6	0.6857	0.76857	0.99998	14973	0.0483
CD22	6	0.68591	0.76865	0.99998	14974	0.0455
LUM	6	0.68598	0.76868	0.99998	14975	0.3303
NCOA4	6	0.6861	0.76873	0.99998	14976	0.2097
SEC11C	6	0.6861	0.76873	0.99998	14977	0.1052
KHDRBS3	6	0.68644	0.76887	0.99998	14978	-0.0793
PDP1	6	0.68644	0.76887	0.99998	14979	-0.1987
RAMP3	6	0.68658	0.76892	0.99998	14980	-0.0461
EXOC6	6	0.68669	0.76896	0.99998	14981	0.0193
TRIML2	6	0.68687	0.76903	0.99998	14982	0.1206

IL31RA	6	0.68687	0.76903	0.99998	14983	-0.0417
OR2B11	6	0.68694	0.76907	0.99998	14984	-0.0496
APOL2	5	0.6871	0.72958	0.99998	14985	-0.0839
PALLD	4	0.68716	0.69826	0.99998	14986	0.1007
TGFA	6	0.68747	0.76928	0.99998	14987	0.1436
ZNF586	6	0.68747	0.76928	0.99998	14988	-0.0254
ATP2B2	6	0.68747	0.76928	0.99998	14989	0.2359
CLDN6	6	0.68782	0.76942	0.99998	14990	0.1518
hsa-mir-548j	4	0.68788	0.69878	0.99998	14991	-0.0017
SLFN13	6	0.68798	0.76949	0.99998	14992	0.2277
ZNF747	6	0.68798	0.76949	0.99998	14993	0.0884
AIF1L	6	0.68798	0.76949	0.99998	14994	0.2971
HLA-DRB5	6	0.68798	0.76949	0.99998	14995	-0.0032
USP32	6	0.68823	0.7696	0.99998	14996	0.1444
ZNF883	6	0.68836	0.76965	0.99998	14997	-0.0556
ARSJ	6	0.6884	0.76966	0.99998	14998	-0.1015
MIP	6	0.68857	0.76973	0.99998	14999	-0.0594
SQSTM1	6	0.68857	0.76973	0.99998	15000	0.2605
hsa-mir-6812	4	0.68868	0.6994	0.99998	15001	0.1191
TMED9	6	0.68878	0.76983	0.99998	15002	-0.0491
ZNF786	6	0.68878	0.76983	0.99998	15003	-0.0555
GTF2IRD2B	2	0.689	0.68884	0.99998	15004	0.2323
CYP4B1	6	0.68902	0.76993	0.99998	15005	0.0845
C6orf141	6	0.68902	0.76993	0.99998	15006	0.1594
COMMD9	6	0.68912	0.76996	0.99998	15007	0.0702
FAM72B	2	0.68916	0.689	0.99998	15008	0.0987
PYROXD1	6	0.6893	0.77004	0.99998	15009	0.222
ZNF449	6	0.68956	0.77014	0.99998	15010	0.2059
hsa-mir-9-3	4	0.68968	0.70018	0.99998	15011	-0.1421
PHF20	6	0.6897	0.7702	0.99998	15012	-0.0693
STAG1	6	0.68979	0.77024	0.99998	15013	-0.0633
MRPL15	6	0.68985	0.77026	0.99998	15014	0.2838
LCN15	6	0.68985	0.77026	0.99998	15015	-0.0484
hsa-mir-3646	4	0.68987	0.70033	0.99998	15016	0.0497
POLL	6	0.68992	0.77029	0.99998	15017	0.1138
HIF1AN	6	0.68998	0.77031	0.99998	15018	0.1266
hsa-mir-6827	4	0.69019	0.70057	0.99998	15019	-0.3524
ZNF766	6	0.69019	0.77041	0.99998	15020	0.1445
TRIM7	6	0.69019	0.77041	0.99998	15021	0.2939
EFEMP1	6	0.69078	0.77063	0.99998	15022	0.1736
SASH3	6	0.69085	0.77067	0.99998	15023	0.3322
FEZ2	6	0.69085	0.77067	0.99998	15024	0.1911
hsa-mir-6843	4	0.69096	0.70118	0.99998	15025	-0.0304
ILVBL	6	0.69103	0.77074	0.99998	15026	0.1176
hsa-mir-526a-	1	0.69107	0.69114	0.99998	15027	0.4961
hsa-mir-5704	4	0.69113	0.70132	0.99998	15028	-0.0609
CDH18	6	0.69116	0.7708	0.99998	15029	-0.0171
ACOT4	6	0.6912	0.77081	0.99998	15030	0.0575
C1orf159	6	0.69145	0.77091	0.99998	15031	0.1302
ITSN2	6	0.69145	0.77091	0.99998	15032	0.2091
OR13C3	6	0.6919	0.77108	0.99998	15033	0.0115
OR51D1	6	0.6919	0.77108	0.99998	15034	0.0765
SEMA4G	6	0.69207	0.77116	0.99998	15035	-0.019
INPP5K	6	0.69212	0.77118	0.99998	15036	0.0352
OR51T1	6	0.69219	0.77121	0.99998	15037	0.0772
DTX3L	6	0.69225	0.77124	0.99998	15038	0.0965
C5orf46	6	0.69232	0.77126	0.99998	15039	0.1764
ASB8	6	0.69232	0.77126	0.99998	15040	0.0499
C7orf29	3	0.69255	0.69243	0.99998	15041	-0.0646
ZNF585A	6	0.69262	0.77139	0.99998	15042	-0.0627
IFIT1B	6	0.69264	0.77139	0.99998	15043	0.1162
DSEL	6	0.69266	0.7714	0.99998	15044	0.2958
SERPIND1	6	0.69268	0.77141	0.99998	15045	0.2826
IDH1	6	0.69294	0.77152	0.99998	15046	0.141
38961	3	0.69317	0.69305	0.99998	15047	0.0558
VEZF1	6	0.69346	0.77174	0.99998	15048	-0.045
KDELC1	6	0.69358	0.77179	0.99998	15049	-0.0013
TAOK2	4	0.69362	0.7033	0.99998	15050	0.0309
ANKRA2	6	0.69383	0.77189	0.99998	15051	0.068
TENM3	6	0.69383	0.77189	0.99998	15052	0.1859
CHRM2	6	0.69383	0.77189	0.99998	15053	0.0886
PTPRC	6	0.69398	0.77195	0.99998	15054	0.0655
ZNF326	6	0.69405	0.77197	0.99998	15055	0.0383
MTIF2	6	0.69425	0.77206	0.99998	15056	0.1658
TATDN2	6	0.69425	0.77206	0.99998	15057	0.0142
ALPPL2	5	0.69434	0.73368	0.99998	15058	0.1851
hsa-mir-30e	4	0.69437	0.70387	0.99998	15059	0.0196
ZNF24	6	0.69444	0.77213	0.99998	15060	-0.0061
CDK5R1	6	0.69444	0.77213	0.99998	15061	0.1667
DGCR14	4	0.69444	0.70393	0.99998	15062	0.2
SLC26A7	4	0.69444	0.70393	0.99998	15063	0.0679

FAM150A	6	0.69462	0.7722	0.99998	15064	0.1845
PACRGL	6	0.69474	0.77224	0.99998	15065	0.0378
TANK	6	0.69483	0.77229	0.99998	15066	0.1133
GRM8	6	0.69497	0.77234	0.99998	15067	-0.0008
CXorf48	6	0.69512	0.7724	0.99998	15068	-0.0295
SLC31A2	6	0.69519	0.77243	0.99998	15069	0.1717
PCDHGA11	2	0.69523	0.69492	0.99998	15070	0.0559
MFHAS1	6	0.69523	0.77244	0.99998	15071	0.1089
TOR1A	6	0.69535	0.7725	0.99998	15072	-0.0397
KIAA1704	3	0.69552	0.69543	0.99998	15073	0.0942
PIK3CD	6	0.69555	0.7726	0.99998	15074	0.1607
MARCH4	6	0.69561	0.77262	0.99998	15075	0.2114
hsa-mir-1282	4	0.69566	0.70483	0.99998	15076	0.0004
hsa-mir-1204	4	0.6957	0.70485	0.99998	15077	-0.0448
MOSPD3	6	0.69571	0.77266	0.99998	15078	0.2071
RNASE8	6	0.69588	0.77273	0.99998	15079	0.0334
NINJ2	6	0.69588	0.77273	0.99998	15080	-0.0362
SORCS2	6	0.69596	0.77277	0.99998	15081	0.1027
C19orf21	1	0.69609	0.69614	0.99998	15082	0.1301
CRYM	6	0.69611	0.77283	0.99998	15083	-0.0091
PNPLA7	6	0.69627	0.7729	0.99998	15084	0.1105
ZNF669	6	0.69636	0.77293	0.99998	15085	-0.0136
ARSH	6	0.69649	0.77299	0.99998	15086	0.2287
KIAA0556	6	0.69656	0.77302	0.99998	15087	0.0598
LYRM1	6	0.69656	0.77302	0.99998	15088	-0.0173
MYBPC2	6	0.69667	0.77306	0.99998	15089	0.0782
CIITA	6	0.69685	0.77313	0.99998	15090	0.1373
TMTC4	6	0.69708	0.77323	0.99998	15091	0.1764
PITX2	6	0.69733	0.77335	0.99998	15092	0.1266
OR1J1	6	0.69738	0.77338	0.99998	15093	-0.0281
GLRA3	6	0.69746	0.77342	0.99998	15094	0.1381
MYH1	5	0.6976	0.73559	0.99998	15095	0.3347
FER	6	0.69784	0.77359	0.99998	15096	0.0963
MON1A	6	0.69784	0.77359	0.99998	15097	0.0555
IL21R	6	0.69807	0.77369	0.99998	15098	0.1326
C20orf26	6	0.69807	0.77369	0.99998	15099	0.0385
CCNDBP1	6	0.69807	0.77369	0.99998	15100	0.1757
hsa-mir-6835	2	0.69812	0.69781	0.99998	15101	0.1002
STS	6	0.69823	0.77375	0.99998	15102	0.0644
GPR62	6	0.69831	0.77379	0.99998	15103	0.1347
NEK6	6	0.6984	0.77383	0.99998	15104	-0.0069
CAMSAP1	6	0.69848	0.77386	0.99998	15105	0.4071
HAMP	6	0.69892	0.77404	0.99998	15106	-0.0177
SLC10A5	6	0.69892	0.77404	0.99998	15107	0.0282
CNBP	6	0.69912	0.77414	0.99998	15108	-0.0327
GJD2	6	0.69912	0.77414	0.99998	15109	0.05
PHOSPHO2-KL	2	0.69914	0.69886	0.99998	15110	0.0532
hsa-mir-4796	4	0.69923	0.70767	0.99998	15111	-0.1025
JOSD2	6	0.69936	0.77424	0.99998	15112	0.3283
GRN	6	0.69936	0.77424	0.99998	15113	0.3036
hsa-mir-8084	4	0.69942	0.70783	0.99998	15114	0.1691
hsa-mir-6068	4	0.69954	0.70793	0.99998	15115	0.1152
PCOLCE	6	0.69969	0.77437	0.99998	15116	0.1475
FXYD7	6	0.69969	0.77437	0.99998	15117	0.132
C10orf107	6	0.69969	0.77437	0.99998	15118	0.1358
NKD1	6	0.69969	0.77437	0.99998	15119	0.1384
hsa-mir-6078	4	0.69991	0.70821	0.99998	15120	0.0728
C10orf111	6	0.69991	0.77446	0.99998	15121	0.2294
CABYR	6	0.69991	0.77446	0.99998	15122	0.0997
FAM25C	1	0.70001	0.70009	0.99998	15123	0.6889
PODN	6	0.7001	0.77455	0.99998	15124	0.0508
SLC34A2	6	0.70019	0.77458	0.99998	15125	0.1036
BCL11B	6	0.70042	0.77467	0.99998	15126	0.2006
hsa-mir-548k	4	0.70044	0.70863	0.99998	15127	0.0079
MAP2K5	6	0.70056	0.77474	0.99998	15128	-0.0731
GNPDA2	6	0.70088	0.77487	0.99998	15129	0.0169
ANKS4B	6	0.70088	0.77487	0.99998	15130	0.2521
hsa-mir-6129	4	0.70088	0.70896	0.99998	15131	0.0462
CHD6	6	0.70101	0.77493	0.99998	15132	0.0969
SLC6A1	6	0.7011	0.77496	0.99998	15133	0.0393
HIST1H2BO	6	0.7011	0.77496	0.99998	15134	-0.1726
TMEM8A	6	0.70118	0.775	0.99998	15135	0.114
RPH3AL	6	0.70126	0.77504	0.99998	15136	0.2442
ADPRHL2	6	0.70135	0.77507	0.99998	15137	-0.0161
ARHGAP1	6	0.70149	0.77513	0.99998	15138	-0.0478
hsa-mir-125a	4	0.70149	0.70944	0.99998	15139	0.0701
hsa-mir-382	3	0.70154	0.70149	0.99998	15140	-0.151
DDO	6	0.70166	0.7752	0.99998	15141	0.2105
LRRTM1	6	0.70166	0.7752	0.99998	15142	0.1043
hsa-mir-1-2	3	0.70183	0.70177	0.99998	15143	0.0103
TEX261	6	0.70184	0.77527	0.99998	15144	0.0224

GDI1	6	0.70202	0.77534	0.99998	15145	-0.049
GPI	6	0.70207	0.77536	0.99998	15146	0.1812
ADCK1	6	0.70215	0.77539	0.99998	15147	0.1092
OR2T5	3	0.70222	0.7022	0.99998	15148	-0.7128
hsa-mir-526b	3	0.70222	0.7022	0.99998	15149	-0.9148
hsa-mir-218-2	3	0.70222	0.7022	0.99998	15150	0.2006
PIK3R3	6	0.70238	0.77549	0.99998	15151	0.1108
PAX7	6	0.70238	0.77549	0.99998	15152	0.1754
PSMB11	6	0.70238	0.77549	0.99998	15153	0.1354
VPS13B	6	0.70262	0.7756	0.99998	15154	0.1123
CRISP3	6	0.70262	0.7756	0.99998	15155	0.1155
ALDH1A3	6	0.70285	0.77571	0.99998	15156	-0.0136
FBXO48	6	0.70285	0.77571	0.99998	15157	0.0787
DNHD1	6	0.70285	0.77571	0.99998	15158	-0.0192
VSTM4	6	0.70311	0.77582	0.99998	15159	0.1839
EBLN2	6	0.70315	0.77584	0.99998	15160	-0.0661
hsa-mir-10b	4	0.70325	0.71085	0.99998	15161	0.1674
MAP3K10	6	0.70325	0.77589	0.99998	15162	-0.0575
GXYLT2	6	0.70325	0.77589	0.99998	15163	0.087
MRGPRX2	6	0.70358	0.77602	0.99998	15164	0.0988
POFUT2	6	0.70386	0.77613	0.99998	15165	0.1256
MT2A	3	0.70389	0.70388	0.99998	15166	1.2443
hsa-mir-4667	4	0.70394	0.71139	0.99998	15167	0.0058
PLEKHA5	6	0.70429	0.77631	0.99998	15168	0.1044
CTSL	3	0.70443	0.70444	0.99998	15169	-0.1
C14orf39	6	0.70459	0.77644	0.99998	15170	0.0591
FAAH2	6	0.70459	0.77644	0.99998	15171	0.1539
ALDH9A1	6	0.70472	0.7765	0.99998	15172	-0.0475
APLP1	6	0.70485	0.77654	0.99998	15173	-0.1113
SLC30A5	4	0.70493	0.71218	0.99998	15174	-0.0112
KLHL33	6	0.70509	0.77665	0.99998	15175	0.0006
SLC22A3	6	0.70509	0.77665	0.99998	15176	0.3036
SERPINB10	6	0.70534	0.77675	0.99998	15177	0.1776
AREG	6	0.70534	0.77675	0.99998	15178	-0.0568
SLFN11	6	0.7056	0.77687	0.99998	15179	0.1872
FGD3	6	0.7056	0.77687	0.99998	15180	0.0224
MCIDAS	6	0.7056	0.77687	0.99998	15181	0.3111
SYNGR4	6	0.70575	0.77693	0.99998	15182	0.0435
37865	3	0.70591	0.70592	0.99998	15183	-0.0062
RBM48	6	0.70607	0.77706	0.99998	15184	0.0115
SLC16A6	6	0.70623	0.77712	0.99998	15185	-0.0309
EAF1	6	0.7064	0.7772	0.99998	15186	-0.0409
ACIN1	6	0.70658	0.77728	0.99998	15187	0.2196
DYRK4	6	0.70658	0.77728	0.99998	15188	0.1253
ABLIM2	6	0.70658	0.77728	0.99998	15189	0.1348
ELMOD1	6	0.70671	0.77733	0.99998	15190	0.0395
DUSP21	6	0.70671	0.77733	0.99998	15191	0.342
CCL4	5	0.70683	0.74101	0.99998	15192	-0.0004
hsa-mir-3974	4	0.70699	0.71389	0.99998	15193	0.0801
NCDN	6	0.70699	0.77744	0.99998	15194	0.0609
PRR5L	6	0.70716	0.77751	0.99998	15195	0.2529
SFTPA1	5	0.70748	0.7414	0.99998	15196	-0.0355
TNKS2	5	0.70748	0.7414	0.99998	15197	0.0397
PAK3	6	0.70753	0.77768	0.99998	15198	0.2475
KIAA1430	6	0.70759	0.7777	0.99998	15199	0.0333
ZDHHC5	6	0.70776	0.77777	0.99998	15200	0.1486
PYCARD	6	0.70792	0.77785	0.99998	15201	-0.0102
ACBD7	6	0.70792	0.77785	0.99998	15202	0.0793
RAI2	6	0.70809	0.77791	0.99998	15203	0.0958
DACT2	6	0.70809	0.77791	0.99998	15204	0.0896
PSG3	5	0.70816	0.74181	0.99998	15205	0.0569
CASP1	5	0.70825	0.74187	0.99998	15206	0.0548
LTBR	6	0.70842	0.77804	0.99998	15207	0.0938
DNM3	6	0.70858	0.77811	0.99998	15208	-0.0627
PTK2B	6	0.70858	0.77811	0.99998	15209	0.216
hsa-mir-665	4	0.70859	0.71518	0.99998	15210	-0.0358
ZNF578	3	0.70862	0.70862	0.99998	15211	0.1932
DERL1	6	0.70876	0.77818	0.99998	15212	-0.1228
PJA1	6	0.70876	0.77818	0.99998	15213	-0.0575
MAGEF1	6	0.70899	0.77827	0.99998	15214	0.2083
VAT1L	6	0.70899	0.77827	0.99998	15215	-0.0021
SLC35A3	6	0.7091	0.77832	0.99998	15216	0.0731
OR5AP2	6	0.70914	0.77834	0.99998	15217	0.0915
CASQ2	6	0.70935	0.77845	0.99998	15218	0.2016
TXLNG	6	0.70935	0.77845	0.99998	15219	0.0542
PER3	6	0.70956	0.77853	0.99998	15220	0.1485
CEACAM16	6	0.70956	0.77853	0.99998	15221	0.0536
GSPT2	6	0.70973	0.77861	0.99998	15222	0.1648
GNG8	6	0.70993	0.77869	0.99998	15223	0.0293
ARX	6	0.70993	0.77869	0.99998	15224	0.0771
MASP2	4	0.71042	0.71663	0.99998	15225	0.0346

UBTD2	6	0.71054	0.77895	0.99998	15226	0.3755
CA12	6	0.71072	0.77901	0.99998	15227	0.1576
C16orf70	6	0.71074	0.77902	0.99998	15228	0.0582
PCDHA6	2	0.71076	0.71056	0.99998	15229	-0.0146
CCDC70	6	0.71086	0.77908	0.99998	15230	0.0519
hsa-mir-20a	1	0.71089	0.71108	0.99998	15231	0.2375
ANKRD22	6	0.71097	0.77912	0.99998	15232	0.2263
CAPN10	6	0.7111	0.77917	0.99998	15233	-0.0415
CLEC6A	6	0.71127	0.77925	0.99998	15234	0.1298
POMGNT2	4	0.7113	0.71733	0.99998	15235	0.3571
BIRC8	6	0.71134	0.77928	0.99998	15236	0.1695
PLG	6	0.71138	0.7793	0.99998	15237	-0.0247
SLC39A2	6	0.71162	0.7794	0.99998	15238	0.1644
STRA8	6	0.71162	0.7794	0.99998	15239	0.2178
KLHL5	6	0.71203	0.77958	0.99998	15240	0.0686
hsa-mir-526a-	3	0.71212	0.71209	0.99998	15241	0.257
CTAGE9	3	0.71212	0.71209	0.99998	15242	-0.1769
hsa-mir-548o-	3	0.71212	0.71209	0.99998	15243	0.2298
hsa-mir-4436t	3	0.71212	0.71209	0.99998	15244	-0.9148
LCE2C	3	0.71212	0.71209	0.99998	15245	0.0701
ORM2	3	0.71212	0.71209	0.99998	15246	0.2502
hsa-mir-548al	3	0.71212	0.71209	0.99998	15247	0.1794
LOC10012921	3	0.71212	0.71209	0.99998	15248	-0.1106
hsa-mir-4661	3	0.71212	0.71209	0.99998	15249	-0.0498
hsa-mir-548at	3	0.71212	0.71209	0.99998	15250	-0.2809
USP17L3	3	0.71212	0.71209	0.99998	15251	-0.9148
MTRNR2L10	3	0.71212	0.71209	0.99998	15252	0.095
MT1G	3	0.71212	0.71209	0.99998	15253	0.8124
hsa-mir-4759	3	0.71212	0.71209	0.99998	15254	0.1932
hsa-mir-525	3	0.71212	0.71209	0.99998	15255	0.1932
CXCL2	3	0.71212	0.71209	0.99998	15256	-0.9148
CXCL1	6	0.71214	0.77963	0.99998	15257	-0.1029
PSG5	6	0.71214	0.77963	0.99998	15258	0.127
ZNF221	4	0.71229	0.71814	0.99998	15259	0.0483
DCAF5	6	0.7126	0.77983	0.99998	15260	0.0627
MSMB	6	0.71278	0.77991	0.99998	15261	0.3753
LCP1	6	0.71278	0.77991	0.99998	15262	0.2164
TDRD5	6	0.71288	0.77995	0.99998	15263	-0.1056
ACP6	6	0.7131	0.78005	0.99998	15264	0.1184
IKZF2	6	0.71337	0.78016	0.99998	15265	-0.0878
LOC730159	6	0.71337	0.78016	0.99998	15266	-0.0261
SLC25A1	6	0.71337	0.78016	0.99998	15267	0.3274
PROSER1	6	0.71364	0.78028	0.99998	15268	0.1022
PGAP3	6	0.71364	0.78028	0.99998	15269	0.2635
BMP6	6	0.71364	0.78028	0.99998	15270	0.1651
BHMT	6	0.71364	0.78028	0.99998	15271	-0.0128
USP47	6	0.71364	0.78028	0.99998	15272	0.0698
OR2W1	4	0.71373	0.71933	0.99998	15273	0.0076
OPRK1	6	0.71385	0.78036	0.99998	15274	0.2574
UBE2QL1	6	0.71404	0.78044	0.99998	15275	0.2801
BCL2L13	6	0.71404	0.78044	0.99998	15276	0.1556
FGF12	6	0.71404	0.78044	0.99998	15277	-0.0625
RBMXL3	6	0.71422	0.78051	0.99998	15278	0.0351
ASB18	6	0.71439	0.78059	0.99998	15279	0.2804
TMEM214	6	0.71439	0.78059	0.99998	15280	-0.0151
MAT1A	6	0.71462	0.78069	0.99998	15281	0.0436
SLC35G5	6	0.71468	0.78072	0.99998	15282	0.1987
hsa-mir-513c	2	0.7147	0.71446	0.99998	15283	-0.077
NPHP4	6	0.71475	0.78076	0.99998	15284	-0.027
CHMP2B	6	0.71527	0.78099	0.99998	15285	0.1133
OLFML3	6	0.71527	0.78099	0.99998	15286	0.0101
RAB38	6	0.71551	0.7811	0.99998	15287	0.0697
MYLK4	6	0.71551	0.7811	0.99998	15288	-0.0962
CSH2	3	0.71568	0.71567	0.99998	15289	-0.118
C15orf52	6	0.7157	0.7812	0.99998	15290	0.0467
SUMO3	6	0.7157	0.7812	0.99998	15291	0.1226
ZNF629	6	0.71594	0.78132	0.99998	15292	0.1871
CASKIN1	6	0.71594	0.78132	0.99998	15293	0.0575
ZNF433	6	0.71594	0.78132	0.99998	15294	0.0494
PCDHGB2	2	0.71627	0.71601	0.99998	15295	0.1851
hsa-mir-563	4	0.71628	0.72148	0.99998	15296	0.017
RP1L1	6	0.71631	0.78148	0.99998	15297	-0.1807
ADCK5	6	0.71631	0.78148	0.99998	15298	-0.02
ZBTB46	6	0.7166	0.78161	0.99998	15299	0.1949
FAM71F1	6	0.7166	0.78161	0.99998	15300	0.1432
RSBN1L	6	0.71677	0.78169	0.99998	15301	0.0932
VSX2	6	0.71685	0.78172	0.99998	15302	0.1654
GBX1	6	0.71697	0.78177	0.99998	15303	0.0627
hsa-mir-297	1	0.71705	0.71724	0.99998	15304	0.2002
AP1S2	4	0.71705	0.72214	0.99998	15305	0.0626
GRHL2	6	0.71706	0.78182	0.99998	15306	0.1408

FAM72A	1	0.71712	0.71731	0.99998	15307	0.1739
WIZ	6	0.71717	0.78187	0.99998	15308	0.0985
hsa-mir-4641	4	0.71738	0.72242	0.99998	15309	-0.1087
HS3ST6	6	0.71745	0.78199	0.99998	15310	0.1732
GLMN	6	0.7176	0.78205	0.99998	15311	0.045
MPDU1	6	0.71771	0.78211	0.99998	15312	0.0099
BTBD3	6	0.71771	0.78211	0.99998	15313	0.2602
hsa-mir-21	4	0.71788	0.72283	0.99998	15314	-0.1115
hsa-mir-138-2	4	0.71788	0.72283	0.99998	15315	0.0942
TRIM74	2	0.71789	0.71766	0.99998	15316	0.0443
C16orf11	6	0.71795	0.78221	0.99998	15317	0.1709
TRIQK	6	0.71795	0.78221	0.99998	15318	0.1194
SORCS3	6	0.71795	0.78221	0.99998	15319	0.1845
C22orf31	6	0.71795	0.78221	0.99998	15320	0.0048
DGCR6L	5	0.71801	0.74778	0.99998	15321	0.1416
ZNF773	5	0.71834	0.74798	0.99998	15322	-0.1653
MPZ	6	0.71835	0.78241	0.99998	15323	0.2011
HOOK2	6	0.71835	0.78241	0.99998	15324	0.1757
LONRF2	6	0.71848	0.78247	0.99998	15325	0.0546
CCNA1	6	0.71848	0.78247	0.99998	15326	0.4467
PLXNA1	6	0.71848	0.78247	0.99998	15327	0.2743
FAM135B	6	0.71861	0.78253	0.99998	15328	0.2396
hsa-mir-548h-	2	0.71869	0.7185	0.99998	15329	0.1787
C7orf10	6	0.71884	0.78263	0.99998	15330	-0.081
hsa-mir-659	4	0.71891	0.72371	0.99998	15331	-0.0433
SNTG1	6	0.71901	0.7827	0.99998	15332	0.0393
MTCH1	6	0.71921	0.78277	0.99998	15333	0.1368
GRIA2	6	0.71921	0.78277	0.99998	15334	-0.1181
SAMD10	6	0.71921	0.78277	0.99998	15335	0.1946
ELF1	6	0.71921	0.78277	0.99998	15336	0.3506
LINGO1	6	0.71942	0.78284	0.99998	15337	0.1194
SMCHD1	6	0.71959	0.78291	0.99998	15338	0.1532
BBS1	6	0.71966	0.78294	0.99998	15339	0.0338
FAM46D	6	0.71966	0.78294	0.99998	15340	0.2257
ITPKC	6	0.71975	0.78299	0.99998	15341	0.0358
hsa-mir-3679	4	0.71983	0.72449	0.99998	15342	-0.0449
TULP2	6	0.71989	0.78305	0.99998	15343	0.1729
NDUFS4	4	0.72	0.72463	0.99998	15344	-0.0539
BSX	6	0.7201	0.78314	0.99998	15345	0.1363
NPL	6	0.7201	0.78314	0.99998	15346	-0.1258
FAM208B	6	0.7201	0.78314	0.99998	15347	0.1649
MR1	6	0.72027	0.78322	0.99998	15348	0.1979
TFAP2A	6	0.72027	0.78322	0.99998	15349	0.1518
VAPB	6	0.72054	0.78334	0.99998	15350	0.2732
NSD1	6	0.7206	0.78336	0.99998	15351	-0.0916
NRN1L	6	0.72087	0.78348	0.99998	15352	-0.0804
RILPL2	6	0.72087	0.78348	0.99998	15353	0.2988
ZNF300	6	0.72087	0.78348	0.99998	15354	-0.0075
LRRC46	6	0.72109	0.78358	0.99998	15355	0.3773
hsa-mir-5193	4	0.72112	0.72559	0.99998	15356	0.1213
PHF6	6	0.72126	0.78366	0.99998	15357	0.0698
MFNG	6	0.72126	0.78366	0.99998	15358	-0.0339
TCF3	6	0.72147	0.78376	0.99998	15359	0.022
BMPR2	6	0.72147	0.78376	0.99998	15360	0.0744
IL4	6	0.72147	0.78376	0.99998	15361	0.1179
CERS1	6	0.72161	0.78382	0.99998	15362	0.0931
CILP	4	0.72161	0.72599	0.99998	15363	-0.0457
HTATIP2	6	0.72178	0.78389	0.99998	15364	0.0467
TTLL5	6	0.72191	0.78395	0.99998	15365	0.0648
ZNF680	6	0.72221	0.78409	0.99998	15366	0.0254
CCL2	6	0.72221	0.78409	0.99998	15367	0.0869
PVRL3	6	0.72221	0.78409	0.99998	15368	0.2068
hsa-mir-199a-	3	0.72231	0.72235	0.99998	15369	0.3731
TRIM64B	3	0.72231	0.72235	0.99998	15370	0.0646
OBP2B	3	0.72231	0.72235	0.99998	15371	0.0953
hsa-mir-33a	4	0.72232	0.72659	0.99998	15372	0.0717
IFNL2	5	0.72232	0.75046	0.99998	15373	-0.3531
hsa-mir-545	4	0.72237	0.72664	0.99998	15374	0.2245
CHID1	6	0.72238	0.78416	0.99998	15375	-0.0249
XKR3	6	0.72238	0.78416	0.99998	15376	0.3125
OR1D5	6	0.72257	0.78425	0.99998	15377	0.1396
hsa-mir-6755	4	0.72272	0.72694	0.99998	15378	0.0151
APOBEC3G	6	0.72279	0.78434	0.99998	15379	0.0694
F2RL3	6	0.72279	0.78434	0.99998	15380	0.1775
CDK5R2	6	0.72279	0.78434	0.99998	15381	0.0156
hsa-mir-3164	4	0.72304	0.72721	0.99998	15382	-0.0727
AMFR	6	0.72307	0.78446	0.99998	15383	0.2032
FH	6	0.72307	0.78446	0.99998	15384	0.1214
OR52N1	6	0.72346	0.78463	0.99998	15385	0.168
MMRN2	6	0.72352	0.78466	0.99998	15386	0.1464
hsa-mir-3123	4	0.72363	0.72772	0.99998	15387	-0.0827

SPSB3	6	0.72366	0.78472	0.99998	15388	0.1102
KRTAP5-6	6	0.72366	0.78472	0.99998	15389	0.0354
CELF2	6	0.72383	0.78478	0.99998	15390	0.1019
TLL1	6	0.72383	0.78478	0.99998	15391	0.0612
TRAK2	6	0.72383	0.78478	0.99998	15392	0.0897
FAM160B2	6	0.72393	0.78483	0.99998	15393	0.3709
MRPS23	6	0.72399	0.78485	0.99998	15394	0.247
CXCR4	6	0.72399	0.78485	0.99998	15395	0.224
DDX11	6	0.72406	0.78489	0.99998	15396	0.1493
TBC1D2	6	0.72411	0.78491	0.99998	15397	0.009
ACPL2	6	0.72422	0.78496	0.99998	15398	0.0178
EDIL3	6	0.72422	0.78496	0.99998	15399	0.0519
BPNT1	6	0.72433	0.785	0.99998	15400	0.0331
CDKL2	6	0.72433	0.785	0.99998	15401	0.1651
OR10AD1	6	0.72444	0.78504	0.99998	15402	0.1493
GRIN2C	6	0.72444	0.78504	0.99998	15403	0.2375
CXorf27	6	0.72477	0.7852	0.99998	15404	0.1999
PQLC3	6	0.72477	0.7852	0.99998	15405	0.2572
CDC42	6	0.72477	0.7852	0.99998	15406	-0.0786
GPR133	6	0.72477	0.7852	0.99998	15407	0.1357
NCMAP	6	0.72492	0.78527	0.99998	15408	-0.0545
ATIC	6	0.72492	0.78527	0.99998	15409	-0.1498
LGR6	6	0.72492	0.78527	0.99998	15410	0.0342
MAF	6	0.72498	0.78529	0.99998	15411	0.101
PLAGL1	6	0.72505	0.78532	0.99998	15412	0.1147
TRMT11	6	0.72513	0.78535	0.99998	15413	-0.0073
PGLS	6	0.72525	0.78541	0.99998	15414	0.2823
UMOD	6	0.72525	0.78541	0.99998	15415	0.2236
PIH1D2	6	0.72544	0.78549	0.99998	15416	-0.059
SMOC1	6	0.72546	0.78549	0.99998	15417	0.0728
MECOM	6	0.72557	0.78554	0.99998	15418	0.1829
SETD6	6	0.72557	0.78554	0.99998	15419	0.0473
BCAS1	6	0.7257	0.7856	0.99998	15420	0.1832
NGB	6	0.72581	0.78566	0.99998	15421	0.3322
CAPSL	6	0.72605	0.78577	0.99998	15422	0.3116
FAM167A	6	0.72617	0.78582	0.99998	15423	-0.1042
ILK	6	0.72629	0.78588	0.99998	15424	0.0064
AWAT1	6	0.72653	0.786	0.99998	15425	0.0959
LIX1	6	0.72682	0.78611	0.99998	15426	0.208
RBMXL2	6	0.72691	0.78615	0.99998	15427	0.1722
C7orf50	6	0.72699	0.78619	0.99998	15428	0.103
hsa-mir-378f	4	0.72706	0.73066	0.99998	15429	0.235
CELA3A	5	0.72713	0.75342	0.99998	15430	-0.0714
AQR	4	0.72726	0.73083	0.99998	15431	0.3424
OR4M1	6	0.7273	0.78632	0.99998	15432	0.0922
C20orf24	3	0.72756	0.72761	0.99998	15433	-0.1189
FAM129C	6	0.72788	0.78657	0.99998	15434	0.1793
ERVFRD-1	6	0.7281	0.78666	0.99998	15435	0.23
CMYA5	6	0.7284	0.78679	0.99998	15436	-0.011
KRTAP5-10	4	0.72849	0.73187	0.99998	15437	0.1793
UGT2B17	4	0.72849	0.73187	0.99998	15438	-0.0185
hsa-mir-5009	4	0.72849	0.73187	0.99998	15439	0.1348
hsa-mir-519d	4	0.72849	0.73187	0.99998	15440	0.9361
MT1H	4	0.72849	0.73187	0.99998	15441	-0.3005
hsa-mir-4729	4	0.72849	0.73187	0.99998	15442	0.0589
OCM2	4	0.72849	0.73187	0.99998	15443	-0.1702
hsa-mir-548as	4	0.72849	0.73187	0.99998	15444	-0.2245
hsa-mir-320d-	4	0.72849	0.73187	0.99998	15445	0.1677
HEMK1	6	0.72861	0.78688	0.99998	15446	0.0362
LRRC40	6	0.72873	0.78693	0.99998	15447	0.0431
TRPS1	6	0.72876	0.78695	0.99998	15448	0.0049
FCRL1	6	0.72884	0.78699	0.99998	15449	-0.0149
SCYL2	6	0.72906	0.78708	0.99998	15450	0.0403
TMEM246	6	0.72916	0.78713	0.99998	15451	0.01
COL9A1	6	0.72925	0.78717	0.99998	15452	0.1517
WRN	6	0.72929	0.78719	0.99998	15453	0.0847
ZNF181	5	0.72937	0.75477	0.99998	15454	-0.2029
hsa-mir-299	4	0.72946	0.73272	0.99998	15455	-0.0331
hsa-mir-4703	1	0.72958	0.72983	0.99998	15456	0.1974
ZNF410	6	0.72958	0.78731	0.99998	15457	0.0427
CCT6B	6	0.72958	0.78731	0.99998	15458	0.3742
CD37	6	0.72958	0.78731	0.99998	15459	-0.1033
DEFB115	6	0.72972	0.78739	0.99998	15460	-0.0339
hsa-mir-3912	4	0.72975	0.73297	0.99998	15461	0.1282
hsa-mir-3934	4	0.72975	0.73297	0.99998	15462	-0.1222
PLCL1	6	0.72977	0.78741	0.99998	15463	0.3122
GMEB1	6	0.72987	0.78746	0.99998	15464	0.0029
TMEM37	6	0.72987	0.78746	0.99998	15465	0.0418
ARHGEF35	6	0.72987	0.78746	0.99998	15466	0.0151
HYDIN	6	0.72987	0.78746	0.99998	15467	-0.0592
hsa-mir-592	3	0.72992	0.72999	0.99998	15468	-0.0883

DDIT4L	6	0.73007	0.78754	0.99998	15469	0.1963
hsa-mir-3609	4	0.73008	0.73326	0.99998	15470	0.2042
SPOCK1	6	0.73021	0.7876	0.99998	15471	0.0253
HDGFRP3	6	0.73021	0.7876	0.99998	15472	0.349
hsa-mir-200c	4	0.7303	0.73346	0.99998	15473	0.1658
LRRC15	4	0.73033	0.73348	0.99998	15474	-0.0848
CCDC41	6	0.73034	0.78766	0.99998	15475	0.1488
RTN1	6	0.7305	0.78774	0.99998	15476	0.0788
NR2F6	6	0.73075	0.78784	0.99998	15477	0.1417
LRRC6	6	0.73085	0.78789	0.99998	15478	-0.0327
MIIP	6	0.73088	0.7879	0.99998	15479	0.1018
CBS	4	0.7309	0.73397	0.99998	15480	0.0666
CYP17A1	6	0.73091	0.78792	0.99998	15481	0.0712
APOBEC3A_B	2	0.73094	0.73078	0.99998	15482	0.156
RP1	6	0.73095	0.78794	0.99998	15483	0.2691
DERA	6	0.73098	0.78795	0.99998	15484	0.1843
hsa-mir-4281	4	0.731	0.73406	0.99998	15485	-0.0329
CHRNA7	4	0.731	0.73406	0.99998	15486	-0.1247
DACH1	6	0.73105	0.78799	0.99998	15487	0.0731
TMEM14B	3	0.73112	0.73119	0.99998	15488	-0.1395
hsa-mir-6828	4	0.73112	0.73415	0.99998	15489	-0.0197
hsa-mir-376c	1	0.73152	0.73183	0.99998	15490	0.3392
ZG16B	6	0.73157	0.78823	0.99998	15491	0.239
hsa-mir-7978	1	0.73185	0.73214	0.99998	15492	0.2523
NEDD8-MDP1	3	0.73194	0.73199	0.99998	15493	-0.0927
GOLIM4	4	0.73196	0.73489	0.99998	15494	-0.0868
APPL1	6	0.73197	0.78843	0.99998	15495	-0.0159
MOGAT2	6	0.73208	0.78848	0.99998	15496	-0.0338
UNC119	6	0.73237	0.78862	0.99998	15497	0.0611
PTPRF	6	0.73237	0.78862	0.99998	15498	0.0486
SH3GL1	6	0.73237	0.78862	0.99998	15499	0.0424
ARL8B	6	0.73237	0.78862	0.99998	15500	0.0261
UBR7	6	0.73237	0.78862	0.99998	15501	0.2284
SUSD1	6	0.73257	0.78872	0.99998	15502	0.034
DYNLL2	6	0.73257	0.78872	0.99998	15503	0.0617
SULT6B1	6	0.73275	0.7888	0.99998	15504	0.1084
CARD9	6	0.73275	0.7888	0.99998	15505	-0.0233
hsa-mir-5195	4	0.73291	0.73572	0.99998	15506	0.0239
CHD3	6	0.73297	0.7889	0.99998	15507	0.2165
HOPX	6	0.73297	0.7889	0.99998	15508	-0.0385
ATXN7L3	6	0.73297	0.7889	0.99998	15509	0.1739
SCGB2A1	6	0.73297	0.7889	0.99998	15510	-0.1513
CDK15	6	0.73297	0.7889	0.99998	15511	0.0892
ABI2	6	0.7331	0.78895	0.99998	15512	0.2899
C17orf98	6	0.73321	0.789	0.99998	15513	0.1267
ZNF771	6	0.73331	0.78905	0.99998	15514	0.036
PTPN7	6	0.73335	0.78907	0.99998	15515	-0.0556
PAFAH1B1	4	0.73338	0.73611	0.99998	15516	0.0346
PNLIPRP3	6	0.73341	0.7891	0.99998	15517	-0.0144
ASAH1	6	0.73355	0.78916	0.99998	15518	0.0784
S100A14	6	0.73355	0.78916	0.99998	15519	0.1307
TTC24	6	0.73387	0.78931	0.99998	15520	0.1302
SPIDR	2	0.73388	0.73375	0.99998	15521	0.0238
PAGE2B	1	0.73396	0.73419	0.99998	15522	0.8124
hsa-mir-3118-	1	0.73396	0.73419	0.99998	15523	0.8124
PLN	1	0.73396	0.73419	0.99998	15524	0.8124
hsa-mir-518a-	1	0.73396	0.73419	0.99998	15525	0.8124
A1CF	6	0.73404	0.78938	0.99998	15526	0.0876
KCNMB4	6	0.73404	0.78938	0.99998	15527	0.0281
ZNF251	6	0.73417	0.78944	0.99998	15528	0.1366
ANK2	6	0.73417	0.78944	0.99998	15529	0.0563
LLGL2	6	0.73417	0.78944	0.99998	15530	0.2818
VSIG2	6	0.73423	0.78947	0.99998	15531	0.0773
hsa-mir-219b	4	0.73423	0.73682	0.99998	15532	0.1723
ZNF561	5	0.73425	0.75789	0.99998	15533	-0.0574
ISG20	6	0.73433	0.78951	0.99998	15534	0.2587
LRP1B	6	0.73433	0.78951	0.99998	15535	0.2288
DPY19L1	5	0.73447	0.75804	0.99998	15536	0.2637
TGFB2	6	0.73454	0.78961	0.99998	15537	0.0851
STC1	6	0.73454	0.78961	0.99998	15538	0.2027
CCDC125	6	0.73454	0.78961	0.99998	15539	-0.0129
KHDC1	6	0.73454	0.78961	0.99998	15540	0.1734
ANO10	6	0.73474	0.78969	0.99998	15541	0.0374
MATN3	6	0.73482	0.78974	0.99998	15542	0.0587
TMEM88B	6	0.73482	0.78974	0.99998	15543	0.0623
NR2F1	6	0.73482	0.78974	0.99998	15544	0.125
RPUSD1	6	0.735	0.78981	0.99998	15545	0.0559
C15orf59	4	0.73519	0.73765	0.99998	15546	-0.0793
hsa-mir-1296	4	0.73519	0.73765	0.99998	15547	-0.0595
RNASE9	6	0.73529	0.78993	0.99998	15548	-0.0534
hsa-mir-608	4	0.73529	0.73774	0.99998	15549	-0.1009

OR10G9	5	0.73533	0.7586	0.99998	15550	0.2994
CBWD3	2	0.73545	0.73536	0.99998	15551	0.0654
STXBP5	6	0.73565	0.7901	0.99998	15552	0.1893
hsa-mir-6507	2	0.73575	0.73568	0.99998	15553	-0.0075
PITPNM1	6	0.73588	0.79023	0.99998	15554	-0.0603
ACTRT2	6	0.73592	0.79025	0.99998	15555	0.3246
IFNA17	5	0.73597	0.75904	0.99998	15556	0.458
CFH	6	0.73602	0.79029	0.99998	15557	-0.0741
STK31	6	0.73611	0.79033	0.99998	15558	0.0684
C14orf93	6	0.73611	0.79033	0.99998	15559	0.0638
IMMP2L	6	0.73627	0.7904	0.99998	15560	0.3546
C8B	4	0.7363	0.7386	0.99998	15561	0.0295
SLCO1C1	6	0.73632	0.79043	0.99998	15562	0.1985
hsa-mir-365a	4	0.73646	0.73873	0.99998	15563	0.2213
ZNF684	6	0.73647	0.79051	0.99998	15564	0.2289
FAM71A	6	0.73659	0.79056	0.99998	15565	0.0795
CASC3	6	0.7367	0.79061	0.99998	15566	0.0313
CADM2	6	0.7367	0.79061	0.99998	15567	0.2262
KRTAP12-4	6	0.73687	0.79069	0.99998	15568	0.1106
DAB2	6	0.73712	0.79081	0.99998	15569	0.3277
NCOA5	6	0.73745	0.79094	0.99998	15570	0.1227
CENPV	6	0.73745	0.79094	0.99998	15571	-0.0176
BLVRB	6	0.73745	0.79094	0.99998	15572	0.0107
TBX20	6	0.73745	0.79094	0.99998	15573	0.0455
C1orf228	6	0.73762	0.79102	0.99998	15574	0.1995
FGF22	6	0.73796	0.79117	0.99998	15575	0.2759
CNTNAP3	4	0.73808	0.7402	0.99998	15576	-0.0552
ZNF177	2	0.73812	0.73802	0.99998	15577	0.2837
BACH2	6	0.73816	0.79126	0.99998	15578	0.0482
MYNN	6	0.73826	0.79131	0.99998	15579	0.2454
RNF141	6	0.73839	0.79137	0.99998	15580	0.0335
LIN54	6	0.73848	0.79143	0.99998	15581	0.1239
RNASE10	6	0.73848	0.79143	0.99998	15582	0.0279
FCGR1B	4	0.73851	0.74058	0.99998	15583	-0.005
IFNA4	5	0.73859	0.76075	0.99998	15584	-0.7622
CCDC74A	5	0.73859	0.76075	0.99998	15585	-0.3257
ZNF468	5	0.73859	0.76075	0.99998	15586	0.1348
TRIM6	4	0.73885	0.74088	0.99998	15587	0.0409
PDK3	6	0.73891	0.79162	0.99998	15588	0.2766
OSGIN2	6	0.73891	0.79162	0.99998	15589	0.0754
TBC1D24	6	0.73891	0.79162	0.99998	15590	0.0848
hsa-mir-1203	4	0.7391	0.74109	0.99998	15591	-0.0012
OR6C70	6	0.73921	0.79176	0.99998	15592	0.1069
ARL6IP6	6	0.73921	0.79176	0.99998	15593	-0.015
hsa-mir-643	4	0.73925	0.74122	0.99998	15594	0.2373
RND3	6	0.73944	0.79188	0.99998	15595	-0.0299
LUZP4	6	0.73969	0.792	0.99998	15596	0.0786
SYT5	6	0.74054	0.79239	0.99998	15597	-0.0446
SUCLG2	6	0.74062	0.79243	0.99998	15598	0.07
B4GALT2	6	0.74066	0.79245	0.99998	15599	0.1334
hsa-mir-4313	4	0.74082	0.74266	0.99998	15600	0.0566
SEMA3F	6	0.74083	0.79254	0.99998	15601	0.3428
ARHGAP6	6	0.74101	0.79262	0.99998	15602	0.1749
INSL6	6	0.74117	0.79269	0.99998	15603	0.0545
FKBP10	6	0.74117	0.79269	0.99998	15604	0.0594
ALDH1B1	6	0.74123	0.79272	0.99998	15605	0.2679
B4GALT5	6	0.74136	0.79278	0.99998	15606	0.3272
PHF21B	6	0.74136	0.79278	0.99998	15607	0.097
TMEM104	6	0.74136	0.79278	0.99998	15608	0.3298
ZDHHC23	6	0.74136	0.79278	0.99998	15609	0.0478
SLC15A2	6	0.74136	0.79278	0.99998	15610	-0.0729
TNFSF12-TNFS	5	0.74149	0.76262	0.99998	15611	0.1766
NWD1	6	0.74161	0.7929	0.99998	15612	0.0147
MRS2	6	0.74161	0.7929	0.99998	15613	-0.0095
LRRC37B	5	0.74172	0.76277	0.99998	15614	0.1421
hsa-mir-6834	4	0.74198	0.7437	0.99998	15615	-0.138
NOTCH2	6	0.74216	0.79316	0.99998	15616	-0.4182
SLC15A1	6	0.74216	0.79316	0.99998	15617	0.2728
GZMB	6	0.74216	0.79316	0.99998	15618	0.3755
DPEP2	6	0.74216	0.79316	0.99998	15619	0.1841
DYNC2H1	6	0.74216	0.79316	0.99998	15620	0.0453
ZNF749	6	0.74216	0.79316	0.99998	15621	0.2567
hsa-mir-891b	4	0.74223	0.74391	0.99998	15622	-0.4872
FEM1A	6	0.74264	0.79337	0.99998	15623	0.1132
KLRB1	6	0.74296	0.79352	0.99998	15624	0.0221
SLC6A7	6	0.7431	0.79359	0.99998	15625	0.3394
NPC1	6	0.7431	0.79359	0.99998	15626	0.2062
hsa-mir-4440	3	0.74319	0.74327	0.99998	15627	0.4098
ZNF654	6	0.74327	0.79367	0.99998	15628	0.073
hsa-mir-222	4	0.74328	0.74486	0.99998	15629	0.1311
hsa-mir-3684	3	0.74328	0.74336	0.99998	15630	0.3298

LOC10013053	6	0.74353	0.79379	0.99998	15631	0.1331
ZNF18	6	0.74353	0.79379	0.99998	15632	0.1888
FAIM2	6	0.74353	0.79379	0.99998	15633	-0.0399
hsa-mir-1249	4	0.74356	0.74511	0.99998	15634	0.3415
PIGP	6	0.74371	0.79388	0.99998	15635	0.1193
hsa-mir-8064	4	0.74371	0.74526	0.99998	15636	0.0441
DPY19L2	6	0.74396	0.79401	0.99998	15637	-0.0143
POGLUT1	6	0.74396	0.79401	0.99998	15638	0.096
MUC20	4	0.74407	0.74559	0.99998	15639	0.1139
hsa-mir-548i-3	2	0.74412	0.74402	0.99998	15640	0.544
MSANTD3-TM	3	0.74413	0.74421	0.99998	15641	0.0407
TEX36	6	0.74417	0.79412	0.99998	15642	0.07
LARP6	6	0.74417	0.79412	0.99998	15643	0.1193
CNPY1	6	0.74417	0.79412	0.99998	15644	0.2429
FCRLA	6	0.7445	0.79429	0.99998	15645	0.0702
ZNF140	6	0.7445	0.79429	0.99998	15646	0.3427
SPDYA	6	0.7445	0.79429	0.99998	15647	0.2003
MPP3	6	0.74463	0.79435	0.99998	15648	0.155
SMIM21	4	0.74472	0.74615	0.99998	15649	0.1392
C18orf63	6	0.74472	0.7944	0.99998	15650	0.0824
RFXAP	6	0.74481	0.79444	0.99998	15651	0.0207
PRM1	6	0.74495	0.7945	0.99998	15652	0.2276
S100A10	6	0.74506	0.79455	0.99998	15653	-0.1479
KMT2D	2	0.74523	0.74512	0.99998	15654	0.0594
BTBD9	6	0.74526	0.79466	0.99998	15655	0.1836
hsa-mir-6130	4	0.74541	0.74677	0.99998	15656	-0.0561
hsa-mir-802	4	0.74547	0.74683	0.99998	15657	0.04
POMT1	6	0.74551	0.79478	0.99998	15658	0.0456
HSF2	6	0.74551	0.79478	0.99998	15659	0.1131
WDR17	6	0.74562	0.79484	0.99998	15660	0.2617
HACE1	6	0.74577	0.79491	0.99998	15661	0.1731
hsa-mir-4714	4	0.74579	0.74713	0.99998	15662	0.4699
DLAT	6	0.74586	0.79496	0.99998	15663	0.149
PIGY	6	0.74598	0.79501	0.99998	15664	0.0798
ZDHHC3	6	0.74598	0.79501	0.99998	15665	0.2873
YTHDC2	6	0.74598	0.79501	0.99998	15666	0.103
PPP1R17	6	0.74598	0.79501	0.99998	15667	0.0953
LYPD6B	6	0.74598	0.79501	0.99998	15668	-0.095
SLC7A14	6	0.74611	0.79508	0.99998	15669	0.0249
EFHB	6	0.74625	0.79514	0.99998	15670	0.2087
IRAK1BP1	6	0.74625	0.79514	0.99998	15671	0.115
hsa-mir-6852	4	0.7464	0.74767	0.99998	15672	-0.0136
RSPO2	6	0.74651	0.79527	0.99998	15673	-0.0326
ZNRF4	6	0.74655	0.79529	0.99998	15674	0.0854
MRPS27	6	0.74666	0.79535	0.99998	15675	0.0894
AGPAT6	6	0.74666	0.79535	0.99998	15676	0.2638
WFDC10A	5	0.74671	0.76611	0.99998	15677	-0.0195
OR52N5	6	0.74687	0.79545	0.99998	15678	-0.0978
hsa-mir-4640	4	0.7469	0.74816	0.99998	15679	0.0931
TMEM70	6	0.74692	0.79548	0.99998	15680	0.0227
PF4	4	0.74703	0.74827	0.99998	15681	-0.0848
ZNF534	6	0.74705	0.79554	0.99998	15682	-0.1022
SSPO	6	0.74705	0.79554	0.99998	15683	0.2151
RSPH10B	6	0.74705	0.79554	0.99998	15684	-0.0736
ZNF579	6	0.74705	0.79554	0.99998	15685	-0.0212
FANCD2	6	0.74722	0.79562	0.99998	15686	0.0842
ABCB4	6	0.74722	0.79562	0.99998	15687	0.087
hsa-mir-4775	3	0.74724	0.74736	0.99998	15688	-0.1273
CEL	6	0.74753	0.79576	0.99998	15689	0.0774
UGT2B15	4	0.74753	0.74871	0.99998	15690	-0.2414
hsa-mir-4708	4	0.74753	0.74871	0.99998	15691	0.0874
C11orf21	6	0.74765	0.79582	0.99998	15692	0.0262
hsa-mir-3115	4	0.74765	0.74883	0.99998	15693	0.0065
UGT2A1	1	0.74768	0.748	0.99998	15694	0.2924
PITHD1	6	0.74772	0.79585	0.99998	15695	0.2498
LZIC	6	0.74793	0.79595	0.99998	15696	0.0588
UBAC2	6	0.74793	0.79595	0.99998	15697	0.1129
GRIPAP1	6	0.74793	0.79595	0.99998	15698	0.2021
LYN	6	0.74793	0.79595	0.99998	15699	0.0759
hsa-mir-7161	4	0.74803	0.74917	0.99998	15700	0.2648
C16orf71	6	0.74803	0.79601	0.99998	15701	0.0451
PPP1R9A	6	0.74811	0.79605	0.99998	15702	0.0901
MTMR12	6	0.74817	0.79608	0.99998	15703	-0.0578
hsa-mir-4503	2	0.74826	0.74814	0.99998	15704	0.332
RAG2	6	0.74828	0.79613	0.99998	15705	0.2218
CCDC137	6	0.74828	0.79613	0.99998	15706	0.1847
ZNF256	6	0.74828	0.79613	0.99998	15707	0.0114
FAM196A	6	0.74828	0.79613	0.99998	15708	0.0402
TRAF6	6	0.74844	0.79621	0.99998	15709	-0.0245
TGM1	6	0.74849	0.79623	0.99998	15710	0.1725
JPH2	6	0.74862	0.79628	0.99998	15711	-0.0472

hsa-mir-559	4	0.74865	0.74971	0.99998	15712	-0.0471
MOK	6	0.74875	0.79633	0.99998	15713	0.162
TLR2	6	0.74875	0.79633	0.99998	15714	-0.0268
ATP13A4	6	0.74893	0.79642	0.99998	15715	-0.0297
KRTAP20-3	6	0.74893	0.79642	0.99998	15716	0.0841
hsa-mir-6079	4	0.74902	0.75004	0.99998	15717	-0.0223
KLC1	6	0.74906	0.79648	0.99998	15718	0.0863
FTSJ1	6	0.74908	0.7965	0.99998	15719	0.0546
IDH3G	6	0.74935	0.79662	0.99998	15720	0.0796
TYSND1	6	0.74935	0.79662	0.99998	15721	-0.0349
MS4A12	6	0.7495	0.79669	0.99998	15722	0.2981
SHANK1	6	0.7495	0.79669	0.99998	15723	0.0222
hsa-mir-5000	4	0.74955	0.75053	0.99998	15724	0.0404
ACACA	6	0.74961	0.79674	0.99998	15725	0.0538
MYBPHL	6	0.74981	0.79684	0.99998	15726	0.1967
OR4C46	6	0.75001	0.79694	0.99998	15727	0.0644
IGFBP3	6	0.75001	0.79694	0.99998	15728	0.138
MTG2	2	0.75012	0.74997	0.99998	15729	0.0532
TEX26	6	0.75022	0.79705	0.99998	15730	0.1299
ADAMTS20	6	0.75033	0.79711	0.99998	15731	0.4192
GDF5	6	0.75059	0.79723	0.99998	15732	0.1835
SLC39A8	4	0.75076	0.75166	0.99998	15733	-0.0934
OSCP1	4	0.75076	0.75166	0.99998	15734	0.0709
FAM118B	4	0.75081	0.75172	0.99998	15735	0.1796
OR10J3	6	0.75086	0.79736	0.99998	15736	0.1229
hsa-mir-4724	4	0.75089	0.75179	0.99998	15737	0.2074
hsa-mir-1225	4	0.75103	0.75191	0.99998	15738	0.3179
hsa-mir-6850	4	0.75103	0.75191	0.99998	15739	0.3545
hsa-mir-5093	4	0.75103	0.75191	0.99998	15740	0.0084
CELA2B	6	0.75113	0.79749	0.99998	15741	0.3817
LOC284385	3	0.75118	0.75129	0.99998	15742	0.085
MYL3	6	0.75132	0.7976	0.99998	15743	0.0315
FBXO42	6	0.75132	0.7976	0.99998	15744	0.1247
NRSN1	6	0.75132	0.7976	0.99998	15745	0.3199
STX10	6	0.75144	0.79766	0.99998	15746	-0.0633
TFIP11	6	0.75157	0.79772	0.99998	15747	-0.0074
DPEP1	6	0.75173	0.79781	0.99998	15748	0.0066
S100A3	6	0.75188	0.79789	0.99998	15749	0.1643
HMG20B	6	0.75188	0.79789	0.99998	15750	0.078
MYL12B	4	0.75213	0.75297	0.99998	15751	0.0369
NEURL1B	6	0.75219	0.79804	0.99998	15752	0.0548
LPHN3	6	0.75219	0.79804	0.99998	15753	0.1331
MBD2	6	0.75241	0.79816	0.99998	15754	-0.0235
UCHL1	6	0.75241	0.79816	0.99998	15755	0.226
PDDC1	6	0.75241	0.79816	0.99998	15756	0.0477
PDE10A	6	0.7525	0.79819	0.99998	15757	0.0489
NECAP1	6	0.75256	0.79823	0.99998	15758	0.0286
PROB1	6	0.7527	0.7983	0.99998	15759	-0.0573
DNAJC10	6	0.7527	0.7983	0.99998	15760	-0.0218
hsa-mir-374a	4	0.75279	0.75356	0.99998	15761	-0.0537
CAMTA1	6	0.75294	0.79842	0.99998	15762	0.0155
hsa-mir-495	4	0.75302	0.75377	0.99998	15763	-0.0518
BUD13	6	0.75313	0.79852	0.99998	15764	-0.0677
TLDC1	6	0.75313	0.79852	0.99998	15765	0.2816
TMEM220	6	0.75313	0.79852	0.99998	15766	-0.0332
FAM69A	6	0.75326	0.79858	0.99998	15767	0.0659
MYOZ1	6	0.75358	0.79873	0.99998	15768	0.2861
USMG5	6	0.75367	0.79878	0.99998	15769	0.2858
HRSP12	6	0.75377	0.79883	0.99998	15770	0.093
SLC6A8	6	0.75396	0.79891	0.99998	15771	0.3578
CCDC101	6	0.75396	0.79891	0.99998	15772	0.1664
hsa-mir-4461	4	0.75398	0.75466	0.99998	15773	0.2847
VWA5A	6	0.75405	0.79894	0.99998	15774	-0.0336
CHRD	6	0.75405	0.79894	0.99998	15775	0.2708
PCDHA9	2	0.75405	0.75384	0.99998	15776	0.1035
SLC35A1	6	0.75413	0.79899	0.99998	15777	0.1698
TMEM240	6	0.75413	0.79899	0.99998	15778	0.1403
hsa-mir-4325	4	0.75424	0.75493	0.99998	15779	0.5599
CXCL3	4	0.75435	0.75503	0.99998	15780	0.5798
UFSP1	6	0.7545	0.79917	0.99998	15781	-0.0221
ODC1	6	0.75464	0.79923	0.99998	15782	-0.0399
CLEC4G	6	0.75464	0.79923	0.99998	15783	0.1799
hsa-mir-613	4	0.75468	0.75533	0.99998	15784	-0.0433
CPB1	6	0.75477	0.7993	0.99998	15785	0.3123
NTAN1	6	0.75477	0.7993	0.99998	15786	0.1863
IFNGR1	6	0.75477	0.7993	0.99998	15787	-0.1414
HSD17B8	6	0.75503	0.79942	0.99998	15788	0.0933
BMP2K	6	0.75503	0.79942	0.99998	15789	0.1662
NXPH1	6	0.75527	0.79955	0.99998	15790	0.1208
SLC6A19	6	0.75527	0.79955	0.99998	15791	0.2259
CHPF2	6	0.75535	0.79958	0.99998	15792	0.1553

hsa-mir-29b-1	1	0.75546	0.75577	0.99998	15793	0.1705
KRTAP1-1	6	0.75549	0.79965	0.99998	15794	-0.0514
PHPT1	6	0.75549	0.79965	0.99998	15795	0.2186
hsa-mir-6806	4	0.75565	0.75622	0.99998	15796	0.1217
ATP12A	6	0.75592	0.79987	0.99998	15797	0.0062
KIF20A	6	0.75617	0.8	0.99998	15798	0.0053
CCL11	6	0.75617	0.8	0.99998	15799	0.0572
CYP8B1	4	0.75624	0.75675	0.99998	15800	0.0655
EPDR1	6	0.75624	0.80003	0.99998	15801	0.1303
FAM184A	6	0.75638	0.8001	0.99998	15802	0.121
DYX1C1	6	0.75648	0.80015	0.99998	15803	0.1648
ST6GAL1	6	0.75648	0.80015	0.99998	15804	-0.0465
CSAD	6	0.75659	0.8002	0.99998	15805	0.2096
hsa-mir-3616	4	0.75668	0.75714	0.99998	15806	0.1586
MAGEB3	6	0.75671	0.80026	0.99998	15807	0.3552
LIME1	6	0.7569	0.80037	0.99998	15808	0.3213
hsa-mir-377	4	0.75697	0.75741	0.99998	15809	0.1636
SCAND1	6	0.757	0.80042	0.99998	15810	0.092
ZC3H12C	6	0.757	0.80042	0.99998	15811	0.1518
hsa-mir-548e	4	0.75715	0.75759	0.99998	15812	-0.1475
hsa-mir-7108	4	0.75728	0.7577	0.99998	15813	0.527
UPK2	6	0.75737	0.8006	0.99998	15814	0.0995
C7orf43	6	0.75737	0.8006	0.99998	15815	-0.0192
DCD	6	0.75737	0.8006	0.99998	15816	0.0165
IPO8	6	0.75737	0.8006	0.99998	15817	0.2733
ANKIB1	6	0.75749	0.80066	0.99998	15818	0.3187
CDCP1	6	0.75757	0.8007	0.99998	15819	0.0199
TIAM2	6	0.75762	0.80072	0.99998	15820	-0.0316
ZNF813	3	0.75763	0.75778	0.99998	15821	0.3233
IL22RA2	6	0.75769	0.80075	0.99998	15822	0.0018
C11orf70	6	0.7579	0.80085	0.99998	15823	0.036
LARP1B	6	0.7579	0.80085	0.99998	15824	0.0399
NLRX1	6	0.7579	0.80085	0.99998	15825	0.0757
CXorf36	6	0.7579	0.80085	0.99998	15826	0.0683
BMP2	6	0.7579	0.80085	0.99998	15827	-0.0627
hsa-mir-3976	4	0.75803	0.75838	0.99998	15828	0.2036
OR1L6	6	0.75814	0.80098	0.99998	15829	0.075
ZNF397	5	0.7582	0.77389	0.99998	15830	0.1845
ZMYND19	6	0.7583	0.80106	0.99998	15831	0.0357
GRXCR1	6	0.7583	0.80106	0.99998	15832	-0.0477
EPHA4	6	0.75856	0.80117	0.99998	15833	0.104
HPN	6	0.75885	0.80135	0.99998	15834	0.1423
TMED5	6	0.75885	0.80135	0.99998	15835	-0.0141
VGLL4	6	0.759	0.80141	0.99998	15836	0.2358
MYLIP	6	0.75908	0.80145	0.99998	15837	0.1461
DST	6	0.75917	0.80149	0.99998	15838	-0.0803
SIGLEC12	6	0.7592	0.8015	0.99998	15839	0.1058
FCRL6	6	0.7593	0.80155	0.99998	15840	0.0901
hsa-mir-6729	4	0.75943	0.75967	0.99998	15841	-0.1806
hsa-mir-4670	4	0.75953	0.75977	0.99998	15842	0.0783
hsa-mir-4288	4	0.75953	0.75977	0.99998	15843	0.0836
hsa-mir-4540	4	0.75962	0.75987	0.99998	15844	-0.1028
hsa-mir-6857	2	0.75963	0.75929	0.99998	15845	0.3706
LYPLAL1	6	0.75974	0.80179	0.99998	15846	0.0367
C4B_2	6	0.75997	0.8019	0.99998	15847	0.108
hsa-mir-876	2	0.76002	0.75967	0.99998	15848	0.1688
NAGS	6	0.76007	0.80195	0.99998	15849	-0.0889
hsa-mir-6869	4	0.76009	0.76031	0.99998	15850	-0.0204
PIK3R1	6	0.76016	0.802	0.99998	15851	0.0873
LRRC8B	6	0.76016	0.802	0.99998	15852	0.1683
CDKN2A	6	0.76029	0.80206	0.99998	15853	0.2051
DNASE2	6	0.76045	0.80215	0.99998	15854	0.0595
HSPA13	6	0.76059	0.80222	0.99998	15855	0.0615
C9orf123	4	0.76062	0.76082	0.99998	15856	0.1975
USH1C	6	0.76071	0.80228	0.99998	15857	0.0042
FOXN1	6	0.76071	0.80228	0.99998	15858	0.0303
40422	3	0.76097	0.76115	0.99998	15859	-0.0715
FCAR	6	0.76098	0.80241	0.99998	15860	0.1172
CPEB4	6	0.76098	0.80241	0.99998	15861	0.0307
C10orf62	6	0.76107	0.80246	0.99998	15862	0.1594
hsa-mir-31	4	0.76125	0.76144	0.99998	15863	-0.0617
APOOL	6	0.76127	0.80254	0.99998	15864	0.0084
MAPRE2	6	0.76127	0.80254	0.99998	15865	-0.0193
TAAR1	6	0.76127	0.80254	0.99998	15866	0.0593
DCDC1	6	0.76151	0.80265	0.99998	15867	0.0296
OR2K2	6	0.76164	0.80271	0.99998	15868	0.0134
DUSP26	6	0.76175	0.80277	0.99998	15869	0.0073
EPHX2	6	0.76175	0.80277	0.99998	15870	0.0757
hsa-mir-4681	4	0.76201	0.76218	0.99998	15871	0.1024
hsa-mir-421	4	0.76201	0.76218	0.99998	15872	0.114
ARHGEF28	6	0.76203	0.80292	0.99998	15873	0.1121

PPFIA3	6	0.7621	0.80295	0.99998	15874	-0.0081
SLC16A9	6	0.7621	0.80295	0.99998	15875	0.0615
hsa-mir-1247	4	0.76216	0.76233	0.99998	15876	0.1866
PRDM13	6	0.76222	0.80301	0.99998	15877	0.1186
EVI5L	6	0.76281	0.80329	0.99998	15878	0.0645
C7orf62	6	0.76286	0.80332	0.99998	15879	0.163
CERS5	6	0.7629	0.80334	0.99998	15880	0.4801
hsa-mir-3941	4	0.76306	0.76316	0.99998	15881	0.1518
HLA-DQA1	6	0.76307	0.80344	0.99998	15882	0.0946
SH3KBP1	6	0.76307	0.80344	0.99998	15883	0.1396
CYP4Z1	6	0.76307	0.80344	0.99998	15884	-0.0159
ZCCHC10	6	0.76307	0.80344	0.99998	15885	0.2688
APITD1-CORT	2	0.76311	0.76274	0.99998	15886	0.0383
hsa-mir-487b	4	0.76339	0.76347	0.99998	15887	0.0996
IRF1	6	0.7634	0.8036	0.99998	15888	0.1887
DEFB129	6	0.76343	0.80361	0.99998	15889	0.0579
SLC16A8	6	0.76355	0.80368	0.99998	15890	0.343
OPRD1	6	0.76375	0.80377	0.99998	15891	0.1447
EMC8	6	0.76375	0.80377	0.99998	15892	0.1451
hsa-mir-1263	4	0.76386	0.76392	0.99998	15893	0.0444
ENPP1	6	0.76388	0.80384	0.99998	15894	0.0344
METTL21B	4	0.76399	0.76404	0.99998	15895	-0.1161
RNF151	6	0.76412	0.80396	0.99998	15896	0.2381
STATH	6	0.76412	0.80396	0.99998	15897	-0.0566
GPALPP1	3	0.76414	0.76433	0.99998	15898	0.3004
GATA1	6	0.76436	0.80408	0.99998	15899	0.1124
CPS1	6	0.76436	0.80408	0.99998	15900	0.1279
FAM105B	6	0.76436	0.80408	0.99998	15901	0.1282
CLDN11	6	0.7645	0.80416	0.99998	15902	-0.0254
PKP4	6	0.76465	0.80423	0.99998	15903	-0.1217
C9orf116	6	0.76483	0.80432	0.99998	15904	0.0949
PROM1	6	0.76509	0.80446	0.99998	15905	0.007
HLA-C	6	0.76509	0.80446	0.99998	15906	0.3493
KCNK10	6	0.76509	0.80446	0.99998	15907	0.0164
SEMA4F	6	0.76526	0.80456	0.99998	15908	0.088
KRT36	6	0.76526	0.80456	0.99998	15909	0.298
MSS51	6	0.76536	0.80462	0.99998	15910	0.099
LAT	4	0.76548	0.76546	0.99998	15911	-0.0942
RALBP1	6	0.76556	0.80472	0.99998	15912	0.1667
RPS6KA1	6	0.76576	0.80482	0.99998	15913	0.1931
CA9	6	0.76576	0.80482	0.99998	15914	0.1186
TCHHL1	6	0.76576	0.80482	0.99998	15915	-0.0451
hsa-mir-4434	4	0.76577	0.76574	0.99998	15916	-0.1486
hsa-mir-520c	2	0.76586	0.76544	0.99998	15917	0.4943
GH2	6	0.76612	0.805	0.99998	15918	-0.1091
hsa-mir-4287	4	0.76622	0.76618	0.99998	15919	0.041
BAHCC1	6	0.76637	0.80512	0.99998	15920	0.1783
OR4C11	6	0.76644	0.80516	0.99998	15921	0.0017
CLCN4	6	0.76658	0.80524	0.99998	15922	0.2915
OR4D5	6	0.76658	0.80524	0.99998	15923	0.2685
XKR9	6	0.76658	0.80524	0.99998	15924	0.052
SSR2	6	0.76658	0.80524	0.99998	15925	-0.0493
hsa-mir-519e	2	0.76693	0.76654	0.99998	15926	0.0965
hsa-mir-596	4	0.76695	0.76692	0.99998	15927	-0.0786
TEK	6	0.76699	0.80546	0.99998	15928	-0.0651
ATP11A	6	0.76699	0.80546	0.99998	15929	0.1417
CSF2	6	0.76712	0.80553	0.99998	15930	0.0232
ACOT2	5	0.76723	0.78029	0.99998	15931	-0.3206
HMX1	6	0.7673	0.80563	0.99998	15932	0.3715
ST8SIA4	6	0.7673	0.80563	0.99998	15933	0.116
MRRF	6	0.7673	0.80563	0.99998	15934	0.2246
DCAF4L2	6	0.7673	0.80563	0.99998	15935	0.0465
COL4A4	6	0.7673	0.80563	0.99998	15936	0.0105
TNIK	6	0.7673	0.80563	0.99998	15937	-0.0269
NKX3-1	6	0.7676	0.80578	0.99998	15938	0.2778
OR4F6	6	0.7676	0.80578	0.99998	15939	0.0511
CKAP2	6	0.7676	0.80578	0.99998	15940	0.1746
hsa-mir-194-1	4	0.76766	0.7676	0.99998	15941	-0.0331
hsa-mir-1285-	4	0.76779	0.76774	0.99998	15942	-0.4525
SKOR1	6	0.76804	0.80601	0.99998	15943	0.0562
SEMA7A	6	0.76804	0.80601	0.99998	15944	0.0186
LMO1	6	0.76804	0.80601	0.99998	15945	0.0152
ACSS3	6	0.76821	0.80609	0.99998	15946	0.0386
GABARAPL2	6	0.76838	0.80619	0.99998	15947	0.0392
FBXO38	6	0.76838	0.80619	0.99998	15948	0.0037
SHROOM3	6	0.76851	0.80626	0.99998	15949	0.1628
PRR18	6	0.76851	0.80626	0.99998	15950	0.2493
ABL1	6	0.76851	0.80626	0.99998	15951	0.0628
FTH1P18	6	0.76868	0.80635	0.99998	15952	0.0914
LCE2B	6	0.76868	0.80635	0.99998	15953	0.1653
RABL2A	4	0.7687	0.76862	0.99998	15954	0.2928

hsa-mir-452	2	0.76876	0.76842	0.99998	15955	0.0755
HACL1	6	0.76878	0.8064	0.99998	15956	0.1245
UNC79	6	0.76878	0.8064	0.99998	15957	0.0137
HOMER3	6	0.76883	0.80643	0.99998	15958	0.1405
TDRP	3	0.76883	0.76902	0.99998	15959	0.1135
CIC	6	0.7689	0.80647	0.99998	15960	-0.0195
ZMPSTE24	6	0.7689	0.80647	0.99998	15961	0.0455
CWC27	6	0.7689	0.80647	0.99998	15962	0.1652
hsa-mir-4792	4	0.76892	0.76883	0.99998	15963	-0.0733
A4GALT	4	0.76907	0.76898	0.99998	15964	0.2557
ZNF621	6	0.76912	0.80659	0.99998	15965	-0.0234
hsa-mir-4678	4	0.76918	0.76909	0.99998	15966	0.1252
KIAA1755	6	0.76922	0.80664	0.99998	15967	0.1376
KLRC4	2	0.76961	0.76927	0.99998	15968	0.0192
ZNF510	6	0.76965	0.80687	0.99998	15969	0.0268
AKR1C3	6	0.76965	0.80687	0.99998	15970	0.1161
RXFP1	6	0.76979	0.80694	0.99998	15971	0.0123
HERPUD2	6	0.76979	0.80694	0.99998	15972	-0.076
TMEM257	6	0.77016	0.80714	0.99998	15973	0.0888
ZNF488	6	0.77027	0.8072	0.99998	15974	0.214
FLJ27352	3	0.77031	0.77048	0.99998	15975	-0.1098
TM9SF2	6	0.77046	0.80731	0.99998	15976	0.3568
GSG1	6	0.77046	0.80731	0.99998	15977	-0.02
PRDX5	6	0.77046	0.80731	0.99998	15978	0.114
hsa-mir-4263	4	0.77054	0.7704	0.99998	15979	0.0066
C15orf56	6	0.77056	0.80736	0.99998	15980	0.2068
BAX	6	0.7706	0.80738	0.99998	15981	0.135
DUOX1	6	0.77069	0.80744	0.99998	15982	0.054
HOOK1	6	0.77077	0.80748	0.99998	15983	0.16
PAEP	6	0.77093	0.80755	0.99998	15984	0.0795
CD300LF	6	0.771	0.80758	0.99998	15985	-0.0318
hsa-mir-3117	4	0.77103	0.77087	0.99998	15986	0.0975
HKR1	6	0.77124	0.80771	0.99998	15987	0.0777
HIST1H4L	6	0.77124	0.80771	0.99998	15988	0.1204
MAP1LC3B2	6	0.77124	0.80771	0.99998	15989	0.0214
RAB2A	6	0.77162	0.80791	0.99998	15990	-0.0386
CEMP1	6	0.77162	0.80791	0.99998	15991	0.1441
NFU1	6	0.77162	0.80791	0.99998	15992	0.0835
IFNL4	6	0.77176	0.80799	0.99998	15993	0.1488
NUDT7	6	0.77176	0.80799	0.99998	15994	0.2323
FAM8A1	6	0.77205	0.80813	0.99998	15995	-0.0087
MRPL32	6	0.77215	0.8082	0.99998	15996	0.1448
CNBD2	6	0.77215	0.8082	0.99998	15997	0.0966
ZNF658	6	0.77215	0.8082	0.99998	15998	-0.0908
PRKAB2	6	0.77215	0.8082	0.99998	15999	-0.0217
hsa-mir-8061	2	0.77226	0.77195	0.99998	16000	0.3399
TNFAIP8L1	6	0.77227	0.80826	0.99998	16001	0.3182
SLC7A10	6	0.77227	0.80826	0.99998	16002	0.0736
ENAH	6	0.77233	0.80829	0.99998	16003	0.2228
TRNP1	6	0.7724	0.80833	0.99998	16004	-0.0303
SCGB3A1	6	0.7724	0.80833	0.99998	16005	0.1018
PCDHA12	2	0.77242	0.77211	0.99998	16006	0.1628
hsa-mir-621	4	0.77255	0.77235	0.99998	16007	0.0208
IMPG1	6	0.77255	0.80841	0.99998	16008	0.2399
OR4K14	6	0.77278	0.80853	0.99998	16009	0.1908
ABCA13	6	0.77278	0.80853	0.99998	16010	-0.0995
GAN	6	0.77278	0.80853	0.99998	16011	-0.0305
GIMAP6	6	0.77278	0.80853	0.99998	16012	0.1043
hsa-mir-4515	4	0.77292	0.77271	0.99998	16013	-0.0562
SLC24A3	6	0.77302	0.80865	0.99998	16014	0.1498
hsa-mir-4449	4	0.77319	0.77296	0.99998	16015	0.1826
ZNF541	6	0.7732	0.80875	0.99998	16016	-0.0206
PIM3	6	0.77342	0.80886	0.99998	16017	0.2024
MAST2	6	0.77342	0.80886	0.99998	16018	-0.0688
PCDHB12	6	0.77352	0.80892	0.99998	16019	0.1939
ANXA3	6	0.77371	0.80902	0.99998	16020	0.0863
INF2	6	0.77388	0.8091	0.99998	16021	-0.013
CLDN7	6	0.77402	0.80918	0.99998	16022	-0.0194
C11orf1	6	0.77407	0.80919	0.99998	16023	0.0497
HPS5	6	0.77407	0.80919	0.99998	16024	0.2582
hsa-mir-455	4	0.77414	0.7739	0.99998	16025	0.04
USP50	6	0.77431	0.80932	0.99998	16026	0.1876
LAMA2	6	0.77443	0.80937	0.99998	16027	-0.0537
PQLC1	6	0.77443	0.80937	0.99998	16028	-0.0212
CCDC9	6	0.77443	0.80937	0.99998	16029	0.1999
C9orf72	6	0.77459	0.80947	0.99998	16030	0.0744
C7orf57	6	0.77473	0.80954	0.99998	16031	0.0041
GRIA3	6	0.77481	0.80959	0.99998	16032	0.0883
GSTZ1	6	0.7751	0.80974	0.99998	16033	0.2655
C1S	6	0.7751	0.80974	0.99998	16034	0.0602
KCTD6	6	0.77523	0.8098	0.99998	16035	0.3192

AMY1C	2	0.77542	0.77509	0.99998	16036	0.2767
ARHGEF15	6	0.7755	0.80995	0.99998	16037	-0.1169
hsa-mir-4764	4	0.77561	0.77537	0.99998	16038	0.158
ZCCHC24	6	0.77566	0.81003	0.99998	16039	0.0138
FOXA3	6	0.77566	0.81003	0.99998	16040	-0.0017
hsa-mir-548b	3	0.77569	0.7758	0.99998	16041	0.3366
SMAD9	6	0.7758	0.81009	0.99998	16042	0.0953
OR1S2	6	0.77583	0.8101	0.99998	16043	0.0268
ALDH3B1	5	0.77587	0.78661	0.99998	16044	0.034
METTL22	6	0.77592	0.81016	0.99998	16045	-0.0537
KIAA2013	6	0.77592	0.81016	0.99998	16046	0.1812
OPN5	6	0.77592	0.81016	0.99998	16047	0.0608
ART5	6	0.77592	0.81016	0.99998	16048	0.0328
TMEM51	6	0.77611	0.81025	0.99998	16049	-0.1424
LPAR5	6	0.77611	0.81025	0.99998	16050	0.0082
CNTN5	6	0.77628	0.81034	0.99998	16051	0.0041
PBK	6	0.77628	0.81034	0.99998	16052	0.2278
C12orf60	6	0.77628	0.81034	0.99998	16053	0.0236
NVL	6	0.77628	0.81034	0.99998	16054	0.0164
ST8SIA2	6	0.77628	0.81034	0.99998	16055	0.2681
ENC1	6	0.77628	0.81034	0.99998	16056	0.074
IVD	6	0.77628	0.81034	0.99998	16057	0.0485
NLRC3	6	0.77642	0.81041	0.99998	16058	0.1538
ODF2L	5	0.77657	0.78713	0.99998	16059	0.0408
TMEM110	4	0.77658	0.77632	0.99998	16060	0.1611
USP34	6	0.77658	0.8105	0.99998	16061	0.0315
WFDC5	6	0.7767	0.81057	0.99998	16062	-0.0569
PRM3	6	0.7767	0.81057	0.99998	16063	0.1665
OR8K5	6	0.77718	0.81081	0.99998	16064	0.015
SLAMF9	6	0.77718	0.81081	0.99998	16065	0.0247
CCDC169	5	0.77726	0.78762	0.99998	16066	0.1508
AKAP10	6	0.77728	0.81086	0.99998	16067	0.0687
C18orf42	6	0.77743	0.81095	0.99998	16068	-0.1199
KCNH7	6	0.77748	0.81098	0.99998	16069	-0.0596
ANAPC1	6	0.77748	0.81098	0.99998	16070	0.1273
SCRT1	6	0.77762	0.81105	0.99998	16071	-0.0169
BAAT	6	0.77779	0.81114	0.99998	16072	0.1057
hsa-mir-3529	2	0.77786	0.77751	0.99998	16073	0.0948
LRSAM1	6	0.77798	0.81126	0.99998	16074	0.0284
MCHR1	6	0.77798	0.81126	0.99998	16075	0.0827
ZBTB4	6	0.77819	0.81138	0.99998	16076	0.0898
hsa-mir-4780	4	0.77819	0.77788	0.99998	16077	0.0157
C17orf78	6	0.77835	0.81147	0.99998	16078	0.294
MLNR	6	0.77835	0.81147	0.99998	16079	0.306
TRIT1	6	0.77855	0.81158	0.99998	16080	0.0149
PLK3	6	0.77867	0.81164	0.99998	16081	0.2106
EXD1	6	0.77879	0.8117	0.99998	16082	0.0284
hsa-mir-591	4	0.77882	0.7785	0.99998	16083	0.0264
MAGED2	6	0.77886	0.81174	0.99998	16084	0.0794
DPAGT1	6	0.77894	0.81178	0.99998	16085	0.0978
TM4SF18	6	0.77898	0.8118	0.99998	16086	0.1077
OR2W3	6	0.77904	0.81184	0.99998	16087	0.0501
GDF2	6	0.7791	0.81187	0.99998	16088	0.0258
WDR85	3	0.77918	0.77925	0.99998	16089	0.1525
OR8H2	6	0.77942	0.81205	0.99998	16090	0.1695
RDH10	6	0.77956	0.81212	0.99998	16091	0.1268
ABCA3	6	0.77956	0.81212	0.99998	16092	0.1052
TMEM101	6	0.77963	0.81216	0.99998	16093	0.2174
C1orf194	6	0.77972	0.81221	0.99998	16094	0.1193
CENPBD1	6	0.77972	0.81221	0.99998	16095	0.0918
FCN2	6	0.77985	0.81228	0.99998	16096	0.0642
RAB11FIP5	6	0.77996	0.81234	0.99998	16097	0.1081
KLHL21	6	0.78006	0.81238	0.99998	16098	-0.0094
HLTF	6	0.78006	0.81238	0.99998	16099	0.1188
PIWIL1	4	0.78022	0.77991	0.99998	16100	0.0093
HSD17B2	6	0.78023	0.81248	0.99998	16101	-0.0331
MDFI	6	0.78033	0.81254	0.99998	16102	-0.0412
CAPNS2	6	0.78033	0.81254	0.99998	16103	0.0193
C5orf20	6	0.78033	0.81254	0.99998	16104	0.1187
hsa-mir-885	4	0.78038	0.78008	0.99998	16105	-0.0192
MIER1	6	0.78043	0.8126	0.99998	16106	0.074
ZNF83	6	0.7805	0.81264	0.99998	16107	0.1748
ANKRD42	6	0.78056	0.81266	0.99998	16108	-0.0712
SULT2A1	6	0.78064	0.81271	0.99998	16109	0.1744
SLC27A6	6	0.78064	0.81271	0.99998	16110	-0.0055
hsa-mir-8080	4	0.78072	0.78045	0.99998	16111	0.0675
ANKRD65	6	0.78082	0.8128	0.99998	16112	0.1793
PRAMEF2	6	0.78099	0.8129	0.99998	16113	0.0217
PLEKHD1	6	0.78117	0.813	0.99998	16114	0.2024
NBN	6	0.7815	0.81317	0.99998	16115	0.0053
SHC2	6	0.7815	0.81317	0.99998	16116	0.1342

CASZ1	6	0.7815	0.81317	0.99998	16117	0.1117
NEURL2	6	0.78156	0.8132	0.99998	16118	-0.0155
ADNP2	6	0.78164	0.81325	0.99998	16119	0.1981
TSSK1B	6	0.78164	0.81325	0.99998	16120	-0.0577
SULT1B1	6	0.78186	0.81337	0.99998	16121	0.0423
ZNF124	6	0.78186	0.81337	0.99998	16122	-0.0204
WNT9A	6	0.78186	0.81337	0.99998	16123	0.0509
TBC1D8B	4	0.78197	0.78171	0.99998	16124	0.0051
hsa-mir-485	4	0.78197	0.78171	0.99998	16125	-0.1632
CDX1	6	0.78206	0.81347	0.99998	16126	0.0871
PAPPA	6	0.78208	0.81348	0.99998	16127	0.0082
NFATC2	6	0.78209	0.81349	0.99998	16128	0.0525
hsa-mir-4437	4	0.7821	0.78185	0.99998	16129	0.4601
TEX22	6	0.78215	0.81352	0.99998	16130	0.2543
ZNF81	6	0.78243	0.81367	0.99998	16131	0.0719
CHRDL1	6	0.78284	0.8139	0.99998	16132	-0.0364
GYPA	5	0.78289	0.79181	0.99998	16133	0.2394
FZD4	6	0.78294	0.81397	0.99998	16134	0.0878
ASL	6	0.78294	0.81397	0.99998	16135	-0.0089
CLCN3	6	0.78294	0.81397	0.99998	16136	0.1821
SCIMP	6	0.78294	0.81397	0.99998	16137	0.4286
SERPING1	6	0.783	0.814	0.99998	16138	-0.0072
SLC39A3	6	0.78309	0.81404	0.99998	16139	0.0591
DFNB59	6	0.78309	0.81404	0.99998	16140	0.3037
DCAF16	6	0.78309	0.81404	0.99998	16141	0.0768
GPR171	4	0.78312	0.78288	0.99998	16142	-0.0341
AMDHD1	6	0.78327	0.81414	0.99998	16143	0.1162
BPIFB1	6	0.78327	0.81414	0.99998	16144	0.038
ATOH7	6	0.78327	0.81414	0.99998	16145	0.2327
hsa-mir-4293	4	0.78336	0.78313	0.99998	16146	-0.001
C12orf4	6	0.7834	0.81422	0.99998	16147	0.1144
WISP2	6	0.78348	0.81426	0.99998	16148	-0.0094
GPR150	4	0.78354	0.78331	0.99998	16149	-0.0103
GNRH2	6	0.78358	0.81432	0.99998	16150	0.0582
CYP4X1	6	0.78358	0.81432	0.99998	16151	0.3065
hsa-mir-660	4	0.78363	0.7834	0.99998	16152	0.0477
HYKK	2	0.78372	0.78333	0.99998	16153	0.0444
RNF181	6	0.78374	0.81443	0.99998	16154	0.1398
DCAF7	6	0.78374	0.81443	0.99998	16155	-0.0611
hsa-mir-4303	4	0.78384	0.78361	0.99998	16156	0.0797
GTF3C2	6	0.78386	0.81448	0.99998	16157	0.1896
CBLN4	6	0.78386	0.81448	0.99998	16158	0.1721
OR6B2	6	0.78393	0.81454	0.99998	16159	-0.0242
DCAF8	6	0.7841	0.81462	0.99998	16160	0.0323
WBP5	6	0.7841	0.81462	0.99998	16161	-0.0099
IFNA6	6	0.7842	0.81468	0.99998	16162	0.0038
XPO7	6	0.78431	0.81473	0.99998	16163	-0.0146
TTC12	6	0.7847	0.81494	0.99998	16164	0.1648
MXD3	6	0.7847	0.81494	0.99998	16165	-0.0037
CDH11	6	0.7847	0.81494	0.99998	16166	0.0158
HAS2	6	0.78486	0.81503	0.99998	16167	-0.0277
hsa-mir-891a	4	0.78507	0.78488	0.99998	16168	0.1502
FCHO2	6	0.78509	0.81515	0.99998	16169	0.1528
ZNF469	6	0.78509	0.81515	0.99998	16170	0.1193
ACAP2	6	0.78509	0.81515	0.99998	16171	0.1526
SGCD	6	0.78531	0.81527	0.99998	16172	0.1328
ING4	6	0.78538	0.81532	0.99998	16173	0.1161
SH3BP5	6	0.78538	0.81532	0.99998	16174	0.1496
MS4A10	6	0.78595	0.81563	0.99998	16175	0.0712
KCNN1	6	0.78604	0.81568	0.99998	16176	0.0407
SLC16A12	6	0.78614	0.81574	0.99998	16177	0.112
LAMB1	6	0.78614	0.81574	0.99998	16178	0.0516
ARL13B	6	0.78629	0.81582	0.99998	16179	0.0192
PRRT2	6	0.78633	0.81584	0.99998	16180	-0.1084
PRAMEF16	4	0.78639	0.78619	0.99998	16181	0.0238
hsa-mir-628	4	0.78647	0.78627	0.99998	16182	-0.0664
EMP1	6	0.7867	0.81605	0.99998	16183	0.1247
GMPPA	6	0.7867	0.81605	0.99998	16184	-0.0028
hsa-mir-4438	4	0.78676	0.78658	0.99998	16185	-0.0351
hsa-mir-1539	4	0.78684	0.78666	0.99998	16186	-0.0124
MICAL1	6	0.787	0.81622	0.99998	16187	0.138
MYOD1	6	0.787	0.81622	0.99998	16188	0.1127
C11orf83	6	0.787	0.81622	0.99998	16189	0.049
hsa-mir-128-1	4	0.78718	0.78699	0.99998	16190	-0.0616
RGMB	6	0.7872	0.81633	0.99998	16191	0.1649
CSMD2	6	0.7872	0.81633	0.99998	16192	-0.0212
hsa-mir-1302-	4	0.78734	0.78714	0.99998	16193	0.1382
hsa-mir-8053	2	0.78739	0.78701	0.99998	16194	0.1956
hsa-mir-4317	4	0.78742	0.78722	0.99998	16195	0.0524
IRG1	6	0.78743	0.81646	0.99998	16196	-0.0225
WFS1	6	0.78743	0.81646	0.99998	16197	0.1237

FBXO47	6	0.78764	0.81658	0.99998	16198	-0.0252
LCE1C	4	0.78775	0.78755	0.99998	16199	0.0729
TRIM62	4	0.78775	0.78755	0.99998	16200	-0.0421
hsa-mir-4324	4	0.78775	0.78755	0.99998	16201	-0.1057
VWA9	6	0.78776	0.81665	0.99998	16202	0.1096
SLC35G3	6	0.78776	0.81665	0.99998	16203	0.2477
HCRTR1	6	0.78776	0.81665	0.99998	16204	-0.0753
CST1	4	0.78789	0.78767	0.99998	16205	0.1493
CEBPA	5	0.78791	0.79561	0.99998	16206	-0.0392
BCL10	6	0.78798	0.81678	0.99998	16207	-0.0657
PACSIN2	6	0.78798	0.81678	0.99998	16208	0.3084
RBAK	2	0.78814	0.78777	0.99998	16209	0.1294
TTLL9	6	0.78817	0.81689	0.99998	16210	-0.0757
hsa-mir-3064	4	0.78819	0.78799	0.99998	16211	0.052
LARGE	6	0.78846	0.81705	0.99998	16212	0.1775
UNCX	6	0.78855	0.81711	0.99998	16213	0.1043
PDLIM7	6	0.78884	0.81726	0.99998	16214	0.0967
NDST4	6	0.78884	0.81726	0.99998	16215	-0.1145
GPR20	6	0.78884	0.81726	0.99998	16216	0.0413
ESYT3	6	0.78884	0.81726	0.99998	16217	0.1102
HSF4	6	0.78899	0.81735	0.99998	16218	0.0351
GPHA2	6	0.78899	0.81735	0.99998	16219	0.2049
hsa-mir-3654	4	0.78906	0.78888	0.99998	16220	-0.1109
ATF7IP2	6	0.78908	0.81739	0.99998	16221	-0.08
PPP1R3G	6	0.78928	0.81751	0.99998	16222	0.0962
ZNF250	6	0.78928	0.81751	0.99998	16223	-0.0612
COQ7	6	0.78941	0.81759	0.99998	16224	-0.0308
CREBZF	6	0.78941	0.81759	0.99998	16225	0.2124
RAPGEF4	6	0.78941	0.81759	0.99998	16226	-0.0112
PPFIBP1	6	0.78963	0.81771	0.99998	16227	0.0206
CYP2C9	4	0.78969	0.78949	0.99998	16228	0.3096
UBE2E3	6	0.78972	0.81775	0.99998	16229	0.0625
TCP11X2	6	0.78972	0.81775	0.99998	16230	0.1408
OR10G7	6	0.78997	0.81788	0.99998	16231	0.0573
AIF1	6	0.78997	0.81788	0.99998	16232	0.0364
GDPD2	6	0.79004	0.81792	0.99998	16233	0.127
PDLIM3	6	0.7901	0.81795	0.99998	16234	0.0392
hsa-mir-636	4	0.79013	0.78993	0.99998	16235	-0.0108
SMIM14	6	0.7902	0.81801	0.99998	16236	0.1916
OR2AT4	6	0.7902	0.81801	0.99998	16237	0.1333
hsa-mir-6875	4	0.79024	0.79003	0.99998	16238	-0.0085
FNIP2	6	0.79031	0.81808	0.99998	16239	0.0602
C22orf15	6	0.7906	0.81824	0.99998	16240	-0.0315
hsa-mir-7151	4	0.79062	0.79041	0.99998	16241	-0.0666
MYL12A	4	0.79062	0.79041	0.99998	16242	-0.0626
SCP2D1	6	0.79068	0.81828	0.99998	16243	0.3014
ANKRD33B	6	0.79078	0.81833	0.99998	16244	0.1307
hsa-mir-6504	4	0.79086	0.79064	0.99998	16245	-0.0588
hsa-mir-4500	4	0.79104	0.79083	0.99998	16246	-0.0593
ANKK1	6	0.79106	0.81849	0.99998	16247	0.0766
MTM1	6	0.79106	0.81849	0.99998	16248	0.15
CCDC180	6	0.79118	0.81855	0.99998	16249	0.0206
ZNF808	4	0.7912	0.79099	0.99998	16250	-0.529
OR52R1	6	0.79135	0.81866	0.99998	16251	0.2438
HNRNPCL1	1	0.79137	0.79178	0.99998	16252	0.2393
BCL6B	6	0.79149	0.81874	0.99998	16253	0.1564
SLC9A9	6	0.79149	0.81874	0.99998	16254	0.0494
PYCRL	6	0.79157	0.81877	0.99998	16255	0.2015
RGCC	6	0.79157	0.81877	0.99998	16256	0.0075
CREG1	6	0.79157	0.81877	0.99998	16257	0.2009
SPATA33	2	0.79165	0.79134	0.99998	16258	0.3001
MORF4L2	6	0.79167	0.81883	0.99998	16259	0.1461
CA4	6	0.79179	0.8189	0.99998	16260	0.2225
hsa-mir-103a-	1	0.79183	0.79222	0.99998	16261	0.2676
RNF5	6	0.79187	0.81894	0.99998	16262	0.1523
FAM228A	6	0.79187	0.81894	0.99998	16263	0.0864
C9orf169	6	0.79199	0.819	0.99998	16264	-0.0725
hsa-mir-302f	3	0.79207	0.79214	0.99998	16265	-0.0863
LSG1	6	0.79211	0.81907	0.99998	16266	-0.033
OR4D2	6	0.7922	0.81913	0.99998	16267	-0.012
CANX	6	0.7922	0.81913	0.99998	16268	0.0383
LOC440335	5	0.7923	0.79901	0.99998	16269	0.0465
TMEM218	6	0.79231	0.81919	0.99998	16270	0.111
ZNF17	6	0.79231	0.81919	0.99998	16271	-0.12
OCA2	6	0.79231	0.81919	0.99998	16272	-0.0052
hsa-mir-379	4	0.79249	0.79229	0.99998	16273	0.0255
BPIFB4	6	0.79252	0.81929	0.99998	16274	0.0731
BFAR	6	0.79262	0.81934	0.99998	16275	0.2959
TUBAL3	6	0.79275	0.81942	0.99998	16276	0.1209
C11orf94	6	0.79279	0.81944	0.99998	16277	0.0531
CHRDL2	6	0.79285	0.81948	0.99998	16278	0.086

MFAP3	6	0.79285	0.81948	0.99998	16279	0.1529
OXER1	6	0.79303	0.81957	0.99998	16280	0.0479
DNAJB3	6	0.79303	0.81957	0.99998	16281	0.3077
EPB49	6	0.79303	0.81957	0.99998	16282	0.2486
SYNGR3	6	0.79326	0.8197	0.99998	16283	0.1279
CREM	4	0.7933	0.7931	0.99998	16284	0.2105
KLHDC4	6	0.79335	0.81975	0.99998	16285	0.1405
GPR27	6	0.79335	0.81975	0.99998	16286	0.2571
ESRRB	6	0.79342	0.81979	0.99998	16287	0.1137
KLKB1	6	0.79352	0.81985	0.99998	16288	0.1152
CTTNBP2NL	6	0.79366	0.81993	0.99998	16289	0.0803
GRK1	6	0.79371	0.81996	0.99998	16290	-0.0002
HSPBP1	6	0.79385	0.82005	0.99998	16291	0.0067
HSD3B1	6	0.79385	0.82005	0.99998	16292	0.0355
MRPL9	6	0.79385	0.82005	0.99998	16293	-0.1439
MORN2	6	0.79385	0.82005	0.99998	16294	0.0349
hsa-mir-593	4	0.79388	0.7937	0.99998	16295	0.0671
FUT11	6	0.79427	0.82029	0.99998	16296	0.1826
ATAD2B	6	0.79427	0.82029	0.99998	16297	0.03
ASXL3	6	0.79427	0.82029	0.99998	16298	0.2543
BDKRB1	6	0.79427	0.82029	0.99998	16299	0.2378
hsa-mir-759	4	0.79429	0.79412	0.99998	16300	0.0819
SPATA31A3	2	0.79432	0.79398	0.99998	16301	0.123
ARHGEF2	4	0.79436	0.79419	0.99998	16302	0.1512
CENPO	6	0.79436	0.82034	0.99998	16303	0.2125
FAM188B	6	0.79445	0.82038	0.99998	16304	0.1875
NPAS1	6	0.79445	0.82038	0.99998	16305	-0.0883
SPANXC	1	0.79458	0.79499	0.99998	16306	0.1985
P4HB	4	0.79467	0.79449	0.99998	16307	0.0461
CRAT	6	0.79479	0.82058	0.99998	16308	-0.0365
USP3	6	0.79479	0.82058	0.99998	16309	0.0361
CLN6	6	0.79479	0.82058	0.99998	16310	0.1616
hsa-mir-216a	4	0.7948	0.79463	0.99998	16311	0.0562
LOC729020	6	0.79489	0.82064	0.99998	16312	0.0442
SPIB	6	0.79499	0.82068	0.99998	16313	0.0996
KHDRBS2	6	0.79511	0.82075	0.99998	16314	0.4073
XPNPEP1	6	0.79511	0.82075	0.99998	16315	0.072
DPH7	3	0.79516	0.79519	0.99998	16316	0.0754
DUS4L	6	0.79525	0.82082	0.99998	16317	0.2328
MICALL1	6	0.79525	0.82082	0.99998	16318	-0.0227
hsa-mir-3176	4	0.79532	0.79512	0.99998	16319	0.117
ABLIM3	6	0.79539	0.8209	0.99998	16320	0.1318
HSPB11	6	0.79539	0.8209	0.99998	16321	0.1048
COL9A2	6	0.79546	0.82096	0.99998	16322	0.1625
ZNF674	5	0.79554	0.80161	0.99998	16323	0.05
ARTN	6	0.79556	0.82101	0.99998	16324	0.0227
PPIG	6	0.79577	0.82113	0.99998	16325	-0.0001
SPANXN5	5	0.7958	0.80181	0.99998	16326	-0.1578
HSPB3	6	0.79586	0.82119	0.99998	16327	0.1777
PPEF1	6	0.79586	0.82119	0.99998	16328	0.317
TNFSF11	6	0.79591	0.82122	0.99998	16329	0.0968
TMLHE	4	0.796	0.7958	0.99998	16330	0.1029
EPHB3	6	0.79612	0.82133	0.99998	16331	-0.0764
ARF3	6	0.79612	0.82133	0.99998	16332	0.029
GLRX2	6	0.79643	0.82152	0.99998	16333	0.0226
AIFM2	6	0.79666	0.82165	0.99998	16334	0.083
LUC7L2	3	0.79685	0.79685	0.99998	16335	0.1828
SORBS3	6	0.79692	0.8218	0.99998	16336	0.2823
NOSTRIN	5	0.79695	0.80274	0.99998	16337	0.0886
RORA	6	0.79723	0.82199	0.99998	16338	0.0583
hsa-mir-548h-	3	0.79728	0.79728	0.99998	16339	0.1932
RFTN1	6	0.79736	0.82206	0.99998	16340	0.0074
CDK7	6	0.79747	0.82213	0.99998	16341	0.1487
A2M	6	0.79747	0.82213	0.99998	16342	0.0909
CLASRP	6	0.7976	0.8222	0.99998	16343	-0.0952
hsa-mir-320d-	2	0.79761	0.7972	0.99998	16344	0.2176
PRAMEF21	2	0.79761	0.7972	0.99998	16345	0.3043
TRIM64	2	0.79761	0.7972	0.99998	16346	0.5549
PAGE2	2	0.79761	0.7972	0.99998	16347	0.1181
LGALS7B	2	0.79761	0.7972	0.99998	16348	0.8847
PPIAL4F	2	0.79761	0.7972	0.99998	16349	0.2649
hsa-mir-500a	2	0.79761	0.7972	0.99998	16350	0.6562
hsa-mir-519b	2	0.79761	0.7972	0.99998	16351	0.2091
TMSB15B	2	0.79761	0.7972	0.99998	16352	0.4686
AASDH	6	0.79771	0.82226	0.99998	16353	0.0571
OR2F2	6	0.79771	0.82226	0.99998	16354	0.0319
CDK5RAP3	4	0.79776	0.79754	0.99998	16355	-0.0326
KIAA1211	6	0.79789	0.82237	0.99998	16356	0.2143
CCDC85A	6	0.79789	0.82237	0.99998	16357	0.2193
C7orf34	6	0.79789	0.82237	0.99998	16358	0.069
hsa-mir-1260l	4	0.79793	0.79773	0.99998	16359	0.0091

KRT4	6	0.798	0.82243	0.99998	16360	-0.0192
WDFY3	6	0.79806	0.82246	0.99998	16361	-0.0569
MAPRE3	6	0.79806	0.82246	0.99998	16362	-0.0978
hsa-mir-4733	4	0.7981	0.79793	0.99998	16363	0.012
KIAA1239	6	0.79815	0.82251	0.99998	16364	0.1691
CGB1	3	0.79821	0.79819	0.99998	16365	0.1523
TAS2R60	6	0.79827	0.82258	0.99998	16366	0.2924
C9	6	0.79827	0.82258	0.99998	16367	-0.0962
GPR31	6	0.79871	0.82282	0.99998	16368	0.0438
VLDLR	6	0.79871	0.82282	0.99998	16369	-0.0157
ZCWPW2	6	0.79874	0.82284	0.99998	16370	0.1227
BRDT	6	0.79885	0.82291	0.99998	16371	-0.0398
RALY	6	0.79885	0.82291	0.99998	16372	0.0228
SATB2	4	0.79898	0.7988	0.99998	16373	0.2218
DNAJC17	6	0.79902	0.82301	0.99998	16374	0.0618
CD14	6	0.79902	0.82301	0.99998	16375	0.0601
WDR92	6	0.79933	0.82317	0.99998	16376	0.2235
hsa-mir-3939	4	0.79934	0.79914	0.99998	16377	0.0659
NCEH1	6	0.79948	0.82326	0.99998	16378	0.2579
TIE1	6	0.79953	0.82329	0.99998	16379	-0.1051
NAV3	6	0.79953	0.82329	0.99998	16380	-0.1032
GAGE12J	1	0.7996	0.79997	0.99998	16381	0.2098
HELZ2	6	0.79962	0.82334	0.99998	16382	0.0585
CTGF	5	0.7998	0.80502	0.99998	16383	0.3023
hsa-mir-1206	1	0.79982	0.80018	0.99998	16384	0.5137
hsa-mir-2053	4	0.79994	0.79975	0.99998	16385	0.1581
hsa-mir-4474	3	0.80006	0.80005	0.99998	16386	0.1932
PRAMEF20	1	0.80009	0.80043	0.99998	16387	0.8027
OR51A2	6	0.80011	0.82362	0.99998	16388	-0.1302
SLC12A7	6	0.80011	0.82362	0.99998	16389	0.0437
HRH3	6	0.80011	0.82362	0.99998	16390	0.3306
ZFP14	6	0.80011	0.82362	0.99998	16391	0.2221
SPICE1	4	0.80019	0.79999	0.99998	16392	0.0466
hsa-mir-3192	4	0.80047	0.80027	0.99998	16393	-0.0659
DYNLRB2	6	0.80075	0.82398	0.99998	16394	-0.0626
hsa-mir-657	4	0.8008	0.8006	0.99998	16395	0.3918
hsa-mir-208b	4	0.80085	0.80065	0.99998	16396	0.0527
KCNC2	6	0.80093	0.82408	0.99998	16397	0.0262
KCNK18	6	0.80104	0.82415	0.99998	16398	0.2312
PCDHA10	2	0.80109	0.80074	0.99998	16399	0.1251
CORO2B	6	0.80114	0.82421	0.99998	16400	0.2343
STXBP5L	6	0.80114	0.82421	0.99998	16401	0.1308
RNF135	6	0.80114	0.82421	0.99998	16402	0.2693
hsa-mir-6125	4	0.80115	0.80098	0.99998	16403	0.1579
FER1L6	6	0.80121	0.82425	0.99998	16404	-0.0526
ZSCAN22	6	0.80124	0.82427	0.99998	16405	0.1445
MEF2BNB-ME	2	0.80132	0.80097	0.99998	16406	0.088
DDI1	6	0.80141	0.82437	0.99998	16407	0.1851
CHCHD10	6	0.80156	0.82445	0.99998	16408	0.0142
ZDHHC2	6	0.80156	0.82445	0.99998	16409	0.0984
MAGEE2	6	0.80156	0.82445	0.99998	16410	0.0409
hsa-mir-4791	4	0.80158	0.80138	0.99998	16411	0.1209
hsa-mir-8055	4	0.80168	0.80146	0.99998	16412	0.0244
PYY	6	0.80168	0.82451	0.99998	16413	-0.0099
DZANK1	6	0.8018	0.82457	0.99998	16414	0.1009
TMEM109	6	0.80196	0.82467	0.99998	16415	0.0851
OR6C68	6	0.80196	0.82467	0.99998	16416	0.0652
AUNIP	6	0.80208	0.82473	0.99998	16417	0.2266
LOC10050700	6	0.80218	0.82479	0.99998	16418	-0.0196
TCEAL5	5	0.8022	0.80696	0.99998	16419	0.2947
FEZ1	6	0.80224	0.82482	0.99998	16420	-0.0402
HIST1H3C	6	0.80232	0.82488	0.99998	16421	0.0982
CIB4	6	0.80245	0.82497	0.99998	16422	0.0367
SH3BP1	6	0.80245	0.82497	0.99998	16423	0.2167
TAF6L	6	0.80245	0.82497	0.99998	16424	0.0407
C4orf48	6	0.80245	0.82497	0.99998	16425	-0.0045
hsa-mir-1269a	4	0.80248	0.80227	0.99998	16426	-0.0043
CHAC1	6	0.8026	0.82507	0.99998	16427	0.3306
RAPGEF6	6	0.8026	0.82507	0.99998	16428	0.1815
ARMC1	6	0.8026	0.82507	0.99998	16429	0.1516
APOBEC3C	6	0.80277	0.82517	0.99998	16430	0.0697
METTL20	6	0.80277	0.82517	0.99998	16431	0.0998
hsa-mir-654	4	0.8028	0.80258	0.99998	16432	0.1608
COMMD3	2	0.80291	0.80255	0.99998	16433	0.43
GABRA6	6	0.80306	0.82535	0.99998	16434	0.1207
CLSTN2	6	0.80313	0.8254	0.99998	16435	0.0836
P2RY1	6	0.80324	0.82546	0.99998	16436	-0.0652
DBF4B	6	0.80343	0.82559	0.99998	16437	-0.0558
hsa-mir-4719	1	0.80345	0.80373	0.99998	16438	0.4387
hsa-mir-1226	4	0.80355	0.80335	0.99998	16439	-0.0311
TEX14	6	0.80364	0.82572	0.99998	16440	0.1013

LMTK3	4	0.8037	0.8035	0.99998	16441	-0.0246
HLA-DPA1	6	0.80375	0.82578	0.99998	16442	0.0462
HYI	6	0.80375	0.82578	0.99998	16443	0.2474
hsa-mir-3165	4	0.8038	0.8036	0.99998	16444	0.0576
MEGF8	6	0.80387	0.82585	0.99998	16445	0.2604
OR13C2	5	0.80401	0.80841	0.99998	16446	0.1513
KRTAP5-8	5	0.80401	0.80841	0.99998	16447	-0.7128
HSPA1A	5	0.80401	0.80841	0.99998	16448	-0.9148
ZNF812	5	0.80401	0.80841	0.99998	16449	0.1426
TXNRD3NB	6	0.80415	0.82603	0.99998	16450	0.0717
SPAG1	6	0.80415	0.82603	0.99998	16451	-0.084
CASP10	6	0.80426	0.82608	0.99998	16452	0.1389
GABRG2	6	0.80447	0.8262	0.99998	16453	0.2776
FANK1	6	0.80447	0.8262	0.99998	16454	0.1199
NAT8	6	0.80447	0.8262	0.99998	16455	-0.0671
BMI1	6	0.80458	0.82628	0.99998	16456	0.138
ITGB6	6	0.80458	0.82628	0.99998	16457	0.1491
RPE65	6	0.80472	0.82636	0.99998	16458	0.0942
USP4	6	0.80472	0.82636	0.99998	16459	-0.0386
OOEP	6	0.80484	0.82643	0.99998	16460	-0.05
IL16	6	0.80484	0.82643	0.99998	16461	0.0428
hsa-mir-2278	4	0.80488	0.80467	0.99998	16462	0.0039
CCNH	6	0.805	0.82652	0.99998	16463	-0.0227
MROH2B	4	0.805	0.80478	0.99998	16464	-0.0408
SMARCA4	6	0.80511	0.82658	0.99998	16465	0.1415
hsa-let-7g	4	0.80519	0.80498	0.99998	16466	0.0173
FGG	6	0.80534	0.82673	0.99998	16467	0.1961
LPCAT4	6	0.8054	0.82676	0.99998	16468	0.069
MKRN1	6	0.80544	0.82678	0.99998	16469	0.1929
ATP5O	6	0.80544	0.82678	0.99998	16470	0.0189
LEPREL4	6	0.80544	0.82678	0.99998	16471	0.0117
hsa-mir-5007	4	0.80547	0.80526	0.99998	16472	0.0791
LPPR2	6	0.80553	0.82683	0.99998	16473	-0.0407
FUBP1	5	0.80556	0.80971	0.99998	16474	-0.1399
PML	6	0.80567	0.82691	0.99998	16475	0.0032
C2orf72	6	0.80567	0.82691	0.99998	16476	-0.0746
hsa-mir-4797	2	0.8057	0.80534	0.99998	16477	0.2456
hsa-mir-15b	4	0.80575	0.80555	0.99998	16478	0.3135
SMCR7	1	0.80596	0.80618	0.99998	16479	0.2311
MPV17L2	6	0.80598	0.82711	0.99998	16480	0.1478
DEFB1	6	0.80598	0.82711	0.99998	16481	0.1025
TRIM73	2	0.80599	0.80564	0.99998	16482	0.4409
C1orf105	4	0.80609	0.80591	0.99998	16483	0.2188
LGALS8	6	0.80616	0.82722	0.99998	16484	0.2793
HIST2H2AC	6	0.80616	0.82722	0.99998	16485	0.2566
HCN2	6	0.80624	0.82726	0.99998	16486	0.0857
CDADC1	6	0.80632	0.82732	0.99998	16487	-0.0322
CCL5	6	0.80641	0.82737	0.99998	16488	0.2337
C6orf48	6	0.80651	0.82744	0.99998	16489	0.0111
DEPDC7	6	0.80662	0.8275	0.99998	16490	0.4901
TENM1	6	0.80668	0.82753	0.99998	16491	-0.0205
SPAG16	6	0.80684	0.82763	0.99998	16492	-0.0052
DLL1	6	0.80684	0.82763	0.99998	16493	0.0773
MAPK12	5	0.80693	0.81087	0.99998	16494	0.332
MOB1B	6	0.80702	0.82774	0.99998	16495	0.1043
KCNS1	6	0.80702	0.82774	0.99998	16496	0.1223
TNIP3	6	0.80715	0.82782	0.99998	16497	-0.0683
GGTLC1	6	0.80715	0.82782	0.99998	16498	0.0652
KIAA1161	6	0.80727	0.82789	0.99998	16499	0.0967
HP1BP3	6	0.80727	0.82789	0.99998	16500	0.1664
hsa-mir-6069	4	0.80739	0.80718	0.99998	16501	0.1721
ACAA2	6	0.80746	0.82799	0.99998	16502	0.0254
CROCC	6	0.80754	0.82803	0.99998	16503	0.1399
SPTBN2	6	0.80754	0.82803	0.99998	16504	0.121
hsa-mir-499b	3	0.80755	0.80743	0.99998	16505	0.2085
MYLK3	4	0.8076	0.80741	0.99998	16506	0.0484
MFSD6	6	0.80767	0.82811	0.99998	16507	-0.1004
ZNF740	6	0.80767	0.82811	0.99998	16508	0.2242
FBXW10	4	0.80775	0.80756	0.99998	16509	-0.0312
MVP	6	0.80782	0.8282	0.99998	16510	0.0474
IGF2BP3	6	0.80794	0.82827	0.99998	16511	0.0071
hsa-mir-3678	3	0.80797	0.80785	0.99998	16512	0.0053
hsa-mir-3686	3	0.80797	0.80785	0.99998	16513	0.1851
PRAMEF22	3	0.80797	0.80785	0.99998	16514	0.1846
CREB3L3	6	0.808	0.8283	0.99998	16515	0.0991
NEUROD4	6	0.808	0.8283	0.99998	16516	0.1068
UBE3D	6	0.808	0.8283	0.99998	16517	-0.1486
EML1	6	0.80822	0.82843	0.99998	16518	0.1056
CASP2	6	0.80858	0.82864	0.99998	16519	0.0687
ZNF804B	6	0.80868	0.82869	0.99998	16520	0.0997
OR10Q1	6	0.80875	0.82873	0.99998	16521	0.1476

MUC6	6	0.80879	0.82876	0.99998	16522	0.1992
ARMCX5	6	0.80888	0.82881	0.99998	16523	0.0274
hsa-mir-320c-	3	0.80891	0.80878	0.99998	16524	0.0665
CPNE3	6	0.80906	0.82892	0.99998	16525	0.2678
C1orf74	6	0.8091	0.82894	0.99998	16526	-0.0642
LHX8	6	0.80919	0.82901	0.99998	16527	0.1457
BOK	6	0.80919	0.82901	0.99998	16528	0.1625
CIDEA	6	0.80919	0.82901	0.99998	16529	0.1036
ZNF454	6	0.8093	0.82907	0.99998	16530	0.0383
GJA4	6	0.8093	0.82907	0.99998	16531	-0.1638
ZNF485	6	0.80936	0.82911	0.99998	16532	-0.0578
MSH3	6	0.80949	0.82918	0.99998	16533	-0.0742
IMPG2	6	0.80949	0.82918	0.99998	16534	-0.1149
CCNJL	6	0.80963	0.82928	0.99998	16535	-0.0253
TMEM33	6	0.80968	0.82931	0.99998	16536	0.0352
KIF20B	6	0.80977	0.82935	0.99998	16537	0.0771
RNF43	6	0.80986	0.82941	0.99998	16538	0.1357
IZUMO1	6	0.80991	0.82943	0.99998	16539	0.1106
ZNF587	4	0.80991	0.80973	0.99998	16540	0.0643
hsa-mir-3689c	4	0.80991	0.80973	0.99998	16541	-0.5289
RFPL4A	4	0.80991	0.80973	0.99998	16542	-0.2536
ZNF765	4	0.80991	0.80973	0.99998	16543	0.3916
ZNF728	4	0.80991	0.80973	0.99998	16544	0.0712
hsa-mir-548x-	4	0.80991	0.80973	0.99998	16545	0.091
hsa-mir-494	4	0.80991	0.80973	0.99998	16546	-0.0203
hsa-mir-181d	4	0.80991	0.80973	0.99998	16547	0.1591
hsa-mir-206	4	0.80991	0.80973	0.99998	16548	0.1019
ZNF626	4	0.80991	0.80973	0.99998	16549	-0.4324
ZNF611	4	0.80991	0.80973	0.99998	16550	0.1373
hsa-mir-548aj	4	0.80991	0.80973	0.99998	16551	-0.084
hsa-mir-4511	4	0.80991	0.80973	0.99998	16552	-0.1093
SPRR1A	4	0.80991	0.80973	0.99998	16553	0.1397
hsa-mir-1283-	4	0.80991	0.80973	0.99998	16554	-0.0085
ROPN1B	4	0.80991	0.80973	0.99998	16555	0.1475
OCM	4	0.80991	0.80973	0.99998	16556	-0.3351
hsa-mir-548a-	4	0.80991	0.80973	0.99998	16557	-0.1583
LCE3D	4	0.80991	0.80973	0.99998	16558	-0.409
SERPINB4	4	0.80991	0.80973	0.99998	16559	0.0228
hsa-mir-487a	4	0.80991	0.80973	0.99998	16560	-0.1352
MTRNR2L7	4	0.80991	0.80973	0.99998	16561	0.1123
hsa-mir-548ay	4	0.80991	0.80973	0.99998	16562	0.376
PGLYRP1	6	0.81009	0.82955	0.99998	16563	0.2241
CLIC2	6	0.81009	0.82955	0.99998	16564	0.1301
TSSC4	6	0.81019	0.8296	0.99998	16565	0.1149
TMED4	6	0.81019	0.8296	0.99998	16566	0.0825
CASP9	6	0.81035	0.82969	0.99998	16567	0.1466
ZNF791	6	0.81035	0.82969	0.99998	16568	0.2798
EFNA3	6	0.81045	0.82977	0.99998	16569	0.0594
NOTO	6	0.81045	0.82977	0.99998	16570	0.0581
HSBP1	6	0.81045	0.82977	0.99998	16571	-0.0407
ZNF25	6	0.81075	0.82996	0.99998	16572	0.0597
NFAT5	6	0.81088	0.83004	0.99998	16573	0.1353
hsa-mir-548f-:	3	0.81088	0.81075	0.99998	16574	-0.0448
MLLT4	6	0.81098	0.8301	0.99998	16575	0.1297
SAA2	3	0.81105	0.81091	0.99998	16576	0.8048
ELF3	6	0.81107	0.83015	0.99998	16577	0.0223
STARD13	6	0.81107	0.83015	0.99998	16578	0.1503
ZNF219	6	0.81107	0.83015	0.99998	16579	0.0511
hsa-mir-2052	2	0.8111	0.81077	0.99998	16580	0.1505
E2F7	6	0.81114	0.8302	0.99998	16581	0.1422
OR9Q1	6	0.81123	0.83024	0.99998	16582	0.1738
PTTG1IP	6	0.81123	0.83024	0.99998	16583	0.0774
ABHD14A	6	0.81138	0.83033	0.99998	16584	0.1608
PEX19	6	0.81138	0.83033	0.99998	16585	0.1018
ZNF679	6	0.81155	0.83043	0.99998	16586	-0.0474
KIF1A	6	0.81155	0.83043	0.99998	16587	-0.0648
TBX19	6	0.81173	0.83054	0.99998	16588	0.2432
PMM1	6	0.81173	0.83054	0.99998	16589	0.0821
40787	3	0.81177	0.81162	0.99998	16590	0.3057
AMT	6	0.81187	0.83063	0.99998	16591	0.0476
LOC10028918	4	0.81211	0.81192	0.99998	16592	0.1169
DPRX	6	0.81219	0.83084	0.99998	16593	0.3026
PKDCC	6	0.81219	0.83084	0.99998	16594	0.1097
CRAMP1L	6	0.81219	0.83084	0.99998	16595	0.0277
KCNK15	6	0.81219	0.83084	0.99998	16596	0.1745
TYMS	6	0.81219	0.83084	0.99998	16597	0.1702
HSBP1L1	6	0.81234	0.83095	0.99998	16598	0.3562
TOM1	6	0.81234	0.83095	0.99998	16599	0.0591
SYPL1	6	0.81234	0.83095	0.99998	16600	0.2824
SPATS2L	6	0.81252	0.83105	0.99998	16601	-0.0015
COQ10A	6	0.81252	0.83105	0.99998	16602	-0.0236

hsa-mir-6770-	2	0.81263	0.81231	0.99998	16603	0.1489
TRABD2B	6	0.81265	0.83113	0.99998	16604	0.2131
FAXC	6	0.81265	0.83113	0.99998	16605	0.028
hsa-mir-6816	4	0.81266	0.81247	0.99998	16606	0.0615
DENND5A	6	0.81283	0.83126	0.99998	16607	0.1684
ARFGAP1	6	0.81283	0.83126	0.99998	16608	0.0384
CRELD2	6	0.81283	0.83126	0.99998	16609	0.2063
DEFB4B	3	0.81292	0.81277	0.99998	16610	0.3985
PPM1B	6	0.81294	0.83134	0.99998	16611	-0.0466
SOBP	6	0.81303	0.83139	0.99998	16612	0.0719
LPIN3	6	0.81327	0.83153	0.99998	16613	0.0588
PRKRA	4	0.81338	0.81322	0.99998	16614	0.0236
TMBIM1	6	0.81342	0.83163	0.99998	16615	0.1033
SEC24B	6	0.81342	0.83163	0.99998	16616	-0.0285
hsa-mir-5687	4	0.81364	0.81346	0.99998	16617	-0.0676
PHF11	6	0.81366	0.83178	0.99998	16618	0.0559
CTBS	6	0.81371	0.83181	0.99998	16619	-0.0743
HIST1H1E	6	0.81384	0.83188	0.99998	16620	-0.1202
KCNQ2	6	0.81384	0.83188	0.99998	16621	0.0322
ANKRD30B	6	0.81384	0.83188	0.99998	16622	0.061
CNIH1	2	0.81393	0.81363	0.99998	16623	0.2731
CAPN12	6	0.81401	0.832	0.99998	16624	0.1171
PRKCB	6	0.81412	0.83206	0.99998	16625	-0.0055
SYPL2	6	0.8142	0.83212	0.99998	16626	0.0178
LALBA	6	0.8142	0.83212	0.99998	16627	0.1512
PRICKLE2	6	0.81434	0.83221	0.99998	16628	0.0581
CACNA2D3	6	0.81434	0.83221	0.99998	16629	0.054
ZKSCAN1	6	0.81434	0.83221	0.99998	16630	0.0339
LCN10	6	0.81446	0.83229	0.99998	16631	0.1349
VAMP1	6	0.81446	0.83229	0.99998	16632	0.2388
TRAPPC6B	6	0.81463	0.8324	0.99998	16633	0.0573
PRKCE	6	0.81473	0.83247	0.99998	16634	0.1297
STRIP1	6	0.81485	0.83254	0.99998	16635	0.0518
ANKS1A	6	0.81491	0.83257	0.99998	16636	0.3202
FOXP2	6	0.81517	0.83273	0.99998	16637	-0.0002
GNRH1	6	0.81522	0.83277	0.99998	16638	0.089
SNX19	6	0.81525	0.83278	0.99998	16639	0.2541
RPS6KA3	6	0.81537	0.83287	0.99998	16640	0.0293
CTSS	6	0.81546	0.83293	0.99998	16641	0.0996
GUCY1B3	6	0.81573	0.83309	0.99998	16642	0.2088
ZNF43	6	0.81585	0.83317	0.99998	16643	0.026
hsa-mir-6790	4	0.81592	0.81572	0.99998	16644	0.364
USP17L13	1	0.81592	0.8162	0.99998	16645	0.2708
CTAGE15	3	0.81603	0.81589	0.99998	16646	-0.0584
hsa-mir-325	4	0.81622	0.81603	0.99998	16647	0.0254
RABGAP1	6	0.81627	0.83344	0.99998	16648	0.0749
ICOSLG	6	0.81643	0.83354	0.99998	16649	-0.1682
CANT1	6	0.81656	0.83362	0.99998	16650	0.0531
KRBA1	6	0.81663	0.83366	0.99998	16651	0.3068
MESDC1	6	0.81666	0.83368	0.99998	16652	0.1555
PPIL3	6	0.81666	0.83368	0.99998	16653	-0.0078
COL4A1	6	0.81676	0.83373	0.99998	16654	-0.0756
ABCC9	6	0.81676	0.83373	0.99998	16655	0.1856
hsa-mir-302b	4	0.81678	0.8166	0.99998	16656	0.0504
hsa-mir-548v	4	0.81678	0.8166	0.99998	16657	0.0718
MAP7	6	0.81687	0.83381	0.99998	16658	0.0004
PPFIA2	6	0.81714	0.83397	0.99998	16659	0.2854
ZNF395	4	0.81716	0.81699	0.99998	16660	0.0217
HAGHL	6	0.81719	0.834	0.99998	16661	0.1464
ZC3HAV1L	6	0.8173	0.83407	0.99998	16662	-0.0101
ARHGAP5	6	0.8173	0.83407	0.99998	16663	0.1822
CAMK2N2	6	0.81744	0.83417	0.99998	16664	0.0833
COBLL1	6	0.81753	0.83423	0.99998	16665	0.1803
CAB39L	6	0.81753	0.83423	0.99998	16666	0.1402
PSMB6	6	0.81763	0.83429	0.99998	16667	0.1169
HSPE1-MOB4	1	0.81769	0.81799	0.99998	16668	0.2587
LYNX1	6	0.81771	0.83435	0.99998	16669	0.0998
hsa-mir-4795	2	0.81775	0.81743	0.99998	16670	0.1118
RPL38	6	0.81782	0.83442	0.99998	16671	-0.0801
DENND2A	6	0.81792	0.83448	0.99998	16672	0.1115
KIF21B	6	0.81792	0.83448	0.99998	16673	0.211
KRTAP9-6	5	0.81797	0.82028	0.99998	16674	0.1932
USP22	6	0.81797	0.83451	0.99998	16675	-0.0626
KRTAP6-2	6	0.81797	0.83451	0.99998	16676	-0.002
ALPK2	6	0.81808	0.83456	0.99998	16677	0.0805
AP5S1	6	0.81808	0.83456	0.99998	16678	0.1307
RLN3	4	0.81819	0.818	0.99998	16679	0.0952
hsa-mir-3162	4	0.81819	0.818	0.99998	16680	0.4602
SAP30L	6	0.81823	0.83465	0.99998	16681	0.1283
FOXH1	6	0.81828	0.83469	0.99998	16682	0.204
METTL5	6	0.81839	0.83477	0.99998	16683	0.1097

CPED1	6	0.81839	0.83477	0.99998	16684	-0.0508
ASTE1	6	0.81849	0.83483	0.99998	16685	0.0032
hsa-mir-1269b	2	0.81856	0.81824	0.99998	16686	0.2537
TNKS	6	0.81865	0.83494	0.99998	16687	0.1294
ADAT2	6	0.81865	0.83494	0.99998	16688	0.1174
OR52A5	6	0.81871	0.83497	0.99998	16689	0.1221
FLG	6	0.81878	0.83502	0.99998	16690	-0.0422
MRGPRF	6	0.81878	0.83502	0.99998	16691	0.0691
hsa-mir-16-1	4	0.81884	0.81866	0.99998	16692	-0.0581
PRKCD	6	0.81903	0.83518	0.99998	16693	-0.0227
OR10G4	6	0.81915	0.83526	0.99998	16694	0.0092
RPTN	6	0.81915	0.83526	0.99998	16695	-0.1314
FAM187B	6	0.81932	0.83537	0.99998	16696	0.1155
TANGO2	6	0.81932	0.83537	0.99998	16697	0.0846
ISLR2	6	0.81932	0.83537	0.99998	16698	-0.113
COL17A1	6	0.81932	0.83537	0.99998	16699	0.0325
PPP3CB	6	0.81948	0.83546	0.99998	16700	0.083
ZNF552	6	0.81948	0.83546	0.99998	16701	-0.1041
PTPN11	6	0.81948	0.83546	0.99998	16702	0.0681
OR10P1	4	0.81962	0.81945	0.99998	16703	0.1605
hsa-mir-3666	4	0.81969	0.81952	0.99998	16704	0.0808
GH1	6	0.81978	0.83566	0.99998	16705	0.0537
TYW1	6	0.81978	0.83566	0.99998	16706	-0.4789
KRTAP19-1	6	0.81978	0.83566	0.99998	16707	0.07
RPS4Y2	6	0.81978	0.83566	0.99998	16708	0.3238
RPL26L1	6	0.81978	0.83566	0.99998	16709	0.1035
hsa-mir-6832	4	0.81984	0.81967	0.99998	16710	-0.0709
hsa-mir-4758	4	0.82018	0.82	0.99998	16711	0.3168
DRD5	6	0.82036	0.83606	0.99998	16712	0.0549
OR4X1	6	0.82044	0.8361	0.99998	16713	0.1783
ZNF333	6	0.82044	0.8361	0.99998	16714	0.2117
ATMIN	6	0.82044	0.8361	0.99998	16715	0.0327
DZIP3	6	0.82044	0.8361	0.99998	16716	0.0097
LHFP	6	0.82053	0.83617	0.99998	16717	0.0978
COL20A1	6	0.82053	0.83617	0.99998	16718	-0.0052
GRAP2	6	0.82082	0.83635	0.99998	16719	0.2808
MAFG	6	0.82089	0.83639	0.99998	16720	0.0151
CYP4F12	5	0.82093	0.82286	0.99998	16721	-0.0738
hsa-mir-300	4	0.82096	0.82078	0.99998	16722	0.0877
PAQR3	6	0.82101	0.83647	0.99998	16723	0.1617
DAGLA	6	0.82101	0.83647	0.99998	16724	-0.0977
ODF3	6	0.82119	0.83658	0.99998	16725	-0.0083
hsa-mir-5194	4	0.82127	0.8211	0.99998	16726	0.1295
C1orf174	6	0.82139	0.83671	0.99998	16727	0.0199
LCE1F	5	0.82148	0.82333	0.99998	16728	-0.0597
SULT1A2	5	0.82148	0.82333	0.99998	16729	0.2665
CLP1	6	0.82151	0.83678	0.99998	16730	0.1256
LRIF1	6	0.82151	0.83678	0.99998	16731	0.1857
CCDC89	6	0.82151	0.83678	0.99998	16732	0.0065
hsa-mir-3667	4	0.82154	0.82137	0.99998	16733	0.2453
hsa-mir-488	4	0.82161	0.82143	0.99998	16734	0.5279
COL28A1	6	0.82168	0.83689	0.99998	16735	0.2663
FANCG	6	0.82183	0.83698	0.99998	16736	0.1857
hsa-mir-551a	4	0.82193	0.82174	0.99998	16737	0.1414
HSD17B6	6	0.82203	0.8371	0.99998	16738	-0.0147
TMIGD2	6	0.82208	0.83713	0.99998	16739	-0.021
TNNI3K	6	0.82219	0.8372	0.99998	16740	0.0765
F2RL2	6	0.82234	0.83731	0.99998	16741	0.0589
TGFB3	6	0.82234	0.83731	0.99998	16742	0.313
hsa-mir-4452	4	0.82235	0.82216	0.99998	16743	0.1428
FXR1	6	0.82249	0.83741	0.99998	16744	0.2069
C16orf55	2	0.82268	0.8224	0.99998	16745	0.3049
OR51F1	6	0.82272	0.83755	0.99998	16746	0.1281
XPOT	4	0.82278	0.82261	0.99998	16747	0.0195
SPPL2C	6	0.8228	0.83761	0.99998	16748	-0.0044
ZMIZ2	6	0.82299	0.83772	0.99998	16749	0.0313
CST3	6	0.82301	0.83773	0.99998	16750	0.1204
SLC25A28	6	0.82307	0.83777	0.99998	16751	0.1125
GLIS3	6	0.82316	0.83782	0.99998	16752	0.0017
KCNK3	6	0.82337	0.83796	0.99998	16753	0.0052
ZSWIM8	4	0.82339	0.82323	0.99998	16754	0.0664
HECTD2	6	0.82349	0.83804	0.99998	16755	0.0526
PALM	6	0.82352	0.83806	0.99998	16756	0.0422
RELL1	6	0.82359	0.8381	0.99998	16757	0.0525
NHLH2	6	0.82359	0.8381	0.99998	16758	0.2292
DDX31	6	0.82359	0.8381	0.99998	16759	-0.0742
hsa-mir-1321	4	0.82365	0.82348	0.99998	16760	0.4177
UQCC2	4	0.82383	0.82367	0.99998	16761	-0.0109
ECT2L	6	0.82386	0.83828	0.99998	16762	0.3004
NET1	6	0.82386	0.83828	0.99998	16763	0.2048
GPAM	6	0.82386	0.83828	0.99998	16764	0.1343

SLC14A1	6	0.82396	0.83835	0.99998	16765	0.1674
FBXL7	6	0.82398	0.83836	0.99998	16766	0.2411
PRRC2B	6	0.82405	0.83841	0.99998	16767	0.014
hsa-mir-5581	4	0.82417	0.82402	0.99998	16768	-0.0506
C8G	6	0.82417	0.83849	0.99998	16769	0.0584
KRTAP12-1	6	0.82429	0.83856	0.99998	16770	-0.1132
ADAD2	6	0.82429	0.83856	0.99998	16771	0.0365
YWHAQ	6	0.82451	0.83871	0.99998	16772	0.2876
SLC47A2	6	0.8246	0.83877	0.99998	16773	-0.0765
SIAE	6	0.82484	0.83892	0.99998	16774	-0.0379
CD163	6	0.82484	0.83892	0.99998	16775	0.1476
SH3GL3	6	0.82502	0.83904	0.99998	16776	0.0173
SNAP23	6	0.82516	0.83913	0.99998	16777	0.0024
MAP10	6	0.82516	0.83913	0.99998	16778	0.0517
hsa-mir-3151	4	0.82518	0.82501	0.99998	16779	0.2456
PRMT3	6	0.82528	0.83919	0.99998	16780	0.1127
SLC17A5	6	0.82528	0.83919	0.99998	16781	-0.0725
TSPYL4	6	0.82528	0.83919	0.99998	16782	0.1692
GLTSCR1	6	0.82535	0.83924	0.99998	16783	0.2113
MBL2	6	0.82542	0.83929	0.99998	16784	-0.0165
B3GNT2	6	0.82542	0.83929	0.99998	16785	0.0867
SGK2	6	0.82542	0.83929	0.99998	16786	0.0636
TMEM120B	6	0.82551	0.83935	0.99998	16787	0.105
NRP1	6	0.82563	0.83942	0.99998	16788	0.0137
RANBP3	6	0.82563	0.83942	0.99998	16789	0.0362
C4orf40	6	0.82563	0.83942	0.99998	16790	0.3591
PCDHA5	2	0.8258	0.82553	0.99998	16791	0.1867
CCRN4L	6	0.8258	0.83954	0.99998	16792	-0.0522
PANK4	6	0.826	0.83968	0.99998	16793	-0.1076
STAB1	6	0.8261	0.83974	0.99998	16794	0.127
HSPA14	6	0.8261	0.83974	0.99998	16795	0.3059
TUBB6	6	0.82622	0.83981	0.99998	16796	0.0983
ADCY3	6	0.8263	0.83987	0.99998	16797	-0.0234
hsa-mir-6821	4	0.82633	0.82614	0.99998	16798	0.0026
EPM2A	6	0.8264	0.83995	0.99998	16799	-0.0373
NISCH	6	0.8264	0.83995	0.99998	16800	0.0561
hsa-mir-937	3	0.82657	0.8265	0.99998	16801	-0.019
MYL6	6	0.82671	0.84013	0.99998	16802	-0.0235
FAM9B	6	0.82677	0.84018	0.99998	16803	0.1009
hsa-mir-3940	4	0.82679	0.82661	0.99998	16804	-0.0052
ABRACL	6	0.82686	0.84023	0.99998	16805	0.077
S1PR5	6	0.82713	0.8404	0.99998	16806	-0.0218
PPID	6	0.82742	0.84059	0.99998	16807	0.1568
FABP4	6	0.82742	0.84059	0.99998	16808	0.0028
TYW3	6	0.82742	0.84059	0.99998	16809	0.0081
PTPLAD2	6	0.82742	0.84059	0.99998	16810	0.0659
ZNF641	6	0.82756	0.8407	0.99998	16811	0.1628
ECM2	6	0.82756	0.8407	0.99998	16812	-0.063
FBXO2	6	0.82773	0.84081	0.99998	16813	0.1325
ANKRD62	6	0.8278	0.84085	0.99998	16814	-0.0408
hsa-mir-1227	4	0.82783	0.82768	0.99998	16815	0.1641
ARNT2	6	0.82783	0.84087	0.99998	16816	0.0606
IQSEC2	6	0.82788	0.84092	0.99998	16817	-0.0387
ZNF827	6	0.82797	0.84098	0.99998	16818	0.0872
C12orf42	6	0.82797	0.84098	0.99998	16819	-0.0335
PATL1	6	0.82804	0.84103	0.99998	16820	0.0765
GRM5	6	0.82814	0.84109	0.99998	16821	-0.1397
TXLNB	6	0.82814	0.84109	0.99998	16822	0.0739
NFIC	6	0.8282	0.84113	0.99998	16823	0.2294
OR4C45	6	0.8283	0.8412	0.99998	16824	-0.0193
TAS2R5	6	0.8285	0.84134	0.99998	16825	0.0559
LRBA	6	0.8285	0.84134	0.99998	16826	0.0549
SMIM10	6	0.8285	0.84134	0.99998	16827	0.0209
SHISA3	6	0.82861	0.84142	0.99998	16828	0.2214
CACNG5	6	0.82869	0.84147	0.99998	16829	0.1101
C5orf58	6	0.8288	0.84153	0.99998	16830	-0.062
OLIG2	6	0.8288	0.84153	0.99998	16831	0.2503
GRAMD3	6	0.82886	0.84158	0.99998	16832	0.2023
PTH2R	6	0.82902	0.84168	0.99998	16833	0.0676
HOXC11	6	0.82902	0.84168	0.99998	16834	0.1023
RAP1GAP2	6	0.82909	0.84172	0.99998	16835	0.1224
STK32A	6	0.82916	0.84176	0.99998	16836	0.1904
OR7C1	6	0.82916	0.84176	0.99998	16837	0.1216
DISP2	6	0.82937	0.8419	0.99998	16838	0.0758
SPATA12	6	0.82937	0.8419	0.99998	16839	0.0589
KRTAP9-9	4	0.82938	0.82922	0.99998	16840	0.5028
CTDSP1	6	0.82976	0.84213	0.99998	16841	0.2391
AK3	6	0.82976	0.84213	0.99998	16842	0.2128
C20orf112	6	0.82993	0.84227	0.99998	16843	0.2624
PFKP	6	0.82993	0.84227	0.99998	16844	0.1407
IFNA14	5	0.82994	0.83088	0.99998	16845	0.3259

ERVW-1	6	0.82998	0.8423	0.99998	16846	0.0855
NEGR1	6	0.83004	0.84234	0.99998	16847	-0.0738
SDR42E1	6	0.83014	0.84241	0.99998	16848	-0.0807
BPTF	6	0.83023	0.84247	0.99998	16849	0.0892
NOG	6	0.83023	0.84247	0.99998	16850	0.1021
FLNB	6	0.8303	0.84252	0.99998	16851	-0.0185
CYP20A1	6	0.8303	0.84252	0.99998	16852	0.1438
TG	6	0.8303	0.84252	0.99998	16853	0.1674
MAP3K2	6	0.8303	0.84252	0.99998	16854	0.0868
KLHL17	6	0.83041	0.84259	0.99998	16855	0.0374
ODAM	6	0.83041	0.84259	0.99998	16856	0.2851
DTX4	6	0.83041	0.84259	0.99998	16857	0.1283
BNC2	6	0.83041	0.84259	0.99998	16858	0.0128
CLCF1	6	0.83041	0.84259	0.99998	16859	0.0313
VAX2	6	0.8305	0.84266	0.99998	16860	0.1697
KCNIP2	6	0.83059	0.84271	0.99998	16861	-0.0075
DPPA2	6	0.83059	0.84271	0.99998	16862	0.3567
STBD1	6	0.83073	0.84279	0.99998	16863	0.0118
RGS6	6	0.83073	0.84279	0.99998	16864	-0.0595
TEAD4	6	0.83082	0.84286	0.99998	16865	-0.0294
ZDHHC22	6	0.83082	0.84286	0.99998	16866	0.2473
HDAC7	6	0.83087	0.8429	0.99998	16867	0.0677
CEPT1	6	0.83087	0.8429	0.99998	16868	-0.0228
GCNT1	6	0.83087	0.8429	0.99998	16869	0.1833
PLEKHA3	6	0.83095	0.84296	0.99998	16870	0.2443
WBSCR17	6	0.83095	0.84296	0.99998	16871	0.0801
GSTP1	6	0.83095	0.84296	0.99998	16872	-0.0348
hsa-mir-554	4	0.83101	0.83087	0.99998	16873	0.0727
GULP1	6	0.83103	0.84301	0.99998	16874	0.277
FAHD1	6	0.83125	0.84315	0.99998	16875	0.1774
PLA2R1	6	0.83129	0.84318	0.99998	16876	0.1785
MNX1	6	0.83136	0.84323	0.99998	16877	0.153
NPY4R	6	0.83145	0.84329	0.99998	16878	0.0342
ENGASE	6	0.83152	0.84333	0.99998	16879	0.1709
NCK2	6	0.83158	0.84338	0.99998	16880	0.1027
GOT1	6	0.83166	0.84343	0.99998	16881	0.1934
NDUFA7	6	0.83166	0.84343	0.99998	16882	0.0399
SFTPB	6	0.83175	0.8435	0.99998	16883	0.0222
GDF15	6	0.83175	0.8435	0.99998	16884	0.1514
RSG1	6	0.83187	0.84357	0.99998	16885	0.2649
SIGLEC14	6	0.83221	0.84379	0.99998	16886	0.1735
RBP1	6	0.83231	0.84385	0.99998	16887	0.2091
SSH3	6	0.83241	0.84392	0.99998	16888	-0.0153
PACSIN3	6	0.83241	0.84392	0.99998	16889	0.1436
FAM175A	6	0.83254	0.84401	0.99998	16890	0.0392
GPBAR1	6	0.83264	0.84408	0.99998	16891	0.2213
RCCD1	6	0.833	0.84435	0.99998	16892	0.2473
HMCN1	6	0.83321	0.84449	0.99998	16893	0.0696
C1orf168	6	0.83321	0.84449	0.99998	16894	0.1788
TLR10	6	0.83331	0.84457	0.99998	16895	0.3169
CAMK1	6	0.83346	0.84466	0.99998	16896	0.184
APPL2	6	0.83346	0.84466	0.99998	16897	0.0365
GALNT18	6	0.83366	0.84478	0.99998	16898	0.331
RAD51B	6	0.83366	0.84478	0.99998	16899	0.1061
FAM83H	6	0.83381	0.8449	0.99998	16900	0.0409
ITGAM	6	0.83389	0.84496	0.99998	16901	0.1736
TMPRSS3	6	0.83408	0.84509	0.99998	16902	0.0858
FGF16	6	0.83413	0.84513	0.99998	16903	-0.0305
UBAP2L	6	0.83421	0.84518	0.99998	16904	-0.0483
KRTAP9-1	6	0.83427	0.84522	0.99998	16905	0.171
MGMT	6	0.83436	0.84529	0.99998	16906	-0.1279
hsa-mir-3141	4	0.83442	0.83427	0.99998	16907	0.5498
hsa-mir-5691	4	0.83448	0.83435	0.99998	16908	0.0866
VPS36	4	0.83451	0.83438	0.99998	16909	0.0246
37500	3	0.8346	0.83457	0.99998	16910	0.0584
TFAP2C	6	0.83461	0.84546	0.99998	16911	0.0562
MLL4	3	0.83469	0.83466	0.99998	16912	0.0228
BCHE	6	0.83474	0.84554	0.99998	16913	0.0271
WDR81	6	0.83505	0.84575	0.99998	16914	0.1314
HDDC2	6	0.83505	0.84575	0.99998	16915	-0.0098
DAZL	6	0.83505	0.84575	0.99998	16916	-0.1387
UPK3A	6	0.83505	0.84575	0.99998	16917	0.0164
hsa-mir-6864	4	0.83511	0.83497	0.99998	16918	0.2832
UBR1	6	0.83521	0.84586	0.99998	16919	-0.0897
MAOB	6	0.83521	0.84586	0.99998	16920	0.1171
TGM7	6	0.8353	0.84592	0.99998	16921	0.1677
TRIM2	6	0.83535	0.84595	0.99998	16922	-0.0052
TMEM190	6	0.83561	0.84614	0.99998	16923	0.0818
MTHFR	6	0.83561	0.84614	0.99998	16924	0.1865
MTNR1A	6	0.83561	0.84614	0.99998	16925	0.0408
KCNK13	6	0.83561	0.84614	0.99998	16926	-0.005

TRIM44	6	0.83574	0.84623	0.99998	16927	0.1948
TTC16	6	0.83574	0.84623	0.99998	16928	-0.0032
POU6F2	6	0.83574	0.84623	0.99998	16929	0.1275
ZNF350	6	0.83574	0.84623	0.99998	16930	-0.0188
CHRFAM7A	4	0.83582	0.83565	0.99998	16931	0.1889
IRX6	6	0.83584	0.8463	0.99998	16932	-0.0305
hsa-mir-4762	2	0.8359	0.83578	0.99998	16933	0.3716
HPX	6	0.83591	0.84634	0.99998	16934	0.053
AGAP9	2	0.83599	0.83588	0.99998	16935	0.7761
hsa-mir-8087	4	0.83602	0.83585	0.99998	16936	0.1825
C17orf53	6	0.83613	0.8465	0.99998	16937	0.026
SLC36A2	6	0.83613	0.8465	0.99998	16938	0.1284
SLC2A14	3	0.83618	0.83615	0.99998	16939	0.0093
CCR9	6	0.83626	0.84658	0.99998	16940	0.142
CCDC158	6	0.83626	0.84658	0.99998	16941	0.0597
CACNG1	6	0.83626	0.84658	0.99998	16942	0.0695
OR2T10	6	0.83626	0.84658	0.99998	16943	-0.0434
UCP2	6	0.83635	0.84665	0.99998	16944	0.0241
FAM111A	6	0.83635	0.84665	0.99998	16945	0.0279
PARP10	6	0.83643	0.84671	0.99998	16946	-0.034
WDR91	6	0.83645	0.84672	0.99998	16947	0.0692
GLRA1	6	0.8366	0.84682	0.99998	16948	0.2902
NR2C2	6	0.8366	0.84682	0.99998	16949	0.1155
NRXN1	6	0.8366	0.84682	0.99998	16950	0.1604
CPOX	6	0.83669	0.84689	0.99998	16951	-0.0761
RARS	6	0.83669	0.84689	0.99998	16952	0.0803
GABRQ	6	0.83669	0.84689	0.99998	16953	0.043
C2orf83	6	0.83683	0.84698	0.99998	16954	0.0804
MAPK11	6	0.83683	0.84698	0.99998	16955	0.184
OR52E6	6	0.83704	0.84712	0.99998	16956	0.0382
LRRC47	6	0.83704	0.84712	0.99998	16957	0.099
KCNV1	6	0.83717	0.84722	0.99998	16958	0.1686
hsa-mir-186	4	0.83737	0.8372	0.99998	16959	0.2005
hsa-mir-1912	2	0.83739	0.83729	0.99998	16960	0.1404
GOLGA4	6	0.83739	0.84737	0.99998	16961	0.18
C2orf88	6	0.83739	0.84737	0.99998	16962	-0.0192
CUX1	6	0.83766	0.84756	0.99998	16963	-0.0152
UCMA	6	0.83766	0.84756	0.99998	16964	0.2083
SLC22A5	6	0.83766	0.84756	0.99998	16965	0.2209
REST	6	0.83775	0.84762	0.99998	16966	0.0072
TARSL2	6	0.83783	0.84768	0.99998	16967	0.1052
PPFIBP2	6	0.83783	0.84768	0.99998	16968	0.3207
TMX3	6	0.83783	0.84768	0.99998	16969	0.1983
FKBP1A	6	0.83791	0.84774	0.99998	16970	0.1245
CTCFL	6	0.83794	0.84776	0.99998	16971	-0.1062
OR13A1	6	0.83797	0.84778	0.99998	16972	0.1176
MTMR6	6	0.83801	0.84782	0.99998	16973	0.1736
NDUFC2	4	0.83805	0.83784	0.99998	16974	0.1075
ICAM2	6	0.83808	0.84787	0.99998	16975	0.0252
RSPH10B2	6	0.83808	0.84787	0.99998	16976	-0.1541
hsa-mir-3195	4	0.83817	0.83796	0.99998	16977	0.1711
PPT1	6	0.83824	0.84798	0.99998	16978	0.2079
FGF20	6	0.83848	0.84813	0.99998	16979	0.1161
hsa-mir-346	4	0.83853	0.83831	0.99998	16980	0.1578
hsa-mir-93	4	0.83853	0.83831	0.99998	16981	0.3261
KRBOX4	5	0.83869	0.83898	0.99998	16982	-0.0965
COL27A1	6	0.83874	0.84831	0.99998	16983	0.1923
DCHS2	6	0.83874	0.84831	0.99998	16984	-0.0045
C11orf40	6	0.83874	0.84831	0.99998	16985	0.0982
ZNF846	6	0.83874	0.84831	0.99998	16986	-0.0359
hsa-mir-491	4	0.83876	0.83854	0.99998	16987	0.0452
GSAP	1	0.83888	0.83927	0.99998	16988	0.2663
FCGRT	6	0.83891	0.84842	0.99998	16989	-0.0472
SLC44A2	6	0.83891	0.84842	0.99998	16990	0.0735
POLDIP3	6	0.83891	0.84842	0.99998	16991	-0.0266
hsa-mir-4737	4	0.83898	0.83875	0.99998	16992	0.104
SOX3	6	0.83906	0.84853	0.99998	16993	-0.0494
CPSF1	6	0.83906	0.84853	0.99998	16994	0.446
ALG10B	5	0.83928	0.83953	0.99998	16995	0.0561
ETS2	6	0.83934	0.84872	0.99998	16996	0.2657
PDHX	6	0.83942	0.84878	0.99998	16997	0.0489
OR13J1	6	0.83942	0.84878	0.99998	16998	0.0077
hsa-mir-6509	4	0.83951	0.83931	0.99998	16999	0.126
LY6G5B	6	0.8396	0.84891	0.99998	17000	0.2679
hsa-mir-4304	4	0.83969	0.83948	0.99998	17001	0.0821
WDR45	6	0.83972	0.84898	0.99998	17002	0.0459
C12orf73	6	0.83972	0.84898	0.99998	17003	-0.0595
C7orf63	6	0.83972	0.84898	0.99998	17004	0.1847
DDT	2	0.8398	0.83976	0.99998	17005	0.2622
hsa-mir-6883	4	0.83986	0.83965	0.99998	17006	-0.073
hsa-mir-6818	4	0.83986	0.83965	0.99998	17007	0.2785

CTIF	6	0.8399	0.84911	0.99998	17008	0.0338
FBXW5	6	0.8399	0.84911	0.99998	17009	0.0428
BBX	6	0.84001	0.84919	0.99998	17010	0.1401
RUNX1T1	6	0.84012	0.84926	0.99998	17011	0.1231
PADI4	6	0.84026	0.84935	0.99998	17012	0.0008
hsa-mir-1236	4	0.84041	0.84016	0.99998	17013	0.0072
ZNF460	6	0.84044	0.84948	0.99998	17014	0.2111
CSRP1	6	0.84044	0.84948	0.99998	17015	0.0986
PFDN4	6	0.84052	0.84953	0.99998	17016	0.138
HNF1B	4	0.84057	0.84031	0.99998	17017	0.1793
PRSS53	6	0.84064	0.84962	0.99998	17018	-0.0718
ZNF577	6	0.84064	0.84962	0.99998	17019	0.183
STEAP1	6	0.84071	0.84967	0.99998	17020	0.2959
FILIP1	6	0.84076	0.8497	0.99998	17021	0.0953
FAM53B	6	0.84082	0.84974	0.99998	17022	0.1926
BAIAP3	6	0.84082	0.84974	0.99998	17023	0.0315
C1orf43	6	0.84087	0.84978	0.99998	17024	0.1409
PRSS48	4	0.84094	0.84069	0.99998	17025	0.1281
LOXL2	6	0.84098	0.84987	0.99998	17026	0.2563
OR8D1	6	0.84107	0.84993	0.99998	17027	0.1321
CHI3L2	3	0.8411	0.84109	0.99998	17028	0.1139
MCOLN3	6	0.84111	0.84997	0.99998	17029	0.1283
PP2D1	6	0.84117	0.85002	0.99998	17030	-0.0731
EMR2	6	0.84126	0.85007	0.99998	17031	0.0083
ACTN4	6	0.84126	0.85007	0.99998	17032	-0.0772
AHI1	6	0.84126	0.85007	0.99998	17033	0.0867
IL17C	6	0.84126	0.85007	0.99998	17034	0.1557
ZBED6CL	3	0.84137	0.84138	0.99998	17035	0.0841
C3orf35	6	0.84139	0.85016	0.99998	17036	-0.0521
USP25	6	0.84148	0.85023	0.99998	17037	0.2678
GM2A	6	0.8417	0.85038	0.99998	17038	0.2776
YTHDF3	6	0.8417	0.85038	0.99998	17039	0.045
WDR89	6	0.84183	0.85046	0.99998	17040	0.165
GPR110	6	0.84185	0.85048	0.99998	17041	0.063
PTPN6	6	0.84203	0.8506	0.99998	17042	0.0621
COPZ2	6	0.84211	0.85066	0.99998	17043	0.1168
41153	3	0.84211	0.84213	0.99998	17044	0.3158
GREM2	6	0.84231	0.85081	0.99998	17045	0.1645
C11orf24	6	0.84239	0.85088	0.99998	17046	0.1559
SKA2	6	0.84239	0.85088	0.99998	17047	0.0378
FAM154A	6	0.84251	0.85096	0.99998	17048	-0.023
RFTN2	6	0.84269	0.85108	0.99998	17049	0.0336
SMARCA5	6	0.84269	0.85108	0.99998	17050	-0.0446
DENND5B	6	0.84269	0.85108	0.99998	17051	-0.0305
CALCOCO2	6	0.84279	0.85115	0.99998	17052	0.0273
FREM1	6	0.84293	0.85127	0.99998	17053	-0.0014
ZNF720	6	0.84302	0.85133	0.99998	17054	0.0531
FOXC2	6	0.84319	0.85145	0.99998	17055	0.1459
hsa-mir-513a-	1	0.8432	0.84356	0.99998	17056	0.3968
TMEM191B	6	0.84326	0.85149	0.99998	17057	0.1589
LYPLA1	6	0.84331	0.85153	0.99998	17058	-0.0235
GCN1L1	6	0.84372	0.85183	0.99998	17059	0.3508
CLDN15	6	0.84382	0.8519	0.99998	17060	-0.006
OR5K3	6	0.84382	0.8519	0.99998	17061	0.0242
KRTAP9-3	5	0.84388	0.84405	0.99998	17062	-0.0305
SLC18A1	6	0.84395	0.85199	0.99998	17063	-0.0223
VTI1A	6	0.84403	0.85204	0.99998	17064	-0.0235
DCTN1	6	0.84403	0.85204	0.99998	17065	-0.1193
BTBD16	6	0.84403	0.85204	0.99998	17066	0.0689
ULBP3	6	0.84403	0.85204	0.99998	17067	-0.0449
MAL2	6	0.84422	0.85218	0.99998	17068	0.0634
hsa-mir-520h	1	0.84432	0.84468	0.99998	17069	0.3269
ZNF362	6	0.84432	0.85225	0.99998	17070	-0.0322
DNAJC9	6	0.84432	0.85225	0.99998	17071	0.1785
LOC283710	6	0.84432	0.85225	0.99998	17072	0.1268
LOC388813	5	0.8444	0.84455	0.99998	17073	0.0408
TTC33	6	0.84453	0.85239	0.99998	17074	-0.0485
MEOX2	6	0.84453	0.85239	0.99998	17075	0.2483
COA1	6	0.84466	0.85248	0.99998	17076	0.044
hsa-mir-5699	4	0.84478	0.84453	0.99998	17077	-0.0018
MEOX1	6	0.84484	0.85262	0.99998	17078	0.0802
PRR5-ARHGAF	4	0.84485	0.84461	0.99998	17079	-0.0289
SLC30A10	6	0.84505	0.85276	0.99998	17080	-0.1071
hsa-mir-6747	4	0.84526	0.84499	0.99998	17081	0.1357
TSPAN14	6	0.84531	0.85295	0.99998	17082	0.0527
CAST	6	0.84531	0.85295	0.99998	17083	0.069
PPP1R3F	6	0.84531	0.85295	0.99998	17084	0.1341
HABP4	6	0.84531	0.85295	0.99998	17085	0.1134
FAM117A	6	0.84531	0.85295	0.99998	17086	0.0906
MT3	6	0.84531	0.85295	0.99998	17087	0.0594
hsa-mir-4751	4	0.84537	0.8451	0.99998	17088	-0.0896

CD164L2	6	0.84544	0.85305	0.99998	17089	-0.1084
ZBTB40	6	0.84544	0.85305	0.99998	17090	0.0575
AKR1B15	5	0.84549	0.84563	0.99998	17091	0.0347
C20orf96	6	0.8455	0.85309	0.99998	17092	0.1134
ACTN1	6	0.84559	0.85316	0.99998	17093	0.1235
TNNI1	6	0.84568	0.85322	0.99998	17094	0.0199
LYZL6	6	0.84568	0.85322	0.99998	17095	0.0639
OR10A5	6	0.84568	0.85322	0.99998	17096	0.1691
TBC1D30	6	0.84582	0.85332	0.99998	17097	0.1773
ZNF385D	6	0.84582	0.85332	0.99998	17098	0.0449
IZUMO4	6	0.84582	0.85332	0.99998	17099	0.1387
TDRKH	6	0.84598	0.85342	0.99998	17100	-0.0061
CFI	6	0.84598	0.85342	0.99998	17101	0.2326
MTUS2	6	0.84598	0.85342	0.99998	17102	-0.0559
MRGPRD	6	0.84598	0.85342	0.99998	17103	0.1583
PKDREJ	6	0.84613	0.85356	0.99998	17104	0.275
FAM134B	6	0.84625	0.85364	0.99998	17105	0.0557
EML6	6	0.84625	0.85364	0.99998	17106	0.1107
POLK	6	0.84638	0.85374	0.99998	17107	-0.0494
RPP40	6	0.84638	0.85374	0.99998	17108	0.0779
MYF6	6	0.8465	0.85381	0.99998	17109	0.0495
OR1N2	6	0.8465	0.85381	0.99998	17110	0.1696
ELAVL4	6	0.8465	0.85381	0.99998	17111	0.0203
RPS6KA5	6	0.84662	0.85389	0.99998	17112	0.1373
SLC25A4	6	0.84662	0.85389	0.99998	17113	0.1056
OPN1LW	6	0.84662	0.85389	0.99998	17114	0.1178
GPM6B	6	0.84662	0.85389	0.99998	17115	0.0504
GPR6	6	0.84662	0.85389	0.99998	17116	0.0532
HEPACAM	6	0.84662	0.85389	0.99998	17117	0.1308
NTHL1	6	0.84673	0.85396	0.99998	17118	-0.002
hsa-mir-509-3	2	0.84677	0.84671	0.99998	17119	0.2591
WDR54	6	0.84707	0.8542	0.99998	17120	0.0232
JUN	6	0.84715	0.85426	0.99998	17121	0.2362
SCAMP1	6	0.84721	0.85431	0.99998	17122	0.1983
VWA1	6	0.84721	0.85431	0.99998	17123	0.0122
IFITM5	6	0.84728	0.85435	0.99998	17124	0.0867
KLK11	6	0.84745	0.85448	0.99998	17125	0.0656
TRIM64C	5	0.84748	0.8476	0.99998	17126	-0.0461
hsa-mir-3152	4	0.84749	0.84722	0.99998	17127	0.1343
KIAA1549L	6	0.84753	0.85455	0.99998	17128	0.1608
NFXL1	6	0.84755	0.85457	0.99998	17129	0.1044
hsa-mir-548t	3	0.84761	0.84768	0.99998	17130	0.0345
TMPRSS12	6	0.8477	0.85468	0.99998	17131	0.0649
UROS	6	0.8477	0.85468	0.99998	17132	0.0493
SPSB4	4	0.84773	0.84747	0.99998	17133	-0.0558
hsa-mir-6746	4	0.84773	0.84747	0.99998	17134	-0.0494
THEMIS	6	0.8478	0.85476	0.99998	17135	0.0261
PTPRG	6	0.84785	0.85479	0.99998	17136	-0.0203
MAGED1	6	0.84789	0.85482	0.99998	17137	0.2201
PDLIM2	6	0.84789	0.85482	0.99998	17138	0.1512
SPATA31C2	5	0.84791	0.84802	0.99998	17139	0.2889
DEFB132	6	0.84802	0.85491	0.99998	17140	0.3059
SEC61G	6	0.8481	0.85498	0.99998	17141	-0.0835
TMEM204	6	0.8481	0.85498	0.99998	17142	0.215
FFAR2	6	0.84822	0.85506	0.99998	17143	0.0692
ZNF343	6	0.84832	0.85515	0.99998	17144	0.2605
OR9Q2	6	0.84832	0.85515	0.99998	17145	-0.0344
NDUFB1	6	0.84832	0.85515	0.99998	17146	0.1031
MYO19	6	0.84843	0.85523	0.99998	17147	0.1443
AP1B1	6	0.84846	0.85525	0.99998	17148	0.1065
MUC2	6	0.84846	0.85525	0.99998	17149	0.0106
hsa-mir-4538	4	0.84848	0.84822	0.99998	17150	0.2504
FNBP1L	6	0.84852	0.85529	0.99998	17151	0.1337
GYPB	2	0.84861	0.84849	0.99998	17152	0.2025
PRKAA2	4	0.84863	0.84838	0.99998	17153	0.2488
ANKDD1A	6	0.84864	0.85538	0.99998	17154	-0.0002
ODF3L2	6	0.84873	0.85546	0.99998	17155	0.0625
HSPB8	6	0.84873	0.85546	0.99998	17156	0.0752
UTS2B	6	0.84873	0.85546	0.99998	17157	0.0855
MORC1	6	0.84873	0.85546	0.99998	17158	-0.0524
CUL2	6	0.84882	0.85553	0.99998	17159	0.1055
THAP9	6	0.84887	0.85556	0.99998	17160	0.1531
GPRC6A	6	0.84887	0.85556	0.99998	17161	0.2303
TNFRSF10C	6	0.84896	0.85562	0.99998	17162	0.0786
AAGAB	6	0.84901	0.85566	0.99998	17163	0.04
ST6GAL2	6	0.84906	0.85569	0.99998	17164	0.2963
PM20D2	6	0.84913	0.85574	0.99998	17165	-0.0432
ARMC6	6	0.84916	0.85576	0.99998	17166	-0.0432
CD2AP	6	0.84916	0.85576	0.99998	17167	0.072
ORAI3	6	0.84924	0.85582	0.99998	17168	0.1799
HIST1H2AB	6	0.84924	0.85582	0.99998	17169	-0.0043

RNF187	6	0.8494	0.85593	0.99998	17170	0.0151
CHST8	6	0.8496	0.85608	0.99998	17171	0.1619
ZNF548	6	0.84976	0.8562	0.99998	17172	0.1677
hsa-mir-125b-	4	0.84979	0.84954	0.99998	17173	0.5272
FLYWCH1	6	0.84988	0.85628	0.99998	17174	-0.0396
MAP3K4	6	0.84988	0.85628	0.99998	17175	-0.0064
DPF1	6	0.84993	0.85632	0.99998	17176	0.0738
TTC39A	6	0.84993	0.85632	0.99998	17177	0.2274
FMOD	6	0.84993	0.85632	0.99998	17178	0.1206
E2F2	6	0.85002	0.85639	0.99998	17179	0.0548
hsa-mir-4439	4	0.85005	0.84981	0.99998	17180	0.2671
POLN	6	0.8502	0.85652	0.99998	17181	0.1155
hsa-mir-548a-	2	0.85023	0.85011	0.99998	17182	0.2428
PDF	6	0.85036	0.85664	0.99998	17183	0.0917
RRM2B	6	0.85036	0.85664	0.99998	17184	0.0299
C15orf61	6	0.85036	0.85664	0.99998	17185	0.2409
LOC10013109	6	0.85036	0.85664	0.99998	17186	0.0647
SLC5A8	6	0.85052	0.85677	0.99998	17187	0.2791
ZNF311	4	0.85054	0.85029	0.99998	17188	0.15
IL12RB1	4	0.85056	0.8503	0.99998	17189	0.2004
SLC27A3	6	0.85065	0.85685	0.99998	17190	0.137
hsa-mir-8072	4	0.8507	0.85043	0.99998	17191	0.3318
C6orf195	6	0.85087	0.85702	0.99998	17192	-0.0634
PHC2	6	0.85087	0.85702	0.99998	17193	0.3828
TRAF3IP1	6	0.85087	0.85702	0.99998	17194	-0.022
ALDH3A1	6	0.85097	0.8571	0.99998	17195	0.062
CRNN	6	0.85116	0.85723	0.99998	17196	0.0314
MTHFSD	6	0.85116	0.85723	0.99998	17197	0.0482
KCNK7	6	0.85138	0.85739	0.99998	17198	0.2119
FAM120B	6	0.85138	0.85739	0.99998	17199	0.1464
IFNA2	6	0.8515	0.85747	0.99998	17200	0.0155
ANKS3	6	0.85155	0.85751	0.99998	17201	0.3431
CAND2	5	0.85157	0.85167	0.99998	17202	-0.0587
CCDC102B	6	0.85158	0.85753	0.99998	17203	0.2654
LAMA4	6	0.85158	0.85753	0.99998	17204	0.0312
TSPAN4	6	0.85171	0.85763	0.99998	17205	0.2178
ABI3	6	0.85171	0.85763	0.99998	17206	0.2366
FXYD4	6	0.8518	0.85771	0.99998	17207	-0.0572
KRTAP10-7	6	0.85184	0.85774	0.99998	17208	-0.0317
SPON2	6	0.85187	0.85776	0.99998	17209	0.127
UGP2	6	0.85191	0.85779	0.99998	17210	-0.0996
WIPF2	6	0.85191	0.85779	0.99998	17211	0.1152
TUBB2A	5	0.85199	0.85207	0.99998	17212	-0.0586
hsa-mir-1246	4	0.85208	0.85179	0.99998	17213	0.2112
C1orf68	6	0.85218	0.85799	0.99998	17214	0.1785
NADK2	6	0.85218	0.85799	0.99998	17215	0.0725
CD63	6	0.85226	0.85805	0.99998	17216	0.0366
NRBP2	6	0.85239	0.85815	0.99998	17217	0.0957
HSD3B7	6	0.85239	0.85815	0.99998	17218	0.3161
CAMK2A	6	0.85251	0.85824	0.99998	17219	0.0644
TMEM185B	6	0.85255	0.85827	0.99998	17220	0.0367
NIPA2	6	0.85257	0.85828	0.99998	17221	-0.0081
TREML1	6	0.85263	0.85833	0.99998	17222	0.0414
SHQ1	6	0.85273	0.8584	0.99998	17223	-0.0077
TOMM34	6	0.85273	0.8584	0.99998	17224	0.2466
AP1AR	4	0.85285	0.8526	0.99998	17225	0.1236
TIGD4	6	0.85289	0.85852	0.99998	17226	0.2817
SLC25A51	6	0.85289	0.85852	0.99998	17227	0.1889
RAD51AP1	6	0.85289	0.85852	0.99998	17228	0.0044
C1QTNF9B-AS	6	0.85289	0.85852	0.99998	17229	-0.0296
C11orf96	6	0.85289	0.85852	0.99998	17230	-0.0681
ANGPTL7	6	0.85299	0.8586	0.99998	17231	-0.028
NUCB2	6	0.85299	0.8586	0.99998	17232	0.066
PPP1R14D	6	0.85312	0.85869	0.99998	17233	0.0927
C11orf85	6	0.85322	0.85876	0.99998	17234	0.0446
hsa-mir-2909	4	0.85325	0.85302	0.99998	17235	-0.0377
APEX2	6	0.85334	0.85885	0.99998	17236	0.1565
OSBPL1A	6	0.85334	0.85885	0.99998	17237	-0.0174
CECR5	6	0.85334	0.85885	0.99998	17238	0.0731
FAM162A	6	0.85334	0.85885	0.99998	17239	0.0701
GPR37	6	0.85334	0.85885	0.99998	17240	-0.1346
ZBTB41	6	0.85346	0.85895	0.99998	17241	-0.0267
OR51B4	6	0.85352	0.85898	0.99998	17242	-0.0022
GPR137B	6	0.8536	0.85904	0.99998	17243	-0.0174
GBF1	6	0.85368	0.85909	0.99998	17244	-0.0271
KCNK2	6	0.85378	0.85918	0.99998	17245	-0.0422
PADI3	6	0.85378	0.85918	0.99998	17246	0.1671
FLVCR2	6	0.85378	0.85918	0.99998	17247	0.156
NRIP1	6	0.85378	0.85918	0.99998	17248	0.1508
GMFG	6	0.85389	0.85927	0.99998	17249	0.0546
OR8B8	6	0.85389	0.85927	0.99998	17250	0.2442

MPO	6	0.85389	0.85927	0.99998	17251	0.0299
TXLNA	6	0.85389	0.85927	0.99998	17252	0.1765
C1orf141	6	0.85413	0.85946	0.99998	17253	-0.0138
TEX37	6	0.85413	0.85946	0.99998	17254	-0.072
hsa-mir-378c	4	0.85414	0.85388	0.99998	17255	0.1951
hsa-mir-450a-	2	0.85432	0.85425	0.99998	17256	0.4223
XCL1	3	0.85439	0.85448	0.99998	17257	0.6975
HIGD1B	6	0.85446	0.8597	0.99998	17258	0.0891
BEND7	6	0.85446	0.8597	0.99998	17259	0.1848
WBP1	6	0.85446	0.8597	0.99998	17260	0.102
C5	6	0.8546	0.85981	0.99998	17261	0.099
PROS1	6	0.85462	0.85982	0.99998	17262	0.1211
S100G	4	0.85471	0.85447	0.99998	17263	-0.0493
MYOZ3	6	0.85471	0.85988	0.99998	17264	0.2259
NAV1	6	0.85471	0.85988	0.99998	17265	0.0035
RPS27L	6	0.85471	0.85988	0.99998	17266	0.0794
ELOVL5	6	0.8548	0.85995	0.99998	17267	0.1779
SCRIB	6	0.85483	0.85998	0.99998	17268	0.0342
MRPS18A	6	0.85496	0.86009	0.99998	17269	0.0006
ZNF277	6	0.85512	0.8602	0.99998	17270	0.2037
hsa-mir-4431	4	0.85513	0.85486	0.99998	17271	0.0644
GRIA1	6	0.8552	0.86027	0.99998	17272	0.2055
PRKCA	6	0.8552	0.86027	0.99998	17273	0.1397
EFCAB1	4	0.8552	0.85494	0.99998	17274	0.2077
PTGES2	6	0.85526	0.86032	0.99998	17275	-0.0156
PAWR	6	0.85535	0.86038	0.99998	17276	0.0375
HDGFRP2	6	0.85535	0.86038	0.99998	17277	0.0837
TMEM66	6	0.85535	0.86038	0.99998	17278	-0.0007
hsa-mir-153-2	3	0.85542	0.85551	0.99998	17279	0.0121
hsa-mir-195	4	0.85545	0.85519	0.99998	17280	0.1292
SLC44A1	6	0.85551	0.86052	0.99998	17281	0.096
NFKBIZ	6	0.85551	0.86052	0.99998	17282	0.1225
NXPE3	6	0.85562	0.8606	0.99998	17283	0.1941
hsa-mir-633	4	0.85579	0.85553	0.99998	17284	0.2397
SLC9A6	6	0.85585	0.86079	0.99998	17285	0.0405
PTCH2	6	0.85592	0.86085	0.99998	17286	0.1514
SLC25A33	6	0.85592	0.86085	0.99998	17287	0.139
EHD1	6	0.85592	0.86085	0.99998	17288	-0.0002
PCDHA3	2	0.856	0.85594	0.99998	17289	0.1783
ETFB	6	0.85607	0.86098	0.99998	17290	-0.1732
C1QTNF2	6	0.85607	0.86098	0.99998	17291	-0.0337
KRT33A	6	0.85607	0.86098	0.99998	17292	0.1225
IQGAP2	6	0.85638	0.86121	0.99998	17293	0.3028
MOB3B	6	0.85638	0.86121	0.99998	17294	0.117
MORN5	6	0.85638	0.86121	0.99998	17295	-0.0037
TNK1	6	0.85647	0.86128	0.99998	17296	-0.0502
CLK4	6	0.85662	0.86141	0.99998	17297	-0.1412
ADRBK2	6	0.85662	0.86141	0.99998	17298	-0.0761
LCE2D	5	0.85667	0.85672	0.99998	17299	0.051
RLN1	5	0.85667	0.85672	0.99998	17300	-0.0306
TECPR1	6	0.85669	0.86146	0.99998	17301	-0.0531
TGIF1	6	0.85669	0.86146	0.99998	17302	0.0598
PROKR1	6	0.85669	0.86146	0.99998	17303	0.0652
HIGD1A	6	0.85681	0.86155	0.99998	17304	-0.0633
RCBTB2	6	0.85695	0.86165	0.99998	17305	-0.0445
CTTN	6	0.85695	0.86165	0.99998	17306	0.197
GZMA	6	0.85701	0.86169	0.99998	17307	-0.0861
ZNF506	6	0.85711	0.86177	0.99998	17308	-0.1508
PIFO	6	0.85711	0.86177	0.99998	17309	0.0353
RTN3	6	0.85711	0.86177	0.99998	17310	0.1607
MTMR4	6	0.85725	0.86188	0.99998	17311	0.1797
GALNT2	6	0.85725	0.86188	0.99998	17312	0.0521
CNTN4	6	0.8573	0.86193	0.99998	17313	-0.0363
PURB	6	0.85741	0.86203	0.99998	17314	0.0042
GPR50	6	0.85741	0.86203	0.99998	17315	-0.0977
KRTAP23-1	6	0.8575	0.86209	0.99998	17316	0.396
HOXA2	6	0.85756	0.86214	0.99998	17317	0.0658
SMCR7L	2	0.85758	0.85753	0.99998	17318	0.1421
FAM151B	6	0.85763	0.86219	0.99998	17319	0.0937
CHD2	6	0.8577	0.86226	0.99998	17320	-0.1093
SH3PXD2B	6	0.85784	0.86236	0.99998	17321	-0.0726
OLFM4	6	0.85784	0.86236	0.99998	17322	-0.0632
SEC63	6	0.85784	0.86236	0.99998	17323	0.1987
RAP1A	6	0.85784	0.86236	0.99998	17324	-0.123
SPATA31A5	1	0.85789	0.85822	0.99998	17325	0.1746
hsa-mir-4290	4	0.85827	0.85798	0.99998	17326	0.0479
DGCR6	6	0.85853	0.8629	0.99998	17327	0.1646
FOXD4L5	6	0.85853	0.8629	0.99998	17328	0.0657
PCDHB15	6	0.85853	0.8629	0.99998	17329	0.3447
ALPI	6	0.85853	0.8629	0.99998	17330	0.1444
UIMC1	6	0.85853	0.8629	0.99998	17331	0.0982

ZFP1	6	0.85853	0.8629	0.99998	17332	0.0058
MAGEA3	6	0.85853	0.8629	0.99998	17333	0.1223
KRTAP4-12	6	0.85853	0.8629	0.99998	17334	-0.3595
SCGB1D4	6	0.85853	0.8629	0.99998	17335	0.0233
SPANXN3	6	0.85853	0.8629	0.99998	17336	0.4964
ARHGAP23	6	0.85853	0.8629	0.99998	17337	0.0839
KRTAP5-9	6	0.85853	0.8629	0.99998	17338	0.3647
GNPNAT1	6	0.85853	0.8629	0.99998	17339	0.0029
TRIM50	6	0.85853	0.8629	0.99998	17340	0.0366
hsa-mir-6731	4	0.85886	0.85853	0.99998	17341	-0.0283
hsa-mir-504	4	0.85899	0.85866	0.99998	17342	0.264
IL1RAPL1	6	0.85917	0.86341	0.99998	17343	0.0411
CLCA2	6	0.85917	0.86341	0.99998	17344	0.1787
NRSN2	6	0.85928	0.86349	0.99998	17345	0.3218
NFE2L3	6	0.85928	0.86349	0.99998	17346	0.1289
OTOP2	6	0.85928	0.86349	0.99998	17347	-0.0565
TALDO1	6	0.85951	0.86366	0.99998	17348	0.0686
VTA1	6	0.85951	0.86366	0.99998	17349	0.2524
NKD2	6	0.85962	0.86374	0.99998	17350	-0.0237
DFNB31	6	0.85963	0.86375	0.99998	17351	0.1532
KIAA1958	6	0.85967	0.86379	0.99998	17352	-0.0293
IL1RN	6	0.85967	0.86379	0.99998	17353	0.0469
hsa-mir-378g	4	0.85969	0.85936	0.99998	17354	0.0441
hsa-mir-489	4	0.85994	0.85961	0.99998	17355	0.0509
hsa-mir-600	4	0.85994	0.85961	0.99998	17356	0.529
TCF19	6	0.86002	0.86405	0.99998	17357	0.0675
SUN5	6	0.86002	0.86405	0.99998	17358	0.1216
C1orf233	6	0.86011	0.86412	0.99998	17359	0.1401
RSPH4A	6	0.86011	0.86412	0.99998	17360	0.013
ERMAP	6	0.86011	0.86412	0.99998	17361	0.0345
ST6GALNAC6	6	0.86011	0.86412	0.99998	17362	0.0733
FAM194B	6	0.86011	0.86412	0.99998	17363	-0.0011
39692	3	0.86016	0.86023	0.99998	17364	0.0272
FAM160A2	6	0.86022	0.86421	0.99998	17365	0.0597
NDEL1	6	0.86032	0.86428	0.99998	17366	0.0551
SESN3	6	0.86041	0.86435	0.99998	17367	0.0965
IFNA8	6	0.86049	0.86441	0.99998	17368	0.0721
PRPS1L1	6	0.86049	0.86441	0.99998	17369	-0.0333
ZNF41	6	0.86049	0.86441	0.99998	17370	0.1542
CDY2A	1	0.86052	0.86086	0.99998	17371	0.2985
WFDC2	6	0.86058	0.86448	0.99998	17372	0.028
TRAPPC11	4	0.86059	0.86026	0.99998	17373	0.2173
HMHB1	5	0.86063	0.86067	0.99998	17374	0.1069
FSTL4	6	0.86066	0.86454	0.99998	17375	-0.047
ABCA6	6	0.86066	0.86454	0.99998	17376	0.1942
PGLYRP2	6	0.86079	0.86464	0.99998	17377	0.0337
IQCF5	6	0.86084	0.86468	0.99998	17378	0.1361
OSBPL9	4	0.86086	0.86053	0.99998	17379	0.7253
CCDC178	6	0.86088	0.86472	0.99998	17380	-0.0843
LILRB1	3	0.86092	0.86096	0.99998	17381	0.2119
TRIM14	6	0.86105	0.86485	0.99998	17382	-0.05
hsa-mir-5589	4	0.86109	0.86076	0.99998	17383	-0.01
TBC1D22B	6	0.86123	0.86499	0.99998	17384	0.1087
HIP1	6	0.86148	0.8652	0.99998	17385	0.2339
SEPP1	6	0.86148	0.8652	0.99998	17386	-0.0467
TYRP1	6	0.86148	0.8652	0.99998	17387	0.3224
TCAIM	6	0.86157	0.86527	0.99998	17388	0.036
BTN3A1	5	0.86158	0.86163	0.99998	17389	0.1677
SERTM1	6	0.86178	0.86544	0.99998	17390	-0.0797
MIA2	6	0.86178	0.86544	0.99998	17391	0.0792
KRTAP19-7	5	0.86178	0.86182	0.99998	17392	0.0829
KRTAP13-3	6	0.86188	0.86552	0.99998	17393	0.0631
TEX29	6	0.86198	0.86559	0.99998	17394	0.067
FUZ	6	0.86204	0.86564	0.99998	17395	0.0758
DSPP	6	0.8621	0.86568	0.99998	17396	0.2517
hsa-mir-4256	4	0.86216	0.86189	0.99998	17397	0.1256
SNX16	6	0.86221	0.86578	0.99998	17398	0.1704
DKK2	6	0.86241	0.86592	0.99998	17399	0.2601
GPX2	6	0.86241	0.86592	0.99998	17400	0.1429
SIRT6	6	0.86256	0.86605	0.99998	17401	0.0919
MSI1	6	0.86256	0.86605	0.99998	17402	0.1006
OR4K13	6	0.86256	0.86605	0.99998	17403	0.1118
SLC8A2	6	0.86256	0.86605	0.99998	17404	0.2145
ABCB10	6	0.86269	0.86616	0.99998	17405	0.0734
ANO8	6	0.86269	0.86616	0.99998	17406	0.1808
SLC25A38	6	0.86269	0.86616	0.99998	17407	0.153
SLC6A5	6	0.8628	0.86625	0.99998	17408	0.1932
hsa-mir-3656	4	0.86296	0.86271	0.99998	17409	0.0793
ATG10	6	0.86299	0.86641	0.99998	17410	0.2703
MMP23B	6	0.86299	0.86641	0.99998	17411	0.0311
41153	3	0.86303	0.86303	0.99998	17412	0.0087

PCDHB4	6	0.86306	0.86647	0.99998	17413	-0.035
MBNL3	6	0.86315	0.86654	0.99998	17414	0.1946
RGMA	6	0.86315	0.86654	0.99998	17415	-0.1048
LRRK2	6	0.86327	0.86663	0.99998	17416	-0.0248
SCG2	6	0.86327	0.86663	0.99998	17417	0.0999
SLC30A7	6	0.86327	0.86663	0.99998	17418	0.1143
VPREB3	6	0.86327	0.86663	0.99998	17419	0.1242
KLRD1	6	0.86327	0.86663	0.99998	17420	-0.033
MYRF	6	0.86327	0.86663	0.99998	17421	-0.0654
RGS20	6	0.86338	0.86673	0.99998	17422	0.1607
STK24	6	0.86345	0.86679	0.99998	17423	0.0681
hsa-mir-2392	4	0.86345	0.86321	0.99998	17424	0.203
NAALADL1	6	0.8636	0.8669	0.99998	17425	0.1872
SLC25A32	6	0.8636	0.8669	0.99998	17426	0.1333
ANO7	6	0.8636	0.8669	0.99998	17427	0.0779
hsa-mir-1302-	4	0.86371	0.86346	0.99998	17428	0.1514
ZNF439	4	0.86371	0.86346	0.99998	17429	0.0804
FBXO16	6	0.86381	0.86707	0.99998	17430	0.034
TBL1X	6	0.86381	0.86707	0.99998	17431	0.1331
CHCHD3	6	0.86387	0.86712	0.99998	17432	0.0368
hsa-mir-6811	4	0.86401	0.86376	0.99998	17433	-0.0106
DOK7	6	0.86412	0.86732	0.99998	17434	0.0412
ANKRD23	6	0.86412	0.86732	0.99998	17435	0.2775
ZNF576	6	0.86418	0.86737	0.99998	17436	0.1216
hsa-mir-99a	4	0.86427	0.86404	0.99998	17437	-0.0078
SPATA3	6	0.86447	0.86759	0.99998	17438	-0.0806
TFAP2D	6	0.86447	0.86759	0.99998	17439	0.034
NSUN6	6	0.86447	0.86759	0.99998	17440	-0.0661
TMEM156	6	0.86459	0.8677	0.99998	17441	0.0234
RSPH3	6	0.86459	0.8677	0.99998	17442	0.0783
PRAMEF10	4	0.86476	0.86453	0.99998	17443	0.0027
hsa-mir-216b	4	0.86487	0.86464	0.99998	17444	0.137
HIST1H2BE	5	0.86491	0.86501	0.99998	17445	0.1112
LHFPL4	6	0.86491	0.86795	0.99998	17446	-0.135
RGS13	6	0.86491	0.86795	0.99998	17447	0.0163
FRS3	6	0.86491	0.86795	0.99998	17448	0.0455
PRIMA1	6	0.8651	0.86811	0.99998	17449	0.096
PEBP4	6	0.86526	0.86824	0.99998	17450	-0.0919
PAGE4	6	0.86526	0.86824	0.99998	17451	0.0905
PIAS4	6	0.86536	0.86832	0.99998	17452	0.1679
STAMBP	6	0.86539	0.86834	0.99998	17453	0.1191
NEU2	6	0.8654	0.86835	0.99998	17454	0.2448
ATXN3	6	0.86547	0.8684	0.99998	17455	0.1248
ADAMTSL1	6	0.86553	0.86844	0.99998	17456	0.2849
ZNF407	6	0.86561	0.8685	0.99998	17457	0.4136
PDLIM4	6	0.86561	0.8685	0.99998	17458	0.0124
NPFFR2	6	0.86561	0.8685	0.99998	17459	0.236
APOB	6	0.86561	0.8685	0.99998	17460	0.0744
ASNS	6	0.86561	0.8685	0.99998	17461	0.1912
VWCE	6	0.86561	0.8685	0.99998	17462	0.122
SLC35E3	6	0.86561	0.8685	0.99998	17463	0.1737
CYP2C8	6	0.86574	0.86862	0.99998	17464	-0.0899
SOHLH2	6	0.86582	0.8687	0.99998	17465	-0.0401
ATXN1	6	0.86582	0.8687	0.99998	17466	-0.0048
hsa-mir-6792	4	0.86589	0.86567	0.99998	17467	0.0124
hsa-mir-5705	4	0.86589	0.86567	0.99998	17468	0.3115
GABRR1	6	0.86605	0.86889	0.99998	17469	0.2114
SPRR3	6	0.86615	0.86897	0.99998	17470	0.0993
CYP4F22	6	0.86615	0.86897	0.99998	17471	0.0353
CNBD1	6	0.86615	0.86897	0.99998	17472	0.0429
LHX3	6	0.86615	0.86897	0.99998	17473	0.1051
DYRK3	6	0.86615	0.86897	0.99998	17474	0.3987
VKORC1L1	6	0.86621	0.86902	0.99998	17475	-0.069
RGS5	6	0.86624	0.86905	0.99998	17476	0.0804
SAMD4B	6	0.86629	0.86908	0.99998	17477	0.0505
METTL15	6	0.86633	0.86911	0.99998	17478	-0.0073
ABI3BP	6	0.86641	0.86918	0.99998	17479	0.1246
RASSF2	6	0.86645	0.86921	0.99998	17480	0.1267
SSR3	6	0.86647	0.86923	0.99998	17481	0.0117
IFNAR2	6	0.86647	0.86923	0.99998	17482	0.125
BAALC	6	0.86661	0.86936	0.99998	17483	0.1335
RGS11	6	0.86664	0.86939	0.99998	17484	-0.0555
hsa-let-7a-2	4	0.86673	0.86646	0.99998	17485	0.3216
FAM181B	6	0.86673	0.86946	0.99998	17486	0.2006
TLX3	6	0.86673	0.86946	0.99998	17487	0.0554
MYO3A	6	0.86685	0.86955	0.99998	17488	0.2398
EN1	6	0.86694	0.86963	0.99998	17489	0.192
hsa-mir-572	4	0.86698	0.86672	0.99998	17490	0.1306
HOXA7	6	0.867	0.86967	0.99998	17491	0.2043
CHD9	6	0.867	0.86967	0.99998	17492	0.0027
VPS4B	6	0.86711	0.86976	0.99998	17493	0.0803

WNT7A	6	0.86711	0.86976	0.99998	17494	0.1958
RAB40AL	5	0.86721	0.86736	0.99998	17495	0.0437
hsa-mir-4713	4	0.86726	0.86699	0.99998	17496	0.1914
MBD3L2	4	0.86726	0.86699	0.99998	17497	0.0722
P2RX4	6	0.86735	0.86995	0.99998	17498	0.2706
GCLM	6	0.86741	0.87	0.99998	17499	0.0931
ASPG	6	0.86741	0.87	0.99998	17500	0.0497
NANOG	6	0.86755	0.87011	0.99998	17501	0.1673
AKAP8	6	0.86755	0.87011	0.99998	17502	0.0634
DEF6	6	0.86755	0.87011	0.99998	17503	0.0021
CSHL1	6	0.86765	0.87019	0.99998	17504	0.2027
F11R	6	0.86765	0.87019	0.99998	17505	0.1531
HOMER1	6	0.86765	0.87019	0.99998	17506	0.0972
ZNF419	6	0.86772	0.87026	0.99998	17507	-0.0807
hsa-mir-8058	4	0.86777	0.86752	0.99998	17508	-0.0003
TMEM105	6	0.86781	0.87033	0.99998	17509	0.1746
SPTY2D1	6	0.86793	0.87044	0.99998	17510	0.1249
SRL	6	0.86793	0.87044	0.99998	17511	0.0334
TERF1	6	0.86805	0.87053	0.99998	17512	0.0175
BCO2	6	0.86805	0.87053	0.99998	17513	0.077
CHRNA5	6	0.86805	0.87053	0.99998	17514	-0.058
NDUFB4	6	0.86805	0.87053	0.99998	17515	-0.1214
hsa-mir-6794	4	0.86815	0.86791	0.99998	17516	0.149
NPY	6	0.86818	0.87064	0.99998	17517	0.2232
ARHGEF18	6	0.86818	0.87064	0.99998	17518	0.4212
MAGEC1	6	0.86818	0.87064	0.99998	17519	0.1128
AFF2	6	0.86828	0.87073	0.99998	17520	0.0794
ARAF	6	0.86832	0.87077	0.99998	17521	0.0761
IL26	6	0.86839	0.87083	0.99998	17522	0.0504
TOR1B	6	0.86844	0.87087	0.99998	17523	0.0766
TTC17	6	0.86844	0.87087	0.99998	17524	0.3671
PDZD4	6	0.86849	0.87091	0.99998	17525	0.01
DENND1C	6	0.86854	0.87095	0.99998	17526	0.1577
POLD1	6	0.86856	0.87097	0.99998	17527	0.1493
TSPY8	1	0.86868	0.86898	0.99998	17528	0.3118
SAFB	6	0.8687	0.87109	0.99998	17529	0.0057
CRABP1	6	0.8687	0.87109	0.99998	17530	0.1163
ERCC6	2	0.86872	0.86872	0.99998	17531	0.107
RHD	6	0.86877	0.87114	0.99998	17532	-0.0917
CABP2	6	0.86886	0.87122	0.99998	17533	0.2246
NREP	6	0.86886	0.87122	0.99998	17534	0.2734
NEU1	6	0.86895	0.87129	0.99998	17535	0.2042
ZNF431	6	0.86913	0.87144	0.99998	17536	-0.0081
RFPL3	6	0.86913	0.87144	0.99998	17537	0.1049
CNR2	6	0.86927	0.87155	0.99998	17538	0.0896
TRIM39-RPP2:	2	0.86927	0.86928	0.99998	17539	0.3169
MAP2	6	0.86939	0.87166	0.99998	17540	0.3397
PIK3C2G	6	0.86955	0.8718	0.99998	17541	0.0714
CX3CL1	6	0.86955	0.8718	0.99998	17542	0.1936
TPO	6	0.86965	0.8719	0.99998	17543	-0.0374
ITPRIP	6	0.86965	0.8719	0.99998	17544	0.0806
TMEM14A	6	0.86972	0.87195	0.99998	17545	0.2493
P2RX2	6	0.86972	0.87195	0.99998	17546	0.0277
CYP2A13	5	0.86973	0.86988	0.99998	17547	0.0584
DMWD	6	0.86986	0.87206	0.99998	17548	0.0601
PGBD3	6	0.87	0.87218	0.99998	17549	0.3483
PPP1R42	6	0.87007	0.87224	0.99998	17550	0.1589
SERINC2	6	0.8701	0.87228	0.99998	17551	0.2061
MBOAT7	6	0.87014	0.8723	0.99998	17552	0.4091
PCDHGA2	2	0.87018	0.87019	0.99998	17553	0.1707
RBP4	6	0.87019	0.87235	0.99998	17554	0.2078
ZNF425	6	0.87019	0.87235	0.99998	17555	0.0078
ZBTB7C	6	0.87042	0.87253	0.99998	17556	0.0395
BEX4	6	0.87042	0.87253	0.99998	17557	0.0882
LCP2	6	0.87053	0.87262	0.99998	17558	0.2189
LATS1	6	0.87053	0.87262	0.99998	17559	-0.03
ANGPT1	6	0.87066	0.87275	0.99998	17560	0.1308
CCDC66	6	0.87066	0.87275	0.99998	17561	-0.0225
KIFC2	6	0.87082	0.8729	0.99998	17562	0.2304
FAM159A	6	0.87093	0.87299	0.99998	17563	-0.0443
ARID3C	6	0.871	0.87304	0.99998	17564	0.0064
ITGB8	6	0.87121	0.87321	0.99998	17565	-0.0273
TMEM176A	6	0.8713	0.87328	0.99998	17566	0.1237
NF1	6	0.8713	0.87328	0.99998	17567	0.2542
WDR90	6	0.8713	0.87328	0.99998	17568	0.2371
hsa-mir-493	4	0.87136	0.87112	0.99998	17569	0.1664
KIAA1407	6	0.87141	0.87336	0.99998	17570	0.3185
TBK1	6	0.87154	0.87346	0.99998	17571	0.0418
UBALD1	6	0.87172	0.87362	0.99998	17572	0.1869
TSPYL5	6	0.87172	0.87362	0.99998	17573	0.0999
CDV3	6	0.87172	0.87362	0.99998	17574	-0.059

CST9	6	0.87178	0.87368	0.99998	17575	0.0033
PLVAP	6	0.87178	0.87368	0.99998	17576	-0.0506
RNF186	6	0.87183	0.87373	0.99998	17577	-0.0336
COL12A1	6	0.87188	0.87378	0.99998	17578	0.0543
DOPEY2	6	0.87194	0.87382	0.99998	17579	0.1491
REPIN1	6	0.87194	0.87382	0.99998	17580	0.3523
FEZF2	6	0.87198	0.87386	0.99998	17581	0.1785
BBIP1	6	0.87212	0.87397	0.99998	17582	0.2377
PGP	6	0.87212	0.87397	0.99998	17583	0.1816
COMMD3-BM	2	0.87221	0.87229	0.99998	17584	0.2004
ASPH	6	0.87229	0.87412	0.99998	17585	0.3521
PCDH19	6	0.87229	0.87412	0.99998	17586	0.0447
hsa-mir-3977	4	0.87232	0.87209	0.99998	17587	0.1302
UBA6	6	0.87261	0.87439	0.99998	17588	0.074
CPE	6	0.8727	0.87446	0.99998	17589	0.0557
ZNF512B	6	0.87277	0.87452	0.99998	17590	0.1265
TEPP	6	0.87277	0.87452	0.99998	17591	0.2495
CSDC2	6	0.87285	0.87459	0.99998	17592	0.052
CCDC159	6	0.87292	0.87465	0.99998	17593	0.0831
SYNGR1	6	0.87292	0.87465	0.99998	17594	0.1437
PCP2	6	0.87301	0.87474	0.99998	17595	0.1561
STUB1	6	0.87314	0.87484	0.99998	17596	0.021
TGIF2	4	0.87318	0.87297	0.99998	17597	-0.0304
SPIC	6	0.87352	0.87517	0.99998	17598	0.1404
EDDM3B	6	0.87352	0.87517	0.99998	17599	0.1522
KRTAP21-1	6	0.87352	0.87517	0.99998	17600	0.212
BDNF	6	0.8738	0.8754	0.99998	17601	-0.13
GGH	6	0.87387	0.87546	0.99998	17602	0.082
SOX8	6	0.87402	0.87559	0.99998	17603	0.078
hsa-mir-3610	4	0.87409	0.8739	0.99998	17604	0.1598
TNKS1BP1	6	0.8741	0.87566	0.99998	17605	-0.0654
RAD51C	6	0.87426	0.87578	0.99998	17606	0.0698
FAM205A	6	0.87426	0.87578	0.99998	17607	0.0219
DNAJB4	6	0.87426	0.87578	0.99998	17608	0.2225
GJC3	6	0.87426	0.87578	0.99998	17609	-0.0401
SHE	6	0.87426	0.87578	0.99998	17610	0.3541
DYNC1LI2	6	0.87426	0.87578	0.99998	17611	0.2448
HEBP2	6	0.87438	0.87588	0.99998	17612	0.0118
TIMD4	6	0.87444	0.87594	0.99998	17613	0.2154
TMPRSS4	6	0.87444	0.87594	0.99998	17614	0.021
AMELX	5	0.87449	0.87472	0.99998	17615	0.3164
ZNF714	5	0.87449	0.87472	0.99998	17616	0.1281
PCDH11X	5	0.87449	0.87472	0.99998	17617	0.1766
CSNK2A3	5	0.87449	0.87472	0.99998	17618	0.0257
GLB1L3	5	0.87449	0.87472	0.99998	17619	0.0399
HIST1H2AL	5	0.87449	0.87472	0.99998	17620	0.1233
OR8B2	5	0.87449	0.87472	0.99998	17621	0.1494
OR5K2	5	0.87449	0.87472	0.99998	17622	-0.4558
ZNF69	5	0.87449	0.87472	0.99998	17623	0.144
ACSM2B	5	0.87449	0.87472	0.99998	17624	0.1074
TMEM14C	5	0.87449	0.87472	0.99998	17625	0.0313
KRT37	5	0.87449	0.87472	0.99998	17626	-0.2882
SFTPA2	5	0.87449	0.87472	0.99998	17627	-0.0575
PCDH11Y	5	0.87449	0.87472	0.99998	17628	-0.0057
S100A7	5	0.87449	0.87472	0.99998	17629	0.1932
ZNF442	6	0.87451	0.876	0.99998	17630	0.0991
hsa-mir-4447	4	0.87453	0.87434	0.99998	17631	0.0973
hsa-mir-151a	3	0.8746	0.87476	0.99998	17632	0.2725
SLC19A2	6	0.87461	0.87609	0.99998	17633	-0.0048
TMEM160	6	0.87461	0.87609	0.99998	17634	0.076
UAP1	6	0.87467	0.87614	0.99998	17635	0.1006
PRDM7	4	0.87479	0.8746	0.99998	17636	0.1485
GALK2	6	0.87486	0.87632	0.99998	17637	0.0966
ZYG11B	6	0.87486	0.87632	0.99998	17638	0.3104
IL22RA1	6	0.87486	0.87632	0.99998	17639	0.1104
MARK1	6	0.87497	0.8764	0.99998	17640	0.1661
XRCC2	6	0.87497	0.8764	0.99998	17641	-0.0508
SEC61B	6	0.87497	0.8764	0.99998	17642	0.117
DLX3	6	0.87497	0.8764	0.99998	17643	0.0516
TMIGD1	6	0.87509	0.8765	0.99998	17644	0.2434
FOXE3	6	0.87509	0.8765	0.99998	17645	0.2362
PPIL6	6	0.87509	0.8765	0.99998	17646	0.0195
OR6K3	6	0.87517	0.87656	0.99998	17647	-0.0686
TFAP2B	6	0.87521	0.87658	0.99998	17648	0.1662
TRMT1	6	0.87527	0.87665	0.99998	17649	-0.1196
hsa-mir-5584	4	0.87533	0.87517	0.99998	17650	0.0066
DEK	6	0.87537	0.87674	0.99998	17651	0.2356
HES1	6	0.87537	0.87674	0.99998	17652	0.032
FAM83B	6	0.87547	0.87683	0.99998	17653	0.3823
DDX43	6	0.87553	0.87688	0.99998	17654	-0.0014
ACSL5	6	0.87553	0.87688	0.99998	17655	0.1996

hsa-mir-3184	1	0.87557	0.87596	0.99998	17656	0.4748
GGA2	6	0.87566	0.87699	0.99998	17657	0.2951
MAFA	6	0.87566	0.87699	0.99998	17658	0.2315
BAG3	6	0.87566	0.87699	0.99998	17659	0.0046
HES6	6	0.87566	0.87699	0.99998	17660	0.0164
SHCBP1L	6	0.87584	0.87714	0.99998	17661	0.01
ULK2	6	0.87587	0.87717	0.99998	17662	-0.056
PBX2	6	0.87593	0.87723	0.99998	17663	0.0329
PCDH18	6	0.87593	0.87723	0.99998	17664	-0.0507
FAM35A	6	0.87601	0.87729	0.99998	17665	0.094
ZNF93	4	0.87602	0.87586	0.99998	17666	0.3138
TMEM98	6	0.87609	0.87737	0.99998	17667	0.3348
SPINK6	6	0.87609	0.87737	0.99998	17668	0.1824
SERPINB7	6	0.87609	0.87737	0.99998	17669	0.0121
hsa-mir-1301	4	0.87617	0.87601	0.99998	17670	0.0612
APLF	6	0.87621	0.87747	0.99998	17671	-0.0058
AKAP5	6	0.87621	0.87747	0.99998	17672	0.0173
DOCK8	6	0.87637	0.87759	0.99998	17673	0.0314
hsa-mir-548ai	3	0.87638	0.87651	0.99998	17674	0.0742
CD276	6	0.8764	0.87762	0.99998	17675	0.0624
VIPR2	6	0.87642	0.87763	0.99998	17676	0.054
hsa-mir-3919	2	0.87643	0.8765	0.99998	17677	0.188
PRAMEF1	6	0.87649	0.8777	0.99998	17678	0.2105
TTC4	6	0.87649	0.8777	0.99998	17679	0.1214
PDE1C	6	0.87649	0.8777	0.99998	17680	0.1802
PEG3	1	0.8765	0.87687	0.99998	17681	0.3933
TACC2	6	0.87659	0.8778	0.99998	17682	0.1986
TMPPE	6	0.8767	0.8779	0.99998	17683	0.0348
hsa-mir-5197	4	0.8767	0.87656	0.99998	17684	0.0893
DDR2	6	0.87678	0.87798	0.99998	17685	0.0557
MMADHC	6	0.87684	0.87803	0.99998	17686	-0.0561
KLF10	6	0.87696	0.87813	0.99998	17687	0.2302
SPRYD7	6	0.87696	0.87813	0.99998	17688	0.1118
EFNA5	6	0.87696	0.87813	0.99998	17689	-0.0518
THAP4	6	0.87696	0.87813	0.99998	17690	0.0074
PDE6D	6	0.87696	0.87813	0.99998	17691	0.2243
ZNF324B	6	0.87696	0.87813	0.99998	17692	-0.0199
PRELID2	6	0.87696	0.87813	0.99998	17693	-0.0719
EXOC5	4	0.87704	0.8769	0.99998	17694	0.3665
HMGN4	6	0.87709	0.87823	0.99998	17695	0.0527
KCTD17	6	0.87709	0.87823	0.99998	17696	-0.0743
DSG4	6	0.87709	0.87823	0.99998	17697	-0.0455
CD1C	6	0.87718	0.87831	0.99998	17698	0.0967
RPA4	6	0.87718	0.87831	0.99998	17699	0.0205
BASP1	6	0.87736	0.87846	0.99998	17700	0.2913
SIRT2	6	0.87736	0.87846	0.99998	17701	-0.0385
DUSP13	6	0.8774	0.87849	0.99998	17702	0.0629
TTC19	4	0.87743	0.87725	0.99998	17703	0.053
PRSS33	6	0.87751	0.87858	0.99998	17704	0.0123
HOXD11	6	0.87751	0.87858	0.99998	17705	-0.0369
TBX4	6	0.87764	0.87868	0.99998	17706	0.0834
ANO4	6	0.8777	0.87873	0.99998	17707	0.0454
GPR35	6	0.8777	0.87873	0.99998	17708	-0.009
PDC	6	0.8777	0.87873	0.99998	17709	0.0143
C1QTNF8	6	0.8777	0.87873	0.99998	17710	0.1438
RNF214	6	0.87776	0.87879	0.99998	17711	0.0881
DRD4	6	0.8778	0.87883	0.99998	17712	0.0692
ZFAND2A	6	0.87787	0.87889	0.99998	17713	-0.0207
SART1	6	0.87791	0.87893	0.99998	17714	-0.1049
LMO4	6	0.87805	0.87905	0.99998	17715	0.4372
NID2	6	0.87817	0.87916	0.99998	17716	0.2695
SNTB1	6	0.87821	0.8792	0.99998	17717	-0.0371
OR6C75	6	0.87828	0.87926	0.99998	17718	0.1304
TMEM200C	6	0.87833	0.8793	0.99998	17719	0.1016
SERPINI2	6	0.8784	0.87937	0.99998	17720	0.057
KRTAP3-3	6	0.87843	0.8794	0.99998	17721	0.5028
CCPG1	6	0.87843	0.8794	0.99998	17722	-0.0604
MPV17L	6	0.87843	0.8794	0.99998	17723	-0.0645
FAM43A	6	0.87849	0.87946	0.99998	17724	0.342
UEVLD	6	0.87849	0.87946	0.99998	17725	-0.0559
SSC5D	6	0.87859	0.87954	0.99998	17726	0.1512
GSKIP	6	0.87859	0.87954	0.99998	17727	0.0755
MKX	6	0.87864	0.87959	0.99998	17728	0.2832
GNB2	6	0.87864	0.87959	0.99998	17729	0.1208
STX1B	6	0.87869	0.87963	0.99998	17730	0.1251
GIPC2	6	0.87869	0.87963	0.99998	17731	0.1061
PRKD3	6	0.87895	0.87986	0.99998	17732	-0.0019
TOP2B	6	0.87895	0.87986	0.99998	17733	0.0403
ATP11C	6	0.87902	0.87992	0.99998	17734	0.1772
TPGS1	4	0.87915	0.87904	0.99998	17735	0.1434
hsa-mir-6086	3	0.8793	0.8794	0.99998	17736	0.1737

ESR1	6	0.87979	0.88062	0.99998	17737	0.0638
RAB30	6	0.87987	0.88067	0.99998	17738	0.0375
GDF10	6	0.87998	0.88077	0.99998	17739	0.2099
ZFP37	6	0.87998	0.88077	0.99998	17740	0.0464
KRT20	6	0.87998	0.88077	0.99998	17741	0.2311
ADAL	6	0.87998	0.88077	0.99998	17742	0.1086
ZNF707	6	0.87998	0.88077	0.99998	17743	-0.0691
ZNF12	6	0.8801	0.88088	0.99998	17744	0.2297
ADAT1	6	0.8801	0.88088	0.99998	17745	-0.0004
PPL	6	0.88019	0.88097	0.99998	17746	0.0505
USP29	6	0.88033	0.88109	0.99998	17747	0.1035
RNF26	6	0.88042	0.88117	0.99998	17748	0.2184
TBC1D4	6	0.8805	0.88125	0.99998	17749	-0.0425
HSF2BP	6	0.88061	0.88136	0.99998	17750	0.227
TP53	6	0.88075	0.88149	0.99998	17751	0.2052
ZDHHC21	6	0.88097	0.88169	0.99998	17752	-0.0356
KIR2DL3	5	0.88104	0.88129	0.99998	17753	0.4185
SLC18B1	6	0.88118	0.88187	0.99998	17754	0.13
TMEM107	6	0.88125	0.88194	0.99998	17755	-0.1246
hsa-mir-6824	4	0.88126	0.88112	0.99998	17756	-0.0594
PRR5	4	0.88126	0.88112	0.99998	17757	0.0346
ESPNL	6	0.88168	0.88232	0.99998	17758	0.1448
ZNF334	6	0.88173	0.88236	0.99998	17759	-0.0139
USP13	6	0.88188	0.8825	0.99998	17760	0.1302
hsa-mir-4639	4	0.88195	0.88183	0.99998	17761	0.1224
IQCF6	6	0.882	0.88262	0.99998	17762	-0.0021
hsa-mir-384	4	0.8821	0.88198	0.99998	17763	0.2079
MSMP	6	0.88221	0.88279	0.99998	17764	0.1763
CLRN3	6	0.88221	0.88279	0.99998	17765	0.079
PPY	6	0.88221	0.88279	0.99998	17766	-0.0192
SYT4	6	0.88228	0.88286	0.99998	17767	0.2383
KCNMB2	6	0.88228	0.88286	0.99998	17768	-0.0333
TBC1D9B	6	0.88228	0.88286	0.99998	17769	0.2299
FBXO44	6	0.88236	0.88292	0.99998	17770	-0.0235
CRTAC1	6	0.88249	0.88304	0.99998	17771	0.1188
C20orf141	6	0.88257	0.88312	0.99998	17772	-0.0507
ILDR2	6	0.88257	0.88312	0.99998	17773	0.1981
BCL2L15	6	0.8826	0.88314	0.99998	17774	0.2497
GJA1	6	0.88263	0.88317	0.99998	17775	0.048
HDLBP	6	0.88266	0.8832	0.99998	17776	0.0244
hsa-mir-4494	4	0.88275	0.88265	0.99998	17777	0.2773
DPCD	6	0.88277	0.8833	0.99998	17778	0.1033
ZSCAN18	6	0.88277	0.8833	0.99998	17779	0.033
STAC2	6	0.88298	0.8835	0.99998	17780	0.0144
OR6Q1	4	0.88301	0.88292	0.99998	17781	0.1124
hsa-mir-3650	4	0.88301	0.88292	0.99998	17782	0.1786
TCF12	6	0.88303	0.88356	0.99998	17783	0.0137
ZNF211	6	0.8831	0.88362	0.99998	17784	-0.0417
ZNF551	6	0.88314	0.88366	0.99998	17785	0.173
ENKUR	6	0.88334	0.88385	0.99998	17786	0.183
hsa-mir-6840	4	0.88345	0.88335	0.99998	17787	0.2478
MSANTD2	6	0.88349	0.88398	0.99998	17788	0.1745
NTSR1	6	0.88349	0.88398	0.99998	17789	-0.0495
PPP2R2C	6	0.88349	0.88398	0.99998	17790	0.274
SERPINB3	4	0.88351	0.88341	0.99998	17791	0.1447
PRKCI	6	0.88354	0.88402	0.99998	17792	0.2192
SPTLC2	6	0.88354	0.88403	0.99998	17793	0.0165
GMNC	6	0.88359	0.88408	0.99998	17794	0.1041
PWP1	6	0.88359	0.88408	0.99998	17795	-0.0508
hsa-mir-4637	3	0.8836	0.88372	0.99998	17796	0.076
UST	6	0.88372	0.88419	0.99998	17797	0.1098
HTR1F	6	0.8839	0.88437	0.99998	17798	0.0751
ACTL8	6	0.8839	0.88437	0.99998	17799	0.0462
CXorf30	6	0.88401	0.88447	0.99998	17800	0.206
MMAA	6	0.88401	0.88447	0.99998	17801	0.1439
KCNE1L	6	0.88401	0.88447	0.99998	17802	0.071
PPP6R3	6	0.88409	0.88453	0.99998	17803	0.0125
KRT32	6	0.88409	0.88453	0.99998	17804	-0.0052
CDC25C	6	0.88419	0.88462	0.99998	17805	0.0637
MAP9	6	0.88419	0.88462	0.99998	17806	0.0342
AQP10	6	0.88431	0.88473	0.99998	17807	0.0289
hsa-mir-3129	3	0.88436	0.88448	0.99998	17808	0.0132
hsa-mir-20b	4	0.88439	0.88427	0.99998	17809	0.2205
hsa-mir-380	4	0.88442	0.88431	0.99998	17810	0.304
UBASH3A	6	0.88444	0.88485	0.99998	17811	0.0678
TLE3	6	0.88447	0.88486	0.99998	17812	0.2711
HS3ST3B1	4	0.88455	0.88444	0.99998	17813	0.2005
PCDHGA9	2	0.88459	0.88458	0.99998	17814	0.2617
ZNF841	6	0.88461	0.88499	0.99998	17815	0.1399
WARS2	6	0.88466	0.88504	0.99998	17816	0.1304
SPATA31E1	6	0.8847	0.88508	0.99998	17817	0.0949

FGF8	6	0.8847	0.88508	0.99998	17818	0.2414
SAMD8	6	0.88474	0.88511	0.99998	17819	0.0559
C11orf49	6	0.8848	0.88517	0.99998	17820	-0.0561
CCDC109B	6	0.8848	0.88517	0.99998	17821	0.1057
PARP11	6	0.8848	0.88517	0.99998	17822	0.3532
RAB8A	6	0.8848	0.88517	0.99998	17823	-0.0049
SSR1	6	0.8849	0.88526	0.99998	17824	0.2614
PARD3	6	0.8849	0.88526	0.99998	17825	0.0524
hsa-mir-544b	4	0.88513	0.88502	0.99998	17826	-0.0235
OR51L1	6	0.88518	0.88552	0.99998	17827	0.0752
ZCRB1	6	0.88525	0.88558	0.99998	17828	-0.0225
PRCP	6	0.8853	0.88563	0.99998	17829	0.0185
ACSBG2	6	0.88535	0.88567	0.99998	17830	0.0388
ASB5	4	0.88539	0.88529	0.99998	17831	0.3121
ZNF789	6	0.88541	0.88572	0.99998	17832	0.0662
ITGA6	6	0.88541	0.88572	0.99998	17833	0.1896
BCAM	6	0.88545	0.88576	0.99998	17834	0.1016
ZNF501	6	0.88549	0.8858	0.99998	17835	0.1765
KRTAP15-1	6	0.88549	0.8858	0.99998	17836	0.145
SERPINB6	6	0.88565	0.88595	0.99998	17837	0.0351
CPLX4	6	0.88569	0.88599	0.99998	17838	0.28
FTO	6	0.88569	0.88599	0.99998	17839	0.3183
GYS1	6	0.88573	0.88602	0.99998	17840	0.0408
hsa-mir-3611	1	0.88575	0.88613	0.99998	17841	0.4381
ZNF610	6	0.88578	0.88606	0.99998	17842	-0.0228
GPR83	6	0.88578	0.88606	0.99998	17843	0.154
DEPDC4	6	0.88578	0.88606	0.99998	17844	0.1449
TULP3	6	0.88596	0.88623	0.99998	17845	0.0973
ADAM12	6	0.88596	0.88623	0.99998	17846	0.0458
hsa-mir-4794	3	0.88596	0.88608	0.99998	17847	0.4294
PZP	6	0.886	0.88627	0.99998	17848	0.2858
SOX1	6	0.88604	0.8863	0.99998	17849	0.3497
SYT3	6	0.88604	0.8863	0.99998	17850	0.0551
hsa-mir-335	4	0.88609	0.886	0.99998	17851	0.0751
DPF2	6	0.88615	0.88641	0.99998	17852	-0.0186
NXPE1	6	0.8862	0.88646	0.99998	17853	0.0988
hsa-mir-4253	4	0.88621	0.88611	0.99998	17854	0.3381
LRIG3	6	0.88628	0.88654	0.99998	17855	0.0406
ARSA	6	0.88628	0.88654	0.99998	17856	0.1748
RDH12	6	0.88628	0.88654	0.99998	17857	0.2069
HEY1	6	0.88628	0.88654	0.99998	17858	-0.1
APBB1	6	0.88628	0.88654	0.99998	17859	0.0393
hsa-mir-101-2	3	0.88638	0.88647	0.99998	17860	0.2008
MEP1B	6	0.88643	0.88668	0.99998	17861	0.1505
TSPAN19	6	0.88643	0.88668	0.99998	17862	-0.043
NECAP2	6	0.88652	0.88678	0.99998	17863	0.0166
FAM214B	6	0.88656	0.88681	0.99998	17864	0.1545
TMED3	4	0.88656	0.88646	0.99998	17865	-0.0471
RAB20	6	0.88675	0.88699	0.99998	17866	-0.0674
BAIAP2	6	0.88686	0.88708	0.99998	17867	0.0804
CAPN7	6	0.88688	0.8871	0.99998	17868	0.0174
SPZ1	6	0.88688	0.8871	0.99998	17869	-0.0542
OR5AS1	6	0.8869	0.88712	0.99998	17870	-0.0395
C11orf57	6	0.88697	0.88719	0.99998	17871	0.1859
TMEM56	1	0.88697	0.88735	0.99998	17872	0.3815
VSTM5	6	0.88701	0.88723	0.99998	17873	-0.0228
COA4	4	0.88705	0.88696	0.99998	17874	0.0123
FCHO1	6	0.88709	0.8873	0.99998	17875	-0.0008
KRTAP4-9	4	0.88718	0.8871	0.99998	17876	-0.1301
BANK1	6	0.88727	0.88749	0.99998	17877	0.2885
MPP6	6	0.88731	0.88752	0.99998	17878	0.0481
GABBR2	6	0.88735	0.88756	0.99998	17879	0.1696
TFDP2	6	0.88741	0.88762	0.99998	17880	0.2981
DPYD	6	0.88741	0.88762	0.99998	17881	0.3308
hsa-mir-7153	4	0.88745	0.88739	0.99998	17882	0.2168
TBXAS1	6	0.88757	0.88778	0.99998	17883	0.2999
GPR88	6	0.88757	0.88778	0.99998	17884	0.1838
RASIP1	6	0.88762	0.88782	0.99998	17885	0.0701
C10orf53	6	0.88768	0.88787	0.99998	17886	-0.0115
EFCAB3	6	0.88768	0.88787	0.99998	17887	0.1872
hsa-mir-517c	1	0.88776	0.88814	0.99998	17888	0.3923
RNF44	6	0.88779	0.88798	0.99998	17889	0.1193
HSD11B1L	6	0.88779	0.88798	0.99998	17890	0.1263
LOC10013034	6	0.88779	0.88798	0.99998	17891	0.2109
CLEC4F	6	0.88779	0.88798	0.99998	17892	0.3611
CLSTN1	6	0.88779	0.88798	0.99998	17893	0.1161
hsa-mir-1182	4	0.88787	0.8878	0.99998	17894	0.0801
hsa-mir-6841	2	0.88787	0.88785	0.99998	17895	0.5329
C3orf33	6	0.88792	0.8881	0.99998	17896	0.0141
LAX1	6	0.8881	0.88826	0.99998	17897	-0.0177
NOX5	5	0.88818	0.88849	0.99998	17898	-0.072

HDX	6	0.8882	0.88836	0.99998	17899	-0.0254
KRTAP13-1	6	0.88829	0.88845	0.99998	17900	0.1227
DVL2	6	0.88829	0.88845	0.99998	17901	-0.0283
ELFN1	6	0.88829	0.88845	0.99998	17902	0.2143
FXYD2	4	0.88835	0.88829	0.99998	17903	0.2008
PTPRH	6	0.88839	0.88856	0.99998	17904	0.0483
JAG2	6	0.88839	0.88856	0.99998	17905	0.0654
hsa-mir-4530	4	0.88843	0.88838	0.99998	17906	0.0145
IRAK1	6	0.8885	0.88866	0.99998	17907	0.071
SCAF11	6	0.8885	0.88866	0.99998	17908	0.0453
TPBG	6	0.88859	0.88875	0.99998	17909	0.1733
GDNF	6	0.88867	0.88882	0.99998	17910	0.2767
ABHD12	6	0.88867	0.88882	0.99998	17911	-0.0027
OR8G2	6	0.88867	0.88882	0.99998	17912	0.1326
PIAS2	6	0.8888	0.88895	0.99998	17913	-0.0796
PKHD1	6	0.88883	0.88899	0.99998	17914	0.0148
TK2	6	0.88892	0.88906	0.99998	17915	0.1547
BFSP1	6	0.88892	0.88906	0.99998	17916	0.1719
hsa-mir-3128	4	0.88893	0.88885	0.99998	17917	0.1031
SIX5	6	0.88902	0.88917	0.99998	17918	-0.1025
VSTM2L	6	0.88911	0.88926	0.99998	17919	-0.0126
GPC2	6	0.88911	0.88926	0.99998	17920	-0.0932
HDAC10	6	0.88918	0.88933	0.99998	17921	0.0258
hsa-mir-548m	4	0.88921	0.88912	0.99998	17922	0.1891
IFNA21	4	0.88921	0.88912	0.99998	17923	0.0729
hsa-let-7b	4	0.88921	0.88912	0.99998	17924	0.0036
ABHD5	6	0.88926	0.8894	0.99998	17925	0.1805
SGK223	6	0.88933	0.88948	0.99998	17926	0.0348
TFPI2	6	0.88943	0.88958	0.99998	17927	0.061
CCDC39	6	0.8895	0.88965	0.99998	17928	-0.049
CES5A	6	0.88959	0.88973	0.99998	17929	0.183
JAGN1	6	0.88959	0.88973	0.99998	17930	0.1438
hsa-mir-3187	4	0.88972	0.88962	0.99998	17931	0.0953
VNN2	6	0.88975	0.8899	0.99998	17932	0.1667
CFTR	6	0.88975	0.8899	0.99998	17933	0.2058
CD99L2	6	0.88981	0.88996	0.99998	17934	-0.0536
RNF149	6	0.88986	0.89002	0.99998	17935	0.1234
TMEM47	6	0.88991	0.89007	0.99998	17936	0.0238
OSTF1	6	0.88991	0.89007	0.99998	17937	0.3129
TMEM59L	6	0.88991	0.89007	0.99998	17938	0.2698
TTC21A	6	0.88997	0.89013	0.99998	17939	0.0885
TTLL3	4	0.89005	0.88995	0.99998	17940	-0.0138
FETUB	6	0.89008	0.89024	0.99998	17941	0.0906
PLCD4	6	0.89008	0.89024	0.99998	17942	0.0488
hsa-mir-106b	4	0.89013	0.89005	0.99998	17943	0.0617
DCDC2B	6	0.8902	0.89035	0.99998	17944	0.1578
STAG2	6	0.8902	0.89035	0.99998	17945	0.3478
LRRC38	6	0.89028	0.89044	0.99998	17946	0.1344
ARG1	6	0.89036	0.8905	0.99998	17947	-0.0382
SLC8A3	6	0.89036	0.8905	0.99998	17948	0.2245
hsa-mir-3713	4	0.89039	0.89031	0.99998	17949	0.0481
OR5AN1	6	0.89043	0.89058	0.99998	17950	0.0616
TWIST2	6	0.89043	0.89058	0.99998	17951	0.1816
ARPC1A	6	0.89043	0.89058	0.99998	17952	0.1005
FAM175B	6	0.89053	0.89066	0.99998	17953	0.3255
INPP5J	6	0.89053	0.89066	0.99998	17954	0.1342
RANBP17	6	0.89061	0.89074	0.99998	17955	-0.0298
CTPS2	6	0.89061	0.89074	0.99998	17956	0.2161
KLC2	6	0.89067	0.8908	0.99998	17957	0.1585
PLEKHS1	6	0.89067	0.8908	0.99998	17958	-0.019
USP35	6	0.8907	0.89082	0.99998	17959	0.1543
IL18R1	6	0.89073	0.89085	0.99998	17960	0.1177
FAM122B	6	0.89073	0.89085	0.99998	17961	0.1536
ADAM29	6	0.89073	0.89085	0.99998	17962	0.2181
FILIP1L	6	0.89083	0.89095	0.99998	17963	0.032
CAPRIN2	6	0.89093	0.89105	0.99998	17964	0.1874
SCARA3	6	0.89093	0.89105	0.99998	17965	0.3101
C22orf24	6	0.89102	0.89114	0.99998	17966	0.2038
DES	6	0.89104	0.89115	0.99998	17967	0.2482
THSD7B	6	0.89107	0.89119	0.99998	17968	0.0914
CXorf21	6	0.89107	0.89119	0.99998	17969	0.342
STK40	6	0.89111	0.89122	0.99998	17970	-0.0495
TSKU	6	0.89113	0.89125	0.99998	17971	0.0134
ILKAP	6	0.89113	0.89125	0.99998	17972	0.262
NRXN2	6	0.89113	0.89125	0.99998	17973	0.1855
CXCL11	6	0.89121	0.89133	0.99998	17974	0.0727
SLC2A8	6	0.89135	0.89146	0.99998	17975	-0.02
ZNF546	6	0.89135	0.89146	0.99998	17976	0.001
C2	6	0.89152	0.89164	0.99998	17977	0.203
OR11H1	3	0.89156	0.89165	0.99998	17978	0.0919
C2CD4A	6	0.89159	0.89173	0.99998	17979	0.1401

ZNF571	6	0.89163	0.89176	0.99998	17980	-0.0534
C1QL2	6	0.89163	0.89176	0.99998	17981	0.2654
ITGA11	6	0.89179	0.89192	0.99998	17982	0.0381
PTPRCAP	6	0.8919	0.89202	0.99998	17983	0.3015
PBOV1	6	0.8919	0.89202	0.99998	17984	0.0945
WWC3	6	0.8919	0.89202	0.99998	17985	0.0117
CLDN17	6	0.8919	0.89202	0.99998	17986	0.17
hsa-mir-892a	4	0.89198	0.89188	0.99998	17987	0.2287
APBB2	6	0.89198	0.89211	0.99998	17988	0.0548
UBAC1	6	0.89207	0.89219	0.99998	17989	-0.0765
TTL	6	0.89217	0.89231	0.99998	17990	0.3669
RAB40C	6	0.89217	0.89231	0.99998	17991	0.1229
PARP16	6	0.89221	0.89235	0.99998	17992	0.2467
CCDC13	6	0.89232	0.89246	0.99998	17993	0.3446
IL27RA	6	0.89232	0.89246	0.99998	17994	0.1454
SPATA31D1	6	0.89232	0.89246	0.99998	17995	0.1825
SHMT2	6	0.89232	0.89246	0.99998	17996	0.1713
ZNF37A	6	0.89232	0.89246	0.99998	17997	0.0288
LLGL1	6	0.89253	0.89268	0.99998	17998	-0.0491
hsa-mir-4282	4	0.89258	0.89247	0.99998	17999	0.122
POLB	6	0.89259	0.89273	0.99998	18000	-0.1155
hsa-mir-574	4	0.89268	0.89257	0.99998	18001	0.0511
hsa-mir-4524b	1	0.89272	0.89313	0.99998	18002	0.3272
NPAS3	6	0.89275	0.89288	0.99998	18003	-0.0913
NTS	4	0.89278	0.89267	0.99998	18004	0.1481
CASS4	6	0.89281	0.89294	0.99998	18005	-0.0017
MCCC2	6	0.89288	0.89302	0.99998	18006	0.0637
QPCT	6	0.89296	0.89311	0.99998	18007	0.0476
COL25A1	6	0.89296	0.89311	0.99998	18008	0.072
TINAG	6	0.89304	0.89319	0.99998	18009	0.3116
VIMP	6	0.89311	0.89326	0.99998	18010	-0.0309
CYP2D6	6	0.89316	0.8933	0.99998	18011	0.242
LMAN2	6	0.89321	0.89336	0.99998	18012	0.1077
ZDHHC11	6	0.89326	0.89341	0.99998	18013	0.0809
hsa-mir-1279	2	0.8933	0.8932	0.99998	18014	0.2304
RLTPR	6	0.89331	0.89346	0.99998	18015	0.1395
CLEC12A	6	0.89331	0.89346	0.99998	18016	0.1002
MRFAP1	6	0.89336	0.8935	0.99998	18017	0.0192
NDFIP2	6	0.89338	0.89353	0.99998	18018	0.2011
SLX4IP	6	0.89351	0.89366	0.99998	18019	0.1301
LRRC39	6	0.89365	0.89379	0.99998	18020	0.0664
EPN1	6	0.89371	0.89384	0.99998	18021	0.0381
KLF16	6	0.89371	0.89384	0.99998	18022	0.1654
hsa-mir-149	4	0.89373	0.89362	0.99998	18023	-0.0664
SYT9	6	0.89387	0.894	0.99998	18024	0.0958
KIF26B	6	0.89387	0.894	0.99998	18025	0.3334
RNF6	6	0.89402	0.89415	0.99998	18026	0.1171
hsa-mir-3664	4	0.8941	0.89399	0.99998	18027	-0.0234
MTERFD3	6	0.89414	0.89426	0.99998	18028	0.2051
FIZ1	6	0.89414	0.89426	0.99998	18029	0.0835
HIVEP3	6	0.89414	0.89426	0.99998	18030	0.2441
STAR	6	0.8943	0.89441	0.99998	18031	0.0126
C1orf192	6	0.89435	0.89445	0.99998	18032	0.2814
IQCG	6	0.89439	0.89449	0.99998	18033	0.0792
GNB1	6	0.89444	0.89454	0.99998	18034	0.1686
RELN	6	0.89449	0.89459	0.99998	18035	0.1245
IFRD2	6	0.89449	0.89459	0.99998	18036	-0.0371
DCLK2	6	0.89454	0.89464	0.99998	18037	-0.0527
BRAP	6	0.89459	0.89468	0.99998	18038	0.1596
CHRNB3	6	0.89462	0.89471	0.99998	18039	0.2891
SRPX	6	0.89471	0.8948	0.99998	18040	0.1999
CUTA	6	0.89471	0.8948	0.99998	18041	-0.0052
hsa-mir-769	4	0.89476	0.89466	0.99998	18042	0.0812
SPINK5	6	0.89477	0.89484	0.99998	18043	0.1272
CDCA4	6	0.89479	0.89486	0.99998	18044	0.4179
EDA	6	0.8949	0.89496	0.99998	18045	0.1958
RMDN1	6	0.8949	0.89496	0.99998	18046	0.1474
FAM150B	6	0.8949	0.89496	0.99998	18047	0.089
ZFP42	6	0.89496	0.89503	0.99998	18048	0.095
SLC11A1	6	0.89506	0.89513	0.99998	18049	0.0468
PTF1A	6	0.89506	0.89513	0.99998	18050	0.2325
BANF2	6	0.89506	0.89513	0.99998	18051	0.2674
SVOPL	6	0.89506	0.89513	0.99998	18052	0.0105
SGK3	5	0.89514	0.89545	0.99998	18053	0.19
ALPL	6	0.89522	0.89529	0.99998	18054	0.0619
CXCL14	6	0.89522	0.89529	0.99998	18055	-0.0433
CCDC14	6	0.89522	0.89529	0.99998	18056	-0.0771
C6orf118	6	0.89536	0.89543	0.99998	18057	0.121
ZSCAN20	6	0.89554	0.89562	0.99998	18058	-0.057
hsa-mir-4725	4	0.8956	0.89549	0.99998	18059	0.2017
TCTN3	6	0.89565	0.89575	0.99998	18060	0.1365

LIMS2	6	0.89565	0.89575	0.99998	18061	0.0829
PKD2L1	6	0.89571	0.89581	0.99998	18062	0.1202
GINM1	6	0.89573	0.89584	0.99998	18063	0.0243
CFHR5	6	0.89576	0.89588	0.99998	18064	-0.0481
FDCSP	6	0.8958	0.89591	0.99998	18065	0.1098
EMX1	6	0.89582	0.89593	0.99998	18066	0.204
SLC1A3	6	0.89586	0.89597	0.99998	18067	0.0426
hsa-mir-507	4	0.89594	0.89582	0.99998	18068	0.0579
P4HA2	6	0.89598	0.89609	0.99998	18069	0.1187
RAB11FIP4	6	0.89598	0.89609	0.99998	18070	0.1106
CST5	6	0.89607	0.89616	0.99998	18071	0.0158
FXYD5	6	0.89611	0.8962	0.99998	18072	0.1782
hsa-mir-571	4	0.89616	0.89605	0.99998	18073	0.0116
CA5B	6	0.89618	0.89626	0.99998	18074	0.1198
SCN3B	6	0.89624	0.89632	0.99998	18075	0.1542
SIRT4	6	0.89624	0.89632	0.99998	18076	-0.0879
hsa-mir-549a	4	0.89625	0.89615	0.99998	18077	0.0191
KIF18B	6	0.89641	0.89649	0.99998	18078	0.0885
MEGF6	6	0.89641	0.89649	0.99998	18079	0.0345
ITPR1	6	0.89641	0.89649	0.99998	18080	0.229
PI4KB	6	0.8965	0.89657	0.99998	18081	0.1715
DNASE1L3	6	0.89669	0.89676	0.99998	18082	0.1554
SPINK1	6	0.89669	0.89676	0.99998	18083	0.0875
ZBTB14	6	0.89675	0.89682	0.99998	18084	0.5376
CD36	4	0.89688	0.89676	0.99998	18085	0.1616
COMMD5	6	0.89696	0.89704	0.99998	18086	0.2721
KRTAP27-1	6	0.89708	0.89715	0.99998	18087	0.1737
TMEM67	6	0.89708	0.89715	0.99998	18088	0.0052
ARHGAP8	6	0.89708	0.89715	0.99998	18089	0.3476
UNC93B1	6	0.89708	0.89715	0.99998	18090	0.0323
KLF14	6	0.89718	0.89725	0.99998	18091	0.1989
MICU3	6	0.89718	0.89725	0.99998	18092	-0.0113
SLC46A3	6	0.89724	0.8973	0.99998	18093	0.1081
hsa-mir-4471	4	0.89728	0.89716	0.99998	18094	0.0262
CLEC2L	6	0.89732	0.89738	0.99998	18095	0.2645
TANC1	6	0.89732	0.89738	0.99998	18096	0.1034
ASB4	6	0.89742	0.89749	0.99998	18097	0.048
OR52B6	6	0.89742	0.89749	0.99998	18098	-0.0177
DBC1	2	0.89745	0.89741	0.99998	18099	0.1769
KRTAP10-10	6	0.89757	0.89765	0.99998	18100	0.159
LRRFIP2	6	0.89757	0.89765	0.99998	18101	-0.0239
hsa-mir-320c-	3	0.89763	0.89776	0.99998	18102	0.1307
C17orf75	6	0.89777	0.89787	0.99998	18103	0.0317
CNTNAP1	6	0.89783	0.89792	0.99998	18104	-0.0589
OR8I2	6	0.89783	0.89792	0.99998	18105	-0.0136
OR2M7	6	0.89783	0.89792	0.99998	18106	-0.0229
PLIN2	6	0.89793	0.89803	0.99998	18107	-0.0541
hsa-mir-5684	4	0.89798	0.89786	0.99998	18108	0.1003
TAP2	6	0.89799	0.8981	0.99998	18109	0.1215
RIT2	4	0.89807	0.89795	0.99998	18110	-0.021
CSTF2	6	0.89808	0.89819	0.99998	18111	0.0565
SPTSSB	6	0.89808	0.89819	0.99998	18112	0.0799
SNX22	6	0.89813	0.89824	0.99998	18113	0.3126
TOR4A	6	0.89819	0.89829	0.99998	18114	0.2141
MRPS18B	6	0.89819	0.89829	0.99998	18115	0.1528
HEATR2	6	0.89826	0.89835	0.99998	18116	0.0569
MRPS17	6	0.89826	0.89835	0.99998	18117	0.0626
C1orf85	6	0.89828	0.89838	0.99998	18118	-0.0258
TCEA1	6	0.89831	0.89841	0.99998	18119	0.1043
ASPSCR1	6	0.89848	0.89857	0.99998	18120	0.0369
ENTHD2	6	0.89848	0.89857	0.99998	18121	0.2743
ALDH2	6	0.89848	0.89857	0.99998	18122	0.0333
UBQLNL	6	0.89854	0.89863	0.99998	18123	0.0043
RCL1	6	0.89859	0.89868	0.99998	18124	0.0276
EGLN3	6	0.89863	0.89871	0.99998	18125	0.0463
PLCH1	6	0.89887	0.89896	0.99998	18126	0.1375
hsa-mir-145	4	0.89891	0.89881	0.99998	18127	0.1465
DHX34	6	0.89892	0.89901	0.99998	18128	0.0954
RHOB	6	0.89892	0.89901	0.99998	18129	0.2435
PIP5K1A	6	0.899	0.89908	0.99998	18130	0.1054
BSPH1	5	0.89905	0.89935	0.99998	18131	0.3523
DHDH	6	0.89907	0.89916	0.99998	18132	0.2041
HPCAL4	6	0.89917	0.89924	0.99998	18133	0.0408
IL17RA	6	0.89917	0.89924	0.99998	18134	0.3341
hsa-mir-8065	4	0.89922	0.89912	0.99998	18135	0.2216
NLRP1	6	0.89924	0.89933	0.99998	18136	0.3779
RARA	6	0.89925	0.89933	0.99998	18137	0.0653
CENPB	6	0.89929	0.89937	0.99998	18138	0.0665
hsa-mir-3935	4	0.89949	0.89942	0.99998	18139	0.0722
RHBDL2	6	0.8995	0.89959	0.99998	18140	0.0674
hsa-mir-6876	4	0.89959	0.89951	0.99998	18141	0.0572

SPANXN4	6	0.89963	0.89972	0.99998	18142	0.0337
CLASP2	6	0.89963	0.89972	0.99998	18143	0.0605
GABRG3	6	0.89963	0.89972	0.99998	18144	0.176
CAMSAP3	6	0.8998	0.89988	0.99998	18145	0.0765
CTNNA2	6	0.89988	0.89996	0.99998	18146	-0.0457
MGAT3	6	0.89994	0.90003	0.99998	18147	0.1406
CAMSAP2	6	0.90001	0.9001	0.99998	18148	0.0665
NR1D1	6	0.90008	0.90017	0.99998	18149	0.3624
IFLTD1	6	0.90008	0.90017	0.99998	18150	-0.0692
PRAMEF12	6	0.90022	0.90031	0.99998	18151	0.1275
ADPRM	6	0.90022	0.90031	0.99998	18152	0.184
RNF17	6	0.90022	0.90031	0.99998	18153	0.3335
SP140L	6	0.90036	0.90044	0.99998	18154	-0.024
LILRB4	6	0.90036	0.90044	0.99998	18155	-0.0063
ANXA7	6	0.90058	0.90065	0.99998	18156	0.1057
FST	6	0.90064	0.90071	0.99998	18157	-0.0503
LPPR4	6	0.90064	0.90071	0.99998	18158	0.0271
STYK1	6	0.90064	0.90071	0.99998	18159	0.1483
ZKSCAN2	6	0.90064	0.90071	0.99998	18160	-0.0587
NDUFB6	6	0.90078	0.90085	0.99998	18161	0.259
hsa-mir-4483	4	0.90078	0.9007	0.99998	18162	0.082
SV2B	6	0.90088	0.90095	0.99998	18163	0.1644
TMCO2	6	0.90098	0.90104	0.99998	18164	0.1985
OSCAR	6	0.90098	0.90104	0.99998	18165	-0.0434
LRRC45	6	0.90102	0.90108	0.99998	18166	0.0986
RUVBL2	6	0.90106	0.90112	0.99998	18167	0.0436
SP5	6	0.90106	0.90112	0.99998	18168	0.1221
PTGDS	6	0.90114	0.90121	0.99998	18169	0.1309
OR6K6	6	0.90114	0.90121	0.99998	18170	0.1327
NUMBL	6	0.90123	0.90129	0.99998	18171	0.1746
PIGO	6	0.90123	0.90129	0.99998	18172	0.0491
ASGR1	6	0.90128	0.90134	0.99998	18173	-0.0831
SLC38A6	6	0.90128	0.90134	0.99998	18174	0.1254
DSC1	6	0.90133	0.9014	0.99998	18175	0.031
hsa-mir-92a-2	3	0.90133	0.90147	0.99998	18176	0.1813
GLA	6	0.90137	0.90144	0.99998	18177	0.0176
SLC25A39	6	0.90143	0.9015	0.99998	18178	0.0472
CDRT15	6	0.90143	0.9015	0.99998	18179	-0.1021
ZBTB10	6	0.90153	0.9016	0.99998	18180	0.0669
SLC6A13	6	0.90159	0.90166	0.99998	18181	-0.1095
ZNF436	6	0.90165	0.90172	0.99998	18182	0.1847
KCNH5	6	0.90172	0.90178	0.99998	18183	0.0218
NRGN	6	0.90176	0.90181	0.99998	18184	-0.0768
hsa-mir-1469	4	0.90177	0.9017	0.99998	18185	0.1106
SLC41A1	6	0.90177	0.90183	0.99998	18186	-0.0051
APOL6	6	0.90179	0.90186	0.99998	18187	0.0855
ZNF74	6	0.90181	0.90188	0.99998	18188	0.1915
KCTD18	6	0.90181	0.90188	0.99998	18189	-0.0128
TMEM128	6	0.90185	0.90192	0.99998	18190	0.2808
SS18L1	6	0.90193	0.90201	0.99998	18191	0.2296
hsa-mir-3133	3	0.90209	0.90221	0.99998	18192	0.3736
HOXA6	6	0.90213	0.90221	0.99998	18193	0.0301
KIAA0825	6	0.90222	0.90231	0.99998	18194	0.3998
DUSP7	6	0.90222	0.90231	0.99998	18195	0.2762
PHKG2	6	0.90232	0.90241	0.99998	18196	0.0406
SULT1A3	4	0.90237	0.9023	0.99998	18197	0.2087
TOM1L1	6	0.90238	0.90247	0.99998	18198	0.1399
CAPN8	5	0.9024	0.90266	0.99998	18199	0.041
SYNE4	6	0.90248	0.90258	0.99998	18200	0.0723
ZNF627	3	0.90261	0.90271	0.99998	18201	0.1144
hsa-mir-154	4	0.90264	0.90259	0.99998	18202	0.124
EDNRA	6	0.90265	0.90273	0.99998	18203	0.1477
MAEL	6	0.90265	0.90273	0.99998	18204	0.399
EBPL	6	0.9027	0.90278	0.99998	18205	0.1133
hsa-mir-6723	4	0.90275	0.90269	0.99998	18206	0.23
ASIC4	6	0.90278	0.90286	0.99998	18207	-0.0648
THOC6	4	0.90291	0.90285	0.99998	18208	0.0941
BMP8A	3	0.90294	0.90301	0.99998	18209	0.0742
SMN2	2	0.90294	0.90291	0.99998	18210	0.5164
KCTD19	6	0.90298	0.90306	0.99998	18211	0.0145
ZNF217	6	0.90303	0.90311	0.99998	18212	0.1094
DEPDC1B	6	0.90318	0.90327	0.99998	18213	-0.0769
EML3	6	0.90327	0.90337	0.99998	18214	-0.0504
GIN1	6	0.90332	0.90341	0.99998	18215	0.0937
SLC2A1	6	0.90349	0.90357	0.99998	18216	0.2368
USP27X	6	0.90355	0.90364	0.99998	18217	0.1444
ATCAY	6	0.90355	0.90364	0.99998	18218	-0.0478
hsa-mir-134	4	0.90359	0.90352	0.99998	18219	0.2524
OR5I1	6	0.9036	0.90369	0.99998	18220	0.0473
FLRT1	6	0.90365	0.90374	0.99998	18221	0.1409
PATE4	6	0.90374	0.90383	0.99998	18222	-0.0013

NRP2	6	0.90379	0.90388	0.99998	18223	0.0681
IQSEC3	6	0.90382	0.90391	0.99998	18224	0.0224
RASSF3	6	0.90386	0.90395	0.99998	18225	0.0048
PANK3	6	0.90393	0.90403	0.99998	18226	0.0664
KLRG2	4	0.90395	0.90386	0.99998	18227	0.1063
GBP7	6	0.9041	0.90419	0.99998	18228	0.047
TLN1	6	0.9041	0.90419	0.99998	18229	0.2623
GLIS1	6	0.9041	0.90419	0.99998	18230	-0.0218
ARMC5	6	0.9041	0.90419	0.99998	18231	0.3514
MYL4	6	0.9041	0.90419	0.99998	18232	0.1324
FREM3	6	0.9041	0.90419	0.99998	18233	0.2136
UCP3	6	0.9041	0.90419	0.99998	18234	0.1747
H2AFY2	6	0.90429	0.9044	0.99998	18235	-0.0418
C1orf54	6	0.90436	0.90445	0.99998	18236	0.0107
DUS2L	2	0.90437	0.9043	0.99998	18237	0.1906
ATXN7L1	6	0.90448	0.90457	0.99998	18238	0.2211
CYP2C18	6	0.90448	0.90457	0.99998	18239	0.1909
NEK11	6	0.90448	0.90457	0.99998	18240	0.0647
VAMP5	6	0.90453	0.90462	0.99998	18241	0.1976
C8orf44-SGK3	4	0.90457	0.90446	0.99998	18242	0.0065
C15orf26	6	0.90466	0.90476	0.99998	18243	0.2026
GALR3	6	0.90466	0.90476	0.99998	18244	-0.0643
TXNDC12	6	0.90466	0.90476	0.99998	18245	0.1239
ERP27	6	0.90472	0.90482	0.99998	18246	0.1626
SLC2A9	6	0.90475	0.90486	0.99998	18247	0.0792
UNC45A	6	0.90484	0.90495	0.99998	18248	0.1097
IL17RE	6	0.90497	0.90508	0.99998	18249	0.0073
CLIP4	6	0.90497	0.90508	0.99998	18250	0.1122
EDN1	6	0.90497	0.90508	0.99998	18251	0.357
TBX18	6	0.90497	0.90508	0.99998	18252	-0.0652
TAF15	6	0.90497	0.90508	0.99998	18253	0.064
OR2A1	1	0.90501	0.90544	0.99998	18254	0.7046
HSPA12B	6	0.9051	0.90521	0.99998	18255	0.1136
hsa-mir-4476	4	0.90512	0.90502	0.99998	18256	0.1143
BRAF	6	0.90515	0.90526	0.99998	18257	0.2588
PRH2	5	0.90518	0.90546	0.99998	18258	0.116
hsa-mir-1972-	1	0.9053	0.90573	0.99998	18259	0.5204
CCDC33	6	0.90534	0.90547	0.99998	18260	0.0101
ARL11	6	0.90538	0.9055	0.99998	18261	0.1256
C19orf33	6	0.90543	0.90555	0.99998	18262	0.2334
NYAP2	6	0.90547	0.90559	0.99998	18263	0.0068
GRID2IP	6	0.90547	0.90559	0.99998	18264	0.1435
RNF138	6	0.90554	0.90565	0.99998	18265	-0.0138
SLC16A7	6	0.90554	0.90565	0.99998	18266	0.1053
C9orf62	6	0.90561	0.90572	0.99998	18267	-0.0634
SMIM7	6	0.90569	0.90579	0.99998	18268	0.1574
TAS2R9	6	0.90575	0.90586	0.99998	18269	0.0765
C20orf173	6	0.90578	0.90589	0.99998	18270	-0.0117
NTNG1	6	0.9058	0.9059	0.99998	18271	0.0231
SLC30A2	6	0.90589	0.90599	0.99998	18272	-0.079
CD80	6	0.90602	0.90613	0.99998	18273	-0.0413
GPR12	6	0.90602	0.90613	0.99998	18274	0.3025
PDZRN3	6	0.90602	0.90613	0.99998	18275	0.2137
B3GALNT2	6	0.90611	0.90622	0.99998	18276	0.0332
C10orf32	6	0.90611	0.90622	0.99998	18277	0.0131
C14orf105	6	0.90619	0.90631	0.99998	18278	0.0456
CCDC122	6	0.90619	0.90631	0.99998	18279	0.1091
FAM173A	6	0.90624	0.90637	0.99998	18280	0.1611
HLA-DQB1	6	0.90629	0.90642	0.99998	18281	0.1076
WWP2	6	0.90634	0.90646	0.99998	18282	0.1527
LRRC23	6	0.90642	0.90654	0.99998	18283	-0.0144
TMEM63A	6	0.90648	0.9066	0.99998	18284	0.2929
ARSE	6	0.9066	0.90671	0.99998	18285	0.0702
EZH1	6	0.90661	0.90672	0.99998	18286	0.0682
ADAM21	6	0.90664	0.90676	0.99998	18287	0.0403
TROVE2	6	0.9067	0.90682	0.99998	18288	-0.0718
NHLRC4	6	0.90679	0.90692	0.99998	18289	-0.0272
FOXI1	6	0.90684	0.90697	0.99998	18290	0.2703
ARHGEF40	6	0.90688	0.90699	0.99998	18291	0.1454
PDGFA	6	0.90696	0.90707	0.99998	18292	0.0309
PSG11	6	0.90696	0.90707	0.99998	18293	0.441
RWDD2A	6	0.90696	0.90707	0.99998	18294	0.1906
ZNF630	6	0.90705	0.90716	0.99998	18295	0.1484
NT5C	6	0.90705	0.90716	0.99998	18296	0.2424
ERO1L	6	0.90705	0.90716	0.99998	18297	0.0053
HNRNPA3	5	0.90707	0.90728	0.99998	18298	0.0013
C7orf69	6	0.90711	0.90722	0.99998	18299	-0.0569
FAM229B	6	0.90722	0.90733	0.99998	18300	0.0196
CLRN1	6	0.9074	0.90751	0.99998	18301	-0.1074
GABRA4	6	0.9074	0.90751	0.99998	18302	0.199
CLINT1	6	0.9074	0.90751	0.99998	18303	-0.0227

SAT1	6	0.90747	0.90758	0.99998	18304	0.1561
FTCD	6	0.90752	0.90764	0.99998	18305	-0.0887
MOBP	6	0.90752	0.90764	0.99998	18306	0.2753
CALCRL	6	0.90752	0.90764	0.99998	18307	0.1424
hsa-mir-200a	4	0.90752	0.90743	0.99998	18308	0.1417
PCDHGB1	2	0.90759	0.90757	0.99998	18309	0.2132
SLC27A2	6	0.90767	0.90778	0.99998	18310	0.1155
SERPINA6	6	0.90775	0.90784	0.99998	18311	0.1324
METTL13	6	0.90781	0.90791	0.99998	18312	0.0873
DNAJC5G	6	0.90781	0.90791	0.99998	18313	0.0072
TRIM54	6	0.90781	0.90791	0.99998	18314	0.0576
MAP3K9	6	0.9079	0.90798	0.99998	18315	0.2452
INS	4	0.90791	0.9078	0.99998	18316	0.072
EVA1C	6	0.90795	0.90803	0.99998	18317	-0.0254
CAMTA2	6	0.908	0.90808	0.99998	18318	0.0689
LPGAT1	6	0.908	0.90808	0.99998	18319	0.1244
HSPA6	6	0.90806	0.90815	0.99998	18320	0.0548
LDB3	6	0.9081	0.90819	0.99998	18321	0.4086
NCAM1	6	0.90813	0.90823	0.99998	18322	0.0399
IGF2R	6	0.90821	0.9083	0.99998	18323	0.1363
KIR3DL1	6	0.90821	0.9083	0.99998	18324	0.193
EIF4E2	6	0.90832	0.90841	0.99998	18325	0.1027
FAM209A	6	0.90837	0.90846	0.99998	18326	-0.0426
SYNPR	6	0.90845	0.90855	0.99998	18327	0.138
DRAM2	6	0.9085	0.9086	0.99998	18328	0.1207
NOX1	6	0.90859	0.90869	0.99998	18329	0.1066
ARIH2	6	0.90861	0.90871	0.99998	18330	0.0006
LINGO2	6	0.90864	0.90874	0.99998	18331	0.089
P2RX5	6	0.90872	0.90881	0.99998	18332	0.1423
PELI1	6	0.90879	0.90888	0.99998	18333	0.1331
OR4A16	6	0.90879	0.90888	0.99998	18334	0.0657
GTPBP10	6	0.90884	0.90893	0.99998	18335	0.1831
PIK3CG	6	0.90888	0.90897	0.99998	18336	0.1535
SLC13A3	6	0.90892	0.90901	0.99998	18337	0.1239
RFPL4AL1	3	0.90895	0.90901	0.99998	18338	0.257
hsa-mir-4715	3	0.90895	0.90901	0.99998	18339	0.1799
hsa-mir-1254-	3	0.90895	0.90901	0.99998	18340	0.215
CBR4	6	0.90898	0.90907	0.99998	18341	0.0716
SCAF8	6	0.90898	0.90907	0.99998	18342	-0.0639
CCDC17	6	0.90898	0.90907	0.99998	18343	0.1803
CD248	6	0.90905	0.90914	0.99998	18344	0.0629
hsa-mir-28	4	0.90927	0.90915	0.99998	18345	0.088
hsa-mir-6848	4	0.90927	0.90915	0.99998	18346	0.0015
DESI2	6	0.90928	0.90936	0.99998	18347	0.0815
USP26	6	0.90928	0.90936	0.99998	18348	0.0606
INSM2	6	0.90937	0.90945	0.99998	18349	0.0041
hsa-mir-3168	4	0.90943	0.90932	0.99998	18350	0.0798
PCDH1	6	0.90948	0.90955	0.99998	18351	-0.0046
RHBDF2	6	0.90948	0.90955	0.99998	18352	0.2103
RRH	6	0.90956	0.90964	0.99998	18353	-0.0798
BEND3	6	0.90963	0.90971	0.99998	18354	0.0143
PHKA1	6	0.90963	0.90971	0.99998	18355	0.1789
CSF3R	5	0.90971	0.90991	0.99998	18356	0.3921
NNAT	6	0.90974	0.90982	0.99998	18357	0.0904
EIF2A	6	0.90974	0.90982	0.99998	18358	0.0405
LOC389895	6	0.90987	0.90994	0.99998	18359	0.0787
USP46	6	0.90987	0.90994	0.99998	18360	0.3324
RRAGB	6	0.90987	0.90994	0.99998	18361	0.1688
MRC1	6	0.90987	0.90994	0.99998	18362	0.1195
AGBL1	6	0.90987	0.90994	0.99998	18363	0.2431
SURF1	6	0.90999	0.91006	0.99998	18364	0.2081
EMR3	6	0.90999	0.91006	0.99998	18365	-0.06
CAPZA1	5	0.91006	0.91027	0.99998	18366	0.5262
CLIP3	6	0.91011	0.91018	0.99998	18367	0.069
ALS2	6	0.91011	0.91018	0.99998	18368	-0.0013
CTH	6	0.9103	0.91037	0.99998	18369	0.2769
CES3	6	0.9103	0.91037	0.99998	18370	0.1625
ERG	6	0.91037	0.91045	0.99998	18371	0.1751
CLDN10	6	0.91041	0.9105	0.99998	18372	0.0668
SCAI	6	0.91044	0.91053	0.99998	18373	0.0576
ZNF212	6	0.91044	0.91053	0.99998	18374	0.0697
HNMT	6	0.91052	0.91062	0.99998	18375	0.101
OR56B1	6	0.91052	0.91062	0.99998	18376	0.3214
PXDNL	5	0.91056	0.91076	0.99998	18377	0.3956
38596	3	0.91059	0.91068	0.99998	18378	0.246
LYAR	6	0.91059	0.91068	0.99998	18379	0.0605
MRPS5	6	0.91064	0.91073	0.99998	18380	0.0351
AP5M1	6	0.91064	0.91073	0.99998	18381	0.0779
hsa-mir-4694	4	0.91066	0.91053	0.99998	18382	0.0428
C1orf65	6	0.91073	0.91082	0.99998	18383	-0.0236
ZNF106	6	0.91082	0.91091	0.99998	18384	0.2183

GPR113	6	0.91082	0.91091	0.99998	18385	0.0915
EFCAB7	6	0.91087	0.91096	0.99998	18386	0.1613
hsa-mir-383	4	0.91092	0.91077	0.99998	18387	0.1469
HOXD4	6	0.91092	0.91101	0.99998	18388	0.0399
ARV1	6	0.91092	0.91101	0.99998	18389	0.0678
CNEP1R1	6	0.911	0.9111	0.99998	18390	0.0249
hsa-mir-7849	2	0.91102	0.911	0.99998	18391	0.1791
C12orf75	6	0.91105	0.91116	0.99998	18392	0.2217
TRMT12	6	0.91114	0.91126	0.99998	18393	-0.0375
FCAMR	6	0.91114	0.91126	0.99998	18394	0.2986
STX6	6	0.91114	0.91126	0.99998	18395	-0.059
FCN1	6	0.91133	0.91145	0.99998	18396	0.1436
FBLIM1	6	0.91133	0.91145	0.99998	18397	-0.0343
PAQR4	6	0.91153	0.91164	0.99998	18398	0.2623
EHMT1	6	0.91153	0.91164	0.99998	18399	0.0037
hsa-let-7e	4	0.91175	0.91162	0.99998	18400	0.0514
CXorf65	6	0.91182	0.91193	0.99998	18401	0.2217
FAM9C	6	0.91191	0.91202	0.99998	18402	0.0886
HPS4	6	0.91196	0.91207	0.99998	18403	0.1431
KIF1C	6	0.91196	0.91207	0.99998	18404	0.3622
CORO7	4	0.91205	0.91192	0.99998	18405	0.1076
PRKD1	6	0.91212	0.91226	0.99998	18406	-0.0164
C6orf223	6	0.91222	0.91236	0.99998	18407	0.2877
SLC22A12	6	0.91243	0.91257	0.99998	18408	-0.0148
BAP1	6	0.91243	0.91257	0.99998	18409	0.1937
FKBP8	6	0.91243	0.91257	0.99998	18410	0.1241
WDR11	6	0.91254	0.91269	0.99998	18411	0.2127
TRIO	6	0.91254	0.91269	0.99998	18412	0.084
KDM1B	6	0.91254	0.91269	0.99998	18413	0.1979
KRT23	4	0.91262	0.91249	0.99998	18414	0.0397
EPB41	6	0.91264	0.91279	0.99998	18415	0.1332
CACNB2	6	0.91274	0.91287	0.99998	18416	0.033
CNTD1	6	0.91274	0.91287	0.99998	18417	0.0856
GJA8	6	0.91279	0.91292	0.99998	18418	0.1983
PRKAR1B	6	0.91279	0.91292	0.99998	18419	0.4056
CD274	6	0.91281	0.91294	0.99998	18420	0.4271
C18orf32	6	0.91293	0.91305	0.99998	18421	0.0351
JPH3	6	0.91293	0.91305	0.99998	18422	0.1495
ZFYVE19	6	0.91293	0.91305	0.99998	18423	-0.0278
SLC37A3	6	0.91303	0.91314	0.99998	18424	0.09
DAPP1	6	0.91303	0.91314	0.99998	18425	-0.0288
REEP1	6	0.91309	0.91321	0.99998	18426	0.2547
CD72	6	0.91318	0.9133	0.99998	18427	0.1618
CALML5	6	0.91325	0.91337	0.99998	18428	0.0411
PARK7	6	0.91334	0.91347	0.99998	18429	0.1228
hsa-mir-6851	4	0.91336	0.91324	0.99998	18430	0.2326
EMCN	6	0.9134	0.91354	0.99998	18431	0.2568
hsa-mir-4319	4	0.91342	0.91331	0.99998	18432	0.0544
TMPRSS6	6	0.91345	0.91358	0.99998	18433	0.0308
FADS2	6	0.91351	0.91365	0.99998	18434	0.1033
ST8SIA3	6	0.91357	0.91371	0.99998	18435	0.0902
GJC2	6	0.91369	0.91381	0.99998	18436	0.1559
WDR48	6	0.91369	0.91381	0.99998	18437	0.2402
RRP12	6	0.91369	0.91381	0.99998	18438	0.0523
hsa-mir-101-1	3	0.91377	0.91383	0.99998	18439	0.3259
LYPD3	6	0.91381	0.91394	0.99998	18440	0.1009
CASK	6	0.91389	0.91401	0.99998	18441	0.0425
ACSBG1	6	0.91397	0.91409	0.99998	18442	0.0274
CEACAM8	6	0.91397	0.91409	0.99998	18443	0.2936
CARD6	6	0.91397	0.91409	0.99998	18444	-0.0736
GUCY2F	6	0.91401	0.91413	0.99998	18445	0.0027
ALS2CR11	6	0.91401	0.91413	0.99998	18446	0.1412
MAP1LC3A	4	0.91403	0.91392	0.99998	18447	0.0442
ACSS1	6	0.9141	0.91422	0.99998	18448	0.0552
PATZ1	6	0.91412	0.91424	0.99998	18449	0.0056
SEMA4C	6	0.9142	0.91432	0.99998	18450	0.417
ABCC4	6	0.91428	0.91439	0.99998	18451	0.1863
ZNF563	5	0.91431	0.91456	0.99998	18452	-0.0152
GBA2	6	0.91438	0.9145	0.99998	18453	0.0449
TMEM209	6	0.91438	0.9145	0.99998	18454	-0.0691
FRRS1L	6	0.91452	0.91463	0.99998	18455	0.1859
ZNF568	6	0.91461	0.91472	0.99998	18456	0.0857
LSM12	6	0.9147	0.91479	0.99998	18457	0.0668
SGSH	6	0.91476	0.91486	0.99998	18458	-0.0287
NEFM	6	0.91482	0.91491	0.99998	18459	0.2272
PABPC5	6	0.915	0.91509	0.99998	18460	0.0263
PRSS22	6	0.9151	0.91519	0.99998	18461	0.3476
TMEM88	6	0.9151	0.91519	0.99998	18462	0.2083
IGSF22	6	0.91517	0.91527	0.99998	18463	0.1917
CALB1	6	0.91517	0.91527	0.99998	18464	0.062
SOWAHA	6	0.91527	0.91538	0.99998	18465	0.1376

ZNF480	6	0.91527	0.91538	0.99998	18466	0.2933
FOXF1	6	0.91527	0.91538	0.99998	18467	0.0635
KLHDC7B	6	0.91539	0.91549	0.99998	18468	0.1347
AKAP12	6	0.91551	0.91562	0.99998	18469	0.0967
GYG2	6	0.91554	0.91566	0.99998	18470	0.2154
hsa-mir-3118-	1	0.91557	0.91599	0.99998	18471	0.8643
SYT11	6	0.91561	0.91572	0.99998	18472	0.1345
NKX2-8	6	0.91561	0.91572	0.99998	18473	0.0976
LRFN2	6	0.91571	0.91582	0.99998	18474	0.076
XRRA1	6	0.91571	0.91582	0.99998	18475	0.141
COLQ	6	0.91577	0.91588	0.99998	18476	0.0982
hsa-mir-1305	4	0.91642	0.91637	0.99998	18477	0.0951
hsa-mir-4283-	4	0.9171	0.91706	0.99998	18478	0.118
hsa-mir-6515	4	0.9171	0.91706	0.99998	18479	0.0956
ARRDC1	6	0.91712	0.91723	0.99998	18480	0.006
OR8B3	6	0.91712	0.91723	0.99998	18481	-0.3109
KRTAP9-4	6	0.91712	0.91723	0.99998	18482	0.2152
XAGE3	6	0.91712	0.91723	0.99998	18483	-0.0397
SLC9A3R1	6	0.91712	0.91723	0.99998	18484	0.0485
UBE2D1	6	0.91712	0.91723	0.99998	18485	0.0778
LYPLA2	6	0.91712	0.91723	0.99998	18486	0.0743
ZNF286B	6	0.91712	0.91723	0.99998	18487	0.1159
ZNF490	6	0.91712	0.91723	0.99998	18488	-0.0032
PRR23A	6	0.91712	0.91723	0.99998	18489	-0.1087
UBB	6	0.91712	0.91723	0.99998	18490	0.1945
ZFP112	6	0.91712	0.91723	0.99998	18491	0.1152
TUBB1	6	0.91712	0.91723	0.99998	18492	0.037
CARD16	6	0.91712	0.91723	0.99998	18493	0.1916
KRTAP10-5	6	0.91712	0.91723	0.99998	18494	-0.0006
ANK3	6	0.91712	0.91723	0.99998	18495	-0.0107
RNASE2	6	0.91712	0.91723	0.99998	18496	0.0078
PCNXL2	6	0.91712	0.91723	0.99998	18497	0.1234
HIST2H3D	6	0.91712	0.91723	0.99998	18498	0.073
CYP11B2	6	0.91712	0.91723	0.99998	18499	-0.157
EVPLL	6	0.91712	0.91723	0.99998	18500	0.3245
SUPT20HL1	6	0.91712	0.91723	0.99998	18501	0.211
ZNF780A	6	0.91712	0.91723	0.99998	18502	-0.1958
CDRT1	6	0.91712	0.91723	0.99998	18503	0.2016
ZNF33A	6	0.91712	0.91723	0.99998	18504	-0.0857
KRTAP5-3	6	0.91712	0.91723	0.99998	18505	0.2506
ZNF708	6	0.91712	0.91723	0.99998	18506	0.2321
ALG10	6	0.91712	0.91723	0.99998	18507	0.3441
NUDT11	6	0.91712	0.91723	0.99998	18508	-0.0063
XAGE2	6	0.91712	0.91723	0.99998	18509	0.2203
OR13C5	6	0.91712	0.91723	0.99998	18510	0.145
ZNF726	5	0.91719	0.91745	0.99998	18511	0.1285
hsa-mir-548i-2	1	0.91732	0.9178	0.99998	18512	0.8834
U2AF1L4	4	0.91738	0.91734	0.99998	18513	0.0857
ZNF479	4	0.91749	0.91745	0.99998	18514	0.2766
hsa-mir-6080	4	0.91799	0.91795	0.99998	18515	0.1266
hsa-mir-5047	4	0.91801	0.91797	0.99998	18516	0.2071
hsa-mir-1261	4	0.91805	0.91801	0.99998	18517	0.4023
ZNF704	4	0.91819	0.91815	0.99998	18518	0.2696
hsa-mir-4495	2	0.91825	0.9182	0.99998	18519	0.4343
RHOC	6	0.91856	0.91864	0.99998	18520	0.3382
IL17F	6	0.91866	0.91876	0.99998	18521	0.1276
KRT1	6	0.91871	0.91883	0.99998	18522	0.053
FGFRL1	6	0.9188	0.91892	0.99998	18523	-0.0348
KIF25	6	0.91883	0.91895	0.99998	18524	-0.0053
OR6C1	6	0.91885	0.91898	0.99998	18525	0.1407
DEFB128	6	0.91892	0.91903	0.99998	18526	0.2118
DNAJC28	6	0.91897	0.91909	0.99998	18527	-0.0018
ZNF606	6	0.919	0.91912	0.99998	18528	0.1345
PDIK1L	6	0.91907	0.91918	0.99998	18529	0.0435
LRRC20	6	0.91907	0.91918	0.99998	18530	0.1657
NAGA	6	0.91907	0.91918	0.99998	18531	0.0905
PRR16	6	0.91907	0.91918	0.99998	18532	-0.0303
TPSG1	6	0.91912	0.91924	0.99998	18533	0.1402
FUOM	6	0.91912	0.91924	0.99998	18534	0.0014
hsa-mir-5571	4	0.91913	0.91908	0.99998	18535	0.508
DRD2	6	0.91931	0.91942	0.99998	18536	0.1974
YY2	6	0.91938	0.91949	0.99998	18537	0.0318
L1CAM	6	0.91946	0.91956	0.99998	18538	0.1496
ZADH2	6	0.91962	0.91971	0.99998	18539	0.1476
CALCA	6	0.91965	0.91973	0.99998	18540	0.2073
GAS2	6	0.91969	0.91978	0.99998	18541	0.0138
LAMB3	6	0.91969	0.91978	0.99998	18542	0.198
CD101	6	0.91983	0.91993	0.99998	18543	0.3252
RRBP1	6	0.91983	0.91993	0.99998	18544	0.0399
BBS10	6	0.91989	0.91999	0.99998	18545	-0.0916
IGFLR1	6	0.91989	0.91999	0.99998	18546	0.0971

hsa-mir-548al	2	0.92002	0.91997	0.99998	18547	0.5464
TMEM26	6	0.92013	0.92023	0.99998	18548	-0.0033
TP53I11	6	0.92013	0.92023	0.99998	18549	0.0132
INIP	6	0.92016	0.92027	0.99998	18550	0.084
TNNT2	6	0.92019	0.92031	0.99998	18551	-0.144
HHEX	6	0.92019	0.92031	0.99998	18552	0.22
MMP2	6	0.92027	0.92039	0.99998	18553	0.1109
MAMSTR	6	0.92037	0.92049	0.99998	18554	0.2182
AIG1	6	0.92037	0.92049	0.99998	18555	0.1117
ALG8	6	0.92045	0.92058	0.99998	18556	0.0022
SIX4	6	0.92051	0.92064	0.99998	18557	0.0831
TRIM59	6	0.92059	0.92071	0.99998	18558	0.1477
MPND	6	0.92059	0.92071	0.99998	18559	0.0443
CPB2	6	0.92066	0.92078	0.99998	18560	0.0388
B3GALNT1	6	0.92069	0.92082	0.99998	18561	0.1045
RAB35	6	0.92073	0.92085	0.99998	18562	0.0821
hsa-mir-4786	4	0.92087	0.92088	0.99998	18563	0.1223
PADI2	6	0.92092	0.92104	0.99998	18564	0.2573
hsa-mir-4271	4	0.92094	0.92095	0.99998	18565	0.1498
SZRD1	6	0.92102	0.92113	0.99998	18566	0.0082
CXCR7	3	0.92103	0.9211	0.99998	18567	0.1656
QSER1	6	0.9211	0.92121	0.99998	18568	0.1011
FNIP1	6	0.92122	0.92133	0.99998	18569	-0.032
ZNF175	6	0.92122	0.92133	0.99998	18570	-0.1039
DIRC2	6	0.92128	0.9214	0.99998	18571	0.2064
PPP1R32	6	0.92133	0.92144	0.99998	18572	0.1815
CABS1	6	0.92133	0.92144	0.99998	18573	0.136
NEFH	6	0.92133	0.92144	0.99998	18574	0.0577
MDH1	6	0.92139	0.92151	0.99998	18575	0.0295
OR2S2	6	0.92144	0.92156	0.99998	18576	0.1153
PCGF5	6	0.92148	0.9216	0.99998	18577	0.0782
TMEM91	6	0.92163	0.92173	0.99998	18578	0.1634
TTLL12	6	0.92163	0.92173	0.99998	18579	0.1089
TBPL1	6	0.92163	0.92173	0.99998	18580	0.1629
NR3C2	6	0.92167	0.92178	0.99998	18581	0.157
hsa-mir-4289	4	0.92168	0.92173	0.99998	18582	0.2322
LGR5	6	0.92172	0.92181	0.99998	18583	0.1559
RFNG	6	0.92182	0.9219	0.99998	18584	0.2004
FERMT2	6	0.92185	0.92193	0.99998	18585	0.0839
RAB9B	6	0.92185	0.92193	0.99998	18586	0.2397
TAS1R3	6	0.92201	0.92208	0.99998	18587	0.2264
IRF2BP1	6	0.92206	0.92214	0.99998	18588	0.1384
hsa-mir-2682	4	0.92209	0.92216	0.99998	18589	0.2515
PROX2	6	0.92216	0.92223	0.99998	18590	0.3458
KIF15	6	0.92216	0.92223	0.99998	18591	0.0526
SYCE1	6	0.92237	0.92244	0.99998	18592	0.171
DCST2	6	0.92237	0.92244	0.99998	18593	0.1172
hsa-mir-4752	4	0.92238	0.92244	0.99998	18594	0.0305
NSMCE2	6	0.92241	0.92249	0.99998	18595	-0.0542
hsa-mir-514a-	1	0.92241	0.92281	0.99998	18596	0.6747
SEMG2	5	0.92244	0.92275	0.99998	18597	-0.0057
PRR12	6	0.92254	0.92263	0.99998	18598	0.1116
CALCR	6	0.92255	0.92264	0.99998	18599	0.2927
ACSM4	6	0.92267	0.92276	0.99998	18600	0.2511
ZNF664-FAM1	4	0.92284	0.9229	0.99998	18601	0.1731
TMEM203	6	0.92289	0.92297	0.99998	18602	0.0578
KRTAP4-4	6	0.92289	0.92297	0.99998	18603	0.4594
PABPC3	6	0.92289	0.92297	0.99998	18604	0.0798
NECAB2	6	0.92289	0.92297	0.99998	18605	0.1955
DUSP9	6	0.92289	0.92297	0.99998	18606	0.1143
OTOR	6	0.92307	0.92315	0.99998	18607	0.199
LOC441155	6	0.92307	0.92315	0.99998	18608	0.1872
CLEC2A	4	0.92309	0.92318	0.99998	18609	0.11
KIF2B	6	0.92311	0.92319	0.99998	18610	0.1058
SLC10A4	6	0.92318	0.92326	0.99998	18611	-0.0127
CDK5	6	0.92321	0.92329	0.99998	18612	0.1451
KBTBD3	6	0.92325	0.92333	0.99998	18613	0.1686
UAP1L1	6	0.92325	0.92333	0.99998	18614	0.1297
TAS2R41	6	0.92343	0.92353	0.99998	18615	0.1278
SLC2A10	6	0.92347	0.92357	0.99998	18616	0.087
SPTBN4	6	0.92347	0.92357	0.99998	18617	0.0812
RYR1	6	0.92347	0.92357	0.99998	18618	0.1394
ZNF705B	3	0.92348	0.92356	0.99998	18619	0.0714
NMU	6	0.92353	0.92362	0.99998	18620	0.2404
POLR3D	6	0.92357	0.92366	0.99998	18621	-0.0533
hsa-mir-3938	4	0.9236	0.92371	0.99998	18622	0.1489
SLC44A5	6	0.9236	0.92369	0.99998	18623	0.1643
KAT2B	6	0.92375	0.92384	0.99998	18624	0.1595
RBAK-LOC389	2	0.92384	0.92373	0.99998	18625	0.3246
PAPD5	6	0.92385	0.92393	0.99998	18626	0.2353
PCDHB9	6	0.92391	0.92399	0.99998	18627	0.3038

KCNIP3	6	0.92391	0.92399	0.99998	18628	0.0003
SNTB2	6	0.92391	0.92399	0.99998	18629	0.1937
SPATA2	6	0.92391	0.92399	0.99998	18630	0.1181
hsa-mir-4801	3	0.92401	0.92409	0.99998	18631	0.1578
AKR1E2	6	0.92403	0.92413	0.99998	18632	-0.0489
DEFB114	6	0.92406	0.92417	0.99998	18633	0.1069
WDPCP	6	0.92406	0.92417	0.99998	18634	0.0949
PROM2	6	0.92418	0.92428	0.99998	18635	-0.0114
PCDHB2	6	0.92418	0.92428	0.99998	18636	0.2196
MEST	6	0.92424	0.92436	0.99998	18637	0.0889
MROH7	6	0.92424	0.92436	0.99998	18638	0.0079
FXYD1	6	0.92424	0.92436	0.99998	18639	0.0284
ANKRD29	6	0.92433	0.92445	0.99998	18640	0.2195
hsa-mir-2467	4	0.92444	0.9245	0.99998	18641	0.0316
MST4	6	0.92444	0.92457	0.99998	18642	0.1143
AADAC	6	0.9245	0.92463	0.99998	18643	0.3313
C19orf44	6	0.92456	0.92468	0.99998	18644	-0.0013
PLEKHA2	6	0.92461	0.92473	0.99998	18645	0.1133
KCNK6	6	0.92465	0.92478	0.99998	18646	0.093
DOLK	6	0.9247	0.92482	0.99998	18647	0.0834
DYNC1I2	6	0.92473	0.92486	0.99998	18648	0.0599
ACY3	6	0.92473	0.92486	0.99998	18649	0.1413
SST	6	0.9248	0.92493	0.99998	18650	0.0609
FAM198B	6	0.9248	0.92493	0.99998	18651	0.2492
ANKDD1B	6	0.92486	0.92499	0.99998	18652	0.1209
NAV2	6	0.92486	0.92499	0.99998	18653	0.2256
ZNF230	6	0.92497	0.92511	0.99998	18654	0.1955
hsa-mir-365b	4	0.92509	0.92516	0.99998	18655	0.3327
PIRT	6	0.92512	0.92527	0.99998	18656	-0.0528
S1PR3	6	0.92516	0.92531	0.99998	18657	0.1357
ARHGAP18	6	0.92516	0.92531	0.99998	18658	0.2363
FBXL2	6	0.92524	0.92537	0.99998	18659	0.2187
hsa-mir-4664	4	0.92525	0.92534	0.99998	18660	0.0537
CSF3	4	0.92525	0.92534	0.99998	18661	0.2155
NACC1	6	0.92531	0.92543	0.99998	18662	0.0776
MYO18A	4	0.92532	0.92539	0.99998	18663	0.2331
SRPRB	6	0.92537	0.9255	0.99998	18664	0.262
TRIM33	6	0.92537	0.9255	0.99998	18665	0.0985
HAPLN4	6	0.92537	0.9255	0.99998	18666	0.1317
STAT3	6	0.92553	0.92566	0.99998	18667	0.1079
NAALAD2	6	0.92553	0.92566	0.99998	18668	0.1147
DCLK3	6	0.92558	0.92572	0.99998	18669	0.2938
CNNM4	6	0.92567	0.92581	0.99998	18670	0.0231
C9orf24	6	0.92572	0.92586	0.99998	18671	0.2374
STARD8	6	0.92578	0.92591	0.99998	18672	0.0529
LOH12CR1	6	0.92583	0.92597	0.99998	18673	-0.0331
GPRIN3	6	0.92583	0.92597	0.99998	18674	0.0497
CLIC3	6	0.92583	0.92597	0.99998	18675	0.3554
ENOPH1	6	0.92588	0.92602	0.99998	18676	0.1647
LMNB2	6	0.92588	0.92602	0.99998	18677	0.134
hsa-mir-664a	4	0.92588	0.92596	0.99998	18678	0.128
COPS7A	6	0.92594	0.92608	0.99998	18679	0.2293
ST3GAL5	6	0.92594	0.92608	0.99998	18680	0.1043
VSX1	6	0.92602	0.92616	0.99998	18681	0.1151
hsa-mir-498	4	0.92603	0.92611	0.99998	18682	0.1559
ADAM30	6	0.92608	0.92621	0.99998	18683	0.097
MOSPD1	6	0.92608	0.92621	0.99998	18684	0.1871
PDXDC1	6	0.92612	0.92624	0.99998	18685	0.0543
C16orf58	6	0.92613	0.92625	0.99998	18686	0.1447
PCDHGB7	2	0.92619	0.92611	0.99998	18687	0.3366
PDE7A	6	0.92627	0.92639	0.99998	18688	-0.0031
HOXA10	6	0.92627	0.92639	0.99998	18689	0.182
IL20	6	0.92632	0.92644	0.99998	18690	0.2166
NLRP2	6	0.92636	0.92648	0.99998	18691	0.0792
TDRD6	6	0.92636	0.92648	0.99998	18692	0.0019
GLYR1	6	0.92636	0.92648	0.99998	18693	0.0671
hsa-mir-4757	4	0.92636	0.92644	0.99998	18694	0.0738
GRB10	6	0.92656	0.9267	0.99998	18695	0.0625
TNFAIP3	6	0.92662	0.92675	0.99998	18696	0.2533
hsa-mir-4676	4	0.92663	0.92674	0.99998	18697	0.1758
CISH	6	0.92666	0.92678	0.99998	18698	0.0239
ECHS1	6	0.9267	0.92683	0.99998	18699	0.1592
AKR1C4	6	0.9267	0.92683	0.99998	18700	0.2372
SLC15A3	6	0.92678	0.92691	0.99998	18701	0.062
NXT2	6	0.92678	0.92691	0.99998	18702	0.1034
C1orf204	6	0.92678	0.92691	0.99998	18703	0.0412
COL24A1	6	0.92691	0.92704	0.99998	18704	0.1039
SCRN2	6	0.92705	0.92718	0.99998	18705	0.0621
SPARC	6	0.92733	0.92744	0.99998	18706	-0.0071
HKDC1	6	0.92733	0.92744	0.99998	18707	0.055
hsa-mir-190b	4	0.92742	0.92751	0.99998	18708	0.1668

MRVI1	6	0.92746	0.92757	0.99998	18709	0.3
OR5D16	6	0.9275	0.92761	0.99998	18710	0.0267
CDIP1	6	0.9275	0.92761	0.99998	18711	0.1623
COX6B2	6	0.9275	0.92761	0.99998	18712	0.0745
SEMA3A	6	0.92756	0.92767	0.99998	18713	0.2175
ZNF207	6	0.92757	0.92768	0.99998	18714	0.1987
SERPINB1	6	0.92758	0.92769	0.99998	18715	0.1954
OR8G5	5	0.92759	0.92792	0.99998	18716	0.1137
AGPAT5	6	0.92763	0.92774	0.99998	18717	0.237
FRMD8	6	0.92763	0.92774	0.99998	18718	0.0929
DCAF12L2	6	0.92763	0.92774	0.99998	18719	0.2543
LCN6	6	0.9277	0.92781	0.99998	18720	0.1928
RAMP2	6	0.9277	0.92781	0.99998	18721	0.3121
ATRNL1	6	0.9277	0.92781	0.99998	18722	-0.1011
GPR82	6	0.92782	0.92794	0.99998	18723	0.1415
POPDC3	6	0.92791	0.92803	0.99998	18724	0.178
OR9G4	6	0.92798	0.9281	0.99998	18725	-0.0132
VCAN	6	0.92806	0.92816	0.99998	18726	0.158
CYLC2	6	0.92806	0.92816	0.99998	18727	0.1854
ZNF329	6	0.92806	0.92816	0.99998	18728	0.0556
hsa-mir-4634	4	0.92807	0.92818	0.99998	18729	0.3476
PHOX2B	6	0.92814	0.92824	0.99998	18730	-0.1582
EXO1	6	0.92825	0.92835	0.99998	18731	0.0036
TM4SF20	6	0.92832	0.92842	0.99998	18732	0.1539
NAPRT1	6	0.92832	0.92842	0.99998	18733	0.1106
REG3A	6	0.92841	0.92853	0.99998	18734	0.2821
CASP5	6	0.92841	0.92853	0.99998	18735	0.1731
SH3RF1	6	0.92853	0.92864	0.99998	18736	-0.0002
MRPS15	6	0.9286	0.92871	0.99998	18737	0.0391
CCDC134	6	0.9286	0.92871	0.99998	18738	0.1558
GABRE	6	0.92863	0.92875	0.99998	18739	0.0347
ANKRD12	6	0.92869	0.92881	0.99998	18740	0.1533
SLC12A5	6	0.92881	0.92893	0.99998	18741	0.1826
ZMYM2	6	0.92898	0.92911	0.99998	18742	0.1338
B3GNT1	6	0.92898	0.92911	0.99998	18743	0.3042
CENPC	2	0.929	0.92894	0.99998	18744	0.3229
ZFPM2	6	0.92902	0.92914	0.99998	18745	0.1343
hsa-mir-3605	4	0.92918	0.92923	0.99998	18746	0.0224
hsa-mir-519c	2	0.92923	0.92918	0.99998	18747	0.9041
hsa-mir-519a-	2	0.92923	0.92918	0.99998	18748	0.5736
PNMAL2	6	0.92924	0.92938	0.99998	18749	-0.0495
SCNN1B	6	0.92926	0.9294	0.99998	18750	0.0893
LHB	5	0.92931	0.92962	0.99998	18751	0.1317
ZNF705D	1	0.92931	0.92954	0.99998	18752	0.448
PYGO1	6	0.92931	0.92946	0.99998	18753	0.162
PITPNM3	6	0.92931	0.92946	0.99998	18754	0.0859
YPEL2	6	0.92952	0.92964	0.99998	18755	0.0501
TACR1	6	0.92952	0.92964	0.99998	18756	0.029
PDLIM1	6	0.92959	0.92972	0.99998	18757	0.0059
CCDC7	6	0.92967	0.92979	0.99998	18758	0.1628
DISC1	4	0.92967	0.92971	0.99998	18759	0.215
C8orf58	6	0.92974	0.92985	0.99998	18760	0.0288
LENG8	6	0.92974	0.92985	0.99998	18761	0.0958
CNKSR1	6	0.92974	0.92985	0.99998	18762	0.0589
LRRC49	6	0.92978	0.9299	0.99998	18763	0.2173
SLCO1B1	6	0.92984	0.92997	0.99998	18764	0.1872
SRGAP3	6	0.92984	0.92997	0.99998	18765	0.2042
BCAT1	6	0.92984	0.92997	0.99998	18766	0.058
CYB5R1	6	0.92984	0.92997	0.99998	18767	0.145
ME3	6	0.92984	0.92997	0.99998	18768	-0.1308
C15orf54	6	0.9299	0.93003	0.99998	18769	-0.0535
SFMBT2	6	0.9299	0.93003	0.99998	18770	0.2162
CHRM3	6	0.92992	0.93004	0.99998	18771	0.0576
PRKCG	6	0.92996	0.93008	0.99998	18772	0.0637
TRPM7	6	0.93004	0.93016	0.99998	18773	0.1985
GAL3ST3	6	0.93004	0.93016	0.99998	18774	0.0568
SCN7A	6	0.93004	0.93016	0.99998	18775	0.1765
MOCS2	6	0.93011	0.93022	0.99998	18776	0.0597
hsa-mir-1275	4	0.93017	0.93021	0.99998	18777	0.1899
OR51E2	6	0.93022	0.93033	0.99998	18778	-0.0289
GCC2	6	0.93023	0.93034	0.99998	18779	-0.0223
BEST3	6	0.93024	0.93035	0.99998	18780	0.158
ZNF215	6	0.93027	0.93038	0.99998	18781	0.3088
GDPD1	6	0.93027	0.93038	0.99998	18782	0.0101
ALDH4A1	6	0.93034	0.93045	0.99998	18783	0.0412
GRM1	6	0.93039	0.93049	0.99998	18784	0.1981
RPS6KB2	6	0.93047	0.93057	0.99998	18785	0.2087
PIKFYVE	6	0.93053	0.93063	0.99998	18786	0.0369
TRMU	6	0.93069	0.9308	0.99998	18787	0.1294
ENTPD5	6	0.93075	0.93086	0.99998	18788	0.0505
CRTC1	6	0.93075	0.93086	0.99998	18789	0.2432

hsa-mir-4496	2	0.93081	0.93077	0.99998	18790	0.2602
TMEM132B	6	0.93086	0.93097	0.99998	18791	0.0321
CRYGN	6	0.93089	0.93101	0.99998	18792	0.0986
SLC35G1	6	0.93089	0.93101	0.99998	18793	-0.0079
AOAH	6	0.93099	0.93111	0.99998	18794	0.0762
KATNBL1	6	0.93108	0.9312	0.99998	18795	0.1237
UBE2D4	6	0.93108	0.9312	0.99998	18796	-0.0796
NPTXR	6	0.93118	0.9313	0.99998	18797	-0.0747
DNAJC7	6	0.93118	0.9313	0.99998	18798	0.0045
PLOD2	6	0.93127	0.93139	0.99998	18799	0.1359
C1orf185	6	0.93127	0.93139	0.99998	18800	0.096
SLC18A2	6	0.93138	0.9315	0.99998	18801	0.1275
ITFG2	6	0.93144	0.93155	0.99998	18802	0.1104
SH3RF3	6	0.93148	0.93159	0.99998	18803	-0.023
POLR2A	6	0.93152	0.93162	0.99998	18804	0.1214
LASP1	6	0.93158	0.93169	0.99998	18805	0.0641
ACTG2	5	0.93164	0.93195	0.99998	18806	0.0529
EFCAB13	6	0.93164	0.93176	0.99998	18807	0.196
CDH8	6	0.93173	0.93186	0.99998	18808	0.0764
GLDC	6	0.93173	0.93186	0.99998	18809	0.1117
ZIC1	6	0.93173	0.93186	0.99998	18810	0.0759
MBD3L1	6	0.9319	0.93203	0.99998	18811	0.1044
hsa-mir-6807	4	0.93195	0.93194	0.99998	18812	0.2422
SAMD9L	6	0.93201	0.93212	0.99998	18813	0.1305
CYTL1	6	0.93211	0.93224	0.99998	18814	0.0272
NES	6	0.93211	0.93224	0.99998	18815	-0.0252
C19orf59	6	0.93211	0.93224	0.99998	18816	0.1434
PLEKHB1	6	0.93216	0.93229	0.99998	18817	0.0168
KLRC3	5	0.9322	0.93248	0.99998	18818	0.2276
OR6K2	6	0.93228	0.93241	0.99998	18819	-0.0545
IER2	6	0.93228	0.93241	0.99998	18820	0.0082
SMTN	6	0.93228	0.93241	0.99998	18821	0.1019
FOXG1	4	0.9323	0.93228	0.99998	18822	0.2519
TARP	4	0.9323	0.93228	0.99998	18823	0.1183
TEX19	6	0.93235	0.93247	0.99998	18824	0.2241
HEMGN	6	0.93235	0.93247	0.99998	18825	0.141
RFX8	6	0.9324	0.93252	0.99998	18826	0.1935
hsa-mir-409	4	0.9324	0.93238	0.99998	18827	0.1904
LRRC3	6	0.93245	0.93257	0.99998	18828	0.0005
FGFR3	6	0.93252	0.93263	0.99998	18829	0.0743
SECTM1	6	0.93255	0.93266	0.99998	18830	0.1759
ENTPD1	6	0.93262	0.93274	0.99998	18831	0.0783
CCDC114	6	0.93268	0.9328	0.99998	18832	-0.0154
hsa-mir-6764	4	0.93276	0.93274	0.99998	18833	0.0671
FAM122C	6	0.93286	0.93297	0.99998	18834	0.0441
PARK2	6	0.9329	0.93301	0.99998	18835	0.3709
PTPN14	6	0.93297	0.93308	0.99998	18836	0.047
NCKAP5L	6	0.93297	0.93308	0.99998	18837	0.1269
OSTN	6	0.93297	0.93308	0.99998	18838	0.0558
CSNK2A2	6	0.93297	0.93308	0.99998	18839	0.283
PSG4	6	0.93307	0.93317	0.99998	18840	0.4511
MDM2	6	0.93312	0.93323	0.99998	18841	0.1479
POLG2	6	0.93321	0.93332	0.99998	18842	0.0707
GRM4	6	0.93321	0.93332	0.99998	18843	0.1074
hsa-mir-558	4	0.93329	0.93328	0.99998	18844	0.1742
C14orf182	6	0.93348	0.93358	0.99998	18845	0.1766
TMX1	6	0.9335	0.9336	0.99998	18846	0.1373
PRKAR2B	6	0.93359	0.93369	0.99998	18847	0.107
DPYSL4	6	0.93359	0.93369	0.99998	18848	0.0686
MTMR11	6	0.93367	0.93378	0.99998	18849	0.1551
IGFN1	6	0.93367	0.93378	0.99998	18850	0.1516
RFPL4B	6	0.93367	0.93378	0.99998	18851	0.1371
hsa-mir-5196	4	0.93369	0.93367	0.99998	18852	0.1884
CTHRC1	6	0.93372	0.93383	0.99998	18853	0.0539
PEX11G	6	0.93377	0.93388	0.99998	18854	0.0911
TP53I13	6	0.93377	0.93388	0.99998	18855	0.0502
FAM120C	6	0.9339	0.93402	0.99998	18856	0.1371
CCDC142	4	0.93406	0.93403	0.99998	18857	0.0736
TXNDC2	6	0.9341	0.9342	0.99998	18858	0.1745
RAB11FIP2	6	0.93413	0.93424	0.99998	18859	0.1757
PRSS42	6	0.93415	0.93426	0.99998	18860	-0.0038
PKNOX1	6	0.93426	0.93437	0.99998	18861	0.0324
MST1R	6	0.93429	0.9344	0.99998	18862	0.2255
NSG1	6	0.93433	0.93443	0.99998	18863	0.0407
ERC1	6	0.93433	0.93443	0.99998	18864	0.016
CRYZL1	6	0.93433	0.93443	0.99998	18865	-0.0546
ZSCAN29	6	0.93437	0.93447	0.99998	18866	0.2319
GALNT16	6	0.93449	0.9346	0.99998	18867	0.2217
ICAM5	6	0.93449	0.9346	0.99998	18868	0.2058
PRTN3	6	0.93453	0.93464	0.99998	18869	0.1892
POC5	6	0.93462	0.93472	0.99998	18870	0.1181

OR5T1	6	0.93462	0.93472	0.99998	18871	0.1409
UBE3B	6	0.93467	0.93477	0.99998	18872	-0.0191
PRSS1	6	0.93467	0.93477	0.99998	18873	0.0474
RNF180	6	0.93471	0.93481	0.99998	18874	0.1259
MT1E	4	0.9348	0.93477	0.99998	18875	0.5971
UACA	6	0.93483	0.93493	0.99998	18876	-0.0403
ROBO4	6	0.9349	0.935	0.99998	18877	0.1462
ARHGEF12	6	0.93501	0.93509	0.99998	18878	-0.0614
RIMS3	6	0.93501	0.93509	0.99998	18879	0.2528
PCCA	5	0.93505	0.9353	0.99998	18880	0.2999
IDS	6	0.93507	0.93514	0.99998	18881	0.0658
hsa-mir-1323	4	0.93516	0.93512	0.99998	18882	0.1908
CAPN15	5	0.93524	0.93548	0.99998	18883	0.1828
MAP1LC3B	5	0.93541	0.93565	0.99998	18884	0.1291
TMC3	6	0.93542	0.93549	0.99998	18885	0.017
hsa-mir-6761	4	0.93545	0.9354	0.99998	18886	0.2451
hsa-mir-4668	4	0.93545	0.9354	0.99998	18887	0.0724
KCTD14	6	0.93549	0.93556	0.99998	18888	0.0088
ISX	6	0.93551	0.93559	0.99998	18889	0.1305
SLC38A10	6	0.93553	0.93561	0.99998	18890	0.1919
hsa-mir-3606	4	0.93557	0.93551	0.99998	18891	0.225
HIST2H4A	1	0.93558	0.93582	0.99998	18892	0.4549
HGF	6	0.9356	0.93567	0.99998	18893	0.2022
AP3S2	4	0.93568	0.93562	0.99998	18894	0.1212
UBE2NL	6	0.93571	0.93578	0.99998	18895	0.1323
RAD1	6	0.93575	0.93582	0.99998	18896	-0.017
RANBP2	3	0.93578	0.93594	0.99998	18897	0.124
TMEM92	6	0.93592	0.93598	0.99998	18898	0.0118
PI15	6	0.93592	0.93598	0.99998	18899	0.1676
RABGEF1	6	0.93597	0.93604	0.99998	18900	0.1087
NABP1	6	0.93597	0.93604	0.99998	18901	0.0893
TRO	6	0.93599	0.93606	0.99998	18902	0.1313
SLC6A18	6	0.93606	0.93613	0.99998	18903	-0.0421
TMPRSS11F	6	0.93606	0.93613	0.99998	18904	0.0007
AKR1C2	5	0.93608	0.93634	0.99998	18905	0.2133
ZNF486	5	0.93608	0.93634	0.99998	18906	0.4491
CYP4A22	5	0.93608	0.93634	0.99998	18907	-0.0551
CYP2A6	5	0.93608	0.93634	0.99998	18908	0.0422
hsa-mir-196b	4	0.93609	0.93602	0.99998	18909	0.1349
YES1	6	0.93619	0.93625	0.99998	18910	0.2821
CNDP1	6	0.93619	0.93625	0.99998	18911	0.2189
PTPN3	6	0.93619	0.93625	0.99998	18912	0.1309
CFP	6	0.93625	0.93633	0.99998	18913	-0.0431
hsa-mir-6082	4	0.9363	0.93623	0.99998	18914	0.1881
STK16	6	0.93633	0.9364	0.99998	18915	0.1822
MGAT4A	6	0.93645	0.93653	0.99998	18916	-0.0262
GK	6	0.93645	0.93653	0.99998	18917	-0.0266
C19orf70	6	0.93657	0.93665	0.99998	18918	0.0693
C8orf4	6	0.93661	0.93668	0.99998	18919	-0.0515
C5orf49	6	0.93661	0.93668	0.99998	18920	0.0486
CCDC23	4	0.93661	0.93655	0.99998	18921	0.1167
ITM2B	6	0.93673	0.9368	0.99998	18922	0.1306
CXCR1	6	0.93678	0.93686	0.99998	18923	0.5309
KCND1	6	0.93687	0.93695	0.99998	18924	0.078
SNRK	6	0.93692	0.937	0.99998	18925	0.2032
ZEB1	4	0.93694	0.93689	0.99998	18926	0.1997
ATF2	6	0.93697	0.93705	0.99998	18927	0.1943
ARMCX3	6	0.93701	0.93709	0.99998	18928	0.0387
PTPN2	6	0.93706	0.93714	0.99998	18929	0.3939
ACSS2	4	0.93713	0.93708	0.99998	18930	0.1036
NUCB1	6	0.93719	0.93728	0.99998	18931	0.2204
AP4M1	6	0.93719	0.93728	0.99998	18932	0.1327
LDB1	6	0.9372	0.93729	0.99998	18933	0.1195
hsa-mir-6772	4	0.93723	0.93719	0.99998	18934	0.2505
SMPD2	6	0.93733	0.93743	0.99998	18935	0.1571
OLR1	6	0.93733	0.93743	0.99998	18936	0.3371
NDUFA4	6	0.93733	0.93743	0.99998	18937	0.0655
MYPN	6	0.93744	0.93754	0.99998	18938	0.0055
LYSMD1	6	0.93744	0.93754	0.99998	18939	0.1173
ACTRT3	4	0.93745	0.93741	0.99998	18940	0.0379
MEMO1	6	0.93751	0.93761	0.99998	18941	0.2158
OR13D1	6	0.93751	0.93761	0.99998	18942	0.3773
hsa-mir-6073	4	0.93757	0.93752	0.99998	18943	0.3311
FMO4	6	0.93759	0.93769	0.99998	18944	-0.0239
C1orf177	6	0.93765	0.93775	0.99998	18945	-0.0305
ATG16L1	6	0.93782	0.93793	0.99998	18946	0.2281
hsa-mir-711	4	0.93782	0.93776	0.99998	18947	0.1142
KLF13	6	0.93805	0.93816	0.99998	18948	0.123
KIF13B	6	0.93809	0.9382	0.99998	18949	0.2282
TESPA1	6	0.93813	0.93824	0.99998	18950	0.2441
CCDC172	6	0.93827	0.93837	0.99998	18951	0.0196

ZFAND5	6	0.93834	0.93844	0.99998	18952	0.1058
DPP9	6	0.93847	0.93856	0.99998	18953	0.0112
KLRC2	5	0.93847	0.93866	0.99998	18954	0.4695
DPY19L3	6	0.93855	0.93866	0.99998	18955	-0.0353
SOX7	6	0.93858	0.93869	0.99998	18956	0.0836
ABCC10	4	0.93877	0.93869	0.99998	18957	0.0578
MFSD7	6	0.93889	0.93899	0.99998	18958	0.066
KAT2A	6	0.93893	0.93903	0.99998	18959	0.0337
SIGLEC9	6	0.93893	0.93903	0.99998	18960	0.2148
NRROS	3	0.93894	0.93914	0.99998	18961	0.2015
hsa-mir-147a	3	0.93903	0.93922	0.99998	18962	0.1717
GPR112	6	0.93907	0.93916	0.99998	18963	0.1026
OR8B12	6	0.93907	0.93916	0.99998	18964	0.2111
SECISBP2L	6	0.93913	0.93922	0.99998	18965	0.1361
WBP11	6	0.93913	0.93922	0.99998	18966	0.0525
CHST9	6	0.9392	0.93927	0.99998	18967	0.1404
ADPRHL1	6	0.9392	0.93927	0.99998	18968	-0.0656
CLPSL1	6	0.9392	0.93927	0.99998	18969	0.0635
KRT222	6	0.93924	0.93931	0.99998	18970	0.2455
MAN1B1	6	0.93931	0.93937	0.99998	18971	0.0883
ENPP2	6	0.93931	0.93937	0.99998	18972	0.1581
FAT2	6	0.9394	0.93946	0.99998	18973	0.0879
CUL5	6	0.93945	0.9395	0.99998	18974	0.3365
CCNY	6	0.93945	0.9395	0.99998	18975	0.081
HINT1	6	0.93945	0.9395	0.99998	18976	0.0273
KCTD20	6	0.93951	0.93956	0.99998	18977	0.0072
SBF1	6	0.93951	0.93956	0.99998	18978	0.1435
BEGAIN	6	0.93957	0.93962	0.99998	18979	0.2252
COX5B	6	0.93959	0.93964	0.99998	18980	0.2273
CD83	6	0.93963	0.93967	0.99998	18981	0.2077
CCDC155	6	0.93968	0.93972	0.99998	18982	0.1448
AZU1	4	0.9397	0.93968	0.99998	18983	0.066
SPARCL1	6	0.9397	0.93975	0.99998	18984	0.1167
TDRD3	6	0.93972	0.93977	0.99998	18985	0.1561
LRRC32	6	0.93974	0.93979	0.99998	18986	0.2561
hsa-mir-4999	4	0.93975	0.93972	0.99998	18987	0.225
CR1L	5	0.93975	0.93995	0.99998	18988	0.179
LOC10013070	6	0.93981	0.93988	0.99998	18989	0.0973
URM1	6	0.93981	0.93988	0.99998	18990	0.1079
SND1	6	0.93981	0.93988	0.99998	18991	0.1044
hsa-mir-6786	4	0.93995	0.93992	0.99998	18992	0.1997
FTL	6	0.94001	0.94008	0.99998	18993	0.0467
PLEC	6	0.94001	0.94008	0.99998	18994	0.2697
ARL6IP1	6	0.94001	0.94008	0.99998	18995	0.1649
DIP2A	6	0.9401	0.94016	0.99998	18996	0.0156
ZNF473	6	0.9401	0.94016	0.99998	18997	0.2863
FBXO45	4	0.94016	0.94011	0.99998	18998	0.1159
LYSMD3	6	0.94016	0.94022	0.99998	18999	0.0161
OTUD7A	4	0.94022	0.94018	0.99998	19000	0.1752
MRO	6	0.94023	0.94028	0.99998	19001	0.1446
DCAF4	6	0.94033	0.94038	0.99998	19002	0.1084
SOCS7	6	0.94039	0.94044	0.99998	19003	0.2199
ACSM3	6	0.94039	0.94044	0.99998	19004	0.196
PEX12	6	0.94044	0.94049	0.99998	19005	0.1046
TM7SF3	6	0.94044	0.94049	0.99998	19006	0.046
UBC	6	0.94044	0.94049	0.99998	19007	0.1153
hsa-mir-4274	4	0.94049	0.94045	0.99998	19008	0.1328
ZNRF2	6	0.94066	0.94071	0.99998	19009	0.0284
CAPS	4	0.94076	0.9407	0.99998	19010	0.0594
MTRNR2L6	6	0.94081	0.94086	0.99998	19011	0.0819
TMEM235	6	0.94092	0.94096	0.99998	19012	0.2284
RASGEF1B	6	0.94095	0.94099	0.99998	19013	0.3674
CLEC5A	6	0.94095	0.94099	0.99998	19014	-0.0062
GRK4	6	0.94102	0.94107	0.99998	19015	0.0667
GPLD1	6	0.94107	0.94112	0.99998	19016	0.1678
DEFB127	6	0.94109	0.94114	0.99998	19017	0.0249
DMRT2	6	0.94111	0.94116	0.99998	19018	0.0575
BTG4	6	0.94116	0.94123	0.99998	19019	0.1756
ZNF366	6	0.94116	0.94123	0.99998	19020	0.0124
PYGL	6	0.94126	0.94134	0.99998	19021	0.1774
GNAQ	6	0.94126	0.94134	0.99998	19022	0.1708
BRCA2	6	0.94135	0.94144	0.99998	19023	-0.0642
CERK	6	0.94142	0.94151	0.99998	19024	0.1762
MDFIC	6	0.94147	0.94155	0.99998	19025	0.251
TRIM13	6	0.94151	0.94159	0.99998	19026	0.12
LEPROT	6	0.94151	0.94159	0.99998	19027	0.0932
S1PR2	6	0.94151	0.94159	0.99998	19028	0.0828
SMPX	6	0.94162	0.9417	0.99998	19029	0.2839
FAM105A	6	0.94165	0.94173	0.99998	19030	0.159
NDNF	6	0.9417	0.94178	0.99998	19031	0.278
ABHD8	6	0.94178	0.94186	0.99998	19032	0.1794

DSCR4	6	0.94184	0.9419	0.99998	19033	-0.0116
SFRP4	6	0.9419	0.94196	0.99998	19034	0.152
ARSB	6	0.9419	0.94196	0.99998	19035	0.1047
SOX30	6	0.94202	0.94208	0.99998	19036	0.1233
ADRA2A	6	0.94202	0.94208	0.99998	19037	0.1754
hsa-mir-4424	4	0.94213	0.94209	0.99998	19038	0.1932
ZNF337	6	0.94216	0.94221	0.99998	19039	0.1903
FGF10	6	0.94218	0.94224	0.99998	19040	0.1299
ZNF730	6	0.94224	0.94229	0.99998	19041	0.1633
CHST11	6	0.94241	0.94248	0.99998	19042	0.2545
OR2D2	6	0.94241	0.94248	0.99998	19043	0.0784
APOBEC4	6	0.94241	0.94248	0.99998	19044	0.2323
FGF13	5	0.94249	0.94267	0.99998	19045	-0.0048
B3GNT4	6	0.94255	0.94261	0.99998	19046	0.0918
MT4	6	0.94255	0.94261	0.99998	19047	0.0588
GSTO2	6	0.94255	0.94261	0.99998	19048	0.1747
RGS8	6	0.94264	0.94269	0.99998	19049	0.0044
ANKRD28	6	0.94264	0.94269	0.99998	19050	0.1784
SH3BP4	6	0.94264	0.94269	0.99998	19051	0.1972
LAMP5	6	0.94264	0.94269	0.99998	19052	0.0371
PAN3	4	0.94267	0.94263	0.99998	19053	0.276
DRAXIN	6	0.94272	0.94278	0.99998	19054	0.2751
CARD8	6	0.94275	0.9428	0.99998	19055	0.0281
CCDC11	6	0.94276	0.94282	0.99998	19056	0.1386
ITGB3BP	5	0.94279	0.94296	0.99998	19057	0.1678
MORN3	6	0.9428	0.94285	0.99998	19058	0.2032
KLHL3	6	0.9428	0.94285	0.99998	19059	0.0415
NOVA1	6	0.94283	0.94288	0.99998	19060	0.0761
AGR3	6	0.94283	0.94288	0.99998	19061	0.0187
CCSER2	6	0.94286	0.94291	0.99998	19062	-0.0176
QKI	6	0.94292	0.94297	0.99998	19063	0.06
hsa-mir-3161	4	0.94307	0.94304	0.99998	19064	0.1727
TSPAN9	6	0.94307	0.94311	0.99998	19065	0.0005
TJP3	6	0.94311	0.94315	0.99998	19066	0.0922
ME1	6	0.94311	0.94315	0.99998	19067	0.1372
ADRB1	6	0.94319	0.94323	0.99998	19068	0.1278
DAP	6	0.94326	0.9433	0.99998	19069	0.153
SGCZ	6	0.94329	0.94333	0.99998	19070	0.1274
GALNT13	6	0.94346	0.9435	0.99998	19071	0.149
NMRK1	6	0.94352	0.94355	0.99998	19072	0.1694
GABRA5	6	0.94355	0.94359	0.99998	19073	0.3023
PRICKLE1	6	0.94355	0.94359	0.99998	19074	-0.0288
FLT3	6	0.94364	0.94367	0.99998	19075	0.0895
LAGE3	6	0.94364	0.94367	0.99998	19076	0.2155
IMPA1	6	0.94369	0.94372	0.99998	19077	0.0298
DOK4	6	0.94369	0.94372	0.99998	19078	0.0766
GPR34	6	0.9439	0.94393	0.99998	19079	0.1147
DMRT1	6	0.9439	0.94393	0.99998	19080	-0.0578
hsa-mir-5092	4	0.94393	0.94391	0.99998	19081	0.2282
CDY2B	1	0.94394	0.94419	0.99998	19082	0.6125
GRM7	6	0.94396	0.94399	0.99998	19083	0.1035
NTNG2	6	0.94401	0.94403	0.99998	19084	0.2149
ERAS	6	0.94406	0.94409	0.99998	19085	0.1579
LRIT1	6	0.94413	0.94415	0.99998	19086	-0.0275
LOC154872	6	0.94413	0.94415	0.99998	19087	0.0439
PTBP2	6	0.94416	0.94418	0.99998	19088	0.3222
SUCNR1	6	0.94416	0.94418	0.99998	19089	0.109
OR14J1	6	0.94421	0.94423	0.99998	19090	0.1476
TMEM115	6	0.94437	0.94439	0.99998	19091	0.1846
TATDN1	4	0.94445	0.94439	0.99998	19092	0.3513
C12orf71	6	0.94456	0.94458	0.99998	19093	0.1603
hsa-mir-3972	4	0.94466	0.94459	0.99998	19094	0.2302
CPD	6	0.94469	0.94471	0.99998	19095	0.0319
FAM110D	6	0.94474	0.94477	0.99998	19096	0.077
MIOX	6	0.94474	0.94477	0.99998	19097	0.4452
hsa-mir-1914	4	0.94481	0.94472	0.99998	19098	0.0781
PLCZ1	6	0.94481	0.94484	0.99998	19099	0.3039
MEDAG	6	0.94488	0.9449	0.99998	19100	0.0625
CA10	6	0.94504	0.94507	0.99998	19101	0.1895
NAT14	6	0.94521	0.94521	0.99998	19102	0.0247
BMPER	6	0.94526	0.94526	0.99998	19103	-0.0023
BSPRY	6	0.94526	0.94526	0.99998	19104	0.075
MAN2B1	6	0.9453	0.94531	0.99998	19105	0.1137
PGAM2	6	0.94534	0.94535	0.99998	19106	0.1004
GCH1	6	0.94539	0.94539	0.99998	19107	0.0128
CD69	6	0.94544	0.94545	0.99998	19108	0.3769
PTK7	6	0.94548	0.94549	0.99998	19109	0.2375
EMX2	6	0.94556	0.94557	0.99998	19110	-0.1008
COPRS	6	0.94556	0.94557	0.99998	19111	0.0997
CUEDC1	6	0.9456	0.9456	0.99998	19112	0.0966
RHOV	6	0.9456	0.9456	0.99998	19113	0.1455

NARR	3	0.94564	0.94584	0.99998	19114	0.3508
BST1	6	0.94574	0.94573	0.99998	19115	0.2624
PHF16	6	0.94574	0.94573	0.99998	19116	0.146
CTDSPL	6	0.94579	0.94578	0.99998	19117	-0.0134
NIPSNAP3B	6	0.94583	0.94582	0.99998	19118	0.1066
hsa-mir-548av	3	0.9459	0.9461	0.99998	19119	0.3802
hsa-mir-524	3	0.9459	0.9461	0.99998	19120	0.3366
C3orf62	6	0.94595	0.94593	0.99998	19121	0.3852
GJA3	6	0.94598	0.94597	0.99998	19122	0.0626
TIGD6	4	0.94602	0.94592	0.99998	19123	0.272
ARF5	6	0.94604	0.94603	0.99998	19124	-0.0352
SFRP1	6	0.94616	0.94614	0.99998	19125	0.1204
KRT12	6	0.9462	0.94619	0.99998	19126	-0.0048
MMP8	6	0.9462	0.94619	0.99998	19127	0.2147
SERPINH1	6	0.94627	0.94626	0.99998	19128	0.0829
THSD4	6	0.94642	0.94641	0.99998	19129	0.1533
hsa-mir-4309	4	0.94646	0.94636	0.99998	19130	0.1617
SERINC1	6	0.9465	0.94651	0.99998	19131	0.0012
LRRTM4	6	0.94664	0.94663	0.99998	19132	0.3152
P2RX7	4	0.94675	0.94666	0.99998	19133	0.1362
hsa-mir-184	4	0.94675	0.94666	0.99998	19134	0.1986
DDX50	6	0.94681	0.94679	0.99998	19135	0.1365
CYLC1	5	0.94685	0.94705	0.99998	19136	0.37
PDCD1LG2	6	0.94691	0.94689	0.99998	19137	0.2939
FOXA1	6	0.94691	0.94689	0.99998	19138	0.0204
FEM1B	6	0.94691	0.94689	0.99998	19139	0.2307
SPRYD4	6	0.94691	0.94689	0.99998	19140	0.0012
COL4A5	4	0.94695	0.94687	0.99998	19141	0.2346
C12orf43	6	0.94698	0.94696	0.99998	19142	-0.0068
hsa-mir-1255b	1	0.94704	0.94729	0.99998	19143	0.7831
SYCE3	6	0.94708	0.94706	0.99998	19144	0.1929
CDYL2	6	0.94712	0.9471	0.99998	19145	0.0876
GPR26	6	0.94717	0.94716	0.99998	19146	0.1024
ZNF180	6	0.94717	0.94716	0.99998	19147	0.225
HMBOX1	6	0.94724	0.94723	0.99998	19148	0.2314
C18orf54	6	0.94729	0.94728	0.99998	19149	0.207
RNF144B	6	0.94739	0.94738	0.99998	19150	0.1867
ST8SIA5	6	0.94739	0.94738	0.99998	19151	0.3042
LEPROTL1	6	0.94739	0.94738	0.99998	19152	0.1238
CHCHD7	6	0.94744	0.94744	0.99998	19153	0.2462
LYZ	6	0.94744	0.94744	0.99998	19154	0.1789
C9orf37	6	0.94748	0.94748	0.99998	19155	0.0381
WDR52	6	0.9475	0.9475	0.99998	19156	0.118
hsa-mir-4740	4	0.9476	0.94754	0.99998	19157	0.1296
CDH9	6	0.94769	0.94768	0.99998	19158	0.2347
PRDM5	6	0.94773	0.94773	0.99998	19159	0.0375
THEMIS2	6	0.94784	0.94785	0.99998	19160	0.0242
CXorf23	6	0.94801	0.94803	0.99998	19161	0.0976
TRIOBP	6	0.9481	0.94811	0.99998	19162	0.2184
REPS2	6	0.9481	0.94811	0.99998	19163	0.1924
P2RY6	6	0.9481	0.94811	0.99998	19164	0.1655
MCF2	6	0.94816	0.94817	0.99998	19165	0.1422
HNRNPF	6	0.94823	0.94824	0.99998	19166	0.1506
hsa-mir-1264	4	0.94824	0.94816	0.99998	19167	0.1967
LUZP6	5	0.94829	0.9485	0.99998	19168	0.1276
SLC26A1	6	0.94829	0.94831	0.99998	19169	0.1253
SLA2	6	0.94838	0.94839	0.99998	19170	0.0222
CES4A	6	0.94843	0.94844	0.99998	19171	0.0232
UCP1	6	0.94848	0.94849	0.99998	19172	0.3919
HS3ST2	6	0.94848	0.94849	0.99998	19173	0.1386
C22orf23	6	0.94852	0.94853	0.99998	19174	0.1361
GPATCH3	6	0.94855	0.94857	0.99998	19175	-0.0231
TEX30	6	0.94855	0.94857	0.99998	19176	-0.0487
TRAF7	6	0.94864	0.94866	0.99998	19177	0.1845
MACF1	6	0.94864	0.94866	0.99998	19178	0.1376
COBL	6	0.94868	0.94869	0.99998	19179	0.0108
WFIKKN1	6	0.94868	0.94869	0.99998	19180	0.2296
ATG4B	6	0.9487	0.94872	0.99998	19181	0.1098
LTK	6	0.94875	0.94875	0.99998	19182	0.063
GLIPR1L1	6	0.94883	0.94882	0.99998	19183	0.2085
STX7	6	0.94883	0.94882	0.99998	19184	0.0765
ARHGAP40	6	0.94889	0.94888	0.99998	19185	0.1295
GMCL1	6	0.94892	0.94892	0.99998	19186	0.1272
WDR41	6	0.94892	0.94892	0.99998	19187	0.1518
hsa-mir-890	4	0.94895	0.94886	0.99998	19188	0.2954
TGFB1	6	0.94895	0.94894	0.99998	19189	-0.0107
GTPBP2	6	0.94901	0.94902	0.99998	19190	-0.0261
RTTN	6	0.94906	0.94907	0.99998	19191	0.1026
OR1A1	6	0.9492	0.9492	0.99998	19192	0.1861
WWOX	6	0.94922	0.94923	0.99998	19193	0.0677
C22orf46	6	0.94924	0.94925	0.99998	19194	-0.0518

KCNE1	6	0.94924	0.94925	0.99998	19195	-0.0673
CSMD1	6	0.94927	0.94928	0.99998	19196	0.1089
GNA15	6	0.94929	0.9493	0.99998	19197	0.1716
SOWAHB	6	0.94938	0.94938	0.99998	19198	0.2016
ARC	6	0.94938	0.94938	0.99998	19199	0.2131
PDE4C	6	0.94948	0.94948	0.99998	19200	0.2191
UCKL1	6	0.94948	0.94948	0.99998	19201	0.057
hsa-mir-4633	4	0.9495	0.94942	0.99998	19202	0.1852
PTRF	6	0.94954	0.94953	0.99998	19203	0.4313
RCAN2	6	0.94954	0.94953	0.99998	19204	0.1577
TSEN15	6	0.9496	0.94958	0.99998	19205	0.1468
PIK3AP1	6	0.9496	0.94958	0.99998	19206	0.236
ENDOD1	6	0.94968	0.94967	0.99998	19207	0.2223
ATL3	6	0.94968	0.94967	0.99998	19208	0.1294
GUCA2A	6	0.94968	0.94967	0.99998	19209	0.0658
DUSP2	6	0.94968	0.94967	0.99998	19210	0.23
HPS3	6	0.94977	0.94976	0.99998	19211	-0.0515
LCN12	6	0.94977	0.94976	0.99998	19212	0.0657
LGI2	6	0.94977	0.94976	0.99998	19213	0.2802
TMEM55A	6	0.94977	0.94976	0.99998	19214	0.1531
PPIAL4G	1	0.94983	0.95011	0.99998	19215	0.5962
FAM46C	6	0.94985	0.94984	0.99998	19216	0.1091
SLC22A25	6	0.94985	0.94984	0.99998	19217	0.0218
METTL18	6	0.94989	0.94989	0.99998	19218	0.2538
DAZAP1	6	0.94989	0.94989	0.99998	19219	0.1169
PARP4	6	0.94992	0.94992	0.99998	19220	0.135
C12orf57	6	0.94995	0.94995	0.99998	19221	-0.0057
ZFP82	6	0.95	0.94999	0.99998	19222	0.3271
PTGFRN	6	0.95003	0.95003	0.99998	19223	0.2104
ZC3H6	6	0.95004	0.95004	0.99998	19224	0.0228
LOXL4	6	0.95007	0.95007	0.99998	19225	0.0676
OR4A15	6	0.95009	0.95009	0.99998	19226	0.0604
MERTK	6	0.95013	0.95013	0.99998	19227	0.1601
IFI27	6	0.95013	0.95013	0.99998	19228	0.0002
CHRM4	6	0.95021	0.95023	0.99998	19229	0.0898
HECW1	6	0.95026	0.95028	0.99998	19230	0.0411
TSPAN12	6	0.95026	0.95028	0.99998	19231	0.2902
IGF2	6	0.95026	0.95028	0.99998	19232	0.2303
FBXO11	6	0.9503	0.95032	0.99998	19233	0.1434
SPEM1	6	0.95036	0.95038	0.99998	19234	0.0422
hsa-mir-548f-:	3	0.95037	0.95061	0.99998	19235	0.1236
NPDC1	6	0.95039	0.95041	0.99998	19236	-0.0133
hsa-mir-5685	4	0.95044	0.95036	0.99998	19237	0.1221
FERMT1	6	0.95055	0.95056	0.99998	19238	0.0739
STC2	6	0.95057	0.95059	0.99998	19239	0.1629
EYA1	6	0.95057	0.95059	0.99998	19240	0.0005
TAF9	6	0.95065	0.95067	0.99998	19241	0.0326
FAM110B	6	0.95068	0.9507	0.99998	19242	0.1167
SLC25A25	6	0.95073	0.95075	0.99998	19243	-0.0181
ATF5	6	0.95083	0.95084	0.99998	19244	0.1389
PLIN3	6	0.95096	0.95098	0.99998	19245	0.1382
KCNJ3	6	0.95113	0.95115	0.99998	19246	0.2098
PTP4A1	6	0.95115	0.95117	0.99998	19247	0.2445
WEE2	6	0.9512	0.95124	0.99998	19248	0.0693
TEX12	6	0.95127	0.95131	0.99998	19249	0.1526
DVL3	6	0.95127	0.95131	0.99998	19250	0.2364
ZNF70	6	0.95141	0.95146	0.99998	19251	0.1076
UBQLN3	4	0.95144	0.95137	0.99998	19252	0.0617
BIRC2	6	0.95149	0.95153	0.99998	19253	0.0984
CDC25A	6	0.95155	0.95159	0.99998	19254	0.0235
DSG1	6	0.95158	0.95162	0.99998	19255	0.2488
MRGPRX4	6	0.95164	0.95167	0.99998	19256	0.1749
KCNA6	6	0.95178	0.95181	0.99998	19257	0.0757
MAP2K1	6	0.95182	0.95185	0.99998	19258	0.115
CLMP	6	0.95188	0.95191	0.99998	19259	0.2498
DGKG	6	0.95192	0.95194	0.99998	19260	0.0861
GAB2	6	0.95195	0.95198	0.99998	19261	0.2186
TPRA1	6	0.95198	0.95201	0.99998	19262	0.0183
PCDHB1	6	0.95198	0.95201	0.99998	19263	0.3144
ME2	6	0.95202	0.95205	0.99998	19264	0.148
CRYBA2	6	0.95206	0.95209	0.99998	19265	0.2051
DKK4	6	0.95206	0.95209	0.99998	19266	0.1451
CCNG1	6	0.95228	0.95231	0.99998	19267	0.1197
DNTT	6	0.95228	0.95231	0.99998	19268	0.1377
hsa-mir-1298	4	0.9524	0.95232	0.99998	19269	0.3505
QRFP	6	0.95245	0.95249	0.99998	19270	-0.0099
hsa-mir-4770	4	0.95253	0.95246	0.99998	19271	0.1581
RAET1E	6	0.95255	0.95259	0.99998	19272	0.315
hsa-mir-744	4	0.95255	0.95248	0.99998	19273	0.1606
LAMA1	6	0.95258	0.95262	0.99998	19274	0.275
BMPR1A	6	0.9526	0.95263	0.99998	19275	0.1717

hsa-mir-4669	4	0.95264	0.95255	0.99998	19276	0.3421
LIPF	6	0.95264	0.95268	0.99998	19277	0.1283
HTATSF1	6	0.95265	0.95268	0.99998	19278	-0.006
FANCB	6	0.9527	0.95273	0.99998	19279	0.0202
PBLD	6	0.95273	0.95276	0.99998	19280	0.3763
ZNF699	6	0.95275	0.95278	0.99998	19281	0.1827
NEIL1	6	0.95275	0.95278	0.99998	19282	0.1049
CSNK1G2	6	0.95275	0.95278	0.99998	19283	0.1974
MYOM2	6	0.95278	0.95281	0.99998	19284	0.1548
DNAJC1	6	0.95286	0.95289	0.99998	19285	0.1705
KCND2	6	0.95286	0.95289	0.99998	19286	0.2327
LRRC56	6	0.95289	0.95292	0.99998	19287	0.3615
TMEM234	6	0.95293	0.95296	0.99998	19288	0.1049
ZNF879	6	0.95296	0.95299	0.99998	19289	0.1624
SPINT4	6	0.95298	0.95301	0.99998	19290	0.1497
PIP5K1B	6	0.95306	0.95308	0.99998	19291	0.1462
hsa-mir-764	4	0.9531	0.95302	0.99998	19292	0.0964
ADORA3	6	0.95311	0.95314	0.99998	19293	0.1637
SERPINE1	4	0.95315	0.95307	0.99998	19294	0.0753
GPC4	6	0.95316	0.95318	0.99998	19295	0.1644
LHX1	6	0.95326	0.95329	0.99998	19296	0.128
SHISA7	6	0.95326	0.95329	0.99998	19297	0.0706
IFI6	6	0.95326	0.95329	0.99998	19298	0.34
KBTBD8	6	0.95326	0.95329	0.99998	19299	0.1462
SLC24A5	6	0.95331	0.95335	0.99998	19300	0.1195
ANO1	6	0.95336	0.95339	0.99998	19301	0.1464
hsa-mir-3668	2	0.9534	0.95352	0.99998	19302	0.346
MKLN1	6	0.95341	0.95344	0.99998	19303	0.1412
FBXL15	6	0.95341	0.95344	0.99998	19304	0.1341
DMPK	6	0.95345	0.95349	0.99998	19305	0.2791
RFX4	6	0.95349	0.95353	0.99998	19306	0.0176
BHLHA9	6	0.95356	0.95359	0.99998	19307	0.1875
PRB3	5	0.95359	0.95377	0.99998	19308	0.1247
MPZL2	6	0.95361	0.95364	0.99998	19309	0.0244
hsa-mir-127	4	0.95364	0.95352	0.99998	19310	0.0792
FUS	6	0.95368	0.9537	0.99998	19311	0.0282
GPR125	6	0.95368	0.9537	0.99998	19312	0.0508
ACAD8	6	0.95368	0.9537	0.99998	19313	0.0435
SPDEF	6	0.95375	0.95378	0.99998	19314	0.0585
AXDND1	6	0.95383	0.95386	0.99998	19315	0.2181
DIP2C	6	0.95383	0.95386	0.99998	19316	0.1596
UGT8	6	0.95384	0.95386	0.99998	19317	0.1498
JAZF1	6	0.95386	0.95389	0.99998	19318	0.0418
MANBAL	6	0.95386	0.95389	0.99998	19319	0.2252
hsa-mir-23b	4	0.95386	0.95376	0.99998	19320	0.1661
HHIPL2	6	0.9539	0.95392	0.99998	19321	0.177
PRR14L	6	0.95393	0.95395	0.99998	19322	0.1587
C17orf50	6	0.95395	0.95396	0.99998	19323	0.2144
HEXB	6	0.95399	0.95401	0.99998	19324	0.0272
OR5W2	6	0.95402	0.95405	0.99998	19325	0.3643
CD38	6	0.95403	0.95405	0.99998	19326	0.232
IFITM10	6	0.95408	0.9541	0.99998	19327	0.0921
L3HYPDH	6	0.95411	0.95413	0.99998	19328	-0.0021
EFEMP2	6	0.95414	0.95417	0.99998	19329	0.1026
MIDN	6	0.95421	0.95423	0.99998	19330	0.0161
ATP4B	6	0.95421	0.95423	0.99998	19331	0.3002
IFT27	6	0.95424	0.95427	0.99998	19332	0.0829
ZDHHC4	6	0.95429	0.95432	0.99998	19333	-0.0245
DCAF8L1	6	0.95429	0.95432	0.99998	19334	-0.0085
ZNF628	6	0.95438	0.95439	0.99998	19335	0.1213
IRX4	6	0.9544	0.95441	0.99998	19336	0.1636
NOS3	6	0.95443	0.95444	0.99998	19337	0.1751
ITK	6	0.95443	0.95444	0.99998	19338	0.2978
BLNK	6	0.95443	0.95444	0.99998	19339	0.0822
SCT	6	0.95445	0.95446	0.99998	19340	0.0191
TP53TG3D	4	0.95449	0.95437	0.99998	19341	0.4142
hsa-mir-4490	4	0.95455	0.95442	0.99998	19342	0.1289
CCS	6	0.95455	0.95458	0.99998	19343	0.1647
PAX3	6	0.95455	0.95458	0.99998	19344	0.258
CDKN2AIPNL	6	0.95455	0.95458	0.99998	19345	0.0998
HOXD1	6	0.95466	0.95467	0.99998	19346	0.1458
TMCO3	6	0.95466	0.95467	0.99998	19347	0.1652
LEPR	6	0.95466	0.95467	0.99998	19348	0.0621
NPY5R	6	0.95466	0.95467	0.99998	19349	0.0491
BRIP1	4	0.95468	0.95456	0.99998	19350	0.1166
SERPINA12	6	0.95473	0.95474	0.99998	19351	0.0245
BRCC3	6	0.95484	0.95484	0.99998	19352	0.0016
LRMP	6	0.95487	0.95487	0.99998	19353	0.0025
hsa-mir-6738	2	0.95498	0.95512	0.99998	19354	0.4994
GPR108	6	0.95499	0.95499	0.99998	19355	0.038
FAM131B	6	0.95509	0.9551	0.99998	19356	0.1122

NAA40	6	0.95509	0.9551	0.99998	19357	0.2461
COL16A1	6	0.95512	0.95514	0.99998	19358	0.0929
TLK2	6	0.9552	0.95522	0.99998	19359	-0.0346
CSRP2BP	6	0.95526	0.95527	0.99998	19360	0.2013
THAP8	6	0.95532	0.95532	0.99998	19361	0.0984
MTMR2	6	0.95532	0.95532	0.99998	19362	0.2266
ITGAV	6	0.95542	0.95541	0.99998	19363	0.0731
CDC34	6	0.95556	0.95554	0.99998	19364	0.2094
NEB	6	0.95556	0.95554	0.99998	19365	0.1255
TPST1	6	0.95558	0.95556	0.99998	19366	0.1635
ATP6V1C2	6	0.95562	0.95561	0.99998	19367	0.1509
ERBB4	6	0.95566	0.95565	0.99998	19368	0.0445
TIAF1	6	0.95566	0.95565	0.99998	19369	0.2764
KIAA0319L	6	0.95575	0.95573	0.99998	19370	0.0501
FOXD1	6	0.95575	0.95573	0.99998	19371	0.1633
ADAMTS16	6	0.95575	0.95573	0.99998	19372	0.126
CDC37	6	0.95575	0.95573	0.99998	19373	0.0831
PRR23B	6	0.95575	0.95573	0.99998	19374	0.2333
TIMP3	6	0.95575	0.95573	0.99998	19375	-0.0168
DAOA	6	0.95575	0.95573	0.99998	19376	0.0307
hsa-mir-4783	4	0.95578	0.95569	0.99998	19377	0.4346
CD53	6	0.95579	0.95578	0.99998	19378	0.1693
COL6A3	6	0.95582	0.95581	0.99998	19379	0.2846
hsa-mir-4254	2	0.95583	0.95599	0.99998	19380	0.3827
WDR49	6	0.95589	0.95587	0.99998	19381	0.1667
SPATA21	6	0.95589	0.95588	0.99998	19382	0.0354
GSTA2	2	0.9559	0.95607	0.99998	19383	0.4915
FKBP14	6	0.95591	0.95589	0.99998	19384	0.0702
WDR13	6	0.95593	0.95592	0.99998	19385	0.093
GPC1	6	0.95598	0.95596	0.99998	19386	0.3117
MANSC1	6	0.95604	0.95602	0.99998	19387	0.1982
AUTS2	6	0.95608	0.95606	0.99998	19388	0.0695
SETDB2	6	0.95617	0.95614	0.99998	19389	0.1027
NRG3	6	0.95637	0.95634	0.99998	19390	0.0309
VSIG4	6	0.95641	0.95638	0.99998	19391	0.1216
EGFR	6	0.9565	0.95648	0.99998	19392	0.0937
XIRP1	6	0.95651	0.95649	0.99998	19393	0.1333
CYP11B1	5	0.95652	0.9567	0.99998	19394	0.1696
CDH23	6	0.95657	0.95655	0.99998	19395	0.0361
EGR3	6	0.95661	0.95659	0.99998	19396	0.1145
NRG1	6	0.95661	0.95659	0.99998	19397	0.1728
ANXA11	6	0.95666	0.95664	0.99998	19398	0.2309
ZCCHC16	6	0.95666	0.95664	0.99998	19399	0.2284
C10orf25	6	0.95671	0.95668	0.99998	19400	0.0306
ZFY	6	0.95677	0.95674	0.99998	19401	0.2695
TNFRSF10D	6	0.95685	0.95683	0.99998	19402	0.364
CMTM5	6	0.95688	0.95686	0.99998	19403	0.1189
HGSNAT	6	0.95691	0.95688	0.99998	19404	-0.0099
RGS9BP	6	0.95695	0.95692	0.99998	19405	0.0262
KLHL23	2	0.95698	0.95713	0.99998	19406	0.4578
TLCD1	6	0.95709	0.95704	0.99998	19407	0.2312
HOXB9	4	0.95715	0.95704	0.99998	19408	0.1135
KIRREL	6	0.9572	0.95715	0.99998	19409	0.0709
QRICH1	6	0.95722	0.95717	0.99998	19410	0.1847
NR1H4	6	0.95728	0.95723	0.99998	19411	0.2652
CD302	6	0.95748	0.95744	0.99998	19412	0.0781
FAM127C	5	0.95752	0.95769	0.99998	19413	0.377
DRC1	6	0.95753	0.95749	0.99998	19414	0.0833
GPR19	6	0.95753	0.95749	0.99998	19415	0.0745
ADAM15	6	0.95753	0.95749	0.99998	19416	0.3809
FAM19A3	6	0.95759	0.95754	0.99998	19417	0.1292
CGREF1	6	0.9576	0.95755	0.99998	19418	0.0798
RNASEH2A	6	0.95769	0.95764	0.99998	19419	0.0911
MEP1A	6	0.9578	0.95776	0.99998	19420	0.2207
TTC3	6	0.9578	0.95776	0.99998	19421	0.077
MAP6D1	6	0.95787	0.95781	0.99998	19422	0.1977
BRD1	6	0.95787	0.95781	0.99998	19423	0.1185
ZNF792	6	0.95787	0.95781	0.99998	19424	0.1367
ACAP1	6	0.95799	0.95794	0.99998	19425	0.1118
PCDHB6	6	0.95804	0.95799	0.99998	19426	0.0109
SIX3	6	0.9581	0.95804	0.99998	19427	0.1572
SSSCA1	6	0.9581	0.95804	0.99998	19428	0.1586
STARD9	6	0.95816	0.9581	0.99998	19429	0.0686
MPL	6	0.9582	0.95814	0.99998	19430	0.1449
40787	3	0.95828	0.95848	0.99998	19431	0.1843
CNTN3	6	0.95831	0.95825	0.99998	19432	0.2302
CNTRL	6	0.95834	0.95829	0.99998	19433	0.1594
ARHGAP20	6	0.95834	0.95829	0.99998	19434	0.3291
PMVK	6	0.95841	0.95836	0.99998	19435	0.1555
TBC1D10A	6	0.95844	0.95839	0.99998	19436	0.0602
ATG2A	6	0.95844	0.95839	0.99998	19437	0.4882

EMB	6	0.95845	0.9584	0.99998	19438	0.1224
UBE2S	6	0.95849	0.95844	0.99998	19439	0.1551
GSX1	6	0.95856	0.95852	0.99998	19440	0.1051
DUSP6	6	0.9586	0.95855	0.99998	19441	0.3564
KDM4D	6	0.9586	0.95855	0.99998	19442	0.23
hsa-mir-221	4	0.9586	0.95853	0.99998	19443	0.2291
KLHL9	6	0.95863	0.95858	0.99998	19444	0.1703
SAR1A	6	0.95868	0.95864	0.99998	19445	0.3167
CYP39A1	6	0.95871	0.95867	0.99998	19446	0.1769
IFIT3	6	0.95873	0.95869	0.99998	19447	0.2768
ZNF649	6	0.95878	0.95874	0.99998	19448	0.206
OR6M1	6	0.95885	0.95881	0.99998	19449	0.2111
GPT	6	0.95885	0.95881	0.99998	19450	0.0399
GLS	6	0.95889	0.95885	0.99998	19451	0.1351
MBIP	6	0.95891	0.95886	0.99998	19452	0.3146
DEGS1	6	0.95895	0.9589	0.99998	19453	0.2217
SPTA1	6	0.95897	0.95892	0.99998	19454	0.1243
SIK1	6	0.95897	0.95892	0.99998	19455	0.2492
SYDE1	6	0.95899	0.95894	0.99998	19456	0.0176
TPMT	6	0.95901	0.95896	0.99998	19457	0.0193
POTEC	4	0.95904	0.95898	0.99998	19458	0.2871
OR5M1	4	0.95904	0.95898	0.99998	19459	0.429
DEFB131	4	0.95904	0.95898	0.99998	19460	0.2979
hsa-mir-501	4	0.95904	0.95898	0.99998	19461	0.1932
C17orf102	6	0.95909	0.95903	0.99998	19462	0.2548
TRIM10	6	0.95918	0.95913	0.99998	19463	0.0617
KLHL8	6	0.95918	0.95913	0.99998	19464	0.0279
RAD50	6	0.95928	0.95923	0.99998	19465	0.1548
C10orf54	6	0.9593	0.95925	0.99998	19466	0.1722
UBE4A	6	0.95941	0.95935	0.99998	19467	0.1944
CEACAM19	6	0.95944	0.95938	0.99998	19468	0.0239
ITPRIPL2	6	0.95948	0.95941	0.99998	19469	0.1137
NKX6-1	6	0.95952	0.95946	0.99998	19470	0.1382
SKIV2L	6	0.95952	0.95946	0.99998	19471	0.0015
MPG	6	0.95957	0.95951	0.99998	19472	0.4961
RPL10L	6	0.95957	0.95951	0.99998	19473	0.1377
NIN	6	0.9596	0.95954	0.99998	19474	0.1027
ACVR1C	6	0.95968	0.95962	0.99998	19475	0.1388
MTHFD2L	6	0.95971	0.95965	0.99998	19476	0.1774
LYRM9	6	0.95976	0.95969	0.99998	19477	-0.027
RTP2	6	0.95976	0.95969	0.99998	19478	0.0284
ZNF597	6	0.95978	0.95971	0.99998	19479	0.0373
DMKN	6	0.95985	0.95979	0.99998	19480	0.1035
HIST1H2BC	6	0.95996	0.95991	0.99998	19481	0.8179
C2orf15	6	0.95997	0.95992	0.99998	19482	0.0629
SPATA9	6	0.95998	0.95994	0.99998	19483	0.2958
MALL	6	0.96006	0.96001	0.99998	19484	0.088
FOXS1	6	0.96006	0.96001	0.99998	19485	0.215
GPR22	6	0.96018	0.96014	0.99998	19486	0.0769
PEF1	6	0.9605	0.96047	0.99998	19487	0.0096
CD19	6	0.9605	0.96047	0.99998	19488	0.1132
HTRA1	6	0.96057	0.96054	0.99998	19489	0.3536
CLC	6	0.96057	0.96054	0.99998	19490	0.2265
MLEC	6	0.96069	0.96066	0.99998	19491	0.1701
KCTD8	6	0.96072	0.9607	0.99998	19492	0.4268
PRRG4	4	0.96072	0.96064	0.99998	19493	0.118
hsa-mir-1252	3	0.96073	0.96096	0.99998	19494	0.4366
CCDC111	6	0.96075	0.96072	0.99998	19495	0.1051
SLC36A4	6	0.96077	0.96075	0.99998	19496	0.3034
RDH14	6	0.96083	0.9608	0.99998	19497	-0.009
PGM1	6	0.96083	0.9608	0.99998	19498	0.2243
IRGC	6	0.96083	0.9608	0.99998	19499	0.1254
C2orf57	6	0.96083	0.9608	0.99998	19500	0.1281
OSBPL3	6	0.96097	0.96095	0.99998	19501	0.255
RBPJ	6	0.96107	0.96105	0.99998	19502	0.2904
ABO	6	0.96107	0.96105	0.99998	19503	0.2193
C2orf62	6	0.96113	0.96111	0.99998	19504	0.3259
RGAG4	6	0.96116	0.96115	0.99998	19505	0.0716
TTC8	6	0.96118	0.96116	0.99998	19506	0.1022
HOXB2	4	0.96118	0.96107	0.99998	19507	0.2495
KIF6	6	0.9612	0.96118	0.99998	19508	0.2686
KLHL6	6	0.96122	0.96121	0.99998	19509	0.0861
ZNF202	6	0.96127	0.96126	0.99998	19510	0.4159
OXR1	6	0.96129	0.96128	0.99998	19511	0.0484
KRT15	6	0.96132	0.96132	0.99998	19512	0.0549
hsa-mir-6501	4	0.96134	0.96123	0.99998	19513	0.1969
hsa-mir-3166	4	0.96136	0.96125	0.99998	19514	0.093
NME1	3	0.96137	0.96157	0.99998	19515	0.2009
GRAMD1B	6	0.9614	0.9614	0.99998	19516	0.1996
FAM47B	6	0.96145	0.96144	0.99998	19517	0.1727
EFCAB4B	6	0.96149	0.96149	0.99998	19518	0.2495

IGFL3	6	0.96156	0.96155	0.99998	19519	0.0631
KLK2	6	0.96171	0.9617	0.99998	19520	0.1608
ALX3	6	0.96171	0.9617	0.99998	19521	0.053
XRCC1	6	0.96177	0.96177	0.99998	19522	0.1163
PKLR	6	0.96177	0.96177	0.99998	19523	0.1463
HOMER2	6	0.9619	0.96191	0.99998	19524	0.1282
HAND1	6	0.96199	0.962	0.99998	19525	0.1468
ARL6	6	0.96201	0.96202	0.99998	19526	0.0819
PTPRZ1	6	0.96204	0.96205	0.99998	19527	0.1044
CARD17	6	0.96211	0.96211	0.99998	19528	0.1537
FGB	6	0.96211	0.96211	0.99998	19529	0.1147
FN3KRP	6	0.96211	0.96211	0.99998	19530	0.1379
USP17L1P	3	0.96222	0.96237	0.99998	19531	0.4184
OBSL1	4	0.96225	0.96219	0.99998	19532	0.3354
MYEOV	6	0.96227	0.96227	0.99998	19533	0.2755
LRRC48	6	0.96228	0.96229	0.99998	19534	0.0542
ZNF836	6	0.96235	0.96237	0.99998	19535	0.1932
CIAPIN1	6	0.96235	0.96237	0.99998	19536	0.1713
ENTPD4	6	0.96258	0.96257	0.99998	19537	0.2923
PATE1	6	0.96258	0.96257	0.99998	19538	0.2062
ERAP2	6	0.96263	0.96263	0.99998	19539	0.0904
NPEPPS	6	0.96263	0.96263	0.99998	19540	0.0241
C19orf47	6	0.96269	0.96268	0.99998	19541	0.1071
GSTT2	1	0.9627	0.9629	0.99998	19542	1.6406
RIPK1	6	0.96276	0.96275	0.99998	19543	0.1185
DOK3	6	0.96278	0.96277	0.99998	19544	0.1182
MAML2	6	0.96282	0.9628	0.99998	19545	0.2621
LRGUK	6	0.96288	0.96287	0.99998	19546	0.0174
RBM23	6	0.96289	0.96287	0.99998	19547	0.1197
LRRC10B	6	0.96292	0.96291	0.99998	19548	0.1168
hsa-mir-193b	4	0.96301	0.96295	0.99998	19549	0.0716
hsa-mir-34c	2	0.96304	0.9632	0.99998	19550	0.2988
ZNF235	6	0.96312	0.96311	0.99998	19551	0.3582
ANKRD53	6	0.96312	0.96311	0.99998	19552	0.271
FAM24B	6	0.96312	0.96311	0.99998	19553	0.0184
SLC39A6	6	0.96329	0.9633	0.99998	19554	0.1123
STOX1	6	0.96333	0.96334	0.99998	19555	0.2667
GNL1	6	0.96333	0.96334	0.99998	19556	0.0342
USP11	6	0.96336	0.96336	0.99998	19557	0.1343
MTMR8	6	0.96341	0.96342	0.99998	19558	0.0405
CCDC126	6	0.96341	0.96342	0.99998	19559	0.102
ALG5	6	0.96348	0.96349	0.99998	19560	0.0739
KIAA0753	6	0.96354	0.96354	0.99998	19561	0.158
ITLN1	6	0.96354	0.96354	0.99998	19562	0.1473
DSCR6	2	0.96355	0.9637	0.99998	19563	0.3532
CNR1	6	0.96371	0.96372	0.99998	19564	0.1921
POU2AF1	6	0.96374	0.96376	0.99998	19565	0.3465
DHCR7	6	0.96377	0.96378	0.99998	19566	0.1004
DPF3	6	0.96386	0.96387	0.99998	19567	0.283
KANK3	6	0.964	0.96401	0.99998	19568	0.214
KCNG3	6	0.964	0.96401	0.99998	19569	0.1201
IL12A	6	0.964	0.96401	0.99998	19570	0.2084
PAK1	6	0.96409	0.96409	0.99998	19571	0.271
DCX	6	0.96417	0.96418	0.99998	19572	0.0903
OR4Q3	6	0.96426	0.96427	0.99998	19573	0.2843
NT5C2	6	0.96436	0.96437	0.99998	19574	0.2155
INPP5E	6	0.96436	0.96437	0.99998	19575	0.0264
IBSP	6	0.96439	0.96439	0.99998	19576	0.2475
MAGEA4	6	0.9645	0.96451	0.99998	19577	0.1209
KCNJ8	6	0.9645	0.96451	0.99998	19578	0.1815
SNN	6	0.96457	0.96458	0.99998	19579	0.35
PRM2	6	0.96462	0.96464	0.99998	19580	0.1354
IFITM2	6	0.96491	0.96492	0.99998	19581	0.0141
OR10T2	6	0.96493	0.96494	0.99998	19582	0.1207
SYN2	6	0.96496	0.96496	0.99998	19583	0.0343
ZNF550	6	0.96496	0.96497	0.99998	19584	0.1244
B3GNTL1	6	0.965	0.96501	0.99998	19585	0.1276
CLEC9A	6	0.965	0.96501	0.99998	19586	0.0727
SYT7	6	0.965	0.96501	0.99998	19587	0.2208
MTRNR2L1	1	0.9651	0.96533	0.99998	19588	1.5941
COX4I2	6	0.96519	0.96519	0.99998	19589	0.1394
MCTS1	6	0.96526	0.96525	0.99998	19590	0.3097
OR2AK2	6	0.9653	0.96529	0.99998	19591	0.2527
BRICD5	6	0.9653	0.96529	0.99998	19592	0.0607
OR1N1	6	0.96536	0.96536	0.99998	19593	0.2391
TRAF1	6	0.96536	0.96536	0.99998	19594	0.0673
AQP9	6	0.96542	0.96542	0.99998	19595	0.0845
SOX6	6	0.96547	0.96547	0.99998	19596	0.2164
SSX2IP	6	0.9655	0.9655	0.99998	19597	0.1146
OR52M1	6	0.96561	0.96561	0.99998	19598	0.0629
RBPJL	6	0.96562	0.96562	0.99998	19599	0.0444

TMEM50B	6	0.96574	0.96575	0.99998	19600	0.2189
PPFIA4	6	0.96574	0.96575	0.99998	19601	0.1338
KCNAB3	6	0.96579	0.96579	0.99998	19602	0.1026
MXRA8	6	0.96585	0.96585	0.99998	19603	0.2612
KIAA0408	6	0.96589	0.96589	0.99998	19604	0.2682
RNF11	6	0.96591	0.96591	0.99998	19605	0.1732
APOA5	6	0.96592	0.96592	0.99998	19606	0.1443
WDFY1	6	0.96594	0.96594	0.99998	19607	0.1987
STXBP4	6	0.96597	0.96597	0.99998	19608	0.1655
ST8SIA1	6	0.96597	0.96597	0.99998	19609	0.1876
LPIN2	6	0.96597	0.96597	0.99998	19610	0.1769
CARD18	6	0.96602	0.96605	0.99998	19611	0.1805
hsa-mir-2114	4	0.96603	0.96598	0.99998	19612	0.0867
DECR1	6	0.96612	0.96615	0.99998	19613	0.1503
PIWIL3	6	0.96618	0.96622	0.99998	19614	0.2494
FGFBP1	6	0.96618	0.96622	0.99998	19615	0.1513
CDC42EP5	6	0.96618	0.96622	0.99998	19616	0.1392
SLC29A1	6	0.96627	0.96629	0.99998	19617	-0.0048
LCA5L	6	0.96632	0.96634	0.99998	19618	0.2145
AMN1	6	0.96634	0.96637	0.99998	19619	0.2368
SNX29	6	0.9664	0.96642	0.99998	19620	0.0423
IGSF10	6	0.9664	0.96642	0.99998	19621	0.2233
APLNR	6	0.96646	0.96649	0.99998	19622	0.3172
EXD3	6	0.96648	0.9665	0.99998	19623	0.1211
ZRANB2	6	0.96651	0.96654	0.99998	19624	0.0684
CACNA1H	6	0.96651	0.96654	0.99998	19625	0.4084
PTX4	6	0.96651	0.96654	0.99998	19626	0.2412
AVPR2	6	0.96654	0.96657	0.99998	19627	0.1981
NECAB1	6	0.9666	0.96662	0.99998	19628	0.3109
ACOT9	6	0.9666	0.96662	0.99998	19629	0.3049
EIF2AK3	6	0.9666	0.96662	0.99998	19630	0.1043
MARCH8	6	0.96662	0.96665	0.99998	19631	0.0582
KRTAP21-3	4	0.96664	0.96659	0.99998	19632	0.1789
AXIN2	6	0.96668	0.9667	0.99998	19633	0.134
METTL24	4	0.96668	0.96663	0.99998	19634	0.2474
GPR155	4	0.96668	0.96663	0.99998	19635	0.1123
hsa-mir-4273	4	0.96671	0.96665	0.99998	19636	0.2403
MB	6	0.96677	0.9668	0.99998	19637	0.276
THNSL1	6	0.96679	0.96681	0.99998	19638	0.0672
MYOM1	6	0.96679	0.96682	0.99998	19639	0.142
MSRB3	6	0.96695	0.96697	0.99998	19640	0.1674
AK8	6	0.967	0.96701	0.99998	19641	0.0065
RBM14	4	0.96702	0.96696	0.99998	19642	0.169
MLL5	6	0.96704	0.96705	0.99998	19643	0.2854
DYNAP	6	0.96708	0.96709	0.99998	19644	0.1698
PRG3	6	0.96708	0.96709	0.99998	19645	0.1499
hsa-mir-595	4	0.96711	0.96705	0.99998	19646	0.2147
ITGBL1	6	0.9672	0.96723	0.99998	19647	0.0493
TGFB1I1	6	0.96727	0.9673	0.99998	19648	0.3296
hsa-mir-4297	4	0.9673	0.96723	0.99998	19649	0.2408
PNKD	6	0.96732	0.96735	0.99998	19650	0.179
hsa-mir-3194	4	0.96734	0.96727	0.99998	19651	0.4214
C19orf25	6	0.96735	0.96739	0.99998	19652	0.0354
GPNMB	6	0.96739	0.96743	0.99998	19653	0.0764
SMIM8	6	0.96752	0.96755	0.99998	19654	0.1159
SNAI2	6	0.96762	0.96766	0.99998	19655	0.248
PRPS1	6	0.96764	0.96769	0.99998	19656	0.2004
C4orf27	6	0.96769	0.96773	0.99998	19657	0.0861
SKAP1	6	0.96772	0.96776	0.99998	19658	0.1022
ANKRD54	6	0.96779	0.96783	0.99998	19659	0.1506
RAB44	6	0.96793	0.96797	0.99998	19660	0.2438
HNF4G	6	0.96793	0.96797	0.99998	19661	0.0851
PHLDB3	6	0.96796	0.96799	0.99998	19662	0.1128
NAP1L2	6	0.96796	0.968	0.99998	19663	0.081
TGDS	6	0.96799	0.96802	0.99998	19664	0.1243
IDH2	6	0.96807	0.9681	0.99998	19665	0.2526
CDK3	6	0.96807	0.9681	0.99998	19666	0.0786
TGM3	6	0.96811	0.96814	0.99998	19667	0.3102
OXCT2	6	0.96818	0.96822	0.99998	19668	0.1379
SLC26A9	6	0.96822	0.96825	0.99998	19669	0.0164
MYRIP	6	0.96824	0.96827	0.99998	19670	0.1321
LMOD2	6	0.96827	0.96829	0.99998	19671	0.3768
AKR7A3	6	0.96831	0.96833	0.99998	19672	0.0404
XRCC3	6	0.96834	0.96836	0.99998	19673	0.2097
MTPN	6	0.96836	0.96838	0.99998	19674	0.1363
TSSK4	6	0.96836	0.96838	0.99998	19675	0.1185
CPXM2	6	0.96839	0.96841	0.99998	19676	0.1274
NPY2R	6	0.96841	0.96844	0.99998	19677	0.1369
IL25	6	0.96844	0.96846	0.99998	19678	0.072
NUDT2	6	0.96847	0.9685	0.99998	19679	0.1268
LBR	6	0.96857	0.9686	0.99998	19680	0.4388

RBM38	6	0.96862	0.96864	0.99998	19681	0.2547
KYNU	6	0.96865	0.96868	0.99998	19682	0.0799
PRR14	6	0.96873	0.96876	0.99998	19683	0.198
SLC25A37	6	0.96875	0.96878	0.99998	19684	0.117
BDH2	6	0.96875	0.96878	0.99998	19685	0.0384
ERAL1	6	0.9689	0.96892	0.99998	19686	0.2357
ZNF518B	6	0.9689	0.96892	0.99998	19687	0.1726
hsa-mir-152	4	0.96894	0.96888	0.99998	19688	0.1882
GRPEL2	6	0.96894	0.96897	0.99998	19689	0.315
PLEKHO2	6	0.96899	0.96902	0.99998	19690	0.2772
hsa-mir-5692b	3	0.96918	0.96928	0.99998	19691	0.8124
IGSF9	6	0.96921	0.96925	0.99998	19692	0.0291
ZACN	6	0.96924	0.96928	0.99998	19693	0.2655
ZMAT1	6	0.96928	0.96932	0.99998	19694	0.3038
AFF1	6	0.96937	0.96941	0.99998	19695	0.2217
PCDHGB3	2	0.96943	0.9696	0.99998	19696	0.5045
RIPPLY1	6	0.96946	0.96951	0.99998	19697	0.1234
REP15	6	0.96951	0.96957	0.99998	19698	0.3448
VWA5B2	6	0.96955	0.9696	0.99998	19699	0.2897
PPP2R5B	6	0.96965	0.9697	0.99998	19700	0.1758
AP5B1	6	0.96967	0.96973	0.99998	19701	0.1481
FAM71C	6	0.96975	0.9698	0.99998	19702	0.0548
hsa-mir-6842	4	0.96975	0.96969	0.99998	19703	0.2276
hsa-mir-5089	4	0.96983	0.96977	0.99998	19704	0.1969
AMOTL1	6	0.96984	0.96989	0.99998	19705	0.2717
ZNF618	6	0.96984	0.96989	0.99998	19706	0.0297
TNFRSF17	6	0.96988	0.96992	0.99998	19707	0.0082
MYADM	6	0.96988	0.96992	0.99998	19708	0.2078
RABEP1	6	0.96988	0.96992	0.99998	19709	0.1152
KCNC1	6	0.96992	0.96996	0.99998	19710	0.3706
HOXA13	6	0.96992	0.96996	0.99998	19711	0.1632
YBX2	6	0.96997	0.97	0.99998	19712	0.2314
OR4M2	5	0.96998	0.97002	0.99998	19713	0.1916
TAS2R8	6	0.97001	0.97004	0.99998	19714	0.1634
RTP3	6	0.97019	0.97021	0.99998	19715	0.1548
CYP3A4	5	0.97022	0.97025	0.99998	19716	0.2671
VDAC1	6	0.97039	0.97043	0.99998	19717	0.2962
GPX3	4	0.97055	0.9705	0.99998	19718	0.1182
ZCCHC17	6	0.97056	0.97061	0.99998	19719	0.1801
AGMO	6	0.9706	0.97066	0.99998	19720	0.2501
hsa-mir-15a	4	0.97062	0.97058	0.99998	19721	0.2475
ZNF845	6	0.97063	0.97068	0.99998	19722	0.4525
EDNRB	6	0.97066	0.97071	0.99998	19723	0.1713
MEX3B	6	0.97066	0.97071	0.99998	19724	0.1521
CLIC6	6	0.97068	0.97073	0.99998	19725	0.2687
DCHS1	6	0.9707	0.97075	0.99998	19726	0.2038
STYX	6	0.97078	0.97082	0.99998	19727	0.4331
EFCAB5	4	0.97086	0.97081	0.99998	19728	0.5709
PAQR7	6	0.97086	0.97091	0.99998	19729	0.3133
NUAK1	6	0.97094	0.971	0.99998	19730	0.2457
CRISPLD2	6	0.97094	0.971	0.99998	19731	0.0449
SNCA	6	0.97094	0.971	0.99998	19732	0.1202
RNFT2	6	0.97094	0.971	0.99998	19733	0.1456
TBC1D21	6	0.97108	0.97114	0.99998	19734	0.0349
BMP3	6	0.97114	0.97121	0.99998	19735	0.1094
PSPH	6	0.97119	0.97126	0.99998	19736	0.1806
FUT2	6	0.97119	0.97126	0.99998	19737	0.1176
FYN	6	0.97119	0.97126	0.99998	19738	0.1656
BCORL1	6	0.97122	0.97129	0.99998	19739	0.0505
TIMM21	6	0.97124	0.97131	0.99998	19740	0.3367
BCAN	6	0.97124	0.97131	0.99998	19741	-0.005
hsa-mir-503	4	0.97125	0.97124	0.99998	19742	0.2144
DCLRE1C	6	0.97132	0.97139	0.99998	19743	0.2979
SLCO1B3	5	0.97133	0.97136	0.99998	19744	0.1806
GCHFR	6	0.97137	0.97145	0.99998	19745	0.2039
HNF4A	6	0.97141	0.97149	0.99998	19746	0.2927
NUP214	6	0.97141	0.97149	0.99998	19747	0.1459
KBTBD13	6	0.97141	0.97149	0.99998	19748	0.3871
EZH2	6	0.97141	0.97149	0.99998	19749	0.1771
hsa-mir-371a	1	0.97151	0.97157	0.99998	19750	0.586
DLGAP5	6	0.97154	0.97163	0.99998	19751	0.1608
PPARGC1B	6	0.97178	0.97187	0.99998	19752	0.0487
GNG12	6	0.97178	0.97187	0.99998	19753	0.2414
AMZ2	6	0.97178	0.97187	0.99998	19754	0.1328
ESCO1	6	0.97184	0.97193	0.99998	19755	0.0934
CEP63	6	0.97198	0.97206	0.99998	19756	0.0824
EPS15	6	0.97207	0.97215	0.99998	19757	0.0708
GCNT3	6	0.9721	0.97217	0.99998	19758	0.0208
hsa-mir-3120	2	0.97211	0.97225	0.99998	19759	0.5574
TPM2	6	0.97213	0.9722	0.99998	19760	0.3345
NIPAL4	6	0.97219	0.97226	0.99998	19761	0.0746

FAM189A2	6	0.9722	0.97226	0.99998	19762	0.167
CBFA2T3	6	0.97233	0.97239	0.99998	19763	0.1809
AFF4	6	0.97233	0.97239	0.99998	19764	0.1034
PPP1R1C	6	0.97238	0.97244	0.99998	19765	0.5475
BAG6	6	0.97243	0.97249	0.99998	19766	0.1526
SLC22A23	6	0.97246	0.97252	0.99998	19767	0.1026
NCR3LG1	6	0.97249	0.97254	0.99998	19768	0.1883
hsa-mir-4632	4	0.9725	0.97252	0.99998	19769	0.514
NPVF	6	0.97253	0.97259	0.99998	19770	0.1272
AHRR	6	0.97253	0.97259	0.99998	19771	0.1741
MB21D2	6	0.97256	0.97263	0.99998	19772	0.367
DNAH1	6	0.97257	0.97264	0.99998	19773	0.2349
MED13L	6	0.97261	0.97267	0.99998	19774	0.2974
C10orf91	6	0.97273	0.97279	0.99998	19775	0.1374
C10orf71	6	0.97275	0.9728	0.99998	19776	0.2639
FBF1	6	0.97278	0.97284	0.99998	19777	0.1147
EBLN1	6	0.97284	0.97289	0.99998	19778	0.1819
FANCF	6	0.97294	0.973	0.99998	19779	0.0547
hsa-mir-648	4	0.97297	0.973	0.99998	19780	0.1824
CDK2AP2	6	0.97299	0.97306	0.99998	19781	0.1666
TBC1D28	3	0.973	0.97306	0.99998	19782	0.6889
NGLY1	6	0.9731	0.97317	0.99998	19783	0.2202
ENTPD8	6	0.97323	0.9733	0.99998	19784	0.1654
ICMT	6	0.97334	0.97341	0.99998	19785	0.086
hsa-mir-4804	4	0.97345	0.97348	0.99998	19786	0.3587
hsa-mir-148b	4	0.9735	0.97353	0.99998	19787	0.1599
APH1B	6	0.97352	0.9736	0.99998	19788	0.1257
DEFB121	6	0.97353	0.97362	0.99998	19789	0.2018
ALKBH2	6	0.97356	0.97365	0.99998	19790	0.1202
TMEM99	6	0.9736	0.97369	0.99998	19791	0.235
WDFY2	6	0.9736	0.97369	0.99998	19792	0.1398
STK33	6	0.97367	0.97377	0.99998	19793	0.0999
SLC26A4	6	0.97371	0.9738	0.99998	19794	0.099
SEC23IP	6	0.97371	0.9738	0.99998	19795	0.1431
GTSF1L	6	0.97373	0.97383	0.99998	19796	0.16
HEPHL1	6	0.97376	0.97386	0.99998	19797	0.2489
KLHL28	6	0.97376	0.97386	0.99998	19798	0.0282
PCSK1	6	0.97376	0.97386	0.99998	19799	0.089
hsa-mir-3139	4	0.97379	0.97382	0.99998	19800	0.2817
C1orf51	6	0.97387	0.97396	0.99998	19801	0.1813
ZC2HC1A	6	0.97387	0.97396	0.99998	19802	0.0967
SEMA6D	6	0.97387	0.97396	0.99998	19803	0.0041
PPIC	6	0.97389	0.97398	0.99998	19804	0.188
PTPN5	6	0.97392	0.97401	0.99998	19805	0.0392
CCDC152	6	0.97398	0.97407	0.99998	19806	0.3949
PDCD1	6	0.97399	0.97409	0.99998	19807	-0.0284
FAAH	6	0.97402	0.97411	0.99998	19808	0.2633
NPFFR1	6	0.97405	0.97414	0.99998	19809	0.1887
PNMA3	6	0.97405	0.97414	0.99998	19810	0.3412
hsa-mir-4485	3	0.9741	0.97418	0.99998	19811	0.2171
KCNG2	6	0.97413	0.97422	0.99998	19812	0.1018
DOK6	6	0.97413	0.97422	0.99998	19813	0.2093
WDR63	6	0.97417	0.97426	0.99998	19814	0.2383
HCFC2	6	0.9742	0.9743	0.99998	19815	0.2935
PDE4D	6	0.97425	0.97434	0.99998	19816	0.0628
CLCNKB	6	0.97425	0.97434	0.99998	19817	0.1006
SLIT1	6	0.97432	0.97442	0.99998	19818	0.1149
AMELY	5	0.97438	0.9744	0.99998	19819	0.4228
MNT	6	0.97441	0.97451	0.99998	19820	0.4471
ARL4C	6	0.97452	0.97463	0.99998	19821	0.2636
BHLHA15	6	0.9747	0.97481	0.99998	19822	0.205
SLFN14	6	0.97477	0.97488	0.99998	19823	0.16
RIIAD1	6	0.97477	0.97488	0.99998	19824	0.0816
PLEKHG5	6	0.9748	0.97491	0.99998	19825	0.1058
OVOL2	6	0.9748	0.97491	0.99998	19826	0.1254
KTN1	6	0.9748	0.97491	0.99998	19827	0.0699
ISL1	6	0.97482	0.97493	0.99998	19828	0.0231
TRIM71	6	0.97486	0.97497	0.99998	19829	0.3485
SCGB2B2	6	0.97497	0.9751	0.99998	19830	0.1175
AP3S1	6	0.97497	0.9751	0.99998	19831	0.168
UBE2T	6	0.97507	0.97519	0.99998	19832	0.2517
DAPK2	6	0.97512	0.97524	0.99998	19833	0.1686
CSF1R	6	0.97512	0.97524	0.99998	19834	0.2293
INPPL1	6	0.97515	0.97528	0.99998	19835	0.1952
WDR78	6	0.97518	0.9753	0.99998	19836	0.1782
SCAPER	6	0.97518	0.9753	0.99998	19837	0.14
BCL2L14	6	0.97523	0.97535	0.99998	19838	0.0914
MANSC4	6	0.97523	0.97535	0.99998	19839	0.2851
AP2A2	6	0.97535	0.97548	0.99998	19840	0.4478
ATP1B1	6	0.97537	0.97551	0.99998	19841	0.1081
XXYLT1	6	0.97537	0.97551	0.99998	19842	0.1919

ANKRD10	6	0.97542	0.97555	0.99998	19843	0.16
ZSWIM6	6	0.97542	0.97555	0.99998	19844	0.1325
KISS1	6	0.97542	0.97555	0.99998	19845	0.1825
PRDX3	6	0.97553	0.97565	0.99998	19846	0.1523
WDR72	6	0.97553	0.97565	0.99998	19847	0.2214
GIMAP5	5	0.97557	0.97564	0.99998	19848	0.1246
GBA	6	0.9756	0.97573	0.99998	19849	0.1164
CDK5RAP2	6	0.97565	0.97578	0.99998	19850	0.2
CCDC74B	5	0.97565	0.97571	0.99998	19851	0.1358
hsa-mir-588	4	0.97577	0.97585	0.99998	19852	0.3385
PLAU	3	0.97583	0.97595	0.99998	19853	0.2375
VPS37D	6	0.97589	0.97603	0.99998	19854	0.0842
TMOD4	6	0.97596	0.97609	0.99998	19855	0.2662
SIRT3	6	0.97598	0.97611	0.99998	19856	0.1842
ANGPT2	6	0.97603	0.97616	0.99998	19857	0.3461
MPPE1	6	0.97604	0.97617	0.99998	19858	0.0786
RNF19B	6	0.97607	0.9762	0.99998	19859	0.1738
PROSC	6	0.97609	0.97622	0.99998	19860	0.1568
CCDC64	6	0.97611	0.97625	0.99998	19861	0.1493
GYG1	6	0.97613	0.97626	0.99998	19862	0.2217
CYSLTR1	6	0.97624	0.97637	0.99998	19863	0.2716
ARHGAP33	6	0.9763	0.97643	0.99998	19864	0.0918
CELA2A	6	0.97635	0.97648	0.99998	19865	0.0908
PGAM4	6	0.97637	0.9765	0.99998	19866	0.2434
hsa-mir-4526	4	0.97639	0.97649	0.99998	19867	0.2311
L3MBTL2	6	0.97639	0.97652	0.99998	19868	0.0075
ARHGAP27	6	0.97652	0.97665	0.99998	19869	0.1185
PLSCR3	6	0.97665	0.97678	0.99998	19870	0.1461
KIAA1456	6	0.97666	0.97679	0.99998	19871	0.2999
DCDC2	5	0.97668	0.97673	0.99998	19872	0.2928
CXorf57	6	0.97672	0.97685	0.99998	19873	0.1195
hsa-mir-4753	4	0.97674	0.97685	0.99998	19874	0.2572
MMP20	6	0.97688	0.97702	0.99998	19875	0.1662
ASZ1	6	0.97688	0.97702	0.99998	19876	0.011
NOXA1	6	0.97691	0.97705	0.99998	19877	0.1449
TRAF4	6	0.97691	0.97705	0.99998	19878	0.392
FAM174B	4	0.97693	0.97704	0.99998	19879	0.2366
POM121L2	6	0.97695	0.97708	0.99998	19880	0.3344
CLEC14A	6	0.97698	0.97711	0.99998	19881	0.0935
ATP13A5	6	0.97705	0.97718	0.99998	19882	0.2497
GAPDHS	6	0.97705	0.97718	0.99998	19883	0.1503
ITPA	6	0.97709	0.97722	0.99998	19884	0.2038
ZNF318	6	0.97714	0.97726	0.99998	19885	0.1659
ZNF713	6	0.97716	0.97728	0.99998	19886	0.2873
PLA2G7	6	0.97723	0.97735	0.99998	19887	0.2719
SLC22A10	6	0.97725	0.97737	0.99998	19888	0.2099
ZNF655	6	0.97737	0.9775	0.99998	19889	0.3544
IL10	6	0.97739	0.97752	0.99998	19890	0.3676
OR56A4	6	0.97742	0.97755	0.99998	19891	0.196
CHN2	6	0.97747	0.9776	0.99998	19892	0.1843
NOX4	6	0.97751	0.97765	0.99998	19893	0.2675
CDR1	6	0.97759	0.97774	0.99998	19894	0.3199
MYF5	6	0.97763	0.97778	0.99998	19895	0.1358
PID1	6	0.97771	0.97785	0.99998	19896	0.1817
GIMAP2	6	0.97781	0.97794	0.99998	19897	0.304
TBX15	6	0.97783	0.97797	0.99998	19898	0.1436
PHEX	6	0.97783	0.97797	0.99998	19899	0.277
PPP1R14B	6	0.97786	0.97799	0.99998	19900	0.076
CNPY3	6	0.9779	0.97804	0.99998	19901	0.1599
NDUFB11	6	0.97799	0.97813	0.99998	19902	0.1525
FZD10	6	0.97805	0.97819	0.99998	19903	0.2578
PLS1	6	0.97812	0.97825	0.99998	19904	0.3955
LOC147646	6	0.97815	0.97829	0.99998	19905	0.2572
SIAH3	6	0.97817	0.97831	0.99998	19906	0.3077
AMOTL2	6	0.97821	0.97835	0.99998	19907	0.0909
SUV420H2	6	0.97823	0.97837	0.99998	19908	0.0546
STON1-GTF2A	3	0.97838	0.97845	0.99998	19909	0.1916
SPATA31A1	3	0.97843	0.97851	0.99998	19910	0.2454
CYR61	6	0.97846	0.9786	0.99998	19911	0.0791
SPATA8	6	0.97846	0.9786	0.99998	19912	0.1342
SNTA1	6	0.97851	0.97865	0.99998	19913	0.1713
DUSP10	6	0.97856	0.97869	0.99998	19914	0.4122
OR56A5	6	0.97856	0.97869	0.99998	19915	0.2368
EGLN2	6	0.97856	0.9787	0.99998	19916	0.1407
KIAA1598	6	0.97863	0.97877	0.99998	19917	0.1504
ZNF330	6	0.97874	0.97888	0.99998	19918	0.2448
hsa-mir-938	4	0.97876	0.97882	0.99998	19919	0.4175
ARNTL	6	0.97883	0.97897	0.99998	19920	0.1593
PGRMC1	6	0.97883	0.97897	0.99998	19921	0.399
CDKN2B	6	0.97885	0.97899	0.99998	19922	0.1875
BPIFA1	6	0.9789	0.97904	0.99998	19923	0.3099

CAMK2N1	6	0.97892	0.97906	0.99998	19924	0.076
AARSD1	6	0.97901	0.97915	0.99998	19925	0.1383
NGFRAP1	6	0.97902	0.97917	0.99998	19926	0.2946
HABP2	6	0.97906	0.9792	0.99998	19927	0.1683
ANHX	6	0.97906	0.9792	0.99998	19928	0.0757
BAI1	6	0.9791	0.97924	0.99998	19929	0.2612
HDAC6	6	0.9791	0.97924	0.99998	19930	0.2823
TAS2R46	6	0.97914	0.97927	0.99998	19931	0.2416
NPHP3	6	0.97916	0.9793	0.99998	19932	0.1117
MYOT	6	0.9792	0.97933	0.99998	19933	0.3487
hsa-mir-2355	4	0.97922	0.97928	0.99998	19934	0.1948
GID8	6	0.97928	0.97941	0.99998	19935	0.1786
CDH22	6	0.97934	0.97948	0.99998	19936	0.1878
CLGN	6	0.97936	0.9795	0.99998	19937	0.1346
hsa-mir-4436b	3	0.97939	0.97947	0.99998	19938	0.4034
GOLGA5	6	0.97944	0.97958	0.99998	19939	0.3352
TMEM155	6	0.97947	0.97961	0.99998	19940	0.2528
GLYATL3	6	0.97947	0.97961	0.99998	19941	0.1618
hsa-mir-1250	4	0.97952	0.97958	0.99998	19942	0.3348
SH3GLB1	6	0.97959	0.97975	0.99998	19943	0.2682
PLEKHH2	5	0.97965	0.97972	0.99998	19944	0.2651
POPDC2	6	0.97966	0.97982	0.99998	19945	0.1273
HSPBAP1	6	0.97968	0.97984	0.99998	19946	0.2206
ATPAF1	6	0.97978	0.97995	0.99998	19947	0.2421
STK25	6	0.97986	0.98002	0.99998	19948	0.0934
KLHL4	6	0.97986	0.98002	0.99998	19949	0.2781
MYCBPAP	6	0.97988	0.98004	0.99998	19950	0.0659
MAT2B	6	0.97991	0.98006	0.99998	19951	0.0986
MYH11	6	0.97994	0.98009	0.99998	19952	0.0973
PHOSPHO1	6	0.97998	0.98013	0.99998	19953	0.1608
TEX101	6	0.9801	0.98025	0.99998	19954	0.3935
GADD45A	6	0.9801	0.98025	0.99998	19955	0.1441
ADAM28	6	0.98015	0.98031	0.99998	19956	0.1543
MRAP2	6	0.98016	0.98032	0.99998	19957	0.1195
BMP8B	3	0.98017	0.98024	0.99998	19958	0.426
CD1A	6	0.98024	0.9804	0.99998	19959	0.1092
CD300LG	6	0.98026	0.98042	0.99998	19960	0.1799
TMEM38B	6	0.9803	0.98046	0.99998	19961	0.3791
HINT2	6	0.98032	0.98047	0.99998	19962	0.1509
ARRDC3	6	0.98034	0.9805	0.99998	19963	0.3194
NKX2-2	6	0.98035	0.9805	0.99998	19964	0.352
C1orf123	6	0.98036	0.98052	0.99998	19965	0.4143
MGAM	6	0.98038	0.98054	0.99998	19966	0.1494
SPATA13	6	0.98045	0.98061	0.99998	19967	0.2526
RPS6KA6	6	0.98045	0.98061	0.99998	19968	0.1823
OTUD1	6	0.98048	0.98065	0.99998	19969	0.3087
ZNF587B	6	0.98052	0.98069	0.99998	19970	0.2414
LIF	6	0.98064	0.98082	0.99998	19971	0.2189
hsa-mir-506	4	0.98073	0.9807	0.99998	19972	0.3802
hsa-mir-4480	4	0.98073	0.9807	0.99998	19973	0.1972
CELF4	6	0.98078	0.98096	0.99998	19974	0.0842
IL17D	6	0.98082	0.98099	0.99998	19975	0.0977
RHPN2	6	0.98087	0.98103	0.99998	19976	0.1986
ANGEL2	6	0.98093	0.9811	0.99998	19977	0.3677
PRR15	6	0.98093	0.9811	0.99998	19978	0.1199
FAM184B	6	0.98099	0.98115	0.99998	19979	0.2673
NR1I3	4	0.98099	0.98096	0.99998	19980	0.2348
BAMBI	6	0.98101	0.98118	0.99998	19981	0.0955
SCNM1	1	0.98103	0.98107	0.99998	19982	0.5524
ERLEC1	6	0.98104	0.98121	0.99998	19983	0.2241
MSN	6	0.98104	0.98121	0.99998	19984	0.0323
PLA2G6	6	0.98109	0.98126	0.99998	19985	0.1014
TMEM39A	6	0.98124	0.98141	0.99998	19986	0.4927
ACVR2A	6	0.98127	0.98144	0.99998	19987	0.0135
IFNB1	6	0.98129	0.98146	0.99998	19988	0.0933
AP1G2	6	0.9813	0.98148	0.99998	19989	0.1442
hsa-mir-4292	4	0.98131	0.98125	0.99998	19990	0.1812
SCML1	6	0.98141	0.98159	0.99998	19991	0.3304
hsa-mir-4761	4	0.98145	0.98139	0.99998	19992	0.332
IGSF1	6	0.98149	0.98165	0.99998	19993	0.1378
ZNF716	5	0.98157	0.98165	0.99998	19994	0.6453
VIL1	6	0.98162	0.98179	0.99998	19995	0.3299
PLK1S1	6	0.98163	0.9818	0.99998	19996	0.308
TRIM16	4	0.98167	0.98162	0.99998	19997	0.6214
FAM102B	6	0.98168	0.98185	0.99998	19998	0.2611
AMIGO2	6	0.9817	0.98187	0.99998	19999	0.4351
CBLN2	6	0.98177	0.98194	0.99998	20000	0.1303
MED25	6	0.98185	0.98203	0.99998	20001	0.0979
MPZL3	6	0.98188	0.98206	0.99998	20002	0.1949
DNAH5	6	0.98188	0.98206	0.99998	20003	0.3168
STAMBPL1	6	0.98193	0.98212	0.99998	20004	0.2074

CLDN3	6	0.98197	0.98215	0.99998	20005	0.2069
CDC42EP3	4	0.98198	0.98193	0.99998	20006	0.2174
MSRA	6	0.98199	0.98218	0.99998	20007	0.1883
AKAP14	6	0.98203	0.9822	0.99998	20008	0.1111
PDCL	6	0.98204	0.98222	0.99998	20009	0.2207
hsa-mir-1180	4	0.98209	0.98204	0.99998	20010	0.1979
ANKAR	6	0.98219	0.98236	0.99998	20011	0.0733
SAP30	6	0.98225	0.98241	0.99998	20012	0.3106
ASCC2	6	0.98226	0.98242	0.99998	20013	0.3451
PRF1	6	0.98227	0.98243	0.99998	20014	0.0854
DNPH1	6	0.9823	0.98247	0.99998	20015	0.2613
ANKRD1	6	0.98232	0.98249	0.99998	20016	0.265
BTLA	6	0.98232	0.98249	0.99998	20017	0.4094
RBMS3	4	0.98238	0.98234	0.99998	20018	0.2454
PPARA	6	0.98238	0.98254	0.99998	20019	0.1798
CD52	6	0.98238	0.98254	0.99998	20020	0.1723
PRKAA1	6	0.98238	0.98254	0.99998	20021	0.1326
GFRA4	6	0.98257	0.98272	0.99998	20022	0.2461
CSGALNACT1	6	0.98257	0.98272	0.99998	20023	0.0811
FAM208A	6	0.98265	0.9828	0.99998	20024	0.2243
hsa-mir-1471	4	0.98268	0.98264	0.99998	20025	0.285
GZMM	6	0.98271	0.98286	0.99998	20026	0.1919
hsa-mir-138-1	4	0.98277	0.98272	0.99998	20027	0.2258
KDM2B	6	0.98277	0.98292	0.99998	20028	0.2747
CD8B	6	0.98279	0.98294	0.99998	20029	0.299
ANGPTL5	6	0.9828	0.98295	0.99998	20030	0.3502
hsa-mir-3180-	2	0.98284	0.98291	0.99998	20031	1.2164
TOB2	6	0.98293	0.98309	0.99998	20032	0.2376
CYP2E1	6	0.98299	0.98314	0.99998	20033	0.2121
GNAT3	6	0.98313	0.98328	0.99998	20034	0.2392
DLX2	6	0.98319	0.98334	0.99998	20035	0.365
WFDC1	6	0.98319	0.98334	0.99998	20036	0.2492
MYOM3	6	0.98321	0.98336	0.99998	20037	0.2667
NAGLU	6	0.98321	0.98336	0.99998	20038	0.3927
AAK1	6	0.98325	0.9834	0.99998	20039	0.2668
C3orf30	6	0.98328	0.98343	0.99998	20040	0.2302
hsa-mir-4275	4	0.9833	0.98325	0.99998	20041	0.3107
DAB1	6	0.98331	0.98347	0.99998	20042	0.2519
GUCA1C	6	0.98335	0.98352	0.99998	20043	0.1418
MYO16	6	0.98337	0.98353	0.99998	20044	0.4646
ACAD10	6	0.98343	0.98359	0.99998	20045	0.1919
TM4SF5	6	0.98345	0.9836	0.99998	20046	0.2651
GPR146	6	0.98353	0.98368	0.99998	20047	0.0783
HOXA5	6	0.98354	0.98369	0.99998	20048	0.1789
SUB1	6	0.98363	0.98376	0.99998	20049	0.2879
AKAP4	6	0.98369	0.98382	0.99998	20050	0.3697
FAHD2A	6	0.9837	0.98384	0.99998	20051	0.142
TH	6	0.98386	0.98399	0.99998	20052	0.163
LIG3	6	0.98386	0.98399	0.99998	20053	0.037
DLC1	6	0.98389	0.98402	0.99998	20054	0.2864
CRB2	6	0.98389	0.98402	0.99998	20055	0.2078
WHSC1	6	0.98395	0.98407	0.99998	20056	0.1368
BCMO1	6	0.98403	0.98415	0.99998	20057	0.2915
TMEM38A	6	0.98407	0.9842	0.99998	20058	0.2836
LDOC1	6	0.98422	0.98436	0.99998	20059	0.1128
NQO1	6	0.98431	0.98444	0.99998	20060	0.1829
RNF7	6	0.98441	0.98454	0.99998	20061	0.274
ARMS2	6	0.9845	0.98463	0.99998	20062	0.1052
F3	6	0.98461	0.98473	0.99998	20063	0.3538
TMPRSS5	6	0.98461	0.98473	0.99998	20064	0.1134
HYAL2	6	0.98462	0.98475	0.99998	20065	0.4106
ZNF296	6	0.98462	0.98475	0.99998	20066	0.1795
ARSK	6	0.98469	0.98482	0.99998	20067	0.2548
NKAPL	6	0.98472	0.98485	0.99998	20068	0.1216
ANKRD50	6	0.98476	0.98489	0.99998	20069	0.2717
FAM221B	6	0.98477	0.9849	0.99998	20070	0.1119
SOX18	6	0.98479	0.98493	0.99998	20071	0.1772
MAF1	6	0.98481	0.98494	0.99998	20072	0.0924
KRT9	6	0.98487	0.98501	0.99998	20073	0.1157
KRTAP10-3	6	0.98487	0.98501	0.99998	20074	0.1863
PGPEP1L	6	0.98498	0.98512	0.99998	20075	0.1756
TLX1NB	6	0.98498	0.98512	0.99998	20076	0.1486
TPST2	6	0.985	0.98513	0.99998	20077	0.2635
INPP5F	6	0.98503	0.98517	0.99998	20078	0.2377
NLRP12	6	0.98505	0.98518	0.99998	20079	0.363
SLC6A2	6	0.98525	0.98537	0.99998	20080	0.1102
CDC25B	6	0.98528	0.98541	0.99998	20081	0.2632
TFE3	6	0.98532	0.98546	0.99998	20082	0.0974
OGFOD1	6	0.98534	0.98547	0.99998	20083	0.1194
TRIM42	6	0.98536	0.98549	0.99998	20084	0.1351
TRPM6	6	0.98538	0.98551	0.99998	20085	0.2852

GATA5	6	0.98538	0.98551	0.99998	20086	0.0927
KIAA1967	4	0.9854	0.98536	0.99998	20087	0.1401
FAM9A	5	0.9854	0.98544	0.99998	20088	0.2604
PLGRKT	6	0.98543	0.98556	0.99998	20089	0.354
CYYR1	6	0.9855	0.98562	0.99998	20090	0.3158
OR52N4	6	0.98554	0.98566	0.99998	20091	0.2971
C1orf115	6	0.98555	0.98567	0.99998	20092	0.2111
PIWIL2	6	0.98557	0.98569	0.99998	20093	0.1641
LY96	6	0.98559	0.98571	0.99998	20094	0.2662
POLR3G	6	0.98566	0.98578	0.99998	20095	0.424
ATP5G3	6	0.9857	0.98581	0.99998	20096	0.1194
ZNF248	6	0.9857	0.98582	0.99998	20097	0.2227
TNFRSF6B	6	0.98577	0.98589	0.99998	20098	0.2635
SORBS1	6	0.98577	0.98589	0.99998	20099	0.1432
ZMAT4	6	0.98578	0.9859	0.99998	20100	0.2122
EPHA10	6	0.98578	0.98591	0.99998	20101	0.3382
FGF9	6	0.9858	0.98592	0.99998	20102	0.1536
HSPB6	6	0.98583	0.98595	0.99998	20103	0.0868
ARMC2	6	0.98586	0.98598	0.99998	20104	0.1516
SYT6	6	0.98597	0.98608	0.99998	20105	0.1816
NCLN	6	0.98598	0.98609	0.99998	20106	0.1945
FLJ45513	6	0.98599	0.9861	0.99998	20107	0.2821
PBDC1	6	0.98608	0.98618	0.99998	20108	0.1368
ADAR	6	0.98609	0.9862	0.99998	20109	0.1901
OR10K1	6	0.98622	0.98632	0.99998	20110	0.1899
LSM14B	6	0.98632	0.98641	0.99998	20111	0.108
GDI2	6	0.98637	0.98645	0.99998	20112	0.2571
FBXO10	6	0.98642	0.9865	0.99998	20113	0.257
SLC4A11	6	0.98643	0.98651	0.99998	20114	0.1618
MEFV	6	0.98643	0.98651	0.99998	20115	0.1568
PPAPDC2	4	0.98647	0.98642	0.99998	20116	0.3778
STAU2	6	0.98654	0.9866	0.99998	20117	0.0785
NKRF	6	0.98656	0.98662	0.99998	20118	0.4422
NUP210L	6	0.9866	0.98666	0.99998	20119	0.1088
SYCP2L	4	0.98663	0.98656	0.99998	20120	0.2902
ANPEP	6	0.98665	0.98671	0.99998	20121	0.1517
CMC1	6	0.98677	0.98683	0.99998	20122	0.0893
RALGAPA2	6	0.98711	0.98717	0.99998	20123	0.146
T	6	0.9872	0.98727	0.99998	20124	0.1218
ALK	6	0.98724	0.98731	0.99998	20125	0.1072
CMPK2	6	0.9873	0.98737	0.99998	20126	0.3026
CHRNB4	5	0.98732	0.98728	0.99998	20127	0.3392
TMEM68	6	0.98734	0.98741	0.99998	20128	0.0732
HERC4	6	0.98746	0.98753	0.99998	20129	0.1964
DOK2	6	0.98747	0.98754	0.99998	20130	0.0929
AQP8	6	0.98748	0.98756	0.99998	20131	0.2989
STAP1	6	0.98752	0.9876	0.99998	20132	0.1209
NRXN3	6	0.98759	0.98767	0.99998	20133	0.1556
SOS1	6	0.98764	0.9877	0.99998	20134	0.1245
IFT74	6	0.98766	0.98773	0.99998	20135	0.3235
SLC23A2	6	0.98766	0.98773	0.99998	20136	0.3069
SIGIRR	6	0.98776	0.98782	0.99998	20137	0.1331
hsa-mir-6718	4	0.98777	0.98776	0.99998	20138	0.3258
hsa-mir-602	4	0.9878	0.9878	0.99998	20139	0.3329
SAMD7	6	0.9878	0.98787	0.99998	20140	0.214
POLI	6	0.98786	0.98793	0.99998	20141	0.2265
CCDC25	6	0.98786	0.98793	0.99998	20142	0.1684
SLC16A3	6	0.9879	0.98796	0.99998	20143	0.3406
ABCA10	6	0.9879	0.98796	0.99998	20144	0.1721
EDEM3	6	0.98798	0.98806	0.99998	20145	0.1275
SLC43A3	6	0.98801	0.98808	0.99998	20146	0.1801
SPO11	6	0.98801	0.98808	0.99998	20147	0.2333
PLAC1L	6	0.98804	0.98811	0.99998	20148	0.251
hsa-mir-3180-	1	0.98805	0.98807	0.99998	20149	0.8076
SRRM5	6	0.98808	0.98815	0.99998	20150	0.1403
FHL5	6	0.9882	0.98828	0.99998	20151	0.2659
C4orf19	6	0.98822	0.98829	0.99998	20152	0.0682
ZNF441	6	0.98823	0.9883	0.99998	20153	0.2372
SLC4A10	6	0.98828	0.98836	0.99998	20154	0.1381
SEMA3D	6	0.98835	0.98842	0.99998	20155	0.2566
SIRPB1	6	0.98842	0.9885	0.99998	20156	0.294
hsa-mir-3140	4	0.98843	0.98845	0.99998	20157	0.4076
KHDC1L	6	0.98844	0.98851	0.99998	20158	0.1914
KAL1	4	0.98847	0.98848	0.99998	20159	0.1633
SLC25A2	6	0.98848	0.98856	0.99998	20160	0.2875
hsa-mir-4423	2	0.98849	0.98851	0.99998	20161	0.3325
ZFYVE9	6	0.98853	0.98861	0.99998	20162	0.2226
TCF4	6	0.98854	0.98862	0.99998	20163	0.2057
ZFP62	6	0.98857	0.98864	0.99998	20164	0.3606
LOC10013037	6	0.9886	0.98867	0.99998	20165	0.0447
PAG1	6	0.98862	0.98869	0.99998	20166	0.3347

OTUD3	6	0.98867	0.98875	0.99998	20167	0.3873
GABRB3	6	0.98871	0.98879	0.99998	20168	0.263
GFI1B	6	0.98872	0.9888	0.99998	20169	0.4131
NIM1	6	0.98873	0.98881	0.99998	20170	0.1209
HBD	6	0.98879	0.98888	0.99998	20171	0.3057
TRIM36	6	0.98882	0.9889	0.99998	20172	0.1572
TMEM225	6	0.98883	0.98891	0.99998	20173	0.1462
PPP1R3C	6	0.98885	0.98893	0.99998	20174	0.0466
GJC1	6	0.98886	0.98894	0.99998	20175	0.0708
GRPR	6	0.98887	0.98895	0.99998	20176	0.2021
TMEM2	6	0.98888	0.98896	0.99998	20177	0.1039
TRAIP	6	0.98892	0.989	0.99998	20178	0.1207
B3GNT6	6	0.98894	0.98902	0.99998	20179	0.3005
LOC339862	6	0.98894	0.98902	0.99998	20180	0.2462
TRPC3	6	0.98895	0.98904	0.99998	20181	0.1889
MGME1	6	0.98895	0.98904	0.99998	20182	0.0677
NOC2L	6	0.98899	0.98907	0.99998	20183	0.0742
ELTD1	6	0.98904	0.98911	0.99998	20184	0.2
SDSL	6	0.98911	0.98917	0.99998	20185	0.252
TSPEAR	6	0.98917	0.98923	0.99998	20186	0.17
NRAP	6	0.98943	0.98951	0.99998	20187	0.2018
GLRX3	6	0.98944	0.98952	0.99998	20188	0.1264
UBE2W	6	0.98947	0.98955	0.99998	20189	0.213
GANC	6	0.98948	0.98956	0.99998	20190	0.3654
OR5M3	6	0.98952	0.98961	0.99998	20191	0.2808
SWAP70	6	0.98955	0.98965	0.99998	20192	0.19
AOX1	6	0.98971	0.9898	0.99998	20193	0.2723
CT62	6	0.98972	0.98981	0.99998	20194	0.1879
DHRS4L1	6	0.98974	0.98982	0.99998	20195	0.1853
HPR	6	0.98974	0.98982	0.99998	20196	0.2006
NOL3	6	0.98989	0.98996	0.99998	20197	0.2666
TCF21	6	0.98994	0.99	0.99998	20198	0.4121
hsa-mir-302c	4	0.98996	0.98993	0.99998	20199	0.4038
hsa-mir-4537	4	0.98998	0.98995	0.99998	20200	0.2254
hsa-mir-518b	4	0.98999	0.98996	0.99998	20201	1.2334
B4GALNT2	6	0.99002	0.99008	0.99998	20202	0.4629
C10orf95	6	0.99005	0.99012	0.99998	20203	0.2116
SLC22A8	6	0.99008	0.99015	0.99998	20204	0.2499
DLGAP4	6	0.9901	0.99017	0.99998	20205	0.1869
WSCD1	6	0.9901	0.99017	0.99998	20206	0.3265
PTPRR	6	0.99015	0.99021	0.99998	20207	0.2176
FSTL5	6	0.99016	0.99023	0.99998	20208	0.162
hsa-mir-1255a	4	0.99019	0.99016	0.99998	20209	0.6017
hsa-mir-6499	4	0.9902	0.99018	0.99998	20210	0.2642
BRD8	6	0.9902	0.99026	0.99998	20211	0.209
TCEA3	6	0.99022	0.99028	0.99998	20212	0.4789
GML	6	0.99029	0.99035	0.99998	20213	0.123
NEDD9	6	0.99029	0.99035	0.99998	20214	0.1836
TCF15	6	0.9903	0.99036	0.99998	20215	0.3136
NLRP14	6	0.99032	0.99037	0.99998	20216	0.1329
TWSG1	6	0.99034	0.9904	0.99998	20217	0.2312
PAK7	6	0.99036	0.99042	0.99998	20218	0.1724
KRTAP10-6	6	0.99041	0.99047	0.99998	20219	0.3004
TMEFF2	6	0.99046	0.99051	0.99998	20220	0.2336
ZNF320	4	0.99048	0.99046	0.99998	20221	0.5292
PDZD7	6	0.99049	0.99054	0.99998	20222	0.2734
ZNF319	6	0.99049	0.99054	0.99998	20223	0.1217
DCAF12L1	6	0.99049	0.99054	0.99998	20224	0.2236
GRK7	6	0.99052	0.99056	0.99998	20225	0.323
ZNF385B	6	0.99053	0.99058	0.99998	20226	0.1933
CCBL1	6	0.99056	0.9906	0.99998	20227	0.3547
SSTR3	6	0.99061	0.99065	0.99998	20228	0.1286
BICC1	6	0.99063	0.99068	0.99998	20229	0.1233
CCDC88B	6	0.9907	0.99074	0.99998	20230	0.2071
PCDH17	6	0.99073	0.99076	0.99998	20231	0.1948
KCP	6	0.99075	0.99078	0.99998	20232	0.1496
CDRT15L2	6	0.99075	0.99079	0.99998	20233	0.2543
IRX1	6	0.99083	0.99086	0.99998	20234	0.3012
VAV1	6	0.99091	0.99094	0.99998	20235	0.222
CAV3	6	0.99094	0.99098	0.99998	20236	0.3171
ONECUT2	6	0.99097	0.99102	0.99998	20237	0.3225
CUL4A	6	0.99099	0.99103	0.99998	20238	0.1028
GABRA1	6	0.99102	0.99107	0.99998	20239	0.2847
RBM10	6	0.99109	0.99115	0.99998	20240	0.1937
SLC22A24	4	0.9911	0.9911	0.99998	20241	0.4779
hsa-mir-6765	4	0.99113	0.99114	0.99998	20242	0.2683
SSTR5	6	0.99113	0.99118	0.99998	20243	0.2321
EXT2	6	0.99122	0.99127	0.99998	20244	0.4449
QRICH2	6	0.99124	0.99128	0.99998	20245	0.2197
NMRK2	6	0.99129	0.99134	0.99998	20246	0.1565
RERGL	6	0.99133	0.99136	0.99998	20247	0.2247

ANKRD9	6	0.99139	0.99142	0.99998	20248	0.3125
hsa-mir-1297	3	0.99141	0.99151	0.99998	20249	0.4661
IMPAD1	6	0.99145	0.99148	0.99998	20250	0.2095
PCK1	6	0.99147	0.9915	0.99998	20251	0.1985
hsa-mir-410	4	0.99155	0.99157	0.99998	20252	0.4777
CLDN20	6	0.99155	0.9916	0.99998	20253	0.1709
RNASE12	6	0.99158	0.99163	0.99998	20254	0.2658
PARP8	6	0.99171	0.99175	0.99998	20255	0.2103
hsa-mir-6865	4	0.99183	0.99183	0.99998	20256	0.3803
MBOAT4	6	0.99185	0.9919	0.99998	20257	0.2627
ZNF790	6	0.99188	0.99193	0.99998	20258	0.2525
TOX3	6	0.99188	0.99193	0.99998	20259	0.2364
WDR19	6	0.99189	0.99194	0.99998	20260	0.2835
SAMD3	6	0.99196	0.99202	0.99998	20261	0.2234
TNFRSF14	6	0.99201	0.99208	0.99998	20262	0.2004
SPAM1	6	0.99204	0.99211	0.99998	20263	0.1372
CDC14A	4	0.99207	0.99206	0.99998	20264	0.3413
SLC41A3	6	0.99209	0.99215	0.99998	20265	0.1857
LEMD3	4	0.99225	0.99225	0.99998	20266	0.3421
FSBP	6	0.99237	0.99243	0.99998	20267	0.369
ZGPAT	6	0.9924	0.99245	0.99998	20268	0.2934
OCRL	6	0.99244	0.9925	0.99998	20269	0.2699
SLC5A4	6	0.99252	0.99258	0.99998	20270	0.1558
DSCAML1	6	0.99254	0.9926	0.99998	20271	0.4016
STAT5B	6	0.99256	0.99262	0.99998	20272	0.1984
ATP6V0E1	6	0.99256	0.99262	0.99998	20273	0.1792
MYH2	6	0.99256	0.99262	0.99998	20274	0.1865
IGSF8	6	0.99257	0.99263	0.99998	20275	0.1697
CLEC18A	2	0.99269	0.99271	0.99998	20276	0.4543
SMR3B	4	0.99275	0.99276	0.99998	20277	0.2687
SETD3	6	0.99276	0.99282	0.99998	20278	0.2524
USP40	6	0.99277	0.99283	0.99998	20279	0.0912
GPR1	6	0.99281	0.99287	0.99998	20280	0.1461
hsa-mir-6085	4	0.99281	0.99282	0.99998	20281	0.233
TNC	6	0.99293	0.993	0.99998	20282	0.2537
SPINK13	6	0.99295	0.99302	0.99998	20283	0.1571
OR51I1	6	0.99296	0.99303	0.99998	20284	0.2401
SMYD4	6	0.99297	0.99305	0.99998	20285	0.3435
ASPA	6	0.99303	0.99311	0.99998	20286	0.1114
TNS4	6	0.99304	0.99312	0.99998	20287	0.3113
MSRB1	6	0.99305	0.99313	0.99998	20288	0.4397
PTH2	6	0.99312	0.9932	0.99998	20289	0.2241
GORAB	4	0.99313	0.99313	0.99998	20290	0.1787
AGXT2L2	1	0.99314	0.99313	0.99998	20291	0.7444
FGD4	6	0.99316	0.99324	0.99998	20292	0.0671
TPRG1	6	0.99316	0.99324	0.99998	20293	0.317
PRAF2	6	0.99317	0.99324	0.99998	20294	0.2147
SGPP2	6	0.99321	0.99329	0.99998	20295	0.3101
SLC25A16	6	0.99326	0.99332	0.99998	20296	0.1523
CCDC77	6	0.99331	0.99337	0.99998	20297	0.335
TMEM54	6	0.99331	0.99337	0.99998	20298	0.1805
GALR2	6	0.99333	0.9934	0.99998	20299	0.423
GIF	6	0.99342	0.99348	0.99998	20300	0.1679
SYNJ2	6	0.99345	0.99351	0.99998	20301	0.27
TMEM186	6	0.99345	0.99351	0.99998	20302	0.6141
TMEM57	6	0.99348	0.99353	0.99998	20303	0.4359
INHBB	6	0.9935	0.99355	0.99998	20304	0.2249
KLHL35	6	0.99351	0.99357	0.99998	20305	0.4548
SIVA1	6	0.99356	0.99362	0.99998	20306	0.3597
FAM19A5	6	0.99364	0.99371	0.99998	20307	0.438
BCOR	6	0.99378	0.99383	0.99998	20308	0.2715
ZC4H2	6	0.99381	0.99386	0.99998	20309	0.3508
PLBD1	6	0.99382	0.99387	0.99998	20310	0.1273
RAPGEF5	6	0.99389	0.99393	0.99998	20311	0.1108
ACSF3	6	0.99397	0.99402	0.99998	20312	0.1082
ARMC8	6	0.99398	0.99403	0.99998	20313	0.2332
OXGR1	6	0.994	0.99405	0.99998	20314	0.2166
METTL4	6	0.99405	0.99409	0.99998	20315	0.3479
VSTM1	6	0.99406	0.9941	0.99998	20316	0.5015
AMER2	6	0.99408	0.99412	0.99998	20317	0.2748
ENPP6	6	0.99408	0.99413	0.99998	20318	0.3092
C16orf13	6	0.99412	0.99415	0.99998	20319	0.2332
FEZF1	6	0.99418	0.99421	0.99998	20320	0.1418
hsa-mir-144	4	0.99423	0.99425	0.99998	20321	0.4369
C10orf90	6	0.99433	0.99436	0.99998	20322	0.192
ZNF536	6	0.99437	0.99439	0.99998	20323	0.2497
ANKRD55	6	0.99438	0.99441	0.99998	20324	0.1677
SHPRH	6	0.99452	0.99455	0.99998	20325	0.2023
TIGIT	6	0.99454	0.99457	0.99998	20326	0.2439
TMEM8C	6	0.99459	0.99462	0.99998	20327	0.2818
TOPORS	6	0.99465	0.99468	0.99998	20328	0.254

SEC14L2	6	0.99467	0.9947	0.99998	20329	0.2125
C17orf112	6	0.99468	0.99471	0.99998	20330	0.2405
hsa-mir-520e	4	0.99499	0.99499	0.99998	20331	0.6876
OR51B2	6	0.99502	0.99506	0.99998	20332	0.3425
ZNF711	6	0.99511	0.99516	0.99998	20333	0.1775
C2orf73	6	0.99518	0.99523	0.99998	20334	0.2207
RBMY1J	2	0.99522	0.99522	0.99998	20335	0.5643
C9orf172	6	0.99526	0.99531	0.99998	20336	0.355
GP9	6	0.99526	0.99532	0.99998	20337	0.5156
TEX38	6	0.99529	0.99535	0.99998	20338	0.2774
ORC2	6	0.99529	0.99535	0.99998	20339	0.1408
RBPMS	6	0.99542	0.99548	0.99998	20340	0.1968
SMPD1	6	0.99552	0.99559	0.99998	20341	0.2219
RAB21	6	0.99553	0.9956	0.99998	20342	0.1449
KCTD9	6	0.99557	0.99563	0.99998	20343	0.3628
TAAR6	6	0.99557	0.99563	0.99998	20344	0.1819
SLC31A1	6	0.99565	0.99571	0.99998	20345	0.4178
DDX53	6	0.9957	0.99576	0.99998	20346	0.2277
ZNF521	6	0.99571	0.99577	0.99998	20347	0.2763
VAT1	6	0.99574	0.9958	0.99998	20348	0.142
ACADL	6	0.9958	0.99586	0.99998	20349	0.1946
hsa-mir-224	4	0.99582	0.99579	0.99998	20350	0.4717
HDHD1	6	0.99585	0.99591	0.99998	20351	0.2051
GSC2	6	0.9959	0.99595	0.99998	20352	0.2629
CBLC	6	0.99592	0.99597	0.99998	20353	0.1827
LCA5	6	0.99593	0.99598	0.99998	20354	0.2275
ZNF862	6	0.99595	0.99599	0.99998	20355	0.1594
YIF1B	6	0.99596	0.99601	0.99998	20356	0.1496
FAM76B	6	0.99597	0.99602	0.99998	20357	0.2812
EDAR	6	0.99601	0.99605	0.99998	20358	0.311
C9orf91	6	0.99603	0.99608	0.99998	20359	0.3491
C4orf45	6	0.99605	0.9961	0.99998	20360	0.2508
LPPR5	6	0.99606	0.9961	0.99998	20361	0.1545
ARNTL2	6	0.9961	0.99614	0.99998	20362	0.2194
OR1L8	6	0.99612	0.99616	0.99998	20363	0.3616
BPIFA3	6	0.99618	0.99622	0.99998	20364	0.3551
ASCL5	6	0.9962	0.99624	0.99998	20365	0.2658
hsa-mir-6785	4	0.9962	0.99618	0.99998	20366	0.5778
CTNNA3	6	0.99624	0.99628	0.99998	20367	0.244
FNDC3A	6	0.99626	0.9963	0.99998	20368	0.2771
TKTL2	6	0.99632	0.99637	0.99998	20369	0.2509
CDK14	6	0.99635	0.99639	0.99998	20370	0.2896
MPP4	6	0.99636	0.99641	0.99998	20371	0.2179
ZNF709	6	0.9964	0.99644	0.99998	20372	0.3074
C3orf20	6	0.99648	0.99652	0.99998	20373	0.1571
HDAC11	6	0.99652	0.99657	0.99998	20374	0.1714
ZNF557	6	0.99669	0.99674	0.99998	20375	0.2422
LIPG	6	0.99673	0.99678	0.99998	20376	0.3147
ELMO1	6	0.99675	0.99679	0.99998	20377	0.3244
NEXN	6	0.99679	0.99684	0.99998	20378	0.41
FYTTD1	6	0.9968	0.99685	0.99998	20379	0.3173
MIXL1	6	0.9968	0.99685	0.99998	20380	0.4421
TMEM135	6	0.99681	0.99687	0.99998	20381	0.3329
DLEU7	6	0.99682	0.99688	0.99998	20382	0.3891
PADI1	6	0.99686	0.99691	0.99998	20383	0.2532
ADCYAP1R1	6	0.99691	0.99697	0.99998	20384	0.2445
AMMECR1	6	0.99692	0.99698	0.99998	20385	0.1759
RGS19	6	0.99696	0.99702	0.99998	20386	0.2826
SOGA1	6	0.99701	0.99707	0.99998	20387	0.4324
SCRN1	6	0.9971	0.99716	0.99998	20388	0.2146
PBXIP1	6	0.99714	0.99718	0.99998	20389	0.4069
TCTEX1D4	6	0.99717	0.99721	0.99998	20390	0.3591
STYXL1	6	0.99721	0.99726	0.99998	20391	0.3342
RB1	6	0.99723	0.99726	0.99998	20392	0.2249
ADAM33	6	0.9973	0.99733	0.99998	20393	0.2376
CDH2	6	0.99733	0.99735	0.99998	20394	0.3621
OR52H1	6	0.99746	0.99748	0.99998	20395	0.2048
STARD6	6	0.9975	0.99752	0.99998	20396	0.2352
RAP2C	6	0.99751	0.99753	0.99998	20397	0.332
MYO7A	6	0.99755	0.99757	0.99998	20398	0.2092
PAFAH2	6	0.99757	0.9976	0.99998	20399	0.2116
CRIP2	6	0.99767	0.99771	0.99998	20400	0.1984
ZNF197	6	0.99767	0.99772	0.99998	20401	0.3981
DHRS9	6	0.99771	0.99774	0.99998	20402	0.333
DSG3	6	0.99771	0.99774	0.99998	20403	0.3351
PKN2	6	0.99777	0.9978	0.99998	20404	0.1821
C19orf82	3	0.9978	0.99784	0.99998	20405	0.4684
ZBTB44	6	0.99787	0.99789	0.99998	20406	0.2834
EXO5	6	0.99788	0.99791	0.99998	20407	0.2502
PDS5B	6	0.99793	0.99795	0.99998	20408	0.3753
PPP1R12C	6	0.99795	0.99797	0.99998	20409	0.3287

MAGEB10	6	0.99804	0.99805	0.99998	20410	0.3998
CHGB	6	0.99817	0.99817	0.99998	20411	0.2501
IRX3	6	0.99817	0.99818	0.99998	20412	0.2663
TRIM35	6	0.99823	0.99824	0.99998	20413	0.4804
LOC10050554	6	0.99823	0.99824	0.99998	20414	0.3012
IL20RA	6	0.99829	0.99829	0.99998	20415	0.3702
GATM	6	0.99831	0.99832	0.99998	20416	0.3312
GPR180	6	0.99834	0.99834	0.99998	20417	0.3106
hsa-mir-4528	2	0.99836	0.99835	0.99998	20418	0.894
ABLIM1	6	0.99837	0.99837	0.99998	20419	0.2325
KCNJ2	6	0.99841	0.99841	0.99998	20420	0.2544
HS2ST1	6	0.99851	0.9985	0.99998	20421	0.3471
ZBTB8B	6	0.99855	0.99853	0.99998	20422	0.3452
SLC23A3	6	0.99858	0.99856	0.99998	20423	0.2643
KCNB2	6	0.99864	0.99863	0.99998	20424	0.223
N4BP2	6	0.99865	0.99865	0.99998	20425	0.2138
CYP3A7-CYP3/	2	0.9987	0.99869	0.99998	20426	0.6734
TAS2R1	6	0.99873	0.99872	0.99998	20427	0.2766
KCNF1	6	0.99877	0.99875	0.99998	20428	0.4318
C9orf173	6	0.99881	0.9988	0.99998	20429	0.3511
C5orf54	6	0.99884	0.99883	0.99998	20430	0.4539
C7orf41	6	0.99885	0.99884	0.99998	20431	0.2605
CYSLTR2	6	0.99885	0.99884	0.99998	20432	0.3304
KRTAP3-1	6	0.99887	0.99886	0.99998	20433	0.411
WRAP73	6	0.99887	0.99886	0.99998	20434	0.2878
PGBD1	6	0.9989	0.99889	0.99998	20435	0.3518
B9D1	6	0.99904	0.99903	0.99998	20436	0.4112
SMAD7	6	0.99904	0.99903	0.99998	20437	0.1991
FAM222B	6	0.9991	0.9991	0.99998	20438	0.3603
KLHL14	6	0.99919	0.9992	0.99998	20439	0.2741
ZNF772	6	0.99921	0.99921	0.99998	20440	0.2837
NEUROD2	6	0.99922	0.99922	0.99998	20441	0.499
CCDC68	6	0.99925	0.99925	0.99998	20442	0.2902
TUBB4B	6	0.99939	0.99939	0.99998	20443	0.915
G6PC2	6	0.99944	0.99944	0.99998	20444	0.203
STX11	6	0.99945	0.99944	0.99998	20445	0.2878
PTPRA	6	0.99949	0.99949	0.99998	20446	0.292
SHROOM2	6	0.99951	0.99951	0.99998	20447	0.3098
HDAC8	6	0.99951	0.99951	0.99998	20448	0.3804
WNT11	6	0.99952	0.99952	0.99998	20449	0.3546
FRMD3	6	0.99961	0.99961	0.99998	20450	0.3209
TGM4	6	0.99962	0.99961	0.99998	20451	0.3441
CST9L	6	0.99967	0.99967	0.99998	20452	0.2626
CCDC166	6	0.99976	0.99976	0.99998	20453	0.3448
LOC10012952	6	0.9998	0.99979	0.99998	20454	0.3385
PCDHGB6	2	0.99981	0.99981	0.99998	20455	0.7348
TNP2	6	0.99984	0.99983	0.99998	20456	0.4284
FZD8	6	0.99985	0.99985	0.99998	20457	0.4389
RTCB	4	0.99991	0.9999	0.99998	20458	0.7722
BRS3	6	0.99993	0.99993	0.99998	20459	0.4266
PPM1F	6	0.99998	0.99999	0.99999	20460	0.3539
