# Supplementary material for: ASH2L drives proliferation and sensitivity to bleomycin and other genotoxins in Hodgkin’s lymphoma and testicular cancer cells
Source: Cell Death Dis. 2020 Nov 30;11(11):1019. doi: 10.1038/s41419-020-03231-0 (PMC7705021; doi:10.1038/s41419-020-03231-0)
Supplement: Supplementary file 3 — Table S3 [file 41419_2020_3231_MOESM3_ESM.pdf]

Information:

In the next sheet in this file we provide the differential gene expression analysis between control (shCtrl) and ASH2L depleted (shASH2L) L1236 cells. Three indpendent experiments were performed.

Row 1 is a header and contains information about the content of the respective column: Gene ID, Fold change, gene description, gene location, fold discovery rate (FDR), etc. The expression levels (represented as normalized counts per million reads). The last 6 columns contain the data regarding gene reads, for all 6 individual experiments.

Gene_ID	P.Value	adj.P.Val (FDR)	log2 Fold changeC	Fold_Change	Gene_Name	Gene_type	Description	Chromosome_Name	Gene_Start	Gene_End	Strand	Source_gene	Average expression all exps	Average expression shCtrl	Average expression shASH2L	shCtr-3	shCtr-2	shCtr-1	shASH2L-3	shASH2L-2	shASH2L-1
ENSG00000205038	4.14E-07	0.004356803	5.78906795	55.29464828	**PKHD1L1**	protein_coding	PKHD1 like 1 [Source:HGNC Symbol;Acc:HGNC:20313]	chr8	109362477	109530330	1	ensembl_havana	-1.153936762	-4.048470737	1.740597213	-4.33318091	-4.389814537	-3.422416761	1.212343926	1.682828385	2.326619329
ENSG00000260314	4.12E-06	0.004356803	4.162838001	17.91179481	**MRC1**	protein_coding	mannose receptor C-type 1 [Source:HGNC Symbol;Acc:HGNC:7228]	chr10	17809344	17911170	1	ensembl_havana	-0.703434846	-2.784853847	1.377984154	-2.32062672	-2.611518056	-3.422416761	1.514288036	1.529563273	1.090101154
ENSG00000240216	1.84E-06	0.004356803	2.102586301	4.294786161	**CPHL1P**	transcribed_unitary_pseudogene	ceruloplasmin and hephaestin-like 1 pseudogene [Source:HGNC Symbol;Acc:HGNC:31714]	chr3	149229356	149291800	-1	havana	-0.720819917	-1.772113067	0.330473234	-2.32062672	-1.72386249	-1.271849989	-0.12879794	0.343769191	0.77644845
ENSG00000069535	3.81E-06	0.004356803	1.659595837	3.159280068	**MAOB**	protein_coding	monoamine oxidase B [Source:HGNC Symbol;Acc:HGNC:6834]	chrX	43766611	43882447	-1	ensembl_havana	1.380504517	0.550706598	2.210302435	0.355307344	0.720251491	0.57656096	2.053540577	2.315517031	2.261849697
ENSG00000122122	3.23E-06	0.004356803	-1.596021279	-3.023084444	**SASH3**	protein_coding	SAM and SH3 domain containing 3 [Source:HGNC Symbol;Acc:HGNC:15975]	chrX	129779979	129795201	1	ensembl_havana	5.027635941	5.82564658	4.229625301	5.975266871	5.838498013	5.663174856	4.378023791	4.2585809	4.052271213
ENSG00000189233	4.37E-06	0.004356803	-1.690526313	-3.227744344	**NUGGC**	protein_coding	nuclear GTPase, germinal center associated [Source:HGNC Symbol;Acc:HGNC:33550]	chr8	28021964	28083871	-1	ensembl_havana	4.264692637	5.109955793	3.41942948	5.292691307	4.884552362	5.152623711	3.669971868	3.203092147	3.385224425
ENSG00000182578	4.55E-06	0.004356803	-1.849037314	-3.602597096	**CSF1R**	protein_coding	colony stimulating factor 1 receptor [Source:HGNC Symbol;Acc:HGNC:2433]	chr5	150053291	150113372	-1	ensembl_havana	3.915927406	4.840446063	2.991408749	5.244799796	4.468781447	4.807756945	3.279095331	2.677309452	3.017821464
ENSG00000102970	3.12E-06	0.004356803	-1.870816288	-3.657394599	**CCL17**	protein_coding	C-C motif chemokine ligand 17 [Source:HGNC Symbol;Acc:HGNC:10615]	chr16	57404767	57416062	1	ensembl_havana	5.859839974	6.795248118	4.92443183	6.86094621	6.80596713	6.718831015	5.012704806	4.84667624	4.913914443
ENSG00000188488	3.34E-06	0.004356803	-1.919910162	-3.783994946	**SERPINA5**	protein_coding	serpin family A member 5 [Source:HGNC Symbol;Acc:HGNC:8723]	chr14	94561442	94593120	1	ensembl_havana	4.106647719	5.0666028	3.146692638	5.547702161	4.775201171	4.876905068	3.535494485	2.85657011	3.048013319
ENSG00000244731	1.50E-06	0.004356803	-1.976497059	-3.935363934	**C4A**	protein_coding	complement C4A (Rodgers blood group) [Source:HGNC Symbol;Acc:HGNC:1323]	chr6	31982024	32002681	1	ensembl_havana	4.365323856	5.353572386	3.377075327	5.628960829	4.9819684	5.449787929	3.607495467	3.024849801	3.498880712
ENSG00000151224	2.25E-06	0.004356803	-2.006109041	-4.016973771	**MAT1A**	protein_coding	methionine adenosyltransferase 1A [Source:HGNC Symbol;Acc:HGNC:6903]	chr10	80271820	80289684	-1	ensembl_havana	0.772522557	1.775577078	-0.230531963	1.968722995	1.909699171	1.448309068	-0.08686711	-0.00684508	-0.597883695
ENSG00000139193	3.10E-06	0.004356803	-2.489789057	-5.616958163	**CD27**	protein_coding	CD27 molecule [Source:HGNC Symbol;Acc:HGNC:11922]	chr12	6444867	6451718	1	ensembl_havana	2.618906196	3.863800725	1.374011667	3.760287894	3.776492281	4.054621999	1.107892607	1.300906777	1.713235619
ENSG00000182621	4.40E-06	0.004356803	-2.605452474	-6.085823415	**PLCB1**	protein_coding	phospholipase C beta 1 [Source:HGNC Symbol;Acc:HGNC:15917]	chr20	8077251	8968360	1	ensembl_havana	-0.67820699	0.624519247	-1.980933226	0.791576534	0.378171619	0.703809589	-1.90365811	-2.00847383	-2.030667746
ENSG00000129691	7.83E-07	0.004356803	-2.947904408	-7.716274187	**ASH2L**	protein_coding	ASH2 like histone lysine methyltransferase complex subunit [Source:HGNC Symbol;Acc:HGNC:744]	chr8	38105242	38144076	1	ensembl_havana	3.906995482	5.380947686	2.433043278	5.32827068	5.450495655	5.364076724	2.246292314	2.566495926	2.486341593
ENSG00000005981	3.06E-06	0.004356803	-5.425341702	-42.9724969	**ASB4**	protein_coding	ankyrin repeat and SOCS box containing 4 [Source:HGNC Symbol;Acc:HGNC:16009]	chr7	95478444	95540232	1	ensembl_havana	-1.967560362	0.745110488	-4.680231213	-0.41068387	1.364499946	1.281515389	-5.3500916	-4.33911973	-4.351482308
ENSG00000226025	6.09E-06	0.00542025	-1.447372553	-2.727109355	**AC005515.1**	transcribed_unprocessed_pseudogene		chr19	39679374	39686373	1	havana	-0.235940093	0.487746183	-0.959626369	0.727424294	0.298820819	0.436993436	-0.75894329	-1.15951152	-0.960424301
ENSG00000167476	6.42E-06	0.00542025	-1.481626664	-2.792634303	**JSRP1**	protein_coding	junctional sarcoplasmic reticulum protein 1 [Source:HGNC Symbol;Acc:HGNC:24963]	chr19	2252252	2269759	-1	ensembl_havana	-0.090513657	0.650299675	-0.831326989	1.075455542	0.592979824	0.28246366	-0.35894771	-0.86978558	-1.265247677
ENSG00000173662	7.78E-06	0.005923552	-1.362472432	-2.571254533	**TAS1R1**	protein_coding	taste 1 receptor member 1 [Source:HGNC Symbol;Acc:HGNC:14448]	chr1	6555181	6579757	1	ensembl_havana	3.854179738	4.535415954	3.172943522	4.498880254	4.530870564	4.576497044	3.090972172	3.210869813	3.21698858
ENSG00000145416	7.84E-06	0.005923552	-1.430219175	-2.69487653	**MARCH1**	protein_coding	membrane associated ring-CH-type finger 1 [Source:HGNC Symbol;Acc:HGNC:26077]	chr4	163524298	164384050	-1	ensembl_havana	2.268634317	2.983743905	1.55352473	3.032160045	2.826996371	3.092075299	1.644455473	1.426455924	1.589662793
ENSG00000187994	8.93E-06	0.006412366	-1.678557737	-3.201077791	**RINL**	protein_coding	Ras and Rab interactor like [Source:HGNC Symbol;Acc:HGNC:24795]	chr19	38867834	38878279	-1	ensembl_havana	3.10519415	3.944473019	2.265915282	4.045427109	3.680623265	4.107368684	2.40164672	2.097907935	2.298191191
ENSG00000113946	1.24E-05	0.007474584	1.89844078	3.728100574	**CLDN16**	protein_coding	claudin 16 [Source:HGNC Symbol;Acc:HGNC:2037]	chr3	190322541	190412143	1	ensembl_havana	1.11581272	0.16659233	2.06503311	-0.03191641	0.09469996	0.436993436	1.966610161	2.089494957	2.138994213
ENSG00000080618	1.36E-05	0.007474584	-1.169621138	-2.24952615	**CPB2**	protein_coding	carboxypeptidase B2 [Source:HGNC Symbol;Acc:HGNC:2300]	chr13	46053195	46105033	-1	ensembl_havana	-0.811695095	-0.226884526	-1.396505664	-0.22998119	0.185737694	-0.636410081	-1.41444944	-1.00738779	-1.767679756
ENSG00000188493	1.53E-05	0.007474584	-1.188015022	-2.278390477	**C19orf54**	protein_coding	chromosome 19 open reading frame 54 [Source:HGNC Symbol;Acc:HGNC:24758]	chr19	40740856	40751553	-1	ensembl_havana	4.470852423	5.064859933	3.876844912	5.076899593	5.026777573	5.090902635	3.942194775	3.836638454	3.851701507
ENSG00000142621	1.16E-05	0.007474584	-1.240038894	-2.362049001	**FHAD1**	protein_coding	forkhead associated phosphopeptide binding domain 1 [Source:HGNC Symbol;Acc:HGNC:29408]	chr1	15247272	15400283	1	ensembl_havana	2.877665775	3.497685222	2.257646327	3.44832903	3.503197858	3.541528776	2.254470464	2.256618822	2.261849697
ENSG00000170476	1.29E-05	0.007474584	-1.438496592	-2.710382738	**MZB1**	protein_coding	marginal zone B and B1 cell specific protein [Source:HGNC Symbol;Acc:HGNC:30125]	chr5	139387480	139390081	-1	ensembl_havana	5.692861658	6.412109954	4.973613362	6.528622774	6.246642252	6.461064836	4.976083394	4.864043597	5.080713097
ENSG00000165029	1.58E-05	0.007474584	-1.544071277	-2.916162828	**ABCA1**	protein_coding	ATP binding cassette subfamily A member 1 [Source:HGNC Symbol;Acc:HGNC:29]	chr9	104781002	104928237	-1	ensembl_havana	1.208813342	1.98084898	0.436777703	2.602259737	1.688874845	1.651412359	0.915012722	0.256279753	0.139040636
ENSG00000205744	1.43E-05	0.007474584	-1.645932768	-3.129501284	**DENND1C**	protein_coding	DENN domain containing 1C [Source:HGNC Symbol;Acc:HGNC:26225]	chr19	6467207	6482557	-1	ensembl_havana	2.747999795	3.570966179	1.925033411	3.97580247	3.380093932	3.357002135	2.294679948	1.614107429	1.866312856
ENSG00000198734	1.67E-05	0.007474584	-1.819109596	-3.528633507	**F5**	protein_coding	coagulation factor V [Source:HGNC Symbol;Acc:HGNC:3542]	chr1	169514166	169586588	-1	ensembl_havana	1.481769938	2.391324736	0.57221514	2.497167313	2.29082961	2.385977285	0.540060475	0.671430284	0.505154661
ENSG00000099337	1.62E-05	0.007474584	-1.829781995	-3.554833515	**KCNK6**	protein_coding	potassium two pore domain channel subfamily K member 6 [Source:HGNC Symbol;Acc:HGNC:6281]	chr19	38319844	38332076	1	ensembl_havana	1.122807854	2.037698852	0.207916856	2.023178671	2.021022296	2.068895587	0.032074365	0.163140205	0.428535999
ENSG00000228607	1.32E-05	0.007474584	-1.906262623	-3.748368064	**CLDN25**	protein_coding	claudin 25 [Source:HGNC Symbol;Acc:HGNC:37218]	chr11	113779747	113780500	1	ensembl_havana	3.019754192	3.972885504	2.066622881	3.950266431	3.980970421	3.987419659	1.841901733	2.179444752	2.178522159
ENSG00000144366	1.42E-05	0.007474584	-2.004380682	-4.012164288	**GULP1**	protein_coding	GULP, engulfment adaptor PTB domain containing 1 [Source:HGNC Symbol;Acc:HGNC:18649]	chr2	188291669	188595931	1	ensembl_havana	1.776410239	2.77860058	0.774219898	2.781444624	2.592024241	2.962332875	0.894226935	0.693803314	0.734629445
ENSG00000166199	1.48E-05	0.007474584	-2.305097016	-4.942006868	**ALKBH3**	protein_coding	alkB homolog 3, alpha-ketoglutaratedependent dioxygenase [Source:HGNC Symbol;Acc:HGNC:30141]	chr11	43880811	43920266	1	ensembl_havana	-0.065345423	1.087203085	-1.21789393	1.516132283	1.062111262	0.683365711	-0.97139419	-1.32958732	-1.352700279
ENSG00000213626	1.96E-05	0.008036359	-1.218675785	-2.327329993	**LBH**	protein_coding	limb bud and heart development [Source:HGNC Symbol;Acc:HGNC:29532]	chr2	30231531	30323730	1	ensembl_havana	3.407403361	4.016741254	2.798065469	3.97580247	3.942327796	4.132093496	2.675796091	2.726970195	2.99143012
ENSG00000168062	1.92E-05	0.008036359	-1.237654446	-2.358148293	**BATF2**	protein_coding	basic leucine zipper ATF-like transcription factor 2 [Source:HGNC Symbol;Acc:HGNC:25163]	chr11	64987943	64997045	-1	ensembl_havana	1.665922841	2.284750064	1.047095618	2.183196095	2.488543365	2.182510732	0.955706889	1.163381334	1.022198632
ENSG00000211890	1.94E-05	0.008036359	-1.524025836	-2.875924568	**IGHA2**	IG_C_gene	immunoglobulin heavy constant alpha 2 (A2m marker) [Source:HGNC Symbol;Acc:HGNC:5479]	chr14	105583731	105588395	-1	havana	2.896574144	3.658587062	2.134561226	3.625980797	3.678012323	3.671768066	2.170524752	1.993561814	2.239597111
ENSG00000228815	2.22E-05	0.008663711	1.506971152	2.842127256	**MARK2P13**	processed_pseudogene	microtubule affinity regulating kinase 2 pseudogene 13 [Source:HGNC Symbol;Acc:HGNC:39804]	chr7	36109024	36110872	-1	havana	0.108982979	-0.644502597	0.862468556	-1.23095973	-0.842251242	0.139703184	0.36983092	0.737534657	1.480040089
ENSG00000253303	2.23E-05	0.008663711	-1.078005918	-2.111116096	**IGHVIII-82**	IG_V_pseudogene	immunoglobulin heavy variable (III)-82 (pseudogene) [Source:HGNC Symbol;Acc:HGNC:5709]	chr14	106879563	106879812	-1	havana	-0.573905341	-0.034902382	-1.1129083	0.206373087	-0.258437325	-0.052642908	-0.82634296	-1.32958732	-1.182794618
ENSG00000227507	2.37E-05	0.008958192	-1.806782385	-3.498611291	**LTB**	protein_coding	lymphotoxin beta [Source:HGNC Symbol;Acc:HGNC:6711]	chr6	31580525	31582522	-1	ensembl_havana	0.45769101	1.361082202	-0.445700183	1.268150092	1.284372232	1.530724282	-0.30988669	-0.62862566	-0.398588198
ENSG00000103888	2.78E-05	0.009297275	1.728129059	3.312979001	**CEMIP**	protein_coding	cell migration inducing hyaluronan binding protein [Source:HGNC Symbol;Acc:HGNC:29213]	chr15	80779343	80951776	1	ensembl_havana	0.269695657	-0.594368873	1.133760187	-1.41179303	-0.569826728	0.198513145	0.457455004	1.241396536	1.70242902
ENSG00000236453	2.68E-05	0.009297275	1.336200881	2.524855601	**AC003092.1**	lincRNA		chr7	94022833	94066661	1	havana	1.163662856	0.495562415	1.831763297	0.004630787	0.570631912	0.911424546	1.386165364	2.002551439	2.106537087
ENSG00000044524	2.76E-05	0.009297275	1.026783668	2.037476851	**EPHA3**	protein_coding	EPH receptor A3 [Source:HGNC Symbol;Acc:HGNC:3387]	chr3	89107524	89482134	1	ensembl_havana	1.917419484	1.40402765	2.430811318	1.206734491	1.364499946	1.640848515	2.246292314	2.433220573	2.612921068
ENSG00000038945	2.60E-05	0.009297275	-1.062896869	-2.089122179	**MSR1**	protein_coding	macrophage scavenger receptor 1 [Source:HGNC Symbol;Acc:HGNC:7376]	chr8	16107878	16567490	-1	ensembl_havana	0.863250632	1.394699067	0.331802198	1.283104003	1.464851682	1.436141515	0.276538753	0.343769191	0.375098651
ENSG00000268655	2.67E-05	0.009297275	-1.587004049	-3.004248285	**AC008687.4**	protein_coding		chr19	49017496	49020523	-1	havana	0.430772428	1.224274452	-0.362729596	1.749374982	1.029710695	0.89373768	0.308311185	-0.74417246	-0.652327512
ENSG00000019144	2.86E-05	0.009329018	-0.996881989	-1.995682186	**PHLDB1**	protein_coding	pleckstrin homology like domain family B member 1 [Source:HGNC Symbol;Acc:HGNC:23697]	chr11	118606440	118658038	1	ensembl_havana	4.818723342	5.317164337	4.320282348	5.563099455	5.315060343	5.073333212	4.606043821	4.277220992	4.07758223
ENSG00000036530	3.35E-05	0.009830711	1.010002088	2.013914014	**CYP46A1**	protein_coding	cytochrome P450 family 46 subfamily A member 1 [Source:HGNC Symbol;Acc:HGNC:2641]	chr14	99684304	99727301	1	ensembl_havana	0.69115804	0.186156996	1.196159084	0.004630787	0.298820819	0.255019383	1.071324589	1.315408538	1.201744126
ENSG00000114805	3.27E-05	0.009830711	-1.072843675	-2.103575611	**PLCH1**	protein_coding	phospholipase C eta 1 [Source:HGNC Symbol;Acc:HGNC:29185]	chr3	155375580	155745067	-1	ensembl_havana	-0.011730507	0.524691331	-0.548152344	0.004630787	0.658018659	0.911424546	-1.13267621	-0.32890884	-0.182871985
ENSG00000150457	3.27E-05	0.009830711	-1.117722349	-2.170041073	**LATS2**	protein_coding	large tumor suppressor kinase 2 [Source:HGNC Symbol;Acc:HGNC:6515]	chr13	20973032	21061547	-1	ensembl_havana	1.031940393	1.590801568	0.473079219	1.802235788	1.558674763	1.411494153	0.785553575	0.37179175	0.261892331
ENSG00000158865	3.16E-05	0.009830711	-1.182599608	-2.269854163	**SLC5A11**	protein_coding	solute carrier family 5 member 11 [Source:HGNC Symbol;Acc:HGNC:23091]	chr16	24845841	24911628	1	ensembl_havana	4.68045697	5.271756774	4.089157166	5.413135017	4.953104074	5.449031232	4.113831997	3.81128221	4.342357293
ENSG00000137868	3.29E-05	0.009830711	-1.296801799	-2.456836411	**STRA6**	protein_coding	stimulated by retinoic acid 6 [Source:HGNC Symbol;Acc:HGNC:30650]	chr15	74179466	74212267	-1	ensembl_havana	4.080848342	4.729249241	3.432447443	4.785479275	4.559523199	4.842745249	3.452619495	3.406428015	3.438294818
ENSG00000160117	3.70E-05	0.010618634	-0.911006416	-1.880356767	**ANKLE1**	protein_coding	ankyrin repeat and LEM domain containing 1 [Source:HGNC Symbol;Acc:HGNC:26812]	chr19	17281645	17287646	1	ensembl_havana	3.978418855	4.433922063	3.522915647	4.305543488	4.450539589	4.545683113	3.416632027	3.542087686	3.610027229
ENSG00000198759	4.65E-05	0.010886609	1.545439084	2.918928929	**EGFL6**	protein_coding	EGF like domain multiple 6 [Source:HGNC Symbol;Acc:HGNC:3235]	chrX	13569605	13633575	1	ensembl_havana	0.001137578	-0.771581964	0.77385712	-1.73427603	-0.18055577	-0.399914092	-0.40973604	1.529563273	1.201744126
ENSG00000155719	4.74E-05	0.010886609	1.366466874	2.578383523	**OTOA**	protein_coding	otoancorin [Source:HGNC Symbol;Acc:HGNC:16378]	chr16	21663971	21761935	1	ensembl_havana	-0.4255257	-1.108759137	0.257707737	-1.51148723	-1.178380098	-0.636410081	0.032074365	0.163140205	0.577908642
ENSG00000105383	5.27E-05	0.010886609	1.028145494	2.039401027	**CD33**	protein_coding	CD33 molecule [Source:HGNC Symbol;Acc:HGNC:1659]	chr19	51225064	51243860	1	ensembl_havana	-0.048373977	-0.562446724	0.46569877	-0.4596317	-0.783495335	-0.444213135	0.457455004	0.315191528	0.624449778
ENSG00000178498	4.57E-05	0.010886609	-0.883457395	-1.844791019	**DTX3**	protein_coding	deltex E3 ubiquitin ligase 3 [Source:HGNC Symbol;Acc:HGNC:24457]	chr12	57604622	57609804	1	ensembl_havana	4.606047531	5.047776228	4.164318834	5.166318578	5.090012847	4.88699726	4.263156055	4.247280137	3.982520308
ENSG00000260219	4.59E-05	0.010886609	-0.901297311	-1.867744756	**AC106782.2**	lincRNA		chr16	30355441	30357104	1	havana	1.023151422	1.473800078	0.572502766	1.369711653	1.464851682	1.586836898	0.428834256	0.554044597	0.734629445
ENSG00000197888	4.44E-05	0.010886609	-0.967640866	-1.955640063	**UGT2B17**	protein_coding	UDP glucuronosyltransferase family 2 member B17 [Source:HGNC Symbol;Acc:HGNC:12547]	chr4	68537184	68568527	-1	ensembl_havana	5.144643772	5.628464204	4.660823339	5.700326094	5.519935527	5.665130992	4.709312628	4.62884954	4.644307848
ENSG00000111186	5.31E-05	0.010886609	-1.002598151	-2.003605047	**WNT5B**	protein_coding	Wnt family member 5B [Source:HGNC Symbol;Acc:HGNC:16265]	chr12	1529891	1647243	1	ensembl_havana	5.917278223	6.418577298	5.415979147	6.570745689	6.305763976	6.37922223	5.465246552	5.337091883	5.445599007
ENSG00000268223	4.42E-05	0.010886609	-1.01081692	-2.015051791	**ARL14EPL**	protein_coding	ADP ribosylation factor like GTPase 14 effector protein like [Source:HGNC Symbol;Acc:HGNC:44201]	chr5	116032324	116060118	1	ensembl_havana	6.13206978	6.63747824	5.62666132	6.904811905	6.65283618	6.354786635	5.803655513	5.666819163	5.409509284
ENSG00000173890	4.70E-05	0.010886609	-1.021269548	-2.029704281	**GPR160**	protein_coding	G protein-coupled receptor 160 [Source:HGNC Symbol;Acc:HGNC:23693]	chr3	170037929	170085403	1	ensembl_havana	2.04120871	2.551843484	1.530573936	2.625913238	2.569661407	2.459955808	1.567772387	1.648877048	1.375072374
ENSG00000170482	4.18E-05	0.010886609	-1.081054639	-2.115582048	**SLC23A1**	protein_coding	solute carrier family 23 member 1 [Source:HGNC Symbol;Acc:HGNC:10974]	chr5	139367196	139384553	-1	ensembl_havana	1.118456574	1.658983894	0.577929255	1.553614465	1.51252579	1.910811426	0.457455004	0.343769191	0.932563571
ENSG00000135525	5.00E-05	0.010886609	-1.196481459	-2.291800493	**MAP7**	protein_coding	microtubule associated protein 7 [Source:HGNC Symbol;Acc:HGNC:6869]	chr6	136342281	136550819	-1	ensembl_havana	2.614265602	3.212506332	2.016024873	3.283361026	3.033096853	3.321061117	2.062883947	1.715999071	2.2691916
ENSG00000065882	5.27E-05	0.010886609	-1.229036525	-2.344103912	**TBC1D1**	protein_coding	TBC1 domain family member 1 [Source:HGNC Symbol;Acc:HGNC:11578]	chr4	37891087	38139175	1	ensembl_havana	0.83131892	1.445837182	0.216800657	1.671956805	1.636045198	1.029509544	0.276538753	0.478736765	-0.104873545
ENSG00000224655	4.52E-05	0.010886609	-1.30491324	-2.470688689	**LINC01823**	lincRNA	long intergenic non-protein coding RNA 1823 [Source:HGNC Symbol;Acc:HGNC:52628]	chr2	121790444	121796702	1	havana	2.335214197	2.987670817	1.682757577	3.435000789	2.698916685	2.829094977	1.976531838	1.541948597	1.529792296
ENSG00000110777	5.15E-05	0.010886609	-1.351684845	-2.552099974	**POU2AF1**	protein_coding	POU class 2 associating factor 1 [Source:HGNC Symbol;Acc:HGNC:9211]	chr11	111352252	111455630	-1	ensembl_havana	2.994626664	3.670469086	2.318784241	3.880693918	3.446030999	3.684682341	2.466446406	1.966251876	2.523654441
ENSG00000167460	3.88E-05	0.010886609	-1.420910025	-2.677543521	**TPM4**	protein_coding	tropomyosin 4 [Source:HGNC Symbol;Acc:HGNC:12013]	chr19	16067021	16103005	1	ensembl_havana	6.846180092	7.556635105	6.13572508	7.802795495	7.556434295	7.310675524	6.570977375	6.034252892	5.801944973
ENSG00000123560	5.28E-05	0.010886609	-1.647978761	-3.133942611	**PLP1**	protein_coding	proteolipid protein 1 [Source:HGNC Symbol;Acc:HGNC:9086]	chrX	103773718	103792619	1	ensembl_havana	0.287388903	1.111378284	-0.536600478	1.565895203	1.214022341	0.554217307	0.106248156	-0.68524259	-1.030806998
ENSG00000143851	3.99E-05	0.010886609	-1.875780061	-3.669999995	**PTPN7**	protein_coding	protein tyrosine phosphatase, non-receptor type 7 [Source:HGNC Symbol;Acc:HGNC:9659]	chr1	202147013	202161588	-1	ensembl_havana	1.505422461	2.443312491	0.56753243	3.014347502	2.344503139	1.971086832	0.955706889	0.37179175	0.375098651
ENSG00000106034	5.18E-05	0.010886609	-1.909200233	-3.756008254	**CPED1**	protein_coding	cadherin like and PC-esterase domain containing 1 [Source:HGNC Symbol;Acc:HGNC:26159]	chr7	120988677	121297444	1	ensembl_havana	0.331986194	1.286586311	-0.622613922	1.516132283	1.062111262	1.281515389	-0.26243933	-1.15951152	-0.445890917
ENSG00000107165	4.96E-05	0.010886609	-1.910239188	-3.758714111	**TYRP1**	protein_coding	tyrosinase related protein 1 [Source:HGNC Symbol;Acc:HGNC:12450]	chr9	12685439	12710290	1	ensembl_havana	-1.031072114	-0.07595252	-1.986191708	0.10902545	0.06303108	-0.399914092	-2.0571346	-1.87077278	-2.030667746
ENSG00000102024	4.45E-05	0.010886609	-2.482650032	-5.589231893	**PLS3**	protein_coding	plastin 3 [Source:HGNC Symbol;Acc:HGNC:9091]	chrX	115561174	115650861	1	ensembl_havana	1.716312134	2.957637151	0.474987118	3.575247577	3.037177367	2.260486508	0.716189844	0.602150945	0.106620565
ENSG00000117115	5.79E-05	0.01089897	0.999145326	1.998815521	**PADI2**	protein_coding	peptidyl arginine deiminase 2 [Source:HGNC Symbol;Acc:HGNC:18341]	chr1	17066761	17119435	-1	ensembl_havana	1.262123848	0.762551185	1.76169651	0.464289132	0.699804047	1.123560375	1.500601289	1.590453245	2.194034997
ENSG00000104998	5.87E-05	0.01089897	-0.81184914	-1.755460021	**IL27RA**	protein_coding	interleukin 27 receptor subunit alpha [Source:HGNC Symbol;Acc:HGNC:17290]	chr19	14031748	14053216	1	ensembl_havana	5.563215729	5.969140299	5.157291159	6.117628764	5.85881344	5.930978693	5.323765864	5.029304794	5.118802818
ENSG00000280208	5.75E-05	0.01089897	-0.848972484	-1.801217605	**GGT4P**	unprocessed_pseudogene	gamma-glutamyltransferase 4 pseudogene [Source:HGNC Symbol;Acc:HGNC:33207]	chr13	18253526	18270822	-1	havana	4.261394811	4.685881053	3.836908569	4.959631449	4.604215712	4.493795999	4.125025993	3.707901612	3.677798104
ENSG00000163219	5.65E-05	0.01089897	-0.935622119	-1.912715259	**ARHGAP25**	protein_coding	Rho GTPase activating protein 25 [Source:HGNC Symbol;Acc:HGNC:28951]	chr2	68679601	68826833	1	ensembl_havana	6.073424642	6.541235702	5.605613583	6.824356258	6.376313214	6.423037634	5.832621987	5.411537248	5.572681514
ENSG00000141449	6.00E-05	0.01089897	-1.01319057	-2.018369863	**GREB1L**	protein_coding	growth regulation by estrogen in breast cancer 1 like [Source:HGNC Symbol;Acc:HGNC:31042]	chr18	21242242	21525417	1	ensembl_havana	0.906943925	1.41353921	0.40034864	1.738566362	1.029710695	1.472340574	0.618191201	0.02879206	0.554062661
ENSG00000246273	5.92E-05	0.01089897	-1.031499706	-2.044148074	**SBF2-AS1**	antisense	SBF2 antisense RNA 1 [Source:HGNC Symbol;Acc:HGNC:27438]	chr11	9758292	9811319	1	havana	2.554352988	3.070102841	2.038603135	2.964197743	3.241966183	3.004144598	1.831005931	2.203033505	2.08176997
ENSG00000104921	6.07E-05	0.01089897	-1.253611685	-2.384375882	**FCER2**	protein_coding	Fc fragment of IgE receptor II [Source:HGNC Symbol;Acc:HGNC:3612]	chr19	7688758	7702146	-1	ensembl_havana	8.884998322	9.511804165	8.258192479	9.712259115	9.280577616	9.542575763	8.276565503	8.101435625	8.39657631
ENSG00000133106	5.88E-05	0.01089897	-1.369915538	-2.584554345	**EPSTI1**	protein_coding	epithelial stromal interaction 1 [Source:HGNC Symbol;Acc:HGNC:16465]	chr13	42886388	42992271	-1	ensembl_havana	4.966365404	5.651323173	4.281407635	5.73947847	5.743675159	5.470815888	4.482676951	4.165594683	4.195951269
ENSG00000225783	6.01E-05	0.01089897	-1.485697222	-2.800524835	**MIAT**	lincRNA	myocardial infarction associated transcript (non-protein coding) [Source:HGNC Symbol;Acc:HGNC:33425]	chr22	26646428	26676475	1	havana	5.593754732	6.336603343	4.850906121	7.088935143	6.149623388	5.771251498	5.385574858	4.852902895	4.31424061
ENSG00000244998	5.61E-05	0.01089897	-1.556004237	-2.940383309	**AC100803.1**	antisense		chr8	141389939	141392574	-1	havana	2.933333614	3.711335733	2.155331495	4.029996525	3.482399	3.621611673	2.286727206	1.881076089	2.298191191
ENSG00000259116	6.79E-05	0.010941725	5.777776535	54.86356766	**AL049869.3**	antisense		chr14	64514154	64540368	-1	havana	-2.461203333	-5.3500916	0.427684935	-5.3500916	-5.3500916	-5.3500916	1.486783452	0.19485955	-0.398588198
ENSG00000183023	7.54E-05	0.010941725	0.990750932	1.987219082	**SLC8A1**	protein_coding	solute carrier family 8 member A1 [Source:HGNC Symbol;Acc:HGNC:11068]	chr2	40097270	40611053	-1	ensembl_havana	5.072830716	4.57745525	5.568206182	4.21876443	4.594556843	4.919044477	5.303217218	5.612805897	5.78859543
ENSG00000008118	9.24E-05	0.010941725	0.839304981	1.789187992	**CAMK1G**	protein_coding	calcium/calmodulin dependent protein kinase IG [Source:HGNC Symbol;Acc:HGNC:14585]	chr1	209583717	209613938	1	ensembl_havana	3.138056549	2.718404059	3.55770904	2.802355359	2.744550137	2.608306682	3.729854637	3.551306511	3.391965972
ENSG00000158828	8.71E-05	0.010941725	0.818602878	1.763697181	**PINK1**	protein_coding	PTEN induced putative kinase 1 [Source:HGNC Symbol;Acc:HGNC:14581]	chr1	20633455	20651511	1	ensembl_havana	5.844094676	5.434793237	6.253396115	5.463234296	5.425807982	5.415337433	6.349806084	6.182526059	6.227856203
ENSG00000237461	7.79E-05	0.010941725	0.789452177	1.72841802	**AL359710.1**	lincRNA		chr9	99585786	99819889	-1	havana	-0.460790445	-0.855516533	-0.066064357	-0.67394004	-1.03440435	-0.85820521	0.106248156	-0.19956768	-0.104873545
ENSG00000196296	8.93E-05	0.010941725	0.742220977	1.672748995	**ATP2A1**	protein_coding	ATPase sarcoplasmic/endoplasmic reticulum Ca2+ transporting 1 [Source:HGNC Symbol;Acc:HGNC:811]	chr16	28878405	28904509	1	ensembl_havana	2.207810673	1.836700184	2.578921161	1.87313074	1.7890623	1.847907512	2.607204823	2.510878368	2.618680293
ENSG00000156453	9.09E-05	0.010941725	-0.76181953	-1.695627806	**PCDH1**	protein_coding	protocadherin 1 [Source:HGNC Symbol;Acc:HGNC:8655]	chr5	141853111	141879246	-1	ensembl_havana	4.699405657	5.080315422	4.318495892	5.035551843	5.076198147	5.129196277	4.320759576	4.301098552	4.333629549
ENSG00000092377	9.28E-05	0.010941725	-0.773397309	-1.709290145	**TBL1Y**	protein_coding	transducin beta like 1 Y-linked [Source:HGNC Symbol;Acc:HGNC:18502]	chrY	6910686	7091683	1	ensembl_havana	2.832350376	3.21904903	2.445651721	3.513183533	2.987432543	3.156531015	2.717806083	2.271570328	2.347578751
ENSG00000227589	8.22E-05	0.010941725	-0.776787357	-1.71331136	**AL136528.1**	antisense		chr1	3658938	3668772	-1	havana	3.569356754	3.957750432	3.180963075	3.875769846	3.987311414	4.010170036	3.063307783	3.203092147	3.276489296
ENSG00000135362	8.58E-05	0.010941725	-0.778328722	-1.715142829	**PRR5L**	protein_coding	proline rich 5 like [Source:HGNC Symbol;Acc:HGNC:25878]	chr11	36296288	36465204	1	ensembl_havana	7.243552878	7.632717239	6.854388517	7.965605379	7.493047307	7.439499031	7.14017721	6.718547168	6.704441173
ENSG00000270030	9.30E-05	0.010941725	-0.806012131	-1.748371942	**AC136475.7**	lincRNA		chr11	325703	326294	-1	havana	0.855817897	1.258823963	0.452811831	1.253039554	1.311578958	1.211853376	0.399634207	0.478736765	0.480064523
ENSG00000204261	7.89E-05	0.010941725	-0.825826584	-1.77255032	**PSMB8-AS1**	lincRNA	PSMB8 antisense RNA 1 (head to head) [Source:HGNC Symbol;Acc:HGNC:39758]	chr6	32844086	32846495	1	havana	3.952045222	4.364958514	3.53913193	4.347050969	4.310901545	4.436923027	3.548855684	3.526590849	3.541949257
ENSG00000273314	9.04E-05	0.010941725	-0.830919115	-1.778818254	**AC005229.4**	antisense		chr7	148696467	148698664	-1	havana	1.395756455	1.811216012	0.980296897	1.92171846	1.798708764	1.713220813	1.01467363	0.975275413	0.950941648
ENSG00000090659	6.56E-05	0.010941725	-0.856832192	-1.8110573	**CD209**	protein_coding	CD209 molecule [Source:HGNC Symbol;Acc:HGNC:1641]	chr19	7739994	7747564	-1	ensembl_havana	4.160608663	4.589024759	3.732192567	4.525847985	4.327656656	4.913569637	3.623370039	3.535908912	4.037298749
ENSG00000102226	7.59E-05	0.010941725	-0.861138463	-1.816471163	**USP11**	protein_coding	ubiquitin specific peptidase 11 [Source:HGNC Symbol;Acc:HGNC:12609]	chrX	47232690	47248328	1	ensembl_havana	6.148830976	6.579400208	5.718261745	6.551189802	6.567243147	6.619767674	5.608511182	5.740537503	5.805736548
ENSG00000172037	7.91E-05	0.010941725	-0.872668295	-1.83104633	**LAMB2**	protein_coding	laminin subunit beta 2 [Source:HGNC Symbol;Acc:HGNC:6487]	chr3	49121114	49133118	-1	ensembl_havana	0.66386768	1.100201827	0.227533532	0.968509893	1.078042694	1.254052895	0.176794179	0.130707766	0.375098651
ENSG00000135457	6.52E-05	0.010941725	-0.87542872	-1.834553172	**TFCP2**	protein_coding	transcription factor CP2 [Source:HGNC Symbol;Acc:HGNC:11748]	chr12	51093663	51173134	-1	ensembl_havana	3.372464306	3.810178666	2.934749946	4.134697477	3.701342418	3.594496101	3.217678921	2.900515694	2.686055222
ENSG00000137747	7.52E-05	0.010941725	-0.919569734	-1.891551077	**TMPRSS13**	protein_coding	transmembrane serine protease 13 [Source:HGNC Symbol;Acc:HGNC:29808]	chr11	117900643	117929459	-1	ensembl_havana	7.200199496	7.659984363	6.740414629	7.874743766	7.477741158	7.627468163	7.051436405	6.542898718	6.626908762
ENSG00000187678	8.37E-05	0.010941725	-0.945765674	-1.926210902	**SPRY4**	protein_coding	sprouty RTK signaling antagonist 4 [Source:HGNC Symbol;Acc:HGNC:15533]	chr5	142310427	142326455	-1	ensembl_havana	0.999264761	1.472147598	0.526381925	1.959443807	1.476918714	0.980080273	0.894226935	0.578298274	0.106620565
ENSG00000143382	6.76E-05	0.010941725	-1.007863742	-2.010931225	**ADAMTSL4**	protein_coding	ADAMTS like 4 [Source:HGNC Symbol;Acc:HGNC:19706]	chr1	150549369	150560937	1	ensembl_havana	2.663262148	3.167194019	2.159330277	3.130521836	2.991644088	3.379416132	2.126646177	2.089494957	2.261849697
ENSG00000134802	7.88E-05	0.010941725	-1.067217668	-2.095388374	**SLC43A3**	protein_coding	solute carrier family 43 member 3 [Source:HGNC Symbol;Acc:HGNC:17466]	chr11	57406954	57427580	-1	ensembl_havana	5.01583401	5.549442844	4.482225176	5.645690218	5.640168091	5.362470221	4.505127529	4.493519334	4.448028664
ENSG00000282890	8.35E-05	0.010941725	-1.076076868	-2.108295175	**AC009975.1**	lincRNA		chr2	48809340	49419013	1	havana	2.731699484	3.269737918	2.19366105	3.727865017	3.131799381	2.949549354	2.50808372	2.187350601	1.885548828
ENSG00000108622	7.78E-05	0.010941725	-1.085021934	-2.121407735	**ICAM2**	protein_coding	intercellular adhesion molecule 2 [Source:HGNC Symbol;Acc:HGNC:5345]	chr17	64002594	64020634	-1	ensembl_havana	3.826098535	4.368609502	3.283587568	4.247639431	4.449008971	4.409180104	3.310812123	3.245359567	3.294591014
ENSG00000165923	8.12E-05	0.010941725	-1.101236406	-2.145384757	**AGBL2**	protein_coding	ATP/GTP binding protein like 2 [Source:HGNC Symbol;Acc:HGNC:26296]	chr11	47659591	47715389	-1	ensembl_havana	2.583004348	3.133622551	2.032386145	3.264647647	3.195256367	2.94096364	2.153133136	1.95703246	1.986992839
ENSG00000086730	8.73E-05	0.010941725	-1.114464488	-2.165146264	**LAT2**	protein_coding	linker for activation of T cells family member 2 [Source:HGNC Symbol;Acc:HGNC:12749]	chr7	74199652	74229834	1	ensembl_havana	4.165487008	4.722719252	3.608254764	4.976979013	4.632810281	4.558368462	3.795780677	3.41318562	3.615797995
ENSG00000168874	8.21E-05	0.010941725	-1.116751378	-2.16858107	**ATOH8**	protein_coding	atonal bHLH transcription factor 8 [Source:HGNC Symbol;Acc:HGNC:24126]	chr2	85751344	85788066	1	ensembl_havana	2.910674467	3.469050156	2.352298778	3.556907091	3.231320443	3.618922935	2.600804468	2.081032632	2.375059235
ENSG00000121895	6.86E-05	0.010941725	-1.127590029	-2.184934497	**TMEM156**	protein_coding	transmembrane protein 156 [Source:HGNC Symbol;Acc:HGNC:26260]	chr4	38966744	39032922	-1	ensembl_havana	0.582759899	1.146554913	0.018964884	1.355633303	0.945375194	1.138656243	0.069637898	-0.11936381	0.106620565
ENSG00000185436	8.40E-05	0.010941725	-1.149157903	-2.217844017	**IFNLR1**	protein_coding	interferon lambda receptor 1 [Source:HGNC Symbol;Acc:HGNC:18584]	chr1	24154157	24187959	-1	ensembl_havana	1.391121536	1.965700487	0.816542584	1.727676152	2.124367304	2.045058006	0.399634207	1.080904991	0.969088554
ENSG00000197471	7.38E-05	0.010941725	-1.166427187	-2.244551482	**SPN**	protein_coding	sialophorin [Source:HGNC Symbol;Acc:HGNC:11249]	chr16	29662979	29670876	1	ensembl_havana	2.342257736	2.925471329	1.759044142	2.921828527	2.774189768	3.080395692	1.580839304	1.682828385	2.013464737
ENSG00000229391	6.73E-05	0.010941725	-1.192574794	-2.285602936	**HLA-DRB6**	transcribed_unprocessed_pseudogene	major histocompatibility complex, class II, DR beta 6 (pseudogene) [Source:HGNC Symbol;Acc:HGNC:4954]	chr6	32552713	32560022	-1	havana	-0.704622	-0.108334603	-1.300909397	-0.36334239	0.125688576	-0.087349994	-1.63841642	-1.15951152	-1.10480025
ENSG00000150275	7.82E-05	0.010941725	-1.202357999	-2.301154738	**PCDH15**	protein_coding	protocadherin related 15 [Source:HGNC Symbol;Acc:HGNC:14674]	chr10	53802771	55627942	-1	ensembl_havana	-0.864619609	-0.26344061	-1.465798609	0.267803752	-0.521012188	-0.537113394	-0.75894329	-1.87077278	-1.767679756
ENSG00000153721	6.97E-05	0.010941725	-1.296536398	-2.456384489	**CNKSR3**	protein_coding	CNKSR family member 3 [Source:HGNC Symbol;Acc:HGNC:23034]	chr6	154387504	154510659	-1	ensembl_havana	-0.060476736	0.587791463	-0.708744936	1.092533711	0.501432867	0.16940781	-0.00649342	-0.93694677	-1.182794618
ENSG00000182667	9.15E-05	0.010941725	-1.347489837	-2.544689864	**NTM**	protein_coding	neurotrimin [Source:HGNC Symbol;Acc:HGNC:17941]	chr11	131370478	132336822	1	ensembl_havana	-0.6008512	0.072893719	-1.274596119	0.490295136	-0.218971072	-0.052642908	-0.63291296	-1.74507732	-1.445798078
ENSG00000128573	7.53E-05	0.010941725	-1.388279732	-2.617663646	**FOXP2**	protein_coding	forkhead box P2 [Source:HGNC Symbol;Acc:HGNC:13875]	chr7	114086327	114693772	1	ensembl_havana	3.824945312	4.519085178	3.130805446	4.448367234	4.424295625	4.684592674	2.911257603	3.264170155	3.21698858
ENSG00000259207	8.20E-05	0.010941725	-1.429549771	-2.693626409	**ITGB3**	protein_coding	integrin subunit beta 3 [Source:HGNC Symbol;Acc:HGNC:6156]	chr17	47253846	47311816	1	ensembl_havana	0.180979485	0.895754371	-0.5337954	1.126095262	0.837200041	0.723967808	-0.08686711	-0.80561234	-0.708906755
ENSG00000061492	8.98E-05	0.010941725	-1.435302713	-2.704389054	**WNT8A**	protein_coding	Wnt family member 8A [Source:HGNC Symbol;Acc:HGNC:12788]	chr5	138083892	138092365	1	ensembl_havana	1.196642305	1.914293661	0.478990949	2.040883175	1.62524309	2.076754718	0.851734776	0.130707766	0.454530303
ENSG00000139626	6.74E-05	0.010941725	-1.572416011	-2.974023426	**ITGB7**	protein_coding	integrin subunit beta 7 [Source:HGNC Symbol;Acc:HGNC:6162]	chr12	53191318	53207307	-1	ensembl_havana	2.447162961	3.233370967	1.660954955	3.380421283	2.940275486	3.379416132	1.936429043	1.504469011	1.541966812
ENSG00000068831	7.51E-05	0.010941725	-1.623508354	-3.081234223	**RASGRP2**	protein_coding	RAS guanyl releasing protein 2 [Source:HGNC Symbol;Acc:HGNC:9879]	chr11	64726911	64745456	-1	ensembl_havana	-0.15447966	0.657274517	-0.966233837	0.931023971	0.378171619	0.662627962	-0.97139419	-1.15951152	-0.767795802
ENSG00000111348	8.61E-05	0.010941725	-1.638953032	-3.114397371	**ARHGDIB**	protein_coding	Rho GDP dissociation inhibitor beta [Source:HGNC Symbol;Acc:HGNC:679]	chr12	14942017	14961728	-1	ensembl_havana	5.395212893	6.214689409	4.575736377	6.504102541	6.145376775	5.994588911	4.565534632	4.62884954	4.53282496
ENSG00000139832	7.92E-05	0.010941725	-1.842962413	-3.587459172	**RAB20**	protein_coding	RAB20, member RAS oncogene family [Source:HGNC Symbol;Acc:HGNC:18260]	chr13	110523066	110561733	-1	ensembl_havana	-0.699148621	0.222332585	-1.620629828	-0.10791135	0.243386951	0.531522158	-1.63841642	-1.87077278	-1.352700279
ENSG00000151067	6.67E-05	0.010941725	-1.845932682	-3.594852761	**CACNA1C**	protein_coding	calcium voltage-gated channel subunit alpha1 C [Source:HGNC Symbol;Acc:HGNC:1390]	chr12	1970786	2697950	1	havana	3.198709383	4.121675724	2.275743042	4.521125456	3.850241744	3.993659972	2.364348155	2.081032632	2.381848339
ENSG00000174469	8.28E-05	0.010941725	-2.695480192	-6.477693431	**CNTNAP2**	protein_coding	contactin associated protein like 2 [Source:HGNC Symbol;Acc:HGNC:13830]	chr7	146116002	148420998	1	ensembl_havana	0.091207788	1.438947884	-1.256532308	2.023178671	1.170104606	1.123560375	-0.21650287	-1.52242631	-2.030667746
ENSG00000250433	9.61E-05	0.010947367	2.186642695	4.552448496	**CLSTN2-AS1**	antisense	CLSTN2 antisense RNA 1 [Source:HGNC Symbol;Acc:HGNC:49095]	chr3	140505611	140508789	-1	havana	-0.95886516	-2.052186508	0.134456187	-1.99786555	-1.72386249	-2.43483148	0.36983092	0.02879206	0.004745582
ENSG00000085982	9.83E-05	0.010947367	1.251790491	2.38136785	**USP40**	protein_coding	ubiquitin specific peptidase 40 [Source:HGNC Symbol;Acc:HGNC:20069]	chr2	233475520	233566782	-1	ensembl_havana	1.168748822	0.542853576	1.794644067	0.490295136	0.855801933	0.28246366	1.905603022	1.900443368	1.577885811

ENSG00000168631	9.61E-05	0.010947367	0.896190053	1.861144477	**DPCR1**	protein_coding	diffuse panbronchiolitis critical region 1 [Source:HGNC Symbol;Acc:HGNC:21666]	chr6	30934523	30954221	1	ensembl_havana	2.174567459	1.726472432	2.622662485	1.602122376	2.06964138	1.507653541	2.44516827	2.905316921	2.517502265
ENSG00000135842	0.0001	0.010947367	-0.732109775	-1.661066434	**FAM129A**	protein_coding	family with sequence similarity 129 member A [Source:HGNC Symbol;Acc:HGNC:16784]	chr1	184790724	184974550	-1	ensembl_havana	3.561675196	3.927730084	3.195620309	3.510009124	4.131689787	4.14149134	2.752865953	3.385963185	3.448031787
ENSG00000105808	9.87E-05	0.010947367	-0.766072496	-1.700633775	**RASA4**	protein_coding	RAS p21 protein activator 4 [Source:HGNC Symbol;Acc:HGNC:23181]	chr7	102573807	102616757	-1	ensembl_havana	-0.065215624	0.317820624	-0.448251871	0.355307344	0.428746719	0.16940781	-0.46237776	-0.28449416	-0.597883695
ENSG00000197646	9.56E-05	0.010947367	-0.911394828	-1.880863078	**PDCD1LG2**	protein_coding	programmed cell death 1 ligand 2 [Source:HGNC Symbol;Acc:HGNC:18731]	chr9	5510570	5571254	1	ensembl_havana	5.485742683	5.941440097	5.030045268	6.293163686	5.840834308	5.690322297	5.273344812	4.942047085	4.874743908
ENSG00000141736	0.000101	0.010947367	-0.963135191	-1.949541939	**ERBB2**	protein_coding	erb-b2 receptor tyrosine kinase 2 [Source:HGNC Symbol;Acc:HGNC:3430]	chr17	39687914	39730426	1	ensembl_havana	1.162216512	1.643784108	0.680648917	1.312554705	1.836662271	1.782135348	0.485518989	0.779979312	0.77644845
ENSG00000154646	9.90E-05	0.010947367	-1.246400533	-2.372487577	**TMPRSS15**	protein_coding	transmembrane serine protease 15 [Source:HGNC Symbol;Acc:HGNC:9490]	chr21	18269116	18485879	-1	ensembl_havana	4.212632186	4.835832452	3.589431919	4.443383695	4.952023854	5.112089807	3.390895783	3.694095415	3.68330456
ENSG00000145936	9.62E-05	0.010947367	-1.47698403	-2.78366197	**KCNMB1**	protein_coding	potassium calcium-activated channel subfamily M regulatory beta subunit 1 [Source:HGNC Symbol;Acc:HGNC:6285]	chr5	170374671	170389677	-1	ensembl_havana	2.837968431	3.576460446	2.099476416	3.697490029	3.714142396	3.317748913	2.487415269	2.055343696	1.755670283
ENSG00000196684	0.0001	0.010947367	-1.538448056	-2.904818565	**HSH2D**	protein_coding	hematopoietic SH2 domain containing [Source:HGNC Symbol;Acc:HGNC:24920]	chr19	16134028	16158575	1	ensembl_havana	2.367727573	3.136951601	1.598503545	3.179296803	3.135610417	3.095947583	1.386165364	1.602328815	1.807016457
ENSG00000046889	0.000106	0.010976366	1.659989799	3.160142903	**PREX2**	protein_coding	phosphatidylinositol-3,4,5-trisphosphate dependent Rac exchange factor 2 [Source:HGNC Symbol;Acc:HGNC:22950]	chr8	67952118	68237030	1	ensembl_havana	1.564661286	0.734666387	2.394656186	-0.14746469	1.029710695	1.321753151	1.644455473	2.881149392	2.658363693
ENSG00000225156	0.000103	0.010976366	1.466543281	2.763589395	**AC012354.1**	lincRNA		chr2	44954664	44968762	1	havana	1.458055121	0.72478348	2.191326761	0.540941683	0.636663181	0.996745577	1.820027213	2.372104732	2.381848339
ENSG00000177337	0.000105	0.010976366	0.857722589	1.812175386	**DLGAP1-AS1**	antisense	DLGAP1 antisense RNA 1 [Source:HGNC Symbol;Acc:HGNC:31676]	chr18	3593732	3598352	1	havana	0.4457621	0.016900805	0.874623394	0.075059705	-0.071090603	0.046733314	0.830009965	0.861296647	0.932563571
ENSG00000235848	0.000104	0.010976366	-0.861959587	-1.81750532	**RMDN2-AS1**	antisense	RMDN2 antisense RNA 1 [Source:HGNC Symbol;Acc:HGNC:41150]	chr2	37949911	38067041	-1	havana	4.235151929	4.666131723	3.804172136	4.837056698	4.698694759	4.462643711	3.996838595	3.735123853	3.680553959
ENSG00000108813	0.000105	0.010976366	-1.167792299	-2.246676334	**DLX4**	protein_coding	distal-less homeobox 4 [Source:HGNC Symbol;Acc:HGNC:2917]	chr17	49968970	49974959	1	ensembl_havana	1.169170739	1.753066888	0.58527459	1.738566362	1.592342839	1.928291464	0.36983092	0.529376201	0.856616648
ENSG00000130208	0.000106	0.010976366	-1.433234832	-2.700515505	**APOC1**	protein_coding	apolipoprotein C1 [Source:HGNC Symbol;Acc:HGNC:607]	chr19	44914247	44919349	1	ensembl_havana	-0.59706606	0.119551356	-1.313683476	-0.14746469	0.52486995	-0.018751196	-1.31428442	-1.08144551	-1.54532049
ENSG00000224389	0.000106	0.010976366	-1.596426602	-3.023933895	**C4B**	protein_coding	complement C4B (Chido blood group) [Source:HGNC Symbol;Acc:HGNC:1324]	chr6	32014762	32035418	1	ensembl_havana	2.944094706	3.742308006	2.145881405	3.878233983	3.386505103	3.962184934	2.044136302	2.081032632	2.31247528
ENSG00000196189	0.000111	0.011076111	-0.7271904	-1.655412091	**SEMA4A**	protein_coding	semaphorin 4A [Source:HGNC Symbol;Acc:HGNC:10729]	chr1	156147366	156177752	1	ensembl_havana	6.24952497	6.61312017	5.88592977	6.781229228	6.501942827	6.556188457	6.033699796	5.823410619	5.800678896
ENSG00000184208	0.00011	0.011076111	-0.731973782	-1.660909864	**C22orf46**	protein_coding	chromosome 22 open reading frame 46 [Source:HGNC Symbol;Acc:HGNC:26294]	chr22	41688939	41698136	1	ensembl_havana	4.256525081	4.622511972	3.89053819	4.596388705	4.674011773	4.597135437	3.88803019	3.890901443	3.892682937
ENSG00000029534	0.000109	0.011076111	-0.853947543	-1.807439734	**ANK1**	protein_coding	ankyrin 1 [Source:HGNC Symbol;Acc:HGNC:492]	chr8	41653220	41896762	-1	ensembl_havana	1.814466394	2.241440166	1.387492623	2.503565738	2.191810142	2.028944618	1.567772387	1.43975459	1.154950891
ENSG00000267060	0.000111	0.011076111	-0.887475955	-1.849936762	**PTGES3L**	protein_coding	prostaglandin E synthase 3 like [Source:HGNC Symbol;Acc:HGNC:43943]	chr17	42968088	42980433	-1	ensembl_havana	1.247997391	1.691735368	0.804259414	1.716703112	1.749818205	1.608684788	0.873137292	0.938274071	0.601366878
ENSG00000186854	0.000108	0.011076111	-1.124005577	-2.17951265	**TRABD2A**	protein_coding	TraB domain containing 2A [Source:HGNC Symbol;Acc:HGNC:27013]	chr2	84821650	84907008	-1	ensembl_havana	1.607019927	2.169022716	1.045017139	2.275818664	2.235081811	1.996167673	1.01467363	1.063828438	1.056549347
ENSG00000221890	0.000114	0.011313451	-0.762180526	-1.696052145	**NPTXR**	protein_coding	neuronal pentraxin receptor [Source:HGNC Symbol;Acc:HGNC:7954]	chr22	38818452	38843982	-1	ensembl_havana	0.175618674	0.556708937	-0.20547159	0.075059705	0.614986832	0.980080273	-0.75894329	-0.11936381	0.261892331
ENSG00000196620	0.000117	0.011496068	-0.927770518	-1.902333934	**UGT2B15**	protein_coding	UDP glucuronosyltransferase family 2 member B15 [Source:HGNC Symbol;Acc:HGNC:12546]	chr4	68646630	68670628	-1	ensembl_havana	2.26384197	2.727727229	1.799956711	2.781444624	2.709182922	2.692554143	1.88468023	1.648877048	1.866312856
ENSG00000151704	0.000118	0.011548923	-1.560483085	-2.949525916	**KCNJ1**	protein_coding	potassium voltage-gated channel subfamily J member 1 [Source:HGNC Symbol;Acc:HGNC:6255]	chr11	128836315	128867373	-1	ensembl_havana	4.237364004	5.017605546	3.457122461	5.371764013	4.910364411	4.770688216	3.571944101	3.297423241	3.502000041
ENSG00000041880	0.00012	0.011596558	-0.867417543	-1.824394269	**PARP3**	protein_coding	poly(ADP-ribose) polymerase family member 3 [Source:HGNC Symbol;Acc:HGNC:273]	chr3	51942345	51948867	1	ensembl_havana	4.66423993	5.097948702	4.230531158	5.037757838	5.098824377	5.15726389	4.061190507	4.243493459	4.386909509
ENSG00000168646	0.000125	0.011790701	1.115018687	2.165978146	**AXIN2**	protein_coding	axin 2 [Source:HGNC Symbol;Acc:HGNC:904]	chr17	65528563	65561647	-1	ensembl_havana	5.698980689	5.141471346	6.256490033	4.855932554	5.34334845	5.225133033	6.16422576	6.317420987	6.287823352
ENSG00000108828	0.000124	0.011790701	0.691166656	1.614588651	**VAT1**	protein_coding	vesicle amine transport 1 [Source:HGNC Symbol;Acc:HGNC:16919]	chr17	43014605	43025123	-1	ensembl_havana	6.136629009	5.791045681	6.482212337	5.744214219	5.804189573	5.82473325	6.458223053	6.461704327	6.526709631
ENSG00000242574	0.000123	0.011790701	-0.791815761	-1.731252028	**HLA-DMB**	protein_coding	major histocompatibility complex, class II, DM beta [Source:HGNC Symbol;Acc:HGNC:4935]	chr6	32934629	32941070	-1	ensembl_havana	2.684804983	3.080712864	2.288897103	3.238034301	3.024901037	2.979203253	2.379383493	2.210811486	2.27649633
ENSG00000197872	0.000123	0.011790701	-0.807720487	-1.75044349	**FAM49A**	protein_coding	family with sequence similarity 49 member A [Source:HGNC Symbol;Acc:HGNC:25373]	chr2	16549459	16666331	-1	ensembl_havana	3.216504105	3.620364349	2.812643862	4.062862309	3.555343125	3.242887613	3.166591014	2.753838306	2.517502265
ENSG00000183287	0.000126	0.011790701	-1.072191922	-2.102625512	**CCBE1**	protein_coding	collagen and calcium binding EGF domains 1 [Source:HGNC Symbol;Acc:HGNC:29426]	chr18	59430940	59697380	-1	ensembl_havana	0.109810192	0.645906153	-0.426285769	0.770507832	0.77991046	0.387300166	-0.26243933	-0.47099802	-0.545419959
ENSG00000229474	0.000128	0.011906082	-0.959263049	-1.944316454	**PATL2**	protein_coding	PAT1 homolog 2 [Source:HGNC Symbol;Acc:HGNC:33630]	chr15	44665732	44711316	-1	ensembl_havana	6.670738094	7.150369618	6.19110657	7.252909338	6.987996144	7.210203373	6.154425948	6.039175865	6.379717896
ENSG00000178202	0.000129	0.011946528	-0.679104967	-1.601146113	**KDELC2**	protein_coding	KDEL motif containing 2 [Source:HGNC Symbol;Acc:HGNC:28496]	chr11	108472105	108498432	-1	ensembl_havana	5.161962136	5.50151462	4.822409653	5.260940923	5.569378219	5.674224717	4.553985209	4.904172218	5.009071532
ENSG00000257017	0.000137	0.011993746	2.361822798	5.140193956	**HP**	protein_coding	haptoglobin [Source:HGNC Symbol;Acc:HGNC:5141]	chr16	72054592	72061055	1	ensembl_havana	-0.347254125	-1.528165524	0.833657274	-2.73705375	-0.727039001	-1.120403822	0.141952326	1.426455924	0.932563571
ENSG00000135218	0.000138	0.011993746	1.270467878	2.412397889	**CD36**	protein_coding	CD36 molecule [Source:HGNC Symbol;Acc:HGNC:1663]	chr7	80369575	80679277	1	ensembl_havana	5.960913829	5.32567989	6.596147768	4.631867438	5.760432415	5.584739816	6.136284886	6.837927688	6.81423073
ENSG00000267493	0.00013	0.011993746	1.114318631	2.164927379	**CIRBP-AS1**	antisense	CIRBP antisense RNA 1 [Source:HGNC Symbol;Acc:HGNC:28588]	chr19	1267814	1270241	-1	havana	-0.667207556	-1.224366871	-0.11004824	-1.41179303	-1.518257998	-0.743049581	-0.17198403	-0.32890884	0.17074815
ENSG00000175155	0.000136	0.011993746	0.667834406	1.588686438	**YPEL2**	protein_coding	yippee like 2 [Source:HGNC Symbol;Acc:HGNC:18326]	chr17	59331689	59401729	1	ensembl_havana	4.079979323	3.74606212	4.413896526	3.789382034	3.766695609	3.682108717	4.477446056	4.413210966	4.351032555
ENSG00000269220	0.000136	0.011993746	-0.682087757	-1.604459926	**LINC00528**	lincRNA	long intergenic non-protein coding RNA 528 [Source:HGNC Symbol;Acc:HGNC:26875]	chr22	17777322	17779481	1	havana	3.163010368	3.504054246	2.82196649	3.519511473	3.512020676	3.480630591	2.853206155	2.790632353	2.822060962
ENSG00000090861	0.000137	0.011993746	-0.700647744	-1.62523433	**AARS**	protein_coding	alanyl-tRNA synthetase [Source:HGNC Symbol;Acc:HGNC:20]	chr16	70252295	70289543	-1	ensembl_havana	7.729830509	8.080154381	7.379506637	8.056939676	8.073046427	8.110477039	7.306483706	7.376750401	7.455285803
ENSG00000255561	0.000136	0.011993746	-0.722004131	-1.649471821	**FDXACB1**	protein_coding	ferredoxin-fold anticodon binding domain containing 1 [Source:HGNC Symbol;Acc:HGNC:25110]	chr11	111874056	111881243	-1	ensembl_havana	2.062748184	2.42375025	1.701746118	2.458169134	2.446207271	2.366874344	1.786580698	1.660283167	1.65837449
ENSG00000100368	0.000136	0.011993746	-0.743601936	-1.67435093	**CSF2RB**	protein_coding	colony stimulating factor 2 receptor beta common subunit [Source:HGNC Symbol;Acc:HGNC:2436]	chr22	36913628	36940449	1	ensembl_havana	5.331769312	5.70357028	4.959968344	5.946202462	5.53299787	5.631510506	5.169051589	4.867738128	4.843115314
ENSG00000162545	0.000134	0.011993746	-1.719017535	-3.292121394	**CAMK2N1**	protein_coding	calcium/calmodulin dependent protein kinase II inhibitor 1 [Source:HGNC Symbol;Acc:HGNC:24190]	chr1	20482391	20486220	-1	ensembl_havana	1.973907955	2.833416722	1.114399187	2.996312281	3.00836839	2.495569495	1.089724456	1.114463419	1.139009687
ENSG00000144476	0.000134	0.011993746	-1.752024887	-3.368309919	**ACKR3**	protein_coding	atypical chemokine receptor 3 [Source:HGNC Symbol;Acc:HGNC:23692]	chr2	236567787	236582358	1	ensembl_havana	0.634782003	1.510794447	-0.24123044	1.82284946	1.298039728	1.411494153	0.399634207	-0.47099802	-0.652327512
ENSG00000255274	0.000157	0.012006638	1.225748349	2.338767338	**SMIM35**	protein_coding	small integral membrane protein 35 [Source:HGNC Symbol;Acc:HGNC:44179]	chr11	118003634	118088299	-1	havana	1.914768721	1.301894546	2.527642895	1.50341865	1.093800119	1.30846487	2.874587014	2.072520377	2.635821295
ENSG00000154975	0.000169	0.012006638	1.043301884	2.060939113	**CA10**	protein_coding	carbonic anhydrase 10 [Source:HGNC Symbol;Acc:HGNC:1369]	chr17	51630313	52160017	-1	ensembl_havana	1.175316949	0.653666008	1.696967891	0.705390237	0.592979824	0.662627962	1.693402841	1.831488934	1.566011899
ENSG00000166268	0.000143	0.012006638	0.971164557	1.960422428	**MYRFL**	protein_coding	myelin regulatory factor-like [Source:HGNC Symbol;Acc:HGNC:26316]	chr12	69825304	69959097	1	ensembl_havana	-0.126445823	-0.612028102	0.359136455	-0.56281016	-0.473795405	-0.799478738	0.56657698	0.37179175	0.139040636
ENSG00000231952	0.000163	0.012006638	0.945166516	1.925411105	**DPY19L1P2**	unprocessed_pseudogene	DPY19L1 pseudogene 2 [Source:HGNC Symbol;Acc:HGNC:22851]	chr7	32812757	32838570	1	havana	1.718221862	1.245638603	2.19080512	1.109412078	1.155163158	1.472340574	2.187709208	2.122856455	2.261849697
ENSG00000167995	0.000153	0.012006638	0.914764687	1.885261548	**BEST1**	protein_coding	bestrophin 1 [Source:HGNC Symbol;Acc:HGNC:12703]	chr11	61949821	61965515	1	ensembl_havana	-0.062904862	-0.520287206	0.394477481	-0.73288918	-0.383759306	-0.444213135	0.106248156	0.45273451	0.624449778
ENSG00000198722	0.000174	0.012006638	0.844421451	1.795544553	**UNC13B**	protein_coding	unc-13 homolog B [Source:HGNC Symbol;Acc:HGNC:12566]	chr9	35161992	35405338	1	ensembl_havana	5.058012932	4.635802206	5.480223657	4.38912643	4.714069201	4.804210988	5.3611681	5.541351573	5.538151299
ENSG00000270820	0.000149	0.012006638	0.78850034	1.727278049	**AC016727.1**	antisense		chr2	61471188	61484130	1	havana	1.04737665	0.65312648	1.44162682	0.683014432	0.699804047	0.57656096	1.444517675	1.413033531	1.467329254
ENSG00000257696	0.000146	0.012006638	0.782094817	1.719625986	**AC010203.2**	antisense		chr12	100143058	100144671	1	havana	-0.136222107	-0.527269515	0.254825301	-0.41068387	-0.428075095	-0.743049581	0.339398945	0.286036324	0.139040636
ENSG00000213906	0.000172	0.012006638	0.780816955	1.718103508	**LTB4R2**	protein_coding	leukotriene B4 receptor 2 [Source:HGNC Symbol;Acc:HGNC:19260]	chr14	24305734	24312053	1	ensembl_havana	2.406207582	2.015799104	2.79661606	1.912130943	1.827267088	2.307999282	2.78144541	2.625878149	2.98252462
ENSG00000095383	0.000168	0.012006638	0.739575252	1.66968419	**TBC1D2**	protein_coding	TBC1 domain family member 2 [Source:HGNC Symbol;Acc:HGNC:18026]	chr9	98198999	98255721	-1	ensembl_havana	2.083959731	1.714172105	2.453747357	1.660549964	1.709476673	1.772489677	2.494337705	2.399587437	2.467316929
ENSG00000128872	0.000162	0.012006638	0.701426474	1.626111826	**TMOD2**	protein_coding	tropomodulin 2 [Source:HGNC Symbol;Acc:HGNC:11872]	chr15	51751561	51816368	1	ensembl_havana	5.85271791	5.502004673	6.203431147	5.274099343	5.616500831	5.615413846	6.047885069	6.280017198	6.282391174
ENSG00000184481	0.000175	0.012006638	0.686741371	1.609643692	**FOXO4**	protein_coding	forkhead box O4 [Source:HGNC Symbol;Acc:HGNC:7139]	chrX	71096197	71103535	1	ensembl_havana	5.246740237	4.903369552	5.590110923	5.138837395	4.904792248	4.666479012	5.770535776	5.658311783	5.34148521
ENSG00000133812	0.000175	0.012006638	0.682999974	1.605474749	**SBF2**	protein_coding	SET binding factor 2 [Source:HGNC Symbol;Acc:HGNC:2135]	chr11	9778667	10294207	-1	havana	4.312687617	3.971187629	4.654187604	3.907479941	3.838598732	4.167484215	4.625091485	4.555916886	4.78155444
ENSG00000203446	0.000174	0.012006638	0.673288545	1.594703869	**AC004988.1**	antisense		chr7	40538127	40546928	-1	havana	0.452136905	0.115492632	0.788781178	0.237415364	0.09469996	0.014362573	0.851734776	0.779979312	0.734629445
ENSG00000156804	0.000148	0.012006638	0.6721988	1.593499758	**FBXO32**	protein_coding	F-box protein 32 [Source:HGNC Symbol;Acc:HGNC:16731]	chr8	123497889	123541206	-1	ensembl_havana	4.479178899	4.143079499	4.815278299	4.292670028	4.144985604	3.991582865	4.939776522	4.86033958	4.645718794
ENSG00000106780	0.000148	0.012006638	0.661241864	1.581443336	**MEGF9**	protein_coding	multiple EGF like domains 9 [Source:HGNC Symbol;Acc:HGNC:3234]	chr9	120600813	120714470	-1	ensembl_havana	5.912036236	5.581415304	6.242657168	5.506882745	5.631422654	5.605940514	6.17557537	6.261413433	6.290982702
ENSG00000150477	0.000166	0.012006638	0.648791208	1.567853987	**KIAA1328**	protein_coding	KIAA1328 [Source:HGNC Symbol;Acc:HGNC:29248]	chr18	36829106	37232172	1	ensembl_havana	2.832565999	2.508170395	3.156961604	2.625913238	2.408903349	2.489694598	3.267019432	3.029255182	3.174610196
ENSG00000104946	0.000174	0.012006638	0.647043969	1.565956317	**TBC1D17**	protein_coding	TBC1 domain family member 17 [Source:HGNC Symbol;Acc:HGNC:25699]	chr19	49877425	49888749	1	ensembl_havana	4.384265752	4.060743767	4.707787736	4.153128786	4.096927042	3.932175474	4.842766089	4.731093453	4.549503667
ENSG00000204264	0.000173	0.012006638	-0.649994158	-1.569161842	**PSMB8**	protein_coding	proteasome subunit beta 8 [Source:HGNC Symbol;Acc:HGNC:9545]	chr6	32840717	32844703	-1	ensembl_havana	7.747484429	8.072481508	7.42248735	8.093186906	8.042416561	8.081841057	7.402295995	7.396066793	7.469099262
ENSG00000138613	0.000158	0.012006638	-0.672224518	-1.593528165	**APH1B**	protein_coding	aph-1 homolog B, gamma-secretase subunit [Source:HGNC Symbol;Acc:HGNC:24080]	chr15	63276018	63309126	1	ensembl_havana	4.521496608	4.857608867	4.185384349	4.737428462	4.958493072	4.876905068	4.028181329	4.273512191	4.254459527
ENSG00000155660	0.000174	0.012006638	-0.674107607	-1.595609488	**PDIA4**	protein_coding	protein disulfide isomerase family A member 4 [Source:HGNC Symbol;Acc:HGNC:30167]	chr7	149003062	149028641	-1	ensembl_havana	8.216872525	8.553926329	7.879818722	8.745831065	8.448256312	8.46769161	8.011293437	7.788435739	7.839726989
ENSG00000186517	0.000153	0.012006638	-0.674571444	-1.59612257	**ARHGAP30**	protein_coding	Rho GTPase activating protein 30 [Source:HGNC Symbol;Acc:HGNC:27414]	chr1	161046946	161069970	-1	ensembl_havana	4.801478063	5.138763785	4.464192341	5.121261956	5.0672469	5.227782498	4.398463935	4.426631717	4.567481372
ENSG00000279662	0.000148	0.012006638	-0.685716634	-1.608500778	**AC131649.2**	TEC		chr16	10445309	10449747	-1	havana	3.743448184	4.086306501	3.400589867	4.47628667	4.085150794	3.697482039	3.764626412	3.372156889	3.064986301
ENSG00000048052	0.00016	0.012006638	-0.685828183	-1.608625152	**HDAC9**	protein_coding	histone deacetylase 9 [Source:HGNC Symbol;Acc:HGNC:14065]	chr7	18086949	19002416	1	ensembl_havana	1.022339262	1.365253353	0.67942517	1.222335231	1.51252579	1.360899039	0.592614899	0.821210834	0.624449778
ENSG00000182749	0.000167	0.012006638	-0.695797416	-1.619779481	**PAQR7**	protein_coding	progestin and adipoQ receptor family member 7 [Source:HGNC Symbol;Acc:HGNC:23146]	chr1	25861210	25871253	-1	ensembl_havana	3.35427695	3.702175658	3.006378242	3.738753807	3.709036025	3.658737143	2.972031052	3.038025793	3.009077882
ENSG00000182389	0.00015	0.012006638	-0.696203618	-1.620235607	**CACNB4**	protein_coding	calcium voltage-gated channel auxiliary subunit beta 4 [Source:HGNC Symbol;Acc:HGNC:1404]	chr2	151832768	152099475	-1	ensembl_havana	2.229788098	2.577889907	1.881686289	2.341580783	2.558348641	2.833740298	1.681320805	1.800897362	2.162840701
ENSG00000101265	0.000158	0.012006638	-0.706749594	-1.632122783	**RASSF2**	protein_coding	Ras association domain family member 2 [Source:HGNC Symbol;Acc:HGNC:9883]	chr20	4780023	4823645	-1	ensembl_havana	4.669218051	5.022592848	4.315843254	5.072602584	5.028828229	4.966347733	4.324648722	4.394551181	4.22832986
ENSG00000104894	0.000153	0.012006638	-0.709444651	-1.635174554	**CD37**	protein_coding	CD37 molecule [Source:HGNC Symbol;Acc:HGNC:1666]	chr19	49335171	49343335	1	ensembl_havana	5.642292627	5.997014953	5.287570302	6.134217744	5.867433235	5.989393879	5.395777777	5.229218631	5.237714498
ENSG00000113368	0.000176	0.012006638	-0.719626355	-1.646755485	**LMNB1**	protein_coding	lamin B1 [Source:HGNC Symbol;Acc:HGNC:6637]	chr5	126776623	126837020	1	ensembl_havana	6.338404118	6.698217295	5.97859094	6.503703618	6.740219681	6.850728587	5.701176434	6.034800719	6.199795667
ENSG00000172716	0.000149	0.012006638	-0.757583811	-1.690656784	**SLFN11**	protein_coding	schlafen family member 11 [Source:HGNC Symbol;Acc:HGNC:26633]	chr17	35350305	35373701	-1	ensembl_havana	5.905634185	6.284426091	5.52684228	6.300072106	6.25191871	6.301287457	5.46083574	5.496716106	5.622974993
ENSG00000181381	0.000158	0.012006638	-0.787368397	-1.725923353	**DDX60L**	protein_coding	DExD/H-box 60 like [Source:HGNC Symbol;Acc:HGNC:26429]	chr4	168356735	168537786	-1	ensembl_havana	-0.470660163	-0.076975964	-0.864344361	-0.67394004	0.030651399	0.41236075	-1.52209115	-0.80561234	-0.265329594
ENSG00000149639	0.000175	0.012006638	-0.822744894	-1.768768078	**SOGA1**	protein_coding	suppressor of glucose, autophagy associated 1 [Source:HGNC Symbol;Acc:HGNC:16111]	chr20	36777442	36863686	-1	ensembl_havana	5.761103278	6.172475724	5.349730831	6.301449829	5.999297534	6.216679809	5.464365468	5.296551041	5.288275983
ENSG00000075651	0.000141	0.012006638	-0.827558013	-1.774678897	**PLD1**	protein_coding	phospholipase D1 [Source:HGNC Symbol;Acc:HGNC:9067]	chr3	171600405	171810950	-1	ensembl_havana	2.686366522	3.100145529	2.272587516	3.503639275	2.868808811	2.9279885	2.607204823	1.993561814	2.21699591
ENSG00000230454	0.000146	0.012006638	-0.869296248	-1.826771577	**U73166.1**	lincRNA		chr3	50260303	50263358	1	havana	0.778761909	1.213410033	0.344113785	1.541228291	1.170104606	0.928897202	0.56657698	0.426254992	0.039509383
ENSG00000135828	0.000153	0.012006638	-0.878755676	-1.838788662	**RNASEL**	protein_coding	ribonuclease L [Source:HGNC Symbol;Acc:HGNC:10050]	chr1	182573634	182589256	-1	ensembl_havana	2.050826192	2.49020403	1.611448354	2.298074107	2.619498965	2.553039018	1.278000209	1.811166797	1.745178056
ENSG00000101336	0.000141	0.012006638	-0.919211945	-1.89108203	**HCK**	protein_coding	HCK proto-oncogene, Src family tyrosine kinase [Source:HGNC Symbol;Acc:HGNC:4840]	chr20	32052188	32101856	1	ensembl_havana	4.107841984	4.567447956	3.648236012	4.334544199	4.689650085	4.678149585	3.552176738	3.753878337	3.63865296
ENSG00000278023	0.000148	0.012006638	-0.948443356	-1.929789323	**RDM1**	protein_coding	RAD52 motif containing 1 [Source:HGNC Symbol;Acc:HGNC:19950]	chr17	35918066	35930773	-1	ensembl_havana	1.930361067	2.404582745	1.456139389	2.032058081	2.541211407	2.640478746	1.212343926	1.614107429	1.541966812
ENSG00000103202	0.000176	0.012006638	-0.970602912	-1.959659379	**NME4**	protein_coding	NME/NM23 nucleoside diphosphate kinase 4 [Source:HGNC Symbol;Acc:HGNC:7852]	chr16	396725	410367	1	ensembl_havana	3.147782415	3.633083871	2.662480959	3.752250231	3.512020676	3.634980708	2.711879034	2.721536001	2.554027843
ENSG00000126803	0.00015	0.012006638	-1.010328328	-2.014369477	**HSPA2**	protein_coding	heat shock protein family A (Hsp70) member 2 [Source:HGNC Symbol;Acc:HGNC:5235]	chr14	64535905	64546173	1	ensembl_havana	-0.362011356	0.143152808	-0.867175519	0.004630787	0.06303108	0.361796557	-0.82634296	-1.00738779	-0.767795802
ENSG00000010610	0.000147	0.012006638	-1.192796984	-2.285954968	**CD4**	protein_coding	CD4 molecule [Source:HGNC Symbol;Acc:HGNC:1678]	chr12	6786858	6820808	1	ensembl_havana	3.098616232	3.695014724	2.50221774	4.300040351	3.51787272	3.2671311	2.936891701	2.271570328	2.298191191
ENSG00000196507	0.000169	0.012006638	-1.259330655	-2.393846516	**TCEAL3**	protein_coding	transcription elongation factor A like 3 [Source:HGNC Symbol;Acc:HGNC:28247]	chrX	103607451	103629690	1	ensembl_havana	-0.689080321	-0.059414993	-1.318745648	-0.41068387	0.06303108	0.16940781	-1.52209115	-1.08144551	-1.352700279
ENSG00000153885	0.000166	0.012006638	-1.285742111	-2.438074332	**KCTD15**	protein_coding	potassium channel tetramerization domain containing 15 [Source:HGNC Symbol;Acc:HGNC:23297]	chr19	33795933	33815763	1	ensembl_havana	1.541039574	2.18391063	0.898168518	2.260788651	2.101167058	2.18977618	0.807953002	0.715834679	1.170717874
ENSG00000204338	0.000163	0.012006638	-1.414411665	-2.665510131	**CYP21A1P**	transcribed_unprocessed_pseudogene	cytochrome P450 family 21 subfamily A member 1, pseudogene [Source:HGNC Symbol;Acc:HGNC:2599]	chr6	32005636	32008451	1	havana	1.693728481	2.400934313	0.986522648	2.57213685	1.953538152	2.677127938	0.995284613	0.841392961	1.12289037
ENSG00000249631	0.000173	0.012006638	-1.644182814	-3.125707576	**AC005699.1**	lincRNA		chr4	11625714	11813958	1	havana	-0.397718287	0.42437312	-1.219809694	1.04068047	0.06303108	0.16940781	-0.97139419	-1.42278721	-1.265247677
ENSG00000124493	0.00017	0.012006638	-2.115379392	-4.333039512	**GRM4**	protein_coding	glutamate metabotropic receptor 4 [Source:HGNC Symbol;Acc:HGNC:4596]	chr6	34018645	34155622	-1	ensembl_havana	1.096977128	2.154666824	0.039287432	2.142776609	1.855271053	2.46595281	-0.04612063	0.19485955	-0.030876629
ENSG00000164070	0.000177	0.012021555	-0.710391285	-1.636247837	**HSPA4L**	protein_coding	heat shock protein family A (Hsp70) member 4 like [Source:HGNC Symbol;Acc:HGNC:17041]	chr4	127781821	127840733	1	ensembl_havana	5.618770697	5.97396634	5.263575055	5.924311445	5.925271013	6.072316563	5.136234527	5.283702404	5.370788233
ENSG00000182378	0.000177	0.012021555	-0.767502177	-1.702319904	**PLCXD1**	protein_coding	phosphatidylinositol specific phospholipase C X domain containing 1 [Source:HGNC Symbol;Acc:HGNC:23148]	chrX	276322	303356	1	ensembl_havana	5.679448749	6.063199837	5.29569766	5.979863317	6.005558658	6.204177537	5.317924627	5.213819317	5.355349037
ENSG00000176746	0.000178	0.012021555	-0.880585965	-1.84112294	**MAGEB6**	protein_coding	MAGE family member B6 [Source:HGNC Symbol;Acc:HGNC:23796]	chrX	26192440	26195646	1	ensembl_havana	0.085293882	0.525586864	-0.354999101	0.075059705	0.699804047	0.80189684	-0.89704638	-0.2414061	0.073455173
ENSG00000184785	0.000185	0.012431641	-0.717781951	-1.644651546	**SMIM10**	protein_coding	small integral membrane protein 10 [Source:HGNC Symbol;Acc:HGNC:41913]	chrX	134990938	134992473	1	ensembl_havana	2.695736944	3.05462792	2.336845969	2.991768026	2.904984633	3.2671311	2.22979561	2.139252294	2.641490002
ENSG00000228005	0.000197	0.012466761	1.3883664	2.617820905	**AC020743.1**	antisense		chr7	50141540	50142823	-1	havana	0.160246634	-0.533936566	0.854429834	-0.85854608	-0.620350959	-0.122912658	0.830009965	0.693803314	1.039476224
ENSG00000122786	0.000201	0.012466761	0.971654725	1.961088612	**CALD1**	protein_coding	caldesmon 1 [Source:HGNC Symbol;Acc:HGNC:1441]	chr7	134744252	134970728	1	ensembl_havana	0.697872363	0.212045	1.183699725	0.355307344	0.477608744	-0.196781087	1.143556601	1.590453245	0.817089329
ENSG00000080709	0.000196	0.012466761	0.854855406	1.808577482	**KCNN2**	protein_coding	potassium calcium-activated channel subfamily N member 2 [Source:HGNC Symbol;Acc:HGNC:6291]	chr5	114360945	114496500	1	ensembl_havana	1.618768785	1.191341082	2.046196488	1.022973463	1.324992307	1.226057475	1.946559732	2.029189031	2.162840701
ENSG00000267257	0.0002	0.012466761	0.79198293	1.731452644	**AC105105.1**	antisense		chr18	58535415	58538552	1	havana	1.102042616	0.706051151	1.498034081	0.637193984	0.679062629	0.80189684	1.514288036	1.343981945	1.635832261
ENSG00000066248	0.000189	0.012466761	0.686658978	1.609551767	**NGEF**	protein_coding	neuronal guanine nucleotide exchange factor [Source:HGNC Symbol;Acc:HGNC:7807]	chr2	232878686	233013272	-1	ensembl_havana	2.030002891	1.686673403	2.37333238	1.738566362	1.558674763	1.762779083	2.408992098	2.187350601	2.523654441
ENSG00000012822	0.000199	0.012466761	0.682673495	1.605111474	**CALCOCO1**	protein_coding	calcium binding and coiled-coil domain 1 [Source:HGNC Symbol;Acc:HGNC:29306]	chr12	53708517	53727745	-1	ensembl_havana	6.870789077	6.52945233	7.212125824	6.690202486	6.54054267	6.357611833	7.387963495	7.147245643	7.101168336
ENSG00000163328	0.000188	0.012466761	0.671416742	1.592636185	**GPR155**	protein_coding	G protein-coupled receptor 155 [Source:HGNC Symbol;Acc:HGNC:22951]	chr2	174431571	174487094	-1	ensembl_havana	3.870752927	3.535044556	4.206461298	3.569159951	3.555343125	3.480630591	4.309028837	4.185467242	4.124887815
ENSG00000156050	0.0002	0.012466761	0.631735135	1.549427376	**FAM161B**	protein_coding	family with sequence similarity 161 member B [Source:HGNC Symbol;Acc:HGNC:19854]	chr14	73931501	73950414	-1	ensembl_havana	2.848638741	2.532771174	3.164506308	2.516278079	2.586465946	2.495569495	3.131498845	3.187409884	3.174610196
ENSG00000186352	0.000187	0.012466761	-0.635588229	-1.553571057	**ANKRD37**	protein_coding	ankyrin repeat domain 37 [Source:HGNC Symbol;Acc:HGNC:29593]	chr4	185396021	185400628	1	ensembl_havana	1.42001725	1.737811364	1.102223136	1.355633303	1.719668303	2.138132487	0.762800879	1.063828438	1.480040089
ENSG00000179222	0.000201	0.012466761	-0.680572737	-1.602775917	**MAGED1**	protein_coding	MAGE family member D1 [Source:HGNC Symbol;Acc:HGNC:6813]	chrX	51803007	51902357	1	ensembl_havana	4.660125218	5.000411587	4.319838849	5.184352789	5.118214983	4.698666988	4.434901752	4.436615978	4.087998817
ENSG00000154760	0.000191	0.012466761	-0.704408608	-1.62947657	**SLFN13**	protein_coding	schlafen family member 13 [Source:HGNC Symbol;Acc:HGNC:26481]	chr17	35435096	35448837	-1	ensembl_havana	5.99532852	6.347532824	5.643124216	6.29131584	6.294728489	6.456554143	5.507773497	5.66398894	5.757610211
ENSG00000213888	0.0002	0.012466761	-0.778806717	-1.715711186	**LINC01521**	lincRNA	long intergenic non-protein coding RNA 1521 [Source:HGNC Symbol;Acc:HGNC:26029]	chr22	31346777	31348719	1	ensembl_havana	0.867047322	1.256450681	0.477643963	1.142587728	1.062111262	1.564653053	0.24405081	0.37179175	0.817089329
ENSG00000169105	0.000195	0.012466761	-0.794462127	-1.734430614	**CHST14**	protein_coding	carbohydrate sulfotransferase 14 [Source:HGNC Symbol;Acc:HGNC:24464]	chr15	40470998	40474571	1	ensembl_havana	4.705746179	5.102977242	4.308515116	5.136780775	5.099800122	5.072350831	4.443869204	4.322794032	4.158882111
ENSG00000177853	0.000196	0.012466761	-0.839347427	-1.789240633	**ZNF518A**	protein_coding	zinc finger protein 518A [Source:HGNC Symbol;Acc:HGNC:29009]	chr10	96129715	96205288	1	ensembl_havana	6.214462442	6.634136155	5.794788728	6.863124813	6.545564416	6.493719236	5.884262275	5.740537503	5.759566407
ENSG00000099840	0.000197	0.012466761	-0.900975004	-1.867327536	**IZUMO4**	protein_coding	IZUMO family member 4 [Source:HGNC Symbol;Acc:HGNC:26950]	chr19	2096429	2099593	1	ensembl_havana	4.483514198	4.9340017	4.033026696	5.572259566	4.831500081	4.398245452	4.532287853	3.919661155	3.647131082
ENSG00000123584	0.000196	0.012466761	-1.098431381	-2.141217548	**MAGEA9**	protein_coding	MAGE family member A9 [Source:HGNC Symbol;Acc:HGNC:6807]	chrX	149781930	149787737	1	ensembl_havana	3.088183256	3.637398946	2.538967565	3.660747824	3.656952914	3.594496101	2.40164672	2.780215171	2.435040805
ENSG00000115041	0.000199	0.012466761	-1.237596394	-2.358053408	**KCNIP3**	protein_coding	potassium voltage-gated channel interacting protein 3 [Source:HGNC Symbol;Acc:HGNC:15523]	chr2	95297304	95386083	1	ensembl_havana	0.386290122	1.005088319	-0.232508075	1.158893785	0.910210399	0.946160774	0.141952326	-0.57414696	-0.265329594
ENSG00000172322	0.0002	0.012466761	-2.206596472	-4.615850417	**CLEC12A**	protein_coding	C-type lectin domain family 12 member A [Source:HGNC Symbol;Acc:HGNC:31713]	chr12	9951316	9995694	1	ensembl_havana	-0.487078013	0.616220224	-1.590376249	1.411140715	0.52486995	-0.087349994	-1.13267621	-1.87077278	-1.767679756
ENSG00000242686	0.000203	0.012523126	-0.670240634	-1.591338372	**AC107464.1**	antisense		chr4	652850	656213	-1	havana	1.823744217	2.158864534	1.4886239	2.214735639	1.927394938	2.334463026	1.619344613	1.210694826	1.635832261
ENSG00000100867	0.000214	0.013088874	0.601821408	1.517631373	**DHRS2**	protein_coding	dehydrogenase/reductase 2 [Source:HGNC Symbol;Acc:HGNC:18349]	chr14	23630115	23645639	1	ensembl_havana	9.434147831	9.133237127	9.735058535	9.08200881	9.107287603	9.210414968	9.678579475	9.735290172	9.791305958
ENSG00000196526	0.000214	0.013088874	-0.877586224	-1.83729874	**AFAP1**	protein_coding	actin filament associated protein 1 [Source:HGNC Symbol;Acc:HGNC:24017]	chr4	7758714	7939926	-1	ensembl_havana	3.184142266	3.622935378	2.745349154	3.905065295	3.307624065	3.656116775	3.015987943	2.578569518	2.641490002
ENSG00000147082	0.000215	0.013097433	-0.902824541	-1.869722989	**CCNB3**	protein_coding	cyclin B3 [Source:HGNC Symbol;Acc:HGNC:18709]	chrX	50202713	50351910	1	ensembl_havana	2.614485298	3.065897569	2.163073027	2.991768026	3.304241881	2.901682799	2.10871413	2.24151074	2.138994213
ENSG00000198216	0.000216	0.013113029	-0.917170508	-1.888408011	**CACNA1E**	protein_coding	calcium voltage-gated channel subunit alpha1 E [Source:HGNC Symbol;Acc:HGNC:1392]	chr1	181317690	181808084	1	ensembl_havana	4.232614277	4.691199532	3.774029023	4.995256012	4.458168414	4.620174169	4.175428345	3.62301021	3.523648515
ENSG00000245522	0.000219	0.013172643	-0.841509963	-1.791924637	**AC026250.1**	lincRNA		chr11	9754770	9759533	-1	havana	0.483234225	0.903989206	0.062479243	0.565613454	0.89230127	1.254052895	-0.17198403	0.097529431	0.261892331
ENSG00000166165	0.000219	0.013172643	-1.111330399	-2.160447838	**CKB**	protein_coding	creatine kinase B [Source:HGNC Symbol;Acc:HGNC:1991]	chr14	103519659	103523111	-1	ensembl_havana	2.843706021	3.399371221	2.288040822	3.652134298	3.234877758	3.311101606	2.632526022	2.271570328	1.960026115
ENSG00000171241	0.000224	0.013355803	-0.601558934	-1.517355291	**SHCBP1**	protein_coding	SHC binding and spindle associated 1 [Source:HGNC Symbol;Acc:HGNC:29547]	chr16	46580554	46621626	-1	ensembl_havana	6.174536079	6.475315546	5.873756612	6.379622001	6.555911329	6.490413308	5.761239148	5.935616522	5.924414164
ENSG00000165899	0.000223	0.013355803	-1.051665392	-2.072921368	**OTOGL**	protein_coding	otogelin like [Source:HGNC Symbol;Acc:HGNC:26901]	chr12	80099537	80380879	1	ensembl_havana	-0.039042894	0.486789802	-0.56487559	0.968509893	0.15602554	0.335833975	0.141952326	-1.00738779	-0.829191301
ENSG00000172005	0.000225	0.013355803	-1.890298119	-3.707118207	**MAL**	protein_coding	mal, T cell differentiation protein [Source:HGNC Symbol;Acc:HGNC:6817]	chr2	95025677	95053996	1	ensembl_havana	-1.009035519	-0.063886459	-1.954184578	0.464289132	-0.299013651	-0.356934859	-1.7649502	-1.74507732	-2.35252622
ENSG00000151718	0.000245	0.013414014	1.038501075	2.054092393	**WWC2**	protein_coding	WW and C2 domain containing 2 [Source:HGNC Symbol;Acc:HGNC:24148]	chr4	183099293	183320777	1	ensembl_havana	0.917750164	0.398499626	1.437000702	0.004630787	0.570631912	0.620236179	0.995284613	1.43975459	1.875962902
ENSG00000141542	0.000249	0.013414014	0.986437634	1.981286666	**RAB40B**	protein_coding	RAB40B, member RAS oncogene family [Source:HGNC Symbol;Acc:HGNC:18284]	chr17	82654973	82698728	-1	ensembl_havana	1.479542923	0.986324106	1.97276174	0.872903038	1.228369434	0.857699845	1.763844323	2.097907935	2.056532963
ENSG00000161912	0.000246	0.013414014	0.749566476	1.681287535	**ADCY10P1**	transcribed_unprocessed_pseudogene	adenylate cyclase 10, soluble pseudogene 1 [Source:HGNC Symbol;Acc:HGNC:44143]	chr6	41101022	41140835	1	havana	3.375417742	3.000634504	3.750200981	3.084314947	2.868808811	3.048779755	3.706198956	3.682954534	3.861449452
ENSG00000139985	0.00024	0.013414014	0.749079004	1.68071954	**ADAM21**	protein_coding	ADAM metallopeptidase domain 21 [Source:HGNC Symbol;Acc:HGNC:200]	chr14	70452157	70459899	1	ensembl_havana	1.25275862	0.878219118	1.627298122	0.931023971	0.740413176	0.963220207	1.631954676	1.614107429	1.635832261
ENSG00000119772	0.000247	0.013414014	0.702845915	1.627712516	**DNMT3A**	protein_coding	DNA methyltransferase 3 alpha [Source:HGNC Symbol;Acc:HGNC:2978]	chr2	25227855	25342590	-1	ensembl_havana	4.098914586	3.747491628	4.450337543	3.559980076	3.756831958	3.925662851	4.37051862	4.41152456	4.56896945
ENSG00000105711	0.000246	0.013414014	0.675121876	1.596731656	**SCN1B**	protein_coding	sodium voltage-gated channel beta subunit 1 [Source:HGNC Symbol;Acc:HGNC:10586]	chr19	35030466	35040449	1	ensembl_havana	1.011985863	0.674424925	1.349546802	0.326725152	0.699804047	0.996745577	0.935503283	1.465989718	1.647147403
ENSG00000145536	0.00025	0.013414014	0.638256436	1.556446983	**ADAMTS16**	protein_coding	ADAM metallopeptidase with thrombospondin type 1 motif 16 [Source:HGNC Symbol;Acc:HGNC:17108]	chr5	5140330	5320304	1	ensembl_havana	2.159921739	1.840793521	2.479049957	1.341416217	1.927394938	2.253569408	2.005893922	2.614195633	2.817060315
ENSG00000175564	0.000251	0.013414014	0.618412622	1.535185108	**UCP3**	protein_coding	uncoupling protein 3 [Source:HGNC Symbol;Acc:HGNC:12519]	chr11	74000281	74009435	-1	ensembl_havana	1.161553134	0.852346823	1.470759445	0.832812769	0.570631912	1.153595787	1.514288036	1.163381334	1.734608964
ENSG00000123094	0.00025	0.013414014	0.614403022	1.530924379	**RASSF8**	protein_coding	Ras association domain family member 8 [Source:HGNC Symbol;Acc:HGNC:13232]	chr12	25959029	26079892	1	ensembl_havana	4.268550692	3.961349181	4.575752203	3.649251657	4.047212239	4.187583646	4.209480398	4.712038443	4.805737767

ENSG00000227630	0.00023	0.013414014	-0.596290366	-1.511824177	LINC01132	lincRNA	long intergenic non-protein coding RNA 1132 [Source:HGNC Symbol;Acc:HGNC:49444]	chr1	234724042	234736720	1	havana	5.78630639	6.084451573	5.488161207	6.153163945	5.910888514	6.189302261	5.545754476	5.292891664	5.625837481
ENSG00000102265	0.000244	0.013414014	-0.596630503	-1.512180654	TIMP1	protein_coding	TIMP metallopeptidase inhibitor 1 [Source:HGNC Symbol;Acc:HGNC:11820]	chrX	47582313	47586789	1	ensembl_havana	5.329822173	5.628137424	5.031506921	5.829451325	5.652855272	5.402105675	5.197961942	5.052214115	4.844344708
ENSG00000138794	0.000248	0.013414014	-0.59708928	-1.512661604	CASP6	protein_coding	caspase 6 [Source:HGNC Symbol;Acc:HGNC:1507]	chr4	109688622	109703583	-1	ensembl_havana	4.34032116	4.6388658	4.04177652	4.505271103	4.728019942	4.683306356	3.949769201	4.093802465	4.081757895
ENSG00000138696	0.000251	0.013414014	-0.626266405	-1.54356518	BMPR1B	protein_coding	bone morphogenetic protein receptor type 1B [Source:HGNC Symbol;Acc:HGNC:1077]	chr4	94757968	95158448	1	ensembl_havana	4.340011615	4.653144818	4.026878413	4.886853659	4.621983834	4.45059696	4.244801141	3.945529874	3.890304222
ENSG00000175189	0.000245	0.013414014	-0.675241626	-1.596864197	INHBC	protein_coding	inhibin beta C subunit [Source:HGNC Symbol;Acc:HGNC:6068]	chr12	57434760	57450828	1	ensembl_havana	0.60895715	0.946577963	0.271336337	0.832812769	0.945375194	1.061545926	0.24405081	0.19485955	0.375098651
ENSG00000112303	0.000235	0.013414014	-0.679813721	-1.601932902	VNN2	protein_coding	vanin 2 [Source:HGNC Symbol;Acc:HGNC:12706]	chr6	132743870	132763459	-1	ensembl_havana	1.685210926	2.025117787	1.345304066	1.968722995	2.169677667	1.936952697	1.261864131	1.426455924	1.347592143
ENSG00000140968	0.000242	0.013414014	-0.689937265	-1.613213367	IRF8	protein_coding	interferon regulatory factor 8 [Source:HGNC Symbol;Acc:HGNC:5358]	chr16	85898803	85922609	1	ensembl_havana	3.411045919	3.756014552	3.066077287	3.481120682	3.826860993	3.96006198	2.78144541	3.055408658	3.361377791
ENSG00000072682	0.000249	0.013414014	-0.690670052	-1.614032974	P4HA2	protein_coding	prolyl 4-hydroxylase subunit alpha 2 [Source:HGNC Symbol;Acc:HGNC:8547]	chr5	132191838	132295315	-1	ensembl_havana	7.288708966	7.634043992	6.94337394	7.784028151	7.481302187	7.636801637	6.993067103	6.82087654	7.016178175
ENSG00000145439	0.000247	0.013414014	-0.7525039	-1.684714236	CBR4	protein_coding	carbonyl reductase 4 [Source:HGNC Symbol;Acc:HGNC:25891]	chr4	168863770	169010275	-1	ensembl_havana	3.655958137	4.032210087	3.279706187	3.987262005	4.001999572	4.107368684	3.127051692	3.241567838	3.470499031
ENSG00000210194	0.000234	0.013414014	-0.806612076	-1.749099154	MT-TE	Mt_tRNA	mitochondrially encoded tRNA glutamic acid [Source:HGNC Symbol;Acc:HGNC:7479]	chrMT	14674	14742	-1	insdc	0.062012964	0.465319002	-0.341293075	0.515840645	0.740413176	0.139703184	-0.17198403	-0.19956768	-0.652327512
ENSG00000197142	0.000248	0.013414014	-0.812972882	-1.756827914	ACSL5	protein_coding	acyl-CoA synthetase long chain family member 5 [Source:HGNC Symbol;Acc:HGNC:16526]	chr10	112374018	112428380	1	ensembl_havana	5.269085549	5.67557199	4.862599108	5.850310141	5.608296132	5.568109699	4.992066932	4.944381572	4.65134882
ENSG00000104951	0.000232	0.013414014	-0.815349155	-1.759723981	IL4I1	protein_coding	interleukin 4 induced 1 [Source:HGNC Symbol;Acc:HGNC:19094]	chr19	49889654	49929539	-1	ensembl_havana	7.201049175	7.608723752	6.793374597	8.005176892	7.317961977	7.503032387	7.07500463	6.64222936	6.662889802
ENSG00000236345	0.00025	0.013414014	-0.98984372	-1.985969849	AL354719.2	antisense		chr6	63806836	63822642	1	havana	0.758780602	1.253702462	0.263858742	1.312554705	1.324992307	1.123560375	0.36983092	0.130707766	0.291037539
ENSG00000226674	0.000249	0.013414014	-0.991791798	-1.988653325	TEX41	lincRNA	testis expressed 41 (non-protein coding) [Source:HGNC Symbol;Acc:HGNC:48667]	chr2	144667967	145262988	1	havana	0.750836541	1.24673244	0.254940642	1.82284946	0.855801933	1.061545926	0.915012722	-0.00684508	-0.143345712
ENSG00000143365	0.000227	0.013414014	-1.041526795	-2.058404897	RORC	protein_coding	RAR related orphan receptor C [Source:HGNC Symbol;Acc:HGNC:10260]	chr1	151806071	151831872	-1	ensembl_havana	0.0351973	0.555960698	-0.485566097	0.589870393	0.592979824	0.485031876	-0.63291296	-0.47099802	-0.352787316
ENSG00000244345	0.000251	0.013414014	-1.11827036	-2.170865524	AC104435.2	lincRNA		chr3	72504806	72550552	-1	havana	0.620741051	1.179876231	0.061605871	1.142587728	1.214022341	1.183018625	0.141952326	-0.15890847	0.201773757
ENSG00000280913	0.000231	0.013414014	-1.162732006	-2.238809858	AL731733.1	transcribed_unprocessed_pseudogene		chr10	46753605	46759740	1	havana	2.59751597	3.178881973	2.016149967	3.018821299	3.213401092	3.304423528	1.841901733	1.831488934	2.375059235
ENSG00000149927	0.000237	0.013414014	-1.644327111	-3.126020222	DOC2A	protein_coding	double C2 domain alpha [Source:HGNC Symbol;Acc:HGNC:2985]	chr16	30005509	30023270	-1	ensembl_havana	-0.887372311	-0.065208756	-1.709535867	-0.27307956	-0.383759306	0.461212593	-1.52209115	-2.16071837	-1.445798078
ENSG00000227070	0.000254	0.013448947	0.597058605	1.512629442	AC104170.1	antisense		chr1	51518309	51561629	1	havana	0.183252	-0.115277303	0.481781302	0.490295136	-0.299013651	-0.537113394	1.052687018	0.286036324	0.106620565
ENSG00000072571	0.000254	0.013448947	-0.622962737	-1.54003457	HMMR	protein_coding	hyaluronan mediated motility receptor [Source:HGNC Symbol;Acc:HGNC:5012]	chr5	163460203	163491945	1	ensembl_havana	5.302422032	5.6139034	4.990940664	5.363873117	5.768431829	5.709405256	4.754702143	5.081144309	5.136975539
ENSG00000097046	0.000267	0.013710643	-0.575573692	-1.490269956	CDC7	protein_coding	cell division cycle 7 [Source:HGNC Symbol;Acc:HGNC:1745]	chr1	91500893	91525764	1	ensembl_havana	5.483541447	5.771328293	5.195754601	5.61720751	5.870295093	5.826482276	5.011498951	5.296551041	5.27921381
ENSG00000129521	0.00026	0.013710643	-0.614956792	-1.531512128	EGLN3	protein_coding	egl-9 family hypoxia inducible factor 3 [Source:HGNC Symbol;Acc:HGNC:14661]	chr14	33924231	34462774	-1	ensembl_havana	0.609379846	0.916858242	0.30190145	1.190963204	0.855801933	0.703809589	0.513047461	0.286036324	0.106620565
ENSG00000104177	0.000264	0.013710643	-0.623903944	-1.541039609	MYEF2	protein_coding	myelin expression factor 2 [Source:HGNC Symbol;Acc:HGNC:17940]	chr15	48134631	48178517	-1	ensembl_havana	5.991866702	6.303818674	5.67991473	6.347970893	6.331328314	6.232156815	5.667120629	5.697590588	5.675032972
ENSG00000183486	0.000266	0.013710643	-0.661275397	-1.581480094	MX2	protein_coding	MX dynamin like GTPase 2 [Source:HGNC Symbol;Acc:HGNC:7533]	chr21	41361943	41409390	1	ensembl_havana	4.181997073	4.512634772	3.851359374	4.338128639	4.590397449	4.609378227	3.591444275	4.004891038	3.95774281
ENSG00000183150	0.000267	0.013710643	-0.762641747	-1.696594449	GPR19	protein_coding	G protein-coupled receptor 19 [Source:HGNC Symbol;Acc:HGNC:4473]	chr12	12660891	12696207	-1	ensembl_havana	3.104705659	3.486026532	2.723384786	3.390813464	3.503197858	3.564068275	2.741273741	2.753838306	2.675042311
ENSG00000106462	0.000265	0.013710643	-0.884285825	-1.845850647	EZH2	protein_coding	enhancer of zeste 2 polycomb repressive complex 2 subunit [Source:HGNC Symbol;Acc:HGNC:3527]	chr7	148807383	148884321	-1	ensembl_havana	5.599810497	6.04195341	5.157667584	6.432974659	5.925271013	5.767614557	5.714576804	4.890919645	4.867506304
ENSG00000091137	0.000266	0.013710643	-0.950053775	-1.931944667	SLC26A4	protein_coding	solute carrier family 26 member 4 [Source:HGNC Symbol;Acc:HGNC:8818]	chr7	107660635	107717809	1	ensembl_havana	4.93435582	5.409382707	4.459328933	5.536045381	5.344172115	5.347930627	4.501696252	4.273512191	4.602778354
ENSG00000176597	0.000261	0.013710643	-1.188124283	-2.278563036	B3GNT5	protein_coding	UDP-GlcNAc:betaGal beta-1,3-N-acetylglucosaminyltransferase 5 [Source:HGNC Symbol;Acc:HGNC:15684]	chr3	183253244	183298504	1	ensembl_havana	2.28109023	2.875152372	1.687028089	2.637596133	2.734533378	3.253327603	1.729053464	1.399485086	1.932545716
ENSG00000104611	0.000264	0.013710643	-1.21659472	-2.323975275	SH2D4A	protein_coding	SH2 domain containing 4A [Source:HGNC Symbol;Acc:HGNC:26102]	chr8	19313617	19396218	1	ensembl_havana	1.900202041	2.508499401	1.291904681	3.323695481	2.256240478	1.945562243	2.126646177	0.779979312	0.969088554
ENSG00000182459	0.00029	0.01377236	1.196557352	2.291921056	TEX19	protein_coding	testis expressed 19 [Source:HGNC Symbol;Acc:HGNC:33802]	chr17	82359247	82363776	1	ensembl_havana	-0.073148133	-0.671426809	0.525130543	-0.85854608	-0.569826728	-0.585907617	0.540060475	0.343769191	0.691561964
ENSG00000175746	0.000272	0.01377236	0.941289417	1.920243701	C15orf54	lincRNA	chromosome 15 open reading frame 54 (putative) [Source:HGNC Symbol;Acc:HGNC:33797]	chr15	39250684	39254845	1	ensembl_havana	-0.118168254	-0.588812962	0.352476455	-0.85854608	-0.672709	-0.235183806	-0.08686711	0.45273451	0.691561964
ENSG00000275223	0.000286	0.01377236	0.876187836	1.83551873	AL121906.2	lincRNA		chr20	33655701	33656423	-1	havana	1.208852568	0.77075865	1.646946486	0.41082706	1.062111262	0.83933763	1.458744294	1.790554303	1.691540863
ENSG00000168283	0.000289	0.01377236	0.665942922	1.586604914	BMI1	protein_coding	BMI1 proto-oncogene, polycomb ring finger [Source:HGNC Symbol;Acc:HGNC:1066]	chr10	22321211	22331484	1	ensembl_havana	7.165151436	6.832179975	7.498122897	6.778263754	6.933488132	6.784788038	7.43639359	7.514705932	7.543269168
ENSG00000008311	0.000288	0.01377236	0.625326123	1.542559483	AASS	protein_coding	aminoadipate-semialdehyde synthase [Source:HGNC Symbol;Acc:HGNC:17366]	chr7	122075647	122144280	-1	ensembl_havana	4.754199216	4.441536154	5.066862277	4.502079217	4.425852674	4.396676572	5.080974089	5.017157896	5.102454846
ENSG00000221887	0.000287	0.01377236	0.624760871	1.541955223	HMSD	protein_coding	histocompatibility minor serpin domain containing [Source:HGNC Symbol;Acc:HGNC:23037]	chr18	63949301	63981774	1	ensembl_havana	6.039985012	5.727604577	6.352365448	6.055297949	5.744299282	5.383216499	6.684661784	6.297471303	6.074963256
ENSG00000183833	0.00027	0.01377236	0.621663924	1.53864875	MAATS1	protein_coding	MYCBP associated and testis expressed 1 [Source:HGNC Symbol;Acc:HGNC:24010]	chr3	119703022	119767102	1	ensembl_havana	4.774991692	4.46415973	5.085823654	4.572242036	4.476314668	4.343922486	5.173371332	5.04787839	5.036221241
ENSG00000137449	0.000284	0.01377236	0.613719973	1.530199729	CPEB2	protein_coding	cytoplasmic polyadenylation element binding protein 2 [Source:HGNC Symbol;Acc:HGNC:21745]	chr4	15002674	15070153	1	ensembl_havana	5.115970499	4.809110512	5.422830485	4.961956545	4.805420659	4.659954333	5.644739761	5.403923997	5.219827697
ENSG00000249859	0.000283	0.01377236	-0.613393939	-1.52985396	PVT1	lincRNA	Pvt1 oncogene (non-protein coding) [Source:HGNC Symbol;Acc:HGNC:9709]	chr8	127794533	128101253	1	ensembl_havana	3.981265359	4.287962329	3.67456839	4.080089315	4.36222147	4.421576202	3.494651998	3.677351664	3.851701507
ENSG00000104804	0.000275	0.01377236	-0.617353274	-1.53405826	TULP2	protein_coding	tubby like protein 2 [Source:HGNC Symbol;Acc:HGNC:12424]	chr19	48880965	48898733	-1	ensembl_havana	1.990028327	2.298704964	1.681351689	2.411297517	2.124367304	2.360450071	1.75234034	1.578479109	1.713235619
ENSG00000154134	0.000289	0.01377236	-0.628750929	-1.546225707	ROBO3	protein_coding	roundabout guidance receptor 3 [Source:HGNC Symbol;Acc:HGNC:13433]	chr11	124865386	124881470	1	ensembl_havana	3.270968487	3.585343951	2.956593022	3.394260955	3.594636359	3.767134538	2.741273741	3.046743406	3.08176192
ENSG00000122861	0.000281	0.01377236	-0.728307185	-1.656694036	PLAU	protein_coding	plasminogen activator, urokinase [Source:HGNC Symbol;Acc:HGNC:9052]	chr10	73909177	73917497	1	ensembl_havana	4.323325103	4.687478696	3.959171511	4.621609293	4.689650085	4.75117671	3.979675389	4.000410511	3.897428633
ENSG00000239713	0.000278	0.01377236	-0.729369805	-1.657914726	APOBEC3G	protein_coding	apolipoprotein B mRNA editing enzyme catalytic subunit 3G [Source:HGNC Symbol;Acc:HGNC:17357]	chr22	39077005	39087743	1	ensembl_havana	3.729416856	4.094101758	3.364731953	4.120197428	4.176774304	3.985333543	3.262971565	3.50461176	3.326612535
ENSG00000157617	0.000285	0.01377236	-0.731544654	-1.660415902	C2CD2	protein_coding	C2 calcium dependent domain containing 2 [Source:HGNC Symbol;Acc:HGNC:1266]	chr21	41885112	41953890	-1	ensembl_havana	2.079972892	2.445745219	1.714200565	2.424845514	2.464503644	2.447886498	1.763844323	1.602328815	1.776428555
ENSG00000110944	0.000284	0.01377236	-0.731739688	-1.660640384	IL23A	protein_coding	interleukin 23 subunit alpha [Source:HGNC Symbol;Acc:HGNC:15488]	chr12	56334174	56340410	1	ensembl_havana	1.403371641	1.769241485	1.037501798	1.613998728	1.836662271	1.857063457	0.762800879	1.210694826	1.139009687
ENSG00000255346	0.000278	0.01377236	-0.84737531	-1.799224623	NOX5	protein_coding	NADPH oxidase 5 [Source:HGNC Symbol;Acc:HGNC:14874]	chr15	68930525	69062743	1	ensembl_havana	4.359413379	4.783101034	3.935725724	4.39257795	4.672700896	5.284024256	3.718075281	3.724296517	4.364805373
ENSG00000204390	0.000285	0.01377236	-0.857185479	-1.811500845	HSPA1L	protein_coding	heat shock protein family A (Hsp70) member 1 like [Source:HGNC Symbol;Acc:HGNC:5234]	chr6	31809619	31815065	-1	ensembl_havana	2.887809072	3.316401811	2.459216332	3.500443773	3.266504942	3.182256719	2.50808372	2.578569518	2.290995759
ENSG00000185340	0.000291	0.01377236	-0.911156186	-1.880551982	GAS2L1	protein_coding	growth arrest specific 2 like 1 [Source:HGNC Symbol;Acc:HGNC:16955]	chr22	29306582	29312785	1	ensembl_havana	-0.654819298	-0.199241205	-1.110397391	0.075059705	-0.036373238	-0.636410081	-0.89704638	-1.08144551	-1.352700279
ENSG00000141404	0.000288	0.01377236	-0.959954048	-1.945247934	GNAL	protein_coding	G protein subunit alpha L [Source:HGNC Symbol;Acc:HGNC:4388]	chr18	11688956	11885685	1	ensembl_havana	0.668813665	1.148790689	0.188836641	0.613726219	1.324992307	1.507653541	-0.30988669	0.163140205	0.713256405
ENSG00000163131	0.000284	0.01377236	-1.134246378	-2.195038703	CTSS	protein_coding	cathepsin S [Source:HGNC Symbol;Acc:HGNC:2545]	chr1	150730196	150765957	-1	ensembl_havana	1.697002177	2.264125366	1.129878989	2.672087675	2.263224966	1.857063457	1.356079599	1.046547334	0.987010032
ENSG00000257523	0.000283	0.01377236	-1.182722658	-2.27004777	LINC02326	lincRNA	long intergenic non-protein coding RNA 2326 [Source:HGNC Symbol;Acc:HGNC:53246]	chr14	28975836	28978924	-1	havana	1.459003393	2.050364721	0.867642064	2.52259249	1.817810321	1.810691353	1.178360195	0.919411336	0.505154661
ENSG00000125810	0.000283	0.01377236	-1.294842214	-2.453501603	CD93	protein_coding	CD93 molecule [Source:HGNC Symbol;Acc:HGNC:15855]	chr20	23079349	23086340	-1	ensembl_havana	3.889568625	4.536989732	3.242147518	4.982715487	4.071289234	4.556964474	3.37972385	3.002618823	3.34409988
ENSG00000178078	0.000288	0.01377236	-1.651947914	-3.142576606	STAP2	protein_coding	signal transducing adaptor family member 2 [Source:HGNC Symbol;Acc:HGNC:30430]	chr19	4324043	4342786	-1	ensembl_havana	0.162193506	0.988167463	-0.663780451	1.075455542	0.89230127	0.996745577	-0.97139419	-0.42206346	-0.597883695
ENSG00000259712	0.000292	0.013784356	-0.551355023	-1.465461456	AC023906.5	antisense		chr15	52056675	52100523	-1	havana	2.406610169	2.682287681	2.130932658	2.464742477	2.734533378	2.847587186	1.91595172	2.17149534	2.305350914
ENSG00000227269	0.000294	0.013784356	-0.61708989	-1.533778221	AL162427.1	lincRNA		chr9	99183373	99184891	1	havana	3.292188365	3.60073331	2.983643421	3.4316494	3.691020036	3.679530495	2.787093985	3.024849801	3.138986475
ENSG00000108691	0.000294	0.013784356	-1.662141428	-3.164859442	CCL2	protein_coding	C-C motif chemokine ligand 2 [Source:HGNC Symbol;Acc:HGNC:10618]	chr17	34255218	34257203	1	ensembl_havana	6.914751948	7.745822662	6.083681234	7.879363855	7.589459574	7.768644556	5.811298363	6.002677782	6.437067557
ENSG00000082482	0.000322	0.013811778	5.881731605	58.96273765	KCNK2	protein_coding	potassium two pore domain channel subfamily K member 2 [Source:HGNC Symbol;Acc:HGNC:6277]	chr1	215005775	215237093	1	ensembl_havana	-1.391690256	-4.332556059	1.549175546	-5.3500916	-3.81833984	-3.829236737	2.126646177	1.625790657	0.895089805
ENSG00000147408	0.000304	0.013811778	1.278738552	2.426267388	CSGALNACT1	protein_coding	chondroitin sulfate N-acetylgalactosaminyltransferase 1 [Source:HGNC Symbol;Acc:HGNC:24290]	chr8	19404161	19758029	-1	ensembl_havana	-0.170735631	-0.810104907	0.468633645	-0.92573229	-0.967469033	-0.537113394	0.032074365	0.478736765	0.895089805
ENSG00000278997	0.00031	0.013811778	0.786758935	1.725194397	AL662907.1	TEC		chr1	33141871	33143230	-1	havana	0.704729698	0.31135023	1.098109165	0.383334258	0.352203286	0.198513145	1.293957805	1.163381334	0.836988357
ENSG00000276728	0.000322	0.013811778	0.782876959	1.720558516	AC142472.1	lincRNA		chr17	45146730	45148470	-1	havana	0.818544473	0.427105993	1.209982952	0.515840645	0.403680778	0.361796557	1.458744294	1.097781778	1.073422785
ENSG00000008382	0.000311	0.013811778	0.71476494	1.641215791	MPND	protein_coding	MPN domain containing [Source:HGNC Symbol;Acc:HGNC:25934]	chr19	4343527	4360086	1	ensembl_havana	3.118517543	2.761135073	3.475900013	2.705773822	2.693756041	2.883875356	3.538846404	3.396231887	3.492621748
ENSG00000099949	0.000314	0.013811778	0.597125856	1.512699954	LZTR1	protein_coding	leucine zipper like transcription regulator 1 [Source:HGNC Symbol;Acc:HGNC:6742]	chr22	20982013	20999038	1	ensembl_havana	3.323976037	3.025413109	3.622538964	3.045376594	2.966188404	3.064674328	3.709177215	3.551306511	3.607133167
ENSG00000111885	0.000307	0.013811778	0.583649674	1.498635648	MAN1A1	protein_coding	mannosidase alpha class 1A member 1 [Source:HGNC Symbol;Acc:HGNC:6821]	chr6	119177209	119349761	-1	ensembl_havana	5.034263884	4.742439047	5.326088721	4.656480562	4.820888145	4.749948434	5.215871954	5.369592622	5.392801588
ENSG00000206417	0.000312	0.013811778	0.575745842	1.490447794	H1FX-AS1	antisense	H1FX antisense RNA 1 [Source:HGNC Symbol;Acc:HGNC:27953]	chr3	129315392	129326225	1	havana	1.230746079	0.942873158	1.518619	0.770507832	0.996565715	1.061545926	1.400976194	1.541948597	1.61293221
ENSG00000110169	0.000311	0.013811778	0.572307148	1.48689951	HPX	protein_coding	hemopexin [Source:HGNC Symbol;Acc:HGNC:5171]	chr11	6431049	6442617	-1	ensembl_havana	2.214948257	1.928794683	2.501101831	1.95010455	1.979216042	1.857063457	2.487415269	2.535862351	2.480027873
ENSG00000154265	0.000319	0.013811778	0.56602077	1.480434611	ABCA5	protein_coding	ATP binding cassette subfamily A member 5 [Source:HGNC Symbol;Acc:HGNC:35]	chr17	69244311	69327244	-1	ensembl_havana	4.938569751	4.655559366	5.221580136	4.283403858	4.78736503	4.89590921	4.897318888	5.339754527	5.427666993
ENSG00000235859	0.000322	0.013811778	0.558753517	1.472996004	AC006978.1	processed_pseudogene		chr7	30371508	30372741	1	havana	2.242873715	1.963496957	2.522250474	1.738566362	1.96214833	2.18977618	2.254470464	2.535862351	2.776418607
ENSG00000132376	0.000303	0.013811778	0.554832766	1.468998341	INPP5K	protein_coding	inositol polyphosphate-5-phosphatase K [Source:HGNC Symbol;Acc:HGNC:33882]	chr17	1494571	1516888	-1	ensembl_havana	4.623969441	4.346553058	4.901385825	4.430009476	4.340922035	4.268727664	4.983482422	4.871423222	4.84925183
ENSG00000196839	0.000314	0.013811778	-0.559769123	-1.474033306	ADA	protein_coding	adenosine deaminase [Source:HGNC Symbol;Acc:HGNC:186]	chr20	44619522	44652233	-1	ensembl_havana	7.064811697	7.344696258	6.784927136	7.520171453	7.238235468	7.275681854	6.922645838	6.683690139	6.74844543
ENSG00000132205	0.000316	0.013811778	-0.575903208	-1.490610377	EMILIN2	protein_coding	elastin microfibril interfacer 2 [Source:HGNC Symbol;Acc:HGNC:19881]	chr18	2847030	2915993	1	ensembl_havana	7.48693998	7.774891584	7.198988376	8.061690085	7.699273937	7.563710729	7.515933452	7.066763812	7.014267864
ENSG00000132635	0.000313	0.013811778	-0.58694354	-1.502061142	PCED1A	protein_coding	PC-esterase domain containing 1A [Source:HGNC Symbol;Acc:HGNC:16212]	chr20	2835314	2841190	-1	ensembl_havana	4.788616327	5.082088097	4.495144557	5.080113972	5.07718931	5.088961009	4.479191796	4.443233971	4.563007904
ENSG00000215784	0.000311	0.013811778	-0.597135763	-1.512710342	FAM72D	protein_coding	family with sequence similarity 72 member D [Source:HGNC Symbol;Acc:HGNC:33593]	chr1	145096000	145112696	1	ensembl_havana	1.232004994	1.530572875	0.933437112	1.109412078	1.62524309	1.857063457	0.485518989	1.097781778	1.21701057
ENSG00000070182	0.000315	0.013811778	-0.601944503	-1.517760867	SPTB	protein_coding	spectrin beta, erythrocytic [Source:HGNC Symbol;Acc:HGNC:11274]	chr14	64746283	64879907	-1	ensembl_havana	8.394278232	8.695250484	8.093305981	8.860640095	8.4797048	8.745406556	8.322992921	7.878170945	8.078754077
ENSG00000121931	0.000309	0.013811778	-0.603802656	-1.519716962	LRIF1	protein_coding	ligand dependent nuclear receptor interacting factor 1 [Source:HGNC Symbol;Acc:HGNC:30299]	chr1	110947185	110964079	-1	ensembl_havana	5.012112157	5.314013485	4.71021083	5.240017832	5.4273634	5.274659223	4.56224422	4.857864942	4.710523326
ENSG00000122884	0.000311	0.013811778	-0.650766584	-1.570002204	P4HA1	protein_coding	prolyl 4-hydroxylase subunit alpha 1 [Source:HGNC Symbol;Acc:HGNC:8546]	chr10	73007217	73096974	-1	ensembl_havana	6.551057736	6.876441028	6.225674444	7.119718846	6.83994187	6.669662369	6.40870151	6.149519985	6.118801837
ENSG00000148344	0.000319	0.013811778	-0.70578966	-1.63103717	PTGES	protein_coding	prostaglandin E synthase [Source:HGNC Symbol;Acc:HGNC:9599]	chr9	129738331	129753047	-1	ensembl_havana	3.827334742	4.180229571	3.474439912	4.239995762	4.193324268	4.107368684	3.470282097	3.423263045	3.529774593
ENSG00000064666	0.000317	0.013811778	-0.781042311	-1.718371905	CNN2	protein_coding	calponin 2 [Source:HGNC Symbol;Acc:HGNC:2156]	chr19	1026581	1039068	1	ensembl_havana	6.569560126	6.960081281	6.17903897	7.119718846	6.928828577	6.831696421	6.497082535	6.072107096	5.96792728
ENSG00000162004	0.000297	0.013811778	-0.914217771	-1.884546994	CCDC78	protein_coding	coiled-coil domain containing 78 [Source:HGNC Symbol;Acc:HGNC:14153]	chr16	722582	726954	-1	ensembl_havana	0.83274686	1.289855746	0.375637975	1.222335231	1.093800119	1.553431888	0.106248156	0.286036324	0.734629445
ENSG00000137869	0.000319	0.013811778	-0.93036688	-1.905760573	CYP19A1	protein_coding	cytochrome P450 family 19 subfamily A member 1 [Source:HGNC Symbol;Acc:HGNC:2594]	chr15	51208057	51338610	-1	ensembl_havana	6.525162059	6.990345499	6.059978619	7.061551454	6.791244267	7.118240775	5.936025816	5.884256568	6.359653472
ENSG00000248161	0.000296	0.013811778	-1.066084199	-2.093742756	AC098487.1	lincRNA		chr4	102418602	102450010	-1	havana	-0.281357055	0.251685044	-0.814399155	-0.03191641	0.325758945	0.461212593	-1.22062457	-0.86978558	-0.352787316
ENSG00000010361	0.000309	0.013811778	-1.073873173	-2.105077244	FUZ	protein_coding	fuzzy planar cell polarity protein [Source:HGNC Symbol;Acc:HGNC:26219]	chr19	49806869	49817376	-1	ensembl_havana	1.284318563	1.82125515	0.747381977	1.770752281	1.817810321	1.875202847	0.457455004	0.779979312	1.004711615
ENSG00000135299	0.000313	0.013811778	-1.092974043	-2.133133183	ANKRD6	protein_coding	ankyrin repeat domain 6 [Source:HGNC Symbol;Acc:HGNC:17280]	chr6	89433170	89633834	1	ensembl_havana	2.266939623	2.813426645	1.720452601	2.50993591	2.913888608	3.016455416	1.631954676	1.578479109	1.950924019
ENSG00000134548	0.000318	0.013811778	-1.144672148	-2.21095881	SPX	protein_coding	spexin hormone [Source:HGNC Symbol;Acc:HGNC:28139]	chr12	21526307	21537377	1	ensembl_havana	1.244899601	1.817235675	0.672563527	2.362851808	1.524202165	1.564653053	0.935503283	0.504278656	0.577908642
ENSG00000275183	0.000314	0.013811778	-1.26486467	-2.403046674	LENG9	protein_coding	leukocyte receptor cluster member 9 [Source:HGNC Symbol;Acc:HGNC:16306]	chr19	54461796	54463711	-1	havana	1.231826146	1.864258481	0.599393811	2.014244272	1.739838083	1.838693089	0.513047461	0.779979312	0.505154661
ENSG00000092621	0.000315	0.013811778	-2.519779984	-5.734946325	PHGDH	protein_coding	phosphoglycerate dehydrogenase [Source:HGNC Symbol;Acc:HGNC:8923]	chr1	119648411	119744226	1	ensembl_havana	-0.176348166	1.083541826	-1.436238158	0.437805725	1.464851682	1.347968071	-1.41444944	-1.24204464	-1.652220391
ENSG00000232104	0.000325	0.013898443	-0.606859677	-1.522940605	RFX3-AS1	lincRNA	RFX3 antisense RNA 1 [Source:HGNC Symbol;Acc:HGNC:51197]	chr9	3526723	3691814	1	havana	3.179887414	3.483317252	2.876457575	3.380421283	3.591864916	3.477665558	2.826026634	3.011552364	2.791793727
ENSG00000204305	0.000326	0.013898988	-0.707037998	-1.632449088	AGER	protein_coding	advanced glycosylation end-product specific receptor [Source:HGNC Symbol;Acc:HGNC:320]	chr6	32180968	32184324	-1	ensembl_havana	4.428815607	4.782334607	4.075296608	5.253367589	4.684456126	4.409180104	4.442080167	3.961753514	3.822056143
ENSG00000108515	0.000331	0.014055735	-0.847447117	-1.799314177	ENO3	protein_coding	enolase 3 [Source:HGNC Symbol;Acc:HGNC:3354]	chr17	4948092	4957131	1	ensembl_havana	4.749477202	5.17320076	4.325753643	5.413135017	5.084108452	5.022358812	4.470441894	4.14544456	4.361374475
ENSG00000164188	0.000342	0.014063009	1.358286414	2.563804782	RANBP3L	protein_coding	RAN binding protein 3 like [Source:HGNC Symbol;Acc:HGNC:26353]	chr5	36248434	36302114	-1	ensembl_havana	0.344915644	-0.334227563	1.024058851	-0.4596317	-0.143136894	-0.399914092	1.071324589	1.399485086	0.601366878
ENSG00000152527	0.000343	0.014063009	1.120411844	2.17409027	PLEKHH2	protein_coding	pleckstrin homology, MyTH4 and FERM domain containing H2 [Source:HGNC Symbol;Acc:HGNC:30506]	chr2	43637273	43767987	1	ensembl_havana	2.724701507	2.164495585	3.284907429	1.578072284	2.704058936	2.211355536	2.947019153	3.584613649	3.323089485
ENSG00000166391	0.000337	0.014063009	1.033497001	2.046979992	MOGAT2	protein_coding	monoacylglycerol O-acyltransferase 2 [Source:HGNC Symbol;Acc:HGNC:23248]	chr11	75717819	75732958	1	ensembl_havana	1.450655861	0.93390736	1.967404361	0.852997155	1.109387295	0.83933763	2.015549972	1.890792227	1.995870885
ENSG00000170369	0.000338	0.014063009	0.766975606	1.701698685	CST2	protein_coding	cystatin SA [Source:HGNC Symbol;Acc:HGNC:2474]	chr20	23823769	23826731	-1	ensembl_havana	-0.49405394	-0.877541743	-0.110566137	-1.61858537	-0.569826728	-0.444213135	-0.69455235	0.130707766	0.232146171
ENSG00000248810	0.000336	0.014063009	0.69199491	1.615515855	LINC02432	lincRNA	long intergenic non-protein coding RNA 2432 [Source:HGNC Symbol;Acc:HGNC:53363]	chr4	141319450	141332618	-1	havana	4.603561951	4.257564496	4.949559406	4.071501525	4.349151381	4.352040582	4.889463255	4.977813878	4.981401085
ENSG00000232692	0.000333	0.014063009	-0.557240079	-1.47145159	AP001596.1	antisense		chr21	26378552	26471698	1	havana	3.656049411	3.93466945	3.377429371	4.105550163	3.810266049	3.888192139	3.508395033	3.293766078	3.330127003
ENSG00000164430	0.000341	0.014063009	-0.562362005	-1.476684893	CGAS	protein_coding	cyclic GMP-AMP synthase [Source:HGNC Symbol;Acc:HGNC:21367]	chr6	73413515	73452276	-1	ensembl_havana	4.090401889	4.371582891	3.809220886	4.380461329	4.489776126	4.244511218	3.767486582	3.968651039	3.691525037
ENSG00000012211	0.000342	0.014063009	-0.564567748	-1.47894433	PRICKLE3	protein_coding	prickle planar cell polarity protein 3 [Source:HGNC Symbol;Acc:HGNC:6645]	chrX	49175264	49186528	-1	ensembl_havana	4.217023519	4.499307393	3.934739646	4.584365888	4.36222147	4.551334821	4.070484623	3.751214005	3.982520308
ENSG00000171680	0.000338	0.014063009	-0.570192842	-1.484722017	PLEKHG5	protein_coding	pleckstrin homology and RhoGEF domain containing G5 [Source:HGNC Symbol;Acc:HGNC:29105]	chr1	6466092	6520061	-1	ensembl_havana	3.652650287	3.937746708	3.367553866	4.082228299	3.83391509	3.897096734	3.528767195	3.304709872	3.269184531
ENSG00000280206	0.000333	0.014063009	-0.71237859	-1.638503313	AC026401.3	lincRNA		chr16	15701237	15702118	1	havana	3.725198783	4.081388078	3.369009488	4.323737133	4.100831193	3.819595909	3.62967128	3.264170155	3.213187029
ENSG00000121858	0.000336	0.014063009	-0.897002045	-1.86219228	TNFSF10	protein_coding	TNF superfamily member 10 [Source:HGNC Symbol;Acc:HGNC:11925]	chr3	172505508	172523507	-1	ensembl_havana	2.392094864	2.840595887	1.943593841	2.853343439	2.635736107	3.032708114	1.75234034	1.861445274	2.21699591
ENSG00000130270	0.000343	0.014063009	-0.901754327	-1.868336512	ATP8B3	protein_coding	ATPase phospholipid transporting 8B3 [Source:HGNC Symbol;Acc:HGNC:13535]	chr19	1782075	1812276	-1	ensembl_havana	3.047578492	3.498455655	2.596701329	3.435000789	3.317723301	3.742642876	2.711879034	2.41986126	2.658363693
ENSG00000072952	0.00038	0.014072736	1.470898455	2.77194466	MRVI1	protein_coding	murine retrovirus integration site 1 homolog [Source:HGNC Symbol;Acc:HGNC:7237]	chr11	10573091	10693988	-1	ensembl_havana	-0.529786147	-1.265235375	0.205663081	-1.73427603	-0.620350959	-1.441079133	-0.00649342	0.45273451	0.17074815
ENSG00000144802	0.000363	0.014072736	1.209706019	2.312905015	NFKBIZ	protein_coding	NFKB inhibitor zeta [Source:HGNC Symbol;Acc:HGNC:29805]	chr3	101827991	101861022	1	ensembl_havana	5.477444567	4.872591558	6.082297577	4.502079217	4.900318971	5.215376485	6.016371058	5.884256568	6.346265105
ENSG00000165471	0.00036	0.014072736	1.065587167	2.093021552	MBL2	protein_coding	mannose binding lectin 2 [Source:HGNC Symbol;Acc:HGNC:6922]	chr10	52765380	52771700	-1	ensembl_havana	1.194324907	0.661531324	1.727118491	0.540941683	0.699804047	0.743848241	1.874103935	1.648877048	1.65837449
ENSG00000042832	0.000397	0.014072736	1.054054019	2.076356285	TG	protein_coding	thyroglobulin [Source:HGNC Symbol;Acc:HGNC:11764]	chr8	132866958	133134903	1	ensembl_havana	0.978155427	0.451128417	1.505182436	0.10902545	0.214850257	1.029509544	1.245545533	1.452931788	1.817069986
ENSG00000272989	0.000396	0.014072736	0.940084635	1.918640791	LINC02012	lincRNA	long intergenic non-protein coding RNA 2012 [Source:HGNC Symbol;Acc:HGNC:52847]	chr3	197505262	197506986	-1	havana	0.180976398	-0.289065919	0.651018716	-0.56281016	-0.473795405	0.16940781	0.308311185	0.693803314	0.950941648
ENSG00000178623	0.000381	0.014072736	0.883069102	1.844294571	GPR35	protein_coding	G protein-coupled receptor 35 [Source:HGNC Symbol;Acc:HGNC:4492]	chr2	240605431	240631259	1	ensembl_havana	0.076239797	-0.365294754	0.517774348	-0.56281016	-0.258437325	-0.274636777	0.457455004	0.693803314	0.402064725
ENSG00000185630	0.00041	0.014072736	0.87171098	1.829831723	PBX1	protein_coding	PBX homeobox 1 [Source:HGNC Symbol;Acc:HGNC:8632]	chr1	164555584	164899296	1	ensembl_havana	2.943303043	2.507447553	3.379158532	2.765560239	2.29082961	2.46595281	3.456169357	3.347673311	3.33363293
ENSG00000226137	0.000382	0.014072736	0.81101459	1.754444841	BAIAP2-AS1	lincRNA	BAIAP2 antisense RNA 1 (head to head) [Source:HGNC Symbol;Acc:HGNC:44342]	chr17	81029130	81034881	-1	havana	3.653276119	3.247768823	4.058783414	3.071451915	3.224179389	3.447675166	3.947248808	4.151519203	4.07758223
ENSG00000163508	0.000388	0.014072736	0.748085199	1.67956217	EOMES	protein_coding	eomesodermin [Source:HGNC Symbol;Acc:HGNC:3372]	chr3	27715949	27722711	-1	ensembl_havana	7.823565872	7.449523273	8.197608472	7.353078209	7.545554133	7.449937476	8.173052819	8.133758354	8.286014242
ENSG00000118762	0.000374	0.014072736	0.655901524	1.57560022	PKD2	protein_coding	polycystin 2, transient receptor potential cation channel [Source:HGNC Symbol;Acc:HGNC:9009]	chr4	88007668	88077777	1	ensembl_havana	4.457412909	4.129462147	4.785363671	3.860895981	4.238324537	4.289165922	4.563890364	4.777658146	5.014542502
ENSG00000243701	0.000367	0.014072736	0.63329941	1.551108289	DUBR	lincRNA	DPPA2 upstream binding RNA [Source:HGNC Symbol;Acc:HGNC:48569]	chr3	107240692	107326964	1	havana	3.62702641	3.310376704	3.943676115	3.062812401	3.405569423	3.46274829	3.69722711	4.129119342	4.004681893
ENSG00000164951	0.00036	0.014072736	0.609490691	1.525720494	PDP1	protein_coding	pyruvate dehyrogenase phosphatase catalytic subunit 1 [Source:HGNC Symbol;Acc:HGNC:9279]	chr8	93857807	93926066	1	ensembl_havana	4.471871076	4.167125731	4.776616421	4.025557302	4.23655122	4.239268669	4.725582535	4.802320534	4.801946194
ENSG00000197363	0.000397	0.014072736	0.608958912	1.525158216	ZNF517	protein_coding	zinc finger protein 517 [Source:HGNC Symbol;Acc:HGNC:27984]	chr8	144798876	144811169	1	ensembl_havana	1.099294906	0.79481545	1.403774362	0.637193984	0.818355163	0.928897202	1.278000209	1.491756274	1.441566602
ENSG00000137075	0.000359	0.014072736	0.601802049	1.517611009	RNF38	protein_coding	ring finger protein 38 [Source:HGNC Symbol;Acc:HGNC:18052]	chr9	36336396	36487548	-1	ensembl_havana	5.690326277	5.389425253	5.991227302	5.515614461	5.335085841	5.317575455	6.050822859	6.017166234	5.905692813
ENSG00000198324	0.000408	0.014072736	0.592097192	1.50743646	FAM109A	protein_coding	family with sequence similarity 109 member A [Source:HGNC Symbol;Acc:HGNC:26509]	chr12	111360651	111369121	-1	ensembl_havana	1.550824077	1.254775481	1.846872673	1.297904502	1.298039728	1.168382212	1.820027213	1.97541275	1.745178056
ENSG00000126107	0.000353	0.014072736	0.587155125	1.50228145	HECTD3	protein_coding	HECT domain E3 ubiquitin protein ligase 3 [Source:HGNC Symbol;Acc:HGNC:26117]	chr1	45002540	45011329	-1	ensembl_havana	6.293533086	5.999955524	6.587110648	5.927883982	6.016967286	6.055015303	6.590490532	6.567338914	6.6035025
ENSG00000241489	0.00037	0.014072736	0.575597067	1.490294101	AC244197.3	protein_coding		chrX	149482543	149533935	-1	havana	0.902105658	0.614307125	1.189904191	1.022973463	0.243386951	0.57656096	1.593788933	0.758913071	1.21701057
ENSG00000089091	0.000349	0.014072736	0.558331078	1.472564756	DZANK1	protein_coding	double zinc ribbon and ankyrin repeat domains 1 [Source:HGNC Symbol;Acc:HGNC:15858]	chr20	18383367	18467281	-1	ensembl_havana	3.837882078	3.558716539	4.117047617	3.528951643	3.605669198	3.541528776	4.058857592	4.139344231	4.152941027
ENSG00000132004	0.000408	0.014072736	0.555922298	1.470108156	FBXW9	protein_coding	F-box and WD repeat domain containing 9 [Source:HGNC Symbol;Acc:HGNC:28136]	chr19	12688053	12696643	-1	ensembl_havana	2.70272473	2.424763581	2.980685879	2.424845514	2.482570884	2.366874344	2.98683266	3.093770837	2.861454141
ENSG00000272150	0.000368	0.014072736	0.547827066	1.461882204	NBPF25P	transcribed_unprocessed_pseudogene	NBPF member 25, pseudogene [Source:HGNC Symbol;Acc:HGNC:45046]	chr1	145572345	145607858	-1	havana	2.645241916	2.371328384	2.919155449	2.305416943	2.283977809	2.524590399	2.900875057	2.821440049	3.035151242
ENSG00000148842	0.000395	0.014072736	0.54735833	1.461407311	CNNM2	protein_coding	cyclin and CBS domain divalent metal cation transport mediator 2 [Source:HGNC Symbol;Acc:HGNC:103]	chr10	102918293	103090221	1	ensembl_havana	4.166616344	3.892937179	4.440295509	3.87082891	3.847920646	3.96006198	4.458102187	4.41152456	4.451259779
ENSG00000183044	0.000387	0.014072736	0.545138554	1.459160472	ABAT	protein_coding	4-aminobutyrate aminotransferase [Source:HGNC Symbol;Acc:HGNC:23]	chr16	8674565	8784575	1	ensembl_havana	5.856465821	5.583896544	6.129035098	5.612035169	5.673318837	5.466335627	6.115108632	6.268417988	6.003578674
ENSG00000175611	0.000371	0.014072736	0.523033289	1.436973334	LINC00476	lincRNA	long intergenic non-protein coding RNA 476 [Source:HGNC Symbol;Acc:HGNC:27858]	chr9	95759231	95875977	-1	havana	2.394000932	2.132484287	2.655517577	2.075653419	2.206378717	2.115420726	2.587917939	2.748504565	2.630130226
ENSG00000008277	0.0004	0.014072736	0.514073347	1.428076582	ADAM22	protein_coding	ADAM metallopeptidase domain 22 [Source:HGNC Symbol;Acc:HGNC:201]	chr7	87934143	88202889	1	ensembl_havana	6.341995722	6.084959049	6.599032396	6.07369042	6.017483722	6.163703004	6.607350692	6.519212342	6.670534154
ENSG00000009790	0.000374	0.014072736	-0.521293729	-1.435241716	TRAF3IP3	protein_coding	TRAF3 interacting protein 3 [Source:HGNC Symbol;Acc:HGNC:30766]	chr1	209756032	209782320	1	ensembl_havana	6.629531135	6.890177999	6.368884271	6.757332985	6.940313196	6.972887817	6.242887539	6.428736283	6.435028989
ENSG00000166681	0.00041	0.014072736	-0.52692018	-1.440850029	BEX3	protein_coding	brain expressed X-linked 3 [Source:HGNC Symbol;Acc:HGNC:13388]	chrX	103376340	103378077	1	ensembl_havana	5.738938235	6.002398325	5.475478145	6.110834623	5.943865115	5.952495237	5.566430817	5.459659614	5.400344005

ENSG00000137944	0.000349	0.014072736	-0.541204279	-1.455186717	**KYAT3**	protein_coding	kynurenine aminotransferase 3 [Source:HGNC Symbol;Acc:HGNC:33238]	chr1	88935773	88992953	-1	ensembl_havana	4.647010057	4.917612197	4.376407917	5.158230437	4.912587263	4.68201889	4.581875053	4.382549869	4.16479883
ENSG00000049449	0.000357	0.014072736	-0.547536526	-1.461587829	**RCN1**	protein_coding	reticulocalbin 1 [Source:HGNC Symbol;Acc:HGNC:9934]	chr11	32090904	32105755	1	ensembl_havana	4.49274455	4.766512813	4.218976287	4.609795683	4.932440306	4.75730245	4.049488025	4.352981311	4.254459527
ENSG00000126583	0.000371	0.014072736	-0.553882676	-1.468031247	**PRKCG**	protein_coding	protein kinase C gamma [Source:HGNC Symbol;Acc:HGNC:9402]	chr19	53879190	53907652	1	ensembl_havana	1.050613347	1.327554685	0.773672009	1.464591378	1.170104606	1.347968071	0.873137292	0.671430284	0.77644845
ENSG00000198498	0.0004	0.014072736	-0.55832045	-1.472553908	**TMA16**	protein_coding	translation machinery associated 16 homolog [Source:HGNC Symbol;Acc:HGNC:25638]	chr4	163494442	163520539	1	ensembl_havana	3.689419953	3.968580178	3.410259728	3.897797013	3.99572295	4.012220572	3.283098272	3.433270566	3.514410346
ENSG00000156869	0.000353	0.014072736	-0.559512974	-1.473771617	**FRRS1**	protein_coding	ferric chelate reductase 1 [Source:HGNC Symbol;Acc:HGNC:27622]	chr1	99708632	99766631	-1	ensembl_havana	4.159256847	4.439013335	3.87950036	4.552320854	4.352430021	4.412289128	3.939661103	3.80615704	3.892682937
ENSG00000110031	0.000374	0.014072736	-0.561230987	-1.475527682	**LPXN**	protein_coding	leupaxin [Source:HGNC Symbol;Acc:HGNC:14061]	chr11	58526871	58578220	-1	ensembl_havana	4.487836732	4.768452225	4.207221238	4.6536066	4.809004826	4.842745249	4.068166701	4.310178017	4.243318997
ENSG00000119408	0.000402	0.014072736	-0.566350109	-1.480772604	**NEK6**	protein_coding	NIMA related kinase 6 [Source:HGNC Symbol;Acc:HGNC:7749]	chr9	124257606	124353307	1	ensembl_havana	7.693163985	7.976339039	7.40998893	8.124659481	7.861667652	7.942689984	7.505049821	7.36612394	7.358793031
ENSG00000213190	0.000401	0.014072736	-0.567258182	-1.481704938	**MLLT11**	protein_coding	MLLT11, transcription factor 7 cofactor [Source:HGNC Symbol;Acc:HGNC:16997]	chr1	151057758	151068497	1	ensembl_havana	6.64824458	6.931873671	6.364615489	6.885650617	7.189570746	6.72039965	6.380021597	6.555934105	6.157890764
ENSG00000110811	0.000354	0.014072736	-0.573016365	-1.487630637	**P3H3**	protein_coding	prolyl 3-hydroxylase 3 [Source:HGNC Symbol;Acc:HGNC:19318]	chr12	6828410	6839851	1	ensembl_havana	2.95592003	3.242428212	2.669411847	3.387357714	3.097039309	3.242887613	2.879883083	2.498222232	2.630130226
ENSG00000154920	0.000393	0.014072736	-0.583105809	-1.498070801	**EME1**	protein_coding	essential meiotic structure-specific endonuclease 1 [Source:HGNC Symbol;Acc:HGNC:24965]	chr17	50373220	50381483	1	ensembl_havana	4.615836231	4.907389135	4.324283326	4.684909615	4.89583178	5.14142601	4.028181329	4.317400672	4.627267977
ENSG00000082512	0.000415	0.014072736	-0.606886496	-1.522968916	**TRAF5**	protein_coding	TNF receptor associated factor 5 [Source:HGNC Symbol;Acc:HGNC:12035]	chr1	211326615	211374946	1	ensembl_havana	5.858819717	6.162262966	5.555376469	6.124391059	6.178543022	6.183854816	5.602917503	5.483131946	5.580079959
ENSG00000187720	0.000353	0.014072736	-0.609246045	-1.525461791	**THSD4**	protein_coding	thrombospondin type 1 domain containing 4 [Source:HGNC Symbol;Acc:HGNC:25835]	chr15	71096952	71783383	1	ensembl_havana	1.074637528	1.37926055	0.770014505	0.660286109	1.657409759	1.820085783	0.106248156	1.080904991	1.12289037
ENSG00000138413	0.000386	0.014072736	-0.615313735	-1.531891093	**IDH1**	protein_coding	isocitrate dehydrogenase (NADP(+)) 1, cytosolic [Source:HGNC Symbol;Acc:HGNC:5382]	chr2	208236227	208266074	-1	ensembl_havana	6.281846727	6.589503594	5.974189859	6.616109214	6.645170331	6.507231238	5.924539883	6.012723726	5.985305968
ENSG00000183742	0.000414	0.014072736	-0.61651496	-1.533167116	**MACC1**	protein_coding	MACC1, MET transcriptional regulator [Source:HGNC Symbol;Acc:HGNC:30215]	chr7	20134655	20217404	-1	ensembl_havana	2.879471729	3.187729209	2.571214249	3.000842267	3.198903614	3.363441747	2.286727206	2.666037677	2.760877864
ENSG00000186301	0.000377	0.014072736	-0.616701392	-1.533365252	**MST1P2**	unprocessed_pseudogene	macrophage stimulating 1 pseudogene 2 [Source:HGNC Symbol;Acc:HGNC:7383]	chr1	16645622	16650289	1	havana	3.121766803	3.4301175	2.813416107	3.599344412	3.124146975	3.566861112	2.957076007	2.602417742	2.880754572
ENSG00000175595	0.000377	0.014072736	-0.620528178	-1.537437943	**ERCC4**	protein_coding	ERCC excision repair 4, endonuclease catalytic subunit [Source:HGNC Symbol;Acc:HGNC:3436]	chr16	13920157	13952345	1	ensembl_havana	4.486986879	4.797250968	4.17672279	4.828162729	4.762933881	4.800656295	4.129479384	4.235890155	4.16479883
ENSG00000130768	0.000353	0.014072736	-0.625241485	-1.54246899	**SMPDL3B**	protein_coding	sphingomyelin phosphodiesterase acid like 3B [Source:HGNC Symbol;Acc:HGNC:21416]	chr1	27934993	27959157	1	ensembl_havana	2.820997749	3.133618491	2.508377006	3.097064305	3.263024855	3.040766314	2.44516827	2.732383996	2.347578751
ENSG00000169136	0.000397	0.014072736	-0.628389041	-1.545837898	**ATF5**	protein_coding	activating transcription factor 5 [Source:HGNC Symbol;Acc:HGNC:790]	chr19	49928702	49933935	1	ensembl_havana	8.114631844	8.428826365	7.800437323	8.612790125	8.414539383	8.259149585	7.981580369	7.693787687	7.725943914
ENSG00000166965	0.000382	0.014072736	-0.63457852	-1.552484129	**RCCD1**	protein_coding	RCC1 domain containing 1 [Source:HGNC Symbol;Acc:HGNC:30457]	chr15	90954870	90963125	1	ensembl_havana	4.730508456	5.047797716	4.413219196	4.820495442	5.060246219	5.262651489	4.293237984	4.358243523	4.588176082
ENSG00000214212	0.000383	0.014072736	-0.695351597	-1.619279017	**C19orf38**	protein_coding	chromosome 19 open reading frame 38 [Source:HGNC Symbol;Acc:HGNC:34073]	chr19	10836575	10869790	1	ensembl_havana	1.951979365	2.299655163	1.604303566	2.464742477	2.311191904	2.123031109	1.644455473	1.726888623	1.441566602
ENSG00000116793	0.000379	0.014072736	-0.699533462	-1.623979546	**PHTF1**	protein_coding	putative homeodomain transcription factor 1 [Source:HGNC Symbol;Acc:HGNC:8939]	chr1	113696831	113759489	-1	ensembl_havana	4.345397953	4.695164684	3.995631222	4.693330227	4.780079028	4.612084796	3.914076604	4.022675282	4.05014178
ENSG00000162241	0.000361	0.014072736	-0.70001636	-1.624523214	**SLC25A45**	protein_coding	solute carrier family 25 member 45 [Source:HGNC Symbol;Acc:HGNC:27442]	chr11	65375192	65383701	-1	ensembl_havana	3.632439306	3.982447486	3.282431126	4.005410009	3.817401554	4.124530893	3.357117048	3.199187534	3.290988795
ENSG00000065320	0.000397	0.014072736	-0.704619812	-1.629715136	**NTN1**	protein_coding	netrin 1 [Source:HGNC Symbol;Acc:HGNC:8029]	chr17	9021542	9244000	1	ensembl_havana	2.076536228	2.428846134	1.724226321	2.490740384	2.494491223	2.301306795	1.644455473	1.881076089	1.647147403
ENSG00000042493	0.000405	0.014072736	-0.714669236	-1.64110692	**CAPG**	protein_coding	capping actin protein, gelsolin like [Source:HGNC Symbol;Acc:HGNC:1474]	chr2	85394748	85418432	-1	ensembl_havana	2.688949553	3.046284171	2.331614935	2.553756523	3.370423439	3.214672551	1.986385747	2.560421027	2.448038032
ENSG00000215788	0.000344	0.014072736	-0.737654271	-1.667462447	**TNFRSF25**	protein_coding	TNF receptor superfamily member 25 [Source:HGNC Symbol;Acc:HGNC:11910]	chr1	6461151	6466195	-1	ensembl_havana	2.494519434	2.86334657	2.125692298	3.138766373	2.887010108	2.564263227	2.270688985	2.155463891	1.950924019
ENSG00000225077	0.000382	0.014072736	-0.742494937	-1.67306667	**LINC00337**	lincRNA	long intergenic non-protein coding RNA 337 [Source:HGNC Symbol;Acc:HGNC:28620]	chr1	6236240	6239444	1	ensembl_havana	2.408058986	2.779306455	2.036811518	2.77087449	2.991644088	2.575400786	2.044136302	2.106272137	1.960026115
ENSG00000120289	0.000411	0.014072736	-0.756164105	-1.688993885	**MAGEB4**	protein_coding	MAGE family member B4 [Source:HGNC Symbol;Acc:HGNC:6811]	chrX	30242052	30244193	1	havana	4.547147131	4.925229183	4.169065079	5.1118699	4.899198481	4.764619169	4.188292236	4.220562267	4.098340733
ENSG00000108511	0.00041	0.014072736	-0.770098408	-1.705386107	**HOXB6**	protein_coding	homeobox B6 [Source:HGNC Symbol;Acc:HGNC:5117]	chr17	48595751	48604992	-1	ensembl_havana	0.450955209	0.836004414	0.065906005	0.705390237	0.679062629	1.123560375	0.106248156	-0.19956768	0.291037539
ENSG00000105559	0.000389	0.014072736	-0.7706106	-1.705991667	**PLEKHA4**	protein_coding	pleckstrin homology domain containing A4 [Source:HGNC Symbol;Acc:HGNC:14339]	chr19	48837097	48868632	-1	ensembl_havana	1.038368207	1.423673507	0.653062907	1.383653947	1.311578958	1.575787614	0.692307165	0.37179175	0.895089805
ENSG00000257002	0.000412	0.014072736	-0.78236767	-1.719951246	**AP000438.1**	antisense		chr11	62909546	62918361	1	havana	-0.41944178	-0.028257945	-0.810625615	-0.18813307	-0.258437325	0.361796557	-0.89704638	-0.93694677	-0.597883695
ENSG00000171236	0.000377	0.014072736	-0.819406176	-1.764679488	**LRG1**	protein_coding	leucine rich alpha-2-glycoprotein 1 [Source:HGNC Symbol;Acc:HGNC:29480]	chr19	4536409	4540474	-1	ensembl_havana	4.106053311	4.515756399	3.696350222	4.623079215	4.61653999	4.307649991	3.776033227	3.65186594	3.6611515
ENSG00000279693	0.000404	0.014072736	-0.822914405	-1.768975914	**AC099521.2**	TEC		chr16	68367325	68370262	-1	havana	2.962011507	3.373468709	2.550554304	3.330909387	3.154516033	3.634980708	2.494337705	2.504564179	2.652761029
ENSG00000137959	0.000385	0.014072736	-0.850969964	-1.803713204	**IFI44L**	protein_coding	interferon induced protein 44 like [Source:HGNC Symbol;Acc:HGNC:17817]	chr1	78619922	78646145	1	ensembl_havana	5.820855207	6.246340189	5.395370225	6.130088348	6.188208409	6.420723811	5.299269788	5.155569952	5.731270935
ENSG00000182050	0.000403	0.014072736	-0.861027087	-1.816330937	**MGAT4C**	protein_coding	MGAT4 family member C [Source:HGNC Symbol;Acc:HGNC:30871]	chr12	85955666	86838904	-1	ensembl_havana	-0.393489741	0.037023802	-0.824003285	-0.03191641	0.378171619	-0.235183806	-0.82634296	-0.68524259	-0.960424301
ENSG00000213654	0.000414	0.014072736	-0.86556794	-1.822056807	**GPSM3**	protein_coding	G protein signaling modulator 3 [Source:HGNC Symbol;Acc:HGNC:13945]	chr6	32190766	32195523	-1	ensembl_havana	6.88077136	7.31355533	6.44798739	7.680356309	7.293224227	6.967085453	6.625593275	6.443252589	6.275116306
ENSG00000168676	0.000382	0.014072736	-0.885331545	-1.847189074	**KCTD19**	protein_coding	potassium channel tetramerization domain containing 19 [Source:HGNC Symbol;Acc:HGNC:24753]	chr16	67289428	67326763	-1	ensembl_havana	1.252201317	1.694867089	0.809535544	1.355633303	1.667974555	2.060993409	0.618191201	0.578298274	1.232117157
ENSG00000167680	0.000411	0.014072736	-0.921956633	-1.894683187	**SEMA6B**	protein_coding	semaphorin 6B [Source:HGNC Symbol;Acc:HGNC:10739]	chr19	4542593	4559808	-1	ensembl_havana	-0.64998071	-0.189002394	-1.110959027	-0.22998119	-0.383759306	0.046733314	-1.22062457	-1.08144551	-1.030806998
ENSG00000167483	0.000413	0.014072736	-0.942386743	-1.92170481	**FAM129C**	protein_coding	family with sequence similarity 129 member C [Source:HGNC Symbol;Acc:HGNC:24130]	chr19	17523301	17553839	1	ensembl_havana	0.65114002	1.122333391	0.179946649	1.327057633	0.962641309	1.077301233	0.24405081	0.256279753	0.039509383
ENSG00000146192	0.000395	0.014072736	-0.99670922	-1.995443209	**FGD2**	protein_coding	FYVE, RhoGEF and PH domain containing 2 [Source:HGNC Symbol;Acc:HGNC:3664]	chr6	37005646	37029070	1	ensembl_havana	1.655191128	2.153545738	1.156836518	1.760103226	2.277093311	2.423440678	1.01467363	1.179325527	1.276510397
ENSG00000105048	0.00036	0.014072736	-1.045779104	-2.064480947	**TNNT1**	protein_coding	troponin T1, slow skeletal type [Source:HGNC Symbol;Acc:HGNC:11948]	chr19	55132794	55149354	-1	ensembl_havana	1.462374883	1.985264435	0.939485331	1.738566362	2.297649024	1.919577919	0.785553575	0.993426194	1.039476224
ENSG00000196167	0.000406	0.014072736	-1.063343041	-2.089768367	**COLCA1**	antisense	colorectal cancer associated 1 [Source:HGNC Symbol;Acc:HGNC:33789]	chr11	111290787	111305045	-1	ensembl_havana	2.849356868	3.381028388	2.317685348	3.497241177	3.213401092	3.432442896	2.699951379	1.947753749	2.305350914
ENSG00000164362	0.000352	0.014072736	-1.104853646	-2.150770585	**TERT**	protein_coding	telomerase reverse transcriptase [Source:HGNC Symbol;Acc:HGNC:11730]	chr5	1253147	1295069	-1	ensembl_havana	-0.544860014	0.007566809	-1.097286837	-0.27307956	-0.258437325	0.554217307	-1.22062457	-1.24204464	-0.829191301
ENSG00000149781	0.00039	0.014072736	-1.187806246	-2.278060791	**FERMT3**	protein_coding	fermitin family member 3 [Source:HGNC Symbol;Acc:HGNC:23151]	chr11	64206678	64223886	1	ensembl_havana	5.582432547	6.17633567	4.988529424	6.334126531	6.057708879	6.1371716	5.321821412	5.009374461	4.634392398
ENSG00000125245	0.000379	0.014072736	-1.243148391	-2.367145508	**GPR18**	protein_coding	G protein-coupled receptor 18 [Source:HGNC Symbol;Acc:HGNC:4472]	chr13	99254714	99261744	-1	ensembl_havana	0.040766998	0.662341193	-0.580807197	1.369711653	0.477608744	0.139703184	0.36983092	-1.08144551	-1.030806998
ENSG00000167964	0.000349	0.014072736	-1.26373021	-2.401157786	**RAB26**	protein_coding	RAB26, member RAS oncogene family [Source:HGNC Symbol;Acc:HGNC:14259]	chr16	2140803	2154165	1	ensembl_havana	2.543837845	3.17570295	1.91197274	3.387357714	3.020785603	3.118965531	2.452295909	1.614107429	1.669514881
ENSG00000139998	0.000412	0.014072736	-1.291300749	-2.447486241	**RAB15**	protein_coding	RAB15, member RAS oncogene family [Source:HGNC Symbol;Acc:HGNC:20150]	chr14	64945814	64973226	-1	ensembl_havana	-0.256748185	0.38890219	-0.90239856	0.770507832	0.592979824	-0.196781087	-0.51701327	-1.00738779	-1.182794618
ENSG00000213853	0.000358	0.014072736	-2.090853157	-4.259999193	**EMP2**	protein_coding	epithelial membrane protein 2 [Source:HGNC Symbol;Acc:HGNC:3334]	chr16	10528422	10580698	-1	ensembl_havana	-0.17544777	0.869978809	-1.220874348	1.022973463	0.945375194	0.641587769	-0.82634296	-0.80561234	-2.030667746
ENSG00000167037	0.000417	0.014119963	0.675033813	1.596634193	**SGSM1**	protein_coding	small G protein signaling modulator 1 [Source:HGNC Symbol;Acc:HGNC:29410]	chr22	24806169	24927578	1	ensembl_havana	-0.438784964	-0.776301871	-0.101268058	-1.070289	-0.672709	-0.585907617	-0.26243933	-0.08087422	0.039509383
ENSG00000233223	0.000419	0.014158387	-0.59116739	-1.506465246	**AC016876.1**	antisense		chr17	7581964	7584072	-1	havana	1.54019574	1.835779435	1.244612045	1.781323307	1.7890623	1.936952697	1.278000209	1.179325527	1.276510397
ENSG00000164308	0.000423	0.014224423	-0.540247424	-1.454221896	**ERAP2**	protein_coding	endoplasmic reticulum aminopeptidase 2 [Source:HGNC Symbol;Acc:HGNC:29499]	chr5	96875939	96919716	1	ensembl_havana	7.132647564	7.402771275	6.862523852	7.255754902	7.446249367	7.506309557	6.770924582	6.860044429	6.956602544
ENSG00000124140	0.000423	0.014224423	-0.684907359	-1.607598748	**SLC12A5**	protein_coding	solute carrier family 12 member 5 [Source:HGNC Symbol;Acc:HGNC:13818]	chr20	46021690	46060152	1	ensembl_havana	5.971639032	6.314092711	5.629185352	6.314700425	6.121073623	6.506504086	5.738825475	5.457209502	5.69152108
ENSG00000171724	0.000426	0.014237212	1.078905972	2.112433568	**VAT1L**	protein_coding	vesicle amine transport 1 like [Source:HGNC Symbol;Acc:HGNC:29315]	chr16	77788530	77980107	1	ensembl_havana	1.666392473	1.126939487	2.205845459	0.660286109	1.452682866	1.267849486	1.831005931	2.721536001	2.064994446
ENSG00000164920	0.000427	0.014237212	0.791634385	1.731034388	**OSR2**	protein_coding	odd-skipped related transciption factor 2 [Source:HGNC Symbol;Acc:HGNC:15830]	chr8	98944403	98952104	1	ensembl_havana	1.05386769	0.658050498	1.449684883	0.705390237	0.760296981	0.508464275	1.681320805	1.465989718	1.201744126
ENSG00000012124	0.000425	0.014237212	-1.343077557	-2.536919176	**CD22**	protein_coding	CD22 molecule [Source:HGNC Symbol;Acc:HGNC:1643]	chr19	35319261	35347355	1	ensembl_havana	2.553166302	3.224705081	1.881627524	3.957275751	2.97897239	2.737867101	2.246292314	1.881076089	1.517514168
ENSG00000159618	0.000427	0.014237212	-1.404750196	-2.647719326	**ADGRG5**	protein_coding	adhesion G protein-coupled receptor G5 [Source:HGNC Symbol;Acc:HGNC:19010]	chr16	57542421	57591681	1	ensembl_havana	1.593519038	2.295894136	0.89114394	2.023178671	1.864486201	3.000017535	0.339398945	0.841392961	1.492639914
ENSG00000121989	0.00043	0.014255558	0.566348703	1.480771161	**ACVR2A**	protein_coding	activin A receptor type 2A [Source:HGNC Symbol;Acc:HGNC:173]	chr2	147844517	147930826	1	ensembl_havana	2.606517026	2.323342674	2.889691377	2.222513956	2.446207271	2.301306795	2.863936192	2.998131222	2.807006718
ENSG00000131459	0.000429	0.014255558	-0.528039345	-1.441968196	**GFPT2**	protein_coding	glutamine-fructose-6-phosphate transaminase 2 [Source:HGNC Symbol;Acc:HGNC:4242]	chr5	180300690	180353387	-1	ensembl_havana	4.303490046	4.567509718	4.039470373	4.611277685	4.49275058	4.598500889	4.070484623	3.929121717	4.118804779
ENSG00000184828	0.000432	0.014303475	-1.423043294	-2.681505651	**ZBTB7C**	protein_coding	zinc finger and BTB domain containing 7C [Source:HGNC Symbol;Acc:HGNC:31700]	chr18	48026673	48410752	-1	ensembl_havana	0.291990119	1.003511765	-0.419531528	1.671956805	1.140065346	0.198513145	0.457455004	-0.68524259	-1.030806998
ENSG00000168952	0.000434	0.014306699	-0.61443648	-1.530959884	**STXBP6**	protein_coding	syntaxin binding protein 6 [Source:HGNC Symbol;Acc:HGNC:19666]	chr14	24809656	25050297	-1	ensembl_havana	4.30244865	4.60966689	3.995230409	4.514804607	4.655550213	4.658645849	3.861504875	3.973231137	4.150955216
ENSG00000212694	0.000434	0.014306699	-0.63332393	-1.551134651	**LINC01089**	lincRNA	long intergenic non-protein coding RNA 1089 [Source:HGNC Symbol;Acc:HGNC:27886]	chr12	121795267	121803906	-1	havana	3.614250882	3.930912846	3.297588917	4.120197428	3.791063673	3.881477438	3.431134914	3.275339896	3.186291941
ENSG00000227115	0.000446	0.014317984	1.256030798	2.38837736	**LINC01630**	lincRNA	long intergenic non-protein coding RNA 1630 [Source:HGNC Symbol;Acc:HGNC:52295]	chr18	51346249	51643939	1	havana	2.232136802	1.604121403	2.860152201	0.892538	1.62524309	2.294583118	2.44516827	2.895698436	3.239589895
ENSG00000108576	0.000448	0.014317984	0.816928241	1.761651122	**SLC6A4**	protein_coding	solute carrier family 6 member 4 [Source:HGNC Symbol;Acc:HGNC:11050]	chr17	30194319	30236002	-1	ensembl_havana	0.620911848	0.212447727	1.029375968	0.14220989	0.185737694	0.309395597	1.125834805	1.01135145	0.950941648
ENSG00000171045	0.000441	0.014317984	0.613532455	1.530000851	**TSNARE1**	protein_coding	t-SNARE domain containing 1 [Source:HGNC Symbol;Acc:HGNC:26437]	chr8	142212080	142403240	-1	ensembl_havana	1.920128647	1.613362419	2.226894874	1.671956805	1.592342839	1.575787614	2.394263752	2.155463891	2.13095698
ENSG00000123353	0.000447	0.014317984	0.518701133	1.43266483	**ORMDL2**	protein_coding	ORMDL sphingolipid biosynthesis regulator 2 [Source:HGNC Symbol;Acc:HGNC:16037]	chr12	55817919	55821879	1	ensembl_havana	4.866521574	4.607171008	5.12587214	4.572242036	4.671388827	4.57788216	5.134019951	5.154562121	5.089034349
ENSG00000161921	0.000438	0.014317984	-0.500742811	-1.414941897	**CXCL16**	protein_coding	C-X-C motif chemokine ligand 16 [Source:HGNC Symbol;Acc:HGNC:16642]	chr17	4733526	4739922	-1	ensembl_havana	2.869719552	3.120090958	2.619348147	3.126381832	3.077360027	3.156531015	2.638787485	2.566495926	2.652761029
ENSG00000171848	0.000442	0.014317984	-0.501171673	-1.41536257	**RRM2**	protein_coding	ribonucleotide reductase regulatory subunit M2 [Source:HGNC Symbol;Acc:HGNC:10452]	chr2	10120698	10131419	1	ensembl_havana	8.377426954	8.628012791	8.126841118	8.508291456	8.737622629	8.638124287	8.018514518	8.218903045	8.143105791
ENSG00000019582	0.000446	0.014317984	-0.551708417	-1.465820471	**CD74**	protein_coding	CD74 molecule [Source:HGNC Symbol;Acc:HGNC:1697]	chr5	150401637	150412929	-1	ensembl_havana	8.102381054	8.378235263	7.826526845	8.370031853	8.37084985	8.393824085	7.884471936	7.81323548	7.78187312
ENSG00000204642	0.000439	0.014317984	-0.575280359	-1.48996698	**HLA-F**	protein_coding	major histocompatibility complex, class I, F [Source:HGNC Symbol;Acc:HGNC:4963]	chr6	29722775	29738528	1	ensembl_havana	5.262631037	5.550271217	4.974990858	5.696142299	5.577806606	5.376864746	5.167969629	5.042440346	4.714562598
ENSG00000111678	0.000444	0.014317984	-0.591095713	-1.506390402	**C12orf57**	protein_coding	chromosome 12 open reading frame 57 [Source:HGNC Symbol;Acc:HGNC:29521]	chr12	6942978	6946003	1	ensembl_havana	6.468051744	6.763599601	6.172503887	6.882892397	6.748020484	6.659885921	6.387477621	6.094351856	6.035682185
ENSG00000186715	0.000441	0.014317984	-0.593976584	-1.50940147	**MST1L**	transcribed_unprocessed_pseudogene	macrophage stimulating 1 like [Source:HGNC Symbol;Acc:HGNC:7390]	chr1	16754910	16770237	-1	havana	1.147246031	1.444234323	0.850257739	1.369711653	1.311578958	1.651412359	0.851734776	0.625615656	1.073422785
ENSG00000270977	0.000446	0.014317984	-0.741072447	-1.671417848	**AC015849.5**	lincRNA		chr17	35893707	35911023	-1	havana	1.598902931	1.969439154	1.228366707	1.553614465	1.900769223	2.453933774	0.955706889	1.163381334	1.566011899
ENSG00000272282	0.000439	0.014317984	-0.833774618	-1.782342527	**LINC02084**	lincRNA	long intergenic non-protein coding RNA 2084 [Source:HGNC Symbol;Acc:HGNC:52934]	chr3	27712910	27714005	1	havana	0.329565302	0.746452611	-0.087322007	0.515840645	0.760296981	0.963220207	-0.51701327	-0.00684508	0.261892331
ENSG00000100206	0.000445	0.014317984	-1.245872247	-2.371618978	**DMC1**	protein_coding	DNA meiotic recombinase 1 [Source:HGNC Symbol;Acc:HGNC:2927]	chr22	38518949	38570286	-1	ensembl_havana	0.44252506	1.065461183	-0.180411064	0.770507832	1.214022341	1.211853376	-0.63291296	0.315191528	-0.223511759
ENSG00000114859	0.000449	0.014325478	0.516424455	1.430405764	**CLCN2**	protein_coding	chloride voltage-gated channel 2 [Source:HGNC Symbol;Acc:HGNC:2020]	chr3	184346185	184361651	-1	ensembl_havana	4.052773271	3.794561043	4.310985498	3.660747824	3.861791544	3.861143762	4.207375531	4.335300678	4.390280286
ENSG00000165118	0.00045	0.014341396	-0.997389877	-1.996384873	**C9orf64**	protein_coding	chromosome 9 open reading frame 64 [Source:HGNC Symbol;Acc:HGNC:28144]	chr9	83938311	83956986	-1	ensembl_havana	3.147182324	3.645877263	2.648487386	3.506827715	3.68322949	3.747574584	2.254470464	2.753838306	2.937153388
ENSG00000158234	0.000454	0.014420453	-0.954010674	-1.937250711	**FAIM**	protein_coding	Fas apoptotic inhibitory molecule [Source:HGNC Symbol;Acc:HGNC:18703]	chr3	138608606	138633376	1	ensembl_havana	1.357157129	1.834162466	0.880151792	1.902479286	1.808291156	1.791716956	0.873137292	1.097781778	0.669536307
ENSG00000105784	0.000457	0.014422984	0.83911721	1.788955139	**RUNDC3B**	protein_coding	RUN domain containing 3B [Source:HGNC Symbol;Acc:HGNC:30286]	chr7	87627548	87832296	1	ensembl_havana	3.026703678	2.607145073	3.446262283	2.175202284	2.744550137	2.901682799	3.221855697	3.472620579	3.644310574
ENSG00000214336	0.000455	0.014422984	-0.50074731	-1.414946309	**FOXI3**	protein_coding	forkhead box I3 [Source:HGNC Symbol;Acc:HGNC:35123]	chr2	88446787	88452656	-1	ensembl_havana	3.481037745	3.7314114	3.23066409	4.067188383	3.608414274	3.518631543	3.542190552	3.114650475	3.035151242
ENSG00000155363	0.000457	0.014422984	-0.621565242	-1.538543508	**MOV10**	protein_coding	Mov10 RISC complex RNA helicase [Source:HGNC Symbol;Acc:HGNC:7200]	chr1	112673141	112700746	1	ensembl_havana	6.670097844	6.980880465	6.359315223	6.895110993	7.030576082	7.016954319	6.37064705	6.306119802	6.401178816
ENSG00000157796	0.00046	0.014444309	0.684932726	1.607627014	**WDR19**	protein_coding	WD repeat domain 19 [Source:HGNC Symbol;Acc:HGNC:18340]	chr4	39182404	39285810	1	ensembl_havana	4.926543175	4.584076812	5.269009538	4.679268445	4.602839827	4.470122166	5.237693459	5.28830435	5.281030804
ENSG00000235863	0.000459	0.014444309	-2.031433366	-4.088108159	**B3GALT4**	protein_coding	beta-1,3-galactosyltransferase 4 [Source:HGNC Symbol;Acc:HGNC:919]	chr6	33277132	33284832	1	ensembl_havana	-1.016923618	-0.001206935	-2.032640301	0.986893817	-0.071090603	-0.91942402	-1.41444944	-2.33094524	-2.35252622
ENSG00000275897	0.000461	0.014460513	1.596911445	3.024950313	**AC021491.4**	lincRNA		chr17	50475819	50478391	1	havana	-0.758415793	-1.556871516	0.040039929	-1.61858537	-1.518257998	-1.533771185	0.428834256	-0.04338487	-0.265329594
ENSG00000154079	0.000462	0.014466963	-0.723586485	-1.65128196	**SDHAF4**	protein_coding	succinate dehydrogenase complex assembly factor 4 [Source:HGNC Symbol;Acc:HGNC:20957]	chr6	70566917	70589569	1	ensembl_havana	1.294244922	1.656038164	0.932451679	1.760103226	1.464851682	1.743159585	0.894226935	0.880929471	1.022198632
ENSG00000166394	0.00047	0.014625385	-0.502944821	-1.417103195	**CYB5R2**	protein_coding	cytochrome b5 reductase 2 [Source:HGNC Symbol;Acc:HGNC:24376]	chr11	7665100	7677222	-1	ensembl_havana	5.040437551	5.291909962	4.788965141	5.252418121	5.37190052	5.251411245	4.718209861	4.896958637	4.751726925
ENSG00000225791	0.000469	0.014625385	-0.66533932	-1.585941241	**TRAM2-AS1**	lincRNA	TRAM2 antisense RNA 1 (head to head) [Source:HGNC Symbol;Acc:HGNC:48663]	chr6	52577307	52583993	1	havana	2.353668557	2.686338218	2.020998897	2.464742477	2.891524778	2.702747398	1.926226713	2.114588126	2.022181853
ENSG00000270393	0.000471	0.014633323	0.712289394	1.638402014	**AC000095.1**	processed_pseudogene		chr22	19018043	19018916	-1	havana	0.060930013	-0.295214684	0.41707471	-0.67394004	-0.258437325	0.046733314	-0.00649342	0.343769191	0.913948357
ENSG00000149679	0.000474	0.014633323	0.572031743	1.486615694	**CABLES2**	protein_coding	Cdk5 and Abl enzyme substrate 2 [Source:HGNC Symbol;Acc:HGNC:16143]	chr20	62388632	62407285	-1	ensembl_havana	5.179488381	4.893472509	5.465504252	4.659348809	4.95956846	5.061500258	5.321821412	5.498305866	5.576385479
ENSG00000184489	0.000473	0.014633323	-0.964861874	-1.951876637	**PTP4A3**	protein_coding	protein tyrosine phosphatase type IVA, member 3 [Source:HGNC Symbol;Acc:HGNC:9636]	chr8	141391993	141432454	1	ensembl_havana	6.71146381	7.193894747	6.229032873	7.427732154	6.868275945	7.285676141	6.613324743	6.008267496	6.065506379
ENSG00000214290	0.000473	0.014633323	-1.243011103	-2.36692026	**COLCA2**	protein_coding	colorectal cancer associated 2 [Source:HGNC Symbol;Acc:HGNC:26978]	chr11	111298555	111308735	1	ensembl_havana	3.474004704	4.095510255	2.852499152	4.575282564	3.924815359	3.786432844	3.487731073	2.329873398	2.739892986
ENSG00000265972	0.000476	0.014658075	0.539091565	1.453057269	**TXNIP**	protein_coding	thioredoxin interacting protein [Source:HGNC Symbol;Acc:HGNC:16952]	chr1	145992435	145996600	-1	ensembl_havana	6.795538939	6.525993157	7.065084722	6.206104081	6.576034612	6.795840778	6.812022689	7.100387277	7.2828442
ENSG00000007944	0.000481	0.014659862	0.618795219	1.535592287	**MYLIP**	protein_coding	myosin regulatory light chain interacting protein [Source:HGNC Symbol;Acc:HGNC:21155]	chr6	16129125	16148248	1	ensembl_havana	6.105073892	5.795676282	6.414471501	6.213928948	5.540922579	5.632177319	6.721264173	6.18153689	6.34061344
ENSG00000112394	0.000482	0.014659862	0.510117337	1.424166021	**SLC16A10**	protein_coding	solute carrier family 16 member 10 [Source:HGNC Symbol;Acc:HGNC:17027]	chr6	111087503	111231194	1	ensembl_havana	2.477551486	2.222492817	2.732610154	2.067039104	2.220801645	2.379637701	2.581431252	2.688493842	2.927905368
ENSG00000165283	0.000478	0.014659862	-0.518221077	-1.432188191	**STOML2**	protein_coding	stomatin like 2 [Source:HGNC Symbol;Acc:HGNC:14559]	chr9	35099776	35103195	-1	ensembl_havana	6.314716858	6.573827397	6.05560632	6.547324033	6.519185416	6.65497274	6.028344152	6.047886553	6.090588254
ENSG00000006695	0.000481	0.014659862	-0.737973199	-1.667831104	**COX10**	protein_coding	COX10, heme A:farnesyltransferase cytochrome c oxidase assembly factor [Source:HGNC Symbol;Acc:HGNC:2260]	chr17	14069496	14208677	1	ensembl_havana	4.003235959	4.372222558	3.634249359	4.371743869	4.449008971	4.295914835	3.538846404	3.634621851	3.729279822
ENSG00000167771	0.000481	0.014659862	-0.741024173	-1.671361922	**RCOR2**	protein_coding	REST corepressor 2 [Source:HGNC Symbol;Acc:HGNC:27455]	chr11	63911221	63916844	-1	ensembl_havana	-0.338253392	0.032258694	-0.708765479	0.206373087	-0.218971072	0.109374069	-0.69455235	-0.93694677	-0.494797317
ENSG00000126264	0.000479	0.014659862	-1.242260132	-2.365688518	**HCST**	protein_coding	hematopoietic cell signal transducer [Source:HGNC Symbol;Acc:HGNC:16977]	chr19	35902480	35904377	1	ensembl_havana	1.70125305	2.322383116	1.080122984	2.77087449	1.855271053	2.341003803	1.245545533	0.938274071	1.056549347
ENSG00000277449	0.000486	0.014679924	0.628578074	1.546040458	**CEBPB-AS1**	antisense	CEBPB antisense RNA 1 [Source:HGNC Symbol;Acc:HGNC:51226]	chr20	50184598	50191498	-1	havana	1.691884235	1.377595198	2.006173272	1.175017601	1.338282095	1.6194859	1.705384535	2.081032632	2.23210265
ENSG00000138002	0.000487	0.014679924	0.503959927	1.418100645	**IFT172**	protein_coding	intraflagellar transport 172 [Source:HGNC Symbol;Acc:HGNC:30391]	chr2	27444371	27489789	-1	ensembl_havana	6.283115652	6.031135688	6.535095615	6.081196161	5.969180386	6.043030518	6.561532956	6.455579932	6.588173957
ENSG00000172081	0.000485	0.014679924	-0.513919093	-1.427923899	**MOB3A**	protein_coding	MOB kinase activator 3A [Source:HGNC Symbol;Acc:HGNC:29802]	chr19	2071038	2096673	-1	ensembl_havana	6.567446444	6.82440599	6.310486897	7.098477203	6.787615648	6.58712512	6.635803171	6.248718889	6.046938632
ENSG00000141753	0.000488	0.014679924	-1.461929744	-2.754765939	**IGFBP4**	protein_coding	insulin like growth factor binding protein 4 [Source:HGNC Symbol;Acc:HGNC:5473]	chr17	40443461	40457731	1	ensembl_havana	-0.927822813	-0.196857941	-1.658787685	0.237415364	-0.428075095	-0.399914092	-1.52209115	-2.00847383	-1.445798078
ENSG00000121742	0.000486	0.014679924	-1.874522639	-3.666802695	**GJB6**	protein_coding	gap junction protein beta 6 [Source:HGNC Symbol;Acc:HGNC:4288]	chr13	20221962	20232395	-1	ensembl_havana	-0.308824694	0.628436625	-1.246086014	0.206373087	0.570631912	1.108304877	-1.13267621	-1.42278721	-1.182794618
ENSG00000228314	0.00049	0.014717769	1.051329559	2.072438886	**CYP4F29P**	transcribed_unprocessed_pseudogene	cytochrome P450 family 4 subfamily F member 29, pseudogene [Source:HGNC Symbol;Acc:HGNC:2647]	chr21	13843133	13848364	-1	havana	0.17982725	-0.34583753	0.705492029	-0.10791135	-0.842251242	-0.087349994	0.668022463	0.426254992	1.022198632
ENSG00000116525	0.000492	0.014736859	0.61122847	1.527559388	**TRIM62**	protein_coding	tripartite motif containing 62 [Source:HGNC Symbol;Acc:HGNC:25574]	chr1	33145402	33182059	-1	ensembl_havana	4.495483998	4.189869763	4.801098233	4.099226952	4.154408151	4.315974186	4.790294097	4.798454392	4.81454621
ENSG00000006459	0.000501	0.01479255	0.607327649	1.523434687	**KDM7A**	protein_coding	lysine demethylase 7A [Source:HGNC Symbol;Acc:HGNC:22224]	chr7	140084746	140177035	-1	ensembl_havana	4.211386022	3.907722197	4.515049847	4.165286793	3.724301235	3.833578563	4.657831492	4.379102505	4.508215543
ENSG00000164403	0.000498	0.01479255	-0.492242107	-1.406629235	**SHROOM1**	protein_coding	shroom family member 1 [Source:HGNC Symbol;Acc:HGNC:24084]	chr5	132822141	132830898	-1	ensembl_havana	3.6688581	3.914979154	3.422737047	3.850894189	3.900383301	3.993659972	3.330286772	3.426606605	3.511317764
ENSG00000188610	0.000497	0.01479255	-0.4992438	-1.413472485	**FAM72B**	protein_coding	family with sequence similarity 72 member B [Source:HGNC Symbol;Acc:HGNC:24805]	chr1	121167646	121185539	-1	ensembl_havana	2.728536009	2.978157908	2.478914109	2.802355359	2.913888608	3.218229759	2.286727206	2.453030417	2.696984702
ENSG00000233621	0.000496	0.01479255	-0.502854846	-1.417014819	**LINC01137**	antisense	long intergenic non-protein coding RNA 1137 [Source:HGNC Symbol;Acc:HGNC:49453]	chr1	37454879	37474411	-1	havana	3.436314474	3.687741897	3.184887051	3.835760276	3.622061853	3.605403563	3.334150354	3.155524498	3.064986301
ENSG00000175793	0.0005	0.01479255	-0.543079843	-1.457079751	**SFN**	protein_coding	stratifin [Source:HGNC Symbol;Acc:HGNC:10773]	chr1	26863138	26864457	1	ensembl_havana	-0.209996384	0.061543538	-0.481536305	0.206373087	0.378171619	-0.399914092	-0.30988669	-0.2414061	-0.893316134
ENSG00000198056	0.000498	0.01479255	-0.639217496	-1.557484165	**PRIM1**	protein_coding	DNA primase subunit 1 [Source:HGNC Symbol;Acc:HGNC:9369]	chr12	56731596	56752373	-1	ensembl_havana	7.497582782	7.81719153	7.177974034	7.582697572	7.839349402	8.029527616	6.819276926	7.297474736	7.417170441
ENSG00000198542	0.0005	0.01479255	-0.645010362	-1.563750518	**ITGBL1**	protein_coding	integrin subunit beta like 1 [Source:HGNC Symbol;Acc:HGNC:6164]	chr13	101452593	101720856	1	ensembl_havana	1.430675484	1.753180665	1.108170303	1.833046887	1.817810321	1.608684788	1.309740823	1.063828438	0.950941648
ENSG00000130755	0.000502	0.01479255	-1.158140199	-2.231695506	**GMFG**	protein_coding	glia maturation factor gamma [Source:HGNC Symbol;Acc:HGNC:4374]	chr19	39328353	39342372	-1	ensembl_havana	0.698326794	1.277396893	0.119256695	1.075455542	1.488885652	1.267849486	-0.04612063	0.02879206	0.375098651
ENSG00000137135	0.000504	0.01482902	-0.617714484	-1.534442393	**ARHGEF39**	protein_coding	Rho guanine nucleotide exchange factor 39 [Source:HGNC Symbol;Acc:HGNC:25909]	chr9	35658875	35675866	-1	ensembl_havana	4.394260808	4.70311805	4.085403566	4.39257795	4.782511786	4.934264414	3.826276378	4.233983051	4.195951269
ENSG00000225217	0.000506	0.014852932	-0.981302191	-1.97424658	**HSPA7**	unprocessed_pseudogene	heat shock protein family A (Hsp70) member 7 [Source:HGNC Symbol;Acc:HGNC:5240]	chr1	161606291	161608217	1	havana	1.967445432	2.458096527	1.476794337	2.907424358	2.206378717	2.260486508	2.135529305	0.975275413	1.319578292
ENSG00000230306	0.000508	0.014894507	0.759255553	1.69261699	**BANF1P2**	processed_pseudogene	barrier to autointegration factor 1 pseudogene 2 [Source:HGNC Symbol;Acc:HGNC:23426]	chr10	133344643	133344906	-1	havana	-0.495402265	-0.875030041	-0.115774488	-0.73288918	-0.620350959	-1.271849989	0.141952326	-0.04338487	-0.445890917
ENSG00000181847	0.00051	0.014902256	0.559088209	1.473337766	**TIGIT**	protein_coding	T cell immunoreceptor with Ig and ITIM domains [Source:HGNC Symbol;Acc:HGNC:26838]	chr3	114276913	114310288	1	ensembl_havana	0.407272425	0.127728321	0.68681653	-0.56281016	0.325758945	0.620236179	0.032074365	0.938274071	1.090101154
ENSG00000188993	0.000514	0.014902256	0.542113489	1.456104088	**LRRC66**	protein_coding	leucine rich repeat containing 66 [Source:HGNC Symbol;Acc:HGNC:34299]	chr4	51993702	52017620	-1	ensembl_havana	0.443784572	0.172727828	0.714841317	0.237415364	0.403680778	-0.122912658	0.785553575	1.01135145	0.347618925
ENSG00000123360	0.000513	0.014902256	-0.62626784	-1.543566715	**PDE1B**	protein_coding	phosphodiesterase 1B [Source:HGNC Symbol;Acc:HGNC:8775]	chr12	54549350	54579239	1	ensembl_havana	3.079968084	3.393102004	2.766834164	3.320074956	3.538169938	3.321061117	2.574915267	2.933793498	2.791793727
ENSG00000141198	0.000512	0.014902256	-0.675308365	-1.59693807	**TOM1L1**	protein_coding	target of myb1 like 1 membrane trafficking protein [Source:HGNC Symbol;Acc:HGNC:11983]	chr17	54899387	54961956	1	ensembl_havana	6.265349463	6.603003646	5.927695281	6.715916082	6.384343694	6.708751162	6.049060903	5.575639848	6.158385092
ENSG00000238005	0.000513	0.014902256	-0.86420162	-1.820332026	**AL391832.2**	lincRNA		chr1	234957342	234970062	-1	havana	0.374017626	0.806118436	-0.058083184	1.43811357	0.403680778	0.57656096	0.513047461	-0.2414061	-0.445890917
ENSG00000164185	0.000517	0.014972124	0.602865086	1.518729658	**ZNF474**	protein_coding	zinc finger protein 474 [Source:HGNC Symbol;Acc:HGNC:23245]	chr5	122129513	122153571	1	ensembl_havana	0.233297873	-0.06813467	0.534730416	-0.27307956	0.15602554	-0.087349994	0.308311185	0.671430284	0.624449778
ENSG00000160469	0.000518	0.014976829	-0.483877779	-1.398497601	**BRSK1**	protein_coding	BR serine/threonine kinase 1 [Source:HGNC Symbol;Acc:HGNC:18994]	chr19	55282072	55312533	1	ensembl_havana	4.847741226	5.089680116	4.605802337	5.391735711	5.038020279	4.839284357	4.923199632	4.532832903	4.361374475

ENSG00000107968	0.000527	0.015043268	0.49224661	1.406633626	**MAP3K8**	protein_coding	mitogen-activated protein kinase kinase kinase 8 [Source:HGNC Symbol;Acc:HGNC:6860]	chr10	30433937	30461833	1	ensembl_havana	6.408797525	6.162674219	6.65492083	6.119192109	6.155266165	6.213564384	6.64749473	6.617573411	6.699694348
ENSG00000160183	0.000526	0.015043268	-0.528214107	-1.442142881	**TMPRSS3**	protein_coding	transmembrane serine protease 3 [Source:HGNC Symbol;Acc:HGNC:11877]	chr21	42371837	42396846	-1	ensembl_havana	4.917794894	5.181901948	4.65368784	5.555421349	5.098824377	4.891460117	4.957418084	4.620114598	4.383530839
ENSG00000250565	0.000526	0.015043268	-0.62039968	-1.537301012	**ATP6V1E2**	protein_coding	ATPase H+ transporting V1 subunit E2 [Source:HGNC Symbol;Acc:HGNC:18125]	chr2	46490750	46542557	-1	ensembl_havana	3.489762564	3.799962404	3.179562724	3.812757512	3.803095076	3.784034626	3.149151625	3.102158989	3.287377559
ENSG00000124602	0.000524	0.015043268	-0.715843412	-1.642443123	**UNC5CL**	protein_coding	unc-5 family C-terminal like [Source:HGNC Symbol;Acc:HGNC:21203]	chr6	41026911	41039217	-1	ensembl_havana	1.19687935	1.554801056	0.838957644	1.705645971	1.428032914	1.530724282	1.089724456	0.779979312	0.647169164
ENSG00000140379	0.000523	0.015043268	-0.949661703	-1.931419706	**BCL2A1**	protein_coding	BCL2 related protein A1 [Source:HGNC Symbol;Acc:HGNC:991]	chr15	79960889	79971446	-1	ensembl_havana	2.753805072	3.228635923	2.27897422	3.547648667	3.227754334	2.910504768	2.408992098	2.218547759	2.209382804
ENSG00000128284	0.000526	0.015043268	-1.025807715	-2.036099007	**APOL3**	protein_coding	apolipoprotein L3 [Source:HGNC Symbol;Acc:HGNC:14868]	chr22	36140330	36166177	-1	ensembl_havana	1.119382986	1.632286844	0.606479129	1.705645971	1.909699171	1.281515389	0.618191201	0.671430284	0.529815901
ENSG00000139946	0.000534	0.015089009	0.903192642	1.870200106	**PELI2**	protein_coding	pellino E3 ubiquitin protein ligase family member 2 [Source:HGNC Symbol;Acc:HGNC:8828]	chr14	56117814	56301526	1	ensembl_havana	3.325603584	2.874007263	3.777199905	2.528879384	3.093124869	3.000017535	3.676073242	3.871404772	3.784121701
ENSG00000197808	0.000536	0.015089009	0.892961389	1.856984006	**ZNF461**	protein_coding	zinc finger protein 461 [Source:HGNC Symbol;Acc:HGNC:21629]	chr19	36637192	36666853	-1	ensembl_havana	0.971682517	0.525201823	1.418163212	0.383334258	0.77991046	0.41236075	1.195452122	1.517070703	1.541966812
ENSG00000205592	0.000532	0.015089009	0.860533102	1.815709124	**MUC19**	protein_coding	mucin 19, oligomeric [Source:HGNC Symbol;Acc:HGNC:14362]	chr12	40393395	40570832	1	havana	3.329120091	2.89885354	3.759386642	2.553756523	2.831702492	3.311101606	3.525391749	3.808721901	3.944046277
ENSG00000114544	0.000537	0.015089009	0.736411749	1.666026963	**SLC41A3**	protein_coding	solute carrier family 41 member 3 [Source:HGNC Symbol;Acc:HGNC:31046]	chr3	126006355	126101561	-1	ensembl_havana	5.167078314	4.798872439	5.535284189	4.605340519	4.810197572	4.981079227	5.520545247	5.44736723	5.63794009
ENSG00000144369	0.000535	0.015089009	0.52783993	1.441768895	**FAM171B**	protein_coding	family with sequence similarity 171 member B [Source:HGNC Symbol;Acc:HGNC:29412]	chr2	186693971	186765965	1	ensembl_havana	3.36465496	3.100734995	3.628574925	2.828074641	3.227754334	3.246376011	3.37972385	3.803587612	3.702413313
ENSG00000124374	0.000536	0.015089009	0.522192332	1.436135956	**PAIP2B**	protein_coding	poly(A) binding protein interacting protein 2B [Source:HGNC Symbol;Acc:HGNC:29200]	chr2	71182739	71227083	-1	ensembl_havana	4.346236071	4.085139905	4.607332237	3.964251181	4.116342995	4.174825539	4.520468078	4.56959204	4.731936594
ENSG00000165832	0.000534	0.015089009	-0.538478057	-1.452439486	**TRUB1**	protein_coding	TruB pseudouridine synthase family member 1 [Source:HGNC Symbol;Acc:HGNC:16060]	chr10	114938193	114977676	1	ensembl_havana	3.79228717	4.061526198	3.523048141	3.991820453	4.098880438	4.093877704	3.41298341	3.520345202	3.635815812
ENSG00000102145	0.000537	0.015089009	-1.023813054	-2.033285855	**GATA1**	protein_coding	GATA binding protein 1 [Source:HGNC Symbol;Acc:HGNC:4170]	chrX	48786554	48794311	1	ensembl_havana	0.449480414	0.961386941	-0.062426114	0.237415364	1.324992307	1.321753151	-0.51701327	0.256279753	0.073455173
ENSG00000167208	0.00054	0.015136325	-0.608691515	-1.524875561	**SNX20**	protein_coding	sorting nexin 20 [Source:HGNC Symbol;Acc:HGNC:30390]	chr16	50666300	50681353	-1	ensembl_havana	5.181422947	5.485768705	4.87707719	5.606101126	5.428140481	5.423064507	4.88551934	4.834141723	4.911570507
ENSG00000212978	0.000541	0.015137436	-0.643430562	-1.562039095	**AC016747.1**	processed_transcript		chr2	61141592	61144969	-1	havana	4.765555681	5.087270962	4.443840399	5.007688468	5.200567061	5.053557356	4.226209692	4.614261782	4.491049724
ENSG00000185818	0.000545	0.015185292	-0.522988173	-1.436928396	**NAT8L**	protein_coding	N-acetyltransferase 8 like [Source:HGNC Symbol;Acc:HGNC:26742]	chr4	2059512	2069089	1	ensembl_havana	3.29865221	3.560146296	3.037158123	3.848382869	3.415007936	3.417048083	3.302948055	2.961718855	2.846807461
ENSG00000111196	0.000544	0.015185292	-0.530508186	-1.444437906	**MAGOHB**	protein_coding	mago homolog B, exon junction complex core component [Source:HGNC Symbol;Acc:HGNC:25504]	chr12	10604190	10613623	-1	ensembl_havana	5.071049128	5.336303221	4.805795035	5.269413672	5.350744554	5.388751436	4.663984316	4.844178034	4.909222755
ENSG00000101310	0.000546	0.015189446	-0.480612112	-1.395335558	**SEC23B**	protein_coding	Sec23 homolog B, coat complex II component [Source:HGNC Symbol;Acc:HGNC:10702]	chr20	18507482	18561415	1	ensembl_havana	8.686544042	8.926850098	8.446237986	8.988311309	8.912596874	8.879642111	8.494417014	8.417862131	8.426434814
ENSG00000182870	0.000548	0.015212871	-1.383775522	-2.609503835	**GALNT9**	protein_coding	polypeptide N-acetylgalactosaminyltransferase 9 [Source:HGNC Symbol;Acc:HGNC:4131]	chr12	132196372	132329349	-1	ensembl_havana	1.325277395	2.017165156	0.633389634	2.101191983	1.987674772	1.962628713	1.089724456	0.578298274	0.232146171
ENSG00000215296	0.000559	0.015305675	1.550327753	2.928836692	**TMCO5B**	transcribed_unitary_pseudogene	transmembrane and coiled-coil domains 5B (pseudogene) [Source:HGNC Symbol;Acc:HGNC:34243]	chr15	33235552	33247621	-1	havana	-0.478027058	-1.253190934	0.297136818	-1.99786555	-0.903502039	-0.85820521	-0.00649342	0.578298274	0.319605599
ENSG00000184995	0.000559	0.015305675	0.921005388	1.893434334	**IFNE**	protein_coding	interferon epsilon [Source:HGNC Symbol;Acc:HGNC:18163]	chr9	21480839	21482313	-1	ensembl_havana	0.347450149	-0.113052546	0.807952843	-0.10791135	0.125688576	-0.356934859	0.785553575	0.841392961	0.796911992
ENSG00000068903	0.000568	0.015305675	0.480652612	1.39537473	**SIRT2**	protein_coding	sirtuin 2 [Source:HGNC Symbol;Acc:HGNC:10886]	chr19	38878555	38899862	-1	ensembl_havana	5.702303188	5.461976882	5.942629494	5.536825437	5.477022698	5.372082511	6.006720521	5.934442638	5.886725323
ENSG00000166908	0.000567	0.015305675	0.470650173	1.38573383	**PIP4K2C**	protein_coding	phosphatidylinositol-5-phosphate 4-kinase type 2 gamma [Source:HGNC Symbol;Acc:HGNC:23786]	chr12	57591174	57603418	1	ensembl_havana	7.494581881	7.259256795	7.729906968	7.213690681	7.276709281	7.287370422	7.67156939	7.759726842	7.758424671
ENSG00000174851	0.00056	0.015305675	-0.481489911	-1.396184801	**YIF1A**	protein_coding	Yip1 interacting factor homolog A, membrane trafficking protein [Source:HGNC Symbol;Acc:HGNC:16688]	chr11	66284580	66289170	-1	ensembl_havana	5.051439164	5.29218412	4.810694209	5.51323832	5.280193633	5.083120406	5.021117684	4.831621697	4.579343245
ENSG00000010292	0.000556	0.015305675	-0.485346363	-1.399921919	**NCAPD2**	protein_coding	non-SMC condensin I complex subunit D2 [Source:HGNC Symbol;Acc:HGNC:24305]	chr12	6493356	6531955	1	ensembl_havana	8.302942176	8.545615357	8.060268995	8.378106878	8.534676312	8.724062882	7.855530272	8.073713445	8.251563267
ENSG00000258738	0.000554	0.015305675	-0.487846524	-1.402350059	**AL121603.2**	antisense		chr14	34874343	34876459	1	havana	3.224449228	3.46837249	2.980525966	3.765621565	3.421266147	3.218229759	3.306885447	2.943162138	2.691530312
ENSG00000059377	0.000562	0.015305675	-0.488528149	-1.403012779	**TBXAS1**	protein_coding	thromboxane A synthase 1 [Source:HGNC Symbol;Acc:HGNC:11609]	chr7	139777051	140020325	1	ensembl_havana	2.558834068	2.803098143	2.314569994	2.982636349	2.693756041	2.73290204	2.50808372	2.233896951	2.20172931
ENSG00000182054	0.000555	0.015305675	-0.553828452	-1.467976071	**IDH2**	protein_coding	isocitrate dehydrogenase (NADP(+)) 2, mitochondrial [Source:HGNC Symbol;Acc:HGNC:5383]	chr15	90083045	90102504	-1	ensembl_havana	6.25807216	6.534986386	5.981157934	6.666875566	6.41602594	6.522057653	6.056095854	5.954848973	5.932528977
ENSG00000243927	0.000558	0.015305675	-0.605867204	-1.521893288	**MRPS6**	protein_coding	mitochondrial ribosomal protein S6 [Source:HGNC Symbol;Acc:HGNC:14051]	chr21	34073224	34143034	1	ensembl_havana	4.679731198	4.9826648	4.376797596	5.025582909	4.993564355	4.928847136	4.29720193	4.454742908	4.37844795
ENSG00000129173	0.000565	0.015305675	-0.607524508	-1.523642576	**E2F8**	protein_coding	E2F transcription factor 8 [Source:HGNC Symbol;Acc:HGNC:24727]	chr11	19224063	19241620	-1	ensembl_havana	4.037230309	4.340992563	3.733468055	4.000894365	4.477816603	4.54426672	3.423901694	3.959446991	3.817055479
ENSG00000007372	0.000567	0.015305675	-0.618509271	-1.535287957	**PAX6**	protein_coding	paired box 6 [Source:HGNC Symbol;Acc:HGNC:8620]	chr11	31784779	31818062	-1	ensembl_havana	1.629111108	1.938365743	1.319856472	1.297904502	2.169677667	2.34751506	0.807953002	1.504469011	1.647147403
ENSG00000156239	0.000556	0.015305675	-0.750415077	-1.682276768	**N6AMT1**	protein_coding	N-6 adenine-specific DNA methyltransferase 1 [Source:HGNC Symbol;Acc:HGNC:16021]	chr21	28872191	28885371	-1	ensembl_havana	2.49123702	2.866444559	2.116029482	2.863328324	2.808016853	2.9279885	2.099663825	2.226242768	2.022181853
ENSG00000228708	0.000562	0.015305675	-0.979615796	-1.971940192	**AL109763.1**	sense_overlapping		chr21	18477358	18486599	-1	havana	0.242621932	0.73242983	-0.247185966	0.565613454	0.720251491	0.911424546	-0.35894771	-0.52165082	0.139040636
ENSG00000153930	0.000568	0.015305675	-1.038847543	-2.054585748	**ANKFN1**	protein_coding	ankyrin repeat and fibronectin type III domain containing 1 [Source:HGNC Symbol;Acc:HGNC:26766]	chr17	55882301	56511659	1	ensembl_havana	-0.773879682	-0.254455911	-1.293303454	0.206373087	-0.569826728	-0.399914092	-0.82634296	-1.87077278	-1.182794618
ENSG00000171446	0.000565	0.015305675	-1.237971567	-2.358666699	**KRT27**	protein_coding	keratin 27 [Source:HGNC Symbol;Acc:HGNC:30841]	chr17	40776808	40782534	-1	ensembl_havana	3.80312982	4.422115603	3.184144036	4.858430778	4.327656656	4.080259376	3.610684386	2.774978237	3.166769484
ENSG00000125871	0.000569	0.015314094	-0.473717849	-1.388683522	**MGME1**	protein_coding	mitochondrial genome maintenance exonuclease 1 [Source:HGNC Symbol;Acc:HGNC:16205]	chr20	17968913	17991122	1	ensembl_havana	5.426196954	5.663055879	5.189338029	5.437566969	5.805386305	5.746214362	4.968646223	5.309286258	5.290081607
ENSG00000197181	0.000576	0.015357802	0.595054222	1.510529356	**PIWIL2**	protein_coding	piwi like RNA-mediated gene silencing 2 [Source:HGNC Symbol;Acc:HGNC:17644]	chr8	22275297	22357563	1	ensembl_havana	2.721685228	2.424158116	3.019212339	2.528879384	2.402591162	2.341003803	3.086398123	3.114650475	2.856588418
ENSG00000182108	0.000575	0.015357802	0.492809929	1.407182971	**DEXI**	protein_coding	Dexi homolog [Source:HGNC Symbol;Acc:HGNC:13267]	chr16	10928891	10942460	-1	ensembl_havana	4.846364141	4.599959177	5.092769106	4.617190518	4.527973752	4.654713261	5.067112011	5.066215902	5.144979405
ENSG00000174564	0.000576	0.015357802	-0.569327217	-1.483831443	**IL20RB**	protein_coding	interleukin 20 receptor subunit beta [Source:HGNC Symbol;Acc:HGNC:6004]	chr3	136946230	137011085	1	ensembl_havana	4.651703955	4.936367564	4.367040347	4.590389821	5.018545676	5.200167194	4.116077755	4.441582317	4.543460969
ENSG00000236200	0.000576	0.015357802	-0.719705694	-1.646846047	**KDM4A-AS1**	antisense	KDM4A antisense RNA 1 [Source:HGNC Symbol;Acc:HGNC:40528]	chr1	43699765	43708138	-1	havana	0.550480501	0.910333348	0.190627654	0.892538	0.89230127	0.946160774	0.106248156	0.063570082	0.402064725
ENSG00000228420	0.000576	0.015357802	-0.784583746	-1.722595236	**LINC01768**	lincRNA	long intergenic non-protein coding RNA 1768 [Source:HGNC Symbol;Acc:HGNC:52558]	chr1	109828355	109871436	1	havana	4.510539889	4.902831762	4.118248015	5.023358204	4.797022811	4.888114269	4.440288909	3.883621033	4.030834104
ENSG00000114626	0.000582	0.015360836	0.870783871	1.828656209	**ABTB1**	protein_coding	ankyrin repeat and BTB domain containing 1 [Source:HGNC Symbol;Acc:HGNC:18275]	chr3	127672935	127680920	1	ensembl_havana	4.52822495	4.092833014	4.963616885	3.947922403	4.032979498	4.297597141	4.96241916	4.99705787	4.931373625
ENSG00000163762	0.000582	0.015360836	0.763598511	1.697719967	**TM4SF18**	protein_coding	transmembrane 4 L six family member 18 [Source:HGNC Symbol;Acc:HGNC:25181]	chr3	149318498	149334414	-1	ensembl_havana	4.011997969	3.630198713	4.393797224	2.781444624	3.866385698	4.242765818	3.700223929	4.448177619	5.032990124
ENSG00000105619	0.000578	0.015360836	-0.482406589	-1.397072209	**TFPT**	protein_coding	TCF3 fusion partner [Source:HGNC Symbol;Acc:HGNC:13630]	chr19	54107013	54115675	-1	ensembl_havana	3.747682981	3.988886276	3.506479686	4.1693168	3.891395322	3.905946705	3.721029149	3.409810774	3.388599136
ENSG00000007968	0.000581	0.015360836	-0.584076968	-1.499079576	**E2F2**	protein_coding	E2F transcription factor 2 [Source:HGNC Symbol;Acc:HGNC:3114]	chr1	23506430	23531220	-1	ensembl_havana	4.706793049	4.998831533	4.414754565	4.835789483	4.977728472	5.182976644	4.303127503	4.456379573	4.484756619
ENSG00000164674	0.000581	0.015360836	-0.820994546	-1.766623425	**SYTL3**	protein_coding	synaptotagmin like 3 [Source:HGNC Symbol;Acc:HGNC:15587]	chr6	158650014	158764876	1	ensembl_havana	4.825459362	5.235956636	4.414962089	5.490870222	5.221337869	4.995661816	4.653199592	4.598043022	3.993643654
ENSG00000162391	0.000583	0.015369567	1.477398842	2.784462459	**FAM151A**	protein_coding	family with sequence similarity 151 member A [Source:HGNC Symbol;Acc:HGNC:25032]	chr1	54609182	54623556	-1	ensembl_havana	1.129643182	0.390943761	1.868342603	0.040274975	0.428746719	0.703809589	1.936429043	1.85152878	1.817069986
ENSG00000005469	0.000586	0.015416709	0.538073238	1.45203199	**CROT**	protein_coding	carnitine O-octanoyltransferase [Source:HGNC Symbol;Acc:HGNC:2366]	chr7	87345681	87399795	1	ensembl_havana	4.531663445	4.262626826	4.800700063	4.153128786	4.354066551	4.280685141	4.648552773	4.84667624	4.906871177
ENSG00000173253	0.000589	0.015438529	-0.893600774	-1.857807181	**DMRT2**	protein_coding	doublesex and mab-3 related transcription factor 2 [Source:HGNC Symbol;Acc:HGNC:2935]	chr9	1049858	1057552	1	ensembl_havana	1.51537418	1.962174567	1.068573792	2.049654613	1.970707426	1.866161661	0.995284613	0.993426194	1.21701057
ENSG00000163565	0.000589	0.015438529	-1.192648686	-2.285720002	**IFI16**	protein_coding	interferon gamma inducible protein 16 [Source:HGNC Symbol;Acc:HGNC:5395]	chr1	158999968	159055155	1	ensembl_havana	3.720269777	4.31659412	3.123945434	4.142918254	4.583438366	4.223425739	3.113627387	3.033647152	3.224561762
ENSG00000187024	0.000595	0.015441401	0.786721179	1.725149248	**PTRH1**	protein_coding	peptidyl-tRNA hydrolase 1 homolog [Source:HGNC Symbol;Acc:HGNC:27039]	chr9	127690348	127724873	-1	ensembl_havana	1.123919775	0.730559185	1.517280365	0.613726219	0.720251491	0.857699845	1.567772387	1.529563273	1.454505434
ENSG00000158062	0.000592	0.015441401	0.531946459	1.445878634	**UBXN11**	protein_coding	UBX domain protein 11 [Source:HGNC Symbol;Acc:HGNC:30600]	chr1	26281328	26318363	-1	ensembl_havana	4.420661567	4.154688338	4.686634796	4.181339713	4.154408151	4.12831715	4.668581774	4.646162349	4.745160266
ENSG00000126351	0.000595	0.015441401	0.492899745	1.407270579	**THRA**	protein_coding	thyroid hormone receptor, alpha [Source:HGNC Symbol;Acc:HGNC:11796]	chr17	40058290	40093867	1	ensembl_havana	4.367615204	4.121165331	4.614065076	4.107651756	4.112480652	4.143363585	4.58835975	4.565048043	4.688787436
ENSG00000088992	0.000595	0.015441401	-0.697822734	-1.622054995	**TESC**	protein_coding	tescalcin [Source:HGNC Symbol;Acc:HGNC:26065]	chr12	117038923	117099479	-1	ensembl_havana	4.300295426	4.649206793	3.951384059	4.845896173	4.613810345	4.487913861	4.184017004	3.753878337	3.916256835
ENSG00000109099	0.000596	0.015441401	-0.71074685	-1.636651154	**PMP22**	protein_coding	peripheral myelin protein 22 [Source:HGNC Symbol;Acc:HGNC:9118]	chr17	15229777	15265357	-1	ensembl_havana	3.652386836	4.007760261	3.297013411	4.058523223	3.942327796	4.022429763	3.192361093	3.237766117	3.460913023
ENSG00000205885	0.000592	0.015441401	-0.743201556	-1.673886324	**C1RL-AS1**	antisense	C1RL antisense RNA 1 [Source:HGNC Symbol;Acc:HGNC:27461]	chr12	7108052	7122501	1	havana	4.855149979	5.226750757	4.483549201	5.351511955	4.925852934	5.402887382	4.473948226	4.36523008	4.611469298
ENSG00000215039	0.000601	0.015542253	-0.842167633	-1.792741694	**CD27-AS1**	processed_transcript	CD27 antisense RNA 1 [Source:HGNC Symbol;Acc:HGNC:43896]	chr12	6439001	6451567	-1	havana	4.605435229	5.026519045	4.184351412	4.954969984	4.852492614	5.272094537	3.916655568	4.212836819	4.42356185
ENSG00000181481	0.000603	0.015580144	-0.735217805	-1.664648764	**RNF135**	protein_coding	ring finger protein 135 [Source:HGNC Symbol;Acc:HGNC:21158]	chr17	30968785	30999911	1	ensembl_havana	4.099173212	4.466782115	3.731564309	4.505271103	4.450539589	4.444535651	3.601096404	3.729720342	3.863876181
ENSG00000106086	0.000609	0.015639908	0.538103798	1.452062748	**PLEKHA8**	protein_coding	pleckstrin homology domain containing A8 [Source:HGNC Symbol;Acc:HGNC:30037]	chr7	30027404	30130483	1	ensembl_havana	5.482154265	5.213102366	5.751206164	5.16430079	5.123022184	5.351984123	5.71605808	5.7324551	5.805105311
ENSG00000188859	0.000609	0.015639908	0.531863372	1.445795367	**FAM78B**	protein_coding	family with sequence similarity 78 member B [Source:HGNC Symbol;Acc:HGNC:13495]	chr1	166057426	166166969	-1	ensembl_havana	5.386928759	5.120997073	5.652860445	5.266594948	5.190526483	4.905869787	5.882944088	5.661153154	5.414484094
ENSG00000108018	0.000609	0.015639908	-0.477792101	-1.392610785	**SORCS1**	protein_coding	sortilin related VPS10 domain containing receptor 1 [Source:HGNC Symbol;Acc:HGNC:16697]	chr10	106573663	107164534	-1	ensembl_havana	6.325823883	6.564719934	6.086927833	6.569983764	6.472488071	6.651687966	6.061349647	5.985777508	6.213656343
ENSG00000236393	0.00061	0.015643587	-0.975457992	-1.966265301	**AC091806.1**	lincRNA		chrX	40262917	40287720	1	havana	3.831420869	4.319149865	3.343691873	4.79594333	4.0890869	4.072419366	3.538846404	3.237766117	3.254463098
ENSG00000123609	0.000611	0.01565235	-0.571527792	-1.486096491	**NMI**	protein_coding	N-myc and STAT interactor [Source:HGNC Symbol;Acc:HGNC:7854]	chr2	151270465	151290057	-1	ensembl_havana	5.899825256	6.185589152	5.61406136	6.276914214	6.205081921	6.07477132	5.643183379	5.584647409	5.614353292
ENSG00000136104	0.000615	0.015656822	-0.490140513	-1.40458167	**RNASEH2B**	protein_coding	ribonuclease H2 subunit B [Source:HGNC Symbol;Acc:HGNC:25671]	chr13	50909678	51024120	1	ensembl_havana	4.011824667	4.256894924	3.766754411	4.10133777	4.266405117	4.402941884	3.555490165	3.798434986	3.946338082
ENSG00000049089	0.000615	0.015656822	-0.561298309	-1.475596537	**COL9A2**	protein_coding	collagen type IX alpha 2 chain [Source:HGNC Symbol;Acc:HGNC:2218]	chr1	40300487	40317813	-1	ensembl_havana	4.777379405	5.058028559	4.496730251	5.267535135	4.944439586	4.962110957	4.763324634	4.277220992	4.449645126
ENSG00000174099	0.000616	0.015656822	-0.585070202	-1.500111984	**MSRB3**	protein_coding	methionine sulfoxide reductase B3 [Source:HGNC Symbol;Acc:HGNC:27375]	chr12	65278643	65491430	1	ensembl_havana	3.491093526	3.783628627	3.198558425	3.625980797	3.893647572	3.831257512	3.100076994	3.368684571	3.126913708
ENSG00000107821	0.000616	0.015656822	-0.73534952	-1.66480075	**KAZALD1**	protein_coding	Kazal type serine peptidase inhibitor domain 1 [Source:HGNC Symbol;Acc:HGNC:25460]	chr10	101061841	101068131	1	ensembl_havana	2.015542678	2.383217438	1.647867918	2.458169134	2.324608835	2.366874344	1.681320805	1.769643034	1.492639914
ENSG00000165304	0.000618	0.01567382	-0.519514196	-1.433472468	**MELK**	protein_coding	maternal embryonic leucine zipper kinase [Source:HGNC Symbol;Acc:HGNC:16870]	chr9	36572862	36677683	1	ensembl_havana	7.29909788	7.558854978	7.039340782	7.526468883	7.653823537	7.496272514	6.995508776	7.071310277	7.051203292
ENSG00000071909	0.000619	0.01567647	-1.017599286	-2.024547211	**MYO3B**	protein_coding	myosin IIIB [Source:HGNC Symbol;Acc:HGNC:15576]	chr2	170178145	170655171	1	ensembl_havana	3.480939431	3.989739074	2.972139788	4.446707967	3.817401554	3.705107701	3.140352234	2.876266922	2.899800207
ENSG00000241769	0.000621	0.015690312	0.653423769	1.57289653	**LINC00893**	antisense	long intergenic non-protein coding RNA 893 [Source:HGNC Symbol;Acc:HGNC:44265]	chrX	149527591	149540959	-1	ensembl_havana	2.396433438	2.069721553	2.723145322	2.26832323	1.817810321	2.123031109	2.879883083	2.620048716	2.669504168
ENSG00000260011	0.000624	0.015699234	0.543398378	1.457401497	**AC132938.1**	lincRNA		chr17	82381110	82382690	-1	havana	-0.087213533	-0.358912722	0.184485656	-0.61730533	-0.473795405	0.014362573	-0.08686711	-0.00684508	0.647169164
ENSG00000135637	0.000624	0.015699234	0.540622785	1.454600306	**CCDC142**	protein_coding	coiled-coil domain containing 142 [Source:HGNC Symbol;Acc:HGNC:25889]	chr2	74471986	74483408	-1	ensembl_havana	3.810832411	3.540521019	4.081143804	3.4316494	3.439879526	3.75003413	4.013800015	4.033680126	4.195951269
ENSG00000015285	0.000622	0.015699234	-0.912863174	-1.882778357	**WAS**	protein_coding	Wiskott-Aldrich syndrome [Source:HGNC Symbol;Acc:HGNC:12731]	chrX	48676596	48691427	1	ensembl_havana	4.927656742	5.384088329	4.471225155	5.568449919	5.310010233	5.273804834	4.468685528	4.333520638	4.611469298
ENSG00000120896	0.000627	0.015732077	0.569532909	1.484043015	**SORBS3**	protein_coding	sorbin and SH3 domain containing 3 [Source:HGNC Symbol;Acc:HGNC:30907]	chr8	22544986	22575788	1	ensembl_havana	2.40290265	2.118136196	2.687669105	2.464742477	1.987674772	1.901991338	3.020790384	2.660368592	2.381848339
ENSG00000198909	0.000629	0.015752877	0.488553948	1.403037868	**MAP3K3**	protein_coding	mitogen-activated protein kinase kinase kinase 3 [Source:HGNC Symbol;Acc:HGNC:6855]	chr17	63622415	63696303	1	ensembl_havana	5.170803367	4.926526393	5.41508034	4.850923091	4.944439586	4.9842165	5.388364636	5.434138874	5.422737512
ENSG00000132821	0.000631	0.015756938	-0.800730796	-1.741983303	**VSTM2L**	protein_coding	V-set and transmembrane domain containing 2 like [Source:HGNC Symbol;Acc:HGNC:16096]	chr20	37903104	37945350	1	ensembl_havana	3.698843529	4.099208927	3.298478131	4.401170796	4.156285298	3.740170686	3.812494555	3.206986221	2.875953617
ENSG00000006757	0.000631	0.015756938	-1.365907603	-2.577384188	**PNPLA4**	protein_coding	patatin like phospholipase domain containing 4 [Source:HGNC Symbol;Acc:HGNC:24887]	chrX	7898247	7927739	-1	ensembl_havana	1.137585198	1.820539	0.454631396	2.067039104	1.970707426	1.423870469	0.540060475	0.343769191	0.480064523
ENSG00000124380	0.000633	0.015788573	-0.714014406	-1.640362202	**SNRNP27**	protein_coding	small nuclear ribonucleoprotein U4/U6.U5 subunit 27 [Source:HGNC Symbol;Acc:HGNC:30240]	chr2	69893560	69905575	1	ensembl_havana	4.379054379	4.736061582	4.022047176	4.698916787	4.89583178	4.613436179	4.028181329	4.007126095	4.030834104
ENSG00000017483	0.000638	0.015879278	-0.525186074	-1.439119181	**SLC38A5**	protein_coding	solute carrier family 38 member 5 [Source:HGNC Symbol;Acc:HGNC:18070]	chrX	48458537	48470256	-1	ensembl_havana	6.238649873	6.50124291	5.976056836	6.522334759	6.574631572	6.406762399	6.066003677	6.060854758	5.801312073
ENSG00000242550	0.00064	0.015908484	1.494083019	2.816850543	**SERPINB10**	protein_coding	serpin family B member 10 [Source:HGNC Symbol;Acc:HGNC:8942]	chr18	63897174	63936111	1	ensembl_havana	-0.543280182	-1.290321692	0.203761328	-1.86005921	-0.569826728	-1.441079133	-0.00649342	0.478736765	0.139040636
ENSG00000196118	0.000642	0.015908484	0.456210596	1.37193354	**CCDC189**	protein_coding	coiled-coil domain containing 189 [Source:HGNC Symbol;Acc:HGNC:28078]	chr16	30757423	30762710	-1	ensembl_havana	2.369862685	2.141757387	2.597967982	2.142776609	2.08549033	2.197005222	2.587917939	2.542041324	2.663944684
ENSG00000242732	0.000643	0.015908484	-0.74558277	-1.676651408	**RTL5**	protein_coding	retrotransposon Gag like 5 [Source:HGNC Symbol;Acc:HGNC:29430]	chrX	72127110	72131901	-1	ensembl_havana	6.406705499	6.779496884	6.033914114	6.514039928	6.813123877	7.011326848	5.957475089	6.019382369	6.124884885
ENSG00000197619	0.000648	0.015970444	0.480738639	1.395457937	**ZNF615**	protein_coding	zinc finger protein 615 [Source:HGNC Symbol;Acc:HGNC:24740]	chr19	51991332	52008230	-1	ensembl_havana	4.022287869	3.78191855	4.262657189	3.762957194	3.852559113	3.730239343	4.291251919	4.329953957	4.16676569
ENSG00000169689	0.000648	0.015970444	0.476159451	1.391035704	**CENPX**	protein_coding	centromere protein X [Source:HGNC Symbol;Acc:HGNC:11422]	chr17	82018702	82024107	-1	ensembl_havana	3.757070158	3.518990433	3.995149883	3.474621708	3.569499592	3.512849998	3.967289729	3.998165018	4.019994903
ENSG00000276231	0.000646	0.015970444	-1.086621328	-2.123760865	**PIK3R6**	protein_coding	phosphoinositide-3-kinase regulatory subunit 6 [Source:HGNC Symbol;Acc:HGNC:27101]	chr17	8802723	8867677	-1	ensembl_havana	1.234574993	1.777885657	0.691264329	1.987104222	1.603393164	1.743159585	1.178360195	0.225896484	0.669536307
ENSG00000242258	0.00065	0.015975019	-1.044132275	-2.062125698	**LINC00996**	lincRNA	long intergenic non-protein coding RNA 996 [Source:HGNC Symbol;Acc:HGNC:27800]	chr7	150433654	150448140	1	havana	0.145268233	0.667334371	-0.376797905	0.637193984	0.453384582	0.911424546	-0.26243933	-0.42206346	-0.445890917
ENSG00000114487	0.000652	0.016016835	0.75337537	1.685732206	**MORC1**	protein_coding	MORC family CW-type zinc finger 1 [Source:HGNC Symbol;Acc:HGNC:7198]	chr3	108958239	109118142	-1	ensembl_havana	0.778033533	0.401345849	1.154721218	0.10902545	0.658018659	0.436993436	1.052687018	1.372000412	1.039476224
ENSG00000110675	0.000654	0.016033013	1.502965091	2.834246212	**ELMOD1**	protein_coding	ELMO domain containing 1 [Source:HGNC Symbol;Acc:HGNC:25334]	chr11	107591091	107666779	1	ensembl_havana	2.156247171	1.404764626	2.907729717	0.727424294	1.979216042	1.507653541	2.619920974	3.046743406	3.05652477
ENSG00000100336	0.000656	0.016055639	-0.602859839	-1.518724135	**APOL4**	protein_coding	apolipoprotein L4 [Source:HGNC Symbol;Acc:HGNC:14867]	chr22	36189124	36204840	-1	ensembl_havana	4.730138632	5.031568551	4.428708712	5.083321205	4.980909585	5.030474863	4.503412911	4.252941584	4.529771642
ENSG00000237248	0.000658	0.016070316	-0.575718502	-1.490419549	**LINC00987**	lincRNA	long intergenic non-protein coding RNA 987 [Source:HGNC Symbol;Acc:HGNC:48911]	chr12	9240003	9243052	1	havana	3.715297869	4.003157121	3.427438618	4.03442213	4.047212239	3.927836994	3.345679402	3.495088671	3.441547782
ENSG00000117593	0.000659	0.016076464	-0.461082162	-1.376573996	**DARS2**	protein_coding	aspartyl-tRNA synthetase 2, mitochondrial [Source:HGNC Symbol;Acc:HGNC:25538]	chr1	173824503	173858546	1	ensembl_havana	6.498424474	6.728965555	6.267883393	6.558890401	6.764727635	6.863278628	6.101005427	6.275388699	6.427256053
ENSG00000125611	0.000664	0.01610066	-0.456868788	-1.372559593	**CHCHD5**	protein_coding	coiled-coil-helix-coiled-coil-helix domain containing 5 [Source:HGNC Symbol;Acc:HGNC:17840]	chr2	112584240	112589275	1	ensembl_havana	2.137754835	2.366189229	1.909320441	2.376860232	2.433879495	2.287827958	1.936429043	1.984515821	1.807016457
ENSG00000135124	0.000662	0.01610066	-0.581383134	-1.496283071	**P2RX4**	protein_coding	purinergic receptor P2X 4 [Source:HGNC Symbol;Acc:HGNC:8535]	chr12	121209857	121234106	1	ensembl_havana	4.705977857	4.996669424	4.41528629	5.32194891	4.9379069	4.730152463	4.670111011	4.304737198	4.271010662
ENSG00000103034	0.000663	0.01610066	-0.762303438	-1.696196648	**NDRG4**	protein_coding	NDRG family member 4 [Source:HGNC Symbol;Acc:HGNC:14466]	chr16	58462846	58513628	1	ensembl_havana	-0.290139746	0.091011973	-0.671291465	0.540941683	-0.18055577	-0.087349994	-0.04612063	-0.93694677	-1.030806998
ENSG00000230628	0.000667	0.016118584	1.263429728	2.400657729	**AL160408.3**	lincRNA		chr1	234669523	234696153	-1	havana	-0.192323124	-0.824037987	0.43939174	-0.41068387	-0.620350959	-1.441079133	0.457455004	0.821210834	0.039509383
ENSG00000135093	0.000671	0.016118584	0.610423466	1.526707269	**USP30**	protein_coding	ubiquitin specific peptidase 30 [Source:HGNC Symbol;Acc:HGNC:20065]	chr12	109023089	109088026	1	ensembl_havana	3.489357621	3.184145887	3.794569354	3.214825476	3.276895115	3.060717071	3.850756671	3.756537757	3.776413633
ENSG00000151012	0.000668	0.016118584	0.46094025	1.376438595	**SLC7A11**	protein_coding	solute carrier family 7 member 11 [Source:HGNC Symbol;Acc:HGNC:11059]	chr4	138164097	138242349	-1	ensembl_havana	2.879618467	2.649148341	3.110088592	2.376860232	2.798432633	2.772152159	2.853206155	3.226300474	3.250759147
ENSG00000140876	0.000668	0.016118584	-0.462149268	-1.377592571	**NUDT7**	protein_coding	nudix hydrolase 7 [Source:HGNC Symbol;Acc:HGNC:8054]	chr16	77722492	77742260	1	ensembl_havana	2.432618045	2.663692679	2.201543411	2.700213803	2.698916685	2.591947549	2.262602515	2.203033505	2.138994213
ENSG00000102048	0.000671	0.016118584	-0.610938795	-1.527252704	**ASB9**	protein_coding	ankyrin repeat and SOCS box containing 9 [Source:HGNC Symbol;Acc:HGNC:17184]	chrX	15235288	15270467	-1	ensembl_havana	1.381790779	1.687260176	1.076321381	1.660549964	1.749818205	1.651412359	1.161063346	1.01135145	1.056549347
ENSG00000184271	0.00067	0.016118584	-0.780741492	-1.718013642	**POU6F1**	protein_coding	POU class 6 homeobox 1 [Source:HGNC Symbol;Acc:HGNC:9224]	chr12	51186936	51217708	-1	ensembl_havana	1.600739129	1.991109875	1.210368383	1.85322802	2.191810142	1.928291464	1.071324589	1.226127349	1.333653212
ENSG00000225032	0.000675	0.01616758	0.763331603	1.697405906	**AL162586.1**	antisense		chr9	127816066	127822520	1	havana	2.212810838	1.831145037	2.594476639	1.341416217	1.759729761	2.392289132	2.270688985	2.548193946	2.964546987
ENSG00000280173	0.000676	0.01616758	0.588683995	1.50387431	**AC104447.1**	TEC		chr3	47346950	47349073	1	havana	1.806750402	1.512408404	2.101092399	1.327057633	1.476918714	1.733248866	1.986385747	2.122856455	2.194034997
ENSG00000168591	0.000677	0.01616758	0.470786714	1.385864986	**TMUB2**	protein_coding	transmembrane and ubiquitin like domain containing 2 [Source:HGNC Symbol;Acc:HGNC:28459]	chr17	44186970	44191731	1	ensembl_havana	5.711091577	5.47569822	5.946484934	5.51956602	5.438204625	5.469324013	6.033699796	5.904780739	5.900974267
ENSG00000165312	0.000678	0.01616758	0.450315759	1.366339271	**OTUD1**	protein_coding	OTU deubiquitinase 1 [Source:HGNC Symbol;Acc:HGNC:27346]	chr10	23439458	23442390	1	ensembl_havana	2.046347706	1.821189826	2.271505585	1.882980075	1.817810321	1.762779083	2.333799072	2.278988373	2.20172931
ENSG00000226954	0.000684	0.016298438	1.278011257	2.42504456	**AL023802.1**	lincRNA		chr22	49718053	49724506	-1	havana	-0.680971047	-1.319976676	-0.041965419	-1.14838881	-1.617400645	-1.194140569	-0.04612063	0.063570082	-0.143345712
ENSG00000148483	0.000687	0.016338255	0.930950849	1.906532136	**TMEM236**	protein_coding	transmembrane protein 236 [Source:HGNC Symbol;Acc:HGNC:23473]	chr10	17752252	17800868	1	ensembl_havana	-0.710927744	-1.176403169	-0.245452319	-1.51148723	-0.967469033	-1.050253243	-0.82634296	0.19485955	-0.104873545
ENSG00000160695	0.000691	0.016397719	0.463871724	1.379238283	**VPS11**	protein_coding	VPS11, CORVET/HOPS core subunit [Source:HGNC Symbol;Acc:HGNC:14583]	chr11	119067692	119081978	1	ensembl_havana	6.421964686	6.190028824	6.653900548	6.162797236	6.194616212	6.212673025	6.665623111	6.6245051	6.671573434
ENSG00000110442	0.000692	0.016403722	0.550740712	1.464837583	**COMMD9**	protein_coding	COMM domain containing 9 [Source:HGNC Symbol;Acc:HGNC:25014]	chr11	36269284	36289449	-1	ensembl_havana	5.126966388	4.851596032	5.402336744	4.730626344	4.998804589	4.825357162	5.379979066	5.459659614	5.367371551
ENSG00000114796	0.000698	0.016498701	0.593785406	1.509201466	**KLHL24**	protein_coding	kelch like family member 24 [Source:HGNC Symbol;Acc:HGNC:25947]	chr3	183635568	183684477	1	ensembl_havana	4.902255605	4.605362902	5.199148308	4.752281178	4.543834716	4.519972811	5.257156287	5.267945066	5.072343571
ENSG00000137941	0.000701	0.016498701	0.548795718	1.462864068	**TTLL7**	protein_coding	tubulin tyrosine ligase like 7 [Source:HGNC Symbol;Acc:HGNC:26242]	chr1	83865028	83999150	-1	ensembl_havana	5.289885392	5.015487534	5.564283251	4.753623874	5.141144845	5.151693882	5.386505383	5.707932607	5.598411764
ENSG00000133687	0.000701	0.016498701	0.478602018	1.393392804	**TMTC1**	protein_coding	transmembrane and tetratricopeptide repeat containing 1 [Source:HGNC Symbol;Acc:HGNC:24099]	chr12	29500840	29784759	-1	ensembl_havana	7.397357746	7.158056737	7.636658755	7.032532194	7.304482797	7.137155219	7.5341023	7.723825306	7.652048659
ENSG00000169740	0.000701	0.016498701	-0.545403553	-1.45942852	**ZNF32**	protein_coding	zinc finger protein 32 [Source:HGNC Symbol;Acc:HGNC:13095]	chr10	43643859	43648856	-1	ensembl_havana	2.97631311	3.249014887	2.703611334	3.421548267	3.093124869	3.232371524	2.826026634	2.654677143	2.630130226
ENSG00000153093	0.000702	0.016505036	-0.455824867	-1.37156678	**ACOXL**	protein_coding	acyl-CoA oxidase like [Source:HGNC Symbol;Acc:HGNC:25621]	chr2	110732573	111118222	1	ensembl_havana	3.926955801	4.154868234	3.699043367	4.086496776	4.133596708	4.244511218	3.607495467	3.710647067	3.778987568
ENSG00000271941	0.000707	0.016511032	0.598175064	1.513800474	**AC104184.1**	antisense		chr3	43346923	43347643	-1	havana	0.041513004	-0.257574528	0.340600536	-0.31750517	-0.258437325	-0.196781087	0.176794179	0.315191528	0.529815901
ENSG00000147883	0.000706	0.016511032	0.57562106	1.490318886	**CDKN2B**	protein_coding	cyclin dependent kinase inhibitor 2B [Source:HGNC Symbol;Acc:HGNC:1788]	chr9	22002903	22009363	-1	ensembl_havana	4.421073929	4.133263399	4.708884459	4.041035185	4.206066476	4.152688536	4.493082186	4.830360032	4.803211159
ENSG00000151176	0.000704	0.016511032	0.485031265	1.399616196	**PLBD2**	protein_coding	phospholipase B domain containing 2 [Source:HGNC Symbol;Acc:HGNC:27283]	chr12	113358566	113391625	1	ensembl_havana	4.708685603	4.46616997	4.951201235	4.411415107	4.464242561	4.522852242	4.953655868	4.949039243	4.950908594
ENSG00000147434	0.000705	0.016511032	-0.570776756	-1.485323063	**CHRNA6**	protein_coding	cholinergic receptor nicotinic alpha 6 subunit [Source:HGNC Symbol;Acc:HGNC:15963]	chr8	42752620	42796392	-1	ensembl_havana	6.834646953	7.120035331	6.549258574	7.064529726	7.048121896	7.247454371	6.39443282	6.588016411	6.665326492
ENSG00000244682	0.000709	0.016538884	-0.864725663	-1.820993361	**FCGR2C**	polymorphic_pseudogene	Fc fragment of IgG receptor IIc (gene/pseudogene) [Source:HGNC Symbol;Acc:HGNC:15626]	chr1	161581339	161605662	1	ensembl_havana	3.460909049	3.893271881	3.028546218	4.620137871	3.535287761	3.52439001	3.538846404	2.666037677	2.880754572
ENSG00000007341	0.000712	0.016571574	-0.519168384	-1.433128909	**ST7L**	protein_coding	suppression of tumorigenicity 7 like [Source:HGNC Symbol;Acc:HGNC:18441]	chr1	112523518	112620825	-1	ensembl_havana	2.725078899	2.984663091	2.465494706	3.023281264	2.877938163	3.052769845	2.548552981	2.406377083	2.441554055
ENSG00000124813	0.000714	0.016584911	0.810650723	1.754002401	**RUNX2**	protein_coding	runt related transcription factor 2 [Source:HGNC Symbol;Acc:HGNC:10472]	chr6	45328157	45664349	1	ensembl_havana	5.511288589	5.105963228	5.91661395	4.502079217	5.177645664	5.638164802	5.507773497	5.998752055	6.243316298
ENSG00000239779	0.000718	0.016639832	0.625011232	1.542222832	**WBP1**	protein_coding	WW domain binding protein 1 [Source:HGNC Symbol;Acc:HGNC:12737]	chr2	74458329	74460891	1	ensembl_havana	3.835963992	3.523458377	4.148469608	3.714137432	3.269976654	3.586261044	4.19042511	3.947858737	4.307124977

ENSG00000160447	0.000718	0.016639832	-0.957361905	-1.941755975	**PKN3**	protein_coding	protein kinase N3 [Source:HGNC Symbol;Acc:HGNC:17999]	chr9	128702523	128720918	1	ensembl_havana	2.171319766	2.650000718	1.692638813	2.643402293	2.61404572	2.692554143	1.966610161	1.452931788	1.65837449
ENSG00000184564	0.000722	0.016676805	0.737470148	1.667249652	**SLITRK6**	protein_coding	SLIT and NTRK like family member 6 [Source:HGNC Symbol;Acc:HGNC:23503]	chr13	85792787	85806683	-1	ensembl_havana	1.36768617	0.998951096	1.736421244	1.142587728	0.996565715	0.857699845	1.786580698	1.625790657	1.796892377
ENSG00000215417	0.000727	0.016676805	0.540921787	1.454901807	**MIR17HG**	processed_transcript	miR-17-92a-1 cluster host gene [Source:HGNC Symbol;Acc:HGNC:23564]	chr13	91347820	91354579	1	havana	6.113012091	5.842551197	6.383472984	5.934410745	5.904201767	5.689041079	6.503101101	6.341533555	6.305784295
ENSG00000244187	0.000724	0.016676805	0.513233169	1.427245159	**TMEM141**	protein_coding	transmembrane protein 141 [Source:HGNC Symbol;Acc:HGNC:28211]	chr9	136791355	136793257	1	ensembl_havana	2.536149046	2.279532462	2.792765631	2.058373044	2.45233188	2.32789246	2.587917939	2.881149392	2.90922956
ENSG00000272927	0.000727	0.016676805	-0.551200215	-1.465304213	**AC107464.3**	antisense		chr4	661209	661945	-1	havana	1.787331536	2.062931643	1.511731429	2.167163933	1.944876279	2.076754718	1.693402841	1.426455924	1.415335521
ENSG00000135502	0.000726	0.016676805	-0.586430448	-1.501527032	**SLC26A10**	protein_coding	solute carrier family 26 member 10 [Source:HGNC Symbol;Acc:HGNC:14470]	chr12	57619527	57626151	1	ensembl_havana	4.192895855	4.486111079	3.899680631	4.646396549	4.477816603	4.334120086	4.122794131	3.759192284	3.817055479
ENSG00000272173	0.000723	0.016676805	-1.546621265	-2.921321753	**U47924.2**	antisense		chr12	6943508	6944604	-1	havana	0.532009469	1.305320101	-0.241301164	1.516132283	1.338282095	1.061545926	-0.08686711	0.256279753	-0.893316134
ENSG00000150054	0.000731	0.016698324	0.64685302	1.565749068	**MPP7**	protein_coding	membrane palmitoylated protein 7 [Source:HGNC Symbol;Acc:HGNC:26542]	chr10	28050993	28334486	-1	ensembl_havana	3.010815904	2.687389394	3.334242415	2.341580783	2.783935853	2.936651547	3.149151625	3.329927104	3.523648515
ENSG00000177732	0.00073	0.016698324	-0.498608565	-1.412850255	**SOX12**	protein_coding	SRY-box 12 [Source:HGNC Symbol;Acc:HGNC:11198]	chr20	325595	330224	1	ensembl_havana	3.533519417	3.7828237	3.284215134	3.87082891	3.693607564	3.784034626	3.409325542	3.237766117	3.205553743
ENSG00000164691	0.000731	0.016698324	-0.8890383	-1.851941208	**TAGAP**	protein_coding	T cell activation RhoGTPase activating protein [Source:HGNC Symbol;Acc:HGNC:15669]	chr6	159034468	159045152	-1	ensembl_havana	0.188211328	0.632730478	-0.256307822	0.565613454	0.403680778	0.928897202	-0.21650287	-0.32890884	-0.223511759
ENSG00000223773	0.000734	0.016728698	-0.47356107	-1.38853262	**CD99P1**	transcribed_unprocessed_pseudogene	CD99 molecule pseudogene 1 [Source:HGNC Symbol;Acc:HGNC:7083]	chrX	2609348	2657229	1	havana	6.14226793	6.379048465	5.905487395	6.613893079	6.107554554	6.415697761	6.082175557	5.675979229	5.958307398
ENSG00000041982	0.000744	0.016919758	1.335480033	2.523594363	**TNC**	protein_coding	tenascin C [Source:HGNC Symbol;Acc:HGNC:5318]	chr9	115019578	115118257	-1	ensembl_havana	2.754373148	2.086633132	3.422113165	1.613998728	2.324608835	2.321291832	3.291070988	3.256675325	3.718593182
ENSG00000274180	0.000754	0.016919758	0.59479657	1.510259613	**NATD1**	protein_coding	N-acetyltransferase domain containing 1 [Source:HGNC Symbol;Acc:HGNC:30770]	chr17	21238870	21253410	-1	ensembl_havana	4.431040256	4.133641971	4.728438541	4.245732307	3.953166093	4.202027512	4.842766089	4.667515227	4.675034306
ENSG00000112096	0.000747	0.016919758	0.53873684	1.452700041	**SOD2**	protein_coding	superoxide dismutase 2 [Source:HGNC Symbol;Acc:HGNC:11180]	chr6	159669057	159745186	-1	ensembl_havana	5.363411381	5.09404296	5.632779801	4.884404214	5.229384864	5.168339803	5.526466923	5.743891853	5.627980626
ENSG00000108187	0.000747	0.016919758	0.523425099	1.437363642	**PBLD**	protein_coding	phenazine biosynthesis like protein domain containing [Source:HGNC Symbol;Acc:HGNC:23301]	chr10	68282660	68333049	-1	ensembl_havana	2.368861524	2.107148975	2.630574074	2.092729261	2.249222012	1.979495653	2.613576909	2.688493842	2.58965147
ENSG00000230918	0.000753	0.016919758	0.522618686	1.436560433	**AC008063.1**	antisense		chr2	162073256	162075169	1	havana	1.904568917	1.643259574	2.16587826	1.85322802	1.592342839	1.484207863	2.278730383	2.179444752	2.039459644
ENSG00000080546	0.000751	0.016919758	0.457531117	1.373189868	**SESN1**	protein_coding	sestrin 1 [Source:HGNC Symbol;Acc:HGNC:21595]	chr6	108986437	109094819	-1	ensembl_havana	3.824344665	3.595579106	4.053110224	3.441680301	3.561022392	3.784034626	3.942194775	4.011585847	4.205550049
ENSG00000107560	0.000753	0.016919758	-0.482554822	-1.397215761	**RAB11FIP2**	protein_coding	RAB11 family interacting protein 2 [Source:HGNC Symbol;Acc:HGNC:29152]	chr10	118004916	118046603	-1	ensembl_havana	3.505009552	3.746286963	3.263732141	3.786761224	3.729353954	3.722745712	3.283098272	3.199187534	3.308910617
ENSG00000139329	0.000746	0.016919758	-0.633013002	-1.550800389	**LUM**	protein_coding	lumican [Source:HGNC Symbol;Acc:HGNC:6724]	chr12	91102629	91111831	-1	ensembl_havana	-0.292507234	0.023999267	-0.609013735	-0.41068387	0.501432867	-0.018751196	-1.13267621	-0.19956768	-0.494797317
ENSG00000072694	0.000754	0.016919758	-0.762510238	-1.696439803	**FCGR2B**	protein_coding	Fc fragment of IgG receptor IIb [Source:HGNC Symbol;Acc:HGNC:3618]	chr1	161663147	161678654	1	ensembl_havana	2.919752135	3.301007254	2.538497016	3.649251657	2.983208668	3.270561436	2.681872935	2.264113944	2.669504168
ENSG00000122584	0.000753	0.016919758	-1.555615431	-2.939590982	**NXPH1**	protein_coding	neurexophilin 1 [Source:HGNC Symbol;Acc:HGNC:20693]	chr7	8433955	8752963	1	ensembl_havana	0.106195857	0.884003572	-0.671611859	0.613726219	1.109387295	0.928897202	-0.46237776	-0.52165082	-1.030806998
ENSG00000232973	0.000756	0.016936912	-0.618214279	-1.534974064	**CYP1B1-AS1**	antisense	CYP1B1 antisense RNA 1 [Source:HGNC Symbol;Acc:HGNC:28543]	chr2	38073447	38231651	1	havana	4.481301293	4.790408432	4.172194153	4.712789269	4.68575637	4.972679659	4.179729066	4.133217998	4.203635396
ENSG00000018189	0.000759	0.016951809	0.574162903	1.488813357	**RUFY3**	protein_coding	RUN and FYVE domain containing 3 [Source:HGNC Symbol;Acc:HGNC:30285]	chr4	70704204	70807315	1	ensembl_havana	5.546395425	5.259313973	5.833476877	5.161268806	5.303249135	5.313423979	5.854307795	5.774391348	5.871731487
ENSG00000154269	0.000759	0.016951809	-0.59869833	-1.51434963	**ENPP3**	protein_coding	ectonucleotide pyrophosphatase/phosphodiesterase 3 [Source:HGNC Symbol;Acc:HGNC:3358]	chr6	131628442	131747418	1	ensembl_havana	5.629422651	5.928771816	5.330073486	6.374394803	5.8662869	5.545633746	5.672471286	5.234951258	5.082797914
ENSG00000246877	0.000765	0.017009831	0.579558983	1.494392358	**DNM1P35**	antisense	dynamin 1 pseudogene 35 [Source:HGNC Symbol;Acc:HGNC:35182]	chr15	75727670	75738623	-1	havana	-0.476681899	-0.76646139	-0.186902408	-0.99620085	-0.383759306	-0.91942402	-0.35894771	0.063570082	-0.265329594
ENSG00000116852	0.000765	0.017009831	0.517115307	1.431090895	**KIF21B**	protein_coding	kinesin family member 21B [Source:HGNC Symbol;Acc:HGNC:29442]	chr1	200969390	201023700	-1	ensembl_havana	2.715430977	2.456873324	2.973988631	2.305416943	2.408903349	2.656299678	2.74708149	3.015998475	3.158885927
ENSG00000136783	0.000769	0.017009831	0.444902058	1.361221702	**NIPSNAP3A**	protein_coding	nipsnap homolog 3A [Source:HGNC Symbol;Acc:HGNC:23619]	chr9	104747688	104760122	1	ensembl_havana	4.371525035	4.149074006	4.593976064	3.97580247	4.163769556	4.307649991	4.400307849	4.640414466	4.741205876
ENSG00000139192	0.000766	0.017009831	-0.505476603	-1.419592249	**TAPBPL**	protein_coding	TAP binding protein like [Source:HGNC Symbol;Acc:HGNC:30683]	chr12	6451690	6466517	1	ensembl_havana	4.497784244	4.750522546	4.245045943	4.605340519	4.819704208	4.82652291	4.179729066	4.30836669	4.247042073
ENSG00000125510	0.000767	0.017009831	-0.708497557	-1.634101454	**OPRL1**	protein_coding	opioid related nociceptin receptor 1 [Source:HGNC Symbol;Acc:HGNC:8155]	chr20	64080173	64100643	1	ensembl_havana	2.850245564	3.204494343	2.495996785	3.214825476	3.100943157	3.297714395	2.607204823	2.485454087	2.395331447
ENSG00000164400	0.000768	0.017009831	-0.746028527	-1.677169532	**CSF2**	protein_coding	colony stimulating factor 2 [Source:HGNC Symbol;Acc:HGNC:2434]	chr5	132073790	132076170	1	ensembl_havana	5.186666826	5.55968109	4.813652562	6.112405344	5.409374036	5.15726389	5.158195301	4.793283367	4.489479019
ENSG00000166069	0.000773	0.017069018	1.293100988	2.450542195	**TMCO5A**	protein_coding	transmembrane and coiled-coil domains 5A [Source:HGNC Symbol;Acc:HGNC:28558]	chr15	37921939	37967724	1	ensembl_havana	0.106189083	-0.540361411	0.752739577	-1.51148723	-0.218971072	0.109374069	0.106248156	1.14725896	1.004711615
ENSG00000088002	0.000774	0.017069018	-1.441872491	-2.716732447	**SULT2B1**	protein_coding	sulfotransferase family 2B member 1 [Source:HGNC Symbol;Acc:HGNC:11459]	chr19	48552075	48599425	1	ensembl_havana	-0.456251718	0.264684528	-1.177187963	0.14220989	0.214850257	0.436993436	-0.82634296	-1.52242631	-1.182794618
ENSG00000107611	0.000788	0.017196248	1.209882153	2.313187407	**CUBN**	protein_coding	cubilin [Source:HGNC Symbol;Acc:HGNC:2548]	chr10	16823964	17129831	-1	ensembl_havana	-0.053228361	-0.658169437	0.551712716	-1.31854494	-0.34076431	-0.315199064	0.276538753	0.602150945	0.77644845
ENSG00000153132	0.000792	0.017196248	0.854687399	1.808366879	**CLGN**	protein_coding	calmegin [Source:HGNC Symbol;Acc:HGNC:2060]	chr4	140388455	140427968	-1	ensembl_havana	2.186826762	1.759483063	2.614170462	1.109412078	2.053616384	2.115420726	2.221475976	2.764446992	2.856588418
ENSG00000105136	0.000801	0.017196248	0.57553244	1.490227344	**ZNF419**	protein_coding	zinc finger protein 419 [Source:HGNC Symbol;Acc:HGNC:20648]	chr19	57487711	57496097	1	ensembl_havana	1.429651075	1.141884854	1.717417295	0.832812769	1.324992307	1.267849486	1.500601289	1.938414976	1.713235619
ENSG00000092009	0.000786	0.017196248	0.539888997	1.453860651	**CMA1**	protein_coding	chymase 1 [Source:HGNC Symbol;Acc:HGNC:2097]	chr14	24505353	24508265	-1	ensembl_havana	6.184238268	5.91429377	6.454182767	5.546927966	6.046619148	6.149334196	6.151691922	6.474279617	6.736576762
ENSG00000114268	0.000795	0.017196248	0.524969764	1.438903423	**PFKFB4**	protein_coding	6-phosphofructo-2-kinase/fructose-2,6-biphosphatase 4 [Source:HGNC Symbol;Acc:HGNC:8875]	chr3	48517684	48562015	-1	ensembl_havana	4.020053093	3.757568211	4.282537975	3.87082891	3.764236011	3.637639713	4.418618523	4.189409115	4.239586287
ENSG00000214922	0.000802	0.017196248	0.470209609	1.385310726	**HLA-F-AS1**	processed_transcript	HLA-F antisense RNA 1 [Source:HGNC Symbol;Acc:HGNC:26645]	chr6	29726601	29749049	-1	havana	3.612139307	3.377034502	3.847244112	3.341663059	3.317723301	3.471717147	3.776033227	3.854126336	3.911572771
ENSG00000254901	0.000783	0.017196248	0.45840491	1.374021815	**BORCS8**	protein_coding	BLOC-1 related complex subunit 8 [Source:HGNC Symbol;Acc:HGNC:37247]	chr19	19176903	19192591	-1	ensembl_havana	3.961469727	3.732267272	4.190672182	3.643469038	3.77893109	3.774401688	4.051836127	4.249169756	4.271010662
ENSG00000156802	0.000789	0.017196248	-0.437139013	-1.353916731	**ATAD2**	protein_coding	ATPase family, AAA domain containing 2 [Source:HGNC Symbol;Acc:HGNC:30123]	chr8	123319850	123416350	-1	ensembl_havana	7.783158839	8.001728345	7.564589333	7.874126635	8.036823263	8.094235139	7.439311114	7.617759001	7.636697883
ENSG00000143226	0.000803	0.017196248	-0.44227899	-1.358749016	**FCGR2A**	protein_coding	Fc fragment of IgG receptor IIa [Source:HGNC Symbol;Acc:HGNC:3616]	chr1	161505430	161524013	1	ensembl_havana	5.822737582	6.043877077	5.601598087	5.964871009	6.012311019	6.154449202	5.54825134	5.535943831	5.720599088
ENSG00000121900	0.000784	0.017196248	-0.517726373	-1.431697174	**TMEM54**	protein_coding	transmembrane protein 54 [Source:HGNC Symbol;Acc:HGNC:24143]	chr1	32894594	32901438	-1	ensembl_havana	3.41578138	3.674644567	3.156918194	3.669310227	3.709036025	3.645587448	3.24666556	3.167564252	3.05652477
ENSG00000260086	0.000784	0.017196248	-0.522554358	-1.436496381	**AC007611.1**	lincRNA		chr16	48623436	48744921	1	havana	0.061482708	0.322759887	-0.199794472	0.613726219	0.214850257	0.139703184	-0.00649342	-0.28449416	-0.308395834
ENSG00000142949	0.000804	0.017196248	-0.620840194	-1.537770484	**PTPRF**	protein_coding	protein tyrosine phosphatase, receptor type F [Source:HGNC Symbol;Acc:HGNC:9670]	chr1	43525187	43623666	1	ensembl_havana	5.776677746	6.087097843	5.46625765	6.18883777	5.886782843	6.185672917	5.649398858	5.341526896	5.407847195
ENSG00000263006	0.0008	0.017196248	-0.649444563	-1.568564183	**ROCK1P1**	transcribed_unprocessed_pseudogene	Rho associated coiled-coil containing protein kinase 1 pseudogene 1 [Source:HGNC Symbol;Acc:HGNC:37832]	chr18	109065	122219	1	havana	0.268021814	0.592744095	-0.056700468	0.355307344	0.760296981	0.662627962	-0.12879794	0.063570082	-0.104873545
ENSG00000156509	0.000797	0.017196248	-0.665934847	-1.586596033	**FBXO43**	protein_coding	F-box protein 43 [Source:HGNC Symbol;Acc:HGNC:28521]	chr8	100133360	100145800	-1	ensembl_havana	3.962688862	4.295656286	3.629721438	4.14496615	4.314268171	4.427734536	3.654604579	3.584613649	3.649946087
ENSG00000132026	0.00079	0.017196248	-0.748789691	-1.680382528	**RTBDN**	protein_coding	retbindin [Source:HGNC Symbol;Acc:HGNC:30310]	chr19	12825478	12835428	-1	ensembl_havana	3.031349033	3.405743878	2.656954187	3.441680301	3.491349538	3.284201795	2.874587014	2.55432044	2.541955109
ENSG00000137720	0.000792	0.017196248	-0.877279617	-1.836908311	**C11orf1**	protein_coding	chromosome 11 open reading frame 1 [Source:HGNC Symbol;Acc:HGNC:1163]	chr11	111878935	111885975	1	ensembl_havana	3.453666235	3.892306044	3.015026427	3.931406694	3.900383301	3.845128136	2.787093985	3.155524498	3.102460798
ENSG00000103056	0.000801	0.017196248	-0.908097971	-1.876569824	**SMPD3**	protein_coding	sphingomyelin phosphodiesterase 3 [Source:HGNC Symbol;Acc:HGNC:14240]	chr16	68358325	68448688	-1	ensembl_havana	7.023839376	7.477888362	6.56979039	7.477132475	7.18773644	7.76879617	6.618482319	6.493935796	6.596953057
ENSG00000154059	0.000798	0.017196248	-0.947832396	-1.92897226	**IMPACT**	protein_coding	impact RWD domain protein [Source:HGNC Symbol;Acc:HGNC:20387]	chr18	24426616	24453535	1	ensembl_havana	4.699695473	5.173611671	4.225779275	5.421606647	5.105640774	4.993587592	4.215776639	4.161587111	4.299974075
ENSG00000204110	0.00079	0.017196248	-0.954625386	-1.938076323	**LINC02520**	lincRNA	long intergenic non-protein coding RNA 2520 [Source:HGNC Symbol;Acc:HGNC:53511]	chr6	37507348	37535616	1	havana	3.212739721	3.690052414	2.735427028	4.352378	3.73438904	2.983390203	3.372227541	2.535862351	2.298191191
ENSG00000103485	0.000796	0.017196248	-1.297369984	-2.457804192	**QPRT**	protein_coding	quinolinate phosphoribosyltransferase [Source:HGNC Symbol;Acc:HGNC:9755]	chr16	29679008	29698699	1	ensembl_havana	1.8127279	2.461412892	1.164042908	2.376860232	2.535453458	2.471924986	1.444517675	0.861296647	1.186314404
ENSG00000149403	0.000821	0.017252337	0.841101307	1.79141713	**GRIK4**	protein_coding	glutamate ionotropic receptor kainate type subunit 4 [Source:HGNC Symbol;Acc:HGNC:4582]	chr11	120511700	120988904	1	ensembl_havana	1.853340074	1.43278942	2.273890727	1.43811357	1.798708764	1.061545926	2.187709208	2.479027387	2.154935587
ENSG00000185189	0.000808	0.017252337	0.540610033	1.454587448	**NRBP2**	protein_coding	nuclear receptor binding protein 2 [Source:HGNC Symbol;Acc:HGNC:19339]	chr8	143833594	143840974	-1	ensembl_havana	5.646597191	5.376292174	5.916902207	5.41737705	5.345818033	5.365681439	6.061932222	5.791349196	5.897425203
ENSG00000274211	0.000817	0.017252337	0.478580502	1.393372023	**SOCS7**	protein_coding	suppressor of cytokine signaling 7 [Source:HGNC Symbol;Acc:HGNC:29846]	chr17	38352228	38405593	1	ensembl_havana	5.763427539	5.524137288	6.00271779	5.494890045	5.477022698	5.600499121	6.002478026	6.000996635	6.004678708
ENSG00000163602	0.000815	0.017252337	0.471481245	1.386532319	**RYBP**	protein_coding	RING1 and YY1 binding protein [Source:HGNC Symbol;Acc:HGNC:10480]	chr3	72371825	72446621	-1	ensembl_havana	7.954415037	7.718674415	8.19015566	7.892221943	7.754222001	7.5095793	8.425127336	8.167997088	7.977342555
ENSG00000115738	0.000815	0.017252337	0.44378588	1.360168966	**ID2**	protein_coding	inhibitor of DNA binding 2 [Source:HGNC Symbol;Acc:HGNC:5361]	chr2	8678845	8684453	1	ensembl_havana	5.309372482	5.087479542	5.531265422	5.047643285	5.002983115	5.211812226	5.507773497	5.470228945	5.615793825
ENSG00000123338	0.000818	0.017252337	-0.44641617	-1.362651058	**NCKAP1L**	protein_coding	NCK associated protein 1 like [Source:HGNC Symbol;Acc:HGNC:4862]	chr12	54497711	54548238	1	ensembl_havana	6.621560846	6.844768931	6.39835276	6.758670989	6.907254368	6.868381435	6.321379185	6.493537315	6.380141782
ENSG00000164967	0.000815	0.017252337	-0.447230934	-1.363420834	**RPP25L**	protein_coding	ribonuclease P/MRP subunit p25 like [Source:HGNC Symbol;Acc:HGNC:19909]	chr9	34610486	34612104	-1	ensembl_havana	4.278232362	4.501847829	4.054616896	4.438382881	4.579246746	4.487913861	4.01138913	4.087479328	4.064982229
ENSG00000132563	0.000821	0.017252337	-0.44753828	-1.363711323	**REEP2**	protein_coding	receptor accessory protein 2 [Source:HGNC Symbol;Acc:HGNC:17975]	chr5	138439017	138446969	1	ensembl_havana	3.825608274	4.049377414	3.601839134	4.181339713	4.041129678	3.925662851	3.778870895	3.578614721	3.448031787
ENSG00000117308	0.000814	0.017252337	-0.44757975	-1.363750523	**GALE**	protein_coding	UDP-galactose-4-epimerase [Source:HGNC Symbol;Acc:HGNC:4116]	chr1	23795599	23800804	-1	ensembl_havana	3.886619569	4.110409444	3.662829694	4.016637667	4.161902128	4.152688536	3.613866272	3.69131825	3.68330456
ENSG00000196678	0.000819	0.017252337	-0.450946948	-1.366937185	**ERI2**	protein_coding	ERI1 exoribonuclease family member 2 [Source:HGNC Symbol;Acc:HGNC:30541]	chr16	20780193	20900349	-1	ensembl_havana	4.268517273	4.493990747	4.0430438	4.325543942	4.619264479	4.53716382	3.842642641	4.151519203	4.134969555
ENSG00000126016	0.000822	0.017252337	-0.462580874	-1.378004762	**AMOT**	protein_coding	angiomotin [Source:HGNC Symbol;Acc:HGNC:17810]	chrX	112774503	112840815	-1	ensembl_havana	7.742487223	7.97377766	7.511196786	8.309125919	7.788817515	7.823389546	7.80196046	7.385582177	7.346047723
ENSG00000064042	0.000822	0.017252337	-0.769850908	-1.705093566	**LIMCH1**	protein_coding	LIM and calponin homology domains 1 [Source:HGNC Symbol;Acc:HGNC:29191]	chr4	41359607	41700044	1	ensembl_havana	2.42976964	2.814695094	2.044844186	2.298074107	2.974723635	3.17128754	1.554586035	2.385911525	2.194034997
ENSG00000137841	0.000813	0.017252337	-1.132288045	-2.192061151	**PLCB2**	protein_coding	phospholipase C beta 2 [Source:HGNC Symbol;Acc:HGNC:9055]	chr15	40278176	40307935	-1	ensembl_havana	0.227118232	0.793262254	-0.33902579	1.04068047	1.029710695	0.309395597	0.106248156	-0.47099802	-0.652327512
ENSG00000203506	0.000827	0.017336322	1.030937731	2.043351969	**RBMS3-AS2**	antisense	RBMS3 antisense RNA 2 [Source:HGNC Symbol;Acc:HGNC:39988]	chr3	29526251	29642809	-1	havana	0.409866792	-0.105602073	0.925335658	-0.4596317	-0.218971072	0.361796557	0.873137292	0.779979312	1.12289037
ENSG00000255121	0.000833	0.017438879	0.608464178	1.524635293	**AP003392.4**	lincRNA		chr11	118994824	118998004	-1	havana	3.371395597	3.067163508	3.675627685	2.812698157	2.953290123	3.435502243	3.473788816	3.660411303	3.892682937
ENSG00000174939	0.000836	0.017465243	-0.896538313	-1.861593803	**ASPHD1**	protein_coding	aspartate beta-hydroxylase domain containing 1 [Source:HGNC Symbol;Acc:HGNC:27380]	chr16	29900375	29919864	1	ensembl_havana	0.013604596	0.461873752	-0.434664561	0.383334258	0.614986832	0.387300166	-0.51701327	-0.52165082	-0.265329594
ENSG00000261659	0.000843	0.017469675	1.312683858	2.48403217	**Z92544.2**	processed_transcript		chr16	689001	692554	1	havana	1.022687676	0.366345747	1.679029605	0.174648163	0.030651399	0.89373768	1.729053464	1.541948597	1.766086754
ENSG00000196136	0.000844	0.017469675	1.143478716	2.209130608	**SERPINA3**	protein_coding	serpin family A member 3 [Source:HGNC Symbol;Acc:HGNC:16]	chr14	94612377	94624055	-1	ensembl_havana	-0.809302221	-1.381041579	-0.237562863	-1.73427603	-1.42549258	-0.983356126	-0.63291296	0.063570082	-0.143345712
ENSG00000140854	0.000844	0.017469675	0.515227058	1.429219059	**KATNB1**	protein_coding	katanin regulatory subunit B1 [Source:HGNC Symbol;Acc:HGNC:6217]	chr16	57735730	57757250	1	ensembl_havana	4.616598979	4.35898545	4.874212508	4.390853222	4.325989874	4.360113253	4.915484041	4.925599042	4.78155444
ENSG00000175662	0.000838	0.017469675	0.461776885	1.377237038	**TOM1L2**	protein_coding	target of myb1 like 2 membrane trafficking protein [Source:HGNC Symbol;Acc:HGNC:11984]	chr17	17843511	17972422	-1	ensembl_havana	4.758261448	4.527373006	4.989149891	4.546135504	4.474811168	4.561172345	5.069431626	4.893338276	5.004679769
ENSG00000205903	0.000841	0.017469675	0.454309085	1.370126487	**ZNF316**	protein_coding	zinc finger protein 316 [Source:HGNC Symbol;Acc:HGNC:13843]	chr7	6637322	6656432	1	ensembl_havana	5.596329534	5.369174992	5.823484077	5.508474265	5.271560182	5.327490528	5.968081312	5.771764666	5.730606253
ENSG00000226979	0.00084	0.017469675	-0.469987879	-1.385097831	**LTA**	protein_coding	lymphotoxin alpha [Source:HGNC Symbol;Acc:HGNC:6709]	chr6	31572054	31574324	1	ensembl_havana	10.16354711	10.39854104	9.928553166	10.52997861	10.37603986	10.28960467	10.01537001	9.881031717	9.889257771
ENSG00000072818	0.000842	0.017469675	-0.609723119	-1.525966318	**ACAP1**	protein_coding	ArfGAP with coiled-coil, ankyrin repeat and PH domains 1 [Source:HGNC Symbol;Acc:HGNC:16467]	chr17	7336529	7351478	1	ensembl_havana	6.360053497	6.664915057	6.055191938	6.795314074	6.619546066	6.579885031	6.333492935	5.976112069	5.855970809
ENSG00000118640	0.000846	0.017475021	-0.998522735	-1.997953124	**VAMP8**	protein_coding	vesicle associated membrane protein 8 [Source:HGNC Symbol;Acc:HGNC:12647]	chr2	85561562	85582031	1	ensembl_havana	1.066381426	1.565642793	0.567120059	1.126095262	1.96214833	1.608684788	0.339398945	0.648704825	0.713256405
ENSG00000171105	0.000847	0.017482024	-0.462529625	-1.377955812	**INSR**	protein_coding	insulin receptor [Source:HGNC Symbol;Acc:HGNC:6091]	chr19	7112255	7294034	-1	ensembl_havana	1.104237296	1.335502109	0.872972484	1.206734491	1.464851682	1.334920154	0.785553575	0.938274071	0.895089805
ENSG00000125266	0.000853	0.017517593	0.465008489	1.380325475	**EFNB2**	protein_coding	ephrin B2 [Source:HGNC Symbol;Acc:HGNC:3227]	chr13	106489731	106535662	-1	ensembl_havana	5.44522826	5.212724015	5.677732504	5.677870992	5.081147168	4.879153887	6.191103288	5.480721407	5.361372817
ENSG00000228863	0.000854	0.017517593	-0.474008468	-1.388963289	**AL121985.1**	antisense		chr1	160670778	160699761	1	havana	1.468475666	1.7054799	1.231471431	1.959443807	1.592342839	1.564653053	1.541278047	1.046547334	1.106588913
ENSG00000253276	0.000852	0.017517593	-0.491098592	-1.405514748	**CCDC71L**	protein_coding	coiled-coil domain containing 71 like [Source:HGNC Symbol;Acc:HGNC:26685]	chr7	106656765	106660996	-1	ensembl_havana	3.653720837	3.899270134	3.408171541	4.016637667	3.93359815	3.747574584	3.575212489	3.35471112	3.294591014
ENSG00000108733	0.000854	0.017517593	-0.834712296	-1.783501335	**PEX12**	protein_coding	peroxisomal biogenesis factor 12 [Source:HGNC Symbol;Acc:HGNC:8854]	chr17	35574795	35578863	-1	ensembl_havana	3.432732559	3.850088707	3.015376412	4.012157084	3.884617399	3.653491639	2.921565963	3.187409884	2.937153388
ENSG00000269069	0.000857	0.01754008	0.546872809	1.460915575	**AC007842.1**	processed_pseudogene		chr19	40023384	40025502	-1	havana	1.229348528	0.955912124	1.502784933	0.911909315	0.910210399	1.045616658	1.47283199	1.343981945	1.691540863
ENSG00000156587	0.000857	0.01754008	-0.548476652	-1.462540577	**UBE2L6**	protein_coding	ubiquitin conjugating enzyme E2 L6 [Source:HGNC Symbol;Acc:HGNC:12490]	chr11	57551656	57568284	-1	ensembl_havana	6.992533589	7.266771915	6.718295263	7.260957237	7.293863856	7.245494652	6.681253313	6.656894348	6.816738129
ENSG00000152749	0.00086	0.017561258	-0.676085485	-1.597798505	**GPR180**	protein_coding	G protein-coupled receptor 180 [Source:HGNC Symbol;Acc:HGNC:28899]	chr13	94601903	94634645	1	ensembl_havana	4.140912432	4.478955174	3.802869689	4.435039348	4.546699886	4.455126288	3.584973459	3.895734719	3.92790089
ENSG00000196872	0.000862	0.017574873	-0.484417292	-1.399020683	**KIAA1211L**	protein_coding	KIAA1211 like [Source:HGNC Symbol;Acc:HGNC:33454]	chr2	98793846	98936259	-1	ensembl_havana	3.103086468	3.345295114	2.860877822	3.210920778	3.252533942	3.572430621	2.64502189	2.790632353	3.146979223
ENSG00000213088	0.000866	0.01764489	0.869784708	1.82739018	**ACKR1**	protein_coding	atypical chemokine receptor 1 (Duffy blood group) [Source:HGNC Symbol;Acc:HGNC:4035]	chr1	159203307	159206500	1	ensembl_havana	0.056484659	-0.378407695	0.491377012	-0.14746469	-0.299013651	-0.688744748	0.975631465	0.529376201	-0.030876629
ENSG00000237181	0.00087	0.017700484	-0.474637926	-1.389569435	**AC147651.4**	antisense		chr7	603185	608482	1	havana	3.88464898	4.121967943	3.647330018	4.230383872	4.171215189	3.964304768	3.729854637	3.646140782	3.565994634
ENSG00000171860	0.000876	0.017791146	-0.484825879	-1.399416957	**C3AR1**	protein_coding	complement C3a receptor 1 [Source:HGNC Symbol;Acc:HGNC:1319]	chr12	8058302	8066471	-1	ensembl_havana	1.390048586	1.632461526	1.147635647	1.87313074	1.62524309	1.399010747	1.340798121	1.226127349	0.875981471
ENSG00000228630	0.000878	0.017804182	-0.958375405	-1.943120546	**HOTAIR**	antisense	HOX transcript antisense RNA [Source:HGNC Symbol;Acc:HGNC:33510]	chr12	53962308	53974956	-1	havana	2.24633396	2.725521662	1.767146257	2.931352026	2.552658819	2.692554143	1.705384535	1.861445274	1.734608964
ENSG00000112195	0.00089	0.017815065	0.959720086	1.9449325	**TREML2**	protein_coding	triggering receptor expressed on myeloid cells like 2 [Source:HGNC Symbol;Acc:HGNC:21092]	chr6	41190277	41201194	-1	ensembl_havana	2.947927302	2.468067258	3.427787345	2.092729261	2.529672436	2.781800078	3.287090137	3.54824011	3.448031787
ENSG00000091428	0.000884	0.017815065	0.687848248	1.610879131	**RAPGEF4**	protein_coding	Rap guanine nucleotide exchange factor 4 [Source:HGNC Symbol;Acc:HGNC:16626]	chr2	172735274	173052893	1	ensembl_havana	0.966023924	0.622099799	1.309948048	-0.06941355	0.874167025	1.061545926	0.785553575	1.566404757	1.577885811
ENSG00000137266	0.000889	0.017815065	0.560750066	1.475035898	**SLC22A23**	protein_coding	solute carrier family 22 member 23 [Source:HGNC Symbol;Acc:HGNC:21106]	chr6	3268962	3457022	-1	ensembl_havana	3.632793823	3.35241879	3.913168856	3.159173914	3.300851749	3.597230708	3.735708444	3.968651039	4.035147085
ENSG00000144724	0.000884	0.017815065	0.513078416	1.427092071	**PTPRG**	protein_coding	protein tyrosine phosphatase, receptor type G [Source:HGNC Symbol;Acc:HGNC:9671]	chr3	61561569	62297613	1	havana	6.257763808	6.0012246	6.514303016	5.86777506	5.955231125	6.180667615	6.462200577	6.485945185	6.594763285
ENSG00000234420	0.000882	0.017815065	0.478913307	1.393693488	**ZNF37BP**	transcribed_processed_pseudogene	zinc finger protein 37B, pseudogene [Source:HGNC Symbol;Acc:HGNC:13103]	chr10	42513510	42552822	-1	havana	6.243225502	6.003768848	6.482682156	5.958480907	5.946036984	6.106788653	6.426001808	6.5010897	6.520954959
ENSG00000106477	0.000888	0.017815065	-0.446708502	-1.362927198	**CEP41**	protein_coding	centrosomal protein 41 [Source:HGNC Symbol;Acc:HGNC:12370]	chr7	130393771	130442433	-1	ensembl_havana	3.343521598	3.566875849	3.120167347	3.397700228	3.523701123	3.779226197	2.90607567	3.072584571	3.381841801
ENSG00000140006	0.000892	0.017815065	-0.507871691	-1.421950941	**WDR89**	protein_coding	WD repeat domain 89 [Source:HGNC Symbol;Acc:HGNC:20489]	chr14	63597039	63641861	-1	ensembl_havana	4.272091144	4.526026989	4.018155298	4.453333619	4.589008315	4.535739035	3.926925567	4.002652514	4.124887815
ENSG00000120162	0.000892	0.017815065	-0.554766677	-1.468931048	**MOB3B**	protein_coding	MOB kinase activator 3B [Source:HGNC Symbol;Acc:HGNC:23825]	chr9	27325209	27529781	-1	ensembl_havana	4.886815755	5.164199093	4.609432416	5.201180564	5.033942152	5.257474564	4.686826487	4.566564299	4.574906464
ENSG00000198042	0.000889	0.017815065	-0.599618176	-1.51531547	**MAK16**	protein_coding	MAK16 homolog [Source:HGNC Symbol;Acc:HGNC:13703]	chr8	33484750	33501260	1	ensembl_havana	5.044255264	5.344064352	4.744446176	5.315599317	5.363800282	5.352793458	4.56224422	4.834141723	4.836952585
ENSG00000064300	0.000888	0.017815065	-0.605016175	-1.520995807	**NGFR**	protein_coding	nerve growth factor receptor [Source:HGNC Symbol;Acc:HGNC:7809]	chr17	49495293	49515017	1	ensembl_havana	5.311758455	5.614266542	5.009250367	5.674330594	5.796386522	5.372082511	5.146158264	5.034792482	4.846800355
ENSG00000169884	0.000884	0.017815065	-0.672674708	-1.594025499	**WNT10B**	protein_coding	Wnt family member 10B [Source:HGNC Symbol;Acc:HGNC:12775]	chr12	48965340	48971763	-1	ensembl_havana	1.86726865	2.203606004	1.530931296	2.142776609	2.101167058	2.366874344	1.656848881	1.358059196	1.577885811
ENSG00000064961	0.000895	0.017823338	0.434730105	1.351657945	**HMG20B**	protein_coding	high mobility group 20B [Source:HGNC Symbol;Acc:HGNC:5002]	chr19	3572777	3579088	1	ensembl_havana	4.281672487	4.064307435	4.499037539	4.191282832	3.9894189	4.012220572	4.667050915	4.408145822	4.421915881
ENSG00000179213	0.000894	0.017823338	-0.600875731	-1.516636901	**SIGLECL1**	protein_coding	SIGLEC family like 1 [Source:HGNC Symbol;Acc:HGNC:26856]	chr19	51246348	51269330	1	ensembl_havana	4.355717752	4.656155617	4.055279887	4.934988992	4.43361281	4.599865049	4.374276086	3.682954534	4.10860904
ENSG00000109854	0.000896	0.017827681	-0.471697943	-1.386740597	**HTATIP2**	protein_coding	HIV-1 Tat interactive protein 2 [Source:HGNC Symbol;Acc:HGNC:16637]	chr11	20363685	20383783	1	ensembl_havana	5.776741318	6.012590289	5.540892346	6.067765535	6.07914118	5.890864153	5.52139269	5.625227347	5.476057001
ENSG00000266903	0.000898	0.017827681	-0.508024502	-1.422101562	**AC243964.2**	antisense		chr19	44632199	44718759	-1	havana	1.706129401	1.960141652	1.452117151	1.565895203	1.909699171	2.404830583	0.995284613	1.504469011	1.856597828
ENSG00000231681	0.000901	0.01786169	0.481864479	1.39654734	**AC020743.2**	lincRNA		chr7	50202001	50262994	-1	havana	3.142619931	2.901687692	3.383552171	2.694632273	2.868808811	3.141621992	3.23844292	3.267903019	3.644310574
ENSG00000156052	0.000904	0.017891687	-0.516113948	-1.430097935	**GNAQ**	protein_coding	G protein subunit alpha q [Source:HGNC Symbol;Acc:HGNC:4390]	chr9	77716087	78031458	-1	ensembl_havana	7.381375948	7.639432922	7.123318974	7.950609741	7.560161018	7.407528006	7.344570254	7.035352456	6.990034212
ENSG00000147138	0.000905	0.017891687	-1.886438975	-3.697215085	**GPR174**	protein_coding	G protein-coupled receptor 174 [Source:HGNC Symbol;Acc:HGNC:30245]	chrX	79144663	79175315	1	ensembl_havana	-0.184627674	0.758591813	-1.127847161	1.268150092	0.570631912	0.436993436	0.032074365	-1.52242631	-1.89318954
ENSG00000273729	0.00091	0.017927768	0.544432986	1.458447027	**AC007686.3**	antisense		chr14	77028086	77031572	1	havana	0.802064109	0.529847615	1.074280602	0.206373087	0.699804047	0.683365711	0.873137292	1.210694826	1.139009687
ENSG00000109265	0.000912	0.017927768	0.453282327	1.369151722	**KIAA1211**	protein_coding	KIAA1211 [Source:HGNC Symbol;Acc:HGNC:29219]	chr4	56049073	56328625	1	ensembl_havana	2.233552666	2.006911502	2.460193829	1.727676152	1.900769223	2.392289132	2.213108088	2.385911525	2.781561875
ENSG00000205611	0.000913	0.017927768	-0.483342758	-1.397979067	**LINC01597**	lincRNA	long intergenic non-protein coding RNA 1597 [Source:HGNC Symbol;Acc:HGNC:51594]	chr20	30278906	30288479	-1	havana	1.739401777	1.981073156	1.497730398	1.95010455	1.900769223	2.092345696	1.386165364	1.426455924	1.680569906
ENSG00000184898	0.000912	0.017927768	-0.83237236	-1.780610983	**RBM43**	protein_coding	RNA binding motif protein 43 [Source:HGNC Symbol;Acc:HGNC:24790]	chr2	151247940	151261879	-1	ensembl_havana	3.140384249	3.55657043	2.724198069	3.652134298	3.563853664	3.453723327	2.581431252	2.7591524	2.832010557
ENSG00000171483	0.00091	0.017927768	-1.319252246	-2.495367405	**SSX6**	transcribed_unprocessed_pseudogene	SSX family member 6, pseudogene [Source:HGNC Symbol;Acc:HGNC:19652]	chrX	48107992	48120210	1	havana	-0.67995876	-0.020332637	-1.339584883	-0.99620085	0.403680778	0.531522158	-2.42392269	-1.24204464	-0.352787316
ENSG00000168765	0.000914	0.017936818	-0.485820769	-1.400382336	**GSTM4**	protein_coding	glutathione S-transferase mu 4 [Source:HGNC Symbol;Acc:HGNC:4636]	chr1	109656081	109674836	1	ensembl_havana	6.486638181	6.729548565	6.243727796	6.702073778	6.642159529	6.844412388	6.304247216	6.12965545	6.297280722
ENSG00000163349	0.000916	0.017950059	0.440630006	1.357196868	**HIPK1**	protein_coding	homeodomain interacting protein kinase 1 [Source:HGNC Symbol;Acc:HGNC:19006]	chr1	113929192	113977869	1	ensembl_havana	7.429226496	7.208911494	7.649541499	7.429203332	7.210499329	6.98703182	7.921537394	7.62632644	7.400760664
ENSG00000204946	0.000919	0.017974101	-0.432690543	-1.349748434	**ZNF783**	protein_coding	zinc finger family member 783 [Source:HGNC Symbol;Acc:HGNC:27222]	chr7	149262171	149297302	1	ensembl_havana	4.684265176	4.900610447	4.467919904	5.123340803	4.703837799	4.874652739	4.732917724	4.247280137	4.42356185
ENSG00000233452	0.000922	0.018004939	0.516023276	1.430008058	**STXBP5-AS1**	antisense	STXBP5 antisense RNA 1 [Source:HGNC Symbol;Acc:HGNC:44183]	chr6	146841901	147204614	-1	havana	3.558436734	3.300425095	3.816448372	3.207005484	3.324417061	3.369852742	3.691214731	3.76448673	3.993643654
ENSG00000180104	0.000924	0.018004939	0.426727628	1.344181197	**EXOC3**	protein_coding	exocyst complex component 3 [Source:HGNC Symbol;Acc:HGNC:30378]	chr5	443158	471937	1	ensembl_havana	6.55735535	6.343991536	6.770719164	6.394330365	6.330081587	6.307562656	6.846244555	6.7645215	6.701391436
ENSG00000068784	0.000925	0.018004939	-0.459106097	-1.374689787	**SRBD1**	protein_coding	S1 RNA binding domain 1 [Source:HGNC Symbol;Acc:HGNC:25521]	chr2	45388680	45612165	-1	ensembl_havana	5.809678746	6.039231794	5.580125698	5.934410745	6.046113038	6.1371716	5.456411402	5.53749097	5.746474722
ENSG00000018408	0.000925	0.018004939	-0.564397328	-1.478769639	**WWTR1**	protein_coding	WW domain containing transcription regulator 1 [Source:HGNC Symbol;Acc:HGNC:24042]	chr3	149517235	149736714	-1	ensembl_havana	4.62258333	4.904781994	4.340384666	5.253367589	4.918129458	4.542848935	4.612421026	4.291961583	4.116771388
ENSG00000175305	0.000931	0.018075938	-0.420125512	-1.338043958	**CCNE2**	protein_coding	cyclin E2 [Source:HGNC Symbol;Acc:HGNC:1590]	chr8	94879770	94896678	-1	ensembl_havana	4.711262024	4.921324781	4.501199268	4.715547829	5.106611921	4.941814591	4.309028837	4.663269859	4.531299108
ENSG00000163492	0.000931	0.018075938	-0.504678896	-1.418807534	**CCDC141**	protein_coding	coiled-coil domain containing 141 [Source:HGNC Symbol;Acc:HGNC:26821]	chr2	178829757	179050086	-1	havana	-0.066406963	0.185932485	-0.318746411	-0.06941355	0.214850257	0.41236075	-0.57379957	-0.19956768	-0.182871985
ENSG00000224789	0.000937	0.018160071	0.703938286	1.628945444	**AC012363.1**	antisense		chr2	120174885	120216544	-1	havana	2.287248113	1.93527897	2.639217257	2.040883175	1.836662271	1.928291464	2.681872935	2.743151031	2.492627803
ENSG00000139433	0.000939	0.018180279	0.514846791	1.428842393	**GLTP**	protein_coding	glycolipid transfer protein [Source:HGNC Symbol;Acc:HGNC:24867]	chr12	109850943	109880488	-1	ensembl_havana	4.893567479	4.636144084	5.150990874	4.587380999	4.728019942	4.59303131	5.146158264	5.132209824	5.174604535
ENSG00000184226	0.000945	0.0181844	0.767026601	1.701758836	**PCDH9**	protein_coding	protocadherin 9 [Source:HGNC Symbol;Acc:HGNC:8661]	chr13	66302834	67230445	-1	ensembl_havana	6.744223893	6.360710593	7.127737194	6.306947603	6.337959391	6.437224785	6.853329157	7.143186702	7.386695723
ENSG00000084676	0.000943	0.0181844	0.486227091	1.400776796	**NCOA1**	protein_coding	nuclear receptor coactivator 1 [Source:HGNC Symbol;Acc:HGNC:7668]	chr2	24491914	24770702	1	ensembl_havana	7.162052935	6.91893939	7.405166481	6.882278742	6.94439529	6.930146638	7.460233301	7.398410046	7.356856094
ENSG00000132623	0.000941	0.0181844	-0.495786163	-1.410088941	**ANKEF1**	protein_coding	ankyrin repeat and EF-hand domain containing 1 [Source:HGNC Symbol;Acc:HGNC:15803]	chr20	9986126	10058303	1	ensembl_havana	6.070445634	6.318338716	5.822552553	6.391314407	6.317974017	6.245727724	5.80016806	5.84543707	5.822052529
ENSG00000023697	0.000946	0.0181844	-0.530841691	-1.444771852	**DERA**	protein_coding	deoxyribose-phosphate aldolase [Source:HGNC Symbol;Acc:HGNC:24269]	chr12	15911172	16037282	1	ensembl_havana	4.80996356	5.075384405	4.544542714	4.845896173	5.152474544	5.227782498	4.431299106	4.532832903	4.669496133
ENSG00000163626	0.000946	0.0181844	-0.606227383	-1.522273287	**COX18**	protein_coding	COX18, cytochrome c oxidase assembly factor [Source:HGNC Symbol;Acc:HGNC:26801]	chr4	73052362	73069755	-1	ensembl_havana	3.857298259	4.160411951	3.554184568	4.187313804	4.143093687	4.150828362	3.423901694	3.649006201	3.589645809

ENSG00000213949	0.000948	0.018195135	0.884503462	1.846129123	**ITGA1**	protein_coding	integrin subunit alpha 1 [Source:HGNC Symbol;Acc:HGNC:6134]	chr5	52787896	52959210	1	ensembl_havana	0.339113719	-0.103138012	0.78136545	-1.31854494	0.477608744	0.531522158	-0.17198403	1.32976598	1.186314404
ENSG00000064601	0.00095	0.018213038	0.505704418	1.419816434	**CTSA**	protein_coding	cathepsin A [Source:HGNC Symbol;Acc:HGNC:9251]	chr20	45890144	45898820	1	ensembl_havana	6.636755646	6.383903437	6.889607855	6.25290387	6.479627244	6.419179198	6.819276926	6.879084165	6.970462475
ENSG00000134516	0.000953	0.018246455	-0.460001496	-1.375543245	**DOCK2**	protein_coding	dedicator of cytokinesis 2 [Source:HGNC Symbol;Acc:HGNC:2988]	chr5	169637247	170083382	1	ensembl_havana	8.116940081	8.346940829	7.886939333	8.366745275	8.269675323	8.404401888	7.837610273	7.867600564	7.955607161
ENSG00000116717	0.000955	0.018267545	0.446623471	1.362846871	**GADD45A**	protein_coding	growth arrest and DNA damage inducible alpha [Source:HGNC Symbol;Acc:HGNC:4095]	chr1	67685061	67688338	1	ensembl_havana	2.296638178	2.073326442	2.519949913	1.912130943	2.162224075	2.145624309	2.386842807	2.643226439	2.529780494
ENSG00000178028	0.000962	0.018365207	0.565630483	1.480034169	**DMAP1**	protein_coding	DNA methyltransferase 1 associated protein 1 [Source:HGNC Symbol;Acc:HGNC:18291]	chr1	44213455	44220681	1	ensembl_havana	6.58817365	6.305358408	6.870988891	6.245765281	6.353178019	6.317131925	6.879673424	6.776690704	6.956602544
ENSG00000186166	0.000964	0.018379995	0.423047637	1.340756862	**CCDC84**	protein_coding	coiled-coil domain containing 84 [Source:HGNC Symbol;Acc:HGNC:30460]	chr11	118998142	119015791	1	ensembl_havana	5.333551481	5.122027662	5.545075299	5.076899593	5.159042677	5.130140715	5.488834672	5.55821656	5.588174665
ENSG00000168404	0.000965	0.018379995	-0.53461162	-1.448552146	**MLKL**	protein_coding	mixed lineage kinase domain like pseudokinase [Source:HGNC Symbol;Acc:HGNC:26617]	chr16	74671855	74700960	-1	ensembl_havana	3.979582589	4.246888399	3.712276779	4.054171047	4.342571664	4.343922486	3.477287033	3.727010979	3.932532325
ENSG00000147654	0.000968	0.018411893	-0.597490918	-1.513082779	**EBAG9**	protein_coding	estrogen receptor binding site associated, antigen, 9 [Source:HGNC Symbol;Acc:HGNC:3123]	chr8	109539711	109565996	1	ensembl_havana	3.028608657	3.327354116	2.729863198	3.320074956	3.350885786	3.311101606	2.607204823	2.716081261	2.866303509
ENSG00000083097	0.000969	0.018414606	0.430297204	1.347511144	**DOPEY1**	protein_coding	dopey family member 1 [Source:HGNC Symbol;Acc:HGNC:21194]	chr6	83067666	83171350	1	ensembl_havana	5.550170068	5.335021466	5.76531867	5.340831717	5.336742153	5.327490528	5.728587894	5.771764666	5.795603451
ENSG00000010379	0.000974	0.018459315	0.920843333	1.893221661	**SLC6A13**	protein_coding	solute carrier family 6 member 13 [Source:HGNC Symbol;Acc:HGNC:11046]	chr12	220621	262873	-1	ensembl_havana	2.868343265	2.407921598	3.328764932	2.084216604	2.580886153	2.558662038	3.287090137	3.264170155	3.435034503
ENSG00000169194	0.000986	0.018459315	0.918316574	1.889908743	**IL13**	protein_coding	interleukin 13 [Source:HGNC Symbol;Acc:HGNC:5973]	chr5	132656263	132661110	1	ensembl_havana	4.890741495	4.431583208	5.349899782	4.088626288	4.529773752	4.678149585	5.277363718	5.429980014	5.342355613
ENSG00000251615	0.00099	0.018459315	0.598300892	1.51393251	**AC104825.1**	lincRNA		chr4	8355090	8358338	-1	havana	2.149073365	1.849922919	2.448223811	1.694503432	1.96214833	1.893116995	2.294679948	2.41986126	2.630130226
ENSG00000273448	0.000976	0.018459315	0.58467112	1.499697076	**AC006480.2**	lincRNA		chr7	67333047	67334383	1	havana	-0.066852495	-0.359188055	0.225483064	-0.31750517	-0.672709	-0.087349994	0.210814386	0.063570082	0.402064725
ENSG00000170456	0.000988	0.018459315	0.508052249	1.422128914	**DENND5B**	protein_coding	DENN domain containing 5B [Source:HGNC Symbol;Acc:HGNC:28338]	chr12	31382223	31591097	-1	ensembl_havana	3.855216445	3.60119032	4.109242569	3.268409792	3.535287761	3.999873408	3.744444859	4.151519203	4.431763645
ENSG00000171488	0.000987	0.018459315	0.507712687	1.421794232	**LRRC8C**	protein_coding	leucine rich repeat containing 8 VRAC subunit C [Source:HGNC Symbol;Acc:HGNC:25075]	chr1	89633072	89769903	1	ensembl_havana	7.061127857	6.807271514	7.314984201	6.827228579	6.871993085	6.722592877	7.402755971	7.269818327	7.272378304
ENSG00000185621	0.000981	0.018459315	0.50052095	1.41472432	**LMLN**	protein_coding	leishmanolysin like peptidase [Source:HGNC Symbol;Acc:HGNC:15991]	chr3	197960200	198043720	1	ensembl_havana	4.046436723	3.796176248	4.296697199	3.646363245	3.756831958	3.985333543	4.181874628	4.326378436	4.381838531
ENSG00000138798	0.000991	0.018459315	0.481375071	1.396073667	**EGF**	protein_coding	epidermal growth factor [Source:HGNC Symbol;Acc:HGNC:3229]	chr4	109912884	110012266	1	ensembl_havana	0.756765777	0.516078242	0.997453313	0.326725152	0.760296981	0.461212593	0.716189844	1.271458479	1.004711615
ENSG00000006432	0.000982	0.018459315	0.431108513	1.348269139	**MAP3K9**	protein_coding	mitogen-activated protein kinase kinase kinase 9 [Source:HGNC Symbol;Acc:HGNC:6861]	chr14	70722526	70809534	-1	ensembl_havana	4.975939972	4.760385715	5.191494228	4.708641512	4.78736503	4.785150603	5.184114441	5.214786604	5.175581638
ENSG00000179406	0.000974	0.018459315	-0.420813163	-1.338681879	**LINC00174**	processed_transcript	long intergenic non-protein coding RNA 174 [Source:HGNC Symbol;Acc:HGNC:27788]	chr7	66376044	66493566	-1	havana	4.461553433	4.671960015	4.251146852	4.608312156	4.590397449	4.81717044	4.166788249	4.203121467	4.383530839
ENSG00000038532	0.000983	0.018459315	-0.523077327	-1.437017197	**CLEC16A**	protein_coding	C-type lectin domain containing 16A [Source:HGNC Symbol;Acc:HGNC:29013]	chr16	10944488	11182189	1	ensembl_havana	7.267745174	7.529283838	7.006206511	7.473470283	7.349684246	7.764696985	7.00614277	6.890630685	7.121846078
ENSG00000135540	0.000991	0.018459315	-0.540088562	-1.454061775	**NHSL1**	protein_coding	NHS like 1 [Source:HGNC Symbol;Acc:HGNC:21021]	chr6	138422043	138692571	-1	ensembl_havana	0.573451892	0.843496173	0.303407611	0.727424294	0.874167025	0.928897202	0.276538753	0.37179175	0.261892331
ENSG00000226990	0.000987	0.018459315	-0.543032541	-1.457031978	**AC025946.1**	lincRNA		chr10	8400860	8461863	1	havana	3.324136344	3.595652614	3.052620073	3.781505278	3.458255814	3.54719675	3.118116049	2.929086265	3.110657906
ENSG00000067182	0.000991	0.018459315	-0.791561892	-1.730947409	**TNFRSF1A**	protein_coding	TNF receptor superfamily member 1A [Source:HGNC Symbol;Acc:HGNC:11916]	chr12	6328757	6342114	-1	ensembl_havana	3.128049667	3.523830613	2.732268721	3.663607608	3.583518497	3.324365734	2.89041725	2.566495926	2.739892986
ENSG00000180353	0.00098	0.018459315	-1.250859013	-2.379830813	**HCLS1**	protein_coding	hematopoietic cell-specific Lyn substrate 1 [Source:HGNC Symbol;Acc:HGNC:4844]	chr3	121631399	121660927	-1	ensembl_havana	0.523591587	1.149021093	-0.10183792	1.464591378	0.874167025	1.108304877	0.032074365	-0.62862566	0.291037539
ENSG00000164418	0.000998	0.018568919	0.528714655	1.442643324	**GRIK2**	protein_coding	glutamate ionotropic receptor kainate type subunit 2 [Source:HGNC Symbol;Acc:HGNC:4580]	chr6	101398788	102070083	1	ensembl_havana	1.593445883	1.329088555	1.85780321	1.022973463	1.603393164	1.360899039	1.669136731	2.037960042	1.866312856
ENSG00000146094	0.001001	0.01859186	0.533702386	1.447639507	**DOK3**	protein_coding	docking protein 3 [Source:HGNC Symbol;Acc:HGNC:24583]	chr5	177501907	177511274	-1	ensembl_havana	3.143137514	2.876286321	3.409988707	2.677756939	2.882481267	3.068620759	3.2548416	3.498270024	3.476854499
ENSG00000128512	0.001007	0.018651701	0.703699438	1.628675782	**DOCK4**	protein_coding	dedicator of cytokinesis 4 [Source:HGNC Symbol;Acc:HGNC:19192]	chr7	111726110	112206411	-1	ensembl_havana	1.730081086	1.378231367	2.081930805	0.872903038	1.719668303	1.542122761	1.729053464	2.218547759	2.298191191
ENSG00000106789	0.001006	0.018651701	-0.919956941	-1.892058822	**CORO2A**	protein_coding	coronin 2A [Source:HGNC Symbol;Acc:HGNC:2255]	chr9	98120975	98192640	-1	ensembl_havana	0.713583471	1.173561942	0.253605	0.464289132	1.415548178	1.640848515	-0.17198403	0.45273451	0.480064523
ENSG00000157379	0.001008	0.018651846	0.454782779	1.370576427	**DHRS1**	protein_coding	dehydrogenase/reductase 1 [Source:HGNC Symbol;Acc:HGNC:16445]	chr14	24290598	24299833	-1	ensembl_havana	4.719817237	4.492425847	4.947208626	4.49406847	4.525071112	4.458137959	5.015113495	4.916115275	4.910397109
ENSG00000268049	0.001012	0.018697377	0.603679519	1.519587257	**AC012313.2**	antisense		chr19	58357999	58359603	1	havana	2.174398349	1.872558589	2.476238108	1.892762624	1.779350901	1.945562243	2.541886448	2.498222232	2.388605644
ENSG00000255847	0.001015	0.01873852	0.578347006	1.493137478	**AP003717.1**	antisense		chr11	73963657	73970287	-1	ensembl_havana	1.064874669	0.775701166	1.354048171	0.749126884	0.874167025	0.703809589	1.386165364	1.300906777	1.375072374
ENSG00000133069	0.00102	0.018805241	0.417462674	1.335576556	**TMCC2**	protein_coding	transmembrane and coiled-coil domain family 2 [Source:HGNC Symbol;Acc:HGNC:24239]	chr1	205228176	205273343	1	ensembl_havana	4.340577947	4.13184661	4.549309284	4.366487997	4.16563457	3.863417264	4.797308319	4.605437797	4.245181736
ENSG00000136960	0.001041	0.018867843	1.009979499	2.013882483	**ENPP2**	protein_coding	ectonucleotide pyrophosphatase/phosphodiesterase 2 [Source:HGNC Symbol;Acc:HGNC:3357]	chr8	119557086	119673453	-1	ensembl_havana	2.305706413	1.800716663	2.810696163	1.142587728	2.331270798	1.928291464	2.473469945	3.068309725	2.890308818
ENSG00000144589	0.001033	0.018867843	0.593316931	1.508711474	**STK11IP**	protein_coding	serine/threonine kinase 11 interacting protein [Source:HGNC Symbol;Acc:HGNC:19184]	chr2	219597860	219616451	1	ensembl_havana	4.659085686	4.36242722	4.955744152	4.380461329	4.422736894	4.284083438	5.051942913	4.854145008	4.961144534
ENSG00000118855	0.001039	0.018867843	0.483190068	1.397831118	**MFSD1**	protein_coding	major facilitator superfamily domain containing 1 [Source:HGNC Symbol;Acc:HGNC:25874]	chr3	158732198	158829719	1	ensembl_havana	5.898039713	5.656444679	6.139634747	5.642069861	5.71657922	5.610684956	6.046119518	6.187954396	6.184830327
ENSG00000180354	0.001027	0.018867843	0.478254286	1.393056996	**MTURN**	protein_coding	maturin, neural progenitor differentiation regulator homolog [Source:HGNC Symbol;Acc:HGNC:25457]	chr7	30134810	30162762	1	ensembl_havana	4.938584448	4.699457305	5.177711591	4.487627691	4.826793299	4.783950926	4.998167601	5.211882794	5.323084377
ENSG00000138434	0.001035	0.018867843	0.447146844	1.363341367	**SSFA2**	protein_coding	sperm specific antigen 2 [Source:HGNC Symbol;Acc:HGNC:11319]	chr2	181891833	181930738	1	ensembl_havana	7.449365074	7.225791652	7.672938496	7.108481374	7.288524931	7.280368651	7.626202205	7.707940356	7.684672927
ENSG00000175061	0.001043	0.018867843	0.427054081	1.344485392	**LRRC75A-AS1**	processed_transcript	LRRC75A antisense RNA 1 [Source:HGNC Symbol;Acc:HGNC:28619]	chr17	16438822	16478678	1	havana	8.13910739	7.92558035	8.35263443	7.71350722	8.072176702	7.991057127	8.152276495	8.449536785	8.456090011
ENSG00000228727	0.001028	0.018867843	-0.407817913	-1.326677686	**SAPCD1**	protein_coding	suppressor APC domain containing 1 [Source:HGNC Symbol;Acc:HGNC:13938]	chr6	31762799	31764851	1	ensembl_havana	0.500570319	0.704479275	0.296661362	0.464289132	0.720251491	0.928897202	0.069637898	0.315191528	0.505154661
ENSG00000116497	0.001039	0.018867843	-0.422137549	-1.339911345	**S100PBP**	protein_coding	S100P binding protein [Source:HGNC Symbol;Acc:HGNC:25768]	chr1	32816767	32858879	1	ensembl_havana	5.684600942	5.895669716	5.473532168	5.77627577	5.944951458	5.965781921	5.314017288	5.524287101	5.582292114
ENSG00000107201	0.001042	0.018867843	-0.472593541	-1.387601727	**DDX58**	protein_coding	DExD/H-box helicase 58 [Source:HGNC Symbol;Acc:HGNC:19102]	chr9	32455705	32526324	-1	ensembl_havana	5.267897271	5.504194041	5.031600501	5.702413451	5.54235877	5.267809903	5.215871954	4.998181916	4.880747632
ENSG00000235821	0.001041	0.018867843	-0.539174292	-1.453140593	**IFITM4P**	processed_pseudogene	interferon induced transmembrane protein 4 pseudogene [Source:HGNC Symbol;Acc:HGNC:21669]	chr6	29750729	29751148	-1	havana	0.724555057	0.994142204	0.454967911	1.075455542	1.013233389	0.89373768	0.513047461	0.37179175	0.480064523
ENSG00000137473	0.001043	0.018867843	-0.553866184	-1.468014465	**TTC29**	protein_coding	tetratricopeptide repeat domain 29 [Source:HGNC Symbol;Acc:HGNC:29936]	chr4	146706638	146945882	-1	ensembl_havana	0.699673934	0.976607026	0.422740842	1.126095262	0.89230127	0.911424546	0.692307165	0.343769191	0.232146171
ENSG00000102302	0.001031	0.018867843	-0.600452832	-1.516192393	**FGD1**	protein_coding	FYVE, RhoGEF and PH domain containing 1 [Source:HGNC Symbol;Acc:HGNC:3663]	chrX	54445454	54496166	-1	ensembl_havana	2.827846728	3.128073144	2.527620312	3.167256782	3.049350114	3.167612535	2.657410455	2.523424442	2.402026039
ENSG00000146216	0.001042	0.018867843	-0.921175945	-1.893658191	**TTBK1**	protein_coding	tau tubulin kinase 1 [Source:HGNC Symbol;Acc:HGNC:19140]	chr6	43243680	43288259	1	ensembl_havana	2.053699377	2.51428735	1.593111405	2.878177262	2.446207271	2.218477517	2.015549972	1.759072628	1.004711615
ENSG00000168209	0.001043	0.018867843	-1.01321194	-2.01839976	**DDIT4**	protein_coding	DNA damage inducible transcript 4 [Source:HGNC Symbol;Acc:HGNC:24944]	chr10	72273920	72276036	1	ensembl_havana	2.6514128	3.15801877	2.14480683	3.218719635	2.991644088	3.263692587	2.50808372	1.97541275	1.950924019
ENSG00000204671	0.001025	0.018867843	-1.456514683	-2.744445486	**IL31**	protein_coding	interleukin 31 [Source:HGNC Symbol;Acc:HGNC:19372]	chr12	122172030	122174199	-1	ensembl_havana	2.960271998	3.68852934	2.232014657	3.218719635	3.810266049	4.036602336	2.278730383	2.155463891	2.261849697
ENSG00000144161	0.001048	0.018911231	-0.42678892	-1.344238305	**ZC3H8**	protein_coding	zinc finger CCCH-type containing 8 [Source:HGNC Symbol;Acc:HGNC:30941]	chr2	112211525	112255136	-1	ensembl_havana	4.252732006	4.466126466	4.039337546	4.31466899	4.458168414	4.625541994	3.939661103	4.011585847	4.16676569
ENSG00000111961	0.001047	0.018911231	-1.320312649	-2.497202213	**SASH1**	protein_coding	SAM and SH3 domain containing 1 [Source:HGNC Symbol;Acc:HGNC:19182]	chr6	148272304	148552050	1	ensembl_havana	-1.193946217	-0.533789892	-1.854102542	0.10902545	-0.727039001	-0.983356126	-1.04978262	-1.74507732	-2.767447692
ENSG00000119711	0.00105	0.01892276	0.407191212	1.326101508	**ALDH6A1**	protein_coding	aldehyde dehydrogenase 6 family member A1 [Source:HGNC Symbol;Acc:HGNC:7179]	chr14	74056850	74084493	-1	ensembl_havana	5.219508869	5.015913263	5.423104476	4.997524442	4.995662733	5.054552614	5.408659816	5.419110534	5.441543077
ENSG00000109927	0.001052	0.018929028	0.493213777	1.407576933	**TECTA**	protein_coding	tectorin alpha [Source:HGNC Symbol;Acc:HGNC:11720]	chr11	121101173	121191493	1	ensembl_havana	2.447429687	2.200822799	2.694036575	2.092729261	2.162224075	2.34751506	2.501227084	2.753838306	2.827044337
ENSG00000168038	0.001058	0.0189375	0.438625992	1.355312926	**ULK4**	protein_coding	unc-51 like kinase 4 [Source:HGNC Symbol;Acc:HGNC:15784]	chr3	41246599	41962430	-1	ensembl_havana	2.751596945	2.532283949	2.970909941	2.528879384	2.470551233	2.59742123	2.911257603	2.966320977	3.035151242
ENSG00000169508	0.001054	0.0189375	-0.78889366	-1.727749019	**GPR183**	protein_coding	G protein-coupled receptor 183 [Source:HGNC Symbol;Acc:HGNC:3128]	chr13	99294530	99307405	-1	ensembl_havana	4.372035606	4.766482436	3.977588776	5.549249306	4.304144633	4.446053367	4.513670186	3.584613649	3.834482493
ENSG00000039068	0.001056	0.0189375	-1.117662012	-2.169950318	**CDH1**	protein_coding	cadherin 1 [Source:HGNC Symbol;Acc:HGNC:1748]	chr16	68737225	68835548	1	ensembl_havana	2.768409347	3.327240353	2.209578341	3.464818021	3.405569423	3.111333615	1.986385747	2.336998362	2.305350914
ENSG00000165682	0.001056	0.0189375	-3.817826472	-14.10198624	**CLEC1B**	protein_coding	C-type lectin domain family 1 member B [Source:HGNC Symbol;Acc:HGNC:24356]	chr12	9985642	10013424	-1	ensembl_havana	-0.933409203	0.975504033	-2.842322439	1.528734855	1.228369434	0.16940781	-2.42392269	-3.7505184	-2.35252622
ENSG00000092068	0.001063	0.019000798	-0.45178837	-1.367734655	**SLC7A8**	protein_coding	solute carrier family 7 member 8 [Source:HGNC Symbol;Acc:HGNC:11066]	chr14	23125295	23183674	-1	ensembl_havana	2.472352616	2.698246801	2.246458431	2.535139	2.693756041	2.865845363	2.005893922	2.278988373	2.454492999
ENSG00000139194	0.001068	0.019032794	0.789213267	1.728131817	**RBP5**	protein_coding	retinol binding protein 5 [Source:HGNC Symbol;Acc:HGNC:15847]	chr12	7123684	7128942	-1	ensembl_havana	-0.319131426	-0.713738059	0.075475208	-0.85854608	-0.967469033	-0.315199064	0.176794179	-0.2414061	0.291037539
ENSG00000163590	0.00107	0.019032794	0.417727976	1.335822182	**PPM1L**	protein_coding	protein phosphatase, Mg2+/Mn2+ dependent 1L [Source:HGNC Symbol;Acc:HGNC:16381]	chr3	160755602	161078907	1	ensembl_havana	6.910924665	6.702060677	7.119788653	6.659373564	6.752990953	6.693817514	7.119618167	7.141152941	7.098594852
ENSG00000214575	0.001067	0.019032794	-0.43133419	-1.348480061	**CPEB1**	protein_coding	cytoplasmic polyadenylation element binding protein 1 [Source:HGNC Symbol;Acc:HGNC:21744]	chr15	82543201	82648861	-1	ensembl_havana	2.706557945	2.92222504	2.49089085	2.812698157	2.991644088	2.962332875	2.438005243	2.517165043	2.517502265
ENSG00000275055	0.00107	0.019032794	-0.495247927	-1.409562967	**AC011468.5**	antisense		chr19	52049007	52049754	1	havana	-0.211449191	0.036174772	-0.459073154	0.14220989	0.125688576	-0.159374151	-0.40973604	-0.42206346	-0.545419959
ENSG00000164758	0.001071	0.019034874	-0.548246693	-1.462307474	**MED30**	protein_coding	mediator complex subunit 30 [Source:HGNC Symbol;Acc:HGNC:23032]	chr8	117520713	117540262	1	ensembl_havana	2.320250219	2.594373565	2.046126872	2.683404011	2.535453458	2.564263227	2.025141822	1.966251876	2.146986918
ENSG00000138640	0.001078	0.019064333	0.892251881	1.856070977	**FAM13A**	protein_coding	family with sequence similarity 13 member A [Source:HGNC Symbol;Acc:HGNC:19367]	chr4	88725955	89111398	-1	ensembl_havana	4.579851433	4.133725493	5.025977374	3.749561028	4.31258584	4.339029611	4.941043813	5.029304794	5.107583515
ENSG00000184900	0.001076	0.019064333	-0.415018106	-1.333315411	**SUMO3**	protein_coding	small ubiquitin-like modifier 3 [Source:HGNC Symbol;Acc:HGNC:11124]	chr21	44805617	44818779	-1	ensembl_havana	5.58660868	5.794117734	5.379099627	5.763003272	5.854773176	5.764576753	5.35832518	5.40052739	5.378446311
ENSG00000226057	0.001078	0.019064333	-0.9518504	-1.934352067	**PHF2P2**	transcribed_unprocessed_pseudogene	PHD finger protein 2 pseudogene 2 [Source:HGNC Symbol;Acc:HGNC:38808]	chr13	18946755	19062718	-1	havana	2.798713554	3.274638754	2.322788354	3.183287918	3.139411412	3.501216933	2.487415269	2.233896951	2.247052842
ENSG00000105369	0.001078	0.019064333	-2.727872076	-6.624777846	**CD79A**	protein_coding	CD79a molecule [Source:HGNC Symbol;Acc:HGNC:1698]	chr19	41877120	41881372	1	ensembl_havana	-1.257154506	0.106781533	-2.621090544	0.040274975	0.298820819	-0.018751196	-3.67188552	-2.16071837	-2.030667746
ENSG00000073464	0.001081	0.019071041	0.740807156	1.671110527	**CLCN4**	protein_coding	chloride voltage-gated channel 4 [Source:HGNC Symbol;Acc:HGNC:2022]	chrX	10156945	10237660	1	ensembl_havana	-0.28312897	-0.653532549	0.087274608	-0.73288918	-0.783495335	-0.444213135	-0.21650287	0.130707766	0.347618925
ENSG00000249105	0.00108	0.019071041	-0.894612262	-1.859110165	**AC096725.1**	processed_pseudogene		chr4	58117758	58118070	1	havana	0.581294267	1.028600398	0.133988136	0.749126884	1.228369434	1.108304877	0.106248156	0.063570082	0.232146171
ENSG00000027869	0.001085	0.019108728	-0.521958186	-1.435902893	**SH2D2A**	protein_coding	SH2 domain containing 2A [Source:HGNC Symbol;Acc:HGNC:10821]	chr1	156806243	156816862	-1	ensembl_havana	6.227175363	6.488154456	5.96619627	6.560809146	6.402250488	6.501403733	6.122946619	5.920281013	5.855361177
ENSG00000204576	0.001087	0.019129342	-0.551445116	-1.465552974	**PRR3**	protein_coding	proline rich 3 [Source:HGNC Symbol;Acc:HGNC:21149]	chr6	30556886	30563723	1	ensembl_havana	6.205730005	6.481452563	5.930007447	6.510073183	6.469471503	6.464813003	5.820967432	6.022147759	5.94690715
ENSG00000180964	0.00109	0.019131147	-0.424072236	-1.341709403	**TCEAL8**	protein_coding	transcription elongation factor A like 8 [Source:HGNC Symbol;Acc:HGNC:28683]	chrX	103252995	103255203	-1	ensembl_havana	3.547517065	3.759553182	3.335480947	3.791998092	3.776492281	3.710169175	3.31472814	3.372156889	3.319557811
ENSG00000174365	0.00109	0.019131147	-0.478396578	-1.393194399	**SNHG11**	processed_transcript	small nucleolar RNA host gene 11 [Source:HGNC Symbol;Acc:HGNC:25046]	chr20	38446578	38450921	1	ensembl_havana	3.375748328	3.614946617	3.136550039	3.620103955	3.616618304	3.608117591	3.153531275	3.046743406	3.209375435
ENSG00000112561	0.001091	0.019135803	0.664215016	1.584705785	**TFEB**	protein_coding	transcription factor EB [Source:HGNC Symbol;Acc:HGNC:11753]	chr6	41683978	41736259	-1	ensembl_havana	2.063516023	1.731408515	2.395623531	1.82284946	1.709476673	1.661899414	2.64502189	2.187350601	2.354498101
ENSG00000280351	0.001096	0.019147422	0.753364972	1.685720057	**AC127496.7**	TEC		chr17	81003335	81005132	-1	ensembl_havana	-0.256542551	-0.633225037	0.120139935	-0.61730533	-0.299013651	-0.983356126	0.069637898	0.286036324	0.004745582
ENSG00000132967	0.001096	0.019147422	-0.511284658	-1.425318816	**HMGB1P5**	transcribed_processed_pseudogene	high mobility group box 1 pseudogene 5 [Source:HGNC Symbol;Acc:HGNC:4997]	chr3	22381819	22382929	1	havana	2.694097928	2.949740257	2.438455598	3.218719635	2.61404572	3.016455416	2.587917939	2.179444752	2.548004104
ENSG00000147535	0.001094	0.019147422	-0.655132021	-1.574760053	**PLPP5**	protein_coding	phospholipid phosphatase 5 [Source:HGNC Symbol;Acc:HGNC:25026]	chr8	38263130	38269243	-1	ensembl_havana	4.123946298	4.451512309	3.796380288	4.693330227	4.553838016	4.107368684	3.999273898	3.751214005	3.63865296
ENSG00000137203	0.0011	0.019194265	-0.480761535	-1.395480085	**TFAP2A**	protein_coding	transcription factor AP-2 alpha [Source:HGNC Symbol;Acc:HGNC:11742]	chr6	10393186	10419659	-1	ensembl_havana	1.291378119	1.531758887	1.050997351	1.553614465	1.390249836	1.651412359	1.161063346	0.821210834	1.170717874
ENSG00000183929	0.001105	0.01925815	-0.864372977	-1.820548249	**DUSP5P1**	processed_pseudogene	dual specificity phosphatase 5 pseudogene 1 [Source:HGNC Symbol;Acc:HGNC:32020]	chr1	228650241	228651379	1	havana	1.265345092	1.69753158	0.833158603	1.613998728	1.603393164	1.875202847	0.807953002	0.956893362	0.734629445
ENSG00000242866	0.001111	0.019318877	0.450314978	1.366338531	**STRC**	protein_coding	stereocilin [Source:HGNC Symbol;Acc:HGNC:16035]	chr15	43599563	43618800	-1	ensembl_havana	1.004316019	0.77915853	1.229473508	0.515840645	0.910210399	0.911424546	0.915012722	1.43975459	1.333653212
ENSG00000204673	0.00111	0.019318877	-0.450596567	-1.366605243	**AKT1S1**	protein_coding	AKT1 substrate 1 [Source:HGNC Symbol;Acc:HGNC:28426]	chr19	49869033	49878459	-1	ensembl_havana	6.327992425	6.553290708	6.102694141	6.788125054	6.37912897	6.492618101	6.292380884	6.008825278	6.006876262
ENSG00000239305	0.001117	0.019327536	0.668609046	1.589539697	**RNF103**	protein_coding	ring finger protein 103 [Source:HGNC Symbol;Acc:HGNC:12859]	chr2	86603393	86623866	-1	ensembl_havana	5.469573321	5.135268798	5.803877845	5.144989722	5.17672119	5.084095483	5.668651414	5.792645426	5.950336693
ENSG00000101040	0.001115	0.019327536	-0.43950899	-1.356142697	**ZMYND8**	protein_coding	zinc finger MYND-type containing 8 [Source:HGNC Symbol;Acc:HGNC:9397]	chr20	47209214	47356889	-1	ensembl_havana	5.928703172	6.148457667	5.708948676	5.975842228	6.122034441	6.347496332	5.510336908	5.753242752	5.863266369
ENSG00000060982	0.001116	0.019327536	-0.536506954	-1.450456425	**BCAT1**	protein_coding	branched chain amino acid transaminase 1 [Source:HGNC Symbol;Acc:HGNC:976]	chr12	24810022	24949459	-1	ensembl_havana	7.073049033	7.34130251	6.804795556	7.268960491	7.367618668	7.387328372	6.610141661	6.9461431	6.858101907
ENSG00000138835	0.001115	0.019327536	-0.894878873	-1.859453761	**RGS3**	protein_coding	regulator of G protein signaling 3 [Source:HGNC Symbol;Acc:HGNC:9999]	chr9	113444731	113597743	1	ensembl_havana	2.579859684	3.027299121	2.132420247	3.159173914	3.061421012	2.861302436	2.08139109	2.046678052	2.2691916
ENSG00000176909	0.001126	0.019358632	0.696427697	1.62048728	**MAMSTR**	protein_coding	MEF2 activating motif and SAP domain containing transcriptional regulator [Source:HGNC Symbol;Acc:HGNC:26689]	chr19	48712742	48719721	-1	ensembl_havana	-0.608965455	-0.957179303	-0.260751607	-1.14838881	-1.03440435	-0.688744748	-0.46237776	-0.52165082	0.201773757
ENSG00000260565	0.001128	0.019358632	0.578103294	1.492885267	**ERVK13-1**	processed_transcript	endogenous retrovirus group K13 member 1 [Source:HGNC Symbol;Acc:HGNC:27548]	chr16	2660349	2673444	-1	havana	5.11078932	4.821737673	5.399840967	4.945601627	4.701268571	4.81834282	5.370604241	5.397974678	5.430943981
ENSG00000179981	0.001128	0.019358632	0.528839152	1.442767822	**TSHZ1**	protein_coding	teashirt zinc finger homeobox 1 [Source:HGNC Symbol;Acc:HGNC:10669]	chr18	75210755	75289950	1	ensembl_havana	5.088715022	4.824295446	5.353134598	4.776260551	4.801827565	4.894798222	5.43678149	5.284623969	5.337998336
ENSG00000114423	0.001127	0.019358632	0.493260109	1.407622138	**CBLB**	protein_coding	Cbl proto-oncogene B [Source:HGNC Symbol;Acc:HGNC:1542]	chr3	105655461	105869552	-1	ensembl_havana	5.149457217	4.902827162	5.396087271	4.817930597	4.944439586	4.946111303	5.379979066	5.479916998	5.32836575
ENSG00000163930	0.001123	0.019358632	0.45365933	1.369509554	**BAP1**	protein_coding	BRCA1 associated protein 1 [Source:HGNC Symbol;Acc:HGNC:950]	chr3	52401013	52410350	-1	ensembl_havana	7.514352375	7.287522709	7.74118204	7.20095152	7.303212643	7.358403965	7.730104912	7.750918224	7.742522983
ENSG00000116983	0.001123	0.019358632	-0.408155327	-1.326988002	**HPCAL4**	protein_coding	hippocalcin like 4 [Source:HGNC Symbol;Acc:HGNC:18212]	chr1	39678648	39691689	-1	ensembl_havana	2.239803133	2.443880797	2.03572547	2.283275357	2.546946467	2.501420566	1.895179555	2.155463891	2.056532963
ENSG00000185101	0.001123	0.019358632	-0.648268686	-1.567286237	**ANO9**	protein_coding	anoctamin 9 [Source:HGNC Symbol;Acc:HGNC:20679]	chr11	417933	442011	-1	ensembl_havana	3.180660999	3.504795342	2.856526656	3.614203075	3.327752331	3.572430621	2.921565963	2.86645213	2.781561875
ENSG00000131019	0.001136	0.019415894	0.523461593	1.437400001	**ULBP3**	protein_coding	UL16 binding protein 3 [Source:HGNC Symbol;Acc:HGNC:14895]	chr6	150063150	150069095	-1	ensembl_havana	2.019776742	1.758045946	2.281507539	1.705645971	1.855271053	1.713220813	2.22979561	2.491852285	2.122874721
ENSG00000182013	0.001135	0.019415894	-0.427327773	-1.344740478	**PNMA8A**	protein_coding	PNMA family member 8A [Source:HGNC Symbol;Acc:HGNC:25578]	chr19	46466491	46471563	-1	ensembl_havana	5.55890568	5.772569567	5.345241794	5.775615037	5.712119685	5.829973978	5.344983642	5.233042912	5.457698827
ENSG00000229980	0.001136	0.019415894	-0.700185127	-1.624713263	**TOB1-AS1**	processed_transcript	TOB1 antisense RNA 1 [Source:HGNC Symbol;Acc:HGNC:44340]	chr17	50866679	50909737	1	havana	0.904759643	1.254852207	0.55466708	1.490591983	1.062111262	1.211853376	0.894226935	0.478736765	0.291037539
ENSG00000276462	0.001147	0.01944376	0.676706162	1.598486059	**BX255923.2**	lincRNA		chr9	41100794	41106229	-1	havana	-0.341068279	-0.67942136	-0.002715197	-0.99620085	-0.299013651	-0.743049581	-0.46237776	0.315191528	0.139040636
ENSG00000240616	0.001146	0.01944376	0.637999083	1.556169364	**RPS6P25**	processed_pseudogene	ribosomal protein S6 pseudogene 25 [Source:HGNC Symbol;Acc:HGNC:35948]	chr19	12894133	12894880	1	havana	0.574045447	0.255045906	0.893044989	0.267803752	0.298820819	0.198513145	1.071324589	0.938274071	0.669536307
ENSG00000002746	0.001146	0.01944376	0.484748891	1.399342281	**HECW1**	protein_coding	HECT, C2 and WW domain containing E3 ubiquitin protein ligase 1 [Source:HGNC Symbol;Acc:HGNC:22195]	chr7	43112599	43566001	1	ensembl_havana	4.252010203	4.009635758	4.494384648	3.815331561	3.983087183	4.230488529	4.40766003	4.419936947	4.655556968
ENSG00000101190	0.00114	0.01944376	0.449386773	1.365459736	**TCFL5**	protein_coding	transcription factor like 5 [Source:HGNC Symbol;Acc:HGNC:11646]	chr20	62841115	62861763	-1	ensembl_havana	4.268914779	4.044221393	4.493608166	3.959604644	4.112480652	4.060578883	4.345853613	4.548263191	4.586707693
ENSG00000092421	0.001142	0.01944376	0.40272837	1.322005675	**SEMA6A**	protein_coding	semaphorin 6A [Source:HGNC Symbol;Acc:HGNC:10738]	chr5	116443616	116574934	-1	ensembl_havana	7.45385771	7.252493526	7.655221895	7.361028207	7.142051588	7.254400782	7.733582871	7.560509714	7.6715731
ENSG00000164056	0.001139	0.01944376	-0.415185539	-1.333470158	**SPRY1**	protein_coding	sprouty RTK signaling antagonist 1 [Source:HGNC Symbol;Acc:HGNC:11269]	chr4	123396795	123403760	1	ensembl_havana	2.265849356	2.473442126	2.058256587	2.477799991	2.476573576	2.46595281	2.072167195	2.097907935	2.004694631
ENSG00000271013	0.001143	0.01944376	-0.957814008	-1.942364566	**LRRC37A9P**	processed_pseudogene	leucine rich repeat containing 37 member A9, pseudogene [Source:HGNC Symbol;Acc:HGNC:43813]	chr17	35912635	35918010	-1	havana	0.860793158	1.339700162	0.381886154	1.190963204	1.046001934	1.782135348	0.24405081	0.37179175	0.529815901
ENSG00000168769	0.00115	0.019460753	0.506163934	1.420268735	**TET2**	protein_coding	tet methylcytosine dioxygenase 2 [Source:HGNC Symbol;Acc:HGNC:25941]	chr4	105145875	105279816	1	ensembl_havana	6.093973871	5.840891904	6.347055838	5.868394913	5.77516609	5.879114708	6.438617944	6.338874196	6.263675373
ENSG00000196338	0.001151	0.019460753	0.424126343	1.341759723	**NLGN3**	protein_coding	neuroligin 3 [Source:HGNC Symbol;Acc:HGNC:14289]	chrX	71144831	71171201	1	ensembl_havana	3.368577907	3.156514736	3.580641079	3.359409615	3.073391752	3.03674284	3.756011721	3.557419829	3.428491686
ENSG00000160703	0.001154	0.019468711	-0.414684403	-1.333007043	**NLRX1**	protein_coding	NLR family member X1 [Source:HGNC Symbol;Acc:HGNC:29890]	chr11	119166568	119184016	1	ensembl_havana	4.774018795	4.981360997	4.566676594	4.878262343	4.96814283	5.097677818	4.463403562	4.596559508	4.640066711
ENSG00000167695	0.001153	0.019468711	-1.241867868	-2.365045383	**FAM57A**	protein_coding	family with sequence similarity 57 member A [Source:HGNC Symbol;Acc:HGNC:29646]	chr17	732412	742972	1	ensembl_havana	2.053133091	2.674067025	1.432199157	2.833163932	2.759446068	2.429591076	1.656848881	1.097781778	1.541966812
ENSG00000258727	0.001158	0.019474648	0.893607101	1.857815329	**AL135999.1**	antisense		chr14	23561097	23568073	1	havana	3.376164524	2.929360973	3.822968075	3.341663059	2.769291924	2.677127938	3.982139812	3.637510218	3.849254195
ENSG00000167646	0.001157	0.019474648	0.508663738	1.422731814	**DNAAF3**	protein_coding	dynein axonemal assembly factor 3 [Source:HGNC Symbol;Acc:HGNC:30492]	chr19	55158661	55166722	-1	ensembl_havana	2.161978054	1.907646185	2.416309923	1.940704441	1.96214833	1.820085783	2.459388507	2.572545352	2.21699591
ENSG00000240344	0.001157	0.019474648	-0.575299226	-1.489986465	**PPIL3**	protein_coding	peptidylprolyl isomerase like 3 [Source:HGNC Symbol;Acc:HGNC:9262]	chr2	200870907	200889303	-1	ensembl_havana	3.427803331	3.715452944	3.140153718	3.605306254	3.793477994	3.747574584	3.053967167	3.068309725	3.298184262
ENSG00000175764	0.001163	0.019488868	0.547572551	1.461624326	**TTLL11**	protein_coding	tubulin tyrosine ligase like 11 [Source:HGNC Symbol;Acc:HGNC:18113]	chr9	121821928	122093606	-1	ensembl_havana	2.099526266	1.82573999	2.373312541	1.738566362	1.836662271	1.901991338	2.179142566	2.351143587	2.58965147
ENSG00000167778	0.001161	0.019488868	0.412395939	1.330894247	**SPRYD3**	protein_coding	SPRY domain containing 3 [Source:HGNC Symbol;Acc:HGNC:25920]	chr12	53064316	53079420	-1	ensembl_havana	5.069528774	4.863330804	5.275726743	4.812787188	4.83970018	4.937505044	5.269314679	5.245402277	5.312463274
ENSG00000132535	0.001163	0.019488868	-0.547424642	-1.461474485	**DLG4**	protein_coding	discs large MAGUK scaffold protein 4 [Source:HGNC Symbol;Acc:HGNC:2903]	chr17	7189890	7219702	-1	ensembl_havana	3.406390519	3.68010284	3.132678198	3.765621565	3.602918888	3.671768066	3.349502035	2.933793498	3.11473906
ENSG00000231105	0.001165	0.019501789	-0.497322371	-1.411591229	**AL031728.1**	antisense		chr1	21293290	21299774	1	havana	2.458217997	2.706879182	2.209556811	2.73325697	2.817537823	2.569842754	2.294679948	2.203033505	2.13095698
ENSG00000162614	0.001169	0.019540117	-0.740706875	-1.670994372	**NEXN**	protein_coding	nexilin F-actin binding protein [Source:HGNC Symbol;Acc:HGNC:29557]	chr1	77888513	77943895	1	ensembl_havana	1.585286767	1.955640204	1.214933329	1.882980075	2.004444884	1.979495653	1.033805522	1.130954377	1.480040089
ENSG00000234171	0.001172	0.019552909	-0.416092211	-1.33430845	**RNASEH1-AS1**	antisense	RNASEH1 antisense RNA 1 [Source:HGNC Symbol;Acc:HGNC:49289]	chr2	3558492	3561745	1	havana	2.43039018	2.638436285	2.222344074	2.444931907	2.68857687	2.781800078	2.053540577	2.301016365	2.31247528
ENSG00000259761	0.001172	0.019552909	-1.683719049	-3.212550313	**AC078905.1**	lincRNA		chr15	87042054	87047161	1	havana	-0.977057863	-0.135198338	-1.818917387	0.383334258	-0.299013651	-0.489915621	-1.90365811	-1.52242631	-2.030667746
ENSG00000121764	0.001176	0.01958544	-0.549133176	-1.463206284	**HCRTR1**	protein_coding	hypocretin receptor 1 [Source:HGNC Symbol;Acc:HGNC:4848]	chr1	31617686	31632518	1	ensembl_havana	2.515998451	2.790565039	2.241431863	2.959551034	2.630344006	2.781800078	2.262602515	2.163501883	2.298191191
ENSG00000184371	0.001179	0.019599933	0.570468413	1.485005643	**CSF1**	protein_coding	colony stimulating factor 1 [Source:HGNC Symbol;Acc:HGNC:2432]	chr1	109910242	109930992	1	ensembl_havana	8.506110351	8.220876145	8.791344558	8.518397611	8.247834186	7.896396637	9.106143472	8.672032143	8.595858058
ENSG00000133874	0.001179	0.019599933	-0.405146026	-1.324222938	**RNF122**	protein_coding	ring finger protein 122 [Source:HGNC Symbol;Acc:HGNC:21147]	chr8	33547755	33567125	-1	ensembl_havana	2.083215847	2.28578886	1.880642835	2.418087419	2.220801645	2.218477517	2.044136302	1.821363648	1.776428555
ENSG00000230533	0.001187	0.019620226	0.970690593	1.959778483	**AL356234.2**	lincRNA		chr6	137657998	137763984	1	havana	1.228469696	0.7431244	1.713814993	0.355307344	0.962641309	0.911424546	1.669136731	1.759072628	1.713235619
ENSG00000169245	0.001187	0.019620226	0.899258238	1.865106791	**CXCL10**	protein_coding	C-X-C motif chemokine ligand 10 [Source:HGNC Symbol;Acc:HGNC:10637]	chr4	76021117	76023497	-1	ensembl_havana	5.5516359	5.102006781	6.001265019	4.848411822	5.34169971	5.115908812	5.437679577	6.431651253	6.134464227
ENSG00000148343	0.001185	0.019620226	0.435352151	1.352240864	**MIGA2**	protein_coding	mitoguardin 2 [Source:HGNC Symbol;Acc:HGNC:23621]	chr9	129036621	129072082	1	ensembl_havana	5.606071758	5.388395683	5.823747834	5.240975494	5.400690482	5.523521073	5.738825475	5.838550639	5.893867387
ENSG00000119686	0.001186	0.019620226	0.401042119	1.320461391	**FLVCR2**	protein_coding	feline leukemia virus subgroup C cellular receptor family member 2 [Source:HGNC Symbol;Acc:HGNC:20105]	chr14	75578617	75663214	1	ensembl_havana	2.506843919	2.30632286	2.707364979	2.175202284	2.396251236	2.34751506	2.607204823	2.769722224	2.745167891
ENSG00000168016	0.001184	0.019620226	-0.397098913	-1.316857204	**TRANK1**	protein_coding	tetratricopeptide repeat and ankyrin repeat containing 1 [Source:HGNC Symbol;Acc:HGNC:29011]	chr3	36826820	36945098	-1	ensembl_havana	4.34280964	4.541359096	4.144260183	4.451680056	4.530870564	4.641526669	4.070484623	4.143413982	4.218881946
ENSG00000229839	0.001191	0.019652928	-0.471942364	-1.386975558	**AC018462.1**	antisense		chr2	62069447	62146881	-1	havana	0.324637404	0.560608586	0.088666222	0.10902545	0.679062629	0.89373768	-0.30988669	0.256279753	0.319605599
ENSG00000258839	0.001194	0.019691805	0.544112927	1.458123509	**MC1R**	protein_coding	melanocortin 1 receptor [Source:HGNC Symbol;Acc:HGNC:6929]	chr16	89912119	89920977	1	ensembl_havana	2.681946056	2.409889592	2.954002519	2.625913238	2.256240478	2.34751506	3.127051692	2.943162138	2.791793727
ENSG00000198807	0.001205	0.019844172	-0.555963936	-1.470150586	**PAX9**	protein_coding	paired box 9 [Source:HGNC Symbol;Acc:HGNC:8623]	chr14	36657568	36679715	1	ensembl_havana	4.648470178	4.926452146	4.37048821	5.442570608	4.865172614	4.471613216	4.780416538	4.266065862	4.064982229
ENSG00000164023	0.00121	0.019907394	0.508193396	1.422268056	**SGMS2**	protein_coding	sphingomyelin synthase 2 [Source:HGNC Symbol;Acc:HGNC:28395]	chr4	107824563	107915047	1	ensembl_havana	4.205122661	3.951025963	4.459219359	3.490814579	4.213297524	4.148965785	4.125025993	4.670338547	4.582293537
ENSG00000122641	0.001212	0.019907697	0.552353307	1.466475844	**INHBA**	protein_coding	inhibin beta A subunit [Source:HGNC Symbol;Acc:HGNC:6066]	chr7	41667168	41705834	-1	ensembl_havana	4.044363104	3.768186451	4.320539757	3.694696689	3.922611281	3.687251381	4.349675785	4.326378436	4.285565051

ENSG00000219665	0.001223	0.020055106	0.508275901	1.422349395	**ZNF433-AS1**	processed_transcript	ZNF433 and ZNF878 antisense RNA 1 [Source:HGNC Symbol;Acc:HGNC:53776]	chr19	11987617	12046275	1	havana	3.222167156	2.968029206	3.476305107	3.00985979	2.948964943	2.945262884	3.431134914	3.419911719	3.577868687
ENSG00000268573	0.001223	0.020055106	-0.634301055	-1.552185577	**AC011815.1**	lincRNA		chr18	35280867	35290201	-1	havana	2.558868062	2.876018589	2.241717534	3.138766373	2.913888608	2.575400786	2.310455111	2.358164528	2.056532963
ENSG00000135679	0.001225	0.020055114	-0.498011547	-1.412265708	**MDM2**	protein_coding	MDM2 proto-oncogene [Source:HGNC Symbol;Acc:HGNC:6973]	chr12	68808176	68850686	1	ensembl_havana	5.61748405	5.866489824	5.368478277	5.941497334	5.837913348	5.820058789	5.344983642	5.450655454	5.309795736
ENSG00000189164	0.001227	0.020073397	0.458188531	1.373815752	**ZNF527**	protein_coding	zinc finger protein 527 [Source:HGNC Symbol;Acc:HGNC:29385]	chr19	37371061	37393066	1	ensembl_havana	1.983441925	1.754347659	2.21253619	1.705645971	1.864486201	1.692910804	2.179142566	2.233896951	2.224569053
ENSG00000080561	0.001232	0.020120215	0.402168744	1.321492965	**MID2**	protein_coding	midline 2 [Source:HGNC Symbol;Acc:HGNC:7096]	chrX	107825755	107927193	1	ensembl_havana	3.306098279	3.105013907	3.507182651	2.959551034	3.195256367	3.160234321	3.398295962	3.596537187	3.526714805
ENSG00000001461	0.001234	0.020131096	0.416138305	1.334351082	**NIPAL3**	protein_coding	NIPA like domain containing 3 [Source:HGNC Symbol;Acc:HGNC:25233]	chr1	24415794	24472976	1	ensembl_havana	5.852819509	5.644750356	6.060888662	5.666511001	5.599354734	5.668385335	6.029536014	6.064615297	6.088514673
ENSG00000272434	0.001237	0.020140576	0.494202511	1.40854193	**AC137630.3**	antisense		chr3	49029316	49029706	1	havana	-0.214069337	-0.461170592	0.033031919	-0.36334239	-0.383759306	-0.636410081	0.210814386	-0.00684508	-0.104873545
ENSG00000160712	0.001237	0.020140576	-1.422184593	-2.679910078	**IL6R**	protein_coding	interleukin 6 receptor [Source:HGNC Symbol;Acc:HGNC:6019]	chr1	154405193	154469450	1	ensembl_havana	0.67222874	1.383321036	-0.038863557	1.87313074	1.199531136	1.077301233	-0.04612063	0.19485955	-0.265329594
ENSG00000186088	0.001238	0.020140576	-1.728279255	-3.313323926	**GSAP**	protein_coding	gamma-secretase activating protein [Source:HGNC Symbol;Acc:HGNC:28042]	chr7	77310751	77416400	-1	ensembl_havana	-1.137409882	-0.273270254	-2.001549509	0.14220989	-0.218971072	-0.743049581	-2.22890346	-1.74507732	-2.030667746
ENSG00000111652	0.001243	0.020193991	0.402512384	1.321807772	**COPS7A**	protein_coding	COP9 signalosome subunit 7A [Source:HGNC Symbol;Acc:HGNC:16758]	chr12	6723741	6731875	1	ensembl_havana	5.723264343	5.522008151	5.924520535	5.484415117	5.458124712	5.623484625	5.843505636	5.87938462	6.050671348
ENSG00000236941	0.001245	0.020195484	-0.703682451	-1.628656606	**AC106872.1**	processed_pseudogene		chr4	164948079	164949438	1	havana	1.156215301	1.508056526	0.804374075	1.222335231	1.558674763	1.743159585	0.457455004	0.693803314	1.261863909
ENSG00000134207	0.001247	0.02020428	1.074917551	2.106601678	**SYT6**	protein_coding	synaptotagmin 6 [Source:HGNC Symbol;Acc:HGNC:18638]	chr1	114089291	114153919	-1	ensembl_havana	-0.582561637	-1.120020412	-0.045102861	-2.15023884	-0.672709	-0.537113394	-0.69455235	0.130707766	0.428535999
ENSG00000198821	0.001255	0.020262293	0.630897764	1.548528316	**CD247**	protein_coding	CD247 molecule [Source:HGNC Symbol;Acc:HGNC:1677]	chr1	167430640	167518610	-1	ensembl_havana	-0.077625235	-0.393074117	0.237823647	-0.27307956	-0.106664058	-0.799478738	0.513047461	0.343769191	-0.143345712
ENSG00000122507	0.001255	0.020262293	0.626400348	1.543708494	**BBS9**	protein_coding	Bardet-Biedl syndrome 9 [Source:HGNC Symbol;Acc:HGNC:30000]	chr7	33129244	33606068	1	ensembl_havana	4.171305311	3.858105137	4.484505485	4.105550163	3.812648474	3.656116775	4.623513749	4.354737515	4.47526519
ENSG00000161267	0.001255	0.020262293	0.557220473	1.471431593	**BDH1**	protein_coding	3-hydroxybutyrate dehydrogenase 1 [Source:HGNC Symbol;Acc:HGNC:1027]	chr3	197509783	197573323	-1	ensembl_havana	4.566565686	4.28795545	4.845175923	3.926652984	4.273340686	4.663872681	4.602844617	4.86033958	5.072343571
ENSG00000180346	0.001256	0.020262293	0.402066879	1.32139966	**TIGD2**	protein_coding	tigger transposable element derived 2 [Source:HGNC Symbol;Acc:HGNC:18333]	chr4	89111500	89114899	1	ensembl_havana	5.686515967	5.485482528	5.887549407	5.432545917	5.52502939	5.498872277	5.8610183	5.883040123	5.918589796
ENSG00000102057	0.00126	0.020262293	-0.401492121	-1.32087333	**KCND1**	protein_coding	potassium voltage-gated channel subfamily D member 1 [Source:HGNC Symbol;Acc:HGNC:6237]	chrX	48961378	48971569	-1	ensembl_havana	4.947035929	5.14778199	4.746289869	5.311958391	5.029852464	5.101535114	4.869634972	4.634643592	4.734591043
ENSG00000139174	0.001259	0.020262293	-0.446884856	-1.363093812	**PRICKLE1**	protein_coding	prickle planar cell polarity protein 1 [Source:HGNC Symbol;Acc:HGNC:17019]	chr12	42456757	42590355	-1	ensembl_havana	4.052394068	4.275836496	3.828951641	3.828133367	4.375174213	4.624201909	3.466766833	3.905352962	4.114735126
ENSG00000147394	0.00126	0.020262293	-0.754964084	-1.687589579	**ZNF185**	protein_coding	zinc finger protein 185 with LIM domain [Source:HGNC Symbol;Acc:HGNC:12976]	chrX	152914442	152973480	1	ensembl_havana	2.384223162	2.761705203	2.00674112	2.614134963	2.918320072	2.752660576	2.044136302	1.919554095	2.056532963
ENSG00000114270	0.001262	0.02026688	0.431618182	1.348745534	**COL7A1**	protein_coding	collagen type VII alpha 1 chain [Source:HGNC Symbol;Acc:HGNC:2214]	chr3	48564073	48595267	-1	ensembl_havana	4.073498951	3.85768986	4.289308043	3.843347077	3.791063673	3.938658831	4.342021288	4.220562267	4.305340572
ENSG00000047056	0.001263	0.02026996	0.443798833	1.360181178	**WDR37**	protein_coding	WD repeat domain 37 [Source:HGNC Symbol;Acc:HGNC:31406]	chr10	1049538	1132297	1	ensembl_havana	4.736822994	4.514923577	4.95872241	4.468131009	4.455121723	4.621517999	4.941043813	4.954840266	4.980283151
ENSG00000145050	0.001267	0.020281627	-0.396955443	-1.316726255	**MANF**	protein_coding	mesencephalic astrocyte derived neurotrophic factor [Source:HGNC Symbol;Acc:HGNC:15461]	chr3	51385047	51389397	1	ensembl_havana	6.07742469	6.275902412	5.878946969	6.378316976	6.17946633	6.269923929	5.973045624	5.754573661	5.909221621
ENSG00000272688	0.001267	0.020281627	-0.489930782	-1.404377494	**AP005329.3**	lincRNA		chr18	3246401	3247086	-1	havana	1.954438941	2.199404332	1.709473551	2.503565738	2.132018545	1.962628713	1.936429043	1.602328815	1.589662793
ENSG00000177034	0.001269	0.020294017	-0.466675754	-1.381921585	**MTX3**	protein_coding	metaxin 3 [Source:HGNC Symbol;Acc:HGNC:24812]	chr5	79976731	79991265	-1	ensembl_havana	5.448149388	5.681487265	5.214811511	5.591909175	5.692849699	5.759702922	5.034239593	5.314709981	5.295484959
ENSG00000283265	0.001275	0.020315425	0.604741994	1.520706772	**AL356234.3**	lincRNA		chr6	137693068	137700415	1	havana	0.048323414	-0.254047583	0.350694412	-0.4596317	-0.143136894	-0.159374151	0.308311185	0.45273451	0.291037539
ENSG00000267796	0.001276	0.020315425	0.468824255	1.383981113	**LIN37**	protein_coding	lin-37 DREAM MuvB core complex component [Source:HGNC Symbol;Acc:HGNC:33234]	chr19	35748361	35754519	1	ensembl_havana	1.969598092	1.735185964	2.204010219	1.892762624	1.864486201	1.448309068	2.318278476	2.271570328	2.022181853
ENSG00000174136	0.001276	0.020315425	0.413463581	1.331879517	**RGMB**	protein_coding	repulsive guidance molecule BMP co-receptor b [Source:HGNC Symbol;Acc:HGNC:26896]	chr5	98768650	98798643	1	ensembl_havana	3.649355071	3.442623281	3.856086862	3.175294617	3.613888808	3.538686417	3.584973459	4.081128356	3.90215877
ENSG00000152208	0.001274	0.020315425	0.392869768	1.313002595	**GRID2**	protein_coding	glutamate ionotropic receptor delta type subunit 2 [Source:HGNC Symbol;Acc:HGNC:4576]	chr4	92303622	93810157	1	ensembl_havana	1.267447598	1.071012714	1.463882482	1.477650254	1.093800119	0.641587769	1.895179555	1.491756274	1.004711615
ENSG00000101096	0.001277	0.020315425	-0.409592152	-1.328310249	**NFATC2**	protein_coding	nuclear factor of activated T cells 2 [Source:HGNC Symbol;Acc:HGNC:7776]	chr20	51386957	51562831	-1	ensembl_havana	6.047181252	6.251977328	5.842385176	6.306947603	6.193702552	6.25528183	5.842149662	5.804259391	5.880746475
ENSG00000123739	0.001282	0.020362683	0.449434235	1.365504658	**PLA2G12A**	protein_coding	phospholipase A2 group XIIA [Source:HGNC Symbol;Acc:HGNC:18554]	chr4	109709989	109730077	-1	ensembl_havana	4.967631939	4.742914821	5.192349056	4.650726902	4.769080564	4.808936996	5.189456144	5.154562121	5.233028904
ENSG00000134184	0.001283	0.020362683	-0.559391128	-1.473647151	**GSTM1**	protein_coding	glutathione S-transferase mu 1 [Source:HGNC Symbol;Acc:HGNC:4632]	chr1	109687814	109709039	1	ensembl_havana	9.532755916	9.81245148	9.253060352	9.979875706	9.761247401	9.696231332	9.356804614	9.106331422	9.29604502
ENSG00000180901	0.001288	0.020371846	0.419774039	1.337718019	**KCTD2**	protein_coding	potassium channel tetramerization domain containing 2 [Source:HGNC Symbol;Acc:HGNC:21294]	chr17	75032575	75065889	1	ensembl_havana	6.080971869	5.87108485	6.290858888	5.819228361	6.007119703	5.786906485	6.281917171	6.360450177	6.230209317
ENSG00000048140	0.001288	0.020371846	0.386223568	1.306967773	**TSPAN17**	protein_coding	tetraspanin 17 [Source:HGNC Symbol;Acc:HGNC:13594]	chr5	176647387	176659054	1	ensembl_havana	6.117880564	5.92476878	6.310992348	6.044916748	5.906991679	5.822397913	6.423737271	6.282325892	6.226913881
ENSG00000111801	0.001287	0.020371846	-0.630057778	-1.547626973	**BTN3A3**	protein_coding	butyrophilin subfamily 3 member A3 [Source:HGNC Symbol;Acc:HGNC:1140]	chr6	26440472	26453415	1	ensembl_havana	4.057331308	4.372360198	3.742302419	4.579831375	4.36059417	4.176655048	3.773189967	3.743191361	3.71052593
ENSG00000128266	0.001298	0.020399921	0.667916984	1.588777376	**GNAZ**	protein_coding	G protein subunit alpha z [Source:HGNC Symbol;Acc:HGNC:4395]	chr22	23070361	23125037	1	ensembl_havana	2.176418675	1.842460183	2.510377167	1.578072284	1.944876279	2.004431986	2.459388507	2.535862351	2.535880644
ENSG00000279355	0.0013	0.020399921	0.546697711	1.460738277	**AGPAT4-IT1**	TEC	AGPAT4 intronic transcript 1 [Source:HGNC Symbol;Acc:HGNC:20988]	chr6	161160105	161161982	-1	havana	-0.261677868	-0.535026724	0.011670987	-0.27307956	-0.473795405	-0.85820521	0.339398945	-0.08087422	-0.223511759
ENSG00000107140	0.001299	0.020399921	0.510787913	1.424828138	**TESK1**	protein_coding	testis-specific kinase 1 [Source:HGNC Symbol;Acc:HGNC:11731]	chr9	35605305	35610041	1	ensembl_havana	4.552188638	4.296794681	4.807582595	4.390853222	4.352430021	4.147100801	5.024708239	4.756557736	4.641481809
ENSG00000164953	0.001301	0.020399921	0.402235935	1.321554512	**TMEM67**	protein_coding	transmembrane protein 67 [Source:HGNC Symbol;Acc:HGNC:28396]	chr8	93754844	93819234	1	ensembl_havana	3.44300184	3.241883872	3.644119807	3.435000789	3.290633293	3.000017535	3.798579805	3.68574783	3.448031787
ENSG00000095585	0.001299	0.020399921	0.40056076	1.320020889	**BLNK**	protein_coding	B cell linker [Source:HGNC Symbol;Acc:HGNC:14211]	chr10	96191702	96271587	-1	ensembl_havana	1.63844384	1.43816346	1.83872422	1.749374982	1.324992307	1.24012309	2.10871413	1.726888623	1.680569906
ENSG00000108861	0.001301	0.020399921	0.395254825	1.315175041	**DUSP3**	protein_coding	dual specificity phosphatase 3 [Source:HGNC Symbol;Acc:HGNC:3069]	chr17	43766121	43778988	-1	ensembl_havana	5.959394938	5.761767526	6.157022351	5.730642052	5.8059843	5.748676226	6.136284886	6.163113864	6.171668302
ENSG00000112343	0.001297	0.020399921	-0.395722397	-1.315601353	**TRIM38**	protein_coding	tripartite motif containing 38 [Source:HGNC Symbol;Acc:HGNC:10059]	chr6	25962802	25991226	1	ensembl_havana	7.042180041	7.24004124	6.844318843	7.418873443	7.26653502	7.034715256	7.034320666	6.832266334	6.666369529
ENSG00000183765	0.001301	0.020399921	-0.448119365	-1.364260705	**CHEK2**	protein_coding	checkpoint kinase 2 [Source:HGNC Symbol;Acc:HGNC:16627]	chr22	28687743	28742422	-1	ensembl_havana	3.202311563	3.426371245	2.978251881	3.283361026	3.415007936	3.580744775	2.89565563	2.876266922	3.162833091
ENSG00000087884	0.001321	0.020440987	0.593879153	1.509299537	**AAMDC**	protein_coding	adipogenesis associated Mth938 domain containing [Source:HGNC Symbol;Acc:HGNC:30205]	chr11	77821109	77918432	1	ensembl_havana	2.843474238	2.546534662	3.140413815	2.52259249	2.575284697	2.541726798	2.972031052	3.147441811	3.301768582
ENSG00000134531	0.001316	0.020440987	0.577022465	1.491767256	**EMP1**	protein_coding	epithelial membrane protein 1 [Source:HGNC Symbol;Acc:HGNC:3333]	chr12	13196716	13219939	1	ensembl_havana	1.095469585	0.806958353	1.383980818	0.749126884	0.927899934	0.743848241	1.371200905	1.625790657	1.154950891
ENSG00000137494	0.001322	0.020440987	0.519638655	1.433596137	**ANKRD42**	protein_coding	ankyrin repeat domain 42 [Source:HGNC Symbol;Acc:HGNC:26752]	chr11	83193739	83260694	1	ensembl_havana	4.058729745	3.798910418	4.318549073	3.914699725	3.688427859	3.79360367	4.353487857	4.347699835	4.254459527
ENSG00000160961	0.001321	0.020440987	0.447140965	1.363335812	**ZNF333**	protein_coding	zinc finger protein 333 [Source:HGNC Symbol;Acc:HGNC:15624]	chr19	14689801	14733746	1	ensembl_havana	4.277114165	4.053543682	4.500684648	4.113938229	3.866385698	4.18030712	4.475698201	4.387705518	4.638650224
ENSG00000122644	0.001308	0.020440987	0.411122678	1.329720175	**ARL4A**	protein_coding	ADP ribosylation factor like GTPase 4A [Source:HGNC Symbol;Acc:HGNC:695]	chr7	12686856	12690934	1	ensembl_havana	5.36565331	5.160091971	5.571214649	5.308308254	5.111457876	5.060509783	5.660211925	5.542892927	5.510539096
ENSG00000172339	0.001321	0.020440987	0.384310558	1.305235886	**ALG14**	protein_coding	ALG14, UDP-N-acetylglucosaminyltransferase subunit [Source:HGNC Symbol;Acc:HGNC:28287]	chr1	94974407	95072945	-1	ensembl_havana	2.021189129	1.82903385	2.213344407	1.843172741	1.900769223	1.743159585	2.221475976	2.271570328	2.146986918
ENSG00000168806	0.001314	0.020440987	-0.40997839	-1.328665912	**LCMT2**	protein_coding	leucine carboxyl methyltransferase 2 [Source:HGNC Symbol;Acc:HGNC:17558]	chr15	43323649	43330605	-1	ensembl_havana	4.616433007	4.821422202	4.411443812	4.811498466	4.864024481	4.788743659	4.345853613	4.499881942	4.388595882
ENSG00000149346	0.001318	0.020440987	-0.428734727	-1.346052544	**SLX4IP**	protein_coding	SLX4 interacting protein [Source:HGNC Symbol;Acc:HGNC:16225]	chr20	10435303	10636829	1	ensembl_havana	3.833104649	4.047472013	3.618737285	3.914699725	4.049234073	4.17848224	3.427522837	3.694095415	3.734593603
ENSG00000148690	0.00131	0.020440987	-0.4759351	-1.390819404	**FRA10AC1**	protein_coding	FRA10A associated CGG repeat 1 [Source:HGNC Symbol;Acc:HGNC:1162]	chr10	93667883	93702572	-1	ensembl_havana	3.131848438	3.369815988	2.893880889	3.481120682	3.220595563	3.40773172	2.89565563	2.821440049	2.964546987
ENSG00000087076	0.001322	0.020440987	-0.481318347	-1.396018778	**HSD17B14**	protein_coding	hydroxysteroid 17-beta dehydrogenase 14 [Source:HGNC Symbol;Acc:HGNC:23238]	chr19	48813017	48836678	-1	ensembl_havana	1.594711373	1.835370546	1.354052199	1.996207752	1.699212533	1.810691353	1.619344613	1.210694826	1.232117157
ENSG00000267074	0.001314	0.020440987	-0.496106656	-1.410402225	**AC015911.3**	sense_intronic		chr17	35499690	35510270	-1	havana	1.625150471	1.873203799	1.377097143	1.833046887	1.798708764	1.987855745	1.229040238	1.300906777	1.601344415
ENSG00000189014	0.001307	0.020440987	-0.774128826	-1.710157058	**FAM35DP**	unprocessed_pseudogene	family with sequence similarity 35 member D, pseudogene [Source:HGNC Symbol;Acc:HGNC:34038]	chr10	47689707	47730436	1	havana	0.384642911	0.771707324	-0.002421502	1.04068047	0.570631912	0.703809589	0.24405081	-0.42206346	0.17074815
ENSG00000131650	0.001316	0.020440987	-1.155078895	-2.226965017	**KREMEN2**	protein_coding	kringle containing transmembrane protein 2 [Source:HGNC Symbol;Acc:HGNC:18797]	chr16	2963944	2968383	1	ensembl_havana	1.495745542	2.073284989	0.918206094	2.275818664	1.739838083	2.204198221	1.458744294	0.648704825	0.647169164
ENSG00000145431	0.001328	0.020489798	0.421897132	1.339688076	**PDGFC**	protein_coding	platelet derived growth factor C [Source:HGNC Symbol;Acc:HGNC:8801]	chr4	156760454	156971394	-1	ensembl_havana	4.549069336	4.33812077	4.760017902	4.329150785	4.445942855	4.239268669	4.780416538	4.795871197	4.703765972
ENSG00000185215	0.001328	0.020489798	-0.745457143	-1.676505415	**TNFAIP2**	protein_coding	TNF alpha induced protein 2 [Source:HGNC Symbol;Acc:HGNC:11895]	chr14	103123442	103137439	1	ensembl_havana	2.734234147	3.106962718	2.361505575	3.062812401	3.116453761	3.141621992	2.062883947	2.491852285	2.529780494
ENSG00000174945	0.001344	0.020701136	-0.93057836	-1.906039952	**AMZ1**	protein_coding	archaelysin family metallopeptidase 1 [Source:HGNC Symbol;Acc:HGNC:22231]	chr7	2679522	2775500	1	ensembl_havana	0.377114546	0.842403726	-0.088174634	1.005046425	0.818355163	0.703809589	-0.26243933	0.02879206	-0.030876629
ENSG00000174500	0.001353	0.02077617	-0.380149033	-1.301476294	**GCSAM**	protein_coding	germinal center associated signaling and motility [Source:HGNC Symbol;Acc:HGNC:20253]	chr3	112120841	112133305	-1	ensembl_havana	4.7218836	4.911958117	4.531809084	5.12748953	4.800627876	4.807756945	4.740215807	4.433295563	4.421915881
ENSG00000166317	0.001353	0.02077617	-0.530787959	-1.444718044	**SYNPO2L**	protein_coding	synaptopodin 2 like [Source:HGNC Symbol;Acc:HGNC:23532]	chr10	73644881	73663803	-1	ensembl_havana	2.120172314	2.385566293	1.854778334	2.040883175	2.523868156	2.591947549	1.656848881	1.938414976	1.969071146
ENSG00000009950	0.001353	0.02077617	-1.547483367	-2.923067951	**MLXIPL**	protein_coding	MLX interacting protein like [Source:HGNC Symbol;Acc:HGNC:12744]	chr7	73593194	73624543	-1	ensembl_havana	-0.885813554	-0.11207187	-1.659555237	-0.56281016	-0.258437325	0.485031876	-1.90365811	-1.42278721	-1.652220391
ENSG00000136305	0.001355	0.020791577	-0.56361792	-1.477970957	**CIDEB**	protein_coding	cell death-inducing DFFA-like effector b [Source:HGNC Symbol;Acc:HGNC:1977]	chr14	24305096	24311430	-1	ensembl_havana	3.039058432	3.320867392	2.757249472	2.950212473	3.549641413	3.46274829	2.50808372	2.826511306	2.937153388
ENSG00000145632	0.001359	0.020808036	1.031329792	2.043907337	**PLK2**	protein_coding	polo like kinase 2 [Source:HGNC Symbol;Acc:HGNC:19699]	chr5	58453982	58460260	-1	ensembl_havana	2.217265271	1.701600375	2.732930166	1.424690178	2.256240478	1.423870469	2.831503656	3.033647152	2.33363969
ENSG00000169241	0.001358	0.020808036	-0.571302852	-1.485864802	**SLC50A1**	protein_coding	solute carrier family 50 member 1 [Source:HGNC Symbol;Acc:HGNC:30657]	chr1	155135344	155138857	1	ensembl_havana	5.434717729	5.720369155	5.149066303	5.890534108	5.68376819	5.586805167	5.148354299	5.194336108	5.104508502
ENSG00000274026	0.001362	0.020825675	0.432234205	1.349321563	**FAM27E3**	transcribed_processed_pseudogene	family with sequence similarity 27 member E3 [Source:HGNC Symbol;Acc:HGNC:28655]	chr9	67717411	67719178	-1	ensembl_havana	0.805764469	0.589647366	1.021881571	0.749126884	0.658018659	0.361796557	1.125834805	1.063828438	0.875981471
ENSG00000235823	0.001363	0.020826478	-0.578811206	-1.493617986	**OLMALINC**	lincRNA	oligodendrocyte maturation-associated long intergenic non-coding RNA [Source:HGNC Symbol;Acc:HGNC:28060]	chr10	100373513	100454043	1	havana	3.098185943	3.387591546	2.80878034	3.655011191	3.373654145	3.134109303	3.153531275	2.620048716	2.652761029
ENSG00000088340	0.001365	0.02083327	0.589868245	1.505109287	**FER1L4**	transcribed_unitary_pseudogene	fer-1 like family member 4, pseudogene [Source:HGNC Symbol;Acc:HGNC:15801]	chr20	35558737	35607562	-1	ensembl_havana	1.235855888	0.940921765	1.530790011	1.005046425	0.679062629	1.138656243	1.430149367	1.286257764	1.875962902
ENSG00000234073	0.001368	0.020857559	-0.785726223	-1.723959907	**AC011816.1**	processed_pseudogene		chr3	36880184	36880729	-1	havana	-0.390206058	0.002657054	-0.783069169	-0.06941355	0.030651399	0.046733314	-0.63291296	-1.00738779	-0.708906755
ENSG00000141576	0.00137	0.020866397	-0.405790011	-1.324814172	**RNF157**	protein_coding	ring finger protein 157 [Source:HGNC Symbol;Acc:HGNC:29402]	chr17	76142453	76240373	-1	ensembl_havana	6.409697	6.612592005	6.206801995	6.671855316	6.49528432	6.67063638	6.266330692	6.140894693	6.213180599
ENSG00000206538	0.001376	0.020924727	0.907358836	1.875608648	**VGLL3**	protein_coding	vestigial like family member 3 [Source:HGNC Symbol;Acc:HGNC:24327]	chr3	86876388	86991119	-1	ensembl_havana	3.367001848	2.913322431	3.820681266	2.683404011	3.077360027	2.979203253	3.829017014	4.064054462	3.568972322
ENSG00000102879	0.001377	0.020924727	-0.898220175	-1.863765273	**CORO1A**	protein_coding	coronin 1A [Source:HGNC Symbol;Acc:HGNC:2252]	chr16	30182827	30189076	1	ensembl_havana	8.783840998	9.232951085	8.334730911	9.337944827	9.002666132	9.358242298	8.59696415	8.273770445	8.133458137
ENSG00000187815	0.001382	0.020981852	-0.480796363	-1.395513773	**ZFP69**	protein_coding	ZFP69 zinc finger protein [Source:HGNC Symbol;Acc:HGNC:24708]	chr1	40477215	40496343	1	ensembl_havana	3.713082951	3.953481132	3.472684769	3.919492919	4.075263288	3.865687188	3.401981866	3.5140724	3.502000041
ENSG00000101400	0.001387	0.020987715	0.860325481	1.815447841	**SNTA1**	protein_coding	syntrophin alpha 1 [Source:HGNC Symbol;Acc:HGNC:11167]	chr20	33407955	33443892	-1	ensembl_havana	3.071263582	2.641100841	3.501426322	2.298074107	2.68857687	2.936651547	3.445493458	3.507772205	3.551013303
ENSG00000228606	0.001385	0.020987715	0.49225312	1.406639973	**AL139011.1**	antisense		chr1	160261744	160262778	1	havana	1.061096757	0.814970197	1.307223317	1.126095262	0.720251491	0.598563838	1.541278047	1.163381334	1.21701057
ENSG00000180902	0.001387	0.020987715	-0.647355866	-1.566294899	**D2HGDH**	protein_coding	D-2-hydroxyglutarate dehydrogenase [Source:HGNC Symbol;Acc:HGNC:28358]	chr2	241734579	241768816	1	ensembl_havana	3.100775405	3.424453338	2.777097472	3.683468931	3.124146975	3.465744108	3.035102527	2.660368592	2.635821295
ENSG00000188092	0.001398	0.021021281	0.604756559	1.520722124	**GPR89B**	protein_coding	G protein-coupled receptor 89B [Source:HGNC Symbol;Acc:HGNC:13840]	chr1	147928393	147993521	1	ensembl_havana	4.800452428	4.498074149	5.102830708	4.679268445	4.530870564	4.284083438	5.161460773	5.070496947	5.076534403
ENSG00000174871	0.001399	0.021021281	0.548909109	1.462979049	**CNIH2**	protein_coding	cornichon family AMPA receptor auxiliary protein 2 [Source:HGNC Symbol;Acc:HGNC:28744]	chr11	66278190	66285301	1	ensembl_havana	0.899477705	0.62502315	1.17393226	0.540941683	0.77991046	0.554217307	1.033805522	1.226127349	1.261863909
ENSG00000115919	0.0014	0.021021281	0.459320893	1.374894474	**KYNU**	protein_coding	kynureninase [Source:HGNC Symbol;Acc:HGNC:6469]	chr2	142877498	143055832	1	ensembl_havana	1.37314217	1.143481724	1.602802616	0.770507832	1.476918714	1.183018625	1.325353043	1.929015357	1.554039449
ENSG00000151229	0.001402	0.021021281	0.412664395	1.331141922	**SLC2A13**	protein_coding	solute carrier family 2 member 13 [Source:HGNC Symbol;Acc:HGNC:15956]	chr12	39755021	40106089	-1	ensembl_havana	4.800526745	4.594194547	5.006858942	4.556942526	4.555261413	4.670379702	4.963666724	5.030404003	5.0265061
ENSG00000159131	0.001402	0.021021281	-0.377906776	-1.299455092	**GART**	protein_coding	phosphoribosylglycinamide formyltransferase, phosphoribosylglycinamide synthetase, phosphoribosylaminoimidazole synthetase [Source:HGNC Symbol;Acc:HGNC:4163]	chr21	33503931	33543491	-1	ensembl_havana	7.803769708	7.992723096	7.61481632	7.945478447	7.962735918	8.069954924	7.550177856	7.593049615	7.70122149
ENSG00000163606	0.001397	0.021021281	-0.452467574	-1.36837872	**CD200R1**	protein_coding	CD200 receptor 1 [Source:HGNC Symbol;Acc:HGNC:24235]	chr3	112921209	112975122	-1	ensembl_havana	5.64116947	5.867403257	5.414935683	5.943851816	5.898044904	5.760313052	5.464365468	5.543662986	5.236778596
ENSG00000163217	0.001397	0.021021281	-0.607477038	-1.523592444	**BMP10**	protein_coding	bone morphogenetic protein 10 [Source:HGNC Symbol;Acc:HGNC:20869]	chr2	68865481	68871517	-1	ensembl_havana	4.046077691	4.34981621	3.742339172	4.855932554	4.146875043	4.046641033	4.056520899	3.634621851	3.535874767
ENSG00000146376	0.001401	0.021021281	-0.708328082	-1.633909505	**ARHGAP18**	protein_coding	Rho GTPase activating protein 18 [Source:HGNC Symbol;Acc:HGNC:21035]	chr6	129576132	129710225	-1	ensembl_havana	0.937106876	1.291270917	0.582942835	1.237769075	1.140065346	1.495978329	0.592614899	0.602150945	0.554062661
ENSG00000248645	0.001395	0.021021281	-0.87601071	-1.835293389	**AC106872.6**	unprocessed_pseudogene		chr4	164938322	164939990	-1	havana	0.050366739	0.488372094	-0.387638616	0.437805725	0.428746719	0.598563838	-0.57379957	-0.62862566	0.039509383
ENSG00000124334	0.001425	0.021049085	0.963527481	1.95007212	**IL9R**	protein_coding	interleukin 9 receptor [Source:HGNC Symbol;Acc:HGNC:6030]	chrX	155997581	156010817	1	ensembl_havana	0.123644087	-0.358119653	0.605407828	-0.67394004	-0.569826728	0.16940781	0.56657698	0.45273451	0.796911992
ENSG00000231213	0.001424	0.021049085	0.888951255	1.851829475	**PLSCR5**	protein_coding	phospholipid scramblase family member 5 [Source:HGNC Symbol;Acc:HGNC:19952]	chr3	146576555	146606216	-1	ensembl_havana	5.962962766	5.518487138	6.407438393	4.665068254	5.816112458	6.074280703	5.848241536	6.679141789	6.694931854
ENSG00000274471	0.001426	0.021049085	0.50183714	1.416015581	**AC242376.2**	unprocessed_pseudogene		chr15	23309607	23313276	1	havana	3.661717947	3.410799377	3.912636517	3.323695481	3.442958541	3.465744108	3.949769201	3.929121717	3.859018634
ENSG00000009830	0.001427	0.021049085	0.478074077	1.392882999	**POMT2**	protein_coding	protein O-mannosyltransferase 2 [Source:HGNC Symbol;Acc:HGNC:19743]	chr14	77274956	77320884	-1	ensembl_havana	4.828705168	4.589668129	5.067742207	4.544585015	4.605590287	4.618829086	5.1306917	4.983500571	5.089034349
ENSG00000246090	0.001413	0.021049085	0.471721048	1.386762806	**AP002026.1**	antisense		chr4	99088857	99301356	1	havana	1.8968531	1.660992576	2.132713624	1.283104003	1.7890623	1.910811426	1.644455473	2.286368471	2.467316929
ENSG00000157017	0.001414	0.021049085	0.439196977	1.355849433	**GHRL**	protein_coding	ghrelin and obestatin prepropeptide [Source:HGNC Symbol;Acc:HGNC:18129]	chr3	10285675	10292947	-1	ensembl_havana	0.024944355	-0.194654133	0.244542843	-0.36334239	-0.299013651	0.078393642	0.141952326	0.163140205	0.428535999
ENSG00000111224	0.00143	0.021049085	0.406850307	1.325788191	**PARP11**	protein_coding	poly(ADP-ribose) polymerase family member 11 [Source:HGNC Symbol;Acc:HGNC:1186]	chr12	3791047	3873448	-1	ensembl_havana	4.547878124	4.344452971	4.751303278	4.255242815	4.375174213	4.402941884	4.659372159	4.72703137	4.867506304
ENSG00000131788	0.001414	0.021049085	0.394625459	1.314601429	**PIAS3**	protein_coding	protein inhibitor of activated STAT 3 [Source:HGNC Symbol;Acc:HGNC:16861]	chr1	145848522	145859836	-1	ensembl_havana	5.814774363	5.617461634	6.012087093	5.662942581	5.650193543	5.539248778	6.072956691	5.995941411	5.967363176
ENSG00000198561	0.001411	0.021049085	0.39253471	1.312697693	**CTNND1**	protein_coding	catenin delta 1 [Source:HGNC Symbol;Acc:HGNC:2515]	chr11	57753243	57819546	1	ensembl_havana	2.836419447	2.640152092	3.032686802	2.654944978	2.651792536	2.613718761	3.090972172	3.038025793	2.969062441
ENSG00000109771	0.001429	0.021049085	-0.391157727	-1.311445384	**LRP2BP**	protein_coding	LRP2 binding protein [Source:HGNC Symbol;Acc:HGNC:25434]	chr4	185363879	185395899	-1	ensembl_havana	-0.021643517	0.173935346	-0.21722238	0.10902545	0.185737694	0.227042895	-0.30988669	-0.15890847	-0.182871985
ENSG00000139579	0.001422	0.021049085	-0.420928637	-1.338789032	**NABP2**	protein_coding	nucleic acid binding protein 2 [Source:HGNC Symbol;Acc:HGNC:28412]	chr12	56222015	56229854	1	ensembl_havana	5.249083529	5.459547848	5.038619211	5.386552298	5.525755619	5.466335627	5.009084214	5.02710386	5.079669558
ENSG00000259248	0.001423	0.021049085	-0.424469928	-1.342079308	**USP3-AS1**	antisense	USP3 antisense RNA 1 [Source:HGNC Symbol;Acc:HGNC:44140]	chr15	63544247	63601589	-1	havana	1.512167237	1.724402201	1.299932273	1.613998728	1.729788441	1.829419434	1.178360195	1.241396536	1.480040089
ENSG00000136040	0.00142	0.021049085	-0.439892401	-1.356503153	**PLXNC1**	protein_coding	plexin C1 [Source:HGNC Symbol;Acc:HGNC:9106]	chr12	94148723	94307675	1	ensembl_havana	3.208406489	3.428352689	2.988460288	3.418165445	3.383303079	3.483589543	2.967063242	3.029255182	2.969062441
ENSG00000146842	0.001416	0.021049085	-0.447099893	-1.363297	**TMEM209**	protein_coding	transmembrane protein 209 [Source:HGNC Symbol;Acc:HGNC:21898]	chr7	130164715	130207770	-1	ensembl_havana	5.335837791	5.559387738	5.112287845	5.545378328	5.50528174	5.627503147	5.013909653	5.144444897	5.178508984
ENSG00000130772	0.001421	0.021049085	-0.747734793	-1.679154282	**MED18**	protein_coding	mediator complex subunit 18 [Source:HGNC Symbol;Acc:HGNC:25944]	chr1	28329002	28335967	1	ensembl_havana	3.711994849	4.085862245	3.338127452	4.19918828	4.077246218	3.981152237	3.209288915	3.535908912	3.269184531
ENSG00000124766	0.001431	0.021049085	-0.915247072	-1.885892017	**SOX4**	protein_coding	SRY-box 4 [Source:HGNC Symbol;Acc:HGNC:11200]	chr6	21592768	21598619	1	havana	1.146919525	1.604543061	0.689295989	1.770752281	1.500754142	1.542122761	0.64332197	0.919411336	0.505154661
ENSG00000231808	0.001417	0.021049085	-0.982461135	-1.975833166	**LINC01388**	lincRNA	long intergenic non-protein coding RNA 1388 [Source:HGNC Symbol;Acc:HGNC:50660]	chr9	112713	113754	-1	havana	0.350722676	0.841953243	-0.140507892	0.727424294	0.818355163	0.980080273	-0.00649342	-0.52165082	0.106620565
ENSG00000229375	0.001421	0.021049085	-1.128874961	-2.186881368	**USP24P1**	unprocessed_pseudogene	ubiquitin specific peptidase 24 pseudogene 1 [Source:HGNC Symbol;Acc:HGNC:42014]	chr13	19015487	19019336	-1	havana	0.691933519	1.256370999	0.127496039	0.911909315	1.184892896	1.672310787	0.210814386	-0.11936381	0.291037539
ENSG00000228242	0.001434	0.021053655	0.668238763	1.589131777	**AC093495.1**	antisense		chr3	14144637	14165978	1	havana	0.627562577	0.293443195	0.961681959	0.14220989	0.325758945	0.41236075	1.033805522	0.900298706	0.950941648
ENSG00000242086	0.001434	0.021053655	0.39878295	1.31839525	**MUC20-OT1**	lincRNA	MUC20 overlapping transcript [Source:HGNC Symbol;Acc:HGNC:53807]	chr3	195658062	195739964	1	havana	3.685335002	3.485943527	3.884726477	3.428290208	3.600163325	3.429377047	3.86417946	3.943197246	3.846802724
ENSG00000253981	0.001435	0.021053655	-0.72845327	-1.656861798	**ALG1L13P**	transcribed_unprocessed_pseudogene	asparagine-linked glycosylation 1-like 13, pseudogene [Source:HGNC Symbol;Acc:HGNC:44382]	chr8	8236003	8244667	-1	havana	-0.071483166	0.292743469	-0.435709801	0.464289132	-0.071090603	0.485031876	-0.51701327	-0.68524259	-0.104873545
ENSG00000199237	0.001455	0.021278018	3.040921835	8.23016774	**RNU6-834P**	snRNA	RNA, U6 small nuclear 834, pseudogene [Source:HGNC Symbol;Acc:HGNC:47797]	chr12	49593104	49593213	1	ensembl	-0.780533439	-2.300994357	0.739927478	-1.86005921	-3.410093826	-1.632830031	1.178360195	0.693803314	0.347618925
ENSG00000178585	0.001463	0.021278018	0.558681153	1.472922122	**CTNNBIP1**	protein_coding	catenin beta interacting protein 1 [Source:HGNC Symbol;Acc:HGNC:16913]	chr1	9848276	9910336	-1	ensembl_havana	4.112484237	3.833143661	4.391824813	3.657882359	3.94667289	3.894875732	4.38549012	4.406453481	4.383530839
ENSG00000005483	0.001452	0.021278018	0.457672314	1.373324268	**KMT2E**	protein_coding	lysine methyltransferase 2E [Source:HGNC Symbol;Acc:HGNC:18541]	chr7	105014179	105114361	1	ensembl_havana	6.609482829	6.380646672	6.838318986	6.407611484	6.375910514	6.358418017	6.962192329	6.743561808	6.80920282
ENSG00000119280	0.001465	0.021278018	0.380011289	1.301352039	**C1orf198**	protein_coding	chromosome 1 open reading frame 198 [Source:HGNC Symbol;Acc:HGNC:25900]	chr1	230837119	230869589	-1	ensembl_havana	5.298972879	5.108967234	5.488978524	4.998657321	5.156231424	5.172012959	5.411405351	5.521944411	5.533585809
ENSG00000164162	0.001464	0.021278018	-0.40607236	-1.325073476	**ANAPC10**	protein_coding	anaphase promoting complex subunit 10 [Source:HGNC Symbol;Acc:HGNC:24077]	chr4	144831908	145098541	-1	ensembl_havana	3.66884252	3.8718787	3.46580634	4.016637667	3.819772235	3.779226197	3.545526967	3.433270566	3.418621485
ENSG00000198805	0.001465	0.021278018	-0.414475341	-1.33281389	**PNP**	protein_coding	purine nucleoside phosphorylase [Source:HGNC Symbol;Acc:HGNC:7892]	chr14	20468954	20477094	1	ensembl_havana	6.794643859	7.001881529	6.587406188	7.084940582	6.999805323	6.920898682	6.598945241	6.601761828	6.561511496
ENSG00000025708	0.00146	0.021278018	-0.414684945	-1.333007544	**TYMP**	protein_coding	thymidine phosphorylase [Source:HGNC Symbol;Acc:HGNC:3148]	chr22	50525752	50530056	-1	ensembl_havana	2.922709573	3.130052045	2.7153671	3.348787899	3.029004765	3.012363471	2.962078267	2.660368592	2.523654441
ENSG00000144824	0.001463	0.021278018	-0.50483936	-1.418965349	**PHLDB2**	protein_coding	pleckstrin homology like domain family B member 2 [Source:HGNC Symbol;Acc:HGNC:29573]	chr3	111732497	111976517	1	ensembl_havana	2.248320152	2.500739832	1.995900473	2.126286315	2.683379039	2.692554143	1.75234034	2.072520377	2.162840701
ENSG00000178700	0.00146	0.021278018	-0.520411517	-1.434364331	**DHFR2**	protein_coding	dihydrofolate reductase 2 [Source:HGNC Symbol;Acc:HGNC:27309]	chr3	94047836	94063389	-1	ensembl_havana	3.200446661	3.46065242	2.940240902	3.519511473	3.40872246	3.453723327	2.874587014	2.881149392	3.064986301
ENSG00000166592	0.001459	0.021278018	-0.580803573	-1.495682104	**RRAD**	protein_coding	RRAD, Ras related glycolysis inhibitor and calcium channel regulator [Source:HGNC Symbol;Acc:HGNC:10446]	chr16	66921679	66925644	-1	ensembl_havana	0.966413298	1.256815084	0.676011511	0.931023971	1.440410536	1.399010747	0.176794179	0.900298706	0.950941648
ENSG00000258284	0.001469	0.021298674	2.439624545	5.425005293	**POLR2KP1**	processed_pseudogene	RNA polymerase II subunit K pseudogene 1 [Source:HGNC Symbol;Acc:HGNC:42650]	chr12	49594091	49594258	1	havana	-0.501038854	-1.720851127	0.718773419	-1.99786555	-1.42549258	-1.739195246	1.400976194	0.715834679	0.039509383
ENSG00000145331	0.00147	0.021298674	0.674857547	1.596439131	**TRMT10A**	protein_coding	tRNA methyltransferase 10A [Source:HGNC Symbol;Acc:HGNC:28403]	chr4	99546709	99564032	-1	ensembl_havana	3.938181386	3.600752612	4.275610159	3.666461735	3.646306798	3.489489303	4.427687441	4.093802465	4.305340572
ENSG00000167941	0.001472	0.021299691	2.077741692	4.22145897	**SOST**	protein_coding	sclerostin [Source:HGNC Symbol;Acc:HGNC:13771]	chr17	43753731	43758788	-1	ensembl_havana	0.576558399	-0.462312447	1.615429245	-1.14838881	-0.521012188	0.28246366	1.47283199	1.831488934	1.541966812
ENSG00000148218	0.001473	0.021299691	0.435238005	1.352133879	**ALAD**	protein_coding	aminolevulinate dehydratase [Source:HGNC Symbol;Acc:HGNC:395]	chr9	113386317	113401333	-1	ensembl_havana	6.446140135	6.228521132	6.663759137	6.128536772	6.328833781	6.228192844	6.64749473	6.687527574	6.656255107
ENSG00000089327	0.001476	0.02131908	-0.617560615	-1.534278747	**FXYD5**	protein_coding	FXYD domain containing ion transport regulator 5 [Source:HGNC Symbol;Acc:HGNC:4029]	chr19	35154730	35169883	1	ensembl_havana	0.612109873	0.92089018	0.303329566	0.770507832	1.046001934	0.946160774	-0.04612063	0.554044597	0.402064725
ENSG00000172340	0.001479	0.021323847	-0.415944816	-1.334172136	**SUCLG2**	protein_coding	succinate-CoA ligase GDP-forming beta subunit [Source:HGNC Symbol;Acc:HGNC:11450]	chr3	67360460	67654614	-1	ensembl_havana	5.303871419	5.511843827	5.095899011	5.512445403	5.485260027	5.537826051	5.074059699	5.144444897	5.069192438
ENSG00000169599	0.001481	0.021323847	-0.431941337	-1.349047678	**NFU1**	protein_coding	NFU1 iron-sulfur cluster scaffold [Source:HGNC Symbol;Acc:HGNC:16287]	chr2	69395750	69437628	-1	ensembl_havana	4.642915871	4.85888654	4.426945203	4.86714065	4.914806697	4.794712273	4.355390123	4.48552641	4.439919076
ENSG00000229972	0.001483	0.021323847	-0.686088996	-1.608915989	**IQCF3**	protein_coding	IQ motif containing F3 [Source:HGNC Symbol;Acc:HGNC:31816]	chr3	51817604	51830856	1	ensembl_havana	1.468701495	1.811745993	1.125656997	1.85322802	1.909699171	1.672310787	1.400976194	1.080904991	0.895089805

ENSG00000253217	0.00148	0.021323847	-0.703373895	-1.628308315	**AP001574.1**	lincRNA		chr8	100337595	100350707	1	havana	2.413675498	2.765362446	2.061988551	3.134649994	2.564016113	2.59742123	2.187709208	1.993561814	2.004694631
ENSG00000198753	0.001483	0.021323847	-1.138592772	-2.201661648	**PLXNB3**	protein_coding	plexin B3 [Source:HGNC Symbol;Acc:HGNC:9105]	chrX	153764196	153779346	1	ensembl_havana	-0.241242826	0.32805356	-0.810539213	0.14220989	0.243386951	0.598563838	-0.89704638	-0.57414696	-0.960424301
ENSG00000168502	0.001489	0.021357409	0.521264205	1.435212346	**MTCL1**	protein_coding	microtubule crosslinking factor 1 [Source:HGNC Symbol;Acc:HGNC:29121]	chr18	8705661	8832778	1	ensembl_havana	7.105654641	6.845022538	7.366286744	6.928782582	6.810444255	6.795840778	7.59333262	7.221550375	7.283977236
ENSG00000117408	0.00149	0.021357409	0.381076287	1.302313051	**IPO13**	protein_coding	importin 13 [Source:HGNC Symbol;Acc:HGNC:16853]	chr1	43946939	43968022	1	ensembl_havana	6.08588178	5.895343637	6.276419924	5.870252878	5.940601168	5.875176865	6.240830886	6.295643941	6.292784944
ENSG00000182264	0.001489	0.021357409	-0.536243341	-1.450191418	**IZUMO1**	protein_coding	izumo sperm-egg fusion 1 [Source:HGNC Symbol;Acc:HGNC:28539]	chr19	48740852	48746909	-1	ensembl_havana	2.202720105	2.470841775	1.934598435	2.52259249	2.331270798	2.558662038	1.863449533	1.769643034	2.170702736
ENSG00000105227	0.001498	0.021441742	0.834149816	1.782806117	**PRX**	protein_coding	periaxin [Source:HGNC Symbol;Acc:HGNC:13797]	chr19	40393768	40413366	-1	ensembl_havana	1.915608172	1.498533264	2.33268308	1.464591378	1.488885652	1.542122761	2.548552981	2.351143587	2.09835267
ENSG00000139318	0.001503	0.021492986	0.462318298	1.377753983	**DUSP6**	protein_coding	dual specificity phosphatase 6 [Source:HGNC Symbol;Acc:HGNC:3072]	chr12	89347232	89353271	-1	ensembl_havana	3.385262624	3.154103475	3.616421773	3.071451915	3.162009452	3.228849057	3.601096404	3.510925742	3.737243172
ENSG00000168710	0.001505	0.021492986	0.400881556	1.32031444	**AHCYL1**	protein_coding	adenosylhomocysteinase like 1 [Source:HGNC Symbol;Acc:HGNC:344]	chr1	109984686	110023741	1	ensembl_havana	9.399897212	9.199456434	9.60033799	9.451148145	9.135395214	9.011825943	9.853482155	9.479878052	9.467653762
ENSG00000155158	0.001506	0.021492986	-0.405674171	-1.324707801	**TTC39B**	protein_coding	tetratricopeptide repeat domain 39B [Source:HGNC Symbol;Acc:HGNC:23704]	chr9	15163622	15307360	-1	ensembl_havana	6.350969428	6.553806514	6.148132342	6.575688413	6.524281924	6.561449204	6.101005427	6.15759107	6.185800531
ENSG00000248367	0.001507	0.021492986	-0.487557284	-1.402068936	**AC008610.1**	antisense		chr5	180293245	180295253	1	havana	2.577635246	2.821413889	2.333856604	2.744105292	3.053384979	2.666751395	2.386842807	2.491852285	2.122874721
ENSG00000134070	0.00151	0.021518472	0.423999264	1.34164154	**IRAK2**	protein_coding	interleukin 1 receptor associated kinase 2 [Source:HGNC Symbol;Acc:HGNC:6113]	chr3	10164865	10243743	1	ensembl_havana	3.222286557	3.010286925	3.434286189	2.931352026	3.120305496	2.979203253	3.368464729	3.611304352	3.323089485
ENSG00000087077	0.001518	0.02154329	0.869158907	1.826597681	**TRIP6**	protein_coding	thyroid hormone receptor interactor 6 [Source:HGNC Symbol;Acc:HGNC:12311]	chr7	100867138	100873454	1	ensembl_havana	0.195946786	-0.238632667	0.630526239	-0.41068387	0.09469996	-0.399914092	0.485518989	0.671430284	0.734629445
ENSG00000117425	0.001519	0.02154329	0.436572258	1.353384957	**PTCH2**	protein_coding	patched 2 [Source:HGNC Symbol;Acc:HGNC:9586]	chr1	44819844	44843063	-1	ensembl_havana	2.201699583	1.983413454	2.419985712	1.912130943	1.953538152	2.084571268	2.37188541	2.459573709	2.428498016
ENSG00000115598	0.001518	0.02154329	0.373060264	1.295097096	**IL1RL2**	protein_coding	interleukin 1 receptor like 2 [Source:HGNC Symbol;Acc:HGNC:5999]	chr2	102186973	102240002	1	ensembl_havana	2.751413089	2.564882957	2.937943221	2.711312495	2.464503644	2.518832732	3.063307783	2.836600654	2.913921226
ENSG00000187123	0.00152	0.02154329	-0.963968424	-1.950668229	**LYPD6**	protein_coding	LY6/PLAUR domain containing 6 [Source:HGNC Symbol;Acc:HGNC:28751]	chr2	149329985	149474148	1	ensembl_havana	1.348846247	1.830830459	0.866862035	2.418087419	1.779350901	1.295053056	1.593788933	0.45273451	0.554062661
ENSG00000147485	0.001515	0.02154329	-2.075571838	-4.215114548	**PXDNL**	protein_coding	peroxidasin like [Source:HGNC Symbol;Acc:HGNC:26359]	chr8	51319578	51809445	-1	ensembl_havana	-1.149773798	-0.111987879	-2.187559717	0.237415364	0.06303108	-0.636410081	-1.04978262	-2.16071837	-3.352178165
ENSG00000104043	0.001535	0.021635524	1.57224863	2.973678399	**ATP8B4**	protein_coding	ATPase phospholipid transporting 8B4 (putative) [Source:HGNC Symbol;Acc:HGNC:13536]	chr15	49858238	50182817	-1	ensembl_havana	-0.387512179	-1.173636493	0.398612136	-1.14838881	-1.178380098	-1.194140569	1.01467363	0.286036324	-0.104873545
ENSG00000226835	0.001549	0.021635524	0.958145756	1.942811264	**AC097059.1**	lincRNA		chr1	94247866	94334848	1	havana	1.461935023	0.982862145	1.941007901	-0.31750517	1.62524309	1.640848515	0.975631465	2.542041324	2.305350914
ENSG00000272693	0.00158	0.021635524	0.706436453	1.631768564	**AC073107.1**	transcribed_unprocessed_pseudogene		chr7	65647010	65770810	-1	havana	0.209537454	-0.143680772	0.56275568	-0.22998119	-0.34076431	0.139703184	0.485518989	0.578298274	0.624449778
ENSG00000090530	0.001548	0.021635524	0.647657981	1.566622932	**P3H2**	protein_coding	prolyl 3-hydroxylase 2 [Source:HGNC Symbol;Acc:HGNC:19317]	chr3	189956728	190122437	-1	ensembl_havana	5.714766672	5.390937682	6.038595663	5.00430841	5.53299787	5.635506766	5.846890007	6.172105806	6.096791176
ENSG00000065675	0.001539	0.021635524	0.636030046	1.554046902	**PRKCQ**	protein_coding	protein kinase C theta [Source:HGNC Symbol;Acc:HGNC:9410]	chr10	6427143	6580301	-1	ensembl_havana	2.346590947	2.028575924	2.664605969	1.222335231	2.45233188	2.411060659	2.072167195	2.989113925	2.932536788
ENSG00000278662	0.001567	0.021635524	0.623549171	1.540660699	**GOLGA6L10**	protein_coding	golgin A6 family-like 10 [Source:HGNC Symbol;Acc:HGNC:37228]	chr15	82339998	82349475	-1	ensembl_havana	-0.11529748	-0.427072066	0.196477105	-0.31750517	-0.473795405	-0.489915621	0.276538753	0.343769191	-0.030876629
ENSG00000214243	0.001559	0.021635524	0.586584821	1.501687709	**AC004980.2**	processed_pseudogene		chr7	76650401	76650897	-1	havana	-0.202179427	-0.495471838	0.091112984	-0.56281016	-0.18055577	-0.743049581	0.210814386	0.286036324	-0.223511759
ENSG00000154229	0.001569	0.021635524	0.531907287	1.445839377	**PRKCA**	protein_coding	protein kinase C alpha [Source:HGNC Symbol;Acc:HGNC:9393]	chr17	66302636	66810743	1	ensembl_havana	8.604337596	8.338383953	8.87029124	8.16381626	8.270000436	8.581335162	8.792960492	8.863444313	8.954468915
ENSG00000154240	0.00158	0.021635524	0.499092736	1.413324489	**CEP112**	protein_coding	centrosomal protein 112 [Source:HGNC Symbol;Acc:HGNC:28514]	chr17	65635538	66192084	-1	ensembl_havana	1.756953619	1.507407251	2.006499987	0.986893817	1.58120722	1.954120714	1.567772387	2.139252294	2.31247528
ENSG00000198169	0.001571	0.021635524	0.494699259	1.409027002	**ZNF251**	protein_coding	zinc finger protein 251 [Source:HGNC Symbol;Acc:HGNC:13045]	chr8	144720907	144756417	-1	ensembl_havana	4.678603582	4.431253952	4.925953212	4.230383872	4.565186067	4.498191918	4.862964519	4.982365024	4.932530093
ENSG00000160445	0.001531	0.021635524	0.482431174	1.397096016	**ZER1**	protein_coding	zyg-11 related cell cycle regulator [Source:HGNC Symbol;Acc:HGNC:30960]	chr9	128729786	128772414	-1	ensembl_havana	5.908884248	5.667668661	6.150099835	5.566159292	5.618544736	5.818301954	6.083323784	6.190906722	6.176068999
ENSG00000151414	0.001562	0.021635524	0.455707066	1.371454791	**NEK7**	protein_coding	NIMA related kinase 7 [Source:HGNC Symbol;Acc:HGNC:13386]	chr1	198156963	198322420	1	ensembl_havana	6.385257333	6.1574038	6.613110866	6.272706027	6.222656431	5.976848943	6.70083647	6.597689015	6.540807112
ENSG00000102908	0.001548	0.021635524	0.45006711	1.366103802	**NFAT5**	protein_coding	nuclear factor of activated T cells 5 [Source:HGNC Symbol;Acc:HGNC:7774]	chr16	69565094	69704666	1	ensembl_havana	7.935924783	7.710891228	8.160958338	8.016965543	7.531521174	7.584186967	8.399543542	8.08816905	7.995162422
ENSG00000174749	0.001561	0.021635524	0.443500897	1.359900311	**FAM241A**	protein_coding	family with sequence similarity 241 member A [Source:HGNC Symbol;Acc:HGNC:26813]	chr4	112145397	112195256	1	ensembl_havana	5.072600835	4.850850386	5.294351283	5.003179961	4.956339888	4.59303131	5.531523371	5.305659081	5.045871397
ENSG00000095209	0.001574	0.021635524	0.423355386	1.341042896	**TMEM38B**	protein_coding	transmembrane protein 38B [Source:HGNC Symbol;Acc:HGNC:25535]	chr9	105694544	105776612	1	ensembl_havana	4.403263826	4.191586133	4.614941519	4.056348776	4.225865299	4.292544325	4.451003248	4.59061015	4.803211159
ENSG00000084444	0.001566	0.021635524	0.421047584	1.338899416	**FAM234B**	protein_coding	family with sequence similarity 234 member B [Source:HGNC Symbol;Acc:HGNC:29288]	chr12	13044284	13142521	1	ensembl_havana	5.321708554	5.111184762	5.532232346	4.889298954	5.145876371	5.298378963	5.388364636	5.560501132	5.64783127
ENSG00000151502	0.001545	0.021635524	0.398521953	1.318156762	**VPS26B**	protein_coding	VPS26, retromer complex component B [Source:HGNC Symbol;Acc:HGNC:28119]	chr11	134224645	134247792	1	ensembl_havana	6.602228226	6.402967249	6.801489202	6.405050469	6.308298684	6.495552594	6.818242837	6.753913364	6.832311407
ENSG00000267317	0.001578	0.021635524	0.383861133	1.304829345	**AC027307.2**	antisense		chr19	1457670	1458580	-1	havana	3.863889605	3.671959039	4.055820171	3.553827547	3.800696809	3.661352761	3.942194775	4.139344231	4.085921508
ENSG00000166900	0.001555	0.021635524	0.376801649	1.298460069	**STX3**	protein_coding	syntaxin 3 [Source:HGNC Symbol;Acc:HGNC:11438]	chr11	59713456	59805882	1	ensembl_havana	5.444946936	5.256546112	5.63334776	5.161268806	5.306633645	5.301735885	5.55074389	5.646890005	5.702409386
ENSG00000151498	0.001556	0.021635524	0.376786957	1.298446847	**ACAD8**	protein_coding	acyl-CoA dehydrogenase family member 8 [Source:HGNC Symbol;Acc:HGNC:87]	chr11	134253495	134265855	1	ensembl_havana	6.520879892	6.332486414	6.709273371	6.317426798	6.330912858	6.349119586	6.715721605	6.723313053	6.688785454
ENSG00000168389	0.001579	0.021635524	-0.371540638	-1.293733657	**MFSD2A**	protein_coding	major facilitator superfamily domain containing 2A [Source:HGNC Symbol;Acc:HGNC:25897]	chr1	39955112	39969968	1	ensembl_havana	6.949245342	7.135015661	6.763475023	7.610751983	7.0642077	6.7300873	7.227673981	6.674579055	6.388172033
ENSG00000125746	0.00157	0.021635524	-0.380868085	-1.302125122	**EML2**	protein_coding	echinoderm microtubule associated protein like 2 [Source:HGNC Symbol;Acc:HGNC:18035]	chr19	45606994	45645629	-1	ensembl_havana	5.604049314	5.794483357	5.413615272	5.934410745	5.662132841	5.786906485	5.509482944	5.311096432	5.42026644
ENSG00000077152	0.001547	0.021635524	-0.383185802	-1.304218692	**UBE2T**	protein_coding	ubiquitin conjugating enzyme E2 T [Source:HGNC Symbol;Acc:HGNC:25009]	chr1	202331657	202341980	-1	ensembl_havana	5.978699086	6.170291987	5.787106186	6.029480675	6.186831597	6.29456369	5.629099866	5.775047272	5.957171419
ENSG00000245937	0.00157	0.021635524	-0.386227357	-1.306971206	**LINC01184**	lincRNA	long intergenic non-protein coding RNA 1184 [Source:HGNC Symbol;Acc:HGNC:49565]	chr5	127940426	128083172	-1	havana	3.956379685	4.149493364	3.763266006	4.136757067	4.202437314	4.109285709	3.735708444	3.780254478	3.773835098
ENSG00000172123	0.001549	0.021635524	-0.386460044	-1.307182019	**SLFN12**	protein_coding	schlafen family member 12 [Source:HGNC Symbol;Acc:HGNC:25500]	chr17	35411060	35433283	-1	ensembl_havana	2.844232497	3.037462519	2.651002475	3.171281297	2.995843375	2.945262884	2.792720531	2.648963151	2.511323741
ENSG00000167550	0.001577	0.021635524	-0.387473308	-1.30810043	**RHEBL1**	protein_coding	RHEB like 1 [Source:HGNC Symbol;Acc:HGNC:21166]	chr12	49064685	49070025	-1	ensembl_havana	4.605792255	4.799528909	4.412055601	4.926680408	4.733059677	4.738846643	4.489622106	4.351222967	4.39532173
ENSG00000198176	0.001534	0.021635524	-0.387736261	-1.308338872	**TFDP1**	protein_coding	transcription factor Dp-1 [Source:HGNC Symbol;Acc:HGNC:11749]	chr13	113584721	113641470	1	ensembl_havana	6.693434904	6.887303034	6.499566774	6.805383636	6.915036128	6.941489339	6.403654565	6.491543256	6.6035025
ENSG00000108469	0.001556	0.021635524	-0.405507705	-1.324554958	**RECQL5**	protein_coding	RecQ like helicase 5 [Source:HGNC Symbol;Acc:HGNC:9950]	chr17	75626845	75667189	-1	ensembl_havana	5.60136553	5.804119382	5.398611677	5.741509981	5.721025012	5.949823153	5.302231373	5.387718417	5.505885242
ENSG00000186814	0.00155	0.021635524	-0.430727407	-1.347913024	**ZSCAN30**	protein_coding	zinc finger and SCAN domain containing 30 [Source:HGNC Symbol;Acc:HGNC:33517]	chr18	35251058	35290245	-1	ensembl_havana	5.713183576	5.92854728	5.497819873	5.927289173	5.921964678	5.936387988	5.439474076	5.455573779	5.598411764
ENSG00000077684	0.00158	0.021635524	-0.500937122	-1.415132483	**JADE1**	protein_coding	jade family PHD finger 1 [Source:HGNC Symbol;Acc:HGNC:30027]	chr4	128809623	128875224	1	ensembl_havana	5.110670913	5.361139474	4.860202351	5.204129902	5.350744554	5.528543966	4.764756719	4.930317649	4.885532687
ENSG00000126458	0.00157	0.021635524	-0.505308491	-1.419426839	**RRAS**	protein_coding	RAS related [Source:HGNC Symbol;Acc:HGNC:10447]	chr19	49635292	49640201	-1	ensembl_havana	3.985524843	4.238179088	3.732870598	4.357685434	4.350791632	4.006060199	3.942194775	3.702394978	3.55402204
ENSG00000240771	0.001555	0.021635524	-0.553674061	-1.467818983	**ARHGEF25**	protein_coding	Rho guanine nucleotide exchange factor 25 [Source:HGNC Symbol;Acc:HGNC:30275]	chr12	57610180	57619379	1	ensembl_havana	5.842553922	6.119390952	5.565716891	6.331881075	6.117705714	5.908586067	5.751159942	5.430812746	5.515177986
ENSG00000152147	0.001572	0.021635524	-0.554492677	-1.468652091	**GEMIN6**	protein_coding	gem nuclear organelle associated protein 6 [Source:HGNC Symbol;Acc:HGNC:20044]	chr2	38751534	38785000	1	ensembl_havana	5.197180201	5.47442654	4.919933863	5.484415117	5.477773493	5.46109101	4.7959082	4.895752859	5.068140529
ENSG00000280734	0.00158	0.021635524	-0.587308414	-1.502441079	**LINC01232**	processed_transcript	long intergenic non-protein coding RNA 1232 [Source:HGNC Symbol;Acc:HGNC:49755]	chr13	99486962	99499306	-1	havana	1.923628499	2.217282706	1.629974292	2.451565702	1.996084195	2.204198221	1.681320805	1.452931788	1.755670283
ENSG00000136147	0.00156	0.021635524	-0.652305058	-1.57167733	**PHF11**	protein_coding	PHD finger protein 11 [Source:HGNC Symbol;Acc:HGNC:17024]	chr13	49495610	49528987	1	ensembl_havana	3.096807413	3.422959942	2.770654884	3.532084689	3.558185553	3.178609585	2.981915645	2.682912485	2.647136522
ENSG00000069667	0.001565	0.021635524	-0.656096477	-1.575813148	**RORA**	protein_coding	RAR related orphan receptor A [Source:HGNC Symbol;Acc:HGNC:10258]	chr15	60488284	61229319	-1	ensembl_havana	2.250530304	2.578578543	1.922482065	2.954889309	2.541211407	2.239634912	2.090556387	2.063957599	1.61293221
ENSG00000067842	0.001588	0.021638848	1.863429062	3.638715011	**ATP2B3**	protein_coding	ATPase plasma membrane Ca2+ transporting 3 [Source:HGNC Symbol;Acc:HGNC:816]	chrX	153517676	153582939	1	ensembl_havana	-0.344509608	-1.276224139	0.587204922	-1.41179303	-1.617400645	-0.799478738	1.071324589	0.315191528	0.375098651
ENSG00000185352	0.001587	0.021638848	0.837999427	1.787569615	**HS6ST3**	protein_coding	heparan sulfate 6-O-sulfotransferase 3 [Source:HGNC Symbol;Acc:HGNC:19134]	chr13	96090839	96839562	1	ensembl_havana	6.291278748	5.872279034	6.710278461	6.329181906	5.807179545	5.480475652	6.89086505	6.655470162	6.584500172
ENSG00000115170	0.001587	0.021638848	-0.511415477	-1.425448065	**ACVR1**	protein_coding	activin A receptor type 1 [Source:HGNC Symbol;Acc:HGNC:171]	chr2	157736444	157875862	-1	ensembl_havana	4.693299272	4.949007011	4.437591533	4.736070601	4.882285857	5.228664573	4.361081916	4.354737515	4.596955169
ENSG00000211777	0.001587	0.021638848	-0.699679058	-1.624143445	**TRAV3**	TR_V_gene	T cell receptor alpha variable 3 (gene/pseudogene) [Source:HGNC Symbol;Acc:HGNC:12128]	chr14	21723713	21724321	1	havana	1.798893566	2.148733095	1.449054037	2.369873023	2.256240478	1.820085783	1.740743887	1.315408538	1.291009686
ENSG00000064763	0.001583	0.021638848	-0.864762303	-1.82103961	**FAR2**	protein_coding	fatty acyl-CoA reductase 2 [Source:HGNC Symbol;Acc:HGNC:25531]	chr12	29149103	29340980	1	ensembl_havana	3.138847138	3.57122829	2.706465987	3.888048657	3.439879526	3.385756687	2.815009813	2.895698436	2.40868971
ENSG00000153044	0.001594	0.021690868	-0.556591658	-1.470790393	**CENPH**	protein_coding	centromere protein H [Source:HGNC Symbol;Acc:HGNC:17268]	chr5	69189548	69210357	1	ensembl_havana	3.228181857	3.506477686	2.949886028	3.283361026	3.68322949	3.552842542	2.675796091	3.007092508	3.166769484
ENSG00000128596	0.001595	0.021694499	0.416473122	1.334660791	**CCDC136**	protein_coding	coiled-coil domain containing 136 [Source:HGNC Symbol;Acc:HGNC:22225]	chr7	128790757	128822132	1	ensembl_havana	5.398805469	5.190568908	5.60704203	5.11813806	5.100775207	5.352793458	5.563965245	5.57111489	5.686045955
ENSG00000166548	0.001597	0.021699858	-0.445279816	-1.361578174	**TK2**	protein_coding	thymidine kinase 2, mitochondrial [Source:HGNC Symbol;Acc:HGNC:11831]	chr16	66508003	66552544	-1	ensembl_havana	3.910632276	4.133272184	3.687992368	4.210965817	4.049234073	4.139616662	3.706198956	3.710647067	3.647131082
ENSG00000240583	0.001602	0.02174747	-1.920720735	-3.786121567	**AQP1**	protein_coding	aquaporin 1 (Colton blood group) [Source:HGNC Symbol;Acc:HGNC:633]	chr7	30911694	30925516	1	ensembl_havana	0.065113497	1.025473864	-0.89524687	1.312554705	1.428032914	0.335833975	-0.63291296	-1.15951152	-0.893316134
ENSG00000053747	0.00161	0.021753919	1.43385982	2.701685647	**LAMA3**	protein_coding	laminin subunit alpha 3 [Source:HGNC Symbol;Acc:HGNC:6483]	chr18	23689443	23955066	1	ensembl_havana	1.207975553	0.491045643	1.924905463	-0.10791135	0.837200041	0.743848241	1.895179555	2.072520377	1.807016457
ENSG00000184203	0.001605	0.021753919	0.420214531	1.338126522	**PPP1R2**	protein_coding	protein phosphatase 1 regulatory inhibitor subunit 2 [Source:HGNC Symbol;Acc:HGNC:9288]	chr3	195514425	195543386	-1	ensembl_havana	4.940723083	4.730615818	5.150830349	4.642053159	4.820888145	4.72890615	5.079823989	5.157583505	5.215083552
ENSG00000134333	0.001607	0.021753919	-0.418883558	-1.336892589	**LDHA**	protein_coding	lactate dehydrogenase A [Source:HGNC Symbol;Acc:HGNC:6535]	chr11	18394388	18408425	1	ensembl_havana	9.403726877	9.613168656	9.194285098	9.565076927	9.625723889	9.648705152	9.082436659	9.290096605	9.210322029
ENSG00000163322	0.001608	0.021753919	-0.553494	-1.467635798	**ABRAXAS1**	protein_coding	abraxas 1, BRCA1 A complex subunit [Source:HGNC Symbol;Acc:HGNC:25829]	chr4	83459517	83523348	-1	ensembl_havana	4.620954973	4.897701973	4.344207973	4.91952048	4.94008775	4.833497689	4.22204552	4.373915965	4.436662434
ENSG00000137558	0.001609	0.021753919	-0.730284252	-1.658965923	**PI15**	protein_coding	peptidase inhibitor 15 [Source:HGNC Symbol;Acc:HGNC:8946]	chr8	74824537	74855029	1	ensembl_havana	1.499667194	1.86480932	1.134525068	1.912130943	1.719668303	1.962628713	1.089724456	0.821210834	1.492639914
ENSG00000164845	0.001614	0.021780386	0.464140796	1.379495544	**FAM86FP**	transcribed_unprocessed_pseudogene	family with sequence similarity 86 member F, pseudogene [Source:HGNC Symbol;Acc:HGNC:42357]	chr12	8232512	8242948	-1	havana	2.047895524	1.815825126	2.279965922	1.671956805	1.779350901	1.996167673	2.254470464	2.163501883	2.42192542
ENSG00000180447	0.001617	0.021781736	0.636387165	1.554431633	**GAS1**	protein_coding	growth arrest specific 1 [Source:HGNC Symbol;Acc:HGNC:4165]	chr9	86944363	86947189	-1	ensembl_havana	1.369090002	1.050896419	1.687283584	0.832812769	1.242575255	1.077301233	1.514288036	1.671599815	1.875962902
ENSG00000224066	0.001625	0.021781736	0.448420056	1.364545079	**AL049795.1**	antisense		chr1	32204769	32206814	-1	havana	3.094859528	2.8706495	3.319069556	3.058473165	2.651792536	2.901682799	3.562094275	3.155524498	3.239589895
ENSG00000274386	0.001623	0.021781736	0.441790918	1.358289421	**TMEM269**	protein_coding	transmembrane protein 269 [Source:HGNC Symbol;Acc:HGNC:52381]	chr1	42785007	42816619	1	ensembl_havana	0.942014314	0.721118855	1.162909773	0.660286109	0.799260866	0.703809589	1.071324589	1.343981945	1.073422785
ENSG00000262580	0.001617	0.021781736	0.370104442	1.292446393	**AC087741.1**	antisense		chr17	80200673	80205949	-1	havana	5.886134615	5.701082394	6.071186836	5.775615037	5.694788321	5.632843823	6.164768247	6.033704856	6.015087405
ENSG00000049883	0.001623	0.021781736	-0.378523826	-1.300010996	**PTCD2**	protein_coding	pentatricopeptide repeat domain 2 [Source:HGNC Symbol;Acc:HGNC:25734]	chr5	72320367	72368395	1	ensembl_havana	4.672437192	4.861699105	4.483175279	4.669343006	4.826793299	5.088961009	4.305097297	4.479100033	4.665328507
ENSG00000024526	0.001626	0.021781736	-0.402962774	-1.322220487	**DEPDC1**	protein_coding	DEP domain containing 1 [Source:HGNC Symbol;Acc:HGNC:22949]	chr1	68474152	68497221	-1	ensembl_havana	5.897593667	6.099075054	5.69611228	5.850937542	6.20099568	6.245291939	5.375299268	5.82214175	5.890895822
ENSG00000203791	0.001625	0.021781736	-0.417782923	-1.33587306	**EEF1AKMT2**	protein_coding	EEF1A lysine methyltransferase 2 [Source:HGNC Symbol;Acc:HGNC:33787]	chr10	124748149	124791870	-1	ensembl_havana	1.650391464	1.859282926	1.441500002	1.912130943	1.817810321	1.847907512	1.554586035	1.315408538	1.454505434
ENSG00000233695	0.001623	0.021781736	-0.707246542	-1.632685078	**GAS6-AS1**	antisense	GAS6 antisense RNA 1 [Source:HGNC Symbol;Acc:HGNC:39826]	chr13	113815630	113845744	1	ensembl_havana	-0.146360084	0.207263186	-0.499983355	0.297565234	0.214850257	0.109374069	-0.30988669	-0.74417246	-0.445890917
ENSG00000116701	0.001628	0.021784007	-0.460569084	-1.37608452	**NCF2**	protein_coding	neutrophil cytosolic factor 2 [Source:HGNC Symbol;Acc:HGNC:7661]	chr1	183555563	183590876	-1	ensembl_havana	8.696907377	8.927191919	8.466622835	9.189343973	8.862238643	8.729993141	8.619103266	8.416391385	8.364373854
ENSG00000261795	0.001629	0.021785363	-0.81347315	-1.757437216	**AC093627.7**	lincRNA		chr7	161765	164972	-1	havana	-0.902556873	-0.495820298	-1.309293449	-1.51148723	-0.258437325	0.28246366	-2.22890346	-0.86978558	-0.829191301
ENSG00000167363	0.001645	0.021857667	0.597351942	1.512937029	**FN3K**	protein_coding	fructosamine 3 kinase [Source:HGNC Symbol;Acc:HGNC:24822]	chr17	82735575	82751197	1	ensembl_havana	1.116884242	0.818208271	1.415560213	0.812341977	0.679062629	0.963220207	1.229040238	1.43975459	1.577885811
ENSG00000166866	0.001639	0.021857667	0.416206761	1.334414398	**MYO1A**	protein_coding	myosin IA [Source:HGNC Symbol;Acc:HGNC:7595]	chr12	57028517	57051198	-1	ensembl_havana	1.439133473	1.231030093	1.647236853	1.175017601	1.170104606	1.347968071	1.514288036	1.590453245	1.836969279
ENSG00000165434	0.001645	0.021857667	0.410915551	1.329529281	**PGM2L1**	protein_coding	phosphoglucomutase 2 like 1 [Source:HGNC Symbol;Acc:HGNC:20898]	chr11	74330318	74398473	-1	ensembl_havana	4.103516981	3.898059205	4.308974756	3.719644151	4.014471486	3.96006198	4.179729066	4.458014382	4.289180821
ENSG00000233539	0.001646	0.021857667	0.405992662	1.325000277	**AC011294.1**	lincRNA		chr7	46673785	46759851	-1	havana	2.860194581	2.65719825	3.063190911	2.464742477	2.61404572	2.892806553	2.792720531	3.059721845	3.337130358
ENSG00000206530	0.001639	0.021857667	0.395603151	1.315492616	**CFAP44**	protein_coding	cilia and flagella associated protein 44 [Source:HGNC Symbol;Acc:HGNC:25631]	chr3	113286947	113441610	-1	havana	3.886676695	3.688875119	4.08447827	3.599344412	3.608414274	3.858866672	3.929481684	4.051115116	4.272838011
ENSG00000198331	0.001641	0.021857667	-0.378720716	-1.300188425	**HYLS1**	protein_coding	HYLS1, centriolar and ciliogenesis associated [Source:HGNC Symbol;Acc:HGNC:26558]	chr11	125883614	125900648	1	ensembl_havana	5.255435206	5.444795564	5.066074848	5.388282172	5.557353082	5.388751436	5.047243228	5.134256222	5.016725093
ENSG00000104368	0.001647	0.021857667	-0.535812866	-1.449758771	**PLAT**	protein_coding	plasminogen activator, tissue type [Source:HGNC Symbol;Acc:HGNC:9051]	chr8	42175233	42207724	-1	ensembl_havana	6.619091517	6.88699795	6.351185084	7.135511693	6.75516016	6.770321998	6.580360368	6.371768956	6.101425928
ENSG00000143178	0.001642	0.021857667	-0.636346554	-1.554387876	**TBX19**	protein_coding	T-box 19 [Source:HGNC Symbol;Acc:HGNC:11596]	chr1	168281040	168314426	1	ensembl_havana	5.370488826	5.688662102	5.052315549	5.508474265	5.685719046	5.871792996	5.091283933	4.947876234	5.117786479
ENSG00000130956	0.001653	0.021908473	0.409596297	1.328314065	**HABP4**	protein_coding	hyaluronan binding protein 4 [Source:HGNC Symbol;Acc:HGNC:17062]	chr9	96450201	96491336	1	ensembl_havana	4.45465987	4.249861722	4.659458019	4.247639431	4.240095677	4.261850058	4.614010932	4.611326446	4.753036678
ENSG00000198826	0.001654	0.021908473	-0.384658996	-1.305551162	**ARHGAP11A**	protein_coding	Rho GTPase activating protein 11A [Source:HGNC Symbol;Acc:HGNC:15783]	chr15	32615144	32639949	1	ensembl_havana	6.811466784	7.003796282	6.619137287	6.850944768	7.059200126	7.101243953	6.415099466	6.679492172	6.762820222
ENSG00000196730	0.001655	0.021908473	-0.637721538	-1.555870018	**DAPK1**	protein_coding	death associated protein kinase 1 [Source:HGNC Symbol;Acc:HGNC:2674]	chr9	87497228	87708633	1	ensembl_havana	1.982812841	2.30167361	1.663952072	2.383813765	2.037411386	2.483795679	1.554586035	1.590453245	1.846816935
ENSG00000157870	0.001658	0.021915141	0.52529719	1.439230025	**FAM213B**	protein_coding	family with sequence similarity 213 member B [Source:HGNC Symbol;Acc:HGNC:28390]	chr1	2586491	2591469	1	ensembl_havana	4.573758231	4.311109636	4.836406826	4.222647985	4.187828682	4.522852242	4.760456192	4.845427678	4.903336608
ENSG00000166432	0.001659	0.021915141	0.501415099	1.415601405	**ZMAT1**	protein_coding	zinc finger matrin-type 1 [Source:HGNC Symbol;Acc:HGNC:29377]	chrX	101882288	101932031	-1	ensembl_havana	3.23825868	2.987551131	3.488966229	2.643402293	2.896025364	3.423225735	3.157897671	3.517212211	3.791788806
ENSG00000145687	0.001665	0.021969055	0.543645194	1.457650851	**SSBP2**	protein_coding	single stranded DNA binding protein 2 [Source:HGNC Symbol;Acc:HGNC:15831]	chr5	81413021	81751797	-1	ensembl_havana	6.430241824	6.158419227	6.702064421	5.938548818	6.367022561	6.169686301	6.651758006	6.805548907	6.648886349
ENSG00000103066	0.001667	0.021976754	0.584457329	1.499474855	**PLA2G15**	protein_coding	phospholipase A2 group XV [Source:HGNC Symbol;Acc:HGNC:17163]	chr16	68245304	68261062	1	ensembl_havana	4.62105841	4.328829745	4.913287074	4.045427109	4.442870209	4.498191918	4.798707081	5.024899564	4.916254578
ENSG00000072121	0.001672	0.022023247	0.364239793	1.287203178	**ZFYVE26**	protein_coding	zinc finger FYVE-type containing 26 [Source:HGNC Symbol;Acc:HGNC:20761]	chr14	67727374	67816590	-1	ensembl_havana	5.946734248	5.764614352	6.128854144	5.705885681	5.787935768	5.800021607	6.069484373	6.174096427	6.142981633
ENSG00000163141	0.001676	0.022049625	0.605206563	1.521196541	**BNIPL**	protein_coding	BCL2 interacting protein like [Source:HGNC Symbol;Acc:HGNC:16976]	chr1	151036570	151047600	1	ensembl_havana	0.729267898	0.426664616	1.031871179	0.206373087	0.453384582	0.620236179	0.830009965	1.226127349	1.039476224
ENSG00000148158	0.001677	0.022049625	0.439377126	1.356018749	**SNX30**	protein_coding	sorting nexin family member 30 [Source:HGNC Symbol;Acc:HGNC:23685]	chr9	112750838	112881671	1	ensembl_havana	5.207462662	4.987774099	5.427151225	4.949121894	4.960643048	5.053557356	5.409575575	5.479916998	5.391961103
ENSG00000118156	0.001678	0.022049625	-0.540410238	-1.454386021	**ZNF541**	protein_coding	zinc finger protein 541 [Source:HGNC Symbol;Acc:HGNC:25294]	chr19	47520685	47555856	-1	ensembl_havana	2.170072384	2.440277503	1.899867265	2.260788651	2.535453458	2.524590399	1.681320805	1.871294071	2.146986918
ENSG00000114446	0.001683	0.022069221	0.380525994	1.3018164	**IFT57**	protein_coding	intraflagellar transport 57 [Source:HGNC Symbol;Acc:HGNC:17367]	chr3	108160812	108222570	-1	ensembl_havana	6.390895503	6.200632505	6.5811585	6.212953158	6.247082695	6.141861664	6.592104777	6.582031645	6.569339077
ENSG00000204348	0.001684	0.022069221	-0.446173049	-1.362421445	**DXO**	protein_coding	decapping exoribonuclease [Source:HGNC Symbol;Acc:HGNC:2992]	chr6	31969810	31972292	-1	ensembl_havana	6.042939893	6.266026417	5.819853369	6.419080124	6.274130823	6.104868305	6.052582667	5.720245956	5.686731483
ENSG00000170899	0.001684	0.022069221	-0.986971427	-1.982019871	**GSTA4**	protein_coding	glutathione S-transferase alpha 4 [Source:HGNC Symbol;Acc:HGNC:4629]	chr6	52977948	52995378	-1	ensembl_havana	1.145757866	1.63924358	0.652272153	1.716703112	1.592342839	1.608684788	1.107892607	0.343769191	0.505154661
ENSG00000163491	0.001688	0.022100614	1.07703932	2.109702133	**NEK10**	protein_coding	NIMA related kinase 10 [Source:HGNC Symbol;Acc:HGNC:18592]	chr3	27110085	27369460	-1	ensembl_havana	1.031623054	0.493103394	1.570142714	0.174648163	0.89230127	0.41236075	1.444517675	1.541948597	1.723961872
ENSG00000163347	0.001693	0.022135808	0.648331453	1.567354426	**CLDN1**	protein_coding	claudin 1 [Source:HGNC Symbol;Acc:HGNC:2032]	chr3	190305701	190322475	-1	ensembl_havana	3.734302303	3.410136577	4.05846803	2.945520426	3.532399813	3.752489491	3.812494555	4.12706564	4.235843895
ENSG00000223749	0.001694	0.022137235	0.398716106	1.318334166	**MIR503HG**	lincRNA	MIR503 host gene [Source:HGNC Symbol;Acc:HGNC:28258]	chrX	134543337	134546632	-1	havana	2.025045939	1.825687886	2.224403992	1.602122376	1.845996665	2.028944618	1.936429043	2.308284917	2.428498016
ENSG00000238133	0.001698	0.022149095	0.501431051	1.415617058	**MAP3K20-AS1**	antisense	MAP3K20 antisense RNA 1 [Source:HGNC Symbol;Acc:HGNC:27935]	chr2	173166446	173282036	-1	havana	3.303046249	3.052330724	3.553761775	3.272162151	3.202541664	2.682288355	3.921799706	3.628827713	3.110657906
ENSG00000122678	0.001697	0.022149095	-0.430214567	-1.347433961	**POLM**	protein_coding	DNA polymerase mu [Source:HGNC Symbol;Acc:HGNC:9185]	chr7	44072247	44082540	-1	ensembl_havana	4.48906897	4.704176253	4.273961686	4.690528814	4.636849344	4.785150603	4.305097297	4.262328235	4.254459527
ENSG00000196421	0.001733	0.022389395	0.858086214	1.812632194	**C20orf204**	protein_coding	chromosome 20 open reading frame 204 [Source:HGNC Symbol;Acc:HGNC:27655]	chr20	64034344	64039962	1	havana	2.72796524	2.298922133	3.157008347	1.95010455	2.169677667	2.776984183	3.072588313	3.068309725	3.330127003
ENSG00000085563	0.001737	0.022389395	0.719829601	1.646987495	**ABCB1**	protein_coding	ATP binding cassette subfamily B member 1 [Source:HGNC Symbol;Acc:HGNC:40]	chr7	87503633	87713323	-1	ensembl_havana	4.329356321	3.96944152	4.689271121	3.506827715	4.077246218	4.324250626	4.445656025	4.817682322	4.804475016
ENSG00000125652	0.001732	0.022389395	0.522906918	1.436847469	**ALKBH7**	protein_coding	alkB homolog 7 [Source:HGNC Symbol;Acc:HGNC:21306]	chr19	6372433	6375251	1	ensembl_havana	0.961711346	0.700257887	1.223164805	0.892538	0.52486995	0.683365711	1.400976194	1.195095435	1.073422785
ENSG00000177822	0.001734	0.022389395	0.479822555	1.39457213	**AC098864.1**	antisense		chr4	181874438	182145249	-1	havana	2.341633403	2.101722125	2.58154468	2.150951608	2.101167058	2.053047709	2.494337705	2.648963151	2.601333184
ENSG00000135916	0.001726	0.022389395	0.451498681	1.367460045	**ITM2C**	protein_coding	integral membrane protein 2C [Source:HGNC Symbol;Acc:HGNC:6175]	chr2	230864639	230879248	1	ensembl_havana	5.823147237	5.597397897	6.048896578	5.313780003	5.690908468	5.78750522	5.855652395	6.16211129	6.128926047
ENSG00000251562	0.001723	0.022389395	0.404996704	1.324085885	**MALAT1**	lincRNA	metastasis associated lung adenocarcinoma transcript 1 (non-protein coding) [Source:HGNC Symbol;Acc:HGNC:29665]	chr11	65497688	65506516	1	havana	7.935519011	7.733020659	8.138017362	7.858455936	7.689422907	7.651183133	8.182325525	8.130558475	8.101168087
ENSG00000186684	0.001728	0.022389395	0.393130534	1.313239941	**CYP27C1**	protein_coding	cytochrome P450 family 27 subfamily C member 1 [Source:HGNC Symbol;Acc:HGNC:33480]	chr2	127184120	127220078	-1	ensembl_havana	1.966874412	1.770309145	2.163439679	1.464591378	1.96214833	1.884187727	1.820027213	2.322713072	2.347578751
ENSG00000167112	0.001729	0.022389395	0.361154115	1.284453012	**TRUB2**	protein_coding	TruB pseudouridine synthase family member 2 [Source:HGNC Symbol;Acc:HGNC:17170]	chr9	128305161	128322742	-1	ensembl_havana	5.253550108	5.072973051	5.434127165	4.908713517	5.117251617	5.192954018	5.288358331	5.454755221	5.559267944
ENSG00000065457	0.001723	0.022389395	-0.385104159	-1.30595407	**ADAT1**	protein_coding	adenosine deaminase, tRNA specific 1 [Source:HGNC Symbol;Acc:HGNC:228]	chr16	75596981	75623300	-1	ensembl_havana	5.091125681	5.28367776	4.898573601	5.175363862	5.247988455	5.427680963	4.778999921	4.920864951	4.995855932
ENSG00000238164	0.001721	0.022389395	-0.395347525	-1.31525955	**TNFRSF14-AS1**	antisense	TNFRSF14 antisense RNA 1 [Source:HGNC Symbol;Acc:HGNC:26966]	chr1	2549920	2557031	-1	havana	3.974328254	4.172002016	3.776654491	4.268453071	3.997818193	4.249734786	3.86417946	3.668906348	3.796877665
ENSG00000179598	0.001724	0.022389395	-0.426938813	-1.344377976	**PLD6**	protein_coding	phospholipase D family member 6 [Source:HGNC Symbol;Acc:HGNC:30447]	chr17	17200995	17206315	-1	ensembl_havana	2.914202825	3.127672232	2.700733419	2.954889309	3.224179389	3.203947998	2.555188851	2.86645213	2.680559275
ENSG00000176105	0.001736	0.022389395	-0.440406399	-1.356986529	**YES1**	protein_coding	YES proto-oncogene 1, Src family tyrosine kinase [Source:HGNC Symbol;Acc:HGNC:12841]	chr18	721588	812546	-1	ensembl_havana	5.27905932	5.499262519	5.058856121	5.609071199	5.622623879	5.26609248	5.214824556	5.072632715	4.889111092
ENSG00000177465	0.001735	0.022389395	-0.469117107	-1.384262075	**ACOT4**	protein_coding	acyl-CoA thioesterase 4 [Source:HGNC Symbol;Acc:HGNC:19748]	chr14	73591706	73596496	1	ensembl_havana	1.487996154	1.722554708	1.253437601	1.613998728	1.678462547	1.875202847	1.212343926	1.271458479	1.276510397
ENSG00000139636	0.001743	0.022444213	0.408117648	1.326953346	**LMBR1L**	protein_coding	limb development membrane protein 1 like [Source:HGNC Symbol;Acc:HGNC:18268]	chr12	49096551	49110900	-1	ensembl_havana	6.405803918	6.201745094	6.609862742	6.448810398	6.145849239	6.010575645	6.773772305	6.580156303	6.475659618
ENSG00000189077	0.001747	0.022481193	0.443871205	1.360249412	**TMEM120A**	protein_coding	transmembrane protein 120A [Source:HGNC Symbol;Acc:HGNC:21697]	chr7	75986837	75994659	-1	ensembl_havana	4.362004535	4.140068933	4.583940138	4.126429589	4.231218154	4.062559057	4.57045622	4.577133707	4.604230486
ENSG00000112320	0.00176	0.022580149	0.74915281	1.680805526	**SOBP**	protein_coding	sine oculis binding protein homolog [Source:HGNC Symbol;Acc:HGNC:29256]	chr6	107489958	107660167	1	ensembl_havana	1.985084099	1.610507694	2.359660504	1.671956805	1.476918714	1.682647563	2.153133136	2.433220573	2.492627803
ENSG00000261338	0.001757	0.022580149	0.446038219	1.362294124	**AC021016.2**	sense_overlapping		chr2	218255319	218257366	1	havana	0.106724733	-0.116294377	0.329743842	0.206373087	0.030651399	-0.585907617	0.540060475	0.554044597	-0.104873545
ENSG00000129103	0.001758	0.022580149	-0.388722052	-1.309233164	**SUMF2**	protein_coding	sulfatase modifying factor 2 [Source:HGNC Symbol;Acc:HGNC:20415]	chr7	56064002	56080670	1	ensembl_havana	6.909678336	7.104039362	6.71531731	7.112411372	7.053419846	7.146286868	6.686551905	6.732122429	6.727277595
ENSG00000128050	0.001762	0.022588979	-0.379845743	-1.30120272	**PAICS**	protein_coding	phosphoribosylaminoimidazole carboxylase and phosphoribosylaminoimidazolesuccinocarboxamide synthase [Source:HGNC Symbol;Acc:HGNC:8587]	chr4	56435741	56464579	1	ensembl_havana	8.29150796	8.481430832	8.101585089	8.32162771	8.454562953	8.668101833	7.913651516	8.132607215	8.258496535
ENSG00000075399	0.001764	0.02260178	0.40505978	1.324143777	**VPS9D1**	protein_coding	VPS9 domain containing 1 [Source:HGNC Symbol;Acc:HGNC:13526]	chr16	89707134	89720986	-1	ensembl_havana	3.352317007	3.149787117	3.554846898	3.275904777	3.085263982	3.088192593	3.67911428	3.563507352	3.421919061

ENSG00000164039	0.001766	0.022603042	-0.415652623	-1.33390195	**BDH2**	protein_coding	3-hydroxybutyrate dehydrogenase 2 [Source:HGNC Symbol;Acc:HGNC:32389]	chr4	103079435	103099883	-1	ensembl_havana	4.350664678	4.55849099	4.142838367	4.402883241	4.563772432	4.708817296	4.077416127	4.120886932	4.230212041
ENSG00000237731	0.001788	0.022727826	1.353800496	2.555845266	**RNGTTP1**	processed_pseudogene	RNA guanylyltransferase and 5'-phosphatase pseudogene 1 [Source:HGNC Symbol;Acc:HGNC:39652]	chr21	25643489	25644216	1	havana	-0.953908352	-1.6308086	-0.277008104	-0.73288918	-2.420341378	-1.739195246	0.069637898	-0.86978558	-0.030876629
ENSG00000117262	0.001784	0.022727826	0.577472671	1.492232847	**GPR89A**	protein_coding	G protein-coupled receptor 89A [Source:HGNC Symbol;Acc:HGNC:31984]	chr1	145607990	145670648	1	ensembl_havana	4.810578668	4.521842333	5.099315004	4.67360513	4.536646796	4.355275073	5.137340541	5.033696613	5.126907856
ENSG00000116711	0.001782	0.022727826	0.472122438	1.387148688	**PLA2G4A**	protein_coding	phospholipase A2 group IVA [Source:HGNC Symbol;Acc:HGNC:9035]	chr1	186828953	186988981	1	ensembl_havana	7.505968869	7.26990765	7.742030088	7.101907495	7.258256239	7.449559217	7.465747009	7.852297796	7.908045459
ENSG00000227741	0.001789	0.022727826	0.434960608	1.35187392	**AL121987.2**	antisense		chr1	160202199	160208869	-1	havana	1.473454563	1.255974259	1.690934867	2.014244272	1.029710695	0.723967808	2.341497201	1.529563273	1.201744126
ENSG00000080493	0.001793	0.022727826	0.393261665	1.313359311	**SLC4A4**	protein_coding	solute carrier family 4 member 4 [Source:HGNC Symbol;Acc:HGNC:11030]	chr4	71186757	71572087	1	ensembl_havana	3.726218329	3.529587496	3.922849161	3.264647647	3.535287761	3.788827082	3.729854637	3.907747537	4.130945309
ENSG00000147065	0.001792	0.022727826	0.370220293	1.292550182	**MSN**	protein_coding	moesin [Source:HGNC Symbol;Acc:HGNC:7373]	chrX	65588377	65741931	1	ensembl_havana	6.157523153	5.972413007	6.3426333	6.108737664	5.99195838	5.816542977	6.500954495	6.314716765	6.212228639
ENSG00000142875	0.00179	0.022727826	0.355750106	1.279650742	**PRKACB**	protein_coding	protein kinase cAMP-activated catalytic subunit beta [Source:HGNC Symbol;Acc:HGNC:9381]	chr1	84078062	84238498	1	ensembl_havana	5.634933295	5.457058242	5.812808348	5.361233198	5.501594918	5.50834661	5.734446832	5.851669064	5.852309148
ENSG00000163297	0.001792	0.022727826	-0.38563832	-1.306437692	**ANTXR2**	protein_coding	anthrax toxin receptor 2 [Source:HGNC Symbol;Acc:HGNC:21732]	chr4	79901149	80125454	-1	ensembl_havana	6.538365142	6.731184302	6.345545982	7.093448981	6.555911329	6.544192596	6.70345248	6.229704417	6.103481048
ENSG00000071575	0.00178	0.022727826	-0.389170925	-1.309640575	**TRIB2**	protein_coding	tribbles pseudokinase 2 [Source:HGNC Symbol;Acc:HGNC:30809]	chr2	12716889	12742734	1	ensembl_havana	5.813654153	6.008239616	5.619068691	6.227037929	5.891298222	5.906382696	5.840792412	5.440768214	5.575645446
ENSG00000140694	0.001778	0.022727826	-0.56324386	-1.4775878	**PARN**	protein_coding	poly(A)-specific ribonuclease [Source:HGNC Symbol;Acc:HGNC:8609]	chr16	14435701	14632728	-1	ensembl_havana	6.052595875	6.334217805	5.770973945	6.486444744	6.330081587	6.186127085	5.812683632	5.691349603	5.808888601
ENSG00000227471	0.001789	0.022727826	-0.881504714	-1.842295793	**AKR1B15**	protein_coding	aldo-keto reductase family 1 member B15 [Source:HGNC Symbol;Acc:HGNC:37281]	chr7	134549136	134579875	1	ensembl_havana	0.120317275	0.561069632	-0.320435083	0.749126884	0.679062629	0.255019383	-0.08686711	-0.52165082	-0.352787316
ENSG00000127585	0.001803	0.022832161	-0.703664868	-1.628636757	**FBXL16**	protein_coding	F-box and leucine rich repeat protein 16 [Source:HGNC Symbol;Acc:HGNC:14150]	chr16	692498	705829	-1	ensembl_havana	1.619992077	1.971824511	1.268159643	2.023178671	1.855271053	2.037023808	1.430149367	1.300906777	1.073422785
ENSG00000197361	0.001807	0.022862556	-0.817880944	-1.762814837	**FBXL22**	protein_coding	F-box and leucine rich repeat protein 22 [Source:HGNC Symbol;Acc:HGNC:27537]	chr15	63597353	63602428	1	ensembl_havana	-0.533994033	-0.125053561	-0.942934505	-0.27307956	0.09469996	-0.196781087	-1.31428442	-0.80561234	-0.708906755
ENSG00000187189	0.001815	0.022909484	0.398782591	1.318394922	**TSPYL4**	protein_coding	TSPY like 4 [Source:HGNC Symbol;Acc:HGNC:21559]	chr6	116249961	116254140	-1	ensembl_havana	6.364545812	6.165154517	6.563937107	6.14959856	6.171135209	6.174729781	6.625987302	6.512934602	6.552889417
ENSG00000064309	0.001816	0.022909484	0.396841759	1.316622502	**CDON**	protein_coding	cell adhesion associated, oncogene regulated [Source:HGNC Symbol;Acc:HGNC:17104]	chr11	125955796	126063335	-1	ensembl_havana	5.403911738	5.205490859	5.602332618	5.065051883	5.0672469	5.484173792	5.496612447	5.507807812	5.802577594
ENSG00000249334	0.001813	0.022909484	-1.250222697	-2.378781394	**AC084048.1**	lincRNA		chr4	10685003	10697661	1	havana	0.239137336	0.864248685	-0.385974012	1.490591983	0.570631912	0.531522158	-0.12879794	-0.80561234	-0.223511759
ENSG00000100379	0.001821	0.022960859	0.519502367	1.433460715	**KCTD17**	protein_coding	potassium channel tetramerization domain containing 17 [Source:HGNC Symbol;Acc:HGNC:25705]	chr22	37051736	37063390	1	ensembl_havana	4.167832598	3.908081415	4.427583782	4.080089315	3.94667289	3.697482039	4.725582535	4.311987074	4.245181736
ENSG00000105976	0.001827	0.02299035	0.565342967	1.47973924	**MET**	protein_coding	MET proto-oncogene, receptor tyrosine kinase [Source:HGNC Symbol;Acc:HGNC:7029]	chr7	116672390	116798386	1	ensembl_havana	6.551151632	6.268480149	6.833823115	5.979863317	6.298133023	6.527444106	6.624016089	6.974118357	6.9033349
ENSG00000146676	0.001828	0.02299035	0.355605163	1.279522186	**PURB**	protein_coding	purine rich element binding protein B [Source:HGNC Symbol;Acc:HGNC:9702]	chr7	44876293	44885361	-1	ensembl_havana	7.391146064	7.213343483	7.568948646	7.140652268	7.283380907	7.215997274	7.488893833	7.661870623	7.556081481
ENSG00000186810	0.001827	0.02299035	-0.685702817	-1.608485374	**CXCR3**	protein_coding	C-X-C motif chemokine receptor 3 [Source:HGNC Symbol;Acc:HGNC:4540]	chrX	71615916	71618517	-1	ensembl_havana	2.363979662	2.70683107	2.021128253	2.584261583	2.523868156	3.012363471	1.88468023	2.063957599	2.114746929
ENSG00000099958	0.001833	0.023014924	-0.424620886	-1.342219745	**DERL3**	protein_coding	derlin 3 [Source:HGNC Symbol;Acc:HGNC:14236]	chr22	23834503	23839128	-1	ensembl_havana	1.849419726	2.061730169	1.637109283	2.348706028	1.759729761	2.076754718	2.015549972	1.32976598	1.566011899
ENSG00000100055	0.001834	0.023014924	-0.55754047	-1.471758001	**CYTH4**	protein_coding	cytohesin 4 [Source:HGNC Symbol;Acc:HGNC:9505]	chr22	37282027	37315345	1	ensembl_havana	2.333482754	2.612252989	2.054712519	2.631766511	2.45233188	2.752660576	1.976531838	2.081032632	2.106573087
ENSG00000146828	0.001839	0.023065765	0.537357992	1.451312293	**SLC12A9**	protein_coding	solute carrier family 12 member 9 [Source:HGNC Symbol;Acc:HGNC:17435]	chr7	100826820	100867009	1	ensembl_havana	4.928159572	4.659480576	5.196838568	4.676439566	4.500159995	4.801842166	5.163633656	5.208001931	5.218880115
ENSG00000188191	0.001844	0.023102856	-0.61218643	-1.528574035	**PRKAR1B**	protein_coding	protein kinase cAMP-dependent type I regulatory subunit beta [Source:HGNC Symbol;Acc:HGNC:9390]	chr7	549197	727650	-1	ensembl_havana	3.738134438	4.044227653	3.432041223	4.230383872	3.800696809	4.101602276	3.487731073	3.403037306	3.405355289
ENSG00000253616	0.001846	0.023113049	-0.389171806	-1.309641375	**AC107959.3**	antisense		chr8	23071377	23074488	-1	havana	0.290993515	0.485579418	0.096407612	0.355307344	0.52486995	0.57656096	-0.00649342	0.063570082	0.232146171
ENSG00000207924	0.001857	0.023228938	0.646273203	1.565119922	**MIR196A2**	miRNA	microRNA 196a-2 [Source:HGNC Symbol;Acc:HGNC:31568]	chr12	53991738	53991847	1	mirbase	6.698077254	6.374940653	7.021213856	6.275980121	6.215022054	6.633819782	7.007655534	7.007713695	7.048272339
ENSG00000231563	0.001861	0.023234609	0.559264661	1.473517977	**AL670729.1**	antisense		chr1	228407381	228409694	1	havana	0.357457448	0.077825118	0.637089779	-0.03191641	0.125688576	0.139703184	0.540060475	0.841392961	0.529815901
ENSG00000181045	0.00186	0.023234609	0.469173071	1.384315774	**SLC26A11**	protein_coding	solute carrier family 26 member 11 [Source:HGNC Symbol;Acc:HGNC:14471]	chr17	80219699	80253500	1	ensembl_havana	4.715172757	4.480586221	4.949759292	4.49406847	4.46272642	4.484963773	5.074059699	4.9432148	4.832003378
ENSG00000185291	0.001867	0.023292246	-1.080390739	-2.114608724	**IL3RA**	protein_coding	interleukin 3 receptor subunit alpha [Source:HGNC Symbol;Acc:HGNC:6012]	chrX	1336616	1382689	1	ensembl_havana	3.33702404	3.87721941	2.796828671	4.086496776	3.643632949	3.901528505	2.581431252	2.881149392	2.927905368
ENSG00000186104	0.001877	0.023396433	-0.595206596	-1.510688903	**CYP2R1**	protein_coding	cytochrome P450 family 2 subfamily R member 1 [Source:HGNC Symbol;Acc:HGNC:20580]	chr11	14877440	14892252	-1	ensembl_havana	4.44121582	4.738819118	4.143612522	4.872094212	4.671388827	4.672974316	4.075109324	4.131170126	4.224558116
ENSG00000106089	0.00188	0.023397922	0.459116896	1.374700078	**STX1A**	protein_coding	syntaxin 1A [Source:HGNC Symbol;Acc:HGNC:11433]	chr7	73699206	73719672	-1	ensembl_havana	3.361100086	3.131541638	3.590658534	2.883093138	3.347603631	3.163928144	3.441917197	3.68574783	3.644310574
ENSG00000080608	0.00188	0.023397922	-0.372789472	-1.294854031	**PUM3**	protein_coding	pumilio RNA binding family member 3 [Source:HGNC Symbol;Acc:HGNC:29676]	chr9	2720469	2844241	-1	ensembl_havana	6.207078577	6.393473313	6.020683842	6.268485529	6.428894845	6.483039567	5.839433884	6.090668133	6.131949508
ENSG00000171053	0.001885	0.023412058	1.614116349	3.061240409	**PATE1**	protein_coding	prostate and testis expressed 1 [Source:HGNC Symbol;Acc:HGNC:24664]	chr11	125746293	125749867	1	ensembl_havana	1.20029412	0.393235946	2.007352295	-1.31854494	1.062111262	1.436141515	0.955706889	2.195213363	2.871136632
ENSG00000235665	0.001887	0.023412058	1.1309969	2.190100236	**LINC00298**	lincRNA	long intergenic non-protein coding RNA 298 [Source:HGNC Symbol;Acc:HGNC:49257]	chr2	7922425	8278084	-1	havana	0.467365124	-0.098133326	1.032863574	-0.73288918	0.15602554	0.28246366	0.739683594	0.841392961	1.517514168
ENSG00000004660	0.001896	0.023412058	0.697396681	1.621576044	**CAMKK1**	protein_coding	calcium/calmodulin dependent protein kinase kinase 1 [Source:HGNC Symbol;Acc:HGNC:1469]	chr17	3860315	3894891	-1	ensembl_havana	2.068314892	1.719616551	2.417013232	1.892762624	1.464851682	1.801235349	2.349154471	2.372104732	2.529780494
ENSG00000119900	0.001895	0.023412058	0.516311314	1.430293591	**OGFRL1**	protein_coding	opioid growth factor receptor like 1 [Source:HGNC Symbol;Acc:HGNC:21378]	chr6	71288803	71308950	1	ensembl_havana	6.187139909	5.928984252	6.445295566	5.656497098	6.123474469	6.006981191	6.31406181	6.494732428	6.527092461
ENSG00000105607	0.001894	0.023412058	0.422916474	1.340634972	**GCDH**	protein_coding	glutaryl-CoA dehydrogenase [Source:HGNC Symbol;Acc:HGNC:4189]	chr19	12891026	12914207	1	ensembl_havana	5.1522516	4.940793363	5.363709837	4.858430778	4.87887941	5.085069902	5.317924627	5.367854419	5.405350465
ENSG00000196743	0.001893	0.023412058	0.355017625	1.279001207	**GM2A**	protein_coding	GM2 ganglioside activator [Source:HGNC Symbol;Acc:HGNC:4367]	chr5	151212150	151270440	1	ensembl_havana	6.307642724	6.130133911	6.485151536	6.063982445	6.159951678	6.166467609	6.443097102	6.487946991	6.524410516
ENSG00000171163	0.00189	0.023412058	-0.421225365	-1.339064417	**ZNF692**	protein_coding	zinc finger protein 692 [Source:HGNC Symbol;Acc:HGNC:26049]	chr1	248850006	248859144	-1	ensembl_havana	5.239625444	5.450238126	5.029012761	5.416529641	5.491221409	5.442963328	4.990843697	4.983500571	5.112694017
ENSG00000159640	0.001884	0.023412058	-0.682631715	-1.605064991	**ACE**	protein_coding	angiotensin I converting enzyme [Source:HGNC Symbol;Acc:HGNC:2707]	chr17	63477061	63498380	1	ensembl_havana	0.496880864	0.838196721	0.155565006	0.683014432	0.818355163	1.013220568	-0.00649342	-0.08087422	0.554062661
ENSG00000185897	0.001889	0.023412058	-1.474139712	-2.778179299	**FFAR3**	protein_coding	free fatty acid receptor 3 [Source:HGNC Symbol;Acc:HGNC:4499]	chr19	35358460	35360485	1	ensembl_havana	-0.84061611	-0.103546254	-1.577685966	0.297565234	-0.071090603	-0.537113394	-1.7649502	-1.42278721	-1.54532049
ENSG00000104889	0.001908	0.0235297	0.804289501	1.746285569	**RNASEH2A**	protein_coding	ribonuclease H2 subunit A [Source:HGNC Symbol;Acc:HGNC:18518]	chr19	12806556	12815201	1	ensembl_havana	-0.207710818	-0.609855568	0.194433933	-1.41179303	-0.143136894	-0.274636777	-0.30988669	0.602150945	0.291037539
ENSG00000119508	0.001917	0.0235297	0.527774887	1.441703896	**NR4A3**	protein_coding	nuclear receptor subfamily 4 group A member 3 [Source:HGNC Symbol;Acc:HGNC:7982]	chr9	99821855	99866891	1	ensembl_havana	5.582410088	5.318522644	5.846297531	5.383086314	5.448964991	5.123516628	6.023566832	5.806827625	5.708498138
ENSG00000166762	0.001912	0.0235297	0.461337554	1.376817704	**CATSPER2**	protein_coding	cation channel sperm associated 2 [Source:HGNC Symbol;Acc:HGNC:18810]	chr15	43628503	43668118	-1	ensembl_havana	4.367727745	4.137058968	4.598396522	3.961929783	4.222285664	4.226961456	4.530605231	4.563530191	4.701054144
ENSG00000177181	0.001916	0.0235297	0.352472464	1.276746819	**RIMKLA**	protein_coding	ribosomal modification protein rimK like family member A [Source:HGNC Symbol;Acc:HGNC:28725]	chr1	42380795	42422578	1	ensembl_havana	4.925028181	4.748791949	5.101264413	4.561549439	4.847853934	4.836972474	4.897318888	5.191390799	5.215083552
ENSG00000104938	0.001916	0.0235297	-0.403865409	-1.323048005	**CLEC4M**	protein_coding	C-type lectin domain family 4 member M [Source:HGNC Symbol;Acc:HGNC:13523]	chr19	7763149	7769605	1	ensembl_havana	0.197565127	0.399497831	-0.004367578	0.075059705	0.52486995	0.598563838	-0.40973604	0.19485955	0.201773757
ENSG00000118507	0.001916	0.0235297	-0.412179044	-1.330694175	**AKAP7**	protein_coding	A-kinase anchoring protein 7 [Source:HGNC Symbol;Acc:HGNC:377]	chr6	131135666	131283535	1	ensembl_havana	3.58757929	3.793668812	3.381489768	3.770935591	3.776492281	3.833578563	3.318633557	3.308339433	3.517496313
ENSG00000137804	0.001914	0.0235297	-0.412786103	-1.331254224	**NUSAP1**	protein_coding	nucleolar and spindle associated protein 1 [Source:HGNC Symbol;Acc:HGNC:18538]	chr15	41332694	41381050	1	ensembl_havana	7.33568736	7.542080411	7.129294308	7.412087248	7.674764652	7.539389334	7.050849575	7.165120685	7.171912665
ENSG00000113532	0.00192	0.023549837	0.474881325	1.389803891	**ST8SIA4**	protein_coding	ST8 alpha-N-acetyl-neuraminide alpha-2,8-sialyltransferase 4 [Source:HGNC Symbol;Acc:HGNC:10871]	chr5	100806935	100903266	-1	ensembl_havana	5.551185874	5.313745211	5.788626536	5.924311445	5.141144845	4.875779343	6.269360601	5.63320814	5.463310868
ENSG00000198440	0.001928	0.023620665	0.452840934	1.368732893	**ZNF583**	protein_coding	zinc finger protein 583 [Source:HGNC Symbol;Acc:HGNC:26427]	chr19	56397966	56436035	1	ensembl_havana	2.409300893	2.182880426	2.635721359	2.167163933	2.213608205	2.16786914	2.501227084	2.764446992	2.641490002
ENSG00000138180	0.001931	0.023636621	-0.366962131	-1.289634399	**CEP55**	protein_coding	centrosomal protein 55 [Source:HGNC Symbol;Acc:HGNC:1161]	chr10	93496632	93529092	1	ensembl_havana	7.007203913	7.190684978	6.823722847	7.022263813	7.187965856	7.361825265	6.651370956	6.777672913	7.042124673
ENSG00000243797	0.001939	0.02369678	0.746544991	1.677770042	**AC004917.1**	sense_overlapping		chr7	106372251	106770207	-1	havana	0.292750403	-0.080522093	0.666022898	-0.06941355	0.06303108	-0.235183806	0.64332197	0.578298274	0.77644845
ENSG00000146221	0.001947	0.02369678	0.614661322	1.5311985	**TCTE1**	protein_coding	t-complex-associated-testis-expressed 1 [Source:HGNC Symbol;Acc:HGNC:11693]	chr6	44278743	44297688	-1	ensembl_havana	1.153041567	0.845710906	1.460372228	0.911909315	0.679062629	0.946160774	1.514288036	1.491756274	1.375072374
ENSG00000181035	0.001943	0.02369678	0.544940768	1.458960443	**SLC25A42**	protein_coding	solute carrier family 25 member 42 [Source:HGNC Symbol;Acc:HGNC:28380]	chr19	19063999	19112888	1	ensembl_havana	4.17738964	3.904919256	4.449860024	3.805007693	3.891395322	4.018354752	4.493082186	4.46453516	4.391962726
ENSG00000279342	0.001943	0.02369678	0.433997126	1.350971393	**AP000866.6**	sense_intronic		chr11	124789240	124792818	-1	ensembl_havana	1.267216836	1.050218273	1.484215399	0.949888679	0.89230127	1.30846487	1.47283199	1.343981945	1.635832261
ENSG00000172331	0.001945	0.02369678	0.36523229	1.28808901	**BPGM**	protein_coding	bisphosphoglycerate mutase [Source:HGNC Symbol;Acc:HGNC:1093]	chr7	134646808	134679813	1	ensembl_havana	4.778588859	4.595972714	4.961205004	4.63478499	4.627407213	4.525725938	5.050769426	4.96292271	4.869922876
ENSG00000101003	0.001947	0.02369678	-0.376672325	-1.29834368	**GINS1**	protein_coding	GINS complex subunit 1 [Source:HGNC Symbol;Acc:HGNC:28980]	chr20	25407727	25452628	1	ensembl_havana	6.090749587	6.27908575	5.902413425	6.00938848	6.419937744	6.407931024	5.653269978	5.979530799	6.074439497
ENSG00000173531	0.001945	0.02369678	-0.896417658	-1.861438121	**MST1**	protein_coding	macrophage stimulating 1 [Source:HGNC Symbol;Acc:HGNC:7380]	chr3	49683947	49689501	-1	ensembl_havana	3.525009831	3.97321866	3.076801002	4.07365327	3.873249614	3.972753095	3.449060876	3.046743406	2.734598724
ENSG00000179409	0.001957	0.023793701	-0.386567494	-1.30727938	**GEMIN4**	protein_coding	gem nuclear organelle associated protein 4 [Source:HGNC Symbol;Acc:HGNC:15717]	chr17	744414	753999	-1	ensembl_havana	6.006087561	6.199371308	5.812803814	6.224135099	6.144904156	6.229074668	5.779772881	5.829738281	5.82890028
ENSG00000256269	0.001965	0.023868901	-0.425776637	-1.343295436	**HMBS**	protein_coding	hydroxymethylbilane synthase [Source:HGNC Symbol;Acc:HGNC:4982]	chr11	119084866	119093549	1	ensembl_havana	5.740508052	5.953396371	5.527619734	5.957898582	5.902525227	5.999765303	5.450194445	5.580150658	5.552514099
ENSG00000177738	0.001967	0.023871342	-0.525780813	-1.439712568	**AC025171.1**	sense_overlapping		chr5	43014414	43067419	-1	havana	2.631022055	2.893912461	2.368131648	3.00985979	2.77907104	2.892806553	2.386842807	2.42655638	2.290995759
ENSG00000250961	0.001977	0.023874261	1.168426773	2.247664604	**AC025470.2**	antisense		chr5	57395060	57533424	1	havana	-0.834771109	-1.418984495	-0.250557722	-1.61858537	-1.104596936	-1.533771185	-0.57379957	-0.28449416	0.106620565
ENSG00000153291	0.001994	0.023874261	0.611266625	1.527599788	**SLC25A27**	protein_coding	solute carrier family 25 member 27 [Source:HGNC Symbol;Acc:HGNC:21065]	chr6	46652915	46678193	1	ensembl_havana	4.419819709	4.114186396	4.725453021	3.998631232	4.158160005	4.185767952	4.491353183	4.666101492	5.018904388
ENSG00000141376	0.00198	0.023874261	0.428149831	1.345506939	**BCAS3**	protein_coding	BCAS3, microtubule associated cell migration factor [Source:HGNC Symbol;Acc:HGNC:14347]	chr17	60677453	61392838	1	ensembl_havana	4.837391219	4.623316304	5.051466135	4.715547829	4.630111276	4.524289806	5.08556534	5.004907857	5.063925207
ENSG00000162415	0.001982	0.023874261	0.41559937	1.333852713	**ZSWIM5**	protein_coding	zinc finger SWIM-type containing 5 [Source:HGNC Symbol;Acc:HGNC:29299]	chr1	45016399	45306209	-1	ensembl_havana	4.933645783	4.725846098	5.141445467	4.541479027	4.746828853	4.889230414	5.036612625	5.067287354	5.320436423
ENSG00000080200	0.001973	0.023874261	0.389518488	1.309956121	**CRYBG3**	protein_coding	crystallin beta-gamma domain containing 3 [Source:HGNC Symbol;Acc:HGNC:34427]	chr3	97822040	97944963	1	ensembl_havana	5.691386001	5.496626757	5.886145245	5.42329502	5.462682856	5.603902396	5.797372015	5.927379202	5.933684518
ENSG00000275111	0.001997	0.023874261	0.385601477	1.306404329	**ZNF2**	protein_coding	zinc finger protein 2 [Source:HGNC Symbol;Acc:HGNC:12991]	chr2	95165432	95184317	1	ensembl_havana	3.548577219	3.35577648	3.741377957	3.3380874	3.418140435	3.311101606	3.709177215	3.876303697	3.63865296
ENSG00000079387	0.001992	0.023874261	-0.347333735	-1.272207269	**SENP1**	protein_coding	SUMO1/sentrin specific peptidase 1 [Source:HGNC Symbol;Acc:HGNC:17927]	chr12	48042898	48106308	-1	ensembl_havana	6.943980737	7.117647604	6.77031387	7.218560562	7.074915486	7.059466766	6.854674669	6.724332269	6.731934671
ENSG00000143374	0.00199	0.023874261	-0.365269137	-1.288121908	**TARS2**	protein_coding	threonyl-tRNA synthetase 2, mitochondrial [Source:HGNC Symbol;Acc:HGNC:30740]	chr1	150487364	150507609	1	ensembl_havana	6.512033487	6.694668055	6.329398918	6.724842878	6.552351804	6.806809484	6.325266663	6.240193375	6.422736717
ENSG00000280832	0.001981	0.023874261	-0.396028908	-1.315880892	**GSEC**	antisense	G-quadruplex forming sequence containing lncRNA [Source:HGNC Symbol;Acc:HGNC:48645]	chr11	126340889	126355587	-1	havana	2.27736256	2.475377015	2.079348106	2.348706028	2.749532556	2.32789246	2.015549972	2.271570328	1.950924019
ENSG00000276085	0.001985	0.023874261	-0.399377125	-1.318938344	**CCL3L1**	protein_coding	C-C motif chemokine ligand 3 like 1 [Source:HGNC Symbol;Acc:HGNC:10628]	chr17	36194869	36196758	-1	ensembl_havana	2.563365852	2.763054414	2.363677289	2.464742477	2.922737966	2.901682799	1.976531838	2.566495926	2.548004104
ENSG00000140743	0.001994	0.023874261	-0.404263516	-1.323413147	**CDR2**	protein_coding	cerebellar degeneration related protein 2 [Source:HGNC Symbol;Acc:HGNC:1799]	chr16	22345936	22437165	-1	ensembl_havana	5.340084055	5.542215813	5.137952297	5.607586927	5.570786374	5.448274139	5.229419377	5.07583045	5.108607065
ENSG00000161558	0.001981	0.023874261	-0.43431116	-1.351265494	**TMEM143**	protein_coding	transmembrane protein 143 [Source:HGNC Symbol;Acc:HGNC:25603]	chr19	48332356	48364237	-1	ensembl_havana	3.248437695	3.465593275	3.031282115	3.461535265	3.439879526	3.495365035	2.911257603	3.059721845	3.122866897
ENSG00000106327	0.001986	0.023874261	-0.521746375	-1.435692095	**TFR2**	protein_coding	transferrin receptor 2 [Source:HGNC Symbol;Acc:HGNC:11762]	chr7	100620416	100642779	-1	ensembl_havana	1.946033119	2.206906306	1.685159931	2.305416943	2.169677667	2.145624309	1.841901733	1.759072628	1.454505434
ENSG00000205309	0.001998	0.023874261	-0.62019626	-1.537084267	**NT5M**	protein_coding	5',3'-nucleotidase, mitochondrial [Source:HGNC Symbol;Acc:HGNC:15769]	chr17	17303335	17347663	1	ensembl_havana	0.341301533	0.651399663	0.031203403	0.613726219	0.636663181	0.703809589	0.106248156	0.130707766	-0.143345712
ENSG00000149922	0.001995	0.023874261	-0.718824687	-1.645840679	**TBX6**	protein_coding	T-box 6 [Source:HGNC Symbol;Acc:HGNC:11605]	chr16	30085793	30091887	-1	ensembl_havana	-0.14043985	0.218972494	-0.499852193	0.41082706	-0.036373238	0.28246366	-0.21650287	-0.57414696	-0.708906755
ENSG00000205045	0.001993	0.023874261	-0.749346275	-1.681030936	**SLFN12L**	protein_coding	schlafen family member 12 like [Source:HGNC Symbol;Acc:HGNC:33920]	chr17	35464249	35537861	-1	ensembl_havana	4.106561285	4.481234422	3.731888148	4.539923521	4.211493157	4.692286589	3.607495467	3.751214005	3.83695497
ENSG00000182053	0.001979	0.023874261	-1.23577082	-2.355071434	**TRIM49B**	protein_coding	tripartite motif containing 49B [Source:HGNC Symbol;Acc:HGNC:42955]	chr11	49027501	49038451	1	ensembl_havana	-0.181360668	0.436524742	-0.799246079	1.142587728	0.185737694	-0.018751196	-0.04612063	-1.52242631	-0.829191301
ENSG00000130711	0.001984	0.023874261	-1.236230741	-2.355822334	**PRDM12**	protein_coding	PR/SET domain 12 [Source:HGNC Symbol;Acc:HGNC:13997]	chr9	130664594	130682981	1	ensembl_havana	-0.503531199	0.114584172	-1.121646569	0.267803752	0.09469996	-0.018751196	-1.41444944	-0.68524259	-1.265247677
ENSG00000211895	0.001978	0.023874261	-2.095550896	-4.273893315	**IGHA1**	IG_C_gene	immunoglobulin heavy constant alpha 1 [Source:HGNC Symbol;Acc:HGNC:5478]	chr14	105703995	105708665	-1	havana	-0.191128297	0.85664715	-1.238903745	0.949888679	0.352203286	1.267849486	-0.26243933	-2.00847383	-1.445798078
ENSG00000108821	0.002001	0.023880096	-0.393936157	-1.313973479	**COL1A1**	protein_coding	collagen type I alpha 1 chain [Source:HGNC Symbol;Acc:HGNC:2197]	chr17	50183289	50201632	-1	ensembl_havana	2.992287502	3.189255581	2.795319424	3.316445323	3.202541664	3.048779755	2.991732974	2.816350902	2.577874395
ENSG00000176485	0.002004	0.023880096	-0.458717981	-1.374320017	**PLA2G16**	protein_coding	phospholipase A2 group XVI [Source:HGNC Symbol;Acc:HGNC:17825]	chr11	63573195	63616883	-1	ensembl_havana	2.556649284	2.786008274	2.327290293	2.727802065	2.764377395	2.865845363	2.135529305	2.379024645	2.467316929
ENSG00000122694	0.002003	0.023880096	-0.506508274	-1.420607763	**GLIPR2**	protein_coding	GLI pathogenesis related 2 [Source:HGNC Symbol;Acc:HGNC:18007]	chr9	36136536	36163913	1	ensembl_havana	6.499071698	6.752325835	6.245817561	6.872733565	6.665412926	6.718831015	6.512934561	6.148507917	6.076010204
ENSG00000124568	0.00201	0.023919339	2.112868032	4.325503369	**SLC17A1**	protein_coding	solute carrier family 17 member 1 [Source:HGNC Symbol;Acc:HGNC:10929]	chr6	25782897	25832059	-1	ensembl_havana	-0.454108525	-1.510542542	0.602325491	-2.51386715	-1.03440435	-0.983356126	0.485518989	0.841392961	0.480064523
ENSG00000166669	0.002009	0.023919339	0.45372913	1.369575814	**ATF7IP2**	protein_coding	activating transcription factor 7 interacting protein 2 [Source:HGNC Symbol;Acc:HGNC:20397]	chr16	10326434	10483638	1	ensembl_havana	0.930847911	0.703983346	1.157712476	0.040274975	0.874167025	1.197508038	0.618191201	1.300906777	1.554039449
ENSG00000143507	0.002016	0.023957024	0.467789247	1.382988584	**DUSP10**	protein_coding	dual specificity phosphatase 10 [Source:HGNC Symbol;Acc:HGNC:3065]	chr1	221701424	221742176	-1	ensembl_havana	4.23859487	4.004700246	4.472489493	4.255242815	4.041129678	3.717728245	4.722637985	4.387705518	4.307124977
ENSG00000197535	0.002018	0.023957024	0.393635747	1.313699901	**MYO5A**	protein_coding	myosin VA [Source:HGNC Symbol;Acc:HGNC:7602]	chr15	52307283	52529050	-1	ensembl_havana	7.013050543	6.816232669	7.209868416	6.80635439	6.767186395	6.875157222	7.222732714	7.220587614	7.186284921
ENSG00000167755	0.00202	0.023957024	-0.712882713	-1.639075958	**KLK6**	protein_coding	kallikrein related peptidase 6 [Source:HGNC Symbol;Acc:HGNC:6367]	chr19	50958631	50969673	-1	ensembl_havana	1.60593522	1.962376577	1.249493864	1.882980075	1.927394938	2.076754718	1.430149367	1.163381334	1.154950891
ENSG00000167779	0.00202	0.023957024	-0.744985631	-1.675957576	**IGFBP6**	protein_coding	insulin like growth factor binding protein 6 [Source:HGNC Symbol;Acc:HGNC:5475]	chr12	53097436	53102345	1	ensembl_havana	1.842421236	2.214914051	1.469928421	1.802235788	2.317915967	2.524590399	1.245545533	1.671599815	1.492639914
ENSG00000197296	0.002028	0.02399127	0.448828548	1.364931497	**FITM2**	protein_coding	fat storage inducing transmembrane protein 2 [Source:HGNC Symbol;Acc:HGNC:16135]	chr20	44302838	44311169	-1	ensembl_havana	4.613475297	4.389061024	4.837889571	4.245732307	4.432064119	4.489386645	4.769044464	4.933846503	4.810777746
ENSG00000057704	0.002027	0.02399127	0.368524808	1.291032043	**TMCC3**	protein_coding	transmembrane and coiled-coil domain family 3 [Source:HGNC Symbol;Acc:HGNC:29199]	chr12	94567124	94650562	-1	ensembl_havana	1.003565735	0.819303331	1.187828139	0.832812769	1.140065346	0.485031876	1.212343926	1.554228498	0.796911992
ENSG00000166598	0.002028	0.02399127	-0.50818087	-1.422255707	**HSP90B1**	protein_coding	heat shock protein 90 beta family member 1 [Source:HGNC Symbol;Acc:HGNC:12028]	chr12	103930107	103953645	1	ensembl_havana	9.695212747	9.949303181	9.441122312	10.08220998	9.95172731	9.813972257	9.419517869	9.469024781	9.434824284
ENSG00000169727	0.002032	0.024012536	0.546685947	1.460726366	**GPS1**	protein_coding	G protein pathway suppressor 1 [Source:HGNC Symbol;Acc:HGNC:4549]	chr17	82050691	82057470	1	ensembl_havana	6.24273917	5.969396196	6.516082143	5.766997858	5.976104762	6.165085969	6.46880551	6.537496783	6.541944137
ENSG00000109171	0.002046	0.024120609	-0.423558673	-1.341231874	**SLAIN2**	protein_coding	SLAIN motif family member 2 [Source:HGNC Symbol;Acc:HGNC:29282]	chr4	48341322	48426212	1	ensembl_havana	5.653625713	5.865405049	5.441846376	5.89540813	5.924720483	5.776086536	5.391149029	5.475888213	5.458501885
ENSG00000128394	0.002045	0.024120609	-0.440710129	-1.357272245	**APOBEC3F**	protein_coding	apolipoprotein B mRNA editing enzyme catalytic subunit 3F [Source:HGNC Symbol;Acc:HGNC:17356]	chr22	39040604	39053910	1	ensembl_havana	2.509608165	2.72996323	2.289253101	2.683404011	2.793616541	2.712869138	2.333799072	2.379024645	2.154935587
ENSG00000231793	0.002044	0.024120609	-0.775689422	-1.712007975	**DOC2GP**	transcribed_unitary_pseudogene	double C2 domain gamma pseudogene [Source:HGNC Symbol;Acc:HGNC:37962]	chr11	67612651	67616257	-1	havana	1.249638163	1.637482874	0.861793452	1.541228291	1.569984981	1.801235349	1.033805522	0.529376201	1.022198632
ENSG00000273419	0.00205	0.02412774	0.465688486	1.380976229	**AC004877.1**	antisense		chr7	149858400	149862492	-1	havana	0.642792989	0.409948746	0.875637232	0.515840645	0.378171619	0.335833975	0.873137292	0.821120834	0.932563571
ENSG00000106415	0.002049	0.02412774	0.376312426	1.298019832	**GLCCI1**	protein_coding	glucocorticoid induced 1 [Source:HGNC Symbol;Acc:HGNC:18713]	chr7	7968794	8094272	1	ensembl_havana	4.708517466	4.520361253	4.896673679	4.312848501	4.562357411	4.685877846	4.671638628	5.006025805	5.012356603
ENSG00000233382	0.002056	0.024137231	0.360978194	1.284296397	**NKAPP1**	transcribed_processed_pseudogene	NFKB activating protein pseudogene 1 [Source:HGNC Symbol;Acc:HGNC:26706]	chrX	120120953	120245267	-1	havana	2.49215876	2.311669663	2.672647857	2.411297517	2.263224966	2.260486508	2.803908217	2.572545352	2.641490002
ENSG00000266338	0.002057	0.024137231	0.34494608	1.270103512	**NBPF15**	protein_coding	NBPF member 15 [Source:HGNC Symbol;Acc:HGNC:28791]	chr1	144421386	144461674	-1	ensembl_havana	4.845320269	4.672847229	5.017793308	4.612758167	4.659526198	4.746257321	4.979787651	4.99705787	5.076534403
ENSG00000008282	0.002056	0.024137231	-0.425678348	-1.343203922	**SYPL1**	protein_coding	synaptophysin like 1 [Source:HGNC Symbol;Acc:HGNC:11507]	chr7	106090503	106112576	-1	ensembl_havana	6.013360214	6.226199388	5.80052104	6.534102395	6.119631196	6.024864572	6.009743254	5.696206034	5.695613831
ENSG00000197859	0.002053	0.024137231	-0.536529441	-1.450479033	**ADAMTSL2**	protein_coding	ADAMTS like 2 [Source:HGNC Symbol;Acc:HGNC:14631]	chr9	133532164	133575519	1	ensembl_havana	3.504214851	3.772479571	3.23595013	3.97580247	3.701342418	3.640293825	3.338003616	3.347673311	3.022173463
ENSG00000204977	0.00206	0.024144829	-0.349250031	-1.273898233	**TRIM13**	protein_coding	tripartite motif containing 13 [Source:HGNC Symbol;Acc:HGNC:9976]	chr13	49995888	50020481	1	ensembl_havana	3.606768487	3.781393503	3.432143472	3.773585281	3.893647572	3.676947656	3.438332049	3.563507352	3.294591014
ENSG00000069493	0.002074	0.024150956	0.52895956	1.442888241	**CLEC2D**	protein_coding	C-type lectin domain family 2 member D [Source:HGNC Symbol;Acc:HGNC:14351]	chr12	9664969	9699555	1	ensembl_havana	1.335897427	1.071417647	1.600377207	0.437805725	1.415548178	1.360899039	1.089724456	1.769643034	1.941764132
ENSG00000095951	0.002068	0.024150956	0.415166312	1.333452387	**HIVEP1**	protein_coding	human immunodeficiency virus type I enhancer binding protein 1 [Source:HGNC Symbol;Acc:HGNC:4920]	chr6	12008762	12164999	1	ensembl_havana	5.307259442	5.099676286	5.514842598	5.345291428	4.948778335	5.004959096	5.730787785	5.466172976	5.347567034
ENSG00000174132	0.002073	0.024150956	0.414769409	1.333085588	**FAM174A**	protein_coding	family with sequence similarity 174 member A [Source:HGNC Symbol;Acc:HGNC:24943]	chr5	100535305	100586741	1	ensembl_havana	1.740150188	1.532765483	1.947534892	1.87313074	1.351450576	1.373715134	2.278730383	1.861445274	1.70242902
ENSG00000178385	0.002071	0.024150956	0.39779614	1.317493771	**PLEKHM3**	protein_coding	pleckstrin homology domain containing M3 [Source:HGNC Symbol;Acc:HGNC:34006]	chr2	207821288	208025560	-1	ensembl_havana	4.188980697	3.990082626	4.387878767	4.041035185	3.931407459	3.997805235	4.494809119	4.36348661	4.305340572
ENSG00000069869	0.002071	0.024150956	0.390811868	1.311131027	**NEDD4**	protein_coding	neural precursor cell expressed, developmentally down-regulated 4, E3 ubiquitin protein ligase [Source:HGNC Symbol;Acc:HGNC:7727]	chr15	55826922	55993746	-1	ensembl_havana	5.472903468	5.277497534	5.668309402	5.065051883	5.347462077	5.419978642	5.51374772	5.655464804	5.835715681
ENSG00000259205	0.00207	0.024150956	0.359031464	1.282564575	**PRKXP1**	processed_pseudogene	protein kinase, X-linked, pseudogene 1 [Source:HGNC Symbol;Acc:HGNC:9442]	chr15	100553529	100558954	-1	havana	1.547675739	1.368160006	1.727191471	1.297904502	1.155163158	1.651412359	1.693402841	1.465989718	2.022181853
ENSG00000204252	0.002068	0.024150956	-0.696658078	-1.620746074	**HLA-DOA**	protein_coding	major histocompatibility complex, class II, DO alpha [Source:HGNC Symbol;Acc:HGNC:4936]	chr6	33004178	33009612	-1	ensembl_havana	1.422209795	1.770538834	1.073880756	2.005254198	1.798708764	1.507653541	1.107892607	1.358059196	0.755690467
ENSG00000225489	0.002068	0.024150956	-0.893930525	-1.858231861	**AL354707.1**	lincRNA		chr9	6716495	6727438	1	havana	2.274139279	2.721104541	1.827174016	2.596285266	2.592024241	2.975004118	1.430149367	2.046678052	2.004694631
ENSG00000247240	0.002076	0.024157026	-0.452024568	-1.367958599	**UBL7-AS1**	antisense	UBL7 antisense RNA 1 (head to head) [Source:HGNC Symbol;Acc:HGNC:48614]	chr15	74461265	74481302	1	havana	3.454905953	3.680918237	3.228893669	3.75493443	3.729353954	3.558466327	3.275081252	3.17950436	3.232095396
ENSG00000188070	0.002081	0.02418229	0.343624383	1.268940464	**C11orf95**	protein_coding	chromosome 11 open reading frame 95 [Source:HGNC Symbol;Acc:HGNC:28449]	chr11	63759892	63768775	-1	ensembl_havana	4.351845525	4.180033333	4.523657716	4.253345723	4.25418706	4.032567216	4.581875053	4.592099791	4.396998304
ENSG00000057935	0.002081	0.02418229	-0.350135502	-1.274680343	**MTA3**	protein_coding	metastasis associated 1 family member 3 [Source:HGNC Symbol;Acc:HGNC:23784]	chr2	42494569	42756947	1	ensembl_havana	7.699251355	7.874319106	7.524183604	7.938263836	7.894229552	7.79046393	7.620083569	7.512347619	7.440119625
ENSG00000109738	0.002084	0.024193632	0.6876344	1.610640371	**GLRB**	protein_coding	glycine receptor beta [Source:HGNC Symbol;Acc:HGNC:4329]	chr4	157076057	157172090	1	ensembl_havana	0.961532981	0.617715781	1.305350181	0.004630787	0.636663181	1.211853376	0.692307165	1.554228498	1.669514881
ENSG00000107959	0.002087	0.024205389	0.346803227	1.271739539	**PITRM1**	protein_coding	pitrilysin metallopeptidase 1 [Source:HGNC Symbol;Acc:HGNC:17663]	chr10	3137728	3172841	-1	ensembl_havana	6.740375041	6.566973428	6.913776654	6.477940356	6.594497202	6.628482725	6.83435902	6.961495733	6.94547521
ENSG00000242612	0.002088	0.024205389	-0.896204144	-1.861162655	**DECR2**	protein_coding	2,4-dienoyl-CoA reductase 2 [Source:HGNC Symbol;Acc:HGNC:2754]	chr16	401826	412487	1	ensembl_havana	1.076482136	1.524584208	0.628380064	1.490591983	1.390249836	1.692910804	0.762800879	0.693803314	0.428535999
ENSG00000138622	0.002092	0.024224009	0.710993532	1.636931024	**HCN4**	protein_coding	hyperpolarization activated cyclic nucleotide gated potassium channel 4 [Source:HGNC Symbol;Acc:HGNC:16882]	chr15	73319859	73369264	-1	ensembl_havana	1.34809824	0.992601474	1.703595006	0.267803752	1.214022341	1.495978329	1.212343926	1.938414976	1.960026115
ENSG00000147044	0.002094	0.024224182	0.425401247	1.342945956	**CASK**	protein_coding	calcium/calmodulin dependent serine protein kinase [Source:HGNC Symbol;Acc:HGNC:1497]	chrX	41514934	41923645	-1	ensembl_havana	4.683886303	4.471185679	4.896586927	4.332748634	4.500159995	4.580648409	4.87096537	4.855386052	4.963409358
ENSG00000122550	0.002095	0.024224182	0.372208982	1.294333132	**KLHL7**	protein_coding	kelch like family member 7 [Source:HGNC Symbol;Acc:HGNC:15646]	chr7	23105758	23177914	1	ensembl_havana	5.88134708	5.695242589	6.067451571	5.541496935	5.804189573	5.740041259	5.964346837	6.112111638	6.125896237

ENSG00000014914	0.002097	0.024227214	0.435232823	1.352129023	**MTMR11**	protein_coding	myotubularin related protein 11 [Source:HGNC Symbol;Acc:HGNC:24307]	chr1	149928651	149936869	-1	ensembl_havana	2.533048521	2.315432109	2.750664932	2.260788651	2.357615216	2.32789246	2.723708883	2.881149392	2.647136522
ENSG00000083812	0.002102	0.024259416	0.483641064	1.398268157	**ZNF324**	protein_coding	zinc finger protein 324 [Source:HGNC Symbol;Acc:HGNC:14096]	chr19	58467045	58475436	1	ensembl_havana	4.942451891	4.700631359	5.184272423	4.819213589	4.609706171	4.672974316	5.266284673	5.240661197	5.045871397
ENSG00000278272	0.002106	0.024287901	-0.652240089	-1.571606553	**HIST1H3C**	protein_coding	histone cluster 1 H3 family member c [Source:HGNC Symbol;Acc:HGNC:4768]	chr6	26045411	26045821	1	ensembl_havana	0.187746116	0.51386616	-0.138373929	0.968509893	0.403680778	0.16940781	0.24405081	-0.00684508	-0.652327512
ENSG00000128710	0.002115	0.024345704	0.699840742	1.624325475	**HOXD10**	protein_coding	homeobox D10 [Source:HGNC Symbol;Acc:HGNC:5133]	chr2	176108790	176119942	1	ensembl_havana	3.975169576	3.625249205	4.325089947	3.159173914	3.638270336	4.078303364	4.120558811	4.275367784	4.579343245
ENSG00000125703	0.002114	0.024345704	-0.372853298	-1.294911318	**ATG4C**	protein_coding	autophagy related 4C cysteine peptidase [Source:HGNC Symbol;Acc:HGNC:16040]	chr1	62784135	62865513	1	ensembl_havana	3.778119302	3.964545951	3.591692653	3.875769846	3.918192999	4.099675007	3.438332049	3.599502737	3.737243172
ENSG00000203907	0.002116	0.024345704	-0.380529257	-1.301819344	**OOEP**	protein_coding	oocyte expressed protein [Source:HGNC Symbol;Acc:HGNC:21382]	chr6	73368555	73395133	-1	ensembl_havana	0.626715529	0.816980158	0.436450901	0.812341977	0.799260866	0.83933763	0.485518989	0.343769191	0.480064523
ENSG00000186160	0.002124	0.024422893	-0.383531076	-1.304530863	**CYP4Z1**	protein_coding	cytochrome P450 family 4 subfamily Z member 1 [Source:HGNC Symbol;Acc:HGNC:20583]	chr1	47067488	47118319	1	ensembl_havana	7.313655789	7.505421327	7.121890252	7.591734477	7.53621401	7.388315495	7.151169416	7.131965371	7.082535968
ENSG00000151151	0.002136	0.024483126	0.544281236	1.458293628	**IPMK**	protein_coding	inositol polyphosphate multikinase [Source:HGNC Symbol;Acc:HGNC:20739]	chr10	58191517	58267934	-1	ensembl_havana	3.910577878	3.63843726	4.182718496	3.746866803	3.670151025	3.498293951	4.463403562	4.199216933	3.885534993
ENSG00000134121	0.002134	0.024483126	0.427135214	1.344561004	**CHL1**	protein_coding	cell adhesion molecule L1 like [Source:HGNC Symbol;Acc:HGNC:1939]	chr3	196596	409417	1	ensembl_havana	5.007129968	4.793562362	5.220697575	5.030022053	4.822071111	4.528593921	5.41323281	5.177565958	5.071293958
ENSG00000138821	0.002135	0.024483126	-0.560557357	-1.474838882	**SLC39A8**	protein_coding	solute carrier family 39 member 8 [Source:HGNC Symbol;Acc:HGNC:20862]	chr4	102251041	102431258	-1	ensembl_havana	7.669155975	7.949434653	7.388877296	8.171998767	7.936760352	7.73954484	7.511675141	7.276087406	7.378869342
ENSG00000260896	0.002131	0.024483126	-0.578758677	-1.493563604	**LINC02170**	lincRNA	long intergenic non-protein coding RNA 2170 [Source:HGNC Symbol;Acc:HGNC:53032]	chr16	80828735	80892595	-1	havana	4.857094325	5.146473664	4.567714987	5.130593265	5.200567061	5.108260665	4.422252934	4.543651409	4.737240617
ENSG00000203288	0.002146	0.024575263	0.345949204	1.270986939	**TDRKH-AS1**	antisense	TDRKH antisense RNA 1 [Source:HGNC Symbol;Acc:HGNC:40578]	chr1	151790804	151794402	1	havana	2.357500256	2.184525654	2.530474859	2.26832323	2.139629423	2.145624309	2.587917939	2.517165043	2.486341593
ENSG00000198420	0.00215	0.024575263	-0.354827777	-1.27883291	**TCAF1**	protein_coding	TRPM8 channel associated factor 1 [Source:HGNC Symbol;Acc:HGNC:22201]	chr7	143851375	143902198	-1	ensembl_havana	3.915697857	4.093111745	3.738283968	4.175340781	3.953166093	4.150828362	3.823530526	3.554366408	3.83695497
ENSG00000196126	0.002149	0.024575263	-0.476559662	-1.391421639	**HLA-DRB1**	protein_coding	major histocompatibility complex, class II, DR beta 1 [Source:HGNC Symbol;Acc:HGNC:4948]	chr6	32578769	32589848	-1	ensembl_havana	4.354402253	4.592682084	4.116122422	4.525847985	4.550987	4.701211268	4.021008592	4.216704714	4.11065396
ENSG00000151632	0.002151	0.024575263	-0.982284617	-1.975591432	**AKR1C2**	protein_coding	aldo-keto reductase family 1 member C2 [Source:HGNC Symbol;Acc:HGNC:385]	chr10	4987400	5018031	-1	ensembl_havana	-0.157789395	0.333352914	-0.648931703	1.058172782	0.298820819	-0.356934859	-0.21650287	-0.28449416	-1.445798078
ENSG00000174705	0.002157	0.024616757	-0.360185833	-1.283591226	**SH3PXD2B**	protein_coding	SH3 and PX domains 2B [Source:HGNC Symbol;Acc:HGNC:29242]	chr5	172325000	172454523	-1	ensembl_havana	6.583940727	6.764033644	6.40384781	6.72175908	6.658476176	6.911865676	6.387942346	6.328634332	6.494966753
ENSG00000122862	0.00216	0.024617913	-0.509151845	-1.423213248	**SRGN**	protein_coding	serglycin [Source:HGNC Symbol;Acc:HGNC:9361]	chr10	69088106	69104811	1	ensembl_havana	6.802995154	7.057571076	6.548419231	7.309237431	7.113591639	6.749884159	6.633453446	6.721272459	6.29053179
ENSG00000178718	0.00216	0.024617913	-0.81275986	-1.756568527	**RPP25**	protein_coding	ribonuclease P and MRP subunit p25 [Source:HGNC Symbol;Acc:HGNC:30361]	chr15	74954416	74957464	-1	ensembl_havana	-0.216301924	0.190078006	-0.622681854	-0.03191641	0.214850257	0.387300166	-0.51701327	-0.80561234	-0.545419959
ENSG00000090674	0.002163	0.024626902	0.521961647	1.435906337	**MCOLN1**	protein_coding	mucolipin 1 [Source:HGNC Symbol;Acc:HGNC:13356]	chr19	7522626	7534009	1	ensembl_havana	3.81079814	3.549817316	4.071778963	3.414774672	3.662246656	3.572430621	4.113831997	4.116753044	3.984751849
ENSG00000038002	0.002168	0.024658814	-0.346117658	-1.271135352	**AGA**	protein_coding	aspartylglucosaminidase [Source:HGNC Symbol;Acc:HGNC:318]	chr4	177430770	177442503	-1	ensembl_havana	3.553763597	3.726822426	3.380704768	3.683468931	3.754355472	3.742642876	3.306885447	3.439903888	3.395324969
ENSG00000231852	0.002169	0.024658814	-1.293152777	-2.450630164	**CYP21A2**	protein_coding	cytochrome P450 family 21 subfamily A member 2 [Source:HGNC Symbol;Acc:HGNC:2600]	chr6	32038265	32041670	1	ensembl_havana	0.666769621	1.313346009	0.020193232	1.158893785	1.078042694	1.703101548	-0.69455235	0.063570082	0.691561964
ENSG00000110844	0.002174	0.024694971	-0.358020913	-1.281666503	**PRPF40B**	protein_coding	pre-mRNA processing factor 40 homolog B [Source:HGNC Symbol;Acc:HGNC:25031]	chr12	49568218	49644666	1	ensembl_havana	3.242671224	3.42168168	3.063660767	3.366447561	3.415007936	3.483589543	3.049274082	3.068309725	3.073398494
ENSG00000198720	0.002177	0.024713172	0.485563937	1.400133058	**ANKRD13B**	protein_coding	ankyrin repeat domain 13B [Source:HGNC Symbol;Acc:HGNC:26363]	chr17	29589769	29614761	1	ensembl_havana	4.041109097	3.798327128	4.283891065	3.683468931	3.701342418	4.010170036	4.285277268	4.220562267	4.34583366
ENSG00000078902	0.002179	0.024713172	0.459301501	1.374875994	**TOLLIP**	protein_coding	toll interacting protein [Source:HGNC Symbol;Acc:HGNC:16476]	chr11	1274371	1309654	-1	ensembl_havana	4.272993976	4.043343225	4.502644727	4.067188383	4.102779313	3.96006198	4.657831492	4.453104385	4.396998304
ENSG00000021355	0.002184	0.024749331	-0.339118451	-1.264983398	**SERPINB1**	protein_coding	serpin family B member 1 [Source:HGNC Symbol;Acc:HGNC:3311]	chr6	2832332	2842006	-1	ensembl_havana	6.068319079	6.237878305	5.898759854	6.183368258	6.269368816	6.260897841	5.859678694	5.933855337	5.902745531
ENSG00000033100	0.002186	0.024751611	-0.336983944	-1.263113205	**CHPF2**	protein_coding	chondroitin polymerizing factor 2 [Source:HGNC Symbol;Acc:HGNC:29270]	chr7	151232489	151238827	1	ensembl_havana	6.579181536	6.747673508	6.410689563	7.096891236	6.656819641	6.489309647	6.751192825	6.330420407	6.150455458
ENSG00000137331	0.002187	0.024751611	-0.600716171	-1.516469173	**IER3**	protein_coding	immediate early response 3 [Source:HGNC Symbol;Acc:HGNC:5392]	chr6	30743199	30744554	-1	ensembl_havana	3.067539804	3.36789789	2.767181719	3.584331121	3.124146975	3.395215574	2.911257603	2.743151031	2.647136522
ENSG00000159374	0.002193	0.024755533	0.476234533	1.3911081	**M1AP**	protein_coding	meiosis 1 associated protein [Source:HGNC Symbol;Acc:HGNC:25183]	chr2	74557883	74648338	-1	ensembl_havana	2.547627855	2.309510588	2.785745121	2.305416943	2.256240478	2.366874344	2.64502189	2.743151031	2.969062441
ENSG00000171792	0.00219	0.024755533	-0.424384157	-1.341999521	**RHNO1**	protein_coding	RAD9-HUS1-RAD1 interacting nuclear orphan 1 [Source:HGNC Symbol;Acc:HGNC:28206]	chr12	2876258	2889523	1	ensembl_havana	5.826769076	6.038961155	5.614576998	5.996403738	6.10172166	6.018758067	5.504348515	5.628860454	5.710522025
ENSG00000157890	0.002193	0.024755533	-0.539901042	-1.45387279	**MEGF11**	protein_coding	multiple EGF like domains 11 [Source:HGNC Symbol;Acc:HGNC:29635]	chr15	65895079	66253747	-1	ensembl_havana	2.841542489	3.111493011	2.571591968	3.397700228	2.864222379	3.072556425	2.705927533	2.301016365	2.707832006
ENSG00000261716	0.002195	0.024763112	0.407589267	1.326467443	**AC239868.1**	sense_overlapping		chr1	149844498	149849024	-1	havana	4.037412313	3.833617679	4.241206946	3.902646601	3.771602261	3.826604176	4.209480398	4.207015462	4.307124977
ENSG00000170989	0.002204	0.024818332	0.79044322	1.729605745	**S1PR1**	protein_coding	sphingosine-1-phosphate receptor 1 [Source:HGNC Symbol;Acc:HGNC:3165]	chr1	101236888	101241518	1	ensembl_havana	1.068743536	0.673521926	1.463965146	0.911909315	0.799260866	0.309395597	1.986385747	1.315408538	1.090101154
ENSG00000171206	0.002203	0.024818332	-0.424937393	-1.342514242	**TRIM8**	protein_coding	tripartite motif containing 8 [Source:HGNC Symbol;Acc:HGNC:15579]	chr10	102642310	102660680	1	ensembl_havana	5.112518355	5.324987052	4.900049659	5.490870222	5.27674645	5.207344484	5.169051589	4.849170128	4.68192726
ENSG00000168061	0.002215	0.024931253	-0.454330562	-1.370146883	**SAC3D1**	protein_coding	SAC3 domain containing 1 [Source:HGNC Symbol;Acc:HGNC:30179]	chr11	65040901	65044828	1	ensembl_havana	0.841466788	1.068632069	0.614301507	1.253039554	0.799260866	1.153595787	0.762800879	0.478736765	0.601366878
ENSG00000204444	0.002219	0.02493157	-0.408364377	-1.3271803	**APOM**	protein_coding	apolipoprotein M [Source:HGNC Symbol;Acc:HGNC:13916]	chr6	31652416	31658210	1	ensembl_havana	2.041515609	2.245697798	1.837333421	2.334420173	2.357615216	2.045058006	1.831005931	1.95703246	1.723961872
ENSG00000078596	0.002218	0.02493157	-2.915966149	-7.547328917	**ITM2A**	protein_coding	integral membrane protein 2A [Source:HGNC Symbol;Acc:HGNC:6173]	chrX	79360384	79367667	-1	ensembl_havana	-1.751415111	-0.293432037	-3.209398186	0.297565234	-0.258437325	-0.91942402	-1.52209115	-4.33911973	-3.766983675
ENSG00000146826	0.002227	0.025007642	0.44615472	1.362404136	**C7orf43**	protein_coding	chromosome 7 open reading frame 43 [Source:HGNC Symbol;Acc:HGNC:25604]	chr7	100154420	100158715	-1	ensembl_havana	4.627522342	4.404444982	4.850599702	4.338128639	4.442870209	4.432336097	4.890775501	4.905370983	4.755652622
ENSG00000162627	0.00223	0.025007642	0.419282949	1.33726274	**SNX7**	protein_coding	sorting nexin 7 [Source:HGNC Symbol;Acc:HGNC:14971]	chr1	98661701	98760500	1	ensembl_havana	5.582285589	5.372644115	5.791927063	5.439236778	5.527932114	5.150763453	5.878320927	5.839805165	5.657655098
ENSG00000255435	0.002231	0.025007642	-0.394871944	-1.314826049	**AP001267.3**	antisense		chr11	118511911	118531094	-1	havana	-0.252114144	-0.054678172	-0.449550116	-0.27307956	0.030651399	0.078393642	-0.57379957	-0.42206346	-0.352787316
ENSG00000164080	0.002237	0.025052351	0.354686187	1.278707409	**RAD54L2**	protein_coding	RAD54 like 2 [Source:HGNC Symbol;Acc:HGNC:29123]	chr3	51541144	51668667	1	ensembl_havana	6.980147683	6.802804589	7.157490777	6.704852885	6.80327417	6.900286714	7.077887767	7.192629817	7.201954746
ENSG00000257557	0.002243	0.025092639	0.602034782	1.517855847	**PPP1R12A-AS1**	bidirectional_promoter_lncRNA	PPP1R12A antisense RNA 1 [Source:HGNC Symbol;Acc:HGNC:51903]	chr12	79934901	79942712	1	havana	0.184717524	-0.116299867	0.485734915	-0.31750517	-0.258437325	0.227042895	0.106248156	0.554044597	0.796911992
ENSG00000135002	0.002244	0.025092639	-0.432113897	-1.349209047	**RFK**	protein_coding	riboflavin kinase [Source:HGNC Symbol;Acc:HGNC:30324]	chr9	76385517	76394517	-1	ensembl_havana	2.655076015	2.871132964	2.439019067	2.749499015	2.944626756	2.919273119	2.302589091	2.413134925	2.601333184
ENSG00000099338	0.002247	0.02511074	0.790889192	1.73014049	**CATSPERG**	protein_coding	cation channel sperm associated auxiliary subunit gamma [Source:HGNC Symbol;Acc:HGNC:25243]	chr19	38335775	38370943	1	ensembl_havana	2.001144073	1.605699477	2.396588669	1.649052215	1.603393164	1.564653053	2.170524752	2.372104732	2.647136522
ENSG00000130734	0.00226	0.025115879	0.491259975	1.405671981	**ATG4D**	protein_coding	autophagy related 4D cysteine peptidase [Source:HGNC Symbol;Acc:HGNC:20789]	chr19	10543895	10553418	1	ensembl_havana	3.596782524	3.351152536	3.842412512	3.404554274	3.213401092	3.435502243	3.921799706	3.831602732	3.773835098
ENSG00000081913	0.00225	0.025115879	0.443409306	1.359813979	**PHLPP1**	protein_coding	PH domain and leucine rich repeat protein phosphatase 1 [Source:HGNC Symbol;Acc:HGNC:20610]	chr18	62715450	62980433	1	ensembl_havana	5.214221233	4.99251658	5.435925886	4.888076825	4.995662733	5.093810182	5.459951957	5.402226693	5.445599007
ENSG00000213976	0.00226	0.025115879	0.411975822	1.330506743	**AC010615.1**	unprocessed_pseudogene		chr19	21382865	21387177	1	havana	1.695150763	1.489162852	1.901138674	1.602122376	1.62524309	1.24012309	1.905603022	2.106272137	1.691540863
ENSG00000104765	0.002257	0.025115879	0.383328931	1.30434809	**BNIP3L**	protein_coding	BCL2 interacting protein 3 like [Source:HGNC Symbol;Acc:HGNC:1085]	chr8	26382898	26505636	1	ensembl_havana	5.708950198	5.517285733	5.900614664	5.588903523	5.623981035	5.338972641	5.941734672	5.953111124	5.806998196
ENSG00000111684	0.002254	0.025115879	0.379882311	1.301235701	**LPCAT3**	protein_coding	lysophosphatidylcholine acyltransferase 3 [Source:HGNC Symbol;Acc:HGNC:30244]	chr12	6976186	7018510	-1	ensembl_havana	5.965723047	5.775781892	6.155664202	5.913540446	5.728614642	5.685190587	6.249551485	6.069970549	6.147470573
ENSG00000239665	0.002259	0.025115879	0.375992966	1.297732439	**AL157392.3**	processed_transcript		chr10	13631143	13668445	1	havana	3.65113019	3.463133707	3.839126673	3.334502858	3.430602854	3.624295409	3.784529539	3.745870536	3.986979943
ENSG00000156521	0.002254	0.025115879	-0.469367665	-1.384502506	**TYSND1**	protein_coding	trypsin domain containing 1 [Source:HGNC Symbol;Acc:HGNC:28531]	chr10	70137981	70146676	-1	ensembl_havana	3.042460404	3.277144236	2.807776572	3.113890058	3.173177104	3.544365547	2.626237264	2.841618992	2.955473459
ENSG00000173212	0.002263	0.025135227	0.839942217	1.789978448	**MAB21L3**	protein_coding	mab-21 like 3 [Source:HGNC Symbol;Acc:HGNC:26787]	chr1	116111755	116135240	1	ensembl_havana	1.011403887	0.591432779	1.431374996	0.174648163	0.855801933	0.743848241	1.356079599	1.590453245	1.347592143
ENSG00000137267	0.002275	0.025145422	0.56947632	1.483984805	**TUBB2A**	protein_coding	tubulin beta 2A class IIa [Source:HGNC Symbol;Acc:HGNC:12412]	chr6	3153669	3157526	-1	ensembl_havana	5.029393244	4.744655084	5.314131404	4.649284895	4.85827007	4.726410287	5.428673449	5.325496637	5.188224127
ENSG00000188997	0.002284	0.025145422	0.481865803	1.396548622	**KCTD21**	protein_coding	potassium channel tetramerization domain containing 21 [Source:HGNC Symbol;Acc:HGNC:27452]	chr11	78171249	78188822	-1	ensembl_havana	3.715721825	3.474788923	3.956654726	3.4316494	3.756831958	3.235885411	4.044780323	4.095904033	3.729279822
ENSG00000131370	0.002275	0.025145422	0.473853747	1.388814338	**SH3BP5**	protein_coding	SH3 domain binding protein 5 [Source:HGNC Symbol;Acc:HGNC:10827]	chr3	15254853	15341368	-1	ensembl_havana	4.762881133	4.52595426	4.999808006	4.31830309	4.623341591	4.636218098	4.924481562	5.098018313	4.976924144
ENSG00000228434	0.002268	0.025145422	0.430475347	1.347677544	**AC004951.1**	lincRNA		chr7	44004046	44007866	-1	havana	0.662390624	0.447152951	0.877628298	0.004630787	0.592979824	0.743848241	0.485518989	0.900298706	1.2470672
ENSG00000198917	0.002275	0.025145422	0.365140499	1.288007058	**SPOUT1**	protein_coding	SPOUT domain containing methyltransferase 1 [Source:HGNC Symbol;Acc:HGNC:26933]	chr9	128819651	128829821	-1	ensembl_havana	6.179566892	5.996996643	6.362137142	5.796609825	6.001909639	6.192470465	6.200657915	6.396276768	6.489476744
ENSG00000136213	0.00228	0.025145422	0.353984854	1.278085945	**CHST12**	protein_coding	carbohydrate sulfotransferase 12 [Source:HGNC Symbol;Acc:HGNC:17423]	chr7	2403560	2448483	1	ensembl_havana	5.156555423	4.979562996	5.33354785	5.095020432	4.87204229	4.971626266	5.500055822	5.218649283	5.281938444
ENSG00000161682	0.002276	0.025145422	0.346170003	1.271181472	**FAM171A2**	protein_coding	family with sequence similarity 171 member A2 [Source:HGNC Symbol;Acc:HGNC:30480]	chr17	44353215	44363875	-1	ensembl_havana	1.167023572	0.993938571	1.340108573	1.04068047	1.029710695	0.911424546	1.430149367	1.358059196	1.232117157
ENSG00000146112	0.002285	0.025145422	-0.353371203	-1.277542427	**PPP1R18**	protein_coding	protein phosphatase 1 regulatory subunit 18 [Source:HGNC Symbol;Acc:HGNC:29413]	chr6	30676389	30687895	-1	ensembl_havana	6.880887407	7.057573009	6.704201805	7.285995405	7.040011232	6.846712389	6.985410133	6.647970031	6.479225252
ENSG00000115541	0.002268	0.025145422	-0.37498039	-1.296821928	**HSPE1**	protein_coding	heat shock protein family E (Hsp10) member 1 [Source:HGNC Symbol;Acc:HGNC:5269]	chr2	197499994	197503457	1	ensembl_havana	4.691022362	4.878512557	4.503532166	4.578316698	5.04917605	5.008044923	4.261128114	4.599525013	4.649943372
ENSG00000121005	0.00228	0.025145422	-0.398393968	-1.31803983	**CRISPLD1**	protein_coding	cysteine rich secretory protein LCCL domain containing 1 [Source:HGNC Symbol;Acc:HGNC:18206]	chr8	74984515	75034558	1	ensembl_havana	5.254963981	5.454160965	5.055766997	5.323757958	5.458124712	5.580600224	5.02351238	5.026002133	5.117786479
ENSG00000267554	0.002284	0.025145422	-0.676940751	-1.598746002	**AC015911.8**	processed_pseudogene		chr17	35470069	35470628	-1	havana	1.588090077	1.926560452	1.249619701	1.82284946	1.864486201	2.092345696	1.356079599	0.938274071	1.454505434
ENSG00000140481	0.00228	0.025145422	-1.355893724	-2.559556272	**CCDC33**	protein_coding	coiled-coil domain containing 33 [Source:HGNC Symbol;Acc:HGNC:26552]	chr15	74202705	74336472	1	ensembl_havana	0.322030161	0.999977023	-0.355916702	1.158893785	0.658018659	1.183018625	-0.75894329	-0.2414061	-0.067400719
ENSG00000128578	0.002294	0.025148959	0.443132006	1.359552635	**STRIP2**	protein_coding	striatin interacting protein 2 [Source:HGNC Symbol;Acc:HGNC:22209]	chr7	129434433	129488399	1	ensembl_havana	4.851806205	4.630240202	5.073372208	4.376980663	4.701268571	4.81247137	4.953655868	5.081144309	5.185316447
ENSG00000099991	0.002292	0.025148959	-0.382713979	-1.303792226	**CABIN1**	protein_coding	calcineurin binding protein 1 [Source:HGNC Symbol;Acc:HGNC:24187]	chr22	24011192	24178628	1	ensembl_havana	7.295856601	7.487213591	7.104499612	7.681062131	7.308497589	7.472081052	7.303777149	6.997913662	7.011808026
ENSG00000131844	0.002293	0.025148959	-0.411584098	-1.330145529	**MCCC2**	protein_coding	methylcrotonoyl-CoA carboxylase 2 [Source:HGNC Symbol;Acc:HGNC:6937]	chr5	71587288	71658704	1	ensembl_havana	6.946946943	7.152738992	6.741154894	7.13936884	7.14276186	7.176086276	6.624410547	6.748579901	6.850474235
ENSG00000169129	0.002292	0.025148959	-0.781379983	-1.718774148	**AFAP1L2**	protein_coding	actin filament associated protein 1 like 2 [Source:HGNC Symbol;Acc:HGNC:25901]	chr10	114294824	114404756	-1	ensembl_havana	0.950455455	1.341145446	0.559765463	0.968509893	1.524202165	1.530724282	0.36983092	0.880929471	0.428535999
ENSG00000274922	0.00229	0.025148959	-1.717443784	-3.288532171	**AL139384.1**	lincRNA		chr13	112686769	112689815	-1	havana	-0.85017892	0.008542972	-1.708900812	-0.14746469	0.09469996	0.078393642	-1.7649502	-2.33094524	-1.030806998
ENSG00000196083	0.002298	0.025174843	0.732986392	1.662076046	**IL1RAP**	protein_coding	interleukin 1 receptor accessory protein [Source:HGNC Symbol;Acc:HGNC:5995]	chr3	190514051	190659750	1	ensembl_havana	3.501165532	3.134672336	3.867658728	2.791937877	3.314364738	3.297714395	3.818023093	3.945529874	3.839423218
ENSG00000080986	0.002304	0.025212124	-0.334158821	-1.260642163	**NDC80**	protein_coding	NDC80, kinetochore complex component [Source:HGNC Symbol;Acc:HGNC:16909]	chr18	2571511	2616635	1	ensembl_havana	5.530467916	5.697547327	5.363388506	5.542274049	5.761665985	5.788701946	5.189456144	5.435799067	5.464910307
ENSG00000167699	0.002306	0.025223656	-0.434610098	-1.351545515	**GLOD4**	protein_coding	glyoxalase domain containing 4 [Source:HGNC Symbol;Acc:HGNC:14111]	chr17	757097	783390	-1	ensembl_havana	6.409574672	6.62687972	6.192269623	6.720729681	6.617844195	6.542065286	6.163683069	6.211890079	6.201235721
ENSG00000115677	0.002309	0.025231295	-0.336770996	-1.262926777	**HDLBP**	protein_coding	high density lipoprotein binding protein [Source:HGNC Symbol;Acc:HGNC:4857]	chr2	241227264	241317061	-1	ensembl_havana	8.566771704	8.735157202	8.398386207	8.78057645	8.699269315	8.725625842	8.421273167	8.386978998	8.386906455
ENSG00000124171	0.002321	0.025347655	0.367799324	1.290382988	**PARD6B**	protein_coding	par-6 family cell polarity regulator beta [Source:HGNC Symbol;Acc:HGNC:16245]	chr20	50731544	50756795	1	ensembl_havana	3.124588429	2.940688767	3.308488091	2.917043099	3.085263982	2.819759219	3.267019432	3.523471405	3.134973434
ENSG00000100490	0.002326	0.025357923	-0.981527255	-1.974554591	**CDKL1**	protein_coding	cyclin dependent kinase like 1 [Source:HGNC Symbol;Acc:HGNC:1781]	chr14	50329404	50416461	-1	ensembl_havana	-0.224957483	0.265806145	-0.71572111	0.565613454	-0.18055577	0.41236075	-0.57379957	-0.74417246	-0.829191301
ENSG00000169946	0.002325	0.025357923	-1.585272272	-3.00064422	**ZFPM2**	protein_coding	zinc finger protein, FOG family member 2 [Source:HGNC Symbol;Acc:HGNC:16700]	chr8	104590733	105804532	1	ensembl_havana	0.091420078	0.884056214	-0.701216057	1.528734855	0.52486995	0.598563838	0.485518989	-0.93694677	-1.652220391
ENSG00000154175	0.002331	0.025397279	0.473335248	1.388315293	**ABI3BP**	protein_coding	ABI family member 3 binding protein [Source:HGNC Symbol;Acc:HGNC:17265]	chr3	100749156	100993515	-1	ensembl_havana	-0.247174614	-0.483842238	-0.01050699	-0.79434999	-0.569826728	-0.087349994	-0.46237776	0.02879206	0.402064725
ENSG00000232907	0.002335	0.025418343	-0.639688253	-1.557992462	**DLGAP4-AS1**	antisense	DLGAP4 antisense RNA 1 [Source:HGNC Symbol;Acc:HGNC:51223]	chr20	36507702	36573391	-1	havana	1.090766601	1.410610728	0.770922475	1.058172782	1.440410536	1.733248866	0.668022463	0.693803314	0.950941648
ENSG00000269982	0.002345	0.025492958	0.450214739	1.366243601	**AC018809.2**	antisense		chr3	9958717	9962539	1	havana	0.188032992	-0.037074378	0.413140361	-0.41068387	-0.036373238	0.335833975	-0.08686711	0.529376201	0.796911992
ENSG00000070610	0.002344	0.025492958	0.362689751	1.285820941	**GBA2**	protein_coding	glucosylceramidase beta 2 [Source:HGNC Symbol;Acc:HGNC:18986]	chr9	35736866	35749228	-1	ensembl_havana	6.70674963	6.525404754	6.888094505	6.584391964	6.48859494	6.503227358	6.962816344	6.785506597	6.915960576
ENSG00000135111	0.002351	0.025499137	0.395977783	1.315834262	**TBX3**	protein_coding	T-box 3 [Source:HGNC Symbol;Acc:HGNC:11602]	chr12	114670254	114684164	-1	ensembl_havana	3.250754065	3.052765174	3.448742957	2.991768026	3.158267607	3.008259887	3.487731073	3.510925742	3.347572056
ENSG00000267002	0.002349	0.025499137	0.357153955	1.280896543	**AC060780.1**	lincRNA		chr17	43148368	43171037	-1	havana	5.715011108	5.53643413	5.893588085	5.490064912	5.572896033	5.546341445	5.895418496	5.871432446	5.913913313
ENSG00000237753	0.002349	0.025499137	-0.372183368	-1.294310153	**AC079922.2**	lincRNA		chr2	112641832	112645690	-1	havana	1.371470636	1.55756232	1.185378952	1.464591378	1.535784795	1.672310787	1.033805522	1.14725896	1.375072374
ENSG00000150093	0.002357	0.025528355	0.421104596	1.338952328	**ITGB1**	protein_coding	integrin subunit beta 1 [Source:HGNC Symbol;Acc:HGNC:6153]	chr10	32900319	33005792	-1	ensembl_havana	6.812738977	6.602186679	7.023291275	6.268955083	6.674610346	6.862994609	6.7928488	7.098030139	7.178994887
ENSG00000176623	0.002357	0.025528355	-0.34944022	-1.274066181	**RMDN1**	protein_coding	regulator of microtubule dynamics 1 [Source:HGNC Symbol;Acc:HGNC:24285]	chr8	86468257	86514357	-1	ensembl_havana	5.118172038	5.292892148	4.943451928	5.324661633	5.2836326	5.27038221	4.999384646	4.881204367	4.949766771
ENSG00000112782	0.002365	0.025537279	1.089955198	2.128674259	**CLIC5**	protein_coding	chloride intracellular channel 5 [Source:HGNC Symbol;Acc:HGNC:13517]	chr6	45880827	46080348	-1	ensembl_havana	0.169315068	-0.375662531	0.714292667	-1.070289	0.030651399	-0.087349994	0.485518989	0.800742365	0.856616648
ENSG00000237595	0.002361	0.025537279	0.439966548	1.356572872	**AL161937.2**	lincRNA		chr20	50162765	50166102	-1	havana	-0.434951542	-0.654934816	-0.214968267	-0.61730533	-0.428075095	-0.91942402	-0.30988669	0.063570082	-0.398588198
ENSG00000198373	0.002364	0.025537279	0.425451593	1.342992821	**WWP2**	protein_coding	WW domain containing E3 ubiquitin protein ligase 2 [Source:HGNC Symbol;Acc:HGNC:16804]	chr16	69762306	69941741	1	ensembl_havana	6.154518567	5.941792771	6.367244364	5.91113593	5.887348039	6.026894343	6.380021597	6.391151713	6.330559782
ENSG00000182986	0.002362	0.025537279	0.402916687	1.322178249	**ZNF320**	protein_coding	zinc finger protein 320 [Source:HGNC Symbol;Acc:HGNC:13842]	chr19	52863790	52897693	-1	havana	0.196329923	-0.005128421	0.397788266	-0.27307956	0.030651399	0.227042895	0.210814386	0.45273451	0.529815901
ENSG00000180263	0.002372	0.025561268	0.471984521	1.387016088	**FGD6**	protein_coding	FYVE, RhoGEF and PH domain containing 6 [Source:HGNC Symbol;Acc:HGNC:21740]	chr12	95076749	95217482	-1	ensembl_havana	2.74972567	2.51373341	2.985717931	2.341580783	2.512189067	2.68743038	2.879883083	3.072584571	3.004686139
ENSG00000157514	0.002373	0.025561268	0.377060983	1.298693498	**TSC22D3**	protein_coding	TSC22 domain family member 3 [Source:HGNC Symbol;Acc:HGNC:3051]	chrX	107713221	107777342	-1	ensembl_havana	8.644909783	8.456379291	8.833440275	8.397240074	8.493041975	8.478855825	8.695052582	8.884492536	8.920775706
ENSG00000113966	0.002372	0.025561268	0.342570486	1.268013835	**ARL6**	protein_coding	ADP ribosylation factor like GTPase 6 [Source:HGNC Symbol;Acc:HGNC:13210]	chr3	97764521	97801242	1	ensembl_havana	4.24796589	4.076680646	4.419251133	4.007662542	4.206066476	4.016312922	4.379893999	4.506216612	4.371642787
ENSG00000255624	0.002375	0.025561268	-0.406435273	-1.325406843	**AC073585.1**	transcribed_processed_pseudogene		chr10	122897681	122898615	1	havana	3.633233747	3.836451383	3.43001611	4.228453786	3.836258812	3.444641551	3.818023093	3.337051803	3.134973434
ENSG00000255769	0.00239	0.025713368	0.445532923	1.361817071	**GOLGA2P10**	transcribed_unprocessed_pseudogene	golgin A2 pseudogene 10 [Source:HGNC Symbol;Acc:HGNC:26229]	chr15	82472993	82513950	-1	havana	5.293787024	5.071020563	5.516553486	4.989569283	4.867466142	5.356026263	5.539911601	5.338867525	5.670881333
ENSG00000188659	0.0024	0.025779908	0.652723799	1.572133574	**SAXO2**	protein_coding	stabilizer of axonemal microtubules 2 [Source:HGNC Symbol;Acc:HGNC:33727]	chr15	82262810	82284930	1	ensembl_havana	1.768434966	1.442073066	2.094796865	1.283104003	1.7890623	1.254052895	1.966610161	2.203033505	2.114746929
ENSG00000172725	0.0024	0.025779908	-1.722506833	-3.300093346	**CORO1B**	protein_coding	coronin 1B [Source:HGNC Symbol;Acc:HGNC:2253]	chr11	67435510	67443821	-1	ensembl_havana	-0.466613146	0.39464027	-1.327866563	0.727424294	0.09469996	0.361796557	-1.22062457	-0.86978558	-1.89318954
ENSG00000176410	0.002406	0.025781744	0.419564065	1.337523338	**DNAJC30**	protein_coding	DnaJ heat shock protein family (Hsp40) member C30 [Source:HGNC Symbol;Acc:HGNC:16410]	chr7	73680969	73683453	-1	ensembl_havana	2.825570077	2.615788044	3.035352109	2.578211954	2.592024241	2.677127938	3.118116049	2.957102004	3.030838274
ENSG00000064933	0.002408	0.025781744	-0.343770611	-1.269069087	**PMS1**	protein_coding	PMS1 homolog 1, mismatch repair system component [Source:HGNC Symbol;Acc:HGNC:9121]	chr2	189784085	189877629	1	ensembl_havana	5.008014098	5.179899403	4.836128792	5.068292738	5.164648804	5.306756667	4.753260037	4.773725255	4.981401085
ENSG00000138085	0.002408	0.025781744	-0.348464182	-1.273204519	**ATRAID**	protein_coding	all-trans retinoic acid induced differentiation factor [Source:HGNC Symbol;Acc:HGNC:24090]	chr2	27212027	27217178	1	ensembl_havana	6.238783571	6.413015662	6.064551479	6.367395614	6.466448614	6.405202758	5.997614135	6.085389302	6.110651001
ENSG00000272047	0.002409	0.025781744	-0.454259071	-1.370078989	**GTF2H5**	protein_coding	general transcription factor IIH subunit 5 [Source:HGNC Symbol;Acc:HGNC:21157]	chr6	158168352	158199344	1	ensembl_havana	3.925605555	4.152735091	3.698476019	4.260919171	4.063308132	4.133977969	3.68819912	3.740507201	3.666721737
ENSG00000281912	0.002402	0.025781744	-0.609165294	-1.525376411	**LINC01144**	lincRNA	long intergenic non-protein coding RNA 1144 [Source:HGNC Symbol;Acc:HGNC:49460]	chr1	45303910	45305619	1	havana	0.201281767	0.505864414	-0.10330088	0.326725152	0.570631912	0.620236179	-0.04612063	-0.15890847	-0.104873545
ENSG00000055211	0.002411	0.025784548	0.333410023	1.259988025	**GINM1**	protein_coding	glycoprotein integral membrane 1 [Source:HGNC Symbol;Acc:HGNC:21074]	chr6	149566294	149591748	1	ensembl_havana	4.488215801	4.321510789	4.654920812	4.305543488	4.402318629	4.256670251	4.631385227	4.7161429	4.61723431
ENSG00000224578	0.002422	0.025873839	-0.387328114	-1.307968788	**HNRNPA1P48**	protein_coding	heterogeneous nuclear ribonucleoprotein A1 pseudogene 48 [Source:HGNC Symbol;Acc:HGNC:48778]	chr16	51553436	51647132	1	havana	1.545990394	1.739654451	1.352326337	1.649052215	1.836662271	1.733248866	1.195452122	1.541948597	1.319578292
ENSG00000164306	0.002425	0.025895102	-0.386181757	-1.306929896	**PRIMPOL**	protein_coding	primase and DNA directed polymerase [Source:HGNC Symbol;Acc:HGNC:26575]	chr4	184649613	184694963	1	ensembl_havana	3.879097227	4.072188105	3.686006348	3.97580247	4.073277629	4.167484215	3.508395033	3.68574783	3.863876181
ENSG00000165671	0.00243	0.025923969	-0.360378885	-1.283762999	**NSD1**	protein_coding	nuclear receptor binding SET domain protein 1 [Source:HGNC Symbol;Acc:HGNC:14234]	chr5	177133025	177300215	1	ensembl_havana	7.234064843	7.414254286	7.053875401	7.463863831	7.305328946	7.473570081	7.044378641	7.006597054	7.110650508
ENSG00000218073	0.00244	0.025934657	0.418604495	1.336634016	**AL021407.3**	processed_pseudogene		chr6	16160926	16161411	1	havana	1.192127134	0.982824887	1.401429382	1.237769075	0.927899934	0.782805653	1.541278047	1.372000412	1.291009686
ENSG00000112144	0.002438	0.025934657	0.411285364	1.32987013	**ICK**	protein_coding	intestinal cell kinase [Source:HGNC Symbol;Acc:HGNC:21219]	chr6	53001279	53061802	-1	ensembl_havana	4.811205014	4.605562332	5.016847696	4.608312156	4.654222446	4.554152393	4.919346995	5.066215902	5.064980193
ENSG00000256128	0.002442	0.025934657	0.400054931	1.319558152	**LINC00944**	lincRNA	long intergenic non-protein coding RNA 944 [Source:HGNC Symbol;Acc:HGNC:48640]	chr12	126729787	126772411	-1	havana	2.473599108	2.273571643	2.673626573	2.23025056	2.45233188	2.138132487	2.717806083	2.861519581	2.441554055
ENSG00000224114	0.00244	0.025934657	0.375540216	1.297325246	**AL591846.1**	processed_pseudogene		chr1	206695837	206696269	-1	havana	1.548398158	1.36062805	1.736168266	1.383653947	1.214022341	1.484207863	1.808964307	1.504469011	1.89507148
ENSG00000172399	0.002441	0.025934657	-0.330587943	-1.257525751	**MYOZ2**	protein_coding	myozenin 2 [Source:HGNC Symbol;Acc:HGNC:1330]	chr4	119135784	119187789	1	ensembl_havana	2.849531578	3.01482555	2.684237606	3.345229877	3.069412532	2.629834241	2.991732974	2.748504565	2.31247528
ENSG00000162997	0.002436	0.025934657	-0.584463192	-1.499480948	**PRORSD1P**	transcribed_unitary_pseudogene	prolyl-tRNA synthetase associated domain containing 1, pseudogene [Source:HGNC Symbol;Acc:HGNC:34379]	chr2	55282319	55284522	1	havana	2.69963612	2.991867716	2.407404524	2.838235332	3.24901995	2.888347866	2.238067541	2.479027387	2.505118643
ENSG00000106077	0.002447	0.025952156	0.56566088	1.480065353	**ABHD11**	protein_coding	abhydrolase domain containing 11 [Source:HGNC Symbol;Acc:HGNC:16407]	chr7	73736094	73738867	-1	ensembl_havana	3.331716104	3.048885664	3.614546544	2.749499015	2.983208668	3.413949308	3.242560098	3.759192284	3.84188725
ENSG00000102218	0.002447	0.025952156	-0.353063669	-1.277270126	**RP2**	protein_coding	RP2, ARL3 GTPase activating protein [Source:HGNC Symbol;Acc:HGNC:10274]	chrX	46836940	46882358	1	ensembl_havana	5.269485105	5.44601694	5.092953271	5.53370268	5.42113164	5.383216499	5.140653506	5.129134774	5.009071532
ENSG00000088970	0.002456	0.026027276	0.398124451	1.317793624	**KIZ**	protein_coding	kizuna centrosomal protein [Source:HGNC Symbol;Acc:HGNC:15865]	chr20	21125983	21246622	1	ensembl_havana	4.650125348	4.451063122	4.849187573	4.423275583	4.467270071	4.462643711	4.731453667	4.958309717	4.857799336
ENSG00000127419	0.002459	0.026027517	0.52419847	1.438134362	**TMEM175**	protein_coding	transmembrane protein 175 [Source:HGNC Symbol;Acc:HGNC:28709]	chr4	932387	958656	1	ensembl_havana	4.361337159	4.099237924	4.623436394	4.103445504	3.920403831	4.273864437	4.637651633	4.603961871	4.628695679
ENSG00000106346	0.00246	0.026027517	0.342842991	1.268253367	**USP42**	protein_coding	ubiquitin specific peptidase 42 [Source:HGNC Symbol;Acc:HGNC:20068]	chr7	6104884	6161564	1	ensembl_havana	5.35025894	5.178837445	5.521680436	5.170345707	5.184100449	5.182066179	5.560671248	5.51332185	5.491048209
ENSG00000204745	0.002468	0.02609378	-0.583158489	-1.498125504	**AC083899.1**	unprocessed_pseudogene		chr2	87125198	87196629	1	havana	0.592022096	0.88360134	0.300442851	0.812341977	0.89230127	0.946160774	0.032074365	0.315191528	0.554062661
ENSG00000251143	0.002474	0.026120188	0.482593973	1.397253678	**AP002490.1**	antisense		chr11	72014291	72020910	1	havana	0.467415413	0.226118426	0.708712399	0.660286109	0.214850257	-0.196781087	1.143556601	0.554044597	0.428535999
ENSG00000165699	0.002473	0.026120188	0.366564221	1.289278754	**TSC1**	protein_coding	TSC complex subunit 1 [Source:HGNC Symbol;Acc:HGNC:12362]	chr9	132891348	132946874	-1	ensembl_havana	6.243325471	6.060043361	6.426607582	5.986159613	6.076665349	6.11730512	6.419197505	6.445314475	6.415310765
ENSG00000196139	0.00248	0.026165686	-1.054414315	-2.076874895	**AKR1C3**	protein_coding	aldo-keto reductase family 1 member C3 [Source:HGNC Symbol;Acc:HGNC:386]	chr10	5035354	5107686	1	ensembl_havana	1.242418781	1.769625939	0.715211624	2.722326456	1.882741771	0.703809589	2.072167195	0.426254992	-0.352787316
ENSG00000279744	0.002489	0.026230983	0.571069444	1.485624429	**AC132938.5**	TEC		chr17	82462601	82464255	1	havana	0.71811782	0.432583098	1.003652542	0.267803752	0.453384582	0.57656096	0.915012722	1.163381334	0.932563571
ENSG00000176714	0.00249	0.026230983	-0.613662534	-1.530138808	**CCDC121**	protein_coding	coiled-coil domain containing 121 [Source:HGNC Symbol;Acc:HGNC:25833]	chr2	27625639	27629012	-1	ensembl_havana	2.30746278	2.614294048	2.000631513	2.424845514	2.904984633	2.513051996	1.996172808	2.063957599	1.941764132
ENSG00000285219	0.002495	0.02627008	-0.498416511	-1.412662186	**AL591485.1**	bidirectional_promoter_lncRNA		chr6	8435568	9294133	1	havana	1.885857109	2.135065365	1.636648854	2.150951608	2.101167058	2.153077428	1.500601289	1.602328815	1.807016457
ENSG00000185513	0.002505	0.026321498	0.452738604	1.368635813	**L3MBTL1**	protein_coding	L3MBTL1, histone methyl-lysine binding protein [Source:HGNC Symbol;Acc:HGNC:15905]	chr20	43507680	43550950	1	ensembl_havana	4.141746584	3.915377282	4.368115886	3.96656885	3.889139551	3.890423445	4.310990596	4.310178017	4.483179044
ENSG00000060971	0.00251	0.026321498	0.333216473	1.259818999	**ACAA1**	protein_coding	acetyl-CoA acyltransferase 1 [Source:HGNC Symbol;Acc:HGNC:82]	chr3	38103129	38137242	-1	ensembl_havana	6.039058421	5.872450185	6.205666657	5.826264424	5.914220301	5.876865828	6.1942952	6.234481581	6.188223192
ENSG00000140577	0.002509	0.026321498	-0.346086124	-1.271107567	**CRTC3**	protein_coding	CREB regulated transcription coactivator 3 [Source:HGNC Symbol;Acc:HGNC:26148]	chr15	90529925	90645345	1	ensembl_havana	6.827752442	7.000795504	6.65470938	7.109530421	6.913927014	6.978929076	6.759117942	6.618304633	6.586705565
ENSG00000184209	0.002513	0.026321498	-0.372965311	-1.29501186	**SNRNP35**	protein_coding	small nuclear ribonucleoprotein U11/U12 subunit 35 [Source:HGNC Symbol;Acc:HGNC:30852]	chr12	123457641	123473154	1	ensembl_havana	3.463414986	3.649897642	3.276932331	3.741463211	3.627484939	3.580744775	3.441917197	3.17950436	3.209375435
ENSG00000100934	0.002511	0.026321498	-0.379872934	-1.301227244	**SEC23A**	protein_coding	Sec23 homolog A, coat complex II component [Source:HGNC Symbol;Acc:HGNC:10701]	chr14	39031919	39109646	-1	ensembl_havana	5.688686315	5.878622782	5.498749848	5.999236466	5.809567068	5.827064813	5.529839857	5.492734025	5.473675663

ENSG00000126749	0.002515	0.026321498	-0.384829159	-1.305705159	**EMG1**	protein_coding	EMG1, N1-specific pseudouridine methyltransferase [Source:NCBI gene;Acc:10436]	chr12	6970893	6979941	1	ensembl_havana	4.753588499	4.946003078	4.561173919	4.852177089	4.942265309	5.043566836	4.374276086	4.625943765	4.683301907
ENSG00000204934	0.002512	0.026321498	-0.428635343	-1.34595982	**ATP6V0E2-AS1**	antisense	ATP6V0E2 antisense RNA 1 [Source:HGNC Symbol;Acc:HGNC:44180]	chr7	149867697	149880610	-1	havana	2.508905225	2.723222896	2.294587553	2.85834452	2.71937662	2.591947549	2.349154471	2.41986126	2.114746929
ENSG00000232818	0.00251	0.026321498	-0.934499028	-1.911226852	**RPS2P32**	processed_pseudogene	ribosomal protein S2 pseudogene 32 [Source:HGNC Symbol;Acc:HGNC:30518]	chr7	23490473	23491364	1	havana	0.272059715	0.739309229	-0.195189799	0.705390237	0.89230127	0.620236179	0.141952326	-0.37473441	-0.352787316
ENSG00000141682	0.002523	0.026369404	0.366851721	1.289535707	**PMAIP1**	protein_coding	phorbol-12-myristate-13-acetate-induced protein 1 [Source:HGNC Symbol;Acc:HGNC:9108]	chr18	59899948	59904306	1	ensembl_havana	7.665914767	7.482488906	7.849340628	7.725873287	7.423048374	7.298545057	8.066769149	7.868522808	7.612729927
ENSG00000111679	0.002523	0.026369404	-0.787981585	-1.726657078	**PTPN6**	protein_coding	protein tyrosine phosphatase, non-receptor type 6 [Source:HGNC Symbol;Acc:HGNC:9658]	chr12	6946468	6961316	1	ensembl_havana	2.26019201	2.654182803	1.866201218	3.155115434	2.546946467	2.260486508	2.213108088	1.578479109	1.807016457
ENSG00000263567	0.002531	0.026435111	1.566589099	2.962035844	**AC007923.1**	lincRNA		chr17	31762440	31769048	1	havana	-1.040479424	-1.823773974	-0.257184875	-2.51386715	-2.420341378	-0.537113394	-0.51701327	-0.57414696	0.319605599
ENSG00000125885	0.002535	0.026454056	-0.351371842	-1.275773168	**MCM8**	protein_coding	minichromosome maintenance 8 homologous recombination repair factor [Source:HGNC Symbol;Acc:HGNC:16147]	chr20	5950652	5998977	1	ensembl_havana	6.443798572	6.619484493	6.268112651	6.438829121	6.617844195	6.801780162	6.144008971	6.263752065	6.396576916
ENSG00000103365	0.00254	0.026483238	-0.366882802	-1.289563488	**GGA2**	protein_coding	golgi associated, gamma adaptin ear containing, ARF binding protein 2 [Source:HGNC Symbol;Acc:HGNC:16064]	chr16	23463542	23521995	-1	ensembl_havana	6.453065277	6.636506678	6.269623876	6.69859235	6.52319133	6.687736355	6.349328909	6.21576051	6.243782209
ENSG00000119698	0.002551	0.026545611	0.819104669	1.764310729	**PPP4R4**	protein_coding	protein phosphatase 4 regulatory subunit 4 [Source:HGNC Symbol;Acc:HGNC:23788]	chr14	94146128	94279735	1	ensembl_havana	1.899993661	1.490441327	2.309545996	0.892538	1.667974555	1.910811426	2.062883947	2.329873398	2.535880644
ENSG00000205959	0.00255	0.026545611	0.443757904	1.36014259	**AC105345.1**	lincRNA		chr4	8482270	8512610	1	havana	0.611990001	0.390111049	0.833868953	0.383334258	0.06303108	0.723967808	0.762800879	0.648704825	1.090101154
ENSG00000198925	0.002556	0.026545611	0.367943841	1.290512254	**ATG9A**	protein_coding	autophagy related 9A [Source:HGNC Symbol;Acc:HGNC:22408]	chr2	219219380	219229717	-1	ensembl_havana	4.165240777	3.981268856	4.349212697	3.97580247	3.966065478	4.001938621	4.398463935	4.359993337	4.289180821
ENSG00000264247	0.002555	0.026545611	-0.325586712	-1.253173973	**LINC00909**	lincRNA	long intergenic non-protein coding RNA 909 [Source:HGNC Symbol;Acc:HGNC:44331]	chr18	74591774	74598508	-1	havana	3.313458836	3.476252192	3.15066548	3.441680301	3.439879526	3.54719675	3.100076994	3.131139307	3.22078014
ENSG00000138614	0.002553	0.026545611	-0.364079842	-1.287060474	**INTS14**	protein_coding	integrator complex subunit 14 [Source:HGNC Symbol;Acc:HGNC:25372]	chr15	65578753	65611289	-1	ensembl_havana	6.185758541	6.367798462	6.003718621	6.385263508	6.360116897	6.358014981	5.971186008	5.96867691	6.071292944
ENSG00000137094	0.002557	0.026545611	-0.475253637	-1.3901626	**DNAJB5**	protein_coding	DnaJ heat shock protein family (Hsp40) member B5 [Source:HGNC Symbol;Acc:HGNC:14887]	chr9	34989641	34998900	1	ensembl_havana	4.893006182	5.130633	4.655379363	5.473863577	5.0672469	4.850788523	4.878922094	4.555916886	4.531299108
ENSG00000165168	0.002559	0.026555189	-0.413956328	-1.332334493	**CYBB**	protein_coding	cytochrome b-245 beta chain [Source:HGNC Symbol;Acc:HGNC:2578]	chrX	37780011	37813461	1	ensembl_havana	1.143050343	1.350028507	0.936072179	1.190963204	1.524202165	1.334920154	0.739683594	1.029056719	1.039476224
ENSG00000139880	0.002563	0.026573017	-0.495284748	-1.409598944	**CDH24**	protein_coding	cadherin 24 [Source:HGNC Symbol;Acc:HGNC:14265]	chr14	23047062	23057538	-1	ensembl_havana	3.557758966	3.805401341	3.310116592	3.895366092	3.678012323	3.842825607	3.302948055	3.358217192	3.269184531
ENSG00000212556	0.002567	0.026573122	0.56351436	1.477864869	**RF00019**	misc_RNA		chr3	127202372	127202473	-1	ensembl	-0.435733299	-0.717490479	-0.153976118	-1.41179303	-0.34076431	-0.399914092	-0.63291296	0.097529431	0.073455173
ENSG00000240498	0.002565	0.026573122	0.422316499	1.340077557	**CDKN2B-AS1**	antisense	CDKN2B antisense RNA 1 [Source:HGNC Symbol;Acc:HGNC:34341]	chr9	21994778	22121097	1	havana	3.265768556	3.054610307	3.476926805	3.477874854	2.983208668	2.702747398	3.773189967	3.479075818	3.178514631
ENSG00000136205	0.002576	0.026583298	0.49772635	1.411986554	**TNS3**	protein_coding	tensin 3 [Source:HGNC Symbol;Acc:HGNC:21616]	chr7	47275154	47582558	-1	ensembl_havana	0.469625029	0.220761854	0.718488204	-0.03191641	0.185737694	0.508464275	0.457455004	0.841392961	0.856616648
ENSG00000177335	0.002577	0.026583298	0.402611381	1.321898477	**C8orf31**	processed_transcript	chromosome 8 open reading frame 31 (putative) [Source:HGNC Symbol;Acc:HGNC:26731]	chr8	143039209	143059942	1	ensembl_havana	2.504225548	2.302919857	2.705531238	2.084216604	2.370609194	2.453933774	2.423571602	2.886015394	2.807006718
ENSG00000175634	0.002574	0.026583298	0.352359953	1.276647253	**RPS6KB2**	protein_coding	ribosomal protein S6 kinase B2 [Source:HGNC Symbol;Acc:HGNC:10437]	chr11	67428460	67435408	1	ensembl_havana	4.947145066	4.77096509	5.123325042	4.803741862	4.770306765	4.738846643	5.212727477	5.064070606	5.093177045
ENSG00000162390	0.002574	0.026583298	-0.352613265	-1.27687143	**ACOT11**	protein_coding	acyl-CoA thioesterase 11 [Source:HGNC Symbol;Acc:HGNC:18156]	chr1	54542257	54639192	1	ensembl_havana	1.639847412	1.816154044	1.463540779	1.833046887	1.678462547	1.936952697	1.541278047	1.271458479	1.577885811
ENSG00000218510	0.002579	0.026583298	-0.457020182	-1.372703634	**LINC00339**	transcribed_processed_pseudogene	long intergenic non-protein coding RNA 339 [Source:HGNC Symbol;Acc:HGNC:25011]	chr1	22024558	22031223	1	havana	3.152436023	3.380946114	2.923925932	3.301834776	3.263024855	3.57797871	2.921565963	2.881149392	2.969062441
ENSG00000125962	0.002575	0.026583298	-0.516550802	-1.43053104	**ARMCX5**	protein_coding	armadillo repeat containing, X-linked 5 [Source:HGNC Symbol;Acc:HGNC:25772]	chrX	102599168	102604159	1	ensembl_havana	3.489682855	3.747958256	3.231407454	3.87082891	3.706476045	3.666569812	3.188097919	3.351196507	3.154927934
ENSG00000136048	0.002582	0.026597496	-0.472544247	-1.387554316	**DRAM1**	protein_coding	DNA damage regulated autophagy modulator 1 [Source:HGNC Symbol;Acc:HGNC:25645]	chr12	101877351	102012130	1	ensembl_havana	2.104022575	2.340294698	1.867750452	2.602259737	2.464503644	1.954120714	2.22979561	1.831488934	1.541966812
ENSG00000125827	0.002584	0.026597727	-0.407049554	-1.325971304	**TMX4**	protein_coding	thioredoxin related transmembrane protein 4 [Source:HGNC Symbol;Acc:HGNC:25237]	chr20	7977348	8019829	-1	ensembl_havana	6.258748807	6.462273585	6.05522403	6.603878128	6.468716374	6.314226252	6.102705109	6.0489717	6.013995283
ENSG00000198945	0.002591	0.026599534	-0.34507431	-1.270216406	**L3MBTL3**	protein_coding	L3MBTL3, histone methyl-lysine binding protein [Source:HGNC Symbol;Acc:HGNC:23035]	chr6	130013699	130141451	1	ensembl_havana	3.088355475	3.260892629	2.91581832	3.301834776	3.276895115	3.203947998	2.957076007	2.881149392	2.90922956
ENSG00000120159	0.002591	0.026599534	-0.403946386	-1.323122269	**CAAP1**	protein_coding	caspase activity and apoptosis inhibitor 1 [Source:HGNC Symbol;Acc:HGNC:25834]	chr9	26840685	26892804	-1	ensembl_havana	4.758594313	4.960567506	4.55662112	5.184352789	4.949860986	4.747488742	4.731453667	4.480709313	4.457700378
ENSG00000221886	0.00259	0.026599534	-0.565529956	-1.479931044	**ZBED8**	protein_coding	zinc finger BED-type containing 8 [Source:HGNC Symbol;Acc:HGNC:30804]	chr5	160393148	160400097	-1	ensembl_havana	2.556041089	2.838806067	2.273276111	2.541371574	2.995843375	2.979203253	2.144358071	2.466087459	2.209382804
ENSG00000196562	0.002586	0.026599534	-0.852144874	-1.80518272	**SULF2**	protein_coding	sulfatase 2 [Source:HGNC Symbol;Acc:HGNC:20392]	chr20	47656348	47786616	-1	ensembl_havana	2.258133972	2.684206409	1.832061535	1.82284946	2.882481267	3.347288501	1.278000209	2.055343696	2.162840701
ENSG00000075391	0.002619	0.026623836	0.497168678	1.411440858	**RASAL2**	protein_coding	RAS protein activator like 2 [Source:HGNC Symbol;Acc:HGNC:9874]	chr1	178094141	178484147	1	ensembl_havana	5.391069595	5.142485256	5.639653934	4.954969984	5.214147108	5.258338676	5.622796123	5.671758732	5.624406947
ENSG00000272419	0.002611	0.026623836	0.423090337	1.340796546	**AC241585.2**	transcribed_unprocessed_pseudogene		chr1	145164099	145216058	-1	havana	1.780304276	1.568759107	1.991849445	1.516132283	1.592342839	1.5978022	1.831005931	2.131077666	2.013464737
ENSG00000115232	0.002616	0.026623836	0.366520818	1.289239967	**ITGA4**	protein_coding	integrin subunit alpha 4 [Source:HGNC Symbol;Acc:HGNC:6140]	chr2	181457202	181536187	1	ensembl_havana	6.558979992	6.375719583	6.742240401	6.323316325	6.198265076	6.605577348	6.663705352	6.646178524	6.916837327
ENSG00000010030	0.00262	0.026623836	0.354949767	1.27894105	**ETV7**	protein_coding	ETS variant 7 [Source:HGNC Symbol;Acc:HGNC:18160]	chr6	36354091	36387800	-1	ensembl_havana	2.156040688	1.978565804	2.333515571	1.85322802	2.037411386	2.045058006	2.22979561	2.322713072	2.448038032
ENSG00000181135	0.002622	0.026623836	0.351889638	1.276231137	**ZNF707**	protein_coding	zinc finger protein 707 [Source:HGNC Symbol;Acc:HGNC:27815]	chr8	143684452	143713898	1	ensembl_havana	4.35874005	4.182795231	4.534684869	4.285261859	4.059300963	4.20382287	4.602844617	4.479100033	4.522109956
ENSG00000123595	0.002615	0.026623836	0.337484659	1.263551668	**RAB9A**	protein_coding	RAB9A, member RAS oncogene family [Source:HGNC Symbol;Acc:HGNC:9792]	chrX	13689125	13710506	1	ensembl_havana	6.262432557	6.093690227	6.431174886	6.288076403	6.090475597	5.902518682	6.593314277	6.41238703	6.287823352
ENSG00000136052	0.002598	0.026623836	0.336721262	1.262883241	**SLC41A2**	protein_coding	solute carrier family 41 member 2 [Source:HGNC Symbol;Acc:HGNC:31045]	chr12	104802553	104958744	-1	ensembl_havana	3.905609421	3.73724879	4.073970052	3.677822113	3.877807482	3.656116775	4.061190507	4.189409115	3.971310534
ENSG00000109787	0.002615	0.026623836	0.32762883	1.254949082	**KLF3**	protein_coding	Kruppel like factor 3 [Source:HGNC Symbol;Acc:HGNC:16516]	chr4	38664196	38701042	1	ensembl_havana	4.969034334	4.805219919	5.132848749	4.881950602	4.780079028	4.753630128	5.238724391	5.085381349	5.074440509
ENSG00000204304	0.002604	0.026623836	-0.331761056	-1.258548711	**PBX2**	protein_coding	PBX homeobox 2 [Source:HGNC Symbol;Acc:HGNC:8633]	chr6	32184741	32190186	-1	ensembl_havana	7.511720859	7.677601387	7.345840331	7.919245496	7.62462993	7.488928735	7.625808237	7.270981324	7.140731432
ENSG00000138378	0.002613	0.026623836	-0.338844188	-1.264742942	**STAT4**	protein_coding	signal transducer and activator of transcription 4 [Source:HGNC Symbol;Acc:HGNC:11365]	chr2	191029576	191151596	-1	ensembl_havana	4.232370499	4.401792593	4.062948405	4.431688049	4.397565399	4.376124331	4.049488025	4.057599296	4.081757895
ENSG00000100350	0.002613	0.026623836	-0.350672759	-1.27515512	**FOXRED2**	protein_coding	FAD dependent oxidoreductase domain containing 2 [Source:HGNC Symbol;Acc:HGNC:26264]	chr22	36487190	36507101	-1	ensembl_havana	4.60721798	4.782554359	4.431881601	4.690528814	4.800627876	4.856506388	4.295221319	4.429967487	4.570455996
ENSG00000256073	0.00262	0.026623836	-0.46161391	-1.377081466	**URB1-AS1**	lincRNA	URB1 antisense RNA 1 (head to head) [Source:HGNC Symbol;Acc:HGNC:23128]	chr21	32393130	32393960	1	havana	1.650542596	1.881349551	1.419735642	1.716703112	1.827267088	2.100078453	1.212343926	1.517070703	1.529792296
ENSG00000122986	0.002603	0.026623836	-0.467636072	-1.382841756	**HVCN1**	protein_coding	hydrogen voltage gated channel 1 [Source:HGNC Symbol;Acc:HGNC:28240]	chr12	110627841	110704950	-1	ensembl_havana	0.256429343	0.490247379	0.022611306	0.237415364	0.15602554	1.077301233	-0.35894771	-0.32890884	0.755690467
ENSG00000099953	0.002617	0.026623836	-0.95931559	-1.944387265	**MMP11**	protein_coding	matrix metallopeptidase 11 [Source:HGNC Symbol;Acc:HGNC:7157]	chr22	23768226	23784316	1	ensembl_havana	-0.297274214	0.182383581	-0.776932009	0.075059705	-0.036373238	0.508464275	-0.46237776	-1.15951152	-0.708906755
ENSG00000171385	0.002601	0.026623836	-1.028302557	-2.039623063	**KCND3**	protein_coding	potassium voltage-gated channel subfamily D member 3 [Source:HGNC Symbol;Acc:HGNC:6239]	chr1	111770662	111989155	-1	ensembl_havana	-0.221449698	0.292701581	-0.735600976	0.749126884	0.325758945	-0.196781087	-0.08686711	-1.15951152	-0.960424301
ENSG00000179750	0.002626	0.026649877	-0.359173135	-1.282690526	**APOBEC3B**	protein_coding	apolipoprotein B mRNA editing enzyme catalytic subunit 3B [Source:HGNC Symbol;Acc:HGNC:17352]	chr22	38982347	38992804	1	ensembl_havana	6.112787555	6.292374123	5.933200988	6.041073185	6.424617952	6.411431231	5.736637815	6.083801879	5.979163271
ENSG00000251364	0.002629	0.02665944	-0.767933694	-1.702829152	**AC107884.1**	antisense		chr11	7427266	7512515	-1	havana	1.834021269	2.217988116	1.450054421	2.327223844	2.242169235	2.084571268	1.245545533	1.715999071	1.38861866
ENSG00000106066	0.002655	0.026673661	1.774310268	3.420744301	**CPVL**	protein_coding	carboxypeptidase, vitellogenic like [Source:HGNC Symbol;Acc:HGNC:14399]	chr7	28995231	29195451	-1	ensembl_havana	-0.585655146	-1.47281028	0.301499987	-2.32062672	-1.178380098	-0.91942402	0.276538753	0.225896484	0.402064725
ENSG00000186376	0.002657	0.026673661	0.45996234	1.375505911	**ZNF75D**	protein_coding	zinc finger protein 75D [Source:HGNC Symbol;Acc:HGNC:13145]	chrX	135248920	135344087	-1	ensembl_havana	4.472947609	4.242966439	4.702928778	4.352378	4.257688505	4.118832811	4.656289177	4.799744258	4.652752901
ENSG00000135482	0.002655	0.026673661	0.404138491	1.323298463	**ZC3H10**	protein_coding	zinc finger CCCH-type containing 10 [Source:HGNC Symbol;Acc:HGNC:25893]	chr12	56118159	56127514	1	ensembl_havana	4.100137459	3.898068214	4.302206704	3.980397213	3.94667289	3.767134538	4.27927777	4.359993337	4.267349007
ENSG00000144567	0.002657	0.026673661	0.370450612	1.292756548	**RETREG2**	protein_coding	reticulophagy regulator family member 2 [Source:HGNC Symbol;Acc:HGNC:28450]	chr2	219176225	219185479	1	ensembl_havana	5.34080463	5.155579324	5.526029936	5.150096696	5.136397752	5.180243524	5.568072193	5.547507131	5.462510483
ENSG00000263528	0.002638	0.026673661	-0.363223174	-1.286296448	**IKBKE**	protein_coding	inhibitor of nuclear factor kappa B kinase subunit epsilon [Source:HGNC Symbol;Acc:HGNC:14552]	chr1	206470476	206496889	1	ensembl_havana	4.37716346	4.558775047	4.195551873	4.359450248	4.52216262	4.794712273	4.058857592	4.171585246	4.356212782
ENSG00000142599	0.00264	0.026673661	-0.365580949	-1.288400342	**RERE**	protein_coding	arginine-glutamic acid dipeptide repeats [Source:HGNC Symbol;Acc:HGNC:9965]	chr1	8352397	8817643	-1	ensembl_havana	6.049842129	6.232632604	5.867051655	6.327830426	6.095865088	6.274202299	5.951828302	5.806827625	5.842499037
ENSG00000141756	0.002635	0.026673661	-0.369158787	-1.291599499	**FKBP10**	protein_coding	FK506 binding protein 10 [Source:HGNC Symbol;Acc:HGNC:18169]	chr17	41812680	41823217	1	ensembl_havana	7.356172145	7.540751539	7.171592752	7.648772864	7.497866764	7.475614988	7.208069746	7.206311737	7.100396772
ENSG00000169750	0.002643	0.026673661	-0.384431709	-1.305345498	**RAC3**	protein_coding	Rac family small GTPase 3 [Source:HGNC Symbol;Acc:HGNC:9803]	chr17	82031624	82034204	1	ensembl_havana	3.435805412	3.628021267	3.243589558	3.855903752	3.583518497	3.444641551	3.525391749	3.245359567	2.960017356
ENSG00000171169	0.002647	0.026673661	-0.456433222	-1.372145264	**NAIF1**	protein_coding	nuclear apoptosis inducing factor 1 [Source:HGNC Symbol;Acc:HGNC:25446]	chr9	128061233	128068206	-1	ensembl_havana	3.420327415	3.648544027	3.192110804	3.680648284	3.514949665	3.75003413	3.077206287	3.093770837	3.405355289
ENSG00000196550	0.002652	0.026673661	-0.55579589	-1.469979351	**FAM72A**	protein_coding	family with sequence similarity 72 member A [Source:HGNC Symbol;Acc:HGNC:24044]	chr1	206186179	206204414	-1	ensembl_havana	1.583648296	1.861546241	1.305750352	1.727676152	1.603393164	2.253569408	0.995284613	1.241396536	1.680569906
ENSG00000277363	0.00265	0.026673661	-0.594481431	-1.509929752	**SRCIN1**	protein_coding	SRC kinase signaling inhibitor 1 [Source:HGNC Symbol;Acc:HGNC:29506]	chr17	38530016	38605930	-1	ensembl_havana	1.37766666	1.674907376	1.080425944	1.50341865	1.558674763	1.962628713	1.143556601	0.821210834	1.276510397
ENSG00000180777	0.00264	0.026673661	-0.712713704	-1.638883954	**ANKRD30B**	protein_coding	ankyrin repeat domain 30B [Source:HGNC Symbol;Acc:HGNC:24165]	chr18	14728272	14852738	1	ensembl_havana	0.119478525	0.475835377	-0.236878327	0.490295136	0.428746719	0.508464275	-0.26243933	-0.52165082	0.073455173
ENSG00000166130	0.002646	0.026673661	-0.723542565	-1.651231691	**IKBIP**	protein_coding	IKBKB interacting protein [Source:HGNC Symbol;Acc:HGNC:26430]	chr12	98613405	98645113	-1	ensembl_havana	3.556882625	3.918653908	3.195111343	4.134697477	3.980970421	3.640293825	3.196611706	3.175535294	3.213187029
ENSG00000107738	0.002658	0.026673661	-1.055536002	-2.078490281	**VSIR**	protein_coding	V-set immunoregulatory receptor [Source:HGNC Symbol;Acc:HGNC:30085]	chr10	71747559	71773498	-1	ensembl_havana	0.831633701	1.359401702	0.3038657	1.327057633	1.37743234	1.373715134	0.339398945	-0.11936381	0.691561964
ENSG00000110975	0.002644	0.026673661	-4.243957123	-18.94778266	**SYT10**	protein_coding	synaptotagmin 10 [Source:HGNC Symbol;Acc:HGNC:19266]	chr12	33374238	33439819	-1	ensembl_havana	-2.367231736	-0.245253174	-4.489210298	-0.61730533	0.325758945	-0.444213135	-5.3500916	-5.3500916	-2.767447692
ENSG00000177565	0.002676	0.026833075	-0.352432016	-1.276711023	**TBL1XR1**	protein_coding	transducin beta like 1 X-linked receptor 1 [Source:HGNC Symbol;Acc:HGNC:29529]	chr3	177019340	177228000	-1	ensembl_havana	7.501179403	7.677395411	7.324963395	7.712817099	7.663259921	7.656109214	7.294140051	7.322817644	7.357932491
ENSG00000147324	0.002687	0.026910936	0.424169928	1.34180026	**MFHAS1**	protein_coding	malignant fibrous histiocytoma amplified sequence 1 [Source:HGNC Symbol;Acc:HGNC:16982]	chr8	8783354	8893645	-1	ensembl_havana	6.871436417	6.659351453	7.083521381	6.696150342	6.660792134	6.621111883	7.230786085	7.093041276	6.926736783
ENSG00000175745	0.002686	0.026910936	0.400065188	1.319567534	**NR2F1**	protein_coding	nuclear receptor subfamily 2 group F member 1 [Source:HGNC Symbol;Acc:HGNC:7975]	chr5	93583224	93594615	1	ensembl_havana	3.243719117	3.043686523	3.443751711	3.387357714	2.961901777	2.781800078	3.703214536	3.337051803	3.290988795
ENSG00000131242	0.00269	0.02692008	-0.396096602	-1.315942637	**RAB11FIP4**	protein_coding	RAB11 family interacting protein 4 [Source:HGNC Symbol;Acc:HGNC:30267]	chr17	31391624	31538217	1	ensembl_havana	3.720508133	3.918556434	3.522459831	3.838293645	3.94884054	3.968535116	3.542190552	3.545167177	3.480021764
ENSG00000110987	0.002694	0.026939553	0.592469351	1.50782537	**BCL7A**	protein_coding	BCL tumor suppressor 7A [Source:HGNC Symbol;Acc:HGNC:1004]	chr12	122019422	122062044	1	ensembl_havana	3.871698483	3.575463808	4.167933159	3.355877725	3.701342418	3.66917128	4.181874628	4.143413982	4.178510866
ENSG00000163754	0.0027	0.026966694	0.374764706	1.296628066	**GYG1**	protein_coding	glycogenin 1 [Source:HGNC Symbol;Acc:HGNC:4699]	chr3	148991341	149027668	1	ensembl_havana	7.192903795	7.005521442	7.380286148	6.842136266	7.130161943	7.044266118	7.305746057	7.434978572	7.400133814
ENSG00000187049	0.0027	0.026966694	-0.395393644	-1.315301595	**TMEM216**	protein_coding	transmembrane protein 216 [Source:HGNC Symbol;Acc:HGNC:25018]	chr11	61391687	61398863	1	ensembl_havana	4.438808321	4.636505143	4.241111499	4.686316468	4.624698072	4.598500889	4.261128114	4.151519203	4.310687181
ENSG00000103811	0.002707	0.027010604	-0.377012012	-1.298649415	**CTSH**	protein_coding	cathepsin H [Source:HGNC Symbol;Acc:HGNC:2535]	chr15	78921058	78949574	-1	ensembl_havana	4.444264345	4.632770351	4.255758339	4.682091787	4.600084112	4.616135152	4.295221319	4.14544456	4.326609138
ENSG00000163923	0.002713	0.027055128	-0.381803023	-1.302969237	**RPL39L**	protein_coding	ribosomal protein L39 like [Source:HGNC Symbol;Acc:HGNC:17094]	chr3	187120948	187180908	-1	ensembl_havana	2.141675543	2.332577054	1.950774031	2.101191983	2.383487184	2.513051996	1.619344613	2.046678052	2.186299428
ENSG00000114573	0.002716	0.027062916	0.320684046	1.248922579	**ATP6V1A**	protein_coding	ATPase H+ transporting V1 subunit A [Source:HGNC Symbol;Acc:HGNC:851]	chr3	113747019	113812056	1	ensembl_havana	7.639133108	7.478791085	7.799475132	7.326257294	7.57321708	7.536898883	7.654095716	7.905836025	7.838493653
ENSG00000204257	0.002719	0.027076472	-0.652954059	-1.572384513	**HLA-DMA**	protein_coding	major histocompatibility complex, class II, DM alpha [Source:HGNC Symbol;Acc:HGNC:4934]	chr6	32948613	32969094	-1	ensembl_havana	3.871924326	4.198401356	3.545447297	4.458282965	4.189662871	3.947258231	3.6265241	3.449797033	3.560020757
ENSG00000214655	0.002726	0.027111978	0.344084896	1.269345579	**ZSWIM8**	protein_coding	zinc finger SWIM-type containing 8 [Source:HGNC Symbol;Acc:HGNC:23528]	chr10	73785582	73801797	1	ensembl_havana	6.21911862	6.047076172	6.391161068	6.203650081	5.967043105	5.97053533	6.510802547	6.373502451	6.289178206
ENSG00000126787	0.002725	0.027111978	-0.356640407	-1.28044067	**DLGAP5**	protein_coding	DLG associated protein 5 [Source:HGNC Symbol;Acc:HGNC:16864]	chr14	55148112	55191678	-1	ensembl_havana	6.413146215	6.591466418	6.234826011	6.421616377	6.741783219	6.610999658	6.040809867	6.333985937	6.32968223
ENSG00000150636	0.002733	0.027155479	-0.509671936	-1.423726409	**CCDC102B**	protein_coding	coiled-coil domain containing 102B [Source:HGNC Symbol;Acc:HGNC:26295]	chr18	68715209	69055189	1	ensembl_havana	1.748108895	2.002944863	1.493272927	2.067039104	1.987674772	1.954120714	1.415636519	1.43975459	1.624427672
ENSG00000119684	0.002739	0.027181412	-0.415545257	-1.333802683	**MLH3**	protein_coding	mutL homolog 3 [Source:HGNC Symbol;Acc:HGNC:7128]	chr14	75013764	75051532	-1	ensembl_havana	5.011763311	5.219535939	4.803990683	5.24384467	5.104668973	5.310094174	4.756142808	4.816408402	4.839420837
ENSG00000180818	0.002738	0.027181412	-0.527903891	-1.441832817	**HOXC10**	protein_coding	homeobox C10 [Source:HGNC Symbol;Acc:HGNC:5122]	chr12	53985065	53990279	1	ensembl_havana	0.32853779	0.592489735	0.064585844	0.637193984	0.679062629	0.461212593	0.308311185	0.02879206	-0.143345712
ENSG00000188522	0.002745	0.027223882	-0.434406804	-1.351355079	**FAM83G**	protein_coding	family with sequence similarity 83 member G [Source:HGNC Symbol;Acc:HGNC:32554]	chr17	18968789	19004804	-1	ensembl_havana	4.629235675	4.846439077	4.412032273	5.035551843	4.85827007	4.645495317	4.64233364	4.520368735	4.073394445
ENSG00000160917	0.002751	0.027261157	0.326573645	1.25403155	**CPSF4**	protein_coding	cleavage and polyadenylation specific factor 4 [Source:HGNC Symbol;Acc:HGNC:2327]	chr7	99438922	99457371	1	ensembl_havana	6.029816264	5.866529441	6.193103087	5.801811129	5.831466344	5.966310852	6.154425948	6.168116305	6.256767008
ENSG00000263069	0.002759	0.02730839	1.209747316	2.312971223	**AC124319.2**	antisense		chr17	80351828	80415168	-1	ensembl_havana	-0.396969044	-1.001842702	0.207904615	-0.4596317	-1.42549258	-1.120403822	0.339398945	0.315191528	-0.030876629
ENSG00000144736	0.00276	0.02730839	0.340910737	1.266555886	**SHQ1**	protein_coding	SHQ1, H/ACA ribonucleoprotein assembly factor [Source:HGNC Symbol;Acc:HGNC:25543]	chr3	72749277	72861914	-1	ensembl_havana	5.662429363	5.491973995	5.832884732	5.404613346	5.476271512	5.595037127	5.68613968	5.854774995	5.95773952
ENSG00000037241	0.002761	0.02730839	-0.551841501	-1.465955694	**RPL26L1**	protein_coding	ribosomal protein L26 like 1 [Source:HGNC Symbol;Acc:HGNC:17050]	chr5	172958729	172969771	1	ensembl_havana	4.022379203	4.298299954	3.746458453	4.350604507	4.345865273	4.19843008	3.706198956	3.67172695	3.861449452
ENSG00000166582	0.002764	0.02731508	-0.32128193	-1.249440266	**CENPV**	protein_coding	centromere protein V [Source:HGNC Symbol;Acc:HGNC:29920]	chr17	16342534	16353656	-1	ensembl_havana	4.278834476	4.439475441	4.118193511	4.350604507	4.568009187	4.399812629	4.025794377	4.226329326	4.10245683
ENSG00000150867	0.002773	0.027348746	0.467650358	1.382855449	**PIP4K2A**	protein_coding	phosphatidylinositol-5-phosphate 4-kinase type 2 alpha [Source:HGNC Symbol;Acc:HGNC:8997]	chr10	22534849	22714555	-1	ensembl_havana	6.541700631	6.307875452	6.77552581	6.058560765	6.407382452	6.457683139	6.689193922	6.77472428	6.862659227
ENSG00000129158	0.002773	0.027348746	0.319383166	1.247796931	**SERGEF**	protein_coding	secretion regulating guanine nucleotide exchange factor [Source:HGNC Symbol;Acc:HGNC:17499]	chr11	17788048	18013162	-1	ensembl_havana	2.8614708	2.701779217	3.021162382	2.744105292	2.608571783	2.752660576	3.063307783	2.910102222	3.090077142
ENSG00000170365	0.002772	0.027348746	-0.517114546	-1.43109014	**SMAD1**	protein_coding	SMAD family member 1 [Source:HGNC Symbol;Acc:HGNC:6767]	chr4	145481194	145558079	1	ensembl_havana	3.774790895	4.033348168	3.516233622	4.163267561	3.972472175	3.964304768	3.613866272	3.304709872	3.630124721
ENSG00000129810	0.002778	0.027374676	-0.396691543	-1.316485419	**SGO1**	protein_coding	shugoshin 1 [Source:HGNC Symbol;Acc:HGNC:25088]	chr3	20160593	20186292	-1	ensembl_havana	4.67310649	4.871452262	4.474760719	4.680680807	5.01026654	4.923409439	4.368636211	4.50463555	4.551010395
ENSG00000118369	0.002784	0.027406626	0.46768206	1.382885836	**USP35**	protein_coding	ubiquitin specific peptidase 35 [Source:HGNC Symbol;Acc:HGNC:20061]	chr11	78188812	78214711	1	ensembl_havana	3.106840632	2.872999602	3.340681662	2.817841883	3.029004765	2.772152159	3.445493458	3.382523978	3.194027549
ENSG00000088876	0.002785	0.027406626	-0.376157531	-1.297880478	**ZNF343**	protein_coding	zinc finger protein 343 [Source:HGNC Symbol;Acc:HGNC:16017]	chr20	2481817	2524702	-1	ensembl_havana	4.647304593	4.835383359	4.459225827	5.018898477	4.807811092	4.679440507	4.547343787	4.496704146	4.333629549
ENSG00000176155	0.002793	0.027452541	0.33862428	1.264550174	**CCDC57**	protein_coding	coiled-coil domain containing 57 [Source:HGNC Symbol;Acc:HGNC:27564]	chr17	82101460	82212830	-1	ensembl_havana	4.458784678	4.289472538	4.628096819	4.383933617	4.116342995	4.368141003	4.713768103	4.509373547	4.661148806
ENSG00000231113	0.002793	0.027452541	0.316327868	1.245157179	**AL035587.1**	antisense		chr6	42940364	42948360	1	havana	0.957632697	0.799468763	1.115796631	0.705390237	0.910210399	0.782805653	1.01467363	1.226127349	1.106588913
ENSG00000258376	0.002798	0.027483396	-0.79361522	-1.733412748	**AC004846.1**	antisense		chr14	73242651	73245979	-1	havana	0.924262078	1.321069688	0.527454468	1.383653947	1.298039728	1.281515389	0.276538753	0.529376201	0.77644845
ENSG00000166578	0.002809	0.027567117	0.678306167	1.600259826	**IQCD**	protein_coding	IQ motif containing D [Source:HGNC Symbol;Acc:HGNC:25168]	chr12	113195441	113221094	-1	ensembl_havana	-0.089441276	-0.428594359	0.249711808	-0.79434999	-0.569826728	0.078393642	-0.04612063	0.315191528	0.480064523
ENSG00000164287	0.002816	0.02760402	0.385873959	1.306651093	**CDC20B**	protein_coding	cell division cycle 20B [Source:HGNC Symbol;Acc:HGNC:24222]	chr5	55112995	55173175	-1	ensembl_havana	0.161754486	-0.031182494	0.354691465	0.040274975	-0.18055577	0.046733314	0.339398945	0.19485955	0.529815901
ENSG00000109586	0.002816	0.02760402	-0.405986901	-1.324994987	**GALNT7**	protein_coding	polypeptide N-acetylgalactosaminyltransferase 7 [Source:HGNC Symbol;Acc:HGNC:4129]	chr4	173168753	173323967	1	ensembl_havana	6.92803343	7.131026881	6.725039979	7.257412227	7.11310849	7.022559925	6.787927212	6.647970031	6.739222695
ENSG00000153317	0.002829	0.027690476	0.400870411	1.320304241	**ASAP1**	protein_coding	ArfGAP with SH3 domain, ankyrin repeat and PH domain 1 [Source:HGNC Symbol;Acc:HGNC:2720]	chr8	130052104	130443660	-1	ensembl_havana	5.637870528	5.437435322	5.838305734	5.293614645	5.504545129	5.514146193	5.813375768	5.852290786	5.849250648
ENSG00000106799	0.002828	0.027690476	0.323320633	1.251207125	**TGFBR1**	protein_coding	transforming growth factor beta receptor 1 [Source:HGNC Symbol;Acc:HGNC:11772]	chr9	99104038	99154192	1	ensembl_havana	5.563143197	5.40148288	5.724803514	5.263770706	5.503808141	5.436869794	5.572985148	5.862202061	5.739223333
ENSG00000204311	0.002831	0.027690476	-0.536496351	-1.450445764	**PJVK**	protein_coding	pejvakin [Source:HGNC Symbol;Acc:HGNC:29502]	chr2	178451346	178461409	1	ensembl_havana	1.582822137	1.851070312	1.314573961	2.040883175	1.739838083	1.772489677	1.371200905	1.130954377	1.441566602
ENSG00000285130	0.002836	0.027720233	0.398519415	1.318154443	**AL358113.1**	protein_coding		chr9	69035750	69255187	1	havana	4.098897579	3.899637871	4.298157286	4.290821549	3.89814155	3.509950514	4.66551843	4.208958525	4.019994903
ENSG00000115042	0.002841	0.027757011	0.3312696	1.258120058	**FAHD2A**	protein_coding	fumarylacetoacetate hydrolase domain containing 2A [Source:HGNC Symbol;Acc:HGNC:24252]	chr2	95402721	95416616	1	ensembl_havana	5.342181779	5.176546979	5.507816579	5.232333568	5.179492837	5.11781453	5.559846573	5.469418663	5.4941845
ENSG00000155858	0.002854	0.027856295	0.340874749	1.266524292	**LSM11**	protein_coding	LSM11, U7 small nuclear RNA associated [Source:HGNC Symbol;Acc:HGNC:30860]	chr5	157743695	157760709	1	ensembl_havana	4.505302701	4.334865326	4.675740075	4.210965817	4.479316977	4.314313185	4.581875053	4.756557736	4.688787436
ENSG00000103018	0.002858	0.027871816	-0.323052282	-1.250974414	**CYB5B**	protein_coding	cytochrome b5 type B [Source:HGNC Symbol;Acc:HGNC:24374]	chr16	69424525	69466266	1	ensembl_havana	7.465355252	7.626881393	7.303829111	7.663847852	7.602598558	7.614197768	7.314817468	7.270283638	7.326386226
ENSG00000164081	0.002863	0.027871816	-0.372701884	-1.294775421	**TEX264**	protein_coding	testis expressed 264 [Source:HGNC Symbol;Acc:HGNC:30247]	chr3	51662693	51704323	1	ensembl_havana	4.917581847	5.103932789	4.731230905	5.178366399	5.054218477	5.079213492	4.715250209	4.696888066	4.78155444
ENSG00000105612	0.002861	0.027871816	-0.391383439	-1.311650578	**DNASE2**	protein_coding	deoxyribonuclease 2, lysosomal [Source:HGNC Symbol;Acc:HGNC:2960]	chr19	12875211	12881468	-1	ensembl_havana	5.271127738	5.466819457	5.075436019	5.604613794	5.438204625	5.357639953	5.3257077	4.992552911	4.908047446
ENSG00000267712	0.002862	0.027871816	-0.649582541	-1.568714206	**LINC01539**	lincRNA	long intergenic non-protein coding RNA 1539 [Source:HGNC Symbol;Acc:HGNC:51307]	chr18	56083356	56137536	-1	havana	3.047667798	3.372459069	2.722876528	3.535210946	3.234877758	3.347288501	2.638787485	2.81124374	2.718598359
ENSG00000127129	0.002873	0.027877523	0.801473569	1.742880396	**EDN2**	protein_coding	endothelin 2 [Source:HGNC Symbol;Acc:HGNC:3177]	chr1	41478775	41484673	-1	ensembl_havana	-0.255195058	-0.655931842	0.145541726	-0.92573229	-0.299013651	-0.743049581	-0.04612063	0.163140205	0.319605599
ENSG00000122733	0.002866	0.027877523	0.583279472	1.49825114	**PHF24**	protein_coding	PHD finger protein 24 [Source:HGNC Symbol;Acc:HGNC:29180]	chr9	34957608	34982544	1	ensembl_havana	6.34234395	6.050704214	6.633983686	5.891144262	6.172527074	6.088441307	6.710900762	6.647970031	6.543080266
ENSG00000130449	0.002879	0.027877523	0.361426753	1.284695769	**ZSWIM6**	protein_coding	zinc finger SWIM-type containing 6 [Source:HGNC Symbol;Acc:HGNC:29316]	chr5	61332273	61546170	1	ensembl_havana	4.928371507	4.74765813	5.109084883	4.899038893	4.570826794	4.773108704	5.233562349	5.017157896	5.076534403
ENSG00000144134	0.002881	0.027877523	0.36106529	1.284373933	**RABL2A**	protein_coding	RAB, member of RAS oncogene family like 2A [Source:HGNC Symbol;Acc:HGNC:9799]	chr2	113627229	113643396	1	ensembl_havana	4.081947411	3.901414766	4.262480056	3.9734996	3.829216193	3.901528505	4.261128114	4.187439524	4.338872529
ENSG00000130363	0.002878	0.027877523	0.360449692	1.283826007	**RSPH3**	protein_coding	radial spoke head 3 homolog [Source:HGNC Symbol;Acc:HGNC:21054]	chr6	158972871	159000187	-1	ensembl_havana	4.057934056	3.87770921	4.238158902	4.090752661	3.852559113	3.689815855	4.491353183	4.2585809	3.964542621
ENSG00000130303	0.002871	0.027877523	-0.371224156	-1.293449883	**BST2**	protein_coding	bone marrow stromal cell antigen 2 [Source:HGNC Symbol;Acc:HGNC:1119]	chr19	17402939	17405648	-1	ensembl_havana	7.082097167	7.267709245	6.896485089	7.443001175	7.291517163	7.068609398	7.012787098	6.88456509	6.792103081
ENSG00000114491	0.002881	0.027877523	-0.379650702	-1.30102682	**UMPS**	protein_coding	uridine monophosphate synthetase [Source:HGNC Symbol;Acc:HGNC:12563]	chr3	124730366	124749273	1	ensembl_havana	5.226356397	5.416181749	5.036531046	5.184352789	5.559482462	5.504709995	4.710799315	5.189423914	5.209369908
ENSG00000211460	0.002868	0.027877523	-0.403609133	-1.322813004	**TSN**	protein_coding	translin [Source:HGNC Symbol;Acc:HGNC:12379]	chr2	121737103	121767853	1	ensembl_havana	7.306815761	7.508620328	7.105011194	7.692483595	7.468138918	7.365238471	7.184989991	7.060589818	7.069453773
ENSG00000137628	0.002874	0.027877523	-0.862832096	-1.818604838	**DDX60**	protein_coding	DExD/H-box helicase 60 [Source:HGNC Symbol;Acc:HGNC:25942]	chr4	168216293	168318807	-1	ensembl_havana	2.720840403	3.152256451	2.289424355	3.387357714	3.154516033	2.914895605	2.459388507	1.993561814	2.415322744
ENSG00000139278	0.002889	0.0279355	-1.400668865	-2.640239611	**GLIPR1**	protein_coding	GLI pathogenesis related 1 [Source:HGNC Symbol;Acc:HGNC:17001]	chr12	75480680	75503853	1	ensembl_havana	-0.801082996	-0.100748564	-1.501417429	0.040274975	0.243386951	-0.585907617	-1.52209115	-1.62946086	-1.352700279
ENSG00000185950	0.002893	0.027945541	0.428337291	1.345681782	**IRS2**	protein_coding	insulin receptor substrate 2 [Source:HGNC Symbol;Acc:HGNC:6126]	chr13	109752698	109786568	-1	ensembl_havana	4.780891653	4.566723007	4.995060298	4.888076825	4.562357411	4.249734786	5.313038798	4.870195902	4.801946194
ENSG00000107485	0.002894	0.027945541	0.369286205	1.291713578	**GATA3**	protein_coding	GATA binding protein 3 [Source:HGNC Symbol;Acc:HGNC:4172]	chr10	8045378	8075203	1	ensembl_havana	6.912377972	6.72773487	7.097021075	6.825952698	6.663434398	6.693817514	7.246759203	6.938258679	7.106045344
ENSG00000163913	0.002899	0.027956818	0.358689444	1.282260552	**IFT122**	protein_coding	intraflagellar transport 122 [Source:HGNC Symbol;Acc:HGNC:13556]	chr3	129440036	129520507	1	ensembl_havana	5.370854235	5.191509513	5.550198957	5.050923438	5.23027621	5.293328889	5.478398647	5.51174856	5.660449663
ENSG00000158158	0.002897	0.027956818	0.313294431	1.242541839	**CNNM4**	protein_coding	cyclin and CBS domain divalent metal cation transport mediator 4 [Source:HGNC Symbol;Acc:HGNC:105]	chr2	96760902	96811891	1	ensembl_havana	5.385667036	5.22901982	5.542314251	5.326467285	5.201476385	5.15911579	5.647071191	5.510173552	5.469698011
ENSG00000073711	0.002903	0.027982196	0.848350967	1.800441803	**PPP2R3A**	protein_coding	protein phosphatase 2 regulatory subunit B''alpha [Source:HGNC Symbol;Acc:HGNC:9307]	chr3	135965673	136147891	1	ensembl_havana	3.591386651	3.167211168	4.015562135	2.649185179	3.389700035	3.46274829	3.756011721	4.351222967	3.939451717
ENSG00000229419	0.002909	0.027995111	0.892454019	1.856331052	**RALGAPA1P1**	processed_pseudogene	Ral GTPase activating protein catalytic alpha subunit 1 pseudogene 1 [Source:HGNC Symbol;Acc:HGNC:23203]	chr9	105520128	105526359	1	havana	1.51457064	1.068343631	1.96079765	0.613726219	1.155163158	1.436141515	1.895179555	1.947753749	2.039459644
ENSG00000010165	0.002911	0.027995111	-0.328013648	-1.255283866	**METTL13**	protein_coding	methyltransferase like 13 [Source:HGNC Symbol;Acc:HGNC:24248]	chr1	171781664	171814023	1	ensembl_havana	6.021546722	6.185553546	5.857539898	6.072076954	6.160419393	6.32416429	5.697431913	5.84543707	6.029750712

ENSG00000168234	0.002907	0.027995111	-0.378341046	-1.299846303	**TTC39C**	protein_coding	tetratricopeptide repeat domain 39C [Source:HGNC Symbol;Acc:HGNC:26595]	chr18	23992773	24135610	1	ensembl_havana	5.784121418	5.973291941	5.594950895	5.860939034	5.985637823	6.073298967	5.379979066	5.651183775	5.753689845
ENSG00000153237	0.002914	0.028008744	0.32056892	1.248822919	**CCDC148**	protein_coding	coiled-coil domain containing 148 [Source:HGNC Symbol;Acc:HGNC:25191]	chr2	158171081	158456753	-1	ensembl_havana	-0.116002112	-0.276286571	0.044282348	-0.56281016	-0.143136894	-0.122912658	-0.26243933	0.163140205	0.232146171
ENSG00000174306	0.002921	0.028010347	0.392819195	1.312956569	**ZHX3**	protein_coding	zinc fingers and homeoboxes 3 [Source:HGNC Symbol;Acc:HGNC:15935]	chr20	41178448	41317672	-1	ensembl_havana	5.439066209	5.242656612	5.635475806	5.093960778	5.237387224	5.396621833	5.575435365	5.649753938	5.681238116
ENSG00000128714	0.002922	0.028010347	0.360702421	1.284050926	**HOXD13**	protein_coding	homeobox D13 [Source:HGNC Symbol;Acc:HGNC:5136]	chr2	176092891	176095938	1	ensembl_havana	5.343364818	5.163013607	5.523716029	4.693330227	5.397519822	5.398190773	5.126242055	5.679486903	5.765419128
ENSG00000123384	0.002919	0.028010347	-0.444255577	-1.360611867	**LRP1**	protein_coding	LDL receptor related protein 1 [Source:HGNC Symbol;Acc:HGNC:6692]	chr12	57128493	57213351	1	ensembl_havana	8.897925968	9.120053757	8.67579818	9.230788512	8.913776365	9.215596394	8.862827909	8.54607689	8.61848974
ENSG00000262898	0.002921	0.028010347	-0.847740807	-1.799680501	**AC139099.2**	lincRNA		chr17	83144131	83177607	1	havana	1.477694106	1.90156451	1.053823703	2.049654613	1.709476673	1.945562243	0.894226935	1.210694826	1.056549347
ENSG00000187601	0.002939	0.028134042	-0.448223841	-1.364359505	**MAGEH1**	protein_coding	MAGE family member H1 [Source:HGNC Symbol;Acc:HGNC:24092]	chrX	55452105	55453566	1	ensembl_havana	3.436807983	3.660919904	3.212696063	3.919492919	3.719230757	3.344036035	3.318633557	3.319173646	3.000280985
ENSG00000104826	0.002937	0.028134042	-1.523374943	-2.874627345	**LHB**	protein_coding	luteinizing hormone beta polypeptide [Source:HGNC Symbol;Acc:HGNC:6584]	chr19	49015980	49017081	-1	ensembl_havana	-1.009292772	-0.247605301	-1.770980244	0.14220989	-0.569826728	-0.315199064	-0.89704638	-1.87077278	-2.545121571
ENSG00000204991	0.002944	0.028165972	0.312285558	1.241673237	**SPIRE2**	protein_coding	spire type actin nucleation factor 2 [Source:HGNC Symbol;Acc:HGNC:30623]	chr16	89818179	89871319	1	ensembl_havana	3.615574093	3.459431313	3.771716872	3.428290208	3.442958541	3.507045191	3.738626465	3.76448673	3.812037421
ENSG00000113657	0.002948	0.028176793	1.02470572	2.034544337	**DPYSL3**	protein_coding	dihydropyrimidinase like 3 [Source:HGNC Symbol;Acc:HGNC:3015]	chr5	147390811	147510056	-1	ensembl_havana	-0.265156356	-0.777509217	0.247196504	-1.31854494	-0.428075095	-0.585907617	-0.30988669	0.19485955	0.856616648
ENSG00000248713	0.002949	0.028176793	0.435925401	1.35277828	**AC083902.2**	protein_coding	Homo sapiens uncharacterized LOC285556 (LOC285556), mRNA. [Source:RefSeq mRNA;Acc:NM_001354435]	chr4	99636529	99654648	-1	havana	6.019641851	5.801679151	6.237604552	5.771644272	5.785512147	5.847881034	6.183092475	6.36001306	6.16970812
ENSG00000044574	0.002953	0.028198548	-0.400904848	-1.320335756	**HSPA5**	protein_coding	heat shock protein family A (Hsp70) member 5 [Source:HGNC Symbol;Acc:HGNC:5238]	chr9	125234853	125241330	-1	ensembl_havana	9.827796073	10.0282485	9.627343648	10.1542873	9.933474379	9.996983809	9.631131158	9.586574169	9.664325619
ENSG00000182473	0.002956	0.028203512	0.321834336	1.249918767	**EXOC7**	protein_coding	exocyst complex component 7 [Source:HGNC Symbol;Acc:HGNC:23214]	chr17	76081017	76121576	-1	ensembl_havana	7.803109728	7.64219256	7.964026896	7.627509544	7.619706374	7.679361763	7.95328495	7.973692002	7.965103737
ENSG00000160185	0.002962	0.028214034	0.645365227	1.564135206	**UBASH3A**	protein_coding	ubiquitin associated and SH3 domain containing A [Source:HGNC Symbol;Acc:HGNC:12462]	chr21	42403447	42447681	1	ensembl_havana	1.514008793	1.19132618	1.836691406	1.075455542	1.124807863	1.373715134	1.874103935	1.890792227	1.745178056
ENSG00000090924	0.002959	0.028214034	-0.323020514	-1.250946868	**PLEKHG2**	protein_coding	pleckstrin homology and RhoGEF domain containing G2 [Source:HGNC Symbol;Acc:HGNC:29515]	chr19	39412585	39428415	1	ensembl_havana	5.385436774	5.546947031	5.223926517	5.768991011	5.407010972	5.46483911	5.46083574	5.110545547	5.100398263
ENSG00000100767	0.002963	0.028214034	-0.491611125	-1.406014161	**PAPLN**	protein_coding	papilin, proteoglycan like sulfated glycoprotein [Source:HGNC Symbol;Acc:HGNC:19262]	chr14	73237497	73274640	1	ensembl_havana	3.99602796	4.241833522	3.750222397	4.325543942	4.035021363	4.364935261	3.895893608	3.663248546	3.691525037
ENSG00000074319	0.002966	0.028217784	-0.389292427	-1.309750876	**TSG101**	protein_coding	tumor susceptibility 101 [Source:HGNC Symbol;Acc:HGNC:15971]	chr11	18468336	18527232	-1	ensembl_havana	5.126639679	5.321285892	4.931993465	5.486839167	5.274155646	5.202862864	4.992066932	4.974391071	4.829522392
ENSG00000102575	0.002967	0.028217784	-0.453026296	-1.368908764	**ACP5**	protein_coding	acid phosphatase 5, tartrate resistant [Source:HGNC Symbol;Acc:HGNC:124]	chr19	11574660	11579008	-1	ensembl_havana	2.535152192	2.761665339	2.308639044	2.931352026	2.734533378	2.619110614	2.600804468	2.131077666	2.194034997
ENSG00000099219	0.002976	0.028226182	-0.335619175	-1.261918882	**ERMP1**	protein_coding	endoplasmic reticulum metallopeptidase 1 [Source:HGNC Symbol;Acc:HGNC:23703]	chr9	5765076	5833117	-1	ensembl_havana	5.994271318	6.162080906	5.826461731	6.097149593	6.022637945	6.36645518	5.823030972	5.653325878	6.003028343
ENSG00000100889	0.002975	0.028226182	-0.344532729	-1.269739663	**PCK2**	protein_coding	phosphoenolpyruvate carboxykinase 2, mitochondrial [Source:HGNC Symbol;Acc:HGNC:8725]	chr14	24094053	24110598	1	ensembl_havana	6.375724894	6.547991258	6.20345853	6.47387296	6.552351804	6.61774901	6.190570615	6.210435986	6.209368987
ENSG00000115866	0.00297	0.028226182	-0.357341995	-1.281063505	**DARS**	protein_coding	aspartyl-tRNA synthetase [Source:HGNC Symbol;Acc:HGNC:2678]	chr2	135906677	135986100	-1	ensembl_havana	7.261563905	7.440234902	7.082892907	7.400348371	7.484480936	7.435875398	6.973693147	7.118331848	7.156653727
ENSG00000115129	0.002976	0.028226182	-0.972967955	-1.962874528	**TP53I3**	protein_coding	tumor protein p53 inducible protein 3 [Source:HGNC Symbol;Acc:HGNC:19373]	chr2	24077433	24085861	-1	ensembl_havana	1.39126695	1.877750927	0.904782972	1.959443807	1.970707426	1.703101548	1.400976194	0.938274071	0.375098651
ENSG00000065413	0.002978	0.02822634	-0.351435476	-1.275829442	**ANKRD44**	protein_coding	ankyrin repeat domain 44 [Source:HGNC Symbol;Acc:HGNC:25259]	chr2	196967017	197311173	-1	havana	5.113700704	5.289418442	4.937982966	5.325564741	5.216847845	5.325842741	4.894705093	4.898163408	5.021080396
ENSG00000161956	0.002985	0.028250574	0.347751905	1.272576076	**SENP3**	protein_coding	SUMO1/sentrin/SMT3 specific peptidase 3 [Source:HGNC Symbol;Acc:HGNC:17862]	chr17	7561875	7571969	1	ensembl_havana	2.551325456	2.377449504	2.725201409	2.383813765	2.331270798	2.417263948	2.681872935	2.753838306	2.739892986
ENSG00000123562	0.002986	0.028250574	-0.370428867	-1.292737062	**MORF4L2**	protein_coding	mortality factor 4 like 2 [Source:HGNC Symbol;Acc:HGNC:16849]	chrX	103675496	103688158	-1	ensembl_havana	8.062345987	8.24756042	7.877131553	8.28796858	8.294497082	8.160215598	7.820498098	7.959046662	7.851849901
ENSG00000127125	0.002984	0.028250574	-0.560480804	-1.474760626	**PPCS**	protein_coding	phosphopantothenoylcysteine synthetase [Source:HGNC Symbol;Acc:HGNC:25686]	chr1	42456117	42473385	1	ensembl_havana	4.575604515	4.855844916	4.295364113	4.918323696	4.910364411	4.738846643	4.168953129	4.335300678	4.381838531
ENSG00000136828	0.002991	0.0282607	0.483008072	1.397654792	**RALGPS1**	protein_coding	Ral GEF with PH domain and SH3 binding motif 1 [Source:HGNC Symbol;Acc:HGNC:16851]	chr9	126914774	127223166	1	ensembl_havana	5.224004133	4.982500097	5.465508169	4.726529627	5.057235496	5.163735169	5.385574858	5.419110534	5.591839116
ENSG00000183060	0.002991	0.0282607	0.349288492	1.273932195	**LYSMD4**	protein_coding	LysM domain containing 4 [Source:HGNC Symbol;Acc:HGNC:26571]	chr15	99715697	99733561	-1	ensembl_havana	4.351988225	4.177343979	4.526632472	4.097113041	4.211493157	4.223425739	4.523855049	4.514096035	4.541946331
ENSG00000167302	0.002999	0.028292275	0.447066646	1.363265582	**TEPSIN**	protein_coding	TEPSIN, adaptor related protein complex 4 accessory protein [Source:HGNC Symbol;Acc:HGNC:26458]	chr17	81228277	81239091	-1	ensembl_havana	4.360549071	4.137015748	4.584082394	4.051990026	4.073277629	4.28577959	4.586741306	4.589118969	4.576386907
ENSG00000183323	0.002997	0.028292275	-0.310518796	-1.240153581	**CCDC125**	protein_coding	coiled-coil domain containing 125 [Source:HGNC Symbol;Acc:HGNC:28924]	chr5	69280175	69332809	-1	ensembl_havana	3.361992976	3.517252373	3.206733578	3.519511473	3.479403105	3.552842542	3.205075544	3.175535294	3.239589895
ENSG00000186716	0.003001	0.028292275	-0.314934589	-1.243955252	**BCR**	protein_coding	BCR, RhoGEF and GTPase activating protein [Source:HGNC Symbol;Acc:HGNC:1014]	chr22	23179704	23318037	1	ensembl_havana	2.753619088	2.911086383	2.596151794	2.964197743	2.944626756	2.824434649	2.632526022	2.620048716	2.535880644
ENSG00000060140	0.003014	0.028396636	-0.810202005	-1.753456943	**STYK1**	protein_coding	serine/threonine/tyrosine kinase 1 [Source:HGNC Symbol;Acc:HGNC:18889]	chr12	10618939	10674318	-1	ensembl_havana	2.294784337	2.69988534	1.889683335	2.614134963	2.803232702	2.682288355	1.619344613	2.358164528	1.691540863
ENSG00000198838	0.003016	0.02839813	1.242118505	2.365456294	**RYR3**	protein_coding	ryanodine receptor 3 [Source:HGNC Symbol;Acc:HGNC:10485]	chr15	33310945	33866121	1	ensembl_havana	2.515358363	1.89429911	3.136417615	1.590147445	1.96214833	2.130601557	3.394600617	3.068309725	2.946342503
ENSG00000068796	0.003025	0.028440137	0.382778362	1.303850412	**KIF2A**	protein_coding	kinesin family member 2A [Source:HGNC Symbol;Acc:HGNC:6318]	chr5	62306162	62537249	1	ensembl_havana	7.521469905	7.330080725	7.712859086	7.06046692	7.360308785	7.569466468	7.476711608	7.817062266	7.844803385
ENSG00000269925	0.003023	0.028440137	0.365504166	1.288331773	**Z98884.2**	sense_intronic		chr1	7776383	7776775	1	havana	0.030089078	-0.152663006	0.212841161	-0.31750517	0.09469996	-0.235183806	0.069637898	0.529376201	0.039509383
ENSG00000116161	0.003026	0.028440137	-0.316118206	-1.244976237	**CACYBP**	protein_coding	calcyclin binding protein [Source:HGNC Symbol;Acc:HGNC:30423]	chr1	174999163	175011715	1	ensembl_havana	7.37171641	7.529775513	7.213657307	7.414635813	7.579176423	7.595514301	7.090505564	7.24067191	7.309794447
ENSG00000124103	0.003032	0.028479584	0.804699493	1.746781907	**FAM209A**	protein_coding	family with sequence similarity 209 member A [Source:HGNC Symbol;Acc:HGNC:16100]	chr20	56517187	56526142	1	ensembl_havana	-0.006348617	-0.408698363	0.39600113	-0.4596317	-0.18055577	-0.585907617	0.210814386	0.399280361	0.577908642
ENSG00000141577	0.003046	0.028533644	0.503035994	1.417192754	**CEP131**	protein_coding	centrosomal protein 131 [Source:HGNC Symbol;Acc:HGNC:29511]	chr17	81189593	81222999	-1	ensembl_havana	2.518139035	2.266621038	2.769657032	2.117969943	2.227959397	2.453933774	2.74708149	2.795812872	2.766076734
ENSG00000047346	0.003047	0.028533644	0.398688596	1.318309029	**FAM214A**	protein_coding	family with sequence similarity 214 member A [Source:HGNC Symbol;Acc:HGNC:25609]	chr15	52581317	52709817	-1	ensembl_havana	6.351134628	6.15179033	6.550478926	6.330532122	6.177156951	5.947681916	6.64282947	6.542898718	6.46570859
ENSG00000164983	0.003043	0.028533644	0.311192718	1.240733026	**TMEM65**	protein_coding	transmembrane protein 65 [Source:HGNC Symbol;Acc:HGNC:25203]	chr8	124306189	124372692	-1	ensembl_havana	3.612197863	3.456601505	3.767794222	3.253301988	3.549641413	3.566861112	3.578473489	3.844159232	3.880749946
ENSG00000119929	0.003048	0.028533644	-0.384920794	-1.305788095	**CUTC**	protein_coding	cutC copper transporter [Source:HGNC Symbol;Acc:HGNC:24271]	chr10	99702558	99756134	1	ensembl_havana	3.872103519	4.064563916	3.679643122	4.14496615	4.061305939	3.987419659	3.68819912	3.637510218	3.713220029
ENSG00000025423	0.00304	0.028533644	-0.455734792	-1.371481149	**HSD17B6**	protein_coding	hydroxysteroid 17-beta dehydrogenase 6 [Source:HGNC Symbol;Acc:HGNC:23316]	chr12	56752161	56787790	1	ensembl_havana	1.983145299	2.211012695	1.755277902	2.253214515	2.249222012	2.130601557	1.669136731	1.769643034	1.827053942
ENSG00000280213	0.003051	0.028545364	0.691209355	1.614636438	**UCKL1-AS1**	antisense	UCKL1 antisense RNA 1 [Source:HGNC Symbol;Acc:HGNC:31967]	chr20	63953384	63956985	1	havana	-0.734788189	-1.080392866	-0.389183511	-1.61858537	-1.178380098	-0.444213135	-0.63291296	-0.57414696	0.039509383
ENSG00000170364	0.003057	0.028556906	0.360923594	1.284247793	**SETMAR**	protein_coding	SET domain and mariner transposase fusion gene [Source:HGNC Symbol;Acc:HGNC:10762]	chr3	4303304	4317567	1	ensembl_havana	4.089594036	3.909132239	4.270055833	3.75493443	4.012400306	3.96006198	4.207375531	4.329953957	4.272838011
ENSG00000134590	0.003055	0.028556906	0.311282824	1.24081052	**RTL8C**	protein_coding	retrotransposon Gag like 8C [Source:HGNC Symbol;Acc:HGNC:2569]	chrX	135032366	135033546	1	ensembl_havana	5.248134302	5.09249289	5.403775713	5.197238709	5.093935728	4.986304232	5.490566694	5.420788101	5.299972345
ENSG00000223508	0.003063	0.028594092	0.363713673	1.286733848	**RPL23AP53**	transcribed_processed_pseudogene	ribosomal protein L23a pseudogene 53 [Source:HGNC Symbol;Acc:HGNC:35921]	chr8	213186	232231	-1	havana	1.35740646	1.175549624	1.539263296	1.158893785	1.046001934	1.321753151	1.567772387	1.315408538	1.734608964
ENSG00000182504	0.003065	0.028603057	0.362146972	1.285337272	**CEP97**	protein_coding	centrosomal protein 97 [Source:HGNC Symbol;Acc:HGNC:26244]	chr3	101723925	101770562	1	ensembl_havana	5.699498969	5.518425483	5.880572455	5.582873369	5.486752684	5.485650398	5.869029922	5.933267798	5.839419647
ENSG00000100027	0.003073	0.028617077	0.489999692	1.404444575	**YPEL1**	protein_coding	yippee like 1 [Source:HGNC Symbol;Acc:HGNC:12845]	chr22	21697544	21735834	-1	ensembl_havana	1.632914266	1.38791442	1.877914112	1.04068047	1.547275176	1.575787614	1.717267538	1.938414976	1.978059821
ENSG00000133030	0.003077	0.028617077	0.375058772	1.296892386	**MPRIP**	protein_coding	myosin phosphatase Rho interacting protein [Source:HGNC Symbol;Acc:HGNC:30321]	chr17	17042545	17217679	1	ensembl_havana	6.469938601	6.282409215	6.657467986	6.262367374	6.185912993	6.398947278	6.705318159	6.596205137	6.670880664
ENSG00000136273	0.003071	0.028617077	0.33571638	1.26200391	**HUS1**	protein_coding	HUS1 checkpoint clamp component [Source:HGNC Symbol;Acc:HGNC:5309]	chr7	47695730	47979581	-1	ensembl_havana	5.142537636	4.974679446	5.310395826	4.843376131	5.048165446	5.032496762	5.242840767	5.363499728	5.324846984
ENSG00000138346	0.003075	0.028617077	-0.385410812	-1.306231688	**DNA2**	protein_coding	DNA replication helicase/nuclease 2 [Source:HGNC Symbol;Acc:HGNC:2939]	chr10	68414064	68472121	-1	ensembl_havana	5.698372482	5.891077888	5.505667075	5.73947847	6.017483722	5.916271471	5.296302111	5.57639263	5.644306485
ENSG00000121552	0.003073	0.028617077	-0.662620398	-1.58295517	**CSTA**	protein_coding	cystatin A [Source:HGNC Symbol;Acc:HGNC:2481]	chr3	122325244	122341972	1	ensembl_havana	2.724620632	3.055930831	2.393310433	2.987209412	3.209790357	2.970792724	2.587917939	2.315517031	2.27649633
ENSG00000146859	0.003082	0.028626382	0.582427182	1.497366291	**TMEM140**	protein_coding	transmembrane protein 140 [Source:HGNC Symbol;Acc:HGNC:21870]	chr7	135148072	135166215	1	ensembl_havana	1.536255243	1.245041652	1.827468834	1.04068047	1.270574017	1.423870469	1.863449533	1.705026699	1.91393027
ENSG00000118096	0.00308	0.028626382	-0.388371899	-1.308915441	**IFT46**	protein_coding	intraflagellar transport 46 [Source:HGNC Symbol;Acc:HGNC:26146]	chr11	118544528	118572970	-1	ensembl_havana	5.059698179	5.253884128	4.865512229	5.200196109	5.342524316	5.218931959	4.827772085	4.84667624	4.922088362
ENSG00000023445	0.003086	0.028640378	0.664446555	1.584960136	**BIRC3**	protein_coding	baculoviral IAP repeat containing 3 [Source:HGNC Symbol;Acc:HGNC:591]	chr11	102317450	102339403	1	ensembl_havana	9.42574973	9.093526453	9.757973008	8.880595154	9.107712076	9.292272129	9.271039284	9.988509935	10.0143698
ENSG00000053254	0.003099	0.028640378	0.450701952	1.366705073	**FOXN3**	protein_coding	forkhead box N3 [Source:HGNC Symbol;Acc:HGNC:1928]	chr14	89124871	89619149	-1	ensembl_havana	5.306369985	5.08101901	5.531720961	5.08971436	4.9840837	5.169258969	5.434083869	5.593599079	5.567479934
ENSG00000187650	0.003098	0.028640378	0.413524535	1.33193579	**VMAC**	protein_coding	vimentin type intermediate filament associated coiled-coil protein [Source:HGNC Symbol;Acc:HGNC:33803]	chr19	5904858	5910853	1	ensembl_havana	2.131608271	1.924846003	2.338370538	1.92171846	1.729788441	2.123031109	2.386842807	2.226242768	2.402026039
ENSG00000135932	0.003091	0.028640378	0.357386227	1.281102782	**CAB39**	protein_coding	calcium binding protein 39 [Source:HGNC Symbol;Acc:HGNC:20292]	chr2	230712845	230821075	1	ensembl_havana	7.274732726	7.096039613	7.453425839	7.158501393	7.081598586	7.048018858	7.443564604	7.429989409	7.486723505
ENSG00000170266	0.003099	0.028640378	0.328255888	1.255494656	**GLB1**	protein_coding	galactosidase beta 1 [Source:HGNC Symbol;Acc:HGNC:4298]	chr3	32996608	33097230	-1	ensembl_havana	7.27026678	7.106138836	7.434394725	6.927890832	7.159704327	7.23082135	7.283938849	7.516472141	7.502773184
ENSG00000157353	0.003101	0.028640378	0.324127105	1.25190675	**FUK**	protein_coding	fucokinase [Source:HGNC Symbol;Acc:HGNC:29500]	chr16	70454421	70480274	1	ensembl_havana	4.372333428	4.210269875	4.53439698	4.325543942	4.16003228	4.145233403	4.602844617	4.523494887	4.476851438
ENSG00000100342	0.003096	0.028640378	-0.357171424	-1.280912053	**APOL1**	protein_coding	apolipoprotein L1 [Source:HGNC Symbol;Acc:HGNC:618]	chr22	36253010	36267530	1	ensembl_havana	6.929013258	7.10759897	6.750427546	7.128542732	7.222419601	6.971834576	6.810636785	6.776363153	6.6642827
ENSG00000148908	0.003095	0.028640378	-0.364754684	-1.287662657	**RGS10**	protein_coding	regulator of G protein signaling 10 [Source:HGNC Symbol;Acc:HGNC:9992]	chr10	119499828	119542708	-1	ensembl_havana	2.59559584	2.777973182	2.413218498	2.964197743	2.788784317	2.580937487	2.514907924	2.42655638	2.298191191
ENSG00000198924	0.003102	0.028640378	-0.399777431	-1.319304362	**DCLRE1A**	protein_coding	DNA cross-link repair 1A [Source:HGNC Symbol;Acc:HGNC:17660]	chr10	113834725	113854383	-1	ensembl_havana	5.174522377	5.374411093	4.974633662	5.336358176	5.340874633	5.44600047	4.81400461	4.978953011	5.130943364
ENSG00000128340	0.003104	0.028640378	-0.520474523	-1.434426974	**RAC2**	protein_coding	Rac family small GTPase 2 [Source:HGNC Symbol;Acc:HGNC:9802]	chr22	37225261	37244448	-1	ensembl_havana	6.644860334	6.905097595	6.384623073	6.948556037	6.842567309	6.92416944	6.482360627	6.467397067	6.204111524
ENSG00000124104	0.003105	0.028640378	-1.037170504	-2.052198815	**SNX21**	protein_coding	sorting nexin family member 21 [Source:HGNC Symbol;Acc:HGNC:16154]	chr20	45833810	45843275	1	ensembl_havana	-0.085354139	0.433231114	-0.603939391	0.515840645	0.298820819	0.485031876	-0.35894771	-0.42206346	-1.030806998
ENSG00000160746	0.003109	0.028641459	0.380096428	1.301428839	**ANO10**	protein_coding	anoctamin 10 [Source:HGNC Symbol;Acc:HGNC:25519]	chr3	43354859	43691594	-1	ensembl_havana	4.739485524	4.54943731	4.929533738	4.587380999	4.542399994	4.518530937	4.857605861	4.981228583	4.949766771
ENSG00000169758	0.003109	0.028641459	-0.580835753	-1.495715466	**TMEM266**	protein_coding	transmembrane protein 266 [Source:HGNC Symbol;Acc:HGNC:26763]	chr15	76059837	76229121	1	ensembl_havana	1.013934947	1.304352824	0.72351707	1.43811357	1.58120722	0.89373768	0.716189844	0.900298706	0.554062661
ENSG00000275023	0.003114	0.028642046	0.331986118	1.258745061	**MLLT6**	protein_coding	MLLT6, PHD finger containing [Source:HGNC Symbol;Acc:HGNC:7138]	chr17	38705542	38729803	1	ensembl_havana	7.655221646	7.489228587	7.821214704	7.549262665	7.384732551	7.533690544	7.847274155	7.778493368	7.837876589
ENSG00000047644	0.003114	0.028642046	0.329700852	1.256752755	**WWC3**	protein_coding	WWC family member 3 [Source:HGNC Symbol;Acc:HGNC:29237]	chrX	10015562	10144478	1	ensembl_havana	6.822120374	6.657269947	6.9869708	6.637721781	6.52609775	6.80799031	7.024490994	6.81482881	7.121592595
ENSG00000272655	0.003122	0.028684876	0.382938345	1.303995007	**POLR2J4**	transcribed_unprocessed_pseudogene	RNA polymerase II subunit J4, pseudogene [Source:HGNC Symbol;Acc:HGNC:28195]	chr7	44013562	44019170	-1	havana	2.313177002	2.121707829	2.504646175	1.92171846	2.108942002	2.334463026	2.196225281	2.572545352	2.745167891
ENSG00000153140	0.003121	0.028684876	-0.563690837	-1.478045659	**CETN3**	protein_coding	centrin 3 [Source:HGNC Symbol;Acc:HGNC:1868]	chr5	90392261	90409786	-1	ensembl_havana	4.252995352	4.534840771	3.971149934	4.643502409	4.46575711	4.495262794	3.848057062	4.000410511	4.064982229
ENSG00000137103	0.003142	0.028725346	0.729383443	1.657930399	**TMEM8B**	protein_coding	transmembrane protein 8B [Source:HGNC Symbol;Acc:HGNC:21427]	chr9	35814451	35865518	1	ensembl_havana	3.334957263	2.970265541	3.699648985	2.843288967	2.84572944	3.221778217	3.898505252	3.426606605	3.773835098
ENSG00000173451	0.003142	0.028725346	0.341830013	1.267363185	**THAP2**	protein_coding	THAP domain containing 2 [Source:HGNC Symbol;Acc:HGNC:20854]	chr12	71663009	71680639	1	ensembl_havana	2.82695705	2.656042043	2.997872056	2.878177262	2.729498798	2.360450071	3.291070988	3.055408658	2.647136522
ENSG00000186162	0.003145	0.028725346	0.335480771	1.261797826	**CIDECP**	transcribed_unprocessed_pseudogene	cell death-inducing DFFA-like effector c pseudogene [Source:HGNC Symbol;Acc:HGNC:24230]	chr3	10014238	10026365	-1	havana	3.898696281	3.730955896	4.066436667	3.584331121	3.824501942	3.784034626	3.984600033	4.143413982	4.071295985
ENSG00000168268	0.003138	0.028725346	-0.325940416	-1.25348125	**NT5DC2**	protein_coding	5'-nucleotidase domain containing 2 [Source:HGNC Symbol;Acc:HGNC:25717]	chr3	52524385	52535054	-1	ensembl_havana	7.080524853	7.243495061	6.917554645	7.174629571	7.289808076	7.266047537	6.89086505	6.972689517	6.889109367
ENSG00000145736	0.003143	0.028725346	-0.329106567	-1.256235172	**GTF2H2**	protein_coding	general transcription factor IIH subunit 2 [Source:HGNC Symbol;Acc:HGNC:4656]	chr5	71032670	71067689	-1	ensembl_havana	2.991118547	3.155671831	2.826565264	2.954889309	3.297453632	3.214672551	2.613576909	2.924363622	2.941755261
ENSG00000185808	0.003144	0.028725346	-0.355701848	-1.279607939	**PIGP**	protein_coding	phosphatidylinositol glycan anchor biosynthesis class P [Source:HGNC Symbol;Acc:HGNC:3046]	chr21	37059170	37073170	-1	ensembl_havana	3.512264197	3.690115121	3.334413273	3.657882359	3.764236011	3.648226993	3.213490017	3.459622798	3.330127003
ENSG00000204568	0.00314	0.028725346	-0.363993519	-1.286983466	**MRPS18B**	protein_coding	mitochondrial ribosomal protein S18B [Source:HGNC Symbol;Acc:HGNC:14516]	chr6	30617709	30626395	1	ensembl_havana	6.856564847	7.038561607	6.674568088	6.954990153	6.991943287	7.168751381	6.54370842	6.724671848	6.755323995
ENSG00000246084	0.003134	0.028725346	-0.378008951	-1.299547125	**LINC02325**	lincRNA	long intergenic non-protein coding RNA 2325 [Source:HGNC Symbol;Acc:HGNC:53245]	chr14	97458816	97581601	1	havana	6.016449987	6.205454463	5.827445512	6.314700425	6.144431383	6.15723158	5.99273379	5.657600565	5.832002181
ENSG00000012174	0.003139	0.028725346	-0.395522438	-1.315419022	**MBTPS2**	protein_coding	membrane bound transcription factor peptidase, site 2 [Source:HGNC Symbol;Acc:HGNC:15455]	chrX	21839636	21885424	1	ensembl_havana	6.074308122	6.272069341	5.876546903	6.300531493	6.201450279	6.314226252	5.781896147	5.864669277	5.983075285
ENSG00000081052	0.003156	0.028754115	1.00731131	2.010161354	**COL4A4**	protein_coding	collagen type IV alpha 4 chain [Source:HGNC Symbol;Acc:HGNC:2206]	chr2	227002711	227164453	-1	ensembl_havana	5.532060586	5.028404931	6.035716241	4.558479799	4.994613926	5.532121069	6.028940206	5.856015498	6.222193019
ENSG00000110841	0.003156	0.028754115	0.571700181	1.486274076	**PPFIBP1**	protein_coding	PPFIA binding protein 1 [Source:HGNC Symbol;Acc:HGNC:9249]	chr12	27523431	27695564	1	ensembl_havana	4.951936544	4.666086454	5.237786635	4.205089066	4.786153249	5.007017047	5.013909653	5.259532745	5.439917506
ENSG00000131069	0.003153	0.028754115	0.506331921	1.42043412	**ACSS2**	protein_coding	acyl-CoA synthetase short chain family member 2 [Source:HGNC Symbol;Acc:HGNC:15814]	chr20	34872146	34927962	1	ensembl_havana	0.039007198	-0.214158763	0.292173158	-0.27307956	-0.383759306	0.014362573	0.428834256	-0.00684508	0.454530303
ENSG00000261534	0.003166	0.028754115	0.498132932	1.412384537	**AL596244.1**	sense_overlapping		chr9	121815674	121819452	-1	havana	2.618735437	2.369668971	2.867801903	2.023178671	2.440056551	2.645771691	2.681872935	2.952470332	2.969062441
ENSG00000189227	0.003163	0.028754115	0.457227096	1.372900524	**C15orf61**	protein_coding	chromosome 15 open reading frame 61 [Source:HGNC Symbol;Acc:HGNC:34453]	chr15	67521068	67530143	1	ensembl_havana	1.213497631	0.984884083	1.442111179	0.911909315	1.013233389	1.029509544	1.527846157	1.343981945	1.454505434
ENSG00000135636	0.003176	0.028754115	0.37269599	1.294770131	**DYSF**	protein_coding	dysferlin [Source:HGNC Symbol;Acc:HGNC:3097]	chr2	71453722	71686768	1	ensembl_havana	6.986179685	6.79983169	7.17252768	6.952361482	7.002677368	6.444456221	7.388428063	7.2707488	6.858406177
ENSG00000171522	0.003164	0.028754115	0.306115924	1.236374603	**PTGER4**	protein_coding	prostaglandin E receptor 4 [Source:HGNC Symbol;Acc:HGNC:9596]	chr5	40679498	40693735	1	ensembl_havana	6.967518967	6.814461005	7.120576929	6.998676887	6.654829285	6.789876841	7.305991982	6.964374224	7.09136458
ENSG00000135372	0.003169	0.028754115	-0.311109715	-1.240661645	**NAT10**	protein_coding	N-acetyltransferase 10 [Source:HGNC Symbol;Acc:HGNC:29830]	chr11	34105602	34147670	1	ensembl_havana	6.709983483	6.86553834	6.554428625	6.814418706	6.836141115	6.9460552	6.500954495	6.501882393	6.660448989
ENSG00000131944	0.003164	0.028754115	-0.329418984	-1.25650724	**FAAP24**	protein_coding	Fanconi anemia core complex associated protein 24 [Source:HGNC Symbol;Acc:HGNC:28467]	chr19	32972209	32978222	1	ensembl_havana	3.061376411	3.226085903	2.896666918	3.045376594	3.386505103	3.246376011	2.669693543	3.046743406	2.973563806
ENSG00000162757	0.003172	0.028754115	-0.387900509	-1.308487832	**C1orf74**	protein_coding	chromosome 1 open reading frame 74 [Source:HGNC Symbol;Acc:HGNC:26319]	chr1	209779208	209784559	-1	ensembl_havana	3.776484021	3.970434276	3.582533767	3.921883558	4.004085726	3.985333543	3.423901694	3.707901612	3.615797995
ENSG00000261167	0.003167	0.028754115	-0.453123319	-1.369000828	**AC107027.3**	sense_overlapping		chr3	131455126	131458598	-1	havana	1.308461543	1.535023202	1.081899884	1.637462096	1.402954459	1.564653053	1.107892607	0.861296647	1.276510397
ENSG00000170545	0.003175	0.028754115	-0.720338448	-1.6475685	**SMAGP**	protein_coding	small cell adhesion glycoprotein [Source:HGNC Symbol;Acc:HGNC:26918]	chr12	51244558	51270890	-1	ensembl_havana	0.537153637	0.897322861	0.176984412	0.355307344	1.124807863	1.211853376	-0.04612063	0.286036324	0.291037539
ENSG00000232650	0.003174	0.028754115	-0.776350426	-1.71279255	**LINC01780**	lincRNA	long intergenic non-protein coding RNA 1780 [Source:HGNC Symbol;Acc:HGNC:52570]	chr1	119328176	119356182	1	havana	1.098403876	1.486579088	0.710228663	1.109412078	1.709476673	1.640848515	0.668022463	0.861296647	0.601366878
ENSG00000162496	0.00317	0.028754115	-1.020062578	-2.028006924	**DHRS3**	protein_coding	dehydrogenase/reductase 3 [Source:HGNC Symbol;Acc:HGNC:17693]	chr1	12567910	12617731	-1	ensembl_havana	0.250769347	0.760800636	-0.259261942	-0.03191641	0.927899934	1.38641838	-0.57379957	-0.37473441	0.17074815
ENSG00000228544	0.003183	0.028782943	0.450747734	1.366748445	**CCDC183-AS1**	antisense	CCDC183 antisense RNA 1 [Source:HGNC Symbol;Acc:HGNC:44105]	chr9	136803927	136808848	-1	havana	4.854799027	4.62942516	5.080172894	4.46649433	4.477816603	4.943964547	4.938508117	5.058693368	5.243317197
ENSG00000115942	0.003181	0.028782943	-0.309207718	-1.239027079	**ORC2**	protein_coding	origin recognition complex subunit 2 [Source:HGNC Symbol;Acc:HGNC:8488]	chr2	200908973	200963680	-1	ensembl_havana	5.447003015	5.601606874	5.292399156	5.597901758	5.570082469	5.636836396	5.266284673	5.278160622	5.332752173
ENSG00000171813	0.003193	0.028859335	-0.700634325	-1.625219213	**PWWP2B**	protein_coding	PWWP domain containing 2B [Source:HGNC Symbol;Acc:HGNC:25150]	chr10	132397168	132417863	1	ensembl_havana	-0.370354651	-0.020037488	-0.720671813	0.004630787	-0.143136894	0.078393642	-0.69455235	-0.57414696	-0.893316134
ENSG00000075407	0.003208	0.028951574	0.338993149	1.264873536	**ZNF37A**	protein_coding	zinc finger protein 37A [Source:HGNC Symbol;Acc:HGNC:13102]	chr10	38094334	38150293	1	ensembl_havana	5.958249728	5.788753153	6.127746303	5.726545379	5.818485256	5.821228825	6.010950576	6.22730989	6.144978442
ENSG00000144843	0.003207	0.028951574	-0.361074171	-1.284381839	**ADPRH**	protein_coding	ADP-ribosylarginine hydrolase [Source:HGNC Symbol;Acc:HGNC:269]	chr3	119579268	119589945	1	ensembl_havana	5.865267359	6.045804445	5.684730274	6.062899738	6.041042151	6.033471446	5.609308512	5.747906805	5.696975505
ENSG00000116750	0.003217	0.029017991	-0.373374113	-1.295378867	**UCHL5**	protein_coding	ubiquitin C-terminal hydrolase L5 [Source:HGNC Symbol;Acc:HGNC:19678]	chr1	193012250	193060080	-1	ensembl_havana	5.759647014	5.946334071	5.572959958	5.939139003	5.921964678	5.977898531	5.46083574	5.619395278	5.638648856
ENSG00000152990	0.003223	0.029035519	0.462540425	1.377966128	**ADGRA3**	protein_coding	adhesion G protein-coupled receptor A3 [Source:HGNC Symbol;Acc:HGNC:13839]	chr4	22345071	22516054	-1	ensembl_havana	4.131145449	3.899875237	4.362415662	3.646363245	3.940150332	4.113112135	4.238630573	4.436615978	4.412000434
ENSG00000173207	0.003222	0.029035519	-0.442217143	-1.358690769	**CKS1B**	protein_coding	CDC28 protein kinase regulatory subunit 1B [Source:HGNC Symbol;Acc:HGNC:19083]	chr1	154974653	154979249	1	ensembl_havana	4.417374051	4.638482622	4.196265479	4.479536069	4.782511786	4.653400012	4.040057209	4.201170521	4.347568708
ENSG00000279407	0.003227	0.029052692	-0.560943544	-1.475233726	**AC007191.1**	TEC		chr19	45714387	45717381	-1	havana	1.094691714	1.375163486	0.814219942	1.477650254	1.199531136	1.448309068	0.692307165	0.693803314	1.056549347
ENSG00000249476	0.003232	0.029062712	0.32206689	1.250120262	**AC008467.1**	lincRNA		chr5	109237120	109326369	-1	havana	-0.348674079	-0.509707524	-0.187640635	-0.85854608	-0.473795405	-0.196781087	-0.51701327	-0.11936381	0.073455173
ENSG00000066422	0.003234	0.029062712	0.312069108	1.24148696	**ZBTB11**	protein_coding	zinc finger and BTB domain containing 11 [Source:HGNC Symbol;Acc:HGNC:16740]	chr3	101648889	101677495	-1	ensembl_havana	6.042453534	5.88641898	6.198488089	5.757659894	5.915329189	5.986267857	6.050822859	6.211890079	6.332751327
ENSG00000197056	0.003233	0.029062712	-0.585652837	-1.500717929	**ZMYM1**	protein_coding	zinc finger MYM-type containing 1 [Source:HGNC Symbol;Acc:HGNC:26253]	chr1	35059786	35115859	1	ensembl_havana	2.784899123	3.077725541	2.492072704	3.036579028	3.085263982	3.111333615	2.254470464	2.466087459	2.755660191
ENSG00000223829	0.003238	0.029082225	-0.660086171	-1.580177003	**AC004870.2**	processed_transcript		chr7	46969702	47027481	1	havana	0.437904746	0.767947831	0.107861661	0.832812769	0.52486995	0.946160774	-0.08686711	-0.11936381	0.529815901
ENSG00000172113	0.003245	0.02908527	0.309162526	1.238988268	**NME6**	protein_coding	NME/NM23 nucleoside diphosphate kinase 6 [Source:HGNC Symbol;Acc:HGNC:20567]	chr3	48290722	48301685	-1	ensembl_havana	5.522403079	5.367821816	5.676984342	5.303732557	5.375127923	5.424604968	5.591664658	5.710678003	5.728610365
ENSG00000183826	0.003244	0.02908527	0.305646612	1.235972473	**BTBD9**	protein_coding	BTB domain containing 9 [Source:HGNC Symbol;Acc:HGNC:21228]	chr6	38168451	38640148	-1	ensembl_havana	5.486109775	5.333286469	5.638933081	5.465694167	5.284491062	5.249674177	5.781896147	5.574886673	5.560016423
ENSG00000213096	0.003244	0.02908527	-0.310003462	-1.239710675	**ZNF254**	protein_coding	zinc finger protein 254 [Source:HGNC Symbol;Acc:HGNC:13047]	chr19	24033405	24129961	1	ensembl_havana	4.390906376	4.545908107	4.235904645	4.539923521	4.624698072	4.473102727	4.244801141	4.329953957	4.132958835
ENSG00000277147	0.00325	0.029110827	0.386959664	1.307634789	**LINC00869**	transcribed_unprocessed_pseudogene	long intergenic non-protein coding RNA 869 [Source:HGNC Symbol;Acc:HGNC:29050]	chr1	149606334	149679523	1	havana	0.935897264	0.742417432	1.129377097	0.727424294	0.837200041	0.662627962	0.995284613	1.286257764	1.106588913
ENSG00000274214	0.003255	0.029110827	0.357814198	1.281482873	**AP005212.2**	processed_pseudogene		chr18	14253548	14254075	1	havana	1.733595438	1.554688339	1.912502537	1.727676152	1.440410536	1.495978329	2.170524752	1.790554303	1.776428555
ENSG00000070540	0.003254	0.029110827	0.343484577	1.268817503	**WIPI1**	protein_coding	WD repeat domain, phosphoinositide interacting 1 [Source:HGNC Symbol;Acc:HGNC:25471]	chr17	68420948	68457513	-1	ensembl_havana	5.10330661	4.931564321	5.275048899	4.952633592	4.993564355	4.848495017	5.312059643	5.261406393	5.25168066
ENSG00000165934	0.003256	0.029110827	-0.345056684	-1.270200888	**CPSF2**	protein_coding	cleavage and polyadenylation specific factor 2 [Source:HGNC Symbol;Acc:HGNC:2325]	chr14	92121937	92172145	1	ensembl_havana	6.773125506	6.945653848	6.600597163	6.924020203	6.933488132	6.979453209	6.497513266	6.629591433	6.674686791
ENSG00000163681	0.003265	0.029174953	0.342252424	1.267734314	**SLMAP**	protein_coding	sarcolemma associated protein [Source:HGNC Symbol;Acc:HGNC:16643]	chr3	57755450	57929168	1	ensembl_havana	6.627719404	6.456593192	6.798845616	6.431717035	6.475122405	6.462940137	6.708298235	6.812594292	6.87564432
ENSG00000103852	0.003269	0.029185449	-0.335468035	-1.261786688	**TTC23**	protein_coding	tetratricopeptide repeat domain 23 [Source:HGNC Symbol;Acc:HGNC:25730]	chr15	99136323	99251223	-1	ensembl_havana	3.356213899	3.523947917	3.188479882	3.366447561	3.630188857	3.575207333	3.011169462	3.245359567	3.308910617
ENSG00000186897	0.00327	0.029185449	-1.083010157	-2.118451585	**C1QL4**	protein_coding	complement C1q like 4 [Source:HGNC Symbol;Acc:HGNC:31416]	chr12	49332411	49337188	-1	ensembl_havana	-0.652635178	-0.111130099	-1.194140256	0.206373087	-0.521012188	-0.018751196	-0.35894771	-1.87077278	-1.352700279
ENSG00000172594	0.003274	0.029201305	0.323689158	1.251526777	**SMPDL3A**	protein_coding	sphingomyelin phosphodiesterase acid like 3A [Source:HGNC Symbol;Acc:HGNC:17389]	chr6	122789049	122809720	1	ensembl_havana	2.908948585	2.747104006	3.070793165	3.005358074	2.734533378	2.501420566	3.334150354	3.007092508	2.871136632
ENSG00000125449	0.00328	0.029237721	0.445054433	1.36136548	**ARMC7**	protein_coding	armadillo repeat containing 7 [Source:HGNC Symbol;Acc:HGNC:26168]	chr17	75109952	75130265	1	ensembl_havana	4.19086935	3.968342133	4.413396566	3.938508019	4.100831193	3.865687188	4.510259191	4.394551181	4.335379327
ENSG00000185163	0.003282	0.029239314	0.327939706	1.255219531	**DDX51**	protein_coding	DEAD-box helicase 51 [Source:HGNC Symbol;Acc:HGNC:20082]	chr12	132136594	132144335	-1	ensembl_havana	4.702018153	4.5380483	4.865988006	4.408008413	4.556683408	4.649453079	4.794506721	4.836657353	4.966799942
ENSG00000136802	0.003287	0.029254075	0.428003085	1.345370086	**LRRC8A**	protein_coding	leucine rich repeat containing 8 VRAC subunit A [Source:HGNC Symbol;Acc:HGNC:19027]	chr9	128882112	128918039	1	ensembl_havana	5.206245108	4.992243565	5.420246651	4.95029341	4.953104074	5.073333212	5.430479178	5.477501077	5.352759697
ENSG00000128563	0.003288	0.029254075	0.320444995	1.248715652	**PRKRIP1**	protein_coding	PRKR interacting protein 1 [Source:HGNC Symbol;Acc:HGNC:21894]	chr7	102363872	102426676	1	ensembl_havana	5.578104174	5.417881676	5.738326671	5.41737705	5.444363229	5.391904749	5.788247262	5.728396855	5.698335895

ENSG00000157933	0.003291	0.02926151	0.311413901	1.24092326	**SKI**	protein_coding	SKI proto-oncogene [Source:HGNC Symbol;Acc:HGNC:10896]	chr1	2228695	2310119	1	ensembl_havana	4.693795317	4.538088367	4.849502267	4.528987771	4.556683408	4.528593921	4.822280851	4.904172218	4.822053734
ENSG00000163001	0.003296	0.029278225	0.414161674	1.332524144	**CFAP36**	protein_coding	cilia and flagella associated protein 36 [Source:HGNC Symbol;Acc:HGNC:30540]	chr2	55519604	55545879	1	ensembl_havana	5.916693098	5.709612261	6.123773935	5.748934472	5.799993175	5.579909136	6.156609444	6.095402623	6.119309738
ENSG00000161981	0.003297	0.029278225	0.331936046	1.258701375	**SNRNP25**	protein_coding	small nuclear ribonucleoprotein U11/U12 subunit 25 [Source:HGNC Symbol;Acc:HGNC:14161]	chr16	53010	57669	1	ensembl_havana	4.70983544	4.543867417	4.875803464	4.51004573	4.598704279	4.522852242	4.893396418	4.866507668	4.867506304
ENSG00000030419	0.0033	0.029285198	0.493258148	1.407620225	**IKZF2**	protein_coding	IKAROS family zinc finger 2 [Source:HGNC Symbol;Acc:HGNC:13177]	chr2	212999691	213152427	-1	ensembl_havana	3.223522462	2.976893387	3.470151536	2.424845514	3.073391752	3.432442896	3.081809525	3.575605876	3.753039206
ENSG00000102962	0.003308	0.029338459	-0.86175407	-1.817246428	**CCL22**	protein_coding	C-C motif chemokine ligand 22 [Source:HGNC Symbol;Acc:HGNC:10621]	chr16	57358772	57366190	1	ensembl_havana	2.871538039	3.302415074	2.440661004	2.936090303	3.238426324	3.732728596	2.459388507	2.081032632	2.781561875
ENSG00000196155	0.003315	0.029385187	-0.407176398	-1.326087891	**PLEKHG4**	protein_coding	pleckstrin homology and RhoGEF domain containing G4 [Source:HGNC Symbol;Acc:HGNC:24501]	chr16	67277510	67289499	1	ensembl_havana	5.738705561	5.942293761	5.535117362	5.875812458	5.852459364	6.09860946	5.573802349	5.467796732	5.563753005
ENSG00000174775	0.00332	0.029412966	0.447676394	1.363841882	**HRAS**	protein_coding	HRas proto-oncogene, GTPase [Source:HGNC Symbol;Acc:HGNC:5173]	chr11	532242	537287	-1	ensembl_havana	3.763342284	3.539504087	3.987180481	3.4316494	3.659602213	3.527260648	4.006555239	3.952505214	4.002480991
ENSG00000275074	0.003322	0.029412966	-0.361104533	-1.28440887	**NUDT18**	protein_coding	nudix hydrolase 18 [Source:HGNC Symbol;Acc:HGNC:26194]	chr8	22106872	22109419	-1	ensembl_havana	2.588775081	2.769327348	2.408222814	2.738691328	2.698916685	2.87037403	2.278730383	2.385911525	2.560026536
ENSG00000247137	0.003325	0.029419785	0.342301091	1.26777708	**AP000873.2**	processed_transcript		chr11	83184491	83193794	-1	havana	1.316537333	1.145386787	1.487687878	1.092533711	1.062111262	1.281515389	1.371200905	1.47893052	1.61293221
ENSG00000165757	0.003329	0.029439123	-0.38173855	-1.30291101	**JCAD**	protein_coding	junctional cadherin 5 associated [Source:HGNC Symbol;Acc:HGNC:29283]	chr10	30012800	30115494	-1	ensembl_havana	5.464883593	5.655752868	5.274014318	6.018352908	5.508224429	5.440681268	5.549082669	5.10325122	5.169709067
ENSG00000121417	0.003332	0.029441578	0.303676966	1.234286207	**ZNF211**	protein_coding	zinc finger protein 211 [Source:HGNC Symbol;Acc:HGNC:13003]	chr19	57630395	57642779	1	ensembl_havana	4.340141481	4.188302998	4.491979964	4.207050644	4.227651792	4.130206559	4.513670186	4.543651409	4.418618298
ENSG00000167207	0.003339	0.029468228	-0.702418649	-1.627230526	**NOD2**	protein_coding	nucleotide binding oligomerization domain containing 2 [Source:HGNC Symbol;Acc:HGNC:5331]	chr16	50693588	50734041	1	ensembl_havana	2.211395886	2.56260521	1.860186561	2.863328324	2.277093311	2.547393995	1.976531838	1.890792227	1.713235619
ENSG00000166689	0.003337	0.029468228	-0.753517472	-1.685898256	**PLEKHA7**	protein_coding	pleckstrin homology domain containing A7 [Source:HGNC Symbol;Acc:HGNC:27049]	chr11	16777317	17014443	-1	ensembl_havana	0.591848056	0.968606793	0.21508932	0.383334258	1.062111262	1.460374858	-0.35894771	0.602150945	0.402064725
ENSG00000060558	0.003346	0.029492556	-0.321907666	-1.2499823	**GNA15**	protein_coding	G protein subunit alpha 15 [Source:HGNC Symbol;Acc:HGNC:4383]	chr19	3136193	3163769	1	ensembl_havana	5.549317984	5.710271817	5.388364151	6.033355204	5.634119209	5.463341038	5.660981195	5.359131852	5.144979405
ENSG00000136449	0.003345	0.029492556	-0.359166335	-1.28268448	**MYCBPAP**	protein_coding	MYCBP associated protein [Source:HGNC Symbol;Acc:HGNC:19677]	chr17	50508384	50531501	1	ensembl_havana	2.819097381	2.998680548	2.639514214	3.049755341	2.749532556	3.196753748	2.594375592	2.453030417	2.871136632
ENSG00000126603	0.003349	0.029507707	-0.310670936	-1.240284369	**GLIS2**	protein_coding	GLIS family zinc finger 2 [Source:HGNC Symbol;Acc:HGNC:29450]	chr16	4314761	4339597	1	ensembl_havana	1.699174475	1.854509943	1.543839007	2.150951608	1.709476673	1.703101548	1.831005931	1.372000412	1.428510677
ENSG00000234617	0.003355	0.029520456	1.056162913	2.079393667	**SNRK-AS1**	antisense	SNRK antisense RNA 1 [Source:HGNC Symbol;Acc:HGNC:41269]	chr3	43349644	43351962	-1	havana	0.510712935	-0.017368521	1.038794392	-0.31750517	0.15602554	0.109374069	1.245545533	0.938274071	0.932563571
ENSG00000164500	0.003354	0.029520456	0.765502625	1.69996215	**SPATA48**	protein_coding	spermatogenesis associated 48 [Source:HGNC Symbol;Acc:HGNC:22564]	chr7	50096036	50159830	1	ensembl_havana	2.042297986	1.659546673	2.425049299	1.912130943	1.535784795	1.530724282	2.40164672	2.249084557	2.624416619
ENSG00000164733	0.003357	0.029522741	-0.317993867	-1.246595895	**CTSB**	protein_coding	cathepsin B [Source:HGNC Symbol;Acc:HGNC:2527]	chr8	11842524	11869448	-1	ensembl_havana	7.165738345	7.324735278	7.006741411	7.379409588	7.270440028	7.324356218	7.011581312	6.968394488	7.040248433
ENSG00000178381	0.003371	0.0296254	0.386970597	1.307644698	**ZFAND2A**	protein_coding	zinc finger AN1-type containing 2A [Source:HGNC Symbol;Acc:HGNC:28073]	chr7	1152071	1160759	-1	ensembl_havana	3.782664798	3.5891795	3.976150097	3.522665065	3.678012323	3.566861112	3.919229929	3.954822853	4.054397508
ENSG00000135537	0.003381	0.029692556	-0.325865034	-1.253415756	**AFG1L**	protein_coding	AFG1 like ATPase [Source:HGNC Symbol;Acc:HGNC:16411]	chr6	108294894	108526796	1	ensembl_havana	1.987348058	2.150280575	1.824415541	2.167163933	2.101167058	2.182510732	1.852715862	1.715999071	1.90453169
ENSG00000074219	0.003384	0.029702381	0.803227877	1.745001017	**TEAD2**	protein_coding	TEA domain transcription factor 2 [Source:HGNC Symbol;Acc:HGNC:11715]	chr19	49340595	49362457	-1	ensembl_havana	0.573769431	0.172155493	0.97538337	-0.18813307	0.243386951	0.461212593	0.851734776	1.179325527	0.895089805
ENSG00000138771	0.003393	0.029722167	0.442170541	1.358646881	**SHROOM3**	protein_coding	shroom family member 3 [Source:HGNC Symbol;Acc:HGNC:30422]	chr4	76435100	76783253	1	ensembl_havana	4.347627084	4.126541813	4.568712354	4.183333827	4.129780342	4.066511271	4.651652314	4.698271967	4.356212782
ENSG00000153561	0.00339	0.029722167	0.312761186	1.242082658	**RMND5A**	protein_coding	required for meiotic nuclear division 5 homolog A [Source:HGNC Symbol;Acc:HGNC:25850]	chr2	86720173	86778041	1	ensembl_havana	6.859984841	6.703604248	7.016365434	6.581370593	6.807162389	6.722279763	6.920720066	7.076906789	7.051469447
ENSG00000140057	0.003394	0.029722167	-0.332628272	-1.259305462	**AK7**	protein_coding	adenylate kinase 7 [Source:HGNC Symbol;Acc:HGNC:20091]	chr14	96392111	96489427	1	ensembl_havana	2.124581444	2.29089558	1.958267308	2.535139	2.184470312	2.153077428	2.238067541	1.780136556	1.856597828
ENSG00000156469	0.003388	0.029722167	-0.460452162	-1.375973001	**MTERF3**	protein_coding	mitochondrial transcription termination factor 3 [Source:HGNC Symbol;Acc:HGNC:24258]	chr8	96239398	96261610	-1	ensembl_havana	4.467278686	4.697504767	4.237052605	4.712789269	4.755523124	4.624201909	4.171114765	4.201170521	4.338872529
ENSG00000171889	0.003397	0.029722873	0.766289614	1.700889731	**MIR31HG**	sense_overlapping	MIR31 host gene [Source:HGNC Symbol;Acc:HGNC:37187]	chr9	21455642	21559669	-1	havana	1.137569404	0.754424597	1.520714211	0.383334258	1.078042694	0.80189684	1.293957805	1.491756274	1.776428555
ENSG00000136490	0.003399	0.029723761	-1.040616157	-2.057106031	**LIMD2**	protein_coding	LIM domain containing 2 [Source:HGNC Symbol;Acc:HGNC:28142]	chr17	63695902	63701172	-1	ensembl_havana	1.760299303	2.280607382	1.239991224	2.005254198	2.482570884	2.353997062	1.356079599	0.975275413	1.38861866
ENSG00000227199	0.003408	0.029783896	1.153387639	2.224355899	**ST7-AS1**	antisense	ST7 antisense RNA 1 [Source:HGNC Symbol;Acc:HGNC:16000]	chr7	116952446	116954334	-1	havana	-0.087818211	-0.664512031	0.488875608	-0.79434999	-0.842251242	-0.356934859	0.176794179	0.861296647	0.428535999
ENSG00000114739	0.00341	0.029783896	0.350764482	1.275236194	**ACVR2B**	protein_coding	activin A receptor type 2B [Source:HGNC Symbol;Acc:HGNC:174]	chr3	38453851	38493142	1	ensembl_havana	4.343658474	4.168276233	4.519040715	3.96656885	4.222285664	4.315974186	4.301155015	4.659011962	4.596955169
ENSG00000172893	0.003416	0.02981794	-0.734702	-1.664053711	**DHCR7**	protein_coding	7-dehydrocholesterol reductase [Source:HGNC Symbol;Acc:HGNC:2860]	chr11	71428193	71452868	-1	ensembl_havana	4.048505115	4.415856115	3.681154115	5.098194734	4.261181472	3.888192139	4.084314488	3.501444376	3.45770348
ENSG00000186153	0.003418	0.029823121	-0.310132314	-1.239821403	**WWOX**	protein_coding	WW domain containing oxidoreductase [Source:HGNC Symbol;Acc:HGNC:12799]	chr16	78099413	79212667	1	ensembl_havana	4.811992267	4.967058424	4.656926109	5.015544611	4.9819684	4.903662261	4.670111011	4.705171625	4.595495693
ENSG00000124151	0.003429	0.029893934	0.341903156	1.26742744	**NCOA3**	protein_coding	nuclear receptor coactivator 3 [Source:HGNC Symbol;Acc:HGNC:7670]	chr20	47501902	47656877	1	ensembl_havana	7.634737072	7.463785494	7.80568865	7.478757137	7.493047307	7.419552037	7.837780374	7.896673151	7.682612425
ENSG00000197934	0.003433	0.029893934	-0.344406418	-1.269628499	**CYYR1-AS1**	antisense	cysteine and tyrosine rich 1 antisense RNA 1 [Source:HGNC Symbol;Acc:HGNC:39560]	chr21	26393635	26569252	1	havana	3.660949436	3.833152645	3.488746227	3.863385632	3.788645305	3.847426997	3.49810005	3.526590849	3.441547782
ENSG00000145014	0.003432	0.029893934	-0.423315208	-1.34100555	**TMEM44**	protein_coding	transmembrane protein 44 [Source:HGNC Symbol;Acc:HGNC:25120]	chr3	194587673	194633689	-1	ensembl_havana	3.014218872	3.225876477	2.802561268	3.023281264	3.191599876	3.46274829	2.741273741	2.743151031	2.923259032
ENSG00000123411	0.003437	0.029906748	0.36309925	1.286185963	**IKZF4**	protein_coding	IKAROS family zinc finger 4 [Source:HGNC Symbol;Acc:HGNC:13179]	chr12	56007659	56038435	1	ensembl_havana	3.536057114	3.354507489	3.717606739	3.494031456	3.180574467	3.388916545	3.831752454	3.64326966	3.677798104
ENSG00000163412	0.003438	0.029906748	0.323732435	1.25156432	**EIF4E3**	protein_coding	eukaryotic translation initiation factor 4E family member 3 [Source:HGNC Symbol;Acc:HGNC:31837]	chr3	71675416	71754773	-1	ensembl_havana	5.795393649	5.633527431	5.957259866	5.361233198	5.714032602	5.825316494	5.746093808	6.016056889	6.109628901
ENSG00000006715	0.003441	0.029908589	0.337324908	1.263411762	**VPS41**	protein_coding	VPS41, HOPS complex subunit [Source:HGNC Symbol;Acc:HGNC:12713]	chr7	38722963	38932394	-1	ensembl_havana	7.063658601	6.894996147	7.232321055	6.847490696	6.884220454	6.95327729	7.106688965	7.255553746	7.334720454
ENSG00000135617	0.003445	0.029929008	0.458307947	1.373929471	**PRADC1**	protein_coding	protease associated domain containing 1 [Source:HGNC Symbol;Acc:HGNC:16047]	chr2	73228006	73233238	-1	ensembl_havana	4.033114718	3.803960745	4.262268692	3.760287894	3.961778485	3.689815855	4.316859918	4.245388041	4.224558116
ENSG00000164841	0.003454	0.029991338	0.362672085	1.285805195	**TMEM74**	protein_coding	transmembrane protein 74 [Source:HGNC Symbol;Acc:HGNC:26409]	chr8	108606850	108787615	-1	ensembl_havana	0.990336126	0.809000084	1.171672169	0.326725152	1.242575255	0.857699845	0.785553575	1.590453245	1.139009687
ENSG00000169439	0.00347	0.030017623	2.123179692	4.356530647	**SDC2**	protein_coding	syndecan 2 [Source:HGNC Symbol;Acc:HGNC:10659]	chr8	96493351	96611780	1	ensembl_havana	-0.924693882	-1.986283728	0.136895964	-3.00120074	-0.842251242	-2.115399201	-0.46237776	0.225896484	0.647169164
ENSG00000250091	0.003464	0.030017623	0.798123225	1.738837634	**DNAH10OS**	protein_coding	dynein axonemal heavy chain 10 opposite strand [Source:HGNC Symbol;Acc:HGNC:37121]	chr12	123926424	123934984	-1	havana	2.563042722	2.16398111	2.962104335	1.996207752	2.154731774	2.341003803	3.162250891	2.710605818	3.013456297
ENSG00000138363	0.003467	0.030017623	-0.338940741	-1.264827588	**ATIC**	protein_coding	5-aminoimidazole-4-carboxamide ribonucleotide formyltransferase/IMP cyclohydrolase [Source:HGNC Symbol;Acc:HGNC:794]	chr2	215311817	215349773	1	ensembl_havana	6.589951389	6.75942176	6.420481019	6.621634703	6.75081848	6.905812097	6.212775236	6.481933222	6.566734599
ENSG00000279794	0.003468	0.030017623	-0.396063163	-1.315912136	**AC024580.1**	TEC		chr19	56149817	56150490	1	havana	0.252397139	0.45042872	0.054365557	0.770507832	0.325758945	0.255019383	0.399634207	0.02879206	-0.265329594
ENSG00000166851	0.003468	0.030017623	-0.399254721	-1.318826445	**PLK1**	protein_coding	polo like kinase 1 [Source:HGNC Symbol;Acc:HGNC:9077]	chr16	23677656	23690367	1	ensembl_havana	7.0011432	7.200770561	6.801515839	7.051215315	7.303424413	7.247671954	6.783342083	6.860662552	6.760542882
ENSG00000231789	0.003461	0.030017623	-0.576672318	-1.491405243	**PIK3CD-AS2**	antisense	PIK3CD antisense RNA 2 [Source:HGNC Symbol;Acc:HGNC:51334]	chr1	9672426	9687555	-1	havana	-0.010945683	0.277390476	-0.299281842	0.515840645	0.403680778	-0.087349994	0.069637898	-0.42206346	-0.545419959
ENSG00000080822	0.003478	0.030070566	0.306398567	1.236616848	**CLDND1**	protein_coding	claudin domain containing 1 [Source:HGNC Symbol;Acc:HGNC:1322]	chr3	98497912	98523066	-1	ensembl_havana	7.961295698	7.808096415	8.114494982	7.73745582	7.799066479	7.887766945	8.009180462	8.122076533	8.21222795
ENSG00000198301	0.003494	0.030174416	-0.319060482	-1.24751787	**SDAD1**	protein_coding	SDA1 domain containing 1 [Source:HGNC Symbol;Acc:HGNC:25537]	chr4	75940950	75990962	-1	ensembl_havana	6.049623027	6.209153268	5.890092786	6.118150067	6.248843122	6.260466615	5.741737204	5.960049993	5.968491162
ENSG00000246582	0.003493	0.030174416	-0.482693566	-1.397350138	**AC100861.1**	processed_transcript		chr8	23224471	23230926	1	havana	1.417534169	1.658880952	1.176187386	1.649052215	1.729788441	1.5978022	1.309740823	1.286257764	0.932563571
ENSG00000182993	0.003501	0.030210812	-0.346033347	-1.271061069	**C12orf60**	protein_coding	chromosome 12 open reading frame 60 [Source:HGNC Symbol;Acc:HGNC:28726]	chr12	14803572	14906586	1	ensembl_havana	2.113967718	2.286984391	1.940951045	2.348706028	2.427675878	2.084571268	1.966610161	2.029189031	1.827053942
ENSG00000238260	0.003504	0.030219937	0.712975279	1.639181127	**AL513320.1**	antisense		chr1	3623190	3624743	-1	havana	0.157640617	-0.198847022	0.514128256	-0.36334239	-0.18055577	-0.052642908	0.592614899	0.602150945	0.347618925
ENSG00000229771	0.003506	0.030222539	-0.763383235	-1.697466656	**AL035665.1**	lincRNA		chr20	40696499	40698616	-1	havana	2.152382756	2.534074374	1.770691139	2.677756939	2.564016113	2.360450071	2.187709208	1.465989718	1.65837449
ENSG00000110881	0.003509	0.03022993	-0.309267042	-1.239078029	**ASIC1**	protein_coding	acid sensing ion channel subunit 1 [Source:HGNC Symbol;Acc:HGNC:100]	chr12	50057548	50083611	1	ensembl_havana	3.282997962	3.437631483	3.128364441	3.461535265	3.344313992	3.507045191	3.157897671	3.068309725	3.158885927
ENSG00000206579	0.003514	0.030242769	0.498233426	1.412482923	**XKR4**	protein_coding	XK related 4 [Source:HGNC Symbol;Acc:HGNC:29394]	chr8	55102389	55542054	1	ensembl_havana	5.469961125	5.220844412	5.719077838	4.834521154	5.53299787	5.295014212	5.534884518	5.917907168	5.704441827
ENSG00000133740	0.003515	0.030242769	-0.364447964	-1.287388926	**E2F5**	protein_coding	E2F transcription factor 5 [Source:HGNC Symbol;Acc:HGNC:3119]	chr8	85177225	85217158	1	ensembl_havana	3.619266993	3.801490974	3.437043011	3.873301493	3.754355472	3.776815959	3.42027144	3.485502303	3.405355289
ENSG00000205726	0.003528	0.030285214	0.540902479	1.454882335	**ITSN1**	protein_coding	intersectin 1 [Source:HGNC Symbol;Acc:HGNC:6183]	chr21	33642400	33899861	1	ensembl_havana	5.262303073	4.991851833	5.532754312	4.749592033	5.093935728	5.132027739	5.517150487	5.552106624	5.529005826
ENSG00000175691	0.003527	0.030285214	0.412179945	1.330695006	**ZNF77**	protein_coding	zinc finger protein 77 [Source:HGNC Symbol;Acc:HGNC:13150]	chr19	2933218	2944971	-1	ensembl_havana	3.554697715	3.348607743	3.760787688	3.348787899	3.276895115	3.420140216	3.617041156	3.775017686	3.890304222
ENSG00000234465	0.003523	0.030285214	0.304119479	1.234664853	**PINLYP**	protein_coding	phospholipase A2 inhibitor and LY6/PLAUR domain containing [Source:HGNC Symbol;Acc:HGNC:44206]	chr19	43576800	43583964	1	ensembl_havana	0.128683311	-0.023376428	0.280743051	-0.36334239	0.09469996	0.198513145	-0.08686711	0.399280361	0.529815901
ENSG00000184378	0.003525	0.030285214	-0.466145538	-1.381413797	**ACTRT3**	protein_coding	actin related protein T3 [Source:HGNC Symbol;Acc:HGNC:24022]	chr3	169766921	169769895	-1	ensembl_havana	2.996659593	3.229732361	2.763586824	3.355877725	3.24901995	3.08429941	2.723708883	2.800974855	2.766076734
ENSG00000234210	0.003543	0.030292884	0.717401956	1.644218414	**AC006372.3**	lincRNA		chr7	157466231	157499716	1	havana	0.496229836	0.137528858	0.854930814	-0.06941355	0.09469996	0.387300166	0.975631465	0.602150945	0.987010032
ENSG00000261008	0.003538	0.030292884	0.622764676	1.539823161	**LINC01572**	lincRNA	long intergenic non-protein coding RNA 1572 [Source:HGNC Symbol;Acc:HGNC:51385]	chr16	72283301	72665009	-1	havana	1.560950371	1.249568033	1.872332709	0.986893817	1.242575255	1.519235028	1.831005931	1.900443368	1.885548828
ENSG00000203877	0.003552	0.030292884	0.433033412	1.350069252	**RIPPLY2**	protein_coding	ripply transcriptional repressor 2 [Source:HGNC Symbol;Acc:HGNC:21390]	chr6	83853266	83857515	1	ensembl_havana	2.254684281	2.038167575	2.471200987	1.833046887	2.344503139	1.936952697	2.408992098	2.643226439	2.361384423
ENSG00000225190	0.003552	0.030292884	0.380291135	1.301604492	**PLEKHM1**	protein_coding	pleckstrin homology and RUN domain containing M1 [Source:HGNC Symbol;Acc:HGNC:29017]	chr17	45435900	45490749	-1	ensembl_havana	5.049643712	4.859498145	5.23978928	4.958467494	4.771531924	4.848495017	5.409575575	5.201185201	5.108607065
ENSG00000160284	0.003549	0.030292884	0.351859023	1.276204054	**SPATC1L**	protein_coding	spermatogenesis and centriole associated 1 like [Source:HGNC Symbol;Acc:HGNC:1298]	chr21	46161148	46184476	-1	ensembl_havana	1.957537141	1.78160763	2.133466653	1.82284946	1.739838083	1.782135348	2.254470464	2.097907935	2.04802156
ENSG00000131373	0.003539	0.030292884	0.346225549	1.271230416	**HACL1**	protein_coding	2-hydroxyacyl-CoA lyase 1 [Source:HGNC Symbol;Acc:HGNC:17856]	chr3	15560704	15601852	-1	ensembl_havana	5.156033832	4.982921057	5.329146606	4.794639466	5.187775973	4.966347733	5.15492242	5.4498341	5.382683299
ENSG00000177042	0.003532	0.030292884	0.324297321	1.252054464	**TMEM80**	protein_coding	transmembrane protein 80 [Source:HGNC Symbol;Acc:HGNC:27453]	chr11	695428	705028	1	ensembl_havana	3.334956177	3.172807516	3.497104837	3.101289176	3.198903614	3.218229759	3.491195686	3.491900287	3.508218538
ENSG00000014919	0.003546	0.030292884	-0.315291107	-1.244262695	**COX15**	protein_coding	COX15, cytochrome c oxidase assembly homolog [Source:HGNC Symbol;Acc:HGNC:2263]	chr10	99711844	99732100	-1	ensembl_havana	5.667006616	5.824652169	5.509361063	5.724492672	5.853038165	5.896425672	5.365893885	5.53206871	5.630120593
ENSG00000214837	0.003552	0.030292884	-0.425022147	-1.342593113	**LINC01347**	transcribed_unprocessed_pseudogene	long intergenic non-protein coding RNA 1347 [Source:HGNC Symbol;Acc:HGNC:50566]	chr1	243056307	243101744	-1	havana	3.002599124	3.215110198	2.79008805	3.179296803	3.131799381	3.334234408	2.613576909	2.721536001	3.035151242
ENSG00000068079	0.003547	0.030292884	-0.473393462	-1.388371314	**IFI35**	protein_coding	interferon induced protein 35 [Source:HGNC Symbol;Acc:HGNC:5399]	chr17	43006725	43014456	1	ensembl_havana	6.095059705	6.331756437	5.858362974	6.352851647	6.368642631	6.273775032	6.021172226	5.718201016	5.835715681
ENSG00000263667	0.003536	0.030292884	-0.491888496	-1.406284506	**AL035696.3**	lincRNA		chr6	181466	205484	-1	havana	1.132653808	1.378598057	0.88670956	1.397462791	1.37743234	1.360899039	0.739683594	1.063828438	0.856616648
ENSG00000106330	0.003559	0.030329363	0.559664267	1.473926177	**MOSPD3**	protein_coding	motile sperm domain containing 3 [Source:HGNC Symbol;Acc:HGNC:25078]	chr7	100612102	100615384	1	ensembl_havana	2.215119722	1.935287588	2.494951856	1.87313074	1.944876279	1.987855745	2.669693543	2.42655638	2.388605644
ENSG00000151702	0.003561	0.030329363	-0.394075587	-1.314100475	**FLI1**	protein_coding	Fli-1 proto-oncogene, ETS transcription factor [Source:HGNC Symbol;Acc:HGNC:3749]	chr11	128686535	128813267	1	ensembl_havana	5.280994565	5.478032358	5.083956771	5.475491922	5.375933647	5.582671505	5.010292088	5.113660433	5.127917792
ENSG00000101442	0.00357	0.030355267	0.358613185	1.282192775	**ACTR5**	protein_coding	ARP5 actin related protein 5 homolog [Source:HGNC Symbol;Acc:HGNC:14671]	chr20	38748442	38772520	1	ensembl_havana	4.648186408	4.468879816	4.827493001	4.394300619	4.482313051	4.530025778	4.689844966	4.802320534	4.990313502
ENSG00000081059	0.003568	0.030355267	-0.361367518	-1.284643023	**TCF7**	protein_coding	transcription factor 7 [Source:HGNC Symbol;Acc:HGNC:11639]	chr5	134114711	134151865	1	ensembl_havana	5.240467213	5.421150972	5.059783454	5.600142575	5.352382995	5.310927346	5.254100611	4.893338276	5.031911475
ENSG00000172009	0.003569	0.030355267	-0.515434622	-1.4294247	**THOP1**	protein_coding	thimet oligopeptidase 1 [Source:HGNC Symbol;Acc:HGNC:11793]	chr19	2785460	2815807	1	ensembl_havana	7.293853607	7.551570919	7.036136296	7.580996833	7.428301224	7.645414699	7.196178048	6.995101382	6.917129459
ENSG00000175550	0.003573	0.030356974	0.323142602	1.251052733	**DRAP1**	protein_coding	DR1 associated protein 1 [Source:HGNC Symbol;Acc:HGNC:3019]	chr11	65919257	65921561	1	ensembl_havana	3.741214484	3.579643184	3.902785785	3.799817921	3.470377911	3.468733719	4.188292236	3.759192284	3.760872836
ENSG00000099954	0.003581	0.030412105	-0.4030779	-1.322326004	**CECR2**	protein_coding	CECR2, histone acetyl-lysine reader [Source:HGNC Symbol;Acc:HGNC:1840]	chr22	17359949	17558149	1	ensembl_havana	1.897420469	2.098959419	1.695881519	1.959443807	2.191810142	2.145624309	1.430149367	1.800897362	1.856597828
ENSG00000139971	0.003589	0.030460025	-0.685069248	-1.607779152	**C14orf37**	protein_coding	chromosome 14 open reading frame 37 [Source:HGNC Symbol;Acc:HGNC:19846]	chr14	57999735	58298139	-1	ensembl_havana	2.656195231	2.998729855	2.313660607	3.155115434	2.891524778	2.949549354	2.170524752	2.271570328	2.498886741
ENSG00000137098	0.003592	0.030468016	0.675846655	1.597534021	**SPAG8**	protein_coding	sperm associated antigen 8 [Source:HGNC Symbol;Acc:HGNC:14105]	chr9	35808045	35812272	-1	ensembl_havana	1.947047863	1.609124535	2.284971191	1.397462791	1.7890623	1.640848515	2.213108088	2.155463891	2.486341593
ENSG00000182197	0.003599	0.030505187	0.46507918	1.380393112	**EXT1**	protein_coding	exostosin glycosyltransferase 1 [Source:HGNC Symbol;Acc:HGNC:3512]	chr8	117794490	118111853	-1	ensembl_havana	3.860017229	3.627477639	4.092556819	3.689093732	3.561022392	3.632316794	4.008974209	4.195301803	4.073394445
ENSG00000138382	0.003601	0.030505187	-0.357408515	-1.281122574	**METTL5**	protein_coding	methyltransferase like 5 [Source:HGNC Symbol;Acc:HGNC:25006]	chr2	169810081	169824931	-1	ensembl_havana	4.661539255	4.840243512	4.482834998	4.76831163	4.881151268	4.871267639	4.318811065	4.531280758	4.59841317
ENSG00000170791	0.003607	0.030526242	0.298764438	1.23009048	**CHCHD7**	protein_coding	coiled-coil-helix-coiled-coil-helix domain containing 7 [Source:HGNC Symbol;Acc:HGNC:28314]	chr8	56211686	56218798	1	ensembl_havana	6.234409537	6.085027318	6.383791756	6.002063643	6.175306783	6.077711527	6.319918676	6.472663145	6.358793446
ENSG00000260231	0.003607	0.030526242	-0.479369487	-1.394134243	**JHDM1D-AS1**	bidirectional_promoter_lncRNA	JHDM1D antisense RNA 1 (head to head) [Source:HGNC Symbol;Acc:HGNC:48959]	chr7	140177184	140179640	1	havana	0.921982958	1.161667702	0.682298215	1.253039554	0.910210399	1.321753151	0.592614899	0.578298274	0.875981471
ENSG00000175215	0.003612	0.030543818	0.324652021	1.252362331	**CTDSP2**	protein_coding	CTD small phosphatase 2 [Source:HGNC Symbol;Acc:HGNC:17077]	chr12	57819927	57846739	-1	ensembl_havana	5.678588292	5.516262282	5.840914302	5.596405944	5.540922579	5.411458321	5.954968134	5.797173085	5.770601689
ENSG00000197380	0.003618	0.030557036	0.744555626	1.675458121	**DACT3**	protein_coding	dishevelled binding antagonist of beta catenin 3 [Source:HGNC Symbol;Acc:HGNC:30745]	chr19	46647612	46661138	-1	ensembl_havana	1.377684984	1.00540717	1.749962797	1.005046425	1.228369434	0.782805653	1.593788933	1.780136556	1.875962902
ENSG00000137038	0.003617	0.030557036	-0.372641185	-1.294720947	**DMAC1**	protein_coding	distal membrane arm assembly complex 1 [Source:HGNC Symbol;Acc:HGNC:30536]	chr9	7796490	7888380	-1	ensembl_havana	4.121166417	4.307487009	3.934845824	4.311025713	4.240095677	4.371339638	4.028181329	3.886051921	3.890304222
ENSG00000122735	0.003622	0.030557036	-0.443219566	-1.359635151	**DNAI1**	protein_coding	dynein axonemal intermediate chain 1 [Source:HGNC Symbol;Acc:HGNC:2954]	chr9	34457414	34520989	1	ensembl_havana	5.035487799	5.257097582	4.813878016	5.153152225	5.054218477	5.563922043	4.593204216	4.77503741	5.073392421
ENSG00000254521	0.00362	0.030557036	-0.996525976	-1.995189774	**SIGLEC12**	protein_coding	sialic acid binding Ig like lectin 12 (gene/pseudogene) [Source:HGNC Symbol;Acc:HGNC:15482]	chr19	51491357	51501789	-1	ensembl_havana	0.485611139	0.983874127	-0.012651849	1.126095262	0.77991046	1.045616658	-0.04612063	-0.52165082	0.529815901
ENSG00000078687	0.003627	0.030564314	0.440745003	1.357305054	**TNRC6C**	protein_coding	trinucleotide repeat containing 6C [Source:HGNC Symbol;Acc:HGNC:29318]	chr17	77959240	78108835	1	ensembl_havana	4.297350837	4.076978335	4.517723338	3.875769846	4.171215189	4.183949971	4.456330725	4.621574099	4.47526519
ENSG00000168310	0.003626	0.030564314	-0.368842657	-1.29131651	**IRF2**	protein_coding	interferon regulatory factor 2 [Source:HGNC Symbol;Acc:HGNC:6117]	chr4	184387713	184474580	-1	ensembl_havana	4.833429582	5.017850911	4.649008254	5.173358694	4.917022721	4.963171318	4.805680618	4.449821744	4.691522399
ENSG00000231873	0.003639	0.030645677	0.732590641	1.661620178	**AC099560.1**	lincRNA		chr3	40766207	40862626	1	havana	1.317507422	0.951212101	1.683802742	0.852997155	1.124807863	0.875831286	1.717267538	1.517070703	1.817069986
ENSG00000167088	0.003643	0.030645677	-0.44210002	-1.35858047	**SNRPD1**	protein_coding	small nuclear ribonucleoprotein D1 polypeptide [Source:HGNC Symbol;Acc:HGNC:11158]	chr18	21612267	21633524	1	ensembl_havana	6.404864629	6.62591464	6.183814619	6.664379231	6.587557915	6.625806774	6.061349647	6.182526059	6.307568153
ENSG00000104413	0.003642	0.030645677	-0.47700149	-1.391847829	**ESRP1**	protein_coding	epithelial splicing regulatory protein 1 [Source:HGNC Symbol;Acc:HGNC:25966]	chr8	94641074	94707466	1	ensembl_havana	2.196803268	2.435304013	1.958302523	2.464742477	2.249222012	2.591947549	1.797815913	1.890792227	2.186299428
ENSG00000186594	0.003647	0.030658861	0.368241955	1.29077895	**MIR22HG**	lincRNA	MIR22 host gene [Source:HGNC Symbol;Acc:HGNC:28219]	chr17	1711493	1717174	-1	havana	3.614324565	3.430203587	3.798445542	3.926652984	3.363940242	3.000017535	4.198925214	3.831602732	3.364808681
ENSG00000136870	0.003655	0.030695566	0.339144367	1.265006122	**ZNF189**	protein_coding	zinc finger protein 189 [Source:HGNC Symbol;Acc:HGNC:12980]	chr9	101398873	101410660	1	ensembl_havana	5.104001498	4.934429315	5.273573682	4.920716271	5.011304034	4.871267639	5.179826793	5.363499728	5.277394524
ENSG00000204131	0.003656	0.030695566	-0.423106602	-1.340811662	**NHSL2**	protein_coding	NHS like 2 [Source:HGNC Symbol;Acc:HGNC:33737]	chrX	71910818	72161750	1	ensembl_havana	1.375624177	1.587177478	1.164070876	1.411140715	1.558674763	1.791716956	1.033805522	1.241396536	1.21701057
ENSG00000154654	0.003657	0.030695566	-0.609421364	-1.52564718	**NCAM2**	protein_coding	neural cell adhesion molecule 2 [Source:HGNC Symbol;Acc:HGNC:7657]	chr21	20998315	21543329	1	ensembl_havana	3.784359043	4.089069725	3.479648361	4.226521114	4.004085726	4.036602336	3.364692078	3.429942433	3.644310574
ENSG00000004866	0.003664	0.030731894	0.314358446	1.243458575	**ST7**	protein_coding	suppression of tumorigenicity 7 [Source:HGNC Symbol;Acc:HGNC:11351]	chr7	116953238	117230103	1	ensembl_havana	7.588229476	7.431050253	7.745408699	7.212470639	7.516619904	7.564060216	7.580988689	7.816743755	7.838493653
ENSG00000269837	0.003679	0.030749835	0.54193562	1.455924578	**IPO5P1**	transcribed_processed_pseudogene	importin 5 pseudogene 1 [Source:HGNC Symbol;Acc:HGNC:49687]	chr19	23255053	23257939	-1	havana	2.927218182	2.656250372	3.198185992	2.383813765	2.812785192	2.772152159	3.127051692	3.151488815	3.31601747
ENSG00000164506	0.003677	0.030749835	0.310682051	1.240293925	**STXBP5**	protein_coding	syntaxin binding protein 5 [Source:HGNC Symbol;Acc:HGNC:19665]	chr6	147204425	147390476	1	ensembl_havana	7.608933356	7.453592331	7.764274382	7.564643402	7.420902829	7.375230761	7.825481559	7.747413086	7.7199285
ENSG00000115841	0.003675	0.030749835	0.300002948	1.231146929	**RMDN2**	protein_coding	regulator of microtubule dynamics 2 [Source:HGNC Symbol;Acc:HGNC:26567]	chr2	37923187	38067142	1	ensembl_havana	4.255725962	4.105724488	4.405727436	4.193263259	4.124036797	3.999873408	4.522162557	4.414895403	4.280124347
ENSG00000184014	0.003679	0.030749835	0.298946317	1.230245566	**DENND5A**	protein_coding	DENN domain containing 5A [Source:HGNC Symbol;Acc:HGNC:19344]	chr11	9138825	9265390	-1	ensembl_havana	7.134695197	6.985222038	7.284168355	7.316296669	6.9230518	6.716317647	7.59413838	7.246832235	7.011534451
ENSG00000047597	0.003672	0.030749835	-0.381149764	-1.302379381	**XK**	protein_coding	X-linked Kx blood group [Source:HGNC Symbol;Acc:HGNC:12811]	chrX	37685756	37732130	1	ensembl_havana	4.8259447	5.016519582	4.635369817	4.816646463	5.057235496	5.175676786	4.506840112	4.728386669	4.670882671
ENSG00000213465	0.003674	0.030749835	-0.593841243	-1.509259878	**ARL2**	protein_coding	ADP ribosylation factor like GTPase 2 [Source:HGNC Symbol;Acc:HGNC:693]	chr11	65014113	65022184	1	ensembl_havana	1.715877232	2.012797853	1.41895661	1.770752281	2.021022296	2.246618983	1.229040238	1.625790657	1.402038935
ENSG00000140470	0.003685	0.030763725	0.635646902	1.553634241	**ADAMTS17**	protein_coding	ADAM metallopeptidase with thrombospondin type 1 motif 17 [Source:HGNC Symbol;Acc:HGNC:17109]	chr15	99971589	100342005	-1	ensembl_havana	0.166182621	-0.15164083	0.484006073	-0.51029874	-0.143136894	0.198513145	0.36983092	0.504278656	0.577908642
ENSG00000138604	0.003685	0.030763725	0.374162739	1.296087158	**GLCE**	protein_coding	glucuronic acid epimerase [Source:HGNC Symbol;Acc:HGNC:17855]	chr15	69160584	69272217	1	ensembl_havana	6.01927334	5.832191971	6.206354709	5.539941449	5.957385964	5.999248499	6.01937366	6.326398625	6.273291843
ENSG00000186831	0.00369	0.03079012	-0.454787359	-1.370580778	**KRT17P2**	transcribed_unprocessed_pseudogene	keratin 17 pseudogene 2 [Source:HGNC Symbol;Acc:HGNC:6429]	chr17	18426861	18431848	1	havana	1.663809577	1.891203256	1.436415898	1.625778111	1.909699171	2.138132487	0.995284613	1.517070703	1.796892377
ENSG00000141664	0.00371	0.030941991	0.369686207	1.292071768	**ZCCHC2**	protein_coding	zinc finger CCHC-type containing 2 [Source:HGNC Symbol;Acc:HGNC:22916]	chr18	62523007	62587709	1	ensembl_havana	5.015939041	4.831095938	5.200782144	4.694728896	4.78129592	5.017262996	5.106047052	5.223463133	5.272836248
ENSG00000205356	0.003717	0.030970464	0.532525235	1.446458804	**TECPR1**	protein_coding	tectonin beta-propeller repeat containing 1 [Source:HGNC Symbol;Acc:HGNC:22214]	chr7	98214624	98252251	-1	ensembl_havana	5.915944787	5.64968217	6.182207404	5.724492672	5.410947263	5.813606574	6.269864968	6.134775054	6.141982191
ENSG00000136114	0.00372	0.030970464	0.467781295	1.382980961	**THSD1**	protein_coding	thrombospondin type 1 domain containing 1 [Source:HGNC Symbol;Acc:HGNC:17754]	chr13	52377167	52406494	-1	ensembl_havana	1.218382709	0.984492061	1.452273356	0.660286109	1.124807863	1.168382212	1.309740823	1.541948597	1.505130649
ENSG00000077454	0.003728	0.030970464	0.431884348	1.34899439	**LRCH4**	protein_coding	leucine rich repeats and calponin homology domain containing 4 [Source:HGNC Symbol;Acc:HGNC:6691]	chr7	100574011	100586153	-1	ensembl_havana	3.003220496	2.787278321	3.21916267	2.631766511	2.882481267	2.847587186	3.196611706	3.329927104	3.1309492
ENSG00000229066	0.003733	0.030970464	-0.315495485	-1.244438975	**AC093459.1**	lincRNA		chr2	175257250	175454640	1	havana	-0.03024738	0.127500362	-0.187995123	0.540941683	-0.071090603	-0.087349994	0.210814386	-0.32890884	-0.445890917
ENSG00000157191	0.003727	0.030970464	-0.348900393	-1.27358954	**NECAP2**	protein_coding	NECAP endocytosis associated 2 [Source:HGNC Symbol;Acc:HGNC:25528]	chr1	16440672	16460078	1	ensembl_havana	7.713564146	7.888014342	7.53911395	8.056123747	7.883929137	7.723990143	7.714071544	7.447582245	7.45568806
ENSG00000107625	0.003728	0.030970464	-0.358507516	-1.282098866	**DDX50**	protein_coding	DExD-box helicase 50 [Source:HGNC Symbol;Acc:HGNC:17906]	chr10	68901278	68946847	1	ensembl_havana	4.897470987	5.076724745	4.718217229	5.006562662	5.157169117	5.066442455	4.549007011	4.815133357	4.790511319
ENSG00000060566	0.003731	0.030970464	-0.366747893	-1.289442904	**CREB3L3**	protein_coding	cAMP responsive element binding protein 3 like 3 [Source:HGNC Symbol;Acc:HGNC:18855]	chr19	4153601	4173054	1	ensembl_havana	0.897170504	1.08054445	0.713796558	0.931023971	0.962641309	1.347968071	0.668022463	0.504278656	0.969088554
ENSG00000172671	0.003732	0.030970464	-0.508371761	-1.422443905	**ZFAND4**	protein_coding	zinc finger AN1-type containing 4 [Source:HGNC Symbol;Acc:HGNC:23504]	chr10	45615501	45672780	-1	ensembl_havana	3.416879466	3.671065346	3.162693586	3.669310227	3.74938971	3.594496101	3.030347571	3.159548924	3.298184262
ENSG00000102886	0.003726	0.030970464	-0.567707504	-1.482166481	**GDPD3**	protein_coding	glycerophosphodiester phosphodiesterase domain containing 3 [Source:HGNC Symbol;Acc:HGNC:28638]	chr16	30104810	30113856	-1	ensembl_havana	3.076305468	3.36015922	2.792451716	3.249500187	3.241966183	3.58901129	2.874587014	2.716081261	2.786686872
ENSG00000141447	0.003739	0.030982068	-0.355371567	-1.279315027	**OSBPL1A**	protein_coding	oxysterol binding protein like 1A [Source:HGNC Symbol;Acc:HGNC:16398]	chr18	24162044	24397880	-1	ensembl_havana	4.971892515	5.149578298	4.794206731	5.311046722	5.073220565	5.064467608	4.880243959	4.694116277	4.808259957
ENSG00000186442	0.003737	0.030982068	-0.366189268	-1.288943717	**KRT3**	protein_coding	keratin 3 [Source:HGNC Symbol;Acc:HGNC:6440]	chr12	52789685	52796117	-1	ensembl_havana	7.511401987	7.694496621	7.328307353	7.634996513	7.759325575	7.689167775	7.376768744	7.309975549	7.298177766
ENSG00000196878	0.003743	0.03099863	0.725890662	1.653921385	**LAMB3**	protein_coding	laminin subunit beta 3 [Source:HGNC Symbol;Acc:HGNC:6490]	chr1	209614870	209652466	-1	ensembl_havana	2.82759419	2.464648859	3.190539521	2.109605353	2.512189067	2.772152159	3.135932331	3.230132489	3.205553743

ENSG00000165572	0.003749	0.031011246	0.341961884	1.267479034	**KBTBD6**	protein_coding	kelch repeat and BTB domain containing 6 [Source:HGNC Symbol;Acc:HGNC:25340]	chr13	41127569	41132746	-1	ensembl_havana	4.596755614	4.425774672	4.767736556	4.414813776	4.510469659	4.352040582	4.829141634	4.864043597	4.610024438
ENSG00000261971	0.003748	0.031011246	0.338684207	1.264602702	**MMP25-AS1**	antisense	MMP25 antisense RNA 1 [Source:HGNC Symbol;Acc:HGNC:51372]	chr16	3051096	3059370	-1	havana	3.393815907	3.224473804	3.56315801	3.183287918	3.202541664	3.287591829	3.571944101	3.578614721	3.538915209
ENSG00000183718	0.003756	0.031013739	0.342564402	1.268008487	**TRIM52**	protein_coding	tripartite motif containing 52 [Source:HGNC Symbol;Acc:HGNC:19024]	chr5	181254417	181261139	-1	ensembl_havana	5.747212492	5.575930291	5.918494693	5.700326094	5.510427513	5.517037266	5.956222156	5.870205135	5.929056787
ENSG00000152932	0.003757	0.031013739	0.31239291	1.241765633	**RAB3C**	protein_coding	RAB3C, member RAS oncogene family [Source:HGNC Symbol;Acc:HGNC:30269]	chr5	58582221	58859394	1	ensembl_havana	5.962002297	5.805805842	6.118198752	5.715563702	5.792167333	5.909686491	5.971806146	6.135286016	6.247504092
ENSG00000257135	0.003758	0.031013739	-0.50494546	-1.419069708	**AC007249.2**	lincRNA		chr2	10448694	10451327	1	havana	1.183903828	1.436376558	0.931431098	1.175017601	1.547275176	1.586836898	0.830009965	0.841392961	1.12289037
ENSG00000176170	0.003752	0.031013739	-0.755112437	-1.687763124	**SPHK1**	protein_coding	sphingosine kinase 1 [Source:HGNC Symbol;Acc:HGNC:11240]	chr17	76376584	76387860	1	ensembl_havana	0.68478866	1.062344878	0.307232441	1.04068047	0.89230127	1.254052895	0.210814386	0.478736765	0.232146171
ENSG00000071553	0.003761	0.031017095	0.310364497	1.240020952	**ATP6AP1**	protein_coding	ATPase H+ transporting accessory protein 1 [Source:HGNC Symbol;Acc:HGNC:868]	chrX	154428632	154436516	1	ensembl_havana	7.693464529	7.53828228	7.848646777	7.598848762	7.518810215	7.497187863	7.935433514	7.775874151	7.834632668
ENSG00000137807	0.003763	0.031017095	-0.329780272	-1.256821941	**KIF23**	protein_coding	kinesin family member 23 [Source:HGNC Symbol;Acc:HGNC:6392]	chr15	69414246	69448427	1	ensembl_havana	7.392508024	7.55739816	7.227617888	7.444456872	7.641312455	7.586425153	7.037878551	7.363945624	7.281029489
ENSG00000234883	0.003766	0.031025511	0.513294786	1.427306117	**MIR155HG**	lincRNA	MIR155 host gene [Source:HGNC Symbol;Acc:HGNC:35460]	chr21	25561909	25575168	1	havana	7.821559075	7.564911682	8.078206468	8.039569918	7.599669947	7.05549518	8.413077249	8.038226098	7.783316056
ENSG00000083093	0.00377	0.031041955	-0.412467669	-1.33096042	**PALB2**	protein_coding	partner and localizer of BRCA2 [Source:HGNC Symbol;Acc:HGNC:26144]	chr16	23603160	23641310	-1	ensembl_havana	4.929344437	5.135578272	4.723110603	5.196251561	5.160913808	5.049569447	4.670111011	4.720235712	4.778985085
ENSG00000179820	0.003779	0.031098178	-0.322474958	-1.25047391	**MYADM**	protein_coding	myeloid associated differentiation marker [Source:HGNC Symbol;Acc:HGNC:7544]	chr19	53866223	53876437	1	ensembl_havana	4.647236263	4.808473742	4.485998784	5.48764628	4.615175813	4.322599132	5.234596236	4.267931051	3.955469065
ENSG00000099940	0.003794	0.031201644	0.298073332	1.229501361	**SNAP29**	protein_coding	synaptosome associated protein 29 [Source:HGNC Symbol;Acc:HGNC:11133]	chr22	20858983	20891218	1	ensembl_havana	5.257952599	5.108915932	5.406989265	5.007688468	5.129725469	5.189333861	5.333449009	5.425809131	5.461709654
ENSG00000123191	0.003804	0.031249946	0.437411079	1.354172079	**ATP7B**	protein_coding	ATPase copper transporting beta [Source:HGNC Symbol;Acc:HGNC:870]	chr13	51930436	52012125	-1	ensembl_havana	4.394039411	4.175333871	4.61274495	4.047618066	4.118270295	4.360113253	4.636087582	4.545190308	4.656956961
ENSG00000087586	0.003802	0.031249946	-0.369057275	-1.291508622	**AURKA**	protein_coding	aurora kinase A [Source:HGNC Symbol;Acc:HGNC:11393]	chr20	56369389	56392337	-1	ensembl_havana	6.481575042	6.66610368	6.297046405	6.366079477	6.810146212	6.82208535	6.085044413	6.463740028	6.342354773
ENSG00000128581	0.003809	0.031254732	0.383907279	1.304871082	**IFT22**	protein_coding	intraflagellar transport 22 [Source:HGNC Symbol;Acc:HGNC:21895]	chr7	101313367	101321823	-1	ensembl_havana	4.508824064	4.316870424	4.700777703	4.155162249	4.430513763	4.364935261	4.660911184	4.7161429	4.725279026
ENSG00000009335	0.003808	0.031254732	-0.29874785	-1.230076337	**UBE3C**	protein_coding	ubiquitin protein ligase E3C [Source:HGNC Symbol;Acc:HGNC:16803]	chr7	157138913	157269372	1	ensembl_havana	7.119159667	7.268533592	6.969785742	7.398847457	7.219280409	7.187472911	7.068351467	6.927678639	6.91332712
ENSG00000104689	0.003811	0.031254732	-0.406710799	-1.325659994	**TNFRSF10A**	protein_coding	TNF receptor superfamily member 10a [Source:HGNC Symbol;Acc:HGNC:11904]	chr8	23190452	23225126	-1	ensembl_havana	3.631122135	3.834477535	3.427766736	4.132634943	3.751874727	3.618922935	3.648411535	3.491900287	3.142988385
ENSG00000214176	0.00382	0.031274115	0.454626161	1.370427646	**PLEKHM1P1**	transcribed_unprocessed_pseudogene	pleckstrin homology and RUN domain containing M1 pseudogene 1 [Source:HGNC Symbol;Acc:HGNC:35411]	chr17	64779259	64837154	-1	havana	5.329002132	5.101689051	5.556315212	5.038859572	4.928052069	5.338155514	5.622796123	5.427478931	5.618670583
ENSG00000175581	0.003818	0.031274115	0.332199434	1.258931193	**MRPL48**	protein_coding	mitochondrial ribosomal protein L48 [Source:HGNC Symbol;Acc:HGNC:16653]	chr11	73787316	73865133	1	ensembl_havana	4.013805687	3.84770597	4.179905404	3.820465923	3.847920646	3.874731339	4.070484623	4.214772063	4.254459527
ENSG00000206535	0.003816	0.031274115	-0.343136889	-1.268511755	**LNP1**	protein_coding	leukemia NUP98 fusion partner 1 [Source:HGNC Symbol;Acc:HGNC:28014]	chr3	100401193	100456319	1	ensembl_havana	1.933645686	2.10521413	1.762077241	1.968722995	2.093349987	2.253569408	1.693402841	1.660283167	1.932545716
ENSG00000007171	0.003824	0.031293298	0.526263696	1.440194534	**NOS2**	protein_coding	nitric oxide synthase 2 [Source:HGNC Symbol;Acc:HGNC:7873]	chr17	27756766	27800499	-1	ensembl_havana	0.288665991	0.025534143	0.551797839	-0.51029874	0.125688576	0.461212593	0.106248156	0.426254992	1.12289037
ENSG00000013306	0.003829	0.031296711	0.353394073	1.277562678	**SLC25A39**	protein_coding	solute carrier family 25 member 39 [Source:HGNC Symbol;Acc:HGNC:24279]	chr17	44319625	44324870	-1	ensembl_havana	7.035992195	6.859295159	7.212689232	6.766007912	6.833210628	6.978666937	7.194585056	7.210924492	7.232558147
ENSG00000255857	0.003831	0.031296711	-0.295506216	-1.227315545	**PXN-AS1**	antisense	PXN antisense RNA 1 [Source:HGNC Symbol;Acc:HGNC:44123]	chr12	120201291	120213138	1	havana	2.557621883	2.705374991	2.409868775	2.62003612	2.714288775	2.781800078	2.349154471	2.413134925	2.467316929
ENSG00000177432	0.00383	0.031296711	-0.980999422	-1.973832302	**NAP1L5**	protein_coding	nucleosome assembly protein 1 like 5 [Source:HGNC Symbol;Acc:HGNC:19968]	chr4	88695915	88697829	-1	havana	1.580406673	2.070906384	1.089906962	2.643402293	2.108942002	1.460374858	1.325353043	0.993426194	0.950941648
ENSG00000104679	0.003838	0.031316964	0.360963172	1.284283024	**R3HCC1**	protein_coding	R3H domain and coiled-coil containing 1 [Source:HGNC Symbol;Acc:HGNC:27329]	chr8	23270120	23296279	1	ensembl_havana	4.613644095	4.433162509	4.794125681	4.456635069	4.383211018	4.45964144	4.919346995	4.731093453	4.731936594
ENSG00000250722	0.00384	0.031316964	0.35896945	1.282509445	**SELENOP**	protein_coding	selenoprotein P [Source:HGNC Symbol;Acc:HGNC:10751]	chr5	42799880	42887392	-1	havana	1.411661465	1.232176739	1.59114619	1.158893785	1.311578958	1.226057475	1.444517675	1.637380031	1.691540863
ENSG00000153208	0.00384	0.031316964	0.301776608	1.232661441	**MERTK**	protein_coding	MER proto-oncogene, tyrosine kinase [Source:HGNC Symbol;Acc:HGNC:7027]	chr2	111898479	112029561	1	ensembl_havana	4.93372994	4.782841636	5.084618244	4.765652227	4.826793299	4.756079381	5.075214401	5.089605981	5.089034349
ENSG00000186665	0.003843	0.03132123	0.380416927	1.301717987	**C17orf58**	protein_coding	chromosome 17 open reading frame 58 [Source:HGNC Symbol;Acc:HGNC:27568]	chr17	67991101	67996431	-1	ensembl_havana	5.889031234	5.698822771	6.079239698	5.847797803	5.762282375	5.486388134	6.200657915	6.0489717	5.98808948
ENSG00000121039	0.00386	0.031418799	0.439633096	1.356259361	**RDH10**	protein_coding	retinol dehydrogenase 10 [Source:HGNC Symbol;Acc:HGNC:19975]	chr8	73294612	73325281	1	ensembl_havana	4.068046449	3.848229901	4.287862997	3.860895981	3.966065478	3.717728245	4.205267587	4.329953957	4.328367446
ENSG00000253658	0.003858	0.031418799	-0.640284191	-1.558636159	**LINC01592**	lincRNA	long intergenic non-protein coding RNA 1592 [Source:HGNC Symbol;Acc:HGNC:51557]	chr8	68911803	69104190	-1	havana	0.701277514	1.02141961	0.381135419	1.327057633	1.013233389	0.723967808	0.399634207	0.45273451	0.291037539
ENSG00000188001	0.003865	0.031442915	-0.488637942	-1.403119555	**TPRG1**	protein_coding	tumor protein p63 regulated 1 [Source:HGNC Symbol;Acc:HGNC:24759]	chr3	188947214	189325304	1	ensembl_havana	5.600980369	5.84529934	5.356661398	5.506882745	5.886217425	6.14279785	4.881564614	5.597312611	5.59110697
ENSG00000168743	0.003874	0.031482226	1.347328584	2.544405454	**NPNT**	protein_coding	nephronectin [Source:HGNC Symbol;Acc:HGNC:27405]	chr4	105894775	106004027	1	ensembl_havana	-0.432505857	-1.10617015	0.241158435	-1.61858537	-0.428075095	-1.271849989	-0.08686711	0.256279753	0.554062661
ENSG00000170145	0.003872	0.031482226	0.295565855	1.227366281	**SIK2**	protein_coding	salt inducible kinase 2 [Source:HGNC Symbol;Acc:HGNC:21680]	chr11	111602391	111730853	1	ensembl_havana	5.780321767	5.63253884	5.928104695	5.553109926	5.654184297	5.690322297	5.872355011	5.924425867	5.987533207
ENSG00000260947	0.003881	0.031492704	1.158279719	2.23191134	**AL356489.2**	lincRNA		chr9	33697459	33700986	1	havana	1.530986935	0.951847076	2.110126795	0.267803752	1.214022341	1.373715134	1.996172808	2.195213363	2.138994213
ENSG00000196358	0.003885	0.031492704	0.641941965	1.560428188	**NTNG2**	protein_coding	netrin G2 [Source:HGNC Symbol;Acc:HGNC:14288]	chr9	132161676	132244534	1	ensembl_havana	3.509175808	3.188204825	3.83014679	2.926598135	3.00836839	3.629647951	3.721029149	3.795851755	3.973559468
ENSG00000128607	0.003884	0.031492704	0.324891954	1.252570628	**KLHDC10**	protein_coding	kelch domain containing 10 [Source:HGNC Symbol;Acc:HGNC:22194]	chr7	130070510	130135720	1	ensembl_havana	6.020150907	5.85770493	6.182596884	5.845910675	5.885651786	5.841552328	6.216966213	6.239718255	6.091106184
ENSG00000172175	0.003887	0.031492704	0.297035386	1.228617115	**MALT1**	protein_coding	MALT1 paracaspase [Source:HGNC Symbol;Acc:HGNC:6819]	chr18	58671386	58754477	1	ensembl_havana	7.778279503	7.62976181	7.926797195	7.774965426	7.609466048	7.504853956	8.039371883	7.91865611	7.822363593
ENSG00000033170	0.003888	0.031492704	-0.42710905	-1.34453662	**FUT8**	protein_coding	fucosyltransferase 8 [Source:HGNC Symbol;Acc:HGNC:4019]	chr14	65410592	65744121	1	ensembl_havana	7.703147009	7.916701534	7.489592484	8.122062831	7.877400489	7.750641281	7.541013769	7.574141607	7.353622077
ENSG00000239569	0.003882	0.031492704	-0.67173531	-1.592987902	**KMT2E-AS1**	antisense	KMT2E antisense RNA 1 (head to head) [Source:HGNC Symbol;Acc:HGNC:40845]	chr7	105013425	105014321	-1	havana	1.87960346	2.215471115	1.543735805	2.253214515	2.139629423	2.253569408	1.88468023	1.241396536	1.505130649
ENSG00000074621	0.003892	0.031507746	-0.77977501	-1.716863105	**SLC24A1**	protein_coding	solute carrier family 24 member 1 [Source:HGNC Symbol;Acc:HGNC:10975]	chr15	65611366	65660995	1	ensembl_havana	1.74085526	2.130742765	1.350967755	1.81257944	2.206378717	2.373270137	0.873137292	1.210694826	1.969071146
ENSG00000140406	0.0039	0.031516745	-0.293687432	-1.225769262	**TLNRD1**	protein_coding	talin rod domain containing 1 [Source:HGNC Symbol;Acc:HGNC:13519]	chr15	81000944	81005788	1	ensembl_havana	2.479174012	2.626017728	2.332330296	2.516278079	2.913888608	2.447886498	2.22979561	2.596492542	2.170702736
ENSG00000178127	0.003896	0.031516745	-0.388348665	-1.308894362	**NDUFV2**	protein_coding	NADH:ubiquinone oxidoreductase core subunit V2 [Source:HGNC Symbol;Acc:HGNC:7717]	chr18	9102630	9134345	1	ensembl_havana	1.076869813	1.271044145	0.882695481	1.625778111	0.89230127	1.295053056	1.325353043	0.37179175	0.950941648
ENSG00000177721	0.003899	0.031516745	-0.490704794	-1.405131151	**ANXA2R**	protein_coding	annexin A2 receptor [Source:HGNC Symbol;Acc:HGNC:33463]	chr5	43039081	43043170	-1	ensembl_havana	1.833297604	2.078650002	1.587945207	2.150951608	1.873642861	2.211355536	1.619344613	1.578479109	1.566011899
ENSG00000153107	0.003913	0.031603832	-0.321617688	-1.249731081	**ANAPC1**	protein_coding	anaphase promoting complex subunit 1 [Source:HGNC Symbol;Acc:HGNC:19988]	chr2	111611639	111884690	-1	ensembl_havana	6.126261707	6.287070551	5.965452864	6.182869999	6.245320117	6.433021537	5.80016806	5.988608005	6.107582527
ENSG00000144893	0.003919	0.031634322	0.297355108	1.228889425	**MED12L**	protein_coding	mediator complex subunit 12 like [Source:HGNC Symbol;Acc:HGNC:16050]	chr3	151085697	151437072	1	ensembl_havana	4.29463337	4.145955816	4.443310924	3.840822574	4.26292479	4.334120086	4.173273167	4.545190308	4.611469298
ENSG00000158859	0.003926	0.03164245	0.834210786	1.782881462	**ADAMTS4**	protein_coding	ADAM metallopeptidase with thrombospondin type 1 motif 4 [Source:HGNC Symbol;Acc:HGNC:220]	chr1	161184308	161199056	-1	ensembl_havana	0.089559757	-0.327545636	0.50666515	-0.61730533	-0.620350959	0.255019383	-0.08686711	0.602150945	1.004711615
ENSG00000168887	0.003924	0.03164245	0.361509435	1.284769398	**C2orf68**	protein_coding	chromosome 2 open reading frame 68 [Source:HGNC Symbol;Acc:HGNC:34353]	chr2	85606654	85612066	-1	ensembl_havana	5.227329903	5.046575186	5.408084621	4.975828975	5.135446456	5.028450127	5.411405351	5.386001929	5.426846581
ENSG00000143498	0.003927	0.03164245	-0.362228713	-1.2854101	**TAF1A**	protein_coding	TATA-box binding protein associated factor, RNA polymerase I subunit A [Source:HGNC Symbol;Acc:HGNC:11532]	chr1	222557902	222589933	-1	ensembl_havana	3.524771697	3.705886054	3.34365734	3.722389648	3.744406796	3.650861717	3.24666556	3.433270566	3.351035895
ENSG00000136279	0.003933	0.031658969	0.343648434	1.268961619	**DBNL**	protein_coding	drebrin like [Source:HGNC Symbol;Acc:HGNC:2696]	chr7	44044640	44069456	1	ensembl_havana	7.665952289	7.494128072	7.837776506	7.40291774	7.486347523	7.593118954	7.811865288	7.859273728	7.842190503
ENSG00000273899	0.003931	0.031658969	0.337441893	1.263514213	**NOL12**	protein_coding	nucleolar protein 12 [Source:HGNC Symbol;Acc:HGNC:28585]	chr22	37681673	37693478	1	ensembl_havana	1.50780617	1.339085224	1.676527116	1.397462791	1.298039728	1.321753151	1.740743887	1.554228498	1.734608964
ENSG00000139116	0.003943	0.031690828	0.314500433	1.24358096	**KIF21A**	protein_coding	kinesin family member 21A [Source:HGNC Symbol;Acc:HGNC:19349]	chr12	39293228	39443390	-1	ensembl_havana	6.34435512	6.187104904	6.501605337	5.949721265	6.263285259	6.348308187	6.328175412	6.554024508	6.62261609
ENSG00000133026	0.003945	0.031690828	0.296854757	1.228463299	**MYH10**	protein_coding	myosin heavy chain 10 [Source:HGNC Symbol;Acc:HGNC:7568]	chr17	8474205	8630761	-1	ensembl_havana	5.346218495	5.197791117	5.494645874	5.112916487	5.202385136	5.278071728	5.44305639	5.472657064	5.568224166
ENSG00000156471	0.003946	0.031690828	-0.306754763	-1.236922202	**PTDSS1**	protein_coding	phosphatidylserine synthase 1 [Source:HGNC Symbol;Acc:HGNC:9587]	chr8	96261715	96336995	1	ensembl_havana	7.497860982	7.651238364	7.3444836	7.82834772	7.516071806	7.609295565	7.502047738	7.175839706	7.355563357
ENSG00000171840	0.003943	0.031690828	-0.504185941	-1.418322823	**NINJ2**	protein_coding	ninjurin 2 [Source:HGNC Symbol;Acc:HGNC:7825]	chr12	564296	663779	-1	ensembl_havana	2.429955565	2.682048535	2.177862595	2.807536027	2.619498965	2.619110614	2.099663825	2.278988373	2.154935587
ENSG00000169951	0.00395	0.031702454	0.291377212	1.22380798	**ZNF764**	protein_coding	zinc finger protein 764 [Source:HGNC Symbol;Acc:HGNC:28200]	chr16	30553764	30558498	-1	ensembl_havana	2.533360761	2.387672155	2.679049367	2.490740384	2.357615216	2.314660866	2.798325219	2.631684122	2.60713876
ENSG00000131016	0.003954	0.031702454	-0.37955746	-1.300942736	**AKAP12**	protein_coding	A-kinase anchoring protein 12 [Source:HGNC Symbol;Acc:HGNC:370]	chr6	151239999	151358557	1	ensembl_havana	6.389781169	6.579559899	6.200002439	6.459957405	6.604843949	6.673878342	6.209098117	6.16211129	6.228797909
ENSG00000151876	0.003954	0.031702454	-0.636591334	-1.55465163	**FBXO4**	protein_coding	F-box protein 4 [Source:HGNC Symbol;Acc:HGNC:13583]	chr5	41925254	41941743	1	ensembl_havana	2.932396786	3.250692453	2.614101119	3.22260331	3.418140435	3.111333615	2.44516827	2.625878149	2.771256937
ENSG00000187210	0.003959	0.031715164	0.464241034	1.379591394	**GCNT1**	protein_coding	glucosaminyl (N-acetyl) transferase 1, core 2 [Source:HGNC Symbol;Acc:HGNC:4203]	chr9	76419850	76507416	1	ensembl_havana	3.308594966	3.076474449	3.540715483	2.666396046	3.241966183	3.321061117	3.318633557	3.587603785	3.715909107
ENSG00000168772	0.00396	0.031715164	0.399979898	1.319489525	**CXXC4**	protein_coding	CXXC finger protein 4 [Source:HGNC Symbol;Acc:HGNC:24593]	chr4	104468312	104494901	-1	ensembl_havana	-0.618128956	-0.818118905	-0.418139007	-1.51148723	-0.783495335	-0.159374151	-0.97139419	-0.42206346	0.139040636
ENSG00000280214	0.00397	0.031740571	0.647091516	1.566007927	**AC027682.7**	TEC		chr16	67550815	67552935	-1	havana	0.136308148	-0.18723761	0.459853906	-0.4596317	0.09469996	-0.196781087	0.428834256	0.779979312	0.17074815
ENSG00000173041	0.003969	0.031740571	-0.318194016	-1.24676885	**ZNF680**	protein_coding	zinc finger protein 680 [Source:HGNC Symbol;Acc:HGNC:26897]	chr7	64519884	64563106	-1	ensembl_havana	5.497471011	5.656568019	5.338374004	5.438402115	5.685719046	5.845582897	5.110559324	5.432476768	5.472085918
ENSG00000111331	0.003968	0.031740571	-0.666130339	-1.586811039	**OAS3**	protein_coding	2'-5'-oligoadenylate synthetase 3 [Source:HGNC Symbol;Acc:HGNC:8088]	chr12	112938352	112973249	1	ensembl_havana	5.396582478	5.729647648	5.063517308	5.448386336	5.840250589	5.900306018	5.006665429	4.871423222	5.312463274
ENSG00000143157	0.003975	0.031765549	-0.292269453	-1.224565083	**POGK**	protein_coding	pogo transposable element derived with KRAB domain [Source:HGNC Symbol;Acc:HGNC:18800]	chr1	166839447	166856344	1	ensembl_havana	7.398828545	7.544963272	7.252693819	7.573793746	7.546806852	7.514289216	7.293892098	7.266556937	7.197632422
ENSG00000104957	0.003977	0.031766005	0.32835205	1.255578343	**CCDC130**	protein_coding	coiled-coil domain containing 130 [Source:HGNC Symbol;Acc:HGNC:28118]	chr19	13731760	13763296	1	ensembl_havana	5.062873773	4.898697748	5.227049798	4.894177143	4.870899613	4.931016489	5.233562349	5.264212311	5.183374733
ENSG00000279879	0.003986	0.031784208	0.704475864	1.629552535	**AC091152.4**	TEC		chr17	44689858	44693340	1	havana	-0.721599722	-1.073837654	-0.36936179	-1.86005921	-0.672709	-0.688744748	-0.89704638	-0.28449416	0.073455173
ENSG00000108100	0.003983	0.031784208	0.35100202	1.275446178	**CCNY**	protein_coding	cyclin Y [Source:HGNC Symbol;Acc:HGNC:23354]	chr10	35247025	35572669	1	ensembl_havana	6.347570828	6.172069818	6.523071838	6.026151348	6.311671382	6.178386724	6.428714569	6.557841179	6.582659766
ENSG00000160050	0.003985	0.031784208	-0.358385353	-1.281990306	**CCDC28B**	protein_coding	coiled-coil domain containing 28B [Source:HGNC Symbol;Acc:HGNC:28163]	chr1	32200386	32205387	1	ensembl_havana	5.332713359	5.511906035	5.153520682	5.550794794	5.567263407	5.417659904	5.235629383	5.27630862	4.948624044
ENSG00000170231	0.003994	0.03182557	-0.350313913	-1.274837986	**FABP6**	protein_coding	fatty acid binding protein 6 [Source:HGNC Symbol;Acc:HGNC:3561]	chr5	160187367	160238735	1	ensembl_havana	0.214418141	0.389575098	0.039261185	0.004630787	0.679062629	0.485031876	-0.30988669	0.225896484	0.201773757
ENSG00000130021	0.004003	0.03185144	0.623716483	1.540839383	**PUDP**	protein_coding	pseudouridine 5'-phosphatase [Source:HGNC Symbol;Acc:HGNC:16818]	chrX	7048920	7148190	-1	ensembl_havana	2.978160474	2.666302233	3.290018716	2.312722596	2.749532556	2.936651547	3.226020416	3.245359567	3.398676164
ENSG00000124356	0.004004	0.03185144	-0.291721489	-1.224100059	**STAMBP**	protein_coding	STAM binding protein [Source:HGNC Symbol;Acc:HGNC:16950]	chr2	73828916	73873659	1	ensembl_havana	6.253897505	6.399758249	6.10803676	6.353737286	6.448943	6.396594462	6.039627286	6.160104052	6.124378943
ENSG00000160190	0.004002	0.03185144	-0.314235107	-1.243352274	**SLC37A1**	protein_coding	solute carrier family 37 member 1 [Source:HGNC Symbol;Acc:HGNC:11024]	chr21	42496008	42581440	1	ensembl_havana	4.640447978	4.797565531	4.483330424	4.853429997	4.699982239	4.839284357	4.602844617	4.368710712	4.478435943
ENSG00000148985	0.004007	0.031859605	0.33368623	1.260229276	**PGAP2**	protein_coding	post-GPI attachment to proteins 2 [Source:HGNC Symbol;Acc:HGNC:17893]	chr11	3797724	3826371	1	ensembl_havana	3.603719043	3.436875928	3.770562157	3.474621708	3.337712124	3.498293951	3.874828493	3.587603785	3.849254195
ENSG00000140835	0.00401	0.031869308	-0.773898552	-1.709884116	**CHST4**	protein_coding	carbohydrate sulfotransferase 4 [Source:HGNC Symbol;Acc:HGNC:1972]	chr16	71525233	71538746	1	ensembl_havana	1.833674821	2.220624098	1.446725545	1.671956805	2.242169235	2.747746253	1.033805522	1.705026699	1.601344415
ENSG00000230989	0.004013	0.031876911	0.342505692	1.267956887	**HSBP1**	protein_coding	heat shock factor binding protein 1 [Source:HGNC Symbol;Acc:HGNC:5203]	chr16	83807843	83819737	1	ensembl_havana	6.441665405	6.270412559	6.612918251	6.192307603	6.358894812	6.260035261	6.559884119	6.6072972	6.671573434
ENSG00000144395	0.004024	0.031939496	-0.294609815	-1.226553204	**CCDC150**	protein_coding	coiled-coil domain containing 150 [Source:HGNC Symbol;Acc:HGNC:26834]	chr2	196639554	196763490	1	ensembl_havana	4.937062268	5.084367176	4.78975736	5.129559428	5.191442156	4.932099943	4.845475607	4.864043597	4.659752877
ENSG00000136738	0.004027	0.031946238	0.376838994	1.298493682	**STAM**	protein_coding	signal transducing adaptor molecule [Source:HGNC Symbol;Acc:HGNC:11357]	chr10	17644125	17715914	1	ensembl_havana	5.052407987	4.86398849	5.240827484	4.628943975	4.979849994	4.9831715	5.129580575	5.322807524	5.270094353
ENSG00000088451	0.004031	0.031962195	-0.393194825	-1.313298464	**TGDS**	protein_coding	TDP-glucose 4,6-dehydratase [Source:HGNC Symbol;Acc:HGNC:20324]	chr13	94574051	94596257	-1	ensembl_havana	4.277910485	4.474507897	4.081313072	4.558479799	4.477816603	4.38722729	4.075109324	4.035871058	4.132958835
ENSG00000131652	0.004037	0.031975054	-0.388028942	-1.308604323	**THOC6**	protein_coding	THO complex 6 [Source:HGNC Symbol;Acc:HGNC:28369]	chr16	3024027	3027755	1	ensembl_havana	4.255422523	4.449436994	4.061408052	4.445046788	4.422736894	4.480527301	4.153730304	4.070480875	3.960012977
ENSG00000254649	0.004035	0.031975054	-0.543195489	-1.457196555	**AP003086.3**	antisense		chr11	78388061	78392405	1	havana	-0.151621114	0.11997663	-0.423218859	-0.18813307	0.06303108	0.485031876	-0.51701327	-0.68524259	-0.067400719
ENSG00000121933	0.004041	0.031988857	-0.406820271	-1.325760589	**TMIGD3**	protein_coding	transmembrane and immunoglobulin domain containing 3 [Source:HGNC Symbol;Acc:HGNC:51375]	chr1	111483348	111563962	-1	ensembl_havana	2.094375419	2.297785555	1.890965283	2.397621087	2.154731774	2.341003803	2.034670322	1.85152878	1.786696749
ENSG00000168101	0.004049	0.032019615	0.521319833	1.435267686	**NUDT16L1**	protein_coding	nudix hydrolase 16 like 1 [Source:HGNC Symbol;Acc:HGNC:28154]	chr16	4693694	4695859	1	ensembl_havana	3.118051104	2.857391187	3.37871102	2.765560239	2.922737966	2.883875356	3.515217767	3.315571274	3.305344019
ENSG00000227354	0.004076	0.032019615	0.393438394	1.313520206	**RBM26-AS1**	antisense	RBM26 antisense RNA 1 [Source:HGNC Symbol;Acc:HGNC:39805]	chr13	79406309	79424328	1	havana	1.38270745	1.185988253	1.579426647	1.109412078	1.324992307	1.123560375	1.593788933	1.578479109	1.566011899
ENSG00000163719	0.004059	0.032019615	0.372260425	1.294379286	**MTMR14**	protein_coding	myotubularin related protein 14 [Source:HGNC Symbol;Acc:HGNC:26190]	chr3	9649433	9702393	1	ensembl_havana	6.267799809	6.081669596	6.453930022	5.983873229	6.153387691	6.10774787	6.480182914	6.45148251	6.430124641
ENSG00000106012	0.004067	0.032019615	0.359369075	1.282864748	**IQCE**	protein_coding	IQ motif containing E [Source:HGNC Symbol;Acc:HGNC:29171]	chr7	2558972	2614734	1	ensembl_havana	5.463911523	5.284226985	5.64359606	5.365630383	5.213245738	5.273804834	5.838074075	5.528961101	5.563753005
ENSG00000165030	0.004062	0.032019615	0.358566813	1.282151563	**NFIL3**	protein_coding	nuclear factor, interleukin 3 regulated [Source:HGNC Symbol;Acc:HGNC:7787]	chr9	91409045	91423862	-1	ensembl_havana	5.117481318	4.938197912	5.296764725	4.883177929	4.907023696	5.024392109	5.260205505	5.159594251	5.470494419
ENSG00000186687	0.004074	0.032019615	0.343723215	1.269027397	**LYRM7**	protein_coding	LYR motif containing 7 [Source:HGNC Symbol;Acc:HGNC:28072]	chr5	131170810	131205426	1	ensembl_havana	4.771833403	4.599971795	4.943695011	4.464855791	4.658202087	4.676857507	4.834606865	4.905370983	5.091107184
ENSG00000080819	0.004082	0.032019615	0.343499298	1.268830449	**CPOX**	protein_coding	coproporphyrinogen oxidase [Source:HGNC Symbol;Acc:HGNC:2321]	chr3	98521132	98593723	-1	ensembl_havana	8.064006941	7.892257292	8.23575659	7.596979981	7.926760201	8.153031695	7.873396364	8.367865862	8.466007544
ENSG00000099622	0.004076	0.032019615	-0.29353088	-1.225636256	**CIRBP**	protein_coding	cold inducible RNA binding protein [Source:HGNC Symbol;Acc:HGNC:1982]	chr19	1259384	1274880	1	ensembl_havana	7.716157447	7.862922887	7.569392007	7.923278095	7.856625554	7.808865013	7.610558056	7.596207927	7.501410038
ENSG00000167325	0.004077	0.032019615	-0.300682741	-1.231727178	**RRM1**	protein_coding	ribonucleotide reductase catalytic subunit M1 [Source:HGNC Symbol;Acc:HGNC:10451]	chr11	4094707	4138876	1	ensembl_havana	7.115071937	7.265413307	6.964730566	7.089998494	7.375093205	7.331148222	6.83367688	7.036720591	7.023794227
ENSG00000148773	0.004054	0.032019615	-0.332799206	-1.259454678	**MKI67**	protein_coding	marker of proliferation Ki-67 [Source:HGNC Symbol;Acc:HGNC:7107]	chr10	128096659	128126385	-1	ensembl_havana	8.467155398	8.633555001	8.300755795	8.460375712	8.616977827	8.823311466	8.154736355	8.343640397	8.403890632
ENSG00000214274	0.004083	0.032019615	-0.378280205	-1.299791488	**ANG**	protein_coding	angiogenin [Source:HGNC Symbol;Acc:HGNC:483]	chr14	20684177	20698971	1	ensembl_havana	1.642979145	1.832119248	1.453839043	2.183196095	1.759729761	1.553431888	1.740743887	1.315408538	1.305364703
ENSG00000179218	0.004059	0.032019615	-0.399776274	-1.319303304	**CALR**	protein_coding	calreticulin [Source:HGNC Symbol;Acc:HGNC:1455]	chr19	12938578	12944489	1	ensembl_havana	10.27650646	10.47639459	10.07661832	10.661484	10.48307001	10.28462977	10.22931636	9.975727866	10.02481073
ENSG00000143971	0.004073	0.032019615	-0.44777511	-1.363935206	**ETAA1**	protein_coding	ETAA1, ATR kinase activator [Source:HGNC Symbol;Acc:HGNC:24648]	chr2	67397319	67412089	1	ensembl_havana	5.549745976	5.773633531	5.325858421	5.833901194	5.791563583	5.695435817	5.206417886	5.413223639	5.357933737
ENSG00000266844	0.004078	0.032019615	-0.466247572	-1.381511501	**AC093330.1**	antisense		chr18	76974690	76984162	1	havana	-0.292231841	-0.059108055	-0.525355627	-0.36334239	-0.299013651	0.485031876	-0.89704638	-0.57414696	-0.104873545
ENSG00000138792	0.00405	0.032019615	-0.525484776	-1.439417173	**ENPEP**	protein_coding	glutamyl aminopeptidase [Source:HGNC Symbol;Acc:HGNC:3355]	chr4	110365733	110565285	1	ensembl_havana	1.907014124	2.169756512	1.644271736	2.275818664	2.220801645	2.012649227	1.593788933	1.625790657	1.713235619
ENSG00000175395	0.00408	0.032019615	-0.528366393	-1.442295117	**ZNF25**	protein_coding	zinc finger protein 25 [Source:HGNC Symbol;Acc:HGNC:13043]	chr10	37949572	37976633	-1	ensembl_havana	2.335787347	2.599970544	2.07160415	2.516278079	2.580886153	2.702747398	1.863449533	2.286368471	2.064994446
ENSG00000236408	0.004071	0.032019615	-0.594854329	-1.510320079	**AC073336.1**	antisense		chr7	154838388	154865483	-1	havana	0.003225037	0.300652201	-0.294202128	0.267803752	0.125688576	0.508464275	-0.57379957	-0.2414061	-0.067400719
ENSG00000135314	0.004091	0.032047193	0.349873734	1.274449081	**KHDC1**	protein_coding	KH domain containing 1 [Source:HGNC Symbol;Acc:HGNC:21366]	chr6	73241314	73310365	-1	ensembl_havana	2.612164701	2.437227835	2.787101568	2.334420173	2.458430599	2.518832732	2.758627316	2.836600654	2.766076734
ENSG00000141569	0.004091	0.032047193	-0.409776047	-1.328479575	**TRIM65**	protein_coding	tripartite motif containing 65 [Source:HGNC Symbol;Acc:HGNC:27316]	chr17	75880335	75897003	-1	ensembl_havana	4.873745188	5.078633211	4.668857164	4.825611491	5.108552257	5.301735885	4.545678644	4.706547606	4.754345243
ENSG00000145087	0.004096	0.032054169	0.333301186	1.259892976	**STXBP5L**	protein_coding	syntaxin binding protein 5 like [Source:HGNC Symbol;Acc:HGNC:30757]	chr3	120908072	121424761	1	ensembl_havana	3.638678321	3.472027728	3.805328915	3.380421283	3.452156355	3.583505546	3.729854637	3.854126336	3.832005771
ENSG00000184743	0.004098	0.032054169	-0.312117905	-1.241528952	**ATL3**	protein_coding	atlastin GTPase 3 [Source:HGNC Symbol;Acc:HGNC:24526]	chr11	63624087	63671921	-1	ensembl_havana	5.85450802	6.010566973	5.698449067	6.00938848	6.031358083	5.990954354	5.635376184	5.769133193	5.690837825
ENSG00000102531	0.004098	0.032054169	-0.365409588	-1.288247317	**FNDC3A**	protein_coding	fibronectin type III domain containing 3A [Source:HGNC Symbol;Acc:HGNC:20296]	chr13	48975912	49209779	1	ensembl_havana	4.951255104	5.133959898	4.76855031	5.399476077	5.033942152	4.968461464	4.938508117	4.778966731	4.588176082
ENSG00000198133	0.004106	0.032096842	-0.558490881	-1.472727877	**TMEM229B**	protein_coding	transmembrane protein 229B [Source:HGNC Symbol;Acc:HGNC:20130]	chr14	67447084	67533739	-1	ensembl_havana	2.010660927	2.289906368	1.731415486	2.214735639	2.494491223	2.160492241	1.841901733	1.693970236	1.65837449
ENSG00000131697	0.004114	0.032138356	0.333288528	1.259881921	**NPHP4**	protein_coding	nephrocystin 4 [Source:HGNC Symbol;Acc:HGNC:19104]	chr1	5862811	5992473	-1	ensembl_havana	4.638604658	4.471960394	4.805248921	4.528987771	4.324321165	4.562572245	4.807071289	4.747228368	4.861447107
ENSG00000260686	0.004118	0.032155875	0.973185269	1.963170219	**AC008669.1**	sense_overlapping		chr5	122832356	122834533	1	havana	1.147368093	0.660775459	1.633960728	0.986893817	0.740413176	0.255019383	1.527846157	1.726888623	1.647147403
ENSG00000260070	0.004126	0.032199651	0.820265075	1.765730391	**AC006960.3**	antisense		chr7	36319775	36320479	1	havana	-0.261470719	-0.671603256	0.148661818	-0.51029874	-1.104596936	-0.399914092	-0.04612063	0.063570082	0.428535999
ENSG00000163629	0.004132	0.03221091	0.370364713	1.292679578	**PTPN13**	protein_coding	protein tyrosine phosphatase, non-receptor type 13 [Source:HGNC Symbol;Acc:HGNC:9646]	chr4	86594315	86815171	1	ensembl_havana	5.563712838	5.378530482	5.748895195	5.339938118	5.322602517	5.473050812	5.74971429	5.775702898	5.721268396
ENSG00000184678	0.004131	0.03221091	0.328364278	1.255588985	**HIST2H2BE**	protein_coding	histone cluster 2 H2B family member e [Source:HGNC Symbol;Acc:HGNC:4760]	chr1	149884459	149886652	-1	havana	4.583020049	4.41883791	4.747202188	4.519547838	4.293949556	4.443016337	4.807071289	4.707922275	4.726613
ENSG00000272143	0.004139	0.032245742	-0.374548409	-1.296433683	**FGF14-AS2**	lincRNA	FGF14 antisense RNA 2 [Source:HGNC Symbol;Acc:HGNC:44368]	chr13	102394630	102395703	1	havana	0.850782404	1.038056609	0.6635082	0.968509893	0.962641309	1.183018625	0.540060475	0.715834679	0.734629445
ENSG00000134461	0.004146	0.032260348	0.335495032	1.261810299	**ANKRD16**	protein_coding	ankyrin repeat domain 16 [Source:HGNC Symbol;Acc:HGNC:23471]	chr10	5861617	5889906	-1	ensembl_havana	2.227641927	2.059894411	2.395389443	1.940704441	2.162224075	2.076754718	2.221475976	2.459573709	2.505118643
ENSG00000205269	0.004145	0.032260348	0.327407719	1.25475676	**TMEM170B**	protein_coding	transmembrane protein 170B [Source:HGNC Symbol;Acc:HGNC:34244]	chr6	11538278	11583524	1	ensembl_havana	5.360246632	5.196542773	5.523950491	5.318323994	5.217746969	5.053557356	5.572167483	5.545201873	5.454482118
ENSG00000104823	0.004147	0.032260348	-0.422970576	-1.340685248	**ECH1**	protein_coding	enoyl-CoA hydratase 1 [Source:HGNC Symbol;Acc:HGNC:3149]	chr19	38815422	38832005	-1	ensembl_havana	6.244921353	6.456406641	6.033436065	6.405050469	6.400271785	6.56389767	6.090194021	6.03151063	5.978603545
ENSG00000055955	0.004182	0.032272607	0.557799509	1.472022282	**ITIH4**	protein_coding	inter-alpha-trypsin inhibitor heavy chain family member 4 [Source:HGNC Symbol;Acc:HGNC:6169]	chr3	52812975	52831479	-1	ensembl_havana	-0.110570074	-0.389469829	0.16832968	-0.18813307	-0.783495335	-0.196781087	0.24405081	0.02879206	0.232146171
ENSG00000127418	0.004171	0.032272607	0.48404256	1.398657343	**FGFRL1**	protein_coding	fibroblast growth factor receptor like 1 [Source:HGNC Symbol;Acc:HGNC:3693]	chr4	1009936	1026897	1	ensembl_havana	1.439164795	1.197143515	1.681186075	1.058172782	1.109387295	1.423870469	1.717267538	1.602328815	1.723961872
ENSG00000067082	0.004177	0.032272607	0.391325359	1.311597775	**KLF6**	protein_coding	Kruppel like factor 6 [Source:HGNC Symbol;Acc:HGNC:2235]	chr10	3775996	3785281	-1	ensembl_havana	7.448067481	7.252404801	7.64373016	7.230663826	7.166938168	7.359612409	7.764154919	7.481333486	7.685702076
ENSG00000130304	0.00418	0.032272607	0.355772117	1.279670266	**SLC27A1**	protein_coding	solute carrier family 27 member 1 [Source:HGNC Symbol;Acc:HGNC:10995]	chr19	17468769	17506168	1	ensembl_havana	2.873784462	2.695898404	3.051670521	2.716829986	2.68857687	2.682288355	3.183822111	2.970908466	3.000280985
ENSG00000100625	0.004171	0.032272607	0.315276286	1.244249913	**SIX4**	protein_coding	SIX homeobox 4 [Source:HGNC Symbol;Acc:HGNC:10890]	chr14	60709528	60724348	-1	ensembl_havana	4.406575679	4.248937536	4.564213822	4.149053241	4.383211018	4.21454835	4.535647221	4.667515227	4.489479019
ENSG00000197119	0.004163	0.032272607	0.297909148	1.229361447	**SLC25A29**	protein_coding	solute carrier family 25 member 29 [Source:HGNC Symbol;Acc:HGNC:20116]	chr14	100291111	100306547	-1	ensembl_havana	5.765283042	5.616328468	5.914237616	5.680697068	5.474767967	5.69352037	5.966215283	5.817055082	5.959442484
ENSG00000152492	0.004172	0.032272607	0.292766223	1.224986817	**CCDC50**	protein_coding	coiled-coil domain containing 50 [Source:HGNC Symbol;Acc:HGNC:18111]	chr3	191329077	191398670	1	ensembl_havana	6.854144283	6.707761171	7.000527395	6.657940177	6.814313224	6.651030113	6.956564012	7.073711441	6.971306732
ENSG00000075188	0.004175	0.032272607	-0.334851787	-1.26124783	**NUP37**	protein_coding	nucleoporin 37 [Source:HGNC Symbol;Acc:HGNC:29929]	chr12	102073103	102120124	-1	ensembl_havana	5.03334209	5.200767984	4.865916197	5.099251284	5.181337648	5.32171502	4.671638628	4.901771696	5.024338267
ENSG00000137309	0.004175	0.032272607	-0.339177093	-1.265034817	**HMGA1**	protein_coding	high mobility group AT-hook 1 [Source:HGNC Symbol;Acc:HGNC:5010]	chr6	34236873	34246231	1	ensembl_havana	7.646089754	7.815678301	7.476501208	7.690381811	7.879816693	7.876836397	7.428960958	7.450664715	7.54987795
ENSG00000173209	0.004153	0.032272607	-0.345857235	-1.270905917	**AHSA2P**	transcribed_unitary_pseudogene	activator of HSP90 ATPase homolog 2, pseudogene [Source:HGNC Symbol;Acc:HGNC:20437]	chr2	61177418	61191203	1	ensembl_havana	7.7005251	7.873453717	7.527596483	8.080670573	7.777275962	7.762414617	7.676333022	7.386655533	7.519800893
ENSG00000272525	0.004155	0.032272607	-0.380267408	-1.301583086	**AC099522.2**	lincRNA		chr5	73497550	73498293	-1	havana	0.465870046	0.65600375	0.275736342	0.660286109	0.799260866	0.508464275	0.339398945	0.286036324	0.201773757
ENSG00000161609	0.00417	0.032272607	-0.459180059	-1.374760265	**CCDC155**	protein_coding	coiled-coil domain containing 155 [Source:HGNC Symbol;Acc:HGNC:26520]	chr19	49388218	49417994	1	ensembl_havana	1.382114989	1.611705019	1.15252496	1.590147445	1.415548178	1.829419434	1.033805522	1.14725896	1.276510397

ENSG00000183508	0.004174	0.032272607	-0.472663719	-1.387669226	**FAM46C**	protein_coding	family with sequence similarity 46 member C [Source:HGNC Symbol;Acc:HGNC:24712]	chr1	117605934	117628372	1	ensembl_havana	3.419649092	3.655980951	3.183317233	3.414774672	3.651639676	3.901528505	2.89041725	3.372156889	3.287377559
ENSG00000075618	0.004181	0.032272607	-0.496519399	-1.410805787	**FSCN1**	protein_coding	fascin actin-bundling protein 1 [Source:HGNC Symbol;Acc:HGNC:11148]	chr7	5592823	5606655	1	ensembl_havana	7.173392242	7.421651941	6.925132542	7.475099072	7.382328746	7.407528006	7.164250456	6.888207506	6.722939664
ENSG00000259438	0.004177	0.032272607	-0.568682789	-1.483168788	**AC090970.2**	lincRNA		chr15	52010999	52019095	-1	havana	0.782816563	1.067157958	0.498475169	1.175017601	1.029710695	0.996745577	0.399634207	0.426254992	0.669536307
ENSG00000114302	0.004189	0.03228954	0.28584001	1.21911989	**PRKAR2A**	protein_coding	protein kinase cAMP-dependent type II regulatory subunit alpha [Source:HGNC Symbol;Acc:HGNC:9391]	chr3	48744597	48847846	-1	ensembl_havana	6.914674159	6.771754154	7.057594165	6.647866398	6.785494731	6.881901334	6.947449727	7.065960005	7.159372761
ENSG00000161999	0.004187	0.03228954	-0.418988063	-1.336989433	**JMJD8**	protein_coding	jumonji domain containing 8 [Source:HGNC Symbol;Acc:HGNC:14148]	chr16	681671	684528	-1	ensembl_havana	3.726078906	3.935572937	3.516584875	4.047618066	3.8686773	3.890423445	3.776033227	3.43659104	3.337130358
ENSG00000166845	0.004195	0.032321375	-0.360711763	-1.28405924	**C18orf54**	protein_coding	chromosome 18 open reading frame 54 [Source:HGNC Symbol;Acc:HGNC:13796]	chr18	54357917	54385218	1	ensembl_havana	5.70402439	5.884380271	5.523668508	5.592659611	5.938421094	6.122060108	5.259189815	5.663280516	5.648535195
ENSG00000080823	0.004202	0.032351464	0.580601866	1.495473002	**MOK**	protein_coding	MOK protein kinase [Source:HGNC Symbol;Acc:HGNC:9833]	chr14	102224500	102305200	-1	ensembl_havana	3.440136971	3.149836038	3.730437904	3.264647647	3.252533942	2.932326526	4.008974209	3.566541508	3.615797995
ENSG00000165724	0.004204	0.032351464	0.564558851	1.478935209	**ZMYND19**	protein_coding	zinc finger MYND-type containing 19 [Source:HGNC Symbol;Acc:HGNC:21146]	chr9	137582079	137590490	-1	ensembl_havana	4.444587454	4.162308029	4.72686688	3.848382869	4.241864646	4.396676572	4.659372159	4.75256682	4.76866166
ENSG00000121440	0.004209	0.032363232	-0.465123853	-1.380435857	**PDZRN3**	protein_coding	PDZ domain containing ring finger 3 [Source:HGNC Symbol;Acc:HGNC:17704]	chr3	73382433	73624940	-1	ensembl_havana	2.480484911	2.713046838	2.247922985	2.77087449	2.586465946	2.781800078	2.379383493	2.24151074	2.122874721
ENSG00000185585	0.00421	0.032363232	-0.838657164	-1.788384769	**OLFML2A**	protein_coding	olfactomedin like 2A [Source:HGNC Symbol;Acc:HGNC:27270]	chr9	124777158	124814885	1	ensembl_havana	3.583718741	4.003047323	3.164390159	3.587346304	3.953166093	4.468629573	3.053967167	3.210869813	3.228333496
ENSG00000197008	0.004213	0.032366333	-0.393916155	-1.313955262	**ZNF138**	protein_coding	zinc finger protein 138 [Source:HGNC Symbol;Acc:HGNC:12922]	chr7	64794388	64833681	1	ensembl_havana	4.35237425	4.549332327	4.155416172	4.382198518	4.572233536	4.693564928	4.037689841	4.271654209	4.156904467
ENSG00000185640	0.004232	0.032426285	1.068699378	2.097541533	**KRT79**	protein_coding	keratin 79 [Source:HGNC Symbol;Acc:HGNC:28930]	chr12	52821410	52834295	-1	ensembl_havana	-0.609911067	-1.144260755	-0.075561378	-1.31854494	-1.42549258	-0.688744748	-0.35894771	0.163140205	-0.030876629
ENSG00000101844	0.004228	0.032426285	0.324384279	1.252129934	**ATG4A**	protein_coding	autophagy related 4A cysteine peptidase [Source:HGNC Symbol;Acc:HGNC:16489]	chrX	108091668	108154671	1	ensembl_havana	5.518612278	5.356420139	5.680804417	5.329171533	5.480023535	5.260065348	5.657130739	5.730427405	5.654855109
ENSG00000196943	0.004231	0.032426285	0.300807177	1.231833422	**NOP9**	protein_coding	NOP9 nucleolar protein [Source:HGNC Symbol;Acc:HGNC:19826]	chr14	24299862	24309124	1	ensembl_havana	6.531094263	6.380690675	6.681497852	6.265664989	6.345383102	6.531023934	6.618482319	6.628503013	6.797508223
ENSG00000184307	0.00423	0.032426285	0.29450073	1.226460466	**ZDHHC23**	protein_coding	zinc finger DHHC-type containing 23 [Source:HGNC Symbol;Acc:HGNC:28654]	chr3	113947901	113965401	1	ensembl_havana	6.290879766	6.143629401	6.438130131	6.036116368	6.147737548	6.247034288	6.359316589	6.401808442	6.553265364
ENSG00000132589	0.004229	0.032426285	-0.477325208	-1.392160173	**FLOT2**	protein_coding	flotillin 2 [Source:HGNC Symbol;Acc:HGNC:3758]	chr17	28879335	28897679	-1	ensembl_havana	7.279543681	7.518206284	7.040881077	7.43465461	7.620216487	7.499747756	7.161263177	7.049518044	6.911862009
ENSG00000060762	0.004238	0.032444097	0.413085266	1.331530307	**MPC1**	protein_coding	mitochondrial pyruvate carrier 1 [Source:HGNC Symbol;Acc:HGNC:21606]	chr6	166364919	166383013	-1	ensembl_havana	5.467362361	5.260819728	5.673904994	5.250517308	5.409374036	5.122567842	5.660211925	5.685779305	5.675723752
ENSG00000108963	0.004239	0.032444097	0.322025523	1.250084417	**DPH1**	protein_coding	diphthamide biosynthesis 1 [Source:HGNC Symbol;Acc:HGNC:3003]	chr17	2030110	2043430	1	ensembl_havana	3.594021476	3.433008715	3.755034238	3.525811778	3.337712124	3.435502243	3.787350561	3.64326966	3.834482493
ENSG00000169249	0.004243	0.032444668	-0.321764211	-1.249858014	**ZRSR2**	protein_coding	zinc finger CCCH-type, RNA binding motif and serine/arginine rich 2 [Source:HGNC Symbol;Acc:HGNC:23019]	chrX	15790472	15823260	1	ensembl_havana	3.759081514	3.91996362	3.598199408	3.980397213	3.9115401	3.867953547	3.588212495	3.590587736	3.615797995
ENSG00000278845	0.004243	0.032444668	-0.322189715	-1.250226697	**MRPL45**	protein_coding	mitochondrial ribosomal protein L45 [Source:HGNC Symbol;Acc:HGNC:16651]	chr17	38297023	38323218	1	ensembl_havana	5.318287334	5.479382192	5.157192477	5.459947931	5.481521616	5.496677028	5.090142026	5.126053155	5.255382248
ENSG00000253746	0.004247	0.03245089	0.53908213	1.453047766	**AC091182.2**	lincRNA		chr8	37405439	37406724	-1	havana	1.135593417	0.866052353	1.405134482	1.222335231	0.799260866	0.57656096	1.554586035	1.554228498	1.106588913
ENSG00000106333	0.004248	0.03245089	-0.556387059	-1.470581825	**PCOLCE**	protein_coding	procollagen C-endopeptidase enhancer [Source:HGNC Symbol;Acc:HGNC:8738]	chr7	100602177	100608175	1	ensembl_havana	3.01893475	3.29712828	2.740741221	3.359409615	3.360687686	3.17128754	3.030347571	2.590542907	2.601333184
ENSG00000149809	0.004257	0.03249797	-0.288714601	-1.221551426	**TM7SF2**	protein_coding	transmembrane 7 superfamily member 2 [Source:HGNC Symbol;Acc:HGNC:11863]	chr11	65111845	65116384	1	ensembl_havana	5.399843852	5.544201152	5.255486552	5.814089585	5.506753835	5.311760037	5.496612447	5.236857083	5.032990124
ENSG00000197594	0.00427	0.032578326	0.34773922	1.272564887	**ENPP1**	protein_coding	ectonucleotide pyrophosphatase/phosphodiesterase 1 [Source:HGNC Symbol;Acc:HGNC:3356]	chr6	131808016	131895155	1	ensembl_havana	2.885928173	2.712058563	3.059797783	2.214735639	2.904984633	3.016455416	2.657410455	3.249141357	3.272841537
ENSG00000184384	0.004272	0.032580945	-0.300883118	-1.231898266	**MAML2**	protein_coding	mastermind like transcriptional coactivator 2 [Source:HGNC Symbol;Acc:HGNC:16259]	chr11	95976598	96343180	-1	ensembl_havana	6.460457332	6.610898891	6.310015773	6.77562265	6.602780528	6.454293496	6.527349298	6.264219337	6.138478683
ENSG00000268895	0.004278	0.03260869	0.346488289	1.27146195	**A1BG-AS1**	antisense	A1BG antisense RNA 1 [Source:HGNC Symbol;Acc:HGNC:37133]	chr19	58347751	58355183	1	havana	4.432653345	4.259409201	4.60589749	4.348828832	4.202437314	4.226961456	4.780416538	4.562010741	4.47526519
ENSG00000163898	0.004282	0.032619209	-0.537125612	-1.451078544	**LIPH**	protein_coding	lipase H [Source:HGNC Symbol;Acc:HGNC:18483]	chr3	185506262	185552613	-1	ensembl_havana	2.421328147	2.689890953	2.152765342	2.490740384	2.754497827	2.824434649	1.740743887	2.42655638	2.290995759
ENSG00000146802	0.004302	0.032735434	0.283476545	1.217124328	**TMEM168**	protein_coding	transmembrane protein 168 [Source:HGNC Symbol;Acc:HGNC:25826]	chr7	112762382	112790592	-1	ensembl_havana	5.721701811	5.579963538	5.863440083	5.648579977	5.515554998	5.575755639	5.943000244	5.791349196	5.855970809
ENSG00000251602	0.0043	0.032735434	-0.359337692	-1.282836842	**AL928654.1**	antisense		chr14	105416884	105419739	-1	havana	1.639952504	1.81962135	1.460283658	1.578072284	1.987674772	1.893116995	1.309740823	1.517070703	1.554039449
ENSG00000173926	0.004308	0.032765629	0.622605606	1.539653391	**MARCH3**	protein_coding	membrane associated ring-CH-type finger 3 [Source:HGNC Symbol;Acc:HGNC:28728]	chr5	126867714	127030808	-1	ensembl_havana	1.104614908	0.793312105	1.415917712	0.437805725	1.013233389	0.928897202	1.309740823	1.372000412	1.566011899
ENSG00000169733	0.004319	0.032832287	0.682483577	1.604900189	**RFNG**	protein_coding	RFNG O-fucosylpeptide 3-beta-N-acetylglucosaminyltransferase [Source:HGNC Symbol;Acc:HGNC:9974]	chr17	82047902	82051831	-1	ensembl_havana	4.333719888	3.9924781	4.674961676	3.83322245	3.991523312	4.152688536	4.842766089	4.562010741	4.620108199
ENSG00000214814	0.004331	0.032872267	1.632770973	3.101080491	**FER1L6**	protein_coding	fer-1 like family member 6 [Source:HGNC Symbol;Acc:HGNC:28065]	chr8	123851987	124120061	1	havana	0.165853002	-0.650532484	0.982238488	-1.31854494	-0.143136894	-0.489915621	0.955706889	0.671430284	1.319578292
ENSG00000198947	0.004331	0.032872267	0.379211793	1.30063107	**DMD**	protein_coding	dystrophin [Source:HGNC Symbol;Acc:HGNC:2928]	chrX	31097677	33339441	-1	ensembl_havana	4.656414688	4.466808792	4.846020585	4.21876443	4.573638908	4.608023037	4.716730794	4.950201315	4.871129645
ENSG00000247735	0.004327	0.032872267	0.316560058	1.245357593	**AC120114.1**	antisense		chr16	29926836	29928933	1	havana	0.66768296	0.509402931	0.825962989	0.297565234	0.428746719	0.80189684	0.56657698	0.821210834	1.090101154
ENSG00000144355	0.004338	0.0329061	-0.609142336	-1.525352137	**DLX1**	protein_coding	distal-less homeobox 1 [Source:HGNC Symbol;Acc:HGNC:2914]	chr2	172084740	172089677	1	ensembl_havana	2.542215761	2.846786929	2.237644593	3.159173914	2.704058936	2.677127938	2.632526022	2.293711009	1.786696749
ENSG00000154767	0.004341	0.032913191	-0.320859183	-1.249074202	**XPC**	protein_coding	XPC complex subunit, DNA damage recognition and repair factor [Source:HGNC Symbol;Acc:HGNC:12816]	chr3	14145147	14178783	-1	ensembl_havana	5.305021141	5.465450733	5.14459155	5.588151131	5.397519822	5.410681246	5.312059643	4.994807149	5.126907856
ENSG00000126522	0.004348	0.032945964	0.369066084	1.291516508	**ASL**	protein_coding	argininosuccinate lyase [Source:HGNC Symbol;Acc:HGNC:746]	chr7	66075798	66093558	1	ensembl_havana	4.694165334	4.509632292	4.878698376	4.497278108	4.455121723	4.576497044	4.968646223	4.839168605	4.828280298
ENSG00000273295	0.004367	0.033024248	0.450113704	1.366147923	**AP000350.6**	lincRNA		chr22	23901432	23907068	-1	havana	1.510971875	1.285915023	1.736028727	1.369711653	1.140065346	1.347968071	1.631954676	1.671599815	1.90453169
ENSG00000140992	0.004361	0.033024248	0.2826538	1.216430419	**PDPK1**	protein_coding	3-phosphoinositide dependent protein kinase 1 [Source:HGNC Symbol;Acc:HGNC:8816]	chr16	2537964	2603188	1	ensembl_havana	6.571108904	6.429782004	6.712435803	6.499308172	6.417983168	6.372054672	6.788983252	6.688224194	6.660099964
ENSG00000143924	0.004363	0.033024248	0.282466988	1.216272916	**EML4**	protein_coding	echinoderm microtubule associated protein like 4 [Source:HGNC Symbol;Acc:HGNC:1316]	chr2	42169350	42332548	1	ensembl_havana	7.853367771	7.712134277	7.994601265	7.655432456	7.730636179	7.750334196	7.94493965	8.001703672	8.037160472
ENSG00000166337	0.004366	0.033024248	-0.334984742	-1.261364069	**TAF10**	protein_coding	TATA-box binding protein associated factor 10 [Source:HGNC Symbol;Acc:HGNC:11543]	chr11	6606296	6612667	-1	ensembl_havana	2.145362668	2.312855039	1.977870297	2.142776609	2.317915967	2.477872542	1.874103935	2.011485396	2.04802156
ENSG00000111670	0.00437	0.033030745	0.296097666	1.227818801	**GNPTAB**	protein_coding	N-acetylglucosamine-1-phosphate transferase alpha and beta subunits [Source:HGNC Symbol;Acc:HGNC:29670]	chr12	101745497	101830938	-1	ensembl_havana	5.653947344	5.505898511	5.801996177	5.44672709	5.499378294	5.57159015	5.697431913	5.801686578	5.906870041
ENSG00000114737	0.004378	0.033073018	-0.462088224	-1.377534283	**CISH**	protein_coding	cytokine inducible SH2 containing protein [Source:HGNC Symbol;Acc:HGNC:1984]	chr3	50606490	50611831	-1	ensembl_havana	6.371748857	6.602792969	6.140704745	6.56157593	6.653500854	6.593302123	6.29535663	6.110033564	6.016724041
ENSG00000105699	0.004381	0.03307897	-0.440323824	-1.356908862	**LSR**	protein_coding	lipolysis stimulated lipoprotein receptor [Source:HGNC Symbol;Acc:HGNC:29572]	chr19	35248330	35267964	1	ensembl_havana	4.649675745	4.869837657	4.429513833	4.947949426	4.798225501	4.863338045	4.621934287	4.423288215	4.243318997
ENSG00000135144	0.004391	0.033088576	-0.390224129	-1.310596995	**DTX1**	protein_coding	deltex E3 ubiquitin ligase 1 [Source:HGNC Symbol;Acc:HGNC:3060]	chr12	113056709	113098028	1	ensembl_havana	3.175657849	3.370769914	2.980545785	3.380421283	3.198903614	3.532984844	2.936891701	2.952470332	3.052275322
ENSG00000259673	0.004387	0.033088576	-0.392921948	-1.313050085	**IQCH-AS1**	lincRNA	IQCH antisense RNA 1 [Source:HGNC Symbol;Acc:HGNC:44104]	chr15	67403619	67521844	-1	havana	2.3766942	2.573155173	2.180233226	2.464742477	2.482570884	2.772152159	2.153133136	2.155463891	2.23210265
ENSG00000215424	0.004392	0.033088576	-0.706866442	-1.632254979	**MCM3AP-AS1**	antisense	MCM3AP antisense RNA 1 [Source:HGNC Symbol;Acc:HGNC:16417]	chr21	46229217	46259390	1	ensembl_havana	1.221921735	1.575354956	0.868488514	1.541228291	1.51252579	1.672310787	0.540060475	1.114463419	0.950941648
ENSG00000178573	0.004389	0.033088576	-0.707608095	-1.633094296	**MAF**	protein_coding	MAF bZIP transcription factor [Source:HGNC Symbol;Acc:HGNC:6776]	chr16	79585843	79600714	-1	ensembl_havana	2.082106154	2.435910201	1.728302106	1.791817439	2.672926856	2.842986308	1.430149367	1.831488934	1.923268018
ENSG00000168781	0.004402	0.033109635	0.422317342	1.34007834	**PPIP5K1**	protein_coding	diphosphoinositol pentakisphosphate kinase 1 [Source:HGNC Symbol;Acc:HGNC:29023]	chr15	43533462	43590253	-1	ensembl_havana	5.757537788	5.546379117	5.96869646	5.445896751	5.656838681	5.536401919	5.998831646	5.920281013	5.986976719
ENSG00000187531	0.004401	0.033109635	0.337150099	1.263258686	**SIRT7**	protein_coding	sirtuin 7 [Source:HGNC Symbol;Acc:HGNC:14935]	chr17	81911939	81921323	-1	ensembl_havana	5.288124417	5.119549367	5.456699466	5.024470986	5.114357655	5.219819461	5.37154447	5.532844567	5.465709362
ENSG00000104635	0.004404	0.033109635	0.315281674	1.24425456	**SLC39A14**	protein_coding	solute carrier family 39 member 14 [Source:HGNC Symbol;Acc:HGNC:20858]	chr8	22367249	22434129	1	ensembl_havana	9.46326431	9.305623473	9.620905147	9.544183271	9.256661902	9.116025245	9.79546259	9.567012531	9.500240319
ENSG00000186889	0.004399	0.033109635	-0.704154245	-1.629189301	**TMEM17**	protein_coding	transmembrane protein 17 [Source:HGNC Symbol;Acc:HGNC:26623]	chr2	62500221	62511894	-1	ensembl_havana	-0.623505635	-0.271428513	-0.975582758	-0.36334239	-0.620350959	0.16940781	-1.41444944	-1.15951152	-0.352787316
ENSG00000132256	0.00441	0.033136859	-0.418984942	-1.336986541	**TRIM5**	protein_coding	tripartite motif containing 5 [Source:HGNC Symbol;Acc:HGNC:16276]	chr11	5663557	5938619	-1	ensembl_havana	3.659946686	3.869439157	3.450454215	4.007662542	3.843267218	3.75738771	3.427522837	3.466136326	3.45770348
ENSG00000249863	0.004423	0.033220476	0.366661522	1.28936571	**AC021106.1**	processed_pseudogene		chr4	37868292	37869978	1	havana	0.494840184	0.311509423	0.678170945	0.355307344	0.243386951	0.335833975	0.762800879	0.693803314	0.577908642
ENSG00000253824	0.004431	0.033262211	-0.379009579	-1.300448781	**AP003472.1**	lincRNA		chr8	100381260	100414459	-1	havana	1.470812159	1.660316948	1.281307369	1.705645971	1.51252579	1.762779083	1.212343926	1.256505808	1.375072374
ENSG00000188315	0.004438	0.033273607	0.315238374	1.244217216	**C3orf62**	protein_coding	chromosome 3 open reading frame 62 [Source:HGNC Symbol;Acc:HGNC:24771]	chr3	49268602	49277909	-1	ensembl_havana	4.461781632	4.304162445	4.619400818	4.259029533	4.320977944	4.332479857	4.55066832	4.712038443	4.595495693
ENSG00000107438	0.00444	0.033273607	-0.404180708	-1.323337188	**PDLIM1**	protein_coding	PDZ and LIM domain 1 [Source:HGNC Symbol;Acc:HGNC:2067]	chr10	95237572	95291024	-1	ensembl_havana	1.569072138	1.771162492	1.366981784	1.694503432	1.827267088	1.791716956	1.400976194	1.271458479	1.428510677
ENSG00000125520	0.004439	0.033273607	-0.426397862	-1.343873984	**SLC2A4RG**	protein_coding	SLC2A4 regulator [Source:HGNC Symbol;Acc:HGNC:15930]	chr20	63739861	63743505	1	ensembl_havana	2.046957584	2.260156515	1.833758652	2.327223844	2.256240478	2.197005222	1.895179555	1.682828385	1.923268018
ENSG00000112984	0.004447	0.033294369	-0.341823283	-1.267357273	**KIF20A**	protein_coding	kinesin family member 20A [Source:HGNC Symbol;Acc:HGNC:9787]	chr5	138178719	138187715	1	ensembl_havana	6.800490146	6.971401788	6.629578505	6.628969256	7.094138372	7.191097735	6.37017672	6.767161391	6.751397403
ENSG00000168907	0.004447	0.033294369	-1.560266704	-2.949083566	**PLA2G4F**	protein_coding	phospholipase A2 group IVF [Source:HGNC Symbol;Acc:HGNC:27396]	chr15	42139034	42156636	-1	ensembl_havana	-0.670306076	0.109827276	-1.450439428	0.892538	0.125688576	-0.688744748	-0.89704638	-2.00847383	-1.445798078
ENSG00000101203	0.004476	0.03337177	0.973822886	1.964038059	**COL20A1**	protein_coding	collagen type XX alpha 1 chain [Source:HGNC Symbol;Acc:HGNC:14670]	chr20	63293186	63334851	1	ensembl_havana	-0.435113271	-0.922024714	0.051798172	-1.23095973	-1.338333324	-0.196781087	-0.12879794	-0.00684508	0.291037539
ENSG00000197063	0.004471	0.03337177	0.308554116	1.238465874	**MAFG**	protein_coding	MAF bZIP transcription factor G [Source:HGNC Symbol;Acc:HGNC:6781]	chr17	81918270	81927714	-1	ensembl_havana	6.497027568	6.34275051	6.651304626	6.450053216	6.34208839	6.236109925	6.738494269	6.718888111	6.496531498
ENSG00000041802	0.004464	0.03337177	0.302829264	1.233561175	**LSG1**	protein_coding	large 60S subunit nuclear export GTPase 1 [Source:HGNC Symbol;Acc:HGNC:25652]	chr3	194640788	194672477	-1	ensembl_havana	6.184831015	6.033416383	6.336245647	5.878891881	6.042057755	6.179299513	6.234643277	6.346837611	6.427256053
ENSG00000157600	0.004471	0.03337177	-0.287362707	-1.220407293	**TMEM164**	protein_coding	transmembrane protein 164 [Source:HGNC Symbol;Acc:HGNC:26217]	chrX	110002631	110182734	1	ensembl_havana	6.7818312	6.925512553	6.638149846	7.103751203	6.891000238	6.781786218	6.781928338	6.614644812	6.517876389
ENSG00000072415	0.004469	0.03337177	-0.342045147	-1.267552187	**MPP5**	protein_coding	membrane palmitoylated protein 5 [Source:HGNC Symbol;Acc:HGNC:18669]	chr14	67241109	67335819	1	ensembl_havana	5.043554449	5.214577022	4.872531875	5.227510036	5.15529312	5.26092791	4.781831765	4.884855267	4.950908594
ENSG00000138685	0.004464	0.03337177	-0.642292962	-1.560807874	**FGF2**	protein_coding	fibroblast growth factor 2 [Source:HGNC Symbol;Acc:HGNC:3676]	chr4	122826708	122898236	1	ensembl_havana	2.658569343	2.979715824	2.337422862	3.142871041	2.953290123	2.842986308	2.221475976	2.329873398	2.460919213
ENSG00000181652	0.004475	0.03337177	-0.78274515	-1.720401328	**ATG9B**	protein_coding	autophagy related 9B [Source:HGNC Symbol;Acc:HGNC:21899]	chr7	151012209	151024499	-1	ensembl_havana	-0.198597808	0.192774767	-0.589970383	0.040274975	0.125688576	0.41236075	-0.40973604	-1.00738779	-0.352787316
ENSG00000283906	0.004467	0.03337177	-0.915933993	-1.886790174	**IGHV4-80**	IG_V_pseudogene	immunoglobulin heavy variable 4-80 (pseudogene) [Source:HGNC Symbol;Acc:HGNC:5656]	chr14	106872783	106873186	-1	havana	0.047580621	0.505547617	-0.410386376	0.727424294	0.453384582	0.335833975	-0.63291296	-0.37473441	-0.223511759
ENSG00000173334	0.00449	0.033383715	0.73637165	1.665980656	**TRIB1**	protein_coding	tribbles pseudokinase 1 [Source:HGNC Symbol;Acc:HGNC:16891]	chr8	125430321	125438405	1	ensembl_havana	1.521944324	1.153758499	1.890130149	1.109412078	1.51252579	0.83933763	2.044136302	1.881076089	1.745178056
ENSG00000163576	0.004496	0.033383715	0.678082378	1.600011615	**EFHB**	protein_coding	EF-hand domain family member B [Source:HGNC Symbol;Acc:HGNC:26330]	chr3	19879472	19947025	-1	ensembl_havana	-0.228820111	-0.5678613	0.110221078	-0.67394004	-0.672709	-0.356934859	0.069637898	0.256279753	0.004745582
ENSG00000072062	0.004483	0.033383715	0.363202714	1.286278206	**PRKACA**	protein_coding	protein kinase cAMP-activated catalytic subunit alpha [Source:HGNC Symbol;Acc:HGNC:9380]	chr19	14091688	14118084	-1	ensembl_havana	6.047257512	5.865656155	6.228858869	5.881350696	5.919756238	5.795861532	6.247504317	6.17310146	6.265970829
ENSG00000104154	0.004481	0.033383715	0.342199583	1.267687882	**SLC30A4**	protein_coding	solute carrier family 30 member 4 [Source:HGNC Symbol;Acc:HGNC:11015]	chr15	45479611	45522807	-1	ensembl_havana	4.394059948	4.222960157	4.56515974	4.155162249	4.324321165	4.189397057	4.598032469	4.586131975	4.511314775
ENSG00000176890	0.004486	0.033383715	-0.309507508	-1.239284574	**TYMS**	protein_coding	thymidylate synthetase [Source:HGNC Symbol;Acc:HGNC:12441]	chr18	657604	673578	1	ensembl_havana	7.555817922	7.710571676	7.401064169	7.531565381	7.811329462	7.788820185	7.286682845	7.442636553	7.473873108
ENSG00000234614	0.004497	0.033383715	-0.400062824	-1.319565372	**AL450992.2**	antisense		chr1	151841877	151850385	1	havana	2.329824599	2.529856011	2.129793187	2.73325697	2.297649024	2.558662038	2.294679948	2.046678052	2.04802156
ENSG00000102362	0.004498	0.033383715	-0.4380447	-1.354766952	**SYTL4**	protein_coding	synaptotagmin like 4 [Source:HGNC Symbol;Acc:HGNC:15588]	chrX	100674491	100732123	-1	ensembl_havana	2.403536726	2.622559076	2.184514376	3.234191999	2.662398396	1.971086832	2.770081475	2.081032632	1.70242902
ENSG00000104870	0.004488	0.033383715	-0.532070007	-1.44600246	**FCGRT**	protein_coding	Fc fragment of IgG receptor and transporter [Source:HGNC Symbol;Acc:HGNC:3621]	chr19	49506816	49526333	1	ensembl_havana	2.01247173	2.278506734	1.746436727	2.23025056	2.283977809	2.321291832	1.874103935	1.811166797	1.554039449
ENSG00000141905	0.004498	0.033383715	-0.5681949	-1.482667296	**NFIC**	protein_coding	nuclear factor I C [Source:HGNC Symbol;Acc:HGNC:7786]	chr19	3314403	3469217	1	ensembl_havana	4.565142022	4.849239472	4.281044572	4.956136763	4.743086637	4.848495017	4.553985209	4.273512191	4.015636317
ENSG00000173068	0.004538	0.033404183	1.22387879	2.335738541	**BNC2**	protein_coding	basonuclin 2 [Source:HGNC Symbol;Acc:HGNC:30988]	chr9	16409503	16870843	-1	ensembl_havana	0.631502107	0.019562712	1.243441502	-0.79434999	0.298820819	0.554217307	1.052687018	1.358059196	1.319578292
ENSG00000172020	0.004536	0.033404183	0.624916414	1.542121477	**GAP43**	protein_coding	growth associated protein 43 [Source:HGNC Symbol;Acc:HGNC:4140]	chr3	115623324	115721490	1	ensembl_havana	2.361413909	2.048955702	2.673872116	2.150951608	2.344503139	1.651412359	2.803908217	2.69405369	2.523654441
ENSG00000150672	0.004543	0.033404183	0.59266228	1.508027021	**DLG2**	protein_coding	discs large MAGUK scaffold protein 2 [Source:HGNC Symbol;Acc:HGNC:2901]	chr11	83455012	85627922	-1	ensembl_havana	-0.022537969	-0.318869109	0.273793171	-0.31750517	-0.620350959	-0.018751196	0.399634207	0.130707766	0.291037539
ENSG00000136856	0.004504	0.033404183	0.493195354	1.407558959	**SLC2A8**	protein_coding	solute carrier family 2 member 8 [Source:HGNC Symbol;Acc:HGNC:13812]	chr9	127397138	127408424	1	ensembl_havana	3.702231469	3.455633792	3.948829146	3.435000789	3.321074063	3.610826524	3.95980697	3.986884828	3.899795641
ENSG00000132024	0.004536	0.033404183	0.349245433	1.273894173	**CC2D1A**	protein_coding	coiled-coil and C2 domain containing 1A [Source:HGNC Symbol;Acc:HGNC:30237]	chr19	13906201	13930879	1	ensembl_havana	5.748641895	5.574019178	5.923264611	5.606101126	5.52502939	5.590927018	6.034293642	5.905978998	5.829521194
ENSG00000133424	0.004527	0.033404183	0.34006094	1.265810061	**LARGE1**	protein_coding	LARGE xylosyl- and glucuronyltransferase 1 [Source:HGNC Symbol;Acc:HGNC:6511]	chr22	33162226	33922841	-1	ensembl_havana	4.621151568	4.451121098	4.791182038	4.345270912	4.458168414	4.549923968	4.75901983	4.826568403	4.78795788
ENSG00000101193	0.004539	0.033404183	0.338686091	1.264604353	**GID8**	protein_coding	GID complex subunit 8 homolog [Source:HGNC Symbol;Acc:HGNC:15857]	chr20	62938119	62948475	1	ensembl_havana	6.592754771	6.423411725	6.762097816	6.289465624	6.48411806	6.496651492	6.728254329	6.747577678	6.810461441
ENSG00000225697	0.004511	0.033404183	0.301577994	1.232491754	**SLC26A6**	protein_coding	solute carrier family 26 member 6 [Source:HGNC Symbol;Acc:HGNC:14472]	chr3	48625723	48635493	-1	ensembl_havana	5.848939823	5.698150826	5.99972882	5.617944904	5.708286224	5.768221351	5.888209623	5.979530799	6.131446038
ENSG00000172086	0.004541	0.033404183	0.28856324	1.221423273	**KRCC1**	protein_coding	lysine rich coiled-coil 1 [Source:HGNC Symbol;Acc:HGNC:28039]	chr2	88027205	88055729	-1	ensembl_havana	3.20829921	3.06401759	3.35258083	2.991768026	3.135610417	3.064674328	3.250759372	3.462883238	3.34409988
ENSG00000133703	0.00452	0.033404183	-0.280641452	-1.21473486	**KRAS**	protein_coding	KRAS proto-oncogene, GTPase [Source:HGNC Symbol;Acc:HGNC:6407]	chr12	25204789	25250936	-1	ensembl_havana	7.237436638	7.377757363	7.097115912	7.485035478	7.374690164	7.273546448	7.191659995	7.091988787	7.007698953
ENSG00000108439	0.004522	0.033404183	-0.288760393	-1.2215902	**PNPO**	protein_coding	pyridoxamine 5'-phosphate oxidase [Source:HGNC Symbol;Acc:HGNC:30260]	chr17	47941506	47949308	1	ensembl_havana	5.059266453	5.203646649	4.914886256	5.053106071	5.231167006	5.32666687	4.735841389	4.981228583	5.027588796
ENSG00000196227	0.004517	0.033404183	-0.290805782	-1.223323345	**FAM217B**	protein_coding	family with sequence similarity 217 member B [Source:HGNC Symbol;Acc:HGNC:16170]	chr20	59933764	59948680	1	ensembl_havana	5.899394975	6.044797866	5.753992084	5.94737635	6.143485371	6.043531877	5.61249343	5.876942463	5.772540359
ENSG00000065485	0.00453	0.033404183	-0.297343257	-1.22887933	**PDIA5**	protein_coding	protein disulfide isomerase family A member 5 [Source:HGNC Symbol;Acc:HGNC:24811]	chr3	123067062	123225227	1	ensembl_havana	4.23231575	4.380987378	4.083644121	4.23231138	4.422736894	4.487913861	3.924364913	4.083248454	4.243318997
ENSG00000268996	0.00452	0.033404183	-0.319126976	-1.24757537	**MAN1B1-AS1**	antisense	MAN1B1 antisense RNA 1 (head to head) [Source:HGNC Symbol;Acc:HGNC:48715]	chr9	137084946	137086817	-1	havana	0.269565919	0.429129407	0.110002431	0.267803752	0.910210399	0.109374069	-0.12879794	0.602150945	-0.143345712
ENSG00000060656	0.004519	0.033404183	-0.324965595	-1.252634566	**PTPRU**	protein_coding	protein tyrosine phosphatase, receptor type U [Source:HGNC Symbol;Acc:HGNC:9683]	chr1	29236516	29326813	1	ensembl_havana	2.458685826	2.621168623	2.296203028	2.73325697	2.500414659	2.629834241	2.333799072	2.256618822	2.298191191
ENSG00000138964	0.004519	0.033404183	-0.415983747	-1.334208138	**PARVG**	protein_coding	parvin gamma [Source:HGNC Symbol;Acc:HGNC:14654]	chr22	44172956	44219533	1	ensembl_havana	1.856876915	2.064868788	1.648885042	1.738566362	1.996084195	2.459955808	1.356079599	1.705026699	1.885548828
ENSG00000143369	0.00453	0.033404183	-0.507260781	-1.421348942	**ECM1**	protein_coding	extracellular matrix protein 1 [Source:HGNC Symbol;Acc:HGNC:3153]	chr1	150508062	150513789	1	ensembl_havana	1.831843622	2.085474012	1.578213231	2.206915158	2.061651132	1.987855745	1.47283199	1.637380031	1.624427672
ENSG00000229162	0.004509	0.033404183	-1.098670314	-2.141572197	**AL445471.1**	antisense		chr1	24961345	24963097	1	havana	0.431867046	0.981202203	-0.117468111	1.158893785	0.945375194	0.83933763	-0.35894771	-0.28449416	0.291037539
ENSG00000128815	0.004557	0.033487127	-0.468867028	-1.384022146	**WDFY4**	protein_coding	WDFY family member 4 [Source:HGNC Symbol;Acc:HGNC:29323]	chr10	48684876	48982956	1	ensembl_havana	9.173779349	9.408212863	8.939345835	9.58873171	9.098527769	9.537379108	9.085161715	8.82167399	8.911201799
ENSG00000178773	0.004565	0.033529992	-0.559870716	-1.47413711	**CPNE7**	protein_coding	copine 7 [Source:HGNC Symbol;Acc:HGNC:2320]	chr16	89575768	89597246	1	ensembl_havana	3.791369441	4.071304799	3.511434082	4.224585849	3.73438904	4.254939508	3.558795999	3.43659104	3.538915209
ENSG00000111181	0.004594	0.033557147	0.722973539	1.650580542	**SLC6A12**	protein_coding	solute carrier family 6 member 12 [Source:HGNC Symbol;Acc:HGNC:11045]	chr12	190077	214570	-1	ensembl_havana	2.370705905	2.009219135	2.732192674	2.005254198	2.14720036	1.875202847	2.379383493	3.131139307	2.686055222
ENSG00000181873	0.004605	0.033557147	0.418225603	1.336283025	**IBA57**	protein_coding	IBA57, iron-sulfur cluster assembly [Source:HGNC Symbol;Acc:HGNC:27302]	chr1	228165815	228182257	1	ensembl_havana	3.86162688	3.652514078	4.070739682	3.666461735	3.54104637	3.75003413	3.937122974	4.1063662	4.168729871
ENSG00000078967	0.004576	0.033557147	0.375418806	1.297216074	**UBE2D4**	protein_coding	ubiquitin conjugating enzyme E2 D4 (putative) [Source:HGNC Symbol;Acc:HGNC:21647]	chr7	43926438	43956136	1	ensembl_havana	4.5590121	4.371302698	4.746721503	4.007662542	4.538087248	4.568158303	4.513670186	4.812579879	4.913914443
ENSG00000107614	0.004587	0.033557147	0.344982077	1.270135203	**TRDMT1**	protein_coding	tRNA aspartic acid methyltransferase 1 [Source:HGNC Symbol;Acc:HGNC:2977]	chr10	17142254	17202054	-1	ensembl_havana	3.055690435	2.883199397	3.228181474	2.716829986	2.896025364	3.03674284	3.035102527	3.347673311	3.301768582
ENSG00000116128	0.004607	0.033557147	0.285494738	1.21882816	**BCL9**	protein_coding	B cell CLL/lymphoma 9 [Source:HGNC Symbol;Acc:HGNC:1008]	chr1	147541412	147626216	1	ensembl_havana	4.872819989	4.73007262	5.015567357	4.665068254	4.775201171	4.749948434	4.982251883	5.05977042	5.004679769
ENSG00000197429	0.004598	0.033557147	-0.280611639	-1.214709758	**IPP**	protein_coding	intracisternal A particle-promoted polypeptide [Source:HGNC Symbol;Acc:HGNC:6108]	chr1	45694324	45750650	-1	ensembl_havana	4.635199449	4.775505269	4.49489363	4.850923091	4.731801393	4.743791322	4.573727977	4.467784529	4.443168384
ENSG00000071539	0.004583	0.033557147	-0.298739858	-1.230069522	**TRIP13**	protein_coding	thyroid hormone receptor interactor 13 [Source:HGNC Symbol;Acc:HGNC:12307]	chr5	892643	919357	1	ensembl_havana	5.633334384	5.782704312	5.483964455	5.517986695	5.898605706	5.931520536	5.265273256	5.544432635	5.642187474
ENSG00000212916	0.004592	0.033557147	-0.314292339	-1.243401599	**MAP10**	protein_coding	microtubule associated protein 10 [Source:HGNC Symbol;Acc:HGNC:29265]	chr1	232804892	232808407	1	ensembl_havana	4.20939556	4.366541729	4.05224939	4.167303204	4.504587447	4.427734536	3.893277228	4.110529916	4.152941027
ENSG00000111602	0.004608	0.033557147	-0.321470294	-1.249603409	**TIMELESS**	protein_coding	timeless circadian regulator [Source:HGNC Symbol;Acc:HGNC:11813]	chr12	56416373	56449403	-1	ensembl_havana	7.149954772	7.310689919	6.989219625	7.164822939	7.329653828	7.43759299	6.913959546	7.018555883	7.035143447
ENSG00000135185	0.004579	0.033557147	-0.338364711	-1.264322677	**TMEM243**	protein_coding	transmembrane protein 243 [Source:HGNC Symbol;Acc:HGNC:21707]	chr7	87196160	87220587	-1	ensembl_havana	3.920095545	4.089277901	3.75091319	4.220707514	4.045187566	4.001938621	3.790166076	3.795851755	3.666721737
ENSG00000122176	0.004605	0.033557147	-0.343260646	-1.268620575	**FMOD**	protein_coding	fibromodulin [Source:HGNC Symbol;Acc:HGNC:3774]	chr1	203340628	203351489	-1	ensembl_havana	5.452171831	5.623802154	5.280541508	5.948549284	5.552372274	5.370484904	5.606116546	5.113660433	5.121847546
ENSG00000160753	0.0046	0.033557147	-0.34413878	-1.26939299	**RUSC1**	protein_coding	RUN and SH3 domain containing 1 [Source:HGNC Symbol;Acc:HGNC:17153]	chr1	155320896	155331114	1	ensembl_havana	2.82867145	3.00074084	2.656602059	3.000842267	3.073391752	2.9279885	2.741273741	2.748504565	2.480027873
ENSG00000104450	0.004571	0.033557147	-0.376551374	-1.298234836	**SPAG1**	protein_coding	sperm associated antigen 1 [Source:HGNC Symbol;Acc:HGNC:11212]	chr8	100157906	100259278	1	ensembl_havana	5.363113905	5.551389592	5.174838217	5.69124582	5.553797118	5.409125838	5.332483614	5.050047881	5.141983157
ENSG00000214194	0.004602	0.033557147	-0.404088987	-1.323253057	**SMIM30**	protein_coding	small integral membrane protein 30 [Source:HGNC Symbol;Acc:HGNC:48953]	chr7	113116718	113118613	-1	havana	4.560617995	4.762662488	4.358573501	4.566141688	4.831500081	4.890345696	4.004132206	4.526614279	4.544974018
ENSG00000085831	0.004604	0.033557147	-0.436589768	-1.353401383	**TTC39A**	protein_coding	tetratricopeptide repeat domain 39A [Source:HGNC Symbol;Acc:HGNC:18657]	chr1	51287258	51345116	-1	ensembl_havana	5.794486071	6.012780955	5.576191187	6.036116368	6.070705961	5.931520536	5.525622456	5.525846784	5.67710432
ENSG00000021826	0.004578	0.033557147	-0.443679564	-1.360068735	**CPS1**	protein_coding	carbamoyl-phosphate synthase 1 [Source:HGNC Symbol;Acc:HGNC:2323]	chr2	210477682	210679107	1	ensembl_havana	4.712913235	4.934753017	4.491073453	4.527418732	5.13354198	5.143298339	4.271239432	4.587626245	4.614354683
ENSG00000279220	0.004608	0.033557147	-1.645140909	-3.12778405	**AC007383.4**	processed_transcript		chr2	206203376	206265326	1	havana	-0.582578617	0.239991838	-1.405149071	0.237415364	-0.002471726	0.485031876	-2.0571346	-0.80561234	-1.352700279
ENSG00000106355	0.004615	0.033580601	-0.364966736	-1.287851935	**LSM5**	protein_coding	LSM5 homolog, U6 small nuclear RNA and mRNA degradation associated [Source:HGNC Symbol;Acc:HGNC:17162]	chr7	32485332	32495283	-1	ensembl_havana	4.919027836	5.101511204	4.736544468	4.972373353	5.263745654	5.068414604	4.552327717	4.825302309	4.832003378
ENSG00000108448	0.004616	0.033580601	-0.367452446	-1.290072769	**TRIM16L**	protein_coding	tripartite motif containing 16 like [Source:HGNC Symbol;Acc:HGNC:32670]	chr17	18697998	18736118	1	ensembl_havana	1.563423865	1.747150088	1.379697643	1.760103226	1.535784795	1.945562243	1.261864131	1.241396536	1.635832261
ENSG00000155666	0.004622	0.033603264	-0.365326505	-1.288173131	**KDM8**	protein_coding	lysine demethylase 8 [Source:HGNC Symbol;Acc:HGNC:25840]	chr16	27203495	27221768	1	ensembl_havana	3.453436138	3.636099391	3.270772886	3.789382034	3.597402489	3.52151365	3.416632027	3.347673311	3.048013319
ENSG00000008256	0.004628	0.033630081	0.439739833	1.356359708	**CYTH3**	protein_coding	cytohesin 3 [Source:HGNC Symbol;Acc:HGNC:9504]	chr7	6161776	6272644	-1	ensembl_havana	6.239886598	6.020016681	6.459756514	5.848426298	5.985637823	6.225985923	6.417832785	6.47589428	6.485542478
ENSG00000184232	0.004632	0.033630081	0.304298432	1.234818012	**OAF**	protein_coding	out at first homolog [Source:HGNC Symbol;Acc:HGNC:28752]	chr11	120210767	120230332	1	ensembl_havana	4.387897823	4.235748607	4.540047039	4.404593656	4.079226426	4.223425739	4.680770516	4.451463998	4.487906603
ENSG00000268061	0.004631	0.033630081	-0.747644254	-1.679048907	**NAPA-AS1**	antisense	NAPA antisense RNA 1 [Source:HGNC Symbol;Acc:HGNC:44118]	chr19	47484282	47501597	1	havana	-0.454863963	-0.081041836	-0.828686089	0.174648163	-0.143136894	-0.274636777	-0.82634296	-1.00738779	-0.652327512
ENSG00000105443	0.004644	0.033694754	0.285191491	1.218571995	**CYTH2**	protein_coding	cytohesin 2 [Source:HGNC Symbol;Acc:HGNC:9502]	chr19	48469032	48482314	1	ensembl_havana	6.748956774	6.606361029	6.891552519	6.792704028	6.62430065	6.402078408	7.078175764	6.877557983	6.718923811
ENSG00000218537	0.004655	0.033741932	0.524529527	1.438464411	**MIF-AS1**	antisense	MIF antisense RNA 1 [Source:HGNC Symbol;Acc:HGNC:27669]	chr22	23894426	23898930	-1	havana	1.119240791	0.856976027	1.381505554	0.770507832	0.837200041	0.963220207	1.444517675	1.452931788	1.2470672

ENSG00000113460	0.004654	0.033741932	-0.333960341	-1.260468741	**BRIX1**	protein_coding	BRX1, biogenesis of ribosomes [Source:HGNC Symbol;Acc:HGNC:24170]	chr5	34915376	34925996	1	ensembl_havana	5.680725572	5.847705742	5.513745401	5.793349462	5.861692431	5.888075333	5.373423091	5.523506627	5.644306485
ENSG00000166398	0.00466	0.033745768	0.331863208	1.258637828	**KIAA0355**	protein_coding	KIAA0355 [Source:HGNC Symbol;Acc:HGNC:29016]	chr19	34254537	34355586	1	ensembl_havana	4.908991415	4.743059811	5.074923019	4.710025423	4.728019942	4.791134069	5.087855498	5.113660433	5.023253127
ENSG00000203414	0.004659	0.033745768	-0.672024665	-1.593307433	**BTBD7P1**	processed_pseudogene	BTB domain containing 7 pseudogene 1 [Source:HGNC Symbol;Acc:HGNC:44875]	chr10	13156684	13158136	-1	havana	-0.443206178	-0.107193845	-0.779218511	-0.06941355	0.06303108	-0.315199064	-0.63291296	-0.93694677	-0.767795802
ENSG00000198026	0.004667	0.033760113	0.377781203	1.299341991	**ZNF335**	protein_coding	zinc finger protein 335 [Source:HGNC Symbol;Acc:HGNC:15807]	chr20	45948653	45972172	-1	ensembl_havana	6.046701851	5.85781125	6.235592453	5.865913899	5.764129965	5.943389886	6.289398988	6.234004576	6.183373795
ENSG00000050628	0.004667	0.033760113	-0.66826979	-1.589165953	**PTGER3**	protein_coding	prostaglandin E receptor 3 [Source:HGNC Symbol;Acc:HGNC:9595]	chr1	70852353	71047808	-1	ensembl_havana	0.977214333	1.311349228	0.643079438	1.749374982	1.155163158	1.029509544	1.033805522	0.225896484	0.669536307
ENSG00000219626	0.004672	0.033776879	0.30413335	1.234676724	**FAM228B**	protein_coding	family with sequence similarity 228 member B [Source:HGNC Symbol;Acc:HGNC:24736]	chr2	24076526	24169640	1	ensembl_havana	2.841412905	2.68934623	2.99347958	2.744105292	2.662398396	2.661535001	2.981915645	3.015998475	2.98252462
ENSG00000103544	0.004675	0.033776879	-0.301411151	-1.232349228	**VPS35L**	protein_coding	VPS35 endosomal protein sorting factor like [Source:HGNC Symbol;Acc:HGNC:24641]	chr16	19555240	19706793	1	ensembl_havana	4.291218715	4.441924291	4.14051314	4.440051745	4.43361281	4.452108316	4.111582737	4.197260696	4.112695986
ENSG00000170927	0.004676	0.033776879	-1.935442894	-3.824955315	**PKHD1**	protein_coding	PKHD1, fibrocystin/polyductin [Source:HGNC Symbol;Acc:HGNC:9016]	chr6	51615300	52087625	-1	ensembl_havana	-0.577455793	0.390265654	-1.54517724	0.565613454	0.243386951	0.361796557	-1.22062457	-0.86978558	-2.545121571
ENSG00000140807	0.004687	0.033836145	0.736682627	1.666339802	**NKD1**	protein_coding	naked cuticle homolog 1 [Source:HGNC Symbol;Acc:HGNC:17045]	chr16	50548330	50649249	1	ensembl_havana	-0.323375487	-0.6917168	0.044965827	-1.14838881	-0.569826728	-0.356934859	-0.04612063	-0.08087422	0.261892331
ENSG00000127870	0.00469	0.033843023	-0.283319456	-1.216991808	**RNF6**	protein_coding	ring finger protein 6 [Source:HGNC Symbol;Acc:HGNC:10069]	chr13	26132115	26222493	-1	ensembl_havana	5.942144563	6.083804291	5.800484835	6.11188196	6.107069379	6.032461534	5.797372015	5.851047075	5.753035414
ENSG00000278864	0.004696	0.033870889	0.516074107	1.430058442	**AC055811.4**	TEC		chr17	17181504	17183257	1	havana	1.376085183	1.11804813	1.634122236	1.005046425	0.740413176	1.608684788	1.580839304	1.426455924	1.89507148
ENSG00000171097	0.0047	0.033879765	0.502580119	1.416745008	**KYAT1**	protein_coding	kynurenine aminotransferase 1 [Source:HGNC Symbol;Acc:HGNC:1564]	chr9	128832942	128882494	-1	ensembl_havana	3.700984161	3.449694102	3.952274221	3.167256782	3.449096928	3.732728596	3.882763999	3.919661155	4.054397508
ENSG00000231770	0.004705	0.0339003	0.316987235	1.245726394	**TMEM44-AS1**	antisense	TMEM44 antisense RNA 1 [Source:HGNC Symbol;Acc:HGNC:44272]	chr3	194584011	194590260	1	havana	2.25219859	2.093704973	2.410692208	2.005254198	2.093349987	2.182510732	2.40164672	2.34408831	2.486341593
ENSG00000228842	0.004709	0.033915902	-0.942995681	-1.922516102	**PCDH9-AS2**	antisense	PCDH9 antisense RNA 2 [Source:HGNC Symbol;Acc:HGNC:39896]	chr13	66825169	66915031	1	havana	0.212784801	0.684282642	-0.25871304	1.126095262	0.243386951	0.683365711	-0.30988669	-0.47099802	0.004745582
ENSG00000088826	0.004714	0.033928696	-0.882903798	-1.844083264	**SMOX**	protein_coding	spermine oxidase [Source:HGNC Symbol;Acc:HGNC:15862]	chr20	4120980	4187747	1	ensembl_havana	-0.823529032	-0.382077133	-1.26498093	-0.92573229	0.09469996	-0.315199064	-1.52209115	-1.24204464	-1.030806998
ENSG00000248476	0.004717	0.033929398	0.415997876	1.334221205	**BACH1-IT1**	sense_intronic	BACH1 intronic transcript 1 [Source:HGNC Symbol;Acc:HGNC:40006]	chr21	29351634	29361894	1	havana	-0.215942145	-0.423941083	-0.007943207	-0.10791135	-0.18055577	-0.983356126	0.24405081	0.130707766	-0.398588198
ENSG00000103356	0.004718	0.033929398	-0.283957349	-1.217530024	**EARS2**	protein_coding	glutamyl-tRNA synthetase 2, mitochondrial [Source:HGNC Symbol;Acc:HGNC:29419]	chr16	23522014	23557731	-1	ensembl_havana	5.141699658	5.283678332	4.999720983	5.280633819	5.149650449	5.420750727	4.972369601	4.854145008	5.172648341
ENSG00000181031	0.00473	0.033993303	-0.951817049	-1.93430735	**RPH3AL**	protein_coding	rabphilin 3A like (without C2 domains) [Source:HGNC Symbol;Acc:HGNC:10296]	chr17	212389	386254	-1	ensembl_havana	0.716687567	1.192596091	0.240779042	1.528734855	0.837200041	1.211853376	0.176794179	0.343769191	0.201773757
ENSG00000182179	0.004737	0.034026116	-0.36353054	-1.286570522	**UBA7**	protein_coding	ubiquitin like modifier activating enzyme 7 [Source:HGNC Symbol;Acc:HGNC:12471]	chr3	49805207	49813946	-1	ensembl_havana	6.849923919	7.031689188	6.668158649	6.938850739	7.00736478	7.148852046	6.704945216	6.572253136	6.727277595
ENSG00000131400	0.00474	0.034033831	-0.909139912	-1.877925607	**NAPSA**	protein_coding	napsin A aspartic peptidase [Source:HGNC Symbol;Acc:HGNC:13395]	chr19	50358477	50365830	-1	ensembl_havana	1.452358413	1.90692837	0.997788457	2.014244272	1.953538152	1.753002685	0.64332197	1.179325527	1.170717874
ENSG00000162913	0.004743	0.034040187	0.925327869	1.899115788	**OBSCN-AS1**	antisense	OBSCN antisense RNA 1 [Source:HGNC Symbol;Acc:HGNC:32047]	chr1	228203506	228213664	-1	havana	1.264914929	0.802250994	1.727578863	0.872903038	0.658018659	0.875831286	1.444517675	2.046678052	1.691540863
ENSG00000132646	0.00476	0.034145198	-0.319516018	-1.247911841	**PCNA**	protein_coding	proliferating cell nuclear antigen [Source:HGNC Symbol;Acc:HGNC:8729]	chr20	5114953	5126626	-1	ensembl_havana	8.067919376	8.227677385	7.908161367	8.238359727	8.252338644	8.192333784	7.845075803	7.926648093	7.952760204
ENSG00000235576	0.004765	0.034160565	-0.478944661	-1.393723777	**LINC01871**	lincRNA	long intergenic non-protein coding RNA 1871 [Source:HGNC Symbol;Acc:HGNC:52690]	chr2	7725801	7730705	1	havana	3.475875303	3.715347634	3.236402972	3.532084689	3.764236011	3.8497222	3.221855697	3.085333629	3.402019592
ENSG00000125458	0.004768	0.034167078	-0.383896226	-1.304861085	**NT5C**	protein_coding	5', 3'-nucleotidase, cytosolic [Source:HGNC Symbol;Acc:HGNC:17144]	chr17	75130225	75131795	-1	ensembl_havana	2.962936253	3.154884367	2.77098814	3.175294617	2.991644088	3.297714395	2.815009813	2.721536001	2.776418607
ENSG00000237289	0.004775	0.034197707	-0.354662066	-1.278686029	**CKMT1B**	protein_coding	creatine kinase, mitochondrial 1B [Source:HGNC Symbol;Acc:HGNC:1995]	chr15	43593054	43604901	1	ensembl_havana	5.215139833	5.392470866	5.0378088	5.556191001	5.432794196	5.1884274	5.237693459	4.958309717	4.917423224
ENSG00000226067	0.004791	0.034298863	0.602383493	1.518222769	**LINC00623**	lincRNA	long intergenic non-protein coding RNA 623 [Source:HGNC Symbol;Acc:HGNC:44252]	chr1	120913275	121009291	1	havana	0.295113108	-0.006078638	0.596304855	-0.41068387	0.030651399	0.361796557	0.339398945	0.779979312	0.669536307
ENSG00000163515	0.004799	0.034307886	1.387506809	2.616261611	**RETNLB**	protein_coding	resistin like beta [Source:HGNC Symbol;Acc:HGNC:20388]	chr3	108743424	108757384	-1	ensembl_havana	-0.566742893	-1.260496297	0.127010512	-1.86005921	-1.178380098	-0.743049581	0.210814386	0.130707766	0.039509383
ENSG00000275437	0.004806	0.034307886	0.563206426	1.477549462	**AL121832.3**	sense_intronic		chr20	62402236	62405935	-1	havana	2.530845832	2.249242618	2.812449045	2.183196095	2.263224966	2.301306795	2.967063242	2.8516036	2.618680293
ENSG00000239521	0.004801	0.034307886	0.402219777	1.321539711	**CASTOR3**	protein_coding	CASTOR family member 3 [Source:HGNC Symbol;Acc:HGNC:29954]	chr7	100200653	100272218	-1	ensembl_havana	3.766935199	3.565825311	3.968045088	3.487590512	3.711591469	3.498293951	3.895893608	3.970942906	4.037298749
ENSG00000178607	0.004798	0.034307886	0.361219926	1.284511607	**ERN1**	protein_coding	endoplasmic reticulum to nucleus signaling 1 [Source:HGNC Symbol;Acc:HGNC:3449]	chr17	64039142	64130819	-1	ensembl_havana	5.080599252	4.899989288	5.261209215	4.784165916	4.851334342	5.064467608	5.179826793	5.302932702	5.30086815
ENSG00000143315	0.004807	0.034307886	0.294176083	1.226184508	**PIGM**	protein_coding	phosphatidylinositol glycan anchor biosynthesis class M [Source:HGNC Symbol;Acc:HGNC:18858]	chr1	160024953	160031991	-1	ensembl_havana	6.042501747	5.895413706	6.189589788	5.79791191	5.943865115	5.944464091	6.12182951	6.180051864	6.26688799
ENSG00000249353	0.004804	0.034307886	-0.587321259	-1.502454456	**NPM1P27**	processed_pseudogene	nucleophosmin 1 pseudogene 27 [Source:HGNC Symbol;Acc:HGNC:45206]	chr5	93682838	93683667	-1	havana	-0.386048157	-0.092387527	-0.679708786	-0.18813307	-0.258437325	0.16940781	-0.51701327	-0.86978558	-0.652327512
ENSG00000196542	0.004834	0.034432815	0.98774224	1.983079121	**SPTSSB**	protein_coding	serine palmitoyltransferase small subunit B [Source:HGNC Symbol;Acc:HGNC:24045]	chr3	161344792	161372880	-1	ensembl_havana	2.142430945	1.648559825	2.636302065	1.355633303	1.827267088	1.762779083	2.853206155	2.660368592	2.395331447
ENSG00000134986	0.004838	0.034432815	0.93306268	1.90932498	**NREP**	protein_coding	neuronal regeneration related protein [Source:HGNC Symbol;Acc:HGNC:16834]	chr5	111662621	111997464	-1	ensembl_havana	4.986271697	4.519740357	5.452803037	4.142918254	4.69740613	4.718896689	5.559846573	5.313807442	5.484755097
ENSG00000165801	0.004832	0.034432815	0.32453523	1.252260953	**ARHGEF40**	protein_coding	Rho guanine nucleotide exchange factor 40 [Source:HGNC Symbol;Acc:HGNC:25516]	chr14	21070270	21090240	1	ensembl_havana	3.907897842	3.745630227	4.070165458	3.703060534	3.766695609	3.767134538	4.042420699	4.165594683	4.002480991
ENSG00000232931	0.004836	0.034432815	0.312122852	1.241533209	**LINC00342**	lincRNA	long intergenic non-protein coding RNA 342 [Source:HGNC Symbol;Acc:HGNC:42470]	chr2	95807118	95816215	-1	havana	4.635358549	4.479297123	4.791419974	4.436712083	4.494235511	4.506943775	4.786069135	4.724316947	4.863873841
ENSG00000157625	0.004827	0.034432815	0.301031369	1.232024862	**TAB3**	protein_coding	TGF-beta activated kinase 1 and MAP3K7 binding protein 3 [Source:HGNC Symbol;Acc:HGNC:30681]	chrX	30827442	30975084	-1	ensembl_havana	6.928155618	6.777639933	7.078671302	6.888709135	6.754230899	6.689979765	7.122134297	7.080891047	7.032988563
ENSG00000136630	0.004841	0.034432815	0.284157227	1.217698718	**HLX**	protein_coding	H2.0 like homeobox [Source:HGNC Symbol;Acc:HGNC:4978]	chr1	220879400	220885059	1	ensembl_havana	0.033178276	-0.108900338	0.175256889	0.206373087	-0.258437325	-0.274636777	0.457455004	0.063570082	0.004745582
ENSG00000186470	0.004841	0.034432815	-0.27739602	-1.21200531	**BTN3A2**	protein_coding	butyrophilin subfamily 3 member A2 [Source:HGNC Symbol;Acc:HGNC:1139]	chr6	26365159	26378320	1	ensembl_havana	5.540233754	5.678931764	5.401535744	5.633343755	5.646191719	5.757259818	5.346897146	5.392000723	5.465709362
ENSG00000184497	0.004844	0.034435605	-1.143286474	-2.208836256	**TMEM255B**	protein_coding	transmembrane protein 255B [Source:HGNC Symbol;Acc:HGNC:28297]	chr13	113759240	113816995	1	ensembl_havana	-0.525863811	0.045779425	-1.097507048	0.770507832	-0.473795405	-0.159374151	-0.63291296	-1.00738779	-1.652220391
ENSG00000130939	0.004849	0.034452388	0.29134384	1.223779672	**UBE4B**	protein_coding	ubiquitination factor E4B [Source:HGNC Symbol;Acc:HGNC:12500]	chr1	10032832	10181239	1	ensembl_havana	6.707492082	6.561820162	6.853164002	6.528230575	6.530084543	6.627145369	6.871388543	6.810356309	6.877747155
ENSG00000235944	0.004857	0.034475093	0.277764219	1.212314673	**ZNF815P**	transcribed_unprocessed_pseudogene	zinc finger protein 815, pseudogene [Source:HGNC Symbol;Acc:HGNC:22029]	chr7	5823160	5854365	1	havana	3.071621441	2.932739332	3.210503551	2.996312281	2.887010108	2.914895605	3.250759372	3.167564252	3.213187029
ENSG00000123575	0.004856	0.034475093	-0.343086675	-1.268467605	**FAM199X**	protein_coding	family with sequence similarity 199, X-linked [Source:HGNC Symbol;Acc:HGNC:25195]	chrX	104166620	104195902	1	ensembl_havana	6.791017009	6.962560347	6.619473672	7.055031987	6.914481677	6.918167375	6.598142162	6.623048562	6.63723029
ENSG00000125648	0.004862	0.034476905	0.303035969	1.233737929	**SLC25A23**	protein_coding	solute carrier family 25 member 23 [Source:HGNC Symbol;Acc:HGNC:19375]	chr19	6436079	6465203	-1	ensembl_havana	6.075179372	5.923661387	6.226697356	6.039422805	5.827937615	5.903623742	6.353141897	6.186968944	6.139981228
ENSG00000137693	0.004861	0.034476905	-0.374129662	-1.296057443	**YAP1**	protein_coding	Yes associated protein 1 [Source:HGNC Symbol;Acc:HGNC:16262]	chr11	102110461	102233423	1	ensembl_havana	3.290839134	3.477903965	3.103774303	3.61124356	3.399242599	3.423225735	3.209288915	2.919625469	3.182408526
ENSG00000172575	0.004869	0.034479224	-0.312205594	-1.241604416	**RASGRP1**	protein_coding	RAS guanyl releasing protein 1 [Source:HGNC Symbol;Acc:HGNC:9878]	chr15	38488103	38565575	-1	ensembl_havana	9.693387563	9.84949036	9.537284766	10.11143515	9.748277113	9.688758814	9.717693376	9.489801449	9.404359472
ENSG00000225335	0.004869	0.034479224	-0.640247232	-1.55859623	**AC016027.1**	antisense		chr22	18076527	18078884	-1	ensembl_havana	-0.12680925	0.193314366	-0.446932866	0.355307344	0.243386951	-0.018751196	-0.51701327	-0.47099802	-0.352787316
ENSG00000073350	0.004865	0.034479224	-0.944519927	-1.924548364	**LLGL2**	protein_coding	LLGL2, scribble cell polarity complex component [Source:HGNC Symbol;Acc:HGNC:6629]	chr17	75525080	75575208	1	ensembl_havana	0.690716313	1.162976276	0.218456349	1.590147445	1.078042694	0.820738689	0.399634207	-0.11936381	0.375098651
ENSG00000049249	0.004878	0.034513907	0.322990366	1.250920727	**TNFRSF9**	protein_coding	TNF receptor superfamily member 9 [Source:HGNC Symbol;Acc:HGNC:11924]	chr1	7915894	7943165	-1	ensembl_havana	6.986286628	6.824791445	7.147781812	7.134739024	6.922776138	6.416859174	7.366665811	7.268421491	6.808258133
ENSG00000168939	0.004881	0.034513907	0.298669355	1.230009412	**SPRY3**	protein_coding	sprouty RTK signaling antagonist 3 [Source:HGNC Symbol;Acc:HGNC:11271]	chrX	155767812	155782459	1	ensembl_havana	2.535267712	2.385933034	2.684602389	2.484284696	2.433879495	2.239634912	2.84781106	2.688493842	2.517502265
ENSG00000253293	0.004881	0.034513907	-0.363835385	-1.286842407	**HOXA10**	protein_coding	homeobox A10 [Source:HGNC Symbol;Acc:HGNC:5100]	chr7	27170591	27180261	-1	ensembl_havana	1.926224267	2.108141959	1.744306575	1.95010455	2.199112819	2.17520851	1.500601289	1.790554303	1.941764132
ENSG00000105246	0.004886	0.034528376	-0.43899515	-1.35565977	**EBI3**	protein_coding	Epstein-Barr virus induced 3 [Source:HGNC Symbol;Acc:HGNC:3129]	chr19	4229498	4237531	1	ensembl_havana	5.920799403	6.140296978	5.701301828	6.311057227	6.088019157	6.02181455	6.006115214	5.698282368	5.399507903
ENSG00000119139	0.004889	0.034533338	0.610563973	1.526855965	**TJP2**	protein_coding	tight junction protein 2 [Source:HGNC Symbol;Acc:HGNC:11828]	chr9	69121264	69255208	1	ensembl_havana	1.138617953	0.833335967	1.443899939	1.283104003	0.77991046	0.436993436	1.926226713	1.114463419	1.291009686
ENSG00000277969	0.004902	0.034610721	-0.782185705	-1.719734325	**AC006449.6**	lincRNA		chr17	38702452	38704747	1	havana	2.255247186	2.646340039	1.864154333	2.873244578	2.383487184	2.682288355	2.08139109	1.95703246	1.554039449
ENSG00000089280	0.004906	0.034616386	-0.298429649	-1.229805061	**FUS**	protein_coding	FUS RNA binding protein [Source:HGNC Symbol;Acc:HGNC:4010]	chr16	31180110	31194871	1	ensembl_havana	9.196984843	9.346199668	9.047770019	9.450682568	9.3337977	9.254118735	9.106637657	9.016341606	9.020330795
ENSG00000205302	0.004913	0.03463074	0.473384525	1.388362713	**SNX2**	protein_coding	sorting nexin 2 [Source:HGNC Symbol;Acc:HGNC:11173]	chr5	122774996	122830108	1	ensembl_havana	8.167674849	7.930982587	8.404367111	8.11894067	7.885485968	7.788521121	8.390063433	8.399581847	8.423456054
ENSG00000188177	0.004922	0.03463074	-0.292004009	-1.224339795	**ZC3H6**	protein_coding	zinc finger CCCH-type containing 6 [Source:HGNC Symbol;Acc:HGNC:24762]	chr2	112275594	112340063	1	ensembl_havana	3.28804532	3.434047324	3.142043315	3.3380874	3.436793925	3.527260648	3.044565681	3.195272324	3.186291941
ENSG00000170627	0.004921	0.03463074	-0.318521575	-1.247051957	**GTSF1**	protein_coding	gametocyte specific factor 1 [Source:HGNC Symbol;Acc:HGNC:26565]	chr12	54455950	54473602	-1	ensembl_havana	5.297396055	5.456656843	5.138135267	5.444234638	5.522848508	5.402887382	5.054287027	5.191390799	5.168727976
ENSG00000234390	0.004916	0.03463074	-0.425198172	-1.342756934	**USP27X-AS1**	lincRNA	USP27X antisense RNA 1 (head to head) [Source:HGNC Symbol;Acc:HGNC:27249]	chrX	49876724	49879241	-1	havana	1.048095497	1.260694582	0.835496411	0.968509893	1.37743234	1.436141515	0.716189844	0.821210834	0.969088554
ENSG00000277075	0.004922	0.03463074	-0.47055235	-1.385639873	**HIST1H2AE**	protein_coding	histone cluster 1 H2A family member e [Source:HGNC Symbol;Acc:HGNC:4724]	chr6	26216975	26217483	1	ensembl_havana	1.564852777	1.800128952	1.329576602	2.023178671	2.029240114	1.347968071	1.693402841	1.625790657	0.669536307
ENSG00000230445	0.004913	0.03463074	-0.566550584	-1.480978384	**LRRC37A6P**	transcribed_processed_pseudogene	leucine rich repeat containing 37 member A6, pseudogene [Source:HGNC Symbol;Acc:HGNC:33746]	chr10	27246350	27259455	-1	havana	1.449390127	1.732665419	1.166114835	1.283104003	1.845996665	2.068895587	0.894226935	1.372000412	1.232117157
ENSG00000163812	0.004942	0.03466109	0.287771428	1.220753089	**ZDHHC3**	protein_coding	zinc finger DHHC-type containing 3 [Source:HGNC Symbol;Acc:HGNC:18470]	chr3	44915257	44976185	-1	ensembl_havana	6.413194214	6.2693085	6.557079928	6.263310319	6.302377424	6.242237756	6.571795714	6.539428369	6.5600157
ENSG00000137411	0.004936	0.03466109	-0.277718682	-1.212276408	**VARS2**	protein_coding	valyl-tRNA synthetase 2, mitochondrial [Source:HGNC Symbol;Acc:HGNC:21642]	chr6	30908242	30926459	1	ensembl_havana	7.244472154	7.383331495	7.105612813	7.442793098	7.28552648	7.421674905	7.191926155	6.984931539	7.139980745
ENSG00000198707	0.004933	0.03466109	-0.286162543	-1.219392471	**CEP290**	protein_coding	centrosomal protein 290 [Source:HGNC Symbol;Acc:HGNC:29021]	chr12	88049014	88142216	-1	ensembl_havana	4.328199784	4.471281055	4.185118512	4.458282965	4.394387857	4.561172345	4.125025993	4.139344231	4.290985313
ENSG00000235363	0.004938	0.03466109	-0.314530545	-1.243606916	**SNRPGP10**	processed_pseudogene	small nuclear ribonucleoprotein polypeptide G pseudogene 10 [Source:HGNC Symbol;Acc:HGNC:39329]	chr1	205351247	205351471	1	havana	0.955761824	1.113027097	0.798496552	1.237769075	0.740413176	1.360899039	0.851734776	0.504278656	1.039476224
ENSG00000170703	0.004941	0.03466109	-0.369495346	-1.291900845	**TTLL6**	protein_coding	tubulin tyrosine ligase like 6 [Source:HGNC Symbol;Acc:HGNC:26664]	chr17	48762235	48817214	-1	ensembl_havana	5.282819239	5.467566912	5.098071567	5.459125168	5.606237645	5.337337923	5.121778643	5.104295527	5.068140529
ENSG00000061656	0.00493	0.03466109	-0.402360574	-1.32166869	**SPAG4**	protein_coding	sperm associated antigen 4 [Source:HGNC Symbol;Acc:HGNC:11214]	chr20	35615892	35621049	1	ensembl_havana	2.873010563	3.07419085	2.671830276	3.134649994	3.108719303	2.979203253	2.879883083	2.69405369	2.441554055
ENSG00000167077	0.004943	0.03466109	-0.570202337	-1.484731788	**MEI1**	protein_coding	meiotic double-stranded break formation protein 1 [Source:HGNC Symbol;Acc:HGNC:28613]	chr22	41699499	41799456	1	ensembl_havana	2.536312087	2.821413256	2.251210919	2.807536027	2.913888608	2.742815133	2.50808372	2.155463891	2.090085146
ENSG00000196968	0.00495	0.034688695	-0.35271424	-1.276960802	**FUT11**	protein_coding	fucosyltransferase 11 [Source:HGNC Symbol;Acc:HGNC:19233]	chr10	73772291	73780251	1	ensembl_havana	4.139456275	4.315813395	3.963099155	4.298201297	4.449008971	4.200229918	4.021008592	3.973231137	3.895057737
ENSG00000165949	0.004969	0.034768697	0.703157044	1.628063582	**IFI27**	protein_coding	interferon alpha inducible protein 27 [Source:HGNC Symbol;Acc:HGNC:5397]	chr14	94104836	94116698	1	ensembl_havana	1.965062815	1.613484293	2.316641337	1.142587728	1.769573688	1.928291464	2.099663825	2.566495926	2.283764259
ENSG00000148341	0.004967	0.034768697	0.41218651	1.330701061	**SH3GLB2**	protein_coding	SH3 domain containing GRB2 like, endophilin B2 [Source:HGNC Symbol;Acc:HGNC:10834]	chr9	129007036	129028303	-1	ensembl_havana	5.541273472	5.335180217	5.747366727	5.182360084	5.289631136	5.533549431	5.764106035	5.640425293	5.837568852
ENSG00000198689	0.004976	0.034768697	0.333999083	1.26050259	**SLC9A6**	protein_coding	solute carrier family 9 member A6 [Source:HGNC Symbol;Acc:HGNC:11079]	chrX	135973841	136047269	1	ensembl_havana	5.731249291	5.564249749	5.898248832	5.474677979	5.654848351	5.563222917	5.880304096	5.883648474	5.930793927
ENSG00000167515	0.004968	0.034768697	0.329732788	1.256780576	**TRAPPC2L**	protein_coding	trafficking protein particle complex 2 like [Source:HGNC Symbol;Acc:HGNC:30887]	chr16	88856220	88862686	1	ensembl_havana	4.796408702	4.631542308	4.961275096	4.576800429	4.690945658	4.626880836	4.850879427	4.973248329	5.059697532
ENSG00000117016	0.004975	0.034768697	0.326721525	1.254160098	**RIMS3**	protein_coding	regulating synaptic membrane exocytosis 3 [Source:HGNC Symbol;Acc:HGNC:21292]	chr1	40620679	40665657	-1	ensembl_havana	6.01598236	5.852621597	6.179343123	5.791389702	5.891861652	5.874613438	6.213824122	6.196793309	6.127411938
ENSG00000200087	0.004971	0.034768697	-0.528444521	-1.442373225	**SNORA73B**	snoRNA	small nucleolar RNA, H/ACA box 73B [Source:HGNC Symbol;Acc:HGNC:10116]	chr1	28508559	28508762	1	ensembl	2.282512321	2.546734582	2.018290061	1.996207752	2.635736107	3.008259887	1.356079599	2.358164528	2.340626055
ENSG00000107371	0.004981	0.034791397	-0.284212032	-1.217744977	**EXOSC3**	protein_coding	exosome component 3 [Source:HGNC Symbol;Acc:HGNC:17944]	chr9	37766978	37801437	-1	ensembl_havana	4.769038277	4.911144293	4.626932261	4.917125919	4.860574589	4.955732372	4.640774659	4.611326446	4.628695679
ENSG00000260630	0.004991	0.034794556	0.516419062	1.430400417	**SNAI3-AS1**	antisense	SNAI3 antisense RNA 1 [Source:HGNC Symbol;Acc:HGNC:28327]	chr16	88663298	88687186	1	havana	1.097545843	0.839336312	1.355755374	0.832812769	0.403680778	1.281515389	1.212343926	1.163381334	1.691540863
ENSG00000099326	0.004991	0.034794556	0.425921032	1.343429889	**MZF1**	protein_coding	myeloid zinc finger 1 [Source:HGNC Symbol;Acc:HGNC:13108]	chr19	58561931	58573575	-1	ensembl_havana	5.084302224	4.871341708	5.29726274	5.034447579	4.78129592	4.798281624	5.2853681	5.251071083	5.355349037
ENSG00000109466	0.00499	0.034794556	0.347050273	1.27195733	**KLHL2**	protein_coding	kelch like family member 2 [Source:HGNC Symbol;Acc:HGNC:6353]	chr4	165207618	165323156	1	ensembl_havana	4.658352146	4.484827009	4.831877283	4.527418732	4.427408044	4.499654252	4.754702143	4.83288226	4.908047446
ENSG00000173581	0.004988	0.034794556	-0.549815192	-1.46389816	**CCDC106**	protein_coding	coiled-coil domain containing 106 [Source:HGNC Symbol;Acc:HGNC:30181]	chr19	55641062	55653161	1	ensembl_havana	3.297006844	3.57191444	3.022099248	3.741463211	3.464329594	3.509950514	3.353314566	3.015998475	2.696984702
ENSG00000185133	0.004999	0.034830411	-0.471230398	-1.386291258	**INPP5J**	protein_coding	inositol polyphosphate-5-phosphatase J [Source:HGNC Symbol;Acc:HGNC:8956]	chr22	31122731	31134696	1	ensembl_havana	1.483590395	1.719205594	1.247975196	1.770752281	1.7890623	1.5978022	1.458744294	1.114463419	1.170717874
ENSG00000279059	0.005005	0.03483559	0.387770748	1.308370148	**AC007485.2**	TEC		chr17	54956760	54958426	-1	havana	4.980705744	4.78682037	5.174591118	4.642053159	4.96600401	4.75240394	5.051942913	5.210913556	5.260916884
ENSG00000175183	0.005003	0.03483559	-0.395637007	-1.315523487	**CSRP2**	protein_coding	cysteine and glycine rich protein 2 [Source:HGNC Symbol;Acc:HGNC:2470]	chr12	76858715	76879060	-1	ensembl_havana	2.746842679	2.944661182	2.549024176	2.744105292	2.974723635	3.11515462	2.50808372	2.491852285	2.647136522
ENSG00000175727	0.005029	0.034851102	0.344102267	1.269360863	**MLXIP**	protein_coding	MLX interacting protein [Source:HGNC Symbol;Acc:HGNC:17055]	chr12	122078722	122147347	1	ensembl_havana	5.944745494	5.772694361	6.116796628	5.838970007	5.660811121	5.818301954	6.169100816	6.113149554	6.068139513
ENSG00000245526	0.005026	0.034851102	0.340468665	1.266167847	**LINC00461**	lincRNA	long intergenic non-protein coding RNA 461 [Source:HGNC Symbol;Acc:HGNC:42810]	chr5	88507546	88691041	-1	havana	4.297618829	4.127384496	4.467853161	4.07580181	4.195151489	4.11120019	4.376151155	4.462907727	4.564500602
ENSG00000114439	0.005017	0.034851102	0.304427376	1.234928381	**BBX**	protein_coding	BBX, HMG-box containing [Source:HGNC Symbol;Acc:HGNC:14422]	chr3	107522936	107811324	1	ensembl_havana	6.656728234	6.504514546	6.808941922	6.430038495	6.483370561	6.600134583	6.740679118	6.850740658	6.835405991
ENSG00000112159	0.005014	0.034851102	-0.274225193	-1.20934443	**MDN1**	protein_coding	midasin AAA ATPase 1 [Source:HGNC Symbol;Acc:HGNC:18302]	chr6	89642499	89819723	-1	ensembl_havana	7.913633742	8.050746339	7.776521146	8.213327587	7.973289445	7.965621984	7.918323873	7.711885201	7.699354363
ENSG00000117153	0.005024	0.034851102	-0.300316625	-1.231414639	**KLHL12**	protein_coding	kelch like family member 12 [Source:HGNC Symbol;Acc:HGNC:19360]	chr1	202891100	202928636	-1	ensembl_havana	7.128618583	7.278776896	6.978460271	7.375054264	7.181758785	7.279517638	7.014894801	6.877557983	7.04292803
ENSG00000167380	0.005024	0.034851102	-0.313640747	-1.242840145	**ZNF226**	protein_coding	zinc finger protein 226 [Source:HGNC Symbol;Acc:HGNC:13019]	chr19	44165073	44178381	1	ensembl_havana	4.839019595	4.995839969	4.682199222	5.165310036	4.887945457	4.934264414	4.781831765	4.654741462	4.610024438
ENSG00000155592	0.005016	0.034851102	-0.315663406	-1.244583828	**ZKSCAN2**	protein_coding	zinc finger with KRAB and SCAN domains 2 [Source:HGNC Symbol;Acc:HGNC:25677]	chr16	25236001	25257931	-1	ensembl_havana	4.944531901	5.102363604	4.786700198	5.235219966	5.093935728	4.977935117	4.838692245	4.785491911	4.735916438
ENSG00000106443	0.005027	0.034851102	-0.317590989	-1.246247827	**PHF14**	protein_coding	PHD finger protein 14 [Source:HGNC Symbol;Acc:HGNC:22203]	chr7	10973336	11169630	1	ensembl_havana	6.891978243	7.050773738	6.733182749	7.148328949	6.976355165	7.027637098	6.753719179	6.654044568	6.7917845
ENSG00000229950	0.005019	0.034851102	-0.924605367	-1.898164949	**TFAP2A-AS1**	antisense	TFAP2A antisense RNA 1 [Source:HGNC Symbol;Acc:HGNC:40579]	chr6	10409340	10416446	1	havana	1.787623613	2.249926296	1.325320929	1.968722995	2.357615216	2.423440678	1.527846157	1.114463419	1.333653212
ENSG00000130787	0.005043	0.034909104	0.401159289	1.320568638	**HIP1R**	protein_coding	huntingtin interacting protein 1 related [Source:HGNC Symbol;Acc:HGNC:18415]	chr12	122834453	122862960	1	ensembl_havana	4.040802769	3.840223125	4.241382414	3.770935591	3.667521044	4.08221274	4.168953129	4.212836819	4.342357293
ENSG00000197050	0.00505	0.034909104	0.302884829	1.233608686	**ZNF420**	protein_coding	zinc finger protein 420 [Source:HGNC Symbol;Acc:HGNC:20649]	chr19	37007857	37130314	1	ensembl_havana	4.181777328	4.030334913	4.333219742	4.069346566	4.104724807	3.916933367	4.301155015	4.46453516	4.233969052
ENSG00000198718	0.005056	0.034909104	0.297232885	1.228785319	**TOGARAM1**	protein_coding	TOG array regulator of axonemal microtubules 1 [Source:HGNC Symbol;Acc:HGNC:19959]	chr14	44962208	45074431	1	ensembl_havana	4.664803019	4.516186576	4.813419461	4.576800429	4.486795527	4.484963773	4.865636402	4.84667624	4.727945741
ENSG00000150773	0.005058	0.034909104	0.288907947	1.221715146	**PIH1D2**	protein_coding	PIH1 domain containing 2 [Source:HGNC Symbol;Acc:HGNC:25210]	chr11	112064010	112074274	-1	ensembl_havana	1.844583496	1.700129523	1.98903747	1.760103226	1.667974555	1.672310787	2.005893922	1.947753749	2.013464737
ENSG00000076053	0.005042	0.034909104	-0.319515557	-1.247911442	**RBM7**	protein_coding	RNA binding motif protein 7 [Source:HGNC Symbol;Acc:HGNC:9904]	chr11	114400030	114414203	1	ensembl_havana	4.81538006	4.975137838	4.655622282	5.1118699	4.977728472	4.835815142	4.700360184	4.687163416	4.579343245
ENSG00000267156	0.005059	0.034909104	-0.364257004	-1.287218534	**TPMTP1**	processed_pseudogene	thiopurine S-methyltransferase pseudogene 1 [Source:HGNC Symbol;Acc:HGNC:17490]	chr18	47630112	47630848	1	havana	1.787200657	1.969329159	1.605072155	1.738566362	2.061651132	2.107769984	1.325353043	1.831488934	1.65837449
ENSG00000258590	0.005046	0.034909104	-0.441742989	-1.358244296	**NBEAP1**	transcribed_unprocessed_pseudogene	neurobeachin pseudogene 1 [Source:HGNC Symbol;Acc:HGNC:1007]	chr15	20657638	20688408	-1	havana	1.412769045	1.633640539	1.191897551	1.882980075	1.709476673	1.30846487	1.580839304	1.080904991	0.913948357
ENSG00000271538	0.005049	0.034909104	-0.461579914	-1.377049016	**LINC02427**	lincRNA	long intergenic non-protein coding RNA 2427 [Source:HGNC Symbol;Acc:HGNC:53358]	chr4	184506460	184537554	-1	havana	-0.025822927	0.204967029	-0.256612884	0.41082706	0.09469996	0.109374069	0.069637898	-0.57414696	-0.265329594
ENSG00000261504	0.005055	0.034909104	-0.615565195	-1.532158123	**LINC01686**	lincRNA	long intergenic non-protein coding RNA 1686 [Source:HGNC Symbol;Acc:HGNC:27643]	chr1	182615254	182616629	1	havana	1.4388575	1.746640098	1.131074903	1.843172741	1.900769223	1.495978329	1.486783452	1.01135145	0.895089805
ENSG00000213281	0.005063	0.034917848	-0.280751229	-1.214827295	**NRAS**	protein_coding	NRAS proto-oncogene, GTPase [Source:HGNC Symbol;Acc:HGNC:7989]	chr1	114704469	114716894	-1	ensembl_havana	7.689013947	7.829389561	7.548638332	7.811685606	7.948588509	7.727894568	7.555152022	7.62705324	7.463709736
ENSG00000136842	0.005067	0.034931253	0.41517924	1.333464336	**TMOD1**	protein_coding	tropomodulin 1 [Source:HGNC Symbol;Acc:HGNC:11871]	chr9	97501180	97601748	1	ensembl_havana	7.595963499	7.388373879	7.803553119	7.478960092	7.524089778	7.162071766	8.01400558	7.768317303	7.628336475
ENSG00000081386	0.005078	0.034962954	0.339874567	1.26564655	**ZNF510**	protein_coding	zinc finger protein 510 [Source:HGNC Symbol;Acc:HGNC:29161]	chr9	96755865	96778129	-1	ensembl_havana	4.212683145	4.042745861	4.382620429	4.0388342	4.133596708	3.955806677	4.275264199	4.4823168	4.390280286
ENSG00000114648	0.005079	0.034962954	0.280272329	1.214424102	**KLHL18**	protein_coding	kelch like family member 18 [Source:HGNC Symbol;Acc:HGNC:29120]	chr3	47282917	47346816	1	ensembl_havana	6.041553126	5.901416961	6.18168929	5.849682467	5.94928867	5.905279747	6.162597074	6.234004576	6.148466221
ENSG00000134330	0.005076	0.034962954	-0.308235282	-1.238192205	**IAH1**	protein_coding	isoamyl acetate hydrolyzing esterase 1 (putative) [Source:HGNC Symbol;Acc:HGNC:27696]	chr2	9473658	9496543	1	ensembl_havana	4.207187719	4.36130536	4.053070078	4.309200618	4.367092387	4.407623076	4.032943419	4.10844956	4.017817256
ENSG00000067141	0.005081	0.034964092	0.319975406	1.248309268	**NEO1**	protein_coding	neogenin 1 [Source:HGNC Symbol;Acc:HGNC:7754]	chr15	73051710	73305206	1	ensembl_havana	7.925562553	7.76557485	8.085550255	7.821641937	7.714801469	7.760281145	8.128279085	7.959768046	8.168603635
ENSG00000261416	0.005088	0.034994756	-0.770622779	-1.706006069	**AC012645.3**	antisense		chr16	30183505	30184957	-1	havana	-0.101855698	0.283455691	-0.487167087	0.297565234	0.325758945	0.227042895	-0.63291296	-0.68524259	-0.143345712
ENSG00000128951	0.005092	0.034999767	-0.322068306	-1.250121489	**DUT**	protein_coding	deoxyuridine triphosphatase [Source:HGNC Symbol;Acc:HGNC:3078]	chr15	48331011	48343373	1	ensembl_havana	5.686313984	5.847348137	5.525279831	5.805052447	5.869723176	5.867268788	5.425055197	5.508596823	5.642187474
ENSG00000100784	0.005103	0.035044839	0.361121183	1.284423693	**RPS6KA5**	protein_coding	ribosomal protein S6 kinase A5 [Source:HGNC Symbol;Acc:HGNC:10434]	chr14	90847862	91060636	-1	ensembl_havana	2.941565326	2.761004735	3.122125918	2.822967335	2.918320072	2.541726798	3.086398123	3.249141357	3.030838274
ENSG00000131797	0.005102	0.035044839	0.270557718	1.20627406	**CLUHP3**	transcribed_unprocessed_pseudogene	clustered mitochondria homolog pseudogene 3 [Source:HGNC Symbol;Acc:HGNC:28447]	chr16	31700590	31711986	1	havana	5.227339352	5.092060493	5.362618211	5.109774447	5.056230524	5.110176506	5.375299268	5.328180747	5.384374617
ENSG00000078124	0.005114	0.035086144	0.278885723	1.213257454	**ACER3**	protein_coding	alkaline ceramidase 3 [Source:HGNC Symbol;Acc:HGNC:16066]	chr11	76860867	77026797	1	ensembl_havana	4.468867648	4.329424786	4.608310509	4.238078501	4.424295625	4.325900232	4.518771605	4.668927578	4.637232345
ENSG00000131885	0.005113	0.035086144	-0.898259736	-1.863816381	**KRT17P1**	transcribed_unprocessed_pseudogene	keratin 17 pseudogene 1 [Source:HGNC Symbol;Acc:HGNC:6428]	chr17	16840895	16845881	-1	havana	0.296997657	0.746127525	-0.152132211	0.326725152	0.699804047	1.211853376	-1.04978262	0.063570082	0.529815901
ENSG00000157954	0.00513	0.035178044	0.285476754	1.218812967	**WIPI2**	protein_coding	WD repeat domain, phosphoinositide interacting 2 [Source:HGNC Symbol;Acc:HGNC:32225]	chr7	5190188	5233826	1	ensembl_havana	6.880058829	6.737320452	7.022797206	6.758670989	6.748020484	6.705269883	7.076446919	7.05249687	6.93944783
ENSG00000125249	0.005147	0.035273062	0.287222193	1.220288436	**RAP2A**	protein_coding	RAP2A, member of RAS oncogene family [Source:HGNC Symbol;Acc:HGNC:9861]	chr13	97434222	97469128	1	ensembl_havana	6.011524814	5.867913717	6.15513591	5.965450526	5.901965947	5.736324678	6.221666555	6.165116922	6.078624255
ENSG00000146733	0.005148	0.035273062	-0.369296565	-1.291722853	**PSPH**	protein_coding	phosphoserine phosphatase [Source:HGNC Symbol;Acc:HGNC:9577]	chr7	56011051	56051604	-1	ensembl_havana	4.385633989	4.570282271	4.200985706	4.383933617	4.698694759	4.628218436	4.127254407	4.199216933	4.276485779
ENSG00000128159	0.005154	0.035284572	0.453308806	1.369176851	**TUBGCP6**	protein_coding	tubulin gamma complex associated protein 6 [Source:HGNC Symbol;Acc:HGNC:18127]	chr22	50217689	50244992	-1	ensembl_havana	6.198854972	5.972200569	6.425509375	6.003757294	5.750525702	6.162318712	6.546208824	6.318321269	6.411998033
ENSG00000134313	0.005155	0.035284572	0.288155142	1.221077816	**KIDINS220**	protein_coding	kinase D interacting substrate 220 [Source:HGNC Symbol;Acc:HGNC:29508]	chr2	8721081	8837630	-1	ensembl_havana	7.679844501	7.53576693	7.823922072	7.584396309	7.463218952	7.55968553	7.832327174	7.806353667	7.833085376
ENSG00000128039	0.00516	0.035303016	-0.356804556	-1.280586367	**SRD5A3**	protein_coding	steroid 5 alpha-reductase 3 [Source:HGNC Symbol;Acc:HGNC:25812]	chr4	55346109	55373096	1	ensembl_havana	4.030008555	4.208410833	3.851606277	4.234236316	4.2990561	4.091940082	3.908904805	3.816389236	3.829524789

ENSG00000125967	0.005166	0.035325435	0.461698974	1.377162664	**NECAB3**	protein_coding	N-terminal EF-hand calcium binding protein 3 [Source:HGNC Symbol;Acc:HGNC:15851]	chr20	33657087	33674463	-1	ensembl_havana	4.436836069	4.205986582	4.667685556	3.959604644	4.167497176	4.490857928	4.575361077	4.675968666	4.751726925
ENSG00000101363	0.005182	0.035417798	0.275504165	1.210417008	**MANBAL**	protein_coding	mannosidase beta like [Source:HGNC Symbol;Acc:HGNC:15799]	chr20	37289638	37317260	1	ensembl_havana	5.006837836	4.869085753	5.144589918	4.79594333	4.886815311	4.924498619	5.101520624	5.136299722	5.19594941
ENSG00000237187	0.005197	0.035432811	0.67189406	1.593163199	**NR2F1-AS1**	antisense	NR2F1 antisense RNA 1 [Source:HGNC Symbol;Acc:HGNC:48622]	chr5	93409359	93585648	-1	havana	1.298555206	0.962608177	1.634502236	0.812341977	1.476918714	0.598563838	1.717267538	1.811166797	1.375072374
ENSG00000197122	0.005204	0.035432811	0.639770234	1.558080997	**SRC**	protein_coding	SRC proto-oncogene, non-receptor tyrosine kinase [Source:HGNC Symbol;Acc:HGNC:11283]	chr20	37344685	37406050	1	ensembl_havana	0.369398123	0.049513006	0.68928324	-0.22998119	0.501432867	-0.122912658	0.36983092	0.880929471	0.817089329
ENSG00000188687	0.005191	0.035432811	0.518330263	1.432296587	**SLC4A5**	protein_coding	solute carrier family 4 member 5 [Source:HGNC Symbol;Acc:HGNC:18168]	chr2	74216242	74343414	-1	ensembl_havana	2.877192677	2.618027546	3.136357809	2.744105292	2.698916685	2.411060659	3.473788816	2.998131222	2.937153388
ENSG00000088325	0.005199	0.035432811	-0.305850746	-1.236147368	**TPX2**	protein_coding	TPX2, microtubule nucleation factor [Source:HGNC Symbol;Acc:HGNC:1249]	chr20	31739271	31801805	1	ensembl_havana	8.242685015	8.395610388	8.089759642	8.17099419	8.501373219	8.514463754	7.939719028	8.129404778	8.20015512
ENSG00000104343	0.005199	0.035432811	-0.309724766	-1.239471214	**UBE2W**	protein_coding	ubiquitin conjugating enzyme E2 W [Source:HGNC Symbol;Acc:HGNC:25616]	chr8	73780097	73878910	-1	ensembl_havana	4.332650814	4.487513197	4.177788431	4.535246918	4.562357411	4.364935261	4.201042438	4.193340247	4.138982607
ENSG00000002586	0.005206	0.035432811	-0.314895187	-1.243921279	**CD99**	protein_coding	CD99 molecule (Xg blood group) [Source:HGNC Symbol;Acc:HGNC:7082]	chrX	2691133	2741309	1	ensembl_havana	5.935621224	6.093068817	5.77817363	6.17787793	6.095375965	6.005952557	5.954968134	5.733130366	5.64642239
ENSG00000167315	0.005206	0.035432811	-0.315250954	-1.244228066	**ACAA2**	protein_coding	acetyl-CoA acyltransferase 2 [Source:HGNC Symbol;Acc:HGNC:83]	chr18	49782167	49813960	-1	ensembl_havana	6.046883505	6.204508982	5.889258028	6.049843472	6.276721671	6.286961804	5.701924173	5.902981482	6.06286843
ENSG00000165732	0.005202	0.035432811	-0.322221696	-1.250254412	**DDX21**	protein_coding	DExD-box helicase 21 [Source:HGNC Symbol;Acc:HGNC:2744]	chr10	68956128	68985073	1	ensembl_havana	8.016936627	8.178047475	7.855825779	8.221477413	8.131109876	8.181555135	7.803006281	7.879238988	7.885232068
ENSG00000198001	0.005199	0.035432811	-0.380351822	-1.301659245	**IRAK4**	protein_coding	interleukin 1 receptor associated kinase 4 [Source:HGNC Symbol;Acc:HGNC:17967]	chr12	43758944	43789543	1	ensembl_havana	5.454867199	5.64504311	5.264691288	5.779574903	5.572193156	5.583361271	5.253080613	5.232087792	5.308905459
ENSG00000169282	0.005211	0.035439855	0.458938345	1.374529953	**KCNAB1**	protein_coding	potassium voltage-gated channel subfamily A member regulatory beta subunit 1 [Source:HGNC Symbol;Acc:HGNC:6228]	chr3	156037701	156539138	1	ensembl_havana	0.496460564	0.266991391	0.725929736	0.174648163	0.547932369	0.078393642	0.64332197	0.821210834	0.713256405
ENSG00000198719	0.005212	0.035439855	-0.285562457	-1.218885372	**DLL1**	protein_coding	delta like canonical Notch ligand 1 [Source:HGNC Symbol;Acc:HGNC:2908]	chr6	170282206	170306565	-1	ensembl_havana	3.873028174	4.015809402	3.730246945	4.275947831	4.016539696	3.754940679	4.01138913	3.677351664	3.502000041
ENSG00000176894	0.00523	0.03551522	0.427653922	1.345044516	**PXMP2**	protein_coding	peroxisomal membrane protein 2 [Source:HGNC Symbol;Acc:HGNC:9716]	chr12	132687606	132704991	1	ensembl_havana	0.504044901	0.29021794	0.717871862	0.297565234	0.403680778	0.16940781	0.540060475	0.900298706	0.713256405
ENSG00000198270	0.005227	0.03551522	-0.36830543	-1.290835741	**TMEM116**	protein_coding	transmembrane protein 116 [Source:HGNC Symbol;Acc:HGNC:25084]	chr12	111931282	112013185	-1	ensembl_havana	2.66116728	2.845319995	2.477014566	2.781444624	2.754497827	3.000017535	2.459388507	2.479027387	2.492627803
ENSG00000279275	0.005231	0.03551522	-0.9724593	-1.962182594	**AP002505.2**	TEC		chr18	13279415	13280110	-1	havana	0.063913246	0.550142896	-0.422316404	0.683014432	0.658018659	0.309395597	-0.75894329	-0.28449416	-0.223511759
ENSG00000154217	0.005235	0.035525792	0.344103326	1.269361795	**PITPNC1**	protein_coding	phosphatidylinositol transfer protein, cytoplasmic 1 [Source:HGNC Symbol;Acc:HGNC:21045]	chr17	67377281	67697261	1	ensembl_havana	4.350653808	4.178602145	4.522705471	3.954943093	4.327656656	4.253206686	4.378023791	4.552860279	4.637232345
ENSG00000128656	0.005241	0.035546436	0.349023338	1.27369808	**CHN1**	protein_coding	chimerin 1 [Source:HGNC Symbol;Acc:HGNC:1943]	chr2	174799363	175005369	-1	ensembl_havana	4.009505711	3.834994042	4.18401738	3.54144321	3.957478716	4.006060199	3.979675389	4.185467242	4.386909509
ENSG00000185485	0.005243	0.035546436	0.333727699	1.260265501	**SDHAP1**	transcribed_unprocessed_pseudogene	succinate dehydrogenase complex flavoprotein subunit A pseudogene 1 [Source:HGNC Symbol;Acc:HGNC:32455]	chr3	195959748	195990318	-1	havana	3.565636326	3.398772477	3.732500176	3.320074956	3.389700035	3.486542439	3.623370039	3.831602732	3.742527757
ENSG00000067836	0.005259	0.035625111	0.438271876	1.3549803	**ROGDI**	protein_coding	rogdi homolog [Source:HGNC Symbol;Acc:HGNC:29478]	chr16	4796968	4802950	-1	ensembl_havana	1.902446995	1.683311057	2.121582933	1.613998728	1.62524309	1.810691353	2.246292314	1.947753749	2.170702736
ENSG00000104518	0.005259	0.035625111	-0.596220847	-1.511751329	**GSDMD**	protein_coding	gasdermin D [Source:HGNC Symbol;Acc:HGNC:25697]	chr8	143553207	143563062	1	ensembl_havana	4.551185749	4.849296172	4.253075325	5.034447579	4.565186067	4.94825487	4.596424846	4.110529916	4.052271213
ENSG00000164442	0.005269	0.035643749	0.354935874	1.278928733	**CITED2**	protein_coding	Cbp/p300 interacting transactivator with Glu/Asp rich carboxy-terminal domain 2 [Source:HGNC Symbol;Acc:HGNC:1987]	chr6	139371807	139374620	-1	ensembl_havana	1.772129079	1.594661142	1.949597016	1.683274163	1.569984981	1.530724282	2.117708014	1.97541275	1.755670283
ENSG00000168301	0.00527	0.035643749	0.32057657	1.248829541	**KCTD6**	protein_coding	potassium channel tetramerization domain containing 6 [Source:HGNC Symbol;Acc:HGNC:22235]	chr3	58492114	58502360	1	ensembl_havana	4.327273916	4.166985631	4.487562201	4.062862309	4.332645478	4.105449108	4.409492236	4.557442765	4.495751603
ENSG00000176510	0.005267	0.035643749	-1.338565272	-2.528996906	**OR10AC1**	polymorphic_pseudogene	olfactory receptor family 10 subfamily AC member 1 (gene/pseudogene) [Source:HGNC Symbol;Acc:HGNC:14758]	chr7	143509256	143519469	-1	havana	-0.902514036	-0.2332314	-1.571796672	0.040274975	0.243386951	-0.983356126	-1.90365811	-1.15951152	-1.652220391
ENSG00000144847	0.005286	0.035692705	0.706853826	1.632240705	**IGSF11**	protein_coding	immunoglobulin superfamily member 11 [Source:HGNC Symbol;Acc:HGNC:16669]	chr3	118900557	119146068	-1	ensembl_havana	2.31820911	1.964782197	2.671636023	1.383653947	2.357615216	2.153077428	2.459388507	2.886015394	2.669504168
ENSG00000070759	0.005287	0.035692705	0.375103372	1.29693248	**TESK2**	protein_coding	testis-specific kinase 2 [Source:HGNC Symbol;Acc:HGNC:11732]	chr1	45343883	45491166	-1	ensembl_havana	4.518401378	4.330849692	4.705953064	4.281543461	4.358965032	4.352040582	4.770470885	4.759212226	4.588176082
ENSG00000198246	0.005282	0.035692705	0.297100215	1.228672325	**SLC29A3**	protein_coding	solute carrier family 29 member 3 [Source:HGNC Symbol;Acc:HGNC:23096]	chr10	71319258	71381423	1	ensembl_havana	2.405738687	2.25718858	2.554288795	2.214735639	2.242169235	2.314660866	2.44516827	2.548193946	2.669504168
ENSG00000149633	0.005285	0.035692705	-0.70860649	-1.634224845	**KIAA1755**	protein_coding	KIAA1755 [Source:HGNC Symbol;Acc:HGNC:29372]	chr20	38210488	38260772	-1	ensembl_havana	0.313609721	0.667912966	-0.040693524	0.075059705	0.760296981	1.168382212	-0.63291296	0.37179175	0.139040636
ENSG00000143164	0.00529	0.035699658	-0.277729089	-1.212285153	**DCAF6**	protein_coding	DDB1 and CUL4 associated factor 6 [Source:HGNC Symbol;Acc:HGNC:30002]	chr1	167935783	168075843	1	ensembl_havana	5.948509631	6.087374176	5.809645087	6.124391059	6.101234519	6.036496949	5.882284543	5.824044636	5.722606082
ENSG00000247828	0.005304	0.035763043	0.360269841	1.283665972	**TMEM161B-AS1**	antisense	TMEM161B antisense RNA 1 [Source:HGNC Symbol;Acc:HGNC:43839]	chr5	88268895	88436685	1	havana	4.025543167	3.845408246	4.205678088	3.909890552	3.711591469	3.914742718	4.136133802	4.159579142	4.321321319
ENSG00000204386	0.005305	0.035763043	0.308789856	1.23866826	**NEU1**	protein_coding	neuraminidase 1 [Source:HGNC Symbol;Acc:HGNC:7758]	chr6	31857659	31862906	-1	ensembl_havana	7.738725701	7.584330773	7.893120629	7.743043418	7.627170019	7.382778883	8.052769241	7.860201309	7.766391338
ENSG00000197208	0.005313	0.035803356	0.377868592	1.2994207	**SLC22A4**	protein_coding	solute carrier family 22 member 4 [Source:HGNC Symbol;Acc:HGNC:10968]	chr5	132294443	132344206	1	ensembl_havana	1.835189604	1.646255308	2.0241239	1.716703112	1.657409759	1.564653053	2.22979561	1.910030375	1.932545716
ENSG00000164051	0.005317	0.035815164	-0.352681852	-1.276932135	**CCDC51**	protein_coding	coiled-coil domain containing 51 [Source:HGNC Symbol;Acc:HGNC:25714]	chr3	48432164	48440456	-1	ensembl_havana	4.267193824	4.44353475	4.090852898	4.243822659	4.408632007	4.678149585	3.834482717	4.185467242	4.252608736
ENSG00000146223	0.005321	0.035819928	-0.319322788	-1.247744711	**RPL7L1**	protein_coding	ribosomal protein L7 like 1 [Source:HGNC Symbol;Acc:HGNC:21370]	chr6	42879618	42889925	1	ensembl_havana	8.195892213	8.355553607	8.036230818	8.404096302	8.284555206	8.378009313	8.0356694	8.063816347	8.009206708
ENSG00000082781	0.00536	0.035826913	0.838558215	1.788262114	**ITGB5**	protein_coding	integrin subunit beta 5 [Source:HGNC Symbol;Acc:HGNC:6160]	chr3	124761948	124901418	-1	ensembl_havana	-0.10442206	-0.523701167	0.314857047	-1.41179303	-0.299013651	0.139703184	-0.12879794	0.256279753	0.817089329
ENSG00000138646	0.005331	0.035826913	0.551163502	1.465266925	**HERC5**	protein_coding	HECT and RLD domain containing E3 ubiquitin protein ligase 5 [Source:HGNC Symbol;Acc:HGNC:24368]	chr4	88457117	88506163	1	ensembl_havana	5.12689413	4.851312379	5.402475881	4.177343198	5.084108452	5.292485489	4.982251883	5.594342551	5.63083321
ENSG00000253669	0.005351	0.035826913	0.498549643	1.412792552	**GASAL1**	lincRNA	growth arrest associated lncRNA 1 [Source:HGNC Symbol;Acc:HGNC:53461]	chr8	102805517	102809971	1	havana	0.893859858	0.644585037	1.14313468	0.613726219	0.636663181	0.683365711	1.195452122	0.900298706	1.333653212
ENSG00000084710	0.005331	0.035826913	0.382073398	1.30321345	**EFR3B**	protein_coding	EFR3 homolog B [Source:HGNC Symbol;Acc:HGNC:29155]	chr2	25042130	25159137	1	ensembl_havana	4.431375334	4.240338635	4.622412034	4.03442213	4.206066476	4.480527301	4.57045622	4.520368735	4.776411146
ENSG00000206560	0.005351	0.035826913	0.30272561	1.23347255	**ANKRD28**	protein_coding	ankyrin repeat domain 28 [Source:HGNC Symbol;Acc:HGNC:29024]	chr3	15667236	15859771	-1	ensembl_havana	6.24788292	6.096520115	6.399245725	5.849682467	6.172527074	6.267350804	6.233092219	6.433729774	6.530915182
ENSG00000188542	0.005352	0.035826913	0.293729667	1.225805147	**DUSP28**	protein_coding	dual specificity phosphatase 28 [Source:HGNC Symbol;Acc:HGNC:33237]	chr2	240560054	240564014	1	ensembl_havana	2.176690287	2.029825454	2.323555121	1.959443807	2.184470312	1.945562243	2.318278476	2.466087459	2.186299428
ENSG00000084731	0.005364	0.035826913	0.278445624	1.212887401	**KIF3C**	protein_coding	kinesin family member 3C [Source:HGNC Symbol;Acc:HGNC:6321]	chr2	25926596	25982749	-1	ensembl_havana	5.565384981	5.426162169	5.704607793	5.48036596	5.441287214	5.356833333	5.802261543	5.687870688	5.623691148
ENSG00000136247	0.005359	0.035826913	-0.278093697	-1.21259157	**ZDHHC4**	protein_coding	zinc finger DHHC-type containing 4 [Source:HGNC Symbol;Acc:HGNC:18471]	chr7	6577434	6589374	1	ensembl_havana	5.498316982	5.637363831	5.359270133	5.564630185	5.69607929	5.651382017	5.311079824	5.373928988	5.392801588
ENSG00000115163	0.005339	0.035826913	-0.285110938	-1.218503958	**CENPA**	protein_coding	centromere protein A [Source:HGNC Symbol;Acc:HGNC:1851]	chr2	26764289	26801067	1	ensembl_havana	5.304389677	5.446945146	5.161834208	5.381350195	5.548803996	5.410681246	5.06246156	5.24161066	5.181430402
ENSG00000121940	0.005345	0.035826913	-0.287267303	-1.220326592	**CLCC1**	protein_coding	chloride channel CLIC like 1 [Source:HGNC Symbol;Acc:HGNC:29675]	chr1	108929508	108963457	-1	ensembl_havana	5.702938599	5.846572251	5.559304948	5.837071289	5.853616734	5.849028731	5.552403201	5.51332185	5.612189792
ENSG00000189283	0.005345	0.035826913	-0.312097285	-1.241511208	**FHIT**	protein_coding	fragile histidine triad [Source:HGNC Symbol;Acc:HGNC:3701]	chr3	59749310	61251459	-1	ensembl_havana	1.708374132	1.864422774	1.552325489	1.578072284	1.96214833	2.053047709	1.178360195	1.671599815	1.807016457
ENSG00000244155	0.005348	0.035826913	-0.327659717	-1.25497595	**CYP4F34P**	unprocessed_pseudogene	cytochrome P450 family 4 subfamily F member 34, pseudogene [Source:HGNC Symbol;Acc:HGNC:39953]	chr13	18741730	18742718	-1	havana	1.132133007	1.295962866	0.968303149	1.477650254	1.256642557	1.153595787	1.052687018	0.938274071	0.913948357
ENSG00000169972	0.005351	0.035826913	-0.332098733	-1.258843322	**PUSL1**	protein_coding	pseudouridylate synthase-like 1 [Source:HGNC Symbol;Acc:HGNC:26914]	chr1	1308567	1311677	1	ensembl_havana	1.471710344	1.63775971	1.305660977	1.671956805	1.558674763	1.682647563	1.325353043	1.32976598	1.261863909
ENSG00000241837	0.00534	0.035826913	-0.333546703	-1.260107402	**ATP5PO**	protein_coding	ATP synthase peripheral stalk subunit OSCP [Source:HGNC Symbol;Acc:HGNC:850]	chr21	33903453	33915980	-1	ensembl_havana	5.191301565	5.358074917	5.024528214	5.428348316	5.345818033	5.3000584	4.983482422	5.084323255	5.005778964
ENSG00000183605	0.005363	0.035826913	-0.415940073	-1.334167749	**SFXN4**	protein_coding	sideroflexin 4 [Source:HGNC Symbol;Acc:HGNC:16088]	chr10	119140767	119165667	-1	ensembl_havana	5.375136109	5.583106145	5.167166072	5.780233825	5.63748283	5.331601781	5.334413759	5.082204736	5.084879722
ENSG00000168495	0.005361	0.035826913	-0.416777004	-1.334941946	**POLR3D**	protein_coding	RNA polymerase III subunit D [Source:HGNC Symbol;Acc:HGNC:1080]	chr8	22245104	22254600	1	ensembl_havana	5.406336477	5.614724978	5.197947975	5.799212821	5.526481482	5.518480632	5.217964471	5.126053155	5.249826298
ENSG00000275713	0.005332	0.035826913	-0.590752122	-1.506031684	**HIST1H2BH**	protein_coding	histone cluster 1 H2B family member h [Source:HGNC Symbol;Acc:HGNC:4755]	chr6	26251651	26253710	1	ensembl_havana	3.076897847	3.372273908	2.781521786	3.784135645	3.439879526	2.892806553	2.941964313	2.836600654	2.566000389
ENSG00000235106	0.005367	0.0358293	0.331366173	1.258204278	**BRD3OS**	protein_coding	BRD3 opposite strand [Source:HGNC Symbol;Acc:HGNC:24742]	chr9	134025439	134034666	1	ensembl_havana	4.100784685	3.935101599	4.266467771	3.760287894	4.014471486	4.030545416	4.201042438	4.273512191	4.324848684
ENSG00000079246	0.005381	0.035889067	-0.304229849	-1.234759312	**XRCC5**	protein_coding	X-ray repair cross complementing 5 [Source:HGNC Symbol;Acc:HGNC:12833]	chr2	216107464	216206303	1	ensembl_havana	9.779379419	9.931494343	9.627264495	10.03305618	9.928986355	9.832440492	9.67029355	9.603060177	9.608439757
ENSG00000101160	0.005381	0.035889067	-0.310365075	-1.240021448	**CTSZ**	protein_coding	cathepsin Z [Source:HGNC Symbol;Acc:HGNC:2547]	chr20	58995185	59007247	-1	ensembl_havana	6.322466374	6.477648912	6.167283837	6.451708643	6.523554953	6.457683139	6.196419224	6.124002823	6.181429463
ENSG00000272079	0.005384	0.035895866	0.72360978	1.651308624	**AC004233.3**	lincRNA		chr16	3006120	3007388	1	havana	-0.087427036	-0.449231926	0.274377854	-1.14838881	-0.18055577	-0.018751196	-0.04612063	0.315191528	0.554062661
ENSG00000185305	0.005392	0.035929382	0.283385526	1.217047542	**ARL15**	protein_coding	ADP ribosylation factor like GTPase 15 [Source:HGNC Symbol;Acc:HGNC:25945]	chr5	53883945	54310582	-1	ensembl_havana	3.399663915	3.257971152	3.541356678	3.264647647	3.24901995	3.26024586	3.584973459	3.479075818	3.560020757
ENSG00000116885	0.005412	0.035960027	0.632689125	1.550452282	**OSCP1**	protein_coding	organic solute carrier partner 1 [Source:HGNC Symbol;Acc:HGNC:29971]	chr1	36415827	36450451	-1	ensembl_havana	2.209652736	1.893308174	2.525997299	2.31999144	1.817810321	1.542122761	2.657410455	2.479027387	2.441554055
ENSG00000088727	0.005412	0.035960027	0.52121354	1.435161944	**KIF9**	protein_coding	kinesin family member 9 [Source:HGNC Symbol;Acc:HGNC:16666]	chr3	47228026	47283451	-1	ensembl_havana	2.421836539	2.161229769	2.68244331	2.369873023	2.101167058	2.012649227	2.64502189	2.800974855	2.601333184
ENSG00000124588	0.00541	0.035960027	0.407581716	1.326460501	**NQO2**	protein_coding	N-ribosyldihydronicotinamide:quinone reductase 2 [Source:HGNC Symbol;Acc:HGNC:7856]	chr6	2987987	3028869	1	ensembl_havana	4.240199376	4.036408518	4.443990234	3.957275751	4.035021363	4.116928438	4.309028837	4.614261782	4.408680083
ENSG00000172824	0.005407	0.035960027	-0.285442983	-1.218784437	**CES4A**	protein_coding	carboxylesterase 4A [Source:HGNC Symbol;Acc:HGNC:26741]	chr16	66988589	67009758	1	ensembl_havana	7.452325806	7.595047297	7.309604314	7.753483162	7.46132218	7.57033655	7.442893833	7.234008163	7.251910946
ENSG00000122034	0.005409	0.035960027	-0.301991511	-1.232845071	**GTF3A**	protein_coding	general transcription factor IIIA [Source:HGNC Symbol;Acc:HGNC:4662]	chr13	27424544	27435823	1	ensembl_havana	5.99717416	6.148169916	5.846178404	6.030588746	6.193245505	6.220675496	5.660981195	5.886686386	5.990867632
ENSG00000137573	0.005414	0.035960027	-0.380320248	-1.301630758	**SULF1**	protein_coding	sulfatase 1 [Source:HGNC Symbol;Acc:HGNC:20391]	chr8	69466624	69660915	1	ensembl_havana	10.08262319	10.27278332	9.89246307	10.75930497	10.08765459	9.971390398	10.24199986	9.714582282	9.720807069
ENSG00000235560	0.00541	0.035960027	-0.427773113	-1.345155644	**AC002310.1**	antisense		chr16	30534752	30537144	1	havana	1.58255188	1.796438437	1.368665324	1.541228291	1.827267088	2.02081993	1.278000209	1.399485086	1.428510677
ENSG00000236849	0.005419	0.035973851	-0.824834018	-1.771331235	**LINC01474**	lincRNA	long intergenic non-protein coding RNA 1474 [Source:HGNC Symbol;Acc:HGNC:51111]	chr9	72871728	72874109	-1	havana	2.512687406	2.925104415	2.100270397	2.912241745	2.974723635	2.888347866	1.644455473	2.358164528	2.298191191
ENSG00000162365	0.005424	0.035990487	-0.558621519	-1.47286124	**CYP4A22**	protein_coding	cytochrome P450 family 4 subfamily A member 22 [Source:HGNC Symbol;Acc:HGNC:20575]	chr1	47137435	47149741	1	ensembl_havana	0.965830505	1.245141264	0.686519745	1.424690178	1.547275176	0.76345844	1.052687018	0.715834679	0.291037539
ENSG00000163166	0.005432	0.036028476	-0.269028001	-1.204995702	**IWS1**	protein_coding	IWS1, SUPT6H interacting protein [Source:HGNC Symbol;Acc:HGNC:25467]	chr2	127436207	127526886	-1	ensembl_havana	6.218751398	6.353265398	6.084237397	6.361243343	6.345383102	6.353169748	6.081026415	6.064615297	6.107070479
ENSG00000108384	0.005436	0.036042389	-0.328645099	-1.25583341	**RAD51C**	protein_coding	RAD51 paralog C [Source:HGNC Symbol;Acc:HGNC:9820]	chr17	58692573	58735611	1	ensembl_havana	5.287405473	5.451728023	5.123082923	5.355054536	5.485260027	5.514869505	4.987167754	5.087495211	5.294585805
ENSG00000120725	0.005445	0.03608328	-0.341304709	-1.266901806	**SIL1**	protein_coding	SIL1 nucleotide exchange factor [Source:HGNC Symbol;Acc:HGNC:24624]	chr5	138946720	139293557	-1	ensembl_havana	5.357666265	5.52831862	5.18701391	5.580605547	5.394342179	5.610008133	5.352622479	5.034792482	5.173626769
ENSG00000104814	0.005449	0.036089476	-0.32756941	-1.254897396	**MAP4K1**	protein_coding	mitogen-activated protein kinase kinase kinase kinase 1 [Source:HGNC Symbol;Acc:HGNC:6863]	chr19	38587641	38618882	-1	ensembl_havana	6.614186512	6.777971217	6.450401807	6.791397232	6.68179598	6.860720441	6.544959164	6.376531066	6.429715192
ENSG00000189423	0.00546	0.036145182	-0.609678291	-1.525918903	**USP32P3**	transcribed_unprocessed_pseudogene	ubiquitin specific peptidase 32 pseudogene 3 [Source:HGNC Symbol;Acc:HGNC:43576]	chr17	20415547	20431008	1	havana	0.685253112	0.990092258	0.380413967	0.968509893	0.720251491	1.281515389	0.668022463	-0.00684508	0.480064523
ENSG00000136531	0.00547	0.03614762	0.937337426	1.914990754	**SCN2A**	protein_coding	sodium voltage-gated channel alpha subunit 2 [Source:HGNC Symbol;Acc:HGNC:10588]	chr2	165194993	165392310	1	ensembl_havana	5.88780552	5.419136807	6.356474233	4.745548893	5.727352473	5.784509055	6.193763704	6.432898725	6.44276027
ENSG00000197381	0.005463	0.03614762	0.284961515	1.218377762	**ADARB1**	protein_coding	adenosine deaminase, RNA specific B1 [Source:HGNC Symbol;Acc:HGNC:226]	chr21	45073853	45226560	1	ensembl_havana	4.221735067	4.079254309	4.364215824	4.043232818	4.028887076	4.165643032	4.368636211	4.256703576	4.467307685
ENSG00000189362	0.005468	0.03614762	-0.321221198	-1.24938767	**NEMP2**	protein_coding	nuclear envelope integral membrane protein 2 [Source:HGNC Symbol;Acc:HGNC:33700]	chr2	190504342	190534722	-1	ensembl_havana	3.179577047	3.340187646	3.018966448	3.283361026	3.259536354	3.477665558	2.967063242	3.024849801	3.064986301
ENSG00000125775	0.005466	0.03614762	-0.494022621	-1.408366309	**SDCBP2**	protein_coding	syndecan binding protein 2 [Source:HGNC Symbol;Acc:HGNC:15756]	chr20	1309909	1329239	-1	ensembl_havana	-0.184930875	0.062080435	-0.431942186	0.326725152	0.09469996	-0.235183806	-0.00649342	-0.32890884	-0.960424301
ENSG00000276449	0.005478	0.0361873	0.511042192	1.42507929	**AC004076.2**	antisense		chr19	57449689	57453011	1	havana	0.311420553	0.055899457	0.566941649	-0.27307956	0.271370116	0.16940781	0.141952326	1.029056719	0.529815901
ENSG00000276334	0.005498	0.036263431	0.376851193	1.298504662	**AL133243.2**	sense_intronic		chr2	32521927	32523547	1	havana	-0.010421426	-0.198847022	0.178004171	-0.36334239	-0.18055577	-0.052642908	-0.12879794	0.315191528	0.347618925
ENSG00000173715	0.0055	0.036263431	0.282431612	1.216243093	**C11orf80**	protein_coding	chromosome 11 open reading frame 80 [Source:HGNC Symbol;Acc:HGNC:26197]	chr11	66744451	66843328	1	ensembl_havana	2.985974451	2.844758645	3.127190257	2.705773822	2.944626756	2.883875356	3.015987943	3.171555278	3.194027549
ENSG00000198952	0.005499	0.036263431	0.27265276	1.208027051	**SMG5**	protein_coding	SMG5, nonsense mediated mRNA decay factor [Source:HGNC Symbol;Acc:HGNC:24644]	chr1	156249224	156282825	-1	ensembl_havana	8.147289583	8.010963203	8.283615963	8.061147972	7.944113375	8.027628262	8.360059698	8.225276826	8.265511366
ENSG00000240356	0.0055	0.036263431	-0.28529095	-1.218656007	**RPL23AP7**	transcribed_processed_pseudogene	ribosomal protein L23a pseudogene 7 [Source:HGNC Symbol;Acc:HGNC:17336]	chr2	113610502	113627090	-1	havana	4.305701305	4.44834678	4.16305583	4.468131009	4.402318629	4.474590701	4.129479384	4.167594302	4.192093803
ENSG00000119636	0.005516	0.036265891	0.449299456	1.365377095	**BBOF1**	protein_coding	basal body orientation factor 1 [Source:HGNC Symbol;Acc:HGNC:19855]	chr14	74019353	74082863	1	ensembl_havana	1.851769887	1.627120159	2.076419615	1.613998728	1.524202165	1.743159585	2.144358071	2.097907935	1.986992839
ENSG00000143363	0.005518	0.036265891	0.304373968	1.234882665	**PRUNE1**	protein_coding	prune exopolyphosphatase 1 [Source:HGNC Symbol;Acc:HGNC:13420]	chr1	151008420	151035713	1	ensembl_havana	5.51531441	5.363127426	5.667501394	5.346181719	5.373515124	5.369685436	5.606116546	5.636821229	5.759566407
ENSG00000110925	0.005504	0.036265891	0.277355435	1.211971215	**CSRNP2**	protein_coding	cysteine and serine rich nuclear protein 2 [Source:HGNC Symbol;Acc:HGNC:16006]	chr12	51061205	51083664	-1	ensembl_havana	6.19035253	6.051674812	6.329030247	6.072076954	6.10609854	5.976848943	6.347418629	6.344188021	6.295484092
ENSG00000131725	0.005517	0.036265891	0.276301861	1.211086458	**WDR44**	protein_coding	WD repeat domain 44 [Source:HGNC Symbol;Acc:HGNC:30512]	chrX	118346073	118449961	1	ensembl_havana	6.97133329	6.833182359	7.109484221	7.162803057	6.864836217	6.471907805	7.421262834	7.114449797	6.792740032
ENSG00000101882	0.005509	0.036265891	-0.338053317	-1.264049812	**NKAP**	protein_coding	NFKB activating protein [Source:HGNC Symbol;Acc:HGNC:29873]	chrX	119920672	119943772	-1	ensembl_havana	4.566783468	4.735810126	4.39775681	4.918323696	4.655550213	4.63355647	4.480935426	4.424960935	4.287374069
ENSG00000233535	0.00551	0.036265891	-0.392018605	-1.312228177	**AC003035.2**	lincRNA		chrX	13955393	13963904	1	havana	1.913299755	2.109309058	1.717290453	2.290693707	2.108942002	1.928291464	1.91595172	1.566404757	1.669514881
ENSG00000010626	0.005514	0.036265891	-0.451623521	-1.36757838	**LRRC23**	protein_coding	leucine rich repeat containing 23 [Source:HGNC Symbol;Acc:HGNC:19138]	chr12	6873569	6914243	1	ensembl_havana	2.837399767	3.063211528	2.611588007	3.005358074	3.184258973	3.000017535	2.613576909	2.5296568	2.691530312
ENSG00000184349	0.005536	0.036348365	0.763044262	1.697067869	**EFNA5**	protein_coding	ephrin A5 [Source:HGNC Symbol;Acc:HGNC:3225]	chr5	107376889	107670895	-1	ensembl_havana	2.074084001	1.69256187	2.455606133	1.237769075	1.739838083	2.100078453	2.302589091	2.590542907	2.4736864
ENSG00000111639	0.005535	0.036348365	-0.365308891	-1.288157403	**MRPL51**	protein_coding	mitochondrial ribosomal protein L51 [Source:HGNC Symbol;Acc:HGNC:14044]	chr12	6491886	6493841	-1	ensembl_havana	6.971316099	7.153970545	6.788661654	7.193550508	7.141814753	7.126546374	6.686174079	6.866521552	6.81328933
ENSG00000137962	0.005541	0.036357785	0.424743328	1.342333665	**ARHGAP29**	protein_coding	Rho GTPase activating protein 29 [Source:HGNC Symbol;Acc:HGNC:30207]	chr1	94148988	94275068	-1	ensembl_havana	6.000127395	5.787755731	6.212499059	5.198225183	5.989854603	6.175187406	5.739553958	6.485945185	6.411998033
ENSG00000177225	0.005542	0.036357785	0.395321121	1.315235479	**GATD1**	protein_coding	glutamine amidotransferase like class 1 domain containing 1 [Source:HGNC Symbol;Acc:HGNC:26616]	chr11	767220	777488	-1	ensembl_havana	5.5918074	5.39414684	5.789467961	5.336358176	5.275883366	5.570198977	5.805743945	5.764516533	5.798143406
ENSG00000132330	0.005551	0.036395907	-0.329377997	-1.256471543	**SCLY**	protein_coding	selenocysteine lyase [Source:HGNC Symbol;Acc:HGNC:18161]	chr2	238060889	238099413	1	ensembl_havana	1.8833607	2.048049699	1.718671702	1.760103226	2.116675269	2.267370602	1.541278047	1.748424202	1.866312856
ENSG00000108556	0.005568	0.036493413	-0.741158939	-1.671518055	**CHRNE**	protein_coding	cholinergic receptor nicotinic epsilon subunit [Source:HGNC Symbol;Acc:HGNC:1966]	chr17	4897774	4903074	-1	ensembl_havana	0.393097246	0.763676715	0.022517776	1.175017601	0.453384582	0.662627962	0.069637898	0.02879206	-0.030876629
ENSG00000278932	0.005573	0.036506314	-0.397339986	-1.317077268	**CR381653.1**	lincRNA		chr21	9325013	9368775	1	havana	0.577034451	0.775704444	0.378364458	1.092533711	0.658018659	0.57656096	0.592614899	0.19485955	0.347618925
ENSG00000111110	0.005591	0.036583578	0.342980566	1.268374314	**PPM1H**	protein_coding	protein phosphatase, Mg2+/Mn2+ dependent 1H [Source:HGNC Symbol;Acc:HGNC:18583]	chr12	62643982	62935037	-1	ensembl_havana	6.264397275	6.092906992	6.435887558	5.920132273	6.071203518	6.287385184	6.388406921	6.354757311	6.564498441
ENSG00000167100	0.005592	0.036583578	-0.466464981	-1.381719705	**SAMD14**	protein_coding	sterile alpha motif domain containing 14 [Source:HGNC Symbol;Acc:HGNC:27312]	chr17	50110040	50129882	-1	ensembl_havana	2.58989832	2.823130811	2.35666583	2.848324962	2.918320072	2.702747398	2.581431252	2.271570328	2.21699591
ENSG00000256667	0.00559	0.036583578	-0.967996775	-1.956122574	**KLRA1P**	transcribed_unprocessed_pseudogene	killer cell lectin like receptor A1, pseudogene [Source:HGNC Symbol;Acc:HGNC:6372]	chr12	10588063	10599669	-1	ensembl_havana	1.294207143	1.77820553	0.810208755	1.912130943	1.739838083	1.682647563	0.513047461	0.715834679	1.201744126
ENSG00000279519	0.005597	0.036597425	0.285763693	1.219055402	**AC007382.1**	TEC		chr2	36839922	36842539	-1	havana	2.383643726	2.240761879	2.526525572	2.348706028	2.162224075	2.211355536	2.687924289	2.392765686	2.498886741
ENSG00000176533	0.005607	0.036651036	0.887510622	1.849981216	**GNG7**	protein_coding	G protein subunit gamma 7 [Source:HGNC Symbol;Acc:HGNC:4410]	chr19	2511219	2702709	-1	ensembl_havana	1.462808858	1.019053547	1.906564169	0.705390237	0.927899934	1.423870469	1.863449533	2.029189031	1.827053942
ENSG00000143353	0.005612	0.036664753	-0.430105348	-1.347331958	**LYPLAL1**	protein_coding	lysophospholipase like 1 [Source:HGNC Symbol;Acc:HGNC:20440]	chr1	219173844	219212865	1	ensembl_havana	5.126309545	5.341362219	4.911256871	5.536045381	5.24446336	5.243577917	4.928320531	4.834141723	4.971308361
ENSG00000067955	0.005617	0.036680612	-0.342337453	-1.267809034	**CBFB**	protein_coding	core-binding factor beta subunit [Source:HGNC Symbol;Acc:HGNC:1539]	chr16	67029116	67101058	1	ensembl_havana	5.971939039	6.143107765	5.800770312	6.110310669	6.140169437	6.17884319	5.798770715	5.677383323	5.926156899
ENSG00000234667	0.005635	0.036707794	0.536656603	1.450606887	**ACTBP13**	unitary_pseudogene	actin, beta pseudogene 13 [Source:HGNC Symbol;Acc:HGNC:44626]	chr3	49873347	49877305	-1	havana	0.561198531	0.292870229	0.829526833	0.267803752	0.501432867	0.109374069	0.830009965	1.256505808	0.402064725
ENSG00000235072	0.005638	0.036707794	0.471296879	1.386355142	**AC012074.1**	lincRNA		chr2	25369136	25375845	-1	havana	-0.397676037	-0.633324476	-0.162027597	-0.61730533	-0.967469033	-0.315199064	-0.35894771	-0.32890884	0.201773757
ENSG00000101935	0.005629	0.036707794	0.325383594	1.25299755	**AMMECR1**	protein_coding	Alport syndrome, mental retardation, midface hypoplasia and elliptocytosis chromosomal region gene 1 [Source:HGNC Symbol;Acc:HGNC:467]	chrX	110194186	110440233	-1	ensembl_havana	5.933494085	5.770802288	6.096185882	5.691946335	5.87486229	5.745598239	6.033105705	6.09855034	6.156901601
ENSG00000092445	0.005634	0.036707794	0.297744006	1.229220733	**TYRO3**	protein_coding	TYRO3 protein tyrosine kinase [Source:HGNC Symbol;Acc:HGNC:12446]	chr15	41557675	41583586	1	ensembl_havana	3.036697788	2.887825785	3.185569791	2.689029065	2.966188404	3.008259887	3.049274082	3.275339896	3.232095396
ENSG00000163808	0.005626	0.036707794	-0.272335338	-1.207761289	**KIF15**	protein_coding	kinesin family member 15 [Source:HGNC Symbol;Acc:HGNC:17273]	chr3	44761717	44873376	1	ensembl_havana	5.159607306	5.295774975	5.023439637	5.016663433	5.387965818	5.482695674	4.757582036	5.076894789	5.235842086
ENSG00000160683	0.005636	0.036707794	-0.296775631	-1.228395925	**CXCR5**	protein_coding	C-X-C motif chemokine receptor 5 [Source:HGNC Symbol;Acc:HGNC:1060]	chr11	118883766	118897799	1	ensembl_havana	2.583115834	2.731503649	2.434728018	2.912241745	3.085263982	2.197005222	2.613576909	2.721536001	1.969071146
ENSG00000204856	0.005639	0.036707794	-0.497470208	-1.411735887	**FAM216A**	protein_coding	family with sequence similarity 216 member A [Source:HGNC Symbol;Acc:HGNC:30180]	chr12	110468364	110490385	1	ensembl_havana	3.563640008	3.812375113	3.314904904	3.760287894	3.788645305	3.888192139	3.020790384	3.406428015	3.517496313
ENSG00000114686	0.005643	0.036713424	-0.27158457	-1.207132943	**MRPL3**	protein_coding	mitochondrial ribosomal protein L3 [Source:HGNC Symbol;Acc:HGNC:10379]	chr3	131462212	131502983	-1	ensembl_havana	6.847417629	6.983209914	6.711625344	6.8618803	6.987469041	7.100280401	6.568929493	6.753913364	6.812033175
ENSG00000165891	0.005651	0.036718784	0.508463143	1.422534008	**E2F7**	protein_coding	E2F transcription factor 7 [Source:HGNC Symbol;Acc:HGNC:23820]	chr12	77021247	77065580	-1	ensembl_havana	5.796092083	5.541860512	6.050323655	5.228476033	5.688964622	5.708140881	5.922616639	6.249191054	5.979163271
ENSG00000229692	0.005649	0.036718784	0.364092349	1.287071632	**SOS1-IT1**	sense_intronic	SOS1 intronic transcript 1 [Source:HGNC Symbol;Acc:HGNC:41385]	chr2	38992279	38993857	-1	havana	3.095994593	2.913948418	3.278040767	2.749499015	2.873380708	3.118965531	3.230173147	3.252913259	3.351035895
ENSG00000279865	0.005646	0.036718784	-0.308714227	-1.238603327	**AC006511.3**	TEC		chr12	8080381	8082163	-1	havana	0.811668664	0.966025777	0.657311551	0.931023971	0.874167025	1.092886337	0.56657698	0.529376201	0.875981471
ENSG00000201457	0.005656	0.036737636	0.275256012	1.210208827	**SNORA55**	snoRNA	small nucleolar RNA, H/ACA box 55 [Source:HGNC Symbol;Acc:HGNC:32649]	chr1	39567374	39567508	-1	ensembl	-0.166928832	-0.304556839	-0.029300826	-0.85854608	-0.036373238	-0.018751196	-0.57379957	0.19485955	0.291037539
ENSG00000186416	0.005659	0.036740016	-0.308640194	-1.238539769	**NKRF**	protein_coding	NFKB repressing factor [Source:HGNC Symbol;Acc:HGNC:19374]	chrX	119588337	119605895	-1	ensembl_havana	5.986247847	6.140567944	5.83192775	6.211976707	6.156204486	6.053522639	5.814759046	5.868362208	5.812661996
ENSG00000143870	0.00567	0.03679058	-0.370857627	-1.293121313	**PDIA6**	protein_coding	protein disulfide isomerase family A member 6 [Source:HGNC Symbol;Acc:HGNC:30168]	chr2	10783391	10837977	-1	ensembl_havana	9.952667254	10.13809607	9.767238441	10.27546452	10.11987586	10.01894782	9.781337566	9.736300965	9.784076791
ENSG00000198915	0.005676	0.036816848	-1.35791998	-2.563153678	**RASGEF1A**	protein_coding	RasGEF domain family member 1A [Source:HGNC Symbol;Acc:HGNC:24246]	chr10	43194535	43266919	-1	ensembl_havana	-0.109380552	0.569579438	-0.788340542	0.613726219	0.658018659	0.436993436	-1.41444944	-0.68524259	-0.265329594
ENSG00000170584	0.005688	0.036876456	-0.419653351	-1.337606118	**NUDCD2**	protein_coding	NudC domain containing 2 [Source:HGNC Symbol;Acc:HGNC:30535]	chr5	163446526	163460140	-1	ensembl_havana	4.282804766	4.492631442	4.072978091	4.487627691	4.405478771	4.584787863	3.984600033	4.173576585	4.060757655
ENSG00000241973	0.005704	0.036933395	0.305364271	1.235730611	**PI4KA**	protein_coding	phosphatidylinositol 4-kinase alpha [Source:HGNC Symbol;Acc:HGNC:8983]	chr22	20707691	20859417	-1	ensembl_havana	6.357548576	6.20486644	6.510230711	6.168848444	6.156673417	6.28907746	6.55492627	6.455579932	6.520185932
ENSG00000177058	0.00571	0.036933395	0.286848356	1.21997227	**SLC38A9**	protein_coding	solute carrier family 38 member 9 [Source:HGNC Symbol;Acc:HGNC:26907]	chr5	55625845	55773194	-1	ensembl_havana	4.812490295	4.669066117	4.955914472	4.569195086	4.840867827	4.597135437	4.880243959	5.061922115	4.925577343
ENSG00000075131	0.005708	0.036933395	-0.269784134	-1.20562742	**TIPIN**	protein_coding	TIMELESS interacting protein [Source:HGNC Symbol;Acc:HGNC:30750]	chr15	66336206	66386746	-1	ensembl_havana	4.790084275	4.924976343	4.655192208	4.850923091	4.933535283	4.990470653	4.545678644	4.67737277	4.742525211
ENSG00000143942	0.005704	0.036933395	-0.28582207	-1.219104731	**CHAC2**	protein_coding	ChaC cation transport regulator homolog 2 [Source:HGNC Symbol;Acc:HGNC:32363]	chr2	53767792	53775196	1	ensembl_havana	4.325142563	4.468053598	4.182231527	4.39257795	4.579246746	4.432336097	4.095738958	4.243493459	4.207462165
ENSG00000124496	0.005708	0.036933395	-0.33980786	-1.26558803	**TRERF1**	protein_coding	transcriptional regulating factor 1 [Source:HGNC Symbol;Acc:HGNC:18273]	chr6	42224931	42452051	-1	ensembl_havana	5.651953772	5.821857702	5.482049842	5.719004464	5.738045873	6.008522768	5.455524904	5.444896135	5.545728488

ENSG00000250585	0.005717	0.036960379	0.577511799	1.49227332	**LINC00604**	lincRNA	long intergenic non-protein coding RNA 604 [Source:HGNC Symbol;Acc:HGNC:43924]	chr5	40240173	40267657	1	havana	3.337890656	3.049134756	3.626646555	2.853343439	3.370423439	2.923637391	3.525391749	3.657568469	3.696979447
ENSG00000106591	0.005719	0.036960495	0.287776407	1.220757301	**MRPL32**	protein_coding	mitochondrial ribosomal protein L32 [Source:HGNC Symbol;Acc:HGNC:14035]	chr7	42932200	42948958	1	ensembl_havana	6.68799824	6.544110036	6.831886443	6.580235946	6.525008529	6.527085634	6.851982388	6.763530294	6.880146647
ENSG00000197930	0.005725	0.036981278	-0.317366167	-1.246053633	**ERO1A**	protein_coding	endoplasmic reticulum oxidoreductase 1 alpha [Source:HGNC Symbol;Acc:HGNC:13280]	chr14	52639916	52695900	-1	ensembl_havana	6.66792062	6.826603704	6.509237537	6.80408828	6.847222992	6.82849984	6.402735038	6.520386383	6.604591189
ENSG00000144043	0.00573	0.037000435	0.304366024	1.234875865	**TEX261**	protein_coding	testis expressed 261 [Source:HGNC Symbol;Acc:HGNC:30712]	chr2	70985938	70994945	-1	ensembl_havana	7.667258655	7.515075643	7.819441666	7.38699991	7.537294808	7.620932211	7.779644163	7.787785875	7.890894961
ENSG00000138375	0.005738	0.037032702	-0.396057198	-1.315906695	**SMARCAL1**	protein_coding	SWI/SNF related, matrix associated, actin dependent regulator of chromatin, subfamily a like 1 [Source:HGNC Symbol;Acc:HGNC:11102]	chr2	216412414	216483053	1	ensembl_havana	4.906540167	5.104568766	4.708511568	5.088650799	5.094914784	5.130140715	4.688336516	4.552860279	4.88433791
ENSG00000092020	0.005748	0.037061629	0.300018096	1.231159856	**PPP2R3C**	protein_coding	protein phosphatase 2 regulatory subunit B''gamma [Source:HGNC Symbol;Acc:HGNC:17485]	chr14	35085467	35122517	-1	ensembl_havana	4.802960074	4.652951026	4.952969122	4.715547829	4.677937271	4.565367978	4.937238596	4.990295146	4.931373625
ENSG00000060339	0.005745	0.037061629	-0.297961313	-1.229405899	**CCAR1**	protein_coding	cell division cycle and apoptosis regulator 1 [Source:HGNC Symbol;Acc:HGNC:24236]	chr10	68721012	68792377	1	ensembl_havana	6.857650678	7.006631335	6.708670022	7.077722383	7.012296137	6.929875483	6.706063755	6.720591619	6.699354691
ENSG00000139645	0.005755	0.037090429	0.266996975	1.203300503	**ANKRD52**	protein_coding	ankyrin repeat domain 52 [Source:HGNC Symbol;Acc:HGNC:26614]	chr12	56237807	56258391	-1	ensembl_havana	8.095473483	7.961974995	8.228971971	7.984883845	7.928820693	7.972220449	8.270025458	8.207164391	8.209726063
ENSG00000089818	0.005768	0.037134241	0.341264768	1.266866732	**NECAP1**	protein_coding	NECAP endocytosis associated 1 [Source:HGNC Symbol;Acc:HGNC:24539]	chr12	8076939	8097859	1	ensembl_havana	5.434294469	5.263662085	5.604926853	5.318323994	5.288775727	5.183886535	5.654042957	5.523506627	5.637230975
ENSG00000159685	0.005766	0.037134241	0.331287124	1.25813534	**CHCHD6**	protein_coding	coiled-coil-helix-coiled-coil-helix domain containing 6 [Source:HGNC Symbol;Acc:HGNC:28184]	chr3	126704220	126960406	1	ensembl_havana	3.116523043	2.950879481	3.282166605	2.887992321	2.896025364	3.068620759	3.326412816	3.214742978	3.305344019
ENSG00000143797	0.005772	0.037134241	0.276414074	1.21118066	**MBOAT2**	protein_coding	membrane bound O-acyltransferase domain containing 2 [Source:HGNC Symbol;Acc:HGNC:25193]	chr2	8852690	9003813	-1	ensembl_havana	6.577678196	6.439471159	6.715885233	6.355064725	6.431609917	6.531738835	6.677456654	6.665410066	6.804788979
ENSG00000114346	0.00577	0.037134241	-0.277959846	-1.212479072	**ECT2**	protein_coding	epithelial cell transforming 2 [Source:HGNC Symbol;Acc:HGNC:3155]	chr3	172750682	172821474	1	ensembl_havana	6.856203344	6.995183267	6.717223421	6.830413354	7.08775916	7.067377288	6.534503015	6.862823901	6.754343349
ENSG00000239672	0.005779	0.037162792	-0.274795893	-1.209822916	**NME1**	protein_coding	NME/NM23 nucleoside diphosphate kinase 1 [Source:HGNC Symbol;Acc:HGNC:7849]	chr17	51153536	51162428	1	ensembl_havana	5.25303583	5.390433776	5.115637883	5.246708152	5.503070777	5.421522399	4.981020293	5.182518623	5.183374733
ENSG00000148187	0.005789	0.037210474	-0.348352682	-1.273106121	**MRRF**	protein_coding	mitochondrial ribosome recycling factor [Source:HGNC Symbol;Acc:HGNC:7234]	chr9	122264603	122331343	1	ensembl_havana	5.025684735	5.199861076	4.851508394	5.138837395	5.241813874	5.218931959	4.679252543	4.886070184	4.989202456
ENSG00000196081	0.005793	0.037222008	-0.43168596	-1.348808899	**ZNF724**	protein_coding	zinc finger protein 724 [Source:HGNC Symbol;Acc:HGNC:32460]	chr19	23221599	23250390	-1	havana	0.797533432	1.013376411	0.581690452	0.911909315	0.874167025	1.254052895	0.56657698	0.554044597	0.624449778
ENSG00000102007	0.005801	0.03725622	-0.386131282	-1.306884172	**PLP2**	protein_coding	proteolipid protein 2 [Source:HGNC Symbol;Acc:HGNC:9087]	chrX	49171926	49175239	1	ensembl_havana	7.227024875	7.420090516	7.033959234	7.293630594	7.576550335	7.390090618	7.060210333	7.061665456	6.980001913
ENSG00000005059	0.005804	0.037258747	-1.076369466	-2.108722809	**MCUB**	protein_coding	mitochondrial calcium uniporter dominant negative beta subunit [Source:HGNC Symbol;Acc:HGNC:26076]	chr4	109560205	109688726	1	ensembl_havana	-0.26917351	0.269011223	-0.807358243	0.660286109	0.636663181	-0.489915621	-0.30988669	-1.00738779	-1.10480025
ENSG00000131381	0.005824	0.037366282	0.299899151	1.231058355	**RBSN**	protein_coding	rabenosyn, RAB effector [Source:HGNC Symbol;Acc:HGNC:20759]	chr3	15070073	15099163	-1	ensembl_havana	5.856663846	5.70671427	6.006613421	5.633343755	5.743050767	5.743748289	5.987223596	6.073174184	5.959442484
ENSG00000155530	0.005837	0.037418745	0.526170209	1.440101211	**LRGUK**	protein_coding	leucine rich repeats and guanylate kinase domain containing [Source:HGNC Symbol;Acc:HGNC:21964]	chr7	134127299	134264591	1	ensembl_havana	1.333889934	1.070804829	1.596975038	0.892538	1.242575255	1.077301233	1.541278047	1.504469011	1.745178056
ENSG00000283196	0.005836	0.037418745	0.464228127	1.379579052	**AC006453.2**	transcribed_unprocessed_pseudogene		chr2	89542842	89585652	-1	havana	1.766569116	1.534455052	1.998683179	1.424690178	1.62524309	1.553431888	2.034670322	1.871294071	2.090085146
ENSG00000203875	0.005841	0.037425079	0.403572649	1.322779552	**SNHG5**	processed_transcript	small nucleolar RNA host gene 5 [Source:HGNC Symbol;Acc:HGNC:21026]	chr6	85660950	85678736	-1	havana	3.105810622	2.904024297	3.307596946	2.59028595	3.093124869	3.028662073	3.153531275	3.485502303	3.283757261
ENSG00000140297	0.005844	0.037433851	-0.465171042	-1.38048101	**GCNT3**	protein_coding	glucosaminyl (N-acetyl) transferase 3, mucin type [Source:HGNC Symbol;Acc:HGNC:4205]	chr15	59594875	59640239	1	ensembl_havana	-0.166984286	0.065601236	-0.399569807	-0.27307956	0.271370116	0.198513145	-0.51701327	-0.32890884	-0.352787316
ENSG00000106771	0.005852	0.037463328	0.262209079	1.199313708	**TMEM245**	protein_coding	transmembrane protein 245 [Source:HGNC Symbol;Acc:HGNC:1363]	chr9	109015152	109119945	-1	ensembl_havana	7.252011574	7.120907034	7.383116113	7.153169805	7.130878091	7.078673207	7.428960958	7.379556578	7.340830801
ENSG00000186871	0.005854	0.037463775	-0.303582112	-1.234205058	**ERCC6L**	protein_coding	ERCC excision repair 6 like, spindle assembly checkpoint helicase [Source:HGNC Symbol;Acc:HGNC:20794]	chrX	72204657	72239047	-1	ensembl_havana	5.959134719	6.110925775	5.807343663	5.863428611	6.212768939	6.256579774	5.628313403	5.925607926	5.86810966
ENSG00000182372	0.005873	0.037464971	0.294504996	1.226464093	**CLN8**	protein_coding	CLN8, transmembrane ER and ERGIC protein [Source:HGNC Symbol;Acc:HGNC:2079]	chr8	1755778	1801711	1	ensembl_havana	3.727951976	3.580699478	3.875204475	3.674990394	3.583518497	3.483589543	3.97225676	3.945529874	3.70782679
ENSG00000116133	0.005861	0.037464971	0.269665765	1.205528506	**DHCR24**	protein_coding	24-dehydrocholesterol reductase [Source:HGNC Symbol;Acc:HGNC:2859]	chr1	54849633	54887218	-1	ensembl_havana	8.5971207	8.462287818	8.731953583	8.430780348	8.484008547	8.472074559	8.72211889	8.713769346	8.759972513
ENSG00000221909	0.005873	0.037464971	-0.266795536	-1.203132502	**FAM200A**	protein_coding	family with sequence similarity 200 member A [Source:HGNC Symbol;Acc:HGNC:25401]	chr7	99546308	99558536	-1	ensembl_havana	3.603262119	3.736659887	3.469864351	3.587346304	3.838598732	3.784034626	3.32252843	3.542087686	3.544976937
ENSG00000186185	0.005873	0.037464971	-0.285389836	-1.21873954	**KIF18B**	protein_coding	kinesin family member 18B [Source:HGNC Symbol;Acc:HGNC:27102]	chr17	44924709	44947711	-1	ensembl_havana	5.426730243	5.569425162	5.284035325	5.313780003	5.63479256	5.759702922	5.029481787	5.407312625	5.415311564
ENSG00000144026	0.00586	0.037464971	-0.303254078	-1.233924462	**ZNF514**	protein_coding	zinc finger protein 514 [Source:HGNC Symbol;Acc:HGNC:25894]	chr2	95147330	95165413	-1	ensembl_havana	4.51683885	4.668465889	4.36521181	4.611277685	4.651563241	4.74255674	4.2324335	4.344168079	4.519033852
ENSG00000162543	0.005867	0.037464971	-0.667626448	-1.588457454	**UBXN10**	protein_coding	UBX domain protein 10 [Source:HGNC Symbol;Acc:HGNC:26354]	chr1	20186085	20196048	1	ensembl_havana	1.643900437	1.977713661	1.310087213	2.191145857	1.979216042	1.762779083	1.278000209	1.210694826	1.441566602
ENSG00000260337	0.005868	0.037464971	-0.708694904	-1.634324999	**AC091544.4**	processed_transcript		chr15	92592574	92596462	-1	havana	3.289578846	3.643926298	2.935231394	3.383893667	3.9894189	3.558466327	2.607204823	2.989113925	3.209375435
ENSG00000228794	0.005879	0.03748636	0.299043849	1.230328738	**LINC01128**	processed_transcript	long intergenic non-protein coding RNA 1128 [Source:HGNC Symbol;Acc:HGNC:49377]	chr1	825138	859446	1	havana	4.101347966	3.951826042	4.250869891	3.914699725	4.059300963	3.881477438	4.289263117	4.354737515	4.10860904
ENSG00000153933	0.005881	0.03748636	-0.388879826	-1.30937635	**DGKE**	protein_coding	diacylglycerol kinase epsilon [Source:HGNC Symbol;Acc:HGNC:2852]	chr17	56834099	56869567	1	ensembl_havana	3.834527697	4.02896761	3.640087784	3.984977368	3.966065478	4.135859983	3.617041156	3.710647067	3.592575128
ENSG00000110063	0.005884	0.03748858	-0.271472064	-1.20703881	**DCPS**	protein_coding	decapping enzyme, scavenger [Source:HGNC Symbol;Acc:HGNC:29812]	chr11	126303752	126345749	1	ensembl_havana	6.050613	6.186349032	5.914876968	6.137307054	6.137321128	6.284418915	5.881624696	5.893951383	5.969054825
ENSG00000086570	0.005893	0.037501251	0.277414494	1.212020831	**FAT2**	protein_coding	FAT atypical cadherin 2 [Source:HGNC Symbol;Acc:HGNC:3596]	chr5	151504093	151568944	-1	ensembl_havana	1.59332564	1.454618393	1.732032887	1.383653947	1.415548178	1.564653053	1.693402841	1.715999071	1.786696749
ENSG00000130559	0.005905	0.037501251	0.276057989	1.210881755	**CAMSAP1**	protein_coding	calmodulin regulated spectrin associated protein 1 [Source:HGNC Symbol;Acc:HGNC:19946]	chr9	135808487	135907228	-1	ensembl_havana	5.50650116	5.368472165	5.644530155	5.337253995	5.286206455	5.481956046	5.589241872	5.615738227	5.728610365
ENSG00000144659	0.0059	0.037501251	0.264695782	1.20138269	**SLC25A38**	protein_coding	solute carrier family 25 member 38 [Source:HGNC Symbol;Acc:HGNC:26054]	chr3	39383324	39397351	1	ensembl_havana	6.306567516	6.174219625	6.438915406	6.137821296	6.196441797	6.188395781	6.40595082	6.483539346	6.427256053
ENSG00000122335	0.0059	0.037501251	0.26353837	1.200419258	**SERAC1**	protein_coding	serine active site containing 1 [Source:HGNC Symbol;Acc:HGNC:21061]	chr6	158109515	158168280	-1	ensembl_havana	3.705776176	3.574006992	3.837545361	3.513183533	3.670151025	3.538686417	3.792976108	3.910138144	3.809521832
ENSG00000117724	0.005897	0.037501251	-0.312627679	-1.241967722	**CENPF**	protein_coding	centromere protein F [Source:HGNC Symbol;Acc:HGNC:1857]	chr1	214603195	214664588	1	ensembl_havana	7.725518788	7.881832628	7.569204949	7.811685606	7.838472832	7.995339445	7.429863926	7.623597674	7.654153247
ENSG00000078081	0.005903	0.037501251	-0.56484055	-1.479224013	**LAMP3**	protein_coding	lysosomal associated membrane protein 3 [Source:HGNC Symbol;Acc:HGNC:14582]	chr3	183122213	183163839	-1	ensembl_havana	4.821382429	5.103802703	4.538962154	4.814074761	5.312537498	5.184795852	4.506840112	4.501468219	4.60857813
ENSG00000213445	0.005893	0.037501251	-0.728681619	-1.657124067	**SIPA1**	protein_coding	signal-induced proliferation-associated 1 [Source:HGNC Symbol;Acc:HGNC:10885]	chr11	65638097	65650930	1	ensembl_havana	2.576927787	2.941268597	2.212586977	2.817841883	2.913888608	3.092075299	2.466446406	2.097907935	2.073406591
ENSG00000145016	0.005914	0.037528917	0.443222643	1.359638051	**RUBCN**	protein_coding	RUN and cysteine rich domain containing beclin 1 interacting protein [Source:HGNC Symbol;Acc:HGNC:28991]	chr3	197671393	197749727	-1	ensembl_havana	5.697353679	5.475742357	5.918965001	5.181362699	5.593824664	5.65203971	5.804351993	6.038629697	5.913913313
ENSG00000137767	0.005914	0.037528917	-0.352776532	-1.277015939	**SQOR**	protein_coding	sulfide quinone oxidoreductase [Source:HGNC Symbol;Acc:HGNC:20390]	chr15	45631148	45691294	1	ensembl_havana	3.312900669	3.489288935	3.136512403	3.54144321	3.503197858	3.423225735	3.058645034	3.256675325	3.094216849
ENSG00000279759	0.005928	0.037543826	0.347118106	1.272017136	**AC118344.2**	TEC		chr19	40355842	40360606	1	havana	1.625632382	1.452073329	1.799191435	1.397462791	1.428032914	1.530724282	1.644455473	1.748424202	2.004694631
ENSG00000071054	0.00592	0.037543826	0.263205647	1.200142443	**MAP4K4**	protein_coding	mitogen-activated protein kinase kinase kinase kinase 4 [Source:HGNC Symbol;Acc:HGNC:6866]	chr2	101696850	101894689	1	ensembl_havana	7.998103727	7.866500903	8.12970655	7.862506275	7.85272386	7.884272574	8.103168363	8.120787029	8.165164259
ENSG00000241878	0.005929	0.037543826	-0.278101623	-1.212598231	**PISD**	protein_coding	phosphatidylserine decarboxylase [Source:HGNC Symbol;Acc:HGNC:8999]	chr22	31618491	31662432	-1	ensembl_havana	4.680033412	4.819084223	4.5409826	4.75496532	4.820888145	4.881399205	4.482676951	4.490327477	4.649943372
ENSG00000067167	0.00593	0.037543826	-0.300577267	-1.231637131	**TRAM1**	protein_coding	translocation associated membrane protein 1 [Source:HGNC Symbol;Acc:HGNC:20568]	chr8	70573442	70608387	-1	ensembl_havana	6.395539294	6.545827927	6.24525066	6.672565308	6.482248585	6.482669889	6.287407627	6.203630774	6.24471358
ENSG00000270194	0.005926	0.037543826	-0.415808129	-1.334045737	**AC097359.2**	antisense		chr3	37241789	37244177	-1	havana	1.556762188	1.764666252	1.348858123	1.882980075	1.891783655	1.519235028	1.356079599	1.399485086	1.291009686
ENSG00000172890	0.005936	0.037565811	0.261217557	1.198489738	**NADSYN1**	protein_coding	NAD synthetase 1 [Source:HGNC Symbol;Acc:HGNC:29832]	chr11	71453109	71524107	1	ensembl_havana	5.980525257	5.849916479	6.111134036	5.953815718	5.734908975	5.861024745	6.209098117	6.011610958	6.112693032
ENSG00000233435	0.005953	0.037657973	0.455528309	1.371284872	**AGGF1P2**	processed_pseudogene	angiogenic factor with G-patch and FHA domains 1 pseudogene 2 [Source:HGNC Symbol;Acc:HGNC:38052]	chr10	133640789	133642894	1	havana	0.802436552	0.574672397	1.030200706	0.14220989	0.77991046	0.80189684	0.807953002	1.080904991	1.201744126
ENSG00000092853	0.005968	0.037736488	-0.282320159	-1.216149138	**CLSPN**	protein_coding	claspin [Source:HGNC Symbol;Acc:HGNC:19715]	chr1	35720218	35769967	-1	ensembl_havana	6.448533206	6.589693285	6.307373126	6.355506933	6.672316482	6.74125644	6.13683798	6.368730317	6.416551081
ENSG00000189058	0.005974	0.037756094	-0.331897373	-1.258667634	**APOD**	protein_coding	apolipoprotein D [Source:HGNC Symbol;Acc:HGNC:612]	chr3	195568702	195584205	-1	ensembl_havana	0.220541809	0.386490496	0.054593123	0.040274975	0.477608744	0.641587769	-0.40973604	0.225896484	0.347618925
ENSG00000043462	0.005996	0.037866466	-0.468848378	-1.384004254	**LCP2**	protein_coding	lymphocyte cytosolic protein 2 [Source:HGNC Symbol;Acc:HGNC:6529]	chr5	170246237	170298227	-1	ensembl_havana	6.312264796	6.546688985	6.077840607	6.632257679	6.447410687	6.560398588	5.947421026	6.023804452	6.262296344
ENSG00000205835	0.005996	0.037866466	-0.816627335	-1.761283729	**GMNC**	protein_coding	geminin coiled-coil domain containing [Source:HGNC Symbol;Acc:HGNC:40049]	chr3	190852877	190892429	-1	ensembl_havana	-1.061620817	-0.65330715	-1.469934485	0.297565234	-0.903502039	-1.353984645	-0.89704638	-1.74507732	-1.767679756
ENSG00000204248	0.006006	0.037892252	0.325591468	1.253178104	**COL11A2**	protein_coding	collagen type XI alpha 2 chain [Source:HGNC Symbol;Acc:HGNC:2187]	chr6	33162681	33192499	-1	ensembl_havana	3.720757416	3.557961682	3.88355315	3.663607608	3.421266147	3.58901129	4.065845049	3.782865762	3.801948638
ENSG00000179930	0.006005	0.037892252	-0.517558262	-1.431530355	**ZNF648**	protein_coding	zinc finger protein 648 [Source:HGNC Symbol;Acc:HGNC:18190]	chr1	182054570	182061712	-1	ensembl_havana	-0.263373733	-0.004594602	-0.522152864	-0.67394004	-0.002471726	0.662627962	-1.13267621	-0.32890884	-0.104873545
ENSG00000198752	0.006013	0.037901689	0.415416025	1.333683212	**CDC42BPB**	protein_coding	CDC42 binding protein kinase beta [Source:HGNC Symbol;Acc:HGNC:1738]	chr14	102932379	103057462	-1	ensembl_havana	4.863068879	4.655360866	5.070776892	4.369994038	4.643556088	4.952532473	4.920632351	5.106381877	5.185316447
ENSG00000163686	0.00601	0.037901689	0.38572814	1.306519032	**ABHD6**	protein_coding	abhydrolase domain containing 6 [Source:HGNC Symbol;Acc:HGNC:21398]	chr3	58237506	58295693	1	ensembl_havana	6.484330495	6.291466424	6.677194565	6.294086722	6.360524029	6.219788522	6.7928488	6.583530166	6.655204729
ENSG00000170017	0.006016	0.037905231	0.290105166	1.222729406	**ALCAM**	protein_coding	activated leukocyte cell adhesion molecule [Source:HGNC Symbol;Acc:HGNC:400]	chr3	105366909	105576900	1	ensembl_havana	8.072515617	7.927463034	8.2175682	7.797109495	8.050135209	7.935144399	8.163177246	8.309071745	8.180455609
ENSG00000115271	0.006021	0.03791859	0.290489437	1.223055131	**GCA**	protein_coding	grancalcin [Source:HGNC Symbol;Acc:HGNC:15990]	chr2	162318840	162371595	1	ensembl_havana	4.233349175	4.088104456	4.378593894	3.947922403	4.176774304	4.139616662	4.215776639	4.414895403	4.505109638
ENSG00000178397	0.006044	0.037988733	-0.263559446	-1.200436795	**FAM220A**	protein_coding	family with sequence similarity 220 member A [Source:HGNC Symbol;Acc:HGNC:22422]	chr7	6329409	6348981	-1	ensembl_havana	5.113842711	5.245622434	4.982062989	5.087586454	5.323438107	5.325842741	4.797308319	5.061922115	5.086958531
ENSG00000156136	0.006045	0.037988733	-0.266420153	-1.202819492	**DCK**	protein_coding	deoxycytidine kinase [Source:HGNC Symbol;Acc:HGNC:2704]	chr4	70992538	71030914	1	ensembl_havana	5.50248653	5.635696606	5.369276453	5.406321712	5.682466152	5.818301954	5.159284613	5.434138874	5.514405873
ENSG00000170312	0.006039	0.037988733	-0.270244689	-1.206012356	**CDK1**	protein_coding	cyclin dependent kinase 1 [Source:HGNC Symbol;Acc:HGNC:1722]	chr10	60778331	60794852	1	ensembl_havana	6.784577498	6.919699842	6.649455154	6.687746223	7.031854714	7.039498591	6.407326821	6.728063246	6.812975394
ENSG00000279846	0.006041	0.037988733	-0.342535571	-1.267983147	**AC015660.5**	TEC		chr15	99497812	99498375	-1	havana	3.007702308	3.178970093	2.836434522	3.279637718	3.024901037	3.232371524	2.820528739	2.688493842	3.000280985
ENSG00000116260	0.006042	0.037988733	-0.372981228	-1.295026149	**QSOX1**	protein_coding	quiescin sulfhydryl oxidase 1 [Source:HGNC Symbol;Acc:HGNC:9756]	chr1	180154834	180204030	1	ensembl_havana	9.363424017	9.549914631	9.176933403	9.796908822	9.498275107	9.354559966	9.455157151	8.976404778	9.099238281
ENSG00000111716	0.006051	0.0380103	-0.331825077	-1.258604562	**LDHB**	protein_coding	lactate dehydrogenase B [Source:HGNC Symbol;Acc:HGNC:6541]	chr12	21635342	21757857	-1	ensembl_havana	10.17379635	10.33970889	10.00788381	10.40142381	10.30269892	10.31500393	9.948617259	10.04191129	10.03312288
ENSG00000110876	0.006055	0.038019806	-0.357555427	-1.28125304	**SELPLG**	protein_coding	selectin P ligand [Source:HGNC Symbol;Acc:HGNC:10722]	chr12	108622277	108633959	-1	ensembl_havana	0.974939953	1.153717667	0.796162239	1.158893785	1.256642557	1.045616658	0.915012722	0.919411336	0.554062661
ENSG00000159593	0.006068	0.038067326	-0.28438164	-1.217888147	**NAE1**	protein_coding	NEDD8 activating enzyme E1 subunit 1 [Source:HGNC Symbol;Acc:HGNC:621]	chr16	66802875	66873256	-1	ensembl_havana	6.706262027	6.848452847	6.564071207	6.873042462	6.878261894	6.794054184	6.531140978	6.588762764	6.572309877
ENSG00000211979	0.006065	0.038067326	-0.492459127	-1.406840845	**IGHV7-81**	IG_V_gene	immunoglobulin heavy variable 7-81 (non-functional) [Source:HGNC Symbol;Acc:HGNC:5669]	chr14	106874583	106875071	-1	ensembl_havana	1.434127133	1.680356697	1.18789757	1.694503432	1.759729761	1.586836898	1.444517675	1.114463419	1.004711615
ENSG00000189136	0.006076	0.03809311	0.469962684	1.385073642	**UBE2Q2P1**	transcribed_unprocessed_pseudogene	ubiquitin conjugating enzyme E2 Q2 pseudogene 1 [Source:HGNC Symbol;Acc:HGNC:37439]	chr15	84526781	84571216	-1	havana	2.256151639	2.021170298	2.491132981	2.183196095	1.827267088	2.053047709	2.574915267	2.523424442	2.375059235
ENSG00000084764	0.006077	0.03809311	0.328805811	1.255973314	**MAPRE3**	protein_coding	microtubule associated protein RP/EB family member 3 [Source:HGNC Symbol;Acc:HGNC:6892]	chr2	26970612	27027196	1	ensembl_havana	5.782880704	5.618477799	5.94728361	5.799862837	5.658164043	5.397406516	6.086762992	5.911358882	5.843728955
ENSG00000237854	0.006084	0.038103382	0.657842049	1.577720938	**LINC00674**	transcribed_unprocessed_pseudogene	long intergenic non-protein coding RNA 674 [Source:HGNC Symbol;Acc:HGNC:44355]	chr17	68101908	68115518	1	havana	0.030376441	-0.298544583	0.359297465	-0.18813307	-0.071090603	-0.636410081	0.276538753	0.426254992	0.375098651
ENSG00000230438	0.006093	0.038103382	0.433469805	1.350477689	**SERPINB9P1**	lincRNA	serpin family B member 9 pseudogene 1 [Source:HGNC Symbol;Acc:HGNC:28590]	chr6	2854657	2881407	-1	havana	-0.103785452	-0.320520354	0.112949451	-0.41068387	-0.106664058	-0.444213135	-0.17198403	0.37179175	0.139040636
ENSG00000105419	0.006085	0.038103382	0.333958903	1.260467485	**MEIS3**	protein_coding	Meis homeobox 3 [Source:HGNC Symbol;Acc:HGNC:29537]	chr19	47403124	47419523	-1	ensembl_havana	4.13391233	3.966932878	4.300891781	3.805007693	4.043160049	4.052630893	4.261128114	4.297450705	4.344096524
ENSG00000167528	0.006087	0.038103382	0.287104686	1.220189047	**ZNF641**	protein_coding	zinc finger protein 641 [Source:HGNC Symbol;Acc:HGNC:31834]	chr12	48337180	48351414	-1	ensembl_havana	5.76176054	5.618208197	5.905312883	5.521931771	5.702516865	5.630175956	5.876335029	5.98180546	5.857798161
ENSG00000188785	0.006095	0.038103382	0.274294092	1.209402187	**ZNF548**	protein_coding	zinc finger protein 548 [Source:HGNC Symbol;Acc:HGNC:26561]	chr19	57389850	57402992	1	ensembl_havana	3.824406524	3.687259478	3.961553571	3.686284073	3.678012323	3.697482039	3.921799706	3.938520645	4.024340361
ENSG00000185164	0.006093	0.038103382	-0.327338591	-1.254696639	**NOMO2**	protein_coding	NODAL modulator 2 [Source:HGNC Symbol;Acc:HGNC:22652]	chr16	18417325	18562211	-1	ensembl_havana	5.380074027	5.543743322	5.216404731	5.765001948	5.41644009	5.449787929	5.320848202	5.161602199	5.166763792
ENSG00000198478	0.006105	0.038107994	0.456536138	1.37224315	**SH3BGRL2**	protein_coding	SH3 domain binding glutamate rich protein like 2 [Source:HGNC Symbol;Acc:HGNC:15567]	chr6	79631283	79703659	1	ensembl_havana	0.396412416	0.168144347	0.624680485	-0.14746469	0.453384582	0.198513145	0.513047461	0.880929471	0.480064523
ENSG00000173083	0.006106	0.038107994	0.280540795	1.214650111	**HPSE**	protein_coding	heparanase [Source:HGNC Symbol;Acc:HGNC:5164]	chr4	83292461	83335153	-1	ensembl_havana	1.578957373	1.438686975	1.71922777	0.949888679	1.603393164	1.762779083	1.293957805	1.841543652	2.022181853
ENSG00000262772	0.0061	0.038107994	-0.550584588	-1.464679072	**LINC01977**	lincRNA	long intergenic non-protein coding RNA 1977 [Source:HGNC Symbol;Acc:HGNC:52804]	chr17	79823452	79827704	1	havana	1.611728163	1.887020457	1.336435869	1.749374982	1.882741771	2.028944618	1.278000209	1.529563273	1.201744126
ENSG00000260083	0.006104	0.038107994	-0.685067489	-1.607777191	**MIR762HG**	antisense	MIR762 host gene [Source:HGNC Symbol;Acc:HGNC:51386]	chr16	30875222	30895220	1	havana	0.14323395	0.485767694	-0.199299795	0.705390237	0.52486995	0.227042895	0.276538753	-0.52165082	-0.352787316
ENSG00000162419	0.006116	0.038151382	0.284479662	1.217970898	**GMEB1**	protein_coding	glucocorticoid modulatory element binding protein 1 [Source:HGNC Symbol;Acc:HGNC:4370]	chr1	28668732	28719353	1	ensembl_havana	4.4761231	4.333883269	4.618362931	4.338128639	4.340922035	4.322599132	4.56224422	4.687163416	4.605681157
ENSG00000092201	0.00613	0.038225572	-0.282962159	-1.216690445	**SUPT16H**	protein_coding	SPT16 homolog, facilitates chromatin remodeling subunit [Source:HGNC Symbol;Acc:HGNC:11465]	chr14	21351472	21384266	-1	ensembl_havana	8.203890348	8.345371428	8.062409269	8.274936622	8.347728846	8.413448814	7.940669638	8.129917646	8.116640522
ENSG00000225439	0.006134	0.038231192	-0.603203047	-1.519085473	**BOLA3-AS1**	antisense	BOLA3 antisense RNA 1 (head to head) [Source:HGNC Symbol;Acc:HGNC:42922]	chr2	74148009	74150061	1	havana	-0.223841691	0.077759833	-0.525443214	-0.14746469	0.271370116	0.109374069	-0.97139419	-0.42206346	-0.182871985
ENSG00000188725	0.006143	0.038268244	-0.264475393	-1.201199178	**SMIM15**	protein_coding	small integral membrane protein 15 [Source:HGNC Symbol;Acc:HGNC:33861]	chr5	61157709	61162474	-1	ensembl_havana	5.727264492	5.859502189	5.595026796	5.794654492	5.907549015	5.87630306	5.546587245	5.607659933	5.63083321
ENSG00000161526	0.006153	0.038314441	0.28092581	1.214974311	**SAP30BP**	protein_coding	SAP30 binding protein [Source:HGNC Symbol;Acc:HGNC:30785]	chr17	75667116	75708062	1	ensembl_havana	6.379008209	6.238545304	6.519471114	6.190325864	6.253673249	6.2716368	6.488440794	6.5812818	6.488690749
ENSG00000187239	0.006157	0.038323653	0.262800044	1.199805079	**FNBP1**	protein_coding	formin binding protein 1 [Source:HGNC Symbol;Acc:HGNC:17069]	chr9	129887187	130043194	-1	ensembl_havana	10.09810548	9.966705458	10.2295055	10.09709491	9.950950243	9.852071225	10.37403376	10.18247123	10.13201152
ENSG00000143375	0.00616	0.038327005	-0.329857553	-1.256889267	**CGN**	protein_coding	cingulin [Source:HGNC Symbol;Acc:HGNC:17429]	chr1	151510510	151538692	1	ensembl_havana	4.700839379	4.865768155	4.535910602	4.764320685	4.887945457	4.945038324	4.538998785	4.558967031	4.509765991
ENSG00000171408	0.006171	0.038331198	0.684867322	1.607554136	**PDE7B**	protein_coding	phosphodiesterase 7B [Source:HGNC Symbol;Acc:HGNC:8792]	chr6	135851696	136195574	1	ensembl_havana	2.320821673	1.978388012	2.663255334	1.477650254	2.304436355	2.153077428	2.535188966	2.790632353	2.663944684
ENSG00000176749	0.006169	0.038331198	0.4695101	1.384639202	**CDK5R1**	protein_coding	cyclin dependent kinase 5 regulatory subunit 1 [Source:HGNC Symbol;Acc:HGNC:1775]	chr17	32486619	32491256	1	ensembl_havana	2.623815803	2.389060753	2.858570852	2.424845514	2.427675878	2.314660866	3.053967167	2.961718855	2.560026536
ENSG00000283125	0.00617	0.038331198	-0.444240124	-1.360597293	**AC022726.2**	antisense		chr18	63207622	63208168	1	havana	2.968485427	3.190605489	2.746365365	3.155115434	3.069412532	3.347288501	2.836959964	2.710605818	2.691530312
ENSG00000113721	0.00617	0.038331198	-1.52369393	-2.875263009	**PDGFRB**	protein_coding	platelet derived growth factor receptor beta [Source:HGNC Symbol;Acc:HGNC:8804]	chr5	150113837	150155872	-1	ensembl_havana	0.179765307	0.941612272	-0.582081658	1.424690178	0.77991046	0.620236179	-0.97139419	-0.42206346	-0.352787316
ENSG00000263606	0.006178	0.038356529	0.562093959	1.476410558	**AP000919.1**	transcribed_processed_pseudogene		chr18	2945550	2946890	-1	havana	0.690835439	0.40978846	0.971882419	0.770507832	0.477608744	-0.018751196	1.178360195	0.900298706	0.836988357
ENSG00000087502	0.006201	0.038463881	-0.332604578	-1.25928478	**ERGIC2**	protein_coding	ERGIC and golgi 2 [Source:HGNC Symbol;Acc:HGNC:30208]	chr12	29337352	29381189	-1	ensembl_havana	6.038478504	6.204780793	5.872176216	6.243855676	6.234254948	6.136231755	5.795271419	5.920873865	5.900383363
ENSG00000126460	0.006201	0.038463881	-0.796188381	-1.736507186	**PRRG2**	protein_coding	proline rich and Gla domain 2 [Source:HGNC Symbol;Acc:HGNC:9470]	chr19	49580646	49591015	1	ensembl_havana	-0.232421996	0.165672194	-0.630516187	0.267803752	0.214850257	0.014362573	-0.97139419	-0.37473441	-0.545419959
ENSG00000129422	0.006206	0.038477522	-0.267609311	-1.203811339	**MTUS1**	protein_coding	microtubule associated scaffold protein 1 [Source:HGNC Symbol;Acc:HGNC:29789]	chr8	17643795	17800917	-1	ensembl_havana	5.713642652	5.847447307	5.579837996	6.035012537	5.820854158	5.686475227	5.764821868	5.592855225	5.381836897
ENSG00000143153	0.00621	0.038485978	0.617385842	1.534092891	**ATP1B1**	protein_coding	ATPase Na+/K+ transporting subunit beta 1 [Source:HGNC Symbol;Acc:HGNC:804]	chr1	169105697	169132722	1	ensembl_havana	3.829059606	3.520366685	4.137752526	3.032160045	3.764236011	3.764703998	3.974733877	4.269793831	4.168729871
ENSG00000177453	0.006222	0.038528091	0.393338979	1.313429696	**NIM1K**	protein_coding	NIM1 serine/threonine protein kinase [Source:HGNC Symbol;Acc:HGNC:28646]	chr5	43192071	43280850	1	ensembl_havana	3.481676636	3.285007146	3.678346125	3.519511473	3.127978252	3.207531715	3.74734528	3.654720022	3.632973073
ENSG00000171450	0.006225	0.038528091	0.319070359	1.247526411	**CDK5R2**	protein_coding	cyclin dependent kinase 5 regulatory subunit 2 [Source:HGNC Symbol;Acc:HGNC:1776]	chr2	218959655	218962162	1	ensembl_havana	0.971504146	0.811968967	1.131039325	0.892538	0.77991046	0.76345844	1.195452122	1.01135145	1.186314404
ENSG00000196659	0.006225	0.038528091	-0.501386175	-1.415573025	**TTC30B**	protein_coding	tetratricopeptide repeat domain 30B [Source:HGNC Symbol;Acc:HGNC:26425]	chr2	177548998	177553014	-1	ensembl_havana	0.317652309	0.568345396	0.066959221	0.540941683	0.679062629	0.485031876	-0.12879794	0.097529431	0.232146171
ENSG00000260409	0.00623	0.038530609	0.332704907	1.259372358	**AC012414.5**	lincRNA		chr15	20729747	20756183	-1	havana	2.387338777	2.220986324	2.553691231	2.341580783	2.213608205	2.107769984	2.792720531	2.453030417	2.415322744
ENSG00000127884	0.006246	0.038530609	0.272112716	1.207574934	**ECHS1**	protein_coding	enoyl-CoA hydratase, short chain 1 [Source:HGNC Symbol;Acc:HGNC:3151]	chr10	133362480	133373689	-1	ensembl_havana	5.510155764	5.374099406	5.646212123	5.321043534	5.403063912	5.398190773	5.645517323	5.638263935	5.654855109
ENSG00000101871	0.006246	0.038530609	0.257855644	1.195700149	**MID1**	protein_coding	midline 1 [Source:HGNC Symbol;Acc:HGNC:7095]	chrX	10445310	10833654	-1	ensembl_havana	5.88222863	5.753300808	6.011156452	5.477118432	5.658826268	6.123957723	5.74971429	5.915529411	6.368225656
ENSG00000166888	0.006241	0.038530609	-0.263063618	-1.200024298	**STAT6**	protein_coding	signal transducer and activator of transcription 6 [Source:HGNC Symbol;Acc:HGNC:11368]	chr12	57095408	57132139	-1	ensembl_havana	9.763950739	9.895482548	9.63241893	10.06382502	9.834258886	9.788363743	9.802277471	9.540446493	9.554532826
ENSG00000138161	0.006243	0.038530609	-0.30233234	-1.233136359	**CUZD1**	protein_coding	CUB and zona pellucida like domains 1 [Source:HGNC Symbol;Acc:HGNC:17937]	chr10	122832149	122850793	-1	ensembl_havana	3.166208374	3.317374544	3.015042204	3.663607608	3.317723301	2.970792724	3.360909534	2.933793498	2.750423579
ENSG00000284968	0.006243	0.038530609	-0.336389302	-1.262592689	**AC093827.4**	antisense		chr4	86924630	86936202	-1	havana	0.467866465	0.636061116	0.299671814	0.41082706	0.658018659	0.83933763	-0.04612063	0.343769191	0.601366878
ENSG00000079263	0.00624	0.038530609	-0.366436751	-1.289164843	**SP140**	protein_coding	SP140 nuclear body protein [Source:HGNC Symbol;Acc:HGNC:17133]	chr2	230203110	230313215	1	ensembl_havana	5.624359	5.807577375	5.441140625	6.071538731	5.796386522	5.554806873	5.589241872	5.399676988	5.334503014
ENSG00000140451	0.006242	0.038530609	-0.443688999	-1.36007763	**PIF1**	protein_coding	PIF1 5'-to-3' DNA helicase [Source:HGNC Symbol;Acc:HGNC:26220]	chr15	64815632	64825668	-1	ensembl_havana	4.526426768	4.748271267	4.304582268	4.544585015	4.687055443	5.013173344	4.201042438	4.356491584	4.356212782
ENSG00000114455	0.006252	0.038531334	0.729723645	1.658321402	**HHLA2**	protein_coding	HERV-H LTR-associating 2 [Source:HGNC Symbol;Acc:HGNC:4905]	chr3	108296490	108378285	1	ensembl_havana	2.765468769	2.400606946	3.130330591	1.902479286	2.729498798	2.569842754	3.011169462	3.151488815	3.228333496
ENSG00000189091	0.006252	0.038531334	-0.277113326	-1.211767843	**SF3B3**	protein_coding	splicing factor 3b subunit 3 [Source:HGNC Symbol;Acc:HGNC:10770]	chr16	70523788	70577670	1	ensembl_havana	8.361326837	8.4998835	8.222770174	8.422784629	8.439322627	8.637543244	8.105006838	8.223235471	8.340068211
ENSG00000278834	0.006256	0.038537203	-0.27954442	-1.213811521	**AC073508.3**	antisense		chr17	40648300	40649718	1	havana	2.744047391	2.883819601	2.604275181	3.018821299	2.822274848	2.810362656	2.711879034	2.608318707	2.492627803
ENSG00000162065	0.006264	0.038548857	0.29515932	1.227020472	**TBC1D24**	protein_coding	TBC1 domain family member 24 [Source:HGNC Symbol;Acc:HGNC:29203]	chr16	2475051	2509560	1	ensembl_havana	4.006494313	3.858914654	4.154073973	3.888048657	3.754355472	3.934339832	4.16027405	4.133217998	4.168729871
ENSG00000112406	0.006262	0.038548857	0.2739449	1.209109496	**HECA**	protein_coding	hdc homolog, cell cycle regulator [Source:HGNC Symbol;Acc:HGNC:21041]	chr6	139135112	139180802	1	ensembl_havana	4.931407809	4.794435358	5.068380259	4.886853659	4.820888145	4.675564271	5.103785613	5.11883701	4.982518152
ENSG00000143977	0.006266	0.038548857	-0.394531087	-1.314515439	**SNRPG**	protein_coding	small nuclear ribonucleoprotein polypeptide G [Source:HGNC Symbol;Acc:HGNC:11163]	chr2	70281362	70293771	-1	ensembl_havana	6.830955638	7.028221182	6.633690095	7.152152026	6.983509621	6.949001898	6.635803171	6.538656045	6.726611069
ENSG00000154889	0.006281	0.038629257	-0.341910482	-1.267433877	**MPPE1**	protein_coding	metallophosphoesterase 1 [Source:HGNC Symbol;Acc:HGNC:15988]	chr18	11882622	11909223	-1	ensembl_havana	4.175287879	4.346243121	4.004332638	4.376980663	4.352430021	4.309318677	3.911493022	4.116753044	3.984751849
ENSG00000174738	0.006285	0.03863378	0.268962817	1.204941259	**NR1D2**	protein_coding	nuclear receptor subfamily 1 group D member 2 [Source:HGNC Symbol;Acc:HGNC:7963]	chr3	23945260	23980618	1	ensembl_havana	5.447224439	5.312743031	5.581705847	5.289917741	5.293900585	5.354410766	5.607713412	5.538263917	5.599140213
ENSG00000165511	0.006314	0.03879552	-0.561420676	-1.4757217	**C10orf25**	antisense	chromosome 10 open reading frame 25 [Source:HGNC Symbol;Acc:HGNC:23509]	chr10	44997698	45000888	-1	ensembl_havana	0.539329566	0.820039904	0.258619228	0.683014432	0.699804047	1.077301233	-0.17198403	0.256279753	0.691561964
ENSG00000134574	0.006325	0.038849033	-0.431067645	-1.348230946	**DDB2**	protein_coding	damage specific DNA binding protein 2 [Source:HGNC Symbol;Acc:HGNC:2718]	chr11	47214465	47239240	1	ensembl_havana	4.708638033	4.924171855	4.49310421	4.495674182	5.107582415	5.169258969	4.265181149	4.614261782	4.5998697
ENSG00000117791	0.006331	0.038867923	-0.412001359	-1.330530294	**MARC2**	protein_coding	mitochondrial amidoxime reducing component 2 [Source:HGNC Symbol;Acc:HGNC:26064]	chr1	220748225	220784815	1	ensembl_havana	3.480574399	3.686575078	3.274573719	3.817901026	3.731873694	3.509950514	3.217678921	3.443209147	3.162833091
ENSG00000137200	0.006336	0.038879805	-0.280806198	-1.214873583	**CMTR1**	protein_coding	cap methyltransferase 1 [Source:HGNC Symbol;Acc:HGNC:21077]	chr6	37433219	37482827	1	ensembl_havana	8.532938288	8.673341387	8.392535189	8.930421154	8.568373889	8.521229119	8.583333151	8.30385403	8.290418386

ENSG00000227906	0.006339	0.038886791	0.444419618	1.360766583	**SNAP25-AS1**	antisense	SNAP25 antisense RNA 1 [Source:HGNC Symbol;Acc:HGNC:44312]	chr20	10021978	10368776	-1	havana	1.022038838	0.799829029	1.244248647	1.142587728	0.636663181	0.620236179	1.386165364	1.114463419	1.232117157
ENSG00000117586	0.006349	0.038914036	0.713027935	1.639240955	**TNFSF4**	protein_coding	TNF superfamily member 4 [Source:HGNC Symbol;Acc:HGNC:11934]	chr1	173183734	173207313	-1	ensembl_havana	0.65115882	0.294644853	1.007672788	0.10902545	0.243386951	0.531522158	0.955706889	1.210694826	0.856616648
ENSG00000196810	0.006349	0.038914036	-0.312813004	-1.242127272	**CTBP1-AS2**	antisense	CTBP1 antisense RNA 2 (head to head) [Source:HGNC Symbol;Acc:HGNC:28307]	chr4	1249300	1288291	1	ensembl_havana	4.205047405	4.361453907	4.048640903	4.399456317	4.281963521	4.402941884	3.991955622	4.059754229	4.094212859
ENSG00000011590	0.006359	0.038958718	-0.47761777	-1.392442516	**ZBTB32**	protein_coding	zinc finger and BTB domain containing 32 [Source:HGNC Symbol;Acc:HGNC:16763]	chr19	35704527	35717038	1	ensembl_havana	4.507199124	4.746008009	4.268390239	5.047643285	4.602839827	4.587540916	4.372398576	4.340627656	4.092144484
ENSG00000159164	0.006371	0.038985618	0.595974159	1.511492855	**SV2A**	protein_coding	synaptic vesicle glycoprotein 2A [Source:HGNC Symbol;Acc:HGNC:20566]	chr1	149903318	149917882	-1	ensembl_havana	0.713069209	0.41508213	1.011056289	0.727424294	0.15602554	0.361796557	1.178360195	0.578298274	1.276510397
ENSG00000148444	0.006372	0.038985618	0.35657007	1.280378246	**COMMD3**	protein_coding	COMM domain containing 3 [Source:HGNC Symbol;Acc:HGNC:23332]	chr10	22315974	22320308	1	ensembl_havana	4.148603862	3.970318827	4.326888897	3.961929783	4.06530755	3.883719146	4.299179827	4.304737198	4.376749666
ENSG00000165240	0.006367	0.038985618	0.264756824	1.201433523	**ATP7A**	protein_coding	ATPase copper transporting alpha [Source:HGNC Symbol;Acc:HGNC:869]	chrX	77910656	78050395	1	ensembl_havana	5.550041223	5.417662811	5.682419635	5.623096139	5.302401766	5.327490528	5.849591799	5.57111489	5.626552217
ENSG00000165185	0.006377	0.038999211	0.315290991	1.244262596	**KIAA1958**	protein_coding	KIAA1958 [Source:HGNC Symbol;Acc:HGNC:23427]	chr9	112486847	112669397	1	ensembl_havana	3.598824053	3.441178558	3.756469549	3.257093797	3.436793925	3.629647951	3.67911428	3.721576939	3.868717429
ENSG00000188385	0.006399	0.039036662	0.510445683	1.424490187	**JAKMIP3**	protein_coding	Janus kinase and microtubule interacting protein 3 [Source:HGNC Symbol;Acc:HGNC:23523]	chr10	132104671	132184809	1	ensembl_havana	0.763191412	0.50796857	1.018414253	0.540941683	0.428746719	0.554217307	0.873137292	1.195095435	0.987010032
ENSG00000165698	0.006396	0.039036662	0.508701015	1.422768575	**SPACA9**	protein_coding	sperm acrosome associated 9 [Source:HGNC Symbol;Acc:HGNC:1367]	chr9	132878027	132890201	1	ensembl_havana	2.202203247	1.947852739	2.456553754	1.863213701	1.827267088	2.153077428	2.386842807	2.55432044	2.428498016
ENSG00000273820	0.006399	0.039036662	0.367364809	1.289994406	**USP27X**	protein_coding	ubiquitin specific peptidase 27, X-linked [Source:HGNC Symbol;Acc:HGNC:13486]	chrX	49879948	49882565	1	ensembl_havana	3.058606868	2.874924463	3.242289272	2.547577338	3.081317417	2.995878633	3.063307783	3.329927104	3.33363293
ENSG00000205485	0.006405	0.039036662	0.281171716	1.21518142	**AC004980.1**	transcribed_unprocessed_pseudogene		chr7	76549360	76627982	1	havana	2.003436132	1.862850274	2.14402199	1.81257944	1.891783655	1.884187727	2.161855151	2.155463891	2.114746929
ENSG00000071205	0.006394	0.039036662	0.270226052	1.205996777	**ARHGAP10**	protein_coding	Rho GTPase activating protein 10 [Source:HGNC Symbol;Acc:HGNC:26099]	chr4	147732063	148072780	1	ensembl_havana	4.455618681	4.320505655	4.590731707	4.17132759	4.290535124	4.499654252	4.459871478	4.595074467	4.717249177
ENSG00000254986	0.006402	0.039036662	-0.296620916	-1.228264198	**DPP3**	protein_coding	dipeptidyl peptidase 3 [Source:HGNC Symbol;Acc:HGNC:3008]	chr11	66480013	66509657	1	ensembl_havana	5.47816879	5.626479248	5.329858332	5.73608625	5.553797118	5.589554376	5.375299268	5.344181374	5.270094353
ENSG00000069702	0.006397	0.039036662	-0.377381772	-1.2989823	**TGFBR3**	protein_coding	transforming growth factor beta receptor 3 [Source:HGNC Symbol;Acc:HGNC:11774]	chr1	91680343	91906335	-1	ensembl_havana	3.768251761	3.956942647	3.579560875	4.161245498	3.915978773	3.79360367	3.776033227	3.399638609	3.563010788
ENSG00000228382	0.006402	0.039036662	-0.488653049	-1.403134249	**ITPKB-IT1**	sense_intronic	ITPKB intronic transcript 1 [Source:HGNC Symbol;Acc:HGNC:41349]	chr1	226656640	226675067	-1	havana	-0.173307798	0.071018726	-0.417634323	0.355307344	-0.34076431	0.198513145	-0.35894771	-0.62862566	-0.265329594
ENSG00000147010	0.006408	0.039043221	-0.26390726	-1.200726239	**SH3KBP1**	protein_coding	SH3 domain containing kinase binding protein 1 [Source:HGNC Symbol;Acc:HGNC:13867]	chrX	19533975	19887601	-1	ensembl_havana	7.065634994	7.197588624	6.933681364	7.172623381	7.171817985	7.248324504	6.880994601	6.948180195	6.971869295
ENSG00000224597	0.006415	0.039051372	0.40184341	1.321194995	**SVIL-AS1**	transcribed_unprocessed_pseudogene	SVIL antisense RNA 1 [Source:HGNC Symbol;Acc:HGNC:51219]	chr10	29409402	29487745	1	havana	4.255509726	4.054588021	4.456431431	3.770935591	4.143093687	4.249734786	4.328527411	4.552860279	4.487906603
ENSG00000130856	0.006421	0.039051372	-0.417538582	-1.335646829	**ZNF236**	protein_coding	zinc finger protein 236 [Source:HGNC Symbol;Acc:HGNC:13028]	chr18	76822607	76970727	1	ensembl_havana	4.662218796	4.870988087	4.453449506	4.753623874	4.813769905	5.045570483	4.511965697	4.384270467	4.464112353
ENSG00000229043	0.006414	0.039051372	-0.523027836	-1.436967902	**AC091729.3**	antisense		chr7	1160374	1165267	1	havana	2.700880408	2.962394326	2.43936649	2.797156021	3.065422305	3.024604652	2.521699999	2.322713072	2.4736864
ENSG00000155974	0.00642	0.039051372	-0.650459789	-1.569668372	**GRIP1**	protein_coding	glutamate receptor interacting protein 1 [Source:HGNC Symbol;Acc:HGNC:18708]	chr12	66347431	67069162	-1	ensembl_havana	2.701911352	3.027141246	2.376681457	2.959551034	2.841068927	3.280803777	2.034670322	2.523424442	2.571949608
ENSG00000105289	0.00643	0.039094538	-0.665204999	-1.58579359	**TJP3**	protein_coding	tight junction protein 3 [Source:HGNC Symbol;Acc:HGNC:11829]	chr19	3708109	3750813	1	ensembl_havana	1.267797082	1.600399582	0.935194583	1.237769075	1.592342839	1.971086832	0.935503283	0.779979312	1.090101154
ENSG00000167113	0.006435	0.039104814	0.261824127	1.198993741	**COQ4**	protein_coding	coenzyme Q4 [Source:HGNC Symbol;Acc:HGNC:19693]	chr9	128322536	128334072	1	ensembl_havana	4.936236655	4.805324592	5.067148719	4.6919302	4.780079028	4.943964547	4.956165102	5.072632715	5.172648341
ENSG00000105953	0.006441	0.039107667	0.26710576	1.20339124	**OGDH**	protein_coding	oxoglutarate dehydrogenase [Source:HGNC Symbol;Acc:HGNC:8124]	chr7	44606572	44709066	1	ensembl_havana	7.247935881	7.114383001	7.381488761	7.105855416	7.066704984	7.170588603	7.389356751	7.365253008	7.389856525
ENSG00000120802	0.006439	0.039107667	-0.287294733	-1.220349794	**TMPO**	protein_coding	thymopoietin [Source:HGNC Symbol;Acc:HGNC:11875]	chr12	98515512	98550379	1	ensembl_havana	7.263169517	7.406816883	7.11952215	7.362348958	7.440869079	7.417232613	7.047911839	7.102479282	7.208175329
ENSG00000176142	0.006444	0.039110401	-0.29751457	-1.229025262	**TMEM39A**	protein_coding	transmembrane protein 39A [Source:HGNC Symbol;Acc:HGNC:25600]	chr3	119429500	119468830	-1	ensembl_havana	6.181713911	6.330471196	6.032956627	6.525875139	6.359709651	6.105828798	6.149500965	6.060854758	5.888514156
ENSG00000168703	0.006465	0.039187542	0.703098847	1.627997909	**WFDC12**	protein_coding	WAP four-disulfide core domain 12 [Source:HGNC Symbol;Acc:HGNC:16115]	chr20	45123425	45124465	-1	ensembl_havana	0.619613722	0.268064299	0.971163146	-0.61730533	0.77991046	0.641587769	0.457455004	1.399485086	1.056549347
ENSG00000140941	0.00646	0.039187542	0.356025311	1.279894868	**MAP1LC3B**	protein_coding	microtubule associated protein 1 light chain 3 beta [Source:HGNC Symbol;Acc:HGNC:13352]	chr16	87383995	87404779	1	ensembl_havana	7.09243253	6.914419874	7.270445185	6.916847027	7.067952007	6.758460589	7.340254328	7.275855703	7.195225524
ENSG00000173517	0.006468	0.039187542	0.279907697	1.214117203	**PEAK1**	protein_coding	pseudopodium enriched atypical kinase 1 [Source:HGNC Symbol;Acc:HGNC:29431]	chr15	77100656	77420144	-1	ensembl_havana	6.583609464	6.443655616	6.723563313	6.405477621	6.399479543	6.526009684	6.65407813	6.749247663	6.767364145
ENSG00000161204	0.006467	0.039187542	0.255350494	1.193625692	**ABCF3**	protein_coding	ATP binding cassette subfamily F member 3 [Source:HGNC Symbol;Acc:HGNC:72]	chr3	184186023	184194012	1	ensembl_havana	6.572040649	6.444365402	6.699715895	6.380925847	6.448943	6.503227358	6.638930196	6.689616426	6.770601064
ENSG00000083817	0.006478	0.039235322	0.261344638	1.198595313	**ZNF416**	protein_coding	zinc finger protein 416 [Source:HGNC Symbol;Acc:HGNC:20645]	chr19	57571566	57578927	-1	ensembl_havana	3.230452714	3.099780395	3.361125033	3.040984517	3.131799381	3.126557287	3.32252843	3.409810774	3.351035895
ENSG00000185686	0.006483	0.039245517	-0.273048377	-1.208358362	**PRAME**	protein_coding	preferentially expressed antigen in melanoma [Source:HGNC Symbol;Acc:HGNC:9336]	chr22	22547701	22559361	-1	ensembl_havana	8.85242612	8.988950308	8.715901931	9.163312657	8.987984793	8.815553476	8.887769143	8.664352339	8.59558431
ENSG00000100023	0.006488	0.039246579	0.342197919	1.26768642	**PPIL2**	protein_coding	peptidylprolyl isomerase like 2 [Source:HGNC Symbol;Acc:HGNC:9261]	chr22	21666009	21700015	1	ensembl_havana	5.236799631	5.065700672	5.407898591	4.87949281	5.107582415	5.21002679	5.35832518	5.37825236	5.487118232
ENSG00000260339	0.006486	0.039246579	-0.292453209	-1.224721067	**HEXA-AS1**	antisense	HEXA antisense RNA 1 [Source:HGNC Symbol;Acc:HGNC:25810]	chr15	72376113	72378788	1	havana	0.984501107	1.130727711	0.838274502	1.04068047	1.338282095	1.013220568	0.692307165	1.130954377	0.691561964
ENSG00000228789	0.006495	0.039246741	1.34603896	2.542132029	**HCG22**	lincRNA	HLA complex group 22 (gene/pseudogene) [Source:HGNC Symbol;Acc:HGNC:27780]	chr6	31053450	31059890	1	ensembl_havana	-1.065243963	-1.738263443	-0.392224482	-1.61858537	-1.617400645	-1.978804318	-1.04978262	0.225896484	-0.352787316
ENSG00000167114	0.006496	0.039246741	0.430300609	1.347514324	**SLC27A4**	protein_coding	solute carrier family 27 member 4 [Source:HGNC Symbol;Acc:HGNC:10998]	chr9	128340646	128361470	1	ensembl_havana	5.298630842	5.083480538	5.513781147	4.900251775	5.106611921	5.243577917	5.52139269	5.510961271	5.508989479
ENSG00000151687	0.006495	0.039246741	-0.311344864	-1.24086388	**ANKAR**	protein_coding	ankyrin and armadillo repeat containing [Source:HGNC Symbol;Acc:HGNC:26350]	chr2	189674290	189761193	1	ensembl_havana	2.11368513	2.269357562	1.958012698	2.305416943	2.242169235	2.260486508	1.936429043	1.881076089	2.056532963
ENSG00000109066	0.006501	0.039259043	0.308725115	1.238612675	**TMEM104**	protein_coding	transmembrane protein 104 [Source:HGNC Symbol;Acc:HGNC:25984]	chr17	74776483	74839779	1	ensembl_havana	4.953697978	4.799335421	5.108060536	4.718301125	4.896954887	4.78275025	5.119541749	5.202160994	5.002478865
ENSG00000047365	0.006507	0.039260788	0.436681588	1.353487522	**ARAP2**	protein_coding	ArfGAP with RhoGAP domain, ankyrin repeat and PH domain 2 [Source:HGNC Symbol;Acc:HGNC:16924]	chr4	35948221	36244509	-1	ensembl_havana	4.181237059	3.962896265	4.399577853	4.185325188	3.99572295	3.707640658	4.572093026	4.279071824	4.347568708
ENSG00000267757	0.006507	0.039260788	-0.778565559	-1.715424415	**EML2-AS1**	processed_transcript	EML2 antisense RNA 1 [Source:HGNC Symbol;Acc:HGNC:48331]	chr19	45641494	45642840	1	ensembl_havana	0.821123524	1.210406303	0.431840744	1.397462791	0.979703225	1.254052895	0.668022463	0.554044597	0.073455173
ENSG00000160219	0.00652	0.039320605	-0.516040165	-1.430024798	**GAB3**	protein_coding	GRB2 associated binding protein 3 [Source:HGNC Symbol;Acc:HGNC:17515]	chrX	154675249	154751583	-1	ensembl_havana	3.713365488	3.97138557	3.455345406	4.041035185	4.0890869	3.784034626	3.466766833	3.375620869	3.523648515
ENSG00000168803	0.006532	0.039377016	-0.307253064	-1.237349504	**ADAL**	protein_coding	adenosine deaminase like [Source:HGNC Symbol;Acc:HGNC:31853]	chr15	43330657	43354555	1	ensembl_havana	5.023381563	5.177008095	4.869755031	5.096079309	5.224025191	5.210919784	4.804288604	4.820226792	4.984749696
ENSG00000183154	0.006545	0.039442434	-0.660318349	-1.580431328	**AC138356.1**	antisense		chr8	37734761	37737426	-1	havana	0.762242963	1.092402138	0.432083788	1.092533711	1.155163158	1.029509544	0.069637898	0.648704825	0.577908642
ENSG00000129480	0.006553	0.039458881	-0.286432281	-1.21962048	**DTD2**	protein_coding	D-tyrosyl-tRNA deacylase 2 (putative) [Source:HGNC Symbol;Acc:HGNC:20277]	chr14	31446036	31457510	-1	ensembl_havana	3.332229607	3.475445748	3.189013466	3.578281784	3.563853664	3.284201795	3.345679402	3.199187534	3.022173463
ENSG00000109805	0.006554	0.039458881	-0.32556497	-1.253155087	**NCAPG**	protein_coding	non-SMC condensin I complex subunit G [Source:HGNC Symbol;Acc:HGNC:24304]	chr4	17810902	17844862	1	ensembl_havana	6.847619907	7.010402392	6.684837422	6.777603931	7.162508828	7.091094416	6.573022354	6.791678237	6.689811674
ENSG00000257267	0.006558	0.039470413	0.310782789	1.240380533	**ZNF271P**	transcribed_unitary_pseudogene	zinc finger protein 271, pseudogene [Source:HGNC Symbol;Acc:HGNC:13065]	chr18	35290282	35328390	1	ensembl_havana	5.704866111	5.549474717	5.860257506	5.568449919	5.489733371	5.59024086	5.773384267	5.870818921	5.93656933
ENSG00000118515	0.006566	0.039497043	0.316647517	1.245433091	**SGK1**	protein_coding	serum/glucocorticoid regulated kinase 1 [Source:HGNC Symbol;Acc:HGNC:10810]	chr6	134169246	134318112	-1	ensembl_havana	7.314101913	7.155778154	7.472425671	7.339283007	7.241112645	6.88693881	7.580785447	7.521366961	7.315124605
ENSG00000103264	0.006568	0.039497043	0.29147587	1.223891673	**FBXO31**	protein_coding	F-box protein 31 [Source:HGNC Symbol;Acc:HGNC:16510]	chr16	87326987	87392142	-1	ensembl_havana	4.537115184	4.391377249	4.682853119	4.456635069	4.371946913	4.345549764	4.826401234	4.656166367	4.565991757
ENSG00000185000	0.006575	0.039519407	0.305588047	1.2359223	**DGAT1**	protein_coding	diacylglycerol O-acyltransferase 1 [Source:HGNC Symbol;Acc:HGNC:2843]	chr8	144314584	144326910	-1	ensembl_havana	4.32403933	4.171245307	4.476833354	4.270330414	4.085150794	4.158254713	4.659372159	4.297450705	4.473677197
ENSG00000107443	0.006578	0.03952148	0.376597822	1.298276633	**CCNJ**	protein_coding	cyclin J [Source:HGNC Symbol;Acc:HGNC:23434]	chr10	96043394	96060870	1	ensembl_havana	5.127064348	4.938765437	5.315363259	5.11918011	5.006109086	4.691007115	5.457297356	5.261406393	5.227386029
ENSG00000266094	0.006587	0.039560627	-0.282133231	-1.215991573	**RASSF5**	protein_coding	Ras association domain family member 5 [Source:HGNC Symbol;Acc:HGNC:17609]	chr1	206507530	206589448	1	ensembl_havana	6.661200338	6.802266954	6.520133723	6.996126692	6.79697098	6.613703189	6.682769183	6.471449601	6.406182385
ENSG00000179988	0.006596	0.039598796	0.310013468	1.239719273	**PSTK**	protein_coding	phosphoseryl-tRNA kinase [Source:HGNC Symbol;Acc:HGNC:28578]	chr10	122954381	122997513	1	ensembl_havana	3.23058905	3.075582317	3.385595784	3.207005484	3.057408591	2.962332875	3.41298341	3.396231887	3.347572056
ENSG00000158321	0.00661	0.039663061	0.481954763	1.396634739	**AUTS2**	protein_coding	AUTS2, activator of transcription and developmental regulator [Source:HGNC Symbol;Acc:HGNC:14262]	chr7	69598296	70793495	1	ensembl_havana	5.753748365	5.512770984	5.994725747	5.166318578	5.570786374	5.801207999	5.865697151	6.042993264	6.075486825
ENSG00000184402	0.006625	0.039736136	0.284759591	1.218207247	**SS18L1**	protein_coding	SS18L1, nBAF chromatin remodeling complex subunit [Source:HGNC Symbol;Acc:HGNC:15592]	chr20	62143795	62182484	1	ensembl_havana	5.617414718	5.475034922	5.759794514	5.40033356	5.550232366	5.47453884	5.620425101	5.906577754	5.752380686
ENSG00000260496	0.00663	0.039753277	1.129679743	2.188101622	**AC009041.1**	lincRNA		chr16	991151	1000926	-1	ensembl_havana	1.994836552	1.429996681	2.559676423	0.791576534	1.535784795	1.962628713	2.528460247	2.584568634	2.566000389
ENSG00000130592	0.006633	0.039755298	-0.543005104	-1.457004269	**LSP1**	protein_coding	lymphocyte-specific protein 1 [Source:HGNC Symbol;Acc:HGNC:6707]	chr11	1852970	1892267	1	ensembl_havana	3.274835078	3.546337631	3.003332526	3.088577264	3.756831958	3.79360367	2.798325219	3.206986221	3.004686139
ENSG00000131845	0.006641	0.039784476	0.292477582	1.224741757	**ZNF304**	protein_coding	zinc finger protein 304 [Source:HGNC Symbol;Acc:HGNC:13505]	chr19	57351307	57359898	1	ensembl_havana	3.438811174	3.292572383	3.585049965	3.369953701	3.373654145	3.134109303	3.581727135	3.674542048	3.498880712
ENSG00000225663	0.00666	0.039866292	0.83344227	1.781931984	**MCRIP1**	protein_coding	MAPK regulated corepressor interacting protein 1 [Source:HGNC Symbol;Acc:HGNC:28007]	chr17	81822361	81833302	-1	ensembl_havana	3.755132333	3.338411198	4.171853469	3.092827025	3.363940242	3.558466327	4.372398576	4.118821469	4.024340361
ENSG00000111358	0.006659	0.039866292	-0.257424895	-1.195343199	**GTF2H3**	protein_coding	general transcription factor IIH subunit 3 [Source:HGNC Symbol;Acc:HGNC:4657]	chr12	123633739	123662606	1	ensembl_havana	5.307257517	5.435969964	5.178545069	5.42666585	5.451260379	5.429983664	5.138445708	5.214786604	5.182402895
ENSG00000152518	0.006664	0.039873129	-0.390230064	-1.310602387	**ZFP36L2**	protein_coding	ZFP36 ring finger protein like 2 [Source:HGNC Symbol;Acc:HGNC:1108]	chr2	43222402	43226609	-1	ensembl_havana	4.933216358	5.128331391	4.738101326	5.026693976	5.316739792	5.041560403	4.766187383	4.755228657	4.692887938
ENSG00000110200	0.006669	0.039886343	0.300716905	1.231756346	**ANAPC15**	protein_coding	anaphase promoting complex subunit 15 [Source:HGNC Symbol;Acc:HGNC:24531]	chr11	72106378	72112780	-1	ensembl_havana	3.378910846	3.228552394	3.529269299	3.130521836	3.347603631	3.207531715	3.452619495	3.554366408	3.580821993
ENSG00000132003	0.006673	0.039893997	0.373762722	1.295727841	**ZSWIM4**	protein_coding	zinc finger SWIM-type containing 4 [Source:HGNC Symbol;Acc:HGNC:25704]	chr19	13795460	13832230	1	ensembl_havana	3.233477198	3.046595837	3.420358559	3.387357714	2.90944349	2.842986308	3.826276378	3.382523978	3.052275322
ENSG00000112290	0.006677	0.039898006	0.257808625	1.195661181	**WASF1**	protein_coding	WAS protein family member 1 [Source:HGNC Symbol;Acc:HGNC:12732]	chr6	110099819	110180004	-1	ensembl_havana	4.535265887	4.406361575	4.6641702	4.277815454	4.535204905	4.406064366	4.565534632	4.765827161	4.661148806
ENSG00000237765	0.006686	0.039903551	0.394798903	1.314759483	**FAM200B**	protein_coding	family with sequence similarity 200 member B [Source:HGNC Symbol;Acc:HGNC:27740]	chr4	15681662	15705565	1	ensembl_havana	3.258384736	3.060985284	3.455784187	3.014347502	3.176880527	2.991727823	3.32252843	3.475851809	3.568972322
ENSG00000164068	0.006688	0.039903551	0.361306184	1.284588409	**RNF123**	protein_coding	ring finger protein 123 [Source:HGNC Symbol;Acc:HGNC:21148]	chr3	49689499	49721529	1	ensembl_havana	6.034243972	5.85359088	6.214897064	5.798562512	5.726720974	6.035489153	6.267341369	6.131705472	6.245644351
ENSG00000160209	0.006691	0.039903551	-0.281737109	-1.215657743	**PDXK**	protein_coding	pyridoxal kinase [Source:HGNC Symbol;Acc:HGNC:8819]	chr21	43719094	43762307	1	ensembl_havana	5.777652971	5.918521526	5.636784416	6.027816972	5.924169743	5.803577862	5.756928108	5.571870036	5.581555106
ENSG00000173171	0.006691	0.039903551	-0.283091261	-1.216799328	**MTX1**	protein_coding	metaxin 1 [Source:HGNC Symbol;Acc:HGNC:7504]	chr1	155208699	155213824	1	ensembl_havana	4.252855708	4.394401339	4.111310078	4.440051745	4.363846937	4.379305334	4.224129108	4.081128356	4.028672769
ENSG00000113552	0.006685	0.039903551	-0.28744537	-1.220477222	**GNPDA1**	protein_coding	glucosamine-6-phosphate deaminase 1 [Source:HGNC Symbol;Acc:HGNC:4417]	chr5	141991749	142013041	-1	ensembl_havana	6.779583638	6.923306324	6.635860953	6.88626284	6.904464965	6.979191166	6.61093809	6.682292173	6.614352596
ENSG00000158850	0.006697	0.03991835	0.267306927	1.203559051	**B4GALT3**	protein_coding	beta-1,4-galactosyltransferase 3 [Source:HGNC Symbol;Acc:HGNC:926]	chr1	161171310	161177968	-1	ensembl_havana	5.956868219	5.823214755	6.090521682	5.837071289	5.838498013	5.794074963	6.132407281	6.051681002	6.087476763
ENSG00000123358	0.006711	0.039985291	-0.406521517	-1.325486078	**NR4A1**	protein_coding	nuclear receptor subfamily 4 group A member 1 [Source:HGNC Symbol;Acc:HGNC:7980]	chr12	52022832	52059507	1	ensembl_havana	3.882255058	4.085515816	3.678994299	4.0388342	4.098880438	4.118832811	3.790166076	3.707901612	3.538915209
ENSG00000145386	0.006718	0.040014342	-0.264893821	-1.201547615	**CCNA2**	protein_coding	cyclin A2 [Source:HGNC Symbol;Acc:HGNC:1578]	chr4	121816444	121823933	-1	ensembl_havana	6.308628704	6.441075615	6.176181793	6.179876831	6.569005731	6.574344281	5.877659265	6.293814262	6.357071854
ENSG00000119203	0.006724	0.040033173	-0.285077303	-1.218475551	**CPSF3**	protein_coding	cleavage and polyadenylation specific factor 3 [Source:HGNC Symbol;Acc:HGNC:2326]	chr2	9423568	9473101	1	ensembl_havana	7.345981058	7.488519709	7.203442406	7.46979877	7.486907028	7.50885333	7.11204334	7.20752705	7.290756828
ENSG00000255151	0.006747	0.040050918	0.931952725	1.907856583	**GLYATL1B**	protein_coding	glycine-N-acyltransferase like 1B [Source:HGNC Symbol;Acc:HGNC:37865]	chr11	59086307	59094786	1	havana	-1.100323647	-1.56630001	-0.634347284	-2.73705375	-0.428075095	-1.533771185	-1.31428442	0.063570082	-0.652327512
ENSG00000160972	0.006739	0.040050918	0.637949183	1.556115541	**PPP1R16A**	protein_coding	protein phosphatase 1 regulatory subunit 16A [Source:HGNC Symbol;Acc:HGNC:14941]	chr8	144477969	144502121	1	ensembl_havana	3.631037803	3.312063211	3.950012395	3.341663059	3.000030474	3.594496101	4.098013036	3.866489156	3.885534993
ENSG00000175931	0.006744	0.040050918	0.327297861	1.254661217	**UBE2O**	protein_coding	ubiquitin conjugating enzyme E2 O [Source:HGNC Symbol;Acc:HGNC:29554]	chr17	76389451	76453206	-1	ensembl_havana	5.899816975	5.736168045	6.063465906	5.586645168	5.71657922	5.905279747	5.988449903	6.094877335	6.107070479
ENSG00000116741	0.006739	0.040050918	0.280380058	1.214514789	**RGS2**	protein_coding	regulator of G protein signaling 2 [Source:HGNC Symbol;Acc:HGNC:9998]	chr1	192809039	192812283	1	ensembl_havana	6.76410927	6.623919241	6.904299299	6.976711456	6.712101361	6.182944905	7.222211594	6.952535736	6.538150565
ENSG00000107833	0.00674	0.040050918	-0.281762962	-1.215679528	**NPM3**	protein_coding	nucleophosmin/nucleoplasmin 3 [Source:HGNC Symbol;Acc:HGNC:7931]	chr10	101781325	101783413	-1	ensembl_havana	4.557440701	4.698322183	4.41655922	4.492460969	4.804223955	4.798281624	4.287271569	4.488728896	4.473677197
ENSG00000143578	0.006745	0.040050918	-0.357123307	-1.280869332	**CREB3L4**	protein_coding	cAMP responsive element binding protein 3 like 4 [Source:HGNC Symbol;Acc:HGNC:18854]	chr1	153967534	153974363	1	ensembl_havana	2.998184767	3.17674642	2.819623113	3.272162151	3.158267607	3.099809502	2.89041725	2.682912485	2.885539604
ENSG00000137124	0.006733	0.040050918	-0.403396649	-1.322618191	**ALDH1B1**	protein_coding	aldehyde dehydrogenase 1 family member B1 [Source:HGNC Symbol;Acc:HGNC:407]	chr9	38392664	38398661	1	ensembl_havana	6.916130282	7.117828607	6.714431958	7.121799915	7.017468894	7.214217012	6.797403862	6.723992611	6.621899402
ENSG00000237984	0.006751	0.040060238	0.600395415	1.516132052	**PTENP1**	transcribed_processed_pseudogene	phosphatase and tensin homolog pseudogene 1 [Source:HGNC Symbol;Acc:HGNC:9589]	chr9	33673504	33677499	-1	havana	-0.534801798	-0.834999506	-0.234604091	-1.61858537	-0.727039001	-0.159374151	-0.69455235	-0.2414061	0.232146171
ENSG00000124201	0.006758	0.040083261	0.259287275	1.196887268	**ZNFX1**	protein_coding	zinc finger NFX1-type containing 1 [Source:HGNC Symbol;Acc:HGNC:29271]	chr20	49237946	49278426	-1	ensembl_havana	8.461225195	8.331581558	8.590868832	8.683443761	8.252338644	8.058962268	8.971855531	8.51960821	8.281142757
ENSG00000135776	0.006776	0.040172912	-0.269782096	-1.205625717	**ABCB10**	protein_coding	ATP binding cassette subfamily B member 10 [Source:HGNC Symbol;Acc:HGNC:41]	chr1	229516582	229558695	-1	ensembl_havana	6.394931393	6.529822441	6.260040344	6.566168095	6.569005731	6.454293496	6.335903527	6.318321269	6.125896237
ENSG00000103174	0.006792	0.040233611	0.260158164	1.197609992	**NAGPA**	protein_coding	N-acetylglucosamine-1-phosphodiester alpha-N-acetylglucosaminidase [Source:HGNC Symbol;Acc:HGNC:17378]	chr16	5024844	5034141	-1	ensembl_havana	2.930405674	2.800326592	3.060484756	2.802355359	2.774189768	2.824434649	3.090972172	3.046743406	3.043738688
ENSG00000059588	0.00679	0.040233611	-0.312389689	-1.241762861	**TARBP1**	protein_coding	TAR (HIV-1) RNA binding protein 1 [Source:HGNC Symbol;Acc:HGNC:11568]	chr1	234391313	234479103	-1	ensembl_havana	8.53527277	8.691467614	8.379077925	8.944525173	8.496658089	8.63321958	8.532221161	8.189805856	8.415206758
ENSG00000231645	0.006798	0.0402574	-0.345271186	-1.270389757	**KRT17P6**	transcribed_unprocessed_pseudogene	keratin 17 pseudogene 6 [Source:HGNC Symbol;Acc:HGNC:50724]	chr17	20512560	20517479	-1	havana	0.43692889	0.609564483	0.264293297	0.075059705	0.740413176	1.013220568	-0.12879794	0.343769191	0.577908642
ENSG00000174744	0.006815	0.040310402	0.254048129	1.192548656	**BRMS1**	protein_coding	breast cancer metastasis suppressor 1 [Source:HGNC Symbol;Acc:HGNC:17262]	chr11	66337333	66345125	-1	ensembl_havana	4.465185881	4.338161817	4.592209945	4.287117471	4.325989874	4.401378105	4.545678644	4.555916886	4.675034306
ENSG00000058804	0.00681	0.040310402	-0.307332366	-1.23741752	**NDC1**	protein_coding	NDC1 transmembrane nucleoporin [Source:HGNC Symbol;Acc:HGNC:25525]	chr1	53765460	53838860	-1	ensembl_havana	6.346280897	6.49994708	6.192614714	6.34039528	6.59415103	6.56529493	6.018173369	6.202168328	6.357502444
ENSG00000111254	0.006816	0.040310402	-0.323323595	-1.251209694	**AKAP3**	protein_coding	A-kinase anchoring protein 3 [Source:HGNC Symbol;Acc:HGNC:373]	chr12	4615508	4649047	-1	ensembl_havana	0.656194866	0.817856664	0.494533069	0.41082706	1.013233389	1.029509544	0.210814386	0.625615656	0.647169164
ENSG00000197879	0.006821	0.040322749	-0.329630667	-1.256691618	**MYO1C**	protein_coding	myosin IC [Source:HGNC Symbol;Acc:HGNC:7597]	chr17	1464186	1492812	-1	ensembl_havana	6.812806361	6.977621695	6.647991028	7.248868491	6.898309227	6.785687367	7.033726831	6.56354732	6.346698933
ENSG00000215908	0.006828	0.040334844	0.413518891	1.33193058	**CROCCP2**	transcribed_unprocessed_pseudogene	ciliary rootlet coiled-coil, rootletin pseudogene 2 [Source:HGNC Symbol;Acc:HGNC:28170]	chr1	16618253	16657232	-1	havana	4.65068091	4.443921464	4.857440356	4.300040351	4.410206043	4.621517999	4.906430054	4.768464666	4.897426347
ENSG00000131018	0.006832	0.040334844	0.365420004	1.288256618	**SYNE1**	protein_coding	spectrin repeat containing nuclear envelope protein 1 [Source:HGNC Symbol;Acc:HGNC:17089]	chr6	152121684	152637801	-1	ensembl_havana	5.252878698	5.070168696	5.4355887	4.894177143	4.994613926	5.32171502	5.37154447	5.403923997	5.531297635
ENSG00000144445	0.00684	0.040334844	0.312675058	1.242008509	**KANSL1L**	protein_coding	KAT8 regulatory NSL complex subunit 1 like [Source:HGNC Symbol;Acc:HGNC:26310]	chr2	210021423	210171383	-1	ensembl_havana	3.562550834	3.406213305	3.718888363	3.383893667	3.452156355	3.382589892	3.694224053	3.864025053	3.598415984
ENSG00000124406	0.006836	0.040334844	0.279721633	1.213960629	**ATP8A1**	protein_coding	ATPase phospholipid transporting 8A1 [Source:HGNC Symbol;Acc:HGNC:13531]	chr4	42408373	42657105	-1	ensembl_havana	5.75534472	5.615483903	5.895205536	5.657214686	5.579906031	5.609330992	5.907137684	5.814505007	5.963973917
ENSG00000149554	0.006839	0.040334844	-0.266155597	-1.202598944	**CHEK1**	protein_coding	checkpoint kinase 1 [Source:HGNC Symbol;Acc:HGNC:1925]	chr11	125625136	125676255	1	ensembl_havana	6.808941141	6.942018939	6.675863342	6.782874083	7.028527911	7.014654824	6.530299244	6.709654251	6.787636531
ENSG00000171208	0.006836	0.040334844	-0.414686924	-1.333009373	**NETO2**	protein_coding	neuropilin and tolloid like 2 [Source:HGNC Symbol;Acc:HGNC:14644]	chr16	47077703	47143997	-1	ensembl_havana	4.492301158	4.69964462	4.284957695	4.718301125	4.654222446	4.726410287	4.104813841	4.354737515	4.39532173
ENSG00000008323	0.006843	0.04034152	0.262897092	1.199885791	**PLEKHG6**	protein_coding	pleckstrin homology and RhoGEF domain containing G6 [Source:HGNC Symbol;Acc:HGNC:25562]	chr12	6310436	6328506	1	ensembl_havana	1.927604404	1.796155858	2.05905295	1.694503432	1.855271053	1.838693089	2.005893922	2.089494957	2.08176997
ENSG00000233016	0.006869	0.040407491	0.505795922	1.41990649	**SNHG7**	antisense	small nucleolar RNA host gene 7 [Source:HGNC Symbol;Acc:HGNC:28254]	chr9	136721366	136728184	-1	havana	3.857163856	3.604265895	4.110061817	3.214825476	3.68322949	3.914742718	3.977206749	4.120886932	4.232091769
ENSG00000119242	0.006862	0.040407491	0.431413723	1.348554403	**CCDC92**	protein_coding	coiled-coil domain containing 92 [Source:HGNC Symbol;Acc:HGNC:29563]	chr12	123918660	123972831	-1	ensembl_havana	3.067825977	2.852119115	3.283532839	2.754872648	2.995843375	2.805641323	3.398295962	3.333493852	3.118808702
ENSG00000148297	0.006864	0.040407491	0.308915939	1.238776517	**MED22**	protein_coding	mediator complex subunit 22 [Source:HGNC Symbol;Acc:HGNC:11477]	chr9	133338323	133348131	-1	ensembl_havana	5.9915953	5.83713733	6.14605327	5.701022215	5.822037152	5.988352624	6.100438421	6.139367215	6.198354174
ENSG00000102078	0.006868	0.040407491	0.295978668	1.22771753	**SLC25A14**	protein_coding	solute carrier family 25 member 14 [Source:HGNC Symbol;Acc:HGNC:10984]	chrX	130339888	130373361	1	ensembl_havana	4.332726766	4.184737433	4.4807161	4.330950831	4.178622592	4.044638874	4.565534632	4.441582317	4.435031352
ENSG00000204217	0.006869	0.040407491	0.281327583	1.215312713	**BMPR2**	protein_coding	bone morphogenetic protein receptor type 2 [Source:HGNC Symbol;Acc:HGNC:1078]	chr2	202376936	202567751	1	ensembl_havana	5.042796351	4.90213256	5.183460142	4.986146455	4.889074717	4.831176508	5.20852415	5.155569952	5.186286325
ENSG00000165092	0.00689	0.04048416	0.510114412	1.424163134	**ALDH1A1**	protein_coding	aldehyde dehydrogenase 1 family member A1 [Source:HGNC Symbol;Acc:HGNC:402]	chr9	72900662	73080442	-1	ensembl_havana	2.03179749	1.776740284	2.286854696	1.987104222	1.882741771	1.460374858	2.364348155	2.256618822	2.239597111
ENSG00000273824	0.006886	0.04048416	0.322281547	1.250306281	**AC008033.3**	lincRNA		chr12	68426331	68427737	-1	havana	2.238978052	2.077837279	2.400118826	2.4315721	1.944876279	1.857063457	2.64502189	2.271570328	2.283764259
ENSG00000276023	0.006888	0.04048416	0.259411368	1.196990223	**DUSP14**	protein_coding	dual specificity phosphatase 14 [Source:HGNC Symbol;Acc:HGNC:17007]	chr17	37489831	37513501	1	ensembl_havana	4.627699896	4.497994212	4.757405581	4.468131009	4.572233536	4.453618091	4.757582036	4.790690888	4.723943818
ENSG00000044446	0.0069	0.040510238	0.274381892	1.209475791	**PHKA2**	protein_coding	phosphorylase kinase regulatory subunit alpha 2 [Source:HGNC Symbol;Acc:HGNC:8926]	chrX	18892300	18984598	-1	ensembl_havana	5.620010506	5.48281956	5.757201453	5.504492167	5.444363229	5.499603285	5.800866225	5.651183775	5.819554357
ENSG00000163795	0.006899	0.040510238	-0.276700235	-1.211420924	**ZNF513**	protein_coding	zinc finger protein 513 [Source:HGNC Symbol;Acc:HGNC:26498]	chr2	27377231	27380790	-1	ensembl_havana	3.862130229	4.000480346	3.723780111	4.226521114	3.955324016	3.819595909	4.018609733	3.663248546	3.489482054
ENSG00000181038	0.006905	0.040520196	-0.408471057	-1.327278442	**METTL23**	protein_coding	methyltransferase like 23 [Source:HGNC Symbol;Acc:HGNC:26988]	chr17	76726830	76733936	1	ensembl_havana	2.810789778	3.015025306	2.60655425	2.968829535	3.165741616	2.910504768	2.50808372	2.620048716	2.691530312
ENSG00000170044	0.006912	0.040528847	0.549038018	1.463109776	**ZPLD1**	protein_coding	zona pellucida like domain containing 1 [Source:HGNC Symbol;Acc:HGNC:27022]	chr3	102099244	102479841	1	ensembl_havana	1.368237467	1.093718458	1.642756476	0.791576534	1.428032914	1.061545926	1.458744294	1.682828385	1.786696749
ENSG00000167193	0.006911	0.040528847	0.253616413	1.192191848	**CRK**	protein_coding	CRK proto-oncogene, adaptor protein [Source:HGNC Symbol;Acc:HGNC:2362]	chr17	1420689	1463162	-1	ensembl_havana	5.698975266	5.572167059	5.825783473	5.603869553	5.685719046	5.426912579	5.884920917	5.925017017	5.667412484
ENSG00000178685	0.006917	0.04054219	-0.413293251	-1.33172228	**PARP10**	protein_coding	poly(ADP-ribose) polymerase family member 10 [Source:HGNC Symbol;Acc:HGNC:25895]	chr8	143977153	144012772	-1	ensembl_havana	5.018556945	5.225203571	4.81191032	5.259996428	5.04512938	5.370484904	5.010292088	4.649027725	4.776411146
ENSG00000070190	0.00692	0.040544261	-0.312706278	-1.242035387	**DAPP1**	protein_coding	dual adaptor of phosphotyrosine and 3-phosphoinositides 1 [Source:HGNC Symbol;Acc:HGNC:16500]	chr4	99816833	99870154	1	ensembl_havana	5.984923931	6.14127707	5.828570792	5.965450526	6.318393215	6.139987468	5.748267187	6.01216745	5.725277738
ENSG00000091732	0.006923	0.040544446	-0.288840916	-1.221658383	**ZC3HC1**	protein_coding	zinc finger C3HC-type containing 1 [Source:HGNC Symbol;Acc:HGNC:29913]	chr7	130018286	130051451	-1	ensembl_havana	5.145979718	5.290400176	5.00155926	5.160256727	5.397519822	5.313423979	4.912902981	5.020480842	5.071293958
ENSG00000109618	0.006926	0.040544446	-0.432723951	-1.34977969	**SEPSECS**	protein_coding	Sep (O-phosphoserine) tRNA:Sec (selenocysteine) tRNA synthase [Source:HGNC Symbol;Acc:HGNC:30605]	chr4	25120014	25160442	-1	ensembl_havana	4.784071135	5.00043311	4.567709159	5.129559428	4.99146292	4.880276983	4.482676951	4.660432658	4.560017867
ENSG00000119227	0.00694	0.040575553	0.532482501	1.446415959	**PIGZ**	protein_coding	phosphatidylinositol glycan anchor biosynthesis class Z [Source:HGNC Symbol;Acc:HGNC:30596]	chr3	196946343	196969060	-1	ensembl_havana	1.585462664	1.319221414	1.851703914	0.911909315	1.415548178	1.630206748	1.729053464	1.821363648	2.004694631
ENSG00000258311	0.006945	0.040575553	0.440585901	1.357155378	**AC009779.3**	protein_coding		chr12	55716036	55724703	1	havana	2.079995795	1.859702845	2.300288746	1.637462096	1.996084195	1.945562243	2.294679948	2.293711009	2.31247528
ENSG00000005882	0.006942	0.040575553	0.256273647	1.194389715	**PDK2**	protein_coding	pyruvate dehydrogenase kinase 2 [Source:HGNC Symbol;Acc:HGNC:8810]	chr17	50094737	50112152	1	ensembl_havana	5.917791572	5.789654749	6.045928396	5.828814508	5.795784536	5.744365203	6.092476849	6.017720587	6.027587752
ENSG00000179331	0.006945	0.040575553	-0.281201759	-1.215206725	**RAB39A**	protein_coding	RAB39A, member RAS oncogene family [Source:HGNC Symbol;Acc:HGNC:16521]	chr11	107928503	107963482	1	ensembl_havana	3.871960439	4.012561318	3.731359559	3.863385632	4.030934738	4.143363585	3.648411535	3.753878337	3.791788806
ENSG00000170522	0.006942	0.040575553	-0.344805868	-1.26998008	**ELOVL6**	protein_coding	ELOVL fatty acid elongase 6 [Source:HGNC Symbol;Acc:HGNC:15829]	chr4	110045846	110199199	-1	ensembl_havana	3.895349907	4.067752841	3.722946973	4.433364672	4.024783013	3.745110838	3.954796815	3.699633758	3.514410346
ENSG00000136636	0.006957	0.040612778	0.263832912	1.200664363	**KCTD3**	protein_coding	potassium channel tetramerization domain containing 3 [Source:HGNC Symbol;Acc:HGNC:21305]	chr1	215567392	215621807	1	ensembl_havana	6.138268295	6.006351839	6.270184752	5.939728948	6.111430089	5.967896481	6.256184791	6.345954955	6.208414509

ENSG00000108561	0.006957	0.040612778	-0.270956625	-1.206607642	**C1QBP**	protein_coding	complement C1q binding protein [Source:HGNC Symbol;Acc:HGNC:1243]	chr17	5432777	5448830	-1	ensembl_havana	6.157446474	6.292924787	6.021968161	6.221226416	6.301953545	6.355594399	5.888866466	6.044082097	6.132955922
ENSG00000182324	0.006972	0.040662569	0.252650621	1.191394019	**KCNJ14**	protein_coding	potassium voltage-gated channel subfamily J member 14 [Source:HGNC Symbol;Acc:HGNC:6260]	chr19	48455509	48466980	1	ensembl_havana	2.419302714	2.292977404	2.545628025	2.084216604	2.42144547	2.373270137	2.341497201	2.69405369	2.601333184
ENSG00000170836	0.006969	0.040662569	0.250371948	1.189513749	**PPM1D**	protein_coding	protein phosphatase, Mg2+/Mn2+ dependent 1D [Source:HGNC Symbol;Acc:HGNC:9277]	chr17	60600183	60666280	1	ensembl_havana	4.256889123	4.131703149	4.382075096	4.080089315	4.211493157	4.103526974	4.345853613	4.458014382	4.342357293
ENSG00000134698	0.006979	0.040689969	0.295183514	1.227041049	**AGO4**	protein_coding	argonaute 4, RISC catalytic component [Source:HGNC Symbol;Acc:HGNC:18424]	chr1	35808172	35857890	1	ensembl_havana	4.807298559	4.659706802	4.954890315	4.781535605	4.666128586	4.531456215	4.999384646	4.950201315	4.915084985
ENSG00000140323	0.006999	0.040705489	0.413649379	1.332051055	**DISP2**	protein_coding	dispatched RND transporter family member 2 [Source:HGNC Symbol;Acc:HGNC:19712]	chr15	40358235	40378639	1	ensembl_havana	4.15446406	3.947639371	4.36128875	3.674990394	3.944501979	4.223425739	4.244801141	4.389419981	4.449645126
ENSG00000196267	0.006997	0.040705489	0.392651602	1.312804056	**ZNF836**	protein_coding	zinc finger protein 836 [Source:HGNC Symbol;Acc:HGNC:34333]	chr19	52153864	52171643	-1	ensembl_havana	3.082733434	2.886407633	3.279059235	2.828074641	3.020785603	2.810362656	3.279095331	3.535908912	3.022173463
ENSG00000174903	0.006996	0.040705489	0.250076921	1.189270522	**RAB1B**	protein_coding	RAB1B, member RAS oncogene family [Source:HGNC Symbol;Acc:HGNC:18370]	chr11	66268533	66277492	1	ensembl_havana	6.48611363	6.36107517	6.611152091	6.461190662	6.348259816	6.273775032	6.720895331	6.604347645	6.508213297
ENSG00000123473	0.006996	0.040705489	-0.253851224	-1.192385903	**STIL**	protein_coding	STIL, centriolar assembly protein [Source:HGNC Symbol;Acc:HGNC:10879]	chr1	47250139	47314147	-1	ensembl_havana	6.375280843	6.502206455	6.248355231	6.368710551	6.557332683	6.580576131	6.138495991	6.308840171	6.29772953
ENSG00000048544	0.007	0.040705489	-0.255810901	-1.194006674	**MRPS10**	protein_coding	mitochondrial ribosomal protein S10 [Source:HGNC Symbol;Acc:HGNC:14502]	chr6	42206801	42217865	-1	ensembl_havana	6.423036449	6.5509419	6.295130999	6.586277112	6.587210073	6.479338515	6.345027217	6.295186739	6.24517904
ENSG00000169189	0.007002	0.040705489	-0.451734578	-1.367683659	**NSMCE1**	protein_coding	NSE1 homolog, SMC5-SMC6 complex component [Source:HGNC Symbol;Acc:HGNC:29897]	chr16	27224991	27268794	-1	ensembl_havana	4.568685402	4.794552691	4.342818114	4.95029341	4.654222446	4.779142217	4.289263117	4.226329326	4.512861898
ENSG00000119669	0.006996	0.040705489	-0.584602681	-1.499625934	**IRF2BPL**	protein_coding	interferon regulatory factor 2 binding protein like [Source:HGNC Symbol;Acc:HGNC:14282]	chr14	77024543	77028699	-1	ensembl_havana	2.107882745	2.400184086	1.815581405	2.477799991	2.42144547	2.301306795	2.025141822	1.517070703	1.90453169
ENSG00000103249	0.007032	0.040794157	0.390820063	1.311138475	**CLCN7**	protein_coding	chloride voltage-gated channel 7 [Source:HGNC Symbol;Acc:HGNC:2025]	chr16	1444934	1475580	-1	ensembl_havana	5.654402012	5.45899198	5.849812043	5.494890045	5.2836326	5.598453296	5.979843779	5.763855802	5.805736548
ENSG00000116761	0.007027	0.040794157	0.377542299	1.299126844	**CTH**	protein_coding	cystathionine gamma-lyase [Source:HGNC Symbol;Acc:HGNC:2501]	chr1	70411218	70439851	1	ensembl_havana	2.727525055	2.538753906	2.916296205	2.497167313	2.683379039	2.435715365	3.00148395	2.895698436	2.851706228
ENSG00000130766	0.00703	0.040794157	0.32067857	1.248917838	**SESN2**	protein_coding	sestrin 2 [Source:HGNC Symbol;Acc:HGNC:20746]	chr1	28259527	28282491	1	ensembl_havana	5.240635207	5.080295922	5.400974492	4.923104885	5.123022184	5.194760696	5.360221082	5.420788101	5.421914291
ENSG00000163743	0.007031	0.040794157	0.255554195	1.193794237	**RCHY1**	protein_coding	ring finger and CHY zinc finger domain containing 1 [Source:HGNC Symbol;Acc:HGNC:17479]	chr4	75479037	75514764	-1	ensembl_havana	5.053358428	4.92558133	5.181135525	4.892959141	4.97241098	4.91137387	5.124012075	5.216719236	5.202675264
ENSG00000139684	0.00702	0.040794157	-0.285122624	-1.218513828	**ESD**	protein_coding	esterase D [Source:HGNC Symbol;Acc:HGNC:3465]	chr13	46771256	46797232	-1	ensembl_havana	6.286230453	6.428791765	6.143669141	6.436740968	6.480376684	6.369257643	6.068904839	6.219620585	6.142481999
ENSG00000166794	0.007034	0.040794157	-0.411658626	-1.330214246	**PPIB**	protein_coding	peptidylprolyl isomerase B [Source:HGNC Symbol;Acc:HGNC:9255]	chr15	64155812	64163205	-1	ensembl_havana	8.521066745	8.726896058	8.315237431	8.943643104	8.673777414	8.563267655	8.334972684	8.332209301	8.27853031
ENSG00000132906	0.007046	0.040846174	0.35799967	1.281647631	**CASP9**	protein_coding	caspase 9 [Source:HGNC Symbol;Acc:HGNC:1511]	chr1	15490832	15526534	-1	ensembl_havana	4.029617859	3.850618024	4.208617694	3.921883558	3.819772235	3.810198279	4.192554836	4.10844956	4.324848684
ENSG00000101417	0.007054	0.040877487	0.348306808	1.273065641	**PXMP4**	protein_coding	peroxisomal membrane protein 4 [Source:HGNC Symbol;Acc:HGNC:15920]	chr20	33702754	33720319	-1	ensembl_havana	4.578696377	4.404542973	4.752849782	4.126429589	4.514865633	4.572333698	4.623513749	4.790690888	4.844344708
ENSG00000096092	0.007061	0.040901401	-0.278487479	-1.21292259	**TMEM14A**	protein_coding	transmembrane protein 14A [Source:HGNC Symbol;Acc:HGNC:21076]	chr6	52671109	52686588	1	ensembl_havana	4.826093376	4.965337116	4.686849637	4.991846665	4.877742138	5.026422545	4.645446557	4.66468637	4.750415982
ENSG00000234771	0.007065	0.040906802	0.261675448	1.198870183	**SLC25A25-AS1**	antisense	SLC25A25 antisense RNA 1 [Source:HGNC Symbol;Acc:HGNC:27844]	chr9	128108581	128118693	-1	havana	5.222077866	5.091240142	5.35291559	5.055285407	5.04917605	5.169258969	5.266284673	5.337979977	5.454482118
ENSG00000243708	0.007083	0.040949241	0.394447579	1.314439353	**PLA2G4B**	protein_coding	phospholipase A2 group IVB [Source:HGNC Symbol;Acc:HGNC:9036]	chr15	41837775	41848147	1	ensembl_havana	0.162993309	-0.034230481	0.360217098	-0.27307956	0.15602554	0.014362573	0.308311185	0.45273451	0.319605599
ENSG00000179943	0.007082	0.040949241	-0.325235537	-1.252868968	**FIZ1**	protein_coding	FLT3 interacting zinc finger 1 [Source:HGNC Symbol;Acc:HGNC:25917]	chr19	55591371	55601970	-1	ensembl_havana	2.919364757	3.081982526	2.756746989	3.088577264	3.112591715	3.044778598	2.869271432	2.671684573	2.729284962
ENSG00000172728	0.00708	0.040949241	-0.338416996	-1.264368498	**FUT10**	protein_coding	fucosyltransferase 10 [Source:HGNC Symbol;Acc:HGNC:19234]	chr8	33370824	33473422	-1	ensembl_havana	3.775155192	3.94436369	3.605946694	4.062862309	3.955324016	3.814904746	3.620209068	3.587603785	3.610027229
ENSG00000232119	0.00708	0.040949241	-0.416258476	-1.334462233	**MCTS1**	protein_coding	MCTS1, re-initiation and release factor [Source:HGNC Symbol;Acc:HGNC:23357]	chrX	120594010	120621074	1	ensembl_havana	5.899414443	6.107543681	5.691285205	6.216365541	6.09928431	6.006981191	5.596498057	5.791997457	5.685360101
ENSG00000151491	0.007093	0.040968923	-0.414988136	-1.333287713	**EPS8**	protein_coding	epidermal growth factor receptor pathway substrate 8 [Source:HGNC Symbol;Acc:HGNC:3420]	chr12	15620158	15882329	-1	ensembl_havana	5.027780903	5.235274971	4.820286835	5.000920413	5.339223061	5.365681439	4.788887151	4.821497347	4.850476007
ENSG00000133256	0.00709	0.040968923	-0.677324668	-1.599171502	**PDE6B**	protein_coding	phosphodiesterase 6B [Source:HGNC Symbol;Acc:HGNC:8786]	chr4	625584	670782	1	ensembl_havana	2.058473191	2.397135525	1.719810857	2.62003612	2.651792536	1.919577919	1.786580698	1.748424202	1.624427672
ENSG00000135503	0.007097	0.040977038	0.249100921	1.188466239	**ACVR1B**	protein_coding	activin A receptor type 1B [Source:HGNC Symbol;Acc:HGNC:172]	chr12	51951667	51997078	1	ensembl_havana	5.00795561	4.883405149	5.13250607	4.885629456	4.870899613	4.893686378	5.121778643	5.11883701	5.156902557
ENSG00000198612	0.007107	0.041022077	0.291983741	1.224322594	**COPS8**	protein_coding	COP9 signalosome subunit 8 [Source:HGNC Symbol;Acc:HGNC:24335]	chr2	237085312	237100466	1	ensembl_havana	6.089573534	5.943581664	6.235565405	5.865292978	6.008159462	5.957292552	6.228429016	6.210920847	6.267346352
ENSG00000153250	0.007112	0.041031002	0.510034832	1.424084578	**RBMS1**	protein_coding	RNA binding motif single stranded interacting protein 1 [Source:HGNC Symbol;Acc:HGNC:9907]	chr2	160272151	160493794	-1	ensembl_havana	3.590499822	3.335482406	3.845517239	2.649185179	3.664886261	3.692375779	3.431134914	4.046776082	4.05864072
ENSG00000116791	0.00712	0.041063209	-0.319203108	-1.247641207	**CRYZ**	protein_coding	crystallin zeta [Source:HGNC Symbol;Acc:HGNC:2419]	chr1	74705482	74733408	-1	ensembl_havana	6.847828735	7.007430289	6.688227181	6.980443449	7.131355326	6.910492091	6.752636997	6.695171957	6.616872587
ENSG00000196776	0.007133	0.041069455	0.247800602	1.187395542	**CD47**	protein_coding	CD47 molecule [Source:HGNC Symbol;Acc:HGNC:1682]	chr3	108043094	108091862	-1	ensembl_havana	6.946955449	6.823055148	7.070855749	6.691954402	6.825268451	6.95194259	6.947449727	7.066763812	7.198353709
ENSG00000260442	0.007125	0.041069455	-0.262747501	-1.199761383	**ATP2A1-AS1**	antisense	ATP2A1 antisense RNA 1 [Source:HGNC Symbol;Acc:HGNC:51370]	chr16	28878957	28879920	-1	havana	0.577816968	0.709190718	0.446443218	0.749126884	0.614986832	0.76345844	0.485518989	0.399280361	0.454530303
ENSG00000143228	0.007131	0.041069455	-0.287949246	-1.220903561	**NUF2**	protein_coding	NUF2, NDC80 kinetochore complex component [Source:HGNC Symbol;Acc:HGNC:14621]	chr1	163266576	163355764	1	ensembl_havana	5.349700916	5.49367554	5.205726293	5.41737705	5.493450591	5.570198977	5.041347011	5.265146406	5.310685463
ENSG00000214050	0.007128	0.041069455	-0.50287712	-1.417036697	**FBXO16**	protein_coding	F-box protein 16 [Source:HGNC Symbol;Acc:HGNC:13618]	chr8	28348287	28490318	-1	ensembl_havana	2.496461062	2.747899622	2.245022501	2.643402293	2.970462332	2.629834241	2.20469138	2.210811486	2.319564638
ENSG00000168826	0.007163	0.041224349	0.319484256	1.247884367	**ZBTB49**	protein_coding	zinc finger and BTB domain containing 49 [Source:HGNC Symbol;Acc:HGNC:19883]	chr4	4290251	4321786	1	ensembl_havana	2.823540093	2.663797965	2.983282221	2.464742477	2.788784317	2.737867101	2.842395714	2.984584053	3.122866897
ENSG00000169031	0.007175	0.041280318	0.740953427	1.671279964	**COL4A3**	protein_coding	collagen type IV alpha 3 chain [Source:HGNC Symbol;Acc:HGNC:2204]	chr2	227164565	227314792	1	ensembl_havana	4.703761241	4.333284527	5.074237954	3.807595594	4.402318629	4.789939359	4.96241916	5.028204747	5.232089956
ENSG00000073670	0.007179	0.041288437	0.462625792	1.378047667	**ADAM11**	protein_coding	ADAM metallopeptidase domain 11 [Source:HGNC Symbol;Acc:HGNC:189]	chr17	44759031	44781846	1	ensembl_havana	3.38539815	3.154085254	3.616711046	3.040984517	3.016658396	3.404612849	3.685177192	3.523471405	3.64148454
ENSG00000248809	0.007183	0.041294347	-0.921981206	-1.894715458	**LINC01095**	lincRNA	long intergenic non-protein coding RNA 1095 [Source:HGNC Symbol;Acc:HGNC:49220]	chr4	146109455	146121913	-1	havana	-0.47725937	-0.016268767	-0.938249972	-0.18813307	-0.143136894	0.28246366	-0.82634296	-0.80561234	-1.182794618
ENSG00000072840	0.007194	0.041337864	0.472162545	1.387187252	**EVC**	protein_coding	EvC ciliary complex subunit 1 [Source:HGNC Symbol;Acc:HGNC:3497]	chr4	5711197	5814305	1	ensembl_havana	4.517102987	4.281021715	4.75318426	4.216818725	4.196976397	4.429270021	4.82502908	4.778966731	4.655556968
ENSG00000166046	0.007225	0.041337864	0.397245796	1.316991282	**TCP11L2**	protein_coding	t-complex 11 like 2 [Source:HGNC Symbol;Acc:HGNC:28627]	chr12	106301929	106347015	1	ensembl_havana	2.664623232	2.466000334	2.86324613	2.722326456	2.415188039	2.260486508	2.941964313	2.961718855	2.686055222
ENSG00000178996	0.007222	0.041337864	0.292778345	1.224997109	**SNX18**	protein_coding	sorting nexin 18 [Source:HGNC Symbol;Acc:HGNC:19245]	chr5	54517759	54546585	1	ensembl_havana	3.986063958	3.839674786	4.132453131	3.873301493	3.86409045	3.781632414	4.09118002	4.141380542	4.16479883
ENSG00000172977	0.007225	0.041337864	0.278413968	1.212860788	**KAT5**	protein_coding	lysine acetyltransferase 5 [Source:HGNC Symbol;Acc:HGNC:5275]	chr11	65711996	65719604	1	ensembl_havana	5.257287337	5.118080353	5.396494321	5.140891087	5.162782514	5.050567458	5.481885717	5.367854419	5.339742827
ENSG00000116903	0.007219	0.041337864	0.275929552	1.210773959	**EXOC8**	protein_coding	exocyst complex component 8 [Source:HGNC Symbol;Acc:HGNC:24659]	chr1	231332753	231337852	-1	ensembl_havana	6.224300487	6.086335711	6.362265263	6.024483799	6.116742008	6.117781325	6.37017672	6.383429831	6.333189237
ENSG00000204084	0.007201	0.041337864	0.264520912	1.201237078	**INPP5B**	protein_coding	inositol polyphosphate-5-phosphatase B [Source:HGNC Symbol;Acc:HGNC:6077]	chr1	37860697	37947057	-1	ensembl_havana	4.644907892	4.512647436	4.777168348	4.382198518	4.579246746	4.576497044	4.703350509	4.803606949	4.824547585
ENSG00000160613	0.007212	0.041337864	0.249062582	1.188434657	**PCSK7**	protein_coding	proprotein convertase subtilisin/kexin type 7 [Source:HGNC Symbol;Acc:HGNC:8748]	chr11	117204337	117232525	-1	ensembl_havana	7.140121509	7.015590218	7.2646528	7.084140341	6.936768193	7.025862119	7.314328577	7.203147122	7.276482701
ENSG00000111906	0.007203	0.041337864	-0.254816119	-1.193183654	**HDDC2**	protein_coding	HD domain containing 2 [Source:HGNC Symbol;Acc:HGNC:21078]	chr6	125219962	125302078	-1	ensembl_havana	5.290301166	5.417709226	5.162893107	5.388282172	5.491964852	5.372880652	5.115057527	5.216719236	5.156902557
ENSG00000124181	0.00721	0.041337864	-0.255828023	-1.194020845	**PLCG1**	protein_coding	phospholipase C gamma 1 [Source:HGNC Symbol;Acc:HGNC:9065]	chr20	41136960	41196801	1	ensembl_havana	7.63240345	7.760317461	7.504489438	7.791727806	7.718458872	7.770765706	7.551215556	7.421635604	7.540617155
ENSG00000214694	0.007217	0.041337864	-0.257482601	-1.195391013	**ARHGEF33**	protein_coding	Rho guanine nucleotide exchange factor 33 [Source:HGNC Symbol;Acc:HGNC:37252]	chr2	38889841	38975449	1	ensembl_havana	3.398399613	3.527140913	3.269658312	3.376940521	3.651639676	3.552842542	3.113627387	3.358217192	3.337130358
ENSG00000004455	0.007212	0.041337864	-0.263783516	-1.200623254	**AK2**	protein_coding	adenylate kinase 2 [Source:HGNC Symbol;Acc:HGNC:362]	chr1	33007940	33080996	-1	ensembl_havana	8.087832115	8.219723873	7.955940356	8.225837484	8.181867358	8.251466776	7.904910737	7.944688461	8.018221872
ENSG00000283098	0.0072	0.041337864	-0.837799762	-1.787322237	**AL132857.2**	bidirectional_promoter_lncRNA		chr14	36320753	36656163	1	havana	-0.509974977	-0.091075096	-0.928874857	-0.31750517	0.06303108	-0.018751196	-1.04978262	-1.24204464	-0.494797317
ENSG00000117748	0.007229	0.041340614	-0.274430875	-1.209516856	**RPA2**	protein_coding	replication protein A2 [Source:HGNC Symbol;Acc:HGNC:10290]	chr1	27891524	27914746	-1	ensembl_havana	6.315152082	6.45236752	6.177936645	6.335920384	6.504892327	6.516289849	6.005509652	6.216726498	6.311573784
ENSG00000112624	0.007243	0.041407347	-0.425319942	-1.342870274	**BICRAL**	protein_coding	BRD4 interacting chromatin remodeling complex associated protein like [Source:HGNC Symbol;Acc:HGNC:21111]	chr6	42746958	42868560	1	ensembl_havana	4.354615025	4.567274996	4.141955054	4.764320685	4.411778365	4.525725938	4.127254407	4.10844956	4.190161196
ENSG00000112812	0.007267	0.041524794	-0.34583131	-1.27088308	**PRSS16**	protein_coding	serine protease 16 [Source:HGNC Symbol;Acc:HGNC:9480]	chr6	27247701	27256624	1	ensembl_havana	3.716569221	3.889484876	3.543653566	3.959604644	3.924815359	3.784034626	3.504971528	3.545167177	3.580821993
ENSG00000140025	0.007272	0.04153981	-0.380690996	-1.301965298	**EFCAB11**	protein_coding	EF-hand calcium binding domain 11 [Source:HGNC Symbol;Acc:HGNC:20357]	chr14	89794669	89954777	-1	ensembl_havana	2.905478228	3.095823726	2.71513273	3.014347502	3.120305496	3.152818179	2.466446406	2.769722224	2.90922956
ENSG00000173261	0.00728	0.041564852	-0.764136001	-1.698352587	**PLAC8L1**	protein_coding	PLAC8 like 1 [Source:HGNC Symbol;Acc:HGNC:31746]	chr5	146084386	146104369	-1	ensembl_havana	-0.513828247	-0.131760246	-0.895896247	0.14220989	-0.18055577	-0.356934859	-0.97139419	-1.00738779	-0.708906755
ENSG00000071127	0.007292	0.041621309	-0.275008881	-1.210001537	**WDR1**	protein_coding	WD repeat domain 1 [Source:HGNC Symbol;Acc:HGNC:12754]	chr4	10074339	10116949	-1	ensembl_havana	8.204265485	8.341769925	8.066761045	8.32411512	8.305111381	8.396083274	8.099768088	8.053310244	8.047204802
ENSG00000261578	0.007304	0.041669201	0.419068051	1.337063562	**AP003119.3**	sense_overlapping		chr11	76800364	76804555	1	havana	-0.001806632	-0.211340658	0.207727393	-0.51029874	-0.036373238	-0.087349994	0.106248156	0.225896484	0.291037539
ENSG00000171161	0.007309	0.041681428	-0.259569503	-1.197121433	**ZNF672**	protein_coding	zinc finger protein 672 [Source:HGNC Symbol;Acc:HGNC:26179]	chr1	248838210	248849517	1	ensembl_havana	5.044805063	5.174589815	4.915020312	5.32827068	5.144931306	5.050567458	5.058963859	4.84667624	4.839420837
ENSG00000064787	0.007317	0.041695837	-0.26210302	-1.199225545	**BCAS1**	protein_coding	breast carcinoma amplified sequence 1 [Source:HGNC Symbol;Acc:HGNC:974]	chr20	53936777	54070594	-1	ensembl_havana	7.263620994	7.394672503	7.132569484	7.353078209	7.309973911	7.52096539	7.096204647	7.093567232	7.207936572
ENSG00000169621	0.007317	0.041695837	-0.302984871	-1.233694233	**APLF**	protein_coding	aprataxin and PNKP like factor [Source:HGNC Symbol;Acc:HGNC:28724]	chr2	68467561	68655862	1	ensembl_havana	3.608105274	3.75959771	3.456612838	3.791998092	3.815026972	3.671768066	3.518617073	3.406428015	3.444793427
ENSG00000169248	0.007324	0.041720568	0.417542083	1.33565007	**CXCL11**	protein_coding	C-X-C motif chemokine ligand 11 [Source:HGNC Symbol;Acc:HGNC:10638]	chr4	76033682	76041415	-1	ensembl_havana	1.479294105	1.270523064	1.688065146	0.791576534	1.535784795	1.484207863	1.415636519	1.831488934	1.817069986
ENSG00000157741	0.007328	0.041727208	0.303584004	1.234206677	**UBN2**	protein_coding	ubinuclein 2 [Source:HGNC Symbol;Acc:HGNC:21931]	chr7	139230356	139308236	1	ensembl_havana	5.571903782	5.42011178	5.723695784	5.554651286	5.320092837	5.385591216	5.764106035	5.719564631	5.687416685
ENSG00000160460	0.007332	0.041729834	-0.337618476	-1.263668874	**SPTBN4**	protein_coding	spectrin beta, non-erythrocytic 4 [Source:HGNC Symbol;Acc:HGNC:14896]	chr19	40466241	40576464	1	ensembl_havana	2.806072584	2.974881822	2.637263345	2.978048743	2.913888608	3.032708114	2.594375592	2.716081261	2.601333184
ENSG00000143811	0.007336	0.041735335	-0.248620171	-1.188070272	**PYCR2**	protein_coding	pyrroline-5-carboxylate reductase 2 [Source:HGNC Symbol;Acc:HGNC:30262]	chr1	225919877	225924340	-1	ensembl_havana	5.235548523	5.359858608	5.111238437	5.310134477	5.355654308	5.41378704	5.03898176	5.125024485	5.169709067
ENSG00000164086	0.007345	0.041774652	0.276604578	1.211340604	**DUSP7**	protein_coding	dual specificity phosphatase 7 [Source:HGNC Symbol;Acc:HGNC:3073]	chr3	52048919	52056550	-1	ensembl_havana	3.182280193	3.043977904	3.320582482	2.892874923	3.14698349	3.092075299	3.217678921	3.453079729	3.290988795
ENSG00000265354	0.007354	0.041805953	-0.274090667	-1.209231669	**TIMM23**	protein_coding	translocase of inner mitochondrial membrane 23 [Source:HGNC Symbol;Acc:HGNC:17312]	chr10	45972449	46003734	1	ensembl_havana	6.355544763	6.492590097	6.218499429	6.548098016	6.548068749	6.381603525	6.251084957	6.22347036	6.180942971
ENSG00000188818	0.00736	0.041823795	0.342477272	1.26793191	**ZDHHC11**	protein_coding	zinc finger DHHC-type containing 11 [Source:HGNC Symbol;Acc:HGNC:19158]	chr5	795606	850986	-1	ensembl_havana	4.452328225	4.281089589	4.623566861	4.189299683	4.174923645	4.479045439	4.647000501	4.539024836	4.684675246
ENSG00000100031	0.007369	0.041839772	-0.323784775	-1.251609727	**GGT1**	protein_coding	gamma-glutamyltransferase 1 [Source:HGNC Symbol;Acc:HGNC:4250]	chr22	24583750	24629005	1	ensembl_havana	6.500293454	6.662185842	6.338401066	6.720043006	6.567595836	6.698918682	6.433674754	6.33309538	6.248433064
ENSG00000159753	0.007367	0.041839772	-0.56286316	-1.477197944	**CARMIL2**	protein_coding	capping protein regulator and myosin 1 linker 2 [Source:HGNC Symbol;Acc:HGNC:27089]	chr16	67644919	67657569	1	ensembl_havana	5.816514279	6.097945859	5.535082699	5.886867755	6.009198471	6.39777135	5.478398647	5.603972974	5.522876476
ENSG00000064012	0.00738	0.041888143	-0.353003303	-1.277216683	**CASP8**	protein_coding	caspase 8 [Source:HGNC Symbol;Acc:HGNC:1509]	chr2	201233443	201287711	1	ensembl_havana	5.258466596	5.434968248	5.081964945	5.499699095	5.414088582	5.391117067	4.993289132	5.16460889	5.087996813
ENSG00000068305	0.007385	0.041897207	-0.27649599	-1.211249433	**MEF2A**	protein_coding	myocyte enhancer factor 2A [Source:HGNC Symbol;Acc:HGNC:6993]	chr15	99565417	99716466	1	ensembl_havana	6.765105168	6.903353163	6.626857173	7.057208418	6.858796888	6.794054184	6.733749048	6.550963884	6.595858586
ENSG00000241288	0.007415	0.041960353	0.745247751	1.676262105	**AC092902.2**	processed_transcript		chr3	125827238	125916384	-1	havana	0.204975499	-0.167648376	0.577599374	-0.41068387	0.030651399	-0.122912658	0.513047461	0.343769191	0.875981471
ENSG00000262001	0.007415	0.041960353	0.586642222	1.501747458	**DLGAP1-AS2**	antisense	DLGAP1 antisense RNA 2 [Source:HGNC Symbol;Acc:HGNC:28146]	chr18	3603000	3608336	1	havana	-0.368882489	-0.6622036	-0.075561378	-0.61730533	-0.569826728	-0.799478738	-0.35894771	0.163140205	-0.030876629
ENSG00000231880	0.007416	0.041960353	0.55624546	1.470437495	**KF459542.1**	antisense		chr11	47577725	47578277	1	havana	-0.544228224	-0.822350954	-0.266105494	-0.67394004	-0.672709	-1.120403822	0.069637898	-0.42206346	-0.445890917
ENSG00000125046	0.007417	0.041960353	0.27461907	1.209674644	**SSUH2**	protein_coding	ssu-2 homolog (C. elegans) [Source:HGNC Symbol;Acc:HGNC:24809]	chr3	8619400	8745040	-1	ensembl_havana	0.362791917	0.225482382	0.500101452	-0.03191641	0.271370116	0.436993436	0.308311185	0.478736765	0.713256405
ENSG00000160602	0.007412	0.041960353	-0.254167443	-1.192647286	**NEK8**	protein_coding	NIMA related kinase 8 [Source:HGNC Symbol;Acc:HGNC:13387]	chr17	28725897	28743455	1	ensembl_havana	4.163632437	4.290716159	4.036548716	4.155162249	4.339270518	4.377715709	3.880123677	4.12706564	4.10245683
ENSG00000159322	0.007419	0.041960353	-0.287056583	-1.220148364	**ADPGK**	protein_coding	ADP dependent glucokinase [Source:HGNC Symbol;Acc:HGNC:25250]	chr15	72751369	72785846	-1	ensembl_havana	7.264222669	7.40775096	7.120694377	7.442168688	7.383130459	7.397953732	7.076446919	7.093304278	7.192331935
ENSG00000196353	0.007412	0.041960353	-0.334423206	-1.260873207	**CPNE4**	protein_coding	copine 4 [Source:HGNC Symbol;Acc:HGNC:2317]	chr3	131533555	132285410	-1	ensembl_havana	1.825557125	1.992768728	1.658345522	1.565895203	2.045536638	2.366874344	1.356079599	1.705026699	1.91393027
ENSG00000188707	0.0074	0.041960353	-0.341689189	-1.267239481	**ZBED6CL**	protein_coding	ZBED6 C-terminal like [Source:HGNC Symbol;Acc:HGNC:21720]	chr7	150329789	150332721	1	havana	5.04011266	5.210957254	4.869268066	5.656497098	5.019577236	4.95679743	5.432282649	4.695502837	4.48001871
ENSG00000185504	0.007428	0.041992218	0.493803146	1.408152074	**FAAP100**	protein_coding	Fanconi anemia core complex associated protein 100 [Source:HGNC Symbol;Acc:HGNC:26171]	chr17	81539885	81553961	-1	ensembl_havana	4.610888402	4.363986829	4.857789975	4.207050644	4.28368192	4.601227922	4.899927955	4.829097263	4.844344708
ENSG00000196152	0.007436	0.041993302	0.339451651	1.265275588	**ZNF79**	protein_coding	zinc finger protein 79 [Source:HGNC Symbol;Acc:HGNC:13153]	chr9	127424374	127445372	1	ensembl_havana	3.990819252	3.821093426	4.160545077	3.784135645	3.854872765	3.824271868	3.999273898	4.19137602	4.290985313
ENSG00000166886	0.007432	0.041993302	0.273732753	1.20893171	**NAB2**	protein_coding	NGFI-A binding protein 2 [Source:HGNC Symbol;Acc:HGNC:7627]	chr12	57088894	57095476	1	ensembl_havana	4.422267109	4.285400733	4.559133486	4.31466899	4.250677097	4.290856112	4.654745211	4.459647342	4.563007904
ENSG00000126653	0.007437	0.041993302	-0.333836419	-1.260360476	**NSRP1**	protein_coding	nuclear speckle splicing regulatory protein 1 [Source:HGNC Symbol;Acc:HGNC:25305]	chr17	30115521	30186475	1	ensembl_havana	4.728837073	4.895755282	4.561918864	4.91232484	4.96600401	4.808936996	4.549007011	4.52817145	4.60857813
ENSG00000183444	0.007442	0.042005611	0.367408519	1.29003349	**OR7E38P**	unprocessed_pseudogene	olfactory receptor family 7 subfamily E member 38 pseudogene [Source:HGNC Symbol;Acc:HGNC:8411]	chr7	97966090	97967074	-1	havana	2.285325421	2.101621161	2.469029681	1.87313074	2.249222012	2.182510732	2.10871413	2.590542907	2.707832006
ENSG00000159200	0.00745	0.042024115	0.285068462	1.218468084	**RCAN1**	protein_coding	regulator of calcineurin 1 [Source:HGNC Symbol;Acc:HGNC:3040]	chr21	34513142	34615142	-1	ensembl_havana	4.828291603	4.685757372	4.970825834	4.563081813	4.771531924	4.722658379	4.878922094	4.967521	5.066034408
ENSG00000112029	0.007451	0.042024115	-0.282627171	-1.216407967	**FBXO5**	protein_coding	F-box protein 5 [Source:HGNC Symbol;Acc:HGNC:13584]	chr6	152970519	152983579	-1	ensembl_havana	5.082002892	5.223316477	4.940689306	5.069371407	5.380758564	5.219819461	4.841409418	5.011602588	4.969055913
ENSG00000272145	0.00746	0.042061795	-0.259718928	-1.197245429	**NFYC-AS1**	antisense	NFYC antisense RNA 1 [Source:HGNC Symbol;Acc:HGNC:49451]	chr1	40690380	40692066	-1	havana	2.636314804	2.766174268	2.50645534	2.807536027	2.798432633	2.692554143	2.528460247	2.504564179	2.486341593
ENSG00000196754	0.007466	0.042062928	1.077028107	2.109685735	**S100A2**	protein_coding	S100 calcium binding protein A2 [Source:HGNC Symbol;Acc:HGNC:10492]	chr1	153561108	153567890	-1	ensembl_havana	-0.568654186	-1.10716824	-0.030140133	-1.61858537	-0.783495335	-0.91942402	0.069637898	-0.19956768	0.039509383
ENSG00000235174	0.007466	0.042062928	0.400655891	1.320107934	**RPL39P3**	processed_pseudogene	ribosomal protein L39 pseudogene 3 [Source:HGNC Symbol;Acc:HGNC:21383]	chr6	73373108	73373263	-1	ensembl_havana	1.418238232	1.217910286	1.618566177	1.424690178	1.199531136	1.029509544	1.75234034	1.47893052	1.624427672
ENSG00000137500	0.007478	0.042111214	-0.336729372	-1.26289034	**CCDC90B**	protein_coding	coiled-coil domain containing 90B [Source:HGNC Symbol;Acc:HGNC:28108]	chr11	83259097	83286407	-1	ensembl_havana	3.784399932	3.952764618	3.616035246	4.012157084	3.927016074	3.919120695	3.532134761	3.660411303	3.655559672
ENSG00000119041	0.007485	0.04212959	-0.255028437	-1.193359265	**GTF3C3**	protein_coding	general transcription factor IIIC subunit 3 [Source:HGNC Symbol;Acc:HGNC:4666]	chr2	196763032	196799725	-1	ensembl_havana	6.26544553	6.392959748	6.137931311	6.409742196	6.386344349	6.3827927	6.119592695	6.124002823	6.170198415
ENSG00000144580	0.007487	0.04212959	-0.270324405	-1.206078997	**CNOT9**	protein_coding	CCR4-NOT transcription complex subunit 9 [Source:HGNC Symbol;Acc:HGNC:10445]	chr2	218568580	218597080	1	ensembl_havana	6.54497971	6.680141912	6.409817507	6.701378165	6.684074832	6.65497274	6.3602642	6.458849511	6.41033881
ENSG00000106258	0.007528	0.042130051	1.224422343	2.336618725	**CYP3A5**	protein_coding	cytochrome P450 family 3 subfamily A member 5 [Source:HGNC Symbol;Acc:HGNC:2638]	chr7	99648194	99680026	-1	ensembl_havana	-0.649222213	-1.261433385	-0.037011041	-1.86005921	-1.338333324	-0.585907617	-0.26243933	0.256279753	-0.104873545
ENSG00000142609	0.007499	0.042130051	0.636711266	1.554780875	**CFAP74**	protein_coding	cilia and flagella associated protein 74 [Source:HGNC Symbol;Acc:HGNC:29368]	chr1	1921951	2003837	-1	ensembl_havana	-0.45855564	-0.776911273	-0.140200007	-0.85854608	-0.672709	-0.799478738	-0.26243933	-0.32890884	0.17074815
ENSG00000273230	0.007506	0.042130051	0.490098944	1.4045412	**AC102953.2**	lincRNA		chr7	1464497	1467522	-1	havana	2.393814761	2.148765289	2.638864234	2.159080544	1.979216042	2.307999282	2.587917939	2.721536001	2.60713876
ENSG00000128709	0.007506	0.042130051	0.398436463	1.318078653	**HOXD9**	protein_coding	homeobox D9 [Source:HGNC Symbol;Acc:HGNC:5140]	chr2	176122720	176124937	1	ensembl_havana	2.120417782	1.921199551	2.319636013	1.738566362	2.045536638	1.979495653	2.22979561	2.249084557	2.480027873
ENSG00000142459	0.007512	0.042130051	0.365812066	1.288606758	**EVI5L**	protein_coding	ecotropic viral integration site 5 like [Source:HGNC Symbol;Acc:HGNC:30464]	chr19	7830233	7864976	1	ensembl_havana	4.022839199	3.839933166	4.205745232	3.890491928	3.756831958	3.872475611	4.376151155	4.165594683	4.075489857
ENSG00000187240	0.007514	0.042130051	0.308574497	1.23848337	**DYNC2H1**	protein_coding	dynein cytoplasmic 2 heavy chain 1 [Source:HGNC Symbol;Acc:HGNC:2962]	chr11	103109431	103479863	1	ensembl_havana	4.297985059	4.14369781	4.452272307	4.136757067	4.041129678	4.253206686	4.368636211	4.46453516	4.523645552
ENSG00000285230	0.007523	0.042130051	0.276785405	1.211492443	**RALY-AS1**	lincRNA	RALY antisense RNA 1 [Source:HGNC Symbol;Acc:HGNC:50743]	chr20	33980687	33994357	-1	havana	4.177508787	4.039116085	4.31590149	3.931406694	3.94667289	4.239268669	4.217869294	4.288290531	4.441544645
ENSG00000168014	0.007517	0.042130051	0.269484135	1.205376743	**C2CD3**	protein_coding	C2 calcium dependent domain containing 3 [Source:HGNC Symbol;Acc:HGNC:24564]	chr11	74012714	74171210	-1	ensembl_havana	5.378602979	5.243860912	5.513345046	5.150096696	5.222234199	5.35925184	5.488834672	5.444071495	5.607128972
ENSG00000151304	0.007521	0.042130051	-0.248484693	-1.18795871	**SRFBP1**	protein_coding	serum response factor binding protein 1 [Source:HGNC Symbol;Acc:HGNC:26333]	chr5	121961961	122075570	1	ensembl_havana	4.771754111	4.895996457	4.647511764	5.045452365	4.94769487	4.694842136	4.819527376	4.671748138	4.451259779
ENSG00000136827	0.007527	0.042130051	-0.262436055	-1.199502409	**TOR1A**	protein_coding	torsin family 1 member A [Source:HGNC Symbol;Acc:HGNC:3098]	chr9	129812944	129824134	-1	ensembl_havana	7.027518552	7.158736579	6.896300525	7.210027453	7.183600703	7.082581582	6.935419043	6.876335874	6.877146657
ENSG00000178966	0.007502	0.042130051	-0.271354833	-1.206940732	**RMI1**	protein_coding	RecQ mediated genome instability 1 [Source:HGNC Symbol;Acc:HGNC:25764]	chr9	83980711	84004074	1	ensembl_havana	4.584229896	4.719907313	4.44855248	4.479536069	4.845528988	4.834656881	4.263156055	4.50463555	4.577865833
ENSG00000120699	0.007517	0.042130051	-0.280742357	-1.214819824	**EXOSC8**	protein_coding	exosome component 8 [Source:HGNC Symbol;Acc:HGNC:17035]	chr13	36998816	37009613	1	ensembl_havana	5.533993203	5.674364381	5.393622025	5.591158349	5.781869063	5.650065731	5.344025937	5.415749536	5.421090601
ENSG00000123610	0.007515	0.042130051	-0.379364846	-1.300769059	**TNFAIP6**	protein_coding	TNF alpha induced protein 6 [Source:HGNC Symbol;Acc:HGNC:11898]	chr2	151357592	151380048	1	ensembl_havana	3.486840355	3.676522778	3.297157932	4.041035185	3.654298741	3.334234408	3.48425812	3.389394213	3.017821464
ENSG00000172967	0.007503	0.042130051	-1.172557667	-2.254109604	**XKR3**	protein_coding	XK related 3 [Source:HGNC Symbol;Acc:HGNC:28778]	chr22	16783412	16821699	-1	ensembl_havana	-0.924715859	-0.338437025	-1.510994693	0.297565234	-0.569826728	-0.743049581	-0.17198403	-2.00847383	-2.35252622
ENSG00000181396	0.007545	0.042156869	0.447582523	1.363753144	**OGFOD3**	protein_coding	2-oxoglutarate and iron dependent oxygenase domain containing 3 [Source:HGNC Symbol;Acc:HGNC:26174]	chr17	82389223	82418637	-1	ensembl_havana	4.417518148	4.193726887	4.64130941	4.113938229	4.083178707	4.384063726	4.659372159	4.66468637	4.5998697
ENSG00000057657	0.007538	0.042156869	0.401719432	1.321081463	**PRDM1**	protein_coding	PR/SET domain 1 [Source:HGNC Symbol;Acc:HGNC:9346]	chr6	106086320	106109939	1	ensembl_havana	4.035028625	3.834168909	4.235888341	3.649251657	3.726829807	4.126425264	4.219958918	4.133217998	4.354488106
ENSG00000253392	0.007545	0.042156869	0.328697333	1.255878879	**AC119403.1**	antisense		chr19	2915146	2926807	-1	havana	-0.154126814	-0.31847548	0.010221852	-0.4596317	-0.299013651	-0.196781087	-0.17198403	0.163140205	0.039509383
ENSG00000065548	0.007544	0.042156869	-0.349748643	-1.274338584	**ZC3H15**	protein_coding	zinc finger CCCH-type containing 15 [Source:HGNC Symbol;Acc:HGNC:29528]	chr2	186486156	186509363	1	ensembl_havana	5.834269361	6.009143683	5.65939504	6.209043382	6.002431493	5.815956174	5.754046908	5.60618628	5.617951931
ENSG00000152127	0.007558	0.042212686	-0.260931666	-1.198252264	**MGAT5**	protein_coding	mannosyl (alpha-1,6-)-glycoprotein beta-1,6-N-acetyl-glucosaminyltransferase [Source:HGNC Symbol;Acc:HGNC:7049]	chr2	134119983	134454621	1	ensembl_havana	7.137706456	7.268172289	7.007240623	7.275284539	7.170425435	7.358806892	7.007050619	6.962935696	7.051735553
ENSG00000214087	0.007562	0.042221214	0.300021649	1.231162888	**ARL16**	protein_coding	ADP ribosylation factor like GTPase 16 [Source:HGNC Symbol;Acc:HGNC:27902]	chr17	81681174	81683924	-1	ensembl_havana	4.585090527	4.435079702	4.735101351	4.238078501	4.579246746	4.487913861	4.640774659	4.797163373	4.767366022
ENSG00000198797	0.007572	0.042257476	1.057546247	2.081388459	**BRINP2**	protein_coding	BMP/retinoic acid inducible neural specific 2 [Source:HGNC Symbol;Acc:HGNC:13746]	chr1	177171497	177282422	1	ensembl_havana	3.123721051	2.594947928	3.652494174	1.660549964	2.803232702	3.321061117	3.318633557	3.748544744	3.890304222
ENSG00000204231	0.007575	0.042257867	0.265016452	1.201649752	**RXRB**	protein_coding	retinoid X receptor beta [Source:HGNC Symbol;Acc:HGNC:10478]	chr6	33193588	33200688	-1	ensembl_havana	7.370315878	7.237807652	7.502824104	7.411874664	7.157597368	7.143950924	7.626596066	7.412179387	7.469696857
ENSG00000141560	0.007579	0.042259849	0.3547603	1.278773099	**FN3KRP**	protein_coding	fructosamine 3 kinase related protein [Source:HGNC Symbol;Acc:HGNC:25700]	chr17	82716683	82730328	1	ensembl_havana	5.096688835	4.919308685	5.274068985	4.680680807	4.918129458	5.15911579	5.145058991	5.319214226	5.357933737
ENSG00000266208	0.007583	0.042259849	0.246341193	1.186194998	**AC080112.1**	antisense		chr17	40360655	40364693	1	havana	3.139506211	3.016335615	3.262676808	3.146964063	2.873380708	3.028662073	3.372227541	3.139313586	3.276489296
ENSG00000100147	0.007584	0.042259849	-0.275806905	-1.210671033	**CCDC134**	protein_coding	coiled-coil domain containing 134 [Source:HGNC Symbol;Acc:HGNC:26185]	chr22	41800679	41826299	1	ensembl_havana	2.470898293	2.608801745	2.33299484	2.477799991	2.635736107	2.712869138	2.170524752	2.439854124	2.388605644
ENSG00000169896	0.007591	0.042266453	0.452807308	1.368700992	**ITGAM**	protein_coding	integrin subunit alpha M [Source:HGNC Symbol;Acc:HGNC:6149]	chr16	31259990	31332892	1	ensembl_havana	3.240419423	3.014015769	3.466823077	2.797156021	2.927142373	3.317748913	3.338003616	3.526590849	3.535874767
ENSG00000158286	0.007592	0.042266453	0.324660692	1.252369858	**RNF207**	protein_coding	ring finger protein 207 [Source:HGNC Symbol;Acc:HGNC:32947]	chr1	6205475	6221299	1	ensembl_havana	5.592246391	5.429916045	5.754576737	5.464874676	5.339223061	5.485650398	5.888209623	5.536717608	5.838802979
ENSG00000244617	0.007594	0.042266453	-1.121934198	-2.176385614	**ASPRV1**	protein_coding	aspartic peptidase retroviral like 1 [Source:HGNC Symbol;Acc:HGNC:26321]	chr2	69960089	69962265	-1	ensembl_havana	-0.02238392	0.538583179	-0.583351019	0.852997155	0.325758945	0.436993436	0.176794179	-0.57414696	-1.352700279
ENSG00000095015	0.007609	0.042319566	0.330223324	1.25720797	**MAP3K1**	protein_coding	mitogen-activated protein kinase kinase kinase 1 [Source:HGNC Symbol;Acc:HGNC:6848]	chr5	56815574	56896152	1	ensembl_havana	4.739926127	4.574814465	4.905037789	4.468131009	4.582042512	4.674269874	4.808460622	5.02710386	4.879548884
ENSG00000103148	0.007609	0.042319566	-0.299026795	-1.230314195	**NPRL3**	protein_coding	NPR3 like, GATOR1 complex subunit [Source:HGNC Symbol;Acc:HGNC:14124]	chr16	84271	138860	-1	ensembl_havana	4.593608878	4.743122275	4.444095481	4.75496532	4.756760897	4.717640609	4.553985209	4.448177619	4.330123614

ENSG00000198860	0.007613	0.042323743	-0.245268356	-1.185313231	**TSEN15**	protein_coding	tRNA splicing endonuclease subunit 15 [Source:HGNC Symbol;Acc:HGNC:16791]	chr1	184051651	184123978	1	ensembl_havana	5.893482426	6.016116604	5.770848248	5.906314844	6.051166164	6.090868804	5.68083957	5.798464096	5.833241077
ENSG00000257497	0.007623	0.042363163	-0.462052276	-1.377499959	**AC121761.1**	antisense		chr12	75483454	75489820	-1	havana	1.446597894	1.677624032	1.215571756	1.738566362	1.845996665	1.448309068	1.125834805	1.625790657	0.895089805
ENSG00000155657	0.007628	0.042374709	-0.307799755	-1.23781847	**TTN**	protein_coding	titin [Source:HGNC Symbol;Acc:HGNC:12403]	chr2	178525989	178830802	-1	ensembl_havana	4.886058145	5.039958023	4.732158268	5.273163425	5.150592429	4.696118214	5.016316334	4.725674797	4.454483673
ENSG00000151617	0.00765	0.042478174	-0.369272745	-1.291701526	**EDNRA**	protein_coding	endothelin receptor type A [Source:HGNC Symbol;Acc:HGNC:3179]	chr4	147480917	147544954	1	ensembl_havana	3.781184686	3.965821058	3.596548313	4.105550163	3.729353954	4.062559057	3.568668292	3.406428015	3.814548632
ENSG00000197021	0.007661	0.042525255	0.29886655	1.230177547	**CXorf40B**	protein_coding	chromosome X open reading frame 40B [Source:HGNC Symbol;Acc:HGNC:17402]	chrX	149929527	149938811	-1	ensembl_havana	4.608793337	4.459360062	4.758226612	4.458282965	4.3912033	4.528593921	4.82365562	4.731093453	4.719930763
ENSG00000125977	0.007667	0.042541259	-0.289521783	-1.222235071	**EIF2S2**	protein_coding	eukaryotic translation initiation factor 2 subunit beta [Source:HGNC Symbol;Acc:HGNC:3266]	chr20	34088298	34112332	-1	ensembl_havana	7.684400825	7.829161717	7.539639934	7.728948313	7.963004051	7.795532787	7.413979824	7.603981302	7.600958675
ENSG00000130724	0.007679	0.042594172	-0.291920706	-1.224269102	**CHMP2A**	protein_coding	charged multivesicular body protein 2A [Source:HGNC Symbol;Acc:HGNC:30216]	chr19	58551566	58555124	-1	ensembl_havana	5.667596094	5.813556447	5.521635742	5.940318651	5.811950647	5.688400044	5.69292562	5.419110534	5.45287107
ENSG00000263513	0.007682	0.042594514	-0.531031402	-1.444961849	**FAM72C**	protein_coding	family with sequence similarity 72 member C [Source:HGNC Symbol;Acc:HGNC:30602]	chr1	143955364	143971965	-1	ensembl_havana	0.484614939	0.75013064	0.219099238	0.683014432	0.570631912	0.996745577	-0.12879794	0.256279753	0.529815901
ENSG00000006282	0.007691	0.042625128	-0.375448274	-1.297242571	**SPATA20**	protein_coding	spermatogenesis associated 20 [Source:HGNC Symbol;Acc:HGNC:26125]	chr17	50543058	50555852	1	ensembl_havana	6.47046435	6.658188487	6.282740214	6.647144143	6.60827644	6.719144878	6.390263726	6.289229886	6.168727029
ENSG00000019485	0.0077	0.042626276	0.32551015	1.25310747	**PRDM11**	protein_coding	PR/SET domain 11 [Source:HGNC Symbol;Acc:HGNC:13996]	chr11	45095806	45235110	1	ensembl_havana	3.076843848	2.914088773	3.239598923	2.596285266	3.085263982	3.060717071	3.030347571	3.426606605	3.261842592
ENSG00000260942	0.007699	0.042626276	0.284302068	1.217820976	**CAPN10-AS1**	antisense	CAPN10 antisense RNA 1 (head to head) [Source:HGNC Symbol;Acc:HGNC:48839]	chr2	240582700	240586699	-1	havana	2.642174752	2.500023719	2.784325786	2.451565702	2.535453458	2.513051996	2.723708883	2.905316921	2.723951555
ENSG00000196132	0.0077	0.042626276	-0.260192882	-1.197638813	**MYT1**	protein_coding	myelin transcription factor 1 [Source:HGNC Symbol;Acc:HGNC:7622]	chr20	64102394	64242253	1	ensembl_havana	2.199188287	2.329284728	2.069091846	2.253214515	2.440056551	2.294583118	1.986385747	2.233896951	1.986992839
ENSG00000126243	0.00771	0.042635985	0.531897544	1.445829612	**LRFN3**	protein_coding	leucine rich repeat and fibronectin type III domain containing 3 [Source:HGNC Symbol;Acc:HGNC:28370]	chr19	35935358	35945767	1	ensembl_havana	1.201693763	0.935744991	1.467642535	0.986893817	0.962641309	0.857699845	1.797815913	1.271458479	1.333653212
ENSG00000138439	0.007713	0.042635985	0.315800632	1.244702216	**FAM117B**	protein_coding	family with sequence similarity 117 member B [Source:HGNC Symbol;Acc:HGNC:14440]	chr2	202635188	202769757	1	ensembl_havana	2.861875444	2.703975128	3.019775761	2.50993591	2.709182922	2.892806553	2.947019153	2.933793498	3.178514631
ENSG00000264575	0.007713	0.042635985	-0.263798614	-1.200635819	**LINC00526**	lincRNA	long intergenic non-protein coding RNA 526 [Source:HGNC Symbol;Acc:HGNC:28278]	chr18	5236724	5238598	-1	havana	1.837013262	1.968912569	1.705113955	2.126286315	1.979216042	1.801235349	1.820027213	1.693970236	1.601344415
ENSG00000196504	0.007714	0.042635985	-0.265749915	-1.202260824	**PRPF40A**	protein_coding	pre-mRNA processing factor 40 homolog A [Source:HGNC Symbol;Acc:HGNC:16463]	chr2	152651593	152717997	-1	ensembl_havana	7.241864053	7.374739011	7.108989096	7.442793098	7.398080682	7.283343251	7.126875046	7.117556273	7.082535968
ENSG00000149657	0.00772	0.042656937	0.246934691	1.186683077	**LSM14B**	protein_coding	LSM family member 14B [Source:HGNC Symbol;Acc:HGNC:15887]	chr20	62122461	62135378	1	ensembl_havana	5.991151825	5.86768448	6.114619171	5.807640268	5.834400372	5.961012799	6.084471098	6.067295408	6.192091007
ENSG00000112319	0.007731	0.042700671	0.519322132	1.433281645	**EYA4**	protein_coding	EYA transcriptional coactivator and phosphatase 4 [Source:HGNC Symbol;Acc:HGNC:3522]	chr6	133240598	133532120	1	ensembl_havana	3.664451949	3.404790883	3.924113015	2.912241745	3.549641413	3.752489491	3.718075281	3.917286289	4.136977476
ENSG00000167487	0.007739	0.042725273	0.311566822	1.241054801	**KLHL26**	protein_coding	kelch like family member 26 [Source:HGNC Symbol;Acc:HGNC:25623]	chr19	18636965	18671714	1	ensembl_havana	2.742635184	2.586851773	2.898418595	2.660681873	2.541211407	2.558662038	3.053967167	2.890865039	2.750423579
ENSG00000165071	0.007754	0.042794167	-0.259525101	-1.19708459	**TMEM71**	protein_coding	transmembrane protein 71 [Source:HGNC Symbol;Acc:HGNC:26572]	chr8	132685007	132760712	-1	ensembl_havana	4.740791469	4.87055402	4.611028918	4.87332995	4.903675229	4.834656881	4.593204216	4.606912214	4.632970326
ENSG00000105426	0.007759	0.042805481	-0.255050046	-1.19337714	**PTPRS**	protein_coding	protein tyrosine phosphatase, receptor type S [Source:HGNC Symbol;Acc:HGNC:9681]	chr19	5158495	5340803	-1	ensembl_havana	3.030812914	3.158337937	2.903287891	3.411375911	2.896025364	3.167612535	3.157897671	2.596492542	2.955473459
ENSG00000134594	0.007764	0.042817297	-0.368944449	-1.291407624	**RAB33A**	protein_coding	RAB33A, member RAS oncogene family [Source:HGNC Symbol;Acc:HGNC:9773]	chrX	130171649	130184870	1	ensembl_havana	3.730788851	3.915261076	3.546316627	4.262806338	3.798294548	3.684682341	3.861504875	3.297423241	3.480021764
ENSG00000108679	0.007776	0.042830989	0.715311159	1.64183729	**LGALS3BP**	protein_coding	galectin 3 binding protein [Source:HGNC Symbol;Acc:HGNC:6564]	chr17	78971238	78980109	-1	ensembl_havana	0.438042188	0.080386608	0.795697767	-0.41068387	0.214850257	0.436993436	0.716189844	0.648704825	1.022198632
ENSG00000278828	0.007776	0.042830989	0.318830931	1.247319391	**HIST1H3H**	protein_coding	histone cluster 1 H3 family member h [Source:HGNC Symbol;Acc:HGNC:4775]	chr6	27810064	27811300	1	ensembl_havana	4.164038198	4.004622733	4.323453664	4.140867447	3.991523312	3.881477438	4.537323976	4.361741031	4.071295985
ENSG00000060688	0.007776	0.042830989	-0.246565394	-1.186379352	**SNRNP40**	protein_coding	small nuclear ribonucleoprotein U5 subunit 40 [Source:HGNC Symbol;Acc:HGNC:30857]	chr1	31259568	31296782	-1	ensembl_havana	6.362939132	6.486221829	6.239656435	6.454188228	6.48710419	6.51737307	6.201186876	6.269349365	6.248433064
ENSG00000168528	0.007796	0.042924822	-0.265299917	-1.201885879	**SERINC2**	protein_coding	serine incorporator 2 [Source:HGNC Symbol;Acc:HGNC:23231]	chr1	31409565	31434680	1	ensembl_havana	1.817130955	1.949780913	1.684480997	1.760103226	1.936162086	2.153077428	1.527846157	1.602328815	1.923268018
ENSG00000272899	0.007808	0.042961108	0.292862087	1.225068217	**AC025594.2**	protein_coding		chr7	128866308	128872044	1	ensembl_havana	1.875973657	1.729542614	2.022404701	1.541228291	2.116675269	1.530724282	1.936429043	2.34408831	1.786696749
ENSG00000279789	0.007806	0.042961108	0.291956286	1.224299295	**AC120114.4**	TEC		chr16	29913144	29915337	-1	havana	3.354643513	3.208665371	3.500621656	3.298158902	3.120305496	3.207531715	3.50153988	3.501444376	3.498880712
ENSG00000255815	0.007812	0.042962629	-1.007391591	-2.010273216	**KRT8P11**	processed_pseudogene	keratin 8 pseudogene 11 [Source:HGNC Symbol;Acc:HGNC:31058]	chr9	99305176	99306611	1	havana	-0.602611529	-0.098915734	-1.106307325	-0.18813307	-0.569826728	0.461212593	-1.13267621	-1.08144551	-1.10480025
ENSG00000100897	0.00782	0.04298621	0.318142651	1.246724462	**DCAF11**	protein_coding	DDB1 and CUL4 associated factor 11 [Source:HGNC Symbol;Acc:HGNC:20258]	chr14	24114195	24125242	1	ensembl_havana	6.366662712	6.207591386	6.525734038	6.386562267	6.176232164	6.059979728	6.606951541	6.469424757	6.500825815
ENSG00000175471	0.007826	0.04298621	0.298455389	1.229827002	**MCTP1**	protein_coding	multiple C2 and transmembrane domain containing 1 [Source:HGNC Symbol;Acc:HGNC:26183]	chr5	94703741	95284575	-1	ensembl_havana	5.983703935	5.83447624	6.132931629	5.91113593	5.805386305	5.786906485	6.107792173	6.118844781	6.172157932
ENSG00000137364	0.007831	0.04298621	0.259467016	1.197036394	**TPMT**	protein_coding	thiopurine S-methyltransferase [Source:HGNC Symbol;Acc:HGNC:12014]	chr6	18128311	18155074	-1	ensembl_havana	6.106050512	5.976317004	6.23578402	5.923715161	5.980879167	6.024356683	6.140703712	6.297927782	6.268720565
ENSG00000143434	0.00783	0.04298621	-0.272523238	-1.207918601	**SEMA6C**	protein_coding	semaphorin 6C [Source:HGNC Symbol;Acc:HGNC:10740]	chr1	151131685	151146664	-1	ensembl_havana	2.667069801	2.80333142	2.530808182	2.892874923	2.683379039	2.833740298	2.693950367	2.399587437	2.498886741
ENSG00000204616	0.007825	0.04298621	-0.792955695	-1.732620504	**TRIM31**	protein_coding	tripartite motif containing 31 [Source:HGNC Symbol;Acc:HGNC:16289]	chr6	30102897	30113106	-1	ensembl_havana	1.604864945	2.001342792	1.208387097	2.237945898	1.855271053	1.910811426	1.309740823	0.737534657	1.577885811
ENSG00000247765	0.007846	0.043051597	0.270722245	1.206411633	**AC068446.2**	lincRNA		chr15	21298233	21325241	-1	havana	4.408554208	4.273193085	4.543915331	4.623079215	4.091050934	4.105449108	4.816768636	4.399664195	4.415313161
ENSG00000131480	0.007858	0.043100873	0.286286234	1.219497021	**AOC2**	protein_coding	amine oxidase, copper containing 2 [Source:HGNC Symbol;Acc:HGNC:549]	chr17	42844600	42850707	1	ensembl_havana	3.838160713	3.695017596	3.981303829	3.705837742	3.648975701	3.730239343	4.008974209	4.009357694	3.925579585
ENSG00000176248	0.007881	0.043118793	0.579917921	1.494764205	**ANAPC2**	protein_coding	anaphase promoting complex subunit 2 [Source:HGNC Symbol;Acc:HGNC:19989]	chr9	137174784	137188549	-1	ensembl_havana	5.375310022	5.085351062	5.665268983	4.903884315	4.9840837	5.368085171	5.681597922	5.69482015	5.619388877
ENSG00000147799	0.007872	0.043118793	0.525818912	1.439750589	**ARHGAP39**	protein_coding	Rho GTPase activating protein 39 [Source:HGNC Symbol;Acc:HGNC:29351]	chr8	144529179	144605816	-1	ensembl_havana	2.647696799	2.384787343	2.910606255	2.50993591	2.184470312	2.459955808	3.011169462	2.975481414	2.745167891
ENSG00000162772	0.007877	0.043118793	0.357253347	1.280984791	**ATF3**	protein_coding	activating transcription factor 3 [Source:HGNC Symbol;Acc:HGNC:785]	chr1	212565334	212620777	1	ensembl_havana	8.011825032	7.833198358	8.190451706	7.836448774	7.869698534	7.793447767	8.315072974	8.068907121	8.187375022
ENSG00000144036	0.007885	0.043118793	0.276778038	1.211486256	**EXOC6B**	protein_coding	exocyst complex component 6B [Source:HGNC Symbol;Acc:HGNC:17085]	chr2	72175984	72826041	-1	ensembl_havana	5.892544387	5.754155368	6.030933406	5.746913397	5.69607929	5.819473415	6.066584376	6.015501897	6.010713945
ENSG00000075336	0.007882	0.043118793	-0.258857018	-1.196530372	**TIMM21**	protein_coding	translocase of inner mitochondrial membrane 21 [Source:HGNC Symbol;Acc:HGNC:25010]	chr18	74148511	74160530	1	ensembl_havana	5.145625149	5.275053658	5.01619664	5.206092784	5.336742153	5.282326038	4.967402959	5.019374044	5.061812918
ENSG00000118242	0.007877	0.043118793	-0.330676077	-1.257602575	**MREG**	protein_coding	melanoregulin [Source:HGNC Symbol;Acc:HGNC:25478]	chr2	215942805	216034096	-1	ensembl_havana	6.28676782	6.452105859	6.121429782	6.779582495	6.597263599	5.979471482	6.50481609	6.125546647	5.733926608
ENSG00000273002	0.00788	0.043118793	-0.435108989	-1.352012967	**AL355388.2**	antisense		chr1	155978799	155982986	1	havana	2.575649155	2.793203649	2.35809466	2.878177262	2.793616541	2.707817144	2.349154471	2.226242768	2.498886741
ENSG00000043039	0.007885	0.043118793	-1.089438567	-2.127912114	**BARX2**	protein_coding	BARX homeobox 2 [Source:HGNC Symbol;Acc:HGNC:956]	chr11	129375940	129452279	1	ensembl_havana	-0.01641112	0.528308163	-0.561130404	0.383334258	0.325758945	0.875831286	-0.46237776	-1.42278721	0.201773757
ENSG00000181004	0.007891	0.043118818	0.4461741	1.362422437	**BBS12**	protein_coding	Bardet-Biedl syndrome 12 [Source:HGNC Symbol;Acc:HGNC:26648]	chr4	122732702	122744943	1	ensembl_havana	2.514756531	2.291669482	2.737843581	2.26832323	2.569661407	2.037023808	2.514907924	3.007092508	2.691530312
ENSG00000224616	0.007888	0.043118818	0.354606795	1.278637042	**RTCA-AS1**	antisense	RTCA antisense RNA 1 [Source:HGNC Symbol;Acc:HGNC:50573]	chr1	100264009	100266120	-1	havana	0.726309162	0.549005765	0.903612559	0.41082706	0.453384582	0.782805653	0.785553575	0.938274071	0.987010032
ENSG00000238287	0.007915	0.043231043	0.850565765	1.80320793	**AL603839.3**	antisense		chr1	40493157	40508661	-1	havana	-0.53110968	-0.956392563	-0.105826797	-1.41179303	-0.967469033	-0.489915621	-0.08686711	-0.52165082	0.291037539
ENSG00000164076	0.007918	0.043231043	-0.850149594	-1.802687838	**CAMKV**	protein_coding	CaM kinase like vesicle associated [Source:HGNC Symbol;Acc:HGNC:28788]	chr3	49857988	49870222	-1	ensembl_havana	-0.478670181	-0.053595384	-0.903744978	0.040274975	-0.34076431	0.139703184	-1.13267621	-0.68524259	-0.893316134
ENSG00000221838	0.007931	0.043256564	0.285903633	1.219173655	**AP4M1**	protein_coding	adaptor related protein complex 4 mu 1 subunit [Source:HGNC Symbol;Acc:HGNC:574]	chr7	100101549	100110345	1	ensembl_havana	4.491546248	4.348594432	4.634498065	4.418204457	4.38481302	4.242765818	4.686826487	4.596559508	4.620108199
ENSG00000112033	0.007928	0.043256564	0.258815908	1.196496277	**PPARD**	protein_coding	peroxisome proliferator activated receptor delta [Source:HGNC Symbol;Acc:HGNC:9235]	chr6	35342558	35428191	1	ensembl_havana	5.378480311	5.249072357	5.507888265	5.297302101	5.249747778	5.200167194	5.54158341	5.465360412	5.516720974
ENSG00000185324	0.007929	0.043256564	0.240627441	1.181506397	**CDK10**	protein_coding	cyclin dependent kinase 10 [Source:HGNC Symbol;Acc:HGNC:1770]	chr16	89680737	89696364	1	ensembl_havana	5.843730524	5.723416804	5.964044244	5.729277786	5.694142403	5.746830222	5.964969921	5.93796143	5.989201383
ENSG00000229816	0.007948	0.043328893	0.500360457	1.414566947	**DDX50P1**	processed_pseudogene	DEAD-box helicase 50 pseudogene 1 [Source:HGNC Symbol;Acc:HGNC:18974]	chr2	32201600	32203999	1	havana	0.78052937	0.530349141	1.030709598	0.727424294	0.243386951	0.620236179	0.955706889	0.919411336	1.21701057
ENSG00000112218	0.007954	0.043328893	0.317065971	1.245794381	**GPR63**	protein_coding	G protein-coupled receptor 63 [Source:HGNC Symbol;Acc:HGNC:13302]	chr6	96794126	96837463	-1	ensembl_havana	3.720360448	3.561827463	3.878893434	3.352337167	3.817401554	3.515743667	3.756011721	4.007126095	3.873542485
ENSG00000206149	0.007951	0.043328893	0.253791647	1.192336664	**HERC2P9**	transcribed_unprocessed_pseudogene	hect domain and RLD 2 pseudogene 9 [Source:HGNC Symbol;Acc:HGNC:30495]	chr15	28589492	28685264	1	havana	4.612652404	4.485756581	4.739548228	4.312848501	4.597323125	4.547098117	4.560596196	4.810021875	4.848026613
ENSG00000154237	0.007971	0.043408794	-0.509678094	-1.423732485	**LRRK1**	protein_coding	leucine rich repeat kinase 1 [Source:HGNC Symbol;Acc:HGNC:18608]	chr15	100919215	101078254	1	ensembl_havana	6.468586339	6.723425386	6.213747292	6.496504081	6.664753718	7.009018359	6.200657915	6.208494913	6.232089049
ENSG00000188010	0.007985	0.043413415	0.427403024	1.344810621	**MORN2**	protein_coding	MORN repeat containing 2 [Source:HGNC Symbol;Acc:HGNC:30166]	chr2	38875962	38929072	1	ensembl_havana	4.580818896	4.367117384	4.794520407	4.530555105	4.467270071	4.103526974	4.784658061	4.86033958	4.738563581
ENSG00000132471	0.007989	0.043413415	0.294974345	1.22686316	**WBP2**	protein_coding	WW domain binding protein 2 [Source:HGNC Symbol;Acc:HGNC:12738]	chr17	75845699	75856507	-1	ensembl_havana	5.911082191	5.763595018	6.058569363	5.891754158	5.810163332	5.588867565	6.148952706	6.036442954	5.990312429
ENSG00000140105	0.00799	0.043413415	-0.291366462	-1.223798861	**WARS**	protein_coding	tryptophanyl-tRNA synthetase [Source:HGNC Symbol;Acc:HGNC:12729]	chr14	100333788	100376805	-1	ensembl_havana	8.194519686	8.340202917	8.048836455	8.349866266	8.384226339	8.286516145	8.039223966	8.013980833	8.093304566
ENSG00000183161	0.007977	0.043413415	-0.296947119	-1.228541949	**FANCF**	protein_coding	Fanconi anemia complementation group F [Source:HGNC Symbol;Acc:HGNC:3587]	chr11	22622519	22626787	-1	ensembl_havana	3.286136325	3.434609885	3.137662765	3.414774672	3.370423439	3.518631543	3.213490017	3.072584571	3.126913708
ENSG00000131732	0.007986	0.043413415	-0.315548045	-1.244484313	**ZCCHC9**	protein_coding	zinc finger CCHC-type containing 9 [Source:HGNC Symbol;Acc:HGNC:25424]	chr5	81301590	81313297	1	ensembl_havana	4.731701273	4.889475295	4.57392725	4.952633592	4.925852934	4.789939359	4.535647221	4.592099791	4.594034738
ENSG00000270504	0.007987	0.043413415	-0.511214862	-1.425249862	**AL391422.3**	antisense		chr6	3751111	3753871	1	havana	2.89761069	3.153218121	2.642003259	3.547648667	3.234877758	2.677127938	3.166591014	2.439854124	2.319564638
ENSG00000186472	0.008004	0.043468856	0.311967196	1.241399264	**PCLO**	protein_coding	piccolo presynaptic cytomatrix protein [Source:HGNC Symbol;Acc:HGNC:13406]	chr7	82754013	83162930	-1	ensembl_havana	4.080754111	3.924770513	4.236737709	3.522665065	3.942327796	4.309318677	3.942194775	4.256703576	4.511314775
ENSG00000172260	0.008043	0.043642616	0.587239917	1.502369747	**NEGR1**	protein_coding	neuronal growth regulator 1 [Source:HGNC Symbol;Acc:HGNC:17302]	chr1	71395940	72282734	-1	havana	-0.354867261	-0.64848722	-0.061247303	-0.92573229	-0.036373238	-0.983356126	-0.63291296	0.554044597	-0.104873545
ENSG00000140995	0.008043	0.043642616	0.27404148	1.209190442	**DEF8**	protein_coding	differentially expressed in FDCP 8 homolog [Source:HGNC Symbol;Acc:HGNC:25969]	chr16	89947925	89968060	1	ensembl_havana	6.716167388	6.579146648	6.853188128	6.628969256	6.489711993	6.618758695	6.959380915	6.789407545	6.810775925
ENSG00000179010	0.008045	0.043642616	0.271183719	1.206797589	**MRFAP1**	protein_coding	Morf4 family associated protein 1 [Source:HGNC Symbol;Acc:HGNC:24549]	chr4	6640091	6642745	1	ensembl_havana	7.504045023	7.368453163	7.639636882	7.355512026	7.369844875	7.380002588	7.677664025	7.669122598	7.572124023
ENSG00000169413	0.008052	0.043665112	-0.668091256	-1.588969305	**RNASE6**	protein_coding	ribonuclease A family member k6 [Source:HGNC Symbol;Acc:HGNC:10048]	chr14	20781051	20782467	1	ensembl_havana	-0.160806041	0.173239587	-0.494851669	0.540941683	-0.002471726	-0.018751196	-0.51701327	-0.52165082	-0.445890917
ENSG00000145730	0.008066	0.043725644	0.323121505	1.251034439	**PAM**	protein_coding	peptidylglycine alpha-amidating monooxygenase [Source:HGNC Symbol;Acc:HGNC:8596]	chr5	102753981	103031105	1	ensembl_havana	5.782611101	5.621050348	5.944171853	5.674330594	5.648860834	5.539959616	6.043172125	5.837295022	5.952048412
ENSG00000168395	0.008078	0.043756118	-0.240674404	-1.181544859	**ING5**	protein_coding	inhibitor of growth family member 5 [Source:HGNC Symbol;Acc:HGNC:19421]	chr2	241687085	241729478	1	ensembl_havana	5.124119392	5.244456595	5.00378219	5.321043534	5.174870462	5.237455787	5.078672972	4.949039243	4.983634356
ENSG00000182405	0.008076	0.043756118	-0.414870922	-1.333179392	**PGBD4**	protein_coding	piggyBac transposable element derived 4 [Source:HGNC Symbol;Acc:HGNC:19401]	chr15	34102073	34108684	1	ensembl_havana	3.850644703	4.058080164	3.643209242	4.10133777	4.158160005	3.914742718	3.538846404	3.682954534	3.70782679
ENSG00000158715	0.008085	0.043775863	-0.291303683	-1.223745609	**SLC45A3**	protein_coding	solute carrier family 45 member 3 [Source:HGNC Symbol;Acc:HGNC:8642]	chr1	205657851	205680459	-1	ensembl_havana	4.30646927	4.452121112	4.160817428	4.390853222	4.513401796	4.452108316	4.19042511	4.131170126	4.160857048
ENSG00000197375	0.00809	0.043788121	0.294082666	1.226105114	**SLC22A5**	protein_coding	solute carrier family 22 member 5 [Source:HGNC Symbol;Acc:HGNC:10969]	chr5	132369752	132395614	1	ensembl_havana	4.565326882	4.418285549	4.712368215	4.487627691	4.380001666	4.38722729	4.676211797	4.706547606	4.754345243
ENSG00000123416	0.008098	0.04381743	-0.244449933	-1.184641008	**TUBA1B**	protein_coding	tubulin alpha 1b [Source:HGNC Symbol;Acc:HGNC:18809]	chr12	49127782	49131397	-1	ensembl_havana	10.58447086	10.70669583	10.46224589	10.61253738	10.74611588	10.76143422	10.37974024	10.46998758	10.53700986
ENSG00000067798	0.008118	0.043891353	0.976443384	1.967608761	**NAV3**	protein_coding	neuron navigator 3 [Source:HGNC Symbol;Acc:HGNC:15998]	chr12	77324641	78213008	1	ensembl_havana	0.124384385	-0.363837306	0.612606077	-1.070289	-0.002471726	-0.018751196	0.457455004	0.900298706	0.480064523
ENSG00000170175	0.008115	0.043891353	-0.292388173	-1.224665858	**CHRNB1**	protein_coding	cholinergic receptor nicotinic beta 1 subunit [Source:HGNC Symbol;Acc:HGNC:1961]	chr17	7445061	7457707	1	ensembl_havana	2.762324797	2.908518884	2.61613071	2.78670079	2.918320072	3.020535788	2.600804468	2.572545352	2.675042311
ENSG00000130024	0.008133	0.043955789	0.243838047	1.184138676	**PHF10**	protein_coding	PHD finger protein 10 [Source:HGNC Symbol;Acc:HGNC:18250]	chr6	169703905	169725566	-1	ensembl_havana	6.667382105	6.545463082	6.789301129	6.59716266	6.577786494	6.461440091	6.848947555	6.791678237	6.727277595
ENSG00000273237	0.00815	0.044032596	0.481469202	1.39616476	**AC004520.1**	antisense		chr7	26173533	26174945	1	havana	0.471458835	0.230724234	0.712193437	0.040274975	0.453384582	0.198513145	0.785553575	0.821210834	0.529815901
ENSG00000171033	0.008157	0.044035246	0.336720524	1.262882595	**PKIA**	protein_coding	cAMP-dependent protein kinase inhibitor alpha [Source:HGNC Symbol;Acc:HGNC:9017]	chr8	78516139	78605267	1	ensembl_havana	2.868227206	2.699866944	3.036587467	2.722326456	2.864222379	2.513051996	3.144758638	3.051082538	2.913921226
ENSG00000157212	0.008154	0.044035246	-0.242942642	-1.183403972	**PAXIP1**	protein_coding	PAX interacting protein 1 [Source:HGNC Symbol;Acc:HGNC:8624]	chr7	154943687	155003084	-1	ensembl_havana	4.992080668	5.113551989	4.870609347	5.21489285	5.109521447	5.01624167	5.000600664	4.851659712	4.759567665
ENSG00000117697	0.008168	0.044045476	-0.2504409	-1.189570603	**NSL1**	protein_coding	NSL1, MIS12 kinetochore complex component [Source:HGNC Symbol;Acc:HGNC:24548]	chr1	212726153	212791782	-1	ensembl_havana	6.019525851	6.144746301	5.8943054	6.090789374	6.149623388	6.193826139	5.861687637	5.851047075	5.970181489
ENSG00000197498	0.008164	0.044045476	-0.299454453	-1.230678951	**RPF2**	protein_coding	ribosome production factor 2 homolog [Source:HGNC Symbol;Acc:HGNC:20870]	chr6	110982015	111028263	1	ensembl_havana	5.039985403	5.18971263	4.890258176	5.107675946	5.223129973	5.238331969	4.737301003	4.881204367	5.052269159
ENSG00000121211	0.008165	0.044045476	-0.457612988	-1.373267797	**MND1**	protein_coding	meiotic nuclear divisions 1 [Source:HGNC Symbol;Acc:HGNC:24839]	chr4	153344649	153415118	1	ensembl_havana	2.766280768	2.995087262	2.537474274	2.887992321	3.093124869	3.004144598	2.246292314	2.584568634	2.781561875
ENSG00000279965	0.008179	0.044089318	0.426363734	1.343842194	**AL390755.1**	TEC		chr13	110123208	110125234	1	havana	3.891764949	3.678583082	4.104946816	3.947922403	3.711591469	3.376235373	4.153730304	4.214772063	3.946338082
ENSG00000247853	0.008186	0.044093578	0.481829864	1.396513833	**AC006064.2**	antisense		chr12	6578622	6584739	1	havana	-0.088484592	-0.329399524	0.15243034	-0.36334239	-0.428075095	-0.196781087	0.176794179	-0.19956768	0.480064523
ENSG00000214113	0.008189	0.044093578	0.31141738	1.240926253	**LYRM4**	protein_coding	LYR motif containing 4 [Source:HGNC Symbol;Acc:HGNC:21365]	chr6	5102593	5260939	-1	ensembl_havana	3.595452402	3.439743712	3.751161092	3.404554274	3.488372191	3.42630467	3.613866272	3.785472329	3.854144675
ENSG00000124657	0.008188	0.044093578	-0.413242182	-1.33167514	**OR2B6**	protein_coding	olfactory receptor family 2 subfamily B member 6 [Source:HGNC Symbol;Acc:HGNC:8241]	chr6	27957241	27958182	1	ensembl_havana	1.950967971	2.157589062	1.744346881	2.214735639	2.270175802	1.987855745	1.996172808	1.682828385	1.554039449
ENSG00000230002	0.008208	0.044150746	-0.284901034	-1.218326686	**ALMS1-IT1**	sense_intronic	ALMS1 intronic transcript 1 [Source:HGNC Symbol;Acc:HGNC:41305]	chr2	73456764	73459482	1	havana	0.474381605	0.616832122	0.331931089	0.705390237	0.52486995	0.620236179	0.513047461	0.163140205	0.319605599
ENSG00000163378	0.008207	0.044150746	-0.310419479	-1.24006821	**EOGT**	protein_coding	EGF domain specific O-linked N-acetylglucosamine transferase [Source:HGNC Symbol;Acc:HGNC:28526]	chr3	68975214	69013961	-1	ensembl_havana	4.989471797	5.144681537	4.834262058	5.061803732	5.237387224	5.134853654	4.850879427	4.810021875	4.841884873
ENSG00000182253	0.008209	0.044150746	-0.330575449	-1.25751486	**SYNM**	protein_coding	synemin [Source:HGNC Symbol;Acc:HGNC:24466]	chr15	99098217	99135593	1	ensembl_havana	6.011050354	6.176338078	5.845762629	6.195769112	6.076665349	6.256579774	5.777646485	5.890323458	5.869317946
ENSG00000148672	0.008215	0.044164899	0.245517874	1.185518251	**GLUD1**	protein_coding	glutamate dehydrogenase 1 [Source:HGNC Symbol;Acc:HGNC:4335]	chr10	87050486	87094866	-1	ensembl_havana	6.748360282	6.625601345	6.871119218	6.580614261	6.644501807	6.651687966	6.85265593	6.853538095	6.907163631
ENSG00000124767	0.008221	0.044164899	-0.259624203	-1.197166823	**GLO1**	protein_coding	glyoxalase I [Source:HGNC Symbol;Acc:HGNC:4323]	chr6	38675925	38703141	-1	ensembl_havana	7.644461016	7.774273117	7.514648914	7.751804433	7.820680722	7.750334196	7.550593026	7.50603987	7.487313846
ENSG00000059691	0.008218	0.044164899	-0.333058562	-1.259681112	**GATB**	protein_coding	glutamyl-tRNA amidotransferase subunit B [Source:HGNC Symbol;Acc:HGNC:8849]	chr4	151670504	151761023	-1	ensembl_havana	3.623806799	3.79033608	3.457277518	3.680648284	3.829216193	3.861143762	3.302948055	3.640392813	3.428491686
ENSG00000168280	0.008228	0.044174525	-0.253911866	-1.192436025	**KIF5C**	protein_coding	kinesin family member 5C [Source:HGNC Symbol;Acc:HGNC:6325]	chr2	148875250	149026759	1	ensembl_havana	3.740979088	3.867935021	3.614023155	4.116027648	3.803095076	3.684682341	3.885399497	3.581617303	3.375052666
ENSG00000120738	0.008229	0.044174525	-0.429241207	-1.346525179	**EGR1**	protein_coding	early growth response 1 [Source:HGNC Symbol;Acc:HGNC:3238]	chr5	138465490	138469315	1	ensembl_havana	3.478900099	3.693520702	3.264279495	4.404593656	3.461295901	3.214672551	4.072798827	2.806118434	2.913921226
ENSG00000163528	0.008232	0.04417547	-0.287903647	-1.220864973	**CHCHD4**	protein_coding	coiled-coil-helix-coiled-coil-helix domain containing 4 [Source:HGNC Symbol;Acc:HGNC:26467]	chr3	14112077	14124870	-1	ensembl_havana	4.276267875	4.420219699	4.132316052	4.268453071	4.483808757	4.50839727	3.924364913	4.149497163	4.32308608
ENSG00000104231	0.008248	0.044244808	-0.334259442	-1.26073009	**ZFAND1**	protein_coding	zinc finger AN1-type containing 1 [Source:HGNC Symbol;Acc:HGNC:25858]	chr8	81701334	81732903	-1	ensembl_havana	5.192606826	5.359736547	5.025477105	5.433383973	5.393546673	5.252278995	5.024708239	4.984635224	5.067087853
ENSG00000100106	0.008255	0.044262935	0.302704265	1.233454301	**TRIOBP**	protein_coding	TRIO and F-actin binding protein [Source:HGNC Symbol;Acc:HGNC:17009]	chr22	37696988	37776556	1	ensembl_havana	3.822399309	3.671047177	3.973751442	3.697490029	3.654298741	3.661352761	4.116077755	3.922032118	3.883144453
ENSG00000224383	0.008262	0.044287167	0.962460158	1.948629968	**PRR29**	protein_coding	proline rich 29 [Source:HGNC Symbol;Acc:HGNC:25673]	chr17	63998351	64004304	1	ensembl_havana	0.07981974	-0.401410339	0.561049819	-0.99620085	-0.569826728	0.361796557	0.106248156	0.821210834	0.755690467
ENSG00000177694	0.008269	0.044304429	0.6052819	1.521275979	**NAALADL2**	protein_coding	N-acetylated alpha-linked acidic dipeptidase like 2 [Source:HGNC Symbol;Acc:HGNC:23219]	chr3	174438573	175810552	1	havana	0.959852346	0.657211396	1.262493296	0.892538	0.570631912	0.508464275	1.325353043	1.529563273	0.932563571
ENSG00000112146	0.008275	0.044312491	0.262246919	1.199345166	**FBXO9**	protein_coding	F-box protein 9 [Source:HGNC Symbol;Acc:HGNC:13588]	chr6	53051991	53100873	1	ensembl_havana	6.421017019	6.289893559	6.552140479	6.277847702	6.312513326	6.279319651	6.539531425	6.514113761	6.602776251
ENSG00000120332	0.008276	0.044312491	-0.31533068	-1.244296826	**TNN**	protein_coding	tenascin N [Source:HGNC Symbol;Acc:HGNC:22942]	chr1	175067858	175148066	1	ensembl_havana	5.891126923	6.048792263	5.733461583	6.171864556	5.847239677	6.127272556	5.809911762	5.661862623	5.728610365
ENSG00000272463	0.008281	0.044321902	-0.545406055	-1.459431051	**AL357054.4**	lincRNA		chr6	708592	711405	-1	havana	-0.075017186	0.197685841	-0.347720214	0.040274975	0.243386951	0.309395597	-0.69455235	-0.42206346	0.073455173
ENSG00000166887	0.008286	0.044330174	-0.253546555	-1.192134121	**VPS39**	protein_coding	VPS39, HOPS complex subunit [Source:HGNC Symbol;Acc:HGNC:20593]	chr15	42158701	42208316	-1	ensembl_havana	6.805072898	6.931846176	6.678299621	6.979009209	6.900550717	6.915978601	6.678596701	6.697249779	6.659052383
ENSG00000141562	0.008299	0.044356704	0.290106862	1.222730843	**NARF**	protein_coding	nuclear prelamin A recognition factor [Source:HGNC Symbol;Acc:HGNC:29916]	chr17	82458180	82490537	1	ensembl_havana	5.729113177	5.584059746	5.874166608	5.599396023	5.656838681	5.495944535	5.845537212	5.885471989	5.891490625
ENSG00000165355	0.0083	0.044356704	0.261316105	1.198571609	**FBXO33**	protein_coding	F-box protein 33 [Source:HGNC Symbol;Acc:HGNC:19833]	chr14	39397669	39432500	-1	ensembl_havana	4.825199227	4.694541174	4.955857279	4.823055734	4.73054201	4.530025778	5.034239593	4.975532909	4.857799336
ENSG00000164458	0.008295	0.044356704	-0.352334157	-1.276624426	**TBXT**	protein_coding	T-box transcription factor T [Source:HGNC Symbol;Acc:HGNC:11515]	chr6	166157656	166168700	-1	ensembl_havana	6.244236492	6.42040357	6.068069413	6.670434284	6.272401002	6.318375424	6.395819845	6.007709498	5.800678896
ENSG00000178035	0.008311	0.044401701	-0.274863048	-1.209879233	**IMPDH2**	protein_coding	inosine monophosphate dehydrogenase 2 [Source:HGNC Symbol;Acc:HGNC:6053]	chr3	49024325	49029408	-1	ensembl_havana	7.831234001	7.968665525	7.693802477	7.719359962	7.970758297	8.215878316	7.428283362	7.778983943	7.874140126
ENSG00000087274	0.008327	0.044467597	0.262055522	1.199186063	**ADD1**	protein_coding	adducin 1 [Source:HGNC Symbol;Acc:HGNC:243]	chr4	2843857	2930076	1	ensembl_havana	7.816908146	7.685880385	7.947935907	7.91969412	7.632911049	7.505035987	8.138947896	7.867600564	7.837259262
ENSG00000161955	0.00833	0.044469455	-0.410555981	-1.329197958	**TNFSF13**	protein_coding	TNF superfamily member 13 [Source:HGNC Symbol;Acc:HGNC:11928]	chr17	7558292	7561608	1	ensembl_havana	0.202968079	0.40824607	-0.002309911	0.727424294	0.214850257	0.28246366	0.308311185	-0.00684508	-0.308395834
ENSG00000276603	0.008358	0.044480944	0.448277031	1.364409808	**AL109614.1**	sense_intronic		chr20	37526642	37527060	-1	havana	1.829600841	1.605462325	2.053739357	1.590147445	1.415548178	1.810691353	1.91595172	2.106272137	2.138994213
ENSG00000183628	0.008361	0.044480944	0.416152529	1.334364238	**DGCR6**	protein_coding	DiGeorge syndrome critical region gene 6 [Source:HGNC Symbol;Acc:HGNC:2846]	chr22	18906028	18914238	1	ensembl_havana	2.441630602	2.233554338	2.649706867	2.067039104	2.191810142	2.441813766	2.613576909	2.69405369	2.641490002
ENSG00000024862	0.008347	0.044480944	0.332653625	1.259327593	**CCDC28A**	protein_coding	coiled-coil domain containing 28A [Source:HGNC Symbol;Acc:HGNC:21098]	chr6	138773509	138793319	1	ensembl_havana	4.166305678	3.999978865	4.33263249	4.058523223	4.112480652	3.82893272	4.265181149	4.43992877	4.292787552
ENSG00000197121	0.00837	0.044480944	0.30235302	1.233154035	**PGAP1**	protein_coding	post-GPI attachment to proteins 1 [Source:HGNC Symbol;Acc:HGNC:25712]	chr2	196833004	196927796	-1	ensembl_havana	4.453494137	4.302317627	4.604670647	4.321928059	4.25418706	4.330837762	4.743124734	4.509373547	4.56151366
ENSG00000164574	0.008363	0.044480944	0.295076708	1.226950212	**GALNT10**	protein_coding	polypeptide N-acetylgalactosaminyltransferase 10 [Source:HGNC Symbol;Acc:HGNC:19873]	chr5	154190730	154420984	1	ensembl_havana	6.347700509	6.200162155	6.495238863	6.185359571	6.222656431	6.192470465	6.52903572	6.40604919	6.550631679
ENSG00000167107	0.008356	0.044480944	0.24016738	1.181129687	**ACSF2**	protein_coding	acyl-CoA synthetase family member 2 [Source:HGNC Symbol;Acc:HGNC:26101]	chr17	50426158	50474845	1	ensembl_havana	5.454611194	5.334527503	5.574694884	5.324661633	5.362174763	5.316746115	5.588433372	5.585395507	5.550255773
ENSG00000101361	0.008352	0.044480944	-0.247555639	-1.187193945	**NOP56**	protein_coding	NOP56 ribonucleoprotein [Source:HGNC Symbol;Acc:HGNC:15911]	chr20	2652145	2658393	1	ensembl_havana	7.91156452	8.03534234	7.787786701	8.009538688	8.025057639	8.071430692	7.71925118	7.811797815	7.832311108
ENSG00000143486	0.008355	0.044480944	-0.257265031	-1.195210752	**EIF2D**	protein_coding	eukaryotic translation initiation factor 2D [Source:HGNC Symbol;Acc:HGNC:6583]	chr1	206571292	206612463	-1	ensembl_havana	5.546185312	5.674817828	5.417552796	5.743538634	5.635465598	5.64544925	5.443950581	5.358256688	5.45045112
ENSG00000198783	0.00836	0.044480944	-0.263842279	-1.200672159	**ZNF830**	protein_coding	zinc finger protein 830 [Source:HGNC Symbol;Acc:HGNC:28291]	chr17	34961530	34963775	1	ensembl_havana	4.231856072	4.363777211	4.099934932	4.430009476	4.310901545	4.350420613	4.142757667	3.998165018	4.158882111
ENSG00000134285	0.008367	0.044480944	-0.271916576	-1.207410771	**FKBP11**	protein_coding	FK506 binding protein 11 [Source:HGNC Symbol;Acc:HGNC:18624]	chr12	48921518	48926474	-1	ensembl_havana	4.607930765	4.743889054	4.471972477	4.666494579	4.835020118	4.730152463	4.468685528	4.562010741	4.385221163
ENSG00000275832	0.008366	0.044480944	-0.452687859	-1.368587674	**ARHGAP23**	protein_coding	Rho GTPase activating protein 23 [Source:HGNC Symbol;Acc:HGNC:29293]	chr17	38419280	38512392	1	ensembl_havana	1.49606323	1.72240716	1.2697193	1.578072284	1.759729761	1.829419434	1.278000209	1.063828438	1.467329254
ENSG00000172752	0.008339	0.044480944	-0.781281634	-1.718656983	**COL6A5**	protein_coding	collagen type VI alpha 5 chain [Source:HGNC Symbol;Acc:HGNC:26674]	chr3	130345516	130484844	1	ensembl_havana	0.067843481	0.458484298	-0.322797336	0.749126884	0.547932369	0.078393642	0.069637898	-0.68524259	-0.352787316
ENSG00000188938	0.008377	0.044505531	0.241374761	1.182118579	**FAM120AOS**	protein_coding	family with sequence similarity 120A opposite strand [Source:HGNC Symbol;Acc:HGNC:23389]	chr9	93446494	93453592	-1	ensembl_havana	4.342666162	4.221978782	4.463353543	4.243822659	4.243631448	4.17848224	4.473948226	4.514096035	4.402016368
ENSG00000148737	0.008403	0.044609436	0.400928378	1.32035729	**TCF7L2**	protein_coding	transcription factor 7 like 2 [Source:HGNC Symbol;Acc:HGNC:11641]	chr10	112950250	113167678	1	ensembl_havana	2.244931002	2.044466814	2.445395191	1.649052215	2.331270798	2.153077428	2.246292314	2.578569518	2.511323741
ENSG00000225914	0.008402	0.044609436	-0.831771803	-1.779869915	**HCG23**	antisense	HLA complex group 23 (non-protein coding) [Source:HGNC Symbol;Acc:HGNC:19713]	chr6	32254640	32407763	1	havana	1.507932216	1.923818118	1.092046315	1.649052215	2.256240478	1.866161661	1.325353043	1.195095435	0.755690467
ENSG00000137877	0.00843	0.044693612	0.652620576	1.572021093	**SPTBN5**	protein_coding	spectrin beta, non-erythrocytic 5 [Source:HGNC Symbol;Acc:HGNC:15680]	chr15	41848144	41894077	-1	ensembl_havana	1.802242229	1.475931942	2.128552517	1.126095262	1.256642557	2.045058006	2.099663825	1.938414976	2.347578751

ENSG00000274354	0.008443	0.044693612	0.295364955	1.227195378	**AC105105.4**	sense_intronic		chr18	58511598	58512310	-1	havana	1.00785121	0.860168733	1.155533688	1.022973463	0.874167025	0.683365711	1.245545533	1.130954377	1.090101154
ENSG00000131437	0.008442	0.044693612	0.285536089	1.218863095	**KIF3A**	protein_coding	kinesin family member 3A [Source:HGNC Symbol;Acc:HGNC:6319]	chr5	132692628	132737638	-1	ensembl_havana	5.881852782	5.739084738	6.024620827	5.794002125	5.737419039	5.68583305	5.982307915	6.044082097	6.047472469
ENSG00000181788	0.008444	0.044693612	0.251616711	1.19054051	**SIAH2**	protein_coding	siah E3 ubiquitin protein ligase 2 [Source:HGNC Symbol;Acc:HGNC:10858]	chr3	150741127	150763477	-1	ensembl_havana	6.685080607	6.559272252	6.810888962	6.596788661	6.577436288	6.503591807	6.8137532	6.809396109	6.809517578
ENSG00000100564	0.008425	0.044693612	0.23772738	1.179133755	**PIGH**	protein_coding	phosphatidylinositol glycan anchor biosynthesis class H [Source:HGNC Symbol;Acc:HGNC:8964]	chr14	67581955	67600287	-1	ensembl_havana	4.569618016	4.450754326	4.688481706	4.453333619	4.514865633	4.384063726	4.676211797	4.760537642	4.628695679
ENSG00000163785	0.008443	0.044693612	-0.257270905	-1.195215618	**RYK**	protein_coding	receptor-like tyrosine kinase [Source:HGNC Symbol;Acc:HGNC:10481]	chr3	134065303	134250744	-1	ensembl_havana	6.169870577	6.29850603	6.041235125	6.282971118	6.339199332	6.273347639	6.059600507	6.017166234	6.046938632
ENSG00000163344	0.008438	0.044693612	-0.277703825	-1.212263925	**PMVK**	protein_coding	phosphomevalonate kinase [Source:HGNC Symbol;Acc:HGNC:9141]	chr1	154924734	154936991	-1	ensembl_havana	5.102949447	5.24180136	4.964097535	5.227510036	5.197835644	5.3000584	4.978553955	4.982365024	4.931373625
ENSG00000028277	0.008428	0.044693612	-0.366437891	-1.289165863	**POU2F2**	protein_coding	POU class 2 homeobox 2 [Source:HGNC Symbol;Acc:HGNC:9213]	chr19	42086110	42196585	-1	ensembl_havana	2.037648325	2.220867271	1.85442938	2.058373044	2.263224966	2.341003803	1.763844323	2.002551439	1.796892377
ENSG00000125733	0.00845	0.044711009	-0.304567073	-1.235047965	**TRIP10**	protein_coding	thyroid hormone receptor interactor 10 [Source:HGNC Symbol;Acc:HGNC:12304]	chr19	6737925	6751526	1	ensembl_havana	6.673077337	6.825360874	6.520793801	7.074234031	6.904464965	6.497383625	6.888239526	6.568096038	6.106045838
ENSG00000130244	0.008455	0.044717494	0.378519839	1.300007403	**FAM98C**	protein_coding	family with sequence similarity 98 member C [Source:HGNC Symbol;Acc:HGNC:27119]	chr19	38403135	38409088	1	ensembl_havana	3.156692927	2.967433007	3.345952846	3.10550171	2.868808811	2.9279885	3.295040884	3.34414149	3.398676164
ENSG00000102030	0.008465	0.044748311	0.253210982	1.191856861	**NAA10**	protein_coding	N(alpha)-acetyltransferase 10, NatA catalytic subunit [Source:HGNC Symbol;Acc:HGNC:18704]	chrX	153929242	153935223	-1	ensembl_havana	5.062432144	4.935826653	5.189037635	4.980423634	4.941176941	4.885879385	5.1915873	5.180539597	5.194986009
ENSG00000094916	0.008467	0.044748311	-0.277543324	-1.212129067	**CBX5**	protein_coding	chromobox 5 [Source:HGNC Symbol;Acc:HGNC:1555]	chr12	54230940	54280133	-1	ensembl_havana	8.102195477	8.240967139	7.963423815	8.139371798	8.224427628	8.359101991	7.794267848	8.03617555	8.059828047
ENSG00000172673	0.00849	0.044826929	0.462369945	1.377803306	**THEMIS**	protein_coding	thymocyte selection associated [Source:HGNC Symbol;Acc:HGNC:21569]	chr6	127708072	127918631	-1	ensembl_havana	4.323818894	4.092633922	4.555003867	3.525811778	4.474811168	4.27727882	4.230361879	4.883639327	4.551010395
ENSG00000165512	0.008485	0.044826929	-0.252971324	-1.191658888	**ZNF22**	protein_coding	zinc finger protein 22 [Source:HGNC Symbol;Acc:HGNC:13012]	chr10	45000475	45005326	1	ensembl_havana	5.809964649	5.936450311	5.683478987	5.89783898	5.962759021	5.948752932	5.60531745	5.692738824	5.752380686
ENSG00000146858	0.008491	0.044826929	-0.859384285	-1.814263852	**ZC3HAV1L**	protein_coding	zinc finger CCCH-type containing, antiviral 1 like [Source:HGNC Symbol;Acc:HGNC:22423]	chr7	139025706	139036029	-1	ensembl_havana	1.024989095	1.454681237	0.595296952	2.058373044	1.228369434	1.077301233	0.785553575	0.861296647	0.139040636
ENSG00000128602	0.008514	0.044918033	0.406067998	1.32506947	**SMO**	protein_coding	smoothened, frizzled class receptor [Source:HGNC Symbol;Acc:HGNC:11119]	chr7	129188872	129213545	1	ensembl_havana	0.937446972	0.734412973	1.140480971	0.565613454	0.874167025	0.76345844	0.873137292	1.491756274	1.056549347
ENSG00000165731	0.008514	0.044918033	-0.695370393	-1.619300114	**RET**	protein_coding	ret proto-oncogene [Source:HGNC Symbol;Acc:HGNC:9967]	chr10	43077027	43130351	1	ensembl_havana	-0.189198875	0.158486322	-0.536884071	0.812341977	0.06303108	-0.399914092	-0.21650287	-0.68524259	-0.708906755
ENSG00000165169	0.008524	0.044950636	0.320119657	1.24843409	**DYNLT3**	protein_coding	dynein light chain Tctex-type 3 [Source:HGNC Symbol;Acc:HGNC:11694]	chrX	37836757	37847637	-1	ensembl_havana	5.439211419	5.279151591	5.599271247	5.353284333	5.390360257	5.093810182	5.738825475	5.571870036	5.487118232
ENSG00000275131	0.008528	0.044951099	0.383907742	1.304871501	**AC241952.1**	transcribed_unprocessed_pseudogene		chr1	120489860	120701289	-1	havana	3.848620384	3.656666513	4.040574255	4.036629851	3.711591469	3.221778217	4.312949691	4.009357694	3.799415379
ENSG00000115977	0.00853	0.044951099	0.243101056	1.183533921	**AAK1**	protein_coding	AP2 associated kinase 1 [Source:HGNC Symbol;Acc:HGNC:19679]	chr2	69457997	69674349	-1	ensembl_havana	7.5198574	7.398306872	7.641407928	7.332110954	7.426552399	7.436257262	7.589297055	7.693093749	7.641832978
ENSG00000213160	0.008547	0.045005342	-0.242502158	-1.18304271	**KLHL23**	protein_coding	kelch like family member 23 [Source:HGNC Symbol;Acc:HGNC:27506]	chr2	169694488	169776989	1	ensembl_havana	4.756842202	4.878093281	4.635591123	4.766982541	4.913697407	4.953599895	4.557294489	4.668927578	4.680551301
ENSG00000137161	0.008544	0.045005342	-0.27944699	-1.213729552	**CNPY3**	protein_coding	canopy FGF signaling regulator 3 [Source:HGNC Symbol;Acc:HGNC:11968]	chr6	42929192	42939287	1	ensembl_havana	4.798403511	4.938127007	4.658680016	4.98386005	4.935722747	4.894798222	4.7959082	4.584636156	4.595495693
ENSG00000004766	0.008572	0.045078817	0.258344153	1.196105092	**VPS50**	protein_coding	VPS50, EARP/GARPII complex subunit [Source:HGNC Symbol;Acc:HGNC:25956]	chr7	93232340	93361121	1	ensembl_havana	5.737964037	5.608791961	5.867136114	5.492479496	5.644855307	5.689041079	5.815450187	5.889717915	5.896240239
ENSG00000150787	0.008569	0.045078817	0.238355551	1.17964728	**PTS**	protein_coding	6-pyruvoyltetrahydropterin synthase [Source:HGNC Symbol;Acc:HGNC:9689]	chr11	112226365	112269955	1	ensembl_havana	4.723186785	4.604009009	4.842364561	4.590389821	4.636849344	4.584787863	4.852227225	4.861575309	4.813291149
ENSG00000270049	0.008573	0.045078817	-0.300113901	-1.231241616	**AC009061.2**	antisense		chr16	67481314	67505063	1	havana	1.824633884	1.974690834	1.674576933	1.968722995	1.855271053	2.100078453	1.729053464	1.614107429	1.680569906
ENSG00000214725	0.008568	0.045078817	-1.247255689	-2.373894284	**CDIPT-AS1**	transcribed_unitary_pseudogene	CDIPT antisense RNA 1 (head to head) [Source:HGNC Symbol;Acc:HGNC:48609]	chr16	29863593	29868053	1	havana	-0.336485447	0.287142398	-0.960113291	0.705390237	0.243386951	-0.087349994	-1.22062457	-1.00738779	-0.652327512
ENSG00000104856	0.008587	0.045134415	-0.261147062	-1.198431178	**RELB**	protein_coding	RELB proto-oncogene, NF-kB subunit [Source:HGNC Symbol;Acc:HGNC:9956]	chr19	45001430	45038198	1	ensembl_havana	4.323297116	4.453870647	4.192723586	4.564612562	4.432064119	4.364935261	4.374276086	4.149497163	4.054397508
ENSG00000267169	0.008598	0.045159016	0.411953167	1.33048585	**AC022098.1**	antisense		chr19	14137179	14171267	1	havana	1.635009474	1.42903289	1.840986058	1.464591378	1.500754142	1.321753151	1.852715862	1.737696596	1.932545716
ENSG00000175105	0.008596	0.045159016	0.249319293	1.188646144	**ZNF654**	protein_coding	zinc finger protein 654 [Source:HGNC Symbol;Acc:HGNC:25612]	chr3	88059274	88144665	1	ensembl_havana	5.013270763	4.888611116	5.137930409	4.942072749	4.887945457	4.835815142	5.178752887	5.099066414	5.135971927
ENSG00000103042	0.008634	0.045266662	0.296937459	1.228533722	**SLC38A7**	protein_coding	solute carrier family 38 member 7 [Source:HGNC Symbol;Acc:HGNC:25582]	chr16	58665109	58685104	-1	ensembl_havana	4.194342869	4.04587414	4.342811599	4.122277808	4.055282633	3.96006198	4.368636211	4.286451496	4.37334709
ENSG00000143093	0.008622	0.045266662	0.265296616	1.201883129	**STRIP1**	protein_coding	striatin interacting protein 1 [Source:HGNC Symbol;Acc:HGNC:25916]	chr1	110031577	110074641	1	ensembl_havana	6.054247693	5.921599385	6.186896001	5.98958241	5.915329189	5.859886555	6.318456687	6.189431314	6.052800001
ENSG00000180979	0.00863	0.045266662	0.251699714	1.190609008	**LRRC57**	protein_coding	leucine rich repeat containing 57 [Source:HGNC Symbol;Acc:HGNC:26719]	chr15	42537820	42548802	-1	ensembl_havana	5.522586131	5.396736274	5.648435988	5.402046998	5.495676335	5.292485489	5.624374643	5.718882985	5.602050337
ENSG00000114742	0.008634	0.045266662	0.238196191	1.179516983	**WDR48**	protein_coding	WD repeat domain 48 [Source:HGNC Symbol;Acc:HGNC:30914]	chr3	39051998	39096671	1	ensembl_havana	6.91027386	6.791175764	7.029371955	6.76401063	6.843441395	6.766075268	6.987251516	7.106393634	6.994470716
ENSG00000115266	0.008626	0.045266662	-0.740894245	-1.671211408	**APC2**	protein_coding	APC2, WNT signaling pathway regulator [Source:HGNC Symbol;Acc:HGNC:24036]	chr19	1446302	1473244	1	ensembl_havana	0.388475102	0.758922224	0.018027979	0.852997155	0.477608744	0.946160774	0.106248156	0.130707766	-0.182871985
ENSG00000165646	0.008644	0.045268268	0.660508452	1.580639593	**SLC18A2**	protein_coding	solute carrier family 18 member A2 [Source:HGNC Symbol;Acc:HGNC:10935]	chr10	117241073	117279430	1	ensembl_havana	0.773189973	0.442935747	1.103444199	0.206373087	0.125688576	0.996745577	0.785553575	1.163381334	1.361397687
ENSG00000134532	0.008642	0.045268268	0.482619607	1.397278506	**SOX5**	protein_coding	SRY-box 5 [Source:HGNC Symbol;Acc:HGNC:11201]	chr12	23529500	24562544	-1	ensembl_havana	1.811816267	1.570506463	2.053126071	1.716703112	1.558674763	1.436141515	2.466446406	1.947753749	1.745178056
ENSG00000011105	0.008643	0.045268268	0.346310252	1.271305054	**TSPAN9**	protein_coding	tetraspanin 9 [Source:HGNC Symbol;Acc:HGNC:21640]	chr12	3077355	3286564	1	ensembl_havana	4.705216645	4.532061519	4.878371771	4.249544037	4.591785246	4.754855274	4.72705256	4.95715416	4.950908594
ENSG00000271855	0.008647	0.04527058	0.348411155	1.273157722	**AC073195.1**	lincRNA		chr2	9555899	9556775	1	havana	-0.317265546	-0.491471124	-0.143059969	-0.18813307	-0.428075095	-0.85820521	0.210814386	-0.2414061	-0.398588198
ENSG00000256525	0.008655	0.045283432	0.242598207	1.183121474	**POLG2**	protein_coding	DNA polymerase gamma 2, accessory subunit [Source:HGNC Symbol;Acc:HGNC:9180]	chr17	64477785	64497036	-1	ensembl_havana	6.428146285	6.306847181	6.549445388	6.269424485	6.281459509	6.36965755	6.492335985	6.504653393	6.651346786
ENSG00000180953	0.008659	0.045283432	-0.267289342	-1.203544381	**ST20**	protein_coding	suppressor of tumorigenicity 20 [Source:HGNC Symbol;Acc:HGNC:33520]	chr15	79898840	79923702	-1	ensembl_havana	3.324506749	3.45815142	3.190862078	3.369953701	3.575123512	3.429377047	3.072588313	3.256675325	3.243322595
ENSG00000139146	0.008657	0.045283432	-0.289259484	-1.222012874	**SINHCAF**	protein_coding	SIN3-HDAC complex associated factor [Source:HGNC Symbol;Acc:HGNC:30702]	chr12	31280584	31327058	-1	ensembl_havana	6.806139418	6.95076916	6.661509676	7.19057944	6.926355647	6.735372393	6.797753656	6.660448674	6.526326699
ENSG00000214248	0.008667	0.0453092	0.289799522	1.22247039	**AC010336.1**	antisense		chr19	7898804	7903542	-1	havana	-0.14872439	-0.293624151	-0.003824629	-0.18813307	-0.569826728	-0.122912658	0.210814386	-0.32890884	0.106620565
ENSG00000183570	0.008703	0.045313327	0.694800079	1.618660113	**PCBP3**	protein_coding	poly(rC) binding protein 3 [Source:HGNC Symbol;Acc:HGNC:8651]	chr21	45643694	45942454	1	ensembl_havana	1.435347926	1.087947886	1.782747965	0.565613454	1.214022341	1.484207863	1.681320805	1.715999071	1.950924019
ENSG00000136854	0.0087	0.045313327	0.407211939	1.32612056	**STXBP1**	protein_coding	syntaxin binding protein 1 [Source:HGNC Symbol;Acc:HGNC:11444]	chr9	127579370	127696027	1	ensembl_havana	5.792028195	5.588422226	5.995634165	5.383086314	5.634119209	5.748061154	6.003691441	5.916124218	6.067086836
ENSG00000088899	0.00869	0.045313327	0.291343389	1.223779289	**LZTS3**	protein_coding	leucine zipper tumor suppressor family member 3 [Source:HGNC Symbol;Acc:HGNC:30139]	chr20	3162617	3173592	-1	ensembl_havana	3.558512209	3.412840515	3.704183903	3.532084689	3.40872246	3.297714395	3.809722321	3.611304352	3.691525037
ENSG00000221978	0.008676	0.045313327	0.254176827	1.192655044	**CCNL2**	protein_coding	cyclin L2 [Source:HGNC Symbol;Acc:HGNC:20570]	chr1	1385711	1399328	-1	ensembl_havana	7.707266997	7.580178584	7.834355411	7.764181013	7.484667704	7.491687035	7.962206533	7.742558614	7.798301086
ENSG00000035499	0.008713	0.045313327	-0.24328544	-1.183685193	**DEPDC1B**	protein_coding	DEP domain containing 1B [Source:HGNC Symbol;Acc:HGNC:24902]	chr5	60596912	60700190	-1	ensembl_havana	5.530135459	5.651778179	5.408492738	5.461592049	5.704442549	5.789299937	5.242840767	5.478306834	5.504330615
ENSG00000175711	0.008705	0.045313327	-0.246203759	-1.186082004	**B3GNTL1**	protein_coding	UDP-GlcNAc:betaGal beta-1,3-N-acetylglucosaminyltransferase like 1 [Source:HGNC Symbol;Acc:HGNC:21727]	chr17	82942155	83051810	-1	ensembl_havana	1.839960985	1.963062865	1.716859106	1.716703112	1.864486201	2.307999282	1.514288036	1.614107429	2.022181853
ENSG00000023902	0.008711	0.045313327	-0.248083891	-1.187628723	**PLEKHO1**	protein_coding	pleckstrin homology domain containing O1 [Source:HGNC Symbol;Acc:HGNC:24310]	chr1	150149183	150164720	1	ensembl_havana	4.007240655	4.1312826	3.88319871	4.323737133	4.184153292	3.885957376	4.028181329	3.940860841	3.680553959
ENSG00000074695	0.008702	0.045313327	-0.272688716	-1.208057158	**LMAN1**	protein_coding	lectin, mannose binding 1 [Source:HGNC Symbol;Acc:HGNC:6631]	chr18	59327823	59359962	-1	ensembl_havana	7.959160288	8.095504645	7.82281593	8.101646952	8.060697029	8.124169956	7.739785738	7.840595758	7.888066293
ENSG00000272711	0.008714	0.045313327	-0.300557922	-1.231620616	**AC019069.1**	lincRNA		chr2	74832655	74833987	-1	havana	2.48966287	2.639941831	2.339383909	2.516278079	2.64110813	2.762439285	2.20469138	2.24151074	2.571949608
ENSG00000116984	0.008716	0.045313327	-0.3095065	-1.239283708	**MTR**	protein_coding	5-methyltetrahydrofolate-homocysteine methyltransferase [Source:HGNC Symbol;Acc:HGNC:7468]	chr1	236795281	236903981	1	ensembl_havana	6.558403643	6.713156893	6.403650393	6.75699829	6.618525184	6.763947206	6.356944834	6.435390438	6.418615908
ENSG00000167635	0.008685	0.045313327	-0.312453421	-1.241817718	**ZNF146**	protein_coding	zinc finger protein 146 [Source:HGNC Symbol;Acc:HGNC:12931]	chr19	36214602	36238774	1	ensembl_havana	7.741278702	7.897505412	7.585051991	7.978869013	7.886758491	7.826888733	7.535151623	7.62214022	7.59786413
ENSG00000169598	0.008711	0.045313327	-0.360095511	-1.283510868	**DFFB**	protein_coding	DNA fragmentation factor subunit beta [Source:HGNC Symbol;Acc:HGNC:2773]	chr1	3857267	3885429	1	ensembl_havana	3.381459139	3.561506895	3.201411383	3.550741415	3.449096928	3.684682341	3.153531275	3.256675325	3.194027549
ENSG00000121988	0.008681	0.045313327	-0.365801125	-1.288596985	**ZRANB3**	protein_coding	zinc finger RANBP2-type containing 3 [Source:HGNC Symbol;Acc:HGNC:25249]	chr2	135136916	135531236	-1	ensembl_havana	3.674779953	3.857680516	3.491879391	3.703060534	3.927016074	3.942964938	3.487731073	3.572590744	3.415316355
ENSG00000174796	0.00869	0.045313327	-0.382516798	-1.303614043	**THAP6**	protein_coding	THAP domain containing 6 [Source:HGNC Symbol;Acc:HGNC:23189]	chr4	75513946	75550473	1	ensembl_havana	4.011683293	4.202941692	3.820424894	4.266573281	4.163769556	4.17848224	3.694224053	3.839149739	3.92790089
ENSG00000282826	0.008678	0.045313327	-1.028767758	-2.040280852	**FRG1CP**	unprocessed_pseudogene	FSHD region gene 1 family member C, pseudogene [Source:HGNC Symbol;Acc:HGNC:51762]	chr20	28580709	28602664	-1	havana	-0.479499095	0.034884784	-0.993882974	0.540941683	-0.036373238	-0.399914092	-1.13267621	-0.74417246	-1.10480025
ENSG00000115255	0.008732	0.045363802	0.628914566	1.546401097	**REEP6**	protein_coding	receptor accessory protein 6 [Source:HGNC Symbol;Acc:HGNC:30078]	chr19	1490747	1497927	1	ensembl_havana	0.62425684	0.309799557	0.938714123	0.267803752	0.06303108	0.598563838	0.873137292	1.029056719	0.913948357
ENSG00000166278	0.008736	0.045363802	0.529772408	1.443701426	**C2**	protein_coding	complement C2 [Source:HGNC Symbol;Acc:HGNC:1248]	chr6	31897785	31945672	1	ensembl_havana	-0.291576676	-0.55646288	-0.026690472	-0.4596317	-0.521012188	-0.688744748	-0.08686711	0.315191528	-0.308395834
ENSG00000169813	0.008738	0.045363802	-0.252940127	-1.19163312	**HNRNPF**	protein_coding	heterogeneous nuclear ribonucleoprotein F [Source:HGNC Symbol;Acc:HGNC:5039]	chr10	43385617	43409166	-1	ensembl_havana	8.520561706	8.64703177	8.394091643	8.666881723	8.561927187	8.7122864	8.391570539	8.369821178	8.420883213
ENSG00000107581	0.00873	0.045363802	-0.254105048	-1.192595706	**EIF3A**	protein_coding	eukaryotic translation initiation factor 3 subunit A [Source:HGNC Symbol;Acc:HGNC:3271]	chr10	119033670	119080823	-1	ensembl_havana	7.66633842	7.793390944	7.539285896	7.857051242	7.729375778	7.793745811	7.539759599	7.511363849	7.56673424
ENSG00000127951	0.008743	0.045373348	0.534298328	1.448237616	**FGL2**	protein_coding	fibrinogen like 2 [Source:HGNC Symbol;Acc:HGNC:3696]	chr7	77193371	77199848	-1	ensembl_havana	3.918636629	3.651487465	4.185785793	2.973446503	3.83391509	4.147100801	3.666911477	4.518803115	4.371642787
ENSG00000086544	0.00875	0.045393122	0.261193519	1.19846977	**ITPKC**	protein_coding	inositol-trisphosphate 3-kinase C [Source:HGNC Symbol;Acc:HGNC:14897]	chr19	40717103	40740860	1	ensembl_havana	4.487534606	4.356937847	4.618131365	4.546135504	4.322650523	4.202027512	4.842766089	4.623032125	4.388595882
ENSG00000261211	0.008765	0.045441687	0.722171094	1.649662725	**AL031123.2**	lincRNA		chr6	6680309	6683633	-1	havana	0.611194601	0.250109054	0.972280148	-0.41068387	0.214850257	0.946160774	-0.12879794	1.179325527	1.866312856
ENSG00000155111	0.008766	0.045441687	0.266049315	1.202510353	**CDK19**	protein_coding	cyclin dependent kinase 19 [Source:HGNC Symbol;Acc:HGNC:19338]	chr6	110609978	110815958	-1	ensembl_havana	5.312100546	5.179075889	5.445125204	5.192296197	5.195099047	5.149832423	5.466127098	5.386860428	5.482388085
ENSG00000139197	0.008782	0.04549364	0.266564204	1.202939598	**PEX5**	protein_coding	peroxisomal biogenesis factor 5 [Source:HGNC Symbol;Acc:HGNC:9719]	chr12	7188685	7218574	1	ensembl_havana	5.833573491	5.700291388	5.966855593	5.709349575	5.642848364	5.748676226	5.895418496	5.933855337	6.071292944
ENSG00000276814	0.008781	0.04549364	-0.349313635	-1.273954397	**AC004801.6**	lincRNA		chr12	48039784	48040761	-1	havana	0.822985153	0.99764197	0.648328335	1.190963204	0.855801933	0.946160774	0.995284613	0.37179175	0.577908642
ENSG00000019169	0.008794	0.045530976	-0.26355962	-1.20043694	**MARCO**	protein_coding	macrophage receptor with collagenous structure [Source:HGNC Symbol;Acc:HGNC:6895]	chr2	118942166	118994660	1	ensembl_havana	7.148473676	7.280253486	7.016693866	7.558894825	7.237349028	7.044516605	7.230008688	6.968681221	6.851391688
ENSG00000101353	0.008797	0.045530976	-0.308671162	-1.238566356	**MROH8**	protein_coding	maestro heat like repeat family member 8 [Source:HGNC Symbol;Acc:HGNC:16125]	chr20	37101226	37179588	-1	ensembl_havana	2.333791391	2.488126972	2.179455809	2.404475508	2.523868156	2.536037252	2.221475976	2.122856455	2.194034997
ENSG00000104213	0.008799	0.045530976	-0.343119064	-1.268496083	**PDGFRL**	protein_coding	platelet derived growth factor receptor like [Source:HGNC Symbol;Acc:HGNC:8805]	chr8	17576433	17644071	1	ensembl_havana	3.332391852	3.503951384	3.16083232	3.532084689	3.458255814	3.52151365	3.349502035	3.055408658	3.077586267
ENSG00000204091	0.008804	0.04554279	1.138690283	2.201810462	**TDRG1**	antisense	testis development related 1 [Source:HGNC Symbol;Acc:HGNC:43642]	chr6	40334954	40379887	1	ensembl_havana	0.388752503	-0.180592638	0.958097645	-0.56281016	-0.34076431	0.361796557	0.618191201	1.399485086	0.856616648
ENSG00000272994	0.008826	0.045640765	0.245904005	1.185835593	**AC012360.3**	lincRNA		chr2	105334027	105337475	-1	havana	4.225102921	4.102150918	4.348054923	4.097113041	4.077246218	4.132093496	4.368636211	4.273512191	4.402016368
ENSG00000250506	0.008876	0.04576269	0.675339255	1.596972263	**CDK3**	protein_coding	cyclin dependent kinase 3 [Source:HGNC Symbol;Acc:HGNC:1772]	chr17	76000906	76005999	1	ensembl_havana	-0.332265509	-0.669935137	0.005404118	-0.36334239	-0.727039001	-0.91942402	-0.08686711	0.063570082	0.039509383
ENSG00000174804	0.00888	0.04576269	0.466868839	1.382106548	**FZD4**	protein_coding	frizzled class receptor 4 [Source:HGNC Symbol;Acc:HGNC:4042]	chr11	86945679	86955391	-1	ensembl_havana	0.805191082	0.571756663	1.038625501	0.14220989	0.592979824	0.980080273	0.399634207	1.300906777	1.415335521
ENSG00000048707	0.008857	0.04576269	0.408825995	1.327605025	**VPS13D**	protein_coding	vacuolar protein sorting 13 homolog D [Source:HGNC Symbol;Acc:HGNC:23595]	chr1	12230032	12512047	1	ensembl_havana	6.617853539	6.413440542	6.822266537	6.477940356	6.171135209	6.591246059	6.760195274	6.796208927	6.910395409
ENSG00000259429	0.008866	0.04576269	0.378557127	1.300041004	**UBE2Q2P2**	transcribed_unprocessed_pseudogene	ubiquitin conjugating enzyme E2 Q2 pseudogene 2 [Source:HGNC Symbol;Acc:HGNC:37440]	chr15	82355142	82420075	1	havana	2.342207609	2.152929045	2.531486173	2.191145857	2.021022296	2.246618983	2.408992098	2.572545352	2.612921068
ENSG00000236397	0.008885	0.04576269	0.319102451	1.247554162	**DDX11L2**	unprocessed_pseudogene	DEAD/H-box helicase 11 like 2 [Source:HGNC Symbol;Acc:HGNC:37103]	chr2	113599036	113601261	-1	havana	2.101402015	1.941850789	2.26095324	2.032058081	1.7890623	2.004431986	2.310455111	2.011485396	2.460919213
ENSG00000140104	0.008883	0.04576269	0.299377413	1.230613234	**CLBA1**	protein_coding	clathrin binding box of aftiphilin containing 1 [Source:HGNC Symbol;Acc:HGNC:20126]	chr14	104985775	105010482	1	ensembl_havana	2.764172285	2.614483579	2.913860992	2.677756939	2.541211407	2.624482391	2.879883083	2.952470332	2.90922956
ENSG00000163611	0.008876	0.04576269	0.282684795	1.216456554	**SPICE1**	protein_coding	spindle and centriole associated protein 1 [Source:HGNC Symbol;Acc:HGNC:25083]	chr3	113442718	113515187	-1	ensembl_havana	4.693829326	4.552486929	4.835171723	4.390853222	4.526523162	4.740084403	4.769044464	4.795871197	4.94059951
ENSG00000179562	0.008883	0.04576269	0.252576031	1.191332423	**GCC1**	protein_coding	GRIP and coiled-coil domain containing 1 [Source:HGNC Symbol;Acc:HGNC:19095]	chr7	127580618	127593611	-1	ensembl_havana	5.554669296	5.428381281	5.680957312	5.445896751	5.450495655	5.388751436	5.653269978	5.687870688	5.701731269
ENSG00000034693	0.008882	0.04576269	-0.251541603	-1.190478531	**PEX3**	protein_coding	peroxisomal biogenesis factor 3 [Source:HGNC Symbol;Acc:HGNC:8858]	chr6	143450807	143490010	1	ensembl_havana	4.782567314	4.908338115	4.656796512	4.982715487	4.948778335	4.793520524	4.695863043	4.671748138	4.602778354
ENSG00000124074	0.008875	0.04576269	-0.274242044	-1.209358555	**ENKD1**	protein_coding	enkurin domain containing 1 [Source:HGNC Symbol;Acc:HGNC:25246]	chr16	67662945	67667265	-1	ensembl_havana	3.277172713	3.414293735	3.140051691	3.477874854	3.427497328	3.337509022	3.271055973	3.175535294	2.973563806
ENSG00000115641	0.008867	0.04576269	-1.318709987	-2.494429658	**FHL2**	protein_coding	four and a half LIM domains 2 [Source:HGNC Symbol;Acc:HGNC:3703]	chr2	105357712	105438513	-1	ensembl_havana	-0.421230153	0.23812484	-1.080585146	-0.79434999	0.614986832	0.89373768	-1.31428442	-1.32958732	-0.597883695
ENSG00000246790	0.008889	0.045765639	0.554183117	1.468336995	**AP000977.1**	antisense		chr11	121447331	121453013	-1	havana	2.795024787	2.517933229	3.072116346	2.78670079	2.169677667	2.59742123	3.109124716	3.038025793	3.069198529
ENSG00000126010	0.008892	0.045766362	0.563942505	1.478303516	**GRPR**	protein_coding	gastrin releasing peptide receptor [Source:HGNC Symbol;Acc:HGNC:4609]	chrX	16123556	16153021	1	ensembl_havana	-0.037107165	-0.319078418	0.244864088	-0.61730533	-0.18055577	-0.159374151	0.276538753	0.256279753	0.201773757
ENSG00000261115	0.008896	0.045770854	0.513250181	1.427261989	**TMEM178B**	protein_coding	transmembrane protein 178B [Source:HGNC Symbol;Acc:HGNC:44112]	chr7	141074232	141480380	1	ensembl_havana	4.835475231	4.57885014	5.092100321	4.481158028	4.36059417	4.894798222	4.956165102	5.147487521	5.172648341
ENSG00000178927	0.008906	0.045796944	-0.257202404	-1.195158869	**C17orf62**	protein_coding	chromosome 17 open reading frame 62 [Source:HGNC Symbol;Acc:HGNC:28672]	chr17	82442589	82450829	-1	ensembl_havana	6.368193373	6.496794575	6.239592172	6.427937571	6.389539637	6.672906518	6.218012057	6.154065525	6.346698933
ENSG00000116663	0.008907	0.045796944	-0.344870477	-1.270036955	**FBXO6**	protein_coding	F-box protein 6 [Source:HGNC Symbol;Acc:HGNC:13585]	chr1	11664124	11674354	1	ensembl_havana	4.390257231	4.56269247	4.217821993	4.585874231	4.543834716	4.558368462	4.405825494	4.114681649	4.132958835
ENSG00000257108	0.008915	0.04580361	0.568388494	1.482866268	**NHLRC4**	protein_coding	NHL repeat containing 4 [Source:HGNC Symbol;Acc:HGNC:26700]	chr16	566996	569495	1	ensembl_havana	-0.057445781	-0.341640028	0.226748466	0.075059705	-0.18055577	-0.91942402	0.935503283	0.097529431	-0.352787316
ENSG00000066739	0.008915	0.04580361	0.24774189	1.187347221	**ATG2B**	protein_coding	autophagy related 2B [Source:HGNC Symbol;Acc:HGNC:20187]	chr14	96279202	96363870	-1	ensembl_havana	6.944127488	6.820256542	7.067998433	6.891761182	6.768414206	6.80059424	7.139349408	7.063545895	7.001099996
ENSG00000013563	0.008924	0.045833682	-0.274252362	-1.209367205	**DNASE1L1**	protein_coding	deoxyribonuclease 1 like 1 [Source:HGNC Symbol;Acc:HGNC:2957]	chrX	154401238	154412112	-1	ensembl_havana	4.46602499	4.603151171	4.328898809	4.628943975	4.699982239	4.480527301	4.29720193	4.518803115	4.170691382
ENSG00000155849	0.008949	0.045853575	0.469079698	1.384226182	**ELMO1**	protein_coding	engulfment and cell motility 1 [Source:HGNC Symbol;Acc:HGNC:16286]	chr7	36854361	37449249	-1	ensembl_havana	6.091575377	5.857035528	6.326115226	5.333667378	6.122994619	6.114444587	6.009743254	6.631041383	6.337561041
ENSG00000143951	0.00895	0.045853575	0.38956193	1.309995567	**WDPCP**	protein_coding	WD repeat containing planar cell polarity effector [Source:HGNC Symbol;Acc:HGNC:28027]	chr2	63121383	63827843	-1	ensembl_havana	4.173979794	3.979198829	4.368760759	3.950266431	3.999910397	3.987419659	4.456330725	4.187439524	4.462512029
ENSG00000134057	0.008944	0.045853575	-0.30109055	-1.232075401	**CCNB1**	protein_coding	cyclin B1 [Source:HGNC Symbol;Acc:HGNC:1579]	chr5	69167010	69178245	1	ensembl_havana	7.332103865	7.48264914	7.181558591	7.220261144	7.671158755	7.556527522	7.014292914	7.253672477	7.276710382
ENSG00000005436	0.008937	0.045853575	-0.380188443	-1.301511846	**GCFC2**	protein_coding	GC-rich sequence DNA-binding factor 2 [Source:HGNC Symbol;Acc:HGNC:1317]	chr2	75652000	75710989	-1	ensembl_havana	4.182429547	4.372523768	3.992335326	4.454985288	4.400735959	4.261850058	3.934580371	3.973231137	4.069194468
ENSG00000164050	0.00895	0.045853575	-0.465368859	-1.38067031	**PLXNB1**	protein_coding	plexin B1 [Source:HGNC Symbol;Acc:HGNC:9103]	chr3	48403854	48430051	-1	ensembl_havana	3.208390085	3.441074515	2.975705655	3.778870107	3.213401092	3.330952345	3.23844292	3.002618823	2.686055222
ENSG00000166863	0.008943	0.045853575	-0.672132137	-1.593426128	**TAC3**	protein_coding	tachykinin 3 [Source:HGNC Symbol;Acc:HGNC:11521]	chr12	57010000	57028883	-1	ensembl_havana	-0.207607744	0.128458324	-0.543673812	0.004630787	0.271370116	0.109374069	-0.51701327	-0.80561234	-0.308395834
ENSG00000103489	0.008935	0.045853575	-1.525137525	-2.878141506	**XYLT1**	protein_coding	xylosyltransferase 1 [Source:HGNC Symbol;Acc:HGNC:15516]	chr16	17101769	17470881	-1	ensembl_havana	-1.325854936	-0.563286174	-2.088423699	-1.31854494	-0.569826728	0.198513145	-1.90365811	-2.33094524	-2.030667746
ENSG00000161040	0.008961	0.045867618	0.414822324	1.333134484	**FBXL13**	protein_coding	F-box and leucine rich repeat protein 13 [Source:HGNC Symbol;Acc:HGNC:21658]	chr7	102813230	103074843	-1	ensembl_havana	1.068181735	0.860770573	1.275592897	0.812341977	1.046001934	0.723967808	1.01467363	1.625790657	1.186314404
ENSG00000135720	0.008963	0.045867618	0.246619838	1.186424124	**DYNC1LI2**	protein_coding	dynein cytoplasmic 1 light intermediate chain 2 [Source:HGNC Symbol;Acc:HGNC:2966]	chr16	66720893	66751798	-1	ensembl_havana	8.026200772	7.902890854	8.149510691	7.973693408	7.876831382	7.858147771	8.17466975	8.169868695	8.103993628
ENSG00000184999	0.008959	0.045867618	-0.483778862	-1.398401718	**SLC22A10**	protein_coding	solute carrier family 22 member 10 [Source:HGNC Symbol;Acc:HGNC:18057]	chr11	63137867	63369718	1	ensembl_havana	1.450209048	1.692098479	1.208319617	1.671956805	1.547275176	1.857063457	1.261864131	1.256505808	1.106588913
ENSG00000011198	0.008981	0.045912124	0.30016693	1.231286874	**ABHD5**	protein_coding	abhydrolase domain containing 5 [Source:HGNC Symbol;Acc:HGNC:21396]	chr3	43690108	43734371	1	ensembl_havana	4.904619076	4.754535611	5.054702541	4.703092555	4.860574589	4.699939689	5.110559324	5.054377101	4.999171199
ENSG00000156265	0.008976	0.045912124	-0.313349748	-1.242589483	**MAP3K7CL**	protein_coding	MAP3K7 C-terminal like [Source:HGNC Symbol;Acc:HGNC:16457]	chr21	29077471	29175889	1	ensembl_havana	2.763172374	2.919847248	2.6064975	2.776169237	3.00836839	2.975004118	2.379383493	2.643226439	2.796882569
ENSG00000107829	0.008981	0.045912124	-0.395342957	-1.315255385	**FBXW4**	protein_coding	F-box and WD repeat domain containing 4 [Source:HGNC Symbol;Acc:HGNC:10847]	chr10	101610664	101695295	-1	ensembl_havana	4.644632819	4.842304297	4.44696134	4.901463638	4.935722747	4.689726507	4.695863043	4.372182967	4.272838011
ENSG00000123080	0.009003	0.045941946	0.256369211	1.194468833	**CDKN2C**	protein_coding	cyclin dependent kinase inhibitor 2C [Source:HGNC Symbol;Acc:HGNC:1789]	chr1	50960745	50974633	1	ensembl_havana	4.5600871	4.431902495	4.688271706	4.426646458	4.439791005	4.429270021	4.667050915	4.644727524	4.753036678
ENSG00000116199	0.008998	0.045941946	0.246676464	1.186470692	**FAM20B**	protein_coding	FAM20B, glycosaminoglycan xylosylkinase [Source:HGNC Symbol;Acc:HGNC:23017]	chr1	179025804	179076562	1	ensembl_havana	6.874603356	6.751265123	6.997941588	6.777273906	6.715924711	6.760596753	6.981104412	7.012171633	7.000548718
ENSG00000114738	0.009001	0.045941946	-0.242073411	-1.182691179	**MAPKAPK3**	protein_coding	mitogen-activated protein kinase-activated protein kinase 3 [Source:HGNC Symbol;Acc:HGNC:6888]	chr3	50611520	50649297	1	ensembl_havana	6.803387139	6.924423845	6.682350434	6.852825327	6.865696918	7.054749289	6.645163986	6.63031659	6.771570726
ENSG00000101333	0.008997	0.045941946	-0.604541417	-1.520495363	**PLCB4**	protein_coding	phospholipase C beta 4 [Source:HGNC Symbol;Acc:HGNC:9059]	chr20	9068763	9481242	1	ensembl_havana	0.399974177	0.702244885	0.097703468	1.206734491	0.760296981	0.139703184	0.716189844	-0.19956768	-0.223511759
ENSG00000196735	0.008993	0.045941946	-0.717366611	-1.644178132	**HLA-DQA1**	protein_coding	major histocompatibility complex, class II, DQ alpha 1 [Source:HGNC Symbol;Acc:HGNC:4942]	chr6	32628179	32647062	1	ensembl_havana	3.067710489	3.426393794	2.709027183	4.0388342	3.321074063	2.919273119	2.89041725	2.737777558	2.498886741
ENSG00000101542	0.009039	0.046109926	0.974770238	1.965328176	**CDH20**	protein_coding	cadherin 20 [Source:HGNC Symbol;Acc:HGNC:1760]	chr18	61333582	61555773	1	ensembl_havana	-0.374348671	-0.86173379	0.113036447	-1.99786555	-0.727039001	0.139703184	-0.40973604	-0.00684508	0.755690467
ENSG00000267340	0.009054	0.046122887	0.379867911	1.301222714	**AC060780.2**	processed_pseudogene		chr17	43169880	43170077	-1	havana	1.373101795	1.18316784	1.563035751	1.158893785	1.029710695	1.360899039	1.580839304	1.554228498	1.554039449
ENSG00000245275	0.009054	0.046122887	0.332313411	1.259030655	**SAP30L-AS1**	antisense	SAP30L antisense RNA 1 (head to head) [Source:HGNC Symbol;Acc:HGNC:26760]	chr5	154329437	154445850	-1	havana	1.07402339	0.907866684	1.240180095	0.660286109	1.242575255	0.820738689	1.125834805	1.43975459	1.154950891
ENSG00000112679	0.009054	0.046122887	-0.335516866	-1.261829396	**DUSP22**	protein_coding	dual specificity phosphatase 22 [Source:HGNC Symbol;Acc:HGNC:16077]	chr6	291630	351355	1	ensembl_havana	5.765777737	5.933536169	5.598019304	6.137821296	5.90308429	5.759702922	5.88689504	5.409848883	5.497313988
ENSG00000133597	0.00905	0.046122887	-0.374237515	-1.296154337	**ADCK2**	protein_coding	aarF domain containing kinase 2 [Source:HGNC Symbol;Acc:HGNC:19039]	chr7	140673153	140696261	1	ensembl_havana	3.967846595	4.154965352	3.780727837	4.142918254	4.06530755	4.256670251	3.95980697	3.590587736	3.791788806
ENSG00000064687	0.009066	0.046143347	0.450452384	1.366468671	**ABCA7**	protein_coding	ATP binding cassette subfamily A member 7 [Source:HGNC Symbol;Acc:HGNC:37]	chr19	1040101	1065572	1	ensembl_havana	5.150512806	4.925286614	5.375738998	4.875798254	4.772756043	5.127305544	5.503490998	5.289222981	5.334503014
ENSG00000261342	0.009074	0.046143347	0.43785877	1.354592366	**AC006538.1**	sense_intronic		chr19	2727743	2729327	-1	havana	3.034574572	2.815645187	3.253503957	2.516278079	2.826996371	3.10366111	3.118116049	3.337051803	3.305344019
ENSG00000173281	0.009067	0.046143347	0.298905476	1.23021074	**PPP1R3B**	protein_coding	protein phosphatase 1 regulatory subunit 3B [Source:HGNC Symbol;Acc:HGNC:14942]	chr8	9136255	9151574	-1	ensembl_havana	4.1511577	4.001704962	4.300610438	4.130569456	4.057293197	3.817252234	4.416797878	4.256703576	4.22832986
ENSG00000100568	0.009069	0.046143347	0.240581141	1.18146848	**VTI1B**	protein_coding	vesicle transport through interaction with t-SNAREs 1B [Source:HGNC Symbol;Acc:HGNC:17793]	chr14	67647075	67674831	-1	ensembl_havana	6.296791355	6.176500785	6.417081926	6.12750146	6.198720536	6.203280358	6.404573505	6.403506237	6.443166034
ENSG00000090971	0.009074	0.046143347	-0.323031101	-1.250956048	**NAT14**	protein_coding	N-acetyltransferase 14 (putative) [Source:HGNC Symbol;Acc:HGNC:28918]	chr19	55485004	55487568	1	ensembl_havana	4.141111031	4.302626582	3.979595481	4.419896814	4.269877069	4.218105863	4.242747216	3.861556733	3.834482493
ENSG00000271870	0.009089	0.046202063	-0.299868077	-1.23103184	**AC024060.1**	antisense		chr3	3152942	3153435	1	havana	3.710955403	3.860889441	3.561021364	3.807595594	3.904856379	3.870216351	3.584973459	3.482292639	3.615797995
ENSG00000053524	0.009094	0.046211687	0.293447786	1.225565666	**MCF2L2**	protein_coding	MCF.2 cell line derived transforming sequence-like 2 [Source:HGNC Symbol;Acc:HGNC:30319]	chr3	183178043	183428778	-1	ensembl_havana	3.59136184	3.444637947	3.738085733	3.428290208	3.439879526	3.465744108	3.623370039	3.727010979	3.863876181
ENSG00000135045	0.009099	0.046219778	-0.319014394	-1.247478018	**C9orf40**	protein_coding	chromosome 9 open reading frame 40 [Source:HGNC Symbol;Acc:HGNC:23433]	chr9	74946581	74952886	-1	ensembl_havana	3.38617235	3.545679547	3.226665153	3.348787899	3.691020036	3.597230708	3.157897671	3.252913259	3.269184531
ENSG00000186017	0.009106	0.046238256	0.27651703	1.211267098	**ZNF566**	protein_coding	zinc finger protein 566 [Source:HGNC Symbol;Acc:HGNC:25919]	chr19	36445119	36489902	-1	ensembl_havana	3.672703377	3.534444862	3.810961892	3.663607608	3.56667939	3.373047587	3.921799706	3.936176647	3.574909323
ENSG00000077782	0.00911	0.046241103	0.406696283	1.325646655	**FGFR1**	protein_coding	fibroblast growth factor receptor 1 [Source:HGNC Symbol;Acc:HGNC:3688]	chr8	38411138	38468834	-1	ensembl_havana	4.121807976	3.918459834	4.325156118	3.468093324	3.953166093	4.334120086	4.047136094	4.389419981	4.538912277
ENSG00000198892	0.009115	0.046252517	0.446143004	1.362393073	**SHISA4**	protein_coding	shisa family member 4 [Source:HGNC Symbol;Acc:HGNC:27139]	chr1	201888680	201892306	1	ensembl_havana	0.22476532	0.001693818	0.447836823	-0.22998119	0.125688576	0.109374069	0.24405081	0.343769191	0.755690467
ENSG00000118785	0.009125	0.046284237	1.049976929	2.070496737	**SPP1**	protein_coding	secreted phosphoprotein 1 [Source:HGNC Symbol;Acc:HGNC:11255]	chr4	87975650	87983426	1	ensembl_havana	2.198748648	1.673760183	2.723737112	0.812341977	2.124367304	2.084571268	2.494337705	3.046743406	2.630130226

ENSG00000125388	0.009129	0.046284237	0.358799931	1.282358757	**GRK4**	protein_coding	G protein-coupled receptor kinase 4 [Source:HGNC Symbol;Acc:HGNC:4543]	chr4	2963608	3040747	1	ensembl_havana	2.720414419	2.541014454	2.899814385	2.649185179	2.575284697	2.398573485	2.879883083	3.106334847	2.713225226
ENSG00000108771	0.009131	0.046284237	-0.442687425	-1.35913374	**DHX58**	protein_coding	DExH-box helicase 58 [Source:HGNC Symbol;Acc:HGNC:29517]	chr17	42101404	42112733	-1	ensembl_havana	4.608793494	4.830137207	4.387449781	4.854681818	4.867466142	4.76826366	4.567177027	4.181514568	4.413657748
ENSG00000165688	0.00914	0.046308341	0.285310271	1.218672327	**PMPCA**	protein_coding	peptidase, mitochondrial processing alpha subunit [Source:HGNC Symbol;Acc:HGNC:18667]	chr9	136410570	136423761	1	ensembl_havana	5.649681247	5.507026112	5.792336383	5.358588439	5.470247905	5.692241991	5.641625317	5.851047075	5.884336756
ENSG00000213047	0.009142	0.046308341	0.245048143	1.185132318	**DENND1B**	protein_coding	DENN domain containing 1B [Source:HGNC Symbol;Acc:HGNC:28404]	chr1	197504748	197775696	-1	ensembl_havana	5.132430394	5.009906322	5.254954465	5.1118699	4.998804589	4.919044477	5.310099338	5.246348626	5.20841543
ENSG00000157601	0.009153	0.046343849	-0.807574019	-1.750265787	**MX1**	protein_coding	MX dynamin like GTPase 1 [Source:HGNC Symbol;Acc:HGNC:7532]	chr21	41420304	41459214	1	ensembl_havana	5.919700881	6.323487891	5.515913872	5.944439837	6.554488573	6.471535263	5.482756169	5.25483795	5.810147496
ENSG00000111981	0.009169	0.046380689	0.473911978	1.388870395	**ULBP1**	protein_coding	UL16 binding protein 1 [Source:HGNC Symbol;Acc:HGNC:14893]	chr6	149964007	149973710	1	ensembl_havana	2.819354348	2.582398359	3.056310337	2.199052053	2.646460224	2.901682799	2.874587014	3.175535294	3.118808702
ENSG00000164535	0.009171	0.046380689	0.341728068	1.267273633	**DAGLB**	protein_coding	diacylglycerol lipase beta [Source:HGNC Symbol;Acc:HGNC:28923]	chr7	6409126	6484190	-1	ensembl_havana	4.713153765	4.542289731	4.884017799	4.298201297	4.681852118	4.646815778	4.804288604	4.936194287	4.911570507
ENSG00000165775	0.009174	0.046380689	0.23765837	1.179077354	**FUNDC2**	protein_coding	FUN14 domain containing 2 [Source:HGNC Symbol;Acc:HGNC:24925]	chrX	155025980	155060303	1	ensembl_havana	6.302331623	6.183502438	6.421160808	6.234750376	6.156673417	6.159083522	6.438169262	6.42121349	6.404099673
ENSG00000235750	0.009167	0.046380689	-0.279856672	-1.214074263	**KIAA0040**	protein_coding	KIAA0040 [Source:HGNC Symbol;Acc:HGNC:28950]	chr1	175156987	175192999	-1	ensembl_havana	5.599682544	5.73961088	5.459754208	5.714874564	5.822628285	5.68132979	5.435882843	5.453116712	5.490263069
ENSG00000132196	0.009176	0.046380689	-0.324369923	-1.252117474	**HSD17B7**	protein_coding	hydroxysteroid 17-beta dehydrogenase 7 [Source:HGNC Symbol;Acc:HGNC:5215]	chr1	162790702	162812817	1	ensembl_havana	4.655871464	4.818056426	4.493686503	4.897824991	4.900318971	4.656025315	4.56224422	4.453104385	4.465710904
ENSG00000254995	0.009187	0.046390233	0.470452539	1.385544012	**STX16-NPEPL1**	protein_coding	STX16-NPEPL1 readthrough (NMD candidate) [Source:HGNC Symbol;Acc:HGNC:41993]	chr20	58651434	58715410	1	havana	1.847242385	1.612016115	2.082468654	1.738566362	1.324992307	1.772489677	2.135529305	2.055343696	2.056532963
ENSG00000169499	0.009191	0.046390233	-0.238285317	-1.179589853	**PLEKHA2**	protein_coding	pleckstrin homology domain containing A2 [Source:HGNC Symbol;Acc:HGNC:14336]	chr8	38901235	38973909	1	ensembl_havana	6.90944544	7.028588099	6.790302782	7.047662198	6.929651945	7.108450153	6.846244555	6.68124281	6.84342098
ENSG00000278970	0.009186	0.046390233	-0.298675913	-1.230015003	**HEIH**	TEC	hepatocellular carcinoma up-regulated EZH2-associated long non-coding RNA [Source:HGNC Symbol;Acc:HGNC:45049]	chr5	180829954	180831605	-1	havana	5.646813293	5.79615125	5.497475336	5.914741202	5.755487562	5.718224984	5.494887673	5.539036451	5.458501885
ENSG00000157426	0.009189	0.046390233	-0.405012934	-1.324100781	**AASDH**	protein_coding	aminoadipate-semialdehyde dehydrogenase [Source:HGNC Symbol;Acc:HGNC:23993]	chr4	56338287	56387508	-1	ensembl_havana	4.409275554	4.611782021	4.206769087	4.776260551	4.562357411	4.496728099	4.16244872	4.208958525	4.248900015
ENSG00000196655	0.009197	0.0463948	0.331722346	1.258514942	**TRAPPC4**	protein_coding	trafficking protein particle complex 4 [Source:HGNC Symbol;Acc:HGNC:19943]	chr11	119018432	119025454	1	ensembl_havana	6.43009103	6.264229857	6.595952202	6.107688041	6.366211844	6.318789685	6.579953679	6.569609096	6.638293832
ENSG00000162073	0.009198	0.0463948	-0.311320952	-1.240843314	**PAQR4**	protein_coding	progestin and adipoQ receptor family member 4 [Source:HGNC Symbol;Acc:HGNC:26386]	chr16	2969245	2973489	1	ensembl_havana	4.593333621	4.748994097	4.437673145	4.623079215	4.761701395	4.86220168	4.445656025	4.406453481	4.460909928
ENSG00000237945	0.009216	0.046444855	0.424961834	1.342536986	**LINC00649**	antisense	long intergenic non-protein coding RNA 649 [Source:HGNC Symbol;Acc:HGNC:44305]	chr21	33915534	33977691	1	ensembl_havana	1.212109753	0.999628836	1.42459067	1.075455542	0.910210399	1.013220568	1.293957805	1.343981945	1.635832261
ENSG00000132740	0.009218	0.046444855	0.349409975	1.274039471	**IGHMBP2**	protein_coding	immunoglobulin mu binding protein 2 [Source:HGNC Symbol;Acc:HGNC:5542]	chr11	68903842	68940602	1	ensembl_havana	4.404633045	4.229928057	4.579338032	4.167303204	4.240095677	4.28238529	4.688336516	4.506216612	4.543460969
ENSG00000226688	0.009211	0.046444855	0.275434204	1.210358313	**ENTPD1-AS1**	antisense	ENTPD1 antisense RNA 1 [Source:HGNC Symbol;Acc:HGNC:45203]	chr10	95753206	96090238	-1	havana	3.964161288	3.826444186	4.101878391	3.791998092	3.71668882	3.970645647	3.999273898	4.087479328	4.218881946
ENSG00000198742	0.009229	0.046468225	0.249792196	1.189035836	**SMURF1**	protein_coding	SMAD specific E3 ubiquitin protein ligase 1 [Source:HGNC Symbol;Acc:HGNC:16807]	chr7	99027438	99144100	-1	ensembl_havana	6.321414394	6.196518296	6.446310492	6.220741065	6.182232713	6.18658111	6.480182914	6.480727454	6.378021108
ENSG00000145220	0.009228	0.046468225	-0.258173532	-1.195963642	**LYAR**	protein_coding	Ly1 antibody reactive [Source:HGNC Symbol;Acc:HGNC:26021]	chr4	4267701	4290169	-1	ensembl_havana	4.251934759	4.381021524	4.122847993	4.179342839	4.45968935	4.504032384	3.93203328	4.131170126	4.305340572
ENSG00000065911	0.009234	0.046476859	-0.254331433	-1.192782861	**MTHFD2**	protein_coding	methylenetetrahydrofolate dehydrogenase (NADP+ dependent) 2, methenyltetrahydrofolate cyclohydrolase [Source:HGNC Symbol;Acc:HGNC:7434]	chr2	74198562	74217565	1	ensembl_havana	8.352565764	8.47973148	8.225400048	8.432772373	8.548946713	8.457475355	8.12214701	8.286707444	8.267345689
ENSG00000174943	0.009249	0.046501386	0.269777851	1.20562217	**KCTD13**	protein_coding	potassium channel tetramerization domain containing 13 [Source:HGNC Symbol;Acc:HGNC:22234]	chr16	29905012	29927035	-1	ensembl_havana	5.032253522	4.897364597	5.167142448	4.901463638	4.911476265	4.879153887	5.215871954	5.089605981	5.19594941
ENSG00000138760	0.009252	0.046501386	0.23116882	1.173785523	**SCARB2**	protein_coding	scavenger receptor class B member 2 [Source:HGNC Symbol;Acc:HGNC:1665]	chr4	76158733	76234536	-1	ensembl_havana	7.733215355	7.617630945	7.848799764	7.606485559	7.603802731	7.642604544	7.828394915	7.843412916	7.874591462
ENSG00000177683	0.009247	0.046501386	-0.255447125	-1.193705643	**THAP5**	protein_coding	THAP domain containing 5 [Source:HGNC Symbol;Acc:HGNC:23188]	chr7	108554543	108569750	-1	ensembl_havana	5.314432257	5.442155819	5.186708694	5.430868342	5.515554998	5.380044118	5.106047052	5.311096432	5.142982598
ENSG00000116157	0.009251	0.046501386	-0.408579339	-1.327378065	**GPX7**	protein_coding	glutathione peroxidase 7 [Source:HGNC Symbol;Acc:HGNC:4559]	chr1	52602372	52609051	1	ensembl_havana	2.612003753	2.816293423	2.407714084	2.833163932	2.948964943	2.666751395	2.587917939	2.329873398	2.305350914
ENSG00000284773	0.009261	0.046531641	-0.243915351	-1.184202128	**AC114490.3**	protein_coding		chr1	34974356	34985313	-1	havana	0.522006286	0.643963961	0.40004861	0.683014432	0.740413176	0.508464275	0.485518989	0.45273451	0.261892331
ENSG00000169231	0.009266	0.046539105	-0.356406056	-1.280232693	**THBS3**	protein_coding	thrombospondin 3 [Source:HGNC Symbol;Acc:HGNC:11787]	chr1	155195588	155209051	-1	ensembl_havana	4.234900408	4.413103436	4.05669738	4.715547829	4.231218154	4.292544325	4.215776639	4.038058667	3.916256835
ENSG00000260855	0.009273	0.04655959	0.451382178	1.367349622	**AL591848.4**	lincRNA		chr1	246772301	246775772	1	havana	1.98788006	1.762188971	2.213571149	1.464591378	1.646767026	2.17520851	2.144358071	2.081032632	2.415322744
ENSG00000111087	0.009282	0.046579079	0.246234463	1.186107247	**GLI1**	protein_coding	GLI family zinc finger 1 [Source:HGNC Symbol;Acc:HGNC:4317]	chr12	57460135	57472262	1	ensembl_havana	3.847965894	3.724848662	3.971083125	3.888048657	3.638270336	3.648226993	4.077416127	3.914907507	3.920925741
ENSG00000065923	0.009283	0.046579079	0.245079043	1.185157702	**SLC9A7**	protein_coding	solute carrier family 9 member A7 [Source:HGNC Symbol;Acc:HGNC:17123]	chrX	46599252	46759172	-1	ensembl_havana	8.076546899	7.954007377	8.199086421	7.854237744	7.940169188	8.067615199	8.105148163	8.229470541	8.262640559
ENSG00000086598	0.00929	0.046579079	-0.260711799	-1.198069664	**TMED2**	protein_coding	transmembrane p24 trafficking protein 2 [Source:HGNC Symbol;Acc:HGNC:16996]	chr12	123584531	123598577	1	ensembl_havana	7.347114096	7.477469995	7.216758196	7.595296005	7.472477254	7.364636727	7.263570728	7.215280908	7.171422952
ENSG00000162746	0.009287	0.046579079	-0.611428445	-1.527771142	**FCRLB**	protein_coding	Fc receptor like B [Source:HGNC Symbol;Acc:HGNC:26431]	chr1	161721563	161728143	1	ensembl_havana	0.676420865	0.982135088	0.370706643	1.022973463	0.740413176	1.183018625	0.56657698	0.343769191	0.201773757
ENSG00000278910	0.0093	0.046615067	-0.379448413	-1.300844407	**BANCR**	lincRNA	BRAF-activated non-protein coding RNA [Source:HGNC Symbol;Acc:HGNC:43877]	chr9	69296682	69311111	-1	havana	8.135780036	8.325504243	7.94605583	8.656152642	8.326316971	7.994043115	8.371501847	7.749083262	7.717582382
ENSG00000074755	0.009316	0.04667611	0.275793266	1.210659588	**ZZEF1**	protein_coding	zinc finger ZZ-type and EF-hand domain containing 1 [Source:HGNC Symbol;Acc:HGNC:29027]	chr17	4004445	4143020	-1	ensembl_havana	6.244862888	6.106966255	6.382759521	6.209532684	5.971847542	6.139518538	6.43277417	6.347278737	6.368225656
ENSG00000280276	0.009338	0.046755368	0.472631053	1.387637807	**AC009229.4**	TEC		chr2	38121935	38123881	-1	havana	1.335755783	1.099440256	1.572071309	1.175017601	1.046001934	1.077301233	1.593788933	1.315408538	1.807016457
ENSG00000135749	0.009336	0.046755368	0.307554652	1.237608192	**PCNX2**	protein_coding	pecanex homolog 2 [Source:HGNC Symbol;Acc:HGNC:8736]	chr1	232983435	233295713	-1	ensembl_havana	5.01728946	4.863512134	5.171066786	4.801147032	4.736827958	5.052561412	5.166886857	5.119870101	5.2264434
ENSG00000163618	0.009372	0.046756539	0.918785123	1.890522635	**CADPS**	protein_coding	calcium dependent secretion activator [Source:HGNC Symbol;Acc:HGNC:1426]	chr3	62398346	62875389	-1	ensembl_havana	1.724064515	1.264671954	2.183457077	0.637193984	1.569984981	1.586836898	2.135529305	2.131077666	2.283764259
ENSG00000236882	0.009351	0.046756539	0.40008546	1.319586076	**LINC01554**	lincRNA	long intergenic non-protein coding RNA 1554 [Source:HGNC Symbol;Acc:HGNC:24687]	chr5	95852232	95860133	1	ensembl_havana	0.84160779	0.64156506	1.04165052	0.14220989	0.570631912	1.211853376	0.716189844	0.993426194	1.415335521
ENSG00000100417	0.009374	0.046756539	0.329164859	1.256285931	**PMM1**	protein_coding	phosphomannomutase 1 [Source:HGNC Symbol;Acc:HGNC:9114]	chr22	41576894	41589890	-1	ensembl_havana	4.249267343	4.084684914	4.413849773	3.94322289	4.171215189	4.139616662	4.413149681	4.373915965	4.454483673
ENSG00000177370	0.009373	0.046756539	0.288343221	1.221237013	**TIMM22**	protein_coding	translocase of inner mitochondrial membrane 22 [Source:HGNC Symbol;Acc:HGNC:17317]	chr17	997117	1003671	1	ensembl_havana	4.689727406	4.545555796	4.833899017	4.617190518	4.482313051	4.53716382	4.981020293	4.807459326	4.713217431
ENSG00000248932	0.009362	0.046756539	0.274658167	1.209707426	**AC097103.2**	antisense		chr3	139389815	139583319	1	havana	1.066941647	0.929612564	1.204270731	0.968509893	0.874167025	0.946160774	1.195452122	1.097781778	1.319578292
ENSG00000152484	0.009364	0.046756539	0.272590565	1.207974973	**USP12**	protein_coding	ubiquitin specific peptidase 12 [Source:HGNC Symbol;Acc:HGNC:20485]	chr13	27066142	27171896	-1	ensembl_havana	6.873732896	6.737437613	7.010028179	6.723473115	6.767186395	6.721653331	7.041132257	7.09093553	6.898016749
ENSG00000164073	0.009381	0.046756539	0.254030401	1.192534002	**MFSD8**	protein_coding	major facilitator superfamily domain containing 8 [Source:HGNC Symbol;Acc:HGNC:28486]	chr4	127917732	127966034	-1	ensembl_havana	4.650133351	4.52311815	4.777148552	4.644950205	4.530870564	4.393533683	4.868303346	4.744551715	4.718590593
ENSG00000117410	0.009379	0.046756539	0.247170948	1.186877425	**ATP6V0B**	protein_coding	ATPase H+ transporting V0 subunit b [Source:HGNC Symbol;Acc:HGNC:861]	chr1	43974487	43978295	1	ensembl_havana	6.376919986	6.253334512	6.500505461	6.339054268	6.22757488	6.193374389	6.644775162	6.456398022	6.400343198
ENSG00000078043	0.009369	0.046756539	0.242892513	1.183362853	**PIAS2**	protein_coding	protein inhibitor of activated STAT 2 [Source:HGNC Symbol;Acc:HGNC:17311]	chr18	46803224	46920160	-1	ensembl_havana	5.094871317	4.97342506	5.216317573	4.89174011	4.944439586	5.084095483	5.093565039	5.234951258	5.320436423
ENSG00000100697	0.00936	0.046756539	0.23747426	1.178926895	**DICER1**	protein_coding	dicer 1, ribonuclease III [Source:HGNC Symbol;Acc:HGNC:17098]	chr14	95086228	95158010	-1	ensembl_havana	6.450345055	6.331607925	6.569082185	6.275512848	6.290888761	6.428422165	6.500954495	6.566581392	6.639710669
ENSG00000186130	0.00937	0.046756539	-0.239178875	-1.180320678	**ZBTB6**	protein_coding	zinc finger and BTB domain containing 6 [Source:HGNC Symbol;Acc:HGNC:16764]	chr9	122908056	122913330	-1	ensembl_havana	4.66563343	4.785222868	4.546043993	4.736070601	4.851334342	4.76826366	4.538998785	4.581639856	4.517493337
ENSG00000044459	0.009352	0.046756539	-0.314842381	-1.243875748	**CNTLN**	protein_coding	centlein [Source:HGNC Symbol;Acc:HGNC:23432]	chr9	17134982	17503923	1	ensembl_havana	3.901114868	4.058536058	3.743693678	4.051990026	4.091050934	4.032567216	3.666911477	3.705150922	3.859018634
ENSG00000005189	0.009345	0.046756539	-0.333633791	-1.26018347	**REXO5**	protein_coding	RNA exonuclease 5 [Source:HGNC Symbol;Acc:HGNC:24661]	chr16	20806429	20849668	1	ensembl_havana	3.168060035	3.33487693	3.001243139	3.207005484	3.411868621	3.385756687	2.717806083	3.183462537	3.102460798
ENSG00000139926	0.009402	0.046784084	0.296651779	1.228290474	**FRMD6**	protein_coding	FERM domain containing 6 [Source:HGNC Symbol;Acc:HGNC:19839]	chr14	51489100	51730727	1	ensembl_havana	3.146226944	2.997901054	3.294552834	3.146964063	3.00836839	2.83837071	3.32252843	3.34414149	3.21698858
ENSG00000123444	0.009399	0.046784084	-0.23833489	-1.179630386	**KBTBD4**	protein_coding	kelch repeat and BTB domain containing 4 [Source:HGNC Symbol;Acc:HGNC:23761]	chr11	47572197	47579015	-1	ensembl_havana	5.755424134	5.87459158	5.636256689	6.063441193	5.891298222	5.669035323	5.83739369	5.612071882	5.459304496
ENSG00000042088	0.009395	0.046784084	-0.293243217	-1.225391898	**TDP1**	protein_coding	tyrosyl-DNA phosphodiesterase 1 [Source:HGNC Symbol;Acc:HGNC:18884]	chr14	89954939	90044768	1	ensembl_havana	5.765510047	5.912131655	5.618888438	5.955566932	5.918097686	5.862730348	5.543253284	5.696898477	5.616513552
ENSG00000179119	0.009392	0.046784084	-0.313498435	-1.242717553	**SPTY2D1**	protein_coding	SPT2 chromatin protein domain containing 1 [Source:HGNC Symbol;Acc:HGNC:26818]	chr11	18606401	18634791	-1	ensembl_havana	5.517375682	5.6741249	5.360626464	5.937367722	5.608981642	5.476025335	5.487100569	5.391145278	5.203633546
ENSG00000130347	0.0094	0.046784084	-0.356430868	-1.280254711	**RTN4IP1**	protein_coding	reticulon 4 interacting protein 1 [Source:HGNC Symbol;Acc:HGNC:18647]	chr6	106571971	106629487	-1	ensembl_havana	2.95793567	3.136151104	2.779720236	3.032160045	3.029004765	3.347288501	2.626237264	2.846619933	2.866303509
ENSG00000182379	0.009406	0.046784703	-0.234956437	-1.176871201	**NXPH4**	protein_coding	neurexophilin 4 [Source:HGNC Symbol;Acc:HGNC:8078]	chr12	57216795	57226449	1	ensembl_havana	2.788977016	2.906455235	2.671498797	2.843288967	2.85962132	3.016455416	2.638787485	2.620048716	2.755660191
ENSG00000233746	0.009425	0.046802979	0.915145474	1.885759213	**LINC00656**	lincRNA	long intergenic non-protein coding RNA 656 [Source:HGNC Symbol;Acc:HGNC:27304]	chr20	23125068	23132621	-1	havana	-0.977415443	-1.43498818	-0.519842705	-1.61858537	-2.100471557	-0.585907617	-1.13267621	-0.62862566	0.201773757
ENSG00000138653	0.009429	0.046802979	0.890908338	1.854343273	**NDST4**	protein_coding	N-deacetylase and N-sulfotransferase 4 [Source:HGNC Symbol;Acc:HGNC:20779]	chr4	114827763	115113876	-1	ensembl_havana	8.758472992	8.313018823	9.203927161	8.573415828	8.213538513	8.152102128	8.927151166	9.216129187	9.46850113
ENSG00000267123	0.009433	0.046802979	0.545342518	1.459366779	**LINC02081**	lincRNA	long intergenic non-protein coding RNA 2081 [Source:HGNC Symbol;Acc:HGNC:52930]	chr17	78617389	78632057	-1	havana	1.05871171	0.786040451	1.331382969	0.683014432	0.89230127	0.782805653	1.245545533	1.14725896	1.601344415
ENSG00000204262	0.009432	0.046802979	0.277317792	1.211939593	**COL5A2**	protein_coding	collagen type V alpha 2 chain [Source:HGNC Symbol;Acc:HGNC:2210]	chr2	189031896	189179879	-1	ensembl_havana	1.743708413	1.605049517	1.882367309	1.82284946	1.351450576	1.640848515	2.025141822	1.726888623	1.89507148
ENSG00000109180	0.009431	0.046802979	-0.321329646	-1.249481591	**OCIAD1**	protein_coding	OCIA domain containing 1 [Source:HGNC Symbol;Acc:HGNC:16074]	chr4	48805212	48861817	1	ensembl_havana	6.195975063	6.356639886	6.03531024	6.432136365	6.427341077	6.210442216	5.98783688	6.09592772	6.022166122
ENSG00000089048	0.009431	0.046802979	-0.324765993	-1.252461271	**ESF1**	protein_coding	ESF1 nucleolar pre-rRNA processing protein homolog [Source:HGNC Symbol;Acc:HGNC:15898]	chr20	13714322	13784886	-1	ensembl_havana	5.079390728	5.241773724	4.917007732	5.325564741	5.221337869	5.178418563	4.853573765	4.939708814	4.957740617
ENSG00000122783	0.009419	0.046802979	-0.341752958	-1.267295497	**C7orf49**	protein_coding	chromosome 7 open reading frame 49 [Source:HGNC Symbol;Acc:HGNC:22432]	chr7	135092363	135170795	-1	ensembl_havana	4.87058119	5.04145767	4.699704711	5.08971436	5.01026654	5.024392109	4.689844966	4.83288226	4.576386907
ENSG00000173456	0.009436	0.046802979	-0.38609354	-1.306849983	**RNF26**	protein_coding	ring finger protein 26 [Source:HGNC Symbol;Acc:HGNC:14646]	chr11	119334527	119337313	1	ensembl_havana	5.724942688	5.917989458	5.531895918	5.747587404	5.840834308	6.165546662	5.528997362	5.472657064	5.594033327
ENSG00000189159	0.009463	0.046881573	-0.235243616	-1.177105488	**JPT1**	protein_coding	Jupiter microtubule associated homolog 1 [Source:HGNC Symbol;Acc:HGNC:14569]	chr17	75135248	75168281	-1	ensembl_havana	6.728637859	6.846259667	6.611016051	6.711086369	6.912261744	6.915430888	6.503958851	6.645102551	6.683986751
ENSG00000133121	0.009464	0.046881573	-0.337960574	-1.263968556	**STARD13**	protein_coding	StAR related lipid transfer domain containing 13 [Source:HGNC Symbol;Acc:HGNC:19164]	chr13	33103135	33350630	-1	ensembl_havana	-0.242919834	-0.073939547	-0.411900121	-0.18813307	0.125688576	-0.159374151	-0.46237776	-0.37473441	-0.398588198
ENSG00000236423	0.009459	0.046881573	-0.374716273	-1.296584537	**LINC01134**	lincRNA	long intergenic non-protein coding RNA 1134 [Source:HGNC Symbol;Acc:HGNC:49449]	chr1	3900372	3917225	1	havana	2.118610118	2.305968255	1.931251982	2.418087419	2.324608835	2.17520851	2.025141822	1.790554303	1.978059821
ENSG00000166803	0.009464	0.046881573	-0.439713218	-1.356334685	**PCLAF**	protein_coding	PCNA clamp associated factor [Source:HGNC Symbol;Acc:HGNC:28961]	chr15	64364311	64387687	-1	ensembl_havana	3.173251195	3.393107804	2.953394586	3.283361026	3.491349538	3.404612849	2.787093985	2.871367873	3.201721902
ENSG00000176476	0.009469	0.046887342	-0.259893948	-1.197390682	**SGF29**	protein_coding	SAGA complex associated factor 29 [Source:HGNC Symbol;Acc:HGNC:25156]	chr16	28553915	28591790	1	ensembl_havana	3.596615862	3.726562836	3.466668888	3.794609414	3.812648474	3.572430621	3.456169357	3.575605876	3.368231431
ENSG00000136731	0.009491	0.046953757	-0.295328995	-1.227164791	**UGGT1**	protein_coding	UDP-glucose glycoprotein glucosyltransferase 1 [Source:HGNC Symbol;Acc:HGNC:15663]	chr2	128091200	128195677	1	ensembl_havana	7.750036646	7.897701143	7.602372148	7.987596624	7.796811929	7.908694878	7.580582176	7.601949463	7.624584805
ENSG00000243943	0.009491	0.046953757	-0.30880147	-1.238678231	**ZNF512**	protein_coding	zinc finger protein 512 [Source:HGNC Symbol;Acc:HGNC:29380]	chr2	27582969	27623215	1	ensembl_havana	5.240175235	5.39457597	5.085774499	5.183356781	5.56655778	5.433813349	4.896012582	5.130160519	5.231150397
ENSG00000271959	0.009492	0.046953757	-0.982913978	-1.976453451	**AC100803.3**	antisense		chr8	141434545	141437954	1	havana	2.062997078	2.554454067	1.571540089	2.245600406	2.14720036	3.270561436	1.7752573	1.226127349	1.713235619
ENSG00000171503	0.009501	0.046963387	0.26669424	1.203048029	**ETFDH**	protein_coding	electron transfer flavoprotein dehydrogenase [Source:HGNC Symbol;Acc:HGNC:3483]	chr4	158672125	158709623	1	ensembl_havana	4.709068895	4.575721775	4.842416014	4.680680807	4.538087248	4.50839727	4.878922094	4.788093741	4.860232208
ENSG00000134077	0.0095	0.046963387	-0.255108863	-1.193425793	**THUMPD3**	protein_coding	THUMP domain containing 3 [Source:HGNC Symbol;Acc:HGNC:24493]	chr3	9362842	9386791	1	ensembl_havana	5.980778842	6.108333274	5.853224411	6.003192965	6.105127047	6.216679809	5.673996407	5.890928746	5.994748079
ENSG00000144061	0.009508	0.046966355	0.387612558	1.308226694	**NPHP1**	protein_coding	nephrocystin 1 [Source:HGNC Symbol;Acc:HGNC:7905]	chr2	110122311	110205066	-1	ensembl_havana	2.736550454	2.542744175	2.930356733	2.390733944	2.724446586	2.513051996	2.91642099	2.910102222	2.964546987
ENSG00000135378	0.009507	0.046966355	-0.259727854	-1.197252837	**PRRG4**	protein_coding	proline rich and Gla domain 4 [Source:HGNC Symbol;Acc:HGNC:30799]	chr11	32829943	32858123	1	ensembl_havana	2.513738163	2.64360209	2.383874236	2.931352026	2.678162413	2.321291832	2.600804468	2.41986126	2.13095698
ENSG00000158201	0.009512	0.046968123	-0.312339749	-1.241719877	**ABHD3**	protein_coding	abhydrolase domain containing 3 [Source:HGNC Symbol;Acc:HGNC:18718]	chr18	21650897	21704805	-1	ensembl_havana	5.159845499	5.316015373	5.003675624	5.376129266	5.285349013	5.28656784	5.04370839	4.862809981	5.104508502
ENSG00000223572	0.009515	0.046968123	-0.532076662	-1.446009131	**CKMT1A**	protein_coding	creatine kinase, mitochondrial 1A [Source:HGNC Symbol;Acc:HGNC:31736]	chr15	43692886	43699222	1	ensembl_havana	3.593046452	3.859084783	3.327008121	4.088626288	3.935785518	3.552842542	3.37972385	3.264170155	3.337130358
ENSG00000261609	0.009544	0.047096477	0.244188846	1.184426641	**GAN**	protein_coding	gigaxonin [Source:HGNC Symbol;Acc:HGNC:4137]	chr16	81314952	81390884	1	ensembl_havana	4.927475271	4.805380848	5.049569695	4.663640517	4.923650443	4.828851585	4.872294542	5.147487521	5.128927021
ENSG00000184445	0.009568	0.047178931	-0.245805802	-1.185754877	**KNTC1**	protein_coding	kinetochore associated 1 [Source:HGNC Symbol;Acc:HGNC:17255]	chr12	122527246	122626396	1	ensembl_havana	6.245310239	6.36821314	6.122407338	6.18585697	6.348259816	6.570522634	5.925180395	6.160104052	6.281937568
ENSG00000156876	0.009566	0.047178931	-0.252915441	-1.19161273	**SASS6**	protein_coding	SAS-6 centriolar assembly protein [Source:HGNC Symbol;Acc:HGNC:25403]	chr1	100083563	100132955	-1	ensembl_havana	5.06902994	5.19548766	4.942572219	5.117095256	5.307478533	5.161889192	4.841409418	5.007142888	4.979164351
ENSG00000268658	0.00957	0.047178931	-0.594092433	-1.50952268	**LINC00664**	lincRNA	long intergenic non-protein coding RNA 664 [Source:HGNC Symbol;Acc:HGNC:44319]	chr19	21483374	21503238	1	havana	0.038431286	0.335477503	-0.25861493	-0.27307956	0.403680778	0.875831286	-0.57379957	-0.52165082	0.319605599
ENSG00000239880	0.009581	0.047198074	0.25976693	1.197285265	**AC068760.1**	processed_pseudogene		chr3	180323107	180325059	1	havana	5.360685852	5.230802387	5.490569317	5.393459385	5.186857969	5.112089807	5.573802349	5.469418663	5.428486938
ENSG00000104973	0.009578	0.047198074	0.259314199	1.196909605	**MED25**	protein_coding	mediator complex subunit 25 [Source:HGNC Symbol;Acc:HGNC:28845]	chr19	49818279	49840383	1	ensembl_havana	6.387018231	6.257361132	6.516675331	6.327379651	6.228913371	6.215790374	6.651370956	6.49831184	6.400343198
ENSG00000250654	0.009594	0.047247046	-0.711986323	-1.638057867	**AC023794.5**	transcribed_unprocessed_pseudogene		chr12	54076838	54081903	-1	havana	0.905359356	1.261352517	0.549366194	1.190963204	1.311578958	1.281515389	0.851734776	0.625615656	0.17074815
ENSG00000168256	0.009599	0.047256551	0.265918857	1.202401619	**NKIRAS2**	protein_coding	NFKB inhibitor interacting Ras like 2 [Source:HGNC Symbol;Acc:HGNC:17898]	chr17	42011382	42025644	1	ensembl_havana	5.844676369	5.711716941	5.977635797	5.665798022	5.774555182	5.694797617	5.988449903	5.95137118	5.993086308
ENSG00000069966	0.00961	0.047271784	0.263416619	1.200317958	**GNB5**	protein_coding	G protein subunit beta 5 [Source:HGNC Symbol;Acc:HGNC:4401]	chr15	52115105	52191369	-1	ensembl_havana	5.478590905	5.346882596	5.610299215	5.207073224	5.292194321	5.541380242	5.488834672	5.620125578	5.721937394
ENSG00000102882	0.009609	0.047271784	-0.256727956	-1.194765891	**MAPK3**	protein_coding	mitogen-activated protein kinase 3 [Source:HGNC Symbol;Acc:HGNC:6877]	chr16	30114105	30123506	-1	ensembl_havana	7.44967992	7.578043898	7.321315942	7.719359962	7.537114731	7.477657001	7.5341023	7.271213811	7.158631715
ENSG00000125726	0.009612	0.047271784	-0.291773409	-1.224144112	**CD70**	protein_coding	CD70 molecule [Source:HGNC Symbol;Acc:HGNC:11937]	chr19	6583183	6604103	-1	ensembl_havana	4.149791995	4.2956787	4.003905291	4.49406847	4.28368192	4.109285709	4.082018697	4.04894723	3.880749946
ENSG00000196597	0.009616	0.047274225	0.25541904	1.193682406	**ZNF782**	protein_coding	zinc finger protein 782 [Source:HGNC Symbol;Acc:HGNC:33110]	chr9	96816472	96875623	-1	ensembl_havana	3.365047924	3.237338404	3.492757444	3.130521836	3.176880527	3.404612849	3.456169357	3.372156889	3.649946087
ENSG00000273301	0.009634	0.047326938	0.98189865	1.975062969	**AC016717.2**	lincRNA		chr2	225698514	225703654	1	havana	-0.065634103	-0.556583428	0.425315222	-0.56281016	-0.569826728	-0.537113394	0.915012722	0.504278656	-0.143345712
ENSG00000180233	0.009637	0.047326938	0.355530207	1.27945571	**ZNRF2**	protein_coding	zinc and ring finger 2 [Source:HGNC Symbol;Acc:HGNC:22316]	chr7	30284307	30367692	1	ensembl_havana	2.246470691	2.068705587	2.424235794	2.134555023	1.979216042	2.092345696	2.326059645	2.351143587	2.59550415
ENSG00000117758	0.009635	0.047326938	0.343672508	1.268982794	**STX12**	protein_coding	syntaxin 12 [Source:HGNC Symbol;Acc:HGNC:11430]	chr1	27773183	27824452	1	ensembl_havana	5.606972557	5.435136303	5.778808811	5.569974985	5.606237645	5.129196277	5.979843779	5.780937208	5.575645446
ENSG00000232759	0.00964	0.047326938	-0.358717373	-1.282285376	**AC002480.2**	antisense		chr7	22563337	22573996	1	havana	2.281624493	2.460983179	2.102265806	2.411297517	2.396251236	2.575400786	1.874103935	2.163501883	2.2691916
ENSG00000138674	0.009653	0.047343645	-0.264240899	-1.201003952	**SEC31A**	protein_coding	SEC31 homolog A, COPII coat complex component [Source:HGNC Symbol;Acc:HGNC:17052]	chr4	82818661	82901166	-1	ensembl_havana	7.275937588	7.408058037	7.143817138	7.556204296	7.369844875	7.298124941	7.208595998	7.121429986	7.101425432
ENSG00000168811	0.009653	0.047343645	-0.289476138	-1.222196401	**IL12A**	protein_coding	interleukin 12A [Source:HGNC Symbol;Acc:HGNC:5969]	chr3	159988750	159996019	1	ensembl_havana	1.979076546	2.123814615	1.834338477	2.084216604	2.242169235	2.045058006	1.7752573	1.861445274	1.866312856
ENSG00000152952	0.009648	0.047343645	-0.746848279	-1.678122785	**PLOD2**	protein_coding	procollagen-lysine,2-oxoglutarate 5-dioxygenase 2 [Source:HGNC Symbol;Acc:HGNC:9082]	chr3	146069440	146163653	-1	ensembl_havana	4.583629927	4.957054067	4.210205788	5.550794794	4.694825412	4.625541994	4.345853613	4.081128356	4.203635396
ENSG00000143575	0.009661	0.047349727	-0.234751985	-1.176704431	**HAX1**	protein_coding	HCLS1 associated protein X-1 [Source:HGNC Symbol;Acc:HGNC:16915]	chr1	154272511	154275875	1	ensembl_havana	6.860282073	6.977658066	6.742906081	7.017521293	6.987205417	6.928247487	6.755881109	6.743561808	6.729275326
ENSG00000068400	0.009661	0.047349727	-0.237140448	-1.178654146	**GRIPAP1**	protein_coding	GRIP1 associated protein 1 [Source:HGNC Symbol;Acc:HGNC:18706]	chrX	48973720	49002264	-1	ensembl_havana	6.848887913	6.967458137	6.730317688	7.069929063	6.925255205	6.907190142	6.797403862	6.684039419	6.709509785
ENSG00000224939	0.009665	0.047354215	-0.40376833	-1.32295898	**LINC00184**	lincRNA	long intergenic non-protein coding RNA 184 [Source:HGNC Symbol;Acc:HGNC:37192]	chr1	234629311	234634780	1	havana	5.327219665	5.52910383	5.1253355	5.835804086	5.340049083	5.411458321	5.211677793	5.060846669	5.103482039
ENSG00000138303	0.009676	0.047387592	-0.342310575	-1.267785414	**ASCC1**	protein_coding	activating signal cointegrator 1 complex subunit 1 [Source:HGNC Symbol;Acc:HGNC:24268]	chr10	72096032	72217134	-1	ensembl_havana	4.880819862	5.05197515	4.709664575	5.232333568	5.041071327	4.882520555	4.728521088	4.73514413	4.665328507
ENSG00000177098	0.009681	0.047400345	-0.739318134	-1.669386644	**SCN4B**	protein_coding	sodium voltage-gated channel beta subunit 4 [Source:HGNC Symbol;Acc:HGNC:10592]	chr11	118133377	118152888	-1	ensembl_havana	-0.441806466	-0.0721474	-0.811465533	-0.51029874	-0.143136894	0.436993436	-1.41444944	-0.42206346	-0.597883695
ENSG00000136108	0.00969	0.047425349	-0.230939846	-1.173599243	**CKAP2**	protein_coding	cytoskeleton associated protein 2 [Source:HGNC Symbol;Acc:HGNC:1990]	chr13	52455429	52476628	1	ensembl_havana	6.484715758	6.600185681	6.369245835	6.460368608	6.66178355	6.678404884	6.235159926	6.449429427	6.423148151
ENSG00000273340	0.00971	0.047433988	0.500781743	1.41498008	**MICE**	transcribed_unprocessed_pseudogene	MHC class I polypeptide-related sequence E (pseudogene) [Source:HGNC Symbol;Acc:HGNC:7094]	chr6	29741731	29748969	-1	havana	2.418987643	2.168596771	2.669378514	2.067039104	2.256240478	2.182510732	2.863936192	2.608318707	2.535880644
ENSG00000198556	0.00971	0.047433988	0.250608856	1.189709099	**ZNF789**	protein_coding	zinc finger protein 789 [Source:HGNC Symbol;Acc:HGNC:27801]	chr7	99472841	99503650	1	ensembl_havana	5.214547939	5.089243511	5.339852368	4.91232484	5.115322954	5.240082739	5.22630432	5.309286258	5.483966524
ENSG00000076108	0.0097	0.047433988	0.240424019	1.181339815	**BAZ2A**	protein_coding	bromodomain adjacent to zinc finger domain 2A [Source:HGNC Symbol;Acc:HGNC:962]	chr12	56595596	56636816	-1	ensembl_havana	8.921424796	8.801212787	9.041636806	8.966258656	8.732205615	8.705174091	9.198648554	9.02049644	8.905765423
ENSG00000133863	0.009711	0.047433988	-0.23595651	-1.177687287	**TEX15**	protein_coding	testis expressed 15, meiosis and synapsis associated [Source:HGNC Symbol;Acc:HGNC:11738]	chr8	30831544	30913003	-1	ensembl_havana	1.827244289	1.945222544	1.709266035	1.253039554	2.116675269	2.46595281	1.052687018	1.881076089	2.194034997
ENSG00000099625	0.009707	0.047433988	-0.396590251	-1.316392992	**CBARP**	protein_coding	CACN beta subunit associated regulatory protein [Source:HGNC Symbol;Acc:HGNC:28617]	chr19	1228287	1238027	-1	ensembl_havana	-0.407512514	-0.209217389	-0.60580764	0.075059705	-0.428075095	-0.274636777	-0.51701327	-0.80561234	-0.494797317
ENSG00000184903	0.009702	0.047433988	-0.500037887	-1.414250702	**IMMP2L**	protein_coding	inner mitochondrial membrane peptidase subunit 2 [Source:HGNC Symbol;Acc:HGNC:14598]	chr7	110663051	111562517	-1	ensembl_havana	1.2375206	1.487539544	0.987501656	1.625778111	1.364499946	1.472340574	0.894226935	0.821210834	1.2470672
ENSG00000127947	0.009721	0.047466916	0.253499152	1.192094951	**PTPN12**	protein_coding	protein tyrosine phosphatase, non-receptor type 12 [Source:HGNC Symbol;Acc:HGNC:9645]	chr7	77537275	77640071	1	ensembl_havana	5.241641239	5.114891663	5.368390815	5.056373842	5.188693392	5.099607755	5.297292015	5.385142919	5.422737512
ENSG00000086300	0.009726	0.047472178	0.234028789	1.17611472	**SNX10**	protein_coding	sorting nexin 10 [Source:HGNC Symbol;Acc:HGNC:14974]	chr7	26291895	26374329	1	ensembl_havana	6.890710363	6.773695969	7.007724758	6.636630615	6.908090139	6.776367152	6.906518637	7.118590281	6.998065357
ENSG00000157343	0.00974	0.047508924	-0.725567121	-1.653550516	**ARMC12**	protein_coding	armadillo repeat containing 12 [Source:HGNC Symbol;Acc:HGNC:21099]	chr6	35737032	35749079	1	ensembl_havana	0.689394643	1.052178203	0.326611083	1.283104003	0.910210399	0.963220207	0.106248156	0.343769191	0.529815901
ENSG00000164099	0.009738	0.047508924	-0.859406079	-1.814291259	**PRSS12**	protein_coding	serine protease 12 [Source:HGNC Symbol;Acc:HGNC:9477]	chr4	118280038	118353003	-1	ensembl_havana	-0.302347221	0.127355818	-0.732050261	0.075059705	0.325758945	-0.018751196	-0.46237776	-1.08144551	-0.652327512
ENSG00000161395	0.009748	0.047530581	0.231303539	1.173895137	**PGAP3**	protein_coding	post-GPI attachment to proteins 3 [Source:HGNC Symbol;Acc:HGNC:23719]	chr17	39671122	39696797	-1	ensembl_havana	3.388138077	3.272486308	3.503789847	3.175294617	3.231320443	3.410843864	3.43473797	3.44650685	3.630124721
ENSG00000130783	0.00978	0.047595434	0.328501143	1.255708106	**CCDC62**	protein_coding	coiled-coil domain containing 62 [Source:HGNC Symbol;Acc:HGNC:30723]	chr12	122774327	122827528	1	ensembl_havana	0.92972203	0.765471459	1.093972601	0.660286109	0.760296981	0.875831286	1.01467363	1.210694826	1.056549347
ENSG00000139718	0.009765	0.047595434	0.32685337	1.254274718	**SETD1B**	protein_coding	SET domain containing 1B [Source:HGNC Symbol;Acc:HGNC:29187]	chr12	121804180	121832584	1	ensembl_havana	4.346264867	4.182838182	4.509691552	4.323737133	4.057293197	4.167484215	4.773319503	4.406453481	4.349301671
ENSG00000153815	0.009782	0.047595434	0.249689561	1.188951249	**CMIP**	protein_coding	c-Maf inducing protein [Source:HGNC Symbol;Acc:HGNC:24319]	chr16	81445170	81711762	1	ensembl_havana	3.988218305	3.863373525	4.113063086	3.860895981	3.843267218	3.885957376	4.086606631	4.04894723	4.203635396
ENSG00000236144	0.009776	0.047595434	-0.253018548	-1.191697896	**TMEM147-AS1**	antisense	TMEM147 antisense RNA 1 [Source:HGNC Symbol;Acc:HGNC:51273]	chr19	35540738	35546029	-1	havana	5.36055446	5.487063734	5.234045185	5.507678725	5.408586778	5.544925699	5.18197221	5.179549065	5.340614281
ENSG00000103187	0.009784	0.047595434	-0.268787056	-1.204794472	**COTL1**	protein_coding	coactosin like F-actin binding protein 1 [Source:HGNC Symbol;Acc:HGNC:18304]	chr16	84565594	84618077	-1	ensembl_havana	2.330652551	2.465046079	2.196259023	2.471286007	2.440056551	2.483795679	2.270688985	2.195213363	2.122874721
ENSG00000238107	0.009776	0.047595434	-0.292447016	-1.224715809	**AC245100.6**	lincRNA		chr1	148295180	148297556	1	havana	1.437275481	1.583498989	1.291051973	1.383653947	1.464851682	1.901991338	0.975631465	1.195095435	1.70242902
ENSG00000237149	0.009777	0.047595434	-0.510309878	-1.424356102	**ZNF503-AS2**	antisense	ZNF503 antisense RNA 2 [Source:HGNC Symbol;Acc:HGNC:23525]	chr10	75401519	75408982	1	havana	-0.151235034	0.103919905	-0.406389973	0.10902545	0.06303108	0.139703184	-0.46237776	-0.15890847	-0.597883695
ENSG00000165650	0.009806	0.047669188	0.287966015	1.220917752	**PDZD8**	protein_coding	PDZ domain containing 8 [Source:HGNC Symbol;Acc:HGNC:26974]	chr10	117277274	117375467	-1	ensembl_havana	5.627973308	5.483990301	5.771956316	5.506086325	5.455077929	5.490806648	5.68008082	5.773735126	5.862053002

ENSG00000166004	0.009806	0.047669188	-0.245488263	-1.185493919	**CEP295**	protein_coding	centrosomal protein 295 [Source:HGNC Symbol;Acc:HGNC:29366]	chr11	93661639	93730358	1	ensembl_havana	5.471562091	5.594306223	5.34881796	5.577576228	5.619225395	5.586117045	5.275355665	5.379978088	5.391120127
ENSG00000172845	0.009818	0.047712852	-0.259254383	-1.196859981	**SP3**	protein_coding	Sp3 transcription factor [Source:HGNC Symbol;Acc:HGNC:11208]	chr2	173906459	173965702	-1	ensembl_havana	6.580621934	6.710249125	6.450994742	6.711086369	6.769947501	6.649713506	6.534503015	6.459257688	6.359223523
ENSG00000242588	0.009832	0.047714628	0.422163408	1.339935363	**AC108010.1**	processed_transcript		chr7	128574751	128622694	1	havana	2.116638935	1.905557231	2.327720639	1.940704441	1.699212533	2.076754718	2.487415269	2.233896951	2.261849697
ENSG00000007314	0.009831	0.047714628	0.364998877	1.287880627	**SCN4A**	protein_coding	sodium voltage-gated channel alpha subunit 4 [Source:HGNC Symbol;Acc:HGNC:10591]	chr17	63938554	63972918	-1	ensembl_havana	0.167766606	-0.014732832	0.350266045	-0.41068387	0.030651399	0.335833975	-0.21650287	0.554044597	0.713256405
ENSG00000166340	0.009825	0.047714628	0.258745098	1.196437552	**TPP1**	protein_coding	tripeptidyl peptidase 1 [Source:HGNC Symbol;Acc:HGNC:2073]	chr11	6612763	6619485	-1	ensembl_havana	6.419699095	6.290326547	6.549071644	6.334575204	6.336718384	6.199686052	6.534922719	6.573761842	6.538530372
ENSG00000162976	0.009826	0.047714628	-0.343405092	-1.268747599	**PQLC3**	protein_coding	PQ loop repeat containing 3 [Source:HGNC Symbol;Acc:HGNC:28503]	chr2	11155198	11178874	1	ensembl_havana	4.491632894	4.66333544	4.319930348	4.718301125	4.654222446	4.617482748	4.330462852	4.189409115	4.439919076
ENSG00000204334	0.009843	0.04775415	-0.614750871	-1.531293546	**ERICH2**	protein_coding	glutamate rich 2 [Source:HGNC Symbol;Acc:HGNC:44395]	chr2	170783786	170798971	1	ensembl_havana	0.135833184	0.44320862	-0.171542251	0.791576534	0.125688576	0.41236075	-0.08686711	-0.11936381	-0.308395834
ENSG00000125434	0.009856	0.047800695	0.31526747	1.24424231	**SLC25A35**	protein_coding	solute carrier family 25 member 35 [Source:HGNC Symbol;Acc:HGNC:31921]	chr17	8287763	8295343	-1	ensembl_havana	3.402953114	3.245319379	3.560586849	3.142871041	3.191599876	3.40148722	3.591444275	3.491900287	3.598415984
ENSG00000143067	0.009861	0.04780534	0.517221183	1.431195924	**ZNF697**	protein_coding	zinc finger protein 697 [Source:HGNC Symbol;Acc:HGNC:32034]	chr1	119619422	119647773	-1	ensembl_havana	1.494489799	1.235879207	1.753100391	0.589870393	1.476918714	1.640848515	1.371200905	2.002551439	1.885548828
ENSG00000198873	0.009877	0.047868955	-0.23423463	-1.176282537	**GRK5**	protein_coding	G protein-coupled receptor kinase 5 [Source:HGNC Symbol;Acc:HGNC:4544]	chr10	119207589	119459742	1	ensembl_havana	5.029843701	5.146961016	4.912726386	5.351511955	5.029852464	5.059518628	5.083271541	4.830360032	4.824547585
ENSG00000255987	0.009883	0.047881169	-0.420221942	-1.338133395	**TOMM20P2**	processed_pseudogene	TOMM20 pseudogene 2 [Source:HGNC Symbol;Acc:HGNC:50519]	chr17	35514766	35515186	-1	havana	0.315581461	0.525692432	0.10547049	0.297565234	0.403680778	0.875831286	0.106248156	-0.08087422	0.291037539
ENSG00000008196	0.009891	0.047902119	0.893741423	1.857988307	**TFAP2B**	protein_coding	transcription factor AP-2 beta [Source:HGNC Symbol;Acc:HGNC:11743]	chr6	50818723	50847613	1	ensembl_havana	-0.638258132	-1.085128843	-0.19138742	-0.67394004	-0.842251242	-1.739195246	0.785553575	-0.32890884	-1.030806998
ENSG00000180098	0.009898	0.047907496	0.262533951	1.199583806	**TRNAU1AP**	protein_coding	tRNA selenocysteine 1 associated protein 1 [Source:HGNC Symbol;Acc:HGNC:30813]	chr1	28553085	28578545	1	ensembl_havana	4.559845118	4.428578142	4.691112093	4.341704195	4.539526263	4.404503969	4.689844966	4.739183467	4.644307848
ENSG00000125841	0.009897	0.047907496	-0.256186212	-1.194317331	**NRSN2**	protein_coding	neurensin 2 [Source:HGNC Symbol;Acc:HGNC:16229]	chr20	346782	359660	1	ensembl_havana	5.565345062	5.693438168	5.437251956	5.777596329	5.724192212	5.578525965	5.563142451	5.506228494	5.242384924
ENSG00000157216	0.009938	0.048027396	0.318785022	1.247279699	**SSBP3**	protein_coding	single stranded DNA binding protein 3 [Source:HGNC Symbol;Acc:HGNC:15674]	chr1	54225432	54413479	-1	ensembl_havana	5.31158346	5.152190949	5.470975971	5.015544611	5.213245738	5.227782498	5.46083574	5.398826084	5.553266089
ENSG00000165948	0.009934	0.048027396	0.262955111	1.199934046	**IFI27L1**	protein_coding	interferon alpha inducible protein 27 like 1 [Source:HGNC Symbol;Acc:HGNC:19754]	chr14	94081282	94103846	1	ensembl_havana	4.411637675	4.28016012	4.543115231	4.277815454	4.28368192	4.278982986	4.596424846	4.458014382	4.574906464
ENSG00000254999	0.009943	0.048027396	0.256624701	1.194680383	**BRK1**	protein_coding	BRICK1, SCAR/WAVE actin nucleating complex subunit [Source:HGNC Symbol;Acc:HGNC:23057]	chr3	10115592	10127190	1	ensembl_havana	7.338738414	7.210426063	7.467050764	7.140139034	7.303212643	7.187926512	7.424890598	7.479926074	7.49633562
ENSG00000180815	0.009943	0.048027396	0.241488368	1.18221167	**MAP3K15**	protein_coding	mitogen-activated protein kinase kinase kinase 15 [Source:HGNC Symbol;Acc:HGNC:31689]	chrX	19360056	19515261	-1	ensembl_havana	2.754374335	2.633630151	2.875118519	2.341580783	2.739550451	2.819759219	2.613576909	3.007092508	3.004686139
ENSG00000177917	0.009935	0.048027396	-0.23714468	-1.178657603	**ARL6IP6**	protein_coding	ADP ribosylation factor like GTPase 6 interacting protein 6 [Source:HGNC Symbol;Acc:HGNC:24048]	chr2	152717893	152761253	1	ensembl_havana	4.284336384	4.402908725	4.165764044	4.481158028	4.414917876	4.31265027	4.201042438	4.181514568	4.114735126
ENSG00000179958	0.009931	0.048027396	-0.275246368	-1.210200737	**DCTPP1**	protein_coding	dCTP pyrophosphatase 1 [Source:HGNC Symbol;Acc:HGNC:28777]	chr16	30423619	30430075	-1	ensembl_havana	5.639257271	5.776880455	5.501634087	5.682108032	5.87486229	5.773671043	5.437679577	5.497511205	5.56971148
ENSG00000171530	0.009947	0.048029121	-0.415982298	-1.334206798	**TBCA**	protein_coding	tubulin folding cofactor A [Source:HGNC Symbol;Acc:HGNC:11579]	chr5	77691166	77868780	-1	ensembl_havana	5.953776076	6.161767225	5.745784927	6.300072106	6.07269516	6.112534409	5.652496585	5.747906805	5.836951393
ENSG00000125207	0.009955	0.048036312	0.552121468	1.466240203	**PIWIL1**	protein_coding	piwi like RNA-mediated gene silencing 1 [Source:HGNC Symbol;Acc:HGNC:9007]	chr12	130337887	130372637	1	ensembl_havana	1.285687624	1.00962689	1.561748358	0.727424294	0.979703225	1.321753151	1.554586035	1.226127349	1.90453169
ENSG00000179818	0.009952	0.048036312	-0.447325184	-1.363509908	**PCBP1-AS1**	processed_transcript	PCBP1 antisense RNA 1 [Source:HGNC Symbol;Acc:HGNC:42948]	chr2	69962263	70103220	-1	havana	5.023942483	5.247605075	4.800279891	5.404613346	5.289631136	5.048570744	4.874949217	4.671748138	4.854142319
ENSG00000188986	0.009961	0.04803716	0.428493493	1.345827488	**NELFB**	protein_coding	negative elongation factor complex member B [Source:HGNC Symbol;Acc:HGNC:24324]	chr9	137255173	137273546	1	ensembl_havana	5.030119008	4.815872261	5.244365754	4.555403613	4.804223955	5.087989216	5.128468593	5.329967383	5.274661287
ENSG00000205755	0.009962	0.04803716	0.382012283	1.303158244	**CRLF2**	protein_coding	cytokine receptor like factor 2 [Source:HGNC Symbol;Acc:HGNC:14281]	chrX	1187549	1212750	-1	ensembl_havana	1.016952398	0.825946257	1.20795854	-0.27307956	1.364499946	1.38641838	0.308311185	1.602328815	1.713235619
ENSG00000176438	0.009979	0.048100564	-0.359214304	-1.28272713	**SYNE3**	protein_coding	spectrin repeat containing nuclear envelope family member 3 [Source:HGNC Symbol;Acc:HGNC:19861]	chr14	95407266	95475836	-1	ensembl_havana	6.091308099	6.270915251	5.911700947	6.410167962	6.185453471	6.217124322	5.985382178	6.010497332	5.739223333
ENSG00000277728	0.009984	0.048108718	0.681561754	1.603875052	**AC097641.2**	antisense		chr17	73202968	73203431	-1	havana	-0.165174663	-0.50595554	0.175606214	-0.73288918	-0.34076431	-0.444213135	0.308311185	-0.04338487	0.261892331
ENSG00000172869	0.009987	0.048109682	0.285884267	1.219157289	**DMXL1**	protein_coding	Dmx like 1 [Source:HGNC Symbol;Acc:HGNC:2937]	chr5	119037772	119249138	1	ensembl_havana	6.5829363	6.439994167	6.725878434	6.411444503	6.380735509	6.527802489	6.664089108	6.780615531	6.732930663
ENSG00000106608	0.009999	0.048112351	0.306137061	1.236392717	**URGCP**	protein_coding	upregulator of cell proliferation [Source:HGNC Symbol;Acc:HGNC:30890]	chr7	43875894	43926411	-1	ensembl_havana	5.916426475	5.763357944	6.069495006	5.636258325	5.776387131	5.877428377	6.050822859	6.10273664	6.054925517
ENSG00000138166	0.010001	0.048112351	-0.294342017	-1.226325548	**DUSP5**	protein_coding	dual specificity phosphatase 5 [Source:HGNC Symbol;Acc:HGNC:3071]	chr10	110497838	110511544	1	ensembl_havana	5.551921334	5.699092342	5.404750326	6.070461682	5.517016653	5.509798693	5.681597922	5.197275416	5.335377639
ENSG00000054793	0.009999	0.048112351	-0.392446139	-1.312617105	**ATP9A**	protein_coding	ATPase phospholipid transporting 9A (putative) [Source:HGNC Symbol;Acc:HGNC:13540]	chr20	51596514	51768634	-1	ensembl_havana	3.919625626	4.115848695	3.723402557	3.96656885	3.803095076	4.57788216	3.41298341	3.614239733	4.142984527
ENSG00000171766	0.009998	0.048112351	-0.859164531	-1.81398752	**GATM**	protein_coding	glycine amidinotransferase [Source:HGNC Symbol;Acc:HGNC:4175]	chr15	45361124	45402327	-1	ensembl_havana	1.523354981	1.952937247	1.093772716	2.092729261	1.855271053	1.910811426	0.692307165	1.372000412	1.21701057
ENSG00000172613	0.010029	0.048170453	0.396213389	1.316049167	**RAD9A**	protein_coding	RAD9 checkpoint clamp component A [Source:HGNC Symbol;Acc:HGNC:9827]	chr11	67317871	67398410	1	ensembl_havana	4.573890792	4.375784097	4.771997486	4.165286793	4.455121723	4.506943775	4.776162508	4.777658146	4.762171804
ENSG00000091622	0.010021	0.048170453	0.338807656	1.264710917	**PITPNM3**	protein_coding	PITPNM family member 3 [Source:HGNC Symbol;Acc:HGNC:21043]	chr17	6451264	6556494	-1	ensembl_havana	2.341978333	2.172574505	2.511382161	2.191145857	2.045536638	2.28104102	2.581431252	2.498222232	2.454492999
ENSG00000171174	0.010024	0.048170453	0.319628213	1.248008892	**RBKS**	protein_coding	ribokinase [Source:HGNC Symbol;Acc:HGNC:30325]	chr2	27781364	27891098	-1	ensembl_havana	2.9666878	2.806873694	3.126501907	2.62003612	2.85962132	2.94096364	2.798325219	3.297423241	3.283757261
ENSG00000103381	0.01003	0.048170453	-0.240596705	-1.181481226	**CPPED1**	protein_coding	calcineurin like phosphoesterase domain containing 1 [Source:HGNC Symbol;Acc:HGNC:25632]	chr16	12659799	12804017	-1	ensembl_havana	4.961690993	5.081989345	4.841392641	5.101362066	5.075206302	5.069399668	4.878922094	4.871423222	4.773832606
ENSG00000255458	0.010018	0.048170453	-0.449303913	-1.365381314	**AC108471.2**	lincRNA		chr4	39639140	39666644	1	havana	-0.274500058	-0.049848101	-0.499152015	0.437805725	-0.143136894	-0.444213135	-0.21650287	-0.62862566	-0.652327512
ENSG00000278467	0.010054	0.048239914	0.357341296	1.281062884	**AC138393.3**	lincRNA		chr1	223994262	223995196	1	havana	0.865338072	0.686667424	1.04400872	0.727424294	0.403680778	0.928897202	1.089724456	0.919411336	1.12289037
ENSG00000100413	0.010049	0.048239914	0.240060733	1.181042379	**POLR3H**	protein_coding	RNA polymerase III subunit H [Source:HGNC Symbol;Acc:HGNC:30349]	chr22	41525804	41544606	-1	ensembl_havana	5.531051273	5.411020906	5.651081639	5.380481351	5.358102933	5.494478434	5.674758363	5.580150658	5.698335895
ENSG00000136122	0.010054	0.048239914	-0.317793609	-1.246422869	**BORA**	protein_coding	bora, aurora kinase A activator [Source:HGNC Symbol;Acc:HGNC:24724]	chr13	72727749	72756198	1	ensembl_havana	4.802007086	4.96090389	4.643110282	4.953802261	4.952023854	4.976885556	4.511965697	4.623032125	4.794333023
ENSG00000089199	0.010061	0.048255711	-0.33799766	-1.264001048	**CHGB**	protein_coding	chromogranin B [Source:HGNC Symbol;Acc:HGNC:1930]	chr20	5911430	5925361	1	ensembl_havana	3.320276752	3.489275582	3.151277923	3.171281297	3.688427859	3.608117591	2.853206155	3.195272324	3.405355289
ENSG00000273045	0.010067	0.048270072	-0.513058522	-1.427072393	**C2orf15**	protein_coding	chromosome 2 open reading frame 15 [Source:HGNC Symbol;Acc:HGNC:28436]	chr2	99141485	99151487	1	ensembl_havana	-0.423100389	-0.166571128	-0.67962965	-0.41068387	-0.258437325	0.16940781	-0.63291296	-1.00738779	-0.398588198
ENSG00000076554	0.010081	0.048316624	-0.353729902	-1.277860103	**TPD52**	protein_coding	tumor protein D52 [Source:HGNC Symbol;Acc:HGNC:12005]	chr8	80034745	80231232	-1	ensembl_havana	6.335967954	6.512832905	6.159103003	6.408037879	6.570062249	6.560398588	6.154425948	6.033704856	6.289178206
ENSG00000257923	0.010085	0.048321428	0.253782313	1.192328949	**CUX1**	protein_coding	cut like homeobox 1 [Source:HGNC Symbol;Acc:HGNC:2557]	chr7	101815904	102283958	1	ensembl_havana	5.857146771	5.730255615	5.984037928	5.588903523	5.731765241	5.87009808	5.918119074	5.966381438	6.067613271
ENSG00000145979	0.010091	0.048335748	-0.239073565	-1.180234523	**TBC1D7**	protein_coding	TBC1 domain family member 7 [Source:HGNC Symbol;Acc:HGNC:21066]	chr6	13266542	13328583	-1	ensembl_havana	4.280508622	4.400045405	4.16097184	4.54768433	4.293949556	4.358502328	4.310990596	4.007126095	4.16479883
ENSG00000184207	0.010102	0.04837178	0.308529932	1.238445114	**PGP**	protein_coding	phosphoglycolate phosphatase [Source:HGNC Symbol;Acc:HGNC:8909]	chr16	2211997	2214807	-1	ensembl_havana	3.98459482	3.830329854	4.138859786	3.705837742	3.859488969	3.925662851	4.136133802	4.044601662	4.235843895
ENSG00000170921	0.01012	0.048407376	0.641177523	1.559601582	**TANC2**	protein_coding	tetratricopeptide repeat, ankyrin repeat and coiled-coil containing 2 [Source:HGNC Symbol;Acc:HGNC:30212]	chr17	63009556	63427699	1	ensembl_havana	4.519828604	4.199239843	4.840417366	3.522665065	4.322650523	4.75240394	4.523855049	4.940878423	5.056518626
ENSG00000163558	0.01012	0.048407376	0.242096224	1.182709882	**PRKCI**	protein_coding	protein kinase C iota [Source:HGNC Symbol;Acc:HGNC:9404]	chr3	170222365	170305981	1	ensembl_havana	6.059866141	5.938818029	6.180914253	5.806346938	5.988801563	6.021305586	6.041991479	6.182526059	6.318225222
ENSG00000160051	0.010118	0.048407376	-0.329268679	-1.25637634	**IQCC**	protein_coding	IQ motif containing C [Source:HGNC Symbol;Acc:HGNC:25545]	chr1	32205661	32208687	1	ensembl_havana	3.15042895	3.31506329	2.98579461	3.341663059	3.488372191	3.11515462	2.947019153	3.064022175	2.946342503
ENSG00000255112	0.010125	0.04841469	0.309619707	1.239380958	**CHMP1B**	protein_coding	charged multivesicular body protein 1B [Source:HGNC Symbol;Acc:HGNC:24287]	chr18	11851396	11854449	1	havana	6.031459336	5.876649482	6.18626919	5.969500643	5.813140961	5.847306843	6.271377011	6.250134922	6.037295636
ENSG00000197451	0.010132	0.048416601	-0.252539121	-1.191301944	**HNRNPAB**	protein_coding	heterogeneous nuclear ribonucleoprotein A/B [Source:HGNC Symbol;Acc:HGNC:5034]	chr5	178204507	178211163	1	ensembl_havana	8.275131977	8.401401537	8.148862417	8.422362572	8.416009066	8.365832974	8.111915494	8.148387069	8.186284687
ENSG00000269343	0.010129	0.048416601	-0.415567488	-1.333823237	**ZNF587B**	protein_coding	zinc finger protein 587B [Source:HGNC Symbol;Acc:HGNC:37142]	chr19	57819741	57846238	1	ensembl_havana	4.831968894	5.039752638	4.62418515	5.363873117	4.769080564	4.986304232	4.713768103	4.46453516	4.694252187
ENSG00000230521	0.010143	0.048454203	-0.60513988	-1.52112623	**HCG4P7**	unprocessed_pseudogene	HLA complex group 4 pseudogene 7 [Source:HGNC Symbol;Acc:HGNC:22926]	chr6	29887294	29888268	-1	havana	1.001748618	1.304318558	0.699178678	1.451413217	1.013233389	1.448309068	0.716189844	0.779979312	0.601366878
ENSG00000165782	0.010148	0.048459913	0.247093086	1.186813371	**PIP4P1**	protein_coding	phosphatidylinositol-4,5-bisphosphate 4-phosphatase 1 [Source:HGNC Symbol;Acc:HGNC:19299]	chr14	20457719	20461612	-1	ensembl_havana	4.878407019	4.754860476	5.001953562	4.844636702	4.783726629	4.636218098	5.060130702	4.993680471	4.952049513
ENSG00000123268	0.010156	0.048486355	-0.269533238	-1.20541777	**ATF1**	protein_coding	activating transcription factor 1 [Source:HGNC Symbol;Acc:HGNC:783]	chr12	50763710	50821122	1	ensembl_havana	4.790936073	4.925702692	4.656169454	5.061803732	4.917022721	4.798281624	4.713768103	4.633197259	4.621543
ENSG00000196396	0.010165	0.048509528	0.275978527	1.210815062	**PTPN1**	protein_coding	protein tyrosine phosphatase, non-receptor type 1 [Source:HGNC Symbol;Acc:HGNC:9642]	chr20	50510321	50585241	1	ensembl_havana	7.379939381	7.241950118	7.517928645	7.154186866	7.298758278	7.272905209	7.536619394	7.535371959	7.481794581
ENSG00000112379	0.010175	0.048541281	0.586446257	1.501543486	**ARFGEF3**	protein_coding	ARFGEF family member 3 [Source:HGNC Symbol;Acc:HGNC:21213]	chr6	138161921	138344663	1	ensembl_havana	3.921093128	3.627869999	4.214316256	2.912241745	3.744406796	4.226961456	3.848057062	4.245388041	4.549503667
ENSG00000084092	0.010184	0.048570096	0.275866303	1.210720879	**NOA1**	protein_coding	nitric oxide associated 1 [Source:HGNC Symbol;Acc:HGNC:28473]	chr4	56963370	56978823	-1	ensembl_havana	4.278646585	4.140713433	4.416579737	3.998631232	4.185992157	4.237516911	4.379893999	4.428300566	4.441544645
ENSG00000269313	0.010216	0.048590824	0.408902562	1.327675486	**MAGIX**	protein_coding	MAGI family member, X-linked [Source:HGNC Symbol;Acc:HGNC:30006]	chrX	49162564	49168483	1	ensembl_havana	0.892784914	0.688333633	1.097236195	0.683014432	0.658018659	0.723967808	0.894226935	1.063828438	1.333653212
ENSG00000109680	0.010194	0.048590824	0.371221747	1.293447724	**TBC1D19**	protein_coding	TBC1 domain family member 19 [Source:HGNC Symbol;Acc:HGNC:25624]	chr4	26576437	26755351	1	ensembl_havana	2.514617778	2.329006904	2.700228652	2.167163933	2.433879495	2.385977285	2.693950367	2.620048716	2.786686872
ENSG00000103502	0.010201	0.048590824	0.364132183	1.28710717	**CDIPT**	protein_coding	CDP-diacylglycerol--inositol 3-phosphatidyltransferase [Source:HGNC Symbol;Acc:HGNC:1769]	chr16	29858357	29863736	-1	ensembl_havana	6.067334593	5.885268502	6.249400685	5.86777506	5.819077846	5.9689526	6.357419497	6.247301464	6.143481095
ENSG00000263753	0.010215	0.048590824	0.294914196	1.226812011	**LINC00667**	lincRNA	long intergenic non-protein coding RNA 667 [Source:HGNC Symbol;Acc:HGNC:27906]	chr18	5237826	5246508	1	havana	5.595676865	5.448219767	5.743133963	5.409732386	5.393546673	5.541380242	5.750437297	5.769791511	5.709173082
ENSG00000084072	0.010204	0.048590824	-0.23542574	-1.177254094	**PPIE**	protein_coding	peptidylprolyl isomerase E [Source:HGNC Symbol;Acc:HGNC:9258]	chr1	39692182	39763914	1	ensembl_havana	5.346469063	5.464181933	5.228756193	5.316508114	5.517016653	5.559021032	5.084418896	5.238760394	5.363089288
ENSG00000184227	0.010214	0.048590824	-0.332921007	-1.259561012	**ACOT1**	protein_coding	acyl-CoA thioesterase 1 [Source:HGNC Symbol;Acc:HGNC:33128]	chr14	73537114	73543794	1	ensembl_havana	1.958757795	2.125218298	1.792297291	2.298074107	2.132018545	1.945562243	2.015549972	1.637380031	1.723961872
ENSG00000231249	0.010202	0.048590824	-0.369085084	-1.291533517	**ITPR1-AS1**	antisense	ITPR1 antisense RNA 1 (head to head) [Source:HGNC Symbol;Acc:HGNC:44470]	chr3	4490891	4493163	-1	havana	1.564157728	1.74870027	1.379615186	1.528734855	1.944876279	1.772489677	1.356079599	1.491756274	1.291009686
ENSG00000144401	0.010207	0.048590824	-0.481233564	-1.39593674	**METTL21A**	protein_coding	methyltransferase like 21A [Source:HGNC Symbol;Acc:HGNC:30476]	chr2	207580631	207625928	-1	ensembl_havana	3.831592368	4.07220915	3.590975586	4.118114044	4.102779313	3.995734092	3.409325542	3.584613649	3.778987568
ENSG00000103363	0.010222	0.048595166	0.283591891	1.217221643	**ELOB**	protein_coding	elongin B [Source:HGNC Symbol;Acc:HGNC:11619]	chr16	2771414	2777297	-1	ensembl_havana	5.211304356	5.069508411	5.353100302	5.065051883	5.157169117	4.986304232	5.396701747	5.324600823	5.337998336
ENSG00000123342	0.010223	0.048595166	-0.459850377	-1.375399167	**MMP19**	protein_coding	matrix metallopeptidase 19 [Source:HGNC Symbol;Acc:HGNC:7165]	chr12	55835433	55842966	-1	ensembl_havana	2.115723348	2.345648537	1.88579816	1.863213701	2.678162413	2.495569495	1.593788933	1.95703246	2.106573087
ENSG00000130749	0.010231	0.048614336	-0.28027917	-1.214429861	**ZC3H4**	protein_coding	zinc finger CCCH-type containing 4 [Source:HGNC Symbol;Acc:HGNC:17808]	chr19	47064187	47113752	-1	ensembl_havana	5.273071328	5.413210913	5.132931743	5.409732386	5.304942383	5.524957971	5.164718872	5.100113754	5.133962604
ENSG00000170325	0.010241	0.048649	-0.233436138	-1.175631677	**PRDM10**	protein_coding	PR/SET domain 10 [Source:HGNC Symbol;Acc:HGNC:13995]	chr11	129899706	130002835	-1	ensembl_havana	4.36014872	4.476866789	4.243430651	4.556942526	4.450539589	4.423118252	4.291251919	4.2585809	4.180459133
ENSG00000115159	0.010264	0.048706439	0.591148296	1.506445308	**GPD2**	protein_coding	glycerol-3-phosphate dehydrogenase 2 [Source:HGNC Symbol;Acc:HGNC:4456]	chr2	156435290	156613735	1	ensembl_havana	-0.129391684	-0.424965832	0.166182464	-1.070289	-0.218971072	0.014362573	-0.12879794	0.097529431	0.529815901
ENSG00000145390	0.010258	0.048706439	0.260196172	1.197641544	**USP53**	protein_coding	ubiquitin specific peptidase 53 [Source:HGNC Symbol;Acc:HGNC:29255]	chr4	119212587	119295517	1	ensembl_havana	6.540911661	6.410813575	6.671009747	6.382228515	6.526460642	6.323751569	6.685796154	6.832266334	6.494966753
ENSG00000253352	0.010261	0.048706439	0.226954257	1.170361533	**TUG1**	bidirectional_promoter_lncRNA	taurine up-regulated 1 (non-protein coding) [Source:HGNC Symbol;Acc:HGNC:26066]	chr22	30969245	30979395	1	ensembl_havana	8.227237157	8.113760029	8.340714286	8.169360259	8.06719676	8.104723067	8.374670695	8.296334627	8.351137536
ENSG00000115956	0.01028	0.048766563	0.224780563	1.168599489	**PLEK**	protein_coding	pleckstrin [Source:HGNC Symbol;Acc:HGNC:9070]	chr2	68365173	68397453	1	ensembl_havana	4.466844738	4.354454456	4.57923502	4.341704195	4.582042512	4.139616662	4.565534632	4.817682322	4.354488106
ENSG00000111875	0.010292	0.048792306	0.245420596	1.185438317	**ASF1A**	protein_coding	anti-silencing function 1A histone chaperone [Source:HGNC Symbol;Acc:HGNC:20995]	chr6	118894220	118909167	1	ensembl_havana	4.756253577	4.633543279	4.878963875	4.561549439	4.664810523	4.674269874	4.800104487	4.855386052	4.981401085
ENSG00000086967	0.010289	0.048792306	-0.408160914	-1.326993141	**MYBPC2**	protein_coding	myosin binding protein C, fast type [Source:HGNC Symbol;Acc:HGNC:7550]	chr19	50432903	50466321	1	ensembl_havana	-0.126885753	0.077194704	-0.33096621	0.355307344	-0.036373238	-0.087349994	-0.26243933	-0.42206346	-0.308395834
ENSG00000188215	0.01032	0.048843947	0.342034469	1.267542806	**DCUN1D3**	protein_coding	defective in cullin neddylation 1 domain containing 3 [Source:HGNC Symbol;Acc:HGNC:28734]	chr16	20854925	20900384	-1	ensembl_havana	3.322745507	3.151728272	3.493762741	3.290778936	3.227754334	2.936651547	3.703214536	3.396231887	3.381841801
ENSG00000128567	0.01031	0.048843947	0.2476394	1.187262874	**PODXL**	protein_coding	podocalyxin like [Source:HGNC Symbol;Acc:HGNC:9171]	chr7	131500262	131558217	-1	ensembl_havana	6.258057052	6.134237352	6.381876751	5.960807856	6.037481895	6.404422305	6.187370443	6.354757311	6.6035025
ENSG00000154813	0.010309	0.048843947	0.24115351	1.181937304	**DPH3**	protein_coding	diphthamide biosynthesis 3 [Source:HGNC Symbol;Acc:HGNC:27717]	chr3	16257978	16264972	-1	ensembl_havana	6.474457811	6.353881056	6.595034567	6.33726431	6.466070307	6.258308552	6.619669915	6.648328066	6.517105719
ENSG00000087053	0.010323	0.048843947	0.227362883	1.170693071	**MTMR2**	protein_coding	myotubularin related protein 2 [Source:HGNC Symbol;Acc:HGNC:7450]	chr11	95832882	95925315	-1	ensembl_havana	5.434165179	5.320483737	5.54784662	5.275034653	5.344995309	5.341421249	5.477525561	5.575639848	5.590374452
ENSG00000112851	0.010313	0.048843947	-0.232474055	-1.174847951	**ERBIN**	protein_coding	erbb2 interacting protein [Source:HGNC Symbol;Acc:HGNC:15842]	chr5	65926475	66082549	1	ensembl_havana	6.659820252	6.77605728	6.543583225	6.770325892	6.798774704	6.759071244	6.498374343	6.599171369	6.533203963
ENSG00000130529	0.010317	0.048843947	-0.594349074	-1.509791233	**TRPM4**	protein_coding	transient receptor potential cation channel subfamily M member 4 [Source:HGNC Symbol;Acc:HGNC:17993]	chr19	49157741	49211836	1	ensembl_havana	0.440569947	0.737744485	0.14339541	0.749126884	0.760296981	0.703809589	-0.00649342	-0.04338487	0.480064523
ENSG00000101161	0.010333	0.048857733	0.271846805	1.20735238	**PRPF6**	protein_coding	pre-mRNA processing factor 6 [Source:HGNC Symbol;Acc:HGNC:15860]	chr20	63981135	64033100	1	ensembl_havana	7.516779023	7.380855621	7.652702425	7.277853077	7.342488804	7.522224982	7.58220754	7.683867416	7.69203232
ENSG00000175548	0.010333	0.048857733	-0.310502088	-1.240139219	**ALG10B**	protein_coding	ALG10B, alpha-1,2-glucosyltransferase [Source:HGNC Symbol;Acc:HGNC:31088]	chr12	38316578	38329728	1	ensembl_havana	4.691057228	4.846308272	4.535806184	4.864657476	4.744335121	4.92993222	4.451003248	4.574121769	4.582293537
ENSG00000135912	0.010341	0.04886753	-0.226526019	-1.170014183	**TTLL4**	protein_coding	tubulin tyrosine ligase like 4 [Source:HGNC Symbol;Acc:HGNC:28976]	chr2	218710845	218755416	1	ensembl_havana	5.582474903	5.695737913	5.469211894	5.689142231	5.656175543	5.741895965	5.482756169	5.40815854	5.516720974
ENSG00000189403	0.010342	0.04886753	-0.26068676	-1.198048871	**HMGB1**	protein_coding	high mobility group box 1 [Source:HGNC Symbol;Acc:HGNC:4983]	chr13	30456704	30617597	-1	ensembl_havana	7.594861658	7.725205038	7.464518278	7.698073427	7.782298772	7.695242915	7.356966442	7.526050416	7.510537975
ENSG00000271856	0.010348	0.048879887	-0.250751085	-1.189826392	**LINC01215**	lincRNA	long intergenic non-protein coding RNA 1215 [Source:HGNC Symbol;Acc:HGNC:49651]	chr3	108125821	108138610	1	havana	6.401285984	6.526661527	6.275910442	6.54072836	6.509305302	6.529950918	6.349328909	6.186968944	6.291433474
ENSG00000233585	0.010374	0.048908707	1.059638241	2.084408787	**AC231533.2**	transcribed_processed_pseudogene		chrX	48775644	48776970	-1	havana	-1.092263958	-1.622083079	-0.562444838	-1.41179303	-2.100471557	-1.353984645	-1.04978262	-0.74417246	0.106620565
ENSG00000187713	0.010364	0.048908707	0.292496445	1.224757771	**TMEM203**	protein_coding	transmembrane protein 203 [Source:HGNC Symbol;Acc:HGNC:28217]	chr9	137204082	137205638	-1	ensembl_havana	4.175593986	4.029345763	4.321842208	3.825582078	4.094970997	4.167484215	4.230361879	4.279071824	4.456092922
ENSG00000114770	0.010374	0.048908707	0.264716467	1.201399915	**ABCC5**	protein_coding	ATP binding cassette subfamily C member 5 [Source:HGNC Symbol;Acc:HGNC:56]	chr3	183919934	184018015	-1	ensembl_havana	5.888797803	5.75643957	6.021156036	5.562333481	5.663453351	6.043531877	5.820278929	6.01439127	6.228797909
ENSG00000205084	0.010363	0.048908707	0.225356904	1.169066425	**TMEM231**	protein_coding	transmembrane protein 231 [Source:HGNC Symbol;Acc:HGNC:37234]	chr16	75536744	75556286	-1	ensembl_havana	3.888931567	3.776253115	4.001610019	3.807595594	3.703911515	3.817252234	4.042420699	3.910138144	4.052271213
ENSG00000156858	0.010367	0.048908707	-0.309208057	-1.23902737	**PRR14**	protein_coding	proline rich 14 [Source:HGNC Symbol;Acc:HGNC:28458]	chr16	30650717	30656440	1	ensembl_havana	5.079150991	5.233755019	4.924546962	5.327369264	5.281054144	5.09284165	5.162547624	4.900569936	4.710523326
ENSG00000264548	0.01037	0.048908707	-0.590633064	-1.505907405	**AC132872.2**	antisense		chr17	82214227	82217352	1	havana	-0.187839127	0.107477405	-0.483155659	-0.14746469	-0.106664058	0.57656096	-0.89704638	-0.32890884	-0.223511759
ENSG00000033867	0.01038	0.048918377	0.252423835	1.191206751	**SLC4A7**	protein_coding	solute carrier family 4 member 7 [Source:HGNC Symbol;Acc:HGNC:11033]	chr3	27372721	27484420	-1	ensembl_havana	4.565212958	4.439001041	4.691424876	4.37349158	4.340922035	4.602589507	4.620353093	4.66468637	4.789235164
ENSG00000272578	0.010387	0.048920888	0.348893407	1.273583374	**AP000347.1**	transcribed_unprocessed_pseudogene		chr22	23658094	23717356	-1	ensembl_havana	1.110146828	0.935700124	1.284593531	0.949888679	0.77991046	1.077301233	1.261864131	1.300906777	1.291009686
ENSG00000204301	0.010387	0.048920888	-0.551848119	-1.465962418	**NOTCH4**	protein_coding	notch 4 [Source:HGNC Symbol;Acc:HGNC:7884]	chr6	32194843	32224067	-1	ensembl_havana	0.179669452	0.455593511	-0.096254607	0.683014432	0.428746719	0.255019383	-0.08686711	-0.2414061	0.039509383
ENSG00000042753	0.010409	0.049005345	0.255805383	1.194002107	**AP2S1**	protein_coding	adaptor related protein complex 2 sigma 1 subunit [Source:HGNC Symbol;Acc:HGNC:565]	chr19	46838136	46850992	-1	ensembl_havana	5.188429689	5.060526998	5.31633238	5.008813397	5.118214983	5.054552614	5.347852947	5.32011339	5.281030804
ENSG00000134317	0.010417	0.049016544	0.340948411	1.266588962	**GRHL1**	protein_coding	grainyhead like transcription factor 1 [Source:HGNC Symbol;Acc:HGNC:17923]	chr2	9951693	10002277	1	ensembl_havana	4.885731327	4.715257121	5.056205532	4.667919496	4.853649958	4.624201909	5.02828988	5.034792482	5.105534235
ENSG00000271122	0.010418	0.049016544	0.249488145	1.18878527	**AC018647.2**	antisense		chr7	35695214	35699413	1	havana	4.594453059	4.469708986	4.719197131	4.201157891	4.477816603	4.730152463	4.449223041	4.793283367	4.915084985
ENSG00000162817	0.010429	0.049039281	0.318485788	1.247021023	**C1orf115**	protein_coding	chromosome 1 open reading frame 115 [Source:HGNC Symbol;Acc:HGNC:25873]	chr1	220689845	220699157	1	ensembl_havana	2.58625036	2.427007466	2.745493254	2.649185179	2.357615216	2.274222003	3.067955511	2.726970195	2.441554055
ENSG00000136770	0.010426	0.049039281	0.244944921	1.185047528	**DNAJC1**	protein_coding	DnaJ heat shock protein family (Hsp40) member C1 [Source:HGNC Symbol;Acc:HGNC:20090]	chr10	21756537	22003769	-1	ensembl_havana	4.318812213	4.196339752	4.441284673	4.120197428	4.245396088	4.223425739	4.316859918	4.472644903	4.534349199
ENSG00000132881	0.010457	0.049153218	0.310022451	1.239726993	**RSG1**	protein_coding	REM2 and RAB like small GTPase 1 [Source:HGNC Symbol;Acc:HGNC:28127]	chr1	16231700	16237162	-1	ensembl_havana	1.818273937	1.663262712	1.973285163	1.578072284	1.415548178	1.996167673	1.808964307	1.871294071	2.239597111
ENSG00000183828	0.010482	0.049255485	0.284371774	1.217879819	**NUDT14**	protein_coding	nudix hydrolase 14 [Source:HGNC Symbol;Acc:HGNC:20141]	chr14	105172938	105181323	-1	ensembl_havana	3.318742516	3.176556629	3.460928403	3.283361026	3.162009452	3.08429941	3.542190552	3.560466802	3.280127855
ENSG00000143793	0.010487	0.049261008	0.382224058	1.303349551	**C1orf35**	protein_coding	chromosome 1 open reading frame 35 [Source:HGNC Symbol;Acc:HGNC:19032]	chr1	228100726	228105411	-1	ensembl_havana	3.585933629	3.394821599	3.777045658	3.260875666	3.442958541	3.480630591	3.848057062	3.76448673	3.718593182
ENSG00000142065	0.010513	0.049304685	0.310289463	1.23995646	**ZFP14**	protein_coding	ZFP14 zinc finger protein [Source:HGNC Symbol;Acc:HGNC:29312]	chr19	36334453	36379199	-1	ensembl_havana	3.493229997	3.338085266	3.648374729	3.312806534	3.363940242	3.337509022	3.487731073	3.790671376	3.666721737
ENSG00000162825	0.01051	0.049304685	0.295640114	1.227429459	**NBPF20**	protein_coding	NBPF member 20 [Source:HGNC Symbol;Acc:HGNC:32000]	chr1	145289900	145405778	-1	ensembl_havana	2.853805648	2.705985591	3.001625705	2.637596133	2.803232702	2.677127938	2.831503656	3.222458254	2.950915205
ENSG00000112367	0.010513	0.049304685	0.278943461	1.21330601	**FIG4**	protein_coding	FIG4 phosphoinositide 5-phosphatase [Source:HGNC Symbol;Acc:HGNC:16873]	chr6	109691312	109825428	1	ensembl_havana	5.560347555	5.420875824	5.699819285	5.430868342	5.433568358	5.398190773	5.773384267	5.598795351	5.727278238
ENSG00000184584	0.010506	0.049304685	-0.2255072	-1.169188221	**TMEM173**	protein_coding	transmembrane protein 173 [Source:HGNC Symbol;Acc:HGNC:27962]	chr5	139475534	139482935	-1	ensembl_havana	7.751134306	7.863887906	7.638380706	7.980160018	7.827175996	7.784327703	7.778581262	7.589511536	7.547049321
ENSG00000272556	0.010508	0.049304685	-0.442130597	-1.358609264	**GTF2IP13**	transcribed_unprocessed_pseudogene	general transcription factor IIi pseudogene 13 [Source:HGNC Symbol;Acc:HGNC:51725]	chr7	45769105	45815263	1	havana	2.879398403	3.100463702	2.658333105	2.982636349	2.817537823	3.501216933	2.568369719	2.584568634	2.822060962
ENSG00000184661	0.010525	0.04934352	-0.371346276	-1.293559375	**CDCA2**	protein_coding	cell division cycle associated 2 [Source:HGNC Symbol;Acc:HGNC:14623]	chr8	25458997	25507920	1	ensembl_havana	4.905610019	5.091283157	4.719936881	4.781535605	5.146820816	5.345493051	4.517073136	4.870195902	4.772541606
ENSG00000106829	0.010542	0.04940446	0.228036621	1.171239911	**TLE4**	protein_coding	transducin like enhancer of split 4 [Source:HGNC Symbol;Acc:HGNC:11840]	chr9	79571773	79726882	1	ensembl_havana	4.051211935	3.937193625	4.165230245	4.007662542	3.970339769	3.833578563	4.267203405	4.193340247	4.035147085
ENSG00000105483	0.010545	0.04940446	-0.238472909	-1.179743243	**CARD8**	protein_coding	caspase recruitment domain family member 8 [Source:HGNC Symbol;Acc:HGNC:17057]	chr19	48180770	48255946	-1	ensembl_havana	5.720571702	5.839808157	5.601335248	5.876428869	5.749904268	5.893091334	5.580323351	5.532844567	5.690837825
ENSG00000105464	0.010561	0.049466078	-0.292705324	-1.224935109	**GRIN2D**	protein_coding	glutamate ionotropic receptor NMDA type subunit 2D [Source:HGNC Symbol;Acc:HGNC:4588]	chr19	48394875	48444931	1	ensembl_havana	3.184270602	3.330623264	3.03791794	3.320074956	3.273440031	3.398354805	2.921565963	3.097971009	3.094216849
ENSG00000187954	0.010579	0.049506337	0.336365661	1.262571999	**CYHR1**	protein_coding	cysteine and histidine rich 1 [Source:HGNC Symbol;Acc:HGNC:17806]	chr8	144449582	144465677	-1	ensembl_havana	5.682829864	5.514647034	5.851012695	5.547702161	5.435115436	5.561123504	5.98783688	5.837922967	5.727278238
ENSG00000166311	0.010588	0.049506337	0.233640638	1.175798333	**SMPD1**	protein_coding	sphingomyelin phosphodiesterase 1 [Source:HGNC Symbol;Acc:HGNC:11120]	chr11	6390431	6394998	1	ensembl_havana	4.443333369	4.32651305	4.560153687	4.394300619	4.383211018	4.202027512	4.656289177	4.568078964	4.456092922
ENSG00000148120	0.010581	0.049506337	-0.226595793	-1.170070771	**C9orf3**	protein_coding	chromosome 9 open reading frame 3 [Source:HGNC Symbol;Acc:HGNC:1361]	chr9	94726701	95087218	1	ensembl_havana	5.040713753	5.15401165	4.927415857	5.073678037	5.084108452	5.30424846	4.874949217	4.852902895	5.054395457
ENSG00000136732	0.010591	0.049506337	-0.262209569	-1.199314116	**GYPC**	protein_coding	glycophorin C (Gerbich blood group) [Source:HGNC Symbol;Acc:HGNC:4704]	chr2	126655933	126696670	1	ensembl_havana	4.630918651	4.762023435	4.499813866	4.917125919	4.677937271	4.691007115	4.596424846	4.509373547	4.393643206
ENSG00000173145	0.010575	0.049506337	-0.291626088	-1.224019115	**NOC3L**	protein_coding	NOC3 like DNA replication regulator [Source:HGNC Symbol;Acc:HGNC:24034]	chr10	94333226	94362959	-1	ensembl_havana	5.561837929	5.707650973	5.416024885	5.72859517	5.763514364	5.630843386	5.392075968	5.390289326	5.465709362
ENSG00000123700	0.01059	0.049506337	-0.316602792	-1.245394482	**KCNJ2**	protein_coding	potassium voltage-gated channel subfamily J member 2 [Source:HGNC Symbol;Acc:HGNC:6263]	chr17	70168673	70180048	1	ensembl_havana	3.162185809	3.320487205	3.003884413	3.421548267	3.467356922	3.072556425	3.025576891	3.076846787	2.90922956
ENSG00000157657	0.010605	0.049525463	0.235800134	1.177559644	**ZNF618**	protein_coding	zinc finger protein 618 [Source:HGNC Symbol;Acc:HGNC:29416]	chr9	113876282	114056591	1	ensembl_havana	3.377122191	3.259222124	3.495022258	2.817841883	3.209790357	3.75003413	3.086398123	3.466136326	3.932532325
ENSG00000183624	0.010605	0.049525463	0.226855575	1.170281482	**HMCES**	protein_coding	5-hydroxymethylcytosine binding, ES cell specific [Source:HGNC Symbol;Acc:HGNC:24446]	chr3	129278828	129306186	1	ensembl_havana	5.412273485	5.298845697	5.525701272	5.193286055	5.337569597	5.365681439	5.451084223	5.545201873	5.580817721
ENSG00000152422	0.010605	0.049525463	-0.276994718	-1.211668225	**XRCC4**	protein_coding	X-ray repair cross complementing 4 [Source:HGNC Symbol;Acc:HGNC:12831]	chr5	83077498	83353787	1	ensembl_havana	3.011976827	3.150474186	2.873479468	3.130521836	3.124146975	3.196753748	2.741273741	2.910102222	2.969062441

ENSG00000076706	0.010614	0.049550131	-0.741622316	-1.672055014	**MCAM**	protein_coding	melanoma cell adhesion molecule [Source:HGNC Symbol;Acc:HGNC:6934]	chr11	119308529	119321521	-1	ensembl_havana	0.958526798	1.329337956	0.58771564	1.464591378	1.228369434	1.295053056	0.276538753	0.554044597	0.932563571
ENSG00000232160	0.010624	0.049580298	0.24239939	1.18295844	**RAP2C-AS1**	antisense	RAP2C antisense RNA 1 [Source:HGNC Symbol;Acc:HGNC:40957]	chrX	132217147	132432862	1	havana	1.930285922	1.809086228	2.051485617	1.770752281	1.855271053	1.801235349	2.053540577	2.122856455	1.978059821
ENSG00000273079	0.010629	0.049589449	-0.606031246	-1.522066346	**GRIN2B**	protein_coding	glutamate ionotropic receptor NMDA type subunit 2B [Source:HGNC Symbol;Acc:HGNC:4586]	chr12	13437942	13981957	-1	ensembl_havana	-0.126566297	0.176449326	-0.42958192	0.267803752	0.06303108	0.198513145	-0.69455235	-0.2414061	-0.352787316
ENSG00000174628	0.010638	0.049598663	0.246928576	1.186678047	**IQCK**	protein_coding	IQ motif containing K [Source:HGNC Symbol;Acc:HGNC:28556]	chr16	19716456	19858467	1	ensembl_havana	2.272572772	2.149108483	2.39603706	2.327223844	2.191810142	1.928291464	2.613576909	2.365151467	2.209382804
ENSG00000143303	0.010636	0.049598663	-0.237155679	-1.178666589	**RRNAD1**	protein_coding	ribosomal RNA adenine dimethylase domain containing 1 [Source:HGNC Symbol;Acc:HGNC:24273]	chr1	156728442	156736960	1	ensembl_havana	5.02397845	5.14255629	4.905400611	5.232333568	5.064250767	5.131084535	4.938508117	4.859102792	4.918590923
ENSG00000231728	0.010704	0.049878758	0.317973766	1.246578526	**AC234782.2**	antisense		chrX	103917411	103919548	-1	havana	1.453693699	1.294706816	1.612680582	1.451413217	0.996565715	1.436141515	1.91595172	1.286257764	1.635832261
ENSG00000163235	0.010705	0.049878758	-0.303945364	-1.234515854	**TGFA**	protein_coding	transforming growth factor alpha [Source:HGNC Symbol;Acc:HGNC:11765]	chr2	70447280	70554193	-1	ensembl_havana	2.997183852	3.149156535	2.84521117	2.828074641	3.224179389	3.395215574	2.657410455	2.895698436	2.98252462
ENSG00000211644	0.010711	0.049887606	0.459627117	1.375186338	**IGLV1-51**	IG_V_gene	immunoglobulin lambda variable 1-51 [Source:HGNC Symbol;Acc:HGNC:5882]	chr22	22322472	22322969	1	ensembl_havana	-0.33258162	-0.562395179	-0.102768062	-0.4596317	-0.428075095	-0.799478738	0.24405081	-0.19956768	-0.352787316
ENSG00000181852	0.010717	0.049901386	0.258585715	1.196305383	**RNF41**	protein_coding	ring finger protein 41 [Source:HGNC Symbol;Acc:HGNC:18401]	chr12	56202175	56221933	-1	ensembl_havana	6.729409298	6.60011644	6.858702156	6.751632583	6.560879955	6.487836783	6.921362275	6.880913454	6.773830738
ENSG00000138758	0.010731	0.049920338	0.235516975	1.177328545	**sept.11**	protein_coding	septin 11 [Source:HGNC Symbol;Acc:HGNC:25589]	chr4	76949703	77040384	1	ensembl_havana	8.185325156	8.067566668	8.303083643	7.940623597	8.12129319	8.140783218	8.23066834	8.325284522	8.353298067
ENSG00000271503	0.010726	0.049920338	-0.236516411	-1.178144429	**CCL5**	protein_coding	C-C motif chemokine ligand 5 [Source:HGNC Symbol;Acc:HGNC:10632]	chr17	35871491	35880793	-1	ensembl_havana	6.037690234	6.15594844	5.919432029	5.826264424	6.347438483	6.294142412	5.614877513	6.054385227	6.089033348
ENSG00000140332	0.010731	0.049920338	-0.270736328	-1.206423409	**TLE3**	protein_coding	transducin like enhancer of split 3 [Source:HGNC Symbol;Acc:HGNC:11839]	chr15	70047790	70098176	-1	ensembl_havana	3.886122654	4.021490818	3.75075449	4.177343198	3.74938971	4.137739545	3.964799786	3.526590849	3.760872836
ENSG00000013503	0.01074	0.049925455	0.25788888	1.195727695	**POLR3B**	protein_coding	RNA polymerase III subunit B [Source:HGNC Symbol;Acc:HGNC:30348]	chr12	106357658	106510198	1	ensembl_havana	5.047798436	4.918853996	5.176742876	4.831981146	4.993564355	4.931016489	5.034239593	5.217684583	5.278304454
ENSG00000196123	0.010743	0.049925455	0.228038983	1.171241829	**KIAA0895L**	protein_coding	KIAA0895 like [Source:HGNC Symbol;Acc:HGNC:34408]	chr16	67175602	67184040	-1	ensembl_havana	5.300637078	5.186617586	5.414656569	5.304648858	5.062249882	5.192954018	5.518848865	5.327286598	5.397834245
ENSG00000134910	0.010739	0.049925455	-0.25634019	-1.194444806	**STT3A**	protein_coding	STT3A, catalytic subunit of the oligosaccharyltransferase complex [Source:HGNC Symbol;Acc:HGNC:6172]	chr11	125591712	125625215	1	ensembl_havana	8.238674129	8.366844224	8.110504034	8.341073035	8.361218706	8.398240932	7.996741932	8.154071212	8.180698958
ENSG00000158109	0.010751	0.04993999	0.26738978	1.203628172	**TPRG1L**	protein_coding	tumor protein p63 regulated 1 like [Source:HGNC Symbol;Acc:HGNC:27007]	chr1	3625002	3630127	1	ensembl_havana	5.254537822	5.120842932	5.388232712	5.196251561	5.095893176	5.07038406	5.37154447	5.448189989	5.344963677
ENSG00000119392	0.010753	0.04993999	-0.248454789	-1.187934087	**GLE1**	protein_coding	GLE1, RNA export mediator [Source:HGNC Symbol;Acc:HGNC:4315]	chr9	128504700	128542288	1	ensembl_havana	7.121086349	7.245313744	6.996858955	7.231145851	7.280371735	7.224423647	6.91557207	7.032612285	7.042392509
ENSG00000133983	0.010772	0.049951643	0.536914638	1.450866359	**COX16**	protein_coding	COX16, cytochrome c oxidase assembly homolog [Source:HGNC Symbol;Acc:HGNC:20213]	chr14	70325081	70359731	-1	ensembl_havana	0.526924283	0.258466964	0.795381601	0.14220989	0.378171619	0.255019383	0.36983092	1.179325527	0.836988357
ENSG00000204271	0.010764	0.049951643	0.239849308	1.180869311	**SPIN3**	protein_coding	spindlin family member 3 [Source:HGNC Symbol;Acc:HGNC:27272]	chrX	56818298	56995827	-1	ensembl_havana	5.506138985	5.386214331	5.626063639	5.449215244	5.331767502	5.377660246	5.631456686	5.623043083	5.623691148
ENSG00000134440	0.010776	0.049951643	-0.223035552	-1.167186864	**NARS**	protein_coding	asparaginyl-tRNA synthetase [Source:HGNC Symbol;Acc:HGNC:7643]	chr18	57600656	57622213	-1	ensembl_havana	7.649761052	7.761278828	7.538243276	7.69178334	7.755151267	7.836901876	7.452256357	7.53110706	7.631366411
ENSG00000177045	0.010772	0.049951643	-0.498315902	-1.412563675	**SIX5**	protein_coding	SIX homeobox 5 [Source:HGNC Symbol;Acc:HGNC:10891]	chr19	45764785	45769226	-1	ensembl_havana	2.332019147	2.581177098	2.082861195	2.705773822	2.402591162	2.63516631	2.270688985	2.131077666	1.846816935
ENSG00000245534	0.010768	0.049951643	-0.588797022	-1.503992135	**RORA-AS1**	antisense	RORA antisense RNA 1 [Source:HGNC Symbol;Acc:HGNC:51410]	chr15	60479178	60630637	1	havana	0.21973377	0.514132281	-0.074664741	0.565613454	0.614986832	0.361796557	-0.04612063	-0.28449416	0.106620565
ENSG00000112655	0.010775	0.049951643	-0.616364743	-1.533007487	**PTK7**	protein_coding	protein tyrosine kinase 7 (inactive) [Source:HGNC Symbol;Acc:HGNC:9618]	chr6	43076268	43161719	1	ensembl_havana	2.226531266	2.534713638	1.918348895	1.996207752	2.850374945	2.757558215	1.717267538	1.890792227	2.146986918
ENSG00000177666	0.010786	0.049977835	0.373806348	1.295767024	**PNPLA2**	protein_coding	patatin like phospholipase domain containing 2 [Source:HGNC Symbol;Acc:HGNC:30802]	chr11	818902	825573	1	ensembl_havana	4.844006418	4.657103244	5.030909592	4.656480562	4.632810281	4.68201889	5.222140422	4.975532909	4.895055446
ENSG00000198771	0.010792	0.04999164	1.055902423	2.079018251	**RCSD1**	protein_coding	RCSD domain containing 1 [Source:HGNC Symbol;Acc:HGNC:28310]	chr1	167630093	167706249	1	ensembl_havana	-0.097010314	-0.624961526	0.430940897	-1.23095973	-0.521012188	-0.122912658	0.399634207	0.602150945	0.291037539
ENSG00000148331	0.010805	0.050036894	0.33954902	1.265360986	**ASB6**	protein_coding	ankyrin repeat and SOCS box containing 6 [Source:HGNC Symbol;Acc:HGNC:17181]	chr9	129634604	129642169	-1	ensembl_havana	6.242337621	6.072563111	6.412112131	5.938548818	6.077656191	6.201484325	6.366880108	6.504653393	6.364802892
ENSG00000177380	0.01082	0.050088999	0.261618571	1.19882292	**PPFIA3**	protein_coding	PTPRF interacting protein alpha 3 [Source:HGNC Symbol;Acc:HGNC:9247]	chr19	49119389	49151026	1	ensembl_havana	5.497313412	5.366504126	5.628122697	5.516405639	5.251504958	5.331601781	5.810605229	5.574886673	5.49887619
ENSG00000124155	0.010843	0.05016121	0.302569076	1.233338724	**PIGT**	protein_coding	phosphatidylinositol glycan anchor biosynthesis class T [Source:HGNC Symbol;Acc:HGNC:14938]	chr20	45416067	45456934	1	ensembl_havana	6.679395183	6.528110645	6.830679721	6.249576923	6.636791427	6.697963586	6.693711867	6.868981399	6.929345897
ENSG00000119547	0.010844	0.05016121	0.279130704	1.213463492	**ONECUT2**	protein_coding	one cut homeobox 2 [Source:HGNC Symbol;Acc:HGNC:8139]	chr18	57435685	57491297	1	ensembl_havana	7.616866821	7.477301469	7.756432173	7.776782531	7.294503201	7.360618674	7.975718199	7.687355986	7.606222334
ENSG00000115504	0.010846	0.05016121	0.244987116	1.185082187	**EHBP1**	protein_coding	EH domain binding protein 1 [Source:HGNC Symbol;Acc:HGNC:29144]	chr2	62673851	63046487	1	ensembl_havana	6.335495775	6.213002217	6.457989332	6.088130985	6.272833652	6.278042013	6.402735038	6.464960072	6.506272887
ENSG00000102172	0.010852	0.050170677	-0.221662423	-1.166076487	**SMS**	protein_coding	spermine synthase [Source:HGNC Symbol;Acc:HGNC:11123]	chrX	21940573	21994835	1	ensembl_havana	7.460846914	7.571678125	7.350015702	7.564643402	7.586156045	7.564234928	7.353401536	7.351466025	7.345179545
ENSG00000164252	0.010855	0.050170677	-0.273972804	-1.209132883	**AGGF1**	protein_coding	angiogenic factor with G-patch and FHA domains 1 [Source:HGNC Symbol;Acc:HGNC:24684]	chr5	77029251	77065234	1	ensembl_havana	3.514645854	3.651632256	3.377659451	3.490814579	3.769151021	3.694931168	3.109124716	3.572590744	3.451262894
ENSG00000149243	0.01087	0.05017236	0.803118925	1.744869239	**KLHL35**	protein_coding	kelch like family member 35 [Source:HGNC Symbol;Acc:HGNC:26597]	chr11	75422394	75430629	-1	ensembl_havana	-0.080953793	-0.482513256	0.320605669	-0.4596317	-0.672709	-0.315199064	-0.00649342	0.648704825	0.319605599
ENSG00000166619	0.010869	0.05017236	0.304721166	1.235179887	**BLCAP**	protein_coding	bladder cancer associated protein [Source:HGNC Symbol;Acc:HGNC:1055]	chr20	37492472	37527931	-1	ensembl_havana	6.647075651	6.494715068	6.799436234	6.260951799	6.538025227	6.685168179	6.698964983	6.823098276	6.876245443
ENSG00000131067	0.010868	0.05017236	0.291714727	1.224094321	**GGT7**	protein_coding	gamma-glutamyltransferase 7 [Source:HGNC Symbol;Acc:HGNC:4259]	chr20	34844720	34872860	-1	ensembl_havana	6.405873267	6.260015903	6.55173063	6.15418101	6.30956437	6.316302329	6.575472508	6.521950287	6.557769096
ENSG00000187514	0.010867	0.05017236	-0.261030447	-1.198334311	**PTMA**	protein_coding	prothymosin alpha [Source:HGNC Symbol;Acc:HGNC:9623]	chr2	231706895	231713541	1	ensembl_havana	7.999393174	8.129908397	7.86887795	8.015846955	8.177368146	8.196510091	7.847949898	7.851520607	7.907163347
ENSG00000134109	0.01088	0.050172364	-0.22743509	-1.170751665	**EDEM1**	protein_coding	ER degradation enhancing alpha-mannosidase like protein 1 [Source:HGNC Symbol;Acc:HGNC:18967]	chr3	5187646	5219957	1	ensembl_havana	6.916859062	7.030576606	6.803141517	7.148584137	6.987469041	6.955676641	6.883303754	6.776690704	6.749430092
ENSG00000126804	0.010877	0.050172364	-0.259462935	-1.197033008	**ZBTB1**	protein_coding	zinc finger and BTB domain containing 1 [Source:HGNC Symbol;Acc:HGNC:20259]	chr14	64503712	64533690	1	ensembl_havana	5.949337509	6.079068977	5.819606042	6.212953158	6.104154899	5.920098874	5.860348652	5.898774468	5.699695004
ENSG00000218052	0.010876	0.050172364	-0.372831646	-1.294891884	**ADAMTS7P4**	transcribed_unprocessed_pseudogene	ADAMTS7 pseudogene 4 [Source:HGNC Symbol;Acc:HGNC:49410]	chr15	85255369	85330334	-1	havana	1.183806683	1.370222506	0.99739086	1.126095262	1.476918714	1.507653541	0.830009965	0.900298706	1.261863909
ENSG00000197632	0.010915	0.050286186	0.299064501	1.230346351	**SERPINB2**	protein_coding	serpin family B member 2 [Source:HGNC Symbol;Acc:HGNC:8584]	chr18	63871692	63903890	1	ensembl_havana	6.679674046	6.530141795	6.829206297	6.178377915	6.695415413	6.716632057	6.54370842	7.066763812	6.877146657
ENSG00000150048	0.01091	0.050286186	-0.423792617	-1.341449382	**CLEC1A**	protein_coding	C-type lectin domain family 1 member A [Source:HGNC Symbol;Acc:HGNC:24355]	chr12	10069554	10111627	-1	ensembl_havana	0.678748125	0.890644433	0.466851816	0.705390237	1.242575255	0.723967808	0.513047461	0.625615656	0.261892331
ENSG00000147592	0.010915	0.050286186	-0.452139688	-1.368067759	**LACTB2**	protein_coding	lactamase beta 2 [Source:HGNC Symbol;Acc:HGNC:18512]	chr8	70635318	70669174	-1	ensembl_havana	3.06392559	3.289995433	2.837855746	3.264647647	3.314364738	3.290973915	2.541886448	2.993629619	2.97805117
ENSG00000146414	0.010919	0.050287853	0.312575575	1.241922868	**SHPRH**	protein_coding	SNF2 histone linker PHD RING helicase [Source:HGNC Symbol;Acc:HGNC:19336]	chr6	145864245	145964423	-1	ensembl_havana	5.734780353	5.578492565	5.89106814	5.491675083	5.482270074	5.761532538	5.843505636	5.90238123	5.927317554
ENSG00000284634	0.010925	0.050299294	0.488698513	1.403178466	**AC092821.3**	processed_transcript		chr12	9448295	9658392	1	havana	0.403256531	0.158907275	0.647605788	0.004630787	-0.036373238	0.508464275	0.428834256	0.737534657	0.77644845
ENSG00000114251	0.010931	0.05030868	-0.357518586	-1.281220321	**WNT5A**	protein_coding	Wnt family member 5A [Source:HGNC Symbol;Acc:HGNC:12784]	chr3	55465715	55490539	-1	ensembl_havana	1.784247996	1.963007289	1.605488703	1.95010455	2.14720036	1.791716956	1.415636519	1.811166797	1.589662793
ENSG00000176845	0.010972	0.050485294	-0.669585689	-1.590616111	**METRNL**	protein_coding	meteorin like, glial cell differentiation regulator [Source:HGNC Symbol;Acc:HGNC:27584]	chr17	83079691	83095119	1	ensembl_havana	2.088008779	2.422801624	1.753215935	2.631766511	1.864486201	2.772152159	1.946559732	1.578479109	1.734608964
ENSG00000244265	0.010981	0.050485677	0.84509656	1.796384975	**SIAH2-AS1**	antisense	SIAH2 antisense RNA 1 [Source:HGNC Symbol;Acc:HGNC:40526]	chr3	150761937	150762538	1	havana	-0.527066808	-0.949615088	-0.104518528	-1.14838881	-0.842251242	-0.85820521	0.24405081	-0.37473441	-0.182871985
ENSG00000267702	0.010983	0.050485677	0.470389931	1.385483886	**AP005131.6**	sense_intronic		chr18	13486462	13490676	1	havana	0.949603606	0.71440864	1.184798572	0.464289132	0.570631912	1.108304877	1.195452122	1.01135145	1.347592143
ENSG00000170919	0.01098	0.050485677	0.358268471	1.281886448	**TPT1-AS1**	processed_transcript	TPT1 antisense RNA 1 [Source:HGNC Symbol;Acc:HGNC:43686]	chr13	45341345	45393413	1	ensembl_havana	2.501469613	2.322335378	2.680603848	2.167163933	2.364126834	2.435715365	2.717806083	2.648963151	2.675042311
ENSG00000227855	0.010996	0.050512279	0.683513152	1.606045931	**DPY19L2P3**	transcribed_unprocessed_pseudogene	DPY19L2 pseudogene 3 [Source:HGNC Symbol;Acc:HGNC:22367]	chr7	29650227	29742594	1	havana	-0.368240763	-0.709997339	-0.026484187	-1.070289	-0.473795405	-0.585907617	-0.82634296	0.37179175	0.375098651
ENSG00000088543	0.010994	0.050512279	-0.227025217	-1.170419099	**C3orf18**	protein_coding	chromosome 3 open reading frame 18 [Source:HGNC Symbol;Acc:HGNC:24837]	chr3	50558025	50571027	-1	ensembl_havana	5.164000618	5.277513226	5.050488009	5.103469765	5.256763693	5.472306222	4.857605861	5.068358012	5.225500155
ENSG00000177483	0.010999	0.050512279	-0.479921484	-1.394667762	**RBM44**	protein_coding	RNA binding motif protein 44 [Source:HGNC Symbol;Acc:HGNC:24756]	chr2	237798389	237842808	1	ensembl_havana	0.24270318	0.482663922	0.002742438	0.613726219	0.52486995	0.309395597	-0.12879794	0.063570082	0.073455173
ENSG00000044090	0.011006	0.050524654	-0.262495348	-1.199551708	**CUL7**	protein_coding	cullin 7 [Source:HGNC Symbol;Acc:HGNC:21024]	chr6	43037617	43053945	-1	ensembl_havana	6.457274651	6.588522325	6.326026977	6.734054918	6.522827615	6.508684443	6.396281891	6.353001131	6.228797909
ENSG00000011332	0.011011	0.050535197	0.400396776	1.319870857	**DPF1**	protein_coding	double PHD fingers 1 [Source:HGNC Symbol;Acc:HGNC:20225]	chr19	38211006	38229714	-1	ensembl_havana	3.176165791	2.975967403	3.376364179	2.883093138	3.000030474	3.044778598	3.50153988	3.35471112	3.272841537
ENSG00000213139	0.01103	0.050536332	0.31677014	1.245538952	**CRYGS**	protein_coding	crystallin gamma S [Source:HGNC Symbol;Acc:HGNC:2417]	chr3	186538441	186546702	-1	ensembl_havana	1.709605152	1.551220082	1.867990222	1.637462096	1.440410536	1.575787614	1.808964307	1.881076089	1.91393027
ENSG00000102053	0.011033	0.050536332	0.285942791	1.219206746	**ZC3H12B**	protein_coding	zinc finger CCCH-type containing 12B [Source:HGNC Symbol;Acc:HGNC:17407]	chrX	65366638	65507887	1	ensembl_havana	2.538811058	2.395839663	2.681782453	2.411297517	2.206378717	2.569842754	2.809469694	2.485454087	2.750423579
ENSG00000105583	0.011029	0.050536332	0.252952325	1.191643195	**WDR83OS**	protein_coding	WD repeat domain 83 opposite strand [Source:HGNC Symbol;Acc:HGNC:30203]	chr19	12668071	12671356	-1	ensembl_havana	5.826447205	5.699971042	5.952923367	5.69264651	5.732394536	5.67487208	5.984153258	5.899376222	5.97524062
ENSG00000169679	0.011027	0.050536332	-0.223363426	-1.167452155	**BUB1**	protein_coding	BUB1 mitotic checkpoint serine/threonine kinase [Source:HGNC Symbol;Acc:HGNC:1148]	chr2	110637698	110678114	-1	ensembl_havana	7.686287441	7.797969154	7.574605728	7.613342772	7.860084891	7.920479798	7.384474458	7.662756964	7.676585763
ENSG00000115339	0.011031	0.050536332	-0.340765803	-1.266428654	**GALNT3**	protein_coding	polypeptide N-acetylgalactosaminyltransferase 3 [Source:HGNC Symbol;Acc:HGNC:4125]	chr2	165747591	165794682	-1	ensembl_havana	2.993244734	3.163627636	2.822861833	3.191237175	3.139411412	3.160234321	2.711879034	2.769722224	2.986984242
ENSG00000131187	0.01102	0.050536332	-0.707907342	-1.63343307	**F12**	protein_coding	coagulation factor XII [Source:HGNC Symbol;Acc:HGNC:3530]	chr5	177402140	177409576	-1	ensembl_havana	0.716496189	1.07044986	0.362542518	1.142587728	0.760296981	1.30846487	0.692307165	0.256279753	0.139040636
ENSG00000214279	0.011041	0.050559829	0.465396254	1.380696527	**SCART1**	protein_coding	scavenger receptor family member expressed on T cells 1 [Source:HGNC Symbol;Acc:HGNC:32411]	chr10	133453928	133523558	1	havana	1.964934718	1.732236591	2.197632845	1.327057633	1.769573688	2.100078453	1.986385747	2.315517031	2.290995759
ENSG00000233429	0.011051	0.050575356	0.563247142	1.477591162	**HOTAIRM1**	antisense	HOXA transcript antisense RNA, myeloid-specific 1 [Source:HGNC Symbol;Acc:HGNC:37117]	chr7	27095647	27100265	1	havana	1.470167192	1.18854362	1.751790763	1.451413217	1.390249836	0.723967808	2.135529305	1.602328815	1.517514168
ENSG00000140836	0.011052	0.050575356	-0.398975985	-1.318571666	**ZFHX3**	protein_coding	zinc finger homeobox 3 [Source:HGNC Symbol;Acc:HGNC:777]	chr16	72782885	73891871	-1	ensembl_havana	9.200258083	9.399746076	9.00077009	9.234639877	9.286374586	9.678223764	8.85293141	9.076643781	9.07273508
ENSG00000198585	0.011058	0.05058491	0.23500866	1.176913802	**NUDT16**	protein_coding	nudix hydrolase 16 [Source:HGNC Symbol;Acc:HGNC:26442]	chr3	131381671	131388830	1	ensembl_havana	5.755998332	5.638494002	5.873502662	5.604613794	5.619905732	5.690962479	5.783309925	5.872658714	5.964539347
ENSG00000135622	0.011065	0.050603172	0.278347128	1.212804598	**SEMA4F**	protein_coding	ssemaphorin 4F [Source:HGNC Symbol;Acc:HGNC:10734]	chr2	74654228	74683853	1	ensembl_havana	4.971426133	4.832252569	5.110599697	4.778900489	4.782511786	4.935345433	5.17444925	4.992552911	5.16479693
ENSG00000121680	0.011075	0.050630419	0.36887563	1.291346023	**PEX16**	protein_coding	peroxisomal biogenesis factor 16 [Source:HGNC Symbol;Acc:HGNC:8857]	chr11	45909669	45918812	-1	ensembl_havana	4.42126251	4.236824694	4.605700325	4.193263259	4.189662871	4.327547954	4.686826487	4.636088476	4.494186012
ENSG00000116205	0.011101	0.05073484	-0.297071434	-1.228647815	**TCEANC2**	protein_coding	transcription elongation factor A N-terminal and central domain containing 2 [Source:HGNC Symbol;Acc:HGNC:26494]	chr1	54053587	54112519	1	ensembl_havana	6.075813416	6.224349133	5.927277698	6.394760703	6.245320117	6.032966578	6.047296792	5.862202061	5.872334242
ENSG00000085840	0.011108	0.050751576	-0.238475477	-1.179745343	**ORC1**	protein_coding	origin recognition complex subunit 1 [Source:HGNC Symbol;Acc:HGNC:8487]	chr1	52372829	52404459	-1	ensembl_havana	6.008377811	6.12761555	5.889140073	5.894799777	6.190958096	6.297088776	5.69142039	5.98180546	5.994194368
ENSG00000117543	0.01112	0.050791762	-0.317059275	-1.2457886	**DPH5**	protein_coding	diphthamide biosynthesis 5 [Source:HGNC Symbol;Acc:HGNC:24270]	chr1	100989623	101026088	-1	ensembl_havana	4.805998244	4.964527881	4.647468606	4.967752944	4.901438591	5.024392109	4.493082186	4.670338547	4.778985085
ENSG00000116990	0.011125	0.050795566	-0.230189027	-1.172988628	**MYCL**	protein_coding	MYCL proto-oncogene, bHLH transcription factor [Source:HGNC Symbol;Acc:HGNC:7555]	chr1	39895426	39902256	-1	ensembl_havana	6.603923332	6.719017846	6.488828819	7.080400003	6.717832593	6.358820941	6.882644373	6.44036099	6.143481095
ENSG00000158458	0.011135	0.050808062	0.662365809	1.582675853	**NRG2**	protein_coding	neuregulin 2 [Source:HGNC Symbol;Acc:HGNC:7998]	chr5	139846779	140043299	-1	ensembl_havana	2.686435365	2.35525246	3.017618269	1.82284946	2.377062558	2.865845363	2.89565563	2.947823742	3.209375435
ENSG00000268543	0.011137	0.050808062	0.62513998	1.542360468	**AC012313.4**	TEC		chr19	58418560	58419310	1	havana	1.014276279	0.701706289	1.326846269	0.355307344	1.046001934	0.703809589	1.229040238	1.271458479	1.480040089
ENSG00000126759	0.011142	0.050808062	0.385916084	1.306689246	**CFP**	protein_coding	complement factor properdin [Source:HGNC Symbol;Acc:HGNC:8864]	chrX	47623172	47630305	-1	ensembl_havana	0.147642494	-0.045315548	0.340600536	0.004630787	-0.218971072	0.078393642	0.176794179	0.315191528	0.529815901
ENSG00000103197	0.01114	0.050808062	0.380151416	1.301478443	**TSC2**	protein_coding	TSC complex subunit 2 [Source:HGNC Symbol;Acc:HGNC:12363]	chr16	2047465	2089487	1	ensembl_havana	6.399434234	6.209358526	6.589509942	6.155705264	6.045100285	6.427270028	6.684283462	6.484742767	6.599503597
ENSG00000113845	0.011147	0.050808062	-0.220132889	-1.164840877	**TIMMDC1**	protein_coding	translocase of inner mitochondrial membrane domain containing 1 [Source:HGNC Symbol;Acc:HGNC:1321]	chr3	119498532	119525090	1	ensembl_havana	5.976990306	6.08705675	5.866923861	6.037219356	6.066719315	6.15723158	5.805048137	5.859730618	5.935992829
ENSG00000126814	0.011149	0.050808062	-0.251686561	-1.190598152	**TRMT5**	protein_coding	tRNA methyltransferase 5 [Source:HGNC Symbol;Acc:HGNC:23141]	chr14	60971451	60981358	-1	ensembl_havana	4.791524756	4.917368036	4.665681476	4.844636702	5.033942152	4.873525255	4.540671651	4.749900063	4.706472713
ENSG00000197465	0.011155	0.05082083	-0.902414057	-1.869191079	**GYPE**	protein_coding	glycophorin E (MNS blood group) [Source:HGNC Symbol;Acc:HGNC:4705]	chr4	143870867	143905563	-1	ensembl_havana	0.075931632	0.527138661	-0.375275396	0.852997155	0.243386951	0.485031876	-0.00649342	-0.15890847	-0.960424301
ENSG00000124596	0.011171	0.050877918	-0.222519729	-1.166769621	**OARD1**	protein_coding	O-acyl-ADP-ribose deacylase 1 [Source:HGNC Symbol;Acc:HGNC:21257]	chr6	41033627	41097787	-1	ensembl_havana	5.43432507	5.545584934	5.323065205	5.537605071	5.578506754	5.520642978	5.310099338	5.381701753	5.277394524
ENSG00000163900	0.011181	0.050885182	0.229029739	1.17204643	**TMEM41A**	protein_coding	transmembrane protein 41A [Source:HGNC Symbol;Acc:HGNC:30544]	chr3	185476496	185499057	-1	ensembl_havana	5.381827982	5.267313113	5.496342852	5.202164347	5.379955531	5.219819461	5.426865457	5.57111489	5.491048209
ENSG00000012048	0.011179	0.050885182	-0.225462853	-1.169152282	**BRCA1**	protein_coding	BRCA1, DNA repair associated [Source:HGNC Symbol;Acc:HGNC:1100]	chr17	43044295	43170245	-1	ensembl_havana	6.500698251	6.613429678	6.387966825	6.370461938	6.651838593	6.817988502	6.17288117	6.391151713	6.599867592
ENSG00000125901	0.011183	0.050885182	-0.333418526	-1.259995452	**MRPS26**	protein_coding	mitochondrial ribosomal protein S26 [Source:HGNC Symbol;Acc:HGNC:14045]	chr20	3045945	3048254	1	ensembl_havana	3.46281234	3.629521603	3.296103077	3.599344412	3.643632949	3.645587448	3.275081252	3.155524498	3.45770348
ENSG00000229956	0.011193	0.050913918	0.635235145	1.553190884	**ZRANB2-AS2**	processed_transcript	ZRANB2 antisense RNA 2 (head to head) [Source:HGNC Symbol;Acc:HGNC:43595]	chr1	71081324	71489976	1	havana	-0.437912452	-0.755530025	-0.12029488	-0.79434999	-0.783495335	-0.688744748	0.24405081	-0.42206346	-0.182871985
ENSG00000143952	0.011212	0.050982362	0.290989622	1.22347924	**VPS54**	protein_coding	VPS54, GARP complex subunit [Source:HGNC Symbol;Acc:HGNC:18652]	chr2	63892146	64019072	-1	ensembl_havana	7.168636411	7.0231416	7.314131221	7.340623806	7.066954475	6.661846518	7.585047528	7.2737687	7.083577437
ENSG00000259985	0.011237	0.051080633	0.469536044	1.384664103	**AC017100.1**	antisense		chr18	31685655	31686823	1	havana	2.528546921	2.293778899	2.763314943	2.191145857	2.500414659	2.18977618	2.729587629	2.69405369	2.866303509
ENSG00000172731	0.011261	0.051175496	-0.307573147	-1.237624058	**LRRC20**	protein_coding	leucine rich repeat containing 20 [Source:HGNC Symbol;Acc:HGNC:23421]	chr10	70298970	70382650	-1	ensembl_havana	3.233501812	3.387288386	3.079715239	3.142871041	3.283780556	3.735213561	2.842395714	3.102158989	3.294591014
ENSG00000103064	0.01127	0.051196265	-0.271584687	-1.20713304	**SLC7A6**	protein_coding	solute carrier family 7 member 6 [Source:HGNC Symbol;Acc:HGNC:11064]	chr16	68264516	68301823	1	ensembl_havana	7.127960206	7.263752549	6.992167863	7.431302415	7.205522218	7.154433015	7.05260935	7.014673183	6.909221054
ENSG00000103740	0.011318	0.051293469	0.43937496	1.356016713	**ACSBG1**	protein_coding	acyl-CoA synthetase bubblegum family member 1 [Source:HGNC Symbol;Acc:HGNC:29567]	chr15	78167468	78245688	-1	ensembl_havana	0.657085711	0.437398231	0.876773191	0.040274975	0.325758945	0.946160774	0.339398945	1.029056719	1.261863909
ENSG00000142408	0.011314	0.051293469	0.248459331	1.187937826	**CACNG8**	protein_coding	calcium voltage-gated channel auxiliary subunit gamma 8 [Source:HGNC Symbol;Acc:HGNC:13628]	chr19	53963040	53990215	1	ensembl_havana	2.311021877	2.186792211	2.435251542	2.298074107	2.162224075	2.100078453	2.494337705	2.491852285	2.319564638
ENSG00000157388	0.01131	0.051293469	0.236016796	1.177736501	**CACNA1D**	protein_coding	calcium voltage-gated channel subunit alpha1 D [Source:HGNC Symbol;Acc:HGNC:1391]	chr3	53328963	53813733	1	ensembl_havana	7.581080556	7.463072159	7.699088954	7.508686822	7.303424413	7.57710524	7.691283542	7.59379337	7.812189951
ENSG00000136144	0.011303	0.051293469	0.223144259	1.167274815	**RCBTB1**	protein_coding	RCC1 and BTB domain containing protein 1 [Source:HGNC Symbol;Acc:HGNC:18243]	chr13	49531946	49585583	-1	ensembl_havana	5.807307479	5.695735349	5.918879608	5.757659894	5.648860834	5.680685319	5.997614135	5.887293202	5.871731487
ENSG00000113648	0.011308	0.051293469	-0.21988601	-1.164641562	**H2AFY**	protein_coding	H2A histone family member Y [Source:HGNC Symbol;Acc:HGNC:4740]	chr5	135333900	135399914	-1	ensembl_havana	8.199929388	8.309872393	8.089986383	8.304209251	8.250582479	8.374825449	8.074440452	8.022015848	8.17350285
ENSG00000168813	0.01131	0.051293469	-0.236613066	-1.178223363	**ZNF507**	protein_coding	zinc finger protein 507 [Source:HGNC Symbol;Acc:HGNC:23783]	chr19	32345594	32387667	1	ensembl_havana	5.972744172	6.091050705	5.854437639	6.020027555	6.124433689	6.128690871	5.728587894	5.942639841	5.892085183
ENSG00000168393	0.011311	0.051293469	-0.267862687	-1.204022779	**DTYMK**	protein_coding	deoxythymidylate kinase [Source:HGNC Symbol;Acc:HGNC:3061]	chr2	241675742	241686991	-1	ensembl_havana	4.297084141	4.431015485	4.163152798	4.241910479	4.414917876	4.636218098	4.070484623	4.057599296	4.361374475
ENSG00000164941	0.01132	0.051293469	-0.336238715	-1.262460908	**INTS8**	protein_coding	integrator complex subunit 8 [Source:HGNC Symbol;Acc:HGNC:26048]	chr8	94813311	94881746	1	ensembl_havana	5.596961838	5.765081195	5.42884248	5.918337487	5.601423058	5.775483041	5.404990956	5.349475722	5.532060763
ENSG00000134198	0.011333	0.051339309	0.512402755	1.426423874	**TSPAN2**	protein_coding	tetraspanin 2 [Source:HGNC Symbol;Acc:HGNC:20659]	chr1	115048011	115089500	-1	ensembl_havana	2.696494636	2.440293259	2.952696013	2.362851808	2.739550451	2.218477517	3.072588313	2.933793498	2.851706228
ENSG00000248632	0.011337	0.051339309	-0.316664516	-1.245447765	**AC106872.5**	processed_pseudogene		chr4	164968587	164970002	-1	havana	1.950431513	2.108763771	1.792099255	2.199052053	2.270175802	1.857063457	2.025141822	1.821363648	1.529792296
ENSG00000069943	0.011344	0.051353425	-0.362114077	-1.285307965	**PIGB**	protein_coding	phosphatidylinositol glycan anchor biosynthesis class B [Source:HGNC Symbol;Acc:HGNC:8959]	chr15	55318960	55355648	1	ensembl_havana	4.737219144	4.918276182	4.556162105	5.099251284	4.89583178	4.759745482	4.540671651	4.598043022	4.529771642
ENSG00000114316	0.011356	0.051360758	0.247306134	1.186988645	**USP4**	protein_coding	ubiquitin specific peptidase 4 [Source:HGNC Symbol;Acc:HGNC:12627]	chr3	49277831	49340712	-1	ensembl_havana	6.087632589	5.963979522	6.211285656	5.82817741	5.909776206	6.15398495	6.096463127	6.214310315	6.323083526
ENSG00000117407	0.011351	0.051360758	0.226066309	1.169641422	**ARTN**	protein_coding	artemin [Source:HGNC Symbol;Acc:HGNC:727]	chr1	43933320	43937241	1	ensembl_havana	1.235401969	1.122368814	1.348435123	1.109412078	0.962641309	1.295053056	1.356079599	1.14725896	1.541966812
ENSG00000239857	0.011355	0.051360758	-0.264581474	-1.201287505	**GET4**	protein_coding	golgi to ER traffic protein 4 [Source:HGNC Symbol;Acc:HGNC:21690]	chr7	876552	896436	1	ensembl_havana	2.805935323	2.93822606	2.673644586	3.005358074	2.788784317	3.020535788	2.820528739	2.542041324	2.658363693
ENSG00000140543	0.011365	0.051384936	0.223324395	1.167420571	**DET1**	protein_coding	DET1, COP1 ubiquitin ligase partner [Source:HGNC Symbol;Acc:HGNC:25477]	chr15	88494440	88546675	-1	ensembl_havana	1.647373741	1.535711544	1.759035939	1.369711653	1.524202165	1.713220813	1.619344613	1.715999071	1.941764132
ENSG00000224660	0.011369	0.051389162	0.223610204	1.167651869	**SH3BP5-AS1**	antisense	SH3BP5 antisense RNA 1 [Source:HGNC Symbol;Acc:HGNC:44501]	chr3	15254184	15264493	1	havana	5.549012255	5.437207153	5.660817357	5.492479496	5.401482059	5.417659904	5.706402489	5.601016608	5.675032972
ENSG00000223797	0.011374	0.051395255	0.233409265	1.175609779	**ENTPD3-AS1**	antisense	ENTPD3 antisense RNA 1 [Source:HGNC Symbol;Acc:HGNC:26710]	chr3	40313802	40453329	-1	havana	1.67801252	1.561307887	1.794717153	1.516132283	1.614359492	1.553431888	1.786580698	1.790554303	1.807016457
ENSG00000179889	0.011384	0.05140918	-0.354256042	-1.278326213	**PDXDC1**	protein_coding	pyridoxal dependent decarboxylase domain containing 1 [Source:HGNC Symbol;Acc:HGNC:28995]	chr16	14974591	15139339	1	ensembl_havana	7.406819956	7.583947977	7.229691935	7.876593577	7.510212437	7.365037917	7.338812812	7.193856706	7.156406288
ENSG00000185955	0.011384	0.05140918	-0.393286363	-1.313381795	**C7orf61**	protein_coding	chromosome 7 open reading frame 61 [Source:HGNC Symbol;Acc:HGNC:22135]	chr7	100456615	100464271	-1	ensembl_havana	0.253239617	0.449882799	0.056596436	0.41082706	0.477608744	0.461212593	0.24405081	-0.04338487	-0.030876629
ENSG00000127837	0.011398	0.051439595	0.217975049	1.163099926	**AAMP**	protein_coding	angio associated migratory cell protein [Source:HGNC Symbol;Acc:HGNC:18]	chr2	218264123	218270257	-1	ensembl_havana	6.787794848	6.678807324	6.896782373	6.716260443	6.650174415	6.669987112	6.929046577	6.867136907	6.894163634
ENSG00000152056	0.011398	0.051439595	-0.288477465	-1.221350656	**AP1S3**	protein_coding	adaptor related protein complex 1 sigma 3 subunit [Source:HGNC Symbol;Acc:HGNC:18971]	chr2	223751686	223838027	-1	ensembl_havana	2.991827967	3.136066699	2.847589235	2.917043099	3.220595563	3.270561436	2.758627316	2.8516036	2.932536788
ENSG00000226779	0.01142	0.051522094	-0.363695187	-1.28671736	**NAALADL2-AS2**	antisense	NAALADL2 antisense RNA 2 [Source:HGNC Symbol;Acc:HGNC:41015]	chr3	175234861	175271096	-1	havana	2.425343627	2.60719122	2.243496034	2.892874923	2.523868156	2.404830583	2.394263752	2.358164528	1.978059821
ENSG00000090776	0.01143	0.051549129	0.413447489	1.331864662	**EFNB1**	protein_coding	ephrin B1 [Source:HGNC Symbol;Acc:HGNC:3226]	chrX	68828997	68842147	1	ensembl_havana	3.306162909	3.099439165	3.512886654	3.040984517	3.053384979	3.203947998	3.65769116	3.482292639	3.398676164
ENSG00000183258	0.011459	0.051665319	0.223901927	1.167888	**DDX41**	protein_coding	DEAD-box helicase 41 [Source:HGNC Symbol;Acc:HGNC:18674]	chr5	177511577	177517469	-1	ensembl_havana	6.238968877	6.127017913	6.350919841	6.041073185	6.12730753	6.212673025	6.281917171	6.371335256	6.399507096
ENSG00000156983	0.01147	0.051696352	0.29345838	1.225574666	**BRPF1**	protein_coding	bromodomain and PHD finger containing 1 [Source:HGNC Symbol;Acc:HGNC:14255]	chr3	9731729	9748018	1	ensembl_havana	6.045345959	5.898616769	6.192075149	5.765001948	5.91311056	6.017737799	6.182556836	6.194833781	6.198834831
ENSG00000213420	0.011474	0.05169998	0.401257025	1.320658103	**GPC2**	protein_coding	glypican 2 [Source:HGNC Symbol;Acc:HGNC:4450]	chr7	100169606	100177372	-1	ensembl_havana	1.988015272	1.78738676	2.188643785	1.749374982	1.719668303	1.893116995	2.153133136	2.322713072	2.090085146
ENSG00000124429	0.011488	0.051747386	-0.491059861	-1.405477016	**POF1B**	protein_coding	POF1B, actin binding protein [Source:HGNC Symbol;Acc:HGNC:13711]	chrX	85277396	85379743	-1	ensembl_havana	3.190729176	3.436259107	2.945199246	3.919492919	3.461295901	2.9279885	3.341846614	2.7591524	2.734598724
ENSG00000149182	0.011497	0.05176043	0.227884692	1.171116576	**ARFGAP2**	protein_coding	ADP ribosylation factor GTPase activating protein 2 [Source:HGNC Symbol;Acc:HGNC:13504]	chr11	47164299	47177125	-1	ensembl_havana	6.841548033	6.727605687	6.955490379	6.778263754	6.734890938	6.669662369	7.005840027	6.921470928	6.939160183
ENSG00000204438	0.011498	0.05176043	-0.250604756	-1.189705717	**GPANK1**	protein_coding	G-patch domain and ankyrin repeats 1 [Source:HGNC Symbol;Acc:HGNC:13920]	chr6	31661229	31666283	-1	ensembl_havana	4.921567384	5.046869762	4.796265006	5.11918011	5.023696113	4.997733062	4.786069135	4.790690888	4.812034995
ENSG00000187535	0.011502	0.051760449	0.262490142	1.199547379	**IFT140**	protein_coding	intraflagellar transport 140 [Source:HGNC Symbol;Acc:HGNC:29077]	chr16	1510427	1612110	-1	ensembl_havana	3.649526262	3.518281191	3.780771333	3.634751416	3.430602854	3.489489303	3.947248808	3.590587736	3.804477455
ENSG00000154174	0.011518	0.051814653	0.240735488	1.181594887	**TOMM70**	protein_coding	translocase of outer mitochondrial membrane 70 [Source:HGNC Symbol;Acc:HGNC:11985]	chr3	100363431	100401398	-1	ensembl_havana	7.782458635	7.662090891	7.902826379	7.59286012	7.739271752	7.6541408	7.806486888	7.935476457	7.96651579
ENSG00000260742	0.011536	0.051832179	1.07696294	2.109590442	**AC009962.1**	antisense		chr2	181887851	181891663	-1	havana	0.447411432	-0.091070038	0.985892902	0.490295136	0.09469996	-0.85820521	1.01467363	1.029056719	0.913948357
ENSG00000156671	0.01154	0.051832179	0.233753927	1.175890668	**SAMD8**	protein_coding	sterile alpha motif domain containing 8 [Source:HGNC Symbol;Acc:HGNC:26320]	chr10	75099586	75182123	1	ensembl_havana	5.428233304	5.31135634	5.545110267	5.479554763	5.307478533	5.147035724	5.675519917	5.601016608	5.358794276
ENSG00000135838	0.011534	0.051832179	-0.237357579	-1.178831551	**NPL**	protein_coding	N-acetylneuraminate pyruvate lyase [Source:HGNC Symbol;Acc:HGNC:16781]	chr1	182789293	182830384	1	ensembl_havana	3.735185345	3.853864134	3.616506556	3.744167537	3.859488969	3.957935897	3.515217767	3.560466802	3.773835098
ENSG00000180879	0.011526	0.051832179	-0.268224305	-1.204324611	**SSR4**	protein_coding	signal sequence receptor subunit 4 [Source:HGNC Symbol;Acc:HGNC:11326]	chrX	153793516	153798505	1	ensembl_havana	6.817866073	6.951978226	6.683753921	7.077454347	6.889308276	6.889172054	6.811676338	6.714791468	6.524793956

ENSG00000135414	0.011532	0.051832179	-0.292147685	-1.224461731	**GDF11**	protein_coding	growth differentiation factor 11 [Source:HGNC Symbol;Acc:HGNC:4216]	chr12	55743280	55757278	1	ensembl_havana	3.515422091	3.661495933	3.369348249	3.490814579	3.824501942	3.66917128	3.267019432	3.396231887	3.444793427
ENSG00000180304	0.011547	0.051851032	0.235610046	1.1774045	**OAZ2**	protein_coding	ornithine decarboxylase antizyme 2 [Source:HGNC Symbol;Acc:HGNC:8096]	chr15	64687573	64703281	-1	ensembl_havana	6.598669566	6.480864543	6.716474589	6.636630615	6.503418331	6.302544682	6.894467361	6.675984511	6.578971895
ENSG00000160336	0.011554	0.05185626	0.350719643	1.27519656	**ZNF761**	protein_coding	zinc finger protein 761 [Source:HGNC Symbol;Acc:HGNC:23179]	chr19	53431993	53458261	1	ensembl_havana	1.889032649	1.713672828	2.064392471	1.602122376	1.709476673	1.829419434	2.144358071	1.97541275	2.073406591
ENSG00000171858	0.011563	0.05185626	0.314527925	1.243604658	**RPS21**	protein_coding	ribosomal protein S21 [Source:HGNC Symbol;Acc:HGNC:10409]	chr20	62387116	62388520	1	ensembl_havana	7.600014097	7.442750135	7.75727806	7.352413728	7.521179305	7.454657372	7.701788319	7.684216653	7.885829209
ENSG00000184205	0.01156	0.05185626	0.242997547	1.183449009	**TSPYL2**	protein_coding	TSPY like 2 [Source:HGNC Symbol;Acc:HGNC:24358]	chrX	53082367	53088540	1	ensembl_havana	4.604670322	4.483171548	4.726169095	4.298201297	4.563772432	4.587540916	4.511965697	4.882422361	4.784119228
ENSG00000001631	0.011559	0.05185626	-0.225290689	-1.16901277	**KRIT1**	protein_coding	KRIT1, ankyrin repeat containing [Source:HGNC Symbol;Acc:HGNC:1573]	chr7	92198969	92246166	-1	ensembl_havana	6.364123451	6.476768796	6.251478106	6.423304739	6.552708152	6.454293496	6.162053769	6.325951067	6.266429483
ENSG00000183638	0.01159	0.05192378	0.597904832	1.51351695	**RP1L1**	protein_coding	RP1 like 1 [Source:HGNC Symbol;Acc:HGNC:15946]	chr8	10606349	10712187	-1	ensembl_havana	-0.513327674	-0.81228009	-0.214375258	-0.85854608	-1.178380098	-0.399914092	-0.51701327	-0.19956768	0.073455173
ENSG00000146700	0.011596	0.05192378	0.270940019	1.206593754	**SSC4D**	protein_coding	scavenger receptor cysteine rich family member with 4 domains [Source:HGNC Symbol;Acc:HGNC:14461]	chr7	76389334	76409695	-1	ensembl_havana	-0.29976584	-0.435235849	-0.16429583	-0.51029874	-0.106664058	-0.688744748	-0.12879794	0.130707766	-0.494797317
ENSG00000127328	0.011586	0.05192378	0.243013763	1.183462312	**RAB3IP**	protein_coding	RAB3A interacting protein [Source:HGNC Symbol;Acc:HGNC:16508]	chr12	69738681	69823204	1	ensembl_havana	5.52491839	5.403411508	5.646425271	5.534484003	5.352382995	5.323367526	5.853635024	5.56430073	5.521340061
ENSG00000135778	0.011595	0.05192378	0.23645415	1.178093586	**NTPCR**	protein_coding	nucleoside-triphosphatase, cancer-related [Source:HGNC Symbol;Acc:HGNC:28204]	chr1	232950605	232983882	1	ensembl_havana	5.07998957	4.961762495	5.198216645	4.951463976	4.943352857	4.990470653	5.149451065	5.245402277	5.199796594
ENSG00000166347	0.011584	0.05192378	-0.278329253	-1.212789571	**CYB5A**	protein_coding	cytochrome b5 type A [Source:HGNC Symbol;Acc:HGNC:2570]	chr18	74250847	74292016	-1	ensembl_havana	5.76091919	5.900083816	5.621754563	5.818587015	6.000865365	5.88079907	5.660981195	5.67527667	5.529005826
ENSG00000283083	0.0116	0.05192378	-0.768097359	-1.703022338	**LINC02498**	lincRNA	long intergenic non-protein coding RNA 2498 [Source:HGNC Symbol;Acc:HGNC:53483]	chr4	10737572	10749586	1	havana	-0.765869542	-0.381820862	-1.149918221	0.326725152	-0.672709	-0.799478738	-0.89704638	-1.00738779	-1.54532049
ENSG00000214135	0.011608	0.051941314	0.436095081	1.352937393	**AC132008.2**	transcribed_unprocessed_pseudogene		chr3	197578213	197627906	-1	havana	4.076521319	3.858473778	4.294568859	3.674990394	4.083178707	3.817252234	4.374276086	4.370447884	4.138982607
ENSG00000115257	0.011611	0.051941314	0.263626832	1.200492867	**PCSK4**	protein_coding	proprotein convertase subtilisin/kexin type 4 [Source:HGNC Symbol;Acc:HGNC:8746]	chr19	1481428	1490752	-1	ensembl_havana	0.742828757	0.611015341	0.874642173	0.705390237	0.325758945	0.80189684	1.052687018	0.602150945	0.969088554
ENSG00000117595	0.011621	0.051972679	-0.528254665	-1.442183424	**IRF6**	protein_coding	interferon regulatory factor 6 [Source:HGNC Symbol;Acc:HGNC:6121]	chr1	209785623	209806175	-1	ensembl_havana	1.500672477	1.764799809	1.236545144	1.902479286	1.827267088	1.564653053	1.089724456	1.286257764	1.333653212
ENSG00000204044	0.011675	0.052196133	-0.273429688	-1.208677779	**SLC12A5-AS1**	antisense	SLC12A5 and MMP9 antisense RNA 1 [Source:HGNC Symbol;Acc:HGNC:53143]	chr20	46013500	46022073	-1	havana	0.946781356	1.0834962	0.810066512	1.327057633	0.910210399	1.013220568	0.995284613	0.578298274	0.856616648
ENSG00000162511	0.011682	0.052209163	-0.610303611	-1.526580439	**LAPTM5**	protein_coding	lysosomal protein transmembrane 5 [Source:HGNC Symbol;Acc:HGNC:29612]	chr1	30732469	30757820	-1	ensembl_havana	0.900594353	1.205746158	0.595442547	0.892538	1.338282095	1.38641838	0.56657698	0.343769191	0.875981471
ENSG00000141627	0.011687	0.052217287	0.217600034	1.162797628	**DYM**	protein_coding	dymeclin [Source:HGNC Symbol;Acc:HGNC:21317]	chr18	49041474	49461347	-1	ensembl_havana	5.971911503	5.863111486	6.08071152	5.814089585	5.877709464	5.897535407	6.034293642	6.108473041	6.099367876
ENSG00000163794	0.011697	0.052246236	0.365819031	1.288612979	**UCN**	protein_coding	urocortin [Source:HGNC Symbol;Acc:HGNC:12516]	chr2	27307400	27308445	-1	ensembl_havana	0.72814453	0.545235015	0.911054045	0.832812769	0.271370116	0.531522158	1.371200905	0.648704825	0.713256405
ENSG00000119878	0.011709	0.052281039	0.332849962	1.259498987	**CRIPT**	protein_coding	CXXC repeat containing interactor of PDZ3 domain [Source:HGNC Symbol;Acc:HGNC:14312]	chr2	46616416	46625742	1	ensembl_havana	5.407995424	5.241570443	5.574420405	5.249565961	5.395931875	5.079213492	5.67932167	5.552106624	5.491832921
ENSG00000254858	0.011724	0.052301358	0.470537229	1.38562535	**MPV17L2**	protein_coding	MPV17 mitochondrial inner membrane protein like 2 [Source:HGNC Symbol;Acc:HGNC:28177]	chr19	18193182	18196948	1	ensembl_havana	4.182390639	3.947122025	4.417659254	3.776230114	4.100831193	3.964304768	4.431299106	4.282766379	4.538912277
ENSG00000107897	0.011719	0.052301358	0.220309927	1.164983827	**ACBD5**	protein_coding	acyl-CoA binding domain containing 5 [Source:HGNC Symbol;Acc:HGNC:23338]	chr10	27195214	27242130	-1	ensembl_havana	5.383880827	5.273725863	5.49403579	5.200196109	5.322602517	5.298378963	5.421427847	5.516463293	5.544216231
ENSG00000123892	0.011724	0.052301358	-0.351393404	-1.275792236	**RAB38**	protein_coding	RAB38, member RAS oncogene family [Source:HGNC Symbol;Acc:HGNC:9776]	chr11	88113242	88175467	-1	ensembl_havana	1.321096035	1.496792737	1.145399333	1.705645971	1.558674763	1.226057475	1.527846157	1.195095435	0.713256405
ENSG00000260917	0.011761	0.052449912	0.232041296	1.174495589	**AL158212.3**	sense_overlapping		chr10	112823490	112827726	1	havana	2.7640524	2.648031752	2.880073048	2.700213803	2.646460224	2.59742123	2.991732974	2.8516036	2.796882569
ENSG00000172602	0.011781	0.052488081	0.258640572	1.196350872	**RND1**	protein_coding	Rho family GTPase 1 [Source:HGNC Symbol;Acc:HGNC:18314]	chr12	48857145	48865898	-1	ensembl_havana	4.821411229	4.692090943	4.950731515	4.769639496	4.835020118	4.471613216	5.103785613	4.999305086	4.749103847
ENSG00000141622	0.01178	0.052488081	-0.24457884	-1.184746862	**RNF165**	protein_coding	ring finger protein 165 [Source:HGNC Symbol;Acc:HGNC:31696]	chr18	46326809	46463140	1	ensembl_havana	5.81398344	5.93627286	5.69169402	6.037219356	5.837913348	5.933685876	5.759803566	5.545970702	5.769307793
ENSG00000272654	0.011778	0.052488081	-0.372425837	-1.294527701	**AL358472.2**	lincRNA		chr1	153977743	153979160	1	havana	1.001573442	1.18778636	0.815360523	1.158893785	1.311578958	1.092886337	0.692307165	0.821210834	0.932563571
ENSG00000159363	0.011799	0.052536268	0.380865944	1.30212319	**ATP13A2**	protein_coding	ATPase 13A2 [Source:HGNC Symbol;Acc:HGNC:30213]	chr1	16985958	17011928	-1	ensembl_havana	5.593166013	5.402733041	5.783598984	5.3735117	5.323438107	5.511249316	5.899335479	5.775047272	5.676414202
ENSG00000170608	0.011797	0.052536268	-0.71947558	-1.646583392	**FOXA3**	protein_coding	forkhead box A3 [Source:HGNC Symbol;Acc:HGNC:5023]	chr19	45863989	45873797	1	ensembl_havana	1.036332163	1.396069953	0.676594373	1.222335231	1.657409759	1.30846487	0.975631465	0.625615656	0.428535999
ENSG00000126062	0.011815	0.05259088	0.512236757	1.426259758	**TMEM115**	protein_coding	transmembrane protein 115 [Source:HGNC Symbol;Acc:HGNC:30055]	chr3	50354749	50359610	-1	ensembl_havana	4.823116451	4.566998072	5.079234829	4.402883241	4.636849344	4.661261632	5.224223873	5.083264384	4.93021623
ENSG00000136715	0.01183	0.052632346	0.243776274	1.184087975	**SAP130**	protein_coding	Sin3A associated protein 130 [Source:HGNC Symbol;Acc:HGNC:29813]	chr2	127941217	128028120	-1	ensembl_havana	6.158545046	6.036656909	6.280433184	5.927883982	6.119631196	6.062455549	6.206992691	6.283709336	6.350597523
ENSG00000175787	0.011831	0.052632346	0.243718631	1.184040666	**ZNF169**	protein_coding	zinc finger protein 169 [Source:HGNC Symbol;Acc:HGNC:12957]	chr9	94259311	94301454	1	ensembl_havana	3.296015419	3.174156103	3.417874735	3.027727485	3.224179389	3.270561436	3.349502035	3.443209147	3.460913023
ENSG00000115486	0.011853	0.05266833	0.292417023	1.224690348	**GGCX**	protein_coding	gamma-glutamyl carboxylase [Source:HGNC Symbol;Acc:HGNC:4247]	chr2	85544723	85561547	-1	ensembl_havana	5.220323688	5.074115177	5.366532199	5.147034682	5.115322954	4.959987894	5.368721944	5.339754527	5.391120127
ENSG00000168763	0.011856	0.05266833	0.251622648	1.190545409	**CNNM3**	protein_coding	cyclin and CBS domain divalent metal cation transport mediator 3 [Source:HGNC Symbol;Acc:HGNC:104]	chr2	96816245	96833911	1	ensembl_havana	4.776498485	4.65068716	4.902309809	4.744198658	4.510469659	4.697393164	4.966158622	4.851659712	4.889111092
ENSG00000165861	0.011856	0.05266833	0.232424174	1.174807331	**ZFYVE1**	protein_coding	zinc finger FYVE-type containing 1 [Source:HGNC Symbol;Acc:HGNC:13180]	chr14	72969451	73027212	-1	ensembl_havana	6.65019919	6.533987104	6.766411277	6.708319256	6.550211866	6.343430189	6.997641854	6.734485022	6.567106956
ENSG00000162374	0.011857	0.05266833	-0.254033701	-1.192536729	**ELAVL4**	protein_coding	ELAV like RNA binding protein 4 [Source:HGNC Symbol;Acc:HGNC:3315]	chr1	50048014	50203786	1	ensembl_havana	2.278052018	2.405068869	2.151035168	2.397621087	2.564016113	2.253569408	2.238067541	2.264113944	1.950924019
ENSG00000204610	0.011858	0.05266833	-0.33118439	-1.258045752	**TRIM15**	protein_coding	tripartite motif containing 15 [Source:HGNC Symbol;Acc:HGNC:16284]	chr6	30163206	30172696	1	ensembl_havana	7.460072794	7.625664989	7.294480599	7.836607165	7.468138918	7.572248885	7.514018767	7.285325142	7.084097889
ENSG00000101350	0.01187	0.052688448	0.25101462	1.190043756	**KIF3B**	protein_coding	kinesin family member 3B [Source:HGNC Symbol;Acc:HGNC:6320]	chr20	32277664	32335011	1	ensembl_havana	7.341437765	7.215930455	7.466945075	7.257648832	7.253222221	7.136920312	7.418308544	7.544441313	7.438085369
ENSG00000146054	0.011868	0.052688448	-0.826000318	-1.772763789	**TRIM7**	protein_coding	tripartite motif containing 7 [Source:HGNC Symbol;Acc:HGNC:16278]	chr5	181193924	181205293	-1	ensembl_havana	1.368784605	1.781784764	0.955784446	2.058373044	1.524202165	1.762779083	0.692307165	0.841392961	1.333653212
ENSG00000196391	0.01188	0.052713461	0.217127123	1.162416529	**ZNF774**	protein_coding	zinc finger protein 774 [Source:HGNC Symbol;Acc:HGNC:33108]	chr15	90352245	90369146	1	ensembl_havana	3.318922713	3.210359151	3.427486274	3.142871041	3.269976654	3.218229759	3.345679402	3.482292639	3.454486781
ENSG00000137133	0.011884	0.052713461	-0.310693332	-1.240303623	**HINT2**	protein_coding	histidine triad nucleotide binding protein 2 [Source:HGNC Symbol;Acc:HGNC:18344]	chr9	35812960	35815354	-1	ensembl_havana	3.800365721	3.955712387	3.645019055	3.909890552	4.071289234	3.885957376	3.654604579	3.605415612	3.675036974
ENSG00000267040	0.011888	0.052713461	-0.405105221	-1.324185485	**AC027097.1**	antisense		chr18	57630302	57669296	1	ensembl_havana	0.878395884	1.080948495	0.675843274	1.058172782	1.155163158	1.029509544	0.592614899	0.578298274	0.856616648
ENSG00000227388	0.01189	0.052713461	-0.408644637	-1.327438145	**AL133410.1**	lincRNA		chr9	35772163	35790432	1	havana	1.054193552	1.25851587	0.849871233	1.142587728	1.298039728	1.334920154	0.540060475	1.114463419	0.895089805
ENSG00000148154	0.011903	0.052734483	0.265343108	1.201921861	**UGCG**	protein_coding	UDP-glucose ceramide glucosyltransferase [Source:HGNC Symbol;Acc:HGNC:12524]	chr9	111896766	111935369	1	ensembl_havana	5.425416029	5.292744476	5.558087583	5.080113972	5.410947263	5.387172191	5.445737301	5.663280516	5.565244932
ENSG00000170004	0.011909	0.052734483	-0.21524923	-1.160904447	**CHD3**	protein_coding	chromodomain helicase DNA binding protein 3 [Source:HGNC Symbol;Acc:HGNC:1918]	chr17	7884806	7912760	1	ensembl_havana	7.450093726	7.557718341	7.342469111	7.832642179	7.461701734	7.37881111	7.616717138	7.251317434	7.159372761
ENSG00000167670	0.011905	0.052734483	-0.266295013	-1.202715164	**CHAF1A**	protein_coding	chromatin assembly factor 1 subunit A [Source:HGNC Symbol;Acc:HGNC:1910]	chr19	4402662	4445018	1	ensembl_havana	6.167706701	6.300854207	6.034559194	6.244810794	6.274562954	6.383188874	6.086762992	5.972113284	6.044801307
ENSG00000143570	0.011909	0.052734483	-0.335376106	-1.261706289	**SLC39A1**	protein_coding	solute carrier family 39 member 1 [Source:HGNC Symbol;Acc:HGNC:12876]	chr1	153959099	153968184	-1	ensembl_havana	6.542673923	6.710361977	6.37498587	6.827547373	6.683098622	6.620439935	6.638149075	6.366991075	6.119817461
ENSG00000172244	0.011918	0.052755466	-0.245655902	-1.18563168	**C5orf34**	protein_coding	chromosome 5 open reading frame 34 [Source:HGNC Symbol;Acc:HGNC:24738]	chr5	43486701	43515145	-1	ensembl_havana	3.642557272	3.765385223	3.519729321	3.510009124	3.918192999	3.867953547	3.306885447	3.713387308	3.538915209
ENSG00000099290	0.011927	0.052766319	0.262356406	1.199436188	**WASHC2A**	protein_coding	WASH complex subunit 2A [Source:HGNC Symbol;Acc:HGNC:23416]	chr10	50067888	50133506	1	ensembl_havana	5.733492269	5.602314066	5.864670472	5.676455875	5.567263407	5.563222917	5.833304626	5.878164058	5.882542733
ENSG00000114405	0.011929	0.052766319	0.245692792	1.185661997	**C3orf14**	protein_coding	chromosome 3 open reading frame 14 [Source:HGNC Symbol;Acc:HGNC:25024]	chr3	62318973	62336213	1	ensembl_havana	6.403833262	6.280986866	6.526679658	6.101374214	6.432771957	6.308814427	6.428262796	6.620860995	6.530915182
ENSG00000277142	0.011931	0.052766319	-0.298357122	-1.229743238	**LINC00235**	lincRNA	long intergenic non-protein coding RNA 235 [Source:HGNC Symbol;Acc:HGNC:14138]	chr16	525155	527407	-1	havana	0.99300412	1.142182681	0.843825559	0.986893817	1.199531136	1.24012309	0.785553575	0.758913071	0.987010032
ENSG00000281920	0.011935	0.052766319	-0.528282086	-1.442210835	**AC007389.5**	lincRNA		chr2	65623272	65628424	1	havana	-0.503181496	-0.239040453	-0.767322538	-0.85854608	0.06303108	0.078393642	-1.41444944	-0.74417246	-0.143345712
ENSG00000277224	0.01195	0.052818495	0.376779532	1.298440165	**HIST1H2BF**	protein_coding	histone cluster 1 H2B family member f [Source:HGNC Symbol;Acc:HGNC:4752]	chr6	26199520	26200715	1	havana	1.118684335	0.930294569	1.307074102	1.022973463	0.910210399	0.857699845	1.580839304	1.300906777	1.039476224
ENSG00000133216	0.01196	0.052843787	0.565689411	1.480094623	**EPHB2**	protein_coding	EPH receptor B2 [Source:HGNC Symbol;Acc:HGNC:3393]	chr1	22710839	22921500	1	ensembl_havana	5.215290772	4.932446067	5.498135478	4.243822659	5.114357655	5.439157887	5.146158264	5.640425293	5.707822878
ENSG00000172986	0.011964	0.05284422	0.416025232	1.334246505	**GXYLT2**	protein_coding	glucoside xylosyltransferase 2 [Source:HGNC Symbol;Acc:HGNC:33383]	chr3	72888073	72998138	1	ensembl_havana	3.624750504	3.416737888	3.83276312	3.290778936	3.370423439	3.58901129	3.466766833	3.968651039	4.062871488
ENSG00000164142	0.011982	0.052867061	0.343636692	1.268951291	**FAM160A1**	protein_coding	family with sequence similarity 160 member A1 [Source:HGNC Symbol;Acc:HGNC:34237]	chr4	151409216	151663632	1	ensembl_havana	1.033734688	0.861916342	1.205553034	0.660286109	0.996565715	0.928897202	1.178360195	1.315408538	1.12289037
ENSG00000050438	0.011983	0.052867061	0.249763112	1.189011866	**SLC4A8**	protein_coding	solute carrier family 4 member 8 [Source:HGNC Symbol;Acc:HGNC:11034]	chr12	51391317	51515763	1	ensembl_havana	4.493826189	4.368944633	4.618707745	4.288970699	4.41805057	4.399812629	4.454557084	4.682975558	4.718590593
ENSG00000130812	0.011983	0.052867061	-0.393711176	-1.313768587	**ANGPTL6**	protein_coding	angiopoietin like 6 [Source:HGNC Symbol;Acc:HGNC:23140]	chr19	10092338	10102796	-1	ensembl_havana	1.619673118	1.816528706	1.42281753	2.040883175	1.855271053	1.553431888	1.693402841	1.241396536	1.333653212
ENSG00000049768	0.011983	0.052867061	-0.451601855	-1.367557843	**FOXP3**	protein_coding	forkhead box P3 [Source:HGNC Symbol;Acc:HGNC:6106]	chrX	49250436	49264826	-1	ensembl_havana	0.856781753	1.08258268	0.630980825	1.237769075	1.013233389	0.996745577	0.56657698	0.821210834	0.505154661
ENSG00000148357	0.011989	0.052875944	0.788884779	1.727738384	**HMCN2**	protein_coding	hemicentin 2 [Source:HGNC Symbol;Acc:HGNC:21293]	chr9	130265882	130434123	1	ensembl_havana	1.341404919	0.94696253	1.735847309	0.237415364	1.155163158	1.448309068	1.554586035	1.748424202	1.90453169
ENSG00000196975	0.012001	0.052891635	-0.217440739	-1.162669245	**ANXA4**	protein_coding	annexin A4 [Source:HGNC Symbol;Acc:HGNC:542]	chr2	69644425	69827100	1	ensembl_havana	5.66897735	5.77769772	5.560256981	5.705885681	5.788541038	5.838666441	5.501774432	5.546739121	5.632257389
ENSG00000101004	0.012004	0.052891635	-0.393300508	-1.313394672	**NINL**	protein_coding	ninein like [Source:HGNC Symbol;Acc:HGNC:29163]	chr20	25452705	25585517	-1	ensembl_havana	-0.239629029	-0.042978775	-0.436279282	0.41082706	-0.521012188	-0.018751196	-0.12879794	-0.68524259	-0.494797317
ENSG00000271151	0.012003	0.052891635	-0.720731856	-1.648017836	**AC016737.1**	lincRNA		chr2	173968351	173969418	1	havana	0.259362609	0.619728537	-0.101003319	0.355307344	0.77991046	0.723967808	-0.17198403	-0.42206346	0.291037539
ENSG00000177383	0.012017	0.052935756	-0.224097414	-1.168046261	**MAGEF1**	protein_coding	MAGE family member F1 [Source:HGNC Symbol;Acc:HGNC:29639]	chr3	184710367	184712002	-1	ensembl_havana	5.054163437	5.166212144	4.94211473	5.205111677	5.198746691	5.094778063	4.937238596	4.985768986	4.903336608
ENSG00000162777	0.01203	0.052972962	-0.618627846	-1.535414147	**DENND2D**	protein_coding	DENN domain containing 2D [Source:HGNC Symbol;Acc:HGNC:26192]	chr1	111187174	111204535	-1	ensembl_havana	2.851757242	3.161071165	2.542443319	3.122229914	3.073391752	3.287591829	2.099663825	2.710605818	2.817060315
ENSG00000222009	0.01204	0.052984719	0.252254579	1.191067007	**BTBD19**	protein_coding	BTB domain containing 19 [Source:HGNC Symbol;Acc:HGNC:27145]	chr1	44808482	44815585	1	ensembl_havana	2.159766701	2.033639411	2.28589399	2.075653419	2.004444884	2.02081993	2.238067541	2.278988373	2.340626055
ENSG00000102572	0.012045	0.052984719	0.233217824	1.17545379	**STK24**	protein_coding	serine/threonine kinase 24 [Source:HGNC Symbol;Acc:HGNC:11403]	chr13	98445185	98577940	-1	ensembl_havana	7.228384628	7.111775716	7.34499354	7.251484449	7.151257964	6.932584735	7.526946591	7.321022126	7.187011902
ENSG00000124641	0.012046	0.052984719	-0.24101326	-1.181822409	**MED20**	protein_coding	mediator complex subunit 20 [Source:HGNC Symbol;Acc:HGNC:16840]	chr6	41905354	41921139	-1	ensembl_havana	5.347744232	5.468250862	5.227237602	5.524293648	5.442826041	5.437632895	5.211677793	5.211882794	5.25815222
ENSG00000136193	0.012047	0.052984719	-0.854345225	-1.807938027	**SCRN1**	protein_coding	secernin 1 [Source:HGNC Symbol;Acc:HGNC:22192]	chr7	29920103	29990289	-1	ensembl_havana	2.523846763	2.951019376	2.096674151	2.327223844	3.191599876	3.334234408	2.053540577	2.089494957	2.146986918
ENSG00000175121	0.012061	0.053007059	0.740260844	1.67047784	**WFDC5**	protein_coding	WAP four-disulfide core domain 5 [Source:HGNC Symbol;Acc:HGNC:20477]	chr20	45109452	45115172	-1	ensembl_havana	0.372434548	0.002304126	0.74256497	-0.27307956	-0.002471726	0.28246366	0.36983092	1.210694826	0.647169164
ENSG00000114744	0.012063	0.053007059	0.249923401	1.189143977	**COMMD2**	protein_coding	COMM domain containing 2 [Source:HGNC Symbol;Acc:HGNC:24993]	chr3	149738470	149752499	-1	ensembl_havana	6.34985518	6.224893479	6.474816881	6.139876434	6.328833781	6.205970223	6.439963152	6.488347019	6.496140471
ENSG00000162600	0.012059	0.053007059	-0.254745722	-1.193125434	**OMA1**	protein_coding	OMA1 zinc metallopeptidase [Source:HGNC Symbol;Acc:HGNC:29661]	chr1	58415384	58546802	-1	ensembl_havana	3.510523825	3.637896686	3.383150964	3.744167537	3.436793925	3.732728596	3.441917197	3.279044008	3.428491686
ENSG00000143195	0.012087	0.053015616	0.484608822	1.399206428	**ILDR2**	protein_coding	immunoglobulin like domain containing receptor 2 [Source:HGNC Symbol;Acc:HGNC:18131]	chr1	166895711	166975482	-1	ensembl_havana	4.63162331	4.389318899	4.873927721	3.694696689	4.565186067	4.908073941	4.484416377	4.923233938	5.214132848
ENSG00000105404	0.012084	0.053015616	0.418076838	1.336145239	**RABAC1**	protein_coding	Rab acceptor 1 [Source:HGNC Symbol;Acc:HGNC:9794]	chr19	41956681	41959390	-1	ensembl_havana	1.591574061	1.382535642	1.80061248	1.649052215	1.390249836	1.108304877	1.976531838	1.648877048	1.776428555
ENSG00000138688	0.012075	0.053015616	0.308362699	1.238301565	**KIAA1109**	protein_coding	KIAA1109 [Source:HGNC Symbol;Acc:HGNC:26953]	chr4	122152333	122362758	1	ensembl_havana	6.297501167	6.143319818	6.451682516	6.187844854	5.956847556	6.285267043	6.438169262	6.419955869	6.496922419
ENSG00000134452	0.012079	0.053015616	0.224704932	1.168538228	**FBXO18**	protein_coding	F-box protein, helicase, 18 [Source:HGNC Symbol;Acc:HGNC:13620]	chr10	5890203	5937594	1	ensembl_havana	6.331885366	6.219532899	6.444237832	6.177377772	6.134943235	6.346277692	6.426454289	6.382999623	6.523259583
ENSG00000166912	0.012081	0.053015616	-0.251318806	-1.190294698	**MTMR10**	protein_coding	myotubularin related protein 10 [Source:HGNC Symbol;Acc:HGNC:25999]	chr15	30938941	30991607	-1	ensembl_havana	7.634496409	7.760155812	7.508837006	7.940770954	7.643656108	7.696040375	7.615725514	7.380634426	7.530151079
ENSG00000198171	0.012074	0.053015616	-0.338103429	-1.26409372	**DDRGK1**	protein_coding	DDRGK domain containing 1 [Source:HGNC Symbol;Acc:HGNC:16110]	chr20	3190350	3204685	-1	ensembl_havana	4.263919848	4.432971563	4.094868134	4.49406847	4.436705215	4.368141003	4.340101302	3.998165018	3.946338082
ENSG00000168071	0.012092	0.053021246	-0.266815834	-1.203149429	**CCDC88B**	protein_coding	coiled-coil domain containing 88B [Source:HGNC Symbol;Acc:HGNC:26757]	chr11	64340223	64357534	1	ensembl_havana	4.655033626	4.788441543	4.521625709	4.860924683	4.658202087	4.846197859	4.676211797	4.416577876	4.472087453
ENSG00000167702	0.012112	0.053041953	0.356484024	1.280301883	**KIFC2**	protein_coding	kinesin family member C2 [Source:HGNC Symbol;Acc:HGNC:29530]	chr8	144466043	144474202	1	ensembl_havana	5.464725716	5.286483704	5.642967728	5.333667378	5.118214983	5.407568751	5.827834449	5.532844567	5.568224166
ENSG00000108840	0.012111	0.053041953	0.298672018	1.230011682	**HDAC5**	protein_coding	histone deacetylase 5 [Source:HGNC Symbol;Acc:HGNC:14068]	chr17	44076746	44123702	-1	ensembl_havana	3.991668981	3.842332972	4.14100499	3.912297142	3.776492281	3.838209494	4.338178756	3.947858737	4.136977476
ENSG00000139182	0.012106	0.053041953	-0.222937044	-1.167107171	**CLSTN3**	protein_coding	calsyntenin 3 [Source:HGNC Symbol;Acc:HGNC:18371]	chr12	7129698	7158945	1	ensembl_havana	6.911647734	7.023116256	6.800179212	7.103224669	6.963279831	7.002844267	6.911052455	6.751915625	6.737569556
ENSG00000123178	0.012109	0.053041953	-0.228372345	-1.171512498	**SPRYD7**	protein_coding	SPRY domain containing 7 [Source:HGNC Symbol;Acc:HGNC:14297]	chr13	49912706	49936490	-1	ensembl_havana	4.044181221	4.158367393	3.929995048	4.228453786	4.141199286	4.105449108	3.954796815	3.964056355	3.871131974
ENSG00000269713	0.012138	0.053141723	0.244909882	1.185018747	**NBPF9**	protein_coding	NBPF member 9 [Source:HGNC Symbol;Acc:HGNC:31991]	chr1	149054027	149103561	-1	ensembl_havana	4.239340479	4.116885538	4.36179542	4.109750292	4.075263288	4.165643032	4.293237984	4.301098552	4.491049724
ENSG00000127946	0.012154	0.053195472	-0.256752766	-1.194786437	**HIP1**	protein_coding	huntingtin interacting protein 1 [Source:HGNC Symbol;Acc:HGNC:4913]	chr7	75533300	75738962	-1	ensembl_havana	6.002487217	6.1308636	5.874110834	6.01779426	6.114329924	6.260466615	5.860348652	5.82023635	5.9417475
ENSG00000124762	0.012161	0.053207185	-0.306247384	-1.236487268	**CDKN1A**	protein_coding	cyclin dependent kinase inhibitor 1A [Source:HGNC Symbol;Acc:HGNC:1784]	chr6	36676460	36687339	1	ensembl_havana	5.62380339	5.776927082	5.470679698	5.848426298	5.764129965	5.718224984	5.58438406	5.565817771	5.261837262
ENSG00000095303	0.012176	0.053260041	-0.636724071	-1.554794674	**PTGS1**	protein_coding	prostaglandin-endoperoxide synthase 1 [Source:HGNC Symbol;Acc:HGNC:9604]	chr9	122370530	122395703	1	ensembl_havana	2.050104838	2.368466873	1.731742802	1.968722995	2.64110813	2.495569495	1.75234034	1.841543652	1.601344415
ENSG00000185100	0.012186	0.05328424	0.351331939	1.275737883	**ADSSL1**	protein_coding	adenylosuccinate synthase like 1 [Source:HGNC Symbol;Acc:HGNC:20093]	chr14	104724186	104747325	1	ensembl_havana	-0.539145163	-0.714811132	-0.363479193	-0.92573229	-0.903502039	-0.315199064	-0.51701327	-0.74417246	0.17074815
ENSG00000197978	0.012189	0.05328424	0.244633854	1.18479204	**GOLGA6L9**	protein_coding	golgin A6 family-like 9 [Source:HGNC Symbol;Acc:HGNC:37229]	chr15	82430018	82439153	1	ensembl_havana	4.342918532	4.220601605	4.465235459	4.054171047	4.276796007	4.330837762	4.310990596	4.443233971	4.641481809
ENSG00000196544	0.012217	0.053320367	0.434385974	1.351335568	**BORCS6**	protein_coding	BLOC-1 related complex subunit 6 [Source:HGNC Symbol;Acc:HGNC:25939]	chr17	8188333	8190907	-1	ensembl_havana	2.119043388	1.901850401	2.336236375	1.87313074	2.06964138	1.762779083	2.294679948	2.278988373	2.435040805
ENSG00000166123	0.012213	0.053320367	0.394816037	1.314775098	**GPT2**	protein_coding	glutamic--pyruvic transaminase 2 [Source:HGNC Symbol;Acc:HGNC:18062]	chr16	46884378	46931297	1	ensembl_havana	5.562118924	5.364710905	5.759526942	5.015544611	5.467226644	5.611361461	5.644739761	5.757895571	5.875945493
ENSG00000272462	0.012211	0.053320367	0.327255775	1.254624617	**U91328.1**	lincRNA		chr6	25992662	26001775	1	havana	3.495292462	3.331664574	3.658920349	3.316445323	3.344313992	3.334234408	3.815261472	3.554366408	3.607133167
ENSG00000160584	0.012231	0.053320367	0.266175433	1.202615479	**SIK3**	protein_coding	SIK family kinase 3 [Source:HGNC Symbol;Acc:HGNC:29165]	chr11	116843402	117098437	-1	ensembl_havana	7.576556105	7.443468389	7.709643822	7.767507888	7.434695509	7.128201771	8.062554313	7.592305477	7.474071676
ENSG00000138617	0.012212	0.053320367	0.257724883	1.19559178	**PARP16**	protein_coding	poly(ADP-ribose) polymerase family member 16 [Source:HGNC Symbol;Acc:HGNC:26040]	chr15	65234460	65300618	-1	ensembl_havana	4.151144106	4.022281664	4.280006547	3.895366092	4.144985604	4.026493296	4.158096097	4.311987074	4.369936469
ENSG00000154803	0.012218	0.053320367	0.252168515	1.190995957	**FLCN**	protein_coding	folliculin [Source:HGNC Symbol;Acc:HGNC:27310]	chr17	17212212	17237188	-1	ensembl_havana	4.383747723	4.257663465	4.509831981	4.259029533	4.290535124	4.223425739	4.58835975	4.456379573	4.484756619
ENSG00000063978	0.012228	0.053320367	-0.217994637	-1.163115718	**RNF4**	protein_coding	ring finger protein 4 [Source:HGNC Symbol;Acc:HGNC:10067]	chr4	2462220	2625320	1	ensembl_havana	6.404644338	6.513641657	6.29564702	6.560809146	6.545564416	6.434551408	6.371587251	6.323262802	6.192091007
ENSG00000116560	0.012227	0.053320367	-0.234618954	-1.176595933	**SFPQ**	protein_coding	splicing factor proline and glutamine rich [Source:HGNC Symbol;Acc:HGNC:10774]	chr1	35176378	35193148	-1	ensembl_havana	8.718671558	8.835981035	8.601362081	8.870497429	8.804458771	8.832986906	8.569162391	8.588766972	8.64615688
ENSG00000163462	0.01223	0.053320367	-0.634687994	-1.552601938	**TRIM46**	protein_coding	tripartite motif containing 46 [Source:HGNC Symbol;Acc:HGNC:19019]	chr1	155173787	155184971	1	ensembl_havana	3.692139779	4.009483776	3.374795782	4.043232818	3.866385698	4.118832811	3.287090137	3.64326966	3.194027549
ENSG00000260196	0.012235	0.053321943	-0.503079607	-1.417235596	**AC124798.1**	antisense		chr11	17380649	17383531	1	havana	0.566788564	0.818328368	0.315248761	0.637193984	1.155163158	0.662627962	0.36983092	0.343769191	0.232146171
ENSG00000134909	0.012245	0.053348396	-0.237186839	-1.178692047	**ARHGAP32**	protein_coding	Rho GTPase activating protein 32 [Source:HGNC Symbol;Acc:HGNC:17399]	chr11	128965060	129279324	-1	ensembl_havana	4.693256834	4.811850253	4.574663414	4.830709463	4.620624797	4.9842165	4.657831492	4.320998484	4.745160266
ENSG00000178074	0.012249	0.053350715	-0.232935342	-1.175223656	**C2orf69**	protein_coding	chromosome 2 open reading frame 69 [Source:HGNC Symbol;Acc:HGNC:26799]	chr2	199911256	199955935	1	ensembl_havana	4.136631859	4.25309953	4.020164189	4.195240971	4.31258584	4.25147178	3.947248808	4.031485863	4.081757895
ENSG00000151690	0.012262	0.053388523	0.224396895	1.168288754	**MFSD6**	protein_coding	major facilitator superfamily domain containing 6 [Source:HGNC Symbol;Acc:HGNC:24711]	chr2	190408355	190509205	1	ensembl_havana	6.542135131	6.429936684	6.654333578	6.484424432	6.448177047	6.357208572	6.720895331	6.625232818	6.616872587
ENSG00000101608	0.012265	0.053388523	-0.306418809	-1.236634199	**MYL12A**	protein_coding	myosin light chain 12A [Source:HGNC Symbol;Acc:HGNC:16701]	chr18	3247481	3256236	1	ensembl_havana	8.285560499	8.438769904	8.132351094	8.681064163	8.44959639	8.185649158	8.241766139	8.139245598	8.016041545
ENSG00000121766	0.012271	0.053393329	0.247540707	1.187181658	**ZCCHC17**	protein_coding	zinc finger CCHC-type containing 17 [Source:HGNC Symbol;Acc:HGNC:30246]	chr1	31296982	31364953	1	ensembl_havana	5.911041507	5.787271153	6.03481186	5.744214219	5.879415075	5.738184165	5.99151112	6.050056032	6.06286843
ENSG00000228701	0.012274	0.053393329	-0.319171374	-1.247613764	**TNKS2-AS1**	antisense	TNKS2 antisense RNA 1 (head to head) [Source:HGNC Symbol;Acc:HGNC:45173]	chr10	91782839	91798291	-1	havana	-0.195979427	-0.03639374	-0.355565114	-0.14746469	-0.071090603	0.109374069	-0.63291296	-0.32890884	-0.104873545
ENSG00000184117	0.012284	0.053405927	-0.229200939	-1.172185534	**NIPSNAP1**	protein_coding	nipsnap homolog 1 [Source:HGNC Symbol;Acc:HGNC:7827]	chr22	29554808	29581337	-1	ensembl_havana	5.346376959	5.460977429	5.23177649	5.596405944	5.373515124	5.413011218	5.309118186	5.184494938	5.201716345
ENSG00000185220	0.01228	0.053405927	-0.303089072	-1.233783341	**PGBD2**	protein_coding	piggyBac transposable element derived 2 [Source:HGNC Symbol;Acc:HGNC:19399]	chr1	248906196	248919946	1	ensembl_havana	3.608853073	3.760397609	3.457308537	3.897797013	3.691020036	3.692375779	3.470282097	3.526590849	3.375052666
ENSG00000165282	0.012299	0.053455471	-0.232482121	-1.17485452	**PIGO**	protein_coding	phosphatidylinositol glycan anchor biosynthesis class O [Source:HGNC Symbol;Acc:HGNC:23215]	chr9	35088688	35096601	-1	ensembl_havana	5.482070803	5.598311864	5.365829743	5.677163607	5.471002233	5.646769753	5.440370489	5.297464437	5.359654302
ENSG00000115540	0.012305	0.053466325	-0.276291573	-1.211077822	**MOB4**	protein_coding	MOB family member 4, phocein [Source:HGNC Symbol;Acc:HGNC:17261]	chr2	197515571	197553699	1	ensembl_havana	3.638280029	3.776425815	3.500134242	3.628910265	3.9115401	3.788827082	3.438332049	3.5140724	3.547998277
ENSG00000225548	0.012314	0.053472043	0.936776905	1.914246879	**LINC01980**	lincRNA	long intergenic non-protein coding RNA 1980 [Source:HGNC Symbol;Acc:HGNC:52808]	chr3	27797563	27931579	1	havana	1.699388245	1.230999793	2.167776698	1.43811357	1.415548178	0.83933763	2.286727206	1.841543652	2.375059235
ENSG00000254420	0.012313	0.053472043	-0.496958494	-1.411235242	**AP003086.2**	antisense		chr11	78324758	78444049	1	havana	0.426588453	0.6750677	0.178109206	0.540941683	0.740413176	0.743848241	0.339398945	-0.00684508	0.201773757
ENSG00000086102	0.012328	0.053514707	-0.230379634	-1.173143612	**NFX1**	protein_coding	nuclear transcription factor, X-box binding 1 [Source:HGNC Symbol;Acc:HGNC:7803]	chr9	33290511	33371157	1	ensembl_havana	6.817932786	6.933122603	6.702742969	6.957031368	6.926905554	6.915430888	6.678976516	6.754911197	6.674341194
ENSG00000204439	0.012343	0.05356647	-0.214786237	-1.160531947	**C6orf47**	protein_coding	chromosome 6 open reading frame 47 [Source:HGNC Symbol;Acc:HGNC:19076]	chr6	31658298	31660772	-1	havana	6.231572816	6.338965934	6.124179697	6.638448767	6.286610375	6.09183866	6.410074891	6.066223961	5.896240239
ENSG00000139597	0.01235	0.053577657	0.327312345	1.254673813	**N4BP2L1**	protein_coding	NEDD4 binding protein 2 like 1 [Source:HGNC Symbol;Acc:HGNC:25037]	chr13	32400723	32428311	-1	ensembl_havana	4.170366518	4.006710346	4.33402269	4.185325188	4.16563457	3.66917128	4.64233364	4.324587347	4.035147085
ENSG00000172318	0.012356	0.053587811	0.655081121	1.574704495	**B3GALT1**	protein_coding	beta-1,3-galactosyltransferase 1 [Source:HGNC Symbol;Acc:HGNC:916]	chr2	167868948	167874041	1	ensembl_havana	3.39259566	3.065055099	3.72013622	3.257093797	3.195256367	2.742815133	3.712149339	3.611304352	3.83695497
ENSG00000227748	0.012369	0.053612664	0.358556435	1.28214234	**AL138916.1**	lincRNA		chr6	146859181	146863081	1	havana	2.617487526	2.438209308	2.796765743	2.348706028	2.357615216	2.608306682	2.587917939	2.631684122	3.170695167
ENSG00000132300	0.012369	0.053612664	-0.223305392	-1.167405194	**PTCD3**	protein_coding	pentatricopeptide repeat domain 3 [Source:HGNC Symbol;Acc:HGNC:24717]	chr2	86106182	86142157	1	ensembl_havana	7.046149042	7.157801738	6.934496346	7.169608857	7.148667237	7.155129121	6.899691082	6.958322745	6.94547521
ENSG00000132849	0.012373	0.053614224	-0.241303357	-1.182060074	**PATJ**	protein_coding	PATJ, crumbs cell polarity complex component [Source:HGNC Symbol;Acc:HGNC:28881]	chr1	61742406	62178675	1	ensembl_havana	3.160021645	3.280673324	3.039369967	3.257093797	3.314364738	3.270561436	3.067955511	2.993629619	3.05652477
ENSG00000136153	0.012378	0.053617715	-0.282294963	-1.216127898	**LMO7**	protein_coding	LIM domain 7 [Source:HGNC Symbol;Acc:HGNC:6646]	chr13	75620434	75859870	1	ensembl_havana	3.1188436	3.259991081	2.977696118	2.86829497	3.300851749	3.610826524	2.711879034	2.989113925	3.232095396
ENSG00000236871	0.012406	0.053722761	0.252996875	1.191679993	**LINC00106**	lincRNA	long intergenic non-protein coding RNA 106 [Source:HGNC Symbol;Acc:HGNC:31843]	chrX	1396427	1399402	1	havana	3.647689895	3.521191457	3.774188332	3.770935591	3.276895115	3.515743667	3.939661103	3.614239733	3.76866416
ENSG00000198208	0.012409	0.053722761	-0.278447967	-1.212889371	**RPS6KL1**	protein_coding	ribosomal protein S6 kinase like 1 [Source:HGNC Symbol;Acc:HGNC:20222]	chr14	74903954	74923396	-1	ensembl_havana	3.542931869	3.682155852	3.403707885	3.840822574	3.608414274	3.597230708	3.648411535	3.368684571	3.194027549
ENSG00000123388	0.012417	0.053741226	-0.494055587	-1.408398491	**HOXC11**	protein_coding	homeobox C11 [Source:HGNC Symbol;Acc:HGNC:5123]	chr12	53973126	53977643	1	ensembl_havana	1.480519743	1.727547536	1.23349195	1.85322802	1.646767026	1.682647563	1.161063346	1.465989718	1.073422785
ENSG00000070476	0.012427	0.053768695	0.25756432	1.195458725	**ZXDC**	protein_coding	ZXD family zinc finger C [Source:HGNC Symbol;Acc:HGNC:28160]	chr3	126437601	126475919	-1	ensembl_havana	5.926294911	5.797512751	6.055077071	5.597154045	5.734908975	6.060475232	5.914252827	6.03589575	6.215082635
ENSG00000131165	0.012473	0.053943484	0.241056272	1.181857644	**CHMP1A**	protein_coding	charged multivesicular body protein 1A [Source:HGNC Symbol;Acc:HGNC:8740]	chr16	89644431	89657845	-1	ensembl_havana	6.441426809	6.320898673	6.561954945	6.452122203	6.233810572	6.276763244	6.645163986	6.555552388	6.485148461
ENSG00000140526	0.012475	0.053943484	0.222566641	1.166807562	**ABHD2**	protein_coding	abhydrolase domain containing 2 [Source:HGNC Symbol;Acc:HGNC:18717]	chr15	89087459	89202360	1	ensembl_havana	6.85374329	6.742459969	6.965026611	6.732012875	6.769334379	6.726032654	6.908139494	7.023532619	6.96340772
ENSG00000229124	0.01249	0.053975451	0.453404276	1.369267459	**VIM-AS1**	antisense	VIM antisense RNA 1 [Source:HGNC Symbol;Acc:HGNC:44879]	chr10	17214239	17229985	-1	havana	1.11869483	0.891992693	1.345396968	0.872903038	0.945375194	0.857699845	1.052687018	1.465989718	1.517514168
ENSG00000166780	0.012487	0.053975451	0.245558716	1.185551814	**C16orf45**	protein_coding	chromosome 16 open reading frame 45 [Source:HGNC Symbol;Acc:HGNC:19213]	chr16	15434295	15625028	1	ensembl_havana	4.20819278	4.085413422	4.330972138	4.051990026	4.16563457	4.038615671	4.383627157	4.352981311	4.256307946
ENSG00000171953	0.012502	0.05398958	0.397086392	1.316845776	**ATPAF2**	protein_coding	ATP synthase mitochondrial F1 complex assembly factor 2 [Source:HGNC Symbol;Acc:HGNC:18802]	chr17	17977409	18039209	-1	ensembl_havana	3.146841198	2.948298002	3.345384394	2.945520426	2.887010108	3.012363471	3.558795999	3.264170155	3.213187029

ENSG00000145022	0.012502	0.05398958	0.292585599	1.224833459	**TCTA**	protein_coding	T cell leukemia translocation altered [Source:HGNC Symbol;Acc:HGNC:11692]	chr3	49412206	49416475	1	ensembl_havana	5.623868512	5.477575712	5.770161312	5.315599317	5.570786374	5.546341445	5.746093808	5.828474967	5.735915159
ENSG00000100578	0.012505	0.05398958	-0.304853562	-1.235293245	**KIAA0586**	protein_coding	KIAA0586 [Source:HGNC Symbol;Acc:HGNC:19960]	chr14	58427385	58551289	1	ensembl_havana	5.346294405	5.498721186	5.193867624	5.536045381	5.463441148	5.496677028	5.076368179	5.220576751	5.284657942
ENSG00000166896	0.012527	0.054070471	-0.303825454	-1.234413251	**ATP23**	protein_coding	ATP23 metallopeptidase and ATP synthase assembly factor homolog [Source:HGNC Symbol;Acc:HGNC:29452]	chr12	57941541	57957269	1	ensembl_havana	2.565659378	2.717572105	2.413746651	2.705773822	2.608571783	2.83837071	2.501227084	2.358164528	2.381848339
ENSG00000063322	0.012535	0.054085232	0.2386472	1.179885776	**MED29**	protein_coding	mediator complex subunit 29 [Source:HGNC Symbol;Acc:HGNC:23074]	chr19	39391303	39400637	1	ensembl_havana	5.76954952	5.65022592	5.88887312	5.636986049	5.60555083	5.708140881	5.859008424	5.918500996	5.889109942
ENSG00000100211	0.01254	0.054091379	-0.290565429	-1.223119556	**CBY1**	protein_coding	chibby family member 1, beta catenin antagonist [Source:HGNC Symbol;Acc:HGNC:1307]	chr22	38656636	38673854	1	ensembl_havana	4.06718463	4.212467345	3.921901916	4.163267561	4.195151489	4.278982986	3.839927791	4.040242964	3.885534993
ENSG00000268006	0.012554	0.05413803	0.418961119	1.336964463	**PTOV1-AS1**	antisense	PTOV1 antisense RNA 1 [Source:HGNC Symbol;Acc:HGNC:44174]	chr19	49838639	49851676	-1	havana	1.790941055	1.581460496	2.000421614	1.528734855	1.719668303	1.495978329	2.072167195	1.881076089	2.04802156
ENSG00000078668	0.012567	0.054176543	-0.242127305	-1.182735362	**VDAC3**	protein_coding	voltage dependent anion channel 3 [Source:HGNC Symbol;Acc:HGNC:12674]	chr8	42391624	42405897	1	ensembl_havana	7.471255708	7.592319361	7.350192055	7.597540869	7.632405406	7.547011808	7.279687836	7.45864845	7.312239881
ENSG00000131471	0.012576	0.054183303	0.214121038	1.159996971	**AOC3**	protein_coding	amine oxidase, copper containing 3 [Source:HGNC Symbol;Acc:HGNC:550]	chr17	42851184	42858130	1	ensembl_havana	3.328546157	3.221485638	3.435606676	3.230339436	3.266504942	3.167612535	3.441917197	3.462883238	3.402019592
ENSG00000222035	0.012573	0.054183303	-0.569221892	-1.483723119	**AC079354.1**	antisense		chr2	202113626	202117058	-1	havana	0.140029991	0.424640937	-0.144580955	-0.03191641	0.325758945	0.980080273	-0.82634296	0.130707766	0.261892331
ENSG00000278389	0.012584	0.054202259	-0.486636165	-1.401174041	**AC099518.6**	lincRNA		chr16	19111035	19111484	-1	havana	2.433222119	2.676540202	2.189904037	3.088577264	2.646460224	2.294583118	2.356771314	2.114588126	2.09835267
ENSG00000105856	0.012593	0.054223984	0.309109325	1.238942579	**HBP1**	protein_coding	HMG-box transcription factor 1 [Source:HGNC Symbol;Acc:HGNC:23200]	chr7	107168961	107202529	1	ensembl_havana	5.321357676	5.166803014	5.475912338	5.522719493	4.985140188	4.99254936	5.681597922	5.32011339	5.426025703
ENSG00000093000	0.012606	0.054245314	-0.22630617	-1.169835901	**NUP50**	protein_coding	nucleoporin 50 [Source:HGNC Symbol;Acc:HGNC:8065]	chr22	45163841	45188015	1	ensembl_havana	6.890361861	7.003514946	6.777208776	7.016403136	7.024679735	6.969461969	6.733383384	6.821151671	6.776731275
ENSG00000258072	0.012611	0.054245314	-0.296924364	-1.228522571	**AC126177.7**	processed_pseudogene		chr12	107903270	107903948	-1	havana	0.252030053	0.400492235	0.103567871	0.490295136	0.298820819	0.41236075	0.24405081	0.097529431	-0.030876629
ENSG00000188428	0.012605	0.054245314	-0.312315219	-1.241698765	**BLOC1S5**	protein_coding	biogenesis of lysosomal organelles complex 1 subunit 5 [Source:HGNC Symbol;Acc:HGNC:18561]	chr6	8013567	8064414	-1	ensembl_havana	4.1493685	4.30552611	3.99321089	4.163267561	4.535204905	4.218105863	3.996838595	4.085365442	3.897428633
ENSG00000174007	0.012613	0.054245314	-0.327489942	-1.254828274	**CEP19**	protein_coding	centrosomal protein 19 [Source:HGNC Symbol;Acc:HGNC:28209]	chr3	196706277	196712293	-1	ensembl_havana	3.537098704	3.700843675	3.373353734	3.538330443	3.664886261	3.899314321	3.349502035	3.365203876	3.405355289
ENSG00000137504	0.012623	0.054269985	0.213566815	1.159551434	**CREBZF**	protein_coding	CREB/ATF bZIP transcription factor [Source:HGNC Symbol;Acc:HGNC:24905]	chr11	85659708	85682908	-1	ensembl_havana	6.304312115	6.197528708	6.411095522	6.262367374	6.175769548	6.154449202	6.45600857	6.403081976	6.374196021
ENSG00000253388	0.012642	0.054287561	0.429967778	1.347203487	**AC124067.1**	lincRNA		chr8	37480089	37480809	1	havana	0.246803785	0.031819896	0.461787673	-0.22998119	0.185737694	0.139703184	0.069637898	0.478736765	0.836988357
ENSG00000104442	0.012641	0.054287561	-0.26062737	-1.197999553	**ARMC1**	protein_coding	armadillo repeat containing 1 [Source:HGNC Symbol;Acc:HGNC:17684]	chr8	65602456	65634217	-1	ensembl_havana	5.620891502	5.751205187	5.490577817	5.736765332	5.786118434	5.730731794	5.427769736	5.465360412	5.578603302
ENSG00000186532	0.012638	0.054287561	-0.27398621	-1.209144118	**SMYD4**	protein_coding	SET and MYND domain containing 4 [Source:HGNC Symbol;Acc:HGNC:21067]	chr17	1779485	1830634	-1	ensembl_havana	4.348414734	4.485407839	4.211421629	4.406302045	4.39915155	4.650769923	4.044780323	4.245388041	4.344096524
ENSG00000245498	0.012638	0.054287561	-0.328813216	-1.255979761	**AP000866.1**	antisense		chr11	124800450	124834487	1	havana	1.88341209	2.047818698	1.719005482	2.049654613	2.191810142	1.901991338	1.874103935	1.705026699	1.577885811
ENSG00000112531	0.012665	0.054371036	0.239722835	1.180765795	**QKI**	protein_coding	QKI, KH domain containing RNA binding [Source:HGNC Symbol;Acc:HGNC:21100]	chr6	163414000	163578596	1	ensembl_havana	6.202018473	6.082157056	6.321879891	6.063982445	6.021093611	6.161395112	6.367822766	6.271210315	6.326606591
ENSG00000162972	0.012669	0.054371861	-0.239564088	-1.180635877	**MAIP1**	protein_coding	matrix AAA peptidase interacting protein 1 [Source:HGNC Symbol;Acc:HGNC:26198]	chr2	199955317	200008540	1	ensembl_havana	3.368360392	3.488142436	3.248578347	3.569159951	3.503197858	3.392069497	3.375980565	3.183462537	3.186291941
ENSG00000185053	0.012684	0.054401423	0.284991477	1.218403066	**SGCZ**	protein_coding	sarcoglycan zeta [Source:HGNC Symbol;Acc:HGNC:14075]	chr8	14089864	15238339	-1	havana	2.200074363	2.057578624	2.342570101	2.52259249	2.08549033	1.564653053	2.879883083	2.233896951	1.91393027
ENSG00000100532	0.01268	0.054401423	0.225996553	1.16958487	**CGRRF1**	protein_coding	cell growth regulator with ring finger domain 1 [Source:HGNC Symbol;Acc:HGNC:15528]	chr14	54509812	54539309	1	ensembl_havana	3.997893371	3.884895094	4.110891647	3.892931069	3.944501979	3.817252234	4.068166701	4.21863478	4.04587346
ENSG00000041357	0.012694	0.05443013	-0.254011566	-1.192518433	**PSMA4**	protein_coding	proteasome subunit alpha 4 [Source:HGNC Symbol;Acc:HGNC:9533]	chr15	78540405	78552419	1	ensembl_havana	8.154195559	8.281201342	8.027189775	8.233796883	8.373171965	8.236635177	7.94730643	8.052769402	8.081493495
ENSG00000139970	0.012702	0.054433068	0.665193737	1.585781211	**RTN1**	protein_coding	reticulon 1 [Source:HGNC Symbol;Acc:HGNC:10467]	chr14	59595976	59870966	-1	ensembl_havana	2.296642839	1.964045971	2.629239708	1.327057633	2.191810142	2.373270137	2.438005243	2.69405369	2.755660191
ENSG00000169021	0.012699	0.054433068	-0.269541902	-1.205425009	**UQCRFS1**	protein_coding	ubiquinol-cytochrome c reductase, Rieske iron-sulfur polypeptide 1 [Source:HGNC Symbol;Acc:HGNC:12587]	chr19	29205321	29213541	-1	ensembl_havana	6.014115553	6.148886503	5.879344602	6.190821554	6.202813216	6.05302474	5.882284543	5.854774995	5.900974267
ENSG00000161082	0.012707	0.054435022	1.005927801	2.008234582	**CELF5**	protein_coding	CUGBP Elav-like family member 5 [Source:HGNC Symbol;Acc:HGNC:14058]	chr19	3224703	3297076	1	ensembl_havana	-0.354464071	-0.857427972	0.148499829	-0.92573229	-0.903502039	-0.743049581	0.618191201	0.225896484	-0.398588198
ENSG00000049130	0.012715	0.05445385	0.355964253	1.279840701	**KITLG**	protein_coding	KIT ligand [Source:HGNC Symbol;Acc:HGNC:6343]	chr12	88492793	88580851	-1	ensembl_havana	6.555980368	6.377998242	6.733962494	6.382662477	6.487849758	6.26348249	6.900016937	6.65368795	6.648182596
ENSG00000184441	0.012758	0.054608297	0.642282356	1.560796401	**AP001062.1**	antisense		chr21	44331234	44335851	1	havana	1.828933657	1.507792479	2.150074835	1.283104003	1.338282095	1.901991338	2.196225281	2.139252294	2.114746929
ENSG00000164109	0.012755	0.054608297	-0.226437909	-1.169942729	**MAD2L1**	protein_coding	mitotic arrest deficient 2 like 1 [Source:HGNC Symbol;Acc:HGNC:6763]	chr4	120055608	120067074	-1	ensembl_havana	7.235987652	7.349206607	7.122768697	7.160527303	7.447974492	7.439118024	6.886596144	7.215280908	7.26642904
ENSG00000101224	0.012763	0.054609403	-0.247725417	-1.187333664	**CDC25B**	protein_coding	cell division cycle 25B [Source:HGNC Symbol;Acc:HGNC:1726]	chr20	3786772	3806121	1	ensembl_havana	6.112364351	6.23622706	5.988501643	6.163302474	6.305763976	6.239614729	6.007930374	6.002677782	5.954896772
ENSG00000198380	0.012766	0.054609403	-0.266430939	-1.202828485	**GFPT1**	protein_coding	glutamine--fructose-6-phosphate transaminase 1 [Source:HGNC Symbol;Acc:HGNC:4241]	chr2	69319769	69387254	-1	ensembl_havana	8.600482019	8.733697489	8.46726655	8.868329939	8.7151153	8.617647226	8.485650692	8.49980512	8.416343837
ENSG00000173653	0.012776	0.054634503	0.309122692	1.238954058	**RCE1**	protein_coding	Ras converting CAAX endopeptidase 1 [Source:HGNC Symbol;Acc:HGNC:13721]	chr11	66842835	66846546	1	ensembl_havana	3.487085809	3.332524463	3.641647155	3.348787899	3.402409479	3.246376011	3.718075281	3.551306511	3.655559672
ENSG00000114650	0.012794	0.054694572	0.249170805	1.18852381	**SCAP**	protein_coding	SREBF chaperone [Source:HGNC Symbol;Acc:HGNC:30634]	chr3	47413694	47477126	-1	ensembl_havana	6.534633422	6.41004802	6.659218825	6.352851647	6.352359482	6.52493293	6.682390365	6.605454435	6.689811674
ENSG00000089177	0.012813	0.05474408	0.279763426	1.213995797	**KIF16B**	protein_coding	kinesin family member 16B [Source:HGNC Symbol;Acc:HGNC:15869]	chr20	16272104	16573434	-1	ensembl_havana	4.874137102	4.734255389	5.014018816	4.854681818	4.701268571	4.646815778	5.102653563	4.886070184	5.0533327
ENSG00000167985	0.01281	0.05474408	-0.215840815	-1.16138058	**SDHAF2**	protein_coding	succinate dehydrogenase complex assembly factor 2 [Source:HGNC Symbol;Acc:HGNC:26034]	chr11	61430042	61447529	1	ensembl_havana	2.220342928	2.328263335	2.11242252	2.334420173	2.389883326	2.260486508	2.144358071	2.179444752	2.013464737
ENSG00000228037	0.012828	0.054792539	-0.400723858	-1.320170127	**AL139246.3**	lincRNA		chr1	2581560	2584533	1	havana	1.264098924	1.464460853	1.063736995	1.327057633	1.524202165	1.542122761	0.692307165	1.179325527	1.319578292
ENSG00000212907	0.012834	0.054802953	0.258563608	1.196287051	**MT-ND4L**	protein_coding	mitochondrially encoded NADH:ubiquinone oxidoreductase core subunit 4L [Source:HGNC Symbol;Acc:HGNC:7460]	chrMT	10470	10766	1	insdc	9.847118402	9.717836598	9.976400206	9.932275945	9.485686453	9.735547395	10.09540945	9.749043413	10.08474775
ENSG00000178878	0.012844	0.054827649	-0.267889695	-1.204045319	**APOLD1**	protein_coding	apolipoprotein L domain containing 1 [Source:HGNC Symbol;Acc:HGNC:25268]	chr12	12725917	12829975	1	ensembl_havana	4.785363903	4.919308751	4.651419056	4.669343006	4.996710779	5.091872468	4.293237984	4.825302309	4.835716874
ENSG00000185046	0.012851	0.054830201	-0.35696445	-1.280728302	**ANKS1B**	protein_coding	ankyrin repeat and sterile alpha motif domain containing 1B [Source:HGNC Symbol;Acc:HGNC:24600]	chr12	98726457	99984654	-1	ensembl_havana	1.562531482	1.741013708	1.384049257	1.882980075	1.699212533	1.640848515	1.371200905	1.300906777	1.480040089
ENSG00000232233	0.012852	0.054830201	-0.510247935	-1.424294948	**LINC02043**	lincRNA	long intergenic non-protein coding RNA 2043 [Source:HGNC Symbol;Acc:HGNC:52883]	chr3	186810880	186825521	-1	havana	0.891071965	1.146195933	0.635947998	0.749126884	1.124807863	1.564653053	0.56657698	0.504278656	0.836988357
ENSG00000113649	0.012856	0.054831754	-0.232462624	-1.174838642	**TCERG1**	protein_coding	transcription elongation regulator 1 [Source:HGNC Symbol;Acc:HGNC:15630]	chr5	146447311	146511961	1	ensembl_havana	7.274695659	7.390926971	7.158464347	7.43465461	7.394506733	7.343619571	7.135756769	7.174348801	7.165287472
ENSG00000079841	0.012883	0.054930253	0.443375954	1.359782544	**RIMS1**	protein_coding	regulating synaptic membrane exocytosis 1 [Source:HGNC Symbol;Acc:HGNC:17282]	chr6	71886703	72403143	1	ensembl_havana	2.948937416	2.727249439	3.170625393	2.376860232	2.961901777	2.842986308	3.100076994	3.252913259	3.158885927
ENSG00000130396	0.012888	0.054932191	0.250955431	1.189994934	**AFDN**	protein_coding	afadin, adherens junction formation factor [Source:HGNC Symbol;Acc:HGNC:7137]	chr6	167826922	167972023	1	ensembl_havana	6.853965535	6.72848782	6.979443251	6.620531297	6.70506546	6.859866703	6.969041697	6.917020437	7.052267618
ENSG00000166436	0.012899	0.054965545	-0.266847256	-1.203175634	**TRIM66**	protein_coding	tripartite motif containing 66 [Source:HGNC Symbol;Acc:HGNC:29005]	chr11	8612037	8682694	-1	ensembl_havana	4.721531763	4.85495539	4.588108135	4.865899597	4.717887347	4.981079227	4.647000501	4.520368735	4.596955169
ENSG00000183397	0.012945	0.05503123	0.608724952	1.524910903	**C19orf71**	protein_coding	chromosome 19 open reading frame 71 [Source:HGNC Symbol;Acc:HGNC:34496]	chr19	3539154	3544030	1	ensembl_havana	0.852375841	0.548013366	1.156738317	0.589870393	0.477608744	0.57656096	1.606623359	0.841392961	1.022198632
ENSG00000105738	0.012931	0.05503123	0.415114262	1.333404279	**SIPA1L3**	protein_coding	signal induced proliferation associated 1 like 3 [Source:HGNC Symbol;Acc:HGNC:23801]	chr19	37907228	38208372	1	ensembl_havana	3.401080338	3.193523207	3.60863747	3.163221009	2.957602376	3.459746237	3.623370039	3.569569297	3.632973073
ENSG00000115904	0.012931	0.05503123	0.235231578	1.177095667	**SOS1**	protein_coding	SOS Ras/Rac guanine nucleotide exchange factor 1 [Source:HGNC Symbol;Acc:HGNC:11187]	chr2	38981396	39124345	-1	ensembl_havana	7.781562573	7.663946784	7.899178362	7.616851419	7.64849874	7.726490192	7.828223704	7.958036117	7.911275265
ENSG00000167130	0.012937	0.05503123	0.233832505	1.175954715	**DOLPP1**	protein_coding	dolichyldiphosphatase 1 [Source:HGNC Symbol;Acc:HGNC:29565]	chr9	129081100	129090438	1	ensembl_havana	4.753720324	4.636804071	4.870636576	4.585874231	4.689650085	4.634887898	4.882884061	4.859102792	4.869922876
ENSG00000164300	0.012943	0.05503123	0.217964917	1.163091758	**SERINC5**	protein_coding	serine incorporator 5 [Source:HGNC Symbol;Acc:HGNC:18825]	chr5	80111651	80256079	-1	ensembl_havana	3.543169795	3.434187336	3.652152254	3.3380874	3.538169938	3.42630467	3.548855684	3.724296517	3.68330456
ENSG00000166197	0.012937	0.05503123	-0.221248755	-1.165742183	**NOLC1**	protein_coding	nucleolar and coiled-body phosphoprotein 1 [Source:HGNC Symbol;Acc:HGNC:15608]	chr10	102152176	102163871	1	ensembl_havana	7.024967488	7.135591866	6.914343111	7.173376029	7.108510492	7.124889075	6.909111134	6.887297764	6.946620433
ENSG00000122483	0.012944	0.05503123	-0.390805996	-1.311125691	**CCDC18**	protein_coding	coiled-coil domain containing 18 [Source:HGNC Symbol;Acc:HGNC:30370]	chr1	93179919	93278730	1	ensembl_havana	3.750053908	3.945456907	3.55465091	3.807595594	4.026836504	4.001938621	3.188097919	3.696867244	3.778987568
ENSG00000184719	0.012931	0.05503123	-0.532577116	-1.446510821	**RNLS**	protein_coding	renalase, FAD dependent amine oxidase [Source:HGNC Symbol;Acc:HGNC:25641]	chr10	88273864	88584530	-1	ensembl_havana	3.197976676	3.464265234	2.931688118	3.773585281	3.500244888	3.118965531	2.952056343	2.938485423	2.904522587
ENSG00000162517	0.012952	0.055044241	0.225978519	1.169570249	**PEF1**	protein_coding	penta-EF-hand domain containing 1 [Source:HGNC Symbol;Acc:HGNC:30009]	chr1	31629862	31644896	-1	ensembl_havana	6.222235325	6.109246065	6.335224584	6.094502908	6.176232164	6.057003124	6.351236663	6.34241892	6.312018169
ENSG00000011523	0.012963	0.055072393	0.395861323	1.315728047	**CEP68**	protein_coding	centrosomal protein 68 [Source:HGNC Symbol;Acc:HGNC:29076]	chr2	65056366	65087004	1	ensembl_havana	5.136547971	4.938617309	5.334478632	4.659348809	4.994613926	5.161889192	5.257156287	5.403923997	5.342355613
ENSG00000255920	0.012968	0.055079494	0.652834352	1.57225405	**CCND2-AS1**	processed_transcript	CCND2 antisense RNA 1 [Source:HGNC Symbol;Acc:HGNC:49398]	chr12	4248765	4276184	-1	havana	-0.356107762	-0.682524938	-0.029690586	-1.070289	-0.620350959	-0.356934859	-0.75894329	-0.04338487	0.713256405
ENSG00000106484	0.012997	0.055184622	-1.586256033	-3.002691031	**MEST**	protein_coding	mesoderm specific transcript [Source:HGNC Symbol;Acc:HGNC:7028]	chr7	130486171	130506465	1	ensembl_havana	-1.249638256	-0.45651024	-2.042766273	0.10902545	-0.620350959	-0.85820521	-1.7649502	-3.01064834	-1.352700279
ENSG00000271327	0.013007	0.055195208	-0.253153273	-1.191809186	**AC010201.2**	lincRNA		chr12	89367807	89369301	1	havana	0.09840164	0.224978277	-0.028174996	0.41082706	0.09469996	0.16940781	0.069637898	-0.15890847	0.004745582
ENSG00000189057	0.013007	0.055195208	-0.671662973	-1.592908031	**FAM111B**	protein_coding	family with sequence similarity 111 member B [Source:HGNC Symbol;Acc:HGNC:24200]	chr11	59107185	59127410	1	ensembl_havana	4.090360349	4.426191835	3.754528863	3.572206975	4.837362047	4.869006484	3.341846614	4.055441139	3.866298836
ENSG00000118420	0.013042	0.055316483	0.248645951	1.188091502	**UBE3D**	protein_coding	ubiquitin protein ligase E3D [Source:HGNC Symbol;Acc:HGNC:21381]	chr6	82892398	83065841	-1	ensembl_havana	3.07682323	2.952500255	3.201146205	2.791937877	2.961901777	3.10366111	3.135932331	3.151488815	3.31601747
ENSG00000123600	0.013043	0.055316483	-0.269801973	-1.205642328	**METTL8**	protein_coding	methyltransferase like 8 [Source:HGNC Symbol;Acc:HGNC:25856]	chr2	171317405	171434802	-1	ensembl_havana	4.051814063	4.186715049	3.916913076	4.009911563	4.309215282	4.241018303	3.806944749	3.917286289	4.026508191
ENSG00000160691	0.013057	0.055356803	-0.268118079	-1.20423594	**SHC1**	protein_coding	SHC adaptor protein 1 [Source:HGNC Symbol;Acc:HGNC:10840]	chr1	154962298	154974395	-1	ensembl_havana	5.820566934	5.954625974	5.686507895	6.214416596	5.854195071	5.795266255	5.826463658	5.635377079	5.597682946
ENSG00000133706	0.013064	0.055370731	-0.225817456	-1.169439685	**LARS**	protein_coding	leucyl-tRNA synthetase [Source:HGNC Symbol;Acc:HGNC:6512]	chr5	146113038	146182660	-1	ensembl_havana	8.50115966	8.614068388	8.388250933	8.581093549	8.577246063	8.683865553	8.296999721	8.366343227	8.50140985
ENSG00000158042	0.013087	0.055450996	-0.27577515	-1.210644386	**MRPL17**	protein_coding	mitochondrial ribosomal protein L17 [Source:HGNC Symbol;Acc:HGNC:14053]	chr11	6680782	6683401	-1	ensembl_havana	4.271237388	4.409124964	4.133349813	4.343488656	4.352430021	4.531456215	3.939661103	4.125009009	4.335379327
ENSG00000141499	0.01311	0.055535376	0.23373436	1.17587472	**WRAP53**	protein_coding	WD repeat containing antisense to TP53 [Source:HGNC Symbol;Acc:HGNC:25522]	chr17	7686071	7703502	1	ensembl_havana	4.773034362	4.656167182	4.889901542	4.538366337	4.746828853	4.683306356	4.846828461	4.938538256	4.88433791
ENSG00000234072	0.013115	0.055538706	-0.219197198	-1.16408564	**AC074117.1**	antisense		chr2	27356246	27367622	1	havana	4.300100667	4.409699267	4.190502068	4.484396486	4.39915155	4.345549764	4.236567839	4.226329326	4.10860904
ENSG00000164659	0.013129	0.055553672	0.519938271	1.433893895	**KIAA1324L**	protein_coding	KIAA1324 like [Source:HGNC Symbol;Acc:HGNC:21945]	chr7	86876906	87059699	-1	ensembl_havana	3.142795938	2.882826803	3.402765074	2.341580783	3.187934094	3.118965531	3.205075544	3.535908912	3.467310767
ENSG00000214029	0.01313	0.055553672	0.27311942	1.208417867	**ZNF891**	protein_coding	zinc finger protein 891 [Source:HGNC Symbol;Acc:HGNC:38709]	chr12	133106817	133130473	-1	ensembl_havana	3.3884481	3.25188839	3.52500781	3.253301988	3.187934094	3.314429088	3.41298341	3.566541508	3.595498512
ENSG00000138160	0.013128	0.055553672	-0.21488366	-1.160610318	**KIF11**	protein_coding	kinesin family member 11 [Source:HGNC Symbol;Acc:HGNC:6388]	chr10	92593286	92655395	1	ensembl_havana	6.890160593	6.997602423	6.782718763	6.894198179	7.035428866	7.063180224	6.650596543	6.852295459	6.845264287
ENSG00000116127	0.013155	0.055643174	0.300749491	1.231784168	**ALMS1**	protein_coding	ALMS1, centrosome and basal body associated protein [Source:HGNC Symbol;Acc:HGNC:428]	chr2	73385758	73610793	1	ensembl_havana	5.907623397	5.757248652	6.057998142	5.72996008	5.581303953	5.960481922	5.954968134	6.038083322	6.180942971
ENSG00000090104	0.013163	0.055658129	0.410179642	1.32885127	**RGS1**	protein_coding	regulator of G protein signaling 1 [Source:HGNC Symbol;Acc:HGNC:9991]	chr1	192575727	192580031	1	ensembl_havana	7.305514906	7.100425085	7.510604727	7.528235093	7.089479417	6.683560744	7.720912121	7.49334103	7.317561029
ENSG00000157259	0.013171	0.055661726	-0.22296283	-1.167128031	**GATAD1**	protein_coding	GATA zinc finger domain containing 1 [Source:HGNC Symbol;Acc:HGNC:29941]	chr7	92447448	92459432	1	ensembl_havana	5.768082612	5.879564027	5.656601197	5.808286499	5.974509777	5.855895805	5.557369713	5.723647762	5.688786115
ENSG00000159176	0.013171	0.055661726	-0.264224436	-1.200990248	**CSRP1**	protein_coding	cysteine and glycine rich protein 1 [Source:HGNC Symbol;Acc:HGNC:2469]	chr1	201483530	201509456	-1	ensembl_havana	7.49653326	7.628645477	7.364421042	7.59548321	7.656478583	7.633974639	7.446913786	7.347723043	7.298626295
ENSG00000169299	0.013176	0.055667496	0.215245189	1.160901195	**PGM2**	protein_coding	phosphoglucomutase 2 [Source:HGNC Symbol;Acc:HGNC:8906]	chr4	37826633	37862937	1	ensembl_havana	5.899855852	5.792233258	6.007478447	5.714874564	5.804788063	5.857037146	5.903241856	6.011610958	6.107582527
ENSG00000105376	0.013201	0.055702335	0.343409055	1.268751084	**ICAM5**	protein_coding	intercellular adhesion molecule 5 [Source:HGNC Symbol;Acc:HGNC:5348]	chr19	10289981	10296778	1	ensembl_havana	5.037255261	4.865550734	5.208959789	5.216841153	4.511936473	4.867874577	5.488834672	5.050047881	5.087996813
ENSG00000010322	0.013204	0.055702335	0.273000304	1.208318098	**NISCH**	protein_coding	nischarin [Source:HGNC Symbol;Acc:HGNC:18006]	chr3	52455118	52493071	1	ensembl_havana	6.775789992	6.63928984	6.912290144	6.592668253	6.52936049	6.795840778	6.935101088	6.859735268	6.942034077
ENSG00000163249	0.013198	0.055702335	0.235932509	1.177667696	**CCNYL1**	protein_coding	cyclin Y like 1 [Source:HGNC Symbol;Acc:HGNC:26868]	chr2	207711540	207761839	1	ensembl_havana	4.119804114	4.00183786	4.237770369	3.959604644	4.075263288	3.970645647	4.184017004	4.249169756	4.280124347
ENSG00000139178	0.013196	0.055702335	-0.249501703	-1.188796442	**C1RL**	protein_coding	complement C1r subcomponent like [Source:HGNC Symbol;Acc:HGNC:21265]	chr12	7089587	7109273	-1	ensembl_havana	5.767995245	5.892746096	5.643244393	5.894799777	5.947663743	5.835774768	5.707892178	5.601016608	5.620824393
ENSG00000143443	0.013191	0.055702335	-0.906320943	-1.874259797	**C1orf56**	protein_coding	chromosome 1 open reading frame 56 [Source:HGNC Symbol;Acc:HGNC:26045]	chr1	151047740	151051986	1	ensembl_havana	0.866262197	1.319422668	0.413101725	1.005046425	1.270574017	1.682647563	0.428834256	0.671430284	0.139040636
ENSG00000106688	0.013214	0.055729128	0.96236451	1.948500781	**SLC1A1**	protein_coding	solute carrier family 1 member 1 [Source:HGNC Symbol;Acc:HGNC:10939]	chr9	4490444	4587469	1	ensembl_havana	1.316963326	0.835781071	1.798145581	-0.36334239	1.351450576	1.519235028	1.278000209	1.993561814	2.122874721
ENSG00000122042	0.013222	0.055729972	0.20988198	1.156593564	**UBL3**	protein_coding	ubiquitin like 3 [Source:HGNC Symbol;Acc:HGNC:12504]	chr13	29764371	29850684	-1	ensembl_havana	4.420806823	4.315865833	4.525747813	4.539923521	4.383211018	4.02446296	4.75901983	4.578637321	4.239586287
ENSG00000162639	0.01322	0.055729972	-0.38306364	-1.30410826	**HENMT1**	protein_coding	HEN methyltransferase 1 [Source:HGNC Symbol;Acc:HGNC:26400]	chr1	108648290	108661526	-1	ensembl_havana	5.092893425	5.284425245	4.901361605	5.311958391	5.247988455	5.293328889	4.701856121	4.936194287	5.066034408
ENSG00000259144	0.013235	0.055768017	-0.846625985	-1.798290362	**RANBP20P**	processed_pseudogene	RAN binding protein 20 pseudogene [Source:HGNC Symbol;Acc:HGNC:19734]	chr14	20720742	20723127	-1	havana	1.01012534	1.433438333	0.586812348	1.683274163	1.109387295	1.507653541	0.24405081	0.529376201	0.987010032
ENSG00000151208	0.013243	0.055785577	-0.36928763	-1.291714854	**DLG5**	protein_coding	discs large MAGUK scaffold protein 5 [Source:HGNC Symbol;Acc:HGNC:2904]	chr10	77790791	77926526	-1	ensembl_havana	6.518011428	6.702655243	6.333367613	6.623838987	6.605874554	6.878252187	6.419652125	6.280479233	6.29997148
ENSG00000111665	0.013248	0.0557891	-0.216864698	-1.162205106	**CDCA3**	protein_coding	cell division cycle associated 3 [Source:HGNC Symbol;Acc:HGNC:14624]	chr12	6844793	6852066	-1	ensembl_havana	6.08709456	6.195526909	5.978662211	5.992997105	6.227128441	6.36645518	5.765537345	6.051139549	6.119309738
ENSG00000120885	0.013261	0.055829341	-0.410084676	-1.328763801	**CLU**	protein_coding	clusterin [Source:HGNC Symbol;Acc:HGNC:2095]	chr8	27596917	27615031	-1	ensembl_havana	8.548833541	8.753855879	8.343771203	8.717471007	8.867404441	8.676692189	8.543319749	8.254380009	8.233613851
ENSG00000092203	0.013268	0.05584257	0.208951595	1.155847925	**TOX4**	protein_coding	TOX high mobility group box family member 4 [Source:HGNC Symbol;Acc:HGNC:20161]	chr14	21476597	21499175	1	ensembl_havana	6.476935761	6.372459964	6.581411559	6.448810398	6.404226481	6.264343012	6.655236795	6.620496077	6.468501804
ENSG00000139269	0.013281	0.055878574	-0.27628118	-1.211069097	**INHBE**	protein_coding	inhibin beta E subunit [Source:HGNC Symbol;Acc:HGNC:24029]	chr12	57452323	57459280	1	ensembl_havana	6.663015874	6.801156464	6.524875284	6.510867405	6.81490753	7.077694456	6.361211189	6.445726499	6.767688164
ENSG00000164120	0.013292	0.055907528	0.812315041	1.756027017	**HPGD**	protein_coding	15-hydroxyprostaglandin dehydrogenase [Source:HGNC Symbol;Acc:HGNC:5154]	chr4	174490177	174523154	-1	ensembl_havana	-0.395040919	-0.80119844	0.011116601	-1.61858537	-0.428075095	-0.356934859	-0.26243933	0.256279753	0.039509383
ENSG00000088538	0.013301	0.0559309	0.397780724	1.317479692	**DOCK3**	protein_coding	dedicator of cytokinesis 3 [Source:HGNC Symbol;Acc:HGNC:2989]	chr3	50675241	51384198	1	ensembl_havana	5.131411149	4.932520786	5.330301511	4.752281178	4.904792248	5.140488933	5.387435309	5.237809053	5.365660171
ENSG00000168411	0.01332	0.055996009	-0.227894573	-1.171124597	**RFWD3**	protein_coding	ring finger and WD repeat domain 3 [Source:HGNC Symbol;Acc:HGNC:25539]	chr16	74621394	74666881	-1	ensembl_havana	7.551955427	7.665902714	7.438008141	7.539954894	7.661774063	7.795979185	7.350304809	7.46211476	7.501604852
ENSG00000179094	0.013327	0.05600423	0.266466951	1.20285851	**PER1**	protein_coding	period circadian regulator 1 [Source:HGNC Symbol;Acc:HGNC:8845]	chr17	8140472	8156506	-1	ensembl_havana	4.58377344	4.450539965	4.717006916	4.744198658	4.248918908	4.358502328	5.050769426	4.593587896	4.506663426
ENSG00000081181	0.01333	0.05600423	-0.231890742	-1.174373031	**ARG2**	protein_coding	arginase 2 [Source:HGNC Symbol;Acc:HGNC:664]	chr14	67619798	67651720	1	ensembl_havana	2.599253103	2.715198475	2.483307732	2.643402293	2.749532556	2.752660576	2.341497201	2.572545352	2.535880644
ENSG00000085788	0.013341	0.056005408	0.234131379	1.176198356	**DDHD2**	protein_coding	DDHD domain containing 2 [Source:HGNC Symbol;Acc:HGNC:29106]	chr8	38225218	38275558	1	ensembl_havana	6.210370283	6.093304593	6.327435972	6.067765535	6.148209239	6.063939005	6.373465816	6.321018749	6.287823352
ENSG00000139618	0.013341	0.056005408	-0.250096815	-1.189286922	**BRCA2**	protein_coding	BRCA2, DNA repair associated [Source:HGNC Symbol;Acc:HGNC:1101]	chr13	32315474	32400266	1	ensembl_havana	6.159476171	6.284524579	6.034427764	6.632622597	6.254111551	5.966839588	6.282417168	6.0489717	5.771894425
ENSG00000158156	0.013342	0.056005408	-0.336731607	-1.262892297	**XKR8**	protein_coding	XK related 8 [Source:HGNC Symbol;Acc:HGNC:25508]	chr1	27959462	27968096	1	ensembl_havana	1.812220443	1.980586247	1.643854639	1.843172741	2.06964138	2.028944618	1.693402841	1.602328815	1.635832261
ENSG00000184486	0.013355	0.056025549	0.782780882	1.720443938	**POU3F2**	protein_coding	POU class 3 homeobox 2 [Source:HGNC Symbol;Acc:HGNC:9215]	chr6	98834592	98839470	1	ensembl_havana	1.421918204	1.030527764	1.813308645	0.297565234	1.298039728	1.495978329	1.606623359	1.947753749	1.885548828
ENSG00000138385	0.013351	0.056025549	-0.252787554	-1.191507104	**SSB**	protein_coding	Sjogren syndrome antigen B [Source:HGNC Symbol;Acc:HGNC:11316]	chr2	169791933	169812064	1	ensembl_havana	5.780043984	5.90643776	5.653650207	5.932040791	5.862267541	5.925004949	5.582761148	5.624499627	5.753689845
ENSG00000180113	0.013366	0.056053175	0.672303387	1.593615282	**TDRD6**	protein_coding	tudor domain containing 6 [Source:HGNC Symbol;Acc:HGNC:21339]	chr6	46687875	46704319	1	ensembl_havana	0.799196946	0.463045252	1.135348639	0.41082706	0.214850257	0.76345844	0.785553575	1.343981945	1.276510397
ENSG00000145545	0.013373	0.056053175	0.24052085	1.181419107	**SRD5A1**	protein_coding	steroid 5 alpha-reductase 1 [Source:HGNC Symbol;Acc:HGNC:11284]	chr5	6633343	6674386	1	ensembl_havana	4.887469888	4.767209463	5.007730313	4.719675806	4.817333415	4.764619169	5.047243228	5.020480842	4.955466869
ENSG00000246982	0.013372	0.056053175	-0.704741571	-1.629852684	**Z84485.1**	antisense		chr6	36146698	36197205	-1	havana	0.375112835	0.72748362	0.02274205	0.660286109	0.818355163	0.703809589	0.210814386	-0.37473441	0.232146171
ENSG00000143621	0.013386	0.056092418	-0.210988351	-1.157480871	**ILF2**	protein_coding	interleukin enhancer binding factor 2 [Source:HGNC Symbol;Acc:HGNC:6037]	chr1	153661788	153671048	-1	ensembl_havana	8.50404641	8.609540586	8.398552235	8.600344196	8.673777414	8.554500147	8.380987587	8.446657766	8.368011352
ENSG00000172794	0.013412	0.056182704	0.484338545	1.398944322	**RAB37**	protein_coding	RAB37, member RAS oncogene family [Source:HGNC Symbol;Acc:HGNC:30268]	chr17	74670578	74747335	1	ensembl_havana	0.770965978	0.528796706	1.01313525	0.637193984	0.185737694	0.76345844	1.245545533	0.861296647	0.932563571
ENSG00000104228	0.013427	0.056195494	0.316087855	1.244950047	**TRIM35**	protein_coding	tripartite motif containing 35 [Source:HGNC Symbol;Acc:HGNC:16285]	chr8	27284887	27311319	-1	ensembl_havana	5.208919133	5.050875205	5.366963061	4.991846665	5.118214983	5.042563968	5.470521786	5.403923997	5.2264434
ENSG00000223501	0.013426	0.056195494	-0.223983494	-1.167954032	**VPS52**	protein_coding	VPS52, GARP complex subunit [Source:HGNC Symbol;Acc:HGNC:10518]	chr6	33250272	33272047	-1	ensembl_havana	6.988721512	7.100713259	6.876729765	7.209538319	7.064457623	7.028143834	7.044083817	6.798468947	6.787636531
ENSG00000163689	0.013424	0.056195494	-0.275986913	-1.2108221	**C3orf67**	protein_coding	chromosome 3 open reading frame 67 [Source:HGNC Symbol;Acc:HGNC:24763]	chr3	58717365	59050084	-1	ensembl_havana	2.63138733	2.769380786	2.493393873	2.362851808	2.900511953	3.044778598	2.099663825	2.504564179	2.875953617
ENSG00000116584	0.013439	0.056229374	-0.208394804	-1.155401925	**ARHGEF2**	protein_coding	Rho/Rac guanine nucleotide exchange factor 2 [Source:HGNC Symbol;Acc:HGNC:682]	chr1	155946851	156007070	-1	ensembl_havana	6.568804669	6.673002071	6.464607267	6.888403575	6.591378653	6.539223984	6.674793035	6.392862088	6.326166678
ENSG00000104312	0.013471	0.056314244	0.297126165	1.228694426	**RIPK2**	protein_coding	receptor interacting serine/threonine kinase 2 [Source:HGNC Symbol;Acc:HGNC:10020]	chr8	89757747	89791063	1	ensembl_havana	4.941229085	4.792666003	5.089792168	5.130593265	4.710240923	4.53716382	5.432282649	4.871423222	4.965670633
ENSG00000204498	0.013467	0.056314244	-0.305248042	-1.235631061	**NFKBIL1**	protein_coding	NFKB inhibitor like 1 [Source:HGNC Symbol;Acc:HGNC:7800]	chr6	31546870	31558829	1	ensembl_havana	2.287347373	2.439971394	2.134723352	2.541371574	2.324608835	2.453933774	2.302589091	2.114588126	1.986992839
ENSG00000271860	0.013466	0.056314244	-0.405605921	-1.324645135	**AL589740.1**	lincRNA		chr6	97283303	98399872	1	havana	1.359363967	1.562166927	1.156561006	1.959443807	1.558674763	1.168382212	1.693402841	0.900298706	0.875981471
ENSG00000154310	0.013492	0.056380527	0.43920069	1.355852923	**TNIK**	protein_coding	TRAF2 and NCK interacting kinase [Source:HGNC Symbol;Acc:HGNC:30765]	chr3	171061339	171460408	-1	ensembl_havana	5.690479453	5.470879108	5.910079798	5.039960465	5.513359736	5.859317124	5.711609682	5.973828406	6.044801307
ENSG00000136840	0.013494	0.056380527	0.347540994	1.272390049	**ST6GALNAC4**	protein_coding	ST6 N-acetylgalactosaminide alpha-2,6-sialyltransferase 4 [Source:HGNC Symbol;Acc:HGNC:17846]	chr9	127907886	127917038	-1	ensembl_havana	2.810721397	2.6369509	2.984491894	2.608209568	2.64110813	2.661535001	3.157897671	2.900515694	2.895062318
ENSG00000164181	0.013532	0.056390408	0.510676847	1.424718452	**ELOVL7**	protein_coding	ELOVL fatty acid elongase 7 [Source:HGNC Symbol;Acc:HGNC:26292]	chr5	60751791	60844389	-1	ensembl_havana	3.249167393	2.99382897	3.504505817	2.191145857	3.300851749	3.489489303	3.039841863	3.67172695	3.801948638
ENSG00000168477	0.013524	0.056390408	0.425432277	1.34297484	**TNXB**	protein_coding	tenascin XB [Source:HGNC Symbol;Acc:HGNC:11976]	chr6	32041154	32115334	-1	ensembl_havana	2.799833646	2.587117507	3.012549785	2.654944978	2.383487184	2.72292036	3.063307783	3.051082538	2.923259032
ENSG00000188641	0.01351	0.056390408	0.332189438	1.25892247	**DPYD**	protein_coding	dihydropyrimidine dehydrogenase [Source:HGNC Symbol;Acc:HGNC:3012]	chr1	97077743	97995000	-1	ensembl_havana	4.479058177	4.312963458	4.645152896	4.062862309	4.287112591	4.588915474	4.456330725	4.743211525	4.735916438
ENSG00000272267	0.013535	0.056390408	0.318771802	1.24726827	**AC021242.3**	antisense		chr8	9555144	9556520	-1	havana	1.128563371	0.969177469	1.287949272	1.158893785	0.927899934	0.820738689	1.325353043	1.399485086	1.139009687
ENSG00000119977	0.013526	0.056390408	0.261342219	1.198593303	**TCTN3**	protein_coding	tectonic family member 3 [Source:HGNC Symbol;Acc:HGNC:24519]	chr10	95663396	95694143	-1	ensembl_havana	5.332038109	5.201367	5.462709219	5.12852485	5.164648804	5.310927346	5.504348515	5.382562814	5.501216326
ENSG00000102181	0.013534	0.056390408	0.215452032	1.161067649	**CD99L2**	protein_coding	CD99 molecule like 2 [Source:HGNC Symbol;Acc:HGNC:18237]	chrX	150766337	150898816	-1	ensembl_havana	5.946118394	5.838392378	6.05384441	5.815375996	5.829703058	5.87009808	6.047296792	6.070504982	6.043731456
ENSG00000196323	0.013542	0.056390408	0.208377938	1.155388418	**ZBTB44**	protein_coding	zinc finger and BTB domain containing 44 [Source:HGNC Symbol;Acc:HGNC:25001]	chr11	130226677	130314686	-1	ensembl_havana	6.29948582	6.195296851	6.403674789	6.164817127	6.195529293	6.225544133	6.385617225	6.403081976	6.422325165
ENSG00000163535	0.013527	0.056390408	-0.214015784	-1.159912344	**SGO2**	protein_coding	shugoshin 2 [Source:HGNC Symbol;Acc:HGNC:30812]	chr2	200510008	200583782	1	ensembl_havana	4.653862921	4.760870813	4.546855029	4.517968493	4.93462943	4.830014515	4.26922283	4.751234058	4.620108199
ENSG00000072864	0.013518	0.056390408	-0.214395106	-1.160217356	**NDE1**	protein_coding	nudE neurodevelopment protein 1 [Source:HGNC Symbol;Acc:HGNC:17619]	chr16	15643267	15726353	1	havana	6.739929542	6.847127095	6.632731989	7.041629224	6.834969634	6.664782426	6.861718095	6.58764309	6.448834781
ENSG00000159445	0.013544	0.056390408	-0.271022023	-1.20666234	**THEM4**	protein_coding	thioesterase superfamily member 4 [Source:HGNC Symbol;Acc:HGNC:17947]	chr1	151873584	151909808	-1	ensembl_havana	4.147730474	4.283241485	4.012219462	4.191282832	4.350791632	4.307649991	4.047136094	4.020464204	3.969058089
ENSG00000181513	0.013541	0.056390408	-0.300668031	-1.231714619	**ACBD4**	protein_coding	acyl-CoA binding domain containing 4 [Source:HGNC Symbol;Acc:HGNC:23337]	chr17	45132600	45144181	1	ensembl_havana	3.672261406	3.822595422	3.521927391	3.964251181	3.635581537	3.867953547	3.648411535	3.479075818	3.438294818
ENSG00000109684	0.013507	0.056390408	-0.772197582	-1.707869311	**CLNK**	protein_coding	cytokine dependent hematopoietic cell linker [Source:HGNC Symbol;Acc:HGNC:17438]	chr4	10486395	10684865	-1	ensembl_havana	4.220895104	4.606993895	3.834796313	5.263770706	4.309215282	4.247995697	3.967289729	3.682954534	3.854144675
ENSG00000228436	0.013552	0.056402846	0.580361465	1.495223828	**AL139260.1**	antisense		chr1	38860000	38919396	1	havana	0.694419011	0.404238278	0.984599743	0.437805725	0.243386951	0.531522158	1.400976194	0.715834679	0.836988357

ENSG00000125744	0.013566	0.056402846	0.454447339	1.370257793	RTN2	protein_coding	reticulon 2 [Source:HGNC Symbol;Acc:HGNC:10468]	chr19	45485289	45497061	-1	ensembl_havana	3.008407588	2.781183919	3.235631258	2.614134963	2.841068927	2.888347866	3.353314566	3.218605774	3.134973434
ENSG00000246067	0.013572	0.056402846	-0.216389628	-1.161822462	RAB30-AS1	lincRNA	RAB30 antisense RNA 1 (head to head) [Source:HGNC Symbol;Acc:HGNC:48672]	chr11	83072066	83106719	1	havana	2.771758161	2.879952975	2.663563347	2.833163932	2.90944349	2.897251502	2.638787485	2.716081261	2.635821295
ENSG00000103035	0.013563	0.056402846	-0.218759787	-1.163732754	PSMD7	protein_coding	proteasome 26S subunit, non-ATPase 7 [Source:HGNC Symbol;Acc:HGNC:9565]	chr16	74296775	74306288	1	ensembl_havana	7.499831364	7.609211258	7.390451471	7.690907544	7.616301002	7.520425228	7.422170631	7.415338351	7.33384543
ENSG00000154928	0.013573	0.056402846	-0.258136343	-1.195932814	EPHB1	protein_coding	EPH receptor B1 [Source:HGNC Symbol;Acc:HGNC:3392]	chr3	134597801	135260467	1	ensembl_havana	8.673098478	8.802166649	8.544030306	8.704685344	8.787905342	8.913909262	8.557228944	8.489351112	8.585510864
ENSG00000125246	0.013574	0.056402846	-0.285263538	-1.218632851	CLYBL	protein_coding	citrate lyase beta like [Source:HGNC Symbol;Acc:HGNC:18355]	chr13	99606669	99897134	1	ensembl_havana	1.844036325	1.986668094	1.701404556	1.863213701	2.004444884	2.092345696	1.669136731	1.578479109	1.856597828
ENSG00000159921	0.013565	0.056402846	-0.344839861	-1.270010003	GNE	protein_coding	glucosamine (UDP-N-acetyl)-2-epimerase/N-acetylmannosamine kinase [Source:HGNC Symbol;Acc:HGNC:23657]	chr9	36214441	36277056	-1	ensembl_havana	6.207387872	6.379807803	6.034967942	6.769662428	6.270669105	6.099091875	6.252616801	5.922650959	5.929636066
ENSG00000275367	0.013599	0.056490523	0.374368635	1.296272144	AC092111.1	lincRNA		chr12	8217758	8221115	1	havana	0.776357882	0.589173564	0.9635422	0.206373087	0.614986832	0.946160774	0.807953002	0.880929471	1.201744126
ENSG00000143919	0.013615	0.056523158	0.26723332	1.203497646	CAMKMT	protein_coding	calmodulin-lysine N-methyltransferase [Source:HGNC Symbol;Acc:HGNC:26276]	chr2	44361950	44772592	1	ensembl_havana	4.4899996	4.35638294	4.623616261	4.31466899	4.395977503	4.358502328	4.682286894	4.551329543	4.637232345
ENSG00000125149	0.013613	0.056523158	0.20879155	1.155719708	C16orf70	protein_coding	chromosome 16 open reading frame 70 [Source:HGNC Symbol;Acc:HGNC:29564]	chr16	67109958	67148539	1	ensembl_havana	4.986402881	4.882007107	5.090798656	4.903884315	4.890203094	4.85193391	5.09470424	5.10325122	5.074440509
ENSG00000243554	0.013628	0.056559002	0.287554758	1.220569764	AC004967.1	transcribed_unprocessed_pseudogene		chr7	97969005	97972254	-1	havana	1.365704039	1.221926661	1.509481418	1.092533711	1.199531136	1.373715134	1.245545533	1.602328815	1.680569906
ENSG00000088808	0.013644	0.056560421	0.264519703	1.201236072	PPP1R13B	protein_coding	protein phosphatase 1 regulatory subunit 13B [Source:HGNC Symbol;Acc:HGNC:14950]	chr14	103733752	103847590	-1	ensembl_havana	4.762146063	4.629886211	4.894405914	4.468131009	4.70640246	4.715125164	4.849530368	4.888496952	4.945190422
ENSG00000152642	0.013636	0.056560421	0.22574824	1.169383581	GPD1L	protein_coding	glycerol-3-phosphate dehydrogenase 1 like [Source:HGNC Symbol;Acc:HGNC:28956]	chr3	32105689	32168713	1	ensembl_havana	4.510509264	4.397635144	4.623383384	3.996364543	4.425852674	4.770688216	4.230361879	4.703794331	4.935993943
ENSG00000153814	0.013639	0.056560421	-0.420149252	-1.338065975	JAZF1	protein_coding	JAZF zinc finger 1 [Source:HGNC Symbol;Acc:HGNC:28917]	chr7	27830573	28180743	-1	ensembl_havana	0.940914766	1.150989392	0.73084014	0.892538	1.199531136	1.360899039	0.428834256	1.029056719	0.734629445
ENSG00000260698	0.013641	0.056560421	-0.42273353	-1.340464981	AL591848.3	lincRNA		chr1	246682108	246685075	1	havana	0.169434991	0.380801756	-0.041931774	0.437805725	0.243386951	0.461212593	-0.00649342	0.063570082	-0.182871985
ENSG00000284719	0.013668	0.056578955	0.335619315	1.261919005	AL033527.5	lincRNA		chr1	39799422	39809450	1	havana	0.991613413	0.823803755	1.15942307	0.832812769	0.799260866	0.83933763	0.975631465	1.226127349	1.276510397
ENSG00000130517	0.013669	0.056578955	0.274216849	1.209337435	PGPEP1	protein_coding	pyroglutamyl-peptidase I [Source:HGNC Symbol;Acc:HGNC:13568]	chr19	18340587	18369950	1	ensembl_havana	6.42777159	6.290663166	6.564880015	6.426254625	6.264155911	6.181578962	6.570568031	6.620131068	6.503940945
ENSG00000065613	0.013676	0.056578955	0.23043444	1.173188179	SLK	protein_coding	STE20 like kinase [Source:HGNC Symbol;Acc:HGNC:11088]	chr10	103967201	104029233	1	ensembl_havana	5.930301065	5.815083845	6.045518285	5.859692633	5.861692431	5.72386647	6.044351805	6.064615297	6.027587752
ENSG00000170185	0.013653	0.056578955	0.226944877	1.170353924	USP38	protein_coding	ubiquitin specific peptidase 38 [Source:HGNC Symbol;Acc:HGNC:20067]	chr4	143184917	143223830	1	ensembl_havana	5.802581517	5.689109078	5.916053955	5.724492672	5.7355369	5.607297662	5.885579258	5.981237131	5.881345476
ENSG00000163597	0.013673	0.056578955	0.210163208	1.156819044	SNHG16	processed_transcript	small nucleolar RNA host gene 16 [Source:HGNC Symbol;Acc:HGNC:44352]	chr17	76557766	76565348	1	havana	5.962650613	5.857569009	6.067732217	5.677163607	5.979819553	5.915723869	5.879643343	6.171607721	6.151945588
ENSG00000187097	0.013658	0.056578955	-0.213883931	-1.159806341	ENTPD5	protein_coding	ectonucleoside triphosphate diphosphohydrolase 5 [Source:HGNC Symbol;Acc:HGNC:3367]	chr14	73958010	74019399	-1	ensembl_havana	4.561750111	4.668692076	4.454808145	4.599378819	4.628759878	4.777937532	4.347765965	4.443233971	4.573424499
ENSG00000018699	0.013665	0.056578955	-0.22757601	-1.170866028	TTC27	protein_coding	tetratricopeptide repeat domain 27 [Source:HGNC Symbol;Acc:HGNC:25986]	chr2	32628032	32821051	1	ensembl_havana	5.600264662	5.714052667	5.486476657	5.577576228	5.833227477	5.731354297	5.388364636	5.536717608	5.534347728
ENSG00000106266	0.01368	0.05658061	0.226104151	1.169672102	SNX8	protein_coding	sorting nexin 8 [Source:HGNC Symbol;Acc:HGNC:14972]	chr7	2251770	2354318	-1	ensembl_havana	5.062681345	4.949629269	5.17573342	4.956136763	4.95956846	4.933182584	5.222140422	5.211882794	5.093177045
ENSG00000148019	0.013694	0.056619848	0.343167649	1.268538802	CEP78	protein_coding	centrosomal protein 78 [Source:HGNC Symbol;Acc:HGNC:25740]	chr9	78236062	78279690	1	ensembl_havana	6.802356691	6.630772867	6.973940515	6.377446303	6.637463533	6.877408764	6.914282195	6.927088578	7.080450772
ENSG00000225778	0.013728	0.056714705	0.718300172	1.645242417	PROSER2-AS1	antisense	PROSER2 antisense RNA 1 [Source:HGNC Symbol;Acc:HGNC:27343]	chr10	11849608	11894700	-1	havana	0.161995268	-0.197154818	0.521145354	-0.73288918	0.06303108	0.078393642	0.308311185	0.286036324	0.969088554
ENSG00000278341	0.013725	0.056714705	0.353670701	1.277807667	AC138028.6	antisense		chr16	88708956	88710437	-1	havana	1.408748507	1.231913156	1.585583857	1.158893785	1.324992307	1.211853376	1.340798121	1.660283167	1.755670283
ENSG00000228594	0.013724	0.056714705	-0.785196376	-1.723326878	FNDC10	protein_coding	fibronectin type III domain containing 10 [Source:HGNC Symbol;Acc:HGNC:42951]	chr1	1598012	1600096	-1	ensembl_havana	-0.524729099	-0.132130911	-0.917327287	-0.67394004	-0.299013651	0.57656096	-1.41444944	-0.62862566	-0.708906755
ENSG00000244462	0.013745	0.056768138	-0.223854978	-1.167849995	RBM12	protein_coding	RNA binding motif protein 12 [Source:HGNC Symbol;Acc:HGNC:9898]	chr20	35648925	35664956	-1	ensembl_havana	7.622054819	7.733982308	7.510127329	7.81586921	7.726693765	7.659383949	7.568333225	7.464149883	7.497898881
ENSG00000144791	0.013774	0.056868728	0.210385717	1.156997476	LIMD1	protein_coding	LIM domains containing 1 [Source:HGNC Symbol;Acc:HGNC:6612]	chr3	45555394	45686338	1	ensembl_havana	6.685769045	6.580576186	6.790961904	6.523122262	6.550568743	6.668037555	6.729721635	6.788108401	6.855055676
ENSG00000120690	0.013781	0.056881668	-0.213129448	-1.159199958	ELF1	protein_coding	E74 like ETS transcription factor 1 [Source:HGNC Symbol;Acc:HGNC:3316]	chr13	40932028	41061440	-1	ensembl_havana	4.872827844	4.979392568	4.76626312	4.989569283	5.033942152	4.914666268	4.761891126	4.859102792	4.677795441
ENSG00000267980	0.013817	0.056884324	0.827051995	1.774056546	AC007292.1	antisense		chr19	4363789	4364640	1	havana	-0.598222995	-1.011748992	-0.184696998	-1.14838881	-0.903502039	-0.983356126	-0.21650287	-0.62862566	0.291037539
ENSG00000118777	0.013812	0.056884324	0.340306197	1.266025266	ABCG2	protein_coding	ATP binding cassette subfamily G member 2 (Junior blood group) [Source:HGNC Symbol;Acc:HGNC:74]	chr4	88090264	88231322	-1	ensembl_havana	3.598421529	3.42826843	3.768574627	2.822967335	3.651639676	3.810198279	3.353314566	3.945529874	4.006879442
ENSG00000232586	0.01381	0.056884324	0.281324235	1.215309893	KIAA1614-AS1	antisense	KIAA1614 antisense RNA 1 [Source:HGNC Symbol;Acc:HGNC:50644]	chr1	180949699	180954887	-1	havana	2.982146418	2.841484301	3.122808536	2.945520426	2.754497827	2.824434649	3.149151625	3.175535294	3.043738688
ENSG00000281404	0.013791	0.056884324	0.265957226	1.202433598	LINC01176	lincRNA	long intergenic non-protein coding RNA 1176 [Source:HGNC Symbol;Acc:HGNC:49548]	chr7	30390885	30412375	1	havana	0.964776023	0.83179741	1.097754636	0.589870393	0.89230127	1.013220568	0.955706889	1.046547334	1.291009686
ENSG00000182173	0.013787	0.056884324	0.227990281	1.171202291	TSEN54	protein_coding	tRNA splicing endonuclease subunit 54 [Source:HGNC Symbol;Acc:HGNC:27561]	chr17	75516060	75524739	1	ensembl_havana	4.479647492	4.365652352	4.593642633	4.277815454	4.397565399	4.421576202	4.525545558	4.674563195	4.580819145
ENSG00000162971	0.013816	0.056884324	-0.207154565	-1.15440909	TYW5	protein_coding	tRNA-yW synthesizing protein 5 [Source:HGNC Symbol;Acc:HGNC:26754]	chr2	199929975	199955736	-1	ensembl_havana	4.579873862	4.683451144	4.476296579	4.749592033	4.679243401	4.621517999	4.555640798	4.462907727	4.410341214
ENSG00000087303	0.013796	0.056884324	-0.22266075	-1.166883677	NID2	protein_coding	nidogen 2 [Source:HGNC Symbol;Acc:HGNC:13389]	chr14	52004803	52069228	-1	ensembl_havana	8.055903506	8.167233881	7.944573131	8.450681376	7.987725048	8.063295221	8.190207366	7.742390926	7.9011211
ENSG00000119650	0.013817	0.056884324	-0.27871568	-1.213114461	IFT43	protein_coding	intraflagellar transport 43 [Source:HGNC Symbol;Acc:HGNC:29669]	chr14	75902136	76084585	1	ensembl_havana	4.341180136	4.480537976	4.201822296	4.468131009	4.538087248	4.43539567	4.244801141	4.122949443	4.237716304
ENSG00000267787	0.013802	0.056884324	-0.503296435	-1.417448614	AC027097.2	antisense		chr18	57639455	57738044	1	havana	0.442904177	0.694552394	0.191255959	0.637193984	0.570631912	0.875831286	0.339398945	0.19485955	0.039509383
ENSG00000164934	0.013827	0.056910082	-0.28507542	-1.21847396	DCAF13	protein_coding	DDB1 and CUL4 associated factor 13 [Source:HGNC Symbol;Acc:HGNC:24535]	chr8	103414714	103443453	1	ensembl_havana	6.646910584	6.789448294	6.504372874	6.848119324	6.740845299	6.779380257	6.427358826	6.481531411	6.604228384
ENSG00000114745	0.013844	0.056963748	0.265599726	1.202135671	GORASP1	protein_coding	golgi reassembly stacking protein 1 [Source:HGNC Symbol;Acc:HGNC:16769]	chr3	39096659	39108363	-1	ensembl_havana	5.936196981	5.803397119	6.068996844	5.716252511	5.804189573	5.889749271	6.10213877	6.030412265	6.074439497
ENSG00000087128	0.013887	0.057107287	1.131817109	2.191345717	TMPRSS11E	protein_coding	transmembrane serine protease 11E [Source:HGNC Symbol;Acc:HGNC:24465]	chr4	68447449	68497604	1	ensembl_havana	1.156489363	0.590580808	1.722397917	0.515840645	1.452682866	-0.196781087	1.681320805	1.861445274	1.624427672
ENSG00000276043	0.013885	0.057107287	-0.348316909	-1.273074554	UHRF1	protein_coding	ubiquitin like with PHD and ring finger domains 1 [Source:HGNC Symbol;Acc:HGNC:12556]	chr19	4903080	4962154	1	ensembl_havana	5.4004733	5.574631754	5.226314845	5.43673134	5.500856421	5.786307501	5.187321836	5.259532745	5.232089956
ENSG00000167291	0.013895	0.057119672	0.362365318	1.285531818	TBC1D16	protein_coding	TBC1 domain family member 16 [Source:HGNC Symbol;Acc:HGNC:28356]	chr17	79932343	80035848	-1	ensembl_havana	3.196084845	3.014902186	3.377267504	2.727802065	2.995843375	3.321061117	3.153531275	3.507772205	3.470499031
ENSG00000164105	0.013898	0.057119672	0.327967554	1.255243761	SAP30	protein_coding	Sin3A associated protein 30 [Source:HGNC Symbol;Acc:HGNC:10532]	chr4	173369969	173377532	1	ensembl_havana	3.582613882	3.418630105	3.746597659	3.535210946	3.297453632	3.423225735	3.676073242	3.724296517	3.839423218
ENSG00000073861	0.013929	0.057212754	0.416539524	1.334722221	TBX21	protein_coding	T-box 21 [Source:HGNC Symbol;Acc:HGNC:11599]	chr17	47733244	47746119	1	ensembl_havana	1.930553609	1.722283848	2.138823371	1.625778111	1.739838083	1.801235349	1.976531838	1.966251876	2.4736864
ENSG00000206527	0.013927	0.057212754	0.234121799	1.176190545	HACD2	protein_coding	3-hydroxyacyl-CoA dehydratase 2 [Source:HGNC Symbol;Acc:HGNC:9640]	chr3	123490820	123585185	-1	ensembl_havana	5.92936169	5.81230079	6.046422589	5.709349575	5.894675507	5.832877289	5.937931279	6.060854758	6.140481729
ENSG00000136816	0.013934	0.057216359	-0.245528135	-1.185526684	TOR1B	protein_coding	torsin family 1 member B [Source:HGNC Symbol;Acc:HGNC:11995]	chr9	129803153	129811281	1	ensembl_havana	7.409337299	7.532101366	7.286573231	7.588352268	7.445290071	7.562661759	7.260528622	7.287395506	7.311795565
ENSG00000092096	0.013939	0.057221765	-0.446107384	-1.362359436	SLC22A17	protein_coding	solute carrier family 22 member 17 [Source:HGNC Symbol;Acc:HGNC:23095]	chr14	23346306	23352912	-1	ensembl_havana	-0.428395783	-0.205342091	-0.651449476	-0.73288918	-0.218971072	0.335833975	-1.31428442	-0.37473441	-0.265329594
ENSG00000100290	0.013949	0.057237736	-0.44714616	-1.363340721	BIK	protein_coding	BCL2 interacting killer [Source:HGNC Symbol;Acc:HGNC:1051]	chr22	43110748	43129712	1	ensembl_havana	-0.158448137	0.065124944	-0.382021217	0.004630787	0.243386951	-0.052642908	-0.40973604	-0.47099802	-0.265329594
ENSG00000140043	0.013951	0.057237736	-0.548792915	-1.462861226	PTGR2	protein_coding	prostaglandin reductase 2 [Source:HGNC Symbol;Acc:HGNC:20149]	chr14	73851844	73886827	1	ensembl_havana	0.886790215	1.161186672	0.612393757	1.126095262	0.996565715	1.360899039	0.210814386	0.693803314	0.932563571
ENSG00000162129	0.013968	0.057290388	-0.257977441	-1.195801098	CLPB	protein_coding	ClpB homolog, mitochondrial AAA ATPase chaperonin [Source:HGNC Symbol;Acc:HGNC:30664]	chr11	72285495	72434680	-1	ensembl_havana	4.787330186	4.916318906	4.658341465	4.907507731	4.830324825	5.011124163	4.553985209	4.674563195	4.746475991
ENSG00000147224	0.013979	0.057306145	0.236154771	1.177849141	PRPS1	protein_coding	phosphoribosyl pyrophosphate synthetase 1 [Source:HGNC Symbol;Acc:HGNC:9462]	chrX	107628424	107651026	1	ensembl_havana	6.999089753	6.881012367	7.117167138	6.710049323	7.005283366	6.927704413	7.019100991	7.166621152	7.165779272
ENSG00000099399	0.013977	0.057306145	-0.237791661	-1.179186294	MAGEB2	protein_coding	MAGE family member B2 [Source:HGNC Symbol;Acc:HGNC:6809]	chrX	30215560	30220089	1	ensembl_havana	7.221206486	7.340102316	7.102310656	7.378321989	7.412484948	7.229500013	7.108945861	7.119623548	7.078362558
ENSG00000167904	0.013983	0.057306358	-0.215230705	-1.16088954	TMEM68	protein_coding	transmembrane protein 68 [Source:HGNC Symbol;Acc:HGNC:26510]	chr8	55696424	55773407	-1	ensembl_havana	4.093070225	4.200685577	3.985454873	4.149053241	4.185992157	4.267011334	3.893277228	3.970942906	4.092144484
ENSG00000103549	0.014024	0.057415533	0.256678533	1.194724962	RNF40	protein_coding	ring finger protein 40 [Source:HGNC Symbol;Acc:HGNC:16867]	chr16	30761745	30776307	1	ensembl_havana	6.99139887	6.863059604	7.119738137	6.824995046	6.787615648	6.976568117	7.15062179	7.087507123	7.121085497
ENSG00000119138	0.01402	0.057415533	0.23625672	1.177932378	KLF9	protein_coding	Kruppel like factor 9 [Source:HGNC Symbol;Acc:HGNC:1123]	chr9	70384597	70414624	-1	ensembl_havana	2.373654294	2.255525934	2.491782654	2.298074107	2.337902139	2.130601557	2.619920974	2.535862351	2.319564638
ENSG00000160563	0.014028	0.057415533	0.224579082	1.168436298	MED27	protein_coding	mediator complex subunit 27 [Source:HGNC Symbol;Acc:HGNC:2377]	chr9	131860107	132079908	-1	ensembl_havana	4.672089249	4.559799708	4.78437879	4.371743869	4.666128586	4.641526669	4.656289177	4.835400086	4.861447107
ENSG00000164038	0.014032	0.057415533	0.215269487	1.160920747	SLC9B2	protein_coding	solute carrier family 9 member B2 [Source:HGNC Symbol;Acc:HGNC:25143]	chr4	103019868	103085829	-1	ensembl_havana	5.073865112	4.966230368	5.181499855	4.89174011	5.016480342	4.990470653	5.149451065	5.192373237	5.202675264
ENSG00000184863	0.014034	0.057415533	-0.244327343	-1.18454035	RBM33	protein_coding	RNA binding motif protein 33 [Source:HGNC Symbol;Acc:HGNC:27223]	chr7	155644451	155781485	1	ensembl_havana	6.646728027	6.768891699	6.524564355	6.822438196	6.67624659	6.80799031	6.622042178	6.485143684	6.466507203
ENSG00000243678	0.014017	0.057415533	-0.344678392	-1.26986787	NME2	protein_coding	NME/NM23 nucleoside diphosphate kinase 2 [Source:HGNC Symbol;Acc:HGNC:7850]	chr17	51165435	51171747	1	ensembl_havana	0.846627673	1.018966869	0.674288477	0.949888679	1.029710695	1.077301233	0.692307165	0.800742365	0.529815901
ENSG00000116731	0.014039	0.057419734	0.237749516	1.179151847	PRDM2	protein_coding	PR/SET domain 2 [Source:HGNC Symbol;Acc:HGNC:9347]	chr1	13700198	13825079	1	ensembl_havana	7.255986149	7.137111392	7.374860907	7.175381173	7.119617381	7.11633562	7.411696322	7.434355869	7.27853053
ENSG00000229056	0.014063	0.057502522	-0.344859458	-1.270027255	AC020571.1	antisense		chr2	196260024	196264204	1	havana	3.108608869	3.281038598	2.93617914	3.584331121	3.283780556	2.975004118	3.039841863	3.002618823	2.766076734
ENSG00000170275	0.014068	0.057504665	-0.222063429	-1.16640065	CRTAP	protein_coding	cartilage associated protein [Source:HGNC Symbol;Acc:HGNC:2379]	chr3	33113979	33147773	1	ensembl_havana	7.383121638	7.494153353	7.272089924	7.40420071	7.521907474	7.556351874	7.126875046	7.302488067	7.386906659
ENSG00000143436	0.014078	0.057531215	-0.228926369	-1.171962469	MRPL9	protein_coding	mitochondrial ribosomal protein L9 [Source:HGNC Symbol;Acc:HGNC:14277]	chr1	151759643	151763564	-1	ensembl_havana	5.095949488	5.210412673	4.981486303	5.19130566	5.251504958	5.1884274	4.978553955	4.951362452	5.014542502
ENSG00000111834	0.014091	0.057533049	1.04379407	2.061642339	RSPH4A	protein_coding	radial spoke head 4 homolog A [Source:HGNC Symbol;Acc:HGNC:21558]	chr6	116616479	116632985	1	ensembl_havana	-1.032310515	-1.554207551	-0.51041348	-0.85854608	-1.178380098	-2.625696473	0.106248156	-0.74417246	-0.893316134
ENSG00000141295	0.014089	0.057533049	0.214858891	1.160590392	SCRN2	protein_coding	secernin 2 [Source:HGNC Symbol;Acc:HGNC:30381]	chr17	47837692	47841333	-1	ensembl_havana	5.059211721	4.951782276	5.166641167	5.053106071	4.877742138	4.924498619	5.298281241	5.108465214	5.093177045
ENSG00000157326	0.014083	0.057533049	-0.355540222	-1.279464591	DHRS4	protein_coding	dehydrogenase/reductase 4 [Source:HGNC Symbol;Acc:HGNC:16985]	chr14	23953586	23969279	1	ensembl_havana	2.053259908	2.231030019	1.875489797	2.383813765	2.06964138	2.239634912	2.044136302	1.890792227	1.691540863
ENSG00000169604	0.014108	0.057577104	0.345768529	1.270827777	ANTXR1	protein_coding	anthrax toxin receptor 1 [Source:HGNC Symbol;Acc:HGNC:21014]	chr2	69013178	69249327	1	ensembl_havana	0.944449008	0.771564743	1.117333273	0.355307344	0.962641309	0.996745577	0.894226935	1.271458479	1.186314404
ENSG00000142784	0.014114	0.057577104	0.207210834	1.154454116	WDTC1	protein_coding	WD and tetratricopeptide repeats 1 [Source:HGNC Symbol;Acc:HGNC:29175]	chr1	27234516	27308633	1	ensembl_havana	6.187773274	6.084167858	6.291378691	6.197250076	6.062221131	5.993032365	6.401814925	6.252020808	6.220300341
ENSG00000115446	0.014111	0.057577104	-0.270359919	-1.206108687	UNC50	protein_coding	unc-50 inner nuclear membrane RNA binding protein [Source:HGNC Symbol;Acc:HGNC:16046]	chr2	98608579	98618515	1	ensembl_havana	5.63431272	5.769492679	5.49913276	5.86280662	5.834986462	5.610684956	5.536562159	5.49113811	5.469698011
ENSG00000115252	0.014123	0.057597882	1.244695761	2.369685764	PDE1A	protein_coding	phosphodiesterase 1A [Source:HGNC Symbol;Acc:HGNC:8774]	chr2	182140036	182523192	-1	ensembl_havana	2.048521868	1.426173988	2.670869749	0.14220989	1.855271053	2.28104102	2.187709208	3.064022175	2.760877864
ENSG00000141873	0.014127	0.057598613	0.258605302	1.196321624	SLC39A3	protein_coding	solute carrier family 39 member 3 [Source:HGNC Symbol;Acc:HGNC:17128]	chr19	2732204	2740152	-1	ensembl_havana	4.344063868	4.214761217	4.473366519	4.216818725	4.196976397	4.230488529	4.58835975	4.375646885	4.456092922
ENSG00000132661	0.014131	0.05759945	-0.319184282	-1.247624927	NXT1	protein_coding	nuclear transport factor 2 like export factor 1 [Source:HGNC Symbol;Acc:HGNC:15913]	chr20	23350736	23354777	1	ensembl_havana	3.995023231	4.154615372	3.83543109	4.071501525	4.271609917	4.120734673	3.850756671	3.772392145	3.883144453
ENSG00000164167	0.014145	0.057640007	-0.345508765	-1.270598979	LSM6	protein_coding	LSM6 homolog, U6 small nuclear RNA and mRNA degradation associated [Source:HGNC Symbol;Acc:HGNC:17017]	chr4	146175685	146200000	1	ensembl_havana	3.481406023	3.654160405	3.30865164	3.711376171	3.563853664	3.687251381	3.175232288	3.379076552	3.37164608
ENSG00000221968	0.014153	0.057657162	0.212174322	1.158432772	FADS3	protein_coding	fatty acid desaturase 3 [Source:HGNC Symbol;Acc:HGNC:3576]	chr11	61873519	61892051	-1	ensembl_havana	7.206259487	7.100172326	7.312346648	7.133450323	7.069197953	7.097868702	7.330616904	7.256258591	7.350164449
ENSG00000072133	0.01417	0.057659945	0.266212825	1.202646649	RPS6KA6	protein_coding	ribosomal protein S6 kinase A6 [Source:HGNC Symbol;Acc:HGNC:10435]	chrX	84058346	84187907	-1	ensembl_havana	4.500507384	4.367400971	4.633613797	4.312848501	4.453595962	4.335758451	4.614010932	4.598043022	4.688787436
ENSG00000204613	0.014169	0.057659945	-0.22756925	-1.170860542	TRIM10	protein_coding	tripartite motif containing 10 [Source:HGNC Symbol;Acc:HGNC:10072]	chr6	30151945	30160934	-1	ensembl_havana	7.979305771	8.093090396	7.865521146	8.209051838	8.014360036	8.055859316	7.923942846	7.776529401	7.896091192
ENSG00000060642	0.014162	0.057659945	-0.25466961	-1.19306249	PIGV	protein_coding	phosphatidylinositol glycan anchor biosynthesis class V [Source:HGNC Symbol;Acc:HGNC:26031]	chr1	26787472	26798398	1	ensembl_havana	4.463066765	4.59040157	4.33573196	4.745548893	4.636849344	4.388806474	4.58997638	4.288290531	4.128928969
ENSG00000150961	0.014159	0.057659945	-0.331179731	-1.258041689	SEC24D	protein_coding	SEC24 homolog D, COPII coat complex component [Source:HGNC Symbol;Acc:HGNC:10706]	chr4	118722823	118838683	-1	ensembl_havana	6.64608436	6.811674226	6.480494494	6.977573542	6.816985675	6.640463459	6.460434142	6.537883307	6.443166034
ENSG00000230910	0.014192	0.05773138	-1.054508109	-2.077009923	AL391807.1	antisense		chr6	68629962	68635027	-1	havana	-0.506026976	0.021227078	-1.033281031	-0.06941355	0.185737694	-0.052642908	-0.46237776	-0.86978558	-1.767679756
ENSG00000203709	0.014204	0.057764019	-0.406742115	-1.325688769	MIR29B2CHG	lincRNA	MIR29B2 and MIR29C host gene [Source:HGNC Symbol;Acc:HGNC:32018]	chr1	207801518	207879096	-1	havana	4.238992137	4.442363195	4.03562108	4.710025423	4.400735959	4.216328203	4.047136094	4.157568375	3.90215877
ENSG00000181163	0.014218	0.057805113	-0.312435072	-1.241801924	NPM1	protein_coding	nucleophosmin 1 [Source:HGNC Symbol;Acc:HGNC:7910]	chr5	171387116	171411137	1	ensembl_havana	9.746900984	9.90311852	9.590683448	9.883971844	9.910962816	9.914420901	9.397300018	9.682078221	9.692672105
ENSG00000116251	0.014228	0.057829113	0.225834022	1.169453114	RPL22	protein_coding	ribosomal protein L22 [Source:HGNC Symbol;Acc:HGNC:10315]	chr1	6181269	6209389	-1	ensembl_havana	7.452198611	7.3392816	7.565115622	7.449643834	7.248390581	7.319810386	7.610558056	7.482739527	7.602049285
ENSG00000261448	0.014234	0.057840155	-0.258416368	-1.196164966	AC109446.3	antisense		chr16	17134504	17138736	1	havana	4.516991993	4.646200177	4.387783809	4.940894536	4.720427175	4.27727882	4.688336516	4.358243523	4.116771388
ENSG00000090520	0.014257	0.057898237	-0.209927915	-1.156630391	DNAJB11	protein_coding	DnaJ heat shock protein family (Hsp40) member B11 [Source:HGNC Symbol;Acc:HGNC:14889]	chr3	186567403	186585800	1	ensembl_havana	5.645326545	5.750290502	5.540362587	5.899659433	5.663453351	5.687758724	5.664054178	5.445720304	5.511313281
ENSG00000108702	0.014256	0.057898237	-0.725797762	-1.653814887	CCL1	protein_coding	C-C motif chemokine ligand 1 [Source:HGNC Symbol;Acc:HGNC:10609]	chr17	34360328	34363231	-1	ensembl_havana	0.992838565	1.355737446	0.629939684	1.142587728	1.500754142	1.423870469	0.032074365	0.671430284	1.186314404
ENSG00000126882	0.014262	0.057901851	-0.27561263	-1.210508013	FAM78A	protein_coding	family with sequence similarity 78 member A [Source:HGNC Symbol;Acc:HGNC:25465]	chr9	131258076	131276547	-1	ensembl_havana	3.992037723	4.129844038	3.854231408	4.0388342	4.146875043	4.20382287	3.853451238	3.914907507	3.794335479
ENSG00000198879	0.014299	0.057920109	0.548026987	1.462084798	SFMBT2	protein_coding	Scm like with four mbt domains 2 [Source:HGNC Symbol;Acc:HGNC:20256]	chr10	7158624	7411486	-1	ensembl_havana	4.328652956	4.054639463	4.60266645	3.424923176	4.337617108	4.401378105	4.347765965	4.724316947	4.735916438
ENSG00000161542	0.014284	0.057920109	0.259228795	1.196838754	PRPSAP1	protein_coding	phosphoribosyl pyrophosphate synthetase associated protein 1 [Source:HGNC Symbol;Acc:HGNC:9466]	chr17	76309486	76384521	-1	ensembl_havana	5.421937063	5.292322665	5.551551461	5.207073224	5.330105466	5.339789306	5.498335162	5.475081104	5.681238116
ENSG00000181744	0.014284	0.057920109	0.2441085	1.18436068	C3orf58	protein_coding	chromosome 3 open reading frame 58 [Source:HGNC Symbol;Acc:HGNC:28490]	chr3	143971798	144048719	1	ensembl_havana	5.193806691	5.071752441	5.315860941	4.878262343	5.237387224	5.099607755	5.211677793	5.40052739	5.335377639
ENSG00000126391	0.014297	0.057920109	0.21668782	1.162062626	FRMD8	protein_coding	FERM domain containing 8 [Source:HGNC Symbol;Acc:HGNC:25462]	chr11	65386599	65413525	1	ensembl_havana	4.91150066	4.80315675	5.01984457	4.802445031	4.831500081	4.775525138	5.057796071	4.99705787	5.004679769
ENSG00000077312	0.014293	0.057920109	-0.21632369	-1.161769363	SNRPA	protein_coding	small nuclear ribonucleoprotein polypeptide A [Source:HGNC Symbol;Acc:HGNC:11151]	chr19	40750637	40765389	1	ensembl_havana	5.738357604	5.846519449	5.630195759	5.740155958	5.826759451	5.972642939	5.57625118	5.582400786	5.731935312
ENSG00000010404	0.014296	0.057920109	-0.228417988	-1.171549562	IDS	protein_coding	iduronate 2-sulfatase [Source:HGNC Symbol;Acc:HGNC:5389]	chrX	149476990	149521096	-1	ensembl_havana	7.198377131	7.312586125	7.084168137	7.510673465	7.358475513	7.068609398	7.212536778	7.139880383	6.900087249
ENSG00000115884	0.014288	0.057920109	-0.615534569	-1.532125598	SDC1	protein_coding	syndecan 1 [Source:HGNC Symbol;Acc:HGNC:10658]	chr2	20200797	20225433	-1	ensembl_havana	2.054513399	2.362280684	1.746746115	1.791817439	2.51804043	2.776984183	1.593788933	1.780136556	1.866312856
ENSG00000232117	0.014271	0.057920109	-0.657067117	-1.576873706	LINC00384	lincRNA	long intergenic non-protein coding RNA 384 [Source:HGNC Symbol;Acc:HGNC:42711]	chr13	30151886	30159621	-1	havana	0.419844077	0.748377636	0.091310519	1.190963204	0.477608744	0.57656096	0.36983092	-0.47099802	0.375098651
ENSG00000137171	0.014315	0.057946071	0.352922503	1.277145153	KLC4	protein_coding	kinesin light chain 4 [Source:HGNC Symbol;Acc:HGNC:21624]	chr6	43040777	43075099	1	ensembl_havana	5.051192641	4.874731389	5.227653892	5.121261956	4.87204229	4.630889922	5.52139269	5.021586792	5.139982195
ENSG00000114480	0.014313	0.057946071	0.207101394	1.154366545	GBE1	protein_coding	1,4-alpha-glucan branching enzyme 1 [Source:HGNC Symbol;Acc:HGNC:4180]	chr3	81489699	81762161	-1	ensembl_havana	6.073021842	5.969471145	6.176572539	5.943851816	6.030334916	5.934226703	6.135178062	6.228268177	6.166271379
ENSG00000168264	0.014317	0.057946071	-0.207329858	-1.154549364	IRF2BP2	protein_coding	interferon regulatory factor 2 binding protein 2 [Source:HGNC Symbol;Acc:HGNC:21729]	chr1	234604269	234609525	-1	ensembl_havana	6.415789829	6.519454758	6.3121249	6.76401063	6.355222332	6.439131312	6.531140978	6.166117409	6.239116312
ENSG00000123570	0.014341	0.058028575	0.381002161	1.30224614	RAB9B	protein_coding	RAB9B, member RAS oncogene family [Source:HGNC Symbol;Acc:HGNC:14090]	chrX	103822322	103832228	-1	ensembl_havana	1.251657974	1.061156893	1.442159054	0.770507832	1.078042694	1.334920154	1.161063346	1.660283167	1.505130649
ENSG00000145088	0.014356	0.058070654	-0.304457679	-1.23495432	EAF2	protein_coding	ELL associated factor 2 [Source:HGNC Symbol;Acc:HGNC:23115]	chr3	121835183	121886526	1	ensembl_havana	4.017614502	4.169843341	3.865385662	4.241910479	4.139302394	4.12831715	3.792976108	3.83412279	3.969058089
ENSG00000187068	0.014373	0.058123616	-0.21388249	-1.159805182	C3orf70	protein_coding	chromosome 3 open reading frame 70 [Source:HGNC Symbol;Acc:HGNC:33731]	chr3	185078050	185153014	-1	ensembl_havana	4.429726452	4.536667697	4.322785207	4.487627691	4.611075527	4.511299874	4.318811065	4.387705518	4.261839039
ENSG00000134046	0.014403	0.058217492	0.265778658	1.202284777	MBD2	protein_coding	methyl-CpG binding domain protein 2 [Source:HGNC Symbol;Acc:HGNC:6917]	chr18	54151601	54224788	-1	ensembl_havana	6.819629195	6.686739866	6.952518524	6.59716266	6.811040157	6.65201678	6.926489688	6.950504786	6.980561097
ENSG00000080189	0.014404	0.058217492	0.210938031	1.1574405	SLC35C2	protein_coding	solute carrier family 35 member C2 [Source:HGNC Symbol;Acc:HGNC:17117]	chr20	46345980	46364458	-1	ensembl_havana	6.652267285	6.54679827	6.757736301	6.527445857	6.487477022	6.62547193	6.724579522	6.737853457	6.810775925
ENSG00000139323	0.014437	0.058329901	0.323924639	1.251731071	POC1B	protein_coding	POC1 centriolar protein B [Source:HGNC Symbol;Acc:HGNC:30836]	chr12	89419718	89526024	-1	ensembl_havana	5.139983076	4.978020756	5.301945395	5.137809451	4.946610592	4.849642226	5.280370568	5.322807524	5.302658092
ENSG00000170606	0.01444	0.058329901	-0.249627503	-1.188900107	HSPA4	protein_coding	heat shock protein family A (Hsp70) member 4 [Source:HGNC Symbol;Acc:HGNC:5237]	chr5	133051962	133106449	1	ensembl_havana	8.233974673	8.358788425	8.109160922	8.347978572	8.363556362	8.364830341	7.992317143	8.154953403	8.180212219
ENSG00000090581	0.014479	0.058425681	0.289941653	1.222590832	GNPTG	protein_coding	N-acetylglucosamine-1-phosphate transferase gamma subunit [Source:HGNC Symbol;Acc:HGNC:23026]	chr16	1351923	1364113	1	ensembl_havana	3.809755539	3.664784712	3.954726366	3.708609614	3.538169938	3.747574584	4.044780323	3.931477196	3.887921578
ENSG00000118058	0.014484	0.058425681	0.280934993	1.214982044	KMT2A	protein_coding	lysine methyltransferase 2A [Source:HGNC Symbol;Acc:HGNC:7132]	chr11	118436490	118526832	1	ensembl_havana	8.670063262	8.529595765	8.810530759	8.60081053	8.433916397	8.55406037	8.92090257	8.814991883	8.695697823
ENSG00000008869	0.014474	0.058425681	0.220337319	1.165005946	HEATR5B	protein_coding	HEAT repeat containing 5B [Source:HGNC Symbol;Acc:HGNC:29273]	chr2	36968383	37084342	-1	ensembl_havana	7.241141999	7.13097334	7.351310659	7.063176728	7.093648663	7.236094629	7.306237865	7.353004434	7.394689677
ENSG00000152464	0.014484	0.058425681	-0.292910354	-1.225109204	RPP38	protein_coding	ribonuclease P/MRP subunit p38 [Source:HGNC Symbol;Acc:HGNC:30329]	chr10	15097180	15139818	1	ensembl_havana	3.604583293	3.75103847	3.458128116	3.680648284	3.940150332	3.632316794	3.383457449	3.5140724	3.476854499
ENSG00000149476	0.014475	0.058425681	-0.367100021	-1.289757666	TKFC	protein_coding	triokinase and FMN cyclase [Source:HGNC Symbol;Acc:HGNC:24552]	chr11	61333210	61353295	1	havana	5.284974828	5.468524839	5.101424818	5.447556952	5.37190052	5.586117045	5.172292608	5.138340331	4.993641514
ENSG00000008083	0.014511	0.05843479	0.293443737	1.225562227	JARID2	protein_coding	jumonji and AT-rich interaction domain containing 2 [Source:HGNC Symbol;Acc:HGNC:6196]	chr6	15246296	15522040	1	ensembl_havana	5.246071197	5.099349329	5.392793066	5.151115925	4.883419555	5.263512506	5.500055822	5.27630862	5.402014756
ENSG00000106080	0.014509	0.05843479	0.270311534	1.206068236	FKBP14	protein_coding	FK506 binding protein 14 [Source:HGNC Symbol;Acc:HGNC:18625]	chr7	30010587	30026684	-1	ensembl_havana	5.652741634	5.517585867	5.7878974	5.556191001	5.606237645	5.390328954	5.818211447	5.76649691	5.778983844
ENSG00000148429	0.014503	0.05843479	0.250380641	1.189520917	USP6NL	protein_coding	USP6 N-terminal like [Source:HGNC Symbol;Acc:HGNC:16858]	chr10	11453946	11611754	-1	ensembl_havana	3.99140727	3.866216949	4.116597591	3.708609614	3.902621574	3.987419659	3.949769201	4.251056902	4.148966669
ENSG00000198746	0.014501	0.05843479	0.228853993	1.171903676	GPATCH3	protein_coding	G-patch domain containing 3 [Source:HGNC Symbol;Acc:HGNC:25720]	chr1	26890488	26900466	-1	ensembl_havana	4.122717045	4.008290048	4.237144041	4.065026968	4.006168868	3.953674309	4.355390123	4.245388041	4.11065396
ENSG00000165424	0.014511	0.05843479	-0.235446997	-1.177271441	ZCCHC24	protein_coding	zinc finger CCHC-type containing 24 [Source:HGNC Symbol;Acc:HGNC:26911]	chr10	79382325	79445627	-1	ensembl_havana	5.736180921	5.85390442	5.618457422	5.996970729	5.840250589	5.724491942	5.840113308	5.531292435	5.483966524
ENSG00000189045	0.014493	0.05843479	-0.529185972	-1.443114701	ANKDD1B	protein_coding	ankyrin repeat and death domain containing 1B [Source:HGNC Symbol;Acc:HGNC:32525]	chr5	75611459	75671846	1	ensembl_havana	2.27148997	2.536082956	2.006896984	2.85834452	2.357615216	2.392289132	2.062883947	1.748424202	2.209382804
ENSG00000115459	0.014537	0.058498129	0.227278954	1.170624967	ELMOD3	protein_coding	ELMO domain containing 3 [Source:HGNC Symbol;Acc:HGNC:26158]	chr2	85354394	85391752	1	ensembl_havana	5.013671148	4.900031672	5.127310625	4.794639466	4.902557343	5.002898206	5.086710873	5.064070606	5.231150397
ENSG00000176834	0.014539	0.058498129	-0.208715244	-1.155658583	VSIG10	protein_coding	V-set and immunoglobulin domain containing 10 [Source:HGNC Symbol;Acc:HGNC:26078]	chr12	118063593	118136026	-1	ensembl_havana	3.932789069	4.037146691	3.828431447	4.097113041	4.010326149	4.004000882	3.901112176	3.767126684	3.817055479
ENSG00000184988	0.014539	0.058498129	-0.386341884	-1.307074962	TMEM106A	protein_coding	transmembrane protein 106A [Source:HGNC Symbol;Acc:HGNC:28288]	chr17	43211835	43220041	1	ensembl_havana	4.445451802	4.638622744	4.25228086	4.49406847	4.591785246	4.830014515	4.342021288	4.147472285	4.267349007
ENSG00000184557	0.014544	0.058503886	-0.308038516	-1.238023343	SOCS3	protein_coding	suppressor of cytokine signaling 3 [Source:HGNC Symbol;Acc:HGNC:19391]	chr17	78356778	78360077	-1	ensembl_havana	4.836632768	4.990652026	4.68261351	5.197238709	4.823253108	4.951464261	4.857605861	4.67737277	4.512861898
ENSG00000107796	0.014571	0.058576183	0.224750361	1.168575025	ACTA2	protein_coding	actin, alpha 2, smooth muscle, aorta [Source:HGNC Symbol;Acc:HGNC:130]	chr10	88935074	88991339	-1	ensembl_havana	2.955926362	2.843551181	3.068301543	3.230339436	2.71937662	2.580937487	3.409325542	2.919625469	2.875953617

ENSG00000204370	0.014567	0.058576183	-0.229002176	-1.172024052	**SDHD**	protein_coding	succinate dehydrogenase complex subunit D [Source:HGNC Symbol;Acc:HGNC:10683]	chr11	112086773	112120013	1	ensembl_havana	6.124309066	6.238810154	6.009807978	6.175375403	6.283178509	6.257876552	5.916831476	6.006034208	6.10655825
ENSG00000050344	0.014578	0.058588224	-0.217834565	-1.162986674	**NFE2L3**	protein_coding	nuclear factor, erythroid 2 like 3 [Source:HGNC Symbol;Acc:HGNC:7783]	chr7	26152240	26187125	1	ensembl_havana	7.830613646	7.939530928	7.721696363	7.879671336	7.867122051	8.071799399	7.616915381	7.703984694	7.844189014
ENSG00000101158	0.014587	0.058589565	0.257567591	1.195461435	**NELFCD**	protein_coding	negative elongation factor complex member C/D [Source:HGNC Symbol;Acc:HGNC:15934]	chr20	58981208	58995133	1	ensembl_havana	7.040334496	6.9115507	7.169118291	6.726895087	6.914481677	7.093275336	7.094212528	7.154069317	7.259073029
ENSG00000101290	0.014588	0.058589565	-0.210963681	-1.157461078	**CDS2**	protein_coding	CDP-diacylglycerol synthase 2 [Source:HGNC Symbol;Acc:HGNC:1801]	chr20	5126786	5197887	1	ensembl_havana	7.762248387	7.867730227	7.656766546	8.057619264	7.763795679	7.78177574	7.828908427	7.594908283	7.546482929
ENSG00000169490	0.014594	0.058589565	-0.211894816	-1.158208361	**TM2D2**	protein_coding	TM2 domain containing 2 [Source:HGNC Symbol;Acc:HGNC:24127]	chr8	38988808	38996824	-1	ensembl_havana	4.648052808	4.754000216	4.542105399	4.777581124	4.744335121	4.740084403	4.568817556	4.56959204	4.487906603
ENSG00000164466	0.014594	0.058589565	-0.219021785	-1.16394411	**SFXN1**	protein_coding	sideroflexin 1 [Source:HGNC Symbol;Acc:HGNC:16085]	chr5	175477062	175529742	1	ensembl_havana	7.074058673	7.183569566	6.964547781	7.188347116	7.17806788	7.184293701	6.924248687	6.9461431	7.023251555
ENSG00000174456	0.01461	0.058634542	0.227724967	1.170986925	**C12orf76**	protein_coding	chromosome 12 open reading frame 76 [Source:HGNC Symbol;Acc:HGNC:33790]	chr12	110027028	110073686	-1	ensembl_havana	3.759990387	3.646127904	3.87385287	3.649251657	3.638270336	3.650861717	3.916655568	3.902954406	3.801948638
ENSG00000251141	0.014618	0.058634542	-0.256696861	-1.194740139	**MRPS30-DT**	antisense	MRPS30 divergent transcript [Source:HGNC Symbol;Acc:HGNC:53420]	chr5	44744900	44808777	-1	havana	0.693612736	0.821961166	0.565264306	1.109412078	0.77991046	0.57656096	0.739683594	0.554044597	0.402064725
ENSG00000101901	0.014616	0.058634542	-0.275634078	-1.21052601	**ALG13**	protein_coding	ALG13, UDP-N-acetylglucosaminyltransferase subunit [Source:HGNC Symbol;Acc:HGNC:30881]	chrX	111665811	111760649	1	ensembl_havana	4.897825362	5.035642401	4.760008323	5.157216224	5.015446565	4.934264414	4.760456192	4.741870088	4.777698689
ENSG00000197816	0.014641	0.058639765	0.257567794	1.195461604	**CCDC180**	protein_coding	coiled-coil domain containing 180 [Source:HGNC Symbol;Acc:HGNC:29303]	chr9	97307304	97378524	1	havana	2.583442713	2.454658816	2.71222661	2.458169134	2.488543365	2.417263948	2.600804468	2.780215171	2.755660191
ENSG00000115649	0.014641	0.058639765	0.236878312	1.178440005	**CNPPD1**	protein_coding	cyclin Pas1/PHO80 domain containing 1 [Source:HGNC Symbol;Acc:HGNC:25220]	chr2	219171897	219178106	-1	ensembl_havana	5.399564102	5.281124946	5.518003258	5.249565961	5.23294695	5.360861928	5.505205523	5.53749097	5.511313281
ENSG00000162734	0.014639	0.058639765	0.225124216	1.168877885	**PEA15**	protein_coding	proliferation and apoptosis adaptor protein 15 [Source:HGNC Symbol;Acc:HGNC:8822]	chr1	160205337	160215376	1	ensembl_havana	7.823402351	7.710840243	7.935964459	7.538981872	7.835547083	7.757991774	7.836589247	8.023396684	7.947907447
ENSG00000133943	0.014642	0.058639765	-0.222871149	-1.167053865	**DGLUCY**	protein_coding	D-glutamate cyclase [Source:HGNC Symbol;Acc:HGNC:20498]	chr14	91060333	91225632	1	ensembl_havana	5.803258601	5.914694175	5.691823026	5.865292978	5.860541524	6.018248023	5.577881428	5.689959044	5.807628606
ENSG00000103550	0.014644	0.058639765	-0.255805503	-1.194002207	**KNOP1**	protein_coding	lysine rich nucleolar protein 1 [Source:HGNC Symbol;Acc:HGNC:34404]	chr16	19701934	19718235	-1	ensembl_havana	5.093301408	5.221204159	4.965398656	5.198225183	5.15529312	5.310094174	4.834606865	4.990295146	5.071293958
ENSG00000231652	0.014638	0.058639765	-0.371519262	-1.293714488	**AL590428.1**	antisense		chr6	73693903	73696131	-1	havana	0.630328175	0.816087806	0.444568543	0.326725152	1.013233389	1.108304877	0.069637898	0.758913071	0.505154661
ENSG00000188021	0.014667	0.05870889	0.26014164	1.197596276	**UBQLN2**	protein_coding	ubiquilin 2 [Source:HGNC Symbol;Acc:HGNC:12509]	chrX	56563639	56567868	1	ensembl_havana	6.297290496	6.167219675	6.427361316	6.234269554	6.256301064	6.011088409	6.588470182	6.403081976	6.29053179
ENSG00000104290	0.014669	0.05870889	0.24321084	1.183623988	**FZD3**	protein_coding	frizzled class receptor 3 [Source:HGNC Symbol;Acc:HGNC:4041]	chr8	28494205	28574268	1	ensembl_havana	6.251144195	6.129538775	6.372749615	6.577205851	6.107069379	5.704341096	6.721264173	6.389010888	6.007973785
ENSG00000272077	0.014688	0.058734197	0.446754226	1.362970395	**AC124045.1**	lincRNA		chr3	44667412	44669364	1	havana	1.012736731	0.789359617	1.236113844	0.267803752	1.242575255	0.857699845	0.995284613	1.465989718	1.2470672
ENSG00000196876	0.014684	0.058734197	0.382436774	1.303541735	**SCN8A**	protein_coding	sodium voltage-gated channel alpha subunit 8 [Source:HGNC Symbol;Acc:HGNC:10596]	chr12	51590266	51812864	1	ensembl_havana	4.978706247	4.78748786	5.169924634	4.502079217	4.701268571	5.15911579	5.006665429	5.203136127	5.299972345
ENSG00000163291	0.014692	0.058734197	0.215383674	1.161012636	**PAQR3**	protein_coding	progestin and adipoQ receptor family member 3 [Source:HGNC Symbol;Acc:HGNC:30130]	chr4	78887127	78939438	-1	ensembl_havana	4.893225283	4.785533446	5.00091712	4.710025423	4.762933881	4.883641034	4.96241916	4.925599042	5.114733159
ENSG00000186205	0.01468	0.058734197	-0.508981713	-1.423045423	**MARC1**	protein_coding	mitochondrial amidoxime reducing component 1 [Source:HGNC Symbol;Acc:HGNC:26189]	chr1	220786759	220819657	1	ensembl_havana	0.025378906	0.279869762	-0.229111951	0.237415364	0.403680778	0.198513145	-0.57379957	0.19485955	-0.308395834
ENSG00000088256	0.014703	0.058747261	0.404312949	1.323458493	**GNA11**	protein_coding	G protein subunit alpha 11 [Source:HGNC Symbol;Acc:HGNC:4379]	chr19	3094410	3124004	1	ensembl_havana	5.029131511	4.826975037	5.231287986	4.889298954	4.68575637	4.905869787	5.449304117	5.193355006	5.051204834
ENSG00000100068	0.014702	0.058747261	0.210384745	1.156996696	**LRP5L**	protein_coding	LDL receptor related protein 5 like [Source:HGNC Symbol;Acc:HGNC:25323]	chr22	25351418	25405377	-1	ensembl_havana	2.755479371	2.650286999	2.860671744	2.807536027	2.476573576	2.666751395	2.991732974	2.671684573	2.918597684
ENSG00000196781	0.014721	0.058801619	0.387675343	1.308283628	**TLE1**	protein_coding	transducin like enhancer of split 1 [Source:HGNC Symbol;Acc:HGNC:11837]	chr9	81583683	81689305	-1	ensembl_havana	1.924660852	1.730823181	2.118498523	1.297904502	1.817810321	2.076754718	1.926226713	2.131077666	2.298191191
ENSG00000181634	0.014736	0.05881332	0.775174155	1.71139663	**TNFSF15**	protein_coding	TNF superfamily member 15 [Source:HGNC Symbol;Acc:HGNC:11931]	chr9	114784635	114806126	-1	ensembl_havana	-0.385146598	-0.772733676	0.002440479	-1.41179303	-0.783495335	-0.122912658	-0.26243933	0.163140205	0.106620565
ENSG00000256628	0.01473	0.05881332	0.404284454	1.323432353	**ZBTB11-AS1**	antisense	ZBTB11 antisense RNA 1 [Source:HGNC Symbol;Acc:HGNC:48573]	chr3	101676475	101679217	1	havana	2.914734107	2.71259188	3.116876334	2.535139	2.714288775	2.888347866	3.104607948	3.210869813	3.035151242
ENSG00000152413	0.014734	0.05881332	0.203809749	1.151735751	**HOMER1**	protein_coding	homer scaffolding protein 1 [Source:HGNC Symbol;Acc:HGNC:17512]	chr5	79372636	79514217	-1	ensembl_havana	4.715716795	4.613811921	4.81762167	4.564612562	4.626053278	4.650769923	4.784658061	4.818955118	4.84925183
ENSG00000226121	0.014752	0.058853313	0.566331629	1.480753636	**AC009487.2**	processed_pseudogene		chr2	161500885	161507705	-1	havana	-0.6921151	-0.975280914	-0.408949286	-1.14838881	-1.03440435	-0.743049581	-0.97139419	-0.32890884	0.073455173
ENSG00000073969	0.014758	0.058853313	0.279621906	1.213876716	**NSF**	protein_coding	N-ethylmaleimide sensitive factor, vesicle fusing ATPase [Source:HGNC Symbol;Acc:HGNC:8016]	chr17	46590669	46757464	1	ensembl_havana	5.949840743	5.810029789	6.089651696	5.671492007	5.936783874	5.821813487	6.010950576	6.073174184	6.184830327
ENSG00000136485	0.014756	0.058853313	0.202745286	1.15088628	**DCAF7**	protein_coding	DDB1 and CUL4 associated factor 7 [Source:HGNC Symbol;Acc:HGNC:30915]	chr17	63550461	63594266	1	ensembl_havana	8.127845657	8.026473014	8.2292183	7.947679809	8.000712275	8.131026957	8.147618619	8.210683906	8.329352375
ENSG00000154114	0.014765	0.058865018	0.274881532	1.209894734	**TBCEL**	protein_coding	tubulin folding cofactor E like [Source:HGNC Symbol;Acc:HGNC:28115]	chr11	121024072	121090775	1	ensembl_havana	5.623420799	5.485980032	5.760861565	5.650743504	5.515554998	5.291641595	5.793869321	5.893951383	5.59476399
ENSG00000259081	0.014785	0.058910936	0.401583409	1.320956913	**AF111169.3**	antisense		chr14	76774284	76781518	-1	havana	0.112199533	-0.088592172	0.312991237	-0.27307956	-0.071090603	0.078393642	0.069637898	0.578298274	0.291037539
ENSG00000163904	0.014781	0.058910936	0.225227227	1.168961347	**SENP2**	protein_coding	SUMO1/sentrin/SMT3 specific peptidase 2 [Source:HGNC Symbol;Acc:HGNC:23116]	chr3	185582496	185633551	1	ensembl_havana	6.5604505	6.447836886	6.673064113	6.347526368	6.540183304	6.455800987	6.646329829	6.73279785	6.640064661
ENSG00000259642	0.014789	0.058910936	-0.219027169	-1.163948454	**ST20-AS1**	antisense	ST20 antisense RNA 1 [Source:HGNC Symbol;Acc:HGNC:27521]	chr15	79922771	79926993	1	ensembl_havana	2.151463507	2.260977092	2.041949923	2.503565738	2.351074074	1.928291464	2.278730383	2.081032632	1.766086754
ENSG00000186918	0.014804	0.05892072	0.231916159	1.17439372	**ZNF395**	protein_coding	zinc finger protein 395 [Source:HGNC Symbol;Acc:HGNC:18737]	chr8	28345585	28402701	-1	ensembl_havana	3.841874638	3.725916559	3.957832718	3.547648667	3.741908871	3.888192139	3.86417946	3.933828835	4.075489857
ENSG00000107960	0.014798	0.05892072	-0.211989293	-1.15828421	**STN1**	protein_coding	STN1, CST complex subunit [Source:HGNC Symbol;Acc:HGNC:26200]	chr10	103882542	103918205	-1	ensembl_havana	3.52810832	3.634102967	3.422113674	3.556907091	3.630188857	3.715212953	3.390895783	3.385963185	3.489482054
ENSG00000114978	0.014801	0.05892072	-0.224961493	-1.168746053	**MOB1A**	protein_coding	MOB kinase activator 1A [Source:HGNC Symbol;Acc:HGNC:16015]	chr2	74152528	74178898	-1	ensembl_havana	8.028110417	8.140591163	7.91562967	8.281932665	8.126090162	8.013750663	7.986498526	7.918507692	7.841882793
ENSG00000143248	0.014809	0.058924761	-0.275937641	-1.210780748	**RGS5**	protein_coding	regulator of G protein signaling 5 [Source:HGNC Symbol;Acc:HGNC:10001]	chr1	163111121	163321791	-1	ensembl_havana	1.504222435	1.642191256	1.366253615	1.694503432	1.759729761	1.472340574	1.325353043	1.43975459	1.333653212
ENSG00000006607	0.01482	0.058949988	0.267577892	1.203785123	**FARP2**	protein_coding	FERM, ARH/RhoGEF and pleckstrin domain protein 2 [Source:HGNC Symbol;Acc:HGNC:16460]	chr2	241356243	241494841	1	ensembl_havana	3.990752674	3.856963728	4.124541619	3.843347077	3.875530348	3.852013758	4.158096097	4.013810563	4.201718198
ENSG00000095794	0.01484	0.05901281	0.273071115	1.208377407	**CREM**	protein_coding	cAMP responsive element modulator [Source:HGNC Symbol;Acc:HGNC:2352]	chr10	35126791	35212958	1	ensembl_havana	4.949503891	4.812968334	5.086039449	4.296359896	4.95956846	5.182976644	4.680770516	5.246348626	5.330999204
ENSG00000175806	0.014844	0.059014833	0.268316656	1.204401705	**MSRA**	protein_coding	methionine sulfoxide reductase A [Source:HGNC Symbol;Acc:HGNC:7377]	chr8	10054268	10428891	1	ensembl_havana	2.719705803	2.585547475	2.853864131	2.471286007	2.783935853	2.501420566	2.775774631	2.924363622	2.861454141
ENSG00000148384	0.01485	0.059014975	0.449293647	1.365371598	**INPP5E**	protein_coding	inositol polyphosphate-5-phosphatase E [Source:HGNC Symbol;Acc:HGNC:21474]	chr9	136428619	136439823	-1	ensembl_havana	4.319328736	4.094681912	4.543975559	3.897797013	4.075263288	4.310985435	4.558946287	4.602484434	4.470495956
ENSG00000241399	0.014852	0.059014975	-0.323626917	-1.251472784	**CD302**	protein_coding	CD302 molecule [Source:HGNC Symbol;Acc:HGNC:30843]	chr2	159768630	159798255	-1	ensembl_havana	3.459043597	3.620857055	3.297230138	3.959604644	3.591864916	3.311101606	3.597886198	3.127034733	3.166769484
ENSG00000137216	0.01487	0.059067929	0.245195949	1.185253743	**TMEM63B**	protein_coding	transmembrane protein 63B [Source:HGNC Symbol;Acc:HGNC:17735]	chr6	44126914	44155519	1	ensembl_havana	6.287528787	6.164930812	6.410126762	6.489268435	6.220415216	5.785108786	6.742860663	6.371768956	6.115750668
ENSG00000099821	0.014885	0.059097015	0.269650525	1.205515771	**POLRMT**	protein_coding	RNA polymerase mitochondrial [Source:HGNC Symbol;Acc:HGNC:9200]	chr19	617224	633604	-1	ensembl_havana	4.988130977	4.853305715	5.12295624	4.849668003	4.690945658	5.019303483	5.147256699	5.05977042	5.1618416
ENSG00000146918	0.014882	0.059097015	-0.20465	-1.152406737	**NCAPG2**	protein_coding	non-SMC condensin II complex subunit G2 [Source:HGNC Symbol;Acc:HGNC:21904]	chr7	158631311	158704829	-1	ensembl_havana	6.829722751	6.932047751	6.727397751	6.889625427	6.975558031	6.930959795	6.709414179	6.759227182	6.713551892
ENSG00000125637	0.014891	0.059103204	-0.538019141	-1.451977544	**PSD4**	protein_coding	pleckstrin and Sec7 domain containing 4 [Source:HGNC Symbol;Acc:HGNC:19096]	chr2	113157325	113209396	1	ensembl_havana	-0.370104743	-0.101095173	-0.639114314	0.075059705	-0.218971072	-0.159374151	-0.75894329	-0.80561234	-0.352787316
ENSG00000140521	0.014899	0.059118109	0.235948137	1.177680453	**POLG**	protein_coding	DNA polymerase gamma, catalytic subunit [Source:HGNC Symbol;Acc:HGNC:9179]	chr15	89305198	89334861	-1	ensembl_havana	6.983687711	6.865713643	7.101661779	6.815383402	6.852153247	6.929604278	7.131322743	7.086186333	7.087476262
ENSG00000144354	0.014915	0.059166452	-0.205147958	-1.152804568	**CDCA7**	protein_coding	cell division cycle associated 7 [Source:HGNC Symbol;Acc:HGNC:14628]	chr2	173354820	173368997	1	ensembl_havana	6.456391596	6.558965575	6.353817617	6.358157342	6.558752639	6.759986743	6.156063879	6.328634332	6.576754639
ENSG00000142186	0.014952	0.059280818	0.256267119	1.19438431	**SCYL1**	protein_coding	SCY1 like pseudokinase 1 [Source:HGNC Symbol;Acc:HGNC:14372]	chr11	65525077	65538704	1	ensembl_havana	5.865418987	5.737285428	5.993552547	5.73608625	5.773332589	5.702437444	6.110610588	5.974399661	5.895647392
ENSG00000148057	0.014952	0.059280818	-0.44355269	-1.359949133	**IDNK**	protein_coding	IDNK, gluconokinase [Source:HGNC Symbol;Acc:HGNC:31367]	chr9	83623049	83644130	1	ensembl_havana	0.872707013	1.094483358	0.650930668	1.005046425	1.124807863	1.153595787	0.513047461	0.45273451	0.987010032
ENSG00000135976	0.014957	0.059284312	0.242523029	1.183059824	**ANKRD36**	protein_coding	ankyrin repeat domain 36 [Source:HGNC Symbol;Acc:HGNC:24079]	chr2	97113496	97264521	1	ensembl_havana	3.973926728	3.852665214	4.095188242	3.912297142	3.589088138	4.056610361	4.136133802	3.926762386	4.222668539
ENSG00000103037	0.014968	0.059311543	0.225329675	1.16904436	**SETD6**	protein_coding	SET domain containing 6 [Source:HGNC Symbol;Acc:HGNC:26116]	chr16	58515479	58521181	1	ensembl_havana	4.782315095	4.669650258	4.894979933	4.536807471	4.660849095	4.811294207	4.715250209	4.960618057	5.009071532
ENSG00000234663	0.014973	0.05931358	0.28548536	1.218820237	**LINC01934**	lincRNA	long intergenic non-protein coding RNA 1934 [Source:HGNC Symbol;Acc:HGNC:52757]	chr2	181086076	181409321	1	havana	1.95077219	1.80802951	2.09351487	1.770752281	1.592342839	2.060993409	1.905603022	1.966251876	2.40868971
ENSG00000182318	0.014978	0.059317031	0.294189307	1.226195748	**ZSCAN22**	protein_coding	zinc finger and SCAN domain containing 22 [Source:HGNC Symbol;Acc:HGNC:4929]	chr19	58327019	58342332	1	ensembl_havana	3.537008212	3.389913558	3.684102865	3.312806534	3.436793925	3.420140216	3.767486582	3.649006201	3.635815812
ENSG00000213246	0.014987	0.059336639	0.291183173	1.223643393	**SUPT4H1**	protein_coding	SPT4 homolog, DSIF elongation factor subunit [Source:HGNC Symbol;Acc:HGNC:11467]	chr17	58345175	58353093	-1	ensembl_havana	5.953371781	5.807780194	6.098963367	5.913540446	5.829114817	5.680685319	6.047296792	6.196793309	6.052800001
ENSG00000140545	0.015024	0.059454084	0.26154157	1.198758936	**MFGE8**	protein_coding	milk fat globule-EGF factor 8 protein [Source:HGNC Symbol;Acc:HGNC:7036]	chr15	88898683	88913411	-1	ensembl_havana	5.834613782	5.703842997	5.965384567	5.774954001	5.847239677	5.489335313	6.147855561	6.003797458	5.744500682
ENSG00000135018	0.015025	0.059454084	-0.218184149	-1.163268514	**UBQLN1**	protein_coding	ubiquilin 1 [Source:HGNC Symbol;Acc:HGNC:12508]	chr9	83659963	83708203	-1	ensembl_havana	7.009306491	7.118398565	6.900214417	7.168602614	7.115522616	7.071070466	6.888567978	6.918802281	6.89327299
ENSG00000198894	0.015046	0.05948619	0.227870332	1.171104918	**CIPC**	protein_coding	CLOCK interacting pacemaker [Source:HGNC Symbol;Acc:HGNC:20365]	chr14	77098097	77117287	1	ensembl_havana	5.154956004	5.04102838	5.26889117	5.050923438	5.069240871	5.002898206	5.351669833	5.289222981	5.165780696
ENSG00000131351	0.015042	0.05948619	-0.209736641	-1.156477054	**HAUS8**	protein_coding	HAUS augmin like complex subunit 8 [Source:HGNC Symbol;Acc:HGNC:30532]	chr19	17049729	17075625	-1	ensembl_havana	4.429310029	4.534178349	4.324441708	4.401170796	4.611075527	4.590288725	4.177580308	4.372182967	4.42356185
ENSG00000125676	0.01504	0.05948619	-0.241558184	-1.182268882	**THOC2**	protein_coding	THO complex 2 [Source:HGNC Symbol;Acc:HGNC:19073]	chrX	123600561	123733056	-1	ensembl_havana	7.462320826	7.583099917	7.341541734	7.681414912	7.487839055	7.580045786	7.342893372	7.316072899	7.365658931
ENSG00000154822	0.015078	0.059598789	0.267630786	1.203829258	**PLCL2**	protein_coding	phospholipase C like 2 [Source:HGNC Symbol;Acc:HGNC:9064]	chr3	16802651	17090594	1	ensembl_havana	5.894278551	5.760463158	6.028093944	5.5615671	5.878846762	5.840975612	5.953713021	6.045170108	6.085398702
ENSG00000177427	0.015101	0.059623589	0.358656704	1.282231454	**MIEF2**	protein_coding	mitochondrial elongation factor 2 [Source:HGNC Symbol;Acc:HGNC:17920]	chr17	18260534	18266552	1	ensembl_havana	2.658569391	2.479241039	2.837897743	2.471286007	2.331270798	2.63516631	2.89565563	2.841618992	2.776418607
ENSG00000136450	0.015097	0.059623589	-0.206371607	-1.153782756	**SRSF1**	protein_coding	serine and arginine rich splicing factor 1 [Source:HGNC Symbol;Acc:HGNC:10780]	chr17	58003360	58007346	-1	ensembl_havana	8.24659019	8.349775994	8.143404387	8.363451193	8.309757046	8.376119741	8.12270555	8.118075313	8.189432298
ENSG00000188811	0.015101	0.059623589	-0.266780211	-1.203119721	**NHLRC3**	protein_coding	NHL repeat containing 3 [Source:HGNC Symbol;Acc:HGNC:33751]	chr13	39038306	39050109	1	ensembl_havana	5.090214876	5.223604982	4.956824771	5.313780003	5.187775973	5.169258969	4.981020293	4.855386052	5.034067967
ENSG00000050555	0.015097	0.059623589	-0.552725487	-1.466854207	**LAMC3**	protein_coding	laminin subunit gamma 3 [Source:HGNC Symbol;Acc:HGNC:6494]	chr9	131009082	131094473	1	ensembl_havana	6.162407677	6.43877042	5.886044934	6.308318779	6.164155668	6.843836815	5.984767849	5.76649691	5.906870041
ENSG00000126870	0.015124	0.059696307	0.217239899	1.1625074	**WDR60**	protein_coding	WD repeat domain 60 [Source:HGNC Symbol;Acc:HGNC:21862]	chr7	158856578	158956747	1	ensembl_havana	3.94044704	3.83182709	4.04906699	3.9734996	3.714142396	3.807839275	4.173273167	3.991407493	3.982520308
ENSG00000106006	0.015151	0.059786284	0.688994882	1.612159944	**HOXA6**	protein_coding	homeobox A6 [Source:HGNC Symbol;Acc:HGNC:5107]	chr7	27145396	27150603	-1	ensembl_havana	1.176690355	0.832192914	1.521187796	0.613726219	0.547932369	1.334920154	1.580839304	1.452931788	1.529792296
ENSG00000076003	0.015171	0.059848707	-0.203664032	-1.151619427	**MCM6**	protein_coding	minichromosome maintenance complex component 6 [Source:HGNC Symbol;Acc:HGNC:6949]	chr2	135839626	135876426	-1	ensembl_havana	8.088207531	8.190039546	7.986375515	8.058162704	8.244198404	8.267757532	7.879192717	8.031928492	8.048005336
ENSG00000167842	0.015225	0.060048236	-0.213308955	-1.159344201	**MIS12**	protein_coding	MIS12, kinetochore complex component [Source:HGNC Symbol;Acc:HGNC:24967]	chr17	5486285	5490814	1	ensembl_havana	5.445355641	5.552010119	5.338701164	5.552338628	5.60555083	5.498140898	5.328615561	5.351236196	5.336251734
ENSG00000149743	0.015232	0.060059499	0.35201308	1.276340341	**TRPT1**	protein_coding	tRNA phosphotransferase 1 [Source:HGNC Symbol;Acc:HGNC:20316]	chr11	64223799	64226254	-1	ensembl_havana	3.126958818	2.950952277	3.302965358	2.716829986	2.983208668	3.152818179	3.23844292	3.368684571	3.301768582
ENSG00000170965	0.01524	0.060073537	-1.176514725	-2.260300716	**PLAC1**	protein_coding	placenta specific 1 [Source:HGNC Symbol;Acc:HGNC:9044]	chrX	134565838	134764322	-1	ensembl_havana	-0.684468553	-0.096211191	-1.272725915	-0.18813307	-0.299013651	0.198513145	-2.22890346	-0.93694677	-0.652327512
ENSG00000067334	0.015248	0.060087486	-0.24210437	-1.182716559	**DNTTIP2**	protein_coding	deoxynucleotidyltransferase terminal interacting protein 2 [Source:HGNC Symbol;Acc:HGNC:24013]	chr1	93866283	93879918	-1	ensembl_havana	6.723465934	6.844518119	6.602413749	6.904509736	6.870278672	6.758765949	6.567699367	6.642588822	6.596953057
ENSG00000134253	0.015254	0.06009552	-0.334031739	-1.260531123	**TRIM45**	protein_coding	tripartite motif containing 45 [Source:HGNC Symbol;Acc:HGNC:19018]	chr1	117111060	117122587	-1	ensembl_havana	1.582474214	1.749490084	1.415458345	1.738566362	1.547275176	1.962628713	1.245545533	1.399485086	1.601344415
ENSG00000177000	0.015264	0.060101714	0.262663607	1.199691618	**MTHFR**	protein_coding	methylenetetrahydrofolate reductase [Source:HGNC Symbol;Acc:HGNC:7436]	chr1	11785723	11806920	-1	ensembl_havana	6.028982226	5.897650422	6.160314029	5.950892295	5.79277083	5.949288142	6.216443006	6.164616418	6.099882665
ENSG00000188786	0.015263	0.060101714	0.244858305	1.184976383	**MTF1**	protein_coding	metal regulatory transcription factor 1 [Source:HGNC Symbol;Acc:HGNC:7428]	chr1	37809567	37859620	-1	ensembl_havana	5.524142326	5.401713174	5.646571479	5.503694426	5.440517184	5.26092791	5.677802171	5.652612197	5.609300069
ENSG00000260838	0.015268	0.060102873	-0.339117027	-1.264982149	**AC022893.2**	lincRNA		chr8	72947150	72950445	1	havana	1.961244636	2.130803149	1.791686122	2.23025056	2.077587619	2.084571268	1.7752573	1.648877048	1.950924019
ENSG00000109674	0.015279	0.060127514	-0.22964439	-1.172545893	**NEIL3**	protein_coding	nei like DNA glycosylase 3 [Source:HGNC Symbol;Acc:HGNC:24573]	chr4	177309836	177362943	1	ensembl_havana	3.794313918	3.909136114	3.679491723	3.497241177	3.884617399	4.345549764	3.32252843	3.682954534	4.032992206
ENSG00000159259	0.015288	0.060142846	-0.207711401	-1.154854742	**CHAF1B**	protein_coding	chromatin assembly factor 1 subunit B [Source:HGNC Symbol;Acc:HGNC:1911]	chr21	36385378	36419015	1	ensembl_havana	5.4323516	5.5362073	5.3284959	5.408028057	5.609666826	5.590927018	5.223182524	5.419949561	5.342355613
ENSG00000120697	0.015291	0.060142846	-0.349640039	-1.274242656	**ALG5**	protein_coding	ALG5, dolichyl-phosphate beta-glucosyltransferase [Source:HGNC Symbol;Acc:HGNC:20266]	chr13	36949775	37000261	-1	ensembl_havana	3.719802002	3.894622021	3.544981983	4.153128786	3.805489363	3.725247916	3.601096404	3.491900287	3.541949257
ENSG00000143390	0.015306	0.060182917	-0.238815425	-1.180023364	**RFX5**	protein_coding	regulatory factor X5 [Source:HGNC Symbol;Acc:HGNC:9986]	chr1	151340640	151347357	-1	ensembl_havana	7.270179788	7.389587501	7.150772076	7.570369165	7.303212643	7.295180694	7.235699893	7.129659307	7.086957027
ENSG00000163536	0.015311	0.060186266	0.41143441	1.330007526	**SERPINI1**	protein_coding	serpin family I member 1 [Source:HGNC Symbol;Acc:HGNC:8943]	chr3	167735243	167825568	1	ensembl_havana	5.303294801	5.097577596	5.509012005	4.736070601	5.364612356	5.19204983	5.354525885	5.521162667	5.651347464
ENSG00000159720	0.015327	0.060233054	0.216456295	1.161876152	**ATP6V0D1**	protein_coding	ATPase H+ transporting V0 subunit d1 [Source:HGNC Symbol;Acc:HGNC:13724]	chr16	67438014	67481237	-1	ensembl_havana	6.665193285	6.556965137	6.773421433	6.524695978	6.604843949	6.541355485	6.797753656	6.771440938	6.751069704
ENSG00000156535	0.015335	0.060249268	0.206568351	1.153940112	**CD109**	protein_coding	CD109 molecule [Source:HGNC Symbol;Acc:HGNC:21685]	chr6	73695785	73828316	1	ensembl_havana	6.737470078	6.634185903	6.840754254	6.657581608	6.656819641	6.588156459	6.826151975	6.879999099	6.816111688
ENSG00000118900	0.015348	0.06028233	0.210274704	1.15690845	**UBN1**	protein_coding	ubinuclein 1 [Source:HGNC Symbol;Acc:HGNC:12506]	chr16	4846665	4882360	1	ensembl_havana	5.991924184	5.886786832	6.097061536	5.894191167	5.794579809	5.971589519	6.104402791	6.046800589	6.139981228
ENSG00000110375	0.015385	0.060300366	0.575721278	1.490422416	**UPK2**	protein_coding	uroplakin 2 [Source:HGNC Symbol;Acc:HGNC:12579]	chr11	118925164	118958559	1	ensembl_havana	-0.319832956	-0.607693595	-0.031972317	-0.4596317	-0.727039001	-0.636410081	-0.08686711	0.256279753	-0.265329594
ENSG00000243885	0.015361	0.060300366	0.539866754	1.453838235	**AC108751.4**	lincRNA		chr3	149384179	149385800	-1	havana	0.246931535	-0.023001842	0.516864911	-0.27307956	0.09469996	0.109374069	0.64332197	0.478736765	0.428535999
ENSG00000085365	0.015367	0.060300366	0.255601644	1.193833501	**SCAMP1**	protein_coding	secretory carrier membrane protein 1 [Source:HGNC Symbol;Acc:HGNC:10563]	chr5	78360583	78479071	1	ensembl_havana	5.081868574	4.954067752	5.209669396	4.944426293	4.902557343	5.015219619	5.087855498	5.251071083	5.290081607
ENSG00000162298	0.01537	0.060300366	0.251580885	1.190510945	**SYVN1**	protein_coding	synoviolin 1 [Source:HGNC Symbol;Acc:HGNC:20738]	chr11	65121780	65134533	-1	ensembl_havana	5.338056941	5.212266499	5.463847384	5.311958391	5.098824377	5.226016729	5.544921227	5.458026668	5.388594255
ENSG00000122591	0.01539	0.060300366	0.22436598	1.16826372	**FAM126A**	protein_coding	family with sequence similarity 126 member A [Source:HGNC Symbol;Acc:HGNC:24587]	chr7	22889371	23014130	-1	ensembl_havana	7.735584387	7.623401397	7.847767377	7.682472739	7.631730938	7.556000514	7.836589247	7.864676228	7.842036656
ENSG00000112306	0.015388	0.060300366	0.206779436	1.15410896	**RPS12**	protein_coding	ribosomal protein S12 [Source:HGNC Symbol;Acc:HGNC:10385]	chr6	132814441	132817564	1	ensembl_havana	8.792466322	8.689076604	8.89585604	8.666970797	8.728504084	8.671754932	8.885713952	8.896824965	8.905029204
ENSG00000123131	0.015382	0.060300366	-0.209607408	-1.156373464	**PRDX4**	protein_coding	peroxiredoxin 4 [Source:HGNC Symbol;Acc:HGNC:17169]	chrX	23664262	23686399	1	ensembl_havana	5.121792175	5.226595879	5.016988471	5.189322542	5.213245738	5.277219358	4.935967957	5.010488955	5.104508502
ENSG00000166801	0.01537	0.060300366	-0.210673931	-1.157228637	**FAM111A**	protein_coding	family with sequence similarity 111 member A [Source:HGNC Symbol;Acc:HGNC:24725]	chr11	59142748	59155039	1	ensembl_havana	7.120314484	7.225651449	7.014977519	7.034190472	7.309973911	7.332789964	6.87304933	7.07664078	7.095242446
ENSG00000248884	0.015376	0.060300366	-0.662403135	-1.582716802	**AC010280.2**	lincRNA		chr5	68430427	68434481	-1	havana	-0.250741839	0.080459728	-0.581943406	-0.03191641	0.547932369	-0.274636777	-0.57379957	-0.57414696	-0.597883695
ENSG00000130640	0.015405	0.060342545	0.337411071	1.263487219	**TUBGCP2**	protein_coding	tubulin gamma complex associated protein 2 [Source:HGNC Symbol;Acc:HGNC:18599]	chr10	133278630	133312337	-1	ensembl_havana	5.612623904	5.443918369	5.78132944	5.408880473	5.36947522	5.553399413	5.907785967	5.735828273	5.700374079
ENSG00000108387	0.01541	0.060346915	0.438604522	1.355292757	**sept.04**	protein_coding	septin 4 [Source:HGNC Symbol;Acc:HGNC:9165]	chr17	58520250	58540818	-1	ensembl_havana	-0.025370052	-0.244672313	0.193932209	-0.4596317	-0.383759306	0.109374069	-0.21650287	0.343769191	0.454530303
ENSG00000108528	0.01542	0.060368477	-0.225247175	-1.168977511	**SLC25A11**	protein_coding	solute carrier family 25 member 11 [Source:HGNC Symbol;Acc:HGNC:10981]	chr17	4937130	4940251	-1	ensembl_havana	5.768166816	5.880790404	5.655543229	5.870871668	5.864565689	5.906933854	5.68083957	5.682286915	5.603503201
ENSG00000179941	0.015466	0.060530169	-0.425646286	-1.343174072	**BBS10**	protein_coding	Bardet-Biedl syndrome 10 [Source:HGNC Symbol;Acc:HGNC:26291]	chr12	76344474	76348442	-1	ensembl_havana	4.195982071	4.408805214	3.983158928	4.530555105	4.329321514	4.366539023	3.820779437	4.051115116	4.07758223
ENSG00000110395	0.015473	0.060541042	-0.263547073	-1.2004265	**CBL**	protein_coding	Cbl proto-oncogene [Source:HGNC Symbol;Acc:HGNC:1541]	chr11	119206276	119313926	1	ensembl_havana	8.33805062	8.469824156	8.206277083	8.551968381	8.288304817	8.56919927	8.29067613	8.136185524	8.191969595
ENSG00000163029	0.01548	0.060553476	-0.239672079	-1.180724256	**SMC6**	protein_coding	structural maintenance of chromosomes 6 [Source:HGNC Symbol;Acc:HGNC:20466]	chr2	17663812	17800242	-1	ensembl_havana	7.169998073	7.289834113	7.050162033	7.224140677	7.364780335	7.280581326	6.998859342	7.032063626	7.119563132
ENSG00000197279	0.015487	0.060562905	0.484064915	1.398679016	**ZNF165**	protein_coding	zinc finger protein 165 [Source:HGNC Symbol;Acc:HGNC:12953]	chr6	28080975	28089563	1	ensembl_havana	1.432867414	1.190834956	1.674899871	1.490591983	1.242575255	0.83933763	1.717267538	1.671599815	1.635832261
ENSG00000206120	0.015515	0.060617274	0.681190106	1.603461935	**EGFEM1P**	transcribed_unitary_pseudogene	EGF like and EMI domain containing 1, pseudogene [Source:HGNC Symbol;Acc:HGNC:25149]	chr3	168249522	168830599	1	havana	1.61703855	1.276443497	1.957633603	0.705390237	1.351450576	1.772489677	1.852715862	1.938414976	2.08176997
ENSG00000186174	0.015513	0.060617274	0.353317379	1.277494765	**BCL9L**	protein_coding	B cell CLL/lymphoma 9 like [Source:HGNC Symbol;Acc:HGNC:23688]	chr11	118893875	118925608	-1	ensembl_havana	5.372523758	5.195865068	5.549182448	5.257159226	5.275883366	5.054552614	5.816140998	5.457209502	5.374196843
ENSG00000159231	0.015508	0.060617274	-0.241478625	-1.182203687	**CBR3**	protein_coding	carbonyl reductase 3 [Source:HGNC Symbol;Acc:HGNC:1549]	chr21	36134912	36146566	1	ensembl_havana	2.488667595	2.609406908	2.367928283	2.596285266	2.66767223	2.564263227	2.37188541	2.329873398	2.402026039
ENSG00000069509	0.015517	0.060617274	-0.331629468	-1.258433924	**FUNDC1**	protein_coding	FUN14 domain containing 1 [Source:HGNC Symbol;Acc:HGNC:28746]	chrX	44523639	44543001	-1	ensembl_havana	4.518663914	4.684478648	4.35284918	4.579831375	4.81495872	4.658645849	4.445656025	4.354737515	4.258154001
ENSG00000151239	0.015532	0.060656388	-0.219247171	-1.164125962	**TWF1**	protein_coding	twinfilin actin binding protein 1 [Source:HGNC Symbol;Acc:HGNC:9620]	chr12	43793723	43806375	-1	ensembl_havana	5.645434692	5.755058278	5.535811107	5.780233825	5.719756183	5.765184825	5.492296639	5.532844567	5.582292114
ENSG00000151689	0.015558	0.060726229	0.237665647	1.179083301	**INPP1**	protein_coding	inositol polyphosphate-1-phosphatase [Source:HGNC Symbol;Acc:HGNC:6071]	chr2	190343470	190371665	1	ensembl_havana	5.362839514	5.244006691	5.481672338	5.11918011	5.344172115	5.268667849	5.449304117	5.507807812	5.487905084
ENSG00000182648	0.015555	0.060726229	-0.340954296	-1.266594128	**LINC01006**	lincRNA	long intergenic non-protein coding RNA 1006 [Source:HGNC Symbol;Acc:HGNC:48971]	chr7	156472196	156640654	-1	ensembl_havana	3.279479204	3.449956353	3.109002056	3.563046529	3.482399	3.304423528	3.144758638	3.011552364	3.170695167
ENSG00000185261	0.015581	0.060765118	0.488123575	1.402619388	**KIAA0825**	protein_coding	KIAA0825 [Source:HGNC Symbol;Acc:HGNC:28532]	chr5	94152966	94618597	-1	havana	0.637335362	0.393237575	0.88139715	0.326725152	0.570631912	0.28246366	0.618191201	0.919411336	1.106588913
ENSG00000164902	0.015573	0.060765118	-0.21621813	-1.161684361	**PHAX**	protein_coding	phosphorylated adaptor for RNA export [Source:HGNC Symbol;Acc:HGNC:10241]	chr5	126600268	126627252	1	ensembl_havana	6.21185803	6.319967095	6.103748965	6.374831127	6.307454276	6.277615883	6.118472985	6.068901088	6.123872823
ENSG00000120093	0.015576	0.060765118	-0.282096775	-1.215960847	**HOXB3**	protein_coding	homeobox B3 [Source:HGNC Symbol;Acc:HGNC:5114]	chr17	48548870	48604912	-1	ensembl_havana	2.623391468	2.764439856	2.48234308	2.660681873	2.826996371	2.805641323	2.487415269	2.399587437	2.560026536
ENSG00000131089	0.015614	0.060873697	-0.21747636	-1.162697953	**ARHGEF9**	protein_coding	Cdc42 guanine nucleotide exchange factor 9 [Source:HGNC Symbol;Acc:HGNC:14561]	chrX	63634967	63809274	-1	ensembl_havana	4.282683542	4.391421722	4.173945362	4.421587188	4.332645478	4.420032502	4.147156741	4.114681649	4.259997696
ENSG00000112308	0.015617	0.060873697	-0.247524585	-1.187168391	**C6orf62**	protein_coding	chromosome 6 open reading frame 62 [Source:HGNC Symbol;Acc:HGNC:20998]	chr6	24704861	24720836	-1	ensembl_havana	7.848987017	7.97274931	7.725224725	8.093186906	8.053538519	7.771522504	7.806486888	7.735330402	7.633856883
ENSG00000283709	0.015629	0.06090312	-0.314219005	-1.243338397	**FAM238C**	transcribed_unprocessed_pseudogene	family with sequence similarity 238 member C (non-protein coding) [Source:HGNC Symbol;Acc:HGNC:24672]	chr10	26931206	26944418	-1	havana	0.340051515	0.497161017	0.182942012	0.832812769	0.271370116	0.387300166	0.485518989	0.130707766	-0.067400719
ENSG00000127526	0.015649	0.060965851	0.235702441	1.177479906	**SLC35E1**	protein_coding	solute carrier family 35 member E1 [Source:HGNC Symbol;Acc:HGNC:20803]	chr19	16549831	16572382	-1	ensembl_havana	5.986542732	5.868691512	6.104393952	5.903293462	5.833814044	5.868967029	6.046708275	6.093826185	6.172647396
ENSG00000110455	0.015689	0.061025931	-0.202715698	-1.150862676	**ACCS**	protein_coding	1-aminocyclopropane-1-carboxylate synthase homolog (inactive) [Source:HGNC Symbol;Acc:HGNC:23989]	chr11	44065925	44084222	1	ensembl_havana	5.972815289	6.074173138	5.87145744	6.117107272	5.969180386	6.136231755	5.932844441	5.777013257	5.904514622
ENSG00000092964	0.015686	0.061025931	-0.230538938	-1.173273159	**DPYSL2**	protein_coding	dihydropyrimidinase like 2 [Source:HGNC Symbol;Acc:HGNC:3014]	chr8	26514022	26658178	1	ensembl_havana	8.071293808	8.186563277	7.956024339	8.188722269	8.133017563	8.237949998	7.904910737	7.990168786	7.972993494
ENSG00000119729	0.015669	0.061025931	-0.279586456	-1.213846889	**RHOQ**	protein_coding	ras homolog family member Q [Source:HGNC Symbol;Acc:HGNC:17736]	chr2	46541806	46583121	1	ensembl_havana	4.751011295	4.890804522	4.611218067	4.988429243	5.031898757	4.652085567	4.620353093	4.692728383	4.520572724
ENSG00000127334	0.015685	0.061025931	-0.291531709	-1.223939044	**DYRK2**	protein_coding	dual specificity tyrosine phosphorylation regulated kinase 2 [Source:HGNC Symbol;Acc:HGNC:3093]	chr12	67648338	67665406	1	ensembl_havana	5.848852063	5.994617918	5.703086208	6.205613615	5.889606611	5.888633528	5.763389847	5.724327162	5.621541616
ENSG00000130035	0.015694	0.061025931	-0.319046895	-1.247506122	**GALNT8**	protein_coding	polypeptide N-acetylgalactosaminyltransferase 8 [Source:HGNC Symbol;Acc:HGNC:4130]	chr12	4720341	4851927	1	ensembl_havana	0.852418017	1.011941464	0.692894569	1.175017601	0.799260866	1.061545926	0.668022463	0.554044597	0.856616648
ENSG00000228451	0.015693	0.061025931	-0.478998784	-1.393776064	**SDAD1P1**	transcribed_processed_pseudogene	SDA1 domain containing 1 pseudogene 1 [Source:HGNC Symbol;Acc:HGNC:31403]	chr8	26379259	26382953	-1	havana	0.663056684	0.902556076	0.423557292	0.892538	1.013233389	0.80189684	0.308311185	0.315191528	0.647169164
ENSG00000155926	0.015694	0.061025931	-1.062791864	-2.088970131	**SLA**	protein_coding	Src like adaptor [Source:HGNC Symbol;Acc:HGNC:10902]	chr8	133036724	133103054	-1	ensembl_havana	2.956973308	3.488369241	2.425577376	2.62003612	3.591864916	4.253206686	2.333799072	2.278988373	2.663944684
ENSG00000250959	0.015706	0.061032223	0.330081256	1.257084175	**GLUD1P3**	transcribed_unprocessed_pseudogene	glutamate dehydrogenase 1 pseudogene 3 [Source:HGNC Symbol;Acc:HGNC:4338]	chr10	73730562	73737311	1	havana	0.955483075	0.790442447	1.120523703	0.791576534	0.740413176	0.83933763	1.161063346	0.880929471	1.319578292

ENSG00000279968	0.015709	0.061032223	0.243570824	1.183919365	**GVQW2**	protein_coding	GVQW motif containing 2 [Source:HGNC Symbol;Acc:HGNC:51715]	chr6	138725211	138773652	-1	ensembl_havana	0.036152932	-0.08563248	0.157938344	-0.4596317	0.06303108	0.139703184	-0.21650287	0.399280361	0.291037539
ENSG00000186056	0.015704	0.061032223	-0.31704486	-1.245776152	**MATN1-AS1**	antisense	MATN1 antisense RNA 1 [Source:HGNC Symbol;Acc:HGNC:40364]	chr1	30718504	30726827	1	havana	0.794690124	0.953212555	0.636167694	0.968509893	0.979703225	0.911424546	0.513047461	0.841392961	0.554062661
ENSG00000134352	0.015741	0.061079241	0.204134044	1.151994671	**IL6ST**	protein_coding	interleukin 6 signal transducer [Source:HGNC Symbol;Acc:HGNC:6021]	chr5	55935095	55994993	-1	ensembl_havana	7.826043801	7.723976779	7.928110822	7.776947609	7.733310879	7.661671848	7.989104493	7.90373821	7.891489764
ENSG00000162704	0.015739	0.061079241	-0.199846132	-1.148575849	**ARPC5**	protein_coding	actin related protein 2/3 complex subunit 5 [Source:HGNC Symbol;Acc:HGNC:708]	chr1	183620846	183635757	-1	ensembl_havana	8.570818205	8.670741271	8.470895139	8.800850403	8.702163379	8.509210032	8.613649927	8.48835153	8.310683961
ENSG00000133313	0.015735	0.061079241	-0.217870009	-1.163015246	**CNDP2**	protein_coding	carnosine dipeptidase 2 [Source:HGNC Symbol;Acc:HGNC:24437]	chr18	74495816	74523454	1	ensembl_havana	7.462196289	7.571131293	7.353261284	7.630618614	7.516619904	7.566155363	7.345288321	7.337323976	7.377171555
ENSG00000253716	0.015742	0.061079241	-0.331783086	-1.258567929	**MINCR**	antisense	MYC-induced long non-coding RNA [Source:HGNC Symbol;Acc:HGNC:51653]	chr8	143280161	143281690	-1	havana	2.334386593	2.500278136	2.16849505	2.464742477	2.68857687	2.34751506	2.179142566	2.163501883	2.162840701
ENSG00000267102	0.015727	0.061079241	-0.725022362	-1.652926257	**AC060766.1**	transcribed_processed_pseudogene		chr17	35409602	35410228	-1	havana	0.06935231	0.431863491	-0.293158871	0.206373087	0.325758945	0.76345844	-0.46237776	0.02879206	-0.445890917
ENSG00000072954	0.015753	0.061103758	0.211588192	1.157962226	**TMEM38A**	protein_coding	transmembrane protein 38A [Source:HGNC Symbol;Acc:HGNC:28462]	chr19	16661127	16690029	1	ensembl_havana	3.636379494	3.530585398	3.74217359	3.575247577	3.427497328	3.58901129	3.756011721	3.694095415	3.776413633
ENSG00000159348	0.015803	0.06126781	0.302879176	1.233603852	**CYB5R1**	protein_coding	cytochrome b5 reductase 1 [Source:HGNC Symbol;Acc:HGNC:13397]	chr1	202961869	202967280	-1	ensembl_havana	4.812200627	4.66076104	4.963640215	4.819213589	4.676629958	4.486439571	4.948624269	5.054377101	4.887919276
ENSG00000169020	0.0158	0.06126781	0.288589672	1.221445651	**ATP5ME**	protein_coding	ATP synthase membrane subunit e [Source:HGNC Symbol;Acc:HGNC:846]	chr4	672436	674338	-1	ensembl_havana	4.087493861	3.943199025	4.231788697	4.051990026	3.843267218	3.934339832	4.3323957	3.998165018	4.364805373
ENSG00000146243	0.015839	0.061353564	0.233048316	1.175315689	**IRAK1BP1**	protein_coding	interleukin 1 receptor associated kinase 1 binding protein 1 [Source:HGNC Symbol;Acc:HGNC:17368]	chr6	78867472	78946440	1	ensembl_havana	3.583504274	3.466980116	3.700028432	3.464818021	3.482399	3.453723327	3.753128688	3.62301021	3.723946397
ENSG00000237161	0.015838	0.061353564	0.213627938	1.159600562	**AC068446.1**	processed_pseudogene		chr15	21293653	21295201	-1	havana	4.828052581	4.721238612	4.93486655	5.022244564	4.556683408	4.584787863	5.22630432	4.826568403	4.751726925
ENSG00000114021	0.015843	0.061353564	-0.220694387	-1.165294322	**NIT2**	protein_coding	nitrilase family member 2 [Source:HGNC Symbol;Acc:HGNC:29878]	chr3	100334701	100361635	1	ensembl_havana	6.071088312	6.181435506	5.960741119	5.987872027	6.274562954	6.281871536	5.695180526	6.076902789	6.110140042
ENSG00000271781	0.015831	0.061353564	-0.313678128	-1.242872347	**AC026740.1**	antisense		chr5	675826	676616	1	havana	4.59242354	4.749262604	4.435584476	4.839587794	4.513401796	4.894798222	4.685314876	4.201170521	4.420268032
ENSG00000123091	0.015855	0.061384852	0.266340464	1.202753055	**RNF11**	protein_coding	ring finger protein 11 [Source:HGNC Symbol;Acc:HGNC:10056]	chr1	51236271	51273455	1	ensembl_havana	7.287965655	7.154795423	7.421135887	7.381365203	7.071437948	7.011583119	7.547060271	7.309749226	7.406598165
ENSG00000147164	0.015877	0.061412078	0.247893758	1.187472216	**SNX12**	protein_coding	sorting nexin 12 [Source:HGNC Symbol;Acc:HGNC:14976]	chrX	71056332	71073426	-1	ensembl_havana	6.135651823	6.011704944	6.259598702	5.909932168	6.06322194	6.061960725	6.269864968	6.269814827	6.239116312
ENSG00000111752	0.015879	0.061412078	0.210886344	1.157399033	**PHC1**	protein_coding	polyhomeotic homolog 1 [Source:HGNC Symbol;Acc:HGNC:3182]	chr12	8913896	8941467	1	ensembl_havana	4.253327717	4.147884545	4.358770889	4.11184578	4.129780342	4.202027512	4.37051862	4.293793612	4.412000434
ENSG00000103540	0.015873	0.061412078	-0.266413593	-1.202814023	**CCP110**	protein_coding	centriolar coiled-coil protein 110 [Source:HGNC Symbol;Acc:HGNC:24342]	chr16	19523811	19553408	1	ensembl_havana	7.497696361	7.630903157	7.364489564	7.940476225	7.544121121	7.408112127	7.542266851	7.316298232	7.234903611
ENSG00000119703	0.01587	0.061412078	-0.46500048	-1.380317813	**ZC2HC1C**	protein_coding	zinc finger C2HC-type containing 1C [Source:HGNC Symbol;Acc:HGNC:20354]	chr14	75064170	75079987	1	ensembl_havana	1.103892082	1.336392322	0.871391842	1.109412078	1.500754142	1.399010747	0.428834256	0.938274071	1.2470672
ENSG00000055130	0.015895	0.061457541	-0.199858449	-1.148585656	**CUL1**	protein_coding	cullin 1 [Source:HGNC Symbol;Acc:HGNC:2551]	chr7	148697914	148801036	1	ensembl_havana	7.033747698	7.133676923	6.933818474	7.216614582	7.113591639	7.070824548	7.000683649	6.912556175	6.888215596
ENSG00000221963	0.015912	0.061505919	-0.245769406	-1.185724963	**APOL6**	protein_coding	apolipoprotein L6 [Source:HGNC Symbol;Acc:HGNC:14870]	chr22	35648395	35668409	1	ensembl_havana	7.27128945	7.394174153	7.148404747	7.346864447	7.442023688	7.393634324	7.205699235	7.175591328	7.063923679
ENSG00000040275	0.015922	0.061526795	-0.204064337	-1.151939012	**SPDL1**	protein_coding	spindle apparatus coiled-coil protein 1 [Source:HGNC Symbol;Acc:HGNC:26010]	chr5	169583634	169604778	1	ensembl_havana	5.63005167	5.732083839	5.528019502	5.545378328	5.864565689	5.786307501	5.348808116	5.625227347	5.610023043
ENSG00000143502	0.015942	0.061588963	-0.303382997	-1.234034729	**SUSD4**	protein_coding	sushi domain containing 4 [Source:HGNC Symbol;Acc:HGNC:25470]	chr1	223220819	223364202	-1	ensembl_havana	3.819689481	3.971380979	3.667997982	4.290821549	4.037060343	3.586261044	3.823530526	3.761841936	3.418621485
ENSG00000005075	0.015948	0.061596528	0.227734301	1.170994501	**POLR2J**	protein_coding	RNA polymerase II subunit J [Source:HGNC Symbol;Acc:HGNC:9197]	chr7	102473118	102478907	-1	ensembl_havana	5.31279821	5.198931059	5.42666536	5.049830882	5.298157437	5.248804859	5.351669833	5.45147634	5.476849907
ENSG00000105655	0.01596	0.061624415	-0.521142575	-1.435091351	**ISYNA1**	protein_coding	inositol-3-phosphate synthase 1 [Source:HGNC Symbol;Acc:HGNC:29821]	chr19	18434388	18438301	-1	ensembl_havana	4.426073727	4.686645015	4.165502439	4.803741862	4.565186067	4.691007115	4.596424846	4.053279749	3.846802724
ENSG00000241684	0.016005	0.061694752	0.69177697	1.615271827	**ADAMTS9-AS2**	antisense	ADAMTS9 antisense RNA 2 [Source:HGNC Symbol;Acc:HGNC:42435]	chr3	64684909	65011468	1	havana	-0.533411979	-0.879300464	-0.187523494	-0.99620085	-0.783495335	-0.85820521	0.141952326	-0.52165082	-0.182871985
ENSG00000118432	0.016	0.061694752	0.549357457	1.463433771	**CNR1**	protein_coding	cannabinoid receptor 1 [Source:HGNC Symbol;Acc:HGNC:2159]	chr6	88139864	88166359	-1	ensembl_havana	1.442468708	1.167789979	1.717147436	0.326725152	1.657409759	1.519235028	1.261864131	1.95703246	1.932545716
ENSG00000160062	0.016008	0.061694752	0.412769431	1.33123884	**ZBTB8A**	protein_coding	zinc finger and BTB domain containing 8A [Source:HGNC Symbol;Acc:HGNC:24172]	chr1	32539427	32605939	1	ensembl_havana	1.454962412	1.248577697	1.661347128	0.727424294	1.657409759	1.360899039	1.415636519	1.910030375	1.65837449
ENSG00000145332	0.016007	0.061694752	-0.206934703	-1.154233175	**KLHL8**	protein_coding	kelch like family member 8 [Source:HGNC Symbol;Acc:HGNC:18644]	chr4	87160103	87240314	-1	ensembl_havana	5.734467398	5.83793475	5.631000047	5.814089585	5.86857866	5.831136004	5.579509836	5.643302075	5.67018823
ENSG00000261061	0.015984	0.061694752	-0.223062623	-1.167208766	**AC092718.4**	sense_intronic		chr16	81030770	81031485	1	havana	1.944677846	2.056209158	1.833146535	2.005254198	2.235081811	1.928291464	1.7752573	1.947753749	1.776428555
ENSG00000170903	0.016008	0.061694752	-0.244145731	-1.184391245	**MSANTD4**	protein_coding	Myb/SANT DNA binding domain containing 4 with coiled-coils [Source:HGNC Symbol;Acc:HGNC:29383]	chr11	105995623	106022403	-1	ensembl_havana	4.669898504	4.791971369	4.547825638	4.844636702	4.771531924	4.759745482	4.491353183	4.586131975	4.565991757
ENSG00000175063	0.01599	0.061694752	-0.407576132	-1.326455367	**UBE2C**	protein_coding	ubiquitin conjugating enzyme E2 C [Source:HGNC Symbol;Acc:HGNC:15937]	chr20	45812576	45816957	1	ensembl_havana	5.698941907	5.902729973	5.495153842	5.575300057	5.917544412	6.215345451	5.405909046	5.504647445	5.574905034
ENSG00000272986	0.016016	0.061707287	0.345175748	1.270305721	**AC009570.1**	antisense		chr4	70703747	70704491	-1	havana	-0.030019572	-0.202607446	0.142568302	-0.03191641	-0.218971072	-0.356934859	0.513047461	0.097529431	-0.182871985
ENSG00000235652	0.016027	0.061722235	0.216519865	1.161927349	**AL356599.1**	antisense		chr6	145799409	145886585	1	havana	1.158145907	1.049885974	1.266405839	1.206734491	0.979703225	0.963220207	1.356079599	1.226127349	1.21701057
ENSG00000118600	0.016028	0.061722235	-0.311836476	-1.241286788	**RXYLT1**	protein_coding	ribitol xylosyltransferase 1 [Source:HGNC Symbol;Acc:HGNC:13530]	chr12	63779803	63809558	1	ensembl_havana	4.392434241	4.548352479	4.236516003	4.597884537	4.617902878	4.429270021	4.109329965	4.349462476	4.250755567
ENSG00000178814	0.016033	0.061724229	-0.542164147	-1.456155218	**OPLAH**	protein_coding	5-oxoprolinase, ATP-hydrolysing [Source:HGNC Symbol;Acc:HGNC:8149]	chr8	144051266	144063965	-1	ensembl_havana	0.537065492	0.808147565	0.265983418	1.126095262	0.477608744	0.820738689	0.739683594	0.163140205	-0.104873545
ENSG00000164543	0.016043	0.061748205	0.224624353	1.168472964	**STK17A**	protein_coding	serine/threonine kinase 17a [Source:HGNC Symbol;Acc:HGNC:11395]	chr7	43582758	43626786	1	ensembl_havana	7.4693344	7.357022224	7.581646577	7.470411338	7.381325977	7.219329356	7.642068191	7.578844936	7.524026603
ENSG00000141338	0.016079	0.061813235	0.510672978	1.424714631	**ABCA8**	protein_coding	ATP binding cassette subfamily A member 8 [Source:HGNC Symbol;Acc:HGNC:38]	chr17	68867292	68955392	-1	ensembl_havana	3.18657002	2.931233531	3.441906509	2.283275357	3.14698349	3.363441747	3.140352234	3.569569297	3.615797995
ENSG00000187607	0.016082	0.061813235	-0.200548469	-1.149135138	**ZNF286A**	protein_coding	zinc finger protein 286A [Source:HGNC Symbol;Acc:HGNC:13501]	chr17	15699577	15720787	1	ensembl_havana	3.623058976	3.72333321	3.522784741	3.825582078	3.831567555	3.512849998	3.63281161	3.605415612	3.330127003
ENSG00000175414	0.016071	0.061813235	-0.226957545	-1.1703642	**ARL10**	protein_coding	ADP ribosylation factor like GTPase 10 [Source:HGNC Symbol;Acc:HGNC:22042]	chr5	176365468	176401865	1	ensembl_havana	3.451807155	3.565285928	3.338328382	3.70027797	3.497285861	3.498293951	3.409325542	3.351196507	3.254463098
ENSG00000143545	0.016076	0.061813235	-0.278224416	-1.212701444	**RAB13**	protein_coding	RAB13, member RAS oncogene family [Source:HGNC Symbol;Acc:HGNC:9762]	chr1	153981617	153986358	-1	ensembl_havana	6.876590553	7.015702761	6.737478345	7.312885222	7.045845377	6.688377685	6.887911	6.815147744	6.509376291
ENSG00000155886	0.01608	0.061813235	-0.515827174	-1.429813693	**SLC24A2**	protein_coding	solute carrier family 24 member 2 [Source:HGNC Symbol;Acc:HGNC:10976]	chr9	19507452	19786928	-1	ensembl_havana	0.711567236	0.969480823	0.453653649	0.931023971	0.77991046	1.197508038	0.399634207	-0.04338487	1.004711615
ENSG00000168542	0.016104	0.06188374	-0.710150015	-1.635974221	**COL3A1**	protein_coding	collagen type III alpha 1 chain [Source:HGNC Symbol;Acc:HGNC:2201]	chr2	188974320	189012746	1	ensembl_havana	-0.050999254	0.304075754	-0.406074261	0.683014432	0.214850257	0.014362573	0.141952326	-1.00738779	-0.352787316
ENSG00000164116	0.016119	0.061890429	0.49944521	1.41366983	**GUCY1A1**	protein_coding	guanylate cyclase 1 soluble subunit alpha 1 [Source:HGNC Symbol;Acc:HGNC:4685]	chr4	155666711	155732349	1	ensembl_havana	0.949977796	0.700255191	1.199700402	0.355307344	0.477608744	1.267849486	0.873137292	1.271458479	1.454505434
ENSG00000119638	0.016115	0.061890429	-0.230040316	-1.172867725	**NEK9**	protein_coding	NIMA related kinase 9 [Source:HGNC Symbol;Acc:HGNC:18591]	chr14	75079353	75127344	-1	ensembl_havana	7.338348651	7.453368809	7.223328493	7.470002988	7.402832218	7.48727122	7.162621798	7.171113212	7.336250468
ENSG00000128595	0.016114	0.061890429	-0.230133648	-1.172943603	**CALU**	protein_coding	calumenin [Source:HGNC Symbol;Acc:HGNC:1458]	chr7	128739292	128771807	1	ensembl_havana	7.821704985	7.936771808	7.706638161	7.964011317	7.917658914	7.928645194	7.657183385	7.684391239	7.778339857
ENSG00000179241	0.016131	0.06190426	0.399092679	1.318678324	**LDLRAD3**	protein_coding	low density lipoprotein receptor class A domain containing 3 [Source:HGNC Symbol;Acc:HGNC:27046]	chr11	35943981	36232136	1	ensembl_havana	3.208633717	3.009087378	3.408180057	2.31999144	3.143202419	3.564068275	2.981915645	3.443209147	3.799415379
ENSG00000129167	0.016128	0.06190426	-0.338038218	-1.264036583	**TPH1**	protein_coding	tryptophan hydroxylase 1 [Source:HGNC Symbol;Acc:HGNC:12008]	chr11	18017564	18042426	-1	ensembl_havana	1.04508564	1.214104749	0.876066531	0.892538	1.338282095	1.411494153	0.739683594	0.993426194	0.895089805
ENSG00000112514	0.016137	0.061908041	0.237280147	1.178768283	**CUTA**	protein_coding	cutA divalent cation tolerance homolog [Source:HGNC Symbol;Acc:HGNC:21101]	chr6	33416442	33418317	-1	ensembl_havana	6.917094161	6.798454088	7.035734235	6.786486178	6.801476067	6.807400018	7.106124189	7.047890634	6.953187881
ENSG00000239467	0.016148	0.061934351	-0.364671584	-1.287588489	**AC007405.3**	lincRNA		chr2	170771113	170778148	1	havana	1.306629763	1.488965555	1.124293971	1.578072284	1.452682866	1.436141515	1.340798121	1.195095435	0.836988357
ENSG00000268902	0.016175	0.062004659	0.424000609	1.341642791	**CSAG2**	protein_coding	CSAG family member 2 [Source:HGNC Symbol;Acc:HGNC:16847]	chrX	152708261	152714549	-1	ensembl_havana	1.276875434	1.064875129	1.488875738	0.565613454	1.402954459	1.226057475	1.01467363	1.566404757	1.885548828
ENSG00000239900	0.016171	0.062004659	0.224048163	1.168006387	**ADSL**	protein_coding	adenylosuccinate lyase [Source:HGNC Symbol;Acc:HGNC:291]	chr22	40346500	40390463	1	ensembl_havana	3.318420473	3.206396392	3.430444555	3.014347502	3.283780556	3.321061117	3.275081252	3.426606605	3.589645809
ENSG00000272034	0.016186	0.062032299	0.494637552	1.408966736	**SNORD14A**	snoRNA	small nucleolar RNA, C/D box 14A [Source:HGNC Symbol;Acc:HGNC:10113]	chr11	17074654	17074744	-1	ensembl	0.418445721	0.171126945	0.665764497	0.206373087	0.325758945	-0.018751196	0.399634207	0.821210834	0.77644845
ENSG00000158406	0.016192	0.062036103	0.207568319	1.154740213	**HIST1H4H**	protein_coding	histone cluster 1 H4 family member h [Source:HGNC Symbol;Acc:HGNC:4788]	chr6	26277609	26285638	-1	ensembl_havana	4.969011176	4.865227017	5.072795336	4.946776004	4.94008775	4.708817296	5.197961942	5.105339079	4.915084985
ENSG00000213918	0.016236	0.062125749	0.424010409	1.341651905	**DNASE1**	protein_coding	deoxyribonuclease 1 [Source:HGNC Symbol;Acc:HGNC:2956]	chr16	3611728	3680143	1	ensembl_havana	4.36607509	4.154069886	4.578080295	3.996364543	3.99572295	4.470122166	4.480935426	4.633197259	4.620108199
ENSG00000110429	0.016226	0.062125749	0.257604714	1.195492197	**FBXO3**	protein_coding	F-box protein 3 [Source:HGNC Symbol;Acc:HGNC:13582]	chr11	33740939	33774543	1	ensembl_havana	5.166666844	5.037864487	5.295469201	5.032236513	5.144931306	4.936425642	5.268305384	5.30475086	5.313351359
ENSG00000197860	0.016236	0.062125749	0.215165642	1.160837187	**SGTB**	protein_coding	small glutamine rich tetratricopeptide repeat containing beta [Source:HGNC Symbol;Acc:HGNC:23567]	chr5	65665928	65723035	-1	ensembl_havana	4.849355151	4.74177233	4.956937972	4.677854699	4.882285857	4.665176435	4.937238596	5.032599911	4.900975408
ENSG00000174748	0.016237	0.062125749	0.213576802	1.159559461	**RPL15**	protein_coding	ribosomal protein L15 [Source:HGNC Symbol;Acc:HGNC:10306]	chr3	23916545	23924374	1	ensembl_havana	9.605321148	9.498532747	9.712109548	9.316811614	9.63138632	9.547400306	9.59168309	9.801051738	9.743593818
ENSG00000063601	0.016224	0.062125749	0.207386828	1.154594956	**MTMR1**	protein_coding	myotubularin related protein 1 [Source:HGNC Symbol;Acc:HGNC:7449]	chrX	150692971	150765103	1	ensembl_havana	6.353375582	6.249682168	6.457068996	6.281110162	6.262414081	6.20552226	6.486272246	6.513720815	6.371213927
ENSG00000101213	0.016258	0.062174103	0.304342918	1.234856089	**PTK6**	protein_coding	protein tyrosine kinase 6 [Source:HGNC Symbol;Acc:HGNC:9617]	chr20	63528001	63537370	-1	ensembl_havana	0.470332311	0.318160852	0.62250377	0.174648163	0.271370116	0.508464275	0.618191201	0.602150945	0.647169164
ENSG00000183597	0.016255	0.062174103	0.24776741	1.187368224	**TANGO2**	protein_coding	transport and golgi organization 2 homolog [Source:HGNC Symbol;Acc:HGNC:25439]	chr22	20017014	20065926	1	ensembl_havana	3.850977061	3.727093356	3.974860766	3.602328412	3.706476045	3.872475611	3.842642641	4.057599296	4.024340361
ENSG00000165935	0.01627	0.062205205	-0.225433165	-1.169128223	**SMCO2**	protein_coding	single-pass membrane protein with coiled-coil domains 2 [Source:HGNC Symbol;Acc:HGNC:34448]	chr12	27466810	27502185	1	ensembl_havana	1.315528721	1.428245304	1.202812138	1.516132283	1.500754142	1.267849486	1.229040238	1.256505808	1.12289037
ENSG00000171160	0.01629	0.062262552	0.245661924	1.185636629	**MORN4**	protein_coding	MORN repeat containing 4 [Source:HGNC Symbol;Acc:HGNC:24001]	chr10	97614553	97633500	-1	ensembl_havana	3.307062177	3.184231215	3.429893139	3.146964063	3.176880527	3.228849057	3.494651998	3.41318562	3.381841801
ENSG00000143554	0.016294	0.062262552	-0.261718774	-1.198906187	**SLC27A3**	protein_coding	solute carrier family 27 member 3 [Source:HGNC Symbol;Acc:HGNC:10997]	chr1	153774354	153780157	1	ensembl_havana	3.660606081	3.791465468	3.529746694	3.677822113	3.786222876	3.910351415	3.330286772	3.654720022	3.604233288
ENSG00000204104	0.016308	0.062268162	0.201620524	1.149989369	**TRAF3IP1**	protein_coding	TRAF3 interacting protein 1 [Source:HGNC Symbol;Acc:HGNC:17861]	chr2	238320441	238400900	1	ensembl_havana	3.891208826	3.790398564	3.992019089	3.749561028	3.891395322	3.730239343	3.97225676	4.059754229	3.944046277
ENSG00000119537	0.016309	0.062268162	-0.201104168	-1.149577849	**KDSR**	protein_coding	3-ketodihydrosphingosine reductase [Source:HGNC Symbol;Acc:HGNC:4021]	chr18	63327726	63367510	-1	ensembl_havana	6.0840879	6.184639984	5.983535816	6.205122982	6.232921407	6.115875563	6.034293642	6.008267496	5.908046311
ENSG00000183230	0.016307	0.062268162	-1.973249685	-3.926515751	**CTNNA3**	protein_coding	catenin alpha 3 [Source:HGNC Symbol;Acc:HGNC:2511]	chr10	65912518	67696169	-1	ensembl_havana	-1.440460165	-0.453835322	-2.427085007	0.490295136	-0.218971072	-1.632830031	-1.7649502	-3.33366493	-2.182639896
ENSG00000159063	0.016315	0.062278037	-0.295894326	-1.227645758	**ALG8**	protein_coding	ALG8, alpha-1,3-glucosyltransferase [Source:HGNC Symbol;Acc:HGNC:23161]	chr11	78100936	78139660	-1	ensembl_havana	4.459627736	4.607574899	4.311680573	4.576800429	4.609706171	4.636218098	4.111582737	4.408145822	4.415313161
ENSG00000242516	0.01634	0.062337915	0.401680994	1.321046266	**LINC00960**	lincRNA	long intergenic non-protein coding RNA 960 [Source:HGNC Symbol;Acc:HGNC:48710]	chr3	75672391	75679303	1	havana	0.889640933	0.688800436	1.09048143	0.727424294	0.740413176	0.598563838	1.052687018	0.956893362	1.261863909
ENSG00000167642	0.016336	0.062337915	-0.199005227	-1.147906573	**SPINT2**	protein_coding	serine peptidase inhibitor, Kunitz type 2 [Source:HGNC Symbol;Acc:HGNC:11247]	chr19	38244035	38292614	1	ensembl_havana	6.440574901	6.540077514	6.341072287	6.562725342	6.512606197	6.544901002	6.372526839	6.290606729	6.360083293
ENSG00000243317	0.016352	0.062367781	-0.294933935	-1.226828796	**STMP1**	protein_coding	short transmembrane mitochondrial protein 1 [Source:HGNC Symbol;Acc:HGNC:41909]	chr7	135662496	135693418	1	ensembl_havana	5.240338001	5.387804968	5.092871033	5.51323832	5.431244625	5.218931959	5.166886857	5.022691895	5.089034349
ENSG00000177426	0.01636	0.062382415	-0.262548964	-1.199596289	**TGIF1**	protein_coding	TGFB induced factor homeobox 1 [Source:HGNC Symbol;Acc:HGNC:11776]	chr18	3411608	3459978	1	ensembl_havana	0.407408813	0.538683295	0.276134331	0.075059705	0.720251491	0.820738689	-0.26243933	0.399280361	0.691561964
ENSG00000270574	0.016387	0.062467836	0.268224127	1.204324462	**AC010680.2**	antisense		chr2	178578790	178580906	1	havana	3.462760618	3.328648554	3.596872682	3.572206975	3.220595563	3.193143125	3.885399497	3.351196507	3.55402204
ENSG00000103254	0.016416	0.062471423	0.702849203	1.627716225	**FAM173A**	protein_coding	family with sequence similarity 173 member A [Source:HGNC Symbol;Acc:HGNC:14152]	chr16	720581	722601	1	ensembl_havana	-0.119053989	-0.470478591	0.232370612	-0.14746469	-1.104596936	-0.159374151	0.428834256	0.097529431	0.17074815
ENSG00000273568	0.016431	0.062471423	0.439813938	1.35642938	**AC131009.3**	sense_intronic		chr12	131934642	131934928	1	havana	0.06551368	-0.154393289	0.285420649	-0.22998119	-0.18055577	-0.052642908	0.36983092	-0.04338487	0.529815901
ENSG00000148175	0.016435	0.062471423	0.301864595	1.232736621	**STOM**	protein_coding	stomatin [Source:HGNC Symbol;Acc:HGNC:3383]	chr9	121338988	121370304	-1	ensembl_havana	4.167042245	4.016109947	4.317974542	3.525811778	4.193324268	4.329193796	3.999273898	4.444883737	4.509765991
ENSG00000121716	0.016424	0.062471423	0.221423989	1.165883786	**PILRB**	protein_coding	paired immunoglobin-like type 2 receptor beta [Source:HGNC Symbol;Acc:HGNC:18297]	chr7	100352176	100367733	1	ensembl_havana	4.849896199	4.739184205	4.960608194	4.639150283	4.712794237	4.865608094	4.800104487	4.920864951	5.160855143
ENSG00000144597	0.016414	0.062471423	0.220112205	1.164824177	**EAF1**	protein_coding	ELL associated factor 1 [Source:HGNC Symbol;Acc:HGNC:20907]	chr3	15427355	15450635	1	ensembl_havana	6.981401133	6.87134503	7.091457236	6.79433586	6.909482014	6.910217217	7.082775915	7.125036091	7.066559701
ENSG00000110074	0.016416	0.062471423	-0.204087009	-1.151957114	**FOXRED1**	protein_coding	FAD dependent oxidoreductase domain containing 1 [Source:HGNC Symbol;Acc:HGNC:26927]	chr11	126269055	126278131	1	ensembl_havana	6.247344862	6.349388366	6.145301357	6.245765281	6.413281352	6.389118465	6.061349647	6.166617393	6.207937033
ENSG00000113013	0.016436	0.062471423	-0.215511764	-1.161115721	**HSPA9**	protein_coding	heat shock protein family A (Hsp70) member 9 [Source:HGNC Symbol;Acc:HGNC:5244]	chr5	138554882	138575444	-1	ensembl_havana	9.620375107	9.728130989	9.512619225	9.673858426	9.760669129	9.749865412	9.410626006	9.526636977	9.600594692
ENSG00000123485	0.01641	0.062471423	-0.219168123	-1.16406218	**HJURP**	protein_coding	Holliday junction recognition protein [Source:HGNC Symbol;Acc:HGNC:25444]	chr2	233833416	233854566	-1	ensembl_havana	5.764519758	5.87410382	5.654935696	5.666511001	5.887913013	6.067887446	5.424149214	5.741880179	5.798777696
ENSG00000065308	0.016412	0.062471423	-0.228963879	-1.17199294	**TRAM2**	protein_coding	translocation associated membrane protein 2 [Source:HGNC Symbol;Acc:HGNC:16855]	chr6	52497402	52576915	-1	ensembl_havana	6.69319949	6.80768143	6.57871755	6.667231833	6.78397788	6.971834576	6.482360627	6.601392047	6.652399976
ENSG00000164949	0.016427	0.062471423	-0.373602936	-1.295584341	**GEM**	protein_coding	GTP binding protein overexpressed in skeletal muscle [Source:HGNC Symbol;Acc:HGNC:4234]	chr8	94249253	94262350	-1	ensembl_havana	2.065735556	2.252537024	1.878934088	2.167163933	2.154731774	2.435715365	1.852715862	1.95703246	1.827053942
ENSG00000240954	0.016404	0.062471423	-0.580293864	-1.495153767	**RPL4P1**	processed_pseudogene	ribosomal protein L4 pseudogene 1 [Source:HGNC Symbol;Acc:HGNC:23551]	chr14	21750269	21751546	1	havana	0.09437849	0.384525422	-0.195768442	0.770507832	0.15602554	0.227042895	0.339398945	-0.15890847	-0.767795802
ENSG00000183340	0.016449	0.062489596	-0.350312891	-1.274837083	**JRKL**	protein_coding	JRK like [Source:HGNC Symbol;Acc:HGNC:6200]	chr11	96389989	96507574	1	ensembl_havana	4.50790637	4.683062815	4.332749925	4.722421242	4.753044389	4.573722815	4.164620116	4.434956726	4.398672932
ENSG00000111799	0.016448	0.062489596	-0.362172965	-1.285360431	**COL12A1**	protein_coding	collagen type XII alpha 1 chain [Source:HGNC Symbol;Acc:HGNC:2188]	chr6	75084326	75206051	-1	ensembl_havana	7.516358913	7.697445396	7.335272431	7.703988989	7.540352678	7.847994521	7.279938243	7.404781461	7.321097589
ENSG00000186815	0.016477	0.062577923	-0.208039088	-1.15511708	**TPCN1**	protein_coding	two pore segment channel 1 [Source:HGNC Symbol;Acc:HGNC:18182]	chr12	113221050	113298585	1	ensembl_havana	3.853036244	3.957055787	3.7490167	3.902646601	3.810266049	4.158254713	3.676073242	3.654720022	3.916256835
ENSG00000106536	0.016493	0.062604982	0.296683042	1.228317091	**POU6F2**	protein_coding	POU class 6 homeobox 2 [Source:HGNC Symbol;Acc:HGNC:21694]	chr7	38977998	39493095	1	ensembl_havana	4.798991377	4.650649857	4.947332898	4.220707514	4.777642161	4.953599895	4.533968514	5.213819317	5.094210863
ENSG00000178691	0.016492	0.062604982	-0.264723288	-1.201405596	**SUZ12**	protein_coding	SUZ12 polycomb repressive complex 2 subunit [Source:HGNC Symbol;Acc:HGNC:17101]	chr17	31937018	32001045	1	ensembl_havana	7.567375364	7.699737008	7.43501372	7.827869768	7.684228129	7.587113128	7.431442759	7.452921014	7.420677386
ENSG00000135835	0.016518	0.062658486	0.429588954	1.346849784	**KIAA1614**	protein_coding	KIAA1614 [Source:HGNC Symbol;Acc:HGNC:29327]	chr1	180913154	180951614	1	ensembl_havana	0.405681677	0.1908872	0.620476154	0.237415364	-0.218971072	0.554217307	0.540060475	0.504278656	0.817089329
ENSG00000271011	0.01652	0.062658486	0.211748667	1.158091037	**AC010680.3**	antisense		chr2	178577103	178577622	1	havana	2.384982687	2.279108353	2.49085702	2.355796255	2.227959397	2.253569408	2.607204823	2.379024645	2.486341593
ENSG00000172578	0.016515	0.062658486	-0.269611355	-1.205483041	**KLHL6**	protein_coding	kelch like family member 6 [Source:HGNC Symbol;Acc:HGNC:18653]	chr3	183487531	183555689	-1	ensembl_havana	4.462207039	4.597012716	4.327401361	4.710025423	4.546699886	4.53431284	4.303127503	4.379102505	4.299974075
ENSG00000147003	0.016545	0.062737148	0.393037973	1.313155689	**CLTRN**	protein_coding	collectrin, amino acid transport regulator [Source:HGNC Symbol;Acc:HGNC:29437]	chrX	15627318	15665031	-1	ensembl_havana	-0.29872094	-0.495239927	-0.102201953	-0.4596317	-0.106664058	-0.91942402	-0.17198403	0.130707766	-0.265329594
ENSG00000095777	0.01655	0.062740685	1.131835439	2.191373559	**MYO3A**	protein_coding	myosin IIIA [Source:HGNC Symbol;Acc:HGNC:7601]	chr10	25934071	26212536	1	ensembl_havana	-0.750335765	-1.316253485	-0.184418046	-1.51148723	-0.903502039	-1.533771185	0.106248156	-0.62862566	-0.030876629
ENSG00000167523	0.016576	0.062820629	0.321160581	1.249335177	**SPATA33**	protein_coding	spermatogenesis associated 33 [Source:HGNC Symbol;Acc:HGNC:26463]	chr16	89657802	89671272	1	ensembl_havana	3.83565877	3.675078479	3.99623906	3.516350972	3.788645305	3.72023916	4.030564339	3.98234794	3.975804901
ENSG00000167552	0.016587	0.062846614	0.23863719	1.179877589	**TUBA1A**	protein_coding	tubulin alpha 1a [Source:HGNC Symbol;Acc:HGNC:20766]	chr12	49184796	49189324	-1	ensembl_havana	8.557513369	8.438194774	8.676831964	8.575125523	8.504139643	8.235319157	8.801357986	8.658498888	8.570639017
ENSG00000104783	0.016592	0.062850443	-1.351508395	-2.551787857	**KCNN4**	protein_coding	potassium calcium-activated channel subfamily N member 4 [Source:HGNC Symbol;Acc:HGNC:6293]	chr19	43766533	43781257	-1	ensembl_havana	-0.808754953	-0.133000755	-1.48450915	0.174648163	-0.299013651	-0.274636777	-2.0571346	-0.74417246	-1.652220391
ENSG00000175137	0.016622	0.062896638	0.236247305	1.177924691	**SH3BP5L**	protein_coding	SH3 binding domain protein 5 like [Source:HGNC Symbol;Acc:HGNC:29360]	chr1	248810446	248826633	-1	ensembl_havana	5.70202092	5.583897268	5.820144573	5.665084692	5.54522687	5.541380242	5.946159327	5.838550639	5.675723752
ENSG00000242372	0.016618	0.062896638	0.23457951	1.176563765	**EIF6**	protein_coding	eukaryotic translation initiation factor 6 [Source:HGNC Symbol;Acc:HGNC:6159]	chr20	35278911	35284985	-1	ensembl_havana	6.419070985	6.30178123	6.536360741	6.184364258	6.37470174	6.346277692	6.516764265	6.577526721	6.514791236
ENSG00000152359	0.01662	0.062896638	-0.206847356	-1.154163295	**POC5**	protein_coding	POC5 centriolar protein [Source:HGNC Symbol;Acc:HGNC:26658]	chr5	75674124	75717481	-1	ensembl_havana	5.175349838	5.278773516	5.071926161	5.208052998	5.34169971	5.28656784	4.998167601	5.091713667	5.125897213
ENSG00000258725	0.016616	0.062896638	-0.380665248	-1.301942062	**PRC1-AS1**	antisense	PRC1 antisense RNA 1 [Source:HGNC Symbol;Acc:HGNC:48587]	chr15	90966345	90988624	1	havana	-0.217768056	-0.027435432	-0.40810068	0.10902545	-0.473795405	0.28246366	-0.04612063	-0.86978558	-0.308395834
ENSG00000179840	0.016627	0.062899132	-0.412576954	-1.331061245	**PIK3CD-AS1**	antisense	PIK3CD antisense RNA 1 [Source:HGNC Symbol;Acc:HGNC:32346]	chr1	9652610	9654586	-1	havana	2.240055378	2.446343855	2.033766901	2.797156021	2.396251236	2.145624309	2.246292314	1.841543652	2.013464737
ENSG00000121289	0.016641	0.0629207	0.20313441	1.151196738	**CEP89**	protein_coding	centrosomal protein 89 [Source:HGNC Symbol;Acc:HGNC:25907]	chr19	32875925	32971991	-1	ensembl_havana	4.743605726	4.642038521	4.845172931	4.566141688	4.69740613	4.662567747	4.781831765	4.929139443	4.824547585
ENSG00000121671	0.016642	0.0629207	0.195725	1.145299565	**CRY2**	protein_coding	cryptochrome circadian regulator 2 [Source:HGNC Symbol;Acc:HGNC:2385]	chr11	45847118	45883248	1	ensembl_havana	5.299978005	5.202115505	5.397840505	5.236180816	5.223129973	5.147035724	5.427769736	5.420788101	5.344963677
ENSG00000101079	0.016677	0.06303773	0.22791102	1.171137948	**NDRG3**	protein_coding	NDRG family member 3 [Source:HGNC Symbol;Acc:HGNC:14462]	chr20	36651766	36746078	-1	ensembl_havana	6.560421392	6.446465882	6.674376902	6.351522169	6.477376582	6.510498894	6.657551335	6.626687153	6.738892218
ENSG00000260293	0.016728	0.063137936	0.534116304	1.448054904	**AC106820.4**	sense_intronic		chr16	2476558	2482173	1	havana	1.138662028	0.871603875	1.40572018	0.515840645	0.945375194	1.153595787	1.415636519	1.399485086	1.402038935
ENSG00000102901	0.016729	0.063137936	0.364506692	1.287441332	**CENPT**	protein_coding	centromere protein T [Source:HGNC Symbol;Acc:HGNC:25787]	chr16	67828157	67847811	-1	ensembl_havana	6.680924484	6.498671139	6.86317783	6.547324033	6.63072834	6.317961043	7.014894801	6.757900561	6.816738129
ENSG00000129355	0.016722	0.063137936	0.322267875	1.250294432	**CDKN2D**	protein_coding	cyclin dependent kinase inhibitor 2D [Source:HGNC Symbol;Acc:HGNC:1790]	chr19	10566462	10569059	-1	ensembl_havana	2.863625854	2.702491916	3.024759791	2.547577338	2.826996371	2.73290204	3.067955511	3.055408658	2.950915205
ENSG00000076321	0.016732	0.063137936	0.261568824	1.198781583	**KLHL20**	protein_coding	kelch like family member 20 [Source:HGNC Symbol;Acc:HGNC:25056]	chr1	173714941	173786702	1	ensembl_havana	5.087085486	4.956301073	5.217869898	4.909918296	5.054218477	4.904766446	5.268305384	5.18646855	5.198835759
ENSG00000196313	0.016717	0.063137936	0.231797529	1.174297156	**POM121**	protein_coding	POM121 transmembrane nucleoporin [Source:HGNC Symbol;Acc:HGNC:19702]	chr7	72879365	72951440	1	ensembl_havana	6.527812566	6.411913802	6.643711331	6.317426798	6.424228514	6.494086094	6.645163986	6.616475883	6.669494124
ENSG00000134602	0.016722	0.063137936	0.195949794	1.145478034	**STK26**	protein_coding	serine/threonine kinase 26 [Source:HGNC Symbol;Acc:HGNC:18174]	chrX	132023265	132075943	1	ensembl_havana	6.58707056	6.489095663	6.685045457	6.363883244	6.522827615	6.580576131	6.548289189	6.724332269	6.782514912
ENSG00000105329	0.016734	0.063137936	-0.398836074	-1.318443798	**TGFB1**	protein_coding	transforming growth factor beta 1 [Source:HGNC Symbol;Acc:HGNC:11766]	chr19	41301587	41353911	-1	ensembl_havana	5.572566537	5.771984574	5.3731485	5.823070467	5.913665537	5.579217716	5.657130739	5.281857508	5.180457254
ENSG00000111859	0.01676	0.063201331	0.22129661	1.165780851	**NEDD9**	protein_coding	neural precursor cell expressed, developmentally down-regulated 9 [Source:HGNC Symbol;Acc:HGNC:7733]	chr6	11183298	11382348	-1	ensembl_havana	6.766913412	6.656265107	6.877561717	6.926403357	6.527186151	6.515205814	7.224815311	6.689616426	6.718253414
ENSG00000131043	0.016757	0.063201331	0.199932559	1.148644659	**AAR2**	protein_coding	AAR2 splicing factor homolog [Source:HGNC Symbol;Acc:HGNC:15886]	chr20	36236459	36270918	1	ensembl_havana	6.734787227	6.634820948	6.834753507	6.588536043	6.682773072	6.633153729	6.816862898	6.853538095	6.833859528
ENSG00000088205	0.016797	0.063291489	-0.244060345	-1.184321149	**DDX18**	protein_coding	DEAD-box helicase 18 [Source:HGNC Symbol;Acc:HGNC:2741]	chr2	117814650	117832379	1	ensembl_havana	7.86054189	7.982572062	7.738511717	7.981736346	8.006056775	7.959923066	7.645376051	7.750918224	7.819240876
ENSG00000082458	0.016796	0.063291489	-0.260244468	-1.197681637	**DLG3**	protein_coding	discs large MAGUK scaffold protein 3 [Source:HGNC Symbol;Acc:HGNC:2902]	chrX	70444861	70505490	1	ensembl_havana	5.137100963	5.267223197	5.006978729	5.377871676	5.287063388	5.136734527	5.02828988	4.985768986	5.006877322
ENSG00000176731	0.016788	0.063291489	-0.304475941	-1.234969953	**C8orf59**	protein_coding	chromosome 8 open reading frame 59 [Source:HGNC Symbol;Acc:HGNC:32235]	chr8	85214076	85220421	-1	ensembl_havana	4.093171207	4.245409177	3.940933236	4.239995762	4.288824872	4.207406897	3.848057062	3.890901443	4.083841203
ENSG00000131015	0.016813	0.063335366	0.640584325	1.558960447	**ULBP2**	protein_coding	UL16 binding protein 2 [Source:HGNC Symbol;Acc:HGNC:14894]	chr6	149942000	149949235	1	ensembl_havana	2.151817812	1.831525649	2.472109974	1.158893785	2.021022296	2.314660866	2.161855151	2.743151031	2.511323741
ENSG00000185453	0.016818	0.063339238	0.282991709	1.216715367	**ZSWIM9**	protein_coding	zinc finger SWIM-type containing 9 [Source:HGNC Symbol;Acc:HGNC:34495]	chr19	48170692	48197620	1	ensembl_havana	2.997827352	2.856331497	3.139323206	2.838235332	2.798432633	2.932326526	3.230173147	3.127034733	3.060761739
ENSG00000148053	0.016824	0.06334478	0.328764757	1.255937574	**NTRK2**	protein_coding	neurotrophic receptor tyrosine kinase 2 [Source:HGNC Symbol;Acc:HGNC:8032]	chr9	84668551	85027070	1	ensembl_havana	5.591228821	5.426846442	5.755611199	5.123340803	5.445898781	5.711299742	5.549913519	5.881822651	5.835097428
ENSG00000048028	0.016848	0.063417362	-0.195523143	-1.145139331	**USP28**	protein_coding	ubiquitin specific peptidase 28 [Source:HGNC Symbol;Acc:HGNC:12625]	chr11	113797874	113875570	-1	ensembl_havana	5.938492716	6.036254288	5.840731144	6.02392752	5.947663743	6.1371716	5.818900937	5.751244084	5.952048412
ENSG00000002726	0.016856	0.063429897	-1.524045547	-2.87596386	**AOC1**	protein_coding	amine oxidase, copper containing 1 [Source:HGNC Symbol;Acc:HGNC:80]	chr7	150824627	150861504	1	ensembl_havana	-1.269567837	-0.507545063	-2.03159061	-1.51148723	-0.18055577	0.16940781	-1.90365811	-2.00847383	-2.182639896
ENSG00000180694	0.016863	0.063442265	-0.248150326	-1.187683414	**TMEM64**	protein_coding	transmembrane protein 64 [Source:HGNC Symbol;Acc:HGNC:25441]	chr8	90621995	90791632	-1	ensembl_havana	3.61227546	3.736350623	3.488200297	3.888048657	3.67277622	3.648226993	3.528767195	3.517212211	3.418621485

ENSG00000170345	0.016893	0.063535526	-0.981759941	-1.974873084	**FOS**	protein_coding	Fos proto-oncogene, AP-1 transcription factor subunit [Source:HGNC Symbol;Acc:HGNC:3796]	chr14	75278774	75282230	1	ensembl_havana	0.159556955	0.650436926	-0.331323016	2.444931907	-0.258437325	-0.235183806	1.527846157	-0.62862566	-1.89318954
ENSG00000062725	0.016903	0.063557291	0.195027529	1.144746004	**APPBP2**	protein_coding	amyloid beta precursor protein binding protein 2 [Source:HGNC Symbol;Acc:HGNC:622]	chr17	60443149	60526219	-1	ensembl_havana	6.654136387	6.556622623	6.751650152	6.51720549	6.620566225	6.532096153	6.706808966	6.813552367	6.734589123
ENSG00000034152	0.016912	0.063562673	0.197102642	1.146393744	**MAP2K3**	protein_coding	mitogen-activated protein kinase kinase 3 [Source:HGNC Symbol;Acc:HGNC:6843]	chr17	21284672	21315240	1	ensembl_havana	5.169995169	5.071443848	5.26854649	5.144989722	5.059243343	5.01009848	5.341149002	5.277234918	5.187255551
ENSG00000137343	0.016913	0.063562673	-0.209802893	-1.156530163	**ATAT1**	protein_coding	alpha tubulin acetyltransferase 1 [Source:HGNC Symbol;Acc:HGNC:21186]	chr6	30626842	30646823	1	ensembl_havana	4.751477369	4.856378815	4.646575922	4.91952048	4.851334342	4.798281624	4.657831492	4.640414466	4.641481809
ENSG00000164649	0.016927	0.063598643	0.199906935	1.148624257	**CDCA7L**	protein_coding	cell division cycle associated 7 like [Source:HGNC Symbol;Acc:HGNC:30777]	chr7	21900900	21946084	-1	ensembl_havana	6.667594779	6.567641311	6.767548246	6.481591234	6.564065059	6.65726764	6.65407813	6.758564024	6.890002584
ENSG00000197822	0.01694	0.063630716	-0.291932321	-1.224278958	**OCLN**	protein_coding	occludin [Source:HGNC Symbol;Acc:HGNC:8104]	chr5	69492292	69558104	1	ensembl_havana	3.41495965	3.56092581	3.268993489	3.628910265	3.526606518	3.527260648	3.192361093	3.382523978	3.232095396
ENSG00000152104	0.016963	0.063679217	0.535433611	1.449377709	**PTPN14**	protein_coding	protein tyrosine phosphatase, non-receptor type 14 [Source:HGNC Symbol;Acc:HGNC:9647]	chr1	214348696	214552449	-1	ensembl_havana	1.642806932	1.375090126	1.910523737	1.222335231	1.338282095	1.564653053	2.135529305	1.759072628	1.836969279
ENSG00000144909	0.016959	0.063679217	0.205583584	1.153152714	**OSBPL11**	protein_coding	oxysterol binding protein like 11 [Source:HGNC Symbol;Acc:HGNC:16397]	chr3	125528858	125595090	-1	ensembl_havana	6.030171897	5.927380105	6.132963689	5.82817741	6.015416866	5.93854604	6.062514562	6.169613661	6.166762843
ENSG00000165417	0.016966	0.063679217	-0.199179596	-1.148045321	**GTF2A1**	protein_coding	general transcription factor IIA subunit 1 [Source:HGNC Symbol;Acc:HGNC:4646]	chr14	81175452	81221377	-1	ensembl_havana	5.656896042	5.75648584	5.557306244	5.816018772	5.772108958	5.68132979	5.586005149	5.598795351	5.487118232
ENSG00000144746	0.016991	0.063718131	0.244061342	1.184321967	**ARL6IP5**	protein_coding	ADP ribosylation factor like GTPase 6 interacting protein 5 [Source:HGNC Symbol;Acc:HGNC:16937]	chr3	69084944	69106066	1	ensembl_havana	8.476500566	8.354469895	8.598531237	8.653278026	8.369636826	8.040494833	8.81981721	8.579409718	8.396366783
ENSG00000067829	0.017003	0.063718131	0.201481805	1.1498788	**IDH3G**	protein_coding	isocitrate dehydrogenase 3 (NAD(+)) gamma [Source:HGNC Symbol;Acc:HGNC:5386]	chrX	153785766	153794523	-1	ensembl_havana	5.955729307	5.854988404	6.056470209	5.928478546	5.804189573	5.832297094	6.16531053	6.006034208	5.99806589
ENSG00000123066	0.017001	0.063718131	0.199341528	1.148174188	**MED13L**	protein_coding	mediator complex subunit 13 like [Source:HGNC Symbol;Acc:HGNC:22962]	chr12	115953872	116277338	-1	ensembl_havana	6.324658584	6.22498782	6.424329348	6.248624956	6.176694632	6.249643872	6.453790683	6.402233078	6.416964283
ENSG00000267698	0.016998	0.063718131	-0.346423419	-1.271404782	**AC002116.2**	antisense		chr19	36014508	36045972	1	havana	0.530668552	0.703880262	0.357456842	0.812341977	0.679062629	0.620236179	0.24405081	0.426254992	0.402064725
ENSG00000138036	0.016998	0.063718131	-0.376181966	-1.29790246	**DYNC2LI1**	protein_coding	dynein cytoplasmic 2 light intermediate chain 1 [Source:HGNC Symbol;Acc:HGNC:24595]	chr2	43774039	43810010	1	ensembl_havana	6.289806518	6.477897501	6.101715535	6.726211342	6.505996837	6.201484325	6.13683798	6.122972689	6.045335935
ENSG00000078269	0.016985	0.063718131	-0.410460409	-1.329109907	**SYNJ2**	protein_coding	synaptojanin 2 [Source:HGNC Symbol;Acc:HGNC:11504]	chr6	157981887	158099176	1	ensembl_havana	4.31564207	4.520872274	4.110411865	4.266573281	4.488286596	4.807756945	4.044780323	4.163592288	4.122862985
ENSG00000105538	0.017018	0.063754628	-0.507949273	-1.422027409	**RASIP1**	protein_coding	Ras interacting protein 1 [Source:HGNC Symbol;Acc:HGNC:24716]	chr19	48720587	48740721	-1	ensembl_havana	1.203032363	1.457007	0.949057727	1.341416217	1.228369434	1.801235349	1.195452122	0.578298274	1.073422785
ENSG00000205572	0.017034	0.063784979	-0.199968191	-1.148673029	**SERF1B**	protein_coding	small EDRK-rich factor 1B [Source:HGNC Symbol;Acc:HGNC:10756]	chr5	70025247	70043113	1	ensembl_havana	1.105622164	1.20560626	1.005638068	1.175017601	1.364499946	1.077301233	0.955706889	1.14725896	0.913948357
ENSG00000256069	0.017032	0.063784979	-0.247971771	-1.187536429	**A2MP1**	transcribed_unprocessed_pseudogene	alpha-2-macroglobulin pseudogene 1 [Source:HGNC Symbol;Acc:HGNC:8]	chr12	9228533	9275817	-1	havana	2.305479512	2.429465397	2.181493627	2.553756523	2.440056551	2.294583118	2.187709208	2.233896951	2.122874721
ENSG00000181804	0.017052	0.06379376	0.266574457	1.202948148	**SLC9A9**	protein_coding	solute carrier family 9 member A9 [Source:HGNC Symbol;Acc:HGNC:20653]	chr3	143265222	143848531	-1	ensembl_havana	3.921108935	3.787821706	4.054396163	3.736039304	3.838598732	3.788827082	3.95980697	4.009357694	4.194023826
ENSG00000168297	0.017051	0.06379376	0.222243194	1.166545997	**PXK**	protein_coding	PX domain containing serine/threonine kinase like [Source:HGNC Symbol;Acc:HGNC:23326]	chr3	58332880	58426126	1	ensembl_havana	5.215657451	5.104535854	5.326779048	4.976979013	5.162782514	5.173846035	5.264261129	5.306566731	5.409509284
ENSG00000186638	0.017055	0.06379376	0.194826759	1.144586709	**KIF24**	protein_coding	kinesin family member 24 [Source:HGNC Symbol;Acc:HGNC:19916]	chr9	34252381	34311371	-1	havana	4.265327531	4.167914151	4.36274091	4.105550163	4.144985604	4.253206686	4.289263117	4.347699835	4.451259779
ENSG00000100632	0.017045	0.06379376	-0.232262203	-1.174675443	**ERH**	protein_coding	ERH, mRNA splicing and mitosis factor [Source:HGNC Symbol;Acc:HGNC:3447]	chr14	69380123	69398627	-1	ensembl_havana	7.174291687	7.290422789	7.058160586	7.246724628	7.379920929	7.24462281	6.960318662	7.10300181	7.111161287
ENSG00000180061	0.017073	0.063844291	-0.387430411	-1.308061535	**TMEM150B**	protein_coding	transmembrane protein 150B [Source:HGNC Symbol;Acc:HGNC:34415]	chr19	55312801	55334048	-1	ensembl_havana	0.422899642	0.616614848	0.229184436	0.749126884	0.298820819	0.80189684	0.176794179	0.163140205	0.347618925
ENSG00000141378	0.017082	0.06386407	-0.356516545	-1.280330743	**PTRH2**	protein_coding	peptidyl-tRNA hydrolase 2 [Source:HGNC Symbol;Acc:HGNC:24265]	chr17	59674636	59707626	-1	ensembl_havana	6.005735373	6.183993645	5.827477101	6.242421812	6.228913371	6.080645754	5.681597922	5.878164058	5.922669321
ENSG00000065427	0.017109	0.063948529	-0.206331193	-1.153750436	**KARS**	protein_coding	lysyl-tRNA synthetase [Source:HGNC Symbol;Acc:HGNC:6215]	chr16	75627474	75648643	-1	ensembl_havana	7.751246402	7.854411999	7.648080806	7.75465711	7.844015463	7.964563423	7.557426129	7.679669965	7.707146322
ENSG00000105829	0.017122	0.063963751	-0.253261165	-1.19189832	**BET1**	protein_coding	Bet1 golgi vesicular membrane trafficking protein [Source:HGNC Symbol;Acc:HGNC:14562]	chr7	93962762	94004382	-1	ensembl_havana	4.551592978	4.678223561	4.424962395	4.603852402	4.806616371	4.624201909	4.27927777	4.498293919	4.497315497
ENSG00000131507	0.01712	0.063963751	-0.262436187	-1.199502519	**NDFIP1**	protein_coding	Nedd4 family interacting protein 1 [Source:HGNC Symbol;Acc:HGNC:17592]	chr5	142108505	142154443	1	ensembl_havana	6.766359455	6.897577549	6.635141361	6.994424055	6.948731679	6.749576913	6.633061453	6.652974448	6.619388184
ENSG00000143367	0.017156	0.064042918	-0.250528681	-1.189642985	**TUFT1**	protein_coding	tuftelin 1 [Source:HGNC Symbol;Acc:HGNC:12422]	chr1	151540305	151583583	1	ensembl_havana	4.386988285	4.512252626	4.261723944	4.471398808	4.604215712	4.461143357	4.236567839	4.239696816	4.308907178
ENSG00000278619	0.017162	0.064042918	-0.46624044	-1.381504671	**MRM1**	protein_coding	mitochondrial rRNA methyltransferase 1 [Source:HGNC Symbol;Acc:HGNC:26202]	chr17	36601572	36608971	1	ensembl_havana	2.897853965	3.130974185	2.664733745	2.950212473	2.918320072	3.52439001	2.587917939	2.671684573	2.734598724
ENSG00000268403	0.017148	0.064042918	-0.491847119	-1.406244174	**AC132192.2**	antisense		chr11	9459556	9460702	-1	havana	0.976081779	1.222005338	0.730158219	1.355633303	1.452682866	0.857699845	0.739683594	0.737534657	0.713256405
ENSG00000284128	0.017165	0.064042918	-0.633430051	-1.551248754	**AP000356.3**	transcribed_unprocessed_pseudogene		chr22	24644791	24653356	1	havana	1.086158929	1.402873954	0.769443903	1.477650254	1.476918714	1.254052895	0.592614899	0.625615656	1.090101154
ENSG00000257556	0.017166	0.064042918	-0.711544301	-1.637556065	**LINC02298**	lincRNA	long intergenic non-protein coding RNA 2298 [Source:HGNC Symbol;Acc:HGNC:53216]	chr14	105093609	105099004	1	havana	0.343273488	0.699045638	-0.012498663	0.637193984	0.818355163	0.641587769	-0.04612063	0.554044597	-0.545419959
ENSG00000146707	0.017172	0.064049809	0.196178566	1.14565969	**POMZP3**	protein_coding	POM121 and ZP3 fusion [Source:HGNC Symbol;Acc:HGNC:9203]	chr7	76609986	76627261	-1	ensembl_havana	4.064051318	3.965962036	4.162140601	3.902646601	4.030934738	3.964304768	4.120558811	4.208958525	4.156904467
ENSG00000221983	0.017178	0.064054845	0.226604408	1.170077758	**UBA52**	protein_coding	ubiquitin A-52 residue ribosomal protein fusion product 1 [Source:HGNC Symbol;Acc:HGNC:12458]	chr19	18571730	18577550	1	ensembl_havana	8.630724679	8.517422475	8.744026883	8.455124104	8.628518052	8.468625268	8.673771288	8.780210878	8.778098482
ENSG00000147889	0.017203	0.064097574	0.236134591	1.177832666	**CDKN2A**	protein_coding	cyclin dependent kinase inhibitor 2A [Source:HGNC Symbol;Acc:HGNC:1787]	chr9	21967753	21995301	-1	ensembl_havana	4.327251157	4.209183861	4.445318452	4.253345723	4.096927042	4.27727882	4.585121045	4.247280137	4.503554175
ENSG00000146263	0.0172	0.064097574	-0.242933907	-1.183396807	**MMS22L**	protein_coding	MMS22 like, DNA repair protein [Source:HGNC Symbol;Acc:HGNC:21475]	chr6	97142161	97283217	-1	ensembl_havana	6.326199406	6.447666359	6.204732452	6.626406402	6.333403802	6.383188874	6.311614414	6.049513968	6.253068975
ENSG00000137177	0.017203	0.064097574	-0.367703693	-1.290297456	**KIF13A**	protein_coding	kinesin family member 13A [Source:HGNC Symbol;Acc:HGNC:14566]	chr6	17759183	17987623	-1	ensembl_havana	-0.207486411	-0.023634564	-0.391338257	-0.56281016	0.352203286	0.139703184	-0.69455235	-0.08087422	-0.398588198
ENSG00000164347	0.017222	0.064150849	0.201303683	1.149736839	**GFM2**	protein_coding	G elongation factor mitochondrial 2 [Source:HGNC Symbol;Acc:HGNC:29682]	chr5	74721204	74767371	-1	ensembl_havana	6.708254825	6.607602983	6.808906666	6.535273896	6.601403272	6.686131781	6.763422453	6.760552584	6.902744961
ENSG00000187775	0.017227	0.064152696	0.66467202	1.585207854	**DNAH17**	protein_coding	dynein axonemal heavy chain 17 [Source:HGNC Symbol;Acc:HGNC:2946]	chr17	78423697	78577394	-1	ensembl_havana	0.080976199	-0.251359811	0.413312209	-1.070289	-0.071090603	0.387300166	0.069637898	0.478736765	0.691561964
ENSG00000114933	0.017236	0.064171913	0.196038062	1.14554812	**INO80D**	protein_coding	INO80 complex subunit D [Source:HGNC Symbol;Acc:HGNC:25997]	chr2	205993721	206086303	-1	ensembl_havana	5.774421536	5.676402505	5.872440567	5.675747796	5.738045873	5.615413846	5.870360877	5.914339061	5.832621762
ENSG00000090661	0.017247	0.064179163	0.199525863	1.148320901	**CERS4**	protein_coding	ceramide synthase 4 [Source:HGNC Symbol;Acc:HGNC:23747]	chr19	8206736	8262421	1	ensembl_havana	5.051220423	4.951457491	5.150983354	5.074752688	4.935722747	4.843897038	5.308136366	5.104295527	5.04051817
ENSG00000101695	0.017243	0.064179163	0.196462981	1.14588557	**RNF125**	protein_coding	ring finger protein 125 [Source:HGNC Symbol;Acc:HGNC:21150]	chr18	32018372	32073213	1	ensembl_havana	5.959206218	5.860974728	6.057437709	5.806993748	5.831466344	5.944464091	6.015769787	6.002117617	6.154425723
ENSG00000140948	0.017259	0.064191297	0.285920979	1.219188314	**ZCCHC14**	protein_coding	zinc finger CCHC-type containing 14 [Source:HGNC Symbol;Acc:HGNC:24134]	chr16	87406246	87492045	-1	ensembl_havana	4.325990206	4.183029717	4.468950696	4.130569456	4.241864646	4.176655048	4.57045622	4.401364514	4.435031352
ENSG00000100714	0.017256	0.064191297	-0.194860687	-1.144613626	**MTHFD1**	protein_coding	methylenetetrahydrofolate dehydrogenase, cyclohydrolase and formyltetrahydrofolate synthetase 1 [Source:HGNC Symbol;Acc:HGNC:7432]	chr14	64388031	64463457	1	ensembl_havana	7.820881999	7.918312342	7.723451655	7.730142388	7.937715638	8.087079001	7.543727404	7.751584905	7.875042656
ENSG00000143776	0.017276	0.064235918	0.346414801	1.271397186	**CDC42BPA**	protein_coding	CDC42 binding protein kinase alpha [Source:HGNC Symbol;Acc:HGNC:1737]	chr1	226989865	227318474	-1	ensembl_havana	4.368434004	4.195226603	4.541641404	4.105550163	4.287112591	4.193017056	4.670111011	4.56959204	4.385221163
ENSG00000187098	0.0173	0.064277566	0.266693757	1.203047626	**MITF**	protein_coding	melanogenesis associated transcription factor [Source:HGNC Symbol;Acc:HGNC:7105]	chr3	69739435	69968337	1	ensembl_havana	3.60706351	3.473716631	3.740410389	3.101289176	3.627484939	3.692375779	3.477287033	3.759192284	3.984751849
ENSG00000104915	0.017297	0.064277566	0.243169132	1.183589769	**STX10**	protein_coding	syntaxin 10 [Source:HGNC Symbol;Acc:HGNC:11428]	chr19	13144058	13150383	-1	ensembl_havana	4.680024881	4.558440315	4.801609447	4.541479027	4.477816603	4.656025315	4.865636402	4.751234058	4.78795788
ENSG00000175166	0.017293	0.064277566	0.210606178	1.157174292	**PSMD2**	protein_coding	proteasome 26S subunit, non-ATPase 2 [Source:HGNC Symbol;Acc:HGNC:9559]	chr3	184298709	184309054	1	ensembl_havana	8.974772327	8.869469238	9.080075416	8.709882385	8.932312856	8.966212473	8.980047016	9.135548151	9.124631081
ENSG00000183918	0.01732	0.064283497	0.276697327	1.211418482	**SH2D1A**	protein_coding	SH2 domain containing 1A [Source:HGNC Symbol;Acc:HGNC:10820]	chrX	124227868	124373197	1	ensembl_havana	5.737667593	5.599318929	5.876016256	5.502896245	5.8249904	5.470070144	5.863693787	5.94730313	5.817051852
ENSG00000068097	0.017317	0.064283497	0.235685122	1.177465771	**HEATR6**	protein_coding	HEAT repeat containing 6 [Source:HGNC Symbol;Acc:HGNC:24076]	chr17	60043194	60078931	-1	ensembl_havana	5.61390575	5.496063189	5.731748311	5.277836953	5.515554998	5.694797617	5.614877513	5.725006242	5.855361177
ENSG00000100219	0.01731	0.064283497	-0.251786988	-1.190681034	**XBP1**	protein_coding	X-box binding protein 1 [Source:HGNC Symbol;Acc:HGNC:12801]	chr22	28794555	28800597	-1	ensembl_havana	7.04631056	7.172204054	6.920417066	7.300996023	7.177374783	7.038241355	6.992456039	6.856640009	6.91215515
ENSG00000272240	0.017317	0.064283497	-0.730602888	-1.659332365	**AC004908.1**	antisense		chr8	234347	234887	-1	havana	0.175813602	0.541115046	-0.189487842	0.832812769	0.378171619	0.41236075	-0.17198403	-0.62862566	0.232146171
ENSG00000183474	0.017343	0.064353987	-0.225948924	-1.169546258	**GTF2H2C**	protein_coding	GTF2H2 family member C [Source:HGNC Symbol;Acc:HGNC:31394]	chr5	69560208	69594723	1	ensembl_havana	3.910460441	4.023434903	3.797485979	3.94322289	4.008249006	4.118832811	3.635945119	3.859084184	3.897428633
ENSG00000170421	0.01736	0.064400109	-0.556550118	-1.470748045	**KRT8**	protein_coding	keratin 8 [Source:HGNC Symbol;Acc:HGNC:6446]	chr12	52897187	52949954	-1	ensembl_havana	0.361497896	0.639772955	0.083222837	0.437805725	0.501432867	0.980080273	-0.04612063	0.256279753	0.039509383
ENSG00000249699	0.017369	0.064415426	-0.604731289	-1.520695488	**LINC02261**	lincRNA	long intergenic non-protein coding RNA 2261 [Source:HGNC Symbol;Acc:HGNC:53173]	chr4	27217479	27282225	1	havana	1.39035976	1.692725405	1.087994116	1.802235788	1.428032914	1.847907512	1.386165364	1.080904991	0.796911992
ENSG00000134779	0.017388	0.064470646	0.213792411	1.159732769	**TPGS2**	protein_coding	tubulin polyglutamylase complex subunit 2 [Source:HGNC Symbol;Acc:HGNC:24561]	chr18	36777647	36829216	-1	ensembl_havana	6.790478202	6.683581997	6.897374408	6.507687891	6.777894474	6.765163626	6.790390105	6.940306929	6.961426191
ENSG00000135269	0.017397	0.064470968	0.218569829	1.163579536	**TES**	protein_coding	testin LIM domain protein [Source:HGNC Symbol;Acc:HGNC:14620]	chr7	116210493	116258783	1	ensembl_havana	6.15690977	6.047624855	6.266194684	5.972386637	6.133990979	6.036496949	6.269864968	6.324607561	6.204111524
ENSG00000198015	0.017397	0.064470968	-0.285336656	-1.218694616	**MRPL42**	protein_coding	mitochondrial ribosomal protein L42 [Source:HGNC Symbol;Acc:HGNC:14493]	chr12	93467488	93516213	1	ensembl_havana	5.521306486	5.663974814	5.378638158	5.694745	5.71657922	5.580600224	5.246945431	5.533620008	5.355349037
ENSG00000204482	0.017413	0.06451209	-0.767336921	-1.70212492	**LST1**	protein_coding	leukocyte specific transcript 1 [Source:HGNC Symbol;Acc:HGNC:14189]	chr6	31586124	31588909	1	ensembl_havana	-0.1304856	0.25318286	-0.514154061	0.437805725	0.09469996	0.227042895	-0.82634296	-0.68524259	-0.030876629
ENSG00000070366	0.017445	0.064547222	0.20714569	1.154401989	**SMG6**	protein_coding	SMG6, nonsense mediated mRNA decay factor [Source:HGNC Symbol;Acc:HGNC:17809]	chr17	2059839	2303771	-1	ensembl_havana	5.315939259	5.212366414	5.419512104	5.155185655	5.213245738	5.268667849	5.305186891	5.436628448	5.516720974
ENSG00000247081	0.017443	0.064547222	-0.209622343	-1.156385435	**BAALC-AS1**	antisense	BAALC antisense RNA 1 [Source:HGNC Symbol;Acc:HGNC:50461]	chr8	103156990	103298772	-1	havana	1.280708116	1.385519288	1.175896945	1.268150092	1.428032914	1.460374858	0.995284613	1.241396536	1.291009686
ENSG00000092199	0.017445	0.064547222	-0.212224041	-1.158472696	**HNRNPC**	protein_coding	heterogeneous nuclear ribonucleoprotein C (C1/C2) [Source:HGNC Symbol;Acc:HGNC:5035]	chr14	21209136	21269494	-1	ensembl_havana	9.325599933	9.431711953	9.219487912	9.487915834	9.387822947	9.41939708	9.207890322	9.221011616	9.229561798
ENSG00000115525	0.017442	0.064547222	-0.213466198	-1.159470567	**ST3GAL5**	protein_coding	ST3 beta-galactoside alpha-2,3-sialyltransferase 5 [Source:HGNC Symbol;Acc:HGNC:10872]	chr2	85837120	85905199	-1	ensembl_havana	7.020215938	7.126949037	6.913482839	7.177383534	7.090706917	7.112756659	6.923607761	6.84637828	6.970462475
ENSG00000028116	0.017427	0.064547222	-0.232824397	-1.175133284	**VRK2**	protein_coding	vaccinia related kinase 2 [Source:HGNC Symbol;Acc:HGNC:12719]	chr2	57907651	58159920	1	ensembl_havana	4.701707911	4.81812011	4.585295713	5.022244564	4.84320029	4.588915474	4.756142808	4.543651409	4.456092922
ENSG00000135587	0.017461	0.064591059	0.350890778	1.275347836	**SMPD2**	protein_coding	sphingomyelin phosphodiesterase 2 [Source:HGNC Symbol;Acc:HGNC:11121]	chr6	109440763	109443919	1	ensembl_havana	3.648316404	3.472871015	3.823761793	3.290778936	3.558185553	3.569648554	3.806944749	3.688535729	3.975804901
ENSG00000118503	0.017468	0.064598896	0.236430112	1.178073958	**TNFAIP3**	protein_coding	TNF alpha induced protein 3 [Source:HGNC Symbol;Acc:HGNC:11896]	chr6	137867188	137883312	1	ensembl_havana	7.127030712	7.008815656	7.245245768	7.485237551	6.854756577	6.686452838	7.643820356	7.066495926	7.025421021
ENSG00000143630	0.017475	0.064610096	0.264559874	1.20126952	**HCN3**	protein_coding	hyperpolarization activated cyclic nucleotide gated potassium channel 3 [Source:HGNC Symbol;Acc:HGNC:19183]	chr1	155277583	155289848	1	ensembl_havana	5.252510799	5.120230863	5.384790736	5.03334247	5.211441306	5.115908812	5.392075968	5.337091883	5.425204358
ENSG00000183762	0.017512	0.064694125	0.346417238	1.271399335	**KREMEN1**	protein_coding	kringle containing transmembrane protein 1 [Source:HGNC Symbol;Acc:HGNC:17550]	chr22	29073078	29168333	1	ensembl_havana	3.210041823	3.036833203	3.383250442	2.887992321	2.931533374	3.290973915	3.37972385	3.35471112	3.415316355
ENSG00000121741	0.017512	0.064694125	-0.202813057	-1.150940344	**ZMYM2**	protein_coding	zinc finger MYM-type containing 2 [Source:HGNC Symbol;Acc:HGNC:12989]	chr13	19958670	20091829	1	ensembl_havana	7.185502838	7.286909366	7.08409631	7.351527276	7.261093803	7.24810702	7.10386287	7.09855428	7.049871779
ENSG00000163596	0.017506	0.064694125	-0.224843142	-1.16865018	**ICA1L**	protein_coding	islet cell autoantigen 1 like [Source:HGNC Symbol;Acc:HGNC:14442]	chr2	202773150	202871985	-1	ensembl_havana	3.865187259	3.977608831	3.752765688	3.929031797	3.993624659	4.010170036	3.660771151	3.721576939	3.875948975
ENSG00000007866	0.017516	0.064694125	-0.405067515	-1.324150876	**TEAD3**	protein_coding	TEA domain transcription factor 3 [Source:HGNC Symbol;Acc:HGNC:11716]	chr6	35473597	35497076	-1	ensembl_havana	0.902068667	1.104602424	0.69953491	1.005046425	1.170104606	1.138656243	0.873137292	0.578298274	0.647169164
ENSG00000100077	0.017522	0.06469799	0.323912185	1.251720266	**GRK3**	protein_coding	G protein-coupled receptor kinase 3 [Source:HGNC Symbol;Acc:HGNC:290]	chr22	25564849	25729294	1	ensembl_havana	4.255687057	4.093730964	4.417643149	3.703060534	4.133596708	4.444535651	4.179729066	4.498293919	4.574906464
ENSG00000255921	0.01754	0.064704086	0.681705227	1.604034561	**AC026310.2**	antisense		chr12	24949163	24960158	1	havana	0.335808618	-0.005043995	0.676661231	-0.51029874	0.325758945	0.16940781	0.668022463	0.648704825	0.713256405
ENSG00000270127	0.017546	0.064704086	0.490425272	1.404858934	**AC027020.2**	lincRNA		chr15	100547765	100550153	-1	havana	2.226898776	1.98168614	2.472111412	1.892762624	2.177092949	1.875202847	2.657410455	2.336998362	2.42192542
ENSG00000240849	0.017532	0.064704086	0.228554597	1.171660501	**TMEM189**	protein_coding	transmembrane protein 189 [Source:HGNC Symbol;Acc:HGNC:16735]	chr20	50118254	50153734	-1	ensembl_havana	4.441941499	4.327664201	4.556218798	4.155162249	4.45968935	4.368141003	4.403988623	4.596559508	4.668108262
ENSG00000110002	0.017543	0.064704086	0.227000515	1.170399059	**VWA5A**	protein_coding	von Willebrand factor A domain containing 5A [Source:HGNC Symbol;Acc:HGNC:6658]	chr11	124115362	124147721	1	ensembl_havana	4.441219727	4.327719469	4.554719984	4.239995762	4.329321514	4.413841131	4.493082186	4.621574099	4.549503667
ENSG00000216915	0.017539	0.064704086	-0.589901051	-1.505143512	**GPR89P**	processed_pseudogene	G protein-coupled receptor 89 pseudogene [Source:HGNC Symbol;Acc:HGNC:13951]	chr6	27737000	27738494	-1	havana	1.254238588	1.549189113	0.959288062	1.716703112	1.37743234	1.553431888	0.692307165	1.046547334	1.139009687
ENSG00000235173	0.017557	0.064726989	0.288363183	1.221253911	**HGH1**	protein_coding	HGH1 homolog [Source:HGNC Symbol;Acc:HGNC:24161]	chr8	144137769	144140843	1	ensembl_havana	4.435343777	4.291162185	4.579525368	4.027778621	4.36222147	4.483486464	4.451003248	4.670338547	4.61723431
ENSG00000198162	0.017569	0.064751578	0.229468389	1.172402857	**MAN1A2**	protein_coding	mannosidase alpha class 1A member 2 [Source:HGNC Symbol;Acc:HGNC:6822]	chr1	117347449	117528872	1	ensembl_havana	6.420609019	6.305874824	6.535343214	6.405904646	6.215472255	6.296247572	6.537857235	6.50069319	6.567479216
ENSG00000149311	0.017572	0.064751578	-0.404728142	-1.323839426	**ATM**	protein_coding	ATM serine/threonine kinase [Source:HGNC Symbol;Acc:HGNC:795]	chr11	108222484	108369102	1	ensembl_havana	6.757739175	6.960103246	6.555375104	7.17563162	6.763188779	6.941489339	6.496220687	6.539428369	6.630476257
ENSG00000004700	0.017581	0.064765923	-0.243459917	-1.183828354	**RECQL**	protein_coding	RecQ like helicase [Source:HGNC Symbol;Acc:HGNC:9948]	chr12	21468911	21501669	-1	ensembl_havana	7.96185901	8.083588968	7.840129051	8.182629418	8.122493929	7.945643557	7.844060049	7.867754312	7.808572793
ENSG00000182400	0.017586	0.064769846	0.226187334	1.169739545	**TRAPPC6B**	protein_coding	trafficking protein particle complex 6B [Source:HGNC Symbol;Acc:HGNC:23066]	chr14	39147811	39170532	-1	ensembl_havana	5.185208646	5.072114979	5.298302313	5.138837395	5.102723404	4.97478414	5.311079824	5.290141027	5.29368609
ENSG00000204822	0.017624	0.064851928	0.421233342	1.339071822	**MRPL53**	protein_coding	mitochondrial ribosomal protein L53 [Source:HGNC Symbol;Acc:HGNC:16684]	chr2	74471958	74473322	-1	ensembl_havana	2.214059434	2.003442763	2.424676105	1.968722995	1.918574186	2.123031109	2.117708014	2.572545352	2.58377495
ENSG00000196584	0.017625	0.064851928	-0.194019458	-1.143946402	**XRCC2**	protein_coding	X-ray repair cross complementing 2 [Source:HGNC Symbol;Acc:HGNC:12829]	chr7	152644779	152676165	-1	ensembl_havana	5.164679001	5.26168873	5.067669271	5.105574388	5.293900585	5.385591216	4.896012582	5.11780318	5.189192052
ENSG00000170464	0.017627	0.064851928	-0.199798101	-1.148537611	**DNAJC18**	protein_coding	DnaJ heat shock protein family (Hsp40) member C18 [Source:HGNC Symbol;Acc:HGNC:28429]	chr5	139408588	139444491	-1	ensembl_havana	5.958151857	6.058050907	5.858252807	6.104534582	6.10172166	5.967896481	5.946790314	5.87755339	5.750414716
ENSG00000105248	0.017613	0.064851928	-0.208573101	-1.155544726	**CCDC94**	protein_coding	coiled-coil domain containing 94 [Source:HGNC Symbol;Acc:HGNC:25518]	chr19	4247079	4269090	1	ensembl_havana	3.242202218	3.346488768	3.137915667	3.468093324	3.304241881	3.2671311	3.196611706	3.122918448	3.094216849
ENSG00000141504	0.017637	0.064872751	0.197835744	1.14697643	**SAT2**	protein_coding	spermidine/spermine N1-acetyltransferase family member 2 [Source:HGNC Symbol;Acc:HGNC:23160]	chr17	7626234	7627876	-1	ensembl_havana	4.217118163	4.118200291	4.316036035	4.277815454	4.112480652	3.964304768	4.45278126	4.297450705	4.197876141
ENSG00000066629	0.017661	0.0649456	-0.218815726	-1.163777877	**EML1**	protein_coding	echinoderm microtubule associated protein like 1 [Source:HGNC Symbol;Acc:HGNC:3330]	chr14	99737693	99942060	1	ensembl_havana	6.420301138	6.529709002	6.310893275	6.637358151	6.448177047	6.503591807	6.478874707	6.247301464	6.206503655
ENSG00000172831	0.017682	0.064954474	0.269467801	1.205363097	**CES2**	protein_coding	carboxylesterase 2 [Source:HGNC Symbol;Acc:HGNC:1864]	chr16	66934444	66945096	1	ensembl_havana	4.229801876	4.095067975	4.364535776	3.94557456	4.141199286	4.19843008	4.357289884	4.302919022	4.433398424
ENSG00000185420	0.017674	0.064954474	0.221563088	1.165996201	**SMYD3**	protein_coding	SET and MYND domain containing 3 [Source:HGNC Symbol;Acc:HGNC:15513]	chr1	245749342	246507312	-1	ensembl_havana	4.512415779	4.401634234	4.623197323	4.355918458	4.427408044	4.421576202	4.651652314	4.650458282	4.567481372
ENSG00000135148	0.017679	0.064954474	-0.216811222	-1.162162027	**TRAFD1**	protein_coding	TRAF-type zinc finger domain containing 1 [Source:HGNC Symbol;Acc:HGNC:24808]	chr12	112125501	112153609	1	ensembl_havana	6.2859109	6.394316511	6.177505289	6.525482193	6.387543412	6.269923929	6.230503412	6.218174267	6.083838189
ENSG00000259448	0.017676	0.064954474	-0.311167394	-1.240711248	**LINC02352**	lincRNA	long intergenic non-protein coding RNA 2352 [Source:HGNC Symbol;Acc:HGNC:53274]	chr15	31216020	31224445	1	havana	0.669638496	0.825222193	0.514054799	0.637193984	0.962641309	0.875831286	0.485518989	0.478736765	0.577908642
ENSG00000172345	0.017688	0.064962416	0.506133274	1.420238552	**STARD5**	protein_coding	StAR related lipid transfer domain containing 5 [Source:HGNC Symbol;Acc:HGNC:18065]	chr15	81309053	81324183	-1	ensembl_havana	4.100442228	3.847375591	4.353508865	3.341663059	4.032979498	4.167484215	4.215776639	4.359993337	4.484756619
ENSG00000132874	0.017702	0.064995642	0.843438122	1.794321142	**SLC14A2**	protein_coding	solute carrier family 14 member 2 [Source:HGNC Symbol;Acc:HGNC:10919]	chr18	45212995	45683686	1	havana	0.197367548	-0.224351513	0.619086609	-0.41068387	-0.34076431	0.078393642	0.935503283	0.602150945	0.319605599
ENSG00000225830	0.017727	0.06506029	0.298850794	1.230164113	**ERCC6**	protein_coding	ERCC excision repair 6, chromatin remodeling factor [Source:HGNC Symbol;Acc:HGNC:3438]	chr10	49455368	49539538	-1	ensembl_havana	5.400183335	5.250757937	5.549608732	5.126453466	5.181337648	5.444482698	5.323765864	5.659733168	5.665327164
ENSG00000121964	0.017728	0.06506029	-0.199496531	-1.148297555	**GTDC1**	protein_coding	glycosyltransferase like domain containing 1 [Source:HGNC Symbol;Acc:HGNC:20887]	chr2	143938068	144332568	-1	ensembl_havana	3.583623097	3.683371363	3.483874831	3.885601241	3.698768738	3.465744108	3.648411535	3.501444376	3.301768582
ENSG00000106692	0.017734	0.065063821	0.195161554	1.144852355	**FKTN**	protein_coding	fukutin [Source:HGNC Symbol;Acc:HGNC:3622]	chr9	105558130	105641118	1	ensembl_havana	5.747696425	5.650115648	5.845277202	5.518776574	5.670037832	5.761532538	5.721968021	5.883648474	5.930215113
ENSG00000259287	0.017765	0.065145354	0.345158228	1.270290294	**AC010809.1**	antisense		chr15	33858602	33864825	-1	havana	1.514107856	1.341528742	1.68668697	1.327057633	1.078042694	1.6194859	1.554586035	1.648877048	1.856597828
ENSG00000012983	0.017765	0.065145354	0.213315722	1.159349638	**MAP4K5**	protein_coding	mitogen-activated protein kinase kinase kinase kinase 5 [Source:HGNC Symbol;Acc:HGNC:6867]	chr14	50418501	50561126	-1	ensembl_havana	5.552665944	5.446008083	5.659323804	5.524293648	5.451260379	5.362470221	5.666354627	5.685779305	5.625837481
ENSG00000276718	0.01777	0.065146468	-0.655839423	-1.5755324	**AC005840.4**	antisense		chr12	6466537	6467135	1	havana	-0.074000571	0.25391914	-0.401920283	0.237415364	0.477608744	0.046733314	-0.89704638	-0.04338487	-0.265329594
ENSG00000258289	0.017775	0.065146799	-0.213040571	-1.159128548	**CHURC1**	protein_coding	churchill domain containing 1 [Source:HGNC Symbol;Acc:HGNC:20099]	chr14	64914361	64944591	1	ensembl_havana	4.905871052	5.012391338	4.799350767	5.066132977	4.96814283	5.002898206	4.801500542	4.825302309	4.77124945
ENSG00000109670	0.017792	0.065184537	0.234017793	1.176105755	**FBXW7**	protein_coding	F-box and WD repeat domain containing 7 [Source:HGNC Symbol;Acc:HGNC:16712]	chr4	152320544	152536063	-1	ensembl_havana	4.861278195	4.744269299	4.978287092	4.854681818	4.701268571	4.676857507	4.984711912	4.972104681	4.978044683
ENSG00000115602	0.017794	0.065184537	-0.243661716	-1.183993956	**IL1RL1**	protein_coding	interleukin 1 receptor like 1 [Source:HGNC Symbol;Acc:HGNC:5998]	chr2	102311502	102352037	1	ensembl_havana	7.218777419	7.340608277	7.096946561	7.537618538	7.223539087	7.260667207	7.1897955	6.983513373	7.11753081
ENSG00000166086	0.0178	0.065184961	0.276313564	1.211096282	**JAM3**	protein_coding	junctional adhesion molecule 3 [Source:HGNC Symbol;Acc:HGNC:15532]	chr11	134068925	134152001	1	ensembl_havana	5.622171673	5.484014891	5.760328455	5.299142301	5.553084872	5.599817502	5.716798148	5.817055082	5.747132135
ENSG00000144021	0.017803	0.065184961	0.2057023	1.153247608	**CIAO1**	protein_coding	cytosolic iron-sulfur assembly component 1 [Source:HGNC Symbol;Acc:HGNC:14280]	chr2	96266132	96273349	1	ensembl_havana	7.323304435	7.220453285	7.426155585	7.249582404	7.174367508	7.237409943	7.413295153	7.365906256	7.499265347
ENSG00000124575	0.017814	0.065205949	-0.47013943	-1.385243339	**HIST1H1D**	protein_coding	histone cluster 1 H1 family member d [Source:HGNC Symbol;Acc:HGNC:4717]	chr6	26234268	26234933	-1	ensembl_havana	-0.642133265	-0.40706355	-0.877202979	0.14220989	-0.620350959	-0.743049581	-0.21650287	-0.86978558	-1.54532049
ENSG00000178695	0.017857	0.065346525	0.396556416	1.316362119	**KCTD12**	protein_coding	potassium channel tetramerization domain containing 12 [Source:HGNC Symbol;Acc:HGNC:14678]	chr13	76880166	76886390	-1	ensembl_havana	1.859896194	1.661617986	2.058174402	0.852997155	1.864486201	2.267370602	1.47283199	2.286368471	2.415322744
ENSG00000275549	0.017866	0.065346525	0.211667926	1.158026226	**STPG3-AS1**	antisense	STPG3 antisense RNA 1 [Source:HGNC Symbol;Acc:HGNC:51176]	chr9	137250219	137253497	-1	havana	0.443931419	0.338097456	0.549765382	0.326725152	0.378171619	0.309395597	0.592614899	0.602150945	0.454530303
ENSG00000138279	0.01788	0.065346525	-0.203607127	-1.151574004	**ANXA7**	protein_coding	annexin A7 [Source:HGNC Symbol;Acc:HGNC:545]	chr10	73375101	73414076	-1	ensembl_havana	7.181103632	7.282907195	7.079300068	7.336821633	7.319219217	7.192680736	7.099045768	7.096194143	7.042660294
ENSG00000091986	0.017867	0.065346525	-0.224427863	-1.168313832	**CCDC80**	protein_coding	coiled-coil domain containing 80 [Source:HGNC Symbol;Acc:HGNC:30649]	chr3	112596794	112649530	-1	ensembl_havana	1.749901398	1.862115329	1.637687466	1.237769075	2.108942002	2.239634912	1.089724456	1.890792227	1.932545716
ENSG00000120889	0.01788	0.065346525	-0.239126058	-1.180277468	**TNFRSF10B**	protein_coding	TNF receptor superfamily member 10b [Source:HGNC Symbol;Acc:HGNC:11905]	chr8	23020133	23069179	-1	ensembl_havana	5.347048294	5.466611323	5.227485265	5.674330594	5.294752961	5.430750414	5.523932037	5.071565226	5.086958531
ENSG00000167004	0.017884	0.065346525	-0.260830915	-1.198168587	**PDIA3**	protein_coding	protein disulfide isomerase family A member 3 [Source:HGNC Symbol;Acc:HGNC:4606]	chr15	43746392	43773279	1	ensembl_havana	9.395423654	9.525839111	9.265008196	9.645525037	9.545859704	9.386132592	9.277888206	9.260658723	9.25647766
ENSG00000234602	0.017874	0.065346525	-0.553017294	-1.467150931	**MCIDAS**	protein_coding	multiciliate differentiation and DNA synthesis associated cell cycle protein [Source:HGNC Symbol;Acc:HGNC:40050]	chr5	55219614	55227315	-1	ensembl_havana	1.937807409	2.214316056	1.661298762	2.014244272	2.199112819	2.429591076	1.7752573	1.715999071	1.492639914
ENSG00000140650	0.017911	0.06542941	-0.364775218	-1.287680984	**PMM2**	protein_coding	phosphomannomutase 2 [Source:HGNC Symbol;Acc:HGNC:9115]	chr16	8788823	8849331	1	ensembl_havana	4.43444063	4.616828239	4.252053021	4.868380636	4.509001353	4.473102727	4.267203405	4.207015462	4.281940196
ENSG00000090565	0.017923	0.06545766	0.319791404	1.248150068	**RAB11FIP3**	protein_coding	RAB11 family interacting protein 3 [Source:HGNC Symbol;Acc:HGNC:17224]	chr16	425619	523011	1	ensembl_havana	4.748849009	4.588953307	4.908744711	4.642053159	4.468781447	4.656025315	5.129580575	4.802320534	4.794333023
ENSG00000243970	0.017944	0.065515145	0.44464015	1.360974608	**PPIEL**	transcribed_unprocessed_pseudogene	peptidylprolyl isomerase E like pseudogene [Source:HGNC Symbol;Acc:HGNC:33195]	chr1	39522280	39559671	-1	havana	0.283524902	0.061204827	0.505844977	0.10902545	0.271370116	-0.196781087	0.716189844	0.399280361	0.402064725
ENSG00000105258	0.017963	0.065550968	0.259178237	1.196796811	**POLR2I**	protein_coding	RNA polymerase II subunit I [Source:HGNC Symbol;Acc:HGNC:9196]	chr19	36113710	36115346	-1	ensembl_havana	3.581728037	3.452138919	3.711317156	3.305501307	3.520789865	3.530125585	3.660771151	3.646140782	3.827039534
ENSG00000198763	0.017962	0.065550968	0.240932038	1.181755876	**MT-ND2**	protein_coding	mitochondrially encoded NADH:ubiquinone oxidoreductase core subunit 2 [Source:HGNC Symbol;Acc:HGNC:7456]	chrMT	4470	5511	1	insdc	11.99824361	11.87777759	12.11870963	11.73390308	11.991518	11.9079117	11.94866917	12.14804867	12.25941105
ENSG00000138376	0.017988	0.065628644	-0.227524269	-1.170824037	**BARD1**	protein_coding	BRCA1 associated RING domain 1 [Source:HGNC Symbol;Acc:HGNC:952]	chr2	214725646	214809711	-1	ensembl_havana	4.329916197	4.443678332	4.216154063	4.311025713	4.540963844	4.479045439	4.104813841	4.220562267	4.32308608

ENSG00000179918	0.017995	0.065636979	-0.299785331	-1.230961236	**SEPHS2**	protein_coding	selenophosphate synthetase 2 [Source:HGNC Symbol;Acc:HGNC:19686]	chr16	30443631	30446181	-1	havana	6.748416439	6.898309104	6.598523773	7.106906373	6.880534725	6.707486214	6.678976516	6.532462525	6.584132279
ENSG00000234911	0.018052	0.06582545	0.285896254	1.219167419	**TEX21P**	transcribed_unitary_pseudogene	testis expressed 21, pseudogene [Source:HGNC Symbol;Acc:HGNC:35455]	chr14	64341673	64387986	-1	havana	-0.091956828	-0.234904955	0.050991299	-0.41068387	-0.34076431	0.046733314	0.032074365	-0.08087422	0.201773757
ENSG00000153187	0.018059	0.065834116	-0.193473146	-1.1435133	**HNRNPU**	protein_coding	heterogeneous nuclear ribonucleoprotein U [Source:HGNC Symbol;Acc:HGNC:5048]	chr1	244840638	244864560	-1	ensembl_havana	9.891489165	9.988225738	9.794752592	9.969801978	9.989760468	10.00511477	9.75981997	9.819649361	9.804788445
ENSG00000269609	0.018065	0.065842446	-0.257942051	-1.195771765	**RPARP-AS1**	processed_transcript	RPARP antisense RNA 1 [Source:HGNC Symbol;Acc:HGNC:45238]	chr10	102449817	102461106	1	havana	4.074776534	4.203747559	3.945805509	4.259029533	4.317626958	4.034586187	3.890656094	4.044601662	3.90215877
ENSG00000127054	0.018074	0.065857035	0.258108219	1.195909501	**INTS11**	protein_coding	integrator complex subunit 11 [Source:HGNC Symbol;Acc:HGNC:26052]	chr1	1311585	1324691	-1	ensembl_havana	6.572612772	6.443558662	6.701666881	6.4570757	6.357671691	6.515928595	6.788983252	6.679141789	6.636875602
ENSG00000240024	0.018083	0.065857601	0.266741573	1.2030875	**LINC00888**	transcribed_unprocessed_pseudogene	long intergenic non-protein coding RNA 888 [Source:HGNC Symbol;Acc:HGNC:48575]	chr3	183447608	183456013	1	havana	3.074310657	2.94093987	3.207681443	2.654944978	3.209790357	2.958084275	2.858581149	3.392817101	3.37164608
ENSG00000151929	0.018082	0.065857601	-0.260675844	-1.198039806	**BAG3**	protein_coding	BCL2 associated athanogene 3 [Source:HGNC Symbol;Acc:HGNC:939]	chr10	119651370	119677819	1	ensembl_havana	3.798713128	3.929051049	3.668375206	3.954943093	3.957478716	3.874731339	3.834482717	3.631727691	3.538915209
ENSG00000164307	0.018098	0.065876991	-0.214564551	-1.160353632	**ERAP1**	protein_coding	endoplasmic reticulum aminopeptidase 1 [Source:HGNC Symbol;Acc:HGNC:18173]	chr5	96760810	96808100	-1	ensembl_havana	7.74276184	7.850044115	7.635479564	7.801822342	7.802666447	7.945643557	7.562581261	7.663996928	7.679860503
ENSG00000236924	0.018095	0.065876991	-0.250961762	-1.190000156	**AL162411.1**	lincRNA		chr9	6645956	6670635	1	havana	0.706402187	0.831883068	0.580921306	0.705390237	0.636663181	1.153595787	0.540060475	0.426254992	0.77644845
ENSG00000155980	0.018106	0.06588843	0.269598465	1.205472271	**KIF5A**	protein_coding	kinesin family member 5A [Source:HGNC Symbol;Acc:HGNC:6323]	chr12	57549998	57586632	1	ensembl_havana	7.273750628	7.138951396	7.408549861	7.028932707	7.156659953	7.231261527	7.448474081	7.363727611	7.41344789
ENSG00000169764	0.018127	0.065950322	-0.212021941	-1.158310422	**UGP2**	protein_coding	UDP-glucose pyrophosphorylase 2 [Source:HGNC Symbol;Acc:HGNC:12527]	chr2	63840940	63891562	1	ensembl_havana	7.819185927	7.925196897	7.713174956	7.89845654	8.025699547	7.851434606	7.668129809	7.761548326	7.709846735
ENSG00000196998	0.018133	0.065956694	0.195122821	1.144821619	**WDR45**	protein_coding	WD repeat domain 45 [Source:HGNC Symbol;Acc:HGNC:28912]	chrX	49071470	49101170	-1	ensembl_havana	6.033118461	5.93555705	6.130679871	6.021142907	5.950911768	5.834616475	6.198010193	6.132217523	6.061811898
ENSG00000154914	0.018166	0.065975822	0.333763941	1.260297161	**USP43**	protein_coding	ubiquitin specific peptidase 43 [Source:HGNC Symbol;Acc:HGNC:20072]	chr17	9644698	9729691	1	ensembl_havana	2.767893657	2.601011686	2.934775627	2.654944978	2.415188039	2.73290204	2.952056343	2.952470332	2.899800207
ENSG00000118985	0.018159	0.065975822	0.224016812	1.167981005	**ELL2**	protein_coding	elongation factor for RNA polymerase II 2 [Source:HGNC Symbol;Acc:HGNC:17064]	chr5	95885098	95962071	-1	ensembl_havana	8.145887229	8.033878823	8.257895634	8.222083767	8.010473832	7.869078869	8.402766438	8.184878187	8.186042278
ENSG00000031003	0.018146	0.065975822	0.202038611	1.150322679	**FAM13B**	protein_coding	family with sequence similarity 13 member B [Source:HGNC Symbol;Acc:HGNC:1335]	chr5	137937960	138051961	-1	ensembl_havana	6.773195525	6.67217622	6.874214831	6.574169377	6.691860827	6.750498455	6.796353969	6.925907731	6.900382792
ENSG00000249087	0.01816	0.065975822	-0.195739582	-1.145311141	**ZNF436-AS1**	antisense	ZNF436 antisense RNA 1 [Source:HGNC Symbol;Acc:HGNC:25122]	chr1	23368997	23371839	1	ensembl_havana	2.258321568	2.356191359	2.160451777	2.290693707	2.476573576	2.301306795	2.099663825	2.308284917	2.073406591
ENSG00000196074	0.018157	0.065975822	-0.206461397	-1.153854567	**SYCP2**	protein_coding	synaptonemal complex protein 2 [Source:HGNC Symbol;Acc:HGNC:11490]	chr20	59863563	59933655	-1	ensembl_havana	5.315753311	5.418984009	5.212522613	5.500499047	5.281914141	5.47453884	5.240784047	5.073699414	5.323084377
ENSG00000116918	0.018162	0.065975822	-0.264598666	-1.201301821	**TSNAX**	protein_coding	translin associated factor X [Source:HGNC Symbol;Acc:HGNC:12380]	chr1	231528653	231566524	1	havana	5.484122741	5.616422074	5.351823408	5.709349575	5.69543395	5.444482698	5.316948784	5.434969209	5.303552231
ENSG00000107679	0.018189	0.066041915	-0.220479424	-1.165120705	**PLEKHA1**	protein_coding	pleckstrin homology domain containing A1 [Source:HGNC Symbol;Acc:HGNC:14335]	chr10	122374696	122442602	1	ensembl_havana	5.882599113	5.992838825	5.772359401	6.096091502	5.946036984	5.936387988	5.792465859	5.791349196	5.733263148
ENSG00000207554	0.018201	0.066067486	0.32824319	1.255483606	**MIR647**	miRNA	microRNA 647 [Source:HGNC Symbol;Acc:HGNC:32903]	chr20	63942631	63942726	-1	mirbase	0.003429418	-0.160692177	0.167551013	-0.36334239	-0.258437325	0.139703184	0.141952326	-0.11936381	0.480064523
ENSG00000183506	0.018229	0.066093051	0.266416118	1.202816128	**PI4KAP2**	transcribed_unitary_pseudogene	phosphatidylinositol 4-kinase alpha pseudogene 2 [Source:HGNC Symbol;Acc:HGNC:33577]	chr22	21473000	21517533	-1	ensembl_havana	3.025800707	2.892592648	3.159008766	2.921828527	2.793616541	2.962332875	3.127051692	3.206986221	3.142988385
ENSG00000004139	0.018232	0.066093051	0.256286892	1.19440068	**SARM1**	protein_coding	sterile alpha and TIR motif containing 1 [Source:HGNC Symbol;Acc:HGNC:17074]	chr17	28364356	28404049	1	ensembl_havana	5.792025833	5.663882387	5.920169279	5.514822849	5.736164553	5.740659759	5.901940905	5.895158667	5.963408266
ENSG00000276550	0.018233	0.066093051	0.222999712	1.167157869	**HERC2P2**	transcribed_unprocessed_pseudogene	hect domain and RLD 2 pseudogene 2 [Source:HGNC Symbol;Acc:HGNC:4870]	chr15	22495570	22590815	1	havana	4.169787133	4.058287277	4.281286989	3.905065295	4.094970997	4.174825539	4.192554836	4.342398954	4.308907178
ENSG00000141644	0.018235	0.066093051	0.211494623	1.157887126	**MBD1**	protein_coding	methyl-CpG binding domain protein 1 [Source:HGNC Symbol;Acc:HGNC:6916]	chr18	50266882	50281774	-1	ensembl_havana	6.597959288	6.492211977	6.7037066	6.497305806	6.463040275	6.516289849	6.77696929	6.662577074	6.671573434
ENSG00000165480	0.018224	0.066093051	-0.199103077	-1.147984431	**SKA3**	protein_coding	spindle and kinetochore associated complex subunit 3 [Source:HGNC Symbol;Acc:HGNC:20262]	chr13	21153595	21176602	-1	ensembl_havana	4.45137532	4.550926858	4.351823781	4.275947831	4.692240069	4.684592674	4.120558811	4.459647342	4.47526519
ENSG00000154642	0.018231	0.066093051	-0.209917527	-1.156622063	**C21orf91**	protein_coding	chromosome 21 open reading frame 91 [Source:HGNC Symbol;Acc:HGNC:16459]	chr21	17788967	17819386	-1	ensembl_havana	6.93627032	7.041229084	6.831311557	7.369372614	6.973430184	6.780884453	7.154723925	6.705531266	6.633679478
ENSG00000156973	0.018249	0.066109642	0.214127668	1.160002301	**PDE6D**	protein_coding	phosphodiesterase 6D [Source:HGNC Symbol;Acc:HGNC:8788]	chr2	231732425	231786272	-1	ensembl_havana	4.083504274	3.976440441	4.190568108	4.071501525	4.028887076	3.82893272	4.328527411	4.165594683	4.07758223
ENSG00000164830	0.018245	0.066109642	-0.358138323	-1.281770813	**OXR1**	protein_coding	oxidation resistance 1 [Source:HGNC Symbol;Acc:HGNC:15822]	chr8	106359476	106752694	1	ensembl_havana	4.432700685	4.611769847	4.253631524	4.868380636	4.689650085	4.27727882	4.318811065	4.301098552	4.140984954
ENSG00000062598	0.018271	0.066171908	0.207830362	1.154949973	**ELMO2**	protein_coding	engulfment and cell motility 2 [Source:HGNC Symbol;Acc:HGNC:17233]	chr20	46366049	46432985	-1	ensembl_havana	7.463127461	7.359212281	7.567042642	7.232350209	7.411895816	7.433390816	7.504835593	7.575648341	7.620643993
ENSG00000198039	0.018294	0.066238354	-0.19053594	-1.141187571	**ZNF273**	protein_coding	zinc finger protein 273 [Source:HGNC Symbol;Acc:HGNC:13067]	chr7	64870172	64930966	1	ensembl_havana	3.712940647	3.808208617	3.617672677	3.544549275	3.985200845	3.894875732	3.357117048	3.788074195	3.70782679
ENSG00000157778	0.018311	0.066282763	-0.194799606	-1.144565167	**PSMG3**	protein_coding	proteasome assembly chaperone 3 [Source:HGNC Symbol;Acc:HGNC:22420]	chr7	1567330	1571005	-1	ensembl_havana	5.280693378	5.378093181	5.183293575	5.408028057	5.362174763	5.364076724	5.1915873	5.15657708	5.201716345
ENSG00000146576	0.018333	0.066343514	0.323644392	1.251487943	**C7orf26**	protein_coding	chromosome 7 open reading frame 26 [Source:HGNC Symbol;Acc:HGNC:21702]	chr7	6590017	6608726	1	ensembl_havana	4.991510717	4.82968852	5.153332913	4.6536066	4.990411054	4.845047907	5.175526364	5.160598574	5.1238738
ENSG00000065029	0.018337	0.066343514	0.222871046	1.167053781	**ZNF76**	protein_coding	zinc finger protein 76 [Source:HGNC Symbol;Acc:HGNC:13149]	chr6	35258909	35295985	1	ensembl_havana	6.179765127	6.068329605	6.29120065	6.16128046	6.050156954	5.9935514	6.355519907	6.211405544	6.3066765
ENSG00000165915	0.018351	0.066375999	0.253624051	1.192198159	**SLC39A13**	protein_coding	solute carrier family 39 member 13 [Source:HGNC Symbol;Acc:HGNC:20859]	chr11	47407132	47416501	1	ensembl_havana	4.379513151	4.252701126	4.506325177	4.357685434	4.18231208	4.218105863	4.718209861	4.451463998	4.349301671
ENSG00000099910	0.018355	0.066375999	-0.203223863	-1.151268119	**KLHL22**	protein_coding	kelch like family member 22 [Source:HGNC Symbol;Acc:HGNC:25888]	chr22	20441519	20495883	-1	ensembl_havana	3.991007124	4.092619055	3.889395192	4.124355192	4.071289234	4.08221274	3.967289729	3.873856314	3.827039534
ENSG00000104960	0.018365	0.066393981	0.234963174	1.176876696	**PTOV1**	protein_coding	prostate tumor overexpressed 1 [Source:HGNC Symbol;Acc:HGNC:9632]	chr19	49850735	49860744	1	ensembl_havana	7.432682585	7.315200998	7.550164172	7.319926676	7.312711651	7.312964668	7.66429841	7.486148503	7.500045603
ENSG00000238113	0.018376	0.066416963	-0.233751269	-1.175888501	**LINC01410**	lincRNA	long intergenic non-protein coding RNA 1410 [Source:HGNC Symbol;Acc:HGNC:50702]	chr9	62801461	62813486	1	havana	4.015977824	4.132853459	3.89910219	4.220707514	4.028887076	4.148965785	3.885399497	3.813837983	3.998069089
ENSG00000077238	0.018388	0.066443869	-0.212061512	-1.158342193	**IL4R**	protein_coding	interleukin 4 receptor [Source:HGNC Symbol;Acc:HGNC:6015]	chr16	27313668	27364778	1	ensembl_havana	8.182937299	8.288968055	8.076906543	8.60778751	8.172159253	8.086957404	8.417408674	7.887300045	7.926010911
ENSG00000160999	0.018398	0.066464273	0.269422593	1.205325326	**SH2B2**	protein_coding	SH2B adaptor protein 2 [Source:HGNC Symbol;Acc:HGNC:17381]	chr7	102285091	102321711	1	ensembl_havana	0.492215209	0.357503912	0.626926505	0.464289132	0.325758945	0.28246366	0.830009965	0.648704825	0.402064725
ENSG00000148337	0.018422	0.066534178	0.315289481	1.244261293	**CIZ1**	protein_coding	CDKN1A interacting zinc finger protein 1 [Source:HGNC Symbol;Acc:HGNC:16744]	chr9	128166064	128204383	-1	ensembl_havana	6.011861066	5.854216325	6.169505806	5.655060849	5.911444347	5.99614378	6.166394485	6.163614889	6.178508043
ENSG00000137575	0.018459	0.06664973	0.318288535	1.246850536	**SDCBP**	protein_coding	syndecan binding protein [Source:HGNC Symbol;Acc:HGNC:10662]	chr8	58552924	58582860	1	ensembl_havana	5.323993457	5.164849189	5.483137725	4.86714065	5.375127923	5.252278995	5.37904432	5.542892927	5.527475928
ENSG00000169188	0.018477	0.066699903	-0.198058698	-1.147153697	**APEX2**	protein_coding	apurinic/apyrimidinic endodeoxyribonuclease 2 [Source:HGNC Symbol;Acc:HGNC:17889]	chrX	55000357	55009057	1	ensembl_havana	5.353015795	5.452045144	5.253986446	5.375257272	5.436660858	5.544217304	5.147256699	5.224423979	5.390278661
ENSG00000138028	0.018486	0.066709383	0.920835281	1.893211093	**CGREF1**	protein_coding	cell growth regulator with EF-hand domain 1 [Source:HGNC Symbol;Acc:HGNC:16962]	chr2	27098889	27119115	-1	ensembl_havana	-0.081702858	-0.542120499	0.378714782	-1.23095973	-0.473795405	0.078393642	0.308311185	0.45273451	0.375098651
ENSG00000154832	0.018489	0.066709383	0.293065398	1.225240872	**CXXC1**	protein_coding	CXXC finger protein 1 [Source:HGNC Symbol;Acc:HGNC:24343]	chr18	50282343	50288304	-1	ensembl_havana	6.053251552	5.906718853	6.199784251	5.816018772	5.836743306	6.067394481	6.232057254	6.179556516	6.187738985
ENSG00000127804	0.018509	0.066763534	-0.209675996	-1.156428441	**METTL16**	protein_coding	methyltransferase like 16 [Source:HGNC Symbol;Acc:HGNC:28484]	chr17	2405562	2511891	-1	ensembl_havana	5.18008919	5.284927188	5.075251192	5.267535135	5.295604834	5.291641595	4.988394109	5.107423922	5.129935545
ENSG00000185947	0.018518	0.066779194	0.270110886	1.20590051	**ZNF267**	protein_coding	zinc finger protein 267 [Source:HGNC Symbol;Acc:HGNC:13060]	chr16	31873758	31917357	1	ensembl_havana	7.066584337	6.931528894	7.201639779	7.202427177	6.880250817	6.711908688	7.324803396	7.259308942	7.020807
ENSG00000134569	0.018522	0.066779194	-0.27170593	-1.207234492	**LRP4**	protein_coding	LDL receptor related protein 4 [Source:HGNC Symbol;Acc:HGNC:6696]	chr11	46856868	46918642	-1	ensembl_havana	5.175949263	5.311802228	5.040096298	5.298222494	5.151533793	5.485650398	5.035426597	5.009374461	5.075487836
ENSG00000188735	0.018534	0.066787795	0.227907031	1.171134709	**TMEM120B**	protein_coding	transmembrane protein 120B [Source:HGNC Symbol;Acc:HGNC:32008]	chr12	121712752	121783001	1	ensembl_havana	4.33602012	4.222066604	4.449973635	4.130569456	4.232998034	4.302632322	4.442080167	4.370447884	4.537392854
ENSG00000115425	0.018532	0.066787795	-0.199705103	-1.148463577	**PECR**	protein_coding	peroxisomal trans-2-enoyl-CoA reductase [Source:HGNC Symbol;Acc:HGNC:18281]	chr2	215996329	216082955	-1	ensembl_havana	4.337200041	4.437052593	4.237347489	4.558479799	4.332645478	4.420032502	4.379893999	4.083248454	4.248900015
ENSG00000172809	0.018548	0.066807553	0.293983882	1.226021163	**RPL38**	protein_coding	ribosomal protein L38 [Source:HGNC Symbol;Acc:HGNC:10349]	chr17	74203582	74210655	1	ensembl_havana	7.036629155	6.889637214	7.183621096	6.736433653	6.985886577	6.946591411	7.195381772	7.137076798	7.218404718
ENSG00000136710	0.018553	0.066807553	0.28663186	1.21978921	**CCDC115**	protein_coding	coiled-coil domain containing 115 [Source:HGNC Symbol;Acc:HGNC:28178]	chr2	130338241	130342349	-1	ensembl_havana	4.800343398	4.657027469	4.943659328	4.67360513	4.600084112	4.697393164	5.033051612	4.984635224	4.813291149
ENSG00000113851	0.018554	0.066807553	0.231688655	1.17420854	**CRBN**	protein_coding	cereblon [Source:HGNC Symbol;Acc:HGNC:30185]	chr3	3144628	3179727	-1	ensembl_havana	6.928889442	6.813045115	7.04473377	6.921633105	6.86713028	6.65037196	7.077887767	7.071844214	6.984469327
ENSG00000109775	0.018576	0.066819961	0.218246089	1.163318458	**UFSP2**	protein_coding	UFM1 specific peptidase 2 [Source:HGNC Symbol;Acc:HGNC:25640]	chr4	185399540	185425985	-1	ensembl_havana	5.197211428	5.088088384	5.306334473	5.011060626	5.143985622	5.109218904	5.157105165	5.357380992	5.40451726
ENSG00000144713	0.018563	0.066819961	0.197197246	1.146468921	**RPL32**	protein_coding	ribosomal protein L32 [Source:HGNC Symbol;Acc:HGNC:10336]	chr3	12834485	12841588	-1	ensembl_havana	9.110794103	9.01219548	9.209392726	8.922608496	9.058122961	9.055854983	9.162911908	9.237881946	9.227384323
ENSG00000145996	0.018571	0.066819961	-0.22291623	-1.167090333	**CDKAL1**	protein_coding	CDK5 regulatory subunit associated protein 1 like 1 [Source:HGNC Symbol;Acc:HGNC:21050]	chr6	20534457	21232404	1	ensembl_havana	5.035235955	5.14669407	4.92377784	5.113962314	5.145876371	5.180243524	4.816768636	5.009374461	4.945190422
ENSG00000147118	0.018572	0.066819961	-0.40342845	-1.322647346	**ZNF182**	protein_coding	zinc finger protein 182 [Source:HGNC Symbol;Acc:HGNC:13001]	chrX	47974851	48003978	-1	ensembl_havana	4.428663628	4.630377854	4.226949403	4.774938769	4.647565219	4.468629573	4.088895138	4.361741031	4.230212041
ENSG00000033050	0.018581	0.066821048	0.262860697	1.199855522	**ABCF2**	protein_coding	ATP binding cassette subfamily F member 2 [Source:HGNC Symbol;Acc:HGNC:71]	chr7	151212490	151227230	-1	ensembl_havana	4.282126076	4.150695727	4.413556424	4.189299683	4.238324537	4.02446296	4.427687441	4.389419981	4.42356185
ENSG00000142530	0.018591	0.066842907	0.387260917	1.307907867	**FAM71E1**	protein_coding	family with sequence similarity 71 member E1 [Source:HGNC Symbol;Acc:HGNC:25107]	chr19	50466785	50476753	-1	ensembl_havana	0.295102872	0.101472413	0.48873333	0.14220989	0.214850257	-0.052642908	0.592614899	0.343769191	0.529815901
ENSG00000166349	0.018603	0.066854568	0.353149176	1.277345831	**RAG1**	protein_coding	recombination activating 1 [Source:HGNC Symbol;Acc:HGNC:9831]	chr11	36510709	36593156	1	ensembl_havana	2.047691488	1.871116901	2.224266076	1.671956805	1.953538152	1.987855745	2.246292314	2.271570328	2.154935587
ENSG00000116670	0.018608	0.066854568	0.22586745	1.169480211	**MAD2L2**	protein_coding	mitotic arrest deficient 2 like 2 [Source:HGNC Symbol;Acc:HGNC:6764]	chr1	11674480	11691650	-1	ensembl_havana	5.221787552	5.108853827	5.334721277	5.000920413	5.173017357	5.152623711	5.323765864	5.363499728	5.31689824
ENSG00000091127	0.018608	0.066854568	-0.191182778	-1.141699342	**PUS7**	protein_coding	pseudouridylate synthase 7 (putative) [Source:HGNC Symbol;Acc:HGNC:26033]	chr7	105439661	105522267	-1	ensembl_havana	5.246898498	5.342489887	5.151307108	5.264712734	5.264616028	5.498140898	5.060130702	5.093818279	5.299972345
ENSG00000228175	0.018638	0.066942432	-0.394608252	-1.314585751	**GEMIN8P4**	processed_pseudogene	gem nuclear organelle associated protein 8 pseudogene 4 [Source:HGNC Symbol;Acc:HGNC:37979]	chr1	89993593	89994321	-1	havana	1.622530528	1.819834654	1.425226402	1.578072284	2.108942002	1.772489677	1.293957805	1.43975459	1.541966812
ENSG00000166171	0.018647	0.066960606	0.423526584	1.341202041	**DPCD**	protein_coding	deleted in primary ciliary dyskinesia homolog (mouse) [Source:HGNC Symbol;Acc:HGNC:24542]	chr10	101570560	101609668	1	ensembl_havana	2.610167425	2.398404133	2.821930717	2.298074107	2.523868156	2.373270137	2.967063242	2.671684573	2.827044337
ENSG00000230606	0.01866	0.066989805	0.467461123	1.382674075	**AC092683.1**	lincRNA		chr2	97416165	97433527	-1	havana	0.336848475	0.103117913	0.570579037	-0.03191641	-0.071090603	0.41236075	0.141952326	0.693803314	0.875981471
ENSG00000168575	0.018678	0.067019094	0.202597436	1.15076834	**SLC20A2**	protein_coding	solute carrier family 20 member 2 [Source:HGNC Symbol;Acc:HGNC:10947]	chr8	42416475	42541926	-1	ensembl_havana	5.000876427	4.899577709	5.102175145	4.954969984	4.847853934	4.89590921	5.165803271	5.095919824	5.04480234
ENSG00000265261	0.018673	0.067019094	-0.398310062	-1.317963177	**AC027575.1**	processed_pseudogene		chr18	76884935	76888724	-1	havana	-0.100719453	0.098435578	-0.299874484	-0.27307956	0.15602554	0.41236075	-0.51701327	-0.52165082	0.139040636
ENSG00000174996	0.018683	0.067022402	0.314694532	1.243748281	**KLC2**	protein_coding	kinesin light chain 2 [Source:HGNC Symbol;Acc:HGNC:20716]	chr11	66257294	66267860	1	ensembl_havana	5.264750342	5.107403077	5.422097608	5.096079309	5.098824377	5.127305544	5.561495453	5.453116712	5.25168066
ENSG00000122966	0.018706	0.067086961	-0.213825163	-1.159759097	**CIT**	protein_coding	citron rho-interacting serine/threonine kinase [Source:HGNC Symbol;Acc:HGNC:1985]	chr12	119685790	119877291	-1	ensembl_havana	5.021883908	5.12879649	4.914971327	4.959631449	5.183180103	5.243577917	4.82502908	4.937366748	4.982518152
ENSG00000076641	0.018731	0.067158741	0.385670629	1.30646695	**PAG1**	protein_coding	phosphoprotein membrane anchor with glycosphingolipid microdomains 1 [Source:HGNC Symbol;Acc:HGNC:30043]	chr8	80967810	81112068	-1	ensembl_havana	4.79975364	4.606918325	4.992588954	4.113938229	4.746828853	4.959987894	4.766187383	5.028204747	5.183374733
ENSG00000123933	0.018745	0.067195421	0.406697372	1.325647656	**MXD4**	protein_coding	MAX dimerization protein 4 [Source:HGNC Symbol;Acc:HGNC:13906]	chr4	2247432	2262294	-1	ensembl_havana	4.326360635	4.123011949	4.529709321	4.301877063	3.918192999	4.148965785	4.888149815	4.429967487	4.271010662
ENSG00000228903	0.018758	0.067222865	-0.279226488	-1.213544059	**RASA4CP**	transcribed_unprocessed_pseudogene	RAS p21 protein activator 4C, pseudogene [Source:HGNC Symbol;Acc:HGNC:44185]	chr7	44026951	44041892	-1	havana	2.520104253	2.659717497	2.380491009	2.672087675	2.427675878	2.879388939	2.386842807	2.293711009	2.460919213
ENSG00000112473	0.018763	0.067224851	-0.311459666	-1.240962625	**SLC39A7**	protein_coding	solute carrier family 39 member 7 [Source:HGNC Symbol;Acc:HGNC:4927]	chr6	33200445	33204439	1	ensembl_havana	8.422350767	8.5780806	8.266620934	8.97879891	8.472000917	8.283441972	8.489661672	8.173729006	8.136472125
ENSG00000236017	0.018775	0.067250169	0.202117921	1.150385918	**ASMTL-AS1**	antisense	ASMTL antisense RNA 1 [Source:HGNC Symbol;Acc:HGNC:25811]	chrX	1401769	1414028	1	havana	3.812035494	3.710976534	3.913094455	3.778870107	3.613888808	3.740170686	4.032943419	3.81128221	3.895057737
ENSG00000272456	0.018795	0.067307043	0.274850089	1.209868365	**AC087045.2**	lincRNA		chr8	134722947	134723949	1	havana	2.765003662	2.627578617	2.902428706	2.383813765	2.575284697	2.923637391	2.657410455	2.989113925	3.060761739
ENSG00000141294	0.018801	0.067309122	0.205027323	1.152708177	**LRRC46**	protein_coding	leucine rich repeat containing 46 [Source:HGNC Symbol;Acc:HGNC:25047]	chr17	47831627	47837713	1	ensembl_havana	2.35404865	2.251534988	2.456562311	2.245600406	2.297649024	2.211355536	2.423571602	2.466087459	2.480027873
ENSG00000214455	0.018807	0.067313517	-0.83604739	-1.785152581	**RCN1P2**	processed_pseudogene	reticulocalbin 1 pseudogene 2 [Source:HGNC Symbol;Acc:HGNC:39204]	chr13	45390353	45391267	-1	havana	-0.40987398	0.008149715	-0.827897675	-0.41068387	0.325758945	0.109374069	-0.97139419	-1.15951152	-0.352787316
ENSG00000091157	0.018815	0.067319238	0.201795203	1.150128616	**WDR7**	protein_coding	WD repeat domain 7 [Source:HGNC Symbol;Acc:HGNC:13490]	chr18	56651343	57036606	1	ensembl_havana	5.769139861	5.66824226	5.870037463	5.744214219	5.564438825	5.696073735	5.927739604	5.82214175	5.860231035
ENSG00000166938	0.018818	0.067319238	-0.19821063	-1.147274511	**DIS3L**	protein_coding	DIS3 like exosome 3'-5' exoribonuclease [Source:HGNC Symbol;Acc:HGNC:28698]	chr15	66293217	66333898	1	ensembl_havana	5.883521193	5.982626508	5.784415878	5.852818111	5.943865115	6.151196297	5.640845654	5.792645426	5.919756553
ENSG00000096060	0.018845	0.067399871	-0.20322852	-1.151271836	**FKBP5**	protein_coding	FK506 binding protein 5 [Source:HGNC Symbol;Acc:HGNC:3721]	chr6	35573585	35728583	-1	ensembl_havana	6.897071945	6.998686205	6.795457685	7.003199467	6.957639636	7.035219513	6.811676338	6.697249779	6.877446937
ENSG00000180385	0.01885	0.067402455	0.271647928	1.207185956	**EMC3-AS1**	transcribed_unprocessed_pseudogene	EMC3 antisense RNA 1 [Source:HGNC Symbol;Acc:HGNC:49223]	chr3	9986893	10006990	1	havana	5.380892013	5.245068049	5.516715977	4.999789311	5.269827274	5.465587562	5.425960611	5.485538464	5.638648856
ENSG00000197472	0.018877	0.067481136	0.209021502	1.155903934	**ZNF695**	protein_coding	zinc finger protein 695 [Source:HGNC Symbol;Acc:HGNC:30954]	chr1	246945547	247008093	-1	ensembl_havana	1.845493347	1.740982596	1.950004098	1.222335231	2.004444884	1.996167673	1.386165364	2.187350601	2.27649633
ENSG00000175556	0.018893	0.067523745	-0.32680637	-1.254233857	**LONRF3**	protein_coding	LON peptidase N-terminal domain and ring finger 3 [Source:HGNC Symbol;Acc:HGNC:21152]	chrX	118974614	119018355	1	ensembl_havana	0.310501973	0.473905158	0.147098788	0.14220989	0.477608744	0.80189684	-0.35894771	0.130707766	0.669536307
ENSG00000261040	0.018924	0.067614614	-0.611467403	-1.527812397	**WFDC21P**	transcribed_unitary_pseudogene	WAP four-disulfide core domain 21, pseudogene [Source:HGNC Symbol;Acc:HGNC:50357]	chr17	60083566	60091885	-1	havana	1.465383466	1.771117167	1.159649764	1.727676152	2.021022296	1.564653053	1.245545533	0.956893362	1.276510397
ENSG00000130165	0.018938	0.067650811	0.264766118	1.201441263	**ELOF1**	protein_coding	elongation factor 1 homolog [Source:HGNC Symbol;Acc:HGNC:28691]	chr19	11551147	11559236	-1	ensembl_havana	4.92780089	4.795417831	5.060183949	4.749592033	4.87204229	4.764619169	5.079823989	4.988033841	5.112694017
ENSG00000173992	0.018964	0.067706063	0.226013171	1.169598342	**CCS**	protein_coding	copper chaperone for superoxide dismutase [Source:HGNC Symbol;Acc:HGNC:1613]	chr11	66592821	66606019	1	ensembl_havana	4.429262128	4.316255543	4.542268713	4.464855791	4.216899503	4.267011334	4.607640767	4.532832903	4.486332471
ENSG00000059378	0.018968	0.067706063	-0.25726413	-1.195210005	**PARP12**	protein_coding	poly(ADP-ribose) polymerase family member 12 [Source:HGNC Symbol;Acc:HGNC:21919]	chr7	140023744	140063721	-1	ensembl_havana	6.074264695	6.20289676	5.94563263	6.21051079	6.22757488	6.17060461	6.022968553	5.829738281	5.984191057
ENSG00000141524	0.018966	0.067706063	-0.540878595	-1.45485825	**TMC6**	protein_coding	transmembrane channel like 6 [Source:HGNC Symbol;Acc:HGNC:18021]	chr17	78110458	78132407	-1	ensembl_havana	1.239375786	1.509815083	0.968936488	1.464591378	1.402954459	1.661899414	1.309740823	0.779979312	0.817089329
ENSG00000227403	0.018984	0.067729309	-0.214574876	-1.160361936	**LINC01806**	lincRNA	long intergenic non-protein coding RNA 1806 [Source:HGNC Symbol;Acc:HGNC:52599]	chr2	161244739	161249050	1	havana	4.139641885	4.246929322	4.032354447	4.062862309	4.435159841	4.242765818	3.861504875	4.143413982	4.092144484
ENSG00000178999	0.018984	0.067729309	-0.216891311	-1.162226545	**AURKB**	protein_coding	aurora kinase B [Source:HGNC Symbol;Acc:HGNC:11390]	chr17	8204733	8210600	-1	ensembl_havana	5.383748281	5.492193937	5.275302626	5.2381006	5.591051659	5.647429551	5.103785613	5.354750712	5.367371551
ENSG00000148835	0.018989	0.067729556	0.202298061	1.150529568	**TAF5**	protein_coding	TATA-box binding protein associated factor 5 [Source:HGNC Symbol;Acc:HGNC:11539]	chr10	103367967	103389065	1	ensembl_havana	3.801736794	3.700587763	3.902885824	3.657882359	3.761772213	3.682108717	3.801373512	4.000410511	3.906873449
ENSG00000167106	0.019015	0.067769934	0.332934664	1.259572936	**FAM102A**	protein_coding	family with sequence similarity 102 member A [Source:HGNC Symbol;Acc:HGNC:31419]	chr9	127940579	127980513	-1	ensembl_havana	2.612045845	2.445578512	2.778513177	2.438267468	2.351074074	2.547393995	2.555188851	2.753838306	3.026512374
ENSG00000104863	0.019024	0.067769934	0.215574102	1.161165894	**LIN7B**	protein_coding	lin-7 homolog B, crumbs cell polarity complex component [Source:HGNC Symbol;Acc:HGNC:17788]	chr19	49114324	49118460	1	ensembl_havana	1.975065019	1.867277968	2.08285207	1.781323307	1.909699171	1.910811426	2.062883947	2.046678052	2.138994213
ENSG00000172167	0.019019	0.067769934	-0.246360114	-1.186210555	**MTBP**	protein_coding	MDM2 binding protein [Source:HGNC Symbol;Acc:HGNC:7417]	chr8	120445400	120542133	1	ensembl_havana	5.150371007	5.273551064	5.027190949	5.18534811	5.373515124	5.261789957	5.031862651	5.004907857	5.04480234
ENSG00000273211	0.019015	0.067769934	-0.285804315	-1.219089727	**AC137630.4**	lincRNA		chr3	48985485	48985963	-1	havana	-0.114159366	0.028742791	-0.257061524	-0.22998119	-0.071090603	0.387300166	-0.46237776	-0.2414061	-0.067400719
ENSG00000197712	0.019011	0.067769934	-0.641078879	-1.559494947	**FAM114A1**	protein_coding	family with sequence similarity 114 member A1 [Source:HGNC Symbol;Acc:HGNC:25087]	chr4	38867677	38945739	1	ensembl_havana	0.06991197	0.39045141	-0.250627469	0.355307344	0.428746719	0.387300166	-0.46237776	-0.52165082	0.232146171
ENSG00000157978	0.019036	0.067797258	0.288375155	1.221264046	**LDLRAP1**	protein_coding	low density lipoprotein receptor adaptor protein 1 [Source:HGNC Symbol;Acc:HGNC:18640]	chr1	25543580	25568886	1	ensembl_havana	3.967384324	3.823196747	4.111571902	3.494031456	3.891395322	4.084163462	3.939661103	4.033680126	4.361374475
ENSG00000226441	0.01908	0.067803646	1.624789028	3.083970634	**PLCL2-AS1**	antisense	PLCL2 antisense RNA 1 [Source:HGNC Symbol;Acc:HGNC:40449]	chr3	17042742	17044192	-1	havana	-1.220356449	-2.032750963	-0.407961935	-1.73427603	-1.518257998	-2.845718858	-0.82634296	-0.47099802	0.073455173
ENSG00000176681	0.01907	0.067803646	0.51228709	1.426309519	**LRRC37A**	protein_coding	leucine rich repeat containing 37A [Source:HGNC Symbol;Acc:HGNC:29069]	chr17	46292733	46337794	1	ensembl_havana	-0.022832102	-0.278975647	0.233311443	-0.03191641	-0.569826728	-0.235183806	0.141952326	-0.04338487	0.601366878
ENSG00000221883	0.019054	0.067803646	0.285552798	1.218877212	**ARIH2OS**	protein_coding	ariadne RBR E3 ubiquitin protein ligase 2 opposite strand [Source:HGNC Symbol;Acc:HGNC:34425]	chr3	48917788	48919385	-1	ensembl_havana	0.674767171	0.531990772	0.817543571	0.683014432	0.271370116	0.641587769	1.071324589	0.625615656	0.755690467
ENSG00000196268	0.019045	0.067803646	0.277940458	1.212462778	**ZNF493**	protein_coding	zinc finger protein 493 [Source:HGNC Symbol;Acc:HGNC:23708]	chr19	21397119	21427573	1	ensembl_havana	3.46568216	3.32671193	3.604652389	3.146964063	3.370423439	3.46274829	3.562094275	3.501444376	3.750418515
ENSG00000211455	0.019073	0.067803646	0.196851068	1.146193856	**STK38L**	protein_coding	serine/threonine kinase 38 like [Source:HGNC Symbol;Acc:HGNC:17848]	chr12	27243968	27325959	1	ensembl_havana	5.940149067	5.841723533	6.038574601	6.081730794	5.793374075	5.650065731	6.232574829	6.020489161	5.862659813
ENSG00000183723	0.019069	0.067803646	0.195599437	1.14519989	**CMTM4**	protein_coding	CKLF like MARVEL transmembrane domain containing 4 [Source:HGNC Symbol;Acc:HGNC:19175]	chr16	66614750	66696707	-1	ensembl_havana	4.525555474	4.427755756	4.623355193	4.285261859	4.483808757	4.51419665	4.520468078	4.674563195	4.675034306
ENSG00000153015	0.019068	0.067803646	0.191711774	1.142118049	**CWC27**	protein_coding	CWC27 spliceosome associated protein homolog [Source:HGNC Symbol;Acc:HGNC:10664]	chr5	64768930	65018763	1	ensembl_havana	4.775905825	4.680049937	4.871761712	4.675023044	4.64623008	4.718896689	4.845475607	4.830360032	4.939449496
ENSG00000174243	0.019069	0.067803646	-0.19315824	-1.143263726	**DDX23**	protein_coding	DEAD-box helicase 23 [Source:HGNC Symbol;Acc:HGNC:17347]	chr12	48829764	48852842	-1	ensembl_havana	8.180899738	8.277478857	8.084320618	8.280769011	8.282730444	8.268937118	8.062990902	8.07983165	8.110139302
ENSG00000152969	0.019077	0.067803646	-0.26741483	-1.203649072	**JAKMIP1**	protein_coding	janus kinase and microtubule interacting protein 1 [Source:HGNC Symbol;Acc:HGNC:26460]	chr4	6026199	6200591	-1	ensembl_havana	3.966629946	4.100337361	3.83292253	4.307373212	3.951004938	4.042633933	3.921799706	3.826549371	3.750418515
ENSG00000188234	0.019101	0.067845192	0.195728434	1.145302291	**AGAP4**	protein_coding	ArfGAP with GTPase domain, ankyrin repeat and PH domain 4 [Source:HGNC Symbol;Acc:HGNC:23459]	chr10	45825594	45853875	-1	ensembl_havana	3.310210377	3.21234616	3.408074594	3.122229914	3.116453761	3.398354805	3.32252843	3.347673311	3.55402204
ENSG00000166847	0.019102	0.067845192	-0.200814703	-1.149347218	**DCTN5**	protein_coding	dynactin subunit 5 [Source:HGNC Symbol;Acc:HGNC:24594]	chr16	23641392	23677455	1	ensembl_havana	7.914749374	8.015156726	7.814342023	8.170868568	7.994954602	7.879647007	7.971848476	7.744066923	7.727110671
ENSG00000082898	0.019108	0.067852704	-0.194446079	-1.144284729	**XPO1**	protein_coding	exportin 1 [Source:HGNC Symbol;Acc:HGNC:12825]	chr2	61477849	61538626	-1	ensembl_havana	8.903727123	9.000950162	8.806504083	8.865848813	9.011118536	9.125883136	8.692421282	8.776612518	8.950478449
ENSG00000244005	0.019115	0.06785887	0.200109069	1.148785201	**NFS1**	protein_coding	NFS1, cysteine desulfurase [Source:HGNC Symbol;Acc:HGNC:15910]	chr20	35668055	35699359	-1	ensembl_havana	5.808951813	5.708897279	5.909006347	5.626031464	5.648194018	5.852466354	5.813375768	5.808110029	6.105533245
ENSG00000131748	0.019138	0.067922926	0.190379738	1.141064021	**STARD3**	protein_coding	StAR related lipid transfer domain containing 3 [Source:HGNC Symbol;Acc:HGNC:17579]	chr17	39637065	39663484	1	ensembl_havana	5.132813255	5.037623386	5.228003124	5.031129708	5.012340782	5.069399668	5.240784047	5.179549065	5.26367626
ENSG00000135315	0.019147	0.067940318	0.217791693	1.162952114	**CEP162**	protein_coding	centrosomal protein 162 [Source:HGNC Symbol;Acc:HGNC:21107]	chr6	84124241	84227635	-1	ensembl_havana	3.571446149	3.462550302	3.680341996	3.290778936	3.558185553	3.538686417	3.588212495	3.702394978	3.750418515
ENSG00000151623	0.019167	0.067942889	0.615347511	1.531926958	**NR3C2**	protein_coding	nuclear receptor subfamily 3 group C member 2 [Source:HGNC Symbol;Acc:HGNC:7979]	chr4	148078762	148444698	-1	ensembl_havana	1.675471462	1.367797706	1.983145217	0.515840645	1.667974555	1.919577919	1.593788933	2.131077666	2.224569053
ENSG00000174652	0.019165	0.067942889	0.19791375	1.147038447	**ZNF266**	protein_coding	zinc finger protein 266 [Source:HGNC Symbol;Acc:HGNC:13059]	chr19	9412598	9435578	-1	ensembl_havana	5.647851695	5.54889482	5.74680857	5.599396023	5.560900304	5.486388134	5.752604147	5.76120985	5.726611713
ENSG00000204569	0.019158	0.067942889	-0.20540832	-1.153012633	**PPP1R10**	protein_coding	protein phosphatase 1 regulatory subunit 10 [Source:HGNC Symbol;Acc:HGNC:9284]	chr6	30600400	30618612	-1	ensembl_havana	6.893176427	6.995880587	6.790472267	7.16658802	6.908090139	6.912963603	6.921041206	6.769138144	6.681237452
ENSG00000002549	0.01916	0.067942889	-0.211726325	-1.158073102	**LAP3**	protein_coding	leucine aminopeptidase 3 [Source:HGNC Symbol;Acc:HGNC:18449]	chr4	17577192	17607972	1	ensembl_havana	7.117155431	7.223018594	7.011292269	7.121799915	7.28788293	7.259372936	6.960943488	6.998756278	7.074177041
ENSG00000173480	0.019232	0.067971514	0.287977568	1.220927529	**ZNF417**	protein_coding	zinc finger protein 417 [Source:HGNC Symbol;Acc:HGNC:20646]	chr19	57900296	57916610	-1	ensembl_havana	4.29507016	4.151081376	4.439058944	4.027778621	4.127868366	4.297597141	4.427687441	4.477488956	4.412000434
ENSG00000170581	0.019218	0.067971514	0.239055161	1.180219468	**STAT2**	protein_coding	signal transducer and activator of transcription 2 [Source:HGNC Symbol;Acc:HGNC:11363]	chr12	56341597	56360155	-1	ensembl_havana	7.065480712	6.945953132	7.185008292	6.864368254	6.998236339	6.975254801	7.183384591	7.120656075	7.250984211

ENSG00000119383	0.019216	0.067971514	0.20374705	1.151685698	**PTPA**	protein_coding	protein phosphatase 2 phosphatase activator [Source:HGNC Symbol;Acc:HGNC:9308]	chr9	129110950	129148946	1	ensembl_havana	6.715175965	6.613302439	6.81704949	6.483615514	6.654829285	6.701462519	6.7446761	6.85446937	6.852002999
ENSG00000156990	0.019227	0.067971514	-0.193697192	-1.143690898	**RPUSD3**	protein_coding	RNA pseudouridylate synthase domain containing 3 [Source:HGNC Symbol;Acc:HGNC:28437]	chr3	9837849	9844602	-1	ensembl_havana	7.884950427	7.981799023	7.788101832	7.976427291	7.984294788	7.984674991	7.821358544	7.767987845	7.774959106
ENSG00000132357	0.019206	0.067971514	-0.20028041	-1.148921644	**CARD6**	protein_coding	caspase recruitment domain family member 6 [Source:HGNC Symbol;Acc:HGNC:16394]	chr5	40841184	40860175	1	ensembl_havana	3.07041765	3.170557855	2.970277445	3.10550171	3.135610417	3.270561436	2.853206155	2.980039912	3.077586267
ENSG00000108389	0.019216	0.067971514	-0.217541759	-1.16275066	**MTMR4**	protein_coding	myotubularin related protein 4 [Source:HGNC Symbol;Acc:HGNC:7452]	chr17	58489529	58517905	-1	ensembl_havana	7.630996746	7.739767626	7.522225867	7.905117489	7.690232904	7.623952484	7.602972166	7.537886213	7.425819221
ENSG00000101966	0.019186	0.067971514	-0.246961941	-1.186705491	**XIAP**	protein_coding	X-linked inhibitor of apoptosis [Source:HGNC Symbol;Acc:HGNC:592]	chrX	123859724	123913979	1	ensembl_havana	5.997397575	6.120878545	5.873916604	6.228969909	6.07269516	6.060970566	5.856324226	5.914339061	5.851086526
ENSG00000152240	0.019207	0.067971514	-0.252264359	-1.191075082	**HAUS1**	protein_coding	HAUS augmin like complex subunit 1 [Source:HGNC Symbol;Acc:HGNC:25174]	chr18	46104332	46128333	1	ensembl_havana	5.364266482	5.490398661	5.238134302	5.350624948	5.633445542	5.487125493	5.196901456	5.251071083	5.266430368
ENSG00000272362	0.019201	0.067971514	-0.267433284	-1.203664468	**AL160408.5**	lincRNA		chr1	234644666	234647571	1	havana	0.18408454	0.317801182	0.050367898	0.637193984	-0.071090603	0.387300166	0.24405081	-0.19956768	0.106620565
ENSG00000228509	0.019227	0.067971514	-0.328049103	-1.255314716	**AC006460.1**	antisense		chr2	190676944	190708716	-1	havana	1.645642982	1.809667534	1.481618431	2.040883175	1.845996665	1.542122761	1.514288036	1.541948597	1.38861866
ENSG00000285399	0.019206	0.067971514	-0.552196624	-1.466316587	**AC104162.2**	bidirectional_promoter_lncRNA		chr3	63863155	63905838	1	havana	1.558919488	1.8350178	1.282821176	1.613998728	1.845996665	2.045058006	1.430149367	0.938274071	1.480040089
ENSG00000167799	0.019221	0.067971514	-0.657152066	-1.576966558	**NUDT8**	protein_coding	nudix hydrolase 8 [Source:HGNC Symbol;Acc:HGNC:8055]	chr11	67627938	67629930	-1	ensembl_havana	1.827711165	2.156287198	1.499135132	1.912130943	2.177092949	2.379637701	1.669136731	1.566404757	1.261863909
ENSG00000165802	0.019241	0.067988353	0.460218691	1.375750346	**NSMF**	protein_coding	NMDA receptor synaptonuclear signaling and neuronal migration factor [Source:HGNC Symbol;Acc:HGNC:29843]	chr9	137447573	137459334	-1	ensembl_havana	3.941627138	3.711517792	4.171736484	3.376940521	3.68322949	4.074383366	4.10707367	4.185467242	4.222668539
ENSG00000130649	0.019264	0.068027525	0.66139476	1.581610945	**CYP2E1**	protein_coding	cytochrome P450 family 2 subfamily E member 1 [Source:HGNC Symbol;Acc:HGNC:2631]	chr10	133520406	133561220	1	ensembl_havana	0.033700112	-0.296997268	0.364397492	-0.31750517	-0.036373238	-0.537113394	-0.08686711	0.602150945	0.577908642
ENSG00000115183	0.019267	0.068027525	0.469261369	1.384400501	**TANC1**	protein_coding	tetratricopeptide repeat, ankyrin repeat and coiled-coil containing 1 [Source:HGNC Symbol;Acc:HGNC:29364]	chr2	158968671	159232659	1	ensembl_havana	2.010116132	1.775485448	2.244746817	1.222335231	1.864486201	2.239634912	1.966610161	2.379024645	2.388605644
ENSG00000183808	0.019265	0.068027525	-0.223050567	-1.167199012	**RBM12B**	protein_coding	RNA binding motif protein 12B [Source:HGNC Symbol;Acc:HGNC:32310]	chr8	93729356	93741017	-1	ensembl_havana	6.089222032	6.200747315	5.977696748	6.186354198	6.186372368	6.229515378	5.875009576	6.042993264	6.015087405
ENSG00000205791	0.019314	0.06814406	0.51052312	1.424566649	**LOH12CR2**	lincRNA	loss of heterozygosity, 12, chromosomal region 2 (non-protein coding) [Source:HGNC Symbol;Acc:HGNC:26524]	chr12	12355406	12357067	-1	havana	0.770878562	0.515617002	1.026140122	0.355307344	0.592979824	0.598563838	1.245545533	0.956893362	0.875981471
ENSG00000224046	0.019313	0.06814406	-0.365595224	-1.288413091	**AC005076.1**	antisense		chr7	87151423	87152420	-1	havana	-0.171623638	0.011173974	-0.354421251	-0.10791135	0.09469996	0.046733314	-0.57379957	-0.42206346	-0.067400719
ENSG00000135736	0.019306	0.06814406	-0.412938408	-1.331394771	**CCDC102A**	protein_coding	coiled-coil domain containing 102A [Source:HGNC Symbol;Acc:HGNC:28097]	chr16	57512178	57536599	-1	ensembl_havana	2.388992417	2.595461621	2.182523214	2.516278079	2.608571783	2.661535001	2.364348155	2.187350601	1.995870885
ENSG00000130717	0.019327	0.068155665	0.309801231	1.23953691	**UCK1**	protein_coding	uridine-cytidine kinase 1 [Source:HGNC Symbol;Acc:HGNC:14859]	chr9	131523801	131531268	-1	ensembl_havana	4.82296967	4.668069054	4.977870285	4.539923521	4.72296254	4.741321101	4.999384646	5.071565226	4.862660984
ENSG00000166326	0.019325	0.068155665	-0.187396047	-1.138706583	**TRIM44**	protein_coding	tripartite motif containing 44 [Source:HGNC Symbol;Acc:HGNC:19016]	chr11	35662805	35818007	1	ensembl_havana	5.586739995	5.680438018	5.493041971	5.330071823	5.759815235	5.951426997	5.146158264	5.568847077	5.764120573
ENSG00000198400	0.019332	0.068159257	-0.417866362	-1.335950323	**NTRK1**	protein_coding	neurotrophic receptor tyrosine kinase 1 [Source:HGNC Symbol;Acc:HGNC:8031]	chr1	156815640	156881850	1	ensembl_havana	5.849015326	6.057948508	5.640082145	6.038321501	5.770271565	6.365252456	5.713094006	5.554400884	5.652751546
ENSG00000235831	0.01935	0.068206338	0.188367002	1.139473206	**BHLHE40-AS1**	antisense	BHLHE40 antisense RNA 1 [Source:HGNC Symbol;Acc:HGNC:44471]	chr3	4896809	4979961	-1	havana	0.598682352	0.504498852	0.692865853	0.515840645	0.214850257	0.782805653	0.692307165	0.399280361	0.987010032
ENSG00000248079	0.019376	0.068278389	0.722366248	1.649885891	**DPH6-AS1**	lincRNA	DPH6 antisense RNA 1 (head to head) [Source:HGNC Symbol;Acc:HGNC:44147]	chr15	35546195	35859001	1	havana	-0.276175895	-0.637359019	0.08500723	-0.56281016	-0.299013651	-1.050253243	-0.17198403	0.19485955	0.232146171
ENSG00000172428	0.019394	0.068315455	0.232909368	1.175202499	**COPS9**	protein_coding	COP9 signalosome subunit 9 [Source:HGNC Symbol;Acc:HGNC:21314]	chr2	240126563	240136807	-1	ensembl_havana	3.569215002	3.452760318	3.685669686	3.640569013	3.370423439	3.347288501	3.770341093	3.617169153	3.669498811
ENSG00000164209	0.019396	0.068315455	-0.275476586	-1.21039387	**SLC25A46**	protein_coding	solute carrier family 25 member 46 [Source:HGNC Symbol;Acc:HGNC:25198]	chr5	110738136	110765161	1	ensembl_havana	6.367537641	6.505275934	6.229799348	6.555815092	6.555200126	6.404812584	6.140703712	6.276315588	6.272378745
ENSG00000132912	0.019405	0.068331122	0.213024424	1.159115575	**DCTN4**	protein_coding	dynactin subunit 4 [Source:HGNC Symbol;Acc:HGNC:15518]	chr5	150708440	150759109	-1	ensembl_havana	6.487936711	6.381424499	6.594448923	6.361683662	6.489711993	6.29287784	6.622437176	6.620860995	6.540048598
ENSG00000035141	0.01941	0.068331122	-0.190916535	-1.141488666	**FAM136A**	protein_coding	family with sequence similarity 136 member A [Source:HGNC Symbol;Acc:HGNC:25911]	chr2	70295975	70302090	-1	ensembl_havana	7.606100026	7.701558293	7.510641759	7.660988544	7.717664573	7.726021763	7.441551356	7.53110706	7.559266859
ENSG00000148399	0.019429	0.068331171	0.239124055	1.180275829	**DPH7**	protein_coding	diphthamide biosynthesis 7 [Source:HGNC Symbol;Acc:HGNC:25199]	chr9	137554904	137578935	-1	ensembl_havana	4.290523436	4.170961408	4.410085464	4.118114044	4.08712019	4.307649991	4.25094543	4.428300566	4.551010395
ENSG00000159352	0.019427	0.068331171	-0.190243303	-1.140956116	**PSMD4**	protein_coding	proteasome 26S subunit, non-ATPase 4 [Source:HGNC Symbol;Acc:HGNC:9561]	chr1	151254703	151267479	1	ensembl_havana	7.62291211	7.718033762	7.527790459	7.686169035	7.747700322	7.720231928	7.501833064	7.530718716	7.550819596
ENSG00000113282	0.019423	0.068331171	-0.204590512	-1.15235922	**CLINT1**	protein_coding	clathrin interactor 1 [Source:HGNC Symbol;Acc:HGNC:23186]	chr5	157785743	157859175	-1	ensembl_havana	7.050158484	7.15245374	6.947863228	7.198488732	7.140155817	7.118716671	6.928727213	6.954853326	6.960009145
ENSG00000090263	0.019428	0.068331171	-0.28118421	-1.215191943	**MRPS33**	protein_coding	mitochondrial ribosomal protein S33 [Source:HGNC Symbol;Acc:HGNC:16634]	chr7	141002610	141015228	-1	ensembl_havana	4.43329468	4.573886785	4.292702575	4.6536066	4.589008315	4.479045439	4.207375531	4.356491584	4.31424061
ENSG00000134901	0.019441	0.0683506	0.263571439	1.200446775	**KDELC1**	protein_coding	KDEL motif containing 1 [Source:HGNC Symbol;Acc:HGNC:19350]	chr13	102784281	102799007	-1	ensembl_havana	3.517864449	3.38607873	3.649650169	3.234191999	3.461295901	3.46274829	3.558795999	3.575605876	3.814548632
ENSG00000116688	0.019444	0.0683506	0.199156668	1.148027076	**MFN2**	protein_coding	mitofusin 2 [Source:HGNC Symbol;Acc:HGNC:16877]	chr1	11980181	12013514	1	ensembl_havana	7.70335726	7.603778926	7.802935594	7.582697572	7.545733159	7.682906046	7.797944439	7.782739481	7.828122861
ENSG00000092470	0.01946	0.068391815	-0.192499365	-1.142741719	**WDR76**	protein_coding	WD repeat domain 76 [Source:HGNC Symbol;Acc:HGNC:25773]	chr15	43826963	43868419	1	ensembl_havana	6.44757674	6.543826422	6.351327057	6.393469306	6.628025434	6.609984526	6.236708764	6.409010314	6.408262094
ENSG00000271851	0.019475	0.068427039	0.627784756	1.545190546	**AC087501.4**	sense_intronic		chr17	9553323	9555696	-1	havana	-0.097201473	-0.411093851	0.216690905	-0.56281016	-0.18055577	-0.489915621	0.513047461	0.063570082	0.073455173
ENSG00000189114	0.019485	0.068443955	0.218706276	1.16368959	**BLOC1S3**	protein_coding	biogenesis of lysosomal organelles complex 1 subunit 3 [Source:HGNC Symbol;Acc:HGNC:20914]	chr19	45178745	45181801	1	ensembl_havana	3.745981524	3.636628386	3.855334662	3.538330443	3.646306798	3.725247916	3.848057062	3.808721901	3.909225024
ENSG00000130826	0.019499	0.068478271	-0.222362243	-1.166642263	**DKC1**	protein_coding	dyskerin pseudouridine synthase 1 [Source:HGNC Symbol;Acc:HGNC:2890]	chrX	154762742	154777689	1	ensembl_havana	8.127525887	8.238707009	8.016344766	8.132808512	8.216577768	8.366734748	7.813423011	8.051145657	8.184465629
ENSG00000197442	0.019505	0.068482166	-0.220642217	-1.165252184	**MAP3K5**	protein_coding	mitogen-activated protein kinase kinase kinase 5 [Source:HGNC Symbol;Acc:HGNC:6857]	chr6	136557047	136792518	-1	ensembl_havana	5.129448492	5.2397696	5.019127383	5.226543391	5.225813962	5.266951447	5.00302963	4.924416975	5.129935545
ENSG00000135535	0.019513	0.068495077	-0.201067522	-1.149548648	**CD164**	protein_coding	CD164 molecule [Source:HGNC Symbol;Acc:HGNC:1632]	chr6	109366514	109382812	-1	ensembl_havana	8.545702302	8.646236062	8.445168541	8.790667289	8.55296001	8.595080889	8.565880253	8.315849224	8.453776145
ENSG00000111860	0.019527	0.0685095	-0.256823307	-1.194844858	**CEP85L**	protein_coding	centrosomal protein 85 like [Source:HGNC Symbol;Acc:HGNC:21638]	chr6	118460772	118710075	-1	ensembl_havana	5.154275821	5.282687474	5.025864167	5.588151131	5.141144845	5.118766446	5.190522115	4.935020873	4.952049513
ENSG00000154096	0.019527	0.0685095	-0.333671195	-1.260216142	**THY1**	protein_coding	Thy-1 cell surface antigen [Source:HGNC Symbol;Acc:HGNC:11801]	chr11	119417378	119424985	-1	ensembl_havana	2.192550007	2.359385605	2.02571441	2.245600406	2.29082961	2.541726798	2.135529305	1.85152878	2.090085146
ENSG00000167280	0.019548	0.068550531	0.324496755	1.252227557	**ENGASE**	protein_coding	endo-beta-N-acetylglucosaminidase [Source:HGNC Symbol;Acc:HGNC:24622]	chr17	79074939	79088599	1	ensembl_havana	5.742609088	5.580360711	5.904857466	5.491675083	5.448199049	5.801207999	5.967459571	5.829106762	5.918006064
ENSG00000186010	0.019544	0.068550531	0.289971455	1.222616087	**NDUFA13**	protein_coding	NADH:ubiquinone oxidoreductase subunit A13 [Source:HGNC Symbol;Acc:HGNC:17194]	chr19	19515736	19529054	1	ensembl_havana	2.369116399	2.224130672	2.514102127	2.183196095	2.029240114	2.459955808	2.302589091	2.453030417	2.786686872
ENSG00000271086	0.019574	0.068604122	0.348358636	1.273111376	**NAMA**	lincRNA	non-protein coding RNA, associated with MAP kinase pathway and growth arrest [Source:HGNC Symbol;Acc:HGNC:42408]	chr9	99355337	99377240	-1	havana	1.807998079	1.633818761	1.982177397	1.613998728	1.636045198	1.651412359	2.170524752	1.780136556	1.995870885
ENSG00000155629	0.019577	0.068604122	-0.226605216	-1.170078414	**PIK3AP1**	protein_coding	phosphoinositide-3-kinase adaptor protein 1 [Source:HGNC Symbol;Acc:HGNC:30034]	chr10	96593312	96720514	-1	ensembl_havana	3.296741636	3.410044245	3.183439028	3.380421283	3.331079908	3.518631543	3.234313959	3.007092508	3.308910617
ENSG00000137168	0.019578	0.068604122	-0.235417055	-1.177247008	**PPIL1**	protein_coding	peptidylprolyl isomerase like 1 [Source:HGNC Symbol;Acc:HGNC:9260]	chr6	36854827	36875024	-1	ensembl_havana	6.097744481	6.215453008	5.980035953	6.254327359	6.215472255	6.176559411	5.946159327	5.93796143	6.055987102
ENSG00000172939	0.019614	0.068713619	0.1875613	1.138837023	**OXSR1**	protein_coding	oxidative stress responsive 1 [Source:HGNC Symbol;Acc:HGNC:8508]	chr3	38165089	38255488	1	ensembl_havana	6.730068701	6.636288051	6.823849351	6.532538911	6.686025274	6.690299968	6.719419016	6.860044429	6.892084609
ENSG00000272106	0.019636	0.068748112	0.207970483	1.155062152	**AL691432.2**	antisense		chr1	1613758	1615795	-1	havana	2.219640126	2.115654884	2.323625367	2.253214515	2.139629423	1.954120714	2.459388507	2.413134925	2.09835267
ENSG00000179051	0.019634	0.068748112	-0.192182918	-1.142491093	**RCC2**	protein_coding	regulator of chromosome condensation 2 [Source:HGNC Symbol;Acc:HGNC:30297]	chr1	17406760	17439724	-1	ensembl_havana	8.598472638	8.694564097	8.502381179	8.646247061	8.733856426	8.703588804	8.465757029	8.504756719	8.536629789
ENSG00000197813	0.019638	0.068748112	-0.431980512	-1.349084311	**AC011450.1**	lincRNA		chr19	49331617	49340303	-1	havana	0.095527586	0.311517841	-0.12046267	0.355307344	0.352203286	0.227042895	0.176794179	-0.04338487	-0.494797317
ENSG00000181097	0.019647	0.068759802	0.275018741	1.210009808	**AC105219.1**	sense_intronic		chr8	143696154	143698413	1	ensembl_havana	2.554863272	2.417353902	2.692372643	2.528879384	2.324608835	2.398573485	2.663565071	2.596492542	2.817060315
ENSG00000204160	0.019651	0.068759802	-0.219180202	-1.164071926	**ZDHHC18**	protein_coding	zinc finger DHHC-type containing 18 [Source:HGNC Symbol;Acc:HGNC:20712]	chr1	26826710	26857602	1	ensembl_havana	6.121892205	6.231482306	6.012302104	6.354179901	6.192330976	6.147936041	6.219057144	5.89576193	5.922087238
ENSG00000117834	0.019677	0.068787755	0.698002942	1.622257619	**SLC5A9**	protein_coding	solute carrier family 5 member 9 [Source:HGNC Symbol;Acc:HGNC:22146]	chr1	48222685	48248644	1	ensembl_havana	-0.038643088	-0.387644559	0.310358383	-0.85854608	-0.473795405	0.16940781	0.339398945	0.163140205	0.428535999
ENSG00000176973	0.019666	0.068787755	0.578622094	1.493422212	**FAM89B**	protein_coding	family with sequence similarity 89 member B [Source:HGNC Symbol;Acc:HGNC:16708]	chr11	65572349	65574198	1	ensembl_havana	0.130041166	-0.159269881	0.419352212	0.10902545	0.271370116	-0.85820521	1.01467363	0.426254992	-0.182871985
ENSG00000176542	0.019671	0.068787755	0.327930324	1.255211369	**USF3**	protein_coding	upstream transcription factor family member 3 [Source:HGNC Symbol;Acc:HGNC:30494]	chr3	113648385	113696646	-1	ensembl_havana	5.067311421	4.903346259	5.231276584	4.811498466	4.864024481	5.034515831	5.069431626	5.406466214	5.217931911
ENSG00000198732	0.019679	0.068787755	-0.292828932	-1.225040064	**SMOC1**	protein_coding	SPARC related modular calcium binding 1 [Source:HGNC Symbol;Acc:HGNC:20318]	chr14	69854131	70032366	1	ensembl_havana	0.332071383	0.478485849	0.185656916	-0.10791135	0.77991046	0.76345844	-0.57379957	0.625615656	0.505154661
ENSG00000152904	0.019683	0.068787755	-0.293889025	-1.225940554	**GGPS1**	protein_coding	geranylgeranyl diphosphate synthase 1 [Source:HGNC Symbol;Acc:HGNC:4249]	chr1	235327350	235344532	1	ensembl_havana	3.715760912	3.862705424	3.5688164	3.890491928	4.06530755	3.632316794	3.700223929	3.587603785	3.418621485
ENSG00000240770	0.019693	0.068794088	0.217384499	1.162623922	**C21orf91-OT1**	lincRNA	C21orf91 overlapping transcript 1 [Source:HGNC Symbol;Acc:HGNC:16729]	chr21	17763315	17792523	-1	havana	1.890971424	1.782279174	1.999663673	1.931242682	1.614359492	1.801235349	2.238067541	1.800897362	1.960026115
ENSG00000172350	0.019694	0.068794088	0.195123011	1.144821769	**ABCG4**	protein_coding	ATP binding cassette subfamily G member 4 [Source:HGNC Symbol;Acc:HGNC:13884]	chr11	119149012	119162653	1	ensembl_havana	1.760426322	1.662864816	1.857987827	1.565895203	1.729788441	1.692910804	1.797815913	1.881076089	1.89507148
ENSG00000166881	0.019706	0.068817892	-0.187686827	-1.138936116	**NEMP1**	protein_coding	nuclear envelope integral membrane protein 1 [Source:HGNC Symbol;Acc:HGNC:29001]	chr12	57055643	57088063	-1	ensembl_havana	8.75766045	8.851503863	8.663817036	8.704077802	8.865110814	8.985322975	8.505059571	8.696041986	8.790349552
ENSG00000006451	0.019732	0.06883258	0.228687444	1.171768396	**RALA**	protein_coding	RAS like proto-oncogene A [Source:HGNC Symbol;Acc:HGNC:9839]	chr7	39623483	39708124	1	ensembl_havana	6.390582574	6.276238852	6.504926296	6.076911949	6.401063592	6.350741015	6.413274372	6.579780941	6.521723576
ENSG00000109971	0.019734	0.06883258	-0.197125117	-1.146411604	**HSPA8**	protein_coding	heat shock protein family A (Hsp70) member 8 [Source:HGNC Symbol;Acc:HGNC:5241]	chr11	123057489	123063230	-1	ensembl_havana	11.06880113	11.16736369	10.97023857	10.97223732	11.24660398	11.28324978	10.73378025	11.09827955	11.07865592
ENSG00000117480	0.019727	0.06883258	-0.227756238	-1.171012307	**FAAH**	protein_coding	fatty acid amide hydrolase [Source:HGNC Symbol;Acc:HGNC:3553]	chr1	46394265	46413848	1	ensembl_havana	2.242682499	2.356560618	2.128804381	2.637596133	2.213608205	2.218477517	2.310455111	2.002551439	2.073406591
ENSG00000121486	0.019733	0.06883258	-0.228163641	-1.171343036	**TRMT1L**	protein_coding	tRNA methyltransferase 1 like [Source:HGNC Symbol;Acc:HGNC:16782]	chr1	185118098	185157072	-1	ensembl_havana	6.183844926	6.297926747	6.069763106	6.300531493	6.251040639	6.342208108	5.966215283	6.064078677	6.178995358
ENSG00000151116	0.019728	0.06883258	-0.252506178	-1.191274742	**UEVLD**	protein_coding	UEV and lactate/malate dehyrogenase domains [Source:HGNC Symbol;Acc:HGNC:30866]	chr11	18529609	18588747	-1	ensembl_havana	3.864950427	3.991203516	3.738697338	3.94322289	4.104724807	3.925662851	3.565385028	3.864025053	3.786681933
ENSG00000238058	0.019741	0.068839494	0.492546987	1.406926525	**AL355574.1**	antisense		chr9	135907812	135913513	1	havana	0.676193621	0.429920128	0.922467115	0.040274975	0.428746719	0.820738689	0.894226935	0.671430284	1.201744126
ENSG00000124802	0.019753	0.068864234	-0.291171283	-1.223633307	**EEF1E1**	protein_coding	eukaryotic translation elongation factor 1 epsilon 1 [Source:HGNC Symbol;Acc:HGNC:3212]	chr6	8073360	8102578	-1	ensembl_havana	3.278916531	3.424502173	3.13333089	3.348787899	3.411868621	3.512849998	2.885159781	3.256675325	3.258157563
ENSG00000184304	0.019778	0.068917514	0.579180564	1.494000431	**PRKD1**	protein_coding	protein kinase D1 [Source:HGNC Symbol;Acc:HGNC:9407]	chr14	29576479	30191898	-1	ensembl_havana	0.370187389	0.080597107	0.659777671	0.705390237	-0.106664058	-0.356934859	0.851734776	0.779979312	0.347618925
ENSG00000085760	0.019775	0.068917514	-0.191294032	-1.141787388	**MTIF2**	protein_coding	mitochondrial translational initiation factor 2 [Source:HGNC Symbol;Acc:HGNC:7441]	chr2	55236595	55269347	-1	ensembl_havana	6.678847237	6.774494253	6.583200221	6.678939559	6.766572098	6.877971101	6.491903705	6.542128251	6.715568706
ENSG00000145907	0.019788	0.068936281	-0.214806077	-1.160547906	**G3BP1**	protein_coding	G3BP stress granule assembly factor 1 [Source:HGNC Symbol;Acc:HGNC:30292]	chr5	151771045	151812785	1	ensembl_havana	8.36665511	8.474058149	8.259252072	8.506500644	8.472471845	8.443201957	8.205447605	8.289464629	8.282843981
ENSG00000156313	0.019805	0.068941745	0.275787768	1.210654974	**RPGR**	protein_coding	retinitis pigmentosa GTPase regulator [Source:HGNC Symbol;Acc:HGNC:10295]	chrX	38269163	38327564	-1	ensembl_havana	3.745012518	3.607118634	3.882906402	3.789382034	3.605669198	3.42630467	3.95980697	4.044601662	3.644310574
ENSG00000163156	0.019809	0.068941745	0.23174261	1.174252455	**SCNM1**	protein_coding	sodium channel modifier 1 [Source:HGNC Symbol;Acc:HGNC:23136]	chr1	151156664	151170297	1	ensembl_havana	4.922480179	4.806608874	5.038351484	4.701701975	4.949860986	4.76826366	4.933423317	5.084323255	5.09730788
ENSG00000172500	0.019808	0.068941745	0.204639548	1.152398388	**FIBP**	protein_coding	FGF1 intracellular binding protein [Source:HGNC Symbol;Acc:HGNC:3705]	chr11	65883741	65888539	-1	ensembl_havana	5.465489349	5.363169575	5.567809123	5.39432045	5.336742153	5.358446121	5.650948553	5.555164827	5.497313988
ENSG00000115808	0.019803	0.068941745	0.186187059	1.137752738	**STRN**	protein_coding	striatin [Source:HGNC Symbol;Acc:HGNC:11424]	chr2	36843640	36966472	-1	ensembl_havana	7.107909384	7.014815854	7.201002913	6.957322735	7.053923399	7.033201429	7.136586634	7.244465991	7.221956114
ENSG00000165630	0.019832	0.069006626	0.26938271	1.205292006	**PRPF18**	protein_coding	pre-mRNA processing factor 18 [Source:HGNC Symbol;Acc:HGNC:17351]	chr10	13586927	13630868	1	ensembl_havana	2.093967072	1.959275717	2.228658428	1.912130943	1.944876279	2.02081993	2.090556387	2.147380863	2.448038032
ENSG00000110057	0.019852	0.06904864	0.195398591	1.145040471	**UNC93B1**	protein_coding	unc-93 homolog B1, TLR signaling regulator [Source:HGNC Symbol;Acc:HGNC:13481]	chr11	67991104	68004982	-1	ensembl_havana	2.084915111	1.987215816	2.182614407	2.092729261	1.864486201	2.004431986	2.278730383	2.106272137	2.162840701
ENSG00000137764	0.019854	0.06904864	-0.213322572	-1.159355143	**MAP2K5**	protein_coding	mitogen-activated protein kinase kinase 5 [Source:HGNC Symbol;Acc:HGNC:6845]	chr15	67542709	67807123	1	ensembl_havana	4.488451543	4.595112829	4.381790257	4.597884537	4.586226029	4.601227922	4.40214941	4.291961583	4.451259779
ENSG00000163006	0.019864	0.06904864	-0.278956038	-1.213316587	**CCDC138**	protein_coding	coiled-coil domain containing 138 [Source:HGNC Symbol;Acc:HGNC:26531]	chr2	108786757	108885477	1	ensembl_havana	4.128236747	4.267714766	3.988758728	4.149053241	4.39915155	4.254939508	3.778870895	4.042423959	4.144981331
ENSG00000255966	0.019862	0.06904864	-0.600646293	-1.516395724	**AC006064.3**	antisense		chr12	6532290	6533498	-1	havana	0.187153669	0.487476815	-0.113169478	0.383334258	0.570631912	0.508464275	-0.21650287	0.37179175	-0.494797317
ENSG00000070061	0.019891	0.069126166	0.186951622	1.138355856	**ELP1**	protein_coding	elongator complex protein 1 [Source:HGNC Symbol;Acc:HGNC:5959]	chr9	108867517	108934116	-1	ensembl_havana	7.397710485	7.304234674	7.491186296	7.286691185	7.299183097	7.326829739	7.467946606	7.474888363	7.530723918
ENSG00000126775	0.019911	0.06917985	0.191288453	1.141782973	**ATG14**	protein_coding	autophagy related 14 [Source:HGNC Symbol;Acc:HGNC:19962]	chr14	55366392	55411858	-1	ensembl_havana	4.872558473	4.776914247	4.9682027	4.812787188	4.850175138	4.667780414	5.027096987	5.055457379	4.822053734
ENSG00000277324	0.019916	0.069179863	-0.351984632	-1.276315173	**AC093462.1**	antisense		chr18	54268346	54270028	-1	havana	-0.126931506	0.04906081	-0.302923823	-0.22998119	0.09469996	0.28246366	-0.35894771	-0.28449416	-0.265329594
ENSG00000072042	0.019923	0.069187189	-0.285044141	-1.218447543	**RDH11**	protein_coding	retinol dehydrogenase 11 (all-trans/9-cis/11-cis) [Source:HGNC Symbol;Acc:HGNC:17964]	chr14	67676801	67695814	-1	ensembl_havana	6.618221084	6.760743155	6.475699014	6.977573542	6.814610407	6.490045515	6.568109526	6.483940598	6.375046918
ENSG00000165359	0.019933	0.069198223	-0.204690088	-1.152438759	**INTS6L**	protein_coding	integrator complex subunit 6 like [Source:HGNC Symbol;Acc:HGNC:27334]	chrX	135520643	135582510	1	ensembl_havana	5.197356891	5.299701934	5.095011847	5.428348316	5.27329101	5.197466477	5.185184365	5.036981726	5.062869449
ENSG00000185668	0.019935	0.069198223	-0.509229894	-1.423290245	**POU3F1**	protein_coding	POU class 3 homeobox 1 [Source:HGNC Symbol;Acc:HGNC:9214]	chr1	38044611	38046794	-1	ensembl_havana	0.689654818	0.944269765	0.435039871	1.253039554	0.855801933	0.723967808	0.716189844	0.693803314	-0.104873545
ENSG00000188706	0.019948	0.069223968	0.254199102	1.192673458	**ZDHHC9**	protein_coding	zinc finger DHHC-type containing 9 [Source:HGNC Symbol;Acc:HGNC:18475]	chrX	129803288	129843909	-1	ensembl_havana	4.7965733	4.669473749	4.923672851	4.753623874	4.600084112	4.654713261	4.86697049	4.91373456	4.990313502
ENSG00000203867	0.019964	0.06922534	0.381523745	1.302717032	**RBM20**	protein_coding	RNA binding motif protein 20 [Source:HGNC Symbol;Acc:HGNC:27424]	chr10	110644397	110839469	1	ensembl_havana	1.707229582	1.516467709	1.897991454	0.892538	1.636045198	2.02081993	1.415636519	2.147380863	2.13095698
ENSG00000125503	0.019972	0.06922534	0.291482239	1.223897076	**PPP1R12C**	protein_coding	protein phosphatase 1 regulatory subunit 12C [Source:HGNC Symbol;Acc:HGNC:14947]	chr19	55090913	55117559	-1	ensembl_havana	5.317498063	5.171756943	5.463239183	5.234258475	5.040055028	5.240957328	5.642404558	5.39541744	5.35189555
ENSG00000082805	0.019972	0.06922534	0.259463368	1.197033367	**ERC1**	protein_coding	ELKS/RAB6-interacting/CAST family member 1 [Source:HGNC Symbol;Acc:HGNC:17072]	chr12	990509	1495933	1	ensembl_havana	6.444843044	6.31511136	6.574574728	6.087598719	6.265460904	6.592274457	6.430520246	6.588762764	6.704441173
ENSG00000169519	0.019969	0.06922534	-0.289126117	-1.221899913	**METTL15**	protein_coding	methyltransferase like 15 [Source:HGNC Symbol;Acc:HGNC:26606]	chr11	28108248	28527041	1	ensembl_havana	2.983951071	3.12851413	2.839388013	3.330909387	3.184258973	2.87037403	2.874587014	2.919625469	2.723951555
ENSG00000174547	0.01997	0.06922534	-0.30965054	-1.239407446	**MRPL11**	protein_coding	mitochondrial ribosomal protein L11 [Source:HGNC Symbol;Acc:HGNC:14042]	chr11	66435075	66466738	-1	ensembl_havana	3.842258414	3.997083685	3.687433144	3.828133367	4.112480652	4.050637035	3.691214731	3.625921893	3.745162808
ENSG00000089163	0.019979	0.06923376	0.259938806	1.197427913	**SIRT4**	protein_coding	sirtuin 4 [Source:HGNC Symbol;Acc:HGNC:14932]	chr12	120302316	120313249	1	ensembl_havana	1.200386073	1.07041667	1.330355476	1.175017601	1.124807863	0.911424546	1.356079599	1.315408538	1.319578292
ENSG00000213585	0.020041	0.069430471	-0.19340892	-1.143462394	**VDAC1**	protein_coding	voltage dependent anion channel 1 [Source:HGNC Symbol;Acc:HGNC:12669]	chr5	133971915	134005133	-1	ensembl_havana	8.224879696	8.321584156	8.128175237	8.316753535	8.307647234	8.340351699	8.078332847	8.13452527	8.171667593
ENSG00000103415	0.020057	0.069457533	0.229428199	1.172370198	**HMOX2**	protein_coding	heme oxygenase 2 [Source:HGNC Symbol;Acc:HGNC:5014]	chr16	4474690	4510347	1	ensembl_havana	5.211880608	5.097166508	5.326594708	5.152134434	5.173017357	4.966347733	5.425055197	5.292891664	5.261837262
ENSG00000160285	0.020063	0.069457533	0.201090597	1.149567035	**LSS**	protein_coding	lanosterol synthase [Source:HGNC Symbol;Acc:HGNC:6708]	chr21	46188141	46228824	-1	ensembl_havana	7.511038518	7.41049322	7.611583817	7.75733684	7.321103025	7.153039796	7.895463128	7.545018277	7.394270045
ENSG00000163820	0.020061	0.069457533	-0.187121406	-1.138489831	**FYCO1**	protein_coding	FYVE and coiled-coil domain containing 1 [Source:HGNC Symbol;Acc:HGNC:14673]	chr3	45917899	45995824	-1	ensembl_havana	4.438781929	4.532342632	4.345221226	4.569195086	4.432064119	4.595768691	4.374276086	4.266065862	4.39532173
ENSG00000155275	0.020073	0.069474742	0.234349703	1.176376365	**TRMT44**	protein_coding	tRNA methyltransferase 44 homolog [Source:HGNC Symbol;Acc:HGNC:26653]	chr4	8436140	8493531	1	ensembl_havana	4.155090426	4.037915574	4.272265277	3.991820453	4.071289234	4.050637035	4.310990596	4.193340247	4.312464989
ENSG00000277161	0.020131	0.069658431	-0.198271093	-1.147322594	**PIGW**	protein_coding	phosphatidylinositol glycan anchor biosynthesis class W [Source:HGNC Symbol;Acc:HGNC:23213]	chr17	36535020	36539310	1	ensembl_havana	4.686752692	4.785888239	4.587617146	4.663640517	4.865172614	4.828851585	4.40766003	4.701035791	4.654155616
ENSG00000152465	0.020137	0.06966195	0.280971375	1.215012684	**NMT2**	protein_coding	N-myristoyltransferase 2 [Source:HGNC Symbol;Acc:HGNC:7858]	chr10	15102584	15168693	-1	ensembl_havana	4.323546607	4.18306092	4.464032294	3.855903752	4.309215282	4.384063726	4.305097297	4.472644903	4.614354683
ENSG00000260032	0.020144	0.069668612	-0.247059898	-1.18678607	**NORAD**	lincRNA	non-coding RNA activated by DNA damage [Source:HGNC Symbol;Acc:HGNC:44311]	chr20	36045622	36050960	-1	havana	8.982527618	9.106057568	8.858997669	9.175072382	9.133967042	9.009133279	8.795152051	8.965886376	8.815954581
ENSG00000101204	0.020149	0.069670478	0.211382788	1.157797372	**CHRNA4**	protein_coding	cholinergic receptor nicotinic alpha 4 subunit [Source:HGNC Symbol;Acc:HGNC:1958]	chr20	63343223	63378401	-1	ensembl_havana	5.556178818	5.450487424	5.661870212	5.529005835	5.418787772	5.403668666	5.673234048	5.620855507	5.69152108
ENSG00000243155	0.020178	0.069754559	0.477477413	1.392307055	**AL162431.2**	antisense		chr1	180944042	180976482	-1	havana	2.120141792	1.881403085	2.358880499	1.694503432	1.953538152	1.996167673	2.528460247	2.293711009	2.25447024
ENSG00000136908	0.020185	0.069762189	0.23763408	1.179057502	**DPM2**	protein_coding	dolichyl-phosphate mannosyltransferase subunit 2, regulatory [Source:HGNC Symbol;Acc:HGNC:3006]	chr9	127935099	127938484	-1	ensembl_havana	5.25644754	5.1376305	5.37526458	4.990708424	5.237387224	5.184795852	5.346897146	5.391145278	5.387751314
ENSG00000085552	0.020214	0.069790065	0.613898986	1.530389612	**IGSF9**	protein_coding	immunoglobulin superfamily member 9 [Source:HGNC Symbol;Acc:HGNC:18132]	chr1	159927039	159945604	-1	ensembl_havana	1.547357758	1.240408264	1.854307251	0.832812769	1.155163158	1.733248866	1.786580698	1.890792227	1.885548828
ENSG00000112182	0.020209	0.069790065	0.489762113	1.404213315	**BACH2**	protein_coding	BTB domain and CNC homolog 2 [Source:HGNC Symbol;Acc:HGNC:14078]	chr6	89926529	90296908	-1	ensembl_havana	2.888303481	2.643422424	3.133184538	2.602259737	2.714288775	2.613718761	3.353314566	3.301071157	2.745167891
ENSG00000159433	0.020217	0.069790065	0.252831078	1.191543051	**STARD9**	protein_coding	StAR related lipid transfer domain containing 9 [Source:HGNC Symbol;Acc:HGNC:19162]	chr15	42575659	42720981	1	ensembl_havana	5.152860432	5.026444893	5.279275971	4.989569283	4.926952921	5.162812476	5.313038798	5.250127827	5.274661287
ENSG00000163214	0.020217	0.069790065	0.193657678	1.143659574	**DHX57**	protein_coding	DExH-box helicase 57 [Source:HGNC Symbol;Acc:HGNC:20086]	chr2	38797729	38875934	-1	ensembl_havana	7.056941328	6.960112489	7.153770167	6.958487613	6.978478705	6.94337115	7.138797277	7.139625737	7.182887489
ENSG00000107816	0.020218	0.069790065	-0.286219624	-1.219440717	**LZTS2**	protein_coding	leucine zipper tumor suppressor 2 [Source:HGNC Symbol;Acc:HGNC:29381]	chr10	100996618	101007836	1	ensembl_havana	2.868838337	3.011948149	2.725728525	3.08004	2.927142373	3.028662073	2.90607567	2.705109515	2.566000389
ENSG00000244509	0.020247	0.06987291	-0.210095216	-1.156764526	**APOBEC3C**	protein_coding	apolipoprotein B mRNA editing enzyme catalytic subunit 3C [Source:HGNC Symbol;Acc:HGNC:17353]	chr22	39014083	39020352	1	ensembl_havana	5.370760499	5.475808107	5.265712891	5.435058627	5.538045901	5.454319794	5.294320263	5.255778132	5.247040279
ENSG00000139350	0.020261	0.069906844	0.243770541	1.18408327	**NEDD1**	protein_coding	neural precursor cell expressed, developmentally down-regulated 1 [Source:HGNC Symbol;Acc:HGNC:7723]	chr12	96907223	96953777	1	ensembl_havana	5.941027586	5.819142316	6.062912856	5.669359395	5.835572315	5.952495237	5.798071534	6.189431314	6.201235721
ENSG00000008735	0.020285	0.069971157	0.330357303	1.25732473	**MAPK8IP2**	protein_coding	mitogen-activated protein kinase 8 interacting protein 2 [Source:HGNC Symbol;Acc:HGNC:6883]	chr22	50600685	50613981	1	ensembl_havana	3.028349868	2.863171216	3.193528519	2.897741056	2.759446068	2.932326526	3.32252843	3.218605774	3.039451355
ENSG00000135241	0.020301	0.07000936	0.236792829	1.178370182	**PNPLA8**	protein_coding	patatin like phospholipase domain containing 8 [Source:HGNC Symbol;Acc:HGNC:28900]	chr7	108470422	108569666	-1	ensembl_havana	5.308894037	5.190497622	5.427290451	5.246708152	5.316739792	5.008044923	5.462601683	5.460475394	5.358794276
ENSG00000175592	0.020308	0.070017923	-0.785950272	-1.724227657	**FOSL1**	protein_coding	FOS like 1, AP-1 transcription factor subunit [Source:HGNC Symbol;Acc:HGNC:13718]	chr11	65892049	65900573	-1	ensembl_havana	-0.294867755	0.098107381	-0.687842891	0.986893817	-0.106664058	-0.585907617	-0.40973604	-0.47099802	-1.182794618
ENSG00000205085	0.02034	0.070090129	0.214875894	1.16060407	**FAM71F2**	protein_coding	family with sequence similarity 71 member F2 [Source:HGNC Symbol;Acc:HGNC:27998]	chr7	128672288	128687872	1	ensembl_havana	2.571517986	2.464080039	2.678955933	2.52259249	2.440056551	2.429591076	2.693950367	2.608318707	2.734598724
ENSG00000213366	0.020335	0.070090129	-0.188446136	-1.139535711	**GSTM2**	protein_coding	glutathione S-transferase mu 2 [Source:HGNC Symbol;Acc:HGNC:4634]	chr1	109668022	109709551	1	ensembl_havana	3.820323995	3.914547063	3.726100927	3.938508019	3.726829807	4.078303364	3.732784509	3.569569297	3.875948975
ENSG00000038382	0.020344	0.070090129	-0.385120351	-1.305968728	**TRIO**	protein_coding	trio Rho guanine nucleotide exchange factor [Source:HGNC Symbol;Acc:HGNC:12303]	chr5	14143702	14532128	1	ensembl_havana	9.189816783	9.382376959	8.997256608	9.786000765	9.100905481	9.260224631	9.124107302	8.850121779	9.017540742
ENSG00000135966	0.02035	0.070093855	0.226267189	1.169804293	**TGFBRAP1**	protein_coding	transforming growth factor beta receptor associated protein 1 [Source:HGNC Symbol;Acc:HGNC:16836]	chr2	105264391	105330034	-1	ensembl_havana	6.026812607	5.913679012	6.139946201	5.865913899	5.843166825	6.031956312	6.162597074	6.104303377	6.152938154
ENSG00000168028	0.020365	0.070102803	0.264770319	1.201444761	**RPSA**	protein_coding	ribosomal protein SA [Source:HGNC Symbol;Acc:HGNC:6502]	chr3	39406689	39412542	1	ensembl_havana	9.667049123	9.534663963	9.799434283	9.152220056	9.705289776	9.746482058	9.571163446	9.911106237	9.916033165
ENSG00000257218	0.020366	0.070102803	0.223564101	1.167614556	**GATC**	protein_coding	glutamyl-tRNA amidotransferase subunit C [Source:HGNC Symbol;Acc:HGNC:25068]	chr12	120446438	120463749	1	ensembl_havana	4.967716941	4.85593489	5.079498991	4.889298954	4.923650443	4.754855274	5.056627338	5.100113754	5.081755882
ENSG00000241990	0.020367	0.070102803	-0.353037275	-1.277246759	**PRR34-AS1**	antisense	PRR34 antisense RNA 1 [Source:HGNC Symbol;Acc:HGNC:50499]	chr22	46053705	46057210	1	havana	1.192629352	1.369147989	1.016110714	1.683274163	1.270574017	1.153595787	1.125834805	0.900298706	1.022198632
ENSG00000100246	0.020385	0.070147221	0.190483707	1.141146256	**DNAL4**	protein_coding	dynein axonemal light chain 4 [Source:HGNC Symbol;Acc:HGNC:2955]	chr22	38778508	38794198	-1	ensembl_havana	3.322174403	3.22693255	3.417416257	3.245688341	3.241966183	3.193143125	3.423901694	3.406428015	3.421919061
ENSG00000205976	0.02039	0.070147221	-1.097241603	-2.139452432	**AC091951.1**	unprocessed_pseudogene		chr5	7299774	7306592	-1	havana	-1.059860701	-0.511239899	-1.608481502	0.206373087	-0.299013651	-1.441079133	-0.89704638	-2.16071837	-1.767679756

ENSG00000234741	0.020398	0.070159194	0.217847896	1.16299742	**GAS5**	processed_transcript	growth arrest specific 5 (non-protein coding) [Source:HGNC Symbol;Acc:HGNC:16355]	chr1	173863900	173868882	-1	havana	8.437669952	8.328746004	8.5465939	8.150499539	8.47623375	8.359504723	8.387625547	8.603798096	8.648358057
ENSG00000003756	0.020432	0.070242378	0.198346932	1.147382908	**RBM5**	protein_coding	RNA binding motif protein 5 [Source:HGNC Symbol;Acc:HGNC:9902]	chr3	50088908	50119021	1	ensembl_havana	7.831086903	7.731913437	7.930260369	7.727069909	7.765948713	7.702721688	7.977108762	7.911813029	7.901859316
ENSG00000168899	0.020431	0.070242378	-0.409639494	-1.328353839	**VAMP5**	protein_coding	vesicle associated membrane protein 5 [Source:HGNC Symbol;Acc:HGNC:12646]	chr2	85584408	85593412	1	ensembl_havana	4.393877685	4.598697432	4.189057938	4.52270135	4.73054201	4.542848935	4.366751342	4.055441139	4.144981331
ENSG00000151117	0.020449	0.070262014	0.423487723	1.341165915	**TMEM86A**	protein_coding	transmembrane protein 86A [Source:HGNC Symbol;Acc:HGNC:26890]	chr11	18693122	18704785	1	ensembl_havana	2.227275205	2.015531344	2.439019067	2.183196095	1.900769223	1.962628713	2.302589091	2.413134925	2.601333184
ENSG00000183255	0.020457	0.070262014	0.220116483	1.16482763	**PTTG1IP**	protein_coding	PTTG1 interacting protein [Source:HGNC Symbol;Acc:HGNC:13524]	chr21	44849585	44873903	-1	ensembl_havana	6.119364183	6.009305941	6.229422424	5.956150198	6.086543283	5.985224343	6.274898961	6.252020808	6.161347503
ENSG00000150764	0.020455	0.070262014	-0.210674462	-1.157229064	**DIXDC1**	protein_coding	DIX domain containing 1 [Source:HGNC Symbol;Acc:HGNC:23695]	chr11	111927144	112022584	1	ensembl_havana	2.878471861	2.983809092	2.77313463	2.802355359	2.970462332	3.178609585	2.619920974	2.677309452	3.022173463
ENSG00000148335	0.020444	0.070262014	-0.248651882	-1.188096386	**NTMT1**	protein_coding	N-terminal Xaa-Pro-Lys N-methyltransferase 1 [Source:HGNC Symbol;Acc:HGNC:23373]	chr9	129608884	129636131	1	ensembl_havana	5.241411003	5.365736944	5.117085062	5.259996428	5.351564007	5.485650398	5.121778643	5.11780318	5.111673363
ENSG00000143669	0.020467	0.070278469	0.237677105	1.179092665	**LYST**	protein_coding	lysosomal trafficking regulator [Source:HGNC Symbol;Acc:HGNC:1968]	chr1	235661041	235883640	-1	ensembl_havana	7.806770638	7.687932086	7.92560919	7.866855499	7.538374796	7.658565962	8.011293437	7.892447674	7.87308646
ENSG00000169718	0.0205	0.070342069	0.426004171	1.34350731	**DUS1L**	protein_coding	dihydrouridine synthase 1 like [Source:HGNC Symbol;Acc:HGNC:30086]	chr17	82057506	82065887	-1	ensembl_havana	6.004728963	5.791726878	6.217731049	5.572259566	5.7523884	6.050532668	6.224271254	6.255785199	6.173136693
ENSG00000110917	0.020496	0.070342069	-0.205332475	-1.152952019	**MLEC**	protein_coding	malectin [Source:HGNC Symbol;Acc:HGNC:28973]	chr12	120686869	120701864	1	ensembl_havana	8.335106908	8.437773145	8.232440671	8.535085457	8.347626167	8.430607811	8.255583069	8.186358256	8.255380687
ENSG00000161940	0.020493	0.070342069	-0.957620456	-1.942103995	**BCL6B**	protein_coding	B cell CLL/lymphoma 6B [Source:HGNC Symbol;Acc:HGNC:1002]	chr17	7023020	7030290	1	ensembl_havana	-0.813854307	-0.335044079	-1.292664535	-0.14746469	0.125688576	-0.983356126	-0.46237776	-1.52242631	-1.89318954
ENSG00000171428	0.020521	0.070362344	0.291764938	1.224136924	**NAT1**	protein_coding	N-acetyltransferase 1 [Source:HGNC Symbol;Acc:HGNC:7645]	chr8	18170477	18223689	1	ensembl_havana	3.611576371	3.465693902	3.757458839	3.497241177	3.656952914	3.242887613	3.753128688	3.808721901	3.71052593
ENSG00000242697	0.020515	0.070362344	-0.277470486	-1.212067871	**RPL5P12**	processed_pseudogene	ribosomal protein L5 pseudogene 12 [Source:HGNC Symbol;Acc:HGNC:36221]	chr4	98025390	98026240	1	havana	1.597268531	1.736003774	1.458533288	1.671956805	1.62524309	1.910811426	1.229040238	1.465989718	1.680569906
ENSG00000240137	0.020518	0.070362344	-0.39185102	-1.312075756	**ERICH6-AS1**	antisense	ERICH6 antisense RNA 1 [Source:HGNC Symbol;Acc:HGNC:41205]	chr3	150703564	150720146	1	havana	-0.133788789	0.062136721	-0.329714299	0.10902545	0.030651399	0.046733314	-0.46237776	-0.08087422	-0.445890917
ENSG00000196428	0.020545	0.070416049	0.199037489	1.147932243	**TSC22D2**	protein_coding	TSC22 domain family member 2 [Source:HGNC Symbol;Acc:HGNC:29095]	chr3	150408335	150466431	1	ensembl_havana	5.771650419	5.672131675	5.871169164	5.67857803	5.7523884	5.585428595	5.939200191	5.929148306	5.745158995
ENSG00000213676	0.020546	0.070416049	0.194622054	1.144424314	**ATF6B**	protein_coding	activating transcription factor 6 beta [Source:HGNC Symbol;Acc:HGNC:2349]	chr6	32115264	32128253	-1	ensembl_havana	7.461900763	7.364589736	7.55921179	7.44528804	7.317332946	7.331148222	7.651582111	7.46211476	7.5639385
ENSG00000101639	0.02056	0.070445345	-0.185127166	-1.136917182	**CEP192**	protein_coding	centrosomal protein 192 [Source:HGNC Symbol;Acc:HGNC:25515]	chr18	12991362	13125052	1	ensembl_havana	6.287870335	6.380433918	6.195306752	6.419503143	6.315036213	6.406762399	6.22790995	6.119361416	6.238648891
ENSG00000228672	0.020587	0.070492618	0.286809122	1.219939094	**PROB1**	protein_coding	proline rich basic protein 1 [Source:HGNC Symbol;Acc:HGNC:41906]	chr5	139390592	139395713	-1	ensembl_havana	3.718781771	3.57537721	3.862186332	3.730594929	3.439879526	3.555657175	4.006555239	3.88847872	3.691525037
ENSG00000197713	0.020583	0.070492618	-0.197802468	-1.146949975	**RPE**	protein_coding	ribulose-5-phosphate-3-epimerase [Source:HGNC Symbol;Acc:HGNC:10293]	chr2	210002565	210022259	1	ensembl_havana	5.437520877	5.536422111	5.338619643	5.494890045	5.521392753	5.592983536	5.243868028	5.326391895	5.445599007
ENSG00000143106	0.020593	0.070492618	-0.217461713	-1.162686148	**PSMA5**	protein_coding	proteasome subunit alpha 5 [Source:HGNC Symbol;Acc:HGNC:9534]	chr1	109399031	109426427	-1	ensembl_havana	7.944520857	8.053251713	7.83579	8.101251494	8.1351607	7.923342946	7.884307252	7.8347876	7.788275149
ENSG00000136932	0.020593	0.070492618	-0.321764704	-1.24985844	**TRMO**	protein_coding	tRNA methyltransferase O [Source:HGNC Symbol;Acc:HGNC:30967]	chr9	97904489	97922570	-1	ensembl_havana	3.243502085	3.404384437	3.082619733	3.458245022	3.512020676	3.242887613	2.931801189	3.230132489	3.085925522
ENSG00000260260	0.020599	0.070496107	0.345870528	1.270917628	**SNHG19**	lincRNA	small nucleolar RNA host gene 19 [Source:HGNC Symbol;Acc:HGNC:49574]	chr16	2154797	2155358	-1	havana	1.448614446	1.275679183	1.62154971	1.237769075	1.228369434	1.360899039	1.681320805	1.715999071	1.467329254
ENSG00000119640	0.020638	0.070611503	0.271651403	1.207188865	**ACYP1**	protein_coding	acylphosphatase 1 [Source:HGNC Symbol;Acc:HGNC:179]	chr14	75053221	75069483	-1	ensembl_havana	3.971152983	3.835327281	4.106978685	3.689093732	3.886880247	3.930007865	4.084314488	4.002652514	4.233969052
ENSG00000106571	0.020674	0.070711709	0.525096348	1.43902968	**GLI3**	protein_coding	GLI family zinc finger 3 [Source:HGNC Symbol;Acc:HGNC:4319]	chr7	41960950	42237870	-1	ensembl_havana	3.9357524	3.673204226	4.198300574	3.10550171	3.859488969	4.054621999	3.982139812	4.382549869	4.230212041
ENSG00000119950	0.020699	0.070711709	0.434897905	1.351815166	**MXI1**	protein_coding	MAX interactor 1, dimerization protein [Source:HGNC Symbol;Acc:HGNC:7534]	chr10	110207605	110287365	1	ensembl_havana	4.249954234	4.032505281	4.467403186	3.843347077	4.264666003	3.989502764	4.482676951	4.379102505	4.540430102
ENSG00000162636	0.020695	0.070711709	0.210848898	1.157368992	**FAM102B**	protein_coding	family with sequence similarity 102 member B [Source:HGNC Symbol;Acc:HGNC:27637]	chr1	108560089	108644900	1	ensembl_havana	7.028494061	6.923069612	7.13391851	7.116069735	6.9230518	6.7300873	7.250086093	7.140134985	7.011534451
ENSG00000151883	0.020699	0.070711709	0.189936109	1.140713197	**PARP8**	protein_coding	poly(ADP-ribose) polymerase family member 8 [Source:HGNC Symbol;Acc:HGNC:26124]	chr5	50665899	50846522	1	ensembl_havana	4.534489843	4.439521789	4.629457898	4.255242815	4.470291242	4.59303131	4.475698201	4.667515227	4.745160266
ENSG00000196230	0.020684	0.070711709	-0.201375375	-1.149793974	**TUBB**	protein_coding	tubulin beta class I [Source:HGNC Symbol;Acc:HGNC:20778]	chr6	30720201	30725426	1	ensembl_havana	11.35246307	11.45315076	11.25177539	11.30328065	11.5030406	11.55313103	11.16162615	11.30004376	11.29365625
ENSG00000101104	0.020701	0.070711709	-0.244064508	-1.184324566	**PABPC1L**	protein_coding	poly(A) binding protein cytoplasmic 1 like [Source:HGNC Symbol;Acc:HGNC:15797]	chr20	44910062	44959035	1	ensembl_havana	5.863789353	5.985821607	5.741757098	5.870871668	5.90866304	6.177930112	5.727119434	5.689263261	5.808888601
ENSG00000089558	0.020698	0.070711709	-0.420566498	-1.338453017	**KCNH4**	protein_coding	potassium voltage-gated channel subfamily H member 4 [Source:HGNC Symbol;Acc:HGNC:6253]	chr17	42156891	42181278	-1	ensembl_havana	1.128301602	1.338584851	0.918018353	1.268150092	1.228369434	1.519235028	0.873137292	1.063828438	0.817089329
ENSG00000156127	0.020753	0.070870168	-0.250505527	-1.189623892	**BATF**	protein_coding	basic leucine zipper ATF-like transcription factor [Source:HGNC Symbol;Acc:HGNC:958]	chr14	75522425	75547015	1	ensembl_havana	5.306296191	5.431548955	5.181043428	5.591158349	5.372708048	5.330780467	5.452862136	5.139359554	4.950908594
ENSG00000165209	0.020759	0.070875192	0.198817228	1.147756997	**STRBP**	protein_coding	spermatid perinuclear RNA binding protein [Source:HGNC Symbol;Acc:HGNC:16462]	chr9	123109500	123268576	-1	ensembl_havana	6.304432518	6.205023904	6.403841131	6.038872258	6.231141432	6.345058022	6.285912301	6.370467466	6.555143628
ENSG00000165943	0.020774	0.070908691	0.196297326	1.145754003	**MOAP1**	protein_coding	modulator of apoptosis 1 [Source:HGNC Symbol;Acc:HGNC:16658]	chr14	93182196	93184928	-1	ensembl_havana	5.228122063	5.129973399	5.326270726	5.151115925	5.142092393	5.096711881	5.404990956	5.312904338	5.260916884
ENSG00000089195	0.020789	0.070935066	-0.189757491	-1.140571976	**TRMT6**	protein_coding	tRNA methyltransferase 6 [Source:HGNC Symbol;Acc:HGNC:20900]	chr20	5937235	5950558	-1	ensembl_havana	5.609868428	5.704747174	5.514989683	5.743538634	5.674629152	5.696073735	5.51374772	5.500687226	5.530534103
ENSG00000113194	0.020791	0.070935066	-0.197451393	-1.146670902	**FAF2**	protein_coding	Fas associated factor family member 2 [Source:HGNC Symbol;Acc:HGNC:24666]	chr5	176447628	176510074	1	ensembl_havana	7.181051022	7.279776719	7.082325326	7.297087822	7.290876492	7.251365842	7.039358418	7.119881749	7.08773581
ENSG00000139364	0.020803	0.07094115	0.697785784	1.622013451	**TMEM132B**	protein_coding	transmembrane protein 132B [Source:HGNC Symbol;Acc:HGNC:29397]	chr12	125186836	125662377	1	ensembl_havana	0.087685816	-0.261207076	0.436578708	-1.31854494	-0.106664058	0.641587769	-0.57379957	1.046547334	0.836988357
ENSG00000070371	0.020802	0.07094115	0.287484415	1.220510253	**CLTCL1**	protein_coding	clathrin heavy chain like 1 [Source:HGNC Symbol;Acc:HGNC:2093]	chr22	19179473	19291716	-1	ensembl_havana	4.630964083	4.487221875	4.77470629	4.301877063	4.468781447	4.691007115	4.721163453	4.799744258	4.803211159
ENSG00000159873	0.020816	0.070951621	-0.19126815	-1.141766904	**CCDC117**	protein_coding	coiled-coil domain containing 117 [Source:HGNC Symbol;Acc:HGNC:26599]	chr22	28772674	28789301	1	ensembl_havana	5.479897751	5.575531826	5.384263677	5.604613794	5.634119209	5.487862476	5.383712004	5.487140582	5.281938444
ENSG00000177990	0.020816	0.070951621	-0.248457861	-1.187936616	**DPY19L2**	protein_coding	dpy-19 like 2 [Source:HGNC Symbol;Acc:HGNC:19414]	chr12	63558913	63668939	-1	ensembl_havana	2.541173271	2.665402202	2.41694434	2.547577338	2.546946467	2.901682799	2.20469138	2.433220573	2.612921068
ENSG00000122547	0.020876	0.071137568	0.207816539	1.154938907	**EEPD1**	protein_coding	endonuclease/exonuclease/phosphatase family domain containing 1 [Source:HGNC Symbol;Acc:HGNC:22223]	chr7	36153149	36301543	1	ensembl_havana	6.180130515	6.076222245	6.284038784	5.968344626	6.007639676	6.252682433	6.190037746	6.277704805	6.384373801
ENSG00000285280	0.020896	0.071189808	-0.422420077	-1.340173771	**AL390957.1**	lincRNA		chr1	192167786	192957713	-1	havana	1.223158345	1.434368384	1.011948307	1.82284946	1.402954459	1.077301233	1.245545533	0.821210834	0.969088554
ENSG00000104408	0.020905	0.071204194	-0.309499898	-1.239278037	**EIF3E**	protein_coding	eukaryotic translation initiation factor 3 subunit E [Source:HGNC Symbol;Acc:HGNC:3277]	chr8	108201216	108435333	-1	ensembl_havana	7.844818647	7.999568596	7.690068699	8.122712432	7.951969545	7.924023812	7.611155247	7.778329807	7.680721042
ENSG00000177614	0.020915	0.071221945	0.257277024	1.195220687	**PGBD5**	protein_coding	piggyBac transposable element derived 5 [Source:HGNC Symbol;Acc:HGNC:19405]	chr1	230314482	230426371	-1	ensembl_havana	4.059246744	3.930608232	4.187885256	3.677822113	4.012400306	4.101602276	4.084314488	4.199216933	4.280124347
ENSG00000121101	0.020928	0.071250105	-0.415311154	-1.333586268	**TEX14**	protein_coding	testis expressed 14, intercellular bridge forming factor [Source:HGNC Symbol;Acc:HGNC:11737]	chr17	58556678	58692055	-1	ensembl_havana	1.224800453	1.43245603	1.017144876	1.637462096	1.199531136	1.460374858	0.915012722	0.919411336	1.21701057
ENSG00000148730	0.02099	0.071435074	0.191855257	1.142231643	**EIF4EBP2**	protein_coding	eukaryotic translation initiation factor 4E binding protein 2 [Source:HGNC Symbol;Acc:HGNC:3289]	chr10	70404379	70428618	1	ensembl_havana	7.175331137	7.079403508	7.271258765	7.058295403	7.048879938	7.131035183	7.206489838	7.280020673	7.327265784
ENSG00000259462	0.020993	0.071435074	0.18698125	1.138379234	**CPEB1-AS1**	antisense	CPEB1 antisense RNA 1 [Source:HGNC Symbol;Acc:HGNC:27523]	chr15	82647770	82692820	1	havana	0.070124461	-0.023366164	0.163615086	-0.36334239	0.214850257	0.078393642	-0.17198403	0.37179175	0.291037539
ENSG00000140284	0.021004	0.071457935	-0.31094505	-1.240520048	**SLC27A2**	protein_coding	solute carrier family 27 member 2 [Source:HGNC Symbol;Acc:HGNC:10996]	chr15	50182196	50236395	1	ensembl_havana	1.63127116	1.786743685	1.475798634	1.671956805	1.476918714	2.211355536	1.161063346	1.315408538	1.950924019
ENSG00000169991	0.021039	0.071542451	0.234823011	1.176762364	**IFFO2**	protein_coding	intermediate filament family orphan 2 [Source:HGNC Symbol;Acc:HGNC:27006]	chr1	18904281	18956686	-1	ensembl_havana	5.834861869	5.717450363	5.952273374	5.833901194	5.59520917	5.723240726	6.13683798	5.883648474	5.836333669
ENSG00000138778	0.021039	0.071542451	-0.209615802	-1.156380193	**CENPE**	protein_coding	centromere protein E [Source:HGNC Symbol;Acc:HGNC:1856]	chr4	103105806	103198409	-1	ensembl_havana	6.464937747	6.569745648	6.360129846	6.44715164	6.567948439	6.694136866	6.16585261	6.433729774	6.480807154
ENSG00000253540	0.021064	0.071609555	0.427702459	1.345089769	**FAM86HP**	transcribed_unprocessed_pseudogene	family with sequence similarity 86 member H, pseudogene [Source:HGNC Symbol;Acc:HGNC:42359]	chr3	130099092	130111472	-1	havana	2.062081487	1.848230258	2.275932717	1.424690178	1.798708764	2.321291832	2.062883947	2.329873398	2.435040805
ENSG00000144827	0.021072	0.07161825	-0.201376351	-1.149794752	**ABHD10**	protein_coding	abhydrolase domain containing 10 [Source:HGNC Symbol;Acc:HGNC:25656]	chr3	111979010	111993363	1	ensembl_havana	5.976474824	6.077163	5.875786649	6.047655893	6.09928431	6.084548796	5.828519357	5.847933099	5.950907492
ENSG00000243789	0.021096	0.07165899	0.395492632	1.315391845	**JMJD7**	protein_coding	jumonji domain containing 7 [Source:HGNC Symbol;Acc:HGNC:34397]	chr15	41828085	41837581	1	ensembl_havana	0.161162792	-0.036583524	0.358909108	0.10902545	-0.473795405	0.255019383	0.24405081	0.163140205	0.669536307
ENSG00000166401	0.021123	0.07165899	0.220043054	1.164768346	**SERPINB8**	protein_coding	serpin family B member 8 [Source:HGNC Symbol;Acc:HGNC:8952]	chr18	63969925	64019779	1	ensembl_havana	5.825732738	5.715711211	5.935754265	6.100846813	5.639497244	5.406789577	6.267341369	5.811311061	5.728610365
ENSG00000134444	0.021119	0.07165899	0.201423735	1.149832517	**KIAA1468**	protein_coding	KIAA1468 [Source:HGNC Symbol;Acc:HGNC:29289]	chr18	62187255	62310217	1	ensembl_havana	6.212058457	6.111346589	6.312770325	6.142953663	6.057206645	6.13387946	6.305231685	6.329081058	6.30399823
ENSG00000166348	0.021118	0.07165899	0.194457067	1.144293444	**USP54**	protein_coding	ubiquitin specific peptidase 54 [Source:HGNC Symbol;Acc:HGNC:23513]	chr10	73497538	73625953	-1	ensembl_havana	5.106435175	5.009206642	5.203663708	5.016663433	4.97241098	5.038545512	5.263248291	5.157583505	5.190159329
ENSG00000104375	0.021122	0.07165899	-0.192130341	-1.142449457	**STK3**	protein_coding	serine/threonine kinase 3 [Source:HGNC Symbol;Acc:HGNC:11406]	chr8	98401403	98942827	-1	ensembl_havana	4.269947644	4.366012815	4.173882474	4.329150785	4.500159995	4.268727664	4.166788249	4.2585809	4.096278272
ENSG00000254635	0.021095	0.07165899	-0.21303502	-1.159124088	**WAC-AS1**	antisense	WAC antisense RNA 1 (head to head) [Source:HGNC Symbol;Acc:HGNC:27347]	chr10	28522652	28532743	-1	havana	4.432123467	4.538640976	4.325605957	4.359450248	4.555261413	4.701211268	4.151542442	4.418258389	4.407017038
ENSG00000131263	0.021105	0.07165899	-0.248632492	-1.188080418	**RLIM**	protein_coding	ring finger protein, LIM domain interacting [Source:HGNC Symbol;Acc:HGNC:13429]	chrX	74585217	74614617	-1	ensembl_havana	6.682088712	6.806404958	6.557772466	6.967772934	6.723224598	6.728217341	6.617293745	6.60840173	6.447621923
ENSG00000113643	0.021105	0.07165899	-0.292540445	-1.224795124	**RARS**	protein_coding	arginyl-tRNA synthetase [Source:HGNC Symbol;Acc:HGNC:9870]	chr5	168486445	168519299	1	ensembl_havana	7.387016527	7.53328675	7.240746305	7.553316008	7.624290913	7.422253328	7.135203261	7.271911048	7.315124605
ENSG00000167992	0.021136	0.071672998	0.473251764	1.388234958	**VWCE**	protein_coding	von Willebrand factor C and EGF domains [Source:HGNC Symbol;Acc:HGNC:26487]	chr11	61258286	61295424	-1	ensembl_havana	1.268059413	1.031433531	1.504685295	1.092533711	0.720251491	1.281515389	1.593788933	1.491756274	1.428510677
ENSG00000253304	0.021138	0.071672998	0.184684141	1.136568109	**TMEM200B**	protein_coding	transmembrane protein 200B [Source:HGNC Symbol;Acc:HGNC:33785]	chr1	29119428	29123935	-1	ensembl_havana	0.131564947	0.039222876	0.223907017	0.040274975	0.06303108	0.014362573	0.24405081	0.225896484	0.201773757
ENSG00000152315	0.021168	0.071746876	0.536648433	1.450598672	**KCNK13**	protein_coding	potassium two pore domain channel subfamily K member 13 [Source:HGNC Symbol;Acc:HGNC:6275]	chr14	90061765	90185857	1	ensembl_havana	2.112673655	1.844349439	2.380997872	1.355633303	2.124367304	2.053047709	2.302589091	2.385911525	2.454492999
ENSG00000165912	0.021169	0.071746876	-0.449515238	-1.365581328	**PACSIN3**	protein_coding	protein kinase C and casein kinase substrate in neurons 3 [Source:HGNC Symbol;Acc:HGNC:8572]	chr11	47177525	47186443	-1	ensembl_havana	1.187462203	1.412219821	0.962704584	1.341416217	1.547275176	1.347968071	1.089724456	0.758913071	1.039476224
ENSG00000105939	0.021191	0.07180342	-0.183744903	-1.13582841	**ZC3HAV1**	protein_coding	zinc finger CCCH-type containing, antiviral 1 [Source:HGNC Symbol;Acc:HGNC:23721]	chr7	139043520	139109719	-1	ensembl_havana	7.36069688	7.452569332	7.268824429	7.48967601	7.454091619	7.413940366	7.324560657	7.264456429	7.217456201
ENSG00000235621	0.021206	0.071829625	0.529833711	1.443762774	**LINC00494**	lincRNA	long intergenic non-protein coding RNA 494 [Source:HGNC Symbol;Acc:HGNC:27657]	chr20	48359950	48370629	1	ensembl_havana	0.901357956	0.6364411	1.166274811	0.040274975	1.029710695	0.83933763	0.785553575	1.14725896	1.566011899
ENSG00000198105	0.021209	0.071829625	0.21009656	1.156765604	**ZNF248**	protein_coding	zinc finger protein 248 [Source:HGNC Symbol;Acc:HGNC:13041]	chr10	37776526	37858106	-1	ensembl_havana	4.496813298	4.391765018	4.601861578	4.155162249	4.500159995	4.519972811	4.442080167	4.706547606	4.656956961
ENSG00000187792	0.021231	0.071856074	0.209514792	1.156299231	**ZNF70**	protein_coding	zinc finger protein 70 [Source:HGNC Symbol;Acc:HGNC:13140]	chr22	23738678	23751092	-1	ensembl_havana	3.374768857	3.270011461	3.479526253	3.092827025	3.266504942	3.450702416	3.383457449	3.459622798	3.595498512
ENSG00000181690	0.021223	0.071856074	0.201267035	1.149707633	**PLAG1**	protein_coding	PLAG1 zinc finger [Source:HGNC Symbol;Acc:HGNC:9045]	chr8	56160904	56211324	-1	ensembl_havana	4.96165426	4.861020742	5.062287778	4.888076825	4.92915038	4.765835022	5.124012075	5.166609875	4.896241383
ENSG00000198298	0.021234	0.071856074	-0.393941575	-1.313978413	**ZNF485**	protein_coding	zinc finger protein 485 [Source:HGNC Symbol;Acc:HGNC:23440]	chr10	43606407	43617903	1	ensembl_havana	2.301571625	2.498542413	2.104600838	2.327223844	2.396251236	2.772152159	1.926226713	2.256618822	2.13095698
ENSG00000271447	0.021237	0.071856074	-0.400883556	-1.32031627	**MMP28**	protein_coding	matrix metallopeptidase 28 [Source:HGNC Symbol;Acc:HGNC:14366]	chr17	35756249	35795707	-1	ensembl_havana	1.263592887	1.464034665	1.063151109	0.986893817	1.476918714	1.928291464	0.428834256	1.372000412	1.38861866
ENSG00000227051	0.021249	0.071864322	0.242270823	1.182853025	**C14orf132**	protein_coding	chromosome 14 open reading frame 132 [Source:HGNC Symbol;Acc:HGNC:20346]	chr14	96039324	96093889	1	ensembl_havana	4.985473331	4.864337919	5.106608742	4.721049177	4.962789824	4.909174756	5.088999215	5.178557852	5.052269159
ENSG00000231584	0.021245	0.071864322	0.211244957	1.157686765	**FAHD2CP**	transcribed_unprocessed_pseudogene	fumarylacetoacetate hydrolase domain containing 2C, pseudogene [Source:HGNC Symbol;Acc:HGNC:44135]	chr2	96010551	96023380	1	havana	3.896893225	3.791270747	4.002515703	3.905065295	3.77893109	3.689815855	4.063519655	3.954822853	3.989204602
ENSG00000218283	0.02126	0.071883062	-0.283007453	-1.216728644	**MORF4L1P1**	processed_pseudogene	mortality factor 4 like 1 pseudogene 1 [Source:HGNC Symbol;Acc:HGNC:20400]	chr1	220253570	220254536	1	havana	0.616758869	0.758262596	0.475255143	0.872903038	0.760296981	0.641587769	0.457455004	0.648704825	0.319605599
ENSG00000154640	0.021273	0.07189373	-0.361996135	-1.285202894	**BTG3**	protein_coding	BTG anti-proliferation factor 3 [Source:HGNC Symbol;Acc:HGNC:1132]	chr21	17593653	17612947	-1	ensembl_havana	4.535124012	4.71612208	4.354125945	4.772291565	4.87204229	4.504032384	4.487888949	4.256703576	4.317785309
ENSG00000183960	0.021268	0.07189373	-0.69472619	-1.618577213	**KCNH8**	protein_coding	potassium voltage-gated channel subfamily H member 8 [Source:HGNC Symbol;Acc:HGNC:18864]	chr3	19148454	19535646	1	ensembl_havana	1.074839453	1.422202548	0.727476358	1.649052215	1.37743234	1.24012309	0.785553575	1.226127349	0.17074815
ENSG00000172006	0.021282	0.071909255	0.229012818	1.172032697	**ZNF554**	protein_coding	zinc finger protein 554 [Source:HGNC Symbol;Acc:HGNC:26629]	chr19	2819874	2835773	1	ensembl_havana	2.59815756	2.483651151	2.712663969	2.305416943	2.494491223	2.651045288	2.587917939	2.608318707	2.941755261
ENSG00000213997	0.021289	0.071915488	0.326116654	1.253634384	**PGAM1P7**	processed_pseudogene	phosphoglycerate mutase 1 pseudogene 7 [Source:HGNC Symbol;Acc:HGNC:42454]	chrX	46646594	46647330	-1	havana	3.10950029	2.946441963	3.272558617	2.828074641	2.873380708	3.137870538	3.258912309	3.319173646	3.239589895
ENSG00000170906	0.021311	0.071972789	0.265209278	1.201810372	**NDUFA3**	protein_coding	NADH:ubiquinone oxidoreductase subunit A3 [Source:HGNC Symbol;Acc:HGNC:7686]	chr19	54102728	54109257	1	ensembl_havana	4.256361246	4.123756607	4.388965885	4.298201297	4.075263288	3.997805235	4.420436873	4.349462476	4.396998304
ENSG00000136935	0.021359	0.07211897	-0.187038882	-1.13842471	**GOLGA1**	protein_coding	golgin A1 [Source:HGNC Symbol;Acc:HGNC:4424]	chr9	124878357	124948492	-1	ensembl_havana	5.667220519	5.76073996	5.573701078	5.833266338	5.781869063	5.667084478	5.643183379	5.56505945	5.512860405
ENSG00000135506	0.021371	0.072130161	0.236092697	1.177798464	**OS9**	protein_coding	OS9, endoplasmic reticulum lectin [Source:HGNC Symbol;Acc:HGNC:16994]	chr12	57693955	57721557	1	ensembl_havana	8.690904301	8.572857953	8.80895065	8.642356314	8.576019509	8.500198034	8.845591113	8.723996442	8.857264394
ENSG00000175104	0.021373	0.072130161	0.193624496	1.14363327	**TRAF6**	protein_coding	TNF receptor associated factor 6 [Source:HGNC Symbol;Acc:HGNC:12036]	chr11	36487027	36510272	-1	ensembl_havana	5.964568859	5.867756611	6.061381107	6.092912564	5.878846762	5.631510506	6.333492935	6.074240483	5.776409902
ENSG00000256087	0.021416	0.072257095	0.223371705	1.167458855	**ZNF432**	protein_coding	zinc finger protein 432 [Source:HGNC Symbol;Acc:HGNC:20810]	chr19	52031379	52095738	-1	ensembl_havana	3.306417829	3.194731977	3.418103682	3.210920778	3.224179389	3.149095762	3.394600617	3.554366408	3.305344019
ENSG00000040341	0.021421	0.072257095	-0.258966391	-1.196621086	**STAU2**	protein_coding	staufen double-stranded RNA binding protein 2 [Source:HGNC Symbol;Acc:HGNC:11371]	chr8	73420369	73747708	-1	ensembl_havana	5.803986224	5.93346942	5.674503029	6.027816972	5.936237721	5.836353567	5.67856212	5.615005703	5.729941264
ENSG00000198727	0.021474	0.072419233	0.194761983	1.144535319	**MT-CYB**	protein_coding	mitochondrially encoded cytochrome b [Source:HGNC Symbol;Acc:HGNC:7427]	chrMT	14747	15887	1	insdc	12.77849032	12.68110933	12.87587131	12.66105403	12.63053519	12.75173876	12.79974551	12.8680592	12.95980922
ENSG00000165475	0.021485	0.072441707	0.231814977	1.174311358	**CRYL1**	protein_coding	crystallin lambda 1 [Source:HGNC Symbol;Acc:HGNC:18246]	chr13	20403666	20525873	-1	ensembl_havana	3.568285093	3.452377604	3.684192581	3.218719635	3.543917077	3.594496101	3.463242983	3.666080221	3.92325454
ENSG00000187866	0.021493	0.072442619	-0.213116972	-1.159189934	**FAM122A**	protein_coding	family with sequence similarity 122A [Source:HGNC Symbol;Acc:HGNC:23490]	chr9	68780034	68784608	1	havana	4.784584002	4.891142489	4.678025516	4.954969984	4.988305018	4.730152463	4.682286894	4.743211525	4.60857813
ENSG00000118308	0.021501	0.072442619	-0.243770388	-1.184083144	**LRMP**	protein_coding	lymphoid restricted membrane protein [Source:HGNC Symbol;Acc:HGNC:6690]	chr12	25004342	25108334	1	ensembl_havana	8.047379981	8.169265175	7.925494787	8.394768137	8.081097508	8.031929881	8.057450988	7.799277665	7.919755708
ENSG00000129055	0.021499	0.072442619	-0.293508721	-1.225617432	**ANAPC13**	protein_coding	anaphase promoting complex subunit 13 [Source:HGNC Symbol;Acc:HGNC:24540]	chr3	134477706	134486716	-1	ensembl_havana	5.338447902	5.485202262	5.191693541	5.455004303	5.646191719	5.354410766	5.091283933	5.233042912	5.250753777
ENSG00000141971	0.021515	0.072454395	0.349234789	1.273884775	**MVB12A**	protein_coding	multivesicular body subunit 12A [Source:HGNC Symbol;Acc:HGNC:25153]	chr19	17405722	17433724	1	ensembl_havana	3.726317801	3.551700406	3.900935195	3.569159951	3.464329594	3.621611673	4.138345138	3.803587612	3.760872836
ENSG00000171100	0.021524	0.072454395	0.296910037	1.228510371	**MTM1**	protein_coding	myotubularin 1 [Source:HGNC Symbol;Acc:HGNC:7448]	chrX	150568619	150673322	1	ensembl_havana	4.405568626	4.257113608	4.554023645	4.359450248	4.26292479	4.148965785	4.461638601	4.640414466	4.560017867
ENSG00000166260	0.021521	0.072454395	0.184408419	1.136350914	**COX11**	protein_coding	COX11, cytochrome c oxidase copper chaperone [Source:HGNC Symbol;Acc:HGNC:2261]	chr17	54951902	54968785	-1	ensembl_havana	7.712066403	7.619862194	7.804270613	7.657585739	7.590674759	7.611326083	7.868242932	7.768482004	7.776086904
ENSG00000257086	0.02151	0.072454395	-0.414197205	-1.332556963	**AP001453.4**	lincRNA		chr11	64246939	64249494	-1	havana	0.574132185	0.781230788	0.367033583	1.158893785	0.501432867	0.683365711	0.56657698	-0.04338487	0.577908642
ENSG00000197971	0.021534	0.072470197	0.197890269	1.147019779	**MBP**	protein_coding	myelin basic protein [Source:HGNC Symbol;Acc:HGNC:6925]	chr18	76978827	77133683	-1	ensembl_havana	8.381789865	8.28284473	8.480735	8.542872265	8.197220128	8.108441798	8.680425837	8.402556261	8.3592229
ENSG00000136156	0.021581	0.072611945	0.240827092	1.181669915	**ITM2B**	protein_coding	integral membrane protein 2B [Source:HGNC Symbol;Acc:HGNC:6174]	chr13	48233158	48270357	1	ensembl_havana	5.995111408	5.874697862	6.115524954	5.907521626	5.965438064	5.751133897	6.098168166	6.125546647	6.122860051
ENSG00000142178	0.021605	0.072640097	0.352297334	1.276591843	**SIK1**	protein_coding	salt inducible kinase 1 [Source:HGNC Symbol;Acc:HGNC:11142]	chr21	43414483	43427189	-1	ensembl_havana	1.653917652	1.477768985	1.830066319	1.760103226	1.351450576	1.321753151	1.91595172	1.938414976	1.635832261
ENSG00000171988	0.0216	0.072640097	-0.205041382	-1.152719411	**JMJD1C**	protein_coding	jumonji domain containing 1C [Source:HGNC Symbol;Acc:HGNC:12313]	chr10	63167221	63521850	-1	ensembl_havana	5.481378794	5.583899485	5.378858103	5.582117824	5.593131912	5.576448719	5.381846742	5.454755221	5.299972345
ENSG00000114126	0.0216	0.072640097	-0.2439857	-1.184259874	**TFDP2**	protein_coding	transcription factor Dp-2 [Source:HGNC Symbol;Acc:HGNC:11751]	chr3	141944428	142149544	-1	ensembl_havana	7.193110352	7.315103202	7.071117502	7.618694653	7.324028513	7.002586439	7.271399939	7.054929525	6.88702304
ENSG00000112167	0.021616	0.072659745	-0.192129549	-1.14244883	**SAYSD1**	protein_coding	SAYSVFN motif domain containing 1 [Source:HGNC Symbol;Acc:HGNC:21025]	chr6	39104064	39115189	-1	ensembl_havana	3.290422736	3.38648751	3.194357961	3.294473638	3.482399	3.382589892	3.135932331	3.237766117	3.209375435
ENSG00000197748	0.021637	0.072689165	0.842726848	1.793436728	**CFAP43**	protein_coding	cilia and flagella associated protein 43 [Source:HGNC Symbol;Acc:HGNC:26684]	chr10	104129888	104232362	-1	ensembl_havana	1.246288923	0.824925499	1.667652347	0.297565234	0.979703225	1.197508038	1.763844323	1.759072628	1.480040089
ENSG00000158604	0.021639	0.072689165	0.214395002	1.160217272	**TMED4**	protein_coding	transmembrane p24 trafficking protein 4 [Source:HGNC Symbol;Acc:HGNC:22301]	chr7	44577894	44582287	-1	ensembl_havana	7.010520132	6.903322631	7.117717633	6.841505024	6.930474845	6.937988023	7.150895629	7.088035101	7.114222168
ENSG00000161905	0.02164	0.072689165	-0.205728789	-1.153268783	**ALOX15**	protein_coding	arachidonate 15-lipoxygenase [Source:HGNC Symbol;Acc:HGNC:433]	chr17	4630902	4642294	-1	ensembl_havana	10.09448135	10.19734574	9.991616955	10.28454946	9.95765975	10.34982802	10.11669946	9.795283932	10.06286747
ENSG00000186111	0.021675	0.07279133	0.397643445	1.317354333	**PIP5K1C**	protein_coding	phosphatidylinositol-4-phosphate 5-kinase type 1 gamma [Source:HGNC Symbol;Acc:HGNC:8996]	chr19	3630183	3700479	-1	ensembl_havana	4.745975666	4.547153944	4.944797389	4.517968493	4.389608382	4.733884958	5.159284613	4.810021875	4.865085678
ENSG00000103061	0.021682	0.072797045	0.202483569	1.150677518	**SLC7A6OS**	protein_coding	solute carrier family 7 member 6 opposite strand [Source:HGNC Symbol;Acc:HGNC:25807]	chr16	68284503	68310946	-1	ensembl_havana	5.276594933	5.175353148	5.377836717	5.112916487	5.195099047	5.218043912	5.383712004	5.335314054	5.414484094
ENSG00000140398	0.021701	0.072844939	0.395449107	1.315352162	**NEIL1**	protein_coding	nei like DNA glycosylase 1 [Source:HGNC Symbol;Acc:HGNC:18448]	chr15	75346955	75357114	1	ensembl_havana	0.686647678	0.488923124	0.884372232	0.297565234	0.614986832	0.554217307	0.399634207	1.163381334	1.090101154
ENSG00000157193	0.021723	0.072900318	-0.245826266	-1.185771696	**LRP8**	protein_coding	LDL receptor related protein 8 [Source:HGNC Symbol;Acc:HGNC:6700]	chr1	53242364	53328070	-1	ensembl_havana	7.479783431	7.602696564	7.356870298	7.745744786	7.521179305	7.5411656	7.36454226	7.370036639	7.336031994
ENSG00000253522	0.021776	0.073054776	-0.425551879	-1.343086179	**MIR3142HG**	lincRNA	MIR3142 host gene [Source:HGNC Symbol;Acc:HGNC:51944]	chr5	160438594	160487426	1	havana	4.102297594	4.315073533	3.889521655	4.490851674	4.255938845	4.19843008	3.744444859	3.968651039	3.955469065
ENSG00000149654	0.021779	0.073054776	-0.797226636	-1.737757337	**CDH22**	protein_coding	cadherin 22 [Source:HGNC Symbol;Acc:HGNC:13251]	chr20	46173733	46308498	-1	ensembl_havana	1.782004377	2.180617695	1.383391059	1.649052215	2.351074074	2.541726798	1.400976194	1.578479109	1.170717874
ENSG00000253251	0.021788	0.073064078	-0.217713891	-1.1628894	**AC008560.1**	protein_coding	Uncharacterized protein FLJ26957 [Source:UniProtKB/Swiss-Prot;Acc:Q6ZNX1]	chr5	65624765	65630891	1	havana	2.548541395	2.65739834	2.439684449	2.76022634	2.698916685	2.513051996	2.44516827	2.55432044	2.319564638
ENSG00000116288	0.021792	0.073064078	-0.234178936	-1.176237128	**PARK7**	protein_coding	Parkinsonism associated deglycase [Source:HGNC Symbol;Acc:HGNC:16369]	chr1	7954291	7985505	1	ensembl_havana	7.368941766	7.486031234	7.251852298	7.50031292	7.498606792	7.45917399	7.168584578	7.246359297	7.34061302
ENSG00000130227	0.021798	0.073066836	0.181344066	1.133939812	**XPO7**	protein_coding	exportin 7 [Source:HGNC Symbol;Acc:HGNC:14108]	chr8	21919671	22006585	1	ensembl_havana	6.966993281	6.876321248	7.057665314	6.773969501	6.837019101	7.017975142	6.943661391	7.021875614	7.207458937
ENSG00000129595	0.021814	0.073103702	0.664656846	1.585191181	**EPB41L4A**	protein_coding	erythrocyte membrane protein band 4.1 like 4A [Source:HGNC Symbol;Acc:HGNC:13278]	chr5	112142441	112419316	-1	ensembl_havana	3.795896401	3.463567978	4.128224824	2.807536027	3.724301235	3.858866672	3.991955622	4.237794741	4.154924108
ENSG00000230091	0.021852	0.073189859	0.30830603	1.238252926	**TMEM254-AS1**	antisense	TMEM254 antisense RNA 1 [Source:HGNC Symbol;Acc:HGNC:27340]	chr10	80046860	80078912	-1	havana	1.77995695	1.625803936	1.934109965	1.671956805	1.709476673	1.495978329	1.786580698	2.046678052	1.969071146
ENSG00000163946	0.02186	0.073189859	-0.215491784	-1.161099641	**FAM208A**	protein_coding	family with sequence similarity 208 member A [Source:HGNC Symbol;Acc:HGNC:30314]	chr3	56620133	56683237	-1	ensembl_havana	7.947472185	8.055218076	7.839726293	8.057483372	8.001887155	8.106283702	7.743785193	7.831638292	7.943755393
ENSG00000005700	0.02186	0.073189859	-0.26583019	-1.202327722	**IBTK**	protein_coding	inhibitor of Bruton tyrosine kinase [Source:HGNC Symbol;Acc:HGNC:17853]	chr6	82169983	82247754	-1	ensembl_havana	5.682797053	5.815712148	5.549881958	5.98844238	5.684418769	5.774275295	5.562319187	5.525067154	5.562259533
ENSG00000160606	0.02186	0.073189859	-0.697182097	-1.621334872	**TLCD1**	protein_coding	TLC domain containing 1 [Source:HGNC Symbol;Acc:HGNC:25177]	chr17	28724348	28727935	-1	ensembl_havana	-0.300839804	0.047751244	-0.649430853	0.174648163	-0.258437325	0.227042895	-0.63291296	-0.42206346	-0.893316134
ENSG00000117054	0.021888	0.073266235	-0.185060992	-1.136865035	**ACADM**	protein_coding	acyl-CoA dehydrogenase medium chain [Source:HGNC Symbol;Acc:HGNC:89]	chr1	75724347	75787575	1	ensembl_havana	6.055935054	6.14846555	5.963404557	6.07852002	6.161354369	6.20552226	5.866364322	5.972113284	6.051736067
ENSG00000171475	0.021893	0.073266834	0.187599415	1.13886711	**WIPF2**	protein_coding	WAS/WASL interacting protein family member 2 [Source:HGNC Symbol;Acc:HGNC:30923]	chr17	40219304	40284136	1	ensembl_havana	6.147161534	6.053361826	6.240961241	6.148067837	6.076169672	5.93584797	6.346940664	6.222508879	6.15343418
ENSG00000270580	0.02192	0.073340443	0.824033821	1.77034903	**AC139256.2**	processed_transcript		chr16	15104723	15131601	-1	havana	-0.242581367	-0.654598277	0.169435544	-0.4596317	-0.383759306	-1.120403822	0.069637898	0.063570082	0.375098651

ENSG00000114248	0.021931	0.073342764	0.229263546	1.172236404	**LRRC31**	protein_coding	leucine rich repeat containing 31 [Source:HGNC Symbol;Acc:HGNC:26261]	chr3	169839179	169869930	-1	ensembl_havana	1.113697532	0.999065759	1.228329305	1.005046425	0.962641309	1.029509544	1.195452122	1.114463419	1.375072374
ENSG00000256262	0.021929	0.073342764	-0.50262026	-1.416784427	**USP30-AS1**	antisense	USP30 antisense RNA 1 [Source:HGNC Symbol;Acc:HGNC:40909]	chr12	109052350	109053952	-1	havana	0.324160326	0.575470456	0.072850197	0.637193984	0.325758945	0.76345844	0.485518989	-0.19956768	-0.067400719
ENSG00000015532	0.021953	0.073397518	-0.212309301	-1.158541161	**XYLT2**	protein_coding	xylosyltransferase 2 [Source:HGNC Symbol;Acc:HGNC:15517]	chr17	50346092	50363138	1	ensembl_havana	5.570284003	5.676438653	5.464129352	5.772969075	5.593824664	5.662522222	5.508628474	5.475081104	5.408678479
ENSG00000177173	0.021986	0.073472622	0.602440178	1.518282422	**NAP1L4P1**	processed_pseudogene	nucleosome assembly protein 1 like 4 pseudogene 1 [Source:HGNC Symbol;Acc:HGNC:39740]	chr1	116532936	116534092	-1	havana	2.194950296	1.893730207	2.496170385	1.882980075	1.476918714	2.321291832	2.561794338	2.453030417	2.4736864
ENSG00000246174	0.021984	0.073472622	-0.240311661	-1.181247816	**KCTD21-AS1**	antisense	KCTD21 antisense RNA 1 [Source:HGNC Symbol;Acc:HGNC:48674]	chr11	78139771	78175323	1	ensembl_havana	0.671008784	0.791164614	0.550852953	0.812341977	0.740413176	0.820738689	0.513047461	0.426254992	0.713256405
ENSG00000116455	0.021994	0.073483856	0.259380205	1.196964367	**WDR77**	protein_coding	WD repeat domain 77 [Source:HGNC Symbol;Acc:HGNC:29652]	chr1	111439890	111449376	-1	ensembl_havana	5.095060877	4.965370774	5.224750979	4.946776004	4.84203453	5.107301789	5.173371332	5.250127827	5.250753777
ENSG00000124177	0.02201	0.073520173	0.229342422	1.172300495	**CHD6**	protein_coding	chromodomain helicase DNA binding protein 6 [Source:HGNC Symbol;Acc:HGNC:19057]	chr20	41402101	41618494	-1	ensembl_havana	7.682691742	7.568020531	7.797362953	7.693708223	7.414839069	7.595514301	7.841687169	7.759892527	7.790509164
ENSG00000146410	0.022042	0.073608668	-0.183832119	-1.135897077	**MTFR2**	protein_coding	mitochondrial fission regulator 2 [Source:HGNC Symbol;Acc:HGNC:21115]	chr6	136231024	136250335	-1	ensembl_havana	3.895601278	3.987517337	3.803685218	3.863385632	4.024783013	4.074383366	3.676073242	3.816389236	3.918593177
ENSG00000164221	0.02208	0.073720044	0.233198975	1.175438433	**CCDC112**	protein_coding	coiled-coil domain containing 112 [Source:HGNC Symbol;Acc:HGNC:28599]	chr5	115267188	115296831	-1	ensembl_havana	2.683502919	2.566903432	2.800102407	2.355796255	2.683379039	2.661535001	2.711879034	2.871367873	2.817060315
ENSG00000149257	0.02209	0.073736604	-0.180290119	-1.133111725	**SERPINH1**	protein_coding	serpin family H member 1 [Source:HGNC Symbol;Acc:HGNC:1546]	chr11	75562056	75572783	1	ensembl_havana	5.276074073	5.366219132	5.185929013	5.233296343	5.386367315	5.478993738	5.048419585	5.203136127	5.306231328
ENSG00000130429	0.022097	0.07374353	-0.52175138	-1.435697075	**ARPC1B**	protein_coding	actin related protein 2/3 complex subunit 1B [Source:HGNC Symbol;Acc:HGNC:704]	chr7	99374249	99394814	1	ensembl_havana	0.723824593	0.984700283	0.462948904	0.911909315	1.062111262	0.980080273	0.56657698	0.130707766	0.691561964
ENSG00000214226	0.022108	0.073760204	-0.357484439	-1.281189997	**C17orf67**	protein_coding	chromosome 17 open reading frame 67 [Source:HGNC Symbol;Acc:HGNC:27900]	chr17	56791913	56838773	-1	ensembl_havana	0.520168762	0.698910982	0.341426542	0.683014432	0.592979824	0.820738689	0.339398945	0.45273451	0.232146171
ENSG00000147905	0.022114	0.073764501	-0.266423983	-1.202822686	**ZCCHC7**	protein_coding	zinc finger CCHC-type containing 7 [Source:HGNC Symbol;Acc:HGNC:26209]	chr9	37120539	37358149	1	ensembl_havana	5.172577503	5.305789495	5.039365512	5.427507328	5.203293316	5.28656784	4.998167601	5.040259376	5.079669558
ENSG00000255571	0.022143	0.073827137	0.198600396	1.147584507	**MIR9-3HG**	lincRNA	MIR9-3 host gene [Source:HGNC Symbol;Acc:HGNC:27388]	chr15	89361579	89398487	1	havana	1.233378976	1.134078778	1.332679174	1.175017601	1.029710695	1.197508038	1.309740823	1.286257764	1.402038935
ENSG00000239482	0.022141	0.073827137	-0.386633916	-1.307339569	**AC112487.1**	lincRNA		chr3	112302478	112332791	1	havana	0.744083732	0.93740069	0.550766774	1.190963204	0.658018659	0.963220207	0.618191201	0.554044597	0.480064523
ENSG00000118193	0.02215	0.073832579	-0.188241761	-1.139374293	**KIF14**	protein_coding	kinesin family member 14 [Source:HGNC Symbol;Acc:HGNC:19181]	chr1	200551497	200620734	-1	ensembl_havana	6.821847509	6.915968389	6.727726629	6.905114009	6.917805186	6.924985972	6.692583707	6.776035527	6.714560652
ENSG00000125735	0.022163	0.073860241	-0.455161968	-1.370936708	**TNFSF14**	protein_coding	TNF superfamily member 14 [Source:HGNC Symbol;Acc:HGNC:11930]	chr19	6661253	6670588	-1	ensembl_havana	3.07011234	3.297693324	2.842531356	3.327306943	3.097039309	3.468733719	2.528460247	2.876266922	3.122866897
ENSG00000233532	0.022169	0.073862021	0.284861334	1.218293161	**LINC00460**	lincRNA	long intergenic non-protein coding RNA 460 [Source:HGNC Symbol;Acc:HGNC:42809]	chr13	106374477	106384315	1	havana	1.772113226	1.629682559	1.914543893	1.705645971	1.500754142	1.682647563	1.863449533	1.966251876	1.91393027
ENSG00000135541	0.022194	0.073928041	0.213601671	1.15957945	**AHI1**	protein_coding	Abelson helper integration site 1 [Source:HGNC Symbol;Acc:HGNC:21575]	chr6	135283532	135497776	-1	ensembl_havana	2.95266446	2.845863625	3.059465296	2.672087675	2.877938163	2.987565036	2.981915645	3.033647152	3.162833091
ENSG00000017260	0.022199	0.073928041	0.180315627	1.13313176	**ATP2C1**	protein_coding	ATPase secretory pathway Ca2+ transporting 1 [Source:HGNC Symbol;Acc:HGNC:13211]	chr3	130850595	131016712	1	ensembl_havana	8.133337686	8.043179872	8.2234955	8.003484908	8.033635565	8.092419144	8.177629427	8.225396815	8.267460256
ENSG00000011451	0.022212	0.073954411	0.338307528	1.264272565	**WIZ**	protein_coding	widely interspaced zinc finger motifs [Source:HGNC Symbol;Acc:HGNC:30917]	chr19	15419980	15449951	-1	ensembl_havana	4.449103557	4.279949793	4.618257321	4.387397568	4.037060343	4.415391467	4.802895247	4.534383379	4.517493337
ENSG00000224032	0.022218	0.073956932	0.248174559	1.187703364	**EPB41L4A-AS1**	lincRNA	EPB41L4A antisense RNA 1 [Source:HGNC Symbol;Acc:HGNC:30749]	chr5	112160526	112164276	1	ensembl_havana	3.636316954	3.512229675	3.760404234	3.249500187	3.430602854	3.856585982	3.441917197	3.821478248	4.017817256
ENSG00000166024	0.022246	0.074031424	-0.189944954	-1.140720191	**R3HCC1L**	protein_coding	R3H domain and coiled-coil containing 1 like [Source:HGNC Symbol;Acc:HGNC:23512]	chr10	98134624	98244897	1	ensembl_havana	4.587667849	4.682640326	4.492695372	4.722421242	4.739334689	4.586165046	4.482676951	4.562010741	4.433398424
ENSG00000197566	0.022253	0.074039023	-0.275311048	-1.210254995	**ZNF624**	protein_coding	zinc finger protein 624 [Source:HGNC Symbol;Acc:HGNC:29254]	chr17	16620737	16653856	-1	ensembl_havana	2.27537179	2.413027314	2.137716266	2.52259249	2.317915967	2.398573485	2.072167195	2.139252294	2.20172931
ENSG00000096384	0.022276	0.074098923	-0.181376524	-1.133965324	**HSP90AB1**	protein_coding	heat shock protein 90 alpha family class B member 1 [Source:HGNC Symbol;Acc:HGNC:5258]	chr6	44246166	44253888	1	ensembl_havana	11.64109896	11.73178722	11.5504107	11.66615958	11.74708875	11.78211333	11.46509151	11.58241188	11.6037287
ENSG00000180992	0.022284	0.074109515	-0.196675849	-1.146054656	**MRPL14**	protein_coding	mitochondrial ribosomal protein L14 [Source:HGNC Symbol;Acc:HGNC:14279]	chr6	44113454	44127457	-1	ensembl_havana	5.139288331	5.237626255	5.040950406	5.387417495	5.236500262	5.088961009	5.242840767	4.976673844	4.903336608
ENSG00000107020	0.022291	0.074114432	-0.3231861	-1.251090454	**PLGRKT**	protein_coding	plasminogen receptor with a C-terminal lysine [Source:HGNC Symbol;Acc:HGNC:23633]	chr9	5357973	5437878	-1	ensembl_havana	4.013367549	4.174960599	3.851774498	4.311025713	4.133596708	4.080259376	3.764626412	3.890901443	3.899795641
ENSG00000283103	0.022302	0.074133737	0.288121061	1.221048971	**AC010642.2**	antisense		chr19	58310449	58326933	-1	havana	2.732088521	2.58802799	2.876149052	2.547577338	2.592024241	2.624482391	3.006334834	2.86645213	2.755660191
ENSG00000074966	0.02231	0.074142605	0.436485203	1.353303294	**TXK**	protein_coding	TXK tyrosine kinase [Source:HGNC Symbol;Acc:HGNC:12434]	chr4	48066393	48134256	-1	ensembl_havana	1.114368337	0.896125736	1.332610939	0.326725152	0.962641309	1.399010747	0.975631465	1.517070703	1.505130649
ENSG00000183979	0.022323	0.07416739	0.406277952	1.32526232	**NPB**	protein_coding	neuropeptide B [Source:HGNC Symbol;Acc:HGNC:30099]	chr17	81900745	81902905	1	ensembl_havana	0.854415422	0.651276446	1.057554398	0.383334258	0.477608744	1.092886337	0.873137292	1.097781778	1.201744126
ENSG00000140350	0.022333	0.074185636	-0.19766014	-1.146836828	**ANP32A**	protein_coding	acidic nuclear phosphoprotein 32 family member A [Source:HGNC Symbol;Acc:HGNC:13233]	chr15	68778535	68820897	-1	ensembl_havana	8.077976688	8.176806758	7.979146618	8.101383325	8.191853784	8.237183165	7.851829288	7.979821746	8.105788822
ENSG00000152784	0.02234	0.074189955	-0.227642597	-1.17092007	**PRDM8**	protein_coding	PR/SET domain 8 [Source:HGNC Symbol;Acc:HGNC:13993]	chr4	80183879	80204329	1	ensembl_havana	3.422435475	3.536256774	3.308614177	3.786761224	3.452156355	3.369852742	3.552176738	3.329927104	3.043738688
ENSG00000162522	0.022349	0.074202609	-0.182477281	-1.134830855	**KIAA1522**	protein_coding	KIAA1522 [Source:HGNC Symbol;Acc:HGNC:29301]	chr1	32741885	32774970	1	ensembl_havana	5.313043415	5.404282056	5.221804774	5.364752017	5.425029643	5.423064507	5.185184365	5.265146406	5.215083552
ENSG00000225975	0.022356	0.074208806	1.062429639	2.088445708	**LINC01534**	lincRNA	long intergenic non-protein coding RNA 1534 [Source:HGNC Symbol;Acc:HGNC:51281]	chr19	36685440	36687449	-1	havana	-0.246294397	-0.777509217	0.284920423	-1.31854494	-0.428075095	-0.585907617	0.618191201	0.097529431	0.139040636
ENSG00000139977	0.022362	0.074214025	0.179823348	1.132745177	**NAA30**	protein_coding	N(alpha)-acetyltransferase 30, NatC catalytic subunit [Source:HGNC Symbol;Acc:HGNC:19844]	chr14	57390544	57415917	1	ensembl_havana	5.856366899	5.766455225	5.946278573	5.959644851	5.819077846	5.520642978	6.145109044	5.988608005	5.705118671
ENSG00000143801	0.022386	0.074255799	0.18907405	1.140031786	**PSEN2**	protein_coding	presenilin 2 [Source:HGNC Symbol;Acc:HGNC:9509]	chr1	226870184	226896105	1	ensembl_havana	5.447285133	5.352748109	5.541822158	5.397759581	5.38156115	5.278923594	5.628313403	5.525067154	5.472085918
ENSG00000204410	0.022391	0.074255799	-0.187720211	-1.138962471	**MSH5**	protein_coding	mutS homolog 5 [Source:HGNC Symbol;Acc:HGNC:7328]	chr6	31739948	31762834	1	havana	3.19576254	3.289622646	3.101902435	3.088577264	3.16946415	3.610826524	2.911527603	3.015998475	3.378451227
ENSG00000125657	0.022386	0.074255799	-0.252225663	-1.191043135	**TNFSF9**	protein_coding	TNF superfamily member 9 [Source:HGNC Symbol;Acc:HGNC:11939]	chr19	6530999	6535928	1	ensembl_havana	3.653850042	3.779962874	3.52773721	4.216818725	3.624775944	3.498293951	3.829017014	3.385963185	3.368231431
ENSG00000167525	0.022414	0.074316262	-0.506281499	-1.420384477	**PROCA1**	protein_coding	protein interacting with cyclin A1 [Source:HGNC Symbol;Acc:HGNC:28600]	chr17	28703197	28711854	-1	ensembl_havana	2.019450178	2.272590927	1.766309428	2.126286315	2.383487184	2.307999282	1.797815913	1.47893052	2.022181853
ENSG00000188580	0.022428	0.074344722	0.694057247	1.617826891	**NKAIN2**	protein_coding	sodium/potassium transporting ATPase interacting 2 [Source:HGNC Symbol;Acc:HGNC:16443]	chr6	123804141	124825657	1	ensembl_havana	1.119551439	0.772522816	1.466580062	0.326725152	1.170104606	0.820738689	1.261864131	1.300906777	1.836969279
ENSG00000113719	0.02244	0.074359582	0.188065356	1.139234985	**ERGIC1**	protein_coding	endoplasmic reticulum-golgi intermediate compartment 1 [Source:HGNC Symbol;Acc:HGNC:29205]	chr5	172834275	172952685	1	ensembl_havana	6.61283113	6.518798451	6.706863808	6.447981257	6.492686579	6.615727518	6.676315705	6.637908817	6.806366901
ENSG00000143633	0.022443	0.074359582	-0.351026573	-1.275467884	**C1orf131**	protein_coding	chromosome 1 open reading frame 131 [Source:HGNC Symbol;Acc:HGNC:25332]	chr1	231223763	231241187	-1	ensembl_havana	4.149799551	4.325312838	3.974286265	4.320116713	4.39915155	4.256670251	3.820779437	3.943197246	4.158882111
ENSG00000128604	0.022455	0.074383005	-0.306232197	-1.236474251	**IRF5**	protein_coding	interferon regulatory factor 5 [Source:HGNC Symbol;Acc:HGNC:6120]	chr7	128937612	128950035	1	ensembl_havana	7.41805994	7.571176038	7.264943841	7.310378357	7.579176423	7.823973334	6.977403538	7.466182138	7.351245848
ENSG00000022840	0.02248	0.074448983	0.184944648	1.136773358	**RNF10**	protein_coding	ring finger protein 10 [Source:HGNC Symbol;Acc:HGNC:10055]	chr12	120533480	120577594	1	ensembl_havana	6.77245477	6.679982446	6.864927094	6.699637661	6.668375639	6.671934039	6.922004199	6.843566912	6.829210171
ENSG00000086232	0.022492	0.074471147	0.192510994	1.142750931	**EIF2AK1**	protein_coding	eukaryotic translation initiation factor 2 alpha kinase 1 [Source:HGNC Symbol;Acc:HGNC:24921]	chr7	6022244	6059230	-1	ensembl_havana	9.386848893	9.290593395	9.48310439	9.348202062	9.291668089	9.231910035	9.507151476	9.458344501	9.483817191
ENSG00000173039	0.022505	0.07449721	0.197410698	1.146638558	**RELA**	protein_coding	RELA proto-oncogene, NF-kB subunit [Source:HGNC Symbol;Acc:HGNC:9955]	chr11	65653596	65663094	-1	ensembl_havana	5.95534256	5.856637211	6.054047909	5.902083136	5.852459364	5.815369133	6.163140173	6.043537783	5.95546577
ENSG00000075240	0.022518	0.074522786	0.251724079	1.190629115	**GRAMD4**	protein_coding	GRAM domain containing 4 [Source:HGNC Symbol;Acc:HGNC:29113]	chr22	46576012	46679790	1	ensembl_havana	3.244801091	3.118939052	3.370663131	3.146964063	2.970462332	3.23939076	3.423901694	3.337051803	3.351035895
ENSG00000197386	0.02253	0.074546744	0.298806288	1.230126163	**HTT**	protein_coding	huntingtin [Source:HGNC Symbol;Acc:HGNC:4851]	chr4	3074681	3243960	1	ensembl_havana	7.077575889	6.928172745	7.226979033	6.885344409	6.799375444	7.099798384	7.200681995	7.26422285	7.216032255
ENSG00000166685	0.022541	0.074564274	0.209057884	1.155933084	**COG1**	protein_coding	component of oligomeric golgi complex 1 [Source:HGNC Symbol;Acc:HGNC:6545]	chr17	73192632	73208507	1	ensembl_havana	5.183247865	5.078718923	5.287776807	4.989569283	5.13354198	5.113045506	5.203252712	5.261406393	5.398671317
ENSG00000143418	0.022569	0.07464207	0.184285986	1.136254483	**CERS2**	protein_coding	ceramide synthase 2 [Source:HGNC Symbol;Acc:HGNC:14076]	chr1	150960583	150975004	-1	ensembl_havana	7.515088524	7.422945531	7.607231517	7.452126966	7.417971934	7.398737692	7.655833343	7.620133813	7.545727394
ENSG00000253729	0.022601	0.074728126	-0.259075988	-1.196711993	**PRKDC**	protein_coding	protein kinase, DNA-activated, catalytic polypeptide [Source:HGNC Symbol;Acc:HGNC:9413]	chr8	47773108	47960183	-1	ensembl_havana	8.724494743	8.854032737	8.594956749	8.741692783	8.712087617	9.10831781	8.400580258	8.611347159	8.77294283
ENSG00000177239	0.022634	0.074768075	0.282718498	1.216484972	**MAN1B1**	protein_coding	mannosidase alpha class 1B member 1 [Source:HGNC Symbol;Acc:HGNC:6823]	chr9	137086927	137109187	1	ensembl_havana	5.401447402	5.260088153	5.542806651	5.081183842	5.195099047	5.503981571	5.508628474	5.505438186	5.614353292
ENSG00000130038	0.022621	0.074768075	0.277490394	1.212084596	**CRACR2A**	protein_coding	calcium release activated channel regulator 2A [Source:HGNC Symbol;Acc:HGNC:28657]	chr12	3606633	3764819	-1	ensembl_havana	4.628958079	4.490212882	4.767703276	4.312848501	4.394387857	4.76340229	4.716730794	4.718872731	4.867506304
ENSG00000051009	0.022624	0.074768075	0.218387396	1.163432407	**FAM160A2**	protein_coding	family with sequence similarity 160 member A2 [Source:HGNC Symbol;Acc:HGNC:25378]	chr11	6211335	6234711	-1	ensembl_havana	4.980790967	4.871597269	5.089984665	4.971219637	4.744335121	4.899237049	5.102653563	5.058693368	5.108607065
ENSG00000131848	0.022638	0.074768075	-0.232450547	-1.174828807	**ZSCAN5A**	protein_coding	zinc finger and SCAN domain containing 5A [Source:HGNC Symbol;Acc:HGNC:23710]	chr19	56219670	56368383	-1	ensembl_havana	3.113694549	3.229919823	2.997469276	3.327306943	3.266504942	3.095947583	2.976981814	3.055408658	2.960017356
ENSG00000168913	0.022639	0.074768075	-0.441566419	-1.358078073	**ENHO**	protein_coding	energy homeostasis associated [Source:HGNC Symbol;Acc:HGNC:24838]	chr9	34521040	34523041	-1	ensembl_havana	0.332559274	0.553342483	0.111776064	0.852997155	0.760296981	0.046733314	0.485518989	-0.00684508	-0.143345712
ENSG00000165672	0.022664	0.074818117	-0.220533236	-1.165164164	**PRDX3**	protein_coding	peroxiredoxin 3 [Source:HGNC Symbol;Acc:HGNC:9354]	chr10	119167703	119178833	-1	ensembl_havana	7.871349658	7.981616276	7.761083041	8.125307913	7.994692343	7.824848572	7.812557817	7.769469814	7.70122149
ENSG00000200879	0.022662	0.074818117	-0.25826607	-1.196040357	**SNORD14E**	snoRNA	small nucleolar RNA, C/D box 14E [Source:HGNC Symbol;Acc:HGNC:30354]	chr11	123058077	123058161	-1	ensembl	0.052575922	0.181708957	-0.076557113	0.075059705	0.592979824	-0.122912658	-0.26243933	0.256279753	-0.223511759
ENSG00000177406	0.022674	0.07483343	-0.443398853	-1.359804126	**AC021054.1**	antisense		chr12	630891	663706	1	havana	3.95025974	4.171959166	3.728560313	4.339917525	4.222285664	3.953674309	3.669971868	3.668906348	3.846802724
ENSG00000134775	0.022682	0.074843209	-0.25275096	-1.191476883	**FHOD3**	protein_coding	formin homology 2 domain containing 3 [Source:HGNC Symbol;Acc:HGNC:26178]	chr18	36297714	36780055	1	ensembl_havana	7.076429869	7.202805349	6.950054389	7.183124889	7.080857547	7.34443361	7.032538427	6.873582337	6.944042402
ENSG00000100038	0.022707	0.07490701	0.188376749	1.139480905	**TOP3B**	protein_coding	DNA topoisomerase III beta [Source:HGNC Symbol;Acc:HGNC:11993]	chr22	21957025	21982816	-1	ensembl_havana	4.780553074	4.686364699	4.874741448	4.593392381	4.749318281	4.716383435	4.735841389	4.942047085	4.946335871
ENSG00000155961	0.022715	0.074911875	-0.235517367	-1.177328865	**RAB39B**	protein_coding	RAB39B, member RAS oncogene family [Source:HGNC Symbol;Acc:HGNC:16499]	chrX	155258241	155264589	-1	ensembl_havana	5.424188866	5.541947549	5.306430182	5.684221895	5.619905732	5.32171502	5.398547914	5.305659081	5.215083552
ENSG00000123636	0.022719	0.074911875	-0.36111026	-1.284413968	**BAZ2B**	protein_coding	bromodomain adjacent to zinc finger domain 2B [Source:HGNC Symbol;Acc:HGNC:963]	chr2	159318979	159616692	-1	ensembl_havana	1.617793083	1.798348213	1.437237954	1.738566362	2.06964138	1.586836898	1.567772387	1.452931788	1.291009686
ENSG00000167186	0.022727	0.074922193	0.23550118	1.177315655	**COQ7**	protein_coding	coenzyme Q7, hydroxylase [Source:HGNC Symbol;Acc:HGNC:2244]	chr16	19067599	19080095	1	ensembl_havana	4.349106341	4.231355752	4.466856931	4.179342839	4.310901545	4.20382287	4.527234088	4.546727568	4.326609138
ENSG00000168961	0.022746	0.074968161	-0.312710776	-1.242039259	**LGALS9**	protein_coding	galectin 9 [Source:HGNC Symbol;Acc:HGNC:6570]	chr17	27629798	27649560	1	ensembl_havana	7.416771243	7.573126631	7.260415855	7.736946782	7.58389137	7.398541742	7.640118842	7.141407318	6.999721406
ENSG00000120254	0.022762	0.074986091	-0.182962814	-1.135212842	**MTHFD1L**	protein_coding	methylenetetrahydrofolate dehydrogenase (NADP+ dependent) 1 like [Source:HGNC Symbol;Acc:HGNC:21055]	chr6	150865549	151101887	1	ensembl_havana	5.383875638	5.475357045	5.292394231	5.189322542	5.557353082	5.679395511	5.04016487	5.361754164	5.475263658
ENSG00000115053	0.022758	0.074986091	-0.211313907	-1.157742096	**NCL**	protein_coding	nucleolin [Source:HGNC Symbol;Acc:HGNC:7667]	chr2	231453531	231483641	-1	ensembl_havana	8.836497641	8.942154594	8.730840687	8.88305107	8.955743052	8.987669661	8.578455369	8.772184047	8.841882645
ENSG00000141425	0.022782	0.075034593	-0.208557722	-1.155532408	**RPRD1A**	protein_coding	regulation of nuclear pre-mRNA domain containing 1A [Source:HGNC Symbol;Acc:HGNC:25560]	chr18	35984387	36067576	-1	ensembl_havana	6.318451086	6.422729947	6.214172225	6.380056747	6.484491664	6.403641429	6.204884188	6.185489502	6.252142984
ENSG00000250571	0.022798	0.075068623	0.235341888	1.177185672	**GLI4**	protein_coding	GLI family zinc finger 4 [Source:HGNC Symbol;Acc:HGNC:4320]	chr8	143267433	143276931	1	ensembl_havana	4.256026155	4.138355211	4.373697099	4.082228299	4.001999572	4.330837762	4.40766003	4.273512191	4.439919076
ENSG00000092098	0.022828	0.075152264	0.22021951	1.164910818	**RNF31**	protein_coding	ring finger protein 31 [Source:HGNC Symbol;Acc:HGNC:16031]	chr14	24146683	24160661	1	ensembl_havana	4.110949347	4.000839591	4.221059102	4.007662542	3.904856379	4.089999853	4.127254407	4.212836819	4.32308608
ENSG00000158813	0.022882	0.075241926	0.470950909	1.386022722	**EDA**	protein_coding	ectodysplasin A [Source:HGNC Symbol;Acc:HGNC:3157]	chrX	69616067	70039469	1	ensembl_havana	1.60809369	1.372618236	1.843569145	0.986893817	1.500754142	1.630206748	1.7752573	1.648877048	2.106573087
ENSG00000102312	0.022871	0.075241926	0.178619657	1.131800482	**PORCN**	protein_coding	porcupine O-acyltransferase [Source:HGNC Symbol;Acc:HGNC:17652]	chrX	48508962	48520814	1	ensembl_havana	3.864078063	3.774768235	3.953387892	4.023332558	3.706476045	3.594496101	4.192554836	3.886051921	3.781556918
ENSG00000075151	0.022874	0.075241926	-0.191149153	-1.141672733	**EIF4G3**	protein_coding	eukaryotic translation initiation factor 4 gamma 3 [Source:HGNC Symbol;Acc:HGNC:3298]	chr1	20806292	21176888	-1	ensembl_havana	7.201549868	7.297124444	7.105975292	7.433817291	7.175062051	7.282493991	7.198034317	7.039179971	7.080711586
ENSG00000124217	0.02288	0.075241926	-0.202900556	-1.15101015	**MOCS3**	protein_coding	molybdenum cofactor synthesis 3 [Source:HGNC Symbol;Acc:HGNC:15765]	chr20	50958826	50963931	1	ensembl_havana	3.544538043	3.645988321	3.443087765	3.394260955	3.721768224	3.821935783	3.234313959	3.43659104	3.658358295
ENSG00000267452	0.022867	0.075241926	-0.633746002	-1.551588515	**LINC02073**	lincRNA	long intergenic non-protein coding RNA 2073 [Source:HGNC Symbol;Acc:HGNC:52919]	chr17	51435427	51445802	1	havana	1.619481263	1.936354264	1.302608262	2.245600406	1.987674772	1.575787614	1.47283199	0.993426194	1.441566602
ENSG00000261067	0.022908	0.075310408	0.411537509	1.330102576	**AC109460.3**	processed_transcript		chr16	28974804	28990775	1	havana	0.005084476	-0.200684279	0.210853231	-0.31750517	0.030651399	-0.315199064	0.210814386	0.130707766	0.291037539
ENSG00000203761	0.022924	0.075345913	-0.210628871	-1.157192495	**MSTO2P**	unprocessed_pseudogene	misato family member 2, pseudogene [Source:HGNC Symbol;Acc:HGNC:26329]	chr1	155745829	155750137	1	havana	4.28548063	4.390795066	4.180166195	4.525847985	4.276796007	4.369741207	4.219958918	4.118821469	4.201718198
ENSG00000052795	0.022948	0.075407599	0.25795897	1.195785788	**FNIP2**	protein_coding	folliculin interacting protein 2 [Source:HGNC Symbol;Acc:HGNC:29280]	chr4	158769138	158908049	1	ensembl_havana	6.005461624	5.876482139	6.134441109	5.7769362	5.933503849	5.919006366	6.104402791	6.280017198	6.018903337
ENSG00000163389	0.022975	0.075480405	-0.189109618	-1.140059893	**POGLUT1**	protein_coding	protein O-glucosyltransferase 1 [Source:HGNC Symbol;Acc:HGNC:22954]	chr3	119468938	119494708	1	ensembl_havana	6.396381212	6.490936021	6.301826403	6.575688413	6.539104669	6.358014981	6.338310098	6.352561752	6.214607361
ENSG00000187778	0.022988	0.075504698	0.19477733	1.144547494	**MCRS1**	protein_coding	microspherule protein 1 [Source:HGNC Symbol;Acc:HGNC:6960]	chr12	49556544	49568153	-1	ensembl_havana	7.087926513	6.990537847	7.185315178	6.942093098	7.032876804	6.99664364	7.202004014	7.209712038	7.144229481
ENSG00000279500	0.023023	0.075602554	0.491802861	1.406201035	**AC108704.2**	TEC		chr12	128813186	128814750	-1	havana	1.103437654	0.857536223	1.349339085	1.175017601	0.185737694	1.211853376	1.430149367	1.063828438	1.554039449
ENSG00000239791	0.023031	0.075611101	0.600903806	1.516666416	**AC002310.2**	antisense		chr16	30572250	30583860	1	havana	-0.182622535	-0.483074438	0.117829368	-0.56281016	-0.727039001	-0.159374151	-0.30988669	0.343769191	0.319605599
ENSG00000144647	0.023068	0.07561224	0.476507562	1.391371391	**POMGNT2**	protein_coding	protein O-linked mannose N-acetylglucosaminyltransferase 2 (beta 1,4-) [Source:HGNC Symbol;Acc:HGNC:25902]	chr3	43079232	43106076	-1	ensembl_havana	4.883731012	4.645477231	5.121984793	4.325543942	4.755523124	4.855364626	5.176602673	5.071565226	5.117786479
ENSG00000184588	0.02305	0.07561224	0.432849165	1.349896844	**PDE4B**	protein_coding	phosphodiesterase 4B [Source:HGNC Symbol;Acc:HGNC:8781]	chr1	65792514	66374579	1	ensembl_havana	2.970673666	2.754249084	3.187098249	2.566036057	2.948964943	2.747746253	3.334150354	3.222458254	3.004686139
ENSG00000145198	0.023057	0.07561224	0.360629158	1.28398572	**VWA5B2**	protein_coding	von Willebrand factor A domain containing 5B2 [Source:HGNC Symbol;Acc:HGNC:25144]	chr3	184230429	184242329	1	ensembl_havana	1.86849383	1.688179251	2.048808409	1.528734855	1.428032914	2.107769984	2.025141822	1.919554095	2.20172931
ENSG00000105323	0.023052	0.07561224	-0.180404925	-1.1332019	**HNRNPUL1**	protein_coding	heterogeneous nuclear ribonucleoprotein U like 1 [Source:HGNC Symbol;Acc:HGNC:17011]	chr19	41262496	41307598	1	ensembl_havana	7.773088143	7.863290606	7.68288568	7.867475747	7.875550069	7.846846001	7.712217159	7.676689365	7.659750518
ENSG00000086619	0.023068	0.07561224	-0.187084401	-1.138460629	**ERO1B**	protein_coding	endoplasmic reticulum oxidoreductase 1 beta [Source:HGNC Symbol;Acc:HGNC:14355]	chr1	236215555	236281985	-1	ensembl_havana	6.61166282	6.70520502	6.518120619	7.080132464	6.75361106	6.281871536	6.874044885	6.536336589	6.143980383
ENSG00000132781	0.023067	0.07561224	-0.249577338	-1.188858768	**MUTYH**	protein_coding	mutY DNA glycosylase [Source:HGNC Symbol;Acc:HGNC:7527]	chr1	45329163	45340470	-1	ensembl_havana	4.634467168	4.759255837	4.509678499	4.566141688	4.702553757	5.009072066	4.440288909	4.462907727	4.625838861
ENSG00000151881	0.023047	0.07561224	-0.288054118	-1.220992313	**TMEM267**	protein_coding	transmembrane protein 267 [Source:HGNC Symbol;Acc:HGNC:26139]	chr5	43444252	43483893	-1	ensembl_havana	3.999551675	4.143578734	3.855524616	4.292670028	4.150646514	3.987419659	3.815261472	3.926762386	3.824549991
ENSG00000223756	0.023138	0.075668175	1.263284094	2.400415406	**TSSC2**	transcribed_unprocessed_pseudogene	tumor suppressing subtransferable candidate 2 pseudogene [Source:HGNC Symbol;Acc:HGNC:12384]	chr11	3380961	3408978	1	havana	0.394714304	-0.236927743	1.026356352	-0.85854608	-0.383759306	0.531522158	1.01467363	1.32976598	0.734629445
ENSG00000125831	0.023132	0.075668175	0.528223496	1.442152266	**CST11**	protein_coding	cystatin 11 [Source:HGNC Symbol;Acc:HGNC:15959]	chr20	23450403	23452857	-1	ensembl_havana	0.324255211	0.060143463	0.588366959	0.004630787	-0.106664058	0.28246366	0.276538753	0.841392961	0.647169164
ENSG00000196922	0.023131	0.075668175	0.193465379	1.143507144	**ZNF252P**	transcribed_unprocessed_pseudogene	zinc finger protein 252, pseudogene [Source:HGNC Symbol;Acc:HGNC:13046]	chr8	144973589	145002895	-1	havana	5.364000191	5.267267502	5.460732881	5.226543391	5.282773626	5.292485489	5.392075968	5.439113732	5.551008941
ENSG00000163539	0.023127	0.075668175	0.184065105	1.136080532	**CLASP2**	protein_coding	cytoplasmic linker associated protein 2 [Source:HGNC Symbol;Acc:HGNC:17078]	chr3	33496245	33718356	-1	ensembl_havana	6.919398167	6.827365614	7.011430719	6.714882504	6.764112289	7.003102049	6.897734403	6.98436444	7.152193314
ENSG00000176024	0.023129	0.075668175	0.183670878	1.135770132	**ZNF613**	protein_coding	zinc finger protein 613 [Source:HGNC Symbol;Acc:HGNC:25827]	chr19	51927147	51948759	1	havana	1.952218819	1.86038338	2.044054258	1.92171846	1.936162086	1.723269594	2.072167195	2.155463891	1.90453169
ENSG00000184922	0.023101	0.075668175	0.182437099	1.134799247	**FMNL1**	protein_coding	formin like 1 [Source:HGNC Symbol;Acc:HGNC:1212]	chr17	45221444	45247320	1	ensembl_havana	5.785091189	5.69387264	5.876309739	5.719004464	5.660811121	5.701802335	5.93666125	5.826577919	5.865690047
ENSG00000145191	0.023127	0.075668175	0.17921943	1.132271104	**EIF2B5**	protein_coding	eukaryotic translation initiation factor 2B subunit epsilon [Source:HGNC Symbol;Acc:HGNC:3261]	chr3	184135038	184145311	1	ensembl_havana	6.085669306	5.996059591	6.175279021	5.832631203	6.042057755	6.113489814	5.997614135	6.21576051	6.312462417
ENSG00000100983	0.023137	0.075668175	-0.179557616	-1.132536554	**GSS**	protein_coding	glutathione synthetase [Source:HGNC Symbol;Acc:HGNC:4624]	chr20	34928433	34956027	-1	ensembl_havana	7.029977221	7.119756029	6.940198412	7.029487059	7.14276186	7.187019167	6.872053088	6.956010726	6.992531424
ENSG00000118197	0.023096	0.075668175	-0.194866281	-1.144618064	**DDX59**	protein_coding	DEAD-box helicase 59 [Source:HGNC Symbol;Acc:HGNC:25360]	chr1	200623896	200669969	-1	ensembl_havana	4.864033621	4.961466761	4.76660048	5.116051698	4.990411054	4.777937532	4.898624011	4.751234058	4.649943372
ENSG00000099994	0.023107	0.075668175	-0.280598402	-1.214698613	**SUSD2**	protein_coding	sushi domain containing 2 [Source:HGNC Symbol;Acc:HGNC:30667]	chr22	24181259	24189110	1	ensembl_havana	1.578857825	1.719157026	1.438558624	1.760103226	1.558674763	1.838693089	1.500601289	1.426455924	1.38861866
ENSG00000110066	0.023164	0.075736432	0.186179774	1.137746994	**KMT5B**	protein_coding	lysine methyltransferase 5B [Source:HGNC Symbol;Acc:HGNC:24283]	chr11	68154863	68213828	-1	ensembl_havana	5.726467302	5.633377414	5.819557189	5.715563702	5.679858546	5.504709995	5.880304096	5.839805165	5.738562305
ENSG00000185608	0.023177	0.075743119	-0.197960431	-1.147075563	**MRPL40**	protein_coding	mitochondrial ribosomal protein L40 [Source:HGNC Symbol;Acc:HGNC:14491]	chr22	19431902	19436075	1	ensembl_havana	3.75500136	3.853981575	3.656021144	3.865870994	3.904856379	3.791217353	3.660771151	3.646140782	3.6611515
ENSG00000115368	0.023172	0.075743119	-0.227907039	-1.171134716	**WDR75**	protein_coding	WD repeat domain 75 [Source:HGNC Symbol;Acc:HGNC:25725]	chr2	189441433	189475565	1	ensembl_havana	7.813511516	7.927465035	7.699557996	7.991585129	7.90877825	7.882031727	7.650807812	7.697944322	7.749921855
ENSG00000128191	0.02319	0.075769643	0.183130414	1.135344728	**DGCR8**	protein_coding	DGCR8, microprocessor complex subunit [Source:HGNC Symbol;Acc:HGNC:2847]	chr22	20080232	20111877	1	ensembl_havana	5.22566518	5.134099973	5.317230387	5.0843887	5.075206302	5.242704916	5.291342377	5.27259747	5.387751314
ENSG00000180616	0.023219	0.075828574	0.438380505	1.355082328	**SSTR2**	protein_coding	somatostatin receptor 2 [Source:HGNC Symbol;Acc:HGNC:11331]	chr17	73165012	73176633	1	ensembl_havana	1.393372967	1.174182714	1.612563219	0.791576534	1.476918714	1.254052895	1.541278047	1.881076089	1.415335521
ENSG00000258122	0.023215	0.075828574	-0.347446369	-1.272306597	**AC044802.2**	antisense		chr16	66841433	66853448	-1	havana	-0.127657214	0.04606597	-0.301380399	0.267803752	-0.299013651	0.16940781	-0.17198403	-0.80561234	0.073455173
ENSG00000253982	0.023225	0.075832533	-0.281873042	-1.21577229	**AC100810.1**	antisense		chr8	1761990	1764502	-1	havana	0.929105712	1.070042233	0.788169191	1.126095262	0.945375194	1.138656243	0.851734776	0.821210834	0.691561964
ENSG00000162607	0.023232	0.075836463	-0.180494615	-1.133272351	**USP1**	protein_coding	ubiquitin specific peptidase 1 [Source:HGNC Symbol;Acc:HGNC:12607]	chr1	62436297	62451804	1	ensembl_havana	7.501793736	7.592041043	7.411546428	7.512855616	7.691366137	7.571901376	7.319453688	7.495332607	7.419852989
ENSG00000186868	0.023254	0.075866323	0.342156919	1.267650395	**MAPT**	protein_coding	microtubule associated protein tau [Source:HGNC Symbol;Acc:HGNC:6893]	chr17	45894382	46028334	1	ensembl_havana	4.962462563	4.791384104	5.133541023	4.553863057	4.803026258	5.017262996	5.119541749	5.129134774	5.151946546
ENSG00000124207	0.02325	0.075866323	-0.183367673	-1.135531457	**CSE1L**	protein_coding	chromosome segregation 1 like [Source:HGNC Symbol;Acc:HGNC:2431]	chr20	49046246	49096960	1	ensembl_havana	8.448575885	8.540259722	8.356892049	8.297550811	8.666469312	8.656759043	8.122984739	8.445113068	8.502578339
ENSG00000235162	0.023257	0.075866323	-0.191295543	-1.141788584	**C12orf75**	protein_coding	chromosome 12 open reading frame 75 [Source:HGNC Symbol;Acc:HGNC:35164]	chr12	105235290	105396097	1	ensembl_havana	6.413623612	6.509271384	6.31797584	6.586277112	6.574280599	6.367256439	6.384220351	6.336209927	6.233497243
ENSG00000135205	0.023301	0.07598649	0.296383896	1.228062424	**CCDC146**	protein_coding	coiled-coil domain containing 146 [Source:HGNC Symbol;Acc:HGNC:29296]	chr7	77122434	77329533	1	ensembl_havana	3.198724965	3.050533017	3.346916913	3.040984517	3.135610417	2.975004118	3.353314566	3.249141357	3.438294818
ENSG00000105221	0.023309	0.07598649	0.194937424	1.14467451	**AKT2**	protein_coding	AKT serine/threonine kinase 2 [Source:HGNC Symbol;Acc:HGNC:392]	chr19	40230317	40285536	-1	ensembl_havana	7.376434513	7.278965802	7.473903225	7.359706246	7.210499329	7.26669183	7.56710259	7.4582401	7.396366985
ENSG00000168405	0.023308	0.07598649	-0.41715874	-1.335295218	**CMAHP**	transcribed_unitary_pseudogene	cytidine monophospho-N-acetylneuraminic acid hydroxylase, pseudogene [Source:HGNC Symbol;Acc:HGNC:2098]	chr6	25061626	25452263	-1	ensembl_havana	5.999672265	6.208251635	5.791092895	6.591167	6.112397349	5.921190556	5.87301911	5.746569729	5.753689845
ENSG00000086289	0.023333	0.075995641	0.232940621	1.175227957	**EPDR1**	protein_coding	ependymin related 1 [Source:HGNC Symbol;Acc:HGNC:17572]	chr7	37683843	37951941	1	ensembl_havana	6.108293005	5.991822694	6.224763315	5.724492672	6.190500178	6.060475232	6.058433235	6.303394294	6.312462417
ENSG00000135931	0.023328	0.075995641	0.2125476	1.158732539	**ARMC9**	protein_coding	armadillo repeat containing 9 [Source:HGNC Symbol;Acc:HGNC:20730]	chr2	231198546	231374837	1	ensembl_havana	3.515337923	3.409064123	3.621611723	3.309158545	3.461295901	3.456737925	3.620209068	3.640392813	3.604233288
ENSG00000160781	0.023318	0.075995641	0.207918249	1.155020333	**PAQR6**	protein_coding	progestin and adipoQ receptor family member 6 [Source:HGNC Symbol;Acc:HGNC:30132]	chr1	156243321	156248117	-1	ensembl_havana	5.20911971	5.105160585	5.313078834	5.23040609	5.020608058	5.064467608	5.517999926	5.147487521	5.273749056
ENSG00000198646	0.023323	0.075995641	0.201462489	1.149863404	**NCOA6**	protein_coding	nuclear receptor coactivator 6 [Source:HGNC Symbol;Acc:HGNC:15936]	chr20	34689097	34825649	-1	ensembl_havana	7.013166214	6.912434969	7.113897459	6.835177372	7.017985151	6.884142384	7.072404871	7.137841953	7.131445552
ENSG00000173611	0.023359	0.076061633	0.210526435	1.157110333	**SCAI**	protein_coding	suppressor of cancer cell invasion [Source:HGNC Symbol;Acc:HGNC:26709]	chr9	124942608	125143506	-1	ensembl_havana	4.564329265	4.459066048	4.669592483	4.323737133	4.453595962	4.599865049	4.475698201	4.760537642	4.772541606
ENSG00000250742	0.023367	0.076071354	0.329772432	1.256815111	**LINC02381**	lincRNA	long intergenic non-protein coding RNA 2381 [Source:HGNC Symbol;Acc:HGNC:53304]	chr12	54126098	54142493	1	havana	6.048200068	5.883313852	6.213086284	6.073152799	6.00867906	5.568109699	6.635411816	6.180547043	5.823299995
ENSG00000137857	0.023379	0.076094134	0.983233617	1.976891397	**DUOX1**	protein_coding	dual oxidase 1 [Source:HGNC Symbol;Acc:HGNC:3062]	chr15	45129933	45165576	1	ensembl_havana	0.382459378	-0.10915743	0.874076187	0.660286109	-0.299013651	-0.688744748	0.894226935	0.758913071	0.969088554
ENSG00000153234	0.023386	0.076098043	0.292506043	1.224765919	**NR4A2**	protein_coding	nuclear receptor subfamily 4 group A member 2 [Source:HGNC Symbol;Acc:HGNC:7981]	chr2	156324432	156342348	-1	ensembl_havana	2.376058122	2.2298051	2.522311143	2.608209568	2.093349987	1.987855745	2.711879034	2.42655638	2.428498016
ENSG00000072310	0.023417	0.076181	-0.190844121	-1.141431372	**SREBF1**	protein_coding	sterol regulatory element binding transcription factor 1 [Source:HGNC Symbol;Acc:HGNC:11289]	chr17	17810399	17837002	-1	ensembl_havana	5.617201364	5.712623425	5.521779304	6.077448172	5.49789865	5.562523452	5.914897921	5.339754527	5.310685463

ENSG00000168918	0.023427	0.076196655	-0.194131903	-1.144035565	**INPP5D**	protein_coding	inositol polyphosphate-5-phosphatase D [Source:HGNC Symbol;Acc:HGNC:6079]	chr2	233059967	233207903	1	ensembl_havana	5.211683905	5.308749856	5.114617954	5.315599317	5.173017357	5.437632895	5.06246156	5.038075104	5.243317197
ENSG00000070010	0.02345	0.076254725	-0.186418518	-1.137935289	**UFD1**	protein_coding	ubiquitin recognition factor in ER associated degradation 1 [Source:HGNC Symbol;Acc:HGNC:12520]	chr22	19449910	19479215	-1	ensembl_havana	6.177573412	6.270782671	6.084364153	6.428358	6.276721671	6.107268341	6.196949742	6.093300323	5.962842393
ENSG00000213741	0.023487	0.076322819	0.222460728	1.166721906	**RPS29**	protein_coding	ribosomal protein S29 [Source:HGNC Symbol;Acc:HGNC:10419]	chr14	49570984	49599164	-1	ensembl_havana	5.939610548	5.828380184	6.050840913	5.92073004	5.8083738	5.756036713	6.127406424	5.939717612	6.085398702
ENSG00000119707	0.02348	0.076322819	-0.183134624	-1.135348042	**RBM25**	protein_coding	RNA binding motif protein 25 [Source:HGNC Symbol;Acc:HGNC:23244]	chr14	73058436	73123898	1	ensembl_havana	7.490796274	7.582363586	7.399228962	7.541510365	7.630381057	7.575199336	7.380042863	7.406475763	7.41116826
ENSG00000139133	0.023489	0.076322819	-0.208897426	-1.155804527	**ALG10**	protein_coding	ALG10, alpha-1,2-glucosyltransferase [Source:HGNC Symbol;Acc:HGNC:23162]	chr12	34022281	34029694	1	ensembl_havana	4.152596355	4.257045068	4.048147643	4.107651756	4.347509263	4.315974186	3.984600033	4.131170126	4.028672769
ENSG00000246465	0.023492	0.076322819	-0.371696	-1.293872985	**AC138904.1**	lincRNA		chr16	28284885	28292064	-1	havana	0.084673597	0.270521597	-0.101174403	-0.31750517	0.271370116	0.857699845	-0.40973604	-0.2414061	0.347618925
ENSG00000136895	0.023518	0.076390788	0.670910515	1.592077444	**GARNL3**	protein_coding	GTPase activating Rap/RanGAP domain like 3 [Source:HGNC Symbol;Acc:HGNC:25425]	chr9	127224265	127393660	1	ensembl_havana	2.159134906	1.823679649	2.494590164	1.142587728	1.936162086	2.392289132	2.349154471	2.459573709	2.675042311
ENSG00000115935	0.023529	0.076407731	-0.1787662	-1.131915452	**WIPF1**	protein_coding	WAS/WASL interacting protein family member 1 [Source:HGNC Symbol;Acc:HGNC:12736]	chr2	174559572	174682916	-1	ensembl_havana	6.902694223	6.992077323	6.813311123	7.121799915	6.9744945	6.879937556	6.966243612	6.783878083	6.689811674
ENSG00000163995	0.023572	0.076529101	0.625659045	1.542915492	**ABLIM2**	protein_coding	actin binding LIM protein family member 2 [Source:HGNC Symbol;Acc:HGNC:19195]	chr4	7965310	8158832	-1	ensembl_havana	1.765600998	1.452771475	2.07843052	0.852997155	1.657409759	1.847907512	1.966610161	1.900443368	2.368238032
ENSG00000250107	0.023638	0.076641549	0.418935598	1.336940813	**CACNA1G-AS1**	antisense	CACNA1G antisense RNA 1 [Source:HGNC Symbol;Acc:HGNC:27377]	chr17	50556207	50562108	-1	havana	2.580232605	2.370764806	2.789700404	2.424845514	2.433879495	2.253569408	3.127051692	2.572545352	2.669504168
ENSG00000142166	0.023627	0.076641549	0.204817869	1.152540836	**IFNAR1**	protein_coding	interferon alpha and beta receptor subunit 1 [Source:HGNC Symbol;Acc:HGNC:5432]	chr21	33324477	33359862	1	ensembl_havana	6.597350938	6.494942004	6.699759873	6.548484852	6.520643399	6.415697761	6.675935188	6.726029364	6.697315066
ENSG00000140829	0.023621	0.076641549	0.179590167	1.132562108	**DHX38**	protein_coding	DEAH-box helicase 38 [Source:HGNC Symbol;Acc:HGNC:17211]	chr16	72093562	72112912	1	ensembl_havana	7.427607366	7.337812283	7.51740245	7.316523812	7.325071894	7.371841142	7.5229323	7.49950599	7.52976906
ENSG00000155307	0.023612	0.076641549	-0.227354033	-1.170685889	**SAMSN1**	protein_coding	SAM domain, SH3 domain and nuclear localization signals 1 [Source:HGNC Symbol;Acc:HGNC:10528]	chr21	14485228	14658821	-1	ensembl_havana	4.265546582	4.379223599	4.151869566	4.927870282	4.281963521	3.927836994	4.580249314	4.093802465	3.781556918
ENSG00000058866	0.023634	0.076641549	-0.300389028	-1.23147644	**DGKG**	protein_coding	diacylglycerol kinase gamma [Source:HGNC Symbol;Acc:HGNC:2853]	chr3	186105668	186362237	-1	ensembl_havana	-0.042317554	0.10787696	-0.192512068	-0.31750517	0.501432867	0.139703184	-0.40973604	0.097529431	-0.265329594
ENSG00000187824	0.023636	0.076641549	-0.341517425	-1.267088616	**TMEM220**	protein_coding	transmembrane protein 220 [Source:HGNC Symbol;Acc:HGNC:33757]	chr17	10699015	10730316	-1	ensembl_havana	1.344481148	1.515239861	1.173722435	1.649052215	1.37743234	1.519235028	1.178360195	1.286257764	1.056549347
ENSG00000111412	0.023715	0.076872707	0.19566964	1.145255618	**C12orf49**	protein_coding	chromosome 12 open reading frame 49 [Source:HGNC Symbol;Acc:HGNC:26128]	chr12	116710185	116738070	-1	ensembl_havana	5.859453709	5.761618889	5.957288529	5.812157816	5.784298807	5.688400044	5.939834229	5.991432959	5.9405984
ENSG00000117751	0.02372	0.076873295	-0.186425067	-1.137940455	**PPP1R8**	protein_coding	protein phosphatase 1 regulatory subunit 8 [Source:HGNC Symbol;Acc:HGNC:9296]	chr1	27830778	27851676	1	ensembl_havana	6.552149581	6.645362114	6.458937047	6.660805528	6.653833077	6.621447739	6.422376841	6.495130579	6.459303722
ENSG00000130518	0.023758	0.07690709	0.383629557	1.304619915	**IQCN**	protein_coding	IQ motif containing N [Source:HGNC Symbol;Acc:HGNC:29350]	chr19	18257097	18274509	-1	ensembl_havana	1.530052766	1.338237988	1.721867544	1.464591378	1.228369434	1.321753151	1.905603022	1.831488934	1.428510677
ENSG00000109689	0.023741	0.07690709	0.249291677	1.188623391	**STIM2**	protein_coding	stromal interaction molecule 2 [Source:HGNC Symbol;Acc:HGNC:19205]	chr4	26857678	27025381	1	ensembl_havana	4.715388537	4.590742699	4.840034376	4.538366337	4.546699886	4.687161874	4.670111011	4.772411905	5.077580212
ENSG00000112305	0.023749	0.07690709	0.206756248	1.154090411	**SMAP1**	protein_coding	small ArfGAP 1 [Source:HGNC Symbol;Acc:HGNC:19651]	chr6	70667776	70862015	1	ensembl_havana	5.990316077	5.886937953	6.093694201	5.961969925	5.968112141	5.730731794	6.1942952	6.084860355	6.00192705
ENSG00000206562	0.023755	0.07690709	-0.198756028	-1.14770831	**METTL6**	protein_coding	methyltransferase like 6 [Source:HGNC Symbol;Acc:HGNC:28343]	chr3	15381275	15440566	-1	ensembl_havana	4.950706884	5.050084897	4.85132887	4.997524442	5.051195136	5.101535114	4.748925052	4.930317649	4.874743908
ENSG00000043143	0.023753	0.07690709	-0.221278614	-1.16576631	**JADE2**	protein_coding	jade family PHD finger 2 [Source:HGNC Symbol;Acc:HGNC:22984]	chr5	134524312	134583230	1	ensembl_havana	7.299863124	7.410502431	7.189223816	7.544616286	7.28209203	7.404798976	7.294883656	7.171362361	7.101425432
ENSG00000180011	0.023797	0.077015962	0.178921214	1.132037079	**ZADH2**	protein_coding	zinc binding alcohol dehydrogenase domain containing 2 [Source:HGNC Symbol;Acc:HGNC:28697]	chr18	75195108	75209348	-1	ensembl_havana	6.062912516	5.973451909	6.152373123	6.057473979	5.938421094	5.924460653	6.210675173	6.137838118	6.108606077
ENSG00000272148	0.023869	0.077040663	0.53453256	1.448472766	**AC013403.2**	antisense		chr2	27062428	27062907	-1	havana	-0.129225443	-0.396491723	0.138040837	-0.18813307	-0.143136894	-0.85820521	0.176794179	0.130707766	0.106620565
ENSG00000145569	0.023839	0.077040663	0.463556119	1.378936592	**FAM105A**	protein_coding	family with sequence similarity 105 member A [Source:HGNC Symbol;Acc:HGNC:25629]	chr5	14581775	14615007	1	ensembl_havana	4.486723038	4.254944978	4.718501097	3.677822113	4.43361281	4.653400012	4.496533987	4.803606949	4.855362355
ENSG00000267809	0.023864	0.077040663	0.215506423	1.161111423	**NDUFV2P1**	processed_pseudogene	NADH:ubiquinone oxidoreductase core subunit V2 pseudogene 1 [Source:HGNC Symbol;Acc:HGNC:7718]	chr19	53223973	53224879	-1	havana	0.976488958	0.868735747	1.08424217	1.005046425	0.818355163	0.782805653	1.309740823	0.938274071	1.004711615
ENSG00000156273	0.023835	0.077040663	0.193448028	1.143493391	**BACH1**	protein_coding	BTB domain and CNC homolog 1 [Source:HGNC Symbol;Acc:HGNC:935]	chr21	29194071	29630751	1	ensembl_havana	6.093442986	5.996718972	6.190167	6.003192965	5.986165595	6.000798357	6.265319307	6.140385713	6.16479598
ENSG00000152926	0.023829	0.077040663	0.179734103	1.132675107	**ZNF117**	protein_coding	zinc finger protein 117 [Source:HGNC Symbol;Acc:HGNC:12897]	chr7	64971776	65006684	-1	ensembl_havana	4.968751495	4.878884444	5.058618547	4.742847158	4.94769487	4.946111303	4.953655868	5.109505756	5.112694017
ENSG00000185787	0.023823	0.077040663	-0.192724828	-1.14292032	**MORF4L1**	protein_coding	mortality factor 4 like 1 [Source:HGNC Symbol;Acc:HGNC:16989]	chr15	78810487	78898133	1	ensembl_havana	8.490118617	8.586481031	8.393756203	8.677708436	8.620126599	8.461608059	8.417863858	8.46567587	8.297728881
ENSG00000105726	0.023853	0.077040663	-0.193101139	-1.143218477	**ATP13A1**	protein_coding	ATPase 13A1 [Source:HGNC Symbol;Acc:HGNC:24215]	chr19	19645198	19663693	-1	ensembl_havana	5.80643868	5.90298925	5.709888111	5.881964745	5.844913741	5.982089263	5.752604147	5.641145026	5.735915159
ENSG00000204815	0.023861	0.077040663	-0.243137207	-1.183563579	**TTC25**	protein_coding	tetratricopeptide repeat domain 25 [Source:HGNC Symbol;Acc:HGNC:25280]	chr17	41930635	41965651	1	ensembl_havana	3.083923969	3.205492573	2.962355366	3.323695481	3.256039396	3.03674284	3.058645034	2.900515694	2.927905368
ENSG00000233705	0.023818	0.077040663	-0.264331664	-1.201079514	**SLC26A4-AS1**	antisense	SLC26A4 antisense RNA 1 [Source:HGNC Symbol;Acc:HGNC:22385]	chr7	107653968	107662151	-1	havana	3.393936438	3.52610227	3.261770606	3.438344411	3.473392586	3.666569812	3.341846614	3.106334847	3.337130358
ENSG00000177685	0.023819	0.077040663	-0.274884895	-1.209897554	**CRACR2B**	protein_coding	calcium release activated channel regulator 2B [Source:HGNC Symbol;Acc:HGNC:28703]	chr11	826144	831991	1	ensembl_havana	2.2012132	2.338655647	2.063770752	2.404475508	2.169677667	2.441813766	2.238067541	1.966251876	1.986992839
ENSG00000141858	0.023864	0.077040663	-0.309838086	-1.239568575	**SAMD1**	protein_coding	sterile alpha motif domain containing 1 [Source:HGNC Symbol;Acc:HGNC:17958]	chr19	14087840	14091036	-1	ensembl_havana	3.92440611	4.079325153	3.769487067	4.060694397	4.045187566	4.132093496	3.969775382	3.620092638	3.718593182
ENSG00000170373	0.02385	0.077040663	-0.336417432	-1.262617307	**CST1**	protein_coding	cystatin SN [Source:HGNC Symbol;Acc:HGNC:2473]	chr20	23747553	23751268	-1	ensembl_havana	5.462702721	5.630911437	5.294494005	4.951463976	5.87486229	6.066408046	4.530605231	5.780937208	5.571939578
ENSG00000150753	0.023902	0.077099594	-0.198421336	-1.147442084	**CCT5**	protein_coding	chaperonin containing TCP1 subunit 5 [Source:HGNC Symbol;Acc:HGNC:1618]	chr5	10249921	10266412	1	ensembl_havana	8.99450964	9.093720309	8.895298972	9.023938736	9.078064589	9.179157601	8.759052656	8.94978203	8.97706223
ENSG00000127311	0.023903	0.077099594	-0.202249651	-1.150490963	**HELB**	protein_coding	DNA helicase B [Source:HGNC Symbol;Acc:HGNC:17196]	chr12	66302545	66347645	1	ensembl_havana	5.090499464	5.19162429	4.989374638	5.259051314	5.107582415	5.20823914	4.976083394	4.970960125	5.021080396
ENSG00000020633	0.023903	0.077099594	-0.209933453	-1.156634831	**RUNX3**	protein_coding	runt related transcription factor 3 [Source:HGNC Symbol;Acc:HGNC:10473]	chr1	24899511	24965121	-1	ensembl_havana	6.534985031	6.639951757	6.430018304	6.733714778	6.660130811	6.526009684	6.602553576	6.359575812	6.327925525
ENSG00000003400	0.023921	0.077140621	-0.253887171	-1.192415614	**CASP10**	protein_coding	caspase 10 [Source:HGNC Symbol;Acc:HGNC:1500]	chr2	201182881	201229406	1	ensembl_havana	3.006066947	3.133010533	2.879123361	3.126381832	3.180574467	3.092075299	2.717806083	3.029255182	2.890308818
ENSG00000127990	0.023952	0.07722311	-0.221703804	-1.166109934	**SGCE**	protein_coding	sarcoglycan epsilon [Source:HGNC Symbol;Acc:HGNC:10808]	chr7	94524204	94656572	-1	ensembl_havana	4.732396884	4.843248786	4.621544982	4.914727377	4.903675229	4.711343754	4.659372159	4.602484434	4.602778354
ENSG00000085377	0.023962	0.077236803	0.227304516	1.170645709	**PREP**	protein_coding	prolyl endopeptidase [Source:HGNC Symbol;Acc:HGNC:9358]	chr6	105277565	105403084	-1	ensembl_havana	6.050222102	5.936569844	6.16387436	5.729277786	5.999297534	6.081134212	6.079300984	6.150025753	6.262296344
ENSG00000253200	0.023988	0.077284792	0.437711007	1.354453633	**AC037459.3**	antisense		chr8	22613908	22616657	-1	havana	2.365479969	2.146624465	2.584335472	1.977942882	2.06964138	2.392289132	2.705927533	2.523424442	2.523654441
ENSG00000177479	0.023993	0.077284792	0.178144945	1.131428129	**ARIH2**	protein_coding	ariadne RBR E3 ubiquitin protein ligase 2 [Source:HGNC Symbol;Acc:HGNC:690]	chr3	48918821	48986382	1	ensembl_havana	7.399513718	7.310441246	7.488586191	7.228975469	7.318381179	7.38396709	7.393991243	7.484144198	7.587623131
ENSG00000165548	0.023989	0.077284792	-0.214226736	-1.16008196	**TMEM63C**	protein_coding	transmembrane protein 63C [Source:HGNC Symbol;Acc:HGNC:23787]	chr14	77116568	77259495	1	ensembl_havana	5.73497703	5.842090398	5.627863662	6.031695966	5.733652303	5.760922924	5.712352035	5.579399835	5.591839116
ENSG00000174206	0.024	0.077292459	-0.3227959	-1.250752122	**C12orf66**	protein_coding	chromosome 12 open reading frame 66 [Source:HGNC Symbol;Acc:HGNC:26517]	chr12	64186316	64222296	-1	ensembl_havana	3.269490462	3.430888412	3.108092512	3.474621708	3.310998338	3.507045191	2.921565963	3.072584571	3.330127003
ENSG00000064225	0.024012	0.07731215	0.89100664	1.854469628	**ST3GAL6**	protein_coding	ST3 beta-galactoside alpha-2,3-sialyltransferase 6 [Source:HGNC Symbol;Acc:HGNC:18080]	chr3	98732236	98821201	1	ensembl_havana	-0.044590851	-0.490094171	0.400912469	-0.36334239	-0.569826728	-0.537113394	-0.21650287	0.602150945	0.817089329
ENSG00000179477	0.024028	0.077326374	0.222766535	1.166969241	**ALOX12B**	protein_coding	arachidonate 12-lipoxygenase, 12R type [Source:HGNC Symbol;Acc:HGNC:430]	chr17	8072636	8087703	-1	ensembl_havana	1.789008752	1.677625485	1.90039202	1.590147445	1.749818205	1.692910804	1.91595172	1.871294071	1.91393027
ENSG00000197948	0.024033	0.077326374	0.194038767	1.143961713	**FCHSD1**	protein_coding	FCH and double SH3 domains 1 [Source:HGNC Symbol;Acc:HGNC:25463]	chr5	141639302	141651419	-1	ensembl_havana	6.435078031	6.338058647	6.532097415	6.408890289	6.323414117	6.281871536	6.674031097	6.481531411	6.440729736
ENSG00000115325	0.024025	0.077326374	-0.176071737	-1.129803392	**DOK1**	protein_coding	docking protein 1 [Source:HGNC Symbol;Acc:HGNC:2990]	chr2	74549026	74557554	1	ensembl_havana	5.383321612	5.471357481	5.295285744	5.530573149	5.458124712	5.425374582	5.344983642	5.291975367	5.248898223
ENSG00000279108	0.024059	0.077350844	0.374677351	1.296549558	**AC008537.3**	TEC		chr19	40840159	40842039	1	havana	0.482774373	0.295435698	0.670113049	0.41082706	0.428746719	0.046733314	0.540060475	0.800742365	0.669536307
ENSG00000152223	0.02405	0.077350844	0.242347816	1.182916152	**EPG5**	protein_coding	ectopic P-granules autophagy protein 5 homolog [Source:HGNC Symbol;Acc:HGNC:29331]	chr18	45847609	45967274	-1	ensembl_havana	5.742180086	5.621006179	5.863353994	5.684925829	5.437432948	5.740659759	5.877659265	5.821506896	5.890895822
ENSG00000172936	0.02406	0.077350844	0.224256286	1.168174895	**MYD88**	protein_coding	myeloid differentiation primary response 88 [Source:HGNC Symbol;Acc:HGNC:7562]	chr3	38138478	38143022	1	ensembl_havana	5.912216527	5.800088384	6.02434467	5.684221895	5.828526336	5.887516921	6.025957469	5.972113284	6.074963256
ENSG00000110934	0.024062	0.077350844	-0.781161597	-1.718513991	**BIN2**	protein_coding	bridging integrator 2 [Source:HGNC Symbol;Acc:HGNC:1053]	chr12	51281038	51324668	-1	ensembl_havana	0.678425168	1.069005966	0.287844369	1.477650254	1.046001934	0.683365711	0.428834256	-0.11936381	0.554062661
ENSG00000196114	0.02407	0.077360285	0.407126756	1.326042262	**AL031577.1**	processed_pseudogene		chr6	34576258	34576656	-1	havana	1.394146299	1.190582921	1.597709676	0.660286109	1.657409759	1.254052895	1.089724456	1.780136556	1.923268018
ENSG00000101638	0.024095	0.077390024	0.748078257	1.679554088	**ST8SIA5**	protein_coding	ST8 alpha-N-acetyl-neuraminide alpha-2,8-sialyltransferase 5 [Source:HGNC Symbol;Acc:HGNC:17827]	chr18	46667821	46759257	-1	ensembl_havana	-0.355101252	-0.72914038	0.018937876	-1.14838881	-0.842251242	-0.196781087	-0.00649342	0.130707766	-0.067400719
ENSG00000164082	0.024087	0.077390024	0.273293677	1.208563835	**GRM2**	protein_coding	glutamate metabotropic receptor 2 [Source:HGNC Symbol;Acc:HGNC:4594]	chr3	51707070	51718613	1	ensembl_havana	0.054002618	-0.08264422	0.190649457	-0.18813307	-0.521012188	0.461212593	0.176794179	-0.15890847	0.554062661
ENSG00000250712	0.02409	0.077390024	-0.320811241	-1.249032695	**AC106872.9**	processed_pseudogene		chr4	165010459	165011851	1	havana	3.875397899	4.03580352	3.714992278	3.61124356	4.018604945	4.477562055	3.275081252	3.905352962	3.964542621
ENSG00000187950	0.024105	0.077399561	0.36084568	1.284178438	**OVCH1**	protein_coding	ovochymase 1 [Source:HGNC Symbol;Acc:HGNC:23080]	chr12	29412474	29497686	-1	ensembl_havana	0.956220822	0.775797982	1.136643662	0.749126884	0.979703225	0.598563838	1.125834805	1.097781778	1.186314404
ENSG00000244687	0.024111	0.077399561	0.197729385	1.146891875	**UBE2V1**	protein_coding	ubiquitin conjugating enzyme E2 V1 [Source:HGNC Symbol;Acc:HGNC:12494]	chr20	50081124	50115959	-1	ensembl_havana	3.08278204	2.983917347	3.181646733	3.023281264	2.961901777	2.966569	3.291070988	3.155524498	3.098344712
ENSG00000162623	0.024115	0.077399561	-0.196995158	-1.146308338	**TYW3**	protein_coding	tRNA-yW synthesizing protein 3 homolog [Source:HGNC Symbol;Acc:HGNC:24757]	chr1	74733152	74766678	1	ensembl_havana	5.396040804	5.494538383	5.297543225	5.499699095	5.523575835	5.46034022	5.22630432	5.354750712	5.311574643
ENSG00000154723	0.024127	0.077403461	-0.21092473	-1.157429829	**ATP5PF**	protein_coding	ATP synthase peripheral stalk subunit F6 [Source:HGNC Symbol;Acc:HGNC:847]	chr21	25716503	25735673	-1	ensembl_havana	7.213196597	7.318658963	7.107734232	7.388513194	7.361733055	7.205730638	7.17209649	7.066227991	7.084878216
ENSG00000145817	0.024124	0.077403461	-0.308235956	-1.238192783	**YIPF5**	protein_coding	Yip1 domain family member 5 [Source:HGNC Symbol;Acc:HGNC:24877]	chr5	144158159	144170714	-1	ensembl_havana	6.323181896	6.477299874	6.169063918	6.734054918	6.519914592	6.177930112	6.239801459	6.205578398	6.061811898
ENSG00000105851	0.024175	0.077542728	0.419975549	1.33790488	**PIK3CG**	protein_coding	phosphatidylinositol-4,5-bisphosphate 3-kinase catalytic subunit gamma [Source:HGNC Symbol;Acc:HGNC:8978]	chr7	106865278	106907145	1	ensembl_havana	1.173041972	0.963054198	1.383029747	0.565613454	1.184892896	1.138656243	1.309740823	1.491756274	1.347592143
ENSG00000187486	0.024188	0.077565659	-0.484711558	-1.39930607	**KCNJ11**	protein_coding	potassium voltage-gated channel subfamily J member 11 [Source:HGNC Symbol;Acc:HGNC:6257]	chr11	17385859	17389331	-1	ensembl_havana	1.479363154	1.721718933	1.237007376	1.528734855	1.873642861	1.762779083	1.430149367	1.241396536	1.039476224
ENSG00000257365	0.024203	0.077597462	0.262364415	1.199442847	**FNTB**	protein_coding	farnesyltransferase, CAAX box, beta [Source:HGNC Symbol;Acc:HGNC:3785]	chr14	64986720	65062652	1	ensembl_havana	3.206605478	3.07542327	3.337787686	3.163221009	3.139411412	2.923637391	3.372227541	3.290099621	3.351035895
ENSG00000105325	0.024225	0.077632116	0.239243976	1.180373941	**FZR1**	protein_coding	fizzy and cell division cycle 20 related 1 [Source:HGNC Symbol;Acc:HGNC:24824]	chr19	3506273	3538330	1	ensembl_havana	5.721645584	5.602023596	5.841267572	5.767662548	5.535884622	5.502523618	6.10553347	5.812589488	5.605679757
ENSG00000174197	0.02423	0.077632116	-0.253004255	-1.191686089	**MGA**	protein_coding	MGA, MAX dimerization protein [Source:HGNC Symbol;Acc:HGNC:14010]	chr15	41621224	41773081	1	ensembl_havana	6.431999952	6.55850208	6.305497825	6.523909334	6.446643919	6.704952986	6.178801784	6.350362846	6.387328845
ENSG00000173040	0.024228	0.077632116	-0.527645781	-1.441574884	**EVC2**	protein_coding	EvC ciliary complex subunit 2 [Source:HGNC Symbol;Acc:HGNC:19747]	chr4	5542772	5709548	-1	ensembl_havana	2.044538945	2.308361835	1.780716054	2.142776609	2.304436355	2.477872542	1.956619779	1.831488934	1.554039449
ENSG00000169683	0.024255	0.077693339	0.267726902	1.203909463	**LRRC45**	protein_coding	leucine rich repeat containing 45 [Source:HGNC Symbol;Acc:HGNC:28302]	chr17	82023302	82031151	1	ensembl_havana	3.616212885	3.482349434	3.750076335	3.067138625	3.586306005	3.79360367	3.49810005	3.826549371	3.925579585
ENSG00000165684	0.024268	0.077701318	0.260927049	1.19824843	**SNAPC4**	protein_coding	small nuclear RNA activating complex polypeptide 4 [Source:HGNC Symbol;Acc:HGNC:11137]	chr9	136375577	136400168	-1	ensembl_havana	4.222625491	4.092161966	4.353089015	4.018872751	4.028887076	4.22872607	4.285277268	4.431632485	4.342357293
ENSG00000186577	0.024268	0.077701318	0.200553142	1.14913886	**SMIM29**	protein_coding	small integral membrane protein 29 [Source:HGNC Symbol;Acc:HGNC:1340]	chr6	34246380	34249470	-1	ensembl_havana	2.98534384	2.88506727	3.085620411	3.058473165	2.868808811	2.727919833	3.200849831	3.151488815	2.904522587
ENSG00000240065	0.024311	0.077820879	-0.274474815	-1.209553695	**PSMB9**	protein_coding	proteasome subunit beta 9 [Source:HGNC Symbol;Acc:HGNC:9546]	chr6	32844136	32859585	1	ensembl_havana	7.082951189	7.220188597	6.945713782	7.087604851	7.344342491	7.228618449	6.945872462	6.894861478	6.996407404
ENSG00000273064	0.024325	0.077832112	0.239722707	1.18076569	**AC017083.1**	antisense		chr2	68252870	68253848	1	havana	0.360853221	0.240991868	0.480714575	0.14220989	0.271370116	0.309395597	0.513047461	0.399280361	0.529815901
ENSG00000168067	0.024325	0.077832112	0.206982728	1.154271599	**MAP4K2**	protein_coding	mitogen-activated protein kinase kinase kinase kinase 2 [Source:HGNC Symbol;Acc:HGNC:6864]	chr11	64784914	64803241	-1	ensembl_havana	4.908353517	4.804862153	5.011844881	4.855932554	4.757997609	4.800656295	5.151642097	4.951362452	4.932530093
ENSG00000104324	0.024355	0.077858076	0.283221468	1.216909152	**CPQ**	protein_coding	carboxypeptidase Q [Source:HGNC Symbol;Acc:HGNC:16910]	chr8	96645227	97149654	1	ensembl_havana	2.823270003	2.681659269	2.964880738	2.711312495	2.709182922	2.624482391	2.842395714	2.966320977	3.085925522
ENSG00000129566	0.024348	0.077858076	0.198048352	1.14714547	**TEP1**	protein_coding	telomerase associated protein 1 [Source:HGNC Symbol;Acc:HGNC:11726]	chr14	20365667	20413429	-1	ensembl_havana	5.490017042	5.390992866	5.589041218	5.488452941	5.215948161	5.468577497	5.656359413	5.493531321	5.617232921
ENSG00000138050	0.024345	0.077858076	-0.213505929	-1.159502499	**THUMPD2**	protein_coding	THUMP domain containing 2 [Source:HGNC Symbol;Acc:HGNC:14890]	chr2	39736060	39779267	-1	ensembl_havana	4.714550094	4.821303059	4.60779713	4.67218582	4.945525497	4.846197859	4.530605231	4.62884954	4.663936618
ENSG00000218018	0.024351	0.077858076	-0.733601919	-1.662785323	**AL109955.1**	antisense		chr20	57384160	57393062	-1	havana	-0.024585808	0.342215151	-0.391386768	0.565613454	-0.18055577	0.641587769	-0.69455235	-0.37473441	-0.104873545
ENSG00000183172	0.024366	0.077869985	0.22782148	1.171065264	**SMDT1**	protein_coding	single-pass membrane protein with aspartate rich tail 1 [Source:HGNC Symbol;Acc:HGNC:25055]	chr22	42079691	42084284	1	ensembl_havana	3.639763393	3.525852653	3.753674133	3.544549275	3.555343125	3.477665558	3.756011721	3.682954534	3.822056143
ENSG00000102119	0.024372	0.077869985	0.177159165	1.1306553	**EMD**	protein_coding	emerin [Source:HGNC Symbol;Acc:HGNC:3331]	chrX	154379197	154381523	1	ensembl_havana	6.155241368	6.066661785	6.24382095	6.032249257	6.144904156	6.022831941	6.232574829	6.302939542	6.19594848
ENSG00000125931	0.024375	0.077869985	-0.479838973	-1.394588	**CITED1**	protein_coding	Cbp/p300 interacting transactivator with Glu/Asp rich carboxy-terminal domain 1 [Source:HGNC Symbol;Acc:HGNC:1986]	chrX	72301638	72307187	-1	ensembl_havana	-0.373672076	-0.133752589	-0.613591563	0.174648163	-0.218971072	-0.356934859	0.069637898	-0.80561234	-1.10480025
ENSG00000273015	0.024387	0.077889831	0.176398963	1.130059679	**AC008124.1**	lincRNA		chr12	45718046	45727775	-1	havana	4.402818702	4.31461922	4.491018183	4.438382881	4.207877638	4.297597141	4.596424846	4.401364514	4.47526519
ENSG00000147274	0.024393	0.077893694	-0.182735069	-1.13503365	**RBMX**	protein_coding	RNA binding motif protein, X-linked [Source:HGNC Symbol;Acc:HGNC:9910]	chrX	136848004	136880764	-1	ensembl_havana	8.636865809	8.728233343	8.545498274	8.717126934	8.751193286	8.716379809	8.489878164	8.580348179	8.566268479
ENSG00000180376	0.024402	0.077904275	-0.208897768	-1.155804801	**CCDC66**	protein_coding	coiled-coil domain containing 66 [Source:HGNC Symbol;Acc:HGNC:27709]	chr3	56557161	56621818	1	ensembl_havana	5.579252517	5.683701401	5.474803633	5.626764363	5.744299282	5.680040559	5.3611681	5.493531321	5.56971148
ENSG00000188130	0.024421	0.077912041	-0.202782199	-1.150915726	**MAPK12**	protein_coding	mitogen-activated protein kinase 12 [Source:HGNC Symbol;Acc:HGNC:6874]	chr22	50245450	50261825	-1	ensembl_havana	5.476232855	5.577623954	5.374841755	5.725861468	5.454315226	5.552695168	5.459067633	5.337091883	5.32836575
ENSG00000214941	0.024416	0.077912041	-0.24489278	-1.185004699	**ZSWIM7**	protein_coding	zinc finger SWIM-type containing 7 [Source:HGNC Symbol;Acc:HGNC:26993]	chr17	15976560	15999717	-1	ensembl_havana	2.001898991	2.124345381	1.879452601	1.931242682	2.311191904	2.130601557	1.740743887	1.919554095	1.978059821
ENSG00000253738	0.024411	0.077912041	-0.332038005	-1.258790333	**OTUD6B-AS1**	antisense	OTUD6B antisense RNA 1 (head to head) [Source:HGNC Symbol;Acc:HGNC:50466]	chr8	91059909	91070189	-1	havana	4.045756978	4.21177598	3.879737976	4.301877063	4.104724807	4.22872607	3.74734528	3.970942906	3.920925741
ENSG00000105072	0.024435	0.077930227	0.277757969	1.212309421	**C19orf44**	protein_coding	chromosome 19 open reading frame 44 [Source:HGNC Symbol;Acc:HGNC:26141]	chr19	16496311	16521352	1	ensembl_havana	3.584335215	3.445456231	3.723214199	3.387357714	3.427497328	3.52151365	3.845352391	3.657568469	3.666721737
ENSG00000280594	0.024437	0.077930227	-0.332711648	-1.259378242	**BTG3-AS1**	processed_transcript	BTG3 antisense RNA 1 [Source:HGNC Symbol;Acc:HGNC:53145]	chr21	17611744	17633199	1	havana	0.085842035	0.252197859	-0.080513789	0.852997155	-0.143136894	0.046733314	0.276538753	-0.37473441	-0.143345712
ENSG00000217801	0.024453	0.07795041	0.260444575	1.197847772	**AL390719.1**	transcribed_unprocessed_pseudogene		chr1	1059734	1069355	1	havana	5.105565657	4.975343369	5.235787944	4.819213589	5.023696113	5.083120406	5.169051589	5.361754164	5.176558081
ENSG00000143401	0.024455	0.07795041	-0.253550923	-1.19213773	**ANP32E**	protein_coding	acidic nuclear phosphoprotein 32 family member E [Source:HGNC Symbol;Acc:HGNC:16673]	chr1	150218417	150236156	-1	ensembl_havana	7.3931587	7.519934161	7.266383238	7.609454841	7.557500056	7.392847585	7.208595998	7.325730603	7.264823114
ENSG00000140044	0.02446	0.07795041	-0.96209461	-1.94813629	**JDP2**	protein_coding	Jun dimerization protein 2 [Source:HGNC Symbol;Acc:HGNC:17546]	chr14	75427716	75474111	1	ensembl_havana	-0.662526767	-0.181479462	-1.143574072	-1.070289	0.243386951	0.28246366	-1.7649502	-1.52242631	-0.143345712
ENSG00000146830	0.024468	0.077959654	0.273291696	1.208562175	**GIGYF1**	protein_coding	GRB10 interacting GYF protein 1 [Source:HGNC Symbol;Acc:HGNC:9126]	chr7	100679507	100694037	-1	ensembl_havana	6.390702741	6.254056894	6.527348589	6.227521167	6.141591485	6.393058029	6.5823921	6.514899332	6.484754336
ENSG00000181029	0.024509	0.077973242	0.489943078	1.404389464	**TRAPPC5**	protein_coding	trafficking protein particle complex 5 [Source:HGNC Symbol;Acc:HGNC:23067]	chr19	7680843	7687703	1	ensembl_havana	0.386598651	0.141627112	0.63157019	0.174648163	0.52486995	-0.274636777	0.716189844	0.648704825	0.529815901
ENSG00000131747	0.024501	0.077973242	-0.19026206	-1.14097095	**TOP2A**	protein_coding	DNA topoisomerase II alpha [Source:HGNC Symbol;Acc:HGNC:11989]	chr17	40388516	40417950	-1	ensembl_havana	9.465797642	9.560928672	9.370666612	9.417130589	9.595647541	9.670007886	9.163319163	9.451026222	9.49765445
ENSG00000156787	0.024491	0.077973242	-0.193177442	-1.143278943	**TBC1D31**	protein_coding	TBC1 domain family member 31 [Source:HGNC Symbol;Acc:HGNC:30888]	chr8	123041968	123152153	1	ensembl_havana	4.209813057	4.306401778	4.113224336	4.266573281	4.371946913	4.280685141	4.065845049	4.120886932	4.152941027
ENSG00000066651	0.024509	0.077973242	-0.232571432	-1.174927252	**TRMT11**	protein_coding	tRNA methyltransferase 11 homolog [Source:HGNC Symbol;Acc:HGNC:21080]	chr6	125986430	126039276	1	ensembl_havana	4.166009503	4.282295219	4.049723787	4.275947831	4.273340686	4.297597141	3.957304068	3.995916025	4.195951269
ENSG00000124215	0.024505	0.077973242	-0.272947554	-1.208273918	**CDH26**	protein_coding	cadherin 26 [Source:HGNC Symbol;Acc:HGNC:15902]	chr20	59958427	60034011	1	ensembl_havana	0.931332354	1.067806131	0.794858578	1.126095262	0.837200041	1.24012309	0.716189844	0.529376201	1.139009687
ENSG00000124613	0.024506	0.077973242	-0.318626567	-1.247142714	**ZNF391**	protein_coding	zinc finger protein 391 [Source:HGNC Symbol;Acc:HGNC:18779]	chr6	27374615	27403904	1	ensembl_havana	3.13122614	3.290539423	2.971912856	3.210920778	3.231320443	3.429377047	2.741273741	3.139313586	3.035151242
ENSG00000185862	0.024511	0.077973242	-0.902455366	-1.869244601	**EVI2B**	protein_coding	ecotropic viral integration site 2B [Source:HGNC Symbol;Acc:HGNC:3500]	chr17	31303766	31314112	-1	ensembl_havana	-0.049242021	0.401985662	-0.500469704	1.222335231	0.298820819	-0.315199064	-0.26243933	-0.74417246	-0.494797317
ENSG00000169696	0.024524	0.077999156	0.300845548	1.231866185	**ASPSCR1**	protein_coding	ASPSCR1, UBX domain containing tether for SLC2A4 [Source:HGNC Symbol;Acc:HGNC:13825]	chr17	81976807	82017406	1	ensembl_havana	3.164798088	3.014375314	3.315220862	2.873244578	3.020785603	3.149095762	3.37972385	3.264170155	3.301768582
ENSG00000187609	0.02455	0.078028882	0.676365046	1.598108152	**EXD3**	protein_coding	exonuclease 3'-5' domain containing 3 [Source:HGNC Symbol;Acc:HGNC:26023]	chr9	137306896	137423262	-1	ensembl_havana	1.193451395	0.855268872	1.531633918	0.490295136	1.046001934	1.029509544	1.729053464	1.385808199	1.480040089
ENSG00000119699	0.024561	0.078028882	0.302095116	1.232933609	**TGFB3**	protein_coding	transforming growth factor beta 3 [Source:HGNC Symbol;Acc:HGNC:11769]	chr14	75958099	75982991	-1	ensembl_havana	2.32725998	2.176212422	2.478307538	1.882980075	2.351074074	2.294583118	2.394263752	2.510878368	2.529780494
ENSG00000100731	0.024553	0.078028882	0.205668049	1.153220229	**PCNX1**	protein_coding	pecanex homolog 1 [Source:HGNC Symbol;Acc:HGNC:19740]	chr14	70907405	71115382	1	ensembl_havana	6.810735919	6.707901894	6.913569943	6.812165238	6.606904424	6.70463602	7.019700876	6.893351904	6.827657049
ENSG00000081019	0.02454	0.078028882	0.189767124	1.140579592	**RSBN1**	protein_coding	round spermatid basic protein 1 [Source:HGNC Symbol;Acc:HGNC:25642]	chr1	113761832	113812476	-1	ensembl_havana	5.084592507	4.989708945	5.179476069	5.173358694	5.053211401	4.74255674	5.329583547	5.211882794	4.996961868
ENSG00000182247	0.024557	0.078028882	0.188222011	1.139358696	**UBE2E2**	protein_coding	ubiquitin conjugating enzyme E2 E2 [Source:HGNC Symbol;Acc:HGNC:12478]	chr3	23203020	23591793	1	ensembl_havana	3.256132794	3.162021788	3.3502438	2.727802065	3.344313992	3.413949308	2.900875057	3.488704842	3.6611515
ENSG00000132334	0.024586	0.078075825	0.209913957	1.156619201	**PTPRE**	protein_coding	protein tyrosine phosphatase, receptor type E [Source:HGNC Symbol;Acc:HGNC:9669]	chr10	127907061	128085855	1	ensembl_havana	2.662713535	2.557756556	2.767670513	2.59028595	2.546946467	2.536037252	2.900875057	2.716081261	2.686055222
ENSG00000144840	0.024584	0.078075825	0.197005987	1.146316943	**RABL3**	protein_coding	RAB, member of RAS oncogene family like 3 [Source:HGNC Symbol;Acc:HGNC:18072]	chr3	120686681	120742993	-1	ensembl_havana	5.001170613	4.90266762	5.099673607	4.844636702	4.997758064	4.865608094	4.978553955	5.184494938	5.135971927
ENSG00000152818	0.024619	0.078162451	-0.20809543	-1.155162192	**UTRN**	protein_coding	utrophin [Source:HGNC Symbol;Acc:HGNC:12635]	chr6	144285701	144853034	1	ensembl_havana	5.585488036	5.689535751	5.481440321	5.655779152	5.636138322	5.776689778	5.414145672	5.525067154	5.505108138
ENSG00000155792	0.024632	0.078185821	0.551626255	1.465736994	**DEPTOR**	protein_coding	DEP domain containing MTOR interacting protein [Source:HGNC Symbol;Acc:HGNC:22953]	chr8	119873717	120050913	1	ensembl_havana	1.433646174	1.157833047	1.709459302	1.190963204	0.740413176	1.542122761	1.593788933	1.737696596	1.796892377
ENSG00000073282	0.024638	0.078187959	-0.357323862	-1.281047404	**TP63**	protein_coding	tumor protein p63 [Source:HGNC Symbol;Acc:HGNC:15979]	chr3	189631416	189897279	1	ensembl_havana	1.853335587	2.031997519	1.674673656	1.902479286	2.108942002	2.084571268	1.763844323	1.780136556	1.480040089
ENSG00000122912	0.024653	0.078218215	0.188097158	1.139260098	**SLC25A16**	protein_coding	solute carrier family 25 member 16 [Source:HGNC Symbol;Acc:HGNC:10986]	chr10	68477999	68527474	-1	ensembl_havana	4.367619617	4.273571038	4.461668196	4.336337532	4.229436075	4.254939508	4.463403562	4.441582317	4.48001871
ENSG00000143314	0.024691	0.078320281	-0.194384	-1.144235492	**MRPL24**	protein_coding	mitochondrial ribosomal protein L24 [Source:HGNC Symbol;Acc:HGNC:14037]	chr1	156737303	156741590	-1	ensembl_havana	5.502930201	5.600122201	5.405738201	5.589655524	5.529381289	5.68132979	5.322793966	5.354750712	5.539669924
ENSG00000130413	0.024717	0.078363287	0.235202198	1.177071695	**STK33**	protein_coding	serine/threonine kinase 33 [Source:HGNC Symbol;Acc:HGNC:14568]	chr11	8391868	8594289	-1	ensembl_havana	4.141171161	4.023570062	4.25877226	3.87082891	4.139302394	4.060578883	4.142757667	4.241596386	4.391962726
ENSG00000151014	0.024719	0.078363287	-0.220465573	-1.165109519	**NOCT**	protein_coding	nocturnin [Source:HGNC Symbol;Acc:HGNC:14254]	chr4	139015789	139045939	1	ensembl_havana	3.191044299	3.301277086	3.080811513	3.44832903	3.195256367	3.26024586	3.113627387	3.055408658	3.073398494
ENSG00000263934	0.02472	0.078363287	-0.293249324	-1.225397085	**SNORD3A**	snoRNA	small nucleolar RNA, C/D box 3A [Source:HGNC Symbol;Acc:HGNC:33189]	chr17	19188016	19188714	1	ensembl_havana	0.791146754	0.937771416	0.644522092	1.022973463	1.170104606	0.620236179	0.894226935	0.900298706	0.139040636
ENSG00000218891	0.02476	0.078450423	0.286457928	1.219642161	**ZNF579**	protein_coding	zinc finger protein 579 [Source:HGNC Symbol;Acc:HGNC:26646]	chr19	55576770	55580845	-1	ensembl_havana	1.2400103	1.096781336	1.383239264	1.022973463	1.428032914	0.83933763	1.415636519	1.517070703	1.21701057
ENSG00000071189	0.024764	0.078450423	0.203710066	1.151656174	**SNX13**	protein_coding	sorting nexin 13 [Source:HGNC Symbol;Acc:HGNC:21335]	chr7	17790761	17940501	-1	ensembl_havana	5.903133102	5.801278068	6.004988135	5.794002125	5.765975192	5.843856889	5.93029428	6.057623619	6.027046505
ENSG00000264107	0.024764	0.078450423	-0.405404059	-1.324459803	**AC138207.2**	antisense		chr17	31090787	31095450	-1	havana	-0.327577924	-0.124875895	-0.530279954	-0.06941355	0.09469996	-0.399914092	-0.30988669	-0.62862566	-0.652327512
ENSG00000173548	0.024787	0.078504478	-0.231727024	-1.17423977	**SNX33**	protein_coding	sorting nexin 33 [Source:HGNC Symbol;Acc:HGNC:28468]	chr15	75647906	75662301	1	ensembl_havana	4.471384274	4.587247786	4.355520762	4.612758167	4.483808757	4.665176435	4.338178756	4.382549869	4.34583366
ENSG00000132676	0.024792	0.078504847	-0.187681556	-1.138931955	**DAP3**	protein_coding	death associated protein 3 [Source:HGNC Symbol;Acc:HGNC:2673]	chr1	155687960	155739010	1	ensembl_havana	7.551401484	7.645242262	7.457560706	7.634814366	7.705056253	7.595856169	7.441775189	7.467197194	7.463709736
ENSG00000147570	0.024831	0.07860877	0.41829224	1.336344748	**DNAJC5B**	protein_coding	DnaJ heat shock protein family (Hsp40) member C5 beta [Source:HGNC Symbol;Acc:HGNC:24138]	chr8	66021560	66100526	1	ensembl_havana	0.041497744	-0.167648376	0.250643864	-0.41068387	0.030651399	-0.122912658	-0.12879794	0.256279753	0.624449778
ENSG00000171444	0.024876	0.078728662	0.35840932	1.282011603	**MCC**	protein_coding	mutated in colorectal cancers [Source:HGNC Symbol;Acc:HGNC:6935]	chr5	113022099	113488830	-1	ensembl_havana	4.779937925	4.600733265	4.959142585	4.018872751	4.797022811	4.986304232	4.632954382	5.109505756	5.134967615

ENSG00000155755	0.02488	0.078728662	-0.207475541	-1.154665956	**TMEM237**	protein_coding	transmembrane protein 237 [Source:HGNC Symbol;Acc:HGNC:14432]	chr2	201620184	201643570	-1	ensembl_havana	4.83849204	4.942229811	4.73475427	4.76831163	5.075206302	4.9831715	4.650103376	4.786793412	4.767366022
ENSG00000139651	0.024895	0.078742496	0.209068228	1.155941372	**ZNF740**	protein_coding	zinc finger protein 740 [Source:HGNC Symbol;Acc:HGNC:27465]	chr12	53180700	53195141	1	ensembl_havana	7.153270752	7.048736638	7.257804866	7.247916056	6.992206046	6.906087811	7.356254165	7.260011955	7.157148478
ENSG00000196505	0.02489	0.078742496	0.200118688	1.148792861	**GDAP2**	protein_coding	ganglioside induced differentiation associated protein 2 [Source:HGNC Symbol;Acc:HGNC:18010]	chr1	117863485	117929630	-1	ensembl_havana	4.857042623	4.756983279	4.957101967	4.719675806	4.851334342	4.699939689	4.924481562	4.974391071	4.972433268
ENSG00000146233	0.024913	0.078782676	0.774269624	1.710323967	**CYP39A1**	protein_coding	cytochrome P450 family 39 subfamily A member 1 [Source:HGNC Symbol;Acc:HGNC:17449]	chr6	46549580	46652830	-1	ensembl_havana	0.354937946	-0.032196866	0.742072758	-0.03191641	0.09469996	-0.159374151	0.106248156	1.046547334	1.073422785
ENSG00000143318	0.024931	0.078804843	-0.256772614	-1.194802875	**CASQ1**	protein_coding	calsequestrin 1 [Source:HGNC Symbol;Acc:HGNC:1512]	chr1	160190556	160201886	1	ensembl_havana	4.255221147	4.383607453	4.12683484	4.513220058	4.264666003	4.372936299	4.10707367	4.022675282	4.250755567
ENSG00000178694	0.024926	0.078804843	-0.267055921	-1.203349668	**NSUN3**	protein_coding	NOP2/Sun RNA methyltransferase family member 3 [Source:HGNC Symbol;Acc:HGNC:26208]	chr3	94062916	94128545	1	ensembl_havana	3.660683375	3.794211335	3.527155415	3.770935591	3.927016074	3.684682341	3.383457449	3.608362986	3.589645809
ENSG00000197275	0.024938	0.078810204	-0.275755186	-1.210627633	**RAD54B**	protein_coding	RAD54 homolog B (S. cerevisiae) [Source:HGNC Symbol;Acc:HGNC:17228]	chr8	94371960	94475109	-1	ensembl_havana	0.891435739	1.029313333	0.753558146	0.705390237	1.199531136	1.183018625	0.36983092	0.800742365	1.090101154
ENSG00000124795	0.024947	0.078820012	-0.216394393	-1.161826299	**DEK**	protein_coding	DEK proto-oncogene [Source:HGNC Symbol;Acc:HGNC:2768]	chr6	18223868	18264823	-1	ensembl_havana	7.339635711	7.447832907	7.231438515	7.339059419	7.559806507	7.444632797	7.184187514	7.231142822	7.278985208
ENSG00000260103	0.024977	0.078897374	0.202462785	1.150660941	**AC012435.1**	transcribed_processed_pseudogene		chr15	74478070	74490286	-1	havana	1.960790038	1.859558646	2.062021431	1.683274163	2.029240114	1.866161661	1.976531838	2.187350601	2.022181853
ENSG00000099800	0.025013	0.078940885	0.266526776	1.20290839	**TIMM13**	protein_coding	translocase of inner mitochondrial membrane 13 [Source:HGNC Symbol;Acc:HGNC:11816]	chr19	2425624	2427894	-1	ensembl_havana	5.415374108	5.282110721	5.548637496	5.32827068	5.199657163	5.318404319	5.610105401	5.610602731	5.425204358
ENSG00000063245	0.025006	0.078940885	0.240232267	1.181182811	**EPN1**	protein_coding	epsin 1 [Source:HGNC Symbol;Acc:HGNC:21604]	chr19	55675226	55709858	1	ensembl_havana	5.096221133	4.976105	5.216337267	4.97927634	4.778861109	5.17017755	5.310099338	5.071565226	5.267347237
ENSG00000025039	0.025013	0.078940885	-0.225941591	-1.169540313	**RRAGD**	protein_coding	Ras related GTP binding D [Source:HGNC Symbol;Acc:HGNC:19903]	chr6	89364636	89412270	-1	ensembl_havana	7.058895343	7.171866138	6.945924547	7.497510783	7.188424578	6.829663053	7.224294943	6.884868976	6.728609723
ENSG00000101447	0.025001	0.078940885	-0.274087538	-1.209229045	**FAM83D**	protein_coding	family with sequence similarity 83 member D [Source:HGNC Symbol;Acc:HGNC:16122]	chr20	38926312	38953106	1	ensembl_havana	4.924769336	5.061813105	4.787725567	4.712789269	5.134494532	5.338155514	4.496533987	4.983500571	4.883142143
ENSG00000102466	0.025028	0.078973072	0.251862697	1.19074352	**FGF14**	protein_coding	fibroblast growth factor 14 [Source:HGNC Symbol;Acc:HGNC:3671]	chr13	101710804	102402457	-1	ensembl_havana	3.564349557	3.438418209	3.690280906	3.226476559	3.791063673	3.297714395	3.613866272	3.902954406	3.55402204
ENSG00000166797	0.025035	0.078975699	-0.19605064	-1.145558108	**FAM96A**	protein_coding	family with sequence similarity 96 member A [Source:HGNC Symbol;Acc:HGNC:26235]	chr15	64072559	64094018	-1	ensembl_havana	5.968041558	6.066066878	5.870016238	6.087598719	6.131130434	5.979471482	5.831939025	5.919094579	5.859015111
ENSG00000135677	0.025047	0.078998235	0.229107534	1.172109646	**GNS**	protein_coding	glucosamine (N-acetyl)-6-sulfatase [Source:HGNC Symbol;Acc:HGNC:4422]	chr12	64713445	64759447	-1	ensembl_havana	7.181848461	7.067294693	7.296402228	7.195033751	7.006844708	7.000005621	7.294140051	7.291068833	7.303997799
ENSG00000217825	0.025065	0.079018332	0.303434722	1.234078974	**AC099552.1**	lincRNA		chr7	155196563	155198455	-1	havana	4.287869283	4.136151922	4.439586644	4.086496776	4.116342995	4.205615997	4.24890024	4.633197259	4.436662434
ENSG00000168214	0.025061	0.079018332	0.180902006	1.133592412	**RBPJ**	protein_coding	recombination signal binding protein for immunoglobulin kappa J region [Source:HGNC Symbol;Acc:HGNC:5724]	chr4	26163455	26435131	1	ensembl_havana	5.936573168	5.846122165	6.027024171	5.86777506	5.910888514	5.759702922	6.006720521	6.09855034	5.975801652
ENSG00000081377	0.025146	0.079142071	0.215990394	1.161500998	**CDC14B**	protein_coding	cell division cycle 14B [Source:HGNC Symbol;Acc:HGNC:1719]	chr9	96490241	96619830	-1	ensembl_havana	3.859208549	3.751213352	3.967203746	3.438344411	3.859488969	3.955806677	3.738626465	4.098002545	4.064982229
ENSG00000160785	0.025138	0.079142071	0.180689438	1.133425399	**SLC25A44**	protein_coding	solute carrier family 25 member 44 [Source:HGNC Symbol;Acc:HGNC:29036]	chr1	156193932	156212796	1	ensembl_havana	6.646570125	6.556225407	6.736914844	6.683525733	6.605531101	6.379619386	6.86506006	6.746909142	6.598775331
ENSG00000278291	0.025119	0.079142071	0.179476195	1.132472639	**AL161772.1**	antisense		chr13	20699307	20703718	-1	havana	1.81612714	1.726389043	1.905865237	1.222335231	1.864486201	2.092345696	1.430149367	2.055343696	2.23210265
ENSG00000144136	0.025148	0.079142071	-0.185814786	-1.137459191	**SLC20A1**	protein_coding	solute carrier family 20 member 1 [Source:HGNC Symbol;Acc:HGNC:10946]	chr2	112645857	112663827	1	ensembl_havana	6.506144313	6.599051705	6.41323692	6.521940847	6.551995368	6.723218901	6.341672567	6.414072429	6.483965763
ENSG00000134954	0.025122	0.079142071	-0.196388628	-1.145826515	**ETS1**	protein_coding	ETS proto-oncogene 1, transcription factor [Source:HGNC Symbol;Acc:HGNC:3488]	chr11	128458761	128587558	-1	ensembl_havana	8.369076658	8.467270971	8.270882344	8.60815867	8.317117184	8.476537061	8.344102244	8.192631663	8.275913124
ENSG00000188878	0.025128	0.079142071	-0.221299912	-1.16578352	**FBF1**	protein_coding	Fas binding factor 1 [Source:HGNC Symbol;Acc:HGNC:24674]	chr17	75909574	75941140	-1	ensembl_havana	2.01074376	2.121393716	1.900093804	2.101191983	2.242169235	2.02081993	1.895179555	1.910030375	1.89507148
ENSG00000185238	0.025138	0.079142071	-0.247582375	-1.187215946	**PRMT3**	protein_coding	protein arginine methyltransferase 3 [Source:HGNC Symbol;Acc:HGNC:30163]	chr11	20387530	20509294	1	ensembl_havana	4.540131835	4.663923023	4.416340648	4.626014575	4.658202087	4.707552406	4.228287279	4.454742908	4.565991757
ENSG00000230798	0.025148	0.079142071	-0.303036886	-1.233738713	**FOXD3-AS1**	antisense	FOXD3 antisense RNA 1 (head to head) [Source:HGNC Symbol;Acc:HGNC:40241]	chr1	63320884	63324441	-1	havana	1.57364467	1.725163113	1.422126227	1.931242682	1.657409759	1.586836898	1.47283199	1.300906777	1.492639914
ENSG00000259153	0.025159	0.079148981	0.189656245	1.140491935	**AC004816.1**	lincRNA		chr14	70810205	70815403	1	havana	2.307801374	2.212973252	2.402629496	2.005254198	2.415188039	2.218477517	2.22979561	2.637466823	2.340626055
ENSG00000100802	0.025161	0.079148981	-0.386130489	-1.306883453	**C14orf93**	protein_coding	chromosome 14 open reading frame 93 [Source:HGNC Symbol;Acc:HGNC:20162]	chr14	22985908	23010166	-1	ensembl_havana	2.697164959	2.890230203	2.504099715	2.817841883	2.836393311	3.016455416	2.246292314	2.748504565	2.517502265
ENSG00000135953	0.025199	0.079241762	0.192599941	1.142821387	**MFSD9**	protein_coding	major facilitator superfamily domain containing 9 [Source:HGNC Symbol;Acc:HGNC:28158]	chr2	102715840	102736888	-1	ensembl_havana	4.643916418	4.547616448	4.740216389	4.553863057	4.582042512	4.506943775	4.786069135	4.80874117	4.625838861
ENSG00000172661	0.02522	0.079241762	0.1908339	1.141423285	**WASHC2C**	protein_coding	WASH complex subunit 2C [Source:HGNC Symbol;Acc:HGNC:23414]	chr10	45727200	45792961	1	ensembl_havana	6.07216249	5.97674554	6.16757944	6.063441193	5.967043105	5.899752321	6.199599409	6.22347036	6.07966855
ENSG00000136824	0.025221	0.079241762	0.18798107	1.13916843	**SMC2**	protein_coding	structural maintenance of chromosomes 2 [Source:HGNC Symbol;Acc:HGNC:14011]	chr9	104094260	104141417	1	ensembl_havana	6.788234196	6.69424366	6.882224731	6.447981257	6.80566816	6.829081564	6.700088167	6.96351128	6.983074746
ENSG00000163069	0.025203	0.079241762	0.173724607	1.127966802	**SGCB**	protein_coding	sarcoglycan beta [Source:HGNC Symbol;Acc:HGNC:10806]	chr4	52020706	52038482	-1	ensembl_havana	4.696277527	4.609415224	4.783139831	4.624547642	4.660849095	4.542848935	4.811235279	4.830360032	4.707824182
ENSG00000169371	0.025228	0.079241762	-0.173888541	-1.128094981	**SNUPN**	protein_coding	snurportin 1 [Source:HGNC Symbol;Acc:HGNC:14245]	chr15	75598083	75626469	-1	ensembl_havana	4.365678244	4.452622515	4.278733973	4.468131009	4.41961437	4.470122166	4.295221319	4.232073423	4.308907178
ENSG00000101574	0.025229	0.079241762	-0.210148906	-1.156807577	**METTL4**	protein_coding	methyltransferase like 4 [Source:HGNC Symbol;Acc:HGNC:24726]	chr18	2537525	2571509	-1	ensembl_havana	4.727868034	4.832942487	4.62279358	4.85967827	4.81495872	4.82419047	4.553985209	4.688556671	4.625838861
ENSG00000164933	0.025227	0.079241762	-0.244010473	-1.184280209	**SLC25A32**	protein_coding	solute carrier family 25 member 32 [Source:HGNC Symbol;Acc:HGNC:29683]	chr8	103398635	103415189	-1	ensembl_havana	5.152543186	5.274548422	5.03053795	5.362113708	5.226707517	5.234824043	5.022315528	4.930317649	5.138980672
ENSG00000101974	0.025239	0.079255806	0.185083004	1.13688238	**ATP11C**	protein_coding	ATPase phospholipid transporting 11C [Source:HGNC Symbol;Acc:HGNC:13554]	chrX	139726346	139945276	-1	ensembl_havana	8.074214677	7.981673176	8.166756179	8.097819639	7.995478978	7.85172091	8.244976293	8.162116946	8.093175298
ENSG00000158169	0.025255	0.079289127	0.200532815	1.14912267	**FANCC**	protein_coding	Fanconi anemia complementation group C [Source:HGNC Symbol;Acc:HGNC:3584]	chr9	95099054	95426796	-1	ensembl_havana	5.1237974	5.023530993	5.224063808	4.840851678	5.020608058	5.209133242	5.036612625	5.30020116	5.335377639
ENSG00000156873	0.025324	0.079453417	0.21169595	1.15804872	**PHKG2**	protein_coding	phosphorylase kinase catalytic subunit gamma 2 [Source:HGNC Symbol;Acc:HGNC:8931]	chr16	30748270	30761176	1	ensembl_havana	5.573931013	5.468083038	5.679778988	5.520355035	5.54737421	5.336519869	5.738825475	5.663280516	5.637230975
ENSG00000169989	0.025318	0.079453417	-0.232069124	-1.174518245	**TIGD4**	protein_coding	tigger transposable element derived 4 [Source:HGNC Symbol;Acc:HGNC:18335]	chr4	152769354	152779764	-1	ensembl_havana	0.853874767	0.969909329	0.737840205	0.589870393	1.093800119	1.226057475	0.485518989	0.758913071	0.969088554
ENSG00000120833	0.025323	0.079453417	-0.347429305	-1.272291548	**SOCS2**	protein_coding	suppressor of cytokine signaling 2 [Source:HGNC Symbol;Acc:HGNC:19382]	chr12	93569814	93583487	1	ensembl_havana	3.302987311	3.476701963	3.129272658	3.341663059	3.744406796	3.344036035	3.170918121	3.139313586	3.077586267
ENSG00000106268	0.025345	0.079501381	0.224909847	1.168704215	**NUDT1**	protein_coding	nudix hydrolase 1 [Source:HGNC Symbol;Acc:HGNC:8048]	chr7	2242222	2251146	1	ensembl_havana	4.393680979	4.281226056	4.506135903	4.090752661	4.378394309	4.374531196	4.422252934	4.491924288	4.604230486
ENSG00000117151	0.025372	0.079567634	-0.311273753	-1.240802719	**CTBS**	protein_coding	chitobiase [Source:HGNC Symbol;Acc:HGNC:2496]	chr1	84549606	84574480	-1	ensembl_havana	3.242733685	3.398370562	3.087096809	3.617156532	3.370423439	3.207531715	3.157897671	2.984584053	3.118808702
ENSG00000273344	0.025393	0.079598827	-0.353590489	-1.277736624	**PAXIP1-AS1**	lincRNA	PAXIP1 antisense RNA 1 (head to head) [Source:HGNC Symbol;Acc:HGNC:27328]	chr7	155003448	155005703	1	havana	2.435578157	2.612373402	2.258782913	2.77087449	2.66767223	2.398573485	2.37188541	2.106272137	2.298191191
ENSG00000279148	0.02539	0.079598827	-0.369274068	-1.291702711	**AC126474.1**	TEC		chr12	100026248	100027540	-1	havana	1.46096787	1.645604904	1.276330836	1.660549964	1.678462547	1.5978022	1.01467363	1.385808199	1.428510677
ENSG00000197085	0.025408	0.079629037	-0.258027584	-1.195842661	**NPSR1-AS1**	processed_transcript	NPSR1 antisense RNA 1 [Source:HGNC Symbol;Acc:HGNC:22128]	chr7	34346512	34871582	-1	havana	0.227512651	0.356526442	0.098498859	1.022973463	0.243386951	-0.196781087	0.762800879	-0.15890847	-0.308395834
ENSG00000140525	0.025418	0.079642382	-0.180311861	-1.133128802	**FANCI**	protein_coding	Fanconi anemia complementation group I [Source:HGNC Symbol;Acc:HGNC:25568]	chr15	89243949	89317261	1	ensembl_havana	8.072419416	8.162575347	7.982263486	7.977433206	8.222189166	8.288103668	7.832156429	8.049655617	8.064978411
ENSG00000197162	0.025435	0.079678165	-0.222274958	-1.166571682	**ZNF785**	protein_coding	zinc finger protein 785 [Source:HGNC Symbol;Acc:HGNC:26496]	chr16	30573740	30585771	-1	ensembl_havana	4.062874258	4.174011737	3.951736779	4.236158688	4.211493157	4.074383366	4.136133802	3.919661155	3.799415379
ENSG00000135476	0.025446	0.079696762	-0.249265095	-1.18860149	**ESPL1**	protein_coding	extra spindle pole bodies like 1, separase [Source:HGNC Symbol;Acc:HGNC:16856]	chr12	53268299	53293643	1	ensembl_havana	7.939868531	8.064501079	7.815235984	7.928191568	8.073543178	8.19176849	7.803703075	7.845131808	7.796873068
ENSG00000128713	0.025454	0.079701805	0.842572317	1.793244638	**HOXD11**	protein_coding	homeobox D11 [Source:HGNC Symbol;Acc:HGNC:5134]	chr2	176104216	176109754	1	ensembl_havana	2.286304618	1.86501846	2.707590777	1.341416217	2.021022296	2.232616866	2.89565563	2.614195633	2.612921068
ENSG00000169964	0.025485	0.079781817	-0.17448457	-1.128561133	**TMEM42**	protein_coding	transmembrane protein 42 [Source:HGNC Symbol;Acc:HGNC:28444]	chr3	44861869	44865670	1	ensembl_havana	3.612750786	3.699993071	3.525508501	3.643469038	3.706476045	3.75003413	3.487731073	3.510925742	3.577868687
ENSG00000120137	0.0255	0.079812672	-0.21169499	-1.15804795	**PANK3**	protein_coding	pantothenate kinase 3 [Source:HGNC Symbol;Acc:HGNC:19365]	chr5	168548495	168579600	-1	ensembl_havana	7.384241545	7.49008904	7.27839405	7.673988328	7.405794	7.390484793	7.369021652	7.287165612	7.178994887
ENSG00000244405	0.025519	0.079855611	-0.229674529	-1.172570388	**ETV5**	protein_coding	ETS variant 5 [Source:HGNC Symbol;Acc:HGNC:3494]	chr3	186046308	186110318	-1	ensembl_havana	4.475076297	4.589913562	4.360239033	4.798547527	4.712794237	4.258398921	4.451003248	4.488728896	4.140984954
ENSG00000114331	0.025531	0.07987482	0.209992378	1.156682073	**ACAP2**	protein_coding	ArfGAP with coiled-coil, ankyrin repeat and PH domains 2 [Source:HGNC Symbol;Acc:HGNC:16469]	chr3	195274736	195443078	-1	ensembl_havana	6.478471158	6.37347497	6.583467347	6.252429061	6.349901081	6.518094766	6.430520246	6.662577074	6.657304721
ENSG00000197329	0.025546	0.079882858	0.252701593	1.191436113	**PELI1**	protein_coding	pellino E3 ubiquitin protein ligase 1 [Source:HGNC Symbol;Acc:HGNC:8827]	chr2	64092652	64144454	-1	ensembl_havana	6.732056753	6.605705956	6.85840755	7.027545894	6.645838546	6.143733429	7.278936355	6.758895641	6.537390653
ENSG00000178971	0.02555	0.079882858	0.177185797	1.130676171	**CTC1**	protein_coding	CST telomere replication complex component 1 [Source:HGNC Symbol;Acc:HGNC:26169]	chr17	8224821	8248095	-1	ensembl_havana	6.009180866	5.920587967	6.097773764	6.001498651	5.891298222	5.868967029	6.16585261	6.045713806	6.081754876
ENSG00000165060	0.025547	0.079882858	-0.305966485	-1.236246542	**FXN**	protein_coding	frataxin [Source:HGNC Symbol;Acc:HGNC:3951]	chr9	69035259	69120427	1	ensembl_havana	4.201298282	4.354281525	4.04831504	4.521125456	4.302450449	4.239268669	3.996838595	4.072616667	4.075489857
ENSG00000099365	0.02558	0.079957548	-0.612595128	-1.529007123	**STX1B**	protein_coding	syntaxin 1B [Source:HGNC Symbol;Acc:HGNC:18539]	chr16	30989256	31010661	-1	ensembl_havana	1.823828433	2.130125997	1.517530869	2.528879384	1.873642861	1.987855745	1.400976194	1.504469011	1.647147403
ENSG00000175213	0.025607	0.080015678	0.363415823	1.286468224	**ZNF408**	protein_coding	zinc finger protein 408 [Source:HGNC Symbol;Acc:HGNC:20041]	chr11	46700818	46705912	1	ensembl_havana	3.545977176	3.364269264	3.727685088	3.287074749	3.227754334	3.57797871	3.776033227	3.745870536	3.6611515
ENSG00000282851	0.025609	0.080015678	-0.231634784	-1.174164695	**BISPR**	lincRNA	BST2 interferon stimulated positive regulator (non-protein coding) [Source:HGNC Symbol;Acc:HGNC:51290]	chr19	17405686	17419324	1	havana	2.843419052	2.959236444	2.72760166	3.062812401	3.033096853	2.781800078	2.764365763	2.732383996	2.686055222
ENSG00000076242	0.025649	0.080121429	-0.190800566	-1.141396913	**MLH1**	protein_coding	mutL homolog 1 [Source:HGNC Symbol;Acc:HGNC:7127]	chr3	36993332	37050918	1	ensembl_havana	6.247971638	6.343371921	6.152571355	6.159762087	6.431222362	6.439131312	5.954340714	6.186475965	6.316897385
ENSG00000116604	0.02568	0.080170165	0.206824951	1.154145371	**MEF2D**	protein_coding	myocyte enhancer factor 2D [Source:HGNC Symbol;Acc:HGNC:6997]	chr1	156463727	156500828	-1	ensembl_havana	5.749081872	5.645669396	5.852494347	5.777596329	5.54737421	5.612037649	6.068325073	5.760547603	5.728610365
ENSG00000104723	0.025675	0.080170165	-0.3044649	-1.234960501	**TUSC3**	protein_coding	tumor suppressor candidate 3 [Source:HGNC Symbol;Acc:HGNC:30242]	chr8	15417215	15766649	1	ensembl_havana	4.742102686	4.894335136	4.589870236	5.421606647	4.789785543	4.471613216	4.966158622	4.421613554	4.381838531
ENSG00000089127	0.025681	0.080170165	-0.678016731	-1.599938812	**OAS1**	protein_coding	2'-5'-oligoadenylate synthetase 1 [Source:HGNC Symbol;Acc:HGNC:8086]	chr12	112906777	112933222	1	ensembl_havana	3.192927027	3.531935393	2.853918661	3.253301988	3.624775944	3.717728245	3.049274082	2.42655638	3.085925522
ENSG00000004059	0.02576	0.080398348	0.200388765	1.149007938	**ARF5**	protein_coding	ADP ribosylation factor 5 [Source:HGNC Symbol;Acc:HGNC:658]	chr7	127588345	127591705	1	ensembl_havana	5.65042673	5.550232347	5.750621112	5.526651665	5.627368348	5.496677028	5.817521627	5.781590163	5.652751546
ENSG00000273702	0.025784	0.080450426	-0.310134407	-1.239823201	**AC091271.1**	lincRNA		chr17	59618553	59619714	-1	havana	0.240336142	0.395403346	0.085268939	-0.06941355	0.679062629	0.57656096	-0.26243933	0.478736765	0.039509383
ENSG00000117069	0.025788	0.080450426	-0.646935547	-1.565838636	**ST6GALNAC5**	protein_coding	ST6 N-acetylgalactosaminide alpha-2,6-sialyltransferase 5 [Source:HGNC Symbol;Acc:HGNC:19342]	chr1	76867441	77065711	1	ensembl_havana	0.079708942	0.403176715	-0.243758832	-0.27307956	0.699804047	0.782805653	-0.75894329	0.426254992	-0.398588198
ENSG00000196670	0.025813	0.080476888	-0.193865556	-1.143824376	**ZFP62**	protein_coding	ZFP62 zinc finger protein [Source:HGNC Symbol;Acc:HGNC:23241]	chr5	180847611	180861285	-1	ensembl_havana	5.101481532	5.198414311	5.004548754	5.144989722	5.185020208	5.265233001	4.872294542	5.034792482	5.106559239
ENSG00000258914	0.025809	0.080476888	-0.231024418	-1.173682686	**AL132712.2**	lincRNA		chr14	103875055	103877478	1	havana	0.127249536	0.242770745	0.011728327	0.040274975	0.352203286	0.335833975	-0.12879794	0.19485955	-0.030876629
ENSG00000173085	0.025812	0.080476888	-0.263338536	-1.200252995	**COQ2**	protein_coding	coenzyme Q2, polyprenyltransferase [Source:HGNC Symbol;Acc:HGNC:25223]	chr4	83261536	83284914	-1	ensembl_havana	3.255419416	3.387088684	3.123750148	3.369953701	3.40872246	3.382589892	3.025576891	3.051082538	3.294591014
ENSG00000143158	0.025821	0.08048253	0.39642278	1.316240191	**MPC2**	protein_coding	mitochondrial pyruvate carrier 2 [Source:HGNC Symbol;Acc:HGNC:24515]	chr1	167916729	167937040	-1	ensembl_havana	5.251543458	5.053332068	5.449754848	4.900251775	5.039038012	5.220706416	5.591664658	5.381701753	5.375898133
ENSG00000248839	0.025852	0.080544866	0.411200636	1.329792029	**AC021660.2**	antisense		chr3	98522570	98525334	1	havana	-0.44260541	-0.648205728	-0.237005092	-0.51029874	-1.03440435	-0.399914092	-0.40973604	-0.37473441	0.073455173
ENSG00000277149	0.02588	0.080544866	0.189984181	1.140751207	**TYW1B**	protein_coding	tRNA-yW synthesizing protein 1 homolog B [Source:HGNC Symbol;Acc:HGNC:33908]	chr7	72558744	72828198	-1	ensembl_havana	3.984961875	3.889969784	4.079953965	3.888048657	3.89814155	3.883719146	4.149351258	4.046776082	4.043734556
ENSG00000163811	0.025863	0.080544866	-0.172031823	-1.12664408	**WDR43**	protein_coding	WD repeat domain 43 [Source:HGNC Symbol;Acc:HGNC:28945]	chr2	28894643	28948222	1	ensembl_havana	7.417679726	7.503695638	7.331663815	7.438833928	7.50819277	7.564060216	7.258242876	7.343528187	7.393220431
ENSG00000237190	0.025866	0.080544866	-0.188061131	-1.139231648	**CDKN2AIPNL**	protein_coding	CDKN2A interacting protein N-terminal like [Source:HGNC Symbol;Acc:HGNC:30545]	chr5	134402087	134411898	-1	ensembl_havana	4.736307518	4.830338084	4.642276953	4.869619557	4.833847726	4.787546968	4.653199592	4.606912214	4.666719054
ENSG00000277283	0.025875	0.080544866	-0.225578343	-1.169245878	**AC004812.2**	antisense		chr12	120116907	120119000	1	havana	1.744256316	1.857045487	1.631467144	1.760103226	1.882741771	1.928291464	1.644455473	1.660283167	1.589662793
ENSG00000014123	0.025879	0.080544866	-0.30903698	-1.238880453	**UFL1**	protein_coding	UFM1 specific ligase 1 [Source:HGNC Symbol;Acc:HGNC:23039]	chr6	96521595	96555276	1	ensembl_havana	5.246005112	5.400523602	5.091486622	5.505289466	5.390360257	5.305921083	5.056627338	4.99705787	5.220774657
ENSG00000177303	0.025864	0.080544866	-0.702006191	-1.626765378	**CASKIN2**	protein_coding	CASK interacting protein 2 [Source:HGNC Symbol;Acc:HGNC:18200]	chr17	75500261	75515583	-1	ensembl_havana	1.085568282	1.436571377	0.734565186	1.04068047	1.535784795	1.733248866	0.873137292	0.800742365	0.529815901
ENSG00000188976	0.025889	0.080556518	0.21250101	1.158695121	**NOC2L**	protein_coding	NOC2 like nucleolar associated transcriptional repressor [Source:HGNC Symbol;Acc:HGNC:24517]	chr1	944204	959309	-1	ensembl_havana	7.448708859	7.342458354	7.554959364	7.25124683	7.289594297	7.486533935	7.544978132	7.526050416	7.593849545
ENSG00000116580	0.02596	0.080728864	-0.176070479	-1.129802406	**GON4L**	protein_coding	gon-4 like [Source:HGNC Symbol;Acc:HGNC:25973]	chr1	155749662	155859400	-1	ensembl_havana	7.454335129	7.542370368	7.36629989	7.658123558	7.501009264	7.467978283	7.458464455	7.314268962	7.326166253
ENSG00000122882	0.025958	0.080728864	-0.185719093	-1.137383746	**ECD**	protein_coding	ecdysoneless cell cycle regulator [Source:HGNC Symbol;Acc:HGNC:17029]	chr10	73130155	73169055	-1	ensembl_havana	5.485684916	5.578544463	5.39282537	5.642069861	5.613087875	5.480475652	5.445737301	5.379115482	5.353623327
ENSG00000138134	0.025961	0.080728864	-0.34854256	-1.27327369	**STAMBPL1**	protein_coding	STAM binding protein like 1 [Source:HGNC Symbol;Acc:HGNC:24105]	chr10	88879734	88975153	1	ensembl_havana	3.624159062	3.798430342	3.449887782	3.674990394	3.953166093	3.767134538	3.494651998	3.340601002	3.514410346
ENSG00000134294	0.026001	0.08081665	0.173144834	1.127513599	**SLC38A2**	protein_coding	solute carrier family 38 member 2 [Source:HGNC Symbol;Acc:HGNC:13448]	chr12	46358189	46372867	-1	ensembl_havana	8.261339111	8.174766694	8.347911528	8.350088185	8.086768168	8.08744373	8.502379457	8.274582415	8.266772713
ENSG00000159409	0.025996	0.08081665	-0.602793555	-1.518654359	**CELF3**	protein_coding	CUGBP Elav-like family member 3 [Source:HGNC Symbol;Acc:HGNC:11967]	chr1	151702404	151716814	-1	ensembl_havana	0.91293889	1.214335668	0.611542113	1.190963204	0.979703225	1.472340574	0.399634207	0.880929471	0.554062661
ENSG00000269335	0.026011	0.08083168	0.186253211	1.13780491	**IKBKG**	protein_coding	inhibitor of nuclear factor kappa B kinase subunit gamma [Source:HGNC Symbol;Acc:HGNC:5961]	chrX	154541199	154565046	1	ensembl_havana	3.042833956	2.94970735	3.135960561	3.097064305	2.850374945	2.901682799	3.330286772	3.051082538	3.026512374
ENSG00000106153	0.026023	0.080850551	-0.324226888	-1.25199334	**CHCHD2**	protein_coding	coiled-coil-helix-coiled-coil-helix domain containing 2 [Source:HGNC Symbol;Acc:HGNC:21645]	chr7	56101569	56106576	-1	ensembl_havana	7.783187845	7.94530129	7.621074401	7.994711237	7.851565773	7.989626858	7.864741409	7.556127796	7.442354
ENSG00000176853	0.026034	0.080865686	-0.212736914	-1.158884601	**FAM91A1**	protein_coding	family with sequence similarity 91 member A1 [Source:HGNC Symbol;Acc:HGNC:26306]	chr8	123768456	123815452	1	ensembl_havana	6.811994376	6.918362833	6.705625919	6.989019073	6.914481677	6.851587749	6.667538324	6.776035527	6.673303906
ENSG00000172382	0.026049	0.080894991	-0.593665885	-1.50907644	**PRSS27**	protein_coding	serine protease 27 [Source:HGNC Symbol;Acc:HGNC:15475]	chr16	2712418	2720551	-1	ensembl_havana	0.596226062	0.893059005	0.299393119	0.986893817	0.679062629	1.013220568	0.739683594	0.225896484	-0.067400719
ENSG00000170260	0.026085	0.080970994	0.192038815	1.142376981	**ZNF212**	protein_coding	zinc finger protein 212 [Source:HGNC Symbol;Acc:HGNC:13004]	chr7	149239651	149255609	1	ensembl_havana	4.197346904	4.101327497	4.293366311	4.228453786	4.063308132	4.012220572	4.496533987	4.208958525	4.174606422
ENSG00000140157	0.02609	0.080970994	-0.201225849	-1.149674812	**NIPA2**	protein_coding	non imprinted in Prader-Willi/Angelman syndrome 2 [Source:HGNC Symbol;Acc:HGNC:17044]	chr15	22838641	22868384	-1	ensembl_havana	6.740034243	6.840647168	6.639421319	6.95060637	6.893253104	6.67808203	6.703079055	6.645102551	6.570082351
ENSG00000224546	0.026083	0.080970994	-0.253312483	-1.191940717	**EIF4BP3**	processed_pseudogene	eukaryotic translation initiation factor 4B pseudogene 3 [Source:HGNC Symbol;Acc:HGNC:37936]	chr9	96146007	96147856	1	havana	1.073609766	1.200266007	0.946953524	0.892538	1.440410536	1.267849486	0.785553575	1.179325527	0.875981471
ENSG00000185359	0.026114	0.080995837	0.277538654	1.212125143	**HGS**	protein_coding	hepatocyte growth factor-regulated tyrosine kinase substrate [Source:HGNC Symbol;Acc:HGNC:4897]	chr17	81683326	81703138	1	ensembl_havana	5.847042898	5.708273571	5.985812225	5.62749689	5.67200733	5.825316494	6.067745074	5.957740743	5.931950858
ENSG00000283929	0.026122	0.080995837	0.26161321	1.198818465	**MIR5010**	miRNA	microRNA 5010 [Source:HGNC Symbol;Acc:HGNC:43484]	chr17	42514188	42514307	1	mirbase	0.139377056	0.008570451	0.270183661	0.237415364	-0.258437325	0.046733314	0.668022463	-0.11936381	0.261892331
ENSG00000139668	0.026114	0.080995837	0.175239472	1.129151816	**WDFY2**	protein_coding	WD repeat and FYVE domain containing 2 [Source:HGNC Symbol;Acc:HGNC:20482]	chr13	51584455	51767707	1	ensembl_havana	5.040145831	4.952526095	5.127765567	4.9642779	4.881151268	5.012149117	5.169051589	5.054377101	5.159868011
ENSG00000169139	0.026126	0.080995837	-0.176844996	-1.130409109	**UBE2V2**	protein_coding	ubiquitin conjugating enzyme E2 V2 [Source:HGNC Symbol;Acc:HGNC:12495]	chr8	48008400	48064708	1	ensembl_havana	5.342364587	5.430787085	5.253942089	5.338149257	5.537325834	5.416886163	5.14285793	5.339754527	5.27921381
ENSG00000111229	0.026125	0.080995837	-0.314917447	-1.243940471	**ARPC3**	protein_coding	actin related protein 2/3 complex subunit 3 [Source:HGNC Symbol;Acc:HGNC:706]	chr12	110434825	110450422	-1	ensembl_havana	6.946834255	7.104292979	6.789375532	7.261665195	7.048627302	7.002586439	6.724211526	6.90568403	6.738231039
ENSG00000047932	0.02614	0.081020876	-0.267717257	-1.203901415	**GOPC**	protein_coding	golgi associated PDZ and coiled-coil motif containing [Source:HGNC Symbol;Acc:HGNC:17643]	chr6	117560269	117602542	-1	ensembl_havana	5.697677376	5.831536005	5.563818747	6.028371753	5.750525702	5.715710559	5.583572832	5.535943831	5.571939578
ENSG00000142197	0.026169	0.081094473	0.24576024	1.18571743	**DOPEY2**	protein_coding	dopey family member 2 [Source:HGNC Symbol;Acc:HGNC:1291]	chr21	36156782	36294274	1	ensembl_havana	4.765248761	4.642368641	4.888128881	4.561549439	4.549559377	4.815997107	4.794506721	4.936194287	4.933685634
ENSG00000161265	0.026267	0.081380001	-0.280987858	-1.215026566	**U2AF1L4**	protein_coding	U2 small nuclear RNA auxiliary factor 1 like 4 [Source:HGNC Symbol;Acc:HGNC:23020]	chr19	35742464	35745445	-1	ensembl_havana	2.384201894	2.524695823	2.243707965	2.52259249	2.597561204	2.453933774	2.438005243	2.203033505	2.090085146
ENSG00000189171	0.026296	0.081452807	-0.281436504	-1.215404471	**S100A13**	protein_coding	S100 calcium binding protein A13 [Source:HGNC Symbol;Acc:HGNC:10490]	chr1	153618787	153631360	-1	ensembl_havana	2.708943493	2.849661745	2.568225241	2.950212473	2.896025364	2.702747398	2.581431252	2.504564179	2.618680293
ENSG00000149115	0.026312	0.081484538	-0.255869153	-1.194054886	**TNKS1BP1**	protein_coding	tankyrase 1 binding protein 1 [Source:HGNC Symbol;Acc:HGNC:19081]	chr11	57299638	57324952	-1	ensembl_havana	5.891466599	6.019401176	5.763532023	6.157735102	6.049147037	5.851321389	6.068904839	5.729074023	5.492617207
ENSG00000169446	0.026335	0.081536754	0.223594399	1.167639077	**MMGT1**	protein_coding	membrane magnesium transporter 1 [Source:HGNC Symbol;Acc:HGNC:28100]	chrX	135962070	135974063	-1	ensembl_havana	6.051819734	5.940022535	6.163616933	6.045464993	6.014899689	5.759702922	6.254656733	6.135796798	6.100397269
ENSG00000251287	0.026365	0.081612836	0.394372091	1.314370578	**ALG1L2**	protein_coding	ALG1, chitobiosyldiphosphodolichol beta-mannosyltransferase like 2 [Source:HGNC Symbol;Acc:HGNC:37258]	chr3	130081831	130113227	1	ensembl_havana	0.28365944	0.086473395	0.480845485	-0.18813307	-0.106664058	0.554217307	0.513047461	0.19485955	0.734629445
ENSG00000130653	0.026393	0.081682533	0.303230986	1.233904711	**PNPLA7**	protein_coding	patatin like phospholipase domain containing 7 [Source:HGNC Symbol;Acc:HGNC:24768]	chr9	137459953	137550534	-1	ensembl_havana	1.679578971	1.527963478	1.831194465	1.43811357	1.270574017	1.875202847	1.88468023	1.648877048	1.960026115
ENSG00000126432	0.026403	0.081695233	-0.243619933	-1.183959666	**PRDX5**	protein_coding	peroxiredoxin 5 [Source:HGNC Symbol;Acc:HGNC:9355]	chr11	64318088	64321811	1	ensembl_havana	6.229510126	6.351320092	6.107700159	6.407611484	6.338786137	6.307562656	6.283916119	6.118844781	5.920339577
ENSG00000164542	0.02641	0.081698197	0.216839628	1.16218491	**KIAA0895**	protein_coding	KIAA0895 [Source:HGNC Symbol;Acc:HGNC:22206]	chr7	36324221	36390125	-1	ensembl_havana	4.849631415	4.741211601	4.958051229	4.647841445	4.87204229	4.703751069	4.982251883	5.017157896	4.874743908
ENSG00000118513	0.026427	0.081698197	0.19359632	1.143610935	**MYB**	protein_coding	MYB proto-oncogene, transcription factor [Source:HGNC Symbol;Acc:HGNC:7545]	chr6	135181315	135219173	1	ensembl_havana	6.617039163	6.520241003	6.713837323	6.536054369	6.482248585	6.542420055	6.686551905	6.637908817	6.817051248
ENSG00000165521	0.02642	0.081698197	0.190380117	1.141064321	**EML5**	protein_coding	echinoderm microtubule associated protein like 5 [Source:HGNC Symbol;Acc:HGNC:18197]	chr14	88612431	88792752	-1	ensembl_havana	4.58200775	4.486817691	4.677197809	4.283403858	4.44440735	4.732641866	4.456330725	4.706547606	4.868715096
ENSG00000245317	0.026425	0.081698197	-0.509708419	-1.423762412	**AC008393.1**	lincRNA		chr5	179859013	179861283	1	havana	1.161866911	1.416721121	0.907012702	1.727676152	1.214022341	1.30846487	0.915012722	1.114463419	0.691561964
ENSG00000214456	0.026439	0.081708396	-0.273721963	-1.208922669	**PLIN5**	protein_coding	perilipin 5 [Source:HGNC Symbol;Acc:HGNC:33196]	chr19	4522531	4535224	-1	ensembl_havana	-0.375901434	-0.239040453	-0.512762416	-0.85854608	0.06303108	0.078393642	-1.13267621	-0.37473441	-0.030876629
ENSG00000088881	0.026441	0.081708396	-0.758335122	-1.691537455	**EBF4**	protein_coding	early B cell factor 4 [Source:HGNC Symbol;Acc:HGNC:29278]	chr20	2692878	2760108	1	ensembl_havana	-0.228778743	0.150388818	-0.607946304	0.515840645	0.09469996	-0.159374151	-0.89704638	-0.32890884	-0.597883695
ENSG00000182185	0.026448	0.081712634	-0.296848611	-1.228458066	**RAD51B**	protein_coding	RAD51 paralog B [Source:HGNC Symbol;Acc:HGNC:9822]	chr14	67819779	68730218	1	ensembl_havana	3.18252784	3.330952146	3.034103535	3.494031456	3.245497377	3.253327603	2.981915645	3.055408658	3.064986301
ENSG00000008086	0.026479	0.081790325	0.211186885	1.157640167	**CDKL5**	protein_coding	cyclin dependent kinase like 5 [Source:HGNC Symbol;Acc:HGNC:11411]	chrX	18425583	18653629	1	ensembl_havana	5.006189593	4.90059615	5.111783036	4.923104885	4.831500081	4.947183485	5.12066063	5.144444897	5.070243581
ENSG00000141837	0.026495	0.081822648	-0.640112959	-1.558451177	**CACNA1A**	protein_coding	calcium voltage-gated channel subunit alpha1 A [Source:HGNC Symbol;Acc:HGNC:1388]	chr19	13206442	13633025	-1	ensembl_havana	0.199189054	0.519245534	-0.120867425	0.660286109	0.614986832	0.28246366	-0.46237776	-0.00684508	0.106620565
ENSG00000111727	0.02651	0.081841482	0.216809962	1.162161013	**HCFC2**	protein_coding	host cell factor C2 [Source:HGNC Symbol;Acc:HGNC:24972]	chr12	104064457	104106524	1	ensembl_havana	4.036888942	3.928483961	4.145293923	3.807595594	4.06530755	3.912548737	4.129479384	4.181514568	4.124887815
ENSG00000146670	0.026516	0.081841482	-0.172397867	-1.126929971	**CDCA5**	protein_coding	cell division cycle associated 5 [Source:HGNC Symbol;Acc:HGNC:14626]	chr11	65066300	65084164	-1	ensembl_havana	7.089869366	7.1760683	7.003670433	7.252671955	7.169960953	7.105571991	7.068061502	6.986631499	6.956318298
ENSG00000248663	0.026519	0.081841482	-0.226079152	-1.169651834	**LINC00992**	lincRNA	long intergenic non-protein coding RNA 992 [Source:HGNC Symbol;Acc:HGNC:48943]	chr5	117415509	117546298	1	havana	2.976931719	3.089971295	2.863892143	3.109701981	3.041246372	3.118965531	2.758627316	2.841618992	2.99143012
ENSG00000204611	0.026537	0.081880786	0.176215522	1.129915999	**ZNF616**	protein_coding	zinc finger protein 616 [Source:HGNC Symbol;Acc:HGNC:28062]	chr19	52113091	52139922	-1	ensembl_havana	4.168981024	4.080873263	4.257088785	3.964251181	4.255938845	4.022429763	4.175428345	4.39796187	4.197876141
ENSG00000073910	0.026556	0.081920066	0.243643294	1.183978837	**FRY**	protein_coding	FRY microtubule binding protein [Source:HGNC Symbol;Acc:HGNC:20367]	chr13	31846713	32299122	1	ensembl_havana	3.531882745	3.410061098	3.653704392	3.394260955	3.485388687	3.350533652	3.62967128	3.599502737	3.731939159
ENSG00000167468	0.026574	0.081942589	-0.271459114	-1.207027975	**GPX4**	protein_coding	glutathione peroxidase 4 [Source:HGNC Symbol;Acc:HGNC:4556]	chr19	1103926	1106791	1	ensembl_havana	5.104833616	5.240563173	4.969104059	5.278769844	5.220440981	5.222478693	5.18197221	4.920864951	4.804475016
ENSG00000144306	0.026574	0.081942589	-0.421991999	-1.339776172	**SCRN3**	protein_coding	secernin 3 [Source:HGNC Symbol;Acc:HGNC:30382]	chr2	174395730	174429575	1	ensembl_havana	2.883774529	3.094770529	2.67277853	3.151045506	2.991644088	3.141621992	2.386842807	2.726970195	2.904522587
ENSG00000075340	0.026595	0.081987809	-0.26289594	-1.199884833	**ADD2**	protein_coding	adducin 2 [Source:HGNC Symbol;Acc:HGNC:244]	chr2	70607618	70768225	-1	ensembl_havana	6.315407166	6.446855136	6.183959196	6.131121806	6.487477022	6.721966581	6.036073715	6.191398189	6.324405684
ENSG00000177352	0.026612	0.08202409	-0.261801395	-1.198974848	**CCDC71**	protein_coding	coiled-coil domain containing 71 [Source:HGNC Symbol;Acc:HGNC:25760]	chr3	49162535	49166321	-1	ensembl_havana	3.552601391	3.683502088	3.421700694	3.279637718	3.781365782	3.989502764	3.183822111	3.488704842	3.592575128
ENSG00000280128	0.026637	0.082082104	0.20715551	1.154409846	**AL662795.1**	TEC		chr6	30282349	30286054	-1	havana	4.971090129	4.867512374	5.074667884	4.923104885	4.81495872	4.864473516	5.025903107	5.065143652	5.132956893
ENSG00000244151	0.026659	0.082100916	0.48272182	1.397377504	**AC010973.2**	antisense		chr7	151074742	151076530	-1	havana	-0.279355108	-0.520716018	-0.037994198	-0.22998119	-0.842251242	-0.489915621	-0.08686711	-0.37473441	0.347618925
ENSG00000092841	0.026649	0.082100916	-0.171196567	-1.125991992	**MYL6**	protein_coding	myosin light chain 6 [Source:HGNC Symbol;Acc:HGNC:7587]	chr12	56158161	56163496	1	ensembl_havana	9.262181483	9.347779766	9.176583199	9.400244918	9.415369574	9.227724807	9.230155978	9.258139139	9.04145448
ENSG00000106028	0.026664	0.082100916	-0.261698462	-1.198889308	**SSBP1**	protein_coding	single stranded DNA binding protein 1 [Source:HGNC Symbol;Acc:HGNC:11317]	chr7	141738321	141787922	1	ensembl_havana	6.440511478	6.571360709	6.309662247	6.594167945	6.567595836	6.552318347	6.156063879	6.386437698	6.386485164
ENSG00000116678	0.026666	0.082100916	-0.280194213	-1.214358348	**LEPR**	protein_coding	leptin receptor [Source:HGNC Symbol;Acc:HGNC:6554]	chr1	65420652	65641559	1	ensembl_havana	3.461897981	3.601995087	3.321800875	3.812757512	3.446030999	3.54719675	3.338003616	3.293766078	3.33363293

ENSG00000273604	0.026697	0.082162456	-0.270006302	-1.205813095	**EPOP**	protein_coding	elongin BC and polycomb repressive complex 2 associated protein [Source:HGNC Symbol;Acc:HGNC:34493]	chr17	38671703	38675421	-1	ensembl_havana	4.478153803	4.613156953	4.343150652	4.760318669	4.725493457	4.353658734	4.538998785	4.275367784	4.215085387
ENSG00000071564	0.026695	0.082162456	-0.293357984	-1.225489382	**TCF3**	protein_coding	transcription factor 3 [Source:HGNC Symbol;Acc:HGNC:11633]	chr19	1609290	1652605	-1	ensembl_havana	6.515593513	6.662272505	6.368914521	6.705546825	6.57568398	6.70558671	6.57220471	6.323262802	6.211276052
ENSG00000135404	0.026725	0.082213376	0.181415556	1.133996004	**CD63**	protein_coding	CD63 molecule [Source:HGNC Symbol;Acc:HGNC:1692]	chr12	55725323	55729707	-1	ensembl_havana	7.777632816	7.686925038	7.868340594	7.703468002	7.748011547	7.609295565	7.864574457	7.894109159	7.846338166
ENSG00000143494	0.026724	0.082213376	-0.194999331	-1.14472363	**VASH2**	protein_coding	vasohibin 2 [Source:HGNC Symbol;Acc:HGNC:25723]	chr1	212950520	212992037	1	ensembl_havana	8.695544289	8.793043954	8.598044623	9.040465602	8.713044414	8.625621847	8.787246809	8.49364144	8.513245622
ENSG00000196914	0.026755	0.082271645	0.206031568	1.153510845	**ARHGEF12**	protein_coding	Rho guanine nucleotide exchange factor 12 [Source:HGNC Symbol;Acc:HGNC:14193]	chr11	120336914	120489936	1	ensembl_havana	8.209857892	8.106842108	8.312873676	8.02143124	8.084551867	8.214543216	8.294894935	8.324948607	8.318777486
ENSG00000167470	0.02675	0.082271645	-0.18854914	-1.139617073	**MIDN**	protein_coding	midnolin [Source:HGNC Symbol;Acc:HGNC:16298]	chr19	1248553	1259140	1	ensembl_havana	5.101342066	5.195616636	5.007067496	5.516405639	5.031898757	5.038545512	5.3257077	4.781580346	4.913914443
ENSG00000135392	0.026771	0.08230301	-0.239813637	-1.180840114	**DNAJC14**	protein_coding	DnaJ heat shock protein family (Hsp40) member C14 [Source:HGNC Symbol;Acc:HGNC:24581]	chr12	55820960	55830824	-1	ensembl_havana	5.900839238	6.020746056	5.780932419	6.095562165	5.998251367	5.968424637	5.755488227	5.711363537	5.875945493
ENSG00000196247	0.026794	0.082346121	-0.173392447	-1.127707133	**ZNF107**	protein_coding	zinc finger protein 107 [Source:HGNC Symbol;Acc:HGNC:12887]	chr7	64666083	64711582	1	ensembl_havana	5.68266018	5.769356403	5.595963956	5.62749689	5.816706023	5.863866297	5.484495499	5.63320814	5.67018823
ENSG00000130590	0.026797	0.082346121	-0.367291089	-1.28992849	**SAMD10**	protein_coding	sterile alpha motif domain containing 10 [Source:HGNC Symbol;Acc:HGNC:16129]	chr20	63974113	63980008	-1	ensembl_havana	4.64957514	4.833220684	4.465929595	4.79072079	4.849015003	4.85992626	4.685314876	4.458014382	4.254459527
ENSG00000237292	0.026804	0.082351324	-0.569756793	-1.484273333	**LINC01732**	lincRNA	long intergenic non-protein coding RNA 1732 [Source:HGNC Symbol;Acc:HGNC:52520]	chr1	181174484	181182208	1	havana	-0.160028748	0.124849649	-0.444907144	-0.18813307	0.125688576	0.436993436	-1.13267621	-0.52165082	0.319605599
ENSG00000151332	0.026831	0.082416896	0.271682883	1.207215206	**MBIP**	protein_coding	MAP3K12 binding inhibitory protein 1 [Source:HGNC Symbol;Acc:HGNC:20427]	chr14	36298558	36320676	-1	ensembl_havana	3.96067205	3.824830609	4.096513492	3.858402026	3.931407459	3.684682341	4.061190507	4.085365442	4.142984527
ENSG00000163683	0.026853	0.08244718	0.330064909	1.257069931	**SMIM14**	protein_coding	small integral membrane protein 14 [Source:HGNC Symbol;Acc:HGNC:27321]	chr4	39546330	39639090	-1	ensembl_havana	4.501274804	4.33624235	4.666307259	4.605340519	4.275069381	4.12831715	4.692857142	4.785491911	4.520572724
ENSG00000112977	0.02685	0.08244718	-0.177919439	-1.131251291	**DAP**	protein_coding	death associated protein [Source:HGNC Symbol;Acc:HGNC:2672]	chr5	10679230	10761272	-1	ensembl_havana	7.466526603	7.555486322	7.377566884	7.619247165	7.523908045	7.523303757	7.485423681	7.308843577	7.338433393
ENSG00000184545	0.026862	0.082457222	-0.363829746	-1.286837377	**DUSP8**	protein_coding	dual specificity phosphatase 8 [Source:HGNC Symbol;Acc:HGNC:3074]	chr11	1554044	1571920	-1	ensembl_havana	0.968397406	1.150312279	0.786482533	1.43811357	0.874167025	1.138656243	1.325353043	0.504278656	0.529815901
ENSG00000145354	0.02689	0.082524989	-0.210165864	-1.156821174	**CISD2**	protein_coding	CDGSH iron sulfur domain 2 [Source:HGNC Symbol;Acc:HGNC:24212]	chr4	102868974	102892807	1	ensembl_havana	5.805470491	5.910553423	5.700387559	5.845281083	6.033912831	5.852466354	5.67856212	5.714102418	5.708498138
ENSG00000151576	0.02691	0.082567099	0.182893825	1.135158557	**QTRT2**	protein_coding	queuine tRNA-ribosyltransferase accessory subunit 2 [Source:HGNC Symbol;Acc:HGNC:25771]	chr3	114005833	114088422	1	ensembl_havana	5.977696948	5.886250036	6.069143861	5.713495299	5.878846762	6.066408046	5.915542727	6.102214017	6.189674838
ENSG00000118162	0.026915	0.082567099	-0.224891885	-1.168689664	**KPTN**	protein_coding	kaptin, actin binding protein [Source:HGNC Symbol;Acc:HGNC:6404]	chr19	47475144	47484268	-1	ensembl_havana	4.287587377	4.400033319	4.175141435	4.430009476	4.368712378	4.401378105	4.257063661	4.222487183	4.04587346
ENSG00000197586	0.02693	0.082595281	0.24874122	1.188169961	**ENTPD6**	protein_coding	ectonucleoside triphosphate diphosphohydrolase 6 (putative) [Source:HGNC Symbol;Acc:HGNC:3368]	chr20	25195693	25226729	1	ensembl_havana	6.550795901	6.426425291	6.675166511	6.307404806	6.349080682	6.622790384	6.668686233	6.642588822	6.714224477
ENSG00000135486	0.026943	0.08261791	-0.196218344	-1.145691279	**HNRNPA1**	protein_coding	heterogeneous nuclear ribonucleoprotein A1 [Source:HGNC Symbol;Acc:HGNC:5031]	chr12	54280193	54287088	1	ensembl_havana	9.53563124	9.633740413	9.437522068	9.679918046	9.61965653	9.601646661	9.402311699	9.435240292	9.475014212
ENSG00000197182	0.026974	0.082640822	0.505281784	1.419400563	**MIRLET7BHG**	lincRNA	MIRLET7B host gene [Source:HGNC Symbol;Acc:HGNC:37189]	chr22	46053869	46113928	1	havana	-0.393741692	-0.646382584	-0.1411008	-0.73288918	-0.620350959	-0.585907617	0.141952326	-0.11936381	-0.445890917
ENSG00000125814	0.026961	0.082640822	0.199355283	1.148185135	**NAPB**	protein_coding	NSF attachment protein beta [Source:HGNC Symbol;Acc:HGNC:15751]	chr20	23374519	23421519	-1	ensembl_havana	5.771246258	5.671568616	5.870923899	5.784837873	5.632097265	5.597770709	5.892146201	5.863436196	5.857189301
ENSG00000054219	0.026978	0.082640822	0.191636095	1.142058138	**LY75**	protein_coding	lymphocyte antigen 75 [Source:HGNC Symbol;Acc:HGNC:6729]	chr2	159803355	159904749	-1	ensembl_havana	7.004837943	6.909019895	7.100655991	6.999525952	6.951166582	6.776367152	7.169125429	7.087507123	7.045335419
ENSG00000185522	0.026967	0.082640822	-0.530038078	-1.443967306	**LMNTD2**	protein_coding	lamin tail domain containing 2 [Source:HGNC Symbol;Acc:HGNC:28561]	chr11	554855	560779	-1	ensembl_havana	1.079581918	1.344600957	0.814562879	1.516132283	1.093800119	1.423870469	0.762800879	1.01135145	0.669536307
ENSG00000174680	0.026979	0.082640822	-0.973758005	-1.963949734	**GRIK1-AS1**	antisense	GRIK1 antisense RNA 1 [Source:HGNC Symbol;Acc:HGNC:16458]	chr21	29748175	29764002	1	ensembl_havana	-1.090930376	-0.604051373	-1.577809379	0.10902545	-0.727039001	-1.194140569	-1.04978262	-2.33094524	-1.352700279
ENSG00000136997	0.027014	0.082694777	0.314752607	1.243798349	**MYC**	protein_coding	MYC proto-oncogene, bHLH transcription factor [Source:HGNC Symbol;Acc:HGNC:7553]	chr8	127735434	127741434	1	ensembl_havana	5.803217437	5.645841134	5.960593741	5.583628518	5.561608703	5.79228618	5.802261543	6.1115924	5.96792728
ENSG00000198356	0.027012	0.082694777	0.244886631	1.184999649	**ASNA1**	protein_coding	arsA arsenite transporter, ATP-binding, homolog 1 (bacterial) [Source:HGNC Symbol;Acc:HGNC:752]	chr19	12737139	12748323	1	ensembl_havana	5.924501531	5.802058215	6.046944847	5.808286499	5.844913741	5.752974406	6.060766836	5.949629134	6.13043857
ENSG00000086475	0.027003	0.082694777	0.181498688	1.13406135	**SEPHS1**	protein_coding	selenophosphate synthetase 1 [Source:HGNC Symbol;Acc:HGNC:19685]	chr10	13317424	13348298	-1	ensembl_havana	6.411251323	6.320501978	6.502000667	6.2899284	6.318812292	6.352765243	6.523547627	6.452302926	6.530151448
ENSG00000116685	0.02704	0.08274071	0.303803869	1.234394782	**KIAA2013**	protein_coding	KIAA2013 [Source:HGNC Symbol;Acc:HGNC:28513]	chr1	11919591	11926428	-1	ensembl_havana	5.227827387	5.075925453	5.379729321	5.059634228	4.932440306	5.235701825	5.446629832	5.375659892	5.31689824
ENSG00000215375	0.027042	0.08274071	-0.3237747	-1.251600986	**MYL5**	protein_coding	myosin light chain 5 [Source:HGNC Symbol;Acc:HGNC:7586]	chr4	673580	682033	1	ensembl_havana	4.089538157	4.251425507	3.927650807	4.469765834	4.096927042	4.187583646	3.921799706	3.973231137	3.887921578
ENSG00000162738	0.027046	0.08274071	-0.9793929	-1.971635551	**VANGL2**	protein_coding	VANGL planar cell polarity protein 2 [Source:HGNC Symbol;Acc:HGNC:15511]	chr1	160400586	160428678	1	ensembl_havana	-0.287406957	0.202289493	-0.777103407	0.41082706	-0.002471726	0.198513145	-1.41444944	-0.42206346	-0.494797317
ENSG00000170634	0.027062	0.082772048	-0.189154889	-1.140095668	**ACYP2**	protein_coding	acylphosphatase 2 [Source:HGNC Symbol;Acc:HGNC:180]	chr2	53970838	54305300	1	ensembl_havana	1.899179097	1.993756541	1.804601652	2.142776609	2.08549033	1.753002685	1.936429043	1.841543652	1.635832261
ENSG00000273136	0.02707	0.08277823	0.271309491	1.2069028	**NBPF26**	protein_coding	NBPF member 26 [Source:HGNC Symbol;Acc:HGNC:49571]	chr1	120723923	120841481	1	ensembl_havana	3.269858685	3.13420394	3.405513431	3.122229914	3.116453761	3.163928144	3.532134761	3.429942433	3.254463098
ENSG00000122707	0.027078	0.082785749	0.173408976	1.127720054	**RECK**	protein_coding	reversion inducing cysteine rich protein with kazal motifs [Source:HGNC Symbol;Acc:HGNC:11345]	chr9	36036433	36124451	1	ensembl_havana	4.282800199	4.19609571	4.369504687	4.116027648	4.156285298	4.315974186	4.267203405	4.3192007	4.522109956
ENSG00000260114	0.02709	0.082803151	0.345452586	1.270549502	**AC120114.2**	sense_intronic		chr16	29919634	29921905	-1	havana	2.657782223	2.48505593	2.830508516	2.451565702	2.389883326	2.613718761	2.78144541	2.975481414	2.734598724
ENSG00000161800	0.027133	0.082905043	-0.171420884	-1.12616708	**RACGAP1**	protein_coding	Rac GTPase activating protein 1 [Source:HGNC Symbol;Acc:HGNC:9804]	chr12	49976923	50033136	-1	ensembl_havana	7.512676049	7.598386491	7.426965607	7.431931545	7.680482795	7.682745131	7.246246692	7.533434932	7.501215198
ENSG00000163293	0.027135	0.082905043	-0.451670898	-1.367623291	**NIPAL1**	protein_coding	NIPA like domain containing 1 [Source:HGNC Symbol;Acc:HGNC:27194]	chr4	47914142	48040173	1	ensembl_havana	-0.568142173	-0.342306724	-0.793977622	-0.73288918	-0.34076431	0.046733314	-1.41444944	-0.42206346	-0.545419959
ENSG00000196172	0.027149	0.082932472	-0.310744406	-1.240347533	**ZNF681**	protein_coding	zinc finger protein 681 [Source:HGNC Symbol;Acc:HGNC:26457]	chr19	23739195	23758891	-1	ensembl_havana	3.32072803	3.476100233	3.165355827	3.253301988	3.572314292	3.602684419	3.011169462	3.419911719	3.064986301
ENSG00000107554	0.027164	0.082957942	0.170180286	1.125199086	**DNMBP**	protein_coding	dynamin binding protein [Source:HGNC Symbol;Acc:HGNC:30373]	chr10	99875577	100009919	-1	ensembl_havana	4.552129865	4.467039722	4.637220008	4.224585849	4.587617843	4.588915474	4.411322118	4.751234058	4.749103847
ENSG00000226124	0.027189	0.083018707	-0.285611334	-1.218926668	**FTCDNL1**	protein_coding	formiminotransferase cyclodeaminase N-terminal like [Source:HGNC Symbol;Acc:HGNC:48661]	chr2	199760544	199851173	-1	ensembl_havana	1.040855549	1.183661216	0.898049882	1.058172782	1.093800119	1.399010747	0.64332197	1.01135145	1.039476224
ENSG00000189221	0.027207	0.083037829	0.272630827	1.208008685	**MAOA**	protein_coding	monoamine oxidase A [Source:HGNC Symbol;Acc:HGNC:6833]	chrX	43654907	43746824	1	ensembl_havana	9.215112895	9.078797482	9.351428309	9.354244139	9.031076653	8.851071653	9.485981263	9.26229702	9.306006645
ENSG00000187446	0.027204	0.083037829	0.169781102	1.124887794	**CHP1**	protein_coding	calcineurin like EF-hand protein 1 [Source:HGNC Symbol;Acc:HGNC:17433]	chr15	41230839	41281890	1	ensembl_havana	6.817145432	6.732254881	6.902035983	6.723130471	6.79426116	6.679373012	6.886267243	6.956878167	6.862962537
ENSG00000279088	0.02722	0.083061122	-0.273185195	-1.208472962	**AC022400.7**	TEC		chr10	73742995	73744230	-1	havana	2.941440426	3.078033024	2.804847828	3.062812401	3.037177367	3.134109303	2.89565563	2.572545352	2.946342503
ENSG00000170525	0.027266	0.08317115	0.346467683	1.271443791	**PFKFB3**	protein_coding	6-phosphofructo-2-kinase/fructose-2,6-biphosphatase 3 [Source:HGNC Symbol;Acc:HGNC:8874]	chr10	6144934	6254644	1	ensembl_havana	4.573010216	4.399776375	4.746244058	4.113938229	4.473306098	4.612084796	4.657831492	4.881204367	4.699696315
ENSG00000139629	0.027268	0.08317115	-0.180529193	-1.133299513	**GALNT6**	protein_coding	polypeptide N-acetylgalactosaminyltransferase 6 [Source:HGNC Symbol;Acc:HGNC:4128]	chr12	51351247	51392867	-1	ensembl_havana	6.517458001	6.607722597	6.427193404	6.592293086	6.550211866	6.68066284	6.391654763	6.425815412	6.464110037
ENSG00000244558	0.027283	0.083180668	0.785728557	1.723962695	**KCNK15-AS1**	antisense	KCNK15 antisense RNA 1 (head to head) [Source:HGNC Symbol;Acc:HGNC:49901]	chr20	44694892	44746021	-1	havana	-0.569743594	-0.962607872	-0.176879315	-0.92573229	-0.521012188	-1.441079133	-0.63291296	0.097529431	0.004745582
ENSG00000091542	0.027279	0.083180668	0.170517218	1.1254619	**ALKBH5**	protein_coding	alkB homolog 5, RNA demethylase [Source:HGNC Symbol;Acc:HGNC:25996]	chr17	18183078	18209954	1	ensembl_havana	5.876698333	5.791439723	5.961956942	5.814732934	5.77088429	5.788701946	5.968081312	5.960049993	5.95773952
ENSG00000132964	0.027313	0.083205682	0.193256253	1.143341399	**CDK8**	protein_coding	cyclin dependent kinase 8 [Source:HGNC Symbol;Acc:HGNC:1779]	chr13	26254104	26405238	1	ensembl_havana	5.096905132	5.000277006	5.193533259	4.91112207	5.046142112	5.043566836	5.188389385	5.194336108	5.197874284
ENSG00000143772	0.027304	0.083205682	-0.212364719	-1.158585664	**ITPKB**	protein_coding	inositol-trisphosphate 3-kinase B [Source:HGNC Symbol;Acc:HGNC:6179]	chr1	226631690	226739323	-1	ensembl_havana	8.351478705	8.457661064	8.245296346	8.60286061	8.394799192	8.375323391	8.500554131	8.156841997	8.078492908
ENSG00000101057	0.027314	0.083205682	-0.293628062	-1.22571882	**MYBL2**	protein_coding	MYB proto-oncogene like 2 [Source:HGNC Symbol;Acc:HGNC:7548]	chr20	43667019	43716496	1	ensembl_havana	7.936328158	8.08314219	7.789514127	7.808459173	8.17783025	8.263137145	7.729738323	7.779637783	7.859166277
ENSG00000069020	0.0273	0.083205682	-0.452721465	-1.368619554	**MAST4**	protein_coding	microtubule associated serine/threonine kinase family member 4 [Source:HGNC Symbol;Acc:HGNC:19037]	chr5	66596361	67169595	1	ensembl_havana	4.728033292	4.954394024	4.50167256	4.513220058	4.962789824	5.387172191	4.38549012	4.510949428	4.60857813
ENSG00000101898	0.027353	0.083306735	-0.394313101	-1.314316836	**MCTS2P**	processed_pseudogene	malignant T cell amplified sequence 2, pseudogene [Source:HGNC Symbol;Acc:HGNC:49760]	chr20	31547504	31548049	1	havana	0.439218072	0.636374622	0.242061521	0.660286109	0.52486995	0.723967808	-0.04612063	0.343769191	0.428535999
ENSG00000198018	0.02736	0.083310568	0.179028779	1.132121486	**ENTPD7**	protein_coding	ectonucleoside triphosphate diphosphohydrolase 7 [Source:HGNC Symbol;Acc:HGNC:19745]	chr10	99659506	99706240	1	ensembl_havana	3.473666384	3.384151995	3.563180774	3.428290208	3.363940242	3.360225534	3.610684386	3.495088671	3.583769265
ENSG00000187837	0.027381	0.083355963	-0.341424223	-1.267006761	**HIST1H1C**	protein_coding	histone cluster 1 H1 family member c [Source:HGNC Symbol;Acc:HGNC:4716]	chr6	26055787	26056428	-1	havana	6.23033273	6.401044841	6.059620619	6.839293502	6.409744907	5.954096115	6.29832625	6.016056889	5.864478717
ENSG00000154065	0.027443	0.083526145	-0.57047373	-1.485011116	**ANKRD29**	protein_coding	ankyrin repeat domain 29 [Source:HGNC Symbol;Acc:HGNC:27110]	chr18	23598926	23662885	-1	ensembl_havana	0.455502035	0.7407389	0.17026517	0.683014432	0.962641309	0.57656096	0.24405081	-0.08087422	0.347618925
ENSG00000276234	0.027486	0.083639451	-0.192540201	-1.142774066	**TADA2A**	protein_coding	transcriptional adaptor 2A [Source:HGNC Symbol;Acc:HGNC:11531]	chr17	37406874	37479730	1	ensembl_havana	5.127271375	5.223541475	5.031001274	5.156201297	5.23294695	5.281476179	4.969888417	4.965223687	5.157891719
ENSG00000237636	0.02751	0.08369692	-0.701933951	-1.626683922	**ANKRD26P3**	unprocessed_pseudogene	ankyrin repeat domain 26 pseudogene 3 [Source:HGNC Symbol;Acc:HGNC:39689]	chr13	19290593	19344825	-1	havana	-0.308360855	0.04260612	-0.659327831	0.237415364	-0.218971072	0.109374069	0.141952326	-1.15951152	-0.960424301
ENSG00000184517	0.027523	0.083716131	0.170610138	1.12553439	**ZFP1**	protein_coding	ZFP1 zinc finger protein [Source:HGNC Symbol;Acc:HGNC:23328]	chr16	75148492	75172236	1	ensembl_havana	5.713298147	5.627993078	5.798603217	5.641344698	5.685719046	5.556915491	5.822343453	5.832261595	5.741204602
ENSG00000170191	0.02757	0.083842155	-0.170449802	-1.125409309	**NANP**	protein_coding	N-acetylneuraminic acid phosphatase [Source:HGNC Symbol;Acc:HGNC:16140]	chr20	25612935	25624175	-1	ensembl_havana	4.338801101	4.424026002	4.2535762	4.329150785	4.507531551	4.43539567	4.181874628	4.329953957	4.248900015
ENSG00000111554	0.027582	0.083861895	0.180247564	1.133078303	**MDM1**	protein_coding	Mdm1 nuclear protein [Source:HGNC Symbol;Acc:HGNC:29917]	chr12	68272443	68332381	-1	ensembl_havana	4.064603002	3.97447922	4.154726784	3.987262005	3.94667289	3.989502764	4.113831997	4.135262968	4.215085387
ENSG00000260404	0.0276	0.08389858	-0.194303399	-1.144171567	**AC110079.1**	transcribed_unprocessed_pseudogene		chr4	118591773	118633729	1	havana	4.446338944	4.543490644	4.349187245	4.527418732	4.44440735	4.658645849	4.285277268	4.337078524	4.425205944
ENSG00000143222	0.027644	0.084012845	-0.190667045	-1.141291282	**UFC1**	protein_coding	ubiquitin-fold modifier conjugating enzyme 1 [Source:HGNC Symbol;Acc:HGNC:26941]	chr1	161152776	161158856	1	ensembl_havana	6.612027684	6.707361206	6.516694161	6.818915111	6.709866385	6.593302123	6.551611546	6.579029926	6.419441012
ENSG00000104133	0.027663	0.08404479	-0.219318481	-1.164183505	**SPG11**	protein_coding	SPG11, spatacsin vesicle trafficking associated [Source:HGNC Symbol;Acc:HGNC:11226]	chr15	44562696	44663678	-1	ensembl_havana	6.628410674	6.738069915	6.518751434	6.811520743	6.613409913	6.789279089	6.466167164	6.448607375	6.641479761
ENSG00000167700	0.027666	0.08404479	-0.255313062	-1.193594723	**MFSD3**	protein_coding	major facilitator superfamily domain containing 3 [Source:HGNC Symbol;Acc:HGNC:25157]	chr8	144509074	144511213	1	ensembl_havana	1.332660434	1.460316965	1.205003903	1.341416217	1.603393164	1.436141515	0.915012722	1.452931788	1.2470672
ENSG00000272405	0.027682	0.084075227	-0.259438514	-1.197012745	**AL365181.3**	antisense		chr1	156641666	156644887	-1	havana	5.818288366	5.948007623	5.688569109	5.913540446	5.861117093	6.069365329	5.500915383	5.755903343	5.808888601
ENSG00000117318	0.027695	0.084099377	-0.597606477	-1.513203982	**ID3**	protein_coding	inhibitor of DNA binding 3, HLH protein [Source:HGNC Symbol;Acc:HGNC:5362]	chr1	23557918	23559794	-1	ensembl_havana	-0.411128043	-0.112324804	-0.709931282	-0.85854608	0.185737694	0.335833975	-0.97139419	-0.80561234	-0.352787316
ENSG00000186635	0.027713	0.084134101	-0.195609177	-1.145207622	**ARAP1**	protein_coding	ArfGAP with RhoGAP domain, ankyrin repeat and PH domain 1 [Source:HGNC Symbol;Acc:HGNC:16925]	chr11	72685069	72793599	-1	ensembl_havana	6.245263298	6.343067887	6.14745871	6.489268435	6.21412123	6.325813994	6.333492935	6.06836606	6.040517135
ENSG00000105639	0.027729	0.084165128	-0.196312485	-1.145766042	**JAK3**	protein_coding	Janus kinase 3 [Source:HGNC Symbol;Acc:HGNC:6193]	chr19	17824780	17848071	-1	ensembl_havana	5.780372856	5.878529098	5.682216613	5.81344595	5.86857866	5.953562686	5.69142039	5.680887588	5.674341862
ENSG00000152409	0.027769	0.08422828	0.274898253	1.209908756	**JMY**	protein_coding	junction mediating and regulatory protein, p53 cofactor [Source:HGNC Symbol;Acc:HGNC:28916]	chr5	79236189	79327215	1	ensembl_havana	5.758422642	5.620973516	5.895871768	5.865292978	5.404644032	5.592983536	5.985996245	5.860966869	5.840652192
ENSG00000139990	0.027773	0.08422828	0.196680167	1.146058086	**DCAF5**	protein_coding	DDB1 and CUL4 associated factor 5 [Source:HGNC Symbol;Acc:HGNC:20224]	chr14	69050881	69153150	-1	ensembl_havana	7.174930919	7.076590835	7.273271002	7.038878601	7.129445438	7.061448467	7.266353701	7.235676987	7.317782318
ENSG00000165661	0.027765	0.08422828	0.191177457	1.141695132	**QSOX2**	protein_coding	quiescin sulfhydryl oxidase 2 [Source:HGNC Symbol;Acc:HGNC:30249]	chr9	136206333	136245841	-1	ensembl_havana	4.967023644	4.871434916	5.062612373	4.697522173	4.801827565	5.114955008	4.905131977	5.054377101	5.228328042
ENSG00000198743	0.027764	0.08422828	-0.429317227	-1.346596133	**SLC5A3**	protein_coding	solute carrier family 5 member 3 [Source:HGNC Symbol;Acc:HGNC:11038]	chr21	34073570	34106262	1	ensembl_havana	6.950374518	7.165033132	6.735715905	7.449643834	6.969965726	7.075489837	6.675173853	6.786157488	6.745816374
ENSG00000231595	0.027783	0.084240277	-0.648815407	-1.567880285	**AC005224.1**	lincRNA		chr17	14210488	14217922	1	havana	0.134539066	0.45894677	-0.189868638	0.437805725	0.89230127	0.046733314	-0.63291296	0.130707766	-0.067400719
ENSG00000142583	0.027811	0.084307599	-0.251817402	-1.190706135	**SLC2A5**	protein_coding	solute carrier family 2 member 5 [Source:HGNC Symbol;Acc:HGNC:11010]	chr1	9035107	9088478	-1	ensembl_havana	-0.281254419	-0.155345718	-0.40716312	-0.51029874	-0.002471726	0.046733314	-0.69455235	-0.42206346	-0.104873545
ENSG00000138398	0.027824	0.084313131	-0.204521719	-1.152304273	**PPIG**	protein_coding	peptidylprolyl isomerase G [Source:HGNC Symbol;Acc:HGNC:14650]	chr2	169584340	169641406	1	ensembl_havana	5.404640511	5.50690137	5.302379651	5.514822849	5.573598568	5.432282695	5.223182524	5.361754164	5.322202266
ENSG00000198739	0.027825	0.084313131	-0.507921352	-1.421999889	**LRRTM3**	protein_coding	leucine rich repeat transmembrane neuronal 3 [Source:HGNC Symbol;Acc:HGNC:19410]	chr10	66926006	67099830	1	ensembl_havana	1.597715767	1.851676443	1.343755091	2.312722596	1.688874845	1.553431888	1.458744294	1.130954377	1.441566602
ENSG00000267216	0.027842	0.084349779	0.383403028	1.304415083	**AC020915.1**	protein_coding		chr19	58278966	58315197	1	havana	2.273174898	2.081473384	2.464876413	2.049654613	1.927394938	2.267370602	2.581431252	2.472571931	2.340626055
ENSG00000269939	0.027869	0.084411992	0.336994418	1.263122374	**PCF11-AS1**	lincRNA	PCF11 antisense RNA 1 [Source:HGNC Symbol;Acc:HGNC:52263]	chr11	83185521	83187036	-1	havana	0.461101382	0.292604173	0.629598591	-0.03191641	0.547932369	0.361796557	0.24405081	0.693803314	0.950941648
ENSG00000266236	0.027928	0.084555002	0.324161937	1.251936976	**NARF-IT1**	sense_intronic	NARF intronic transcript 1 [Source:HGNC Symbol;Acc:HGNC:43651]	chr17	82482098	82483388	1	havana	0.415442506	0.253361538	0.577523474	-0.06941355	0.125688576	0.703809589	0.24405081	0.671430284	0.817089329
ENSG00000246985	0.027923	0.084555002	-0.236426893	-1.178071329	**SOCS2-AS1**	processed_transcript	SOCS2 antisense RNA 1 [Source:HGNC Symbol;Acc:HGNC:27054]	chr12	93542463	93571768	-1	havana	0.778261341	0.896474787	0.660047894	1.022973463	0.962641309	0.703809589	0.64332197	0.758913071	0.577908642
ENSG00000235641	0.027946	0.084592014	0.611616597	1.527970402	**LINC00484**	lincRNA	long intergenic non-protein coding RNA 484 [Source:HGNC Symbol;Acc:HGNC:27862]	chr9	91159573	91165658	1	havana	-0.70979243	-1.015600729	-0.403984132	-1.51148723	-1.178380098	-0.356934859	-1.41444944	-0.19956768	0.402064725
ENSG00000151287	0.027955	0.084601023	-0.200690472	-1.149248252	**TEX30**	protein_coding	testis expressed 30 [Source:HGNC Symbol;Acc:HGNC:25188]	chr13	102765990	102773811	-1	ensembl_havana	4.129136447	4.229481683	4.028791211	4.107651756	4.329321514	4.25147178	3.837207823	4.102190434	4.146975376
ENSG00000172172	0.027962	0.084605014	-0.202939487	-1.15104121	**MRPL13**	protein_coding	mitochondrial ribosomal protein L13 [Source:HGNC Symbol;Acc:HGNC:14278]	chr8	120380761	120445402	-1	ensembl_havana	5.340543349	5.442013092	5.239073605	5.464874676	5.506753835	5.354410766	5.175526364	5.298377255	5.243317197
ENSG00000049246	0.027974	0.084621771	-0.249206717	-1.188553395	**PER3**	protein_coding	period circadian regulator 3 [Source:HGNC Symbol;Acc:HGNC:8847]	chr1	7784320	7845177	1	ensembl_havana	4.080316801	4.204920159	3.955713442	4.209009558	4.133596708	4.272154211	3.801373512	4.007126095	4.05864072
ENSG00000115204	0.027984	0.084635355	0.186861642	1.13828486	**MPV17**	protein_coding	MPV17, mitochondrial inner membrane protein [Source:HGNC Symbol;Acc:HGNC:7224]	chr2	27309492	27325680	-1	ensembl_havana	6.200445069	6.107014247	6.29387589	6.016676315	6.128264206	6.176102221	6.250573981	6.241617797	6.389435891
ENSG00000107175	0.028028	0.084733	-0.196430162	-1.145859503	**CREB3**	protein_coding	cAMP responsive element binding protein 3 [Source:HGNC Symbol;Acc:HGNC:2347]	chr9	35732335	35737004	1	ensembl_havana	6.506810906	6.605025988	6.408595825	6.940620202	6.644501807	6.229955954	6.652918535	6.480727454	6.092141487
ENSG00000166133	0.028025	0.084733	-0.240991897	-1.18180491	**RPUSD2**	protein_coding	RNA pseudouridylate synthase domain containing 2 [Source:HGNC Symbol;Acc:HGNC:24180]	chr15	40569300	40574943	1	ensembl_havana	3.750445222	3.870941171	3.629949273	3.907479941	3.786222876	3.919120695	3.568668292	3.699633758	3.621545769
ENSG00000169992	0.028038	0.084746554	0.208391123	1.155398978	**NLGN2**	protein_coding	neuroligin 2 [Source:HGNC Symbol;Acc:HGNC:14290]	chr17	7404874	7419860	1	ensembl_havana	4.739017272	4.63482171	4.843212833	4.86714065	4.553838016	4.483486464	5.134019951	4.650458282	4.745160266
ENSG00000115970	0.028099	0.084911565	-0.191023039	-1.141572937	**THADA**	protein_coding	THADA, armadillo repeat containing [Source:HGNC Symbol;Acc:HGNC:19217]	chr2	43230836	43596046	-1	ensembl_havana	6.507171702	6.602683222	6.411660182	6.603505865	6.518090961	6.686452838	6.333975376	6.342861398	6.558143773
ENSG00000167685	0.02812	0.084956703	-0.173131395	-1.127503096	**ZNF444**	protein_coding	zinc finger protein 444 [Source:HGNC Symbol;Acc:HGNC:16052]	chr19	56132599	56160893	1	ensembl_havana	4.082460151	4.169025849	3.995894454	4.339917525	4.014471486	4.152688536	4.203156559	3.808721901	3.975804901
ENSG00000148296	0.028154	0.085041829	0.193277819	1.14335849	**SURF6**	protein_coding	surfeit 6 [Source:HGNC Symbol;Acc:HGNC:11478]	chr9	133328774	133336398	-1	ensembl_havana	4.976021083	4.879382173	5.072659992	4.703092555	4.826793299	5.108260665	4.939776522	5.064070606	5.214132848
ENSG00000109220	0.028162	0.085048368	0.233324668	1.175540845	**CHIC2**	protein_coding	cysteine rich hydrophobic domain 2 [Source:HGNC Symbol;Acc:HGNC:1935]	chr4	54009789	54064690	-1	ensembl_havana	3.069241979	2.952579645	3.185904313	3.071451915	3.053384979	2.73290204	3.209288915	3.237766117	3.110657906
ENSG00000126254	0.02818	0.085065947	0.301216327	1.232182821	**RBM42**	protein_coding	RNA binding motif protein 42 [Source:HGNC Symbol;Acc:HGNC:28117]	chr19	35629030	35637686	1	ensembl_havana	4.804193465	4.653585302	4.954801629	4.556942526	4.667445445	4.736367933	5.082123272	4.884855267	4.897426347
ENSG00000169252	0.028191	0.085065947	0.233144107	1.175393729	**ADRB2**	protein_coding	adrenoceptor beta 2 [Source:HGNC Symbol;Acc:HGNC:286]	chr5	148825245	148828687	1	ensembl_havana	3.221265699	3.104693645	3.337837752	3.014347502	3.143202419	3.156531015	3.330286772	3.439903888	3.243322595
ENSG00000116704	0.028178	0.085065947	0.18531429	1.137064655	**SLC35D1**	protein_coding	solute carrier family 35 member D1 [Source:HGNC Symbol;Acc:HGNC:20800]	chr1	66999332	67054099	-1	ensembl_havana	6.160300814	6.067643669	6.252957959	5.983873229	6.132561415	6.086496364	6.232057254	6.316970635	6.20984599
ENSG00000103047	0.028189	0.085065947	-0.190984818	-1.141542694	**TANGO6**	protein_coding	transport and golgi organization 6 homolog [Source:HGNC Symbol;Acc:HGNC:25749]	chr16	68843604	69085180	1	ensembl_havana	4.171952471	4.26744488	4.076460062	4.311025713	4.150646514	4.340662414	4.068166701	3.929121717	4.232091769
ENSG00000082213	0.028198	0.085068123	-0.186924937	-1.1383348	**C5orf22**	protein_coding	chromosome 5 open reading frame 22 [Source:HGNC Symbol;Acc:HGNC:25639]	chr5	31532266	31555058	1	ensembl_havana	5.581863315	5.675325783	5.488400846	5.741509981	5.679205908	5.605261461	5.484495499	5.503064662	5.477642378
ENSG00000233296	0.028225	0.085132511	-0.625980609	-1.543259432	**AC092159.2**	antisense		chr2	677186	697371	1	havana	-0.376732596	-0.063742291	-0.689722901	-0.03191641	-0.299013651	0.139703184	-0.40973604	-0.62862566	-1.030806998
ENSG00000141497	0.028249	0.085186343	-0.729436971	-1.657991915	**ZMYND15**	protein_coding	zinc finger MYND-type containing 15 [Source:HGNC Symbol;Acc:HGNC:20997]	chr17	4740015	4746119	1	ensembl_havana	-0.256005596	0.10871289	-0.620724081	0.14220989	-0.071090603	0.255019383	-1.22062457	-0.57414696	-0.067400719
ENSG00000161692	0.028268	0.085207378	0.275001	1.209994928	**DBF4B**	protein_coding	DBF4 zinc finger B [Source:HGNC Symbol;Acc:HGNC:17883]	chr17	44708608	44752264	1	ensembl_havana	4.925799625	4.788299125	5.063300125	4.490851674	4.923650443	4.950395257	4.948624269	5.042440346	5.198835759
ENSG00000213859	0.028265	0.085207378	-0.19343562	-1.143483557	**KCTD11**	protein_coding	potassium channel tetramerization domain containing 11 [Source:HGNC Symbol;Acc:HGNC:21302]	chr17	7351889	7354944	1	ensembl_havana	3.428169801	3.524887611	3.331451991	3.719644151	3.543917077	3.311101606	3.487731073	3.301071157	3.205553743
ENSG00000185813	0.028277	0.085216109	0.196748918	1.146112702	**PCYT2**	protein_coding	phosphate cytidylyltransferase 2, ethanolamine [Source:HGNC Symbol;Acc:HGNC:8756]	chr17	81900965	81911464	-1	ensembl_havana	4.805521283	4.707146825	4.903895742	4.762987913	4.667445445	4.691007115	5.053115446	4.826568403	4.832003378
ENSG00000028528	0.028309	0.085294078	0.170041115	1.125090548	**SNX1**	protein_coding	sorting nexin 1 [Source:HGNC Symbol;Acc:HGNC:11172]	chr15	64094123	64146090	1	ensembl_havana	7.610206883	7.525186325	7.695227441	7.567509124	7.492675912	7.51537394	7.763080541	7.638272067	7.684329714
ENSG00000214517	0.028343	0.085344134	0.181407899	1.133989985	**PPME1**	protein_coding	protein phosphatase methylesterase 1 [Source:HGNC Symbol;Acc:HGNC:30178]	chr11	74171099	74254703	1	ensembl_havana	5.745434535	5.654730586	5.836138485	5.548475941	5.683117318	5.732598499	5.793167761	5.842310949	5.872936745
ENSG00000156675	0.028339	0.085344134	0.173481375	1.127776647	**RAB11FIP1**	protein_coding	RAB11 family interacting protein 1 [Source:HGNC Symbol;Acc:HGNC:30265]	chr8	37858618	37899467	-1	ensembl_havana	7.679890026	7.593149339	7.766630714	8.060605656	7.40421514	7.314627221	8.216478733	7.552880411	7.530532997
ENSG00000169504	0.028334	0.085344134	-0.192653059	-1.142863465	**CLIC4**	protein_coding	chloride intracellular channel 4 [Source:HGNC Symbol;Acc:HGNC:13518]	chr1	24745357	24844324	1	ensembl_havana	6.901142682	6.997469212	6.804816153	7.017241835	7.092423664	6.882742136	6.897734403	6.822463843	6.694250212
ENSG00000066279	0.02835	0.085348472	-0.177851833	-1.131198281	**ASPM**	protein_coding	abnormal spindle microtubule assembly [Source:HGNC Symbol;Acc:HGNC:19048]	chr1	197084128	197146694	-1	ensembl_havana	7.369196698	7.458122615	7.280270782	7.275985503	7.503039005	7.595343337	7.06050188	7.379340912	7.400969553
ENSG00000082438	0.028383	0.085428117	0.918581865	1.890256303	**COBLL1**	protein_coding	cordon-bleu WH2 repeat protein like 1 [Source:HGNC Symbol;Acc:HGNC:23571]	chr2	164653624	164843679	-1	ensembl_havana	1.569906832	1.110615899	2.029197764	-0.27307956	1.667974555	1.936952697	1.415636519	2.439854124	2.23210265
ENSG00000171862	0.02839	0.085431162	-0.219467217	-1.164303533	**PTEN**	protein_coding	phosphatase and tensin homolog [Source:HGNC Symbol;Acc:HGNC:9588]	chr10	87863113	87971930	1	ensembl_havana	7.146439358	7.256172967	7.03670575	7.380061754	7.203027208	7.185429938	7.067191257	6.953115483	7.08981051
ENSG00000239789	0.028406	0.085461413	0.189216026	1.140143983	**MRPS17**	protein_coding	mitochondrial ribosomal protein S17 [Source:HGNC Symbol;Acc:HGNC:14047]	chr7	55951819	55956501	1	ensembl_havana	2.842950295	2.748342282	2.937558308	2.649185179	2.877938163	2.717903502	2.89565563	2.989113925	2.927905368
ENSG00000102781	0.028432	0.085506512	0.18144476	1.134018959	**KATNAL1**	protein_coding	katanin catalytic subunit A1 like 1 [Source:HGNC Symbol;Acc:HGNC:28361]	chr13	30202630	30307484	-1	ensembl_havana	3.948153277	3.857430897	4.038875657	3.686284073	3.904856379	3.981152237	3.893277228	4.125009009	4.098340733
ENSG00000149428	0.028433	0.085506512	-0.177208412	-1.130693896	**HYOU1**	protein_coding	hypoxia up-regulated 1 [Source:HGNC Symbol;Acc:HGNC:16931]	chr11	119044189	119057202	-1	ensembl_havana	9.890965771	9.979569977	9.802361565	10.05948883	9.974115359	9.905105745	9.936080056	9.764154225	9.706850413
ENSG00000171314	0.02844	0.085509156	-0.176309042	-1.129989245	**PGAM1**	protein_coding	phosphoglycerate mutase 1 [Source:HGNC Symbol;Acc:HGNC:8888]	chr10	97426160	97433441	1	ensembl_havana	6.251434044	6.339588565	6.163279523	6.324220276	6.362151408	6.332394012	6.180948725	6.134775054	6.174114791
ENSG00000261652	0.028473	0.085572751	0.377772825	1.299334446	**C15orf65**	protein_coding	chromosome 15 open reading frame 65 [Source:HGNC Symbol;Acc:HGNC:44654]	chr15	55408548	55418764	1	ensembl_havana	0.898830923	0.70994451	1.087717335	0.892538	0.927899934	0.309395597	1.178360195	1.097781778	0.987010032
ENSG00000102763	0.028471	0.085572751	0.174739355	1.128760458	**VWA8**	protein_coding	von Willebrand factor A domain containing 8 [Source:HGNC Symbol;Acc:HGNC:29071]	chr13	41566837	41961120	-1	ensembl_havana	5.445704302	5.358334625	5.533073979	5.362113708	5.264616028	5.448274139	5.518848865	5.486339745	5.594033327
ENSG00000172197	0.02849	0.085605941	0.181859957	1.134345368	**MBOAT1**	protein_coding	membrane bound O-acyltransferase domain containing 1 [Source:HGNC Symbol;Acc:HGNC:21579]	chr6	20102145	20212399	-1	ensembl_havana	3.815352785	3.724422806	3.906282763	3.691897931	3.611154138	3.870216351	3.939661103	3.759192284	4.019994903
ENSG00000176884	0.028526	0.085614358	0.334120488	1.260608668	**GRIN1**	protein_coding	glutamate ionotropic receptor NMDA type subunit 1 [Source:HGNC Symbol;Acc:HGNC:4584]	chr9	137138390	137168762	1	ensembl_havana	-0.20878831	-0.375848555	-0.041728066	-0.06941355	-0.783495335	-0.274636777	0.069637898	-0.19956768	0.004745582
ENSG00000160953	0.028539	0.085614358	0.257340904	1.195273611	**MUM1**	protein_coding	melanoma associated antigen (mutated) 1 [Source:HGNC Symbol;Acc:HGNC:29641]	chr19	1354711	1378431	1	ensembl_havana	5.756185109	5.627514657	5.884855561	5.532138763	5.511161128	5.83924408	5.867031184	5.868362208	5.919173293
ENSG00000101474	0.028531	0.085614358	0.202575328	1.150750707	**APMAP**	protein_coding	adipocyte plasma membrane associated protein [Source:HGNC Symbol;Acc:HGNC:13238]	chr20	24962925	24992979	-1	ensembl_havana	7.515529765	7.414242101	7.616817429	7.244577574	7.435661872	7.562486857	7.534312226	7.644566962	7.6715731
ENSG00000105402	0.028514	0.085614358	0.183648815	1.135752763	**NAPA**	protein_coding	NSF attachment protein alpha [Source:HGNC Symbol;Acc:HGNC:7641]	chr19	47487637	47515240	-1	ensembl_havana	6.525777795	6.433953387	6.617602202	6.504102541	6.513338705	6.284418915	6.675173853	6.643666671	6.533966083
ENSG00000122729	0.02854	0.085614358	0.170153403	1.125178119	**ACO1**	protein_coding	aconitase 1 [Source:HGNC Symbol;Acc:HGNC:117]	chr9	32384603	32454769	1	ensembl_havana	6.481338903	6.396262201	6.566415604	6.478346466	6.358079513	6.352360625	6.629528713	6.516076887	6.553641212
ENSG00000091483	0.028532	0.085614358	-0.222238006	-1.166541802	**FH**	protein_coding	fumarate hydratase [Source:HGNC Symbol;Acc:HGNC:3700]	chr1	241497603	241519761	-1	ensembl_havana	7.349383707	7.46050271	7.238264704	7.643350535	7.428107015	7.310050581	7.315306194	7.188450573	7.211037346

ENSG00000128694	0.028505	0.085614358	-0.316950603	-1.245694763	**OSGEPL1**	protein_coding	O-sialoglycoprotein endopeptidase like 1 [Source:HGNC Symbol;Acc:HGNC:23075]	chr2	189746660	189763227	-1	ensembl_havana	3.626019001	3.784494303	3.467543699	3.72512993	3.870965269	3.75738771	3.262971565	3.456354973	3.68330456
ENSG00000267560	0.028508	0.085614358	-0.677274978	-1.599116423	**AC027514.2**	antisense		chr18	62300036	62300998	-1	havana	0.731060386	1.069697875	0.392422897	0.812341977	1.228369434	1.168382212	0.64332197	0.02879206	0.505154661
ENSG00000163527	0.028559	0.085653761	0.167920604	1.123438075	**STT3B**	protein_coding	STT3B, catalytic subunit of the oligosaccharyltransferase complex [Source:HGNC Symbol;Acc:HGNC:30611]	chr3	31532638	31637622	1	ensembl_havana	7.678891364	7.594931062	7.762851666	7.523913866	7.591715532	7.669163787	7.677283864	7.755910838	7.855360294
ENSG00000089094	0.028573	0.085663118	-0.172660941	-1.127135484	**KDM2B**	protein_coding	lysine demethylase 2B [Source:HGNC Symbol;Acc:HGNC:13610]	chr12	121429097	121581015	-1	ensembl_havana	4.974508643	5.060839114	4.888178173	5.129559428	5.055224852	4.997733062	4.982251883	4.898163408	4.784119228
ENSG00000237667	0.028574	0.085663118	-1.180641181	-2.266774976	**LINC01115**	lincRNA	long intergenic non-protein coding RNA 1115 [Source:HGNC Symbol;Acc:HGNC:49258]	chr2	779840	868426	-1	havana	-0.707583974	-0.117263383	-1.297904564	0.174648163	-0.473795405	-0.052642908	-2.0571346	-1.00738779	-0.829191301
ENSG00000123104	0.028621	0.085784916	0.17443937	1.128525776	**ITPR2**	protein_coding	inositol 1,4,5-trisphosphate receptor type 2 [Source:HGNC Symbol;Acc:HGNC:6181]	chr12	26336515	26833198	-1	ensembl_havana	7.542370046	7.455150361	7.629589731	7.422676696	7.414446982	7.528327405	7.638557465	7.588019218	7.662192512
ENSG00000135108	0.028629	0.085791241	0.200613086	1.149186608	**FBXO21**	protein_coding	F-box protein 21 [Source:HGNC Symbol;Acc:HGNC:13592]	chr12	117141988	117190531	-1	ensembl_havana	4.683497158	4.583190615	4.783803701	4.639150283	4.589008315	4.521413245	4.763324634	4.769781613	4.818304855
ENSG00000170100	0.028664	0.085824277	0.180792079	1.13350604	**ZNF778**	protein_coding	zinc finger protein 778 [Source:HGNC Symbol;Acc:HGNC:26479]	chr16	89217703	89237071	1	ensembl_havana	4.903482774	4.813086735	4.993878814	4.816646463	4.832674381	4.789939359	4.937238596	5.06299676	4.981401085
ENSG00000196305	0.028654	0.085824277	-0.175901384	-1.129669993	**IARS**	protein_coding	isoleucyl-tRNA synthetase [Source:HGNC Symbol;Acc:HGNC:5330]	chr9	92210207	92293756	-1	ensembl_havana	8.047412581	8.135363273	7.959461889	8.066965039	8.12848268	8.210642101	7.839650161	7.972834615	8.065900892
ENSG00000186806	0.028661	0.085824277	-0.191421304	-1.141888119	**VSIG10L**	protein_coding	V-set and immunoglobulin domain containing 10 like [Source:HGNC Symbol;Acc:HGNC:27111]	chr19	51331536	51342124	-1	ensembl_havana	2.436416093	2.532126745	2.340705441	2.50993591	2.608571783	2.477872542	2.394263752	2.34408831	2.283764259
ENSG00000228477	0.028657	0.085824277	-0.246209944	-1.186087089	**AL663070.1**	processed_pseudogene		chr1	39962680	39963404	1	havana	1.762042317	1.885147289	1.638937345	2.451565702	1.719668303	1.484207863	2.10871413	1.637380031	1.170717874
ENSG00000283586	0.028699	0.08584123	0.38686657	1.307550412	**GKN3P**	polymorphic_pseudogene	gastrokine 3, pseudogene [Source:HGNC Symbol;Acc:HGNC:37701]	chr2	68921248	68926834	1	havana	2.310136277	2.116702991	2.503569562	2.471286007	2.077587619	1.801235349	2.711879034	2.293711009	2.505118643
ENSG00000179832	0.028683	0.08584123	0.343246405	1.268608053	**MROH1**	protein_coding	maestro heat like repeat family member 1 [Source:HGNC Symbol;Acc:HGNC:26958]	chr8	144148016	144261940	1	ensembl_havana	4.959122652	4.78749945	5.130745855	4.839587794	4.514865633	5.008044923	5.270323268	5.038075104	5.083839194
ENSG00000214706	0.02869	0.08584123	0.250652811	1.189745346	**IFRD2**	protein_coding	interferon related developmental regulator 2 [Source:HGNC Symbol;Acc:HGNC:5457]	chr3	50287732	50292918	-1	ensembl_havana	5.22684667	5.101520264	5.352173075	4.937354111	5.007149573	5.360057109	5.11617989	5.427478931	5.512860405
ENSG00000137522	0.028683	0.08584123	0.168922443	1.124218486	**RNF121**	protein_coding	ring finger protein 121 [Source:HGNC Symbol;Acc:HGNC:21070]	chr11	71928701	71997597	1	ensembl_havana	5.118342977	5.033881755	5.202804198	5.081183842	5.056230524	4.9642309	5.23666179	5.211882794	5.159868011
ENSG00000085185	0.028711	0.08584123	-0.17628961	-1.129974025	**BCORL1**	protein_coding	BCL6 corepressor like 1 [Source:HGNC Symbol;Acc:HGNC:25657]	chrX	129981107	130058083	1	ensembl_havana	6.457173685	6.54531849	6.369028881	6.548098016	6.605187566	6.482669889	6.415555379	6.380846658	6.310684605
ENSG00000169955	0.028711	0.08584123	-0.22036557	-1.16502876	**ZNF747**	protein_coding	zinc finger protein 747 [Source:HGNC Symbol;Acc:HGNC:28350]	chr16	30530367	30535347	-1	ensembl_havana	3.440013018	3.550195802	3.329830233	3.522665065	3.580725593	3.54719675	3.170918121	3.426606605	3.391965972
ENSG00000171466	0.028702	0.08584123	-0.274083283	-1.209225479	**ZNF562**	protein_coding	zinc finger protein 562 [Source:HGNC Symbol;Acc:HGNC:25950]	chr19	9641808	9675086	-1	ensembl_havana	5.334056168	5.47109781	5.197014527	5.407175137	5.44359484	5.562523452	4.946101873	5.319214226	5.32572748
ENSG00000014138	0.028768	0.085976029	-0.19374239	-1.143726729	**POLA2**	protein_coding	DNA polymerase alpha 2, accessory subunit [Source:HGNC Symbol;Acc:HGNC:30073]	chr11	65261762	65305589	1	ensembl_havana	6.071839345	6.16871054	5.97496815	6.083333506	6.219517755	6.203280358	5.970565603	5.95021005	6.004128796
ENSG00000174695	0.028763	0.085976029	-0.23461786	-1.17659504	**TMEM167A**	protein_coding	transmembrane protein 167A [Source:HGNC Symbol;Acc:HGNC:28330]	chr5	83052846	83077863	-1	ensembl_havana	6.963013405	7.080322335	6.845704475	7.131644205	7.048121896	7.061200903	6.746489256	6.886994389	6.903629778
ENSG00000223960	0.028811	0.086064001	-0.169919798	-1.124995942	**AC009948.1**	antisense		chr2	178413939	178440243	1	havana	4.966538729	5.051498628	4.88157883	4.810208591	5.146820816	5.197466477	4.670111011	4.973248329	5.001377152
ENSG00000185684	0.028816	0.086064001	-0.201770696	-1.150109079	**EP400P1**	transcribed_unprocessed_pseudogene	EP400 pseudogene 1 [Source:HGNC Symbol;Acc:HGNC:26602]	chr12	132084283	132131639	1	havana	3.65357896	3.754464308	3.552693611	3.885601241	3.670151025	3.707640658	3.581727135	3.501444376	3.574909323
ENSG00000157429	0.028812	0.086064001	-0.251710087	-1.190617568	**ZNF19**	protein_coding	zinc finger protein 19 [Source:HGNC Symbol;Acc:HGNC:12981]	chr16	71464555	71565089	-1	ensembl_havana	1.611865377	1.73772042	1.486010333	1.760103226	1.729788441	1.723269594	1.541278047	1.315408538	1.601344415
ENSG00000037280	0.028823	0.086067128	-0.370044395	-1.2923926	**FLT4**	protein_coding	fms related tyrosine kinase 4 [Source:HGNC Symbol;Acc:HGNC:3767]	chr5	180601506	180649624	-1	ensembl_havana	1.691261842	1.876284039	1.506239644	1.516132283	1.646767026	2.46595281	1.107892607	1.554228498	1.856597828
ENSG00000181523	0.028858	0.086153056	0.349541592	1.274155707	**SGSH**	protein_coding	N-sulfoglucosamine sulfohydrolase [Source:HGNC Symbol;Acc:HGNC:10818]	chr17	80206716	80220923	-1	ensembl_havana	6.163523522	5.988752726	6.338294318	6.190325864	5.734908975	6.041023339	6.490606087	6.347719728	6.176557138
ENSG00000137100	0.028912	0.08626262	0.179837837	1.132756553	**DCTN3**	protein_coding	dynactin subunit 3 [Source:HGNC Symbol;Acc:HGNC:2713]	chr9	34613545	34620523	-1	ensembl_havana	5.498447319	5.408528401	5.588366238	5.396900567	5.456602125	5.372082511	5.61090185	5.573379143	5.580817721
ENSG00000138386	0.028903	0.08626262	-0.172931141	-1.127346604	**NAB1**	protein_coding	NGFI-A binding protein 1 [Source:HGNC Symbol;Acc:HGNC:7626]	chr2	190646746	190692766	1	ensembl_havana	5.589212224	5.675677795	5.502746653	5.696840441	5.634119209	5.696073735	5.492296639	5.503856271	5.51208705
ENSG00000204314	0.028912	0.08626262	-0.544049605	-1.458059512	**PRRT1**	protein_coding	proline rich transmembrane protein 1 [Source:HGNC Symbol;Acc:HGNC:13943]	chr6	32148359	32153083	-1	ensembl_havana	1.265501195	1.537525998	0.993476392	1.716703112	1.614359492	1.281515389	1.356079599	0.602150945	1.022198632
ENSG00000263004	0.028934	0.086294413	-0.223087067	-1.167228542	**AC007114.1**	lincRNA		chr17	57078298	57085024	-1	havana	0.512440444	0.623983977	0.400896911	0.770507832	0.592979824	0.508464275	0.428834256	0.37179175	0.402064725
ENSG00000251666	0.028935	0.086294413	-0.251795335	-1.190687923	**ZNF346-IT1**	sense_intronic	ZNF346 intronic transcript 1 [Source:HGNC Symbol;Acc:HGNC:41423]	chr5	177051714	177052963	1	havana	-0.107076013	0.018821654	-0.232973681	0.14220989	-0.34076431	0.255019383	-0.17198403	-0.42206346	-0.104873545
ENSG00000169760	0.028983	0.086382056	0.306682092	1.236859898	**NLGN1**	protein_coding	neuroligin 1 [Source:HGNC Symbol;Acc:HGNC:14291]	chr3	173396284	174286644	1	ensembl_havana	1.924340239	1.770999193	2.077681285	1.738566362	1.891783655	1.682647563	2.238067541	2.218547759	1.776428555
ENSG00000104763	0.028978	0.086382056	-0.18111997	-1.133763689	**ASAH1**	protein_coding	N-acylsphingosine amidohydrolase 1 [Source:HGNC Symbol;Acc:HGNC:735]	chr8	18055992	18084998	-1	ensembl_havana	7.341738626	7.432298611	7.251178641	7.548876038	7.404807415	7.343212379	7.330616904	7.194592339	7.228326678
ENSG00000180817	0.02898	0.086382056	-0.22346132	-1.167531375	**PPA1**	protein_coding	pyrophosphatase (inorganic) 1 [Source:HGNC Symbol;Acc:HGNC:9226]	chr10	70202830	70233911	-1	ensembl_havana	7.470208426	7.581939085	7.358477766	7.603696296	7.619536296	7.522584663	7.25264003	7.417860552	7.404932716
ENSG00000229325	0.029014	0.086426052	0.349341286	1.273978814	**ACAP2-IT1**	sense_intronic	ACAP2 intronic transcript 1 [Source:HGNC Symbol;Acc:HGNC:41426]	chr3	195280723	195282741	-1	havana	-0.152465371	-0.327136014	0.022205272	-0.51029874	-0.383759306	-0.087349994	0.106248156	-0.2414061	0.201773757
ENSG00000197180	0.029014	0.086426052	-0.212963614	-1.159066719	**AC244090.1**	lincRNA		chrX	154424380	154428479	-1	havana	1.665059435	1.771541242	1.558577627	1.902479286	1.688874845	1.723269594	1.567772387	1.541948597	1.566011899
ENSG00000176919	0.029015	0.086426052	-0.615758671	-1.53236361	**C8G**	protein_coding	complement C8 gamma chain [Source:HGNC Symbol;Acc:HGNC:1354]	chr9	136945246	136946974	1	ensembl_havana	0.94650143	1.254380765	0.638622094	1.175017601	1.046001934	1.542122761	0.935503283	0.578298274	0.402064725
ENSG00000148832	0.02903	0.086451859	-0.261657873	-1.198855578	**PAOX**	protein_coding	polyamine oxidase [Source:HGNC Symbol;Acc:HGNC:20837]	chr10	133379234	133391694	1	ensembl_havana	2.664351844	2.79518078	2.533522907	2.991768026	2.769291924	2.624482391	2.831503656	2.590542907	2.178522159
ENSG00000240857	0.029069	0.08654404	-0.193028537	-1.143160948	**RDH14**	protein_coding	retinol dehydrogenase 14 [Source:HGNC Symbol;Acc:HGNC:19979]	chr2	18554723	18560680	-1	ensembl_havana	3.94027309	4.036787359	3.843758822	4.029996525	4.152528558	3.927836994	3.801373512	3.905352962	3.824549991
ENSG00000171928	0.029073	0.08654404	-0.259886815	-1.197384762	**TVP23B**	protein_coding	trans-golgi network vesicle protein 23 homolog B [Source:HGNC Symbol;Acc:HGNC:20399]	chr17	18780995	18806714	1	ensembl_havana	4.710055063	4.839998471	4.580111655	4.978128134	4.84320029	4.698666988	4.535647221	4.670338547	4.534349199
ENSG00000225675	0.029087	0.086566848	-0.331668715	-1.258468159	**LINC01771**	lincRNA	long intergenic non-protein coding RNA 1771 [Source:HGNC Symbol;Acc:HGNC:52561]	chr1	53328233	53336509	1	havana	1.234165474	1.399999832	1.068331117	1.237769075	1.078042694	1.884187727	1.052687018	0.861296647	1.291009686
ENSG00000075142	0.029115	0.086633914	-0.302376255	-1.233173895	**SRI**	protein_coding	sorcin [Source:HGNC Symbol;Acc:HGNC:11292]	chr7	88205118	88226993	-1	ensembl_havana	5.915348961	6.066537089	5.764160834	6.091320465	6.115777658	5.992513143	5.804351993	5.60618628	5.881944229
ENSG00000243335	0.02913	0.086659784	0.271214	1.206822919	**KCTD7**	protein_coding	potassium channel tetramerization domain containing 7 [Source:HGNC Symbol;Acc:HGNC:21957]	chr7	66628767	66649067	1	ensembl_havana	6.314195012	6.178588012	6.449802013	6.038321501	6.310407544	6.187034991	6.502242842	6.45394236	6.393220836
ENSG00000169554	0.029174	0.08677183	0.282539925	1.216334408	**ZEB2**	protein_coding	zinc finger E-box binding homeobox 2 [Source:HGNC Symbol;Acc:HGNC:14881]	chr2	144364364	144524583	-1	ensembl_havana	6.828296945	6.687026982	6.969566907	7.147052336	6.619886199	6.294142412	7.296122147	6.829743028	6.782835547
ENSG00000198626	0.029183	0.086782507	-1.044768467	-2.063035243	**RYR2**	protein_coding	ryanodine receptor 2 [Source:HGNC Symbol;Acc:HGNC:10484]	chr1	237042205	237833988	1	ensembl_havana	0.191104921	0.713489154	-0.331279313	1.43811357	0.592979824	0.109374069	-0.12879794	-0.86978558	0.004745582
ENSG00000235245	0.029242	0.086813368	0.755885613	1.68866788	**AL360181.2**	antisense		chr10	133295187	133295977	-1	havana	0.280864721	-0.097078085	0.658807528	-0.31750517	-0.143136894	0.16940781	0.873137292	0.841392961	0.261892331
ENSG00000269293	0.029231	0.086813368	0.361507277	1.284767476	**ZSCAN16-AS1**	antisense	ZSCAN16 antisense RNA 1 [Source:HGNC Symbol;Acc:HGNC:48982]	chr6	28121795	28137293	-1	havana	2.668309804	2.487556166	2.849063443	2.547577338	2.535453458	2.379637701	3.006334834	2.608318707	2.932536788
ENSG00000115365	0.029235	0.086813368	0.184663279	1.136551675	**LANCL1**	protein_coding	LanC like 1 [Source:HGNC Symbol;Acc:HGNC:6508]	chr2	210431249	210477652	-1	ensembl_havana	6.727251388	6.634919749	6.819583028	6.513247451	6.700891695	6.690620099	6.772705068	6.842315655	6.843728362
ENSG00000101473	0.029241	0.086813368	0.184177359	1.136168932	**ACOT8**	protein_coding	acyl-CoA thioesterase 8 [Source:HGNC Symbol;Acc:HGNC:15919]	chr20	45841721	45857406	-1	ensembl_havana	5.417649513	5.325560833	5.509738192	5.225576098	5.36947522	5.381631181	5.483626096	5.521944411	5.52364407
ENSG00000227953	0.029226	0.086813368	0.172778385	1.127227244	**LINC01341**	processed_transcript	long intergenic non-protein coding RNA 1341 [Source:HGNC Symbol;Acc:HGNC:49457]	chr1	246776013	246792385	1	havana	2.096012339	2.009623147	2.182401532	1.649052215	2.14720036	2.232616866	1.863449533	2.315517031	2.368238032
ENSG00000147419	0.029211	0.086813368	-0.228052865	-1.171253099	**CCDC25**	protein_coding	coiled-coil domain containing 25 [Source:HGNC Symbol;Acc:HGNC:25591]	chr8	27733311	27772653	-1	ensembl_havana	4.99518998	5.109216413	4.881163548	5.174361627	5.171161869	4.982125743	4.87096537	4.875098927	4.897426347
ENSG00000169856	0.029221	0.086813368	-0.565848478	-1.480257823	**ONECUT1**	protein_coding	one cut homeobox 1 [Source:HGNC Symbol;Acc:HGNC:8138]	chr15	52756989	52791078	-1	ensembl_havana	-0.01531515	0.267609089	-0.298239389	0.14220989	0.298820819	0.361796557	0.032074365	-0.32890884	-0.597883695
ENSG00000134489	0.029241	0.086813368	-0.621521784	-1.538497164	**HRH4**	protein_coding	histamine receptor H4 [Source:HGNC Symbol;Acc:HGNC:17383]	chr18	24460629	24479957	1	ensembl_havana	-0.555750353	-0.244989461	-0.866511245	0.791576534	-0.783495335	-0.743049581	-0.30988669	-0.93694677	-1.352700279
ENSG00000214796	0.029296	0.086887687	0.275737319	1.210612639	**AC098934.1**	transcribed_unprocessed_pseudogene		chr1	202851828	202861620	-1	havana	1.08882572	0.95095706	1.226694379	0.727424294	1.214022341	0.911424546	1.178360195	1.315408538	1.186314404
ENSG00000166987	0.029297	0.086887687	0.202619818	1.150786194	**MBD6**	protein_coding	methyl-CpG binding domain protein 6 [Source:HGNC Symbol;Acc:HGNC:20445]	chr12	57520710	57530148	1	ensembl_havana	5.699302264	5.597992355	5.800612174	5.734727125	5.604176218	5.455073722	6.041400794	5.7324551	5.627980626
ENSG00000136231	0.02928	0.086887687	0.196488099	1.14590552	**IGF2BP3**	protein_coding	insulin like growth factor 2 mRNA binding protein 3 [Source:HGNC Symbol;Acc:HGNC:28868]	chr7	23310209	23470467	-1	ensembl_havana	6.706243349	6.6079993	6.804487398	6.596788661	6.652171199	6.57503804	6.866061142	6.756572719	6.790828334
ENSG00000102225	0.029288	0.086887687	0.192547306	1.142779693	**CDK16**	protein_coding	cyclin dependent kinase 16 [Source:HGNC Symbol;Acc:HGNC:8749]	chrX	47217860	47229997	1	ensembl_havana	6.803321005	6.707047353	6.899594658	6.685989186	6.746775187	6.688377685	6.955310287	6.859735268	6.883738419
ENSG00000113638	0.029285	0.086887687	-0.229582971	-1.172495976	**TTC33**	protein_coding	tetratricopeptide repeat domain 33 [Source:HGNC Symbol;Acc:HGNC:29959]	chr5	40512333	40755975	-1	ensembl_havana	4.659280528	4.774072014	4.544489043	4.946776004	4.874324932	4.501115106	4.598032469	4.631749475	4.403685184
ENSG00000108961	0.029309	0.086902252	0.230372036	1.173137434	**RANGRF**	protein_coding	RAN guanine nucleotide release factor [Source:HGNC Symbol;Acc:HGNC:17679]	chr17	8288497	8290092	1	ensembl_havana	4.59014006	4.474954042	4.705326078	4.366487997	4.577846829	4.480527301	4.743124734	4.724316947	4.648536554
ENSG00000151611	0.02932	0.086902252	-0.308899265	-1.2387622	**MMAA**	protein_coding	methylmalonic aciduria (cobalamin deficiency) cblA type [Source:HGNC Symbol;Acc:HGNC:18871]	chr4	145618263	145660035	1	ensembl_havana	2.553960168	2.708409801	2.399510536	2.738691328	2.822274848	2.564263227	2.333799072	2.372104732	2.492627803
ENSG00000103024	0.029315	0.086902252	-0.625022405	-1.542234776	**NME3**	protein_coding	NME/NM23 nucleoside diphosphate kinase 3 [Source:HGNC Symbol;Acc:HGNC:7851]	chr16	1770286	1771730	-1	ensembl_havana	-0.11345246	0.199058743	-0.425963663	0.613726219	-0.299013651	0.28246366	0.24405081	-0.62862566	-0.893316134
ENSG00000145740	0.029336	0.086931298	-0.283916158	-1.217495262	**SLC30A5**	protein_coding	solute carrier family 30 member 5 [Source:HGNC Symbol;Acc:HGNC:19089]	chr5	69093646	69131069	1	ensembl_havana	6.359905723	6.501863802	6.217947643	6.684230006	6.488967387	6.332394012	6.222187871	6.195813878	6.235841182
ENSG00000187626	0.029403	0.087111096	-0.218980288	-1.163910631	**ZKSCAN4**	protein_coding	zinc finger with KRAB and SCAN domains 4 [Source:HGNC Symbol;Acc:HGNC:13854]	chr6	28244623	28252224	-1	ensembl_havana	3.598779499	3.708269643	3.489289356	3.727865017	3.7593042	3.637639713	3.398295962	3.657568469	3.412003636
ENSG00000064547	0.029431	0.087175281	-0.178045887	-1.131350447	**LPAR2**	protein_coding	lysophosphatidic acid receptor 2 [Source:HGNC Symbol;Acc:HGNC:3168]	chr19	19623668	19628930	-1	ensembl_havana	3.540308819	3.629331763	3.451285876	3.791998092	3.520789865	3.575207333	3.591444275	3.304709872	3.45770348
ENSG00000143376	0.02944	0.087183912	0.172883284	1.127309207	**SNX27**	protein_coding	sorting nexin family member 27 [Source:HGNC Symbol;Acc:HGNC:20073]	chr1	151612006	151699091	1	ensembl_havana	5.703780137	5.617338495	5.790221779	5.497296574	5.678552974	5.676165937	5.673996407	5.808750804	5.887918125
ENSG00000138814	0.029453	0.087205295	0.173948535	1.128141892	**PPP3CA**	protein_coding	protein phosphatase 3 catalytic subunit alpha [Source:HGNC Symbol;Acc:HGNC:9314]	chr4	101023409	101348278	-1	ensembl_havana	6.426714584	6.339740317	6.513688852	6.190821554	6.403436409	6.424962989	6.401354649	6.528966949	6.610744957
ENSG00000103245	0.029479	0.087214655	0.399718635	1.319250596	**NARFL**	protein_coding	nuclear prelamin A recognition factor like [Source:HGNC Symbol;Acc:HGNC:14179]	chr16	729753	741329	-1	ensembl_havana	4.109630131	3.909770814	4.309489449	3.794609414	3.880081028	4.054621999	4.420436873	4.379102505	4.128928969
ENSG00000221539	0.029477	0.087214655	0.373711789	1.295682098	**SNORD99**	snoRNA	small nucleolar RNA, C/D box 99 [Source:HGNC Symbol;Acc:HGNC:32762]	chr1	28578749	28578822	-1	ensembl	-0.031050982	-0.217906876	0.155804913	0.004630787	-0.258437325	-0.399914092	0.141952326	0.063570082	0.261892331
ENSG00000272449	0.02948	0.087214655	0.348857076	1.273551302	**AL139246.5**	lincRNA		chr1	2530064	2547460	1	havana	-0.244815217	-0.419243755	-0.070386679	-0.14746469	-0.620350959	-0.489915621	0.428834256	-0.2414061	-0.398588198
ENSG00000162910	0.029471	0.087214655	0.265228716	1.201826564	**MRPL55**	protein_coding	mitochondrial ribosomal protein L55 [Source:HGNC Symbol;Acc:HGNC:16686]	chr1	228106679	228109312	-1	ensembl_havana	4.082658196	3.950043838	4.215272554	3.994094287	3.847920646	4.008116581	4.368636211	4.193340247	4.083841203
ENSG00000170035	0.029497	0.087225157	0.190143925	1.140877526	**UBE2E3**	protein_coding	ubiquitin conjugating enzyme E2 E3 [Source:HGNC Symbol;Acc:HGNC:12479]	chr2	180967248	181076585	1	ensembl_havana	6.204468001	6.109396038	6.299539963	5.995836524	6.222208466	6.110143124	6.213824122	6.327293324	6.357502444
ENSG00000165527	0.029495	0.087225157	-0.170274505	-1.125272573	**ARF6**	protein_coding	ADP ribosylation factor 6 [Source:HGNC Symbol;Acc:HGNC:659]	chr14	49893092	49897054	1	ensembl_havana	5.694167929	5.779305182	5.609030677	5.890534108	5.785512147	5.661869291	5.751159942	5.579399835	5.496532252
ENSG00000070367	0.029502	0.087225157	-0.200512538	-1.149106519	**EXOC5**	protein_coding	exocyst complex component 5 [Source:HGNC Symbol;Acc:HGNC:10696]	chr14	57200507	57269008	-1	ensembl_havana	7.189246284	7.289502553	7.088990015	7.395410922	7.283810277	7.189286461	7.097909991	7.098030139	7.071029915
ENSG00000101126	0.029514	0.087240932	-0.1869612	-1.138363413	**ADNP**	protein_coding	activity dependent neuroprotector homeobox [Source:HGNC Symbol;Acc:HGNC:15766]	chr20	50888916	50931421	-1	ensembl_havana	7.227206401	7.320687001	7.133725801	7.186608473	7.370653554	7.404798976	7.076158576	7.16787035	7.157148478
ENSG00000114554	0.029522	0.087246705	0.431909435	1.349017847	**PLXNA1**	protein_coding	plexin A1 [Source:HGNC Symbol;Acc:HGNC:9099]	chr3	126988594	127037392	1	ensembl_havana	5.726617005	5.510662287	5.942571722	5.362113708	5.311695568	5.858177585	5.916831476	5.980668578	5.930215113
ENSG00000280237	0.029541	0.087254005	0.176849797	1.130412871	**MIR4697HG**	TEC	MIR4697 host gene [Source:HGNC Symbol;Acc:HGNC:27448]	chr11	133896438	133901601	-1	havana	5.007717609	4.91929271	5.096142507	5.050923438	4.824434137	4.882520555	5.169051589	5.029304794	5.090071139
ENSG00000132801	0.029531	0.087254005	-0.271539113	-1.207094909	**ZSWIM3**	protein_coding	zinc finger SWIM-type containing 3 [Source:HGNC Symbol;Acc:HGNC:16157]	chr20	45857617	45879122	1	ensembl_havana	4.012887382	4.148656939	3.877117825	4.234236316	4.234775721	3.976958779	3.795780677	3.954822853	3.880749946
ENSG00000253957	0.029542	0.087254005	-0.771427101	-1.706957455	**IGHV3-22**	IG_V_pseudogene	immunoglobulin heavy variable 3-22 (pseudogene) [Source:HGNC Symbol;Acc:HGNC:5587]	chr14	106257762	106258223	-1	havana	-0.610492389	-0.224778838	-0.996205939	0.355307344	-0.672709	-0.356934859	-0.97139419	-1.52242631	-0.494797317
ENSG00000185088	0.029588	0.087371346	0.226390601	1.169904365	**RPS27L**	protein_coding	ribosomal protein S27 like [Source:HGNC Symbol;Acc:HGNC:18476]	chr15	63125872	63158021	-1	ensembl_havana	4.927528464	4.814333164	5.040723764	4.939715359	4.877742138	4.625541994	5.152736367	4.988033841	4.981401085
ENSG00000167723	0.029641	0.087508112	-0.453931627	-1.369768061	**TRPV3**	protein_coding	transient receptor potential cation channel subfamily V member 3 [Source:HGNC Symbol;Acc:HGNC:18084]	chr17	3510502	3557995	-1	ensembl_havana	2.264070246	2.49103606	2.037104433	2.50993591	2.42144547	2.541726798	1.740743887	1.929015357	2.441554055
ENSG00000139631	0.029652	0.087524047	0.204460814	1.152255628	**CSAD**	protein_coding	cysteine sulfinic acid decarboxylase [Source:HGNC Symbol;Acc:HGNC:18966]	chr12	53157663	53180909	-1	ensembl_havana	4.744721495	4.642491088	4.846951902	4.807625377	4.510469659	4.609378227	4.946101873	4.670338547	4.924415286
ENSG00000198000	0.029672	0.087564615	-0.223909683	-1.167894279	**NOL8**	protein_coding	nucleolar protein 8 [Source:HGNC Symbol;Acc:HGNC:23387]	chr9	92297358	92325636	-1	ensembl_havana	5.637061162	5.749016003	5.52510632	5.696142299	5.713395245	5.837510467	5.333449009	5.569603411	5.672266541
ENSG00000206561	0.029699	0.087607203	0.315900307	1.244788215	**COLQ**	protein_coding	collagen like tail subunit of asymmetric acetylcholinesterase [Source:HGNC Symbol;Acc:HGNC:2226]	chr3	15450133	15521751	-1	ensembl_havana	1.902797736	1.744847582	2.06074789	1.355633303	1.882741771	1.996167673	1.808964307	2.308284917	2.064994446
ENSG00000139625	0.029693	0.087607203	0.234226342	1.17627578	**MAP3K12**	protein_coding	mitogen-activated protein kinase kinase kinase 12 [Source:HGNC Symbol;Acc:HGNC:6851]	chr12	53479669	53500063	-1	ensembl_havana	5.286886829	5.169773658	5.404	5.068292738	5.213245738	5.227782498	5.455524904	5.368723782	5.387751314
ENSG00000115364	0.02972	0.087652059	-0.219278987	-1.164151635	**MRPL19**	protein_coding	mitochondrial ribosomal protein L19 [Source:HGNC Symbol;Acc:HGNC:14052]	chr2	75646783	75690851	1	ensembl_havana	5.403727379	5.513366872	5.294087885	5.577576228	5.470247905	5.492276484	5.225264472	5.312904338	5.344094846
ENSG00000215456	0.029731	0.087667371	-0.223744884	-1.167760878	**BCRP4**	unprocessed_pseudogene	breakpoint cluster region pseudogene 4 [Source:HGNC Symbol;Acc:HGNC:1017]	chr22	22630065	22636153	1	havana	1.943382955	2.055255397	1.831510512	2.023178671	2.154731774	1.987855745	1.797815913	1.811166797	1.885548828
ENSG00000141101	0.029768	0.087741346	-0.224573954	-1.168432145	**NOB1**	protein_coding	NIN1/PSMD8 binding protein 1 homolog [Source:HGNC Symbol;Acc:HGNC:29540]	chr16	69741867	69754940	-1	ensembl_havana	6.183100213	6.29538719	6.070813236	6.336368498	6.242230415	6.307562656	5.979227087	6.043537783	6.189674838
ENSG00000273449	0.029769	0.087741346	-0.519480486	-1.433438974	**AC093788.1**	lincRNA		chr4	163529771	163530697	1	havana	-0.062673067	0.197067176	-0.32241331	0.206373087	-0.002471726	0.387300166	-0.26243933	-0.93694677	0.232146171
ENSG00000099785	0.029788	0.087763487	0.23940156	1.180502879	**MARCH2**	protein_coding	membrane associated ring-CH-type finger 2 [Source:HGNC Symbol;Acc:HGNC:28038]	chr19	8413270	8439017	1	ensembl_havana	3.222612703	3.102911923	3.342313483	3.179296803	3.041246372	3.088192593	3.470282097	3.385963185	3.170695167
ENSG00000018236	0.029787	0.087763487	0.232979364	1.175259518	**CNTN1**	protein_coding	contactin 1 [Source:HGNC Symbol;Acc:HGNC:2171]	chr12	40692442	41072418	1	ensembl_havana	3.914701215	3.798211533	4.031190897	3.468093324	4.116342995	3.810198279	3.848057062	4.30836669	3.937148938
ENSG00000138182	0.029838	0.087890731	-0.224138038	-1.168079151	**KIF20B**	protein_coding	kinesin family member 20B [Source:HGNC Symbol;Acc:HGNC:7212]	chr10	89701610	89774939	1	ensembl_havana	5.285636927	5.397705946	5.173567908	5.313780003	5.377543746	5.501794088	5.031862651	5.292891664	5.19594941
ENSG00000163918	0.029853	0.087918684	-0.171621106	-1.126323384	**RFC4**	protein_coding	replication factor C subunit 4 [Source:HGNC Symbol;Acc:HGNC:9972]	chr3	186789880	186807058	-1	ensembl_havana	5.923431902	6.009242455	5.837621349	5.729277786	6.119150067	6.179299513	5.594083382	5.953111124	5.965669542
ENSG00000010810	0.02988	0.087941366	0.445093759	1.36140259	**FYN**	protein_coding	FYN proto-oncogene, Src family tyrosine kinase [Source:HGNC Symbol;Acc:HGNC:4037]	chr6	111660332	111873452	-1	ensembl_havana	3.752024494	3.529477614	3.974571373	2.838235332	3.870965269	3.879232242	3.663844581	4.120886932	4.138982607
ENSG00000136867	0.029885	0.087941366	0.339343733	1.265180945	**SLC31A2**	protein_coding	solute carrier family 31 member 2 [Source:HGNC Symbol;Acc:HGNC:11017]	chr9	113150942	113164137	1	ensembl_havana	4.025515641	3.855843775	4.195187508	3.669310227	4.150646514	3.747574584	4.19042511	4.222487183	4.17265023
ENSG00000143322	0.029869	0.087941366	0.217843374	1.162993774	**ABL2**	protein_coding	ABL proto-oncogene 2, non-receptor tyrosine kinase [Source:HGNC Symbol;Acc:HGNC:77]	chr1	179099327	179229684	-1	ensembl_havana	6.107087463	5.998165776	6.21600915	5.9718099	5.896361189	6.126326238	6.209098117	6.217209249	6.221720082
ENSG00000039523	0.029876	0.087941366	-0.415319917	-1.333594369	**RIPOR1**	protein_coding	RHO family interacting cell polarization regulator 1 [Source:HGNC Symbol;Acc:HGNC:25836]	chr16	67518418	67546788	1	ensembl_havana	3.586455632	3.794115591	3.378795673	3.605306254	3.795888281	3.981152237	3.441917197	3.466136326	3.228333496
ENSG00000125870	0.029911	0.087999536	-0.290356223	-1.222942203	**SNRPB2**	protein_coding	small nuclear ribonucleoprotein polypeptide B2 [Source:HGNC Symbol;Acc:HGNC:11155]	chr20	16729961	16742563	1	ensembl_havana	6.045697573	6.190875684	5.900519461	6.282971118	6.269368816	6.020287118	5.811298363	5.961203234	5.929056787
ENSG00000123374	0.029924	0.088014059	-0.216712782	-1.162082732	**CDK2**	protein_coding	cyclin dependent kinase 2 [Source:HGNC Symbol;Acc:HGNC:1771]	chr12	55966769	55972784	1	ensembl_havana	8.549272933	8.657629324	8.440916542	8.621549138	8.699832505	8.651506328	8.530538462	8.449639501	8.342571662
ENSG00000158373	0.029928	0.088014059	-0.436749199	-1.353550955	**HIST1H2BD**	protein_coding	histone cluster 1 H2B family member d [Source:HGNC Symbol;Acc:HGNC:4747]	chr6	26158146	26171349	1	ensembl_havana	2.891979907	3.110354507	2.673605308	3.330909387	2.931533374	3.068620759	2.587917939	2.8315648	2.601333184
ENSG00000177963	0.029937	0.088021751	0.175742459	1.129545557	**RIC8A**	protein_coding	RIC8 guanine nucleotide exchange factor A [Source:HGNC Symbol;Acc:HGNC:29550]	chr11	207511	215113	1	ensembl_havana	6.669780752	6.581909522	6.757651981	6.586277112	6.582680477	6.576770978	6.817553032	6.716841244	6.738561666
ENSG00000075420	0.029958	0.088065945	-0.210893949	-1.157405134	**FNDC3B**	protein_coding	fibronectin type III domain containing 3B [Source:HGNC Symbol;Acc:HGNC:24670]	chr3	172039628	172401665	1	ensembl_havana	6.5991241	6.704571074	6.493677126	6.861569004	6.579885948	6.672258271	6.703825809	6.437463583	6.339741985
ENSG00000057019	0.029977	0.088102868	0.180508575	1.133283317	**DCBLD2**	protein_coding	discoidin, CUB and LCCL domain containing 2 [Source:HGNC Symbol;Acc:HGNC:24627]	chr3	98795941	98901689	-1	ensembl_havana	7.313156282	7.222901994	7.40341057	7.21295878	7.228231468	7.227515735	7.325046094	7.433525181	7.451660434
ENSG00000169756	0.029984	0.08810597	-0.19325849	-1.143343171	**LIMS1**	protein_coding	LIM zinc finger domain containing 1 [Source:HGNC Symbol;Acc:HGNC:6616]	chr2	108534355	108687246	1	ensembl_havana	5.684573698	5.781202943	5.587944453	5.931447694	5.773332589	5.638828546	5.647071191	5.605448889	5.511313281
ENSG00000270344	0.029994	0.088117046	0.266272175	1.202696125	**POC1B-AS1**	antisense	POC1B antisense RNA 1 [Source:HGNC Symbol;Acc:HGNC:52949]	chr12	89525654	89548005	1	havana	3.151050056	3.017913969	3.284186144	3.122229914	2.831702492	3.099809502	3.275081252	3.290099621	3.287377559
ENSG00000169918	0.030093	0.088389102	0.580544477	1.495413515	**OTUD7A**	protein_coding	OTU deubiquitinase 7A [Source:HGNC Symbol;Acc:HGNC:20718]	chr15	31475398	31870789	-1	ensembl_havana	1.874201557	1.583929318	2.164473795	0.852997155	1.936162086	1.962628713	1.946559732	2.24151074	2.305350914
ENSG00000066455	0.030123	0.088458163	-0.212928019	-1.159038122	**GOLGA5**	protein_coding	golgin A5 [Source:HGNC Symbol;Acc:HGNC:4428]	chr14	92794231	92839963	1	ensembl_havana	5.886546918	5.993010927	5.780082909	6.119712848	6.009717696	5.849602237	5.781896147	5.849179496	5.709173082
ENSG00000270696	0.03015	0.088521629	-0.297753486	-1.22922881	**AC005034.3**	antisense		chr2	75660462	75662208	1	havana	2.736625843	2.885502586	2.5877491	2.954889309	2.769291924	2.932326526	2.521699999	2.699592193	2.541955109
ENSG00000136169	0.030158	0.088526348	0.263900682	1.200720765	**SETDB2**	protein_coding	SET domain bifurcated 2 [Source:HGNC Symbol;Acc:HGNC:20263]	chr13	49444374	49495003	1	ensembl_havana	3.708647013	3.576696673	3.840597354	3.528951643	3.473392586	3.727745788	3.623370039	3.898145298	4.000276726
ENSG00000100266	0.030172	0.088550023	-0.187698236	-1.138945123	**PACSIN2**	protein_coding	protein kinase C and casein kinase substrate in neurons 2 [Source:HGNC Symbol;Acc:HGNC:8571]	chr22	42835412	43015145	-1	ensembl_havana	6.302008256	6.395857374	6.208159138	6.476314774	6.304494951	6.406762399	6.252106367	6.20119254	6.171178506
ENSG00000122299	0.030202	0.08861924	-0.168806358	-1.124128031	**ZC3H7A**	protein_coding	zinc finger CCCH-type containing 7A [Source:HGNC Symbol;Acc:HGNC:30959]	chr16	11750586	11797267	-1	ensembl_havana	6.93507997	7.01948315	6.850676791	7.150369184	7.049637582	6.858442682	6.964375203	6.860044429	6.727610742
ENSG00000163798	0.030212	0.088629885	0.177988693	1.131305597	**SLC4A1AP**	protein_coding	solute carrier family 4 member 1 adaptor protein [Source:HGNC Symbol;Acc:HGNC:13813]	chr2	27663471	27694976	1	ensembl_havana	6.601421587	6.51242724	6.690415934	6.564638997	6.536944977	6.435697748	6.749747206	6.65582634	6.665674255
ENSG00000127838	0.030224	0.088631251	0.22303595	1.167187186	**PNKD**	protein_coding	paroxysmal nonkinesigenic dyskinesia [Source:HGNC Symbol;Acc:HGNC:9153]	chr2	218270392	218346793	1	ensembl_havana	4.118888193	4.007370218	4.230406168	3.83322245	4.100831193	4.088057012	4.19042511	4.222487183	4.27830621
ENSG00000196092	0.030225	0.088631251	-0.360827652	-1.28416239	**PAX5**	protein_coding	paired box 5 [Source:HGNC Symbol;Acc:HGNC:8619]	chr9	36833275	37034185	-1	ensembl_havana	7.505591217	7.686005043	7.325177391	7.622190324	7.516437227	7.919387578	7.240597022	7.422473164	7.312461988
ENSG00000100804	0.030314	0.088874015	-0.181069605	-1.13372411	**PSMB5**	protein_coding	proteasome subunit beta 5 [Source:HGNC Symbol;Acc:HGNC:9542]	chr14	23016543	23035230	-1	ensembl_havana	6.730161501	6.820696303	6.639626698	6.8618803	6.864836217	6.735372393	6.609743282	6.709997302	6.59913951
ENSG00000096080	0.030321	0.088877657	-0.178098893	-1.131392015	**MRPS18A**	protein_coding	mitochondrial ribosomal protein S18A [Source:HGNC Symbol;Acc:HGNC:14515]	chr6	43671303	43687791	-1	ensembl_havana	4.635916927	4.724966374	4.54686748	4.826887674	4.703837799	4.644173647	4.689844966	4.461278455	4.489479019
ENSG00000114982	0.030341	0.088917417	0.168505764	1.123893836	**KANSL3**	protein_coding	KAT8 regulatory NSL complex subunit 3 [Source:HGNC Symbol;Acc:HGNC:25473]	chr2	96593170	96642787	-1	ensembl_havana	6.472956611	6.388703729	6.557209493	6.365640498	6.375507702	6.424962989	6.521007602	6.5812818	6.569339077
ENSG00000196557	0.030355	0.088941212	-0.331660128	-1.258460669	**CACNA1H**	protein_coding	calcium voltage-gated channel subunit alpha1 H [Source:HGNC Symbol;Acc:HGNC:1395]	chr16	1153121	1221772	1	ensembl_havana	0.410681109	0.576511173	0.244851046	0.931023971	0.09469996	0.703809589	0.339398945	-0.15890847	0.554062661
ENSG00000100979	0.030375	0.088982073	-0.289383915	-1.222118276	**PLTP**	protein_coding	phospholipid transfer protein [Source:HGNC Symbol;Acc:HGNC:9093]	chr20	45898621	45912155	-1	ensembl_havana	4.945568567	5.090260525	4.80087661	4.929059175	5.060246219	5.281476179	4.66551843	4.947876234	4.789235164
ENSG00000179399	0.030385	0.088984974	0.449132823	1.365219402	**GPC5**	protein_coding	glypican 5 [Source:HGNC Symbol;Acc:HGNC:4453]	chr13	91398607	92873682	1	ensembl_havana	3.820591424	3.596025012	4.045157835	3.359409615	3.847920646	3.580744775	4.051836127	3.914907507	4.168729871
ENSG00000267680	0.030401	0.088984974	0.242113734	1.182724236	**ZNF224**	protein_coding	zinc finger protein 224 [Source:HGNC Symbol;Acc:HGNC:13017]	chr19	44094339	44109886	1	ensembl_havana	5.773713768	5.652656901	5.894770635	5.835170068	5.614454027	5.50834661	5.945528064	5.836038311	5.902745531
ENSG00000105778	0.030396	0.088984974	0.184676176	1.136561834	**AVL9**	protein_coding	AVL9 cell migration associated [Source:HGNC Symbol;Acc:HGNC:28994]	chr7	32495426	32588721	1	ensembl_havana	6.844416278	6.75207819	6.936754366	6.759339526	6.717514788	6.779380257	6.894467361	6.980957158	6.934838578
ENSG00000212588	0.030396	0.088984974	-0.469192019	-1.384333955	**SNORA26**	snoRNA	small nucleolar RNA, H/ACA box 26 [Source:HGNC Symbol;Acc:HGNC:32616]	chr4	52713249	52713370	1	ensembl	-0.091262383	0.143333626	-0.325858393	-0.41068387	0.453384582	0.387300166	-0.69455235	-0.42206346	0.139040636
ENSG00000103966	0.030417	0.089014371	0.187036915	1.138423158	**EHD4**	protein_coding	EH domain containing 4 [Source:HGNC Symbol;Acc:HGNC:3245]	chr15	41895939	41972578	-1	ensembl_havana	5.08413413	4.990615672	5.177652587	4.93024709	5.005067848	5.036532078	5.1915873	5.189423914	5.151946546

ENSG00000142227	0.03044	0.089045064	-0.22644994	-1.169952485	**EMP3**	protein_coding	epithelial membrane protein 3 [Source:HGNC Symbol;Acc:HGNC:3335]	chr19	48321509	48330553	1	ensembl_havana	6.882944392	6.996169362	6.769719422	7.268725734	7.012296137	6.707486214	6.902946329	6.820241129	6.585970809
ENSG00000162722	0.030435	0.089045064	-0.24068881	-1.181556657	**TRIM58**	protein_coding	tripartite motif containing 58 [Source:HGNC Symbol;Acc:HGNC:24150]	chr1	247857199	247878205	1	ensembl_havana	3.277371452	3.397715857	3.157027047	3.334502858	3.357427779	3.501216933	2.941964313	3.245359567	3.283757261
ENSG00000087269	0.030451	0.089057586	-0.195801474	-1.145360276	**NOP14**	protein_coding	NOP14 nucleolar protein [Source:HGNC Symbol;Acc:HGNC:16821]	chr4	2937933	2963385	-1	ensembl_havana	6.054131344	6.152032081	5.956230607	6.11188196	6.137796237	6.206418047	5.8509408	6.040267582	5.97748344
ENSG00000168970	0.030518	0.089236265	0.349200952	1.273854897	**JMJD7-PLA2G4B**	protein_coding	JMJD7-PLA2G4B readthrough [Source:HGNC Symbol;Acc:HGNC:34449]	chr15	41828095	41848155	1	ensembl_havana	2.61944976	2.444849284	2.794050236	2.26832323	2.116675269	2.949549354	2.803908217	2.560421027	3.017821464
ENSG00000103353	0.030549	0.089308856	-0.168552864	-1.123930529	**UBFD1**	protein_coding	ubiquitin family domain containing 1 [Source:HGNC Symbol;Acc:HGNC:30565]	chr16	23557362	23574389	1	ensembl_havana	6.704044859	6.788321292	6.619768427	6.88626284	6.754230899	6.724470136	6.678596701	6.608769719	6.571938862
ENSG00000168036	0.030579	0.089326522	0.178595635	1.131781637	**CTNNB1**	protein_coding	catenin beta 1 [Source:HGNC Symbol;Acc:HGNC:2514]	chr3	41194741	41260096	1	ensembl_havana	8.483109239	8.393811422	8.572407057	8.322758883	8.436332396	8.422342987	8.557125639	8.619952646	8.540142885
ENSG00000110583	0.03057	0.089326522	0.163973871	1.120368923	**NAA40**	protein_coding	N(alpha)-acetyltransferase 40, NatD catalytic subunit [Source:HGNC Symbol;Acc:HGNC:25845]	chr11	63938959	63957328	1	ensembl_havana	5.584846741	5.502859805	5.666833676	5.475491922	5.474767967	5.558319526	5.625951438	5.652612197	5.721937394
ENSG00000254233	0.03058	0.089326522	-0.271933109	-1.207424608	**LINC02365**	lincRNA	long intergenic non-protein coding RNA 2365 [Source:HGNC Symbol;Acc:HGNC:53285]	chr4	184584093	184625030	-1	havana	2.357937225	2.493903779	2.22197067	2.711312495	2.482570884	2.287827958	2.326059645	2.122856455	2.21699591
ENSG00000151651	0.030571	0.089326522	-0.503982386	-1.418122721	**ADAM8**	protein_coding	ADAM metallopeptidase domain 8 [Source:HGNC Symbol;Acc:HGNC:215]	chr10	133262403	133276868	-1	ensembl_havana	2.949260538	3.201251731	2.697269345	3.138766373	2.887010108	3.57797871	2.657410455	2.753838306	2.680559275
ENSG00000145103	0.030587	0.089327393	-0.218036478	-1.163149451	**ILDR1**	protein_coding	immunoglobulin like domain containing receptor 1 [Source:HGNC Symbol;Acc:HGNC:28741]	chr3	121987323	122022258	-1	ensembl_havana	4.27882681	4.387845049	4.169808571	4.239995762	4.41805057	4.505488814	4.155914852	4.12706564	4.22644522
ENSG00000143537	0.030622	0.089393181	0.169943055	1.125014078	**ADAM15**	protein_coding	ADAM metallopeptidase domain 15 [Source:HGNC Symbol;Acc:HGNC:193]	chr1	155050566	155062775	1	ensembl_havana	6.477732978	6.39276145	6.562704505	6.39088304	6.343324789	6.444076522	6.576288302	6.541742862	6.570082351
ENSG00000171219	0.030616	0.089393181	-0.334201066	-1.260679078	**CDC42BPG**	protein_coding	CDC42 binding protein kinase gamma [Source:HGNC Symbol;Acc:HGNC:29829]	chr11	64823387	64844569	-1	ensembl_havana	0.826596898	0.993697431	0.659496366	1.327057633	0.760296981	0.89373768	0.935503283	0.225896484	0.817089329
ENSG00000268471	0.030639	0.089425822	0.250447085	1.189575702	**MIR4453HG**	bidirectional_promoter_lncRNA	MIR4453 host gene [Source:HGNC Symbol;Acc:HGNC:25288]	chr4	152536264	152539263	1	havana	3.045788809	2.920565266	3.171012351	2.744105292	3.085263982	2.932326526	3.113627387	3.163562154	3.235847512
ENSG00000160593	0.030659	0.089456893	0.254218882	1.192689811	**JAML**	protein_coding	junction adhesion molecule like [Source:HGNC Symbol;Acc:HGNC:19084]	chr11	118193740	118225094	-1	ensembl_havana	0.643032267	0.515922826	0.770141708	0.589870393	0.403680778	0.554217307	0.955706889	0.478736765	0.875981471
ENSG00000188452	0.030662	0.089456893	0.247695059	1.187308679	**CERKL**	protein_coding	ceramide kinase like [Source:HGNC Symbol;Acc:HGNC:21699]	chr2	181536676	181680665	-1	ensembl_havana	2.760580035	2.636732506	2.884427565	2.283275357	2.783935853	2.842986308	2.632526022	3.102158989	2.918597684
ENSG00000111335	0.030738	0.089661381	-0.505409124	-1.419525853	**OAS2**	protein_coding	2'-5'-oligoadenylate synthetase 2 [Source:HGNC Symbol;Acc:HGNC:8087]	chr12	112978395	113011723	1	ensembl_havana	4.667735053	4.920439615	4.415030491	4.669343006	5.020608058	5.071367781	4.433101554	4.074749302	4.737240617
ENSG00000101577	0.030769	0.089697291	0.215315884	1.160958083	**LPIN2**	protein_coding	lipin 2 [Source:HGNC Symbol;Acc:HGNC:14450]	chr18	2916994	3013315	-1	ensembl_havana	7.952118269	7.844460326	8.059776211	8.156094849	7.750654257	7.626631873	8.2566013	7.968970053	7.953757278
ENSG00000165886	0.03076	0.089697291	0.201141675	1.149607736	**UBTD1**	protein_coding	ubiquitin domain containing 1 [Source:HGNC Symbol;Acc:HGNC:25683]	chr10	97498868	97571209	1	ensembl_havana	0.991966049	0.891395212	1.092536887	0.931023971	0.979703225	0.76345844	1.089724456	1.114463419	1.073422785
ENSG00000125821	0.030768	0.089697291	0.173452437	1.127754026	**DTD1**	protein_coding	D-tyrosyl-tRNA deacylase 1 [Source:HGNC Symbol;Acc:HGNC:16219]	chr20	18587893	18766644	1	ensembl_havana	4.745357251	4.658631033	4.832083469	4.52270135	4.740586423	4.712605325	4.731453667	4.859102792	4.90569395
ENSG00000196663	0.030777	0.089701189	0.186132661	1.137709839	**TECPR2**	protein_coding	tectonin beta-propeller repeat containing 2 [Source:HGNC Symbol;Acc:HGNC:19957]	chr14	102362963	102502481	1	ensembl_havana	4.725074136	4.632007805	4.818140466	4.640602451	4.701268571	4.554152393	4.906430054	4.870195902	4.677795441
ENSG00000225541	0.030787	0.0897118	-0.184897088	-1.136735883	**AC002480.1**	sense_overlapping		chr7	22571607	22661792	-1	havana	1.932248628	2.024697172	1.839800084	1.968722995	1.944876279	2.160492241	1.863449533	1.705026699	1.950924019
ENSG00000148303	0.030806	0.089714081	0.208798108	1.155724962	**RPL7A**	protein_coding	ribosomal protein L7a [Source:HGNC Symbol;Acc:HGNC:10364]	chr9	133348214	133351426	1	ensembl_havana	10.00808226	9.903683203	10.11248131	9.765765109	9.89151441	10.05377009	9.977929843	10.20385079	10.1556633
ENSG00000116668	0.030794	0.089714081	0.188679194	1.13971981	**SWT1**	protein_coding	SWT1, RNA endoribonuclease homolog [Source:HGNC Symbol;Acc:HGNC:16785]	chr1	185157080	185291765	1	ensembl_havana	3.971753642	3.877414044	4.066093239	4.086496776	3.99572295	3.550022408	4.203156559	4.175565179	3.819557978
ENSG00000249509	0.030806	0.089714081	-0.309238478	-1.239053497	**AC023886.1**	antisense		chr4	112515385	112546881	1	havana	1.566310996	1.720930235	1.411691757	1.863213701	1.488885652	1.810691353	1.309740823	1.300906777	1.624427672
ENSG00000042286	0.030826	0.089734406	-0.165821108	-1.12180437	**AIFM2**	protein_coding	apoptosis inducing factor, mitochondria associated 2 [Source:HGNC Symbol;Acc:HGNC:21411]	chr10	70098223	70132934	-1	ensembl_havana	5.308424581	5.391335135	5.225514027	5.221700428	5.477022698	5.475282279	5.084418896	5.30475086	5.287372323
ENSG00000134882	0.030822	0.089734406	-0.199654621	-1.148423391	**UBAC2**	protein_coding	UBA domain containing 2 [Source:HGNC Symbol;Acc:HGNC:20486]	chr13	99200774	99386434	1	ensembl_havana	5.181625424	5.281452734	5.081798113	5.250517308	5.287919812	5.305921083	5.158195301	5.040259376	5.046939663
ENSG00000271361	0.030881	0.08987777	-0.218410915	-1.163451374	**HTATSF1P2**	processed_pseudogene	HIV-1 Tat specific factor 1 pseudogene 2 [Source:HGNC Symbol;Acc:HGNC:38586]	chr6	3023142	3023772	-1	havana	0.032790087	0.141995544	-0.076415371	0.075059705	-0.036373238	0.387300166	-0.21650287	-0.11936381	0.106620565
ENSG00000168434	0.030916	0.089958984	0.211145531	1.157606984	**COG7**	protein_coding	component of oligomeric golgi complex 7 [Source:HGNC Symbol;Acc:HGNC:18622]	chr16	23388493	23453180	-1	ensembl_havana	4.574529579	4.468956813	4.680102344	4.614237131	4.394387857	4.398245452	4.923199632	4.62448868	4.49261872
ENSG00000259943	0.030927	0.089974331	-0.168839603	-1.124153935	**AL050341.2**	antisense		chr1	40256427	40257967	-1	havana	3.673925458	3.758345259	3.589505656	3.817901026	3.73438904	3.722745712	3.676073242	3.523471405	3.568972322
ENSG00000004777	0.030934	0.089976539	0.364109027	1.287086511	**ARHGAP33**	protein_coding	Rho GTPase activating protein 33 [Source:HGNC Symbol;Acc:HGNC:23085]	chr19	35774532	35788822	1	ensembl_havana	3.501948666	3.319894152	3.684003179	3.214825476	3.206170563	3.538686417	3.792976108	3.649006201	3.610027229
ENSG00000206418	0.030952	0.090010695	0.251327824	1.190302138	**RAB12**	protein_coding	RAB12, member RAS oncogene family [Source:HGNC Symbol;Acc:HGNC:31332]	chr18	8609445	8639381	1	ensembl_havana	4.402501484	4.276837572	4.528165396	4.368242082	4.370330552	4.091940082	4.565534632	4.501468219	4.517493337
ENSG00000086061	0.030963	0.090023548	-0.163837192	-1.120262786	**DNAJA1**	protein_coding	DnaJ heat shock protein family (Hsp40) member A1 [Source:HGNC Symbol;Acc:HGNC:5229]	chr9	33025211	33039907	1	ensembl_havana	9.031830752	9.113749348	8.949912156	9.114703449	9.124169844	9.10237475	8.956585401	8.973978759	8.919172307
ENSG00000119471	0.030978	0.0900499	0.166201052	1.122099844	**HSDL2**	protein_coding	hydroxysteroid dehydrogenase like 2 [Source:HGNC Symbol;Acc:HGNC:18572]	chr9	112379937	112472410	1	ensembl_havana	5.867691747	5.784591221	5.950792273	5.510858259	5.822628285	6.020287118	5.708636445	5.965232331	6.178508043
ENSG00000169242	0.031001	0.090096696	-0.773708356	-1.709658709	**EFNA1**	protein_coding	ephrin A1 [Source:HGNC Symbol;Acc:HGNC:3221]	chr1	155127460	155134857	1	ensembl_havana	0.351612929	0.738467107	-0.035241249	1.126095262	0.77991046	0.309395597	-0.30988669	0.130707766	0.073455173
ENSG00000135437	0.031017	0.090111149	0.253200695	1.191848363	**RDH5**	protein_coding	retinol dehydrogenase 5 [Source:HGNC Symbol;Acc:HGNC:9940]	chr12	55720367	55724705	1	ensembl_havana	1.889082206	1.762481859	2.015682554	1.565895203	1.873642861	1.847907512	1.936429043	2.187350601	1.923268018
ENSG00000256234	0.031018	0.090111149	-0.312194305	-1.241594701	**AC022509.2**	antisense		chr12	26211164	26335856	-1	havana	3.516354212	3.672451364	3.360257059	3.996364543	3.886880247	3.134109303	3.542190552	3.469382096	3.069198529
ENSG00000176406	0.03103	0.090128769	-0.314633158	-1.243695372	**RIMS2**	protein_coding	regulating synaptic membrane exocytosis 2 [Source:HGNC Symbol;Acc:HGNC:17283]	chr8	103500748	104256094	1	ensembl_havana	2.938386033	3.095702612	2.781069454	2.838235332	3.104836469	3.344036035	2.699951379	2.85657011	2.786686872
ENSG00000149634	0.031058	0.090161168	0.275956655	1.210796706	**SPATA25**	protein_coding	spermatogenesis associated 25 [Source:HGNC Symbol;Acc:HGNC:16158]	chr20	45886489	45887635	-1	ensembl_havana	0.41959452	0.281616192	0.557572847	0.490295136	0.214850257	0.139703184	0.592614899	0.478736765	0.601366878
ENSG00000143373	0.031058	0.090161168	-0.170956448	-1.1258046	**ZNF687**	protein_coding	zinc finger protein 687 [Source:HGNC Symbol;Acc:HGNC:29277]	chr1	151281618	151292180	1	ensembl_havana	5.013749491	5.099227715	4.928271267	5.12748953	5.009228299	5.160965317	4.95491103	4.882422361	4.947480411
ENSG00000171681	0.03106	0.090161168	-0.232414856	-1.174799743	**ATF7IP**	protein_coding	activating transcription factor 7 interacting protein [Source:HGNC Symbol;Acc:HGNC:20092]	chr12	14365632	14502935	1	ensembl_havana	5.815019164	5.931226591	5.698811736	5.977566924	5.926921343	5.889191508	5.633809663	5.656888996	5.805736548
ENSG00000230790	0.031074	0.090181529	0.616070418	1.53269477	**AC012456.2**	antisense		chr2	11740997	11745301	-1	havana	0.526025057	0.217989848	0.834060266	-0.06941355	0.52486995	0.198513145	1.052687018	0.693803314	0.755690467
ENSG00000269921	0.031131	0.090328483	-0.166823905	-1.122584391	**AC068620.1**	lincRNA		chr4	56387625	56388153	1	havana	0.203551887	0.286963839	0.120139935	0.267803752	0.453384582	0.139703184	0.069637898	0.286036324	0.004745582
ENSG00000163444	0.031145	0.09035289	0.17739494	1.130840094	**TMEM183A**	protein_coding	transmembrane protein 183A [Source:HGNC Symbol;Acc:HGNC:20173]	chr1	203007386	203024848	1	ensembl_havana	4.964916859	4.876219389	5.053614329	4.826887674	4.918129458	4.883641034	5.050769426	5.030404003	5.079669558
ENSG00000175182	0.031199	0.090488949	-0.306118742	-1.236377017	**FAM131A**	protein_coding	family with sequence similarity 131 member A [Source:HGNC Symbol;Acc:HGNC:28308]	chr3	184335926	184348421	1	ensembl_havana	2.316424681	2.469484052	2.16336531	2.438267468	2.351074074	2.619110614	2.221475976	2.195213363	2.073406591
ENSG00000198416	0.031217	0.090505819	-0.235694138	-1.17747313	**ZNF658B**	transcribed_unprocessed_pseudogene	zinc finger protein 658B (pseudogene) [Source:HGNC Symbol;Acc:HGNC:32033]	chr9	39443815	39508885	-1	havana	1.294490308	1.412337377	1.176643239	1.553614465	1.415548178	1.267849486	1.195452122	1.32976598	1.004711615
ENSG00000155438	0.031216	0.090505819	-0.262217945	-1.199321079	**NIFK**	protein_coding	nucleolar protein interacting with the FHA domain of MKI67 [Source:HGNC Symbol;Acc:HGNC:17838]	chr2	121726945	121736923	-1	ensembl_havana	6.342770582	6.473879555	6.211661609	6.677171757	6.459623865	6.284843041	6.248016381	6.19826121	6.188707236
ENSG00000152767	0.031231	0.09052972	0.577536161	1.492298519	**FARP1**	protein_coding	FERM, ARH/RhoGEF and pleckstrin domain protein 1 [Source:HGNC Symbol;Acc:HGNC:3591]	chr13	98142562	98455176	1	ensembl_havana	0.477070385	0.188302304	0.765838465	0.174648163	0.477608744	-0.087349994	0.762800879	0.602150945	0.932563571
ENSG00000057757	0.031248	0.090541193	0.194300422	1.144169206	**PITHD1**	protein_coding	PITH domain containing 1 [Source:HGNC Symbol;Acc:HGNC:25022]	chr1	23778405	23788232	1	ensembl_havana	5.259232581	5.16208237	5.356382792	5.141916838	5.213245738	5.131084535	5.267295382	5.379115482	5.422737512
ENSG00000172765	0.031246	0.090541193	0.182336619	1.134720214	**TMCC1**	protein_coding	transmembrane and coiled-coil domain family 1 [Source:HGNC Symbol;Acc:HGNC:29116]	chr3	129647792	129893576	-1	ensembl_havana	4.799919356	4.708751047	4.891087666	4.588886194	4.771531924	4.765835022	4.82090477	4.981228583	4.871129645
ENSG00000102393	0.031265	0.090571376	0.17067947	1.125588481	**GLA**	protein_coding	galactosidase alpha [Source:HGNC Symbol;Acc:HGNC:4296]	chrX	101397803	101407925	-1	ensembl_havana	4.97181575	4.886476016	5.057155485	4.852177089	4.970278484	4.836972474	5.072904072	5.11158459	4.986977794
ENSG00000144642	0.031275	0.090573031	1.231797602	2.34859443	**RBMS3**	protein_coding	RNA binding motif single stranded interacting protein 3 [Source:HGNC Symbol;Acc:HGNC:13427]	chr3	28574791	30010391	1	ensembl_havana	-0.473803368	-1.089702169	0.142095433	-3.00120074	-0.18055577	-0.087349994	-0.63291296	0.554044597	0.505154661
ENSG00000165138	0.031291	0.090573031	0.373501308	1.295493079	**ANKS6**	protein_coding	ankyrin repeat and sterile alpha motif domain containing 6 [Source:HGNC Symbol;Acc:HGNC:26724]	chr9	98731329	98796965	-1	ensembl_havana	5.186947491	5.000196837	5.373698146	4.663640517	5.018545676	5.318404319	5.349762652	5.262342306	5.508989479
ENSG00000137812	0.031289	0.090573031	-0.168478307	-1.123872447	**KNL1**	protein_coding	kinetochore scaffold 1 [Source:HGNC Symbol;Acc:HGNC:24054]	chr15	40594020	40664342	1	ensembl_havana	6.456872423	6.541111576	6.372633269	6.387427458	6.580934531	6.65497274	6.243914767	6.430819005	6.443166034
ENSG00000115107	0.031287	0.090573031	-0.2004771	-1.149078293	**STEAP3**	protein_coding	STEAP3 metalloreductase [Source:HGNC Symbol;Acc:HGNC:24592]	chr2	119223831	119265652	1	ensembl_havana	8.487608009	8.587846559	8.387369459	8.925291891	8.415519338	8.422728449	8.69035046	8.323043613	8.148714305
ENSG00000281332	0.031339	0.090684691	0.338689309	1.264607174	**LINC00997**	lincRNA	long intergenic non-protein coding RNA 997 [Source:HGNC Symbol;Acc:HGNC:48952]	chr7	32760279	32762924	1	havana	1.488828166	1.319483511	1.65817282	1.397462791	1.452682866	1.108304877	1.786580698	1.529563273	1.65837449
ENSG00000215712	0.031352	0.090684691	0.171295456	1.126069175	**TMEM242**	protein_coding	transmembrane protein 242 [Source:HGNC Symbol;Acc:HGNC:17206]	chr6	157289386	157323601	-1	ensembl_havana	3.902122736	3.816475008	3.987770464	3.802415141	3.909315632	3.737694252	3.93203328	4.129119342	3.90215877
ENSG00000104331	0.031354	0.090684691	0.167872797	1.123400848	**IMPAD1**	protein_coding	inositol monophosphatase domain containing 1 [Source:HGNC Symbol;Acc:HGNC:26019]	chr8	56957933	56993844	-1	ensembl_havana	6.910692766	6.826756368	6.994629165	6.817310861	6.889308276	6.773649967	6.945241073	7.066495926	6.972150495
ENSG00000083454	0.031348	0.090684691	-0.191873569	-1.142246141	**P2RX5**	protein_coding	purinergic receptor P2X 5 [Source:HGNC Symbol;Acc:HGNC:8536]	chr17	3672199	3696404	-1	ensembl_havana	2.825296956	2.92123374	2.729360171	2.765560239	2.769291924	3.228849057	2.669693543	2.535862351	2.98252462
ENSG00000129534	0.031366	0.090700952	-0.176002528	-1.129749194	**MIS18BP1**	protein_coding	MIS18 binding protein 1 [Source:HGNC Symbol;Acc:HGNC:20190]	chr14	45203190	45253540	-1	ensembl_havana	4.356408867	4.444410131	4.268407603	4.362973414	4.427408044	4.542848935	4.127254407	4.302919022	4.375049381
ENSG00000076650	0.031417	0.090773093	0.189375649	1.140270138	**GPATCH1**	protein_coding	G-patch domain containing 1 [Source:HGNC Symbol;Acc:HGNC:24658]	chr19	33080880	33130542	1	ensembl_havana	4.510258629	4.415570805	4.604946454	4.275947831	4.455121723	4.51564286	4.456330725	4.728386669	4.630121969
ENSG00000228649	0.031413	0.090773093	-0.211852924	-1.158174729	**SNHG26**	processed_transcript	small nucleolar RNA host gene 26 [Source:HGNC Symbol;Acc:HGNC:53131]	chr7	22854178	22861579	1	havana	2.758961222	2.864887684	2.65303476	2.418087419	3.061421012	3.11515462	2.099663825	2.841618992	3.017821464
ENSG00000095139	0.031407	0.090773093	-0.223649476	-1.167683654	**ARCN1**	protein_coding	archain 1 [Source:HGNC Symbol;Acc:HGNC:649]	chr11	118572390	118603033	1	ensembl_havana	8.808940944	8.920765682	8.697116206	9.106188463	8.884137465	8.771971117	8.746423397	8.686921686	8.658003534
ENSG00000134146	0.031423	0.090773093	-0.228608286	-1.171704104	**DPH6**	protein_coding	diphthamine biosynthesis 6 [Source:HGNC Symbol;Acc:HGNC:30543]	chr15	35217345	35546193	-1	ensembl_havana	3.062013022	3.176317165	2.947708879	3.155115434	3.069412532	3.304423528	2.775774631	2.924363622	3.142988385
ENSG00000136943	0.031423	0.090773093	-0.258436282	-1.196181476	**CTSV**	protein_coding	cathepsin V [Source:HGNC Symbol;Acc:HGNC:2538]	chr9	97029679	97039643	-1	ensembl_havana	3.460692106	3.589910247	3.331473965	3.424923176	3.622061853	3.722745712	3.353314566	3.249141357	3.391965972
ENSG00000114790	0.03144	0.090804264	0.169892411	1.124974587	**ARHGEF26**	protein_coding	Rho guanine nucleotide exchange factor 26 [Source:HGNC Symbol;Acc:HGNC:24490]	chr3	154121003	154257827	1	ensembl_havana	4.926047723	4.841101517	5.010993928	4.878262343	4.818519299	4.82652291	5.00302963	4.994807149	5.035145006
ENSG00000173894	0.031479	0.090879831	0.295436671	1.227256383	**CBX2**	protein_coding	chromobox 2 [Source:HGNC Symbol;Acc:HGNC:1552]	chr17	79778132	79787983	1	ensembl_havana	4.702749667	4.555031332	4.850468003	4.332748634	4.575042912	4.75730245	4.784658061	4.939708814	4.827037133
ENSG00000178057	0.031477	0.090879831	0.179119044	1.132192321	**NDUFAF3**	protein_coding	NADH:ubiquinone oxidoreductase complex assembly factor 3 [Source:HGNC Symbol;Acc:HGNC:29918]	chr3	49020459	49023495	1	ensembl_havana	4.023537547	3.933978025	4.113097069	3.952606657	3.843267218	4.006060199	4.086606631	4.093802465	4.158882111
ENSG00000102043	0.031489	0.090882879	0.248090181	1.187633901	**MTMR8**	protein_coding	myotubularin related protein 8 [Source:HGNC Symbol;Acc:HGNC:16825]	chrX	64268081	64395431	-1	ensembl_havana	0.955158833	0.831113743	1.079203924	0.589870393	0.77991046	1.123560375	0.995284613	0.880929471	1.361397687
ENSG00000234062	0.031497	0.090882879	0.21616812	1.161644093	**AL390879.1**	transcribed_unprocessed_pseudogene		chrX	136909395	137021630	1	havana	1.473318183	1.365234123	1.581402243	1.451413217	1.270574017	1.373715134	1.593788933	1.426455924	1.723961872
ENSG00000134759	0.031511	0.090882879	-0.171359597	-1.126119241	**ELP2**	protein_coding	elongator acetyltransferase complex subunit 2 [Source:HGNC Symbol;Acc:HGNC:18248]	chr18	36129444	36180556	1	ensembl_havana	6.808955661	6.89463546	6.723275863	7.03667432	6.857355218	6.789876841	6.812715143	6.720591619	6.636520827
ENSG00000183665	0.031509	0.090882879	-0.203358513	-1.151375575	**TRMT12**	protein_coding	tRNA methyltransferase 12 homolog [Source:HGNC Symbol;Acc:HGNC:26091]	chr8	124450820	124462150	1	ensembl_havana	4.214292321	4.315971577	4.112613064	4.292670028	4.339270518	4.315974186	4.049488025	4.061905948	4.22644522
ENSG00000012779	0.031512	0.090882879	-0.412799545	-1.331266627	**ALOX5**	protein_coding	arachidonate 5-lipoxygenase [Source:HGNC Symbol;Acc:HGNC:435]	chr10	45374176	45446119	1	ensembl_havana	0.185433398	0.39183317	-0.020966374	0.464289132	0.428746719	0.28246366	-0.26243933	0.02879206	0.17074815
ENSG00000179364	0.031542	0.090951238	0.389139857	1.309612373	**PACS2**	protein_coding	phosphofurin acidic cluster sorting protein 2 [Source:HGNC Symbol;Acc:HGNC:23794]	chr14	105300563	105398147	1	ensembl_havana	5.077557117	4.882987188	5.272127046	4.823055734	4.677937271	5.14796856	5.334413759	5.27259747	5.209369908
ENSG00000163697	0.031576	0.09101466	0.3778552	1.299408637	**APBB2**	protein_coding	amyloid beta precursor protein binding family B member 2 [Source:HGNC Symbol;Acc:HGNC:582]	chr4	40810027	41216714	-1	ensembl_havana	5.696335667	5.507408067	5.885263267	4.89174011	5.600044505	6.030439586	5.57625118	5.902981482	6.176557138
ENSG00000105755	0.031572	0.09101466	0.175658312	1.129479677	**ETHE1**	protein_coding	ETHE1, persulfide dioxygenase [Source:HGNC Symbol;Acc:HGNC:23287]	chr19	43506719	43527244	-1	ensembl_havana	3.646960469	3.559131313	3.734789625	3.602328412	3.494320753	3.580744775	3.758889004	3.625921893	3.819557978
ENSG00000157500	0.031622	0.091126886	0.178128598	1.13141531	**APPL1**	protein_coding	adaptor protein, phosphotyrosine interacting with PH domain and leucine zipper 1 [Source:HGNC Symbol;Acc:HGNC:24035]	chr3	57227737	57273468	1	ensembl_havana	6.721511256	6.632446957	6.810575555	6.515623577	6.62937752	6.752339775	6.632277147	6.86836683	6.931082688
ENSG00000182325	0.031634	0.09114427	0.180987682	1.133659733	**FBXL6**	protein_coding	F-box and leucine rich repeat protein 6 [Source:HGNC Symbol;Acc:HGNC:13603]	chr8	144355431	144359376	-1	ensembl_havana	4.775331187	4.684837346	4.865825027	4.698916787	4.658202087	4.697393164	4.956165102	4.793283367	4.848026613
ENSG00000132470	0.031644	0.091154237	-0.921412396	-1.893968578	**ITGB4**	protein_coding	integrin subunit beta 4 [Source:HGNC Symbol;Acc:HGNC:6158]	chr17	75721328	75757818	1	ensembl_havana	1.078500568	1.539206766	0.61779437	1.464591378	1.390249836	1.762779083	1.089724456	0.758913071	0.004745582
ENSG00000144591	0.031661	0.09118624	-0.170258196	-1.125259852	**GMPPA**	protein_coding	GDP-mannose pyrophosphorylase A [Source:HGNC Symbol;Acc:HGNC:22923]	chr2	219498867	219506989	1	ensembl_havana	5.137519553	5.222648651	5.052390455	5.222670322	5.297307071	5.14796856	5.049594984	5.084323255	5.023253127
ENSG00000039650	0.031697	0.091252493	0.279830359	1.21405212	**PNKP**	protein_coding	polynucleotide kinase 3'-phosphatase [Source:HGNC Symbol;Acc:HGNC:9154]	chr19	49859882	49878351	-1	ensembl_havana	5.626944534	5.487029354	5.766859713	5.473863577	5.352382995	5.634841491	5.862356664	5.737848397	5.700374079
ENSG00000269556	0.031697	0.091252493	0.168658456	1.124012793	**TMEM185A**	protein_coding	transmembrane protein 185A [Source:HGNC Symbol;Acc:HGNC:17125]	chrX	149596556	149631912	-1	ensembl_havana	5.018200166	4.933870938	5.102529394	5.021130064	4.911476265	4.869006484	5.15492242	5.12399508	5.028670681
ENSG00000073169	0.031738	0.091318358	0.486871238	1.401402367	**SELENOO**	protein_coding	selenoprotein O [Source:HGNC Symbol;Acc:HGNC:30395]	chr22	50200979	50217616	1	ensembl_havana	3.517491317	3.274055698	3.760926936	3.163221009	3.143202419	3.515743667	3.86417946	3.846657471	3.571943877
ENSG00000149577	0.031735	0.091318358	0.180054481	1.132926667	**SIDT2**	protein_coding	SID1 transmembrane family member 2 [Source:HGNC Symbol;Acc:HGNC:24272]	chr11	117178733	117197445	1	ensembl_havana	5.657564023	5.567536783	5.747591263	5.653623169	5.511161128	5.537826051	5.827149217	5.629585979	5.786038595
ENSG00000188486	0.031739	0.091318358	-0.231746791	-1.174255858	**H2AFX**	protein_coding	H2A histone family member X [Source:HGNC Symbol;Acc:HGNC:4739]	chr11	119093854	119095467	-1	ensembl_havana	5.2463806	5.362253995	5.130507204	5.204129902	5.435115436	5.447516647	5.128468593	5.127081093	5.135971927
ENSG00000273466	0.031749	0.091328049	0.188390126	1.139491471	**AC012510.1**	antisense		chr2	218633256	218634014	-1	havana	1.530456737	1.436261674	1.6246518	1.327057633	1.798708764	1.183018625	1.606623359	1.947753749	1.319578292
ENSG00000264230	0.031794	0.091421128	0.167100778	1.122799851	**ANXA8L1**	protein_coding	annexin A8 like 1 [Source:HGNC Symbol;Acc:HGNC:23334]	chr10	46375627	46391784	1	ensembl_havana	5.049147749	4.96559736	5.132698138	4.990708424	4.99251402	4.913569637	5.13180197	5.144444897	5.121847546
ENSG00000245281	0.031789	0.091421128	-0.251530566	-1.190469423	**AC124242.1**	antisense		chr8	18084868	18096394	1	havana	-0.019827223	0.10593806	-0.145592506	0.004630787	0.298820819	0.014362573	-0.30988669	0.225896484	-0.352787316
ENSG00000102743	0.031817	0.091469492	-0.245697486	-1.185665855	**SLC25A15**	protein_coding	solute carrier family 25 member 15 [Source:HGNC Symbol;Acc:HGNC:10985]	chr13	40789412	40810111	1	ensembl_havana	2.925503393	3.048352136	2.80265465	3.027727485	2.877938163	3.23939076	2.687924289	2.806118434	2.913921226
ENSG00000160959	0.031859	0.091527671	0.247241627	1.186935573	**LRRC14**	protein_coding	leucine rich repeat containing 14 [Source:HGNC Symbol;Acc:HGNC:20419]	chr8	144517992	144525178	1	ensembl_havana	5.115061442	4.991440628	5.238682255	4.909918296	4.919235346	5.145168242	5.312059643	5.161602199	5.242384924
ENSG00000136720	0.031851	0.091527671	0.221602551	1.166028095	**HS6ST1**	protein_coding	heparan sulfate 6-O-sulfotransferase 1 [Source:HGNC Symbol;Acc:HGNC:5201]	chr2	128236716	128318577	-1	ensembl_havana	4.683831783	4.573030507	4.794633058	4.497278108	4.510469659	4.711343754	4.800104487	4.793283367	4.790511319
ENSG00000112130	0.031869	0.091527671	-0.182783979	-1.13507213	**RNF8**	protein_coding	ring finger protein 8 [Source:HGNC Symbol;Acc:HGNC:10071]	chr6	37353972	37394738	1	ensembl_havana	5.499540063	5.590932053	5.408148074	5.677870992	5.575002611	5.519922556	5.415057956	5.419949561	5.389436704
ENSG00000106868	0.031864	0.091527671	-0.217983231	-1.163106522	**SUSD1**	protein_coding	sushi domain containing 1 [Source:HGNC Symbol;Acc:HGNC:25413]	chr9	112040785	112175408	-1	ensembl_havana	5.569300018	5.678291633	5.460308402	5.403758404	5.767818061	5.863298434	5.320848202	5.458026668	5.602050337
ENSG00000128564	0.031861	0.091527671	-0.82477541	-1.771259278	**VGF**	protein_coding	VGF nerve growth factor inducible [Source:HGNC Symbol;Acc:HGNC:12684]	chr7	101162509	101165593	-1	ensembl_havana	-0.315778889	0.096608816	-0.728166594	-0.03191641	0.09469996	0.227042895	-0.40973604	-0.42206346	-1.352700279
ENSG00000183077	0.031885	0.091556341	-0.18477252	-1.136637737	**AFMID**	protein_coding	arylformamidase [Source:HGNC Symbol;Acc:HGNC:20910]	chr17	78187317	78207701	1	ensembl_havana	4.776084757	4.868471017	4.683698497	4.758982193	4.962789824	4.883641034	4.55066832	4.718872731	4.78155444
ENSG00000113971	0.031931	0.091668382	0.308058064	1.238040117	**NPHP3**	protein_coding	nephrocystin 3 [Source:HGNC Symbol;Acc:HGNC:7907]	chr3	132680609	132722442	-1	ensembl_havana	3.112016436	2.957987404	3.266045468	2.838235332	3.116453761	2.919273119	3.131498845	3.218605774	3.448031787
ENSG00000154165	0.031946	0.091694126	-0.313713013	-1.242902401	**GPR15**	protein_coding	G protein-coupled receptor 15 [Source:HGNC Symbol;Acc:HGNC:4469]	chr3	98531899	98533150	1	ensembl_havana	5.259768515	5.416625021	5.102912008	4.990708424	5.218645532	6.040521107	4.56224422	5.157583505	5.5889083
ENSG00000071073	0.031959	0.091694339	0.18376698	1.135845791	**MGAT4A**	protein_coding	mannosyl (alpha-1,3-)-glycoprotein beta-1,4-N-acetylglucosaminyltransferase, isozyme A [Source:HGNC Symbol;Acc:HGNC:7047]	chr2	98619106	98731126	-1	ensembl_havana	5.041460164	4.949576674	5.133343654	4.838322801	5.105640774	4.904766446	5.075214401	5.207030082	5.117786479
ENSG00000066117	0.031954	0.091694339	0.175226401	1.129141586	**SMARCD1**	protein_coding	SWI/SNF related, matrix associated, actin dependent regulator of chromatin, subfamily d, member 1 [Source:HGNC Symbol;Acc:HGNC:11106]	chr12	50084972	50100712	1	ensembl_havana	7.798421958	7.710808757	7.886035158	7.73745582	7.733625224	7.661345227	7.966413367	7.849809314	7.841882793
ENSG00000116016	0.031991	0.091755542	0.181673652	1.134198892	**EPAS1**	protein_coding	endothelial PAS domain protein 1 [Source:HGNC Symbol;Acc:HGNC:3374]	chr2	46293667	46386703	1	ensembl_havana	6.917585904	6.826749078	7.008422731	6.75699829	6.823792867	6.899456078	7.008864604	7.002681993	7.013721595
ENSG00000136425	0.031993	0.091755542	-0.523078854	-1.437018719	**CIB2**	protein_coding	calcium and integrin binding family member 2 [Source:HGNC Symbol;Acc:HGNC:24579]	chr15	78104606	78131544	-1	ensembl_havana	0.433421017	0.694960444	0.171881589	0.683014432	0.658018659	0.743848241	0.339398945	-0.32890884	0.505154661
ENSG00000145725	0.032016	0.091802875	0.193346955	1.143413283	**PPIP5K2**	protein_coding	diphosphoinositol pentakisphosphate kinase 2 [Source:HGNC Symbol;Acc:HGNC:29035]	chr5	103120149	103212799	1	ensembl_havana	6.615171731	6.518498253	6.711845208	6.473465589	6.499726737	6.582302433	6.573839534	6.773740061	6.787956029
ENSG00000108349	0.032046	0.09186997	0.192560245	1.142789943	**CASC3**	protein_coding	cancer susceptibility 3 [Source:HGNC Symbol;Acc:HGNC:17040]	chr17	40140318	40172183	1	ensembl_havana	6.682364786	6.586084664	6.778644909	6.501707348	6.605187566	6.651359077	6.759117942	6.699670127	6.877146657
ENSG00000169410	0.032098	0.092002444	0.161910028	1.118767328	**PTPN9**	protein_coding	protein tyrosine phosphatase, non-receptor type 9 [Source:HGNC Symbol;Acc:HGNC:9661]	chr15	75463251	75579289	-1	ensembl_havana	6.41018799	6.329232976	6.491143004	6.27036283	6.372684863	6.344651236	6.444884879	6.515684475	6.512859659
ENSG00000281357	0.032106	0.092005155	0.253570276	1.192153722	**ARRDC3-AS1**	lincRNA	ARRDC3 antisense RNA 1 [Source:HGNC Symbol;Acc:HGNC:44145]	chr5	91380349	91439085	1	havana	1.815903132	1.689117994	1.94268827	1.727676152	1.646767026	1.692910804	1.808964307	1.929015357	2.090085146
ENSG00000107643	0.032128	0.092012989	0.188863867	1.13986571	**MAPK8**	protein_coding	mitogen-activated protein kinase 8 [Source:HGNC Symbol;Acc:HGNC:6881]	chr10	48306639	48439360	1	ensembl_havana	5.722017891	5.627585957	5.816449825	5.719691633	5.609666826	5.553399413	5.820278929	5.815780608	5.813289937
ENSG00000160014	0.032119	0.092012989	0.18627391	1.137821234	**CALM3**	protein_coding	calmodulin 3 [Source:HGNC Symbol;Acc:HGNC:1449]	chr19	46601074	46610793	1	ensembl_havana	7.90985814	7.816721185	8.002995095	7.734398894	7.87626205	7.839502611	8.002064185	7.991015936	8.015905165
ENSG00000257122	0.032126	0.092012989	-0.190338435	-1.141031353	**RRN3P3**	transcribed_unprocessed_pseudogene	RRN3 homolog, RNA polymerase I transcription factor pseudogene 3 [Source:HGNC Symbol;Acc:HGNC:37620]	chr16	22418672	22437715	-1	havana	3.748403305	3.843572522	3.653234088	4.051990026	3.783796374	3.694931168	3.957304068	3.554366408	3.448031787
ENSG00000261618	0.032145	0.092024926	0.234040394	1.17612418	**AC083837.1**	lincRNA		chr8	78837529	78840522	1	havana	1.327370148	1.210349951	1.444390345	1.383653947	1.093800119	1.153595787	1.729053464	1.372000412	1.232117157
ENSG00000272916	0.032144	0.092024926	-0.36547059	-1.288301789	**AC022400.6**	protein_coding	bifunctional heparan sulfate N-deacetylase/N-sulfotransferase 2 isoform 2 [Source:RefSeq peptide;Acc:NP_001317036]	chr10	73796514	73811651	-1	havana	-0.464095631	-0.281360336	-0.646830926	0.10902545	-0.967469033	0.014362573	-0.04612063	-1.24204464	-0.652327512
ENSG00000049239	0.032156	0.092038401	-0.181235939	-1.133854829	**H6PD**	protein_coding	hexose-6-phosphate dehydrogenase/glucose 1-dehydrogenase [Source:HGNC Symbol;Acc:HGNC:4795]	chr1	9234775	9271337	1	ensembl_havana	6.439410654	6.530028623	6.348792684	6.63626671	6.419937744	6.533881415	6.417377591	6.317871198	6.311129263
ENSG00000087074	0.032183	0.092099453	0.329145885	1.256269409	**PPP1R15A**	protein_coding	protein phosphatase 1 regulatory subunit 15A [Source:HGNC Symbol;Acc:HGNC:14375]	chr19	48872392	48876057	1	ensembl_havana	6.533126416	6.368553474	6.697699359	6.411019115	6.150094464	6.544546842	6.872385245	6.617573411	6.603139421
ENSG00000095739	0.032203	0.092136669	0.318922471	1.247398536	**BAMBI**	protein_coding	BMP and activin membrane bound inhibitor [Source:HGNC Symbol;Acc:HGNC:30251]	chr10	28677342	28682939	1	ensembl_havana	3.620185412	3.460724177	3.779646648	3.407969125	3.68322949	3.290973915	3.890656094	3.745870536	3.702413313
ENSG00000233903	0.03221	0.092139687	-0.16750474	-1.123114285	**Z83851.1**	lincRNA		chr22	42276355	42277052	1	havana	0.007732528	0.091484898	-0.076019841	-0.27307956	0.185737694	0.361796557	-0.46237776	0.063570082	0.17074815
ENSG00000159399	0.032227	0.092169696	0.217845207	1.162995252	**HK2**	protein_coding	hexokinase 2 [Source:HGNC Symbol;Acc:HGNC:4923]	chr2	74833981	74893359	1	ensembl_havana	6.632598601	6.523675997	6.741521204	6.749615287	6.389539637	6.431873068	6.842519641	6.722293117	6.659750855
ENSG00000171943	0.032245	0.09220216	-0.202814604	-1.150941578	**SRGAP2C**	protein_coding	SLIT-ROBO Rho GTPase activating protein 2C [Source:HGNC Symbol;Acc:HGNC:30584]	chr1	121184810	121392822	1	ensembl_havana	3.268382588	3.36978989	3.166975286	3.272162151	3.386505103	3.450702416	3.166591014	3.19134646	3.142988385
ENSG00000140320	0.032294	0.092323509	0.189319724	1.140225937	**BAHD1**	protein_coding	bromo adjacent homology domain containing 1 [Source:HGNC Symbol;Acc:HGNC:29153]	chr15	40439721	40468242	1	ensembl_havana	6.23682635	6.142166488	6.331486212	6.071538731	6.201904734	6.153055998	6.353141897	6.364378273	6.276938465
ENSG00000244560	0.032335	0.092423792	0.184439866	1.136375684	**AC004890.2**	transcribed_unprocessed_pseudogene		chr7	149285281	149297312	1	havana	3.436112088	3.343892155	3.528332021	3.418165445	3.227754334	3.385756687	3.65151138	3.326351517	3.607133167
ENSG00000147684	0.032365	0.092490267	-0.185347703	-1.13709099	**NDUFB9**	protein_coding	NADH:ubiquinone oxidoreductase subunit B9 [Source:HGNC Symbol;Acc:HGNC:7704]	chr8	124539103	124568510	1	ensembl_havana	5.44803508	5.540708932	5.355361229	5.548475941	5.579906031	5.493744824	5.308136366	5.36262721	5.395320111
ENSG00000120526	0.032392	0.092549341	-0.220666453	-1.16527176	**NUDCD1**	protein_coding	NudC domain containing 1 [Source:HGNC Symbol;Acc:HGNC:24306]	chr8	109240919	109334385	-1	ensembl_havana	5.25141941	5.361752637	5.141086183	5.37438475	5.37190052	5.338972641	5.027096987	5.15657708	5.239584483
ENSG00000282988	0.032413	0.092588834	0.202860607	1.150978279	**AL031777.3**	protein_coding		chr6	26195595	26199293	-1	havana	1.848525469	1.747095165	1.949955772	1.892762624	1.739838083	1.608684788	1.996172808	2.046678052	1.807016457
ENSG00000149761	0.032434	0.092612654	0.17648796	1.130129392	**NUDT22**	protein_coding	nudix hydrolase 22 [Source:HGNC Symbol;Acc:HGNC:28189]	chr11	64225941	64230686	1	ensembl_havana	3.576897023	3.488653043	3.665141003	3.575247577	3.455209308	3.435502243	3.758889004	3.688535729	3.547998277
ENSG00000186468	0.032433	0.092612654	0.172761571	1.127214106	**RPS23**	protein_coding	ribosomal protein S23 [Source:HGNC Symbol;Acc:HGNC:10410]	chr5	82273358	82278577	-1	ensembl_havana	9.441736244	9.355355458	9.52811703	9.37232927	9.386774025	9.30696308	9.503510591	9.541314251	9.539526247

ENSG00000239605	0.032465	0.092663642	0.409006347	1.327771	**STPG4**	protein_coding	sperm-tail PG-rich repeat containing 4 [Source:HGNC Symbol;Acc:HGNC:26850]	chr2	47045538	47155378	-1	ensembl_havana	3.365875579	3.161372406	3.570378753	2.843288967	3.127978252	3.512849998	3.50153988	3.631727691	3.577868687
ENSG00000123810	0.032458	0.092663642	0.320229695	1.248529315	**B9D2**	protein_coding	B9 domain containing 2 [Source:HGNC Symbol;Acc:HGNC:28636]	chr19	41354421	41364173	-1	ensembl_havana	1.733364444	1.573249596	1.893479292	1.760103226	1.440410536	1.519235028	2.221475976	1.881076089	1.577885811
ENSG00000183185	0.032502	0.092753004	0.211206089	1.157655576	**GABRR3**	protein_coding	gamma-aminobutyric acid type A receptor rho3 subunit (gene/pseudogene) [Source:HGNC Symbol;Acc:HGNC:17969]	chr3	97986673	98035304	-1	ensembl_havana	-0.200227999	-0.305831044	-0.094624955	0.14220989	-0.473795405	-0.585907617	0.36983092	-0.15890847	-0.494797317
ENSG00000076770	0.032532	0.092819464	-0.36350399	-1.286546846	**MBNL3**	protein_coding	muscleblind like splicing regulator 3 [Source:HGNC Symbol;Acc:HGNC:20564]	chrX	132369317	132489968	-1	ensembl_havana	4.184178176	4.365930171	4.002426181	4.230383872	4.241864646	4.625541994	3.613866272	4.157568375	4.235843895
ENSG00000144711	0.032548	0.092846031	-0.256490266	-1.194569064	**IQSEC1**	protein_coding	IQ motif and Sec7 domain 1 [Source:HGNC Symbol;Acc:HGNC:29112]	chr3	12897220	13073117	-1	ensembl_havana	4.749080943	4.877326076	4.620835809	4.9642779	4.762933881	4.904766446	4.744577001	4.649027725	4.468902702
ENSG00000062716	0.032575	0.092885729	0.239397466	1.180499529	**VMP1**	protein_coding	vacuole membrane protein 1 [Source:HGNC Symbol;Acc:HGNC:29559]	chr17	59707192	59842255	1	ensembl_havana	7.490362387	7.370663655	7.61006112	7.472451355	7.369844875	7.269694734	7.61770808	7.535371959	7.677103321
ENSG00000175854	0.032574	0.092885729	0.190606376	1.141243289	**SWI5**	protein_coding	SWI5 homologous recombination repair protein [Source:HGNC Symbol;Acc:HGNC:31412]	chr9	128275379	128288990	1	ensembl_havana	4.221010241	4.125707052	4.316313429	4.077947155	4.131689787	4.167484215	4.171114765	4.380827217	4.396998304
ENSG00000180229	0.032602	0.092945215	0.263045434	1.200009173	**HERC2P3**	transcribed_unprocessed_pseudogene	hect domain and RLD 2 pseudogene 3 [Source:HGNC Symbol;Acc:HGNC:4871]	chr15	20379495	20506180	-1	havana	4.367834212	4.236311495	4.499356928	4.173335581	4.112480652	4.423118252	4.475698201	4.523494887	4.498877698
ENSG00000100600	0.032636	0.092987302	0.377063439	1.298695708	**LGMN**	protein_coding	legumain [Source:HGNC Symbol;Acc:HGNC:9472]	chr14	92703807	92748702	-1	ensembl_havana	1.861104057	1.672572337	2.049635776	1.175017601	1.719668303	2.123031109	1.7752573	2.187350601	2.186299428
ENSG00000122515	0.032635	0.092987302	0.221481964	1.165930638	**ZMIZ2**	protein_coding	zinc finger MIZ-type containing 2 [Source:HGNC Symbol;Acc:HGNC:22229]	chr7	44748581	44769881	1	ensembl_havana	6.797392983	6.686652001	6.908133965	6.590415787	6.618525184	6.851015031	6.902295867	6.8930498	6.929056228
ENSG00000002822	0.032624	0.092987302	0.215068131	1.16075873	**MAD1L1**	protein_coding	mitotic arrest deficient 1 like 1 [Source:HGNC Symbol;Acc:HGNC:6762]	chr7	1815793	2233243	-1	ensembl_havana	3.113023984	3.005489918	3.220558049	2.802355359	2.974723635	3.23939076	3.153531275	3.122918448	3.385224425
ENSG00000198837	0.032652	0.09300529	-0.167592062	-1.123182266	**DENND4B**	protein_coding	DENN domain containing 4B [Source:HGNC Symbol;Acc:HGNC:29044]	chr1	153929501	153946696	-1	ensembl_havana	6.571991979	6.65578801	6.488195948	6.701726013	6.54054267	6.725095346	6.49406381	6.415755862	6.55476817
ENSG00000231154	0.032655	0.09300529	-0.295110007	-1.226978532	**MORF4L2-AS1**	antisense	MORF4L2 antisense RNA 1 [Source:HGNC Symbol;Acc:HGNC:27991]	chrX	103687284	103691772	1	havana	1.716858714	1.864413717	1.56930371	1.81257944	1.719668303	2.060993409	1.325353043	1.637380031	1.745178056
ENSG00000235385	0.032711	0.093146111	-0.426296949	-1.343779986	**LINC02154**	lincRNA	long intergenic non-protein coding RNA 2154 [Source:HGNC Symbol;Acc:HGNC:53015]	chrX	13266048	13303452	-1	havana	0.903223037	1.116371511	0.690074562	0.41082706	1.351450576	1.586836898	0.210814386	0.554044597	1.305364703
ENSG00000185829	0.032738	0.09320215	0.192000916	1.142346972	**ARL17A**	protein_coding	ADP ribosylation factor like GTPase 17A [Source:HGNC Symbol;Acc:HGNC:24096]	chr17	46516702	46579682	-1	ensembl_havana	1.869007207	1.773006749	1.965007665	1.738566362	1.678462547	1.901991338	1.986385747	1.769643034	2.138994213
ENSG00000059758	0.032807	0.093381462	0.206523678	1.15390438	**CDK17**	protein_coding	cyclin dependent kinase 17 [Source:HGNC Symbol;Acc:HGNC:8750]	chr12	96278261	96400560	-1	ensembl_havana	5.668361482	5.565099643	5.771623321	5.68703557	5.644186636	5.364076724	5.84756593	5.773735126	5.693568907
ENSG00000166682	0.032817	0.093391935	-0.178088008	-1.131383478	**TMPRSS5**	protein_coding	transmembrane serine protease 5 [Source:HGNC Symbol;Acc:HGNC:14908]	chr11	113687546	113706373	-1	ensembl_havana	-0.156606902	-0.067562898	-0.245650905	0.174648163	-0.18055577	-0.196781087	-0.00649342	-0.42206346	-0.308395834
ENSG00000091972	0.032843	0.09342949	0.627887251	1.545300327	**CD200**	protein_coding	CD200 molecule [Source:HGNC Symbol;Acc:HGNC:7203]	chr3	112332347	112362812	1	ensembl_havana	0.817510374	0.503566749	1.131454	1.126095262	0.699804047	-0.315199064	1.261864131	1.315408538	0.817089329
ENSG00000162434	0.032839	0.09342949	0.160519862	1.117689815	**JAK1**	protein_coding	Janus kinase 1 [Source:HGNC Symbol;Acc:HGNC:6190]	chr1	64833229	64966504	-1	ensembl_havana	7.712710702	7.632450771	7.792970633	7.651116225	7.608438006	7.63779808	7.800390304	7.763036922	7.815484672
ENSG00000271425	0.032871	0.093488781	0.168783197	1.124109984	**NBPF10**	protein_coding	NBPF member 10 [Source:HGNC Symbol;Acc:HGNC:31992]	chr1	146064699	146144804	-1	ensembl_havana	3.777842998	3.693451399	3.862234596	3.691897931	3.688427859	3.700028408	3.861504875	3.81128221	3.913916704
ENSG00000102678	0.032884	0.093506392	-0.211222787	-1.157668975	**FGF9**	protein_coding	fibroblast growth factor 9 [Source:HGNC Symbol;Acc:HGNC:3687]	chr13	21671383	21704498	1	ensembl_havana	1.763423871	1.869035264	1.657812477	1.43811357	1.979216042	2.18977618	1.245545533	1.871294071	1.856597828
ENSG00000104691	0.032894	0.09351738	-0.367946825	-1.290514923	**UBXN8**	protein_coding	UBX domain protein 8 [Source:HGNC Symbol;Acc:HGNC:30307]	chr8	30732247	30767006	1	ensembl_havana	3.260864202	3.444837615	3.07689079	3.513183533	3.373654145	3.447675166	2.836959964	3.106334847	3.287377559
ENSG00000267321	0.032932	0.093607074	0.407407531	1.32630036	**LINC02001**	lincRNA	long intergenic non-protein coding RNA 2001 [Source:HGNC Symbol;Acc:HGNC:52836]	chr17	35568109	35574843	1	havana	2.927570794	2.723867029	3.13127456	2.711312495	2.896025364	2.564263227	3.295040884	2.947823742	3.150959053
ENSG00000102804	0.032979	0.093722474	0.184753424	1.136622693	**TSC22D1**	protein_coding	TSC22 domain family member 1 [Source:HGNC Symbol;Acc:HGNC:16826]	chr13	44432143	44577147	-1	ensembl_havana	4.930274224	4.837897512	5.022650936	4.88072223	4.887945457	4.745024849	5.049594984	5.004907857	5.013449966
ENSG00000114026	0.033048	0.093899779	0.301829987	1.23270705	**OGG1**	protein_coding	8-oxoguanine DNA glycosylase [Source:HGNC Symbol;Acc:HGNC:8125]	chr3	9749944	9788219	1	ensembl_havana	4.852795066	4.701880072	5.003710059	4.486012993	4.729281527	4.890345696	5.011498951	5.002669359	4.996961868
ENSG00000181610	0.033057	0.093906884	-0.169509378	-1.124675948	**MRPS23**	protein_coding	mitochondrial ribosomal protein S23 [Source:HGNC Symbol;Acc:HGNC:14509]	chr17	57834781	57850056	-1	ensembl_havana	6.055341783	6.140096472	5.970587093	6.083333506	6.136370441	6.200585469	5.862356664	6.010497332	6.038907284
ENSG00000247746	0.033064	0.093906884	-0.339913998	-1.265681142	**USP51**	protein_coding	ubiquitin specific peptidase 51 [Source:HGNC Symbol;Acc:HGNC:23086]	chrX	55484616	55489202	-1	ensembl_havana	1.283926344	1.453883343	1.113969345	1.312554705	1.440410536	1.608684788	0.692307165	1.195095435	1.454505434
ENSG00000267761	0.033097	0.093984034	0.203478081	1.151471003	**MIR4527HG**	lincRNA	MIR4527 host gene [Source:HGNC Symbol;Acc:HGNC:31724]	chr18	47285725	47594550	1	havana	4.580792287	4.479053246	4.682531327	4.060694397	4.597323125	4.779142217	4.349675785	4.831621697	4.866296499
ENSG00000071894	0.033127	0.094049228	0.256368965	1.19446863	**CPSF1**	protein_coding	cleavage and polyadenylation specific factor 1 [Source:HGNC Symbol;Acc:HGNC:2324]	chr8	144393229	144409349	-1	ensembl_havana	7.036659574	6.908475091	7.164844057	6.932936806	6.789128682	7.003359785	7.336647834	7.077172749	7.080711586
ENSG00000204713	0.033155	0.094111146	-0.196942287	-1.14626633	**TRIM27**	protein_coding	tripartite motif containing 27 [Source:HGNC Symbol;Acc:HGNC:9975]	chr6	28903002	28923989	-1	ensembl_havana	7.018969204	7.117440347	6.92049806	7.154441019	7.191173853	7.00670617	6.858368371	7.00156145	6.90156436
ENSG00000243650	0.033163	0.094114686	-0.278933161	-1.213297348	**RN7SL834P**	misc_RNA	RNA, 7SL, cytoplasmic 834, pseudogene [Source:HGNC Symbol;Acc:HGNC:46850]	chr2	230507101	230507386	1	ensembl	0.11262779	0.25209437	-0.026838791	0.565613454	-0.036373238	0.227042895	0.106248156	-0.11936381	-0.067400719
ENSG00000075826	0.033238	0.094309399	0.165928385	1.121887789	**SEC31B**	protein_coding	SEC31 homolog B, COPII coat complex component [Source:HGNC Symbol;Acc:HGNC:23197]	chr10	100486642	100519864	-1	ensembl_havana	4.977339347	4.894375155	5.06030354	4.967752944	4.789785543	4.925586978	5.167969629	4.912542727	5.100398263
ENSG00000124787	0.03326	0.094351377	-0.2186038	-1.163606935	**RPP40**	protein_coding	ribonuclease P/MRP subunit p40 [Source:HGNC Symbol;Acc:HGNC:20992]	chr6	4994732	5004063	-1	ensembl_havana	4.085153414	4.194455314	3.975851514	3.959604644	4.273340686	4.350420613	3.648411535	4.195301803	4.083841203
ENSG00000109083	0.033336	0.094550341	0.229891935	1.172747101	**IFT20**	protein_coding	intraflagellar transport 20 [Source:HGNC Symbol;Acc:HGNC:30989]	chr17	28328325	28335489	-1	ensembl_havana	4.215536507	4.10059054	4.330482474	4.311025713	4.118270295	3.872475611	4.405825494	4.344168079	4.241453849
ENSG00000197903	0.033358	0.094593019	-0.240262617	-1.18120766	**HIST1H2BK**	protein_coding	histone cluster 1 H2B family member k [Source:HGNC Symbol;Acc:HGNC:13954]	chr6	27146418	27146798	-1	ensembl_havana	6.770280557	6.890411865	6.650149248	7.14116532	6.813123877	6.716946399	6.910082121	6.716841244	6.32352438
ENSG00000265962	0.033378	0.094613806	0.603925282	1.519846141	**GACAT2**	antisense	gastric cancer associated transcript 2 (non-protein coding) [Source:HGNC Symbol;Acc:HGNC:50516]	chr18	8695856	8707621	-1	havana	0.815333694	0.513371052	1.117296335	0.968509893	0.52486995	0.046733314	1.786580698	0.578298274	0.987010032
ENSG00000172493	0.033377	0.094613806	0.179214438	1.132267187	**AFF1**	protein_coding	AF4/FMR2 family member 1 [Source:HGNC Symbol;Acc:HGNC:7135]	chr4	86935002	87141054	1	ensembl_havana	6.68864416	6.599036941	6.778251379	6.551961714	6.561588364	6.683560744	6.754440182	6.794915895	6.78539806
ENSG00000185811	0.033413	0.094694622	-0.164425595	-1.120719778	**IKZF1**	protein_coding	IKAROS family zinc finger 1 [Source:HGNC Symbol;Acc:HGNC:13176]	chr7	50304068	50405101	1	ensembl_havana	8.760110151	8.842322949	8.677897354	8.844663916	8.75862626	8.923678671	8.689596697	8.624418215	8.719677151
ENSG00000139546	0.033439	0.094748123	0.195620053	1.145216255	**TARBP2**	protein_coding	TARBP2, RISC loading complex RNA binding subunit [Source:HGNC Symbol;Acc:HGNC:11569]	chr12	53500921	53506431	1	ensembl_havana	5.265239138	5.167429111	5.363049164	5.116051698	5.208730419	5.177505216	5.410490753	5.314709981	5.363946758
ENSG00000147789	0.033455	0.09477463	-0.228289893	-1.171445546	**ZNF7**	protein_coding	zinc finger protein 7 [Source:HGNC Symbol;Acc:HGNC:13139]	chr8	144827464	144847509	1	ensembl_havana	5.32854795	5.442692896	5.214403003	5.477118432	5.372708048	5.47825221	5.11617989	5.282780251	5.244248869
ENSG00000169855	0.033473	0.094806744	0.265671955	1.202195858	**ROBO1**	protein_coding	roundabout guidance receptor 1 [Source:HGNC Symbol;Acc:HGNC:10249]	chr3	78597240	79767815	-1	ensembl_havana	4.629361116	4.496525139	4.762197093	4.608312156	4.400735959	4.480527301	4.671638628	4.784189234	4.830763418
ENSG00000178105	0.033507	0.094884183	-0.206583816	-1.153952481	**DDX10**	protein_coding	DEAD-box helicase 10 [Source:HGNC Symbol;Acc:HGNC:2735]	chr11	108665025	108940930	1	ensembl_havana	5.380315857	5.483607766	5.277023949	5.382218516	5.450495655	5.618109125	5.063625579	5.249183955	5.518262313
ENSG00000092108	0.033526	0.09491784	-0.239486757	-1.180572595	**SCFD1**	protein_coding	sec1 family domain containing 1 [Source:HGNC Symbol;Acc:HGNC:20726]	chr14	30622112	30735812	1	ensembl_havana	5.782264313	5.902007692	5.662520935	5.92192483	6.038500008	5.745598239	5.705657068	5.640425293	5.641480444
ENSG00000107537	0.033539	0.09491784	-0.258266822	-1.196040981	**PHYH**	protein_coding	phytanoyl-CoA 2-hydroxylase [Source:HGNC Symbol;Acc:HGNC:8940]	chr10	13277796	13302412	-1	ensembl_havana	4.510702231	4.639835643	4.38156882	4.630406447	4.648899124	4.640201357	4.177580308	4.48552641	4.481599743
ENSG00000077585	0.033535	0.09491784	-0.262482802	-1.199541276	**GPR137B**	protein_coding	G protein-coupled receptor 137B [Source:HGNC Symbol;Acc:HGNC:11862]	chr1	236142505	236221865	1	ensembl_havana	4.185433112	4.316674513	4.054191712	4.584365888	4.285398275	4.080259376	4.122794131	4.135262968	3.904518036
ENSG00000146834	0.033549	0.094928416	-0.198682157	-1.147649545	**MEPCE**	protein_coding	methylphosphate capping enzyme [Source:HGNC Symbol;Acc:HGNC:20247]	chr7	100428790	100434126	1	ensembl_havana	6.236726598	6.336067676	6.137385519	6.171362308	6.450091168	6.386749552	6.036666585	6.301574424	6.073915548
ENSG00000240731	0.033575	0.094983739	0.250604365	1.189705394	**AL139287.1**	sense_intronic		chr1	1317581	1318689	-1	havana	1.354913988	1.229611806	1.480216171	1.158893785	1.093800119	1.436141515	1.309740823	1.529563273	1.601344415
ENSG00000167548	0.033586	0.094996338	0.235474846	1.177294166	**KMT2D**	protein_coding	lysine methyltransferase 2D [Source:HGNC Symbol;Acc:HGNC:7133]	chr12	49018975	49059774	-1	ensembl_havana	7.839325833	7.72158841	7.957063256	7.801660087	7.550558495	7.812546648	8.021213132	7.937382169	7.912594467
ENSG00000065357	0.033593	0.094996935	-0.159321555	-1.116761843	**DGKA**	protein_coding	diacylglycerol kinase alpha [Source:HGNC Symbol;Acc:HGNC:2849]	chr12	55927319	55954027	1	ensembl_havana	6.105231934	6.184892711	6.025571157	6.178377915	6.173917598	6.202382621	6.036073715	6.006034208	6.034605547
ENSG00000106554	0.033602	0.095003759	-0.162431025	-1.119171419	**CHCHD3**	protein_coding	coiled-coil-helix-coiled-coil-helix domain containing 3 [Source:HGNC Symbol;Acc:HGNC:21906]	chr7	132784868	133082088	-1	ensembl_havana	6.521090282	6.602305794	6.439874769	6.550417476	6.645838546	6.61066136	6.367822766	6.469019447	6.482782095
ENSG00000169084	0.033614	0.095018177	-0.159806682	-1.117137434	**DHRSX**	protein_coding	dehydrogenase/reductase X-linked [Source:HGNC Symbol;Acc:HGNC:18399]	chrX	2219516	2502805	-1	ensembl_havana	3.574026902	3.653930243	3.494123561	3.584331121	3.662246656	3.715212953	3.445493458	3.491900287	3.544976937
ENSG00000215030	0.033665	0.09514479	0.310768663	1.240368388	**RPL13P12**	processed_pseudogene	ribosomal protein L13 pseudogene 12 [Source:HGNC Symbol;Acc:HGNC:35701]	chr17	17383377	17384012	-1	havana	1.677190746	1.521806414	1.832575077	1.565895203	1.170104606	1.829419434	1.986385747	1.625790657	1.885548828
ENSG00000171962	0.033681	0.095171283	0.228871129	1.171917595	**DRC3**	protein_coding	dynein regulatory complex subunit 3 [Source:HGNC Symbol;Acc:HGNC:25384]	chr17	17972813	18016889	1	ensembl_havana	1.240362898	1.125927333	1.354798462	1.175017601	0.962641309	1.24012309	1.400976194	1.32976598	1.333653212
ENSG00000171320	0.03369	0.095178235	-0.160094242	-1.117360125	**ESCO2**	protein_coding	establishment of sister chromatid cohesion N-acetyltransferase 2 [Source:HGNC Symbol;Acc:HGNC:27230]	chr8	27771949	27812640	1	ensembl_havana	4.558368481	4.638415602	4.47832136	4.443383695	4.73054201	4.741321101	4.267203405	4.563530191	4.604230486
ENSG00000074660	0.033725	0.095258469	-0.617195919	-1.533890949	**SCARF1**	protein_coding	scavenger receptor class F member 1 [Source:HGNC Symbol;Acc:HGNC:16820]	chr17	1633858	1645747	-1	ensembl_havana	1.790222079	2.098820039	1.481624119	2.101191983	2.012757401	2.182510732	1.905603022	1.517070703	1.022198632
ENSG00000124772	0.03376	0.09533772	0.434792577	1.351716477	**CPNE5**	protein_coding	copine 5 [Source:HGNC Symbol;Acc:HGNC:2318]	chr6	36740775	36840002	-1	ensembl_havana	-0.338939196	-0.556335484	-0.121542907	-0.92573229	-0.428075095	-0.315199064	-0.26243933	0.163140205	-0.265329594
ENSG00000116273	0.033818	0.095464104	-0.173063408	-1.127449964	**PHF13**	protein_coding	PHD finger protein 13 [Source:HGNC Symbol;Acc:HGNC:22983]	chr1	6613685	6624033	1	ensembl_havana	5.883807871	5.970339575	5.797276167	6.20315878	5.919756238	5.788103707	6.035480601	5.802330212	5.554017688
ENSG00000175906	0.033817	0.095464104	-0.273341391	-1.208603807	**ARL4D**	protein_coding	ADP ribosylation factor like GTPase 4D [Source:HGNC Symbol;Acc:HGNC:656]	chr17	43398959	43401137	1	ensembl_havana	1.538196104	1.6748668	1.401525409	1.602122376	1.547275176	1.875202847	1.430149367	1.385808199	1.38861866
ENSG00000135956	0.033848	0.095529558	0.179519731	1.132506814	**TMEM127**	protein_coding	transmembrane protein 127 [Source:HGNC Symbol;Acc:HGNC:26038]	chr2	96248516	96265994	-1	ensembl_havana	6.292012259	6.202252393	6.381772124	6.330532122	6.258487259	6.017737799	6.478001909	6.389010888	6.278303576
ENSG00000145592	0.033876	0.095591185	0.196034058	1.145544941	**RPL37**	protein_coding	ribosomal protein L37 [Source:HGNC Symbol;Acc:HGNC:10347]	chr5	40825262	40835335	-1	ensembl_havana	8.980719872	8.882702843	9.078736901	8.962925118	8.911624803	8.773558609	9.158764942	9.011479127	9.065966634
ENSG00000114383	0.033931	0.095658772	0.218258849	1.163328748	**TUSC2**	protein_coding	tumor suppressor candidate 2 [Source:HGNC Symbol;Acc:HGNC:17034]	chr3	50320027	50328251	-1	ensembl_havana	4.464813585	4.35568416	4.57394301	4.323737133	4.38481302	4.358502328	4.599638302	4.459647342	4.662543385
ENSG00000196284	0.033916	0.095658772	0.186186319	1.137752155	**SUPT3H**	protein_coding	SPT3 homolog, SAGA and STAGA complex component [Source:HGNC Symbol;Acc:HGNC:11466]	chr6	44809317	45377953	-1	ensembl_havana	4.042931426	3.949838267	4.136024586	3.762957194	3.963923574	4.122634032	4.042420699	4.116753044	4.248900015
ENSG00000134222	0.033934	0.095658772	-0.205132	-1.152791818	**PSRC1**	protein_coding	proline and serine rich coiled-coil 1 [Source:HGNC Symbol;Acc:HGNC:24472]	chr1	109279556	109283186	-1	ensembl_havana	5.075336644	5.177902644	4.972770644	5.10662555	5.281914141	5.145168242	4.961170515	4.951362452	5.005778964
ENSG00000214026	0.033917	0.095658772	-0.230308766	-1.173085986	**MRPL23**	protein_coding	mitochondrial ribosomal protein L23 [Source:HGNC Symbol;Acc:HGNC:10322]	chr11	1947278	1984522	1	ensembl_havana	3.879333775	3.994488157	3.764179392	3.964251181	3.929213438	4.089999853	3.750239882	3.829078264	3.713220029
ENSG00000168517	0.033928	0.095658772	-0.365760618	-1.288560806	**HEXIM2**	protein_coding	hexamethylene bisacetamide inducible 2 [Source:HGNC Symbol;Acc:HGNC:28591]	chr17	45160700	45170040	1	ensembl_havana	3.103444219	3.286324528	2.92056391	3.187268021	3.324417061	3.347288501	3.104607948	2.938485423	2.718598359
ENSG00000158805	0.033942	0.095663075	0.29438887	1.226365375	**ZNF276**	protein_coding	zinc finger protein 276 [Source:HGNC Symbol;Acc:HGNC:23330]	chr16	89720400	89740903	1	ensembl_havana	5.642341956	5.495147521	5.789536391	5.413135017	5.374321749	5.697985797	5.882944088	5.720245956	5.765419128
ENSG00000163002	0.033976	0.095721422	-0.180980654	-1.133654211	**NUP35**	protein_coding	nucleoporin 35 [Source:HGNC Symbol;Acc:HGNC:29797]	chr2	183117513	183161680	1	ensembl_havana	4.507425824	4.597916151	4.416935497	4.517968493	4.648899124	4.626880836	4.257063661	4.532832903	4.460909928
ENSG00000137563	0.03397	0.095721422	-0.255949699	-1.194121552	**GGH**	protein_coding	gamma-glutamyl hydrolase [Source:HGNC Symbol;Acc:HGNC:4248]	chr8	63015079	63039171	-1	ensembl_havana	5.701502167	5.829477017	5.573527317	5.683517618	6.006079194	5.798834237	5.556543148	5.561261852	5.602776952
ENSG00000243302	0.033984	0.095726532	0.214008563	1.159906539	**AC018638.4**	processed_pseudogene		chr7	128651185	128652334	1	havana	3.49963083	3.392626548	3.606635112	3.380421283	3.337712124	3.459746237	3.732784509	3.488704842	3.598415984
ENSG00000078018	0.034004	0.095750361	0.36629021	1.289033904	**MAP2**	protein_coding	microtubule associated protein 2 [Source:HGNC Symbol;Acc:HGNC:6839]	chr2	209424058	209734118	1	ensembl_havana	2.791804195	2.60865909	2.9749493	2.199052053	2.85962132	2.767303895	2.809469694	2.929086265	3.186291941
ENSG00000112419	0.034006	0.095750361	0.357343692	1.281065012	**PHACTR2**	protein_coding	phosphatase and actin regulator 2 [Source:HGNC Symbol;Acc:HGNC:20956]	chr6	143536845	143831185	1	ensembl_havana	2.687901679	2.509229833	2.866573525	2.049654613	2.66767223	2.810362656	2.711879034	2.905316921	2.98252462
ENSG00000118363	0.034046	0.095843838	-0.192900909	-1.143059822	**SPCS2**	protein_coding	signal peptidase complex subunit 2 [Source:HGNC Symbol;Acc:HGNC:28962]	chr11	74949247	74979031	1	ensembl_havana	4.641629893	4.738080347	4.545179438	4.773615774	4.728019942	4.712605325	4.494809119	4.523494887	4.61723431
ENSG00000108064	0.034106	0.095982322	-0.158486135	-1.116115348	**TFAM**	protein_coding	transcription factor A, mitochondrial [Source:HGNC Symbol;Acc:HGNC:11741]	chr10	58385022	58399221	1	ensembl_havana	5.933328762	6.012571829	5.854085694	5.990152087	6.030846591	6.016716808	5.827149217	5.860966869	5.874140997
ENSG00000215251	0.03412	0.095982322	-0.185840774	-1.137479681	**FASTKD5**	protein_coding	FAST kinase domains 5 [Source:HGNC Symbol;Acc:HGNC:25790]	chr20	3146519	3159897	-1	ensembl_havana	6.246995575	6.339915962	6.154075188	6.568458707	6.335890452	6.115398729	6.304247216	6.143436901	6.014541447
ENSG00000026103	0.034116	0.095982322	-0.18857708	-1.139639144	**FAS**	protein_coding	Fas cell surface death receptor [Source:HGNC Symbol;Acc:HGNC:11920]	chr10	88990582	89015785	1	ensembl_havana	7.646536035	7.740824575	7.552247495	8.032120513	7.674437215	7.515915996	7.745055426	7.523515432	7.388171626
ENSG00000087085	0.034122	0.095982322	-0.409754221	-1.328459477	**ACHE**	protein_coding	acetylcholinesterase (Cartwright blood group) [Source:HGNC Symbol;Acc:HGNC:108]	chr7	100889994	100896974	-1	ensembl_havana	2.499853946	2.704731057	2.294976836	2.643402293	2.61404572	2.856745158	2.034670322	2.566495926	2.283764259
ENSG00000167987	0.034141	0.096018247	0.190240759	1.140954104	**VPS37C**	protein_coding	VPS37C, ESCRT-I subunit [Source:HGNC Symbol;Acc:HGNC:26097]	chr11	61130256	61161617	-1	ensembl_havana	4.052193628	3.957073249	4.147314008	4.012157084	3.920403831	3.938658831	4.303127503	4.044601662	4.094212859
ENSG00000143514	0.034176	0.096096204	-0.172091669	-1.126690817	**TP53BP2**	protein_coding	tumor protein p53 binding protein 2 [Source:HGNC Symbol;Acc:HGNC:12000]	chr1	223779899	223845972	-1	ensembl_havana	8.638023776	8.724069611	8.551977941	8.986385287	8.711608981	8.474214563	8.870746394	8.480631463	8.304555966
ENSG00000127337	0.034211	0.096177198	-0.186448889	-1.137959245	**YEATS4**	protein_coding	YEATS domain containing 4 [Source:HGNC Symbol;Acc:HGNC:24859]	chr12	69359703	69390796	1	ensembl_havana	3.586804043	3.680028487	3.493579598	3.631833797	3.678012323	3.730239343	3.459710505	3.566541508	3.454486781
ENSG00000203778	0.03425	0.096227567	0.260257821	1.197692722	**FAM229B**	protein_coding	family with sequence similarity 229 member B [Source:HGNC Symbol;Acc:HGNC:33858]	chr6	112087599	112102790	1	ensembl_havana	2.894850729	2.764721818	3.024979639	2.863328324	2.900511953	2.530325178	2.981915645	3.110498652	2.98252462
ENSG00000177542	0.034237	0.096227567	0.259136237	1.196761971	**SLC25A22**	protein_coding	solute carrier family 25 member 22 [Source:HGNC Symbol;Acc:HGNC:19954]	chr11	790475	798333	-1	ensembl_havana	3.936325509	3.80675739	4.065893627	3.83322245	3.791063673	3.795986047	4.277272381	4.018249731	3.90215877
ENSG00000137547	0.034245	0.096227567	-0.220594485	-1.165213632	**MRPL15**	protein_coding	mitochondrial ribosomal protein L15 [Source:HGNC Symbol;Acc:HGNC:14054]	chr8	54135210	54147901	1	ensembl_havana	5.057210015	5.167507258	4.946912773	5.055285407	5.192357248	5.254879118	4.801500542	5.113660433	4.925577343
ENSG00000135052	0.034256	0.096227567	-0.235190274	-1.177061967	**GOLM1**	protein_coding	golgi membrane protein 1 [Source:HGNC Symbol;Acc:HGNC:15451]	chr9	86026146	86100173	-1	ensembl_havana	5.209083769	5.326678906	5.091488633	5.181362699	5.356470978	5.442203042	4.988394109	4.959464348	5.32660744
ENSG00000177106	0.034297	0.096323297	0.469190336	1.38433234	**EPS8L2**	protein_coding	EPS8 like 2 [Source:HGNC Symbol;Acc:HGNC:21296]	chr11	694438	727727	1	ensembl_havana	-0.099961158	-0.334556326	0.13463401	-0.61730533	-0.299013651	-0.087349994	0.276538753	-0.04338487	0.17074815
ENSG00000196967	0.034358	0.096477908	-0.206083475	-1.153552348	**ZNF585A**	protein_coding	zinc finger protein 585A [Source:HGNC Symbol;Acc:HGNC:26305]	chr19	37106734	37172741	-1	ensembl_havana	2.344093903	2.44713564	2.241052165	2.438267468	2.311191904	2.591947549	2.135529305	2.226242768	2.361384423
ENSG00000243753	0.034428	0.096556662	0.201408809	1.14982062	**HLA-L**	transcribed_unprocessed_pseudogene	major histocompatibility complex, class I, L (pseudogene) [Source:HGNC Symbol;Acc:HGNC:4970]	chr6	30259584	30293014	1	ensembl_havana	3.662146933	3.561442529	3.762851338	3.708609614	3.439879526	3.535838447	3.795780677	3.663248546	3.829524789
ENSG00000124702	0.034409	0.096556662	0.168048076	1.123537343	**KLHDC3**	protein_coding	kelch domain containing 3 [Source:HGNC Symbol;Acc:HGNC:20704]	chr6	43014103	43021298	1	ensembl_havana	6.271863735	6.187839697	6.355887773	6.216365541	6.166485935	6.180667615	6.436373139	6.329527646	6.301762534
ENSG00000163939	0.03443	0.096556662	0.164410071	1.120707719	**PBRM1**	protein_coding	polybromo 1 [Source:HGNC Symbol;Acc:HGNC:30064]	chr3	52545352	52685917	-1	ensembl_havana	7.455363736	7.3731587	7.537568771	7.281813662	7.391322439	7.44634	7.471239725	7.577341538	7.564125051
ENSG00000164970	0.034434	0.096556662	-0.158005683	-1.115743717	**FAM219A**	protein_coding	family with sequence similarity 219 member A [Source:HGNC Symbol;Acc:HGNC:19920]	chr9	34398184	34458570	-1	ensembl_havana	6.881115991	6.960118833	6.80211315	6.885956761	6.899430407	7.094969331	6.742860663	6.728063246	6.935415541
ENSG00000125944	0.034418	0.096556662	-0.167134644	-1.122826208	**HNRNPR**	protein_coding	heterogeneous nuclear ribonucleoprotein R [Source:HGNC Symbol;Acc:HGNC:5047]	chr1	23303771	23344336	-1	ensembl_havana	7.969084255	8.052651577	7.885516933	8.066694997	8.057436149	8.033823585	7.878531454	7.856952163	7.921067183
ENSG00000113356	0.034427	0.096556662	-0.196056577	-1.145562822	**POLR3G**	protein_coding	RNA polymerase III subunit G [Source:HGNC Symbol;Acc:HGNC:30075]	chr5	90471748	90514553	1	ensembl_havana	4.889457614	4.987485902	4.791429325	5.006562662	5.039038012	4.916857033	4.72705256	4.818955118	4.828280298
ENSG00000186812	0.034394	0.096556662	-0.224843178	-1.168650209	**ZNF397**	protein_coding	zinc finger protein 397 [Source:HGNC Symbol;Acc:HGNC:18818]	chr18	35241030	35267133	1	ensembl_havana	5.538252766	5.650674355	5.425831177	5.639893277	5.654184297	5.657945492	5.264261129	5.475081104	5.538151299
ENSG00000196912	0.034444	0.096566551	0.306541922	1.236739733	**ANKRD36B**	protein_coding	ankyrin repeat domain 36B [Source:HGNC Symbol;Acc:HGNC:29333]	chr2	97492663	97589965	-1	ensembl_havana	2.943331967	2.790061006	3.096602928	2.863328324	2.494491223	3.012363471	3.100076994	3.046743406	3.142988385
ENSG00000106772	0.034452	0.096571424	-0.162168462	-1.118967754	**PRUNE2**	protein_coding	prune homolog 2 [Source:HGNC Symbol;Acc:HGNC:25209]	chr9	76611376	76906087	-1	ensembl_havana	1.840823039	1.921907269	1.759738808	1.369711653	2.220801645	2.17520851	1.245545533	2.046678052	1.986992839
ENSG00000054277	0.034491	0.096662161	0.278811391	1.213194944	**OPN3**	protein_coding	opsin 3 [Source:HGNC Symbol;Acc:HGNC:14007]	chr1	241590102	241677376	-1	ensembl_havana	5.399255681	5.259849986	5.538661376	5.682812997	5.281914141	4.814822818	5.877659265	5.424973505	5.313351359
ENSG00000121210	0.034564	0.096847387	0.171562401	1.126277553	**TMEM131L**	protein_coding	transmembrane 131 like [Source:HGNC Symbol;Acc:HGNC:29146]	chr4	153466346	153636711	1	ensembl_havana	5.687926653	5.602145453	5.773707854	5.555421349	5.558773018	5.692241991	5.694429282	5.797173085	5.829521194
ENSG00000131899	0.034571	0.096847665	0.320606759	1.248855674	**LLGL1**	protein_coding	LLGL1, scribble cell polarity complex component [Source:HGNC Symbol;Acc:HGNC:6628]	chr17	18225587	18244875	1	ensembl_havana	4.72931648	4.5690131	4.88961986	4.579831375	4.40705625	4.720151676	5.107176444	4.773725255	4.78795788
ENSG00000159388	0.034597	0.096900294	0.191627798	1.14205157	**BTG2**	protein_coding	BTG anti-proliferation factor 2 [Source:HGNC Symbol;Acc:HGNC:1131]	chr1	203305491	203309602	1	ensembl_havana	6.053626095	5.957812196	6.149439994	6.050389849	5.986693174	5.836353567	6.347418629	6.135796798	5.965104555
ENSG00000101191	0.03462	0.096922037	0.236651201	1.178254508	**DIDO1**	protein_coding	death inducer-obliterator 1 [Source:HGNC Symbol;Acc:HGNC:2680]	chr20	62877738	62937952	-1	ensembl_havana	7.023599376	6.905273775	7.141924976	6.891151289	6.845478871	6.979191166	7.213585838	7.167120961	7.04506813
ENSG00000166451	0.034619	0.096922037	-0.205336343	-1.15295511	**CENPN**	protein_coding	centromere protein N [Source:HGNC Symbol;Acc:HGNC:30873]	chr16	81006498	81033114	1	ensembl_havana	5.474468997	5.577137169	5.371800826	5.41737705	5.679858546	5.634175909	5.324737109	5.470228945	5.320436423
ENSG00000179743	0.034625	0.096922037	-0.388275997	-1.308828435	**AL450998.2**	antisense		chr1	15834474	15848147	-1	havana	1.043447263	1.237585261	0.849309264	1.411140715	0.927899934	1.373715134	0.830009965	0.880929471	0.836988357
ENSG00000270012	0.034632	0.09692288	-0.23342191	-1.175620084	**AC232271.1**	lincRNA		chrX	49273054	49275768	1	havana	3.088762374	3.205473329	2.972051418	3.199142872	3.206170563	3.211106551	3.049274082	2.806118434	3.060761739
ENSG00000214021	0.034663	0.096934959	0.183937744	1.135980243	**TTLL3**	protein_coding	tubulin tyrosine ligase like 3 [Source:HGNC Symbol;Acc:HGNC:24483]	chr3	9808086	9855138	1	ensembl_havana	5.752688847	5.660719975	5.844657719	5.704497792	5.654848351	5.622813782	5.813375768	5.829106762	5.891490625
ENSG00000011114	0.034654	0.096934959	0.166523519	1.12235068	**BTBD7**	protein_coding	BTB domain containing 7 [Source:HGNC Symbol;Acc:HGNC:18269]	chr14	93237550	93333092	-1	ensembl_havana	5.643072419	5.55981066	5.726334178	5.669359395	5.467982553	5.542090031	5.823030972	5.686476769	5.669494794
ENSG00000137802	0.034654	0.096934959	-0.193592489	-1.143607898	**MAPKBP1**	protein_coding	mitogen-activated protein kinase binding protein 1 [Source:HGNC Symbol;Acc:HGNC:29536]	chr15	41774434	41827855	1	ensembl_havana	7.462963498	7.559759743	7.366167254	7.641538554	7.463408492	7.574332182	7.491923383	7.33465684	7.271921539
ENSG00000274523	0.034658	0.096934959	-0.250577702	-1.189683407	**RCC1L**	protein_coding	RCC1 like [Source:HGNC Symbol;Acc:HGNC:14948]	chr7	75027122	75074228	-1	ensembl_havana	6.361642648	6.486931499	6.236353797	6.401200402	6.404621355	6.65497274	6.249039965	6.243988714	6.216032713
ENSG00000102837	0.03468	0.096951424	0.381766262	1.302936037	**OLFM4**	protein_coding	olfactomedin 4 [Source:HGNC Symbol;Acc:HGNC:17190]	chr13	53028759	53052057	1	ensembl_havana	2.510500854	2.319617723	2.701383985	2.150951608	2.575284697	2.232616866	2.65122947	2.625878149	2.827044337
ENSG00000127914	0.034703	0.096951424	0.204422398	1.152224946	**AKAP9**	protein_coding	A-kinase anchoring protein 9 [Source:HGNC Symbol;Acc:HGNC:379]	chr7	91940867	92110673	1	ensembl_havana	6.520064842	6.417853644	6.622276041	6.429618555	6.378727055	6.445215321	6.53240266	6.699324612	6.635100852
ENSG00000012963	0.034696	0.096951424	0.15754666	1.115388778	**UBR7**	protein_coding	ubiquitin protein ligase E3 component n-recognin 7 (putative) [Source:HGNC Symbol;Acc:HGNC:20344]	chr14	93207056	93229215	1	ensembl_havana	6.205345644	6.126572314	6.284118975	5.974115467	6.183153664	6.222447811	6.140703712	6.347278737	6.364374475
ENSG00000057252	0.034697	0.096951424	-0.161897068	-1.118757278	**SOAT1**	protein_coding	sterol O-acyltransferase 1 [Source:HGNC Symbol;Acc:HGNC:11177]	chr1	179293714	179358680	1	ensembl_havana	6.814048904	6.894997438	6.73310037	6.846547239	6.905860341	6.932584735	6.695590177	6.771112192	6.732598742
ENSG00000247708	0.034694	0.096951424	-0.188904048	-1.139897457	**STX18-AS1**	antisense	STX18 antisense RNA 1 (head to head) [Source:HGNC Symbol;Acc:HGNC:48877]	chr4	4542131	4710938	1	havana	1.793440368	1.887892392	1.698988344	1.565895203	2.093349987	2.004431986	1.458744294	1.811166797	1.827053942
ENSG00000138030	0.034724	0.096970392	0.344729132	1.269912532	**KHK**	protein_coding	ketohexokinase [Source:HGNC Symbol;Acc:HGNC:6315]	chr2	27086747	27100772	1	ensembl_havana	3.949672152	3.777307587	4.122036718	3.503639275	3.761772213	4.066511271	4.113831997	4.087479328	4.16479883
ENSG00000070413	0.034725	0.096970392	0.254402561	1.192841669	**DGCR2**	protein_coding	DiGeorge syndrome critical region gene 2 [Source:HGNC Symbol;Acc:HGNC:2845]	chr22	19036282	19122454	-1	ensembl_havana	4.956127677	4.828926396	5.083328957	4.701701975	4.761701395	5.023375819	5.098116458	5.058693368	5.093177045
ENSG00000054523	0.03473	0.096970392	0.223147695	1.167277595	**KIF1B**	protein_coding	kinesin family member 1B [Source:HGNC Symbol;Acc:HGNC:16636]	chr1	10210805	10381603	1	ensembl_havana	7.37868703	7.267113183	7.490260878	7.350640279	7.142525142	7.308174127	7.528633483	7.51509861	7.42705054
ENSG00000204070	0.03474	0.096979653	0.165675345	1.121691034	**SYS1**	protein_coding	SYS1, golgi trafficking protein [Source:HGNC Symbol;Acc:HGNC:16162]	chr20	45361937	45376798	1	ensembl_havana	5.373828302	5.290990629	5.456665975	5.369138507	5.383164985	5.120668396	5.574619088	5.499893876	5.295484959
ENSG00000162736	0.034751	0.096990712	-0.15983201	-1.117157047	**NCSTN**	protein_coding	nicastrin [Source:HGNC Symbol;Acc:HGNC:17091]	chr1	160343272	160358952	1	ensembl_havana	7.870340883	7.950256888	7.790424878	8.085210302	7.865975469	7.899584893	7.911390468	7.689445087	7.770439079
ENSG00000256771	0.03478	0.097036155	0.165378101	1.121459951	**ZNF253**	protein_coding	zinc finger protein 253 [Source:HGNC Symbol;Acc:HGNC:13497]	chr19	19865886	19894674	1	ensembl_havana	3.549977566	3.467288516	3.632666617	3.36293288	3.555343125	3.483589543	3.477287033	3.751214005	3.669498811
ENSG00000082146	0.034779	0.097036155	-0.15998352	-1.117274376	**STRADB**	protein_coding	STE20-related kinase adaptor beta [Source:HGNC Symbol;Acc:HGNC:13205]	chr2	201387858	201480846	1	ensembl_havana	4.919516277	4.999508037	4.839524517	4.905093132	5.07718931	5.01624167	4.737301003	4.894546073	4.886726475
ENSG00000236778	0.034812	0.097106515	-0.235850619	-1.177600851	**INTS6-AS1**	antisense	INTS6 antisense RNA 1 [Source:HGNC Symbol;Acc:HGNC:42691]	chr13	51452367	51552364	1	havana	1.125813412	1.243738721	1.007888102	1.022973463	1.284372232	1.423870469	0.618191201	1.114463419	1.291009686
ENSG00000176896	0.034849	0.097171856	0.158351369	1.116011095	**TCEANC**	protein_coding	transcription elongation factor A N-terminal and central domain containing [Source:HGNC Symbol;Acc:HGNC:28277]	chrX	13653189	13681964	1	ensembl_havana	2.538537815	2.45936213	2.6177135	2.516278079	2.389883326	2.471924986	2.65122947	2.560421027	2.641490002

ENSG00000077514	0.034843	0.097171856	-0.174419153	-1.128509961	**POLD3**	protein_coding	DNA polymerase delta 3, accessory subunit [Source:HGNC Symbol;Acc:HGNC:20932]	chr11	74493851	74669117	1	ensembl_havana	4.663563959	4.750773536	4.576354382	4.714169208	4.712794237	4.825357162	4.479191796	4.531280758	4.718590593
ENSG00000272617	0.034868	0.097187572	0.233277339	1.175502281	**COG8**	protein_coding	component of oligomeric golgi complex 8 [Source:NCBI gene;Acc:84342]	chr16	69326913	69339667	-1	ensembl_havana	3.159570084	3.042931415	3.276208753	2.978048743	2.957602376	3.193143125	3.157897671	3.35471112	3.31601747
ENSG00000225648	0.034866	0.097187572	-0.16406388	-1.120438824	**SBDSP1**	transcribed_unprocessed_pseudogene	SBDS, ribosome maturation factor pseudogene 1 [Source:HGNC Symbol;Acc:HGNC:21646]	chr7	72829425	72836701	1	havana	4.19208926	4.2741212	4.110057319	4.15719285	4.339270518	4.325900232	4.037689841	4.167594302	4.124887815
ENSG00000013288	0.034883	0.097190104	0.274375127	1.209470119	**MAN2B2**	protein_coding	mannosidase alpha class 2B member 2 [Source:HGNC Symbol;Acc:HGNC:29623]	chr4	6575175	6623362	1	ensembl_havana	5.145247268	5.008059705	5.282434832	4.968909434	4.892457205	5.162812476	5.414145672	5.198253856	5.234904968
ENSG00000169221	0.034881	0.097190104	0.247557795	1.187195719	**TBC1D10B**	protein_coding	TBC1 domain family member 10B [Source:HGNC Symbol;Acc:HGNC:24510]	chr16	30357102	30370264	-1	ensembl_havana	6.003522352	5.879743454	6.127301249	5.850310141	5.874292181	5.914628042	6.266836119	6.103259075	6.011808554
ENSG00000161551	0.03492	0.097200504	0.254908475	1.19326004	**ZNF577**	protein_coding	zinc finger protein 577 [Source:HGNC Symbol;Acc:HGNC:28673]	chr19	51804816	51890950	-1	ensembl_havana	0.848656025	0.721201787	0.976110263	0.637193984	0.614986832	0.911424546	0.807953002	1.063828438	1.056549347
ENSG00000198265	0.034904	0.097200504	0.209393691	1.156202175	**HELZ**	protein_coding	helicase with zinc finger [Source:HGNC Symbol;Acc:HGNC:16878]	chr17	67070438	67245989	-1	ensembl_havana	7.194069503	7.089372657	7.298766348	7.148839279	6.985886577	7.133392116	7.288924057	7.329754245	7.277620743
ENSG00000173681	0.034903	0.097200504	0.185423559	1.137150779	**BCLAF3**	protein_coding	BCLAF1 and THRAP3 family member 3 [Source:HGNC Symbol;Acc:HGNC:27413]	chrX	19912860	19970298	-1	ensembl_havana	4.369354588	4.276642808	4.462066367	4.298201297	4.269877069	4.261850058	4.39476902	4.467784529	4.523645552
ENSG00000184162	0.034909	0.097200504	-0.284823676	-1.21826136	**NR2C2AP**	protein_coding	nuclear receptor 2C2 associated protein [Source:HGNC Symbol;Acc:HGNC:30763]	chr19	19201416	19203424	-1	ensembl_havana	4.386279514	4.528691352	4.243867676	4.228453786	4.591785246	4.765835022	4.166788249	4.243493459	4.321321319
ENSG00000099364	0.034918	0.097200504	-0.320175069	-1.248482042	**FBXL19**	protein_coding	F-box and leucine rich repeat protein 19 [Source:HGNC Symbol;Acc:HGNC:25300]	chr16	30923055	30948783	1	ensembl_havana	3.82873828	3.988825815	3.668650745	4.189299683	3.880081028	3.897096734	4.10707367	3.551306511	3.347572056
ENSG00000138101	0.034932	0.097213448	0.291484823	1.223899267	**DTNB**	protein_coding	dystrobrevin beta [Source:HGNC Symbol;Acc:HGNC:3058]	chr2	25377198	25673647	-1	ensembl_havana	3.96001214	3.814269729	4.105754551	3.655011191	3.8686773	3.919120695	4.131700936	3.938520645	4.247042073
ENSG00000109929	0.034973	0.097309391	0.19309854	1.143216417	**SC5D**	protein_coding	sterol-C5-desaturase [Source:HGNC Symbol;Acc:HGNC:10547]	chr11	121292453	121308694	1	ensembl_havana	6.833216903	6.736667633	6.929766173	6.849061754	6.836726499	6.524214648	6.964998275	7.001000852	6.823299393
ENSG00000121542	0.034996	0.097353733	0.22537978	1.169084962	**SEC22A**	protein_coding	SEC22 homolog A, vesicle trafficking protein [Source:HGNC Symbol;Acc:HGNC:20260]	chr3	123201927	123274130	1	ensembl_havana	4.343722403	4.231032513	4.456412293	4.181339713	4.325989874	4.185767952	4.255027132	4.592099791	4.522109956
ENSG00000173465	0.03506	0.097439133	0.407875958	1.326731064	**SSSCA1**	protein_coding	Sjogren syndrome/scleroderma autoantigen 1 [Source:HGNC Symbol;Acc:HGNC:11328]	chr11	65570430	65573942	1	ensembl_havana	2.878946927	2.675008948	3.082884905	2.853343439	2.389883326	2.781800078	3.267019432	3.110498652	2.871136632
ENSG00000177830	0.035059	0.097439133	0.24163248	1.182329769	**CHID1**	protein_coding	chitinase domain containing 1 [Source:HGNC Symbol;Acc:HGNC:28474]	chr11	867860	915058	-1	ensembl_havana	5.407075517	5.286259277	5.527891757	5.211965455	5.173017357	5.473795018	5.519697305	5.525067154	5.538910811
ENSG00000135597	0.035039	0.097439133	0.19864885	1.14762305	**REPS1**	protein_coding	RALBP1 associated Eps domain containing 1 [Source:HGNC Symbol;Acc:HGNC:15578]	chr6	138903493	138988261	-1	ensembl_havana	5.567506204	5.468181779	5.66683063	5.3735117	5.582002406	5.449031232	5.641625317	5.662571744	5.696294829
ENSG00000086848	0.035053	0.097439133	0.161753925	1.118646282	**ALG9**	protein_coding	ALG9, alpha-1,2-mannosyltransferase [Source:HGNC Symbol;Acc:HGNC:15672]	chr11	111782195	111871581	-1	ensembl_havana	3.570406266	3.489529303	3.651283228	3.290778936	3.619342646	3.558466327	3.431134914	3.761841936	3.760872836
ENSG00000174442	0.035042	0.097439133	-0.200491506	-1.149089767	**ZWILCH**	protein_coding	zwilch kinetochore protein [Source:HGNC Symbol;Acc:HGNC:25468]	chr15	66504959	66550128	1	ensembl_havana	5.577262834	5.677508587	5.477017081	5.468967484	5.816112458	5.747445819	5.374361485	5.510961271	5.545728488
ENSG00000115657	0.035072	0.09745263	-0.474713453	-1.389642183	**ABCB6**	protein_coding	ATP binding cassette subfamily B member 6 (Langereis blood group) [Source:HGNC Symbol;Acc:HGNC:47]	chr2	219209766	219219017	-1	ensembl_havana	0.865283251	1.102639978	0.627926524	1.075455542	1.155163158	1.077301233	0.851734776	0.861296647	0.17074815
ENSG00000112149	0.035086	0.097471646	-0.189317695	-1.140224334	**CD83**	protein_coding	CD83 molecule [Source:HGNC Symbol;Acc:HGNC:1703]	chr6	14117256	14136918	1	ensembl_havana	5.308420759	5.403079607	5.213761911	5.473863577	5.471002233	5.26437301	5.382779675	5.204110602	5.054395457
ENSG00000106305	0.03513	0.097575539	-0.221861272	-1.16623722	**AIMP2**	protein_coding	aminoacyl tRNA synthetase complex interacting multifunctional protein 2 [Source:HGNC Symbol;Acc:HGNC:20609]	chr7	6009245	6023834	1	ensembl_havana	5.337950151	5.448880787	5.227019515	5.171350736	5.429693388	5.745598239	5.047243228	5.260469873	5.373345445
ENSG00000013441	0.035156	0.097628515	0.172288267	1.126844363	**CLK1**	protein_coding	CDC like kinase 1 [Source:HGNC Symbol;Acc:HGNC:2068]	chr2	200853009	200864744	-1	ensembl_havana	6.31755687	6.231412736	6.403701003	6.361683662	6.38594444	5.946610105	6.5361811	6.495926552	6.178995358
ENSG00000062485	0.035197	0.097725021	0.17079831	1.125681204	**CS**	protein_coding	citrate synthase [Source:HGNC Symbol;Acc:HGNC:2422]	chr12	56271699	56300392	-1	ensembl_havana	8.147490298	8.062091143	8.232889453	7.984026117	8.112859916	8.089387397	8.21791706	8.231622571	8.249128729
ENSG00000277938	0.03524	0.097768109	0.489557572	1.404014244	**AL035252.3**	lincRNA		chr20	25229150	25231933	1	havana	0.813207049	0.568428263	1.057985835	-0.03191641	1.013233389	0.723967808	0.592614899	1.063828438	1.517514168
ENSG00000011009	0.035222	0.097768109	0.213524908	1.159517752	**LYPLA2**	protein_coding	lysophospholipase II [Source:HGNC Symbol;Acc:HGNC:6738]	chr1	23790970	23795539	1	ensembl_havana	6.002047178	5.895284724	6.108809632	5.917738729	5.883953537	5.884161906	6.231021545	6.13426391	5.96114344
ENSG00000149798	0.035237	0.097768109	0.198962464	1.147872548	**CDC42EP2**	protein_coding	CDC42 effector protein 2 [Source:HGNC Symbol;Acc:HGNC:16263]	chr11	65314818	65322429	1	ensembl_havana	3.859086372	3.75960514	3.958567604	4.153128786	3.656952914	3.468733719	4.287271569	3.975515744	3.612915497
ENSG00000066697	0.035231	0.097768109	0.191303804	1.141795122	**MSANTD3**	protein_coding	Myb/SANT DNA binding domain containing 3 [Source:HGNC Symbol;Acc:HGNC:23370]	chr9	100427156	100451711	1	ensembl_havana	4.113536146	4.017884244	4.209188048	3.817901026	4.171215189	4.064536518	4.021008592	4.264198259	4.342357293
ENSG00000112282	0.035254	0.097787131	0.160893713	1.117979484	**MED23**	protein_coding	mediator complex subunit 23 [Source:HGNC Symbol;Acc:HGNC:2372]	chr6	131573966	131628229	-1	ensembl_havana	6.683224391	6.602777534	6.763671247	6.602015853	6.635446274	6.570870476	6.794602449	6.799436445	6.696974848
ENSG00000186260	0.035268	0.097808773	-0.157631195	-1.115454135	**MKL2**	protein_coding	MKL1/myocardin like 2 [Source:HGNC Symbol;Acc:HGNC:29819]	chr16	14071321	14266773	1	ensembl_havana	5.39865808	5.477473677	5.319842482	5.525080082	5.418005636	5.489335313	5.373423091	5.27630862	5.309795736
ENSG00000279058	0.035289	0.097833949	0.439038902	1.355700883	**AGAP14P**	unprocessed_pseudogene	ArfGAP with GTPase domain, ankyrin repeat and PH domain 14, pseudogene [Source:HGNC Symbol;Acc:HGNC:23660]	chr10	46337224	46358714	1	ensembl_havana	-0.522919445	-0.742438897	-0.303399994	-0.67394004	-0.967469033	-0.585907617	0.106248156	-0.52165082	-0.494797317
ENSG00000111707	0.035291	0.097833949	0.244145188	1.184390799	**SUDS3**	protein_coding	SDS3 homolog, SIN3A corepressor complex component [Source:HGNC Symbol;Acc:HGNC:29545]	chr12	118376380	118418031	1	ensembl_havana	6.262460227	6.140387633	6.384532821	6.250528263	6.215472255	5.955162381	6.408243426	6.363942346	6.381412691
ENSG00000157693	0.035328	0.097888658	0.21425386	1.160103771	**TMEM268**	protein_coding	transmembrane protein 268 [Source:HGNC Symbol;Acc:HGNC:24513]	chr9	114611206	114646422	1	ensembl_havana	4.765571592	4.658444662	4.872698522	4.47628667	4.738081868	4.760965448	4.7959082	4.816408402	5.005778964
ENSG00000117676	0.035329	0.097888658	-0.181907185	-1.134382503	**RPS6KA1**	protein_coding	ribosomal protein S6 kinase A1 [Source:HGNC Symbol;Acc:HGNC:10430]	chr1	26529761	26575030	1	ensembl_havana	7.98787997	8.078833563	7.896926377	8.266731462	8.000712275	7.969056952	8.091232171	7.894260108	7.705286853
ENSG00000167969	0.035331	0.097888658	-0.40599244	-1.325000074	**ECI1**	protein_coding	enoyl-CoA delta isomerase 1 [Source:HGNC Symbol;Acc:HGNC:2703]	chr16	2239395	2252300	-1	ensembl_havana	3.63597776	3.83897398	3.43298154	3.689093732	3.724301235	4.103526974	3.565385028	3.375620869	3.357938723
ENSG00000107249	0.035352	0.097927024	-0.691044099	-1.614451498	**GLIS3**	protein_coding	GLIS family zinc finger 3 [Source:HGNC Symbol;Acc:HGNC:28510]	chr9	3824127	4348392	-1	ensembl_havana	2.07735378	2.42287583	1.73183173	2.005254198	2.535453458	2.727919833	1.936429043	1.705026699	1.554039449
ENSG00000109919	0.035372	0.097963124	-0.164943046	-1.121121818	**MTCH2**	protein_coding	mitochondrial carrier 2 [Source:HGNC Symbol;Acc:HGNC:17587]	chr11	47617315	47642623	-1	ensembl_havana	6.752637751	6.835109274	6.670166227	6.809908244	6.853310863	6.842108715	6.606552279	6.676335661	6.727610742
ENSG00000157306	0.0354	0.098021267	0.42281034	1.34053635	**ZFHX2-AS1**	processed_transcript	ZFHX2 antisense RNA 1 [Source:HGNC Symbol;Acc:HGNC:52658]	chr14	23511760	23560778	1	havana	0.347958319	0.136553149	0.559363489	0.727424294	-0.299013651	-0.018751196	0.851734776	0.478736765	0.347618925
ENSG00000104853	0.035422	0.098065318	0.179936537	1.132834051	**CLPTM1**	protein_coding	CLPTM1, transmembrane protein [Source:HGNC Symbol;Acc:HGNC:2087]	chr19	44954585	44993341	1	ensembl_havana	6.505318951	6.415350682	6.595287219	6.37002429	6.36661726	6.509410497	6.634237113	6.525073018	6.626551527
ENSG00000262468	0.035451	0.09812516	-0.411037134	-1.329641331	**LINC01569**	lincRNA	long intergenic non-protein coding RNA 1569 [Source:HGNC Symbol;Acc:HGNC:51380]	chr16	4245825	4253789	-1	havana	1.656913792	1.862432359	1.451395226	1.833046887	1.749818205	2.004431986	1.669136731	1.452931788	1.232117157
ENSG00000132406	0.03546	0.098132547	-0.232474821	-1.174848575	**TMEM128**	protein_coding	transmembrane protein 128 [Source:HGNC Symbol;Acc:HGNC:28201]	chr4	4235542	4248212	-1	ensembl_havana	3.370399527	3.486636937	3.254162116	3.532084689	3.600163325	3.327662798	3.144758638	3.389394213	3.228333496
ENSG00000138119	0.035501	0.098148631	0.265920922	1.20240334	**MYOF**	protein_coding	myoferlin [Source:HGNC Symbol;Acc:HGNC:3656]	chr10	93306429	93482317	-1	ensembl_havana	9.107149436	8.974188975	9.240109897	9.089072699	8.864177986	8.969316241	9.178643134	9.316188111	9.225498447
ENSG00000161714	0.035498	0.098148631	0.204655339	1.152411002	**PLCD3**	protein_coding	phospholipase C delta 3 [Source:HGNC Symbol;Acc:HGNC:9061]	chr17	45108967	45133354	-1	ensembl_havana	3.976853893	3.874526223	4.079181562	3.83322245	3.877807482	3.912548737	4.166788249	4.024882977	4.04587346
ENSG00000008517	0.035519	0.098148631	0.189926494	1.140705595	**IL32**	protein_coding	interleukin 32 [Source:HGNC Symbol;Acc:HGNC:16830]	chr16	3065297	3082192	1	ensembl_havana	5.836512965	5.741549718	5.931476212	5.679991067	5.831466344	5.713191744	5.954340714	5.923834473	5.91625345
ENSG00000204650	0.035521	0.098148631	0.15633191	1.114450015	**LINC02210**	transcribed_unitary_pseudogene	long intergenic non-protein coding RNA 2210 [Source:HGNC Symbol;Acc:HGNC:26327]	chr17	45620328	45655156	1	havana	5.106638111	5.028472156	5.184804066	5.03334247	5.019577236	5.032496762	5.196901456	5.160598574	5.196912168
ENSG00000117090	0.035517	0.098148631	-0.185479188	-1.137194627	**SLAMF1**	protein_coding	signaling lymphocytic activation molecule family member 1 [Source:HGNC Symbol;Acc:HGNC:10903]	chr1	160608100	160647295	-1	ensembl_havana	8.35293019	8.445669784	8.260190596	8.778516807	8.380015568	8.178476976	8.538932403	8.150663414	8.090975969
ENSG00000180881	0.035508	0.098148631	-0.228211684	-1.171382043	**CAPS2**	protein_coding	calcyphosine 2 [Source:HGNC Symbol;Acc:HGNC:16471]	chr12	75275979	75390928	-1	ensembl_havana	1.382327798	1.49643364	1.268221956	1.553614465	1.428032914	1.507653541	1.356079599	1.046547334	1.402038935
ENSG00000100575	0.03548	0.098148631	-0.289950321	-1.222598177	**TIMM9**	protein_coding	translocase of inner mitochondrial membrane 9 [Source:HGNC Symbol;Acc:HGNC:11819]	chr14	58408494	58427614	-1	ensembl_havana	4.38542005	4.530395211	4.24044489	4.428328947	4.666128586	4.496728099	3.982139812	4.299275781	4.439919076
ENSG00000239911	0.035507	0.098148631	-0.917717833	-1.889124564	**PRKAG2-AS1**	antisense	PRKAG2 antisense RNA 1 [Source:HGNC Symbol;Acc:HGNC:40468]	chr7	151877042	151879223	1	havana	-0.46604436	-0.007185444	-0.924903276	-0.03191641	-0.036373238	0.046733314	-0.08686711	-1.24204464	-1.445798078
ENSG00000026950	0.035555	0.098225527	-0.22595547	-1.169551564	**BTN3A1**	protein_coding	butyrophilin subfamily 3 member A1 [Source:HGNC Symbol;Acc:HGNC:1138]	chr6	26402237	26415216	1	ensembl_havana	5.189489137	5.302466872	5.076511403	5.401190533	5.242697577	5.263512506	5.035426597	5.020480842	5.173626769
ENSG00000108424	0.03557	0.098247647	-0.163596207	-1.120075675	**KPNB1**	protein_coding	karyopherin subunit beta 1 [Source:HGNC Symbol;Acc:HGNC:6400]	chr17	47649476	47685505	1	ensembl_havana	9.547693393	9.629491496	9.465895289	9.632265036	9.660362223	9.595847229	9.426094421	9.496180438	9.475411007
ENSG00000130159	0.03558	0.09825544	0.267169093	1.203444069	**ECSIT**	protein_coding	ECSIT signalling integrator [Source:HGNC Symbol;Acc:HGNC:29548]	chr19	11505916	11529174	-1	ensembl_havana	4.125608434	3.992023888	4.259192981	3.926652984	3.845595809	4.20382287	4.291251919	4.226329326	4.259997696
ENSG00000239887	0.03562	0.098348145	0.325709655	1.25328077	**C1orf226**	protein_coding	chromosome 1 open reading frame 226 [Source:HGNC Symbol;Acc:HGNC:34351]	chr1	162366908	162386818	1	ensembl_havana	2.659137315	2.496282488	2.821992143	1.940704441	2.646460224	2.901682799	2.50808372	2.970908466	2.986984242
ENSG00000100243	0.035652	0.098416457	0.19871206	1.147673332	**CYB5R3**	protein_coding	cytochrome b5 reductase 3 [Source:HGNC Symbol;Acc:HGNC:2873]	chr22	42617840	42649568	-1	ensembl_havana	5.577884571	5.478528541	5.6772406	5.430868342	5.52720698	5.4775103	5.74971429	5.657600565	5.624406947
ENSG00000158486	0.035663	0.098427881	0.534697814	1.448638692	**DNAH3**	protein_coding	dynein axonemal heavy chain 3 [Source:HGNC Symbol;Acc:HGNC:2949]	chr16	20933111	21159441	-1	ensembl_havana	0.661773352	0.394424445	0.929122259	-0.14746469	0.547932369	0.782805653	0.739683594	1.210694826	0.836988357
ENSG00000247095	0.035684	0.098430907	0.326316925	1.253808422	**MIR210HG**	lincRNA	MIR210 host gene [Source:HGNC Symbol;Acc:HGNC:39524]	chr11	565660	568457	-1	havana	0.730998222	0.567839759	0.894156684	0.490295136	0.636663181	0.57656096	1.052687018	0.715834679	0.913948357
ENSG00000132467	0.035691	0.098430907	0.180523495	1.133295037	**UTP3**	protein_coding	UTP3, small subunit processome component [Source:HGNC Symbol;Acc:HGNC:24477]	chr4	70688479	70690551	1	ensembl_havana	6.425598978	6.33533723	6.515860725	6.381794423	6.347027641	6.277189626	6.510375765	6.490345497	6.546860913
ENSG00000120837	0.03569	0.098430907	-0.208571754	-1.155543647	**NFYB**	protein_coding	nuclear transcription factor Y subunit beta [Source:HGNC Symbol;Acc:HGNC:7805]	chr12	104117077	104138289	-1	ensembl_havana	4.467428134	4.571714011	4.363142257	4.573763101	4.553838016	4.587540916	4.230361879	4.401364514	4.457700378
ENSG00000186666	0.035677	0.098430907	-0.311264257	-1.240794552	**BCDIN3D**	protein_coding	BCDIN3 domain containing RNA methyltransferase [Source:HGNC Symbol;Acc:HGNC:27050]	chr12	49836039	49843129	-1	ensembl_havana	3.671372627	3.827004756	3.515740498	3.838293645	3.940150332	3.70257029	3.275081252	3.69131825	3.580821993
ENSG00000108799	0.035722	0.098495963	0.168882981	1.124187736	**EZH1**	protein_coding	enhancer of zeste 1 polycomb repressive complex 2 subunit [Source:HGNC Symbol;Acc:HGNC:3526]	chr17	42700275	42745049	-1	ensembl_havana	6.279163976	6.194722486	6.363605467	6.242899925	6.092927862	6.24833967	6.349806084	6.308840171	6.432170146
ENSG00000162521	0.035737	0.098499047	-0.155654433	-1.113926801	**RBBP4**	protein_coding	RB binding protein 4, chromatin remodeling factor [Source:HGNC Symbol;Acc:HGNC:9887]	chr1	32651142	32686211	1	ensembl_havana	8.655948354	8.73377557	8.578121137	8.691347526	8.735740756	8.774238428	8.543632621	8.582878979	8.607851811
ENSG00000137507	0.035736	0.098499047	-0.224954714	-1.168740562	**LRRC32**	protein_coding	leucine rich repeat containing 32 [Source:HGNC Symbol;Acc:HGNC:4161]	chr11	76657524	76670747	-1	ensembl_havana	5.365605073	5.47808243	5.253127716	5.407175137	5.42113164	5.605940514	5.320848202	5.193355006	5.245179939
ENSG00000124222	0.035769	0.098530765	0.177322974	1.130783686	**STX16**	protein_coding	syntaxin 16 [Source:HGNC Symbol;Acc:HGNC:11431]	chr20	58651253	58679526	1	ensembl_havana	6.968515095	6.879853608	7.057176582	6.815704824	6.941674343	6.882181656	7.057583753	7.040817232	7.073128761
ENSG00000077254	0.035768	0.098530765	0.156224846	1.114367313	**USP33**	protein_coding	ubiquitin specific peptidase 33 [Source:HGNC Symbol;Acc:HGNC:20059]	chr1	77695987	77759852	-1	ensembl_havana	6.677997633	6.59988521	6.756110056	6.556584534	6.57568398	6.667387116	6.710900762	6.747911829	6.809517578
ENSG00000213516	0.035756	0.098530765	-0.165642697	-1.12166565	**RBMXL1**	protein_coding	RNA binding motif protein, X-linked like 1 [Source:HGNC Symbol;Acc:HGNC:25073]	chr1	88979456	88992960	-1	ensembl_havana	4.864148085	4.946969433	4.781326736	4.901463638	5.001939617	4.937505044	4.729988122	4.790690888	4.823301198
ENSG00000237807	0.035781	0.098544158	-0.173596664	-1.127866775	**AC022034.2**	lincRNA		chr8	53515171	53523931	-1	havana	5.665862658	5.75266099	5.579064326	5.892973177	5.657501514	5.707508279	5.647847497	5.541351573	5.547993907
ENSG00000273356	0.035803	0.098560075	0.775616304	1.711921209	**LINC02019**	lincRNA	long intergenic non-protein coding RNA 2019 [Source:HGNC Symbol;Acc:HGNC:52854]	chr3	50669989	50672048	1	havana	-0.343034286	-0.730842438	0.044773866	-0.41068387	-0.34076431	-1.441079133	0.032074365	0.02879206	0.073455173
ENSG00000146067	0.035796	0.098560075	0.245743932	1.185704027	**FAM193B**	protein_coding	family with sequence similarity 193 member B [Source:HGNC Symbol;Acc:HGNC:25524]	chr5	177519788	177554541	-1	ensembl_havana	5.392960276	5.27008831	5.515832242	5.175363862	5.304942383	5.329958685	5.59004992	5.399676988	5.557769819
ENSG00000122481	0.03581	0.098560075	0.196331047	1.145780784	**RWDD3**	protein_coding	RWD domain containing 3 [Source:HGNC Symbol;Acc:HGNC:21393]	chr1	95234155	95247225	1	ensembl_havana	1.595410685	1.497245161	1.693576208	1.312554705	1.603393164	1.575787614	1.541278047	1.881076089	1.65837449
ENSG00000210151	0.035814	0.098560075	-0.265432805	-1.201996592	**MT-TS1**	Mt_tRNA	mitochondrially encoded tRNA serine 1 (UCN) [Source:HGNC Symbol;Acc:HGNC:7497]	chrMT	7446	7514	-1	insdc	1.93932606	2.072042462	1.806609657	2.058373044	2.220801645	1.936952697	1.926226713	1.748424202	1.745178056
ENSG00000100284	0.035828	0.098580212	0.192228834	1.142527455	**TOM1**	protein_coding	target of myb1 membrane trafficking protein [Source:HGNC Symbol;Acc:HGNC:11982]	chr22	35299275	35347994	1	ensembl_havana	4.230353187	4.13423877	4.326467604	4.122277808	4.124036797	4.156401706	4.318811065	4.40983618	4.250755567
ENSG00000107338	0.035841	0.098596292	-0.264645394	-1.201340731	**SHB**	protein_coding	SH2 domain containing adaptor protein B [Source:HGNC Symbol;Acc:HGNC:10838]	chr9	37919134	38069211	-1	ensembl_havana	3.735471752	3.867794449	3.603149055	3.550741415	3.829216193	4.223425739	3.456169357	3.584613649	3.76866416
ENSG00000148300	0.035852	0.098609305	0.191476193	1.141931564	**REXO4**	protein_coding	REX4 homolog, 3'-5' exonuclease [Source:HGNC Symbol;Acc:HGNC:12820]	chr9	133406059	133418096	-1	ensembl_havana	5.65297566	5.557237563	5.748713756	5.458301936	5.54307633	5.670334423	5.705657068	5.788103514	5.752380686
ENSG00000110324	0.035912	0.098736504	0.173631193	1.127893769	**IL10RA**	protein_coding	interleukin 10 receptor subunit alpha [Source:HGNC Symbol;Acc:HGNC:5964]	chr11	117986348	118003037	1	ensembl_havana	8.228715915	8.141900318	8.315531511	8.420673107	8.014230664	7.990797183	8.670432468	8.145346343	8.130815723
ENSG00000214562	0.035908	0.098736504	-0.329118027	-1.256245151	**NUTM2D**	protein_coding	NUT family member 2D [Source:HGNC Symbol;Acc:HGNC:23447]	chr10	87357668	87370695	1	ensembl_havana	0.06235706	0.226916074	-0.102201953	0.355307344	0.185737694	0.139703184	-0.17198403	0.130707766	-0.265329594
ENSG00000248092	0.03592	0.09873775	-0.217400638	-1.162636928	**NNT-AS1**	antisense	NNT antisense RNA 1 [Source:HGNC Symbol;Acc:HGNC:49005]	chr5	43571594	43603230	-1	havana	4.8104535	4.919153819	4.701753181	4.736070601	5.027803265	4.993587592	4.663984316	4.710667691	4.730607536
ENSG00000173011	0.035941	0.098759286	0.195436055	1.145070206	**TADA2B**	protein_coding	transcriptional adaptor 2B [Source:HGNC Symbol;Acc:HGNC:30781]	chr4	7041899	7057952	1	ensembl_havana	4.283249416	4.185531388	4.380967443	4.247639431	4.171215189	4.137739545	4.473948226	4.448177619	4.220776485
ENSG00000151292	0.035936	0.098759286	0.160410857	1.117605369	**CSNK1G3**	protein_coding	casein kinase 1 gamma 3 [Source:HGNC Symbol;Acc:HGNC:2456]	chr5	123512099	123617045	1	ensembl_havana	5.473644019	5.393438591	5.553849448	5.455004303	5.441287214	5.284024256	5.635376184	5.561261852	5.464910307
ENSG00000132326	0.035951	0.098766353	-0.210719262	-1.157265	**PER2**	protein_coding	period circadian regulator 2 [Source:HGNC Symbol;Acc:HGNC:8846]	chr2	238244038	238290102	-1	ensembl_havana	3.533706086	3.639065717	3.428346455	3.880693918	3.461295901	3.575207333	3.565385028	3.382523978	3.337130358
ENSG00000169682	0.035983	0.098799822	0.238700951	1.179929737	**SPNS1**	protein_coding	sphingolipid transporter 1 (putative) [Source:HGNC Symbol;Acc:HGNC:30621]	chr16	28974221	28984548	1	ensembl_havana	0.541703512	0.422353037	0.661053988	0.14220989	0.570631912	0.554217307	0.276538753	0.737534657	0.969088554
ENSG00000102543	0.035979	0.098799822	0.203911935	1.151817331	**CDADC1**	protein_coding	cytidine and dCMP deaminase domain containing 1 [Source:HGNC Symbol;Acc:HGNC:20299]	chr13	49247925	49293485	1	ensembl_havana	3.4555315	3.353575533	3.557487467	3.369953701	3.389700035	3.301072861	3.67911428	3.475851809	3.517496313
ENSG00000244879	0.035984	0.098799822	-0.187406175	-1.138714577	**GABPB1-AS1**	antisense	GABPB1 antisense RNA 1 [Source:HGNC Symbol;Acc:HGNC:44157]	chr15	50354959	50372202	1	havana	5.898396843	5.99209993	5.804693755	6.081730794	5.778216757	6.116352239	5.855652395	5.692738824	5.865690047
ENSG00000153922	0.036013	0.098823579	-0.172855733	-1.12728768	**CHD1**	protein_coding	chromodomain helicase DNA binding protein 1 [Source:HGNC Symbol;Acc:HGNC:1915]	chr5	98853985	98928957	-1	ensembl_havana	7.456289886	7.542717753	7.369862019	7.637544166	7.513694307	7.476914786	7.38982087	7.375237119	7.344528069
ENSG00000008441	0.036	0.098823579	-0.175652319	-1.129474985	**NFIX**	protein_coding	nuclear factor I X [Source:HGNC Symbol;Acc:HGNC:7788]	chr19	12995608	13098796	1	ensembl_havana	6.445306973	6.533133132	6.357480813	6.685989186	6.437410781	6.475999431	6.569748995	6.279555016	6.223138428
ENSG00000115484	0.036009	0.098823579	-0.181647813	-1.134178579	**CCT4**	protein_coding	chaperonin containing TCP1 subunit 4 [Source:HGNC Symbol;Acc:HGNC:1617]	chr2	61868089	61888804	-1	ensembl_havana	9.187810671	9.278634578	9.096986765	9.133841508	9.251567433	9.450494792	9.015140982	9.097312091	9.17850722
ENSG00000094975	0.036063	0.098931115	0.188530931	1.139602689	**SUCO**	protein_coding	SUN domain containing ossification factor [Source:HGNC Symbol;Acc:HGNC:1240]	chr1	172532349	172611833	1	ensembl_havana	6.870634953	6.776369488	6.964900419	6.874277387	6.824678398	6.630152677	6.96188022	7.038360644	6.894460393
ENSG00000154945	0.036066	0.098931115	0.156562332	1.114628025	**ANKRD40**	protein_coding	ankyrin repeat domain 40 [Source:HGNC Symbol;Acc:HGNC:28233]	chr17	50693190	50707924	-1	ensembl_havana	6.010331166	5.93205	6.088612333	5.95498343	5.952533043	5.888633528	6.089051251	6.124517615	6.052268132
ENSG00000270164	0.036111	0.099037528	-0.258151212	-1.19594514	**LINC01480**	lincRNA	long intergenic non-protein coding RNA 1480 [Source:HGNC Symbol;Acc:HGNC:51124]	chr19	41535183	41536904	1	havana	0.164999648	0.294075255	0.035924042	0.040274975	0.243386951	0.598563838	-0.12879794	0.097529431	0.139040636
ENSG00000257337	0.036187	0.099225184	0.198325811	1.14736611	**AC068888.1**	antisense		chr12	53014596	53054438	-1	havana	2.266866505	2.1677036	2.366029411	2.075653419	2.139629423	2.287827958	2.37188541	2.351143587	2.375059235
ENSG00000155380	0.036226	0.099312689	-0.16797644	-1.123481556	**SLC16A1**	protein_coding	solute carrier family 16 member 1 [Source:HGNC Symbol;Acc:HGNC:10922]	chr1	112911847	112957013	-1	ensembl_havana	7.122502076	7.206490296	7.038513856	7.294322703	7.243542706	7.08160548	7.065739681	7.087243062	6.962558826
ENSG00000163430	0.03627	0.099376647	0.440672553	1.357236894	**FSTL1**	protein_coding	follistatin like 1 [Source:HGNC Symbol;Acc:HGNC:3972]	chr3	120392293	120451253	-1	ensembl_havana	0.990444716	0.77010844	1.210780993	0.613726219	1.013233389	0.683365711	0.935503283	1.179325527	1.517514168
ENSG00000158552	0.036265	0.099376647	0.313300436	1.24254701	**ZFAND2B**	protein_coding	zinc finger AN1-type containing 2B [Source:HGNC Symbol;Acc:HGNC:25206]	chr2	219195237	219209651	1	ensembl_havana	3.176168913	3.019518695	3.332819131	2.892874923	3.089199779	3.076481383	3.452619495	3.19134646	3.354491438
ENSG00000140386	0.036257	0.099376647	0.167403065	1.123035136	**SCAPER**	protein_coding	S-phase cyclin A associated protein in the ER [Source:HGNC Symbol;Acc:HGNC:13081]	chr15	76347904	76905444	-1	ensembl_havana	5.163055482	5.079353949	5.246757014	4.995256012	4.986195903	5.256609934	5.100386794	5.176573382	5.463310868
ENSG00000129187	0.03628	0.099385178	-0.161012725	-1.118071713	**DCTD**	protein_coding	dCMP deaminase [Source:HGNC Symbol;Acc:HGNC:2710]	chr4	182890060	182917936	-1	ensembl_havana	6.437631926	6.518138288	6.357125563	6.556199864	6.531531558	6.466683442	6.360737772	6.363506286	6.34713263
ENSG00000179428	0.036316	0.099464597	0.192992462	1.143132363	**AC073072.1**	antisense		chr7	22725395	22727620	-1	havana	3.428124112	3.331627881	3.524620343	3.366447561	3.24901995	3.379416132	3.452619495	3.462883238	3.658358295
ENSG00000156697	0.036374	0.099587261	-0.163626714	-1.12009936	**UTP14A**	protein_coding	UTP14A, small subunit processome component [Source:HGNC Symbol;Acc:HGNC:10665]	chrX	129906121	129929761	1	ensembl_havana	5.305341365	5.387154722	5.223528008	5.260940923	5.414872844	5.485650398	5.088999215	5.209943667	5.371641141
ENSG00000183386	0.036374	0.099587261	-0.233346852	-1.175558922	**FHL3**	protein_coding	four and a half LIM domains 3 [Source:HGNC Symbol;Acc:HGNC:3704]	chr1	37996770	38005606	-1	ensembl_havana	1.754499537	1.871172963	1.637826111	1.968722995	1.96214833	1.682647563	1.905603022	1.660283167	1.347592143
ENSG00000197965	0.036464	0.099813014	0.171116106	1.125929196	**MPZL1**	protein_coding	myelin protein zero like 1 [Source:HGNC Symbol;Acc:HGNC:7226]	chr1	167721192	167791919	1	ensembl_havana	6.484178856	6.398620803	6.569736909	6.422038653	6.382340261	6.391483494	6.546208824	6.528966949	6.634034953
ENSG00000033011	0.036473	0.099818172	-0.240831039	-1.181673147	**ALG1**	protein_coding	ALG1, chitobiosyldiphosphodolichol beta-mannosyltransferase [Source:HGNC Symbol;Acc:HGNC:18294]	chr16	5033960	5087379	1	ensembl_havana	3.469545157	3.589960677	3.349129638	3.569159951	3.552495086	3.648226993	3.221855697	3.491900287	3.33363293
ENSG00000083720	0.036502	0.099820053	0.196014301	1.145529254	**OXCT1**	protein_coding	3-oxoacid CoA-transferase 1 [Source:HGNC Symbol;Acc:HGNC:8527]	chr5	41730065	41870519	-1	ensembl_havana	6.509195871	6.411188721	6.607203022	6.345746898	6.368642631	6.519176634	6.442202383	6.558603303	6.820803381
ENSG00000136319	0.036493	0.099820053	0.171967371	1.126593749	**TTC5**	protein_coding	tetratricopeptide repeat domain 5 [Source:HGNC Symbol;Acc:HGNC:19274]	chr14	20256558	20305994	-1	ensembl_havana	4.426657901	4.340674216	4.512641587	4.371743869	4.334304592	4.315974186	4.540671651	4.443233971	4.554019139
ENSG00000100412	0.036495	0.099820053	0.170692128	1.125598357	**ACO2**	protein_coding	aconitase 2 [Source:HGNC Symbol;Acc:HGNC:118]	chr22	41469125	41528989	1	ensembl_havana	6.667916805	6.582570742	6.753262869	6.58740702	6.522827615	6.63747759	6.814444823	6.703809872	6.741533912
ENSG00000132603	0.036515	0.099820053	-0.157503708	-1.11535557	**NIP7**	protein_coding	NIP7, nucleolar pre-rRNA processing protein [Source:HGNC Symbol;Acc:HGNC:24328]	chr16	69339430	69343111	1	ensembl_havana	6.377138409	6.455890263	6.298386556	6.437576592	6.498987284	6.431106914	6.268351338	6.321916789	6.304891539
ENSG00000223745	0.03651	0.099820053	-0.159541887	-1.116932411	**CCDC18-AS1**	processed_transcript	CCDC18 antisense RNA 1 [Source:HGNC Symbol;Acc:HGNC:52262]	chr1	93262186	93346025	-1	havana	5.411400328	5.491171271	5.331629385	5.69264651	5.367856086	5.413011218	5.51374772	5.187454344	5.29368609
ENSG00000159840	0.036488	0.099820053	-0.34461388	-1.269811087	**ZYX**	protein_coding	zyxin [Source:HGNC Symbol;Acc:HGNC:13200]	chr7	143381080	143391111	1	ensembl_havana	4.88762846	5.0599354	4.71532152	5.14294186	5.022667496	5.014196845	5.063625579	4.670338547	4.412000434
ENSG00000101464	0.036533	0.0998372	0.194689303	1.144477661	**PIGU**	protein_coding	phosphatidylinositol glycan anchor biosynthesis class U [Source:HGNC Symbol;Acc:HGNC:15791]	chr20	34560542	34698790	-1	ensembl_havana	4.926610075	4.829265423	5.023954727	4.701701975	4.913697407	4.872396888	4.853573765	5.035887519	5.182402895
ENSG00000160058	0.036545	0.0998372	0.161949046	1.118797586	**BSDC1**	protein_coding	BSD domain containing 1 [Source:HGNC Symbol;Acc:HGNC:25501]	chr1	32365103	32394731	-1	ensembl_havana	7.282807329	7.201832806	7.363781851	7.380496368	7.1615746	7.063427449	7.495162286	7.336879796	7.259303473
ENSG00000230524	0.036548	0.0998372	-0.198334839	-1.14737329	**COL6A4P1**	transcribed_unprocessed_pseudogene	collagen type VI alpha 4 pseudogene 1 [Source:HGNC Symbol;Acc:HGNC:33484]	chr3	15151833	15205959	-1	ensembl_havana	0.911636078	1.010803497	0.812468658	0.832812769	1.046001934	1.153595787	0.64332197	0.737534657	1.056549347
ENSG00000146592	0.036549	0.0998372	-1.131571563	-2.190972783	**CREB5**	protein_coding	cAMP responsive element binding protein 5 [Source:HGNC Symbol;Acc:HGNC:16844]	chr7	28299321	28825894	1	ensembl_havana	0.021962507	0.587748288	-0.543823275	-0.06941355	0.679062629	1.153595787	-0.21650287	-0.52165082	-0.893316134
ENSG00000258559	0.036571	0.099863273	0.214768116	1.16051737	**AC005519.1**	sense_overlapping		chr14	74289127	74294425	-1	havana	1.409449266	1.302065208	1.516833324	1.43811357	1.270574017	1.197508038	1.619344613	1.625790657	1.305364703
ENSG00000174238	0.036572	0.099863273	0.167819488	1.123359338	**PITPNA**	protein_coding	phosphatidylinositol transfer protein alpha [Source:HGNC Symbol;Acc:HGNC:9001]	chr17	1517718	1562816	-1	ensembl_havana	6.756763384	6.672853641	6.840673128	6.746246845	6.646172538	6.626141539	6.850971486	6.831320612	6.839727287
ENSG00000188283	0.036589	0.099888257	0.286920056	1.220032902	**ZNF383**	protein_coding	zinc finger protein 383 [Source:HGNC Symbol;Acc:HGNC:18609]	chr19	37217926	37248738	1	ensembl_havana	3.028679866	2.885219838	3.172139893	3.075752348	2.966188404	2.613718761	3.153531275	3.260427607	3.102460798
ENSG00000163605	0.036605	0.099907751	-0.174521908	-1.128590342	**PPP4R2**	protein_coding	protein phosphatase 4 regulatory subunit 2 [Source:HGNC Symbol;Acc:HGNC:18296]	chr3	72996785	73069199	1	ensembl_havana	7.192014984	7.279275938	7.104754029	7.361688734	7.364577383	7.111561696	7.161535003	7.136821656	7.015905428
ENSG00000203880	0.03661	0.099907751	-0.234484012	-1.176485886	**PCMTD2**	protein_coding	protein-L-isoaspartate (D-aspartate) O-methyltransferase domain containing 2 [Source:HGNC Symbol;Acc:HGNC:15882]	chr20	64255695	64287821	1	ensembl_havana	7.370230991	7.487472998	7.252988985	7.754992349	7.442023688	7.265402956	7.368079777	7.212135928	7.178751251
ENSG00000157036	0.036628	0.099939628	0.165037209	1.121194995	**EXOG**	protein_coding	exo/endonuclease G [Source:HGNC Symbol;Acc:HGNC:3347]	chr3	38496127	38542161	1	ensembl_havana	5.141595335	5.05907673	5.22411394	4.897824991	5.062249882	5.217155317	5.079823989	5.170603539	5.421914291
ENSG00000250286	0.036649	0.09997614	-0.385126836	-1.305974598	**AC021491.2**	processed_transcript		chr17	50537294	50538754	-1	havana	0.619135535	0.811698953	0.426572117	0.872903038	0.760296981	0.80189684	0.210814386	0.693803314	0.375098651
ENSG00000101868	0.036672	0.10001966	-0.155659477	-1.113930696	**POLA1**	protein_coding	DNA polymerase alpha 1, catalytic subunit [Source:HGNC Symbol;Acc:HGNC:9173]	chrX	24693919	24996986	1	ensembl_havana	6.600787551	6.678617289	6.522957813	6.552347515	6.640819375	6.842684978	6.38095571	6.507024307	6.680893421
ENSG00000161010	0.036688	0.100045616	0.176174397	1.12988379	**MRNIP**	protein_coding	MRN complex interacting protein [Source:HGNC Symbol;Acc:HGNC:30817]	chr5	179835133	179862173	-1	ensembl_havana	5.725769771	5.637682572	5.813856969	5.490064912	5.625336915	5.79764589	5.730054861	5.817055082	5.894460966
ENSG00000100567	0.036714	0.100097547	-0.172725109	-1.127185618	**PSMA3**	protein_coding	proteasome subunit alpha 3 [Source:HGNC Symbol;Acc:HGNC:9532]	chr14	58244831	58272012	1	ensembl_havana	7.552923018	7.639285572	7.466560464	7.697375882	7.635604826	7.58487601	7.450255211	7.517256429	7.432169751
ENSG00000144118	0.036731	0.100124874	-0.215014155	-1.160715303	**RALB**	protein_coding	RAS like proto-oncogene B [Source:HGNC Symbol;Acc:HGNC:9840]	chr2	120240064	120294713	1	ensembl_havana	9.333631044	9.441138122	9.226123967	9.545734193	9.356835189	9.420844984	9.19666045	9.262063092	9.219648358
ENSG00000250801	0.036763	0.100194123	0.615994252	1.532613854	**AC138965.1**	antisense		chr5	175972611	175985856	1	havana	-0.337329917	-0.645327043	-0.029332791	-0.85854608	-0.842251242	-0.235183806	0.308311185	-0.32890884	-0.067400719

ENSG00000261455	0.03679	0.100248336	-0.407317929	-1.326217989	**LINC01003**	lincRNA	long intergenic non-protein coding RNA 1003 [Source:HGNC Symbol;Acc:HGNC:48957]	chr7	152463786	152465549	1	havana	0.699523507	0.903182471	0.495864542	1.022973463	0.740413176	0.946160774	0.762800879	0.554044597	0.17074815
ENSG00000102904	0.036821	0.100314119	0.305220593	1.235607551	**TSNAXIP1**	protein_coding	translin associated factor X interacting protein 1 [Source:HGNC Symbol;Acc:HGNC:18586]	chr16	67806765	67832148	1	ensembl_havana	0.934778885	0.782168588	1.087389181	0.791576534	0.89230127	0.662627962	1.033805522	1.01135145	1.21701057
ENSG00000223855	0.036862	0.100368422	0.283665824	1.217284023	**AC147651.1**	lincRNA		chr7	520391	525232	1	havana	1.483754603	1.341921691	1.625587515	1.327057633	1.324992307	1.373715134	1.797815913	1.637380031	1.441566602
ENSG00000120784	0.03685	0.100368422	0.200338888	1.148968215	**ZFP30**	protein_coding	ZFP30 zinc finger protein [Source:HGNC Symbol;Acc:HGNC:29555]	chr19	37613749	37692337	-1	ensembl_havana	3.08954259	2.989373146	3.189712034	2.765560239	3.202541664	3.000017535	3.006334834	3.279044008	3.283757261
ENSG00000210195	0.036857	0.100368422	-0.231935398	-1.174409381	**MT-TT**	Mt_tRNA	mitochondrially encoded tRNA threonine [Source:HGNC Symbol;Acc:HGNC:7499]	chrMT	15888	15953	1	insdc	3.146442355	3.262410054	3.030474656	3.092827025	3.327752331	3.366650805	2.770081475	3.015998475	3.305344019
ENSG00000244038	0.036889	0.100422473	-0.158551718	-1.116166087	**DDOST**	protein_coding	dolichyl-diphosphooligosaccharide--protein glycosyltransferase non-catalytic subunit [Source:HGNC Symbol;Acc:HGNC:2728]	chr1	20651767	20661544	-1	ensembl_havana	8.20581405	8.285089909	8.126538191	8.270488184	8.253435162	8.331346383	8.115567921	8.126195218	8.137851434
ENSG00000196787	0.0369	0.100431844	0.30002114	1.231162454	**HIST1H2AG**	protein_coding	histone cluster 1 H2A family member g [Source:HGNC Symbol;Acc:HGNC:4737]	chr6	27133042	27135291	1	ensembl_havana	3.390792815	3.240782244	3.540803385	3.203079534	3.344313992	3.174953208	3.744444859	3.590587736	3.287377559
ENSG00000157782	0.036944	0.100490662	0.473424182	1.388400877	**CABP1**	protein_coding	calcium binding protein 1 [Source:HGNC Symbol;Acc:HGNC:1384]	chr12	120640552	120667324	1	ensembl_havana	0.30101299	0.064300899	0.53772508	0.326725152	-0.18055577	0.046733314	0.36983092	0.426254992	0.817089329
ENSG00000006015	0.036956	0.100490662	0.234013813	1.17610251	**REX1BD**	protein_coding	required for excision 1-B domain containing [Source:HGNC Symbol;Acc:HGNC:26098]	chr19	18588685	18592336	1	ensembl_havana	3.007187269	2.890180362	3.124194175	2.802355359	2.953290123	2.914895605	3.192361093	3.175535294	3.004686139
ENSG00000101407	0.036948	0.100490662	0.165122186	1.121261037	**TTI1**	protein_coding	TELO2 interacting protein 1 [Source:HGNC Symbol;Acc:HGNC:29029]	chr20	37983007	38033468	-1	ensembl_havana	6.962383637	6.879822544	7.04494473	6.719699546	6.847222992	7.072545095	6.928088274	7.026290073	7.180455844
ENSG00000165733	0.036954	0.100490662	-0.180758633	-1.133479763	**BMS1**	protein_coding	BMS1, ribosome biogenesis factor [Source:HGNC Symbol;Acc:HGNC:23505]	chr10	42782801	42834937	1	ensembl_havana	7.417351974	7.507731291	7.326972657	7.505303239	7.490817501	7.527073132	7.231045124	7.350366156	7.399506692
ENSG00000198053	0.03695	0.100490662	-0.304963151	-1.235387083	**SIRPA**	protein_coding	signal regulatory protein alpha [Source:HGNC Symbol;Acc:HGNC:9662]	chr20	1894167	1940592	1	ensembl_havana	0.875904199	1.028385775	0.723422624	0.297565234	1.351450576	1.436141515	0.24405081	0.975275413	0.950941648
ENSG00000146411	0.03698	0.100526851	0.411675232	1.330229557	**SLC2A12**	protein_coding	solute carrier family 2 member 12 [Source:HGNC Symbol;Acc:HGNC:18067]	chr6	133988697	134052636	-1	ensembl_havana	0.846944792	0.641107175	1.052782408	0.41082706	0.636663181	0.875831286	1.178360195	0.975275413	1.004711615
ENSG00000010295	0.036984	0.100526851	0.218650375	1.163644501	**IFFO1**	protein_coding	intermediate filament family orphan 1 [Source:HGNC Symbol;Acc:HGNC:24970]	chr12	6538375	6556083	-1	ensembl_havana	5.167548323	5.058223135	5.276873511	5.003179961	5.143039318	5.028450127	5.351669833	5.226343752	5.252606948
ENSG00000240898	0.036996	0.100541193	-0.293525403	-1.225631603	**AC132942.1**	processed_pseudogene		chr17	3665220	3665702	1	havana	1.938555192	2.085317893	1.79179249	2.711312495	1.882741771	1.661899414	2.270688985	1.491756274	1.61293221
ENSG00000232850	0.037017	0.100559949	0.653848861	1.573360054	**PTGES2-AS1**	lincRNA	PTGES2 antisense RNA 1 (head to head) [Source:HGNC Symbol;Acc:HGNC:48711]	chr9	128128529	128130628	1	havana	-0.618543875	-0.945468306	-0.291619445	-0.85854608	-1.178380098	-0.799478738	-0.57379957	0.097529431	-0.398588198
ENSG00000278376	0.037016	0.100559949	-0.242539658	-1.183073461	**AP004609.3**	lincRNA		chr11	118791254	118793137	1	havana	0.374126223	0.495396052	0.252856394	0.852997155	0.378171619	0.255019383	0.428834256	0.256279753	0.073455173
ENSG00000113387	0.037063	0.100667948	-0.245082682	-1.185160691	**SUB1**	protein_coding	SUB1 homolog, transcriptional regulator [Source:HGNC Symbol;Acc:HGNC:19985]	chr5	32531633	32604079	1	ensembl_havana	7.631888333	7.754429674	7.509346993	7.864837847	7.759325575	7.639125602	7.477585187	7.478517287	7.571938504
ENSG00000277462	0.03711	0.100776351	-0.308874083	-1.238740577	**ZNF670**	protein_coding	zinc finger protein 670 [Source:HGNC Symbol;Acc:HGNC:28167]	chr1	247036784	247078755	-1	ensembl_havana	4.03106742	4.185504462	3.876630379	4.10133777	4.278520569	4.176655048	3.908904805	4.116753044	3.604233288
ENSG00000271270	0.037145	0.100836052	0.183931893	1.135975636	**TMCC1-AS1**	antisense	TMCC1 antisense RNA 1 (head to head) [Source:HGNC Symbol;Acc:HGNC:49060]	chr3	129893871	129918575	1	havana	3.147714215	3.055748269	3.239680161	2.654944978	3.187934094	3.324365734	2.936891701	3.340601002	3.441547782
ENSG00000143126	0.037146	0.100836052	-0.21849323	-1.163517758	**CELSR2**	protein_coding	cadherin EGF LAG seven-pass G-type receptor 2 [Source:HGNC Symbol;Acc:HGNC:3231]	chr1	109250019	109275750	1	ensembl_havana	7.000722492	7.109969107	6.891475877	7.358383072	6.876554919	7.094969331	7.187928593	6.760221348	6.726277691
ENSG00000181619	0.037171	0.100878118	-0.254536371	-1.192952311	**GPR135**	protein_coding	G protein-coupled receptor 135 [Source:HGNC Symbol;Acc:HGNC:19991]	chr14	59429022	59465342	-1	havana	2.368642962	2.495911148	2.241374776	2.26832323	2.552658819	2.666751395	2.10871413	2.097907935	2.517502265
ENSG00000161249	0.037176	0.100878118	-0.295157303	-1.227018757	**DMKN**	protein_coding	dermokine [Source:HGNC Symbol;Acc:HGNC:25063]	chr19	35497220	35513658	-1	ensembl_havana	3.572617796	3.720196448	3.425039145	3.230339436	3.893647572	4.036602336	3.183822111	3.472620579	3.618674744
ENSG00000188677	0.037307	0.101213904	-0.162559784	-1.119271308	**PARVB**	protein_coding	parvin beta [Source:HGNC Symbol;Acc:HGNC:14653]	chr22	43999211	44172949	1	ensembl_havana	4.555988149	4.637268041	4.474708257	4.646396549	4.602839827	4.662567747	4.520468078	4.387705518	4.515951176
ENSG00000156853	0.03733	0.1012567	-0.194893415	-1.144639592	**ZNF689**	protein_coding	zinc finger protein 689 [Source:HGNC Symbol;Acc:HGNC:25173]	chr16	30602558	30624012	-1	ensembl_havana	4.331158551	4.428605258	4.233711844	4.382198518	4.489776126	4.413841131	4.305097297	4.251056902	4.144981331
ENSG00000197226	0.037351	0.101262543	0.201941141	1.150244965	**TBC1D9B**	protein_coding	TBC1 domain family member 9B [Source:HGNC Symbol;Acc:HGNC:29097]	chr5	179862066	179907859	-1	ensembl_havana	6.915088671	6.8141181	7.016059241	6.764343703	6.705386019	6.972624579	7.02866934	6.974974982	7.044533402
ENSG00000138399	0.037345	0.101262543	-0.243468758	-1.183835609	**FASTKD1**	protein_coding	FAST kinase domains 1 [Source:HGNC Symbol;Acc:HGNC:26150]	chr2	169529749	169573875	-1	ensembl_havana	4.967802273	5.089536652	4.846067894	5.038859572	5.063250672	5.166499713	4.612421026	4.944381572	4.981401085
ENSG00000105173	0.037353	0.101262543	-0.25074506	-1.189821423	**CCNE1**	protein_coding	cyclin E1 [Source:HGNC Symbol;Acc:HGNC:1589]	chr19	29811898	29824308	1	ensembl_havana	5.286454634	5.411827164	5.161082104	5.495692667	5.502333036	5.237455787	5.136234527	5.16460889	5.182402895
ENSG00000198728	0.037369	0.101286594	0.195771591	1.145336553	**LDB1**	protein_coding	LIM domain binding 1 [Source:HGNC Symbol;Acc:HGNC:6532]	chr10	102107560	102120453	-1	ensembl_havana	5.807237251	5.709351456	5.905123047	5.724492672	5.725457147	5.678104549	5.948681622	5.80554408	5.96114344
ENSG00000182154	0.03743	0.101433728	-0.247168713	-1.186875586	**MRPL41**	protein_coding	mitochondrial ribosomal protein L41 [Source:HGNC Symbol;Acc:HGNC:14492]	chr9	137551199	137552555	1	ensembl_havana	0.352973882	0.476558239	0.229389526	0.540941683	0.501432867	0.387300166	0.485518989	0.163140205	0.039509383
ENSG00000166170	0.03747	0.101523431	-0.181015997	-1.133681983	**BAG5**	protein_coding	BCL2 associated athanogene 5 [Source:HGNC Symbol;Acc:HGNC:941]	chr14	103556544	103562831	-1	ensembl_havana	6.538273984	6.628781983	6.447765986	6.749615287	6.585469603	6.551261058	6.473192059	6.444902334	6.425203564
ENSG00000119844	0.037502	0.10158976	0.154243375	1.112837834	**AFTPH**	protein_coding	aftiphilin [Source:HGNC Symbol;Acc:HGNC:25951]	chr2	64524305	64593005	1	ensembl_havana	7.267157354	7.190035666	7.344279041	7.37439983	7.138969694	7.056737476	7.50825942	7.304988215	7.219589488
ENSG00000258890	0.037515	0.101596168	0.176149236	1.129864085	**CEP95**	protein_coding	centrosomal protein 95 [Source:HGNC Symbol;Acc:HGNC:25141]	chr17	64506588	64542461	1	ensembl_havana	6.040085453	5.952010835	6.128160072	5.897231651	5.991432723	5.967368131	6.046119518	6.110033564	6.228327133
ENSG00000137265	0.037519	0.101596168	-0.153309458	-1.112117681	**IRF4**	protein_coding	interferon regulatory factor 4 [Source:HGNC Symbol;Acc:HGNC:6119]	chr6	391739	411447	1	ensembl_havana	9.458718557	9.535373286	9.382063828	9.749580012	9.463210786	9.39332906	9.605835189	9.298789293	9.241567001
ENSG00000029364	0.037525	0.101596168	-0.176823498	-1.130392264	**SLC39A9**	protein_coding	solute carrier family 39 member 9 [Source:HGNC Symbol;Acc:HGNC:20182]	chr14	69398015	69462388	1	ensembl_havana	6.471918723	6.560330472	6.383506974	6.641353053	6.582331457	6.457306905	6.396281891	6.391579497	6.362659534
ENSG00000152133	0.037543	0.101625158	0.166866558	1.12261758	**GPATCH11**	protein_coding	G-patch domain containing 11 [Source:HGNC Symbol;Acc:HGNC:26768]	chr2	37084451	37099244	1	ensembl_havana	6.600670174	6.517236896	6.684103453	6.529406852	6.603124636	6.419179198	6.679735848	6.722633176	6.649941336
ENSG00000085721	0.037576	0.101695588	-0.165061296	-1.121213714	**RRN3**	protein_coding	RRN3 homolog, RNA polymerase I transcription factor [Source:HGNC Symbol;Acc:HGNC:30346]	chr16	15060022	15094317	-1	ensembl_havana	6.442129194	6.524659842	6.359598546	6.682468678	6.457341756	6.434169092	6.455565265	6.321467839	6.301762534
ENSG00000133119	0.037622	0.101799774	-0.167684853	-1.123254509	**RFC3**	protein_coding	replication factor C subunit 3 [Source:HGNC Symbol;Acc:HGNC:9971]	chr13	33818049	33966558	1	ensembl_havana	5.892625047	5.976467473	5.808782621	5.765001948	6.046619148	6.117781325	5.651722776	5.887899762	5.886725323
ENSG00000101246	0.037639	0.101826254	0.256296671	1.194408776	**ARFRP1**	protein_coding	ADP ribosylation factor related protein 1 [Source:HGNC Symbol;Acc:HGNC:662]	chr20	63698642	63708025	-1	ensembl_havana	5.9080101	5.779861764	6.036158435	5.756990578	5.632771561	5.949823153	6.137390862	5.966955648	6.004128796
ENSG00000147874	0.03765	0.101835283	-0.158210552	-1.115902169	**HAUS6**	protein_coding	HAUS augmin like complex subunit 6 [Source:HGNC Symbol;Acc:HGNC:25948]	chr9	19053143	19103119	-1	ensembl_havana	6.826288915	6.905394191	6.747183639	6.879514062	6.910594549	6.926073963	6.69521471	6.792974172	6.753362035
ENSG00000111877	0.037656	0.101835283	-0.221053878	-1.165584726	**MCM9**	protein_coding	minichromosome maintenance 9 homologous recombination repair factor [Source:HGNC Symbol;Acc:HGNC:21484]	chr6	118813442	118935162	-1	ensembl_havana	3.73853189	3.849058829	3.628004951	3.843347077	3.793477994	3.910351415	3.558795999	3.729720342	3.595498512
ENSG00000119487	0.037719	0.101984303	0.182522854	1.134866703	**MAPKAP1**	protein_coding	mitogen-activated protein kinase associated protein 1 [Source:HGNC Symbol;Acc:HGNC:18752]	chr9	125437393	125707234	-1	ensembl_havana	6.478795144	6.387533717	6.570056571	6.2294525	6.441265106	6.491883544	6.509094664	6.592860819	6.60821423
ENSG00000267248	0.03774	0.10200479	0.178215972	1.131483834	**AC025048.2**	processed_transcript		chr17	60126535	60135644	-1	havana	4.88925886	4.800150874	4.978366847	4.865899597	4.862875433	4.671677592	5.096979948	4.950201315	4.887919276
ENSG00000099901	0.037738	0.10200479	-0.180700908	-1.133434411	**RANBP1**	protein_coding	RAN binding protein 1 [Source:HGNC Symbol;Acc:HGNC:9847]	chr22	20115938	20127357	1	ensembl_havana	4.957333997	5.047684452	4.866983543	4.982715487	5.079169596	5.081168271	4.81400461	4.810021875	4.976924144
ENSG00000167216	0.037787	0.102110349	-0.20605647	-1.153530755	**KATNAL2**	protein_coding	katanin catalytic subunit A1 like 2 [Source:HGNC Symbol;Acc:HGNC:25387]	chr18	46917492	47102243	1	ensembl_havana	1.251108981	1.354137216	1.148080746	0.812341977	1.270574017	1.979495653	0.716189844	1.080904991	1.647147403
ENSG00000171552	0.037807	0.102146671	0.168260095	1.12370247	**BCL2L1**	protein_coding	BCL2 like 1 [Source:HGNC Symbol;Acc:HGNC:992]	chr20	31664452	31723989	-1	ensembl_havana	9.826457119	9.742327072	9.910587167	10.07665535	9.749249082	9.401076785	10.25454096	9.852144145	9.625076392
ENSG00000173020	0.037841	0.102205819	-0.158705202	-1.116284839	**GRK2**	protein_coding	G protein-coupled receptor kinase 2 [Source:HGNC Symbol;Acc:HGNC:289]	chr11	67266410	67286556	1	ensembl_havana	6.527171637	6.606524238	6.447819035	6.728944381	6.53514276	6.555485572	6.614119418	6.353440377	6.375897312
ENSG00000127124	0.037843	0.102205819	-0.216230968	-1.161694699	**HIVEP3**	protein_coding	human immunodeficiency virus type I enhancer binding protein 3 [Source:HGNC Symbol;Acc:HGNC:13561]	chr1	41506365	42035925	-1	havana	5.781167279	5.889282764	5.673051795	5.993565436	5.644855307	6.029427548	5.834668935	5.521944411	5.66254204
ENSG00000123200	0.037854	0.10221525	-0.166305035	-1.122180722	**ZC3H13**	protein_coding	zinc finger CCCH-type containing 13 [Source:HGNC Symbol;Acc:HGNC:20368]	chr13	45954465	46052759	-1	ensembl_havana	6.443996835	6.527149352	6.360844317	6.559658205	6.469471503	6.552318347	6.330111325	6.351242811	6.401178816
ENSG00000162923	0.037874	0.102243177	0.191053979	1.14159742	**WDR26**	protein_coding	WD repeat domain 26 [Source:HGNC Symbol;Acc:HGNC:21208]	chr1	224385143	224437033	-1	ensembl_havana	8.594396247	8.498869257	8.689923237	8.707978977	8.467377663	8.321251132	8.789534998	8.684741662	8.59549305
ENSG00000213967	0.037878	0.102243177	0.179808876	1.132733814	**ZNF726**	protein_coding	zinc finger protein 726 [Source:HGNC Symbol;Acc:HGNC:32462]	chr19	23914876	23945159	1	ensembl_havana	2.890740147	2.800835709	2.980644586	2.484284696	2.822274848	3.095947583	2.638787485	3.093770837	3.209375435
ENSG00000096093	0.037906	0.10229922	0.237679969	1.179095007	**EFHC1**	protein_coding	EF-hand domain containing 1 [Source:HGNC Symbol;Acc:HGNC:16406]	chr6	52362123	52529886	1	ensembl_havana	6.37365023	6.254810245	6.492490214	6.244333314	6.3568557	6.16324172	6.62519914	6.427067938	6.425203564
ENSG00000168002	0.037934	0.102353816	0.228720154	1.171794963	**POLR2G**	protein_coding	RNA polymerase II subunit G [Source:HGNC Symbol;Acc:HGNC:9194]	chr11	62761544	62766710	1	ensembl_havana	6.052276048	5.937915971	6.166636125	6.007701431	5.954152498	5.851893985	6.175036933	6.076902789	6.247968653
ENSG00000203666	0.037951	0.102380103	0.385002443	1.305861998	**EFCAB2**	protein_coding	EF-hand calcium binding domain 2 [Source:HGNC Symbol;Acc:HGNC:28166]	chr1	244969705	245127164	1	ensembl_havana	1.205588897	1.013087675	1.398090118	0.565613454	1.428032914	1.045616658	1.261864131	1.517070703	1.415335521
ENSG00000101546	0.03798	0.10242004	0.371748186	1.293919788	**RBFA**	protein_coding	ribosome binding factor A [Source:HGNC Symbol;Acc:HGNC:26120]	chr18	80034358	80046397	1	ensembl_havana	2.86110959	2.675235497	3.046983683	2.602259737	2.440056551	2.983390203	2.858581149	3.143383423	3.138986475
ENSG00000282608	0.037975	0.10242004	-0.337376731	-1.263457145	**ADORA3**	protein_coding	adenosine A3 receptor [Source:HGNC Symbol;Acc:HGNC:268]	chr1	111499429	111503760	-1	ensembl_havana	0.99911767	1.167806035	0.830429305	1.222335231	1.013233389	1.267849486	1.178360195	0.578298274	0.734629445
ENSG00000152503	0.038004	0.102441218	0.341454334	1.267033205	**TRIM36**	protein_coding	tripartite motif containing 36 [Source:HGNC Symbol;Acc:HGNC:16280]	chr5	115124762	115180546	-1	ensembl_havana	3.068789382	2.898062215	3.239516548	2.245600406	3.269976654	3.178609585	2.89041725	3.453079729	3.375052666
ENSG00000170448	0.038009	0.102441218	0.154405064	1.112962562	**NFXL1**	protein_coding	nuclear transcription factor, X-box binding like 1 [Source:HGNC Symbol;Acc:HGNC:18726]	chr4	47847233	47914667	-1	ensembl_havana	4.519589033	4.442386502	4.596791565	4.325543942	4.432064119	4.569551444	4.480935426	4.608385126	4.701054144
ENSG00000102390	0.037998	0.102441218	-0.160223987	-1.117460617	**PBDC1**	protein_coding	polysaccharide biosynthesis domain containing 1 [Source:HGNC Symbol;Acc:HGNC:28790]	chrX	76172936	76178204	1	ensembl_havana	4.290928432	4.371040426	4.210816439	4.230383872	4.532316791	4.350420613	4.100283535	4.322794032	4.20937175
ENSG00000123136	0.038029	0.102475395	-0.16131015	-1.118302237	**DDX39A**	protein_coding	DExD-box helicase 39A [Source:HGNC Symbol;Acc:HGNC:17821]	chr19	14408819	14419383	-1	ensembl_havana	6.664870127	6.745525202	6.584215052	6.597536563	6.780026585	6.859012459	6.439066486	6.663285844	6.650292827
ENSG00000243406	0.038065	0.102524519	0.577074189	1.49182074	**MRPS31P5**	transcribed_unprocessed_pseudogene	mitochondrial ribosomal protein S31 pseudogene 5 [Source:HGNC Symbol;Acc:HGNC:39253]	chr13	52167709	52194465	-1	havana	0.270147492	-0.018389602	0.558684587	0.004630787	-0.521012188	0.461212593	0.592614899	0.529376201	0.554062661
ENSG00000147533	0.038083	0.102524519	0.205563729	1.153136843	**GOLGA7**	protein_coding	golgin A7 [Source:HGNC Symbol;Acc:HGNC:24876]	chr8	41490396	41510980	1	ensembl_havana	5.426330473	5.323548609	5.529112337	5.223639564	5.44359484	5.303411421	5.554888594	5.538263917	5.4941845
ENSG00000105193	0.038073	0.102524519	0.187475978	1.138769673	**RPS16**	protein_coding	ribosomal protein S16 [Source:HGNC Symbol;Acc:HGNC:10396]	chr19	39433207	39435948	-1	ensembl_havana	8.741955608	8.648217619	8.835693597	8.547040372	8.770317371	8.627295113	8.775395935	8.84996614	8.881718714
ENSG00000178567	0.038078	0.102524519	0.160079481	1.117348693	**EPM2AIP1**	protein_coding	EPM2A interacting protein 1 [Source:HGNC Symbol;Acc:HGNC:19735]	chr3	36985043	36993168	-1	havana	6.797215123	6.717175383	6.877254864	6.669723242	6.741783219	6.740019686	6.802641893	6.951375543	6.877747155
ENSG00000163915	0.038061	0.102524519	-0.365720946	-1.288525373	**IGF2BP2-AS1**	antisense	IGF2BP2 antisense RNA 1 [Source:HGNC Symbol;Acc:HGNC:32674]	chr3	185712528	185729787	1	havana	1.577842554	1.760703027	1.394982081	1.477650254	1.808291156	1.996167673	1.325353043	1.554228498	1.305364703
ENSG00000168078	0.038121	0.102607721	-0.235304391	-1.177155076	**PBK**	protein_coding	PDZ binding kinase [Source:HGNC Symbol;Acc:HGNC:18282]	chr8	27809620	27838095	-1	ensembl_havana	5.801837638	5.919489833	5.684185442	5.636258325	6.129698032	5.992513143	5.535723582	5.725685002	5.791147742
ENSG00000249846	0.038173	0.102729787	0.236381199	1.178034017	**LINC02021**	lincRNA	long intergenic non-protein coding RNA 2021 [Source:HGNC Symbol;Acc:HGNC:52856]	chr3	130112550	130120579	1	havana	1.866041131	1.747850531	1.98423173	1.705645971	1.699212533	1.838693089	1.763844323	2.002551439	2.186299428
ENSG00000197714	0.038222	0.102842788	-0.182461194	-1.134818201	**ZNF460**	protein_coding	zinc finger protein 460 [Source:HGNC Symbol;Acc:HGNC:21628]	chr19	57280051	57293569	1	ensembl_havana	2.908255618	2.999486215	2.817025021	2.78670079	2.944626756	3.2671311	2.632526022	2.8315648	2.986984242
ENSG00000124275	0.03823	0.102844075	-0.172056139	-1.126663069	**MTRR**	protein_coding	5-methyltetrahydrofolate-homocysteine methyltransferase reductase [Source:HGNC Symbol;Acc:HGNC:7473]	chr5	7851186	7906025	1	ensembl_havana	5.793722077	5.879750146	5.707694007	5.855321729	5.81075935	5.973169361	5.61090185	5.705181976	5.806998196
ENSG00000120053	0.038255	0.102872324	0.207426848	1.154626984	**GOT1**	protein_coding	glutamic-oxaloacetic transaminase 1 [Source:HGNC Symbol;Acc:HGNC:4432]	chr10	99396870	99430624	-1	ensembl_havana	6.093897808	5.990184384	6.197611231	5.859692633	6.09977211	6.011088409	6.205411603	6.216243585	6.171178506
ENSG00000117009	0.038255	0.102872324	-0.448619443	-1.364733678	**KMO**	protein_coding	kynurenine 3-monooxygenase [Source:HGNC Symbol;Acc:HGNC:6381]	chr1	241532134	241595642	1	ensembl_havana	3.751515669	3.97582539	3.527205947	4.798547527	3.935785518	3.193143125	4.299179827	3.151488815	3.1309492
ENSG00000126945	0.038271	0.102895416	-0.206963283	-1.154256041	**HNRNPH2**	protein_coding	heterogeneous nuclear ribonucleoprotein H2 [Source:HGNC Symbol;Acc:HGNC:5042]	chrX	101408295	101414133	1	ensembl_havana	7.2562478	7.359729441	7.152766159	7.559854513	7.288952772	7.230381039	7.214110082	7.166871078	7.077317317
ENSG00000166507	0.038303	0.102952038	0.436800074	1.353598687	**NDST2**	protein_coding	N-deacetylase and N-sulfotransferase 2 [Source:HGNC Symbol;Acc:HGNC:7681]	chr10	73801911	73811798	-1	ensembl_havana	-0.113748155	-0.332148192	0.104651882	-0.22998119	-0.18055577	-0.585907617	-0.17198403	0.315191528	0.17074815
ENSG00000008130	0.038306	0.102952038	0.17764602	1.131036917	**NADK**	protein_coding	NAD kinase [Source:HGNC Symbol;Acc:HGNC:29831]	chr1	1751232	1780457	-1	ensembl_havana	5.586724855	5.497901845	5.675547864	5.457478234	5.461165074	5.575062226	5.723441731	5.616470379	5.686731483
ENSG00000231528	0.038327	0.102971297	-0.273505402	-1.208741213	**FAM225A**	lincRNA	family with sequence similarity 225 member A (non-protein coding) [Source:HGNC Symbol;Acc:HGNC:27855]	chr9	113113073	113119928	1	havana	1.275654815	1.412407516	1.138902114	1.222335231	1.603393164	1.411494153	1.089724456	1.413033531	0.913948357
ENSG00000231752	0.038327	0.102971297	-0.274901555	-1.209911526	**EMBP1**	transcribed_unprocessed_pseudogene	embigin pseudogene 1 [Source:HGNC Symbol;Acc:HGNC:38661]	chr1	121519112	121571892	1	havana	0.05850069	0.195951468	-0.078950087	0.040274975	0.378171619	0.16940781	-0.00649342	-0.00684508	-0.223511759
ENSG00000277972	0.038363	0.103046818	0.17360292	1.127871665	**CISD3**	protein_coding	CDGSH iron sulfur domain 3 [Source:HGNC Symbol;Acc:HGNC:27578]	chr17	38730235	38735044	1	ensembl_havana	3.40134346	3.314542	3.48814492	3.245688341	3.337712124	3.360225534	3.441917197	3.429942433	3.592575128
ENSG00000183722	0.038377	0.103050401	0.295575479	1.227374469	**LHFPL6**	protein_coding	LHFPL tetraspan subfamily member 6 [Source:HGNC Symbol;Acc:HGNC:6586]	chr13	39342892	39603528	-1	havana	3.787970655	3.640182915	3.935758395	3.677822113	3.591864916	3.650861717	4.063519655	4.046776082	3.696979447
ENSG00000126106	0.038378	0.103050401	0.226158856	1.169716455	**TMEM53**	protein_coding	transmembrane protein 53 [Source:HGNC Symbol;Acc:HGNC:26186]	chr1	44635238	44674555	-1	ensembl_havana	1.974896098	1.86181667	2.087975527	1.882980075	1.827267088	1.875202847	2.262602515	1.919554095	2.08176997
ENSG00000204624	0.038415	0.103130532	0.396303514	1.316131384	**DISP3**	protein_coding	dispatched RND transporter family member 3 [Source:HGNC Symbol;Acc:HGNC:29251]	chr1	11479166	11537584	1	ensembl_havana	1.8825093	1.684357543	2.080661057	1.327057633	1.7890623	1.936952697	2.062883947	2.131077666	2.04802156
ENSG00000184470	0.038468	0.103253347	0.318609755	1.247128182	**TXNRD2**	protein_coding	thioredoxin reductase 2 [Source:HGNC Symbol;Acc:HGNC:18155]	chr22	19875517	19941992	-1	ensembl_havana	5.120817726	4.961512848	5.280122603	4.806332033	4.943352857	5.134853654	5.337304143	5.312904338	5.190159329
ENSG00000156928	0.038479	0.103263493	0.176610801	1.130225622	**MALSU1**	protein_coding	mitochondrial assembly of ribosomal large subunit 1 [Source:HGNC Symbol;Acc:HGNC:21721]	chr7	23298739	23311729	1	ensembl_havana	4.0935649	4.0052595	4.1818703	3.848382869	4.091050934	4.076434696	4.051836127	4.179534163	4.31424061
ENSG00000152217	0.038523	0.103360842	0.167902423	1.123423917	**SETBP1**	protein_coding	SET binding protein 1 [Source:HGNC Symbol;Acc:HGNC:15573]	chr18	44680173	45068510	1	ensembl_havana	6.666319513	6.582368302	6.750270725	6.644251504	6.354813701	6.748039701	6.808555428	6.586896157	6.855360589
ENSG00000176531	0.038552	0.103377167	0.367702626	1.290296502	**PHLDB3**	protein_coding	pleckstrin homology like domain family B member 3 [Source:HGNC Symbol;Acc:HGNC:30499]	chr19	43474954	43504935	-1	ensembl_havana	1.809812444	1.625961131	1.993663757	1.541228291	1.749818205	1.586836898	2.22979561	2.037960042	1.713235619
ENSG00000184305	0.038561	0.103377167	0.324327529	1.252080681	**CCSER1**	protein_coding	coiled-coil serine rich protein 1 [Source:HGNC Symbol;Acc:HGNC:29349]	chr4	90127535	91601913	1	ensembl_havana	0.297637155	0.135473391	0.45980092	-0.18813307	0.06303108	0.531522158	0.106248156	0.648704825	0.624449778
ENSG00000145819	0.038546	0.103377167	0.216694681	1.162068152	**ARHGAP26**	protein_coding	Rho GTPase activating protein 26 [Source:HGNC Symbol;Acc:HGNC:17073]	chr5	142770377	143229011	1	ensembl_havana	4.816574671	4.70822733	4.924922011	4.788102413	4.679243401	4.657336177	4.862964519	4.91373456	4.998066956
ENSG00000136235	0.038554	0.103377167	-0.259104475	-1.196735623	**GPNMB**	protein_coding	glycoprotein nmb [Source:HGNC Symbol;Acc:HGNC:4462]	chr7	23235967	23275108	1	ensembl_havana	3.814698185	3.944250423	3.685145948	3.44832903	4.185992157	4.19843008	3.383457449	3.898145298	3.773835098
ENSG00000104835	0.038565	0.103377167	-0.298677569	-1.230016415	**SARS2**	protein_coding	seryl-tRNA synthetase 2, mitochondrial [Source:HGNC Symbol;Acc:HGNC:17697]	chr19	38915266	38930896	-1	ensembl_havana	0.989966966	1.139305751	0.840628181	1.04068047	1.109387295	1.267849486	0.851734776	0.956893362	0.713256405
ENSG00000236655	0.038611	0.103481767	0.689188168	1.612375948	**AC023347.2**	processed_pseudogene		chr2	126311466	126311927	-1	havana	-0.154762671	-0.499356755	0.189831413	-1.14838881	-0.428075095	0.078393642	0.210814386	-0.04338487	0.402064725
ENSG00000280311	0.038623	0.103495259	0.432937481	1.349979483	**AC131212.4**	TEC		chr12	132621999	132622973	-1	havana	-0.175045083	-0.391513823	0.041423658	-0.41068387	-0.842251242	0.078393642	-0.21650287	-0.00684508	0.347618925
ENSG00000060138	0.038653	0.103554244	0.170359629	1.125338969	**YBX3**	protein_coding	Y-box binding protein 3 [Source:HGNC Symbol;Acc:HGNC:2428]	chr12	10699089	10723312	-1	ensembl_havana	7.253900384	7.16872057	7.339080199	7.144495715	7.2228675	7.138798495	7.341934278	7.316298232	7.359008085
ENSG00000115295	0.0387	0.103660828	-0.178754368	-1.131906168	**CLIP4**	protein_coding	CAP-Gly domain containing linker protein family member 4 [Source:HGNC Symbol;Acc:HGNC:26108]	chr2	29097705	29189643	1	ensembl_havana	3.076235465	3.165612649	2.986858281	3.101289176	3.220595563	3.174953208	3.015987943	2.980039912	2.964546987
ENSG00000099889	0.03871	0.103669655	0.20982288	1.156546185	**ARVCF**	protein_coding	ARVCF, delta catenin family member [Source:HGNC Symbol;Acc:HGNC:728]	chr22	19969896	20016808	-1	ensembl_havana	3.07439158	2.969480141	3.17930302	2.926598135	2.803232702	3.178609585	3.213490017	3.081096449	3.243322595
ENSG00000173905	0.038729	0.103681672	0.242388795	1.182949753	**GOLIM4**	protein_coding	golgi integral membrane protein 4 [Source:HGNC Symbol;Acc:HGNC:15448]	chr3	168008677	168095975	-1	ensembl_havana	8.292119205	8.170924808	8.413313603	8.285301962	8.205175733	8.022296727	8.445250329	8.341317124	8.453373355
ENSG00000152944	0.038724	0.103681672	0.154735182	1.11321726	**MED21**	protein_coding	mediator complex subunit 21 [Source:HGNC Symbol;Acc:HGNC:11473]	chr12	27022546	27066343	1	ensembl_havana	5.324283875	5.246916284	5.401651466	5.244799796	5.262003329	5.233945727	5.370604241	5.444071495	5.390278661
ENSG00000247626	0.038738	0.103686071	0.16546761	1.121529532	**mars.02**	protein_coding	methionyl-tRNA synthetase 2, mitochondrial [Source:HGNC Symbol;Acc:HGNC:25133]	chr2	197705369	197708387	1	ensembl_havana	3.969333737	3.886599932	4.052067542	3.760287894	3.891395322	4.008116581	3.977206749	4.068341917	4.11065396
ENSG00000156531	0.038753	0.103706568	0.172313214	1.126863848	**PHF6**	protein_coding	PHD finger protein 6 [Source:HGNC Symbol;Acc:HGNC:18145]	chrX	134373253	134428791	1	ensembl_havana	7.059282478	6.973125871	7.145439085	7.026713166	6.954676424	6.937988023	7.117658139	7.155833161	7.162825956
ENSG00000261485	0.038768	0.103729048	-0.198366913	-1.147398799	**PAN3-AS1**	antisense	PAN3 antisense RNA 1 [Source:HGNC Symbol;Acc:HGNC:39932]	chr13	28136843	28138193	-1	havana	1.983384266	2.082567722	1.884200809	2.283275357	2.053616384	1.910811426	1.956619779	1.919554095	1.776428555
ENSG00000183439	0.038796	0.103763146	-0.272257203	-1.20769588	**TRIM61**	protein_coding	tripartite motif containing 61 [Source:HGNC Symbol;Acc:HGNC:24339]	chr4	164954446	164977668	-1	havana	0.115998463	0.252127064	-0.020130138	0.41082706	0.298820819	0.046733314	0.24405081	-0.19956768	-0.104873545
ENSG00000259237	0.038795	0.103763146	-0.788261499	-1.726992119	**LINC02490**	lincRNA	long intergenic non-protein coding RNA 2490 [Source:HGNC Symbol;Acc:HGNC:53471]	chr15	53116365	53129698	1	havana	-1.215633191	-0.821502441	-1.60976394	-1.14838881	-1.42549258	0.109374069	-1.63841642	-1.74507732	-1.445798078
ENSG00000280367	0.038816	0.103798286	-0.262919387	-1.199904334	**AP002364.1**	TEC		chr11	90223153	90226538	1	havana	0.231609057	0.363068751	0.100149363	0.613726219	0.428746719	0.046733314	0.141952326	0.225896484	-0.067400719
ENSG00000111843	0.038836	0.103819053	-0.185043037	-1.136850886	**TMEM14C**	protein_coding	transmembrane protein 14C [Source:HGNC Symbol;Acc:HGNC:20952]	chr6	10722915	10731129	1	ensembl_havana	6.397602685	6.490124203	6.305081167	6.270831774	6.577085996	6.62245484	6.186836391	6.371335256	6.357071854
ENSG00000173141	0.038838	0.103819053	-0.252548785	-1.191309924	**MRPL57**	protein_coding	mitochondrial ribosomal protein L57 [Source:HGNC Symbol;Acc:HGNC:14514]	chr13	21176645	21179084	1	ensembl_havana	3.264110977	3.39038537	3.137836585	3.275904777	3.350885786	3.544365547	3.196611706	3.102158989	3.11473906
ENSG00000179361	0.038863	0.103856316	0.238852867	1.18005399	**ARID3B**	protein_coding	AT-rich interaction domain 3B [Source:HGNC Symbol;Acc:HGNC:14350]	chr15	74541177	74598131	1	ensembl_havana	7.054141371	6.934714937	7.173567805	6.877975835	6.888179199	7.037989777	7.289172866	7.099078231	7.132452317
ENSG00000187091	0.038867	0.103856316	-0.299759692	-1.230939361	**PLCD1**	protein_coding	phospholipase C delta 1 [Source:HGNC Symbol;Acc:HGNC:9060]	chr3	38007496	38029762	-1	ensembl_havana	1.403230599	1.553110445	1.253350752	1.578072284	1.440410536	1.640848515	1.143556601	1.399485086	1.21701057
ENSG00000170631	0.038964	0.104051772	0.277068473	1.21173017	**ZNF16**	protein_coding	zinc finger protein 16 [Source:HGNC Symbol;Acc:HGNC:12947]	chr8	144930358	144950888	-1	ensembl_havana	3.619931081	3.481396844	3.758465317	3.578281784	3.461295901	3.404612849	3.642191792	3.76448673	3.868717429
ENSG00000267064	0.038967	0.104051772	0.252719963	1.191451283	**UXT-AS1**	antisense	UXT antisense RNA 1 [Source:HGNC Symbol;Acc:HGNC:49239]	chrX	47658833	47660377	1	havana	-0.132772202	-0.259132184	-0.006412221	-0.4596317	-0.299013651	-0.018751196	-0.00649342	-0.11936381	0.106620565
ENSG00000100226	0.038953	0.104051772	0.205669407	1.153221315	**GTPBP1**	protein_coding	GTP binding protein 1 [Source:HGNC Symbol;Acc:HGNC:4669]	chr22	38705723	38738299	1	ensembl_havana	5.664838743	5.562004039	5.767673447	5.609071199	5.447432701	5.629508218	5.907785967	5.696898477	5.698335895
ENSG00000153560	0.038998	0.104051772	0.181426992	1.134004993	**UBP1**	protein_coding	upstream binding protein 1 [Source:HGNC Symbol;Acc:HGNC:12507]	chr3	33388336	33441371	-1	ensembl_havana	7.276958952	7.186245456	7.367672448	7.159514704	7.220850859	7.178370804	7.273161961	7.371555383	7.4583
ENSG00000137822	0.038989	0.104051772	0.165248832	1.121359471	**TUBGCP4**	protein_coding	tubulin gamma complex associated protein 4 [Source:HGNC Symbol;Acc:HGNC:16691]	chr15	43369221	43409771	1	ensembl_havana	6.762035976	6.67941156	6.844660392	6.666162768	6.668704454	6.703367457	6.893813064	6.824683138	6.815484974
ENSG00000164303	0.038983	0.104051772	-0.155596255	-1.113881882	**ENPP6**	protein_coding	ectonucleotide pyrophosphatase/phosphodiesterase 6 [Source:HGNC Symbol;Acc:HGNC:23409]	chr4	184088706	184221230	-1	ensembl_havana	0.434459015	0.512257143	0.356660888	0.355307344	0.720251491	0.461212593	0.176794179	0.602150945	0.291037539
ENSG00000257093	0.038992	0.104051772	-0.186789215	-1.138227716	**KIAA1147**	protein_coding	KIAA1147 [Source:HGNC Symbol;Acc:HGNC:29472]	chr7	141656728	141702153	-1	ensembl_havana	6.189783357	6.283177965	6.09638875	6.541117177	6.138271189	6.170145528	6.247504317	6.028213022	6.013448911
ENSG00000121903	0.03899	0.104051772	-0.261771624	-1.198950107	**ZSCAN20**	protein_coding	zinc finger and SCAN domain containing 20 [Source:HGNC Symbol;Acc:HGNC:13093]	chr1	33472645	33496507	1	ensembl_havana	2.912309111	3.043194923	2.781423299	2.892874923	3.065422305	3.17128754	2.501227084	3.015998475	2.827044337
ENSG00000243696	0.039028	0.10411163	0.58826096	1.5034334	**AC006254.1**	protein_coding		chr3	52813282	52835729	-1	havana	0.853563689	0.559433209	1.147694168	0.004630787	0.910210399	0.76345844	1.143556601	1.097781778	1.201744126
ENSG00000131116	0.039042	0.10411163	0.462644327	1.378065371	**ZNF428**	protein_coding	zinc finger protein 428 [Source:HGNC Symbol;Acc:HGNC:20804]	chr19	43607219	43619874	-1	ensembl_havana	2.53362691	2.302304747	2.764949073	2.032058081	2.408903349	2.46595281	2.921565963	2.743151031	2.630130226
ENSG00000151025	0.039042	0.10411163	0.445707697	1.361982057	**GPR158**	protein_coding	G protein-coupled receptor 158 [Source:HGNC Symbol;Acc:HGNC:23689]	chr10	25175062	25602226	1	ensembl_havana	2.260988578	2.03813473	2.483842427	1.109412078	2.213608205	2.791383906	1.681320805	2.943162138	2.827044337
ENSG00000278952	0.039061	0.10414458	0.360116562	1.283529595	**AP003068.4**	TEC		chr11	65118310	65119111	1	havana	0.185669251	0.00561097	0.365727532	0.660286109	0.15602554	-0.799478738	1.125834805	0.19485955	-0.223511759
ENSG00000204186	0.039077	0.104165381	-0.292294891	-1.224586675	**ZDBF2**	protein_coding	zinc finger DBF-type containing 2 [Source:HGNC Symbol;Acc:HGNC:29313]	chr2	206274663	206314428	1	ensembl_havana	6.167670475	6.31381792	6.02152303	6.468159272	6.159483811	6.313810678	5.939834229	6.05870147	6.06603339
ENSG00000070882	0.0391	0.104208756	-0.197479669	-1.146693376	**OSBPL3**	protein_coding	oxysterol binding protein like 3 [Source:HGNC Symbol;Acc:HGNC:16370]	chr7	24796539	24981634	-1	ensembl_havana	6.102611652	6.201351486	6.003871817	6.059103851	6.245760963	6.299189644	5.989062667	5.97668242	6.045870365
ENSG00000159069	0.039121	0.104242486	0.636803477	1.554880253	**FBXW5**	protein_coding	F-box and WD repeat domain containing 5 [Source:HGNC Symbol;Acc:HGNC:13613]	chr9	136940435	136944696	-1	ensembl_havana	3.253475676	2.935073937	3.571877414	2.689029065	2.597561204	3.518631543	3.691214731	3.485502303	3.538915209

ENSG00000272391	0.039171	0.104242486	0.206577721	1.153947606	**POM121C**	protein_coding	POM121 transmembrane nucleoporin C [Source:HGNC Symbol;Acc:HGNC:34005]	chr7	75416787	75486271	-1	ensembl_havana	6.29975537	6.19646651	6.40304423	6.077984195	6.246642252	6.264773081	6.424643512 6.401383681	6.383105498
ENSG00000121057	0.039163	0.104242486	0.196660625	1.146042563	**AKAP1**	protein_coding	A-kinase anchoring protein 1 [Source:HGNC Symbol;Acc:HGNC:367]	chr17	57085092	57121349	1	ensembl_havana	6.174332475	6.076002162	6.272662787	5.953815718	5.945494323	6.328696445	6.095894333 6.268417988	6.453676041
ENSG00000176871	0.03914	0.104242486	0.188970851	1.13995024	**WSB2**	protein_coding	WD repeat and SOCS box containing 2 [Source:HGNC Symbol;Acc:HGNC:19222]	chr12	118032694	118062430	-1	ensembl_havana	5.646692229	5.552206803	5.741177654	5.734727125	5.615136618	5.306756667	5.892801254 5.724327162	5.606404547
ENSG00000143753	0.039182	0.104242486	-0.157460773	-1.115322377	**DEGS1**	protein_coding	delta 4-desaturase, sphingolipid 1 [Source:HGNC Symbol;Acc:HGNC:13709]	chr1	224175756	224193441	1	ensembl_havana	6.77116723	6.849897617	6.692436844	6.976998875	6.870278672	6.702415302	6.779096684 6.714449556	6.583764291
ENSG00000126457	0.03914	0.104242486	-0.158432421	-1.116073795	**PRMT1**	protein_coding	protein arginine methyltransferase 1 [Source:HGNC Symbol;Acc:HGNC:5187]	chr19	49675786	49689029	1	ensembl_havana	8.354117695	8.433333906	8.274901485	8.421095659	8.428975177	8.449930882	8.308950983 8.260950533	8.254802939
ENSG00000205581	0.039178	0.104242486	-0.18486329	-1.136709253	**HMGN1**	protein_coding	high mobility group nucleosome binding domain 1 [Source:HGNC Symbol;Acc:HGNC:4984]	chr21	39342315	39349647	-1	ensembl_havana	6.967747866	7.060179511	6.875316221	6.991866324	7.104384036	7.084288172	6.764496577 6.855400044	7.006052041
ENSG00000119969	0.039176	0.104242486	-0.191366172	-1.141844483	**HELLS**	protein_coding	helicase, lymphoid specific [Source:HGNC Symbol;Acc:HGNC:4861]	chr10	94501434	94613905	1	ensembl_havana	6.026273312	6.121956399	5.930590226	5.892363796	6.241788488	6.231716911	5.818900937 5.999313528	5.973556214
ENSG00000171621	0.039185	0.104242486	-0.228069512	-1.171266614	**SPSB1**	protein_coding	splA/ryanodine receptor domain and SOCS box containing 1 [Source:HGNC Symbol;Acc:HGNC:30628]	chr1	9292880	9369532	1	ensembl_havana	1.603959797	1.717994553	1.489925041	1.716703112	1.636045198	1.801235349	1.386165364 1.578479109	1.505130649
ENSG00000156384	0.039179	0.104242486	-0.247883376	-1.18746367	**SFR1**	protein_coding	SWI5 dependent homologous recombination repair protein 1 [Source:HGNC Symbol;Acc:HGNC:29574]	chr10	104122058	104126385	1	ensembl_havana	1.980272905	2.104214593	1.856331217	2.049654613	2.242169235	2.02081993	1.786580698 1.831488934	1.950924019
ENSG00000223546	0.039209	0.10428637	0.202028099	1.150314298	**LINC00630**	lincRNA	long intergenic non-protein coding RNA 630 [Source:HGNC Symbol;Acc:HGNC:44263]	chrX	102769161	102885406	1	havana	1.753679894	1.652665844	1.854693943	1.671956805	1.603393164	1.682647563	1.740743887 1.890792227	1.932545716
ENSG00000170385	0.039217	0.104286931	0.166557071	1.122376783	**SLC30A1**	protein_coding	solute carrier family 30 member 1 [Source:HGNC Symbol;Acc:HGNC:11012]	chr1	211571568	211578742	-1	ensembl_havana	6.276276694	6.192998159	6.35955523	6.386129477	6.283178509	5.909686491	6.481489936 6.487546852	6.109628901
ENSG00000196372	0.039258	0.104361947	0.233498198	1.17568225	**ASB13**	protein_coding	ankyrin repeat and SOCS box containing 13 [Source:HGNC Symbol;Acc:HGNC:19765]	chr10	5638867	5666595	-1	ensembl_havana	2.681301646	2.564552547	2.798050745	2.444931907	2.476573576	2.772152159	2.561794338 2.886015394	2.946342503
ENSG00000122565	0.039259	0.104361947	-0.151707869	-1.110883764	**CBX3**	protein_coding	chromobox 3 [Source:HGNC Symbol;Acc:HGNC:1553]	chr7	26201162	26213356	1	ensembl_havana	8.074173906	8.15002784	7.998319971	8.024076222	8.221629007	8.204378291	7.855362252 8.072379961	8.067217701
ENSG00000269729	0.039273	0.104379602	0.160868772	1.117960157	**AC006262.2**	lincRNA		chr19	46163893	46180647	-1	havana	1.500645825	1.420211439	1.581080211	1.749374982	1.402954459	1.108304877	1.966610161 1.529563273	1.2470672
ENSG00000242338	0.039313	0.104466144	-0.221863306	-1.166238864	**BMS1P4**	transcribed_unprocessed_pseudogene	BMS1, ribosome biogenesis factor pseudogene 4 [Source:HGNC Symbol;Acc:HGNC:23652]	chr10	73715843	73730469	-1	havana	-0.036531145	0.074400508	-0.147462797	0.297565234	-0.383759306	0.309395597	0.069637898 -0.74417246	0.232146171
ENSG00000255693	0.039321	0.104467261	0.402408822	1.321712891	**LINC02389**	lincRNA	long intergenic non-protein coding RNA 2389 [Source:HGNC Symbol;Acc:HGNC:53316]	chr12	64883394	64977522	1	havana	0.978894027	0.777689616	1.180098438	0.812341977	1.184892896	0.335833975	1.089724456 1.343981945	1.106588913
ENSG00000274605	0.039335	0.104485483	0.473430393	1.388406855	**AL355338.1**	lincRNA		chr13	100086031	100088848	-1	havana	0.817205853	0.580490657	1.05392105	0.075059705	0.720251491	0.946160774	0.995284613 0.919411336	1.2470672
ENSG00000040531	0.039344	0.104489595	-0.183098392	-1.135319528	**CTNS**	protein_coding	cystinosin, lysosomal cystine transporter [Source:HGNC Symbol;Acc:HGNC:2518]	chr17	3636468	3661542	1	ensembl_havana	7.448632004	7.540181199	7.357082808	7.93279206	7.360715863	7.327035675	7.697295634 7.282329361	7.091623429
ENSG00000176946	0.039356	0.104503801	-0.15832321	-1.115989312	**THAP4**	protein_coding	THAP domain containing 4 [Source:HGNC Symbol;Acc:HGNC:23187]	chr2	241584405	241637449	-1	ensembl_havana	4.044051358	4.123212964	3.964889753	4.181339713	4.073277629	4.115021548	4.065845049 3.914907507	3.913916704
ENSG00000171992	0.039368	0.104514441	-0.167794301	-1.123339726	**SYNPO**	protein_coding	synaptopodin [Source:HGNC Symbol;Acc:HGNC:30672]	chr5	150601080	150659220	1	ensembl_havana	4.785869985	4.869767136	4.701972835	4.971219637	4.830324825	4.807756945	4.731453667 4.681576899	4.692887938
ENSG00000141699	0.039409	0.104604083	0.163988303	1.120380131	**RETREG3**	protein_coding	reticulophagy regulator family member 3 [Source:HGNC Symbol;Acc:HGNC:27258]	chr17	42579513	42610623	-1	ensembl_havana	5.621499756	5.539505604	5.703493908	5.594159312	5.540922579	5.483434922	5.823718163 5.674573768	5.612189792
ENSG00000076924	0.039439	0.104625936	0.291474708	1.223890687	**XAB2**	protein_coding	XPA binding protein 2 [Source:HGNC Symbol;Acc:HGNC:14089]	chr19	7619525	7629565	-1	ensembl_havana	6.58079627	6.435058916	6.726533624	6.404195786	6.373895328	6.527085634	6.849959875 6.759558647	6.570082351
ENSG00000156976	0.039437	0.104625936	0.167916949	1.123435229	**EIF4A2**	protein_coding	eukaryotic translation initiation factor 4A2 [Source:HGNC Symbol;Acc:HGNC:3284]	chr3	186783205	186789900	1	ensembl_havana	9.133874999	9.049916525	9.217833473	8.97174942	9.035991479	9.142008674	9.064742552 9.24553395	9.343223918
ENSG00000143344	0.039427	0.104625936	-0.213970963	-1.159876309	**RGL1**	protein_coding	ral guanine nucleotide dissociation stimulator like 1 [Source:HGNC Symbol;Acc:HGNC:30281]	chr1	183636085	183928531	1	ensembl_havana	5.367673123	5.474658604	5.260687641	5.750280285	5.370284107	5.303411421	5.399470112 5.165609729	5.216983083
ENSG00000118246	0.039482	0.104710379	-0.174853324	-1.128849631	**FASTKD2**	protein_coding	FAST kinase domains 2 [Source:HGNC Symbol;Acc:HGNC:29160]	chr2	206765357	206792509	1	ensembl_havana	6.175227795	6.262654457	6.087801133	6.272706027	6.298133023	6.217124322	6.056680555 6.074773338	6.131949508
ENSG00000163121	0.039485	0.104710379	-0.292648165	-1.224886579	**NEURL3**	protein_coding	neuralized E3 ubiquitin protein ligase 3 [Source:HGNC Symbol;Acc:HGNC:25162]	chr2	96497643	96508109	-1	ensembl_havana	1.331122654	1.477446737	1.184798572	1.424690178	1.547275176	1.460374858	1.195452122 1.01135145	1.347592143
ENSG00000130684	0.039508	0.104731136	0.176174915	1.129884195	**ZNF337**	protein_coding	zinc finger protein 337 [Source:HGNC Symbol;Acc:HGNC:15809]	chr20	25674215	25696841	-1	ensembl_havana	5.802520044	5.714432586	5.890607501	5.725177232	5.661472132	5.756648395	5.811298363 5.864669277	5.995854864
ENSG00000125868	0.039507	0.104731136	0.169194569	1.12443056	**DSTN**	protein_coding	destrin, actin depolymerizing factor [Source:HGNC Symbol;Acc:HGNC:15750]	chr20	17569863	17609919	1	ensembl_havana	6.600725313	6.516128029	6.685322598	6.522728564	6.577786494	6.447869028	6.64282947 6.718888111	6.694250212
ENSG00000162341	0.039549	0.104763593	0.16862498	1.123986712	**TPCN2**	protein_coding	two pore segment channel 2 [Source:HGNC Symbol;Acc:HGNC:20820]	chr11	69048897	69162440	1	ensembl_havana	3.823692527	3.739380037	3.908005017	3.781505278	3.638270336	3.798364496	3.969775382 3.861556733	3.892682937
ENSG00000112081	0.03954	0.104763593	-0.163333733	-1.119871915	**SRSF3**	protein_coding	serine and arginine rich splicing factor 3 [Source:HGNC Symbol;Acc:HGNC:10785]	chr6	36594353	36605600	1	ensembl_havana	8.853230987	8.934897854	8.771564121	9.014379104	8.946348783	8.843965675	8.803275548 8.769471052	8.741945762
ENSG00000067704	0.039537	0.104763593	-0.171344102	-1.126107146	**IARS2**	protein_coding	isoleucyl-tRNA synthetase 2, mitochondrial [Source:HGNC Symbol;Acc:HGNC:29685]	chr1	220094102	220148041	1	ensembl_havana	7.963640032	8.049312083	7.877967982	8.088539248	8.008266994	8.051130008	7.83437454 7.878170945	7.92135846
ENSG00000277258	0.039549	0.104763593	-0.213921881	-1.15983685	**PCGF2**	protein_coding	polycomb group ring finger 2 [Source:HGNC Symbol;Acc:HGNC:12929]	chr17	38733897	38749817	-1	ensembl_havana	4.366734747	4.473695688	4.259773806	4.630406447	4.513401796	4.27727882	4.449223041 4.153538413	4.176559965
ENSG00000139343	0.039568	0.104795597	-0.166146531	-1.122057439	**SNRPF**	protein_coding	small nuclear ribonucleoprotein polypeptide F [Source:HGNC Symbol;Acc:HGNC:11162]	chr12	95858928	95903828	1	ensembl_havana	4.862307042	4.945380307	4.779233776	4.949121894	4.86975603	5.017262996	4.713768103 4.722957818	4.900975408
ENSG00000183401	0.039591	0.104835485	0.216227772	1.161692125	**CCDC159**	protein_coding	coiled-coil domain containing 159 [Source:HGNC Symbol;Acc:HGNC:26996]	chr19	11344684	11354944	1	ensembl_havana	3.582489881	3.474375995	3.690603767	3.686284073	3.357427779	3.379416132	3.74734528 3.668906348	3.655559672
ENSG00000163517	0.039608	0.104856474	0.184713641	1.13659135	**HDAC11**	protein_coding	histone deacetylase 11 [Source:HGNC Symbol;Acc:HGNC:19086]	chr3	13479724	13506424	1	ensembl_havana	4.775415014	4.683058193	4.867771834	4.665068254	4.634157892	4.749948434	4.937238596 4.830360032	4.835716874
ENSG00000184575	0.039613	0.104856474	-0.157134864	-1.115070451	**XPOT**	protein_coding	exportin for tRNA [Source:HGNC Symbol;Acc:HGNC:12826]	chr12	64404350	64451127	1	ensembl_havana	7.913456521	7.992023953	7.834889089	7.869489716	8.03835086	8.068231284	7.668129809 7.89063296	7.945904499
ENSG00000185252	0.039622	0.104860904	-0.24556562	-1.185557487	**ZNF74**	protein_coding	zinc finger protein 74 [Source:HGNC Symbol;Acc:HGNC:13144]	chr22	20394115	20408461	1	ensembl_havana	2.524341406	2.647124216	2.401558596	2.917043099	2.619498965	2.404830583	2.480459456 2.433220573	2.290995759
ENSG00000114841	0.039657	0.104932346	0.277586018	1.212164938	**DNAH1**	protein_coding	dynein axonemal heavy chain 1 [Source:HGNC Symbol;Acc:HGNC:2940]	chr3	52316319	52400491	1	ensembl_havana	4.770745583	4.631952574	4.909538592	4.75496532	4.38481302	4.756079381	4.928320531 4.896958637	4.903336608
ENSG00000178440	0.039672	0.104933598	0.343747305	1.269048587	**LINC00843**	lincRNA	long intergenic non-protein coding RNA 843 [Source:HGNC Symbol;Acc:HGNC:45009]	chr10	49972763	49982053	1	havana	-0.059549592	-0.231423245	0.11232406	-0.06941355	-0.428075095	-0.196781087	0.141952326 0.225896484	-0.030876629
ENSG00000054392	0.039668	0.104933598	-0.415603176	-1.333856233	**HHAT**	protein_coding	hedgehog acyltransferase [Source:HGNC Symbol;Acc:HGNC:18270]	chr1	210328252	210676296	1	ensembl_havana	3.598538662	3.80634025	3.390737073	4.307373212	3.861791544	3.249855994	3.744444859 3.206986221	3.22078014
ENSG00000265763	0.039694	0.104944607	0.592367298	1.507718713	**ZNF488**	protein_coding	zinc finger protein 488 [Source:HGNC Symbol;Acc:HGNC:23535]	chr10	47365496	47384273	-1	ensembl_havana	0.204007684	-0.092175965	0.500191333	-0.79434999	0.15602554	0.361796557	0.36983092 0.529376201	0.601366878
ENSG00000173320	0.039698	0.104944607	0.452266555	1.36818807	**STOX2**	protein_coding	storkhead box 2 [Source:HGNC Symbol;Acc:HGNC:25450]	chr4	183797692	184023526	1	ensembl_havana	1.162220173	0.936086895	1.38835345	0.297565234	1.256642557	1.254052895	1.052687018 1.821363648	1.291009686
ENSG00000005810	0.03969	0.104944607	0.201379226	1.149797043	**MYCBP2**	protein_coding	MYC binding protein 2, E3 ubiquitin protein ligase [Source:HGNC Symbol;Acc:HGNC:23386]	chr13	77044655	77327050	-1	ensembl_havana	6.567350775	6.466661162	6.668040388	6.466113173	6.258050285	6.675820028	6.614913654 6.593604671	6.795602838
ENSG00000214076	0.039712	0.104964202	0.168709646	1.124052677	**CPSF1P1**	processed_pseudogene	cleavage and polyadenylation specific factor 1 pseudogene 1 [Source:HGNC Symbol;Acc:HGNC:19251]	chr22	32269381	32273110	1	havana	1.589950454	1.50559563	1.674305277	1.528734855	1.390249836	1.5978022	1.717267538 1.614107429	1.691540863
ENSG00000284747	0.039736	0.105006263	-0.20796299	-1.155056153	**AL034417.4**	antisense		chr1	7991134	8005312	1	havana	1.905718987	2.009700482	1.801737492	1.683274163	1.953538152	2.392289132	1.554586035 1.811166797	2.039459644
ENSG00000173559	0.03977	0.105077684	0.224979993	1.16876104	**NABP1**	protein_coding	nucleic acid binding protein 1 [Source:HGNC Symbol;Acc:HGNC:26232]	chr2	191678068	191696659	1	ensembl_havana	7.702033785	7.589543788	7.814523781	7.87705566	7.433341512	7.458234193	7.986345087 7.609508181	7.847718075
ENSG00000142687	0.039786	0.105101335	0.152705688	1.111652356	**KIAA0319L**	protein_coding	KIAA0319 like [Source:HGNC Symbol;Acc:HGNC:30071]	chr1	35433490	35557950	-1	ensembl_havana	6.936544736	6.860191892	7.01289758	6.860011514	6.825268451	6.895295711	7.035804184 6.981809733	7.021078822
ENSG00000160007	0.039814	0.105151773	0.203895454	1.151804173	**ARHGAP35**	protein_coding	Rho GTPase activating protein 35 [Source:HGNC Symbol;Acc:HGNC:4591]	chr19	46918676	47005077	1	ensembl_havana	7.096332418	6.994384692	7.198280145	6.92967378	6.939768378	7.113711917	7.196708654 7.219624209	7.178507573
ENSG00000260220	0.03982	0.105151773	-0.493788028	-1.408137317	**CCDC187**	protein_coding	coiled-coil domain containing 187 [Source:HGNC Symbol;Acc:HGNC:30942]	chr9	136249971	136306901	-1	ensembl_havana	0.880561925	1.127455939	0.633667911	1.190963204	0.77991046	1.411494153	0.428834256 0.758913071	0.713256405
ENSG00000267080	0.039855	0.105225354	0.154349241	1.112919498	**ASB16-AS1**	antisense	ASB16 antisense RNA 1 [Source:HGNC Symbol;Acc:HGNC:25442]	chr17	44175973	44186717	-1	ensembl_havana	3.824359689	3.747185069	3.901534309	3.736039304	3.71668882	3.788827082	3.926925567 3.859084184	3.918593177
ENSG00000262228	0.039863	0.105226124	-0.58542589	-1.500481873	**AC087392.3**	lincRNA		chr17	763565	765319	-1	ensembl_havana	-0.522204385	-0.22949144	-0.814917329	0.206373087	-0.258437325	-0.636410081	-0.63291296 -1.15951152	-0.652327512
ENSG00000172164	0.039877	0.105227468	0.482331962	1.396999944	**SNTB1**	protein_coding	syntrophin beta 1 [Source:HGNC Symbol;Acc:HGNC:11168]	chr8	120535745	120813273	-1	ensembl_havana	3.997248703	3.756082722	4.238414684	3.260875666	4.045187566	3.962184934	4.205267587 4.310178017	4.199798448
ENSG00000187953	0.039878	0.105227468	0.178032452	1.131339911	**PMS2CL**	transcribed_unprocessed_pseudogene	PMS2 C-terminal like pseudogene [Source:HGNC Symbol;Acc:HGNC:30061]	chr7	6710128	6753862	1	ensembl_havana	3.455594667	3.366578441	3.544610892	3.369953701	3.337712124	3.392069497	3.452619495 3.54824011	3.632973073
ENSG00000170846	0.039902	0.105252006	0.237977341	1.179338069	**AC093323.1**	protein_coding	Putative MORF4 family-associated protein 1-like protein UPP [Source:UniProtKB/Swiss-Prot;Acc:B2RBV5]	chr4	6663396	6676755	1	havana	3.709112662	3.590123991	3.828101332	3.563046529	3.514949665	3.692375779	3.617041156 3.871404772	3.995858068
ENSG00000121749	0.039896	0.105252006	-0.210686244	-1.157238515	**TBC1D15**	protein_coding	TBC1 domain family member 15 [Source:HGNC Symbol;Acc:HGNC:25694]	chr12	71839707	71927248	1	ensembl_havana	5.459191652	5.564534774	5.353848529	5.716940992	5.647526894	5.329136435	5.379979066 5.430812746	5.250753777
ENSG00000130520	0.03993	0.105306343	0.173131318	1.127503036	**LSM4**	protein_coding	LSM4 homolog, U6 small nuclear RNA and mRNA degradation associated [Source:HGNC Symbol;Acc:HGNC:17259]	chr19	18306230	18323274	-1	ensembl_havana	5.318822088	5.232256429	5.405387747	5.069371407	5.258512353	5.368885525	5.323765864 5.36262721	5.529770166
ENSG00000126705	0.039948	0.105334641	0.358610034	1.282189975	**AHDC1**	protein_coding	AT-hook DNA binding motif containing 1 [Source:HGNC Symbol;Acc:HGNC:25230]	chr1	27534035	27604431	-1	ensembl_havana	2.819950951	2.640645934	2.999255968	2.596285266	2.558348641	2.767303895	3.196611706 2.989113925	2.812042274
ENSG00000213930	0.039968	0.105368171	0.209733177	1.156474277	**GALT**	protein_coding	galactose-1-phosphate uridylyltransferase [Source:HGNC Symbol;Acc:HGNC:4135]	chr9	34638133	34651035	1	ensembl_havana	5.096464554	4.991597966	5.201331143	4.868380636	5.106611921	4.999801339	5.212727477 5.195316543	5.19594941
ENSG00000242265	0.039996	0.105402768	0.156134124	1.11429724	**PEG10**	protein_coding	paternally expressed 10 [Source:HGNC Symbol;Acc:HGNC:14005]	chr7	94656325	94669695	1	ensembl_havana	6.937964811	6.859897749	7.016031873	6.831367417	6.876270226	6.872055602	6.980488259 7.074244491	6.993362868
ENSG00000064651	0.039993	0.105402768	0.151510533	1.110731824	**SLC12A2**	protein_coding	solute carrier family 12 member 2 [Source:HGNC Symbol;Acc:HGNC:10911]	chr5	128083766	128189688	1	ensembl_havana	6.132017514	6.056262247	6.20777278	6.115541665	6.115777658	5.937467418	6.251595753 6.259539793	6.112182795
ENSG00000245532	0.040008	0.105415873	0.215418757	1.161040869	**NEAT1**	lincRNA	nuclear paraspeckle assembly transcript 1 (non-protein coding) [Source:HGNC Symbol;Acc:HGNC:30815]	chr11	65422774	65445540	1	havana	9.509614994	9.401905615	9.617324372	9.620354732	9.34402484	9.241337274	9.714964188 9.533824731	9.603184197
ENSG00000271141	0.040017	0.105420468	0.283749152	1.217354333	**AC010680.4**	lincRNA		chr2	178616581	178617123	1	havana	2.720320492	2.578445916	2.862195068	2.528879384	2.734533378	2.471924986	2.787093985 2.821440049	2.97805117
ENSG00000075624	0.040056	0.10550213	-0.192220669	-1.142520989	**ACTB**	protein_coding	actin beta [Source:HGNC Symbol;Acc:HGNC:132]	chr7	5527147	5563784	-1	ensembl_havana	12.17393858	12.27004891	12.07782824	12.25581941	12.22423287	12.33009446	12.18381807 11.98924364	12.06042302
ENSG00000259345	0.040094	0.105582933	0.810119855	1.7533571	**AC013652.1**	lincRNA		chr15	38865322	39427195	-1	havana	0.076455703	-0.328604225	0.48151563	-0.99620085	0.06303108	-0.052642908	0.618191201 0.478736765	0.347618925
ENSG00000110344	0.040118	0.105589485	0.155980083	1.114178269	**UBE4A**	protein_coding	ubiquitination factor E4A [Source:HGNC Symbol;Acc:HGNC:12499]	chr11	118359585	118399211	1	ensembl_havana	7.914689925	7.836699883	7.992679966	7.885041663	7.811776138	7.813281849	7.997046591 7.982237967	7.998755342
ENSG00000077044	0.040115	0.105589485	-0.177359621	-1.13081241	**DGKD**	protein_coding	diacylglycerol kinase delta [Source:HGNC Symbol;Acc:HGNC:2851]	chr2	233354507	233472104	1	ensembl_havana	7.726320427	7.815000237	7.637640616	7.950317016	7.619536296	7.8751474	7.714256852 7.537692965	7.660972031
ENSG00000161642	0.040109	0.105589485	-0.273141211	-1.208436119	**ZNF385A**	protein_coding	zinc finger protein 385A [Source:HGNC Symbol;Acc:HGNC:17521]	chr12	54369133	54391298	-1	ensembl_havana	4.379944774	4.51651538	4.243374169	4.657915398	4.580645306	4.310985435	4.510259191 4.076878789	4.142984527
ENSG00000105204	0.04016	0.1056603	0.370478884	1.292781881	**DYRK1B**	protein_coding	dual specificity tyrosine phosphorylation regulated kinase 1B [Source:HGNC Symbol;Acc:HGNC:3092]	chr19	39825350	39834201	-1	ensembl_havana	2.258014989	2.072775547	2.443254431	2.298074107	2.177092949	1.743159585	2.981915645 2.510878368	1.836969279
ENSG00000155115	0.040157	0.1056603	-0.172937085	-1.127351248	**GTF3C6**	protein_coding	general transcription factor IIIC subunit 6 [Source:HGNC Symbol;Acc:HGNC:20872]	chr6	110958560	110967890	1	ensembl_havana	4.56283216	4.649300702	4.476363617	4.573763101	4.70896257	4.665176435	4.479191796 4.531280758	4.418618298
ENSG00000162650	0.040187	0.105673177	0.18442964	1.136367629	**ATXN7L2**	protein_coding	ataxin 7 like 2 [Source:HGNC Symbol;Acc:HGNC:28713]	chr1	109483479	109492804	1	ensembl_havana	4.744296891	4.652082071	4.836511711	4.68772195	4.671388827	4.597135437	4.967402959 4.761861841	4.780270335
ENSG00000130193	0.040185	0.105673177	-0.183661613	-1.135762838	**THEM6**	protein_coding	thioesterase superfamily member 6 [Source:HGNC Symbol;Acc:HGNC:29656]	chr8	142727203	142736927	1	ensembl_havana	2.86125411	2.953084917	2.769423304	3.032160045	2.798432633	3.028662073	2.879883083 2.688493842	2.739892986
ENSG00000248685	0.040175	0.105673177	-0.471112399	-1.386177877	**LINC02484**	lincRNA	long intergenic non-protein coding RNA 2484 [Source:HGNC Symbol;Acc:HGNC:53459]	chr4	34120894	34269747	-1	havana	0.383812055	0.619368255	0.148255856	0.705390237	0.428746719	0.723967808	-0.12879794 0.37179175	0.201773757
ENSG00000001629	0.040234	0.105778702	0.160179851	1.117426431	**ANKIB1**	protein_coding	ankyrin repeat and IBR domain containing 1 [Source:HGNC Symbol;Acc:HGNC:22215]	chr7	92246234	92401384	1	ensembl_havana	7.307283597	7.227193672	7.387373523	7.26402255	7.168799093	7.248759373	7.366665811 7.366559209	7.428895549
ENSG00000169967	0.04025	0.105800377	0.150727625	1.110129226	**MAP3K2**	protein_coding	mitogen-activated protein kinase kinase kinase 2 [Source:HGNC Symbol;Acc:HGNC:6854]	chr2	127298730	127388465	-1	ensembl_havana	7.247661205	7.172297392	7.323025018	7.220261144	7.156894364	7.13973667	7.360759324 7.327967345	7.280348384
ENSG00000182004	0.040286	0.105861787	-0.227451528	-1.170765005	**SNRPE**	protein_coding	small nuclear ribonucleoprotein polypeptide E [Source:HGNC Symbol;Acc:HGNC:11161]	chr1	203861603	203870550	1	ensembl_havana	6.248312644	6.362038408	6.13458688	6.350635169	6.367022561	6.368457494	5.952456816 6.197772076	6.253531748
ENSG00000243004	0.040288	0.105861787	-0.241826178	-1.18248852	**AC005062.1**	sense_overlapping		chr7	19918981	20140453	-1	havana	1.341242533	1.462155621	1.220329444	1.490591983	1.614359492	1.281515389	1.430149367 1.226127349	1.004711615
ENSG00000103145	0.040349	0.106002575	-0.222240913	-1.166544152	**HCFC1R1**	protein_coding	host cell factor C1 regulator 1 [Source:HGNC Symbol;Acc:HGNC:21198]	chr16	3022620	3024286	-1	ensembl_havana	1.133600008	1.244720465	1.022479552	1.142587728	1.351450576	1.24012309	1.052687018 0.975275413	1.039476224
ENSG00000136754	0.040363	0.106020828	-0.151012097	-1.110348144	**ABI1**	protein_coding	abl interactor 1 [Source:HGNC Symbol;Acc:HGNC:11320]	chr10	26746593	26861087	-1	ensembl_havana	7.013770809	7.089276857	6.93826476	7.198242222	7.137545058	6.932043292	7.057875831 6.963223517	6.793694932
ENSG00000115548	0.040379	0.106041695	0.166920948	1.122659905	**KDM3A**	protein_coding	lysine demethylase 3A [Source:HGNC Symbol;Acc:HGNC:20815]	chr2	86440647	86492716	1	ensembl_havana	6.023183052	5.939722578	6.106643526	6.029480675	5.939511543	5.850175516	6.142357288 6.087503153	6.090070138
ENSG00000024048	0.040411	0.106107736	0.167525141	1.123130167	**UBR2**	protein_coding	ubiquitin protein ligase E3 component n-recognin 2 [Source:HGNC Symbol;Acc:HGNC:21289]	chr6	42564062	42693504	1	ensembl_havana	7.15897167	7.0752091	7.242734241	7.168099229	7.090706917	6.966821154	7.29537918 7.221309745	7.211513798
ENSG00000260641	0.040427	0.106130237	0.301274042	1.232232115	**AC114811.2**	antisense		chr4	98658904	98664550	1	havana	2.852891897	2.702254876	3.003528918	2.73325697	2.877938163	2.495569495	3.058645034 2.938485423	3.013456297
ENSG00000110536	0.040444	0.106156422	0.260520232	1.19791059	**PTPMT1**	protein_coding	protein tyrosine phosphatase, mitochondrial 1 [Source:HGNC Symbol;Acc:HGNC:26965]	chr11	47565430	47573461	1	ensembl_havana	3.482589881	3.352329765	3.612849997	3.3380874	3.455209308	3.263692587	3.80416182 3.554366408	3.480021764
ENSG00000138801	0.040453	0.10615999	0.150687917	1.110098672	**PAPSS1**	protein_coding	3'-phosphoadenosine 5'-phosphosulfate synthase 1 [Source:HGNC Symbol;Acc:HGNC:8603]	chr4	107590276	107720452	-1	ensembl_havana	7.058221875	6.982877916	7.133565834	6.899666418	7.003198944	7.045768386	7.071247917 7.137841953	7.19160763
ENSG00000187231	0.040467	0.106177883	0.480931852	1.395644837	**SESTD1**	protein_coding	SEC14 and spectrin domain containing 1 [Source:HGNC Symbol;Acc:HGNC:18379]	chr2	179101692	179264790	-1	ensembl_havana	3.514152965	3.273687039	3.754618891	2.484284696	3.503197858	3.833578563	3.423901694 3.81128221	4.028672769
ENSG00000084070	0.040548	0.106369466	-0.166210982	-1.122107567	**SMAP2**	protein_coding	small ArfGAP2 [Source:HGNC Symbol;Acc:HGNC:25082]	chr1	40344850	40423326	1	ensembl_havana	6.981164941	7.064270432	6.89805945	7.313340548	7.024165868	6.85530488	7.072983 6.887601075	6.733594276
ENSG00000140740	0.04057	0.106396131	-0.212536607	-1.158723711	**UQCRC2**	protein_coding	ubiquinol-cytochrome c reductase core protein 2 [Source:HGNC Symbol;Acc:HGNC:12586]	chr16	21952660	21983660	1	ensembl_havana	7.371051607	7.477319911	7.264783303	7.615559752	7.478491579	7.3379084	7.330858626 7.184506076	7.278985208
ENSG00000184979	0.040573	0.106396131	-0.451109191	-1.367090917	**USP18**	protein_coding	ubiquitin specific peptidase 18 [Source:HGNC Symbol;Acc:HGNC:12616]	chr22	18149899	18177397	1	ensembl_havana	3.730921779	3.956476375	3.505367183	3.646363245	4.150646514	4.072419366	3.477287033 3.275339896	3.763474622
ENSG00000130311	0.040581	0.106398471	0.237733554	1.179138802	**DDA1**	protein_coding	DET1 and DDB1 associated 1 [Source:HGNC Symbol;Acc:HGNC:28360]	chr19	17309518	17323301	1	ensembl_havana	5.205365237	5.08649846	5.324232014	5.100307061	5.129725469	5.02946285	5.522239636 5.307473809	5.142982598
ENSG00000177311	0.04059	0.106401786	-0.167225672	-1.122897057	**ZBTB38**	protein_coding	zinc finger and BTB domain containing 38 [Source:HGNC Symbol;Acc:HGNC:26636]	chr3	141324213	141449792	1	ensembl_havana	8.363159331	8.446772167	8.279546495	8.633449626	8.391416333	8.315450541	8.426032705 8.187344125	8.225262653
ENSG00000100105	0.040615	0.106434662	-0.223817359	-1.167819543	**PATZ1**	protein_coding	POZ/BTB and AT hook containing zinc finger 1 [Source:HGNC Symbol;Acc:HGNC:13071]	chr22	31325804	31346232	-1	ensembl_havana	4.750065389	4.861974068	4.638156709	4.766982541	4.853649958	4.965289705	4.673164629 4.659011962	4.582293537
ENSG00000171451	0.040617	0.106434662	-0.737606358	-1.66740707	**DSEL**	protein_coding	dermatan sulfate epimerase-like [Source:HGNC Symbol;Acc:HGNC:18144]	chr18	67506582	67516980	-1	ensembl_havana	-0.513031403	-0.144228224	-0.881834582	0.10902545	0.09469996	-0.636410081	-0.69455235 -1.24204464	-0.708906755
ENSG00000176102	0.04064	0.106474738	-0.200231042	-1.148882329	**CSTF3**	protein_coding	cleavage stimulation factor subunit 3 [Source:HGNC Symbol;Acc:HGNC:2485]	chr11	33077188	33162371	-1	ensembl_havana	5.293393341	5.393508862	5.19327782	5.457478234	5.404644032	5.318404319	5.179826793 5.146474026	5.253532641
ENSG00000110693	0.040659	0.106483506	0.149737793	1.109367829	**SOX6**	protein_coding	SRY-box 6 [Source:HGNC Symbol;Acc:HGNC:16421]	chr11	15966449	16739591	-1	ensembl_havana	1.070614453	0.995745556	1.145483349	0.911909315	1.029710695	1.045616658	1.071324589 1.163381334	1.201744126
ENSG00000103653	0.040662	0.106483506	-0.152511273	-1.111502561	**CSK**	protein_coding	C-terminal Src kinase [Source:HGNC Symbol;Acc:HGNC:2444]	chr15	74782057	74803198	1	ensembl_havana	6.945607478	7.021863114	6.869351841	7.075845088	6.912539422	7.077204832	6.946503575 6.769467339	6.892084609
ENSG00000224927	0.040665	0.106483506	-0.372410457	-1.2945139	**NDUFA5P10**	processed_pseudogene	NADH:ubiquinone oxidoreductase subunit A5 pseudogene 10 [Source:HGNC Symbol;Acc:HGNC:48852]	chr1	109810642	109810998	-1	havana	1.159436663	1.345641892	0.973231435	0.911909315	1.688874845	1.436141515	0.592614899 0.993426194	1.333653212
ENSG00000271009	0.040709	0.106551176	0.329858534	1.256890122	**AC116667.1**	lincRNA		chr16	73060245	73062316	1	havana	0.589198307	0.42426904	0.754127574	-0.36334239	0.89230127	0.743848241	0.141952326 1.343981945	0.77644845
ENSG00000152207	0.040721	0.106551176	0.212787031	1.15892486	**CYSLTR2**	protein_coding	cysteinyl leukotriene receptor 2 [Source:HGNC Symbol;Acc:HGNC:18274]	chr13	48653711	48711226	1	ensembl_havana	8.063265333	7.956871817	8.169658848	8.305239708	7.905991794	7.659383949	8.401270989 8.089619858	8.018085698
ENSG00000198736	0.040705	0.106551176	0.171999689	1.126618986	**MSRB1**	protein_coding	methionine sulfoxide reductase B1 [Source:HGNC Symbol;Acc:HGNC:14133]	chr16	1938210	1943326	-1	ensembl_havana	5.045709464	4.959709619	5.131709309	4.926680408	5.053211401	4.899237049	5.178752887 5.154562121	5.061812918
ENSG00000082516	0.040717	0.106551176	-0.151047762	-1.110375593	**GEMIN5**	protein_coding	gem nuclear organelle associated protein 5 [Source:HGNC Symbol;Acc:HGNC:20043]	chr5	154887416	154938209	-1	ensembl_havana	6.232760344	6.308284225	6.157236463	6.197743394	6.269802376	6.457306905	6.061932222 6.12965545	6.280121716
ENSG00000132170	0.04075	0.106608551	0.610579568	1.52687247	**PPARG**	protein_coding	peroxisome proliferator activated receptor gamma [Source:HGNC Symbol;Acc:HGNC:9236]	chr3	12287368	12434356	1	ensembl_havana	0.347843824	0.04255404	0.653133607	-0.27307956	0.453384582	-0.052642908	0.807953002 0.504278656	0.647169164
ENSG00000099849	0.040818	0.106693294	0.42587018	1.343382537	**RASSF7**	protein_coding	Ras association domain family member 7 [Source:HGNC Symbol;Acc:HGNC:1166]	chr11	560404	564021	1	ensembl_havana	2.362965259	2.150030169	2.575900349	1.738566362	2.351074074	2.360450071	2.521699999 2.764446992	2.441554055
ENSG00000150556	0.040827	0.106693294	0.387425356	1.308056952	**LYPD6B**	protein_coding	LY6/PLAUR domain containing 6B [Source:HGNC Symbol;Acc:HGNC:27018]	chr2	149038107	149215262	1	ensembl_havana	2.245650004	2.051937326	2.439362682	2.833163932	2.139629423	1.183018625	3.525391749 2.203033505	1.589662793
ENSG00000237037	0.040805	0.106693294	0.226603105	1.170076702	**NDUFA6-AS1**	processed_transcript	NDUFA6 antisense RNA 1 (head to head) [Source:HGNC Symbol;Acc:HGNC:45273]	chr22	42090931	42137742	1	havana	0.670007662	0.556706109	0.783309215	0.613726219	0.52486995	0.531522158	0.915012722 0.578298274	0.856616648
ENSG00000251474	0.040803	0.106693294	0.205348004	1.152964429	**RPL32P3**	transcribed_unprocessed_pseudogene	ribosomal protein L32 pseudogene 3 [Source:HGNC Symbol;Acc:HGNC:27024]	chr3	129382922	129399655	-1	havana	5.409952254	5.307278252	5.512626257	5.218786827	5.25326	5.449787929	5.465246552 5.557454232	5.515177986
ENSG00000176124	0.040806	0.106693294	-0.212443159	-1.158648659	**DLEU1**	processed_transcript	deleted in lymphocytic leukemia 1 (non-protein coding) [Source:HGNC Symbol;Acc:HGNC:13747]	chr13	50082171	50723236	1	ensembl_havana	1.195303687	1.301525266	1.089082107	1.355633303	1.214022341	1.334920154	1.071324589 0.919411336	1.276510397
ENSG00000100429	0.040824	0.106693294	-0.312439445	-1.241805688	**HDAC10**	protein_coding	histone deacetylase 10 [Source:HGNC Symbol;Acc:HGNC:18128]	chr22	50245183	50251405	-1	ensembl_havana	0.376285881	0.532505603	0.220066158	0.660286109	0.52486995	0.41236075	0.141952326 0.478736765	0.039509383
ENSG00000197136	0.040841	0.106709752	0.29023815	1.22284212	**PCNX3**	protein_coding	pecanex homolog 3 [Source:HGNC Symbol;Acc:HGNC:18760]	chr11	65615773	65637439	1	ensembl_havana	5.538435937	5.393316861	5.683555012	5.455004303	5.197835644	5.527110637	5.773384267 5.692044381	5.585236388
ENSG00000133935	0.04087	0.106757862	0.225547786	1.169221113	**ERG28**	protein_coding	ergosterol biosynthesis 28 homolog [Source:HGNC Symbol;Acc:HGNC:1187]	chr14	75649791	75661189	-1	ensembl_havana	4.551671192	4.438897299	4.664445085	4.482778166	4.565186067	4.268727664	4.771895897 4.620114598	4.60132476
ENSG00000163520	0.040874	0.106757862	-0.265615821	-1.202149083	**FBLN2**	protein_coding	fibulin 2 [Source:HGNC Symbol;Acc:HGNC:3601]	chr3	13549131	13638422	1	ensembl_havana	5.069618678	5.202426588	4.936810767	5.181362699	5.064250767	5.361666299	5.084418896 4.841675494	4.88433791
ENSG00000170379	0.040884	0.106762859	-0.16263341	-1.11932843	**TCAF2**	protein_coding	TRPM8 channel associated factor 2 [Source:HGNC Symbol;Acc:HGNC:26878]	chr7	143620952	143730409	1	ensembl_havana	2.175725969	2.257042675	2.094409264	2.167163933	2.488543365	2.115420726	1.976531838 2.392765686	1.91393027
ENSG00000143674	0.040903	0.106775629	0.167323138	1.12297292	**MAP3K21**	protein_coding	mitogen-activated protein kinase kinase kinase 21 [Source:HGNC Symbol;Acc:HGNC:29798]	chr1	233327768	233385148	1	ensembl_havana	5.215032557	5.131370988	5.298694126	5.157216224	5.166512682	5.07038406	5.359273442 5.255778132	5.281030804
ENSG00000004961	0.0409	0.106775629	-0.187051069	-1.138434327	**HCCS**	protein_coding	holocytochrome c synthase [Source:HGNC Symbol;Acc:HGNC:4837]	chrX	11111301	11123078	1	ensembl_havana	6.028798437	6.122323972	5.935272903	6.142953663	6.190042115	6.033976137	5.873682904 5.973828406	5.958307398
ENSG00000119328	0.040929	0.106822462	0.247147923	1.186858483	**FAM206A**	protein_coding	family with sequence similarity 206 member A [Source:HGNC Symbol;Acc:HGNC:1364]	chr9	108934181	108950744	1	ensembl_havana	3.818079781	3.69450582	3.941653743	3.714137432	3.766695609	3.602684419	3.848057062 3.926762386	4.05014178
ENSG00000107281	0.040968	0.106905677	0.323486181	1.251350708	**NPDC1**	protein_coding	neural proliferation, differentiation and control 1 [Source:HGNC Symbol;Acc:HGNC:7899]	chr9	137039470	137046203	-1	ensembl_havana	4.436442826	4.274699735	4.598185916	4.130569456	4.061305939	4.632223812	4.552327717 4.574121769	4.668108262
ENSG00000129515	0.041018	0.107016161	-0.217077588	-1.162376619	**SNX6**	protein_coding	sorting nexin 6 [Source:HGNC Symbol;Acc:HGNC:14970]	chr14	34561094	34630183	-1	ensembl_havana	5.953147398	6.061686192	5.844608604	6.102955263	6.076665349	6.005437965	5.784016294 5.802330212	5.947479307
ENSG00000185920	0.041046	0.107070205	0.295623629	1.227415434	**PTCH1**	protein_coding	patched 1 [Source:HGNC Symbol;Acc:HGNC:9585]	chr9	95442980	95517057	-1	ensembl_havana	3.916394594	3.76858278	4.064206409	3.605306254	3.678012323	4.022429763	4.063519655 4.068341917	4.060757655
ENSG00000177463	0.041058	0.107080692	0.182670864	1.134983138	**NR2C2**	protein_coding	nuclear receptor subfamily 2 group C member 2 [Source:HGNC Symbol;Acc:HGNC:7972]	chr3	14947584	15053600	1	ensembl_havana	6.587304406	6.495968974	6.678639838	6.437576592	6.461143269	6.589187061	6.704945216 6.660093635	6.670880664
ENSG00000115694	0.041103	0.107180211	0.190656792	1.141283171	**STK25**	protein_coding	serine/threonine kinase 25 [Source:HGNC Symbol;Acc:HGNC:11404]	chr2	241492674	241509730	-1	ensembl_havana	5.822872923	5.727544527	5.918201319	5.772306826	5.738045873	5.672280881	6.039035631 5.808110029	5.907458296
ENSG00000163738	0.041125	0.10721693	0.149391005	1.109101196	**MTHFD2L**	protein_coding	methylenetetrahydrofolate dehydrogenase (NADP+ dependent) 2 like [Source:HGNC Symbol;Acc:HGNC:31865]	chr4	74114174	74303099	1	ensembl_havana	3.587397752	3.512702249	3.662093254	3.383893667	3.635581537	3.518631543	3.515217767 3.793263891	3.677798104
ENSG00000204394	0.041136	0.107226963	-0.209943581	-1.156642951	**VARS**	protein_coding	valyl-tRNA synthetase [Source:HGNC Symbol;Acc:HGNC:12651]	chr6	31777518	31795953	-1	ensembl_havana	7.908710688	8.013682479	7.803738898	8.007429788	7.90082262	8.132795028	7.826681889 7.804909123	7.779625682
ENSG00000260948	0.041193	0.1073562	0.175894141	1.129664322	**AL390195.2**	sense_overlapping		chr1	111431046	111433068	-1	havana	1.114878804	1.026931733	1.202825874	1.206734491	0.927899934	0.946160774	1.47283199 1.029056719	1.106588913
ENSG00000070761	0.041216	0.1073562	-0.157772595	-1.115563467	**CFAP20**	protein_coding	cilia and flagella associated protein 20 [Source:HGNC Symbol;Acc:HGNC:29523]	chr16	58113588	58129450	-1	ensembl_havana	5.406022915	5.484909212	5.327136617	5.466513193	5.555931748	5.432282695	5.359273442 5.353872886	5.268263524
ENSG00000123552	0.041209	0.1073562	-0.180967653	-1.133643995	**USP45**	protein_coding	ubiquitin specific peptidase 45 [Source:HGNC Symbol;Acc:HGNC:20080]	chr6	99432379	99521728	-1	ensembl_havana	4.507924117	4.598407944	4.417440291	4.430009476	4.689650085	4.675564271	4.345853613 4.423288215	4.483179044

ENSG00000163449	0.041208	0.1073562	-0.276411362	-1.211178383	**TMEM169**	protein_coding	transmembrane protein 169 [Source:HGNC Symbol;Acc:HGNC:25130]	chr2	216081866	216102783	1	ensembl_havana	1.574033727	1.712239408	1.435828047	1.464591378	1.979216042	1.692910804	1.400976194	1.504469011	1.402038935
ENSG00000080845	0.041248	0.107372889	0.222440982	1.166705937	**DLGAP4**	protein_coding	DLG associated protein 4 [Source:HGNC Symbol;Acc:HGNC:24476]	chr20	36306336	36528637	1	ensembl_havana	5.519728996	5.408508505	5.630949487	5.41737705	5.395137246	5.413011218	5.731520337	5.69482015	5.466507974
ENSG00000223768	0.04123	0.107372889	0.153189034	1.112024854	**LINC00205**	bidirectional_promoter_lncRNA	long intergenic non-protein coding RNA 205 [Source:HGNC Symbol;Acc:HGNC:16420]	chr21	45288050	45297806	1	havana	4.815145627	4.738551111	4.891740144	4.750937232	4.638193189	4.82652291	4.869634972	4.825302309	4.980283151
ENSG00000233834	0.041252	0.107372889	-0.176513044	-1.130149041	**AC005083.1**	processed_transcript		chr7	20217577	20221700	1	havana	1.475242663	1.563499185	1.386986141	1.283104003	1.798708764	1.608684788	1.052687018	1.578479109	1.529792296
ENSG00000228403	0.041239	0.107372889	-0.570438083	-1.484974423	**AC035139.1**	sense_intronic		chr10	48878022	48878649	1	havana	-0.15554658	0.129672461	-0.440765622	0.10902545	-0.002471726	0.28246366	-0.97139419	-0.52165082	0.17074815
ENSG00000145675	0.04129	0.107452491	0.17290714	1.127327849	**PIK3R1**	protein_coding	phosphoinositide-3-kinase regulatory subunit 1 [Source:HGNC Symbol;Acc:HGNC:8979]	chr5	68215720	68301821	1	ensembl_havana	7.077450144	6.990996574	7.163903714	6.839609641	7.042043177	7.091336904	6.940496827	7.299071777	7.252142537
ENSG00000167703	0.041314	0.107497002	0.365568101	1.288388868	**SLC43A2**	protein_coding	solute carrier family 43 member 2 [Source:HGNC Symbol;Acc:HGNC:23087]	chr17	1569267	1628886	-1	ensembl_havana	3.5159963	3.33321225	3.698780351	3.36293288	3.162009452	3.474694418	3.815261472	3.769761817	3.511317764
ENSG00000249776	0.041356	0.107584332	-0.153751805	-1.112458722	**AC124854.1**	lincRNA		chr5	92410256	92660863	-1	havana	4.225828713	4.302704616	4.148952811	4.569195086	4.248918908	4.089999853	4.431299106	4.053279749	3.962279577
ENSG00000066379	0.041369	0.107598606	-0.193914564	-1.143863232	**ZNRD1**	protein_coding	zinc ribbon domain containing 1 [Source:HGNC Symbol;Acc:HGNC:13182]	chr6	30058899	30064909	1	ensembl_havana	5.10977969	5.206736972	5.012822408	5.325564741	5.231167006	5.063479169	5.100386794	4.9455474	4.99253303
ENSG00000176261	0.041404	0.107672548	-0.177442698	-1.130877529	**ZBTB8OS**	protein_coding	zinc finger and BTB domain containing 8 opposite strand [Source:HGNC Symbol;Acc:HGNC:24094]	chr1	32600172	32650903	-1	ensembl_havana	4.549434206	4.638155555	4.460712857	4.649284895	4.553838016	4.711343754	4.378023791	4.453104385	4.551010395
ENSG00000182899	0.04142	0.107693459	0.16355806	1.120046058	**RPL35A**	protein_coding	ribosomal protein L35a [Source:HGNC Symbol;Acc:HGNC:10345]	chr3	197949980	197956610	1	ensembl_havana	8.029961437	7.948182407	8.111740467	7.964881024	7.976347536	7.903318661	8.130502484	8.07730771	8.127411206
ENSG00000143933	0.041447	0.107743319	0.170659677	1.125573039	**CALM2**	protein_coding	calmodulin 2 [Source:HGNC Symbol;Acc:HGNC:1445]	chr2	47160082	47176601	-1	ensembl_havana	10.35955318	10.27422334	10.44488302	10.2425218	10.39660745	10.18354079	10.43174246	10.48449779	10.41840881
ENSG00000110171	0.041493	0.107845524	0.225945583	1.169543549	**TRIM3**	protein_coding	tripartite motif containing 3 [Source:HGNC Symbol;Acc:HGNC:10064]	chr11	6448613	6474459	-1	ensembl_havana	3.34716817	3.234195379	3.460140962	3.142871041	3.241966183	3.317748913	3.525391749	3.456354973	3.398676164
ENSG00000212719	0.041503	0.107851236	0.196878466	1.146215624	**C17orf51**	protein_coding	chromosome 17 open reading frame 51 [Source:HGNC Symbol;Acc:HGNC:27904]	chr17	21524790	21574458	-1	ensembl_havana	4.072880106	3.974440873	4.171319339	3.776230114	4.020667242	4.126425264	4.100283535	4.120886932	4.292787552
ENSG00000105219	0.041514	0.10786062	0.767750341	1.702612752	**CNTD2**	protein_coding	cyclin N-terminal domain containing 2 [Source:HGNC Symbol;Acc:HGNC:25805]	chr19	40222208	40226697	-1	ensembl_havana	-0.441064273	-0.824939444	-0.057189103	-0.99620085	-1.03440435	-0.444213135	0.176794179	0.097529431	-0.445890917
ENSG00000107669	0.04153	0.107880637	-0.156589647	-1.114649128	**ATE1**	protein_coding	arginyltransferase 1 [Source:HGNC Symbol;Acc:HGNC:782]	chr10	121740421	121928801	-1	ensembl_havana	5.567975923	5.646270746	5.489681099	5.767662548	5.646191719	5.524957971	5.570530762	5.480721407	5.417791129
ENSG00000166333	0.041569	0.107927849	0.164008095	1.120395501	**ILK**	protein_coding	integrin linked kinase [Source:HGNC Symbol;Acc:HGNC:6040]	chr11	6603708	6610874	1	ensembl_havana	6.689091982	6.607087935	6.771096029	6.672210356	6.653500854	6.495552594	6.870391153	6.744901674	6.697995262
ENSG00000034713	0.041562	0.107927849	0.162361828	1.11911774	**GABARAPL2**	protein_coding	GABA type A receptor associated protein like 2 [Source:HGNC Symbol;Acc:HGNC:13291]	chr16	75566351	75577881	1	ensembl_havana	6.500257297	6.419076383	6.581438211	6.438411732	6.443572767	6.37524465	6.639710894	6.537496783	6.567106956
ENSG00000204929	0.04157	0.107927849	-0.5656208	-1.480024235	**AC007389.1**	processed_transcript		chr2	65436711	66084639	1	havana	-0.54036687	-0.25755647	-0.82317727	-0.51029874	-0.34076431	0.078393642	-0.69455235	-0.74417246	-1.030806998
ENSG00000090238	0.041607	0.108003304	-0.319960864	-1.248296686	**YPEL3**	protein_coding	yippee like 3 [Source:HGNC Symbol;Acc:HGNC:18327]	chr16	30092314	30096915	-1	ensembl_havana	2.083233519	2.243213951	1.923253087	2.390733944	2.004444884	2.334463026	2.144358071	1.890792227	1.734608964
ENSG00000170264	0.041633	0.10805139	0.148215311	1.108197725	**FAM161A**	protein_coding	family with sequence similarity 161 member A [Source:HGNC Symbol;Acc:HGNC:25808]	chr2	61824854	61854143	-1	ensembl_havana	3.953802605	3.879694949	4.02791026	3.672153106	3.944501979	4.022429763	3.831752454	4.083248454	4.168729871
ENSG00000000419	0.04168	0.108154091	-0.263218733	-1.200153329	**DPM1**	protein_coding	dolichyl-phosphate mannosyltransferase subunit 1, catalytic [Source:HGNC Symbol;Acc:HGNC:3005]	chr20	50934867	50958555	-1	ensembl_havana	6.608475253	6.740084619	6.476865886	6.86592105	6.74739797	6.606934837	6.382355747	6.565065153	6.483176759
ENSG00000079156	0.041744	0.108242517	0.282509914	1.216309106	**OSBPL6**	protein_coding	oxysterol binding protein like 6 [Source:HGNC Symbol;Acc:HGNC:16388]	chr2	178194481	178402891	1	ensembl_havana	4.364960055	4.223705098	4.506215012	3.72512993	4.38481302	4.561172345	4.246852146	4.599525013	4.672267877
ENSG00000178188	0.041738	0.108242517	0.157774898	1.115565249	**SH2B1**	protein_coding	SH2B adaptor protein 1 [Source:HGNC Symbol;Acc:HGNC:30417]	chr16	28846600	28874212	1	ensembl_havana	5.504292532	5.425405083	5.583179982	5.548475941	5.310853146	5.416886163	5.741737204	5.489540428	5.518262313
ENSG00000215012	0.04173	0.108242517	0.155901583	1.114117646	**RTL10**	protein_coding	retrotransposon Gag like 10 [Source:HGNC Symbol;Acc:HGNC:26112]	chr22	19846138	19854896	-1	ensembl_havana	5.066034003	4.988083211	5.143984794	4.973526147	5.123981705	4.866741781	5.179826793	5.246348626	5.005778964
ENSG00000167553	0.041738	0.108242517	-0.15696453	-1.114938807	**TUBA1C**	protein_coding	tubulin alpha 1c [Source:HGNC Symbol;Acc:HGNC:20768]	chr12	49188736	49274603	1	ensembl_havana	9.164644233	9.243126498	9.086161968	9.347868734	9.257700498	9.123810262	9.17528176	9.06321331	9.019990833
ENSG00000225969	0.041766	0.108280177	-0.469211755	-1.384352892	**ABHD11-AS1**	transcribed_unitary_pseudogene	ABHD11 antisense RNA 1 (tail to tail) [Source:HGNC Symbol;Acc:HGNC:18289]	chr7	73735038	73736158	1	havana	-0.430872892	-0.196267014	-0.665478769	0.206373087	0.06303108	-0.85820521	-0.35894771	-0.74417246	-0.893316134
ENSG00000164002	0.041784	0.108306359	0.160885338	1.117972994	**EXO5**	protein_coding	exonuclease 5 [Source:HGNC Symbol;Acc:HGNC:26115]	chr1	40508741	40516556	1	ensembl_havana	4.615497256	4.535054587	4.695939925	4.375237177	4.634157892	4.595768691	4.599638302	4.777658146	4.710523326
ENSG00000230513	0.0418	0.108327847	-0.3208806	-1.249092744	**THAP7-AS1**	antisense	THAP7 antisense RNA 1 [Source:HGNC Symbol;Acc:HGNC:41013]	chr22	21001886	21010342	1	havana	0.363493538	0.523933838	0.203053238	0.41082706	0.477608744	0.683365711	0.339398945	0.163140205	0.106620565
ENSG00000138031	0.041825	0.108369266	-0.187650251	-1.138907241	**ADCY3**	protein_coding	adenylate cyclase 3 [Source:HGNC Symbol;Acc:HGNC:234]	chr2	24819169	24919839	-1	ensembl_havana	6.858355897	6.952181022	6.764530771	6.976136445	6.787615648	7.092790972	6.866728144	6.638269357	6.788594814
ENSG00000173960	0.041831	0.108369266	-0.214151825	-1.160021725	**UBXN2A**	protein_coding	UBX domain protein 2A [Source:HGNC Symbol;Acc:HGNC:27265]	chr2	23927285	24004909	1	ensembl_havana	5.761270337	5.868346249	5.654194425	5.940318651	5.968646362	5.696073735	5.772672671	5.59953615	5.590374452
ENSG00000162384	0.041923	0.108589502	-0.178596563	-1.131782365	**C1orf123**	protein_coding	chromosome 1 open reading frame 123 [Source:HGNC Symbol;Acc:HGNC:26059]	chr1	53214099	53220617	-1	ensembl_havana	5.200927898	5.29022618	5.111629617	5.227510036	5.383966234	5.25920227	5.09470424	5.102206156	5.137978454
ENSG00000271857	0.041979	0.108590352	0.681977163	1.604336937	**AL096865.1**	antisense		chr6	45421079	45422005	-1	havana	0.079667976	-0.261320606	0.420656557	-0.79434999	0.06303108	-0.052642908	0.513047461	0.19485955	0.554062661
ENSG00000008513	0.041983	0.108590352	0.229775841	1.172652734	**ST3GAL1**	protein_coding	ST3 beta-galactoside alpha-2,3-sialyltransferase 1 [Source:HGNC Symbol;Acc:HGNC:10862]	chr8	133454848	133571940	-1	ensembl_havana	5.678915291	5.564027371	5.793803212	5.457478234	5.492707913	5.741895965	5.867697737	5.642583417	5.87112848
ENSG00000105254	0.041989	0.108590352	0.203601083	1.15156918	**TBCB**	protein_coding	tubulin folding cofactor B [Source:HGNC Symbol;Acc:HGNC:1989]	chr19	36114289	36125947	1	ensembl_havana	4.727480111	4.62567957	4.829280652	4.606827103	4.690945658	4.579265947	4.915484041	4.756557736	4.81580018
ENSG00000103942	0.04197	0.108590352	0.158559816	1.116172352	**HOMER2**	protein_coding	homer scaffolding protein 2 [Source:HGNC Symbol;Acc:HGNC:17513]	chr15	82836946	82986153	-1	ensembl_havana	7.206324154	7.127044245	7.285604062	7.213934566	7.109964076	7.057234094	7.353401536	7.228750683	7.274659966
ENSG00000130921	0.041945	0.108590352	-0.162175019	-1.11897284	**C12orf65**	protein_coding	chromosome 12 open reading frame 65 [Source:HGNC Symbol;Acc:HGNC:26784]	chr12	123232916	123257959	1	ensembl_havana	3.922113442	4.003200952	3.841025933	3.987262005	4.039096445	3.983244405	3.848057062	3.808721901	3.866298836
ENSG00000140382	0.041988	0.108590352	-0.162694192	-1.11937559	**HMG20A**	protein_coding	high mobility group 20A [Source:HGNC Symbol;Acc:HGNC:5001]	chr15	77420412	77485607	1	ensembl_havana	6.814867023	6.89621412	6.733519927	6.893893779	6.908368621	6.886379958	6.683147902	6.724671848	6.792740032
ENSG00000070814	0.041969	0.108590352	-0.183448805	-1.135595317	**TCOF1**	protein_coding	treacle ribosome biogenesis factor 1 [Source:HGNC Symbol;Acc:HGNC:11654]	chr5	150357639	150400308	1	ensembl_havana	6.586401096	6.678125499	6.494676694	6.498907921	6.67788098	6.857587594	6.424643512	6.461704327	6.597682243
ENSG00000116120	0.041947	0.108590352	-0.197086663	-1.146381047	**FARSB**	protein_coding	phenylalanyl-tRNA synthetase beta subunit [Source:HGNC Symbol;Acc:HGNC:17800]	chr2	222570536	222656337	-1	ensembl_havana	5.756975334	5.855518666	5.658432003	5.607586927	5.901965947	6.057003124	5.287362276	5.812589488	5.875344245
ENSG00000249743	0.041992	0.108590352	-0.616767919	-1.533435962	**AC116345.1**	lincRNA		chr5	73213947	73294934	1	havana	0.978682625	1.287066584	0.670298665	0.770507832	1.078042694	2.012649227	0.762800879	0.225896484	1.022198632
ENSG00000123240	0.042024	0.108610681	0.213841559	1.159772278	**OPTN**	protein_coding	optineurin [Source:HGNC Symbol;Acc:HGNC:17142]	chr10	13099449	13138308	1	ensembl_havana	5.78849237	5.68157159	5.895413149	5.609071199	5.829703058	5.605940514	5.911669561	5.884864407	5.889705481
ENSG00000156110	0.042021	0.108610681	-0.172183343	-1.126762412	**ADK**	protein_coding	adenosine kinase [Source:HGNC Symbol;Acc:HGNC:257]	chr10	74151185	74709303	1	ensembl_havana	5.223335373	5.309427044	5.137243701	5.310134477	5.339223061	5.278923594	5.055457657	5.242559499	5.113713948
ENSG00000175449	0.04203	0.108610681	-0.184312811	-1.13627561	**RFESD**	protein_coding	Rieske Fe-S domain containing [Source:HGNC Symbol;Acc:HGNC:29587]	chr5	95646754	95684773	1	ensembl_havana	3.006428712	3.098585117	2.914272306	3.130521836	3.116453761	3.048779755	2.863936192	2.905316921	2.973563806
ENSG00000147649	0.042015	0.108610681	-0.219839109	-1.164603701	**MTDH**	protein_coding	metadherin [Source:HGNC Symbol;Acc:HGNC:29608]	chr8	97644179	97728770	1	ensembl_havana	7.684108465	7.794028019	7.57418891	8.029213103	7.719728842	7.633142113	7.650807812	7.532659392	7.539099527
ENSG00000169567	0.042072	0.108698861	-0.221765522	-1.166159821	**HINT1**	protein_coding	histidine triad nucleotide binding protein 1 [Source:HGNC Symbol;Acc:HGNC:4912]	chr5	131159027	131171735	-1	ensembl_havana	7.399614326	7.510497087	7.288731565	7.60462665	7.510212437	7.416652174	7.221168791	7.323266174	7.32175973
ENSG00000139437	0.042116	0.108795188	0.187350419	1.138670569	**TCHP**	protein_coding	trichoplein keratin filament binding [Source:HGNC Symbol;Acc:HGNC:28135]	chr12	109900264	109983841	1	ensembl_havana	4.297082285	4.203407075	4.390757494	4.047618066	4.167497176	4.395105983	4.305097297	4.403062832	4.464112353
ENSG00000059728	0.042128	0.10880554	0.179197101	1.13225358	**MXD1**	protein_coding	MAX dimerization protein 1 [Source:HGNC Symbol;Acc:HGNC:6761]	chr2	69897688	69942945	1	ensembl_havana	5.930344952	5.840746402	6.019943503	6.066145426	5.781869063	5.674224717	6.188971417	5.912551693	5.958307398
ENSG00000175048	0.042163	0.108836191	0.215098624	1.160783264	**ZDHHC14**	protein_coding	zinc finger DHHC-type containing 14 [Source:HGNC Symbol;Acc:HGNC:20341]	chr6	157381133	157678146	1	ensembl_havana	5.35818702	5.250637708	5.465736331	5.224608156	5.271560182	5.255744786	5.599711351	5.4233008	5.374196843
ENSG00000174953	0.042152	0.108836191	-0.1831712	-1.135376825	**DHX36**	protein_coding	DEAH-box helicase 36 [Source:HGNC Symbol;Acc:HGNC:14410]	chr3	154272546	154324497	-1	ensembl_havana	7.243249808	7.334835408	7.151664208	7.391103708	7.338360952	7.275041564	7.095920227	7.213346347	7.145726052
ENSG00000112312	0.042161	0.108836191	-0.196983533	-1.146299102	**GMNN**	protein_coding	geminin, DNA replication inhibitor [Source:HGNC Symbol;Acc:HGNC:17493]	chr6	24774931	24786099	1	ensembl_havana	5.646477889	5.744969655	5.547986122	5.588903523	5.876571269	5.769434174	5.464365468	5.545201873	5.634391026
ENSG00000032444	0.042187	0.108841289	0.357126074	1.280871789	**PNPLA6**	protein_coding	patatin like phospholipase domain containing 6 [Source:HGNC Symbol;Acc:HGNC:16268]	chr19	7534004	7561764	1	ensembl_havana	6.325258467	6.14669543	6.503821503	6.134217744	5.980879167	6.324989378	6.667538324	6.495926552	6.347999634
ENSG00000204681	0.042182	0.108841289	0.169203723	1.124437694	**GABBR1**	protein_coding	gamma-aminobutyric acid type B receptor subunit 1 [Source:HGNC Symbol;Acc:HGNC:4070]	chr6	29555629	29633976	-1	ensembl_havana	6.447819025	6.363217164	6.532420886	6.49570191	6.279307875	6.314641706	6.623621523	6.403930374	6.569710762
ENSG00000272140	0.042173	0.108841289	-0.31943013	-1.247837551	**AC022400.5**	lincRNA		chr10	73703735	73713581	-1	havana	0.545522033	0.705237098	0.385806968	0.968509893	0.271370116	0.875831286	0.36983092	0.163140205	0.624449778
ENSG00000213398	0.042198	0.108849153	-0.238240669	-1.179553348	**LCAT**	protein_coding	lecithin-cholesterol acyltransferase [Source:HGNC Symbol;Acc:HGNC:6522]	chr16	67939750	67944131	-1	ensembl_havana	1.699321984	1.818442319	1.58020165	1.912130943	1.891783655	1.651412359	1.863449533	1.529563273	1.347592143
ENSG00000235947	0.042215	0.108873045	1.110483933	2.159180621	**EGOT**	lincRNA	eosinophil granule ontogeny transcript (non-protein coding) [Source:HGNC Symbol;Acc:HGNC:37129]	chr3	4749192	4751590	-1	havana	-0.697596516	-1.252838483	-0.142354549	-1.23095973	-1.838810968	-0.688744748	-0.75894329	0.399280361	-0.067400719
ENSG00000172954	0.042244	0.108915104	-0.173377301	-1.127695295	**LCLAT1**	protein_coding	lysocardiolipin acyltransferase 1 [Source:HGNC Symbol;Acc:HGNC:26756]	chr2	30447226	30644225	1	ensembl_havana	5.84931286	5.936001511	5.76262421	5.979289561	5.863991495	5.964723478	5.721230601	5.769133193	5.797508836
ENSG00000171867	0.042246	0.108915104	-0.209180516	-1.156031345	**PRNP**	protein_coding	prion protein [Source:HGNC Symbol;Acc:HGNC:9449]	chr20	4686236	4701590	1	ensembl_havana	6.193272266	6.297862523	6.088682008	6.369148597	6.375104778	6.149334196	6.228429016	6.013279788	6.024337219
ENSG00000138138	0.042266	0.108946586	-0.154365133	-1.112931758	**ATAD1**	protein_coding	ATPase family, AAA domain containing 1 [Source:HGNC Symbol;Acc:HGNC:25903]	chr10	87751512	87841343	-1	ensembl_havana	5.991384363	6.06856693	5.914201797	6.132154523	6.091457003	5.982089263	5.949941118	5.91671878	5.875945493
ENSG00000204396	0.042326	0.109077642	0.449364437	1.365438596	**VWA7**	protein_coding	von Willebrand factor A domain containing 7 [Source:HGNC Symbol;Acc:HGNC:13939]	chr6	31765590	31777294	-1	ensembl_havana	0.939496893	0.714814674	1.164179112	0.326725152	0.679062629	1.138656243	1.161063346	1.114463419	1.21701057
ENSG00000188672	0.042332	0.109077642	-0.221048178	-1.165580121	**RHCE**	protein_coding	Rh blood group CcEe antigens [Source:HGNC Symbol;Acc:HGNC:10008]	chr1	25362249	25430192	-1	ensembl_havana	0.322759988	0.433284077	0.212235899	0.297565234	0.614986832	0.387300166	0.24405081	0.286036324	0.106620565
ENSG00000020256	0.042381	0.109163999	0.274220746	1.209340702	**ZFP64**	protein_coding	ZFP64 zinc finger protein [Source:HGNC Symbol;Acc:HGNC:15940]	chr20	52051663	52204308	-1	ensembl_havana	4.293008685	4.155898312	4.430119058	3.964251181	4.184153292	4.319290462	4.39476902	4.535932191	4.359655962
ENSG00000174842	0.042377	0.109163999	-0.202073171	-1.150350236	**GLMN**	protein_coding	glomulin, FKBP associated protein [Source:HGNC Symbol;Acc:HGNC:14373]	chr1	92246402	92298987	-1	ensembl_havana	4.486393624	4.587430209	4.385357038	4.65216747	4.659526198	4.45059696	4.403988623	4.36348661	4.388595882
ENSG00000144560	0.042397	0.109185755	0.14801771	1.10804595	**VGLL4**	protein_coding	vestigial like family member 4 [Source:HGNC Symbol;Acc:HGNC:28966]	chr3	11556070	11771350	-1	ensembl_havana	5.206608078	5.132599222	5.280616933	4.971219637	5.166512682	5.260065348	5.125127496	5.327286598	5.389436704
ENSG00000153558	0.04242	0.109226865	0.202589412	1.15076194	**FBXL2**	protein_coding	F-box and leucine rich repeat protein 2 [Source:HGNC Symbol;Acc:HGNC:13598]	chr3	33277025	33403662	1	ensembl_havana	4.420124193	4.318829487	4.521418899	4.124355192	4.344219408	4.487913861	4.470441894	4.469406474	4.624408329
ENSG00000128923	0.042471	0.109336229	0.176990053	1.130522772	**MINDY2**	protein_coding	MINDY lysine 48 deubiquitinase 2 [Source:HGNC Symbol;Acc:HGNC:26954]	chr15	58771192	58861900	1	ensembl_havana	5.444836657	5.356341631	5.533331684	5.281564904	5.36054741	5.426912579	5.434083869	5.635377079	5.530534103
ENSG00000267419	0.042518	0.109380961	0.412481475	1.330973157	**AC011477.1**	transcribed_unprocessed_pseudogene		chr19	19776602	19836073	1	havana	1.082326687	0.87608595	1.288567425	0.749126884	1.155163158	0.723967808	1.229040238	1.195095435	1.441566602
ENSG00000140718	0.042514	0.109380961	0.151876888	1.111013916	**FTO**	protein_coding	FTO, alpha-ketoglutarate dependent dioxygenase [Source:HGNC Symbol;Acc:HGNC:24678]	chr16	53701692	54158512	1	ensembl_havana	6.398344603	6.322406159	6.474283047	6.44922479	6.296431761	6.221561926	6.562768349	6.456398022	6.403682769
ENSG00000135316	0.042507	0.109380961	-0.159006417	-1.116517928	**SYNCRIP**	protein_coding	synaptotagmin binding cytoplasmic RNA interacting protein [Source:HGNC Symbol;Acc:HGNC:16918]	chr6	85607785	85643792	-1	ensembl_havana	7.724011699	7.803514907	7.644508491	7.752476159	7.801917194	7.856151369	7.530949744	7.666827135	7.735748592
ENSG00000111364	0.042506	0.109380961	-0.18017136	-1.133018455	**DDX55**	protein_coding	DEAD-box helicase 55 [Source:HGNC Symbol;Acc:HGNC:20085]	chr12	123602077	123620941	1	ensembl_havana	5.42787722	5.517962901	5.33779154	5.665084692	5.464199043	5.424604968	5.379979066	5.315611956	5.3177836
ENSG00000115514	0.042559	0.109465284	-0.15893281	-1.116460964	**TXNDC9**	protein_coding	thioredoxin domain containing 9 [Source:HGNC Symbol;Acc:HGNC:24110]	chr2	99318982	99340702	-1	ensembl_havana	5.66377353	5.743239935	5.584307125	5.774292663	5.798791959	5.656635183	5.552403201	5.674573768	5.525944406
ENSG00000135709	0.042571	0.109476085	-0.20858298	-1.155552638	**KIAA0513**	protein_coding	KIAA0513 [Source:HGNC Symbol;Acc:HGNC:29058]	chr16	85027751	85094230	1	ensembl_havana	5.036990251	5.141281741	4.932698761	5.261884801	5.01026654	5.151693882	4.963666724	4.953681926	4.880747632
ENSG00000100376	0.042598	0.109527172	0.151872542	1.11101057	**FAM118A**	protein_coding	family with sequence similarity 118 member A [Source:HGNC Symbol;Acc:HGNC:1313]	chr22	45308968	45341955	1	ensembl_havana	4.980277226	4.904340955	5.056213497	4.923104885	4.851334342	4.938583638	5.060130702	5.043529596	5.064980193
ENSG00000010818	0.042644	0.109625231	0.26973543	1.205586719	**HIVEP2**	protein_coding	human immunodeficiency virus type I enhancer binding protein 2 [Source:HGNC Symbol;Acc:HGNC:4921]	chr6	142751467	142956698	-1	ensembl_havana	3.574277596	3.439409881	3.70914531	3.22260331	3.421266147	3.674360185	3.721029149	3.617169153	3.789237629
ENSG00000160055	0.042675	0.109685686	0.176425668	1.130080596	**TMEM234**	protein_coding	transmembrane protein 234 [Source:HGNC Symbol;Acc:HGNC:28837]	chr1	32214472	32222359	-1	ensembl_havana	4.49881812	4.410605286	4.587030954	4.539923521	4.395977503	4.295914835	4.645446557	4.545190308	4.570455996
ENSG00000066557	0.042741	0.109834601	0.152470823	1.111471397	**LRRC40**	protein_coding	leucine rich repeat containing 40 [Source:HGNC Symbol;Acc:HGNC:26004]	chr1	70144805	70205620	-1	ensembl_havana	5.2580894	5.181853989	5.334324811	5.098194734	5.222234199	5.225133033	5.292335689	5.342412265	5.368226481
ENSG00000120616	0.042781	0.10991987	0.151529151	1.110746158	**EPC1**	protein_coding	enhancer of polycomb homolog 1 [Source:HGNC Symbol;Acc:HGNC:19876]	chr10	32267751	32378798	-1	ensembl_havana	5.410934809	5.335170234	5.486699385	5.266594948	5.470247905	5.268667849	5.430479178	5.582400786	5.447218192
ENSG00000118260	0.04281	0.109974038	-0.151729545	-1.110900454	**CREB1**	protein_coding	cAMP responsive element binding protein 1 [Source:HGNC Symbol;Acc:HGNC:2345]	chr2	207529737	207603431	1	ensembl_havana	5.717497709	5.793362481	5.641632936	5.936776811	5.818485256	5.624825377	5.750437297	5.664697016	5.509764495
ENSG00000144837	0.042845	0.11004406	0.311880135	1.241324353	**PLA1A**	protein_coding	phospholipase A1 member A [Source:HGNC Symbol;Acc:HGNC:17661]	chr3	119597842	119629811	1	ensembl_havana	1.387457835	1.231517768	1.543397903	1.424690178	1.256642557	1.013220568	1.656848881	1.372000412	1.601344415
ENSG00000105991	0.04287	0.110087841	0.216658646	1.162039126	**HOXA1**	protein_coding	homeobox A1 [Source:HGNC Symbol;Acc:HGNC:5099]	chr7	27092993	27095996	-1	ensembl_havana	3.630003535	3.521674212	3.738332858	3.394260955	3.675396647	3.495365035	3.726918802	3.732424628	3.755655145
ENSG00000096746	0.042902	0.11015054	-0.155637581	-1.11391379	**HNRNPH3**	protein_coding	heterogeneous nuclear ribonucleoprotein H3 [Source:HGNC Symbol;Acc:HGNC:5043]	chr10	68331174	68343191	1	ensembl_havana	7.645295441	7.723114231	7.56747665	7.692483595	7.69508336	7.78177574	7.564432609	7.484545282	7.653452059
ENSG00000176171	0.042914	0.110161905	0.180797539	1.13351033	**BNIP3**	protein_coding	BCL2 interacting protein 3 [Source:HGNC Symbol;Acc:HGNC:1084]	chr10	131966455	131982013	-1	ensembl_havana	4.472982132	4.382583362	4.563380901	4.312848501	4.509001353	4.325900232	4.544011576	4.58013937	4.565991757
ENSG00000103266	0.042967	0.110278057	0.252147831	1.190978881	**STUB1**	protein_coding	STIP1 homology and U-box containing protein 1 [Source:HGNC Symbol;Acc:HGNC:11427]	chr16	680224	682870	1	ensembl_havana	4.655812518	4.529738603	4.781886433	4.453333619	4.509001353	4.626880836	4.894705093	4.749900063	4.701054144
ENSG00000119943	0.043019	0.110391584	0.289227115	1.221985456	**PYROXD2**	protein_coding	pyridine nucleotide-disulphide oxidoreductase domain 2 [Source:HGNC Symbol;Acc:HGNC:23517]	chr10	98383565	98415184	-1	ensembl_havana	1.749745955	1.605132398	1.894359513	1.158893785	1.817810321	1.838693089	1.656848881	1.871294071	2.154935587
ENSG00000149926	0.043067	0.110496257	-0.381710555	-1.302885728	**FAM57B**	protein_coding	family with sequence similarity 57 member B [Source:HGNC Symbol;Acc:HGNC:25295]	chr16	30024427	30052978	-1	ensembl_havana	0.115076639	0.305931917	-0.075778638	0.237415364	0.243386951	0.436993436	-0.30988669	0.225896484	-0.143345712
ENSG00000080298	0.043103	0.110549221	0.165442532	1.121510037	**RFX3**	protein_coding	regulatory factor X3 [Source:HGNC Symbol;Acc:HGNC:9984]	chr9	3218297	3526004	-1	ensembl_havana	5.198175131	5.115453864	5.280896397	5.489259151	5.048165446	4.808936996	5.597302052	5.291058488	4.954328649
ENSG00000115875	0.043096	0.110549221	-0.187044064	-1.138428799	**SRSF7**	protein_coding	serine and arginine rich splicing factor 7 [Source:HGNC Symbol;Acc:HGNC:10789]	chr2	38743599	38751494	-1	ensembl_havana	8.134016492	8.227538524	8.04049446	8.230063846	8.180830323	8.271721403	7.927623433	8.020357098	8.17350285
ENSG00000132356	0.043147	0.110641172	-0.157521995	-1.115369708	**PRKAA1**	protein_coding	protein kinase AMP-activated catalytic subunit alpha 1 [Source:HGNC Symbol;Acc:HGNC:9376]	chr5	40759379	40798374	-1	ensembl_havana	6.859677222	6.938438219	6.780916224	6.996693792	6.911706226	6.906914638	6.786165425	6.807153155	6.749430092
ENSG00000100911	0.043191	0.110734051	-0.170831361	-1.125706993	**PSME2**	protein_coding	proteasome activator subunit 2 [Source:HGNC Symbol;Acc:HGNC:9569]	chr14	24143362	24147570	-1	ensembl_havana	7.392344335	7.477760016	7.306928655	7.468777245	7.554834176	7.409668627	7.285935	7.311106634	7.323744331
ENSG00000196275	0.043236	0.110830493	0.238500482	1.179765791	**GTF2IRD2**	protein_coding	GTF2I repeat domain containing 2 [Source:HGNC Symbol;Acc:HGNC:30775]	chr7	74796144	74851551	-1	ensembl_havana	1.296940808	1.177690567	1.416191049	1.158893785	1.37743234	0.996745577	1.580839304	1.465989718	1.201744126
ENSG00000182923	0.043263	0.110878089	-0.183730862	-1.135817356	**CEP63**	protein_coding	centrosomal protein 63 [Source:HGNC Symbol;Acc:HGNC:25815]	chr3	134485743	134575017	1	ensembl_havana	4.878272924	4.970138355	4.786407493	4.934988992	4.89133059	5.084095483	4.645446557	4.801032971	4.912742951
ENSG00000163704	0.043289	0.110906857	0.183524996	1.135655291	**PRRT3**	protein_coding	proline rich transmembrane protein 3 [Source:HGNC Symbol;Acc:HGNC:26591]	chr3	9945542	9952394	-1	ensembl_havana	5.395156207	5.303393709	5.486918705	5.553880812	5.274155646	5.082144669	5.811298363	5.497511205	5.151946546
ENSG00000149823	0.043286	0.110906857	0.163873843	1.120291246	**VPS51**	protein_coding	VPS51, GARP complex subunit [Source:HGNC Symbol;Acc:HGNC:1172]	chr11	65089324	65111860	1	ensembl_havana	4.378683592	4.29674667	4.460620514	4.352378	4.243631448	4.294230564	4.525545558	4.469406474	4.386909509
ENSG00000142230	0.043318	0.110961057	-0.147248122	-1.107455034	**SAE1**	protein_coding	SUMO1 activating enzyme subunit 1 [Source:HGNC Symbol;Acc:HGNC:30660]	chr19	47113274	47210636	1	ensembl_havana	8.032832511	8.106456572	7.95920845	8.003061601	8.151015877	8.165292237	7.872068208	7.983799251	8.02175789
ENSG00000228695	0.043332	0.110976376	0.42104283	1.338895005	**CES1P1**	transcribed_unprocessed_pseudogene	carboxylesterase 1 pseudogene 1 [Source:HGNC Symbol;Acc:HGNC:18546]	chr16	55760566	55793960	1	havana	1.269360248	1.058838833	1.479881663	0.464289132	1.170104606	1.542122761	1.261864131	1.541948597	1.635832261
ENSG00000226002	0.043369	0.111052901	-0.358028278	-1.281673045	**GTF2IP14**	unprocessed_pseudogene	general transcription factor IIi pseudogene 14 [Source:HGNC Symbol;Acc:HGNC:51726]	chr7	65084103	65100232	1	havana	-0.31110856	-0.132094422	-0.490122699	-0.10791135	-0.428075095	0.139703184	-0.46237776	-1.08144551	0.073455173
ENSG00000161036	0.043391	0.111088724	0.301934789	1.232796601	**LRWD1**	protein_coding	leucine rich repeats and WD repeat domain containing 1 [Source:HGNC Symbol;Acc:HGNC:21769]	chr7	102464929	102473168	1	ensembl_havana	4.577203998	4.426236603	4.728171392	4.338128639	4.365470575	4.575110596	4.88551934	4.650458282	4.648536554
ENSG00000172915	0.043428	0.111163467	0.265185449	1.201790522	**NBEA**	protein_coding	neurobeachin [Source:HGNC Symbol;Acc:HGNC:7648]	chr13	34942287	35673022	1	ensembl_havana	4.190560625	4.0579679	4.32315335	4.355918458	3.731873694	4.08611155	4.45278126	4.216704714	4.299974075
ENSG00000185909	0.043437	0.111165886	-0.633959799	-1.551818466	**KLHDC8B**	protein_coding	kelch domain containing 8B [Source:HGNC Symbol;Acc:HGNC:28557]	chr3	49171611	49176486	1	ensembl_havana	-0.132536626	0.184443274	-0.449516525	0.040274975	0.403680778	0.109374069	-0.00649342	-0.74417246	-0.597883695
ENSG00000004846	0.04348	0.111257927	-0.951664806	-1.93410324	**ABCB5**	protein_coding	ATP binding cassette subfamily B member 5 [Source:HGNC Symbol;Acc:HGNC:46]	chr7	20615207	20777038	1	ensembl_havana	-0.656501555	-0.180669152	-1.132333958	0.237415364	-0.036373238	-0.743049581	-0.63291296	-1.87077278	-0.893316134
ENSG00000092820	0.04351	0.111313591	-0.185916289	-1.137539221	**EZR**	protein_coding	ezrin [Source:HGNC Symbol;Acc:HGNC:12691]	chr6	158765741	158819412	-1	ensembl_havana	7.280436567	7.373394711	7.187478423	7.403559368	7.419340426	7.29728434	7.333514897	7.202415839	7.026504532
ENSG00000088298	0.043529	0.111325985	0.151597215	1.110798562	**EDEM2**	protein_coding	ER degradation enhancing alpha-mannosidase like protein 2 [Source:HGNC Symbol;Acc:HGNC:15877]	chr20	35115357	35147364	-1	ensembl_havana	5.504239649	5.428441042	5.580038257	5.385686583	5.384767039	5.514869505	5.578695862	5.503064662	5.658354247
ENSG00000151065	0.04353	0.111325985	-0.238610847	-1.179856046	**DCP1B**	protein_coding	decapping mRNA 1B [Source:HGNC Symbol;Acc:HGNC:24451]	chr12	1946054	2004535	-1	ensembl_havana	3.41134919	3.530654614	3.292043767	3.4316494	3.630188857	3.530125585	3.044565681	3.35471112	3.476854499
ENSG00000198198	0.043644	0.111498044	0.213811957	1.159748481	**SZT2**	protein_coding	SZT2, KICSTOR complex subunit [Source:HGNC Symbol;Acc:HGNC:29040]	chr1	43389882	43454247	1	ensembl_havana	6.647599605	6.540693627	6.754505584	6.590040034	6.301953545	6.7300873	6.727887269	6.678440769	6.857188713
ENSG00000211643	0.043655	0.111498044	0.199068753	1.14795712	**IGLV5-52**	IG_V_gene	immunoglobulin lambda variable 5-52 [Source:HGNC Symbol;Acc:HGNC:5926]	chr22	22318727	22319226	1	havana	1.313742184	1.214207808	1.413276561	1.477650254	1.184892896	0.980080273	1.619344613	1.300906777	1.319578292
ENSG00000168090	0.04366	0.111498044	0.185053927	1.136859468	**COPS6**	protein_coding	COP9 signalosome subunit 6 [Source:HGNC Symbol;Acc:HGNC:21749]	chr7	100088954	100092200	1	ensembl_havana	6.546751185	6.454224221	6.639278149	6.300990734	6.567595836	6.494086094	6.55492627	6.634298453	6.728609723
ENSG00000050393	0.043654	0.111498044	0.183764042	1.135843479	**MCUR1**	protein_coding	mitochondrial calcium uniporter regulator 1 [Source:HGNC Symbol;Acc:HGNC:21097]	chr6	13786557	13814568	-1	ensembl_havana	5.353477211	5.261595189	5.445359232	5.091839131	5.362987751	5.329958685	5.386505383	5.52272573	5.426846581
ENSG00000225377	0.043645	0.111498044	0.169701981	1.124826104	**NRSN2-AS1**	antisense	NRSN2 antisense RNA 1 [Source:HGNC Symbol;Acc:HGNC:51222]	chr20	320313	348224	-1	havana	1.980128874	1.895277884	2.064979864	2.067039104	1.798708764	1.820085783	2.170524752	2.055343696	1.969071146
ENSG00000125995	0.043653	0.111498044	0.161217838	1.118230684	**ROMO1**	protein_coding	reactive oxygen species modulator 1 [Source:HGNC Symbol;Acc:HGNC:16185]	chr20	35699272	35700984	1	ensembl_havana	4.727767871	4.647158952	4.808376789	4.718301125	4.612443584	4.610732146	4.860287678	4.722957818	4.841884873
ENSG00000178425	0.043656	0.111498044	-0.146738731	-1.10706408	**NT5DC1**	protein_coding	5'-nucleotidase domain containing 1 [Source:HGNC Symbol;Acc:HGNC:21556]	chr6	116100849	116249497	1	ensembl_havana	5.107899314	5.18126868	5.034529948	5.195263736	5.223129973	5.12541233	5.031862651	5.074765326	4.996961868
ENSG00000230623	0.043626	0.111498044	-0.421013206	-1.338867512	**AC104461.1**	lincRNA		chr1	200333193	200478293	1	havana	0.734883513	0.945390116	0.52437691	1.022973463	1.109387295	0.703809589	0.428834256	0.45273451	0.691561964
ENSG00000158769	0.043688	0.11155129	-0.187019532	-1.138409441	**F11R**	protein_coding	F11 receptor [Source:HGNC Symbol;Acc:HGNC:14685]	chr1	160995211	161021348	-1	ensembl_havana	5.344775468	5.438285234	5.251265702	5.508474265	5.525755619	5.280625819	5.341149002	5.224423979	5.188224127
ENSG00000170178	0.043704	0.11156044	0.791744534	1.731166556	**HOXD12**	protein_coding	homeobox D12 [Source:HGNC Symbol;Acc:HGNC:5135]	chr2	176099730	176101193	1	ensembl_havana	-0.266049768	-0.661922034	0.129822499	-1.51148723	-0.521012188	0.046733314	0.069637898	0.02879206	0.291037539
ENSG00000215302	0.043707	0.11156044	-0.275552331	-1.21045742	**AC127502.1**	transcribed_unprocessed_pseudogene		chr15	30470779	30507623	1	havana	-0.024257625	0.113518541	-0.162033791	0.464289132	-0.071090603	-0.052642908	-0.00649342	-0.37473441	-0.104873545
ENSG00000270959	0.043741	0.111625538	-0.188398416	-1.139498018	**LPP-AS2**	antisense	LPP antisense RNA 2 [Source:HGNC Symbol;Acc:HGNC:27952]	chr3	188151206	188154057	-1	havana	0.987100739	1.081299946	0.892901531	0.852997155	1.109387295	1.281515389	0.716189844	0.993426194	0.969088554
ENSG00000035687	0.043753	0.111638158	-0.181562414	-1.134111444	**ADSS**	protein_coding	adenylosuccinate synthase [Source:HGNC Symbol;Acc:HGNC:292]	chr1	244408494	244452134	-1	ensembl_havana	5.507040526	5.597821733	5.416259318	5.462413406	5.545943005	5.785108786	5.22630432	5.477501077	5.544972558
ENSG00000265728	0.0438	0.111697937	0.629842684	1.547396251	**AP001099.1**	lincRNA		chr18	10594590	10604798	1	havana	-0.657622167	-0.972543509	-0.342700825	-0.99620085	-1.178380098	-0.743049581	-0.82634296	0.063570082	-0.265329594
ENSG00000135407	0.043794	0.111697937	0.36937288	1.291791185	**AVIL**	protein_coding	advillin [Source:HGNC Symbol;Acc:HGNC:14188]	chr12	57797376	57818704	-1	ensembl_havana	0.856645762	0.671959322	1.041332202	1.109412078	0.570631912	0.335833975	1.143556601	1.01135145	0.969088554
ENSG00000164828	0.043792	0.111697937	0.147750777	1.107840954	**SUN1**	protein_coding	Sad1 and UNC84 domain containing 1 [Source:HGNC Symbol;Acc:HGNC:18587]	chr7	816615	896435	1	ensembl_havana	7.925221538	7.851346149	7.999096926	8.008695498	7.748322706	7.797020244	8.17372676	7.906434841	7.917129177
ENSG00000133731	0.043808	0.111698917	-0.195934416	-1.145465825	**IMPA1**	protein_coding	inositol monophosphatase 1 [Source:HGNC Symbol;Acc:HGNC:6050]	chr8	81657961	81686693	-1	ensembl_havana	5.486505769	5.584472977	5.38853856	5.738122539	5.600733946	5.414562445	5.47139912	5.338867525	5.355349037
ENSG00000214900	0.043816	0.111699689	0.33487432	1.261267529	**LINC01588**	lincRNA	long intergenic non-protein coding RNA 1588 [Source:HGNC Symbol;Acc:HGNC:27503]	chr14	49927571	50092643	-1	ensembl_havana	1.971080904	1.803643744	2.138518064	1.727676152	1.614359492	2.068895587	2.034670322	2.233896951	2.146986918
ENSG00000110851	0.043839	0.111738371	0.15213762	1.111214723	**PRDM4**	protein_coding	PR/SET domain 4 [Source:HGNC Symbol;Acc:HGNC:9348]	chr12	107732866	107761272	-1	ensembl_havana	4.883835323	4.807766513	4.959904133	4.847154546	4.801827565	4.774317427	5.046065911	4.925599042	4.908047446
ENSG00000143393	0.043875	0.111786329	0.170730304	1.125628142	**PI4KB**	protein_coding	phosphatidylinositol 4-kinase beta [Source:HGNC Symbol;Acc:HGNC:8984]	chr1	151291797	151327715	-1	ensembl_havana	7.111604933	7.026239781	7.196970084	7.102171026	7.037212627	6.939335689	7.311146741	7.114968007	7.164795505
ENSG00000163832	0.043882	0.111786329	-0.148517066	-1.108429541	**ELP6**	protein_coding	elongator acetyltransferase complex subunit 6 [Source:HGNC Symbol;Acc:HGNC:25976]	chr3	47495640	47513761	-1	ensembl_havana	5.461527999	5.535786532	5.387269466	5.447556952	5.555220555	5.604582089	5.331517573	5.392000723	5.438290102
ENSG00000100528	0.043866	0.111786329	-0.176589887	-1.130209239	**CNIH1**	protein_coding	cornichon family AMPA receptor auxiliary protein 1 [Source:HGNC Symbol;Acc:HGNC:19431]	chr14	54423560	54441431	-1	ensembl_havana	5.95600443	6.044299373	5.867709486	6.076375527	6.003995923	6.05252667	5.829203939	5.795880917	5.978043601
ENSG00000126453	0.04389	0.111787103	0.223093103	1.167233426	**BCL2L12**	protein_coding	BCL2 like 12 [Source:HGNC Symbol;Acc:HGNC:13787]	chr19	49665142	49673916	1	ensembl_havana	5.724495634	5.612949083	5.836042186	5.48764628	5.721025012	5.630175956	5.886237299	5.870818921	5.751070337
ENSG00000101544	0.043917	0.111816219	-0.148704096	-1.108573246	**ADNP2**	protein_coding	ADNP homeobox 2 [Source:HGNC Symbol;Acc:HGNC:23803]	chr18	80109031	80147523	1	ensembl_havana	6.050858977	6.125211025	5.976506929	6.174373175	6.080624642	6.120635256	5.99273379	5.954269922	5.982517074

ENSG00000163104	0.043912	0.111816219	-0.172794775	-1.127240049	**SMARCAD1**	protein_coding	SWI/SNF-related, matrix-associated actin-dependent regulator of chromatin, subfamily a, containing DEAD/H box 1 [Source:HGNC Symbol;Acc:HGNC:18398]	chr4	94207611	94291292	1	ensembl_havana	6.965264136	7.051661523	6.878866749	7.064800173	7.035683824	7.054500573	6.794602449	6.906582256	6.935415541
ENSG00000119986	0.043939	0.111853288	0.57189222	1.48647193	**AVPI1**	protein_coding	arginine vasopressin induced 1 [Source:HGNC Symbol;Acc:HGNC:30898]	chr10	97677424	97687323	-1	ensembl_havana	0.246931911	-0.0390142	0.532878021	-0.27307956	0.243386951	-0.087349994	0.851734776	0.399280361	0.347618925
ENSG00000164011	0.043953	0.111868788	0.161086184	1.118128644	**ZNF691**	protein_coding	zinc finger protein 691 [Source:HGNC Symbol;Acc:HGNC:28028]	chr1	42846573	42852477	1	ensembl_havana	4.501673349	4.421130257	4.582216441	4.316487184	4.520706172	4.426197415	4.547343787	4.62884954	4.570455996
ENSG00000267478	0.044068	0.112141292	0.54530245	1.459326249	**AP002414.3**	processed_pseudogene		chr18	12091674	12093141	-1	havana	0.027679915	-0.24497131	0.30033114	-0.14746469	-0.428075095	-0.159374151	0.540060475	0.504278656	-0.143345712
ENSG00000042445	0.044105	0.112215473	0.160753976	1.117871204	**RETSAT**	protein_coding	retinol saturase [Source:HGNC Symbol;Acc:HGNC:25991]	chr2	85342088	85354620	-1	ensembl_havana	6.185228356	6.104851367	6.265605344	6.161786229	6.157142197	5.995625677	6.272887471	6.29701468	6.226913881
ENSG00000043093	0.044202	0.1123902	0.149244008	1.108988195	**DCUN1D1**	protein_coding	defective in cullin neddylation 1 domain containing 1 [Source:HGNC Symbol;Acc:HGNC:18184]	chr3	182938074	182985953	-1	ensembl_havana	6.245569837	6.170947833	6.320191841	6.095032634	6.243996769	6.173814095	6.273893566	6.398406845	6.288275111
ENSG00000181915	0.044195	0.1123902	0.1476239	1.10774353	**ADO**	protein_coding	2-aminoethanethiol dioxygenase [Source:HGNC Symbol;Acc:HGNC:23506]	chr10	62804857	62808483	1	ensembl_havana	4.548416263	4.474604313	4.622228213	4.558479799	4.471799458	4.393533683	4.677732971	4.625943765	4.563007904
ENSG00000162604	0.044205	0.1123902	-0.148095195	-1.108105463	**TM2D1**	protein_coding	TM2 domain containing 1 [Source:HGNC Symbol;Acc:HGNC:24142]	chr1	61681046	61725423	-1	ensembl_havana	4.254909727	4.328957324	4.180862129	4.450024596	4.287112591	4.249734786	4.303127503	4.110529916	4.128928969
ENSG00000149929	0.044191	0.1123902	-0.184806166	-1.136664246	**HIRIP3**	protein_coding	HIRA interacting protein 3 [Source:HGNC Symbol;Acc:HGNC:4917]	chr16	29992321	29996436	-1	ensembl_havana	5.051239958	5.143643041	4.958836875	4.965437178	5.156231424	5.309260521	4.853573765	5.017157896	5.005778964
ENSG00000128944	0.04423	0.112415139	-0.18501975	-1.136832536	**KNSTRN**	protein_coding	kinetochore localized astrin/SPAG5 binding protein [Source:HGNC Symbol;Acc:HGNC:30767]	chr15	40382721	40394246	1	ensembl_havana	5.918124415	6.01063429	5.82561454	5.732005029	6.131130434	6.168767407	5.666354627	5.874496164	5.935992829
ENSG00000168126	0.044225	0.112415139	-0.487467678	-1.401981856	**OR2W6P**	unprocessed_pseudogene	olfactory receptor family 2 subfamily W member 6 pseudogene [Source:HGNC Symbol;Acc:HGNC:15072]	chr6	27937465	27938403	1	havana	0.104602766	0.348336605	-0.139131073	0.437805725	0.271370116	0.335833975	0.339398945	-0.15890847	-0.597883695
ENSG00000108106	0.044294	0.112558565	-0.182458499	-1.134816081	**UBE2S**	protein_coding	ubiquitin conjugating enzyme E2 S [Source:HGNC Symbol;Acc:HGNC:17895]	chr19	55399745	55407777	-1	ensembl_havana	5.535929882	5.627159132	5.444700633	5.609812762	5.638154614	5.63351002	5.542418588	5.410693312	5.380989997
ENSG00000130119	0.044369	0.112727819	-0.19284391	-1.143014662	**GNL3L**	protein_coding	G protein nucleolar 3 like [Source:HGNC Symbol;Acc:HGNC:25553]	chrX	54530211	54561071	1	ensembl_havana	5.30510479	5.401526745	5.208682835	5.28435456	5.34169971	5.578525965	5.046065911	5.280010249	5.299972345
ENSG00000137942	0.044379	0.112733757	0.179571508	1.132547459	**FNBP1L**	protein_coding	formin binding protein 1 like [Source:HGNC Symbol;Acc:HGNC:20851]	chr1	93448131	93554661	1	ensembl_havana	5.414520799	5.324735045	5.504306553	5.107675946	5.352382995	5.514146193	5.394853215	5.502272618	5.615793825
ENSG00000059122	0.044395	0.112734235	0.264256956	1.201017319	**FLYWCH1**	protein_coding	FLYWCH-type zinc finger 1 [Source:HGNC Symbol;Acc:HGNC:25404]	chr16	2911937	2951208	1	ensembl_havana	4.349265147	4.217136669	4.481393625	4.21876443	4.061305939	4.371339638	4.673164629	4.344168079	4.426848166
ENSG00000066027	0.044391	0.112734235	0.152330326	1.111363162	**PPP2R5A**	protein_coding	protein phosphatase 2 regulatory subunit B'alpha [Source:HGNC Symbol;Acc:HGNC:9309]	chr1	212285537	212361863	1	ensembl_havana	6.525831795	6.449666632	6.601996958	6.539561281	6.455056031	6.354382583	6.678216785	6.57263046	6.555143628
ENSG00000265491	0.044417	0.112770394	0.165391517	1.12147038	**RNF115**	protein_coding	ring finger protein 115 [Source:HGNC Symbol;Acc:HGNC:18154]	chr1	145738868	145824077	-1	ensembl_havana	6.414208042	6.331512284	6.4969038	6.439663537	6.371473382	6.183399932	6.624804897	6.456398022	6.409508482
ENSG00000112699	0.044448	0.112830096	0.176032018	1.129772288	**GMDS**	protein_coding	GDP-mannose 4,6-dehydratase [Source:HGNC Symbol;Acc:HGNC:4369]	chr6	1623806	2245692	-1	ensembl_havana	4.078114046	3.990098036	4.166130055	3.919492919	3.877807482	4.172993708	4.118320023	4.131170126	4.248900015
ENSG00000169908	0.044459	0.112836925	0.506706819	1.420803282	**TM4SF1**	protein_coding	transmembrane 4 L six family member 1 [Source:HGNC Symbol;Acc:HGNC:11853]	chr3	149369022	149377865	-1	ensembl_havana	1.610363513	1.357010104	1.863716922	0.872903038	1.749818205	1.448309068	1.874103935	1.910030375	1.807016457
ENSG00000196205	0.044559	0.11307031	0.206066806	1.15353902	**EEF1A1P5**	processed_pseudogene	eukaryotic translation elongation factor 1 alpha 1 pseudogene 5 [Source:HGNC Symbol;Acc:HGNC:3200]	chr9	133019486	133020874	1	havana	2.47027476	2.367241358	2.573308163	2.484284696	2.206378717	2.411060659	2.735442517	2.517165043	2.467316929
ENSG00000237463	0.044579	0.113100678	0.847711265	1.79964365	**AL157714.2**	antisense		chr1	165476841	165582155	-1	havana	0.48046162	0.056605988	0.904317253	-0.56281016	0.477608744	0.255019383	1.071324589	0.602150945	1.039476224
ENSG00000163126	0.04459	0.113108431	0.369146335	1.291588352	**ANKRD23**	protein_coding	ankyrin repeat domain 23 [Source:HGNC Symbol;Acc:HGNC:24470]	chr2	96824526	96857934	-1	ensembl_havana	0.826720956	0.642147788	1.011294124	0.383334258	0.799260866	0.743848241	1.107892607	0.993426194	0.932563571
ENSG00000080603	0.044602	0.113120903	0.231141164	1.173763022	**SRCAP**	protein_coding	Snf2 related CREBBP activator protein [Source:HGNC Symbol;Acc:HGNC:16974]	chr16	30698209	30741409	1	ensembl_havana	2.103431499	1.987860917	2.219002081	2.214735639	1.891783655	1.857063457	2.270688985	2.271570328	2.114746929
ENSG00000197555	0.044621	0.113147869	0.146723516	1.107052404	**SIPA1L1**	protein_coding	signal induced proliferation associated 1 like 1 [Source:HGNC Symbol;Acc:HGNC:20284]	chr14	71320449	71741229	1	ensembl_havana	6.001787425	5.928425667	6.075149183	5.975266871	5.924169743	5.885840389	6.126849702	6.091721585	6.006876262
ENSG00000284954	0.044657	0.113219799	-0.300195307	-1.231311092	**AL662884.3**	bidirectional_promoter_lncRNA		chr6	32152802	32154365	-1	havana	1.023223427	1.17332108	0.873125774	1.355633303	1.324992307	0.83933763	1.125834805	0.758913071	0.734629445
ENSG00000257242	0.044676	0.113247257	-0.241713301	-1.182396005	**LINC01619**	processed_transcript	long intergenic non-protein coding RNA 1619 [Source:HGNC Symbol;Acc:HGNC:27409]	chr12	91984976	92142914	-1	havana	-0.434452043	-0.313595393	-0.555308693	0.383334258	-0.34076431	-0.983356126	-0.00649342	-0.62862566	-1.030806998
ENSG00000168890	0.044708	0.113308759	-0.269048296	-1.205012653	**TMEM150A**	protein_coding	transmembrane protein 150A [Source:HGNC Symbol;Acc:HGNC:24677]	chr2	85598548	85603196	-1	ensembl_havana	1.382795731	1.517319879	1.248271584	1.490591983	1.452682866	1.608684788	1.161063346	1.413033531	1.170717874
ENSG00000270362	0.044792	0.113481349	0.366532832	1.289250703	**HMGN3-AS1**	antisense	HMGN3 antisense RNA 1 [Source:HGNC Symbol;Acc:HGNC:48984]	chr6	79233718	79236797	1	havana	0.959009082	0.775742666	1.142275498	0.613726219	0.855801933	0.857699845	1.325353043	1.114463419	0.987010032
ENSG00000274528	0.044787	0.113481349	-0.260488347	-1.197884115	**AC090970.3**	sense_intronic		chr15	52017167	52018032	-1	havana	2.129975385	2.260219558	1.999731211	2.362851808	2.220801645	2.197005222	2.090556387	1.769643034	2.138994213
ENSG00000118402	0.04482	0.113511293	0.253895384	1.192422402	**ELOVL4**	protein_coding	ELOVL fatty acid elongase 4 [Source:HGNC Symbol;Acc:HGNC:14415]	chr6	79914812	79947580	-1	ensembl_havana	4.531946362	4.40499867	4.658894054	4.54768433	4.589008315	4.078303364	4.683801679	4.741870088	4.551010395
ENSG00000063169	0.044817	0.113511293	0.205386266	1.152995008	**BICRA**	protein_coding	BRD4 interacting chromatin remodeling complex associated protein [Source:HGNC Symbol;Acc:HGNC:4332]	chr19	47608196	47703277	1	ensembl_havana	3.431099334	3.328406201	3.533792467	3.390813464	3.227754334	3.366650805	3.744444859	3.392817101	3.464115441
ENSG00000108639	0.044898	0.113689649	-0.173962054	-1.128152464	**SYNGR2**	protein_coding	synaptogyrin 2 [Source:HGNC Symbol;Acc:HGNC:11499]	chr17	78168558	78173527	1	ensembl_havana	7.530498682	7.617479709	7.443517655	7.643893686	7.657472967	7.551072473	7.564021404	7.471047904	7.295483658
ENSG00000176108	0.044949	0.113798114	0.476405974	1.39127342	**CHMP6**	protein_coding	charged multivesicular body protein 6 [Source:HGNC Symbol;Acc:HGNC:25675]	chr17	80991598	81009517	1	ensembl_havana	3.747272542	3.509069555	3.985475529	3.3380874	3.367185482	3.821935783	4.044780323	4.089590121	3.822056143
ENSG00000215421	0.044993	0.113889822	0.148677694	1.108552959	**ZNF407**	protein_coding	zinc finger protein 407 [Source:HGNC Symbol;Acc:HGNC:19904]	chr18	74597870	75065671	1	ensembl_havana	4.577875197	4.50353635	4.652214044	4.500480622	4.514865633	4.495262794	4.614010932	4.695502837	4.647128362
ENSG00000166913	0.045045	0.113941244	0.148296511	1.108260101	**YWHAB**	protein_coding	tyrosine 3-monooxygenase/tryptophan 5-monooxygenase activation protein beta [Source:HGNC Symbol;Acc:HGNC:12849]	chr20	44885676	44908532	1	ensembl_havana	9.279404953	9.205256698	9.353553209	9.100131959	9.26135696	9.254281173	9.265612506	9.372208229	9.422838891
ENSG00000185591	0.045042	0.113941244	-0.151250265	-1.110531461	**SP1**	protein_coding	Sp1 transcription factor [Source:HGNC Symbol;Acc:HGNC:11205]	chr12	53380176	53416446	1	ensembl_havana	7.057707242	7.133332375	6.98208211	7.169860308	7.111658074	7.118478743	6.969973188	6.981525597	6.994747545
ENSG00000138442	0.045031	0.113941244	-0.156540262	-1.114610973	**WDR12**	protein_coding	WD repeat domain 12 [Source:HGNC Symbol;Acc:HGNC:14098]	chr2	202874782	203014798	-1	ensembl_havana	6.2948556	6.373125731	6.216585469	6.405904646	6.346616682	6.366855865	6.191635764	6.243988714	6.21413193
ENSG00000099251	0.04504	0.113941244	-0.311945616	-1.241380695	**HSD17B7P2**	transcribed_unprocessed_pseudogene	hydroxysteroid 17-beta dehydrogenase 7 pseudogene 2 [Source:HGNC Symbol;Acc:HGNC:28120]	chr10	38356380	38378505	1	ensembl_havana	2.026835642	2.18280845	1.870862834	2.23025056	2.389883326	1.928291464	2.025141822	1.790554303	1.796892377
ENSG00000168237	0.04508	0.11401051	0.301954333	1.232813301	**GLYCTK**	protein_coding	glycerate kinase [Source:HGNC Symbol;Acc:HGNC:24247]	chr3	52287089	52293476	1	ensembl_havana	3.344052429	3.193075263	3.495029595	3.027727485	3.165741616	3.385756687	3.37972385	3.680155819	3.425209116
ENSG00000237649	0.045213	0.114326889	-0.199103711	-1.147984936	**KIFC1**	protein_coding	kinesin family member C1 [Source:HGNC Symbol;Acc:HGNC:6389]	chr6	33391536	33409924	1	ensembl_havana	7.054194536	7.153746391	6.954642681	6.948556037	7.191631557	7.32105158	6.86506006	7.01022298	6.988645002
ENSG00000235703	0.045222	0.114327781	-0.160205373	-1.117446199	**LINC00894**	antisense	long intergenic non-protein coding RNA 894 [Source:HGNC Symbol;Acc:HGNC:48579]	chrX	149938628	150224580	1	havana	2.713631102	2.793733788	2.633528415	2.897741056	2.608571783	2.874888526	2.663565071	2.491852285	2.745167891
ENSG00000179833	0.045241	0.114356949	-0.145267554	-1.105935735	**SERTAD2**	protein_coding	SERTA domain containing 2 [Source:HGNC Symbol;Acc:HGNC:30784]	chr2	64631621	64751005	-1	ensembl_havana	7.045029508	7.117663285	6.97239573	7.207090159	7.058447488	7.087452208	7.042608792	6.913152209	6.961426191
ENSG00000141510	0.045279	0.114433474	0.18530685	1.137058791	**TP53**	protein_coding	tumor protein p53 [Source:HGNC Symbol;Acc:HGNC:11998]	chr17	7661779	7687550	-1	ensembl_havana	4.719648427	4.626995002	4.812301852	4.572242036	4.584832871	4.7239101	4.738759141	4.884855267	4.813291149
ENSG00000066322	0.045299	0.114463062	0.160500718	1.117674984	**ELOVL1**	protein_coding	ELOVL fatty acid elongase 1 [Source:HGNC Symbol;Acc:HGNC:14418]	chr1	43363397	43368074	-1	ensembl_havana	6.51392728	6.433676921	6.594177639	6.412294904	6.417200595	6.471535263	6.597338636	6.627050508	6.558143773
ENSG00000108468	0.045308	0.114465511	-0.147627744	-1.107746481	**CBX1**	protein_coding	chromobox 1 [Source:HGNC Symbol;Acc:HGNC:1551]	chr17	48070052	48101521	-1	ensembl_havana	7.241379014	7.315192886	7.167565142	7.306038031	7.304905933	7.334634694	7.143208425	7.136821656	7.222665345
ENSG00000237298	0.045329	0.114482435	0.187099698	1.138472701	**TTN-AS1**	antisense	TTN antisense RNA 1 [Source:HGNC Symbol;Acc:HGNC:44124]	chr2	178521183	178779963	1	havana	7.000409446	6.906859597	7.093959295	7.246486224	6.635446274	6.838646294	7.320427839	6.927383639	7.034066407
ENSG00000096401	0.045339	0.114482435	-0.16192837	-1.118781552	**CDC5L**	protein_coding	cell division cycle 5 like [Source:HGNC Symbol;Acc:HGNC:1743]	chr6	44387525	44450426	1	ensembl_havana	7.35800055	7.438964735	7.277036365	7.548102473	7.445673866	7.323117866	7.312371355	7.297018114	7.221719626
ENSG00000102931	0.045331	0.114482435	-0.194903116	-1.144647289	**ARL2BP**	protein_coding	ADP ribosylation factor like GTPase 2 binding protein [Source:HGNC Symbol;Acc:HGNC:17146]	chr16	57245098	57253635	1	ensembl_havana	6.262591222	6.36004278	6.165139664	6.324672039	6.436252473	6.319203828	6.114547143	6.12965545	6.251216398
ENSG00000157653	0.045405	0.114566974	0.232082746	1.174529335	**C9orf43**	protein_coding	chromosome 9 open reading frame 43 [Source:HGNC Symbol;Acc:HGNC:23570]	chr9	113410054	113429684	1	ensembl_havana	1.13509492	1.019053547	1.251136293	0.705390237	0.927899934	1.423870469	1.125834805	1.097781778	1.529792296
ENSG00000198964	0.045387	0.114566974	0.232028553	1.174485216	**SGMS1**	protein_coding	sphingomyelin synthase 1 [Source:HGNC Symbol;Acc:HGNC:29799]	chr10	50305586	50625163	-1	ensembl_havana	5.045998768	4.929984492	5.162013044	4.864657476	5.046142112	4.879153887	5.287362276	5.16460889	5.034067967
ENSG00000167615	0.045392	0.114566974	0.205973062	1.153464067	**LENG8**	protein_coding	leukocyte receptor cluster member 8 [Source:HGNC Symbol;Acc:HGNC:15500]	chr19	54448887	54462037	1	ensembl_havana	8.742473302	8.639486771	8.845459833	8.763766532	8.419725575	8.734968206	9.054533339	8.69612857	8.785717591
ENSG00000123154	0.045412	0.114566974	0.1826029	1.134929671	**WDR83**	protein_coding	WD repeat domain 83 [Source:HGNC Symbol;Acc:HGNC:32672]	chr19	12666800	12675832	1	ensembl_havana	3.229978273	3.138676823	3.321279723	3.075752348	3.150754678	3.189523443	3.302948055	3.406428015	3.254463098
ENSG00000174428	0.045402	0.114566974	0.156658493	1.114702321	**GTF2IRD2B**	protein_coding	GTF2I repeat domain containing 2B [Source:HGNC Symbol;Acc:HGNC:33125]	chr7	75092573	75149817	1	ensembl_havana	2.421654121	2.343324875	2.499983368	2.312722596	2.402591162	2.314660866	2.521699999	2.498222232	2.480027873
ENSG00000228327	0.04543	0.114592682	-0.158972645	-1.116491792	**AL669831.1**	transcribed_unprocessed_pseudogene		chr1	725885	778626	-1	havana	2.198211293	2.277697616	2.118724971	2.383813765	2.06964138	2.379637701	2.294679948	1.890792227	2.170702736
ENSG00000244668	0.045479	0.114635782	1.263520776	2.400809239	**AC093001.1**	antisense		chr3	149284782	149333653	1	havana	-0.356509711	-0.988270099	0.275250677	-2.51386715	-0.620350959	0.16940781	0.069637898	-0.08087422	0.836988357
ENSG00000100024	0.045469	0.114635782	0.725565424	1.653548572	**UPB1**	protein_coding	beta-ureidopropionase 1 [Source:HGNC Symbol;Acc:HGNC:16297]	chr22	24494107	24528390	1	ensembl_havana	-0.408803803	-0.771586515	-0.046021091	-0.41068387	-1.104596936	-0.799478738	-0.21650287	0.343769191	-0.265329594
ENSG00000177125	0.045467	0.114635782	0.143536432	1.104609494	**ZBTB34**	protein_coding	zinc finger and BTB domain containing 34 [Source:HGNC Symbol;Acc:HGNC:31446]	chr9	126860665	126885878	1	ensembl_havana	6.102290886	6.03052267	6.174059102	6.072614977	6.098308216	5.920644818	6.21748923	6.24446243	6.060225648
ENSG00000105771	0.045476	0.114635782	-0.171873119	-1.12652015	**SMG9**	protein_coding	SMG9, nonsense mediated mRNA decay factor [Source:HGNC Symbol;Acc:HGNC:25763]	chr19	43727992	43754990	-1	ensembl_havana	6.014128278	6.100064838	5.928191718	6.113974357	6.109977983	6.076242173	6.041400794	5.89576193	5.847412431
ENSG00000173914	0.045541	0.114770949	0.149244629	1.108988672	**RBM4B**	protein_coding	RNA binding motif protein 4B [Source:HGNC Symbol;Acc:HGNC:28842]	chr11	66664998	66677921	-1	ensembl_havana	4.251901982	4.177279667	4.326524296	4.094996029	4.229436075	4.207406897	4.26922283	4.335300678	4.375049381
ENSG00000183943	0.045564	0.114808646	-0.146517302	-1.106894177	**PRKX**	protein_coding	protein kinase, X-linked [Source:HGNC Symbol;Acc:HGNC:9441]	chrX	3604370	3713608	-1	ensembl_havana	4.642099657	4.715358308	4.568841005	4.757644478	4.701268571	4.687161874	4.640774659	4.549797182	4.515951176
ENSG00000131584	0.045578	0.114810673	0.381812311	1.302977626	**ACAP3**	protein_coding	ArfGAP with coiled-coil, ankyrin repeat and PH domains 3 [Source:HGNC Symbol;Acc:HGNC:16754]	chr1	1292376	1309609	-1	ensembl_havana	4.646063605	4.45515745	4.836969761	4.454985288	4.187828682	4.722658379	5.007875329	4.771097359	4.731936594
ENSG00000111728	0.045595	0.114810673	0.342418901	1.26788061	**ST8SIA1**	protein_coding	ST8 alpha-N-acetyl-neuraminide alpha-2,8-sialyltransferase 1 [Source:HGNC Symbol;Acc:HGNC:10869]	chr12	22063773	22437041	-1	ensembl_havana	2.209875814	2.038666364	2.381085264	1.477650254	2.364126834	2.274222003	2.135529305	2.625878149	2.381848339
ENSG00000183137	0.045596	0.114810673	0.236162278	1.17785527	**CEP57L1**	protein_coding	centrosomal protein 57 like 1 [Source:HGNC Symbol;Acc:HGNC:21561]	chr6	109095110	109163932	1	ensembl_havana	3.151156065	3.033074926	3.269237203	2.754872648	3.195256367	3.149095762	3.100076994	3.233954352	3.473680264
ENSG00000144357	0.045588	0.114810673	0.162523006	1.119242776	**UBR3**	protein_coding	ubiquitin protein ligase E3 component n-recognin 3 (putative) [Source:HGNC Symbol;Acc:HGNC:30467]	chr2	169827458	170084131	1	ensembl_havana	6.350567513	6.269306009	6.431829016	6.354179901	6.276721671	6.177016456	6.438617944	6.495926552	6.360942551
ENSG00000182481	0.045607	0.114817614	-0.147681431	-1.107787704	**KPNA2**	protein_coding	karyopherin subunit alpha 2 [Source:HGNC Symbol;Acc:HGNC:6395]	chr17	68035519	68046842	1	ensembl_havana	9.394858352	9.468699068	9.321017637	9.359544725	9.556115643	9.490436835	9.249336491	9.390247884	9.323468536
ENSG00000255224	0.045643	0.114886871	0.643567037	1.562186867	**AC109322.1**	antisense		chr8	144078002	144079265	-1	havana	-0.637386932	-0.959170451	-0.315603413	-1.23095973	-0.903502039	-0.743049581	0.069637898	-0.52165082	-0.494797317
ENSG00000167065	0.045652	0.114889004	0.244552324	1.184725087	**DUSP18**	protein_coding	dual specificity phosphatase 18 [Source:HGNC Symbol;Acc:HGNC:18484]	chr22	30652051	30667890	-1	ensembl_havana	2.817714895	2.695438733	2.939991057	2.614134963	2.831702492	2.640478746	3.011169462	2.876266922	2.932536788
ENSG00000148200	0.04566	0.114889448	0.151683535	1.110865026	**NR6A1**	protein_coding	nuclear receptor subfamily 6 group A member 1 [Source:HGNC Symbol;Acc:HGNC:7985]	chr9	124517275	124771310	-1	ensembl_havana	5.666771811	5.590930044	5.742613579	5.652184055	5.577806606	5.54279947	5.769109419	5.780937208	5.67779411
ENSG00000204308	0.045672	0.114900353	0.161665654	1.118577839	**RNF5**	protein_coding	ring finger protein 5 [Source:HGNC Symbol;Acc:HGNC:10068]	chr6	32178354	32180793	1	ensembl_havana	5.925117128	5.844284301	6.005949955	5.792696505	5.956847556	5.783308844	6.031916789	6.066759784	5.919173293
ENSG00000233927	0.045688	0.114921015	0.230108902	1.172923484	**RPS28**	protein_coding	ribosomal protein S28 [Source:HGNC Symbol;Acc:HGNC:10418]	chr19	8321158	8323340	1	ensembl_havana	5.498635668	5.383581217	5.613690119	5.337253995	5.311695568	5.501794088	5.698930888	5.357380992	5.784758476
ENSG00000249534	0.045705	0.114942424	0.171307016	1.126078198	**LINC01258**	lincRNA	long intergenic non-protein coding RNA 1258 [Source:HGNC Symbol;Acc:HGNC:49898]	chr4	38420662	38523180	-1	havana	3.22319547	3.137541962	3.308848978	2.987209412	3.00836839	3.417048083	3.153531275	3.267903019	3.50511264
ENSG00000280332	0.045722	0.114966135	0.321486637	1.249617564	**AC020917.4**	TEC		chr19	16356329	16358327	-1	havana	0.155467605	-0.005275713	0.316210924	0.004630787	-0.218971072	0.198513145	0.032074365	0.315191528	0.601366878
ENSG00000162174	0.045766	0.115056875	0.195288688	1.144953246	**ASRGL1**	protein_coding	asparaginase like 1 [Source:HGNC Symbol;Acc:HGNC:16448]	chr11	62337448	62393412	1	ensembl_havana	3.811697029	3.714052685	3.909341372	3.345229877	3.877807482	3.919120695	3.65769116	4.081128356	3.989204602
ENSG00000089876	0.045826	0.11514542	0.210057784	1.156734514	**DHX32**	protein_coding	DEAH-box helicase 32 (putative) [Source:HGNC Symbol;Acc:HGNC:16717]	chr10	125836337	125896436	-1	ensembl_havana	4.780931414	4.675902521	4.885960306	4.737428462	4.668761104	4.621517999	4.831876837	4.837913526	4.988090554
ENSG00000260063	0.045824	0.11514542	0.186908363	1.138321723	**AL512408.1**	antisense		chr1	26692132	26694131	-1	havana	2.2323523	2.138898118	2.325806482	2.117969943	2.206378717	2.092345696	2.20469138	2.510878368	2.261849697
ENSG00000106244	0.045814	0.11514542	0.146324177	1.106746013	**PDAP1**	protein_coding	PDGFA associated protein 1 [Source:HGNC Symbol;Acc:HGNC:14634]	chr7	99392048	99408829	-1	ensembl_havana	6.622996633	6.549834545	6.696158721	6.471834954	6.544848093	6.632820587	6.633845333	6.659027994	6.795602838
ENSG00000111912	0.045834	0.115147491	0.173655634	1.127912877	**NCOA7**	protein_coding	nuclear receptor coactivator 7 [Source:HGNC Symbol;Acc:HGNC:21081]	chr6	125781161	125932030	1	ensembl_havana	4.529624235	4.442796418	4.616452052	4.277815454	4.583438366	4.467135434	4.549007011	4.67737277	4.622976376
ENSG00000166153	0.045915	0.115329432	-0.198622761	-1.147602297	**DEPDC4**	protein_coding	DEP domain containing 4 [Source:HGNC Symbol;Acc:HGNC:22952]	chr12	100203669	100267079	-1	ensembl_havana	0.311883477	0.411194857	0.212572096	0.14220989	0.428746719	0.662627962	0.032074365	0.286036324	0.319605599
ENSG00000128524	0.045968	0.115421161	0.18857969	1.139641205	**ATP6V1F**	protein_coding	ATPase H+ transporting V1 subunit F [Source:HGNC Symbol;Acc:HGNC:16832]	chr7	128862826	128865844	1	ensembl_havana	6.060858912	5.966569068	6.155148757	5.945027618	6.072198117	5.882481467	6.216966213	6.132729392	6.115750668
ENSG00000228878	0.045965	0.115421161	-0.367606185	-1.290210251	**SEPT7-AS1**	lincRNA	SEPT7 antisense RNA 1 (head to head) [Source:HGNC Symbol;Acc:HGNC:51153]	chr7	35751856	35800616	-1	havana	1.715205918	1.899009011	1.531402826	1.912130943	1.739838083	2.045058006	1.325353043	1.726888623	1.541966812
ENSG00000161920	0.046007	0.115499377	-0.162443536	-1.119181124	**MED11**	protein_coding	mediator complex subunit 11 [Source:HGNC Symbol;Acc:HGNC:32687]	chr17	4731428	4733610	1	ensembl_havana	4.260790793	4.34201256	4.179569025	4.394300619	4.305836831	4.325900232	4.287271569	4.167594302	4.083841203
ENSG00000269894	0.046054	0.115598066	0.21095206	1.157451755	**AC018809.1**	sense_intronic		chr3	9935706	9936258	1	havana	1.345175602	1.239699573	1.450651632	1.383653947	1.109387295	1.226057475	1.444517675	1.452931788	1.454505434
ENSG00000225484	0.046077	0.115615656	0.191494732	1.141946238	**NUTM2B-AS1**	antisense	NUTM2B antisense RNA 1 [Source:HGNC Symbol;Acc:HGNC:51204]	chr10	79663088	79826594	-1	havana	3.246318098	3.150570732	3.342065464	3.134649994	3.213401092	3.10366111	3.431134914	3.279044008	3.31601747
ENSG00000061936	0.046077	0.115615656	0.150258654	1.109768419	**SFSWAP**	protein_coding	splicing factor SWAP [Source:HGNC Symbol;Acc:HGNC:10790]	chr12	131711081	131799737	1	ensembl_havana	5.29230595	5.217176623	5.367435277	5.245754289	5.143985622	5.261789957	5.360221082	5.282780251	5.459304496
ENSG00000168944	0.046092	0.115632338	-0.142981718	-1.104184855	**CEP120**	protein_coding	centrosomal protein 120 [Source:HGNC Symbol;Acc:HGNC:26690]	chr5	123344885	123423592	-1	ensembl_havana	5.354339121	5.42582998	5.282848262	5.343509198	5.452024698	5.481956046	5.197961942	5.30475086	5.345831985
ENSG00000187266	0.046161	0.115764025	-0.150482677	-1.109940759	**EPOR**	protein_coding	erythropoietin receptor [Source:HGNC Symbol;Acc:HGNC:3416]	chr19	11377205	11384342	-1	ensembl_havana	1.570977984	1.646219323	1.495736645	1.590147445	1.547275176	1.801235349	1.47283199	1.413033531	1.601344415
ENSG00000187764	0.046158	0.115764025	-0.199280584	-1.148125686	**SEMA4D**	protein_coding	semaphorin 4D [Source:HGNC Symbol;Acc:HGNC:10732]	chr9	89360787	89498130	-1	ensembl_havana	4.847257336	4.946897628	4.747617044	4.885629456	4.955262089	4.999801339	4.816768636	4.631749475	4.794333023
ENSG00000180425	0.046191	0.115819145	-0.246030613	-1.185939664	**C11orf71**	protein_coding	chromosome 11 open reading frame 71 [Source:HGNC Symbol;Acc:HGNC:25937]	chr11	114391443	114400511	-1	ensembl_havana	3.433025068	3.556040374	3.310009762	3.550741415	3.482399	3.634980708	3.283098272	3.429942433	3.21698858
ENSG00000284602	0.046269	0.115996041	0.177843763	1.131191953	**AL031432.4**	bidirectional_promoter_lncRNA		chr1	25232586	25234775	1	havana	1.199954529	1.111032648	1.288876411	1.04068047	1.199531136	1.092886337	1.125834805	1.47893052	1.261863909
ENSG00000118971	0.046288	0.116022309	0.287281174	1.220338325	**CCND2**	protein_coding	cyclin D2 [Source:HGNC Symbol;Acc:HGNC:1583]	chr12	4273772	4305350	1	ensembl_havana	7.404566178	7.260925591	7.548206766	6.792050778	7.388730015	7.601995981	7.336888548	7.496327365	7.811404384
ENSG00000105698	0.046317	0.11607132	0.151644401	1.110834893	**USF2**	protein_coding	upstream transcription factor 2, c-fos interacting [Source:HGNC Symbol;Acc:HGNC:12594]	chr19	35268978	35279821	1	ensembl_havana	4.822435737	4.746613537	4.898257938	4.864657476	4.71915782	4.656025315	5.069431626	4.855386052	4.769956135
ENSG00000115211	0.046323	0.11607132	0.145378797	1.106021014	**EIF2B4**	protein_coding	eukaryotic translation initiation factor 2B subunit delta [Source:HGNC Symbol;Acc:HGNC:3260]	chr2	27364352	27370486	-1	ensembl_havana	6.378304908	6.30561551	6.450994307	6.271300565	6.334647662	6.310898301	6.428262796	6.450251011	6.474469113
ENSG00000117640	0.046339	0.116077124	0.179090711	1.132170086	**MTFR1L**	protein_coding	mitochondrial fission regulator 1 like [Source:HGNC Symbol;Acc:HGNC:28836]	chr1	25818640	25832942	1	ensembl_havana	5.899831891	5.810286536	5.989377247	5.935594263	5.825580325	5.669685019	6.034293642	5.973828406	5.960009692
ENSG00000076826	0.04635	0.116077124	0.145381765	1.10602329	**CAMSAP3**	protein_coding	calmodulin regulated spectrin associated protein family member 3 [Source:HGNC Symbol;Acc:HGNC:29307]	chr19	7595902	7618304	1	ensembl_havana	1.725522408	1.652831525	1.798213291	1.660549964	1.636045198	1.661899414	1.786580698	1.811166797	1.796892377
ENSG00000127561	0.046346	0.116077124	-0.328954756	-1.256102988	**SYNGR3**	protein_coding	synaptogyrin 3 [Source:HGNC Symbol;Acc:HGNC:11501]	chr16	1989660	1994275	1	ensembl_havana	2.72810161	2.892578988	2.563624232	3.22260331	2.983208668	2.471924986	3.175232288	2.392765686	2.122874721
ENSG00000138459	0.046418	0.116207945	-0.146559639	-1.10692666	**SLC35A5**	protein_coding	solute carrier family 35 member A5 [Source:HGNC Symbol;Acc:HGNC:20792]	chr3	112561709	112585577	1	ensembl_havana	6.579962122	6.653241941	6.506682302	6.667588012	6.695415413	6.596722398	6.549120496	6.55478865	6.416137761
ENSG00000067057	0.046411	0.116207945	-0.309055581	-1.238896426	**PFKP**	protein_coding	phosphofructokinase, platelet [Source:HGNC Symbol;Acc:HGNC:8878]	chr10	3066333	3137712	1	ensembl_havana	6.330010359	6.48453815	6.175482569	6.36958651	6.274562954	6.809464985	6.103837119	6.192871587	6.229739001
ENSG00000080839	0.046444	0.1162516	0.143145729	1.10431039	**RBL1**	protein_coding	RB transcriptional corepressor like 1 [Source:HGNC Symbol;Acc:HGNC:9893]	chr20	36996349	37095995	-1	ensembl_havana	6.027975011	5.956402146	6.099547876	5.738122539	5.99720444	6.13387946	5.87301911	6.155073703	6.270550815
ENSG00000229180	0.046473	0.116304321	0.187645881	1.138903791	**AC006001.3**	transcribed_unprocessed_pseudogene		chr7	66526088	66592397	1	havana	5.157040114	5.063217174	5.250863054	4.942072749	5.080158721	5.167420051	5.257156287	5.224423979	5.271008897
ENSG00000135643	0.046488	0.116322074	0.387326287	1.307967132	**KCNMB4**	protein_coding	potassium calcium-activated channel subfamily M regulatory beta subunit 4 [Source:HGNC Symbol;Acc:HGNC:6289]	chr12	70366276	70434292	1	ensembl_havana	1.964115841	1.770452697	2.157778984	1.369711653	1.996084195	1.945562243	2.126646177	2.114588126	2.23210265
ENSG00000187951	0.046529	0.116382835	-0.15583157	-1.11406358	**ARHGAP11B**	processed_transcript	Rho GTPase activating protein 11B [Source:HGNC Symbol;Acc:HGNC:15782]	chr15	30658717	30772993	1	havana	4.550338541	4.628254326	4.472422756	4.38912643	4.636849344	4.858787203	4.285277268	4.491924288	4.640066711
ENSG00000163867	0.046523	0.116382835	-0.161299585	-1.118294048	**ZMYM6**	protein_coding	zinc finger MYM-type containing 6 [Source:HGNC Symbol;Acc:HGNC:13050]	chr1	34986165	35031968	-1	ensembl_havana	4.273703317	4.354353109	4.193053524	4.414813776	4.355701227	4.292544325	4.184017004	4.264198259	4.130945309
ENSG00000110427	0.046549	0.11641336	-0.266026363	-1.202491222	**KIAA1549L**	protein_coding	KIAA1549 like [Source:HGNC Symbol;Acc:HGNC:24836]	chr11	33376466	33674102	1	ensembl_havana	5.542603694	5.675616875	5.409590512	5.233296343	5.789146053	6.004408229	5.161460773	5.545970702	5.521340061
ENSG00000186020	0.046588	0.11647604	0.151866646	1.111006029	**ZNF529**	protein_coding	zinc finger protein 529 [Source:HGNC Symbol;Acc:HGNC:29328]	chr19	36534774	36605276	-1	ensembl_havana	4.870008189	4.794074866	4.945941513	4.833251709	4.833847726	4.715125164	4.957418084	4.95715416	4.923252293
ENSG00000146083	0.04659	0.11647604	-0.182707325	-1.135011823	**RNF44**	protein_coding	ring finger protein 44 [Source:HGNC Symbol;Acc:HGNC:19180]	chr5	176526697	176538025	-1	ensembl_havana	5.118440684	5.209794347	5.027087022	5.302815673	5.004025858	5.322541509	5.132911387	4.919678997	5.028670681
ENSG00000132478	0.046631	0.116537935	0.15878212	1.116344355	**UNK**	protein_coding	unkempt family zinc finger [Source:HGNC Symbol;Acc:HGNC:29369]	chr17	75784771	75825799	1	ensembl_havana	4.546506609	4.467115549	4.625897669	4.506864402	4.425852674	4.468629573	4.719687413	4.602484434	4.555521161
ENSG00000175216	0.046625	0.116537935	-0.155104749	-1.113502463	**CKAP5**	protein_coding	cytoskeleton associated protein 5 [Source:HGNC Symbol;Acc:HGNC:28959]	chr11	46743048	46846308	-1	ensembl_havana	8.607882078	8.685434453	8.530329703	8.591173488	8.66811481	8.79701506	8.407931055	8.578376705	8.604681351
ENSG00000256223	0.046667	0.116605901	0.209481005	1.156272152	**ZNF10**	protein_coding	zinc finger protein 10 [Source:HGNC Symbol;Acc:HGNC:12879]	chr12	133130575	133159465	1	ensembl_havana	3.726220916	3.621480414	3.830961419	3.744167537	3.756831958	3.363441747	3.834482717	3.929121717	3.729279822
ENSG00000137834	0.046675	0.116606499	-0.20317858	-1.151231985	**SMAD6**	protein_coding	SMAD family member 6 [Source:HGNC Symbol;Acc:HGNC:6772]	chr15	66702228	66782848	1	ensembl_havana	1.215955961	1.317545251	1.114366671	1.477650254	1.140065346	1.334920154	1.125834805	1.046547334	1.170717874
ENSG00000250067	0.046699	0.116646217	0.162189619	1.118984163	**YJEFN3**	protein_coding	YjeF N-terminal domain containing 3 [Source:HGNC Symbol;Acc:HGNC:24785]	chr19	19528861	19537581	1	ensembl_havana	3.506771736	3.425676927	3.587866545	3.352337167	3.234877758	3.689815855	3.584973459	3.399638609	3.778987568
ENSG00000172663	0.046719	0.116674977	0.210084807	1.15675618	**TMEM134**	protein_coding	transmembrane protein 134 [Source:HGNC Symbol;Acc:HGNC:26142]	chr11	67461710	67469272	-1	ensembl_havana	2.084858517	1.979816113	2.18990092	2.023178671	1.970707426	1.945562243	2.135529305	2.34408831	2.090085146
ENSG00000116675	0.04676	0.116756688	0.14279638	1.104043013	**DNAJC6**	protein_coding	DnaJ heat shock protein family (Hsp40) member C6 [Source:HGNC Symbol;Acc:HGNC:15469]	chr1	65248219	65415869	1	ensembl_havana	6.583929079	6.512530889	6.655327269	6.604622365	6.594843292	6.338127011	6.733017627	6.746240296	6.486723885
ENSG00000240219	0.046794	0.116821571	0.499474734	1.41369876	**AL512306.2**	lincRNA		chr1	204626775	204629712	1	havana	-0.159479206	-0.409216574	0.090258161	-0.41068387	-0.18055577	-0.636410081	-0.40973604	0.478736765	0.201773757
ENSG00000260589	0.046832	0.116855149	0.167172588	1.12285574	**STAM-AS1**	antisense	STAM antisense RNA 1 (head to head) [Source:HGNC Symbol;Acc:HGNC:49568]	chr10	17641284	17643878	-1	havana	1.457258983	1.373672688	1.540845277	1.092533711	1.592342839	1.436141515	1.229040238	1.85152878	1.541966812
ENSG00000136021	0.046817	0.116855149	0.151088018	1.110406577	**SCYL2**	protein_coding	SCY1 like pseudokinase 2 [Source:HGNC Symbol;Acc:HGNC:19286]	chr12	100267140	100341724	1	ensembl_havana	6.791525932	6.715981923	6.867069941	6.771320517	6.75081848	6.625806774	6.889224658	6.880913454	6.831071712
ENSG00000116771	0.04683	0.116855149	-0.475695009	-1.390587965	**AGMAT**	protein_coding	agmatinase [Source:HGNC Symbol;Acc:HGNC:18407]	chr1	15572353	15585110	-1	ensembl_havana	1.780787699	2.018635203	1.542940195	2.312722596	1.7890623	1.954120714	1.356079599	1.517070703	1.755670283
ENSG00000269404	0.046858	0.116901466	0.444727832	1.361057325	**SPIB**	protein_coding	Spi-B transcription factor [Source:HGNC Symbol;Acc:HGNC:11242]	chr19	50418938	50431313	1	ensembl_havana	0.738975429	0.516611513	0.961339345	-0.06941355	0.77991046	0.83933763	0.762800879	1.226127349	0.895089805
ENSG00000086504	0.046876	0.116924394	0.25854166	1.196268852	**MRPL28**	protein_coding	mitochondrial ribosomal protein L28 [Source:HGNC Symbol;Acc:HGNC:14484]	chr16	367384	370527	-1	ensembl_havana	5.306769408	5.177498578	5.436040238	5.155185655	5.125898835	5.251411245	5.527310896	5.500687226	5.280122593
ENSG00000076258	0.046937	0.117036166	0.342646564	1.268080703	**FMO4**	protein_coding	flavin containing monooxygenase 4 [Source:HGNC Symbol;Acc:HGNC:3772]	chr1	171314208	171342084	1	ensembl_havana	-0.020385422	-0.191708704	0.15093786	0.040274975	-0.34076431	-0.274636777	0.069637898	0.063570082	0.319605599
ENSG00000137285	0.046934	0.117036166	-0.331392101	-1.258226891	**TUBB2B**	protein_coding	tubulin beta 2B class IIb [Source:HGNC Symbol;Acc:HGNC:30829]	chr6	3224261	3231730	-1	ensembl_havana	3.727978363	3.893674414	3.562282313	3.608277961	4.129780342	3.942964938	3.594668832	3.660411303	3.431766804
ENSG00000165632	0.046999	0.117171308	0.223666657	1.16769756	**TAF3**	protein_coding	TATA-box binding protein associated factor 3 [Source:HGNC Symbol;Acc:HGNC:17303]	chr10	7818504	8016627	1	ensembl_havana	4.08599223	3.974158902	4.197825559	3.689093732	4.073277629	4.160105343	4.086606631	4.157568375	4.349301671

ENSG00000164163	0.047064	0.117312928	-0.19635689	-1.145801308	**ABCE1**	protein_coding	ATP binding cassette subfamily E member 1 [Source:HGNC Symbol;Acc:HGNC:69]	chr4	145097932	145129179	1	ensembl_havana	7.271369942	7.369548387	7.173191498	7.352635255	7.363359072	7.392650834	7.020000725	7.193120698	7.30645307
ENSG00000156711	0.047075	0.117319314	-0.326718628	-1.254157579	**MAPK13**	protein_coding	mitogen-activated protein kinase 13 [Source:HGNC Symbol;Acc:HGNC:6875]	chr6	36127809	36144524	1	ensembl_havana	0.803028579	0.966387893	0.639669265	1.397462791	0.699804047	0.80189684	0.762800879	0.578298274	0.577908642
ENSG00000172159	0.047138	0.117455618	0.356256696	1.28010016	**FRMD3**	protein_coding	FERM domain containing 3 [Source:HGNC Symbol;Acc:HGNC:24125]	chr9	83242990	83538546	-1	ensembl_havana	0.403932261	0.225803913	0.58206061	0.10902545	0.15602554	0.41236075	0.399634207	0.841392961	0.505154661
ENSG00000078699	0.04715	0.117466898	0.17029863	1.125291389	**CBFA2T2**	protein_coding	CBFA2/RUNX1 translocation partner 2 [Source:HGNC Symbol;Acc:HGNC:1536]	chr20	33490075	33650036	1	ensembl_havana	5.939879926	5.854730611	6.025029241	5.786150622	5.751146869	6.026894343	5.859678694	5.993688946	6.221720082
ENSG00000226824	0.047209	0.117562922	0.315450903	1.24440052	**AC006001.2**	sense_intronic		chr7	66654538	66669855	1	havana	1.684954656	1.527229204	1.842680107	1.092533711	1.678462547	1.810691353	1.693402841	1.769643034	2.064994446
ENSG00000111790	0.047219	0.117562922	0.16498017	1.121150668	**FGFR1OP2**	protein_coding	FGFR1 oncogene partner 2 [Source:HGNC Symbol;Acc:HGNC:23098]	chr12	26938383	26966650	1	ensembl_havana	6.406797367	6.324307281	6.489287452	6.421193978	6.346205606	6.20552226	6.496220687	6.550963884	6.420677784
ENSG00000130821	0.047247	0.117562922	0.159962663	1.117258223	**SLC6A8**	protein_coding	solute carrier family 6 member 8 [Source:HGNC Symbol;Acc:HGNC:11055]	chrX	153688099	153696593	1	ensembl_havana	3.883558664	3.803577333	3.963539996	3.914699725	3.826860993	3.66917128	4.109329965	3.907747537	3.873542485
ENSG00000108474	0.047254	0.117562922	-0.141911954	-1.103366401	**PIGL**	protein_coding	phosphatidylinositol glycan anchor biosynthesis class L [Source:HGNC Symbol;Acc:HGNC:8966]	chr17	16217191	16351797	1	ensembl_havana	2.758934345	2.829890322	2.687978368	2.738691328	2.935911052	2.815068588	2.587917939	2.800974855	2.675042311
ENSG00000155561	0.047252	0.117562922	-0.160880607	-1.117969328	**NUP205**	protein_coding	nucleoporin 205 [Source:HGNC Symbol;Acc:HGNC:18658]	chr7	135557919	135648757	1	ensembl_havana	7.394352164	7.474792468	7.313911861	7.465913121	7.428883695	7.529580588	7.288426311	7.345295929	7.308013342
ENSG00000081237	0.047231	0.117562922	-0.172878106	-1.127305162	**PTPRC**	protein_coding	protein tyrosine phosphatase, receptor type C [Source:HGNC Symbol;Acc:HGNC:9666]	chr1	198638457	198757476	1	ensembl_havana	6.679493153	6.765932206	6.5930541	6.988163801	6.754850473	6.554782345	6.709785968	6.640070706	6.429305626
ENSG00000074201	0.047215	0.117562922	-0.182548838	-1.134887143	**CLNS1A**	protein_coding	chloride nucleotide-sensitive channel 1A [Source:HGNC Symbol;Acc:HGNC:2080]	chr11	77514936	77637805	-1	ensembl_havana	5.914371348	6.005645767	5.823096929	6.013877657	6.027261054	5.975798591	5.708636445	5.909567816	5.851086526
ENSG00000062282	0.047237	0.117562922	-0.186648641	-1.138116814	**DGAT2**	protein_coding	diacylglycerol O-acyltransferase 2 [Source:HGNC Symbol;Acc:HGNC:16940]	chr11	75759512	75801535	1	ensembl_havana	4.481922864	4.575247185	4.388598544	4.587380999	4.546699886	4.591660669	4.328527411	4.302919022	4.534349199
ENSG00000269973	0.047319	0.117704424	-0.177929063	-1.131258837	**AC010969.2**	lincRNA		chr2	9936360	9939590	1	havana	1.939781371	2.028745902	1.850816839	2.067039104	2.199112819	1.820085783	1.7752573	2.063957599	1.713235619
ENSG00000235706	0.047334	0.117719692	-0.198698276	-1.147662368	**DICER1-AS1**	antisense	DICER1 antisense RNA 1 [Source:HGNC Symbol;Acc:HGNC:43017]	chr14	95157645	95179933	1	havana	2.513861758	2.613210896	2.41451262	2.705773822	2.564016113	2.569842754	2.37188541	2.379024645	2.492627803
ENSG00000223891	0.047352	0.117744216	0.231862336	1.174349908	**OSER1-AS1**	lincRNA	OSER1 antisense RNA 1 (head to head) [Source:HGNC Symbol;Acc:HGNC:48585]	chr20	44210960	44226027	1	havana	3.827365051	3.711433883	3.943296218	3.587346304	3.909315632	3.637639713	3.906311936	3.945529874	3.978046845
ENSG00000157538	0.047368	0.117763367	0.145201958	1.105885452	**VPS26C**	protein_coding	VPS26 endosomal protein sorting factor C [Source:HGNC Symbol;Acc:HGNC:3044]	chr21	37223420	37267919	-1	ensembl_havana	5.995643945	5.923042966	6.068244924	6.020027555	5.907549015	5.841552328	6.16531053	6.022700201	6.016724041
ENSG00000154556	0.047387	0.117789549	0.491823969	1.406221608	**SORBS2**	protein_coding	sorbin and SH3 domain containing 2 [Source:HGNC Symbol;Acc:HGNC:24098]	chr4	185585444	185956652	-1	ensembl_havana	0.44580889	0.199896905	0.691720874	-0.92573229	0.271370116	1.254052895	-0.12879794	0.956893362	1.2470672
ENSG00000141026	0.047424	0.117861581	0.154034126	1.11267644	**MED9**	protein_coding	mediator complex subunit 9 [Source:HGNC Symbol;Acc:HGNC:25487]	chr17	17476986	17493226	1	ensembl_havana	3.504490081	3.427473019	3.581507144	3.387357714	3.506144797	3.388916545	3.584973459	3.596537187	3.563010788
ENSG00000103269	0.04751	0.117907252	0.601537874	1.517333141	**RHBDL1**	protein_coding	rhomboid like 1 [Source:HGNC Symbol;Acc:HGNC:10007]	chr16	675666	678268	1	ensembl_havana	0.551086794	0.250317857	0.851855731	0.41082706	-0.258437325	0.598563838	1.089724456	0.841392961	0.624449778
ENSG00000143603	0.047532	0.117907252	0.408345722	1.327163139	**KCNN3**	protein_coding	potassium calcium-activated channel subfamily N member 3 [Source:HGNC Symbol;Acc:HGNC:6292]	chr1	154697455	154870280	-1	ensembl_havana	1.99826288	1.794090019	2.202435741	1.109412078	1.7890623	2.483795679	1.486783452	2.566495926	2.554027843
ENSG00000185561	0.047525	0.117907252	0.368441711	1.290957683	**TLCD2**	protein_coding	TLC domain containing 2 [Source:HGNC Symbol;Acc:HGNC:33522]	chr17	1702790	1710438	-1	ensembl_havana	0.553804975	0.36958412	0.73802583	0.705390237	0.760296981	-0.356934859	0.785553575	1.080904991	0.347618925
ENSG00000119655	0.047504	0.117907252	0.227275531	1.17062219	**NPC2**	protein_coding	NPC intracellular cholesterol transporter 2 [Source:HGNC Symbol;Acc:HGNC:14537]	chr14	74476192	74494177	-1	ensembl_havana	5.074685713	4.961047948	5.188323479	4.933804977	5.123981705	4.825357162	5.1808999	5.177565958	5.206504578
ENSG00000020426	0.047467	0.117907252	0.184267621	1.136240019	**MNAT1**	protein_coding	MNAT1, CDK activating kinase assembly factor [Source:HGNC Symbol;Acc:HGNC:7181]	chr14	60734742	60969953	1	ensembl_havana	4.415888693	4.323754882	4.508022503	4.270330414	4.458168414	4.242765818	4.449223041	4.535932191	4.538912277
ENSG00000089916	0.047457	0.117907252	0.180355033	1.133162711	**GPATCH2L**	protein_coding	G-patch domain containing 2 like [Source:HGNC Symbol;Acc:HGNC:20210]	chr14	76151916	76254342	1	ensembl_havana	6.498814938	6.408637422	6.588992455	6.411869766	6.338786137	6.475256361	6.561120924	6.634659896	6.571196544
ENSG00000163161	0.047527	0.117907252	0.156804743	1.114815327	**ERCC3**	protein_coding	ERCC excision repair 3, TFIIH core complex helicase subunit [Source:HGNC Symbol;Acc:HGNC:3435]	chr2	127257290	127294176	-1	ensembl_havana	7.016574707	6.938172335	7.094977078	6.942976115	6.94846088	6.923080011	7.104428532	7.040271685	7.140231017
ENSG00000142188	0.047494	0.117907252	-0.167754391	-1.123308651	**TMEM50B**	protein_coding	transmembrane protein 50B [Source:HGNC Symbol;Acc:HGNC:1280]	chr21	33432485	33480011	-1	ensembl_havana	6.093719819	6.177597014	6.009842623	6.262838923	6.21096391	6.058988209	6.01937366	6.029313062	5.980841147
ENSG00000149100	0.047525	0.117907252	-0.187802281	-1.139027265	**EIF3M**	protein_coding	eukaryotic translation initiation factor 3 subunit M [Source:HGNC Symbol;Acc:HGNC:24460]	chr11	32583798	32606262	1	ensembl_havana	7.074563143	7.168464283	6.980662002	7.186608473	7.187277498	7.131506878	6.870391153	7.036720591	7.034874262
ENSG00000168374	0.047512	0.117907252	-0.260501975	-1.197895431	**ARF4**	protein_coding	ADP ribosylation factor 4 [Source:HGNC Symbol;Acc:HGNC:655]	chr3	57571363	57598220	-1	ensembl_havana	8.106771509	8.237022497	7.976520522	8.446328719	8.311337383	7.953401389	8.000545544	8.019942113	7.909073909
ENSG00000224810	0.047477	0.117907252	-0.459036986	-1.374623936	**AL355482.1**	lincRNA		chr1	182062677	182069253	-1	havana	-0.040834631	0.188683862	-0.270353124	-0.41068387	0.378171619	0.598563838	-0.75894329	0.256279753	-0.308395834
ENSG00000253106	0.047543	0.117912988	0.406181095	1.32517335	**AC090198.1**	antisense		chr8	124488510	124491643	1	havana	0.175930658	-0.02715989	0.379021206	-0.03191641	-0.218971072	0.16940781	0.540060475	0.426254992	0.17074815
ENSG00000196683	0.047567	0.117952218	0.180339761	1.133150716	**TOMM7**	protein_coding	translocase of outer mitochondrial membrane 7 [Source:HGNC Symbol;Acc:HGNC:21648]	chr7	22812628	22822852	-1	ensembl_havana	7.26668576	7.176515879	7.356855641	7.228734114	7.151728506	7.149085018	7.332308108	7.290839524	7.447419291
ENSG00000113712	0.047593	0.117995503	-0.145352485	-1.106000843	**CSNK1A1**	protein_coding	casein kinase 1 alpha 1 [Source:HGNC Symbol;Acc:HGNC:2451]	chr5	149492197	149551552	-1	ensembl_havana	6.337673225	6.410349467	6.264996982	6.490879496	6.340438209	6.399730698	6.309162859	6.215277274	6.270550815
[truncated: 2,951,326 more chars]
