# Supplementary material for: ASH2L drives proliferation and sensitivity to bleomycin and other genotoxins in Hodgkin’s lymphoma and testicular cancer cells
Source: Cell Death Dis. 2020 Nov 30;11(11):1019. doi: 10.1038/s41419-020-03231-0 (PMC7705021; doi:10.1038/s41419-020-03231-0)
Supplement: Supplementary file 4 — Table S4 [file 41419_2020_3231_MOESM4_ESM.pdf]

This table provides the sequences and their functions of the primers used for amplifying the sgRNA sequences from the genomic DNA of cells transduced with the GEKO-V2 library

Flow cell anneal sequences: sequences that bind to flow cells in Illumina sequencers
Index primer sequences: sequences that function as annealing site for the illumina sequencing primers
Stagger sequences: random sequence of different lengths. They are used to provide sequence diversity across a flow cell
Priming site sequences: these sequences anneal donwstream (FWD) and upstream (REV) of the sgRNA sequences integrated into the cell's genome via lentiviral transduction. They function as primers when amplifying the sgRNA sequences from genomic DNA of virus-transduced cells
Barcode sequences: sequences that are unique for each sample (e.g. "untreated cells"). They function as identifiers

Note: the forward primers were all mixed toghether into a FWD-primer-pool. This pool was used as FWD primer in all PCR reactions. The reverse primers (carying the barcodes) were unique to each condition. For example, the NGS-Lib-KO-Rev-1 in combination with the FWD primer pool were used to amplify the sgRNA sequences integrated in the gDNA of "Untreated-Time Zero" cells

FORWARD PRIMERS

Primer name	Flow cell
•NGS-Lib-Fwd-1	5′–AATGATACGGCGACCACCGAGATCTACACTCTTTCCCTACACGACGCTCTTCCGATCTTAAGTAGAGGCTTTATATATCTTGTGGAAAGGACGAAACACC–3′
•NGS-Lib-Fwd-2	5′–AATGATACGGCGACCACCGAGATCTACACTCTTTCCCTACACGACGCTCTTCCGATCTATCATGCTTAGCTTTATATATCTTGTGGAAAGGACGAAACACC–3′
•NGS-Lib-Fwd-3	5′–AATGATACGGCGACCACCGAGATCTACACTCTTTCCCTACACGACGCTCTTCCGATCTGATGCACATCTGCTTTATATATCTTGTGGAAAGGACGAAACACC–3′
•NGS-Lib-Fwd-4	5′–AATGATACGGCGACCACCGAGATCTACACTCTTTCCCTACACGACGCTCTTCCGATCTCGATTGCTCGACGCTTTATATATCTTGTGGAAAGGACGAAACACC–3′
•NGS-Lib-Fwd-5	5′–AATGATACGGCGACCACCGAGATCTACACTCTTTCCCTACACGACGCTCTTCCGATCTTCGATAGCAATTCGCTTTATATATCTTGTGGAAAGGACGAAACACC–3′
•NGS-Lib-Fwd-6	5′–AATGATACGGCGACCACCGAGATCTACACTCTTTCCCTACACGACGCTCTTCCGATCTATCGATAGTTGCTTGCTTTATATATCTTGTGGAAAGGACGAAACACC–3′
•NGS-Lib-Fwd-7	5′–AATGATACGGCGACCACCGAGATCTACACTCTTTCCCTACACGACGCTCTTCCGATCTGATCGATCCAGTTAGGCTTTATATATCTTGTGGAAAGGACGAAACACC–3′
•NGS-Lib-Fwd-8	5′–AATGATACGGCGACCACCGAGATCTACACTCTTTCCCTACACGACGCTCTTCCGATCTCGATCGATTTGAGCCTGCTTTATATATCTTGTGGAAAGGACGAAACACC–3′
•NGS-Lib-Fwd-9	5′–AATGATACGGCGACCACCGAGATCTACACTCTTTCCCTACACGACGCTCTTCCGATCTACGATCGATACACGATCGCTTTATATATCTTGTGGAAAGGACGAAACACC–3′
•NGS-Lib-Fwd-10	5′–AATGATACGGCGACCACCGAGATCTACACTCTTTCCCTACACGACGCTCTTCCGATCTTACGATCGATGGTCCAGAGCTTTATATATCTTGTGGAAAGGACGAAACACC–3′

REVERSE PRIMERS

Primer name	Flow cell
•NGS-Lib-KO-Rev-1	5′–CAAGCAGAAGACG[illegible]
•NGS-Lib-KO-Rev-2	5′–CAAGCAGAAGACGGCATACGAGATATAGCGTCGTGACTGGAGTTCAGACGTGTGCTCTTCCGATCTCCGACTCGGTGCCACTTTTTCAA–3′
•NGS-Lib-KO-Rev-3	5′–CAAGCAGAAGACGGCATACGAGATGAAGAAGTGTGACTGGAGTTCAGACGTGTGCTCTTCCGATCTCCGACTCGGTGCCACTTTTTCAA–3′

PCR Program

Step	*Action*	*Temp.*	*Time*
1	Initial denaturation	95 °C	300 sec
2	Denaturation	98 °C	40 sec
3	Annealing	60 °C	30 sec
4	Extension	72°C	30 sec
5	Go to step 2 25X		
6	Final extension	72°C	30 sec
7	4C	4°C	Hold
