# Supplementary material for: ASH2L drives proliferation and sensitivity to bleomycin and other genotoxins in Hodgkin’s lymphoma and testicular cancer cells
Source: Cell Death Dis. 2020 Nov 30;11(11):1019. doi: 10.1038/s41419-020-03231-0 (PMC7705021; doi:10.1038/s41419-020-03231-0)
Supplement: Supplementary file 5 — Supplementary figure legends [file 41419_2020_3231_MOESM5_ESM.docx]

**Legends to supplementary figures**

**Figure S1. CRISPR/Cas9 whole genome knockout screen results in L1236 cells, and SH2L knockdown effect on cell cycle.**

**A.** Graph showing the prevalence of individual sgRNAs targeting ASH2L in DNA extracted from untreated (x axis) and bleomycin treated cells (y axis). **B.** Graph showing the prevalence of individual sgRNAs targeting ASH2L in DNA extracted from cells harvested at “time zero” (x axis) and cells left to grow untreated for 10 days (y axis). **C.** MaGeCK-based gene depletion analysis of the CRISPR/Cas9 screen results highlighting in color the top 5 depleted genes after 10 days of growth in normal medium (the analysis compared the sgRNA expression between cells harvested at “time zero” and cells left to grow untreated for 10 days). **D**. Left, representative flow cytometry cell cycle profile of L1236 cells and the gating strategy used for distinguishing the various phases of the cell cycle. Quantitation of cell cycle phases of L1236 control and ASH2L knockdown cells left untreated (middle panel) or incubated with 4 µg/ml bleomycin for 24 hours (right panel).

**Figure S2. The impact of ASH2L knockdown on closed chromatin markers, localization of endogenous ASH2L at DSBs, and effect of ASH2L ectopic expression on genotoxic stress response.**

**A-B.** Western blot depicting shRNA-mediated knockdown of ASH2L and the corresponding levels of H3K9me3 (panel A) and H3K27me3 (panel B) in L1236 cells. **C.** Wild-type U2OS cells were laser-micro-irradiated and then immuno-stained with the indicated antibodies. DAPI was used to stain the nuclei. Three examples are shown. The white arrowheads mark the boundaries of the irradiated stripes in the nuclei. Scale bars: 10 µm. **D.** Top, the effects of lentivirus-mediated ASH2L expression, compared to empty virus (E.V.) transduced L1236 cells, challenged for 72 hours with 4 µg/ml bleomycin, 4 µM etoposide, or left untreated. Bottom, western blot showing the levels of ASH2L expression and corresponding H3K4me3 levels. **E.** Top, NT2D1 cells transiently transfected with either pcDNA3 empty vector (E.V.) or pcDNA3-ASH2L, challenged for 72 hours with 4 µg/ml bleomycin, or left untreated. Bottom, western blot depicting the levels of ASH2L in these cells.

**Figure S3. Transient nature of KDM5A ectopic expression**

HEK293T cells were transfected with an empty pcDNA3 vector (-) or with a pcDNA3 plasmid encoding a FLAG-tagged version of the KDM5A protein (+). The cells were lysed at the indicated post-transfection times and the lysates analyzed by western blotting using the indicated antibodies.

**Figure S4. ATM inhibitor functionality test**

L1236 control (shCtrl) and ASH2L-depleted (shASH2L) cells were pre-incubated for one hour with 2.5 µM of the KU-060019 ATM inhibitor (ATMi) before the addition, or not, of bleomycin (2 µg/ml; Bleo) for 2 more hours (still in the presence of KU-060019). The cells were then lysed and the lysates analyzed by western blotting using the indicated antibodies. Phosphorylation of ATM at position S1918 is indicative of an active kinase. The asterisk points to a non-specific band.

**Figure S5. Comparison of ASH2L levels between the NT2D1 and L1236 cell lines**

Left, representative western blot of ASH2L levels in NT2D1 and L1236 cells. Right, quantitation of ASH2L protein levels, from 3 independent western blot experiments. Statistical analysis, unpaired t-test.

**Figure S6. Schematic representation of the impact of ASH2L decrease on DNA repair**

ASH2L is a core component of the H3K4 methylation complex involved in the addition of the third methyl on lysine 4 of histone 3 (H3K4). The other core components of the complex are RbBP5, WDR5, and DPY-30. There are six H3K4 methylation complexes that are defined by the catalytic subunit they express (Set1A, Set1B, MLL1, MLL2, MLL3, or MLL4). The trimethylation of H3K4 reduces the cell’s capacity to repair its DNA upon genotoxin-induced damage ^1^. In contrast, ASH2L silencing and the corresponding decrease in H3K4 trimethylation favors the ability of the cells to repair their damaged DNA (this study).

^1^ Bayo, J. et al. Jumonji inhibitors overcome radioresistance in cancer through changes in H3K4 methylation at double-strand breaks. Cell Reports 25, 1040-1050.e1045 (2018).
