# Supplementary figures and images for: ASH2L drives proliferation and sensitivity to bleomycin and other genotoxins in Hodgkin’s lymphoma and testicular cancer cells
Source: Cell Death Dis. 2020 Nov 30;11(11):1019. doi: 10.1038/s41419-020-03231-0 (PMC7705021; doi:10.1038/s41419-020-03231-0)

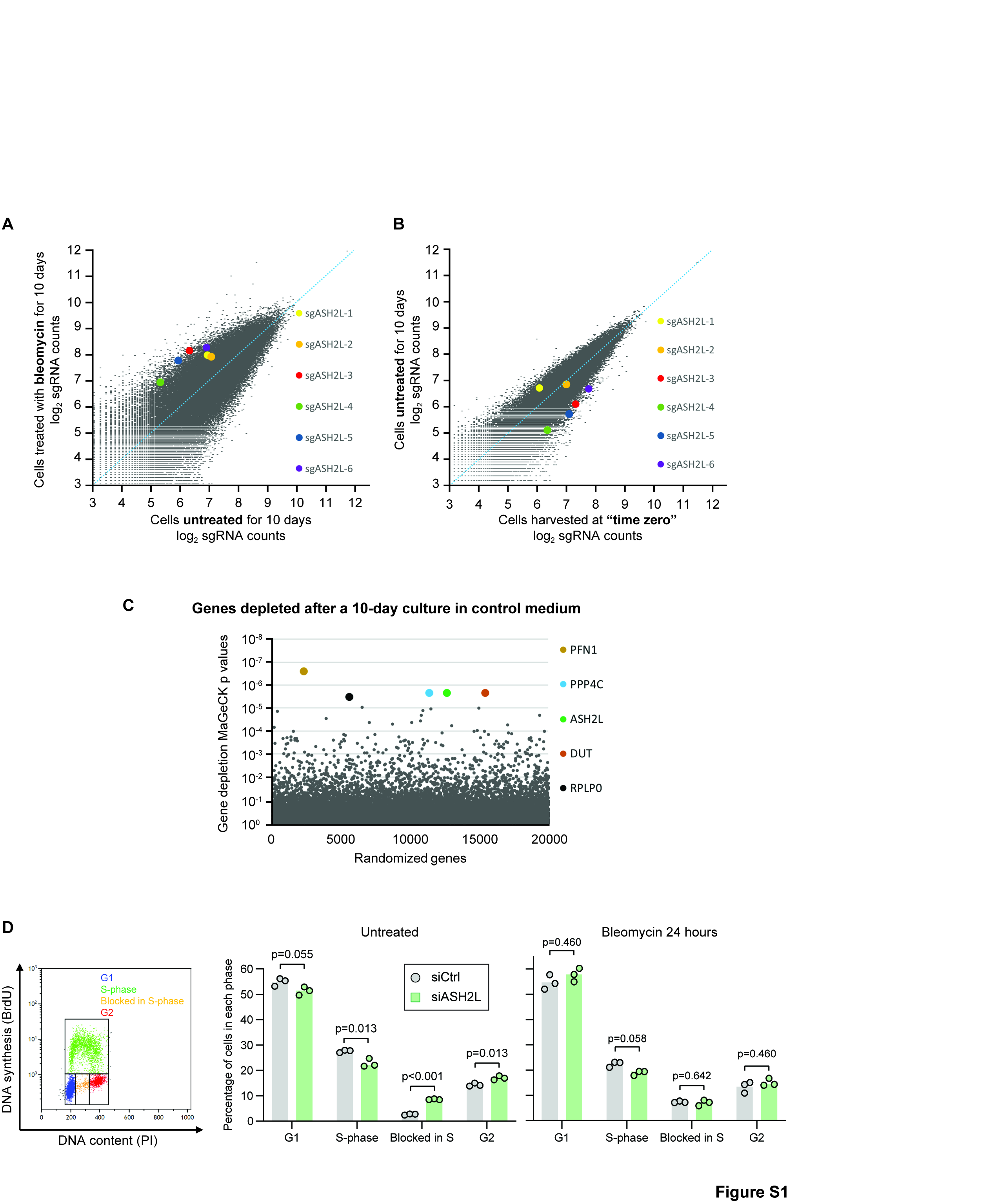

Supplement: Supplementary file 6 — Figure S1 [file 41419_2020_3231_MOESM6_ESM.tif]

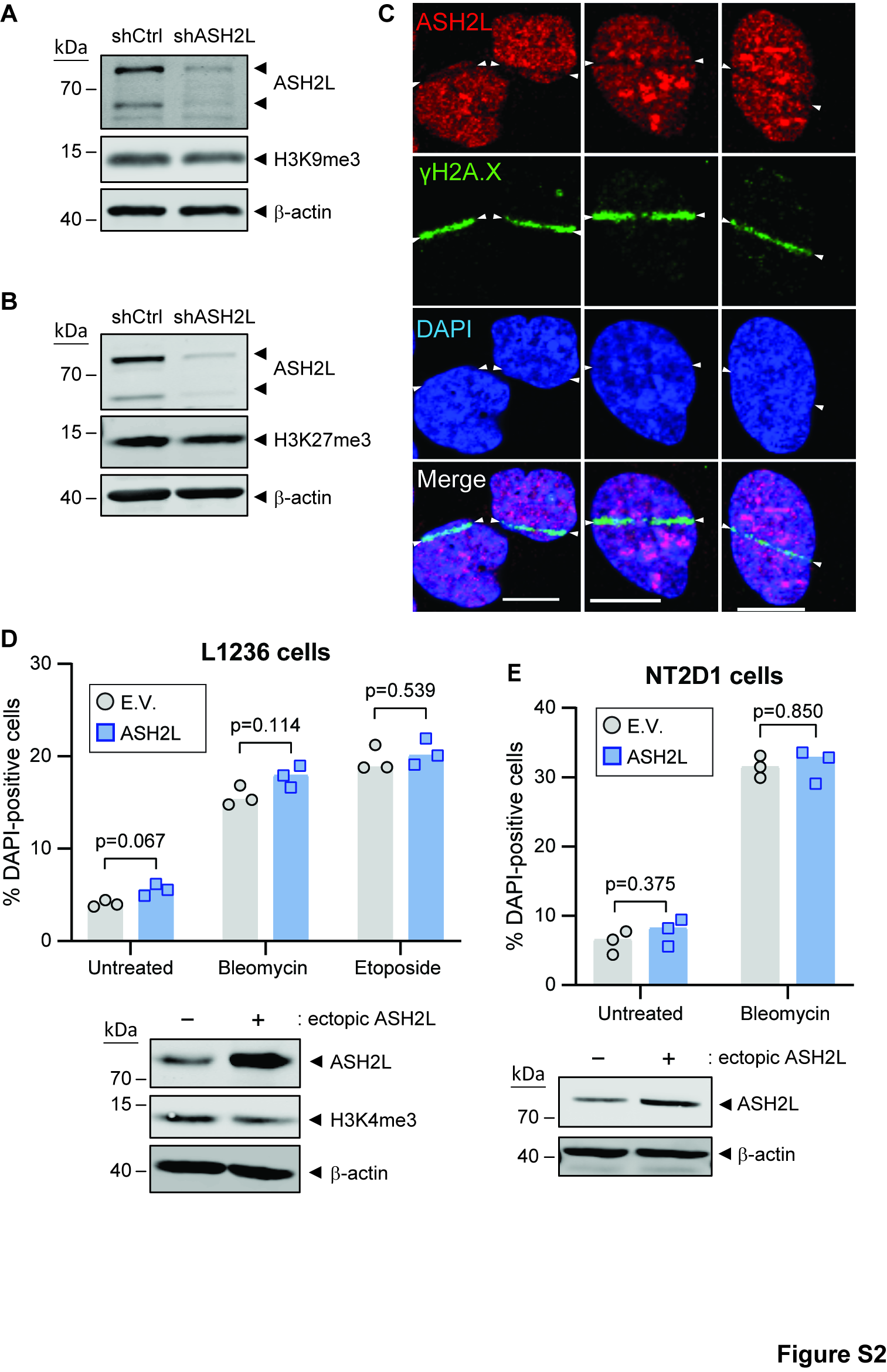

Supplement: Supplementary file 7 — Figure S2 [file 41419_2020_3231_MOESM7_ESM.tif]

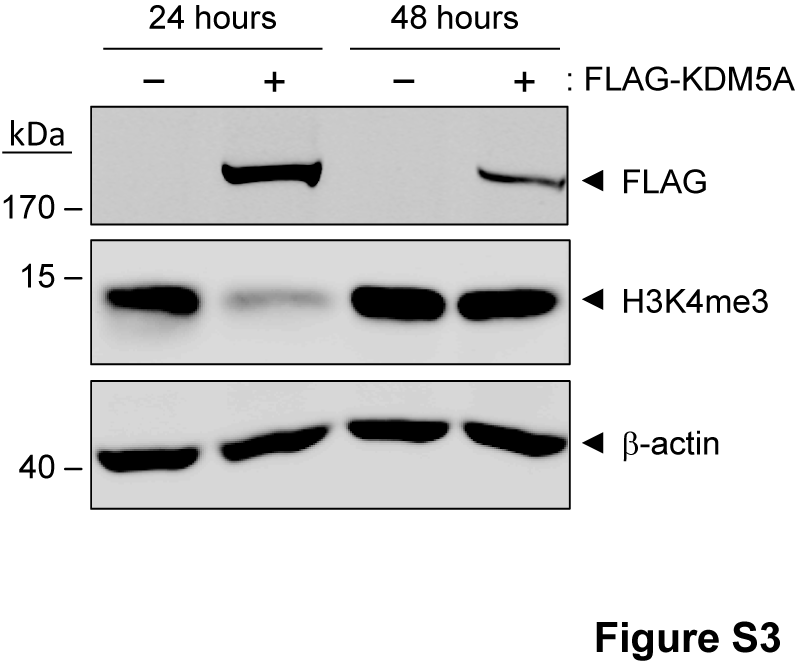

Supplement: Supplementary file 8 — Figure S3 [file 41419_2020_3231_MOESM8_ESM.tif]

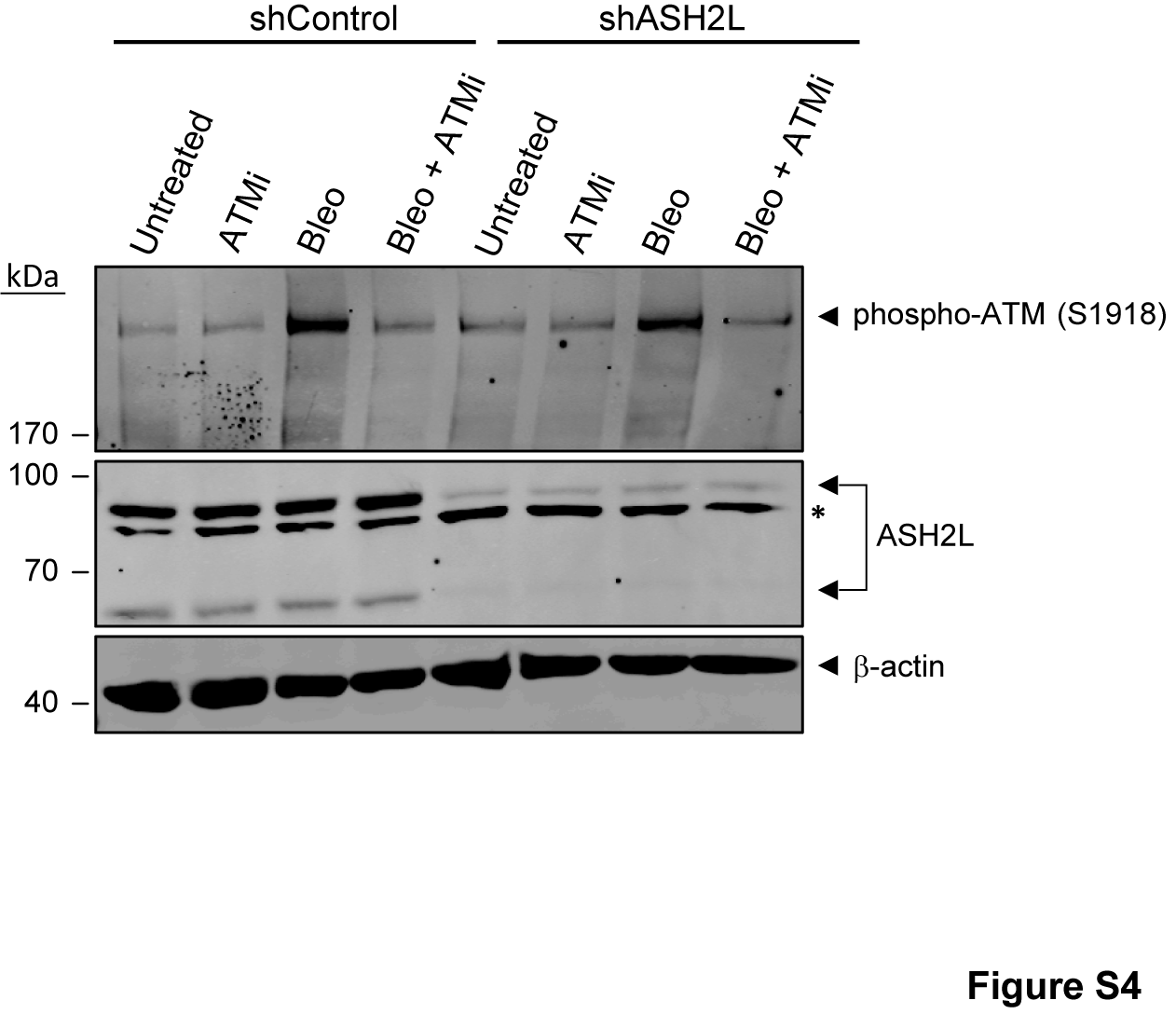

Supplement: Supplementary file 9 — Figure S4 [file 41419_2020_3231_MOESM9_ESM.tif]

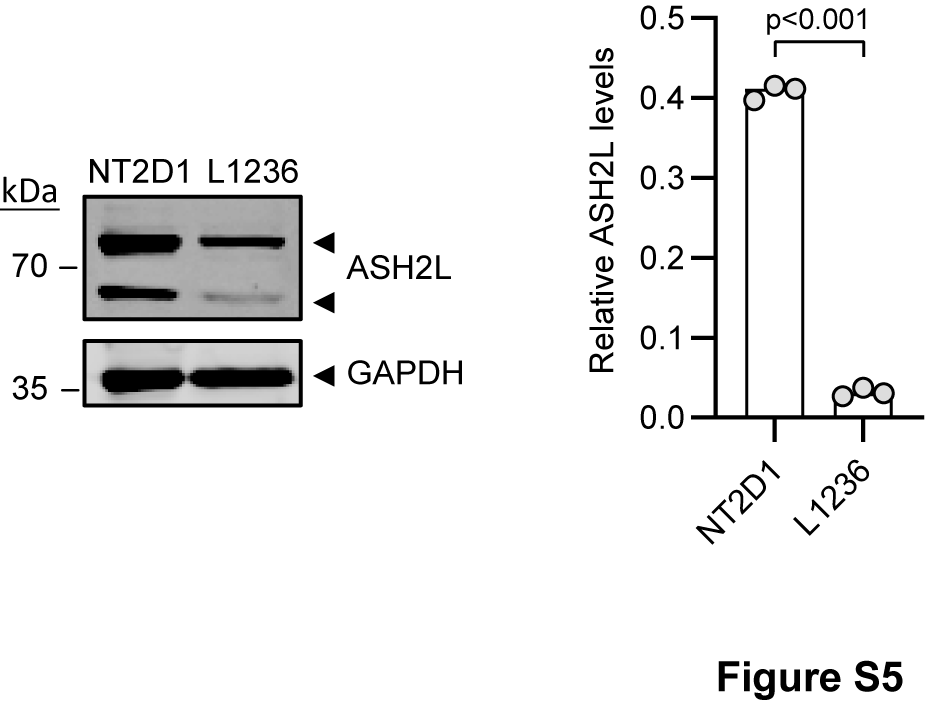

Supplement: Supplementary file 10 — Figure S5 [file 41419_2020_3231_MOESM10_ESM.tif]

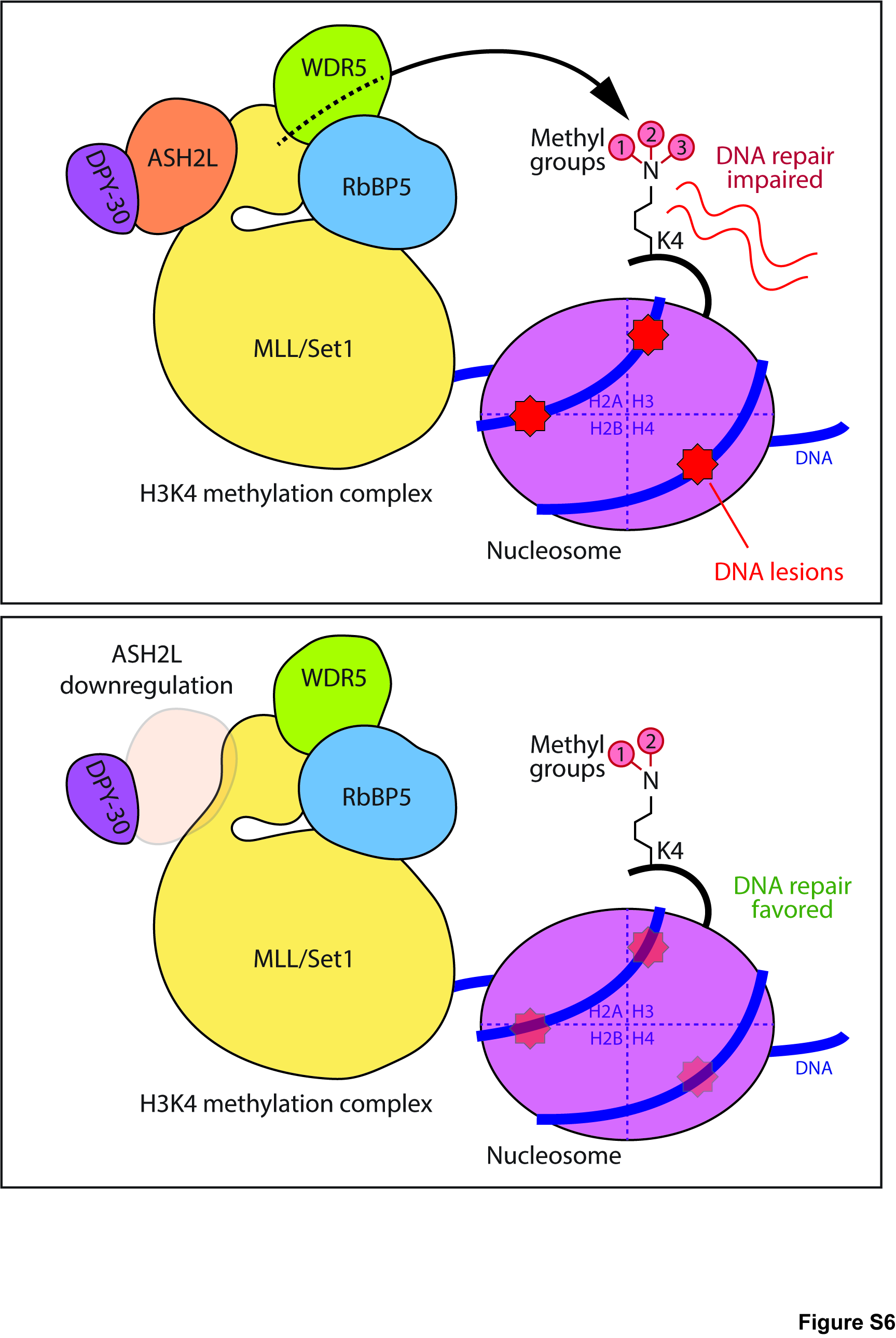

Supplement: Supplementary file 11 — Figure S6 [file 41419_2020_3231_MOESM11_ESM.tif]
